# Collaborative intelligence in AI: Evaluating the performance of a council of AIs on the USMLE

**DOI:** 10.1371/journal.pdig.0000787

**Published:** 2025-10-09

**Authors:** Yahya Shaikh, Zainab Asiyah Jeelani-Shaikh, Muzamillah Mushtaq Jeelani, Aamir Javaid, Tauhid Mahmud, Shiv Gaglani, Michael Christopher Gibbons, Minahil Cheema, Amanda Cross, Denisa Livingston, Morgan Cheatham, Elahe Nezami, Ronald Dixon, Ashwini Niranjan-Azadi, Saad Zafar, Zishan Siddiqui

**Affiliations:** 1 Department of Health Policy & Management, Johns Hopkins Bloomberg School of Public Health, Baltimore, Maryland, United States of America,; 2 Independent Researcher, Baltimore, Maryland, United States of America; 3 International Institute of Islamic Thought and Civilization, International Islamic University of Malaysia, Gombak, Selangor, Malaysia; 4 Division of Cardiology, University of California San Francisco, San Francisco, California, United States of America; 5 Department of Biomedical Informatics, Stony Brook University Renaissance School of Medicine, Stony Brook, New York, United States of America; 6 Ganglia Ventures, LLC, Melbourne Beach, Florida, United States of America; 7 The Greystone Group Inc, Greenbelt, Maryland, United States of America; 8 University of Maryland School of Medicine, Baltimore, Maryland, United States of America; 9 Wahayenta Consulting LLC, Portland, Oregon, United States of America; 10 Multi-Epistemic Researcher, Diné Community Advocacy Alliance, Navajo Nation, United States of America; 11 Division of Genetics and Genomics, Department of Pediatrics, Boston Children’s Hospital, Harvard Medical School, Boston, Massachusetts, United States of America; 12 Public Health Sciences, University of Miami, Florida, United States of America; 13 CareHive Health Inc, Austin, Texas, United States of America; 14 Johns Hopkins School of Medicine, Department of Medicine, Baltimore, Maryland, United States of America; 15 Riphah Institute of Systems Engineering, Riphah International University, Islamabad, Pakistan; 16 Johns Hopkins School of Medicine, Baltimore, Maryland, United States of America; Instituto Politécnico Nacional Escuela Superior de Medicina: Instituto Politecnico Nacional Escuela Superior de Medicina, MEXICO

## Abstract

The stochastic nature of next-token generation and resulting response variability in Large Language Models (LLMs) outputs pose challenges in ensuring consistency and accuracy on knowledge assessments. This study introduces a novel multi-agent framework, referred to as a “Council of AIs”, to enhance LLM performance through collaborative decision-making. The Council consists of multiple GPT-4 instances that iteratively discuss and reach consensus on answers facilitated by a designated “Facilitator AI.” This methodology was applied to 325 United States Medical Licensing Exam (USMLE) questions across all three exam stages: Step 1, focusing on biomedical sciences; Step 2 evaluating clinical knowledge (CK)\; and Step 3, evaluating readiness for independent medical practice. The Council achieved consensus that were correct 97%, 93%, and 94% of the time for Step 1, Step 2 CK, and Step 3, respectively, outperforming single-instance GPT-4 models. In cases where there wasn’t an initial unanimous response, the Council deliberations achieved a consensus that was the correct answer 83% of the time, with the Council correcting over half (53%) of the responses that majority vote had gotten incorrect. The odds of a majority voting response changing from incorrect to correct were 5 (95% CI: 1.1, 22.8) times higher than the odds of changing from correct to incorrect after discussion. This study provides the first evidence that the semantic entropy of the response space can consistently be reduced to zero—demonstrated here through Council deliberation, and suggesting the possibility of other mechanisms to achieve the same outcome.. This study revealed that in a Council model, response variability, often considered a limitation, can be transformed into a strength that supports adaptive reasoning and collaborative refinement of answers. These findings suggest new paradigms for AI implementation and reveal the heightened strength that emerges when AIs begin to collaborate as a collective rather than operate alone.

## Author summary

In our study, we explored how collaboration among multiple artificial intelligence (AI) systems could improve accuracy on medical licensing exams. While individual AI models like GPT-4 often produce varying answers to the same question, a phenomenon or limitation known as “response variability”, we designed a “Council of AIs” to turn this variability into a strength. The Council consists of several AI models working together, discussing their answers through an iterative process until they reach consensus. When tested on 325 medical exam questions, the Council achieved 97%, 93%, and 94% respectively on the Step 1 (basic biomedical sciences), Step 2 clinical knowledge (CK) (diagnosis and clinical management), and Step 3 (readiness for independent practice). This improvement was most notable when answers required debate: in cases where initial responses disagreed, the collaborative process corrected errors 83% of the time. Our findings suggest that collective decision-making even among AIs can enhance accuracy and AI collaboration can potentially lead to more trustworthy tools for healthcare, where accuracy is critical. By demonstrating that diverse AI perspectives can refine answers, we challenge the notion that consistency alone defines a “good” AI. Instead, embracing variability through teamwork might unlock new possibilities for AI in medicine and beyond. This approach could inspire future systems where AIs and humans collaborate (e.g., on Councils with both humans and AIs), combining strengths to solve complex problems. While technical challenges remain, our work highlights a promising path toward more robust, adaptable AI solutions.

## Introduction

Since the release of OpenAI’s Generative Pretrained Transformer 3.5 (GPT 3.5) in December 2022, many studies have evaluated the performance of Large Language Models (LLMs) on medical knowledge and licensing exams, [1–21] While performance has improved across GPT model updates, varying performance has been noted when the same question is asked to a LLM multiple times. [22,23] This is due to the probabilistic token-by-token generation of LLM content, which can generate a variety of responses to the same question, some of which are incorrect or ‘hallucinations’.[24] Response variability represents the presence of multiple linguistic, though not necessarily factual, reasoning paths for a given question. This suggests the possibility that intersecting multiple reasoning paths may result in re-shaping of each other’s reasoning. And it raises the question of how well an artificial intelligence collective of intersecting reasoning paths might perform on medical knowledge and licensing exams.

In this study, we developed a method to create a Council of AI agents (a multi-agent Council, or ensemble of AI models) using instances of OpenAI’s Generative Pretrained Transformer 4 (GPT4) and evaluate the Council’s performance on the United States Medical Licensing Exams (USMLE).

## Methods

### Sampling the response space and adjudicating diverse responses

OpenAI’s GPT-4 was selected as the base LLM given its accessibility, support of Application Programming Interfaces (APIs), extensive documentation and community of support. Our goal was to use a ‘Council’ approach where each LLM instance is a member of the Council, and diverse responses from each LLM instance would undergo a coordinated and iterative exchanges designed to arrive at a consensus response among the models (hitherto referred as “deliberation” or “deliberative process”). To facilitate a deliberative process when there are divergent responses, a Facilitator algorithm (which includes instantiations of the LLM) summarizes the reasoning in each response, formulates a question to help the Council deliberate divergent reasonings, and presents the summary and question to the Council with a request to re-answer the original test question.

**Fig 1:**
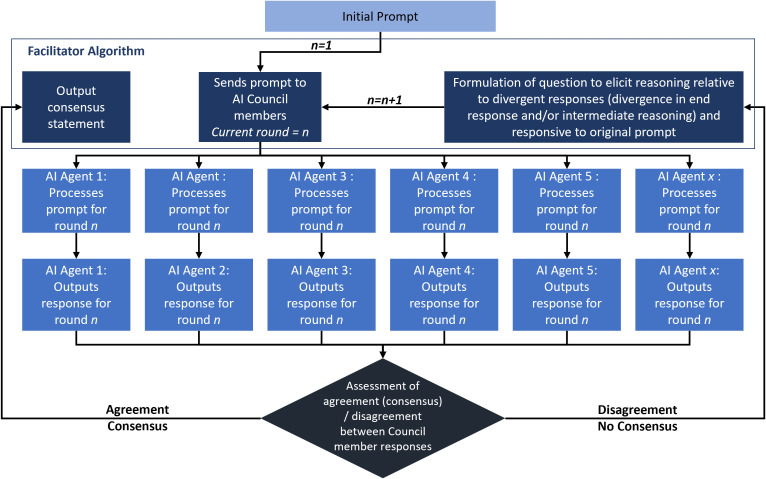
Council of AI Agents Architecture. The User (Human) presents a query, which is passed by the Facilitator algorithm to each of the Council members, whose response is then assessed for agreement. If there is agreement on the answer, a consensus is reached and a consensus statement is output summarizing explanations and identifying the selected response. If there are differing opinions about the correct response, then the Facilitator algorithm summarizes the reasoning presented by each council member, formulates a question that can help clarify reasoning behind difference of opinions, and asks the council members to also respond to the initial prompt (i.e., the USMLE question).

The process for the Council’s discussions is summarized below and in Fig 1:

1. The multiple-choice question is copy and pasted into an interface.
2. The question is transmitted to a facilitating algorithm. The facilitating algorithm sends the same question to LLM-communicating functions that represent unique instantiations of the LLM (i.e., unique AI agents), thereby eliciting a response from each LLM instance/ AI agent.
3. Each LLM-communicating function communicates uniquely with the LLM through an API.
4. Each instantiation of the LLM sends a response back, generated from its sampling of the response space.
5. Once all the responses are received, they are passed to the Facilitator algorithm, which includes an instantiation of the LLM which assesses Council member response for agreement.
6. If there is no consensus in the responses received, then the Facilitator algorthm does the following: [1] summarizes the responses from the various AI Agents, [2] formulates questions to elicit reasoning relating to the divergent responses, and [3] requests a response to the original question.
7. The output of the Facilitator algorithm is a prompt, which includes a summary of each AI agent’s response and reasoning, a clarifying question relating to differences in response selection, and the initial USMLE question along with multiple choices from which the LLM is required to make a selection.

Steps 2–7 are iterated until a consensus response is reached amongst the various instantiations of the LLM. Once a consensus is reached, the transcript of the Council’s deliberations is saved, the browser is refreshed to clear user interface elements (e.g., question input box and deliberation transcript), and the server is reset to ensure any stateful variables (such as round counters) are reinitialized before the next interaction.

For a question requiring deliberation, Table 1 provides a specific example of the Facilitator algorithm’s summarization of Council members’ responses, formulation of question to elicit reasoning related to divergent responses, and a request to respond to the original question. Table 1 does not include the details of the responses from Council members, which can be found as question 25–1 in S2 (S2 Data Raw data files - transcripts of deliberations) as part of transcripts generated by the deliberations conducted by the Council for each question. For details of the API prompts, please refer to the supplemental methods (S1 File details on methods), which includes prompts for each AI agent, analyzing responses, re-prompting from the Facilitator algorithm to the Council if there is disagreement, and synthesizing responses when there is agreement. All LLMs instances were instantiated with a temperature setting of 1. Code used in this paper is publicly available at: https://github.com/councilofai/project-saru.

**Table 1:** Example of Facilitator AI’s Question to Council for Discussion in Case of a Divergent Response.

**Original Test Question**	A healthy 30-year-old woman participates in a study of the relationship between cardiovascular function and posture. Placed in a room with a comfortable temperature (72°F) and humidity (40%), she moves from a standing to a supine position. Which of the following sets of changes is most likely in the reflex control of this woman’s cardiovascular system? (A) Carotid Sinus Baroreceptor Activity, Atrial Stretch Receptor Activity, and Vagal Efferent Activity to the Heart increase (B) Carotid Sinus Baroreceptor Activity and Atrial Stretch Receptor Activity increase, while Vagal Efferent Activity to the Heart decrease (C) Carotid Sinus Baroreceptor Activity and Vagal Efferent Activity to the Heart increase, while Atrial Stretch Receptor Activity decrease (D) Carotid Sinus Baroreceptor Activity increase, while Atrial Stretch Receptor Activity and Vagal Efferent Activity to the Heart decrease (E) Carotid Sinus Baroreceptor Activity decrease, while Atrial Stretch Receptor Activity and Vagal Efferent Activity to the Heart increase (F) Carotid Sinus Baroreceptor Activity and Vagal Efferent Activity to the Heart decrease, while Atrial Stretch Receptor Activity increase (G) Carotid Sinus Baroreceptor Activity and Atrial Stretch Receptor Activity decrease, while Vagal Efferent Activity to the Heart increase (H) Carotid Sinus Baroreceptor Activity, Atrial Stretch Receptor Activity, and Vagal Efferent Activity to the Heart decrease
**Facilitator’s Prompt to Council for Discussion of Divergent Responses**	AI_A, AI_B, and AI_C all agree that transitioning from a standing to a supine position would result in an increase in carotid sinus baroreceptor activity, atrial stretch receptor activity, and vagal efferent activity to the heart. They argue that the increase in blood volume and pressure due to the change in position would stimulate the carotid sinus baroreceptors and atrial stretch receptors. This would then trigger an increase in vagal efferent activity to slow the heart rate and maintain blood pressure. AI_D, however, suggests that while atrial stretch receptor activity and vagal efferent activity would increase, carotid sinus baroreceptor activity could potentially remain stable or decrease if blood pressure is successfully maintained by these changes. AI_E proposes a similar argument to AI_D, suggesting that carotid sinus baroreceptor activity would decrease due to less demand for compensation in blood pressure, while atrial stretch receptor activity and vagal efferent activity would increase. Key Differences: The key difference in perspectives lies in the expected change in carotid sinus baroreceptor activity. AI_A, AI_B, and AI_C predict an increase due to the rise in blood pressure from the positional change. In contrast, AI_D and AI_E predict a decrease or stability in carotid sinus baroreceptor activity, arguing that the body’s successful maintenance of blood pressure would reduce the need for these baroreceptors to compensate. Question to Clarify Disagreements: Can the body’s maintenance of blood pressure during a transition from a standing to a supine position potentially reduce the need for carotid sinus baroreceptor activity, or would the increase in blood pressure inherently stimulate these baroreceptors regardless of the body’s compensatory mechanisms? Based on your preceding response, how would you answer the original question?

This table demonstrates how the Facilitator AI synthesizes divergent responses from Council members and formulates clarifying questions to guide deliberation. When Council members provide different answers to a USMLE question, the Facilitator AI summarizes each member’s reasoning, identifies key differences in their perspectives, and poses a targeted question to help resolve the disagreement.

### Constructing the API prompt

To create a context to facilitate the emergence of responses related to the medical domain, the following instruction was added to prompts presented to the LLM:

You get your knowledge from the following domain spaces: anatomy, physiology, pathology, pathogenesis, pathophysiology, histology, pharmacology, microbiology, endocrinology, immunology, hematology, oncology, genetics, embryology, internal medicine, surgery, pediatrics, obstetrics, gynecology, psychiatry, family medicine.

For ordering an explanation in a way that can simulate reasoning, we specified the construction of content that included scientific problem solving and clinical reasoning: [25–27]

Your sentence completion follows the following pattern: [1] RESTATING THE QUESTION: Restating the question in your own words and defining technical terms and concepts; [2] PLANNING: Identifying assumptions, generating alternate assumption by questioning each assumption, and then evaluating and hypothesize which assumption should be clinically prioritized to pursue; [3] CLINICAL REASONING: Connect each sentence to its subsequent sentence using a clinical thought process to provide a step-by-step explanation to support the alternate assumption; [4] CONCLUDING: Use a step-by-step explanation that connects one sentence to the next with a clinical thought process to generate your final response

The final prompt identifies Council members, focuses their attention on a domain knowledge and reasoning, and ensures that subsequent responses in the discussion are on topic. For each of the members of the AI Council the temperature setting was set at 1 and the top_p (nucleus sampling) set to 1. The JSON used to specify these parameters was input to as the “system message” in the API call, and is detailed below for AI Council member named AI_A (“thisModel” refers to the language model, thisTemp refers to the temperature parameters, thisTopP refers to the top_p parameter, and “thisPrompt” refers to the prompt that initiates the discussion or subsequent prompt that maintains the discussion through facilitator input):

AI_A:{thisModel:“gpt-4”, thisTemp:1, thisTopP:1, thisSysMsg:”Your ‘name’ is ‘AI_A’. You are part of a council that includes ‘AI_B’, ‘AI_C’, ‘AI_D’, ‘AI_E’, and ‘AI_F’. You get your knowledge from the following domain spaces: anatomy, physiology, pathology, pathogenesis, pathophysiology, histology, pharmacology, microbiology, endocrinology, immunology, hematology, oncology, genetics, embryology, internal medicine, surgery, pediatrics, obstetrics, gynecology, psychiatry, family medicine. Your sentence completion follows the following pattern: [1] RESTATING THE QUESTION: Restating the question in your own words and defining technical terms and concepts; [2] PLANNING: Identifying assumptions, generating alternate assumption by questioning each assumption, and then evaluating and hypothesize which assumption should be clinically prioritized to pursue; [3] CLINICAL REASONING: Connect each sentence to its subsequent sentence using a clinical thought process to provide a step-by-step explanation to support the alternate assumption; [4] CONCLUDING: Use a step-by-step explanation that connects one sentence to the next with a clinical thought process to generate your final response; [5] RESPONSE: You always try to connect your response to answer the following: [“+thisPrompt+”]”}

The code for implementing the above is available in the public domain: https://github.com/councilofai/project-saru.

### Selecting the number of council members

There are several technical constraints that determine the number of instantiated AI agents. In the implemented architecture, the LLM’s synthesis of responses from the members of the Council means that all of their responses are passed together within a single API call. The size of the content passed in a single API call is constrained by token limits of a given LLM. Another constraint is the time taken to respond to each call increases linearly by the number of API calls that are made. The latency time between call and response may be quite large for models like GPT-4. Cost is an additional constraint that increases linearly with the number of instantiated AI entities. We found that generating five AI agents as part of the Council allowed us to stay within token limits during Council deliberations. The study was conducted on a standard workstation with internet access, without local inference, relying solely on cloud-based inference from OpenAI.

### USMLE questions

The USMLE questions used in this study have previously been used in single-AI evaluations of OpenAI’s GPT LLM, [2,4] initially sourced from the June 2022 release of the sample exam by the Federation of State Medical Boards and the National Board of Medical Examiners.[28] The ChatGPT-4 model used in this study was released in March 2023 and was trained on web data available up until September 2021.[29] Because the training cut-off for GPT-4 version used (September, 2021) was earlier than the release of the sample exam questions (June, 2022), it ensured that the multiple-choice questions used to assess the Council of AI were not previously seen in the training data of the underlying LLM. From the original 376 questions that were made publicly available, 51 questions containing images or tables were removed from the questions bank, leaving 325 multiple choice questions (Step 1: 94, Step 2CK: 109, Step 3: 122). No sample size calculations were conducted, as the study included the entire set of publicly available questions without images or tables. These questions had been used in prior studies evaluating LLM performance on the USMLE, allowing for direct comparison of results.

### Analysis of AI council performance

We used the USMLE provided answer keys to assess the accuracy of the individual Council members’ initial responses and the Council’s final consensus responses. We also evaluated the relationship between the number of incorrect initial responses and the accuracy of the consensus responses, and the mean number of rounds needed to reach a correct consensus. We calculated semantic entropy of the response space as suggested by Farquhar et al, which measures the uncertainty in a model’s output by evaluating the diversity of meanings among its possible responses.[30] A higher semantic entropy indicates greater uncertainty, suggesting a wider range of potential meanings in the model’s response space. Because we did not have direct access to a model’s output probabilities, we approximated semantic entropy by calculating discrete semantic entropy. To calculate discrete semantic entropy, we identified multiple or diverging responses by identifying the multiple-choice selection of each AI Council member. Multiple responses to a single question are grouped into clusters based on whether they identified the same answer selection as correct, even if the reasoning they offered to reach that response may differ. Semantic clustering groups together outputs that express the same essential idea, which, in the case of multiple-choice questions, is given by the selection of the same multiple-choice response. Discrete semantic entropy method treats each generated response equally, assuming a uniform distribution across all responses. This approach constructs an empirical probability distribution over the semantic clusters by simply counting how many of the responses fall into each cluster and dividing by the total number of varying responses produced. Once this distribution is determined, the Shannon entropy formula was applied to calculate the semantic entropy: [30]


$$H=\sum {\mathrm{\backslash nolimits}}_{i=\text{1}}^{N}p_{i}\;\text{log}\;p_{i\;}$$

where 𝑁 is the total number of clusters, and 𝑝_𝑖_ is the probability of the 𝑖-th cluster.

The resulting entropy value reflects the semantic diversity of the responses. A low entropy score indicates that most responses were semantically similar, suggesting confidence or consistency in the model’s answer. On the other hand, a higher score indicates greater semantic variability, which can be a sign of model uncertainty or hallucination.

We calculated semantic entropy at the end of each round to assess if and how it changes over rounds, and if there is a relationship between number of rounds to reach consensus (semantic entropy of 0) and magnitude of semantic entropy at the end of the initial round.

Because a consensus in round 1 of the Council’s responses indicate an unanimous majority response, we further analyzed the effectiveness of group discussion compared to majority voting for questions where the majority response was not initially unanimous. For each question that was not initially unanimous, we conducted a contingency analysis for the correctness of the majority vote at the end of round 1 versus the post-discussion consensus. For these paired observations, we calculated the matched pairs odds ratio and utilized the McNemar’s test with continuity correction to assess the significance of changes in correctness before and after discussion.

We conducted a preliminary analysis of question characteristics between USMLE questions that resulted in correct versus incorrect final consensus after multi-round AI deliberation. Variables examined include: question word count, number of options in multiple choice, diversity of initial responses (i.e., number of differing responses given in the first round of deliberation, and number of rounds needed to reach consensus. Questions were also disaggregated by USMLE step, medical specialty category of the questions (e.g., cardiology, endocrinology), and question type (e.g., pathophysiology, diagnosis). Independent t-tests were used to compare continuous variables, and chi-square tests for categorical variables.

## Results

The Council’s consensus response was correct 97%, 93%, and 94% of the time on Step 1, Step 2-CK and Step 3, respectively. In examining initial responses to questions (i.e., before any rounds of discussion), all council members provided an initial response that was correct on 79%, 78%, and 77%, respectively. (Table 2) 22% of all questions asked (21% of Step 1, 22% of Step 2CK, 23% of Step 3) required discussion because at least one instance of the LLM suggested an incorrect response. (Table 2)

**Table 2:** Council of AI Performance Overview.

	Step 1		Step 2		Step 3		All Questions	
	N	%	N	%	N	%	N	%
Total Number of Questions	94		109		122		325	
**Final Consensus Response Accuracy**								
Correct	91	96.8	101	92.7	116	94.3	308	94.7
Incorrect	3	3.2	8	7.3	6	5.7	17	5.2
**Members’ Initial Response Accuracy**								
All Council members answer correctly	74	78.7	85	78.0	94	77.0	254	78.2
Majority Vote Correct	88	93.6	97	90.0	113	92.6	298	91.7
At least 1 Council member answered incorrectly (initial round)	20	21.3	24	22.0	28	23.0	72	21.8
All Council members answer incorrectly (initial round)	1	5.0	4	16.7	5	17.9	10	13.9
All Council members unanimously agreed on incorrect response (initial round)	1	5.0	3	12.5	3	10.7	7	9.7

Table showing performance metrics of the Council’s final consensus accuracy and summarized member accuracy across all three USMLE exam steps. The Council achieved 94.7% overall accuracy (308/325 questions correct) compared to 78.2% accuracy when all members agreed initially. Approximately 22% of questions required deliberation due to at least one incorrect initial response. The Council’s deliberative process improved accuracy beyond simple majority voting across all exam steps.

Compared to a simple majority voting approach (i.e., a correct response given by majority of Council members in the first round), the Council performed better overall (95% for Council vs 91% for majority vote) and for each Step (97% vs 93%; 94% vs 90%; 94% vs 93% for Council vs simple majority for Step 1, 2-CK, 3 respectively) (Table 2). Transcripts of Council deliberations for each question are provided within the supplemental materials (S1 File and S2 File). In a contingency analysis comparing the accuracy of the initial majority vote in Round 1 with the Council’s final consensus (Table 3), the Council rarely reverted from a correct initial majority to an incorrect consensus (occurring in only one instance). The odds of a majority voting response changing from incorrect to correct were 5 (95% CI: 1.1,22.8) times higher than the odds of changing from correct to incorrect after discussion.

**Table 3:** Accuracy of majority response versus council’s consensus response.

		Consensus after deliberation			
		Correct	Incorrect	OR (95% CI)	p-value*
**Majority vote at the end of round 1**	**Correct**	44	2	Ref	<0.05
	**Incorrect**	10	9	5.0 (1.1, 22.8)	

*McNemar’s test with continuity correction.

Table of statistical comparison of initial majority vote accuracy versus final consensus accuracy after deliberation. Among 65 questions requiring deliberation, the Council improved accuracy significantly (p < 0.05). When the initial majority was wrong (19 cases), deliberation corrected 10 responses (53%). The odds of changing from incorrect to correct were 5 times higher than changing from correct to incorrect (OR: 5.0, 95% CI: 1.1-22.8), demonstrating the value of structured discussion over simple voting.

**Fig 2:**
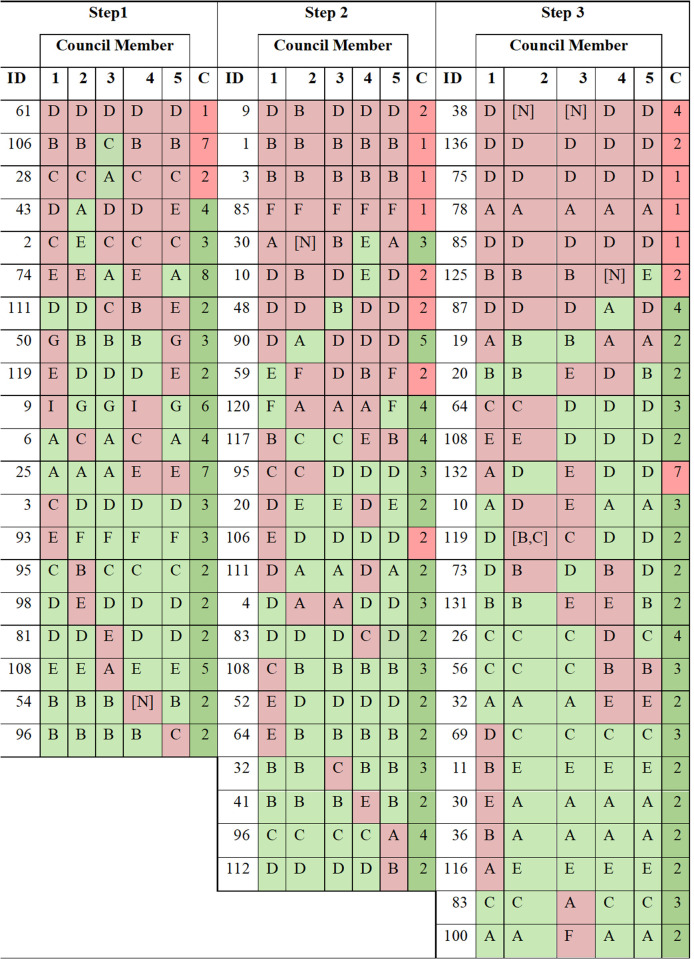
Questions Where at Least One LLM Instance Responded Incorrectly in Round 1. Numbered columns indicate the respective LLM instance responding to the question. The letters in each of the boxes indicate the option that was selected for a multiple-choice question by the corresponding LLM instance. **ID:** Question ID; **C:** indicates if consensus response was correct (green: correct; red: incorrect), and the number of rounds of discussion before reaching consensus.

Among questions where at least one member suggested an incorrect response, there were frequently other members that suggested a correct response (95%, 83%, and 93% of Step 1, Step 2CK, and Step 3, respectively), resulting in a discussion within the Council. (Table 2) An example of the Facilitator algorithm’s prompt to the Council for deliberation of divergent responses is shown in Table 1. For questions that resulted in a deliberation, the Council reached a consensus that was correct 85% of the time for Step 1, 67% of the time for Step 2, 75% of the time for Step 3, and 75% of the time overall. Regardless of the number of members who proposed an initial response that was incorrect, the final consensus response of the Council could still be correct as long as one member proposed a correct response (Fig 2). When no member initially proposed a correct response, there were no instances where the Council’s consensus response was correct. This was true even if there was a diversity of incorrect responses leading to a discussion to reach a consensus (Fig 2).

This table shows a detailed breakdown of all questions requiring deliberation, showing individual Council member responses (columns 1–5), those in green being a correct initial response and incorrect initial response shown in red; and final consensus outcomes in column C for each step, with those in green being correct final consensus and incorrect final consensus shown in red. Numbers in column C for each step show rounds needed to reach consensus. Responses indicated as [N] were initial responses where the AI did not commit to a multiple choice selection. Multiple responses indicated in square brackets (e.g., question ID 119) were those where the AI proposed more than one multiple choice responses as being correct.

For the 72 questions requiring deliberation, individual member responses varied significantly. Notably, the Council never achieved a correct consensus when all members initially answered incorrectly (red rows), highlighting the importance of having at least one correct initial response to guide successful deliberation. Most questions had at least one member with the correct initial answer. In one instance (question ID 60 in step 3) the Facilitator AI flagged initial council responses as divergent based on diverging reasonings even though the selected multiple-choice response by all members was correct.

**Fig 3:**
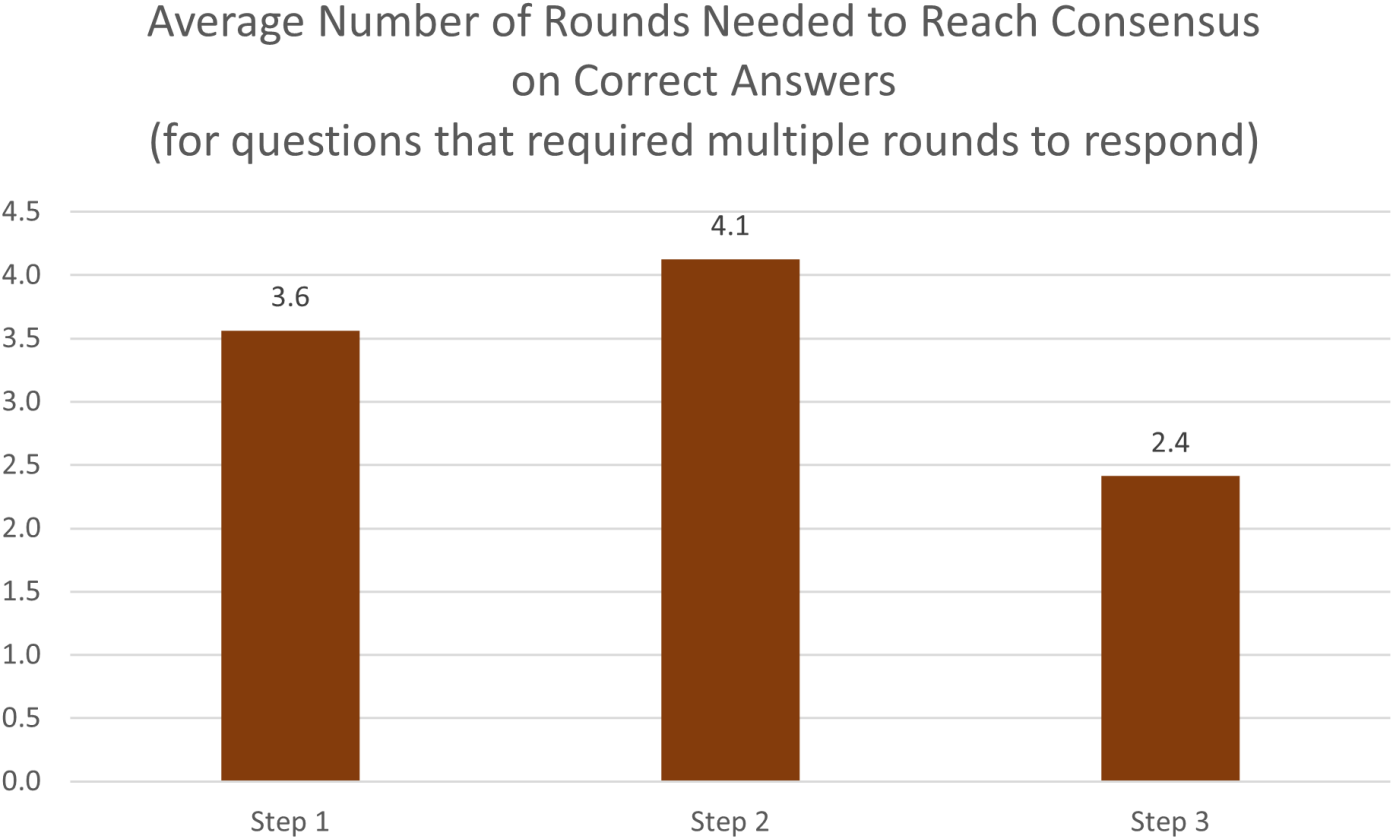
Average number of rounds needed to reach a correct consensus answer. This bar chart shows mean deliberation rounds required across USMLE exam steps. Questions requiring deliberation needed an average of 3.6 rounds (Step 1), 4.1 rounds (Step 2CK), and 2.4 rounds (Step 3) to reach consensus. Step 2CK questions, focusing on clinical diagnosis and management, required the most extensive deliberation, possibly reflecting greater complexity in clinical reasoning compared to basic science (Step 1) or practice-readiness (Step 3) questions.

**Fig 4:**
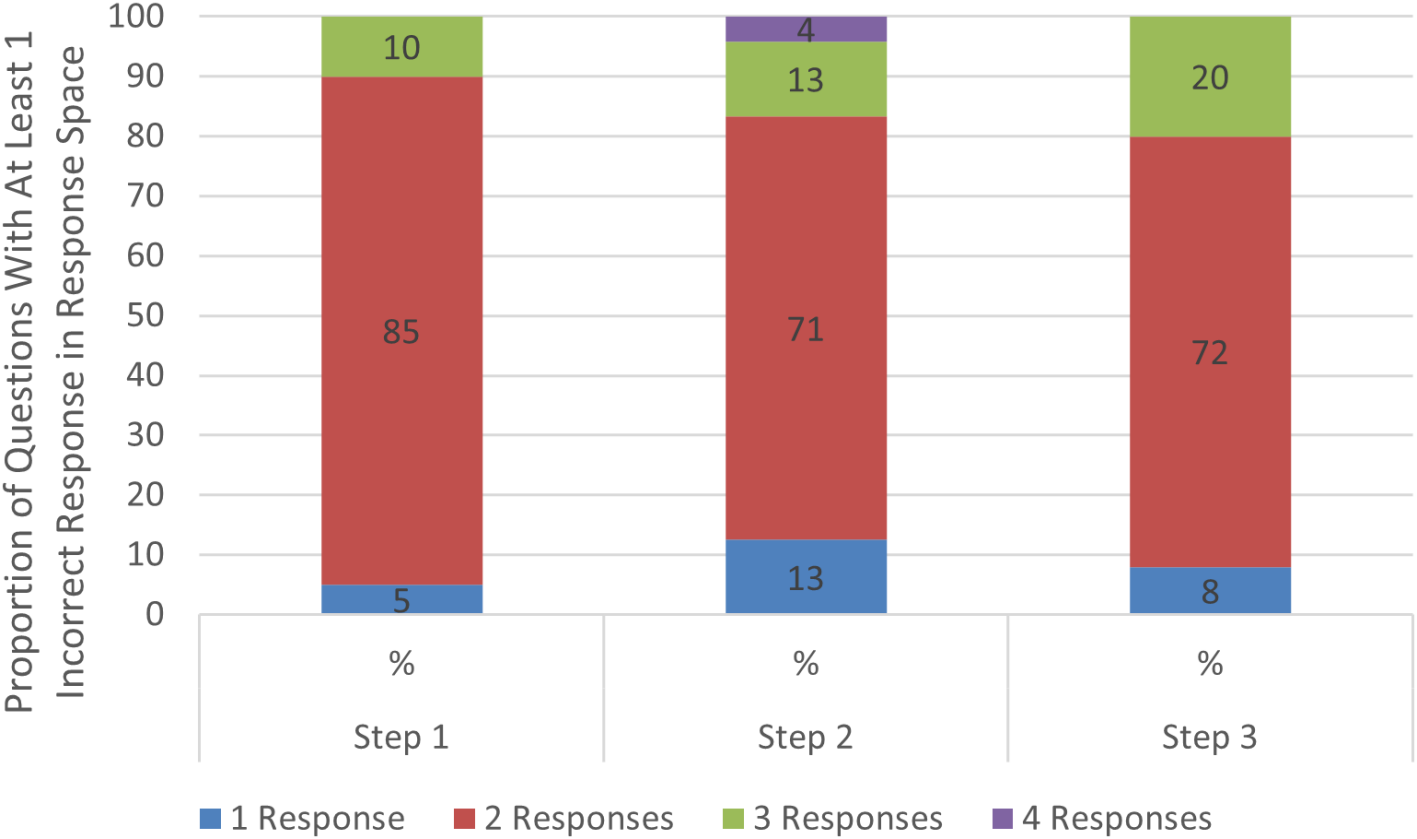
Distribution of incorrect responses among council members. This bar chart visualizes number Council members providing incorrect responses for those questions that required deliberation (i.e., did not have an initial consensus response). For Step 1, 5% of members provided an incorrect response, while majority of incorrect responses (85%) had 2 members providing an incorrect response, with 10% of incorrect responses having 3 members responding incorrectly. For Step 2, 13% of members provided an incorrect response, while majority of incorrect responses (71%) had 2 members providing an incorrect response, with 13% of incorrect responses having 3 members responding incorrectly, and 4% of incorrect responses having 4 members responding incorrectly. For Step 3, 8% of members provided an incorrect response, while majority of incorrect responses (72%) had 2 members providing an incorrect response, with 20% of incorrect responses having 3 members responding incorrectly.

**Fig 5:**
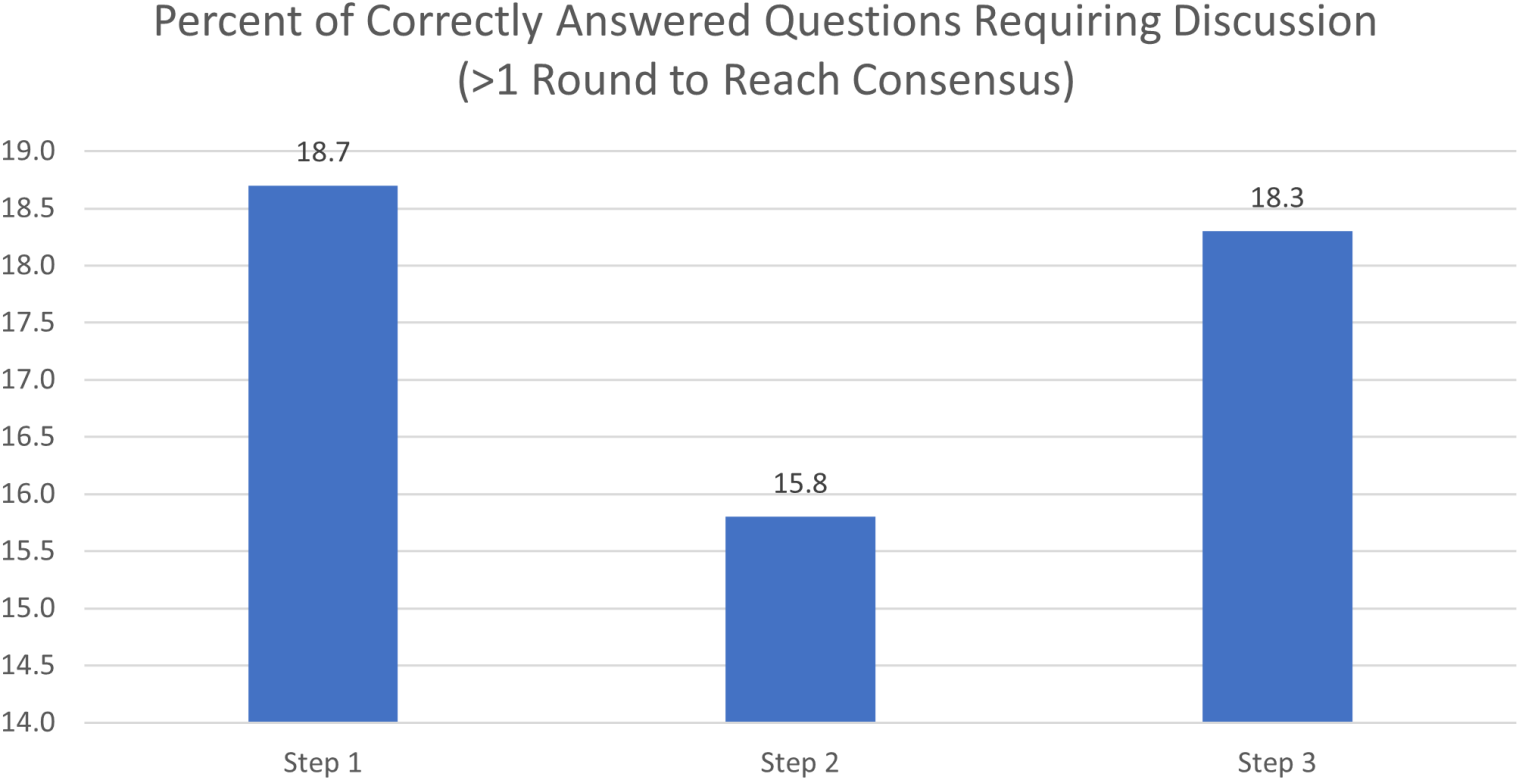
Percent of correct consensus answered questions requiring discussion. This bar chart visualizes what proportion of correctly answered questions required deliberation versus immediate unanimous agreement, stratified by USMLE exam step. Of all correctly answered questions, 16–19% required deliberation before reaching the right answer, indicating that the Council’s collaborative process rescued correct responses that might have been otherwise missed.

**Fig 6:**
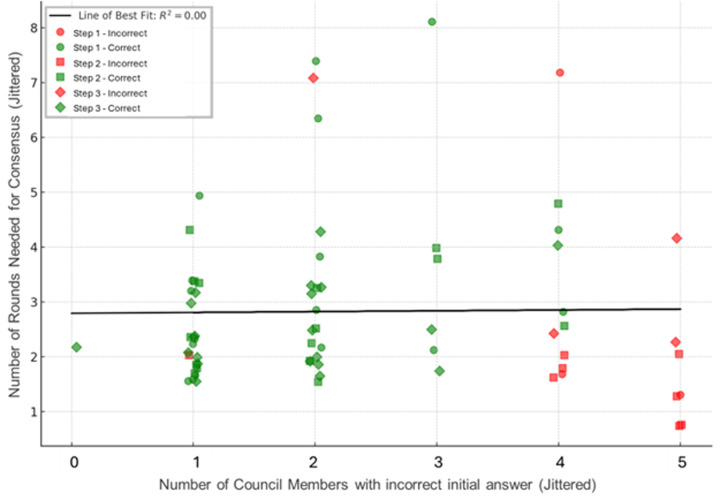
Relationship of number of rounds of deliberation and the number of AI Council Members with an incorrect initial answer. There was no significant association (r^2^ = 0) of the number of correct initial answers and the number of rounds of deliberation needed for consensus. A correct consensus answer was only possible when at least one of the initial answers was correct. This scatter plot with visualizes the relationship between initial disagreement level (x-axis: number of members answering incorrectly) and deliberation complexity (y-axis: rounds needed for consensus). The plot shows “jittered” data, meaning data points are slightly randomly displaced horizontally to prevent overlapping and improve visibility when multiple questions have identical values. Surprisingly, there was no correlation (r^2^ = 0) between the extent of initial disagreement and the number of deliberation rounds required. Questions with 1–4 members initially incorrect required similar deliberation time, suggesting that factors other than the degree of initial disagreement drive consensus difficulty. Green dots represent questions ultimately answered correctly; red dots represent incorrect final consensus.

Achieving a consensus response across diverging suggestions required an average of 3.6 rounds of discussion for Step 1, 4.1 rounds of discussion for Step 2CK, and 2.4 rounds of discussion for Step 3. (Fig 3) Of questions requiring discussion, about 85% of Step 1, 71% of Step 2CK, and 72% of Step 3 questions required 2 rounds before reaching consensus. (Fig 4). Of all correctly answered questions, 19% of Step 1 questions, 16% of Step 2 questions, and 18% of Step 3 questions required a discussion before reaching a consensus that was the correct response (Fig 5). There was no significant association (r^2^ = 0) noted in the number of rounds needed for discussion and the proportion of initial responses that were incorrect (Fig 6).

**Fig 7:**
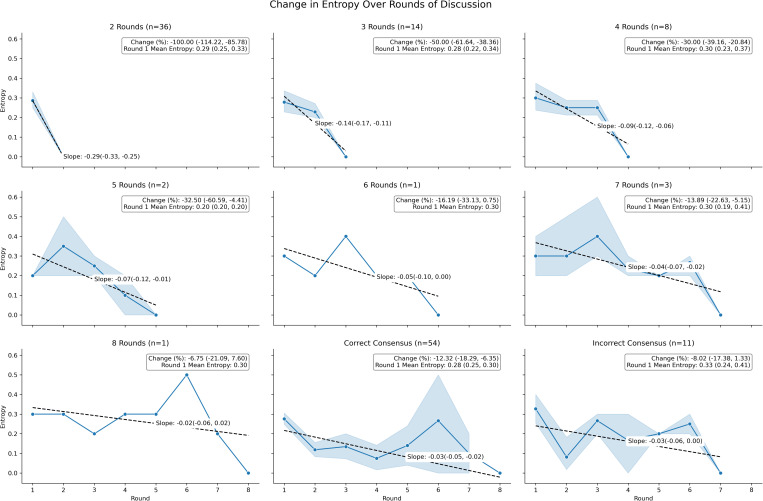
Change in semantic entropy over time by number of rounds of Council deliberation needed for consensus. This matrix of line plots visualize how semantic entropy (measure of response diversity/uncertainty) decreases across deliberation rounds, with separate line plots for questions requiring different total numbers of rounds to reach consensus. Semantic entropy generally decreased with each deliberation round regardless of question complexity, consistently reaching zero (complete consensus) and signifying the final round. This demonstrates that the deliberative process systematically reduces uncertainty and aligns Council member reasoning. Semantic entropy trajectories reveal distinct deliberation patterns: fast-converging questions (2-3 rounds, steep slopes ≤ -0.25) versus slow-converging questions (5 + rounds, gradual slopes ≤ -0.05). Incorrect consensus outcomes show more erratic entropy patterns compared to smooth, predictable decreases in correct consensus cases. Initial entropy levels do not predict deliberation length, but trajectory shape may indicate reasoning complexity.

We quantified the Council’s degree of internal disagreement at each round of deliberation by calculating semantic entropy, where higher entropy indicates greater divergence in multiple‐choice responses and lower entropy indicates growing consensus.[30] Across questions, entropy consistently decreased with each additional re‐prompt, reflecting the Council’s steady progression toward unanimity (Fig 7). Regardless of the total number of rounds needed, entropy generally approached zero by the final round. Notably, even in instances where the final consensus was incorrect, entropy still converged toward zero. This suggests that deliberation seems to lead to decreases in entropy of the response space in every case studied, but it does not guarantee accuracy in every case (though it can improve overall accuracy compared to majority vote or single instances of the LLM (Table 2; Table 3)). Furthermore, the number of rounds of deliberation needed to achieve a consensus is not determined by the amount of disagreement in the initial round of responses to a question (Fig 6): entropy of round 1 responses range from 0.2 to 0.3 regardless of the number of rounds needed to reach consensus (Fig 7). Further complexity on the influence of deliberation on final consensus is given in Fig 7: there appears to be a critical transition point around 4–5 rounds of deliberation, where the character of the process fundamentally changes. Questions requiring 2–4 rounds show steep, predictable linear entropy declines with consistent slopes ranging from -0.29 to -0.09, suggesting straightforward consensus-building. In contrast, questions requiring 5 or more rounds exhibit shallow, erratic patterns with slopes between -0.07 to -0.02, indicating a shift from easy alignment to genuinely difficult reasoning challenges.

Many longer deliberations reveal a distinct two-phase pattern: an initial rapid entropy drop during rounds 1–2, followed by slow convergence in subsequent rounds. Particularly striking are the plateau phenomena visible in the 6 and 8-round panels, where entropy stalls around rounds 3–4 before final convergence, and some cases even show temporary entropy increases mid-deliberation. These non-monotonic behaviors suggest that Councils can temporarily become more divergent before resolving disagreements, potentially indicating either productive “cognitive conflict” or unproductive “reasoning drift.”

Of the 72 USMLE questions analyzed, 54 (75.0%) resulted in correct final consensus and 18 (25.0%) resulted in incorrect consensus after AI deliberation. No statistically significant differences were observed between correct and incorrect consensus groups across any of the examined question characteristics.(Table 4)

**Table 4:** Question Characteristics by Final AI Consensus Accuracy.

Variable	Category	Correct (n = 54)	Incorrect (n = 18)	P-value
Word count		175.9 (59.4)	170.4 (48.8)	0.726
Number of options		5.3 (1.4)	5.2 (0.9)	0.788
Initial disagreement		2.1 (0.5)	1.8 (0.9)	0.068
Rounds to consensus		2.9 (1.3)	2.3 (1.9)	0.121
USMLE Step	STEP 1	17 (31.5%)	3 (16.7%)	0.376
	STEP 2	16 (29.6%)	8 (44.4%)	
	STEP 3	21 (38.9%)	7 (38.9%)	
Topic category	Cardiology	9 (16.7%)	2 (11.1%)	0.576
	Endocrinology	5 (9.3%)	0 (0.0%)	
	Gastroenterology	9 (16.7%)	3 (16.7%)	
	Infectious Disease	1 (1.9%)	0 (0.0%)	
	Nephrology	6 (11.1%)	3 (16.7%)	
	Neurology	1 (1.9%)	0 (0.0%)	
	Obstetrics and Gynecology	2 (3.7%)	0 (0.0%)	
	Pathology	2 (3.7%)	0 (0.0%)	
	Pharmacology	8 (14.8%)	7 (38.9%)	
	Psychiatry	3 (5.6%)	0 (0.0%)	
	Pulmonology	7 (13.0%)	3 (16.7%)	
	Statistics	1 (1.9%)	0 (0.0%)	
Question type	Diagnosis	16 (29.6%)	6 (33.3%)	0.683
	Next step	18 (33.3%)	8 (44.4%)	
	Other	3 (5.6%)	0 (0.0%)	
	Pathophysiology	10 (18.5%)	4 (22.2%)	
	Prevention	2 (3.7%)	0 (0.0%)	
	Statistics	1 (1.9%)	0 (0.0%)	
	Treatment	4 (7.4%)	0 (0.0%)	

Comparison of question characteristics between USMLE questions that resulted in correct versus incorrect final consensus after multi-round AI deliberation. Data are presented as mean (standard deviation) for continuous variables and n (%) for categorical variables. Statistical comparisons used independent t-tests for continuous variables and chi-square tests for categorical variables. Initial disagreement represents the number of unique responses provided by the 5 AI models in round 1. Rounds to consensus indicates the number of deliberation rounds required to reach final agreement. P-values < 0.05 were considered statistically significant.

## Discussion

Collaborative multi-agent approaches are similar to the current practice of medicine, in which input from multiple team members helps to make the best clinical decision for a patient. In this study, we demonstrate the highest ever performance on the USMLE by an AI system through use of a collaborative Council of AI Agents. While a single instance of a LLM (GPT-4 in this case) may potentially provide incorrect answers for at least 20% of questions, a collective process of deliberation within the Council may refine their reasoning pathways, [31] enabling an LLM to correct its incorrect responses 80–90% of the time. This suggests that a multi-agent AI approach can achieve a problem-solving capability on the USMLE unlikely to be achieved by solitary instances and underscores the potential of collaborative AI strategies in medicine.[32] Our review of discussions between AI Council members suggests that collaborative LLMs may better approach the construction of concepts that can be adjusted towards the correct answer, rather than resembling regurgitation from rote memorization as has been previously noted by others.[33] Review of deliberations facilitates human interpretation of linguistic constructions underlying AI decision-making.

### Comparison of USMLE performance with prior studies

Although the exact questions have varied between prior studies of LLMs on USMLE questions, the Council of AI seems to achieve a superior performance to any solitary state-of-the-art LLM to date. In a comparable study utilizing the same USMLE practice questions, GPT-4 answered 88%, 86%, and 90% of Step 1, 2CK, and 3 questions correctly, respectively.[4] In a dataset of USMLE questions known as MedQA, Google’s Med-Gemini-L 1.0 achieved 91% accuracy overall.[34] Prior studies in MedQA have shown an accuracy of 86% by GPT-4 base and 86.5% by MedPaLM 2.[34] By optimizing prompt engineering, Nori et al. were able to increase the performance of GPT-4–90% in this dataset.[3] A comparison of the Council of AI to prior zero-shot tests of GPT-4 is shown in Table 5.

**Table 5:** Comparison of Council of AI accuracy on USMLE questions compared to prior studies using GPT-4 (zero-shot).

Studies using GPT-4 Models (zero shot)	Step 1	Step 2	Step 3
Council of AI	97%	93%	94%
Mihalache et al	88%	86%	90%
Nori et al	81%	82%	90%

AI: Artificial Intelligence. USMLE: United States Medical Licensing Exam.

Table showing performance comparison between the Council of AIs and previous single-instance GPT-4 studies using similar USMLE question sets. The Council of AIs outperformed all prior single-instance GPT-4 evaluations across all USMLE steps. Improvements ranged from 4-16 percentage points, with the largest gains in Steps 1 and 2, demonstrating that collaborative AI approaches can exceed individual model performance on standardized medical examinations.

The lower accuracy observed in previous single-instance GPT-4 studies can be attributed to several inherent limitations of solitary LLM deployment. First, single instances are subject to the stochastic nature of token-by-token generation, which introduces response variability that can lead to incorrect answers on questions the model has the underlying knowledge to answer correctly.[22,23] Second, traditional single-instance approaches lack mechanisms for self-correction or iterative refinement, meaning that initial reasoning errors cannot be identified and corrected through subsequent deliberation. Third, isolated reasoning processes miss the benefits of collaborative error-checking, where multiple perspectives can identify flaws in logic or knowledge gaps that a single instance might overlook. Finally, single-shot responses provide no opportunity for the kind of adaptive reasoning demonstrated by the Council approach, where initial disagreement can trigger deeper analysis and ultimately lead to more accurate conclusions. The Council architecture specifically addresses these limitations by embracing response variability as a feature rather than a bug, providing structured mechanisms for collaborative reasoning, and enabling iterative refinement through deliberation. This explains why the Council approach achieves 4–16 percentage point improvements over prior single-instance studies, with the largest gains occurring in the most knowledge-intensive domains (Steps 1 and 2) where collaborative reasoning provides the greatest advantage over isolated decision-making.

### The value of response variability

The observation that 22% of questions needed multiple rounds of deliberation highlights the stochastic nature of LLMs’ content generation and suggests that a single response from an LLM might not suffice for precise decision-making. This is consistent with findings from other studies, such as Yaneva et al., who reported variability in correct answers across three replications, observing inconsistencies in 20% of USMLE questions.[35]

This study suggests that variability in LLM responses, a limitation in isolation, can be a strength in collaboration. This unexpected finding suggests new approaches to evaluating generative LLMs, which usually favor predictability and consistency, especially in contexts like professional exams where questions have only one correct answer. By valuing the inherent variability in responses across different LLM instances and leveraging consensus-building as a method, this finding suggests we may need to rethink what optimal behavior means for generative LLMs. Evaluation should not only consider the performance of a single LLM instance but also prioritize the system’s capacity for collaborative engagement with other AI instances to identify a best response. A collaborative approach could significantly improve accuracy and reliability in critical decision-making areas like healthcare.

Interestingly, the Council never achieved a correct consensus response without at least one member offering a correct initial response. This underscores the significance of having a Council with sufficient diversity in reasoning to ensure at least one LLM instance can propose a correct response. While this study employed multiple instances of GPT-4 to control for variability and focus on the effects of deliberative coordination, future research could explore the benefits of heterogeneity by constructing Councils composed of diverse language models, such as Claude, or Bard. Each model is trained on distinct corpora and incorporates different architectural and alignment strategies, which may yield varied perspectives, reasoning styles, or domain-specific strengths. This diversity could enrich deliberations by introducing alternative viewpoints or counterfactual reasoning paths, potentially surfacing edge cases or mitigating blind spots specific to a single model family. For example, a model fine-tuned on medical literature might challenge a general-purpose model’s heuristic, or a model with stronger multilingual grounding could raise issues missed by monolingual training. Such cross-model deliberation could potentially improve accuracy, while also increasing robustness, fairness, and generalizability of ensemble reasoning frameworks, particularly in high-stakes domains like clinical decision-making or public policy. Exploring how different models negotiate disagreements or reinforce consensus offers a promising direction for advancing deliberative AI systems. Future research could explore the identification of the optimal characteristics, number, and composition of council members to generate a diverse range of responses to increase the probability that at least one member can suggest a correct answer.

An unexpected finding in this study was that the number of rounds of deliberation needed to achieve a consensus was not associated by the extent of disagreement in the initial round of responses to a question (Fig 7). This suggests that a factor other than the number of divergent responses is driving the amount of deliberation. A preliminary analysis of question characteristics revealed no statistically significant predictors of AI consensus accuracy (Table 4). Future studies can explore the characteristics of questions or responses which increase or decrease the amount of deliberation before consensus; and can explore the characteristics of questions for which the Council failed to achieve a correct consensus. A more in-depth analysis to characterize questions that yield correct versus incorrect responses after deliberation may involve extracting quantitative text-based metrics such as word count, sentence complexity, readability scores (e.g., Flesch-Kincaid), and information density (e.g., ratio of medical terms to total words), as well as indicators of clinical complexity, such as multi-system involvement, presence of diagnostic uncertainty language (e.g., “most likely,” “suggests”), and case timeline intricacy. Features of answer choices themselves, such as semantic similarity may also be relevant. Additionally, patterns of AI behavior in the initial round can be analyzed, including the number of unique responses, semantic entropy of initial answers, and majority/minority voting splits (e.g., 3–2 vs. 4–1). Stratifying questions by USMLE Step, medical domain (e.g., cardiology, pharmacology), and reasoning type (e.g., factual recall vs. clinical synthesis) may identify content-specific performance trends.

When considering divergent responses for a given question, the reduction in semantic entropy across rounds of deliberation until consensus is reached represents an alignment of reasoning pathways among model instances (Fig 7). In this study, declining entropy corresponded with increased epistemic coherence and was associated with improved accuracy, particularly in cases where initial responses were divided. This suggests that entropy may serve as a useful proxy for internal disagreement and reliability in multi-agent AI systems. Beyond medical question answering, entropy tracking has potential applications in clinical decision support, where persistently high entropy could flag ambiguous cases for human oversight; in legal reasoning, where it may help surface interpretive plurality in complex cases; and in public policy simulations, where entropy could serve as a signal for model uncertainty and prompt additional stakeholder consultation. In each context, semantic entropy provides a quantifiable means of monitoring internal disagreement within AI collectives, offering insights into both system confidence and the potential need for external validation.

### Self-correction and collective intelligence

If a LLM produced an incorrect initial response, we found it subsequently generated a correct response 85% of the time through interaction with other instances of itself. This aligns with recent studies on self-correction mechanisms in LLMs.[36–38] This phenomenon, where multiple AI instances engage in a dialogue and reconcile their differences to achieve a consensus, reflects a simulation of collective intelligence, [39] enabling a group of AIs to ‘change their minds’, a level of complexity and sophistication often attributed to human group dynamics.[40] The necessity for LLM instances to explore new reasoning paths to arrive at a correct consensus underscores the flexibility and adaptive reasoning capabilities present in contemporary LLMs. This flexibility is particularly valuable in dynamic, real-world settings where issues often require more than a singular, predetermined solution strategy.

The results suggest that discussion-based consensus provides a statistically significant improvement in the correctness of responses compared to a simple majority vote. Specifically, in comparing the initial majority vote in Round 1 with the Council’s final consensus (Table 3), there were only two instances where a correct initial majority ultimately resulted in an incorrect consensus after discussion. In contrast, for the 19 questions where the initial majority was wrong, in 10 of those cases the Council consensus overturned the initial majority, resulting in a correct final answer. The odds of a majority vote response changing from incorrect to correct were 5 (95% CI:1.1, 22.8) times higher than the odds of it changing from correct to incorrect after discussion, demonstrating a statistically significant improvement in accuracy from pre-discussion majority voting to post-discussion consensus. Taken together, these findings highlight the Council’s deliberative process as a strategy that preserved correct majority opinions and “rescued” those instances in which the majority initially erred. In other words, the multi‐agent Council approach has the potential to both preserve correct majorities and significantly increase accuracy when the majority begins in error. By iteratively reconciling divergent reasoning paths, the Council demonstrates that structured dialogue can leverage variability in AI‐generated responses to achieve superior reliability and performance compared to relying solely on a single, one‐shot majority vote. Future research might explore *why* discussion can improve accuracy (e.g., type of question, quality of explanations, number of conflicting perspectives), as well as any conditions that optimize its effectiveness.

The high performance of the Council of AI Agents is supported by studies of ensemble systems and multi-agent systems that show that AI collaborations can have a superior performance compared to single AI instances [32,41–51] and can be useful for applications such as drug-discovery.[52,53] Ensemble approaches have also been used to improve performance for LLMs across different knowledge domains. For medical question-answering in particular, Yang et al employs boosting-based weighted majority voting which significantly outperformed individual models on various datasets; [54] Jiang et al uses specific algorithms to merge potential candidate outputs from various LLM in a pair-wise fashion improving performance by measuring against with known benchmarks; [55] Pitis et al introduces a “prompt ensemble” method for a “chain-of-thought” language model reasoning; [56] while Naderi et al uses ensemble techniques for named entity recognition in health and life science domains.[57]

It has been suggested that the Council of AI may resemble a Mixture-of-Experts (MoE) system. While our framework shares some similarities with MoEs and multi-agent LLM systems, it represents a distinct architectural and epistemic paradigm. Traditional MoE architectures, such as those implemented in large-scale LLMs by Google and NVIDIA, operate at the level of latent token processing. A learned gating network dynamically routes input tokens to a subset of computationally efficient “experts”, which are sub-networks trained jointly within a single model. These experts produce hidden representations rather than human-interpretable outputs, and their contributions are fused numerically. The emphasis in MoE is on computational efficiency and learned internal specialization, not on deliberation or transparency.

A different, increasingly popular approach connects LLMs in agentic systems, where each LLM instance may be fine-tuned or prompted for domain-specific expertise and tasked with planning, reasoning, or acting autonomously in coordination with others. In such systems (e.g., as seen in platforms like Auto-GPT, LangGraph, or CrewAI), each agent may have a persistent role or memory and interact with external tools or APIs to complete a task. These frameworks emphasize goal-directed autonomy and role-based collaboration, often oriented around real-world actions and long-horizon planning.

The Council of AIs model differs fundamentally from both. In our framework, each LLM instance serves not as a tool or planner, but as an epistemic contributor that is a self-contained reasoner offering a complete response to a shared question. These agents are not trained to specialize nor endowed with memory or external tools; they reason independently from a base model (e.g., GPT-4) and deliberate through natural language exchanges facilitated by a central agent. The Facilitator is not a gating mechanism (as in MoE) nor a task planner (as in agentic systems), but a meta-reasoner that curates, critiques, and synthesizes responses. The deliberation is transparent, auditable, and designed to surface epistemic disagreement and convergence but not necessarily to execute tasks or maximize efficiency.

In this sense, the Council architecture is best understood as a deliberative ensemble, optimized for epistemic robustness rather than efficiency (as in MoE) or autonomy (as in agentic systems). It borrows the modularity of MoE, the multi-agent structure of agentic systems, and the interpretability of human deliberation, but combines them toward a unique end: reasoning through dialogue to improve reliability, not just to act or compute.

A common communication architecture in a multi-agent system is that of agents that are connected to a middle agent. AI agents that are completing a task may have varying levels of information, with the middle agent (e.g., facilitator, mediator) coordinating between other agents and providing each agent with services that it may need.[42–46] In contrast, an alternative multi-agent architecture, studied by others [58–60] and developed for this study, provides all AI agents with full information, equal guidance for reasoning (i.e., same CoT prompts), and awareness of each other’s responses communicated to them through the Facilitator agent. This architecture simulates a council, where each AI member can respond to a query, and if there is variability in responses, deliberate divergent responses through the Facilitator to reach a consensus response. Because the deliberation of the Council occurs through a number of different steps, future directions of research can explore the mechanisms of the Council’s response generation, including characterizing and evaluating each stage of the algorithm (i.e., initial response to the USMLE question, the response of the LLM to compare responses for agreement, the response of the LLM to summarization, the response of the LLM to generating a clarifying question, and the subsequent responses of the Council of AI Agents to the new prompt). Additionally, the current study held all LLM attributes (i.e., prompt, temperature, maximum tokens, and top_p) constant between each instantiation to focus the evaluation on the presence of interaction between Council members and the resulting performance. However, a future direction of research can evaluate the impact of modifications to different parameters individually or in concert, with all or part of the Council embodying the changes.

While our study employed five AI agents due to token limitations and system constraints, varying the number of council members may meaningfully influence performance. Increasing the number of agents could enhance response diversity and improve the likelihood of arriving at a correct consensus, especially when agents disagree or offer complementary rationales. However, as more agents are added, the benefit may diminish if additional responses become redundant or if the Facilitator struggles to synthesize increasingly complex deliberations. Larger councils may also introduce greater computational costs and longer deliberation times. Future work could explore how performance scales with different council sizes and whether an optimal number of agents exists for balancing accuracy, efficiency, and reasoning quality.

### Limitations

The Council of AIs approach, while effective, requires significant computational resources. Each deliberation involves multiple full forward passes through large-scale LLMs, significantly increasing both time and resource demands compared to single-instance querying. On average, answering a single USMLE question using the Council architecture (consisting of five GPT-4 agents and a Facilitator) required approximately 3–7 minutes, depending on the number of deliberation rounds, with some questions taking up to 15 minutes to achieve a consensus. Latency time increases with each additional instantiation of the LLM. This presents a significant challenge in real-time decision-making scenarios, especially when multiple rounds of sampling are required. It is possible to eliminate additional latency time while increasing the number of Council members by employing a strategy of synchronous sampling through multiple instantiations of the LLM API, each with different API keys. This approach would allow for parallel processing of responses, substantially reducing the overall time taken to reach a consensus. Through parallel computing, it would be feasible to maintain the depth and quality of analysis without sacrificing response time. For now, this system would work best for non-emergent scenarios that have a tolerance for the time needed by the Council for deliberation and achieving consensus. Future studies could focus on optimizing the efficiency of such systems and exploring their applicability in different domains.

Token limits in LLMs are a technical constraint that can limit the depth of analysis, particularly when dealing with complex or lengthy prompts. In our study token limits imposed practical constraints on both the number of Council members and the structure of deliberations, as each response needed to be passed through the Facilitator AI for summarization and reflection. To conserve tokens, Council members did not receive each other’s raw outputs directly; instead, they interacted through a Facilitator who synthesized responses for iterative feedback. While this strategy was motivated by efficiency, it remains uncertain whether full message broadcasting among agents would have improved performance or introduced unnecessary redundancy. In fact, it is possible that the Facilitator-mediated structure may have supported more coherent deliberation by filtering and organizing contributions in a focused manner. Additionally, while it is possible that token limits exerted question-specific effects, we did not stratify performance by item length or complexity, as no standardized metric currently exists for assessing difficulty across USMLE questions. However, future studies might explore whether token constraints disproportionately impact more detailed or multi-step questions and evaluate whether expanded token contexts (e.g., via more recent LLM versions or hierarchical deliberation models) can mitigate such effects.

The exclusive use of OpenAI’s GPT-4 in our Council of AI approach was a strategic choice in this study. Future studies can explore the inclusion of different LLMs, each with unique training datasets, algorithms, and probabilistic models, which may possibly lead to more robust and well-rounded consensus. The variation in the success rate of achieving consensus across different exam steps (Step 1, 2, and 3) suggests challenges in maintaining AI consistency across different contexts and that AI systems might require tailored approaches depending on the specific nature and complexity of the tasks at hand. Testing in other datasets, such as MedQA, may help with more direct comparison to other LLMs in the future. While this study focused exclusively on multiple-choice questions without images or tables, future research is needed to determine whether the Council of AIs framework generalizes to other question formats such as clinical vignettes or visual question-answering tasks. These formats may involve more complex reasoning, ambiguity in correct responses, or multimodal inputs, which could present new challenges for consensus-building and response clustering. The current findings do not provide evidence for performance in those contexts, but they suggest that exploring deliberative multi-agent strategies in more diverse settings may be a valuable direction for future investigation. While we selected the present test question set to ensure all questions were from after GPT-4’s training period, a possible limitation is that sample exam questions could be highly similar to older sample exam questions, in which case the LLM may already have encountered highly similar questions in its training data. It is important to note that the GPT-4 model used in this study was the initial version which later came to be known as ‘gpt-4-0314’ to distinguish it from subsequent updates to GPT-4. According to the GPT -4 system card and OpenAI’s technical documentation, GPT-4’s training data cutoff occurred in September 2021, and the model “generally lacks knowledge of events that have occurred after the vast majority of its data cuts off” and “does not learn from its experience”.[29,61] While OpenAI notes that gpt-4–0314 was released in March 2023 and remained unchanged through its deprecation in June 2024, and while there is no indication from OpenAI that such materials were part of the model’s post-training updates, it is not possible to definitively confirm whether specific documents (such as the June 2022 USMLE sample exam used in this study) were included in any fine-tuning processes. Given that there may be a possibility that despite OpenAI documentation later text corpora may have been included in fine-tuning processes, the purpose of this study was not to assess GPT-4’s memorization or static knowledge. Rather, the objective of this study was to evaluate how a deliberative architecture (i.e., a Council of AIs) can improve accuracy by integrating diverse reasoning paths, even among instances of the same model. Thus, even if agent responses reflected memorized content, the observed performance gains are attributable to the structure of deliberation rather than to any individual agent’s latent knowledge.

### Ethical considerations

Ethical considerations around the use of AI in professional and decision-making contexts need to be addressed, ensuring that the deployment of such technologies is responsible and beneficial to society. A Council of AI approach may facilitate integration of voices of underrepresented communities as council members to try to reduce bias, which may be represented within purposefully trained smaller language models or through fine tuning of LLMs. However, LLMs themselves have an inherent limitation in that they are trained on a written corpus, which excludes communities whose knowledge may be codified in a diversity of non-written media including experiential transmissions of knowledge, oral histories, performative representations, and more. Future studies can explore the extent to which rapidly adopted AI systems are disconnecting communities from their own realities and reshaping community knowledge systems toward more hegemonic representations.[62] Additionally, studies also need to be conducted to formalize AI impact assessment on knowledge systems.

The Council of AIs framework offers a novel avenue for mitigating bias in LLMs, particularly in multicultural and multilingual contexts. By facilitating structured deliberation among independently reasoning agents, the architecture enables disagreement to surface and be explicitly negotiated, potentially counterbalancing the influence of any single model’s latent cultural or epistemic biases. Importantly, beyond the content of reasoning itself, non-reasoning attributes, such as the degree of epistemic rigidity, or resistance to updating a prior position, can also shape group consensus. In some cases, rigidity may steer the Council toward suboptimal or biased outcomes if a less flexible agent dominates. However, in other contexts, especially when the training corpus underrepresents marginalized voices, epistemic rigidity may act as a protective feature preserving minority-informed perspectives from being prematurely overwritten during deliberation.

Recognizing these dynamics is essential for designing equitable systems. To ensure transparency and accountability in high-stakes applications such as healthcare, Council-based architectures should provide access to deliberation transcripts, rationales for final decisions, and support external auditability. Further, intentionally including models with diverse training data, cultural grounding, or alignment objectives may help surface a broader range of perspectives, mitigate systemic bias, and help make AI systems both reliable and ethically robust.

### Future research priorities for council of AIs implementation

While this study demonstrates the potential of collaborative AI approaches in medical decision-making, several critical research questions must be addressed to optimize the framework for clinical deployment. These research priorities span technical optimization, clinical integration challenges, and real-world implementation considerations. Table 6 outlines specific research questions and corresponding studies needed to advance the Council of AIs from experimental validation to practical clinical application, with particular emphasis on addressing computational efficiency, real-time decision-making requirements, and human-AI collaboration protocols.

**Table 6:** Priority Research Questions for Council of AIs Clinical Implementation.

Research Domain	Key Research Question	Specific Studies Needed	Expected Clinical Impact
**Council Scale Optimization**	What is the optimal number of Council members for balancing computational cost and diagnostic accuracy?	Systematic evaluation of 3, 5, 7, 10, 15, and 20-member Councils; cost-benefit modeling across medical specialties; identification of diminishing returns threshold	Optimized resource allocation; standardized deployment protocols for different clinical contexts
**Heterogeneous Architecture**	How does mixing different LLM models (GPT-4, Claude, Gemini) affect Council performance compared to homogeneous composition?	Comparative trials of homogeneous vs. mixed Councils; analysis of reasoning diversity and blind spot identification; specialty-specific model combinations	Enhanced diagnostic accuracy through complementary AI perspectives; reduced single-model bias
**Real-Time Implementation**	How can deliberation time be reduced from 3-15 minutes to <60 seconds for emergency scenarios while maintaining accuracy?	Development of adaptive stopping criteria based on entropy convergence; parallel processing architectures; emergency department simulation studies	Feasible integration into time-sensitive clinical workflows; emergency medicine applications
**Clinical Triage**	Which clinical scenarios benefit most from Council deliberation versus single-instance AI responses?	Machine learning classifiers for automatic case routing; validation studies in primary care, emergency, and specialty settings; cost-effectiveness analysis	Intelligent resource allocation; targeted deployment strategies; workflow optimization
**Human-AI Collaboration**	How does incorporating human physicians into AI Councils affect accuracy and clinical acceptance?	Randomized controlled trials of pure AI vs. hybrid human-AI Councils; optimal interaction protocol development; physician trust and workflow integration studies	Enhanced clinical acceptance; improved diagnostic accuracy through human expertise integration
**Semantic Entropy Applications**	Can entropy trajectories reliably flag cases requiring human expert review?	Prospective validation of entropy-based escalation protocols; development of uncertainty threshold algorithms; subspecialty validation studies	Automated quality assurance; expert consultation triage; reduced diagnostic errors
**Bias Detection**	How effectively can Council deliberation identify and correct demographic, cultural, and socioeconomic biases?	Systematic evaluation across diverse patient populations; bias-detection metrics development; equity intervention testing	Improved healthcare equity; reduced AI bias in underrepresented populations; enhanced diagnostic fairness
**Multimodal Integration**	How can Councils incorporate medical imaging, lab results, and other non-textual data sources?	Development of multimodal Council architectures; validation in radiology, pathology, and laboratory medicine; integration protocol optimization	Comprehensive diagnostic support; enhanced accuracy in image-dependent specialties; holistic patient assessment
**Adaptive Learning**	Can Council systems improve performance over time through deliberation analysis and outcome feedback?	Longitudinal performance tracking; feedback mechanism implementation; continuous model updating protocols while maintaining reliability	Self-improving diagnostic systems; adaptation to emerging medical knowledge; enhanced long-term accuracy

## Conclusions

By designing an algorithm that embraces the variability inherent in LLM responses, the Council of AIs model leverages a multi-agent AI framework to achieve superior performance on the USMLE. This study highlights the importance of collective intelligence and collaborative decision-making in AI systems, offering a model for understanding and maximizing AI capabilities. The Council of AI Agents emphasizes the value of collaboration over individual accuracy and demonstrates the dynamic, evolving nature of AI cognition. This study may provide a framework for one possible future of medical AI, in which multidisciplinary teams of AIs and humans work together to improve health outcomes across the world.

## Supporting information

S1 File Supplemental details on methods. (PDF)

S2 File Raw data files containing unprocessed question inputs and LLM outputs including transcripts of deliberations. (ZIP)
