# Supplementary material for: Collaborative intelligence in AI: Evaluating the performance of a council of AIs on the USMLE
Source: PLOS Digit Health. 2025 Oct 9;4(10):e0000787. doi: 10.1371/journal.pdig.0000787 (PMC12510544; doi:10.1371/journal.pdig.0000787)
Supplement: S1 File — (PDF) [file pdig.0000787.s001.pdf]

Supplemental Methods

Selecting a Large Language Model (LLM)

There are a number of state-of-the-art LLMs currently available, pretrained on large, unlabeled text data using self-attention mechanisms within transformer models.(1) Publicly available versions of these cutting-edge LLMs include the public UL2,(2) GPT-3.5,(3) GPT-4,(4) LaMDA,(5) and PaLM.(6) Of these, OpenAI's models, particularly GPT-4, have exhibited human-level performance on a variety of benchmarks and tests, including passing a simulated bar exam at the top 10% of test takers and achieving scores between 86%, 83%, and 91% respectively on the Step 1, Step 2, and Step 3 USMLE exams.(7) OpenAI's GPT are transformer-based,(1) autoregressive, neural network models that undergo unsupervised pretraining on a large corpus of data, and subsequent finetuning using a variety of algorithms including reinforcement learning from human feedback (RLHF).(3) The combination of large amounts of training data and improved training algorithms yields final models with remarkable ability to simulate human reasoning.(3,8–10) OpenAI's GPT-3 model was trained on datasets totaling almost 500 billion tokens, consisting of 175 billion trainable parameters across 96 neural network layers.(3) OpenAI's models, being the most capable models available to the public at this time, are additionally accessible through Application Programming Interfaces (APIs), have extensive documentation, and a large community of support. For these reasons, we selected GPT-4 as the base LLM for our current study.

Conceptualizing the Architecture as a Council of AI

A conceptualization of this process is that each of the API calls to prompt the LLM is initialized in code as a member of an AI Council. Each member of the Council has an identity (e.g. name) and sees their own response history and the responses of the other Council members through another API call initialized as the Facilitator of the discussion. The Facilitator AI presents the initial prompt from the user to each of the Council's members. Each of the members respond to the same prompt using a reasoned approach in their response. Once all the members have responded, the Facilitator AI summarizes the responses, identifies the differences in reasoning that is evident in the members' responses, then re-presents the Council with the summary, differences in their reasoning, a question that serves to clarify their reasoning, and a request to re-answer the question in light of this context of reasonings and responses from other Council members. Once the Council has reached a consensus the Facilitator AI then synthesizes and summarizes the Council's reasoning and presents the consensus response to the user. (See S Fig 1).

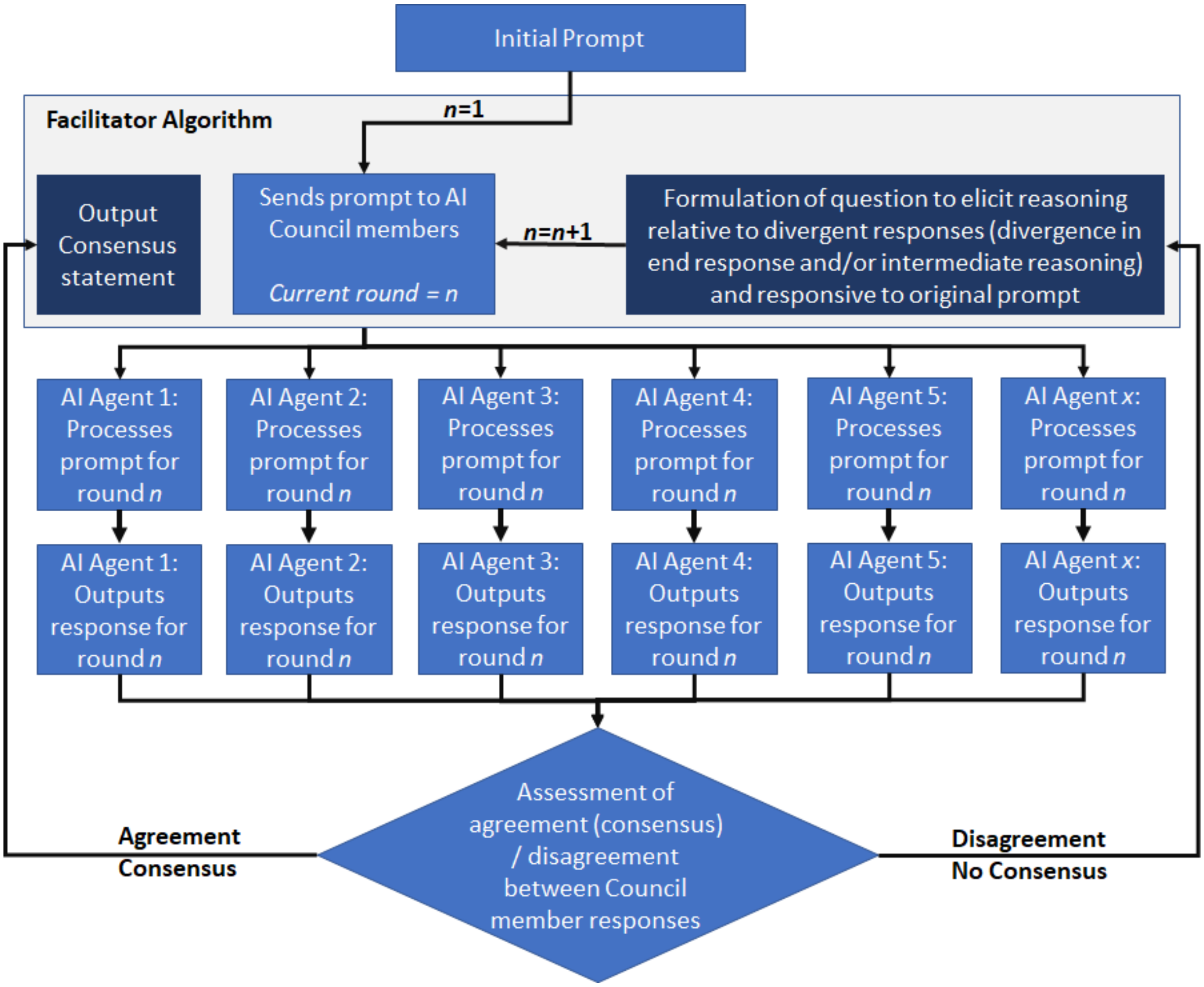

Supplemental Figure 1. Council of AI Agents Architecture

Constructing the API Prompt

To create a context to facilitate the emergence of responses related to the medical domain, the following instruction was added to prompts presented to the LLM:

> "You get your knowledge from the following domain spaces: anatomy, physiology, pathology, pathogenesis, pathophysiology, histology, pharmacology, microbiology, endocrinology, immunology, hematology, oncology, genetics, embryology, internal medicine, surgery, pediatrics, obstetrics, gynecology, psychiatry, family medicine."

For ordering an explanation in a way that can simulate reasoning, we specified the construction of content that included scientific problem solving and clinical reasoning:(11–13)

> "Your sentence completion follows the following pattern: (1) RESTATING THE QUESTION: Restating the question in your own words and defining technical terms and concepts; (2) PLANNING: Identifying assumptions, generating alternate assumption by questioning each assumption, and then evaluating and hypothesize which assumption should be clinically prioritized to pursue; (3) CLINICAL REASONING: Connect each sentence to its subsequent sentence using a clinical thought process to provide a step-by-step explanation to support the alternate assumption; (4) CONCLUDING: Use a step-by-

step explanation that connects one sentence to the next with a clinical thought process to generate your final response"

The final prompt identifies Council members, focuses their attention on a domain knowledge and reasoning, and ensures that subsequent responses in the discussion are on topic. For each of the members of the AI Council the temperature setting was set at 1 and the top_p (nucleus sampling) set to 1. The JSON used to specify these parameters was input to as the "system message" in the API call, and is detailed below for AI Council member named AI_A ("thisModel" refers to the language model, thisTemp refers to the temperature parameters, thisTopP refers to the top_p parameter, and "thisPrompt" refers to the prompt that initiates the discussion or subsequent prompt that maintains the discussion through facilitator input):

> AI_A:{thisModel:"gpt-4", thisTemp:1, thisTopP:1, thisSysMsg:"Your 'name' is 'AI_A'. You are part of a council that includes 'AI_B', 'AI_C', 'AI_D', 'AI_E', and 'AI_F'. You get your knowledge from the following domain spaces: anatomy, physiology, pathology, pathogenesis, pathophysiology, histology, pharmacology, microbiology, endocrinology, immunology, hematology, oncology, genetics, embryology, internal medicine, surgery, pediatrics, obstetrics, gynecology, psychiatry, family medicine. Your sentence completion follows the following pattern: (1) RESTATING THE QUESTION: Restating the question in your own words and defining technical terms and concepts; (2) PLANNING: Identifying assumptions, generating alternate assumption by questioning each assumption, and then evaluating and hypothesize which assumption should be clinically prioritized to pursue; (3) CLINICAL REASONING: Connect each sentence to its subsequent sentence using a clinical thought process to provide a step-by-step explanation to support the alternate assumption; (4) CONCLUDING: Use a step-by-step explanation that connects one sentence to the next with a clinical thought process to generate your final response; (5) RESPONSE: You always try to connect your response to answer the following: ["+thisPrompt+"]"}

The code for implementing the above is available in the public domain: GitHub repository / docker package.

Open Sourcing Code

As part of this study we developed code to: instantiate multiple AI / LLM instances to generate a Council of AIs; allow each Council member to independently consider and present a response from its response space; and allow the Council to conduct a discussion amongst themselves until there is an attainment of consensus. We make the Council of AI code openly available for use by others: https://github.com/councilofai/project-saru.

References

1. Ashish V. Attention is all you need. Adv Neural Inf Process Syst. 2017;30:I.
2. Tay Y, Dehghani M, Tran VQ, Garcia X, Wei J, Wang X, et al. Ul2: Unifying language learning paradigms. arXiv preprint arXiv:220505131. 2022;
3. Brown T, Mann B, Ryder N, Subbiah M, Kaplan JD, Dhariwal P, et al. Language models are few-shot learners. Adv Neural Inf Process Syst. 2020;33:1877–901.
4. OpenAI. Gpt-4 technical report. arXiv preprint arXiv:230308774. 2023;
5. Thoppilan R, De Freitas D, Hall J, Shazeer N, Kulshreshtha A, Cheng HT, et al. Lamda: Language models for dialog applications. arXiv preprint arXiv:220108239. 2022;
6. Chowdhery A, Narang S, Devlin J, Bosma M, Mishra G, Roberts A, et al. Palm: Scaling language modeling with pathways. Journal of Machine Learning Research. 2023;24(240):1–113.
7. Nori H, King N, McKinney SM, Carignan D, Horvitz E. Capabilities of gpt-4 on medical challenge problems. arXiv preprint arXiv:230313375. 2023;
8. Kojima T, Gu SS, Reid M, Matsuo Y, Iwasawa Y. Large language models are zero-shot reasoners. Adv Neural Inf Process Syst. 2022;35:22199–213.
9. Wei J, Wang X, Schuurmans D, Bosma M, Xia F, Chi E, et al. Chain-of-thought prompting elicits reasoning in large language models. Adv Neural Inf Process Syst. 2022;35:24824–37.
10. Häggström O. Are Large Language Models Intelligent? Are Humans? In: Computer Sciences & Mathematics Forum. MDPI; 2023. p. 68.
11. Polya G. How To Solve It. Princeton University Press. New Jersey; 1945.
12. Schwartz A, Elstein AS. Clinical reasoning in medicine. Clinical reasoning in the health professions. 2008;3:223–34.
13. Shin HS. Reasoning processes in clinical reasoning: from the perspective of cognitive psychology. Korean J Med Educ. 2019;31(4):299.
