# Supplementary material for: Collaborative intelligence in AI: Evaluating the performance of a council of AIs on the USMLE
Source: PLOS Digit Health. 2025 Oct 9;4(10):e0000787. doi: 10.1371/journal.pdig.0000787 (PMC12510544; doi:10.1371/journal.pdig.0000787)
Supplement: S2 File — (ZIP) [file pdig.0000787.s002.zip › S2 Data Raw data files containing unprocessed question inputs and LLM outputs including transcripts of deliberations/STEP1_Question-1-1_Type-MC-NJ.docx]

**Facilitator** **[Round 1]:** Serum LDL-cholesterol concentrations are maeasured in blood samples collected from 25 healthy volunteers. The data follow a normal distribution. The mean and standard deviation for this group are 130 mg/dL and 25 mg/dL, respectively. The standard error of the mean is 5.0. With a 95% confidence level, the true mean for the population from which this sample was drawn falls within which of the following ranges (in mg/dL)? (A) 105-155 (B) 120-140 (C) 125-135 (D) 128-132 (E) 129-131

**AI_D:** (1) RESTATING THE QUESTION: In essence, this query requires understanding the concept of calculating a confidence interval to predict the true population mean of LDL-cholesterol concentrations based on the data from a sample of 25 healthy volunteers. LDL-cholesterol is a type of blood lipid that indicates cardiovascular health, and is normally distributed in this scenario. The sample group's mean is 130 mg/dL, the standard deviation is 25 mg/dL, and the standard error of the mean is 5.0. Confidence interval, commonly set at 95%, gives a range where the true population mean is likely to be found. (2) PLANNING: To establish the confidence interval, the formula used is (mean - Z*SEM to mean + Z*SEM), where Z represents the Z score (for 95% confidence level, it is approximately 1.96) and SEM refers to the standard error of the mean. Given the available information, the ranges in the proposed answers can be checked to see which falls within the calculated confidence interval. (3) CLINICAL REASONING: Identifying the values in this case: the mean equals 130, Z is 1.96 for the 95% confidence level, and we know the standard error of the mean (SEM) is given as 5. Applying these in the confidence interval formula, we get the range as (130 - 1.96*5 to 130 + 1.96*5). (4) CONCLUDING: Calculating the above values yields (130 - 9.8 to 130 + 9.8), which sums up to the range (120.2 to 139.8). This interval would be the estimated range within which the true population mean is likely to appear with a 95% confidence level. (5) RESPONSE: Therefore, based on this calculation, the true mean for the population from which the sample was drawn falls within the range of 120-140 mg/dL. The answer should be choice (B) 120-140 mg/dL.

**AI_C:** (1) RESTATING THE QUESTION: The question here revolves around estimation of the true mean LDL-cholesterol level in a population, based on a sample of 25 volunteers. In this context, some key statistical concepts like mean (average value), standard deviation (measure of data dispersion), normal distribution (a type of frequency distribution where data is symmetrically dispersed around the mean), and standard error of the mean (an estimate of how much the sample mean is likely to vary from the true population mean) are brought up. A 95% confidence level (indicates percentage of all possible samples that can be expected to include the true population parameter) is used to establish a range within which the true population mean is likely to fall. (2) PLANNING: We need to answer which of the given ranges is the correct 95% confidence interval for the true population mean. We know that the 95% confidence interval is calculated by using the formula: mean ± 1.96*standard error. Considering the mean as 130 mg/dL, standard deviation as 25 mg/dL and standard error of means as 5 mg/dL, we should hypothesize that the interval calculated would be the valid range. (3) CLINICAL REASONING: Let's calculate the 95% confidence interval which will give us the range. Confidence interval = mean ± 1.96*standard error. Substitute the given data into the formula: 130 ± 1.96*5. Simplifying this we would get: 130 ± 9.8. This gives a range of roughly 120.2 to 139.8 mg/dL. (4) CONCLUDING: Upon calculation, we find that the range that falls within the 95% confidence interval is around 120.2 to 139.8 mg/dL. (5) RESPONSE: Given the provided alternatives, option (B) 120-140 mg/dL is the closest estimation to the calculated 95% confidence interval, and thus, it is reasonable to suggest that the true mean for the population from which this sample was drawn likely falls within this range.

**AI_E:** Restating your question, it appears you're asking about inferential statistics, in particular, 95% confidence intervals in relation to a sample of LDL cholesterol levels from 25 healthy individuals. Your dataset is normally distributed, with a sample mean of 130mg/dL and a standard deviation of 25mg/dL. You want to infer the true population mean from this sample data, using the standard error of 5mg/dL. The aim here is to identify the range within which the true mean LDL cholesterol level of the population lies, with 95% confidence. The calculation of a 95% confidence interval relies on several assumptions. One such assumption is that the sample is representative of the population and that the data is normally distributed. As per the given information, the data is normally distributed and the sample is taken from healthy volunteers. It is also critical that the standard error is correct. As per the above, I would assert that the key clinical detail to prioritize is the standard error. This is because a 95% confidence interval is calculated as the mean plus or minus two times the standard error (since 95% of data falls within two standard deviations from the mean in a normal distribution). When it comes to clinical reasoning, it's important to understand that the process of deriving a confidence interval involves using statistical techniques to infer population parameters from sample data. In this case, the given mean (130 mg/dL) and the standard error (5 mg/dL) represents the building blocks for this inference. In normal distribution data, a 95% confidence interval lies within two standard deviations of the mean (to either side). Hence, the 95% confidence lies between (mean - 2*standard error) and (mean + 2*standard error). Consequent to this reasoning, one could calculate the 95% confidence interval as follows: the lower end of the interval would be (130 - 2*5) = 120 mg/dL and the upper end would be (130 + 2*5) = 140 mg/dL. With this information, the final response would be that with a 95% confidence level, the true mean LDL cholesterol level for the total population from which this sample was drawn falls within the range of 120-140mg/dL. Referring to your answer options, this corresponds to choice (B) (120-140mg/dL).

**AI_B:** (1) RESTATING THE QUESTION: In simpler terms, this question is asking which range of values is likely to contain the true mean LDL-cholesterol concentration in the population from which the 25 healthy volunteers were sampled. The dataset we have follows a normal or bell-shaped distribution. This distribution gives us an average, or mean, of 130 mg/dL and a standard deviation of 25 mg/dL. The standard error of the mean, which measures the precision with which the sample mean estimates the population mean, is 5.0, and we want to apply a 95% confidence level to pinpoint the likely range for the true mean. (2) PLANNING: Stats-wise, the 95% confidence level indicates how likely it is that the true population mean lies within an established range. To calculate this, we generally use the mean ± (Z value corresponding to 95% confidence level multiplied by the standard error). The Z value for a 95% confidence level, from a standard statistical table, is roughly 1.96. An obvious assumption is to multiply 5.0 (standard error) by the Z value and add and subtract the result from the mean. However, this is under the premise that the Z value, standard deviation, standard error and mean are all correctly provided and understood. (3) CLINICAL REASONING: If we rely on these assumptions and take the mean LDL-cholesterol concentration (130 mg/dL) and add and subtract the product of 1.96 (the Z value that corresponds to a 95% confidence level) and the standard error (5.0) we'd have an equation like this: 130 ± (1.96 x 5). (4) CONCLUDING: Following this calculation, the result should provide the confidence interval, which is the range within which we can reasonably expect the true population mean to be located 95% of the time. (5) RESPONSE: With the application of the formula and mathematics, the true mean for the population from which this sample was drawn can be expected to fall within the range 120 to 140 mg/dL (Option B).

**AI_A:** (1) RESTATING THE QUESTION: This question is asking us to use data obtained from a sample of 25 healthy volunteers, whose LDL-cholesterol concentrations have been measured, to estimate the true average or mean LDL-cholesterol concentration for the larger population that these volunteers represent. LDL is a form of cholesterol that's often labelled "bad" because high levels can lead to plaque buildup in your arteries and result in heart disease and stroke. The data are normally distributed, which means that they are distributed in a way that the majority of the measurements are close to the mean (130 mg/dL in this case), and follow a symmetric bell-shaped pattern. The standard deviation, which measures the amount of variation or dispersion of a set of values, is 25 mg/dL. The standard error of the mean, a measure of how spread out the means of samples from the same population are likely to be, is 5.0. The question wants us to identify, with 95% confidence, the range within which the true mean LDL level for the entire population would fall. (2) PLANNING: To address this question, we need to use the concept of a 95% confidence interval, which is a range within which we can be 95% certain that the true population mean lies. The formula for calculating a 95% confidence interval is mean ± (1.96 * standard error). The assumption being made is that the data follow a normal distribution and that the sample is representative of the population, therefore, we can calculate the confidence interval and infer from the sample to the population. If these assumptions are correct, then the 95% confidence interval should be the correct range for the population mean. (3) CLINICAL REASONING: Using the provided data, we can calculate the 95% confidence interval. Given that the mean is 130 mg/dL and the standard error is 5.0 mg/dL, applying this to the formula we previously discussed gives us 130 ± (1.96 * 5), which translates to 130 ± 9.8. Rounding gives us a range of 120.2 to 139.8, which can be roughly rounded to 120 to 140 mg/dL. (4) CONCLUDING: Based on calculations and using the assumption that the data is normally distributed and our sample accurately represents the population, the best estimation for the population mean LDL-cholesterol concentration would fall within the 120 mg/dL - 140 mg/dL range. (5) RESPONSE: So, the most fitting answer would be (B) 120-140.

**Facilitator** The question asks for the estimation of the true mean LDL-cholesterol level in a population, based on a sample of 25 healthy volunteers. The data is normally distributed, with a mean of 130 mg/dL, a standard deviation of 25 mg/dL, and a standard error of the mean of 5.0. The task is to identify the range within which the true population mean is likely to fall, with a 95% confidence level. The 95% confidence interval is calculated using the formula: mean ± 1.96*standard error. This formula is based on the assumption that the sample is representative of the population and that the data is normally distributed. The Z score for a 95% confidence level is approximately 1.96. Substituting the given data into the formula, we get: 130 ± 1.96*5, which simplifies to 130 ± 9.8. This gives a range of roughly 120.2 to 139.8 mg/dL. Therefore, based on these calculations and assumptions, the true mean for the population from which the sample was drawn is likely to fall within the range of 120-140 mg/dL. This corresponds to choice (B) 120-140 mg/dL.
