# Supplementary material for: Collaborative intelligence in AI: Evaluating the performance of a council of AIs on the USMLE
Source: PLOS Digit Health. 2025 Oct 9;4(10):e0000787. doi: 10.1371/journal.pdig.0000787 (PMC12510544; doi:10.1371/journal.pdig.0000787)
Supplement: S2 File — (ZIP) [file pdig.0000787.s002.zip › S2 Data Raw data files containing unprocessed question inputs and LLM outputs including transcripts of deliberations/STEP1_Question-10-1_Type-MC-NJ.docx]

**Facilitator** **[Round 1]:** In informing a couple that their newborn has Down syndrome, there is a specific, relatively limited amount of information that the consulting physician should give immediately. The rest can be discussed at a later time. Which of the following best explains the purpose of using this approach to disclosure? (A) Allowing the couple's primary care physician to discuss most of the information with them (B) Allowing the parents time to tell other family members (C) Delaying parental distress until the information is completely disclosed (D) Disclosing the most important information so that it can be understood as fully as possible (E) Influencing the parents' course of action about what is medically most appropriate

**AI_D:** 1) RESTATING THE QUESTION: The question is asking which reason best explains the strategy of initially sharing a limited amount of information with a couple whose newborn was diagnosed with Down Syndrome. The remaining information will be subsequently disclosed at a future time. 2) PLANNING: The core assumption in the question is that providing information in a phased manner serves a crucial purpose. Alternative assumptions could be: it's necessary to avoid an overload of information which can cause confusion and overwhelm the parents; or, the immediacy of information delivery is related to the necessity of immediate decision-making regarding the newborn's health care. 3) CLINICAL REASONING: As clinicians, our primary objective is to ensure the well-being of our patients. Care, compassion, and effective communication play a vital role when families are faced with complex diagnoses like Down Syndrome. Disclosing such information requires delicacy and sensitivity, especially when dealing with parents who may be emotionally vulnerable after the birth. Overloading them with comprehensive medical information right away could potentially increase their distress. Furthermore, understanding clinical information needs time, and it is crucial that the most important details are understood first. 4) CONCLUDING: Considering the aforementioned reasons, the disclosure strategy can be seen as a method to limit the initial emotional trauma for the parents, facilitate better understanding of the most important points, and provide room for more detailed discussions at a later stage. 5) RESPONSE: Thus, it seems that option D: "Disclosing the most important information so that it can be understood as fully as possible" best explains the purpose of using this approach to disclosure. It is designed to provide parents the opportunity to assimilate and understand the most critical information regarding their newborn's health condition first, which can support their coping process and decision-making capacity.

**AI_E:** (1) RESTATING THE QUESTION: The question is seeking to uncover the rationale behind the practice of initially divulging only a limited amount of information to parents when their newborn has been diagnosed with Down syndrome. The rest of the information can be supplied at a subsequent meeting. To be clear, Down syndrome is a chromosomal disorder that is often associated with physical growth issues, distinctive facial features, and a degree of intellectual disability. (2) PLANNING: The implicit hypothesis here is that this sort of measured disclosure is done for a purpose. What that purpose could be may revolve around patient-centered care principles, issues of health communication between physicians and patients, or parental involvement in decision making, among others. Alternatives might include focusing on the role of primary care physicians, providing time for family involvement, managing parental distress, effective communication, or steering parents towards preferable medical decisions. (3) CLINICAL REASONING: Down syndrome is a lifelong condition that requires comprehensive management and can significantly impact the family. While it's essential for physicians to communicate the diagnosis to parents, the scope and timing of the information provided is also crucial. We don't want to overwhelm them with too much information at an already stressful time. The first discussion should focus on the most immediate information that the parents need while future meetings can delve into more nuances of the condition. The role of primary care physicians is important, but the responsibility of conveying the diagnosis usually lies with the physician handling the case. As regards managing the distress of the parents, providing measured information might provide a buffer, but the aim is not to 'delay' distress. Rather, it's about managing it. Parents discussing with family may happen, but it does not stand as the primary reason for the phased disclosure. And finally, while optimal medical decisions are sought in every case, influencing parental actions is not necessarily the main aim of phased information disclosure. (4) CONCLUDING: The goal of phased disclosure in these contexts is primarily to help the parents grasp the situation. Giving them the most crucial, immediate details first allows for better understanding and assimilation of the rest at a later time. (5) RESPONSE: The best explanation for implementing a phased approach to disclosure is (D) Disclosing the most important information so that it can be understood as fully as possible.

**AI_A:** 1) RESTATING THE QUESTION: This question pertains to the ethics and communication skills needed in medical practice, especially when dealing with sensitive matters such as genetic conditions like Down syndrome. The scenario involves a consultation in which a physician is providing information to parents about their newborn's Down syndrome diagnosis. The question asks for the rationale behind the strategy of giving just some of the information at first and discussing the rest later. 2) PLANNING: In evaluating the choices, several assumptions can be made. Assumption (A) assumes that the couple's primary care physician is more appropriate to disclose detailed information about the condition. Assumption (B) implies the parents need time to relay the information to other family members. Assumption (C) suggests that delaying complete disclosure is intended to postpone any distress. Assumption (D) proposes the purpose is to ensure the most important information is comprehended by the parents, and (E) infers the information might be used to guide parental action towards the most appropriate medical pathway. Among these choices, prioritizing a real-world clinical scenario, it's safe to hypothesize that the disclosure of information about the condition would be arranged in a way that ensures the parents understand and absorb the key points, making (D) a plausible priority assumption. 3) CLINICAL REASONING: Processing a newborn's Down syndrome diagnosis is emotionally daunting for parents. Clinicians need to approach this carefully, taking into account the parents' emotional state and the complexity of the information being disclosed. Initially sharing the most crucial information allows them to understand the diagnosis and implications fully. Delaying the rest of the information until later could allow the parents time to process, absorb, and fully comprehend the initial disclosure without being overwhelmed by the volume and complexity of it all at once. This is better for their psychological health and improves their understanding, facilitating effective mutual decision making subsequently. 4) CONCLUDING: The step-by-step explanation of the clinical reasoning indicates that sharing the most important part of the information about Down syndrome right away would enable the parents to understand it fully without overwhelming them with details. It also allows them to take time to absorb the news, making this a more compassionate, effective approach. 5) RESPONSE: Thus, the purpose of this approach to disclosure might be best explained by option (D), "Disclosing the most important information so that it can be understood as fully as possible." This approach allows for a better understanding, fosters a healthy relationship between the parents and medical team, and ensures informed decision-making for the baby's future care.

**AI_C:** RESTATING THE QUESTION: The question inquires about the rationale behind the approach where a doctor shares some key details with parents about their newborn being diagnosed with Down Syndrome and opts to deliver the remainder of the information at a later stage. This approach involves providing a limited amount of information immediately and deferring the rest for a future discussion. The provided choices include enabling their primary care physician to continue the conversation, facilitating time for family disclosure, delaying the inevitable emotional distress, providing information in an easily digestible manner, or influencing the course of action regarding medical decisions. PLANNING: We need to examine the question from the perspective of why doctors might choose to convey information step-by-step, rather than all at once. Is it to allow another physician to continue the conversation? This seems unlikely, as the care team would want to ensure a consistent message. Is it to allow parents to tell other family members? While this might be a consideration, it does not seem to be the primary motivating factor. Is it to prolong distress until all details are known? This seems counterintuitive, as it could lead to increased anxiety for the parents. Is it to provide information in a comprehensible manner? This seems plausible, as it may help parents absorb the news better. Is it to influence the parents' decisions about medical care? This could be a factor, but it would not necessarily require limited initial information. CLINICAL REASONING: It is unlikely that the consulting physician would limit their initial information to allow the couple's primary care physician to continue the discussion. The idea of having consistent information and emotional support would be crucial in such stressful situations. The option of allowing parents time to inform other family members may be a consideration but it doesn't seem like the primary intention of limiting initial information. Delaying parental distress until the information is completely disclosed seems counterproductive as it would arguably increase anxiety and stress of the unknown. Influencing the parents' course of action seems to be a potential factor, but doesn't necessarily equate to providing limited information initially. CONCLUDING: The idea of providing important information in a way that allows the parents to fully understand appears to be the primary reason for this approach. In such a stressful situation, it makes sense that information should be broken down and delivered at a pace that allows for best comprehension and retention. RESPONSE: Therefore, the best option seems to be (D) Disclosing the most important information so that it can be understood as fully as possible. This approach to breaking down complex, emotional news would allow for better parental understanding and subsequent decision-making.

**AI_B:** RESTATING THE QUESTION: The question wants us to determine the best reason for gently breaking the news of a baby born with Down Syndrome to the parents—providing them with only a certain amount of information at first, and filling in the rest of the details at a later stage. PLANNING: The first assumption would be that this approach is meant to lessen the burden of distress on the parents, as it would prevent them from being overwhelmed by an onslaught of complex and possibly distressing information all at once. Another assumption could be that this method is aimed at ensuring that the parents fully understand the implications of their child's condition, as compact, limited information might be easier to digest and comprehend. Yet another assumption is that this approach is intended to allow space for the primary physician to contribute to the discussion or for the parents to disclose the situation to other family members. We could also assume that this approach might be a strategy to influence medical decisions made by the parents. Clinically speaking, it seems most important to prioritize the ability of parents to understand the most significant aspects of their child's condition. CLINICAL REASONING: When a child is born with a disorder as serious as Down Syndrome, the parents can easily be overwhelmed. Notwithstanding that, it is crucial that they comprehend the key facts about their child's state. Moreover, the way information is disclosed to patients or their kin—especially about life-altering conditions—plays a pivotal role in their ability to absorb and understand it. Oversaturation might render them numb or confused, hindering their capacity to grasp the essential points or ask relevant questions. CONCLUDING: Given our understanding that the initial disclosure of such critical information should aim to facilitate comprehension of the condition without overwhelming the patient or their kin, we can identify the idea that the staged approach of revealing Down syndrome to new parents aims to provide them crucial information about the condition that they can understand fully—as the most clinically relevant reason. RESPONSE: So, looking at the options listed, (D) Disclosing the most important information so that it can be understood as fully as possible seems to best explain the purpose of using this approach to disclosure. The immediate understanding of essential information can aid in the initial adaptation to the diagnosis and enable the parents to make any necessary immediate decisions.

**Facilitator** The consensus among the AI responses is that the strategy of initially providing a limited amount of information to parents about their newborn's Down syndrome diagnosis serves a crucial purpose. This approach is not primarily intended to allow the primary care physician to continue the discussion, to give parents time to inform other family members, to delay parental distress until all information is disclosed, or to influence the parents' course of action regarding medical decisions. Instead, the primary rationale behind this strategy is to ensure that the most important information is understood as fully as possible by the parents. This approach allows for better comprehension and absorption of the news, which can be emotionally overwhelming. By focusing on the most critical information first, parents are less likely to be confused or overwhelmed by the volume and complexity of the information. This facilitates effective mutual decision-making for the baby's future care, supports the parents' coping process, and fosters a healthy relationship between the parents and the medical team. Therefore, the consensus is that option (D) "Disclosing the most important information so that it can be understood as fully as possible" best explains the purpose of using this approach to disclosure.
