# Supplementary material for: Collaborative intelligence in AI: Evaluating the performance of a council of AIs on the USMLE
Source: PLOS Digit Health. 2025 Oct 9;4(10):e0000787. doi: 10.1371/journal.pdig.0000787 (PMC12510544; doi:10.1371/journal.pdig.0000787)
Supplement: S2 File — (ZIP) [file pdig.0000787.s002.zip › S2 Data Raw data files containing unprocessed question inputs and LLM outputs including transcripts of deliberations/STEP1_Question-101-1_Type-MC-NJ.docx]

**Facilitator** **[Round 1]:** A 15-year-old girl is brought to the rehabilitation facility for physical therapy. Two months ago, she was involved in a motor vehicle collision in which she sustained a crush injury to the ulnar nerve at the right elbow. Neurologic examination shows severe weakness and atrophy of the intrinsic muscles of the right hand. When the patient is asked to grasp the therapist’s fingers, right handgrip is noticeably weaker than left handgrip. Sensation to pinprick and fine touch is decreased at the palmar aspect of the ring and small fingers of the right hand. No other deficits are noted. Which of the following cell types is critical for recovery of neurologic function in this patient? (A) Astrocytes (B) Oligodendrocytes (C) Satellite cells (D) Schwann cells (E) Tanycytes

**AI_A:** (1) RESTATING THE QUESTION: The query outlines a scenario where a 15-year-old girl has sustained an ulnar nerve injury at the right elbow due to a vehicular accident and is currently experiencing significant weakness and sensation loss in the right hand. The question aims to understand which cell type would be crucial for the neurological recovery of this patient. The cell types listed include astrocytes, oligodendrocytes, satellite cells, Schwann cells, and tanycytes. (2) PLANNING: The recovery of neurological function after an injury depends on the regeneration of nerve cells, which is facilitated by certain supportive cells. The assumption here is which cell type plays a role in this recovery. To evaluate this, we should think about the location and role of each of the cells listed. Astrocytes, oligodendrocytes, satellite cells, tanycytes are found in the central nervous system. Schwann cells, however, are part of the peripheral nervous system. Given that the ulnar nerve is part of the peripheral nervous system, it may be more accurate to hypothesize Schwann cells play a key role in this patient's recovery. (3) CLINICAL REASONING: Astrocytes are responsible for maintaining the blood-brain barrier and modulate signal transmission in the central nervous system. Oligodendrocytes provide myelin for neurons within the central nervous system. Satellite cells are found surrounding neurons in the peripheral nervous system, specifically in sensory, sympathetic, and parasympathetic ganglia, providing structural support. Tanycytes are specialized ependymal cells in the third ventricle of the brain, involved in monitoring cerebrospinal fluid or controlling the flow of molecules between blood and the brain. None of these would directly relate to a peripheral nerve injury. On the other hand, Schwann cells, specific to the peripheral nervous system, not only provide myelination but also cleanliness and regeneration of axons; these features are fundamental for nerve recovery. (4) CONCLUDING: After evaluating all these cells, it is clear that the ones most directly involved in the process of nerve recovery, particularly in the context of a peripheral nerve like the ulnar nerve, are the Schwann cells. (5) RESPONSE: Therefore, in this patient, the critical cell type for recovery of neurological function following a crush injury to the ulnar nerve at the right elbow is 'Schwann cells' (D).

**AI_C:** RESTATING THE QUESTION: The question is asking the type of cell that plays a critical role in restoring the functionality of the nervous system in a 15-year-old girl who sustained damage to her ulnar nerve, evidenced by weakness and atrophy of the right hand's intrinsic muscles as well as decreased sensation in certain fingers. PLANNING: Among the listed cell types, the one to consider clinically in relation to nerve injury and recovery would be Schwann cells, oligodendrocytes, astocytes, satellite cells, and tanycytes. Schwann cells and oligodendrocytes are known for their role in the myelination of peripheral and central nervous system neurons, respectively. In contrast, astrocytes, among numerous roles, help maintain blood-brain barrier integrity and regulate ions, neurotransmitters, and energy metabolites. Satellite cells, which surround peripheral nerve ganglia, provide nutritional support, while tanycytes, a type of ependymal cell, are mostly associated with neuroendocrine functions. CLINICAL REASONING: Examining each type's relation to neurologic function recovery, Astrocytes primarily function within the central nervous system and are not directly involved in peripheral nerve repair. Oligodendrocytes help form the myelin sheath around neurons in the central nervous system; however, the crush injury in this case affected the ulnar nerve, part of the peripheral nervous system. Therefore, the oligodendrocytes are less likely to be directly involved. Satellite cells, while they do support peripheral nerves, primarily provide nutritional support and don't play a direct role in the regeneration of injured nerves. Tanycytes, although a part of the nervous system, are associated with hypothalamic function and are not related to peripheral nerve function or repair. Schwann cells, on the other hand, are associated with the peripheral nervous system and play a direct role in the repair and regeneration process following nerve injury by promoting re-myelination of the damaged nerve fibers. They can proliferate after nerve injuries, organizing themselves into structures known as bands of Büngner, which guide the regrowing axon back to its original target cells. CONCLUDING: Within the context of a peripheral nerve injury such as the one presented in the case of the young girl with the ulnar nerve crush injury, the cell type most critical for recovery would be the Schwann cells. RESPONSE: Therefore, the correct answer is (D) Schwann cells. They are vital for the regeneration and recovery of neurologic function in this patient following the injury to her ulnar nerve.

**AI_D:** RESTATING THE QUESTION: The question is asking which specific type of cell would be critical in the recovery of neurological functionality for a 15-year-old female patient who, after a motor vehicle collision, has resulted in crush injury to the ulnar nerve at the right elbow, causing substantial weakness and atrophy of the intricate muscles of her right hand. PLANNING: The assumption made in this scenario is that a specific type of cell plays a key role in nerve recovery following injury. We need to evaluate the roles of each listed cell type in neurologic function. Astrocytes, part of the central nervous system (CNS), help in maintaining the blood-brain barrier. Oligodendrocytes, also part of the CNS, are responsible for myelination of neurons. Satellite cells surround neuron cell bodies in the peripheral nervous system (PNS) and provide support. Schwann cells, in the PNS, contribute to nerve myelination and regeneration. Finally, Tanycytes are ependymal cells involved in neurogenesis in the adult hypothalamus. In the context of the peripheral nerve injury described, the key assumption that can be made based on provided information would be that Schwann cells play a significant role in this context. CLINICAL REASONING: In response to peripheral nerve injury such as the crush injury to the ulnar nerve at the right elbow outlined here. Schwann cells are vital for a couple of reasons. Firstly, they reorganize themselves in order to form a pathway (a band of Bunger) along which new nerve sprouts can grow, lending physical support. Secondly, they secrete chemical factors that promote the survival and growth of neurons. It's important to note that other cells, particularly astrocytes and oligodendrocytes, despite being involved in some nerve repair processes, are predominantly associated with the central nervous system (CNS) rather than the peripheral nervous system (PNS), which is where the injury here occurred. CONCLUDING: Using this step-by-step clinical reasoning methodology, it becomes apparent that it's the Schwann cells which play the most significant role in recovery from injuries such as the one described. While all of the listed cell types are known to be involved in aspects of neurologic function, Schwann cells are the ones most specifically equipped to promote nerve regeneration in the peripheral nervous system (PNS) following injury. RESPONSE: (D) Schwann cells are the cell type critical for recovery of neurological function in this patient, given their role in both physical and chemical support of nerve regeneration in the peripheral nervous system.

**AI_B:** (1) RESTATING THE QUESTION: You're asking me about a teenage girl who had a motor vehicle collision around two months ago, which resulted in a crush injury to the ulnar nerve at her right elbow. This has led to severe weakness and atrophy of her right hand's intrinsic muscles, showing a noticeable difference in strength from her left hand. Apart from diminished sensation (both pin-prick and fine touch) on her right hand's ring and small fingers, no other deficits have been recorded. You'd like to know which among the cell types listed would have a crucial part in the recovery of her neurologic function. The cell types are: (A) Astrocytes: star-shaped glial cells in the brain and spinal cord (B) Oligodendrocytes: glial cells in the central nervous system that form the myelin sheath around nerve fibers (C) Satellite cells: glial cells located in the peripheral nervous system (D) Schwann cells: glial cells in the peripheral nervous system that form the myelin sheath around nerve fibers (E) Tanycytes: ependymal cells located in the third ventricle of the brain involved in neuroendocrine functions. (2) PLANNING: The primary question here is regarding which cell type would be critical for the girl's neurological recovery. An assumption that could be made is that the cell type particularly associated with peripheral nerve damage is relevant; further, the contribution of that cell type to the regenerative process after peripheral nerve injury could be hypothesized as significant. (3) CLINICAL REASONING: The damage caused by the accident to the patient's right ulnar nerve, a component of the peripheral nervous system, led to deficits in strength and sensation. Glial cells, which provide support and protection for nerve cells, are essential for nerve function and recovery from injury. While all mentioned cells are types of glial cells, not all are found in the peripheral nervous system. Astrocytes and oligodendrocytes are mainly found in the central nervous system, while tanycytes are found in the brain. Satellite cells are found in the peripheral nervous system, but they primarily support sensory neuron cell bodies located in the ganglia of the peripheral nervous system. On the other hand, Schwann cells are responsible for the formation and maintenance of myelin sheath in the peripheral nerves, aiding in efficient nerve signals. Moreover, Schwann cells are known for their ability to reproduce and form cellular pathways, serving to guide regrowing axons back to their original junctions post-injury, which is critical for the recovery process. (4) CONCLUDING: As the question asks for a crucial cell type for recovery in peripheral nerve injury, the focus is specifically on the cells active in the peripheral nervous system and involved in its repair mechanism. With their unique ability to foster repair and regeneration in the peripheral nerves, Schwann cells seem to be the best fit in this context. (5) RESPONSE: The cell type that is most critical for the recovery of neurologic function in this patient is (D) Schwann cells.

**AI_E:** RESTATING THE QUESTION: The question is asking which types of cells will be crucial for this 15-year-old girl's recovery from a crush injury to the ulnar nerve at her right elbow. This nerve damage has caused significant impairment of her right hand, leading to loss of strength and grip, muscle atrophy, and diminished pinprick and fine touch sensation in parts of her hand. The available options consist of various nerve and glial cells, and the objective is to identify the one most important for nerve repair and function restoration. PLANNING: The main assumptions here revolve around the understanding of the function and role of each cell type mentioned. Astrocytes, found in the central nervous system (CNS), are critical for maintaining the blood brain-barrier, supplying nutrients to nervous tissue, and a series of other tasks but are not directly involved in peripheral nerve repair. Oligodendrocytes are also CNS cells, and are responsible for the myelination of axons within that system. Satellite cells are a type of glial cell found in the peripheral nervous system (PNS), mainly around sensory, sympathetic, and parasympathetic neurons, providing structural support. Schwann cells also reside in the PNS, but they play a crucial role in forming myelin sheaths around neurons. Finally, tanycytes are specialized ependymal cells, mainly involved in metabolic functions in the region of the third ventricle of the brain. This would imply the Schwann cells would be the logical choice due to their importance in nerve regeneration and repair. CLINICAL REASONING: The crush injury mentioned, without other mention of central nervous system damage, suggests the damage is localized to the peripheral nervous system, specifically the ulnar nerve. Schwann cells are the principal glial cells of the PNS and play a significant role in nerve regeneration by forming myelin, which helps speed up nerve signal transmission. In peripheral nerve injury, Schwann cells become activated, proliferate, and differentiate into a phenotype that promotes repair via clearance of debris, production of growth factors, and guidance of axonal regrowth through formation of a structure called Bands of Büngner. This indicates the vital importance of Schwann cells in the series of events required in the repair process following a peripheral nerve injury. CONCLUDING: Considering the role of Schwann cells in promoting debris clearance, growth factor production, and axonal regrowth following peripheral nerve injury denotes a paramount part in the repair mechanism. Without these cells, efficient and effective nerve recovery wouldn't take place. This emphasizes the crucial nature of these cells in the repair and thus recovery from peripheral nerve injuries such as the one presented by this young patient. RESPONSE: Examining all these facts and perspectives, we can state that the cell type which is crucial for the recovery of a crush injury to the ulnar nerve in this case is (D) Schwann cells.

**Facilitator** The question asks which cell type is critical for the recovery of neurological function in a 15-year-old girl who sustained a crush injury to the ulnar nerve at her right elbow, resulting in severe weakness and atrophy of the intrinsic muscles of her right hand, as well as decreased sensation in certain fingers. The cell types listed are astrocytes, oligodendrocytes, satellite cells, Schwann cells, and tanycytes. Astrocytes, oligodendrocytes, and tanycytes are primarily found in the central nervous system and are not directly involved in peripheral nerve repair. Satellite cells, while present in the peripheral nervous system, mainly provide nutritional support and do not play a direct role in nerve regeneration. On the other hand, Schwann cells, which are part of the peripheral nervous system, play a crucial role in nerve recovery. They are responsible for the formation and maintenance of the myelin sheath in peripheral nerves, aiding in efficient nerve signal transmission. Moreover, Schwann cells have the ability to reproduce and form cellular pathways, guiding regrowing axons back to their original junctions post-injury, which is critical for the recovery process. Therefore, the consensus among the responses is that the cell type most critical for the recovery of neurologic function in this patient is Schwann cells.
