# Supplementary material for: Collaborative intelligence in AI: Evaluating the performance of a council of AIs on the USMLE
Source: PLOS Digit Health. 2025 Oct 9;4(10):e0000787. doi: 10.1371/journal.pdig.0000787 (PMC12510544; doi:10.1371/journal.pdig.0000787)
Supplement: S2 File — (ZIP) [file pdig.0000787.s002.zip › S2 Data Raw data files containing unprocessed question inputs and LLM outputs including transcripts of deliberations/STEP1_Question-102-1_Type-MC-NJ.docx]

**Facilitator** **[Round 1]:** A 3-year-old boy is brought to the office because of a 2-day history of bulging of his left eye. He says his eye hurts. He has no history of major medical illness or recent trauma to the area, and he receives no medications. Vital signs are within normal limits. Physical examination shows exophthalmos of the left eye. MRI of the brain shows a 2-cm mass involving the ocular muscles of the left eye. A biopsy specimen of the mass shows malignant cells, some of which have striations. Which of the following is the most likely diagnosis? (A) Neuroblastoma (B) Pheochromocytoma (C) Retinoblastoma (D) Rhabdomyosarcoma (E) Thyroid cancer

**AI_B:** (1) RESTATING THE QUESTION: The patient is a young child who has a rapidly developing, painful exophthalmos. Exophthalmos, or protrusion of the eye, is noted upon physical examination. This is due to a mass located in the ocular muscles, containing malignant cells with striations as determined by biopsy. We must determine the most likely diagnosis from the given options. The list of diagnoses provided include Neuroblastoma typically occurs in adrenal glands or along the spine; Pheochromocytoma originates from adrenal gland; Retinoblastoma arises from the retina; Rhabdomyosarcoma typically presents in striated (skeletal) muscle tissue; and Thyroid cancer, often associated with exophthalmos, but it typically forms within the thyroid gland itself. (2) PLANNING: Let's consider that the presence of striations in the malignant cells suggests the origin of these cells is from some type of muscle tissue. Given the location of the mass and the presence of striations in the cells, one might hypothesize that the mass originated from striated muscle, which would suggest a diagnosis of Rhabdomyosarcoma. (3) CLINICAL REASONING: If the mass indeed originated from the striated muscle which makes up the ocular muscles, this would explain the physical sign of exophthalmos. Furthermore, Rhabdomyosarcoma often presents in those under the age of 6, which aligns with the patient's age. The rapid progression, location, and characteristics of the malignant cells would lead us to deprioritize conditions such as Neuroblastoma, Pheochromocytoma, Retinoblastoma, and Thyroid cancer which do not align as closely with the patient's signs and symptoms. (4) CONCLUDING: Keeping in mind the age of the patient, the presentation of the exophthalmos, the rapid progression of symptoms, the location, and histological evidence, the clinical information points towards a diagnosis of Rhabdomyosarcoma. (5) RESPONSE: Therefore, the most likely diagnosis for the 3-year-old boy exhibiting exophthalmos and having a mass in his ocular muscles showing cells with striations is likely to be (D) Rhabdomyosarcoma.

**AI_E:** (1) RESTATING THE QUESTION: I see that we're talking about a young child who's presenting with bulging, or exophthalmos, of his left eye. There's a mass found in his ocular muscles and a biopsy shows that there are cancerous cells present - some of which have striations. And we're being asked to determine the most probable diagnosis from a selection of cancers: neuroblastoma, pheochromocytoma, retinoblastoma, rhabdomyosarcoma, or thyroid cancer. (2) PLANNING: The critical piece here is to narrow down which of these cancers is most likely given the patient's age, symptoms, physical findings, and biopsy results. Each of these cancers is known for presenting in specific ways and in specific locations. Our assumptions here are that the child is otherwise healthy, and the striations seen in the cancer cells are significant. An alternate assumption could be that the location of the mass is primary to the diagnosis, given it is located in the ocular muscles. (3) CLINICAL REASONING: Neuroblastoma is typically found in the adrenal glands or along the sympathetic chain, so that's an unlikely choice here. Pheochromocytoma also mostly affects the adrenal glands and is uncommon in children. Retinoblastoma presents typically with leukocoria (white pupillary reflex) rather than exophthalmos, and doesn't usually invade ocular muscles. Thyroid cancer rarely presents in children or with exophthalmos, and striations in the malignant cells are atypical. However, Rhabdomyosarcoma, a malignant tumor of striated muscle, is common in children and can involve the eye. Striated muscle fibers in the biopsy would strongly suggest a muscle origin for the tumor. (4) CONCLUDING: Seeing striations in the cancer cells from the biopsy — along with the child's reported symptoms, the fact a mass has been found in his ocular muscles, and his age — suggests the malignant tumor growing in his eye muscles is of the type commonly found in children in those muscles. (5) RESPONSE: Therefore, the most likely diagnosis is (D) Rhabdomyosarcoma.

**AI_D:** RESTATING THE QUESTION: To distill, we're presented with a case of a 3-year-old boy who is without any major prior medical history, and who has been brought in due to two days of painful bulging in his left eye known as exophthalmos. There's a detected 2-cm mass in the ocular muscles of his left eye as per an MRI scan, and a biopsy of this mass reveals malignancy with some striated cells. The puzzle to solve here is: What would be the most likely diagnosis for this child from these given choices? PLANNING: Our assumptions are based on the boy's symptoms and physical exam findings, specifically his age, his exophthalmos with pain, the mass in the ocular muscles, and the biopsy findings. One assumption could be an orbital malignancy, more specifically a rhabdomyosarcoma, due to the striated malignant cells found in the biopsy. We could also consider other types of ocular tumors like retinoblastoma. However, retinoblastoma usually presents as a white pupillary reflex (leukocoria) and not exophthalmos. Neuroblastoma can lead to orbital metastases but is less likely as primary findings do not suggest an adrenal or sympathetic nervous system tumor. Pheochromocytoma in children is comparatively rarer, and its characteristics, like hypertension, are not present here. Thyroid cancer also seems unlikely due to the patient's age and the lack of associated symptoms such as neck masses. Therefore, rhabdomyosarcoma appears to be the most plausible assumption to pursue. CLINICAL REASONING: orbital Rhabdomyosarcoma is the most common soft tissue sarcoma in children. It originates from the muscle tissues and can involve any muscle group, but in the head and neck area, the orbit is a common site. The presentation of a painful, suddenly appearing mass with eye bulging and motion limitation is typical. The findings on MRI and the presence of striations in the malignant cells in the biopsy support a diagnosis of rhabdomyosarcoma. Striated cells are connected to muscle tissue, which is consistent with a rhabdomyosarcoma, but not with neuroblastoma, retinoblastoma, pheochromocytoma or thyroid cancer. CONCLUDING: Given all these reasons, rhabdomyosarcoma is the most probable diagnosis, as it fits the clinical findings, radiological findings, and histology. RESPONSE: Therefore, the most likely diagnosis for this 3-year-old boy with exophthalmos, a mass involving the ocular muscles identified by MRI, and the presence of striated malignant cells upon biopsy is option (D) Rhabdomyosarcoma.

**AI_A:** (1) RESTATING THE QUESTION: A three-year-old boy came presenting with a bulging left eye of two days’ duration and pain associated with it. He has no significant prior health problems, no recent injury, and takes no medications. His vital parameters are in the reference range. Upon examination, there's a protrusion of the left eyeball, which is medically termed "exophthalmos". His brain MRI revealed a mass, 2 cm in size, located in the left eye's muscular structures. A pathological investigation of tissue from this mass revealed malignant cells, some of which displayed striations. The query is to identify the most probable diagnosis from the given choices which are; neuroblastoma, pheochromocytoma, retinoblastoma, rhabdomyosarcoma, and thyroid cancer. (2) PLANNING: The foundational assumption here rests on the location of the mass, the patient's age, and the cellular evidence of malignancy with striations. The differential diagnoses for a child with a bulging eye include a range of malignant and benign conditions that can affect the orbit, retina, or thyroid. However, given the MRI finding showing involvement of the ocular muscles, the orbital focus needs to be emphasized. When we add the pathological finding of 'malignant cells with striations', it is apparent that the tumor consists of muscle cells, indicating a sarcoma. Considering this assumption, the most likely diagnosis to be considered here is orbital rhabdomyosarcoma, which is relatively more common in this age group. (3) CLINICAL REASONING: The given symptoms and examination findings suggest a local process affecting the child's left eye. The lack of past significant medical history or recent trauma suggests a primary disorder rather than a secondary one due to an underlying condition or recent injury. The MRI finding emphasizes this fact, showing a localized mass in the ocular muscles, ruling out widespread neurological or systemic diseases. The pathological investigation revealing malignant cells with striations points towards a malignancy of muscle origin, typically sarcomas. Among given diagnoses, condition (D) Rhabdomyosarcoma, a malignant disease of striated muscle, fits best with these clinical, radiological, and pathological findings. (4) CONCLUDING: Our step-by-step analysis commenced with a symptomatic, radiological, and pathological presentation suggestive of a primary orbital malignancy of muscle origin. The questioning approach to each underlying presumption and fact has led us to advocate a diagnosis of a muscle-originating malignancy as the primary cause. As considering the child's age, lack of trauma history or another significant disease, and the biopsy findings coincide with this, this young patient is likely to have an orbital rhabdomyosarcoma. (5) RESPONSE: Given the available information and the clinical reasoning process, the answer is (D) rhabdomyosarcoma.

**AI_C:** (1) RESTATING THE QUESTION: We're asked to analyze the case of a three-year-old boy who has been suffering from a bulging, painful eye for two days. He doesn't have any records of severe disease or injury, and doesn't take any medications. His vitals are normal. Examination has shown an outward bulging of his left eye, a condition known as exophthalmos, and an MRI revealed a 2-cm mass in the ocular muscles of the same eye. A biopsy of this mass demonstrated malignant cells, with some displaying striations. We need to select the most suitable diagnosis from five options: Neuroblastoma, Pheochromocytoma, Retinoblastoma, Rhabdomyosarcoma, or Thyroid cancer. (2) PLANNING: Several assumptions can be inferred from this case presentation. For instance, we could conjecture that this case might be ophthalmologic in nature given the eye's involvement. However, taking into account the other elements, particularly the presence of a mass involving ocular muscles and the finding of malignant cells with striations in the biopsy, we might question whether this could be indicative of a systemic disease or a specific type of tumor. The nature and location of the mass, together with the age of the patient, can provide us with clues towards a focused diagnostic approach. (3) CLINICAL REASONING: Each of the options presented, namely Neuroblastoma, Pheochromocytoma, Retinoblastoma, Rhabdomyosarcoma, and Thyroid cancer, represents different categories of childhood cancers, affecting different tissues and displaying varying symptoms. Neuroblastoma, for instance, usually presents with generalized symptoms and an abdominal mass, rather than ocular changes. Additionally, Pheochromocytoma is primarily an adrenal tumor mainly linked to hypertension, which is not evident in our case. Similarly, Thyroid cancer in children doesn't usually present with eye symptoms. Retinoblastoma, a tumor of the eye's retina, might initially seem plausible. However, retinoblastomas generally don't involve the eye muscles; moreover, it would not typically present with striated muscle fibers upon biopsy. Given the mass's location, the patient's age, and the presence of striations in the biopsy, the presentation points towards Rhabdomyosarcoma, a malignant soft tissue tumor found in striated muscles. (4) CONCLUDING: Following the clinical reasoning detailed above, the tumor affecting this child's ocular muscles is most consistent with a diagnosis of Rhabdomyosarcoma. The location and histological features of the mass, as well as the age of the patient, align with the typical clinical presentation of this disease. (5) RESPONSE: The most likely diagnosis in this case is Rhabdomyosarcoma (D). This type of cancer tends to strike children and is associated with a mass in striated muscle tissue, like the one seen in this three-year-old patient's ocular muscles.

**Facilitator** The consensus statement synthesizes the responses of AI_A, AI_B, AI_C, AI_D, and AI_E as follows: The case presents a three-year-old boy with a two-day history of painful bulging in his left eye, known as exophthalmos. An MRI reveals a 2-cm mass in the ocular muscles of the left eye, and a biopsy of this mass shows malignant cells, some of which have striations. The task is to identify the most probable diagnosis from the given options: Neuroblastoma, Pheochromocytoma, Retinoblastoma, Rhabdomyosarcoma, or Thyroid cancer. The clinical reasoning process involves considering the patient's age, symptoms, physical findings, and biopsy results. The presence of striations in the malignant cells suggests the origin of these cells is from some type of muscle tissue. Given the location of the mass and the presence of striations in the cells, the mass likely originated from striated muscle, suggesting a diagnosis of Rhabdomyosarcoma. Each of the other options represents different categories of childhood cancers, affecting different tissues and displaying varying symptoms. Neuroblastoma and Pheochromocytoma primarily affect the adrenal glands and are less likely given the absence of related symptoms. Retinoblastoma, a tumor of the eye's retina, might initially seem plausible. However, retinoblastomas generally don't involve the eye muscles and would not typically present with striated muscle fibers upon biopsy. Thyroid cancer in children doesn't usually present with eye symptoms. Given the mass's location, the patient's age, and the presence of striations in the biopsy, the presentation points towards Rhabdomyosarcoma, a malignant soft tissue tumor found in striated muscles. The location and histological features of the mass, as well as the age of the patient, align with the typical clinical presentation of this disease. Therefore, the consensus among the AI responses is that the most likely diagnosis for this three-year-old boy with exophthalmos, a mass involving the ocular muscles identified by MRI, and the presence of striated malignant cells upon biopsy is Rhabdomyosarcoma (D). This type of cancer tends to strike children and is associated with a mass in striated muscle tissue, like the one seen in this patient's ocular muscles.
