# Supplementary material for: Collaborative intelligence in AI: Evaluating the performance of a council of AIs on the USMLE
Source: PLOS Digit Health. 2025 Oct 9;4(10):e0000787. doi: 10.1371/journal.pdig.0000787 (PMC12510544; doi:10.1371/journal.pdig.0000787)
Supplement: S2 File — (ZIP) [file pdig.0000787.s002.zip › S2 Data Raw data files containing unprocessed question inputs and LLM outputs including transcripts of deliberations/STEP1_Question-103-1_Type-MC-NJ.docx]

**Facilitator** **[Round 1]:** An 11-year-old girl is brought to the emergency department by her parents because of a 1-week history of breast enlargement. She has not had pain or nipple discharge. She has asthma treated with inhaled albuterol as needed. She does not smoke cigarettes, drink alcoholic beverages, or use illicit drugs. She is at the 50th percentile for height and weight. Vital signs are within normal limits. Examination of the breasts shows minimal enlargement under the areolae and mild enlargement of the diameter of the areolae without nipple discharge; no masses are palpated. There is scant pubic hair. Which of the following best describes the sexual maturity rating for this patient? (A) 1 (B) 2 (C) 3 (D) 4 (E) 5

**AI_D:** 1) RESTATING THE QUESTION: This question discusses a young, 11-year-old girl presenting with minimal breast enlargement and scant pubic hair without any symptoms of pain or nipple discharge. The sexual maturity rating, also known as the Tanner staging, is a scale that measures physical development in children, adolescents, and adults. The scale defines physical measurements of development based on external primary and secondary sexual characteristics, such as the size of the breasts and the development of pubic hair. 2) PLANNING: There is the assumption that the onset of puberty corresponds with certain physical changes which are reflected in the Tanner stages. Another assumption here is that this girl's physical development corresponds to one of these stages. She has minimal breast enlargement and a small amount of pubic hair, both signs of puberty, and these are important clues. 3) CLINICAL REASONING: A step-wise analysis of the Tanner stages would be helpful here. Stage 1 is the child stage where there's no pubic hair and the breast buds have not started developing. This clearly isn't the case. Stage 2 is the beginning of puberty which is marked by the appearance of breast buds and a small amount of pubic hair starting to grow. This seems to match the patient's presentation. Stage 3 to 5 are subsequent stages of puberty characterized by increased breast size, pubic hair development, and menstrual periods in girls, none of which seems to be applicable to this girl based on the case information provided. 4) CONCLUDING: Given that this girl has minimal breast enlargement under the areolae and scant pubic hair, her signs are most consistent with the onset of puberty, making stage 2 the most likely Tanner stage. 5) RESPONSE: The sexual maturity rating for this patient best correlates with Tanner stage (B) 2.

**AI_E:** 1) RESTATING THE QUESTION: The question asks for the sexual maturity rating (Tanner staging) of an 11-year-old girl who has a week-long history of mild breast enlargement and some pubic hair, with no signs of discomfort or nipple discharge. It's worth noting that Tanner staging is a scale from 1 to 5 used to measure the development of secondary sexual characteristics during puberty. 2) PLANNING: In order to answer this question, I would need to have a clear understanding of the Tanner staging specifics. Stage 1 is pre-pubertal, Stage 2 is the onset of breast budding and sparse pubic hair, Stage 3 is further breast enlargement and pubic hair growth, Stage 4 is secondary mound formation and increased pubic hair, and Stage 5 is full adult development. Based on the description, we can conclude that the patient is beyond Stage 1 since there is breast enlargement and pubic hair. The assumption here is that the girl’s characteristics match one of these stages. 3) CLINICAL REASONING: The 11-year old girl presented with mild breast enlargement and scant pubic hair, which is suggestive of the starting of puberty. There are no signs indicating the presence of a higher stage such as the formation of a secondary mound in the breast (Stage 4 characteristic) or full adult development (Stage 5). Given her age and the signs she is showing, it can be reasoned that she is in the early stages of puberty. Thus we can hypothesize that she is likely in Tanner Stage 2 or perhaps 3, since these stages are characterized by the start of breast development and the appearance of pubic hair. 4) CONCLUDING: In reference to the Tanner staging of sexual maturation, the described characteristics (minimal enlargement under the areolae, mild enlargement of the diameter of the areolae, and scant pubic hair) align well with those typically seen in Stage 2. There isn't sufficient evidence to suggest that the girl has progressed to Stage 3, which would typically present with more substantial breast and pubic hair growth. 5) RESPONSE: Based on the patient's presentation and corresponding Tanner stages, the best description of the sexual maturity rating for this patient would be (B) 2.

**AI_B:** 1) RESTATING THE QUESTION: This clinical scenario describes an 11-year-old girl with a week history of breast enlargement, but without pain or nipple discharge. The girl is in overall good health, without any vices and with controlled asthma. She is within the normal range of height and weight corresponding to 50th percentile. The physical examination indicates minimal breast enlargement and the presence of scant pubic hair. The question asks us to identify the girl's sexual maturity rating (also known as Tanner stage), which is a systematic way to assess and document the growth and development of children and adolescents. 2) PLANNING: Key aspects to help determine the Tanner stage include breast development and pubic hair. The description points to initial stages of breast and pubic hair growth. Tanner stage 1 indicates preadolescent girls with no pubic hair and no breast tissue except for the papilla. Tanner stage 2 typically shows the beginning of breast bud development and the presence of scant, long, downy pubic hair. More advanced stages involve more significant changes. The scenario seems to suggest this patient is in one of the initial stages. 3) CLINICAL REASONING: The Tanner stages are a classification system used to quantify the physical changes that occur during puberty. Each stage is associated with specific changes in the development of secondary sexual characteristics. The features described - minimal enlargement under the areolae and scant pubic hair - suggest the initiation of puberty. Tanner stage 1 is characterized by a prepubertal state with no secondary sexual characteristics, which does not match this case. 4) CONCLUDING: Based on evaluation of the features described in the scenario, the girl doesn't seem to be in prepuberty (stage 1) or in advanced stages of puberty (stages 3, 4 or 5). Stage 2 is the most likely option, as it aligns with described features indicative of early puberty, such as the onset of breast development (including areolar enlargement) and the presence of scant pubic hair. 5) RESPONSE: Therefore, the Tanner stage (Sexual Maturity Rating) that best describes the sexual maturity rating for this 11-year old patient is (B) 2.

**AI_A:** (1) RESTATING THE QUESTION: The question is asking us to determine the sexual maturity rating, also known as Tanner stage, for an 11-year-old girl who has experienced minimal enlargement under the areolae, mild enlargement of the diameter of the areolae, and scant pubic hair. The Tanner stages range from 1 to 5, with 1 being preadolescent and 5 being adult maturity. (2) PLANNING: The key assumptions to clarify in the case are the physical signs relating to puberty onset, namely the onset of breast development and pubic hair growth. The Tanner staging considers both these aspects. Stage 1 is preadolescent, meaning no puberty changes. Stage 2 is the start of puberty, signaled by the appearance of breast buds and sparse, downy pubic hair. Stage 3 marks further enlargement of breasts and darkening and coarsening of pubic hair. Stage 4 shows continued growth with a secondary mound forming on the breast and dense but not fully adult-like pubic hair. Stage 5 is the final stage where breast and pubic hair growth reach adult patterns. (3) CLINICAL REASONING: Given the patient's age and the signs of minimal breast development under the areolae and scant pubic hair, it suggests the early stages of puberty. Breast budding ("thelarche") is often the earliest sign of puberty in girls, followed by sparse pubic hair ("pubarche"). With these signs present, it can be deduced that the girl would be beyond stage 1 Tanner staging, which marks preadolescence with no observable changes. However, detailed breast changes such as secondary mound formation associated with later stages (stage 4) are absent. Thus, she is not likely to be in the mid to late stages of puberty (stages 3-5). (4) CONCLUDING: By comparing the signs observed in the girl with the characteristics of each Tanner stage, it can be concluded that the girl is likely in the early stages of puberty. The minimal breast enlargement and scant pubic hair suggest that puberty has started but is not yet entirely underway. (5) RESPONSE: Therefore, the sexual maturity rating of this patient would be estimated to be Tanner Stage (B) 2, which represents the onset of breast budding and the start of pubic hair growth. This stage is consistent with the physical signs observed in the girl, suggesting that she is in the early phase of puberty.

**AI_C:** 1) RESTATING THE QUESTION: The case is about an 11-year-old girl who has shown signs of breast enlargement for a week. The enlargement is mild and localized under the areolae with a slight increase in the diameter of the areolae. No nipple discharge or painful sensation is reported. She also has minimal pubic hair. Based on this, the inquiry pertains to which stage her sexual maturity rating (SMR) or Tanner stages falls into. The Tanner stages, ranging from 1 to 5, are a recognized system for evaluating the development of secondary sexual characteristics in children and adolescents. 2) PLANNING: In this case, the key assumptions revolve around the girl's age, the signs of her breast development and the presence of pubic hair. Primary assumption could be that she is going through the normal phases of puberty which typically starts at this age, as suggested by the breast enlargement and appearance of pubic hair. An alternate assumption could be a pathophysiological alteration causing these changes, but given no other signs or symptoms, the probability of this is low. Therefore, a clinical prioritization should be towards normal physiological puberty-related changes. 3) CLINICAL REASONING: Usually, puberty in girls starts between ages 8 and 13 and during this transition period, multiple changes manifest based on hormone-driven developments. Tanner Stage 1 is considered pre-pubertal where no secondary sexual characteristics are seen. Tanner Stage 2 is usually the onset of puberty, characterized by breast budding or 'thelarche' and a small amount of pubic hair ('pubarche'). Here, the enlargement of breast tissue typically occurs under the nipple causing a small mound which, along with the minor enlargement of the areolae distinguish Stage 2. Tanner Stages 3,4 and 5 are characterized by more prominent changes including further enlargement of breasts and pubic hair, changes in shape and contour of breasts and, finally, mature stage where the areola and papilla form secondary mound above level of the breast. Keeping this patient's clinical signs in mind, Stage 3, 4, or 5 can be ruled out as the breasts are minimally enlarged, the areolae are mildly enlarged, and there is only scant pubic hair. 4) CONCLUDING: Based on a systematic approach using a clinical thought process, considering the girl’s age, recent onset of breast enlargement and minimal pubic hair; and by ruling out the more advanced Tanner stages, we can infer that this patient is likely to be in the initial phases of puberty which is characterized by Tanner Stage 2. 5) RESPONSE: Thus, the most suitable answer to the question posed is (B) 2, which corresponds to Tanner Stage 2 in the Sexual Maturity Rating scale.

**Facilitator** The question presents a case of an 11-year-old girl who has experienced a week of mild breast enlargement and the appearance of scant pubic hair, with no pain or nipple discharge. The task is to determine her sexual maturity rating, also known as Tanner stage, which is a scale used to assess the development of secondary sexual characteristics during puberty. The Tanner stages range from 1 (prepubescent) to 5 (adult maturity). The key factors to consider in this case are the girl's age, the onset of breast development, and the presence of pubic hair. The girl's symptoms suggest the early stages of puberty, as indicated by the minimal enlargement of the breasts under the areolae and the presence of scant pubic hair. These signs indicate that she has moved beyond Tanner Stage 1, which is characterized by a prepubescent state with no secondary sexual characteristics. However, the girl's symptoms do not align with the more advanced Tanner stages (3-5), which are characterized by further enlargement of the breasts and pubic hair, changes in the shape and contour of the breasts, and the formation of a secondary mound above the level of the breast. Therefore, based on the girl's age, the onset of breast enlargement, and the presence of scant pubic hair, it can be concluded that she is likely in the early stages of puberty, which corresponds to Tanner Stage 2. This stage is characterized by the onset of breast budding and the start of pubic hair growth. In conclusion, the sexual maturity rating that best describes this 11-year-old patient is Tanner Stage (B) 2. This stage aligns with the physical signs observed in the girl, suggesting that she is in the early phase of puberty.
