# Supplementary material for: Collaborative intelligence in AI: Evaluating the performance of a council of AIs on the USMLE
Source: PLOS Digit Health. 2025 Oct 9;4(10):e0000787. doi: 10.1371/journal.pdig.0000787 (PMC12510544; doi:10.1371/journal.pdig.0000787)
Supplement: S2 File — (ZIP) [file pdig.0000787.s002.zip › S2 Data Raw data files containing unprocessed question inputs and LLM outputs including transcripts of deliberations/STEP1_Question-104-1_Type-MC-NJ.docx]

**Facilitator** **[Round 1]:** A 5-year-old boy is brought to the emergency department by his mother because of an episode of bloody stool 3 hours ago. The mother says the stool was hard ""like pebbles"" and she noted bright red blood on the tissue when the patient cleaned himself. His previous bowel movement was 5 days ago. The patient has no abdominal or rectal pain now, but he did have abdominal pain during his bowel movement 5 days ago. He has no history of major medical illness and receives no medications. Vaccinations are up-to-date. The patient has no recent history of travel. He is at the 5th percentile for height and the 10th percentile for weight; BMI is at the 50th percentile. Vital signs are within normal limits. Abdominal examination shows hypoactive bowel sounds and a soft, slightly distended abdomen that is not tender to palpation. Rectal examination shows 1 cm of bright red rectal mucosa protruding from the right side of the anus; there is no rectal bleeding. The remainder of the examination shows no abnormalities. Which of the following is the most likely cause of this patient's physical findings? (A) Constipation (B) Cystic fibrosis (C) Hirschsprung disease (D) Hookworm infestation (E) Intussusception

**AI_C:** RESTATING THE QUESTION: The inquiry here effectively asks, what is the most probably reason for the symptoms experienced by a 5-year-old boy who arrived in the ER for an episode of bloody stool. The symptoms include hard "pebble-like" stool, lack of bowel movement for five days, no abdominal or rectal pain currently (though there was abdominal pain during his last bowel movement), a soft but slightly distended abdomen with hypoactive bowel sounds, and a red rectal mucosa protruding from one side of the anus. The listed possible explanations for this scenario are constipation, cystic fibrosis, Hirschsprung disease, hookworm infestation, and intussusception. PLANNING: Assumptions that could be made from this scenario could include that the kid has a digestive problem induced by diet, a genetic or congenital disorder, or a parasitic infection. However, bearing in mind the history of infrequent and hard stools, the absence of major illness or medication, up-to-date vaccinations, low percentile for height and weight but normal BMI, normal vital signs, and hypoactive bowel sounds with signs of rectal mucosa protrusion, the leading assumption is constipation. CLINICAL REASONING: Constipation, characterized by hard, infrequent bowel movements, as well as potential associated abdominal pain, can lead to straining during defecation. This straining can, in turn, result in rectal lining (mucosa) protrusion, or rectal prolapse, which fits with the physical signs seen here. Also, constipation could cause the abdominal distension observed. Cystic fibrosis often presents other symptoms such as respiratory problems or failure to thrive, none of which are witnessed here. Hirschsprung disease is usually diagnosed in infancy and causes pronounced abdominal distension with bilious vomiting, which also does not match this case. Hookworm infestation would likely come with a history of travel to endemic areas and present gastrointestinal distress or anemia, which is not reported here. Lastly, intussusception would present with an acute abdomen, including severe pain, vomiting, and "currant jelly" stools, and thus is not consistent with the current case. CONCLUDING: Given the current status, history, and physical examination of the child, who presents with a history of infrequent, hard bowel movements, a rectal mucosa protrusion, and no current or previous severe pain or acute abdomen, the assumption of constipation provides the best explanation. RESPONSE: Therefore, the most likely cause of this patient's physical findings is (A) Constipation.

**AI_D:** (1) RESTATING THE QUESTION: The kid in our case is a 5-year-old boy, who was brought to the ER due to experiencing an episode of bloody, hard, and pebble-like stool. His last bowel movement was five days prior to this incident, which was accompanied by abdominal pain. Currently, he doesn't complain of any abdominal or rectal pain. It is also important to note that he has no significant medical history nor is he on any medications. His vaccination status is up to date. Physical examination reveals a slightly distended abdomen with hypoactive bowel sounds and the protrusion of a bright red rectal mucosa from his anus. We need to determine the most likely cause of these symptoms, with the alternatives being constipation, cystic fibrosis, Hirschsprung's disease, hookworm infestation, or intussusception. (2) PLANNING: We should consider a few important assumptions: The boy's stool is characterized as hard and pebble-like; his last bowel movement happened five days ago, and it was associated with abdominal pain; the abdominal examination suggests a sluggish bowel (hypoactive bowel sounds) and distension; there is a protrusion of the rectal mucosa, indicative of possible rectal or lower intestinal trauma. (3) CLINICAL REASONING: The child's infrequent, hard, and painful bowel movements, together with hypoactive bowel sounds, strongly indicate constipation. Furthermore, the bright red blood seen in the stool could be due to anal fissures caused by passing hard stool, consistent with the physical findings of protruding red rectal mucosa. It's unlikely to be cystic fibrosis as there's no history of respiratory or chronic digestive issues, and he would likely be much sicker if this was the case. (4) CONCLUDING: The prevalence of Hirschsprung's disease or hookworm infestation would certainly warrant consideration if the boy had other respective symptoms and history, such as chronic constipation since infancy or exposure to unsanitary environments. However, given the current information, these options are less likely. Lastly, intussusception typically presents with intermittent severe abdominal pain and "currant jelly" stools, neither of which the boy has, ruling out this option. (5) RESPONSE: Evaluating the clinical picture and the presenting features, the most likely cause of this patient's pebble-like stool, prolonged interval between bowel movements, and rectal bleeding is (A) Constipation.

**AI_B:** RESTATING THE QUESTION: We have an underweight five-year-old boy exhibiting symptoms that include hard, pebble-like stools, a bright red rectal mucosa protruding from the anus, and a slightly distended abdomen. His bowel movements have been infrequent (about once every five days), and there was an episode of blood in his stool. This boy does not present with any other health complications, is not on medication, and has no recent travel history. We need to determine which medical condition is most likely from the provided options: Constipation, Cystic fibrosis, Hirschsprung disease, Hookworm infestation, or Intussusception based on the patient's results and symptoms. PLANNING: Each diagnosis presents with specific clinical signs and symptoms. For instance, constipation can cause infrequent bowel movements and hard, pebble-like stools due to slow passage of stool in the colon. Cystic fibrosis patients typically exhibit pancreatic insufficiency, recurrent lung infections, and failure to thrive, including growth abnormalities. Hirschsprung disease often manifests in the early days of life with symptoms like inability to pass meconium, constipation, or abdomen distention. Hookworm infestation typically presents with iron-deficiency anemia due to chronic intestinal blood loss. Lastly, Intussusception presents with drone-like attacks of abdominal pain, palpable "sausage-shaped" mass in the abdomen, and bloody stool (typically jelly-like). To identify the leading assumption in this case, it would be crucial to identify which disease's clinical manifestations align most closely to the boy's symptoms. CLINICAL REASONING: We can exclude Hookworm infestation and Cystic fibrosis based on the absence of clinical cues. The boy has not recently traveled nor suffered consistent gastrointestinal symptoms or growth abnormalities. Hirschsprung disease’s classic presentations such as failure to pass meconium typically appear shortly after birth and wouldn't dramatically affect a five-year-old. Despite the symptoms of Intussusception, such as severe paroxysmal abdominal pain and "currant jelly" stools, the boy doesn't record such severity. The most coherent diagnosis is constipation, which presents with infrequent bowel movements, hard pebble-like stools, and even bright red blood from anal fissures due to straining. It also explains the slight abdominal distention due to retained fecal matter. CONCLUDING: After investigating each option, constipation appears to exhibit the strongest alignment with this patient's symptoms of infrequent bowel movements, pebble-like hard stools, and occasional rectal bleeding - likely due to strain-induced anal fissures. RESPONSE: Therefore, the most likely cause of this patient's physical findings is (A) Constipation.

**AI_A:** RESTATING THE QUESTION: This is an inquiry about a young boy who has presented with symptoms including hard, "pebble-like" stools, a history of abdominal pain during bowel movements, and evidence of blood in his stool. It has been noted that there are periods of 5 days between bowel movements. The child does not have any significant health history, is not on any medications, is somewhat small for his age, but he seems otherwise physically healthy. His vital signs are normal, although the physical examination shows some unusual findings: decreased bowel sounds, a slightly distended non-tender abdomen, and a small portion of red rectal mucosa extending out from the anus. The question is aimed at identifying the most probable cause for these observed symptoms and physical findings. PLANNING: The boy's symptoms of delayed, painful, hard stools, along with rectal bleeding, point to a digestive system problem. Given his period of 5 days without a bowel movement and his hard stool, constipation (Option A) is an obvious consideration. However, other possibilities are also in play such as cystic fibrosis (Option B), an inherited disorder that can lead to thickened intestinal secretions causing digestive issues. Hirschsprung disease (Option C), an absence of ganglion cells in a segment of the colon, could also cause constipation but is generally accompanied by symptoms right from birth. Hookworm infestations (Option D) may cause abdominal discomfort and blood in the stool, but the patient hasn't traveled recently, minimizing the probability of an infection. Finally, intussusception (Option E), a telescoping of the intestine, could provoke abdominal pain, but commonly causes 'currant jelly' stools and acute abdominal pain, which isn't congruent with this patient's presentation. CLINICAL REASONING: Assessing these options, the most plausible assumption is constipation. Let me explain why. Constipation in children is often a result of functional or idiopathic issues rather than pathological causes. The boy's 'pebble-like' stool texture is indicative of type 1 on the Bristol Stool Chart, which is associated with constipation. Also, his infrequent bowel movements (every 5 days) further supports this diagnosis. This condition can indeed result in streaks of bright red blood observed on toilet paper - a sign of a small anal tear (fissure) due to straining and hard stools. The presence of bright red rectal mucosa protruding from the anus could be a rectal prolapse, another complication associated with chronic straining and constipation. CONCLUDING: Considering the above, while other choices represent possible gastrointestinal pathologies, they seem less likely given the child’s overall presentation, the nature of his symptoms, his lack of medical history, and the absence of other suggestive signs. Constipation appears to be the most feasible explanation and should be clinically prioritized for further evaluation and confirmation. This diagnosis also fits with the primary care context, where the most common causes should be considered first. RESPONSE: The most likely cause of this patient's physical findings is (A) Constipation.

**AI_E:** RESTATING THE QUESTION: A 5-year-old boy is presented with symptoms suggestive of a digestive issue, including bloody stool and discomfort. It also seems that he suffers from infrequent bowel movements, as his previous one was five days back. The physical examination reveals not only a swollen abdomen but also a protrusion of the rectal mucosa from the side of his anus. In simple terms, we are trying to determine the most probable cause of his symptoms from the options of constipation, cystic fibrosis, Hirschsprung disease, hookworm infestation, and intussusception. PLANNING: From the provided clinical picture, a few assumptions can be made; firstly, the boy is experiencing hard, infrequent stools indicating possible constipation; secondly, based on the bright red blood in his stool, the problem seems to originate in the lower gastrointestinal tract (since blood in the stool from higher within the GI tract would usually be digested and appear black); and thirdly, the presence of a rectal mucosal protrusion might suggest a form of prolapse related to straining. Each of these assumptions could be questioned. Could the hard, pebble-like stools be a chronically presenting symptom of a different disease? Is there a possibility of bleeding from higher in the GI tract despite the blood appearing bright red? Is there an alternate explanation for the rectal mucosal protrusion? Investigating these assumptions further will help identify the primary clinical concern. CLINICAL REASONING: The boy's symptoms can be traced to hard, infrequent stools, often described as "pebble-like," which point towards constipation. Coupled with the absence of other systemic symptoms, more severe diseases such as cystic fibrosis or Hirschsprung disease would be lower on the list of differentials. Cystic fibrosis usually presents with a broad range of symptoms affecting multiple organs, such as lung symptoms and failure to thrive besides constipation, none of which are seen in the clinical picture. Hirschsprung's disease often presents in neonates or infants with bowel obstruction, which again doesn't match the timeline or symptomatology of this clinical picture. The mention of bright red, undigested blood is suggestive of a lower GI bleed, probably a result of straining. Prolonged straining due to constipation can lead to a range of problems in the anorectal area, including anal fissures. The soft, slightly distended abdomen on the exam may be due to accumulated stool owing to constipation. Hypoactive bowel sounds further strengthen the hypothesis that abnormal bowel movements are a major issue. The rectal mucosal protrusion could be a rectal prolapse, which is known to occur due to excessive straining during defecation, such as in chronic constipation. CONCLUDING: Upon evaluating the possibilities, the symptoms could well be in consonance with chronic constipation. Constipation could cause straining, which could then lead to various manifestations such as anal fissures, lower GI bleeding, and rectal prolapse. The prolapsed rectal mucosa could explain the bright red blood on tissue after cleaning. RESPONSE: The most likely cause of this patient's physical findings is constipation (A). This case presents a typical symptomatology of constipation in children, including reduced frequency of bowel movement (with last being 5 days ago), hard stool consistency ('like pebbles'), abdominal pain related to the bowel movement, and rectal mucosal protrusion possibly due to straining and resulting in rectal prolapse. The rectal prolapse might also be the source of bright red, undigested blood in stool as the tissue can easily bleed from minor trauma.

**Facilitator** The consensus among the AI responses is that the most likely cause of the 5-year-old boy's symptoms is constipation (Option A). This conclusion is based on the boy's presentation of hard, "pebble-like" stools, infrequent bowel movements (every 5 days), and evidence of blood in his stool. The physical examination findings of decreased bowel sounds, a slightly distended non-tender abdomen, and a small portion of red rectal mucosa extending out from the anus further support this diagnosis. The presence of bright red blood on the tissue after cleaning himself suggests a lower gastrointestinal bleed, likely due to an anal fissure caused by straining and passing hard stools. The rectal mucosa protrusion could be a rectal prolapse, another complication associated with chronic straining and constipation. While other options such as cystic fibrosis, Hirschsprung disease, hookworm infestation, and intussusception represent possible gastrointestinal pathologies, they are considered less likely given the child’s overall presentation, the nature of his symptoms, his lack of significant medical history, and the absence of other suggestive signs. For instance, cystic fibrosis often presents with respiratory problems or failure to thrive, which are not witnessed here. Hirschsprung disease is usually diagnosed in infancy and causes pronounced abdominal distension with bilious vomiting, which also does not match this case. Hookworm infestation would likely come with a history of travel to endemic areas and present gastrointestinal distress or anemia, which is not reported here. Lastly, intussusception would present with an acute abdomen, including severe pain, vomiting, and "currant jelly" stools, and thus is not consistent with the current case. Therefore, the consensus is that constipation appears to be the most feasible explanation for the boy's symptoms and should be clinically prioritized for further evaluation and confirmation.
