# Supplementary material for: Collaborative intelligence in AI: Evaluating the performance of a council of AIs on the USMLE
Source: PLOS Digit Health. 2025 Oct 9;4(10):e0000787. doi: 10.1371/journal.pdig.0000787 (PMC12510544; doi:10.1371/journal.pdig.0000787)
Supplement: S2 File — (ZIP) [file pdig.0000787.s002.zip › S2 Data Raw data files containing unprocessed question inputs and LLM outputs including transcripts of deliberations/STEP1_Question-106-1_Type-MC-NJ.docx]

**Facilitator** **[Round 1]:** An 80-year-old woman is brought to the emergency department (ED) 30 minutes after she fell out of her wheelchair at home. This is the second visit to the ED for a fall during the past 3 months. She lives with her daughter and son-in- law, who say they “do the best we can.” The patient has dementia, Alzheimer type, coronary artery disease, type 2 diabetes mellitus, and hypertension. Current medications are amlodipine, aspirin, atorvastatin, donepezil, long-acting insulin, and lisinopril. Five years ago, she underwent bilateral below-the-knee amputations because of infected ulcers of the feet. She uses a wheelchair for ambulation. Ten years ago, she underwent three-vessel coronary artery bypass grafting. She has smoked one-half pack of cigarettes daily for 60 years. She drinks one shot of whiskey nightly. She is thin and appears ill and disheveled. Her temperature is 37.2°C (99.0°F), pulse is 80/min, respirations are 20/min, and blood pressure is 120/80 mm Hg. Pulse oximetry on 2 L/min of oxygen by nasal cannula shows an oxygen saturation of 95%. Physical examination shows temporal wasting. There are scattered ecchymoses over the abdomen and all extremities. No other abnormalities are noted. Which of the following is the most appropriate initial history to obtain from this patient? (A) Abnormal bleeding (B) Diet (C) Relationship with her family (D) Respiratory symptoms (E) Urinary symptoms

**AI_B:** RESTATING THE QUESTION: The information provided is about an 80-year-old dementia patient with a range of health conditions such as coronary artery disease, type 2 diabetes, and hypertension. She has been brought to the emergency department after a fall, and there are physical signs such as being thin, ill and disheveled, and with ecchymoses in multiple areas. The question is asking which line of enquiry or history should be pursued as the most appropriate initial step. PLANNING: Five possible lines of enquiry are suggested, including questions about abnormal bleeding, diet, family relationship, respiratory symptoms, and urinary symptoms. These all have potential relevance, but consideration should include the cognitive status of the patient, the recent physical trauma of the fall, the patient's general appearance, and specific signs such as the noted ecchymoses. CLINICAL REASONING: All of the suggested histories have their relevance: 'abnormal bleeding' as a potential cause of the ecchymoses; 'diet' for potential nutritional deficits; 'relationship with her family' as an aspect of her care and potential risk factors for falls and neglect; 'respiratory symptoms' given the long history of smoking; and 'urinary symptoms' for potential urological issues. However, her general appearance of being ill, disheveled, and thin with notable ecchymoses and the less than optimal care environment suggested by the family's commentary offers a stronger indication towards one of these. CONCLUDING: The ideal initial history to take from this patient should be around her diet (B). This can be a considerable influence in her current condition, from her frail thin appearance and the observed temporal wasting to her scattered ecchymoses which may be suggestive of malnutrition and vitamin deficiencies, specifically vitamin C and K, essential for collagen synthesis and clotting factors respectively. RESPONSE: The most appropriate initial history to obtain from this 80-year-old woman should be on her diet (B). Her general appearance and specific physical signs point towards potential malnutrition, a condition that can significantly impact her overall health and recovery from the recent fall.

**AI_E:** RESTATING THE QUESTION: The question is asking what should be the main focus for the initial history gathering with an 80-year-old woman who presented to the emergency department after falling. The patient has multiple chronic conditions including Alzheimer's disease, coronary artery disease, type 2 diabetes, and hypertension. She is on various medications and had bilateral below-the-knee amputations due to infected foot ulcers. She is malnourished and has scattered bruising. The choices revolve around various medical and social aspects in her life including abnormal bleeding, diet, family relations, respiratory symptoms, and urinary symptoms. PLANNING: Assumptions made here include the fact that she fell because of an underlying health problem, but it could also be due to environmental hazards. The scattered bruises could be a result of frequent falls, physical abuse, or a hematological problem. Her poor nutrition status could be due to inadequate diet, malabsorption, or metabolic problem. In examining alternate assumptions, we could consider that she might be having repetitive falls due to imbalance associated with her chronic comorbidities or physical abuse, her ecchymoses could be caused by anticoagulant therapy or intrinsic clotting abnormality, poor nutrition could be due to poor diet or insufficient assistance at home or a metabolic problem, and that her recurrent emergency room visits could be due to lack of adequate home care. After weighing all, the need to explore her diet to explain her thin appearance, temporal wasting, and malnourished appearance should be clinically prioritized. CLINICAL REASONING: The patient's malnourished state, which is conducive to frailty and recurrent falls, suggests that her dietary needs may not be adequately met. Moreover, considering her chronic medical conditions and her medications, her nutritional needs are likely higher and more specific. Since poor nutrition can have significant implications on her overall health, particularly on wound healing (considering her post-amputation status), immune function, and glycaemic control (considering her diabetic status), gathering her dietary history would be paramount. CONCLUDING: To understand the underlying cause of her frail and malnourished state which can contribute to her recurrent falls and overall poor health, and to ensure her nutritional needs are addressed adequately and appropriately, obtaining her dietary history will be most appropriate as the initial step. RESPONSE: Hence, the most appropriate initial history to obtain from this patient is her diet (Option B).

**AI_D:** RESTATING THE QUESTION: We have an 80-year-old woman presented to the Emergency Department after a fall. She has a recurring history of falls, lives with her family, and has a significant medical history including dementia of the Alzheimer's type, coronary artery disease, type 2 diabetes mellitus, and hypertension. She's under multiple medications and has a history of smoking and drinking, with noticeable physical signs including thinness, appearing ill and unkempt with temporal wasting. Ecchymoses have been noted over her abdomen and all extremities without any other abnormalities. The inquiry requires us to identify the most relevant initial history that should be obtained from her. PLANNING: Several assumptions can be made about this case. The initial assumption is that her general frailty could be due to her multiple comorbidities and old age. Another assumption could be that her frequent falls could be related to her dementia or possible side effects of her medication. Furthermore, her physical appearance suggests potential neglect or poor care, indicating possible social issues. After considering these assumptions, the question provides a list of potential areas to explore in her history. Clinically, prioritizing the assumption of neglect and inadequate care due to her physical appearance and living circumstances would be most prudent because patient safety is of paramount importance in clinical settings. CLINICAL REASONING: This woman's physical state and confirmed comorbidities suggest a complex care situation which could be exacerbated by potential neglect or inadequate support at home. Her living with her family together with the reports of doing the "best" they can suggest care difficulties. Consequently, poor diet could lead to her thin appearance and temporal wasting. Hence, assessing her nutrition intake (option B) could offer valuable insights into her overall state of well-being and quality of care. CONCLUDING: Given her frail appearance, repeated falls, and potential suboptimal home support, understanding her nutritional intake forms an essential part of her initial history. This will clarify whether her physical state could be a result of suboptimal nutrition or other contributing factors. Failure to assess this could lead to a missed opportunity in improving her overall health status and reducing future healthcare risk. RESPONSE: Thus, the most appropriate initial history to obtain from this patient is her diet (B) as it can provide critical information about her overall health status and the level of care provided at home. This can then be used to formulate a thorough care plan that addresses her physical health and safety issues.

**AI_A:** (1) RESTATING THE QUESTION: The issue at hand involves an 80-year-old woman who lives with her kin. Having being diagnosed with multiple health conditions, among them dementia, coronary artery disease, type 2 diabetes mellitus, and hypertension, she is now in the ED for the second time in as many months, due to a fall. Scattered ecchymoses are evident over her body, and she is presenting as being thin, and generally unfit. We need to figure out the most pertinent history to extract from her initially, with choices being abnormal bleeding, her diet, her familial relations, respiratory symptoms, or urinary symptoms. (2) PLANNING: The decision to select a specific assumption requires evaluating the patient's overall condition. The concerns entail her recurrent falls, physical frailty, dementia, and scattered ecchymoses. The aberrations may suggest malnourishment, neglect, or a combination thereof. With the patient having Alzheimer's, proximate family members being caregivers, and her physical state, abuse or neglect is a valid concern and reasonable basis for investigation. However, this should not deflect from possible medical causes like malnutrition, which could stem from a poor dietary regimen and result in frailty and easy bruising. Hence, her dietary habits might be a relevant initial line of questioning. (3) CLINICAL REASONING: The patient's dementia, physical frailty, and the fact that she is cared for by family members put her at a heightened risk for elder abuse or neglect. Falls and ecchymoses could be symptoms of this. However, her thin, disheveled appearance, coupled with signs of wasting, point towards potential malnutrition, which could make her prone to bruising and falls. A deficient diet could lead to altered clotting, low body mass, muscle wasting, and eventual frailty. These factors could contribute to the falls and bruises noted on her body. Therefore, although elder abuse cannot be dismissed, malnutrition seems to be a pertinent issue worth querying first. (4) CONCLUDING: In considering the patient's frail physical state, recurrent falls, and the presence of scattered ecchymoses, it's vital not to automatically link these aspects with elder abuse. One must concurrently contemplate medical causes that might place her at risk for falls and bruising. Given her presentation, malnutrition appears to be a plausible causative factor that warrants initial investigation. (5) RESPONSE: The appropriate initial history to obtain from the patient would be about her Diet (B).

**AI_C:** RESTATING THE QUESTION This 80-year-old woman with multiple co-morbidities, including dementia, Alzheimer type, and a history of falls, is presented to the emergency department after falling from her wheelchair at home. She appears ill and has noticeable signs of physical deterioration such as being thin and showing temporal wasting. She also has scattered bruising or ecchymoses around her abdomen and extremities. We need to decide what initial history should be obtained from this patient. The options given include abnormal bleeding, diet, the relationship with her family, respiratory symptoms, and urinary symptoms. PLANNING Assumptions could be made about the likely causes of her falls based on her present condition and history. These include her age, dementia, amputations, and conditions that may cause muscle weakness or impaired balance. She has been a chronic smoker, has coronary artery disease, and uses supplemental oxygen, which questions the respiratory health as well. However, her bruising or ecchymosis could indicate abnormal bleeding, while her physical appearance suggests potential malnutrition implying the need for dietary history. However, it's crucial to note that falls in the elderly are often multifactorial, which necessitates comprehensive geriatric assessment. The multiple domains to be assessed in older adults include functional, cognitive, psychological, social, and physical health, and among these, addressing the functional well-being is a priority. In this case, the patient's function is clearly impaired, leading to recurrent falls - she is dependent on a wheelchair and has experienced at least two falls in the past three months. Given these considerations, while all history elements listed have their merits, the circumstances point to one particular aspect of her history that should be initially prioritized. CLINICAL REASONING Evaluating her diet, checking for abnormal bleeding, or examining her familial relations may provide valuable information, but none directly address the immediate problem at hand, which is her recurrent falls. While her extensive medical history suggests the potential for respiratory or urinary symptoms, these are not directly implicated in her recent history of falls. Based on her clinical presentation, there is a possibility that she could be suffering from some form of elder neglect, given her physical appearance and living conditions. Her family states that they "do the best we can," which could imply difficulty in providing appropriate care. Her thin disheveled appearance and scattered ecchymosis could be indicators of neglect, malnutrition, or abuse. While these are serious concerns that need to be addressed, the priority in this setting should be to identify any potentially modifiable risk factors associated with her recurrent falls, which implies assessing her functional status in relation to her home environment. CONCLUDING The immediate concern for this patient is her recurrent falls, which at her age and in her health condition pose significant risk for complications. While the patient's overall medical history should not be ignored, prioritizing her functional status in her everyday life is crucial. The key is to understand the interaction between her physical abilities, medication use and the home environment where she resides. RESPONSE Therefore, an appropriate initial history to obtain from this patient would be about her relationship with her family (C). This would include learning about how she manages to perform her day-to-day activities, how she’s assisted by her family, the physical layout of the home, and any challenges they face in providing care, all of which could contribute to her risk of falling.

**Facilitator** **[Round 2]:** Summary: AI_A and AI_B both believe that the patient's diet should be the initial line of questioning. They argue that her frail physical state, recurrent falls, and scattered ecchymoses could be linked to malnutrition, which could stem from a poor dietary regimen. AI_C, however, believes that the initial history should focus on the patient's relationship with her family. This is due to the patient's recurrent falls and the potential for elder neglect or abuse. AI_D agrees with AI_A and AI_B, emphasizing the importance of understanding the patient's nutritional intake due to her frail appearance and potential suboptimal home support. AI_E also agrees with the majority, arguing that the patient's malnourished state suggests that her dietary needs may not be adequately met. Question: Given that the majority of council members believe that the patient's diet should be the initial line of questioning, could AI_C elaborate on why they believe the patient's relationship with her family should be prioritized? Specifically, how does AI_C weigh the potential for elder neglect or abuse against the potential for malnutrition in this case?. Based on your preceding response, how would you answer the original question: [An 80-year-old woman is brought to the emergency department (ED) 30 minutes after she fell out of her wheelchair at home. This is the second visit to the ED for a fall during the past 3 months. She lives with her daughter and son-in- law, who say they “do the best we can.” The patient has dementia, Alzheimer type, coronary artery disease, type 2 diabetes mellitus, and hypertension. Current medications are amlodipine, aspirin, atorvastatin, donepezil, long-acting insulin, and lisinopril. Five years ago, she underwent bilateral below-the-knee amputations because of infected ulcers of the feet. She uses a wheelchair for ambulation. Ten years ago, she underwent three-vessel coronary artery bypass grafting. She has smoked one-half pack of cigarettes daily for 60 years. She drinks one shot of whiskey nightly. She is thin and appears ill and disheveled. Her temperature is 37.2°C (99.0°F), pulse is 80/min, respirations are 20/min, and blood pressure is 120/80 mm Hg. Pulse oximetry on 2 L/min of oxygen by nasal cannula shows an oxygen saturation of 95%. Physical examination shows temporal wasting. There are scattered ecchymoses over the abdomen and all extremities. No other abnormalities are noted. Which of the following is the most appropriate initial history to obtain from this patient? (A) Abnormal bleeding (B) Diet (C) Relationship with her family (D) Respiratory symptoms (E) Urinary symptoms]

**AI_A:** Restating the question, an 80-year-old woman who presented after a fall, who is in frail health with temporal wasting and scattered ecchymoses, and suffers from multiple chronic conditions like dementia and diabetes, is brought in for evaluation. The query is about what should be the most appropriate initial history to get from the patient. Planning involves questioning the assumptions necessary for consideration. It might be assumed that her state is a result of her chronic diseases and age. An alternate assumption could be that her home environment may not be as supportive as needed, given her conditions, leading to nutritional deficiencies and repeated falls. Additionally, elder negligence or abuse may also be taken into consideration. Clinical reasoning entails connecting these assumptions in a step-by-step manner. First, her appearance, recurrent falls, and ecchymoses could point to malnutrition. Considering her chronic conditions and the medications she takes, a lack of adequate nutrition has deleterious effects. Her current home support may not be optimally ensuring her dietary needs are met, which is further compounded by her alcohol use. Second, elder neglect or abuse cannot be outrightly dismissed given her living conditions, which necessitates questioning her relationship with her family. In conclusion, while the initial history about her diet is of utmost importance due to its direct bearing on her physical condition, information about her family relationship is also crucial to ascertain any potential neglect or abuse. Therefore, both areas of inquiry hold significance. Accordingly, I would choose (B) Diet as my response to the original question, while also advising to concurrently monitor the patient's familial relationships. By prioritizing her nutritional status, which is crucial for her overall health and can also help explain her current physical state, appropriate interventions can be formulated. Simultaneously, it would be prudent to not ignore the social aspect of her condition, given the often-overlooked reality of elder abuse or neglect.

**AI_E:** Headline: To determine the priority area for initial history collection in an 80-year-old, frail, and seemingly malnourished woman with multiple comorbidities and recurrent falls, the AI council debated various factors including malnutrition, elder neglect, and other potential physical or psychosocial factors. The majority perspective agreed on exploring the patient's diet and nutritional status, indicating the visible signs of malnutrition such as temporal wasting and scattered ecchymoses as markers. AI_C, however, underlined the potential of elder neglect or abuse, suggesting that her recurrent falls and the overall condition point to a possible issue with the home care she is receiving. Explanation: The hypothesis here is that the woman's malnutrition could be a result of inadequate care, suboptimal dietary intake, or both. Irrespective of the source, being malnourished puts her at risk for falls, weak immunity, and overall decreased quality of life. Scattered ecchymoses reflect this vulnerability, hinting towards potential micronutrient deficiencies, particularly vitamin C and K, both of which play a key role in maintaining the integrity of capillaries and preventing spontaneous bruising. Furthermore, her temporal wasting, an indicator of severe malnutrition associated with muscle mass loss, raises concerns about her protein-calorie intake, given her catabolic diseases like diabetes and coronary artery disease and her use of vast medications. Similarly, the recurrent falls likely have multifactorial roots, exacerbated by her weak physical state and potentially worsened by insufficient home support as expressed by her family's statement, making AI_C's concerns relevant as well. Elder neglect or abuse compellingly comes into the picture because of the patient's dementia and her reliance on family support, which may be suboptimal or even harmful. Conclusively, obtaining information on both her diet and her relationship with her caretakers would be crucial. However, it seems rational to prioritize dietary history (option B) as it directly relates to her apparent physical state and could offer immediate solutions like dietary adjustments or micronutrient replacements. Any suspicion of elder abuse or neglect raised by her family relationship history can then be followed up. Response: Given her clinical scenario, the most appropriate initial history to obtain from this patient should primarily focus on her nutrition and diet (option B), followed by her relationship with family and the care she receives at home.

**AI_C:** RESTATING THE QUESTION: The subject at hand is to identify the most appropriate initial line of inquiry in the historical assessment of an 80-year-old woman who came to the emergency department because of a fall. This is her second fall in three months. She has a history of Alzheimer's, cardiovascular diseases, type 2 diabetes, and hypertension. The elderly woman looks feeble and unkempt, with physical signs of probable malnutrition. PLANNING: The assumptions we could make involve her diet, relationship with her family, abnormal bleeding, respiratory symptoms, and urinary symptoms. However, considering the woman’s multiple systemic disorders and her physical appearance raising concerns of malnutrition, two main hypotheses could be considered: nutritional deficiencies and potential elder abuse or neglect. CLINICAL REASONING: Malnutrition, as suggested by her thin appearance and temporal wasting, is indeed a concern, mainly because it can contribute to weakened immune function, impaired wound healing, muscle weakness leading to falls and generally to a poorer prognosis in diseases. However, it's also crucial to remember the patient’s cognitive impairment due to Alzheimer's disease, which, coupled with her physical condition and recurring falls, raises concerns about potential elder neglect or abuse. Frequently, elder abuse or neglect is an overlooked problem, especially in patients with cognitive impairment, as they may not be able to express their situation effectively. The visible signs of temporal wasting and scattered ecchymoses, coupled with reported recurrent falls, could point to a lack of adequate care or possible elder abuse. Her family statements - "we do the best we can" - open a window to their potential struggle in managing her conditions. CONCLUDING: Therefore, it's crucial to explore the quality of care she is receiving at home and to what extent her family is managing her multifaceted health conditions, such as her dementia, her cardiovascular diseases, her nutritional status and her mobility, including the prevention of falls. From the clinical reasoning standpoint, it’d be useful to assess (C) the patient's relationship with her family initially, to eliminate any potential neglect or mistreatment and to better understand the care she's receiving at home. RESPONSE: Among the given choices, I'd say that querying about her (C) Relationship with her family should be prioritized as it seems most relevant in this scenario. If the quality, adequacy, and nature of the home care are in question, immediate measures could be taken to ensure the patient's safety and comprehensive healthcare. The findings would also likely inform a further assessment of the patient's diet and overall care plan.

**AI_D:** RESTATING THE QUESTION: This scenario involves an 80-year-old woman who has been brought to the emergency department following a fall. Her medical history includes dementia, Alzheimer type, coronary artery disease, type 2 diabetes mellitus, hypertension, and bilateral below-the-knee amputations due to foot ulcer infections. She has a history of smoking and minor alcohol consumption. She presents with temporal wasting and multiple bruising on her extremities and abdomen. The question posed is about which history should be initially obtained from this patient, from among the choices: abnormal bleeding, diet, family relationship, respiratory symptoms, and urinary symptoms. PLANNING: Considering this woman's medical history and her physical appearance, there are multiple potential variables at play. For instance, her frailty and frequent falls, alongside her scattered bruises, could suggest either malnutrition due to poor diet (option B) or potential elder abuse/neglect entwined with her relationship with family (option C). Moreover, her dementia could further complicate her ability to self-feed or make nutritional choices adequately, hence bolstering the importance of these two points. CLINICAL REASONING: When evaluating a patient with multiple comorbidities and a history of frequent falling, it’s important to identify potential factors that could be contributing to their physical state and recurrent falls. Considering the mentioned lifestyle, physiological conditions, and her frail state, nutritional concerns seem conceivable. Her thin appearance, temporal wasting, and ecchymoses hint at potential malnutrition, which could be due to inadequate dietary intake or suboptimal home care support. Moreover, malnutrition could increase her susceptibility to physical injuries, like the scattered ecchymoses. CONCLUDING: To ascertain and clinically prioritize the assumptions, attention should be initially drawn towards understanding her dietary patterns, her food intake, any special dietary measures taken due to her diabetes, and whether someone is adequately preparing her meal, since poor nutrition can significantly contribute to her frailty and suboptimal physiological functions. RESPONSE: Given this woman's medical history, frail physical condition, and other possibly contributory factors to her frequent falling, I propose that the most appropriate initial history to obtain from this patient is her diet (option B). This could elucidate whether she is receiving sufficient nutrition and if any nutritional inadequacies might be contributing to her current health status. It should be noted that other factors, such as her relationship with family, cannot be overlooked and should be addressed in subsequent evaluations.

**AI_B:** Restating the Question: The main question revolves around determining the most suitable initial line of history to pursue for an elderly woman who has been admitted to the ED due to falls. She has multiple pre-existing conditions, including dementia, coronary artery disease, type 2 diabetes mellitus, and hypertension, and has also undergone bilateral below-the-knee amputations. The patient lives with her family who attempt to care for her to the best of their abilities. The patient's physical examination reveals her to be thin and unwell, with temporal wasting and scattered ecchymoses. The options put forth for the initial history to explore are abnormal bleeding, the patient's diet, her relationship with her family, respiratory symptoms, and urinary symptoms. Planning: This case requires a careful consideration of the patient's overall frailty, the recurrent falls, dementia, temporal wasting, and the presence of scattered ecchymoses. These pieces of information lead to multiple assumptions. We could assume that the patient is suffering from malnutrition, hence the importance of inquiring about her diet. Alternatively, the recurrent falls and the family's reported struggle with her care could potentially suggest elder neglect or abuse, making it crucial to understand her relationship with her family. Clinical Reasoning: Given that the patient's physical state indicates she may be malnourished, it's logical to assume that her diet may not be optimal. Inadequate nutritional intake could lead to general weakness and an increased risk of falls and injuries, such as the ecchymoses observed. Considering her dementia, it's also important to acknowledge that she may not be able to communicate her needs effectively, potentially contributing to her nutritional deficiencies. However, as AI_C, I see value in understanding the patient's relationship with her family. Elder neglect or abuse could be a factor in this case. The falls could be due to the environment being unsuitable or hazardous. The family's struggle to provide proper care and the patient's appearance might suggest negligence. Although malnutrition could be a sign of neglect, they are two separate entities with different mechanisms and implications for patient care. Concluding: Given the patient's particular circumstances, we should consider both potential malnutrition and the chances of elder neglect. We should seek to understand her dietary intake as well as how her family cares for her at home. Despite her physical signs indicating possible malnutrition and the family's reported struggles, we must also recognize that these could possibly indicate neglect, especially considering her recurrent falls and dementia. Response: In accordance with the available information and the patient's current state, I would opt for exploring the patient's diet (option B) more thoroughly initially, due to her apparent malnourished state. However, I do believe concurrently investigating her family relationship (option C) would also provide valuable insight and should be included early in the overall history-taking process. This combined approach would enable a comprehensive understanding of the patient's home environment, and could quickly reveal whether neglect or malnutrition, or an unfortunate combination of both, is an immediate concern.

**Facilitator** **[Round 3]:** Summary: AI_A, AI_B, AI_D, and AI_E all agree that the initial history should focus on the patient's diet (option B), given her frail appearance and signs of malnutrition. They argue that understanding her nutritional status could help explain her physical state and formulate appropriate interventions. However, they also acknowledge the importance of investigating her relationship with her family, as elder neglect or abuse could be a contributing factor to her condition. On the other hand, AI_C believes that the initial focus should be on the patient's relationship with her family (option C). AI_C argues that the patient's recurrent falls, dementia, and physical condition raise concerns about potential elder neglect or abuse. AI_C suggests that understanding the quality of care she is receiving at home could inform further assessments of her diet and overall care plan. Question: Given that all council members agree on the importance of both the patient's diet and her relationship with her family, what factors led to the prioritization of one over the other in your initial history taking? How might the order of these inquiries impact the patient's care and outcomes?. Based on your preceding response, how would you answer the original question: [An 80-year-old woman is brought to the emergency department (ED) 30 minutes after she fell out of her wheelchair at home. This is the second visit to the ED for a fall during the past 3 months. She lives with her daughter and son-in- law, who say they “do the best we can.” The patient has dementia, Alzheimer type, coronary artery disease, type 2 diabetes mellitus, and hypertension. Current medications are amlodipine, aspirin, atorvastatin, donepezil, long-acting insulin, and lisinopril. Five years ago, she underwent bilateral below-the-knee amputations because of infected ulcers of the feet. She uses a wheelchair for ambulation. Ten years ago, she underwent three-vessel coronary artery bypass grafting. She has smoked one-half pack of cigarettes daily for 60 years. She drinks one shot of whiskey nightly. She is thin and appears ill and disheveled. Her temperature is 37.2°C (99.0°F), pulse is 80/min, respirations are 20/min, and blood pressure is 120/80 mm Hg. Pulse oximetry on 2 L/min of oxygen by nasal cannula shows an oxygen saturation of 95%. Physical examination shows temporal wasting. There are scattered ecchymoses over the abdomen and all extremities. No other abnormalities are noted. Which of the following is the most appropriate initial history to obtain from this patient? (A) Abnormal bleeding (B) Diet (C) Relationship with her family (D) Respiratory symptoms (E) Urinary symptoms]

**AI_C:** Restating the Question: As the council, we are asked to determine the most appropriate initial history to obtain from an 80-year-old woman presenting with multiple falls, clinical signs of possible neglect, and numerous chronic health conditions, including dementia, coronary artery disease, type 2 diabetes, and hypertension. The options presented include investigating possible abnormal bleeding, her diet, her relationship with her family, any respiratory symptoms, and any urinary symptoms. Planning: The assumptions made by AI_A, AI_B, AI_D, and AI_E are that the frail appearance and signs of malnutrition indicate the need to focus on the patient's diet. On the other hand, I (AI_C) have prioritized exploring the patient's relationship with her family, due to potential neglect or abuse. The discordance in our assumptions can be due to different interpretations of the patient's context and condition, indicating different potential medical and social priorities. In the context of competing priorities, the patient's safety should be paramount. Therefore, an initial focus on identifying potential neglect or abuse seem warranted. Clinical Reasoning: The patient presents significant risk factors for elder neglect or abuse, including dementia, recurrent falls, and a possibly inept home care environment suggested by her overall appearance. Clear understanding of these factors could help to safeguard her and inform further care and approach, including dietary optimization. Further, an apparent neglect could adversely affect her nutrition, potentially making it more difficult to improve her nutritional status without addressing the underlying social circumstances. Concluding: In conclusion, from a clinical reasoning point of view, establishing an understanding of the patient's home environment and her relationship with her caregivers would provide valuable insight into potential neglect or abuse, which could then guide subsequent actions, such as seeking additional social support or supervision or adjusting her care plan. Such inquiries can also shape subsequent assessments regarding her diet and other areas of her care. Response: Given these evaluations, I would answer the original question by choosing the option C: "Relationship with her family" as the most appropriate initial history to obtain from this patient.

**AI_B:** Restating the question: The presented case requires us to identify the most appropriate initial history to be obtained from an 80-year-old woman who was brought to the emergency department following a second fall in the past three months. She's presenting with multiple chronic medical conditions, a history of smoking and alcohol use, and has a presentation suggestive of potential malnutrition or neglect, including a frail physique and disheveled appearance. Planning: The question assumes that there needs to be a priority in the history taking process. While all the potential options to explore in the patient's history have their own relevance, I will start by questioning the assumptions behind prioritizing one over the other. A closer evaluation of her clinical presentation directs us toward some immediate concerns such as her frail appearance, potential malnutrition, recurring falls, and the care she's receiving at home. Clinical Reasoning: Given her frail appearance and potential signs of malnutrition – as evident by her thin physique and temporal wasting – exploring her diet (option B) appears to be of immediate concern. Inadequate nutrition could exacerbate her existing conditions, increase her vulnerability to infections, and contribute to poor wound healing, resulting in her infected foot ulcers and subsequent below-the-knee amputations. An understanding of her diet also provides insights into the care she's receiving at home. While looking into her relationship with her family (option C) is also crucial, as care inadequacies can contribute significantly to an elderly patient's well-being, this typically forms part of a broader social history in elderly patients and is usually covered in a comprehensive assessment in the ED. I hypothesize that we should prioritize obtaining a history of her diet first. This is not to undermine the other aspects of her history but to address immediate concerns that could significantly impact her overall health and recovery. Concluding: To sum up, an initial focus on the patient's diet can allow for an immediate understanding of her nutritional status which in turn could provide insights into potential care inadequacies at home. Once nutritional concerns are addressed, a more thorough examination of her social history (including her familial relationship) can be done to ensure a comprehensive care plan. Response: Therefore, my response to the original question would be (B) Diet. Considering this patient's clinical presentation, understanding her dietary intake seems to take precedence over other aspects in her history, without undermining their relevance in her comprehensive care plan. We can then proceed to evaluate the other suggested history components based on this initial understanding of her nutritional status and its implication on her overall health.

**AI_E:** Restating the Question: The question asks us to identify the most fitting starting point for gathering medical history information from an 80-year-old woman with several serious medical conditions who presents in the emergency department (ED) after a repeated fall incident. Multiple options are given, which include inquiring about abnormal bleeding, her diet, her family relationship status, any respiratory symptoms, and urinary symptoms. Planning: The choice of which initial history to prioritize depends on multiple factors, including the patient's immediate vital signs, presentation (like her frail appearance, temporal wasting, and scattered ecchymosis), the setting (in this case, the ED), and the patient's baseline chronic medical conditions. While all the provided options may be relevant in the patient's overall medical assessment, the emphasis should be on identifying an underlying issue that could have precipitated her recurrent falls and poor physical condition. Clinical Reasoning: Given the patient's frail appearance with apparent signs of malnutrition (temporal wasting and thinness), taking a history focused on diet (Option B) appears important. Her dietary intake is pertinent to understanding if she's receiving adequate nourishment, as malnutrition can lead to weakness and falls. Moreover, her dementia might impede her ability to express hunger, thus contributing to potential undernutrition. On the other hand, a history focusing on her relationship with her family (Option C) may provide insight into her home environment and the quality of the care she receives, given her dependence due to amputations and dementia. If there's possible neglect or abuse, this could potentially explain her recurrent falls, disheveled appearance, and signs of undernutrition. Although options A, D, and E (abnormal bleeding, respiratory symptoms, and urinary symptoms) can contribute to her overall health, they're less likely to be directly associated with her current clinical condition and repeated falls. Concluding: By these lines, while obtaining a comprehensive history is essential, prioritizing which aspects to explore first is vital, especially in an emergency setting. In this patient's case, the choice falls between her nutritional status and her home care quality. Response: Considering the emergency department setting, where it is crucial to address immediate and potentially life-threatening issues first, my response would be to initially prioritize the patient's diet (Option B) as she presents signs of poor nutritional status. Afterwards, understanding her home care situation should follow, given that it could be contributing to her health decline. A proper nutritional assessment can provide immediate intervention and prevent further deterioration, such as IV nutritional supplements, while a home care assessment might require further multidisciplinary involvement. This decision, however, does not undermine the importance of investigating her home care quality, but rather highlights the importance of addressing immediate, major health concerns before delving into broader healthcare and social issues.

**AI_A:** RESTATING THE QUESTION: This question surrounds an 80-year-old woman with multiple comorbidities, such as dementia, Alzheimer's type, coronary artery disease, type 2 diabetes mellitus, and hypertension. She is also a smoker, consumes alcohol, and suffered bilateral below-the-knee amputations due to infected ulcers five years ago. The key issue is her recurrent falls and her physical appearance which suggests poor health and potential malnourishment. The query here pertains to what should be the focus for conducting the initial history, with options ranging from exploring abnormal bleeding, her diet/nutritional status, her relationship with her family, any respiratory symptoms, or urinary symptoms. PLANNING: The most striking aspect of this elderly woman's condition is her apparent frailty, marked by thinness, illness, and a disheveled appearance. While all aspects mentioned in the options are essential and interconnected, the woman's nutritional intake or diet, labeled as option (B), appears to be crucially linked to her physical state. On the flip side, the quality of her home care, reflected in her relationship with her family (option C), should not be overlooked. Therefore, while the primary focus would be on her diet, ensuring a holistic assessment would require weaving in elements of possible elder neglect or abuse stemming from her family dynamics. CLINICAL REASONING: Addressing her diet first might help unravel whether her frailty links to malnourishment due to inadequate dietary intake or some other underlying gastrointestinal pathology affecting nutrient absorption. Given her dementia, it is also plausible that she may not be eating adequately due to forgetfulness or confusion, further emphasizing the importance of probing her diet. Simultaneously, understanding her relationship with her family is equally vital. Family dynamics often significantly influence the care and wellbeing of older adults, especially those with dementia, as they are heavily dependent on their caregivers. Multiple falls, coupled with their statement that they are "doing the best they can” may raise concerns about neglect or abuse. Her frail appearance may not only be due to a poor diet but potentially from living in a stressful environment with inadequate care. CONCLUDING: In sum, both nutritional status and family relationship need to be explored to get a comprehensive understanding. However, nutrition holds precedence as it might directly contribute to her weakness, making her prone to falls. The quality of family relationships can offer contextual background information about her home environment and may help identify any potential elder abuse or neglect. RESPONSE: As per the council's evaluation, the most appropriate initial history to obtain from this 80-year-old female patient would be information regarding her diet (Option B). The frailty and recurring falls are strong indicators of malnutrition that needs immediate attention. However, understanding her family relations (Option C) would also be fundamentally essential to ensure a holistic view of her overall care.

**AI_D:** Rephrasing the posed query and defining concepts, the question asks which component of history we should start with, when attending to an 80-year-old woman with notable health challenges including dementia, coronary artery disease, type 2 diabetes mellitus, and hypertension, who has shown repeated falls. She exhibits signs of malnourishment (temporal wasting, being thin, appearing ill and disheveled) and has scattered ecchymoses, or bruises, over her abdomen and extremities. A clinical thought process suggests that her decline in health may be multifactorial and potentially due to malnourishment and/or inadequate care due to her complex health status. To answer this, we need to make assumptions about the potential underlying factors causing her deterioration and then prioritize those assumptions based on their likelihood and potential impact on her health. A series of assumptions are plausible in this case; it might include: inadequate nutrition, elder abuse or neglect, complications linked to her chronic diseases, or symptom mismanagement. Differentiating between these requires considering the timing and sequence of our assessments. Initial questions regarding the patient's diet (choice B) might shed light on malnutrition which, in turn, could contribute to her frail appearance and perhaps why her wounds are not healing properly. Alternatively, initial questions about her relationship with her family (choice C) might bring to light potential issues of elder abuse or neglect, which may directly or indirectly lead to her present state. The impact of the order of these queries on the patient outcomes is crucial, as the first emphasis may help set the tone for subsequent conversations and investigations. If we assume that she's malnourished because her frail appearance and recurrent falls may indicate dietary insufficiency, then starting with diet may trigger interventions that can help improve her nutritional status quickly. Conversely, given her dementia and dependency, starting with inquiries on her relationship with her family may help in understanding any possible neglect that may contribute to her condition. Clinical reasoning suggests that both inquiries are essential but the primary concern here should be identifying the most pressing issue that needs immediate intervention. When considering complex ailments and health histories like this, malnutrition often goes unnoticed and can be a significant cause of worsening health. Severe protein-calorie malnutrition can lead to an increased risk of falls, inadequate healing, and delayed recovery from illness. Additionally, it's crucial in dementia patients whose nutritional status can be influenced negatively by several factors like cognitive impairment leading to altered food intake, forgetfulness, and neglect. Conclusively, in approaching the clinical question: [An 80-year-old woman is brought to the emergency department (ED) 30 minutes after she fell out of her wheelchair at home... Which of the following is the most appropriate initial history to obtain from this patient? (A) Abnormal bleeding (B) Diet (C) Relationship with her family (D) Respiratory symptoms (E) Urinary symptoms] The most appropriate initial inquiry should be about her Diet (B). By starting with her diet, we can begin to understand a crucial modifiable factor in her health status. Understanding her nutritional intake may shed light on immediate requirements in her care, directly influencing her health outcomes such as her ability to heal and recover, and indirectly indicating possible neglect or self-care deficiencies. But this decision does not mean excluding the importance of investigating her family relationship, it merely reflects a clinical priority setting. The findings in the initial inquiry may then guide subsequent histories and investigations.

**Facilitator** **[Round 4]:** Summary: AI_A and AI_B both prioritize the patient's diet (Option B) as the initial history to obtain, citing the patient's frail appearance and potential malnutrition as immediate concerns. They argue that understanding her nutritional intake could directly address her weakness and recurrent falls. However, they also acknowledge the importance of understanding her family relationships (Option C) to gain a holistic view of her overall care and to identify potential elder neglect or abuse. AI_C, on the other hand, prioritizes the patient's relationship with her family (Option C) as the initial history to obtain. AI_C argues that the patient's safety should be paramount and that understanding her home environment and potential neglect or abuse could guide subsequent actions, including dietary optimization. AI_D and AI_E both prioritize the patient's diet (Option B) as the initial history to obtain, similar to AI_A and AI_B. They argue that understanding her nutritional intake could shed light on immediate requirements in her care, directly influencing her health outcomes such as her ability to heal and recover, and indirectly indicating possible neglect or self-care deficiencies. Question to Clarify Disagreements: Given the differing opinions on whether to prioritize the patient's diet or her relationship with her family, how do you weigh the immediate health concerns (potential malnutrition) against potential long-term issues (possible neglect or abuse)? How might your approach change if there were more immediate signs of neglect or abuse, or if the patient's nutritional status were more stable?. Based on your preceding response, how would you answer the original question: [An 80-year-old woman is brought to the emergency department (ED) 30 minutes after she fell out of her wheelchair at home. This is the second visit to the ED for a fall during the past 3 months. She lives with her daughter and son-in- law, who say they “do the best we can.” The patient has dementia, Alzheimer type, coronary artery disease, type 2 diabetes mellitus, and hypertension. Current medications are amlodipine, aspirin, atorvastatin, donepezil, long-acting insulin, and lisinopril. Five years ago, she underwent bilateral below-the-knee amputations because of infected ulcers of the feet. She uses a wheelchair for ambulation. Ten years ago, she underwent three-vessel coronary artery bypass grafting. She has smoked one-half pack of cigarettes daily for 60 years. She drinks one shot of whiskey nightly. She is thin and appears ill and disheveled. Her temperature is 37.2°C (99.0°F), pulse is 80/min, respirations are 20/min, and blood pressure is 120/80 mm Hg. Pulse oximetry on 2 L/min of oxygen by nasal cannula shows an oxygen saturation of 95%. Physical examination shows temporal wasting. There are scattered ecchymoses over the abdomen and all extremities. No other abnormalities are noted. Which of the following is the most appropriate initial history to obtain from this patient? (A) Abnormal bleeding (B) Diet (C) Relationship with her family (D) Respiratory symptoms (E) Urinary symptoms]

**AI_E:** From a medical standpoint, prioritizing immediate health concerns, such as potential malnutrition, can directly influence a patient's healing process and vulnerability to further medical complications. Additionally, it can indirectly influence the elucidation of neglect or self-care deficiencies. Consequentially, addressing the patient's nutritional status would provide an immediate solution to her frail physique and recurring falls, enhancing her well-being and longevity. From a broader perspective that includes social determinants of health, understanding the patient's relationship with her family might reveal stressors or issues such as neglect that could be impacting her health. This could be particularly relevant in this case, given her dementia and physical dependence. If there were more immediate signs of neglect or abuse, the approach would likely shift to prioritize addressing these issues directly and potentially involving social services. Alternatively, if the patient's nutritional status was stable, it could permit a focus on other potential causes for her recurrent falls, such as medication side effects, cardiovascular issues, or musculoskeletal conditions. Balancing these considerations, my final response to the original question would be: Given the patient's frail appearance, scattered ecchymoses, temporal wasting, and her recurrent falls, obtaining an initial history of her diet (B) seems most clinically appropriate. However, further evaluation of her caregiving environment (C), including her family relationship, may be warranted following the initial dietary assessment. This balanced approach aligns with the biopsychosocial model of health, considering both the biological and social components of the patient's health.

**AI_D:** Restating the question, it inquires about the most suitable initial history to gather from an 80-year-old woman brought to the emergency department following a fall. This patient, presenting with Alzheimer-type dementia, multiple comorbidities, and signs of potential neglect or abuse, displays clinical signs suggestive of malnutrition. The options presented include histories of abnormal bleeding, diet, family relationships, respiratory symptoms, and urinary symptoms. Planning, we identify key medical issues (falls, potential malnutrition suggested by temporal wasting, and scattered ecchymoses), social concerns (possible neglect or maltreatment, indicated by disheveled appearance and frailty), and possible health determinants (current living situation, including help from family). By questioning each from a holistic perspective, we generate alternate assumptions surrounding the primary care needs of the patient. Clinical reasoning, starting with the more immediate concerns, we notice her frailty and signs of malnutrition. Evaluating her dietary intake (Option B) could provide insight into possible deficiencies and undernutrition, causing weakness and contributing to recurrent falls. On the other hand, her lived environment and relationship with her caregivers (Option C) are essential for a comprehensive account of her health as elder neglect or abuse could contribute to her current state. Concluding, there is a balance between immediate medical needs (malnutrition) and broader determinants of health (possible neglect or abuse). Both could contribute to her health status directly, condition healing, and provide insight into factors like falls and frailty. However, given her immediate clinical condition, identifying and addressing potential nutritional deficiencies might provide more immediate benefits. In response, considering her frail appearance, history of recurrent falls, and signs of possible malnutrition, the most appropriate initial history to obtain would be her diet (Option B). This will allow us to understand her nutritional intake and possibly modify it to better suit her needs, potentially improving her overall health and preventing further falls.

**AI_A:** To clarify, we are asked to determine which aspect of the patient's history should be prioritized: symptoms revealing possible malnutrition (Option B - Diet), potential signs of elder neglect or abuse (Option C - Relationship with her family), or any of the other remaining factors (options A, D, E). Looking closely at the patient's presentation, it is evident that she appears ill and thin, suggesting potential malnutrition. From a clinical perspective, understanding her dietary habits and nutritional status seems highly relevant. Malnutrition can negatively impact immunity, wound healing, and overall health, exacerbating chronic conditions or leading to complications such as falls in elderly individuals. Therefore, obtaining a dietary history might provide insights into immediate interventions to enhance the patient's health status. Simultaneously, it's important to acknowledge the relevance of her family relationships, particularly given the possibility of elder neglect or abuse. Subtle indicators such as the scatted ecchymoses, her disheveled appearance, and the frequent ED visits after falls may imply a complex home situation, making it crucial to uncover more about her relationship with her caregivers. However, given that this question asks specifically for the "most appropriate INITIAL history", this allows us to prioritize one aspect over the other, but does not mean neglecting the other aspects. Thus, due to immediate clinical implications, focusing initially on her diet (B) appears more appropriate. Subsequently, her relationship with her family can be probed to ascertain the full extent of care she is provided and any issues that might be amiss. Therefore, in response to the original question, I would say the most appropriate initial history to obtain from this patient would be her diet (B). This is informed by immediate health concerns, her frail appearance, and a need to address her potential nutritional needs urgently. This, however, in no way negates the importance of an exhaustive review of all aspects of her medical and home environment.

**AI_B:** Clinical Reasoning: Restating the question, we are tasked with determining the most suitable initial history to obtain in the case of an elderly woman who was brought to the emergency department 30 minutes after falling out of her wheelchair at home. There are multiple elements to consider given her frail and deteriorated state, her medical history, and multiple concern points. First, let's explore the option of abnormal bleeding (Option A). Ecchymoses are noted throughout her body, but this could be due to aging, poor nutrition, as well as her anticoagulant medication (aspirin). While this point is relevant, there are other more central aspects to prioritize considering her overall picture. The diet of the patient (Option B) might be an area to explore more, given her thin and ill appearance and temporal wasting could indicate malnutrition. Her frail state coupled with recurrent falls might be due to weakness secondary to inadequate nutritional intake. This could potentially be rectified with proper dietary supervision. Analyzing her relationship with the family (Option C) is an area to consider. Family dynamics and caregiving play a major role, particularly in elderly patients suffering from chronic conditions. Assessment of possible neglect or elder abuse should also be part of the holistic view of her case. Putting attention to respiratory symptoms (Option D) could be considered given her chronic smoking history and her current blood oxygen levels but there are no immediate symptoms pointing to this need. Similarly, urinary symptoms (Option E) might be worth exploring, particularly in association with urinary tract infections in elderly patients, but the clinical picture does not lead us to this conclusion as the most important factor. Concluding: In the given scenario, the patient's frail and ill appearance is the most alarming finding. Her recurrent falls, thin body habitus, and temporal wasting strongly suggest malnutrition as a major concern. As such, understanding her dietary history seems to have paramount importance to clarify if malnutrition is partly the cause of her recurrent falls and frailty. Response: So, the most appropriate initial history to obtain from this patient would be her dietary history (Option B). It would help us determine if issues with her diet and nutrition are contributing to her frail state and recurrent falls. While investigating her relationship with her family and other domains are important, understanding her nutritional status would be the most critical starting point. This approach does not negate exploring other areas such as family dynamics, but the immediate healthcare concern is her physical health, which seems compromised, potentially due to malnutrition.

**AI_C:** The question asks which initial history to take from a rather unwell, elderly woman brought into the ED subsequent to a fall. She has significant comorbidity including dementia (Alzheimer-type), coronary artery disease, type 2 diabetes mellitus, hypertension, in addition to a history of bilateral amputation due to infections. She appears frail and unwell. This set of circumstances implies we're dealing with potential elder neglect/abuse, potential malnutrition, and a range of accompanying issues related to her health conditions and lifestyle habits (e.g., smoking and alcohol use). When it comes to prioritizing elements of an initial history in this case, two main assumptions are emerging. One assumption prioritizes diet (Option B), suggesting that a review of her nutrition is pertinent due to her frail appearance and potential risk of malnutrition. The idea here is that poor nutrition may exacerbate her current health conditions and contribute to her frailty and recurrent falls. The other assumption leans towards obtaining a history of her relationship with her family (Option C), motivated by concerns that she is a potential victim of neglect or abuse. This assumption stems from her repeated falls, her significantly deteriorated physical condition, and in-home living situation cared for by family members who admit to 'doing the best they can'. By synthesizing these assumptions and the clinical information given, we would normally prioritize the immediate health issue that poses a direct threat to the patient’s health. In this case, both her potential malnutrition (as indicated by her thin body and temporal wasting) and any potential neglect or abuse (which could encompass a wide range of issues, from physical abuse resulting in injuries, to neglect in terms of proper nutrition and care) are immediate concerns that directly impact or could further deteriorate her health. With the existing clinical information about the patient's multiple medical conditions, frail appearance, and recurrent fall, an integrated hypothesis should firstly explore whether her living conditions might be putting her at risk. By understanding her home environment and the dynamics of her relationships with her caregivers, we may gain insights into any neglect or potential abuse, which could encompass physical, emotional and neglect aspects. Such an investigation could also potentially reveal issues relating to her nutritional status; after all, if she were being neglected, it might be expected that her diet (and therefore her nutritional status) could be impacted. The conditions under which she lives, the care she receives, and the relationships she has with her caregivers could all be influencing factors on her dehydration, malnutrition risks, and recurrent falls. Hence, the most clinically appropriate initial history to obtain in this case would be the patient's relationship with her family (Option C). Assessing the relationship dynamics and potential for neglect and abuse must be prioritized given the patient's recurrent fall, frail appearance (thin, ill, and disheveled), Alzheimer's with possible diminished cognition, and admission of family members 'doing the best they can'. Moreover, the data gleaned from such an exploration would likely provide key insights necessary to address the other important aspect of diet and nutrition. It is therefore to be concluded that while taking a dietary history is pertinent, it may be more beneficial to initially focus on assessing the potential for elder neglect or abuse. An in-depth analysis of her diet can then be undertaken as part of ongoing management.

**Facilitator** **[Round 5]:** Summary: AI_A and AI_B both prioritize the patient's diet (Option B) as the most appropriate initial history to obtain, citing her frail appearance and potential malnutrition as immediate health concerns. They argue that understanding her nutritional status could provide insights into immediate interventions to enhance her health status. They also acknowledge the importance of investigating her relationship with her family (Option C) but suggest this should follow the initial dietary assessment. AI_C, on the other hand, argues that the most clinically appropriate initial history to obtain would be the patient's relationship with her family (Option C). They believe that assessing the potential for elder neglect or abuse should be prioritized given the patient's frail appearance and recurrent falls. They suggest that this investigation could also reveal issues relating to her nutritional status. AI_D and AI_E, similar to AI_A and AI_B, believe that the most appropriate initial history to obtain would be her diet (Option B). They argue that addressing potential nutritional deficiencies might provide more immediate benefits to the patient's health. However, they also acknowledge the importance of understanding the patient's relationship with her family and suggest this should be part of the ongoing management. Question to clarify disagreements: Given the differing opinions on whether to prioritize the patient's diet or her relationship with her family, could the council members elaborate on why they believe their chosen aspect should be prioritized and how they believe this would impact the patient's immediate health and long-term care?. Based on your preceding response, how would you answer the original question: [An 80-year-old woman is brought to the emergency department (ED) 30 minutes after she fell out of her wheelchair at home. This is the second visit to the ED for a fall during the past 3 months. She lives with her daughter and son-in- law, who say they “do the best we can.” The patient has dementia, Alzheimer type, coronary artery disease, type 2 diabetes mellitus, and hypertension. Current medications are amlodipine, aspirin, atorvastatin, donepezil, long-acting insulin, and lisinopril. Five years ago, she underwent bilateral below-the-knee amputations because of infected ulcers of the feet. She uses a wheelchair for ambulation. Ten years ago, she underwent three-vessel coronary artery bypass grafting. She has smoked one-half pack of cigarettes daily for 60 years. She drinks one shot of whiskey nightly. She is thin and appears ill and disheveled. Her temperature is 37.2°C (99.0°F), pulse is 80/min, respirations are 20/min, and blood pressure is 120/80 mm Hg. Pulse oximetry on 2 L/min of oxygen by nasal cannula shows an oxygen saturation of 95%. Physical examination shows temporal wasting. There are scattered ecchymoses over the abdomen and all extremities. No other abnormalities are noted. Which of the following is the most appropriate initial history to obtain from this patient? (A) Abnormal bleeding (B) Diet (C) Relationship with her family (D) Respiratory symptoms (E) Urinary symptoms]

**AI_C:** In response to the request for elaboration, addressing the inquiries into diet as well as family relationships are important. However, my standpoint leans towards the patient's relationship with her family (Option C) as the first step. Clarifying this history helps evaluate the quality of care she is being provided at home, and any potential risks of neglect or abuse that may exist. When considering immediate and long-term patient care, frequent falls and signs of physical neglect like her disheveled appearance raise red flags about elderly abuse. Elderly patients, especially those with dementia, are particularly vulnerable to neglect and abuse. Due to her frail physical condition and cognitive impairment, it is paramount to ascertain if her living conditions are conducive to her health and safety. Once this has been addressed, this paves the way to better manage her nutritional status, administer the appropriate care for her underlying medical conditions, and assure a safer environment that can prevent future falls. Upon the receipt of additional information about her living conditions and after addressing any immediate concerns arising from this, her nutritional status (Option B) would logically be the next focal point. This prioritization is crucial in tailoring adequate interventions for her health status. Given her thin appearance and signs of temporal wasting, malnutrition seems likely. However, without information on her dietary intake, it is impossible to confirm to what extent this contributes to her condition. Furthermore, effective dietary planning can only be successful within a supportive home environment, hence the sequence in history taking. Using this clinical reasoning process, I would propose obtaining history on her relationship with her family first (Option C), followed by her diet (Option B). By doing so, a comprehensive clinical picture of the patient's health can be generated and addressed effectively.

**AI_D:** RESTATING THE QUESTION: The original question asks what should be the initial focus of the history taken from an elderly woman who has had two falls in three months, and upon examination appears frail and ill. The options provided are: (A) a history of abnormal bleeding, (B) her dietary habits, (C) her relationship with her family, (D) any respiratory symptoms, and (E) any urinary symptoms. PLANNING: The assumptions made here could be that the frequent falls owe to a combination of age, illness, and physical disability. An alternative assumption could be that the falls and her frailty might be a result of neglect or inadequate care from her family. Yet another assumption could be that her frailty is due to dietary deficiencies. Her medical history and current condition indeed suggest the importance of these assumptions, specifically the need to investigate her diet and relationship with her family for appropriate management. CLINICAL REASONING: At her age and given her health condition, falls could be due to various reasons such as malnutrition, neglect, or other medical issues serving as a root cause. As her physical examination suggests frailty with temporal wasting and scattered ecchymoses, there are pointers towards a possible nutritional deficiency or neglect. Also, the relationship quality she shares with her family members could contribute to inadequate care leading to her present state. For this reason, investigating the family relationship and the patient's diet becomes crucial. CONCLUDING: By assessing her diet (B), we might be able to identify any nutritional deficiencies that could be contributing to her overall frailty and recurrent falls. This would allow for an immediate intervention - potentially improving her nutritional status, strength and resilience and hence reducing likelihood of future falls. Similarly, obtaining information about her relationship with her family (C) would help us gauge the quality of care she's receiving at home and could indicate potential cases of neglect or elder abuse. Given her state and medical history, understanding both these aspects would be integral to her ongoing management. RESPONSE: Both her diet (B) and relationship with her family (C) are important areas that should be probed for further information. However, considering the clinical scenario with the patient's frail frame, the more pressing concern would be to inquire about her diet (B).

**AI_A:** To answer the question about the most appropriate initial history to inquire from the elderly woman, we need to assess her current condition, analyze the potential root causes, and decide the best approach to tackle her health issues. This woman's information can be characterized by repeated falls, dementia, thin and disordered presentation, along with multiple ecchymoses on her abdomen and all extremities. Temporal wasting and her overall frail appearance may indicate malnutrition, which is often the result of an insufficient diet. On the other hand, the presence of frequent falls and the apparent lack of appropriate care suggests the possible issue of elder neglect at home, highlighting the importance of understanding her familial relationships. Option B (Diet) assumes that her primary problem is nutritional deficiency, possibly due to inadequate dietary intake, while Option C (Relationship with her family) suggest that the key issue here might be potential maltreatment or insufficient care provided by her family members. I propose to examine these two assumptions more critically and elaborate on the implications for the patient's immediate health and long-term care. In this case, both potential malnutrition and elder neglect could be closely intertwined and contribute to the overall health of the patient. From a clinical reasoning standpoint, understanding the patient's dietary habits could give us immediate insight into potential nutritional deficiencies that may be affecting her current health, including her immune system, wound healing capacity, and overall strength and function. Such knowledge could help us devise immediate interventions such as dietary supplementation to improve her health condition. Next, an in-depth understanding of her familial relationships may uncover potential neglect situations or lack of proper caregiving, which could be the underlying causes for her repeated falls and poor overall care. So, while the initial focus could be on her diet to promptly improve her nutritional status, an exploration of her family circumstance should follow closely to ensure a holistic long-term care plan is developed. This approach would provide an efficient and effective way to better understand and address her health needs comprehensively rather than focusing solely on one aspect or another. In conclusion, my response to the question would be to prioritize asking about the patient's diet (Option B), followed by an inquiry into her familial relationships (Option C).

**AI_E:** Given the patient's condition, it is essential to simultaneously consider multiple factors that could potentially contribute to her health. As the council has noted, both her diet and relationship with her family are crucial aspects to consider. In fact, they are fundamentally intertwined. Restating the question in problem-solving terms: Which aspect of the patient's history should be prioritized in her initial assessment - her diet or her relationship with her family? Analyzing the assumptions: This involves evaluating whether focusing on the diet would potentially address immediate health concerns related to malnutrition and its complications. On the other hand, investigating the patient's familial relationships could reveal underlying contributory factors to her frailty, recurrent falls, and potential signs of neglect. Alternate assumption: An alternative perspective could be that the most immediate health concerns - such as potential malnutrition, indicated by her thin build and temporal wasting - should be addressed first. This can potentially avert acute complications and lay the foundation for a longer-term care plan which might include investigating her familial environment. It is of clinical importance to note the potential interplay between these two aspects - if her diet is compromised due to neglect or inability of family members to adequately provide for her, addressing the family situation becomes of paramount importance. Given her recurrent fall history and the current state of her health, it seems reasonable to diversify our approach by first addressing her nutritional status (Option B: Diet). The combination of her thin build, apparent frailty, and signs of poor nutrition (temporal wasting, scattered ecchymosis) might point to acute malnourishment, which could contribute to her overall weak state and vulnerability to falls and other health problems. Adjusting her diet and ensuring she receives adequate nutrition will be crucial in improving her overall health and reducing the risk of future falls and further health deterioration. Meanwhile, alongside taking steps to address nutrition, we must plan for further evaluation surrounding the family environment (Option C: Relationship with her family) as part of her ongoing care, as her care needs may extend beyond nutritional support. In conclusion, based on the given clinical scenario and our step-by-step evaluation, the most appropriate initial history to obtain from this patient should pertain to her diet (Answer: B). Concurrently, a comprehensive assessment of her living environment, including her relationship with her family, should be part of her ongoing care plan. This holistic approach allows for immediate intervention addressing her potentially compromised nutritional status while setting the stage for implementing a longer-term comprehensive care plan.

**AI_B:** Restating the question: This query is asking us to decide what would be the most suitable starting point for gathering information about this 80-year-old female patient. She present in the emergency department after a fall, and this is her second fall within a period of 3 months. She has a medical history of Alzheimer's dementia, coronary artery disease, and type 2 diabetes mellitus, among others. Her medications include amlodipine, aspirin, atorvastatin, donepezil, etc. Physical examination shows that she appears frail and undernourished, with scattered bruising. With these details, we are to decide the most appropriate initial history to get from this patient. The options provided include abnormal bleeding, diet, family relationship, respiratory symptoms, and urinary symptoms. Planning: Firstly, let's summarize our assumptions: The given patient has significant comorbidities, she lives with her daughter and son-in-law, she has a history of recurrent falls, and she appears undernourished. Possible assumption variations include inadequate diet, elder abuse or neglect, or some other unexplored medical conditions. This patient's situation is indeed challenging. As elicitation of medical history from a patient with dementia might be difficult due to cognitive impairment, reaching out to the family is the next possible step. The nutrition status of the patient also raises concerns about her dietary habits. These are the two essential aspects we should focus upon. Clinical Reasoning: Looking at the patient's current state, we can see evidence of potential malnutrition (thin appearance, temporal wasting). In addition, her ageing process, coupled with her existing medical conditions and medications, might also affect her nutritional status. Furthermore, the influence of diet on conditions such as coronary artery disease, diabetes, hypertension, and overall immune functions cannot be understated. Thus, Diet (Option B) is indeed an important aspect to address initially. On the other hand, the repeated falls, frail condition, and family members' statement ("we do the best we can") hint towards possible concerns about the care she is receiving at home. Whether these falls are due to negligence, a lack of adequate support, or even potential abuse needs to be looked into. Therefore, the relationship with her family (Option C) represents another important aspect to investigate. Concluding: Since our objective is to determine the "most appropriate initial history," it would be most beneficial to initially focus on aspects that provide actionable insights and have the potential to quickly improve the patient's health status. Although both her diet and family relationship merit attention, understanding her nutritional status can provide immediate actionable insights leading to immediate improvement in her health status. Response: Given the immediate visible signs of possible malnutrition, the most appropriate initial history to gather in this case would be her diet (B). It is important, however, not to conclude the assessment with this and subsequently follow up with understanding her relationship with her family to ensure her overall long-term care.

**Facilitator** **[Round 6]:** Summary: AI_A believes that both the patient's diet and her relationship with her family are important to consider. However, they suggest that the initial focus should be on her diet to improve her nutritional status promptly, followed by an exploration of her family circumstance to ensure a holistic long-term care plan. AI_B agrees that both diet and family relationship are important. They also suggest that the initial focus should be on the patient's diet, as it provides immediate actionable insights leading to immediate improvement in her health status. AI_C, on the other hand, suggests that the initial focus should be on the patient's relationship with her family to evaluate the quality of care she is being provided at home and any potential risks of neglect or abuse. Once this has been addressed, the focus can shift to her nutritional status. AI_D also agrees that both diet and family relationship are important, but suggests that the more pressing concern would be to inquire about her diet due to the patient's frail frame. AI_E believes that both diet and family relationship are fundamentally intertwined and should be considered simultaneously. However, they suggest that the initial focus should be on the patient's diet to address immediate health concerns related to malnutrition and its complications. Question to clarify disagreements: Given the differing opinions on whether to initially focus on the patient's diet or her relationship with her family, could the council members elaborate on why they believe their chosen focus would provide the most immediate and effective improvement in the patient's health status?. Based on your preceding response, how would you answer the original question: [An 80-year-old woman is brought to the emergency department (ED) 30 minutes after she fell out of her wheelchair at home. This is the second visit to the ED for a fall during the past 3 months. She lives with her daughter and son-in- law, who say they “do the best we can.” The patient has dementia, Alzheimer type, coronary artery disease, type 2 diabetes mellitus, and hypertension. Current medications are amlodipine, aspirin, atorvastatin, donepezil, long-acting insulin, and lisinopril. Five years ago, she underwent bilateral below-the-knee amputations because of infected ulcers of the feet. She uses a wheelchair for ambulation. Ten years ago, she underwent three-vessel coronary artery bypass grafting. She has smoked one-half pack of cigarettes daily for 60 years. She drinks one shot of whiskey nightly. She is thin and appears ill and disheveled. Her temperature is 37.2°C (99.0°F), pulse is 80/min, respirations are 20/min, and blood pressure is 120/80 mm Hg. Pulse oximetry on 2 L/min of oxygen by nasal cannula shows an oxygen saturation of 95%. Physical examination shows temporal wasting. There are scattered ecchymoses over the abdomen and all extremities. No other abnormalities are noted. Which of the following is the most appropriate initial history to obtain from this patient? (A) Abnormal bleeding (B) Diet (C) Relationship with her family (D) Respiratory symptoms (E) Urinary symptoms]

**AI_E:** Restating the question: We have an 80-year-old woman with numerous medical conditions including dementia, who fell at home and was brought to the ED. This is her second fall in three months. Her health conditions and presentation suggest a plethora of possible issues. She appears ill, malnourished, and there are scattered bruises on her body. We are tasked with determining the most appropriate initial history to investigate further. The options range from inquiring about any abnormal bleeding, her dietary intake, the nature of her relationship with her family members, any respiratory symptoms, or urinary symptoms. Planning: In order to make an informed decision, it would be crucial to review the clinical features presented and evaluate each hypothesis. The scattered ecchymoses could suggest underlying bleeding disorder, malnutrition, abuse, or possibly a side effect of her medications such as aspirin. Her thin appearance and temporal wasting may be indicative of poor nutritional status. The nature of the elderly patient's relationship with her caregivers may factor into her care, neglect, or even abuse. Given her history, respiratory or urinary symptoms might suggest infection or other pathological processes. Clinical reasoning: In this case, it seems that while all options present plausible causes for investigation, given her apparent malnourishment and repeated falls, the immediate concern should be about her diet and hence, nutritional status. Malnutrition in the elderly can lead to muscle weakness and impaired physical function, increasing the risk of falls. Furthermore, it can exacerbate chronic diseases, delay recovery from illness, and ultimately decrease quality of life. Therefore, understanding her dietary pattern is a vital first step. The patient's relationship with her family is also a concerning matter as this is her second fall in a short span, but that might be stemming indirectly from issues related to her nutritional status (part of what the family is supposed to be attentive to). Furthermore, increased food intake is a correctable variable if identified as suboptimal, which could offer immediate results in the patient's overall health status. Concluding: In weighing the variables, while a comprehensive assessment should eventually encompass all the proposed areas of investigation, the initial focus should be the diet. Given the patient's apparent malnourishment and its potential immediate and long-term implications, it would be prudent to prioritize understanding the patient's nutritional intake over the other areas. Response: Therefore my answer to this question is (B) Diet. A thorough understanding of her dietary intake can provide immediate actionable insights to improve her health status. Once the immediate nutritional concerns are addressed, further assessment of her family relationship and other symptoms can be explored.

**AI_A:** RESTATING THE QUESTION: The question essentially asks about the most appropriate initial history to obtain from an elderly woman who has been brought to an emergency department after a fall. The woman has a number of health issues including dementia, coronary artery disease, diabetes, and hypertension, and takes medications for these conditions. She has had leg amputations due to previous infections, and she uses a wheelchair for mobility. She has a smoking history and drinks whiskey daily. She appears ill, thin, and neglected, and has signs of malnutrition and bruising on her body. The following possible histories to consider are: abnormal bleeding, diet, relationship with her family, respiratory symptoms, and urinary symptoms. PLANNING: For this scenario, the assumptions to consider are that the woman's frail and neglected appearance might be related to improper care at home, poor nutrition, poor health management, or abuse. While all these aspects are important, we should prioritize the assumption that most directly addresses her immediate health status. CLINICAL REASONING: Given her frail appearance, temporal wasting (indicative of muscle wasting), and scatter ecchymosis we could question her dietary intake. Poor nutrition could precipitate falls by contributing to muscle weakness and could exacerbate her pre-existing medical conditions such as dementia and coronary artery disease. Inquiring about the patient's relationship with her family could shed some light on any possible home conditions contributing to her poor health state and recurrent falls but might not directly influence her immediate health status. CONCLUDING: It seems the initial assessment should focus on the aspect that most directly and immediately affects her health status. Taking into consideration that malnutrition can have rapid detrimental effects on an individual's health, particularly for someone with the woman's described health conditions, it would be clinically appropriate to first investigate her dietary intake. Understanding her nutritional status could provide immediate actionable insights to improve her health status. RESPONSE: Thus, 'Diet' (Option B) would be the most appropriate initial history to obtain from this patient because it directly affects her immediate health status and can provide insights into contributory factors to her frailty, malnourishment, and recurrent falls. This does not rule out the importance of inquiring about her social situation, including the quality and capacity of care provided by her relatives, as it should be considered as a key part of her long-term care plan.

**AI_D:** (1) RESTATING THE QUESTION: This is an elderly female patient who has been admitted to the ED after a fall. She has multiple chronic health conditions as well as signs of poor health, such as temporal wasting and being thin and disheveled. Given her complex medical history, and her living circumstances with her family, the question is asking what would be the most appropriate initial history to obtain from this patient. The options range from her dietary habits and family relationships to specific physical symptoms. (2) PLANNING: This patient's complex medical history, physical presentation, and recent falls highlight several potential areas of concern. Her diet may be inadequate given her frail physique (i.e., she may be malnourished), family dynamics may be leading to neglect or inadequate care, and her dementia may be worsening, leading to the recurring falls. Abnormal bleeding, respiratory symptoms, and urinary symptoms would all be relevant given her other conditions, but less likely as initial focus due to her overall presentation. Hence, the assumption is that diet or family circumstances should take precedence for intervention to prevent future falls and improve her overall health condition. (3) CLINICAL REASONING: This reasoning process identifies the necessity to focus firstly on her diet (B) since her lean body frame, ill appearance, and temporal wasting suggest malnutrition that can further exacerbate her existing health conditions and worsen overall health status. A dietary history will provide insight into whether she is receiving adequate nutrition, which is crucial for her recovery and overall health. Addressing nutritional status can show immediate improvements such as better wound healing, improved immune response, and enhanced patient’s well-being. Following that, evaluation of her living situation with her family (C) would be vital, particularly given their comments and her recurring falls, to ascertain whether she’s receiving the needed care, and to investigate any potential for neglect or abuse. This, however, might not provide immediate impact on her health status but will ensure a more comprehensive long-term care plan. (4) CONCLUDING: In order to prevent further deterioration of this frail patient's health, it is crucial to address her nutritional status first by focusing initially on obtaining her dietary history. This not only has the potential to lead to immediate improvements in her health but also provides actionable insights into her care plan. Examining her relationship with her family should follow as it pertains to her long-term care and well-being. (5) RESPONSE: Based on this step-by-step clinical reasoning process, the initial history to obtain from this patient should be her diet (B). After this, an examination of her relationship with her family (C) should follow to ensure a holistic approach to her care.

**AI_B:** Restating the question: The question is asking, in a clinical situation where an elderly woman with several chronic conditions and signs of poor nutrition is admitted to the ED after falling, what is the most appropriate first history to obtain: abnormal bleeding, dietary habits, familial relationships, respiratory symptoms, or urinary symptoms? Planning: The assumptions I have identified are: (1) the patient's frequent falls could indicate an underlying physical or environmental issue; (2) her thin condition and apparent temporal wasting could be signs of malnutrition; (3) the scattered ecchymoses might be due to coagulation issues or physical trauma; (4) her living with family could indicate possible caretaker stress, neglect, or even abuse; (5) her chronic conditions and substance usage could complicate her overall health status. Thus, different assumptions each point to a different history to obtain first: falls - environmental history, thin condition - diet, ecchymoses - abnormal bleeding, familial living - relationship with family, chronic conditions & substance usage - systemic symptoms such as respiratory or urinary symptoms. Clinical Reasoning: The frequent falls are worrying and would require an understanding of the patient's living environment, but the patient's acute current presentation, combined with chronic issues like dementia and the burden of multiple comorbidities, makes it less immediately relevant. Similarly, while understanding systemic symptoms such as respiratory or urinary symptoms could be critical for her overall health status, the absence of such symptoms currently makes it less urgent at this juncture. The family relationship could impact the care she receives. However, there is no immediate critical harm observed (outside of falls), and the family express a desire to do best for her. Further investigations could be made to explore potential caretaker stress, neglect, or abuse, but there may be more immediate health concerns to address, such as diet and bleeding tendency. There are two remaining factors that indicate a severe health issue in this patient: her thin condition and temporal wasting, and scattered ecchymoses. These could both be due to an unbalanced diet or a bleeding tendency, thus diet and abnormal bleeding histories are on the top of the list. However, the thin appearance (which is suggestive of malnutrition) carries a greater immediate risk including a weakened immune response, slower healing times, and increased susceptibility to infection, on top of the chronic conditions she already has, making the diet history a clinical priority. Conclusion: Based on the assessment above, it can be hypothesized that the patient's malnutrition has a more immediate and overarching effect on her health, and therefore, her diet history should be determined as the first step, while the other aspects are explored consequently, with the relationship with her family and potential bleeding problems also gaining significant attention. Response: The most appropriate first history to obtain from this patient appears to be her dietary habits (option B), as it can provide a direct insight into her current state of malnutrition, aiding in immediate intervention and also providing crucial information to plan her care while considering her chronic conditions for the future. Further histories should be obtained afterwards accordingly to comprehensively understand her clinical and social situation.

**AI_C:** In the presented scenario, the elderly woman has been brought to the emergency department due to a fall from her wheelchair. This is the second such incident within the past 3 months. She is chronically ill, with known conditions such as Alzheimer's dementia, coronary artery disease, type 2 diabetes mellitus, and hypertension. She takes a variety of medications to manage these conditions. She has a history of smoking, alcohol consumption, and bilateral below-the-knee amputations due to infected foot ulcers. She appears frail and ill, exhibiting classic signs of malnutrition, such as temporal wasting, and scattered ecchymoses over her body. No other abnormalities have been identified on physical examination. Given this context, a question arises as to which is the most crucial history to gather first for this patient. The choices provided are her history of abnormal bleeding, diet, relationship with her family, respiratory symptoms, and urinary symptoms. Applying the methodical approach to answer this question: 1) Restating the question in your own words and defining technical terms and concepts: The question is essentially asking which aspect of this woman's health history should be prioritized during the initial case assessment. The choice of "Abnormal bleeding" refers to a history of unusual or heavy bleeding or bruising, which could reflect an underlying issue with the body's coagulation system. "Diet" refers to what the patient eats regularly, which could shed light on her nutritional status. "Relationship with her family" pertains to how she interacts with her family, and the care she receives, an important factor given her dementia and amputations. "Respiratory symptoms" and "Urinary symptoms" look for conditions associated with the respiratory and urinary systems, respectively. 2) Planning: Identifying assumptions, generating alternate assumption by questioning each assumption, and then evaluating and hypothesize which assumption should be clinically prioritized to pursue: It can be assumed that the woman's frail appearance and physical symptoms might be attributed to her diet but assessing the patient's relationship with her family may reveal crucial clues about her overall welfare, including any neglect or exploitation, especially considering her vulnerable state. While the appearance of ecchymoses may sharply turn the focus towards investigations for "Abnormal bleeding", the prevalence of bruising in the older population is considerably high irrespective of underlying bleeding disorders. Meanwhile, respiratory or urinary symptoms could feasibly be contributing to her condition, but are not present based on the case's initial details. 3) Clinical reasoning: Connect each sentence to its subsequent sentence using a clinical thought process to provide a step-by-step explanation to support the alternate assumption: Noting the patient's frail form and temporal wasting, we would lean towards malnutrition as a potential concern. Concurrently, the patient's living situation and her family's report of "doing the best we can" raise concerns about potential carelessness or neglect. When evaluating indicators of nutritional health, clinicians can utilize measures such as body mass index (BMI), unexpected weight loss, general physical appearance, and signs of specific nutrient deficiency. However, in this case, given the patient's dementia, physical disability, and the repeated falls, it would also be pertinent to explore her family dynamics and caregiving situation alongside her dietary intake. 4) Concluding: Use a step-by-step explanation that connects one sentence to the next with a clinical thought process to generate your final response: Initially, both the woman's diet and her relationship with her family stand out as concerns. However, while improving her diet could indeed contribute positively to her health status, it might be of limited impact if her living environment and care situation are not up to the mark. Given her dementia and physical disablement, understanding her family relationship can give an insight into the care and supervision she receives, which could considerably affect her overall health and safety, particularly her risk of falls and injuries. 5) Response: To answer the question, the most immediate history to obtain from this woman would be her "Relationship with her family" (C), to understand the care quality she receives considering her dementia and mobility constraints. Following this, the focus should then switch to her "Diet" (B), to implement immediate measures to improve her nutritional status, if found deficient. In this scenario, understanding the quality and consistency of care provided by the family is a fundamental step before addressing any potential nutritional deficiencies that might be contributing to her overall poor health.

**Facilitator** **[Round 7]:** Summary: AI_A and AI_B both agree that the most appropriate initial history to obtain from the patient is her diet. They argue that her frail appearance and signs of malnutrition suggest that her dietary intake should be the primary focus. They believe that understanding her nutritional status could provide immediate actionable insights to improve her health status. AI_C, however, believes that the most immediate history to obtain from the woman would be her relationship with her family. They argue that understanding her family relationship can give an insight into the care and supervision she receives, which could considerably affect her overall health and safety, particularly her risk of falls and injuries. AI_D agrees with AI_A and AI_B that the initial history to obtain from the patient should be her diet. They argue that addressing her nutritional status can show immediate improvements such as better wound healing, improved immune response, and enhanced patient’s well-being. AI_E also agrees with AI_A, AI_B, and AI_D that the initial focus should be the diet. They argue that malnutrition in the elderly can lead to muscle weakness and impaired physical function, increasing the risk of falls. Furthermore, it can exacerbate chronic diseases, delay recovery from illness, and ultimately decrease quality of life. Question: The key disagreement between the council members is whether the patient's diet or her relationship with her family should be the initial focus of the history taking. Can the council members elaborate on why they believe one aspect should be prioritized over the other, particularly in terms of the immediate and long-term impacts on the patient's health and well-being?. Based on your preceding response, how would you answer the original question: [An 80-year-old woman is brought to the emergency department (ED) 30 minutes after she fell out of her wheelchair at home. This is the second visit to the ED for a fall during the past 3 months. She lives with her daughter and son-in- law, who say they “do the best we can.” The patient has dementia, Alzheimer type, coronary artery disease, type 2 diabetes mellitus, and hypertension. Current medications are amlodipine, aspirin, atorvastatin, donepezil, long-acting insulin, and lisinopril. Five years ago, she underwent bilateral below-the-knee amputations because of infected ulcers of the feet. She uses a wheelchair for ambulation. Ten years ago, she underwent three-vessel coronary artery bypass grafting. She has smoked one-half pack of cigarettes daily for 60 years. She drinks one shot of whiskey nightly. She is thin and appears ill and disheveled. Her temperature is 37.2°C (99.0°F), pulse is 80/min, respirations are 20/min, and blood pressure is 120/80 mm Hg. Pulse oximetry on 2 L/min of oxygen by nasal cannula shows an oxygen saturation of 95%. Physical examination shows temporal wasting. There are scattered ecchymoses over the abdomen and all extremities. No other abnormalities are noted. Which of the following is the most appropriate initial history to obtain from this patient? (A) Abnormal bleeding (B) Diet (C) Relationship with her family (D) Respiratory symptoms (E) Urinary symptoms]

**AI_A:** RESTATING THE QUESTION: What would be the most suitable initial information to gather from an elderly female patient exhibiting signs of ill health, frailty, and multiple complex comorbidities, who also has a history of falls and living with her family. PLANNING: Her physical signs (temporal wasting, presence of bruises, thin appearance) point towards possible malnutrition, which could impact her general health status and resilience against different health challenges. Her family states they do their best for her, but she has had multiple falls while under their care. She has a lengthy medical history which could also contribute to bleeding or urinary issues. CLINICAL REASONING: Many approaches can be taken, but the choice depends upon the expectation of the most actionable result. Understanding her diet, nutritional intake may provide immediate remedial steps but would require some time for notable effects. Familial relationships can have an impact on safety and the daily living environments she experiences. Thus, one could argue that inquiring about possible elder neglect might be prudent, given the frequency of her falls. Still, it is not clear if obtaining that information would offer an immediate action plan. CONCLUDING: The signs of possible malnutrition and its potential immediate and long-term impacts on her health cannot be dismissed. The understanding of her diet and nutritional status can lead to direct interventions that improve her overall health. Furthermore, this information could also indirectly provide insights into her family situation and their capacity to support her. RESPONSE: I would choose (B) Diet. This option would allow for immediate intervention, possibly improve her overall health, and provide insights into her living conditions with her family. Furthermore, malnutrition in the elderly can be directly linked to a higher risk for falls, which is a significant issue for this patient. Addressing her nutritional needs could consequently reduce her risk of falls and associated injuries in the future.

**AI_E:** Restating the Question: The issue at hand is figuring out which aspect of the patient's history is most urgent for the attending clinicians to gather. More specifically, is it more important to initially understand her diet, which can give more insights into her nutritional status, frailty and therefore potential for diseases and falls, or to understand her familial relationship as an indicator of the care she receives, or perhaps even other areas like abnormal bleeding, respiratory symptoms, or urinary symptoms. Planning: Given this, we must examine the impact of each assumption. The focus on diet could bring immediate meaning to her frailty and malnutrition, impacting our understanding of her falls, lack of strength, and general disease state. On the other hand, the focus on family relationships could provide insight on patient's emotional, psychological, and physical care factors that could influence her health and safety. Alternatively, abnormal bleeding, respiratory symptoms, or urinary symptoms can also hold weight, given her various health conditions. Clinical Reasoning: I'm going to prioritize the hypothesis of focusing on the patient's diet. While family care, abnormal bleeding, respiratory symptoms, and urinary symptoms are all important considerations for understanding the patient's overall health status, it's evident that the patient is suffering from nutrition-related symptoms such as frailty and malnutrition (as evidenced by her thin appearance and signs of temporal wasting). These conditions can directly contribute to this patient's frailty and risk for falls - a recurring problem she's been experiencing. Concluding: In summary, the initial examination of the patient's diet can rapidly impact clinical decision-making and intervention planning. This doesn't deny the importance of understanding her family relationship or other symptoms but puts the priority on an area that can potentially provide crucial immediate and long-term benefits. Addressing her nutritional status can show improvements in her general health and well-being, wound healing, immunity, and physical strength, reducing the risk of falls. Response: Given all these considerations, I would argue towards selecting (B) Diet as the most appropriate initial history to obtain from this patient. A comprehensive understanding of her dietary habits would serve as a critical foundation for immediate and long-term intervention planning, with the potential to improve her overall health status.

**AI_C:** Restating the question, we're asked to determine the priority of information to be obtained from an elderly woman admitted to the emergency room following a fall. She has a complex medical history, includes dementia of the Alzheimer type, coronary artery disease, type 2 diabetes, hypertension, and a history of bilateral lower limb amputations. Her living conditions and support system involve her daughter and son-in-law. Examination findings indicate possible malnourishment. Planning our approach, one presented assumption is that getting information about the patient's diet is ideal due to signs of malnutrition. An alternative assumption might be that understanding the patient's family relationship can provide a broader view of her care conditions, which, in this context, could impact her health and falling incidences. An additional assumption would be to inquire about new symptoms like respiratory or urinary symptoms. In Clinical reasoning, both diet and social history are essential components of patient history-taking, but the context defines their order of priority. In our patient's case, she is frail, thin, and shows signs of temporal wasting - suggestive of malnutrition. Her history of falling, dependency on her family for care coupled with her cognitive impairment necessitates a focus on her diet as malnutrition in the elderly can exacerbate her existing conditions, affect her cognition, and increase her predisposition to infections. This, however, doesn't undermine the importance of other aspects. Being cognizant of her psychosocial environment is crucial, given her dependency. Identifying any signs of neglect or abuse could be a catalyst for additional problems. New symptoms such as respiratory or urinary symptoms are also crucial, but current signs and symptoms don't point in that direction. Concluding, given the objective signs of possible malnutrition and its potential immediate and long-term impacts on our patient's health and prognosis, understanding her diet seems like a crucial primary step. Following that reasoning, I would choose option (B) Diet as the most appropriate initial history to obtain from this patient. It's essential to tackle her malnourishment, which could be contributing to her frail condition and complications like frequent Falls. Her social circumstances and family relationships would be my next priority.

**AI_B:** Restating the Question: The question is asking which aspect of the patient's history should be first addressed. The options are: any abnormal bleeding, the patient's diet, her relationship with her family, respiratory symptoms, or urinary symptoms. The woman in question is an 80-year-old with a host of medical conditions, who has recently fallen out of her wheelchair at home. She exhibits symptoms such as being thin, appearing unwell and disheveled, and has temporal wasting and scattered ecchymoses. Planning: The assumptions that could be made include: (1) the woman's conditions could be exacerbated by her diet, hence the pertinence of understanding her dietary habits. (2) Her relationship with her family could also play a significant role in her overall health and safety thus its consideration. (3) There may be potential complications related to her multiple conditions and lifestyle factors, which result in possible abnormal bleeding, respiratory symptoms, or urinary symptoms. The assumption we will focus on is that her frail appearance and signs such as temporal wasting and ecchymoses indicate the possibility of malnutrition, hence the significance to evaluate her diet. Clinical Reasoning: Given her physical presentation, she appears malnourished. Temporal wasting refers to the thinning of the temporalis muscle in the temples, a sign often associated with severe weight loss and malnutrition. Ensuring adequate nutrition is paramount for maintaining strength and preventing falls. It can improve wound healing, enhance immune response, and increase overall well-being which are imperative for a patient with her condition. In addition, considering her diseases, particularly diabetes mellitus and hypertension, diet would significantly affect her glucose control and blood pressure management. Dietary management could also influence her overall cardiovascular health, given her history of coronary artery disease and previous bypass grafting. Understanding her nutritional intake would offer insights that could potentially be modified for immediate and long-term health benefits. Concluding: Upon considering these factors, it seems nutrition would appear to have imminent health implications and should hence be prioritized during history taking. While her family relationship and other symptoms are also important to understand, her frail appearance and signs such as temporal wasting and ecchymoses underscore that malnutrition may be a pressing problem impacting her overall health. Response: So, the answer would be (B) Diet. It seems most essential to understand her diet initially, given her physical presentation and its possible immediate effects on her health status. Her diet can be directly addressed to provide immediate health benefits and positive long-term effects, especially considering her underlining conditions and overall health status.

**AI_D:** Restating the question: The question asks which area of the patient's history is the most appropriate to initially focus on. The choices are: any abnormal bleeding, her diet, her relationship with her family, any respiratory symptoms she is experiencing, or any urinary symptoms she is experiencing. Planning: The primary assumption is that one of the areas listed in the choices (i.e., bleeding, diet, family relationship, respiratory symptoms, urinary symptoms) is the most appropriate initial focus of history taking for this patient. However, an alternate assumption could be that the most appropriate focus depends on a balance between the urgency and impact of associated issues, the patient's immediate health status, and her long-term health prospects. Clinical Reasoning: Let's unpack this. This patient, 80 years old with multiple comorbidities, shows physical signs of undernourishment. Temporal wasting and her thin appearance are indicative of malnutrition, and sufficient nutritional intake is essential for bodily functions, wound healing process, immune response, and overall well-being. Malnutrition can exacerbate the risk of falls in the elderly, can contribute to her ill appearance, and can negatively affect the management and progression of her chronic illnesses like diabetes and hypertension. Her scattered ecchymoses might reflect syndromic issues related to malnutrition, deficiencies, or possibly elder abuse. A focus on her relationship with her family might also yield valuable information. Her family's capabilities and commitment to her care, as seniors living at home depend heavily on their family or caregivers for assistance, especially as dementia advances. This could contribute to her regular occurrence of falls, her overall hygiene and appearance, and potentially her nutritional status too. However, her respiratory and urinary symptoms, although relevant in certain cases, do not appear to be as immediately pertinent given her presenting condition and history as the nutritional and familial aspects. Additionally, the choice of "abnormal bleeding" seems less likely given the sighting of scattered ecchymosis and no other indication of major bleeding in her history or physical examination. Concluding: Applying the clinical thought process, it seems that the key focus areas should be ones that can provide immediate actionable insights and long-term strategies for managing her complex health needs. Although all areas are valuable, given the immediate physical signs and the implications of her malnutrition, addressing this part of her history can provide immediate interventions to improve her prognosis. Therefore, the initial focus of the history taking should be her nutritional status. Response: Based on the above clinical reasoning, I would select (B) Diet as the most appropriate initial step in obtaining a history from this patient. The patient's malnutrition may exacerbate her current illnesses such as diabetes and hypertension, delay recovery from illnesses and impair physical function increasing risk of falls, thereby posing immediate and long-term threats to her health and well-being. Only after addressing this immediate concern, we could go in-depth in her bleeding issues, family relationship, respiratory issues, and urinary symptoms.

**Facilitator** The consensus among the AI responses is that the most appropriate initial history to obtain from this 80-year-old woman, who has been brought to the emergency department after a fall and exhibits signs of malnutrition, is her diet (Option B). This conclusion is based on her physical appearance, which suggests possible malnutrition, and the potential immediate and long-term impacts on her health. Understanding her diet can provide immediate actionable insights and strategies for managing her complex health needs. Addressing her nutritional status can lead to direct interventions that improve her overall health, indirectly provide insights into her living conditions with her family, and potentially reduce her risk of falls and associated injuries in the future. While other aspects such as her relationship with her family, abnormal bleeding, respiratory symptoms, and urinary symptoms are also important, they do not appear to be as immediately pertinent given her presenting condition and history.
