# Supplementary material for: Collaborative intelligence in AI: Evaluating the performance of a council of AIs on the USMLE
Source: PLOS Digit Health. 2025 Oct 9;4(10):e0000787. doi: 10.1371/journal.pdig.0000787 (PMC12510544; doi:10.1371/journal.pdig.0000787)
Supplement: S2 File — (ZIP) [file pdig.0000787.s002.zip › S2 Data Raw data files containing unprocessed question inputs and LLM outputs including transcripts of deliberations/STEP1_Question-107-1_Type-MC-NJ.docx]

**Facilitator** **[Round 1]:** An investigator is studying the impact of glucocorticoids on leukocyte dynamics in an experimental animal model. After dexamethasone administration, the total number of circulating neutrophils increases. Most of the additional neutrophils in circulation are detached; these neutrophils were previously loosely adherent and rolling along the endothelium. The expression of which of the following cell adhesion molecules is most likely decreased in this animal model after dexamethasone administration? (A) Cadherin (B) Intercellular adhesion molecule-1 (C) L-selectin (D) MAC-1 (E) Very late antigen-4

**AI_A:** Restating: An investigator is studying the effects of glucocorticoids such as dexamethasone on leukocyte behavior in lab animals. Post-treatment, there is an increase in the total number of neutrophils circulating, which are found to be detached from their normal loosely adherent state in the endothelium. We need to identify which cell adhesion molecule expression would likely decrease as a result of dexamethasone administration. Planning: Adhesion molecules play a crucial role in the immune response by assisting in the trafficking of immune cells and their attachment to the endothelium. The decreased adhesion of neutrophils to the endothelium after dexamethasone administration suggests a downregulation of these molecules. While all options (A-E) are categories of adhesion molecules, we should focus on those primarily responsible for neutrophil-endothelial interaction during an immune response. Clinical Reasoning: The leukocyte-endothelial cell interaction process occurs in several steps including rolling, activation, and adhesion. L-selectin is critical in the initial slow-rolling of leukocytes along the endothelial surfaces. Therefore, if L-selectin (Option C) expression is decreased, the neutrophils might be more likely to detach from the endothelium, leading to an increased number of neutrophils in free circulation. Concluding: Glucocorticoids like dexamethasone have numerous effects on immune function, including the modulation of adhesion molecule expression. Thus, the administration of dexamethasone likely reduces the expression of a particular adhesion molecule that results in increased circulating neutrophils. Response: The cell adhesion molecule whose expression is most likely decreased after dexamethasone administration in this animal model is (C) L-selectin.

**AI_D:** (1) RESTATING THE QUESTION: This question is looking at the effect of glucocorticoids, specifically dexamethasone, on leukocyte dynamics within a test animal. The underlying model here shows a rise in circulating neutrophils, many of which were previously attached only loosely to the endothelium and rolling along it. We're tasked with determining which cell adhesion molecule - molecules that help cells stick to each other or to their surroundings - might see reduced expression with dexamethasone administration in this context. (2) PLANNING: We need to consider the roles of the listed cell adhesion molecules. Cadherin typically modulates cell-cell adhesion in solid tissues. Intercellular adhesion molecule-1 (ICAM-1) plays a role in the trans-endothelial migration of leukocytes. L-selectin helps with tethering and rolling of leukocytes on vessels. MAC-1 is a leukocyte molecule that interacts with ICAM-1 to promote trans-endothelial migration. Very late antigen-4 (VLA-4) binds to VCAM-1 on endothelial cells and supports stable adhesion and rolling of leukocytes. (3) CLINICAL REASONING: Following the administration of dexamethasone, neutrophils are observed to be detached and circulating, suggesting that they are not adhering to the endothelium as strongly as before. This indicates a potential decrease in the expression of molecules that promote adherence. Considering the options provided, ICAM-1, L-selectin, MAC-1 and VLA-4, all have a role in adherence or migration of neutrophils across the endothelium. However, L-selectin is specifically involved in the initial steps of the adhesion cascade which includes the tethering and rolling of neutrophils along the endothelial surface. (4) CONCLUDING: With the breakdown and examination of the various molecules and their roles, it's feasible to hypothesize that L-selectin, with its role in tethering and rolling of neutrophils on the endothelial surface, may see a decrease in expression since dexamethasone seems to be reducing this process. (5) RESPONSE: Therefore, the most likely cell adhesion molecule whose expression is decreased in this animal model after dexamethasone administration is (C) L-selectin.

**AI_E:** (1) RESTATING THE QUESTION: The query focuses on understanding the impact of a glucocorticoid named dexamethasone on leukocyte dynamics, particularly neutrophils, within an experimental animal model. Following the administration of dexamethasone, a noted increase in the number of neutrophils that are loosely adherent and rolling along the endothelium is observed. The question asks about which cell adhesion molecule's expression is most likely decreased in this scenario. Cell adhesion molecules (CAMs) are proteins located on the cell surface involved in the binding with other cells or with the extracellular matrix in the process called cell adhesion. (2) PLANNING: There are several assumptions we can make here. One is that glucocorticoids such as dexamethasone can modulate the expression of cell adhesion molecules. Another assumption is that the increased neutrophils in circulation are related to the decreased expression of a particular cell adhesion molecule. For answering this question, we should consider the roles of the mentioned molecules: Cadherin, ICAM-1, L-selectin, MAC-1, and VLA-4. (3) CLINICAL REASONING: Neutrophils engage in a multi-step process of adhesion and migration to reach the site of inflammation. This process includes rolling, tight adhesion, and transmigration. 'Rolling' is mediated by selectins (like L-selectin), while 'tight adhesion' and 'transmigration' are guided primarily by integrins (like MAC-1 and VLA-4) and their receptors (like ICAM-1). Dexamethasone, a potent anti-inflammatory glucocorticoid, inhibits many aspects of the immune response, including leukocyte adhesion to the endothelial lining of blood vessels. This inhibition disrupts the migratory pattern of leukocytes, including neutrophils, reducing their adhesion and causing them to remain in circulation. Given the clinical scenario, it would be reasonable to focus on the cell adhesion molecule that primarily binds neutrophils during the 'rolling' phase, which is L-selectin. (4) CONCLUDING: In the presented scenario, where dexamethasone administration leads to an increased number of circulating neutrophils, primarily detached and rolling, it would be reasonable to infer that a cell adhesion molecule involved in the 'rolling' phase of leukocyte migration is downregulated. This downregulation would lessen leukocyte adhesion, allowing more cells to enter circulation. Among the choices, L-selectin, a molecule involved in the rolling step, is most likely to have its expression decreased. (5) RESPONSE: The expression of (C) L-selectin is most likely decreased in this animal model after dexamethasone administration.

**AI_C:** (1) RESTATING THE QUESTION: The question seeks to understand which adhesion molecule is most likely to decrease in this animal model after dexamethasone administration - a glucocorticoid - given that the total number of circulating neutrophils has increased and most of these additional neutrophils have detached from their previous state of loosely adhering and rolling (a key step in the leukocyte extravasation process) along the endothelial wall. Adhesion molecules like Cadherin, ICAM-1 (Intercellular adhesion molecule-1), L-selectin, MAC-1 (Macrophage-1 antigen), and VLA-4 (Very Late Antigen-4) facilitate interactions between leukocytes and the endothelial cells of blood vessels, enhancing their migration towards sites of inflammation. (2) PLANNING: The assumption is that an adhesion molecule that promotes leukocyte-endothelial binding is likely suppressed after dexamethasone administration, as shown by the loss of adherence of the neutrophils in the animal model. Glucocorticoids like dexamethasone can indeed curtail the inflammatory response and hamper leukocyte extravasation by affecting the expression of certain adhesion molecules. (3) CLINICAL REASONING: Let's evaluate the potential role of each adhesion molecule. Cadherins are involved in homotypic cell-cell adhesion, which might not directly impact leukocyte extravasation. ICAM-1 binds to LFA-1 (MAC-1 is a kind of LFA-1) on leukocytes, mediating tight binding and allowing leukocyte transmigration. L-selectin is expressed on leukocytes and promotes initial tethering/rolling interactions with the endothelium. Lastly, VLA-4 is a leukocyte protein that binds VCAM-1 on endothelial cells, mediating strong adhesion. In the context of the question, the detachment of loosely adherent, rolling neutrophils after dexamethasone administration indicates suppression of adhesion molecules involved in mediating the rolling phase. So, we can hypothesize that L-selectin, which facilitates rolling along endothelium, could be affected. (4) CONCLUDING: Given the clinical scenario of increased circulating neutrophils that have detached and are no longer rolling along the endothelium following dexamethasone administration, it's plausible to assume that the expression of the adhesion molecule facilitating the rolling action, L-selectin, has most likely decreased. (5) RESPONSE: Therefore, the cell adhesion molecule that likely experienced a decrease in this animal model after dexamethasone administration is (C) L-selectin.

**AI_B:** (1) RESTATING THE QUESTION: The question is asking which cell adhesion molecule, from the given options, would likely decrease its expression after the administration of dexamethasone in this animal model. To clarify, glucocorticoids like dexamethasone are steroid hormones that can alter the behavior of immune cells such as neutrophils. Neutrophils are a type of leukocyte or white blood cell that primarily respond to infections. One behavior altered is adhesion, where these cells can "stick" to the endothelium, the layer of cells lining the blood vessels, before moving to the site of infection or inflammation. The molecules that govern this process are known as cell adhesion molecules. (2) PLANNING: The role of glucocorticoids in decreasing cell adhesion, and thereby increasing circulation of neutrophils, implies that a cell adhesion molecule involved in neutrophil attachment to the endothelium may be downregulated by these hormones. Different adhesion molecules, such as Cadherin, ICAM-1, L-selectin, MAC-1, and VLA-4, perform various functions in the immune response. By understanding these functions, we can find which molecule's downregulation can lead to increased circulation of neutrophils. (3) CLINICAL REASONING: Normally, before moving to the site of infection or inflammation, neutrophils attach "loosely" and roll along the endothelium, a process known as margination. This is possible due to interactions between selectins (L-selectin on the neutrophil and E/P-selectin on the endothelium) and integrins (like MAC-1 on the neutrophils and ICAM-1 on the endothelium). Continued firm adhesion and transmigration are further facilitated by interactions between integrins and ICAMs. Hence, MAC-1 and ICAM-1 play crucial roles in firm adhesion and transmigration but not in initial loose adhesion and rolling. Therefore, their decrease would typically present as impaired firm adhesion and transmigration. Meanwhile, L-selectin on neutrophils is primarily involved in the initial loose adhesion and rolling phase. If L-selectin gets decreased, one would expect less rolling and loose adhesion, which can result in more circulating neutrophils as observed. (4) CONCLUDING: Hence, based on the behavior of neutrophils observed in this animal model (increased circumferential leukocytes, most of which detached or rolling along the endothelium), it appears the adhesion molecule likely downregulated by dexamethasone administration is the one involved in initial loose adhesion and rolling of neutrophils, i.e., L-selectin. (5) RESPONSE: Therefore, the cell adhesion molecule's expression that is most likely decreased in this animal model after dexamethasone administration is L-selectin (C).

**Facilitator** The consensus statement synthesizing the responses of AI_A, AI_B, AI_C, AI_D, and AI_E is as follows: The question at hand involves understanding the effects of glucocorticoids, specifically dexamethasone, on leukocyte behavior in an experimental animal model. Post-dexamethasone administration, there is an observed increase in the total number of circulating neutrophils, which were previously loosely adherent and rolling along the endothelium. The task is to identify which cell adhesion molecule's expression is likely decreased as a result of this treatment. Cell adhesion molecules play a crucial role in the immune response, aiding in the trafficking of immune cells and their attachment to the endothelium. The decreased adhesion of neutrophils to the endothelium after dexamethasone administration suggests a downregulation of these molecules. The options provided are all categories of adhesion molecules, but the focus should be on those primarily responsible for neutrophil-endothelial interaction during an immune response. The leukocyte-endothelial cell interaction process occurs in several steps including rolling, activation, and adhesion. L-selectin is critical in the initial slow-rolling of leukocytes along the endothelial surfaces. Therefore, if L-selectin expression is decreased, the neutrophils might be more likely to detach from the endothelium, leading to an increased number of neutrophils in free circulation. Glucocorticoids like dexamethasone have numerous effects on immune function, including the modulation of adhesion molecule expression. Thus, the administration of dexamethasone likely reduces the expression of a particular adhesion molecule that results in increased circulating neutrophils. In conclusion, based on the behavior of neutrophils observed in this animal model (increased circulating leukocytes, most of which detached or rolling along the endothelium), it appears the adhesion molecule likely downregulated by dexamethasone administration is the one involved in initial loose adhesion and rolling of neutrophils, i.e., L-selectin. Therefore, the cell adhesion molecule whose expression is most likely decreased after dexamethasone administration in this animal model is (C) L-selectin.
