# Supplementary material for: Collaborative intelligence in AI: Evaluating the performance of a council of AIs on the USMLE
Source: PLOS Digit Health. 2025 Oct 9;4(10):e0000787. doi: 10.1371/journal.pdig.0000787 (PMC12510544; doi:10.1371/journal.pdig.0000787)
Supplement: S2 File — (ZIP) [file pdig.0000787.s002.zip › S2 Data Raw data files containing unprocessed question inputs and LLM outputs including transcripts of deliberations/STEP1_Question-108-1_Type-MC-NJ.docx]

**Facilitator** **[Round 1]:** A 67-year-old man comes to the office because he is concerned about memory loss. He says he sometimes forgets the names of acquaintances he sees while he is out shopping. He also has occasional word-finding difficulty and forgets to buy some items when he goes shopping unless he makes a list. He lives alone and is able to manage his finances, cook, and shop without help. He works part-time as an accountant. He has gastroesophageal reflux disease and hypertension. Current medications are hydrochlorothiazide and omeprazole. Vital signs are within normal limits. Physical and neurologic examinations show no abnormalities. On mental status examination, he is fully oriented. His speech is normal, and thoughts are organized. His mood is euthymic, and he has a full range of affect. His concentration is intact, and he is able to perform calculations quickly and accurately. He can name objects accurately and follow written and verbal commands. He recalls three of four objects after 5 minutes. Which of the following is the most appropriate physician response to this patient’s concern? (A) “I am concerned about your memory loss. Let’s discuss how to further evaluate your memory.” (B) “There's no need to worry right now, but let's meet again in 6 months.” (C) “Unfortunately, your memory loss will likely increase significantly during the next 5 years; let’s discuss some ways to plan for the future.” (D) “Your episodes of forgetfulness are likely just ‘Senior Moments,’ but we should obtain in-depth laboratory test results and an MRI to be certain.” (E) “Your examination findings indicate that your memory loss is likely consistent with the normal aging process.”

**AI_A:** The question is about a 67-year-old man who is experiencing memory issues that are affecting his daily life. These memory issues include forgetting names, having difficulty finding the right words occasionally, and forgetting to buy items unless he makes a list. However, he still retains his ability to manage his finances, cook, and shop without assistance, and he performs his occupational duties as an accountant without any observable struggle. Additionally, his physical and neurological examinations come back normal, and his mental status is intact. The query invites us to suggest the most suitable decision from the given options that the doctor should make. Firstly, we must observe that the man has a fully intact mental status and no observable neurological deficits, suggesting his overall cognitive function is acceptable for his age. He also actively engages in complex tasks such as accounting, without any noticeable difficulty. Moreover, his memory deficits do not interfere with his daily life significantly, as he still manages his affairs independently. The alternate assumption could be that this condition falls under "senior moments," a term colloquially used to refer to temporary minor memory slips or attention lapses, which are common and often normal aging experiences. To further clarify our suspicions, it's crucial to note that serious cognitive diseases often present with more pronounced and pervasive memory deficits, impacting the person's ability to manage their affairs independently. Given the patient’s resources and coping strategies, like making a list to avoid forgetting things, it may be inferred that the memory issues are not causing major life disruptions, and he can efficiently manage them. Based on these assessments, we can hypothesize that the patient's memory loss is more consistent with the normal aging process, rather than a clinical cognitive disorder. However, as with any health concerns, we should closely monitor his condition over time, considering the ages variability and uniqueness of each individual aging process. Hence, the conclusion would be to reassure the patient while not dismissing his concern. Therefore, the most appropriate response would be: (E) “Your examination findings indicate that your memory loss is likely consistent with the normal aging process.” While continuing to monitor the situation closely, regular check-ups will be beneficial.

**AI_B:** RESTATING THE QUESTION: A 67-year-old man is concerned about his memory as he forgets the names of people he knows and has difficulty finding the right words while speaking. He also tends to forget items while shopping unless he has a list. Despite living alone, he's able to handle his daily activities independently. He carries a diagnosis of gastroesophageal reflux disease (GERD) and hypertension for which he is on hydrochlorothiazide and omeprazole. Clinical and neurological examinations are within normal limits. His mental ability is unaltered with normal speech, organization of thoughts, effective concentration and arithmetic skills. He is able to name objects correctly, follow directions and recalls most of the items after a brief period of time. The influence of his existing conditions, age, and medications on memory are the factors in consideration, with the challenge being what type of physician response would fit best under these circumstances. PLANNING: The assumptions are that his concern about his memory could be linked to his existing conditions, the impact of his medications or natural aging. Further assumptions could include whether this could represent a pre-dementia state, or 'Senior Moments' which are often benign and not indicative of a progressive memory disorder. Each of these assumptions needs to be examined through clinical reasoning. CLINICAL REASONING: Starting with the first assumption, both GERD and hypertension are not typically associated with impairment in memory or cognition. His medications, hydrochlorothiazide and omeprazole, do not have memory impairments as common side effects. With regards to the second assumption, this doesn't seem like a pre-dementia state given his high functioning status in multiple domains including finance management, work as an accountant, ability to live alone and absence of neurological signs on examination. Additionally, his affect, concentration, language, and object recall were also intact. Lastly, the concept of 'Senior Moments' refer to occasional forgetfulness common in ageing without being associated with significant cognitive impairment or being indicative of dementia. CONCLUDING: Given the above clinical reasoning, it appears that the patient's memory concerns are consistent with normal age-related forgetfulness or 'Senior Moments', and not secondary to his existing conditions, side effects of medications, or a significant cognitive disorder. RESPONSE: Therefore, the most appropriate response to this patient's concern would be (E) "Your examination findings indicate that your memory loss is likely consistent with the normal aging process.”

**AI_D:** The question asks for the most suitable response that a physician can give to a 67-year-old patient who reports episodes of forgetfulness, such as not remembering names of people and forgetting to buy items when shopping if he does not make a list. Although these issues seem concerning to him, he is able to manage his daily life activities and job as an accountant without any external help. Findings from his physical and neurological examinations, including mental status examination, are all within normal limits. Now that we have established the scenario, the task is to evaluate the given responses from both medical and compassionate perspectives to determine how best to acknowledge his concerns while accurately interpreting his symptoms and examination findings. Assumptions about the patient's condition could include the onset of dementia, normal aging, or potentially a more severe neurological condition. Since the patient’s examination and mental status examination were unremarkable and he can live independently, severe neurological diseases may seem less likely. Another proposition points towards dementia, but his ability to perform complex tasks and maintain his life independently would contradict this diagnosis. Evaluating these assumptions leads us to examine the more likely scenario: normal brain aging. Clinically, normal aging could cause some forgetfulness but wouldn't interfere significantly with daily activities. In this patient's case, he can manage his finances, cook, and shopping without help and performs well on mental status examinations, suggesting relatively preserved cognitive function. His complaint about forgetting acquaintances' names, losing words, and needing to make a list before shopping can be interpreted as part of the normal aging process rather than a disease progression. Consequently, the physician should consider this interpretation but also validate the patient's concerns. Older adults may experience minor forgetfulness as part of normal aging, but it's essential to differentiate normal aging from the early stages of dementia, which requires ongoing clinical judgment. Therefore, based on the patient's concerns and symptoms, and the evaluation of potential conditions, the ideal response to this patient should acknowledge his symptoms as potentially natural while promising a regular follow-up to monitor any significant changes. Maintaining a regular check will allow early detection if a cognitive disorder does develop over time. Connecting the clinical reasoning process to the provided choices, answer choice (E) “Your examination findings indicate that your memory loss is likely consistent with the normal aging process.” seems the most appropriate, as it validates the patient's symptoms and interprets them as potentially normal phenomena.

**AI_E:** RESTATING THE QUESTION: In this case, a 67-year-old man has come to the office because he is worried about forgetting names of acquaintances, occasional difficulty finding the right words, and forgetting to buy certain items while shopping unless he writes them down. Despite this, he's able to live independently and works part-time as an accountant. His physical and neurological examination, as well as a mental status examination, does not reveal any abnormalities. Even though he recalls only three out of four objects after 5 minutes, his other cognitive functions appear to be intact. The query pertains to the best response from a physician concerning this situation. PLANNING: Key assumptions include the patient's experienced memory loss, his ability to live independently, and the possible impact of his current condition on his future cognitive health. An alternative assumption is that his memory loss might be part of the normal aging process rather than an early sign of a significant cognitive disorder such as dementia. The priority would be to determine the degree and impact of his memory loss on his daily activities and to assess whether his symptoms are appropriate for his age or indicative of a serious cognitive impairment. CLINICAL REASONING: This patient's ability to maintain independence in his daily living activities suggests his cognitive function is adequate. Simply forgetting names or needing to write lists isn't necessarily indicative of a significant cognitive problem, particularly in an elderly person. While he is only able to recall three out of four objects after 5 minutes, other cognitive functions appear normal. Various aspects of cognitive function, including memory, decrease with age, and the rate of decline can vary significantly between individuals. The absence of any abnormalities in his physical, neurologic, and mental status examinations also suggest his memory loss might be part of normal aging, rather than indicative of a pathologic process. Additionally, significant cognitive impairment such as dementia typically impacts other cognitive domains beyond memory function. CONCLUDING: Although the memory loss is concerning to the patient, the current information suggests that it may be a part of the normal aging process, and does not indicate a pathologic cognitive decline. Considering patient's concern though, ongoing monitoring and reassessment of his cognitive status to track any significant changes would be advisable. RESPONSE: In light of the above, the most appropriate physician response to this patient's concern would be: (E) "Your examination findings indicate that your memory loss is likely consistent with the normal aging process."

**AI_C:** RESTATING THE QUESTION: A man in his late sixties is experiencing some memory issues, such as occasionally forgetting acquaintances' names and needed shopping items unless he makes a list, and having occasional difficulty with word-finding. Despite this, he is capable of living independently, including work as an accountant, financial management, and cooking. His medical conditions include gastroesophageal reflux disease and hypertension, for which he is on hydrochlorothiazide and omeprazole. His vitals are normal, and there are no abnormalities found during physical and neurologic examination. His mental status examination is largely normal, including orientation, speech, thought organization, mood, and range of affect. He can correctly name objects, can follow verbal and written commands, and has maintained concentration and calculation abilities. However, after 5 minutes, he is only able to recall three of four objects. We are asked to identify the most appropriate physician response toward the man's concern about memory loss. PLANNING: We must first understand the context and significance of the patient's symptoms. Generally, the occurrence of some memory loss with age is expected and could be considered a normal part of aging. Let's call this "normal age-related memory impairment". But this needs to be differentiated from "mild cognitive impairment (MCI)", a state of cognitive decline greater than expected for an individual's age and education level but does not interfere significantly with daily activities. Deterioration in memory, language, and other cognitive domains can all be part of MCI. It is generally seen as a precursor for dementia, yet some people with MCI remain stable or even improve. It's also not to be confused with dementia, a syndrome of progressive cognitive and functional decline significant enough to interfere with daily activities. Since this man doesn't have significant functional impairment that would suggest a diagnosis of dementia, our primary clinical task lies in differentiating normal age-related memory impairment versus MCI. CLINICAL REASONING: This man's symptoms of forgetfulness can be concerning, but it is essential to weigh these issues in the larger context of his cognitive and functional abilities. He is working, has no difficulties managing his finances, and lives independently - all of which suggest no significant impairment in everyday functioning. His mental status examination, which includes elements of orientation, speech, affect along with the ability to follow commands and naming objects, is largely intact, indicating preserved cognition. However, his recall of three out of four objects after 5 minutes may be mildly impaired. Considering this, along with the subjective forgetfulness he reports but with preserved functional independence, the picture leans towards MCI. But we should consider another perspective - that these memory issues may be an expected part of normal aging, the so-called "senior moments". Making this differentiation is significant because the diagnostic approach and potential future therapeutic interventions differ. Yet, this determination can be challenging in clinical practice, because a clear boundary between normal aging and MCI is not easy to identify and may require more detailed cognitive testing. CONCLUDING: The hallmark of MCI is cognitive decline reported by the patient or informant (in this case, the patient himself reports forgetfulness), coupled with objective evidence (impaired recall of three out of four objects), yet with preservation of independence in functional abilities. It seems that a detailed cognitive testing to reliably identify any subtle cognitive deficit that may be missed in routine examination, and further observation might be of value here. Should the testing support a diagnosis of MCI versus normal age-related memory impairment, the patient and physician could decide on the next steps, including discussing potential interventions to slow cognitive decline, if appropriate. RESPONSE: Therefore, evaluating these matters in this patient's context, without creating undue anxiety, the most appropriate physician response to this patient’s concern would be "(A) I am concerned about your memory loss. Let’s discuss how to further evaluate your memory." This response acknowledges and validates the patient's concerns, indicating a willingness to further investigate his memory issues.

**Facilitator** **[Round 2]:** Summary: AI_A and AI_B both believe that the patient's memory loss is likely consistent with the normal aging process, often referred to as "senior moments". They note that the patient's ability to manage his daily life independently, his intact mental status, and the absence of any observable neurological deficits suggest that his overall cognitive function is acceptable for his age. They recommend reassuring the patient while continuing to monitor his condition. AI_C, however, leans towards the possibility of mild cognitive impairment (MCI), a state of cognitive decline greater than expected for an individual's age and education level but does not interfere significantly with daily activities. AI_C suggests further evaluation and detailed cognitive testing to reliably identify any subtle cognitive deficit that may be missed in routine examination. AI_D agrees with AI_A and AI_B, suggesting that the patient's memory loss is likely a part of the normal aging process. However, AI_D emphasizes the importance of regular follow-ups to monitor any significant changes and early detection if a cognitive disorder does develop over time. AI_E aligns with AI_A, AI_B, and AI_D, suggesting that the patient's memory loss might be part of normal aging, rather than indicative of a pathologic process. AI_E also recommends ongoing monitoring and reassessment of the patient's cognitive status. Question: The main disagreement seems to be between AI_C's suggestion of possible mild cognitive impairment (MCI) and the other council members' belief in normal age-related memory loss. Could the council members elaborate on their reasoning behind their respective diagnoses and how they would approach monitoring the patient's condition over time?. Based on your preceding response, how would you answer the original question: [A 67-year-old man comes to the office because he is concerned about memory loss. He says he sometimes forgets the names of acquaintances he sees while he is out shopping. He also has occasional word-finding difficulty and forgets to buy some items when he goes shopping unless he makes a list. He lives alone and is able to manage his finances, cook, and shop without help. He works part-time as an accountant. He has gastroesophageal reflux disease and hypertension. Current medications are hydrochlorothiazide and omeprazole. Vital signs are within normal limits. Physical and neurologic examinations show no abnormalities. On mental status examination, he is fully oriented. His speech is normal, and thoughts are organized. His mood is euthymic, and he has a full range of affect. His concentration is intact, and he is able to perform calculations quickly and accurately. He can name objects accurately and follow written and verbal commands. He recalls three of four objects after 5 minutes. Which of the following is the most appropriate physician response to this patient’s concern? (A) “I am concerned about your memory loss. Let’s discuss how to further evaluate your memory.” (B) “There's no need to worry right now, but let's meet again in 6 months.” (C) “Unfortunately, your memory loss will likely increase significantly during the next 5 years; let’s discuss some ways to plan for the future.” (D) “Your episodes of forgetfulness are likely just ‘Senior Moments,’ but we should obtain in-depth laboratory test results and an MRI to be certain.” (E) “Your examination findings indicate that your memory loss is likely consistent with the normal aging process.”]

**AI_E:** Restating the question: The question presents a 67-year-old man experiencing some memory concerns. Although he reports occasional forgetfulness and difficulties with words, he manages his day-to-day activities independently, holds a part-time job, and performs well on mental status examination. The inquiry aims to identify the most appropriate response from the physician, given the man's clinical picture. Planning: To answer this question, we must consider potential causes of the patient's reported memory loss. These could range from normal age-associated memory impairment to mild cognitive impairment, or even early stages of dementia syndromes like Alzheimer's. It's important to evaluate the patient's functional capability, cognitive performance, and potential risk factors for cognitive diseases. Clinical reasoning: Although this patient has reported memory loss, his ability to perform daily activities independently, continue working, and lack of notable findings on mental status examination suggest that his cognitive function is predominantly preserved. Moreover, he does not present significant cognitive deficits, typically patterned as memory, language, visuospatial, or executive dysfunction syndromes that correlate with early signs of dementia. Given these facts, it's highly plausible that the patient is experiencing age-related cognitive changes - a common phenomenon where memory slightly declines in late adulthood but does not significantly interfere with daily life. This assumption aligns with the patient's absence of observable cognitive deficits and retains functional competence. Concluding: Considering all factors, it appears that the patient's memory loss aligns more with age-related cognitive changes than with a pathological cognitive disorder like dementia or mild cognitive impairment. However, it's crucial to underline the importance of ongoing monitoring and reassessment, given that older age is a risk factor for developing dementia syndromes. Response: Hence, the most suitable physician response seems to be (E) "Your examination findings indicate that your memory loss is likely consistent with the normal aging process." This response affirms the likelihood of regular age-associated memory changes while tactfully reassuring the patient about his current cognitive status. Additionally, it would be helpful to encourage regular follow-up examinations to keep an eye on possible future changes in the patient's cognitive state.

**AI_C:** Recapitulation: The patient, a 67-year-old man, reported concerns about memory loss, manifesting as forgetting names of acquaintances, occasional word finding difficulty, and forgetting to buy items during shopping unless he has a list. Importantly, these symptoms do not interfere with his ability to live independently and maintain part-time employment. His vital signs and physical examination findings, including neurological evaluation and mental status examination, all lie within normal bounds. Assumptions identification and generation: The main assumption here revolves around whether the patient's symptoms can be attributed to elderly cognitive decline (normal aging process) or if there is a sign of a subtle cognitive impairment, such as mild cognitive impairment (MCI). Notably, the key difference lies in whether these cognitive changes significantly interfere with the individual's normal cognitive abilities considering their age and education level. Clinical reasoning: Cognitive changes are a normal part of the aging process and can include episodic memory loss, slower thinking speed, and slight difficulty in coming up with names or words. In contrast, MCI is a stage between expected cognitive decline from normal aging and the more serious decline of dementia. It involves problems with memory, language, thinking, or judgment that are greater than normal age-related changes - but importantly, these changes aren't severe enough to interfere significantly with daily life and usual activities, as we observe in this patient. Concluding: Considering the entirety of this patient's presentation – the described memory issues, preserved independence with daily activities, no noticable neurologic deficits, and normal mental status examination findings – it leans more towards cognitive changes associated with normal aging. However, it is very important to continuously reassess and monitor any potential cognitive deterioration over time due to the risk of progression to MCI or dementia. Hence, regular follow-ups should be planned. Response Choice: I would opt for response choice (E) “Your examination findings indicate that your memory loss is likely consistent with the normal aging process.” Nevertheless, due to potential progression risk, regular follow-ups are paramount to monitor any cognitive changes over time as per the clinical reasoning approach delineated above.

**AI_B:** Restating the question: The patient, a 67-year-old man, is exhibiting memory loss, primarily forgetting acquaintances' names and requiring lists for grocery shopping. Although these memory lapses cause him concern, other cognitive functions are well-preserved. He is capable of performing tasks indicative of intact executive functions, such as managing his finances, working as an accountant, and living independently. His verbal comprehension, attention, calculation ability, and mental orientation are unaffected. Our task is to identify which response would be clinically optimal for addressing the concerns about his memory. Planning: I assume, considering his age and subjective complaints of memory loss, that the intensifying forgetfulness may either be (a) normal cognitive ageing, or (b) a sign of mild cognitive impairment. Both of these conditions involve memory problems that are noticeable by the affected individual or those close to them, although they do not interfere with daily activities. However, mild cognitive impairment (MCI) has a higher risk of progressing to dementia, especially Alzheimer's disease. Clinical reasoning: Considering the man's overall cognitive functioning, it appears his cognitive ability exceeds what one would expect in someone with mild cognitive impairment. His professional work and day-to-day management indicate intact executive functions, which are typically impaired in MCI. On mental status examination, his concentration and ability to execute tasks successfully negate the suspicion of cognitive impairment beyond normal ageing. Further, his ability to recall three out of four items after 5 minutes suggests that his short-term memory, the primary cognitive domain impacted in MCI, is also functioning appropriately. A recall of fewer than one or two items would have raised more significant concerns about MCI. Concluding: Weighing these clinical observations against the possibility of normal ageing and mild cognitive impairment, it becomes apparent that the patient’s symptoms align more with normal cognitive ageing than with an impending cognitive disorder. However, given his concerns and the nature of his memory loss, it would be appropriate to reassure him but emphasize the importance of continuity of care to monitor any potential cognitive decline. Response: The most appropriate response in this case would be: “Your examination findings indicate that your memory loss is likely consistent with the normal aging process.” (E). However, it would be important to reassure him that if his symptoms change or worsen, he should return for a re-evaluation, which may include further cognitive testing or referral to a specialist if necessary.

**AI_A:** Restating the question: The concern revolves around a 67-year-old man who experiences mild memory loss, such as difficulty remembering names and occasional forgetfulness while shopping. Despite these issues, he maintains independence, supports his daily living activities, and exhibits no abnormalities upon physical and neurological examinations. The dilemma is to identify the most appropriate response by the physician to address the man's concern about his memory loss. Planning: There are two set of assumptions primarily at play here. One leaning towards the idea that the patient's memory issues could be a part of the normal aging process. The other raises the possibility of a more severe underlying condition such as Mild Cognitive Impairment (MCI). To derive at a concrete hypothesis, it might be helpful to weigh these assumptions based on the man's overall cognitive function, his ability to perform daily activities, and his memory testing result in which he recalled three out of four objects. Clinical reasoning: Let's begin with analyzing the aspects of the patient's cognitive function which seem intact. He is able to live independently which includes managing his finances, cooking, and shopping. His mental status is intact and exhibits an organized thought process. Also, he is able to perform calculations quickly and accurately, which indicates preserved executive function. On the other hand, he does perceive some memory deficits, which are bothersome but not obstructive to his daily functioning. Additionally, it's important to note that the absence of somatic diseases commonly associated with cognitive deficits,such as stroke or depression, also supports a less severe diagnosis. The capability of recalling 3 out of 4 objects after five minutes does show some impairment, however, it might not be inherently indicative of a pathological process. This could indeed be seen in context of normal age-related cognitive changes but equally a manifestation of early cognitive decline such as MCI. Concluding: It remains a diagnostic dilemma where complete reliance on current cognitive tests may not be good enough to draw a definitive conclusion. Time and monitoring for progressive changes should play an essential role in the diagnostic process. Assessing the patient after a period of time would provide a better understanding of the progression of the memory loss and yield more information on whether this is simply age-related or potentially pathological. Response: In line with the reasoning provided above, the most suitable response seems to be option (B): "There's no need to worry right now, but let's meet again in 6 months," as it combines the need for reassurance with a plan to monitor the situation closely.

**AI_D:** Restating the question: This hypothetical case represents a 67-year-old man with reported memory loss. This patient's symptoms include forgetting names of acquaintances, occasional difficulty finding words, and forgetting to purchase items unless he creates a list. Despite these complaints, he lives independently, manages his finances, and performs his job effectively, which suggests his cognitive impairment is not significant. His physical and neurological examinations are normal, and his cognitive abilities are otherwise unimpaired. The question requests advice on the most suitable response by a care provider to address this patient's concern about his memory loss. Planning: There seems to be a disagreement on whether to attribute this patient's cognitive issues to age-related changes, often expressed as "senior moments," or to a condition like mild cognitive impairment (MCI). MCI is a state where cognitive decline is more than one would anticipate for a person's age and educational level but doesn't significantly interfere with the person's daily functioning. In this case, the primary assumption to evaluate is whether the patient's cognitive impairment is severe enough to suggest a pathological condition such as MCI or whether it falls within the range of normal, age-related cognitive changes. Clinical Reasoning: The clinical evidence drawn from this case favors the interpretation that this man is likely experiencing normal cognitive aging. This conclusion is based on his preserved independent living skills, operative occupation, normal physical and neurological examinations, and satisfactory cognitive performance, barring the specified memory concerns. While he does have some memory lapses, these don't appear to affect his ability to manage daily activities and occupational tasks independently. While periodically observing such patients is valuable to detect any significant cognitive decline as early as possible, proposing an in-depth neurological examination and imaging at this stage might cause undue anxiety and burden. This approach is not indicated unless the memory problems become more frequent or start to interfere with daily life. Concluding: After considering the question context, the patient's described symptoms, the results of his physical and neurological examinations, and the descriptions of the potential diagnoses (normal cognitive aging and MCI), I deduce that the proper response to the patient's worry should be to reassure him that memory loss could be a part of the typical aging process, while also ensuring him of regular follow-ups to keep an eye on any potential cognitive disorder. Response: So, to answer the posed question, I opine that option (E) “Your examination findings indicate that your memory loss is likely consistent with the normal aging process” would be the most appropriate response for the physician to provide to the patient.

**Facilitator** **[Round 3]:** Summary: AI_A believes that the patient's memory loss could be a part of the normal aging process or a sign of Mild Cognitive Impairment (MCI). They suggest that the patient's ability to live independently and perform daily activities, along with the absence of diseases commonly associated with cognitive deficits, supports a less severe diagnosis. However, they also note that the patient's ability to recall only 3 out of 4 objects after five minutes could indicate some impairment. They recommend monitoring the patient over time and suggest response (B): "There's no need to worry right now, but let's meet again in 6 months." AI_B, AI_C, AI_D, and AI_E all lean towards the idea that the patient's memory loss is likely consistent with the normal aging process. They all highlight the patient's preserved cognitive function and ability to live independently as evidence. They also note that the patient's memory loss does not interfere with his daily activities, which is not typically the case with MCI. They all recommend response (E): "Your examination findings indicate that your memory loss is likely consistent with the normal aging process." Key Differences: The key difference between AI_A's perspective and the others is the interpretation of the patient's ability to recall 3 out of 4 objects after five minutes. AI_A sees this as potential evidence of some impairment, while the others do not view it as a significant concern. AI_A also suggests a more cautious approach, recommending a follow-up in 6 months, while the others are more reassuring in their responses. Question for Council Members: How do you interpret the significance of the patient's ability to recall 3 out of 4 objects after five minutes, and how does this influence your recommended response?. Based on your preceding response, how would you answer the original question: [A 67-year-old man comes to the office because he is concerned about memory loss. He says he sometimes forgets the names of acquaintances he sees while he is out shopping. He also has occasional word-finding difficulty and forgets to buy some items when he goes shopping unless he makes a list. He lives alone and is able to manage his finances, cook, and shop without help. He works part-time as an accountant. He has gastroesophageal reflux disease and hypertension. Current medications are hydrochlorothiazide and omeprazole. Vital signs are within normal limits. Physical and neurologic examinations show no abnormalities. On mental status examination, he is fully oriented. His speech is normal, and thoughts are organized. His mood is euthymic, and he has a full range of affect. His concentration is intact, and he is able to perform calculations quickly and accurately. He can name objects accurately and follow written and verbal commands. He recalls three of four objects after 5 minutes. Which of the following is the most appropriate physician response to this patient’s concern? (A) “I am concerned about your memory loss. Let’s discuss how to further evaluate your memory.” (B) “There's no need to worry right now, but let's meet again in 6 months.” (C) “Unfortunately, your memory loss will likely increase significantly during the next 5 years; let’s discuss some ways to plan for the future.” (D) “Your episodes of forgetfulness are likely just ‘Senior Moments,’ but we should obtain in-depth laboratory test results and an MRI to be certain.” (E) “Your examination findings indicate that your memory loss is likely consistent with the normal aging process.”]

**AI_C:** RESTATING THE QUESTION: In simpler terms, we're asked to determine the most suitable response of the doctor to the patient's concern about his forgetfulness. The patient is a 67-year-old man who notices occasional memory lapses. However, we must consider that his overall cognition and daily functionality remain intact. PLANNING: Assumptions here include that the patient's memory might be deteriorating due to age, but considering his age, his activities, and his cognitive test results, another assumption could be that his memory loss is part of the normal aging process. Since the patient is still functionally independent and there are no significant pathological findings, the normal aging process assumption seems clinically plausible. CLINICAL REASONING: Essentially, we need to recognize that some degree of memory decline is expected in the normal aging process. This patient displays relatively minor memory issues, such as forgetting the names of acquaintances, word-finding difficulty, or forgetting shopping items. However, the patient's ability to live independently, manage his finances, successfully execute his job responsibilities, and his well-preserved cognition on examination all suggest that his memory concern might be due to normal age-related cognitive changes rather than the onset of a pathological memory disorder like Alzheimer's disease. CONCLUDING: The clinical presentations and examinations conducted on this patient indicate that his memory loss is more consistent with normal aging rather than a clinical pathological memory disorder. It's essential to reassure the patient without preventing him from seeking help in case of any noticeable increase in his forgetfulness. RESPONSE: Based on a step-by-step examination, the most suitable response would be "Your examination findings indicate that your memory loss is likely consistent with the normal aging process"(E).

**AI_B:** Restating the question: The council is concerned with how to approach a 67-year-old patient expressing worry about his forgetfulness issues. He basically presents normal function in all areas of his life, with minor forgetfulness such as forgetting the names of acquaintances, word-finding difficulty, and a tendency to forget items at the store unless he uses a list. He recalls three out of four objects after a five-minute wait – a factor leading to differing opinions among the council members regarding whether this signifies a normal aging process or a possible cognitive impairment. The question aims to find the best response to allay the patient's concerns and handle his problem. Planning: The assumptions made are that this patient's forgetfulness is either an attribute of the normal aging process or an indication of an underlying cognitive impairment. We could question these assumptions by bringing into focus the variables representing his current lifestyle, his personal history, the nature of his forgetfulness, and his ability to recall objects after a few minutes. Like AI_A, prioritizing an investigation into the nature of his forgetfulness could be a prudent approach. Following AI_B, AI_C, AI_D, and AI_E, believing it is an outcome of the aging process could be the most viable assumption. Clinical reasoning: The process of forgetting names of acquaintances, word-finding difficulty, and occasionally forgetting items to buy unless a list is used can be seen in both cases – normal aging process or cognitive impairment. However, the fact that the patient is living independently, managing his finances, working part-time, and is functionally normal on neurologic examination leans more towards the normal aging process. Moreover, his cognitive skills, like quick and accurate calculations, following commands, and mostly successful recall, support this hypothesis. Concluding: Following the clinical thought process, the forgetfulness issue mentioned by the patient appears to reflect aspects of the normal aging process. Context clues, such as his apparent functional normality in daily life and preserved cognitive skills, lend more weight to this diagnosis than an impairment. Response: Based on the preceding considerations, the most appropriate and comforting response for the physician to give this patient is: “Your examination findings indicate that your memory loss is likely consistent with the normal aging process” (option E). This statement clearly neutralizes the patient's heightened concern while accurately reflecting the most probable underlying cause of his memory-related issues.

**AI_E:** This 67-year-old man's occasional difficulties with memory, such as forgetting names of acquaintances and needing a list for shopping, may be concern-inducing for him, but it's important to approach these symptoms with a balanced view. Evaluating an individual's cognitive health would require us to consider the extent and impact of these perceived difficulties on their daily activities and overall function. The fact that he is able to live without assistance, perform his occupation as an accountant, manage finances, and cook—all activities that require complex cognitive processes—suggests that his cognitive function remains largely intact. However, forgetting one of four objects after a five-minute period represents a slight impairment in delayed recall. It's crucial to recognize that memory alteration is not always a defining factor for cognitive impairment, as it can be influenced by a multitude of factors and to some degree can be a result of natural aging. The first approach is to differentiate normal aging from a pathological condition like Mild Cognitive Impairment (MCI) or dementia. MCI is a stage between the expected cognitive decline of normal aging and the more serious decline of dementia. In MCI, cognitive changes have started to affect the individual's life but not to the point where it interferes significantly with daily activities. MCI often, but not always, progresses to dementia. To the contrary, the patient can well manage his social and occupational functions; moreover, his physical, neurologic, and mental status examinations are normal. This fact lowers the likelihood of a pathological cognitive impairment and appears more similar to cerebral aging. However, it is also important to maintain a watchful wait approach where we follow up the patient after a specific period to observe whether there is any progression or improvement in his cognitive function. This strategy allows for the early detection of any possible underlying cognitive impairment that may be evolving slowly. Applying this conceptual thought process to the provided choices, I would prioritize a response that reflects not just an acknowledgement of the patient's concerns but also gives a more long term and prudent approach to handle his cognitive health. In this case, I believe the most appropriate response maybe (B) "There's no need to worry right now, but let's meet again in 6 months." This approach acknowledges the patient's concerns but also presents a balanced view without causing undue worry.

**AI_D:** RESTATING THE QUESTION: The question seeks the most appropriate response for a 67-year-old man who presented to the clinic due to memory loss, characterized by forgetfulness of acquaintances' names while out shopping, word-finding difficulty, and needing a list for shopping to not forget items. Despite these episodes, the patient is able to live independently, manage his finances, cook, shop, work part-time as an accountant, and shows no abnormal findings in the physical and neurologic examinations. Notably, his mental status examination revealed he could recall three of four objects after 5 minutes. PLANNING: Key assumptions here include: the patient's memory loss is related to aging, the forgetfulness is indicative of a more severe cognitive impairment condition, or, the patient's medication might be having side effects that cause memory loss. Let's consider each of these assumptions. Firstly, memory loss is common with aging, but doesn't interfere with everyday life which aligns with this patient's condition. Secondly, the forgetfulness could indicate an early stage of a neurological disorder like Alzheimer's or MCI, however, the independent living and intact mental status examination could suggest otherwise. Lastly, the medication's side effects could be causing this, however, both hydrochlorothiazide and omeprazole aren't typically associated with cognitive side effects. CLINICAL REASONING: Mild forgetfulness can be a normal part of aging, and given that this patient's day-to-day functionality isn't disrupted by the memory loss, it is more likely these episodes are instances of that, rather than a more severe underlying condition like MCI. Independent living and absence of abnormal neurological findings support this view. Though the patient could only recall three of four objects after 5 minutes, this alone doesn't necessitate an immediate concern as in more severe cognitive conditions, forgetfulness would be more profound and affect daily life. CONCLUDING: Considering a step-by-step clinical reasoning process, the alternate assumption states that these episodes are instances of normal aging related forgetfulness is the most suitable. This conclusion arises from the understanding of normal aging process memory loss and the judgement that the patient's symptoms don't disrupt his day-to-day life significantly enough to suggest a more serious underlying condition. The medication side effects assumption was less suitable due to lack of evidence aligning hydrochlorothiazide & omeprazole with significant cognitive side effects. RESPONSE: Given all these considerations, the most appropriate physician response to this patient’s concern would be: (E) “Your examination findings indicate that your memory loss is likely consistent with the normal aging process.”

**AI_A:** RESTATING THE QUESTION: The question aims to ascertain the physician's best response to the concerns of a 67-year-old man experiencing episodes of memory loss. The patient also occasionally has difficulty finding words and requires a list to carry out his shopping. Despite living alone, he manages his daily living tasks and maintains his part-time profession with no help. His history includes gastroesophageal reflux and hypertension, for which he is on medication. Both physical and neurologic examinations show no abnormalities. The main aim is to determine whether the noted memory loss is a symptom of a more severe cognitive disorder or whether it's part of the normal aging process. PLANNING: The primary assumption is that the patient's memory loss is a symptom of cognitive decline, linked to a disorder such as Alzheimer's disease or mild cognitive impairment (MCI). An alternative assumption to explore could be that the memory loss observed is consistent with the cognitive changes normally seen in the aging process. The key detail supporting this alternate assumption is the patient's ability to manage his daily tasks independently and his normal physical and neurological examination results, including a mental status examination that shows he is fully oriented with intact speech and organized thoughts. CLINICAL REASONING: The patient is 67 years old and demonstrates some memory loss, particularly forgetting names and sporadically having difficulty finding words. These symptoms alone do not indicate a severe cognitive disorder, as they do not significantly interfere with his work or daily activities. Other factors should be considered, such as the patient's general health, absence of neurological examination findings, and normal mental status examination, which all broadly fall within expected parameters for aging seniors. It should also be noted that the patient's memory loss does not substantially affect his independence as he handles his finances, cooks, and shops without assistance all by himself. He is also still part-time employed, demonstrating a reasonably intact cognitive function. However, also keeping in perspective the patient's slight memory irregularity, wherein he could only recall three of four objects after 5 minutes, some level of mild cognitive impairment cannot be entirely ruled out. CONCLUDING: Weighing these considerations, some level of memory loss can be a normal part of the aging process, especially as individuals reach their mid-sixties and older, when such cognitive changes are common. Based on the patient's overall cognitive function, it might be premature to label the memory loss as indicative of a severe cognitive disorder. However, in lightening the patient's anxiety while keeping a close watch on potential memory deterioration, it might be crucial to maintain a balanced view. RESPONSE: Therefore, while the patient's slight memory lapses might be an aspect of normal aging, it would be prudent to continue monitoring these episodes closely. Hence, I would recommend response (B) “There's no need to worry right now, but let's meet again in 6 months.” This response addresses the patient's concerns without causing undue alarm and ensures continued oversight of his situation. Providing reassurance to the patient is essential, but maintaining an ongoing observation of his cognitive changes can afford early detection of any significant cognitive disorders that may subsequently manifest.

**Facilitator** **[Round 4]:** Summary: AI_A believes that the patient's memory loss could be a symptom of cognitive decline, but also considers it could be part of the normal aging process. The patient's ability to manage daily tasks independently and normal physical and neurological examination results support the latter assumption. AI_A recommends monitoring the patient's condition and meeting again in 6 months. AI_B also considers the patient's forgetfulness as either a normal aging process or an indication of cognitive impairment. However, AI_B leans more towards the normal aging process due to the patient's functional normality in daily life and preserved cognitive skills. AI_B suggests reassuring the patient that his memory loss is likely consistent with the normal aging process. AI_C agrees with AI_B, suggesting that the patient's memory loss is more consistent with normal aging rather than a clinical pathological memory disorder. AI_C also recommends reassuring the patient that his memory loss is likely consistent with the normal aging process. AI_D considers three assumptions: the patient's memory loss is related to aging, the forgetfulness is indicative of a more severe cognitive impairment condition, or the patient's medication might be causing memory loss. AI_D concludes that the patient's memory loss is likely due to normal aging and recommends reassuring the patient that his memory loss is likely consistent with the normal aging process. AI_E suggests a balanced view, acknowledging the patient's concerns but also considering the possibility of normal aging or a slowly evolving cognitive impairment. AI_E recommends a watchful wait approach and suggests meeting again in 6 months. Question to clarify disagreements: Given that all council members agree on the possibility of the patient's memory loss being part of the normal aging process, the main disagreement lies in the approach to the patient's concerns. Some members suggest reassuring the patient immediately, while others recommend a watchful wait approach with a follow-up meeting. Could the council members elaborate on why they chose their respective approaches and how they believe their approach would benefit the patient?. Based on your preceding response, how would you answer the original question: [A 67-year-old man comes to the office because he is concerned about memory loss. He says he sometimes forgets the names of acquaintances he sees while he is out shopping. He also has occasional word-finding difficulty and forgets to buy some items when he goes shopping unless he makes a list. He lives alone and is able to manage his finances, cook, and shop without help. He works part-time as an accountant. He has gastroesophageal reflux disease and hypertension. Current medications are hydrochlorothiazide and omeprazole. Vital signs are within normal limits. Physical and neurologic examinations show no abnormalities. On mental status examination, he is fully oriented. His speech is normal, and thoughts are organized. His mood is euthymic, and he has a full range of affect. His concentration is intact, and he is able to perform calculations quickly and accurately. He can name objects accurately and follow written and verbal commands. He recalls three of four objects after 5 minutes. Which of the following is the most appropriate physician response to this patient’s concern? (A) “I am concerned about your memory loss. Let’s discuss how to further evaluate your memory.” (B) “There's no need to worry right now, but let's meet again in 6 months.” (C) “Unfortunately, your memory loss will likely increase significantly during the next 5 years; let’s discuss some ways to plan for the future.” (D) “Your episodes of forgetfulness are likely just ‘Senior Moments,’ but we should obtain in-depth laboratory test results and an MRI to be certain.” (E) “Your examination findings indicate that your memory loss is likely consistent with the normal aging process.”]

**AI_A:** The most appropriate physician response to this patient's concern is (E) "Your examination findings indicate that your memory loss is likely consistent with the normal aging process." This conclusion is derived based on the patient's functional independence, the normal physical and neurological examinations, and the only slightly impaired short-term memory. It's important to note that memory changes can be a part of the normal aging process, characterized by mild forgetfulness. The intact higher cognitive function and ability to carry on daily tasks independently point towards mild cognitive changes associated with aging rather than a pathological cognitive decline. A watch-and-wait approach is appropriate given the current clinical presentation. More intensive methods of monitoring or intervention could be considered if the patient's condition worsens or significantly interferes with his daily life in the future. His concerns should be addressed with empathy and proper explanation to alleviate any anxiety he might be experiencing.

**AI_E:** Restating the question: The case presents a 67-year-old male patient who visits his physician due to memory loss. Normal aging includes some changes in cognitive functions, especially regarding memory and retrieval of information, which could be the situation with this patient. It's necessary to differentiate such changes from pathological cognitive decline, such as Alzheimer's Disease, considering this patient's age. The pharmacology of his current medications, hydrochlorothiazide and omeprazole, suggests no direct link to memory loss. Planning: There are two primary assumptions in this case. One is that this patient's memory loss is merely a manifestation of the normal aging process. The other one is that his forgetfulness suggests the early onset of a cognitive impairment condition. The latter should be clinically prioritized as it implies a significantly different management strategy. Clinical Reasoning: Mild memory loss, such as forgetting acquaintance's names or occasional word-finding difficulty, can be considered a typical symptom of aging, especially when there are no other cognitive function impairments. The patient revealed no difficulty in managing daily tasks, suggesting preserved functional status. Moreover, his mental status examination results were within the normal range, further indicating no severe memory or cognitive abnormalities. On the contrary, early-stage cognitive impairment diseases like Alzheimer's could present very subtly and might not significantly impact daily functioning initially, making it a valid concern. Concluding: Given the patient's age, symptoms, and the impact of memory loss on his functional independence, it seems most plausible that this could be an early sign of cognitive impairment. As a healthcare provider, it's crucial to listen to the patient's concerns seriously, embark on objective assessment tools, and provide reassurance when appropriate. Response: Therefore, the most suitable response would be: (B) "There's no need to worry right now, but let's meet again in 6 months." This response acknowledges the patient's concerns, suggests a potential non-pathological cause for his mild memory lapses, and plans for a follow-up session that would allow for monitoring possible disease progression if it exists.

**AI_D:** RESTATING THE QUESTION: We are asked to choose the most appropriate response by a physician to a 67-year-old man who experiences some memory difficulties but manages to live alone, work part-time, and maintain a full range of daily activities. His mental status examination shows no abnormalities other than recalling three out of four objects after 5 minutes. PLANNING: The doctor's response requires an evaluation of the patient's current condition, considering whether his forgetfulness is due to normal aging or something more clinically significant such as a cognitive impairment. We should also consider whether the patient's medications, hydrochlorothiazide and omeprazole, could be contributing to the memory loss. Arguably, the most relevant assumption is that the man's memory loss is related to normal aging since the bulk of his mental status examination remains intact and he is able to maintain a high level of functionality in his daily life. However, we should also examine the explanation of possible cognitive impairment due to the patient's memory concern. CLINICAL REASONING: The patient demonstrates an overall preservation of his cognitive capabilities except for minor instances of forgetfulness. His ability to perform calculations quickly, accurately name objects, and follow written and verbal commands, coupled with his ability to live independently, suggests an intact overall cognitive function. Per these findings, it renders the possibility of a significant cognitive impairment less probable. Moreover, the medications he's taking, namely hydrochlorothiazide for hypertension and omeprazole for gastroesophageal reflux disease, aren't commonly associated with causing memory loss. This reduces the likelihood of medication-induced memory issues. Considering all these factors, it becomes increasingly probable that the patient's memory issues are better attributed to normal aging — a physiological change rather than a pathological one. CONCLUDING: Given the patient's preserved cognitive abilities and functionality, his forgetfulness can be comprehension of as part of the normal aging process rather than a severe cognitive impairment. This assumption stems from an analysis of his medications, daily functional abilities, mental status examination findings, and knowledge-bearing on the general aging process and its effect on memory. RESPONSE: Based on the above clinical reasoning, the appropriate response is to reassure the patient about his memory loss being part of the natural aging process. Thus, the answer is "Your examination findings indicate that your memory loss is likely consistent with the normal aging process" (Option E).

**AI_C:** Restating the question: A 67-year-old gentleman is concerned about his perceived memory deficits, which include difficulty remembering names and occasional trouble finding the right words. Despite these concerns, he is generally able to carry out his daily tasks effectively. His medical history includes gastroesophageal reflux disease and hypertension, but otherwise, his physical and neurological exams, including an evaluation of his mental state, are within normal limits. The question asks what the most appropriate response from the physician would be given these findings. Planning: Several assumptions can be made from the information provided. The first is that these memory disturbances are part of the normal ageing process. Alternatively, the patient's memory loss could be an early indicator of a pathological disease like Alzheimer's. A third assumption might be that his symptoms could be related to medication side effects. Hypertension and GERD medication side effects, although rare, can include cognitive effects. The best assumption to investigate further would likely be whether this patient's memory loss is part of normal ageing or a harbinger of a more serious condition. Clinical Reasoning: Age-related memory loss (ARML) typically presents with patients forgetting names or where they left keys, difficulty finding words, or forgetting an appointment but remember it later. This could apply to our 67-year-old patient. However, if the symptoms were part of early dementia, it could include memory loss that disrupts daily life or challenges in planning or solving problems which, as per the patient's performance at work and in daily life activities, he does not seem to present. Finally, knowing that hydrochlorothiazide and omeprazole do not typically cause cognitive effects, it's less likely that the symptoms are drug-induced. Concluding: Given the clinical assessment and the patient's generally effective performance in daily life activities, it's reasonable to think that the patient might be experiencing Age-Related Memory Loss rather than an early stage of a pathological cognitive disorder, such as Alzheimer's disease. Response: By connecting with the clinical reasoning and conclusion process, the most fitting response to the patient would be (E) "Your examination findings indicate that your memory loss is likely consistent with the normal aging process." Should the symptoms progress negatively, then further evaluation should be conducted. At this stage, it's important to reassure the patient and alleviate his concerns, but also staying vigilant on monitoring any potential progression.

**AI_B:** Restating the question: This case study presents a 67-year-old man who is experiencing some forgetfulness and memory loss. Despite his concerns about his memory, he is able to independently manage his daily life tasks and his mood, concentration, and cognitive skills seem unaffected according to mental status examination. The scenario begs the question: how should the physician address the man's concerns about his memory loss? Planning: There are a few assumptions that could be made about this man's memory loss. One assumption is that his memory loss is related to normal aging. Another assumption is that this could be the beginning stages of a more serious cognitive decline like dementia. A third assumption could be that his memory loss is the side effect of his medication. The question that arises from these assumptions is: which of these are more likely, and what is the ideal method of communication to address the patient's concerns? Clinical Reasoning: Several factors favor the assumption that the man's memory loss is part of the normal aging process. Firstly, the man is able to independently manage his finances, cook, and shop, indicating that his forgetfulness has not significantly impacted his daily life routine. Secondly, the mental status examination shows intact cognitive skills and mood. It's important to consider the patient's self-perceived cognitive decline. This could also simply be relative to his past cognitive functioning, which, by his account and professional occupation as an accountant, can be inferred to have been above average. His regular medications, hydrochlorothiazide and omeprazole, are not typically associated with cognitive side-effects, which makes the assumption of medicine-induced memory loss unlikely. Meanwhile, the severity and speed of memory loss could give us some indication of whether it's due to normal aging or the early stages of dementia. For example, Alzheimer's Disease, one type of dementia, typically shows slow progression of memory loss with a pattern that affects recent events rather than old memories. However, the clinical vignette suggests a different pattern where the man forgets the names of acquaintances, suggesting memory loss is not severely affecting his daily life or progressively decreasing his cognitive abilities. Concluding: Given that his daily life activities are unaffected, his cognitive skills and orientation are intact with organized thoughts, it is reasonable to conclude that the memory loss experienced by the patient is most likely consistent with the normal aging process. Response: Therefore, the most appropriate physician response to this patient's concern would be: (E) “Your examination findings indicate that your memory loss is likely consistent with the normal aging process.”

**Facilitator** **[Round 5]:** Summary: AI_A, AI_B, AI_C, and AI_D all agree that the patient's memory loss is likely a part of the normal aging process. They base this conclusion on the patient's functional independence, normal physical and neurological examinations, and only slightly impaired short-term memory. They also consider the patient's medications, which are not typically associated with cognitive side-effects. AI_E, however, suggests that the memory loss could be an early sign of cognitive impairment and recommends a follow-up session in 6 months. Key Differences: The key difference lies in the interpretation of the patient's symptoms. AI_A, AI_B, AI_C, and AI_D interpret the patient's memory loss as a part of the normal aging process, while AI_E considers it as a potential early sign of cognitive impairment. AI_E's perspective is more cautious and recommends a follow-up session to monitor possible disease progression. Question for Council Members: What specific factors led you to conclude that the patient's memory loss is either a part of the normal aging process or a potential early sign of cognitive impairment? How did you weigh the patient's functional independence and normal physical and neurological examinations against his reported memory loss in your decision-making process?. Based on your preceding response, how would you answer the original question: [A 67-year-old man comes to the office because he is concerned about memory loss. He says he sometimes forgets the names of acquaintances he sees while he is out shopping. He also has occasional word-finding difficulty and forgets to buy some items when he goes shopping unless he makes a list. He lives alone and is able to manage his finances, cook, and shop without help. He works part-time as an accountant. He has gastroesophageal reflux disease and hypertension. Current medications are hydrochlorothiazide and omeprazole. Vital signs are within normal limits. Physical and neurologic examinations show no abnormalities. On mental status examination, he is fully oriented. His speech is normal, and thoughts are organized. His mood is euthymic, and he has a full range of affect. His concentration is intact, and he is able to perform calculations quickly and accurately. He can name objects accurately and follow written and verbal commands. He recalls three of four objects after 5 minutes. Which of the following is the most appropriate physician response to this patient’s concern? (A) “I am concerned about your memory loss. Let’s discuss how to further evaluate your memory.” (B) “There's no need to worry right now, but let's meet again in 6 months.” (C) “Unfortunately, your memory loss will likely increase significantly during the next 5 years; let’s discuss some ways to plan for the future.” (D) “Your episodes of forgetfulness are likely just ‘Senior Moments,’ but we should obtain in-depth laboratory test results and an MRI to be certain.” (E) “Your examination findings indicate that your memory loss is likely consistent with the normal aging process.”]

**AI_C:** RESTATING THE QUESTION: The question aims to figure out the optimal approach or response to the patient's concern about his memory loss. Despite occasional forgetfulness, the patient remains functionally independent and is well oriented with calculated thoughts and intact concentration. Their physical and neurological examination shows no irregularities. PLANNING: The initial assumption is that the patient's forgetfulness might be a symptom of an underlying cognitive disorder, such as Alzheimer's or dementia. An alternative assumption is that considering the patient's age, his forgetfulness could be attributed to the normal aging process rather than to a significant cognitive disorder. It would be important here to weigh the patient's functional independence, intact concentration, and ability to perform normal daily life activities against his reported forgetfulness. CLINICAL REASONING: In the field of neurology, mild cognitive impairment, defined by memory loss more severe than typically expected for an individual's age, often raises a question of possible early dementia. However, this patient is still functioning independently in daily life, his vital signs and physical examination are normal, and his mental status examination is largely intact. The slight deviation in the recall might be related to age-associated memory impairment, known to occur in individuals over 60 years and is a normal cognitive aging process distinguished from dementia by the preservation of daily functional abilities. CONCLUDING: Upon analyzing the patient's age, ability to handle daily activities independently, and his mental status examination, the patient's symptoms might be considered a part of the normal aging process rather than a significant cognitive disorder. RESPONSE: Therefore, the most appropriate physician response to the patient’s concern would likely be: “Your examination findings indicate that your memory loss is likely consistent with the normal aging process” (Option E). However, it would also be beneficial to reassure the patient and propose periodic evaluations to monitor his memory condition, ensuring nothing is missed.

**AI_D:** Restating the question, the clinical team is evaluating whether or not a 67-year-old man's reported memory loss is symptomatic of a disease or a normal part of the aging process. The patient shows slight impairment in his ability to recall names and shopping items, but is fully cognitively functional and independent in his daily life, his physical and neurological health, and his emotional status are all normal and well-managed. Upon careful consideration, numerous assumptions can be made. One is that the patient is experiencing the natural, age-associated cognitive decline due to the slight impairment in his memory. Alternatively, the forgetfulness could be an early sign of a cognitive disorder such as Alzheimer's disease. Additionally, the memory loss may also be due to an underlying psychological factor such as stress or anxiety. Clinical prioritization would dictate addressing the latter two more serious possibilities first. Applying clinical reasoning, it's clear that the patient's memory concerns do not significantly interfere with his daily function. He is able to live alone, managing finances, shopping, and cooking without help. He can perform his work as an accountant successfully, indicating intact executive and calculative abilities. No signs of mood disorders are evident, reducing the likelihood of psychological contributions to memory loss. Combining these factors with his normal physical and neurological examination results, this incident appears to be a mild age-associated memory impairment. Therefore, using a systematic thought process, it seems that the slight memory impairment observed in the patient can be attributed to the normal aging process. This conclusion is made on the basis of the patient's overall health, functional status, the lack of significant memory abnormalities, and the absence of mood disorders. Given all this, there doesn't seem to be an urgent or immediate concern that would warrant invasive investigational procedures or therapeutic interventions. Connecting this reasoning to the original question, the most appropriate response seems to be: "(E) Your examination findings indicate that your memory loss is likely consistent with the normal aging process." This conclusion is supported by the patient's state of independence and the absence of any significant physical or neurological abnormalities. The physician can also provide reassurance and educational support to help the patient understand and cope with his memory concerns.

**AI_E:** Restating the Question: The question presents a 67-year-old man who is concerned about his experiences of memory loss, including forgetting names, experiencing occasional difficulty finding words, and forgetting to purchase items unless he has a list. Despite his concerns, he lives independently, performing tasks such as cooking, shopping, and managing his finances, and continues to work part-time. His physical and neurological health appears normal. The options provided as potential responses for the physician range from expressing concern and suggesting further evaluation, proposing a follow-up in six months, predicting a significant increase in memory loss and planning for the future, attributing the memory loss to 'Senior Moments' and suggesting in-depth testing, or reassuring the patient that the memory loss aligns with normal aging. Planning: To answer the question, we need to consider several factors, including the patient's functional independence, his cognitive performance in his day-to-day life indicated by his ability to work and manage his finances, the normal range of his physical and neurological examinations, the medications he's currently taking, and the slight impairment observed in his short-term memory. Furthermore, we must keep in mind that there might be different interpretations of these factors, leading to different physician responses. It would be essential to weigh these factors and determine which assumptions should clinically take precedence. Clinical Reasoning: The aging process often sees a slowdown in cognitive functions, including memory. However, if these changes interfere with a person's daily life, it could indicate a cognitive impairment that requires further medical attention. In this case, despite the patient's memory lapses, he has maintained his functional independence, sustains his abilities in mathematics as an accountant, and his physical and neurological exams showed no abnormalities. There is a slight impairment in his short-term memory retention (recalling three out of four items after a delay). Still, it doesn't appear to interfere noticeably with his quality of life. However, the concern the patient manifests should not be dismissed lightly, and it would be beneficial to monitor the situation over time to pick up any developing cognitive impairment early. Concluding: Given that the patient’s functional life and cognitive abilities such as calculations and following commands are not affected, and keeping the slight impairment in mind, indicating a possible beginning but not functionally significant cognitive decline, an approach observing the progression would be apt. Hence, the physician can convey that current findings indicate that the memory loss is likely consistent with the normal aging process, while also keeping the door open for follow-ups to monitor any change. Response: So, based on the clinical reasoning, the most appropriate response for the physician would be "(E) Your examination findings indicate that your memory loss is likely consistent with the normal aging process." The physician could also add, to acknowledge the patient's concerns, that they could schedule follow-up appointments to monitor the situation, reassuring the patient his concerns are taken seriously.

**AI_B:** RESTATING THE QUESTION: The main question here centers around understanding the nature of the concern voiced by a 67-year-old man regarding his occasional forgetfulness and issues with word finding. His living and working circumstances, medical history, current medications, and the results of various physical and mental examinations have been provided. The task is to decide which of the given five responses is the most appropriate for a physician to provide in light of the man's circumstance and examination findings. PLANNING : The first assumption is to consider the patient's symptom of memory loss as part of the natural aging process, given his age and the description of his forgetfulness. An alternate assumption could be to consider the possibility of cognitive impairment because memory loss and word-finding difficulty, even if occasional, could signify the early stages of diseases such as dementia. Another alternate assumption is a side effect of his current medications, but this is less likely as hydrochlorothiazide and omeprazole are not typically associated with cognitive impairments. However, it is important to evaluate these three assumptions based on the clinical indicators available from his physical and mental examination results. CLINICAL REASONING: Starting with the initial assumption of memory loss being a part of the natural aging process, it is supported by the man's functional independence, normal physical and neurological examinations, and the slight issue with short-term memory. This slight impairment isn't necessarily indicative of a cognitive impairment disease process. The alternate assumption of early-stage cognitive impairment does not find strong support in the evidence available. Although memory loss and word-finding difficulties are symptoms, they are outweighed by clear cognitive capacities he continues to demonstrate in his lifestyle and the results of his cognitive testing. For instance, he works as an accountant, lives independently, and doesn't express difficulty managing his everyday life (besides the occasional task that can be aided by things like lists). Additionally, his cognitive capacities are well demonstrated in his mental status examination — he follows commands accurately, has intact concentration, performs quick and accurate calculations, and retains a significant measure of short-term memory (recalling 3 out of 4 objects after five minutes). The last assumption of medication side effect is least likely given the nature of the medications he's taking. CONCLUDING: Through this sequential clinical reasoning process, it can be concluded that the most clinically prioritized assumption is that his memory concerns are likely part of the normal aging process. RESPONSE: Thus, the most appropriate physician response to this patient’s concern would be (E) “Your examination findings indicate that your memory loss is likely consistent with the normal aging process.”.

**AI_A:** In restating the patient's concern, a 67-year-old man is worried about his memory loss. He sometimes forgets acquaintances' names, has word-finding difficulty and forgets items while shopping unless he makes a list. Despite this, he's functionally independent, managing his finances, cooking, and shopping himself without aid. His career as an accountant also demands mental agility, suggesting this kind of deficit isn't reflective of his daily function. He has reflux disease and hypertension, treated with hydrochlorothiazide and omeprazole. His physical, neurological, and mental state examinations show no abnormalities except for recalling three out of four objects after five minutes. Now, the question is about the most suitable response for the physician to give to this patient. The assumptions that principally come to mind are that his memory loss is part of the normal aging process or could indicate the onset of cognitive impairment, particularly a condition such as Mild Cognitive Impairment (MCI). We could also consider that his memory loss might be attributable to another cause like medications, associated with conditions such as depression or metabolic disturbances, but these are less likely given his physical health, mood, and the drugs he's using are not characteristically linked to cognition-related side effects. In evaluating the likelihood of these assumptions, normal aging-related memory loss typically involves minor difficulties such as forgetting names or where items were placed. Nonetheless, that doesn't usually extend to a stage where it negatively impacts daily function or independence - such as the need for list-making so as to avoid forgetting to purchase items during shopping. Basic word-finding difficulties are also typical as people age. Consequently, despite the patient's minimal recall impairment, his functional independence, in association with absence of neurological, physical, and mental status changes, strongly signals towards normal aging-associated changes rather than pathological cognitive impairment. Let's remember that conditions such as MCI usually manifest with more notable impairments in memory and cognitive abilities, impacting on daily life activities or hobbies, which is not exemplified in his case. With these clinical findings in mind, it would be reasonable to inform him that the memory troubles he's experiencing are likely correlated to the normal aging process while staying observant for any future changes or advancements in memory loss as age progresses. It would also be beneficial to explain to him that such changes are typical as people age, which may alleviate any fears or worries he may harbor as a result of not understanding why it's happening. Therefore, finally, I would answer the initial question with answer E: “Your examination findings indicate that your memory loss is likely consistent with the normal aging process.” This reassuring statement addresses his immediate concern while also acknowledging the normalcy of some memory changes with aging. It allows the practitioner to validate the patient's experience and reassure him without proposing unnecessary additional testing or planning for a more severe cognitive decline scenario that is not currently clinically supported.

**Facilitator** The consensus among the AI responses is that the 67-year-old man's memory loss is likely consistent with the normal aging process. This conclusion is based on several factors: the patient's functional independence, his ability to perform daily tasks and work as an accountant, the absence of significant abnormalities in his physical and neurological examinations, and the slight impairment in his short-term memory. While the memory loss could potentially indicate the onset of a cognitive disorder, the evidence strongly suggests that it is more likely a part of the normal aging process. The patient's medications are not typically associated with cognitive impairments, making this a less likely cause. Therefore, the most appropriate response for the physician would be: "Your examination findings indicate that your memory loss is likely consistent with the normal aging process" (Option E). However, it would also be beneficial to reassure the patient and propose periodic evaluations to monitor his memory condition, ensuring nothing is missed.
