# Supplementary material for: Collaborative intelligence in AI: Evaluating the performance of a council of AIs on the USMLE
Source: PLOS Digit Health. 2025 Oct 9;4(10):e0000787. doi: 10.1371/journal.pdig.0000787 (PMC12510544; doi:10.1371/journal.pdig.0000787)
Supplement: S2 File — (ZIP) [file pdig.0000787.s002.zip › S2 Data Raw data files containing unprocessed question inputs and LLM outputs including transcripts of deliberations/STEP1_Question-109-1_Type-MC-NJ.docx]

**Facilitator** **[Round 1]:** A 30-year-old woman comes to the office because of a 4-day history of an increasingly severe, painful rash over her body and in her mouth. The rash began over her trunk area but spread within a day to her face and extremities. Two days before development of the rash, she had flu-like symptoms with muscle aches and fatigue as well as a nonproductive cough, sore throat, and runny nose. Ten days ago, she began treatment with trimethoprim- sulfamethoxazole for a urinary tract infection; she takes no other medications. Temperature is 39.0°C (102.2°F), pulse is 120/min, respirations are 25/min, and blood pressure is 165/105 mm Hg. Physical examination shows diffuse brownish red macular exanthema with bullous lesions. Epidermis at an uninvolved site can be removed with mild tangential pressure. Examination of a biopsy specimen of one of the lesions shows necrosis of keratinocytes throughout the epidermis. There is minimal lymphocytic infiltration within the superficial dermis. Which of the following is the most likely diagnosis? (A) Erythema multiforme (B) Linear IgA bullous dermatosis (C) Pemphigus vulgaris (D) Staphylococcal scalded skin syndrome (E) Toxic epidermal necrolysis

**AI_B:** RESTATING THE QUESTION: A woman has presented with a rapidly progressing and painful skin rash, along with flu-like symptoms that occurred prior to the rash. She has had some recent use of medication - specifically, trimethoprim-sulfamethoxazole for a urinary tract infection - but is not on any other form of medication. Her skin lesions are necrotic with keratinocytes throughout the epidermis, which can be dislodged with gentle manipulative pressure. Given all these indicators, we have to determine the most probable reason for her condition. PLANNING: The important assumptions here include consideration of what the etiological factors might be: an allergic reaction to the new medication (trimethoprim-sulfamethoxazole), a bacterial or viral infection, or an immune disorder. Each carries corresponding diseases: Toxic epidermal necrolysis or a drug eruption for a medication reaction; Staphylococcal scalded skin syndrome, for instance, for an infection; and diseases such as Pemphigus vulgaris or Erythema multiforme for autoimmune disorders. CLINICAL REASONING: Her symptoms seem to correlate with a severe drug reaction given the timeframe of her trimethoprim-sulfamethoxazole therapy initiation and the onset of her symptoms. The presence of necrotic keratinocytes, widespread rash, and blistering, and the ability to dislodge the epidermis with light pressure is consistent with severe drug reactions such as Stevens-Johnson Syndrome or its more extreme form, Toxic Epidermal Necrolysis (TEN). Moreover, the flu-like prognosis she experienced before the rash is also more consistent with a severe hypersensitivity reaction. CONCLUDING: While some of the other listed conditions might partially match the woman's symptoms, they don't fit as well as Toxic Epidermal Necrolysis, which typically follows an episode of medication usage by 1-3 weeks and is characterized by widespread mucocutaneous tenderness, blistering, and necrosis. Necrosis of keratinocytes throughout the epidermis seen in the biopsy findings are similarly indicative of TEN. RESPONSE: Taking into consideration the clinical and histological findings, as well as the timeline after initiating trimethoprim-sulfamethoxazole treatment, the most likely diagnosis is (E) Toxic epidermal necrolysis.

**AI_A:** RESTATING THE QUESTION: The question pertains to a 30-year-old woman presenting with a rapidly developing, painful rash that started from her trunk and spread all over her body and into her mouth. She had flu-like symptoms prior to the rash and also began medication for a urinary tract infection. Her temperature, pulse, respiration, and blood pressure are all elevated. The physical examination reveals a brownish red rash with a bullous nature, and the skin over an unaffected site can be removed with mild pressure. A skin biopsy reveals dead keratinocytes and minimal inflammation within the dermal layer. The task is to identify the most likely medical condition she is suffering from out of five mentioned options. PLANNING: The patient's history, symptoms, and physical examination findings point toward a severe, rapidly spreading skin condition likely related to the recently initiated medication. The assumptions here are that the condition is immune-mediated, most likely drug-induced, leads to predominant keratinocyte death, and is characterized by mild dermal inflammation. By questioning each assumption, one can present alternative assumptions like an infectious cause or an auto-immune blistering disease. To prioritize which assumption to pursue, one needs to consider the timeframe of disease onset, distribution and characteristic of the rash, presence of systemic symptoms, histological findings, and the initiation of the new medication. CLINICAL REASONING: The severe nature of the rash spreading all over the body together with systemic symptoms and the initiation of a new drug point towards a drug reaction. While many drugs can cause skin reactions, trimethoprim-sulfamethoxazole is known for causing severe drug reactions, including Steven-Johnson syndrome (SJS) and toxic epidermal necrolysis (TEN), making this a plausible explanation for her symptoms. Another clue is the necrosis of keratinocytes throughout the epidermis with minimal lymphocytic infiltration, a finding characteristic of TEN. The rash spreading rapidly to involve more than 30% of the body's surface, and the mucosal involvement which can lead to desquamation of the skin (skin coming off with mild pressure), again directs towards TEN. CONCLUDING: Following the line of thought in the clinical reasoning process, it seems most likely the woman is suffering from a severe drug reaction to trimethoprim-sulfamethoxazole leading to TEN. The other options seem less plausible as Erythema multiforme, Pemphigus vulgaris, and Staphylococcal scalded skin syndrome usually present differently, and Linear IgA bullous dermatosis often has a higher infiltration of inflammatory cells. RESPONSE: Based on the history of drug ingestion, evolution, appearance of the rash, systemic symptoms, and the histological findings, the most likely diagnosis is (E) toxic epidermal necrolysis.

**AI_E:** RESTATING THE QUESTION: In simpler words, there is a 30-year-old woman who came to the clinic with a four-day history of a deteriorating, painful rash over her entire body, including her mouth. The rash started on her torso but quickly spread to her face and limbs. Before the rash appeared, she had symptoms similar to the flu - muscle pains, fatigue, a dry cough, a sore throat, and a runny nose. Ten days before her visit, she started taking trimethoprim-sulfamethoxazole to treat a urinary tract infection. No other medications are being taken. She is febrile, with a rapid pulse, high respiratory rate, and elevated blood pressure. A physical examination revealed a widespread reddish-brown skin rash with blistering lesions. She is able to remove the skin at a healthy site with minimal pressure. A biopsy of one of the lesions showed widespread cell death of the skin cells. There is very little white blood cell infiltration into the uppermost layer of the skin. What is her diagnosis? PLANNING: Assumption of a hypersensitivity reaction to the medication she has been taking recently could be made. Alternate assumptions could be infection (bacterial or viral), autoimmune diseases or other dermatological conditions. In this case, it should be prioritized to consider the medication trimethoprim-sulfamethoxazole which the patient started ingesting recently and if it might have caused this reaction. CLINICAL REASONING: On the basis of the information provided, the woman presented with a progressing rash on her body post ingestion of the drug trimethoprim-sulfamethoxazole. This drug is known to have a high risk of inducing serious cutaneous reactions (Severe cutaneous adverse reactions, SCARs), such as Drug Reaction with Eosinophilia and Systemic Symptom (DRESS), Stevens-Johnson Syndrome (SJS), and Toxic Epidermal Necrolysis (TEN). The symptoms of widespread bullous skin lesions, systemic symptoms like fever, positive Nikolsky sign (epidermis at an uninvolved site can be removed with mild tangential pressure), and histology finding of necrosis of keratinocytes throughout the epidermis are all highly suggestive of TEN, a drug-induced condition caused by keratinocyte death leading to detachment of the epidermis from the dermis. CONCLUDING: During the examination of factual details, symptoms and clinical signs, it is crucial to keep in mind the timing of the symptoms in relation to the medication intake and the manifestation of the skin reaction. Furthermore, consideration of the histologic results and type of drug reaction substantiates the conclusion. RESPONSE: The most likely diagnosis for this patient is therefore (E) Toxic epidermal necrolysis.

**AI_D:** RESTATING THE QUESTION: A woman in her third decade of life experienced a painful, progressively worsening rash over her body, including her mouth over four days. Initially, the rash was localized to the trunk and later spread to the face and limbs. Prior to the rash, she had various symptoms resembling the flu and soon after starting treatment with trimethoprim-sulfamethoxazole for a urinary tract infection. She is hyperthermic and tachycardic, with elevated blood pressure. Physical examination reveals brownish-red macular rash with blistering lesions. An interesting feature is that the skin can be easily removed with gentle pressure. A biopsy from the affected skin shows the death of keratinocytes throughout the epidermal layer with minimal lymphocytic infiltration in the superficial dermal layer. The task is to identify the most likely disease process. PLANNING: Understanding the timeline and presentation of her symptoms along with the physical exam findings of necrotic keratinocytes, ease of skin removal, and skin biopsy findings are key to determining the diagnosis. Given her recent intense antibiotic treatment, the assumption is that the symptoms are likely an adverse drug reaction. However, it's important to consider that the symptoms could also be autoimmune or infectious in nature. Weighing these possibilities, the recent initiation of antibiotic treatment and the positive Nikolsky sign (skin detachment with pressure) lean more towards a severe drug reaction. CLINICAL REASONING: Given the rapid onset of the rash after starting trimethoprim-sulfamethoxazole, a history of flu-like symptoms preceding the rash, the physical manifestations, and the histopathology, the clinical evidence points towards a severe drug reaction known as "Toxic Epidermal Necrolysis". This condition is characterized by widespread keratinocyte necrosis leading to separation of the epidermis from the dermis. This results in the skin's loss of barrier function, leading to systemic complications such as hypotension and high temperature as seen in the patient. The minimal lymphocytic infiltration can be seen during early stages of the condition which are presented in this scenario. CONCLUDING: Connecting the information, we established that the woman's symptoms began after initiation of an antibiotic course and progressed rapidly. Physical signs such as easily detached skin further suggest a severe skin reaction to a medication. Considering all aspects, the pathological examination aligning with keratinocyte necrosis and minimal lymphocyte infiltration further substantiates a drug-induced condition—Toxic Epidermal Necrolysis. RESPONSE: Therefore, the most likely diagnosis for this patient's condition is Toxic Epidermal Necrolysis (Choice E).

**AI_C:** RESTATING THE QUESTION: A woman aged 30 has come in for a consultation due to a severe rash that has been worsening over the past four days, spreading across her body and into her mouth. Prior to the occurrence of the rash, she experienced symptoms akin to the flu, and she started a regimen of trimethoprim-sulfamethoxazole for a urinary tract infection ten days ago. Currently, she has a raised temperature, elevated pulse, increased respiratory rate, and high blood pressure. The physical examination reveals a diffuse reddish-brown rash with blistering, and detachment of the epidermis with mild scraping (positive Nikolsky sign). Lesion biopsy shows keratinocyte necrosis with minimal lymphocytic infiltration in the dermal layer. We are attempting to identify the most probable diagnosis from the following options: Erythema multiforme, Linear IgA bullous dermatosis, Pemphigus vulgaris, Staphylococcal scalded skin syndrome, and Toxic epidermal necrolysis. PLANNING: In order to make a diagnosis, we are assuming that the appearance of symptoms like a rash, influenza-like symptoms before rash, the use of certain medication, skin manifestations like necrosis of keratinocytes, are significant. For instance, we can ask whether the initiation of the antibiotic trimethoprim-sulfamethoxazole has any connection to the chief complaints. Secondly, whether the presentation of her rash, along with the presence of necrosis in the epidermis, is indicative of a specific skin pathology. Other assumptions can be regarding whether the lesion's histopathological features match any of the given options or she has a history that could indicate one disease over the others. CLINICAL REASONING: The timeline and the presentation of the symptoms might be suggestive of a drug reaction as the woman had started trimethoprim-sulfamethoxazole just days before the onset of symptoms. Drug reactions often present with systemic symptoms such as fever, and this woman had a temperature of 102.2°F. The presence of necrosis of keratinocytes in the epidermis and the Nikolsky’s sign are indicators of severe disruptions in the epidermal integrity. Toxic epidermal necrolysis (TEN), one option, often manifests as an adverse drug reaction characterized by extensive necrosis and detachment of the epidermis, thus explaining the histopathological features and the Nikolsky's sign. CONCLUDING: Considering the clinical features, including a severe and rapidly progressing rash, flu-like symptoms preceding the rash, recent start of trimethoprim-sulfamethoxazole, necrosis of keratinocytes, and positive Nikolsky's sign, these can point towards a drug-induced reaction. Out of the given options, Toxic epidermal necrolysis (TEN) best fits this patient's scenario. TEN is characterized by widespread erythema, necrosis, and bullous detachment of the epidermis and mucous membranes, resulting in exfoliation and possible sepsis and/or death, commonly precipitated by drug exposure. RESPONSE: The most likely diagnosis for this patient is Toxic epidermal necrolysis (E).

**Facilitator** The consensus statement synthesizes the responses of AI_A, AI_B, AI_C, AI_D, and AI_E as follows: The question pertains to a 30-year-old woman who presented with a rapidly developing, painful rash that started from her trunk and spread all over her body and into her mouth. She had flu-like symptoms prior to the rash and also began medication for a urinary tract infection. Her temperature, pulse, respiration, and blood pressure are all elevated. The physical examination reveals a brownish red rash with a bullous nature, and the skin over an unaffected site can be removed with mild pressure. A skin biopsy reveals dead keratinocytes and minimal inflammation within the dermal layer. The patient's history, symptoms, and physical examination findings point toward a severe, rapidly spreading skin condition likely related to the recently initiated medication. The assumptions here are that the condition is immune-mediated, most likely drug-induced, leads to predominant keratinocyte death, and is characterized by mild dermal inflammation. The severe nature of the rash spreading all over the body together with systemic symptoms and the initiation of a new drug point towards a drug reaction. While many drugs can cause skin reactions, trimethoprim-sulfamethoxazole is known for causing severe drug reactions, including Steven-Johnson syndrome (SJS) and toxic epidermal necrolysis (TEN), making this a plausible explanation for her symptoms. The rash spreading rapidly to involve more than 30% of the body's surface, and the mucosal involvement which can lead to desquamation of the skin (skin coming off with mild pressure), again directs towards TEN. The necrosis of keratinocytes throughout the epidermis with minimal lymphocytic infiltration, a finding characteristic of TEN, further supports this diagnosis. Following the line of thought in the clinical reasoning process, it seems most likely the woman is suffering from a severe drug reaction to trimethoprim-sulfamethoxazole leading to TEN. The other options seem less plausible as Erythema multiforme, Pemphigus vulgaris, and Staphylococcal scalded skin syndrome usually present differently, and Linear IgA bullous dermatosis often has a higher infiltration of inflammatory cells. Therefore, based on the history of drug ingestion, evolution, appearance of the rash, systemic symptoms, and the histological findings, the most likely diagnosis is (E) toxic epidermal necrolysis.
