# Supplementary material for: Collaborative intelligence in AI: Evaluating the performance of a council of AIs on the USMLE
Source: PLOS Digit Health. 2025 Oct 9;4(10):e0000787. doi: 10.1371/journal.pdig.0000787 (PMC12510544; doi:10.1371/journal.pdig.0000787)
Supplement: S2 File — (ZIP) [file pdig.0000787.s002.zip › S2 Data Raw data files containing unprocessed question inputs and LLM outputs including transcripts of deliberations/STEP1_Question-110-1_Type-MC-NJ.docx]

**Facilitator** **[Round 1]:** A 45-year-old woman comes to the office for a follow-up examination 2 weeks after she sustained a vertebral fracture at L1. The fracture occurred spontaneously and there is no history of trauma to the area or other fractures. She gained 27 kg (60 lb) during the 6 months before the fracture occurred. Her only medication is hydromorphone as needed for pain. She is 163 cm (5 ft 4 in) tall and now weighs 100 kg (220 lb); BMI is 38 kg/m2. Temperature is 37.0°C (98.6°F), pulse is 86/min, respirations are 12/min, and blood pressure is 145/98 mm Hg. Physical examination shows central obesity and purple striae over the abdomen bilaterally. The lower extremities appear thin. Results of laboratory studies are shown: Plasma Renin activity 5.0 ng/mL/hr (N=0.6–4.0) Metanephrine 0.3 nmol/L (N<0.4) Serum Cortisol, random 43 μg/dL Adrenocorticotropic hormone 120 pg/mL (N<120) Aldosterone 8 ng/dL (N=2–9) Urine 24-hour free cortisol excretion 340 μg/24 h (N=3.5–45) The most likely cause of the fracture in this patient is an increase in which of the following processes? (A)  Calcium absorption (B)  Calcium excretion (C)  Osteoblast proliferation (D)  Osteoclast proliferation (E)  Phosphorus absorption (F)  Phosphorus excretion

**AI_B:** (1) RESTATING THE QUESTION: This 45-year-old woman presents for a follow-up visit after suffering a spontaneous vertebral fracture at L1. She experienced a significant weight gain in the previous six months and shows signs of central obesity and purple striae on her abdomen. The patient's lab results reveal elevated levels of plasma renin activity, serum cortisol, and 24-hour free urinary cortisol excretion, among others. We are tasked with determining the likely cause of her fracture, specifically considering several processes that involve key minerals like calcium and phosphorus, or cell actions such as osteoblast or osteoclast proliferation. (2) PLANNING: To analyze this case appropriately, we must acknowledge she exhibits several features suggestive of Cushing’s syndrome. The clinical findings consistent with this diagnosis include central obesity, purple striae, thin extremities, and the significant gain in weight. Furthermore, the lab results reveal elevated cortisol levels and ACTH, both typical findings in Cushing's syndrome. Cortisol hypersecretion affects bone metabolism, favoring bone resorption rather than formation. This assumption is applicable to determining which of the choices listed will most likely answer the question about the cause of her fracture. (3) CLINICAL REASONING: Hypersecretion of cortisol can affect bone metabolism in several ways. Specifically, it inhibits osteoblast function, causing a decrease in new bone formation. It also stimulates osteoclast activity, leading to an increase in bone resorption. The net result is a decrease in bone mineral density, which leads to a higher risk for fractures. Therefore, the link between elevated cortisol levels (as seen in Cushing's syndrome) and increased fracture risk is primarily via the stimulation of osteoclast proliferation. (4) CONCLUDING: In this patient's case, who presents with typical symptoms of Cushing's syndrome (including high cortisol levels), the most likely cause of the spontaneous L1 vertebral fracture would be secondary to increased osteoclast activity related to her hypercortisolism. (5) RESPONSE: Therefore, based on the presented details and clinical reasoning, the most likely cause of the fracture in this patient is an increase in osteoclast proliferation (Choice D).

**AI_C:** (1) RESTATING THE QUESTION: The task is to determine the most probable underlying process causing the fractures in a 45-year-old woman presenting with the spontaneous vertebral fracture, recent rapid weight gain, signs of central obesity, high blood pressure, and purple striae. Lab results indicate elevated levels of cortisol, ACTH, and renin activity. Key concepts related to this scenario include understanding the roles of calcium and phosphorus in bone health and metabolism; the function and impact of osteoblasts and osteoclasts on bone formation and resorption; the physiology and pathophysiology of cortisol and ACTH; and the clinical presentation, diagnosis, and implications of Cushing's syndrome. (2) PLANNING: The assumptions I'd make include that the woman's clinical presentation and laboratory results are indicative of excessive cortisol production or Cushing's syndrome, and that excessive cortisol levels can impact bone health. It might be questioned whether the elevated ACTH levels are due to a pituitary issue, or if cortisol directly affects calcium and phosphorus metabolism. However, the clinical priority appears to be understanding the role of cortisol on bone metabolism and predicting its effect, because eliminating suspect options would indicate that Osteoclast proliferation is a likely cause. (3) CLINICAL REASONING: Excessive levels of cortisol, as indicated by the lab findings, are typically associated with conditions like Cushing's syndrome. The woman's signs - purple striae, central obesity, thin extremities - are common with the syndrome, as well as the high-blood pressure caused by cortisol's effect of increasing renal absorption of sodium and water content. Increased cortisol levels are known to decrease bone formation by inhibiting osteoblast function and proliferation. They also enhance osteoclastogenesis, leading to increased osteoclast proliferation and hence, the resorption of bone tissues. This increase in bone resorption and decrease in bone formation is likely to weaken the bones and cause spontaneous fractures, like in the case of this woman. (4) CONCLUDING: The main effect of excessive cortisol on the skeleton is an increase in bone resorption due to osteoclast proliferation and decreased bone formation due to inhibition of osteoblast function and proliferation. This leads to weaker bones and higher risk of fractures. (5) RESPONSE: The most likely cause of the fracture in this patient is an increase in (D) Osteoclast proliferation.

**AI_D:** (1) RESTATING THE QUESTION: The question asks what metabolic alteration is most likely the cause for a spontaneous vertebral fracture in a 45-year-old woman who presents symptoms and lab results suggestive of a possible endocrine disorder. The woman has experienced significant weight gain and displays key features such as central obesity, purple striae, and thinner lower extremities. Her lab results show increased Renin activity and free cortisol excretion, and elevated levels of cortisol and Adrenocorticotropic hormone (ACTH). (2) PLANNING: From the details given, we can conclude this woman has a form of Cushing's syndrome due to the elevated ACTH and cortisol levels. Cushing's can lead to various metabolic changes, among those affecting bone metabolism. However, we have six assumptions to consider about bone metabolism: (A) Increased calcium absorption, (B) Increased calcium excretion, (C) Increased osteoblast proliferation, (D) Increased osteoclast proliferation, (E) Increased phosphorus absorption, and (F) Increased phosphorus excretion. A quick evaluation of these options, considering the metabolic changes in Cushing's syndrome, would lead to prioritizing the option (D) increased osteoclast proliferation. (3) CLINICAL REASONING: Cushing's syndrome is characterized by hypercortisolism and ACTH levels, leading to various metabolic changes, including alterations in glucose, lipid, and bone metabolisms. Bone remodeling involves both the bone-building activity of osteoblasts and bone-resorbing activity of osteoclasts. In conditions of chronic glucocorticoid excess, such as this case, the impact on bone homeostasis is predominantly negative. There are two main factors contributing to this. Firstly, glucocorticoids decrease calcium absorption in the intestine, leading to lower calcium levels in the blood. Secondly, long-term glucocorticoid exposure leads to increased osteoclast activity, leading to increased bone resorption and subsequently enhancing bone fragility and the risk of fractures. (4) CONCLUDING: The clinical features paired with pathological findings suggest this patient has developed Cushing's syndrome. Given the alterations in bone metabolism due to elevated cortisol levels, it is more plausible that the cause for spontaneous vertebral fracture in this patient is increased osteoclast proliferation which is facilitated by chronic glucocorticoid exposure. (5) RESPONSE: Therefore, the most likely cause of the vertebral fracture in this patient is an increase in the process of osteoclast proliferation (D).

**AI_A:** (1) RESTATING THE QUESTION: The patient in question, a 45-year-old woman, has presented with a spontaneous vertebral fracture at the L1 level and shows signs of rapid weight gain, central obesity, and purple striae on her abdomen. There is no history of trauma or other fractures. Her laboratory results show increased levels of renin, cortisol, ACTH, and a significantly increased 24-hour free cortisol excretion. We are tasked with identifying the most likely reason for the occurrence of her fracture. The answer choices refer to different biological processes associated with bone health and mineral homeostasis: (A and B) Calcium absorption/excretion that handles the uptake/loss of calcium from the body; (C and D) Osteoblast/osteoclast proliferation referring to the development and activity of cells responsible for bone formation/resorption; and (E and F) Phosphorus absorption/excretion that manages phosphate handling in the body. (2) PLANNING: The patient's signs and symptoms, notably rapid weight gain, central obesity, purple striae and thin extremities, together with elevated cortisol levels, suggest Cushing's syndrome, a condition characterized by excessive cortisol production. An important aspect of cortisol's effect is its impact on bone health. It has a suppressive effect on osteoblasts (bone-forming cells) and a stimulative effect on osteoclasts (bone-resorbing cells). Therefore, the assumption I would like to make is that due to the excess of cortisol in this patient's body, the balance between bone formation and resorption could be skewed towards resorption, leading to the weakening of bones and spontaneous fractures. These hypotheses will be based on increased osteoclast proliferation (D). (3) CLINICAL REASONING: Cortisol is known to have a catabolic effect on various tissues, including bone, as it reduces the formation of bone by osteoblasts and enhances the resorption of bone by osteoclasts. This is especially relevant in cases of Cushing's syndrome where the chronic overexposure to cortisol leads to osteoporosis, resulting in a greater fragility of the bone structure. This situation results in increased susceptibility to fractures, even without significant trauma, thus explaining the spontaneous vertebral fracture experienced by this patient. (4) CONCLUDING: In sum, the presence of accelerated weight gain, central obesity, and abdominal striae, combined with elevated cortisol, ACTH, and renin levels provide clinical evidence for Cushing's syndrome. The resulting excess of cortisol has a suppressive effect on osteoblasts and an enhancing effect on osteoclasts, thereby leading to increased bone resorption, reduced bone mass, and increased susceptibility to spontaneous fractures. (5) RESPONSE: Therefore, the most likely cause of the fracture in this patient is an increase in (D) Osteoclast proliferation.

**AI_E:** (1) RESTATING THE QUESTION: A woman has presented with vertebral fracture that occurred spontaneously and abruptly gained substantial weight over the presiding six months. She shows signs of central obesity and purple striae over the abdomen, her limbs appear thin and her biochemistry reports indicate a relatively high cortisol level. Given this scenario, our aim is to elucidate the process that has most likely increased leading to this fracture. Vertebral fractures, particularly spontaneous ones, often suggest an underlying condition that impacts the resilience of bone tissue. Osteoblasts and osteoclasts play key roles in bone remodeling, wherein osteoblasts are responsible for bone creation while osteoclasts handle bone resorption. Calcium and phosphorus are significant components of this bone matrix and their absorption and excretion processes impact bone health. (2) PLANNING: Given the patient's symptoms, cortisol levels, and absence of trauma, there's a high inclination towards a diagnosis of Cushing's syndrome which typically presents with spontaneous fractures, abdominal striae, central obesity, and muscular atrophy. The underlying assumption is that this is a case of Cushing's syndrome, caused by excessive cortisol leading to a metabolic condition conducive for bone fractures. However, an alternate assumption would differ explaining the spontaneous fracture. If there's an increase in calcium or phosphorus absorption or excretion, it could disrupt bone metabolism and cause fragility. Yet, only excessive excretion would align with decreased bone density and spontaneous fracture. Increases in osteoblasts could signify an attempt at healing and not the cause of fracture. Meanwhile, increased osteoclast proliferation could potentially augment bone resorption, weakening bone strength. (3) CLINICAL REASONING: Cortisol plays a vital role in our alternate assumption. It enhances bone resorption by increasing osteoclast activity which can lead to decreased bone density, making them weak and prone to fracture. Moreover, Cushing's syndrome typically presents central obesity, muscle wasting, and easy bruising — all observed in the patient. Cushing's syndrome also increases the renal excretion of calcium, but her laboratory data doesn't provide any information about calcium levels for us to make this assumption. An increase in calcium absorption would be evident by hypercalcemia, which is generally not seen in Cushing's syndrome. Osteoblast proliferation would typically enhance bone formation - in opposition to the presentation. As for phosphorus, variations in absorption or excretion are usually balanced by changes in calcium homeostasis and don't typically cause bone loss alone. (4) CONCLUDING: The most reasonable conclusion is that this patient's vertebral fracture has occurred as a collateral consequence of increased osteoclast proliferation due to high cortisol levels. The patient shows typical signs of Cushing's syndrome, which results in excessive cortisol and favors bone resorption over bone creation, resulting in decreased bone density. (5) RESPONSE: The most likely cause of the fracture in this patient is an increase in osteoclast proliferation (Option D).

**Facilitator** The consensus among the AI responses is that the most likely cause of the spontaneous vertebral fracture in the 45-year-old woman is an increase in osteoclast proliferation (Choice D). The woman's symptoms, including rapid weight gain, central obesity, purple striae, and thin extremities, along with her laboratory results showing elevated levels of renin, cortisol, ACTH, and a significantly increased 24-hour free cortisol excretion, suggest a diagnosis of Cushing's syndrome. This condition is characterized by excessive cortisol production, which has a suppressive effect on osteoblasts (bone-forming cells) and a stimulative effect on osteoclasts (bone-resorbing cells). The excess of cortisol in the patient's body likely skews the balance between bone formation and resorption towards resorption, leading to the weakening of bones and spontaneous fractures. Therefore, the consensus is that the most likely cause of the fracture in this patient is an increase in osteoclast proliferation.
