# Supplementary material for: Collaborative intelligence in AI: Evaluating the performance of a council of AIs on the USMLE
Source: PLOS Digit Health. 2025 Oct 9;4(10):e0000787. doi: 10.1371/journal.pdig.0000787 (PMC12510544; doi:10.1371/journal.pdig.0000787)
Supplement: S2 File — (ZIP) [file pdig.0000787.s002.zip › S2 Data Raw data files containing unprocessed question inputs and LLM outputs including transcripts of deliberations/STEP1_Question-111-1_Type-MC-NJ.docx]

**Facilitator** **[Round 1]:** A 25-year-old woman comes to the emergency department because of a 3-hour history of fever, severe headache, light-headedness, dizziness, shaking chills, and muscle aches. Five hours ago, she was diagnosed with Lyme disease and began doxycycline therapy. She has no other history of serious illness and takes no other medications. Menses occur at regular 28-day intervals. She is currently menstruating and using a tampon. She appears anxious. Temperature is 37.0°C (98.6°F), pulse is 120/min, respirations are 30/min, and blood pressure is 90/60 mm Hg. Pulse oximetry on room air shows an oxygen saturation of 94%. Physical examination shows flushing and diaphoresis. Cardiopulmonary examination shows no other abnormalities. Which of the following is the most likely mechanism of this patient’s current condition? (A) Exacerbation of infection by Borrelia burgdorferi (B) Infection-mediated sepsis (C) IgE-mediated allergic reaction to doxycycline (D) Release of bacterial products producing acute inflammation (E) Secretion of bacterial endotoxins

**AI_B:** RESTATING THE QUESTION: To rephrase, a young woman arrived at the emergency department, exhibiting a range of symptoms like anxiety, fever, severe headache, dizziness, chills, and muscle aches. Earlier, she was diagnosed with Lyme disease and was put on doxycycline therapy. She is currently menstruating, uses tampons, and has no past serious illness or medication use. Given all her symptoms and her recent start of doxycycline therapy, we are to determine the most probable cause of her current medical condition from the options provided. PLANNING: Here are the initial assumptions: each of the listed options could potentially cause her symptoms. However, her recent diagnosis of Lyme disease, use of doxycycline, tampon use, and the rapid onset of her symptoms might tell us something crucial about her current condition. CLINICAL REASONING: First, addressing exacerbation of a Borrelia burgdorferi infection, Lyme disease typically produces a slow, progressive illness, not the rapid onset of severe symptoms this patient is experiencing. Infection-mediated sepsis from Lyme disease is also unlikely due to the rapidity of symptom onset. Regarding an IgE-mediated allergic reaction to doxycycline, although possible, it is less likely. Allergic reactions typically present with symptoms such as itching, rash, wheezing which are absent in the vignette. As for the release of bacterial products causing acute inflammation or secretion of bacterial endotoxins, these mechanisms are more consistent with gram-negative infections which again, Lyme disease is not. She was also recently diagnosed with Lyme disease and started on doxycycline therapy, reducing the likelihood of these options. However, given her recent commencement of menstruation and tampon use along with her rapid onset of symptoms after starting doxycycline therapy, an alternate hypothesis comes to mind: Toxic Shock Syndrome (TSS). TSS is often associated with tampon use and can present rapidly with high fever, severe systemic symptoms, and relatively low blood pressure. CONCLUDING: Now, we have a probable situation with TSS that would best explain her current condition. In TSS, toxin-releasing strains of Staphylococcus aureus or Streptococcus pyogenes infiltrate the bloodstream, leading to a deluge of inflammatory cytokines that can cause rapid onset of systemic symptoms. RESPONSE: So, the most likely mechanism of this patient’s current condition seems to be not explicitly listed in the given options, which would be a toxin-mediated inflammatory response associated with Toxic Shock Syndrome due to tampon use. However, amongst the presented options, the closest would be (D) Release of bacterial products producing acute inflammation, as it could indirectly hint towards bacterial toxins that lead to TSS.

**AI_E:** (1) RESTATING THE QUESTION: This question pertains to a 25-year-old woman who, five hours ago, was diagnosed with Lyme disease and started doxycycline treatment. Since the treatment started, she's experienced fever, severe headache, light-headedness, dizziness, shaking chills, and muscle aches, among some other symptoms. The question wants to know the most likely reason for her current condition. The options provide an array of possibilities: the exacerbation of her Lyme disease infection, infection-mediated sepsis, an allergic reaction to the medication, an inflammatory response to bacterial products, or the secretion of bacterial endotoxins. (2) PLANNING: She was diagnosed with Lyme disease, but only five hours have passed since treatment initiation. It seems improbable that the exacerbation of her Lyme disease infection is at play given the relatively short time. Although her symptoms are nonspecific, they seem to indicate a systemic process. She has tachycardia and tachypnea and mild hypotension, which can point towards a shock-like state. However, she is not experiencing respiratory distress, which makes the IgE-mediated allergic reaction to doxycycline less likely. Given her tampon use, another alternative could be the toxic shock syndrome, which is typically caused by the release of bacterial toxins, often from Staphylococcus aureus or Streptococcus pyogenes. (3) CLINICAL REASONING: Considering the given information, we can say with some certainty that this is likely not an allergic reaction as the symptoms profile and the pulmonary examination does not support that. While Lyme disease can present with systemic symptoms, the short time since the initiation of treatment makes it less likely to be an exacerbation of her infection. Sepsis could fit her symptom profile; however, there is no direct indication of a new infection. This would leave the possibility of a toxic shock sequence, possibly from tampon use, as the most likely option. This would align with her systemic symptom profile and the release of bacterial toxins (superantigens) that trigger a massive immunological response in the host. (4) CONCLUDING: Assessing the available data and considering the clinical picture of this patient, it seems reasonable to suggest the most likely mechanism behind her current condition is the release of bacterial products inducing an acute inflammatory response in the body. This conclusion aligns with the pathophysiology of toxic shock syndrome. (5) RESPONSE: So, the most likely mechanism causing her current condition appears to be option (E) Secretion of bacterial endotoxins, what fits the profile of a toxic shock syndrome.

**AI_C:** (1) RESTATING THE QUESTION: The clinical situation presents a 25-year-old woman seen in the emergency department who displays several symptoms including fever, severe headache, light-headedness, dizziness, chills, and muscle aches. These symptoms arose three hours after she began doxycycline therapy for her newly diagnosed Lyme disease. Her blood pressure is comparatively low, and she displays both flushing and diaphoresis (excessive sweating), all indicative of some form of systemic reaction. The question seeks to identify the likely underlying mechanism from five possible options, which include: exacerbation of her Lyme disease, a systemic infection (sepsis), an allergic reaction to the medication, release of bacterial products leading to inflammation, or bacteria releasing endotoxins. (2) PLANNING: Let's scrutinize each assumption: (A) Exacerbation of Lyme infection is possible, but her response seems too acute and severe for this typically gradual condition. (B) Sepsis is a systemic response to infection, but the lack of a clear infection source makes this less likely. (C) An allergic reaction to doxycycline is a plausible cause given the immediacy of her symptoms after initiating therapy. (D) The release of bacterial products causing acute inflammation suggests an immune response, but this usually requires a large bacterial load. (E) Secretion of bacterial endotoxins generally leads to septic syndrome, but, again, there is no clear source of infection. I would prioritize (C), an allergic reaction to her new medication, for further investigation. (3) CLINICAL REASONING: The patient's presentation is characterized by systemic effects (fever, chills, flushing, low blood pressure, tachycardia) which started within a few hours after she began her new medication. These symptoms strongly suggest a systemic reaction to a foreign substance introduced into her body. An allergic reaction (e.g., IgE-mediated reaction to the doxycycline) is a possible explanation. This type of allergic reaction, known as anaphylaxis, can occur rapidly after exposure to the allergen and can manifest with these systemic effects. (4) CONCLUDING: Given the timing of events and the sudden onset, as well as the systemic nature of her symptoms, a likely explanation for this patient's current condition is an IgE-mediated allergic reaction to doxycycline, also known as anaphylaxis. (5) RESPONSE: Thus, the most likely mechanism of this patient’s current condition is (C) IgE-mediated allergic reaction to doxycycline.

**AI_A:** (1) RESTATING THE QUESTION: The query here pertains to a 25-year-old female with recent doxycycline therapy for Lyme disease, now presenting with symptoms of fever, severe headache, light-headedness, dizziness, shaking chills, muscle aches, and menstruation accompanied by light anxiety. There are no clear signs of respiratory or cardiovascular dysfunction, beyond an elevated pulse. The hijab seeks to identify the most likely cause of her condition from among: Borrelia infection exacerbation, infection-mediated sepsis, doxycycline-induced allergic response, acute inflammation due to bacterial by-products, and bacterial secretion of endotoxins. (2) PLANNING: There are several assumptions that can be made here. First, her symptoms could be related to an adverse reaction to doxycycline. However, doxycycline adverse reactions typically manifest with gastrointestinal symptoms, which our patient lacks. She just started the doxycycline, meaning an allergic response is also possible but usually, it would not cause systemic symptoms this severe so quickly. Secondly, her current presentation might be due to an exacerbation of her Lyme disease or perhaps secondary to a bacterial sepsis or a bacteria-induced acute inflammation. However, her infection was only diagnosed five hours ago, and as such, it is unlikely that her condition could worsen this drastically in such a short amount of time. Lastly, there's an assumption that her symptoms could be related to her menstrual status — there's a possibility that she could be experiencing toxic shock syndrome (TSS), a complication of bacterial infection associated with tampon use. After evaluating these assumptions, it seems most clinically apropos to focus on the possibility of TSS, given her presentation and menstrual status. (3) CLINICAL REASONING: Toxic shock syndrome (TSS) is a high-priority diagnostic consideration in a menstruating woman who presents with acute-onset fever, flushing, and systemic symptoms. TSS may be caused by toxins produced by bacteria such as Staphylococcus aureus and group A Streptococcus. These bacteria release superantigens, which can cause a massive activation of T cells and a storm of cytokine release, leading to the abrupt onset of systemic symptoms including high fever, hypotension, rash (diffuse macular erythroderma), and multi-organ involvement. Most cases occur in women who use high-absorbency tampons. These tampons, particularly when left in place for prolonged periods, can facilitate bacterial growth and toxin production. Menstruating women, including those handling menses with tampons, are at higher risk due to vaginal colonization of these bacteria. (4) CONCLUDING: While Lyme disease, allergic reactions, and bacterial infections playing off each other can all lead to symptoms similar to those the patient is experiencing, they are less likely given the context of a menstruating female using a tampon and the acute onset of her systemic symptoms. Toxic shock syndrome is the condition that aligns best with her presentation, making the most likely cause her current condition, the bacterial superantigens inciting a massive activation of T cells and leading to her symptoms. (5) RESPONSE: The most likely mechanism of this patient's current condition would be similar to that seen in cases of toxic shock syndrome. It involves the release of bacterial superantigens, leading to a massive T-cell activation and subsequent cytokine storm. However, among the choices given, none matches this exact mechanism. If forced to choose from the available options and keeping in mind the classic presentation of TSS, option (D) Release of bacterial products producing acute inflammation, is the closest and would be my identified choice.

**AI_D:** RESTATING THE QUESTION: A previously healthy woman aged 25 is brought to the emergency department presenting with symptoms such as fever, headache, dizziness, chills, and muscle aches for the last three hours. It's interesting to note that she was diagnosed with Lyme disease five hours earlier and started on doxycycline therapy. She also happens to be menstruating and is using a tampon. The clinical findings including high pulse rate, high respiratory rate, anxiety, low blood pressure, an oxygen saturation of 94% on room air, and symptoms of flushing and diaphoresis point towards a condition that needs to be identified. A question is thus posed concerning the most likely mechanism underpinning her current condition. PLANNING: The absence of other illnesses and medications excludes other possible confounding factors. Considering her vital signs and symptomatology (i.e., fever, flushing, diaphoresis, anxiety, tachypnea, tachycardia, and hypotension), it's plausible to think about an acute systemic response. This acute systemic response could be due to an infection, an allergic drug reaction or systemic toxin release from bacteria. In such cases, exacerbation of the Lyme disease, infection-mediated sepsis, an allergic reaction to doxycycline, release of bacterial products causing acute inflammation, and secretion of bacterial endotoxins could all be responsible for her condition. CLINICAL REASONING: A correlation should be drawn among the sequence of events, clinical presentation, and the timing of symptom onset following treatment initiation with doxycycline. The manifestations observed in this patient including flushing, diaphoresis, anxiety, fever, tachypnea, tachycardia, hypotension, and lightheadedness, although nonspecific, are commonly seen in systemic reactions. The reactions often manifest within minutes to a few hours after the occurrence of the triggering event (in this case, the initiation of therapy with doxycycline). Therefore, among the given choices, an IgE-mediated allergic reaction to doxycycline would usually have presented sooner than three hours and likely with symptoms like urticaria or bronchospasm, which this patient lacks. Conditions like systemic infection, exacerbation of Lyme disease, release of bacterial products, and secretion of bacterial endotoxins might not manifest as quickly or as dramatically given the prompt initiation of appropriate antibiotic therapy and her previously healthy status. CONCLUDING: Therefore, the provided options don't appear to capture entirely the potential underlying mechanism. If we consider the pattern and timing of symptom onset following doxycycline treatment, along with her menstrual status, another possibility that fits this clinical scenario well and should not be overlooked is toxic shock syndrome (TSS). TSS is an acute, toxin-mediated illness mainly caused by toxin-secreting strains of Staphylococcus aureus and Streptococcus pyogenes, and presents clinically with high fever, rash, hypotension, and multiorgan involvement. Tampon use is a well-recognized risk factor. Given that this possibility isn't listed among the choices, among the given options, the most likely diagnosis would be an infection-mediated sepsis (B). RESPONSE: The most likely mechanism of this patient’s current condition is an infection-mediated sepsis (B), although toxic shock syndrome should also be considered based on her clinical presentation, the timing of symptom onset, and her menstrual status.

**Facilitator** **[Round 2]:** Summary: AI_A and AI_B both believe that the patient's symptoms are most likely due to Toxic Shock Syndrome (TSS), caused by the release of bacterial superantigens due to tampon use. They both chose option (D) Release of bacterial products producing acute inflammation as the closest match from the given options. AI_C, however, believes that the patient's symptoms are most likely due to an IgE-mediated allergic reaction to doxycycline, also known as anaphylaxis, and chose option (C) as the most likely mechanism. AI_D suggests that the patient's symptoms could be due to TSS, but among the given options, they chose option (B) Infection-mediated sepsis as the most likely mechanism. AI_E also suggests that the patient's symptoms could be due to TSS, but they chose option (E) Secretion of bacterial endotoxins as the most likely mechanism. Question to clarify disagreements: Given the patient's symptoms and the timing of their onset, why do some council members believe that an allergic reaction to doxycycline is more likely than TSS, and vice versa? Additionally, why do some council members choose different options as the most likely mechanism, even though they agree on TSS as the most likely cause?. Based on your preceding response, how would you answer the original question: [A 25-year-old woman comes to the emergency department because of a 3-hour history of fever, severe headache, light-headedness, dizziness, shaking chills, and muscle aches. Five hours ago, she was diagnosed with Lyme disease and began doxycycline therapy. She has no other history of serious illness and takes no other medications. Menses occur at regular 28-day intervals. She is currently menstruating and using a tampon. She appears anxious. Temperature is 37.0°C (98.6°F), pulse is 120/min, respirations are 30/min, and blood pressure is 90/60 mm Hg. Pulse oximetry on room air shows an oxygen saturation of 94%. Physical examination shows flushing and diaphoresis. Cardiopulmonary examination shows no other abnormalities. Which of the following is the most likely mechanism of this patient’s current condition? (A) Exacerbation of infection by Borrelia burgdorferi (B) Infection-mediated sepsis (C) IgE-mediated allergic reaction to doxycycline (D) Release of bacterial products producing acute inflammation (E) Secretion of bacterial endotoxins]

**AI_D:** Restating the question: The query concerns the reason for the array of symptoms experienced by a woman recently diagnosed and medicated for Lyme disease. She's also currently menstruating and using a tampon. Given her signs and symptoms alongside specific clinical measures provided, the task is to identify the most probable cause of her current condition from a list of five possible mechanisms. Planning: The assumptions made here could be attributed to either her recent Lyme diagnosis, the subsequent doxycycline therapy, or the fact that she's menstruating and using a tampon. One could hypothesize a number of scenarios, such as an adverse reaction to the medication, Lyme disease exacerbation, or a new infection unrelated to Lyme. Clinical Reasoning: Let's link these symptoms and signs with potential causes. The patient presents with fever, severe headache, dizziness, and other symptoms that could be consistent with an anaphylactic reaction to the antibiotic. However, the symptoms also coincide with Toxic Shock Syndrome (TSS), a potentially fatal condition often associated with tampon use. TSS is caused by the overgrowth of bacteria normally present on the skin or mucosa, resulting in the release of superantigens that trigger widespread inflammation. Concluding: Given the timing of the symptoms following initiation of therapy, the absence of other identifiable triggers such as rash or breathing difficulties, and the fact that she's currently menstruating, the likeliest cause of her symptoms might not be an allergic reaction to doxycycline, but rather Toxic Shock Syndrome caused by superantigen release from Staphylococcus aureus or Streptococcus pyogenes, bacteria that can multiply in the tampon and then get absorbed into bloodstream. Response: On this basis, an answer that matches this explanation most closely is (D) Release of bacterial products producing acute inflammation. This choice aligns with the clinical picture and history, considering the important link between the use of tampons and development of TSS. However, it's also possible that this case may illustrate antibiotic-associated anaphylaxis, infection-mediated sepsis, or other conditions not represented in the options. Therefore, while (D) seems to be the most logical answer, some diagnostic tests would be required for a definitive diagnosis.

**AI_E:** RESTATING THE QUESTION: The question asks for the most likely cause behind the clinical presentation of a 25-year-old woman showing symptoms such as fever, severe headache, lightheadedness, dizziness, shaking chills, and muscle aches, along with vital signs consistent with systemic illness. She has recently been diagnosed with Lyme disease and is on doxycycline. PLANNING: The various options given suggest possibilities like exacerbation of the Lyme disease, systemic infection or sepsis, an allergic reaction to the drug doxycycline, or reactions due to bacterial products or endotoxins. However, the provided symptoms significant to consider are her recent menstruation currently utilizing a tampon and the onset of symptoms after starting doxycycline. CLINICAL REASONING: The patient's symptoms, particularly high pulse, low blood pressure, fever, and flushing, suggest a systemic inflammatory response in the body. Also, concurrent tampon use suggests the potential for Staphylococcal Toxic Shock Syndrome (TSS). The occurrence of TSS is often linked with tampon use, especially if left for a prolonged period, as it encourages bacterial growth and the production of supper antigens that result in a massive immune response, ignition of inflammation, and ultimately lead to the symptoms the patient is presenting with. On the other hand, if it were an allergic reaction to doxycycline, the symptoms would likely include urticarial rash, bronchoconstriction, and facial swelling. Furthermore, an exacerbation of Lyme disease would present differently, generally including symptoms like erythema migrans, joint pains, and neuropathies. CONCLUDING: After careful consideration of all factors, the scenario most aligns with Staphylococcal Toxic Shock Syndrome caused by the use of tampons. A release of bacterial products (Superantigens) produced by the Staphylococcus bacteria activates the body's inflammatory response at a very high scale, inducing the systemic inflammatory disease symptoms. RESPONSE: The answer to this clinical vignette would be (D) Release of bacterial products producing acute inflammation. This explanation highlights the mechanism of Toxic Shock Syndrome and fits with the woman's clinical picture and history of tampon use in menstruation. An allergic reaction to Doxycycline or Sepsis are not the likeliest scenarios according to her listed symptoms.

**AI_A:** Part 1 - Restating the Question: The 25-year-old woman, currently on her menstrual period and using a tampon, has just started treatment for Lyme disease with doxycycline. She has rapidly developed fever, headache, light-headedness, dizziness, chills, and muscle aches, along with an elevated pulse, respiration rate, and signs of flushing and diaphoresis. There are no evident cardiopulmonary abnormalities. We are tasked to identify the most likely underlying mechanism for her current condition considering several options including an exacerbated Lyme disease infection, sepsis, an allergic reaction to doxycycline, a response to bacterial factors leading to inflammation, or the secretion of bacterial endotoxins. Part 2 - Planning: We need to consider: the timing of symptom onset relative to doxycycline initiation; the patient's use of a tampon during her menstrual period, which raises concern for Toxic Shock Syndrome (TSS); and the nature and timing of her symptoms, which are not typical for an allergic reaction to a drug but could be seen in sepsis or in response to bacterial toxins. Part 3 - Clinical Reasoning: Doxycycline, an antibiotic, would typically cause an allergic reaction within minutes to an hour of intake, while this patient's symptoms developed about two hours after drug administration. Fever, headache, light-headedness, dizziness, chills, and muscle aches are all systemic indications that could be interpreted as an immune response to bacterial invasion, an exacerbation of the Borrelia burgdorferi infection (Lyme disease), or a reaction to bacterial superantigens seen in TSS. The patient's hypotension and tachycardia may indicate septic shock, which can result from either TSS or a systemic allergic response (anaphylaxis). Her anxiousness, flushing, and diaphoresis can be understood as a part of this response. Part 4 - Concluding: Considering the timing of symptoms onset, the use of a tampon, and the constellation of the symptoms, the most likely diagnosis is Toxic Shock Syndrome (TSS) caused by the release of bacterial superantigens - products that induce a significant inflammatory response, as described in option (D) Release of bacterial products producing acute inflammation. Part 5 - Response: The patient's current clinical scenario suggests that the primary mechanism behind her condition is the release of bacterial products that trigger acute inflammation (Option D). Though the patient has started doxycycline for Lyme disease, the constellation and timing of her symptoms align more closely with TSS, which can rapidly progress in a patient using a tampon, rather than an involvement of Lyme disease or an allergic response to doxycycline.

**AI_C:** RESTATING THE QUESTION: The question presents a scenario where a 25-year-old woman has come to the emergency department with fever, severe headache, dizziness and other symptoms. Her current condition appears after she begins doxycycline therapy for Lyme disease. The patient is menstruating and using a tampon. The task is to identify the most likely mechanism causing her present condition based on the provided options. PLANNING: The assumed cause behind the patient's condition could be an allergic reaction to the recently-administered doxycycline, infection-mediated sepsis, toxic shock syndrome (TSS) due to tampon use, or some other cause related to her Lyme disease or menstruation. These assumptions are influenced by the fact the patient has Lyme disease, is taking doxycycline, is menstruating and using a tampon, and does not have any other significant medical history or medication use. One alternate assumption could be that the timing of her symptoms coinciding with the beginning of doxycycline therapy and tampon usage could have resulted in an atypical reaction or complication. Based on this, we can hypothetically prioritize the assumption of doxycycline-triggered reaction or tampon usage leading to TSS. CLINICAL REASONING: The key to solving the question resides in tracing the possible pathological pathways arising from the administration of doxycycline/ tampon usage and the eventual symptoms. If TSS is the suspected cause, it would arise from the growth of Staphylococcus aureus or Streptococcus pyogenes, primarily as a result of tampon usage. These bacteria release superantigens that trigger the immune system’s release of inflammatory cytokines, leading to fever, rash, and hypotension—symptoms consistent with the patient's. The quick onset of her symptoms after tampon usage supports the assumption of TSS. If an allergic reaction to doxycycline is suspected, we would expect to see symptoms of anaphylaxis such as urticaria, difficulty breathing, or cardiovascular symptoms. But her presentation is not typical of an anaphylactic reaction, thus making this assumption less likely. CONCLUDING: The patient's rapid onset of fever, headache, dizziness, and hypotension soon after using a tampon during menstruation closely aligns with TSS' clinical picture. With no significant past medical history or any indication of allergy to medication, it is then highly probable that the cause of her condition is due to the release of bacterial superantigens and the resultant systemic inflammatory response, rather than an allergic reaction. RESPONSE: Given her clinical manifestation, the most likely mechanism of this patient’s current condition is (D) Release of bacterial products producing acute inflammation, suggestive of Toxic Shock Syndrome.

**AI_B:** RESTATING THE QUESTION: The query involves a 25-year-old woman who has presented with symptoms such as fever, severe headache, light-headedness, dizziness, shaking chills, and muscle aches. These symptoms arose three hours following the commencement of doxycycline therapy for Lyme disease she was diagnosed with 5 hours ago. Her menses are regular at 28-day intervals and she is currently on her period using a tampon. She is anxious, and her physical examination reveals flushing and diaphoresis, with no cardiopulmonary abnormalities. She has a normal temperature, rapid pulse, rapid breathing, slightly low blood pressure, and a slightly low oxygen saturation on room air. The question requires an evaluation of the most likely cause of the woman's condition. PLANNING: The symptoms presented by the woman in the question are suggestive of several possible conditions, which can be linked to the provided options. These include: an exacerbation of Lyme disease due to Borrelia burgdorferi; infection-mediated sepsis; IgE-mediated allergic reaction to doxycycline; an inflammatory response to bacterial products; and a response to bacterial endotoxins. CLINICAL REASONING: Given the onset of symptoms shortly after starting doxycycline therapy, an IgE-mediated allergic reaction could be plausible. However, while it fits with her tachycardia and hypotension, the remainder of her presentation (fever, chills) is atypical for an anaphylactic reaction. Exacerbation of her Lyme disease is less likely given the short duration between starting treatment and the onset of her severe symptoms. The symptoms also don't correlate well with a primary Lyme infection. The presence of sepsis could lead to many of her symptoms, but she doesn't have a clear source of a bacterial infection other than her Lyme disease, and infection-mediated sepsis typically requires more time to occur. The secretion of bacterial endotoxins is plausible, given her use of a tampon and current menstruation – a risk factor for Toxic Shock Syndrome (TSS), which is caused by toxin-producing strains of Staphylococcus aureus or group A Streptococcus. This scenario fits best with her rapid onset of systemic symptoms. However, secreted bacterial endotoxins are not the causative mechanisms in TSS; instead, TSS is due to bacterial superantigens, which bypass the normal antigen presentation pathway causing polyclonal T cell activation and subsequent massive cytokine release leading to shock. So while E matches the clinical scenario best, in terms of pathophysiology D, "Release of bacterial products producing acute inflammation" would match better. CONCLUDING: A majority of her symptoms are most consistent with TSS caused by tampon use, especially given the timing of her menstruation. Considering the pathophysiology of TSS, the most accurate among the given options would be D) Release of bacterial products producing acute inflammation. RESPONSE: The most likely mechanism of this patient's current condition is the release of bacterial products causing acute inflammation (Option D).

**Facilitator** The consensus among the AI responses is that the most likely mechanism of the patient's current condition is (D) Release of bacterial products producing acute inflammation. This conclusion is based on the patient's symptoms, which include fever, severe headache, light-headedness, dizziness, shaking chills, and muscle aches, along with her vital signs and the fact that she is currently menstruating and using a tampon. These symptoms and circumstances align closely with Toxic Shock Syndrome (TSS), a condition often associated with tampon use. TSS is caused by the overgrowth of bacteria, such as Staphylococcus aureus or Streptococcus pyogenes, which release superantigens that trigger a widespread inflammatory response. The timing of the symptoms' onset, shortly after the initiation of doxycycline therapy and tampon usage, further supports this conclusion. While other possibilities like an allergic reaction to doxycycline, exacerbation of Lyme disease, or infection-mediated sepsis were considered, they were deemed less likely due to the specific constellation of symptoms and their timing.
