# Supplementary material for: Collaborative intelligence in AI: Evaluating the performance of a council of AIs on the USMLE
Source: PLOS Digit Health. 2025 Oct 9;4(10):e0000787. doi: 10.1371/journal.pdig.0000787 (PMC12510544; doi:10.1371/journal.pdig.0000787)
Supplement: S2 File — (ZIP) [file pdig.0000787.s002.zip › S2 Data Raw data files containing unprocessed question inputs and LLM outputs including transcripts of deliberations/STEP1_Question-112-1_Type-MC-NJ.docx]

**Facilitator** **[Round 1]:** A 2-week-old male newborn is brought to the office for a well-child examination. He was delivered following an uncomplicated, spontaneous vaginal delivery at 41 weeks' gestation. The mother has no history of serious illness and did not receive prenatal care. Her only medication is a prenatal vitamin. She has consumed large amounts of vodka nightly for 10 years. Which of the following examination findings is most likely to be present in this patient? (A) Hypospadias (B) Limb hypoplasia (C) Neck webbing (D) Short palpebral fissures (E) Spasticity

**AI_B:** RESTATING THE QUESTION: The doctor wants us to identify the most probable physical finding on examination of a 2-week-old male baby whose mother consumed large amounts of vodka daily for 10 years, and did not avail prenatal medical supervision, apart from taking prenatal vitamins. PLANNING: We make the assumption that the mother's alcohol consumption might affect the development of the fetus. Yet, we could also consider the effect of prenatal vitamins or the lack of prenatal care. The high intake of alcohol could lead to Fetal Alcohol Spectrum Disorders (FASDs). FASDs consist of various physical and mental disabilities that can occur in a child whose mother drank alcohol during pregnancy. CLINICAL REASONING: Considering the high consumption of alcohol by the mother, one of the foremost possibilities would be Fetal Alcohol Syndrome (FAS). The baby's development during pregnancy would have been exposed to the effects of alcohol causing problems with growth, facial features, and the nervous system. Not only does it cause physical complications, but FAS is also known to cause behavioral issues and intellectual disabilities. Of the options provided, short palpebral fissures, which means shorter than average openings of the eyes, is a feature commonly associated with FAS. CONCLUDING: Hence, the baby whose mother consumed large amounts of alcohol during pregnancy would likely exhibit signs and symptoms associated with Fetal Alcohol Syndrome (FAS), especially in terms of facial features and cognitive abilities. RESPONSE: The most likely examination finding in this newborn is (D) Short palpebral fissures.

**AI_D:** 1) RESTATING THE QUESTION: The inquiry pertains to a newborn, 14 days old, that has been brought to the doctor's office for a routine health check. Despite the lack of complication during birth, one crucial aspect is the reported regular consumption of vodka by the mother over the last decade. The query is centered on ascertaining the most probable manifestation in the child given the mother's history of alcohol consumption. 2) PLANNING: To facilitate the planning aspect, assumptions generated from the scenario need to be discussed. First is that the alcohol consumption of the mother has not had an effect on the child's health. Secondly, it could be that the mother's alcohol intake, particularly during pregnancy, might have resulted in certain fetal development problems. Both aspects need exploration but the latter assumption, considering the mother's substantial history of alcohol consumption, should be clinically prioritized. 3) CLINICAL REASONING: With our focus on the alternate assumption that prenatal alcohol exposure potentially led to fetal abnormalities, we can start to connect the dots. Prenatal alcohol exposure is a known cause of Fetal Alcohol Syndrome (FAS). FAS is a condition with an array of manifestations, some of which include alterations in facial features and growth problems. In the list of symptoms, 'Short palpebral fissures', a feature of FAS, stands out. Palpebral fissures are the elliptical open space between the eyelids that appear shorter in FAS. 4) CONCLUDING: Given the substantial history of alcohol consumption of the mother and not receiving prenatal care, it is reasonable to predict that exposure to high levels of alcohol during pregnancy could have led to Fetal Alcohol Syndrome in the newborn. Of the options provided, Short palpebral fissures would be the most likely finding linked to this condition. 5) RESPONSE: Consequently, considering the provided information and with an analytical approach, the most likely examination finding in the male newborn would be (D) Short palpebral fissures.

**AI_E:** 1) RESTATING THE QUESTION: In simpler terms, the question is asking which physical symptom or condition is likely to be found in a newborn whose mother has been consuming large amounts of alcohol every night for a decade, did not go for prenatal care during pregnancy, and her sole medication is a prenatal vitamin. 2) PLANNING: Let's clarify some assumptions: The child was born naturally without complications, mother has no history of previous illness. The only variable is the mother's heavy alcohol consumption habit. Consequently, we might consider the conditions linked to prenatal alcohol exposure. Neurological abnormalities, birth defects, developmental delays, and a specific group of conditions known as Fetal Alcohol Spectrum Disorders (FASDs) could potentially occur. Our alternate assumption will be the clinical presentations of conditions associated with FASDs. 3) CLINICAL REASONING: In evaluating the choices - (A) Hypospadias is a birth defect where the opening of the urethra is on the underside of the penis instead of the tip, mostly linked to genetic factors; (B) Limb hypoplasia, underdevelopment or absence of limb components, often associated with genetic disorders; (C) Neck webbing or pterygium colli condition is a symptom of Turner syndrome, again a genetic disorder; (D) Short palpebral fissures (short eyelid openings), a characteristic feature of FASDs; and (E) Spasticity is generally a condition of the nervous system and is not a standard feature of FASDs. From these, short palpebral fissures align with our assumption. 4) CONCLUDING: Given the circumstances, the condition most likely to be observed in this 2-week-old male newborn is an abnormality linked to the mother's chronic and heavy alcohol usage during pregnancy. 5) RESPONSE: Hence, the clinical finding most likely to be seen in this newborn would be D) Short palpebral fissures, as they are a distinctive feature of Fetal Alcohol Spectrum Disorders caused by prenatal alcohol exposure.

**AI_C:** 1) RESTATING THE QUESTION: The question presents a scenario of a 2-week-old newborn who was born from a mother that has a long-term history of consuming lots of vodka during her pregnancy. It wants to determine the most probable physical examination finding in this infant. It provides options including Hypospadias (a congenital condition where the opening of the urethra is on the underside of the penis, rather than at the end), Limb hypoplasia (underdevelopment or absence of any limb), Neck webbing (excessive skin or folds in the neck), Short palpebral fissures (abnormally short eye openings), and Spasticity (muscle stiffness usually linked with neurological conditions). 2) PLANNING: There's an assumption that the excessive alcohol intake by the mother during pregnancy could cause a certain impact on her baby. Alcohol can interfere with the baby's development and lead to a range of physical, behavioral, and learning problems. The condition caused by prenatal exposure to alcohol is generally referred to as Fetal Alcohol Syndrome (FAS). One possible assumption could be that the newborn might have anomalies associated with FAS. 3) CLINICAL REASONING: Fetal Alcohol Syndrome (FAS) is characterized by pre- and postnatal growth retardation, CNS neurodevelopment abnormalities, and specific facial dysmorphisms. These dysmorphisms include a smooth philtrum, thin vermillion (upper lip border), and small palpebral fissures. The birth history and the maternal history of this case strongly suggest a high risk of FAS. Therefore, we should consider the clinical manifestations of FAS when we choose the most likely examination findings in this patient. 4) CONCLUDING: Based on the mother's heavy habitual consumption of vodka during pregnancy and the understanding of how alcohol can affect fetal development, it is reasonable to conclude that the newborn may exhibit symptoms related to Fetal Alcohol Syndrome. 5) RESPONSE: The most likely examination finding in this 2-week old infant, given the significant and prolonged alcohol consumption by the mother during pregnancy, would be (D) Short palpebral fissures, a common feature of Fetal Alcohol Syndrome.

**AI_A:** (1) RESTATING THE QUESTION: To put it into simpler terms, the question asks about the most probable physical examination finding in a 2-week-old baby boy that was born at 41 weeks' gestation without complications. Notably, the mother consumed large quantities of vodka every night for the past decade and didn't pursue any prenatal care apart from taking prenatal vitamins. The exam findings listed in the question are: Hypospadias (birth defect affecting the opening of the urethra in males), Limb hypoplasia (underdeveloped limbs), Neck webbing (additional skin folds at the neck), Short palpebral fissures (eyelid openings smaller than average), and Spasticity (muscle stiffness and involuntary muscle spasms). (2) PLANNING: Initially, one would think the child may have issues related to prenatal vitamin deficiency due to inadequate prenatal care by the mother. However, we're told that the mother did take prenatal vitamins. Therefore, an alternate assumption to consider is the known effects of chronic alcohol consumption in pregnancy, known as fetal alcohol syndrome. This line of reasoning should be followed clinically due to the mother's consistent vodka intake throughout the last 10 years. (3) CLINICAL REASONING: Fetal alcohol syndrome is a disorder that results in birth defects, including characteristic facial deformations, growth problems, and nervous system abnormalities after prenatal exposure to alcohol. Based on the mother's significant consumption of vodka and the listed examination findings, it seems likely that the newborn could be exhibiting signs of this condition. This includes Short palpebral fissures, a common symptom of fetal alcohol syndrome. (4) CONCLUDING: In conclusion, the clinical thought process suggests that due to the mother's history of chronic alcohol intake, it is likely that the unborn child was exposed to alcohol in utero which may have resulted in the development of fetal alcohol syndrome. (5) RESPONSE: The proposed answer to the question would be: (D) Short palpebral fissures. The baby could be expected to have short palpebral fissures, an expected finding in fetal alcohol syndrome - a condition that can arise from the mother's heavy alcohol consumption during pregnancy.

**Facilitator** The consensus statement synthesizes the responses of AI_A, AI_B, AI_C, AI_D, and AI_E as follows: The question pertains to a 2-week-old male newborn, born at 41 weeks' gestation without complications, whose mother consumed large amounts of vodka nightly for a decade and did not receive prenatal care, apart from taking prenatal vitamins. The question asks for the most probable physical examination finding in this newborn. Given the mother's significant history of alcohol consumption, it is reasonable to assume that the newborn may have been exposed to alcohol in utero, potentially leading to Fetal Alcohol Syndrome (FAS). FAS is a disorder that results from prenatal exposure to alcohol and can cause birth defects, growth problems, and nervous system abnormalities. Among the examination findings listed, short palpebral fissures, which are shorter than average openings of the eyes, are a common feature associated with FAS. Therefore, considering the mother's heavy alcohol consumption during pregnancy and the clinical manifestations of FAS, the most likely examination finding in this newborn would be short palpebral fissures. In conclusion, the newborn's exposure to high levels of alcohol during pregnancy due to the mother's chronic alcohol intake could have resulted in the development of FAS. Consequently, the most probable physical examination finding in this newborn is short palpebral fissures, a characteristic feature of FAS.
