# Supplementary material for: Collaborative intelligence in AI: Evaluating the performance of a council of AIs on the USMLE
Source: PLOS Digit Health. 2025 Oct 9;4(10):e0000787. doi: 10.1371/journal.pdig.0000787 (PMC12510544; doi:10.1371/journal.pdig.0000787)
Supplement: S2 File — (ZIP) [file pdig.0000787.s002.zip › S2 Data Raw data files containing unprocessed question inputs and LLM outputs including transcripts of deliberations/STEP1_Question-115-1_Type-MC-NJ.docx]

**Facilitator** **[Round 1]:** A 15-year-old boy is brought to the office by his parents to discuss results of a biopsy done 3 days ago for a rapidly enlarging neck mass. He first noted the mass 1 month ago. Two weeks ago, he was evaluated for an episode of prolonged epistaxis and was found to have a right ear effusion, which was treated with amoxicillin-clavulanic acid. He has no other history of serious illness and takes no medications. Height is at the 10th percentile, weight is at the 50th percentile, and BMI is at the 75th percentile. Vital signs are within normal limits. Physical examination shows a right ear effusion and a 4-cm, firm mass in the right posterior triangle of the neck. Results of a biopsy specimen show squamous epithelium with indistinct cell margins, enlarged atypical nuclei, and absent keratin formation. Which of the following infectious agents is the most likely underlying cause of this patient's current condition? (A) Cytomegalovirus (B) Epstein-Barr virus (C) HIV (D) Human herpesvirus 8

**AI_C:** RESTATING THE QUESTION: The question is asking us to identify the virus which is most likely responsible for the symptom patterns and biopsy results seen in the patient. The patient is a young adolescent boy who has been experiencing rapid growth of a mass in his neck, prolonged nosebleeds, and a flora imbalance in his right ear, the latter of which was treated with an antibiotic. The biopsy results also show atypical signs within the squamous epithelium of the mass. The options provided are all viruses known to cause various symptoms and conditions. PLANNING: The assumption here is that an infectious agent is responsible for the patient's condition. To evaluate this, we need to consider both the symptoms exhibited by the patient and the molecular changes observed in the biopsy. A common virus amongst the options provided that affects the epithelial cells and manifest with the presenting clinical features is the Epstein-Barr virus. CLINICAL REASONING: Biopsy results of squamous epithelium with indistinct cell margins, enlarged atypical nuclei, and absent keratin formation, along with the presence of a neck mass and prolonged epistaxis in a teenager are highly suggestive of infectious mononucleosis, commonly caused by Epstein-Barr virus (EBV). EBV is known for its capacity to infect and transform B lymphocytes leading to lymphadenopathy, represented here by the neck mass. It also has a tropism for nasopharyngeal epithelial cells leading to epistaxis and ear effusion. CONCLUDING: From my understanding of these symptoms and the cellular and organ level processes that cause them, it seems plausible that the Epstein-Barr virus, with its impact on B cells and epithelial cells, could be a strong candidate for the causative agent of this neural and lymphatic manifestation. RESPONSE: The most likely underlying cause of this patient's condition, taking into consideration both his symptoms and his biopsy results, is option (B) Epstein-Barr virus.

**AI_B:** RESTATING THE QUESTION: This question refers to a 15-year-old boy who noticed a rapidly enlarging neck mass. He was also found to have a right ear effusion two weeks ago that was treated with antibiotics, but he, as yet, has no known serious medical history. His physical development and vital signs are within expected ranges. The biopsy of the neck mass shows atypical squamous cells without keratin formation. We are asked to choose from four viruses to determine which might be the main cause of his symptoms. PLANNING: There are several assumptions in this case. The first one is that the neck mass, prolonged epistaxis, and ear effusion are connected, which is suggestive but not proven. The second assumption is that these changes are caused by an infectious agent, specifically a virus, based on the provided options. We need to look at each possibility individually. CLINICAL REASONING: Anatomically, the ear and neck are interconnected, which might support the first assumption of connectedness. Furthermore, a rapidly enlarging neck mass in the context of atypical squamous cells raised suspicion for malignancy, specifically one of viral etiology given the options. Let's examine the viruses: (A) Cytomegalovirus: CMV usually causes mononucleosis-like symptoms (fever, sore throat, lymphadenopathy) primarily in immunocompromised individuals. Neoplasms associated with CMV are unusual. (B) Epstein-Barr Virus: EBV is associated with several malignancies including nasopharyngeal carcinoma and Burkitt's lymphoma. Nasopharyngeal carcinoma could explain the ear effusion and neck mass, given the anatomical proximity of the nasopharynx to the ear and neck. The atypical squamous cells could potentially represent undifferentiated carcinoma. (C) HIV: This infection could predispose the patient to various cancers, but the patient's lack of other severe illnesses or infections opposed this idea. (D) Human Herpesvirus 8: HHV-8 is mostly associated with Kaposi’s sarcoma, primary effusion lymphoma, and multicentric Castleman's disease, none of which fit this patient's presentation. CONCLUDING: Considering the clinical presentation, the cellular changes observed from the biopsy, and the likelihood of the proposed infectious agents, the EBV appears to be the most plausible cause of the changes observed in this patient. RESPONSE: The infectious agent most likely underlying the patient's current symptoms is (B) Epstein-Barr Virus.

**AI_D:** RESTATING THE QUESTION: The question is asking what infectious agent is most likely the cause of the medical issues that the 15-year-old boy is experiencing, given the information from his biopsy and medical history. The biopsy notes squamous epithelium with unclear cell margins, unusually large, atypical nuclei, and no keratin formation. It's important to note that squamous epithelium refers to a type of epithelium that is found in areas of the body which undergo frequent physical stress, such as the skin, mouth, and throat, and the atypical nuclei and absent keratin formation suggest abnormal cell behavior. PLANNING: The infectious agents presented as options are all viruses. Cytomegalovirus (CMV) is a herpesvirus that can cause a syndrome similar to mononucleosis in adolescents but is typically associated with opportunistic infections. Epstein-Barr virus (EBV) is another herpesvirus that is known to cause mononucleosis and is associated with several types of cancer. HIV is a retrovirus that targets the immune system and in its advanced stage (AIDS) can lead to various opportunistic infections and cancers. Human herpesvirus 8 (HHV-8) is associated with Kaposi's sarcoma, primarily in people who are immunocompromised. CLINICAL REASONING: Given the patient's age and the fact that he has a rapidly enlarging neck mass, prolonged epistaxis, and a right ear infection - all signs suggestive of a generalized upper respiratory tract illness - among these options, Epstein-Barr virus (EBV) appears to be the most likely infectious agent. EBV infections can lead to mononucleosis and are known to be associated with the development of malignant tumors such as nasopharyngeal carcinoma and Burkitt lymphoma, which could account for the abnormal cells observed in the biopsy and the presenting symptoms. Furthermore, while the other listed viruses can cause significant illness, they are usually not associated with the specific presentation seen in this case. CONCLUDING: Leveraging the known pathogenesis and disease presentation associated with the viruses suggested, the combination of an enlarged neck mass, signs of an upper respiratory tract infection and, importantly, the histological findings suggestive of a malignancy points most strongly toward Epstein-Barr virus (EBV) as the underlying causative infectious agent. RESPONSE: Thus, the most likely underlying infectious agent of this patient's current condition is (B) Epstein-Barr virus.

**AI_A:** RESTATING THE QUESTION: The main question asks us to identify the infectious agent that is the most likely cause of the current condition in a 15-year-old boy. The boy is dealing with a rapidly enlarging neck mass, a recent episode of prolonged epistaxis, and had a right ear effusion. His biopsy results showed squamous epithelium with indistinct cell margins, enlarged atypical nuclei, and lack of keratin formation. Given these findings, the choices we're asked to evaluate are Cytomegalovirus, Epstein-Barr virus, HIV, and Human herpesvirus 8. PLANNING: Let's begin by understanding that the key features mentioned here are a rapidly enlarging neck mass, epistaxis, and right ear effusion, as these symptoms may be linked to the cause of pathology. We can piece together the fact that the mass is malignant, given the biopsy results, which showed features of malignancy such as enlarged atypical nuclei, and lack of keratin formation. This indicates a poorly differentiated squamous cell carcinoma. Based on this information and the boy's age, my initial thought is to consider Epstein-Barr virus (EBV), which can cause various conditions, including malignancies like nasopharyngeal carcinoma and lymphomas, especially Burkitt's lymphoma. CLINCAL REASONING: Let’s now dismiss the other options. While I am thinking of childhood cancers, especially in the context of Epstein-Barr virus, CMV generally presents with non-specific symptoms in immunocompetent individuals and does not typically lead to such malignancies. HIV is also associated with various malignancies (like Kaposi's Sarcoma and B cell lymphomas) but the patient has no other signs and symptoms commonly seen in patients with HIV like recurrent severe infections, significant weight loss, or general malaise. Furthermore, Human Herpes Virus 8 is principally associated with Kaposi’s Sarcoma which does not fit with the pathological findings in our case, as Kaposi’s Sarcoma generally appears as a cutaneous or mucosal mass lesion with a characteristic histology, not squamous cell carcinoma. Also, Kaposi's sarcoma is more common in the HIV population. CONCLUDING: After considering and dismissing the other choices, I return to my original hypothesis, which is Epstein-Barr virus (EBV). EBV is known to be associated with the development of several types of malignancies, including nasopharyngeal carcinoma and certain lymphomas, notably Burkitt's lymphoma. RESPONSE: Therefore, based on this 15-year-old patient's presentation, pathological findings suggestive of malignancy, and the exclusion of the other options, the most likely underlying infectious agent causing this patient's current condition is (B) Epstein-Barr virus.

**AI_E:** RESTATING THE QUESTION: This inquiry refers to the diagnosis of an infectious disease causing several symptoms in a 15-year-old male patient. The symptoms are a rapid increase in the size of a neck mass, prolonged nosebleeds, and a right ear effusion, and the biopsy of the neck mass showed abnormal squamous epithelial cells with altered nuclear features and lack of keratin formation. The question seeks the most plausible infectious agent responsible for these signs and symptoms. PLANNING: Here we're considering four viral agents: cytomegalovirus (CMV), Epstein-Barr virus (EBV), HIV, and human herpesvirus 8 (HHV8). Each of these viruses is known to induce various diseases that may theoretically involve the presentation outlined. Questioning Assumptions: Each virus choice would lead to different disease presentations: CMV usually causes mononucleosis-like symptoms but rarely manifests as a neck mass. EBV can contribute to several conditions, including mononucleosis, and is known to be associated with some forms of lymphoma, which could include neck masses. HIV generally manifests with more systemic signs of immune suppression, though it could theoretically facilitate the development of a neoplastic neck mass. HHV8 is primarily associated with Kaposi sarcoma, which primarily affects the skin and mucosa and is less likely to cause a neck mass. Thus, after considering the different presentations, our focus should be on EBV which is known to cause clinical presentations similar to the case described. CLINICAL REASONING: Let's break down the presenting symptoms and connect them with the most likely cause: 1. Rapidly enlarging neck mass: This suggests some form of growth, which could be inflammatory or neoplastic. Based on the biopsy findings, this is likely a malignant process given the atypical nuclei and lack of keratin formation typically seen in squamous cells. 2. Prolonged epistaxis: This could be a sign of coagulation abnormalities, but given the context, it may indicate local spread of the disease process (perhaps involving the nasopharynx). 3. Right ear effusion: This could be an unrelated symptom, although it might also suggest local spread of the disease process, potentially blocking Eustachian tube drainage. Given the above considerations, EBV seems to be the most plausible cause out of the options, as it's known to cause nasopharyngeal carcinoma, which could explain all of these symptoms. CONCLUDING: Based on the patient's symptoms, age, and pathologic findings, combined with what is known about the possible viral agents presented, the Epstein-Barr virus stands out as the most probable cause. This virus can cause nasopharyngeal carcinoma, which could lead to the clinical symptoms seen in the patient and the pathological findings observed in the biopsy. RESPONSE: The most likely infectious agent underlying this patient's current condition is (B) Epstein-Barr virus.

**Facilitator** The consensus among the AI responses is that the most likely infectious agent underlying the 15-year-old boy's current condition is the Epstein-Barr virus (EBV). This conclusion is based on the patient's symptoms, which include a rapidly enlarging neck mass, prolonged epistaxis, and a right ear effusion, as well as the biopsy results showing squamous epithelium with indistinct cell margins, enlarged atypical nuclei, and absent keratin formation. These findings suggest a malignant process, likely a poorly differentiated squamous cell carcinoma. EBV is known to be associated with various conditions, including malignancies like nasopharyngeal carcinoma and lymphomas, especially Burkitt's lymphoma, which could explain the patient's symptoms and biopsy results. The other options - Cytomegalovirus, HIV, and Human herpesvirus 8 - were considered but dismissed due to their typical disease presentations not aligning with the patient's symptoms and biopsy findings. Therefore, the consensus is that the Epstein-Barr virus is the most likely cause of the patient's current condition.
