# Supplementary material for: Collaborative intelligence in AI: Evaluating the performance of a council of AIs on the USMLE
Source: PLOS Digit Health. 2025 Oct 9;4(10):e0000787. doi: 10.1371/journal.pdig.0000787 (PMC12510544; doi:10.1371/journal.pdig.0000787)
Supplement: S2 File — (ZIP) [file pdig.0000787.s002.zip › S2 Data Raw data files containing unprocessed question inputs and LLM outputs including transcripts of deliberations/STEP1_Question-116-1_Type-MC-NJ.docx]

**Facilitator** **[Round 1]:** A 14-year-old boy is brought to the emergency department by his parents because of a 1-month history of intermittent right knee pain that has worsened during the past day. He rates his current pain as a 6 on a 10-point scale and says that it worsens when he walks and lessens when he sits. During the past 2 weeks, he has been walking 1 mile daily in preparation for participation in the school marching band. He has not taken any medications for his pain. He sustained a right tibia and fibula fracture at the age of 8 years after a skateboarding accident, which was treated with internal fixation and casting. He has asthma treated with inhaled budesonide daily and inhaled albuterol as needed. His mother has type 2 diabetes mellitus, and his maternal grandmother has osteoporosis. The patient is 170 cm (5 ft 7 in; 77th percentile) tall and weighs 88 kg (195 lb; >95th percentile); BMI is 31 kg/m² (98th percentile). Temperature is 37.0°C (98.6°F), pulse is 95/min, and blood pressure is 130/80 mm Hg. Physical examination shows hyperpigmented, thickened skin at the nape of the neck. There is tenderness to palpation of the anterior aspect of the right hip and limited range of motion on abduction, internal rotation, and flexion of the right hip. The left hip and knees are nontender; range of motion is full in all directions. The remainder of the examination discloses no abnormalities. Which of the following factors in this patient’s history most increased his risk for developing this condition? (A) BMI (B) Family history (C) Medication use (D) Previous fractures (E) Recent physical activity

**AI_D:** RESTATING THE QUESTION: The question is asking for the primary contributing factor in the patient’s history that led to the onset of the condition he is currently displaying. PLANNING: It's crucial to consider each possible risk factor and how it may influence the patient's condition. His BMI is noticeably high (31 kg/m², 98th percentile), which might contribute to various health issues. Family history often plays a significant role in the development of particular diseases; in his case, his mother has type 2 diabetes and his grandmother osteoporosis. Medication use could be a potential factor, given his regular use of inhaled budesonide and albuterol for his asthma. His history of fractures could have a bearing, especially since the pain he experiences is in a region that previously sustained an injury. Finally, his recent increase in physical activity for the marching band could put added pressure on his joints, possibly leading to the problem. CLINICAL REASONING: Starting with his BMI, high BMI can predispose one to a variety of health conditions including certain musculoskeletal issues. His family history includes type 2 diabetes and osteoporosis. Both can affect bone health, but they usually exert their influence later in life and not typically seen at his age. His medication use, specifically inhaled budesonide, can induce bone demineralization, but it is usually in higher doses and longer use. His past fractures might have damaged the integrity of his bones at the injured site. However, there seems to be no acute indication related to a past injury. Lastly, any recent increase in physical activity might stress his joints but does not usually result in persistent pain unless there is an acute injury, which is not indicated here. CONCLUDING: With several factors to consider, the most compelling appears to be the patient's BMI. High BMI in adolescence can cause an array of health problems, among them slips in the epiphysis of the femoral head, as could be indicated by pain upon hip rotation and abduction. It’s called Slipped Capital Femoral Epiphysis (SCFE) which is common in overweight adolescents. It is a result of shear stress on the hip joint which is increased in overweight adolescents because of the resultant greater force across the hip. RESPONSE: (A) BMI. This patient's high BMI most significantly increased his risk for developing his current condition.

**AI_B:** Restating the question, we are asked to identify the factor from the patient's history that most likely contributed to the development of his condition. Factors include: BMI (Body Mass Index, an indicator of body fat percentage that is calculated from the weight and height), family's medical history (e.g., type 2 diabetes and osteoporosis), daily use of inhaled budesonide and albuterol as needed for his asthma, a prior history of fractures to the right tibia and fibula, and recent increase in physical activity (marching band). Let's evaluate each assumption associated with these factors. Firstly, it could be hypothesized that the patient's high BMI, in the overweight range, could be exerting undue stress on his joints. Secondly, the medications the patient is using primarily target the respiratory system and do not typically impact joint health or adiposity directly, so we can question if medications really have a significant impact in this scenario. Thirdly, the patient's history of previous fractures may have caused anatomical changes that predispose to joint issues. Fourthly, recent exercise could potentially exacerbate any underlying joint condition. Lastly, the family history of type 2 diabetes and osteoporosis might indicate a genetic predisposition to certain metabolic or bone diseases that could affect joint health. Clinical reasoning suggests that factors like prior fractures and family history are both significant, but in this case, we should pay more attention to the patient's high BMI. This young patient's significantly elevated BMI, which is in fact classified as obesity, has been shown in multiple studies to associate with an increased risk for musculoskeletal disorders, including hip and knee issues. The combination of excess body mass and the increased physical activity from training for the school marching band are likely exacerbating stress on his joints. In conclusion, of all the factors presented, the patient's high BMI is most likely the primary risk factor leading to the presentation of his symptoms. It seems probable that the additional weight is contributing to increased wear and tear on his joints, leading to his discomfort. Therefore, in this case, we can say that the patient's BMI has most likely increased his risk for developing this condition. Hence, the response to the question "Which of the following factors in this patient’s history most increased his risk for developing this condition?" would be (A) BMI.

**AI_C:** RESTATING THE QUESTION: The boy in question presents with a history of sustained knee pain that worsens during mobility and decreases during rest. The pain scale is identified as 6 out of 10. His medical history includes a previous leg fracture, asthma, obesity indicated by his BMI of 31 kg/m², and a family history of type 2 diabetes and osteoporosis. The objective is to deduce which of these factors could have amplified his risk for his current condition based on the listed choices. PLANNING: Upon reviewing each assumption, (A) his BMI suggests a factor of obesity which can put an increased burden on the musculoskeletal system, (B) his family history might suggest a genetic predisposition to particular conditions but doesn't provide a direct link to the boy's condition, (C) the medication in use is for asthma and unlikely to influence a musculoskeletal issue heavily, (D) previous fractures could increase his risk only if it has led to chronic conditions or complications related to his musculoskeletal system, (E) his recent physical activity can be a contributing factor if it was excessive enough to cause a musculoskeletal issue. CLINICAL REASONING: As we bridge the sentence of each assumption with the next, it becomes clear that the boy's pain is related to mobility, and he hasn't used pain medication. His weight could add extra pressure on his musculoskeletal system, which might exacerbate preexisting conditions or catalyze new ones. Furthermore, the boy shows no signs of genetics-related conditions, implying that the family history in this case might be less of a contributing factor. The asthma medication seems unrelated to the symptoms. The previous fracture has long been treated and doesn't indicate an ongoing issue linked to his current condition. Conversely, the recent physical activity undergone by the boy - marching band preparations - could cause the intermittent pain, but usually would present with a quicker onset than observed here. CONCLUDING: To tie all these evaluations together, his elevated BMI seems to be the most direct influencing factor to his current condition as it directly adds more burden on his musculoskeletal system compared to the other parameters. RESPONSE: Therefore, factor (A) - BMI would be the one in this patient's history that would most significantly increase his risk for developing this condition.

**AI_E:** RESTATING THE QUESTION: The clinical scenario presents a 14-year-old boy experiencing worsening right knee pain. Multiple contributing factors in the patient's history could potentially be associated with the current pain: (1) BMI-he is overweight with a BMI of 31 kg/m²; (2) Family history- his mother has type 2 diabetes and his grandmother has osteoporosis; (3) Medication use- he regularly uses inhaled budesonide and albuterol for asthma; (4) Previous fractures- he had a fracture in his right tibia and fibula when he was 8 years old; (5) Recent physical activity-he has been walking 1 mile daily for the last 2 weeks. The question seeks to identify the factor that played the most significant role in causing his current situation. PLANNING: Factors known to contribute to knee pain include obesity (reflected in the patient’s high BMI), physical activity, medication side effects, and complications of previous injuries. Although there is a family history of type 2 diabetes and osteoporosis, osteoporosis would be an unlikely cause of knee pain in a young male. The boy also has a high BMI, which could exert additional pressure on his joints, leading to discomfort and pain during physical activities such as walking. The physical activity involved in training for marching band could have exacerbated a latent condition. The medications used for asthma are unlikely to directly cause knee pain. A previous fracture might raise the risk of post-traumatic arthritis. When considering these factors, it seems plausible to think that his high BMI may be a contributing factor to his knee pain. CLINICAL REASONING: This patient's obesity (BMI >95th percentile) has placed him at risk for slipped capital femoral epiphysis (SCFE), a condition characterized by displacement of the proximal femoral epiphysis due to weakness of the growth plate. It is most common in obese adolescents in their growth spurt and is associated with endocrine disorders (hypothyroidism, growth hormone treatment). The presenting symptoms usually include limping and knee/hip/thigh pain because afferent nerve fibers from the hip joint travel along the obturator nerve, which also innervates the knee. The limited range of motion, particularly internal rotation and abduction of the hip, support the diagnosis of SCFE. It is pertinent to note that while his recent physical activity, previous fracture, and medication use (especially steroids) might contribute to some degree, the patient's BMI poses the most significant risk for developing SCFE. CONCLUDING: Based on the overall consideration of multiple factors, along with understanding the high prevalence of SCFE in obese adolescents, the patient’s high BMI could be deemed the most influential factor contributing to his current condition. RESPONSE: The factor in this patient's history that most increased his risk for developing this condition was his (A) BMI.

**AI_A:** RESTATING THE QUESTION: The customarily issue here pertains to a teenager who is brought to the emergency department due to worsening, intermittent knee pain. The briefings include walking 1 mile daily for 2 weeks, a past fracture treated with internal fixation and casting, daily use of inhaled budesonide for asthma, and a family history of type 2 diabetes and osteoporosis. The patient's body mass index (BMI) indicates obesity. A physical examination reveals tender and limited range of motion in the hip along with hyperpigmented, thickened skin at the back of the neck. The inquiry requires recognizing the potentially most significant risk factor for the patient's condition. PLANNING: Factors to consider include the patient's high BMI, family medical history, medication usage, previous fractures, and recent physical activity. Each potential risk factor has certain implications on the patient's overall health. Obesity (BMI) can impact joint health and mobility. Family history might point to genetic factors or shared environmental conditions. Medication usage could lead to side effects, interactions or long-term effects. Previous fractures could affect skeletal structure and function. Finally, sudden changes or increases in physical activity might stress musculoskeletal system. Hypothetically, any of these could increase the risk for joint pain, but from a clinical perspective, one is to be determined to be the most significantly correlating risk factor. CLINICAL REASONING: Each option needs scrutinized independently. High BMI, specifically in obese range, puts additional stress on weight-bearing joints, leading to joint damage and pain, but usually reflects in knee and ankle joint. Family history of diabetes mellitus and osteoporosis may predispose the patient to certain conditions, but the immediate relevance to his present condition remains unclear. Although the use of budesonide, an inhaled corticosteroid, could potentially have systemic effects contributing to joint issues, the medicine is primarily localized to treat his asthma. The history of fracture comes as a possible risk factor, though it happened six years ago, and the patient's current pain is not localized to the previously treated area. Lastly, recent physical activity could potentially exacerbate an existing condition, but the recent onset of walking 1 mile daily is not physically demanding enough to directly cause significant knee pain. CONCLUDING: After collaborating each of the aforementioned factors, it appears that the patient's BMI outweighs other potential risk factors presented in the options. This conclusion is reached considering that his BMI is in obese range (>95 percentile) and acanthosis nigricans (hyperpigmented, thickened skin at nape of neck) is often associated with insulin resistance, which occurs frequently in obese patients. Condition like Slipped capital femoral epiphysis (SCFE) often affects overweight adolescents and presents with knee or hip pain with limping. The pain onset can be insidious and symptoms may be present for weeks to months, which aligns with this patient's pain history. SCFE is notorious for referred knee pain despite the pathology being in hip joint. RESPONSE: Therefore, the most significant risk factor for the development of this patient's condition appears to be option (A) BMI.

**Facilitator** The consensus statement synthesizes the responses of AI_A, AI_B, AI_C, AI_D, and AI_E as follows: The question at hand pertains to a 14-year-old boy who presents with worsening, intermittent right knee pain. His medical history includes a high BMI indicating obesity, a family history of type 2 diabetes and osteoporosis, daily use of inhaled budesonide for asthma, a past fracture treated with internal fixation and casting, and recent physical activity in preparation for participation in the school marching band. The task is to identify the most significant risk factor for the patient's current condition. Upon evaluating each potential risk factor, it is clear that the patient's high BMI, specifically in the obese range, is the most significant. Obesity can put additional stress on weight-bearing joints, leading to joint damage and pain. Although the patient's family history, medication use, previous fractures, and recent physical activity could potentially contribute to his condition, these factors do not appear to be as directly influential as his BMI. The patient's family history of diabetes mellitus and osteoporosis may predispose him to certain conditions, but their immediate relevance to his present condition remains unclear. The use of budesonide, an inhaled corticosteroid, could potentially have systemic effects contributing to joint issues, but the medicine is primarily localized to treat his asthma. The history of fracture is a possible risk factor, but it occurred six years ago, and the patient's current pain is not localized to the previously treated area. Lastly, the recent onset of walking 1 mile daily is not physically demanding enough to directly cause significant knee pain. The patient's high BMI is associated with conditions like Slipped Capital Femoral Epiphysis (SCFE), which often affects overweight adolescents and presents with knee or hip pain. The pain onset can be insidious, and symptoms may be present for weeks to months, aligning with this patient's pain history. SCFE is notorious for referred knee pain despite the pathology being in the hip joint. Therefore, the consensus is that the most significant risk factor for the development of this patient's condition is his high BMI (option A).
