# Supplementary material for: Collaborative intelligence in AI: Evaluating the performance of a council of AIs on the USMLE
Source: PLOS Digit Health. 2025 Oct 9;4(10):e0000787. doi: 10.1371/journal.pdig.0000787 (PMC12510544; doi:10.1371/journal.pdig.0000787)
Supplement: S2 File — (ZIP) [file pdig.0000787.s002.zip › S2 Data Raw data files containing unprocessed question inputs and LLM outputs including transcripts of deliberations/STEP1_Question-117-1_Type-MC-NJ.docx]

**Facilitator** **[Round 1]:** A 38-year-old woman comes to the clinic to discuss results of a biopsy specimen of a mass in the right breast obtained 1 week ago. She has no history of serious illness and takes no medications. Vital signs are within normal limits. Examination of the right breast shows a well-healing biopsy incision. The remainder of the examination discloses no abnormalities. Results of the biopsy specimen disclose ductal carcinoma in situ. The physician informs the patient he has some bad news to share and tells her the diagnosis. He explains her treatment options, including mastectomy, breast-conserving surgery, radiation therapy, chemotherapy, and any combination of these options. The patient appears afraid but listens attentively before responding, ""You're the doctor. My children are still little. Tell me what I should do."" In addition to expressing empathy, which of the following is the most appropriate physician response? (A) ""As the patient, you really should make any decision you feel is best for you."" (B) ""Before I make recommendations, can you tell me what you understand about your options?"" (C) ""Given the stage of the cancer, I recommend mastectomy."" (D) ""How important is it to you to keep your breasts?"" (E) ""Would you like to discuss these options further with your family?"" (F) ""Would you like to think your options over and schedule another visit to discuss them further?"" (G) ""You said you have young children. How old are they?""

**AI_E:** RESTATING THE QUESTION: A woman aged 38 who recently underwent a biopsy for a breast mass and was diagnosed with ductal carcinoma in situ seeks advice from her physician. Having been informed of the various possible therapies including mastectomy and less invasive options, she entrusts the doctor with the decision because she is concerned about her children. The query focuses on the most suitable response from the doctor while ensuring empathy is part of the communication. PLANNING: It seems critical to address the woman's feelings of fright and the role of her children in her decision-making process. Thus any assumption should include these two factors. One probable assumption may suggest the doctor making a unilateral decision but this is debatable as it excludes the woman's thoughts and preferences. A more equitable assumption may be the doctor facilitating dialogue to understand the woman's knowledge and take on the possible options. This will aid in a jointly reached conclusion regarding care and respects the importance of autonomy in medical decision-making. CLINICAL REASONING: In medicine, patient autonomy is a primary ethical principle, and it's important for patients to be active participants in their own health decisions. Physicians are responsible for providing information and advice but ultimately the choice rests on the patient. In this case the patient is handing over the decision power to the doctor which can place more emotional burden on the doctor and also may not be in the best psychological benefit for the patient. The emotional tone of the meeting can be acknowledged and the patient should also be included in the decision-making process by understanding her current grasp of the suggested treatment options. CONCLUDING: The response respecting patient autonomy, acknowledging her emotional state, and attempting to understand her perspective of the situation would be most appropriate. RESPONSE: Hence, the most suitable physician response is (B) "Before I make recommendations, can you tell me what you understand about your options?" This respects patient autonomy, acknowledges her emotional state, attempts to understand her cognition of the situation and incorporates her into the decision-making process.

**AI_C:** (1) RESTATING THE QUESTION: This question revolves around a case wherein a 38-year-old woman has been diagnosed with ductal carcinoma in situ, a type of breast cancer. Various treatment options, including mastectomy, breast-conserving surgery, radiation therapy, chemotherapy, or any combination of these, have been laid out for her. Amidst her fear, the woman appeals to the physician to guide her on what to do mainly because she has young children and seems unsure of how to proceed. The question now is about the most appropriate response for the physician to make in such a setting, considering the delicate nature of the situation and potential psychological impact on the patient. (2) PLANNING: The possible assumptions underpinning this question could include: (i) the woman doesn't understand her options, (ii) the woman wants a definitive recommendation from the physician, (iii) the woman may need input from her family, (iv) the woman may want to keep her breasts, (v) the woman's decision may be influenced by the age of her children. By questioning each of these assumptions, alternative assumption can be generated. In this context, the woman's statement "tell me what I should do" implies she seeks guidance, nonetheless, in such a scenario it should be clinically prioritized to ensure she understands her options and actively participates in the decision-making process. (3) CLINICAL REASONING: Breast cancer treatment requires a careful evaluation of the clinical elements in combination with the patient's personal values and understanding to ensure a shared decision-making process. While some patients might want a specific recommendation from their physician, it is important for the patient to fully understand each procedure's consequences, risks, and benefits. Hence, before making definitive treatment recommendations, it's critical to understand the patient's comprehension of the provided information. (4) CONCLUDING: Harmonizing the clinical priorities and the woman's implicit appeal for guidance, the physician should display empathy (as suggested) as well as encourage the woman to partake in making the decision based on well-understood options. This can be achieved by verifying her grasp of the options. (5) RESPONSE: Therefore, the most appropriate physician response would be: (B) ""Before I make recommendations, can you tell me what you understand about your options?" This approach ensures a shared decision-making process while simultaneously confirming the woman's awareness and understanding of the treatment options, enabling her to make a well-informed decision.

**AI_B:** This scenario involves a 38-year-old woman who has been diagnosed with ductal carcinoma in situ following a biopsy of a mass in her right breast. She has been informed about it and different treatment options have been provided. These include mastectomy (surgical removal of the breast), breast-conserving surgery, radiation therapy, chemotherapy, or a combination of these treatments. At this point, she looks fearful, asks the physician to decide for her considering her young kids. The situation is asking the optimal response for the physician to make. Restated in simpler terms, the question might be: In the context of shared decision-making in medicine and respect for patient autonomy, what should be the ideal course of action or response from the clinician in response to a patient with early stage breast cancer who asked the doctor to choose the treatment for her? The assumptions were that the physician must make the decision on behalf of the patient, as she asked him to do so; the patient must be involved in the decision-making process to facilitate shared decision-making; understanding the patient's insight and preferences is crucial in making a medical decision; and the patient's fear and emotional state need to be taken into account when suggesting a treatment decision. Evaluating these assumptions, it is understood that making a decision by the physician may undermine the patient's autonomy, even if she requested it. The patient's understanding of the situation and her values and preferences should guide the decision-making process. Exploring her expectations, fears, and understanding of the disease is needed for a shared decision in this given clinical context. By clinical reasoning, a good answer would not just make a decision for the patient but would engage her in an open, understanding dialogue about her condition and potential treatment paths. Breast cancer isn't only a physical disease but also impacts the psychosocial aspects of a woman's life. The question above addresses a dilemma that physicians may encounter in clinical practice-- respecting patient autonomy and establishing shared decision-making in situations where patients ask the doctor to choose for them. In such scenarios, it's important to explore the patients' perspectives and understand their insight regarding the disease, treatments, and related effects. Realizing this, the most appropriate response would be to ensure her understanding of the situation and clarification of her perspective on the proposed options. Hence, the ideal response would be (B) "Before I make any recommendations, can you tell me what you understand about your options?" This option strikes a balance between understanding the patient's perspective and involving her in the decision-making process, thus promoting shared decision-making.

**AI_D:** RESTATING THE QUESTION: In essence, the query is about a suitable response from a physician to a 38-year-old woman who is grappling with the recent diagnosis of ductal carcinoma in situ (DCIS), a pre-invasive or non-invasive form of breast cancer confined within the walls of the milk duct, who seeks the doctor's advice on the management of her condition. The patient's treatment options include mastectomy (complete removal of the breast), breast-conserving surgery (partial removal of the breast), radiation therapy (using high-energy beams to kill cancer cells), chemotherapy (using drugs to eliminate cancer cells), or a combination thereof. PLANNING: To answer this question, I hypothesize that the best course of action wouldn't be decided solely based on the diagnosis of ductal carcinoma in situ. There will be assumptions considering the patient's understanding of the situation and preferences, her emotional well-being, and the involvement of her family in the decision-making process. CLINICAL REASONING: Ensuring the patient fully understands her diagnosis and treatment options is vital as she will need to be an active participant in her healthcare decisions. Answer (B) supports this assumption by encouraging an open dialogue with the patient about her understanding of the potential steps forward. Alleviating the patient's fears and uncertainties is of utmost importance; hence, the doctor should ascertain her readiness to make a decision. Having her family involved in the decision-making process might also aid in emotional support, represented in answer (E) and (F). Answer (C), although providing a concrete recommendation, might potentially disregard the patient's preferences or emotional readiness. Answer (D) seems to address the potential cosmetic implications of the treatment options somewhat crudely. Answer (A) may place too much decision-making burden on the patient alone, and answer (G)'s relevance is unclear as it deviates from the critical matter at hand. CONCLUDING: A patient's understanding, preparedness, emotional readiness, and family support are all key elements that need to be considered before proceeding with treatment decisions for ductal carcinoma in situ. Balancing between clinical recommendations and patient preferences can be a delicate task requiring tactful and empathetic communication. RESPONSE: In response to the patient's plea, "You're the doctor. My children are still little. Tell me what I should do," the most appropriate physician response would be, (B) "Before I make recommendations, can you tell me what you understand about your options?" This response acknowledges her emotions, encourages her participation, and ensures patient-centered care by considering her understanding and expectations about the potential treatments.

**AI_A:** RESTATING THE QUESTION: A 38-year-old woman has recently been diagnosed with ductal carcinoma in situ following a breast biopsy, and she is seeking guidance from her doctor about what she should do next. The doctor has provided her with several treatment options and she expressed her fear and concern about the well-being of her young children. We are being asked to determine the most appropriate response for the physician to give that goes beyond just showing empathy. PLANNING: The assumptions underlying this scenario include that the patient understands her diagnosis and the treatment options she has been given, that she is capable of making her own decisions, and that she wants the doctor to tell her what to do. We could question each of these assumptions to generate alternative possibilities. For instance, the patient might not fully understand her diagnosis or the implications of the treatment options. She might not feel equipped to make such a major decision on her own. She might also not actually want the doctor to dictate her treatment, but instead she could be looking for more information or needing reassurance. Given the subjectivity of these assumptions and her emotional state, it is reasonable to prioritize the assumption that the patient could benefit most from further discussion and clarification about her diagnosis and treatment options for ductal carcinoma in situ. CLINICAL REASONING: Ductal carcinoma in situ (DCIS) is a non-invasive breast cancer, where the abnormal cells are confined in the lining of the breast milk duct. The treatment options include mastectomy, breast-conserving surgery, radiation, and chemotherapy. A patient's input is essential to determine the best treatment plan as it largely depends on a variety of personal factors such as the patient's understanding of the condition and its treatments, her personal comfort with risk, her ability to cope with treatment, and her personal, social, and emotional situation. CONCLUDING: Given the patient's fear, her responsibilities towards her young children, and her plea for guidance, the physician needs to tread a delicate balance between providing necessary information and being sensitive to her emotional state. In this context, checking her understanding of her condition and potential treatments before suggesting a course of action carries utmost importance. Any assumptions made without confirming her understanding could potentially lead to informed discontent or confusion about therapeutic choices. RESPONSE: The most appropriate response would hence be (B) "Before I make recommendations, can you tell me what you understand about your options?" This option ensures that the patient has a clear understanding of her situation before she is guided to take any critical decisions related to her treatment pathway. Furthermore, it gives the physician an opportunity to fill in any knowledge gaps and correct any misconceptions she might have. This enables her to actively participate in the decision-making process, which is an ethical practice in patient care.

**Facilitator** The question presents a scenario where a 38-year-old woman has been diagnosed with ductal carcinoma in situ, a non-invasive form of breast cancer. She has been informed about various treatment options, including mastectomy, breast-conserving surgery, radiation therapy, chemotherapy, or a combination of these. The woman, expressing fear and concern for her young children, asks the physician to guide her on what to do next. The question asks for the most appropriate response from the physician, one that goes beyond just showing empathy. The consensus among the AI responses is that the most appropriate response from the physician would be (B) "Before I make recommendations, can you tell me what you understand about your options?" This response respects the patient's autonomy, acknowledges her emotional state, and attempts to understand her cognition of the situation. It also incorporates her into the decision-making process, which is an ethical practice in patient care. The reasoning behind this consensus is that the patient's understanding, preparedness, emotional readiness, and family support are all key elements that need to be considered before proceeding with treatment decisions for ductal carcinoma in situ. Balancing between clinical recommendations and patient preferences can be a delicate task requiring tactful and empathetic communication. This approach ensures a shared decision-making process while simultaneously confirming the woman's awareness and understanding of the treatment options, enabling her to make a well-informed decision.
