# Supplementary material for: Collaborative intelligence in AI: Evaluating the performance of a council of AIs on the USMLE
Source: PLOS Digit Health. 2025 Oct 9;4(10):e0000787. doi: 10.1371/journal.pdig.0000787 (PMC12510544; doi:10.1371/journal.pdig.0000787)
Supplement: S2 File — (ZIP) [file pdig.0000787.s002.zip › S2 Data Raw data files containing unprocessed question inputs and LLM outputs including transcripts of deliberations/STEP1_Question-118-1_Type-MC-NJ.docx]

**Facilitator** **[Round 1]:** A 36-year-old woman with hypertension comes to the office because she thinks she may be “going through early menopause.” She has not had a menstrual period since her most recent office visit 6 months ago. During this time, she also has been “gaining weight around the middle” despite increased exercise; she has had a 6.3-kg (14-lb) weight gain. She has no other history of major medical illness. Her only medication is lisinopril. She does not smoke, drink alcohol, or use illicit drugs. She is 168 cm (5 ft 6 in) tall and weighs 107 kg (236 lb); BMI is 38 kg/m². Vital signs are within normal limits. Examination shows a uterus consistent in size with a 24-week gestation. Pelvic ultrasonography shows oligohydramnios and a fetus with a misshapen cranium, pericardial effusion, small bladder, and echogenic bowel. The most likely cause of the fetal abnormalities in this patient’s pregnancy is interference with which of the following? (A) Fetal lung/epithelial differentiation (B) Fetal lung/surfactant development (C) Fetal renal hemodynamics (D) Maternal placental perfusion (E) Maternal prostaglandin synthesis

**AI_E:** RESTATING THE QUESTION: The query is seeking to understand the underlying cause for the fetal abnormalities in the patient's pregnancy. The patient, a 36-year-old woman with hypertension who has been experiencing changes such as missed periods and weight gain despite an increase in activity, has been found to be pregnant, with the fetus showing various abnormalities. The abnormalities include a misshapen cranium, pericardial effusion, a small bladder, and an echogenic bowel. PLANNING: In terms of assumptions, we can make several. One is that the abnormalities are linked to the mother's health status, notably her hypertension. Another assumption is that the fetal abnormalities could be linked to medication the mother is taking, in this case, lisinopril. Alternatively, the abnormalities could be inherent to the fetus itself, not necessarily linked to maternal health status. A last notion to consider is the possibility that the abnormalities are a consequence of poor placental perfusion, disrupting essential nutrient and waste exchange. Upon evaluating these assumptions, the one that appears to be clinically prioritized revolves around the fetus's own development, particularly relating to renal function, considering the oligohydramnios and small bladder observed. CLINICAL REASONING: Oligohydramnios refers to a condition in which there is less amniotic fluid than expected for a fetus's gestational age. This can be due to factors such as rupture of membranes or decreased fetal urine production. The latter can be related to issues with fetal renal hemodynamics, which is vital in both urine production and the establishment of the amniotic fluid volumes. This is further supported by the patient's ultrasound results that indicate a small bladder. The other abnormalities observed in the fetus, including the misshapen cranium and pericardial effusion, could be secondary to the primary renal issue leading to a reduced fluid space and leading to compression abnormalities. CONCLUDING: Based on the above reasoning, it seems probable that the central underlying issue is linked to the fetal renal system, affecting renal hemodynamics and thereby impacting overall fetal development across systems. RESPONSE: Therefore, the most likely cause of the fetal abnormalities in this patient's pregnancy appears to be an interference with (C) Fetal renal hemodynamics.

**AI_D:** (1) RESTATING THE QUESTION: This woman who is experiencing hypertension and weight gain suspects early menopause because she hasn't had a menstrual cycle in 6 months. However, physical examination and ultrasound findings suggest a mid-stage pregnancy with a fetus presenting various abnormalities such as abnormal head shape, excess fluid around the heart, underdeveloped bladder, and bright bowel on ultrasound, traditionally suggestive of bowel wall thickening or inflammation. The question is asking what would likely account for these fetal anomalies. (2) PLANNING: The primary assumptions surrounding this case arise from the observed fetal abnormalities. The misshapen cranium may suggest neural development problems, the pericardial effusion could indicate cardiac pathologies, the small bladder might imply renal dysfunction, and the echogenic bowel may signal gastrointestinal issues. We could ask whether these symptoms have a single common cause or are the result of multiple disturbances. Past medical history, present health status, medications, and lifestyle factors of the mother should also be considered. Placing the focus on the fetal anomalies, I'd hypothesize that interference with fetal renal hemodynamics (Option C) may account for these findings. (3) CLINICAL REASONING: Fetal renal dysfunction could explain most of the observed abnormalities. Kidneys in the fetus produce most of the amniotic fluid from around the 16th week of gestation. Impaired urine production or excretion could result in oligohydramnios, as seen in this case. Given that amniotic fluid acts as a cushion for the fetus, protecting it from physical bumps and injury, its reduction could result in the physical deformity of the cranium. Additionally, decreased urine output could explain the small fetal bladder. Pericardial effusion and echogenic bowel are less directly explained by fetal renal dysfunction, but could be secondary manifestations due to the primary issue of reduced amniotic fluid volume, causing compression and potential secondary anomalies. (4) CONCLUDING: Therefore, impaired fetal renal hemodynamics could result in most of the observed findings. It would cause a deficiency in urine production, leading to oligohydramnios, which is likely responsible for the fetus's misshapen cranium and could indirectly influence other observed abnormalities. (5) RESPONSE: The most likely cause of the fetal abnormalities in this woman's pregnancy is interference with fetal renal hemodynamics (Option C).

**AI_B:** RESTATING THE QUESTION: This query pertains to a pregnant 36-year-old hypertensive woman who came in with concerns about possible early menopause. She has not had a menstrual period for over six months and has paradoxically gained weight (6.3 kg), despite increased exercise. The physical examination reveals her uterus is of a size that's consistent with a 24-week pregnancy. Ultrasound examination shows features suggestive of fetal abnormalities such as oligohydramnios, a misshapen cranium, pericardial effusion, a small bladder, and an echogenic bowel. The question is seeking to identify the most likely cause of these fetal abnormalities. PLANNING: Among the given options, the potential causes that could be responsible for the fetal anomalies include issues with fetal lung/epithelial differentiation, fetal lung/surfactant development, fetal renal hemodynamics, maternal placental perfusion, or maternal prostaglandin synthesis. All these are crucial for normal fetal development. However, given the constellation of findings in this case, one must consider: firstly, if a renal issue could have led to oligohydramnios, as the kidneys of the fetus produce the amniotic fluid after the 16th week of gestation; secondly, whether the abnormalities could have been caused by an issue with maternal placental perfusion or maternal prostaglandin synthesis. CLINICAL REASONING: Given that ultrasonography reveals oligohydramnios (abnormally low amniotic fluid), it indicates an issue with fetal renal function as the fetus’s kidneys contribute significantly to the amniotic fluid volume in the later half of the pregnancy. Additionally, the other observable abnormalities - a misshapen cranium, pericardial effusion, a small bladder, and an echogenic bowel - could be possibly linked to the oligohydramnios, highlighting compression effects due to the lack of sufficient amniotic fluid. This sequence of events seems clinically plausible, thereby making a renal disorder a probable cause. Considering the other options, while maternal placental perfusion and prostaglandin synthesis are critical to fetal development, the direct signs of a dysfunction or abnormality in these systems are not evident in this case. CONCLUDING: In summary, using clinical reasoning on the provided information, interference with fetal renal hemodynamics appears to be the most clinically plausible cause of the fetal abnormalities observed in the ultrasound examination. The insufficient amniotic fluid volume or oligohydramnios, suggestive of renal dysfunction, and the concurrent suite of anomalies likely due to compression effects make this a more probable cause compared to the others. RESPONSE: Interference with (C) Fetal renal hemodynamics is the most likely cause of the fetal abnormalities in this pregnant patient.

**AI_A:** (1) RESTATING THE QUESTION: The question presents a clinical scenario involving a 36-year-old hypertensive woman, who initially suspected early menopause due to 6 months of amenorrhea. However, this pregnant woman, whose only current medication is lisinopril, exhibits considerable weight gain and an enlarged abdomen. Her unborn baby shows evidence of multiple abnormalities, identified through ultrasonography, which suggests a pathological condition affecting fetal development. The question prompts the identification of which organ system's functioning, when disturbed, is the most likely cause of the observed fetal abnormalities. (2) PLANNING: To solve this query, we need to examine the presented options, each of which implies a distinct pathophysiological mechanism that could impact fetal development. Given the information provided, it seems necessary to consider the patient's hypertension, the drugs she's been using, and the evident fetal abnormalities. (3) CLINICAL REASONING: A process of elimination based on the patient's medical history and present condition and the terminology used in the options can guide our response. The fetal abnormalities are oligohydramnios (reduced amniotic fluid), misshapen cranium, pericardial effusion, a small bladder, and an echogenic bowel, which indicates increased brightness in the fetal bowel area in an ultrasound image. Importantly, Lisinopril, an ACE inhibitor, is known to cause renal tubular dysgenesis, a condition that can result in a series of fetal anomalies, including oligohydramnios due to diminished fetal kidney function. A reduction in amniotic fluid can lead to fetal compression, affecting the cranium's shape and causing limb deformities. Inability to excrete urine due to decreased kidney function also explains the small bladder. The kidneys' reduced function could also lead to hypertension and fluid retention in tissues, as seen in the pericardial effusion. (4) CONCLUDING: Considering the significant effects of hypertension and the impact of Lisinopril, a drug known to be teratogenic and can specifically interfere with fetal renal function, the clinical signs presented in the infant strongly suggest an effect on fetal renal hemodynamics. (5) RESPONSE: Therefore, the most likely cause of the fetal abnormalities in this patient’s pregnancy is interference with fetal renal hemodynamics (C). This is based on the fetal abnormalities found, the impact of the medication the mother is taking, and the mother's current hypertensive status.

**AI_C:** RESTATING THE QUESTION: The patient, a 36-year-old hypertensive woman, is pregnant even though she believed that she might be starting menopause early. She has not had a menstrual period over the past six months and has noticed a weight gain, particularly around the midsection, despite increased exercise. Her only medication is lisinopril, a drug used to treat high blood pressure. Upon examination, the patient's uterus is consistent with a 24-week gestation, and ultrasonography has shown that the fetus has several abnormalities. The ultrasound findings include oligohydramnios (reduced amount of amniotic fluid), a deformed cranium, pericardial effusion, a small bladder, and an abnormally bright bowel. The question aims to identify the most likely cause that led to these fetal abnormalities. PLANNING: It is possible to systematically evaluate the given choices based on the fetal abnormalities observed. Oligohydramnios could suggest problems with fetal renal function, as the urine of the fetus contributes to amniotic fluid volume in the second half of pregnancy (Option C). The small bladder could support this hypothesis. Misshaped cranium and pericardial effusion do not directly relate to any of the given options but can be seen in a variety of pathological states, including chromosomal abnormalities, viral infections, or severe intrauterine growth restriction, all of which could potentially affect multiple organ systems. Echogenic bowel (bright bowel upon ultrasound) is often associated with cystic fibrosis, but it could also indicate fetal distress or another intercurrent pregnancy complication. CLINICAL REASONING: Although the abnormalities found in this fetus could be related to a variety of causes, the most common and likely cause of this set of abnormalities would be related to the kidneys. Oligohydramnios is caused by decreased urine production, which in turn leads to compression of the fetus and resultant deformities (Potter syndrome). Furthermore, amniotic fluid, which is basically fetal urine in the later part of pregnancy, plays an integral role in the development of the lungs by expanding the lung tissue. Lung hypoplasia and resultant neonatal respiratory distress are common in severe oligohydramnios. Hence, the primary problem begins with the dysfunction of the fetal kidneys leading to reduced urine output. CONCLUDING: Given that the urine of the fetus contributes to the amniotic fluid volume, and considering the presentation of oligohydramnios along with the deformities, the most probable cause of this fetus’ abnormalities lies with an issue in the fetal renal hemodynamics. RESPONSE: Interference with fetal renal hemodynamics (Option C) is the most likely cause of the fetal abnormalities seen in this patient.

**Facilitator** The consensus statement synthesizes the responses of AI_A, AI_B, AI_C, AI_D, and AI_E as follows: The question presents a clinical scenario involving a 36-year-old hypertensive woman who initially suspected early menopause due to 6 months of amenorrhea. However, she is found to be pregnant, and her unborn baby shows evidence of multiple abnormalities, identified through ultrasonography. These abnormalities include oligohydramnios (reduced amniotic fluid), a misshapen cranium, pericardial effusion, a small bladder, and an echogenic bowel. The question prompts the identification of which organ system's functioning, when disturbed, is the most likely cause of the observed fetal abnormalities. To solve this query, we need to examine the presented options, each of which implies a distinct pathophysiological mechanism that could impact fetal development. Given the information provided, it seems necessary to consider the patient's hypertension, the drugs she's been using, and the evident fetal abnormalities. A process of elimination based on the patient's medical history and present condition and the terminology used in the options can guide our response. Importantly, Lisinopril, an ACE inhibitor, is known to cause renal tubular dysgenesis, a condition that can result in a series of fetal anomalies, including oligohydramnios due to diminished fetal kidney function. A reduction in amniotic fluid can lead to fetal compression, affecting the cranium's shape and causing limb deformities. Inability to excrete urine due to decreased kidney function also explains the small bladder. The kidneys' reduced function could also lead to hypertension and fluid retention in tissues, as seen in the pericardial effusion. Considering the significant effects of hypertension and the impact of Lisinopril, a drug known to be teratogenic and can specifically interfere with fetal renal function, the clinical signs presented in the infant strongly suggest an effect on fetal renal hemodynamics. Therefore, the most likely cause of the fetal abnormalities in this patient’s pregnancy is interference with fetal renal hemodynamics (Option C). This is based on the fetal abnormalities found, the impact of the medication the mother is taking, and the mother's current hypertensive status.
