# Supplementary material for: Collaborative intelligence in AI: Evaluating the performance of a council of AIs on the USMLE
Source: PLOS Digit Health. 2025 Oct 9;4(10):e0000787. doi: 10.1371/journal.pdig.0000787 (PMC12510544; doi:10.1371/journal.pdig.0000787)
Supplement: S2 File — (ZIP) [file pdig.0000787.s002.zip › S2 Data Raw data files containing unprocessed question inputs and LLM outputs including transcripts of deliberations/STEP1_Question-119-1_Type-MC-NJ.docx]

**Facilitator** **[Round 1]:** A 25-year-old woman, gravida 1, para 1, comes to the office because of a 2-week history of palpitations and heat intolerance. She delivered her child 3 months ago following an uncomplicated pregnancy and delivery. She is breast- feeding. She has no history of serious illness and takes no medications. She is 163 cm (5 ft 4 in) tall and weighs 54 kg (120 lb); BMI is 21 kg/m². Temperature is 37°C (98.6°F), pulse is 106/min, respirations are 20/min, and blood pressure is 124/68 mm Hg. Examination shows moist palms and bilateral lid lag. No exophthalmos is noted. The thyroid gland is enlarged and nontender. No murmurs are heard on cardiac examination. Deep tendon reflexes are 3+. Serum studies show an undetectable TSH concentration, thyroxine (T₄) concentration of 20 μg/dL, and triiodothyronine (T₃) concentration of 275 ng/dL. Which of the following is the most likely mechanism of this patient's symptoms? (A) Activation of mutations of TSH receptors (B) Increased serum thyroglobulin concentration (C) Ischemic injury to the hypothalamus (D) Lymphocytic infiltration of the thyroid (E) Presence of TSH receptor autoantibodies

**AI_B:** 1) RESTATING THE QUESTION: This question is asking for the most likely cause of a series of symptoms in a 25-year-old woman who recently gave birth. She is experiencing palpitations and heat intolerance, along with signs of thyroid abnormalities such as an enlarged thyroid gland, undetectable TSH concentration, and high concentrations of thyroxine and triiodothyronine. Terms to understand include TSH (thyroid-stimulating hormone), which regulates the production of thyroid hormones; thyroxine and triiodothyronine, the primary hormones produced by the thyroid gland; and exophthalmos, which is a bulging of the eyes that can be caused by diseases affecting the thyroid. 2) PLANNING: The question is based on the assumption that the patient's symptoms are caused by her recent pregnancy and the changes in hormonal levels associated with it. However, the questioning of each assumption can generate alternative assumptions such as a pre-existing thyroid condition or a newly developed thyroid disorder post-pregnancy. Considering the patient's labs and symptoms, the hyperthyroidism, characterized by an undetectable TSH and elevated thyroid hormones, is most likely driving her symptoms. The priorities lie in understanding the most common causes of hyperthyroidism in a recent postpartum individual. 3) CLINICAL REASONING: Hyperthyroidism can be caused by several conditions, including Graves' disease, toxic nodular goiter, and thyroiditis. Graves' disease typically presents with exophthalmos, which the patient does not have. Toxic nodular goiter usually happens in older individuals and would be less likely in a healthy young woman. However, there's another condition, called postpartum thyroiditis, which commonly presents months after delivery, that initially causes hyperthyroidism due to inflammation and subsequent release of thyroid hormone, followed by a hypothyroid phase as the stored hormones become depleted. The involvement of TSH receptor-activating antibodies is exclusive to Graves' Disease, and the other process listed in the answer choices (mutation of TSH receptors, elevated serum thyroglobulin, ischemic injury to the hypothalamus) may result in hyperthyroidism but are not typically associated with postpartum thyroiditis. 4) CONCLUDING: Given the patient's recent pregnancy, lack of clinical features typical for Graves' disease, and laboratory findings suggestive of hyperthyroidism, postpartum thyroiditis due to lymphocytic infiltration of the thyroid (option D) becomes the most clinically plausible explanation for her symptoms. 5) RESPONSE: The most likely mechanism of this patient's symptoms is (D) Lymphocytic infiltration of the thyroid.

**AI_D:** RESTATING: A patient who had an uncomplicated pregnancy and delivery about three months prior has been experiencing palpitations and heat intolerance for the past two weeks. Her physical examination reveals moist palms, bilateral lid lag, enlarged and nontender thyroid gland, and increased deep tendon reflexes. Lab tests highlight undetectable TSH levels, increased thyroxine, and increased triiodothyronine levels. We were tasked to identify the most likely mechanism causing this clinical presentation. PLANNING: Given the primary healthcare information provided and the symptomatology, the most likely diagnosis is postpartum thyroiditis. However, we need to consider alternative assumptions and mechanisms, such as mutant activation of TSH receptors or some form of ischemic injury to the hypothalamus. While increased serum thyroglobulin concentration plays a role in thyroid conditions, typically the measurement of this marker is used to follow patients with thyroid cancers. Lymphocytic infiltration of the thyroid is seen in Hashimoto's thyroiditis, but this typically presents with hypothyroidism, not the symptoms the patient is displaying. Presence of TSH receptor autoantibodies is a significant indicator, found in patients suffering from Graves' disease - an autoimmune disorder that can result in hyperthyroidism. CLINICAL REASONING: Despite having multiple possibilities to account for hyperthyroidism, the woman's recent pregnancy and the subsequent onset of her symptoms align more closely with postpartum thyroiditis. This disease occurs in approximately 5-10% of all women postpartum and, interestingly, the hyperthyroid phase of postpartum thyroiditis typically occurs from 1 to 6 months following childbirth, which fits with our patient's timeline. Features of hyperthyroidism in this phase can include heat intolerance and palpitations as noted in our patient. Furthermore, the low TSH and relatively high circulating thyroid hormone (both T4 and T3) are consistent with the hyperthyroid phase of postpartum thyroiditis. CONCLUDING: Postpartum thyroiditis presents in phases, with a hyperthyroid phase followed frequently by a hypothyroid phase and then eventual return to normal function. These phases result from an initial thyroid inflammation causing leakage of thyroid hormone resulting in a transient hyperthyroidism (causing the low TSH and high T3 and T4 phase, as seen in this patient), followed by hypothyroidism as the gland has depleted its hormone stores. RESPONSE: Therefore, the most likely mechanism producing this patient's symptoms is (D) Lymphocytic infiltration of the thyroid, which is consistent with the pattern normally seen in postpartum thyroiditis. This inflammatory event in the thyroid gland results in excessive release of stored thyroid hormone, leading to transient hyperthyroidism. Hence, these symptoms are followed by a hypothyroid phase where hormone levels are depleted and must be rebuilt. Most women will ultimately return to normal thyroid function. It is worth mentioning that although Graves' disease also presents with hyperthyroidism, the timing of symptom development following childbirth in this patient, and her lack of exophthalmos, make postpartum thyroiditis much more likely.

**AI_E:** RESTATING THE QUESTION: The information provided introduces a 25-year-old woman experiencing symptoms of palpitations and heat intolerance, 3 months post childbirth. She has no significant personal medical history. Physical and laboratory findings indicate an overproduction of the thyroid hormones T₄ and T₃, and an undetectable TSH level. The underlying cause of these symptoms and findings is being questioned. The possible mechanisms presented for scrutiny include: (A) Activation of mutations of TSH receptors; (B) Increased serum thyroglobulin concentration; (C) Ischemic injury to the hypothalamus; (D) Lymphocytic infiltration of the thyroid, and (E) Presence of TSH receptor autoantibodies. PLANNING: The observed symptoms can be attributed to a hyperactive thyroid state (hyperthyroidism), supported by the high levels of T₄ and T₃ and concurrent suppression of TSH. In the context of the patient’s recent pregnancy, her age, and the presented physical and laboratory findings, the primary assumption would be that she might be experiencing Postpartum Thyroiditis (PPT), an autoimmune condition. Discrepancies in the assumption arise considering the absence of symptoms related to the typically transient hypothyroid phase of PPT. Postpartum thyroiditis usually has a biphasic pattern of thyrotoxicosis followed by hypothyroidism before recovery to the euthyroid state. The possible mechanisms given in the answers warrant careful examination. CLINICAL REASONING: On evaluating the hypotheses, (A) is plausible, predicting that mutated TSH receptors get activated, causing an abnormal increase in thyroid hormone synthesis. Graves' disease, which is known to cause such mutation, is however inconsistent with the observed lack of exophthalmos, a common feature of Graves' disease. Considering the increased T₄ and T₃ levels, (B) suggests that an increased thyroglobulin concentration could be responsible, as thyroglobulin acts as a substrate for these hormones. Nevertheless, this mechanism isn't the primary driver; thyroglobulin concentration increases as a secondary response to high thyroid-stimulating activity rather than being a cause. (C) seems unlikely because an ischemic injury would typically depress function rather than enhance it. (D) Lymphocytic infiltration of the thyroid, while prevalent in Hashimoto’s thyroiditis, doesn't correlate with the presentation; Hashimoto's generally leads to hypothyroidism. Lastly, (E) raises the presence of TSH receptor autoantibodies as a mechanism. In the context of an enlarged, nontender thyroid and suppressed TSH levels against elevated thyroxine and triiodothyronine levels, the presence of such autoantibodies could lead to an overstimulation of thyroid hormone synthesis and release, even in the absence of TSH. CONCLUDING: Analyzing the clinical progression sequentially, a postpartum woman developed palpitations and heat intolerance along with physical and laboratory findings pointing towards hyperthyroidism. This differential consideration process negates the possibilities of conditions caused by TSH receptor mutations, increased thyroglobulin concentration, ischemic hypothalamic injury, or lymphocytic thyroid infiltration. This leaves the probability of the presence of TSH receptor autoantibodies, known to contribute to an aberrant thyroid hormone production even when TSH is suppressed or low. RESPONSE: In this context, the most likely mechanism of this patient's symptoms is (E) Presence of TSH receptor autoantibodies.

**AI_A:** RESTATING THE QUESTION: In this case, we're focusing on a 25-year-old woman who has been experiencing palpitations and increased heat sensitivity for 2 weeks. These symptoms started three months after she gave birth and started breastfeeding. She has an enlarged, non-tender thyroid gland, increased heart rate, and a decrease in her TSH (thyroid-stimulating hormone), but an increase in her T₄ (thyroxine) and T₃ (triiodothyronine) levels. We're trying to figure out the most probable cause for her symptoms from the options given. PLANNING: Looking at her symptoms and test results, we can assume they're related to thyroid function, given the enlargement of her thyroid gland and altered hormone levels. Let's evaluate each listed mechanism. (A) Mutations in TSH receptors could theoretically lead to changes in TSH, T₄, and T₃ levels, but they are not typically associated with childbirth and breastfeeding. (B) Increased thyroglobulin would also cause changes in hormone levels, but it would not explain the physical changes in the thyroid gland. (C) Ischemic injury to the hypothalamus would affect the regulation of thyroid hormones but would not lead to gland enlargement. (D) Lymphocytic infiltration would account for enlargement and hormonal change, however, it's usually painful. (E) Presence of TSH receptor antibodies would not only explain the hormone alterations but also the physical thyroid changes. These antibodies could have been provoked by the immune system resetting after childbirth. CLINICAL REASONING: Generally, postpartum thyroiditis is a two-phase disease that occurs in the first year after delivery or miscarriage. The first, hyperthyroid phase occurs 1-4 months postpartum and lasts for 1-3 months. The diagnosis is made by laboratory testing that shows high levels of free T₄ and T₃, suppressed TSH, and low radioactive iodine uptake by the thyroid gland. The second phase, usually occurring within 5-12 months postpartum, often takes a clinical picture of hypothyroidism. Nonetheless, the first hyperthyroid phase of postpartum thyroiditis can also mimic Graves' disease which is the leading cause of hyperthyroidism and often presents with an enlarged thyroid and ophthalmopathy in the form of exophthalmos and lid lag. However, our said patient does not show exophthalmos. An important distinguishing feature in this case is the absence of the typical pain and tenderness that distinguishes postpartum thyroiditis from subacute thyroiditis. CONCLUDING: Using the above details, while we can somewhat correlate each possible mechanism with the patient's symptoms, the only one that contains all the elements—altered hormone levels, thyroid enlargement, recent pregnancy—is the presence of TSH receptor autoantibodies or option (E). RESPONSE: Given the clinical reasoning and the process of elimination for the presented symptoms and scenarios, the most likely mechanism in this patient's case seems to be the "Presence of TSH receptor autoantibodies" (E). This could have been induced by an immunological reset after childbirth-a situation often linked with postpartum thyroid changes or disorders such as Graves' disease.

**AI_C:** RESTATING THE QUESTION: A woman in her child-bearing years came in complaining of an increased heart rate and not tolerating heat, which she's experienced for the past 2 weeks. She gave birth 3 months prior, experienced no complications during her pregnancy or delivery, and is currently breastfeeding. Her medical history is unremarkable, and she's not currently on any medication. Her body measurements and vitals are within the normal range, except for a slightly high pulse at 106 beats per minute. On physical examination, her palms are moist, she's observed to have bilaterally delayed relaxation of eyelid closure, but there is no protrusion of the eyes. Her thyroid gland is noticeably enlarged but is not painful on examination. No abnormal heart sounds are detected. Her deep tendon reflexes are overactive. Her lab results reveal an undetectable thyroid-stimulating hormone (TSH) level, making it lower than normal, and increased levels of thyroxine (T4) and triiodothyronine (T3), which are thyroid hormones. The question asks about the most probable cause of her symptoms from the options given. PLANNING: The clinical symptoms, physical exam findings, and lab results suggest a state of hyperthyroidism, a condition in which the thyroid gland is overactive and produces excess thyroid hormones T3 and T4, leading to an increased metabolic rate. In this condition, TSH is usually suppressed due to the inhibitory feedback from the high levels of T3 and T4 on the hypothalamic-pituitary-thyroid axis. The options provided as potential causes of this woman's symptoms all edge towards scenarios that can potentially lead to hyperthyroidism. CLINICAL REASONING: Here are the possibilities I am considering based on the provided options: - Activation of mutations of TSH receptors: Mutations in TSH receptors can cause uncontrolled activation of the receptors, leading to excessive thyroid hormone production. However, these are usually associated with hyperfunctioning thyroid nodules and not a generalized enlarged thyroid as seen in this patient. - Increased serum thyroglobulin concentration: Thyroglobulin is a large glycoprotein secreted by the follicular cells of the thyroid that serves as a precursor for thyroid hormone synthesis. An increased thyroglobulin level can indicate thyroid gland damage rather than an overactive thyroid gland, leading to the release of this protein into the bloodstream. - Ischemic injury to the hypothalamus: Ischemia of the hypothalamus can disrupt the proper functioning of the hypothalamic-pituitary-thyroid axis and possibly lead to increased production of releasing hormones. But this possibility is less likely because the patient does not show any other signs of a hypothalamic disorder. - Lymphocytic infiltration of the thyroid: Lymphocytic infiltration suggests an inflammatory or autoimmune process, such as Hashimoto’s thyroiditis. However, this condition usually initially presents with hypothyroidism rather than hyperthyroidism, making this option less likely in this case. - Presence of TSH receptor autoantibodies: The presence of TSH receptor autoantibodies will mimic the effect of TSH, leading to unregulated overproduction of thyroid hormones and a suppressed TSH level due to feedback inhibition. This mechanism is the pathogenesis of autoimmune disorders like Graves’ disease. However, in this patient, the absence of exophthalmos, unique to Graves', makes it unlikely. Given the patient's recent postpartum period, the most probable diagnosis might be postpartum thyroiditis, an autoimmune inflammation of the thyroid after childbirth. This condition typically begins with a hyperthyroid phase, followed by a hypothyroid phase before a return to euthyroid state. Known pathophysiology involves the rebound of the immune system after its relative immuno-suppression during pregnancy, which may lead to autoimmunity against a variety of tissues, including endocrine glands like the thyroid. In fact, lymphocytic infiltration is a characteristic histopathological finding in postpartum thyroiditis, pertaining to the rebound autoimmunity. CONCLUDING: Though the presence of TSH receptor autoantibodies and activation of mutations of TSH receptors can also lead to hyperthyroidism, these scenarios are far less likely, given her clinical setting, which uniquely fits with postpartum thyroiditis. Therefore, lymphocytic (autoimmune) infiltration of the thyroid, an indication of postpartum thyroiditis, is likely the most potential cause of her hyperthyroid symptoms. RESPONSE: (D) Lymphocytic infiltration of the thyroid is the most likely mechanism of this patient's symptoms.

**Facilitator** **[Round 2]:** SUMMARIES: AI_A: The council member believes that the patient's symptoms are most likely due to the presence of TSH receptor autoantibodies (option E). They argue that this could have been induced by an immunological reset after childbirth, a situation often linked with postpartum thyroid changes or disorders such as Graves' disease. AI_B: This council member suggests that the most likely cause of the patient's symptoms is lymphocytic infiltration of the thyroid (option D). They argue that the patient's recent pregnancy, lack of clinical features typical for Graves' disease, and laboratory findings suggestive of hyperthyroidism point towards postpartum thyroiditis. AI_C: Similar to AI_B, AI_C also believes that the most likely cause of the patient's symptoms is lymphocytic infiltration of the thyroid (option D). They argue that the patient's recent postpartum period and the absence of exophthalmos, unique to Graves', make postpartum thyroiditis the most probable diagnosis. AI_D: This council member also agrees with AI_B and AI_C that the most likely cause of the patient's symptoms is lymphocytic infiltration of the thyroid (option D). They argue that the timing of symptom development following childbirth in this patient, and her lack of exophthalmos, make postpartum thyroiditis much more likely. AI_E: Contrary to the other council members, AI_E believes that the most likely cause of the patient's symptoms is the presence of TSH receptor autoantibodies (option E). They argue that the presence of such autoantibodies could lead to an overstimulation of thyroid hormone synthesis and release, even in the absence of TSH. QUESTION TO CLARIFY DISAGREEMENTS: Given that the majority of council members believe that the most likely cause of the patient's symptoms is lymphocytic infiltration of the thyroid (option D), while two members believe it is the presence of TSH receptor autoantibodies (option E), could the council members elaborate on the key factors that led them to their respective conclusions? Specifically, could they discuss the role of TSH receptor autoantibodies and lymphocytic infiltration in the context of postpartum thyroid changes and how these mechanisms might present differently in a patient?. Based on your preceding response, how would you answer the original question: [A 25-year-old woman, gravida 1, para 1, comes to the office because of a 2-week history of palpitations and heat intolerance. She delivered her child 3 months ago following an uncomplicated pregnancy and delivery. She is breast- feeding. She has no history of serious illness and takes no medications. She is 163 cm (5 ft 4 in) tall and weighs 54 kg (120 lb); BMI is 21 kg/m². Temperature is 37°C (98.6°F), pulse is 106/min, respirations are 20/min, and blood pressure is 124/68 mm Hg. Examination shows moist palms and bilateral lid lag. No exophthalmos is noted. The thyroid gland is enlarged and nontender. No murmurs are heard on cardiac examination. Deep tendon reflexes are 3+. Serum studies show an undetectable TSH concentration, thyroxine (T₄) concentration of 20 μg/dL, and triiodothyronine (T₃) concentration of 275 ng/dL. Which of the following is the most likely mechanism of this patient's symptoms? (A) Activation of mutations of TSH receptors (B) Increased serum thyroglobulin concentration (C) Ischemic injury to the hypothalamus (D) Lymphocytic infiltration of the thyroid (E) Presence of TSH receptor autoantibodies]

**AI_A:** RESPONSE: Before answering the question, it's crucial to understand the mechanisms underlying the options provided. Postpartum thyroiditis, favored by the majority of the council members, is characterized by lymphocytic infiltration of the thyroid (option D). This is a form of destructive thyroiditis, where inflammatory damage leads to unregulated release of preformed thyroid hormone, causing the hyperthyroid phase. In contrast, for those who favored the presence of TSH receptor autoantibodies (option E), as in Graves' disease, the pathophysiology involves stimulating antibodies against the TSH receptor on thyrocytes, resulting in unregulated overproduction of thyroid hormones. Considering the clinical presentation of our patient, the only distinctive sign of Graves’ disease present is bilateral lid lag while classic symptoms such as exophthalmos are absent. While some Graves' disease cases may lack exophthalmos, our patient's recent delivery supports a diagnosis of postpartum thyroiditis, which often manifests within one year postpartum and features hyperthyroidism followed by a hypothyroid phase before eventual recovery to a euthyroid state. In response to the original question, lymphocytic infiltration of the thyroid (option D) seems to be the most likely mechanism of this patient's symptoms given the context of her recent delivery and absence of hallmark Graves’ symptoms. However, this would ideally be confirmed through further diagnostic evaluation, which could include anti-TPO antibody testing or perhaps a thyroid ultrasound, to distinguish between these two differential diagnoses.

**AI_D:** 1) RESTATING THE QUESTION: This clinical scenario involves a 25-year-old woman, who recently gave birth and is now experiencing hyperthyroid symptoms, such as palpitations and heat intolerance. She has physical signs of hyperthyroidism and laboratory findings of an undetectable TSH level and elevated T₃ and T₄ levels. The question is asking for the most likely underlying mechanism causing these symptoms and findings, considering five options: mutations triggering TSH receptors (A), elevated serum thyroglobulin concentrations (B), ischemic damage to the hypothalamus (C), lymphocytic infiltration into the thyroid (D), or presence of TSH receptor autoantibodies (E). 2) PLANNING: This lady is manifesting signs of hyperthyroidism in the postpartum period, a time when thyroiditis is common. Option D, involving lymphocytic infiltration of the thyroid, can cause postpartum thyroiditis, a condition frequently seen during the first year after childbirth, often starting with a hyperthyroid phase. Simultaneously, option E, involving TSH receptor autoantibodies, can lead to Graves' disease, another possibility characterized by hyperthyroidism, and sometimes seen postpartum. Notably, Graves' disease often features exophthalmos, which this patient does not have. 3) CLINICAL REASONING: Both postpartum thyroiditis and Graves' disease can present with an overactive thyroid, exhibits signs such as tachycardia, heat intolerance, and a large, nontender thyroid gland. However, the timing of symptom onset, typically within a few months after delivery, and the lack of exophthalmos in the patient's clinical portrait, prompt a more plausible assumption towards postpartum thyroiditis. This condition is an autoimmune disorder where the patient's immune system mistakenly attacks the thyroid gland, causing lymphocytic infiltration, leading to temporary hyperthyroidism. 4) CONCLUDING: On weighing the clinical presentation, timing, and distinctive features of each condition, it appears more probable that this patient is experiencing hyperthyroid symptoms due to postpartum thyroiditis rather than Graves' disease. Therefore, the lymphocytic infiltration of the thyroid appears to be the most likely mechanism of this patient's symptoms. 5) RESPONSE: Hence, considering her presentation, symptom onset following childbirth, thyroid gland enlargement, absence of exophthalmos, hyperthyroid laboratory findings, and common postpartum thyroid conditions, 'lymphocytic infiltration of the thyroid' (D) is the most likely mechanism of her symptoms.

**AI_E:** RESTATING THE QUESTION: The question is asking for the most probable cause behind the presenting symptoms in a 25-year-old woman who has recently given birth. She presents with, among other features, an enlarged and nontender thyroid, a palpable increase in heart rate (palpitations), and an intolerance to heat—all indicating a hyperthyroid state. Her blood work shows suppressed or undetectable levels of TSH, thyroxine (T₄) concentration of 20 μg/dL, and triiodothyronine (T₃) concentration of 275 ng/dL. TSH or Thyroid Stimulating Hormone is normally responsible for stimulating the thyroid to produce and release thyroid hormones (T3 and T4). The clinical features, her recent pregnancy, and blood test results require an explanation. PLANNING: The key clinical features are increased heart rate (palpitations), heat intolerance, a noticeable swelling in the thyroid gland, suppressed TSH level, and increased T3 and T4 levels. The options provided all connect to thyroid function, but with different underlying mechanisms. We need to examine each option in light of the patient's presentation and her recent childbirth. CLINICAL REASONING: Interestingly, the patient has a suppressed TSH level, indicating minimal to no stimulus to thyroid for producing T3 and T4. However, the presence of high T3 and T4 points at an alternative stimulation pathway. Options (A), (E) indicate direct stimulation of TSH receptors, bypassing the need of TSH itself. Options (B), (D) indicate thyroid pathologies causing hyperthyroidism. Option (C) implies a loss of upstream control on TSH release. The absence of serious illnesses, recent pregnancy, and typical clinical signs (excluding exophthalmos) guide us towards an auto-immune cause. This narrows our options to either Graves' disease, linked with presence of TSH receptor autoantibodies (E), or postpartum thyroiditis, associated with lymphocytic infiltration (D). Although both could present similarly, lack of exophthalmos, a signature sign of Graves', along with recent childbirth, makes postpartum thyroiditis a more fitting explanation. CONCLUDING: Given the facts, it seems more plausible that the patient is experiencing postpartum thyroiditis, characterized by an initial hyperthyroid state resulting from the structural damage to the thyroid gland due to lymphocytic infiltration. This would allow for thyroid hormone to leak into the circulation, causing transient hyperthyroidism even in the absence of TSH stimulation. RESPONSE: Therefore, in response to the original question, the most likely mechanism of this patient's symptom is (D) Lymphocytic infiltration of the thyroid.

**AI_C:** 1) RESTATING THE QUESTION: Our patient is a 25-year-old woman with recent palpitations and heat intolerance. She gave birth three months ago, is currently breastfeeding, and has no prior history of significant illness or medication use. Her thyroid gland is enlarged and non-tender, with no exophthalmos. Laboratory findings show elevated thyroxine (T4) and triiodothyronine (T3) concentrations with undetectable TSH levels. The question asks us to identify the most likely cause for these findings, given the options of TSH receptor mutations, high thyroglobulin levels, hypothalamic injury, lymphocytic thyroid infiltration, or TSH receptor autoantibodies. 2) PLANNING: The primary assumption is that the patient's symptoms are due to hyperthyroidism, evidenced by elevated T4 and T3 levels, palpitations, heat intolerance, and an enlarged thyroid gland. Alternate assumptions can be drawn from each of the provided options. If we evaluate each assumption based on relevance and typical clinical presentations, our clinical priority would be to differentiate between postpartum thyroiditis, typically characterized by lymphocytic infiltration of the thyroid, versus Graves' disease, known to involve TSH receptor autoantibodies. 3) CLINICAL REASONING: Postpartum thyroiditis typically occurs within the first year following childbirth and can present with hyperthyroid symptoms, similar to what we observe in our patient. However, it is notably an inflammatory condition often characterized by painless swelling or lymphocytic infiltration of the thyroid. Conversely, Graves' disease, a common cause of hyperthyroidism, is caused by autoantibodies against the TSH receptor, leading to increased synthesis and release of thyroid hormones. A key distinguishing feature of Graves' disease is exophthalmos, which our patient lacks. 4) CONCLUDING: Based on the timing of symptom onset post-childbirth, the absence of pain (likely indicating inflammation rather than infection or injury), and the lack of exophthalmos, I consider postpartum thyroiditis to be the more probable cause of this patient's symptoms. The likely pathological mechanism in this scenario would be lymphocytic infiltration of the thyroid. 5) RESPONSE: Given the patient's history, clinical presentation, and laboratory values, the most probable mechanism behind her symptoms is lymphocytic infiltration of the thyroid (option D). Though autoantibodies targeting the TSH receptor (option E) can cause similar symptoms, the lack of exophthalmos and the postpartum time frame support lymphocytic infiltration, characteristic of postpartum thyroiditis, as the most likely cause.

**AI_B:** COMPLETING THE TASK: In the clinical scenario presented, this 25-year-old woman, who's a new mother three months postpartum, has been experiencing distressing symptoms like palpitations and heat intolerance. She has no prior history of serious disease and takes no medications. Her physical examination reveals some significant clinical findings including tachycardia (fast heart rate), moist palms, bilateral lid lag, an enlarged but non-tender thyroid gland, and brisk deep tendon reflexes, without any notable ophthalmopathy. Blood tests also selectively report an undetectable TSH level, and elevated levels of thyroid hormones T₄ and T₃, corroborating the clinical symptoms of hyperthyroidism. Beginning with an exploration of possible diagnostic elements, the symptoms and findings seen in this patient are quite compatible with a thyroid disease, that's evoking an abnormal increase in her metabolism due to excessive thyroid hormone production and release, a condition known as thyrotoxicosis. Hyperthyroidism describes the overactive thyroid gland which is the cause of thyrotoxicosis. This could potentially stem from a diversity of events or conditions, even beyond the options provided. In considering the choices, for (A), a mutation of TSH receptors is unlikely as the patient has no prior history of this rare genetic disorder and such a sudden postpartum onset isn't typical for it. Additionally, the sweatiness observed is a common feature of thyroid disease but doesn't specifically point towards this option. For (B), while a thyroglobulin rise can occur in different thyroid disorders, there aren't explicit grounds to attribute it as the cause in this case. Regarding (C), this woman doesn't display any evidence of a recent event of severe hypoxia, trauma, bleeding, or of typical hypothalamic dysfunction that could indicate such brain injury, making it unlikely. Between the remaining options, (D) and (E), for (D), lymphocytic infiltration of the thyroid is synonymous with a thyroiditis caused by an autoimmune response (often represented by Hashimoto's thyroiditis), which can lead to both hypothyroidism and hyperthyroidism at different stages, but this condition doesn't usually present or exacerbate postpartum, and there isn't an accompanying goiter (enlarged thyroid) reported. Conversely, for (E), a TSH receptor autoantibody presence is indicative of Grave's disease, an autoimmune hyperthyroidism characterized by such antibodies that mimic TSH, leading to unregulated synthesis and secretion of thyroid hormones. Although this disease also involves ophthalmopathy (exophthalmos) due to similar infiltration in tissues behind the eyes, not all patients present it, and this condition often involves other noticeable features like dermopathy or acropachy. However, considering the patient's recent postpartum status, neither Grave's nor Hashimoto's being regulated by pregnancy is a usual occurrence. Instead, the onset of thyroid disorders following an uncomplicated, term birth could be related to a Postpartum Thyroiditis, an inflammation of the thyroid which typically occurs within a year after delivery. This condition can initially present as a transient thyrotoxic phase (for a few weeks to 2 months) due to the leakage of preformed thyroid hormones from the inflamed thyroid into circulation, followed by a hypothyroid phase lasting about a year. The signs and symptoms during the hyperthyroid phase could range from being minimal (or even asymtomatic) to severe forms mimicking Grave's disease, with common symptoms including palpitations, fatigue, weight loss, and nervousness, and sometimes, as seen here, a painless thyroid enlargement without any other Graves-associated features. This is often seen in women with a previous history of autoimmune thyroid disease or other immune disorders, and has also been linked to the presence of autoantibodies against thyroid peroxidase. In conclusion, therefore, while both (D) lymphocytic infiltration of the thyroid, and (E) presence of TSH receptor autoantibodies could be plausible in triggering these signs and symptoms, a postpartum thyroiditis precipitated by (D) lymphocytic infiltration of the thyroid seems the most likely underlying mechanism in this patient. Thus, for the clinical question asked, I would select (D) as the most likely answer. However, further tests including anti-thyroid peroxidase antibodies assessment, thyroid scintigraphics, and possibly a follow-up consultation in a few weeks' time should be taken into account for a comprehensive evaluation.

**Facilitator** The consensus among the AI responses is that the most likely mechanism of the patient's symptoms is lymphocytic infiltration of the thyroid (option D). This conclusion is based on the patient's clinical presentation, which includes palpitations, heat intolerance, an enlarged and non-tender thyroid gland, and laboratory findings of suppressed TSH levels with elevated T₄ and T₃ concentrations. These symptoms and findings are indicative of a hyperthyroid state. The patient's recent childbirth is a significant factor in this diagnosis. Postpartum thyroiditis, which often manifests within the first year after delivery, is characterized by an initial hyperthyroid phase due to the unregulated release of preformed thyroid hormone caused by inflammatory damage to the thyroid gland. This is followed by a hypothyroid phase before eventual recovery to a euthyroid state. The lymphocytic infiltration of the thyroid associated with postpartum thyroiditis aligns with the patient's clinical presentation and the timing of symptom onset. While the presence of TSH receptor autoantibodies (option E), as seen in Graves' disease, could also cause similar symptoms, the absence of exophthalmos, a hallmark symptom of Graves' disease, and the postpartum timeframe make this diagnosis less likely. However, further diagnostic evaluation, including anti-TPO antibody testing or a thyroid ultrasound, would ideally be conducted to confirm the diagnosis and distinguish between these two differential diagnoses.
