# Supplementary material for: Collaborative intelligence in AI: Evaluating the performance of a council of AIs on the USMLE
Source: PLOS Digit Health. 2025 Oct 9;4(10):e0000787. doi: 10.1371/journal.pdig.0000787 (PMC12510544; doi:10.1371/journal.pdig.0000787)
Supplement: S2 File — (ZIP) [file pdig.0000787.s002.zip › S2 Data Raw data files containing unprocessed question inputs and LLM outputs including transcripts of deliberations/STEP1_Question-12-1_Type-MC-NJ.docx]

**Facilitator** **[Round 1]:** A 62-year-old man comes to the physician because of a 6-month history of urinary hesitancy and dribbling after urination. He has to urinate two to three times nightly. Physical examination shows a diffusely enlarged, firm, and nontender prostate. Which of the following is most likely to have contributed to the development of this patient's condition? (A) Activation of the α1-adrenergic receptor (B) Conversion of testosterone to dihydrotestosterone (C) Conversion of testosterone to estradiol (D) Inhibition of the α1-adrenergic receptor (E) Production of prostate-specific antigen

**AI_A:** 1) RESTATING THE QUESTION: The patient, a 62-year-old man, is experiencing symptoms pointing towards potential urinary tract issues, such as a diffusely enlarged, firm, and nontender prostate, as well as urinary hesitancy, dribbling after urination, and frequent urination during the night. The query concerns which of the given options is most likely the contributing factor to this patient's condition. 2) PLANNING: The first step in identifying key assumptions is to note the patient's symptoms, which seem indicative of benign prostatic hyperplasia (BPH). BPH is a condition often associated with aging in men, characterized by an enlarged prostate gland that can lead to urinary symptoms. This condition is primarily driven by the hormonal changes in men as they age, particularly changes in the testosterone and estradiol levels. Possible assumptions include (A) Activation of the α1-adrenergic receptor, (B) Conversion of testosterone to dihydrotestosterone, (C) Conversion of testosterone to estradiol, (D) Inhibition of the α1-adrenergic receptor, (E) Production of prostate-specific antigen. Each potential assumption needs to be examined and connected to the available evidence and existing knowledge about BPH. 3) CLINICAL REASONING: Activation and inhibition of the α1-adrenergic receptor in (A) and (D) are associated with contraction and relaxation of prostate smooth muscle, respectively, and these processes are related to urinary symptoms rather than prostate enlargement. Production of prostate-specific antigen (E) is a consequence, not a cause, of BPH. Estradiol (C), while affecting the prostate, doesn't contribute to the prostate enlargement as much as dihydrotestosterone does. The conversion of testosterone to dihydrotestosterone in the prostate gland by the enzyme 5α-reductase (B) is a major component in the pathogenesis of BPH. Dihydrotestosterone acts within the prostate gland to induce growth of prostate cells, leading to prostate enlargement. 4) CONCLUDING: From the analysis of these possible contributors, we can conclude that the most plausible factor that could have contributed towards the progression of this patient's condition is option B: the Conversion of testosterone to dihydrotestosterone. 5) RESPONSE: The most likely contributor to the development of this patient's condition, based on his symptoms and the provided options, is (B) Conversion of testosterone to dihydrotestosterone.

**AI_E:** (1) RESTATING THE QUESTION: The case presents a 62-year-old man with urinary symptoms such as hesitancy, dribbling post-urination, and increased nocturnal frequency (needing to urinate two to three times at night). His physical examination reveals diffusely enlarged, firm, and painless prostate. The query is about the prime factor that likely contributed to the development of this patient's clinical state. In the options, we see biological processes associated with hormones and neurotransmitters crucial for prostate function and growth. (2) PLANNING: The assumptions here are that these symptoms indicate a disorder of the prostate, likely benign prostatic hyperplasia (BPH) given the age of the patient and symptoms. BPH involves prostate enlargement that can impinge on the urethra leading to voiding symptoms. We could question which biological mechanism(s) contribute most directly to the development of BPH. The clinical priority is to establish the major contributing factor from the options given, which all have various influences on prostate physiology. (3) CLINICAL REASONING: Activation of α1-adrenergic receptor (Option A) and production of prostate-specific antigen (Option E) are associated with prostate physiology and pathology, but they may not directly result in prostate growth. Inhibition of α1-adrenergic receptor (Option D) can ease urinary symptoms, but it does not contribute to prostate enlargement itself. Conversion of testosterone to estradiol (Option C) can be involved in prostatic diseases, but its role in BPH is less significant compared to the role of dihydrotestosterone, which is a stronger, more biologically active form of testosterone. (4) CONCLUDING: With these factors in mind, it appears that conversion of testosterone to dihydrotestosterone (Option B) is the most likely contributing factor to the development of this patient's condition. Dihydrotestosterone is produced in the prostate by the enzymatic action of 5-α-reductase on testosterone. It has a key role in prostatic cell proliferation and growth, and therefore is essential in the pathophysiology of BPH. (5) RESPONSE: Therefore, the most likely contributing factor to the development of this patient's condition is the conversion of testosterone to dihydrotestosterone (Option B).

**AI_C:** (1) RESTATING THE QUESTION: The issue at hand revolves around a 62-year-old male who is experiencing urinary difficulties, specifically hesitancy and dribbling after urination, along with needing to urinate multiple times during the night. Upon physical examination, it's determined that he has a notably enlarged, dense, and nonpainful prostate. From a list of options, we are required to identify the most likely factor that could contribute to the development of this patient's condition. (2) PLANNING: The patient's symptoms and the physical examination results point towards the clinical spectrum of Benign Prostatic Hyperplasia (BPH). The potential causes of this condition cover a range of biochemical processes, such as the manipulation of testosterone and the activation or inhibition of specific receptors, to the production of specific antigens. (3) CLINICAL REASONING: The α1-adrenergic receptors when activated can cause the contraction of prostate smooth muscle and can contribute to symptoms of BPH but not to the development of BPH (that is, gland overgrowth) itself. Inhibition of these receptors .. with α1 blockers .. reduce prostatic smooth muscle tone and thus alleviate symptoms of BPH but it does not influence the development of BPH either. The conversion of testosterone to estradiol does not prominently influence the development of BPH. Production of prostate-specific antigen is a consequence of BPH and not a cause for it. Now, the conversion of testosterone to dihydrotestosterone (DHT) by the enzyme 5α-reductase located within the prostate gland is indeed an important element in prostate physiology. DHT is an androgen hormone and is more potent than testosterone. It acts locally and causes the prostate to increase in size. Hence, the pathophysiology of BPH is related to the local effect of DHT. (4) CONCLUDING: Consequently, based on the patient's symptoms and the link between dihydrotestosterone and prostate physiology, it would appear that the enlargement of the prostate and the resulting issues with urination could be associated with the conversion of testosterone to dihydrotestosterone. (5) RESPONSE: Thus, the correct answer is (B) Conversion of testosterone to dihydrotestosterone.

**AI_D:** RESTATING THE QUESTION: The question is asking what factor has primarily contributed to the patient's condition. The patient is a 62-year-old man who presented with urinary hesitation and dribbling, frequent nocturnal urination, and a physical examination confirming a diffusely enlarged, firm, and non-tender prostate. The options provided are all related to hormonal activities or signaling, suggesting that alterations in hormonal dynamics, prostate-specific antigen production, or receptor signaling associated with the prostate might be implicated in his condition. PLANNING: The assumption here is that one of these hormonal activities or processes is responsible for the patient's symptoms. The specific changes relevant to an enlarged prostate could include activation or inhibition of the α1-adrenergic receptor, which is associated with contraction of the prostate and bladder neck muscles, impacting urinary flow. Another assumption could be the conversion of testosterone to either dihydrotestosterone or estradiol. Dihydrotestosterone is the androgen responsible for prostate enlargement. Alternatively, the patient's enlarged prostate could be due to increased production of prostate-specific antigen (PSA), a protein produced by cells of the prostate gland. Each assumption will be evaluated against the patient's clinical picture. CLINICAL REASONING: The provided clinical picture fits findings commonly associated with benign prostatic hyperplasia (BPH), a condition common in men as they age. In considering each alternative, although adrenergic receptor activation or inhibition alters urinary flow, they wouldn't directly cause prostate enlargement. The production of PSA is increased in BPH, but it is more a result rather than a cause. Conversion of testosterone to estradiol doesn't directly lead to prostate enlargement. However, the conversion of testosterone to dihydrotestosterone, catalyzed by the enzyme 5α-reductase, is a key element in the pathophysiology of BPH. Dihydrotestosterone leads to prostatic cell proliferation and thus contributes to prostate enlargement. CONCLUDING: Therefore, based on the patient's age, presentation, and physical examination, the underlying cause of his condition which is most consistent with BPH is likely due to the conversion of testosterone to dihydrotestosterone. RESPONSE: The most likely factor contributing to the development of this patient's condition is (B) Conversion of testosterone to dihydrotestosterone.

**AI_B:** RESTATING THE QUESTION: Our patient appears to have symptoms of benign prostatic hyperplasia (BPH), an age-related enlargement of the prostate, which is causing urinary symptoms. We are asked to determine what physiological process most likely contributed to his condition. The given choices seem to focus on hormonal mechanisms and production of specific compounds related to the prostate. PLANNING: We can reason that the prostate's enlargement in BPH is influenced by hormonal control, more specifically androgens. The initiation and maintenance of prostate growth is governed by the interplay of androgens, their receptors, and the locally produced compounds. Hence, we could logically dismiss the options that related to α1-adrenergic receptors because they play a role in prostate smooth muscle contraction and thereby factor in symptom progression but not in the actual enlargement of the prostate. This leaves us with options related to hormones and prostate-specific antigen. CLINICAL REASONING: Upon closer examination, we can also eliminate the option related to the production of prostate-specific antigen. Although it is produced in increased amounts in both BPH and prostate cancer, it is a marker and not known to contribute to the development of these conditions. This brings us to the role of hormones in the development of BPH. The key lies in understanding the conversion of testosterone. In the prostate, testosterone is converted into a more potent androgen, dihydrotestosterone (DHT), by the enzyme 5α-reductase. DHT is the major intraprostatic androgen and is essential in the development and growth of the prostate gland. CONCLUDING: Therefore, among the given options, the process of converting testosterone to dihydrotestosterone (DHT) plays a crucial role in the development of BPH which manifests as increased prostate size causing this patient's urinary symptoms. RESPONSE: The most likely contributing factor to the development of this patient's condition is (B) Conversion of testosterone to dihydrotestosterone.

**Facilitator** The consensus statement synthesizes the responses of AI_A, AI_B, AI_C, AI_D, and AI_E as follows: The patient, a 62-year-old man, presents with symptoms indicative of benign prostatic hyperplasia (BPH), including urinary hesitancy, dribbling after urination, and frequent nocturnal urination. His physical examination reveals a diffusely enlarged, firm, and non-tender prostate. The question asks us to identify the most likely contributing factor to the development of this patient's condition from a list of options related to hormonal activities, receptor signaling, and antigen production. The patient's symptoms and the physical examination results suggest BPH, a condition often associated with aging in men and characterized by an enlarged prostate gland. The development and growth of the prostate gland are influenced by the interplay of androgens, their receptors, and locally produced compounds. The options related to the activation or inhibition of the α1-adrenergic receptor are associated with contraction and relaxation of prostate smooth muscle, respectively. These processes are related to urinary symptoms rather than prostate enlargement. The production of prostate-specific antigen is a consequence, not a cause, of BPH. The conversion of testosterone to estradiol, while affecting the prostate, doesn't contribute to the prostate enlargement as much as dihydrotestosterone does. The conversion of testosterone to dihydrotestosterone in the prostate gland by the enzyme 5α-reductase is a major component in the pathogenesis of BPH. Dihydrotestosterone is a potent androgen hormone that acts within the prostate gland to induce growth of prostate cells, leading to prostate enlargement. Therefore, the consensus among the AI responses is that the most likely contributing factor to the development of this patient's condition, based on his symptoms and the provided options, is the conversion of testosterone to dihydrotestosterone.
