# Supplementary material for: Collaborative intelligence in AI: Evaluating the performance of a council of AIs on the USMLE
Source: PLOS Digit Health. 2025 Oct 9;4(10):e0000787. doi: 10.1371/journal.pdig.0000787 (PMC12510544; doi:10.1371/journal.pdig.0000787)
Supplement: S2 File — (ZIP) [file pdig.0000787.s002.zip › S2 Data Raw data files containing unprocessed question inputs and LLM outputs including transcripts of deliberations/STEP1_Question-13-1_Type-MC-NJ.docx]

**Facilitator** **[Round 1]:** An 18-year-old woman with sickle cell disease is brought to the emergency department by her parents because of a 2-hour history of severe abdominal pain and nausea. Her parents say that she had a cheeseburger, milk shake, and chocolate bar for lunch. Her temperature is 37°C (98.6°F). Physical examination shows tenderness over the right upper quadrant of the abdomen, radiating to the right shoulder. Ultrasonography of the right upper quadrant of the abdomen shows gallstones. Which of the following is the most likely underlying cause of this patient's current condition? (A) Decreased hepatic secretion of lecithin (B) Decreased reabsorption of bile salts (C) High ratio of cholesterol to bile acids in bile (D) Infestation with parasites secreting β-glucuronidase (E) Overload of unconjugated bilirubin

**AI_B:** RESTATING THE QUESTION: The given scenario describes an 18-year-old female with pre-existing sickle cell disease presenting with recent-onset severe abdominal pain, nausea, and findings suggestive of gallbladder pathology upon examination and ultrasonography. The question asks what the most probable cause for these symptoms is from the given options, which range from altered bile composition to parasitic infection. PLANNING: The identified assumptions include the premise of gallstones being related to her sickle cell disease, that her meal directly led to this event, and that her abdominal pain might be caused by something other than gallstones. Alternative assumptions may include the idea that the gallstones could be incidental findings unrelated to her sickle cell disease. Or that her diet caused an acute change in the bile composition, leading to her current symptoms. Lastly, she could also be suffering from another abdominal pathology unrelated to the gallstones. Upon review, the assumption most likely to be pursued clinically would involve her sickle cell disease's connection to the development of gallstones. CLINICAL REASONING: Sickle cell disease is a genetic condition where abnormal hemoglobin (HbS) causes red blood cells to become rigid, sticky, and sickle-shaped. These cells can block blood vessels, causing episodes known as pain crises. Additionally, the sickled cells are prone to hemolysis, the breakdown of red blood cells, leading to an increase in bilirubin levels. This overloaded bilirubin can accumulate in the gallbladder and precipitate into gallstones. Pain and tenderness in the right upper quadrant can suggest complications from gallstones, such as acute cholecystitis. While the patient's consumption of a fat-rich meal may cause the gallbladder to contract, it is unlikely the root cause of her condition as gallstones typically take time to form. CONCLUDING: Given the patient’s sickle cell disease, a chronic condition marked by increased bilirubin level due to excessive hemolysis, it is reasonable to prioritize the assumption that her presentation might be due to complications from gallstones due to bilirubin overload in the gallbladder. RESPONSE: Hence, the most likely explanation for the patient's current condition would be an "Overload of unconjugated bilirubin" (E). The other given options are less probable in this case due to the clear connection between sickle cell disease, increased bilirubin levels, and the formation of gallstones.

**AI_E:** RESTATING THE QUESTION: We are asked to help diagnose the most probable underlying cause of the symptoms exhibited by an 18-year-old patient with sickle cell disease. Her symptoms include severe abdominal pain, nausea, and tenderness in the right upper portion of her abdomen, subsequently confirmed as gallstones by an ultrasonography. The answer choices present possibilities related to various disruptions or variants in bile production and utilization: lecithin secretion, bile salt reabsorption, cholesterol to bile acids ratio in bile, presence of parasites, and overload of unconjugated bilirubin. PLANNING: Let's examine our assumptions here. One, that the symptoms and gallstones are directly linked to her lunch consumption, two, that her existing sickle cell disease could be contributing to her condition, and three, that the attendant pain is mainly caused by the highlighted abnormalities in her bile system. A possible alternate assumption is that the sickle cell disease could have effected gallbladder function, indirectly causing the gallstones. CLINICAL REASONING: Sickle cell disease causes an increase in the turnover of red blood cells. This increase in red blood cell turnover leads to an overload of unconjugated bilirubin, which is a byproduct of the breakdown of red blood cells. Given the patient's sickle cell condition, this overload might further convert into bilirubin gallstones in the gallbladder, accounting for our ultrasonography results. CONCLUDING: The symptoms this young patient is showing, coupled with her existing sickle cell disease and identified gallstones, point us towards gallbladder compromise. This is likely due to an excess of unconjugated bilirubin produced by her body's increased breakdown of red blood cells. RESPONSE: Thus, the most likely underlying cause of this patient's current condition is an (E) Overload of unconjugated bilirubin.

**AI_C:** RESTATING THE QUESTION: To simplify, the inquiry is seeking the most likely cause for the formation of gallstones in an eighteen-year-old woman with sickle cell disease who's been experiencing severe abdominal pain and nausea for the past 2 hours. Gallstones are hard, pebble-like deposits that form inside the gallbladder. PLANNING: In understanding the causes of gallstones, it's crucial to consider that there are two types - cholesterol stones and pigment stones. Sick cell disease often causes increased turnover of red blood cells, leading to the production of pigmented gallstones; this might be the case with our patient. We might also consider the intake of high-fat foods, which are associated with cholesterol stones, though this seems to be an acute instance, not necessarily a long-term dietary pattern. Both assumption areas should be further dissected. CLINICAL REASONING: Starting with the implications of sickle cell disease on gallstone formation, this condition causes hemolysis, or the breakdown of red blood cells. Hemolysis boosts the level of bilirubin, a component of red blood cells, in the bloodstream. Consequently, this overwhelming bile composition can lead to unconjugated bilirubin precipitation, hence the formation of pigment gallstones. In terms of cholesterol gallstone formation, any imbalance in the composition of bile, mainly a high cholesterol to bile acid ratio, could lead to supersaturation and eventual precipitation of cholesterol stones. However, in the case of the patient, no history of prolonged high-fat diet intake, which could promote such an imbalance, was reported. CONCLUDING: Given the patient's sickle cell disease condition, the rate of red blood cell breakdown is high, leading to high levels of bilirubin in the bloodstream that can cause the formation of pigment gallstones. In contrast, the high-fat diet eaten prior to this episode was most likely incidental - and an acute occurrence - rather than a long-term pattern that could lead to cholesterol gallstone formation. RESPONSE: Hence, the most likely underlying cause of this patient's current condition is (E) Overload of unconjugated bilirubin.

**AI_D:** 1) RESTATING THE QUESTION: The question is asking us to identify the most likely underlying cause behind the development of gallstones in an 18-year-old woman with sickle cell disease who is experiencing severe abdominal pain along with nausea. Gallstones are caused by the super saturation and subsequent precipitation of certain substances in the bile. The possible options indicate disruptions in the balance of these substances. 2) PLANNING: The presence of gallstones can be the result of various underlying causes detailed in options A through E. Option A, decreased hepatic secretion of lecithin, B, decreased reabsorption of bile salts, and C, high ratio of cholesterol to bile acids in bile, all revolve around abnormalities in the composition of bile. Option D, infestation with parasites secreting β-glucuronidase, suggests an infection. Option E, overload of unconjugated bilirubin, speaks to disruption in the metabolism or processing of bilirubin. In this case, with the patient's history of sickle cell disease, we need to consider the special physiological/pathophysiological implications associated with that condition. 3) CLINICAL REASONING: Sickle cell disease is a genetic condition that affects the shape and function of the hemoglobin in red blood cells, leading to hemolytic anemia. Hemolysis leads to increased turnover of hemoglobin, and subsequently, increased production of bilirubin which is the degradation product of heme. The hepatocytes conjugate the bilirubin and it is excreted in the bile. In chronic hemolysis, such as in sickle cell disease, the liver may not be able to adequately conjugate the increased amount of bilirubin produced, leading to accumulation of unconjugated bilirubin, some of which can precipitate in the gallbladder leading to black pigment stones. 4) CONCLUDING: Given the pathophysiology of sickle cell disease and the presentations of chronic hemolysis, it's reasonable to conclude that the increased hemoglobin turnover and subsequent bilirubin production would overwhelm the liver's ability to properly conjugate and excrete bilirubin. This leads to the accumulation of unconjugated bilirubin in the body and deposition into the gallbladder, forming gallstones. 5) RESPONSE: Therefore, the most likely underlying cause of this patient's current condition is overload of unconjugated bilirubin (E).

**AI_A:** RESTATING THE QUESTION: An 18-year-old female patient with sickle cell disease exhibits severe abdominal pain and nausea and has been found to have gallstones via ultrasonography. Sickle cell disease is an inherited disorder where there are insufficient healthy red blood cells to carry adequate oxygen throughout the body, and instead, there are abnormal, "sickle" shaped cells. Gallstones are hardened deposits that form in the gallbladder, which can lead to pain and other complications. The task at hand is to identify the most likely cause of her condition among five choices related to gallbladder and bile function and abnormalities. PLANNING: Each of the listed potential causes pertains to alterations in different aspects of bile composition or metabolism, each of which could theoretically contribute to gallstone formation. However, in the context of this patient's sickle cell disease and symptoms, certain assumptions become more clinically relevant. One key assumption is that the patient's sickle cell condition is directly related to her presentation with gallstones. Sickle cell disease can lead to increased turnover of red blood cells, resulting in an elevated bilirubin level, a byproduct of red blood cell breakdown. Increased bilirubin can subsequently cause pigmented gallstones to form. CLINICAL REASONING: Gallstones broadly fall under two categories: cholesterol stones, which result from imbalances in the composition of bile, and pigmented stones, which are associated with conditions that increase bilirubin levels. Looking at the clinical picture, the patient's symptoms such as abdominal pain, nausea, and especially tenderness in the right upper quadrant which may be due to cholecystitis-- inflammation of the gallbladder often caused by gallstones-- are suggestive of gallstone pathology. Given the patient's sickle cell disease, which increases red blood cell turnover and hence bilirubin levels, it's reasonable to hypothesize that her gallstones are of the pigmented variety, resulting from an overload of unconjugated bilirubin. CONCLUDING: With sickle cell disease, there is an increased rate of hemolysis or red blood cell breakdown. One of the byproducts of this process is unconjugated bilirubin, which, in vast quantities, can form black, pigmented gallstones. The patient's signs, symptoms, and the ultrasound confirmation of gallstones make this the most likely scenario out of the choices given. Let's keep in mind that while other options may contribute to gallstone development theoretically, the clinical context specifically points towards a bilirubin overload. RESPONSE: The most likely underlying cause of this patient's gallstone condition, given her sickle cell disease, signs, symptoms, and examination findings, is (E) Overload of unconjugated bilirubin.

**Facilitator** The consensus statement synthesizes the responses of AI_A, AI_B, AI_C, AI_D, and AI_E as follows: The question presents an 18-year-old female patient with sickle cell disease who is experiencing severe abdominal pain and nausea. Ultrasonography has confirmed the presence of gallstones. The task is to identify the most likely cause of her condition from five options related to gallbladder and bile function and abnormalities. Sickle cell disease is a genetic disorder that results in abnormal, "sickle" shaped red blood cells. This condition often leads to increased turnover of red blood cells, resulting in elevated levels of bilirubin, a byproduct of red blood cell breakdown. Gallstones can be broadly categorized into cholesterol stones, which result from imbalances in the composition of bile, and pigmented stones, which are associated with conditions that increase bilirubin levels. Given the patient's sickle cell disease, which increases red blood cell turnover and hence bilirubin levels, it's reasonable to hypothesize that her gallstones are of the pigmented variety, resulting from an overload of unconjugated bilirubin. The patient's symptoms such as abdominal pain, nausea, and especially tenderness in the right upper quadrant, which may be due to cholecystitis-- inflammation of the gallbladder often caused by gallstones-- are suggestive of gallstone pathology. While other options may contribute to gallstone development theoretically, the clinical context specifically points towards a bilirubin overload. Therefore, the most likely underlying cause of this patient's gallstone condition, given her sickle cell disease, signs, symptoms, and examination findings, is an overload of unconjugated bilirubin.
