# Supplementary material for: Collaborative intelligence in AI: Evaluating the performance of a council of AIs on the USMLE
Source: PLOS Digit Health. 2025 Oct 9;4(10):e0000787. doi: 10.1371/journal.pdig.0000787 (PMC12510544; doi:10.1371/journal.pdig.0000787)
Supplement: S2 File — (ZIP) [file pdig.0000787.s002.zip › S2 Data Raw data files containing unprocessed question inputs and LLM outputs including transcripts of deliberations/STEP1_Question-14-1_Type-MC-NJ.docx]

**Facilitator** **[Round 1]:** A 45-year-old man is brought to the emergency department 30 minutes after the sudden onset of crushing chest pain. His father, maternal aunt, and paternal uncle all died of myocardial infarctions under the age of 50 years. Physical examination shows tendinous xanthomas on the hands and thickened Achilles tendons. Serum lipid studies show a total cholesterol concentration of 410 mg/dL, HDL-cholesterol concentration of 30 mg/dL, and triglyceride concentration of 140 mg/dL. The diagnosis of myocardial infarction is made. This patient most likely has a deficiency of which of the following? (A) Apo B48 (B) Apo C (C) HMG-CoA reductase activity (D) LDL receptor (E) Lipoprotein lipase activity

**AI_B:** 1) RESTATING THE QUESTION: Our patient is a 45-year-old man who has been brought to the emergency room with severe chest pain. His dad, aunt, and uncle have all died young due to heart attacks. On examination, he has a type of lipid buildup in his tendons and thickening of his Achilles tendons. Blood tests show an abnormally high total cholesterol level, low high-density lipoprotein (good cholesterol) level, and a normal triglyceride level. The diagnosis of a myocardial infarction, or heart attack, has been made. We are asked to identify the likely deficiency he has in relation to lipid metabolism. 2) PLANNING: The key things to consider here are the patient's familial history of heart disease, his clinical symptoms, and the labs showing increased levels of cholesterol, suggestive of a lipid metabolism disorder. The options provided include two types of Apolipoproteins - B48 and C, HMG-CoA reductase, LDL receptors, and Lipoprotein lipase. Deficiencies or dysfunction in any of these factors can lead to dyslipidemias, depending on the specific factor and its role in lipid metabolism. 3) CLINICAL REASONING: This patient's clinical presentation and laboratory results strongly suggest a hereditary disorder leading to severely elevated levels of bad (LDL) cholesterol. This results in premature atherosclerosis and myocardial infarction, and the presence of tendinous xanthomas which are often seen in familial hypercholesterolemia. Familial hypercholesterolemia is caused by a genetic abnormality in the LDL receptor, leading to a significant elevation in serum LDL cholesterol levels secondary to decreased receptor-mediated LDL uptake and clearance. Therefore, a deficiency in LDL Receptor is a logical clinical assumption given the patient case. 4) CONCLUDING: The most likely diagnosis for this patient, given the familial history of early heart disease along with symptoms of elevated cholesterol and tendinous xanthomas, would be familial hypercholesterolemia, a disorder caused by impaired LDL receptor function. 5) RESPONSE: This patient most likely has a deficiency of the (D) LDL receptor.

**AI_D:** RESTATING THE QUESTION: The main focus here is to identify the likely deficiency in a middle-aged man who presented with symptoms of a heart attack and has a history of familial early deaths due to myocardial infarction. His physical examination and serum lipid profile point towards a lipid metabolism disorder. The options provided are components or enzymes involved in the metabolism of lipids. PLANNING: We need to understand what role each of these components plays. Apo B48 is primarily involved in chylomicron formation while Apo C activates lipoprotein lipase. HMG-CoA reductase activity refers to the primary enzyme involved in cholesterol synthesis, and the LDL receptor is responsible for the uptake of LDL into cells. Lipoprotein Lipase is needed for the breakdown of triglycerides. CLINICAL REASONING: The presence of tendinous xanthomas and elevated serum cholesterol levels suggest a disorder related to increased LDL, the cholesterol-carrying lipoprotein. Apo B48, Apo C, and lipoprotein lipase deficiencies wouldn't cause this. A deficiency in HMG-CoA reductase would cause decreased endogenous cholesterol synthesis, not hypercholesterolemia. The remaining and most probable option is a deficiency in the LDL receptor. This causes Familial Hypercholesterolemia, a genetic disorder characterized by high cholesterol levels, specifically increased LDL, from birth, which may lead to early cardiovascular disease, as seen in this patient's family. CONCLUDING: The clinical features, blood lipid profile, and family history of this patient point towards a disorder in the uptake of LDL, suggesting a deficiency in LDL receptor activity. RESPONSE: Thus, the most likely deficiency this patient has is (D) LDL receptor.

**AI_E:** Restating the question, the clinical case presented exhibits a middle-aged man with a sudden onset of severe chest pain, suggestive of a cardiac event. His family history is indicative of early cardiac deaths. His physical signs of tendinous xanthomas and Achilles tendon thickening, along with his elevated cholesterol levels, suggest he might have a lipid disorder. The diagnostic query pertains to determining the likely deficiency in this patient's condition linked to his lipoproteins or lipid metabolism enzymes. Let's begin planning a thoughtful approach. Each of these choices in this case indicates different components involved in lipid transport and metabolism. Apo B48 and Apo C are apolipoproteins involved in the formation and metabolism of various lipid particles. HMG-CoA reductase is the rate-limiting enzyme in cholesterol synthesis, while LDL receptors are responsible for LDL (low-density lipoprotein) uptake from the blood. Lipoprotein lipase breaks down triglycerides in lipoproteins. The patient's family history, clinical signs, and lipid profile collectively indicate a problem in cholesterol regulation. Given the patient's high cholesterol concentrations along with the physical examination findings, there are possible implications of a condition known as Familial Hypercholesterolemia (FH). This condition is typically characterized by elevated LDL cholesterol levels, premature coronary heart disease, and physical manifestations like tendinous xanthomas. Considering the clinical details provided, the most apparent issue seems to be the inability to properly metabolize and remove LDL-cholesterol from the system, which commonly results from deficient LDL receptor function. Further clinical reasoning suggests that if the receptors for LDL (which carries cholesterol to tissues) are deficient, this would result in the high serum cholesterol levels observed in this patient. Tendinous xanthomas, a sign of FH, are cholesterol deposits in tendons - another clue that the body is not handling cholesterol appropriately. In light of these factors, the best conclusion yielded by this combination of high cholesterol, xanthomas, and familial early heart disease leads to an assumption of Familial Hypercholesterolemia caused primarily by mutations affecting the LDL receptor gene. Therefore, the response to this question is that the patient most likely has a deficiency in LDL receptor (D).

**AI_C:** 1) RESTATING THE QUESTION: The question pertains to a 45-year-old man presenting with sudden, severe chest pain, subsequently diagnosed with myocardial infarction. He exhibits familial history of early myocardial infarctions and displays physical manifestations of high cholesterol, including tendinous xanthomas and thickened Achilles tendons. His serum lipid studies validate the high cholesterol levels. The inquiry is focused on determining the deficiency most likely responsible for his condition from five given options. 2) PLANNING: The primary assumption here is that the patient's symptoms can be traced back to a deficiency of one of numerous components critical to the metabolism and transport of cholesterol and lipoproteins in the body. Alternative assumptions may consider the possibilities that each listed component (Apo B48, Apo C, HMG-CoA reductase activity, LDL receptor, or Lipoprotein lipase activity) could be deficient, each leading to different implications for lipid metabolism and potential clinical manifestations. 3) CLINICAL REASONING: - Apo B48 is essential for chylomicron formation and any deficiency can lead to abetalipoproteinemia which primarily presents as lipid malabsorption rather than hyperlipidemia. Hence, Apo B48 deficiency seems an unlikely cause in this case. - Apo C is an apolipoprotein found in chylomicrons and VLDLs, important for lipids transportation. However, a deficiency doesn't directly lead to increased total cholesterol levels. - Decreased HMG-CoA reductase activity wouldn't fit this case because this enzyme catalyses the production of cholesterol. Reduced activity would lead to decreased cholesterol production, not increased. - Lipoprotein lipase activity deficiency causes high levels of chylomicrons leading to hypertriglyceridemia. However, this patient's triglyceride levels are not exceptionally high. - LDL receptor deficiency fits the bill as these receptors are crucial for uptake and clearance of circulating LDL particles. A deficiency would make it difficult for cells to take in and break down LDL, consequently leading to high serum cholesterol levels. Plus, the familial pattern of early-onset myocardial infarctions indicates this could be a case of Familial Hypercholesterolemia, primarily caused by mutations in the LDL receptor. 4) CONCLUDING: After the step-by-step evaluation of each option, it appears that the deficiency of LDL receptor provides the most consistent explanation for the patient's presentation. 5) RESPONSE: The patient most likely has a deficiency in (D) LDL receptor.

**AI_A:** RESTATING THE QUESTION: In simple terms, this 45-year-old male came to the emergency room with severe chest pain, was diagnosed with a heart attack, and has a strong family history of early heart disease. His body shows signs of excessive cholesterol deposits and blood cholesterol levels are significantly high. We need to understand which protein or enzyme related to fat metabolism might be missing or not functioning correctly, leading to his increased cholesterol levels and cardiovascular disease. PLANNING: To plan, it is important to recognize the clues given in the patient's history. First, the family history of heart disease and his young age suggest a genetic predisposition. Second, the presence of tendinous xanthomas and elevated cholesterol levels indicate an abnormality in the cholesterol processing pathways. The possible proteins involved are Apo B48, Apo C, HMG-CoA reductase, LDL receptor, and Lipoprotein lipase. We'll individually assess each of these proteins to see which deficiency would most likely lead to this patient's condition. CLINICAL REASONING: Apo B48 is involved in the secretion of chylomicrons, but its deficiency typically leads to abetalipoproteinemia, characterized by fat malabsorption, which is not described in this case. Apo C acts as a co-factor for lipoprotein lipase, but deficiencies are not generally associated with hypercholesterolemia. HMG-CoA reductase activity is the target of statin drugs to lower cholesterol levels. Decreased activity would actually result in lowered cholesterol levels, which is contrary to this patient's presentation. Lipoprotein lipase deficiency causes hypertriglyceridemia leading to pancreatitis, which does not align with this patient's symptoms. However, a deficiency in the LDL receptor would prevent the normal uptake and removal of LDL ("bad" cholesterol) from the bloodstream, leading to high cholesterol levels and deposition in the form of xanthomas, which is consistent with the patient's presentation. Furthermore, familial hypercholesterolemia, a condition caused by defects in the LDL receptor, is known to lead to early onset atherosclerosis and heart disease, which is also consistent with the patient's history and presentation. CONCLUDING: Based on a step-by-step examination of each protein/enzyme possibility and matching their outcome with the patient's presentation, it would appear that the patient's symptoms stem from a deficiency in the LDL receptor, since this deficiency would lead to an accumulation of cholesterol in the blood and tissues, which aligns with the patient's symptoms and presentation. RESPONSE: Thus, the patient most likely has a deficiency of (D) LDL receptor.

**Facilitator** The consensus statement synthesizes the responses of AI_A, AI_B, AI_C, AI_D, and AI_E as follows: The patient in question is a 45-year-old male who presented with severe chest pain and was diagnosed with a myocardial infarction. His family history of early heart disease, physical signs of tendinous xanthomas, and elevated cholesterol levels suggest a lipid metabolism disorder. The task is to identify the likely deficiency related to lipid metabolism in this patient. The options provided are Apo B48, Apo C, HMG-CoA reductase, LDL receptors, and Lipoprotein lipase, all of which play crucial roles in lipid metabolism and transport. However, the patient's symptoms and history align most closely with a deficiency in the LDL receptor. Apo B48 and Apo C deficiencies do not typically result in hypercholesterolemia. Decreased HMG-CoA reductase activity would lead to lower cholesterol levels, contrary to the patient's presentation. Lipoprotein lipase deficiency would result in hypertriglyceridemia, which is not evident in this case. On the other hand, a deficiency in the LDL receptor would prevent the normal uptake and removal of LDL ("bad" cholesterol) from the bloodstream, leading to high cholesterol levels and deposition in the form of xanthomas. Familial hypercholesterolemia, a condition caused by defects in the LDL receptor, is known to lead to early onset atherosclerosis and heart disease, which aligns with the patient's history and presentation. Therefore, the consensus is that the patient most likely has a deficiency in the LDL receptor (D). This conclusion is based on a step-by-step examination of each protein/enzyme possibility and matching their outcome with the patient's presentation.
