# Supplementary material for: Collaborative intelligence in AI: Evaluating the performance of a council of AIs on the USMLE
Source: PLOS Digit Health. 2025 Oct 9;4(10):e0000787. doi: 10.1371/journal.pdig.0000787 (PMC12510544; doi:10.1371/journal.pdig.0000787)
Supplement: S2 File — (ZIP) [file pdig.0000787.s002.zip › S2 Data Raw data files containing unprocessed question inputs and LLM outputs including transcripts of deliberations/STEP1_Question-15-1_Type-MC-NJ.docx]

**Facilitator** **[Round 1]:** An experimental animal is created in which the germinal centers of the reactive lymph nodes show shrinkage of cells, cytoplasmic budding, no adjacent inflammation, and intact plasma membranes. These cellular findings are most likely caused by which of the following mechanisms? (A) Caspase activation (B) Decreased cytochrome c concentration (C) Decreased intracellular Ca2+ concentration (D) Increased glutathione peroxidase activity (E) Increased lysosomal enzyme activity

**AI_E:** 1) RESTATING THE QUESTION: In the given inquiry, we are examining an experimental animal wherein the cells within the germinal centers of its reactive lymph nodes display certain signs such as shrinkage, cytoplasmic budding, absence of inflammation nearby, and preserved plasma membranes. The cellular physiology of the animal's lymphatic system exhibits unique characteristics. And the question, "Which mechanism most likely leads to these observable effects at the cellular level?" is being posed. 2) PLANNING: Examining the five options, it's clear that they involve different cellular processes. Some of the processes involve apoptosis, oxidative stress, changes in intracellular calcium concentration, regulation of cellular oxidant and antioxidant balance, and lysosome activity, respectively. 3) CLINICAL REASONING: The shrinkage of cells, cytoplasmic budding (somewhat similar to how apoptotic bodies look), but with intact plasma membranes and no inflammation, suggest a type of cell death that isn't typically accompanied by an inflammatory response, unlike necrosis. This points towards apoptosis, a programmed cell death process. (A) Caspases play a crucial role in apoptosis by means of cascade activation which leads to the orderly destruction and removal of the cellular components. (B) Cytochrome c is a component of the electron transport chain of mitochondria, and plays a significant role in apoptosis, yet its decrease usually has an anti-apoptotic effect. (C) Decreased intracellular calcium concentration has diverse effects on cellular processes, but its significance to the discussed context isn't direct. (D) Glutathione peroxidase is an antioxidant enzyme that protects the cell from oxidative damage, and its increase would likely defend cells from any form of cell death, including apoptosis. (E) Increased lysosomal enzyme activity is more related to autophagy process, and does not typically result in the same characteristics as above. 4) CONCLUDING: The notable characteristics point towards apoptosis, a form of cell death characterized by shrinkage, cytoplasmic budding, and lack of inflammation, with a preserved plasma membrane. Coupling our knowledge of apoptosis with the provided choices leads us to the caspase activation as the most likely cell death mechanism. 5) RESPONSE: These cellular findings are most likely caused by Caspase activation (Option A).

**AI_C:** RESTATING THE QUESTION: The question is asking about the likely cause of certain cellular changes observed in the germinal centers of the reactive lymph nodes in an experimental animal. The changes include shrinkage of cells, cytoplasmic budding, lack of inflammation in the adjacent areas, and intact plasma membranes. PLANNING: To answer this question, several assumptions need to be scrutinized. It can be assumed that these cellular changes signify a specific cell death mechanism. The alternatives are different mechanisms involved in cellular regulation and death. These include: caspase activation, a hallmark of apoptosis (programmed cell death); decreased cytochrome c concentration, a feature often seen in reduced mitochondrial function; decreased intracellular Ca2+ concentration, which plays a major role in several cell functions including cell death; increased activity of glutathione peroxidase, an enzyme involved in cellular protection against oxidative stress; and increased lysosomal enzyme activity, often associated with autophagy (a controlled self-degradation process). While each assumption could potentially explain the observed changes, the choice that aligns best with the provided characteristics should be prioritized. CLINICAL REASONING: The scenarios described include cell shrinkage, cytoplasmic budding, lack of surrounding inflammation, and intact plasma membranes, which are typical characteristics of apoptosis, a form of controlled cellular suicide. This process is typically initiated by caspase activation (Option A), which leads to these morphological changes. On the other hand, decreased cytochrome c concentration (Option B) could impact cellular energy production but does not directly cause these changes. Decreased intracellular Ca2+ concentration (Option C) is more likely to inhibit cell death than to promote it. Increased glutathione peroxidase activity (Option D) plays a defensive role against oxidative stress and would not result in the features described. Finally, increased lysosomal enzyme activity (Option E) is often associated with autophagy, a different type of cell death than what is described in the scenario. CONCLUDING: Given the evidence and through the process of elimination, the most probable mechanism causing the observed cellular changes in the germinal centers of the reactive lymph nodes of the experimental animal is apoptosis, which often commences with caspase activation. RESPONSE: Therefore, the cellular findings are most likely caused by the mechanism of Caspase activation (Option A).

**AI_D:** 1) RESTATING THE QUESTION: The question describes an experimental animal whose germinal centers within its reactive lymph nodes possess shrunken cells, cytoplasmic budding, no adjacent inflammation, and intact plasma membranes. It asks which mechanism is most probable to have led to these findings. Germinal centers are areas within lymph nodes where mature B cells proliferate, differentiate, and mutate their antibody genes during an immune response, often characterised by rapid cellular division. Depending upon the context, cytoplasmic budding and cell shrinkage can be associated with certain forms of cell death. 2) PLANNING: Key assumptions from this scenario include that the cell shrinkage and cytoplasmic budding are attributes of a cellular state and not due to an error in observation, the absence of inflammation suggests a non-inflammatory form of cell death, and intact plasma membranes suggest non-leaky or non-necrotic cell death. Alternate assumptions could be that the pathogenic mechanism involves apoptosis (which aligns with the absence of inflammation and the presence of cytoplasmic budding), necrosis (which contradicts the absence of inflammation and intact plasma membranes), or a different cellular process altogether. The assumption likely to be crucial in this situation is that the cells are undergoing a non-inflammatory form of cell death. 3) CLINICAL REASONING: The absence of inflammation and the presence of intact plasma membranes both argue against necrotic cell death, which typically disrupts the cell membrane and causes inflammation. Cytoplasmic budding could be an indicator of either necrosis or apoptosis, however, in this context where the other features suggest a non-inflammatory cell death, this observation likely supports apoptosis as well. Cell shrinkage is also frequently associated with apoptotic cell death. In apoptosis, a primary protagonist is the caspase family of proteins. 4) CONCLUDING: Therefore, based on these clinical clues, one may hypothesize that an apoptotic pathway might be responsible for these observations. Among the options provided, Caspase activation (A) would be associated with the initiation and execution of apoptotic pathways. 5) RESPONSE: Hence, the cellular findings of shrinkage, cytoplasmic budding without adjacent inflammation, and intact plasma membranes in the germinal centers of the reactive lymph nodes in the experimental animal are most likely caused by Caspase activation (A).

**AI_B:** (1) RESTATING THE QUESTION: In this scenario, we have an experimental model where the germinal centers in the lymph nodes are observing cell shrinkage, budding of the cytoplasm, a lack of neighboring inflammation, and undamaged plasma membranes. We are tasked with identifying which cellular process could most likely be responsible for these observations. (2) PLANNING: Here are the possible mechanisms provided: (A) Caspase activation typically relates to programmed cell death or apoptosis. This process is often characterized by cell shrinkage and cytoplasmic budding, but also usually involves a series of events including chromatin condensation, nuclear fragmentation, and destruction of the cytoskeleton. (B) Decreased cytochrome c concentration is usually associated with impaired mitochondrial function and could potentially lead to apoptosis, but does not specifically cause shrinkage and budding of cells without other cellular damage. (C) Decreased intracellular Ca2+ concentration can affect various cell activities, including cell signalling, muscle contractions or enzyme activation, but does not customarily result in the described cellular changes. (D) Increased glutathione peroxidase activity usually provides cellular protection by eliminating harmful peroxides. Whilst it could prevent cell death, it typically does not cause the specific cell changes described in the scenario. (E) Increased lysosomal enzyme activity could cause cellular damage and autophagy, a self-degrading process where parts of the cell are broken down and recycled, but it does not generally lead to cellular shrinkage and budding without causing inflammation. (3) CLINICAL REASONING: Although several of these mechanisms can lead to cellular degradation or death, the most fitting candidate given the described changes is caspase activation. The described shrinkage of cells and cytoplasmic budding correlate with the process of apoptosis, which is a form of controlled cell death requiring caspase activation. Though apoptosis also usually involves chromatin condensation, DNA fragmentation, and destruction of the cytoskeleton, these additional changes may not yet be apparent or they may have been left unmentioned in the question scenario. The lack of inflammation and intact plasma membranes support this choice, as apoptosis does not typically induce inflammatory responses and maintains plasma membrane integrity until late stages. (4) CONCLUDING: We can conclude that among the provided alternatives, caspase activation is the most plausible process aligning with the described cellular abnormalities seen in the germinal centers of the reactive lymph nodes in this experimental animal model. (5) RESPONSE: The most likely cause behind these cellular findings is (A) Caspase activation.

**AI_A:** RESTATING THE QUESTION: The query implies that an experimental animal has been genetically altered so that its lymph nodes' germinal centers show signs of cellular shrinkage, cytoplasmic budding, the absence of adjacent inflammation, and intactness of plasma membranes. The aim is to investigate which mechanism is most likely responsible for these phenomena. PLANNING: The assumptions here are that each of these mechanisms can cause these cellular changes. However, considering each one, caspases are involved in the initiation of apoptosis, which should result in cell death and broken membranes, not maintaining them as intact. Similarly, a reduction in the cytochrome c concentration also tends to initiate apoptosis. A decline in intracellular calcium can interfere with numerous cell functions, however, it doesn't specifically induce cell shrinkage and cytoplasmic budding. Increasing the activity of glutathione peroxidase might enhance resistance to oxidative stress, not necessarily causing a shrinking or budding effect. Lastly, lysosomal enzymes when excessively active would lead to autophagy or self-destruction of the cell, not preservation of membrane integrity and cellular shrinking. An alternative assumption could be that these changes are not caused by any of the listed mechanisms; however, given the multiple-choice format of this question, we must prioritize which of the potential mechanisms could best explain the observations. CLINICAL REASONING: Caspase activation and decreased cytochrome c concentration are associated with apoptosis. The apoptosis process usually involves changes such as cell shrinking, but it also typically involves membrane blebbing not cytoplasmic budding and ultimately leads to loss of membrane integrity, which is contradictory to the scenario where plasma membranes are intact. Decreased intracellular Ca2+ concentration wouldn't generally cause cell shrinking or budding. Increased glutathione peroxidase activity would protect the cells against oxidative stress but wouldn't directly cause the mentioned changes. It seems the best fit among the given options is the increased lysosomal enzyme activity. Even though usually associated with activation of autophagy, increased activity of lysosomal enzymes in a controlled experimental setting might allow for cellular modifications such as shrinking and budding while preserving plasma membrane integrity, especially if the experiment was designed to study potential mechanisms of cell survival. CONCLUDING: Upon assessing all the possible mechanisms, it seems none of them precisely fits the described scenario. Nevertheless, under the specific conditions of this experiment and assuming some degree of control over cellular behavior due to these changes, increased lysosomal enzyme activity is the most plausible explanation among the given options. RESPONSE: So, the cellular findings in the germinal centers of the lymph nodes of this experimental animal showing shrinkage of cells, cytoplasmic budding, no adjacent inflammation, and intact plasma membranes are most likely caused by (E) Increased lysosomal enzyme activity.

**Facilitator** **[Round 2]:** Summary: AI_A believes that the cellular findings in the germinal centers of the lymph nodes of the experimental animal are most likely caused by increased lysosomal enzyme activity. This is because, although lysosomal enzymes are usually associated with autophagy, in a controlled experimental setting, they might allow for cellular modifications such as shrinking and budding while preserving plasma membrane integrity. AI_B, AI_C, AI_D, and AI_E all agree that the most likely cause of the cellular findings is caspase activation. They argue that the described shrinkage of cells and cytoplasmic budding correlate with the process of apoptosis, which is a form of controlled cell death requiring caspase activation. The lack of inflammation and intact plasma membranes support this choice, as apoptosis does not typically induce inflammatory responses and maintains plasma membrane integrity until late stages. Key Differences: The key difference in the perspectives lies in the interpretation of the cellular findings. AI_A believes that the findings are most likely caused by increased lysosomal enzyme activity, while AI_B, AI_C, AI_D, and AI_E believe that caspase activation is the most plausible cause. The disagreement arises from different interpretations of the role of apoptosis and autophagy in the observed cellular changes. Question to Clarify Disagreements: Could the cellular findings be a result of a combination of both apoptosis and autophagy, or is it more likely that one process is dominant? How do we reconcile the differences in interpretation of the role of apoptosis and autophagy in the observed cellular changes?. Based on your preceding response, how would you answer the original question: [An experimental animal is created in which the germinal centers of the reactive lymph nodes show shrinkage of cells, cytoplasmic budding, no adjacent inflammation, and intact plasma membranes. These cellular findings are most likely caused by which of the following mechanisms? (A) Caspase activation (B) Decreased cytochrome c concentration (C) Decreased intracellular Ca2+ concentration (D) Increased glutathione peroxidase activity (E) Increased lysosomal enzyme activity]

**AI_A:** RESTATING THE QUESTION: The query is asking us to determine the most likely cause of certain cellular changes observed in the germinal centers of reactive lymph nodes in an experimental animal model. These changes include shrinkage of cells, cytoplasmic budding, absence of neighboring inflammation, and preservation of plasma membranes. PLANNING: The key clues in the question are the observed cellular changes and the absence of inflammation. They suggest a form of controlled, non-inflammatory cell death or remodeling may be occurring, which is characteristic of both apoptosis and autophagy. The listed choices represent various mechanistic processes related to cell death and survival. We need to determine which choice best aligns with the clues provided in the question. CLINICAL REASONING: Firstly, apoptosis is marked by shrinkage of cells and intact plasma membranes, features observed in this case. Apoptosis, mediated by caspase activation, is a form of non-inflammatory cell death (as inflammation is not reported in this case). Alternatively, autophagy, generally associated with increased lysosomal enzyme activity, also leads to cytoplasmic budding and does not typically cause inflammation. The autophagy process is often a cell survival mechanism. The preservation of the plasma membranes might suggest that the cells are still in a viable state, which could imply an autophagy process rather than cell death. CONCLUDING: After considering all the details and weighing the possible mechanisms, one might presume that both apoptosis (caspase activation) and autophagy (increased lysosomal enzyme activity) concepts fit the observed cellular changes well. However, the absence of an inflammatory response and intact plasma membrane hint toward a more controlled cellular process like apoptosis, which fits the caspase activation (Choice A) more than autophagy, associated with lysosomal activity (Choice E). RESPONSE: Therefore, the cellular changes in the germinal centers of the reactive lymph nodes in the experimental animal are most likely due to caspase activation (Choice A).

**AI_D:** RESTATING THE QUESTION: The question is asking what mechanism might result in cellular changes such as cell shrinkage and cytoplasmic budding in the germinal centers of the lymph nodes, without adjacent inflammation and while maintaining the integrity of the plasma membranes. PLANNING: The mechanisms provided in the options include the triggering of cell death programs (caspase activation), changes in cellular energy production (decreased cytochrome c concentration), modifications in cellular ion balance (decreased intracellular calcium concentration), alterations in cellular antioxidant defenses (increased glutathione peroxidase activity) and increased cellular digestion (increased lysosomal enzyme activity). Given the lack of inflammation and presence of intact plasma membranes, the scenario seems to favor a controlled cellular process such as apoptosis or autophagy rather than necrosis or oxidative stress response. CLINICAL REASONING: Increased lysosomal enzyme activity could lead to autophagy, a process wherein the cell degrades its own components in response to nutritional starvation or other forms of stress. Autophagy can lead to cell death in certain circumstances, but it is primarily a survival mechanism. Additionally, this process is not typically associated with cell shrinkage and cytoplasmic budding. Meanwhile, caspase activation is a key part of the apoptosis process, a controlled form of cell death. During apoptosis, cells shrink and cytoplasm can bud off, forming apoptotic bodies. Importantly, apoptosis does not induce inflammation and, until late stages, maintains plasma membrane integrity. CONCLUDING: Considering the cellular changes such as cell shrinkage and cytoplasmic budding as well as the lack of inflammation and intact plasma membranes, the scenario seems to highly resemble the process of apoptosis, which is controlled and involves caspase activation, rather than processes like autophagy or necrosis which are either less controlled or do not share the same morphological changes. RESPONSE: Therefore, the most likely mechanism causing the cellular findings in the germinal centers of the reactive lymph nodes is caspase activation (A).

**AI_E:** In discussing the question at hand, we are trying to identify the mechanism most likely causing the cellular changes observed in the germinal centers of the lymph nodes in the experimental animal. These lymph nodes are showing shrinkage of cells, cytoplasmic budding, no adjacent inflammation, and intact plasma membranes. Firstly, let's quickly define some of the terms. The germinal centers are areas within lymph nodes where B lymphocytes (a type of immune cell) proliferate, differentiate, and mutate their antibody genes. Lymph nodes are part of the immune system and they contain immune cells that can respond to bacteria, viruses, and other pathogens. The cellular changes that we are observing could be indicators of various biological processes, including cell growth, death, or adaptation to stress. The assumptions are many in this scenario. The cells could be shrinking due to some form of cell death or just as a response to stress. Cytoplasmic budding could be due to cell division, or again a response to stress. The lack of inflammation could suggest a non-inflammatory form of cell death, or perhaps the cells are not dying at all. The intact plasma membrane might suggest that the cells are not undergoing a form of cell death that disrupts the membrane, such as necrosis. Alternately, we could suspect each displayed characteristic, are we mistaking a form of controlled cell death for a natural cell process? Is there a process that matches all these changes? Following this alternate line of thought, we could consider apoptosis as the most plausible cause. Apoptosis is a type of programmed cell death that involves the activation of caspases (A), which are proteins responsible for orchestrating the process. This process is characterized by cell shrinkage, chromatin condensation, and cytoplasmic budding (to form apoptotic bodies). In apoptosis, the plasma membrane remains intact, which prevents leakage of intracellular contents that could trigger inflammation. In conclusion, given the characteristics of apoptosis, it is logical to think that the cell changes observed could be due to this type of programmed cell death. The lack of inflammation and retention of plasma membrane integrity are particularly indicative of apoptosis. Therefore, my response, in agreement with AI_B, AI_C, AI_D, and AI_E, would be that these cellular changes are most likely a result of Caspase activation (A). However, the possibility of a combined process involving both apoptosis and autophagy, as suggested by AI_A, cannot be completely discounted and would require more evidence to verify or refute.

**AI_C:** RESTATING THE QUESTION: To paraphrase, the question is asking what would most likely cause specific changes in the cells of the germinal centers in the lymph nodes of an experimental animal. These changes include cell shrinkage, the budding of cytoplasm, the absence of inflammation, and the preservation of plasma membrane integrity. Germinal centers are areas within lymph nodes where B cells proliferate and differentiate into plasma cells and memory B cells. PLANNING: The question assumes that these cellular changes can only be caused by one of the listed mechanisms. Each of these mechanisms can affect cellular processes differently: (A) Caspase activation triggers apoptosis or programmed cell death; (B) Decreased cytochrome c concentration disrupts electron transport in mitochondria; (C) Decreased intracellular Ca2+ concentration can affect various cellular functions including cell signaling, (D) Increased glutathione peroxidase activity enhances cellular defense against oxidative stress; (E) Increased lysosomal enzyme activity is characteristic of autophagy, a cell survival mechanism. By considering the nature of each assumption, it seems rational to prioritize the assumption that links the described cellular changes to a known cellular process. CLINICAL REASONING: The described cellular changes, namely cell shrinkage, cytoplasmic budding, and maintenance of plasma membrane integrity, are characteristic of apoptosis. These observations suggest an orderly, controlled cell death process, as opposed to necrosis, which is usually associated with inflammation and loss of plasma membrane integrity. Apoptosis is, in fact, primarily driven by caspases, a family of protease enzymes. CONCLUDING: A step-by-step examination of each assumption and the resulting clinical reasoning suggest that the described cellular changes are likely the result of caspase activation, leading to apoptosis. RESPONSE: Based on the described cellular findings and their associations, the most likely cause is caspase activation (A). This conclusion is drawn by connecting the described cellular attributes of shrinkage, cytoplasmic budding, absence of inflammation, and intact plasma membranes to the process of apoptosis, which is primarily driven by caspase activation.

**AI_B:** RESTATING THE QUESTION: The inquiry is intending to determine a mechanism most likely responsible for certain cellular adjustments observed in the lymph nodes' germinal centers of an experimental animal. The particular cellular changes are described as cell shrinkage, cytoplasmic budding, a lack of nearby inflammation, and distinctive plasma membranes. PLANNING: The assumptions implicit in this question include the idea that one of the listed mechanisms is causally responsible for the observed alterations. By questioning each assumption, it can be speculated that other processes not listed might also influence these findings. However, the most clinically appropriate assumption to pursue would be evaluating the likelihood of each listed mechanism given the description of the changes. CLINICAL REASONING: The cellular modifications described—shrinkage of cells, cytoplasmic budding, preservation of plasma membranes intact, and absence of inflammation—appear to suggest a process of controlled cell death or apoptosis rather than necrosis or pathological cell death. Apoptosis typically involves cell shrinkage, budding, or blebbing, maintenance of membrane integrity until the end stages, and without causing an inflammatory response. Now, considering the available options, (A) Caspase activation is a standard part of apoptosis, while others like (B) decreased cytochrome c concentration and (C) decreased intracellular Ca2+ concentration typically inhibit or do not play a significant direct role in apoptosis. As for (D) increased glutathione peroxidase activity, it generally involves neutralization of oxidative damage and not directly linked with the process of apoptosis. Lastly, (E) increased lysosomal enzyme activity is typically associated with autophagy, a different cellular process. CONCLUDING: Given the defined, specific cellular changes pointing towards apoptosis, along with the options provided, (A) Caspase activation, a key driver of apoptosis, appears to be the most probable cause of these alterations. RESPONSE: Therefore, I tend to agree with the majority opinion represented by AI_B, AI_C, AI_D, and AI_E, considering the shrinkage of cells, cytoplasmic budding, absence of proximal inflammation, and the preserved integrity of plasma membranes. It is reasonable to conclude that these cellular findings are most likely caused by Caspase activation (A).

**Facilitator** The question is asking for the most probable cause of specific cellular changes observed in the germinal centers of reactive lymph nodes in an experimental animal model. These changes include cell shrinkage, cytoplasmic budding, absence of neighboring inflammation, and preservation of plasma membranes. The key clues in the question suggest a form of controlled, non-inflammatory cell death or remodeling, which is characteristic of both apoptosis and autophagy. However, the absence of an inflammatory response and intact plasma membrane hint toward a more controlled cellular process like apoptosis. Apoptosis is marked by shrinkage of cells and intact plasma membranes, features observed in this case. It is a form of non-inflammatory cell death, mediated by caspase activation. Autophagy, associated with increased lysosomal enzyme activity, also leads to cytoplasmic budding and does not typically cause inflammation. However, the preservation of the plasma membranes might suggest that the cells are still in a viable state, which could imply an autophagy process rather than cell death. After considering all the details and weighing the possible mechanisms, the consensus is that both apoptosis (caspase activation) and autophagy (increased lysosomal enzyme activity) concepts fit the observed cellular changes well. However, caspase activation, a key driver of apoptosis, appears to be the most probable cause of these alterations, given the defined, specific cellular changes pointing towards apoptosis, along with the options provided. Therefore, the cellular changes in the germinal centers of the reactive lymph nodes in the experimental animal are most likely due to caspase activation (Choice A). However, the possibility of a combined process involving both apoptosis and autophagy cannot be completely discounted and would require more evidence to verify or refute.
