# Supplementary material for: Collaborative intelligence in AI: Evaluating the performance of a council of AIs on the USMLE
Source: PLOS Digit Health. 2025 Oct 9;4(10):e0000787. doi: 10.1371/journal.pdig.0000787 (PMC12510544; doi:10.1371/journal.pdig.0000787)
Supplement: S2 File — (ZIP) [file pdig.0000787.s002.zip › S2 Data Raw data files containing unprocessed question inputs and LLM outputs including transcripts of deliberations/STEP1_Question-16-1_Type-MC-NJ.docx]

**Facilitator** **[Round 1]:** Investigators conduct a study that evaluates the effect of finasteride on the incidence of prostate cancer in 500 patients. The investigators recruit an additional 1000 patients for the study. Which of the following effects will this have on the research study? (A) Greater chance of a Type I error (B) Greater chance of a Type II error (C) Less chance of a Type I error (D) Less chance of a Type II error (E) Impossible to predict

**AI_B:** 1) RESTATING THE QUESTION: In this example, investigators are conducting a research study to evaluate the impact of finasteride, a medication used to treat and potentially prevent prostate cancer, on 500 patients. The study is then extended to include an additional 1000 patients. The question posed is what effect will this population enlargement have on the study. For further comprehension, let's define Type I and Type II errors. In statistical hypothesis testing, a type I error is the rejection of a null hypothesis when it's actually true (a false positive), while a Type II error is failing to reject a null hypothesis when it's false (a false negative). 2) PLANNING: The assumptions we may start with are that the enlargement of the sample size will either increase or decrease the potential for either a Type I or Type II error, or that it's unpredictable. Upon questioning each assumption, we see that larger sample sizes typically influence the power of a study or its ability to correctly reject a false null hypothesis, thus could potentially decrease the likelihood of a Type II error. Our clinical priority would now shift towards evaluating the impact of a larger sample size on the occurrence of Type II errors. 3) CLINICAL REASONING: Increasing the sample size doesn't affect the probability of a Type I error, which is determined by the level of significance set for the study (commonly 0.05). A larger sample size can, however, detect smaller effects and hence decrease the likelihood of making a Type II error, because it increases the study's power. 4) CONCLUDING: Therefore, by expanding the sample size in our research study to include an additional 1000 patients, we're more likely to correctly reject a false null hypothesis should it be present. We'd be increasing the statistical power of our study thereby reducing the chance of a Type II error. 5) RESPONSE: In light of the above, it is most likely that increasing the subject pool to 1500 from 500 would result in (D) Less chance of a Type II error.

**AI_C:** The question is asking us about the impact of introducing additional subjects into a study that is looking at the effect of a drug called finasteride on the incidence of prostate cancer. Specifically, it asks how including more patients - a total of 1500 instead of the initial 500 - influences the likelihood of producing a Type I or Type II error in the analysis. A Type I error (false positive) arises when the null hypothesis (no difference or no effect) is incorrectly rejected, thus falsely concluding that something is effective or different when it really isn't. A Type II error (false negative) occurs when the null hypothesis is incorrectly not rejected, concluding that something is not effective or different when it actually is. Assumptions include that the study aims to detect a significant difference or effect, and that the additional patients recruited will be similar in relevant characteristics to the initial set. If we question the assumption that only the patient number changes and other factors stay constant, then recruiting more participants might potentially bring in more variability or confounding factors, exposing the study to errors. However, drawing on conventional statistical understanding, increasing the sample size in research often decreases the chances of committing a Type II error. This is because the larger the sample size, the greater the power of the study, i.e., the ability to correctly reject a false null hypothesis and, hence, detecting an actual effect if it exists. Assuming that the experiment's conduct and the data's quality remain the same with the increase in sample size, it is likely that the recruited extra 1000 patients will enhance the study's ability to discern the effect of finasteride accurately, if such an effect exists. In conclusion, introducing an additional 1000 patients into the study will likely reduce the risk of a Type II error, without necessarily altering the probability of a Type I error. So, the answer to this question should be: (D) Less chance of a Type II error.

**AI_D:** RESTATING THE QUESTION: This inquiry is about the outcome of adding more subjects to a study that aims to assess the impact of finasteride on the rate of prostate cancer. The options provided are concerned with the probability of Type I and Type II errors in the study. To clarify, a Type I error is the incorrect rejection of a true null hypothesis (usually a "false positive"), while a Type II error is incorrectly accepting a false null hypothesis (usually a "false negative"). PLANNING: Here, the primary assumption is that the number of subjects in a study can affect the likelihood of committing Type I or Type II errors. An alternate assumption could be investigating how this increase in subject number can lessen Type I or Type II errors due to the statistical power that comes with bigger sample sizes. We can weigh these assumptions and hypothesize that the addition of more subjects to the study should decrease the likelihood of a Type II error, considering that larger samples generally provide more statistical power and decrease the likelihood of false negatives. CLINICAL REASONING: The concept of study power, or the probability that a study will detect a difference between groups when one actually exists, can be connected to the prevalence of Type II errors. Type II errors occur when researchers accept a null hypothesis that is actually false — effectively a "false negative." Boosting the sample size of a study can result in increased statistical power, reducing the likelihood of these types of errors. When the sample size is small, studies might not detect a difference even when one exists, leading to a Type II error. However, larger sample sizes are more likely to catch these differences, leading to fewer Type II errors. CONCLUDING: Increasing the number of patients in the study evaluating the effect of finasteride on the incidence of prostate cancer should reduce the chance of a Type II error. This is because the larger sample size would yield more statistical power, increasing the likelihood of accurately identifying real differences between finasteride use and prostate cancer incidence. RESPONSE: The expected outcome of recruiting an additional 1000 patients for the research study evaluating the effect of finasteride on the incidence of prostate cancer should be (D) Less chance of a Type II error.

**AI_A:** 1) RESTATING THE QUESTION: The Investigators established a research study to assess the impact of finasteride on the occurrence rate of prostate cancer in 500 patients. Then they expand this number to include another 1000 participants. The question is asking what this increase in participant count would do to the possibilities of Type I and Type II errors in the research study. In statistics, a Type I error is when a true null hypothesis (no effect or relationship) is rejected, it is often called a "false positive". Type II error is when a false null hypothesis (there is an effect or relationship) is accepted, it is often referred to as a "false negative". 2) PLANNING: In statistical analysis, increase in sample size often has intriguing implications on Type I and Type II errors. One assumption could be that the addition of more participants could lead to more chances of false positives (Type I error) or negatives (Type II error). Another assumption could be that with more data coming in, the accuracy of the conclusions drawn would increase, leading to a decrease in both types of errors. Given these assumptions, it is likely that increased sample size would either result in an increase in one of the error types or a decrease in them. 3) CLINICAL REASONING: Generally in clinical studies, an increase in the sample size tends to reduce the chance of a Type II error. This is because having a larger sample size increases the power of a study. In statistical terms, "power" is the probability that a study will reject the null hypothesis when it is false, meaning the study correctly identifies a real effect. By increasing the sample size, investigators are more likely to find a true difference if one exists, thereby reducing the chance of 'falsely' accepting a null hypothesis or in other words, decreasing the risk of a Type II error. 4) CONCLUDING: Therefore, increasing the sample size in a study typically increases its power and reduces the chance of a Type II error. This is because it allows for more accurate data representation and thus, the true effect (if one is present) is more likely to be detected. However, it does not have a direct influence on the chance of a Type I error as it is more related to the threshold for rejecting the null hypothesis. 5) RESPONSE: Thus, recruiting additional patients for the study will likely lead to a (D) Less chance of a Type II error.

**AI_E:** (1) RESTATING THE QUESTION: The research question posed involves a study where the effect of a drug called finasteride on prostate cancer incidence is being evaluated. The study initially started with 500 patients, but an additional 1000 patients have been recruited. We are to determine the likely effect of this increase in sample size on the chances of obtaining either a Type I or Type II error in the research study. Type I error (also known as a "false positive") means making the incorrect assumption that a particular treatment effect or association exists when it does not. Type II error (also known as a "false negative") is the opposite; it occurs when one wrongly concludes that a treatment effect or association does not exist when it does. (2) PLANNING: The key assumption here is based on the sample size of the research study. The increase in the number of patients from 500 to 1500 could impact the Type I or Type II error. An alternate assumption could be that larger sample size increases the statistical power of the study, thus potentially reducing the risk of a Type II error (assuming that an effect or association does indeed exist). (3) CLINICAL REASONING: Statistically, increasing the sample size in a research study typically decreases the probability of committing a Type II error. The larger the sample size, the greater the study's power, which is the ability to detect a true effect or association if it exists. Conversely, a larger sample size does not affect the probability of a Type I error. The Type I error rate (alpha) is usually set a priori before a study begins and does not depend on sample size. (4) CONCLUDING: If we increase the sample size in our study, there is a less chance for obtaining a Type II error, which could result in incorrectly missing an actual effect of the drug. The sample size increase does not influence the chance of a Type I error. (5) RESPONSE: Therefore, the enlargement of the study sample size from 500 to 1500 patients will likely have the effect of providing a (D) Less chance of a Type II error.

**Facilitator** The question asks about the impact of increasing the sample size in a study evaluating the effect of finasteride on the incidence of prostate cancer. The study initially included 500 patients, but an additional 1000 patients were recruited, bringing the total to 1500. The question specifically asks how this increase in sample size might affect the likelihood of Type I and Type II errors. Type I error, often referred to as a "false positive," occurs when a true null hypothesis (no effect or relationship) is incorrectly rejected. On the other hand, Type II error, often referred to as a "false negative," occurs when a false null hypothesis (there is an effect or relationship) is incorrectly accepted. The consensus among the responses is that increasing the sample size in a study typically decreases the likelihood of a Type II error. This is because a larger sample size increases the power of the study, which is the probability that the study will correctly reject a false null hypothesis when it is false. In other words, with a larger sample size, the study is more likely to detect a true effect if one exists, thereby reducing the chance of incorrectly accepting a null hypothesis, which is a Type II error. However, the increase in sample size does not directly influence the likelihood of a Type I error. The probability of a Type I error is determined by the significance level set for the study, which is typically 0.05, and is not affected by the sample size. Therefore, the consensus response to the question is that increasing the sample size from 500 to 1500 patients in the study will likely result in (D) Less chance of a Type II error.
