# Supplementary material for: Collaborative intelligence in AI: Evaluating the performance of a council of AIs on the USMLE
Source: PLOS Digit Health. 2025 Oct 9;4(10):e0000787. doi: 10.1371/journal.pdig.0000787 (PMC12510544; doi:10.1371/journal.pdig.0000787)
Supplement: S2 File — (ZIP) [file pdig.0000787.s002.zip › S2 Data Raw data files containing unprocessed question inputs and LLM outputs including transcripts of deliberations/STEP1_Question-17-1_Type-MC-NJ.docx]

**Facilitator** **[Round 1]:** A 76-year-old man dies of a massive cerebral infarction shortly after admission to the hospital. During the past 10 years, he has had several smaller cerebral infarctions and two transient ischemic attacks. Examination of a cerebral cortex specimen obtained at autopsy shows extensive gliosis. This finding is most likely caused by the growth and proliferation of which of the following? (A) Astrocytes (B) Ependymal cells (C) Fibroblasts (D) Microglial cells (E) Oligodendrocytes

**AI_D:** (1) RESTATING THE QUESTION: The query involves a patient who had a history of multiple cerebral infarctions and transient ischemic attacks, eventually dying of a major cerebral infarction. Postmortem examination of the cerebral cortex showed significant gliosis, a pathological state in the brain indicating past injury. Our task is to identify which cells are responsible for the observed gliosis. (2) PLANNING: To clarify, gliosis is a nonspecific reactive response to injury in the central nervous system, characterized by the proliferation of astrocytes, perhaps microglia too. On the other hand, ependymal cells line the cerebral ventricles and spinal cord but are not typically associated with gliosis. Fibroblasts, which are primarily associated with the growth of connective tissue, are not usual residents of the brain. Oligodendrocytes, while a part of the CNS, are primarily involved in myelination of axons rather than response to injury. Hence, the hypothesis to pursue here would be that astrocytes, known for their role in gliosis, are the most likely culprits. (3) CLINICAL REASONING: Upon neural damage, such as from cerebral infarctions or ischemic attacks, the brain's response is somewhat similar to the scar formation process observed in the rest of the body. Astrocytes, being the most abundant cells in the brain, play a key role in this reparative process by proliferating and hypertrophying at the site of injury to form a glial scar. This process forms the basis of gliosis. Meanwhile, microglial cells, while they do respond to injury, their role is more of an immune nature rather than structural repair. Ependymal cells, oligodendrocytes, and fibroblasts are less likely as they do not typically participate in the gliosis process. (4) CONCLUDING: Given the patterns of brain injury and repair, and the roles of different cells within the brain, it seems most probable that astrocytes are responsible for the extensive gliosis observed in the patient's cerebral cortex. (5) RESPONSE: Therefore, the growth and proliferation causing the observed gliosis is most likely due to Astrocytes (Option A).

**AI_A:** RESTATING THE QUESTION: The given information outlines a clinical history of a 76-year-old man who experienced several smaller cerebral infarctions and two transient ischemic attacks over the past decade, culminating in a fatal massive cerebral infarction. An examination of his cerebral cortex during the autopsy revealed extensive gliosis, a phenomenon usually observed in response to damage in the central nervous system. Based on the aforementioned information, the question is asking which of the listed cell types is most likely responsible for this observed proliferation in response to repeated ischemic events. PLANNING: The listed options are all cells that can be found in the CNS. Astrocytes aid in maintaining the blood-brain barrier and respond to CNS injury, Ependymal cells help produce cerebrospinal fluid, Fibroblasts are often associated with connective tissue repair, Microglia act as phagocytes in the CNS, and Oligodendrocytes function in the maintenance and creation of myelin sheaths around neurons in the CNS. The increased proliferation and growth associated with gliosis generally involved the action of supportive or 'glial' cells of the CNS, leading to the resulting fibrous scar tissue. CLINICAL REASONING: The occurrence of gliosis is most often linked to astrocytes. In response to injury within the CNS, astrocytes proliferate and become hypertrophic, a process known as 'reactive gliosis'. These reactive astrocytes are crucial in limiting inflammation and further damage, acting as a seal or barrier around the injured area. This process led to a fibrous scar that is typical of gliosis. Ependymal cells, Microglia, Oligodendrocytes, and Fibroblasts also have roles in the CNS, but they are not typically associated with the formation of gliosis. CONCLUDING: Thus, based on the clinical reasoning given, extensive gliosis seen in the cerebral cortex of this patient, who suffered from multiple cerebral infarctions over the years, is most likely caused by the growth and proliferation of astrocytes which is the body's response to limit the ischemic damage. RESPONSE: Therefore, the response to the question is (A) Astrocytes.

**AI_B:** (1) RESTATING THE QUESTION: The question pertains to a 76-year-old man who passed away due to a massive cerebral infarction. Over the last decade, he had several smaller cerebral infarctions and a couple of transient ischemic attacks. Upon autopsy, the specimen of his cerebral cortex exhibited extensive gliosis. We are to identify the cell type most likely responsible for this finding, marked by growth and proliferation. (2) PLANNING: From the given options, we can infer that we are examining different types of CNS cells that might proliferate in response to repetitive ischemic insults. For each cell type, we can evaluate its role within the CNS, particularly its relationship to injury and subsequent repair processes. Setting these conditions, we need to assess each possibility. (A) Astrocytes are known to proliferate and form a scar following CNS injury, a process known as astrocytosis or gliosis. (B) Ependymal cells line the ventricles in the brain, and are not known for pathologically significant proliferation after CNS injury. (C) Fibroblasts are key components in the repair process where a breach in the blood-brain barrier occurs, but they are otherwise not involved in reaction to localized cerebral damage. (D) Microglial cells are CNS-resident macrophages that proliferate and become phagocytic following CNS injury, a process called microgliosis. (E) Oligodendrocytes provide myelination to neurons within the CNS, and their proliferation is not typically in response to injury. Our alternate assumption should clinically prioritize the process of gliosis, as the question's stem and the extensive findings suggest its significance. (3) CLINICAL REASONING: An assessment of the types of cells points toward astrocytes. Understanding that gliosis refers to the process of astrocyte proliferation forming a glial scar following CNS injury provides a direct link to the findings. This reparative reaction is typically activated upon neuronal death, aiming to isolate the affected area and prevent the spread of potential harmful substances throughout the CNS. Our patient's history of repeated cerebral infarctions and ischemic attacks indeed suggests repeated neuronal damage, which would trigger such an astrocytic response. (4) CONCLUDING: Given these points, it is reasonable to assume that the growth and proliferation leading to extensive gliosis are largely due to astrocytes. The repetitive cerebral insults due to infarctions and transitory ischemic phenomena would instigate this neuroprotective response, consistent with our patient's neurological history and the autopsy findings. (5) RESPONSE: The growth and proliferation causing extensive gliosis observed in this patient's cerebral cortex are most likely attributable to (A) Astrocytes.

**AI_C:** RESTATING THE QUESTION: The question specifically wants us to identify which cell from the listed options would be responsible for the observed pathology; that is, extensive gliosis in the brain. Gliosis is a process that involves the overproliferation of glial cells in the central nervous system (CNS) in response to injury. PLANNING: Understanding what gliosis indicates is the key to answering this question. This reaction is usually a sign of CNS injury or disease such as a stroke. It should be noted that the glial cells in the CNS include astrocytes, oligodendrocytes, and microglial cells. So, we can hypothesize that the proliferation might have been caused by one of these cells. CLINICAL REASONING: Given the options and their known roles in the brain, astrocytes (A) are a key player in the process of reactive gliosis. Following a CNS injury like the cerebral infarctions that the individual went through, astrocytes begin to proliferate and hypertrophy, which leads to the formation of a glial scar, a crucial part of the brain's response to damage. Ependymal cells (B), fibroblasts (C), and oligodendrocytes (E) are not primarily involved in gliosis, while microglial cells (D) are more linked to neural inflammation rather than to gliosis process. CONCLUDING: Based on the clinical reasoning process, the most probable cells to contribute to the extensive gliosis in the cerebral cortex of this patient, would be astrocytes. They have a significant role in the process of reactive gliosis following a CNS injury, and their proliferation is consistent with the pathology reported in the question. RESPONSE: Therefore, the extensive gliosis observed in this patient's cerebral cortex specimen obtained at autopsy is most likely caused by the growth and proliferation of Astrocytes (A).

**AI_E:** 1) RESTATING THE QUESTION: The query entails discovering which cell type is responsible for the condition known as extensive gliosis, observed in the cerebral cortex of an elderly male patient who succumbed to a substantial cerebral infarction. He had a history of several smaller cerebral infarctions and transient ischemic attacks over the last decade. Gliosis is essentially a process of scarring in the central nervous system triggered usually by injury or disease. 2) PLANNING: Several assumptions could be taken into account here. Every cell type listed is linked with some form of brain function or pathology, but not all lead to gliosis. Astrocytes, for instance, are star-shaped glial cells located in the brain and spinal cord that form part of the blood-brain barrier and provide nutrients to nervous tissue. Ependymal cells line the ventricles in the brain and aid in the production, circulation, and absorption of cerebrospinal fluid. Fibroblasts are not typically found in the brain; they're more generally connected with wound healing in the skin and generate core connective tissue matrices in other parts of the body. Microglial cells are the primary form of immune defense in the central nervous system. Lastly, Oligodendrocytes help in the production of the myelin sheath covering nerve fibers in the central nervous system. 3) CLINICAL REASONING: Gliosis is a reactive process, where glial cells proliferate to form scar tissue in response to damage in the central nervous system. This process typically involves the proliferation of astrocytes, making them a prime candidate to be the cause of extensive gliosis observed in this patient. Ependymal cells, while being crucial for the circulation of cerebrospinal fluid, are not directly associated with gliosis. Fibroblasts are primarily involved in the formation of scar tissue in the periphery, not the central nervous system. Microglial cells, the immune cells of the central nervous system, while they might increase in certain pathological conditions, do not directly contribute to gliosis. As for Oligodendrocytes, their predominant function is the production and maintenance of the myelin sheath and do not proliferate extensively in response to injury or disease. 4) CONCLUDING: With the above reasoning, it can be stated that the pathological finding of extensive gliosis seen in the patient's cerebral cortex post-mortem would most likely be attributed to the proliferation and growth of astrocytes, considering their role in providing structural, nutritive, and regulatory support to neurons, and reactive changes to injury such as infarction. 5) RESPONSE: Therefore, extensive gliosis in cortical tissue is most likely caused by the growth and proliferation of (A) Astrocytes.

**Facilitator** The question pertains to a 76-year-old man who passed away due to a massive cerebral infarction. Over the last decade, he had several smaller cerebral infarctions and a couple of transient ischemic attacks. Upon autopsy, the specimen of his cerebral cortex exhibited extensive gliosis. We are to identify the cell type most likely responsible for this finding, marked by growth and proliferation. The listed options are all cells that can be found in the CNS. Astrocytes aid in maintaining the blood-brain barrier and respond to CNS injury, Ependymal cells help produce cerebrospinal fluid, Fibroblasts are often associated with connective tissue repair, Microglia act as phagocytes in the CNS, and Oligodendrocytes function in the maintenance and creation of myelin sheaths around neurons in the CNS. The occurrence of gliosis is most often linked to astrocytes. In response to injury within the CNS, astrocytes proliferate and become hypertrophic, a process known as 'reactive gliosis'. These reactive astrocytes are crucial in limiting inflammation and further damage, acting as a seal or barrier around the injured area. This process led to a fibrous scar that is typical of gliosis. Ependymal cells, Microglia, Oligodendrocytes, and Fibroblasts also have roles in the CNS, but they are not typically associated with the formation of gliosis. Given these points, it is reasonable to assume that the growth and proliferation leading to extensive gliosis are largely due to astrocytes. The repetitive cerebral insults due to infarctions and transitory ischemic phenomena would instigate this neuroprotective response, consistent with our patient's neurological history and the autopsy findings. Therefore, the response to the question is (A) Astrocytes. The growth and proliferation causing extensive gliosis observed in this patient's cerebral cortex are most likely attributable to Astrocytes.
