# Supplementary material for: Collaborative intelligence in AI: Evaluating the performance of a council of AIs on the USMLE
Source: PLOS Digit Health. 2025 Oct 9;4(10):e0000787. doi: 10.1371/journal.pdig.0000787 (PMC12510544; doi:10.1371/journal.pdig.0000787)
Supplement: S2 File — (ZIP) [file pdig.0000787.s002.zip › S2 Data Raw data files containing unprocessed question inputs and LLM outputs including transcripts of deliberations/STEP1_Question-19-1_Type-MC-NJ.docx]

**Facilitator** **[Round 1]:** A previously healthy 52-year-old woman comes to the physician because of a 2-month history of fatigue, constipation, and frequent urination. Her temperature is 37.1°C (98.8°F), pulse is 80/min, respirations are 14/min, and blood pressure is 140/90 mm Hg. Diffuse crackles are heard bilaterally. Her serum calcium concentration is 11.1 mg/dL, and serum parathyroid hormone concentration is decreased. A chest x-ray shows bilateral hilar lymphadenopathy and interstitial infiltrates. Which of the following is the most likely cause of this patient's hypercalcemia? (A) Calcitriol production by activated macrophages (B) Local resorption of bone by metastases (C) Parathyroid hormone-related peptide secretion (D) Secretion of parathyroid hormone (E) Secretion of thyroid-stimulating hormone

**AI_D:** (1) RESTATING THE QUESTION: The issue at hand is determining the cause of hypercalcemia in a 52 year old woman who has been experiencing various uncomfortable symptoms including constipation and frequent urination. She has been found to have an elevated calcium level in her blood serum, decreased parathyroid hormone and abnormal results on her chest X-ray. Hypercalcemia is an abnormally high level of calcium in the blood, which can cause a variety of health problems. (2) PLANNING: We know that the woman's hypercalcemia is not due the secretion of parathyroid hormone as her serum parathyroid hormone concentration is decreased. The question remains whether this could be a result of Calcitriol production (A) by macrophages, Bone metastases leading to resorption of bone (B), parathyroid hormone-related peptide secretion (C), or Secretion of thyroid-stimulating hormone (E). (3) CLINICAL REASONING: Her symptoms such as fatigue, constipation, and frequent urination can be attributed to hypercalcemia. Bone metastases(B) often produces localized symptoms and the patient does not have any such symptoms reported. The secretion of thyroid-stimulating hormone(E) typically leads to hyperthyroidism and the patient does not exhibit such symptoms. Given the chest findings of bilateral hilar lymphadenopathy and interstitial infiltrates, it suggests a granulomatous disease such as sarcoidosis, which causes activated macrophages to produce elevated levels of calcitriol (A). (4) CONCLUDING: Therefore, after performing the step-by-step clinical reasoning to evaluate this case, it can be suggested that sarcoidosis causing increased Calcitriol production by macrophages appears to be the most likely cause. (5) RESPONSE: Accordingly, the most likely cause of this patient's hypercalcemia is likely due to (A) Calcitriol production by activated macrophages.

**AI_B:** RESTATING THE QUESTION: What we have here is a 52-year-old woman who has been experiencing fatigue, constipation, and frequent urination for the past two months. Her vitals are generally within normal limits, but she does have high blood pressure, and bilateral crackles are heard in her lungs. More importantly, her serum calcium concentration is elevated and her serum parathyroid hormone concentration is decreased. A chest x-ray reveals bilateral hilar lymphadenopathy and interstitial infiltrates. From these findings, we need to identify what is likely causing her hypercalcemia, or high blood calcium levels. PLANNING: The assumptions that would be commonplace with such a presentation would point towards primary hyperparathyroidism or metastases. However, both of these assumptions are contradicted by her decreased parathyroid hormone and the absence of any mention of cancer in her medical history, leading to alternate assumptions such as secretion of calcitriol by activated macrophages, secretion of parathyroid hormone-related peptide (PTHrP) or secretion of thyroid-stimulating hormone (TSH). CLINICAL REASONING: This patient is presenting with hypercalcemia without increased PTH levels, which usually suggests an extrarenal source of calcium level control, such as malignancies or granulomatous diseases. Due to the presence of bilateral hilar lymphadenopathy and interstitial infiltrates on chest X-ray, this patient's presentation is characteristic of sarcoidosis, a multisystem granulomatous disease commonly affecting the lungs. In sarcoidosis, activated macrophages in granulomas can produce calcitriol, the activated form of vitamin D, coming to our initial alternate assumption of calcitriol production by activated macrophages. CONCLUDING: Calcitriol would in turn enhance gastrointestinal absorption of calcium and release of calcium from the bone, leading to hypercalcemia. Decreased parathyroid hormone production is a compensatory response to the hypercalcemia, explaining the abnormal PTH level in this patient. Therefore, the hypercalcemia in this instance is most likely due to calcitriol production by activated macrophages in the lung granulomas. RESPONSE: (A) Calcitriol production by activated macrophages is the most likely cause of this patient's hypercalcemia.

**AI_A:** (1) RESTATING THE QUESTION: The patient, a 52-year-old woman, has been experiencing various symptoms including fatigue, constipation, and frequent urination for two months. She also presents with a slightly elevated blood pressure, bilateral crackles in her lungs, increased serum calcium and decreased serum parathyroid hormone. A chest X-ray revealed bilateral hilar lymphadenopathy and interstitial infiltrates. The key question here is: What could be causing her heightened levels of calcium, also known as hypercalcemia? (2) PLANNING: There are several potential causes for hypercalcemia. Some assumptions based on the choices provided include (A) An increase in Calcitriol production by activated macrophages, (B) Local resorption of bone owing to metastases, (C) Release of parathyroid hormone-related peptide (PTH-RP), (D) Overproduction of parathyroid hormone, or (E) An increase in secretion of thyroid-stimulating hormone (TSH). Parathyroid hormone (PTH) and PTH-RP enhance renal calcium reabsorption, while Calcitriol is the active form of vitamin D and promotes intestinal absorption of calcium. However, the fact that the patient's serum PTH level is decreased would argue against options D and E, while hilar lymphadenopathy and interstitial infiltrates could both be indicative of a granulomatous disease (such as sarcoidosis), which might point to option A. (3) CLINICAL REASONING: Granulomatous diseases, characterized by the presence of granulomas, trigger macrophages activation and as a result, increased production of Calcitriol. The increased Calcitriol promotes intestinal absorption of calcium, causing hypercalcemia. This is consistent with the findings of bilateral hilar lymphadenopathy and interstitial infiltrates in the patient's chest x-ray, which are typical findings in individuals with sarcoidosis. Additionally, the decrease of parathyroid hormone can be explained by the physiological response to elevated calcium levels where high serum calcium suppresses PTH secretion. (4) CONCLUDING: With all this in mind, it is likely that the heightened levels of serum calcium (hypercalcemia) in this patient are due to the increased Calcitriol production by activated macrophages, caused by a granulomatous disease such as sarcoidosis. In this scenario, the increased Calcitriol levels would work to promote absorption of calcium from the gastrointestinal tract. (5) RESPONSE: Therefore, the most likely cause of this patient's hypercalcemia is (A) Calcitriol production by activated macrophages.

**AI_E:** RESTATING THE QUESTION: A 52-year-old woman, who has been healthy up until now, has visited her doctor because she's been feeling weak, her defecation patterns have changed, and she's urinating more frequently for the past two months. Her vitals, including temperature, pulse, respirations, and blood pressure, fall within or slightly above the normal range, but are not alarmingly high. Upon examination, crackles can be heard throughout her lungs, and her blood tests reveal a high level of calcium and a low level of parathyroid hormone. Imaging of her chest shows swelling in the lymph nodes near her lungs and changes in the tissue pattern within the lung itself. We're trying to figure out what might be causing this woman's high calcium level. The possible culprits include increased production of calcitriol from macrophages, bone being broken down due to metastases, the release of a hormone similar to parathyroid hormone, the release of parathyroid hormone itself, or the release of thyroid-stimulating hormone. PLANNING: The immediate assumption given her symptoms and serum calcium concentration would point towards a condition involving excessive release of parathyroid hormone, but this does not correlate with her decreased parathyroid hormone concentration. Another possible assumption here could be overactivity of another source leading to the increased levels of calcium. With the findings of bilateral hilar lymphadenopathy and interstitial infiltrates, it's appropriate to explore a lung-involved pathology causing non-parathyroid hormone mediated hypercalcemia. CLINICAL REASONING: Bilateral hilar lymphadenopathy and interstitial infiltrates may suggest sarcoidosis, a systemic disease involving growth of tiny collections of inflammatory cells. In sarcoidosis, activated macrophages may produce excess calcitriol, causing raised calcium levels. However, the body in conditions of high calcium typically reduces parathyroid hormone secretion. This scenario aligns with the decrease in parathyroid hormone concentration and increased calcium concentration witnessed in this patient. Therefore, calcitriol production by activated macrophages due to possible sarcoidosis seems to fit this clinical presentation better than the other options listed, like hormone secretion by the thyroid or parathyroid or local resorption of bone by metastases (which seems unlikely without any other clinical signs of malignancy). CONCLUDING: In light of her decreased parathyroid hormone concentration, bilateral hilar lymphadenopathy, interstitial infiltrates, and symptoms, the most probable cause of her hypercalcemia would appear to be the excessive production of calcitriol, due to the systemic response to an inflammatory disease such as sarcoidosis. RESPONSE: (A) Calcitriol production by activated macrophages is the most likely cause of this patient's hypercalcemia.

**AI_C:** (1) RESTATING THE QUESTION: This clinical scenario describes a 52-year-old woman who presents with fatigue, constipation, and frequent urination, alongside bilateral crackles in the chest and bilateral hilar lymphadenopathy with interstitial infiltrates. Despite decreased parathyroid hormone levels, her serum calcium concentration is elevated (a condition called hypercalcemia). The question asks what the most probable cause of this hypercalcemia is. Hypercalcemia refers to an unusually high calcium level in the blood, which can result in several symptoms, as seen in this patient. An obvious source of this high calcium level could be the increased production or action of parathyroid hormone (PTH) that triggers calcium release from the bones and enhanced calcium absorption in the gut and kidneys. However, this case shows a decreased PTH level, suggesting other causes of hypercalcemia. (2) PLANNING: When considering the options, it's important to note that all the choices relate somehow to calcium metabolism. Calcitriol, the active form of vitamin D, can increase calcium absorption in the gut; Local bone resorption by metastases could result in increased calcium levels; secretion of PTH and PTH-related peptide (PTHrP) could increase calcium levels through the same mechanisms as PTH; and Thyroid-stimulating hormone is usually associated with calcium homeostasis indirectly, especially through its regulation of calcitonin which decreases calcium levels. Given the patient's decreased PTH levels and the presence of interstitial lung infiltrate, it might be more reasonable to consider mechanisms relating to the lung pathology. (3) CLINICAL REASONING: The clinical presentation possesses two main pathology findings: hypercalcemia despite decreased PTH level, and lung findings denoted by bilateral hilar lymphadenopathy with interstitial infiltrates. Among the choices, (A) Calcitriol production by activated macrophages and (C) PTHrP secretion could plausibly match these criteria. Macroactivated cells in granulomatous diseases like sarcoidosis can secrete calcitriol and induce hypercalcemia. The chest X-ray findings in this patient suggest some form of infiltrative lung disease, possibly sarcoidosis. On the other hand, PTH-related peptide (PTHrP) is commonly secreted by malignant tumors, and can mimic PTH action causing hypercalcemia of malignancy. The crackles and lymphadenopathy may suggest a malignancy; however, this usually causes increased rather than decreased PTH levels. (4) CONCLUDING: Comparing these two hypotheses, sarcoidosis provides a more consistent explanation for this patient's symptoms and findings. (5) RESPONSE: Thus, the most likely cause of this patient's hypercalcemia is (A) Calcitriol production by activated macrophages.

**Facilitator** The consensus among the AI responses is that the most likely cause of the 52-year-old woman's hypercalcemia is Calcitriol production by activated macrophages (Option A). This conclusion is based on her symptoms, including fatigue, constipation, and frequent urination, as well as her clinical findings, such as slightly elevated blood pressure, bilateral crackles in her lungs, increased serum calcium, decreased serum parathyroid hormone, and chest X-ray revealing bilateral hilar lymphadenopathy and interstitial infiltrates. The decreased parathyroid hormone levels argue against options D and E, which involve overproduction of parathyroid hormone and an increase in secretion of thyroid-stimulating hormone, respectively. The absence of any mention of cancer in her medical history makes option B, local resorption of bone owing to metastases, less likely. The presence of bilateral hilar lymphadenopathy and interstitial infiltrates in the patient's chest X-ray suggests a granulomatous disease such as sarcoidosis. In such diseases, activated macrophages produce increased amounts of Calcitriol, the active form of vitamin D, which promotes intestinal absorption of calcium, leading to hypercalcemia. This aligns with the patient's increased serum calcium levels. The decrease in parathyroid hormone can be explained by the physiological response to elevated calcium levels, where high serum calcium suppresses PTH secretion. Therefore, the consensus is that the most likely cause of this patient's hypercalcemia is Calcitriol production by activated macrophages, likely due to a granulomatous disease such as sarcoidosis.
