# Supplementary material for: Collaborative intelligence in AI: Evaluating the performance of a council of AIs on the USMLE
Source: PLOS Digit Health. 2025 Oct 9;4(10):e0000787. doi: 10.1371/journal.pdig.0000787 (PMC12510544; doi:10.1371/journal.pdig.0000787)
Supplement: S2 File — (ZIP) [file pdig.0000787.s002.zip › S2 Data Raw data files containing unprocessed question inputs and LLM outputs including transcripts of deliberations/STEP1_Question-2-1_Type-MC-NJ.docx]

**Facilitator** **[Round 1]:** A 48-year-old man dies suddenly of a cardiac arrhythmia. Six weeks ago, he was resuscitated from a cardiac arrest caused by coronary artery disease and developed oliguric renal failure. Five weeks ago (1 week after being resuscitated), his serum urea nitrogen (BUN) concentration was 40 mg/dL, his serum creatinine concentration was 3.5 mg/dL, and his urine contained granular casts. Four weeks ago, his oliguria resolved, and his BUN and serum creatinine concentrations returned to normal. Examination of his kidneys at autopsy is most likely to show which of the following? (A) Acute inflammation (B) Fibrinous exudate (C) Fibrous scar (D) Granulation tissue (E) Normal kidney

**AI_D:** RESTATING THE QUESTION: The question asks us to predict the likely autopsy findings of the kidneys of a patient who had a history of cardiac arrest leading to oliguric renal failure, with subsequent return of renal function to normal. PLANNING: We have several assumptions here. Assumptions include acute inflammation, fibrinous exudate, fibrous scar, granulation tissue, or a normal kidney. However, given the course of events, acute inflammation and fibrinous exudate would have been likely at the time of acute renal injury or shortly thereafter, not after several weeks of renal recovery. A normal kidney could be another assumption, but the initial very high concentrations of BUN and creatinine indicate severe injury that is unlikely to resolve without any morphological evidence. Therefore, it is reasonable to hypothesize that the kidney may have undergone tissue remodeling or repair, associated with granulation tissue formation or fibrous scar formation. CLINICAL REASONING: The renal injury was undoubtedly serious, indicated by a high BUN and creatinine concentration and the presence of granular casts in the urine. However, the patient's renal function returned to normal, suggesting substantial recovery or repair. Fibrous scars and granulation tissue are both seen in tissue regeneration, but granulation tissue is an early phase in the wound healing process, mainly seen in the first to third week post-injury. As it's already been six weeks, this eliminates granulation tissue as a possibility (option D). Scar formation is a final product of the wound healing process, apparent at the late stage, even weeks or months after the initial injury. Therefore, it is highly likely we will see a fibrous scar. CONCLUDING: The entire clinical picture brings us to the conclusion that the most probable finding on kidney autopsy will be a fibrous scar. This is based upon the timing, the severe initial injury indicated by high BUN and creatinine concentrations, and the full return of renal function suggesting completion of the wound healing process. RESPONSE: The most likely autopsy finding in this 48-year-old man's kidney is (C) Fibrous scar.

**AI_C:** (1) RESTATING THE QUESTION: Given the scenario, we have a middle-aged man who had a cardiac arrest due to coronary artery disease, experienced renal failure that was characterized by high BUN and creatinine concentrations along with the presence of granular casts in his urine. However, these symptoms resolved, and his kidney function measures — BUN and creatinine — returned to normal. After his sudden demise due to a cardiac arrhythmia, his kidney health at the time of death is being questioned, specifically inquiring about what the autopsy of the kidneys would most likely reveal. (2) PLANNING: Considering the data, it seems that he had acute kidney injury (AKI) secondary to cardiac arrest, which resulted in oliguric renal failure. Oliguria and elevated BUN and creatinine levels suggest kidney damage where waste products were not effectively removed by the kidneys. Furthermore, granular casts are indicative of tubular damage frequently seen in acute tubular necrosis (ATN), a common cause of AKI. However, returning to normal kidney function markers suggests recovery. In such recovery scenarios from ATN, for instance, a scar tissue — fibrous scar — can often form in place of the damaged part of the kidney. Therefore, my initial hypothesis is a fibrous scar (C). (3) CLINICAL REASONING: In analyzing the clinical scenario, starting with a cardiac event leading to oliguric renal failure is indicative of a prerenal type of AKI, possibly developing into intrinsic renal failure due to acute tubular necrosis post prolonged renal hypoperfusion. It is crucial to note that the BUN and serum creatinine concentration normalized, which suggests improvement of renal function, supportive recovery post-ATN. During the recovery phase of ATN, tubular epithelial cells proliferate and differentiate to replace the necrotic cells. Over time, the granulation tissue organizes and a fibrous scar may form. His urine containing granular casts further validates this claim as they signify tubular cell necrosis and are present in abundance in ATN. (4) CONCLUDING: With a step-by-step explanation, based on the given history of the renal injury he faced and its subsequent recovery as demonstrated by normalized BUN and creatinine levels, it seems most likely that the kidney at autopsy will present evidence of such a healing process. (5) RESPONSE: Thus, considering the clinical picture and reasoning, my final response is that the autopsy of his kidney is most likely to show Fibrous scar (C).

**AI_A:** (1) RESTATING THE QUESTION: The question describes a scenario where a middle-aged adult male suffered a sudden cardiac arrest due to coronary artery disease six weeks prior and was successfully resuscitated. However, post-resuscitation, he developed a condition of oliguric renal failure characterized by reduced urine output. The patient's laboratory findings a week after resuscitation indicate elevated BUN and creatinine levels and presence of granular casts in the urine, all of which point towards renal impairment. Four weeks prior to his death, his urine output returned to normal, and his BUN and creatinine levels normalized. We are asked to predict the likely findings on kidney examination post mortem. (2) PLANNING: The sudden cardiac arrest, followed by oliguric renal failure, suggests that the patient had suffered from acute kidney injury (AKI), likely secondary to ischemia following cardiogenic shock. Four options provided (A, B, D, and E) reflect stages or elements of inflammation and healing - acute inflammation, fibrinous exudate, granulation tissue, and a normal kidney, while one option (C) represents a chronic process - fibrous scar. Considering the timeline of events, it's unlikely that acute inflammation or fibrinous exudate would still be present, as his lab findings normalized. Similarly, granulation tissue represents an intermediate phase of healing, likely not seen at this point due to normalization of renal function. A normal kidney might also not be correct, as some residual damage might be present. The remaining option, fibrous scar, represents a chronic healing process from the renal injury, providing an assumption to evaluate. (3) CLINICAL REASONING: In situations of severe renal ischemia, such as AKI after cardiogenic shock, tubular cells in the kidney can undergo necrosis, leading to the presence of granular casts in the urine, a sign of tubular injury. When the ischemic insult is removed, as in this case, where the patient's cardiac status had presumably stabilized, the renal function can recover with regeneration of tubular cells. This recovery is indicated by the normalization of renal function tests and resolution of oliguria. However, this process is often not perfect, and areas where tubular necrosis had occurred can get replaced with fibrous scar tissue. (4) CONCLUDING: Thus, considering the severity of the initial event, the clinical progression, and the normalization of parameters, the likely kidney autoptic finding would not be entirely normal tissue, nor would it show signs of acute or active healing processes. Residual tissue changes after the healing from severe ischemic injury would accrue as fibrous scar tissue. (5) RESPONSE: Based on the above reasoning, the most likely kidney autoptic finding is (C) fibrous scar.

**AI_B:** (1) RESTATING THE QUESTION: Let's first comprehend the presented clinical case. A middle-aged man passed away due to a cardiac arrhythmia. He suffered a cardiac arrest six weeks prior to his sudden death which was attributed to coronary artery disease. Following his resuscitation, he had developed oliguric renal failure - a condition characterized by low urine output usually less than 400 mL/day. His renal function tests manifested with elevated serum urea nitrogen (40 mg/dL) and creatinine (3.5 mg/dL) levels, both of which were indicative of kidney impairment. His urine test also showed the presence of granular casts, which are cylindrical aggregates of protein and cell fragments often found in conditions having severe kidney damage. However, his oliguria, along with his abnormal BUN and creatinine levels, got resolved in a week. The query here raises the possible finding that we might encounter in the autopsy of his kidneys. (2) PLANNING: The case calls for recognizing the possible changes that kidney underwent during the described timeline. A few assumptions load the context: acute inflammation, fibrinous exudate, fibrous scar, granulation tissue and normal kidney. Inflammation, fibrinous exudates, and granulation tissue are commonly observed in acute renal disorders. However, his kidney function appeared to return to normal over time, which argues against these assumptions. Fibrous scars are seen in chronic renal disease setting where there is persistent inflammation or repeated injury. While normal kidney suggests complete renal recovery after the initial harm ensued by the cardiac arrest. (3) CLINICAL REASONING: Given this man's history, his kidneys may have initially suffered from acute kidney injury (AKI), possibly due to ischemic insult during his cardiac arrest. AKI primarily causes cellular injury leading to the formation of granular casts that were found in his urine. That may result in renal tubular epithelial cell death and subsequent release of tubular epithelial cell debris along with specific proteins into the tubular lumen - a condition known as acute tubular necrosis (ATN). It is characterised by two phases - oliguric phase, as signalled by the man's decreased urine output initially, followed by a diuretic phase where the oliguria resolves and kidney function tends to improve - a clear match for the patient's clinical course. At this point in ATN, the damaged tubular cells begin to regenerate and repopulate the cavities of the tubules. These changes, however, do not give rise to scar tissue formation as the parenchymal architecture of the kidney is generally preserved in ATN. (4) CONCLUDING: Therefore, considering the acute renal failure episode following the cardiac arrest, its subsequent resolution as witnessed by the normalization of urine output and kidney function tests, and given the two-week point from the insult, it's likely that the man's kidneys were in a state of recovery from acute tubular necrosis. (5) RESPONSE: Consequently, one should expect to see that the kidneys looked relatively normal on autopsy (Option E), assuming the recovery process from acute tubular necrosis was underway and no additional renal insult ensued.

**AI_E:** RESTATING THE QUESTION: The scenario is about a 48-year-old man who died due to a cardiac arrhythmia. His prior medical history includes a cardiac arrest six weeks ago due to coronary artery disease, subsequent resuscitation, and oliguric renal failure. Elevated serum urea nitrogen (BUN) and serum creatinine concentration, along with urinary granular casts, confirmed the renal failure. Fortunately, he recovered from the acute renal failure, as suggested by his returning to a normal urine output (oliguria resolved) and decreased levels of BUN and serum creatinine to normal ranges. We are asked to predict what would be the most likely histological findings in the man's kidneys at autopsy. PLANNING: A cardiac arrest could have led to renal ischemia resulting in acute tubular necrosis (ATN). The oliguria, elevated BUN and creatinine, and granular casts in the urine, all support this assumption. Granular casts are usually seen in ATN as they represent disrupted renal tubular cells. Renal recovery from ATN could lead to several histological appearances, which include acute inflammation, fibrinous exudate, fibrous scar, granulation tissue or a fully recovered kidney with normal appearance. CLINICAL REASONING: Acute inflammation and fibrinous exudate are rather early processes in tissue damage and repair and would likely be seen soon after an ischemic injury to the kidneys, but less likely after weeks of recovery. Fibrous scar and granulation tissue both represent healing and repair. However, granulation tissue, which is an important part of the proliferative phase of wound healing, arises in the early stages of healing and is typically replaced by a fibrous scar as the healing process continues over weeks. So, granulation tissue might be more likely to be seen a week or two after the ATN episode and less likely after four weeks of recovery. A fibrous scar, on the other hand, is the result of the healing of severe tissue injury like ATN and it might still be seen weeks after the recovery from ATN. Finally, if the kidneys had fully recovered histologically, they could look normal. CONCLUDING: Given the severity of the man's initial insult (cardiac arrest and subsequent resuscitation) and the timing of his recovery, it is less likely that his kidneys had fully recovered to look completely normal again within this timeframe. Therefore, considering the time frame of several weeks between the onset of ATN and the man's death, the kidneys would most likely show signs of the later stages of healing, namely fibrous scar tissue. RESPONSE: Thus, based on this clinical scenario, and following the pathogenesis and wound healing process, the kidneys at autopsy are more likely to show (C) Fibrous scar.

**Facilitator** **[Round 2]:** Summary: AI_A, AI_C, AI_D, and AI_E all agree that the most likely autopsy finding in the patient's kidney would be a fibrous scar (C). They base their reasoning on the patient's history of cardiac arrest leading to acute kidney injury (AKI), followed by oliguric renal failure. They argue that the normalization of the patient's renal function suggests recovery, but the severe initial injury likely resulted in some residual damage, which would manifest as fibrous scar tissue. AI_B, however, believes that the most likely autopsy finding would be a normal kidney (E). AI_B argues that the patient's kidneys likely suffered from acute tubular necrosis (ATN) following the cardiac arrest, but the normalization of renal function and urine output suggests that the kidneys were in a state of recovery from ATN at the time of the patient's death. AI_B further argues that ATN does not typically result in scar tissue formation, as the parenchymal architecture of the kidney is generally preserved in ATN. Question to Clarify Disagreements: Can you provide more information on the likelihood and timeline of fibrous scar formation following acute tubular necrosis (ATN) and acute kidney injury (AKI)? Specifically, under what conditions might ATN lead to fibrous scar formation, and how might this process differ from the recovery process in AKI?. Based on your preceding response, how would you answer the original question: [A 48-year-old man dies suddenly of a cardiac arrhythmia. Six weeks ago, he was resuscitated from a cardiac arrest caused by coronary artery disease and developed oliguric renal failure. Five weeks ago (1 week after being resuscitated), his serum urea nitrogen (BUN) concentration was 40 mg/dL, his serum creatinine concentration was 3.5 mg/dL, and his urine contained granular casts. Four weeks ago, his oliguria resolved, and his BUN and serum creatinine concentrations returned to normal. Examination of his kidneys at autopsy is most likely to show which of the following? (A) Acute inflammation (B) Fibrinous exudate (C) Fibrous scar (D) Granulation tissue (E) Normal kidney]

**AI_C:** Restating the question: The question seeks to identify the most likely pathological finding in the kidneys of a man with a history of coronary artery disease and cardiac arrest. This patient had an episode of acute kidney injury marked by high serum BUN and creatinine levels, oliguria, and granular casts in the urine. However, kidney function returned to normal after several weeks. The options provided include acute inflammation, fibrinous exudate, fibrous scar, granulation tissue, and normal kidney. PLANNING: A logical assumption would be that the acute kidney injury (AKI) in this patient was due to acute tubular necrosis (ATN), caused by renal ischemia subsequent to the cardiac arrest. ATN is characterized initially by acute inflammation, progressing to granulation tissue, and then fibrous scar tissue as the damage heals. Another assumption could be that if full recovery took place, then it may resemble a normal kidney at autopsy. The key considerations to evaluate here are the likelihood and timeline of fibrous scar formation (a sign of healing) following ATN and if full recovery to a completely normal state without any signs of prior injury is possible. Clinical reasoning: Considering the clinical course of AKI due to ATN, initial damage to renal tissue caused by ischemia would involve acute inflammation and necrosis. As healing and cellular recovery occur, a stage of granulation tissue formation follows the inflammation. Granular casts detected in the urine reflect this tubular injury and recovery process. Finally, fibrosis or scarring may occur as part of the late healing process in severe cases where injury extends beyond simple tubular cell death, leading to chronic kidney disease. However, as renal function (based on BUN and creatinine levels) and urine output have returned to normal, one could argue that it reflects the kidney's capacity for regeneration and repair following ATN, possibly signifying complete recovery. Concluding: The likelihood of finding a fibrous scar in such a patient depends on the extent of initial injury and the kidney's capacity for regeneration. Despite full functional recovery, if severe initial kidney damage had occurred, some fibrous scar tissue may still be present. Meanwhile, if the renal recovery was complete with restoration of tubular cell architecture, when few to no fibroblastic activities occurred (which form the fibrous scar), the kidney's histology could appear normal at autopsy. So, the response would be dependent on the individual patient's kidney response, but the most likely finding at autopsy given the clinical context would be (C) Fibrous scar, even though (E) Normal kidney remains a possibility. The complete functional recovery does not necessarily equate to the lack of residual histological changes.

**AI_E:** RESTATING THE QUESTION: Our case is that of a 48-year-old man who died of a cardiac arrhythmia. Six weeks prior to his death, he suffered from a cardiac arrest resulting from coronary artery disease leading to subsequent oliguric renal failure, which is a form of acute kidney injury (AKI) characterized by low urine output. After his resuscitation, abnormal kidney function tests and the presence of granular casts in his urine confirmed this AKI. These granular casts represent a breakdown of renal tube cells which is a sign of acute tubular necrosis (ATN). His renal function normalized after four weeks, indicating a recovery. With this background, we are to predict the most likely state of his kidneys at autopsy. PLANNING: Some council members postulate that fibrous scar (C) would be present, given the severe initial kidney injury. Scar tissue often forms during the process of healing, which might have occurred given the duration of his normalized renal function before death. Conversely, some argue that his kidneys would appear normal (E), taking into account that his renal functions, evidenced by BUN and serum creatinine levels, had returned to normal before death, representing recovery from ATN which might have spared structural kidney damage. CLINICAL REASONING: We need to understand the timeline and features of kidney recovery from ATN. This process begins with the proliferation of viable renal tubular cells attempting to replace dead cells sloughed off into the tubule, forming granular casts. By four weeks, as exhibited in this case, renal function normalizes, suggesting that renal tubular cell regeneration has occurred successfully. Given that ATN, particularly when it's prerenal (as in this case, due to reduced kidney perfusion after cardiac arrest), is often reversible, in most cases no lasting structural damage is incurred. This is unlike postrenal causes of kidney injury (like urinary tract obstructions), where prolonged damage often results in fibrotic changes. CONCLUDING: Taking into consideration the aforementioned clinical reasoning, it becomes clear that while possible, the formation of fibrous scar tissue (C) in ATN is not the rule. Moreover, considering the cardiac arrest instigated a prerenal cause of ATN, the restoration of renal function markers and resolution of oliguria indicate a recovery of the tubular epithelium. Given this recovery, fibrous scar tissue is less likely to have formed. RESPONSE: Given the man's clinical course, his kidneys would most likely appear normal (E) at autopsy despite his history of oliguric renal failure following cardiac arrest because his return to normal renal function suggests successful recovery from acute tubular necrosis.

**AI_D:** Restating the question in my own words, the inquiry here is about the most likely finding on a renal autopsy for a patient who presented a cardiac arrest which caused coronary artery disease and resulted in kidney injury, followed by oliguric renal failure, then restoration of renal function and output. It is crucial to note that urea nitrogen and serum creatinine returned to normal in the timeline and granular casts were found in his urine. To identify assumptions, one could presume that the original cardiac arrest and resultant oliguric renal failure led to either permanent or temporary kidney damage. A second assumption might be that once the renal parameters return to normal and the oliguria is resolved, the kidney's structure and function also returns to normal. By reviewing the discussion of the other AIs, we have the opportunity to evaluate these assumptions. In terms of clinical reasoning, acute kidney injury (often used interchangeably with acute renal failure) commonly arises when there has been a significant decrease in blood flow to the kidneys, perhaps brought on by a cardiac arrest. In this setting, acute tubular necrosis can occur as a complication, signified by the presence of granular casts in the urine. The fact that the test results returned to normal along with resolved oliguria implies that kidneys were indeed recovering from ATN, which can spontaneously recover over time. Regarding the conclusion, scar formation in the kidneys post-AKI is a contentious issue. Most recessive damages can heal without substantial scar tissue, especially in conditions like ATN, where tubular cell regeneration can occur without permanent scarring. However, it's important to recognize that some cases might have residual damage manifesting as fibrous scar tissue post-ATN, but this is typically not the predominant feature. Conclusively responding to the question, I tend towards the viewpoint of a "normal kidney" (E) as the most likely autopsy finding. While the initial injury following his cardiac arrest likely caused ATN (evidenced by oliguria and granular casts in his urine), the return of his BUN and serum creatinine concentrations to normal suggest the kidneys were regenerating and recovering. While minor fibrous scarring could be detected in some cases, it might not be extensive enough to be the primary pathological finding on autopsy. Thus, this patient's kidneys could very much resemble normal kidneys in autopsy, despite his clinical history.

**AI_A:** 1. RESTATING THE QUESTION: The question asks for the most likely autopsy finding in a 48-year-old man's kidneys who initially suffered a cardiac arrest derived from coronary artery disease. This led to acute kidney injury (AKI), more specifically oliguric renal failure, as evidenced by an increased serum urea nitrogen concentration, an elevated serum creatinine concentration, and granular casts present in the urine. However, his renal function returned to normal after the resolution of the oliguria. The options provided for the autopsy report include acute inflammation, fibrinous exudate, fibrous scar, granulation tissue, and a normal kidney. 2. PLANNING: The clinical history suggests that the patient suffered from acute tubular necrosis (ATN) following cardiac arrest. This condition occurs when the renal tubular cells become necrotic due to ischemia or hypoxia and subsequently slough off, causing blockages that result in diminished renal filtration and oliguria. The presence of granular casts in the patient's urine further supports this conjecture. Although the renal function returned to normal, it's plausible to hypothesize that this severe initial damage resulted in unavoidable residual renal parenchymal damage. Therefore, the key assumption is that the renal injury was severe enough to cause residual damage but sufficiently managed to allow renal function recovery. 3. CLINICAL REASONING: Given that the renal function normalized a week after the event, it's suggestive of recovery from ATN. In the case of ATN, acute damage to the renal tubules is rapidly reversible as these cells have a robust capacity for regeneration due to their rapid turnover rate. However, in scenarios where severe, prolonged ischemia leads to the death of tubular cells, the basement membrane can be compromised, and attempts to organize and replace the dead tubular tissue can lead to interstitial fibrosis and tubular atrophy, resulting in fibrosis scar tissue. 4. CONCLUDING: Considering the severity and timeline of the patient's renal condition, along with the capacity of renal tubules for regeneration, it would be plausible to expect some degree of scarring from the organization and repair process of the tubular structure. Yet, given that the renal function tests returned to normal values relatively quickly after the oliguria event, it suggests that the tubular structure was preserved enough to allow for tubular regeneration, minimizing the extent of potential scarring. 5. RESPONSE: Therefore, although the formation of fibrous scar tissue (Option C) could be a possible outcome of severe acute tubular necrosis, the quick return to normal renal function in this case suggests a higher likelihood of a Normal Kidney (Option E) upon autopsy examination.

**AI_B:** RESTATING THE QUESTION: Our debate revolves around two possible pathology findings in a kidney autopsy: fibrous scar, indicative of permanent damage following an acute kidney injury (AKI), or normal kidney indicating complete recovery from such an injury. We observe these two interpretations within the context of our patient who suffered from a cardiac arrhythmia, causing a cardiac arrest and an ensuing AKI, which presented typical features such as an increased concentration in blood urea nitrogen (BUN) and serum creatinine, along with oliguria and the presence of granular casts in the urine. However, his renal parameters returned to normal, and the oliguria resolved, which usually hints towards recovery. PLANNING: There seem to be two contrasting hypotheses here: 1) Our patient's kidneys have sustained severe damage leading to fibrous scar formation despite normalization of BUN and serum creatinine. 2) The kidneys have completely recovered and appear normal in the autopsy. Typically, given the patient's history of cardiac arrest, AKI, and eventual recovery of renal function, the renal tissue could go through various stages of patchy necrosis and tubular damage called acute tubular necrosis (ATN). Again, in ATN, although the functional recovery is evident, the structural recovery depends on the severity of the initial injury. Therefore, if the injury caused by ischemia was severe enough to disrupt the renal parenchymal architecture, fibrous scar tissue could form. Conversely, with preservation of renal architecture, despite ATN, a complete recovery might ensue, leading to normal kidney histology. CLINICAL REASONING: As we know, renal impairment in the setting of severe cardiac disease is commonly ischemic in nature due to the reduction of renal blood flow. Ischemia triggers renal parenchymal injury manifesting as an increase in BUN, serum creatinine, and oliguria. In this context, it's pertinent to understand the pathology of ischemic AKI, usually acute tubular necrosis. In ATN, damage is predominantly to the renal tubules, while the glomeruli and blood vessels remain relatively spared. Luckily, tubular cells have robust regenerative capabilities, such that mild to moderate ischemic injury allows complete tubular recovery without permanent damage. Hence, normalization of renal function (BUN, Creatinine) with resolution of oliguria could denote such a recovery. However, severe impairment with extensive damage could lead to disruption of the basement membrane, leading to fibrous scarring. CONCLUDING: Taking into account the timeline of events, where the patient's renal function normalized within a couple of weeks post-injury, the severity of the injury might not have been sufficient to cause permanent scarring. This could potentially favor a full recovery, yielding a normal kidney on autopsy, rather than fibrous scarring. RESPONSE: Judging from the presented history and the physiological recovery of our patient, it is more likely that the examination of his kidneys at autopsy would show a normal kidney (Option E). However, keep in mind the interpretation of the pathology findings would be contingent on the severity and extent of the initial ischemic insult.

**Facilitator** **[Round 3]:** Summary: AI_A believes that while fibrous scar tissue could be a possible outcome of severe acute tubular necrosis, the quick return to normal renal function in this case suggests a higher likelihood of a Normal Kidney (Option E) upon autopsy examination. AI_B also leans towards a Normal Kidney (Option E) as the most likely autopsy finding. They argue that the severity of the injury might not have been sufficient to cause permanent scarring, favoring a full recovery. AI_C, on the other hand, suggests that the most likely finding at autopsy given the clinical context would be (C) Fibrous scar, even though (E) Normal kidney remains a possibility. They argue that complete functional recovery does not necessarily equate to the lack of residual histological changes. AI_D also supports the viewpoint of a "normal kidney" (E) as the most likely autopsy finding. They argue that while minor fibrous scarring could be detected in some cases, it might not be extensive enough to be the primary pathological finding on autopsy. AI_E concludes that the kidneys would most likely appear normal (E) at autopsy despite the patient's history of oliguric renal failure following cardiac arrest. They argue that the return to normal renal function suggests successful recovery from acute tubular necrosis. Question to clarify disagreements: Given the patient's quick recovery and return to normal renal function, what specific factors or evidence lead you to believe that there would be residual fibrous scar tissue (Option C) present in the autopsy, as opposed to a normal kidney (Option E)?. Based on your preceding response, how would you answer the original question: [A 48-year-old man dies suddenly of a cardiac arrhythmia. Six weeks ago, he was resuscitated from a cardiac arrest caused by coronary artery disease and developed oliguric renal failure. Five weeks ago (1 week after being resuscitated), his serum urea nitrogen (BUN) concentration was 40 mg/dL, his serum creatinine concentration was 3.5 mg/dL, and his urine contained granular casts. Four weeks ago, his oliguria resolved, and his BUN and serum creatinine concentrations returned to normal. Examination of his kidneys at autopsy is most likely to show which of the following? (A) Acute inflammation (B) Fibrinous exudate (C) Fibrous scar (D) Granulation tissue (E) Normal kidney]

**AI_A:** Restating the Question: The query seeks to predict the pathological state of the kidneys in a 48-year-old male patient who experienced a cardiac arrhythmia and subsequent cardiac arrest. This individual suffered from coronary artery disease which led to severe cardiac events and subsequent renal complications depicted by oliguric renal failure. Following treatment, his renal function normalized as shown by his laboratory parameters (urea nitrogen and creatinine concentrations). The crucial question here is what would we most likely observe upon autopsy examination of the kidneys given this clinical history? PLANNING: This situation necessitates the consideration of two particular competing hypotheses: (A) the possibility of finding a 'Fibrous scar' or (B) that the kidneys might appear 'Normal'. A Fibrous Scar (Option C) represents the end-stage of tissue damage and repair and implies that a permanent damage occurred to the kidney structures due to the renal failure. On the other hand, a Normal Kidney (Option E) would imply that the initial renal damage was reversible and completely recovered. Clinical Reasoning: After a severe ischemic insult such as a cardiac arrest, the kidneys can develop an acute condition known as acute tubular necrosis (ATN). ATN is characterized by granular casts in the urine and a rise in creatinine and BUN levels, all of which were observed in the case. Following such an event, it's expected that the kidneys might undergo morphological changes. Our patient, however, showed signs of kidney recovery indicated by the resolution of oliguria and normalization of BUN and creatinine levels. This hints towards a reversible inflammatory condition rather than irreversible scarring or fibrosis. Since fibrosis is an indication of long-standing injury and is generally associated with a permanent loss of function, the rapid recovery indicates renal adaptability and repair. Concluding: Despite the patient’s previous renal affliction and oliguric renal failure, the quick return to normal renal function is more indicative of intact renal structure. Although fibrous scarring might be detected in some cases with severe ATN, the patient's recovery suggests successful renal repair without extensive scarring. Therefore, it would be reasonable to anticipate a 'Normal Kidney' (Option E) upon autopsy examination. Response: Examining the autopsy of a 48-year-old man who was resuscitated from a cardiac arrest six weeks ago, developed oliguric renal failure, and then seemed to recover (based on normalization of renal function tests), it is most likely that we would observe a 'Normal kidney' (Option E) despite his past renal affliction.

**AI_E:** Restatement: The question presents the case of a 48-year-old man who died abruptly from a cardiac rhythm disturbance. About six weeks back, he was successfully revived from a cardiac arrest related to coronary artery disease and subsequently developed low urine output renal failure. His kidney function tests at that time demonstrated increased concentrations of serum urea nitrogen and creatinine, and the presence of granular casts in his urine - suggestive of kidney damage. Importantly, he experienced a restoration of urine output and normalization of his kidney function tests four weeks ago. The inquiry requires us to predict the most probable finding in his kidneys upon autopsy. PLANNING: Looking at the defined variables in this question, several assumptions can be made. One significant assumption is that permanent pathophysiological changes occurred in the kidneys following the bout of renal failure. Another hypothesis is that despite the restoration of kidney function, microscopic changes may still be visible, such as a fibrous scar. Alternatively, because kidney function normalized so quickly, it is conceivable that no substantial histologic changes are present, suggesting a normal kidney. Clinical reasoning: The pathology these kidneys underwent typically follows an episode of hypoxic injury due to a cardiac arrest. This led to acute tubular necrosis - a reversible condition shown by the elevations in serum urea nitrogen and creatinine levels and the presence of granular casts in the urine. Following this, the kidneys underwent healing resulting in restoration of function - shown by the normalization of renal function tests and the return of urine output. Now, the changes that could be seen in the autopsy would depend on two factors - (a) the severity of the initial damage, and (b) the nature and completeness of the repair process. Concluding: We know that the patient quickly regained renal function, suggesting a full recovery. The mechanism of healing in acute tubular necrosis involves regeneration of the tubular epithelial cells, which can lead to complete restoration of the tubular architecture. Therefore, the absence of any residual changes in kidney function or urine output suggests that the kidneys did not undergo severe enough damage to cause substantial fibrosis/scarring. Consequently, I would hypothesize that in this rapid and complete recovery, it would be more likely to find a "Normal kidney"(E) on autopsy. Response: Examining the patient's kidneys at autopsy after his sudden death due to a cardiac arrhythmia is most likely to show a normal state (Option E). This conclusion is based on the patient's rapid and full recovery from acute renal failure, suggesting complete renal tubular cell regeneration without significant scar formation.

**AI_D:** Restating the Question: The query discusses a man with a recent history of cardiac arrest and renal failure, who has died from a cardiac arrhythmia. Following his cardiac arrest, his laboratory testing indicated an increased BUN and serum creatinine level, alongside granular casts in his urine - findings consistent with acute tubular necrosis (ATN). ATN, a type of acute kidney injury (AKI), is often caused by ischemia or toxins, which result in damage to the renal tubules. However, four weeks before his death, his renal function appeared to have fully recovered. The primary point of contention in the council is what the post-mortem examination of his kidneys is most likely to reveal. PLANNING: There are two main hypotheses under consideration: 1. Fibrous scar (option C) - When kidneys recover from ATN, there could be residual fibrous scar tissues left behind after the repair process. 2. Normal kidney (option E) - The kidneys might appear histologically normal following a full functional recovery from ATN, despite the past damage. I believe the second hypothesis is better supported scientifically and should be pursued. Clinical Reasoning: The renal tubules have excellent regenerative potential. Consequently, after an episode of ATN, the repair process includes the regeneration of tubular cells. If the injury is not too severe or prolonged, these cells can often fully regenerate, restoring the normal tubular structure. Granular casts that were initially present in the patient's urine are fragments of necrotic tubular cells, which suggest the initiation of regeneration is already underway. Furthermore, the normalization of the patient's BUN and serum creatinine concentrations, coupled with the resolution of his oliguria, strongly suggests a return to normal renal function, which would not occur if there was significant fibrosis (which replaces functional renal tissue with non-functional scar tissue). Consequently, an extensive degree of fibrosis resulting in a clearly identifiable fibrous scar on histological exam is unlikely. Concluding: Based on the rapid recovery of renal function, it's probable that only mild and reversible damage resulted from the patient's earlier episode of ATN. Therefore, it's also likely the compensatory regeneration of renal tubular cells resulted in an almost complete histologic recovery of the kidneys. Response: Given the above rationale, the most likely autopsy finding in this patient's kidneys would be a "Normal Kidney" (option E). Even though minor fibrous scarring may be present, it would not be the primary pathological finding on autopsy owing to an excellent recovery from the patient's episode of AKI due to ATN.

**AI_C:** Reflecting on the question, it poses the inquiry of what the likely autopsy findings would be for a man of 48 years who unfortunately succumbed to a cardiac arrhythmia. To set the clinical background, this man had a history of cardiac arrest due to coronary artery disease that subsequently led to oliguric renal failure. His lab results one week post-resuscitation revealed a BUN concentration of 40 mg/dL, a serum creatinine concentration of 3.5 mg/dL, and the presence of granular casts in his urine. Yet, in time, his oliguria was resolved and kidney function tests normalized. Given this information, we're asked to hypothesize which the most likely histological change on autopsy - Acute inflammation, Fibrinous exudate, Fibrous scar, Granulation tissue, or a Normal kidney. To hypothesize, we need to consider the patient's clinical progress and history. In this case, we're presented with a clinical course of renal failure, likely acute tubular necrosis (ATN), in the aftermath of cardiac arrest. This leads to a transient increase in BUN and creatinine and the appearance of granular casts in his urine. The renal failure, however, resolved with time and kidney function normalized, making it prudent to question whether the histological changes associated with ATN could be still present or if the kidney would appear histologically normal. The key assumptions here: whether the onset of renal failure due to cardiac arrest would leave discernible traces of damage, even after recovery; and whether these histological scars would be retained. When considering these assumptions, we must bear in mind that the kidney has a remarkable ability to recover from ATN. The resolving phase of ATN is characterized by the regeneration of tubular cells, leading to the resolution of cellular casts, and normalization of kidney function (BUN, creatinine). Nonetheless, a short phase of oliguria followed by rapid clinical recovery (within 2 weeks) does not absolutely rule out residual histological changes even if the renal function has returned to normal. Fibrous scar (C), being indicative of healing with fibrosis following severe ATN, is a reasonable expectation. However, Normal kidney (E), representing complete recovery both structurally and functionally, should also be considered. To culminate the reasoning process, the most likely autopsy finding in the given clinical scenario could be a Normal kidney (E). This patient history is indicative of a course of resolving ATN where recovery of renal function is both clinically apparent and swift. Normal kidney histology is largely suggestive of a complete recovery from the acute insult. Although fibrous scars are a conceivable sequelae of ATN, these are more frequently observed in cases of more severe and protracted injury. Therefore, my response to this clinical inquiry would be that the most likely autopsy finding of the man's kidneys would be a Normal kidney (E). The swift resolution of the kidney injury and the return of normal renal function imply that the tubular damage was likely not severe enough to result in significant fibrosis or scarring that would be histologically evident.

**AI_B:** Restating the question: What we're asked here is essentially this: given that this 48-year-old man recently suffered a cardiac arrest, leading to oliguric renal failure, and then his kidney function returned to normal before he died of another cardiac event, what would we expect to find during a post-mortem examination of his kidneys? Before we delve into the answer, let's understand a few concepts. Cardiac arrest, such as the one this man had, can cause a sudden decrease in blood flow to the kidneys which can lead to acute tubular necrosis (ATN). ATN is a condition where the kidney tubular epithelial cells are injured, causing them to die off and slough into the tubular lumen, forming granular casts. "Granular casts" are essentially clumps of dead cells, cellular debris, or protein material formed in the kidney tubules. They are often found in the urine when there is significant kidney damage. PLANNING: Considering the timing of the man's oliguric renal failure to the normalization of his kidney function, this suggests an acute and potentially reversible process like acute tubular necrosis from ischemia after cardiac arrest. The possible findings in the kidneys at autopsy could include (A) acute inflammation, (B) fibrinous exudate, (C) fibrous scar, (D) granulation tissue, or (E) normal kidney. Acute inflammation and fibrinous exudate would likely occur acutely following kidney injury, while fibrous scars and granulation tissue may take more time to form. Considering that the renal failure resolved within two weeks, it seems unlikely that irreversible damage and fibrosis have occurred, although some may argue that minimal scarring could exist. Clinical Reasoning: If we look closely at the sequence of events, we notice that the oliguric renal failure resolved four weeks before his death, which indicates that whatever damage was done to his kidneys was likely reversible, supporting the hypothesis of acute tubular necrosis. During ATN, if ischemia (lack of oxygen due to reduced blood flow) is severe or prolonged, permanent damage in the form of fibrous scarring may occur. However, scarring usually follows a more chronic process than a single episode of renal failure. The patient's kidney function returned to normal, as evidenced by the normalization of his BUN and serum creatinine levels, suggesting that the injury may have been reversible and therefore not severe enough to cause significant fibrosis. Concluding: Taken together, the clinical history of a rapid decline in renal function followed by a recovery to normal kidney function within a short period, supports a reversible and less severe injury such as acute tubular necrosis. The most likely finding upon autopsy would therefore be a normal kidney, given that the patient's kidney apparently recovered from this acute event and his renal function markers normalized. However, it can't be completely ruled out that there might be minor fibrous scars not significant enough to be readily seen on gross examination. Response: Given the clinical scenario, the most likely finding upon examination of the kidneys at autopsy would be (E) Normal kidney. The damage incurred to the kidney tubular cells was likely reversible with no significant microscopic fibrosis since the renal function fully recovered.

**Facilitator** The consensus statement synthesizes the responses of AI_A, AI_B, AI_C, AI_D, and AI_E as follows: The question presents the case of a 48-year-old man who suffered a cardiac arrest due to coronary artery disease, leading to oliguric renal failure. His laboratory tests indicated acute tubular necrosis (ATN), a condition characterized by kidney tubular epithelial cell injury. However, his renal function normalized four weeks before his sudden death from a cardiac arrhythmia. The query seeks to predict the most likely autopsy findings of his kidneys given this clinical history. Two primary hypotheses emerge from the responses: (1) the presence of a fibrous scar, indicative of permanent damage and repair, or (2) a normal kidney, suggesting that the initial renal damage was reversible and completely recovered. The clinical reasoning behind these hypotheses revolves around the nature and severity of the initial kidney damage and the subsequent recovery process. The rapid recovery of renal function, indicated by the normalization of BUN and creatinine levels and the resolution of oliguria, suggests a reversible inflammatory condition rather than irreversible scarring or fibrosis. Fibrosis, which represents the end-stage of tissue damage and repair, is generally associated with a permanent loss of function. However, the patient's swift return to normal renal function indicates renal adaptability and repair, suggesting that the tubular damage was likely not severe enough to result in significant fibrosis or scarring that would be histologically evident. Therefore, despite the initial renal affliction and oliguric renal failure, the consensus is that the most likely autopsy finding would be a 'Normal Kidney' (Option E). This conclusion is based on the patient's rapid and complete recovery from acute renal failure, suggesting complete renal tubular cell regeneration without significant scar formation. However, it is acknowledged that minor fibrous scarring may be present but not significant enough to be the primary pathological finding on autopsy.
