# Supplementary material for: Collaborative intelligence in AI: Evaluating the performance of a council of AIs on the USMLE
Source: PLOS Digit Health. 2025 Oct 9;4(10):e0000787. doi: 10.1371/journal.pdig.0000787 (PMC12510544; doi:10.1371/journal.pdig.0000787)
Supplement: S2 File — (ZIP) [file pdig.0000787.s002.zip › S2 Data Raw data files containing unprocessed question inputs and LLM outputs including transcripts of deliberations/STEP1_Question-20-1_Type-MC-NJ.docx]

**Facilitator** **[Round 1]:** A 66-year-old man who was recently diagnosed with Parkinson disease comes to the physician for a follow-up examination. Carbidopa-levodopa therapy was initiated at the time of diagnosis. The patient tells the physician that he still has episodes during which he ""freezes."" He has a clumsy gait, and there is rigidity of his upper extremities and hands. An adjunct therapy designed to inhibit which of the following enzymes is most likely indicated in this patient? (A) Aromatic L-amino acid decarboxylase (B) Dopamine β-hydroxylase (C) Monoamine oxidase B (D) Phenylethanolamine N-methyltransferase (E) Tyrosine hydroxylase

**AI_E:** RESTATING THE QUESTION: This question is asking which enzyme needs to be inhibited as a part of adjunct therapy for a Parkinson's disease patient who is still showing symptoms despite being on Carbidopa-Levodopa therapy. These enzymes that are being mentioned are all involved in the synthesis or degradation pathways of neurotransmitters in the body, particularly dopamine. PLANNING: Let's consider what each of these enzymes does. Aromatic L-amino acid decarboxylase is involved in the production of dopamine in the central nervous system. Dopamine β-hydroxylase is involved in the production of norepinephrine from dopamine. Monoamine oxidase B breaks down dopamine in the brain. Phenylethanolamine N-methyltransferase also takes part in the synthesis of adrenaline from noradrenaline. Lastly, Tyrosine hydroxylase mediates the conversion of the amino acid tyrosine to DOPA (a precursor for dopamine), and is the rate-limiting enzyme in dopamine synthesis. For the patient, we should find an alternative pathway to either increase dopamine or inhibit its degradation to counter the Parkinson disease symptoms. In this scenario, Carbidopa-Levodopa is unable to manage symptoms alone. We need to augment it with a drug that can inhibit the enzyme that is causing dopamine degradation. CLINICAL REASONING: Given all these, it seems reasonable to increase the lifespan of dopamine that has already been produced. This could be achieved by inhibiting an enzyme involved in its degradation. CONCLUDING: Therefore, in this case I think that inhibition of an enzyme that degrades dopamine (instead of promoting its synthesis which is already targeted by Carbidopa-Levodopa) should be the next step in this patient's treatment plan. RESPONSE: Hence, 'Monoamine oxidase B' (Choice C) would be the correct answer because this enzyme is key in the breakdown of dopamine in the brain. By inhibiting it, we could increase the amount of dopamine available for use in the body and improve the symptoms of Parkinson's disease.

**AI_A:** (1) RESTATING THE QUESTION: The query pertains to a 66-year-old male patient, recently diagnosed with Parkinson's disease and initiated on carbidopa-levodopa medication. He still experiences episodes of 'freezing', rigidity in upper extremities and hands and has a clumsy gait. The question asks for a more suitable enzyme inhibiting adjunct therapy for this patient from a given list. (2) PLANNING: The key underlying assumption would be that the current therapy, carbidopa-levodopa, isn't competently managing the patient's Parkinson symptoms. Let's consider each of the enzyme inhibitors in turn. Aromatic L-amino acid decarboxylase is essentially the enzyme that converts levodopa to dopamine; Dopamine β-hydroxylase catalyzes the conversion of dopamine to norepinephrine; Monoamine oxidase B (MAO-B) degrades dopamine in the brain; Phenylethanolamine N-methyltransferase (PNMT) catalyzes conversion of norepinephrine to epinephrine; Tyrosine hydroxylase is the enzyme converting L-tyrosine into L-3,4-dihydroxyphenylalanine (L-Dopa), the precursor to dopamine. We can hypothesize that inhibiting an enzyme more efficient in managing the patient's symptoms would be one that prevents premature degradation of dopamine, which is the main deficit in Parkinson's disease. (3) CLINICAL REASONING: Carbidopa is a peripheral dopa-decarboxylase inhibitor, which prevents the conversion of levodopa to dopamine in the peripheral tissues, allowing more levodopa to cross the blood-brain barrier. However, in the brain, we want levodopa to be converted into dopamine; hence, inhibiting Aromatic L-amino acid decarboxylase would not be advantageous here. On similar lines, inhibiting Dopamine β-hydroxylase might lead to elevated dopamine levels but would decrease norepinephrine, which could potentially exacerbate orthostatic hypotension, a common non-motor symptom in Parkinson's disease. Phenylethanolamine N-methyltransferase is not directly involved in the metabolic pathway of dopamine, and Tyrosine hydroxylase inhibition would limit dopamine synthesis. (4) CONCLUDING: Thus, based on the primary deficit in Parkinson's disease- the lack of dopamine - and the considerations around other neurotransmitters, the most logical choice would be to inhibit the enzyme which breaks down dopamine, protecting the available dopamine concentrations in the brain for a longer period. (5) RESPONSE: In the context of this patient, an adjunct therapy that inhibits enzyme Monoamine oxidase B (C) is most likely indicated to enhance the Parkinson's disease management by inhibiting dopamine degradation.

**AI_B:** RESTATING THE QUESTION: This scenario presents a 66-year-old patient who was recently diagnosed with Parkinson's disease, and it seems his initial therapy with carbidopa-levodopa is not managing his symptoms adequately. Hence, an additional treatment strategy is needed. This leads to the question regarding which enzyme inhibition would merit consideration as a potential adjunct therapy. The given options are enzymes associated with various biochemical pathways and understanding how these pathways relate to the treatment of Parkinson's can help answer this question. PLANNING: Several enzymes play key roles in the synthesis, modulation, and degradation of neurotransmitters which are significant in managing Parkinson’s disease. It's important to understand the functional role of these enzymes: Aromatic L-amino acid decarboxylase converts levodopa into dopamine, dopamine β-hydroxylase participates in the production of norepinephrine from dopamine; monoamine oxidase B (MAO-B) breaks down dopamine in the brain; phenylethanolamine N-methyltransferase is involved in the synthesis of epinephrine, and tyrosine hydroxylase is an enzyme necessary for dopamine synthesis. CLINICAL REASONING: In Parkinson's disease, the primary pathology is the loss of dopaminergic neurons in the substantia nigra, leading to decreased dopamine in the striatum. Treatment seeks to increase the dopamine levels or mimic its effects. Levodopa is converted to dopamine to increase its level. But, our patient already is on levodopa-carbidopa (which is a peripheral decarboxylase inhibitor to prevent premature conversion of levodopa to dopamine) but still continues to exhibit symptoms. So, inhibiting any enzyme that enhances formation of dopamine may not be ideally beneficial. An alternate strategy is to prevent the degradation of dopamine which is being formed. Amongst the above-mentioned enzymes, monoamine oxidase B plays a significant role in breaking down dopamine in the brain. Hence, inhibiting MAO-B would potentially prolong the effect of the present dopamine by preventing its breakdown. CONCLUDING: Based on the profile of these enzymes, the best adjunct therapy would involve the inhibition of an enzyme that participates in the degradation of dopamine. RESPONSE: Therefore, monoamine oxidase B (MAO-B) inhibitors would be most likely indicated in this patient. Inhibition of MAO-B increases the availability of dopamine by reducing its metabolism, thereby enhancing the therapeutic efficiency of current treatment. [C] Monoamine oxidase B inhibitor is most likely indicated in this patient.

**AI_D:** 1) RESTATING THE QUESTION: A 66-year-old man with a recent Parkinson's disease diagnosis continues to experience mobility problems, even while taking carbidopa-levodopa. The patient reports episodes of "freezing", a clumsy gait, and rigidity in his upper limbs. We are asked to determine which enzyme's inhibition would be most beneficial as an addition to his current therapy. Parkinson's disease is a neurodegenerative disorder characterized by the insufficient formation of dopamine in the substantia nigra area of the brain. Levodopa (L-DOPA) is a precursor of dopamine, and carbidopa is added to prevent its premature conversion to dopamine outside the brain. Despite this therapy, the patient still exhibits Parkinson's typical symptoms, suggesting the need for additional treatment. 2) PLANNING: It seems reasonable to assume that the patient's ongoing symptoms indicate that the carbidopa-levodopa therapy alone isn't sufficient to manage his Parkinson's disease. Therefore, a drug capable of inhibiting an enzyme involved in dopamine metabolism could further increase dopamine levels in the brain and thereby reduce his symptoms. 3) CLINICAL REASONING: Dopamine metabolism involves multiple enzymes, but not all would be helpful targets for drug therapy in this context. For instance, Aromatic L-amino acid decarboxylase helps convert L-DOPA to dopamine; thus, its inhibition would counteract the effects of the patient's existing treatment. Dopamine β-hydroxylase and Phenylethanolamine N-methyltransferase catalyze the conversion of dopamine to other neurotransmitters, thereby reducing available dopamine levels, and thus, are not desired targets. Tyrosine hydroxylase is the rate-limiting enzyme in dopamine synthesis, but inhibiting it would reduce dopamine production. On the other hand, Monoamine oxidase B (MAO-B) breaks down dopamine in the brain. Inhibiting this enzyme could, therefore, help increase dopamine levels in the brain and potentially ameliorate the patient's symptoms. 4) CONCLUDING: In this case, it seems that the best course of action would be to inhibit an enzyme that degrades dopamine, rather than one involved in its synthesis or conversion. This would increase the overall amount of dopamine in the patient's brain, potentially reducing the Parkinson's symptoms that his current treatment hasn't addressed effectively. 5) RESPONSE: In this scenario, an adjunct therapy designed to inhibit Monoamine oxidase B (C) is most likely indicated for this patient.

**AI_C:** RESTATING THE QUESTION: The question is asking which enzyme should be targeted by adjunct therapy to enhance the treatment of Parkinson's disease in a 66-year-old man who is not fully responding to carbidopa-levodopa therapy. PLANNING: The provided information suggests that the current Parkinson's disease treatment (carbidopa-levodopa) is not completely effective, hence the need for an adjunct therapy that targets an enzyme involved in the dopaminergic system. The enzymes listed are: Aromatic L-amino acid decarboxylase, Dopamine β-hydroxylase, Monoamine oxidase B, Phenylethanolamine N-methyltransferase, and Tyrosine hydroxylase. Each of these enzymes has specific roles in neurotransmitter metabolism. CLINICAL REASONING: The primary pathology in Parkinson's disease is the loss of dopaminergic neurons in the substantia nigra, resulting in decreased dopamine levels in the basal ganglia. Carbidopa-levodopa therapy is designed to increase dopamine synthesis and availability. (A) Aromatic L-amino acid decarboxylase: This is the enzyme that converts levodopa to dopamine in the CNS. Inhibiting this would decrease dopamine levels, which is counterproductive in a Parkinson's disease treatment. (B) Dopamine β-hydroxylase: This is the enzyme that converts dopamine to norepinephrine. Inhibiting this would result in increased dopamine and decreased norepinephrine. However, the effect on Parkinson's symptoms is uncertain. (C) Monoamine oxidase B (MAO-B): This enzyme is involved in the breakdown of dopamine. Inhibiting MAO-B would increase dopamine levels, which could potentially improve Parkinson's symptoms. (D) Phenylethanolamine N-methyltransferase: This enzyme converts norepinephrine to epinephrine. Its inhibition would, theoretically, have little effect on Parkinson's symptoms, as it doesn't directly affect dopamine metabolism. (E) Tyrosine hydroxylase: This is the rate-limiting enzyme in the synthesis of catecholamines (dopamine included). However, this would be ineffective here because the main problem in Parkinson's disease is neurodegeneration, not the synthesis capacity of remaining neurons. CONCLUDING: Given the need to enhance dopamine availability in the brain, inhibiting an enzyme involved in the degradation of dopamine seems to be a viable option. Out of the choices provided, the enzyme that best fits these criteria is Monoamine oxidase B (MAO-B) which is responsible for dopamine breakdown. RESPONSE: The most likely indicated enzyme to inhibit as an adjunct therapy in this Parkinson's disease patient is (C) Monoamine oxidase B.

**Facilitator** The consensus statement synthesizes the responses of AI_A, AI_B, AI_C, AI_D, and AI_E as follows: The question pertains to a 66-year-old male patient recently diagnosed with Parkinson's disease. Despite being on carbidopa-levodopa therapy, he continues to experience symptoms such as 'freezing', rigidity in upper extremities and hands, and a clumsy gait. The query asks for a suitable enzyme inhibiting adjunct therapy for this patient from a given list. The enzymes listed are involved in various biochemical pathways related to the synthesis, modulation, and degradation of neurotransmitters significant in managing Parkinson's disease. The primary pathology in Parkinson's disease is the loss of dopaminergic neurons in the substantia nigra, leading to decreased dopamine levels in the striatum. The current treatment, carbidopa-levodopa, is designed to increase dopamine synthesis and availability. However, the patient's ongoing symptoms suggest that this therapy alone isn't sufficient to manage his Parkinson's disease. Given this context, it seems reasonable to assume that the best course of action would be to inhibit an enzyme that degrades dopamine, rather than one involved in its synthesis or conversion. This would increase the overall amount of dopamine in the patient's brain, potentially reducing the Parkinson's symptoms that his current treatment hasn't addressed effectively. Among the enzymes listed, Monoamine oxidase B (MAO-B) breaks down dopamine in the brain. Inhibiting this enzyme could, therefore, help increase dopamine levels in the brain and potentially ameliorate the patient's symptoms. Therefore, an adjunct therapy designed to inhibit Monoamine oxidase B (C) is most likely indicated for this patient. This approach would enhance the therapeutic efficiency of the current treatment by reducing dopamine metabolism, thereby increasing its availability and improving the symptoms of Parkinson's disease.
