# Supplementary material for: Collaborative intelligence in AI: Evaluating the performance of a council of AIs on the USMLE
Source: PLOS Digit Health. 2025 Oct 9;4(10):e0000787. doi: 10.1371/journal.pdig.0000787 (PMC12510544; doi:10.1371/journal.pdig.0000787)
Supplement: S2 File — (ZIP) [file pdig.0000787.s002.zip › S2 Data Raw data files containing unprocessed question inputs and LLM outputs including transcripts of deliberations/STEP1_Question-22-1_Type-MC-NJ.docx]

**Facilitator** **[Round 1]:** A 46-year-old woman comes to the physician because of a 3-day history of intermittent pain with urination and increased urinary frequency. She says that she had one similar episode during the past 6 months. She also has had irregular menses, and her last menstrual period occurred 2 months ago. She has not had fever, nausea, vomiting, or blood in her urine. She is sexually active with one male partner. Physical examination shows no abnormalities. Urinalysis shows: RBC 3–5/hpf WBC 10–20/hpf Nitrites positive Leukocyte esterase positive Bacteria positive Which of the following is the strongest predisposing risk factor for the development of this patient's condition? (A) Leiomyomata uteri (B) Perimenopause (C) Pregnancy (D) Sexual intercourse

**AI_B:** RESTATING THE QUESTION: We are attempting to identify the strongest risk factor likely to have predisposed this 46-year-old woman to her current condition. The patient's complaints include intermittent pain upon urination, increased frequency of urination, and irregular menstrual cycles. She also shared with us that she had a similar experience about six months ago. Her physical examination revealed no abnormalities, however, her urinalysis showed presence of red and white blood cells in the urine, positive nitrites, leukocyte esterase, and bacteria. PLANNING: First, I would assume that the prominent symptoms indicate a urinary tract infection (UTI). In this condition, the urinary tract becomes infected, typically by bacteria, resulting in pain during urination and increased frequency of urination. Supportive findings include her positive urinalysis for nitrites, leukocyte esterase and bacteria, all indicative of an UTI. However, other aspects of her medical history, such as irregular menses, lend themselves to alternative explanations such as perimenopause or leiomyomata uteri (uterine fibroids). We must evaluate the strength of each risk factor to determine which is most likely to contribute to the development of her symptoms. CLINICAL REASONING: In the context of a UTI, sexual intercourse is a known risk factor because it can contribute to the introduction of bacteria into the urethra. Compared to men, women are at increased risk due to their shorter urethra. Pregnancy also increases the risk of UTIs, owing majorly to the physiological changes and the pressure exerted by the growing fetus on the bladder which could affect normal urinary flow. Leiomyomata uteri, or uterine fibroids, could potentially interfere with urinary function in severe cases, but wouldn't typically lead to bacteria in urine. Lastly, despite perimenopause often causing irregular menses, it would not typically contribute to the development of UTI. CONCLUDING: While all presented conditions have some relation to the symptoms of the patient, in terms of clinical reasoning and commonality, sexual intercourse has a direct line of causation to UTIs. It should be considered as a significant predisposing factor in the development of UTIs, especially when presented in combination with the patient's urinalysis results. RESPONSE: In the context of this patient's clinical presentation and urinalysis results, the strongest predisposing risk factor for the development of her current condition is likely (D) Sexual intercourse.

**AI_A:** 1) RESTATING THE QUESTION: The question is about a 46-year-old woman who exhibits signs of a urinary tract infection (UTI), including urinary pain and frequency, as well as laboratory findings which confirm the diagnosis (red and white blood cells, nitrites, leukocyte esterase, and bacteria in the urine). However, her menstrual cycle has been irregular and her last period was two months ago, indicating potential changes in her reproductive health. The question asks about the main factor predisposing her to UTIs from the given options: leiomyomata uteri (benign fibroids in the uterus), perimenopause (the phase before menopause), pregnancy, or sexual intercourse. 2) PLANNING: The woman's age and irregular periods could suggest perimenopausal hormonal changes that alter the urinary tract. Pregnancy might increase UTI risks due to urinary stasis, but she has irregular periods and no other pregnancy symptoms. Leiomyomata uteri could potentially cause urinary symptoms and UTIs if large enough to compress the urinary tract, but there's no physical evidence of fibroids. However, UTIs are commonly associated with sexual activity, as intercourse can introduce bacteria into the urinary tract. 3) CLINICAL REASONING: Though all options could potentially increase UTI risk, we have to consider the highest risk factor. Though perimenopause can affect immunity and urinary tract health, it's not directly associated with UTIs. Pregnancy can increase UTI risk due to urinary stasis and hormonal changes, but there's no evidence of pregnancy in the case. Leiomyomata uteri could cause mechanical changes affecting urine flow, potentially increasing UTI risk, but there's no mention of fibroids in the physical exam. Sexual intercourse is a well-established risk factor for UTIs, as physical contact can introduce bacteria into the urethra and ascend into the urinary tract. 4) CONCLUDING: Upon analyzing each condition and its potential to increase UTI risk, it seems the woman's active sexual life is the most likely predisposing factor. This conclusion doesn't ignore the possibility of interaction between her irregular periods (hormonal changes) or undiagnosed conditions (leiomyomata, pregnancy) and infection risk, but emphasizes the most appropriate answer considering current information. 5) RESPONSE: The strongest predisposing risk factor for the development of this patient's urinary tract infection condition is (D) Sexual intercourse.

**AI_D:** (1) RESTATING THE QUESTION: In simpler terms, the question asks: What factor is most likely to have caused the condition the woman is currently experiencing? The patient, a 46-year-old woman, presents symptoms including pain during urination (dysuria) and increased frequency of urination (polyuria), without the presence of fever, nausea, vomiting, or blood in urine (hematuria). Her urinalysis reveals an increased number of red and white blood cells (indicating inflammation or infection), and is also positive for nitrites, leukocyte esterase, and bacteria, which are indicative of a bacterial urinary tract infection (UTI). She also has irregular menstrual cycles and her last period was 2 months ago. (2) PLANNING: There are a number of assumptions we could make with the information given, some of which include: (A) Leiomyomata uteri (benign tumors in the uterus, more commonly known as fibroids), (B) Perimenopause (the transitional period before menopause, characterized by changes in menstrual cycles), (C) Pregnancy, or (D) Sexual intercourse. Each of these conditions can potentially increase the risk of developing UTIs, but we need to determine which is most clinically relevant in this case. (3) CLINICAL REASONING: When examining the patient's symptoms, it's important to keep in mind that UTIs are mostly caused by the movement of bacteria from the genital area into the urinary tract. This can potentially be facilitated by sexual intercourse, as it could enable the transfer of pathogens from the genital region to the urinary tract. Moreover, compared to other risk factors listed, sexual intercourse can more directly contribute to the pathogenesis of UTIs. Leiomyomata uteri could predispose to UTIs by causing compression or distortion of the urinary tract, but there is no indication of such abnormality in the physical examination. Pregnancy could cause anatomical and physiological changes predisposing to UTIs, but the patient's last menstrual period was 2 months ago and there were no mentioning of pregnancy symptoms. Perimenopause could cause hormone changes which in turn might alter the urinary tract environment, but this indirect link is not as strong as the connection between sexual intercourse and UTIs. (4) CONCLUDING: When we connect these facts, it is reasonable to conclude that, in the patient's case, though there can be multiple risk factors contributing to urinary tract infections, including leiomyomata uteri, perimenopause, and pregnancy, the most direct and significant predisposing risk factor is likely to be sexual intercourse. (5) RESPONSE: Therefore, the strongest predisposing risk factor for the development of this patient's urinary tract infection is (D) Sexual intercourse.

**AI_E:** 1) RESTATING THE QUESTION: The case presented is of a middle-aged woman who is exhibiting symptoms of a urinary tract infection (UTI), such as painful and frequent urination. Her urinalysis results support this diagnosis due to the presence of red blood cells (RBCs), white blood cells (WBCs), nitrites, leukocyte esterase, and bacteria. In addition to these, she is also experiencing irregular menses and reports her last period to have been two months ago. Despite presenting with UTI symptoms, she does not have systemic symptoms such as fever, nausea, vomiting, or hematuria (blood in urine). We are tasked with identifying the strongest predisposing risk factor for her condition from the given options: leiomyomata uteri (uterine fibroids), perimenopause, pregnancy, or sexual intercourse. 2) PLANNING: Here, we assume that each of the given options could potentially predispose the patient to a UTI. Leiomyomata uteri or uterine fibroids can cause urinary symptoms such as frequency and urgency, but they typically do not directly result in a UTI. Perimenopause, a period before menopause with hormonal changes leading to menstrual irregularity is associated with atrophic changes in the urinary tract, which might increase susceptibility to UTIs. Pregnancy could increase the risk because of the urinary stasis and ureteral dilation caused by progesterone and mechanical compression of the bladder. Sexual intercourse can facilitate the transmission of bacteria into the urinary tract, especially the female urinary tract, given its anatomical structure. 3) CLINICAL REASONING: With the assumptions made, while all the alternatives can be linked with UTIs, we have to focus on which one is most correlating with the patient's personal, medical, and lab findings. Leiomyomata uteri are not reported in the patient's history, and apart from the frequency and urgency, the patient does not show other significant symptoms related to this condition, like heavy and painful periods. This alternative seems less plausible. The patient shows irregular menses, which could suggest perimenopause, however, UTI is not a common presentation during this period, making this option less likely. Pregnancy presents with high progesterone levels leading to urinary stasis but the patient's condition including intermittent pain, urinalysis findings, and report of similar past episode while not being reported pregnant makes this option least likely. The most plausible assumption is "Sexual intercourse" as it is a well-known risk factor for UTIs, termed as "honeymoon cystitis". The female urethra's proximity to the vagina and anus can allow bacteria to be introduced into the urinary tract during intercourse. 4) CONCLUDING: The most probable predisposing factor given the patient's history, sexual activity, and absence of systemic symptoms and pregnancy is sexual intercourse. 5) RESPONSE: Therefore, the strongest predisposing risk factor for the development of this patient's condition is (D) Sexual intercourse.

**AI_C:** RESTATING THE QUESTION: This scenario involves a 46-year-old woman who has sought medical attention due to three days of periodic instances of painful urination and a heightened frequency of urination. Such symptoms had been experienced once before over the past half-year. She also experiences irregular menstruation, with her last menstrual cycle happening two months ago. Apart from these issues, the patient reports no further symptoms of fever, nausea, vomiting or blood in urine. Her lifestyle includes sexual activity with a single male partner. Physical assessment reveals no anomalies. Yet, urinalysis results show evidence of red and white blood cells, nitrites, leukocyte esterase, and bacteria, suggestive of a urinary tract infection. The question seeks to identify which among the listed factors is the most influential in the emergence of this patient's condition: leiomyomata uteri (uterine fibroids), perimenopause (the transitional phase towards the cessation of menses), pregnancy, or sexual intercourse. PLANNING: An important assumption is that the urinalysis findings which indicate a urinary tract infection (UTI) are correct. Another assumption might be that her sexual activity has exposed her to bacteria that could have caused the UTI. Yet another assumption could be that her abnormal menses triggered the UTI. A further assumption is that despite the absence of physical abnormalities from the examination, there still could be hidden anomalies that contributed to the UTI, such as the possibility of uterine fibroids being present. Each assumption needs to be critically evaluated against her symptoms and the findings of the laboratory work. Based on the overall clinical history, the hypothesis that her sexual activity is the strongest predisposing risk factor to her UTI symptoms is the most compelling. CLINICAL REASONING: UTIs are common among sexually active women due to the closer proximity of the urethra to the anus and the shorter urethral length, which provide easier access to bacteria leading to infections. This patient has the clinical symptomatology of a uncomplicated UTI, evidenced by her discomfort during urination (dysuria) and increased urinary frequency without systemic signs like fever or nausea. The results of her urinalysis are consistent with this diagnosis, showing both leukocyte esterase and nitrites, which are indicative of an active infection, and presence of bacteria. While irregular menses could be associated with hormonal changes in perimenopause or other conditions like polycystic ovarian syndrome (PCOS), there's no direct link with UTI. Uterine fibroids or leiomyomata uteri could cause irregular menses but they are not known to be a risk factor for UTI. Pregnancy increases the risk of UTI due to changes in the urinary tract, but there's no information supporting this patient's pregnancy. CONCLUDING: Considering this woman's sexual activity, as well as the urinalysis results indicative of a UTI and her symptoms aligning with the condition, it seems that sexual activity is the most probable predisposing risk factor for her UTIs. None of the other suggested options - leiomyomata uteri, perimenopause or pregnancy - provide a strong link to her condition based on the presented information. RESPONSE: Therefore, (D) Sexual intercourse is likely the strongest predisposing risk factor for the development of this patient's condition.

**Facilitator** The question pertains to a 46-year-old woman who is experiencing symptoms indicative of a urinary tract infection (UTI), such as urinary pain and increased frequency. Her urinalysis confirms this diagnosis, showing the presence of red and white blood cells, nitrites, leukocyte esterase, and bacteria. Additionally, she has irregular menstrual cycles, with her last period occurring two months ago. The task is to identify the most significant predisposing risk factor for her condition from the given options: leiomyomata uteri (uterine fibroids), perimenopause, pregnancy, or sexual intercourse. All the options could potentially increase the risk of UTIs. Leiomyomata uteri could cause urinary symptoms and UTIs if large enough to compress the urinary tract, but there's no physical evidence of fibroids. Perimenopause can lead to hormonal changes that alter the urinary tract, but it's not directly associated with UTIs. Pregnancy might increase UTI risks due to urinary stasis and hormonal changes, but the patient has irregular periods and no other pregnancy symptoms. Sexual intercourse is a well-established risk factor for UTIs, as it can introduce bacteria into the urinary tract. Given the woman's active sexual life and the absence of other symptoms or conditions, it seems the most likely predisposing factor. This conclusion doesn't ignore the possibility of interaction between her irregular periods (hormonal changes) or undiagnosed conditions (leiomyomata, pregnancy) and infection risk, but emphasizes the most appropriate answer considering current information. Therefore, the consensus statement is that the strongest predisposing risk factor for the development of this patient's urinary tract infection is (D) Sexual intercourse.
