# Supplementary material for: Collaborative intelligence in AI: Evaluating the performance of a council of AIs on the USMLE
Source: PLOS Digit Health. 2025 Oct 9;4(10):e0000787. doi: 10.1371/journal.pdig.0000787 (PMC12510544; doi:10.1371/journal.pdig.0000787)
Supplement: S2 File — (ZIP) [file pdig.0000787.s002.zip › S2 Data Raw data files containing unprocessed question inputs and LLM outputs including transcripts of deliberations/STEP1_Question-23-1_Type-MC-NJ.docx]

**Facilitator** **[Round 1]:** A 28-year-old man comes to the physician because of a 2-month history of a rash on his wrists and hands. He is a first-year mortuary science student. He also works on his grandfather's farm each weekend. His hobbies include raising homing pigeons and repairing vintage motorcycles. He recently changed to a different type of laundry detergent to save money. Physical examination shows a diffuse erythematous rash involving both hands up to the wrist creases. The rash does not extend over any other parts of the body. Which of the following is the most likely cause of this patient's rash? (A) Change in laundry detergent (B) Exposure to chemicals during motorcycle repair (C) Handling pigeons (D) Pesticide exposure (E) Use of latex gloves

**AI_C:** RESTATING THE QUESTION: A 28-year-old male has presented with a 2-month long rash on his wrists and hands. He has multiple activities that could contribute to his rash such as his job as a student in mortuary science where he could be using latex gloves. Additionally, he could be exposed to chemicals repairing motorcycles, potentially exposed to pesticides on his grandfather's farm, and to allergens from raising pigeons. His recent change in laundry detergent is another factor to consider in this case. The physical exam shows an erythematous rash limited to his hands. We aim to identify the most likely cause of his rash. PLANNING: Our assumptions are (1) the change in laundry detergent caused the rash (2) exposure to chemicals during motorcycle repairs (3) handling pigeons (4) exposure to pesticides on the farm and (5) the use of latex gloves. An important factor to consider here is the fact that the rash is only present on the hands and wrists, and nowhere else on the body. This could suggest that the rash might be a result of an allergen or irritant that primarily comes into contact with these parts of the body. CLINICAL REASONING: If the laundry detergent was causing the rash, it would more likely not be limited to the hands and wrists as the detergent residue remains in all of the patient's clothes. Exposure to chemicals during motorcycle repair could cause a rash, but typically, those chemicals would also contact other parts of the body such as arms, potentially causing a rash there as well. Handling pigeons may cause a systemic allergic reaction or respiratory symptoms, not just localized rashes. Exposure to pesticides on the farm may cause systemic issues, including a rash all over the body not just localized. Lastly, latex gloves, used primarily in healthcare settings and labs for example, as in mortuary science studies, usually contact only the hands and up to the wrists which matches the area of rash in this patient, suggesting the potential of a localized contact dermatitis. CONCLUDING: Weighing the given choices and their respective assumptions, rash due to the use of latex gloves seems most plausible due to the distribution of the rash and the potential for frequent contact with this material in the course of his mortuary science studies. RESPONSE: Hence, the most likely cause of this patient's rash appears to be the (E) use of latex gloves.

**AI_E:** RESTATING THE QUESTION: This problem concerns a 28-year-old man who is in his first year of mortuary science studies and has developed a skin rash specifically located on his hands and wrists. The man also spends time helping out on a farm, raises homing pigeons, fixes vintage motorcycles, and has recently switched to a new laundry detergent brand. The skin eruption is erythematous and diffused, and it is located up to the wrist creases. We're asked to identify what might be the most likely cause of his rash from a list of possibilities: a change in laundry detergent, exposure to chemicals during motorcycle repair, handling pigeons, exposure to pesticides, or the use of latex gloves. PLANNING: It's important to consider the positioning and limitation of the rash on the patient's hands and wrists. If the rash was due to laundry detergent, direct exposure is more likely to cause a more widespread rash rather than isolated to hands. The same applies to exposure to pigeons or pesticides. The exposure to chemicals during motorcycle repair assumes that the patient doesn't use gloves, which might not be the case. The use of latex gloves would limit contact dermatitis to the hands and wrists but would require the assumption that as a mortuary science student, he uses gloves frequently, which is plausible. CLINICAL REASONING: Contact dermatitis is an inflammation of the skin caused by direct contact with an allergen or irritant. In this case, the patient has several potential exposures due to his various activities and recent changes in personal care products. However, the distribution of the rash is key here. The fact it is localized to the hands and wrists, area commonly shielded by gloves. Given this exam finding, the hypothesis that this dermatitis is a result of the use of latex gloves should be given priority. This makes sense considering his field of study, mortuary science, where the frequent use of latex gloves can lead to an allergy. CONCLUDING: Based on the location and description of the patient's rash and the understanding of contact dermatitis, the most probable cause of this patient's symptoms is the use of latex gloves. The reasoning is that latex gloves, routinely used in mortuary sciences, cause localized skin reaction in the areas they cover, if the person develops sensitivity to latex. RESPONSE: (E) Use of latex gloves is the most likely cause of this patient's rash.

**AI_D:** (1) RESTATING THE QUESTION: This 28-year-old man presents with a case of localized rash. The rash is only witnessed on his hands up to the wrists. He's exposed to various possible irritants or allergens in his lifestyle - these include latex (used in gloves common in mortuary science), laundry detergent (which he has recently changed), chemicals (potentially present in his motorcycle repair hobby), pigeons (which he raises), and pesticides (possibly used on his grandfather's farm). Despite the myriad of potential triggers, the rash is exclusively confined to his wrists and hands; it's not present anywhere else on his body. (2) PLANNING: Let's examine each of these activities and associated potential triggers in turn. A change in laundry detergent could indeed cause a rash; however, a detergent-related rash would likely affect more areas of the body, not just the wrists and hands. Exposure to recreational or vocational-related chemicals could cause a localized rash, especially in the absence of proper safety gear. As for the handling of pigeons, allergic reactions to birds typically present respiratory symptoms rather than rashes. Pesticide exposure could potentially induce a rash, but this would more likely occur if the skin was directly exposed without protection. The final possibility, the use of latex gloves, can indeed lead to allergic reactions, presenting as a localized rash to the area of contact. (3) CLINICAL REASONING: With this in mind, we can rule out the change in laundry detergent due to the rash's localized distribution. Pesticide exposure is unlikely as well as the rash is not widespread or generalized, which is commonly seen with pesticide-induced dermatitis. Bird allergies typically cause respiratory symptoms, not rashes, so pigeon handling is less likely. The only exposures that are consistently localized to the hands and wrists would be the gloves he uses for his mortuary science school and possibly the motorcycle repair. However, it is crucial to mention that rashes related to motorcycle repair (which might involve contact with oil, lubricants, or other chemicals), typically occur at the point of contact and can also extend beyond the wrists depending on the use of protective clothing. On the other hand, a latex allergy could induce a rash that stops at the wrist as gloves typically cover only up to the wrist crease. (4) CONCLUDING: With these factors considered, since the gloves he likely uses in his mortuary science school may contain latex and the rash is limited to the area the gloves cover, it is plausible to hypothesize that the latex in the gloves could be the main cause of the rash. (5) RESPONSE: (E) Use of latex gloves is the most likely cause of this patient's rash.

**AI_A:** RESTATING THE QUESTION: Let's initially paraphrase the information given. A 28-year-old man is experiencing a rash on his hands and wrists that has persisted for two months. As a mortuary science student, he might be coming into contact with a variety of chemicals. Additionally, he works on a farm and has hobbies that include raising homing pigeons and repairing motorcycles. Recently, he switched to a more economical laundry detergent. The rash, characterized as diffuse and erythematous, is restricted to his hands and wrists. We need to determine what is the most likely cause of his rash. PLANNING: Identifying the most probable cause of the rash would require considering all possible allergens or irritants this individual may have been exposed to. The change in laundry detergent might have introduced a new set of chemicals that are causing allergic reactions. Working on a farm might have exposed him to pesticides. Repairing motorcycles could expose him to chemicals as well. His use of latex gloves, as a common requirement in the field of mortuary science and possibly while working on motorcycles, could be another possible cause. Finally, the birds might carry mites or other disease-carrying organisms that cause skin reactions. CLINICAL REASONING: It's crucial to note that the rash is localized on the hands and wrists. If the laundry detergent was to blame, it's likely that the rash would be widespread since clothes generally come into contact with more areas of the body. Dealing with chemicals from farming or motorcycle repair might have caused it, yet the lack of rash elsewhere suggests otherwise unless he uses gloves for these activities. Handling pigeons could be to blame, as birds can carry mites or other rash-inducing organisms, but then again, unless he handles these birds only with his bare hands, the rash should have spread elsewhere. CONCLUDING: Considering all these factors, the most plausible assumption might be latex exposure, given its common allergic properties and the localized nature of the rash. We know that he's a mortuary science student where latex gloves usage is prevalent, and he likely could use them as well for his other hobbies for protection. RESPONSE: The most likely cause of this man's rash on his hands and wrists is the (E) use of latex gloves, due to the localized dermatitis, the frequent usage of latex gloves in his field of study, and the known allergic properties of latex.

**AI_B:** RESTATING THE QUESTION: The query is about identifying the cause of a dermatological condition—a rash on the hands and wrists—in a 28-year-old man. This individual has a diverse set of activities: mortuary science studies (which likely involves the use of latex gloves), farming (with potential pesticide exposure), pigeon handling, vintage motorcycle repair (with chemical exposure), and a recent switch in laundry detergent. The rash is erythematous (red and inflamed), localized specifically to his hands and wrists, and has been present for two months. PLANNING: Assumptions could be made for each potential cause of rash. The rash could be an allergic reaction or contact dermatitis, resulting from exposure to a new substance. Key factors to consider are the timing, localization of the rash, and the patient's wide array of hobbies and occupations. (A) Change in laundry detergent: An allergic reaction could result to new laundry detergent, however, the rash would likely be widespread and not limited to hands. (B) Exposure to chemicals during motorcycle repair: Certain chemicals used in repairing motorcycles could cause skin irritation or contact dermatitis, but the specificity to only the hands and wrists is not very typical. (C) Handling pigeons: While pigeon fancier's lung, an allergic alveolitis, is associated with close interaction with pigeons, it’s rare for it to cause localized skin reactions. (D) Pesticide exposure: Chronic pesticide exposure might cause a range of health effects, including skin rash, however, the rash would likely affect more exposed areas of skin and not only hands and wrists. (E) Use of latex gloves: Repeated use of latex gloves could potentially induce a localized allergic contact dermatitis, especially given the specificity of the rash to his hands and wrists CLINICAL REASONING: Since the rash is limited to the hands and wrists—the parts of the body that would be in contact with latex gloves if used regularly or frequently—it suggests a local cause. Given the individual’s occupation as a mortuary science student, frequent latex glove usage seems likely. Furthermore, latex allergy tends to manifest as contact dermatitis, producing symptoms similar to what the patient is experiencing. CONCLUDING: After considering all potential causes and their alignment with the patient's symptoms and exposure, the most plausible cause for the rash is the repeated use of latex gloves, given the professional requirements related to his study and practice in mortuary sciences. RESPONSE: Therefore, the most likely cause of the 28-year-old man's rash is the use of latex gloves (E).

**Facilitator** The consensus statement is as follows: The 28-year-old man's rash on his hands and wrists is most likely caused by the use of latex gloves. This conclusion is based on the fact that the rash is localized to the hands and wrists, areas typically covered by gloves. Given his occupation as a mortuary science student, it is plausible that he frequently uses latex gloves, which are known to cause allergic reactions in some individuals. Other potential causes such as a change in laundry detergent, exposure to chemicals during motorcycle repair, handling pigeons, and pesticide exposure were considered. However, these were deemed less likely due to the rash's specific localization and the nature of these activities. For instance, a rash caused by laundry detergent or pesticide exposure would likely be more widespread, and handling pigeons typically causes respiratory symptoms rather than localized skin reactions. Therefore, the use of latex gloves is the most plausible cause of the rash.
