# Supplementary material for: Collaborative intelligence in AI: Evaluating the performance of a council of AIs on the USMLE
Source: PLOS Digit Health. 2025 Oct 9;4(10):e0000787. doi: 10.1371/journal.pdig.0000787 (PMC12510544; doi:10.1371/journal.pdig.0000787)
Supplement: S2 File — (ZIP) [file pdig.0000787.s002.zip › S2 Data Raw data files containing unprocessed question inputs and LLM outputs including transcripts of deliberations/STEP1_Question-24-1_Type-MC-NJ.docx]

**Facilitator** **[Round 1]:** A 10-year-old boy is brought to the emergency department in the middle of summer because of fever, headache, and photophobia. Several of his camp mates have had a similar illness. Physical examination shows mild nuchal rigidity. A lumbar puncture is performed. Laboratory studies show: Serum glucose 90 mg/dL Cerebrospinal fluid Pressure, opening 50 mm H2O Glucose 65 mg/dL Total protein 70 mg/dL Leukocyte count 43/mm³ (95% lymphocytes) Which of the following infectious agents is the most likely cause of these findings? (A) Adenovirus (B) Enterovirus (C) Herpes simplex virus (D) Neisseria meningitidis (E) Streptococcus pneumoniae

**AI_C:** 1) RESTATING THE QUESTION: A 10-year-old child has been admitted to the emergency wing of the hospital during summer with symptoms of fever, headache, discomfort with bright light (photophobia), and stiffness in the neck (nuchal rigidity). Many of his friends at the summer camp have also reported similar illness symptoms. Upon conducting a lumbar puncture, his cerebrospinal fluid result indicated a glucose elevated level, a mildly increased protein concentration, and an increased leukocyte count with lymphocytic predominance. We are therefore asked to identify the most probable infectious causative agent from the given list: Adenovirus, Enterovirus, Herpes simplex virus, Neisseria meningitidis, and Streptococcus pneumoniae. 2) PLANNING: To identify the most likely causative agent from the list, let's analyze each assumption. These can be either a viral or bacterial infection. However, because the leukocytosis in CSF is predominantly lymphocytic, this suggests that a viral agent is more probable. Bacterial infections are primarily associated with high neutrophilic counts. Further, the timing (occurring in the middle of summer) and the fact that this condition appears to be a cluster (affecting several camp friends) are additional supportive clues. 3) CLINICAL REASONING: Let's question the assumptions. Given that the inflammation seems likely to be viral, it helps to exclude bacterial organisms: Neisseria meningitidis and Streptococcus pneumoniae. Although Herpes simplex virus and Adenovirus can cause meningitis, the typical presentations and mode of transmission do not align well with this scenario. Herpes simplex encephalitis would present with more severe symptoms (like altered mental status or seizures), and adenoviruses are usually associated with respiratory or ocular infections. 4) CONCLUDING: Therefore, considering all the clinical and laboratory findings, the remaining infectious agent is Enterovirus. Enteroviruses are a common cause of viral meningitis, especially during the summer months, and can easily spread in a communal environment like a summer camp. 5) RESPONSE: The most likely cause of these findings is (B) Enterovirus.

**AI_E:** RESTATING THE QUESTION: The query above poses a clinical scenario where a 10-year-old boy presented to the ED during the summer with a trio of symptoms - fever, headache, and photophobia (an aversion to light due to eye discomfort). His peers at the camp have been experiencing similarly. On the physical exam, he exhibited mild nuchal rigidity (stiffness of the neck). A lumbar puncture revealed cerebrospinal fluid (CSF) with an opening pressure of 50 mm H2O, glucose at 65 mg/dL, total protein at 70 mg/dL, and a leukocyte count of 43/mm³ with 95% lymphocytes. The question requires identification of the causative agent from several given options, including Adenovirus, Enterovirus, Herpes simplex virus, Neisseria meningitidis, and Streptococcus pneumoniae. PLANNING: Clearly, with the above symptoms and CSF finding, we are dealing with a case of meningitis, an inflammation of the meninges, the membranes that surround the brain and spinal cord. Each of the options listed falls under the category of pathogens capable of causing meningitis. We need to consider the most likely pathogen under the given clinical scenario. Enteroviruses are known to cause aseptic meningitis, which is characterized by normal glucose and elevated protein in CSF, which aligns with our case. Additionally, Enteroviruses are infamous for their outbreaks in settings like a summer camp. CLINICAL REASONING: Enterovirus deserves a spotlight due to the patient's clinical scenario as it causes aseptic meningitis which manifests as fever, headache, photophobia, and mild neck stiffness - symptoms our patient is currently facing. A lumbar puncture typically reveals lymphocytic pleocytosis (higher count of lymphocytes in CSF), which matches our case too. Enteroviruses primarily spread from person to person through fecal-oral transmission, which could be a likely scenario in a camp setting where hygiene practices might not be the best. Therefore, given the presentation, lab findings, and potential exposure, Enterovirus appears to be the clinical priority in this case. CONCLUDING: Recapitulating, considering the child's presentation in the middle of summer, the camp venue, characteristic CSF findings indicative of aseptic meningitis, and the known epidemiology of Enterovirus, it is rational to connect the causative factor to the Enterovirus. RESPONSE: The most likely causative agent of these findings is Enterovirus (B).

**AI_B:** RESTATING THE QUESTION: The clinical situation is related to a 10-year old boy who has been brought to the hospital with symptoms suggestive of a CNS infection, as evidenced by fever, headache, photophobia, and mild neck stiffness. Additionally, other children in his camp have displayed similar symptoms. The boy has undergone a lumbar puncture and the cerebrospinal fluid (CSF) analysis results are suggestive of lymphocytic pleocytosis. The question is asking what the most likely cause of the illness could be based on the symptoms and CSF findings. PLANNING: To solve this clinical case, we need to think about the etiology of meningitis related to age, as well as seasonality, epidemiology (several camp mates with similar symptoms), symptomatology, and CSF study. The principle assumption is that the patient has a CNS infection caused by an infectious agent. We can consider the following infectious agents: Adenovirus, Enterovirus, Herpes simplex virus, Neisseria meningitidis, and Streptococcus pneumoniae. The clinical priority is to identify the most likely causative agent based on the clinical and laboratory findings. CLINICAL REASONING: Given the season (summer), age of the patient (10 years), and epidemiological background (similar illness in several camp mates), enteroviruses should be strongly considered. Enteroviruses are prevalent in the summer and early fall and can spread easily in group settings like a camp. The CSF findings (lymphocytic pleocytosis with normal glucose) are also more suggestive of viral than bacterial meningitis. Adenovirus could also cause meningitis, but the epidemiology is less typical, and it tends more towards respiratory infections. Herpes simplex virus could cause encephalitis with a different presentation than meningitis (temporal lobe involvement with altered consciousness, seizures). Neisseria meningitidis and Streptococcus pneumoniae typically cause a purulent meningitis, not usually a lymphocytic one, with a more acute, severe presentation, often with rash (Neisseria) or pneumonia (Streptococcus). CONCLUDING: The most likely causative agent, given the time of year, patient's age, the nature of the contact setting, and the CSF findings, is likely an Enterovirus. Enteroviruses are common causes of summer meningitis outbreaks in children, particularly in communal settings like a camp, and typically present with fever, headache, photophobia, and nuchal rigidity. RESPONSE: Based on the available information and my clinical reasoning, the most likely cause of these findings is (B) Enterovirus.

**AI_A:** RESTATING THE QUESTION: We are considering a situation in which a 10-year-old boy, along with various of his camp mates, has come down with symptoms of fever, headache, and photophobia (an intolerance to visual perception of light). A mild nuchal rigidity (stiffness in the neck) has been observed in a physical examination. A lumbar puncture has been performed, revealing a set of specific laboratory findings namely, serum glucose of 90 mg/dL, cerebrospinal fluid (CSF) pressure of 50 mm H2O, glucose level in CSF is 65 mg/dL, total protein of 70 mg/dL and leucocyte count of 43/mm³ with 95% being lymphocytes. We are asked to identify the most probably infectious agent causing this ailment in context with these data points considering options like adenovirus, enterovirus, herpes simplex virus, Neisseria meningitidis, and Streptococcus pneumoniae. PLANNING: One assumption is that the cause of illness could be any of the possible infectious agents listed. However, certain aspects like the age of patient, timing of disease occurrence in summer, symptoms like fever, headache, and photophobia and the lumbar puncture results suggesting a viral rather than a bacterial cause of meningitis guide us to reconsider this assumption. Viral causes of meningitis are more common in children with enteroviruses being the most common aetiologic agents especially during the summer (non-winter) months. CLINICAL REASONING: Well, let's connect the dots step by step. The child's symptoms are consistent with meningitis. Meningitis is inflammation of the meninges, the membranes cover the brain and spinal cord. Causes include infections by bacteria, viruses, or other microbes. The CSF findings with a predominant lymphocytic response as well as the comparatively normal glucose and slightly elevated levels of protein are more indicative of a viral rather than bacterial etiology. Further, considering the known epidemiology of viral meningitis, the summer camp setting, and the fact that several other camp mates are ill with similar symptoms, there’s a good chance we are dealing with an enterovirus, which are common causes of meningitis in summer and early fall. CONCLUDING: Considering the child's presentation and CSF analysis, the most likely cause of these symptoms, which include fever, headache, and photophobia, along with mild nuchal rigidity found in the physical examination, points towards a case of viral meningitis. RESPONSE: The most probable infectious agent in this scenario given the patient's age, the seasonal timing of their symptoms, and the results of their lumbar puncture procedure, is most likely (B) Enterovirus. This diagnosis is also affirmed by the fact that his camp mates also show symptoms of a similar illness, which suggests the presence of a shared infectious agent that can spread easily similar to enteroviruses in a close-contact environment like a camp.

**AI_D:** RESTATING THE QUESTION: So, we have a 10-year-old boy who has been admitted to the emergency department during the summer due to symptoms of fever, headache, and photophobia (which means aversion to light). Furthermore, some of his peers from camp have shown similar symptoms. Observations from the physical examination includes mild nuchal rigidity, i.e., resistance and pain on attempting to flex the neck. Lab results from a lumbar puncture, a procedure used to collect cerebrospinal fluid (CSF), show parameters pertaining to glucose level, protein level, and leukocyte count, majority of which are lymphocytes. Our task is to deduce the most probable infectious agent causing these symptoms and findings, out of the given options. PLANNING: The given choices include viral agents (Adenovirus, Enterovirus, Herpes simplex virus) and bacterial agents (Neisseria meningitidis, Streptococcus pneumoniae), all of which can cause meningitis. However, the patient's summer presentation and the lymphocyte predominance in the CSF are more common in viral than in bacterial meningitis. Viral meningitis often presents with less severe symptoms and has a relatively milder disease course compared to bacterial meningitis. Here, we are challenging the assumption that all possible infectious agents are equally likely, to prioritize the hypothesis of viral etiology given the clinical and laboratory data. CLINICAL REASONING: Seasonality is a major hint pointing towards Enterovirus, as they typically cause outbreaks of meningitis in the summer and early fall in young populations such as at camps or schools. This might be due to the close quarters and community pools fostering easy transmission. The mild nuchal rigidity, while indicative of meningitis, is less severe than that usually seen in bacterial meningitis, further supporting a viral etiology. Moreover, in the CSF results, the ratio of glucose to serum glucose is normal, which is more typical of viral meningitis. An elevated total protein level in the CSF is seen in both bacterial and viral meningitis. Lastly, a lymphocytic pleocytosis (which is a leukocytosis with a lymphocyte predominance) is consistent with viral rather than bacterial meningitis, which typically shows a predominance of neutrophils. CONCLUDING: By aggregating all the information, the clinical and laboratory findings seem to align with the suspicions of viral meningitis more than bacterial meningitis. By specifically evaluating each potential infectious agent, an enterovirus infection might be the most plausible cause of the presented symptoms given the circumstances, i.e., the summer season, the lymphocytic predominance, and the absence of severely impaired health status. RESPONSE: Based on the clinical presentation, examination findings, lab results and their interconnections, the most likely cause of the symptoms experienced by the 10-year-old boy is an Enterovirus infection (Option B). This is supported by the summertime presentation, the lymphocytic predominance in the CSF, the milder manifestation observed, and the fact that the disease has been seen among the boy's camp peers.

**Facilitator** The consensus statement synthesizes the responses of the five AI opinions as follows: The clinical scenario presents a 10-year-old boy who has been admitted to the emergency department during the summer with symptoms of fever, headache, photophobia, and mild nuchal rigidity. Similar symptoms have been observed among his camp mates. The lumbar puncture results reveal a lymphocytic pleocytosis in the cerebrospinal fluid (CSF), which is more indicative of a viral rather than bacterial etiology. Given the patient's age, the seasonal timing of the symptoms, the communal setting of a summer camp, and the CSF findings, the most probable infectious agent causing these symptoms is likely an Enterovirus. Enteroviruses are common causes of viral meningitis, especially during the summer months, and can easily spread in communal environments. The clinical and laboratory findings, including the lymphocytic predominance in the CSF, the mild manifestation of symptoms, and the fact that the disease has been seen among the boy's camp peers, further support this conclusion. Therefore, the most likely cause of these findings is (B) Enterovirus.
