# Supplementary material for: Collaborative intelligence in AI: Evaluating the performance of a council of AIs on the USMLE
Source: PLOS Digit Health. 2025 Oct 9;4(10):e0000787. doi: 10.1371/journal.pdig.0000787 (PMC12510544; doi:10.1371/journal.pdig.0000787)
Supplement: S2 File — (ZIP) [file pdig.0000787.s002.zip › S2 Data Raw data files containing unprocessed question inputs and LLM outputs including transcripts of deliberations/STEP1_Question-25-1_Type-MC-NJ.docx]

**Facilitator** **[Round 1]:** A healthy 30-year-old woman participates in a study of the relationship between cardiovascular function and posture. Placed in a room with a comfortable temperature (72°F) and humidity (40%), she moves from a standing to a supine position. Which of the following sets of changes is most likely in the reflex control of this woman's cardiovascular system? (A) Carotid Sinus Baroreceptor Activity, Atrial Stretch Receptor Activity, and Vagal Efferent Activity to the Heart increase (B) Carotid Sinus Baroreceptor Activity and Atrial Stretch Receptor Activity increase, while Vagal Efferent Activity to the Heart decrease (C) Carotid Sinus Baroreceptor Activity and Vagal Efferent Activity to the Heart increase, while Atrial Stretch Receptor Activity decrease (D) Carotid Sinus Baroreceptor Activity increase, while Atrial Stretch Receptor Activity and Vagal Efferent Activity to the Heart decrease (E) Carotid Sinus Baroreceptor Activity decrease, while Atrial Stretch Receptor Activity and Vagal Efferent Activity to the Heart increase (F) Carotid Sinus Baroreceptor Activity and Vagal Efferent Activity to the Heart decrease, while Atrial Stretch Receptor Activity increase (G) Carotid Sinus Baroreceptor Activity and Atrial Stretch Receptor Activity decrease, while Vagal Efferent Activity to the Heart increase (H) Carotid Sinus Baroreceptor Activity, Atrial Stretch Receptor Activity, and Vagal Efferent Activity to the Heart decrease

**AI_C:** RESTATING THE QUESTION: This question considers how the body's reflex control mechanisms for the cardiovascular system might be expected to respond when a woman transitions from a standing to a supine, or lying down, position. The mechanisms under consideration include Carotid Sinus Baroreceptor Activity, which monitor blood pressure; Atrial Stretch Receptor Activity, which help regulate blood volume; and Vagal Efferent Activity to the Heart, which facilitates a parasympathetic response to slow the heart rate. PLANNING: In order to form an educated and informed hypothesis, we must consider the impacts from the standing to the supine position on the pressure and volume systems of the body. When assuming a supine position, blood that was pooling in the legs while standing is redistributed which leads to an increase in venous return to the heart. This rise in venous return will enhance the Atrial Stretch Receptor Activity. The increase in venous return should also raise blood pressure, therefore increasing the Carotid Sinus Baroreceptor Activity. On the other hand, the Vagal Efferent Activity to the Heart, one of the primary pathways for parasympathetic output to the heart that serves mainly to decrease heart rate, should increase as it is responsive to the increased blood pressure to keep the cardiovascular system stable. CLINICAL REASONING: As we transition from standing to a supine position, gravity no longer influences blood to pool in the lower extremities. This results in an increase in venous return and a corresponding increase in the activity of the Atrial Stretch Receptors, which sense changes in blood volume and venous return. At the same time, the increased volume is causing increased carotid pressure leading to increase in the Carotid Sinus Baroreceptor Activity - these receptors monitor blood pressure changes in the carotid arteries. Vagal Efferent Activity to the Heart, which is often increased to moderate heart rate during times of increased blood pressure, will likely also increase in response to enhanced Carotid Sinus Baroreceptor Activity and to maintain stability in the cardiovascular system. CONCLUDING: Collectively, visibility into the physiological impacts of transitioning from a standing to a supine position helps inform the likely combination of reflex control changes in this scenario. RESPONSE: Given the context, the most likely changes in the reflex control of the woman's cardiovascular system would be: (A) Carotid Sinus Baroreceptor Activity, Atrial Stretch Receptor Activity, and Vagal Efferent Activity to the Heart increase.

**AI_D:** (1) RESTATING THE QUESTION: We are asked to understand the most likely changes in a healthy 30-year-old woman's reflex control of her cardiovascular system as she transitioned from a standing to a supine position. The variables are carotid sinus baroreceptor activity, atrial stretch receptor activity, and vagal efferent activity to the heart. The carotid sinus baroreceptors are involved in maintaining blood pressure and respond to changes in arterial pressure. Atrial stretch receptors detect changes in atrial pressure, typically related to blood volume. Vagal efferent activity refers to output from the vagus nerve to the heart, which is primarily responsible for reducing the heart rate. (2) PLANNING: Since the woman is shifting from a standing to a supine position, it's reasonable to assume that blood distribution in her body will adjust due to gravity; consequently, there will be less blood in the lower parts of the body, causing an increase in venous return to the heart. This would normally cause atrial stretch and hence increased atrial stretch receptor activity. The assumption that vagal efferent activity to the heart and carotid sinus baroreceptor activity may also change with posture should be considered. (3) CLINICAL REASONING: With an increase in venous return, the heart would attempt to adjust output to prevent changes in blood pressure that could be detected by the carotid sinus. While activity at the carotid baroreceptors could decrease due to maintenance of mean arterial pressure, vagal efferent activity would likely increase to lower the heart rate, a mechanism of compensation. (4) CONCLUDING: Considering the physiology of the cardiovascular reflexes and the surrounding neural control, the expected changes when moving from a standing to a supine position would include an increase in atrial stretch receptor activity due to increased venous return, and an increase in vagal efferent activity to maintain blood pressure by slowing the heart rate. Carotid sinus baroreceptor activity could potentially remain stable or decrease if blood pressure is successfully maintained by these changes. (5) RESPONSE: I lean towards "(E) Carotid Sinus Baroreceptor Activity decrease, while Atrial Stretch Receptor Activity and Vagal Efferent Activity to the Heart increase" as the most likely response, because the body will attempt to maintain homeostasis in response to the change in postural position, triggering various cardiovascular reflexes.

**AI_E:** RESTATING THE QUESTION: This problem is fundamentally concerned with understanding how the body's internal cardiovascular reflex control systems might be affected by a person's change in posture -- specifically, moving from standing up to lying down (supine position). Three key elements of this reflex control system are the carotid sinus baroreceptor activity, atrial stretch receptor activity, and vagal efferent activity to the heart. PLANNING: Now, in order to solve this, I need to first understand how each of these components typically responds to a positional change from standing to lying down. 1. Carotid Sinus Baroreceptors: These are sensors located in the carotid sinus (an enlargement of the carotid artery) that detect changes in blood pressure. If blood pressure drops (e.g., as could happen when standing up), they trigger a compensatory increase in heart rate. Thus, if a person lies down (where gravity no longer pushes blood away from the heart), the reduced demand for compensation might reduce the activity level of these baroreceptors. 2. Atrial Stretch Receptors: These are sensors located in the atria of the heart that respond to the degree of stretch of the heart wall (reflective of blood volume). If a person lies down, the pooling blood in the vertebrae vessels will return to the heart, causing the atria to stretch more, potentially increasing the activity of these receptors. 3. Vagal Efferent Activity to the Heart: This reflects the degree of parasympathetic nervous system input to the heart, particularly via the vagus nerve. Increased vagal activity (via acetylcholine) usually slows the heart rate and decreases its force of contraction. Standing up often increases sympathetic activity (to compensate for blood pressure changes and maintain brain perfusion), so transitioning to a lying down position might lead to a relative increase in vagal activity (and less "fight against gravity"). CLINICAL REASONING: Thus, if a person transitions from standing to lying down, it seems reasonable to expect the carotid sinus baroreceptor activity to decrease (as less compensation for dropping blood pressure is needed), the atrial stretch receptor activity to increase (due to returned blood pooling in the heart), and the vagal efferent activity to the heart to increase (due to less need for 'fighting against gravity'). CONCLUDING: Combining these deductions together, the most likely set of changes would be a decrease in carotid sinus baroreceptor activity, an increase in atrial stretch receptor activity, and an increase in vagal efferent activity to the heart. RESPONSE: Based on the physiology of the cardiovascular reflex control system in response to the change in posture from standing to supine position, the most fitting answer would be (E) Carotid Sinus Baroreceptor Activity decrease, while Atrial Stretch Receptor Activity and Vagal Efferent Activity to the Heart increase.

**AI_B:** RESTATING THE QUESTION: The scenario describes a healthy 30-year-old woman who is participating in a study investigating the interplay between cardiovascular function and body posture. While situated in a room with a stable, comfortable temperature and humidity, she transitions from a standing to a supine (lying face upward) position. The question is asking how this posture change is likely to reflect on certain aspects of her cardiovascular reflex control, specifically: the activity of her carotid sinus baroreceptors, atrial stretch receptors, and vagal efferent activity to the heart. PLANNING: Here, we need to understand the roles that the carotid sinus baroreceptors, atrial stretch receptors, and vagal efferences play in cardiovascular control. Carotid sinus baroreceptors, pressure-sensor neurons located in the walls of the carotid sinus, provide the brain with information about blood pressure changes. Higher activity thereof corresponds to higher experienced blood pressure. Atrial stretch receptors detect changes in blood volume and pressure inside the heart, with increased activity signaling higher blood volume. Vagal efferent activity to the heart, on the other hand, exerts a direct effect on heart rate: an enhanced vagal (parasympathetic) activity would decrease the heart rate. CLINICAL REASONING: Transitioning from a standing to a supine position is associated with gravitational changes. In a supine position, gravity doesn't oppose blood return to the heart as much as while standing. This tends to increase venous return to the heart, elevating the activity of the atrial stretch receptors, signaling the increased volume. In response, the parasympathetic activity (vagal efferents) acts to reduce the heart rate in an attempt to counter the volume increase, i.e., avoid potential hypertension. Therefore, when the woman goes from standing to supine, more blood fills the heart which leads to an increase in the stretch of the atria leading to increased atrial stretch receptor activity, and more blood pushes against the wall of the carotid sinus leading to an induced increase in the carotid sinus baroreceptor activity. As a reflex response to this increase in blood pressure, the vagal efferent activity (parasympathetic system) to the heart will also increase in order to reduce the heart rate and cardiac output, thereby lowering the blood pressure. CONCLUDING: Therefore, the effects of a standing-to-supine postural transition on the reflex control of the cardiovascular system in this woman would most likely manifest as an increase in both carotid sinus baroreceptor and atrial stretch receptor activities due to enhanced blood volume and pressure. Simultaneously, there would be an increase in vagal efferent activity to reduce the heart rate in response to the increased volume and pressure, aiming to maintain homeostasis. RESPONSE: Based on the above, the most probable set of changes in the woman's cardiovascular reflex control would be: (A) Carotid Sinus Baroreceptor Activity, Atrial Stretch Receptor Activity, and Vagal Efferent Activity to the Heart increase.

**AI_A:** RESTATING THE QUESTION: This question involves the body's cardiac reflex response to the transition from a standing to a supine position in a healthy, young woman. The question specifically looks at changes in the activity of carotid sinus baroreceptors, atrial stretch receptors, and vagal (parasympathetic) efferent signals to the heart. Carotid sinus baroreceptors are pressure-sensitive sensory receptors located in the carotid sinus (on the carotid artery) that help modulate heart rate in response to changes in blood pressure. Atrial stretch receptors are found in the atria of the heart and respond to changes in blood volume. Vagal efferent activity pertains to parasympathetic signals sent via the vagus nerve to the heart, generally leading to a decrease in heart rate. PLANNING: The primary assumption is that position changes, like transitioning from standing to supine, alter cardiovascular parameters such as blood pressure, blood volume, and consequently, heart rate. To understand these changes, we must consider the physiological responses in terms of the carotid sinus baroreceptor activity, atrial stretch receptor activity, and vagal efferent activity to the heart. CLINICAL REASONING: Carotid sinus baroreceptors are activated by increases in blood pressure, whereas they are deactivated by a decrease in blood pressure. When a person assumes a standing position, gravity will tend to pool blood in the lower extremities, decreasing venous return. As a result, there will be less filling of the heart, which leads to lower stroke volume and cardiac output, and consequently lower blood pressure. This elicits reflexes to increase sympathetic activity and decrease parasympathetic activity that results in an increase in heart rate and contractility to maintain blood pressure. On the other hand, when a person moves from a standing to a supine position, venous return to the heart increases due to the elimination of gravity. This increase in preload (end diastolic volume) will stretch the atria, increasing activity of the atrial stretch receptors, and subsequently increase stroke volume due to the Frank-Starling mechanism. The increased stroke volume results in an increased blood pressure. The carotid sinus baroreceptors will sense this increase in blood pressure, leading to their stimulation. As a reflex, there should be increased parasympathetic activity and decreased sympathetic activity, aiming to decrease heart rate and preserve normal blood pressure. CONCLUDING: Given the above, when this woman moves from a standing to a supine position, an increase in carotid sinus baroreceptor activity as well as atrial stretch receptor activity can be expected, as these receptors respond to an increase in blood pressure and blood volume respectively. As a compensatory reflex response to the increased blood pressure, there should be an increase in parasympathetic (vagal) activity to the heart to help bring down the heart rate and maintain blood pressure. RESPONSE: Therefore, the most likely cardiovascular response in a healthy 30-year-old woman moving from a standing to a supine position would be an increase in carotid sinus baroreceptor activity, an increase in atrial stretch receptor activity, and finally an increase in vagal efferent activity to the heart. This corresponds to option (A).

**Facilitator** **[Round 2]:** Summary: AI_A, AI_B, and AI_C all agree that transitioning from a standing to a supine position would result in an increase in carotid sinus baroreceptor activity, atrial stretch receptor activity, and vagal efferent activity to the heart. They argue that the increase in blood volume and pressure due to the change in position would stimulate the carotid sinus baroreceptors and atrial stretch receptors. This would then trigger an increase in vagal efferent activity to slow the heart rate and maintain blood pressure. AI_D, however, suggests that while atrial stretch receptor activity and vagal efferent activity would increase, carotid sinus baroreceptor activity could potentially remain stable or decrease if blood pressure is successfully maintained by these changes. AI_E proposes a similar argument to AI_D, suggesting that carotid sinus baroreceptor activity would decrease due to less demand for compensation in blood pressure, while atrial stretch receptor activity and vagal efferent activity would increase. Key Differences: The key difference in perspectives lies in the expected change in carotid sinus baroreceptor activity. AI_A, AI_B, and AI_C predict an increase due to the rise in blood pressure from the positional change. In contrast, AI_D and AI_E predict a decrease or stability in carotid sinus baroreceptor activity, arguing that the body's successful maintenance of blood pressure would reduce the need for these baroreceptors to compensate. Question to Clarify Disagreements: Can the body's maintenance of blood pressure during a transition from a standing to a supine position potentially reduce the need for carotid sinus baroreceptor activity, or would the increase in blood pressure inherently stimulate these baroreceptors regardless of the body's compensatory mechanisms?. Based on your preceding response, how would you answer the original question: [A healthy 30-year-old woman participates in a study of the relationship between cardiovascular function and posture. Placed in a room with a comfortable temperature (72°F) and humidity (40%), she moves from a standing to a supine position. Which of the following sets of changes is most likely in the reflex control of this woman's cardiovascular system? (A) Carotid Sinus Baroreceptor Activity, Atrial Stretch Receptor Activity, and Vagal Efferent Activity to the Heart increase (B) Carotid Sinus Baroreceptor Activity and Atrial Stretch Receptor Activity increase, while Vagal Efferent Activity to the Heart decrease (C) Carotid Sinus Baroreceptor Activity and Vagal Efferent Activity to the Heart increase, while Atrial Stretch Receptor Activity decrease (D) Carotid Sinus Baroreceptor Activity increase, while Atrial Stretch Receptor Activity and Vagal Efferent Activity to the Heart decrease (E) Carotid Sinus Baroreceptor Activity decrease, while Atrial Stretch Receptor Activity and Vagal Efferent Activity to the Heart increase (F) Carotid Sinus Baroreceptor Activity and Vagal Efferent Activity to the Heart decrease, while Atrial Stretch Receptor Activity increase (G) Carotid Sinus Baroreceptor Activity and Atrial Stretch Receptor Activity decrease, while Vagal Efferent Activity to the Heart increase (H) Carotid Sinus Baroreceptor Activity, Atrial Stretch Receptor Activity, and Vagal Efferent Activity to the Heart decrease]

**AI_E:** 1) RESTATING THE QUESTION: The question is asking us to predict the changes in a healthy 30-year-old woman's cardiovascular system's reflex control as she moves from a standing to a supine position. Specifically, we need to anticipate changes in her carotid sinus baroreceptor activity, atrial stretch receptor activity, and vagal efferent activity to the heart. Carotid sinus baroreceptors are sensors located in the internal carotid artery that detect changes in blood pressure, sending signals to the brain to adjust heart rate and blood vessel dilation. Atrial stretch receptors are sensors in the atria of the heart that monitor blood volume and trigger responses that regulate blood pressure. Vagal efferent activity refers to signals sent from the vagus nerve (a major component of the parasympathetic nervous system) to the heart, helping regulate heart rate based on the body's current needs. 2) PLANNING: To answer this question, we need to consider the impact of body position on blood distribution and volume, as well as the roles of our three key components in maintaining cardiovascular homeostasis. 3) CLINICAL REASONING: When a person moves from standing to a supine position, gravity's effects are reduced, leading to an increased blood volume returning to the heart. This results in expansion of the atria, activating the atrial stretch receptors. Furthermore, this increase in blood volume and pressure could lead to an increase in carotid sinus baroreceptor activity, as they would likely detect the change in blood pressure associated with the position shift. However, the body also has mechanisms to prevent any drastic changes in blood pressure with a change in posture. Therefore, the increase in vagal efferent activity would help curb the heart rate and maintain blood pressure. 4) CONCLUDING: Considering the physiological effects of moving from a standing to a supine position and the role of each component (carotid sinus baroreceptors, atrial stretch receptors, and the vagal nerve) in maintaining cardiovascular homeostasis, it is highly probable that each component would increase its activity to bring about a dynamic balance in the body's blood pressure that could otherwise be affected by the change in posture. 5) RESPONSE: Thus, I would suggest option A – Carotid Sinus Baroreceptor Activity, Atrial Stretch Receptor Activity, and Vagal Efferent Activity to the Heart would most likely all increase in response to the shift from a standing to the supine position.

**AI_D:** Based on my domain knowledge, the carotid sinus baroreceptors and atrial stretch receptors are both sensitive to changes in blood pressure and volume. When the woman moves from a standing to a supine position, blood that was previously opposed by gravity in the lower body now floods back to the heart, leading to an increase in venous return. The atrial stretch receptors, located in the walls of the atria, detect this increase in blood volume and signal the brain, which in turn increases vagal efferent activity to the heart. Increased vagal efferent activity slows the heart rate and decreases the force of cardiac contraction, lowering cardiac output, and indirectly decreasing blood pressure. This is known as the Bainbridge reflex, or atrial reflex. The carotid sinus baroreceptors, on the other hand, respond to changes in blood pressure. When there's an increase in blood pressure, as might be induced by the increase in venous return, the carotid sinus baroreceptor activity increases. This sends signals to the brain to initiate reflex mechanisms to lower the blood pressure. This is conducted via various pathways, one of them being an increase in vagal efferent activity to the heart, similar to the atrial reflex. Considering these points, an increase in both atrial stretch receptor activity and vagal efferent activity to the heart is expected. However, it's debatable whether the carotid sinus baroreceptor activity would increase or stay the same. It might increase due to the initial increase in venous return and blood pressure. Alternatively, if the reflex actions initiated by the atrial stretch receptors and the vagal efferent activity are successful in quickly decreasing the blood pressure, the carotid baroreceptors might not need to ramp up their activity significantly, and they could remain at the same level or even slightly decrease. Therefore, to answer the original question, I believe the most likely scenario is option (E) -- Carotid Sinus Baroreceptor Activity decrease, while Atrial Stretch Receptor Activity and Vagal Efferent Activity to the Heart increase. This suggests a compromise between the position of AI_D and AI_E: while the carotid sinus baroreceptor activity might not significantly increase, there'll likely be a noticeable rise in atrial stretch receptor activity and vagal efferent activity, responding to increased venous return and the task of maintaining the woman's cardiovascular homeostasis during the positional change. However, since biological systems often have redundant and overlapping mechanisms, this answer doesn't completely rule out some increase in carotid sinus baroreceptor activity.

**AI_C:** Restating the Question: The question revolves around the physiological effects, particularly focusing on the cardiovascular system, when a healthy woman transitions from a standing position to a supine position in a controlled environment. The main points of consideration are the changes in the Carotid Sinus Baroreceptor Activity, Atrial Stretch Receptor Activity, and the Vagal Efferent Activity to the Heart. Planning: Baroreceptors are sensors located in the blood vessels of the body. They sense the blood pressure and send signals to the brain to help regulate it. Specifically, the Carotid Sinus Baroreceptors respond to changes in blood pressure within the carotid arteries. The Atrial stretch receptors are specialized sensory nerves located within the atria of the heart that react to the stretching of the atrial wall due to variations in blood volume. Lastly, Vagal Efferent Activity to the heart refers to the signals sent via the vagus nerve, which exert a parasympathetic influence, typically slowing the heart rate. Clinical Reasoning: When a person shifts from a standing to a supine position, this postural transition leads to a redistribution of blood volume. In a standing position, gravity promotes pooling of blood in the lower part of the body. In contrast, in a supine position, the blood would be more evenly distributed throughout the body. With the increase in blood volume and pressure towards the upper part of the body, including the neck and head in a supine position, the Carotid Sinus Baroreceptors would 'sense' an increase in blood pressure. Simultaneously, due to the improved venous return to the heart because of a shift to supine position, the atrial walls would stretch more – thus leading to an increase in Atrial Stretch Receptor Activity. Considering the Carotid Sinus Baroreceptors and Atrial Stretch Receptor Activity, the body, in attempt to maintain homeostasis, will try to decrease the heart rate and stabilise blood pressure. This is achieved through enhanced vagal efferent activity, which decreases heart rate and thus blood pressure. Concluding: Thus, taking the redistribution of blood volume, its consequent impact on the cardiac receptors activity and the body's reflexive response mechanisms into consideration, it seems likely that when moving from standing to a supine position, Carotid Sinus Baroreceptor Activity and Atrial Stretch Receptor Activity would increase, and so would the Vagal Efferent Activity to the Heart. Response: Therefore, my hypothesis and final response to the original question is option (A): Carotid Sinus Baroreceptor Activity, Atrial Stretch Receptor Activity, and Vagal Efferent Activity to the Heart increase when transitioning from a standing to a supine position.

**AI_B:** (1) RESTATING THE QUESTION: The query wants us to identify changes in cardiovascular reflex control in a healthy woman, who changes her body posture from standing to supine. Here, the key factors in consideration are the activities of three cardiovascular regulators: the Carotid Sinus Baroreceptors (pressure sensors in our carotid arteries), the Atrial Stretch Receptors (sensors in our atria that measure blood volume), and the Vagal Efferent Activity to the Heart (part of the parasympathetic nervous system that modulates the heart rate). (2) PLANNING: The assumptions inherent to answering this question are: (a) that transitioning from a standing to a supine position causes changes in blood pressure and volume, and (b) these changes should trigger responses from carotid sinus baroreceptors, atrial stretch receptors, and vagal efferent fibers. These assumptions are indexed on the body's reflex mechanisms - designed to maintain cardiovascular stability. Our challenge here is to decide how the body might prioritize its response to maintain stability and homeostasis. (3) CLINICAL REASONING: When a person moves from standing to lying position, the blood, earlier pooled in the lower extremities, will now be redistributed more evenly across the body due to removed effects of gravity. This action leads to a higher venous return to the heart, enabling an increased preload (volume of blood in the heart just before contraction) alongside higher stroke volume (volume of blood ejected by the heart). The baroreceptors in the carotid sinus and stretch receptors in the atria are likely to sense this change and react. The carotid sinus baroreceptors, sensitive to distension (stretch) of the arterial walls caused by blood pressure increases, would increase their activity. Concurrently, the increased blood volume would also trigger the atrial stretch receptors. Following which, a compensatory response will be activated to prevent a potentially hypertensive state. Via the parasympathetic pathway (Vagus nerve), efferent vagal activity to the heart would be increased, reducing the heart rate (a response known as the Bainbridge reflex). This reaction is in line with our body's overarching goal to preserve homeostasis. (4) CONCLUDING: Therefore, the clinical reasoning process tracks a cascade of events starting with the positional change leading to increased venous return. This causes a rise in preload, triggering carotid sinus and atrial stretch receptors. To maintain stability, the body bumps up efferent vagal activity, causing heart rate reduction and overall cardiovascular stability. (5) RESPONSE: Consequently, the most likely changes in the reflex control of this woman's cardiovascular system upon transitioning from a standing to supine position would be: Carotid Sinus Baroreceptor Activity, Atrial Stretch Receptor Activity, and Vagal Efferent Activity to the Heart increase (Option A).

**AI_A:** The question here pertains to the change in the woman's cardiovascular system in response to her shift from a standing to a supine position. The options involve various combinations of increases or decreases in three key components: Carotid Sinus Baroreceptor Activity, Atrial Stretch Receptor Activity, and Vagal Efferent Activity to the Heart. To begin with, it is important to define these terms. Baroreceptors are pressure-sensitive sensory neurons that are located in the heart and vascular system, specifically in the carotid sinus and aortic arch. These play an essential role in maintaining blood pressure homeostasis, by detecting changes in blood pressure and sending the information to the brain, which then adjusts our heart rate, force of heart contraction, and blood vessel diameter. Atrial Stretch Receptors are found in the atria-part of the heart and they respond to increased venous return by signaling the brain to reduce blood volume (by reducing reabsorption of fluid in the kidneys) and reduce sympathetic nervous system output. Lastly, Vagal efferent activity refers to the impulses sent from the brain to the heart via the vagus nerve, part of the parasympathetic nervous system. Such impulses cause a decrease in heart rate. The assumption here is that these three factors are instrumental in our bodies’ response to posture changes, and the implication is that the shift from a standing to a supine position influences the cardiovascular reflex control of a healthy 30-year-old woman. One important alternative assumption to be aware of here is that other factors that affect cardiovascular function, such as hormonal changes, aren’t being considered. For the purpose of the question though, it makes sense to focus on the three given options given the anatomical and physiological changes that occur with shifts in body position. Given that when someone stands up from a supine position, the blood rushes down due to gravity reducing venous return to the heart and hence reducing the stroke volume (blood pumped by the heart in each beat). As a result, less blood flows to the Carotid Sinus, causing a decrease in Carotid Sinus Baroreceptor activity. This reduction signals through the glossopharyngeal nerve to the medullar centers in the brain to reduce the parasympathetic stimulation (Vagal Efferent Activity) and increase the sympathetic stimulation to the heart. However, when going from standing to supine, the change in blood flow is reversed, resulting in increased venous return to the heart and therefore, increased stroke volume and blood flow to the Carotid sinus causing an increase in Carotid Sinus Baroreceptor activity. This would then result in an increase of the Vagal Efferent Activity to the Heart because the baroreceptors sense increased blood pressure and aim to bring the heart rate down via parasympathetic stimulation. The increased blood volume being returned to the heart would also stretch the atria, leading to increased Atrial Stretch Receptor Activity. So, in conclusion, in the described scenario of this question, the most likely sequence that happens in this woman’s cardiovascular system, while shifting from standing to a supine position, would be an increase in Carotid Sinus Baroreceptor Activity, Atrial Stretch Receptor Activity, and Vagal Efferent Activity to the Heart. Therefore, I would choose option (A) as the correct answer.

**Facilitator** **[Round 3]:** Summary: AI_A, AI_B, AI_C, and AI_E all agree that the transition from a standing to a supine position would lead to an increase in Carotid Sinus Baroreceptor Activity, Atrial Stretch Receptor Activity, and Vagal Efferent Activity to the Heart. They all base their reasoning on the fact that the shift in position would lead to an increased venous return to the heart, which would trigger these responses to maintain cardiovascular homeostasis. AI_D, however, suggests that while Atrial Stretch Receptor Activity and Vagal Efferent Activity to the Heart would increase, Carotid Sinus Baroreceptor Activity might decrease or remain the same. AI_D argues that if the reflex actions initiated by the atrial stretch receptors and the vagal efferent activity are successful in quickly decreasing the blood pressure, the carotid baroreceptors might not need to ramp up their activity significantly. Question to Clarify Disagreements: Given that the body has redundant and overlapping mechanisms to maintain homeostasis, could the Carotid Sinus Baroreceptor Activity remain the same or even decrease, as suggested by AI_D, despite the increased venous return to the heart? Or would the increased blood pressure inevitably lead to an increase in Carotid Sinus Baroreceptor Activity, as argued by AI_A, AI_B, AI_C, and AI_E?. Based on your preceding response, how would you answer the original question: [A healthy 30-year-old woman participates in a study of the relationship between cardiovascular function and posture. Placed in a room with a comfortable temperature (72°F) and humidity (40%), she moves from a standing to a supine position. Which of the following sets of changes is most likely in the reflex control of this woman's cardiovascular system? (A) Carotid Sinus Baroreceptor Activity, Atrial Stretch Receptor Activity, and Vagal Efferent Activity to the Heart increase (B) Carotid Sinus Baroreceptor Activity and Atrial Stretch Receptor Activity increase, while Vagal Efferent Activity to the Heart decrease (C) Carotid Sinus Baroreceptor Activity and Vagal Efferent Activity to the Heart increase, while Atrial Stretch Receptor Activity decrease (D) Carotid Sinus Baroreceptor Activity increase, while Atrial Stretch Receptor Activity and Vagal Efferent Activity to the Heart decrease (E) Carotid Sinus Baroreceptor Activity decrease, while Atrial Stretch Receptor Activity and Vagal Efferent Activity to the Heart increase (F) Carotid Sinus Baroreceptor Activity and Vagal Efferent Activity to the Heart decrease, while Atrial Stretch Receptor Activity increase (G) Carotid Sinus Baroreceptor Activity and Atrial Stretch Receptor Activity decrease, while Vagal Efferent Activity to the Heart increase (H) Carotid Sinus Baroreceptor Activity, Atrial Stretch Receptor Activity, and Vagal Efferent Activity to the Heart decrease]

**AI_C:** Restating the question: The problem inquires about the physiological changes associated with transitioning from standing to supine in relation to cardiovascular function. The key terms in question are Carotid Sinus Baroreceptor Activity, Atrial Stretch Receptor Activity, and Vagal Efferent Activity to the Heart, all of which contribute to cardiovascular response. Planning: To accurately provide an answer, I must consider the physiological impacts of the posture shift. Key assumptions seem to include a change in blood pressure variation and a corresponding change in these three physiological activities. However, I question the assumption that Carotid Sinus Baroreceptor Activity would decrease or increase in this circumstance- I suspect it might remain unchanged until a more significant blood pressure fluctuation occurs. Considering assumptions and alternate assumptions, my initial instinct is to prioritize the assumption that all these parameters increase as a response to postural change. Clinical Reasoning: Moving from a standing to a supine position substantially increases venous return to the heart due to gravity's impact lessening. Increased venous return, consequently, provokes an elevation in atrial pressure, inducing an increase in the Atrial Stretch Receptor Activity. Further, the Carotid Sinus Baroreceptor is sensitive to changes in blood pressure - an increase in blood pressure due to augmented venous return would then typically stimulate the Carotid Sinus Baroreceptor. Lastly, the Vagal Efferent Activity to the heart would also rise in response to higher blood pressure to reduce it by decreasing the heart rate. Despite this reasoning, the body's homeostatic mechanisms are quite dynamic and adept at quickly restoring balance, potentially minimizing the necessity of a heightened response from the Carotid Sinus Baroreceptor. Concluding: On balance, the Atrial Stretch Receptor Activity and Vagal Efferent Activity to the Heart should increase due to the increase in venous return and atrial pressure when the posture shifts from standing to supine. However, while the Carotid Sinus Baroreceptor Activity may increase due to increased blood pressure, it could also remain relatively stable if the other mechanisms successfully mitigate the blood pressure rise. Response: Therefore, the most likely set of changes in the reflex control of this woman's cardiovascular system after transitioning from the standing to the supine position would be that the Carotid Sinus Baroreceptor Activity either remains at baseline or increases, while both the Atrial Stretch Receptor and the Vagal Efferent Activity to the Heart increase. This corresponds to a slight modification to option (A), which assumes an increase in Carotid Sinus Baroreceptor Activity when it could also maintain baseline levels.

**AI_E:** The question is asking us to understand the changes in the cardiovascular system that would occur when a 30-year-old woman shifts from a standing to a supine position. Specifically, we need to determine how Carotid Sinus Baroreceptor activity, Atrial Stretch Receptor activity, and Vagal Efferent activity to the heart would likely change. Before we answer the question, we need to understand the function of these three physiological systems. The Carotid Sinus Baroreceptors (CSB) are pressure-sensitive sensory receptors located in the carotid arteries that monitor changes in blood pressure. When blood pressure increases, the CSB activity would increase leading to a reduction in heart rate and blood pressure. The Atrial Stretch Receptors (ASR) detect changes in blood volume and pressure within the heart's atria. Increase in blood volume and pressure would lead to increased ASR activity. The Vagal Efferent activity to the heart is primarily responsible for reducing heart rate. When it increases, it would result in a decrease in heart rate. We initially hypothesize that the shift from a standing position to a supine position can increase venous return to the heart due to gravity. The heart could be filled more easily with blood, leading to an increase in blood pressure. Consequently, to maintain homeostasis, reflex mechanisms should be triggered. In applying our clinical reasoning, when the woman transitions from standing to lying down, the gravity no longer pools blood in the lower extremities, enhancing venous return to the heart, and hence increasing the preload (initial stretching of the cardiac myocytes prior to contraction). This increased preload and venous return stretch the atrial walls leading to increased Atrial Stretch Receptor activity. This mechanism, known as the Bainbridge reflex, then brings about a rise in heart rate to accommodate the increased blood volume. Considering the increase in venous return, the increased stroke volume from the heart leads to a rise in the blood pressure. The Carotid Sinus Baroreceptors, detecting this rise, would increase their activity to induce compensatory mechanisms to counter this effect. This includes increasing Vagal Efferent activity to the heart, which slows down the heart rate, hence decreasing the blood pressure. After connecting all these dots, we conclude that following the shift from standing to supine position, we expect an increase in Carotid Sinus Baroreceptor activity due to the increased blood pressure, an increase in Atrial Stretch Receptor activity due to the enhanced venous return and myocardial stretching, and an increase in Vagal Efferent activity to the heart due to the increased activity of the Carotid sinus baroreceptors, aiming to decrease the heart rate and control the blood pressure. As for the question at hand, the correct option would therefore be (A) Carotid Sinus Baroreceptor Activity, Atrial Stretch Receptor Activity, and Vagal Efferent Activity to the Heart increase.

**AI_A:** Restating the Question: In simpler terms, we're considering a healthy, young woman and how her cardiovascular system may respond reflexively when shifting from standing to lying down. This involves understanding changes in carotid sinus baroreceptor activity, atrial stretch receptor activity, and vagal efferent activity to the heart. It's asking about changes in specific mechanisms that are part of the overall cardiovascular reflex control structure. Planning: First, let’s define the terms used in the question. The Carotid Sinus Baroreceptors are pressure-sensitive sensory neurons in the carotid body, near the bifurcation of the common carotid artery, which monitor changes in arterial blood pressure. Atrial Stretch Receptors monitor the stretch in atrial walls, often due to changes in blood volume in the heart; these receptors are intrinsic to the heart's atria. Vagal Efferent activity refers to the signals sent from the brain via the vagus nerve (a cranial nerve) which modulate heart rate and contraction. Consideration needs to be given to whether the activity of each of these mechanisms are directly related to each other or if they're independently modulated by the change in posture. Clinical Reasoning: There will be an increased venous return when going from standing to the supine position. The baroreceptors and stretch receptors measure changes in pressure and stretch respectively and as the venous return, and subsequent stroke volume, increase so will the blood pressure in the carotid and the stretch in the atria thus increasing their activity. The baroreceptors act to modulate blood pressure so increased pressure will stimulate increased activity to counteract this change and bring the pressure back towards homeostatic levels. The increased stretch in the atria will also stimulate a reflex rise in heart rate and force of contraction to shift the increased volume of blood, this is achieved by enhanced sympathetic activity and reduced parasympathetic (Vagal) activity to the heart. Concluding: Therefore, based on the predicted changes in baroreceptor and atrial stretch receptor activity, and understanding of the interconnected cardiovascular control systems, it's likely that Carotid Sinus Baroreceptor Activity and Atrial Stretch Receptor Activity would increase due to increased blood pressure and atrial stretch respectively, while Vagal Efferent Activity to the Heart would decrease in response to the effort to increase heart rate and cardiac output. Response: Consequently, answer (B) Carotid Sinus Baroreceptor Activity and Atrial Stretch Receptor Activity increase, while Vagal Efferent Activity to the Heart decrease appears to be the most plausible given the current understanding of cardiovascular reflex controls in response to posture changes.

**AI_D:** Restating the Question: The question is enquiring what changes are expected to occur in a healthy woman's cardiovascular system, specifically in terms of Carotid Sinus Baroreceptor Activity, Atrial Stretch Receptor Activity, and Vagal Efferent Activity to the Heart, when she changes her posture from standing to lying down (supine) in a room of optimal temperature and humidity. Planning: The assumption of the majority, AI_A, AI_B, AI_C, AI_E, is that shifting from standing to supine position will cause increased venous return to the heart. This would in turn result in increased activity of all three components: Carotid Sinus Baroreceptor Activity, Atrial Stretch Receptor Activity, and Vagal Efferent Activity to the Heart. AI_D's alternative assumption is that if the Atrial Stretch Receptors and Vagal Efferent Activities respond efficiently to increased venous return, the Carotid Sinus Baroreceptors might not need to increase their activity response. Clinical Reasoning: When one shifts from a standing to a supine position, the venous return to the heart increases due to the effect of gravity. This increased venous return distends the atria, increasing Atrial Stretch Receptor Activity. Increased stretch of the atria triggers the release of atrial natriuretic peptide (ANP) that promotes salt and water excretion to help reduce blood volume and pressure. The Carotid Sinus Baroreceptors, located in the carotid artery, monitor the pressure of blood flowing to the brain. An increase in blood pressure would generally cause an increase in Carotid Sinus Baroreceptor Activity. However, increased blood pressure (like when shifting position) also stimulates the parasympathetic nervous system, specifically Vagal Efferent Activity to the heart, to reduce heart rate (via the release of acetylcholine). A reduced heart rate can lower the blood pressure. Concluding: When considering the rapid response of the body to maintain homeostasis, it seems plausible that Carotid Sinus Baroreceptor Activity might not increase as expected. The efficient functioning of Atrial Stretch Receptors and Vagal Efferent Activity may prevent an excessive increase in blood pressure and therefore potential increase in Carotid Sinus Baroreceptor Activity might not occur. Response: Given these considerations, it seems plausible that the most likely change would be (E) Carotid Sinus Baroreceptor Activity decrease, while Atrial Stretch Receptor Activity and Vagal Efferent Activity to the Heart increase. This change reflects quick and efficient cardiovascular reflex controls. However, the actual response of Carotid Sinus Baroreceptor Activity could depend on individual variability and might stay the same or increase slightly in some individuals. Therefore, continuous monitoring of these parameters might provide more accurate answers.

**AI_B:** Restating the Question: The question is about how the cardiovascular reflexes of a healthy adult woman would adjust in response to a change from a standing position to a supine position, considering the comfortable room temperature and humidity. This involves the understanding of carotid sinus baroreceptor activity, atrial stretch receptor activity, and vagal efferent activity. Carotid sinus baroreceptors monitor changes in blood pressure and trigger adjustments via the autonomic nervous system to maintain a stable blood pressure. Atrial stretch receptors are found in the heart walls and respond to blood volume changes. Vagal efferent activity refers to signals sent from the brain via the vagus nerve that influences heart rate. Planning: Emerging from the dispute between AI_A, AI_B, AI_C, and AI_E, and AI_D is the question of whether a transition from standing to supine position would increase, decrease, or leave unchanged the carotid sinus baroreceptor activity. While we all agree that the position change would increase blood return to the heart, leading to increased atrial stretch receptor activity and vagal efferent activity to the heart, the effect on carotid sinus baroreceptor activity is disputed. The assumption here is that increased venous return and blood pressure would necessitate an increase in carotid sinus baroreceptor activity. However, AI_D challenges this assumption, arguing that successful homeostatic actions by the atrial stretch receptors and vagal efferent system might prevent the need for increased carotid sinus baroreceptor activity. Clinical reasoning: Our bodies are designed with several overlapping and redundant mechanisms for maintaining homeostasis. In the context of our disagreement, the body has both direct and indirect responses to changes in body posture. Direct responses include changes in heart rate, stroke volume and systemic vascular resistance, while indirect responses could involve renal, hormonal, and neural mechanisms. For this reason, the body's response to postural changes is complex and involves many interacting systems. Therefore, it's reasonable to theorize that the body could maintain homeostasis through the action of one system (in this case, the atrial stretch receptor and vagal efferent activity), and not need to engage another system (the carotid sinus baroreceptor activity). Concluding: Given the extensive and complex interactions of different body systems in maintaining cardiovascular homeostasis, it is plausible to expect various system responses after such a change in body posture. Although the increased venous return will trigger an increased atrial stretch receptor and vagal efferent activity, the ultimate effect on carotid sinus baroreceptor activity may depend on how quickly and efficiently the body succeeds in stabilizing the blood pressure through the initial responses. Response: Thus, while there's a basis for expecting that carotid sinus baroreceptor activity might increase (as suggested by options A to D), we must also consider the possibility that effective quick responses by the body through increased atrial stretch receptor and vagal efferent activity may curtail the need for increased carotid sinus baroreceptor activity. However, the hypothesis that carotid sinus baroreceptor activity could decrease (as shown in option E) seems less plausible, originating from standing position to a supine posture wouldn’t likely decrease the carotid baroreceptor activity as blood pressure in this area should remain constant or increase due to augmented venous return. Therefore, based on our clinical reasoning process, the most appropriate sets of changes would be one of the ones where: Carotid Sinus Baroreceptor Activity and Atrial Stretch Receptor Activity increase, with either an increase or no significant change in Vagal Efferent Activity to the Heart, possibly answering the original question as (A) or (B). However, this is a hypothetical situation, and the real-world response may depend on individual variations and the efficiency of different systems in maintaining homeostasis.

**Facilitator** **[Round 4]:** Summary: AI_A believes that the Carotid Sinus Baroreceptor Activity and Atrial Stretch Receptor Activity would increase due to increased blood pressure and atrial stretch respectively, while Vagal Efferent Activity to the Heart would decrease in response to the effort to increase heart rate and cardiac output. AI_B suggests that while Carotid Sinus Baroreceptor Activity might increase, the body's quick responses through increased Atrial Stretch Receptor and Vagal Efferent Activity may curtail the need for increased Carotid Sinus Baroreceptor Activity. AI_B also considers the possibility of no significant change in Vagal Efferent Activity to the Heart. AI_C proposes that Carotid Sinus Baroreceptor Activity could either remain at baseline or increase, while both the Atrial Stretch Receptor and the Vagal Efferent Activity to the Heart increase. AI_D argues that Carotid Sinus Baroreceptor Activity might not increase as expected due to the efficient functioning of Atrial Stretch Receptors and Vagal Efferent Activity. AI_D suggests that Carotid Sinus Baroreceptor Activity might decrease, while Atrial Stretch Receptor Activity and Vagal Efferent Activity to the Heart increase. AI_E concludes that all three activities, Carotid Sinus Baroreceptor Activity, Atrial Stretch Receptor Activity, and Vagal Efferent Activity to the Heart, would increase due to the shift from standing to supine position. Question to clarify disagreements: Given the differing opinions on the potential change in Carotid Sinus Baroreceptor Activity, can the council members provide more insight into the factors that might influence this activity and how it interacts with the Atrial Stretch Receptor and Vagal Efferent Activity in response to a change in posture?. Based on your preceding response, how would you answer the original question: [A healthy 30-year-old woman participates in a study of the relationship between cardiovascular function and posture. Placed in a room with a comfortable temperature (72°F) and humidity (40%), she moves from a standing to a supine position. Which of the following sets of changes is most likely in the reflex control of this woman's cardiovascular system? (A) Carotid Sinus Baroreceptor Activity, Atrial Stretch Receptor Activity, and Vagal Efferent Activity to the Heart increase (B) Carotid Sinus Baroreceptor Activity and Atrial Stretch Receptor Activity increase, while Vagal Efferent Activity to the Heart decrease (C) Carotid Sinus Baroreceptor Activity and Vagal Efferent Activity to the Heart increase, while Atrial Stretch Receptor Activity decrease (D) Carotid Sinus Baroreceptor Activity increase, while Atrial Stretch Receptor Activity and Vagal Efferent Activity to the Heart decrease (E) Carotid Sinus Baroreceptor Activity decrease, while Atrial Stretch Receptor Activity and Vagal Efferent Activity to the Heart increase (F) Carotid Sinus Baroreceptor Activity and Vagal Efferent Activity to the Heart decrease, while Atrial Stretch Receptor Activity increase (G) Carotid Sinus Baroreceptor Activity and Atrial Stretch Receptor Activity decrease, while Vagal Efferent Activity to the Heart increase (H) Carotid Sinus Baroreceptor Activity, Atrial Stretch Receptor Activity, and Vagal Efferent Activity to the Heart decrease]

**AI_B:** RESTATING THE QUESTION: The essential inquiry at hand involves understanding the changes in three key components of the cardiovascular reflex system: the Carotid Sinus Baroreceptor activity, the Atrial Stretch Receptor activity, and the Vagal Efferent activity to the heart. These changes occur in response to a change in posture; in this case, from standing to a supine position. PLANNING: A clinician would need to consider the physiological principles operating when a healthy individual moves from a standing to a supine position. This paradigm shift alters blood distribution, gravitational forces, venous return to the heart, and ultimately affects the body's homeostatic response mechanisms, which include baroreceptor activity, atrial stretch receptor activity, and vagal activity to the heart. CLINICAL REASONING: When this 30-year-old woman moves from a standing to a supine position, the gravity-dependent blood pooling in the lower body which happens while standing gets redistributed. This shift results in increased venous return to the heart, and consequently increased cardiac output, which is often called postural tachycardia. The increased Venous return enhances the stretch on atria walls, activating the Atrial Stretch Receptors. Simultaneously, the enhanced blood circulation results in an increased pressure at the level of the carotids, leading to increased Carotid Sinus Baroreceptor activity. Body's compensation response to these changes comes through the parasympathetic drive (to counter this sudden increase in heart rate and blood pressure), which is mediated by Vagal Efferent Activity to the heart, resulting in its increase. CONCLUDING: Therefore, based on the physiological principles, when this healthy woman transitions from a standing to a supine position, one can anticipate increased Atrial Stretch Receptor activity (due to enhanced venous return), heightened Carotid Sinus Baroreceptor activity (due to increased pressure at carotids), and increased Vagal Efferent Activity to the heart (a compensatory mechanism to counter elevated blood pressure and heart rate). RESPONSE: Based on this clinical reasoning, the most likely change in the reflex control of this woman's cardiovascular system would be: "Carotid Sinus Baroreceptor Activity, Atrial Stretch Receptor Activity, and Vagal Efferent Activity to the Heart increase" (Option A).

**AI_A:** 1) RESTATING THE QUESTION: The study is focused on understanding changes in the reflex control of a healthy woman's cardiovascular system when she transitions from a standing to supine position. The variables of interest include Carotid Sinus Baroreceptor Activity, which measures pressure changes in the carotid sinus and influences heart rate and blood pressure; Atrial Stretch Receptor Activity, which senses changes in the atrium's blood volume; and Vagal Efferent Activity to the Heart, which is related to the parasympathetic nervous system's influence on heart rate and rhythm. 2) PLANNING: This transition would result in a shift of blood from the lower body to the chest. This would lead to increased venous return to the heart, distention of the right atrium, increased atrial pressure, and increased stroke volume. The anticipated immediate effect should be an increase in the Carotid Sinus Baroreceptor Activity and Atrial Stretch Receptor Activity due to this increased blood volume and pressure. However, there is a potential for the Vagal Efferent Activity to either increase or decrease. 3) CLINICAL REASONING: The increased venous return would increase the activity of the Atrial stretch receptors. This information feeds back to the brain and results in an increased Carotid Sinus Baroreceptor Activity, as the body tries to mitigate the increased blood pressure caused by the augmented venous return. The body might respond to this increased blood pressure by increasing vagal efferent activity to the heart, aiming to reduce the heart rate via increased parasympathetic stimulation. This is a counteracting measure to handle the increased blood pressure. 4) CONCLUDING: Based on the physiological reflexes and feedback mechanisms, I anticipate that Carotid Sinus Baroreceptor Activity and Atrial Stretch Receptor Activity will increase due to the increased venous return and blood pressure. Yet, the body might try to balance this impact by increasing the Vagal Efferent Activity to the heart, though the net effect could be dependant on multiple factors including the prevailing autonomic system influence. 5) RESPONSE: Therefore, I would hypothesize that "Carotid Sinus Baroreceptor Activity and Atrial Stretch Receptor Activity increase, while Vagal Efferent Activity to the Heart decreases" (Option B) as the most likely set of changes in this situation. However, further studies or more specific patient data might be needed to firmly establish this.

**AI_C:** RESTATING THE QUESTION: In this scenario, a healthy 30-year-old woman's cardiovascular reflex control responses are being studied in relation to a change in posture from standing to supine. Three specific reflex controls are being investigated: Carotid Sinus Baroreceptor Activity, Atrial Stretch Receptor Activity, and Vagal Efferent Activity to the Heart. Baroreceptors are sensory neurons that detect changes in blood pressure, atrial stretch receptors detect changes and stretch in the atria of the heart, and vagal efferent activity contributes to heart rate control. PLANNING: Let's consider the body's physiologic responses to the change in posture. Moving from standing to supine facilitates the return of venous blood to the heart along with gravity providing increased preload, potentially causing an increased Atrial Stretch Receptor Activity due to volume overload. This increase is sensed by the carotid Sinus Baroreceptors which then signals the cardiovascular center in the brainstem. This center regulates the heart rate and vascular resistance. We must also consider the role of the Vagus nerve. Increased efferent vagal activity to the heart would generally decrease heart rate, assuming the homeostasis principle of the body acts towards maintaining a constant blood pressure. An alternate assumption could be that in this state of increased preload, the body might not drastically increase vagal activity to the heart given the compensatory reflex actions in play. CLINICAL REASONING: When the woman transitions from a standing to the supine position, gravity no longer opposes blood return to the heart from the lower parts of her body, leading to increased venous return. This results in increased atrial filling, hence triggering the Atrial Stretch Receptors. The activity of the Carotid Sinus Baroreceptors will likely increase as well, as this transition would be seen as a challenge to maintaining a constant blood pressure due to the augmented venous return. However, unlike the stretch and baroreceptors, a decrease in Vagal Efferent Activity would increase heart rate, providing more cardiac output to match with the increased venous return and maintain blood pressure. CONCLUDING: Given the changes in bodily condition from standing to supine, an increase in both Carotid Sinus Baroreceptor and Atrial Stretch Receptor Activity is reasonable. The Vagal Efferent Activity to the Heart will likely decrease as part of a counterbalance to accommodate the changes and keep the circulatory system and blood pressure in a homeostatic state. RESPONSE: This leads to the conclusion that answer choice (B) Carotid Sinus Baroreceptor Activity and Atrial Stretch Receptor Activity increase, while Vagal Efferent Activity to the Heart decrease is most likely in this scenario. This is in line with the reflex control of cardiovascular function which involves maintaining homeostasis while adjusting to changes in body position.

**AI_D:** Restating the question: The query seeks to understand the adaptive cardiovascular reflex mechanisms that come into play when a healthy, young woman transitions from a standing to a supine position under comfortable environmental conditions. Planning: Let's consider the primary baroreceptors involved in the regulation of blood pressure: the carotid sinus baroreceptors, which monitor blood pressure in the large arteries, and the atrial stretch receptors, which respond to changes in blood volume and pressure within the atria of the heart. The parasympathetic activity, namely the vagal efferent activity to the heart, controls the heart rate. Now, we need to generate assumptions about how these might act in response to the change in posture. When moving from a standing to a supine position, blood flow towards the head and upper body increases due to gravity. This might lead to an increase in both carotid sinus activity and atrial stretch receptor activity. However, the body also needs to maintain a stable heart rate and blood pressure, which is mediated by the autonomic nervous system. This could potentially lead to a decrease in vagal activity to temporarily increase the heart rate, and allow the body to adjust to the new posture. Clinical reasoning: The change in posture from standing to supine causes an increase in venous return. This is due to reduced gravitational resistance, leading to an increase in cardiac preload. The increased preload stretches the atrial walls and increases atrial stretch receptor activity. Concurrently, the increased venous return and thus cardiac output lead to an increase in pressure at the carotid sinus, leading to an increase in carotid sinus baroreceptor activity. Traditionally, the body responds to increased pressure by decreasing heart rate via the vagus nerve in what is known as the baroreceptor reflex. Hence, it would be reasonable to assume that the vagal efferent activity to the heart could decrease. Concluding, the shift from standing to supine position in the mentioned woman is likely to increase her atrial stretch receptor activity and carotid sinus baroreceptor activity due to increased venous return and cardiac preload. The vagal efferent activity to the heart may decrease as part of the baroreceptor reflex in response to the increase in pressure. Response: Therefore, I also agree with AI_A's perspective that option (B) Carotid Sinus Baroreceptor Activity and Atrial Stretch Receptor Activity increase, while Vagal Efferent Activity to the Heart decreases, would be the most likely set of changes in the reflex control of this woman's cardiovascular system when transitioning from a standing to a supine position.

**AI_E:** 1) RESTATING THE QUESTION: We're hypothesizing on the likely changes in certain physiological parameters - namely Carotid Sinus Baroreceptor Activity, Atrial Stretch Receptor Activity, and Vagal Efferent Activity to the Heart - of a healthy young woman, as she changes her posture from standing to supine position. To understand this fully, let's define these terms. Firstly, Carotid Sinus Baroreceptor Activity refers to the actions of receptors in the carotid sinus (an area in the carotid artery) that detect and respond to changes in blood pressure. Secondly, Atrial Stretch Receptor Activity pertains to the actions of receptors in the right atrium of the heart that respond to changes in blood volume by causing reflex responses like changes in heart rate. Lastly, Vagal Efferent Activity to the Heart signifies the influence of the vagus nerve (parasympathetic nervous system "output") onto the heart, primarily causing a decrease in heart rate and force of contraction. 2) PLANNING: We need to understand the physiological responses that occur as someone transitions from a standing to a supine position. When Changing from standing to a supine position there is an increase in venous return to the heart due to gravity, leading to increased pre-load. From this, we can infer several assumptions and generate alternate ones: a. First, we could assume that Carotid Sinus Baroreceptor Activity would decrease because of the reduced demand to counterbalance orthostatic hypotension. b. A second assumption might be that the Atrial Stretch Receptor becomes more active due to increased atrial volume and blood pressure. c. Thirdly, we may assess if the Vagal Efferent Activity to the Heart decreases to allow the heart to respond to increased venous return. 3) CLINICAL REASONING: If we analyze these assumptions - The Carotid Sinus Baroreceptor fires in response to an increased blood pressure - moving to a supine position from standing will increase venous return because of gravity drawing more blood back to the heart, potentially increasing the Carotid Sinus Baroreceptor activity. This would contradict assumption a. As for assumption b, the Atrial Stretch Receptor responds to increased atrial volume or pressure, which would be in line with increased venous return. As for assumption c, the Vagal Efferent Activity could decrease in the face of the increased venous return and atrial stretch receptor activity, in an effort to increase heart rate and cardiac output to handle the increased volume. 4) CONCLUDING: The transition from standing to supine position most likely increases venous return, which can stimulate both the carotid sinus baroreceptors and the atrial stretch receptors. The body would attempt to compensate for the increased venous return by decreasing vagal efferent activity to the heart. 5) RESPONSE: Putting all these observations together, in the given scenario, the most likely changes in reflex control of the woman's cardiovascular system would be - an increase in Carotid Sinus Baroreceptor Activity and Atrial Stretch Receptor Activity, while Vagal Efferent Activity to the Heart decreases. Therefore, the correct response would be (B) Carotid Sinus Baroreceptor Activity and Atrial Stretch Receptor Activity increase, while Vagal Efferent Activity to the Heart decrease.

**Facilitator** **[Round 5]:** Summary: AI_A, AI_C, AI_D, and AI_E all agree that the transition from standing to supine position would likely result in an increase in Carotid Sinus Baroreceptor Activity and Atrial Stretch Receptor Activity due to increased venous return and blood pressure. They also hypothesize that the body might try to balance this impact by decreasing the Vagal Efferent Activity to the heart. This is based on the assumption that the body would try to reduce the heart rate via increased parasympathetic stimulation to handle the increased blood pressure. On the other hand, AI_B believes that all three activities (Carotid Sinus Baroreceptor Activity, Atrial Stretch Receptor Activity, and Vagal Efferent Activity to the Heart) would increase. This is based on the assumption that the body's compensation response to the increased heart rate and blood pressure comes through the parasympathetic drive, which is mediated by Vagal Efferent Activity to the heart. Key Differences: The key difference in the perspectives lies in the predicted response of the Vagal Efferent Activity to the Heart. AI_A, AI_C, AI_D, and AI_E predict a decrease in this activity as a counteracting measure to handle the increased blood pressure. In contrast, AI_B predicts an increase in this activity as a compensatory mechanism to counter elevated blood pressure and heart rate. Question for Clarification: To clarify the disagreements between the council members' opinions, we could ask: "What evidence or physiological principles lead you to predict an increase or decrease in Vagal Efferent Activity to the Heart in response to the transition from standing to supine position?". Based on your preceding response, how would you answer the original question: [A healthy 30-year-old woman participates in a study of the relationship between cardiovascular function and posture. Placed in a room with a comfortable temperature (72°F) and humidity (40%), she moves from a standing to a supine position. Which of the following sets of changes is most likely in the reflex control of this woman's cardiovascular system? (A) Carotid Sinus Baroreceptor Activity, Atrial Stretch Receptor Activity, and Vagal Efferent Activity to the Heart increase (B) Carotid Sinus Baroreceptor Activity and Atrial Stretch Receptor Activity increase, while Vagal Efferent Activity to the Heart decrease (C) Carotid Sinus Baroreceptor Activity and Vagal Efferent Activity to the Heart increase, while Atrial Stretch Receptor Activity decrease (D) Carotid Sinus Baroreceptor Activity increase, while Atrial Stretch Receptor Activity and Vagal Efferent Activity to the Heart decrease (E) Carotid Sinus Baroreceptor Activity decrease, while Atrial Stretch Receptor Activity and Vagal Efferent Activity to the Heart increase (F) Carotid Sinus Baroreceptor Activity and Vagal Efferent Activity to the Heart decrease, while Atrial Stretch Receptor Activity increase (G) Carotid Sinus Baroreceptor Activity and Atrial Stretch Receptor Activity decrease, while Vagal Efferent Activity to the Heart increase (H) Carotid Sinus Baroreceptor Activity, Atrial Stretch Receptor Activity, and Vagal Efferent Activity to the Heart decrease]

**AI_C:** RESTATING THE QUESTION: The question asks us to consider the reflex control of a healthy woman's cardiovascular system when she shifts from a standing to a supine position. We're considering the effect of this change on three physiological markers: Carotid Sinus Baroreceptor Activity, Atrial Stretch Receptor Activity, and Vagal Efferent Activity to the Heart. PLANNING: The first two indicators shortlisted: Carotid Sinus Baroreceptor Activity and Atrial Stretch Receptor Activity, are reflective of blood pressure and cardiac volume respectively. The third indicator: Vagal Efferent Activity to the Heart, is an indicator of heart rate and could either decrease, indicating less parasympathetic influence or increase, indicating more. When moving from standing to supine, gravitational forces on the body are altered, these changes are deemed to affect blood distribution throughout the body, possibly leading to an augmentation in venous return, as there is much less opposition to blood flow back to the heart. CLINICAL REASONING: Now, an increase in venous return would be met with an increased Atrial Stretch Receptor Activity, signifying augmented blood volume in the heart. Baroreceptors, located at the carotid sinus, are sensitive to changes in blood pressure. As blood volume and hence the blood pressure increases, it is expected that Carotid Sinus Baroreceptor Activity would increase to reflect this. The Vagal Efferent Activity to the Heart might however be altered to maintain homeostatic balance. An escalation in blood pressure and cardiac volume could suggest a potential overdrive of the cardiovascular system, necessitating a counteractive measure. The parasympathetic nervous system, of which the vagus nerve is a significant part, plays a role in reducing heart rate, allowing the heart muscles enough time to relax and the chambers to adequately fill with blood. It's therefore plausible that Vagal Efferent Activity to the Heart might decrease, presenting an increase in heart rate, in an attempt to quickly pump out the augmented blood volume and regulate pressure. CONCLUDING: Based on the physiological principles concerning the transition from standing to supine, it would seem probable that Carotid Sinus Baroreceptor Activity and Atrial Stretch Receptor Activity would increase due to increased volume and pressure, while Vagal Efferent Activity to the Heart would potentially decrease as a compensatory response. RESPONSE: My selection would thus be choice (B): Carotid Sinus Baroreceptor Activity and Atrial Stretch Receptor Activity increase, while Vagal Efferent Activity to the Heart decrease.

**AI_B:** Restatement: The question is asking us to consider which cardiovascular responses will be initiated as a healthy young woman switches from standing to lying down (supine position). Key responses to consider include Carotid Sinus Baroreceptor Activity, Atrial Stretch Receptor Activity, and Vagal Efferent Activity to the heart. The Carotid Sinus Baroreceptors monitor blood pressure changes in the carotid artery, Atrial Stretch Receptors are monitored for changes in blood volume, particularly increased venous return and Vagal Efferent Activity to the heart influences heart rate. Planning: The transition from standing to supine position permits greater venous return due to the elimination of blood pooling in the lower extremities. This increased venous return would be detected by the Atrial Stretch Receptors and thus an increase in their activity would be expected to be seen. The increased arterial pressure due to the increased venous return would also stimulate the Carotid Sinus Baroreceptors to increase their activity. The point of contention lies with the output of the Vagal Efferent Activity to the heart, which principally controls the heart rate via parasympathetic innervation. Clinical Reasoning: Should the body perceive the increased blood volume and pressure as a potential threat, it might engage mechanisms to decrease the heart rate - that's where role of the Vagal Efferent Activity to the heart becomes crucial. This would support the hypothesis that the Vagal Efferent Activity to the heart would decrease in response to the context at hand. However, Vagal Efferent Activity could also be expected to increase as a means of compensation for the elevated blood pressure and heart rate, a notion supported by homeostatic principles and the body's built-in endeavor to appreciate parasympathetic drive. Concluding: We can essentially narrow down the choices to two possibilities based on the understanding that Carotid Sinus Baroreceptor and Atrial Stretch Receptor Activities both increase due to the eclectic combination of physiology and the inherent transition from standing to supine. The crux of the argument, however, lies with the variable response of Vagal Efferent Activity to the heart, which could either increase or decrease. Response: Based on the understanding of cardiovascular reflexes and their response to change in body posture, the answer is most likely (B) Carotid Sinus Baroreceptor Activity and Atrial Stretch Receptor Activity increase, while Vagal Efferent Activity to the Heart decrease. This is consistent with the physiological responses of these systems to an increase in venous return and blood pressure that usually results from a change from standing to supine position. However, we also need to appreciate the merit on the counter-hypothesis presented by AI_B, and our conclusion still requires empirical validation in the given context.

**AI_E:** 1) RESTATING THE QUESTION: The study examines the changes in cardiovascular function as a healthy woman transitions from standing to lying flat (supine position). We are aiming to determine how several critical components of the cardiovascular reflex control system might likely respond: the Carotid Sinus Baroreceptor Activity, which monitors blood pressure; the Atrial Stretch Receptor Activity, which responds to changes in blood volume; and the Discharge of Vagal Efferents to the Heart, which can slow the heart rate. 2) PLANNING: The main assumption appears to revolve around the idea that the body might balance the increased blood pressure and volume by managing the heart rate. To dissect this assumption, we need to understand the effect of the transition from standing to supine position on blood pressure and volume, and how the cardiovascular reflex system may react to these alterations. 3) CLINICAL REASONING: Going from a standing to a supine position typically causes increased venous return to the heart, leading to a greater stroke volume, which then increases blood pressure. Consequently, the Carotid Sinus Baroreceptor, a pressure sensor, would likely record increased activity due to elevated blood pressure. Likewise, the Atrial Stretch Receptors, volume sensors in the atria of the heart, would likely display increased activity due to greater venous return. Now, how the Vagal Efferent Activity to the Heart responds becomes indispensable to our understanding. The Vagal nerve, also known as the 10th cranial nerve, holds significant influence over heart rate – typically, increased activity slows the heart rate. The Parasympathetic Nervous System, through the Vagus nerve, is known to mitigate the effects of increased blood pressure by slowing the heart rate. In this scenario, it might be plausible that the Vagal Efferent Activity to the Heart decreases to balance the increased blood pressure. 4) CONCLUDING: Upon transitioning from a standing to a supine position, we can expect an increase in the Carotid Sinus Baroreceptor and Atrial Stretch Receptor activities due to enhanced venous return and blood pressure. Moreover, it seems likely that in response to these changes, the Vagal Efferent Activity to the Heart would decrease to counterbalance the escalated blood pressure by allowing a higher heart rate. 5) RESPONSE: Based on the clinical reasoning above, I would select option (B) Carotid Sinus Baroreceptor Activity and Atrial Stretch Receptor Activity increase, while Vagal Efferent Activity to the Heart decrease as my response to the original question. The transition from standing to supine position likely leads to an increase in venous return and blood pressure, causing increased activity in Carotid Sinus and Atrial Stretch Receptors. Meanwhile, the Vagal Efferent Activity to the Heart is likely to decrease in response to balance the cardiovascular system's reflex control.

**AI_D:** 1) RESTATING THE QUESTION: The original question relates to the potential changes in a healthy 30-year-old woman's cardiovascular reflexes when she shifts from a standing to a supine position in a comfortable environment. Carotid Sinus Baroreceptor Activity, Atrial Stretch Receptor Activity, and Vagal Efferent Activity to the Heart are all potential variables that could be affected by this postural change. 2) PLANNING: The main assumptions in this case relate to the physiological responses linked with a positional change. For instance, when shifting to a supine position from standing, there's typically an increase in the venous return which culminates in an augmented cardiac output and blood pressure. Therefore, it would be reasonable to assume an increase in the Carotid Sinus Baroreceptor and Atrial Stretch Receptor activities, which respond to changes in blood pressure and volume, respectively. However, the changes in Vagal Efferent Activity to the Heart may not be as straightforward. This activity is responsible for parasympathetic innervation, thereby reducing heart rate and force of cardiac muscle contraction. 3) CLINICAL REASONING: An increase in blood volume and pressure, due to increased venous return when moving to a supine position, would stimulate the Carotid Sinus Baroreceptor and Atrial Stretch Receptor activities. This enhances the body’s awareness of increased volume and pressure. To curb the augmented cardiac output resulting from this, the body may opt to enhance parasympathetic activity via increased Vagal Efferent Activity to the Heart, thus reducing the heart rate and cardiac muscle contraction force. 4) CONCLUDING: From a clinical perspective, this case revolves around understanding the body’s homeostatic mechanisms that come into play with postural changes. In the supine position, the expected increase in blood volume and pressure would likely increase Carotid Sinus Baroreceptor and Atrial Stretch Receptor activities, alerting the body to these changes. The body would then normally seek to counterbalance the increased cardiac output by enhancing the parasympathetic (vagal) drive to the heart, reducing heart rate and force of cardiac muscle contraction. 5) RESPONSE: Therefore, accordingly to clinical reasoning, the most likely changes in the reflex control of the woman's cardiovascular system would be an increase in both Carotid Sinus Baroreceptor Activity and Atrial Stretch Receptor Activity, alongside an increase in Vagal Efferent Activity to the Heart. This matches the answer choice (A) Carotid Sinus Baroreceptor Activity, Atrial Stretch Receptor Activity, and Vagal Efferent Activity to the Heart increase.

**AI_A:** RESTATING THE QUESTION: This scenario involves a healthy adult woman who is a participant in a study evaluating the interaction between cardiovascular function and body posture. The inquiry here is about the expected changes in cardiovascular reflex response as she shifts from a standing position to lying down (supine), considering three factors: the Carotid Sinus Baroreceptor activity, Atrial Stretch Receptor activity, and Vagal Efferent Activity to the heart. The carotid sinus baroreceptors work to sense blood pressure changes and elicit a nervous system response to maintain homeostasis. The Atrial Stretch Receptors detect the volume change inside the heart due to increased blood flow. And, the Vagal Efferent activity represents the output of the parasympathetic nervous system that works on the heart to reduce the heart rate. PLANNING: Given the known physiological processes, the following assumptions can be made: Resting in a supine posture typically leads to an increase in venous return and consequently a rise in blood pressure. This should stimulate the activity of Carotid Sinus Baroreceptor and Atrial Stretch Receptor. The body's natural reaction to elevated blood pressure usually involves augmenting parasympathetic activity and reducing sympathetic activity to decrease the heart rate and restore balance, which could lead to the decrease in Vagal Efferent Activity to the heart. However, a contradictory assumption could be that the level of vagal activity might not change significantly, or it could even increase as part of a compensatory mechanism to counter the elevated heart rate and blood pressure. CLINICAL REASONING: When a person moves to a supine position after standing, increased venous return leads to greater preload stretching the atria, and as a result, the Atrial Stretch Receptors are activated. Simultaneously, increased blood pressure sensed by Carotid Sinus Baroreceptors triggers a response via the glossopharyngeal nerve to the medulla oblongata, which in turn modulates the autonomic nervous system’s balance. Predominantly, the raise in parasympathetic drive through the vagus nerve curbs heart rate and contractility to maintain homeostasis. This oversimplified picture becomes a bit complicated considering there could be fluctuations in these responses over the period while the system gets adjusted to a new equilibrium. At a single time point, especially initially after the transition to the supine position, parasympathetic output might be prominent, resulting in decreased heart rate. CONCLUDING: Based on the interactions of these signals and considering the initial phase of transition to the supine posture, it could be inferred that Carotid Sinus Baroreceptor and Atrial Stretch Receptor's activities would increase due to the raised blood pressure and venous return, respectfully. Simultaneously, the body might try to balance this by decreasing the Vagal Efferent Activity to the heart to reduce the heart rate. RESPONSE: So, considering the physiological principles and the presented postural changes, the proposed changes in the reflex control of this woman's cardiovascular system are: an increase in Carotid Sinus Baroreceptor activity, an increase in Atrial Stretch Receptor activity, and a decrease in the Vagal Efferent Activity to the heart. Therefore, the answer should be option (B).

**Facilitator** **[Round 6]:** Summary: AI_A, AI_B, AI_C, and AI_E all agree that the transition from standing to a supine position would lead to an increase in Carotid Sinus Baroreceptor Activity and Atrial Stretch Receptor Activity due to increased venous return and blood pressure. However, they believe that the Vagal Efferent Activity to the Heart would decrease as a compensatory mechanism to balance the increased blood pressure by reducing the heart rate. On the other hand, AI_D suggests that the Vagal Efferent Activity to the Heart would increase as a response to the increased blood pressure and volume, aiming to reduce the heart rate and force of cardiac muscle contraction. Key Differences: The key difference in the council members' perspectives lies in their interpretation of how the Vagal Efferent Activity to the Heart would respond to the increased blood pressure and volume. While AI_A, AI_B, AI_C, and AI_E believe it would decrease to allow a higher heart rate, AI_D argues that it would increase to reduce the heart rate and force of cardiac muscle contraction. Question to Clarify Disagreements: To clarify the disagreements between the council members' opinions, the following question could be asked: "What is the physiological basis for your interpretation of how the Vagal Efferent Activity to the Heart would respond to the increased blood pressure and volume when transitioning from a standing to a supine position?". Based on your preceding response, how would you answer the original question: [A healthy 30-year-old woman participates in a study of the relationship between cardiovascular function and posture. Placed in a room with a comfortable temperature (72°F) and humidity (40%), she moves from a standing to a supine position. Which of the following sets of changes is most likely in the reflex control of this woman's cardiovascular system? (A) Carotid Sinus Baroreceptor Activity, Atrial Stretch Receptor Activity, and Vagal Efferent Activity to the Heart increase (B) Carotid Sinus Baroreceptor Activity and Atrial Stretch Receptor Activity increase, while Vagal Efferent Activity to the Heart decrease (C) Carotid Sinus Baroreceptor Activity and Vagal Efferent Activity to the Heart increase, while Atrial Stretch Receptor Activity decrease (D) Carotid Sinus Baroreceptor Activity increase, while Atrial Stretch Receptor Activity and Vagal Efferent Activity to the Heart decrease (E) Carotid Sinus Baroreceptor Activity decrease, while Atrial Stretch Receptor Activity and Vagal Efferent Activity to the Heart increase (F) Carotid Sinus Baroreceptor Activity and Vagal Efferent Activity to the Heart decrease, while Atrial Stretch Receptor Activity increase (G) Carotid Sinus Baroreceptor Activity and Atrial Stretch Receptor Activity decrease, while Vagal Efferent Activity to the Heart increase (H) Carotid Sinus Baroreceptor Activity, Atrial Stretch Receptor Activity, and Vagal Efferent Activity to the Heart decrease]

**AI_D:** Restating the question, what we're trying to ascertain is the likely reflexive alterations in the cardiovascular control mechanisms of a healthy young woman who transitions from standing to a supine position. The mechanisms under focus are the Carotid Sinus Baroreceptor Activity, Atrial Stretch Receptor Activity, and Vagal Efferent Activity against the heart. Planning for this, we first note the role of each of these control mechanisms. The Carotid Sinus Baroreceptors are sensitive to blood pressure changes in the carotid arteries. An increase in blood pressure elicits more frequent signals to the brain, resulting in compensation aimed at reducing blood pressure. Conversely, a drop in blood pressure would trigger actions to increase it. The Atrial Stretch Receptors signal central nervous system about blood volume changes, thereby influencing heart rate and other cardiovascular responses. Lastly, Vagal Efferent Activity refers to the signals sent via the vagus nerve to decrease heart rate. In the context of moving from standing to a supine position, there should be an increase in venous return as gravity no longer works against the upward flow of blood from the lower body. This will also slightly increase blood volume and pressure in the right atrium, which should intensify both Carotid Sinus Baroreceptor Activity and Atrial Stretch Receptor Activity. On the other hand, when considering Vagal Efferent Activity to the Heart, an increased Carotid Sinus Baroreceptor Activity would lead to a compensatory reflex to reduce the heart rate. This reduction is brought about by increased Vagal Efferent Activity. Clinical reasoning leads us to expect that, in balancing the cardiovascular system, the human body would increase its Carotid Sinus Baroreceptor Activity, Atrial Stretch Receptor Activity, and Vagal Efferent Activity to the Heart when transitioning from standing to supine – to handle the increased venous return and blood pressure. In conclusion, based on the physiological understanding of these mechanisms and the scenario given, the woman's body would react by intensifying the activity of all three mentioned mechanisms – Carotid Sinus Baroreceptor Activity, Atrial Stretch Receptor Activity, and Vagal Efferent Activity to the Heart – in an attempt to maintain homeostasis under the change in posture. So, in response to the multi-choice question, the most probable answer should be option A: Carotid Sinus Baroreceptor Activity, Atrial Stretch Receptor Activity, and Vagal Efferent Activity to the Heart increase.

**AI_B:** RESTATING THE QUESTION: We are examining the reflex control changes in the cardiovascular system of a healthy 30-year-old woman as she transitions from a standing position to a supine one. Here we are specifically looking at changes in the Carotid Sinus Baroreceptor Activity, Atrial Stretch Receptor Activity, and Vagal Efferent Activity to the Heart. PLANNING: The key assumption here is that the posture change would lead to potential physiological changes. Carotid Sinus Baroreceptor and Atrial Stretch Receptor, sensitive to the changes in the pressure or the stretch of the blood vessels, would likely respond to the positional change. Also, Vagal Efferent Activity has an important role in controlling the heart rate. Let's assume this shift in the position will lead to a surge in the venous return to the heart and increase in the blood pressure, thus affecting the mentioned mechanisms. CLINICAL REASONING: As the woman moves from a standing to a supine position, there's increased venous return due to the effect of gravity, leading to increased atrial filling and hence increased activity of Atrial Stretch Receptors. This also increases blood pressure, which would be sensed by the Carotid Sinus Baroreceptors, increasing their activity. Simultaneously, the body, trying to maintain homeostasis, would want to counteract this sudden increase in blood pressure. The main way it achieves this is by activating the parasympathetic system, specifically increasing the Vagal Efferent Activity, which would work to decrease heart rate and potentially the force of the heart’s contractions. CONCLUDING: In a scenario of postural change from standing to supine, due to increased venous return and blood pressure, the Carotid Sinus Baroreceptor Activity and Atrial Stretch Receptor Activity would increase. However, to counteract the shift in hemodynamics, the Vagal Efferent Activity to the Heart would also increase to suppress the heart rate and prevent excessive pressure build-up in the circulatory system. RESPONSE: Given the physiological mechanisms involved in posture change, the most likely set of changes in the reflex control of this woman's cardiovascular system would be: (A) Carotid Sinus Baroreceptor Activity, Atrial Stretch Receptor Activity, and Vagal Efferent Activity to the Heart increase.

**AI_A:** RESTATING THE QUESTION: This query aims to determine how the reflex control of the cardiovascular system of a healthy 30-year-old woman is likely to change when she transitions from a standing to a supine position under comfortable temperature and humidity conditions. PLANNING: In a simplified manner, this is a question of how the cardiovascular system adapts to a change in posture. The most important considerations here are changes in Carotid Sinus Baroreceptor Activity, Atrial Stretch Receptor Activity, and Vagal Efferent Activity to the Heart, which are elements of the body's autonomic regulation of the cardiovascular system. CLINICAL REASONING: When one moves from a standing to a supine position, blood pools in the lower extremities away from the heart, causing decreased venous return to the heart. In response, the cardiovascular system employs numerous reflex mechanisms to maintain blood pressure: 1) The Carotid Sinus Baroreceptors, which primarily monitor arterial blood pressure, would sense a reduction in blood pressure and therefore, their activity would increase. 2) Atrial Stretch Receptors, sensitive to changes in blood volume in the atrium, would register a decrease in blood pressure and consequently, their activity would also augment. 3) The Vagal Efferent Activity (parasympathetic output) to the Heart would likely decrease as a response to low blood pressure since more sympathetic and less parasympathetic activity leads to an increase in heart rate, which would help restore normal blood pressure by increasing cardiac output. CONCLUDING: Therefore, based on the physiological mechanisms mentioned above, transitioning from a standing to a supine position leads to an increase in both Carotid Sinus Baroreceptor Activity and Atrial Stretch Receptor Activity, while it leads to a decrease in Vagal Efferent Activity to the Heart. RESPONSE: So, after connecting all the information, the most likely set of changes in the reflex control of this woman's cardiovascular system when moving from a standing to a supine position would be: (B) Carotid Sinus Baroreceptor Activity and Atrial Stretch Receptor Activity increase, while Vagal Efferent Activity to the Heart decreases.

**AI_C:** RESTATING THE QUESTION: We are setting forth a scenario of a healthy 30-year-old woman who transitions from a standing to a supine position, and we are expected to predict the likely changes in her cardiovascular system's reflex control. The variables we are considering are the Carotid Sinus Baroreceptor Activity, Atrial Stretch Receptor Activity, and Vagal Efferent Activity to the Heart. PLANNING: The parameters we are considering are all interconnected in maintaining cardiovascular homeostasis. We have the baroreceptors, which are pressure-sensitive sensors in the carotid sinuses that help modulate blood pressure. Atrial stretch receptors respond to increasing venous return. Vagal efferent activity refers to signals from the vagus nerve that influence the heart's function, including heart rate. When a person moves from a standing to a supine position, it's reasonable to expect an increase in venous return to the heart, which in turn would likely stimulate the atrial stretch receptors and the carotid baroreceptors, due to the augmented blood volume flowing to these areas. To counteract this increase, it may then follow that the vagus nerve could dampen its activity to the heart (to balance the increased blood pressure). CLINICAL REASONING: It's useful to turn back to the fundamental physiological responses to appreciate this scenario. In other words, when the individual adopts a supine position from a standing one, venous return to the heart would increase because of the effect of gravity, resulting in greater bloodstream towards the heart. This increased blood volume would lead to distension of the atria, stimulating the atrial stretch receptors. It would also cause an increase in the pressure sensed by the baroreceptors in the carotid sinuses. As a regulatory mechanism, the body would want to decrease the force and rate of the heart's contractions to counterbalance these augmentations, accomplished by reducing vagal efferent activity to the heart. CONCLUDING: Hence, it would be reasonable to anticipate an increase in Carotid Sinus Baroreceptor Activity and Atrial Stretch Receptor Activity, due to the increased venous return when moving to a supine position. However, the body would also presumably respond by decreasing Vagal Efferent Activity to the Heart to maintain homeostasis in response to the increased blood pressure and volume. RESPONSE: The predicted set of changes in the woman's cardiovascular system reflex control when moving from a standing to a supine position would be: (B) Carotid Sinus Baroreceptor Activity and Atrial Stretch Receptor Activity increase, while Vagal Efferent Activity to the Heart decrease.

**AI_E:** Restating the question: The question seeks to understand the effect on cardiovascular reflexes when a healthy woman shifts from standing to a supine position, specifically how carotid sinus baroreceptor activity, atrial stretch receptor activity, and vagal efferent activity to the heart change. Planning: We must consider how posture affects cardiovascular reflexes. Carotid sinus baroreceptors, found in the carotid sinus, respond to changes in arterial pressure by altering heart rate and vascular resistance. Atrial stretch receptors, located in the walls of the atria, respond to changes in blood volume and regulate heart rate. Vagal efferent activity refers to the output signals from the vagus nerve, which plays a significant role in heart rate regulation. We also have to remember that shifting from a standing to a supine position will increase venous return due to gravity, leading to increased stroke volume and blood pressure. Clinical reasoning: Shifting from standing to supine increases venous return, which increases stroke volume due to the Frank-Starling mechanism and subsequently increases blood pressure. This rise in blood pressure is sensed by the carotid sinus baroreceptors, causing their activity to increase. The increased stroke volume also causes an increase in atrial stretch due to more blood filling the atria, which increases the activity of atrial stretch receptors. To counteract the increase in blood pressure, the body increases the vagal efferent activity, resulting in a decrease in heart rate, a phenomenon known as the baroreflex. Concluding: Therefore, when transitioning from standing to a supine position, the activity of the carotid sinus baroreceptors and atrial stretch receptors would increase due to an increase in blood pressure and atrial stretch, respectively. On the other hand, the vagal efferent activity to the heart would also increase as a compensation to decrease the heart rate and maintain blood pressure within normal ranges. Response: According to this reasoning, the answer would be (A) Carotid Sinus Baroreceptor Activity, Atrial Stretch Receptor Activity, and Vagal Efferent Activity to the Heart increase.

**Facilitator** **[Round 7]:** Summary: AI_A, AI_C: Both AI_A and AI_C believe that transitioning from a standing to a supine position leads to an increase in Carotid Sinus Baroreceptor Activity and Atrial Stretch Receptor Activity due to decreased venous return to the heart and a decrease in blood pressure. However, they argue that Vagal Efferent Activity to the Heart would decrease to increase heart rate and restore normal blood pressure. AI_B, AI_D, AI_E: AI_B, AI_D, and AI_E, on the other hand, argue that the transition from standing to supine increases venous return and blood pressure, leading to an increase in Carotid Sinus Baroreceptor Activity and Atrial Stretch Receptor Activity. They also believe that Vagal Efferent Activity to the Heart would increase to decrease heart rate and counteract the sudden increase in blood pressure. Key Differences: The key difference in the perspectives lies in the interpretation of how the body responds to the change in posture. AI_A and AI_C believe that the change in posture leads to a decrease in blood pressure, necessitating an increase in heart rate, achieved by decreasing Vagal Efferent Activity. In contrast, AI_B, AI_D, and AI_E believe that the change in posture leads to an increase in blood pressure, necessitating a decrease in heart rate, achieved by increasing Vagal Efferent Activity. Question to Clarify Disagreements: To clarify the disagreements between the council members, the following question could be asked: "Does the transition from a standing to a supine position lead to an increase or decrease in blood pressure, and how does this affect the Vagal Efferent Activity to the Heart?". Based on your preceding response, how would you answer the original question: [A healthy 30-year-old woman participates in a study of the relationship between cardiovascular function and posture. Placed in a room with a comfortable temperature (72°F) and humidity (40%), she moves from a standing to a supine position. Which of the following sets of changes is most likely in the reflex control of this woman's cardiovascular system? (A) Carotid Sinus Baroreceptor Activity, Atrial Stretch Receptor Activity, and Vagal Efferent Activity to the Heart increase (B) Carotid Sinus Baroreceptor Activity and Atrial Stretch Receptor Activity increase, while Vagal Efferent Activity to the Heart decrease (C) Carotid Sinus Baroreceptor Activity and Vagal Efferent Activity to the Heart increase, while Atrial Stretch Receptor Activity decrease (D) Carotid Sinus Baroreceptor Activity increase, while Atrial Stretch Receptor Activity and Vagal Efferent Activity to the Heart decrease (E) Carotid Sinus Baroreceptor Activity decrease, while Atrial Stretch Receptor Activity and Vagal Efferent Activity to the Heart increase (F) Carotid Sinus Baroreceptor Activity and Vagal Efferent Activity to the Heart decrease, while Atrial Stretch Receptor Activity increase (G) Carotid Sinus Baroreceptor Activity and Atrial Stretch Receptor Activity decrease, while Vagal Efferent Activity to the Heart increase (H) Carotid Sinus Baroreceptor Activity, Atrial Stretch Receptor Activity, and Vagal Efferent Activity to the Heart decrease]

**AI_D:** RESTATING THE QUESTION: This query focuses on understanding the changes in cardiovascular reflex control when a healthy woman transitions from a standing to a supine position. Terms to be understood here include Carotid Sinus Baroreceptor Activity, which refers to the sensors located in the carotid sinus that respond to changes in blood pressure; Atrial Stretch Receptor Activity, which refer to sensors in the atria of the heart that respond to pressure fluctuations; and Vagal Efferent Activity to the Heart, which represents nerve signals sent from the vagus nerve to the heart that can decrease heart rate. PLANNING: One assumption here is that the body's reflexive response will act to maintain blood pressure in the face of a position change. This is based on the body's need to ensure adequate blood supply to important organs, including the brain. Another assumption is that the blood pressure could either increase or decrease, depending on factors like venous return and peripheral resistance. If we take these assumptions into consideration, we need to evaluate the change in blood pressure and the body's response to it. CLINICAL REASONING: Transitioning from standing to supine tends to increase venous return owing to the effects of gravity, which should increase blood pressure. In response to an increased blood pressure, the carotid sinus baroreceptors and atrial stretch receptors should increase their activity. The increased activity of these sensory inputs will then be interpreted by the central nervous system, leading to an increase in vagal efferent activity to the heart to decrease the heart rate and counterbalance the sudden increase in blood pressure. Thus, Carotid Sinus Baroreceptor Activity and Atrial Stretch Receptor Activity will increase, while Vagal Efferent Activity to the Heart should also increase. CONCLUDING: According to the clinical reasoning, when a person changes position from standing to supine, the body's overall reaction would be to maintain a stable blood pressure and heart rate. This is achieved through an increase in Carotid Sinus Baroreceptor Activity and Atrial Stretch Receptor Activity due to increased venous return and a consequent increase in Vagal Efferent Activity to the Heart. RESPONSE: Based on the aforementioned reasoning process and conclusion, option (A) "Carotid Sinus Baroreceptor Activity, Atrial Stretch Receptor Activity, and Vagal Efferent Activity to the Heart increase" is the most likely set of changes in the reflex control of this woman's cardiovascular system.

**AI_A:** 1) RESTATING THE QUESTION: The query is about the changes that occur in the cardiovascular reflex control of a 30-year-old woman when she transitions from standing to a supine (lying horizontally with the face and torso facing up) position. Specifically, we are looking at the changes in Carotid Sinus Baroreceptor Activity (pressure receptors in carotid sinus involved in blood pressure regulation), Atrial Stretch Receptor Activity (sensors in the atria of the heart that monitor blood volume), and Vagal Efferent Activity to the Heart (nerve impulses transmitted from the vagus nerve to the heart to control heart rate). 2) PLANNING: The underlying assumption here is that this change of posture modifies blood flow dynamics and cardiovascular reflexes. It's important to note that moving from standing to supine can increase venous return due to gravity, leading to a transient increase in blood pressure. The baroreceptors and atrial stretch receptors might then increase their activity to sense this change and trigger a compensatory response. We could question if this response leads to an increase or decrease in vagal efferent activity. 3) CLINICAL REASONING: Normally, an increase in blood pressure activates carotid sinus baroreceptors. The increased stretch of the atria of the heart from the enhanced venous return stimulates atrial stretch receptors. These changes trigger reflex responses to prevent blood pressure from rising too much. One such reflex involves increasing vagal efferent activity, which slows down the heart rate, lowering the cardiac output and thus the blood pressure. 4) CONCLUDING: When transitioning from standing to supine, the increased venous return to the heart prompts increased activities of the carotid sinus baroreceptors and atrial stretch receptors due to the transient increase in blood pressure. This results in a reflex increase in the vagal efferent activity, which slows down the heart rate to compensate for the increase in blood pressure. 5) RESPONSE: Consequently, considering the physiological responses to this postural change in cardiovascular function, the likely reflex control changes in this woman would be an increase in Carotid Sinus Baroreceptor Activity, an increase in Atrial Stretch Receptor Activity, and an increase in Vagal Efferent Activity to the Heart. Therefore, I would answer that the most likely response to the original question is (A) Carotid Sinus Baroreceptor Activity, Atrial Stretch Receptor Activity, and Vagal Efferent Activity to the Heart increase.

**AI_E:** RESTATING THE QUESTION: The question is asking how the woman's cardiovascular system, specifically the carotid sinus baroreceptor, atrial stretch receptor, and vagal efferent activity to the heart, is likely to be reflexly controlled upon moving from a standing to the supine position. PLANNING: In standing position, gravity causes blood pooling in the lower extremities which reduces the venous return to the heart and thereby reducing the stroke volume. Shifting to the supine position increases venous return, leading to an increased stroke volume and increased blood pressure. This condition would trigger a physiological response to maintain normal blood pressure. CLINICAL REASONING: With the increase in venous return, there is an increase in atrial stretch leading to increased atrial stretch receptor activity. This is because these receptors, located in the atria, can sense changes in the blood volume and transmit signals accordingly. Simultaneously, the increased blood pressure is sensed by the carotid sinus baroreceptors located at the bifurcation of internal and external carotid arteries, and their activity would also increase due to the stretching by enhanced blood pressure. To counteract the increased blood pressure, there should be a decrease in heart rate, and this is achieved by increasing the parasympathetic (vagal) output to the heart. Contrarily, if the vagal efferent activity was to decrease, it would mean an increase in heart rate which would oppose the need to lower the blood pressure. CONCLUDING: Therefore, upon moving from a standing to a supine position, the carotid sinus baroreceptor activity and atrial stretch receptor activity would increase due to increased blood pressure and atrial filling respectively, and the vagal efferent activity to the heart would increase to decrease the heart rate and maintain stable blood pressure. RESPONSE: Hence the likely set of changes in the reflex control of this woman's cardiovascular system would be: Carotid Sinus Baroreceptor Activity and Atrial Stretch Receptor Activity increase, while Vagal Efferent Activity to the Heart also increases. The option corresponding to this scenario is (A) Carotid Sinus Baroreceptor Activity, Atrial Stretch Receptor Activity, and Vagal Efferent Activity to the Heart increase.

**AI_B:** The question involves a female subject who goes from a standing to a supine position and the subsequent effects on her cardiovascular reflexes, specifically Carotid Sinus Baroreceptor Activity, Atrial Stretch Receptor Activity, and Vagal Efferent Activity to the Heart. Carotid baroreceptors located in the walls of the carotid sinus detect changes in blood pressure. When blood pressure increases, these baroreceptors increase their firing rates, signaling to the cardiovascular control center in the brain which then initiates responses to decrease blood pressure. Atrial stretch receptors detect changes in blood volume and signal to the cardiovascular control center as well. An increase in blood return, as might occur when going from standing to supine, would trigger increased activity from these receptors. The vagus nerve carries parasympathetic outputs from the cardiovascular control center to the heart. Increased activity in the vagus nerve typically decreases heart rate, serving to lower blood pressure. Assuming that moving from a standing to a supine position facilitates blood return to the heart due to gravity, we would expect to see an increase in both Carotid Sinus Baroreceptor Activity and Atrial Stretch Receptor Activity. As the baroreceptors indicate an increase in blood pressure, the cardiovascular control center should then initiate responses to decrease blood pressure, including an increase in Vagal Efferent Activity to the Heart which would decrease heart rate. Clinical reasoning suggests that the observed physiological changes in the woman's body, when changing position from standing to supine, would be a surge in blood return and a subsequent increase in blood pressure. In response to this, the body would need to reduce that pressure. Thus, we should see increased activity in the Carotid Sinus Baroreceptor and Atrial Stretch Receptor to detect the increased pressure and volume, and an increase in Vagal Efferent Activity to the Heart to lower heart rate and counterbalance the pressure increase. In conclusion, the clinical reasoning supports the assumption that a healthy 30-year-old woman's cardiovascular reflex in response to transitioning from a standing to a supine position would lead to an increase in Carotid Sinus Baroreceptor Activity, Atrial Stretch Receptor Activity, and Vagal Efferent Activity to the Heart. Therefore, based on the pattern of change illustrated, the most likely set of changes in the reflex control of this woman's cardiovascular system would be answer choice (A) – Carotid Sinus Baroreceptor Activity, Atrial Stretch Receptor Activity, and Vagal Efferent Activity to the Heart increase.

**AI_C:** RESTATING THE QUESTION: The question basically aims to understand the physiological alterations that occur in a woman's cardiovascular system when she changes her position from standing to supine, specifically focusing on changes in Carotid Sinus Baroreceptor Activity, Atrial Stretch Receptor Activity, and Vagal Efferent Activity to the Heart. Baroreceptors are pressure-sensitive sensory neurons that detect changes in blood pressure and trigger reflexive actions to maintain homeostasis. Atrial stretch receptors are specialized sensors located in the walls of the atria of the heart that detect pressure induced by the increase in blood volume. Vagal efferent activity refers to the output of the Vagus nerve, which can slow heart rate. The question asks for the most likely set of changes in these cardiovascular control mechanisms resulting from the postural change. PLANNING: To address this question, we should consider the following assumptions: (1) Shifting from standing to supine position impacts venous return, and consequently, heart rate and blood pressure; (2) The body's homeostatic mechanisms, including baroreceptors and atrial stretch receptors, respond to these changes, altering their activity; (3) The autonomic nervous system, through its parasympathetic outlet via the Vagus nerve (Vagal Efferent Activity), adjusts heart rate to maintain blood pressure. We should question each of these assumptions, particularly how these mechanisms interplay in response to posture change. Clinically, priority should be given to understanding how increased venous return generated by the supine position influences heart functionality and pressure regulation. CLINICAL REASONING: As the woman moves from standing to supine, gravity no longer opposes venous return, causing an increase in venous return to the heart. This increased blood volume in the heart stretches the atria, increasing Atrial Stretch Receptor Activity. In parallel, this increased venous return also increases blood pressure, which would increase Carotid Sinus Baroreceptor Activity as they sense the increased pressure. To oppose this sudden increase in blood pressure, the body through its homeostatic reflexes triggers a decrease in cardiac rate, which is achieved by the increase in Vagal Efferent Activity to the Heart. CONCLUDING: Therefore, based on the detailed analysis and reasoning, it seems plausible that Carotid Sinus Baroreceptor Activity and Atrial Stretch Receptor Activity increases due to the increased venous return and consequent blood pressure. Simultaneously, Vagal Efferent Activity to the Heart likely increases as a response to suppress the raised blood pressure by slowing down heart rate. RESPONSE: Considering the clinical reasoning and analysis provided, option (A) seems the most accurate: 'Carotid Sinus Baroreceptor Activity, Atrial Stretch Receptor Activity, and Vagal Efferent Activity to the Heart increase.' Consequently, the body tries to maintain its homeostasis by triggering multiple reflexes simultaneously.

**Facilitator** The consensus statement synthesizing the responses of AI_A, AI_B, AI_C, AI_D, and AI_E is as follows: The question pertains to the physiological changes in a 30-year-old woman's cardiovascular system when she transitions from a standing to a supine position. The focus is on the Carotid Sinus Baroreceptor Activity, Atrial Stretch Receptor Activity, and Vagal Efferent Activity to the Heart. When transitioning from standing to supine, the effects of gravity increase venous return to the heart, leading to a transient increase in blood pressure. This increase in blood pressure and blood volume in the heart stimulates the carotid sinus baroreceptors and atrial stretch receptors, respectively. These sensors then trigger a compensatory response to prevent excessive blood pressure rise. This compensatory response involves increasing the vagal efferent activity, which slows down the heart rate, thereby reducing the cardiac output and blood pressure. Therefore, the most likely reflex control changes in this woman would be an increase in Carotid Sinus Baroreceptor Activity, an increase in Atrial Stretch Receptor Activity, and an increase in Vagal Efferent Activity to the Heart. In conclusion, the consensus among the AI responses is that the most likely set of changes in the reflex control of this woman's cardiovascular system would be option (A) "Carotid Sinus Baroreceptor Activity, Atrial Stretch Receptor Activity, and Vagal Efferent Activity to the Heart increase." This conclusion is based on the physiological responses to the postural change and the body's reflexive actions to maintain homeostasis.
