# Supplementary material for: Collaborative intelligence in AI: Evaluating the performance of a council of AIs on the USMLE
Source: PLOS Digit Health. 2025 Oct 9;4(10):e0000787. doi: 10.1371/journal.pdig.0000787 (PMC12510544; doi:10.1371/journal.pdig.0000787)
Supplement: S2 File — (ZIP) [file pdig.0000787.s002.zip › S2 Data Raw data files containing unprocessed question inputs and LLM outputs including transcripts of deliberations/STEP1_Question-26-1_Type-MC-NJ.docx]

**Facilitator** **[Round 1]:** A 31-year-old woman with a 5-year history of fatigue comes to the physician for an initial visit. She has seen four other physicians for the same condition within the past 6 months, but no abnormalities were found. She gives the physician a large folder that contains her medical records. She says, ""I can barely get out of bed most mornings, but nobody can figure out why because all of my tests turn out normal. I really think I have chronic fatigue syndrome. What do you know about this condition?"" The physician has not treated any patient with chronic fatigue syndrome before. Which of the following initial statements by the physician is most appropriate to establish rapport with this patient? (A) ""From the size of the folder you brought, it looks like you've had very thorough examinations in the past."" (B) ""I don't have much experience with chronic fatigue syndrome, but I'm committed to learning as much as I can about it."" (C) ""I'm not familiar with chronic fatigue syndrome, except that many physicians don't think it's a real disease."" (D) ""Let's start over from scratch. We'll need to repeat complete testing."" (E) ""When nothing abnormal is found during thorough examinations and laboratory studies, there is often an underlying psychological cause of the symptoms.""

**AI_C:** RESTATING THE QUESTION: The patient, a 31-year-old woman, has visited multiple physicians due to persistent fatigue over the last five years. Despite extensive investigations, her condition still remains unclear. She thinks she might be suffering from chronic fatigue syndrome (CFS), a disorder characterized by extreme fatigue that can't be explained by any underlying medical condition. The problem herein lies in identifying the most effective way for the physician (who has no prior experience with this condition) to build a positive and trusting relationship with the patient from the outset. PLANNING: The assumptions made here include the belief that the patient's previous tests were comprehensive and accurate, that the patient might have CFS despite the lack of concrete evidence, and that the physician's experience with CFS is essential to diagnose and treat the patient. CLINICAL REASONING: It is crucial to establish patient rapport, which can be achieved through empathy, validation of patient's concerns, and a commitment to help. While the patient may indeed have undergone extensive tests (Option A), highlighting the size of the medical records folder may not address her frustration or concern. Discrediting the existence of Chronic Fatigue Syndrome would likely alienate her (Option C). Proposing to repeat all testing would be exhausting and potentially unnecessary (Option D), and quickly attributing her symptoms to a psychological cause without thorough assessment (Option E) may seem dismissive. CONCLUDING: Therefore, it's more suitable for the physician to express commitment to understanding the condition as much as possible (Option B). The physician emphasizes humility (acknowledging limited experience with CFS) and determination to learn more, which can instill trust and hope in the patient. RESPONSE: Hence, "(B) "I don't have much experience with chronic fatigue syndrome, but I'm committed to learning as much as I can about it."" would be the most appropriate initial statement by the physician to establish rapport with this patient.

**AI_B:** RESTATING THE QUESTION: The question asks about a 31-year-old female patient who believes she is suffering from chronic fatigue syndrome but has not yet found a physician who can confirm this. Despite numerous tests with normal results, she continues to experience significant fatigue. The physician, unfamiliar with chronic fatigue syndrome, is tasked with deciding upon the most appropriate initial response to foster a positive patient-provider relationship. PLANNING: In evaluating the options, assumption is that all the options could be true but could not necessarily be the best approach to build rapport. The key to building rapport is to show empathy, validate the patient's concerns, convey readiness to assist, and avoid judgment or unnecessary medical procedures. Thus, the two options which seem best to pursue are (A) acknowledging the patient's extensive past investigations and (B) committing to learn about chronic fatigue syndrome despite a lack of personal experience. CLINICAL REASONING: Beginning with option (A), while acknowledging the patient's extensive prior investigations could reassure her that her efforts are recognized, it could also unintentionally signal that nothing more can be done, which may not help establish rapport. Conversely, option (B) which shows a willingness to learn about the patient's supposed condition, not only validates her concerns but also expresses a commitment to help her. This communicates empathy and a willingness to grow professionally for the patient's sake, which can be instrumental in fostering a patient-provider relationship. CONCLUDING: Since the main goal here is to establish rapport, it's crucial to validate the patient's concerns and demonstrate dedication to addressing her health issue. Between option (A) and (B), the latter appears to the better approach, as it assures the patient that the physician is not dismissing her concerns and is ready to learn more about her supposed condition as an initial step in its management. RESPONSE: Hence, the most appropriate initial statement by the physician to establish rapport with this patient would be: (B) "I don't have much experience with chronic fatigue syndrome, but I'm committed to learning as much as I can about it."

**AI_A:** RESTATING THE QUESTION: The question pertains to how a physician might best build rapport with a new patient who has a persistent, undiagnosed fatigue condition she believes to be Chronic Fatigue Syndrome (CFS). Rapport, in this scenario, refers to a connection or relationship marked by mutual understanding and trust. Chronic Fatigue Syndrome is a complicated disorder characterized by extreme fatigue that lasts for at least six months and that can't be fully explained by an underlying medical condition. The fatigue is severe enough to interfere with daily activities. It's crucial for the council to choose the option which validates the patient's concerns while maintaining professional integrity. PLANNING: To determine the best course of action, we need to take into consideration the patient's long history of undiagnosed fatigue, her belief in the diagnosis of CFS, the physician's lack of experience with the syndrome, and the importance of establishing a trusting rapport. We should avoid assumptions that may undermine the patient's experiences, such as the notion that comprehensive previous examinations have been inadequate or that the symptoms are purely psychological. CLINICAL REASONING: With an approach that validates her self-assumed diagnosis of Chronic Fatigue Syndrome, the physician signals empathy and willingness to understand the patient's concerns, thereby establishing rapport. The assumption here is that the physician should show willingness to investigate and learn more about the presented condition. However, without suggesting that previously performed tests were inadequate or that the cause of her fatigue might be psychological in nature, as these could potentially threaten the rapport with the patient. CONCLUDING: It is important to keep in mind that the statement should neither suggest a lack of respect for the expertise of previous physicians (which might undermine trust in healthcare providers) nor invalidate patient's symptoms by suggesting they are purely psychological unless there is clear supporting evidence. The physician should aim to reassure the patient about their commitment to understanding and managing her condition within their scope of practice. RESPONSE: Therefore, the most appropriate statement by the physician to establish rapport with this patient would be: (B) "I don't have much experience with chronic fatigue syndrome, but I'm committed to learning as much as I can about it." This response acknowledges the patients concerns and assures her that the physician is dedicated to helping her in her ailment.

**AI_D:** RESTATING THE QUESTION: A woman in her early thirties, who's been persistently fatigued for the last five years, is seeking a physician's opinion about her condition. Despite visiting several physicians and reportedly undergoing several tests, no discernible cause for her fatigue has been found. She proposes the idea of chronic fatigue syndrome (CFS), a complex disorder characterized by extreme fatigue that can't be explained by any underlying medical condition, while admitting that the current physician lacks experience in dealing with it. The question is, what would be the most appropriate initial response from the physician to foster a good doctor-patient relationship? PLANNING: Several assumptions can be identified within the given scenarios - (1) the patient's previously conducted diagnostic tests are comprehensive and accurate, limiting the need for repeating them, (2) the patient might doubt the physician's ability due to a lack of experience with CFS, (3) stigmas around CFS could affect the physician's approach, and (4) there might be a possibility of an underlying psychological issue that's causing the symptoms. However, given the subjective feeling of fatigue and the time duration of the symptoms, the assumption of CFS as a possibility should not be discarded without thoroughly considering the previous assumptions. CLINICAL REASONING: Firstly, the physician must ensure to rule out all other potential causes before diagnosing CFS, as it is a diagnosis of exclusion. The physician should revisit the previous tests and examinations since completely negating their credibility could be detrimental to rapport building. Secondly, being transparent about the lack of experience dealing with CFS, while still offering supportive communication, can help foster trust. Discrediting CFS as a potential diagnosis could alienate the patient as she might feel her concerns are not being taken seriously, which wouldn't foster a beneficial patient-doctor relationship. Even the proposition of a psychological cause should be delicately handled, as imposing this prematurely may induce feelings of not being believed or recognized. CONCLUDING: In building a strong rapport, the physician should demonstrate empathy towards the patient’s plight and provide reassurance. It is crucial to respect the patient’s perception of her condition, be honest about their expertise level, and convey commitment towards attempting to understand and manage her condition. RESPONSE: Option (B) ""I don't have much experience with chronic fatigue syndrome, but I'm committed to learning as much as I can about it."" would be the most appropriate initial statement. This response shows respect for the patient's belief about her condition, validation of her experiences, commitment to her care, and honesty about the physician's limitations. This approach would potentially facilitate a trusting and mutually respectful physician-patient relationship.

**AI_E:** RESTATING THE QUESTION: The question is asking me to identify the most effective initial response a physician should take to establish rapport with a new patient. The patient is a 31-year-old woman who has been experiencing fatigue for five years and has consulted with several doctors but has been unable to determine the cause of her symptoms. Despite numerous tests turning up normal, she thinks she has chronic fatigue syndrome, a persistent condition characterized by extreme fatigue not attributable to any underlying medical condition. She is seeking confirmation from the doctor and wants to know what he knows about it. The doctor, however, has never treated a patient with chronic fatigue syndrome before and needs to communicate this to the patient effectively. PLANNING: Several assumptions can be made here. For example, the patient might think that since the physician hasn't treated someone with chronic fatigue syndrome before, he might not be able to help her. An alternative assumption could be that the patient may feel dismissed if the physician expresses doubt about the reality of chronic fatigue syndrome. Another assumption might be that the patient would feel frustrated if the doctor suggested repeating all the tests she has already undergone. Besides, suggesting a psychological cause for her symptoms might seem insensitive as it may give the impression that the doctor is dismissing her concerns. Of these assumptions, the one that needs first to be prioritized is establishing a good rapport and trust with the patient, which would require acknowledging her concerns and being open to learning and working together to diagnose and treat her symptoms. CLINICAL REASONING: Essentially, the underlying question hinges on how a physician should communicate empathy, sincerity, and dedication effectively to a patient in a stressful situation. Of the options presented, some are clearly inappropriate for maintaining trust and rapport. For example, downplaying the legitimacy of chronic fatigue syndrome (C), suggesting re-dos of all testing (D), or implying a psychological basis for her issues (E) would probably be received poorly by the patient. Option B offers an open admission of the physician's lack of personal experience with CFS but also expresses his commitment to learning about the issue, thus validating the patient's concerns and indicating a willingness to grow in knowledge for her benefit. CONCLUDING: After thorough consideration of the alternate assumption (that maintaining rapport and trust requires empathetic communication), the best answer to this question is one that takes into account both the emotional and intellectual needs of the patient. As such, it is crucial for the physician to acknowledge the patient's concerns, stress the commitment to learning more about her suspected condition, and find a way forward together, even if the doctor lacks previous experience with chronic fatigue syndrome. RESPONSE: The most appropriate statement for the physician to make in this scenario is (B) "I don't have much experience with chronic fatigue syndrome, but I'm committed to learning as much as I can about it."

**Facilitator** The consensus among the AI responses is that the most appropriate initial statement by the physician to establish rapport with the patient would be: (B) "I don't have much experience with chronic fatigue syndrome, but I'm committed to learning as much as I can about it." This response is seen as the most effective way to build a positive and trusting relationship with the patient. It acknowledges the patient's concerns, validates her experiences, and demonstrates the physician's commitment to understanding and managing her condition, despite their lack of experience with Chronic Fatigue Syndrome (CFS). The physician's honesty about their limitations and their willingness to learn more about CFS can instill trust and hope in the patient. This approach avoids undermining the patient's experiences or the expertise of previous physicians, and it does not prematurely suggest a psychological cause for the symptoms. Therefore, it is likely to foster a mutually respectful physician-patient relationship.
