# Supplementary material for: Collaborative intelligence in AI: Evaluating the performance of a council of AIs on the USMLE
Source: PLOS Digit Health. 2025 Oct 9;4(10):e0000787. doi: 10.1371/journal.pdig.0000787 (PMC12510544; doi:10.1371/journal.pdig.0000787)
Supplement: S2 File — (ZIP) [file pdig.0000787.s002.zip › S2 Data Raw data files containing unprocessed question inputs and LLM outputs including transcripts of deliberations/STEP1_Question-27-1_Type-MC-NJ.docx]

**Facilitator** **[Round 1]:** A 3-week-old girl delivered at term with no complications is brought to the physician by her mother because of a 1- week history of yellow eyes and skin, tan-colored stools, and dark brown urine. The newborn has been breast-feeding without difficulty. She is alert and appears to be in no distress. She is at the 50th percentile for length and weight. Physical examination shows scleral icterus and jaundice. There is mild hepatomegaly; the spleen is not palpable. Laboratory studies show: Hemoglobin 14.4 g/dL Hematocrit 43% Leukocyte count 8000/mm³ Serum Albumin 3.5 g/dL Serum Total Bilirubinc14 mg/dL Direct Bilirubin 12.5 mg/dL AST 50 U/L ALT 45 U/L Which of the following is the most likely diagnosis? (A) Biliary atresia (B) Crigler-Najjar syndrome, type I (C) Gilbert syndrome (D) Hemolytic disease of the newborn (E) Physiologic jaundice

**AI_A:** RESTATING THE QUESTION: The query pertains to a 3-week-old infant exhibiting a week-long history of jaundice, evidenced by yellowish skin and eyes, tan stools, and dark brown urine. Even though the newborn baby is breastfeeding normally, alert, and not visibly uncomfortable, a mild hepatomegaly is detected during the physical examination. The reported lab results include higher Total and Direct Bilirubin levels. The goal is to identify the most likely diagnosis from the provided options: Biliary atresia, Crigler-Najjar syndrome type I, Gilbert syndrome, Hemolytic disease of the newborn, or Physiologic jaundice. PLANNING: Jaundice in neonatal patients can be a result of myriad of factors, including the conditions mentioned above. In this scenario, it could be due to an excess of bilirubin (a waste product from the liver) in the bloodstream, alterations in bilirubin metabolism, an obstruction in the bile flow pathway, or hemolysis (breakdown of red blood cells). The overall health, age, and associated symptoms can provide valuable clues to determine clinical prioritization. CLINICAL REASONING: Upon examining the symptoms and lab results, the presenting symptoms of hyperbilirubinemia (high levels of bilirubin in the blood), dark brown urine, and mild hepatomegaly, but without hemodynamic instability, suggest abnormal bilirubin metabolism or an obstructive problem rather than hemolysis, ruling out Hemolytic disease of newborn. Also, because the baby is over 1 week old, it is unlikely to be Physiological jaundice which typically resolves by 1 week. Furthermore, although both Crigler-Najjar syndrome type I and Gilbert syndrome result in jaundice, these are generally less severe conditions that wouldn't be expected to cause hepatomegaly. This leaves us to consider Biliary Atresia, a condition where bile ducts are narrow, blocked, or entirely absent, leading to impaired bilirubin clearance. CONCLUDING: Based on the clinical history, the patient's age, manifestations along with the laboratory findings, the most probable diagnosis is Biliary atresia. This was concluded after carefully ruling out the other options via a process of targeted elimination, arguing in favor of a more severe, obstructive pathology. RESPONSE: The most likely diagnosis is Biliary Atresia (A). The findings of visible jaundice, dark urine, tan stools, elevated direct bilirubin, and mild hepatomegaly in a 3-week-old are indicative of this condition.

**AI_B:** RESTATING THE QUESTION: The question pertains to a 3-week-old female infant who presents symptoms including jaundice, dark brown urine, and tan-colored stools. She has been breast-fed without issues, is active, and shows no signs of serious distress. Physical examination reveals scleral icterus, a yellowing of the white part of eye, jaundice (a yellowish discoloration of the skin and eyes, often due to liver diseases), mild hepatomegaly (an enlarged liver), and unpalpable spleen. Her laboratory results include increased levels of total and direct bilirubin, which is indicative of jaundice. The baby's symptoms need to be diagnosed, and the options involve various types of liver diseases or conditions that may cause jaundice. PLANNING: The question requires a diagnosis based on the presented symptoms and lab results. The elevated direct bilirubin level signifies a posthepatic cause of jaundice, indicating that the problem lies in the excretion of bilirubin, which could be due to biliary obstruction. However, the absence of an enlarged spleen and normal hemoglobin level rules out hemolytic disease. Gilbert's syndrome generally presents later in life with mild symptoms, and although Crigler-Najjar syndrome presents with jaundice at birth, it's due to a lack of the enzyme that converts indirect bilirubin to direct. Physiological jaundice of the newborn typically resolves within 1-2 weeks and is not associated with dark urine or pale stools. So, by excluding other possibilities, Biliary atresia appears to be the most probable condition. CLINICAL REASONING: The baby's symptoms suggest an obstruction in the biliary system that's preventing the excretion of bilirubin, evident from the elevated direct bilirubin levels. The tan-colored stools indicate a lack of bilirubin reaching the gut, which would typically color stools brown, and dark urine from an excess of conjugated or direct bilirubin being excreted through the kidney since it can't excrete via normal route (biliary system). Biliary atresia could be the obstruction causing these symptoms. It is a severe liver disease that occurs shortly after birth and can cause both the scleral icterus and jaundice observed in the physical examination. CONCLUDING: Through a process of elimination based on the symptoms, physical examination and the biochemical profile, biliary atresia appears to be the most likely diagnosis. It ties together the clinical features of jaundice, dark urine, light stools, direct (conjugated) hyperbilirubinemia and hepatomegaly in a 3-week-old newborn. RESPONSE: Given the listed symptoms, physical findings, and laboratory results, the most probable diagnosis for the baby would be (A) Biliary Atresia.

**AI_C:** (1) RESTATING THE QUESTION: This 3-week-old girl, with a history of no difficulties during birth or feeding, presents with certain symptoms including yellowing of eyes and skin (jaundice), tan-colored stools, dark brown urine, and slightly enlarged liver (mild hepatomegaly). Her blood tests show elevated levels of total and direct bilirubin with normal liver enzyme values. We are tasked with identifying the most likely cause of her symptoms among options of biliary atresia, Crigler-Najjar syndrome type I, Gilbert syndrome, hemolytic disease of the newborn and physiologic jaundice. (2) PLANNING: In order to establish an accurate diagnosis, let’s consider each option: (a) Biliary Atresia would show conjugated hyperbilirubinemia and could also present with acholic stools and dark urine. (b) Crigler-Najjar Syndrome Type I is associated with a long-standing, unconjugated hyperbilirubinemia because of decreased conjugation of bilirubin. (c) Gilbert Syndrome typically exhibits mild, unconjugated hyperbilirubinemia. (d) Hemolytic Diseases of the Newborn would present with unconjugated hyperbilirubinemia due to excessive breakdown of red blood cells. (e) Physiologic Jaundice usually shows mild unconjugated hyperbilirubinemia shortly after birth and typically resolves within two weeks. Focusing on this neonate's bilirubin levels (total 14 mg/dL and direct 12.5 mg/dL) indicates a high level of conjugated jaundice. It suggests the diagnosis hypothesized is a disease causing conjugated jaundice, making (a) Biliary Atresia a plausible candidate. (3) CLINICAL REASONING: Biliary atresia is a condition characterized by inflammation and obstruction of the biliary tree, leading to progressive fibrosis and cirrhosis of the liver. Total and direct (conjugated) bilirubin levels rise due to blockage of bile flow from the liver into the biliary tree. The newborn's jaundice, dark urine, and pale stools suggest a deficit in the normal excretion pathway for bilirubin, as bile can't enter the intestinal lumen to be converted into urobilinogen and stercobilinogen. Consequently, less bilirubin reaches the intestine, leading to light-colored stools. In contrast, excess conjugated bilirubin spills over into the blood causing dark-colored urine. (4) CONCLUDING: Given the elevated levels of conjugated bilirubin, and considering the presence of yellow skin and eyes, alongside the changes in stool and urine color, the hypothesis of Biliary Atresia as the main cause of her symptoms seems the most plausible. (5) RESPONSE: The most likely diagnosis for this newborn is (A) Biliary Atresia.

**AI_E:** (1) RESTATING THE QUESTION: To comprehend the prevailing situation, a 3-week old newborn girl, who was born full term without any complications, has been afflicted by a week-long presence of jaundice (hyperbilirubinemia characterized by yellowing of skin and sclera), tan-colored stools (acholic stool), and dark urine (due to excess bilirubin excretion). The child is breastfeeding without difficulty, is awake and not in distress. Her physical size and weight are right in the middle for her age. However, on examination, she has yellowing of eyes (scleral icterus), jaundice, and a mildly enlarged liver (hepatomegaly), but no enlarged spleen (splenomegaly). Her lab tests indicate high total and direct (conjugated) bilirubin, while other parameters like hemoglobin, hematocrit, leukocyte count, albumin, AST, ALT are within a reasonable range for her age. The question here is , what is the most probable diagnosis, given the options: Biliary atresia; Crigler-Najjar syndrome, type I; Gilbert syndrome; Hemolytic disease of the newborn; or Physiologic jaundice. (2) PLANNING: The case mentions jaundice, scleral icterus, mildly increased liver size, and direct hyperbilirubinemia, which are signs of a conjugated bilirubinopathy. Her symptoms, along with the presence of dark urine and pale stools, indicate a hepatobiliary disorder. The scenarios we ought to contemplate are: a biliary obstruction, a congenital absence of the biliary tree, an inherited bilirubin-UGT mutation, a mild UGT enzyme dysfunction, a hemolytic process due to blood-group incompatibility, or a simple physiological function of the newborn's liver needing more time to fully mature. (3) CLINICAL REASONING: Physiologic jaundice and Gilbert's syndrome are ruled out because they usually present with indirect (unconjugated) hyperbilirubinemia, not direct (conjugated). Hemolytic disease of the newborn mostly manifests with splenomegaly and could probably manifest earlier than 3 weeks, so it too is unlikely. Crigler-Najjar syndrome, type I, a rare autosomal recessive disorder, causing an almost complete lack of the enzyme responsible for processing bilirubin, also usually presents with unconjugated hyperbilirubinemia, so it's not the likely diagnosis either. This leaves biliary atresia, a congenital condition involving obliteration or discontinuity of the extrahepatic biliary system, resulting in obstruction to bile flow. It usually presents in the first few weeks of life with jaundice, acholic stools, dark urine, and hepatomegaly, very similar to the case described. (4) CONCLUDING: Taking into account all the clinical findings, as well as the normal hemoglobin and leukocyte count, there seems to be no hemolysis or infection that could explain the infant's symptoms. A diagnosis of a condition associated with conjugated hyperbilirubinemia is more likely. Given the infant's age, clinical presentation, and laboratory findings, the most probable diagnosis is biliary atresia. (5) RESPONSE: Therefore, the most likely diagnosis for this 3-week-old girl given her presentation and the laboratory findings is (A) Biliary atresia.

**AI_D:** RESTATING THE QUESTION: The provided clinical scenario and laboratory data refer to a 3-week-old neonate who is demonstrating clear signs of jaundice (yellowing of eyes and skin) and changes in her stool and urine color. Moreover, the baby exhibits mild hepatomegaly but no enlargement of the spleen. There are five potential diagnosis, including Biliary atresia, Crigler-Najjar syndrome, type 1, Gilbert syndrome, Hemolytic disease of the newborn, and Physiological jaundice. Each of these conditions presents with an increase in bilirubin levels, leading to jaundice, however, they can be differentiated based on the lab values and clinical presentation. PLANNING: The first assumption would be that the elevated total and direct bilirubin, mild hepatomegaly, scleral icterus, and presence of jaundice, along with tan colored stools and dark brown urine, could be due to an obstructive process, such as biliary atresia. However, this condition typically presents in the first two weeks of life and is more severe with progressive jaundice that worsens, acholic (pale) stools, and dark urine. In contrast, the baby has tan-colored stools and the jaundice only began a week ago. We could also consider a condition related to abnormal bilirubin metabolism or conjugation, like Crigler-Najjar syndrome, type I or Gilbert syndrome. However, these conditions typically present with unconjugated hyperbilirubinemia, while this baby has a high conjugated (direct) bilirubin level. Alternatively, we could hypothesize a blood disorder related issue, like Hemolytic disease of the newborn, however, this usually presents with a decrease in hemoglobin level and increase in reticulocyte count, neither of which is seen here. Finally, Physiological jaundice is a common cause of jaundice in newborns and the timing corresponds to this case, but it is usually self-limiting and resolves within 1 to 2 weeks of life. In addition, physiological jaundice often presents with an increase in indirect bilirubin with normal liver enzymes and the absence of hepatomegaly. CLINICAL REASONING: Given the clinical presentation, the age at presentation, and features such as tan-colored stools, hepatomegaly, and direct hyperbilirubinemia, a diagnosis of Biliary atresia is clinically prioritized even though it might not perfectly align with the typical time of presentation. This condition is caused by the destruction or absence of all or a portion of the intrahepatic and/or extrahepatic bile ducts, leading to obstruction of bile flow. The direct hyperbilirubinemia indicates that the bilirubin is being conjugated but cannot be excreted, causing it to back up into the blood. The mild hepatomegaly may be due to the liver attempting to cope with the increased workload and obstruction. CONCLUDING: Hence, based on the clinical presentation and lab values, a clinical thought process leads to the prioritization of a diagnosis of Biliary atresia, even though it typically presents in the first two weeks and some features, such as the color of the stool may not be perfectly aligned with the classic presentation. RESPONSE: The most likely diagnosis for this 3-week-old girl given the clinical presentation and lab values is most likely (A) Biliary atresia.

**Facilitator** The consensus statement synthesizes the responses of AI_A, AI_B, AI_C, AI_D, and AI_E as follows: The question pertains to a 3-week-old female infant who presents with symptoms of jaundice, dark brown urine, and tan-colored stools. She has been breast-fed without issues, is active, and shows no signs of serious distress. Physical examination reveals scleral icterus, jaundice, mild hepatomegaly, and unpalpable spleen. Her laboratory results include increased levels of total and direct bilirubin. The baby's symptoms need to be diagnosed, and the options involve various types of liver diseases or conditions that may cause jaundice. The elevated direct bilirubin level signifies a posthepatic cause of jaundice, indicating that the problem lies in the excretion of bilirubin, which could be due to biliary obstruction. However, the absence of an enlarged spleen and normal hemoglobin level rules out hemolytic disease. Gilbert's syndrome generally presents later in life with mild symptoms, and although Crigler-Najjar syndrome presents with jaundice at birth, it's due to a lack of the enzyme that converts indirect bilirubin to direct. Physiological jaundice of the newborn typically resolves within 1-2 weeks and is not associated with dark urine or pale stools. So, by excluding other possibilities, Biliary atresia appears to be the most probable condition. Biliary atresia is a condition characterized by inflammation and obstruction of the biliary tree, leading to progressive fibrosis and cirrhosis of the liver. Total and direct (conjugated) bilirubin levels rise due to blockage of bile flow from the liver into the biliary tree. The newborn's jaundice, dark urine, and pale stools suggest a deficit in the normal excretion pathway for bilirubin, as bile can't enter the intestinal lumen to be converted into urobilinogen and stercobilinogen. Consequently, less bilirubin reaches the intestine, leading to light-colored stools. In contrast, excess conjugated bilirubin spills over into the blood causing dark-colored urine. Through a process of elimination based on the symptoms, physical examination, and the biochemical profile, biliary atresia appears to be the most likely diagnosis. It ties together the clinical features of jaundice, dark urine, light stools, direct (conjugated) hyperbilirubinemia, and hepatomegaly in a 3-week-old newborn. Therefore, the most likely diagnosis for this 3-week-old girl given the clinical presentation and lab values is Biliary atresia.
