# Supplementary material for: Collaborative intelligence in AI: Evaluating the performance of a council of AIs on the USMLE
Source: PLOS Digit Health. 2025 Oct 9;4(10):e0000787. doi: 10.1371/journal.pdig.0000787 (PMC12510544; doi:10.1371/journal.pdig.0000787)
Supplement: S2 File — (ZIP) [file pdig.0000787.s002.zip › S2 Data Raw data files containing unprocessed question inputs and LLM outputs including transcripts of deliberations/STEP1_Question-28-1_Type-MC-NJ.docx]

**Facilitator** **[Round 1]:** "A 65-year-old woman comes to the physician for a follow-up examination after blood pressure measurements were 175/105 mm Hg and 185/110 mm Hg 1 and 3 weeks ago, respectively. She has well-controlled type 2 diabetes mellitus. Her blood pressure now is 175/110 mm Hg. Physical examination shows no other abnormalities. Antihypertensive therapy is started, but her blood pressure remains elevated at her next visit 3 weeks later. Laboratory studies show increased plasma renin activity; the erythrocyte sedimentation rate and serum electrolytes are within the reference ranges. Angiography shows a high-grade stenosis of the proximal right renal artery; the left renal artery appears normal. Which of the following is the most likely diagnosis? (A) Atherosclerosis (B) Congenital renal artery hypoplasia (C) Fibromuscular dysplasia (D) Takayasu arteritis (E) Temporal arteritis

**AI_E:** RESTATING THE QUESTION: The query involves a 65-year-old woman who has persistently high blood pressure, even in spite of antihypertensive therapy. She also has well-controlled type 2 diabetes. Laboratory studies reveal increased plasma renin activity while her erythrocyte sedimentation rate and serum electrolytes are within normal ranges. Additionally, an angiography has shown significant stenosis (narrowing) of her right renal artery. We're tasked with identifying the most likely diagnosis out of the five options presented. PLANNING: To proceed in determining the most probable diagnosis, we shall consider the patient's age, the increased plasma renin activity, the high blood pressure resistant to antihypertensive treatment, the existence of a single renal artery stenosis, as well as ruling out the possible diagnoses. CLINICAL REASONING: The presence of a single renal artery stenosis associated with refractory high blood pressure suggests a condition that results in the release of renin from the affected kidney due to reduced blood flow, a process referred to as the "renin-angiotensin-aldosterone system (RAAS)". This increased renin release often increases blood pressure. Considering the provided choices, the most likely diagnosis is fibromuscular dysplasia (FMD), which predominantly affects middle-aged women and results in stenosis of medium-sized arteries. This differs from conditions such as atherosclerosis, which tends to be bilateral and impacts older patients, and Takayasu arteritis that typically (though not exclusively) affects younger women. Congenital renal artery hypoplasia would not result in an isolated stenosis as seen here. Neither would temporal arteritis, which affects more cranial vessels. CONCLUDING: Applying the clinical detail, the patient's demographics, the refractory hypertension, the increased renin activity, and the angiographic evidence of a stenosed renal artery; the bigger picture strongly aligns with fibromuscular dysplasia. RESPONSE: The most likely diagnosis for this patient is fibromuscular dysplasia (C).

**AI_A:** RESTATING THE QUESTION: The question concerns a 65-year-old woman with consistently high blood pressure despite antihypertensive treatments, as well as increased plasma renin activity and a high-grade stenosis (narrowing) in her proximal right renal artery. The remaining components of her physical examination and lab results appear normal. The query is regarding the most likely diagnosis for this patient given the presented options: atherosclerosis, congenital renal artery hypoplasia, fibromuscular dysplasia, Takayasu arteritis, or temporal arteritis. PLANNING: The significant clues in this case in terms of assumptions include the elderly patient's gender, her controlled type 2 diabetes, consistently high blood pressure, increased plasma renin activity, and high-grade stenosis of the proximal right renal artery. With these, I question their possible link and infer that a cardiovascular issue affecting renal blood flow might be the cause. The uncontrolled hypertension, despite antihypertensive therapy, suggests a secondary cause like renal artery stenosis. The stenosis, coupled with the normal appearance of the other renal artery and increased plasma renin, further supports this assumption. CLINICAL REASONING: The right renal artery's stenosis causes decreased blood flow to the kidney, triggering the renin-angiotensin-aldosterone system, which can result in volume retention and hypertension. Then, going through the differential diagnoses: Atherosclerosis, a common cause of renal artery stenosis, often affects both renal arteries and not mainly one, contradicting the assumptions. Congenital renal artery hypoplasia usually presents earlier in life and doesn't fit the patient's demographic. On the other hand, fibromuscular dysplasia, an idiopathic, non-inflammatory, non-atherosclerotic disease, mostly affects young to middle-aged women, but not limited to them, causing renovascular hypertension. Takayasu arteritis and Temporal arteritis are both forms of vasculitis that would have presented with many more systemic symptoms and signs of inflammation, which are not evident here. CONCLUDING: While considering these diseases' epidemiology, clinical manifestations, this patient's history, and presentation of consistent hypertension, increased plasma renin activity, and high-grade stenosis of the right renal artery, fibromuscular dysplasia seems most likely. It typically presents with hypertension due to renal artery stenosis and may disproportionately affect one artery. RESPONSE: The most fitting diagnosis for the patient's symptoms and findings, given the potential choices, is fibromuscular dysplasia (option C).

**AI_B:** (1) RESTATING THE QUESTION: This clinical scenario involves a 65-year-old woman with a history of well-controlled type 2 diabetes mellitus, who is experiencing persistently elevated blood pressure despite antihypertensive therapy. Additionally, she has increased plasma renin activity and angiography reveals that she has a high-grade stenosis of the proximal right renal artery, while the left renal artery appears normal. The question asks for the most likely diagnosis from a list that includes Atherosclerosis (hardening and narrowing of arteries due to fat buildup), Congenital renal artery hypoplasia (a congenital condition where the renal artery is underdeveloped), Fibromuscular dysplasia (abnormal growth of cells in the artery walls), Takayasu arteritis (a rare type of vasculitis that affects large vessels such as aorta and its branches), and Temporal arteritis (an inflammation of the arteries in the scalp and forehead). (2) PLANNING: To answer this question, it is necessary to rule out those conditions that do not fit this patient's current clinical scenario and identify the most appropriate ailment. Atherosclerosis is common in this age group, but having only one renal artery affected and the presence of increased plasma renin activity may not fully align with this condition. Congenital renal artery hypoplasia is usually present since birth and would likely have manifested much earlier in life. Takayasu arteritis and Temporal arteritis are systemic diseases, which we could expect to show symptoms elsewhere, too, and labs such as the sedimentation rate (ESR) would typically be elevated, which is not the case here. Fibromuscular dysplasia typically affects middle-aged women, can cause renovascular hypertension due to renal artery stenosis (which fits our clinical scenario), and triggers an elevated renin level as a response to decreased blood flow to the kidney. (3) CLINICAL REASONING: Renovascular hypertension, which is secondary hypertension due to the narrowing of the kidney arteries, is suggested by the finding of elevated blood pressure that is resistant to medication, coupled with increased plasma renin activity. The stenotic renal artery has reduced blood flow leading to increased renin secretion from the affected kidney to raise the blood pressure. The resulting systemic hypertension becomes refractory to treatment as it is driven by this ongoing renin-angiotensin-aldosterone system activation. Therefore, the cause of refractory hypertension in this lady is likely due to the unilateral renal artery stenosis. (4) CONCLUDING: Given the age, gender, increased plasma renin activity, hypertension resistant to treatment, and the unilateral renal artery stenosis visualized on angiography, fibromuscular dysplasia, not typically associated with systemic inflammatory or metabolic derangements and often presenting with these clinical features, is the most likely cause for this clinical scenario compared to the other given possibilities in this setting. (5) RESPONSE: In light of the clinical findings and reasoning, the most likely diagnosis in this case would be (C) Fibromuscular dysplasia.

**AI_C:** 1) RESTATING THE QUESTION: The query gives a clinical synopsis of a 65-year-old woman who has had consistent high blood pressure readings over a few weeks, even despite antihypertensive therapy. Laboratory results show increased plasma renin activity which could be a body's response to decrease renal blood flow, and angiography demonstrates a high-grade stenosis in the proximal right renal artery. The challenge here is to determine the most probable cause of her conditions from a given selection of possibilities. 2) PLANNING: Possible causes of her condition include Atherosclerosis (A), Congenital renal artery hypoplasia (B), Fibromuscular dysplasia (C), Takayasu arteritis (D), Temporal arteritis (E). We need to evaluate and clinically prioritize the most likely assumption. Information that could help in this assessment includes patient's age, consistent hypertension, failed response to antihypertensive therapy, and evidence of high-grade stenosis in the renal artery. 3) CLINICAL REASONING: (A) Atherosclerosis is an accumulation of fats, cholesterol, and other substances in and on the artery walls (plaques), which can limit blood flow. It could lead to renal artery stenosis and hypertension, especially in the elderly population. (B) Congenital renal artery hypoplasia is a rare condition where the renal artery is underdeveloped at birth. Hypertension resulting from this condition usually appears early in life, which doesn't match the patient's age and late onset of symptoms. (C) Fibromuscular dysplasia is a non-atherosclerotic, non-inflammatory disease of the arterial wall, leading to stenosis, aneurysm, or dissections. This condition usually affects the renal and carotid arteries and primarily affects middle-aged women. Hypertension is often the only clinical characteristic. (D) Takayasu arteritis is a chronic inflammation of large vessels, especially the aorta and its primary branches, causing narrowing of these arteries. This condition often affects younger women, not those as aged as our patient. (E) Temporal arteritis is a vascular disease that results in inflammation of the vessels in and around the brain. Symptoms often include headache and visual disturbances, which are not reported by our patient. 4) CONCLUDING: Given the patient's age, gender, consistent hypertension, lack of response to antihypertensive therapy, increased plasma renin activity, and the presence of a significant stenosis in one renal artery, both atherosclerosis and fibromuscular dysplasia are potential diagnosis. However, considering that the patient's elevated plasma renin levels suggest an activation of the renin-angiotensin-aldosterone system likely due to a decrease in blood flow to the kidneys, and considering that fibromuscular dysplasia is more common in younger women, the most likely diagnosis for this woman is (A) Atherosclerosis. 5) RESPONSE: The most probable diagnosis for this 65-year-old woman with a history of controlled type 2 diabetes mellitus, persistent hypertension unresponsive to therapy, increase in plasma renin activity, and angiographic evidence of stenosis of the proximal right renal artery, is (A) Atherosclerosis.

**AI_D:** (1) RESTATING THE QUESTION: The clinical scenario presents a 65-year-old woman with persistently high blood pressure or hypertension, well-controlled type 2 diabetes mellitus, and an increased plasma renin level. Additionally, angiography revealed a high-grade stenosis (narrowing) of the proximal right renal artery, but the left renal artery is normal. The question is asking us to identify the most likely diagnosis out of five possible choices: atherosclerosis, congenital renal artery hypoplasia, fibromuscular dysplasia, Takayasu arteritis, and temporal arteritis. (2) PLANNING: We should consider the woman's age, the prolonged, uncontrolled hypertension, the metabolic context due to diabetes, and the structural abnormality shown by angiography. The mentioned conditions all involve vascular pathology in some form. The assumption is that the hypertension in this patient is likely secondary to a vascular issue. By questioning this assumption, we could consider whether the high blood pressure is a primary or an intrinsic renal disease. However, the normality of the left renal artery and other clinical features allow us to prioritize the assumption of a vascular cause as more logical. (3) CLINICAL REASONING: Unilateral renal artery stenosis may increase renin secretion by the affected kidney due to decreased renal perfusion, leading to secondary hypertension. Several factors suggest that this case may not result from atherosclerosis, a common vascular aging-related condition: the nondiscriminate high-grade stenosis and the well-controlled diabetes (atherosclerosis is often associated with uncontrolled diabetes). Takayasu arteritis and temporal arteritis, both forms of vasculitis, are usually associated with a systemic inflammatory response, which we don't have evidence for, since the erythrocyte sedimentation rate (a marker of inflammation) is normal. Renal artery hypoplasia is usually congenital and would likely present much earlier in life. This leaves us with fibromuscular dysplasia, a non-atherosclerotic, non-inflammatory vascular disease that affects medium-sized vessels like renal arteries and can lead to hypertension. (4) CONCLUDING: That fibromuscular dysplasia is an assumption derived from the presented scenario considering the age, the hypertension, the increased renin levels, the unilateral renal artery stenosis, and also the absence of other systemic symptoms that might have led us in the direction of arteritis. Its propensity to affect medium-sized arteries, such as renal arteries, and lead to hypertension due to increased renin release, would most comfortably fit the clinical portrait we have. (5) RESPONSE: Therefore, considering all the information provided, the most likely diagnosis for this 65-year-old woman with hypertension, increased plasma renin activity, and high-grade stenosis of the proximal right renal artery is (C) Fibromuscular dysplasia.

**Facilitator** **[Round 2]:** Summary: AI_A, AI_B, AI_D, and AI_E all agree that the most likely diagnosis for the patient is fibromuscular dysplasia. They all base their reasoning on the patient's age, gender, consistent hypertension, increased plasma renin activity, and high-grade stenosis of the right renal artery. They also rule out other potential diagnoses such as atherosclerosis, congenital renal artery hypoplasia, Takayasu arteritis, and temporal arteritis based on the patient's symptoms and lab results. AI_C, however, believes that the most likely diagnosis is atherosclerosis. AI_C acknowledges that fibromuscular dysplasia is a potential diagnosis but ultimately concludes that atherosclerosis is more likely given the patient's age and the fact that fibromuscular dysplasia is more common in younger women. Key Differences: The key difference in the perspectives lies in the interpretation of the patient's age and the typical demographic affected by fibromuscular dysplasia. While AI_A, AI_B, AI_D, and AI_E believe that fibromuscular dysplasia can affect women of the patient's age, AI_C believes that the patient's age makes atherosclerosis a more likely diagnosis. Question for Council Members: Given that fibromuscular dysplasia typically affects younger women, can you provide more insight into why you believe this diagnosis is more likely than atherosclerosis, which is more common in the patient's age group?. Based on your preceding response, how would you answer the original question: ["A 65-year-old woman comes to the physician for a follow-up examination after blood pressure measurements were 175/105 mm Hg and 185/110 mm Hg 1 and 3 weeks ago, respectively. She has well-controlled type 2 diabetes mellitus. Her blood pressure now is 175/110 mm Hg. Physical examination shows no other abnormalities. Antihypertensive therapy is started, but her blood pressure remains elevated at her next visit 3 weeks later. Laboratory studies show increased plasma renin activity; the erythrocyte sedimentation rate and serum electrolytes are within the reference ranges. Angiography shows a high-grade stenosis of the proximal right renal artery; the left renal artery appears normal. Which of the following is the most likely diagnosis? (A) Atherosclerosis (B) Congenital renal artery hypoplasia (C) Fibromuscular dysplasia (D) Takayasu arteritis (E) Temporal arteritis]

**AI_A:** Restating the question, the inquiry looks into the possible diagnosis for a patient, a 65-year-old woman with well-controlled type 2 diabetes, marked hypertension, stable blood pressure but not responding to anti-hypertensive therapy, increased plasma renin activity, normal erythrocyte sedimentation rate and serum electrolytes, and angiography showing high-grade stenosis of the proximal right renal artery. Planning involves considering the possibilities. The raised blood pressure could suggest a systemic issue like atherosclerosis. However, the normal left renal artery could argue against a systemic issue. The high plasma renin activity may infer a renal cause, like fibromuscular dysplasia. Congenital renal artery hypoplasia, Takayasu arteritis, and temporal arteritis are the other alternatives. But their appearances don't line up with our patient's clinical picture. Clinical reasoning is influenced by several elements. Atherosclerosis is indeed common in this age group, but it tends to have a systemic effect, and the patient's left renal artery is normal. Furthermore, her diabetes is well controlled, not favoring the aggression of atherosclerosis. Fibromuscular dysplasia, while more common in younger women, can occur in this age as well and should not be discounted due to age. It's associated with hypertension, increased renin activity due to renal artery stenosis, as seen in our patient. Concluding, while fibromuscular dysplasia is not as common in women of this age, the clinical picture, including hypertension, increased plasma renin activity, and high-grade stenosis of the proximal right renal artery, is most congruent with this diagnosis. The fact that the patient's left renal artery appears normal is key, leading us away from systemic diagnoses like atherosclerosis and towards a localized renal issue like fibromuscular dysplasia. In response to the query, I believe the most likely diagnosis is Fibromuscular dysplasia (C). Although it's traditionally more common in younger women, the symptoms and lab results align most closely with it, as it can explain the persistent hypertension, the increased plasma renin activity, and the angiographic evidence of unilateral renal artery stenosis.

**AI_B:** RESTATING THE QUESTION: This is a case of a 65-year-old woman who has been showing persistently high blood pressure despite antihypertensive therapy. Her labs show high plasma renin activity, with normal electrolytes and ESR, and her angiography reveals significant stenosis in her right renal artery. We are asked to consider the best possible diagnosis from these options: Atherosclerosis, Congenital renal artery hypoplasia, Fibromuscular dysplasia, Takayasu arteritis, and Temporal arteritis. PLANNING: Given the patient's history, clinical findings and lab results, one might initially consider the possibility of atherosclerosis because of her age, hypertension, and the presence of stenotic lesion in the renal artery. However, other options need to be considered to get a more comprehensive view. The increased plasma-renin activity and the presence of unilateral high-grade stenosis of the renal artery, especially in the context of refractory hypertension, are suggestive of renovascular hypertension, which could be possibly due to fibromuscular dysplasia. CLINICAL REASONING: While atherosclerosis is a common cause of renal artery stenosis, it usually affects the ostia of the renal arteries and it more commonly bilateral. The elevated renin levels also indicates renovascular hypertension due to a decrease in renal perfusion, which is typically seen in fibromuscular dysplasia. Although fibromuscular dysplasia does have a peak incidence in younger women, it can also occur in older women like our patient. CONCLUDING: Despite its lower incidence in the patient's age group, the clinical findings and lab results in this case more strongly suggest fibromuscular dysplasia. RESPONSE: Considering all the above factors, therefore, the most likely diagnosis for this patient is (C) Fibromuscular dysplasia. It is due to significant stenosis in the right renal artery leading to increased release of renin and consequent hypertension. This is more likely than atherosclerosis which usually shows bilateral renal artery involvement and does not cause such drastic increases in renin levels.

**AI_E:** Restating the question: The inquiry demands a diagnosis for a 65-year-old woman. She has a history of well-controlled type 2 diabetes and persistent hypertension despite being on medication. Lab results show increased plasma renin activity, the angiogram displays right renal artery stenosis while other tests indicate normal values. Planning: The assumptions here revolve around the persistent hypertension, the stenosis seen in the right renal artery and the increased plasma renin activity. The elevated blood pressure may suggest secondary hypertension caused by a condition affecting the kidneys, adrenal glands, heart, or endocrine system. Renovascular disease like fibromuscular dysplasia (FMD), which typically seen in women and can cause stenosis of renal arteries, hypertension, and increased renin secretion, could be a potential diagnosis, but the frequency of FMD in women decreases with age, suggesting the possibility of other differential diagnoses such as atherosclerosis. Clinical Reasoning: A significant factor taking precedent is the increased plasma renin activity coupled with the stenosis of the right renal artery seen on angiography. Renal artery stenosis can cause decreased blood flow to the kidneys, which leads to inappropriate activation of the renin-angiotensin-system, manifesting as increased plasma renin activity and hypertension. While both FMD and atherosclerosis can cause renal artery stenosis, the angiogram displaying a high-grade stenosis of the proximal right renal artery is more typically seen in fibromuscular dysplasia than in atherosclerosis. Moreover, given the absence of peripheral vascular disease or other systemic signs and symptoms, atherosclerosis becomes less likely despite her age and well-controlled diabetes. Concluding: Although fibromuscular dysplasia is more common in younger females, it doesn't mean we should completely rule it out in a 65-year-old woman. Given the clinical findings which include uncontrolled hypertension despite antihypertensive therapy, increased plasma renin activity, and the focal lesion visualized in the proximal right renal artery, fibromuscular dysplasia seems the most likely diagnosis in this patient. Thus, in response to the original question, the most likely diagnosis for this patient is (C) Fibromuscular dysplasia.

**AI_C:** The question essentially asks, "Given a 65-year-old woman with consistently elevated blood pressure, well-controlled type 2 diabetes, normal physical examination aside from hypertension, persistent hypertension despite medication, increased plasma renin activity, normal erythrocyte sedimentation rate and serum electrolytes, and angiography showing a high-grade stenosis of the proximal right renal artery with a normal left renal artery, what would be the most likely diagnosis among atherosclerosis, congenital renal artery hypoplasia, fibromuscular dysplasia, Takayasu arteritis, and temporal arteritis?" To answer this question, it would be important to review the pathophysiological aspects involved. Persistent hypertension could be partly from a renin-angiotensin-aldosterone system activation, triggered by renal artery stenosis. On the other hand, the lack of other abnormalities points away from a systemic process, making diseases like Takayasu arteritis or temporal arteritis less likely. Based on these key points, some alternate assumptions can be generated. One is that the unilateral high-grade renal artery stenosis is causing the hypertension. If this is the case, the issue might be more anatomical than pathological. Another assumption is that the patient's type 2 diabetes has led to atherosclerosis, contributing to the stenosis. This would create a compounded systemic issue, beyond just an anatomical abnormality. In terms of likelihood, given the age and gender of the patient, as well as the stenosis of only one renal artery detected by angiography, fibromuscular dysplasia could be a plausible diagnosis. Although fibromuscular dysplasia typically affects younger women, it would be unwise to dismiss it solely based on age. On the contrary, if we emphasize more on the age and the potential commonality of atherosclerosis in older adults with type 2 diabetes, we might be led to conclude atherosclerosis as the most probable diagnosis. However, considering that the abnormality seems confined to the right renal artery alone and is not systemic, suspicion for atherosclerosis might be less compelling. Upon comparing these two assumptions, it seems that the focal abnormality in one renal artery, as opposed to a more widespread atherosclerotic disease, might tilt the scales towards fibromuscular dysplasia, despite the rare incidence in this age group. So, in response to the original question, given the patient's medical features and the lack of systemic involvement, the diagnosis of fibromuscular dysplasia (option C) could indeed be considered as the most likely.

**AI_D:** Restating the question: This appears to be a case of uncontrolled hypertension in a 65-year-old woman with well-managed type 2 diabetes, despite treatment. She has an angiogram that shows a high-grade stenosis in her right renal artery (not in the left one) and an increase in plasma renin activity, but normal electrolytes and erythrocyte sedimentation rate. The question is asking for the most probable diagnosis based on the provided findings, options being atherosclerosis, congenital renal artery hypoplasia, fibromuscular dysplasia, Takayasu arteritis, or temporal arteritis. Planning: We're assuming that the high-grade stenosis of the right renal artery is causing this resistant hypertension. Let's question this assumption - could there be {other factors causing her hypertension} despite the stenosis? However, the increased renin activity suggests a renal cause. Alternatively, if the renal artery stenosis is due to {atherosclerosis versus fibromuscular dysplasia}, it might be noteworthy to consider her demographic (a 65-year-old woman), even though fibromuscular dysplasia is more common in younger women. Clinical reasoning: The main factor here is her resistant hypertension, which is likely caused by the high-grade renal artery stenosis, leading to increased renin secretion — a classic symptom of renal artery stenosis due to the kidney sensing reduced blood flow (ischemia). As a response, renal juxtaglomerular cells release renin, which ultimately leads to the formation of angiotensin II, causing vasoconstriction and thereby increasing blood pressure. The patient's age and gender make fibromuscular dysplasia less likely than atherosclerosis since fibromuscular dysplasia usually occurs in younger women. However, her well-controlled diabetes argues against atherosclerosis since diabetes is a significant risk factor. Concluding: While the demographic preference might lean towards atherosclerosis, the other findings suggest fibromuscular dysplasia — a disease known to cause renal artery stenosis. The angiographic finding of unilateral renal artery stenosis with well-controlled diabetes and no other signs of systemic atherosclerosis (e.g., coronary, cerebral) also favors non-atherosclerotic disease. Response: I, therefore, conclude the most likely diagnosis for this patient is Fibromuscular dysplasia (C), as the patient's presentation of refractory hypertension, increased plasma renin activity, and unilateral renal artery stenosis seen on angiography are classic for this condition. Atherosclerosis (A) is less likely given her controlled diabetes and lack of other manifestations of systemic atherosclerosis. The other potential diagnoses (congenital renal artery hypoplasia, Takayasu arteritis, and Temporal arteritis) also do not fit the patient's clinical picture as neatly as fibromuscular dysplasia.

**Facilitator** The consensus among the AI responses is that the most likely diagnosis for the 65-year-old woman in question is Fibromuscular dysplasia (C). Despite the fact that this condition is more common in younger women, the patient's clinical picture aligns most closely with this diagnosis. Key factors leading to this conclusion include her persistent hypertension despite antihypertensive therapy, increased plasma renin activity, and high-grade stenosis of the proximal right renal artery. The normal appearance of the left renal artery is also significant, as it suggests a localized renal issue rather than a systemic one, such as atherosclerosis. Although the patient's age and well-controlled type 2 diabetes might initially suggest atherosclerosis, the lack of systemic involvement and the unilateral nature of the renal artery stenosis make fibromuscular dysplasia a more fitting diagnosis. The other potential diagnoses, including congenital renal artery hypoplasia, Takayasu arteritis, and temporal arteritis, do not align as closely with the patient's clinical presentation.
