# Supplementary material for: Collaborative intelligence in AI: Evaluating the performance of a council of AIs on the USMLE
Source: PLOS Digit Health. 2025 Oct 9;4(10):e0000787. doi: 10.1371/journal.pdig.0000787 (PMC12510544; doi:10.1371/journal.pdig.0000787)
Supplement: S2 File — (ZIP) [file pdig.0000787.s002.zip › S2 Data Raw data files containing unprocessed question inputs and LLM outputs including transcripts of deliberations/STEP1_Question-29-1_Type-MC-NJ.docx]

**Facilitator** **[Round 1]:** Results of a study that examined the impact of risk factors on cardiovascular health are being evaluated. In the study, serum LDL-cholesterol (LDL) concentration is found to have a correlation of 0.6 with serum high-sensitivity C-reactive protein (hs-CRP) concentration. Which of the following statements best describes the relationship between LDL concentration and hs-CRP concentration based on this finding? (A) Higher LDL concentrations are associated with higher hs-CRP concentrations (B) Higher LDL concentrations are associated with lower hs-CRP concentrations (C) Higher LDL concentrations cause higher hs-CRP concentrations (D) Higher LDL concentrations cause lower hs-CRP concentrations

**AI_D:** RESTATING THE QUESTION: The question is asking about the relationship between LDL cholesterol, commonly known as "bad cholesterol" and high-sensitivity C-reactive protein (hs-CRP), a marker of inflammation in the body. The correlation coefficient between these two parameters in the study is 0.6. The correlation coefficient ranges from -1 to +1 where -1 denotes a strong negative relationship, 0 denotes no relationship, and +1 signifies a strong positive relationship. PLANNING: To address this question, we need to consider the meaning of correlation in statistical terms. The correlation of 0.6 suggests a moderate positive relationship between serum LDL cholesterol and hs-CRP levels. Additionally, it's important to remember that correlation does not imply causation. So, we can't infer from this data alone that greater LDL levels cause higher hs-CRP levels or vice versa. CLINICAL REASONING: Based on the correlation factor, as LDL levels increase, hs-CRP levels are also expected to increase. However, this relationship is a correlation, not a cause-and-effect relationship. That means that we cannot assert that higher LDL levels cause higher hs-CRP levels based on this correlation alone. CONCLUDING: Hence, the knowledge from the correlation coefficient indicates that as LDL levels become higher, it's expected that hs-CRP would also be higher due to the positive correlation. RESPONSE: Therefore option (A) "Higher LDL concentrations are associated with higher hs-CRP concentrations" best describes the relationship between LDL concentration and hs-CRP concentration based on this finding.

**AI_C:** (1) RESTATING THE QUESTION: We are examining the findings of a study that looked into how various risk factors affect cardiovascular health. A key outcome of this study is that there is a correlation of 0.6 between LDL-cholesterol (often called "bad" cholesterol due to its role in artery plaque build-up) and high-sensitivity C-reactive protein (hs-CRP, a protein that increases in the blood with inflammation). Correlation, a statistical tool, informs us about the relationship between two variables. A correlation of 0.6 suggests a moderate positive relationship, implying that when one variable increases, the other tends to go up as well. (2) PLANNING: Many might jump to the conclusion that the relationship between these two variables, LDL and hs-CRP, is causal; that is, when LDL concentrations rise, they cause hs-CRP concentrations to increase. However, it's important to remember that correlation does not imply causation. All we confidently know from this statistic is that LDL and hs-CRP mirror each other's movements to some extent - both might increase or decrease at the same time, but neither necessarily causes the other's behavior. (3) CLINICAL REASONING: LDL concentration might vary independently of hs-CRP, increasing due to diet or other genetic and environmental factors; meanwhile, hs-CRP can also increase or decrease in response to inflammation or infection, irrespective of LDL levels. What is significant here is that the two concentrations are related—they tend to increase or decrease together—but this does not confirm a cause-and-effect relationship. (4) CONCLUDING: Considering these factors, it would be most accurate to conclude that higher concentrations of LDL are associated with higher concentrations of hs-CRP, reflecting the positive correlation described in the study. (5) RESPONSE: Thus, the correct answer is (A) Higher LDL concentrations are associated with higher hs-CRP concentrations.

**AI_B:** RESTATING THE QUESTION: The objective of this inquiry is to decipher the association between serum Low-Density Lipoprotein cholesterol (LDL) and high-sensitivity C-reactive protein (hs-CRP) based on a study's outcomes that identified a correlation coefficient of 0.6 between them. LDL, often labelled as 'bad cholesterol', contributes to plaque buildup in arteries. hs-CRP is a measure of systemic inflammation and can be associated with cardiovascular disease risk. A correlation coefficient ranges from -1 to 1. A correlation coefficient closer to 1 or -1 indicates a strong relationship, with positive values indicating a positive association (as one increases, so does the other), and negative values indicating a negative association (as one increases, the other decreases). PLANNING: To elucidate this query, one must understand the concept of correlation and correlation coefficients. An assumption here is that a correlation of 0.6 signifies a considerable relationship between the two variables. We could hypothesize that because the correlation value is positive, an increase in LDL levels is likely to be associated with a rise in hs-CRP levels. CLINICAL REASONING: Evaluating this, we understand the value of 0.6, which is positive, indicating a positive correlation. This suggests that as the LDL concentration increases, the hs-CRP concentration also increases. This connection doesn't provide direct evidence of causation. It simply indicates an association. CONCLUDING: Using the given information and medical knowledge, a correlation of 0.6 indicates a positive relationship. While one could assume that higher LDL levels could be linked to higher hs-CRP levels, we can't infer causation from this correlation alone, only an association. RESPONSE: Therefore, based on the data provided, the best statement is: (A) Higher LDL concentrations are associated with higher hs-CRP concentrations. However, it's crucial to remember that correlation doesn't imply causation, and further research would be needed to determine any causal relationships.

**AI_A:** (1) RESTATING THE QUESTION: The inquiry evaluates the association between Low-Density Lipoprotein cholesterol (LDL) and high-sensitivity C-reactive protein (hs-CRP) based on a statistical finding. LDL is a type of cholesterol often referred to as "bad" cholesterol as high levels can lead to plaque buildup in your arteries and cause heart diseases. On the other hand, hs-CRP is a protein found in blood in response to inflammation and can be used as a marker of heart disease risk. (2) PLANNING: The correlation coefficient here is 0.6, which indicates a moderate positive relationship between LDL concentrations and hs-CRP concentrations. Therefore, the initial assumption would be as LDL concentration increases, the hs-CRP concentration also increases. A secondary assumption could be that higher LDL concentrations cause higher hs-CRP concentrations. However, a correlation coefficient (r) does not infer causality; causality can only be inferred from carefully controlled experiments. (3) CLINICAL REASONING: A correlation of 0.6 (moderate, positive correlation) means that as LDL goes up, so does hs-CRP, but not exactly at the same rate or consistency. As such, it's an association, not direct causation. From this point, the clinical reasoning would guide us to identify the relationship as associative rather than causative as the given study only provides correlational data. (4) CONCLUDING: Considering the correlation provided and the understanding of LDL and hs-CRP, it can be concluded that there is an association between these two variables. However, since the study does not offer experimental control necessary to infer causality, we cannot state that higher LDL concentrations cause higher hs-CRP concentrations. (5) RESPONSE: Therefore, based on this finding, we can best describe the relationship by stating that "(A) Higher LDL concentrations are associated with higher hs-CRP concentrations".

**AI_E:** RESTATING THE QUESTION: To clarify, this question refers to a statistical analysis from a cardiovascular health study. The study found a relationship between two biochemical parameters: the serum concentration of LDL-cholesterol (or 'bad' cholesterol), and the serum concentration of high-sensitivity C-reactive protein (hs-CRP), a marker of inflammation in the body. The correlation between these two factors is given as 0.6. This means that as one factor changes, the other tends to change in a predictable way. The question is seeking an interpretation of this correlation. PLANNING: Fundamentally, the question is asking if the LDL concentrations are directly or inversely correlated with hs-CRP concentrations, or if LDL concentrations cause specific changes in hs-CRP concentrations. These assumptions are based on the interpretation of the correlation coefficient mentioned in the study - 0.6. Higher positive values (close to +1) suggest strong direct correlations, while higher negative values (closer to -1) suggest strong inverse correlations. Therefore, it is plausible to assume that higher LDL concentrations are associated with higher hs-CRP concentrations (since 0.6 is a relatively high positive value). Meanwhile, claiming causality without further information would be inappropriate, as correlation does not imply causation. CLINICAL REASONING: Inflammatory processes like those indicated by high hs-CRP concentrations can be stimulated by the presence of LDL, a kind of fat transported in the bloodstream. The correlation coefficient of 0.6 suggests that, generally, an increase in LDL might be associated with an increase in hs-CRP, while a decrease in LDL could be associated with a decrease in hs-CRP. However, without more specific study results demonstrating a causal relationship, it's problematic to say definitively that changes in LDL levels "cause" corresponding changes in hs-CRP levels. CONCLUDING: The relationship between LDL concentration and hs-CRP concentration, as defined by the correlation coefficient of 0.6, best matches the description given in option (A): higher LDL concentrations are associated with higher hs-CRP concentrations. This interpretation adheres to the guidelines of correlational analysis, emphasizing association rather than causality. RESPONSE: Given this analysis, 'Higher LDL concentrations are associated with higher hs-CRP concentrations' is the best description of the relationship between LDL concentration and hs-CRP concentration in the context of this particular study.

**Facilitator** The consensus statement synthesizing the responses of AI_A, AI_B, AI_C, AI_D, and AI_E is as follows: The question at hand pertains to the interpretation of a study's findings that examined the impact of risk factors on cardiovascular health. The study found a correlation of 0.6 between serum LDL-cholesterol (LDL) concentration and serum high-sensitivity C-reactive protein (hs-CRP) concentration. LDL, often referred to as "bad cholesterol", contributes to plaque buildup in arteries, while hs-CRP is a marker of inflammation and can be associated with cardiovascular disease risk. The correlation coefficient of 0.6 indicates a moderate positive relationship between LDL and hs-CRP concentrations. This suggests that as LDL concentrations increase, hs-CRP concentrations also tend to increase, and vice versa. However, it's crucial to understand that this correlation does not imply causation. While the data suggests an association, it does not provide evidence that higher LDL concentrations cause higher hs-CRP concentrations. Therefore, based on the given correlation and understanding of LDL and hs-CRP, the best description of the relationship between LDL concentration and hs-CRP concentration based on this finding is: "(A) Higher LDL concentrations are associated with higher hs-CRP concentrations". This statement reflects the positive correlation described in the study, emphasizing association rather than causality. Further research would be needed to determine any causal relationships.
