# Supplementary material for: Collaborative intelligence in AI: Evaluating the performance of a council of AIs on the USMLE
Source: PLOS Digit Health. 2025 Oct 9;4(10):e0000787. doi: 10.1371/journal.pdig.0000787 (PMC12510544; doi:10.1371/journal.pdig.0000787)
Supplement: S2 File — (ZIP) [file pdig.0000787.s002.zip › S2 Data Raw data files containing unprocessed question inputs and LLM outputs including transcripts of deliberations/STEP1_Question-3-1_Type-MC-NJ.docx]

**Facilitator** **[Round 1]:** In a sample of 100 individuals, the mean leukocyte count is 7500/mm³, with a standard deviation of 1000/mm³. If the leukocyte counts in this population follow a normal (gaussian) distribution, approximately 50% of individuals will have which of the following total leukocyte counts? (A) 5500–9500/mm³ (B) <6500/mm3 or >8500/mm³ (C) 6500–8500/mm³ (D) <7500/mm³ (E) >9500/mm³

**AI_E:** RESTATING THE QUESTION: We are asked to estimate which range of leukocyte counts would encompass approximately half of a population of 100 individuals, given that the mean leukocyte count is 7500/mm³, the standard deviation is 1000/mm³, and the distribution of counts follows a normal (gaussian) pattern. PLANNING: The main assumption here is that the distribution of leukocyte counts is normal, which implies that about 68% of the values lie within one standard deviation from the mean, 95% are within two standard deviations and 99.7% fall within three standard deviations. Thus we need to find out which of the given ranges can include about 50% of the counts. CLINICAL REASONING: The mean leukocyte count of the population is 7500/mm³ and the counts follow a normal distribution. In a normal distribution, 50% of values will be less than the mean and 50% will be greater than the mean because the distribution is a symmetric bell curve. Therefore, approximately half of the individuals in this population will have leukocyte counts less than the mean. CONCLUDING: So, given that the mean is 7500/mm³, about 50% of individuals would have leukocyte counts that are less than this value. RESPONSE: Therefore, the answer is option D (<7500/mm³). Approximately 50% of the individuals will have a leukocyte count less than 7500/mm³.

**AI_D:** (1) RESTATING THE QUESTION: The question inquires about the normal distribution of leukocyte counts in a hypothetical population, where the mean is 7500/mm³ and the standard deviation is 1000/mm³. In other words, on average, the blood of individuals in this population have 7500 white blood cells (leukocytes) per cubic millimeter (mm³), and the values deviate from this mean by 1000 leukocytes/mm³. A "normal distribution" (or gaussian distribution) refers to a type of statistical distribution where data is symmetrically distributed around the mean - most values cluster around the average, and fewer values are found as we move away from it, represented graphically by the well-known "bell curve". (2) PLANNING: The Gaussian distribution has specific characteristics - approximately 68% of values fall within one standard deviation of the mean, around 95% within two standard deviations, and nearly all (or 99.7%) within three standard deviations. To answer the question, our assumption needs to focus on the percentage of individuals with leukocyte counts that fall within the middle of this distribution, specifically half, or 50%. (3) CLINICAL REASONING: The mean is the center of the normal distribution, therefore, about 50% of the values lie below and 50% above the mean. Hence, for this scenario where the mean is 7500/mm³, it is logical to assume that approximately half the individuals will have leukocyte values less than the mean. (4) CONCLUDING: Using the principles of a normal distribution, we understand that 50% of the individuals will have counts less than or equal to the mean leukocyte count. Thus, the leukocyte count for these individuals should be less than or equal to 7500/mm³. (5) RESPONSE: Your answer will be: (D) <7500/mm³.

**AI_B:** (1) RESTATING THE QUESTION: This question wants us to determine the leukocyte (white blood cell) count of approximately 50% of the population, given certain statistics. The mean leukocyte count is 7500 cells/mm³, which means this is the average number of leukocytes per cubic millimeter of blood in the population. The standard deviation is 1000 cells/mm³, representing the variability or dispersion of the leukocyte counts around this mean. We also know that the leukocyte counts follow a normal or Gaussian distribution -- a statistical distribution where the most frequent values are around the mean, and values become increasingly less frequent the further they are from the mean. (2) PLANNING: The assumption we have to work with here involves the properties of a normal distribution. In a normal distribution, nearly 68% of the data falls within one standard deviation of the mean, 95% falls within two standard deviations, and 99.7% falls within three standard deviations. Since the question is asking for around 50% of the individuals, we'll need to focus on values close to the mean. (3) CLINICAL REASONING: According to our assumption of a normal distribution, approximately 68% of values (or leukocyte counts) lie within one standard deviation of the mean. This translates as roughly 68% of individuals having a leukocyte count between 6500 (7500-1000) and 8500 (7500+1000) per mm³. This range is more than 50%, meaning values that are even closer to the mean would have to be considered to target the approximate 50% range. (4) CONCLUDING: By the design of the normal distribution, the closest to 50% would be values below the mean, given that it will cover approximately 50% of the individuals in the distribution. Considering a value greater than the mean would incorporate a larger segment of the percentage, increasing it beyond 50%. (5) RESPONSE: So, in a group of 100 individuals with leukocyte counts following a normal distribution and given the provided mean and standard deviation, roughly 50% of individuals will have a leukocyte count less than the mean, that is, (D) <7500/mm³.

**AI_C:** (1) RESTATING THE QUESTION: The question is asking me to identify, from a given list, the total leukocyte count that approximately 50% of individuals in a sample population will have, given that their leukocyte counts follow a normal distribution. The mean leukocyte count for this population is 7500/mm³ and the standard deviation is 1000/mm³. The leukocyte count, also known as white blood cell count, is a measure of the number of white blood cells in the blood. A normal distribution, also known as a Gaussian distribution, is a type of statistical distribution where the data is symmetrically distributed about the mean. (2) PLANNING: The assumptions here is that the total leukocyte counts in this population follow a normal distribution. Normal distribution is symmetrical and the mean, median, and mode are all equal. Therefore, 50% of the population will have leukocyte counts less than the mean and 50% will have counts more than the mean. The alternate assumption is identifying which of the standard deviations will contain approximately 50% of leukocyte counts. (3) CLINICAL REASONING: In a normal distribution, about 68% of values lie within one standard deviation of the mean, 95% within two standard deviations, and 99.7% within three standard deviations. Therefore, approximately 34% of the population will have leukocyte counts between the mean minus one standard deviation and the mean (7500/mm³ - 1000/mm³ = 6500/mm³ to 7500/mm³), and another 34% will have leukocyte counts between the mean and the mean plus one standard deviation (7500/mm³ to 7500/mm³ + 1000/mm³ = 8500/mm³). Therefore, about 68% of the population will have leukocyte counts between 6500/mm³ and 8500/mm³. But since we are interested in only half of the population (50%), we would need to look either below or above the mean. (4) CONCLUDING: Using the logic of normal distribution, the mean of 7500/mm³ would divide the population into two equal halves with approximately 50% of individuals having leukocyte counts less than 7500/mm³ and 50% of individuals having counts more than 7500/mm³. (5) RESPONSE: Thus, the answer to the question is (D) < 7500/mm³. This range is the one that contains approximately 50% of the leukocyte counts in this particular population based on the mean and the rules of a normal distribution.

**AI_A:** (1) RESTATING THE QUESTION: This query ponders on the range of total leukocyte counts for roughly half or 50% of a population of 100 individuals falling in a normal distribution, given that their mean leukocyte count is 7500/mm³ with a standard deviation of 1000/mm³. Leukocyte count, which refers to the total number of white blood cells in a person's body, is a crucial marker for the immune system's functioning. In a normal (gaussian) distribution, most of the data will cluster around the central peak and the likelihood of each occurred observation decreases as it moves further away from the mean. (2) PLANNING: The question assumes that the leukocyte counts follow a normal distribution. In such a distribution, 68% of the readings fall within one standard deviation of the mean, 95% fall within two, and 99.7% fall within three. This empirical rule, also known as the "68-95-99.7" rule, could potentially be used to compute an accurate range of leukocyte counts for approximately 50% of the study sample. In turn, this could imply that about half of the individuals will present leukocyte counts within plus or minus one-half of the standard deviation from the mean, given the symmetric nature of the Gaussian distribution. (3) CLINICAL REASONING: Given the above, approximately 68% of individuals should have leukocyte counts between the mean subtract one standard deviation and the mean add one standard deviation, or in other words, in the range of (7500 - 1000) to (7500 + 1000), which is 6500 to 8500/mm³. However, that range represents 68% of the total population. Meanwhile, we are looking for the range that includes approximately 50% of individuals. Considering the symmetry of the normal distribution, to estimate the range covering approximately 50% of individuals, we would be looking at the values that lie between the median (which is equal to the mean in a gaussian distribution) and half a standard deviation on either side of the mean. (4) CONCLUDING: Therefore, in a normally distributed set of leukocyte counts with a mean of 7500/mm³ and a standard deviation of 1000/mm³, approximately 50% of individuals are expected to exhibit leukocyte counts that fall within a range halfway within the first standard deviation on either side of the mean. (5) RESPONSE: Thus, the anticipated range of leukocyte counts covering approximately half the individuals would be (7500 - 500) to (7500 + 500), which amounts to 7000/mm³ to 8000/mm³. The given options in the question don't contain this exact range. However, option (C) 6500–8500/mm³ is the closest match, considering that the target range (7000 to 8000) falls within this selection.

**Facilitator** **[Round 2]:** Summary: AI_A's opinion: AI_A suggests that approximately 50% of individuals will have leukocyte counts within half a standard deviation from the mean, which is 7000/mm³ to 8000/mm³. However, since this range is not available in the options, AI_A chooses the closest match, which is 6500–8500/mm³. AI_B's opinion: AI_B argues that approximately 50% of individuals will have leukocyte counts less than the mean, which is <7500/mm³. AI_C's opinion: AI_C agrees with AI_B, stating that approximately 50% of individuals will have leukocyte counts less than the mean, which is <7500/mm³. AI_D's opinion: AI_D also concurs with AI_B and AI_C, suggesting that approximately 50% of individuals will have leukocyte counts less than or equal to the mean, which is <7500/mm³. AI_E's opinion: AI_E aligns with AI_B, AI_C, and AI_D, stating that approximately 50% of individuals will have leukocyte counts less than the mean, which is <7500/mm³. Key premises and logic that distinguish differing perspectives: The key premise that distinguishes AI_A's perspective from the others is the interpretation of the normal distribution. AI_A uses the concept of standard deviation to estimate the range that would cover approximately 50% of the population, while AI_B, AI_C, AI_D, and AI_E use the property of symmetry in a normal distribution to conclude that approximately 50% of the population will have leukocyte counts less than the mean. Question to clarify disagreements: Can you explain why you chose to interpret the normal distribution in the way you did, and how this influenced your estimation of the leukocyte count range for approximately 50% of the population?. Based on your preceding response, how would you answer the original question: [In a sample of 100 individuals, the mean leukocyte count is 7500/mm³, with a standard deviation of 1000/mm³. If the leukocyte counts in this population follow a normal (gaussian) distribution, approximately 50% of individuals will have which of the following total leukocyte counts? (A) 5500–9500/mm³ (B) <6500/mm3 or >8500/mm³ (C) 6500–8500/mm³ (D) <7500/mm³ (E) >9500/mm³]

**AI_B:** 1) RESTATING THE QUESTION: We seem to be contemplating which range of white blood cell count, or leukocyte counts, would encompass approximately half of a population of 100 individuals. The leukocyte counts are said to follow a normal, or gaussian, distribution with an average of 7500 cells per cubic millimeter and a standard deviation of 1000 cells per cubic millimeter. 2) PLANNING: The normal distribution principle, where data is symmetrically distributed around the mean, is the central concept here. According to this principle, roughly 50% of data will fall below the mean and 50% above the mean. However, we can also consider the role of standard deviation to delve further into the data distribution. This will need further investigation and clinical reasoning. 3) CLINICAL REASONING: I see two assumptions we could consider. First, the assumption based on the normal distribution principle where about 50% of leukocyte counts will be less than the average. Second, using the concept of standard deviation where about 68% of leukocyte counts would lie within one standard deviation from the mean (6500 to 8500 cells/mm³). Though the second assumption could be valid, it covers more than 50% of the population, not exactly half. Therefore, considering the normal distribution principle seems clinically more relevant for this data. 4) CONCLUDING: Through clinical reasoning, following the normal distribution principle, I would deduce that approximately 50% of individuals will have leukocyte counts less than the mean. 5) RESPONSE: Based on this, the closest answer to the question would be (D) <7500/mm³.

**AI_C:** Restating the question, we are asked to determine the leukocyte count range for approximately 50% of a population given that the leukocyte count follows a normal distribution with a mean of 7500/mm³ and a standard deviation of 1000/mm³. For clarification, a normal distribution, also known as a Gaussian distribution, is a type of continuous probability distribution for a real-valued random variable. It is symmetric about the mean, indicating that data near the mean are more frequent in occurrence than data far from the mean. A standard deviation is a measure of the amount of variation or dispersion of a set of values. Identifying assumptions, we could consider that the range covering 50% of the population would be less than or equal to the given mean of 7500/mm³. Generating an alternate assumption would be that the range covering 50% of the population could be a specific interval around the mean. Evaluating these assumption, we would consider the properties of a normal distribution. We know that about 68% of values drawn from a normal distribution are within one standard deviation away from the mean; about 95% are within two standard deviations, and about 99.7% lie within three standard deviations. In the absence of the exact parameters for a 50% interval, we can approximate that to be less than the mean, hence the majority of our council (AI_B, AI_C, AI_D, and AI_E) believes in this hyposthesis. The clinical reasoning behind this assumption is that in a normal distribution, about half of the measurements fall below the mean and about half fall above. This is because the normal distribution curve is symmetric about the mean. Therefore, we would expect approximately 50% of the leukocyte count measurements to be less than the mean of 7500. Concluding this reasoning process, we can infer that approximately 50% of individuals will have a leukocyte count that is less than the mean leukocyte count. Hence, out of the available options, the most representative range would be total leukocyte counts that are less than 7500/mm³. Our final response would then be: In a sample of 100 individuals, if the leukocyte counts follow a normal (gaussian) distribution and the mean leukocyte count is 7500/mm³, we can expect that approximately 50% of individuals will have total leukocyte counts less than 7500/mm³. Therefore, the most fitting answer option to this question is (D) <7500/mm³.

**AI_A:** 1) RESTATING THE QUESTION: The question is asking for an approximation of the leukocyte (white blood cell) count in which 50% of a particular population will fall within, assuming a normal distribution. A normal (or Gaussian) distribution is a statistical concept where data is evenly distributed around the mean, forming a bell-shaped curve when graphed. The mean, in this case, is 7500/mm³, and standard deviation is another statistical term which tells us about the dispersion or spread of the data points from the mean, and it's 1000/mm³. 2) PLANNING: The assumption for a perfectly symmetrical normal distribution curve is that 50% of data points lay below the mean and 50% above it. However, another assumption could be that the 50% of individuals are referring to those around the mean, which could be visualized as a band on either side of the mean within some standard deviation. 3) CLINICAL REASONING: Reflecting on these assumptions, we could first explore the initial assumption that 50% of the population lies beneath the mean. This would lead us to select (D) <7500/mm³. However, we need to consider that white blood cell count is a quantitative continuous value with a certain degree of variation, represented by the standard deviation. This variation has significant clinical implications, as it reflects the dynamism of our immune response and the fluctuation of leukocyte count in different health and disease states. Therefore, the second assumption - that this 50% of individuals lies within a certain range around the mean - may provide a more clinically relevant answer. According to the empirical rule, ~68% of data in a normal distribution lies within ±1 standard deviation from the mean. In our context, that would be the range from 6500/mm³ to 8500/mm³, giving us option (C). 4) CONCLUDING: While keeping a normal distribution’s perfect symmetry in mind, clinically it may be prudent to consider variability around the mean while interpreting this data. Therefore, while there is a strategic reason that could lead us to select (D), considering the reality of clinical measurements leads us toward option (C). 5) RESPONSE: In light of the above clinical reasoning, the total leukocyte counts for approximately 50% of individuals in this population would lie within the range of 6500–8500/mm³, which is option (C).

**AI_E:** The question essentially asks for the leukocyte counts that roughly half of the individuals will have in a population where leukocyte counts follow a normal distribution. The key concept at play here is the Gaussian, or normal distribution, which is a specific distribution pattern of data that is symmetric around the mean. The assumption is that given this distribution, approximately half the population will have leukocyte counts below the mean, and the other half above the mean. Looking at the options provided, the one that best captures the state of leukocyte counts of approximately half the population being less than the mean would be (D) <7500/mm³, which translates to counts that are less than the given mean. If we were to assume that close to 50% of the individuals would have counts above this mean value (7500), then, logically, close to 50% of individuals would be expected to have counts below the mean. This then leads us to prioritize (D) <7500/mm³, not just because this is the general trend with data following a normal distribution but also because no other option captures this relationship. For example, options (B) and (E) deal with population extremes, whereas options (A) and (C) comprise larger ranges that would theoretically include more than just 50% of our population. Supporting our choice of (D) <7500/mm³, we connect the concept of the normal distribution and its properties of symmetry and bell shape to our current clinical problem. By deduction, if the leukocytes follow a normal distribution, approximately 50% of the 'counts' would fall below the mean, which is 7500/mm³ in this scenario. To sum up, option (D) <7500/mm³ appears to be the best answer to the question "[In a sample of 100 individuals, the mean leukocyte count is 7500/mm³, with a standard deviation of 1000/mm³. If the leukocyte counts in this population follow a normal (gaussian) distribution, approximately 50% of individuals will have which of the following total leukocyte counts?" This conclusion is in line with the property of a normal distribution, which states that approximately half the data falls below the mean and half above.

**AI_D:** RESTATING THE QUESTION: The question asks about the probable leukocyte count range for roughly half (50%) of the individuals in a given sample, where the mean leukocyte count is found to be 7500/mm³, standard deviation 1000/mm³, and the counts follow a normal distribution or gaussian curve. In the normal distribution, the data is symmetrically distributed around the mean, where roughly 68% of the data falls within one standard deviation from the mean, about 95% within two standard deviations, and about 99.7% within three standard deviations. PLANNING: The following assumptions are made: 1) Leukocyte counts follow a normal distribution. This implies that the data is symmetrically distributed around the mean. However, this assumption could come under scrutiny because it's not explicitly stated that our leukocyte count data conforms precisely to these normal distribution proportions. 2) The standard deviation is 1000/mm³, with the mean leukocyte count as 7500/mm³. This information will help us predict where about 50% of our data will fall on our gaussian curve based on these values. This is an assumption as it's contingent on leukocyte counts adhering to the standard proportions of a normal distribution. Therefore, the assumption that we should prioritize clinically is the normal distribution of leukocyte counts. CLINICAL REASONING: If leukocyte counts follow a normal distribution, approximately 68% of the data points fall within one standard deviation of the mean. This would imply a leukocyte count between 6500 (mean - 1sd) and 8500 (mean + 1sd) for 68% of individuals. Since we are looking for the leukocyte counts for around 50% of individuals, and our data is symmetrically distributed around the mean, this would imply that approximately half of our data values, or 50%, would fall below the mean itself. CONCLUDING:Therefore, in a standard gaussian curve, 50% of the data falls below the mean value, and since our leukocyte counts follow this distribution, the notion that approximately 50% of individuals will have a leukocyte count less than 7500/mm³ could be considered the most probable. RESPONSE: Consequently, the correct answer seems to be: (D) <7500/mm³.

**Facilitator** **[Round 3]:** Summary: AI_A's Opinion: AI_A believes that the leukocyte count for approximately 50% of individuals would lie within the range of 6500–8500/mm³, which is option (C). This is based on the assumption that the 50% of individuals lies within a certain range around the mean, considering the standard deviation. AI_B's Opinion: AI_B, on the other hand, argues that approximately 50% of individuals will have leukocyte counts less than the mean, which is option (D). This is based on the principle of normal distribution where data is symmetrically distributed around the mean. AI_C's Opinion: AI_C agrees with AI_B, stating that approximately 50% of individuals will have a leukocyte count that is less than the mean leukocyte count, which is option (D). This is based on the properties of a normal distribution. AI_D's Opinion: AI_D also agrees with AI_B and AI_C, stating that approximately 50% of individuals will have a leukocyte count less than 7500/mm³, which is option (D). This is based on the assumption that leukocyte counts follow a normal distribution. AI_E's Opinion: AI_E concurs with AI_B, AI_C, and AI_D, stating that approximately 50% of individuals will have leukocyte counts less than the mean, which is option (D). This is based on the properties of a normal distribution. Key Differences: The key difference in the council members' perspectives lies in their interpretation of the 50% population reference. AI_A interprets it as a range around the mean, considering the standard deviation, while AI_B, AI_C, AI_D, and AI_E interpret it as less than the mean, based on the properties of a normal distribution. Question to Clarify Disagreements: To clarify the disagreements between the council members' opinions, the following question could be asked: "How does the interpretation of the 50% population reference in the context of a normal distribution and standard deviation influence your choice of answer, and why do you believe your interpretation is more clinically relevant?". Based on your preceding response, how would you answer the original question: [In a sample of 100 individuals, the mean leukocyte count is 7500/mm³, with a standard deviation of 1000/mm³. If the leukocyte counts in this population follow a normal (gaussian) distribution, approximately 50% of individuals will have which of the following total leukocyte counts? (A) 5500–9500/mm³ (B) <6500/mm3 or >8500/mm³ (C) 6500–8500/mm³ (D) <7500/mm³ (E) >9500/mm³]

**AI_E:** In the present question, we're being asked to identify the leukocyte count range that approximately 50% of individuals will have in a sample of 100 individuals with a mean leukocyte count of 7500/mm³, a standard deviation of 1000/mm³ and assuming a normal distribution of leukocyte counts. In a normal distribution, about 68% of values fall within one standard deviation of the mean, meaning that 68% of people would have leukocyte counts between 6500 and 8500/mm³. However, we need to identify the leukocyte count for approximately 50% of individuals. The mean leukocyte count of the population is 7500/mm³. Since we are assuming the distribution is normal (symmetrical), about 50% of individuals will have a count below the mean and 50% above the mean. Therefore, logically, 50% of individuals will have leukocyte counts that are less than the mean, 7500/mm³. The best answer matching this reasoning is option (D), i.e., less than 7500/mm³. In conclusion, taking into account the principles of normal distribution and mean leukocyte count, I would answer the original question as follows: If the leukocyte counts in this population follow a normal (gaussian) distribution, approximately 50% of individuals will have leukocyte counts less than 7500/mm³.

**AI_C:** (1) RESTATING THE QUESTION: I understand that the query revolves around a sample of a hundred individuals, wherein the mean leukocyte count is given to be 7500/mm³ with a standard deviation of 1000/mm³. The leukocyte counts are stated to follow a Gaussian or normal distribution. We are tasked with determining the range of counts in which approximately 50% of individuals would lie. (2) PLANNING: To answer this question, one must have an understanding of normal distribution. In a normal distribution, about 68% of values lie within one standard deviation of the mean, 95% lie within 2 standard deviations, and 99.7% are within 3 standard deviations. The standard deviation here is used as a measuring unit to define the spread of values around the mean. The assumption could be that the 50% of values we're interested in lie directly below the mean in a perfectly symmetrical distribution. Another assumption could be that this 50% is split equally on either side of the mean. These alternate hypotheses must be evaluated according to our understanding of standard distribution theory. (3) CLINICAL REASONING: Considering our knowledge of normal distribution, we know that the median (the value below which 50% of observations fall) of a perfectly symmetrical normal distribution is the same as the mean. Therefore, if the leukocyte counts are normally distributed, about half of the individuals should have counts below the mean of 7500/mm³. It should be remembered that this is a theoretical interpretation and real-world medical data can be influenced by a variety of demographic, pathological, and physiological factors. However, in the context of this question, the concept of normal distribution is crucial to arriving at a hypothetical answer. (4) CONCLUDING: To clarify, the option that specifies "less than the mean leukocyte count" will contain approximately 50% of individuals in a normal (Gaussian) distribution. Therefore, using the information provided and the knowledge of standard distribution theory and normal distribution properties, (5) RESPONSE: I agree with AI_B, AI_C, AI_D, and AI_E. The answer to the question would be option (D), "<7500/mm³".

**AI_D:** RESTATING THE QUESTION: With a sample population of 100 individuals, the average leukocyte count is 7500/mm³, and the standard deviation (which is a measure of the amount of variation or dispersion of a set of values) is 1000/mm³. The leukocyte counts are assumed to follow a normal or gaussian distribution (a statistical method wherein data is evenly distributed around the mean). The question is asking for the leukocyte count range that would be found in approximately half of the individuals. PLANNING: I'm making an assumption that the mention of a "normal distribution" indicates that the leukocyte counts are symmetrically distributed around the mean. This suggests that, in a perfectly normal distribution, 50% of the data will be below the mean and 50% above it. However, there is also an assumption that the standard deviation might influence where the 50% of data lies. CLINICAL REASONING: In a normal, gaussian distribution, the mean, median, and mode are all the same, and the data is symmetrically distributed around the mean. This is an important point since in this distribution, about 68% of data falls within one standard deviation of the mean (both above and below), about 95% falls within two standard deviations, and almost all (or about 99.7%) falls within three standard deviations. Therefore, when we talk about 50% of data, we are essentially talking about the data that falls below the mean. CONCLUDING: After analyzing the assumptions about the properties of a normal distribution and the role of standard deviation, it is clear that the likely answer would be the option indicating that approximately half the individuals will have leukocyte counts less than the mean. RESPONSE: Thus, based on the evidence collected and the discussion, I would choose option (D) <7500/mm³, which represents the leukocyte count of approximately 50% of individuals in a population following a normal distribution. This choice reflects the understanding of the characteristic of a normal distribution regarding symmetry around the mean.

**AI_B:** To understand this question, we must know what the terms 'mean', 'standard deviation', 'normal distribution', 'leukocyte count' and 'gaussian distribution' imply. The 'mean' refers to the average leukocyte count which is 7500/mm³ in this question. A 'leukocyte' is a type of white blood cell that helps fight infections. 'Standard deviation' is a statistical measurement of the amount of variation or dispersion from the average (mean), and in this case, it is 1000/mm³. A 'normal' or 'gaussian distribution' is a pattern of data distribution where most observed values cluster around the mean and the further away these values from the mean, the less they occur. In generating the possible assumptions, we can look into different patterns of data distribution around the mean. We can assume firstly that data symmetrically distributes around the mean, giving us a bell curve. An alternative assumption is that the distribution is skewed, with a significantly higher or lower frequency of values on either side of the mean. However, we know from the question that the distribution is normal (gaussian), favouring the first assumption. Clinically speaking, a normal distribution in a medical context often implies that the majority of individuals will have values (like leukocyte counts) that are around the average, with fewer individuals having extraordinarily low or high counts. Given that this is a normal distribution and assuming the data is symmetric around the mean, we can infer that approximately 50% of individuals would likely have leukocyte counts above the mean (7500) and approximately 50% would have counts below the mean. In conclusion, considering the normal distribution - which suggests data is symmetrically distributed around the mean - it is reasonable to infer that about half of the population would be expected to record leukocyte counts that are less than the mean. Therefore, to answer the question: Approximately 50% of individuals would have total leukocyte counts that are less than the mean of 7500/mm³. Hence, the answer is option (D) "<7500/mm³".

**AI_A:** Restating the question, it is asking to determine which range or threshold of total leukocyte counts would approximately half of the individuals from a sample population have, given that their leukocyte counts follow a normal distribution with a mean count of 7500/mm³ and a standard deviation of 1000/mm³. A normal distribution, also known as a Gaussian distribution, is a type of data distribution where most of the data points cluster around the mean, and the further you go from the mean, the fewer data points you have. Identifying assumptions, one would assume that since leukocyte counts follow a normal distribution, roughly 50% of the individuals would have leukocyte counts below the mean and 50% would have counts above it. By generating alternate assumptions, it could be hypothesized that approximately half the population might fall within one standard deviation from the mean, which would give a range of leukocyte count. By connecting each sentence to its subsequent sentence, using a clinical thought process to evaluate which assumption should be prioritized, it would make sense to follow the rules of a normal distribution: In a normal distribution, roughly 68% of values lie within one standard deviation of the mean, 95% lie within two standard deviations, and 99.7% lie within three standard deviations. Therefore, for 50% of the population, we would look at the mean count and determine that half of the population would fall below it. In conclusion, using a step-by-step explanation that connects one sentence to the next with a clinical thought process to generate a final response, if we have a normal distribution and the mean leukocyte count is 7500/mm³, we would logically expect approximately 50% of individuals to have a leukocyte count of less than the mean. So, my response to [In a sample of 100 individuals, the mean leukocyte count is 7500/mm³, with a standard deviation of 1000/mm³. If the leukocyte counts in this population follow a normal (gaussian) distribution, approximately 50% of individuals will have which of the following total leukocyte counts? (A) 5500–9500/mm³ (B) <6500/mm3 or >8500/mm³ (C) 6500–8500/mm³ (D) <7500/mm³ (E) >9500/mm³] Would be (D) <7500/mm³.

**Facilitator** The consensus statement synthesizing the responses of AI_A, AI_B, AI_C, AI_D, and AI_E is as follows: The question asks to identify the range of leukocyte counts that approximately 50% of individuals from a sample population would have, given that their leukocyte counts follow a normal distribution with a mean count of 7500/mm³ and a standard deviation of 1000/mm³. Understanding the terms 'mean', 'standard deviation', 'normal distribution', 'leukocyte count' and 'gaussian distribution' is crucial to answer this question. The 'mean' refers to the average leukocyte count, which is 7500/mm³. 'Standard deviation' is a statistical measurement of the amount of variation or dispersion from the average, and in this case, it is 1000/mm³. A 'normal' or 'gaussian distribution' is a pattern of data distribution where most observed values cluster around the mean and the further away these values from the mean, the less they occur. In a normal distribution, about 68% of values lie within one standard deviation of the mean, 95% lie within two standard deviations, and 99.7% are within three standard deviations. However, the question asks for the leukocyte count range that would be found in approximately half of the individuals. Since we are assuming the distribution is normal (symmetrical), about 50% of individuals will have a count below the mean and 50% above the mean. Therefore, logically, 50% of individuals will have leukocyte counts that are less than the mean, 7500/mm³. In conclusion, considering the normal distribution, which suggests data is symmetrically distributed around the mean, it is reasonable to infer that about half of the population would be expected to record leukocyte counts that are less than the mean. Therefore, the answer to the question: "In a sample of 100 individuals, the mean leukocyte count is 7500/mm³, with a standard deviation of 1000/mm³. If the leukocyte counts in this population follow a normal (gaussian) distribution, approximately 50% of individuals will have which of the following total leukocyte counts?" would be option (D) "<7500/mm³".
