# Supplementary material for: Collaborative intelligence in AI: Evaluating the performance of a council of AIs on the USMLE
Source: PLOS Digit Health. 2025 Oct 9;4(10):e0000787. doi: 10.1371/journal.pdig.0000787 (PMC12510544; doi:10.1371/journal.pdig.0000787)
Supplement: S2 File — (ZIP) [file pdig.0000787.s002.zip › S2 Data Raw data files containing unprocessed question inputs and LLM outputs including transcripts of deliberations/STEP1_Question-31-1_Type-MC-NJ.docx]

**Facilitator** **[Round 1]:** A 72-year-old man comes to the physician for a health maintenance examination. He has no personal or family history of major medical illnesses. He has never smoked cigarettes. Pulse oximetry on room air shows an oxygen saturation of 98%. Physical examination shows plethoric skin and splenomegaly. Laboratory studies show: Hemoglobin 21.1 g/dL Hematocrit 61% Leukocyte count 15,000/mm³ Segmented neutrophils 68% Basophils 4% Lymphocytes 28% Platelet count 501,000/mm³ Leukocyte alkaline phosphatase increased A peripheral blood smear shows occasional giant platelets. The primary hematologic defect in this patient most likely occurred in which of the following cells? (A) Erythroid progenitor (B) Hematopoietic stem (C) Lymphatic progenitor (D) Megakaryocyte progenitor (E) Pluripotent stem

**AI_C:** RESTATING THE QUESTION: We are asked to identify the most probable site of hematologic disorder in a 72-year-old man presenting for a routine health check-up. He exhibits high levels of hemoglobin and hematocrit, an enlarged spleen, plethoric skin, and increased platelet count. In general, the cells listed in the answer choices are components of hematopoiesis, the process of blood cell formation. PLANNING: The assumption I initially make when looking at the laboratory results is that this man has a myeloproliferative disorder. The elevated levels of hemoglobin, hematocrit, and platelet count suggest overproduction of blood cells. The observation of giant platelets and increased leukocyte alkaline phosphatase point towards increased activity in the bone marrow. We should question if this disorder occurred at the level of a specific cell precursor or if it’s a more global problem affecting all lines of hematopoiesis. CLINICAL REASONING: Myeloproliferative disorders tend to occur due to a defect in the hematopoietic stem cells, which give rise to all the blood cells in the body. In this patient, we see signs of overproduction across multiple lines—erythrocytes, thrombocytes, and leukocytes. This overproduction suggests a problem with a stem cell that gives rise to all these cell lines. An erythroid progenitor defect would primarily affect the production of red blood cells. A lymphoid progenitor defect would primarily affect the production of lymphocytes. A megakaryocyte progenitor defect would primarily affect the production of platelets. A pluripotent stem cell defect would affect the production of all blood cells, but these are usually associated with diseases like aplastic anemia or leukemia, where we see a decrease in outcomes, not an increase. CONCLUDING: Looking at the laboratory results and physical examination, it is indicative of a myeloproliferative disorder, and considering that more than one type of blood cell is overproduced, the most likely defect is at the level of the hematopoietic stem cell. RESPONSE: Therefore, the answer is (B) Hematopoietic stem cell.

**AI_A:** RESTATING THE QUESTION: The question asks us to identify the primary cell where a blood-related abnormality has occurred in a 72-year-old man who has high red blood cell and platelet counts and splenomegaly (expanded spleen), but no major illnesses or family history of such. The choice of cells presented are all in the blood development pathway, as erythroid, hematopoietic, lymphatic, and megakaryocyte progenitors along with pluripotent stem cells, all play roles in the formation and maturation of different blood cells. PLANNING: I notice a few abnormal test results: a high hemoglobin level, high hematocrit, high white blood cell count, high platelet count, and the presence of large platelets. I need to question if these abnormalities imply a particular cell involved in blood cell formation is responsible for the abnormalities. I'll consider the possibilities presented in the question--an erythroid progenitor cell (responsible for red blood cell formation), a hematopoietic stem cell (which can give rise to all types of blood cells), a lymphatic progenitor (births cells of the immune system), a megakaryocyte progenitor (precursor to the cells that form platelets), or a pluripotent stem cell (can differentiate into any cell type). CLINICAL REASONING: Given the elevated hemoglobin and hematocrit, it could infer a problem with the erythroid progenitor, the cell responsible for red blood cell production. However, this would not explain the increase in platelets or leukocytes. A problem with the lymphatic progenitor would likely cause an abnormality in lymphocytes, but the percentage of lymphocytes in this patient's sample is within normal range. Megakaryocyte progenitors are responsible for creating platelets, and an error here could explain the increased and abnormally large platelets. Again, however, it doesn't account for the increased hemoglobin, hematocrit, or leukocytes. This leads me to believe that the defect must be in a cell that can give rise to all these cell types - either a hematopoietic stem cell or a pluripotent stem cell. CONCLUDING: Considering the abnormalities in various blood cell types, we must be looking at an issue arising early in the hematopoietic pathway. This rules out erythroid, lymphatic, and megakaryocyte progenitor cells, which differentiate later in the process. RESPONSE: Given this patient's signs, symptoms, and lab findings, the most likely primary hematologic defect occurred in (B) Hematopoietic stem cell, as it's responsible for the genesis of all blood cell types - erythrocytes, leukocytes, and platelets, all of which are elevated in the patient.

**AI_D:** (1) RESTATING THE QUESTION: The case presents a 72-year-old healthy man who exhibits abnormally high hemoglobin and hematocrit levels, increased leukocyte count, platelet count, leukocyte alkaline phosphatase, plethoric skin, and splenomegaly. His peripheral blood smear also reveals identifiable giant platelets. Based on these findings, the question is asking us to identify the cell where the fundamental hematologic defect likely lies. (2) PLANNING: The assumptions are that the abnormal findings in the patient's laboratory results and physical examination reflect a disorder within a specific cell line involved in blood cell production. We might question whether these abnormalities resulted from erythroid progenitor (responsible for red cell production), hematopoietic stem cell (source of all blood cells), lymphatic progenitor (initiate lymphocyte formation), megakaryocyte progenitor (mediate platelet formation), or a pluripotent stem cell (origin of all blood cells). Given the range of abnormalities in the patient — notably high hemoglobin and hematocrit, high leukocyte count and alkaline phosphatase, splenomegaly, and giant platelets, the clinical priorities might suggest a disorder originating in a progenitor cell that gives rise to multiple cell lines. (3) CLINICAL REASONING: The patient's clinical and laboratory findings are consistent with a myeloproliferative disease known as polycythemia vera (PV), which involves overproduction of red cells, white cells, and platelets, evident in the increased hemoglobin, hematocrit, leukocyte count, and platelet concentration. The increased erythrocytes lead to hyperviscosity manifesting as plethoric skin due to engorged vessels. Splenomegaly is a common physical finding due to extramedullary hematopoiesis. The presence of giant platelets is also a feature of PV and other myeloproliferative disorders. PV originates from a mutation within the hematopoietic stem cell that leads to uncontrolled multiplication of all myeloid lines (erythroid, granulocytic, and megakaryocytic). (4) CONCLUDING: Based on the wide gamut of abnormalities involving various cell lines, it is reasonable to infer that the hematologic defect in this patient most likely arose in a progenitor cell responsible for giving rise to multiple blood cell lines. (5) RESPONSE: Hence, the primary hematologic defect in this patient most likely occurred in the hematopoietic stem cell (Option B).

**AI_E:** RESTATING THE QUESTION: The question is asking us to identify in which progenitor cell line the principal hematological abnormality in this patient likely occurred. The terms relate to cell development: "Erythroid progenitors" give rise to red blood cells, "Hematopoietic stem cells" are multipotent stromal cells that give rise to all the types of blood cells, "Lymphatic progenitors" lead to lymphocyte development, "Megakaryocyte progenitors" ascend to platelets, and "Pluripotent stem cells" can give rise to any cell type in the body. PLANNING: Looking at the lab results, we can observe increased Hemoglobin and Hematocrit, increased platelet count, and splenomegaly with the presence of giant platelets in the blood smear. These findings might indicate a hematological disorder where an overproduction of certain blood cells (red blood cells, platelets) occurs. So, the assumption we should clinically prioritize is that the primary hematologic defect probably lies within a cell line that is responsible for the production of red blood cells and platelets. CLINICAL REASONING: The patient's plethoric skin, raised hemoglobin, hematocrit, leukocyte count, and platelet count along with increased leukocyte alkaline phosphatase are highly suggestive of a myeloproliferative disorder, more specifically Polycythemia Vera. In Polycythemia Vera, there is an uncontrolled production of erythroid cells, leukocytes, and platelets, which can explain nearly all the laboratory findings. The finding of giant platelets further supports the likelihood of increased platelet production. The progenitor cell for both the erythroid lineage (which would give rise to an excess of red blood cells) and the megakaryocyte lineage (which would give rise to the excess and giant platelets) is the same: the hematopoietic stem cell. CONCLUDING: Thus, the unchecked proliferation of blood cells in this patient is most likely due to a mutation or defect in the hematopoietic stem cell, leading to the overproduction of multiple cell lineages. RESPONSE: Therefore, the cell where the primary hematologic defect in this patient most likely occurred is the (B) Hematopoietic stem cell.

**AI_B:** 1) RESTATING THE QUESTION: The question revolves around a 72-year-old man who has arrived for a routine health check-up. He appears to be generally healthy with no past or family history of significant medical conditions, and he doesn't smoke. However, his vital signs and lab results suggest some potentially concerning findings including high hemoglobin and hematocrit, elevated leukocyte count, increased platelet count, and the presence of giant platelets in his blood smear. We are being asked to identify the primary hematologic defect in this patient by determining the specific cell in which the anomaly most likely occurred. 2) PLANNING: The first assumption could be that the issue emerged in the erythroid progenitor cells, given the elevated levels of hemoglobin and hematocrit. An alternative assumption could be that the problem initiated in the hematopoietic stem cells, which are the primary source of all blood cells, including erythrocytes, leukocytes, and platelets. Another could be that the lymphatic progenitors are faulty, as these cells give rise to different types of leukocytes. As well, a defect in the megakaryocyte progenitors which are responsible for the production of platelets could be a likelihood. Lastly, we could assume that the issue has taken place at the level of pluripotent stem cells, which are the forerunner of all other cells in the blood. 3) CLINICAL REASONING: Starting with the erythroid progenitors, while the patient does have high levels of hemoglobin and hematocrit, indicating excessive red blood cell production, this wouldn't explain the high white blood cell and platelet counts. Considering the leukocyte and platelet elevations, a defect in megakaryocyte or lymphatic progenitor wouldn't match the clinical picture, which shows aberrations in all blood components, not just one specific lineage. A defect at the level of pluripotent stem cells would affect all blood cell lines; however, this seems less likely due to the specific findings of elevated leukocyte alkaline phosphatase and presence of giant platelets. Hematopoietic stem cells are multipotent and will form all the blood cells, with the defect likely to present across the various cell lines, matching the patient's findings. 4) CONCLUDING: Analyzing the patient’s health condition, the elevation in the number of erythrocytes, leucocytes, and platelets, with an increase in leukocyte alkaline phosphatase and presence of giant platelets, leads to a strong suspicion towards a myeloproliferative disorder. Polycythemia vera, a type of myeloproliferative disorder, characterized by the overproduction of all three hematopoietic cell lines (RBCs, WBCs, Platelets), increased leukocyte alkaline phosphatase, and giant platelets is a common occurrence. This condition arises from a mutation in the hematopoietic stem cells. 5) RESPONSE: The primary hematologic defect in this patient most likely occurred in the 'Hematopoietic stem cells' (option B), which gave rise to the disease condition known as polycythemia vera.

**Facilitator** The consensus statement synthesizes the responses of AI_A, AI_B, AI_C, AI_D, and AI_E as follows: The question presents a 72-year-old man with no significant personal or family medical history, who exhibits high levels of hemoglobin and hematocrit, increased leukocyte count, platelet count, leukocyte alkaline phosphatase, plethoric skin, and splenomegaly. His peripheral blood smear also reveals occasional giant platelets. We are asked to identify the cell where the primary hematologic defect likely occurred. The abnormal findings in the patient's laboratory results and physical examination suggest a disorder within a specific cell line involved in blood cell production. The elevated levels of hemoglobin, hematocrit, and platelet count indicate overproduction of blood cells, suggesting a myeloproliferative disorder. The observation of giant platelets and increased leukocyte alkaline phosphatase point towards increased activity in the bone marrow. Given the range of abnormalities in the patient, the clinical priorities suggest a disorder originating in a progenitor cell that gives rise to multiple cell lines. The patient's clinical and laboratory findings are consistent with a myeloproliferative disease known as polycythemia vera (PV), which involves overproduction of red cells, white cells, and platelets. PV originates from a mutation within the hematopoietic stem cell that leads to uncontrolled multiplication of all myeloid lines (erythroid, granulocytic, and megakaryocytic). Therefore, based on the wide gamut of abnormalities involving various cell lines, it is reasonable to infer that the hematologic defect in this patient most likely arose in a progenitor cell responsible for giving rise to multiple blood cell lines. Hence, the primary hematologic defect in this patient most likely occurred in the hematopoietic stem cell (Option B).
