# Supplementary material for: Collaborative intelligence in AI: Evaluating the performance of a council of AIs on the USMLE
Source: PLOS Digit Health. 2025 Oct 9;4(10):e0000787. doi: 10.1371/journal.pdig.0000787 (PMC12510544; doi:10.1371/journal.pdig.0000787)
Supplement: S2 File — (ZIP) [file pdig.0000787.s002.zip › S2 Data Raw data files containing unprocessed question inputs and LLM outputs including transcripts of deliberations/STEP1_Question-33-1_Type-MC-NJ.docx]

**Facilitator** **[Round 1]:** A 21-year-old man comes to the emergency department in the winter because of headache, nausea, and dizziness for 4 hours. He has not had fever, chills, or shortness of breath. He has no history of major medical illness. He lives in a basement apartment of a three-story house. He smokes 1½ packs of cigarettes daily. Physical examination shows no abnormalities. It is most appropriate for the physician to ask the patient for more information about which of the following historical factors? (A) Occupational exposure (B) Substance use (C) Travel (D) Type of home heating (E) Use of over-the-counter medication

**AI_D:** RESTATING THE QUESTION: The query involves a 21-year-old male patient who has been feeling unwell with symptoms like headache, nausea, and dizziness. The symptoms have lasted for 4 hours, and his medical condition doesn't include fever, chills, or breathing difficulties. His medical record doesn't reveal any significant past diseases. He is a heavy smoker and resides in the basement of a large apartment building. His physical checkups don't hint at any abnormalities. The primary issue here is determining the most relevant factor in this patient's history that the doctor needs to further explore for making an appropriate medical judgement. PLANNING: The assumptions that can be made from this case include: 1) The patient's symptoms are related to his smoking habits. 2) The symptoms are caused by a problem linked to his environment or living conditions. 3) His symptoms are possibly due to a medical problem not yet diagnosed. 4) The symptoms are caused by substance use. After questioning each assumption, it could be inferred that the alternate assumption prioritized here should focus on exploring more about his local environment or living condition because of his symptoms and his living in a basement. CLINICAL REASONING: The patient's symptoms of headache, nausea, and dizziness, without fever, chills, or breathing problems bring a clinical thought process toward an environmental issue rather than an infectious disease, as there is no fever or chills. As he lives in a basement—an enclosed environment with potentially poor ventilation—the inquiry about the type of home heating used is critical, as it might be linked to carbon monoxide (CO) exposure. CO exposure would lead to hypoxia and symptoms such as headaches, dizziness, and nausea. The lack of abnormal findings in the physical exam reinforces this, as CO poisoning usually doesn't show any distinctive physical signs. Smoking can also increase CO in the blood, which may exacerbate the exposure from a faulty heating system. CONCLUDING: In this case, the symptoms of headache, nausea, and dizziness in a person residing in a basement without any significant medical history suggest an environmental factor is likely playing a role. Understanding the possible cause of the symptoms helps to focus the clinical inquiry. The fact that CO poisoning could potentially result from faulty home heating systems, especially in enclosed spaces like basements, makes investigating the type of heating used a clinical priority. RESPONSE: Therefore, (D) the type of home heating is the most appropriate historical factor for the physician to ask the patient for more information about.

**AI_A:** RESTATING THE QUESTION: In essence, we are being asked to recognize the most relevant historical factors that a physician should dig into when presented with a 21-year-old man who comes to the emergency room in the winter, complaining of a 4-hour duration of headache, nausea, and dizziness but no fever, chills, or shortness of breath. He has no prior significant medical conditions, lives in a basement apartment, smokes excessively, and doesn't show any abnormal physical signs. PLANNING: An underlying assumption is that the young man's symptoms could be caused by various factors including occupational exposure to harmful substances, substance misuse, recent local or international travel that may have exposed him to disease vectors, his home heating type possibly producing harmful fumes, or the use of over-the-counter medications that may have side effects. Now, we can generate an alternate assumption by considering his symptom pattern, personal habits, and living conditions. We can then hypothesize that a possible cause of the symptoms could be carbon monoxide poisoning resulting from improper home heating. CLINICAL REASONING: Clinically, carbon monoxide poisoning often presents like flu without the fever—headache, dizziness, nausea, and vomiting are all symptoms. His living conditions (a basement apartment) can be a place where carbon monoxide may accumulate especially in winter. Moreover, he smokes cigarettes daily—smoking is another source of carbon monoxide. Given these conditions, his symptoms may suggest carbon monoxide poisoning which is a serious condition that can be fatal. CONCLUDING: Assuming the patient's symptoms could be due to carbon monoxide exposure is not only based on his clinical presentation, but also accounts for his smoking habit and living conditions, especially the type of home heating used. RESPONSE: I conclude that it is most appropriate for the physician to inquire more about the type of home heating 'D'. This factor is crucial in this case given his living conditions, symptoms and habits. This can help establish whether the patient might be experiencing carbon monoxide poisoning, a potentially lethal condition that may often be overlooked due to its non-specific symptoms.

**AI_E:** To understand this scenario, the key focus is on the symptoms of a healthy 21-year-old man, who presents with onset of headache, nausea, and dizziness in the winter months. These symptoms may suggest various causes, including carbon monoxide poisoning, illicit drug use, or others. He is a heavy smoker and lives in a basement apartment. His physical examination is unremarkable. The possible areas for further questioning include: his workplace exposure, his substance use, any recent travel, the home heating system in use, and over-the-counter medication use. The assumptions made here are multiple. For one, it is assumed that his tobacco use is not connected to his current symptoms directly as he has no respiratory complaints. Second, as his physical examination is normal, it is assumed that the cause of his symptoms might be non-physical, making factors like recent travel, type of home heating, and use of over-the-counter medication or occupational exposure highly relevant. Uncertainty exists in each of these assumptions. For instance, might he have been exposed to certain adverse conditions at work, including the inhalation of toxic fumes or chemicals? Or could he have used certain substances recently that might lead to these symptoms? Although he doesn't have respiratory symptoms, could his tobacco use be contributing in ways not immediately obvious? Did he recently travel to areas where he might have caught an infection? After examination and hypothesis generation, it becomes clinically important to focus on the assumption about his living situation and its related home heating system. It supports the alternate assumption that the man might be suffering from carbon monoxide poisoning, a common cause of headache, nausea, and dizziness, particularly in colder seasons when the use of heating systems is more prevalent. Since carbon monoxide is a colorless, odorless gas produced from incomplete combustion of carbon-containing materials, it can build up in poorly ventilated spaces like a basement. Combining this with the man's heavy cigarette smoking, which also emits small amounts of carbon monoxide, it can increase his risk of exposure. His symptoms notably fit those of carbon monoxide poisoning, which are often nonspecific and can be mistaken for other conditions. Nonetheless, the absence of fever, chills, or shortness of breath, and no abnormal findings on physical examination, make other pathologies less likely. The clinical priority is to investigate the type of home heating system in use (an assumption D), as this could be shedding light on whether this is a case of carbon monoxide poisoning. Therefore, in response to the posed question, the physician should inquire about the type of home heating.

**AI_B:** RESTATING THE QUESTION: The question suggests a scenario where a young adult man has presented with complaints of a headache, nausea, and dizziness. Despite the absence of significant medical history or physical abnormalities, his symptomology and lifestyle factors, such as living in a basement apartment and heavy smoking, are considered important. The query asks which historical factor the doctor should inquire about for additional insight. PLANNING: Several assumptions can be inferred from the presented data. It could include exposure-related symptoms due to his work (A), poisoning or side effects due to substance abuse (B), acquired diseases from recent travels (C), health implications due to the type of heating system used in his basement home (D), or side effects from over-the-counter (OTC) medications (E). However, given the lack of fever, breath shortness, and unremarkable physical examination, inhalation of a noxious substance becomes more likely. Among the available options, it leads to the prioritization of the type of home heating system used in the patient's residence. CLINICAL REASONING: In winter, due to limited ventilation, individuals living in basement apartments or poorly ventilated spaces may be exposed to carbon monoxide (CO) from inefficient home heating systems. This odorless and colorless gas typically results from incomplete burning of organic substances, common in many home heating systems. The patient's symptoms such as headache, nausea, and dizziness are suggestive of CO poisoning. This presentation could be amplified by his heavy smoking habit which already predisposes to elevated carboxyhemoglobin levels, thus reducing available hemoglobin for oxygen transport. Carboxyhemoglobin formation occurs with increased CO exposure, which binds to hemoglobin with a higher affinity than oxygen, leading to decreasing oxygen supply to tissues. CONCLUDING: In light of the patient’s symptoms, which match those of CO poisoning, and his living circumstances, it is most important to ask him about the kind of home heating system used in his basement apartment. This could involve poor ventilation, which could lead to an increased concentration of CO. It will be very important to remove him from the potential CO source pending confirmation and treat as appropriate. RESPONSE: Considering the symptomology and the living conditions of the individual, the physician should primarily seek information about the type of home heating system (Answer D) the patient uses in his basement apartment.

**AI_C:** 1) RESTATING THE QUESTION: This scenario presents a 21-year-old male patient who is exhibiting symptoms of headache, nausea, and dizziness without any presentation of fever, chills, or shortness of breath. These symptoms have persisted for about four hours. Despite these symptoms, he appears to be in good physical health based on a physical examination. He has no significant medical history and resides in a basement apartment of a three-story house. Our patient is a heavy smoker with a daily consumption of 1½ packs of cigarettes. The main question here involves identifying the most relevant additional historical information that the physician would need to better understand the patient's current condition. The options given revolve around potential environmental exposures, behaviors, and factors. 2) PLANNING: Looking at the available options, each represents a potential cause for his symptoms, and it is essential to weigh each one carefully. We could make assumptions based on occupational exposure, use of substances, recent travel history, type of home heating system, and usage of over-the-counter (OTC) medication. The assumption here is that one of these factors plays a significant role in his symptoms. Given his symptoms and living conditions, the heating system of the house could potentially be implicated, as improper home heating, especially in a basement, can lead to carbon monoxide (CO) exposure. 3) CLINICAL REASONING: Carbon monoxide poisoning can be a prevalent issue during the winter, particularly in poorly ventilated spaces like basements. Individuals exposed to CO often present with nonspecific symptoms such as headache, nausea, and dizziness — symptoms that our patient is currently experiencing. Moreover, this patient’s smoking habit can also increase his CO levels. 4) CONCLUDING: By clinically reasoning, we deduce that the type of home heating, especially in a basement apartment, seems to be the most apparent factor that could be causing these symptoms. This hypothesis lays more emphasis on home heating conditions as a possible source of CO poisoning, which seems to match with the patient's symptoms and the season during which these symptoms emerged. 5) RESPONSE: Thus, I believe that it is most appropriate for the physician to ask the patient for more information about the 'type of home heating' (Option D) to investigate the potential cause of these symptoms further. This information may be crucial for an effective diagnosis and treatment plan.

**Facilitator** The consensus statement synthesizes the responses as follows: The scenario presents a 21-year-old man who has been experiencing headache, nausea, and dizziness for 4 hours during winter. He has no history of major medical illness, lives in a basement apartment, and is a heavy smoker. His physical examination shows no abnormalities. Given these circumstances, several assumptions can be made about potential causes of his symptoms, including occupational exposure, substance use, recent travel, the type of home heating system he uses, and the use of over-the-counter medication. However, considering his symptoms, living conditions, and personal habits, the most plausible cause of his symptoms could be carbon monoxide poisoning. This is due to the fact that carbon monoxide often accumulates in poorly ventilated spaces like basements, especially during winter when heating systems are in use. The symptoms of carbon monoxide poisoning, which include headache, nausea, and dizziness, align with the patient's complaints. Additionally, his heavy smoking habit could contribute to elevated carbon monoxide levels in his blood. Therefore, the physician should prioritize inquiring about the type of home heating system the patient uses (Option D). This information could be crucial in diagnosing potential carbon monoxide poisoning, a serious and potentially fatal condition that often presents with non-specific symptoms and can be overlooked.
