# Supplementary material for: Collaborative intelligence in AI: Evaluating the performance of a council of AIs on the USMLE
Source: PLOS Digit Health. 2025 Oct 9;4(10):e0000787. doi: 10.1371/journal.pdig.0000787 (PMC12510544; doi:10.1371/journal.pdig.0000787)
Supplement: S2 File — (ZIP) [file pdig.0000787.s002.zip › S2 Data Raw data files containing unprocessed question inputs and LLM outputs including transcripts of deliberations/STEP1_Question-35-1_Type-MC-NJ.docx]

**Facilitator** **[Round 1]:** A 25-year-old woman comes to the physician because of a long history of pain with menses. The pain occurs on the first day of her 5-day menstrual period and lasts all day. She rates the pain as 10 on a 10-point scale. The most appropriate initial pharmacotherapy to relieve this patient's pain has which of the following mechanisms of action? (A) Inhibition of estrogen synthesis (B) Inhibition of 11β-hydroxylase activity (C) Inhibition of prostaglandin synthesis (D) Stimulation of follicle-stimulating hormone synthesis (E) Stimulation of luteinizing hormone synthesis

**AI_C:** The question is asking about a young woman who has been enduring severe pain during her menses (defined as dysmenorrhea). The primary objective here is to decide the most suitable initial pharmacological therapy to ease her discomfort, specifically based on the mechanism of action the drug exhibits. The mechanisms in the options cover a broad spectrum concerning women's reproductive endocrinology, focusing on hormonal regulation, steroid metabolism, and prostaglandin synthesis. Pointing at the potential routes for the intervention. Assumptions here may be that every mechanism listed could potentially have an effect on the pain experienced during menses. However, it is essential to note the details given regarding the timing and severity of her pain. Menses-specific pain that is severe in nature, typically suggests a high probability of involvement of prostaglandins, pivotal biochemical compounds in the pain pathway. Prostaglandins are known in the physiology of menses to cause contraction of the smooth muscles of the uterus which can lead to pain. Increased levels of prostaglandins have been found in women with dysmenorrhea. Inhibition of prostaglandin synthesis will, therefore, lead to less uterine contractions, and subsequently, less menstrual pain. In comparison, while the hormones estrogen, follicle-stimulating hormone (FSH), and luteinizing hormone (LH) all play crucial roles in the menstrual cycle, there's no strong linkage suggesting their direct involvement in menses pain. Inhibiting 11β-hydroxylase activity primarily affects cortisol and aldosterone biosynthesis in the adrenal glands, which is not directly linked to menstrual pain either. Concluding, the clinical priority would be to address the most probable cause of the severe menstrual pain. Hence, looking through the lens of pathophysiology, primary dysmenorrhea is feasibly linked to excess prostaglandin secretion, causing uterine hypercontractility. Therefore, my final response is (C) Inhibition of prostaglandin synthesis should be the most suitable initial treatment approach for this patient's pain chronology because it directly addresses the likely underlying cause.

**AI_A:** 1) RESTATING THE QUESTION: The query pertains to a 25-year-old female patient who suffers from severe menstrual pain (dysmenorrhea), characteristically beginning on day one of her menstrual cycle and persisting until its fifth day, making her discomfort very high. Based on this, the question is inquiring about the most suitable initial pharmacology to relieve this severe pain with respect to its mechanism of action. 2) PLANNING: To address this concern, we have to appreciate the alterations in biochemical and physiological processes occurring during the menstrual cycle, especially those related to pain onset and magnification. The given options entertain concepts around hormonal regulation during the menstrual cycle such as estrogen synthesis, effects of 11β-hydroxylase, prostaglandin synthesis, FSH synthesis, and LH synthesis. By generating alternate assumptions around each of these possibilities, we can hypothesize that the most clinically relevant factor here is associated with the mechanism that directly influences pain progression and severity in the menstrual cycle. 3) CLINICAL REASONING: Let's explore these options based on clinical reasoning. (A) Inhibiting estrogen synthesis could affect the menstrual cycle, but estrogen doesn't directly contribute to the pain. (B) Inhibition of 11β-hydroxylase could affect cortisol and aldosterone production, which are not directly related to menstrual pain. (D) and (E) Stimulation of FSH or LH synthesis respectively might influence ovulation and menstrual cycle, but neither directly cause the pain. (C) Inhibition of prostaglandin synthesis, however, could directly lessen the pain, as prostaglandins are responsible for uterine contractions and mediating inflammatory response, connecting them to the pain during menses. 4) CONCLUDING: Evaluating our assumptions pointed us to focus on pain mediation and prostaglandins primarily stand responsible for the pain during menses. Other factors like hormonal regulation through FSH, LH, or estrogen would rather impact cycle regularity and reproductive aspects rather than directly causing pain during menses. 5) RESPONSE: Thus, in alignment with our clinical thought process, the most appropriate initial pharmacotherapy for a patient suffering from dysmenorrhea would focus on inhibiting prostaglandin synthesis (Option C) to potentially reduce the severity of pain.

**AI_E:** (1) RESTATING THE QUESTION: This query is about a 25-year-old woman experiencing dysmenorrhea, which is severe, painful cramps during menstruation. This pain is rated as severe (10 out of 10) and commences on the first day of her 5-day menstrual cycle, lasting all day. The question is asking for the most appropriate initial pharmacotherapy to alleviate her pain, and specifically, the mechanism of action of such a medication. (2) PLANNING: The primary assumption here is that the woman’s pain correlates with her menstrual cycle, hinting at a process mediated by the hormonal and chemical changes that occur during the menstrual cycle. The alternatives to this assumption can be various pharmacological action mechanisms such as inhibitive actions on estrogen synthesis, 11β-hydroxylase activity, and prostaglandin synthesis or stimulative actions on follicle-stimulating hormone (FSH) and luteinizing hormone (LH) syntheses. Evaluating these alternatives reveals that the inhibition of prostaglandin synthesis could be clinically prioritized since prostaglandins are strongly linked to pain and inflammation regulation. (3) CLINICAL REASONING: Since the pain occurs with menstruation, it's reasonable to presume it might be related to changes in hormone levels or chemical mediators that occur during this time. During menstruation, the endometrium breaks down and releases substances called prostaglandins, which cause inflammation and uterine contractions. Hence, higher levels of prostaglandins have been noticed in women with dysmenorrhea, which can lead to painful menstrual cramps. Medications that inhibit prostaglandin synthesis, such as nonsteroidal anti-inflammatory drugs (NSAIDs), can therefore be effective in treating dysmenorrhea. Other options like inhibiting estrogen synthesis, reducing 11β-hydroxylase activity, or promoting FSH or LH synthesis don't have a direct relationship to the pain experienced during menses. (4) CONCLUDING: Hence, addressing the symptomatology of severe menstrual pain in this context would logically involve diminishing the patient's prostaglandin production using pharmacotherapy, resulting in reduced inflammation and uterine contractions, thus alleviating the woman's menstrual pain. (5) RESPONSE: Therefore, given the clinical context of this patient's symptoms and the implications of underlying pathophysiology, an appropriate initial pharmacotherapy for this patient's severe dysmenorrhea would exhibit an "Inhibition of prostaglandin synthesis" (C).

**AI_B:** (1) RESTATING THE QUESTION: Our 25-year-old patient is experiencing painful menstruation, a condition commonly referred to as dysmenorrhea. This pain is severe enough to rate as 10 out of 10 on a pain scale. The purpose of this question is to identify what pharmacological intervention would be the most appropriate for managing this patient's symptoms by understanding the underlying mechanisms of menstrual pain. (2) PLANNING: The primary assumption is that the pain is due to hormonal fluctuations or imbalances related to menstrual cycle, which affect different substances like prostaglandins, estrogens, and other hormones like follicle-stimulating hormone (FSH) and luteinizing hormone (LH). Alternatively, pain could result from more direct enzymatic pathways, such as inhibition of the enzyme 11β-hydroxylase. The key is to identify which specific process exacerbates the patient’s dysmenorrhea. Given her symptoms, the generation of pain-inducing factors such as prostaglandins - natural substances in the endometrium of the uterus that contract the muscle and cause pain - may be at play. (3) CLINICAL REASONING: During menstruation, the endometrial cells produce prostaglandins, which provoke uterine contractions and can be responsible for the pain. If the production of prostaglandins is stemmed, the amount of uterine contractions may decrease, alleviating the menstrual pain. This reasoning suggests that an intervention targeting prostaglandin synthesis is more likely than the other options. (4) CONCLUDING: While modulation of estrogens, FSH and LH can influence the menstrual cycle, reducing prostaglandin synthesis would be more directly connected to relieving menstrual pain. Further, while 11β-hydroxylase is an important enzyme in steroid synthesis pathways, its inhibition has little relevance to menstrual pain and might lead to adrenal insufficiency as it is a key enzyme in cortisol synthesis. (5) RESPONSE: The most appropriate initial pharmacotherapy for this patient, considering her dysmenorrhea, likely involves inhibition of prostaglandin synthesis (Option C).

**AI_D:** (1) RESTATING THE QUESTION: A woman aged 25 consults a doctor as she has experienced pain during her menstrual cycle for an extended period of time. This pain typically commences on the first day of her 5-day menstrual cycle and is present throughout the day. She defines the severity of the pain as 10 on a scale of 1 to 10. We are asked to identify the mechanism of action for the most suited initial pharmacotherapy for relieving the patient's pain. (2) PLANNING: We can make the assumption that the patient's pain is associated to her menstrual cycle due to its timing and persistence. Therefore, the pharmacotherapy would likely target a hormone or compound that's elevated during the menstrual cycle and is understood to induce pain. To generate alternate assumptions, we must consider the potential hormones or compounds and their related pharmacotherapies. A) Estrogen synthesis inhibition: Estrogen helps in the proliferation of the endometrium during the menstrual cycle. However, it's not directly linked to the pain experienced during menses. B) 11β-hydroxylase inhibition: 11β-Hydroxylase is an enzyme involved in the cortisol synthesis, but is not directly associated with menstrual pain. C) Prostaglandin synthesis inhibition: Prostaglandins are known to induce pain and inflammation, and they can be overproduced during menstruation. D) FSH (Follicle-Stimulating Hormone) stimulation: While FSH plays a role in the menstrual cycle, it’s not directly tied to pain experienced during menses. E) LH (Luteinizing Hormone) stimulation: Although LH regulates the menstrual cycle, it doesn't directly cause menstrual pain. Given these alternatives, the best clinical assumption to prioritize for managing this patient's pain would be the prospect that the pain is due to an overproduction of prostaglandins during menstruation. (3) CLINICAL REASONING: Prostaglandins, which are lipids, are generated at the site of tissue damage or infection, serving as potent mediators of inflammation. These compounds are known to increase uterine contractions, constrict blood vessels in the uterus leading to ischemia, and stimulate pain fibers, causing the pain and cramps experienced during menstruation. Evidence suggests that a higher level of prostaglandins will lead to more severe menstrual cramps. This is why prostaglandin synthesis inhibitors, such as NSAIDs (Non-Steroidal Anti-Inflammatory Drugs), are often recommended for relief from dysmenorrhea. These drugs help by reducing the production of prostaglandins, lessening the contraction of the uterus and thus, the severity of pain. (4) CONCLUDING: Therefore, given that prostaglandins play a dominant role in the commencement and persistence of menstrual pain and cramps, the most appropriate pharmacotherapy for this 25-year-old woman suffering from severe menstrual pain would likely function by inhibiting prostaglandin synthesis. (5) RESPONSE: Thus, the most appropriate initial pharmacotherapy to relieve this patient's pain is (C) Inhibition of prostaglandin synthesis.

**Facilitator** The consensus statement synthesizing the responses of AI_A, AI_B, AI_C, AI_D, and AI_E is as follows: The question pertains to a 25-year-old female patient suffering from severe menstrual pain, or dysmenorrhea, which begins on the first day of her menstrual cycle and persists until its fifth day. The query seeks to identify the most suitable initial pharmacological intervention to alleviate this severe pain, focusing on the mechanism of action of the potential treatment. The primary assumption is that the patient's pain is associated with her menstrual cycle, suggesting a process mediated by hormonal and chemical changes that occur during this period. The alternatives to this assumption involve various pharmacological action mechanisms such as inhibiting estrogen synthesis, 11β-hydroxylase activity, and prostaglandin synthesis, or stimulating follicle-stimulating hormone (FSH) and luteinizing hormone (LH) syntheses. Upon evaluating these alternatives, it becomes clear that the inhibition of prostaglandin synthesis could be clinically prioritized. Prostaglandins are known to induce pain and inflammation, and they can be overproduced during menstruation, leading to painful uterine contractions. In contrast, other options like inhibiting estrogen synthesis, reducing 11β-hydroxylase activity, or promoting FSH or LH synthesis do not have a direct relationship to the pain experienced during menses. Therefore, given the clinical context of this patient's symptoms and the implications of underlying pathophysiology, the most appropriate initial pharmacotherapy for this patient's severe dysmenorrhea would likely function by inhibiting prostaglandin synthesis. This approach directly addresses the likely underlying cause of the pain, potentially reducing the severity of the patient's discomfort.
