# Supplementary material for: Collaborative intelligence in AI: Evaluating the performance of a council of AIs on the USMLE
Source: PLOS Digit Health. 2025 Oct 9;4(10):e0000787. doi: 10.1371/journal.pdig.0000787 (PMC12510544; doi:10.1371/journal.pdig.0000787)
Supplement: S2 File — (ZIP) [file pdig.0000787.s002.zip › S2 Data Raw data files containing unprocessed question inputs and LLM outputs including transcripts of deliberations/STEP1_Question-37-1_Type-MC-NJ.docx]

**Facilitator** **[Round 1]:** A 3800-g (8-lb 6-oz) newborn is delivered vaginally at 39 weeks' gestation after an uncomplicated pregnancy. Apgar scores are 9 and 9 at 1 and 5 minutes, respectively. The newborn is crying, has pink skin, and appears vigorous. Physical examination shows a vagina and also a structure that appears to be a penis, with the urethra located at the base near the opening of the vagina. Chromosomal analysis is ordered. Which of the following is the most likely cause of the intersex findings in this newborn if the karyotype is found to be 46,XX? (A) 17α-Hydroxyprogesterone deficiency (B) Increased concentration of müllerian-inhibiting substance (C) Maternal androgen exposure (D) Presence of the sex-determining region Y gene (E) 5α-Reductase deficiency

**AI_E:** RESTATING THE QUESTION: The inquiry pertains to a newborn of 39 weeks' gestation, in good health and with female sex organs and also a structure resembling a penis, with the urethra near the vaginal opening. The question wants to ascertain what could be the most probable cause behind these intersex findings, assuming chromosomal testing reveals a 46,XX karyotype. PLANNING: The key assumption would be that the newborn is exhibiting signs of intersex due to an interplay of genetic, hormonal, and environmental factors. While all the options listed influence sexual development, we need to consider which one is the most likely cause given the 46,XX karyotype, which is typically associated with a female birth. CLINICAL REASONING: It's important to note that the sexual differentiation process is complex, and multiple factors come into play. The 46,XX karyotype usually corresponds to a female phenotype. If there's a presence of a structure appearing like a penis in a 46,XX individual, it suggests exposure to androgens. Given the options, the source of these masculinizing hormones could either be due to maternal androgen exposure (C) or the presence of the sex-determining region Y (SRY) gene (D). The latter would generally result in the individual becoming a phenotypic male despite the 46,XX karyotype, but there's no phallic enlargement. In this case, it's more plausible that the exterior genitalia masculinization is due to increased exposure to androgens during the critical period of sexual differentiation, which likely occurred due to maternal sources. CONCLUDING: The clinical manifestation of an infant with a 46,XX karyotype and ambiguous genitalia suggests an abnormal exposure to androgens during fetal development. Given the options listed, it is more plausible that the intersex findings are due to maternal androgen exposure (C). RESPONSE: (C) Maternal androgen exposure.

**AI_A:** RESTATING THE QUESTION: The clinical scenario describes a newborn with physical characteristics of both male and female, specifically a vagina and a structure resembling a penis, a condition referred to as intersex. The question asks what could be the possible cause of these findings if the chromosomal analysis shows a karyotype of 46,XX, which is typically associated with female characteristics. The options given are conditions or substances related to hormonal and genetic factors that may affect sexual development. PLANNING: We can assume that an abnormality intrinsic to the child could be the cause, which might include conditions like 17α-Hydroxyprogesterone deficiency or 5α-Reductase deficiency, or presence of the sex-determining region Y gene in an XX karyotype. Alternatively, it could be extrinsic factors such as maternal androgen exposure or an increased concentration of a substance like müllerian-inhibiting substance. CLINICAL REASONING: Let's analyze each of these assumptions. - 17α-Hydroxyprogesterone deficiency is a condition that leads to congenital adrenal hyperplasia, where there is impaired cortisol production leading to excessive adrenal androgen production, which can cause virilization in XX individuals. However, this does not usually result in the formation of a structure resembling a penis. - Increased concentration of müllerian-inhibiting substance is more likely to lead to regression of female structures in XY males rather than male characteristics in XX females. - Maternal androgen exposure during pregnancy could potentially lead to virilization of an XX fetus as the excessive androgens could stimulate development of male characteristics. - Presence of the sex-determining region Y gene in an XX karyotype could result in male characteristics, but it would be rare for an XX individual to have this gene. - 5α-Reductase deficiency results in ambiguous genitalia in XY individuals due to decreased conversion of testosterone to dihydrotestosterone, but would not cause the observed phenotype in an XX individual. CONCLUDING: Given these considerations, the presence of a structure resembling a penis in an infant with a 46,XX karyotype is most likely due to maternal androgen exposure. RESPONSE: Hence, the most likely cause of the intersex findings in this newborn if the karyotype is found to be 46,XX is maternal androgen exposure (C).

**AI_B:** RESTATING THE QUESTION: The query is about a newborn with ambiguous genitalia: presence of both female (vagina) and male (structure similar to a penis) sex characteristics, despite being genomically female (46, XX karyotype). The question seeks the most likely cause of the intersex presentation. Several potential causes are given, including (A) 17α-Hydroxyprogesterone deficiency—a disorder of steroidogenesis, (B) Increased concentration of müllerian-inhibiting substance—implicated in the regression of female structures in XY individuals, (C) Maternal androgen exposure—maternal use of substances that mimic male hormones, (D) Presence of the sex-determining region Y (SRY) gene—normally responsible for initiating male sex determination, and (E) 5α-Reductase deficiency—disrupts conversion of testosterone to dihydrotestosterone. PLANNING: The presence of both male and female physical characteristics despite a 46, XX karyotype suggests that the development of the sex organs was influenced by additional factors. It could be due to hormonal influence at a critical fetal development stage or a genetic anomaly, where male determining genes might be present even without a Y chromosome. I'll need to evaluate each possibility and relate it to the pathophysiology of sexual differentiation. CLINICAL REASONING: In general, female development is the default developmental pathway in mammals. In fact, the 46, XX genetic makeup typically produces female characteristics, and any deviation from this requires the influence of male-determining factors. With this newborn, the presence of a structure resembling a penis indicates exposure to some form of androgenic influence during development. However, since the karyotype indicates chromosomal femaleness, the source of this Androgenic influence is presumably external. CONCLUDING: Examining the possible causes listed, "Presence of the sex-determining region Y gene" is not likely considering the 46, XX karyotype. "Increased concentration of müllerian-inhibiting substance", responsible for the development of male internal sex organs and regression of female internal sex organs, is less fitting since a vagina is present. "17α-Hydroxyprogesterone deficiency" and "5α-Reductase deficiency" can lead to ambiguous genitalia, but in males (46, XY). Given that "Maternal androgen exposure" can indeed lead to masculinization in genetic females (46, XX), it's the most plausible cause given the options provided. RESPONSE: Therefore, the most likely cause of the intersex findings in this newborn, assuming a 46,XX karyotype, would be (C) Maternal androgen exposure.

**AI_D:** 1) RESTATING THE QUESTION: The query is about a newborn with an ambiguous genitalia which appears to have both female (vagina) and male (structure resembling a penis) anatomical characteristics. This condition is considered intersex. Additionally, a chromosomal analysis is planned for this newborn. The question asks for the most probable reason behind such a presentation if the newborn is found to have 46,XX karyotype, which is typically associated with a female phenotype. The multiple choices include a variety of possibilities related to hormonal or genetic causes that could have potentially led to the intersex condition. 2) PLANNING: To answer this, let's evaluate the assumptions behind each choice. First, let's define the terms. 17α-Hydroxyprogesterone deficiency and 5α-Reductase deficiency are enzymatic deficiencies involved in steroidogenesis, but these typically affect individuals with XY karyotype, leading to forms of male pseudohermaphroditism. Müllerian-inhibiting substance (MIS), also known as anti-müllerian hormone (AMH), is released by Sertoli cells in males to inhibit the development of female internal genital structures. Presence of SRY gene (sex determining region Y) implies an XY karyotype, which could result in a male phenotype. Maternal androgen exposure implies that the fetus was exposed to high levels of androgens in utero. 3) CLINICAL REASONING: Given the assumption that the karyotype is 46,XX, the infant should phenotypically be female. However, the presence of a penis-like structure implies some factors influenced the phenotypic display. Enzymatic deficiencies could be ruled out due to the XX karyotype. Similarly, high concentration of MIS or presence of SRY gene would require an XY karyotype, which isn't provided in the scenario. The most logical clinical reasoning would point towards maternal androgen exposure, as excess androgens could masculinize the female external genitalia, leading to clitoromegaly that could resemble a penis, therefore causing the observed intersex condition. 4) CONCLUDING: Weighing all possibilities, considering the 46,XX karyotype and the intersex presentation, the most likely cause becomes maternal androgen exposure which could have resulted in masculinization of the female external genitalia and consequently, the current presentation. 5) RESPONSE: The most probable cause of the intersex findings in this newborn, considering the 46,XX karyotype, is (C) maternal androgen exposure.

**AI_C:** (1) RESTATING THE QUESTION: The query is about the most probable cause of a newborn presenting with both male and female genitalia (a condition known as intersex) given that the baby's chromosomal analysis reveals a 46,XX karyotype. Chromosomal analysis, also known as karyotyping, is a lab procedure that allows the visualization of an individual's chromosomes to check for numerical or structural abnormalities. '46,XX' signifies a typically 'female' chromosomal pattern. (2) PLANNING: There can be various reasons for the intersex condition. It could be due to a deficiency in 17α-Hydroxyprogesterone, an increased concentration of müllerian-inhibiting substance, exposure to androgens during gestation, presence of the sex-determining region Y gene, or a deficiency in 5α-Reductase. Now we need to evaluate each case. (3) CLINICAL REASONING: For a 46,XX female to present with masculinized genitals, it suggests exposure to androgenic substances in utero. Let's examine the options - (A) 17α-Hydroxyprogesterone deficiency might lead to congenital adrenal hyperplasia (CAH), but in utero, it would often result in virilization of a female fetus. However, unlike our case, the infant usually presents with salt-losing crisis. (B) Increased concentration of müllerian-inhibiting substance (MIS) wouldn't apply here because MIS prevents development of the female internal genitalia but has no impact on the external genitalia. (C) Maternal androgen exposure during pregnancy can cause virilized external genitalia in an XX fetus. The internal reproductive organs remain female as they are not affected by androgens. (D) Presence of the sex-determining region Y (SRY) gene could lead to a male phenotype, but the karyotype would be expected to show some form of Y chromosome material, which is not the case here. (E) 5α-Reductase deficiency is associated with undervirilized male infants, not female infants with virilization. (4) CONCLUDING: Thus, considering these possibilities and specifically the normal 46,XX karyotype, the most likely cause in this case is exposure to androgens during pregnancy. (5) RESPONSE: The most probable cause of the intersex findings in this newborn, given the karyotype is 46,XX, is (C) Maternal androgen exposure.

**Facilitator** The question pertains to a newborn with intersex characteristics, specifically the presence of both female (vagina) and male (structure resembling a penis) genitalia, despite having a 46,XX karyotype typically associated with female characteristics. The query seeks to identify the most probable cause of these findings. The options provided include conditions or substances related to hormonal and genetic factors that may affect sexual development, such as 17α-Hydroxyprogesterone deficiency, increased concentration of müllerian-inhibiting substance, maternal androgen exposure, presence of the sex-determining region Y gene, and 5α-Reductase deficiency. Upon analyzing each of these assumptions, it is clear that the presence of a structure resembling a penis in an infant with a 46,XX karyotype suggests exposure to androgenic substances during development. The source of this androgenic influence is presumably external, as the karyotype indicates chromosomal femaleness. 17α-Hydroxyprogesterone deficiency, increased concentration of müllerian-inhibiting substance, and 5α-Reductase deficiency are less likely to be the cause, as these conditions typically affect individuals with an XY karyotype or lead to different manifestations. The presence of the sex-determining region Y gene could result in male characteristics, but it would be rare for an XX individual to have this gene. Given these considerations, the most likely cause of the intersex findings in this newborn, assuming a 46,XX karyotype, is maternal androgen exposure. This is because maternal androgen exposure during pregnancy can lead to virilization of an XX fetus, as the excessive androgens could stimulate the development of male characteristics. Therefore, the consensus statement is that the most probable cause of the intersex findings in this newborn, given the karyotype is 46,XX, is maternal androgen exposure.
