# Supplementary material for: Collaborative intelligence in AI: Evaluating the performance of a council of AIs on the USMLE
Source: PLOS Digit Health. 2025 Oct 9;4(10):e0000787. doi: 10.1371/journal.pdig.0000787 (PMC12510544; doi:10.1371/journal.pdig.0000787)
Supplement: S2 File — (ZIP) [file pdig.0000787.s002.zip › S2 Data Raw data files containing unprocessed question inputs and LLM outputs including transcripts of deliberations/STEP1_Question-38-1_Type-MC-NJ.docx]

**Facilitator** **[Round 1]:** A 65-year-old man is admitted to the hospital because of a 12-hour history of chest pain. Physical examination shows no abnormalities. Serum studies show an increased troponin I concentration. The diagnosis of myocardial infarction is made. It is suspected that this patient's condition deteriorated as a result of ischemia-reperfusion injury. The increased serum troponin I concentration in this patient most likely occurred as a result of which of the following processes? (A) Increased enzyme secretion (B) Increased enzyme synthesis (C) Karyolysis (D) Lysis of the endoplasmic reticulum (E) Lysis of the plasma membrane (F) Mitochondrial swelling

**AI_C:** 1) RESTATING THE QUESTION: The query is about a 65-year-old man who is experiencing a myocardial infarction (more colloquially known as a heart attack). This heart attack happened due to an ischemia-reperfusion injury - a process that involves tissue damage caused when blood supply returns to tissue (reperfusion) after a period of ischemia or lack of oxygen. Troponin I is a heart-specific protein in muscle cells that is usually found in the blood only when there has been heart damage. Hence, the presence of increased troponin I in his blood signals heart muscle damage. The question is asking about the specific process that led to the release of this troponin I into the patient's bloodstream. 2) PLANNING: The assumptions made here include that the heart attack is due to ischemia-reperfusion, that there is an increase in troponin I in the blood because of some cardiac cellular process, and that this process somehow involves the release of troponin I into the blood. The alternate assumptions could be that each of the listed processes could be the cause of the increased troponin. 3) CLINICAL REASONING: Myocardial infarction occurs when blood flow to a part of the heart is blocked, often by a blood clot. This can injure the heart muscle, causing a part of it to die due to lack of oxygen, which eventually leads to necrosis. This necrosis affects the integrity of the cell membrane, causing it to rupture or lyse. The rupture of the plasma membrane allows leakage of cell contents, such as troponin I into the bloodstream. 4) CONCLUDING: Clinical reasoning, therefore, centers around the integrity of the heart cell membranes. Given the options, increased enzyme secretion or synthesis, while possible in cells, do not directly account for the release of troponin. Karyolysis, or nucleus disintegration, and mitochondrial swelling are end-stage cellular events post-death and may not directly leak troponin. Finally, lysis of the endoplasmic reticulum would certainly affect cell functioning but again does not explain troponin release. 5) RESPONSE: Therefore, in this clinical scenario, the increased serum Troponin I concentration in this patient most likely occurred as a result of lysis of the plasma membrane (E).

**AI_D:** (1) RESTATING THE QUESTION: What this question appears to be asking is about the reason behind the elevated levels of troponin I, a heart-specific protein, in the patient's serum following his myocardial infarction (heart attack). It suggests some possible processes that may be the result of myocardial infarction. An ischemia-reperfusion injury, which is suspected to have deteriorated the patient's condition, is damage caused when blood supply returns to the tissue after a period of ischemia or lack of oxygen. (2) PLANNING: The assumptions in this case are that the troponin I in the serum is a result of myocardial cell destruction due to the ischemia-reperfusion injury, and that this destruction can be attributed to one of several physiological processes. Given the options, the processes include increased enzyme secretion (A), increased enzyme synthesis (B), karyolysis (C), lysis of the endoplasmic reticulum (D), lysis of the plasma membrane (E) or mitochondrial swelling (F). The plausible alternatives are either processes related to the breakdown of the myocardial cells (C, D, E) or changes in the functioning of these cells (A, B, F). (3) CLINICAL REASONING: Troponin I is a protein, highly specific to cardiac muscle tissue. It's usually undetectable in the blood but its levels will increase significantly in the blood following a myocardial infarction due to damage and subsequent death of heart muscle cells. This happens due to reduced blood flow (ischemia), leading to a cascade of cellular events that culminate in cell death (necrosis). When the ischemic tissue is reperfused, re-oxygenation causes the production of reactive oxygen species which can cause further cellular damage. This process is known as ischemia-reperfusion injury. The final step leading to necrosis is the lysis or breakdown of the cellular membrane that releases the cell contents into the blood, including troponin I, a component of the contractile apparatus of the heart muscle cells. (4) CONCLUDING: The rising serum levels of troponin I could be attributed to the lysis of the plasma membrane of the cardiac muscle cells. This process releases the cell contents into the blood circulation including the intracellular protein, troponin I. (5) RESPONSE: Thus, the elevated serum concentration of troponin I in this patient most likely occurred as a result of lysis of the plasma membrane (E).

**AI_B:** RESTATING THE QUESTION: This task concerns understanding the pathological events leading to the rise in troponin I levels in the blood stream of a patient diagnosed with myocardial infarction due to suspected ischemia-reperfusion injury. Troponin I is a protein found within heart muscles and its concentration increases in the blood in response to heart injury. PLANNING: One assumption could be that increased enzyme secretion or synthesis leads to higher troponin I levels. Another alternate assumption could be that damage to cellular structures, such as karyolysis (destruction of the cell nucleus), lysis of the endoplasmic reticulum, lysis of the plasma membrane, or mitochondrial swelling occurs, leading to the release of troponin I. I would hypothesize that damage to the plasma membrane, a structure that contains troponin I, is the most likely cause of increased troponin I in this patient's serum. CLINICAL REASONING: In the event of a heart attack or ischemia, blood supply to part of the heart is cut off, causing oxygen deprivation and metabolic stress to the cardiac muscles. When reperfusion (resupplying of blood) occurs, there's a paradoxical exacerbation of injury or cell death, known as ischemia-reperfusion injury. Troponin I is released into the bloodstream, not due to active secretion or increased production, but as a result of cellular damage. Karyolysis and mitochondrial swelling, while part of cell death processes, wouldn't specifically lead to the release of troponin. Enzyme synthesis does not significantly change and enzyme does not significantly secretes out of cell in response to cell damage in heart attack. At the contrary, the plasma membrane, which keeps the troponin I contained within the cells, when lysed or damaged, allows for leakage of its contents, including troponin I, into the bloodstream. CONCLUDING: Therefore, in the context of myocardial infarction and ischemia-reperfusion injury, the most probable mechanism for the elevated troponin I levels in this patient's serum is the lysis or damage of the plasma membrane, causing the release of intracellular troponin I into the bloodstream. RESPONSE: Hence, I am inclining towards option (E) Lysis of the plasma membrane, as the most probable reason behind the raised serum troponin I concentration in this patient.

**AI_A:** (1) RESTATING THE QUESTION: So, we're trying to identify the underlying process that likely caused an elevation in serum troponin I in a 65-year-old male patient who suffered a myocardial infarction, thought to have been worsened by ischemia-reperfusion injury. Troponins are part of the contractile apparatus in cardiac cells, and Troponin I in the bloodstream is a well-established marker for myocardial injury as it's released into the blood following the death of heart muscle cells, or cardiomyocytes. (2) PLANNING: Looking at the options, each represents a different physiological or pathological process. Increased enzyme secretion (A) or synthesis (B) would mean that the body is producing and releasing more proteins, but troponin I isn't produced in response to injury but is released when myocardial cells are damaged. Karyolysis (C) is a form of cell death where the cell's nucleus dissolves, and while this can occur in myocardial infarction, it is a downstream event. Lysis of the endoplasmic reticulum (D) can occur during cell death, but it isn't directly connected to troponin I release. Mitochondrial swelling (F) is a sign of cell injury, but it isn't the process that releases troponin I. Among the options, lysis of the plasma membrane (E) seems the most clinical relevant. If the myocardial cells' plasma membranes were lysed or damaged, the contents of the cells, including troponin I, could be released into the blood. (3) CLINICAL REASONING: After myocardial infarction, there is loss of blood supply and subsequent oxygen deprivation (ischemia) in the affected area of the heart. This ischemia leads to tissue damage, cellular dysfunction, and ultimately cell death (necrosis). The cell membrane integrity gets disrupted during this necrosis and cell contents, including troponin I, are released into the systemic circulation where they can be detected in blood tests. The reperfusion injury mentioned just exacerbates this process, causing more cell death and subsequent troponin release. (4) CONCLUDING: Based on the clinical context and pathophysiology of myocardial infarction and ischemia-reperfusion injury, the most likely process responsible for the increase in serum troponin I concentration is the lysis of the plasma membrane of the myocardial cells, leading to the release of intracellular contents into the bloodstream. (5) RESPONSE: Therefore, the increased serum troponin I concentration in this patient most likely occurred as a result of (E) Lysis of the plasma membrane.

**AI_E:** Restating the question in my own terms and defining the technical terms, we are trying to figure out why this 65-year-old man, diagnosed with myocardial infarction, has a higher concentration of troponin I in his blood serum. Troponin I is a protein that’s a part of the cardiac muscle contractile apparatus and its increased level in serum is a sensitive and specific marker of myocardial injury. The suspected cause of the patient's condition deterioration is ischemia-reperfusion injury which is the tissue damage caused when blood supply returns to the tissue (reperfusion) after a period of ischemia or lack of oxygen. With different assumptions in front of us, we need to identify which one is most clinically relevant to the increased Troponin I presence in this patient. Those assumptions being (A) Increased enzyme secretion, (B) Increased enzyme synthesis, (C) Karyolysis - the dissolution of the nucleus of a cell undergoing necrosis or apoptosis, (D) Lysis of the endoplasmic reticulum - break down of the organelle involved in protein and lipid synthesis, (E) Lysis of the plasma membrane - breakdown of the cell’s outer covering, and (F) Mitochondrial swelling. Upon questioning, we can note that Troponin I is not an enzyme, hence scenarios (A) and (B) don’t hold. Karyolysis (C) indicates an overall pathologic cellular process and would not specifically result in troponin I release. Lysis of endoplasmic reticulum (D) might disrupt protein synthesis but doesn't specifically lead to Troponin I release. Mitochondrial Swelling (F) is a result of irreversible cellular injury, but it wouldn't directly lead to troponin I release. Evaluating the remaining assumption, Lysis of the plasma membrane (E) would allow the release of contents from the cell into the extracellular space. As Troponin I is located within cardiac muscle cells, it would make sense clinically that damage to the myocardium, specifically, the lysis or disintegration of the plasma membrane, would result in an increase of Troponin I in the bloodstream. In terms of clinical reasoning, ischemia-reperfusion injury results in pathologic alteration of the cellular membrane permeability, leading to lysis of the plasma membrane. When the plasma membrane undergoes lysis, the components within the cell, including Troponin I, escape into the bloodstream. Therefore, the ischemia-reperfusion injury suffered by this patient due to myocardial infarction has most likely deteriorated the plasma membrane of some cardiac cells, causing the release of Troponin I into the patient's bloodstream. In conclusion, the increased serum troponin I concentration in this patient most likely occurred as a result of lysis of the plasma membrane, a process which would have been accelerated by the ischemia-reperfusion injury that's suspected in this patient's case. Thus, the answer is (E) Lysis of the plasma membrane.

**Facilitator** The consensus statement synthesizing the responses of AI_A, AI_B, AI_C, AI_D, and AI_E is as follows: The question pertains to a 65-year-old male patient who has been diagnosed with a myocardial infarction, commonly known as a heart attack, and has an elevated concentration of troponin I in his serum. Troponin I is a protein specific to cardiac muscle tissue and its presence in the blood is indicative of heart muscle damage. The patient's condition is suspected to have deteriorated due to ischemia-reperfusion injury, a process where tissue damage occurs when blood supply returns to the tissue after a period of ischemia or lack of oxygen. The task is to identify the most likely process that led to the release of troponin I into the patient's bloodstream. The options provided include increased enzyme secretion, increased enzyme synthesis, karyolysis, lysis of the endoplasmic reticulum, lysis of the plasma membrane, and mitochondrial swelling. Upon clinical reasoning, it is established that troponin I is not an enzyme, thus ruling out increased enzyme secretion and synthesis. Karyolysis, lysis of the endoplasmic reticulum, and mitochondrial swelling, while part of cell death processes, do not directly lead to the release of troponin I. The most plausible process is the lysis of the plasma membrane. During a myocardial infarction, ischemia leads to tissue damage, cellular dysfunction, and ultimately cell death or necrosis. The integrity of the cell membrane is disrupted during this process, leading to the release of cell contents, including troponin I, into the systemic circulation. This process is exacerbated by ischemia-reperfusion injury, causing further cell death and subsequent troponin release. Therefore, the consensus is that the increased serum troponin I concentration in this patient most likely occurred as a result of the lysis of the plasma membrane of the myocardial cells, leading to the release of intracellular contents, including troponin I, into the bloodstream. Hence, the answer to the question is (E) Lysis of the plasma membrane.
