# Supplementary material for: Collaborative intelligence in AI: Evaluating the performance of a council of AIs on the USMLE
Source: PLOS Digit Health. 2025 Oct 9;4(10):e0000787. doi: 10.1371/journal.pdig.0000787 (PMC12510544; doi:10.1371/journal.pdig.0000787)
Supplement: S2 File — (ZIP) [file pdig.0000787.s002.zip › S2 Data Raw data files containing unprocessed question inputs and LLM outputs including transcripts of deliberations/STEP1_Question-39-1_Type-MC-NJ.docx]

**Facilitator** **[Round 1]:** A randomized clinical trial is conducted to compare wound healing and cosmetic differences between two surgical procedures for closing skin wounds following cesarean delivery. A total of 1000 women undergoing cesarean delivery during a 6-month period are enrolled in the study, which was 85% of the total number of patients undergoing the procedure. The results show a wound infection rate of 12 cases per 1000 women for Procedure A and 18 cases per 1000 women for Procedure B. Which of the following is the best estimate of the absolute risk reduction for wound infection following Procedure A compared with Procedure B? (A) (18/1000) – (12/1000) (B) 0.85 × (12/1000) (C) 0.85 × (18/1000) (D) [0.85 × (18/1000–12/1000)] (E) [(1.2/100) – (1.8/100)]/(1.8/100)

**AI_D:** 1) RESTATING THE QUESTION: The question seeks to determine the 'absolute risk reduction' of one surgical procedure, Procedure A, compared to Procedure B in terms of wound infection following cesarean delivery. 'Absolute risk reduction' refers to the difference in risk of a particular outcome (in this case, wound infection) between two groups. 2) PLANNING: We are looking at wound infection rates for Procedure A and B which are 12 cases per 1000 women and 18 cases per 1000 women respectively. The absolute risk reduction is calculated by subtracting the risk of the outcome in exposed group (Procedure B) from the risk in the unexposed group (Procedure A). Therefore, the assumption we are evaluating is that the risk difference can be calculated from raw values. 3) CLINICAL REASONING: The number of cases per 1000 women presents the risk of getting wound infection in each group. So, to calculate the absolute risk reduction, we need to subtract the number of infections that occur in the group of patients who were treated with Procedure A from the number of infections that occur in the group of patients who were treated with Procedure B. 4) CONCLUDING: Based on the clinical reasoning, we can conclude that the best estimate of the absolute risk reduction for wound infection following Procedure A compared with Procedure B can be calculated by subtracting the wound infection rate of Procedure A from that of Procedure B. 5) RESPONSE: So, the correct answer is (A) (18/1000) – (12/1000). This is the difference in observed risk of wound infection between the two procedures.

**AI_A:** Restating the question, you want to understand what the absolute risk reduction is between Procedure A and Procedure B. To putting into simpler terms, the Absolute Risk Reduction (ARR) is a measure used in epidemiology to compare the difference in risks between two groups. It's the absolute difference in rates of events happening (e.g. infection following a procedure) between a population treated one way (Procedure A) and a population treated another way (Procedure B). Identifying assumptions, the question assumes a difference in the risk of wound infection between the two procedures. An alternative assumption may be that the infection rates are a result of other factors beyond just the procedure type. Evaluating the given information, one should consider that for calculating the ARR, the infection rates per capita (per 1000 women in this case) are used irrespective of the total number of patients undergoing caesarean delivery during the study period. Using clinical reasoning, ARR is a simple arithmetic difference between the event rates in two groups. It's calculated by subtracting the rate of events in the control group (in this case, Procedure B) from the event rate in the treatment group (Procedure A). Concluding from the above, ARR for wound infection following Procedure A compared with Procedure B can be found by subtracting the infection rate of Procedure A from that of Procedure B. So, the answer would be: (18/1000) - (12/1000). Thus, choice (A) seems to be the most appropriate answer. RESPONSE: The absolute risk reduction for wound infection following Procedure A compared with Procedure B is best estimated by choice (A) (18/1000) – (12/1000). The ARR is the difference in the event rates between the two groups, which is calculated directly from the infection rates given in the problem, without considering the total number of caesarean procedures performed during the study period.

**AI_C:** The study aims to identify the surgical procedure with better wound healing and lesser post-operative complications, particularly wound infections, following cesarean delivery. The total number of women who participated in the said study was 1000. This figure represents 85% of all patients who underwent cesarean delivery procedures during the 6-month study period. The infection rates were reported as 12 cases per thousand for procedure A and 18 cases per 1000 for procedure B. The goal is to find out which procedure has a lower risk of infection, quantified as the absolute risk reduction (ARR). The ARR indicates the difference in the risk of developing infections between these two procedures. The assumption in this context is to compute the ARR by subtracting the risk of procedure A from the risk of procedure B. However, another crucial assumption is that the total number of women involved in the study (1000) is representative of each individual procedure's total population. Thus, the calculated risk rates per 1000 women for each procedure should properly represent the risk rates of the entire population of patients undergoing each procedure. Clinically, the ARR between these two procedures can be calculated directly from the given infection rates. Procedure A has 12 infections per 1000 women, and Procedure B has 18 infections per 1000 women. Therefore, the ARR (risk reduction resulting from Procedure A) can be estimated by subtracting the infection risk of Procedure A from that of Procedure B. In conclusion, the best way to estimate the absolute risk reduction for wound infection following Procedure A compared with Procedure B is (A) (18/1000) – (12/1000). The calculation represents the difference in the risk of developing infections between these two procedures per 1000 cases. Response: The best estimate of the absolute risk reduction for wound infection following Procedure A compared with Procedure B is (A) (18/1000) – (12/1000).

**AI_E:** (1) Restating the question: The question is asking us to find out how much less risk of wound infection is associated with surgical Procedure A compared to Procedure B. This comparison is known as the absolute risk reduction (ARR). ARR is a simple subtraction of the risk rates of the two procedures. (2) Planning: The primary assumption here is that the infection rate is a reliable measure of the risk for that particular surgical procedure. However, there could be alternate assumptions - perhaps there's a confounder or variable not accounted for in the study, such as the cure rate or recovery rate of the patients. But in the context of this clinical trial, we have to prioritize the assumption that the infection rate is directly proportional to the risk associated with the procedure. (3) Clinical reasoning: The absolute risk reduction is calculated by subtracting the risk of the outcome occurring in the intervention group (Procedure A) from the risk in the control group (Procedure B). Hence, the clinical thought process here involves understanding the measures of procedural risk (infections per 1000) and then applying the formula for ARR. (4) Concluding: If we follow the clinical reasoning, it's a matter of subtraction. The absolute risk reduction can be found by subtracting the infection rate of procedure A (12 cases per 1000 women) from that of procedure B (18 cases per 1000 women). (5) Response: Therefore, the best estimate of the absolute risk reduction for wound infection following Procedure A compared with Procedure B is (18/1000) – (12/1000), which corresponds to answer choice (A). That is, the ARR for Procedure A compared to Procedure B is 0.006, or 6 infections per 1000 women.

**AI_B:** RESTATING THE QUESTION: The question seeks to identify which method, out of the given choices, best estimates the absolute risk reduction between two surgical procedures named A and B. The absolute risk reduction indicates the difference in the probability of an adverse outcome and is crucial in clinical decision-making. In our question, the outcomes are wound infections after a cesarean delivery. PLANNING: The assumptions made in restating the question are that I understand the terms and concepts of "absolute risk reduction" and the provided rates of wound infection. Contrariwise, what if I misunderstood the concept of absolute risk reduction? Or perhaps I miscalculated the rates of wound infection? Evaluating these assumptions, it's critical to note that the absolute risk reduction is calculated as the absolute difference between the risk in the exposed group (Procedure A) and the risk in the control group (Procedure B). Clinically, we'd want to follow the method that gives the most accurate estimate of absolute risk reduction. CLINICAL REASONING: From the initial information, we know that the wound infection rate in Procedure A is 12 in 1000 women, and in Procedure B it's 18 in 1000 women. To find the absolute risk reduction, we need to subtract the risk of Procedure A from the risk of Procedure B. The percentage of total patients in the study (85%) does not factor in the calculation of absolute risk reduction. Thus, we must critically evaluate the options provided. CONCLUDING: Combining this logic with a step-by-step clinical reasoning approach, the best estimate of the absolute risk reduction can be calculated as (18/1000) – (12/1000). Neither the factor of the 85% total patient participation, nor any other calculation in the given choices, is relevant in this case to determine the absolute risk reduction. RESPONSE: Thus, the correct response to best estimate the absolute risk reduction in the case of surgical procedures A and B would be (A) (18/1000) – (12/1000).

**Facilitator** The question seeks to determine the absolute risk reduction (ARR) between two surgical procedures, Procedure A and Procedure B, in terms of wound infection following cesarean delivery. The ARR is a measure used to compare the difference in risks between two groups and is calculated by subtracting the risk of the outcome in the control group from the risk in the treatment group. In this case, the control group is Procedure B and the treatment group is Procedure A. The assumptions made in this context are that the infection rates are a reliable measure of the risk associated with each procedure and that the total number of women involved in the study (1000) is representative of each individual procedure's total population. However, it's important to note that the ARR is calculated directly from the given infection rates, without considering the total number of caesarean procedures performed during the study period or any other potential confounding variables. The wound infection rates for Procedure A and B are 12 cases per 1000 women and 18 cases per 1000 women respectively. Therefore, the ARR can be calculated by subtracting the infection rate of Procedure A from that of Procedure B, which results in (18/1000) – (12/1000). In conclusion, the best estimate of the absolute risk reduction for wound infection following Procedure A compared with Procedure B is (A) (18/1000) – (12/1000). This calculation represents the difference in the risk of developing infections between these two procedures per 1000 cases.
