# Supplementary material for: Collaborative intelligence in AI: Evaluating the performance of a council of AIs on the USMLE
Source: PLOS Digit Health. 2025 Oct 9;4(10):e0000787. doi: 10.1371/journal.pdig.0000787 (PMC12510544; doi:10.1371/journal.pdig.0000787)
Supplement: S2 File — (ZIP) [file pdig.0000787.s002.zip › S2 Data Raw data files containing unprocessed question inputs and LLM outputs including transcripts of deliberations/STEP1_Question-4-1_Type-MC-NJ.docx]

**Facilitator** **[Round 1]:** A 55-year-old woman comes to the clinic because of a 2-month history of increasingly severe vaginal pain and itching during sexual intercourse. She avoids intercourse with her husband because of the symptoms. She has been in a monogamous relationship with her husband for the past 25 years. She has type 2 diabetes mellitus. Her vital signs are within normal limits. Pelvic examination shows edematous and erythematous vaginal mucosa with white discharge. A photomicrograph of a vaginal smear is shown. Which of the following is the most likely causal infectious agent? (A) Candida albicans (B) Chlamydia trachomatis (C) Herpes simplex virus (D) Human papillomavirus (E) Trichomonas vaginalis

**AI_D:** 1) RESTATING THE QUESTION: We are presented with the case of a 55-year-old woman who has been experiencing worsening vaginal pain and itching during sexual intercourse for the past two months. She is avoiding sexual contact with her husband due to these symptoms, and she also has type 2 diabetes mellitus. Upon pelvic examination, her vaginal mucosa is found to be swollen (edematous), red (erythematous), and yielding a white discharge. A photomicrograph of a vaginal smear test has been taken. The question requires us to identify the most likely infectious agent responsible for her symptoms. 2) PLANNING: A few assumptions could be made from the case history. The first assumption is that this woman may be suffering from a sexually transmitted infection (STI). However, this seems unlikely given her strict monogamous relationship. Our second assumption could be that her symptoms, including the white discharge, are a result of a vaginal yeast infection, common among women who have diabetes. To decide on the most likely cause, we need to series the probable affecting infectious agents, namely Candida albicans, Chlamydia trachomatis, Herpes simplex virus, Human papillomavirus and Trichomonas vaginalis. 3) CLINICAL REASONING: Evaluating the mentioned infectious agents, Chlamydia trachomatis and Trichomonas vaginalis are typically associated with sexually transmitted infections, which is less likely in this woman's case due to her monogamous relationship for the last 25 years. Herpes simplex virus generally causes painful ulcers rather than a white discharge, making it a less likely candidate. Human papillomavirus often presents with genital warts and does not usually cause the described symptoms. On the other hand, Candida albicans, a type of yeast, can cause a white, cottage cheese-like discharge as well as swelling and redness of the vagina and vulva, symptoms which align closely with this woman’s situation. People with diabetes are more susceptible to yeast infections due to higher amounts of sugar in the mucus of the vagina, which can fuel the growth of yeast. 4) CONCLUDING: In light of these considerations, it seems clinically justified to conclude that this patient's symptoms are most likely due to a yeast infection caused by Candida albicans. 5) RESPONSE: Therefore, the most likely causal infectious agent in this case is Candida albicans (A).

**AI_C:** (1) RESTATING THE QUESTION: This 55-year-old woman is experiencing severe itching and pain in her vagina during intercourse. Her symptoms have been getting worse over the past two months, causing her to avoid sex. Her only known health condition currently is type 2 diabetes. Physical examination of the pelvic region revealed swelling and redness of the vaginal mucosa, accompanied by a white discharge. My task is to determine the most probable infectious agent causing her symptoms from the list provided which includes Candida albicans, Chlamydia trachomatis, Herpes simplex virus, Human papillomavirus, and Trichomonas vaginalis. (2) PLANNING: The clinical findings point towards some form of vaginal infection. Factors to consider include her age, the presence of type 2 diabetes, and her symptoms of pruritus, dyspareunia, and vaginal discharge. Each of these potential agents causes a specific set of symptoms and pathological changes that we can compare to those of the patient. While type 2 diabetes can lead to compromised immune function and increase the risk for infections, its exact role needs further exploration. The monogamous relationship suggests a lower probability for sexually transmitted infections, yet it does not entirely exclude them. (3) CLINICAL REASONING: The presence of an edematous and erythematous vaginal mucosa with a white discharge is highly suggestive of a yeast infection, most commonly caused by Candida albicans. This is often seen in immunocompromised individuals or people with diabetes who have higher levels of blood glucose that provide a conducive environment for Candida growth. Chlamydia trachomatis, while a prevalent sexually transmitted infection, often presents asymptomatically or with a mucopurulent discharge, thus making this less likely. The Herpes simplex virus typically presents with vesicular lesions and ulcers of the genital area accompanied by burning or tingling sensations, symptoms not present in this patient. Human papillomavirus is associated with skin lesions like warts and not typically associated with these symptoms. Trichomonas vaginalis typically has a diffuse, malodorous, greenish-yellow discharge rather than a white one. (4) CONCLUDING: Given the clinical presentation including vaginal pain and itching during sexual intercourse, edematous and erythematous vaginal mucosa, white discharge, and a patient history of type 2 diabetes, a yeast infection caused by Candida albicans appears to be the most likely culprit. The patient's monogamous relationship and the lack of other specific signs of sexually transmitted infections further support this assessment. (5) RESPONSE: The most likely causal infectious agent in this clinical scenario is (A) Candida albicans.

**AI_B:** RESTATING THE QUESTION: The question presents a clinical scenario of a 55-year-old woman who has been experiencing pain and itching during intercourse for the past two months. She has a long-term monogamous relationship with her husband and has a history of type 2 diabetes mellitus. On examination, there's redness and swelling of vaginal mucosa with a white discharge. A microscopic examination of a vaginal swab was done and the most probable causative microorganism is to be identified among the given options. PLANNING: Considering the symptoms, the established risk factors, the nature of the relationship (monogamous), and her vital signs, the diagnosis points towards a form of vaginal infection. The presence of an edematous and erythematous vaginal mucosa with a white discharge suggests an ongoing infection or inflammation, potentially due to a fungal, parasitic, or bacterial agent. Furthermore, her diabetes makes her more susceptible to infections. Each of the microorganisms given as an option needs to be evaluated properly. CLINICAL REASONING: (A) Candida albicans: This fungus often causes vaginal yeast infections, which can lead to itching, inflammation, and a white discharge. Women with diabetes are at a higher risk due to their immune system's compromised ability to fight yeast. (B) Chlamydia trachomatis: A bacterial infection usually transmitted through sex, unlikely as patient has been in a monogamous relationship. (C) Herpes simplex virus: This can cause genital herpes, but usually presents with vesicular lesions, which aren't mentioned in the patient's case. (D) Human papillomavirus: Can cause genital warts and cervical cancer, but not typically associated with the symptoms presented by the patient. (E) Trichomonas vaginalis: A parasitic infection generally contracted through sex, it is less likely considering the patient's monogamous relationship. CONCLUDING: Given the patient's symptoms, elevated risk for yeast infection due to her diabetes, and presentation, Candida albicans is the most likely infectious agent. RESPONSE: The most likely causal infectious agent in this case is (A) Candida albicans.

**AI_A:** (1) RESTATING THE QUESTION: The question is asking which infectious agent is most likely to be the cause of an elderly woman's vaginal discomfort, marked by pain, itching during sexual intercourse, and the presence of vaginal discharge. This woman has been monogamous for 25 years and also has type 2 diabetes mellitus. Upon examination, it is observed that her vaginal mucosa is inflamed and discharging a white substance. Information from her vaginal smear is being considered in the diagnosis. A brief explanation of each infectious agent: (A) Candida albicans: This fungus typically thrives in moist environments, like the mouth or vagina, and can cause yeast infections marked by a white discharge. (B) Chlamydia trachomatis: A bacteria that can cause a sexually transmitted disease, although in many cases symptoms are not visible. (C) Herpes simplex virus: A viral infection that can result in sores on the mouth or genitals. (D) Human papillomavirus (HPV): This includes a group of viruses that can cause warts or lead to cancer. (E) Trichomonas vaginalis: A parasitic protozoan that accounts for a sexually transmitted infection, generally marked by inflammation and a yellowish-green discharge. (2) PLANNING: Noting the patient's long-term monogamous relationship, the new symptoms are not likely due to a newly acquired sexually transmitted infection, including Chlamydia trachomatis, Herpes simplex virus, Human papillomavirus, or Trichomonas vaginalis. However, given her diabetes, her immune status might be diminished, potentially making her more susceptible to infection by Candida albicans, a common opportunistic pathogen. Assessment of the vaginal smear would be crucial to ascertain this. (3) CLINICAL REASONING: Considering the patient’s symptoms (vaginal pain and itching), the physical findings (erythematous and edematous mucosa along with white discharge), and her medical history (diabetes, and a long-term monogamous relationship), it seems that Candida albicans could be the more likely option. A basic understanding of these infections shows that Candida infections often result in white vaginal discharge. Chlamydia trachomatis, Herpes simplex virus, HPV, and Trichomonas vaginalis are less likely, considering her long term monogamous relationship and lack of high-risk sexually transmitted disease symptoms. (4) CONCLUDING: Given the combination of data including the patient's symptoms, medical history, and physical examination findings, the vaginal smear results should be interpreted in that context. Specifically, the white vaginal discharge and the inflammation of the mucosal lining of the vagina could be indicative of a Candida infection. (5) RESPONSE: According to the information provided and with a process of elimination based on the patient's history and symptoms, the most likely infectious agent causing this woman's discomfort and discharge would be Candida albicans.

**AI_E:** RESTATING THE QUESTION: This 55-year-old woman, who has type 2 diabetes mellitus, has presented with two months of worsening vaginal pain and itchiness most notably during intercourse, resulting in her avoiding such activities. Upon examination, her vagina revealed swollen, reddish mucosa accompanied by a white discharge. We are tasked with identifying the most likely infectious agent causing her symptoms from the given options that include Candida albicans, Chlamydia trachomatis, Herpes simplex virus, Human papillomavirus, and Trichomonas vaginalis. PLANNING: Cases like these require understanding the nature of the symptoms, the risk factors predisposing the patient to infection, and the physical findings on examination. Knowing this patient's monogamous relationship status and her diabetes can influence the cause of infection. Each infectious agent in the choices manifests differently. Candida albicans is a fungus causing vaginal yeast infection that presents with intense itching and white, cottage cheese-like discharge. Chlamydia trachomatis is a bacterial sexually transmitted infection with often times mild or absent symptoms. Herpes simplex virus causes clear vesicular eruptions that can be painful. Human papillomavirus often causes changes in the cervical epithelium, leading to warts, but can remain asymptomatic as well. Trichomonas vaginalis is a parasite causing sexually transmitted infection that presents with greenish-yellow, frothy discharge and itching. But given her clinical manifestations and associated factors, Candida albicans or Trichomonas vaginalis seem the most plausible. CLINICAL REASONING: Candida albicans and Trichomonas vaginalis could both cause itching and discomfort. However, the presence of an edematous and reddish vaginal mucosa along with a whitish discharge leans more towards a Candida albicans infection. Moreover, in a Trichomonas vaginalis infection, the discharge is often described as greenish-yellow and frothy, which doesn't match with our patient. Another crucial clue ties the patient’s diabetes to a possible Candida infection, as uncontrolled blood glucose levels which often occur in diabetes can provide an ideal environment for Candida to thrive. CONCLUDING: The patient’s symptoms, examination findings, and the consistency of those findings with the characteristics of a Candida infection, along with her diabetes, make Candida albicans the most likely infectious agent responsible. RESPONSE: So, the most likely causal infectious agent in this patient is likely to be (A) Candida albicans.

**Facilitator** The consensus statement synthesizes the responses of AI_A, AI_B, AI_C, AI_D, and AI_E as follows: The question presents a 55-year-old woman with a 2-month history of increasingly severe vaginal pain and itching during sexual intercourse, leading her to avoid such activities. She has been in a monogamous relationship for the past 25 years and has type 2 diabetes mellitus. Upon pelvic examination, her vaginal mucosa is found to be edematous and erythematous, accompanied by a white discharge. The task is to identify the most likely infectious agent causing her symptoms from the given options: Candida albicans, Chlamydia trachomatis, Herpes simplex virus, Human papillomavirus, and Trichomonas vaginalis. Given her symptoms, medical history, and physical examination findings, the most likely infectious agent causing this woman's discomfort and discharge would be Candida albicans. This fungus often causes vaginal yeast infections, which can lead to itching, inflammation, and a white discharge. Women with diabetes are at a higher risk due to their immune system's compromised ability to fight yeast. The other infectious agents, namely Chlamydia trachomatis, Herpes simplex virus, Human papillomavirus, and Trichomonas vaginalis, are less likely considering her long-term monogamous relationship and the absence of their typical symptoms. Therefore, the consensus among the AI responses is that the most likely causal infectious agent in this case is Candida albicans.
