# Supplementary material for: Collaborative intelligence in AI: Evaluating the performance of a council of AIs on the USMLE
Source: PLOS Digit Health. 2025 Oct 9;4(10):e0000787. doi: 10.1371/journal.pdig.0000787 (PMC12510544; doi:10.1371/journal.pdig.0000787)
Supplement: S2 File — (ZIP) [file pdig.0000787.s002.zip › S2 Data Raw data files containing unprocessed question inputs and LLM outputs including transcripts of deliberations/STEP1_Question-41-1_Type-MC-NJ.docx]

**Facilitator** **[Round 1]:** A 4-year-old girl with type 1 diabetes mellitus is brought to the emergency department by her father because of a 4- hour episode of restlessness, sweating, and confusion that occurred during the night. Yesterday, he allowed her to eat cupcakes and cotton candy at a county fair. At her bedtime that evening, he increased her dose of subcutaneous intermediate-acting and long-acting insulin. Her symptoms began 6 hours later, then resolved spontaneously. After being informed this morning of this nighttime episode, the mother insisted the father bring the patient to the hospital. On arrival, the patient is alert. Her vital signs are within normal limits. Examination shows no abnormalities. Her fingerstick blood glucose concentration is 72 mg/dL. Urinalysis is negative for glucose and ketones. Which of the following is the most likely explanation for this patient's nighttime symptoms? (A) Hyperglycemia caused by increased glucose consumption (B) Hyperglycemia caused by increased glycogen metabolism (C) Hyperglycemia caused by insufficient exogenous insulin (D) Hypoglycemia caused by excess exogenous insulin (E) Hypoglycemia caused by excessive renal glucose loss (F) Hypoglycemia caused by increased glucagon secretion (G) Nightmare disorder (H) Sleep terror disorder

**AI_C:** 1) RESTATING THE QUESTION: The question presents the case of a 4-year-old child with type 1 diabetes mellitus who came in with symptoms of restlessness, sweating, and confusion during the night which resolved spontaneously. The episode happened after eating sugary food and an increased dose of insulin. On examination, her physical findings are within normal limits, with a blood glucose level of 72 mg/dL and urinalysis negative for glucose and ketones. We're asked to identify the most likely explanation for this nocturnal episode. 2) PLANNING: Several possible explanations are given, focusing on different metabolic processes. The presence of hyperglycemia or hypoglycemia might have caused her symptoms due to various potential metabolic alterations such as increased glucose consumption, increased glycogen metabolism, insufficient or excess exogenous insulin, excessive renal glucose loss, or increased glucagon secretion. Other possible assumptions can be drawn towards nightmare disorder or sleep terror disorder. 3) CLINICAL REASONING: The patient's symptoms of restlessness, sweating, and confusion could be indicative of hypoglycemia, as these are common symptoms of low blood sugar, and her blood glucose level at the hospital supports this. Given her history, it's rational to assume that her hypoglycemia could have been caused by the increased dose of insulin administered by her father, especially considering that she had consumed a high amount of carbohydrates previously at the county fair which could magnify the action of insulin. Hyperglycemia options can be ruled out as her blood glucose level is within normal range (70-100mg/dL). Other hypoglycemia causes seem less likely because excessive renal glucose loss would likely have shown glucose in the urine, and increased glucagon secretion doesn't fit the pattern as glucagon's role is to increase blood glucose concentrations. As for the psychiatric disorders, although they can cause similar symptoms, the complete resolution of symptoms and the correlation with the increased insulin dosage makes this likelihood less probable. 4) CONCLUDING: Based on the available facts, the high sugar intake at the fair, the increased insulin dosage at bedtime, the timing of symptoms onset, the spontaneous resolution of symptoms, and the normal glucose levels in the morning, it's reasonable to infer that the child had a hypoglycemic episode likely due to excess exogenous insulin. 5) RESPONSE: Therefore, the most likely explanation for this patient's nighttime symptoms is (D) Hypoglycemia caused by excess exogenous insulin.

**AI_B:** (1) RESTATING THE QUESTION: The prompt presents a case of a 4-year-old girl with type 1 diabetes mellitus who exhibits restlessness, sweating, and confusion during nighttime. These symptoms started several hours after she consumed high-sugar food and after her father increased her subcutaneous insulin dose. The symptoms resolved on their own and the patient's blood glucose level is now 72 mg/dL, within the normal range. Upon hospital examination, tests found no excessive glucose or ketones in her urine. We are asked to identify the cause of the symptoms she experienced during the night. Type 1 diabetes mellitus is a condition where the pancreas produces little to no insulin, a hormone responsible for regulating the amount of glucose in the bloodstream. Hyperglycemia refers to an abnormally high blood glucose level, often resulting from increased glucose consumption, increased glycogen breakdown, or insufficient exogenous insulin. Hypoglycemia, on the other hand, is an abnormally low blood glucose level, often caused by excess exogenous insulin, excess renal glucose loss, or increased glucagon secretion, a hormone that typically raises blood glucose levels. Nightmare disorder and sleep terror disorder are psychiatric conditions, manifesting in frightening dreams and disruptive sleep behaviours, respectively. (2) PLANNING: Given the information provided, we should question the cause of her hypoglycemic symptoms. The starting point is her consumption of lots of sugar and the increase in her insulin dose. Both factors could have upset her glycemic balance. As a diabetic patient, the insulin increase may lead to hypoglycemia, embarked by excessive insulin administration. (3) CLINICAL REASONING: Considering her high-sugar diet during the day and the increased insulin dose, her blood glucose should have been maintained at an adequate level. However, the excess insulin administration might have moved more glucose than needed from her bloodstream into her cells, leading to a potential hypoglycemic status. This is evidenced by her early symptoms of restlessness, sweating, and confusion known as signs of a hypoglycemic episode. These symptoms may have appeared due to her brain cells' insufficient supply of glucose, substituting glucose deficiency. Her symptoms spontaneously resolving could result from her body's hormones, like cortisol, adrenaline, and glucagon, triggering glucose release from she liver to balance the glucose level, hence, the normal blood glucose concentration of 72 mg/dL at the hospital. (4) CONCLUDING: In this context, the high sugary foods she took should have raised her blood glucose levels, but the overreaction to counter the high-sugar food by increasing the insulin dosage might have led to an excessive removal of glucose from the blood into cells than was required. This, eventually, could have plunged the girl into a hypoglycemic episode resulting in her symptoms. (5) RESPONSE: Therefore, the most likely explanation for this patient's nighttime symptoms is (D) Hypoglycemia caused by excess exogenous insulin.

**AI_A:** (1) RESTATEMENT OF QUESTION: Let's break down the presented situation. This 4-year-old girl, diagnosed with type 1 diabetes (an autoimmune disorder where there is an absence of insulin due to immune-mediated destruction of beta cells from the pancreas), was brought to the emergency department due to an episode of restlessness, sweating, and confusion which began during the night and lasted for about 4 hours. These symptoms began a few hours after her father increased her dose of intermediate-acting and long-acting insulin, which are injected subcutaneously to manage her blood sugar levels. She also was allowed to consume sweets like cupcakes and cotton candy, which are high in glucose, earlier in the day. Upon hospital arrival, she was found to have a blood glucose concentration of 72 mg/dL, which is a normal level, and showed no abnormalities. Urinalysis also revealed no evidence of glucose or ketones, which are signs of poorly controlled diabetes. We are then asked to determine the most plausible cause of her symptoms from the provided list. (2) PLANNING: If we take into account the given symptoms and background information, we can see various possible assumptions about what might have caused them: increased glucose consumption, increased glycogen metabolism, insufficient exogenous insulin, excess exogenous insulin, excessive renal glucose loss, increased glucagon secretion, or mental conditions (nightmare or sleep terror disorders). We have to question each of these assumptions to generate an alternate hypothesis. (3) CLINICAL REASONING: Hyperglycemia, or high blood sugar, can be caused by increased glucose consumption, increased glycogen metabolism or insufficient exogenous insulin. However, the patient's blood glucose concentration is at a normal level, so we can eliminate A, B, and C. On the other hand, Hypoglycemia, or low blood sugar, can be induced by excess exogenous insulin, excessive renal glucose loss, or increased glucagon secretion. Given that the symptoms appeared after the father increased the girl's insulin dosage, and resolved on their own, it's reasonable to consider excess exogenous insulin (D) as the primary cause of these symptoms. Factors E and F can also be excluded as they're not suggested by any presented observations. Her normal vitals and no abnormalities on examination also make mental health issues, such as nightmares (G) or sleep terror disorders (H), unlikely. (4) CONCLUSION: Integrating all the information implies that the probable cause of this girl's nighttime symptoms is the father's decision to increase her intermediate-acting and long-acting insulin dose, after she consumed a large amount of glucose in the form of cupcakes and cotton candy. Therefore, the answer is (D) Hypoglycemia caused by excess exogenous insulin. (5) RESPONSE: Our explanation suggests that the symptoms the 4-year-old girl experienced during the night were likely due to hypoglycemia (low blood sugar) caused by an excess of exogenous insulin. This occurred as a result of her father increasing her usual dose of insulin without recognizing the time-activeness of her insulin regimen, and the potential risk for nocturnal hypoglycemia. The symptoms were self-resolving, as the hypoglycemic effects of the excessive insulin dose eventually wore off. Therefore, the most likely explanation for this patient's nighttime symptoms is (D) Hypoglycemia caused by excess exogenous insulin.

**AI_D:** 1) RESTATING THE QUESTION: We need to discern the most likely cause for an episode of restlessness, sweating, and confusion experienced at night by a 4-year-old with type 1 diabetes. The little girl had consumed sugary foods such as cupcakes and cotton candy the previous day and her father had increased her dosage of intermediate and long-acting insulin, an exogenous form of this hormone, which is not present in sufficient amounts in type 1 diabetes. Typically, insulin allows cells to absorb glucose from the bloodstream usable as an energy source. The episode started 6 hours post insulin dose increase, resolved on its own, only to be followed by normal alertness, vital signs, and physical exam results. Her current blood glucose is 72 mg/dL, which falls within a normal range. In addition, urinalysis did not reveal any presence of glucose or ketones, byproducts of fat metabolism often found when the body cannot process glucose properly. 2) PLANNING: The key assumptions here are that her nighttime symptoms are linked to either hyperglycemia or hypoglycemia. First, we need to consider the timing of her symptoms, occurring 6 hours after an increased insulin dosage taken after a day of higher than usual sugar consumption. Could these symptoms be a result of too much sugar, insufficient insulin to balance it, or too much insulin? Other options to consider lie outside a direct connection to diabetes, such as nightmare and sleep terror disorders. We also need to take into account her urinalysis results being, negative for glucose and ketones, as these could indicate hyperglycemia if present. 3) CLINICAL REASONING: Let's consider the symptoms of hyperglycemia versus hypoglycemia. Hyperglycemia generally takes a while to develop and is usually characterized by polyuria, polydipsia, weight loss, and the absence of spontaneous resolution. Given that our patient's symptoms resolved spontaneously and there were no other mentioned symptoms of hyperglycemia, it seems less likely that her symptoms were due to hyperglycemia. Therefore, we can likely rule out options (A), (B), and (C). On the other hand, hypoglycemia may occur within a relatively short time frame post-insulin administration and is characterized by symptoms related to neuroglycopenia (confusion, altered consciousness) and adrenergic overactivity (sweating, restlessness), which were seen in our patient. It can also resolve spontaneously through the action of counter-regulatory hormones like glucagon and epinephrine. Thus, it would seem her symptoms may be linked to hypoglycemic events. (E) and (F) are less likely since her urine glucose was negative (ruling out significant renal loss of glucose), and type 1 diabetes patients typically retain glucagon response to hypoglycemia, at least initially. Lastly, a sleep disorder, like those suggested in (G) and (H), seems unlikely given her diabetic status and their lack of correlation with her high sugar consumption or increased insulin dosage. 4) CONCLUDING: The confounding factors, clinical reasoning, and the fact that symptoms appeared after a change in insulin dose, lead us to believe that hypoglycemia may be the most plausible explanation. Restlessness, sweating, and confusion may well align with the consequences of a drop in blood sugar level—a topic especially relevant to a youngster with type 1 diabetes that had eaten a considerable amount of sugary food and subsequently had her insulin dosage upped. 5) RESPONSE: So, we can consider (D) Hypoglycemia caused by excess exogenous insulin as the most likely explanation for this patient's nighttime symptoms.

**AI_E:** (1) RESTATING THE QUESTION: In simpler terms, a 4-year-old girl with type 1 diabetes mellitus (an autoimmune condition where the body's immune system mistakenly destroys the insulin-producing beta cells in the pancreas leading to reduced insulin and increased blood sugar levels) experienced restlessness, sweating, and confusion during the night. These symptoms occurred after the girl consumed extra sugary foods and her night insulin dose was increased. The symptoms eventually resolved on their own. The next morning, she was brought to the hospital where her blood sugar level was found to be 72 mg/dL (normal is 70-140 mg/dL), and urinalysis showed no excess glucose or ketones. Note that hyperglycemia refers to high blood sugar, while hypoglycemia refers to low blood sugar. Glycogen metabolism refers to the process where the body's stored form of glucose is broken down or built up. Exogenous insulin refers to artificial insulin administered via subcutaneous injections. Glucagon is a hormone that increases blood glucose levels. Nightmare disorder and sleep terror disorder are sleep disorders characterized by frequent nightmares or episodes of intense fear during sleep. The question asks what likely caused the girl's nighttime symptoms. (2) PLANNING: The child's symptoms including sweating, restlessness, confusion are suggestive of a disturbance in glucose metabolism likely related to her diabetes management. We know she consumed a significant amount of sugar and her insulin dose was increased. It's also noteworthy that her symptoms resolved spontaneously. The fact that her blood glucose is normal upon presentation and her urinalysis is negative for glucose and ketones helps us rule out certain possibilities. For example, hyperglycemia (increased blood sugar) scenarios (options A, B, and C) seem less likely considering her blood glucose is within the normal range. The possibilities of hypoglycemia due to excessive renal glucose losses (option E, a condition occurring in conditions such as renal tubular disease which the girl does not have) and increased glucagon secretion (option F, a response to low blood glucose levels not caused by excess insulin) seem less likely since she has normal blood glucose level in the morning and her urinalysis did not show glucose. Sleep-related issues (options G and H) also become less likely as the symptoms resemble hypoglycemic episodes in a diabetic patient. Therefore, hypoglycemia caused by excess exogenous insulin (option D) seems like a possible cause we should consider further. (3) CLINICAL REASONING: The presentation of restlessness, confusion, and sweating are classic symptoms of hypoglycemia caused by an insulin overdose. When a patient with diabetes consumes sugary foods, this will naturally increase their blood glucose levels. However, if the patient then receives an increased dose of insulin (a hormone that lowers blood glucose levels) and does not have more glucose coming into the system, then the body's glucose levels can drop too much, resulting in hypoglycemia. Hypoglycemic episodes can be temporary if the body manages to release enough glucose (either from glycogen stores or from new glucose production) to counteract the excess insulin. These processes were likely at play in this patient, leading to the hypoglycemic symptoms at night which then resolved spontaneously once her blood glucose levels increased sufficiently again. (4) CONCLUDING: Connecting these clinical reasoning steps, it's probable that the combination of high sugar consumption, the increased insulin dose, and perhaps a delay in the action of insulins (with the long-acting insulin continuing its effects much longer than the sugar from the foods consumed) precipitated a hypoglycemic episode in the night, evidenced by the symptoms. By the morning, physiological compensatory mechanisms would have acted to normalize the blood sugar levels, hence the normal blood sugar level at presentation. (5) RESPONSE: Therefore, the most likely explanation for this patient's nighttime symptoms is hypoglycemia caused by excess exogenous insulin (option D).

**Facilitator** The consensus among the AI responses is that the most likely explanation for the 4-year-old girl's nighttime symptoms is hypoglycemia caused by excess exogenous insulin (option D). This conclusion is based on the following reasoning: 1) The girl's symptoms of restlessness, sweating, and confusion are indicative of hypoglycemia, a condition characterized by low blood sugar levels. These symptoms began a few hours after her father increased her dose of intermediate-acting and long-acting insulin, which are used to manage her blood sugar levels. 2) The girl's blood glucose concentration was found to be 72 mg/dL upon arrival at the hospital, which is within the normal range. This suggests that her symptoms were not caused by hyperglycemia, or high blood sugar, ruling out options A, B, and C. 3) The urinalysis revealed no evidence of glucose or ketones, which are signs of poorly controlled diabetes. This rules out options E and F, which suggest hypoglycemia caused by excessive renal glucose loss or increased glucagon secretion. 4) The girl's normal vitals and lack of abnormalities on examination make mental health issues, such as nightmares (option G) or sleep terror disorders (option H), unlikely. 5) The symptoms resolved spontaneously, which is consistent with the effects of an excessive insulin dose wearing off. Therefore, the consensus is that the girl's symptoms were likely caused by hypoglycemia due to an excess of exogenous insulin, which occurred as a result of her father increasing her usual dose of insulin without recognizing the time-activeness of her insulin regimen and the potential risk for nocturnal hypoglycemia.
