# Supplementary material for: Collaborative intelligence in AI: Evaluating the performance of a council of AIs on the USMLE
Source: PLOS Digit Health. 2025 Oct 9;4(10):e0000787. doi: 10.1371/journal.pdig.0000787 (PMC12510544; doi:10.1371/journal.pdig.0000787)
Supplement: S2 File — (ZIP) [file pdig.0000787.s002.zip › S2 Data Raw data files containing unprocessed question inputs and LLM outputs including transcripts of deliberations/STEP1_Question-43-1_Type-MC-NJ.docx]

**Facilitator** **[Round 1]:** A male newborn is delivered at term to a 28-year-old primigravid woman. Physical examination shows a broad, flat nose; low-set ears; a receding chin; prominent infraorbital folds; and bilateral abdominal masses. The newborn develops cyanosis, difficulty breathing, and polyuria and dies 24 hours after birth. At autopsy, examination shows pulmonary hypoplasia and enlarged, polycystic kidneys. Genetic testing shows two copies of a gene having a pathogenic mutation with a disease incidence rate in the general population of 1 in 10,000. Which of the following best describes the mutation occurrence rate for this newborn's disease? (A) 1/100 (B) 1/400 (C) 1/1000 (D) 1/4000 (E) 1/10,000

**AI_C:** RESTATING THE QUESTION: We are asked to determine the mutation occurrence rate, representing the probability of a mutation appearing in a specific site of the gene in this newborn's disease. The baby presented with distinct physical features suggestive of a genetic disorder, experienced symptoms such as breathing difficulty and cyanosis, and had autopsy findings of pulmonary hypoplasia and polycystic kidneys. Genetic testing revealed two copies of a mutated gene. In the general population, this disease has an incidence rate of 1 in 10,000. PLANNING: The underlying assumption is that the mutation occurrence rate would be the same as the disease incidence rate in the general population (1 in 10,000). Let's question this assumption by considering two aspects: 1) The disorder is likely autosomal recessive, given the child's diagnosis with two pathogenic gene copies, and the parents are likely carriers. 2) The probability of a child inheriting an autosomal recessive disorder from carrier parents is 1 in 4, or 1/400 when considering both alleles. CLINICAL REASONING: Genetic disorders with an autosomal recessive inheritance pattern, like the one describing this baby, necessitate that the individual inherits two copies of the mutated gene - one from each parent. Both parents would have been carriers of the mutation, meaning they each carry one mutant allele and one normal allele. If the parents are carriers, the probability distribution for their offspring's genotypes would follow a pattern of 1 normal (25%), 2 carriers (50%), and 1 affected (25%). Squaring the probability of a person in the general population being a carrier of the mutated allele (1/100) results in 1/10,000 – equivalent to the chances of two carrier parents producing an affected offspring (1/4000). CONCLUDING: Therefore, the mutation occurrence rate for this newborn's disease is associated with the probability of inherited pathogenic alleles from carrier parents, rather than directly equivalent to the disease incidence rate in the general population. RESPONSE: The mutation occurrence rate for this newborn's disease best corresponds to option (D) 1/4000.

**AI_B:** RESTATING THE QUESTION: A newborn male, who showed multiple physical abnormalities and health complications and died shortly after birth, was found to have pulmonary hypoplasia and polycystic kidneys at autopsy. Genetic testing provided evidence of a pathogenic mutation found in both copies of a certain gene. The occurrence of this specific disease in the general population is 1 in 10,000 individuals. The question is asking us to calculate the mutation occurrence rate (the rate at which the mutant gene occurs) in the general population for the disease the newborn had. PLANNING: The first assumption is that we need to rely on epidemiologic data about the disease, telling us it has an incidence rate of 1 in 10,000 in the population. Since the disease is genetic and present at birth, it indicates that the disorder is likely autosomal recessive. This is because recessive disorders often require a homozygous state or two copies of the mutant gene for the disease to manifest. This suggests that both parents were likely carriers of the allele. As genetical calculations suggest, the possibility of each parent transmitting the recessive gene to the child is 1 in 2 (or 0.5). Hence, the chance of this child inheriting both copies of the defective gene would be (0.5 from the mother) multiplied by (0.5 from the father), leading to a 1 in 4 chance or a frequency of 0.25. CLINICAL REASONING: To find the actual gene frequency in the population, we have to consider both the affected individuals and the carriers. Although the affected individuals (homozygous recessive) have a frequency of 1/10,000, the heterozygous carriers who do not express the disease are more common in the population. If q represents the frequency of the disease-causing allele, then the frequency of affected individuals is q^2. So if q^2 = 1/10,000, then q (the allele frequency in the population) is the square root of 1/10,000. CONCLUDING: By calculating the square root of 1/10,000, we get a frequency of 0.01 or 1/100 for the disease-causing allele in the population. This represents the possibility of an individual being a carrier of the gene, and hence, the mutation occurrence rate in the general population for this disease. RESPONSE: The mutation occurrence rate for this newborn's disease is (A) 1/100.

**AI_A:** RESTATING THE QUESTION: The question provides clinical features of a male newborn that point towards a genetic disorder. The infant showed signs such as a broad, flat nose, low-set ears, a receding chin, prominent infraorbital folds, and bilateral abdominal masses. He also developed cyanosis, difficulty breathing, and polyuria, and passed away 24 hours after birth. Post-mortem examination revealed pulmonary hypoplasia and enlarged, polycystic kidneys. Genetic testing confirmed the presence of two copies of a pathogenic gene mutation. The question asks what the mutation occurrence rate for the newborn's disease would be, given that the disease rate in general population is 1 in 10,000. PLANNING: The assumption here is that the disorder is genetic, and the presence of two copies of the gene implies it is likely an autosomal recessive disorder. In autosomal recessive conditions, both parents must be carriers of the disorder for the child to manifest the disease. If both parents are carriers, the likelihood of them having an affected child is 1 in 4 or 1/4. Another assumption is that the disease incidence rate in the population (1 in 10,000 or 0.01%) suggests the frequency of affected individuals, not the carrier frequency. The carrier frequency can be calculated using Hardy-Weinberg equilibrium, a principle that states that allele and genotype frequencies in a population will remain constant from generation to generation in the absence of other evolutionary influences. In this situation, carrier frequency (2pq) for a rare autosomal recessive disease can be approximated as √2q, with q representing the frequency of the disease in the population. CLINICAL REASONING: In this case, the disease frequency q is given as 1 in 10,000 or 0.0001. So, the carrier frequency 2pq can be approximated as √2q = √2*(0.0001) = 0.014 or roughly 1 in 100. This means that approximately 1 in 100 people in the population are carriers of this recessive genetic disorder. However, since we're looking for the occurrence rate for this newborn's disease, we should calculate the likelihood of both parents being carriers and then passing on the disorder, which would be (1/100)*(1/100)*(1/4) = 1/40000. CONCLUDING: By connecting the concepts of genetics, population genetics, and mathematical calculations it can be concluded that the mutation occurrence rate for this newborn's disease is 1 in 40000. RESPONSE: So, the best choice is (D) 1/4000.

**AI_D:** (1) RESTATING THE QUESTION: The question essentially wants to know the frequency of a particular disease-causing mutation in a newborn who presents with a unique set of symptoms and physical features. The newborn was found to have pathogenic mutation in both copies of a specific gene, and this disease condition is known to occur once in every 10,000 people in the general population. Now, we need to determine the mutation rate for the newborn's condition. Concepts to define here are mutation occurrence rate (frequency at which a specific mutation causing a disease occurs in the population) and disease incidence rate (the rate at which new cases of a disease occur in a population). (2) PLANNING: Assumptions that can be made include: the incidence rate of 1/10,000 directly correlates with the mutation occurrence rate. An alternate assumption could be that since this condition is a genetic disease and the newborn has mutation in both copies of the gene, the culprit disease here could be a recessive disorder. If so, then the mutation rate would not directly be the same as the disease incidence rate. (3) CLINICAL REASONING: If we look at the genetic basis of the disease, the baby having mutations in both copies of the gene indicates it is likely an autosomal recessive condition. For such conditions, the mutation occurrence rate would be higher than the disease incidence rate because carriers who have just one copy of the mutant allele would be unaffected but considered part of the mutation population. This reasoning makes our alternate assumption - that the mutation occurrence rate is not the same as the incidence rate of the disease - more probable. (4) CONCLUDING: To get the mutation occurrence rate, we need to consider the Hardy-Weinberg principle, which looks at allele frequencies in a population. According to this principle, if 'q' refers to the frequency of the mutant allele (recessive), then 2pq + q^2 = the population. Since this is a rare disease, q^2 (i.e. 1/10,000 = incidence rate of the disease which is homozygous recessive) gives 'q' as 1/100. This tells frequency of the mutant allele in the population. However, as we're considering an individual carrying one or two copies of the mutant allele- this becomes 2pq + q^2. Assuming p and q are almost equal (since it's a rare disorder), we get the frequency ≈ 2* 1/100 * 1/100 + (1/100)^2 = 1/5000. (5) RESPONSE: In this scenario, the existing options do not fall in line with our mathematical calculation of the mutation occurrence rate. Given this, the closest option that fits the estimated value would be (D) 1/4000. The assumption taken here is that the probability of a mutation may vary slightly from the theoretical calculation depending on the specific conditions of a population. The occurrence rate does not perfectly align with the expected result, probably due to genetic drift, selection, or population structures that can cause fluctuations in gene frequency.

**AI_E:** The question is asking us to name the mutation rate for a newborn who had a disease that is likely the result of a pathogenic genetic mutation. This baby showed signs typically associated with a genetic disorder, such as facial abnormalities and enlarged, polycystic kidneys. He also had difficulty breathing suggesting pulmonary issues and died 24 hours after birth. This disease is noted to occur in approximately 1 in 10,000 individuals in the general population. A pathogenic mutation means a mutation that has a harmful effect, and in this case, it leads to the disease. Before we can get to the answer, we need to establish a few assumptions. First, we need to understand that the likelihood of a mutation occurring is not the same as the occurrence rate of the disease caused by that mutation in the general population. The mutation rate depends on the nature of the mutation and how it's inherited. Second, we have to realize that the infant inherited two copies of a gene having a pathogenic mutation, which suggests a recessive inheritance pattern. Recessive inheritance means an individual must inherit two copies of the mutated gene, one from each parent, in order for the disease to manifest. Hence, we can hypothesize that the occurrence rate of the mutation in the population would be evaluated based on the Hardy-Weinberg equation, which describes the genetic variation in a population at equilibrium. Based on this principle, the frequency of people being carriers for recessive conditions (heterozygous) can be estimated by 2pq (where p and q are the frequencies of two alternative genetic alleles: "p" being the normal allele and "q" being the mutant allele), and the frequency of those homozygous recessive (affected) is calculated by q^2. Clinical reasoning leads us to use the Hardy-Weinberg equilibrium principle. If the general disease occurrence is 1/10,000, this equals to q^2. Therefore, we would take the square root of 1/10,000 to find 'q' which equals 1/100. This gives the frequency of the mutant allele in the population. Let's proceed to the conclusion using simple math. If 'p' (the normal allele frequency) is approximately 1 (since 'q' is much smaller), 2pq becomes 2 * 1 * 1/100 = 1/50, representing the frequency of the population being carriers (heterozygous). However, we're interested in the occurrence of homozygous mutation individuals. And since both parents would have needed to pass on the mutant gene, this would be calculated by squaring the carrier frequency (1/100)^2 = 1/10,000. Therefore, the mutation occurrence rate for this newborn's disease is E) 1/10,000.

**Facilitator** **[Round 2]:** Summary: AI_A believes that the mutation occurrence rate for the newborn's disease is 1 in 40,000. This is based on the assumption that the disorder is autosomal recessive and the carrier frequency can be calculated using the Hardy-Weinberg equilibrium. AI_B, on the other hand, calculates the mutation occurrence rate as 1 in 100. This is based on the assumption that the disease is autosomal recessive and the frequency of affected individuals and carriers should be considered to find the gene frequency in the population. AI_C suggests that the mutation occurrence rate is 1 in 4,000. This is based on the assumption that the mutation occurrence rate is associated with the probability of inherited pathogenic alleles from carrier parents, rather than directly equivalent to the disease incidence rate in the general population. AI_D calculates the mutation occurrence rate as 1 in 5,000, but suggests that the closest option is 1 in 4,000. This is based on the assumption that the mutation occurrence rate would be higher than the disease incidence rate because carriers who have just one copy of the mutant allele would be unaffected but considered part of the mutation population. AI_E concludes that the mutation occurrence rate for this newborn's disease is 1 in 10,000. This is based on the assumption that the likelihood of a mutation occurring is not the same as the occurrence rate of the disease caused by that mutation in the general population. Question to clarify disagreements: How are you defining the mutation occurrence rate and what assumptions are you making about the relationship between the mutation occurrence rate and the disease incidence rate in the general population?. Based on your preceding response, how would you answer the original question: [A male newborn is delivered at term to a 28-year-old primigravid woman. Physical examination shows a broad, flat nose; low-set ears; a receding chin; prominent infraorbital folds; and bilateral abdominal masses. The newborn develops cyanosis, difficulty breathing, and polyuria and dies 24 hours after birth. At autopsy, examination shows pulmonary hypoplasia and enlarged, polycystic kidneys. Genetic testing shows two copies of a gene having a pathogenic mutation with a disease incidence rate in the general population of 1 in 10,000. Which of the following best describes the mutation occurrence rate for this newborn's disease? (A) 1/100 (B) 1/400 (C) 1/1000 (D) 1/4000 (E) 1/10,000]

**AI_A:** 1) RESTATING THE QUESTION: The question asks to determine the mutation occurrence rate for a newborn's disease described as presenting with characteristic physiognomic features and enlarged, polycystic kidneys, leading to death within 24 hours. A pathogenic mutation was identified upon genetic testing, which possesses a disease incidence rate of 1 in 10,000 in the population. 2) PLANNING: The key assumption to work through here is that the disease is autosomal recessive since the rare disease is expressed in the child, and we estimate its incidence as 1 in 10,000. Using Hardy-Weinberg equilibrium can help to figure the occurrence (i.e., carrier frequency) of this mutation in population. 3) CLINICAL REASONING: The Hardy-Weinberg equilibrium can be mathematically expressed as p^2 + 2pq + q^2 = 1, where 'p' is the frequency of the normal allele in the population, 'q' is the frequency of the pathogenic/mutant allele, and 'q^2' is the frequency of the disease in the population. Given that the disease incidence is 1 in 10,000 (which corresponds to q^2), we can determine the value of q (i.e., the frequency of the pathogenic allele), and 2pq would provide us the frequency of individuals who are carriers of the pathogenic allele (but likely not expressing the disease due to its presumed autosomal recessive nature). 4) CONCLUDING: Using the above reasoning, if 'q^2' is 1/10,000, then 'q' would be square root of (1/10,000) which equals 1/100. But we are asked about '2pq'. Here, 'p' is almost 1, considering it's a rare disease. Therefore, the mutation occurrence (i.e., carrier frequency) can be approximated as twice 'q' i.e., 2 * 1/100 = 2/100 = 1/50. However, there's no provided option exactly equaling this value. The closest value is 1/100. 5) RESPONSE: By using clinical reasoning based on principles of genetic epidemiology and Hardy-Weinberg equilibrium, we can infer that the newborn's disease's mutation occurrence rate is likely closest to 1/100 (A).

**AI_B:** RESTATING THE QUESTION: The query inquires about the mutation rate for a severe neonatal disease. The newborn was born to a primigravid mother and possessed many physical anomalies; the infant subsequently developed critical symptoms and died shortly after. Autopsy revealed deficient lung development and enlarged, cystic kidneys. A pathogenic mutation found in the deceased infant occurs at a rate of 1 in 10,000 in the general population. The correct mutation rate for this disease needs to be identified. PLANNING: In this scenario, the assumption is that the disease is autosomal recessive, which would necessitate both parents being carriers of the faulty gene. This can be questioned, as the gene could be an autosomal dominant with de novo mutation, but the prevalence of such diseases generally is significantly lower than 1 in 10,000. Therefore, the most plausible clinical priority is to evaluate it as an autosomal recessive disease. The mutation occurrence rate can be calculated using the Hardy-Weinberg equation for autosomal recessive traits, which estimates the frequency of carriers in the population. CLINICAL REASONING: The Hardy-Weinberg Principle predicts the frequency of different genotypes in a sexually reproducing population, assuming no influence from genetic drift, migration, mutation, or selection. For autosomal recessive diseases, since carriers of the disease who are outwardly normal are included in the mutation occurrence rate, this frequency is expected to be higher than the disease incidence rate. Given that the carrier rate is usually around twice the square root of the incidence rate, and the disease incidence in this situation is 1 in 10,000, the approximate carrier rate would be 1 in the square root of 10,000 (which is roughly equal to 100). As such, the overall occurrence rate for the disease in question would involve individuals who are affected and those who are carriers. CONCLUDING: Given the autosomal recessive assumption paired with the incidences observed, the mutation occurrence rate would encompass a higher number of people in the population than the disease incidence does, due to carriers being included in the frequency count. This is the clinical reasoning behind why the occurrence rate would likely be higher than simply the reported disease incidence. RESPONSE: Thus, we can reasonably estimate that the mutation occurrence rate for this newborn's disease is likely to be closest to answer (A) 1/100.

**AI_C:** As there appears to be a lack of consensus among our group, it would prove fruitful to reassess the problem more deeply. The situation at hand involves a neonate with signs of a genetic pathology, who unfortunately passed away shortly after birth. Postmortem examination revealed disease characteristics such as bilateral polycystic kidneys and pulmonary hypoplasia. Genetic testing further confirmed that the baby carried two copies of pathogenic mutation, giving rise to these symptoms. The critical element here is that this particular mutation is known to have a disease incidence rate of 1 in 10,000 in the general population. The question then asks us to deduce the mutation occurrence rate for the newborn's disease. General assumptions could be initially made here, such as living organisms have a genetic predisposition to a wide variety of diseases, the occurrence of which is often dependent upon a multitude of factors, including inheritance patterns and environment. However, in this case, the baby's genetic testing revealed the pathognomonic mutation for the disease implying an autosomal recessive inheritance pattern. This leads us to assume that both parents were carriers of the mutated gene without displaying signs of the disease since autosomal recessive disorders only occur when both copies of the gene are defective. Given these assumptions, we can revise our original hypotheses by focusing on the mutation occurrence rate in autosomal recessive conditions. In autosomal recessive inheritance, the frequency of affected individuals (q^2) is given by the square root of the mutant allele frequency (q) in the population. When it comes to clinical reasoning, we must bear in mind that the frequency of disease occurrence is not equivalent to the mutation occurrence rate. By carefully considering the autosomal recessive inheritance pattern, in which both parents were likely carriers, and the Hardy-Weinberg equilibrium principle, we would calculate the mutant allele frequency by taking the square root of 1/10,000 which gives us 1/100. The mutation occurrence rate, therefore, would be 2pq (2*1/100*99/100) or approximately 1/50. Given the provided options, the best fit to this result appears to be (A) 1/100. Although it's not exactly equivalent to our calculation, 1/100 is the closest available selection. In summary, defining the question and employing the relevant medical and scientific principles, the best estimate of the mutation occurrence rate, given the options provided, would be (A) 1/100.

**AI_E:** Restating the question: The inquiry is directed towards the understanding of the newborn's disease mutation occurrence rate, which is a measure of the frequency of the pathogenic mutation in the population. The newborn presented with specific physical traits and complications before passing away 24 hours post-delivery, and upon autopsy had established conditions like pulmonary hypoplasia and polycystic kidney disease. A pathogenic mutation in both gene copies was identified, and its incidence of disease manifestation in the general population was revealed to be 1 in 10,000. The challenge is to select the correct option which most accurately reflects the disease’s mutation occurrence rate. Planning: The assumptions here encompass the possibility of the disease being autosomal recessive, as it needs two copies of the mutated gene for the disease to manifest. However, we must question this assumption, is the mutation rate supposed to be equivalent to the disease incidence rate? If not, should we consider factors like the carrier frequency? Unlike a disease incidence rate that accounts for new cases in a specific population within a specified period, a mutation rate refers to the likelihood of a given mutation occurring. Clinical Reasoning: In the light of these assumptions, among which the autosomal recessive nature of the disease seems very likely, we need to assess the relation between a genetic mutation and its resultant disease occurrence. The newborn's condition with that mutation might be considerably rare (1/10,000), but the actual genetic mutation could be relatively more common, as carriers of a single copy of the mutation would be phenotypically normal but could still pass on the mutation to their offspring. Concluding: Eventually, the mutation occurrence rate might not directly mirror the disease incidence rate due to the potential prevalence of heterozygous carriers. Consequently, the frequency of the disease cannot be extrapolated directly to the mutation occurrence rate. Response: Presuming the disease is an autosomal recessive disorder and taking into account the Hardy-Weinberg principle, we can calculate the frequency of carriers: assuming q^2 is 1/10,000 (q being the allele frequency for this particular mutation and q^2 the frequency of individuals affected in the population), then 2pq will give us the carrier frequency, with p being the frequency of the normal allele and p + q = 1. Thus, we can figure out p to be approximately 0.05 and q as roughly 0.995. Therefore, using 2pq, we can deduce the carrier frequency to be close to 1 in 100. However, the answer choices seem to require the recurrence risk, for which siblings of affected individuals would carry a 1/4 or 25% chance of being affected, so from the options provided, the closest option would be (C) 1/1000. We should also note that the exact numbers might differ slightly due to approximation in calculations.

**AI_D:** RESTATING THE QUESTION: The query is regarding the occurrence rate of a pathogenic mutation in a newborn who manifested cyanosis, breathing difficulties and polycystic kidneys, and subsequently died. Postmortem examination confirmed pulmonary hypoplasia alongside the polycystic kidneys. Genetic studies indicated the presence of two copies of a mutant gene known to cause a disease occurring in 1 in 10,000 people in the general population. The aim is to infer the overall mutation occurrence in such a scenario. PLANNING: Here, we see that the disease's incidence in the general population is 1 in 10,000, and many might deduce that the mutation occurrence rate mirrors this figure. However, the occurrence rate of a pathogenic mutation does not necessarily equate one-to-one with the disease incidence rate. A pertinent aspect to consider is the disease's inheritance pattern, not explicit in the information provided but vital to ascertain the genetic mutation frequency. The disease traits, characterized by congenital anomalies and early neonatal demise, suggest an autosomal recessive disorder. In autosomal recessive conditions, carriers, being heterozygotes, will not develop the disease but can transmit the mutated gene. CLINICAL REASONING: Given these considerations, let's employ the Hardy-Weinberg equilibrium principles (p2+2pq+q2=1) to provide a quantifiable estimation. In this formula, p represents the dominant allele frequency, q the recessive allele frequency, p2 the homozygous dominant frequency, 2pq the heterozygous frequency (carriers in our case), and q2 the homozygous recessive which, in our case, represents the disease incidence 1/10,000 (1/100^2). From this, we can derive the recessive allele (q) frequency as 1/100 and the carrier frequency (2pq) as approximately 1/50. CONCLUDING: In conclusion, careful interpretation of all provided data enables us to gauge the mutation occurrence rate more effectively. Given the autosomal recessive nature of our disease, the occurrence rate of the mutation in the gene is related to the frequency of carriers and individuals with the disease. The collective occurrence rate of the pathogenic mutation (people with the mutant gene) would be the sum of homozygous recessive individuals and heterozygous carriers. As the carrier frequency is substantially higher than the frequency of affected individuals, the overall mutation rate will be higher than the disease incidence rate. RESPONSE: In the provided options, no exact figure accurately represents our calculated carrier frequency. But, the closest to our derived value is 1/100 (Option A). Hence, I would select option A i.e., 1/100 as a response to the question - "Which of the following best describes the mutation occurrence rate for this newborn's disease?".

**Facilitator** **[Round 3]:** Summary: AI_A, AI_B, AI_C, and AI_D all agree that the disease is likely autosomal recessive and use the Hardy-Weinberg equilibrium to calculate the mutation occurrence rate. They all conclude that the mutation occurrence rate is approximately 1/50, but given the options provided, they all choose 1/100 as the closest answer. AI_E, however, while also assuming the disease is autosomal recessive and using the Hardy-Weinberg equilibrium, concludes that the mutation occurrence rate is 1/100 and chooses 1/1000 as the closest answer, considering the recurrence risk for siblings of affected individuals. Key Differences: The key difference lies in the interpretation of the mutation occurrence rate. AI_A, AI_B, AI_C, and AI_D interpret it as the frequency of individuals who carry the pathogenic allele (including both affected individuals and carriers), while AI_E interprets it as the recurrence risk for siblings of affected individuals. Question to Clarify Disagreements: How should we interpret the mutation occurrence rate in this context? Should it be considered as the frequency of individuals who carry the pathogenic allele (including both affected individuals and carriers), or should it be considered as the recurrence risk for siblings of affected individuals?. Based on your preceding response, how would you answer the original question: [A male newborn is delivered at term to a 28-year-old primigravid woman. Physical examination shows a broad, flat nose; low-set ears; a receding chin; prominent infraorbital folds; and bilateral abdominal masses. The newborn develops cyanosis, difficulty breathing, and polyuria and dies 24 hours after birth. At autopsy, examination shows pulmonary hypoplasia and enlarged, polycystic kidneys. Genetic testing shows two copies of a gene having a pathogenic mutation with a disease incidence rate in the general population of 1 in 10,000. Which of the following best describes the mutation occurrence rate for this newborn's disease? (A) 1/100 (B) 1/400 (C) 1/1000 (D) 1/4000 (E) 1/10,000]

**AI_D:** In phraseological terms, the question seems to be asking about the "mutation occurrence rate," or the frequency at which the pathogenic mutation arises within this newborn's population. This rate can differ substantially from the disease incidence rate depending on the genetic model of the disease, namely whether it is dominant or recessive. In this case, the newborn's clinical features such as pulmonary hypoplasia, polycystic kidneys, and early death point to an autosomal recessive disorder. The disease incidence rate stated in the question is 1 in 10,000. In the case of autosomal recessive disorders, the allele frequency can be calculated by taking the square root of the disease incidence rate because individuals must inherit two copies of the pathogenic allele to manifest the disease. The square root of 1/10,000, or 0.0001, gives an allele frequency, p, of 0.01 or 1 in 100. Once we have the allele frequency (p), mutation occurrence rate can be calculated based on Hardy-Weinberg equilibrium by determining the frequency of heterozygous carriers (2pq). This requires also determining q, the frequency of the normal allele, which is 1-p. Given that p for the pathogenic allele is 0.01, q for the normal allele is 0.99. Thus, 2pq gives an estimated carrier rate of about 1/50. While this precise value is not provided as one of the multiple choice options, the closest approximation offered is 1/100. Therefore, I would select (A) 1/100 as the most suitable answer to this question, reflecting the approximation of the mutation occurrence rate, or otherwise conceptualized as the carrier rate, in the general population for this disease.

**AI_A:** The question asked is about the mutation occurrence rate for a newborn's disease, whose manifestation is characterized by pathologies including craniofacial abnormalities, respiratory distress, and polycystic kidneys. We can define the mutation occurrence rate as the frequency at which a specific mutation appears in a population. Our first assumption is that the newborn's disease is genetic in nature, given genetic testing identified a pathogenic mutation. An alternate assumption could be that it's a multi-factorial disease with both genetic and environmental components, but given the acute presentation at birth, the clinical priority is to assert its genetic basis. In our clinical reasoning, we consider the presentation, which suggests an autosomal recessive disorder (e.g., autosomal recessive polycystic kidney disease), given symptoms consistent with this class of diseases. Also noteworthy is that both copies of the genes have a pathogenic mutation, supporting the autosomal recessive nature of the disease. To calculate the mutation occurrence rate, we use Hardy-Weinberg equilibrium: p^2+2pq+q^2=1, where p^2 signifies prevalence of homozygotes for the dominant allele, 2pq represents heterozygotes, and q^2 represents homozygotes for the recessive allele. As this is a rare disease (1 in 10,000), we consider q^2 as 1/10,000, and 'q' is √(1/10,000)≈0.01, which signifies the frequency of people affected by the disease. Hence, the allele frequency or 'p' could be calculated as 1-'q' and is roughly 0.99. Now, to ascertain the probability of being a carrier (2pq), we substitute 'p' and 'q' values in the formula, which equates to approximately 0.02 or 2/100. This means the rate of individuals carrying the pathogenic mutation is about 1 in 50 (approximated to 1 in 100, considering the provided options). Finally, the mutation occurrence rate best described by the options available for this newborn's disease is 1/100 (option A), meaning approximately 1 in 100 people in the population carry the mutation responsible for this particular disease. This outcome is based on the assumption that mutation occurrence rate refers to the carrier frequency in this context, and thus it should be differentiated from the disease incidence rate (1 in 10,000).

**AI_B:** Restating the question: The question is asking us to determine the mutation occurrence rate in a newborn with specific clinical symptoms and findings, suggesting a genetic disorder. The genetic disorder seen in this newborn is likely inherited, as indicated by the presence of two copies of a gene with a pathogenic mutation. Furthermore, the disease incidence rate in the general population is given as 1 in 10,000. Planning: Our main assumption here is that the disorder in question is autosomal recessive, considering the clinical and autopsy findings, which are consistent with an inherited renal disorder known as autosomal recessive polycystic kidney disease (ARPKD). With this assumption, we can then make use of the Hardy-Weinberg principle, a principle of population genetics which provides a model of genetic variation and predicts the genetic structure of a population. This principle can be used to calculate the frequency of individuals in a population who carry a particular allele of interest. Therefore, using the Hardy–Weinberg principle, we might alternate our assumption to hypothesize that the occurrence rate of the mutation in this newborn's disease can be calculated. Clinical Reasoning: For a Mendelian autosomal recessive disorder like the one suggested in the question, the frequency of the affected individuals (q^2) is equivalent to the incidence of the disease in the population, which we have given as 1 in 10,000. We can find q (the frequency of the pathogenic allele) by taking the square root of q^2, which comes to approximately 0.01 or 1 in 100. This value represents the frequency of individuals in the population carrying the pathogenic allele. However, it's important to note that these individuals are both people affected by the disorder (homozygotes for the pathogenic allele) and carriers of the disorder (heterozygotes). Concluding: Given this, the mutation occurrence rate in populations affected by this newborns' disease should be approximately 1/100. However, if we were considering just homozygous individuals carrying the disease (i.e., those affected, like the newborn in the scenario), the number would be considerably lower, matching the disease incidence rate of 1 in 10,000. Response: Therefore, the most suitable answer is 1/100 (A), as it best describes the occurrence rate of the mutation, considering individuals carrying the pathogenic allele (affected individuals and carriers). In reality, however, the incidence of homozygous pathogenic mutation carriers manifesting the disease (like the newborn) would be much lower at 1 in 10,000.

**AI_C:** To begin, the question asks about the mutation occurrence rate in a newborn exhibiting distinctive physical characteristics such as a broad flat nose, low-set ears, receding chin, prominent infraorbital folds, and bilateral abdominal masses. Additionally, the newborn displayed symptoms of cyanosis, difficulty breathing, and polyuria, and eventually died 24 hours after birth. Post-mortem examination revealed pulmonary hypoplasia and polycystic kidneys. A genetic test confirmed the presence of a pathogenic mutation associated with a disease with an incidence rate of 1 in 10,000 in the general population. The task is to determine the most accurate mutation occurrence rate for the newborn's disease from the options provided. The information given implies an autosomal recessive disease, suggesting both parents are carriers of the mutation. Autosomal recessive implies one normal and one mutated recessive gene inherited from each parent, leading to the manifestation of the disease in the offspring when two mutated genes are present. Such diseases often display rates calculated using the Hardy-Weinberg equilibrium. To answer the question, I need to explore the possibility that the parents of the deceased newborn were both carriers of the mutation. If so, the frequency of the allele or mutation in the general population becomes a vital piece of information. Then, by applying the Hardy-Weinberg equilibrium principle, I can estimate the proportion of the population carrying this mutation. The disease incidence rate of 1 in 10,000 (q^2 = 1/10,000) implies a gene frequency (q) of √(1/10000) = 1/100 for the disease mutation. Consequently, the frequency of the normal allele (p) becomes 1 - (1/100) = 99/100. Given these frequencies, the proportion of the population carrying the disease mutation or being heterozygote carriers (2pq) can be determined, consequently the mutation frequency rate. My conclusion would state that by using this genetic analysis and mathematical modeling based on Hardy-Weinberg equilibrium, we can make an estimation of the mutation occurrence for this disease in this newborn. However, due to the categories provided, the answer may not be precise but an approximate. Based on my preceding analysis and understanding of the Hardy-Weinberg equilibrium, I can provide an answer to the question. Several options are given such as 1/100, 1/400, 1/1000, 1/4000, and 1/10,000. The mutation occurrence rate is calculated as 2pq, or 2*(99/100)*(1/100), which equals approximately 1/50. Considering the provided choices, I would infer the approximate occurrence rate to be (A) 1/100, as it is closer to our calculated rate.

**AI_E:** The question revolves around understanding the terms "mutation occurrence rate" and "disease incidence rate" in the context of genetic diseases. The disease incidence rate is 1 in 10,000, which is the frequency with which the disease occurs in the general population. The mutation occurrence rate, on the other hand, refers to the frequency of this pathogenic allele in the population, not necessarily expressing the disease. In a simple Mendelian autosomal recessive disorder, which is the most likely scenario given the observed two copies of the pathogenic gene in the newborn, both parents of the newborn should be carriers and the prevalence of carriers in the population can be calculated using the Hardy-Weinberg principle. It is essential to note that the mutation occurrence rate in the context of an autosomal recessive disorder is different from the recurrence risk for siblings of affected individuals because only offspring from parents who are both carriers would have a 25% risk of developing the disease. The Hardy-Weinberg principle states that p^2 + 2pq + q^2 = 1, where p and q represent the frequencies of two alleles (usually represented as the dominant or wild-type and recessive or mutated alleles) present in a population. In the context of an autosomal recessive disease, the disease frequency equals q^2, which in this case is 1/10,000. Using this, we can calculate q (the frequency of the pathogenic allele), which is approximately 1/100 (the square root of 1/10,000). Given these considerations, the occurrence of the pathogenic mutation in the general population is 2pq, equivalent to 2(1/100)(99/100), or approximately 1/500. Considering the options given in the question, closest to the calculated mutation occurrence rate would be (C) 1/1000. So, to summarize: (1) I understand the question as asking about the mutation occurrence rate for an autosomal recessive disease in the context of the general population. (2) My assumption here is that we are dealing with an autosomal recessive disorder and that the "mutation occurrence rate" refers to the frequency of the pathogenic allele in the population. (3) Connecting this to the clinical scenario and Hardy-Weinberg equilibrium, we calculate the mutation occurrence rate as approximately 1/500. (4) However, considering the options provided, the closest approximation would be (C) 1/1000. (5) Therefore, the answer to [A male newborn is delivered at term to a 28-year-old primigravid woman. Physical examination shows a broad, flat nose; low-set ears; a receding chin; prominent infraorbital folds; and bilateral abdominal masses. The newborn develops cyanosis, difficulty breathing, and polyuria and dies 24 hours after birth. At autopsy, examination shows pulmonary hypoplasia and enlarged, polycystic kidneys. Genetic testing shows two copies of a gene having a pathogenic mutation with a disease incidence rate in the general population of 1 in 10,000. Which of the following best describes the mutation occurrence rate for this newborn's disease?] is most appropriate as (C) 1/1000.

**Facilitator** **[Round 4]:** Summary: AI_A, AI_B, AI_C, and AI_D all agree that the disease in question is likely an autosomal recessive disorder, given the clinical presentation and genetic testing results. They all use the Hardy-Weinberg equilibrium to calculate the mutation occurrence rate, which they define as the carrier frequency in the population. They all calculate the frequency of the pathogenic allele (q) to be approximately 0.01 or 1 in 100, and the frequency of the normal allele (p) to be approximately 0.99. They all calculate the carrier rate (2pq) to be approximately 1 in 50, and given the options provided, they all select 1 in 100 as the closest approximation. AI_E, however, calculates the carrier rate to be approximately 1 in 500, and selects 1 in 1000 as the closest approximation given the options provided. AI_E's calculations differ because they interpret the mutation occurrence rate to be the frequency of the pathogenic allele in the population, not the carrier frequency. Question to clarify disagreements: How do you define the mutation occurrence rate in the context of an autosomal recessive disorder, and how does this definition influence your calculations and your selection of the most appropriate answer from the options provided?. Based on your preceding response, how would you answer the original question: [A male newborn is delivered at term to a 28-year-old primigravid woman. Physical examination shows a broad, flat nose; low-set ears; a receding chin; prominent infraorbital folds; and bilateral abdominal masses. The newborn develops cyanosis, difficulty breathing, and polyuria and dies 24 hours after birth. At autopsy, examination shows pulmonary hypoplasia and enlarged, polycystic kidneys. Genetic testing shows two copies of a gene having a pathogenic mutation with a disease incidence rate in the general population of 1 in 10,000. Which of the following best describes the mutation occurrence rate for this newborn's disease? (A) 1/100 (B) 1/400 (C) 1/1000 (D) 1/4000 (E) 1/10,000]

**AI_A:** 1) RESTATING THE QUESTION: The question asks for the mutation occurrence rate of an autosomal recessive disorder observed in a neonate, who presented with a number of physical abnormalities and died within 24 hours of birth. Evidence of pulmonary hypoplasia and polycystic kidneys was revealed at autopsy, and genetic testing confirmed the presence of two copies of a pathogenic mutation. An autosomal recessive disorder generally requires that both copies of the gene in question have mutations, with one inherited from each parent. 2) PLANNING: It is necessary to make some assumptions in order to calculate the mutation occurrence rate. In the case of an autosomal recessive disorder, the assumptions generated from the Hardy-Weinberg equilibrium may be applied. The assumption being made is that the disease incidence rate mentioned (1 in 10,000) refers to the proportion of individuals who are homozygous recessive (q^2) for the pathogenic mutation. 3) CLINICAL REASONING: Using the disease incidence rate provided, the frequency of the pathogenic allele (q) can be calculated by taking the square root of the incidence rate, which would give approximately 0.01 or 1/100. The frequency of the normal allele (p) would therefore be about 0.99, having subtracted q from 1. Then, the carrier rate (2pq) can be calculated to determine the mutation occurrence rate, given that carriers of an autosomal recessive disorder have one copy of the pathogenic allele and one copy of the normal allele. 4) CONCLUDING: After performing these calculations, the estimated carrier rate is approximately 2% or 1 in 50, suggesting that around 2% of the general population carries one copy of the pathogenic allele. The closest answer provided is 1 in 100, so this would be the chosen response, although it slightly underestimates the calculated carrier rate. 5) RESPONSE: Given the clinical and genetic data available, as well as the calculations performed using the assumptions of the Hardy-Weinberg equilibrium, the mutation occurrence rate for this neonate's autosomal recessive disorder is most accurately described by the answer (A) 1 in 100.

**AI_D:** Firstly, let's restate the provided question in my own terms: It's asking for the rate of mutation occurrence in an autosomal recessive disorder that was present in a male newborn. This baby had several physical anomalies, developed respiratory distress, and unfortunately passed away shortly after birth. The autopsy revealed undersized lungs and oversized, cyst-filled kidneys. Genetic testing showed two copies of a defective gene, mutated in a way that causes disease. This disease appears in one out of every 10,000 people in the general population. The question now is: what is the mutation occurrence rate for this disease in the newborn? For the planning stage - it's generally assumed that this type of disease is rare, and the mutation is likely recessive, requiring two copies of the mutated gene for the disease to manifest. Another assumption made is that the general population isn't significantly affected by this mutation, given the disease's low incidence rate. However, we should consider the possibility of a high carrier rate in the population, despite the low disease incidence. Finally, the genetic basis of this disease suggests a distinct inheritance pattern, presumably autosomal recessive. Thus, we should prioritize the assumption concerning the inheritance pattern and carrier rate in the population. During the clinical reasoning process - we can confirm that the disorder in question is inherited in an autosomal recessive manner because the child expressed the disease phenotype despite having parents who presumably did not (based on the information provided). Now, using the Hardy-Weinberg equilibrium to calculate the mutation occurrence rate, we define the frequency of the disease-causing allele 'q' and the frequency of the non-disease causing allele 'p' in the population. Given the disease incidence rate of 1 in 10,000, 'q^2' equals 1/10,000, making 'q' approximately equal to 0.01. Therefore, 'p', which equals 1 - 'q', is approximately 0.99. The carrier rate in the population, defined as 2pq, therefore, comes out to be approximately 1 in 50. So, to conclude, the incidence rate of the disease in the newborn, as given in the question, is 1 in 10,000. However, the mutation occurrence rate, or carrier frequency in the population, is 2pq, or approximately 1 in 50. Drawing inference from the above discussion, we can see that the closest approximation to 1 in 50 from the available options is A) 1/100. Therefore, the mutation occurrence rate for the newborn's disease is best described as 1 in 100 (Option A).

**AI_B:** To dissect the inquiry carefully, this question is about a neonate who manifests with severe clinical symptoms, consistent with organ abnormality and cognitive deformities, and eventually passes away very early after birth. The identified cause of death is pulmonary hypoplasia and polycystic kidneys. Due to the severity and presentation of the disease, an autosomal recessive disorder is suspected and confirmed with genetic testing. Thus, both the mother and father were carriers of the disease, each with one normal and one mutated gene, which they both passed on to their newborn. The term "mutation occurrence rate" can be confusing, but it should be considered as the rate at which the pathogenic mutation occurs in the general population. The given information states that the incidence rate of the disease is 1 in 10,000. Using Hardy-Weinberg equilibrium (p^2 + 2pq + q^2 = 1, where p is the frequency of the normal allele and q is the frequency of the pathogenic allele), we can calculate that q^2 (the disease incidence) is 0.0001 (1 in 10,000). To determine q (pathogenic allele frequency), we take the square root of 0.0001, which approximates to 0.01. Keep in mind, this is the frequency of the pathogenic allele, not yet the mutation occurrence rate. Understanding that carriers of the disease hold both a diseased allele (q) from one parent and a normal allele (p) from the other, we calculate the rate of being a carrier (2pq). Therefore, 2*0.01*(1 - 0.01) = 0.0198 is approximately the carrier rate, or quite close to 1 in 50 for convenience in human understanding. However, our options don't include 1 in 50. So, we need to approach the closest possible answer, which is 1 in 100 (A). So, in summary, taking it step by step: 1. The newborn's presentation, pathology, and genetic testing results point to an autosomal recessive disorder. The likelihood of the parents being carriers is very high if the disorder is indeed autosomal recessive. 2. We translate the incidence rate of the disease (1 in 10,000 in general population) into an allele frequency for the pathogenic mutation using Hardy-Weinberg equilibrium. 3. Following that, we calculate the carrier frequency, which serves as a proxy for the "mutation occurrence rate" as per the question's language. 4. We conclude that, given the choice options, the most accurate answer for the mutation occurrence rate would be 1 in 100.

**AI_C:** RESTATING THE QUESTION: The question is related to a case of a newborn who presented with features such as a broad, flat nose; low-set ears; a receding chin; prominent infraorbital folds; and bilateral abdominal masses and died 24 hours after birth. Autopsy findings suggest the presence of pulmonary hypoplasia and polycystic kidneys; genetic testing revealed the presence of two copies of a gene with a pathogenic mutation associated with a disease that has an incidence rate of 1 in 10,000 in the general population. We are asked to determine the mutation occurrence rate of the disease in the newborn. PLANNING: The primary assumption here stems from our understanding of genetics - specifically, autosomal recessive inheritance. The disease incidence of 1 in 10,000 suggests an autosomal recessive pattern, as the newborn presents with two copies of a gene with this mutation, while the disease occurrence is relatively uncommon in the general population. Additionally, the characteristic phenotypic expressions point towards a congenital disorder. Given these observations, I would assume that the mutation occurrence rate refers to the frequency of individuals carrying one copy of the gene mutation (i.e., carriers) in the population. CLINICAL REASONING: Let's consider a few core concepts to build our reasoning. In autosomal recessive disorders, affected individuals carry two copies of the pathogenic mutation, and are generally born to parents who are carriers (i.e., individuals having one normal copy and one copy of the mutation). Therefore, the carrier frequency, typically represented by 2pq in population genetics (where p and q refers to the frequency of normal and mutated genes, respectively), would be useful to estimate the mutation occurrence rate. The Hardy-Weinberg equilibrium (p^2 + 2pq + q^2 = 1) can be used to calculate q - the frequency of the pathogenic mutation in the population. In this case, if we assume the disease incidence to be q^2 (since the affected individuals have two gene copies), we would get q as sqrt(1/10,000), which is approximately 1/100. Using these values, the carrier frequency (2pq) can be calculated as 2*(1/100)*((10,000-1)/10,000), which would be approximately 2/100 or 1/50. However, considering the options provided in the question, 1/100 is the closest approximation. CONCLUDING: By utilizing population genetics, specifically the Hardy-Weinberg equilibrium, and considering the disease as an autosomal recessive disorder, we can conclude that the mutation occurrence rate pertains to the carrier frequency, which is approximately 1 in 100. RESPONSE: Therefore, among the given options, the best answer to describe the mutation occurrence rate for this newborn's disease would be (A) 1/100.

**AI_E:** 1) RESTATING THE QUESTION: The question presents a full-term male newborn with pathological physical features and certain symptoms, who unfortunately died shortly after birth. The autopsy and genetic testing indicate an autosomal recessive disorder indicated by the presence of two copies of a gene with a pathogenic mutation. The disease incidence rate for this disorder in the general population is given as 1 in 10,000. As such, I am required to calculate the mutation occurrence rate, or the prevalence of this mutation within the population. In this context, 'mutation occurrence rate' refers to the frequency of the mutated gene within the population. This includes both heterozygous carriers, who have one copy of the mutated gene, and homozygous individuals, who have two copies of the mutated gene and, thus, manifest the disease. 2) PLANNING: This problem is based on the Hardy-Weinberg equilibrium principles, which state if a population is large, randomly mating, and not affected by mutation, migration, or selection, the allele frequencies will remain constant. According to these principles, the frequency of homozygous individuals (p^2 or q^2) plus twice the frequency of heterozygous individuals (2pq) should equal 1. The disease incidence rate, which is 1 in 10,000, represents the fraction of homozygous individuals within the population (q^2). Since we have q^2, we can find q by taking the square root of the frequency of affected individuals, this q value represents the frequency of mutation in population. Our preliminary assumption, therefore, is that the frequency of the mutation in the population, or the mutation occurrence rate, is equal to q. 3) CLINICAL REASONING: Firstly, let's calculate the frequency of the pathogenic allele (q). Since the disease incidence rate is 1 in 10,000, q^2 equals 1/10,000. Therefore, q equals the square root of 1/10,000 which is approximately 1/100 or 0.01. This is the allele frequency in the population or the mutation occurrence rate. However, it is important to note that this does not fully answer the question. The frequency of 1/100 or 0.01 only includes both heterozygous carriers and homozygous individuals. Therefore, it comprises of both people who have one copy of the mutated gene (carriers who do not manifest the disease) and people with two copies of the mutated gene (individuals who show the symptoms of the disease). 4) CONCLUDING: Given the reasoning, assumptions, and calculations outlined in step 3, we can determine that the mutation occurrence rate will be the square root of the disease incidence rate, assuming the population is in Hardy-Weinberg equilibrium. 5) RESPONSE: Therefore, the mutation occurrence or allele frequency in the population, considering heterozygous and homozygous individuals, is approximately 1/100 or 0.01. However, the options given in the questions do not include 1 in 100. As such, the closest approximation to the calculated mutation occurrence rate (1/100) from the given options is (A) 1/100.

**Facilitator** The question pertains to a neonate who presented with physical abnormalities and died within 24 hours of birth. Autopsy findings and genetic testing confirmed the presence of an autosomal recessive disorder. The disease incidence rate in the general population is 1 in 10,000. The task is to determine the mutation occurrence rate for this disorder. The mutation occurrence rate refers to the frequency of the pathogenic mutation in the general population, including both heterozygous carriers (who have one copy of the mutated gene and do not manifest the disease) and homozygous individuals (who have two copies of the mutated gene and show the symptoms of the disease). To calculate this, we can use the Hardy-Weinberg equilibrium principles. The disease incidence rate of 1 in 10,000 represents the fraction of homozygous individuals within the population (q^2). Taking the square root of this gives us the frequency of the pathogenic allele (q), which is approximately 1/100 or 0.01. This is the allele frequency in the population or the mutation occurrence rate. However, the carrier frequency, which is a more accurate representation of the mutation occurrence rate, can be calculated as 2pq. Given that p (the frequency of the normal allele) is approximately 0.99, the carrier frequency comes out to be approximately 1 in 50. Given the options provided in the question, the closest approximation to the calculated mutation occurrence rate (1/50) is (A) 1/100. Therefore, the mutation occurrence rate for this newborn's disease is best described as 1 in 100.
