# Supplementary material for: Collaborative intelligence in AI: Evaluating the performance of a council of AIs on the USMLE
Source: PLOS Digit Health. 2025 Oct 9;4(10):e0000787. doi: 10.1371/journal.pdig.0000787 (PMC12510544; doi:10.1371/journal.pdig.0000787)
Supplement: S2 File — (ZIP) [file pdig.0000787.s002.zip › S2 Data Raw data files containing unprocessed question inputs and LLM outputs including transcripts of deliberations/STEP1_Question-45-1_Type-MC-NJ.docx]

**Facilitator** **[Round 1]:** A previously healthy 64-year-old man is brought to the emergency department 3 hours after the sudden onset of severe flank pain. He has no history of similar pain or serious illness. He takes no medications. He appears to be in distress. His pulse is 100/min, and blood pressure is 168/92 mm Hg. Physical examination shows a soft abdomen with left sided tenderness. Urinalysis shows microscopic hematuria. A CT scan of the abdomen shows a small ureteric calculus. Analgesic therapy is initiated and the pain resolves 1 hour later. The urine is then strained and a uric acid calculus is found. Which of the following processes is most likely impaired in this patient? (A) Bile salt metabolism (B) Cholesterol metabolism (C) Cytochrome P450 activity (D) Purine metabolism (E) Urea cycle

**AI_D:** 1) RESTATING THE QUESTION: The question asks us to identify the most likely impaired metabolic process in a 64-year-old man who experienced sudden severe flank pain. The patient has a history of good health and was found to have a uric acid-based kidney stone (ureteric calculus). We need to choose between (A) Bile salt metabolism, (B) Cholesterol metabolism, (C) Cytochrome P450 activity, (D) Purine metabolism, and (E) Urea cycle. 2) PLANNING: Connecting the symptom of severe flank pain with a diagnosis of kidney stone, the puzzle becomes the composition of the stone, which in this case is composed of uric acid. The chemistry of uric acid indicates an anomaly in purine metabolism. The other options, although involved in other metabolic functions, do not directly connect to the presence of uric acid in the urinary system. 3) CLINICAL REASONING: Kidney stones can be formed from a variety of substances, including calcium oxalate, struvite, cysteine, and uric acid. The fact that this patient's stone is composed of uric acid suggests a metabolic dysfunction involving purines. Purine metabolism is responsible for the breakdown and recycling of purines, which are components of our DNA and RNA. Uric acid is a product of purine metabolism and typically excreted in the urine. Overproduction or inadequate excretion can lead to the formation of kidney stones. 4) CONCLUDING: Considering the composition of the stone (uric acid) and understanding that uric acid is a by-product of purine metabolism, the reasonable conclusion is that this patient has an issue related to purine metabolism. 5) RESPONSE: Therefore, the most likely impaired process in this patient is (D) Purine metabolism.

**AI_A:** RESTATING THE QUESTION: The clinical scenario maps the journey of a 64-year-old man who experienced sudden severe flank pain. His vital signs show a mildly elevated pulse rate and blood pressure, signs of distress. The findings of a soft abdomen with left-sided tenderness, urinalysis revealing microscopic hematuria, and a CT scan showing a small ureteric calculus suggest some urinary system issue. The uric acid calculus found in his urine confirms this assumption. Based on this, the question seeks to identify the specific impaired biological process in this individual, with options ranging from bile salt metabolism to the urea cycle. PLANNING: Primary assumption based on the facts is that a urinary problem is at play, given the left-sided flank pain, the findings of the CT scan, and the uric acid calculus found in the patient's urine. The major biological processes linked to the urinary system include urea cycle, purine metabolism, and to some extent cytochrome P450 activity. Bile salt metabolism and cholesterol metabolism are more associated with the hepatic system and lipid regulation respectively. CLINICAL REASONING: Recognizing the uric acid calculus as a byproduct of purine metabolism would suggest that the patient could have an impairment in this process. Uric acid is the end product of purine metabolism. Flank pain and microscopic hematuria depicted in the scenario are characteristic of a kidney stone, specifically, a uric acid stone. Overproduction or under excretion of uric acid leads to the formation of this type of kidney stone. CONCLUDING: Using the above clinical thought process, we can conclude that the leading assumption that connects these clinical features (flank pain, microscopic hematuria, CT revealing ureteric calculus and strained urine revealing uric acid calculus) is purine metabolism impairment. RESPONSE: Based on this thorough investigation of the patient's symptoms, signs, examination findings, and the evidence presented, we can conclude that the most likely impaired biological process in this patient is (D) Purine metabolism.

**AI_C:** RESTATING THE QUESTION: The query pertains to finding out the probable underlying metabolic dysfunction in a 64-year-old man who presented with severe flank pain, left-sided abdominal tenderness and was found to have a uric acid calculus in his urinary system on investigation. The choices hint toward different metabolic functions, which include the process of bile salt formation, cholesterol metabolism, the functioning of Cytochrome P450 (a heme-containing protein involved in drug biotransformation), the metabolism of purines (precursors of nucleic acids), and the urea cycle (the process by which ammonia is converted to urea in the body). PLANNING: With respect to this patient's clinical presentation, an impairment in the metabolism of purines could lead to an increase in uric acid excretion and, consequently, the formation of uric acid stones. Alternatively, the patient's condition could be the result of an abnormality in the Cytochrome P450 system. However, although alterations in bile salt, cholesterol or urea metabolism can result in different disease phenotypes, these are not typically associated with the formation of uric acid-based calculi. CLINICAL REASONING: With sudden onset severe flank pain, a small ureteric calculus, and presence of a uric acid calculus found on straining urine, the clinical picture is consistent with a urinary calculi event, often caused by a concentration of certain substances in the urine. Uric acid stones form when there is an overexcretion of uric acid, typically through an imbalance in purine metabolism. They do not usually form due to imbalances in cholesterol metabolism, bile salt metabolism, Cytochrome P450 activity, or in the urea cycle. Therefore, the formation of uric acid kidney stones can likely be related to impaired purine metabolism. CONCLUDING: Given the patient's symptoms of flank pain, urinalysis showing microscopic hematuria, the finding of urinary calculus on the CT scan, and the validation of a uric acid calculus, it is likely that the origin of this clinical scenario lies in an imbalance in the metabolism of purines. RESPONSE: So, the most plausible metabolic impairment in this patient would be (D) Purine metabolism.

**AI_E:** RESTATING THE QUESTION: The patient in this question is a 64-year-old man who, despite having been previously healthy, presented to the Emergency Department with severe flank pain. Relevant findings include left-sided tenderness and the presence of microscopic hematuria noted during urinalysis. A CT scan confirmed the presence of a small ureteric calculus. Although the pain was managed successfully with analgesics, upon straining the urine, a uric acid calculus was discovered. The issue here is to identify which metabolic process is most likely defective in this patient. Options provided range from bile salt metabolism, cholesterol metabolism, cytochrome P450 function, purine metabolism to the urea cycle. PLANNING: To effectively answer this question, it is vital to understand the basis of each metabolic function provided in the option list and how it might connect to the formation of a uric acid stone. In essence, this requires an understanding of the chemical constitution of uric acid and how its overproduction or under-excretion can lead to stone formation. CLINICAL REASONING: Cholesterol metabolism affects the formation of cholesterol stones, usually found in the gallbladder. Disorders with bile salt metabolism also majorly impact the gallbladder rather than the urinary system. Cytochrome P450 activity mostly deals with drug metabolism in the liver. The urea cycle relates to how the body disposes of ammonia, which does not have any direct link with uric acid. On the other hand, purine metabolism leads to the production of uric acid as a waste product. When the rate of purine breakdown exceeds the rate of uric acid excretion by the kidneys, levels of uric acid in the urine can rise, causing urate crystals to form and potentially leading to uric acid stones. CONCLUDING: Based on the clinical details provided and the reasoning above, it can be concluded that the chances of impairment in purine metabolism are higher in this patient compared to the other metabolic functions listed. An overproduction (or under-excretion) of uric acid as a result of this impairment can explain the formation of uric acid stones observed in the patient. RESPONSE: The most likely impaired process in this patient is (D) Purine metabolism.

**AI_B:** RESTATING THE QUESTION: The question is asking for the biochemical pathway most likely to be affected in a 64-year-old man who presents with sudden severe flank pain and left sided tenderness. His urine analysis reveals microscopic hematuria and a uric acid calculus. The uric acid calculus (or stone), formed in the urinary system, strongly suggests that there is an overproduction or under-excretion of uric acid in the body. PLANNING: Various processes could cause an increase in uric acid formation which include: (A) Bile salt metabolism, (B) Cholesterol metabolism, (C) Cytochrome P450 activity, (D) Purine metabolism, and (E) Urea cycle. Considering these possibilities, we need to think about where and how uric acid is made and removed in the body. Process of exclusion helps here: (A) and (B) lead to gallstone formation, while (C) Cytochrome P450 activity is most involved with drug metabolism in the liver. (E) Urea cycle occurs in the liver and its malfunction leads to hyperammonemia. Given the presented symptoms, none of these seem to be the immediate causes. This leaves us with (D) Purine metabolism, which is the biochemical pathway responsible for the production and breakdown of purines, which are nitrogen-containing compounds found in DNA, RNA, ATP, and other molecules. In humans, excess purines are converted to uric acid for excretion. CLINICAL REASONING: In normal circumstances, purines obtained from the diet, along with those synthesized endogenously, are degraded and excreted as uric acid to prevent their accumulation. This patient’s uric acid stone suggests that he might have an impaired purine metabolism, leading to an overproduction of uric acid which then precipitates and forms a calculus when its solubility limit in urine is exceeded. CONCLUDING: Given the presence of a uric acid calculus, one can infer a disturbance in purine metabolism. This fits in well with the clinical scenario presented, as calciuli associated with disorders of bile salt, cholesterol, cytochrome P450 or urea metabolisms would not typically manifest as a uric acid stone. RESPONSE: The process most likely impaired in this patient is (D) Purine metabolism.

**Facilitator** The consensus statement synthesizes the responses of AI_A, AI_B, AI_C, AI_D, and AI_E as follows: The question pertains to a 64-year-old man who presented with sudden severe flank pain and was found to have a uric acid calculus in his urinary system. The options provided hint towards different metabolic functions, including bile salt metabolism, cholesterol metabolism, cytochrome P450 activity, purine metabolism, and the urea cycle. Upon analyzing the patient's symptoms and the nature of the calculus, it becomes clear that the issue lies within the urinary system. The presence of a uric acid calculus suggests an overproduction or under-excretion of uric acid. The metabolic processes directly linked to the urinary system and the formation of uric acid are purine metabolism and, to a lesser extent, cytochrome P450 activity. However, cytochrome P450 is primarily involved in drug metabolism in the liver, making it less likely to be the cause in this case. Bile salt metabolism and cholesterol metabolism are more associated with the hepatic system and lipid regulation, respectively, and are not typically associated with the formation of uric acid-based calculi. Similarly, the urea cycle, which occurs in the liver and is responsible for converting ammonia to urea, does not directly connect to the presence of uric acid in the urinary system. Given the patient's symptoms, the presence of a uric acid calculus, and the understanding that uric acid is a by-product of purine metabolism, the consensus is that the patient likely has an impairment in purine metabolism. This conclusion is supported by the fact that purine metabolism is responsible for the breakdown and recycling of purines, components of our DNA and RNA, with uric acid being a product of this process. Overproduction or inadequate excretion of uric acid can lead to the formation of kidney stones. Therefore, the consensus response to the question, "Which of the following processes is most likely impaired in this patient?" is (D) Purine metabolism.
