# Supplementary material for: Collaborative intelligence in AI: Evaluating the performance of a council of AIs on the USMLE
Source: PLOS Digit Health. 2025 Oct 9;4(10):e0000787. doi: 10.1371/journal.pdig.0000787 (PMC12510544; doi:10.1371/journal.pdig.0000787)
Supplement: S2 File — (ZIP) [file pdig.0000787.s002.zip › S2 Data Raw data files containing unprocessed question inputs and LLM outputs including transcripts of deliberations/STEP1_Question-46-1_Type-MC-NJ.docx]

**Facilitator** **[Round 1]:** A 32-year-old woman with hypothyroidism comes to the physician because of a 6-week history of cold intolerance, constipation, and severe fatigue. She also has had a 4.5-kg (10-lb) weight gain during this period. Her current medication is daily levothyroxine. She also started taking calcium carbonate supplements 2 months ago for osteopenia of the lumbar spine. At that time, serum concentrations of thyroid-stimulating hormone (TSH) and free thyroxine (FT4) were within the reference ranges. Today, physical examination shows coarse, dry skin. Serum studies show a TSH concentration of 16.6 μU/mL and FT4 concentration of 0.3 ng/dL. Which of the following pharmacokinetic changes of levothyroxine is the most likely cause of this patient’s current condition? (A) Decreased absorption (B) Decreased distribution (C) Decreased elimination (D) Increased distribution (E) Increased elimination (F) Increased metabolism

**AI_B:** RESTATING THE QUESTION: This question is asking about a 32-year-old woman who has hypothyroidism and has been experiencing symptoms of this disease despite being on the standard treatment, levothyroxine. Recently, she also started taking calcium supplements. To understand the cause of her symptoms, we need to consider the potential effects of her medication and supplement regime on the pharmacokinetics of levothyroxine. In simpler terms, we are looking for alterations that may have affected how levothyroxine is absorbed, distributed, metabolized, and eliminated in her body. PLANNING: The patient's symptomatology suggests an inadequate level of thyroid hormone in her system despite the levothyroxine treatment. The likely contributors to this would be either decreased absorption, decreased distribution, increased metabolism, or increased elimination of levothyroxine from her system. However, the new event here - taking calcium carbonate supplements - can interfere with the absorption of levothyroxine. CLINICAL REASONING: The calcium carbonate started two months ago is the new variable in this equation. Calcium, by nature, binds to levothyroxine in the gut, which forms an insoluble complex and thus decreases the drug's absorption into her system. Her symptoms, along with the increased TSH level and decreased FT4 level, strongly point toward this. CONCLUDING: Given the patient's history, symptoms, and timeline, it's likely that the calcium carbonate supplements are inhibiting the absorption of levothyroxine, leading to insufficient thyroid hormone in her system despite the treatment regime she's on. RESPONSE: Therefore, the most likely pharmacokinetic change of levothyroxine causing this patient's current condition is (A) Decreased absorption.

**AI_C:** (1) RESTATING THE QUESTION: This is a scenario where a 32-year-old woman with hypothyroidism, who has been regularly taking levothyroxine, has started experiencing symptoms consistent with worsening of her condition. Her symptoms suggest an insufficient level of thyroid hormones despite her medication, as evidenced by her elevated thyroid-stimulating hormone (TSH) level and decreased free thyroxine (FT4) level. The question implies that pharmacokinetics of her medication, levothyroxine, might be under the influence of her recent addition of calcium carbonate supplements in her regimen. Pharmacokinetics refers to the process by which a drug is absorbed, distributed, metabolized, and eliminated by the body. (2) PLANNING: The assumption is that calcium carbonate somehow affects the pharmacokinetics of levothyroxine. There are several ways calcium carbonate could influence levothyroxine: It could decrease its absorption, distribution, or increase its elimination or metabolism. One alternate assumption to note is that the calcium carbonate impacts the absorption of levothyroxine - as it is often the case with calcium supplements interfering with the absorption of other medicines when taken together. (3) CLINICAL REASONING: The symptoms of hypothyroidism in this patient are concurrent with her usage of calcium carbonate and despite her continuous dosage of levothyroxine, indicating a less effective drug level in her body. Calcium carbonate is known to bind to levothyroxine in the gut, resulting in a decrease in the absorption of levothyroxine, leading to lower levels of the hormone in serum, and thus symptoms of hypothyroidism despite the ongoing therapy. This would cause an increase in TSH level as the feedback mechanism in a hypothyroid patient would try to stimulate more thyroid hormone production, exactly what we're seeing in this case. (4) CONCLUDING: Considering the above, it indicates that the pharmacokinetic change that is most likely causing this woman's symptomatology, despite her continuous levothyroxine therapy, is decreased drug absorption due to her concurrent usage of calcium carbonate supplements. (5) RESPONSE: So, the answer would be (A) Decreased absorption, the issue being that calcium carbonate is binding to the levothyroxine in the gastrointestinal tract and thereby reducing the amount of the drug that is being absorbed into the bloodstream, which in turn reduces its therapeutic efficacy.

**AI_A:** (1) RESTATING THE QUESTION: A 32-year-old woman with known hypothyroidism is showing symptoms that indicate her thyroid levels are not being adequately controlled, despite taking daily levothyroxine. She has recently been taking calcium carbonate supplements as well. Her physical symptoms and current blood results suggest her thyroid condition has worsened. The question is asking what pharmacokinetic change has most likely taken place to explain her condition worsening despite her medication. (2) PLANNING: The standard assumptions are that the patient has been appropriately taking her medicine, that the medication has been absorbing, distributing, and being metabolized and eliminated as expected. An altered pharmacokinetic process is one possible explanation for the decline in her condition. Another assumption is that the initiation of the calcium carbonate did not affect the levothyroxine's pharmacokinetics. A potential alternate assumption is the interference of levothyroxine absorption by calcium carbonate, which is known to bind to levothyroxine and reduce its bioavailability. (3) CLINICAL REASONING: To evaluate our assumptions, let's consider the pharmacokinetics of levothyroxine and the implications of the newly introduced calcium carbonate supplement. Levothyroxine is a thyroid hormone replacement therapy, typically given to patients with hypothyroidism. Calcium carbonate, an antacid, and mineral supplement can bind to certain medications in the stomach, reducing their absorption and effectiveness. Hypothyroidism's symptoms include fatigue, weight gain, constipation, and dry skin, which this patient is displaying, suggesting her treatment is currently ineffective. The elevated TSH (thyroid-stimulating hormone) and decreased FT4 (free thyroxine) in the serum support this assumption, as TSH levels increase when the body senses low thyroid hormone levels, while FT4 represents the available active thyroid hormone in the body. (4) CONCLUDING: Thus, even though our patient has been regularly taking her levothyroxine, the calcium carbonate supplement she started taking could have altered the absorption of levothyroxine, decreasing its availability, leading to an increased TSH and decreased FT4—thus worsening her hypothyroid state. (5) RESPONSE: Therefore, (A) Decreased absorption of levothyroxine due to the interaction with calcium carbonate is the most likely cause of the presented symptoms and blood results in this patient's current condition.

**AI_D:** RESTATING THE QUESTION: We have a 32-year-old woman, diagnosed with hypothyroidism, and shows symptoms like fatigue, constipation, weight gain, cold intolerance, and coarse, dry skin, which are typical signs of decreased thyroid function. She's currently on levothyroxine (a thyroid hormone replacement therapy) for these issues. Recently, however, she also started taking calcium carbonate supplements for osteopenia. After starting the calcium supplements, her thyroid-stimulating hormone (TSH) and free thyroxine (FT4) have shown abnormal levels suggesting her hypothyroidism symptoms have worsened. We're asked to consider which pharmacokinetic factor (how the body affects the drug) in the mechanism of levothyroxine might have precipitated this change in the patient's condition. PLANNING: There are six possibilities: (A) Decreased absorption, (B) Decreased distribution, (C) Decreased elimination, (D) Increased Distribution, (E) Increased elimination, and (F) Increased metabolism of levothyroxine. Each of these assumptions concerns a specific aspect of the levothyroxine activity in her body. These include how well her body takes in the drug, how effectively it's dispersed throughout her system, how fast it's removed, and how quickly it's broken down. Given the recent addition of calcium carbonate in her regimen, we need to consider that her recent symptoms might be due to an interaction between the two drugs. CLINICAL REASONING: Absorption is the first pharmacokinetic phase where the drug enters the body usually via the digestive tract. If absorption decreased, less levothyroxine would be taken in, which could explain her heightened hypothyroid symptoms. Drugs such as calcium carbonate, which she has recently started using, can bind with levothyroxine in the gastrointestinal tract, reducing the amount of levothyroxine that can be absorbed into the bloodstream. Distribution pertains to how well the drug can reach necessary areas within the body. This seems unlikely to have changed recently, especially without any indicators such as alterations in body weight or protein levels which could change its volume of distribution. Elimination and metabolism refer to how the body gets rid of the drug or breaks it down, respectively. Increased elimination or metabolism would lessen the amount of levothyroxine in her body, possibly highlighting her hypothyroid symptoms. However, there haven't been any indications of changes in kidney or liver function that may suggest this. CONCLUDING: The patient had a normal TSH and FT4 when she was only on levothyroxine. Her deterioration coincided with the introduction of calcium carbonate, which is known to bind with levothyroxine in the gut and reduce its absorption. This, therefore, suggests an absorption issue rather than a change in distribution, metabolism, or elimination. RESPONSE: The most likely cause of this patient's current condition may be related to a (A) Decreased absorption of levothyroxine probably due to her newly initiated calcium carbonate intake.

**AI_E:** RESTATING THE QUESTION: The information provided describes a 32-year-old woman, diagnosed with hypothyroidism, presenting with symptoms related to hypothyroidism, such as cold intolerance, constipation, fatigue, weight gain, and coarse, dry skin. She is currently taking levothyroxine, a synthetic form of thyroxine or T4, used in treating hypothyroidism. Furthermore, she recently started on calcium carbonate supplements for osteopenia. The tests reveal elevated TSH and decreased FT4 indicating inadequate thyroid hormone levels, despite being on levothyroxine. We're asked to identify the change in levothyroxine's pharmacokinetics, including absorption, distribution, elimination, and metabolism, causing her current condition. PLANNING: Let's look at each assumption initially: 1. Decreased absorption of levothyroxine, which would result in lower levels of the drug reaching the bloodstream. 2. Decreased distribution of levothyroxine, suggesting the drug isn't sufficiently reaching the tissues. 3. Decreased elimination, meaning the drug is not removed from the body effectively, leading to potentially high drug levels. 4. Increased distribution suggesting the drug is spread too thin. 5. Increased elimination, which implies the drug is removed from the body too quickly, reducing its levels. 6. Increased metabolism, meaning the drug is broken down and used more quickly, lowering its levels. The fact that she started taking calcium carbonate two months ago and it's around that time the symptoms aggravated is important. Calcium carbonate is known to bind to levothyroxine and significantly reduce its absorption. Thus, alternate assumption: it is the decreased absorption of levothyroxine that is most likely causing her hypothyroid symptoms. CLINICAL REASONING: Levothyroxine is generally well absorbed in the body, and its distribution, elimination, and metabolism don't significantly alter under normal physiological conditions. The fact that her symptoms, as well as TSH and FT4, were within the normal range before starting calcium carbonate implies that levothyroxine was being properly absorbed, distributed, metabolized, and eliminated. The only recent change in her regimen is the addition of calcium carbonate. Calcium supplements, including calcium carbonate, form insoluble complexes with levothyroxine, reducing its absorption in the gastrointestinal tract, leading to lower levels of the drug in the bloodstream, and therefore, making the treatment less effective. This decrease in absorption results in inadequate thyroid hormone levels and elevates TSH concentration, indicative of enhanced pituitary effort to stimulate the thyroid gland. CONCLUDING: In light of the above deduction, it is the decreased absorption, caused by calcium carbonate supplementation, which is the reason for the unsuccessful control of her hypothyroidism, despite taking daily levothyroxine. RESPONSE: (A) Decreased absorption is the most likely cause for this patient's current condition of exacerbated hypothyroidism despite being on levothyroxine therapy because calcium carbonate, which she started taking two months ago, binds to levothyroxine in the gut, significantly reducing its absorption. This interferes with the therapeutic effect of levothyroxine by reducing its availability in the bloodstream, leading to lower FT4 levels and increased TSH concentration.

**Facilitator** The consensus among the responses is that the most likely pharmacokinetic change of levothyroxine causing the patient's current condition is (A) Decreased absorption. This conclusion is based on the patient's symptoms of hypothyroidism, which have worsened despite her ongoing levothyroxine therapy. The key factor in this situation is the recent introduction of calcium carbonate supplements into her regimen. Calcium carbonate is known to bind to levothyroxine in the gut, forming an insoluble complex that reduces the absorption of levothyroxine into the bloodstream. This decrease in absorption leads to lower levels of the drug in the body, making the treatment less effective. The resulting inadequate thyroid hormone levels are reflected in the patient's elevated TSH concentration and decreased FT4 level. Therefore, despite the patient's regular intake of levothyroxine, the interaction with calcium carbonate has likely decreased its bioavailability, leading to the exacerbation of her hypothyroid symptoms.
