# Supplementary material for: Collaborative intelligence in AI: Evaluating the performance of a council of AIs on the USMLE
Source: PLOS Digit Health. 2025 Oct 9;4(10):e0000787. doi: 10.1371/journal.pdig.0000787 (PMC12510544; doi:10.1371/journal.pdig.0000787)
Supplement: S2 File — (ZIP) [file pdig.0000787.s002.zip › S2 Data Raw data files containing unprocessed question inputs and LLM outputs including transcripts of deliberations/STEP1_Question-48-1_Type-MC-NJ.docx]

**Facilitator** **[Round 1]:** A 19-year-old man undergoes splenectomy because of crush injuries sustained in a motor vehicle collision. During the operation, two small accessory spleens are seen adjacent to the ruptured spleen. The most likely origin of this patient's congenital anomaly is thickening of an embryologic layer at which of the following sites? (A) Adjacent to the ventral pancreas (B) Along the lesser curvature of the stomach (C) At the origin of the celiac vessels (D) In a portion of the hepatic diverticulum (E) Within the dorsal mesogastrium

**AI_A:** RESTATING THE QUESTION: The question essentially inquires about the embryonic origin of accessory spleens, a congenital anomaly. The existence of accessory spleens, also known as splenunculi, derives usually from a process during embryonic development wherein the splenic anlage, or precursor, doesn't completely coalesce into a single, well-defined spleen. The proposed options for this anomaly's origin are various sites associated with embryonic development of the abdominal and thoracic organs. PLANNING: The first assumption is that the accessory spleens are a result of an ordinary splenic development process that went awry at some point. An alternate assumption could be that the accessory spleens may have actually appeared as a response to the trauma, which would be speculative and highly unlikely given the nature of accessory spleens as congenital formations. As such, it becomes essential to focus on the embryological development of the spleen to answer this question correctly, which hypothesizes a failure in the process of the spleen coalescing into a singular organ during development. CLINICAL REASONING: The spleen normaly develops from the dorsal mesogastrium during the fifth week of embryogenesis. During this process, small islets of splenic tissue may separate and remain within the abdomen. These separate islets of splenic tissue form the accessory spleens. So, the site where the issue would have occurred is with the dorsal mesogastrium, leading to the presence of accessory spleens. CONCLUDING: Therefore, considering the embryological development of the spleen from the dorsal mesogastrium and the fact that errors in this process result in accessory spleens, the most likely origin of this patient's congenital anomaly is within the dorsal mesogastrium. RESPONSE: (E) The most likely origin of this patient's congenital anomaly, which caused the presence of accessory spleens, is thickening within the dorsal mesogastrium.

**AI_B:** RESTATING THE QUESTION: This question describes a situation where a young male patient underwent a splenectomy due to injuries following a car accident. During surgery, two smaller, accessory spleens were found near the injured spleen. These accessory spleens are a kind of congenital anomaly. The question here seeks to understand the embryological site that most likely gave rise to these accessory spleens, choosing from five named locations within the embryological anatomy. PLANNING: Firstly, we'd need to understand the development of the spleen, usually forming from dorsal mesogastrium in the upper left quadrant of the embryo. An assumption is that abnormal splenic tissue could have formed due to an error in this embryological process, like thickening at the site of formation. For alternate perspective: could the accessory spleens have originated elsewhere, due to the wandering of splenic tissue during fetal development? Clinically, the most prioritized assumption would be to ascertain the usual site for the development of the spleen. CLINICAL REASONING: In embryology, the spleen develops from the mesenchymal tissue within the dorsal mesogastrium, which later forms the greater omentum, gastrosplenic ligament, and splenorenal (lienorenal) ligament. Aberrations in spleen development could lead to formations of accessory spleens. Locations like the ventral pancreas, lesser curvature of the stomach, the origin of the celiac vessels, or hepatic diverticulum don't inherently contribute to spleen development. CONCLUDING: Given the embryological basis of the spleen’s development, it is most likely that the anomalous accessory spleens in this patient originated from an irregularity during development within the dorsal mesogastrium. Other sites present in the question are less likely to relate to spleen development and the formation of accessory spleens. RESPONSE: The most likely origin of the patient's congenital anomaly, presented as accessory spleens, is thickening of an embryologic layer within the dorsal mesogastrium (E).

**AI_D:** RESTATING THE QUESTION: The query is about a young man who had an emergency surgery - a splenectomy, due to injuries from a car crash. During the surgery, two extra small spleens, known as accessory spleens, were discovered. These accessory spleens suggest a congenital anomaly — the presence of multiple spleens "poly splenia" that brings into focus one's embryologic development. The question requires us to identify where the embryological thickening that resulted in these abnormalities likely occurred. PLANNING: The spleen is formed primarily from mesenchymal cells in the dorsal mesogastrium during embryological development. Any irregularities at this stage may result in anomalies like accessory spleens. Let us explore all the options and prioritize one: (A) Thickening adjacent to the ventral pancreas primarily leads to pancreas development. (B) The lesser curvature of the stomach's thickening is typically involved in the stomach's formation. (C) The celiac vessels originate from the mesenchyme surrounding the primitive gut tube and are not directly associated with the spleen's development. (D) Hepatic diverticulum is instrumental in hepatic and biliary system development. (E) The dorsal mesogastrium is indeed associated with the spleen's embryologic development. ACLINICAL REASONING: Exploring each possibility, As the spleen development occurs from mesenchymal cells in the dorsal mesogastrium, any anomalies in this area may result in the spleen's congenital anomalies such as accessory spleens as seen in this case. CONCLUDING: The thickenings adjacent to the ventral pancreas or in the hepatic diverticulum are tied to other organ developments and not specifically the spleen. The celiac vessels' origin doesn't seem to pertain to the spleen's formation directly. The lesser curvature of the stomach's thickening would influence stomach development rather than spleen development. Hence, in this clinical context, thickening within the dorsal mesogastrium, which is related to the spleen's embryologic development, should be prioritized for further analysis. RESPONSE: Therefore, this patient's congenital anomaly, the presence of accessory spleens, is most likely a result of the thickening of an embryological layer within the dorsal mesogastrium (E).

**AI_C:** (1) RESTATING THE QUESTION: This question pertains to a 19-year-old man who had a splenectomy due to a motor vehicle accident. During his surgery, two additional small spleens, known as accessory spleens, are found close to the ruptured spleen. We are being asked to determine the most probable site of the embryologic layer thickening that has led to this congenital anomaly. (2) PLANNING: An accessory spleen develops due to the abnormal partition of the embryonic splenic mass. This is a common congenital anomaly where the splenic tissue does not fuse completely during developmental stages, creating one or more miniature spleens. Although it is typically asymptomatic, it could become clinically significant during splenectomy, trauma, or pathological entities. Thus, the embryologic origin of the spleen is the primary assumption. Accordingly, other possibilities include the ventral pancreas, lesser curvature of the stomach, origin of the celiac vessels, hepatic diverticulum, and the dorsal mesogastrium. However, the splenic bud arises from the dorsal mesogastrium during embryological development. Therefore, this assumption holds a higher clinical priority as the abnormal thickening of the embryologic layer could lead to the mentioned anomaly. (3) CLINICAL REASONING: The spleen normally develops from the dorsal mesogastrium, which later rotates to the left side of the abdomen. The splenic anlage or primordium divides into several nodules during embryonic development, and these nodules usually coalesce to form a single spleen. In some instances, some nodules do not fuse and continue to develop separately, thus forming accessory spleens. (4) CONCLUDING: Therefore, an abnormal thickening in the embryological layer within the dorsal mesogastrium during embryonic development could potentially prevent full fusion of the splenic nodules, leading to the formation of the accessory spleens as seen in this patient. (5) RESPONSE: The most likely origin of this patient's congenital anomaly is thickening of an embryologic layer within the dorsal mesogastrium (E).

**AI_E:** RESTATING THE QUESTION: A young man needed to have his spleen removed due to damage resulting from a car accident. During the surgery, two additional smaller spleens were seen near the damaged spleen. This error in the development of the spleen is probably due to an abnormal thickening of an embryological layer, but where? The options are: close to the ventral pancreas, along the reduced curve of the stomach, at the origin of the celiac vessels, in a part of the hepatic diverticulum, or inside the dorsal mesogastrium. PLANNING: The normal development of the spleen begins within the dorsal mesogastrium during embryogenesis, which involves thickening and migration of the mesodermal tissue. However, in the context of this case it's crucial to understand accessory spleen formation, which is likely due to fragmentation during embryogenesis or vascular isolation of portions of the splenic mass. Now we can generate alternate assumptions: (A) The anomaly originated near the ventral pancreas, (B) It began on the lesser curvature of the stomach, (C) It started at the origin of the celiac vessels, (D) It began in part of the hepatic diverticulum, or (E) It originated in the dorsal mesogastrium. Considering the embryologic development, we can assume that option E, "within the dorsal mesogastrium," should be clinically prioritized. CLINICAL REASONING: The spleen arises from the dorsal mesogastrium in the embryonic period. If there's any disruption during this process or the tissue becomes fragmented in some way, it can lead to the creation of accessory spleens. These accessory spleens, or splenunculi, are most often found in close proximity to the 'original' spleen, explaining why the two accessory spleens in our case were seen adjacent to the ruptured spleen. Anomalies in the development near the ventral pancreas, along the lesser curvature of the stomach, at the origin of the celiac vessels or in the hepatic diverticulum would lead to different types of anomalies, but not to the formation of accessory spleens. CONCLUDING: It is therefore reasonable to conclude that the thickening of the embryological layer leading to this patient’s congenital anomaly, i.e., the formation of accessory spleens, most likely occurred within the dorsal mesogastrium. RESPONSE: The most likely origin of this patient's congenital anomaly that led to the formation of accessory spleens can be attributed to the thickening of an embryological layer within the dorsal mesogastrium (E).

**Facilitator** The consensus statement synthesizes the responses of AI_A, AI_B, AI_C, AI_D, and AI_E as follows: The question pertains to a 19-year-old man who underwent a splenectomy due to injuries from a motor vehicle accident. During the surgery, two accessory spleens were discovered near the ruptured spleen. These accessory spleens, also known as splenunculi, are a type of congenital anomaly. The question seeks to identify the most likely embryological site that gave rise to these accessory spleens. The spleen normally develops from the dorsal mesogastrium during the fifth week of embryogenesis. During this process, small islets of splenic tissue may separate and remain within the abdomen. These separate islets of splenic tissue form the accessory spleens. Therefore, the site where the issue would have occurred is with the dorsal mesogastrium, leading to the presence of accessory spleens. The development of the spleen occurs from mesenchymal cells in the dorsal mesogastrium. Any irregularities at this stage may result in anomalies like accessory spleens. Other sites such as the ventral pancreas, lesser curvature of the stomach, the origin of the celiac vessels, or hepatic diverticulum are less likely to relate to spleen development and the formation of accessory spleens. Therefore, considering the embryological development of the spleen from the dorsal mesogastrium and the fact that errors in this process result in accessory spleens, the most likely origin of this patient's congenital anomaly is within the dorsal mesogastrium. Hence, the consensus response to the prompt is (E) Within the dorsal mesogastrium.
