# Supplementary material for: Collaborative intelligence in AI: Evaluating the performance of a council of AIs on the USMLE
Source: PLOS Digit Health. 2025 Oct 9;4(10):e0000787. doi: 10.1371/journal.pdig.0000787 (PMC12510544; doi:10.1371/journal.pdig.0000787)
Supplement: S2 File — (ZIP) [file pdig.0000787.s002.zip › S2 Data Raw data files containing unprocessed question inputs and LLM outputs including transcripts of deliberations/STEP1_Question-49-1_Type-MC-NJ.docx]

**Facilitator** **[Round 1]:** A 48-year-old man comes to the physician requesting treatment for alcohol withdrawal. He reports a 30-year history of consuming 6 to 10 beers daily. He has had two citations for driving while intoxicated. He has previously experienced alcohol-associated seizures and withdrawal symptoms. His vital signs are within normal limits. Physical examination shows palmar erythema. The most appropriate pharmacotherapy in this patient most likely has which of the following mechanisms? (A) Blockade of dopamine receptors (B) Decreased activity of dopamine transporters (C) Enhancement of the effect of postsynaptic γ-aminobutyric acid (GABA) (D) Increased GABA transaminase activity (E) Inhibition of glutamate release (F) Inhibition of serotonin reuptake (G) Opening of glutamate channels (H) Stimulation of 5-hydroxytryptophan receptors

**AI_D:** RESTATING THE QUESTION: The query asks for the best pharmacological treatment for a 48-year-old man who is experiencing alcohol withdrawal. This medication should ideally function via one of the listed physiological mechanisms. PLANNING: A key consideration in managing withdrawal symptoms in an alcohol-dependent individual is to manage the hyperexcitability that arises when alcohol consumption is ceased. This hyperexcitability is primarily due to alterations in GABA and glutamate mediated neurotransmission caused by chronic alcohol abuse. Thus, a medicine that could reverse these changes would be appropriate. CLINICAL REASONING: Alcohol enhances the inhibitory neurotransmitter GABA and decreases the excitatory neurotransmitter glutamate, leading to a net depressant effect. This leads to upregulation of excitatory systems and downregulation of inhibitory systems during chronic use to compensate for this effect. When alcohol is abruptly withdrawn, it results in a state of neuronal hyperexcitability because of the underactivity of GABAergic system and overactivity of the glutamatergic system. This can manifest in symptoms of withdrawal like seizures and agitation. Thus, a pharmacological agent that could enhance the activity of the GABAergic system or inhibit the glutamatergic system would be beneficial. CONCLUDING: After considering these factors, we would ideally opt for a medication that somehow amplifies the effects of GABA (an inhibitory neurotransmitter that gets suppressed with chronic alcohol use) or diminishes the effects of glutamate (an excitatory neurotransmitter that gets overstimulated with chronic alcohol use). RESPONSE: The most effective therapeutic option in this scenario would likely function via Enhancement of the effect of postsynaptic γ-aminobutyric acid (GABA) (Option C), like benzodiazepines, which are most commonly used to manage alcohol withdrawal symptoms.

**AI_E:** RESTATING THE QUESTION: The main inquiry here pertains to the most suitable pharmacological approach for a 48-year-old chronic alcoholic undergoing alcohol withdrawal, as evidenced by a history of seizures and withdrawal symptoms. The goal is to prevent further complications, such as alcohol withdrawal-related seizures. Several pharmacological options are suggested, each relating to a distinct biochemical and neurologic mechanism. PLANNING: No assumptions are immediately apparent, however, the question implies that one of the mentioned mechanisms of action will be more effective in treating alcohol withdrawal for this patient. Pharmacologically, alcohol is a depressant which acts mainly by enhancing the effects of GABA, an inhibitory neurotransmitter. As a result, chronic alcohol users develop tolerance to its effects, leading to an imbalance in their neurological system when alcohol is abruptly withdrawn. CLINICAL REASONING: It follows that the most appropriate therapeutic approach to this case would most likely resemble the action of alcohol in the brain in order to cater to the imbalance created by its sudden absence. Alcohol is known to enhance the effects of GABA, which plays a major role in reducing neuron excitability and fostering relaxation and sleep. When alcohol is withdrawn, the body experiences a compensatory surge in neuron excitability which can lead to potentially life-threatening conditions such as seizures. CONCLUDING: Given these insights into the mechanisms by which alcohol functions in the brain and the body's responses upon its absence, the management of this patient should ideally rebalance the disrupted homeostasis. Of the options presented, the one that involves enhancement of the effect of postsynaptic GABA will help maintain neural calm, prevent seizures, and address the physiologic chaos resulting from the sudden cessation of heavy drinking. RESPONSE: Therefore, the best choice for the pharmacotherapy in this patient would most likely be the Enhancement of the effect of postsynaptic γ-aminobutyric acid (GABA) or mechanism (C).

**AI_C:** 1) RESTATING THE QUESTION: This 48-year-old gentleman is seeking medical help for alcohol withdrawal. His alcohol consumption over the years has been substantial and he has previously suffered from alcohol-associated seizures and withdrawal symptoms. The physical indication of his chronic alcohol abuse is seen in the form of palmar erythema, a redness of the palms. We're being asked to identify the most effective mechanism of pharmacotherapy for managing his alcohol withdrawal symptoms. 2) PLANNING: The fundamental assumptions revolve around the neurochemical effects of alcohol in the brain. Chronic alcohol use affects different neurotransmitter systems, including dopamine, GABA, glutamate, and serotonin. Notably, it enhances the inhibitory GABAergic system and inhibits the excitatory glutamatergic system, leading to a state of neurochemical balance. When alcohol use is abruptly discontinued, this balance is disrupted, leading to withdrawal symptoms. Therefore, the treatment should aim towards restoring this balance. 3) CLINICAL REASONING: The alternate hypothesis should ideally reestablish the neurochemical balance disrupted by chronic alcohol use. Given the chronic consumption history, the enhancement of GABA-mediated inhibition may alleviate symptoms, which is achieved through benzodiazepines, medications commonly used in this scenario. They enhance the effect of postsynaptic GABA, thus restoring some inhibitory function and reducing withdrawal symptoms. The other suggested mechanisms can affect neurological function, but they don't specifically target the perturbed GABA-glutamate balance common in alcohol withdrawal. 4) CONCLUDING: After considering the man's history of chronic alcohol use, the withdrawal symptoms arising from dysregulated GABA and glutamate activity, and the pharmacological mechanisms available, it seems that a drug enhancing the effect of postsynaptic GABA will be most beneficial. 5) RESPONSE: Therefore, the most effective pharmacotherapy for managing alcohol withdrawal symptoms in this patient would likely involve the (C) Enhancement of the effect of postsynaptic γ-aminobutyric acid (GABA).

**AI_A:** RESTATING THE QUESTION: The question is asking us to identify the most effective pharmacological treatment mechanism, among the provided options, for managing alcohol withdrawal in a 48-year-old man who has been drinking heavily for about 30 years. Palmar erythema, often associated with chronic alcohol abuse, is observed on his examination. PLANNING: To answer this question correctly, we have to understand the neurochemical basis of alcohol dependency and withdrawal, especially the role of γ-aminobutyric acid (GABA) and glutamate, which are the major inhibitory and excitatory neurotransmitters in the brain, respectively. The key assumptions for this question include: 1) that treatments blocking dopamine receptors or inhibiting serotonin reuptake are used primarily in substance use disorders like schizophrenia or depression, not typically in alcohol withdrawal; 2) that agents which increase GABA transaminase activity would decrease GABA levels, potentially worsening withdrawal symptoms; 3) that enhancing glutamate activity, by either opening glutamate channels or inhibiting glutamate release, might exacerbate withdrawal since chronic alcohol abuse often leads to upregulation of glutamate channels as a form of compensation for alcohol’s inhibitory effects. CLINICAL REASONING: Chronic alcohol consumption results in the adaptation of the central nervous system to the constant presence of alcohol. This manifests predominantly as an upregulation of excitatory neurotransmission (glutamate) and downregulation of inhibitory neurotransmission (GABA). When alcohol is abruptly discontinued, as is the case in withdrawal, the depressant effects of alcohol are no longer present, and the increased activity of excitatory neural circuits goes unchecked. Symptoms of withdrawal, including seizures, are due to this overactivity of the nervous system. CONCLUDING: Therefore, the therapeutic goal in alcohol withdrawal is to attenuate this overactivity. Agents such as benzodiazepines, which enhance the effect of postsynaptic GABA, are first-line treatment for alcohol withdrawal. This allows for increased inhibitory activity within the nervous system, thereby normalizing function and reducing symptoms. RESPONSE: On account of the above reasoning, the most effective pharmacological treatment mechanism for alcohol withdrawal in this patient would be (C) Enhancement of the effect of postsynaptic γ-aminobutyric acid (GABA).

**AI_B:** RESTATING THE QUESTION: The question presents a 48-year-old male with a long history of alcohol abuse who is seeking treatment for alcohol withdrawal. This person has signs of liver damage (palmar erythema) and a history of alcohol-associated seizures and withdrawal symptoms. The query aims to discern the likely mechanism of action of the most suitable drug to aid his withdrawal. PLANNING: Concepts and terms to be defined and understood include alcohol withdrawal, dopamine receptors, dopamine transporters, γ-aminobutyric acid (GABA), GABA transaminase, glutamate, serotonin reuptake, 5-hydroxytryptophan receptors, and palmar erythema. Key assumptions include an understanding of alcoholism pathophysiology and the mechanism of action of potential pharmacotherapy options for alcohol withdrawal. Assumption 1: Blockade of dopamine receptors or decreased activity of dopamine transporters could be beneficial, as these actions could theoretically reduce the reward sensation garnered from alcohol consumption. Assumption 2: Enhancement of GABA’s effects or increased GABA transaminase activity might be desirable given GABA’s inhibitory effects in the CNS, which could help manage withdrawal symptoms like anxiety. Assumption 3: Inhibition of glutamate release may prevent potential neurotoxicity. Assumption 4: Inhibition of serotonin reuptake or stimulation of 5-hydroxytryptophan receptors might potentially help in managing depressive symptoms frequently associated with alcohol withdrawal. CLINICAL REASONING: In a patient with alcohol withdrawal, the focus is typically on managing acute symptoms and preventing complications such as seizures or alcohol withdrawal syndrome. Alcohol enhances the effect of GABA, an inhibitory neurotransmitter in the CNS. During withdrawal, the decrease in GABA's inhibitory effect can trigger overexcitation, leading to withdrawal symptoms such as agitation and anxiety, and at severe stages, seizures and delirium tremens. CONCLUDING: Thus, the immediate focus of pharmacotherapy is to restore inhibitory control and temper excitability, which can be done most directly by enhancing the effect of postsynaptic GABA. RESPONSE: The most appropriate pharmacotherapy in this patient likely involves the enhancement of the effect of postsynaptic γ-aminobutyric acid (GABA) (Option C). Drugs such as benzodiazepines, which enhance GABA's effect in the CNS, are typically first-line treatment for alcohol withdrawal. They mitigate the CNS overactivity that results from withdrawal and reduce the risk of severe complications such as seizures and delirium tremens.

**Facilitator** The question asks for the most effective pharmacological treatment for a 48-year-old man experiencing alcohol withdrawal. The patient has a long history of heavy alcohol consumption, and has previously suffered from alcohol-associated seizures and withdrawal symptoms. The physical examination reveals palmar erythema, a sign of chronic alcohol abuse. The key to answering this question lies in understanding the neurochemical effects of alcohol in the brain. Chronic alcohol use enhances the inhibitory neurotransmitter γ-aminobutyric acid (GABA) and inhibits the excitatory neurotransmitter glutamate, creating a neurochemical balance. When alcohol use is abruptly discontinued, this balance is disrupted, leading to withdrawal symptoms. The goal of pharmacotherapy in this case is to restore this balance and manage the hyperexcitability that arises when alcohol consumption is ceased. This can be achieved by enhancing the effect of postsynaptic GABA, which would increase inhibitory activity within the nervous system, thereby normalizing function and reducing symptoms. Therefore, the consensus among the responses is that the most effective pharmacological treatment mechanism for alcohol withdrawal in this patient would be the enhancement of the effect of postsynaptic γ-aminobutyric acid (GABA) (Option C). This is typically achieved through the use of benzodiazepines, which are considered first-line treatment for alcohol withdrawal. They mitigate the central nervous system overactivity that results from withdrawal and reduce the risk of severe complications such as seizures and delirium tremens.
