# Supplementary material for: Collaborative intelligence in AI: Evaluating the performance of a council of AIs on the USMLE
Source: PLOS Digit Health. 2025 Oct 9;4(10):e0000787. doi: 10.1371/journal.pdig.0000787 (PMC12510544; doi:10.1371/journal.pdig.0000787)
Supplement: S2 File — (ZIP) [file pdig.0000787.s002.zip › S2 Data Raw data files containing unprocessed question inputs and LLM outputs including transcripts of deliberations/STEP1_Question-5-1_Type-MC-NJ.docx]

**Facilitator** **[Round 1]:** A 39-year-old man comes to the physician because of a 6-month history of progressive shortness of breath. He has had a cough productive of white sputum for 2 years. He smoked 1 pack of cigarettes daily for 16 years but quit 10 years ago. He is in mild respiratory distress with pursed lips and a barrel chest; he is using the accessory muscles of respiration. Breath sounds are distant and crackles are present in the lower lung fields bilaterally. Pulmonary function tests show a decreased FEV1:FVC ratio, increased residual volume, and decreased diffusion capacity. An x-ray of the chest shows hyperinflation and hypertranslucency of the lower lobes of both lungs. Which of the following is the most likely diagnosis? (A) Asthma (B) Bronchiectasis (C) Chronic pulmonary fibrosis (D) Cystic fibrosis (E) Emphysema

**AI_B:** RESTATING THE QUESTION: The query presents details of a 39-year-old male's prolonged health ailments, inclusive of progressive shortness of breath spanning six months, a cough productive of white sputum lasting two years, and a cycling specialization of complex respiratory characteristics, quantitatively evidenced by pulmonary function tests and an X-ray report. The man is a former smoker, but ceased the habit a decade ago. The issue is to identify his condition from five provided possible respiratory disorders. PLANNING: The indicated symptoms, along with test results, suggest that the patient could have any of the five diseases. However, some facts such as a history of smoking, progressive shortness of breath, hyperinflated chest on X-ray, decreased FEV1:FVC ratio, and crackles in the lower lung fields are notably suggestive of one disease. The primary disease conditions from the options provided that these symptoms could suggest include Chronic Obstructive Pulmonary Disease (COPD), specifically emphysema or chronic bronchitis. CLINICAL REASONING: A history of smoking is a primary risk factor for COPD, which compresses emphysema and chronic bronchitis. The symptoms of pursed lips and barrel chest hint at an emphysematous change in the lungs, established with hyperinflation evident in the X-ray. Furthermore, presence of distant breath sounds, increased residual volume along with decreased FEV1:FVC ratio are hallmark signs of obstructive lung disorders, pointing towards emphysema again. Chronic Pulmonary Fibrosis usually presents with diffused bilateral infiltrates on X-ray, Cystic fibrosis often has recurrent infections, Bronchiectasis has chronic cough with copious purulent sputum and Asthma features reversible airway obstruction. These symptoms and signs are inconsistent with the patient's test results and clinical picture, narrowing down the diagnosis to emphysema. CONCLUDING: By correlating the clinical symptoms, relevant medical history and the findings from the different diagnostic tests, it could be deduced that the pathology most consistent with the patient's overall clinical picture is Emphysema. RESPONSE: The most likely diagnosis for this 39-year-old male patient, considering his symptoms, medical history and test results, is (E) Emphysema.

**AI_D:** RESTATING THE QUESTION: We have a 39-year-old gentleman, who is exhibiting distressing signs and symptoms in the form of progressive shortness of breath and a persistent cough for quite some time. The indication of his history as a smoker, his current breathing pattern, pseudogynecomastia, and notable physical characteristics such as distant breath sounds and lower lung field crackles could point to a potential respiratory disease. The laboratory findings, including a decreased FEV1:FVC ratio, increased residual volume, and decreased diffusion capacity plus x-ray results showing hyperinflation and hypertranslucency of the lower lobes suggest a chronic obstructive pulmonary disorder. The question asks us to determine the most likely diagnosis from a list that includes: Asthma, Bronchiectasis, Chronic Pulmonary Fibrosis, Cystic Fibrosis, and Emphysema. PLANNING: Considering the patient's clinical profile, it's crucial to consider the effect of his prior smoking status on his current respiratory health. The majority of the listed diseases can develop in individuals of the patient's demographic, however, the combination of a significant smoking history, productive cough, and noted physical examination and radiographic findings suggest a specific pattern relating to chronic obstructive pulmonary disorders (COPD), namely emphysema. To support this assumption, we have to consider the possible discrepancies between presenting factors and symptoms of the diseases listed in the question. CLINICAL REASONING: Asthma primarily presents with a reversible airflow obstruction and bronchial hyper-responsiveness, which doesn't align with the patient's irreversible PFT changes and x-ray findings. Bronchiectasis generally yields more purulent and copious sputum production accompanied by recurrent infections, which hasn't been mentioned in this case. Chronic pulmonary fibrosis usually presents with bibasal crackles and shows reticulonodular shadowing in x-ray, thus it doesn't match the symptoms nor the radiographic images of this patient. Cystic fibrosis, a congenital disorder primarily found in younger populations, mostly presents with recurrent infections, malabsorption, and failure to thrive, none of which are present in this case. CONCLUDING: After a step-by-step elimination of the other diagnoses, it leaves us with Emphysema. This disease typically presents in those with significant smoking history, with symptoms such as progressive shortness of breath, a chronic cough, and physical findings resembling a 'barrel chest' due to hyperinflation of lungs. The physiological changes in patients with Emphysema reduce the lungs' elasticity and cause small airway collapse, leading to obstructive pathology, which is characterized by a decreased FEV1:FVC ratio, and increased residual volume, matching our patient's PFT results. The hypertranslucency on the chest x-ray is also typical of emphysema due to the destruction of lung parenchyma. RESPONSE: Thus, given the clinical presentation, physical examination, history of smoking, pulmonary function tests, and x-ray findings, the most likely diagnosis for this 39-year-old man is (E) Emphysema.

**AI_E:** 1) RESTATING THE QUESTION: This patient, a former smoker, is presenting with symptoms of progressive shortness of breath, a productive cough, and physical signs such as pursed lips, barrel chest, use of accessory respiratory muscles, and crackles heard in the lower lung fields. The Lung Function Tests (LFTs) show a decreased Forced Expiratory Volume in 1 second (FEV1) to Forced Vital Capacity (FVC) ratio, increased residual volume, and decreased diffusion capacity. The radiological findings indicate hyperinflation and increased transparency in the lower lobes. 2) PLANNING: Let's discuss each assumption corresponding to the provided choices. Asthma is characterized by episodes of wheezing, breathlessness, chest tightness and coughing, mostly at night or early morning, but may lack the productive cough and pursed lips sign seen in this patient. Bronchiectasis presents with a chronic cough with large amounts of sputum, and breath sounds would likely show wheezing or crackles, although the increased residual volume, decreased diffusion capacity, and hypertranslucency on radiography may not be present. Chronic pulmonary fibrosis typically presents with dry cough, but the radiography findings may show interstitial markings rather than hyperinflation and lower lobe transparency. Cystic fibrosis is unlikely in a patient of this age that's presenting with these symptoms for the first time. Emphysema, a type of chronic obstructive pulmonary disease (COPD), fits with the smoking history and presents with dyspnea, cough, use of accessory muscles for breathing, barrel chest, decreased breath sounds, and crackles. Radiography and LFTs would show the hyperinflation, hypertranslucency, increased residual volume and decreased diffusion capacity. 3) CLINICAL REASONING: The productive cough and progressive dyspnea, especially in a former smoker, point to the likelihood of a chronic obstructive lung disease. The physical signs and symptoms fit most closely with emphysema, which is a form of COPD, and a condition that's often related to a history of smoking. The presence of a barrel chest is an alteration caused by air being trapped in the lungs over time, leading to increased anterior-posterior diameter of the chest. In terms of differential diagnosis, the LFTs and x-ray results provide critical information. The decreased FEV1:FVC ratio indicates obstructive rather than restrictive pattern of the lung disease, narrowing our choices to either asthma or COPD. The distinction comes in the presentation; asthma is characterized by episodic attacks rather than progressive worsening. Additionally, the hyperinflation and residual volume would not be seen in asthma. This goes along with the increased translucency seen on radiography, indicative of emphysema. 4) CONCLUDING: By systematically analyzing the presented physical signs and symptoms, history of smoking, and diagnostic findings such as LFTs and x-ray results in this patient, it was hypothesized that the most likely assumption would be the presence of a chronic obstructive pulmonary disease. The disease course, clinical presentation, and diagnostic findings are most consistent with emphysema. 5) RESPONSE: Therefore, the most likely diagnosis for this patient is (E) Emphysema.

**AI_C:** 1) RESTATING THE QUESTION: In a nutshell, a 39-year-old man presents with shortness of breath that has been progressively worsening over the past six months and a cough producing white sputum which has been ongoing for two years. Despite ceasing his smoking habit a decade ago, the patient exhibits symptoms suggestive of respiratory distress. His lung sounds are distant and there are crackles bilaterally in the lower lung fields. The results of his pulmonary function tests and chest x-ray point to significant lung pathology. We need to determine, among the options provided, the most likely diagnosis given these findings. 2) PLANNING: The number of potential causes for the patient's symptoms is vast. Still, taking into account the pathological findings and his smoking history, conditions like asthma, bronchiectasis, chronic pulmonary fibrosis, cystic fibrosis, and emphysema should be considered. Although the symptoms can be caused by either of these conditions, the distinctive features of each diagnosis must be matched with the patient's findings. 3) CLINICAL REASONING: While this patient's symptoms could be attributable to any of the conditions, there are several characteristics that help narrow down the diagnosis. First, the history of his extended tobacco use, even though he has quit a decade ago, is a major risk factor for developing emphysema. Secondly, the physical findings suggestive of hyperinflation (barrel chest and pursed lip breathing), together with distant breath sounds and bilateral lower lobe crackles, lean towards emphysema. Finally, the pulmonary function tests show obstructive pattern (decreased FEV1:FVC ratio, increased residual volume) in conjunction with decreased diffusion capacity, which are classical indicators of emphysema. The chest x-ray findings of hyperinflation and hypertranslucency are also supportive of this diagnosis. 4) CONCLUDING: Emphysema, a type of chronic obstructive pulmonary disease (COPD), is primarily caused by smoking and is characterized by irreversible enlargement and damage to the alveolar sacs of the lungs. Over time, this can lead to symptoms such as breathlessness, coughing, and other signs of respiratory distress, as observed in this patient. The presence of a decreased FEV1:FVC ratio and increased residual volume on pulmonary function tests, hyperinflation and hypertranslucency on the chest X-ray, and the patient's history of long-term smoking suggest that this is the most likely diagnosis. 5) RESPONSE: The patient's presenting symptoms coupled with his history and test findings align most accurately with the clinical profile of emphysema (E). Although the other diseases listed may share some similar features, the combination of this patient's smoking history, physical examination findings, and specific test results point most clearly to emphysema.

**AI_A:** RESTATING THE QUESTION: The inquiry in question concerns the diagnosis of a 39-year-old male patient who presents with a chronic and progressive respiratory issue. He has a history of producing white sputum coupled with shortness of breath for six months. His respiratory profile includes a history of smoking that ceased approximately a decade ago and current symptoms such as pursed lips, use of accessory muscles of respiration, and a barrel chest, suggesting a compensatory response to chronic respiratory stress. Physical examination and test results, such as distant breath sounds, crackles in the bilateral lower lung fields, and abnormal pulmonary function tests (PFTs) with a decreased FEV1:FVC ratio, increased residual volume, and decreased diffusion capacity, provide a clue to the underlying pathology. The chest x-ray indicates exacerbation of the condition given the hyperinflation and hypertranslucency seen. Given this clinical scenario, we are required to identify the most likely diagnosis from the provided options. PLANNING: We could make several assumptions based on the information given. The first assumption we could consider is that exposure to long-term smoking damage has resulted in significant lung disease despite cessation. The second assumption is that the patient's current clinical picture shows a chronic restrictive lung disease due to the low FEV1:FVC ratio and decreased diffusion capacity on PFTs. The third assumption would be that the patient has a lungs disorder triggered by an allergic response, given his symptom of white sputum production. These assumptions guide us to consider diseases like asthma, bronchiectasis, chronic pulmonary fibrosis, cystic fibrosis, and emphysema as possible diagnoses. However, assuming that the patient's long smoking history and the duration of symptoms have contributed to his current disease status seems most clinically appropriate. CLINICAL REASONING: To make a clinical decision, we need to correlate the patient's exposure history, clinical symptoms, physical examination findings, and radiological and PFT results to the typical pathophysiology of the lung diseases mentioned. Asthma is often an episodic disease with symptoms such as wheezing, chest tightness, and shortness of breath triggered by specific allergens or irritants, less likely in this case given the progression and chronicity of symptoms. Bronchiectasis is characterized typically by periodically intense episodes of cough coupled with the creation of copious purulent sputum, not fitting the scenario either. Chronic pulmonary fibrosis, usually progressive, leads to restrictive rather than obstructive disease, unlike our patient's obstructive disease pattern on PFTs. Cystic fibrosis often manifests earlier in life with the presence of other systemic symptoms such as gastrointestinal and nutritional abnormalities. Emphysema, part of Chronic Obstructive Pulmonary Disease (COPD) spectrum, is related to tobacco smoking, leads to progressive symptoms, evident in obstructive abnormality in PFTs, hyperinflation, decreased lung sounds, and is associated with the pathognomic "barrel chest." CONCLUDING: The patient likely suffers from a chronic, progressive obstructive respiratory disease linked to his past smoking history. The negative pulmonary function test results showing obstructive pattern, clinical symptoms including use of accessory muscles of respiration, distant breath sounds, and compensate signs like pursed lips and a barrel chest, all suggest a diagnosis associated with long-term smoke exposure. Emphysema matches best with this description; it is a disease characterized by permanent enlargement and destruction of the airspaces distal to the terminal bronchioles, often linked to tobacco smoking. RESPONSE: (E) Emphysema is the most likely diagnosis in this case.

**Facilitator** The consensus statement synthesizes the responses of AI_A, AI_B, AI_C, AI_D, and AI_E as follows: The question presents a 39-year-old male patient with a history of smoking, who has been experiencing progressive shortness of breath for six months and a cough producing white sputum for two years. His physical examination reveals signs of respiratory distress, including pursed lips, a barrel chest, and the use of accessory muscles of respiration. His pulmonary function tests show a decreased FEV1:FVC ratio, increased residual volume, and decreased diffusion capacity, while his chest x-ray shows hyperinflation and hypertranslucency of the lower lobes of both lungs. The task is to identify the most likely diagnosis from the provided options: Asthma, Bronchiectasis, Chronic Pulmonary Fibrosis, Cystic Fibrosis, and Emphysema. Given the patient's history of smoking, his clinical symptoms, and the results of his physical examination and tests, the consensus is that the patient likely suffers from a chronic, progressive obstructive respiratory disease linked to his past smoking history. The symptoms and signs of the other diseases listed in the options do not align with the patient's clinical picture. Asthma is characterized by episodic symptoms and reversible airway obstruction, Bronchiectasis typically presents with copious purulent sputum and recurrent infections, Chronic Pulmonary Fibrosis leads to restrictive rather than obstructive disease, and Cystic Fibrosis often manifests earlier in life with systemic symptoms such as gastrointestinal and nutritional abnormalities. On the other hand, Emphysema, a type of Chronic Obstructive Pulmonary Disease (COPD), is associated with tobacco smoking and leads to progressive symptoms. It is characterized by permanent enlargement and destruction of the airspaces distal to the terminal bronchioles. The patient's clinical symptoms, including the use of accessory muscles of respiration, distant breath sounds, and compensatory signs like pursed lips and a barrel chest, along with the obstructive pattern on pulmonary function tests and hyperinflation seen on the chest x-ray, all suggest a diagnosis of Emphysema. Therefore, the consensus is that the most likely diagnosis for this 39-year-old male patient, considering his symptoms, medical history, and test results, is (E) Emphysema.
