# Supplementary material for: Collaborative intelligence in AI: Evaluating the performance of a council of AIs on the USMLE
Source: PLOS Digit Health. 2025 Oct 9;4(10):e0000787. doi: 10.1371/journal.pdig.0000787 (PMC12510544; doi:10.1371/journal.pdig.0000787)
Supplement: S2 File — (ZIP) [file pdig.0000787.s002.zip › S2 Data Raw data files containing unprocessed question inputs and LLM outputs including transcripts of deliberations/STEP1_Question-50-1_Type-MC-NJ.docx]

**Facilitator** **[Round 1]:** A 48-year-old woman with type 2 diabetes mellitus comes to the physician for a follow-up examination. Current medications are metformin and once-daily insulin. She travels frequently and works long hours. She says that her meals are usually fast food. She leads a sedentary lifestyle. She often forgets to measure her blood glucose concentration. Her last hemoglobin A1c was measured as 8.4%. Which of the following is the most appropriate action by the physician to help this patient improve her diabetic control? (A) Create an exercise regimen for the patient (B) Explore barriers to diet adherence (C) Increase the patient's insulin dosage (D) Increase the patient's metformin dosage (E) Measure the patient's blood glucose concentration (F) Order measurement of the patient's microalbumin concentration (G) Refer the patient to a nutritionist

**AI_A:** RESTATING THE QUESTION: The query here is about the best course of action for a physician to manage a 48-year-old female patient with type 2 diabetes mellitus. Information includes a lifestyle involving frequent travel, long working hours, and poor dietary habits, i.e., fast food and sedentary habits. Her current treatment includes metformin and once-daily insulin, but compliance with measuring her blood glucose levels seems inconsistent. Her HbA1c values suggest her blood sugar control isn't adequate. PLANNING: Multiple factors could be contributing to her inadequate diabetic control: lack of exercise, fast food diet, poor medication administration, and irregular blood glucose monitoring. We could consider modifying any of these factors; however, real-world changes require addressing the patient's lifestyle, complexity, and practicality. CLINICAL REASONING: Given the patient's hectic schedule, frequent travels, and preference for quick meals, her adherence to a set exercise regimen or accurate insulin dosage could be challenging. Increasing metformin wouldn't address the root issue of her lifestyle choices. Regular glucose determination might reveal fluctuating levels, but it won't facilitate control. Microalbumin measurement is more of an investigation tool for renal involvement in diabetes, not directly helping in controlling blood sugar. Looking at these assumptions, modifications to her diet appears to be the most practical and high-yield approach. CONCLUDING: Based on the patient's lifestyle and the potential impact on her diabetes, the most clinically prioritized step would be to address her dietary habits. Her hectic schedule leads her to eat fast food more often, which is likely high in carbohydrates, fats, and sugars - notoriously poor for diabetic control. RESPONSE: Therefore, the most appropriate action by the physician would be (B) to explore barriers to diet adherence so we can formulate a practical and sustainable dietary plan which could significantly improve her diabetic control. (G) Referring her to a nutritionist could further reinforce this focus, providing her with expert guidance and dietary plans suitable for her lifestyle and diabetes control.

**AI_D:** RESTATING THE QUESTION: According to the query, a 48-year-old female, diagnosed with Type 2 diabetes mellitus, visits a doctor for a routine check-up. Her treatment regimen includes metformin and a daily dose of insulin. She has a busy schedule, frequent travels, and relies mostly on fast food. She leads a rather inactive lifestyle and often neglects to monitor her blood sugar levels. The previous measurement of her Hemoglobin A1c, a reliable measure of long-term blood glucose control was 8.4%, which is higher than the recommended target level of less than 7%. The question at large is: What would be the most suitable course of action for the physician to help the patient improve control over her diabetes? PLANNING: Permit me to establish the parameters that must be considered here. The efficacy of the current medication, the patient's proactive role in managing her health, her lifestyle inclining towards physical inactivity, and an improper diet are key factors to consider. Assumptions can revolve around modifying the patient's medication, incorporating an active lifestyle, altering dietary habits, and ensuring regular monitoring of blood glucose levels. Upon studying these issues, the priority seems to be rooted in the exploration of barriers to diet adherence. CLINICAL REASONING: The patient's story reveals a pattern of poor dietary habits, exacerbated by her busy schedule and frequent travel, making her dependent on fast food. A major factor contributing to the uncontrolled diabetes, as indicated by high HbA1c levels, could be this unhealthy diet. The efficacy of medications, though crucial, can only go as far as the extent of dietary control allows. A well-balanced diet, curated for her diabetic condition, is key in managing blood glucose levels. Therefore, it is imperative to understand what's stopping her from adhering to an appropriate diet. CONCLUDING: The path to improved diabetic control for this patient would require a comprehensive approach, entailing appropriate medication, regular monitoring, physical activity, and a suitable diet. However, addressing barriers to diet adherence should take precedence, as it could be the linchpin obstructing effective diabetic management. RESPONSE: Bearing the aforementioned clinical reasoning in mind, an apt approach here would be to (B) Explore barriers to diet adherence. Understanding these barriers could allow tailored solutions, potentially fostering better adherence to a suitable diet, leading to improved diabetic control.

**AI_B:** (1) RESTATING THE QUESTION: The situation involves a 48-year-old woman with type 2 diabetes mellitus struggling to manage her condition. Her current glycaemic control, as reflected by the hemoglobin A1c of 8.4%, is less than optimal. She leads a busy life crammed with work and travel and often relies on fast food. She also doesn't keep up with regular blood glucose concentration monitoring. The question now is figuring out the best strategy the doctor can use to optimize her diabetes control. (2) PLANNING: To address this, we need to analyze various assumptions. These include the efficacy of her current medication dosage, her exercise regimen, diet, adherence to blood glucose measurements, and potential for renal complications. Each of these elements has potential impacts on diabetes management. However, given her lifestyle patterns—reliance on fast food and a lack of regular physical activity—it seems that a crucial concern here is her dietary habits. Exploring the barriers to healthier eating habits may render more substantial results than adjusting medication or implementation of exercise regimens for a patient who leads such a busy life. (3) CLINICAL REASONING: Diabetes is strongly influenced by diet, as high carbohydrate and sugar intake can contribute to hyperglycemia. Fast food often contains high levels of sugar, salt, and unhealthy fats, all of which can exacerbate this condition. Insufficient physical activity also compounds this problem as exercise helps in burning excess glucose. The fact that she seems to have inconsistent self-monitoring only makes the situation worse. She might not realize the extent to which such habits are affecting her control over diabetes if she is not frequently checking her blood glucose levels. So, addressing diet may have a more immediate and significant impact compared to the other aspects. (4) CONCLUDING: Improving her eating habits, given her sedentary lifestyle and frequent consumption of fast food, should be an immediate line of action. Enlisting professional assistance for this, such as a nutritionist who could tailor a healthy, easy-to-follow diet plan suitable to her lifestyle, may be beneficial. However, it's crucial to first understand what obstructs her from adhering to a healthier diet before proceeding to such steps. (5) RESPONSE: Thus, I believe the most appropriate action by the physician is to (B) Explore barriers to diet adherence. Taking time to understand her challenges might pave the way to more comprehensive and realistic strategies to help her take control of her diabetes.

**AI_E:** RESTATING THE QUESTION: The current case concerns a 48-year old woman who is medically managed for type 2 diabetes with metformin and insulin. Her overall lifestyle, which includes frequent travelling, long work hours, fast food diet, and sedentary habits, has led to inadequate glucose management with an elevated hemoglobin A1c level of 8.4% (the target for most patients is less than 7%). Additionally, she is inconsistent about monitoring her blood glucose concentrations. Given this context, it's necessary to identify the most impactful interventions the attending physician can make to improve her diabetes control. PLANNING: The options given range from lifestyle interventions such as diet and exercise modification, medication adjustments in the form of increasing dosages, more consistent monitoring of blood glucose, assessing for early signs of nephropathy, or referring the patient to a nutritionist. Each of these assumptions holds its own merit, but it's important to first identify what is contributing most prominently to the suboptimal control of her diabetes. CLINICAL REASONING: It's noteworthy that the patient works long hours, frequently travels, and relies heavily on fast food for her meals. Furthermore, there's no mention of hi-fidelity to a diet suitable for a diabetic patient. In conjunction, these factors suggest significant dietary obstacles that might be exacerbating her diabetic control problems. Before making assumptions about the adequacy or efficiency of her medication dosages, it's essential to secure a baseline of healthy dietary behavior because medications alone often cannot manage Type 2 Diabetes optimally in the backdrop of unhealthy eating habits. CONCLUDING: Based on these facts, the priority is to initially address the root cause of her poor diabetes control which appears to lie in her dietary behaviors and habits. Once the diet component is addressed, and if the blood glucose control remains suboptimal, then other avenues such as adjusting her medication regimen or directing her to consistent blood glucose monitoring can be pursued. In her case, since she is often consuming fast food and has erratic dietary patterns due to her work-travel lifestyle, an expert opinion in the form of a nutritionist could prove valuable to devise a personalized meal plan taking her lifestyle into consideration. RESPONSE: Therefore, the most appropriate action by the physician, under these circumstances, to help this patient improve her diabetic control would be (G) Refer the patient to a nutritionist. A nutritionist can provide a comprehensive and individualized dietary plan that takes into account her busy lifestyle and frequent travels. This would be the most impactful first step towards better control of her diabetes.

**AI_C:** (1) RESTATING THE QUESTION: In simpler terms, a 48-year-old female patient with type 2 diabetes is struggling with her disease management. Despite taking suitable medications, her hemoglobin A1c (measure of average blood glucose for the past two to three months) remains high (8.4% whereas an ideal value would be less than 7%). She has a busy and travel-intense job, implying an irregular schedule. Moreover, she relies on fast food for her meals and doesn't maintain active habits. Crucially, she often skips checking her blood glucose levels. The query here is about the most effective step a doctor can take to help improve the patient's control over her diabetes. (2) PLANNING: There are multiple factors contributing to this patient's poorly controlled diabetes, ranging from non-adherence to dietary guidelines and a lack of exercise to the irregular monitoring of blood glucose. While it might seem reasonable to increase medication dosage, adjusting treatment without addressing these lifestyle factors may not improve the situation. Increasing insulin dosage or metformin amounts may lead to unwanted side effects and doesn't necessarily address the root problems. Rather, aspects like diet (Patient mostly consumes fast food) and exercise (Sedentary lifestyle) that strongly impact diabetic control need attention. (3) CLINICAL REASONING: It's essential to address the salient cause of poorly controlled diabetes in our case, which is the patient's lifestyle. Her diet is primarily composed of fast food, which is high in carbohydrates and fats. This can cause a significant spike in blood glucose. She also has a sedentary lifestyle, which doesn't help metabolize this glucose. In addition to these, she's frequently travelling and has long work hours, which not only cause stress (another blood glucose-elevating factor) but also make medication adherence difficult. Hence, to improve her diabetic control, the best assumption that prioritizes the factor contributing to her poorly controlled diabetes is exploration and mitigation of barriers to healthier lifestyle choices. (4) CONCLUDING: Going with the prioritized assumption, the most effective step would be to understand what's hindering the patient from adhering to a healthier lifestyle, aiming for appropriate diet and sufficient exercise. For this, exploring barriers to diet adherence (Option B) would be a major initial step. Once these barriers are identified and managed, her overall lifestyle can be improved, which would reflect better in her diabetic control than merely adjusting her medication. (5) RESPONSE: Therefore, in response to the question about helping this 48-year-old woman with type 2 diabetes mellitus improve her diabetic control, the physician should "Explore barriers to diet adherence" (Item B).

**Facilitator** **[Round 2]:** SUMMARIES: AI_A: The council member suggests that the physician should explore barriers to diet adherence and consider referring the patient to a nutritionist. They believe that the patient's lifestyle and dietary habits are the main contributors to her poor diabetes control. AI_B: This council member also recommends exploring barriers to diet adherence. They emphasize the importance of understanding the patient's challenges to develop comprehensive and realistic strategies for managing her diabetes. AI_C: The council member suggests that the physician should explore barriers to diet adherence. They believe that addressing the patient's lifestyle, particularly her dietary habits, is the most effective way to improve her diabetes control. AI_D: This council member also suggests exploring barriers to diet adherence. They believe that understanding these barriers could lead to tailored solutions and better adherence to a suitable diet, leading to improved diabetes control. AI_E: The council member recommends referring the patient to a nutritionist. They believe that a nutritionist can provide a comprehensive and individualized dietary plan that takes into account the patient's busy lifestyle and frequent travels, which would be the most impactful first step towards better control of her diabetes. KEY DIFFERENCES: The key difference in the council members' perspectives lies in the approach to addressing the patient's dietary habits. While AI_A, AI_B, AI_C, and AI_D suggest that the physician should explore barriers to diet adherence, AI_E directly recommends referring the patient to a nutritionist. QUESTION TO CLARIFY DISAGREEMENTS: Why do you believe that exploring barriers to diet adherence (or referring the patient to a nutritionist, in the case of AI_E) is the most effective first step in improving the patient's diabetes control? How do you see this approach addressing the patient's lifestyle and dietary habits?. Based on your preceding response, how would you answer the original question: [A 48-year-old woman with type 2 diabetes mellitus comes to the physician for a follow-up examination. Current medications are metformin and once-daily insulin. She travels frequently and works long hours. She says that her meals are usually fast food. She leads a sedentary lifestyle. She often forgets to measure her blood glucose concentration. Her last hemoglobin A1c was measured as 8.4%. Which of the following is the most appropriate action by the physician to help this patient improve her diabetic control? (A) Create an exercise regimen for the patient (B) Explore barriers to diet adherence (C) Increase the patient's insulin dosage (D) Increase the patient's metformin dosage (E) Measure the patient's blood glucose concentration (F) Order measurement of the patient's microalbumin concentration (G) Refer the patient to a nutritionist]

**AI_D:** RESPONSE: Given the information provided and the factors contributing to the patient's suboptimal diabetes control, the most appropriate action by the physician would be to explore barriers to diet adherence (Option B). The patient's lifestyle factors such as frequent travel, long work hours, reliance on fast food, sedentary lifestyle, and irregular monitoring of blood glucose concentration could be barriers preventing her from adhering to an appropriate diet and lifestyle regimen. Thus, understanding and addressing these barriers will be crucial in aiding the management of her diabetes. It's also important to keep in mind that several alternate plans could be derived from understanding these barriers, one of which might include referral to a nutritionist (Option G). Still, the first step should be recognizing and discussing the existing barriers with the patient.

**AI_C:** AI_A: In this case, exploring barriers to diet adherence is a crucial first step because it deals with the root of the problem. The patient consumes fast food regularly, indicating a problem with diet adherence. A diet high in sugar and poor in nutritional quality can exacerbate blood glucose levels and increase the risk of complications in type 2 diabetes. Additionally, it is important to understand the reasons for this dietary pattern, whether it's due to work-related stress, convenience, lack of education about food choices, or other factors. Once these barriers are identified, proper steps can be taken, which may include referral to a nutritionist or improving meal planning strategies. AI_E: Referring the patient to a nutritionist is beneficial because it provides personalized and practical solutions to manage diabetes more effectively. Nutritionists can help understand the dietary patterns and personal choices that led to the current situation, plan meals that are convenient yet nutritious, and help implementing dietary changes without drastically altering the patient's lifestyle. As meals profoundly impact blood sugar levels and overall diabetes management, working with a nutritionist would help the patient make necessary adjustments that are conducive to long hours of work and frequent travel. Therefore, based on these responses, both AI_A and AI_E would choose option (B) Explore barriers to diet adherence and option (G) Refer the patient to a nutritionist, respectively, as the most appropriate action to help this patient improve her diabetic control. They both highlight the importance of tackling the dietary issues at hand to enhance diabetes management effectively.

**AI_A:** RESPONSE: The key to managing type 2 diabetes mellitus is in our patient's hands. The patient's lifestyle, specifically her dietary habits and sedentary behavior, indicate a strong need for lifestyle intervention. Her elevated hemoglobin A1c shows that her glucose levels are fluctuating and are not well-controlled. Additionally, her reliance on fast food, irregular meal schedules due to her busy lifestyle and forgetfulness in checking her blood glucose levels indicates several barriers to diet adherence. As a Matryoshka doll with layers within layers, each barrier needs to be addressed in detail; why does she often forget to check her blood glucose levels? Is it because she doesn't value its importance, or is it due to her busy schedule? Why does she rely on fast food? Is it due to lack of time or lack of knowledge in choosing healthier meal options? Exploring these barriers can provide crucial information that the physician can use to tailor lifestyle interventions to the patient's particular challenges and preferences, empowering the patient and improving her overall control of the disease. This is where a nutritionist can play an important role as they have the expertise to develop a realistic and manageable meal plan for patients with busy lifestyles like ours. Therefore, my answer is (B) Explore barriers to diet adherence and I also strongly recommend (G) Refer the patient to a nutritionist. It's important for the patient to understand her dietary habits and improve her diet with the help of a nutritionist. This way, we're not only administering a band-aid solution via increased medication dosage, but we're addressing the root cause of the problem – lifestyle.

**AI_E:** AI_A: In my opinion, exploring barriers to diet adherence provides a broader and more in-depth understanding of the patient's lifestyle, food choices, and their associated factors. This translates into a bigger impact on her diabetes control. It also helps the physician develop a comprehensive and realistic plan that fits into her busy lifestyle and frequent travels. My response to the question is (B) Explore barriers to diet adherence. AI_B: I agree with AI_A, understanding and overcoming barriers to diet adherence is pivotal. Considering her busy life and frequent travels, the physician could use this information to help in tailoring customized strategies such as meal prepping or choosing healthier options when having fast food. Thus, my answer remains (B) Explore barriers to diet adherence. AI_C: While referring to a nutritionist (as AI_E suggested) may also be beneficial, the physician actively exploring these barriers creates a platform for direct and open communication with the patient. This may provide more immediate results, making the intervention (B) Explore barriers to diet adherence the preferable option. AI_D: Exploring barriers to diet adherence engages the patient in her own treatment. It helps the physician to personalize her dietary interventions. Drastic dietary changes may not be sustainable for her lifestyle. Tailored, smaller changes will be more acceptable and sustainable. I endorse the action (B) Explore barriers to diet adherence. AI_E: While I agree with the importance of exploring barriers to diet adherence, referring the patient to a nutritionist offers specific expertise to create a dietary plan considering her busy lifestyle, preferences, and travels. Therefore, my answer to the original question is (G) Refer the patient to a nutritionist.

**AI_B:** RESTATING THE QUESTION: This 48-year-old female has type 2 diabetes and she's been taking metformin and insulin. Despite using these medications, her blood sugar levels are not well-controlled, as shown by a high hemoglobin A1c of 8.4%. Her lifestyle includes frequent travel, long working hours, fast food meals, sedentary behavior, and inconsistent monitoring of blood sugar levels. In order to assist the patient in achieving better control of her diabetes, various options are presented including creating an exercise program, exploring barriers to diet adherence, adjusting medication dosages, monitoring blood glucose concentration, checking the patient's microalbumin level, or referring the patient to a nutritionist. PLANNING: We need to think about the significant elements that contribute to poor diabetic control in this case. This patient's lifestyle, diet, lack of physical exercise, and inconsistent monitoring of glucose levels appear to be the key factors. Medical strategies like increasing metformin or insulin doses may not be effective without addressing these underlying issues. Additionally, measuring the patient's blood glucose or ordering a microalbumin level will only give us more data about the disease status, but not necessarily result in better control. CLINICAL REASONING: Type 2 diabetes is a chronic disease managed mainly with lifestyle modifications and adherence to medication. A patient's lifestyle, including diet and physical activities, plays a significant part in managing this disease. Given the patient's current lifestyle – long working hours, frequent travel, fast food consumption, and sedentary lifestyle – it appears her diet and lack of exercise could be major contributors to her poorly controlled diabetes. Adjusting her insulin or metformin dosage wouldn't necessarily be effective without addressing these core issues, as the root problem is not purely medication dosage but lifestyle. CONCLUDING: Based on the clinical reasoning process, understanding barriers to diet adherence or referring the patient to a nutritionist, can provide pragmatic and tailored solutions suitable for the patient's lifestyle. This coupled with regular follow-ups to monitor both implementation and progress can ensure better diabetes control. RESPONSE: Given the context and facts present, exploring barriers to diet adherence will assist in tailoring a suitable diet plan that takes into account the patient's lifestyle and preferences, allowing for better compliance and subsequently better diabetes control. Hence, the most appropriate action by the physician to help this patient improve her diabetic control is to (B) Explore barriers to diet adherence.

**Facilitator** **[Round 3]:** Summary: AI_A believes that exploring barriers to diet adherence is crucial, as it addresses the root cause of the patient's problem. They also recommend referring the patient to a nutritionist to help her understand and improve her dietary habits. AI_B also supports exploring barriers to diet adherence, emphasizing that the patient's lifestyle and diet are major contributors to her poorly controlled diabetes. They believe that understanding these barriers can provide tailored solutions suitable for the patient's lifestyle. AI_C agrees with AI_A and AI_E, stating that exploring barriers to diet adherence and referring the patient to a nutritionist are both important steps. They highlight the importance of understanding the reasons behind the patient's dietary patterns and making necessary adjustments. AI_D suggests that exploring barriers to diet adherence is the most appropriate action, as it helps understand and address the factors contributing to the patient's suboptimal diabetes control. They also mention that referral to a nutritionist could be a possible plan derived from understanding these barriers. AI_E believes that exploring barriers to diet adherence provides a comprehensive understanding of the patient's lifestyle and food choices. However, they also suggest that referring the patient to a nutritionist offers specific expertise to create a dietary plan considering her busy lifestyle, preferences, and travels. Key Differences: The main difference in the council members' opinions lies in the emphasis they place on the role of a nutritionist. While all members agree on the importance of exploring barriers to diet adherence, AI_A, AI_C, and AI_E also highlight the value of referring the patient to a nutritionist. AI_B and AI_D, on the other hand, focus more on understanding and addressing the patient's lifestyle and diet barriers. Question to Clarify Disagreements: To what extent do you believe that the role of a nutritionist is crucial in this case, and how do you think their involvement would complement the exploration of diet adherence barriers?. Based on your preceding response, how would you answer the original question: [A 48-year-old woman with type 2 diabetes mellitus comes to the physician for a follow-up examination. Current medications are metformin and once-daily insulin. She travels frequently and works long hours. She says that her meals are usually fast food. She leads a sedentary lifestyle. She often forgets to measure her blood glucose concentration. Her last hemoglobin A1c was measured as 8.4%. Which of the following is the most appropriate action by the physician to help this patient improve her diabetic control? (A) Create an exercise regimen for the patient (B) Explore barriers to diet adherence (C) Increase the patient's insulin dosage (D) Increase the patient's metformin dosage (E) Measure the patient's blood glucose concentration (F) Order measurement of the patient's microalbumin concentration (G) Refer the patient to a nutritionist]

**AI_B:** The question is asking about the most appropriate action the physician can take to help a 48-year-old female patient with type 2 diabetes mellitus improve her diabetic control. She is currently on metformin and daily insulin, but her latest HbA1c level, which is a good indicator of long-term blood glucose control, is 8.4%, which is above the target level for most adults with diabetes. The patient's lifestyle is largely sedentary and she often consumes fast food due to her busy schedule and traveling requirements. She also tends to forget to measure her blood glucose concentration. When planning a possible course of action, it's important to consider the aspects of her situation that are contributing to her elevated A1c level. This includes her unhealthy diet, sedentary lifestyle, and inconsistent monitoring of blood glucose concentration. While all these factors could be addressed, her dietary habits appear to be the primary issue, as she mentions that her meals are usually comprised of fast food. Yet, it's also possible that other barriers hindering proper diet adherence are present, such as time constraints due to her busy schedule and frequent traveling. Through clinical reasoning, it becomes apparent that exploring the barriers to her diet adherence could be the most beneficial step to take initially. This is based on the fact that dietary habits directly influence blood glucose levels and therefore A1c values. By exploring these barriers, the physician may uncover ways to work around them or reduce their impact. Once these barriers are better understood, the physician could guide her towards healthier dietary choices or possibly refer her to a nutritionist for further dietary advice tailored to her specific needs and routine. Concluding, that the patient's poor dietary habits are likely contributing significantly to her poor diabetic control, the most effective action for the physician would likely be to explore the barriers to her diet adherence before taking further steps, such as adjusting her medication dosage or referring her to a nutritionist. Therefore, my response to the question would be (B) 'Explore barriers to diet adherence' as it addresses the underlying cause of the patient's poor diabetic control, and could provide the foundation for future interventions, including change in diet, lifestyle modifications, and possibly adjustment of medication.

**AI_E:** To summarize, the question asks about the most appropriate way a doctor can help a 48-year-old woman with a diagnosis of type 2 diabetes mellitus improve control of her condition. She takes metformin and insulin once daily, eats fast food, leads a sedentary lifestyle, often forgets to measure blood glucose and has a high hemoglobin A1c value. The options include creating an exercise regimen, understanding her dietary barriers, increasing insulin and metformin dosage, monitoring her blood glucose and microalbumin levels, and referring to a nutritionist. Firstly, various assumptions could be made. One could be that she lacks knowledge about the impact of diet and lifestyle on her condition. Another could be that her busy lifestyle prevents her from making healthier choices. Or it's possible that she doesn't fully understand the importance of regular glucose monitoring. On analyzing, the assumption of lifestyle barriers seems crucial. Irrespective of her understanding of the condition, if her lifestyle doesn't align with the necessary changes, she may not be able to adhere to them. The assumption should thus be clinically explored. Delving into it, barriers to diet adherence commonly include lack of time and knowledge, cultural and personal preferences, and access to healthy food options. By understanding these, the physician can provide personalized advice tailored to her situation. Moreover, while medications are vital for managing diabetes, lifestyle modifications are equally important. This is corroborated by research showing that healthy diet and exercise can significantly improve diabetes control. Summarizing, if the physician explores the patient's dietary barriers, they can provide more effective, personalized advice, addressing her lifestyle. Referring her to a nutritionist could also help create a diet plan suitable for her busy lifestyle. My conclusion aligns with the assumption. By exploring dietary barriers, working on lifestyle modifications and possibly working with a nutritionist, the patient can adopt a healthier lifestyle conducive to better diabetes control. So, my response to the clinical scenario is: The most appropriate action by the physician to help this patient improve her diabetic control would be to (B) explore barriers to diet adherence.

**AI_D:** RESTATING THE QUESTION: The query presented here asks what is the most appropriate step a physician can take to help this particular patient, a 48-year-old woman with poorly controlled type 2 diabetes mellitus, improve her diabetic control. It takes into account her lifestyle factors, including frequent travel, long work hours, sedentary lifestyle, fast food consumption, and inconsistent glucose monitoring, as well as her current medications - metformin and insulin. PLANNING: The main assumption typically made in cases like this is that the medications provided are not sufficiently controlling her diabetes and may require alteration. However, given the patient's lifestyle and behaviors, it can be hypothesized that the issue lies more in her diet and insufficient self-monitoring of glucose levels rather than medication efficacy. Thus, clinically, it might be better to focus on aiding the patient in addressing these lifestyle factors, rather than altering the medication regimen. CLINICAL REASONING: The patient's lifestyle is a clear contributor to her uncontrolled diabetes. her sedentary lifestyle and frequent consumption of fast food - generally high in carbohydrates and unhealthy fats - undoubtedly contribute to elevated glucose levels. Furthermore, her inconsistent self-monitoring likely prevents her from fully understanding and managing her glucose levels. Therefore, exploring her barriers to diet adherence, as well as improving self-monitoring, should take precedence in the clinical management plan. CONCLUDING: It becomes clear that addressing the patient's lifestyle, particularly her dietary habits and monitoring of glucose levels, is likely to provide a more sustainable and effective management plan than adjusting her medication. Addressing her barriers to diet adherence and involving a nutritionist can provide her with an understanding of how her lifestyle affects glucose levels, while also creating strategies that are personalized and suitable for her. The same goes for improving her glucose monitoring. RESPONSE: Given these considerations, the most appropriate action by the physician is likely to be (B) to explore barriers to diet adherence, which will directly address her lifestyle factors contributing to poorly controlled diabetes. In conjunction with this, a referral to a nutritionist (G) could potentially be beneficial as a part of a comprehensive management plan. However, given the specific options provided and their mutual exclusivity, option (B) alone provides the most direct and comprehensive response. Physicians can explore barriers with the patient while also encouraging more congenial changes that can help improve her disease management. By addressing these factors, she can gain greater control over her diabetes and potentially reduce her HbA1c levels.

**AI_A:** To clarify the question: a 48-year-old woman with a known diagnosis of type 2 diabetes mellitus is having a problem with controlling her blood glucose levels. This is evidenced by her high hemoglobin A1c level, which is a measure of long-term blood glucose control. She has stated that she often forgets to check her blood glucose levels, she regularly eats fast food, and she leads a sedentary lifestyle. Planning this situation, it's important to note that good blood glucose control is essential in diabetes management and involves multiple factors, including appropriate medication, exercise, regular blood glucose monitoring, and a healthful diet. There are several assumptions in this case, one being that her sedentary lifestyle is negatively affecting her diabetes control, and another being that her diet is contributing to her high glucose levels. It is also assumed that her forgetting to regularly check her glucose levels could be impacting her control. The assumption about her inactive lifestyle and her diet seem to be the most important as diet and physical activity are two of the most crucial aspects of managing diabetes beyond medication, and she has a substantial challenge in both of these aspects. However, considering her busy schedule and travelling pattern, the change in physical activity might not be as feasible as dietary modification. Here, clinical reasoning also plays a part. Diabetes is a chronic disease that requires ongoing management. Her diabetes has been poorly controlled, given her elevated hemoglobin A1c of 8.4% (Normal is less than 6.5%), which can lead to various complications, including neuropathy, nephropathy, and retinopathy, among others. Dietary modification is a crucial part of diabetes management, and given her lifestyle, it seems suitable to focus on this aspect first. To conclude, focusing on exploring her diet and barriers to her adherence seems to be the most appropriate action. Given her busy lifestyle and frequent travels, she might be facing challenges in planning a balanced diet. By understanding these barriers and providing specific strategies to overcome these, her glucose control might improve. Response: The most appropriate action would be for the physician to explore barriers to diet adherence (Option B). If necessary, referring the patient to a nutritionist (Option G) could also be a subsequent step, depending on the outcome of the barrier exploration. These steps might help the patient plan a balanced diet more efficiently, even with her busy schedule, which could lead to better glucose control.

**AI_C:** Restating the Question: The question asks for the best immediate course of action for a 48-year-old female with poorly controlled type 2 diabetes mellitus despite on metformin and insulin. Her lifestyle is sedentary, and her diet consists mainly of fast food. Also, she often neglects to monitor her blood glucose levels. Planning: The assumptions that we could take into account include: her diet has a substantial impact on her diabetes control, her lifestyle could be improved, her insulin and metformin doses could be insufficient due to her diet, and she might require help in managing her diet. Exploring each of these assumptions will help guide the appropriate care plan. Clinical Reasoning: Her last measured hemoglobin A1c was 8.4%, indicating that her blood sugar levels have been chronically high. While increasing her insulin dosage or metformin could potentially lower her blood glucose levels in the short term, it wouldn't address the cause of her poorly controlled diabetes. Her preference for fast food and her sedentary lifestyle most likely contribute significantly to her high blood glucose levels. Creating an exercise regimen could help enhance her insulin sensitivity, but without dietary changes, it might not be sufficient to control her diabetes. Furthermore, it appears that her current lifestyle might not allow time for regular exercise. Even though measuring her blood glucose concentration would provide valuable information about her current state, it would not directly impact her management as she already has poorly controlled diabetes indicated by her high HbA1c. Hence, I presume that understanding her barriers to diet adherence is a necessary focus. It may help identify obstacles in her lifestyle or beliefs that make it difficult for her to eat healthily. Once these barriers are identified, interventions can be developed to help her improve her diet significantly. Concluding: Ultimately, without changing her diet, which seems to be the source of her problem, effective control of her blood glucose levels is likely to remain elusive. Therefore, exploring her barriers to diet adherence and addressing them would be an essential first step in helping her manage her diabetes better. Response: Given the information available, the most appropriate action by the physician would be to explore barriers to diet adherence (B). This approach, directed at the root cause of her uncontrolled diabetes, is most likely to result in a sustainable and effective improvement in her diabetic control.

**Facilitator** The question presents a scenario of a 48-year-old woman with type 2 diabetes mellitus who is struggling to control her blood glucose levels, as evidenced by her high hemoglobin A1c level of 8.4%. Her lifestyle is sedentary, she frequently consumes fast food due to her busy schedule and travel requirements, and she often forgets to monitor her blood glucose levels. The question asks for the most appropriate action the physician can take to help improve her diabetic control. The consensus among the responses is that the most appropriate action would be to explore the barriers to diet adherence (Option B). This approach is based on the assumption that her diet and lifestyle are significant contributors to her poor diabetes control. By exploring these barriers, the physician can understand the challenges she faces in maintaining a healthy diet and provide personalized strategies to overcome them. This could lead to better glucose control and a reduction in her hemoglobin A1c levels. While all responses agree on the importance of exploring dietary barriers, some also suggest that referring the patient to a nutritionist (Option G) could be a beneficial subsequent step, depending on the outcome of the barrier exploration. This could help the patient plan a balanced diet more efficiently, even with her busy schedule. In conclusion, the consensus is that the physician should explore barriers to diet adherence as the most appropriate immediate action. This approach addresses the root cause of her uncontrolled diabetes and provides a foundation for future interventions, including dietary changes, lifestyle modifications, and possibly medication adjustments.
