# Supplementary material for: Collaborative intelligence in AI: Evaluating the performance of a council of AIs on the USMLE
Source: PLOS Digit Health. 2025 Oct 9;4(10):e0000787. doi: 10.1371/journal.pdig.0000787 (PMC12510544; doi:10.1371/journal.pdig.0000787)
Supplement: S2 File — (ZIP) [file pdig.0000787.s002.zip › S2 Data Raw data files containing unprocessed question inputs and LLM outputs including transcripts of deliberations/STEP1_Question-51-1_Type-MC-NJ.docx]

**Facilitator** **[Round 1]:** A 19-year-old man who is a college freshman comes to the office because of a 4-day history of tender, swollen glands. He also has a 6-day history of fever, malaise, and decreased appetite. His temperature is 38.7°C (101.7°F). Physical examination shows swelling of the parotid glands. Which of the following infectious agents is the most likely cause of these findings? (A) Epstein-Barr virus (B) Hepatitis B virus (C) Measles virus (D) Mumps virus (E) Rubella virus

**AI_C:** 1) RESTATING THE QUESTION: The question is about a 19-year-old male university student who is reportedly experiencing swollen, tender glands, fever, exhaustion, and decreased appetite over a particular period. Furthermore, an examination has noticed swelling in his parotid glands. The scenario requested us to identify the infectious pathogen most likely causing these symptoms, with options for Epstein-Barr virus, Hepatitis B virus, Measles virus, Mumps virus, and Rubella virus. 2) PLANNING: This clinical situation assumes that the patient's symptoms are most likely caused by an infectious disease, given that all the options provided are infectious agents. These symptoms, especially parotid gland inflammation, are associated with various diseases, one of which we must identify. The options given cover a broad range of viral conditions, each with distinguishing features. A careful examination of these features in context with the symptoms provided will aid in forming an alternate hypothesis. 3) CLINICAL REASONING: The patient's presentation of tender, swollen salivary glands and fever hints at a viral illness. Epstein-Barr virus usually causes glandular fever or infectious mononucleosis, primarily seen in adolescents and young adults, but it typically involves lymphadenopathy and tonsil inflammation. Hepatitis B affects the liver and not parotid glands. Measles and Rubella would usually include a rash, which is not mentioned here. Mumps virus, however, mainly targets the salivary glands, particularly the parotids, causing swelling and tenderness. 4) CONCLUDING: Based on the patient's age, the mention of the university environment (mumps can spread swiftly in close clusters), the specific involvement of the parotid glands, and the absence of other distinguishing features for the other choices (like rashes or tonsil inflammation), the Mumps virus is the most likely causative agent for these clinical findings. 5) RESPONSE: The most probable cause of this patient's symptoms is choice (D) Mumps virus.

**AI_E:** RESTATING THE QUESTION: The question is asking about the most probable infectious cause behind the symptoms presented by a 19-year-old college freshman. He's experiencing tender and swollen glands for four days, alongside fever, malaise, and a decreased appetite for six days. A physical exam revealed parotid gland swelling. The infectious agents provided for consideration are the Epstein-Barr virus, Hepatitis B virus, Measles virus, Mumps virus, and Rubella virus. PLANNING: The clinical presentation is indicative of an infectious disease with symptoms including fever, malaise, lost appetite, and most crucially, the enlargement of the parotid glands. When analyzing the suggestive diagnosis relative to the given organisms, the Mumps virus is known to cause parotitis, that majorly leads to the swelling of the parotid gland. None of the other given viruses (Epstein-Barr virus, Hepatitis B virus, Measles virus, Rubella virus) are typically associated with parotiditis. CLINICAL REASONING: The primary symptom that leans towards one of the listed infectious agents is the swelling of the parotid glands. Parotitis, or inflammation of the parotid gland, is one of the most common presentations of a mumps infection. The prodromal symptoms, which are those experienced by the patient (fever, malaise, decreased appetite), are non-specific and can be seen in a variety of viral infections. However, when associated with bilateral parotitis, they strongly suggest mumps. CONCLUDING: Connecting the dots, the young gentleman's exhibiting signs hint at an infectious disease, and the swelling and tenderness of the parotid glands narrow the possibilities to diseases causing parotitis. Among the provided options, the Mumps virus is primarily known for causing such symptoms, thereby heightening its likelihood as the causative agent in this case. RESPONSE: Given the symptoms presented, especially the swelling of the parotid glands, the most likely infectious agent causing the findings in the patient is the (D) Mumps virus.

**AI_D:** (1) RESTATING THE QUESTION: A 19-year-old college student has presented with symptoms of tender and swollen glands, specifically the parotid glands, accompanied by fever, malaise, and a decreased appetite over the past 4 to 6 days. We are tasked with determining the most probable infectious agent causing his symptoms from the following options: Epstein-Barr virus, Hepatitis B virus, Measles virus, Mumps virus, and Rubella virus. (2) PLANNING: First, let's evaluate the possible causes for these symptoms. Epstein-Barr virus primarily results in mononucleosis with tonsillar pharyngitis and atypical lymphocytosis, Hepatitis B virus primarily affects the liver, Measles virus tends to cause rash, Koplik spots, and often involves the respiratory tract, Mumps virus is known to cause parotitis and sometimes orchitis, and Rubella virus causes rash and lymphadenopathy. It's critical to consider the patient's clinical picture: the parotid gland swelling, fever, malaise. The assumption that correlates best with these symptoms is the Mumps virus infection. (3) CLINICAL REASONING: In this case, the swollen parotid glands are a critical clue. Mumps virus is the most common cause of viral parotitis, which would cause the parotid glands to become inflamed and swollen, as observed in this patient. Also, prodromal symptoms for mumps often include fever, malaise, and a decrease in appetite, consistent with this patient's presentation. Furthermore, college campuses often have outbreaks of mumps due to the close living conditions. On the contrary, while the other infectious agents listed can cause fever and malaise, they do not typically cause parotitis, which set mumps virus apart clinically. (4) CONCLUDING: Upon combining the patient's specific symptoms, including his tender and swollen parotid glands, the fever, malaise, and decreased appetite, and considering the infectious agents, mumps virus most correctly matches his clinical representation. (5) RESPONSE: The most likely cause of these findings in this 19-year-old man is (D) Mumps virus.

**AI_A:** (1) RESTATING THE QUESTION: This situation involves a young college freshman presenting with a combination of symptoms which include fever, decreased appetite, malaise, and swollen, tender glands - evidenced by physical examination of the parotid glands. The task at hand is to determine which infectious agent is most likely the cause of these symptoms from the given list: Epstein-Barr virus, Hepatitis B virus, Measles virus, Mumps virus, or Rubella virus. (2) PLANNING: On first glance, these are all viral infections, each with their unique symptomatology. However, the patient's symptoms do not provide a definite diagnosis. It is necessary to consider each virus and its associated symptoms to infer which is most likely causing the patient's current condition. Given the patient's age, lifestyle (college freshman living likely in close quarters with others), and specific symptoms, it would suggest a certain prioritization of these viral causes. (3) CLINICAL REASONING: Starting with Epstein-Bar Virus (EBV), this virus usually presents as infectious mononucleosis with symptoms that may include fever and lymphadenopathy but hallmark presentation is posterior cervical lymphadenopathy which is not represented in this case. Hepatitis B primarily affects the liver, and does not usually cause parotitis. Measles virus could present with fever and malaise; however key symptoms like a maculopapular rash and Koplik spots are not reported. Rubella is also accompanied by a maculopapular rash which seems to be absent. In contrast, Mumps Virus often causes acute onset of unilateral or bilateral tender, swollen parotid glands, fever, malaise, and muscle pain. Decreased appetite can also be seen due to associated discomfort. (4) CONCLUDING: Given the presence of fever, parotid gland swelling (parotitis), decreased appetite, and malaise, along with the absence of symptoms specific to other listed viruses, the Mumps virus remains the most plausible cause in this case. (5) RESPONSE: Out of the given alternatives, 'Mumps virus' (D) is the most likely causative infectious agent for the patient's symptoms.

**AI_B:** 1) RESTATING THE QUESTION: The patient is a 19-year-old man, a college freshman, who has been experiencing tender and swollen glands for the past 4 days. He also has symptoms like fever, malaise, and decreased appetite, which he has had for 6 days. A physical check-up reveals that his parotid glands are swollen. The parotid glands are major salivary glands located in the face, accounting for most saliva production. The question is asking us to identify the infectious agent causing his symptoms. 2) PLANNING: Initial assumptions to be made from the clinical presentation could include a common virus like influenza. However, after further consideration of the signs and symptoms and their duration, it is worth considering a few alternatives: Epstein-Barr Virus (commonly causing mononucleosis), Hepatitis B (which could cause generalized symptoms but less likely with swollen parotid glands), Measles (which could lead to fever and malaise but usually associated with a rash), Rubella (which could also lead to fever and malaise with rash and lymphadenopathy) and Mumps (commonly known to cause parotitis). Evaluating these options, Mumps is a more fitting cause, due to the symptom of swollen parotid glands. However, other symptoms and historical data points, such as vaccination history, would aid in making a more accurate diagnosis. 3) CLINICAL REASONING: The primary clinical feature here is swelling of the parotid glands. While fever, malaise, and decreased appetite could be attributed to many infections, swollen parotid glands significantly narrow down the list of probable causes. Mumps virus, a paramyxovirus, causes an illness characterized by febrile parotitis, orchitis, meningitis, and in certain cases, pancreatitis. In the absence of vaccination, mumps is a common affliction, especially in younger individuals. As measles and rubella usually present with more pronounced cutaneous symptoms, and Epstein-Barr Virus and Hepatitis B Virus would not typically cause parotitis, the clinical presentation fits best with a mumps infection. 4) CONCLUDING: So, based on the patient's symptoms, especially the prominent parotitis, and the fact that mumps is commonly seen in young individuals in a community setting such as a college, it is reasonable to conclude that mumps virus is the most likely cause of this patient's symptoms. 5) RESPONSE: The most plausible infectious agent, considering the given patient's symptoms of fever, malaise, decreased appetite, and notably, swollen parotid glands, is the mumps virus (D). It typically causes such symptoms, with swelling of the parotid glands being a characteristic feature, especially in younger individuals in communities such as a university setting.

**Facilitator** The consensus statement is as follows: The question presents a 19-year-old college freshman who has been experiencing tender, swollen glands, fever, malaise, and decreased appetite. A physical examination reveals swelling of the parotid glands. The task is to identify the most likely infectious agent causing these symptoms from the options provided: Epstein-Barr virus, Hepatitis B virus, Measles virus, Mumps virus, and Rubella virus. Upon analyzing the symptoms and the potential infectious agents, the Mumps virus emerges as the most plausible cause. The primary symptom that points towards Mumps is the swelling of the parotid glands, a condition known as parotitis, which is a common presentation of a mumps infection. The other symptoms, such as fever, malaise, and decreased appetite, are non-specific and can be seen in various viral infections. However, when associated with parotitis, they strongly suggest mumps. The other infectious agents listed, while capable of causing fever and malaise, do not typically cause parotitis. Epstein-Barr virus primarily results in mononucleosis, which typically involves lymphadenopathy and tonsillar pharyngitis. Hepatitis B virus primarily affects the liver. Measles and Rubella viruses usually present with a rash, which is not mentioned in the patient's symptoms. Given the patient's age, the university environment, and the specific involvement of the parotid glands, along with the absence of other distinguishing features for the other choices, the Mumps virus is the most likely causative agent for these clinical findings. Therefore, the most probable cause of this patient's symptoms is the Mumps virus (D).
