# Supplementary material for: Collaborative intelligence in AI: Evaluating the performance of a council of AIs on the USMLE
Source: PLOS Digit Health. 2025 Oct 9;4(10):e0000787. doi: 10.1371/journal.pdig.0000787 (PMC12510544; doi:10.1371/journal.pdig.0000787)
Supplement: S2 File — (ZIP) [file pdig.0000787.s002.zip › S2 Data Raw data files containing unprocessed question inputs and LLM outputs including transcripts of deliberations/STEP1_Question-52-1_Type-MC-NJ.docx]

**Facilitator** **[Round 1]:** A 38-year-old woman who is a veteran comes to the emergency department because of a 4-hour history of a sensation of tightness in her throat and swelling of her face, lips, and tongue. She has not had itching. During the past 3 weeks, she has had two similar, milder episodes that resolved without treatment within 24 to 72 hours. Six weeks ago, she began treatment with an ACE inhibitor for hypertension. She has seasonal allergies. Her temperature is 37.1°C (98.8°F), pulse is 80/min, respirations are 20/min, and blood pressure is 138/81 mm Hg. Physical examination shows angioedema of the lips and tongue. There is no evidence of urticaria. Which of the following is the most likely cause of the angioedema in this patient? (A) Bradykinin (B) Histamine (C) Leukotriene B₄ (D) Nitric oxide (E) Prostaglandin E₂

**AI_A:** RESTATING THE QUESTION: The question seeks help in determining the most likely agent causing the angioedema in this 38-year-old patient who has a history of treatment with an ACE inhibitor and experiences of similar episodes in the past. PLANNING: There are assumptions we have to make: (1) The angioedema is a reaction to the ACE inhibitor. (2) Histamine, involved in allergies, and other mediators may be contributing. (3) The angioedema could be related to her seasonal allergies. By questioning each assumption, we may generate alternate hypotheses, such as her symptoms being part of a different disease process rather than a direct result of the ACE inhibitor or seasonal allergies. However, the clinical priority suggests considering her recent medication history with the ACE inhibitor. CLINICAL REASONING: Angioedema is characterized by a rapid swelling of the dermis and subcutaneous tissues, and sometimes the mucosa. The primary pathophysiological mechanism is the localized increase of vascular permeability. Cases of angioedema that lack accompanying urticaria, like this patient's, often involve the vasoactive peptide bradykinin rather than histamine. ACE inhibitors can lead to angioedema attacks due to excessive bradykinin, as these medications block the degradation of bradykinin. CONCLUDING: We can conclude that her symptoms are more likely related to her recent commencement of an ACE inhibitor for hypertension, which can cause an upregulation in bradykinin, leading to angioedema. Histamine, Leukotriene B₄, Nitric oxide, and Prostaglandin E₂ can all cause similar symptoms but are less likely given the patient's history and clinical presentation. RESPONSE: Likely, the cause of the angioedema in this patient is Bradykinin (A), given her recent commencement on the ACE inhibitor used to treat her hypertension, which prevents the degradation of Bradykinin, resulting in her symptoms.

**AI_D:** RESTATING THE QUESTION: The question inquires about the biochemical that is primarily responsible for the angioedema (swelling beneath the skin) experienced by a 38-year-old woman who has recently initiated treatment with an Angiotensin-Converting Enzyme (ACE) inhibitor for hypertension. PLANNING: Typically, angioedema could be caused by various agents, such as histamine or bradykinin, or even by leukotrienes, nitric oxide, or prostaglandins. In this case, the woman's clinical history suggests the condition has occurred in conjunction with the incorporation of an ACE inhibitor into her therapeutic regimen. This can lead to the hypothesis that the angioedema might be associated with the pharmacological action of this drug. CLINICAL REASONING: ACE inhibitors are typically used to treat high blood pressure. They work by blocking the conversion of angiotensin I to angiotensin II, a potent vasoconstrictor. Additionally, inhibiting this enzyme enhances the activity of bradykinin, a potent vasodilator, as ACE also degrades bradykinin. When bradykinin is not broken down, as in the case of ACE inhibitor usage, it accumulates and can cause angioedema. Hence, the action of an ACE inhibitor doesn't elevate the levels of other biochemicals such as histamine, leukotriene B₄, nitric oxide or prostaglandin E₂, all of which can also cause inflammation or dilation of blood vessels but are not implicated in this case. CONCLUDING: Therefore, in this presented clinical scenario where drug intervention coincides with the appearance of angioedema symptoms, it's plausible that the angioedema is a result of the pharmacological effects of the ACE inhibitor. Specifically, the enhancement of bradykinin activity due to the inhibition of its degradation could in turn cause the uncomfortable swelling the woman is facing. RESPONSE: The most likely cause of the angioedema in this patient is Bradykinin (A).

**AI_E:** 1) RESTATING THE QUESTION: The inquiry here is related to a 38-year-old female patient who exhibits angioedema symptoms, which include a sensation of throat tightness and swelling of the face, lips, and tongue. It is noteworthy that there is no itching or urticaria (hives). Recurrence of minor versions of these episodes have been observed for the past three weeks, self-resolving within a day to three days. The patient has been taking ACE inhibitors for hypertension for the past six weeks and has a history of seasonal allergies. The purpose of the question is to determine the most probable cause of the angioedema from the options given. 2) PLANNING: The starting assumption is that the angioedema in this patient is a result of her medication, the ACE inhibitor, or her allergies. Bradykinin, histamine, leukotriene B₄, nitric oxide, and prostaglandin E₂ are all biological agents that can cause inflammation and are associated with some form of allergic reactions or drug-induced side effects. However, based on the patient's symptoms, it is crucial to hypothesize which agent is likely at play in this case. 3) CLINICAL REASONING: ACE inhibitors are often associated with angioedema side effects, which can involve the vocal cords, lips, throat, and tongue. However, angioedema induced by this class of drug differs from typical allergic reactions because it's not triggered by histamine or leukotriene release hence the absence of urticaria (hives) or itching. Instead, ACE inhibitors can increase the levels of bradykinin, a potent vasodilator that increases vascular permeability, leading to swelling or angioedema. 4) CONCLUDING: It seems reasonable that the new symptom onset coinciding with the ACE inhibitor treatment initiation points towards the drug as the likely cause. Therefore, it is plausible that elevated bradykinin levels due to the ACE inhibitor's interference with its degradation could be the cause of her condition. 5) RESPONSE: A likely cause of angioedema in this patient is (A) Bradykinin.

**AI_B:** RESTATING THE QUESTION: The query presented revolves around understanding the most likely cause of angioedema in a 38-year-old woman with hypertension and seasonal allergies. She recently began ACE inhibitor treatment and is currently experiencing swelling of her face, lips, and tongue but no itching or urticaria. Angioedema is a condition characterized by deep dermal, subcutaneous, or submucosal swelling, most commonly affecting areas with loose connective tissue such as the face, lips, and tongue. Potential causative factors such as bradykinin, histamine, leukotriene B₄, nitric oxide, and prostaglandin E₂ are proposed. PLANNING: The immediate assumption might be to attribute the swelling to an allergic reaction given the patient's history of seasonal allergies. However, the absence of itching and urticaria is suspicious. This leads us to consider other underlying factors. The patient's recent history of starting an ACE inhibitor for hypertension becomes critical because ACE inhibitors are known to cause drug-induced angioedema, primarily mediated by bradykinin, a potent vasodilator peptide. CLINICAL REASONING: Investigating each contributing factor, histamines typically result in urticaria and itching, but the patient's symptoms are inconsistent with histamine-mediated angioedema. Leukotriene B₄ is involved in inflammation, but angioedema caused by leukotriene B₄ usually presents with other classic allergic reactions absent in this case. Nitric oxide and prostaglandin E₂ are involved in vasodilation, but their role in angioedema is less defined than that of bradykinin. The timing of the onset of the patient's signs and symptoms following initiation of her ACE inhibitor therapy makes it a likely causative factor. ACE inhibitors can lead to increased levels of bradykinin because they inhibit its degradation, leading to vasodilation and increased vascular permeability, thus accounting for the angioedema. CONCLUDING: Upon clinically correlating the detailed history, absence of allergic signs of urticaria, recent initiation of ACE inhibitors, recurrence of symptoms, and the proposed options for the causative agent, it seems most plausible that the excess of bradykinin resulting from the use of the ACE inhibitor is the underlying cause of the patient's angioedema. RESPONSE: Therefore, the most likely cause of the angioedema in this patient is (A) Bradykinin.

**AI_C:** (1) RESTATING THE QUESTION: The question describes a 38-year-old woman with a history of hypertension, who has been treated with an ACE inhibitor for six weeks. She exhibits symptoms of angioedema, specifically facial swelling, tongue swelling, and a sensation of throat tightness. She has no sign of itching, which generally accompanies allergic reactions mediated by histamine. Her episodes of angioedema have been recurring, albeit with spontaneous resolution within 24 to 72 hours over the past three weeks. We are asked to identify the most likely underlying cause of the angioedema in this context. Angioedema refers to swelling of the deep layers of the skin, often occurring in the face and throat, which can result from various triggers, including medications like ACE inhibitors, and allergic reactions. Substances like bradykinin, histamine, leukotriene B₄, nitric oxide, and prostaglandin E₂ are mediators of inflammation and vasodilation, which contribute to the symptoms of angioedema. (2) PLANNING: The assumptions in this case include a link between the patient's new medication -- the ACE inhibitor -- and the onset of her angioedema. Another assumption could be the role of her seasonal allergies, although the absence of itching and urticaria makes this less likely. Among the listed substances, only histamine and leukotriene B₄ are typically involved in allergic reactions. This leaves bradykinin, nitric oxide, and prostaglandin E₂ as potential mediators related to ACE inhibitors. (3) CLINICAL REASONING: ACE inhibitors, such as the one the patient is taking, have a known side effect of causing angioedema. This results from the accumulation of bradykinin, a potent vasodilator peptide, when ACE, which is responsible for bradykinin degradation, is inhibited. A build-up of bradykinin leads to increased vascular permeability and resultant angioedema. The lack of itching in this patient is a significant clue towards differentiating bradykinin-mediated angioedema from a histamine- or leukotriene B₄-triggered event (often associated with allergies). Itching is a typical feature of histaminergic responses and is often accompanied by urticaria, which this patient lacks. Similarly, nitric oxide and prostaglandin E₂ aren’t typically associated with angioedema related to ACE inhibitors or allergies. (4) CONCLUDING: The absence of itching and urticaria, the recent start of ACE inhibitor therapy, and the recurrent, self-resolving nature of the angioedema episodes point towards a bradykinin-mediated process rather than a response mediated by histamine, leukotriene B₄, nitric oxide, or prostaglandin E₂. This evidence suggests that ACE inhibitors' inhibition of bradykinin degradation, leading to increased levels of bradykinin and subsequent angioedema, is the most plausible explanation for this patient’s symptoms. (5) RESPONSE: Hence, the most likely cause of the angioedema in this patient is (A) Bradykinin.

**Facilitator** The question seeks to identify the most likely cause of angioedema in a 38-year-old woman who recently began treatment with an ACE inhibitor for hypertension and has a history of seasonal allergies. The patient has experienced similar episodes in the past, which resolved without treatment. The angioedema is characterized by swelling of the face, lips, and tongue, and a sensation of throat tightness, but no itching or urticaria. The consensus among the responses is that the most probable cause of the angioedema in this patient is Bradykinin (A). This conclusion is based on several factors. Firstly, the patient's recent commencement of an ACE inhibitor for hypertension is significant, as ACE inhibitors are known to cause drug-induced angioedema, primarily mediated by bradykinin, a potent vasodilator peptide. ACE inhibitors block the degradation of bradykinin, leading to its accumulation and increased vascular permeability, which results in angioedema. Secondly, the absence of itching and urticaria in the patient's symptoms suggests that the angioedema is not likely to be mediated by histamine or leukotriene B₄, which are typically involved in allergic reactions. Similarly, the roles of nitric oxide and prostaglandin E₂ in angioedema are less defined than that of bradykinin and are not typically associated with angioedema related to ACE inhibitors or allergies. Therefore, considering the patient's clinical history, the timing of the onset of symptoms following the initiation of ACE inhibitor therapy, and the nature of her symptoms, the consensus is that the most likely cause of the angioedema in this patient is Bradykinin (A).
