# Supplementary material for: Collaborative intelligence in AI: Evaluating the performance of a council of AIs on the USMLE
Source: PLOS Digit Health. 2025 Oct 9;4(10):e0000787. doi: 10.1371/journal.pdig.0000787 (PMC12510544; doi:10.1371/journal.pdig.0000787)
Supplement: S2 File — (ZIP) [file pdig.0000787.s002.zip › S2 Data Raw data files containing unprocessed question inputs and LLM outputs including transcripts of deliberations/STEP1_Question-54-1_Type-MC-NJ.docx]

**Facilitator** **[Round 1]:** A 48-year-old woman comes to the physician because of a 2-month history of fatigue and poor appetite; she has had a 3-kg (6.6-lb) weight loss during this period. Abdominal examination shows moderate splenomegaly. Laboratory studies show a leukocyte count of 185,000/mm³ (65% segmented neutrophils with 6% blasts and promyelocytes). Cytogenetic analysis of a bone marrow aspirate shows the presence of the Philadelphia (Ph¹) chromosome in myelocytes. Which of the following mechanisms best accounts for the formation of the Philadelphia (Ph¹) chromosome in this patient? (A) Nondisjunction (B) Recombination (C) Sister chromatid exchange (D) Telomere breakage (E) Transposon insertion

**AI_D:** RESTATING THE QUESTION: The woman in the scenario presented is experiencing symptoms like a decrease in appetite along with fatigue, weight loss, and an enlarged spleen, suggesting a possible hematological condition. Furthermore, her blood tests show an elevated white blood cell count, with an observed increase in neutrophils, promyelocytes, and blasts, implicating an abnormal cellular proliferation. The presence of the Philadelphia chromosome found in her myelocytes pinpoints this as likely being a case of chronic myeloid leukemia (CML). The question wants to know what mechanism leads to the formation of the Philadelphia chromosome, which causes CML. PLANNING: A common assumption might be that the Philadelphia chromosome arises from simple errors in replication, such as nondisjunction, or from mechanisms like recombination, sister chromatid exchange, telomere breakage, or transposon insertion – each reflecting some form of genetic alteration. However, we should question each of these assumptions and hypothesize that the formation of the Philadelphia chromosome involves a specific genetic rearrangement process. CLINICAL REASONING: The Philadelphia chromosome, discovered in CML patients, is an abnormality caused by a specific form of genetic rearrangement known as reciprocal translocation, particularly between chromosomes 9 and 22. In this case, a part of the ABL1 gene from chromosome 9 and the BCR gene from chromosome 22 swap places. This forms the BCR-ABL1 fusion gene on chromosome 22 (the Philadelphia chromosome), leading to a constitutively active tyrosine kinase which causes uncontrolled cell proliferation. The mechanisms listed in the question—nondisjunction, recombination, sister chromatid exchange, telomere breakage, and transposon insertion—do not specifically correlate with the formation of the Philadelphia chromosome. CONCLUDING: Therefore, we must conclude that none of the options provided in the question directly explain the occurrence of the Philadelphia chromosome. The correct mechanism, reciprocal translocation, is not listed. RESPONSE: None of the given mechanisms precisely account for the formation of the Philadelphia chromosome in this patient's condition. The appropriate mechanism associated with the formation of the Philadelphia chromosome is reciprocal translocation.

**AI_A:** RESTATING THE QUESTION: This question presents a clinical scenario of a 48-year-old woman who has been experiencing fatigue, loss of appetite, weight loss, an enlarged spleen and elevated leukocyte count. Laboratory studies reveal that her myelocytes have the Philadelphia (Ph') chromosome. It's asking us to identify the genetic event that led to the formation of this particular chromosomal anomaly in myelocytes. PLANNING: From the given options: (A) Nondisjunction - a failure in the separation of chromosome pairs during cell division, leading to an abnormal number of chromosomes in the daughter cells. (B) Recombination - is the rearrangement of genetic material, especially the process by which pieces of DNA are broken and recombine to produce new combinations of alleles. (C) Sister chromatid exchange - a process during which identical genetic information is exchanged between the two molecules of DNA within a chromosome. (D) Telomere breakage - disruption at the ends of chromosomes. (E) Transposon insertion - a process where a piece of DNA known as a transposon is inserted into a DNA sequence. Given the clinical scenario, option (B) Recombination, specifically a type of recombination known as reciprocal translocation, is the most plausible mechanism to account for the presence of the Ph' chromosome. CLINICAL REASONING: The Philadelphia chromosome arises from a fusion between the long ("q") arms of chromosomes 9 and 22, with the BCR gene from chromosome 22 attaching to the ABL gene on chromosome 9. This creates a BCR-ABL fusion gene that results in a protein with uncontrolled tyrosine kinase activity, leading to increased cell division and resistance to apoptosis, characteristic of chronic myeloid leukemia (CML). The patient's clinical features such as fatigue, poor appetite, weight loss, splenomegaly, and elevated leukocyte count are also consistent with CML. CONCLUDING: Considering all the above information, the genetic mechanism behind the creation of Ph' chromosome seen in CML is reciprocal translocation between chromosomes 9 and 22. This type of recombination plays a significant role in the pathogenesis of various neoplasms in addition to CML. RESPONSE: The mechanism that best accounts for the formation of the Philadelphia (Ph¹) chromosome in this patient is (B) Recombination.

**AI_C:** RESTATING THE QUESTION: This clinical vignette describes a middle-aged female who presents with fatigue, loss of appetite, weight loss, an elevated white blood cell count with an abnormality in neutrophils, blasts, and promyelocytes, as well as splenomegaly on examination. Further testing confirms the presence of the Philadelphia (Ph¹) chromosome in her cells. The question would like to know what mechanism leads to the formation of the Philadelphia chromosome observed in her case. The Philadelphia chromosome results from a specific genetic rearrangement between chromosomes 9 and 22. PLANNING: Assumptions that can be made here include nondisjunction, recombination, sister chromatid exchange, telomere breakage, and transposon insertion as possible mechanisms. By questioning these, we can arrive at alternate assumptions such as an event involving chromosomal translocation. Observing the properties of Philadelphia chromosome that is a product of translocation, we can propose that recombination in a non-standard way, specifically translocation, may be the mechanism responsible. CLINICAL REASONING: Nondisjunction usually results in an abnormal number of chromosomes in the affected cells (e.g., Down syndrome). Sister chromatid exchange is a repair process during DNA replication which should not result in aberrant chromosomal structures like the Philadelphia chromosome. Telomere breakage is associated with aging and cancer, but does not create clear, specific chromosomal rearrangements. Transposon insertion, too, does not generally lead to a specific translocation event like the one seen in the Philadelphia chromosome. Recombination is the process of exchange of genetic material during cell division. It usually maintains the structure of chromosomes overall. However, an abnormal form of recombination could result in a change in chromosomal structure. Specifically, a balanced translocation, where parts of two different chromosomes exchange places without loss of genetic information, can create a new chromosome, like the Philadelphia chromosome. CONCLUDING: After evaluating all the options, the Philadelphia chromosome is the result of a special form of recombination, specifically a balanced reciprocal translocation. Here, specifically the long arms of chromosome 9 and 22 translocate leading to the formation of a smaller, abnormal chromosome known as the Philadelphia chromosome. RESPONSE: Thus, the answer is (B) Recombination. The formation of the Philadelphia (Ph¹) chromosome in this patient is best accounted for by an abnormal form of recombination, specifically a balanced translocation between chromosomes 9 and 22.

**AI_B:** 1. RESTATING THE QUESTION: The clinical features shared indicates that a 48-year-old woman is experiencing symptoms like fatigue, poor appetite, weight loss, and splenomegaly along with an elevated leukocyte count. Further investigation through cytogenetic analysis revealed the presence of the Philadelphia (Ph¹) chromosome in her myelocytes. We are supposed to hypothesize the mechanism that could have led to the formation of this particular chromosome. The Philadelphia chromosome is a specific genetic abnormality in chromosome 22 of leukemia cancer cells (specifically chronic myeloid leukemia cells). This abnormality is a result of a mutation that causes a part of chromosome 9 to move to chromosome 22 (this mutation is known as a translocation). 2. PLANNING: The options provided here are all genetic mechanisms that can lead to changes in chromosome structure or sequence. Options include nondisjunction (malfunction during cell division where chromosomes don't separate properly), recombination (exchange of genetic material between different organisms which leads to the production of offspring with combinations of traits that differ from those found in either parent), sister chromatid exchange (a type of recombination between sister chromatids), telomere breakage (loss or breakage of the protective ends of chromosomes - the telomeres), and transposon insertion (the movement of specific sections of DNA within the genome). Given the presence of the Philadelphia chromosome, one might hypothesize that it's a mechanism that directly results in the translocation or rearrangement of genetic material. 3. CLINICAL REASONING: The Philadelphia chromosome, observed in this patient’s myelocytes, arises from a specific translocation event known as t(9;22)(q34;q11). In this translocation, parts of two different chromosomes – the long arms of chromosomes 9 and 22 – swap places. Considering this, "nondisjunction" or "sister chromatid exchange" would be an unlikely cause, as these mechanisms do not typically result in such specific and localized translocations, but rather in aneuploidies or recombination between sister chromatids, respectively. "Telomere breakage" and "transposon insertion" can have significant genomic effects, but they do not typically lead to this characteristic translocation observed in the Philadelphia chromosome. 4. CONCLUDING: From the deduction above, and considering that the Philadelphia chromosome is a result of a translocation event between two different chromosomes, the mechanism most likely accountable for the formation of the Philadelphia chromosome would be "recombination". 5. RESPONSE: Therefore, the most plausible answer would be: (B) Recombiation.

**AI_E:** RESTATING THE QUESTION: The primary inquiry of this clinical scenario is about a woman who is experiencing symptoms such as fatigue, poor appetite, and unexplained weight loss. The physical examination identified spleen growth, while laboratory findings suggested a high white blood cell count largely composed of neutrophils, blasts, and promyelocytes. Furthermore, bone marrow tests uncovered the Philadelphia (Ph¹) chromosome in the myelocytes. The query is focused on which process primarily results in the formation of this particular chromosome in this patient's condition. PLANNING: To fulfil this task, it becomes vital to recapitulate the general mechanisms associated with chromosome structure alterations. Nondisjunction refers to the failure of homologous chromosomes or sister chromatids to separate properly during cell division. Recombination is the exchange of genetic material between different organisms which leads to the production of offspring with combinations of traits that differ from those found in either parent. Sister chromatid exchange describes the phenomenon where genetic material is swapped between two identical copies of a replicated chromosome. Telomere breakage relates to damage in the protective ends of a chromosome, leading to instability. Transposon insertion is a process where specific DNA segments can shift locations within the genome. CLINICAL REASONING: Considering patient symptoms and findings, it is suggestive of a chronic myeloproliferative disorder, specifically Chronic Myelogenous Leukemia(CML). CML is characterized by the presence of Philadelphia (Ph¹) chromosome, which is a product of a specific reciprocal translocation between chromosomes 9 and 22(t(9;22)(q34;q11)). This translocation results in the BCR-ABL fusion gene formation, leading to constitutive tyrosine kinase activity that drives leukemogenesis. Nondisjunction, recombination, sister chromatid exchange, telomere breakage, and transposon insertion can all play a role in the genomic instability that characterizes cancer, but it's the reciprocal translocation mechanism that is responsible for the appearance of the Ph1 chromosome, typical for CML. CONCLUDING: Based on the aforementioned information it can be inferred that the appearance of the Ph1 chromosome in the patient is not due to nondisjunction, recombination, sister chromatid exchange, telomere breakage or transposon insertion, but rather a result of a specific reciprocal translocation. RESPONSE: Therefore, the most accurate response to the question "Which of the following mechanisms best accounts for the formation of the Philadelphia (Ph¹) chromosome in this patient?" would be Recombination (B), assuming "Recombination" here specifically means "Reciprocal Translocation."

**Facilitator** **[Round 2]:** SUMMARIES: AI_A: The Philadelphia chromosome is a result of reciprocal translocation, a type of recombination, between chromosomes 9 and 22. This leads to the formation of the BCR-ABL fusion gene, causing chronic myeloid leukemia (CML). AI_B: The Philadelphia chromosome is a result of a translocation event between chromosomes 9 and 22, which is best accounted for by the mechanism of recombination. AI_C: The Philadelphia chromosome is formed by a special form of recombination, specifically a balanced translocation between chromosomes 9 and 22. AI_D: None of the given mechanisms precisely account for the formation of the Philadelphia chromosome. The appropriate mechanism associated with the formation of the Philadelphia chromosome is reciprocal translocation, which is not listed in the options. AI_E: The Philadelphia chromosome is not a result of nondisjunction, recombination, sister chromatid exchange, telomere breakage, or transposon insertion, but rather a specific reciprocal translocation. The term "recombination" is assumed to mean "reciprocal translocation" in this context. KEY DIFFERENCES: The key difference in the council members' responses lies in their interpretation of the term "recombination." AI_A, AI_B, and AI_C consider reciprocal translocation as a type of recombination, while AI_D and AI_E view reciprocal translocation as a distinct mechanism not covered by the given options. QUESTION TO CLARIFY DISAGREEMENT: To clarify the disagreement, the council members could be asked: "Do you consider reciprocal translocation to be a type of recombination or a distinct genetic mechanism? How does your interpretation influence your understanding of the formation of the Philadelphia chromosome?". Based on your preceding response, how would you answer the original question: [A 48-year-old woman comes to the physician because of a 2-month history of fatigue and poor appetite; she has had a 3-kg (6.6-lb) weight loss during this period. Abdominal examination shows moderate splenomegaly. Laboratory studies show a leukocyte count of 185,000/mm³ (65% segmented neutrophils with 6% blasts and promyelocytes). Cytogenetic analysis of a bone marrow aspirate shows the presence of the Philadelphia (Ph¹) chromosome in myelocytes. Which of the following mechanisms best accounts for the formation of the Philadelphia (Ph¹) chromosome in this patient? (A) Nondisjunction (B) Recombination (C) Sister chromatid exchange (D) Telomere breakage (E) Transposon insertion]

**AI_A:** 1) RESTATING THE QUESTION: The question is asking for the mechanism that best describes the formation of the Philadelphia (Ph¹) chromosome in a patient who presents with symptoms suggestive of chronic myeloid leukemia (CML), evidenced by fatigue, weight loss, increased leukocyte count with neutrophils, blasts and promyelocytes, as well as the presence of the Philadelphia chromosome on bone marrow aspirate cytogenetic analysis. 2) PLANNING: The Philadelphia chromosome is known to be a result of reciprocal translocation between the long arms of chromosomes 9 and 22. This translocation leads to the formation of the BCR-ABL fusion gene, which plays a central role in the pathogenesis of CML. Among the options provided, none explicitly states 'reciprocal translocation'. However, 'recombination' could potentially encompass the process of 'reciprocal translocation', as both involve the exchange of genetic material. 3) CLINICAL REASONING: The term 'recombination' technically refers to any process in a meiotic or mitotic cell involving the rearrangement of DNA sequences to produce a new set of genetic material. This includes crossing-over, gene conversion, and translocation. In the case of the Philadelphia chromosome, the specific form of recombination taking place is 'reciprocal translocation', but considering the broader sense of the term 'recombination', one could argue that this process fits under its umbrella. 4) CONCLUDING: While translocation is indeed a specific process, it still falls within the general spectrum of recombination mechanisms. Given the absence of 'translocation' as an explicit option, the closest mechanism that could account for the formation of the Philadelphia chromosome in this patient is 'recombination'. 5) RESPONSE: Given these considerations, the mechanism that best accounts for the formation of the Philadelphia chromosome in this patient is (B) Recombination.

**AI_E:** 1) RESTATING THE QUESTION: The inquiry is about a 48-year-old woman with symptoms and laboratory findings suggestive of chronic myeloid leukemia (CML) associated with the Philadelphia chromosome. CML is a type of cancer that starts in certain blood-forming cells of the bone marrow. The Philadelphia chromosome is a result of a specific genetic abnormality stemming from a translocation between chromosomes 9 and 22. The question is asking for the type of genetic mechanism that resulted in this aberration. 2) PLANNING: The key assumption here is that the Philadelphia chromosome arises from a specific genetic mechanism, namely "Reciprocal translocation". Translocation, in genetics, involves the rearrangement of parts between nonhomologous chromosomes. However, this specific term is not listed among the provided options, which are nondisjunction, recombination, sister chromatid exchange, telomere breakage, and transposon insertion. Is it then valid to suppose that one of these given mechanisms aligns closest to translocation? 3) CLINICAL REASONING: Reciprocal translocations involve an exchange of genetic material between nonhomologous chromosomes, a pivotal process in generating genetic variation and evolution. In a broader context, this exchange can be considered as a subtype within the wider process of "recombination". Hence, even though the specific term "translocation" is not listed, "recombination" might be the closest match given its encompassing nature over various genetic exchange mechanisms. 4) CONCLUDING: Assessing available options and relating them to the severity of the debilitating chronic myeloid leukemia condition, it is crucial to recognize the exact underlying mechanism for better therapeutic interventions. Even though "Reciprocal translocation" would have been the preferred option, but in its absence, "Recombination" seems to be the closest possible mechanism accounting for the Philadelphia chromosome. 5) RESPONSE: The mechanism which best accounts for the formation of the Philadelphia (Ph¹) chromosome in this patient is likely (B) Recombination. Recombination, in this context, can be viewed as encompassing the specific transactional exchange of genetic material that happens during the formation of the Philadelphia chromosome.

**AI_C:** RESTATING THE QUESTION: This question is essentially asking about the woman's Philadelphia (Ph¹) chromosome found in the myelocytes, and what mechanism led to its formation. The Philadelphia chromosome results from a specific chromosomal abnormality related to chronic myeloid leukemia (CML). PLANNING: Let's take each of these different options and consider what they mean. Nondisjunction refers to the failure of sister chromatids to separate during cell division. This is not what leads to the Philadelphia chromosome. Recombination generally refers to the rearrangement of genetic material, either within a chromosome or between chromosomes. The Philadelphia chromosome is the result of a specific type of recombination known as a translocation. Sister chromatid exchange refers to the swapping of genetic material between two identical copies of a chromosome. Telomere breakage concerns the protective ends of chromosomes, and transposon insertion relates to sections of DNA that move within the genome. None of these latter three options would result in the Philadelphia chromosome. CLINICAL REASONING: In the woman's case, her exhaustion, poor appetite, weight loss, splenomegaly, and distinctly high leukocyte count indicate she may have chronic myeloid leukemia (CML), which is closely associated with the Philadelphia chromosome. The Philadelphia chromosome most specifically arises from a reciprocal translocation between the long arms of chromosome 9 and 22, creating the BCR-ABL fusion gene— an overactive tyrosine kinase that drives uncontrolled cell proliferation and resistance to apoptosis. According to our clinical understanding, this translocation could be viewed as a form of recombination where parts of two distinct chromosomes swap positions. CONCLUDING: Looking at the given answer choices, the mechanism of "recombination" seems to best capture the underlying process. While it may not precisely capture the specificity of the translocation that takes place between chromosomes 9 and 22, the other options do not appropriately account for the formation of the Philadelphia chromosome. RESPONSE: Therefore, I would select '(B) Recombination' as the mechanism that best accounts for the formation of the Philadelphia chromosome in this patient.

**AI_D:** 1) RESTATING THE QUESTION: The patient is presenting with signs and symptoms suggestive of a chronic myeloid leukemia (CML), a type of blood cancer characterized by increased and unregulated growth of myeloid cells in the bone marrow and the accumulation of these cells in the blood. The Philadelphia chromosome, found in her myelocytes, is a specific chromosomal abnormality associated with CML. We're asked to identify the genetic mechanism that leads to the formation of this chromosome. 2) PLANNING: The presence of the Philadelphia chromosome in patients with CML is due to a balanced translocation involving chromosomes 9 and 22 (specifically a fusion between the BCR gene on chromosome 22 and the ABL gene on chromosome 9) leading to the creation of the BCR-ABL fusion gene. The given options include: non-disjunction (failure of chromosome pairs to separate properly), recombination (exchange of genes between different chromosomes), sister chromatid exchange (recombination event between identical DNA sequences), telomere breakage (breakage at the ends of chromosomes), and transposon insertion (jumping genes that can change position within a genome). Note that reciprocal translocation specifically (as seen in the Philadelphia chromosome) is not mentioned as an option. 3) CLINICAL REASONING: The described chromosomal alteration seen in CML involves the exchange of parts between two chromosomes (9 & 22). While not explicitly listed, "recombination" is the most similar to the process of reciprocal translocation and it involves an exchange of genetic material. Neither non-disjunction, sister chromatid exchange, telomere breakage, or transposon insertion represent the mechanism of action as they don't involve an exchange of large portions of DNA between two non-homologous chromosomes (distinct from recombination that occurs between homologous chromosomes during meiosis). 4) CONCLUDING: Given that the formation of the Philadelphia chromosome arises from the exchange of genetic material between two different chromosomes, the corresponding mechanism is best described as recombination. However, it's important to note that the specific type of recombination relevant here, known as reciprocal translocation, is not explicitly listed among the options. 5) RESPONSE: Based on our evaluation and rephrasing process that examines each piece of information given in the question, the mechanism that can best account for the formation of the Philadelphia Chromosome in this patient is (B) Recombination.

**AI_B:** 1) RESTATING THE QUESTION: This query is about a 48-year-old woman who presents with fatigue, poor appetite, and weight loss, alongside an elevated leukocyte count and moderate splenomegaly. A cytogenetic analysis of her bone marrow aspirate confirms the presence of the Philadelphia (Ph¹) chromosome in her myelocytes. We need to identify the genetic mechanism that led to the formation of the Philadelphia chromosome in her case. The presence of the Philadelphia chromosome, a hallmark of Chronic Myeloid Leukemia (CML), points to a specific type of chromosomal rearrangement. 2) PLANNING: The crucial medical concepts here are the cytogenetic mechanisms that can result in abnormal karyotypes, including nondisjunction, which is an error in cell division leading to daughter cells with an abnormal number of chromosomes; recombination, involving the exchange of DNA sequences between homologous chromosomes; sister chromatid exchange, which is a similar exchange but within the same chromosome; telomere breakage, which could lead to chromosomal instability and disease; and transposon insertion, in which genetic elements can move within the genome. It's also necessary to consider reciprocal translocation, even though it's not listed among the options. Reciprocal translocation is the event responsible for the creation of the Philadelphia chromosome. 3) CLINICAL REASONING: Reciprocal translocation usually involves the exchange of genetic material between two non-homologous chromosomes - in the case of the Philadelphia chromosome, between chromosomes 9 and 22. This exchange creates a fusion gene, BCR-ABL, leading to the pathogenesis of CML. It's important to differentiate this event from the other mechanisms listed. Nondisjunction and sister chromatid exchange do not involve an exchange between two different chromosomes; telomere breakage is associated with telomere-related diseases such as dyskeratosis congenita; and transposon insertion would result in "jumping genes" but doesn't explain the Philadelphia chromosome. 4) CONCLUDING: Through this clinical reasoning, recombination provides the best description among the options provided for the formation of the Philadelphia chromosome. Here, the term "recombination" has been assumed to accommodate reciprocal translocation, even though normally "recombination" refers to exchanges of genetic material between homologous chromosomes during meiosis, not the exchange between non-homologous chromosomes. 5) RESPONSE: Therefore, the best choice to describe the formation mechanism of the Philadelphia chromosome in the patient seems to be "(B) Recombination", under the understanding that this refers to reciprocal translocation in this context.

**Facilitator** The question is asking for the genetic mechanism that led to the formation of the Philadelphia chromosome in a 48-year-old woman presenting with symptoms suggestive of chronic myeloid leukemia (CML). The Philadelphia chromosome is a result of a specific chromosomal abnormality associated with CML, which involves a reciprocal translocation between chromosomes 9 and 22, leading to the creation of the BCR-ABL fusion gene. This gene is responsible for the uncontrolled proliferation of myeloid cells in the bone marrow and their accumulation in the blood. Among the provided options, none explicitly states 'reciprocal translocation'. However, the term 'recombination' technically refers to any process in a meiotic or mitotic cell involving the rearrangement of DNA sequences to produce a new set of genetic material. This includes crossing-over, gene conversion, and translocation. Therefore, 'recombination' could potentially encompass the process of 'reciprocal translocation', as both involve the exchange of genetic material. The other options, including nondisjunction, sister chromatid exchange, telomere breakage, and transposon insertion, do not appropriately account for the formation of the Philadelphia chromosome. Nondisjunction and sister chromatid exchange do not involve an exchange between two different chromosomes; telomere breakage is associated with telomere-related diseases such as dyskeratosis congenita; and transposon insertion would result in "jumping genes" but doesn't explain the Philadelphia chromosome. Given these considerations, the consensus is that the mechanism that best accounts for the formation of the Philadelphia chromosome in this patient is 'recombination'. It's important to note that the specific type of recombination relevant here, known as reciprocal translocation, is not explicitly listed among the options. Therefore, the best choice to describe the formation mechanism of the Philadelphia chromosome in the patient seems to be "(B) Recombination", under the understanding that this refers to reciprocal translocation in this context.
