# Supplementary material for: Collaborative intelligence in AI: Evaluating the performance of a council of AIs on the USMLE
Source: PLOS Digit Health. 2025 Oct 9;4(10):e0000787. doi: 10.1371/journal.pdig.0000787 (PMC12510544; doi:10.1371/journal.pdig.0000787)
Supplement: S2 File — (ZIP) [file pdig.0000787.s002.zip › S2 Data Raw data files containing unprocessed question inputs and LLM outputs including transcripts of deliberations/STEP1_Question-55-1_Type-MC-NJ.docx]

**Facilitator** **[Round 1]:** A 2-year-old boy is brought to the physician because of failure to thrive. He also has had loose, fatty, foul-smelling stools and a cough during the past 2 weeks. He is at the 30th percentile for height and 10th percentile for weight. Physical examination shows no other abnormalities. Laboratory studies show steatorrhea and a sweat chloride concentration of 80 mmol/L. A chest x-ray shows hyperinflation. Sputum culture grows Haemophilus influenzae and Staphylococcus aureus. Secretion of which of the following substances is most likely to be decreased in this patient? (A) Bicarbonate (B) Gastric acid (C) Glucagon (D) Insulin (E) Intrinsic factor

**AI_B:** RESTATING THE QUESTION: The question wants us to determine which substance may be experiencing decreased secretion in a two-year-old boy showing various symptoms which include poor growth, steatorrhea (presence of excess fat in feces), a cough, and a laboratory result with high sweat chloride concentration and evidence of lung infection. PLANNING: The presenting symptoms and laboratory findings suggest this child may have cystic fibrosis, a genetic disorder that affects primarily the lungs and digestive system. Here are the possibilities for decreased secretion: (A) Bicarbonate - involved in neutralizing stomach acid and necessary for pancreatic function (B) Gastric acid - secreted by the stomach for digestion (C) Glucagon - a hormone involved in regulating blood sugar levels (D) Insulin - also involved in blood sugar regulation (E) Intrinsic factor - secreted by the stomach to help with vitamin B12 absorption. CLINICAL REASONING: Cystic fibrosis (CF) is a genetic disorder that affects mucous-secreting tissues of the body, causing them to produce thicker, sticky mucus. While CF can affect secretion of multiple substances, the clinical picture here points most heavily to malfunctioning of the pancreas. The steatorrhea (fatty, foul-smelling stool) results from impaired fat digestion and malabsorption which suggests a decrease in pancreatic enzymes including lipase. The pancreas, under normal circumstances, secretes bicarbonate to neutralize the acidity in the duodenum for optimal enzyme function. In the setting of CF, duct plugging obstruct the secretion of bicarbonate and other pancreatic enzymes, resulting in the hindrance of effective digestion and absorption. CONCLUDING: With failure to thrive, steatorrhea and the clear indication of CF (sweat chloride concentration > 60mmol/L), the key dysfunction is likely in the secretion of pancreatic enzymes and bicarbonate. Even though the other options may exhibit some decrease, when considering the clinical priority and the direct pathological outcomes of CF, bicarbonate secretion is likely to be the most decreased. RESPONSE: Therefore, the secretion of Bicarbonate (A) is most likely to be decreased in this patient.

**AI_E:** (1) RESTATING THE QUESTION: The question asks which of the stated biological substances would most likely observe diminished secretion in this 2-year-old patient. From the description, we learn the patient is failing to thrive, with implications such as being in the low spectrum of height and weight percentiles. Signs like loose, fatty, foul-smelling stools (steatorrhea), and a chronic cough with confirmed bacterial presence in the sputum are noted. The child also has an elevated sweat chloride level which is indicative of an aberration in normal chloride transport. (2) PLANNING: The distinctive presence of steatorrhea, failure to thrive, persistent cough, and sweat chloride concentration at 80 mmol/L, combined with the observation of lung hyperinflation on chest X-ray, and growth of Haemophilus influenzae and Staphylococcus aureus in the sputum may suggest Cystic Fibrosis (CF), an autosomal recessive disorder. CF affects the secretory glands, chiefly involved with mucus and sweat production and is often characterized by thickened secretions that lead to obstructions in the lungs, liver, and pancreas. CF usually decreases the secretion of bicarbonate within the pancreas which can potentially impact digestion and absorption of nutrients leading to the symptoms exhibited by the child. (3) CLINICAL REASONING: Had the pathology impacted insulin or glucagon (C&D options), the patient would display symptoms relevant to sugar metabolism besides other pancreatic symptoms. Intrinsic factor secretion diminishment (Option E) primarily affects vitamin B12 absorption and would lead to signs and symptoms associated with B12 deficiency like anemia, fatigue, etc. Gastric acid decline (Option B) would generally impact protein digestion and its consequences like protein malabsorption, which is not present in the case. Bicarbonate secretion failure in the pancreas, on the contrary, impairs enzyme activity necessary to digest fats and proteins, leading to fat malabsorption, thereby explaining the observed steatorrhea and failure to thrive in this patient. (4) CONCLUDING: Given the symptoms of fat malabsorption, failure to thrive, and high sweat chloride levels, a CF diagnosis seems plausible, and hence it is reasonable to suggest that the secretion of bicarbonate (Option A) - which comes from the pancreas and aids in the digestion process - is decreased in this patient. (5) RESPONSE: So, considering the given case, it is likely that the secretion of "Bicarbonate" is most likely to be decreased in this patient.

**AI_A:** RESTATING THE QUESTION: The question at hand revolves around a young boy presenting with failure to thrive, characterized by fatty and malodorous feces (steatorrhea), a persisting cough, as well as growth below the typical percentiles for his age. Additionally, lab results indicate elevated chloride levels in his sweat and a chest x-ray reveals lung hyperinflation. Analysis of his sputum presents growth of Haemophilus influenzae and Staphylococcus aureus. Connected to this clinical presentation, we need to determine which biochemical substance's secretion is most likely reduced in this patient's body. The choices include bicarbonate, gastric acid, glucagon, insulin, and intrinsic factor. PLANNING: Assumptions made based on described symptoms hint at cystic fibrosis, a genetic disorder that negatively affects glands that produce sweat and mucus. This disease often leads to chest infections because of thick, sticky mucus build-up in the lungs and pancreatic insufficiency due to blockages in the organ, hampering the digestive process by reducing the amount of enzymes reaching the intestine, and could result in malabsorption of dietary fat, consistent with the symptoms of steatorrhea. The sweat test outcome, a typically high sweat chloride concentration, similarly hints at cystic fibrosis as it is a conventional diagnostic measure for this disease. CLINICAL REASONING: The correlation between cystic fibrosis, mucus build-up and respiratory infections might explain the sputum cultures bearing Haemophilus influenzae and Staphylococcus aureus. Additionally, pancreatic duct blockage could cause a decreased pancreatic secretion. Following this line of thought, let's consider the functions of the given substances. Bicarbonate aids in neutralizing stomach acid and is a component of pancreatic secretions, while gastric acid aids digestion. Glucagon, a hormone produced by the pancreas, raises glucose levels in the blood. Insulin, also a pancreatic hormone, assists glucose in entering cells. Lastly, the intrinsic factor, produced by the stomach, aids in vitamin B12 absorption. In this context of cystic fibrosis, which would be more clinically relevant to pursue due to pancreatic dysfunction would be bicarbonate, given its major role in neutralizing stomach acid and aiding digestion by breaking down fats and proteins. CONCLUDING: Given the symptoms and lab results aligning with cystic fibrosis, this disease results in the body producing thick, sticky mucus that can obstruct the functioning of vital organs. Within the choices, bicarbonate, critical in the digestion process because it neutralizes stomach acid and aids in the digestive process by breaking down fats and proteins, is the most likely substance to have its secretion reduced in the patient's body. RESPONSE: The substance most likely to have a decreased secretion in this patient is (A) Bicarbonate.

**AI_C:** RESTATING THE QUESTION: You're asking me to examine the symptoms, physical findings, and lab results for a 2-year-old boy who is falling behind peers in physical growth, has unusually fatty and foul-smelling stools, a persistent cough, some abnormal lab results and a couple of bacteria identified in his sputum culture. Using this information, you're asking me to ascertain which among the listed substances (bicarbonate, gastric acid, glucagon, insulin, intrinsic factor) would most probably have a decreased secretion in this child's system. PLANNING: The symptoms are indicative of two possible medical conditions - malnutrition and a continual lung infection. The steatorrhea, which involves fatty, loose, and foul-smelling stools, suggests that there is an absorption issue in the body, leading to the failed digestion of fat. The low percentiles for weight and height further hint at malnutrition. The increased sweat chloride concentration and inflated lungs, along with the growth of Haemophilus influenzae and Staphylococcus aureus in the sputum, suggests continued lung infection, possibly Cystic Fibrosis. From these symptoms and indications, it can be assumed that a substance, the lack of which would lead to these conditions, is not being adequately produced in the boy's system. CLINICAL REASONING: Rationally, for improved digestion and absorption of fat, the body depends on pancreatic enzymes, one of which is pancreatic lipase. However, for these enzymes to work effectively, they require a certain level of alkalinity in the small intestine, which is provided by the bicarbonate ions secreted by the pancreatic duct cells. If the secretion of bicarbonate is compromised, the intestine will maintain an acidic environment, thus inhibiting the function of the pancreatic enzymes and leading to malabsorption and ultimately malnutrition. Also, a critical component of cystic fibrosis is a defect in the CFTR gene, which manages the movement of chloride and bicarbonate across epithelial membranes. A defect in this gene would cause a decrease in bicarbonate secretion, leading to cystic fibrosis, which could explain the child's respiratory symptoms and the organisms grown in the sputum culture. CONCLUDING: Other substances listed like gastric acid, glucagon, insulin, and intrinsic factor can have various consequences if their secretion is declined, but they do not quite fit the array of symptoms seen in the boy. Gastric acid and intrinsic factor are essential for digestion and absorption of certain nutrients, but their decreased secretion wouldn't explain the respiratory symptoms. The failure of insulin and glucagon wouldn't directly result in malabsorption. RESPONSE: Conclusively, considering the symptoms, physical examination findings, and lab results for the child in question, the substance whose secretion is most likely to be reduced in his system is (A) Bicarbonate.

**AI_D:** RESTATING THE QUESTION: The young patient in the scenario is exhibiting symptoms consistent with a decrease in a certain substance secretion, including failure to prosper shown through his place at the 30th percentile for height and 10th percentile for weight, loose fatty and foul-smelling stools (steatorrhea), a persistent cough, and chronic lung changes indicated by the hyperinflation on the chest x-ray. Additionally, the sweat chloride concentration of 80 mmol/L and the isolated bacteria (Haemophilus influenzae and Staphylococcus aureus) from the sputum culture support this conclusion. The inquiry now is to determine the most probable substance showing a reduction in its secretion. PLANNING: The clinical presentation of the child in conjunction with the laboratory findings suggests a disorder affecting fat digestion and absorption, resulting in steatorrhea, alongside respiratory system involvement. This pattern could point to us a possibility of Cystic Fibrosis (CF), a disorder affecting the CFTR gene that produces a protein involved in ion and water transport in the body. An increase in the sweat chloride levels is a typical diagnostic indicator of CF. Cystic fibrosis is known to cause thick secretions in the respiratory and digestive systems. The substances mentioned (Bicarbonate, Gastric acid, Glucagon, Insulin, and Intrinsic factor) can be related in different organ functions. Considering the given symptoms, we could hypothesize that the disorder might've caused a decrease in bicarbonate secretion, as it plays a key role in neutralizing stomach acid and promoting enzymatic fat digestion in the small intestine. CLINICAL REASONING: In cystic fibrosis, the function of the CFTR protein, which acts as a chloride channel, is disrupted, leading to abnormal ion and water exchange in epithelial surfaces resulting in viscous mucus secretions. This impacts multiple organs, notably the respiratory and digestive systems. Repeat respiratory infections (as indicated by the organisms cultured) and hyperinflation on chest x-ray represent the chest involvement in CF. In terms of digestion, CF affects bicarbonate secretion. Pancreas secretes bicarbonate in pancreatic juice that helps in neutralizing the gastric acid in the small intestine. Bicarbonate also provides the optimal pH for pancreatic enzymes to function, which is crucial for fat digestion. If bicarbonate secretion is reduced, it would lead to a more acidic and stodgy environment that is not conducive for the activity of digestive enzymes. This would reduce fat digestion and absorption leading to the condition steatorrhea, that the patient is suffering from. CONCLUDING: Relating back to our initial hypothesized assumption, considering the patient's complete set of symptoms and how cystic fibrosis affects various organ systems, it's plausible that there is a decrease in the secretion of bicarbonate, brought about by the effects of the disorder on pancreatic function. RESPONSE: In this 2-year-old boy who exhibits symptoms of failure to thrive, steatorrhea, respiratory symptoms, has high sweat chloride levels and sputum culture positive for Haemophilus influenzae and Staphylococcus aureus, a diagnosis of Cystic Fibrosis can be contemplated. Due to this, the secretion of 'Bicarbonate' (Option A) is most likely to be decreased in this patient.

**Facilitator** The consensus statement synthesizes the responses as follows: The question pertains to a 2-year-old boy exhibiting symptoms of failure to thrive, steatorrhea, a persistent cough, and elevated sweat chloride levels. The boy's growth is below average for his age, and his chest x-ray reveals lung hyperinflation. His sputum culture shows the presence of Haemophilus influenzae and Staphylococcus aureus. The task is to identify which biochemical substance's secretion is most likely reduced in this patient's body, with the options being bicarbonate, gastric acid, glucagon, insulin, and intrinsic factor. The symptoms and lab results suggest a diagnosis of cystic fibrosis, a genetic disorder that affects the glands that produce sweat and mucus. This disease often leads to respiratory infections due to mucus build-up in the lungs and pancreatic insufficiency due to blockages in the organ, which can result in malabsorption of dietary fat, consistent with the symptoms of steatorrhea. The high sweat chloride concentration is a typical diagnostic indicator of cystic fibrosis. Given the symptoms and the nature of cystic fibrosis, the substance most likely to have its secretion reduced is bicarbonate. Bicarbonate plays a crucial role in neutralizing stomach acid and aiding digestion by breaking down fats and proteins. In the context of cystic fibrosis, bicarbonate secretion can be compromised, leading to an acidic environment in the intestine that inhibits the function of pancreatic enzymes, resulting in malabsorption and malnutrition. Therefore, the consensus is that the substance most likely to have a decreased secretion in this patient is bicarbonate.
