# Supplementary material for: Collaborative intelligence in AI: Evaluating the performance of a council of AIs on the USMLE
Source: PLOS Digit Health. 2025 Oct 9;4(10):e0000787. doi: 10.1371/journal.pdig.0000787 (PMC12510544; doi:10.1371/journal.pdig.0000787)
Supplement: S2 File — (ZIP) [file pdig.0000787.s002.zip › S2 Data Raw data files containing unprocessed question inputs and LLM outputs including transcripts of deliberations/STEP1_Question-57-1_Type-MC-NJ.docx]

**Facilitator** **[Round 1]:** A 10-year-old boy is brought to the physician because of a 3-week history of nosebleeds and easy bruisability. His older brother has had similar episodes. He is at the 30th percentile for height and weight. Physical examination shows nasal and gingival bleeding and several ecchymoses over the trunk and upper and lower extremities in various stages of healing. Laboratory studies show a platelet count of 300,000/mm³ (N=150,000–400,000). Platelet adhesion testing shows a normal response to ristocetin, but aggregation does not occur in response to thrombin; platelet morphology is normal. Prothrombin time and activated partial thromboplastin time are within the reference ranges. A defect in which of the following is the most likely cause of the findings in this patient? (A) Factor VII (proconvertin) (B) Fibrinogen (C) Glycoprotein IIb-IIIa (D) Granule storage pool (E) von Willebrand factor

**AI_B:** To rephrase the question: A 10-year-old boy with a 3-week history of easy bleeding and bruising, similar to his older brother's, has a physical exam showcasing nasal and gum bleeding, with several bruises on his extremities. Despite having a normal platelet count and morphology, and normal coagulation times, his platelets do not aggregate in response to thrombin. We are asked to decipher which of the following factors could be compromised, causing such symptoms: Factor VII, fibrinogen, glycoprotein IIb-IIIa, granule storage pool, or von Willebrand factor. So let's unpack the respective roles each factor plays: Factor VII plays a crucial role in coagulation and if deficient this often leads to prolonged prothrombin time, which this patient does not have. Fibrinogen acts as a foundation for creating a fibrin clot, and if faulty the overall clotting time (PT and aPTT) would also be prolonged. The von Willebrand factor mediates platelet adhesion -- but our patient's platelets demonstrate normal adhesion. It leaves glycoprotein IIb-IIIa, which facilitates platelet aggregation, and granule storage pool, which are intracellular structures stocked with substances enabling platelets to adhere and aggregate. However, considering the patient's normal platelet adhesion, a defect in glycoprotein IIb-IIIa would cause disruption in platelet aggregation without affecting adhesion, aligning with our patient's laboratory findings. Looking at it logically, given that the patient's platelets do not aggregate in response to thrombin, a critical question is determining which factor(s) could directly be involved in platelet aggregation. Given that glycoprotein IIb-IIIa is a critical mediator for platelet aggregation, a defect would directly affect this process. In conclusion, given the signs of bleeding, the sibling's similar experiences, and the normal platelet count, adhesion, prothrombin time, and aPTT, with abnormal platelet aggregation in response to thrombin but not ristocetin, it suggests a possible defect in the glycoprotein IIb-IIIa, which brings platelets together once adhesion has occurred. A problem in this area could explain the displayed symptoms. Therefore, the most likely cause of the patient's symptoms is a defect in the Glycoprotein IIb-IIIa (C).

**AI_C:** Restating the question: A 10-year-old boy experiencing persistent nosebleeds and bruising, similar to episodes his older brother has experienced, is also found to have multiple instances of ecchymoses all over his body in different stages of healing. Lab results show his platelet count is normal, his response to ristocetin is normal, but there is no aggregation in response to thrombin. His prothrombin time and activated partial thromboplastin time are also within the normal ranges. We need to hypothesize the most likely underlying defect causing these symptoms. Planning: The boy's normal findings in platelet count and adhesion, as well as prothrombin time and activated partial thromboplastin time, can be paired with the abnormal finding of no platelet aggregation in response to thrombin which implies that the defect likely lies in the final common pathway of platelet aggregation. The choices provided all have roles within the hemostatic system. Clinical Reasoning: Factor VII and von Willebrand factor primarily function in the initiation of the coagulation cascade and platelet adhesion respectively. Normal prothrombin time and activated partial thromboplastin time suggests intact coagulation pathways. Fibrinogen is a coagulation factor that is converted into fibrin as part of the clotting process, which is indirectly evaluated through prothrombin time and activated partial thromboplastin time and hence not linked with the primary defect in this context. A granule storage pool usually houses various granules within a platelet including glycoprotein IIb/IIIa, although a defect in storage might cause more systemic manifestations, including those beyond hemostasis. Finally, glycoprotein IIb/IIIa is the platelet membrane receptor for fibrinogen and mediates platelet-platelet interaction and aggregation, which fits with the child's platelet dysfunction. Concluding: Therefore, the defect in platelet aggregation without any defects in platelet count, coagulation pathways, and platelet adhesion strongly suggest a problem at the level of platelet-platelet interaction, which takes place principally through the action of the glycoprotein IIb/IIIa receptor. Family history suggests that the condition could be congenital. Response: The boy's condition is most likely due to a defect in (C) Glycoprotein IIb-IIIa. This would account for his symptoms and lab findings. For example, the absence of platelet aggregation in response to thrombin, irrespective of normal platelet counts, point towards a platelet function disorder, which could be attributable to defects in glycoprotein IIb/IIIa.

**AI_E:** RESTATING THE QUESTION: The clinical query seeks to identify the most likely biological culprit behind the patient's condition. The young boy presents with nosebleeds and easy bruising, along with appearance of ecchymoses - signs suggesting a bleeding disorder. He has a normal platelet count and morphology, and his clotting profiles (prothrombin time and activated partial thromboplastin time) are normal. His platelets respond normally to ristocetin - an agent that causes platelet agglutination or clumping, but fail to aggregate in response to thrombin - an enzyme that converts fibrinogen to fibrin in the clotting process. Given these findings, the question aims to identify which physical or functional component is likely defect. PLANNING: Initial assumptions might lead us to think that there could be something wrong with the clotting factors such as Factor VII or fibrinogen, both of which are pivotal in the coagulation cascade leading to bleeding control. But normal prothrombin time (which tests the extrinsic pathway including Factor VII) and activated partial thromboplastin time (which tests the intrinsic pathway) suggest these components are functional. A possibility could be a platelet dysfunction affecting aggregation, but not adhesion, given the selective aggregation failure in response to thrombin, implying a probable defect in the platelets' glycoprotein IIb-IIIa complex or the granule storage pool. CLINICAL REASONING: Considering the normal physical aspects of platelets, and the detected defect specifically in platelet aggregation following thrombin stimulation, this leads us more towards a defect in the platelet function at a molecular level, rather than a gross physical defect such as a defect in the granule storage pool. The function that's compromised is the final common pathway of the platelet aggregation, implicating possibly the glycoprotein IIb-IIIa complex - a receptor on platelets that binds fibrinogen, bridging adjacent platelets together and enabling clot formation. CONCLUDING: Based on the clinical history and the laboratory findings, it can be concluded that the chances of having a defect in the glycoprotein IIb-IIIa complex - the receptor responsible for fibrinogen binding and thereby platelet aggregation - is most likely in this child's case. The inherited disorder associated with such a defect is Glanzmann thrombasthenia - characterized by normal platelet count and morphology, normal clotting profile, normal platelet adhesion (as evidenced by normal ristocetin response), but impaired platelet aggregation, particularly in response to thrombin. RESPONSE: Therefore, the most likely cause of the findings in this 10-year-old patient is a defect in (C) Glycoprotein IIb-IIIa.

**AI_A:** The question is asking what the most likely cause of the symptoms in the young boy. This boy, who has been experiencing frequent nosebleeds and bruisability, has normal platelet counts, and his blood clotting times (prothrombin time and activated partial thromboplastin time) are within the normal range. Additionally, his platelets show a normal response to ristocetin, a drug that assists in the attraction of platelets to damaged capillaries, but they fail to aggregate, or clump together, in response to thrombin. Thrombin is a crucial factor in blood clotting as it converts fibrinogen, a soluble protein, into fibrin, a non-soluble protein that forms the framework of blood clots. Given this information, I'll assess each of the provided options: (A) Factor VII (Proconvertin) is a coagulation factor that participates in the extrinsic pathway of coagulation but, given that clotting times are normal, a defect here might not be the priority. (B) Fibrinogen is converted by thrombin into fibrin during clot formation. But since prothrombin time and activated partial thromboplastin time are normal, which evaluate the extrinsic and intrinsic pathways of coagulation where fibrinogen acts, it's less likely to be the cause. (C) Glycoprotein IIb-IIIa is a receptor present on the surface of platelets that allows them to bind to fibrinogen and aggregate, forming a platelet plug. The defect of this could explain the lack of platelet aggregation in response to thrombin. (D) The granule storage pool in platelets store substances necessary for platelet function. A defect here generally leads to disorders with abnormal platelet function, generally accompanied by abnormal response to various aggregating agents, not only to thrombin. (E) von Willebrand factor is crucial for platelet adhesion to the sub-endothelium, and its defect could cause abnormal platelet adhesion, but our patient shows a normal response to ristocetin, used to evaluate von Willebrand factor function, which makes this less likely. Given the boy's symptomatic picture and the related findings, the priority could be a defect in glycoprotein IIb-IIIa, which is crucial for platelet aggregation. Glycoprotein IIb-IIIa assists in the platelets' ability to clump together - a process called aggregation. This clumping is crucial in forming a sufficient plug that stops bleeding. In response to various signals such as thrombin or ADP (adenosine diphosphate), activated platelets raise the affinity of the glycoprotein IIb-IIIa complex on their surface which allows fibrinogen to bind and through this, platelets become cross-linked in an aggregation process beneficial in clot formation. Therefore, a defect in this complex would disrupt the formation of stable platelet plugs, leading to issues like nosebleeds and easy bruising. So, my answer is (C) Glycoprotein IIb-IIIa.

**AI_D:** RESTATING THE QUESTION: We are faced with a clinical scenario revolving around a 10-year-old boy who has been experiencing frequent nosebleeds and easily acquiring bruises for the past 3 weeks. These symptoms have also presented in his older brother. Additionally, he falls within the 30th percentile for both height and weight for his age. Nasal and gingival bleeding, as well as multiple bruises in various healing stages over his body, are found during his physical examination. Laboratory results indicate that his platelet count is within the normal range, and platelet adhesion in response to ristocetin (a drug used to induce platelet aggregation) is regular. However, the platelets do not aggregate in response to thrombin. Furthermore, the shape and structural characteristics (morphology) of the platelets are normal. The clotting times when measured by Prothrombin Time and Activated Partial Thromboplastin Time are also within normal limits. Given these observations, we are asked to determine the most likely cause of the boy's symptoms by identifying which factor has a defect. PLANNING: This scenario depicts an individual with an apparent bleeding disorder, with normal platelet count, normal Prothrombin Time, and Activated Partial Thromboplastin Time. However, a lack of platelet aggregation in response to thrombin suggests a defect in platelet function rather than coagulation cascade. In this context, our assumption becomes that there might be a component within the platelet activation pathway that is malfunctioning. The five options given, Factor VII, Fibrinogen, Glycoprotein IIb-IIIa, Granule storage pool, and von Willebrand factor, are all involved either in the coagulation cascade or platelet function. However, Factor VII, Fibrinogen, and von Willebrand factor are major components of the coagulation pathway, while Glycoprotein IIb-IIIa and Granule storage pool defects are more related to platelet function. Given that coagulation times are within normal limits, a defect in the coagulation pathway (Factor VII, Fibrinogen, or von Willebrand factor) seems less likely. CLINICAL REASONING: Considering our focus lies within the platelet pathway as suggested by the lack of response in platelet aggregation to thrombin, we should look more closely at Glycoprotein IIb-IIIa and Granule storage pool. Glycoprotein IIb-IIIa is pivotal in platelet aggregation as it binds fibrinogen to allow the linking of platelets together. Defects in this receptor would result in the inability of platelets to aggregate properly despite normal adhesion, consistent with our patient's presentation. In comparison, defects in Granule storage pools often affect both platelet adhesion and aggregation as granules contain both adhesion molecules and secretion products necessary for attracting and activating other platelets. CONCLUDING: Upon reflecting on the alternate assumption that the defect might be present within the platelet function rather than the coagulation pathway, and considering our patient's platelet function test results, the defect most likely lies in the Glycoprotein IIb-IIIa receptor, making it the most probable cause of the patient's symptoms. RESPONSE: Hence, the most likely cause of the findings in this patient is a defect in (C) Glycoprotein IIb-IIIa.

**Facilitator** The consensus statement, synthesizing the responses of AI_A, AI_B, AI_C, AI_D, and AI_E, is as follows: The question presents a 10-year-old boy with a history of nosebleeds and easy bruising, symptoms also experienced by his older brother. His physical examination reveals nasal and gingival bleeding, along with several ecchymoses in various stages of healing. Laboratory studies show a normal platelet count, normal platelet morphology, and normal clotting times (prothrombin time and activated partial thromboplastin time). His platelets respond normally to ristocetin, a drug used to evaluate platelet adhesion, but fail to aggregate in response to thrombin, an enzyme crucial in the clotting process. Given these findings, the question asks us to identify the most likely defect causing these symptoms. The options provided are Factor VII, Fibrinogen, Glycoprotein IIb-IIIa, Granule storage pool, and von Willebrand factor. Each of these factors plays a role in the coagulation cascade or platelet function. Factor VII and Fibrinogen are major components of the coagulation pathway, while von Willebrand factor mediates platelet adhesion. However, the boy's normal clotting times and platelet adhesion suggest that these factors are likely functioning correctly. The Granule storage pool houses various substances necessary for platelet function, but a defect here would likely affect more than just the response to thrombin. Glycoprotein IIb-IIIa, on the other hand, is a receptor on the surface of platelets that allows them to bind to fibrinogen and aggregate, forming a platelet plug. A defect in this receptor could explain the boy's lack of platelet aggregation in response to thrombin, aligning with his laboratory findings. This defect would disrupt the formation of stable platelet plugs, leading to issues like nosebleeds and easy bruising. Therefore, the consensus among the AI responses is that the most likely cause of the boy's symptoms is a defect in Glycoprotein IIb-IIIa (Option C). This conclusion is based on the boy's clinical history, his laboratory findings, and the understanding of the roles each of the provided options plays in coagulation and platelet function.
