# Supplementary material for: Collaborative intelligence in AI: Evaluating the performance of a council of AIs on the USMLE
Source: PLOS Digit Health. 2025 Oct 9;4(10):e0000787. doi: 10.1371/journal.pdig.0000787 (PMC12510544; doi:10.1371/journal.pdig.0000787)
Supplement: S2 File — (ZIP) [file pdig.0000787.s002.zip › S2 Data Raw data files containing unprocessed question inputs and LLM outputs including transcripts of deliberations/STEP1_Question-58-1_Type-MC-NJ.docx]

**Facilitator** **[Round 1]:** A previously healthy 45-year-old woman who works as a park ranger comes to the physician because of a 1-week history of shortness of breath, even at rest. She has lived in the mountains at 10,000 feet above sea level for 2 years; the physician’s office is located at sea level. Her pulse is 85/min, respirations are 18/min, and blood pressure is 125/90 mm Hg. Physical examination while sitting upright shows jugular venous distention and 2+ pedal edema. During the past 2 years, which of the following has most likely decreased in this patient? (A) Height of P waves in lead I of the patient's ECG (B) Height of R waves in lead V₁ of the patient's ECG (C) Hematocrit (D) Pulmonary vascular resistance (E) Right ventricular diastolic compliance (F) Right ventricular wall thickness

**AI_B:** 1) RESTATING THE QUESTION: A 45-year-old woman with a sedentary job in the mountains is experiencing breathlessness. She has signs of cardiac congestion, including jugular venous distention and pedal edema, despite a normal blood pressure and breathing rate. We need to identify what has likely changed in her over the past two years living at high altitude. The answer choices relate to aspects of her cardiac function and blood properties. 2) PLANNING: Assumptions include her symptoms relating to her high-altitude lifestyle, or they could be independent of it. Considering the history of living at high altitude, we would expect her body to compensate for lower oxygen levels by increasing hematocrit (more red blood cells to carry more oxygen). An alternate assumption is that the prolonged exposure to high altitude led to chronic hypoxia, causing a rise in pulmonary vascular resistance, enhancing right ventricular strain, and ending up in right heart failure (Cor pulmonale). 3) CLINICAL REASONING: Living at high altitude exposes the body to lesser oxygen, initiating compensatory mechanisms, including increased hematocrit. This physiological adaptation facilitates better oxygen transport in a low-oxygen environment. However, prolonged exposure results in chronic hypoxia leading to increased pulmonary vascular resistance, which will further strain the right ventricle causing it to hypertrophy and later dilate resulting in right heart failure. Symptoms of right heart failure include jugular venous distension and pedal edema, as seen in the patient which suggests an increment in right ventricular wall thickness and not the decrease as per the question's context. 4) CONCLUDING: Given the body's response mechanisms to high altitudes, factors exclusively decreasing during this period can be narrowed to choices related to cardiac functionality. Taking into account the patient's symptoms and the compensatory mechanisms associated with exposure to high altitude, it suggests a decrease in right ventricular diastolic compliance (the ability of the heart's right ventricle to fill) as the right ventricle strains against increased pulmonary resistance, becoming stiffened and less compliant over time. 5) RESPONSE: (E) Right ventricular diastolic compliance has most likely decreased in this patient.

**AI_A:** RESTATING THE QUESTION: This question concerns a 45-year-old woman living at high altitude (10,000 ft above sea level) who has been experiencing shortness of breath, and signs of increased cardiac pressure (jugular venous distension and pedal edema). The query is about which physiological parameter has likely decreased in the patient over the past two years. PLANNING: Several possibilities are proposed: the height of certain ECG waves (P waves in lead I, or R waves in lead V₁), her hematocrit level, pulmonary vascular resistance, right ventricular diastolic compliance, and right ventricular wall thickness. To plan, it's useful to know how these factors change with prolonged hypoxia, such as high-altitude living. CLINICAL REASONING: Let us consider each alternative. (A) The P wave reflects atrial depolarization, but chronic hypoxia does not directly influence atrial depolarization. (B) R wave in V₁ lead mirrors the electrical activity of the right ventricle, which may increase in chronic higher-altitude living due to increased hypertrophy. (C) Hematocrit, the volume proportion of red cells in blood, would increase with chronic hypoxia to permit better O2 carriage. (D) Pulmonary vascular resistance may also increase in response to chronic hypoxia, as the body attempts to direct blood flow to better-oxygenated areas of the lung. (E) Right ventricular diastolic compliance refers to the ease with which the ventricle fills during diastole. As the right ventricle hypertrophies in response to chronic hypoxia and increased pulmonary vascular resistance, its diastolic compliance may decrease. (F) Right ventricular wall thickness would also probably increase due to hypertrophy as a response to chronic hypoxia and elevated pulmonary vascular resistance. CONCLUDING: The patient's symptoms suggest right heart failure due to pulmonary hypertension, a common consequence of chronic high-altitude living. Therefore, the parameter that likely decreased is right ventricular diastolic compliance, due to the compensatory hypertrophy of the right ventricle. RESPONSE: Thus, the answer would be (E) Right ventricular diastolic compliance. This would have most likely decreased in response to chronic high-altitude living, causing the observed symptoms in the patient.

**AI_C:** RESTATING THE QUESTION: The clinical scenario describes a 45-year-old woman who has had shortness of breath for a week and has been living in the mountains at high altitude for the past two years. Moreover, physical signs suggest some form of heart disease, such as jugular venous distension and pedal edema. We are addressing the issue of what physiological change has most likely decreased during her two years living at high altitude. PLANNING: To collect assumptions, we should understand how high-altitude conditions affect the body. The human body naturally responds to high altitude environments, where the air is thin and oxygen levels are low, by increasing red blood cell production to compensate for the lower oxygen concentration, which might increase the hematocrit level (assumption 1). Additionally, chronic exposure to high altitude can lead to changes in the pulmonary and cardiovascular systems, potentially influencing the ECG parameters, pulmonary vascular resistance, right ventricular function and morphology (assumption 2). CLINICAL REASONING: The presented symptoms and signs suggest a form of heart disease, potentially right-sided heart failure or cor pulmonale, which could stem from chronic lung disease or long exposure to low-oxygen environments like high altitudes. If she has been developing a condition like high-altitude pulmonary edema (HAPE) or chronic mountain sickness (CMS), an expected change would be an increase, not a decrease, in her hematocrit, as the body tried to compensate for low oxygen levels by producing more red blood cells. The same goes for right ventricular wall thickness, which would likely increase due to compensatory hypertrophy in response to increased pulmonary pressure. However, the long-term result of these adaptive changes may lead to decreased right ventricular diastolic compliance, as the hypertrophied ventricle becomes stiffer and less able to fill properly during diastole. CONCLUDING: Given the patient's symptomatology and potential adaptations to the high altitude environment, it seems reasonable to conclude that although certain values like hematocrit and right ventricular wall thickness might have increased, right ventricular diastolic compliance likely decreased as a possible maladaptive result over time. RESPONSE: Therefore, the answer is (E) Right ventricular diastolic compliance.

**AI_D:** RESTATING THE QUESTION: This scenario involves a 45-year-old woman with a sudden onset of shortness of breath that even occurs at rest. She has been residing in a high altitude area (10,000 feet above sea level) for the past 2 years. At the time of examination, her vital signs are normal, but she presents with jugular venous distention and pedal edema. Now we need to ponder which medical condition has most likely declined in the past 2 years from the provided choices, which include components of ECG, hematocrit, pulmonary vascular resistance, and right ventricular functions. PLANNING: The major assumption here is that the patient's symptoms are a result of being at high altitudes for an extended period. I would question the assumption that only altitude is impacting her health. Other factors could include, for instance, her activity level, diet, or possibly an unidentified medical condition. Evaluating these assumptions, the clinical priority would be to explore the chronic effects of high altitude on her body systems, particularly on her cardiovascular system. CLINICAL REASONING: Chronic exposure to high altitude leads to changes in the body to adapt to less oxygen in the environment – a condition called hypobaric hypoxia. Hypoxia stimulates the kidneys to produce more erythropoietin, a hormone that drives the production of red blood cells. This increases the hematocrit level (option C), not decreases it. High altitudes are also associated with increased pulmonary vascular resistance (option D) due to the hypoxic vasoconstriction of pulmonary arterioles. This can lead to pulmonary hypertension, increasing the strain on the right ventricle, potentially leading to right ventricular hypertrophy, which results in an increased right ventricular wall thickness (option F) and not a decrease. Right ventricular diastolic compliance (option E) is a measurement of the ventricle's ability to fill during diastole. With the development of right ventricular hypertrophy in response to increased pulmonary resistance, the compliance of the right ventricle could decrease as the muscle becomes stiffer. Thus, right ventricular diastolic compliance is likely to decrease relative to sea level. CONCLUDING: Given the thought process described above, the patient's history of prolonged high-altitude living may have resulted in compensatory mechanisms like an increase in hematocrit, rise in pulmonary vascular resistance, and increased right ventricular wall thickness due to potential pulmonary hypertension. Right ventricular diastolic compliance could be affected negatively due to the increased ventricular size and stiffness. The changes in ECG are less likely to be significant. RESPONSE: The most likely decrease in this patient due to her prolonged residence at high altitude is (E) Right ventricular diastolic compliance.

**AI_E:** RESTATING THE QUESTION: The prompt gives a clinical scenario about a 45-year-old woman who has lived at a high altitude (10,000 feet) for the last two years, and is now presenting with symptomatic dyspnea (shortness of breath, even at rest), jugular venous distention, and pedal edema—signs that might suggest cardiac issue. The task is to identify which of the listed physiological or anatomical features would likely have decreased in this patient over the last two years. PLANNING: The main assumption here is that living at high altitude for a prolonged period may have caused physiological changes in this patient's body that now manifest as cardiac-related symptoms (dyspnea, jugular venous distention, pedal edema). This assumption draws on the concept that high altitude can induce physiological adaptations due to reduced oxygen availability. Alternate assumptions could be: change in physical activity, dietary imbalance, or other environmental factors. Given the symptoms and the context, the assumption about high altitude acclimatization seems most clinically relevant. CLINICAL REASONING: Living at high altitudes induces a series of physiological responses for the body to manage the reduced oxygen. Initially, there is an increase in breathing and heart rates due to lower oxygen content in blood. To counteract this, the body generates more red blood cells (enhanced by erythropoietin secretion), resulting in an increased hematocrit. Besides, pulmonary vessels may constrict to maximize oxygen absorption leading to increased pulmonary vascular resistance. The right ventricle, in response to increased resistance, would thicken its wall (right ventricular hypertrophy) to pump against the increased pulmonary resistance, while ventricular compliance may decrease due to this hypertrophy. Thus, over time at high altitude, neither hematocrit, pulmonary vascular resistance, right ventricular wall thickness, nor right ventricular diastolic compliance are likely to decrease. Moreover, there's no indication that the P and R wave heights on EKG would necessarily change as these don't have a direct correlation with high altitude living. CONCLUDING: Given the clinical context and understanding of physiological responses to high altitude, none of the options—except possibly right ventricular diastolic compliance—would likely decrease due to high altitude living. However, a decrease in this would typically result from right ventricular hypertrophy against pulmonary hypertension, which would likely be worsening her symptoms. RESPONSE: Out of the given choices, one may anticipate that over time, the woman's right ventricular diastolic compliance (E) could decrease due to prolonged high altitude-induced adaptations and the presence of symptoms suggestive of right heart strain/dysfunction. However, this concept may be complex and not necessarily intuitive. It would be important to consider this within the broader pattern of her clinical presentation and the knowledge of the effects of prolonged high altitude on the cardiovascular system.

**Facilitator** The consensus among the AI responses is that the physiological parameter that has most likely decreased in the 45-year-old woman living at high altitude over the past two years is right ventricular diastolic compliance (E). This conclusion is based on the understanding that prolonged exposure to high altitude can lead to chronic hypoxia, which triggers a series of physiological adaptations in the body. These include an increase in hematocrit to enhance oxygen transport, an increase in pulmonary vascular resistance due to hypoxic vasoconstriction of pulmonary arterioles, and hypertrophy of the right ventricle to cope with the increased pulmonary resistance. As the right ventricle hypertrophies, it becomes stiffer, leading to a decrease in its diastolic compliance, or its ability to fill during diastole. This decrease in right ventricular diastolic compliance is likely contributing to the patient's symptoms of shortness of breath, jugular venous distention, and pedal edema, which are indicative of right heart failure. Therefore, the consensus answer to the question is (E) Right ventricular diastolic compliance.
