# Supplementary material for: Collaborative intelligence in AI: Evaluating the performance of a council of AIs on the USMLE
Source: PLOS Digit Health. 2025 Oct 9;4(10):e0000787. doi: 10.1371/journal.pdig.0000787 (PMC12510544; doi:10.1371/journal.pdig.0000787)
Supplement: S2 File — (ZIP) [file pdig.0000787.s002.zip › S2 Data Raw data files containing unprocessed question inputs and LLM outputs including transcripts of deliberations/STEP1_Question-59-1_Type-MC-NJ.docx]

**Facilitator** **[Round 1]:** A 25-year-old woman comes to the physician because of a 4-month history of cough and unintentional weight loss. Rhonchi are heard posteriorly over the right upper thorax. A chest x-ray shows an 8-cm cavity in the right upper lobe and hilar lymphadenopathy. Ziehl-Neelsen staining of an induced sputum specimen shows acid-fast bacilli. The patient begins treatment with four antimycobacterial drugs, including rifampin. Synthesis of which of the following components of the suspected pathogen is most likely directly inhibited by the rifampin in this patient's medication regimen? (A) ATP (B) Folic acid (C) Peptidoglycan (D) Protein (E) RNA

**AI_D:** RESTATING THE QUESTION: In simpler terms, the question is asking, "What component synthesis of the suspected pathogen, an acid-fast bacillus, is most likely directly inhibited by the drug rifampin, which is part of the patient's treatment plan?" PLANNING: The question presupposes a component of the pathogen is being directly affected by rifampin, an antibiotic often used to treat bacterial infections. To generate an alternate assumption, we could hypothesize that rifampin disrupts the synthesis of a critical cellular component of the suspected pathogen. Evaluating this assumption, it's necessary to recall the mechanisms of action of rifampin in infectious diseases. CLINICAL REASONING: Rifampin is a well-known antimycobacterial drug used for different types of bacterial infections, specifically tuberculosis, as suggested by the patient's clinical symptoms and the presence of acid-fast bacilli in the sputum specimen. Rifampin inhibits the RNA polymerase in bacteria, preventing RNA synthesis. Therefore, it directly interferes with protein synthesis, as RNA is required for this process. However, it specifically inhibits the synthesis of messenger RNA (mRNA), rather than the protein itself. CONCLUDING: Rifampin, included in this patient's medication regimen, disrupts the disease-causing bacterial growth not by inhibiting ATP, folic acid, peptidoglycan, or protein synthesis, but the appropriate response should be the process that is directly linked to RNA synthesis. RESPONSE: Therefore, the correct answer to the question "Synthesis of which of the following components of the suspected pathogen is most likely directly inhibited by the rifampin in this patient's medication regimen?" is (E) RNA.

**AI_B:** RESTATING THE QUESTION: The inquiry is asking about the mechanism of action of rifampin, an antimicrobial medication that is utilized to treat this woman's condition, which seems to be caused by a pathogen identifiable by Ziehl-Neelsen staining. This type of staining is typically used to detect acid-fast bacilli, particularly mycobacteria that cause tuberculosis. The question requires us to identify which component synthesis, out of the given list, rifampin directly disrupts in the suspected bacteria. PLANNING: Does rifampin inhibit the synthesis of ATP, folic acid, peptidoglycan, protein, or RNA? We need to scrutinize each possibility based on what we know about the drug's mode of action. CLINICAL REASONING: Rifampin is a significant part of the antituberculosis regimen and its primary role is to inhibit RNA synthesis by blocking the bacterial enzyme RNA polymerase. This disrupts the production of vital proteins and thus halts the growth and reproduction of the mycobacteria. ATP is primarily associated with energy production in the cell, and while important, it is not the primary target of rifampin. Folic acid synthesis interference is the mechanism of action of some other antimicrobials like sulfamethoxazole and trimethoprim but not rifampin. Peptidoglycan is involved in cell wall synthesis, inhibition of this process is characteristic of drugs like penicillins and cephalosporins, not rifampin. Protein synthesis would generally refer to the activity at the ribosome level, which is inhibited by a range of drugs, but rifampin's specific action is before this step at the RNA synthesis stage. CONCLUDING: Therefore, based on the mechanism of action of rifampin, it primarily disrupts the synthesis of RNA in mycobacteria. RESPONSE: The answer is (E) RNA.

**AI_A:** RESTATING THE QUESTION: The query seeks to identify which component synthesis of a presumed pathogen, indicated by the presence of acid-fast bacilli in the patient's sputum, is likely to be directly obstructed by rifampin, which is included in her antimycobacterial treatment. PLANNING: The situation suggests an infection by a Mycobacterium species due to the clinical presentation, the radiographic findings, and the identification of acid-fast bacilli in the sputum specimen. The rifampin the patient is taking is an antibiotic that is particularly effective against several bacterial species including those of the Mycobacterium genus. With this in mind, we must consider what rifampin's specific mode of action is - which bacterial component synthesis it inhibits. CLINICAL REASONING: Rifampin acts by binding to the bacterial RNA polymerase, thereby obstructing RNA transcription. RNA is crucial in protein synthesis, acting as a template for the creation of proteins. Thus, if RNA synthesis is interfered with, it can greatly disrupt the normal functioning and replication of the offending bacteria. CONCLUDING: Given these arguments, it emerges that the synthesis affected by rifampin would be that of RNA. This ensures that protein creation is damaged, disrupting the microorganism's ability to replicate and perform its essential functions. RESPONSE: Therefore, amongst the given options, RNA (Option E) is the component whose synthesis is most likely directly inhibited by the rifampin in this patient's medication regimen.

**AI_C:** (1) RESTATING THE QUESTION: This question focuses on a 25-year-old woman with a 4-month history of cough and unintentional weight loss. She has an abnormal finding on her chest x-ray and evidence of acid-fast bacilli on her sputum smear, suggesting a mycobacterial infection such as tuberculosis. She is initiated on a regimen of antimycobacterial drugs, including rifampin. The question asks which component of mycobacterium's physiological processes does rifampin directly inhibit. (2) PLANNING: Rifampin directly acts on the mycobacterium. It is important to understand the mechanism of action of rifampin, which involves the inhibition of a key component in the life cycle of mycobacteria. The options given are ATP, folic acid, peptidoglycan, protein, and RNA. (3) CLINICAL REASONING: Rifampin inhibits the RNA polymerase of mycobacteria and prevents transcription, thus inhibiting RNA synthesis. It does not inhibit ATP, since ATP is a form of energy used by the cell, not a component of bacteria specifically targeted by this drug. Folic acid synthesis is inhibited by drugs like sulfamethoxazole and trimethoprim, not rifampin. Peptidoglycan synthesis is blocked by drugs like penicillins and cephalosporins, not rifampin. While rifampin does inhibit protein synthesis indirectly (as proteins are the product of translation of mRNA, which is transcribed from DNA), rifampin's main action is on the RNA polymerase enzyme, thus primarily blocking RNA synthesis. (4) CONCLUDING: After connecting the dots in terms of drug action and bacterial physiology, it becomes clear that rifampin's primary inhibition is on the synthesis of RNA within the mycobacterium. (5) RESPONSE: Therefore, the answer to the question is (E) RNA. The antibiotic rifampin works by inhibiting the synthesis of RNA in the bacteria.

**AI_E:** RESTATING THE QUESTION: The question simply asks which part of the mycobacterium suspected to be causing this lady's cough and weight loss would rifampin, one of the medications she’s taking, prevent the development of? Rifampin is a drug often used in the treatment of tuberculosis, caused by mycobacteria. PLANNING: Let's break down the options. (A) ATP: This is the energy currency of the cell, and its synthesis is crucial to cell survival. (B) Folic acid: Crucial B vitamin needed for DNA synthesis and cytokinesis. (C) Peptidoglycan: This is a structural component of the bacteria cell wall. (D) Protein: A large biomolecule that performs a vast array of functions within organisms. (E) RNA: A molecule which performs various pivotal roles in coding, decoding, regulation, and expression of genes. Based on the characteristics of Rifampin, we should look at inhibiting RNA synthesis. So the option (E) RNA would be a potential answer. CLINICAL REASONING: Rifampin is a well-known antimicrobial drug utilized in the treatment of tuberculosis. Its mechanism of action is by the inhibition of bacterial DNA-dependent RNA polymerase, which effectively blocks bacterial RNA synthesis. Since the patient's clinical features, the findings on Ziehl-Neelsen staining, and the start of four antimycobacterial drugs point towards tuberculosis, it would be fair to assume that the part of the mycobacteria that is being inhibited by the rifampin is the synthesis of RNA. CONCLUDING: By ruling out the components that are not affected by Rifampin, such as ATP, Folic acid, Peptidoglycan and Protein, and focusing on the known mode of action of Rifampin, which is inhibition of bacterial DNA-dependent RNA polymerase, one can come to the assumption that synthesis of RNA in the infectious agent (Mycobacterium tuberculosis), is likely being inhibited by Rifampin in this patient's treatment regimen. RESPONSE: The correct answer is (E) RNA. Rifampin inhibits RNA synthesis by blocking the action of bacterial DNA-dependent RNA polymerase. Thus, it directly prevents the production of this essential component in the suspected pathogen, Mycobacterium tuberculosis, contributing to its antimycobacterial effect.

**Facilitator** The question is asking which component synthesis of the suspected pathogen, an acid-fast bacillus, is most likely directly inhibited by the drug rifampin, which is part of the patient's treatment plan. Rifampin is a well-known antimicrobial drug utilized in the treatment of tuberculosis, caused by mycobacteria. Its mechanism of action is by the inhibition of bacterial DNA-dependent RNA polymerase, which effectively blocks bacterial RNA synthesis. Therefore, it directly interferes with protein synthesis, as RNA is required for this process. However, it specifically inhibits the synthesis of messenger RNA (mRNA), rather than the protein itself. By ruling out the components that are not affected by Rifampin, such as ATP, Folic acid, Peptidoglycan and Protein, and focusing on the known mode of action of Rifampin, one can come to the assumption that synthesis of RNA in the infectious agent (Mycobacterium tuberculosis), is likely being inhibited by Rifampin in this patient's treatment regimen. Therefore, the correct answer to the question "Synthesis of which of the following components of the suspected pathogen is most likely directly inhibited by the rifampin in this patient's medication regimen?" is (E) RNA. Rifampin inhibits RNA synthesis by blocking the action of bacterial DNA-dependent RNA polymerase. Thus, it directly prevents the production of this essential component in the suspected pathogen, Mycobacterium tuberculosis, contributing to its antimycobacterial effect.
