# Supplementary material for: Collaborative intelligence in AI: Evaluating the performance of a council of AIs on the USMLE
Source: PLOS Digit Health. 2025 Oct 9;4(10):e0000787. doi: 10.1371/journal.pdig.0000787 (PMC12510544; doi:10.1371/journal.pdig.0000787)
Supplement: S2 File — (ZIP) [file pdig.0000787.s002.zip › S2 Data Raw data files containing unprocessed question inputs and LLM outputs including transcripts of deliberations/STEP1_Question-6-1_Type-MC-NJ.docx]

**Facilitator** **[Round 1]:** A previously healthy 33-year-old woman is brought to the emergency department by the Secret Service for stalking the president of the USA for 2 months. She claims to be married to the president's twin brother and states that the president just had his twin kidnapped to avoid competition. She speaks rapidly and is difficult to interrupt. Her associations are often loose. She says, ""I haven't slept for days, but I won't even try to sleep until my husband is rescued. God has been instructing me to take over the White House. I can't wait to be reunited with my husband. I hear his voice telling me what to do."" When asked about drug use, she says she uses only natural substances. She refuses to permit blood or urine tests, saying, ""I don't have time to wait for the results."" Which of the following is the most likely diagnosis? (A) Bipolar disorder, manic, with psychotic features (B) Brief psychotic disorder (C) Delusional disorder (D) Psychotic disorder due to general medical condition (E) Schizophrenia

**AI_E:** RESTATING THE QUESTION: The question presents a situation where a 33-year-old woman without a previous health record exhibits signs of delusion, believes that she's been married to the president's twin, speaks rapidly, is hard to interrupt, has loosened associations, hasn't slept for days, claims divine instructions, and hears voices. No substance abuse is reported. She doesn't want to undergo any lab tests. The question requires us to pinpoint the most likely diagnosis from the given list which comprises of different types of psychotic disorders. PLANNING: It would be necessary to evaluate her symptoms to find out whether they match any known symptomatology of the listed disorders. The reported symptoms are manic behavior, insomnia, delusions, rapid speech, and auditory hallucination. This needs to be assessed against the typical features of Bipolar disorder, Brief psychotic disorder, Delusional disorder, Psychotic disorder due to a general medical condition, or Schizophrenia. CLINICAL REASONING: Bipolar disorder usually presents with depressive and manic episodes. Although the patient's rapid speech, insomnia, and delusions could fall under a manic episode with psychotic features, the absence of depressive episodes makes this diagnosis less likely. Brief psychotic disorder is characterized by sudden onset psychotic symptoms lasting from one day to one month, which is inconsistent with the 2-month duration of symptoms. Delusional disorder involves holding false beliefs in the face of contradictory evidence, but typically doesn't involve rapid speech or sleep deprivation. A psychotic disorder due to a general medical condition would need a medical history or present signs suggesting a medical source of symptoms, which are not given. Finally, Schizophrenia typically involves chronic symptoms (more than 6 months), making it less likely. CONCLUDING: The most likely diagnosis based on this woman's symptoms and their duration would be Bipolar disorder, manic, with psychotic features. RESPONSE: (A) Bipolar disorder, manic, with psychotic features is the most likely diagnosis in this case.

**AI_A:** RESTATING THE QUESTION: A 33-year-old woman who has been following the US president for two months is brought in. She believes she's married to the president's twin, who she thinks has been kidnapped by the president. She talks very fast and it's hard to interrupt her. Her thought associations are often not directly related to each other. She says she hasn't slept for days and won't sleep until her 'husband' is 'rescued'. She believes that God is directing her to occupy the White House. She says she hears her 'husband's' voice guiding her, and claims she only uses natural substances and refuses to permit blood or urine tests. We need to determine the most likely diagnosis from the following options: (A) Bipolar disorder, manic, with psychotic features, (B) Brief psychotic disorder, (C) Delusional disorder, (D) Psychotic disorder due to general medical condition, (E) Schizophrenia. PLANNING: We must identify this woman's specific symptoms and match them to the most fitting diagnosis. For example, 'rapid speech' and 'difficulty interrupting' might indicate hyperactivity or hypomania, 'lack of sleep' might suggest altered mood or energy level, 'loose associations' and 'auditory hallucinations' could suggest a thought disorder or psychosis. Considering that the symptoms have lasted for at least 2 months, and the level of function, observance of any mood disturbance, and timing of onset will be key to the diagnostic process. CLINICAL REASONING: Hyperactive behavior, excessive energy despite lack of sleep, and rapid, diffuse speech all indicate a manic or hypomanic state. The beliefs about being married to the president's kidnapped twin and receiving instructions from God suggest delusions and psychotic features. Uninterrupted symptoms for two months, coupled with significant interpersonal and occupational disturbance, are consistent with an ongoing major episode rather than a brief, detached incident. CONCLUDING: The coordination of these symptoms—manic behavior, psychotic experiences, and enduring functional disturbances—leads to a narrowed diagnosis. Brief psychotic disorder is typically shorter in duration, less than one month. Delusional disorder involves non-bizarre delusions without other psychotic symptoms. Psychotic disorder due to a general medical condition has no identifiable direct cause in this case. Schizophrenia requires two or more core symptoms (delusions, hallucinations, disorganized speech, etc.) for a significant portion of one month, but also at least six months of disturbance, which we do not have evidence for. RESPONSE: Thus, the symptoms and timeline described here seem to relate most closely to (A) Bipolar disorder, manic, with psychotic features. This diagnosis would encompass the manic episodes, the hallucinations of hearing her husband's voice, and the grandiose delusions of being chosen by God.

**AI_C:** The case describes a 33-year-old woman who presents with symptoms suggestive of a severe psychiatric illness. These symptoms include her stalking behavior, delusions, rapidly speaking demeanor that makes her difficult to interrupt, her loose associations, an altered sleep pattern (insomnia), a belief in divine instruction suggesting a grandiose self-view, auditory hallucinations, and refusal to do necessary testings. Having a diagnosis is essential to understand her condition better and determine the suitable treatment intervention, so we ought to comprehend which psychiatric illness best fits her clinical presentation. The plausible alternatives include: Bipolar disorder, manic, with psychotic features; Brief psychotic disorder; Delusional disorder; Psychotic disorder due to general medical condition; and Schizophrenia. One might suppose that the patient's refusal for testings could favor a Delusional disorder or Psychotic disorder due to general medical condition over other options. However, these assumptions overlook essential clinical criteria of these disorders. For instance, Delusional disorder generally involves non-bizarre, plausible delusions with no accompanying psychotic symptoms, and this individual exhibits multiple types of bizarre delusions and possible hallucinations. On the other hand, a Psychotic disorder due to a general medical condition typically requires evidence of a causative medical condition, which is tentatively ruled out due to her previous health status and refusal for medical testing. Her symptoms of grandiosity, decreased need for sleep, talkativeness, and potentially goal-directed activity (in stalking the president), along with her delusions and hallucinations, favor a manic episode. However, the duration needs to be either continuously present for a week unless hospitalization is necessary for a less duration. From the vignette, it's clear that her symptoms lasted for at least two months, a duration much longer than one week needed. Hence, whilst acknowledging these observations, we can critically examine that a diagnosis of Bipolar disorder, manic, with psychotic features could be plausible considering this clinical scenario. In this disorder, the manic episodes can have added psychotic features when delusions or hallucinations are also present. In conclusion, considering the above evidence and clinical manifestations in this case, it is feasible that the woman is suffering from Bipolar disorder, manic, with psychotic features. However, a thorough psychiatric evaluation and observation are needed, that is outside the scope of an emergency department, to confirm the diagnosis and initiate proper management. As a result, the most probable diagnosis for this 33-year-old female who has presented with manic symptoms, delusions, hallucinations, and has been displaying such behaviors for about two months, would likely be (A) Bipolar disorder, manic, with psychotic features.

**AI_B:** 1) RESTATING THE QUESTION: A 33-year-old woman, who was previously healthy, is brought into the emergency department after allegedly stalking the US president for 2 months. She holds certain delusions including a supposed marriage to the president's twin brother and his subsequent kidnapping. She has exhibited other alarming behaviours such as rapid speech that's hard to interrupt, loose associations, and claims of not having slept for many days. She hears voices instructing her with tasks. When asked about substance abuse, she only admits to the use of natural substances. This scenario is asking for a possible diagnosis, given the options of: a) Bipolar disorder, manic, with psychotic features; b) Brief psychotic disorder; c) Delusional disorder; d) Psychotic disorder due to a general medical condition; or e) Schizophrenia. 2) PLANNING: This case presents several assumptions: i) The patient is experiencing a psychiatric disorder; ii) the patient does not abuse substances that may influence her behaviours; iii) these symptoms have been present for a minimum of 2 months. Alternate assumptions include: i) The patient may be lying about her substance use; ii) the patient’s symptoms may not be due to a psychiatric illness, but rather a general medical condition. Given these details, however, the symptoms appear to align with a significantly severe psychiatric disorder. 3) CLINICAL REASONING: The patient demonstrates symptoms of mania such as elevated activity (stalking, rapid speech), decreased need for sleep, and grandiosity (beliefs about her role in an elaborate kidnapping story). She also demonstrates psychotic symptoms evidenced by her belief in being married to the president's twin brother, believing that God is instructing her, and hearing voices. Therefore, a diagnosis within the sphere of psychotic disorders seems most fitting. However, these symptoms appear delusional and lack any affective components, ruling out bipolar disorder. Schizophrenia also seems unlikely given the lack of 'negative symptoms' like blunted affect or social withdrawal and the fact that symptoms have only been present for 2 months. A brief psychotic disorder would resolve within a month, which is not the case here. This woman doesn't have a known medical condition that would suggest her symptoms are a result of that specifically. The symptoms have been present for more than a month but less than six, pointing towards Delusional Disorder. 4) CONCLUDING: After considering all possibilities and reviewing her signs and symptoms, the patient’s behavior leans towards an unwavering belief in the delusion that she is involved in a dramatic political event, and the fact that these beliefs have not fluctuated over a period longer than a month suggests a chronic condition. 5) RESPONSE: Given the information at hand and the details of the case, the most likely diagnosis for this 33-year-old woman would be (C) Delusional Disorder.

**AI_D:** (1) RESTATING THE QUESTION: A 33-year-old woman, seemingly physically healthy, is brought in for psychological evaluation. She has been obsessively following the US president for two months, believes she's married to his twin, and that the president has kidnapped his brother. She talks continuously, presenting disjointed thoughts and claims to only use natural substances. Her sleep patterns are disrupted inevitably affects her mental state. She also hears voices directing her actions. The need is to diagnose her mental disorder, choosing from Bipolar disorder, manic, with psychotic features; Brief psychotic disorder; Delusional disorder; Psychotic disorder due to a general medical condition or Schizophrenia. (2) PLANNING: The woman's symptoms suggest that she's experiencing mania, delusions, and psychosis. In ruling out or confirming each option, several assumptions are made. Is she suffering from a manic episode characteristic of bipolar disorder, but is it accompanied by psychosis? Could it be a brief psychotic disorder, defined as psychosis lasting a month or less? Perhaps her obsession with the president and belief of marriage to his twin can be called a delusional disorder, or could she be having a psychotic episode brought on by a physical health complication? Or ultimately, can her symptoms be attributed to schizophrenia? To determine the probable diagnosis, you should prioritize understanding her symptom duration because duration criteria are significant when diagnosing mental disorders. (3) CLINICAL REASONING: Bipolar disorder with manic, psychotic features is marked by not only periods of mania but also depression as in highs and lows. But the patient doesn't exhibit any depressive symptoms. Brief psychotic disorder, while it may seem plausible, is unlikely because her symptoms have been ongoing for at least two months, exceeding the one-month limit. A general medical condition that induces psychosis could conceivably cause her symptoms, but shes' been reported as physically healthy until now, making this improbable. Schizophrenia characteristically presents itself with hallucinations, disorganized speech, delusions, and negative symptoms. The woman is exhibiting some of these signs but to qualify for this diagnosis, the symptoms have to be present for at least six months, hence this can be ruled out. Delusional disorder, on the other hand, is a psychiatric condition in which a person experiences non-bizarre delusions, including persistent threats and conspiracy theories. There's no specified duration to this unlike schizophrenia or brief psychotic disorder, making it a plausible option considering her beliefs. (4) CONCLUDING: The diagnosis is based on her persistent delusion of being entwined in the president's life, which has been ongoing for two months without presenting any significant physical health issues or depressive symptoms and failing to fulfill the duration criteria of schizophrenia or brief psychotic disorder. (5) RESPONSE: Considering the criteria for each mental disorder, the patient's refusal for any tests, the absence of depressive symptoms, and her specific persistent delusion, the most likely diagnosis is (C) Delusional disorder.

**Facilitator** **[Round 2]:** Summary: AI_A believes the woman's symptoms align with Bipolar disorder, manic, with psychotic features. They argue that her hyperactive behavior, excessive energy despite lack of sleep, rapid speech, and delusions suggest a manic or hypomanic state with psychotic features. The symptoms' duration also fits the diagnosis. AI_B, however, diagnoses the woman with Delusional disorder. They argue that her symptoms are delusional and lack any affective components, ruling out bipolar disorder. They also note the absence of 'negative symptoms' like blunted affect or social withdrawal, making schizophrenia unlikely. AI_C agrees with AI_A, diagnosing the woman with Bipolar disorder, manic, with psychotic features. They argue that her symptoms of grandiosity, decreased need for sleep, talkativeness, and potentially goal-directed activity, along with her delusions and hallucinations, favor a manic episode. AI_D diagnoses the woman with Delusional disorder, arguing that her persistent delusion of being entwined in the president's life, which has been ongoing for two months, fits this diagnosis. They also note the absence of depressive symptoms and her refusal for any tests. AI_E, like AI_A and AI_C, diagnoses the woman with Bipolar disorder, manic, with psychotic features. They argue that her rapid speech, insomnia, and delusions could fall under a manic episode with psychotic features, and the absence of depressive episodes does not rule out this diagnosis. Question to clarify disagreements: How do you interpret the absence of depressive symptoms and the presence of delusions in this case? Do you believe that the presence of delusions alone is sufficient for a diagnosis of Delusional disorder, or do you think that the manic symptoms are more indicative of Bipolar disorder, manic, with psychotic features?. Based on your preceding response, how would you answer the original question: [A previously healthy 33-year-old woman is brought to the emergency department by the Secret Service for stalking the president of the USA for 2 months. She claims to be married to the president's twin brother and states that the president just had his twin kidnapped to avoid competition. She speaks rapidly and is difficult to interrupt. Her associations are often loose. She says, ""I haven't slept for days, but I won't even try to sleep until my husband is rescued. God has been instructing me to take over the White House. I can't wait to be reunited with my husband. I hear his voice telling me what to do."" When asked about drug use, she says she uses only natural substances. She refuses to permit blood or urine tests, saying, ""I don't have time to wait for the results."" Which of the following is the most likely diagnosis? (A) Bipolar disorder, manic, with psychotic features (B) Brief psychotic disorder (C) Delusional disorder (D) Psychotic disorder due to general medical condition (E) Schizophrenia]

**AI_E:** In the context of the presenting symptoms, I interpret the absence of depressive symptoms as not necessarily indicative of a lack of a bipolar condition. Bipolar Disorder is characterized by periods of mood changes, usually switching between manic or hypomanic episodes and depressive episodes. However, the absence of a depressive episode in the patient's history does not rule out the diagnosis, especially if the patient is experiencing a manic episode currently. The presence of delusions, on the other hand, is a common feature in various psychiatric disorders, including Bipolar Disorder, Schizophrenia, and Delusional Disorder. However, in order to accurately diagnose Delusional Disorder, the delusions must be non-bizarre and her functioning in daily life must not be notably impaired or markedly odd or bizarre apart from the delusions. In contrast, the presence of marked hyperactivity, increased talkativeness, and decreased need for sleep, which are classic features of a manic episode, are more in line with a diagnosis of Bipolar Disorder, manic, with psychotic features. The psychotic features are accounted for by her delusions and hallucinations. Given the above reasoning, I would be inclined to support Option (A) Bipolar disorder, manic, with psychotic features as the most likely diagnosis. This decision is primarily driven by the presence of distinct manic symptoms alongside ongoing delusions, indicating a manic episode with psychotic features.

**AI_B:** RESTATING THE QUESTION: The query refers to a 33-year-old woman showing signs of unusual behavior. Her behavior includes stalking, speaking rapidly, expressing loose associations, a lack of need for sleep, hearing voices, and commitment towards an unfeasible goal. Her refusal of blood or urine tests recommends a psychiatric evaluation over substance-induced psychosis. The clinical challenge here is to correctly diagnose her condition from the provided choices. PLANNING: Among the various assumptions, I'm especially considering her clear consciousness, non-bizarre, and persistent delusion of conspiracy and spiritual communication, her refusal to do tests, a duration of two months, her persistent and high energy even after not sleeping for multiple days, pressured speech, and the absence of any affective symptoms like mood swings. Depending on how these symptoms are interpreted, different diagnoses can arise. For instance, her manic symptoms like decreased need for sleep, increased talkativeness, and increased goal-directed activities can suggest Bipolar disorder, manic, with psychotic features. On the other hand, her belief of being married to the president's twin or hearing god's voice suggests the possibility of Delusional disorder. CLINICAL REASONING: In comparison, Brief psychotic disorder lasts less than one month, and Schizophrenia includes elements like disorganized speech, negative symptoms which are absent here. Psychotic disorder due to a general medical condition was ruled out by her lack of past medical history. Additionally, while experiencing grandiosity and hearing voices could be experienced in both Bipolar disorder and Delusional disorder; the absence of depressive symptoms, affective symptoms, or a previous history of mania makes Bipolar disorder less likely. Conversely, Delusional disorder consists of having one or more delusions in the absence of any other significant psychiatric symptoms; the woman's clear consciousness combined with her persistent non-bizarre delusion supports this diagnosis. CONCLUDING: Considering the clinical manifestations stemming from the provided case, it can be inferred that the woman's persistent, non-bizarre delusions and lack of other affective or psychiatric symptoms are hallmark symptoms of delusion disorder. RESPONSE: Hence, given the available symptomatology, the most probable diagnosis would be (C) Delusional disorder.

**AI_D:** The council's viewpoints center on a woman exhibiting symptoms such as rapid speech, insomnia, and delusions. The crux of the debate lies in whether the symptoms point towards Bipolar disorder, manic, with psychotic features or a Delusional disorder. Under the banner of Bipolar disorder, one can expect mood symptoms (manic or depressive), in addition to periods of normal mood. Manic episodes entail elevated, expansive, or irritable mood and abnormally increased activity or energy, whereas Delusional disorder is characterized by the presence of one or more delusions that persist for at least 1 month. This disorder does not have the mood component seen in Bipolar disorder. Assuming we have a bipolar scenario, we need to ask whether the reported symptoms genuinely represent a manic phase. Are the observed behaviors attributable to an abnormally elevated mood and increased energy/activity? Further, are these changes sufficiently drastic and sustained to meet the required criteria for a manic episode? From a delusional disorder perspective, however, the continuous two-month timeframe of consistent delusional belief clearly fits. Yet, the decision hinges on whether the other symptoms can be sufficiently explained by the overarching delusion or if they must be classified separately, suggesting a different disorder. With these assumptions further clarified and explored, my reasoning would follow. On the one hand, the woman's symptoms are undoubtedly consistent with a severe manic episode found in Bipolar disorder; still, I observe no reported history of depressive episodes, which usually alternate with manic phases in Bipolar disorder. Conversely, the woman's strong and consistent delusions could be indicative of Delusional disorder. Finally, when connecting all the argument threads from the various council members, I come to my conclusion that the persistent and firm belief in her delusional claims coupled with an absence of reported depressive episodes leans more towards a diagnosis of Delusional disorder (choice C). This diagnosis does not exclude every symptom of mania, but I find it a more plausible fit given the dominating feature of unrelenting delusions. This conclusion aligns with council members AI_B and AI_D's viewpoints. Therefore, in response to the presented patient case, I consider Delusional disorder the most likely diagnosis.

**AI_C:** 1) RESTATING THE QUESTION: What is the most likely diagnosis for a 33-year-old woman exhibiting abnormal behaviors such as obsessive stalking, rapid speech, sleeping issues, and delusions including the belief that she is receiving divine instructions and hearing voices? The options presented are (A) Bipolar disorder, manic, with psychotic features, (B) Brief psychotic disorder, (C) Delusional disorder, (D) Psychotic disorder due to general medical condition, or (E) Schizophrenia. 2) PLANNING: Assumptions should be made based on the clinical presentations of the patient, and these assumptions should align with the core features of the listed mental disorders. The first assumption is that her symptoms indicate a mental disorder. Another assumption can be made from her refusal to permit blood or urine tests, implying she is fearful or doesn’t want something found in her system to be discovered. The next assumption is that she is mentally coherent and honest about her use of "only natural substances." 3) CLINICAL REASONING: The mention of God giving her instructions and hearing voices, although not specifically characterized, are auditory hallucinations which are considered psychotic symptoms. This suggests that the woman is experiencing a psychotic disorder. Her symptoms also include grandiose delusions about her supposed marriage to the president's twin and the kidnapping plot. Moreover, the fact that she hasn’t slept for days but still shows high energy, speaks rapidly without appropriate pauses, and the difficulty to interrupt her speech suggest a manic state. Therefore, the patient's symptoms align more with a manic episode with psychotic features. 4) CONCLUDING: The duration of her symptoms is another critical factor. She has been exhibiting these symptoms for at least two months, which is more extended than a brief psychotic disorder but less than the duration for schizophrenia (which requires symptoms be present for a minimum of six months). Delusional disorder usually lacks the manic symptoms the woman is displaying. The absence of evidence for a general medical condition that may cause psychosis further guides the diagnosis. 5) RESPONSE: Since there is a presence of manic and psychotic features over an appropriate duration without evidence of a general medical condition causing the psychosis, the most probable diagnosis is (A) Bipolar disorder, manic, with psychotic features.

**AI_A:** 1) RESTATING THE QUESTION: The query seeks to understand the most likely psychiatric diagnosis for a woman who exhibits symptoms such as rapid and incessant talking, loose associative thinking, auditory hallucinations, and persistent delusions about being married to the president's twin brother and being instructed by a deity to take over the White House. Furthermore, she demonstrates hyperactive behavior, lack of sleep, and a refusal to permit tests for potential drug use. Among the given options are bipolar disorder, manic with psychotic features; brief psychotic disorder; delusional disorder; psychotic disorder due to general medical condition; and schizophrenia. 2) PLANNING: In this scenario, we need to consider a few key assumptions. First, the woman's hyperactivity, grandiosity, excessively graphorrheic speech, decreased need for sleep, and potential goal-directed activity could be indicative of either a manic episode or a state of hyperarousal secondary to delusional symptoms. Second, the persistence and specificity of the delusion involving the president's twin, coupled with the absence of any depressive symptoms, could suggest a delusional disorder. Third, the fact that she denies any drug use and refuses tests could mean she is not suffering from a substance-induced psychoactive state. 3) CLINICAL REASONING: The key to distinguishing between these potential diagnoses is that while both bipolar disorder, manic with psychotic features and delusional disorder can present with comparable delusions, mania is additionally characterized by hyperactivity, decreased need for sleep, pressurized speech, and probably goal-directed activity. A state of hyperarousal as seen in delusional disorder could give superficially similar symptoms but is not associated with a wider set of manic symptoms. Furthermore, if the woman were experiencing a manic episode, we would expect to see affective symptoms, which are notably absent in this case. 4) CONCLUDING: In conclusion, given her symptoms of hyperactivity and the lack of any other psychiatric, substance-related or medical conditions, and considering that psychotic symptoms are not limited to the context of mood episodes, it's clear that this patient is most likely suffering from a manic episode with psychotic features rather than a delusional disorder. 5) RESPONSE: Hence, the answer should be (A) Bipolar disorder, manic, with psychotic features. The presentation of manic symptoms such as decreased need for sleep and rapid, difficult to interrupt speech, along with delusions and auditory hallucinations, are enough grounds for this diagnosis. The absence of depressive symptoms and refusal for any tests further solidify this diagnosis.

**Facilitator** **[Round 3]:** Summary: AI_A believes the woman is most likely suffering from a manic episode with psychotic features, which is indicative of Bipolar disorder, manic, with psychotic features. This conclusion is based on the woman's symptoms of hyperactivity, decreased need for sleep, rapid speech, and delusions. The absence of depressive symptoms and refusal for any tests further solidify this diagnosis. AI_B, however, suggests that the woman's persistent, non-bizarre delusions and lack of other affective or psychiatric symptoms are hallmark symptoms of Delusional disorder. The absence of depressive symptoms, affective symptoms, or a previous history of mania makes Bipolar disorder less likely in AI_B's opinion. AI_C agrees with AI_A, stating that the woman's symptoms align more with a manic episode with psychotic features, indicative of Bipolar disorder, manic, with psychotic features. The duration of her symptoms, lack of evidence for a general medical condition causing the psychosis, and the presence of manic and psychotic features over an appropriate duration support this diagnosis. AI_D believes that the woman's persistent and firm belief in her delusional claims, coupled with an absence of reported depressive episodes, leans more towards a diagnosis of Delusional disorder. This diagnosis does not exclude every symptom of mania, but AI_D finds it a more plausible fit given the dominating feature of unrelenting delusions. AI_E supports the diagnosis of Bipolar disorder, manic, with psychotic features, primarily driven by the presence of distinct manic symptoms alongside ongoing delusions, indicating a manic episode with psychotic features. The absence of a depressive episode in the patient's history does not rule out the diagnosis, especially if the patient is experiencing a manic episode currently. Question to clarify disagreements: Given the woman's symptoms, how do we differentiate between a manic episode with psychotic features (indicative of Bipolar disorder) and persistent, non-bizarre delusions (indicative of Delusional disorder)? How much weight should be given to the absence of depressive symptoms or a previous history of mania in diagnosing Bipolar disorder?. Based on your preceding response, how would you answer the original question: [A previously healthy 33-year-old woman is brought to the emergency department by the Secret Service for stalking the president of the USA for 2 months. She claims to be married to the president's twin brother and states that the president just had his twin kidnapped to avoid competition. She speaks rapidly and is difficult to interrupt. Her associations are often loose. She says, ""I haven't slept for days, but I won't even try to sleep until my husband is rescued. God has been instructing me to take over the White House. I can't wait to be reunited with my husband. I hear his voice telling me what to do."" When asked about drug use, she says she uses only natural substances. She refuses to permit blood or urine tests, saying, ""I don't have time to wait for the results."" Which of the following is the most likely diagnosis? (A) Bipolar disorder, manic, with psychotic features (B) Brief psychotic disorder (C) Delusional disorder (D) Psychotic disorder due to general medical condition (E) Schizophrenia]

**AI_B:** When deciphering between Bipolar disorder, manic, with psychotic features, and Delusional disorder, it's significant to analyze the variety and intensity of symptoms. Both conditions encompass delusional beliefs; however, Bipolar disorder is characterized by manic episodes which respectively manifest as hyperactivity, diminished sleep requirement, elevated or irritable mood, and speedy speech. In contrast, Delusional disorder is marked by long-lasting, non-bizarre delusions without the presence of other psychiatric symptoms. The absence of a depressive history or previous manic episodes should be noted but it should not preclude the diagnosis of Bipolar disorder. The initial presentation of Bipolar disorder can manifest as a manic episode without the occurrence of any depressive episodes. Thorough history-taking, observation, and medical tests can provide a more precise diagnosis. To answer the question, the patient’s presentation of rapid speech, decreased need for sleep, hyperactivity, and loose associations are symptomatic of a manic episode. In tandem with her delusional beliefs and auditory hallucinations, hence, the most probable diagnosis is (A) Bipolar disorder, manic, with psychotic features. This conclusion is derived from the episode of manic symptoms in correlation with the delusional belief system and the auditory hallucinations in the absence of a diagnosed general medical condition causing the psychosis.

**AI_E:** Restating the Question: We're trying to arrive at a diagnosis for the 33-year-old woman who's exhibiting behavior such as speech that is rapidly paced, often difficult to interrupt, and loose associations. She hasn't slept for several days straight and is preoccupied with a belief of her husband's forceful abduction, hearing his voice, and divine intervention involved in her current situation. However, she declines to undergo lab tests for evaluation. We have to decide if her symptoms best fit (A) Bipolar disorder, manic, with psychotic features, (B) Brief psychotic disorder, (C) Delusional disorder, (D) Psychotic disorder due to general medical condition, or (E) Schizophrenia. Planning: To efficiently address this problem, we should consider the following assumptions: 1. The woman’s behavior is entirely related to a psychiatric condition and not influenced by substance intake, taking into account her claim of utilizing only 'natural substances'. 2. The claimed perceptions and beliefs aren't based on any undisclosed information but are hallucinations and delusions, which are pathological and need intervention. We need to question these assumptions in order to decipher if they're valid or if there are any alternative possibilities that should be clinically prioritized. Clinical Reasoning: Firstly, her report of consuming only 'natural substances' doesn’t provide assurance that she is free from psychoactive substance influence since several natural substances can also generate psychosis-like effects, although her refusal to take the tests leaves us unable to confirm or disqualify this potential factor. Secondly, we must decide whether her reported communicative phenomenons and steadfast beliefs are reality-based or delusions. If these experiences are delusions, it could suggest a psychotic disorder. Given her consistent beliefs presented over a longer duration and the symptom set she exhibits—pressured speech, minimal need for sleep, and grandiose beliefs—affective disorder with manic and psychotic features can be considered, such as Bipolar disorder, manic, with psychotic features. Concluding: After careful consideration, it appears reasonable to consider bipolar disorder, manic with psychotic features as the woman's presentations align with the core features of manic phase—decreased need for sleep and increased goal-directed activity (her quest for her supposedly kidnapped husband)—and she presents with grandiosity about her divine mission, along with hallucinations of hearing her husband's voice instructing her. The psychosis, in this case, is not out of mood context and aligns with her overall manic presentation. Response: Given the available information, a potential diagnosis for the woman could be (A) Bipolar disorder, manic, with psychotic features. Nevertheless, this is based on the available behavioral observation and reported symptoms, and the definitive diagnosis may necessitate further psychiatric evaluation and management.

**AI_C:** RESTATING THE QUESTION: Essentially, this scenario describes a woman brought to the emergency department for stalking the president. She exhibits signs such as rapid speech, loose associations, lack of sleep, and delusions of grandeur and persecution. Moreover, she refuses to permit blood or urine tests for drug use, insisting that she only uses natural substances. The question requires determining the most likely psychiatric diagnosis based on these symptoms: (A) Bipolar disorder, manic, with psychotic features; (B) Brief psychotic disorder; (C) Delusional disorder; (D) Psychotic disorder due to general medical condition; or (E) Schizophrenia. PLANNING: Key factors to consider in this case include her increased energy levels displayed through hyperactivity, rapid speech, and minimal sleep, which are common in manic episodes. Additionally, the presence of delusions and refusal to allow tests may suggest possible Psychotic features. A diagnosis of Short Psychotic Disorder might come to mind due to the relatively brief two-month timeframe. However, this diagnosis typically applies to severe psychotic symptoms lasting between a day and a month, which doesn't fit here. Delusional Disorder could be an option, as the individual experiences persistent one or more non-bizarre delusions lasting for one month or more. However, her otherwise normal functioning and behavior might contradict this diagnosis. The psychotic symptoms do not appear to be directly attributable to a general medical condition or substance use, thus ruling out a Psychotic Disorder due to a General Medical Condition. Lastly, Schizophrenia requires at least six months of symptom duration, which does not apply here. CLINICAL REASONING: Cardinally, mania is characterized by an elevated, expansive or irritable mood, and increased activity or energy, a clear change from the person’s usual behavior, and these symptoms are present in the woman. Psychotic features are marked by delusions and hallucinations that do not have insight—the belief in the hallucination or delusion is steadfast and unwavering, which is congruent with her delusional claim of being married to the president's twin brother and the perception of his voice instructing her. Typically, patients with a Delusional Disorder function perfectly well and might not exhibit an odd or bizarre behavior aside from the delusion. This woman's rapid-fire speech, decreased need for sleep implies an elevated mood, which might not be seen usually in Delusional disorder. Her symptoms have been lasting for two months, which is longer than the one-day to one-month range typical of Brief Psychotic Disorder. CONCLUDING: Weighing her symptoms against the criteria for diagnosing mental disorders, namely the presence of mania and persistent delusions, the most probable diagnosis given her clinical picture is Bipolar disorder, manic, with psychotic features. Although Delusional Disorder vaguely fits the brief of her symptoms, her hyperactivity, rapid speech, and significantly decreased need for sleep lean more toward a manic episode with psychotic features. RESPONSE: Therefore, I believe the most likely diagnosis for the described woman would be (A) Bipolar disorder, manic, with psychotic features.

**AI_D:** This question is concerned with the diagnosis of a 33-year-old woman who has exhibited erratic behavior, including stalking the president for extended periods. Additionally, the woman holds firmly delusional beliefs, insists on hearing voices, demonstrates speech problems such as talkativeness, and displays a decreased need for sleep. There is no reported history of depression or manic episodes, and the woman has rejected any form of medical testing. The controversy that has arisen among our group is whether this woman ought to be diagnosed with Bipolar disorder, manic, with psychotic features(A) or Delusional disorder (C). This distinction is vital because the correct diagnosis will likely inform the most effective therapeutic strategy. Bipolar disorder with psychotic features usually involves experiencing manic or depressive episodes accompanied by psychotic symptoms such as delusions or hallucinations. In contrast, Delusional disorder is characterized mainly by the presence of one or more delusions with a duration of 1 month or longer, and the absence of other significant psychiatric symptoms. Upon closer examination, let's consider the fundamental characteristics of this case again. The woman's firm adherence to her delusional beliefs, rapid speech, decreased need for sleep, all point to this being a manic episode. The refusal of testing also alludes to a certain degree of paranoia. However, a key point here is that Bipolar disorder invariably involves alternating episodes of depression and mania. Yet, we have no historical information on any previous depressive or manic episodes in this woman. Furthermore, the presence of psychotic features (delusions and hearing voices), while it could fit either diagnoses, tends to point more towards a diagnosis of Delusional disorder given the landscape of her symptoms. Personality disorders such as Borderline, Narcissistic, Histrionic, or Antisocial personality disorders may also present with delusional beliefs, however, the lack of enduring pattern of long-term maladaptive behavior reduces the likelihood of these diagnoses. One could potentially argue for Schizophrenia (E), but the relative short duration of these symptoms and the lack of significant disorganized behavior or negative symptoms, make this diagnosis less likely. Putting all the pieces together, taking into account the symptomatology presented and with the currently available information, I would lean towards diagnosing this woman with Delusional disorder (C) as it provides a more consistent picture of her condition. However, this conclusion could change with additional medical and psychiatric history data. I would maintain a differential diagnosis of Bipolar disorder and carefully monitor the woman’s symptom progression, especially for any emerging depressive symptoms or established cycling pattern between depression and mania.

**AI_A:** 1) RESTATING THE QUESTION: The inquiry here requires us to identify the most fitting psychiatric diagnosis for this 33-year-old woman. Her symptoms include, but are not limited to, rapid speech, loose associations, an apparent lack of need for sleep, the belief that she is receiving divine instructions, claims of hearing voices, and suspect behavior such as stalking. Her substance use is limited to ‘natural substances’ and she refused to be screened for drug use, stating her urgency due to her belief system. Within the context of this question, the terms refer to different psychiatric conditions listed as response options, each with its own diagnostic criteria according to the Diagnostic and Statistical Manual of Mental Disorders (DSM-5). 2) PLANNING: We assume initially that the woman's symptoms are not caused by drug use or an underlying medical condition, as she has not admitted to drug use beyond ‘natural substances’ and she refuses diagnostic testing which could reveal a possible medical cause for her symptoms. If we challenge this assumption, and hypothesize that her symptoms are indeed due to a psychiatric illness, we must identify the most likely among the options given. These options include: (A) Bipolar disorder, manic, with psychotic features (B) Brief psychotic disorder (C) Delusional disorder (D) Psychotic disorder due to general medical condition (E) Schizophrenia 3) CLINICAL REASONING: In order to identify the most likely diagnosis, it's essential to examine the criteria for the mentioned disorders. Beginning with the option (D) Psychotic disorder due to general medical condition, it seems less likely as we're missing crucial medical history or testing to indicate a general medical condition that could explain the presentation. Option (B) Brief psychotic disorder is primarily defined by a sudden onset of psychotic symptoms that last at least a day but less than a month, this does not align with our patient's presentation as her behavior has been ongoing for 2 months. Option (E) Schizophrenia is marked by symptoms such as delusions, hallucinations, disorganized speech, and behavior, along with negative symptoms. However, these must be present for six months or more which is beyond the timeframe of our patient's behaviours. The symptoms and behavior of this woman, as described, appears to align more with option (A) Bipolar disorder, manic, with psychotic features, and option (C) Delusional disorder. The presence of elevated, expansive or irritable mood, increased activity or energy levels, more goal-oriented activity or psychomotor agitation, and excessive involvement in potentially dangerous activities, decreased need for sleep (feeling rested despite few hours of sleep), are symptoms clearly indicative for a manic episode which could be part of a Bipolar disorder. Additionally, she also displays signs of psychosis i.e., her delusions of being married to the president's twin brother, receiving divine instructions, and hearing voices that align with her delusion. However, if we place prominence on her fixed, nonbizarre delusion involving situations that occur in real life (being married, having a husband kidnapped) which has lasted for the past 2 months, and the absence of notable mood symptoms or hallucinations not related to the delusion, the clinical picture could align with a Delusional disorder. 4) CONCLUDING: Given the complexity of the presentation and our analysis, the differentiating factor will likely come down to the relationship between the reported manic symptoms and the delusions. Bipolar disorder would be more likely if the delusions are only present during episodes of mania, while a Delusional disorder would imply that delusions persist irrespective of the mood state. 5) RESPONSE: At the core of our patient's presentation is a persistent, fixed delusion held for 2 months, which is the threshold duration for Delusional disorder, without clear evidence of a mood episode for that same duration. This makes it difficult to primarily diagnose Bipolar disorder. Therefore, taking into account all the presented symptoms, as well as the rules laid out by DSM-5, option (C) Delusional disorder is the most fitting diagnosis.

**Facilitator** **[Round 4]:** Summary: AI_A believes that the woman's symptoms align most closely with Delusional disorder, as her persistent, fixed delusion has lasted for 2 months, which is the threshold duration for this disorder. AI_B, on the other hand, argues that the woman's rapid speech, decreased need for sleep, hyperactivity, and loose associations are symptomatic of a manic episode, and in tandem with her delusional beliefs and auditory hallucinations, the most probable diagnosis is Bipolar disorder, manic, with psychotic features. AI_C also supports the diagnosis of Bipolar disorder, manic, with psychotic features, citing the woman's elevated mood, increased activity, and unwavering belief in her delusions. AI_D leans towards diagnosing the woman with Delusional disorder, but maintains a differential diagnosis of Bipolar disorder and suggests monitoring the woman’s symptom progression. AI_E suggests that the woman's symptoms align with the core features of a manic phase of Bipolar disorder, including decreased need for sleep, increased goal-directed activity, and grandiosity about her divine mission. Key Differences: The key differences in the council members' perspectives revolve around the interpretation of the woman's symptoms. AI_A and AI_D place more emphasis on the woman's persistent delusion, which has lasted for 2 months, and see this as indicative of Delusional disorder. AI_B, AI_C, and AI_E, however, focus more on the woman's manic symptoms, such as rapid speech, decreased need for sleep, and hyperactivity, and see these as indicative of a manic episode in Bipolar disorder. Question for Clarification: To clarify the disagreements between the council members' opinions, we could ask: "How should we weigh the woman's persistent delusion against her manic symptoms in determining a diagnosis? Should the duration of the delusion or the intensity of the manic symptoms be given more weight?". Based on your preceding response, how would you answer the original question: [A previously healthy 33-year-old woman is brought to the emergency department by the Secret Service for stalking the president of the USA for 2 months. She claims to be married to the president's twin brother and states that the president just had his twin kidnapped to avoid competition. She speaks rapidly and is difficult to interrupt. Her associations are often loose. She says, ""I haven't slept for days, but I won't even try to sleep until my husband is rescued. God has been instructing me to take over the White House. I can't wait to be reunited with my husband. I hear his voice telling me what to do."" When asked about drug use, she says she uses only natural substances. She refuses to permit blood or urine tests, saying, ""I don't have time to wait for the results."" Which of the following is the most likely diagnosis? (A) Bipolar disorder, manic, with psychotic features (B) Brief psychotic disorder (C) Delusional disorder (D) Psychotic disorder due to general medical condition (E) Schizophrenia]

**AI_A:** Given both the duration of the patient's symptoms as well as their nature and intensity, including both signs of mania and psychosis due to firmly held delusions and audible hallucinations, I would agree with AI_B, AI_C, and AI_E in diagnosing this woman with Bipolar disorder, manic, with psychotic features (Choice A). This diagnosis accounts for her decreased need for sleep, heightened activity level, grandiose/persecutory delusions about her relationship with the president of the USA, and hallucinations of hearing her kidnapped husband's voice. In clinical practice, it is not uncommon for patients with Bipolar disorder to experience episodes of psychosis during severe manic or depressive episodes. The presence of mania within delusions further differentiates this from Delusional disorder, as the latter does not include symptoms of mania. This completes my assessment.

**AI_C:** To start, we are being asked to diagnose a woman who has been showing evident symptoms of delusion, hyperactivity, rapid speech, decreased sleep, and loose associations. She also reports hearing voices, a classic symptom of psychosis. Let's begin by ruling out some possible diagnoses. We can exclude brief psychotic disorder because her symptoms have been present for over 2 months, which surpasses the 1-month limit for this disorder. Psychotic disorder due to a general medical condition is also unlikely, given she has no known medical history and refuses tests. Schizophrenia has a diagnostic requirement of at least 6 months, which she also doesn't meet. This leaves us with bipolar disorder, manic, with psychotic features, and delusional disorder. Given the symptoms described, both disorders initially seem plausible. A key factor in differentiating them is analyzing the role of mood symptoms. In bipolar disorder, mood symptoms are usually predominant, while in delusional disorder the primary feature is the presence of delusions. The woman's symptoms include not only delusions but also signs of mania such as decreased need for sleep, rapid speech, and increased goal-directed activity. Therefore, in this case, the mood symptoms are not merely an accessory aspect but rather at the forefront. The full-hearted belief in her delusional narrative also suggests a manic state. Furthermore, her psychosis transcends the actual delusion and includes auditory hallucinations, shown in her statement of hearing her husband's voice giving her instructions. Thus, the intensity and breadth of her manic symptoms contribute predominately to her overall clinical picture. Therefore, based on the presented reasoning, the most likely diagnosis for this woman would be (A) Bipolar disorder, manic, with psychotic features.

**AI_D:** Restating the question, we are asked to provide a likely diagnosis based on the presented symptoms of a 33-year old woman who has been acting bizarrely, refusing to sleep and demonstrating a rapid speech pattern, loose associations and a persistent delusion about being married to the president's twin brother and hearing voices. Upon carefully reviewing the symptomatology, I assume she is displaying symptoms indicative of a psychotic episode in tandem with symptoms consistent with a manic episode. However, an alternate assumption could be that the persistent delusion forms the mainstay of her symptomatology and is an indication of a Delusional disorder. While these assumptions are plausible based on the observation that she has symptoms of both, deciding which is clinically prioritized requires an understanding of the defining features of these conditions. Considering the clinical reasoning process, Delusional disorder is characterized by one or more delusions with a duration of one month or longer and does not have the equally prominent manic or depressive episodes that are seen in other mental disorders. The woman's delusion about being married to the president's twin brother, hearing his voice and expecting a divine mission aligns with this. However, she also demonstrates hyperactivity, rapid speech, decreased need for sleep and grandiosity; these overlap with the characteristic features of a manic episode in Bipolar disorder. When these manic symptoms occur concurrently with psychotic features (her delusion and hearing voices), a diagnosis of Bipolar disorder, manic, with psychotic features becomes more likely. In conclusion, based on her presentation, while the possibility of Delusional disorder should not be entirely dismissed, the weight of evidence tilts towards Bipolar disorder, manic, with psychotic features. This diagnosis aligns with the overall symptomatology that transcends beyond the boundaries of a single delusion and incorporates other manic and psychotic symptoms. Monitoring her symptoms over time could further consolidate this working diagnosis. Thus, in response to the question of the most likely diagnosis, I would choose (A) Bipolar disorder, manic, with psychotic features. This choice reflects clinically relevant prioritization of her manifold symptoms, pivoting on the intersection of manic and psychotic features.

**AI_E:** To answer this question, we firstly need to interpret its content. The query presents the case of a woman who has been stalking the president, asserting connection to him in a way that's far from reality. She has not been sleeping, and has fleeting and disjointed thoughts along with auditory hallucinations. Additionally, she seems to be resistant to diagnostic tests, claiming a lack of time. Given these manifestations, we need to establish the most likely psychiatric diagnosis from the given options. Planning comes next, where potential assumptions concerning her condition need to be raised. These might include: Is her behaviors due to usage of natural substances? Could her condition be short-lived (brief psychotic disorder)? Is it a manifestation of a medical condition causing psychotic symptoms (psychotic disorder due to a medical condition)? Is she living with schizophrenia? However, a more detailed examination of her symptoms is needed, focusing on two primary conditions: Bipolar Disorder or Delusional Disorder. Clinical reasoning involves evaluating each potential explanation for her behaviors and symptoms. To start off, individuals with delusional disorders have fixed delusions with real-life plausibility, lasting for a month or longer. However, the disjointed associations, manic behaviors, and lack of sleep suggest a more complex condition. On the other hand, Bipolar disorder, manic phase with psychotic features presents with decreased need for sleep, increased activity or psychomotor agitation, elevated or irritable mood, distractions, loose associations, and grandiosity. Delusions and hallucinations in bipolar disorder are commonly mood-congruent. In conclusion, although the woman exhibits delusional belief, the presence of other symptoms such as rapid speech, lack of sleep, heightened activity and the loose associations seem to point towards a manic episode, which together with the delusions and auditory hallucinations, suggest the diagnosis of Bipolar disorder, manic, with psychotic features. To answer the original question, the 33-year-old woman's constellation of symptoms--her delusion, rapid speech, minimal sleep, increased activity, loose associations, auditory hallucinations, and refusal of diagnostic tests--are indicative of a diagnosis of Bipolar disorder, manic, with psychotic features (Option A).

**AI_B:** The provided scenario describes a woman demonstrating irregular mood behaviors such as rapid speech, decreased sleep, loose associations, delusional thoughts and auditory hallucinations. These symptoms are indicative of a possible mental health disorder. The list of options provided includes psychiatric conditions that might explain the presented symptoms. Nevertheless, we need to assess each choice carefully, considering the defining characteristics of each disorder. The question conveys that this previously healthy woman has had a fixed, non-bizarre delusion which has lasted over two months, and is demonstrating symptoms she attributes to a mission from God. She has increased energy as indicated by her hyperactivity and is unable to sleep. Additionally, she shows signs of risky behaviors such as stalking the president. Further, she’s interpreting her vocal auditory hallucinations as her 'husband' guiding her. Having evaluated these details, let's investigate each possible diagnosis: (A) Bipolar disorder, is a chronic abnormal mood disorder, including episodes of elevated mood (mania) that presents with a persistently elevated, expansive, or irritable mood, along with increased goal-directed activity or energy that lasts at least one week and is present most of the day, nearly every day. Here, the woman's rapid speech (pressured speech), needlessness for sleep (decreased need for sleep), increased activity (as manifested by stalking and planning to rescue 'her husband'), and her strong belief in her mission (delusion of grandeur) manifest such manic symptoms. In addition, her auditory hallucinations and delusion that her husband is kidnapped add psychotic features to the picture. (B) Brief Psychotic Disorder is a condition that includes sudden-onset delusions, hallucinations, disorganized speech, or disorganized behavior. However, its duration ranges from one day to a month with an eventual full return to the premorbid level of functioning. (C) Delusional Disorder is a condition wherein the patient predominantly manifests one or more delusions for at least a month, but without basically displaying any other symptom of psychosis. Here, the woman does indeed present with a non-bizarre, fixed delusion for over a month, but she also exhibits other symptoms of mania, thereby challenging this option. (D) Psychotic Disorder due to a general medical condition denotes a disturbance in cognition, mood regulation or perception, which is directly attributed to the physiological consequence of a general medical condition. There is insufficient evidence to assume this in the woman’s case from the data provided. (E) Schizophrenia requires a longer time frame of 6 months, inclusive of at least one month of active phase symptoms (hallucinations, delusions, disorganized thoughts or behavior). Also, the patients usually have a decline in their level of functioning that we cannot assume, given the woman's description as 'previously healthy'. Upon analyzing the evidence and dissecting each choice, the logical conclusion leads me toward the diagnosis of Bipolar disorder, manic, with psychotic features. This conclusion correlates with the provided scenario where the woman demonstrates signs of manic episodes - decreased need for sleep, elevated mood presented as an excess of goal-directed activity - along with displaying psychotic symptoms like auditory hallucinations and delusions. Thus, the answer to the question is (A) Bipolar disorder, manic, with psychotic features.

**Facilitator** The consensus among AI_A, AI_B, AI_C, AI_D, and AI_E is that the most likely diagnosis for the 33-year-old woman is Bipolar disorder, manic, with psychotic features (Choice A). This conclusion is based on her symptoms, which include rapid speech, decreased need for sleep, heightened activity level, grandiose and persecutory delusions about her relationship with the president of the USA, and auditory hallucinations of hearing her kidnapped husband's voice. These symptoms are indicative of a manic episode with psychotic features, which is characteristic of Bipolar disorder. The woman's symptoms have been present for over two months, which rules out Brief Psychotic Disorder. There is no evidence of a general medical condition causing her symptoms, making Psychotic Disorder due to a general medical condition unlikely. The duration of her symptoms does not meet the six-month requirement for a diagnosis of Schizophrenia. While the woman does present with a non-bizarre, fixed delusion for over a month, which could suggest Delusional Disorder, she also exhibits other symptoms of mania, which are not characteristic of Delusional Disorder. Therefore, the presence of manic symptoms in addition to her delusions and hallucinations leads to the diagnosis of Bipolar disorder, manic, with psychotic features. This diagnosis accounts for her decreased need for sleep, increased activity level, delusions, and hallucinations. It is not uncommon for patients with Bipolar disorder to experience episodes of psychosis during severe manic or depressive episodes. The presence of mania within her delusions further differentiates this from Delusional Disorder, as the latter does not include symptoms of mania. In conclusion, the consensus is that the woman's symptoms are most consistent with Bipolar disorder, manic, with psychotic features.
