# Supplementary material for: Collaborative intelligence in AI: Evaluating the performance of a council of AIs on the USMLE
Source: PLOS Digit Health. 2025 Oct 9;4(10):e0000787. doi: 10.1371/journal.pdig.0000787 (PMC12510544; doi:10.1371/journal.pdig.0000787)
Supplement: S2 File — (ZIP) [file pdig.0000787.s002.zip › S2 Data Raw data files containing unprocessed question inputs and LLM outputs including transcripts of deliberations/STEP1_Question-60-1_Type-MC-NJ.docx]

**Facilitator** **[Round 1]:** A 34-year-old man is brought to the emergency department 30 minutes after being hit by a motor vehicle while walking. His temperature is 36°C (96.8°F), pulse is 140/min, respirations are 24/min, and blood pressure is 90/60 mm Hg. During an exploratory laparotomy, a large laceration is found that includes the right and left lobes of the liver. Active bleeding is present. Compression of which of the following ligaments is most likely to decrease bleeding from the liver in this patient? (A) Coronary (B) Falciform (C) Gastrohepatic (D) Hepatoduodenal (E) Triangular

**AI_D:** RESTATING THE QUESTION: The scenario presents a 34-year-old man who has just been in an accident and is experiencing hemorrhage from a liver injury. In an attempt to control the bleeding, the question is asking which ligament, when compressed, is likely to decrease the blood loss. PLANNING: Assumptions to consider in this scenario include understanding the blood supply of the liver and the anatomical relationship between these vessels and the surrounding ligaments. A potential alternate assumption to question is whether some of these ligaments serve as anatomical landmarks to important vascular structures of the liver. A clinically pivotal assumption to pursue is identifying which ligament, when compressed, can help manage the bleeding from the liver. CLINICAL REASONING: The liver, being a highly vascular organ, receives its blood supply from the hepatic artery and the portal vein which together account for about 75% and 25% of blood supply respectively. The hepatoduodenal ligament, a part of the lesser omentum, is a thickened fold of peritoneum connecting the liver to the first part of the duodenum. It contains the hepatic artery proper, portal vein, and the common bile duct. This makes it a crucial anatomical point of control for bleeding from the liver. CONCLUDING: Compression of the hepatoduodenal ligament can control blood flow into the liver via the hepatic artery and portal vein, thus decreasing active bleeding from a lacerated liver. RESPONSE: Thus, compression of the Hepatoduodenal ligament (D) is most likely to decrease bleeding from the liver in this patient.

**AI_B:** RESTATING THE QUESTION: The question is asking us to identify the ligament whose compression could most likely help reduce bleeding from the liver in a patient who has sustained severe liver lacerations due to a motor vehicle accident. PLANNING: We're assuming the bleeding can be controlled by applying pressure to one of the ligaments stated in the options. These include the Coronary, Falciform, Gastrohepatic, Hepatoduodenal, and Triangular ligaments. Another assumption could be some tissue or ligament, when compressed, may apply pressure on a bleeding vessel in the liver to reduce the hemorrhage. If this is the case, we would want to prioritize the ligament that is in close proximity to major blood vessels in the liver. CLINICAL REASONING: The Hepatoduodenal ligament, an extension of the lesser omentum, contains the common bile duct, hepatic artery and the portal vein, collectively known as the portal triad. If we apply direct pressure or compression to the Hepatoduodenal ligament, it could potentially compress these major vessels and help control the bleeding. None of the other ligaments mentioned in the options house notable vessels that, when compressed, could lead to control of a substantial hemorrhage in the liver. CONCLUDING: Therefore, in this scenario of severe liver lacerations with active bleeding, compression of the hepatoduodenal ligament may help slow or stop the bleeding by applying pressure to the portal triad. RESPONSE: (D) Hepatoduodenal ligament

**AI_E:** (1) RESTATING THE QUESTION: This question is asking which ligament, when compressed, will most likely reduce bleeding from a traumatic laceration involving both lobes of the liver in a 34-year-old male patient. Ligaments in this context refer to anatomical structures that connect and support organs. (2) PLANNING: We should explore the anatomical structures listed in the options and their relation to hepatic blood supply. The assumption here is that by compressing a certain ligament, we could help control the blood supply (and hence, bleeding) to the liver. (3) CLINICAL REASONING: Analysis of the ligaments reveals that some don't necessarily play a significant role in the blood supply of the liver. For example, the falciform ligament primarily attaches the liver to the anterior abdominal wall and diaphragm and doesn't contain any major vascular structures that would impact active bleeding. The coronary and triangular ligaments also have no relevant vascular relationships. The gastrohepatic ligament contains the gastric arteries, which don't supply the liver. The hepatoduodenal ligament, however, contains the portal triad - the hepatic artery, portal vein, and common bile duct. The hepatic artery and portal vein both significantly contribute to the liver's blood supply. Compressing this ligament could effectively decrease the blood flow to the liver and hence control the bleeding. (4) CONCLUDING: Given the anatomical relationships and the need to control active bleeding from the liver, compressing a ligament that restricts the major blood supply to the liver would be the most effective approach. (5) RESPONSE: Therefore, in this scenario, compression of the hepatoduodenal ligament (D) is most likely to decrease bleeding from the liver in this patient.

**AI_C:** RESTATING THE QUESTION: The inquiry is revolving around a clinical scenario: A 34-year-old male is admitted to the emergency room following a road traffic accident. His vital signs indicate a potential shock (increased heart rate, increased respiratory rate, and decreased blood pressure) possibly due to blood loss. There is active bleeding from a significant liver laceration observed during an exploratory laparotomy. The question is asking which ligament, if compressed, could help control the bleeding from the liver. PLANNING: First, let's define the ligaments given in the available options: 1. Coronary ligament: These are two small ligaments that extend from the liver to the diaphragm. 2. Falciform ligament: This thin, crescent-shaped ligament separates the right and left lobes of the liver anteriorly and attaches the liver to the anterior abdominal wall and diaphragm. 3. Gastrohepatic ligament: This is an intraperitoneal fold that attaches part of the liver to the lesser curvature of the stomach. 4. Hepatoduodenal ligament: This ligament contains the hepatic artery proper, portal vein, and common bile duct—collectively known as the portal triad. 5. Triangular ligament: This thin ligament extends from the liver to the diaphragm. Given these definitions, in the setting of active bleeding from a liver injury, the assumption would be that compressing the ligament enclosing the primary blood supply to the liver would be most beneficial in controlling the bleeding. CLINICAL REASONING: While all given ligaments are associated with the liver, their roles vary. In the context of active bleeding, the focus should be on which ligament could facilitate control of the primary blood supply to the liver. The liver receives about 75% of its blood supply from the portal vein and about 25% from the hepatic artery. Both structures are found within the hepatoduodenal ligament, making it a key focus in this situation. CONCLUDING: By compressing the hepatoduodenal ligament, there can be a likely decrease in blood flow from both the hepatic artery and the portal vein. This could result in decreased active bleeding from the wounded liver in this patient subjected to a motor vehicle accident. RESPONSE: Compression of the Hepatoduodenal ligament (D) is most likely to decrease bleeding from the liver in this patient.

**AI_A:** RESTATING THE QUESTION: The question is asking to identify the ligament, from the given options, that when compressed would most likely help to control the bleeding in a liver laceration in a patient who was hit by a vehicle. Here, a brief description of the liver ligaments may help in understanding the question better: The coronary ligament attaches the liver to the diaphragm; the falciform ligament connects the liver to the anterior abdominal wall; the gastrohepatic ligament connects the liver to the lesser curvature of the stomach; the hepatoduodenal ligament contains the portal triad (proper hepatic artery, portal vein, and common bile duct); and the triangular ligaments are extensions of the coronary ligament. PLANNING: A crucial assumption is that the bleeding is coming from the liver's lobes directly and there's potential for control by compressing a specific ligament. We also assume here that ligament compression would not cause further trauma or damage. We also question if hepatic bleeding could be controlled by compressing the portal structures, considering the large vascular supply to the liver. CLINICAL REASONING: Given that the liver has a dual blood supply, from the hepatic artery (which comprises about 25% of blood flow) and portal vein (which carries about 75% of blood), stopping blood flow through these vessels could theoretically help stop liver bleeding. Moreover, considering that hepatoduodenal ligament encloses the portal triad of structures including both these blood suppliers, compression of this ligament would help control liver bleeding. CONCLUDING: Therefore, in a clinical situation like this, where bleeding from the liver is actively happening due to a traumatic laceration, compressing the hepatoduodenal ligament could be beneficial in ceasing the hemorrhage because it encompasses the structures carrying the bulk of the blood flow to the liver. RESPONSE: In this particular case of a 34-year-old man with a laceration in both lobes of the liver after a motor vehicle accident, compression of the Hepatoduodenal ligament (Choice D) is most likely to decrease bleeding from the liver.

**Facilitator** The question presents a scenario where a 34-year-old man has sustained severe liver lacerations due to a motor vehicle accident, resulting in active bleeding. The question asks which ligament, when compressed, would most likely help control the bleeding from the liver. The liver is a highly vascular organ, receiving its blood supply from the hepatic artery and the portal vein, which together account for about 75% and 25% of blood supply respectively. The hepatoduodenal ligament, a part of the lesser omentum, is a thickened fold of peritoneum connecting the liver to the first part of the duodenum. It contains the hepatic artery proper, portal vein, and the common bile duct. This makes it a crucial anatomical point of control for bleeding from the liver. Given the anatomical relationships and the need to control active bleeding from the liver, compressing a ligament that restricts the major blood supply to the liver would be the most effective approach. Therefore, in this scenario, compression of the hepatoduodenal ligament (D) is most likely to decrease bleeding from the liver in this patient.
