# Supplementary material for: Collaborative intelligence in AI: Evaluating the performance of a council of AIs on the USMLE
Source: PLOS Digit Health. 2025 Oct 9;4(10):e0000787. doi: 10.1371/journal.pdig.0000787 (PMC12510544; doi:10.1371/journal.pdig.0000787)
Supplement: S2 File — (ZIP) [file pdig.0000787.s002.zip › S2 Data Raw data files containing unprocessed question inputs and LLM outputs including transcripts of deliberations/STEP1_Question-61-1_Type-MC-NJ.docx]

**Facilitator** **[Round 1]:** A 1-month-old boy is brought to the emergency department because of a 1-day history of fever and cough. His temperature is 38.2°C (100.8°F), pulse is 140/min, and respirations are 54/min. Physical examination shows intercostal retractions. Wheezes are heard in all lung fields. A blood culture grows coagulase-negative, catalase-positive, gram-positive cocci in clusters. Which of the following best represents the infectious agent in this patient's blood culture? (A) Staphylococcus aureus, contaminant (B) Staphylococcus aureus, pathogen (C) Staphylococcus epidermidis, contaminant (D) Staphylococcus epidermidis, pathogen (E) Streptococcus pneumoniae, contaminant (F) Streptococcus pneumoniae, pathogen (G) Streptococcus sanguis, contaminant (H) Streptococcus sanguis, pathogen

**AI_B:** RESTATING THE QUESTION: The inquiry here is about the identification of the specific microbial agent responsible for the symptoms exhibited by a 1-month-old boy who has presented with symptoms of fever, cough, intercostal retractions, and wheezing in all lung fields. His blood culture result showed the growth of coagulase-negative, catalase-positive, gram-positive cocci in clusters. PLANNING: Let's evaluate the lab findings and symptoms. The lab results show coagulase-negative, catalase-positive, gram-positive cocci in clusters which is characteristic of Staphylococcus bacteria, specifically Staphylococcus epidermidis. Coagulase-negative indicates that it's not Staphylococcus aureus. Furthermore, the clinical symptoms point towards a respiratory infection. Assuming that the bacteria is a contaminant or pathogen is required, although the presence of symptoms suggests a pathogen over a contaminant. CLINICAL REASONING: An infant presenting with increased fever, cough, wheezes, and intercostal retractions suggests possible respiratory distress which could very likely be due to an infection. Considering his age, it's important to note that neonates and infants have relatively underdeveloped immune systems. Staphylococcus epidermidis, although normal flora on the skin, can act as a pathogen in this case. Coagulase-negative bacteria could still be pathogenic in immunocompromised individuals, which include infants. CONCLUDING: They are normal flora of the skin, naturally present on the body. But in certain conditions, like if a patient is immunocompromised, these bacteria can access the bloodstream and cause an infection. In this case, the infant's symptoms and the bacterial growth in blood culture most likely suggests that Staphylococcus epidermidis is indeed a pathogen, rather than a mere contaminant. RESPONSE: Thus, the most appropriate option would seem to be (D) Staphylococcus epidermidis, pathogen.

**AI_C:** (1) RESTATING THE QUESTION: The information presents the case of a 1-month-old boy who has been brought to the hospital with symptoms of high fever, cough, and some physical signs indicative of respiratory distress (increased pulse and respirations, intercostal retractions, and wheezing). A blood culture from this patient has grown coagulase-negative, catalase-positive, gram-positive cocci in clusters. Given these findings, we need to identify the most likely responsible pathogen for his condition. Key terms here include coagulase-negative and catalase-positive staphylococci -- these are gram-positive cocci bacteria that do not produce the enzyme coagulase but do produce catalase. Staphylococcus epidermidis is in this category, while Staphylococcus aureus is coagulase-positive. Cluster formation is typically associated with Staphylococcus species. The term contaminant implies the organism was not the pathogen causing the symptoms, but instead incidentally present. (2) PLANNING: Assuming that the identified bacteria are causing the symptoms, Staphylococcus epidermidis would appear to be the best match because it is coagulase-negative and catalase-positive. However, we must also consider whether Staphylococcus epidermidis could be a contaminant. In infants and immunocompromised individuals, Staphylococcus epidermidis can be a significant pathogen, especially in those with indwelling devices. Hence, this assumption needs to be clinically prioritized. (3) CLINICAL REASONING: The fact that the boy is displaying symptoms of respiratory distress suggests a state of systemic infection. This, combined with the results of the blood culture, increases the likelihood of this being a true infection rather than a contamination event. Furthermore, Staphylococcus epidermidis, while commonly a benign skin commensal, is known to turn pathogenic, particularly in individuals with immature or compromised immune systems, like this 1-month-old boy. (4) CONCLUDING: Given these considerations, the clinical reasoning most strongly supports the assumption that the Staphylococcus epidermidis detected in this boy's blood culture is likely pathogenic rather than a contaminant. (5) RESPONSE: Consequently, I suggest that the most suitable answer would be (D) Staphylococcus epidermidis, pathogen.

**AI_E:** (1) RESTATING THE QUESTION: The case describes an infant with fever, cough, and physical signs of respiratory distress, such as intercostal retractions and wheezing. Intercostal retractions are an indication of severe respiratory distress. Wheezing is a high-pitched sound which is usually caused by obstruction or narrowing lower in the windpipe. The blood culture shows coagulase-negative, catalase-positive, gram-positive cocci clusters. We're asked to identify the pathogen based on these laboratory findings. (2) PLANNING: The primary assumption here is that the bacteria growing in his blood culture is responsible for his symptoms. We then question this assumption. Is it actually the same organism causing his respiratory symptoms or could it be a contaminant? If the organism is a pathogenic one, it could be causing both systemic and respiratory symptoms. To hypothesize, we'd need to understand the potential organisms and whether they are likely contaminants or pathogens. (3) CLINICAL REASONING: The bacteria described is coagulase-negative, catalase-positive, gram-positive cocci in clusters. Among the options provided, Staphylococcus epidermidis and Staphylococcus aureus share these characteristics. However, Staphylococcus aureus is usually coagulase-positive, ruling it out as a cause in this case. That leaves us with Staphylococcus epidermidis. This organism can be a contaminant, normally being a part of our skin flora without causing diseases. However, it can become pathogenic especially in cases of immunocompromised patients or in the presence of foreign bodies. Although the baby is young, there isn't sufficient evidence to indicate that he is immunocompromised or there's any foreign body involved, which could argue that Staphylococcus epidermidis is a contaminant here. However, given the systemic symptoms (fever) and respiratory involvement for which Staphylococcus epidermidis could be a potential cause, it is plausible that it acts as a pathogen in this specific case. (4) CONCLUDING: Although Staphylococcus epidermidis is usually considered a contaminant, the systemic symptoms and signs of respiratory distress in this case suggest that it's acting as a pathogen. (5) RESPONSE: Therefore, the best answer is: (D) Staphylococcus epidermidis, pathogen.

**AI_D:** (1) RESTATING THE QUESTION: The question is asking us to identify the microorganism that matches the characteristics described in the patient's blood culture results (coagulase-negative, catalase-positive, gram-positive cocci in clusters), and whether this microorganism is acting as a contaminant or a pathogen in the patient's bloodstream. (2) PLANNING: To each microorganism provided as options, we need to associate it with the appropriate defining characteristics including gram-staining traits, coagulase, and catalase reaction. Next, we have to decide if it could be a contaminant or a pathogen. A 'contaminant' is an organism not causing disease, and might be present due to unsanitary sample collection; 'pathogen' is causing the disease. (3) CLINICAL REASONING: Considering the patient's symptoms, the presence of fever, and cough suggests a respiratory infection, as indicated by the physical examination findings of intercostal retractions and wheezes in all lung fields, symptoms common in severe respiratory infections. The organism seen in the blood culture is coagulase-negative, catalase-positive, gram-positive cocci in clusters. These characteristics suggest a Staphylococci species, as both Staphylococcus aureus and Staphylococcus epidermidis share these traits. The important piece of information is the "coagulase-negative" result which differentiate them - S. aureus is usually coagulase-positive while S. epidermidis is coagulase-negative. It's crucial to note that although S. epidermidis is a commensal bacterium and often a contaminant, in certain situations it can be a pathogen. Staphylococcus epidermidis is a common cause of sepsis in neonates, particularly those with indwelling central venous lines, prosthetic devices, or who are in the intensive care unit. Despite its usual role as a skin contaminant, in the appropriate setting such as this case, it can indeed cause disease. (4) CONCLUDING: On evaluating the above information, the most likely possibility is Staphylococcus epidermidis causing an infection (pathogen), based on the provided laboratory results and the patient's clinical presentation. (5) RESPONSE: The best representation of the infectious agent in this patient's blood culture is (D) Staphylococcus epidermidis, pathogen.

**AI_A:** RESTATING THE QUESTION: We're essentially trying to evaluate the likely origin of a bacterial infection in a one-month-old baby presenting with fever and respiratory symptoms. Specifically, we're looking at bacteria found in the blood culture, which are coagulase-negative, catalase-positive, gram-positive cocci in clusters, or what this bacterium might be and whether it is a contaminant or a pathogen. Contaminants are bacteria that are not causing disease and are often present as a result of a standard microbiological operation, whereas pathogens are bacteria that cause disease. PLANNING: Based on the identified bacterium's characteristics as coagulase-negative, catalase-positive, gram-positive cocci in clusters, one of the main assumptions would be that the bacterium is a staphylococcus species. The other assumption (required to answer the question) is whether the bacterium is a pathogen or a contaminant. To verify these assumptions, the pathophysiology of the baby's condition, the characteristics of the bacterial species proposed, and the epidemiology of these bacteria in similar patient populations are examined. CLINICAL REASONING: The symptoms of high fever, cough, and physical evidence of respiratory distress (intercostal retractions and wheezing) point to a possible severe infection, probably of the lower respiratory tract. The fact that the patient is one month old also raises the seriousness of these symptoms, given the undeveloped state of a baby's immune system. Considering the characteristics from the lab test, Staphylococcus aureus (coagulase-positive) can be ruled out. That leaves us with either Staphylococcus epidermidis or Staphylococi other than Staphylococcus aureus, which are coagulase negative. Since staphylococci are arranged in clusters and are catalase positive-gram positive, our criteria effectively point towards Staphylococcus epidermidis more than Streptococcus spp., which are gram positive but arranged in chains. CONCLUDING: Though Staphylococcus epidermidis is often a skin contaminant, it can act as an opportunistic pathogen, especially in individuals with weakened immune systems, such as infants. Given this patient's serious symptoms, which are consistent with systemic infection, it would be more plausible to consider Staphylococcus epidermidis as a pathogen (D) rather than a contaminant (C) in this case. RESPONSE: The infectious agent in this patient's blood culture best represents Staphylococcus epidermidis, pathogen (D).

**Facilitator** The consensus statement synthesizes the responses of AI_A, AI_B, AI_C, AI_D, and AI_E as follows: The question at hand involves identifying the likely origin of a bacterial infection in a one-month-old baby presenting with fever and respiratory symptoms. The blood culture reveals coagulase-negative, catalase-positive, gram-positive cocci in clusters. The main assumption is that the bacterium is a staphylococcus species, specifically Staphylococcus epidermidis, based on these characteristics. The baby's symptoms of high fever, cough, and physical evidence of respiratory distress suggest a severe infection, likely of the lower respiratory tract. Given the baby's young age, the seriousness of these symptoms is heightened due to the undeveloped state of the infant's immune system. Staphylococcus aureus can be ruled out due to its coagulase-positive nature, leaving us with Staphylococcus epidermidis as the most likely candidate. Although Staphylococcus epidermidis is often a skin contaminant, it can act as an opportunistic pathogen, especially in individuals with weakened immune systems, such as infants. Given the patient's serious symptoms, which are consistent with systemic infection, it is more plausible to consider Staphylococcus epidermidis as a pathogen rather than a contaminant in this case. Therefore, the consensus among the AI responses is that the infectious agent in this patient's blood culture best represents Staphylococcus epidermidis, pathogen (D).
