# Supplementary material for: Collaborative intelligence in AI: Evaluating the performance of a council of AIs on the USMLE
Source: PLOS Digit Health. 2025 Oct 9;4(10):e0000787. doi: 10.1371/journal.pdig.0000787 (PMC12510544; doi:10.1371/journal.pdig.0000787)
Supplement: S2 File — (ZIP) [file pdig.0000787.s002.zip › S2 Data Raw data files containing unprocessed question inputs and LLM outputs including transcripts of deliberations/STEP1_Question-63-1_Type-MC-NJ.docx]

**Facilitator** **[Round 1]:** A new drug, Drug X, is being investigated to determine its pharmacokinetic properties in humans. A male participant who weighs 85 kg (187 lb) with 50 L of total body water begins treatment with an intravenous infusion of Drug X at the rate of 100 mg/h. This drug is known to distribute in total body water. The plasma concentrations of the drug are measured. Results show that the steady-state plasma concentration of Drug X is 50 mg/L. Based on this information, which of the following is the clearance rate of this drug in this man? (A) 20 mg/h (B) 100 mg/h (C) 0.22 L/h (D) 2 L/h (E) 22 L/h

**AI_C:** RESTATING THE QUESTION: In simpler terms, the question is asking us to calculate the clearance rate of Drug X. Clearance rate is a measure of the body's efficiency in removing a drug. It is derived from the volume of blood plasm from which the drug is completely removed per unit time and is measured in volume per time (commonly L/h). Here, we know the infusion rate of the drug, the volume in which the drug is distributed, and the steady-state plasma concentration. PLANNING: Let us question the assumptions and generate alternate ideas. The assumptions are that the infusion rate is 100 mg/h, the steady-state plasma concentration is 50mg/L, and that the drug distributes in the total body water, which in this instance is given as 50 L. We also have the formula for calculating clearance: Clearance = Rate of infusion/Steady-state concentration. Evaluating these, our priority is to calculate the clearance using the details provided. CLINICAL REASONING: Using the formula for clearance, we're told that Drug X has an infusion rate of 100 mg/h and a steady-state plasma concentration of 50 mg/L. This information is essential in calculating the clearance, as it is equal to the rate of infusion divided by the steady-state concentration. CONCLUDING: So, from the data given, the clearance rate for the drug is calculated by dividing the rate of infusion (100 mg/h) by the steady-state concentration (50 mg/L), which gives us a value of 2 L/h. RESPONSE: The clearance rate of Drug X in this man is therefore 2 L/h (Option D).

**AI_B:** RESTATING THE QUESTION: The study is examining the pharmacokinetics—or the ways in which the body alters the drug—of a new medicine we'll call Drug X. Specifically, we're investigating how quickly this drug gets cleared from the body of a human male participant who weighs 85kg, has 50L of total body water, and is being treated with Drug X at a rate of 100mg/h through an intravenous infusion. This drug is understood to distribute throughout all body water. Tests reveal that the steady-state plasma concentration of Drug X is 50mg/L. The question asks to determine the clearance rate of this drug in the man. PLANNING: The clearance rate is used to calculate how quickly a drug is removed from the body and is determined by the ratio of the rate of drug administration to the steady-state plasma drug concentration. In this case, the rate of drug administration is 100mg/h, and the concentration is 50mg/L. With these values and the knowledge that drug clearance is clinically significant as it can influence dosing regimens, the clearance rate can be calculated. CLINICAL REASONING: Drug clearance (Cl) is a pharmacokinetic parameter that describes the volume of plasma from which a drug is completely removed per unit time. It can be calculated using the formula Cl = Rate of administration / Steady-state concentration. In this case, the rate of administration is 100 mg/h and the steady-state concentration is 50 mg/L. Substituting these values into the formula would give us the clearance rate. CONCLUDING: After calculations, the clearance rate for this medication in this man will be obtained. This will shed more light on the drug's pharmacokinetic properties and may influence how it is administered in the future. RESPONSE: When calculated, Cl = 100 mg/h / 50 mg/L = 2 L/h confirms The clearance rate of the drug Drug X in the patient is 2 L/h, corresponding to answer (D).

**AI_D:** (1) RESTATING THE QUESTION: This question wants to know the clearance rate of a new drug, Drug X, based on various parameters mentioned. It's given that the drug is intravenously infused at a rate of 100 mg/h, it distributes in the total body water which in this case is 50 L, and the steady-state concentration of the drug in plasma is 50 mg/L. Clearance rate is a measure of the volume of plasma from which a substance is completely removed per unit time. This rate indicates the body's efficiency in eliminating the drug. (2) PLANNING: The key assumption made here is that the concentration of the drug in the plasma after reaching a steady state can be used to find the clearance rate. Drug clearance is calculated using the formula: CL = Rate of elimination/Plasma drug concentration. We will use this formula to calculate clearance, taking 100 mg/hour as the rate of infusion (which equals the rate of elimination at steady state) and 50 mg/L as the plasma concentration at steady state. (3) CLINICAL REASONING: At steady state, the rate of drug infusion equals the rate of drug elimination since the drug has achieved a stable concentration in the body. Therefore, we can substitute the rate of infusion (100 mg/h) as the rate of elimination in the clearance equation. By substitifying these values into our formula, we can calculate the clearance rate. (4) CONCLUDING: So, we calculate Clearance (CL) = (Rate of elimination)/(Plasma drug concentration), substituting the given values will be 100 mg/h divided by 50 mg/L which equals 2 L/h. (5) RESPONSE: Based on the given data and our calculations, the clearance rate of Drug X in this man is 2 L/h. Therefore, the correct answer is (D) 2 L/h.

**AI_E:** 1) RESTATING THE QUESTION: We're examining the pharmacokinetics of a new drug, Drug X, in humans. It's being administered via an IV at a rate of 100 mg/h to a man who weighs 85 kg and has 50 L of total body water. It's known that the drug distributes throughout this total body water. Now we have steady-state plasma drug concentration data from this participant that shows a concentration of 50 mg/L. Using this data, we aim to calculate the clearance rate of this drug for this man. The clearance rate, in pharmacokinetics, refers to the volume of blood completely cleared of the drug per unit time and is given by the formula: Clearance = (Rate of Drug Administration) / (Steady State Plasma Concentration). 2) PLANNING: Based on the given data, we can make the assumption that the administration rate and the plasma concentration at steady state are reliable. If we question this assumption, we could consider potential errors in dosage administration or concentration measurements. However, given the scenario, it is reasonable to prioritize the clinical assumption that the data provided is accurate and represents a typical pharmacokinetic behaviour for Drug X in this patient. 3) CLINICAL REASONING: The aforementioned formula can be applied to this case. The drug administration rate is 100 mg/h and the steady-state plasma concentration is 50 mg/L. Clearance is the rate of removal of a drug from the body relative to its concentration in the plasma. 4) CONCLUDING: We can then proceed to calculate the clearance rate using these given values and the formula: Clearance = (Rate of Drug Administration) / (Steady State Plasma Concentration), which essentially calculates how quickly the body is able to remove the drug. 5) RESPONSE: Applying the formula Clearance = (Rate of Drug Administration) / (Steady State Plasma Concentration), it becomes Clearance = (100 mg/h) / (50 mg/L) which simplifies to 2 L/h. So the correct answer is (D) 2 L/h.

**AI_A:** (1) RESTATING THE QUESTION: The question is concerning the pharmacokinetic property, specifically the clearance rate, of a new drug called Drug X that's being tested in a male participant of specific physical attributes. The clearance rate, in pharmacology, is a measurement of the body's efficiency at eliminating a drug from the bloodstream, measured in volume over time (L/h). In this case, we're given information including the infusion rate of the drug (100 mg/h), the steady-state plasma concentration of the drug (50 mg/L), and the fact that the drug distributes in total body water. (2) PLANNING: Given the assumption that the drug distributes freely in the total body water, it's reasonable to assume that the volume of distribution (Vd) is the total body water which is given as 50 L. Additionally, the plasma concentration of the drug at steady-state level helps in calculating the rate of drug clearance. One might assume that the clearance rate could be calculated using the dose rate (mg/h) or the plasma concentration (mg/L), but the most clinically relevant assumption to pursue would be using the formula for clearance that incorporates both: clearance (CL) equals rate of infusion divided by steady-state concentration (CL = rate of infusion/ steady-state concentration). (3) CLINICAL REASONING: The steady-state concentration is the point at which the body is eliminating the drug at the same rate that it's being administered, hence, the plasma concentration of the drug remains consistent. In this scenario, the steady-state plasma concentration of Drug X is 50 mg/L, and the drug is being infused at a rate of 100 mg per hour. Plugging these into the clearance formula, the calculation would be CL = 100mg/h divided by 50mg/L. (4) CONCLUDING: If you calculate the clearance rate using the information given, the mg units cancel out, leaving you with an answer in L/h. It indicates how much volume of plasma from which the drug is completely removed per hour. (5) RESPONSE: Therefore, the clearance of the drug in this man is (B) 2 L/h.

**Facilitator** The question is asking for the clearance rate of a new drug, Drug X, in a male participant with specific physical attributes. The clearance rate is a measure of the body's efficiency in eliminating a drug from the bloodstream, measured in volume over time (L/h). The drug is being administered at a rate of 100 mg/h, and it distributes in the total body water, which is 50 L in this case. The steady-state plasma concentration of the drug is 50 mg/L. The clearance rate can be calculated using the formula: Clearance = Rate of infusion / Steady-state concentration. Given the data, the clearance rate for the drug is calculated by dividing the rate of infusion (100 mg/h) by the steady-state concentration (50 mg/L). This calculation results in a clearance rate of 2 L/h. Therefore, the consensus among the responses is that the clearance rate of Drug X in this man is 2 L/h, corresponding to answer (D). This clearance rate is clinically significant as it can influence dosing regimens and sheds light on the drug's pharmacokinetic properties.
