# Supplementary material for: Collaborative intelligence in AI: Evaluating the performance of a council of AIs on the USMLE
Source: PLOS Digit Health. 2025 Oct 9;4(10):e0000787. doi: 10.1371/journal.pdig.0000787 (PMC12510544; doi:10.1371/journal.pdig.0000787)
Supplement: S2 File — (ZIP) [file pdig.0000787.s002.zip › S2 Data Raw data files containing unprocessed question inputs and LLM outputs including transcripts of deliberations/STEP1_Question-64-1_Type-MC-NJ.docx]

**Facilitator** **[Round 1]:** A 78-year-old woman is admitted to the intensive care unit because of diverticulitis complicated by Escherichia coli sepsis. Treatment with ciprofloxacin is started. Three days later, her serum creatinine concentration has increased from 0.7 mg/dL on admission to 1.3 mg/dL. Urinalysis shows muddy brown casts. The most likely cause of the findings in this patient is ischemia of which of the following structures? (A) Bowman capsule (B) Glomerulus (C) Interstitium (D) Proximal tubule (E) Renal vein

**AI_C:** (1) RESTATING THE QUESTION: The question is about a 78-year-old woman who has been admitted to the ICU due to diverticulitis complicated by E. coli sepsis. This question is asking us to determine which part of the kidney is likely suffering from ischemia (restricted blood flow), resulting in the woman's raised serum creatinine concentration and the muddy brown casts found in her urine. (2) PLANNING: The patient was started on ciprofloxacin for an E. coli infection associated with her diverticulitis. The increase of her creatinine levels from 0.7 mg/dL on admission to 1.3 mg/dL suggests renal dysfunction, which is further corroborated by the presence of muddy brown casts in the urine, indicative of acute tubular necrosis (ATN), a type of kidney injury characterized by damage to the tubule cells of the kidneys. The structures listed are all parts of the kidney that could potentially be affected. (3) CLINICAL REASONING: Acute tubular necrosis typically occurs due to prolonged ischemia or exposure to nephrotoxic substances. In the context of our patient, her renal insufficiency is likely attributable to the ischemia-tubular cell damage-tubular obstruction pathway. Ischemia, in this scenario, would most likely affect the highly metabolically active proximal tubules of the kidney, which are particularly susceptible to damage due to their high oxygen requirement and location in the outer medulla where oxygen supply is limited. (4) CONCLUDING: The renal dysfunction, indicated by rising creatinine, in conjunction with the presence of muddy brown casts, indicates acute tubular necrosis due to ischemia. Among the given options, the proximal tubules are the most likely affected structures in this clinical scenario. (5) RESPONSE: Thus, the most likely cause of the findings in this patient is ischemia of the "Proximal tubule" (D).

**AI_D:** (1) RESTATING THE QUESTION: The query is about a 78-year-old woman with a complex case of diverticulitis that has given rise to an E. coli sepsis. She has been given a course of ciprofloxacin. Despite treatment, her condition has deteriorated, marked by an increase in creatinine concentration from 0.7 mg/dL to 1.3 mg/dL over three days. Analysis of her urine presents muddy brown casts. Subsequently, it's hinted that her current condition may be due to ischemia, or a decreased blood supply to a particular part of her kidneys. (2) PLANNING: A few assumptions can be made in this case. The woman's renal function is worsening, as indicated by raised creatinine levels and presence of muddy brown casts, which are granular casts typically associated with acute tubular necrosis (ATN). ATN from ischemia is a common cause of renal injury in sepsis, primarily impacting the proximal tubules and segments of the thick ascending limb in the medulla. (3) CLINICAL REASONING: While all options listed (Bowman capsule, Glomerulus, Interstitium, Proximal tubule, Renal vein) are part of the kidney's complex system, the proximal tubules are particularly vulnerable to hypoxic conditions due to their high energy requirements. This need for oxygen and nutrients demonstrates why they may be affected during sepsis, where there's systemic inflammation and often reduced flow of oxygenated blood to various tissues. As the presence of muddy brown casts in urine strongly hints towards ATN, a situation that is commonly associated with ischemic or toxic insult to the renal tubules, it's clinically reasonable to assume ischemia of the proximal tubules. (4) CONCLUDING: After clinically reasoning through the aforementioned circumstances, it is hypothesized that the most likely structure that the ischemic condition could be affecting is the proximal tubule. (5) RESPONSE: Therefore, the raised creatinine levels and muddy brown casts found in the urine study, which indicate poor renal function and acute tubular necrosis, point towards ischemia of the proximal tubule (Option D). Hence, in such a clinical scenario, the proximal tubule is the most likely structure to be adversely affected by ischemia due to sepsis.

**AI_B:** RESTATING THE QUESTION: The case is about a 78-year-old woman with diverticulitis complicated by E. coli sepsis. After starting treatment with ciprofloxacin, there was a diagnosis of an increase in her serum creatinine levels (from 0.7 to 1.3 mg/dL) and urinalysis revealed the presence of muddy brown casts. The challenge is to determine which renal structure's ischemia most likely caused these observations. PLANNING: It's important to identify the assumptions that: 1. Muddy brown casts in the urine typically indicate acute tubular necrosis (ATN), a condition frequently preceded by renal ischemia. 2. Ischemia to the mentioned structures could have different effects, and the observed symptoms might not exclusively point to one over the others. Questioning these assumptions would lead to the hypothesis that the observed symptoms are because of ischemia in the renal proximal tubule, that's typically more susceptible to ischemia due to its high metabolic demand and the fact that it's located primarily in the renal cortex, which is the final structure to receive blood flow. This would likely result in the onset of acute tubular necrosis. CLINICAL REASONING: Acute tubular necrosis is typically represented by a sudden decline in kidney function, often evidenced by a rise in serum creatinine and the appearance of muddy brown casts consisting of renal tubular epithelial cells in the urine. These findings fit with the information provided for the patient. Since ATN is primarily associated with severe renal ischemia, it raises consideration of which sections of the kidney would be the most vulnerable. The proximal tubule, due to its high metabolic demands, is more vulnerable to damage compared to the other structures in the list. CONCLUDING: Therefore, if the renal proximal tubule was indeed the structure that underwent ischemia, it would synchronize with the patient's clinical picture consisting of increased serum creatinine levels and urinalysis showing muddy brown casts, pointing towards decimated renal function and acute tubular necrosis. It isn't likely the other mentioned structures underwent ischemia as their involvement wouldn't typically result in the clinical picture described with this patient. RESPONSE: The most likely cause of the findings in this patient is ischemia of the 'Proximal tubule' (Answer D).

**AI_A:** (1) RESTATING THE QUESTION: A 78-year-old woman who is in the ICU due to diverticulitis which has been complicated by E. coli sepsis is under treatment. Despite the therapy, her serum creatinine concentration showed an increase from 0.7 mg/dL to 1.3 mg/dL over a period of three days. The presence of muddy brown casts was detected in the urinalysis. Based on this, it is asked which kidney structure is possibly experiencing ischemia. (2) PLANNING: In this scenario, it is assumed that the increase in creatinine, in combination with the presentation of muddy brown casts, is indicative of Acute Tubular Necrosis (ATN), a condition often caused by ischemia or toxicity. In considering whether other kidney structures could be involved in the pathology, we should recall that the Bowman capsule (option A) is involved in initial filtration, the glomerulus (option B) functions in further filtration, the interstitium (option C) helps in supporting these structures, the renal vein (option E) is involved in blood outflow from the kidney. Hypoxic ischemic injury or nephrotoxic injury generally affects the proximal tubule (option D), leading to ATN. (3) CLINICAL REASONING: The patient was diagnosed with E. coli sepsis secondary to diverticulitis. The process of severe infection could possibly lead to hypotension, and subsequently, ischemia of the kidneys. The ciprofloxacin prescribed, while targeting the E. coli, can potentially be nephrotoxic and contribute to ATN. This, together with the ischemic insult, could majorly affect the proximal tubules. An increase in the serum creatinine concentration within a matter of days points towards compromised renal function, supporting this hypothesis. The presence of muddy brown casts in the urine is a characteristic finding in ATN, correlating with the damaged proximal tubules as these casts are composed of sloughed tubular cells. (4) CONCLUDING: While the patient's generalized sepsis, and potentially nephrotoxic medications, could lead to a wide range of complications, the specific findings of increased creatinine and muddy-brown casts in the urine point to a likely cause being Acute Tubular Necrosis (ATN). This condition specifically affects the proximal tubules of the nephrons in kidneys. (5) RESPONSE: Therefore, the most likely cause of the clinical findings in this patient is ischemia of the proximal tubule (Option D).

**AI_E:** (1) RESTATING THE QUESTION: The question presents a case study of a 78-year-old woman who has undergone hospitalization due to complications from diverticulitis and an Escherichia coli (E. coli) sepsis. Following her admission to the ICU, she had been put on a treatment regimen involving the antibiotic, ciprofloxacin. However, her condition has deteriorated, with marked increases in her serum creatinine levels and evidence of muddy brown casts, as shown in the urinalysis results. The task at hand is to identify which renal structure has been affected by ischemia, as suggested by these clinical indicators. (2) PLANNING: There are multiple assumptions to consider in this case: a) that the antibiotic treatment has caused a negative side-effect, b) that the sepsis or diverticulitis has caused renal damage, or c) that the increase in serum creatinine and appearance of muddy brown casts are indicative of Acute Tubular Necrosis (ATN), i.e., ischemia to a particular structure of the kidney. Analyzing the plausible assumptions, it is credible to hypothesize that the ischemia of a specific renal structure due to ATN is clinically prioritized. (3) CLINICAL REASONING: The initial presentation of diverticulitis and E. coli sepsis reflects a patient with significant infectious and inflammatory burden, increasing the potential risks of complications such as septic chronic kidney disease or drug-induced injury. Sepsis and particular medications (like antibiotics) can inflict an injury on the kidney, causing ATN. In ATN, the proximal tubules and thick ascending limb are most often affected due to their high metabolic demands and susceptibility to insults. They are known to be the most oxygen-consuming segments due to active solute reabsorption. During ATN, damaged tubule cells slough off and appear in the urine as 'muddy brown casts,' a characteristic feature of this condition. Furthermore, a gradual increase in serum creatinine levels often signifies a decline in kidney function, which supports the diagnosis of ATN. (4) CONCLUDING: In consideration of the detailed step-by-step connections between the patient's illness, its complications, the treatment administered, and the function and vulnerability of different kidney structures, it could be inferred that the most probable cause of her current condition and her lab results would be the ischemia of renal tubules, specifically the proximal tubules. (5) RESPONSE: Consequently, the most likely cause of the symptoms and findings in this elderly woman is the ischemia of the (D) Proximal tubule, which is a hallmark feature of Acute Tubular Necrosis (ATN).

**Facilitator** The consensus statement synthesizes the responses of AI_A, AI_B, AI_C, AI_D, and AI_E as follows: The case involves a 78-year-old woman admitted to the ICU due to diverticulitis complicated by E. coli sepsis. Despite treatment with ciprofloxacin, her serum creatinine concentration increased from 0.7 mg/dL to 1.3 mg/dL over three days, and urinalysis revealed the presence of muddy brown casts. These findings suggest renal dysfunction, likely due to ischemia of a specific kidney structure. The increase in creatinine levels and the presence of muddy brown casts in the urine are indicative of Acute Tubular Necrosis (ATN), a condition often caused by ischemia or toxicity. ATN primarily affects the proximal tubules of the nephrons in the kidneys due to their high metabolic demand and location in the renal cortex, which is the final structure to receive blood flow. The proximal tubules are particularly susceptible to damage due to their high oxygen requirement and location in the outer medulla where oxygen supply is limited. The patient's sepsis and potentially nephrotoxic medications could lead to a wide range of complications. However, the specific findings of increased creatinine and muddy-brown casts in the urine point to a likely cause being ATN. The presence of muddy brown casts in the urine is a characteristic finding in ATN, correlating with the damaged proximal tubules as these casts are composed of sloughed tubular cells. Therefore, the consensus among the AI responses is that the most likely cause of the clinical findings in this patient is ischemia of the proximal tubule (Option D).
