# Supplementary material for: Collaborative intelligence in AI: Evaluating the performance of a council of AIs on the USMLE
Source: PLOS Digit Health. 2025 Oct 9;4(10):e0000787. doi: 10.1371/journal.pdig.0000787 (PMC12510544; doi:10.1371/journal.pdig.0000787)
Supplement: S2 File — (ZIP) [file pdig.0000787.s002.zip › S2 Data Raw data files containing unprocessed question inputs and LLM outputs including transcripts of deliberations/STEP1_Question-65-1_Type-MC-NJ.docx]

**Facilitator** **[Round 1]:** A 34-year-old woman with myasthenia gravis comes to the emergency department because of a 2-day history of increasing weakness, shortness of breath, and abdominal cramping. Current medications are prednisone and pyridostigmine. Her temperature is 37°C (98.6°F), pulse is 45/min, and respirations are 25/min and shallow. Her voice is soft and hypernasal, and she coughs weakly when swallowing water. Breath and heart sounds are normal. Pulmonary testing shows inability to generate a normal negative inspiratory force during forced inspiration. The abdomen is soft and nontender, with increased bowel sounds. Muscle strength is 4/5 diffusely, with severe, continuous, and diffuse fasciculations. Deep tendon reflexes are sluggish, but symmetric. Which of the following is the most likely cause of this patient's weakness? (A) Aspiration pneumonia (B) Guillain-Barré syndrome (C) Insufficient dose of prednisone (D) Motor neuron disease (E) Pyridostigmine overdose

**AI_A:** RESTATING THE QUESTION: We're being asked to determine the most prevalent cause of the patient's weakness. The patient is a 34-year-old woman with myasthenia gravis and is currently experiencing increased weakness, shortness of breath, and abdominal cramping. She's been on prednisone and pyridostigmine. Her symptoms indicate a more pronounced muscle weakness than usual, including respiratory difficulties and difficulty swallowing, leading to a hypernasal voice. Fasciculations, or muscular twitching, have also been observed, which might indicate an issue at the neuromuscular junction or within the muscle itself. PLANNING: Let's begin by eliminating improbable assumptions. This patient's myasthenia gravis, a neuromuscular disorder leading to fluctuating muscle weakness and fatigue, might cause these symptoms. However, Guillain-Barré syndrome and motor neuron disease do not typically manifest with these specific types of symptoms. While aspiration pneumonia is a possible complication of myasthenia gravis because of weakening of the bulbar muscles, the normal breath sounds and vital signs suggest this is unlikely. Now, we analyze the remaining options: insufficient dose of prednisone which functions as immunosuppression and is commonly used to improve symptoms of myasthenia gravis, and an overdose of pyridostigmine, a medication that affects the parasympathetic nervous system, which may cause symptoms similar to those the patient presents. CLINICAL REASONING: Myasthenia gravis is a disorder that interferes with acetylcholine's ability to signal muscle contraction. Pyridostigmine, a cholinesterase inhibitor, counteracts this by inhibiting the enzyme that breaks down acetylcholine, so the neurotransmitter has a longer lifespan at the neuromuscular junction. This aids in improving muscle strength in myasthenic patients. However, excessive amounts of pyridostigmine can result in a cholinergic crisis, which would present with symptoms such as abdominal cramps, increased salivation, and muscular twitching. Symmetric muscle weakness and bradycardia seen in pyridostigmine overdose could potentially be mistaken for a myasthenic crisis, which is caused by insufficient medication; however, the presence of a cholinergic toxidrome (which includes abdominal cramps and fasciculations), in this case, suggests drug toxicity. CONCLUDING: Given the patient's symptoms such as abdominal cramping and fasciculations, as well as being on pyridostigmine therapy, her clinical indications are characteristic of a cholinergic crisis. Her current manifestation is likely due to pyridostigmine overdose, which would result in continuous, unregulated signaling at the neuromuscular junction, leading to the symptoms presented. RESPONSE: So, the most likely cause of this patient's weakness is (E) Pyridostigmine overdose.

**AI_C:** 1) RESTATING THE QUESTION: The clinical scenario represents a 34-year old woman diagnosed with myasthenia gravis who presents with symptoms of increasing muscle weakness, shallow respirations, and abdominal cramping over the last two days. Physically, the patient exhibits a weak, hypernasal voice, reduced inspiratory force, and 4/5 diffuse muscle strength along with increased bowel sounds and slowed tendon reflexes. Her current treatments include prednisone and pyridostigmine. We are to determine which of the listed conditions or factors – aspiration pneumonia, Guillain-Barré syndrome, insufficient dose of prednisone, motor neuron disease, or pyridostigmine overdose – are most likely contributing to her ongoing weakness. 2) PLANNING: Assumptions; the prominent assumption is that her symptoms are a continuation or exacerbation of her myasthenia gravis, which is inherently characterized by fluctuating muscle weakness, hypernasal speech could be a sign of weak palate muscles and the difficulty in swallowing water could be due to weakened swallowing muscles both characteristic of myasthenia gravis. The other assumption could be that there may be an overdose of pyridostigmine or insufficient dosage of prednisone leading to these symptoms as a result of sub-optimal treatment. 3) CLINICAL REASONING: The question's core revolves around identifying the cause of this patient's present weakness. Her symptoms, including the increased weakness, shortness of breath, and hypernasal speech, point to a possible myasthenic crisis – an acute exacerbation of myasthenia gravis. Notably, a myasthenic crisis can result from medication changes, e.g., having an insufficient dose of pyridostigmine, a drug used in myasthenia gravis to inhibit the breakdown of acetylcholine and therefore enhance neuromuscular transmission. Alternatively, her symptoms could result from an overdose of pyridostigmine, as this can also cause severe weakness due to depolarizing neuromuscular blockade. That said, the patient's symptoms also show some signs of a cholinergic crisis; the abdominal cramping and increased bowel sounds reflect possible overstimulation of the muscarinic receptors. 4) CONCLUDING: Given her myasthenia gravis diagnosis, her symptoms could potentially be attributed to an under- or overdose of her current medications, particularly pyridostigmine. This might result in a myasthenic crisis due to a lack of efficacy or a cholinergic crisis due to excess stimulation of acetylcholine receptors. However, other conditions listed in the answers, like Guillain-Barré syndrome or motor neuron disease, are less likely given the acute onset and specific symptoms. 5) RESPONSE: Consequently, the most probable cause of this woman's weakness is proposed to be 'Pyridostigmine overdose' (option E). However, patient management should differentiate between a myasthenic and a cholinergic crisis, given the overlapping symptoms and possible treatment implications.

**AI_D:** RESTATING THE QUESTION: The question presents a case of a 34-year-old woman with diagnosed myasthenia gravis who is currently experiencing increasing weakness, shortness of breath, and abdominal cramping along with other specific symptoms and is on medication - prednisone and pyridostigmine. It’s required to determine the most likely cause of her increased weakness from the given options which include/aspects such as aspiration pneumonia, Guillain-Barré syndrome, an insufficient dose of prednisone, a motor neuron disease, or an overdose of pyridostigmine. PLANNING: The key symptoms listed in the question stem - profound weakness, shortness of breath, abdominal cramping, decreased voice strength, difficulty swallowing, and abnormal respiratory function tests and fasciculations - are indicators for both an exacerbation of myasthenia gravis (i.e., myasthenic crisis) and a cholinergic crisis due to overdose of pyridostigmine. Besides, other options presented, such as Guillain-Barré syndrome, motor neuron disease, and insufficient dosage of Prednisone, need to be assessed too. CLINICAL REASONING: As a myasthenia gravis patient, her symptoms could theoretically worsen due to insufficient steroids (prednisone), but in such patients, inadequate prednisone usually leads to general symptoms like fatigue, lethargy before it could lead to a severe exacerbation of muscle weakness. Acute exacerbations of neuromuscular diseases like Guillain-Barré syndrome could theoretically present with muscle weakness, but the presence of increased weakness in a patient with known myasthenia gravis should point toward a problem related to the underlying disease itself or its treatment. Moreover, Guillan-Barre syndrome would typically also present with areflexia in contrast to the preserved reflexes in this case. Motor neuron disease is unlikely as the disease characteristically spares the ocular and bulbar muscles until late disease stages, and this patient has bulbar symptoms. Aspiration pneumonia is a complication to consider given the patient's difficulty swallowing, but she has no other signs of infection such as fever, and lung sounds are normal on examination. Because of the fasciculation and the clinical history and symptoms of abdominal cramping and shortness of breath, as well as the coincidental administration of medication right before the onset and increased severity of symptoms, pyridostigmine overdose can be considered. CONCLUDING: The fasciculations, along with the symptoms of hypernasal voice suggestive of palatal muscle weakness, and evidence of poor respiratory force, indicate muscle overactivation related to excessive acetylcholine, supportive of pyridostigmine overdose. Moreover, the presence of increased bowel sounds and abdominal cramping also indicates a cholinergic crisis related to this. RESPONSE: Therefore, based on the analysis, (E) Pyridostigmine overdose is the most likely cause of this patient's weakness.

**AI_E:** RESTATING THE QUESTION: The question is asking us to identify the most likely condition responsible for the specific symptoms this patient is presenting. Myasthenia gravis is an autoimmune disorder that affects the neuromuscular junctions, leading to muscle weakness and fatigue. The patient's current symptoms include difficulty swallowing, shallow respiration, and overall weakness, particularly that she is on two medications: prednisone and pyridostigmine - the former is an anti-inflammatory steroid while the latter is an acetylcholinesterase inhibitor used to increase neuromuscular transmission. Given these factors, we need to determine if her symptoms are being caused by one of the listed conditions. PLANNING: Our primary assumption is that her symptoms are due to the progression of her known Myasthenia gravis. However, we need to generate alternate assumptions. These assumptions include an adverse effect of the medications she is on (overdosage of Pyridostigmine, or insufficient dosage of Prednisone), other neurological diseases, possible pneumonia due to her weak cough reflex or something else entirely. Given that the question emphasizes her medication, Pyridostigmine, and she shows signs of increased muscarinic activity (abdominal cramps, increased bowel sounds), we should prioritize the hypothesis that her symptoms might be due to Pyridostigmine overdose. CLINICAL REASONING: The first step involves understanding that the Myasthenia gravis is managed by medications that increase the availability of acetylcholine at the neuromuscular junction. Pyridostigmine, an acetylcholinesterase inhibitor, does this by preventing the enzyme acetylcholinesterase from breaking down acetylcholine, thereby increasing both its level and duration of action. A potential issue, therefore, is that if too much Pyridostigmine is administered, we can see an overactivity of acetylcholine both at nicotinic and muscarinic receptors. These can lead to excess muscle contractions and resultant fasciculations, and after persistent contractions, the patient might experience overall weakness. This could further explain the hypernasal voice, shallow respiration, and difficulty in generating negative inspiratory force - all signs of muscle weakness. Furthermore, over-stimulation of the muscarinic receptors could explain the patient's abdominal cramps and increased bowel sounds - classic signs of over-active parasympathetic activity, especially in the gastrointestinal tract. CONCLUDING: Pyridostigmine overdose would typically present with both muscarinic and nicotinic symptoms such as abdominal cramping, increased salivation, generalized weakness, and fasciculations. This fits our patient's picture adequately. Other options can produce similar symptoms, but given her medication history and symptom profile, Pyridostigmine overdose is more compelling. RESPONSE: The most likely cause of this patient's increased weakness, shortness of breath, abdominal cramping, and increased bowel sounds is Pyridostigmine overdose (Choice E).

**AI_B:** RESTATING THE QUESTION: A female patient with a history of myasthenia gravis, who is currently medicated with prednisone and pyridostigmine, is demonstrating significant symptoms including weakness, breathing issues, abdominal discomfort, and difficulties with speech and swallowing. Complementary to these are her physical findings which include shallow respirations, weakness in muscle as reflected by 4/5 diffusely and difficulty in generating a normal negative inspiratory force. She is also noted to have sluggish but symmetric deep tendon reflexes. We need to pin down what is likely causing her ongoing weakness from a given list of options including aspiration pneumonia, Guillain-Barré syndrome, an insufficient dosage of prednisone, motor neuron disease, or an overdose of pyridostigmine. PLANNING: I will construct my assumptions based on the fundamental characteristics of each of the potential causes. For instance, aspiration pneumonia often presents with abnormal lung findings; Guillain-Barré syndrome typically involves asymmetric reflexes and progressive weakness; insufficient dose of prednisone could worsen the patient's myasthenia gravis; motor neuron diseases typically present with muscle atrophy and weakness; and pyridostigmine overdose may cause excess acetylcholine, leading to an output more similar to cholinergic crisis. CLINICAL REASONING: In aspiration pneumonia, we usually find chest discomfort, fever, cough and expectoration; there would also be abnormal lung sounds, which is not the case with this patient. Guillain-Barré syndrome typically starts with weakness in the lower limbs and progresses upwards, the reflexes are often abolished, and there's usually a history of gastrointestinal or respiratory infection preceding the symptoms. This doesn't match with our patient's presentation. The patient's myasthenia gravis should have been controlled by prednisone, but it's worth considering if the dose might be insufficient. However, we should also remember worsening of symptoms could be due to a myasthenic crisis or cholinergic crisis. It is unlikely to be motor neuron disease as the onset is often slow and progressive and doesn't match the patient's rapid deterioration. Pyridostigmine overdose may induce a cholinergic crisis, which might present as muscle weakness, increased salivation, abdominal cramping, and features of parasympathetic over-activity like increased bowel sounds. CONCLUDING: Given the clinical features, it seems that this patient's symptomatology matches most closely with the signs of a cholinergic crisis secondary to possible Pyridostigmine overdose, for instance, the shallow respirations, soft and hypernasal voice, and increased bowels sounds, among others. Overnight, her myasthenia gravis has not likely changed to motor neuron disease. The signs are not pointing towards a Guillain-Barré syndrome or an aspiration pneumonia. It's worth remembering in clinical practice that a sort of dichotomy might be necessitated when it comes to assessing myasthenic versus cholinergic crisis; the key difference often lies in the observation of bowel or bladder incontinence, or significantly increased salivation, which tends to indicate the latter, as both conditions might present with increased weakness and breathing difficulty. RESPONSE: Hence, the most likely cause of this patient's weakness seems to be Pyridostigmine overdose (Option E).

**Facilitator** The consensus among the AI responses is that the most likely cause of the patient's weakness is a Pyridostigmine overdose (Option E). The patient's symptoms, including increased weakness, shortness of breath, abdominal cramping, and fasciculations, are characteristic of a cholinergic crisis, which can occur due to an overdose of Pyridostigmine. This medication is an acetylcholinesterase inhibitor used to manage Myasthenia Gravis by increasing the availability of acetylcholine at the neuromuscular junction. However, an overdose can lead to overactivity of acetylcholine at both nicotinic and muscarinic receptors, resulting in excessive muscle contractions and overactive parasympathetic activity. Other conditions such as Guillain-Barré syndrome, motor neuron disease, and aspiration pneumonia were considered but deemed less likely given the patient's symptom profile and medication history. Therefore, the patient's clinical indications suggest that her current manifestation is likely due to Pyridostigmine overdose, which would result in continuous, unregulated signaling at the neuromuscular junction, leading to the symptoms presented.
