# Supplementary material for: Collaborative intelligence in AI: Evaluating the performance of a council of AIs on the USMLE
Source: PLOS Digit Health. 2025 Oct 9;4(10):e0000787. doi: 10.1371/journal.pdig.0000787 (PMC12510544; doi:10.1371/journal.pdig.0000787)
Supplement: S2 File — (ZIP) [file pdig.0000787.s002.zip › S2 Data Raw data files containing unprocessed question inputs and LLM outputs including transcripts of deliberations/STEP1_Question-66-1_Type-MC-NJ.docx]

**Facilitator** **[Round 1]:** A 35-year-old woman comes to the office because she has had three first-trimester spontaneous abortions during the past 3 years. Physical examination shows no abnormalities. Laboratory studies show no endocrine abnormalities. Chromosomal analysis shows a paracentric inversion of the long arm of chromosome 1. Which of the following best describes this patient’s risk for early spontaneous abortions and a liveborn child with aneuploidy? (A) High Risk for Early Spontaneous Abortions: high Risk for Liveborn Child With Aneuploidy (B) High Risk for Early Spontaneous Abortions: low Risk for Liveborn Child With Aneuploidy (C) Low Risk for Early Spontaneous Abortions: high Risk for Liveborn Child With Aneuploidy (D) Low Risk for Early Spontaneous Abortions: low Risk for Liveborn Child With Aneuploidy

**AI_D:** RESTATING THE QUESTION: This inquiry is about a 35-year-old woman who has experienced three first-trimester spontaneous abortions (miscarriages) in the past three years. Her physical and endocrine examinations are reported normal. Still, a chromosomal analysis revealed a paracentric inversion of the long arm of chromosome 1. The task is to discern the patient's risk for early spontaneous abortions (miscarriages) and giving birth to a live child with aneuploidy (an abnormal number of chromosomes). PLANNING: This question relies on the understanding of chromosomal abnormalities, especially paracentric inversion, and their impact on pregnancy outcomes. Paracentric inversion is a form of chromosomal rearrangement where a segment of chromosome - not including the centromere - is reversed end to end. This inversion does not cause an increased risk for aneuploidy but can often lead to problems during meiosis and ultimately, increased risk of miscarriage. CLINICAL REASONING: This patient's paracentric inversion likely involves a nonessential gene, permitting her life without any apparent phenotypic anomalies. During meiosis, chromosomes pair along their entire length to facilitate genetic recombination. If this woman generates a gamete (egg) with the inverted segment recombined, it could result in duplication or deletion of genetic material in the fertilized eggs, leading to abnormal development and miscarriage. However, if she produces a gamete without recombination within the inverted segment, a normal chromosomal set will be expected, leading to chromosomally normal offspring. Hence, while the risk of miscarriages is increased, the risk of a liveborn child with aneuploidy remains low because an affected fetus typically does not survive. CONCLUDING: Given the clinical information, this woman has the potential to conceive a chromosomally normal offspring. Still, with the chromosomal anomaly, there exists a higher risk of miscarriages due to possible abnormal gametes resultant from abnormal chromosomal recombination within the inverted segment. However, should a fertilized egg possess the correct chromosomal number – potentially from a gamete without recombination within the inverted segment - the likelihood of live birth of a child with aneuploidy should remain low. RESPONSE: The best answer is (B) High Risk for Early Spontaneous Abortions: low Risk for Liveborn Child With Aneuploidy.

**AI_E:** RESTATING THE QUESTION: Our focal point is a 35-year-old woman who has experienced three first-trimester spontaneous abortions within the last three years. Upon detailed physical and laboratory examinations, there were no abnormal findings, and no endocrine issues were indicated. However, a chromosomal analysis revealed a paracentric inversion on the long arm of chromosome 1. We need to determine the potential risks this poses for the woman regarding future early spontaneous abortions, and the risk of her eventual child being born with aneuploidy. PLANNING: The key assumption here is that the chromosomal inversion is likely the cause of her recurrent spontaneous abortions. The genetic abnormalities can affect embryonic development and survival rate. Regarding aneuploidy risks for a liveborn child, it's necessary to understand the specific mechanics of paracentric inversion versus pericentric inversion. Paracentric inversion involves non-centromere chromosome segments and when affected alleles are involved in reproduction, homologous recombination during meiosis could lead to genetically nonviable gametes. CLINICAL REASONING: The first layer of reasoning revolves around understanding that a paracentric inversion in the chromosome can contribute to repeated early spontaneous abortions. This is believed to be due to the fact that such inversions can lead to the creation of genetically abnormal gametes during meiosis, which increases the risk of embryonic loss. For liveborn children, however, assuming proper embryonic development and survival through birth, the risk of aneuploidy (an abnormal number of chromosomes) is typically low with this type of chromosomal inversion. These inversions do not generally predispose to offspring's chromosomal imbalances since it doesn’t involve the centromere. CONCLUDING: Given her history of repeated early spontaneous abortions, the presence of a paracentric chromosomal inversion, and the general characteristics of such inversions, the conclusions could be the following: High risk for early spontaneous abortions and low risk for a liveborn child with aneuploidy. RESPONSE: Thus, the most clinically fitting choice, based on my clinical reasoning process, is option (B) High Risk for Early Spontaneous Abortions: low Risk for Liveborn Child With Aneuploidy.

**AI_B:** RESTATING THE QUESTION: The inquiry pertains to a 35-year-old woman who has experienced three first-trimester miscarriages over the last three years. The patient presents with no physical abnormalities or endocrine anomalies according to diagnostic assessments. Chromosomal examination has detected a paracentric inversion on the long arm of chromosome 1. With these details in mind, the aim is to correctly identify her risk for early spontaneous miscarriages and the probability of giving birth to a child with aneuploidy, a condition where there is an abnormal number of chromosomes. PLANNING: The information presented imposes three major assumptions: 1) The woman's recurrent miscarriages are attributed to the chromosomal inversion observed. 2) This chromosomal anomaly has also an effect on the probability of having a liveborn child with aneuploidy. 3) No other factors are contributing to her recurrent miscarriages and potential for a liveborn child with aneuploidy. An alternate assumption could be that even though the woman has a paracentric inversion, it might only be contributing to the risk of spontaneous abortions but not necessarily to the risk a liveborn child with aneuploidy. CLINICAL REASONING: A paracentric inversion is a type of chromosomal rearrangement that involves a segment of a chromosome which does not encompass the centromere. This condition can occur without causing any viable effects in carriers, but can lead to complications during meiosis and contribute to an increased risk for miscarriages. Nevertheless, most paracentric inversions lead to the production of abnormal gametes that are not likely to result in live born with aneuploidy due to loss of important genetic content. CONCLUDING: Therefore, upon connecting one sentence to the next with a clinical thought process, it is reasonable to suggest that this patient with the paracentric inversion on chromosome 1, who has already experienced spontaneous abortions, would have an increased risk for early pregnancy losses. However, her risk of giving birth to a live child with aneuploidy would likely be low due to the unviability of abnormal gametes produced as a result of this chromosomal inversion. RESPONSE: Hence, based on the available data and clinical reasoning, the most accurate description of this patient’s risk would be: (B) High Risk for Early Spontaneous Abortions: Low Risk for Liveborn Child with Aneuploidy.

**AI_A:** 1) RESTATING THE QUESTION: A 35-year-old female patient has experienced three miscarriages in the first trimester over the last three years. Noel abnormalities were found on physical examination or in laboratory studies examining her endocrine system. However, a chromosomal analysis revealed a paracentric inversion on the long arm of chromosome 1. Now the task at hand is to determine the patient's risk of experiencing further early spontaneous abortions and the chance of having a child with a chromosomal abnormality, referred to as aneuploidy. 2) PLANNING: The assumption in this situation is that her miscarriages could be related to the chromosomal abnormality detected - a paracentric inversion. Generally speaking, inversions do not cause any phenotypic effects in carriers because they involve no loss or gain of genetic material, but they can lead to increased risk of miscarriages and chromosomally abnormal offspring due to abnormal segregation during meiosis. It would be hypothesized that paracentric inversions may increase the risk for early miscarriages but have less risk for producing liveborns with aneuploidy. 3) CLINICAL REASONING: In the case of paracentric inversions, they do not usually disrupt the gene sequence, only the order of genes. During meiosis, chromosomes with a paracentric inversion often form a loop to align themselves with the normal homolog. This may lead to the formation of genetically unbalanced gametes if crossovers occur in the loop. When these unbalanced gametes are involved in conception, it may result in embryos with duplications and deletions of genetic material, leading to early pregnancy loss (miscarriages). However, viable liveborn children would generally be from conception events that did not involve crossovers, hence would not possess genetic imbalances (aneuploidies) caused by paracentric inversions. 4) CONCLUDING: From the established knowledge, it's clear that the patient's chromosomal aberration - a paracentric inversion - increases her likelihood of experiencing more early spontaneous abortions due to the potential formation of genetically unbalanced gametes from meiotic crossovers. However, the chance for a livebirth with aneuploidy is low, as viable pregnancies would likely only happen with balanced gametes that didn't undergo crossovers within the inversion loop. 5) RESPONSE: Therefore, the best choice that defines this patient’s risk for early spontaneous abortions and a liveborn child with aneuploidy is (B) High Risk for Early Spontaneous Abortions: low Risk for Liveborn Child With Aneuploidy.

**AI_C:** (1) RESTATING THE QUESTION: This case is about a woman in her mid-thirties who has experienced three first-trimester spontaneous abortions over the past few years. No abnormalities were detected during her physical examination or endocrine laboratory tests. However, her chromosomal analysis revealed a paracentric inversion in the long arm of her first chromosome. We are asked to consider the possible risks she faces in terms of early spontaneous abortions and the likelihood of her delivering a liveborn child affected by aneuploidy. Aneuploidy is a condition where the number of chromosomes in the offspring is not an exact multiple of the haploid number mainly due to errors in meiosis process. A paracentric inversion is a chromosomal rearrangement where a segment of a particular chromosome (excluding the centromere) has flipped and reinserted back into the chromosome in the reversed orientation. (2) PLANNING: We must first understand that the paracentric inversion mostly doesn't affect the gene number or function in the person who carries the inversion. However, potential difficulties may arise during meiosis when the chromosomes attempt to align and exchange information (recombination). One possibility is that a gamete could end up with a duplication or deletion of genes, leading to a chromosomally unbalanced state which could cause early spontaneous abortions as the embryo might not be viable. On the other hand, if the affected gamete with a balanced chromosomal content (i.e., no loss or extra genetic material) is fertilized, it can result in a viable birth but this newborn might possess the same inversion chromosomal structure. (3) CLINICAL REASONING: As per our discussion, there's definitively an increased risk of miscarriages in the early trimesters which aligns with her previous history of spontaneous abortions. These abortions can be attributable to the development of unbalanced gametes when meiotic recombination occurs within the inverted section of the chromosome. But, when it comes to assessing the risk for a newborn showing aneuploidy, it should be recognized that aneuploid conditions are generally a result of an improper distribution of chromosomes during cell division (either in meiosis or mitosis). If a gamete forms with a balanced genetic material and it gets fertilized, it would likely lead to a liveborn child with a normal or balanced chromosomal structure (including the same inversion). So, the risk of the child having aneuploidy, in this case, is relatively low since the paracentric inversion she has is less likely to lead to aneuploidy, per se. (4) CONCLUDING: In summary, While the woman's chromosomal inversion increases her risk of early spontaneous abortions due to the possibility of generating chromosomally-unbalanced gametes during meiosis, the risk of having a liveborn child with aneuploidy is relatively low as long as the fertilized gamete has balanced genetic content. (5) RESPONSE: Therefore, the best answer choice that matches the scenario outlined above is: (B) High Risk for Early Spontaneous Abortions: low Risk for Liveborn Child With Aneuploidy.

**Facilitator** The consensus statement synthesizes the responses of AI_A, AI_B, AI_C, AI_D, and AI_E as follows: The case in question involves a 35-year-old woman who has experienced three first-trimester spontaneous abortions over the past three years. Despite no abnormalities found in physical examination or endocrine laboratory studies, a chromosomal analysis revealed a paracentric inversion on the long arm of chromosome 1. The task is to determine the patient's risk for early spontaneous abortions and the likelihood of her having a liveborn child with aneuploidy, a condition characterized by an abnormal number of chromosomes. The consensus among the AI responses is that the woman's recurrent miscarriages could be attributed to the detected chromosomal abnormality - a paracentric inversion. This type of chromosomal rearrangement, which involves a segment of a chromosome excluding the centromere, does not typically disrupt the gene sequence or cause phenotypic effects in carriers. However, it can lead to complications during meiosis, potentially increasing the risk of miscarriages due to the formation of genetically unbalanced gametes if crossovers occur within the inversion loop. This could result in embryos with duplications and deletions of genetic material, leading to early pregnancy loss. On the other hand, the risk of having a liveborn child with aneuploidy is generally low in this case. Viable pregnancies would likely result from conception events that did not involve crossovers within the inversion loop, hence would not possess genetic imbalances caused by the paracentric inversion. Therefore, while the woman's chromosomal inversion increases her risk of early spontaneous abortions, the risk of having a liveborn child with aneuploidy remains low as long as the fertilized gamete has balanced genetic content. In conclusion, the consensus among the AI responses is that the most accurate description of this patient’s risk is (B) High Risk for Early Spontaneous Abortions: Low Risk for Liveborn Child with Aneuploidy.
