# Supplementary material for: Collaborative intelligence in AI: Evaluating the performance of a council of AIs on the USMLE
Source: PLOS Digit Health. 2025 Oct 9;4(10):e0000787. doi: 10.1371/journal.pdig.0000787 (PMC12510544; doi:10.1371/journal.pdig.0000787)
Supplement: S2 File — (ZIP) [file pdig.0000787.s002.zip › S2 Data Raw data files containing unprocessed question inputs and LLM outputs including transcripts of deliberations/STEP1_Question-67-1_Type-MC-NJ.docx]

**Facilitator** **[Round 1]:** A 12-year-old boy is brought to the office by his parents for genetic counseling because of a family history of genetic disorders. He has no symptoms or history of major medical illness. The patient’s maternal grandfather died of Huntington disease at the age of 35 years. Genetic testing of the mother showed 19 triplet repeats on each huntingtin (HTT) allele (N=10–20). The patient’s paternal grandfather had colon cancer at the age of 30 years, and the father had numerous colon polyps at the age of 20 years treated with a colectomy. Genetic testing of the father shows heterozygosity for a frame-shift mutation in the APC gene. Physical examination of the patient shows abnormalities. Based on this patient’s risk for genetic disease, it is most appropriate for the physician to order which of the following genetic tests at this time? (A) Analysis of triplet repeat sizes of HTT (B) Full sequencing and rearrangement analysis of APC (C) Full sequencing of HTT (D) Specific testing for the APC frame-shift mutation (E) It is not recommended to do predictive genetic testing on a minor

**AI_B:** (1) RESTATING THE QUESTION: This question assesses the most suitable genetic test to perform on a boy whose parents are bringing him in for genetic counseling. The boy's family history discloses a potential risk for Huntington disease from his mother's side and colon cancer from his father's side, based on their genetic makeup. The huntingtin (HTT) gene is associated with Huntington disease, with the abnormal number of triplet repeats (>26) known to cause the disease. His mother's HTT allele has 19 repeats, which is considered normal (10-20). On the father's side, a frameshift mutation in the APC gene is central to colon cancer, as it plays a role in familial adenomatous polyposis, a syndrome that could lead to colon cancer. Here the father is heterozygous for a frameshift APC mutation. (2) PLANNING: It's crucial to explore assumptions in the parent's genetic makeup and identify which ones may have direct implications for the patient. The mother so far does not show any mutation that could potentially pass on Huntington's disease. However, the father carries a frameshift mutation, which puts the boy at a 50% risk of inheriting this mutation. (3) CLINICAL REASONING: Although the boy's mother showed a normal range of triplet repeats in the HTT gene, Huntington's disease is often an inherited genetic mutation passed on from generation to generation. But his mother's confirmed testing result makes it unlikely to inherit the disease from her. On the other hand, his father's confirmed APC gene mutation increases the chance for him to inherit this mutation, subsequently increasing the risk for him to develop familial adenomatous polyposis or even colorectal cancer later in life. (4) CONCLUDING: Given the circumstances, it appears a gene test specialized for identifying the presence of the APC frameshift mutation, which the father carries, would be the most beneficial. It allows us to determine if this specific mutation has been inherited by the boy, which could provide significant insight for preventive measures or early-stage interventions. (5) RESPONSE: Based on each familial genetic risk factor and their inheritance patterns, the most appropriate genetic test to order in this boy would be (D) Specific testing for the APC frame-shift mutation.

**AI_D:** (1) RESTATING THE QUESTION: The physician is considering ordering a genetic test for a 12-year-old boy, based on his family's history of genetic disorders such as Huntington's disease and colon cancer. Huntington's disease, which the maternal grandfather had, is associated with huntingtin (HTT) gene and a specific type of mutation known as triplet repeats, with a normal range of 10-20 triplet repeats. The patient's mother has 19 triplet repeats. The paternal grandfather's colon cancer and the father's numerous colon polyps, which necessitated a colectomy, are related to mutations in the APC gene, particularly a frame-shift mutation that the father has. (2) PLANNING: We might assume both risk factors, the HTT and APC genes, have an equal chance to express in the child. However, it is essential to think about the nature of these diseases and the implications of testing. An alternative assumption could be to prioritize the disease that poses a higher immediate threat to the child's health. The reality of testing minors for such diseases that could not manifest until much later in life or have no treatments introduces numerous ethical and psychological considerations. (3) CLINICAL REASONING: Huntington's disease usually develops in adulthood, often not until the age of 30 or 40, and currently has no cure. Therefore, knowing about it at the age of 12 may cause unnecessary anxiety and concern. On the other hand, the existence of colorectal polyps (a precursor to cancer) in the father at the age of 20 years indicates that the disease related to the APC gene mutation can present itself at a much earlier age, which justifies the reasoning to prioritize testing for this gene. Additionally, unlike Huntington's disease, interventions such as routine colonoscopy, removal of polyps, and other preventative strategies can be used to effectively manage the risk of colon cancer. (4) CONCLUDING: Based on the above thought process, it would be more clinically beneficial and ethically responsible at this stage to conduct a genetic test that targets the disease with more immediate health implications and those where potential interventions and treatments exist. (5) RESPONSE: Hence, for this 12-year-old boy, it would be most appropriate for the physician to order an 'Analysis for the APC frame-shift mutation' (D) at this time.

**AI_C:** RESTATING THE QUESTION: This specific case presents a 12-year-old boy whose medical history seems benign but has a considerable family history of genetic disorders -- Huntington's disease on the maternal side, and colon cancer and polyposis on the paternal side. Hence, we are being asked to decide the most relevant genetic test to order for this boy to help assess his risk of developing genetic disease(s). PLANNING: We must consider the following interlocked suppositions: 1. The boy could have inherited the Huntington disease gene from his mother, despite her escaping the phenotype due to having a number of triplet repeats within the normal range. 2. The boy could have inherited the mutation in the APC gene from his father that might contribute to colon polyposis and, potentially, early-onset colon cancer. 3. The forthrightness of conducting predictive genetic testing on a minor. The best route toward a plausible conclusion, in this case, would be to assess the ethical considerations of genetic testing on a minor and then prioritize what test, if any, should be ordered in light of this patient's family history. CLINICAL REASONING: To start, it's worth mentioning that predictive genetic testing for adult-onset conditions, particularly non-treatable ones like Huntington Disease, is generally avoided in minors due to psychosocial implications and the principles of future autonomy. That is unless an intervention begun in childhood may reduce morbidity or mortality in adulthood. Similarly, testing for a condition like Familial Adenomatous Polyposis (FAP), caused by mutations in the APC gene and characterized by early colon polyps and cancer, can be beneficial because of potential early interventions like increased cancer surveillance or prophylactic surgeries. In this situation, it is alarming that the boy's father developed numerous colonic polyps at an unusually young age, so early detection through genetic testing can be advantageous in developing an individual management plan for the boy. CONCLUDING: As per the available evidence and considerations mentioned above, testing for the APC frame-shift mutation seems to be the most appropriate choice in this context, given early intervention potential, this might help reduce morbidity and possibly mortality as compared to Huntington's disease, increasing the overall quality of life. RESPONSE: Thus, the correct choice in this case would be (D) Specific testing for the APC frame-shift mutation, which could provide appropriate assessment and risk management for this boy considering his clinical and family history.

**AI_E:** (1) RESTATING THE QUESTION: The question is asking which genetic test should be ordered for a 12-year-old boy who has been brought in for genetic counseling due to a family history of genetic disorders that include Huntington's disease and colon cancer. The boy's mother carries the huntingtin (HTT) gene with 19 triplet repeats, which is within the normal range, while his father has a frame-shift mutation in the APC gene associated with colon cancer. The boy, although not symptomatic, exhibits some physical anomalies. (2) PLANNING: The question assumes that ordering a genetic test for the boy is essential due to the family history of inherited diseases. On one hand, the boy's maternal side carries the huntingtin (HTT) gene characteristic of Huntington's disease, but his mother's gene remains below the disease threshold. Alternatively, from his paternal side, he could have inherited a mutation in the APC gene, which is associated with a high risk of colon cancer. As the patient shows some physical abnormalities, another assumption here is that a genetic test could reveal the cause for these. Each assumption should be critically evaluated. (3) CLINICAL REASONING: In Huntington's disease, an expanded CAG triplet repeat (>35) in the HTT gene predicts disease onset, but the mother's 19 repeats is within the normal range implying she is not manifesting nor will pass on the disease. Now considering the APC gene mutation on the father's side, this mutation leads to disrupted control of cell growth that can result in numerous colon polyps and cancer, as seen in the boy's father and grandfather. However, the boy is asymptomatic in terms of gut health making it less likely for colonic polyposis. (4) CONCLUDING: A clinical thought process involves considering both the immediate health of the boy, and the long-term implications of knowledge about inherited disease states. By this analysis, the boy is more likely to have inherited the APC mutation than the HTT mutation. Testing for the APC mutation might reveal the cause of his physical anomalies and would also serve an important role in early surveillance and management for colon polyps and cancer. (5) RESPONSE: Therefore, based on the patient's risk for genetic disease and given the character of the inherited conditions within his family, the most appropriate genetic test for the physician to order at this time would be (D) Specific testing for the APC frame-shift mutation.

**AI_A:** (1) RESTATING THE QUESTION: The question raised concerns about a 12-year-old boy whose family has a significant history of genetic disorders. His maternal grandmother died from Huntington's disease (a neurodegenerative genetic disorder caused by an increase in CAG triplet repeats on the HTT gene) and his mother tested within the normal range of triplet repetitions. His paternal grandfather had colon cancer early in life, and his father showed evidence of multiple colon polyps, leading to surgical removal of the colon, and was found to have a frame-shift mutation in the Adenomatous Polyposis Coli (APC) gene, which is associated with familial adenomatous polyposis and colon cancer. Considering the boy's genetic risk in the context of these two genetic disorders, the question pondered what type of genetic testing would be best for him, given his family history. (2) PLANNING: The primary assumption here is that since the boy is in a family with a significant history of two different genetic disorders (Huntington's disease and familial adenomatous polyposis), he is potentially at risk for these diseases and therefore appropriate testing should be conducted. An alternative view could be prizing the ethical consideration of predictive genetic testing on a minor, based on the question of whether it is morally correct to test a child for adult-onset diseases, particularly when there is no current cure. With regards to clinical priority, we're led to consider which test would be of more significance to the boy's future health either by providing early detection or prevention. (3) CLINICAL REASONING: Huntington's disease is a trinucleotide repeat disorder that shows complete penetrance when a person has over 39 repeats. The boy's mother, however, only has 19 repeated triplets which falls within the normal range (10–20), suggesting that he might not be at risk. Conversely, the boy's father, who had colon polyps early in life leading to a colectomy, has been shown to have a frame-shift mutation in the APC gene. This mutation leads to the condition known as familial adenomatous polyposis. This condition put individuals at a very high risk of developing colorectal cancer early in life if untreated. This is evident in the boy's paternal family history. Precise intervention such as colectomy can prevent colon cancer in these patients. (4) CONCLUDING: Given the aplenty evidence of risk for familial adenomatous polyposis from the patient’s father’s side, including early-onset multiple polyps leading to colectomy and confirmation of a frame-shift mutation in APC gene in his father, it would be more clinically significant to investigate the presence of the identified APC mutation in the boy, as this would drastically influence potential clinical management and future disease prevention. (5) RESPONSE: Therefore, the most appropriate genetic test to order at this time for the patient would be (D) Specific testing for the APC frame-shift mutation.

**Facilitator** The consensus statement synthesizes the responses of AI_A, AI_B, AI_C, AI_D, and AI_E as follows: The question at hand involves determining the most appropriate genetic test for a 12-year-old boy with a significant family history of genetic disorders, specifically Huntington's disease on his mother's side and colon cancer on his father's side. The boy's mother has been tested and found to have 19 triplet repeats on each huntingtin (HTT) allele, which is within the normal range, thus making it unlikely for the boy to inherit Huntington's disease from her. On the other hand, his father has a frame-shift mutation in the APC gene, which is associated with familial adenomatous polyposis and colon cancer. This mutation puts the boy at a 50% risk of inheriting it, subsequently increasing his risk for developing these conditions later in life. While the ethical considerations of predictive genetic testing on a minor are acknowledged, the consensus leans towards the clinical significance of early detection and prevention. Given the boy's father's history of early-onset multiple colon polyps leading to colectomy and the confirmation of a frame-shift mutation in the APC gene, it is deemed more clinically significant to investigate the presence of this identified APC mutation in the boy. This information could drastically influence potential clinical management and future disease prevention, especially considering the potential for early interventions like increased cancer surveillance or prophylactic surgeries in the case of familial adenomatous polyposis. Therefore, the consensus among the responses is that the most appropriate genetic test to order at this time for the patient would be (D) Specific testing for the APC frame-shift mutation. This test could provide an appropriate assessment and risk management for the boy considering his clinical and family history.
