# Supplementary material for: Collaborative intelligence in AI: Evaluating the performance of a council of AIs on the USMLE
Source: PLOS Digit Health. 2025 Oct 9;4(10):e0000787. doi: 10.1371/journal.pdig.0000787 (PMC12510544; doi:10.1371/journal.pdig.0000787)
Supplement: S2 File — (ZIP) [file pdig.0000787.s002.zip › S2 Data Raw data files containing unprocessed question inputs and LLM outputs including transcripts of deliberations/STEP1_Question-69-1_Type-MC-NJ.docx]

**Facilitator** **[Round 1]:** A 25-year-old man comes to the office because of a 4-hour history of irritability, restlessness, tremor, and palpitations. He is a known user of amphetamines. His pulse is 120/min, respirations are 25/min, and blood pressure is 150/100 mm Hg. Physical examination shows no abnormalities. The most likely cause of this patient's symptoms is sympathomimetic activity arising from which of the following? (A) Decreased intracellular metabolism of biogenic amines (B) Decreased monoamine oxidase activity (C) Decreased presynaptic receptor activation (D) Increased intracellular metabolism of biogenic amines (E) Increased presynaptic receptor activation (F) Increased release of biogenic amines

**AI_A:** (1) RESTATING THE QUESTION: The young man's symptoms are likely due to excessive sympathetic activity, likely triggered by his reported use of amphetamines. We're asked to consider what mechanism underlies this overactivity. The listed responses involve various biochemical pathways that could facilitate hyperactivity of the sympathetic nervous system. The term "biogenic amines" refers to a class of neurotransmitters that includes dopamine, serotonin, and norepinephrine, among others. (2) PLANNING: The central task here is to determine whether his symptoms arise from decreased metabolism or diminished activity of specific enzymes (options A and B), decreased stimulation of presynaptic receptors (option C), increased metabolism of biogenic amines (option D), increased activation of presynaptic receptors (option E), or increased release of biogenic amines (option F). We know that amphetamines have a stimulating effect on the nervous system, specifically by increasing the release and preventing the reuptake of biogenic amines. (3) CLINICAL REASONING: If we consider how amphetamines work, we find that they increase the release of biogenic amines while also inhibiting their reuptake. This effect would lead to increased sympathetic activity, consistent with the patient's symptoms. If the metabolism of biogenic amines were decreased (options A and B), this would also increase their concentration, but it is not the primary mechanism of action of amphetamines. Similarly, while decreased presynaptic receptor activation (option C) would make neurons less responsive to the biogenic amines, this would presumably lead to less, not more, sympathetic activity. The same goes for increased metabolism of biogenic amines (option D), which would reduce their concentration. Increased presynaptic receptor activation (option E) could potentially explain the patient's symptoms, however, this is not the known mechanism of action of amphetamines. (4) CONCLUDING: Given the known pharmacological action of amphetamines, the most likely cause of this patient’s symptoms is consistent with increased release of biogenic amines. (5) RESPONSE: This patient's symptoms suggest amphetamine-induced sympathomimetic activity, most likely arising from increased release of biogenic amines (F).

**AI_B:** The question essentially asks, "What could be the pathway that led to the effects of amphetamines in the patient, considering his symptoms of irritability, restlessness, tremor, and palpitations along with vital sign abnormalities?" Biogenic amines refer to certain types of neurotransmitters including catecholamines (epinephrine, norepinephrine) and monoamine oxidase (MAO) is an enzyme that metabolizes these neurotransmitters. Presynaptic receptors typically control the release of neurotransmitters into the synaptic cleft, and their activation or deactivation impact the amount of neurotransmitter available to bind to postsynaptic receptors. The considerations we are given including decreased or increased metabolism of biogenic amines, decreased MAO activity, decreased or increased presynaptic receptor activation, and increased release of biogenic amines. We could consider the effect of each of these alterations and their potential impact on the patient's symptoms. Amphetamines are known to cause stimulative effects on the body, primarily on the cardiovascular and nervous systems. They act primarily as sympathomimetics, meaning they stimulate the sympathetic nervous system ('fight or flight' response). One way they do this is by increasing the release of biogenic amines, such as norepinephrine and dopamine, in the synaptic cleft. This would result in an increased sympathetic activity which matches the symptoms seen in the patient. Decreased metabolism (whether intracellular or by MAO) might also increase available neurotransmitters, but given the acute onset of symptoms (which fits amphetamine use better than changes in long-term metabolic processes), and the patient's known usage of amphetamines, it's more likely that they're experiencing an acute increase in biogenic amine release. The patient's tachycardia, hypertension, and other symptoms reflect an overactivity of the sympathetic nervous system, likely stemming from an increased availability of neurotransmitters. This leads us to hypothesize that options A, B, and C are less likely because decreased metabolism or receptor activation would reduce the availability of neurotransmitters, not increase it. Clinical reasoning connects these ideas to propose that the most probable cause of the patient's symptoms is the increased release of neurotransmitters (biogenic amines), which is congruent with the known actions of amphetamines. Given the comprehensive analysis above, it can be concluded that (F) Increased release of biogenic amines is the most likely cause of this patient's symptoms due to sympathomimetic activity.

**AI_D:** (1) RESTATING THE QUESTION: The given patient is presenting with symptoms typically associated with sympathetic stimulation, including irritability, restlessness, tremor, tachycardia (increased pulse), tachypnea (increased breathing rate), and hypertension (elevated blood pressure). These signs and symptoms can be traced back to the increased activity of certain neurotransmitters collectively known as 'biogenic amines' (like epinephrine, norepinephrine, dopamine etc.). The question is focusing on the possible mechanisms responsible for these symptoms, associated with amphetamine use. (2) PLANNING: It is well established that amphetamines act by increasing the amount of biogenic amines available in the synaptic cleft. How do they accomplish this? They achieve this either by enhancing release, blocking reuptake, or inhibiting the metabolic degradation of these biogenic amines. Let's evaluate each of these possibilities in the context of the answer choices provided. (3) CLINICAL REASONING: Although (A) Decreased intracellular metabolism and (B) Decreased monoamine oxidase activity would increase the lifespan of biogenic amines, the overall concentrations would only sustain high levels if management of these amines in the synaptic cleft is compromised as well. Meanwhile, (C) Decreased presynaptic receptor activation could potentially increase the release of biogenic amines, but amphetamines do not primarily function via this mechanism. Conversely, enhancement of release by (E) Increased presynaptic receptor activation would likely be offset by standard breakdown pathways. Finally, (D) Increased intracellular metabolism of biogenic amines, eventually tends to decrease the concentration of these neurotransmitters, which contradicts with the features of this case. However, if the amphetamines led to an (F) Increased release of biogenic amines, this would result in an overall higher concentration in the synaptic cleft and sustain a sympathomimetic state, aligning with our patient's presentation. (4) CONCLUDING: Amphetamines primarily exert their sympathetic effects by increasing the release of biogenic amines. Despite the possibility of altered metabolism or receptor activity, the large increase in release best explains the prolonged sympathomimetic state experienced by our patient. (5) RESPONSE: Therefore, the most likely cause of this patient's symptoms is sympathomimetic activity arising from (F) Increased release of biogenic amines.

**AI_C:** 1) RESTATING THE QUESTION: This question is about a 25-year-old male who presented to the clinic with a short history of irritability, restlessness, tremors, and palpitations. He has a known history of amphetamine use. His vital signs show tachycardia, tachypnea, and hypertension, all of which are symptoms of a sympathetic nervous system response. The question is asking which underlying mechanism is likely responsible for the excessive sympathomimetic activity observed in this patient. 2) PLANNING: To better understand this situation, it is important to consider the mechanisms of amphetamine action in the body. Amphetamines primarily influence the synaptic activity of biogenic amines, particularly monoamines such as dopamine, norepinephrine, and serotonin. These effects can occur through several mechanisms: modulation of intracellular metabolism, alteration in monoamine oxidase activity, changes in presynaptic receptor activation, and changes in the release of these neurotransmitters. 3) CLINICAL REASONING: Amphetamines are known to increase the release of biogenic amine neurotransmitters and decrease their reuptake, leading to higher levels of these neurotransmitters in the synaptic cleft. This, in turn, stimulates the sympathetic nervous system, which can explain the clinical presentation of restlessness, tremor, and palpitations due to increased heart rate (tachycardia) and high blood pressure (hypertension). Furthermore, decreased monoamine oxidase activity resulting from amphetamine use would lead to an increase, rather than a decrease, in biogenic amine levels. However, excessive sympathomimetic activity from the use of amphetamines would be related to the disruption of reuptake and the increased release of these neurotransmitters rather than just the inhibition of monoamine oxidase. 4) CONCLUDING: Taking these considerations into account, it becomes apparent that the most probable scenario in the context of amphetamine use would be related to the increased release of biogenic amines into the synaptic cleft, which would intensify postsynaptic receptor stimulation and lead to an increased sympathetic response. 5) RESPONSE: Therefore, based on the clinical reasoning, the most likely cause of the patient's symptoms of irritability, restlessness, tremor, and palpitations is due to the sympathomimetic activity stemming from the increased release of biogenic amines (F).

**AI_E:** RESTATING THE QUESTION: The question asks about a 25-year-old man with symptoms such as irritability, restlessness, tremors, and a fast rate of heartbeat, who also has a high breathing rate and elevated blood pressure. These symptoms are characteristic of heightened sympathomimetic activity, a system responsible for our body's "fight or flight" responses. He is known to use amphetamines, a powerful stimulant that can significantly increase sympathomimetic activity. Biogenic amines are important neurotransmitters in this system, and abnormalities in their production, release, and metabolism can lead to varied clinical manifestations. The question wants me to identify which factor amidst given choices could most likely be contributing to his symptoms. PLANNING: I need to make some assumptions to identify the most likely underlying mechanism. The alternatives include changes in intracellular metabolism, enzyme activity, receptor activation, and release of biogenic amines. I'll examine each of the given options, hypothesize their consequences under normal and abnormal conditions, and then clinically prioritize which assumption seems the most plausible in this scenario. CLINICAL REASONING: Starting with decreased intracellular metabolism of biogenic amines, it's reasonable to suggest that if metabolism is reduced, more amines will be available in the synaptic cleft for receptor activation, potentially provoking increased sympathomimetic activity. However, this doesn't take into account amphetamine use, which is known to affect the release of biogenic amines, not their metabolism. The same thought process can be applied to decreased monoamine oxidase activity. Monoamine oxidase is an enzyme that metabolizes amines. Decreased activity would increase amine availability but doesn't directly tie in amphetamine use. Decreased presynaptic receptor activation would potentially cause less release of neurotransmitters because presynaptic receptors typically modulate neurotransmitter release. But, this doesn't typically result in overactivation of the sympathetic system. Increased intracellular metabolism of biogenic amines would mostly lead to a decrease in neurotransmitter availability, opposing the pathophysiology expected in this scenario. Increasing presynaptic receptor activation can increase neurotransmitter release and heighten sympathomimetic responses, but generally, amphetamines don't work directly at this level. Finally, increased release of biogenic amines fits well with the profile of amphetamine action. Amphetamines are known to cause the release of biogenic amines like norepinephrine, dopamine, and serotonin resulting in heightened sympathomimetic activity. CONCLUDING: Upon reflection, based on the pharmacology of amphetamines and the patient's symptoms it seems reasonable to conclude that increased release of biogenic amines resultant from amphetamine use constitutes the best alternate assumption to pursue. RESPONSE: The most likely cause of this patient's symptoms is sympathomimetic activity arising from (F) 'Increased release of biogenic amines'.

**Facilitator** The question presents a 25-year-old man with symptoms of irritability, restlessness, tremor, and palpitations, along with tachycardia, tachypnea, and hypertension. These symptoms are indicative of heightened sympathetic nervous system activity, likely due to his known use of amphetamines. The task is to identify the most probable mechanism underlying this overactivity from the given options, which involve various biochemical pathways related to biogenic amines, a class of neurotransmitters that includes dopamine, serotonin, and norepinephrine. Amphetamines are known to stimulate the nervous system, primarily by increasing the release and preventing the reuptake of biogenic amines. This effect would lead to increased sympathetic activity, consistent with the patient's symptoms. While decreased metabolism or diminished activity of specific enzymes (options A and B) would also increase the concentration of biogenic amines, this is not the primary mechanism of action of amphetamines. Similarly, decreased presynaptic receptor activation (option C) would make neurons less responsive to the biogenic amines, leading to less, not more, sympathetic activity. Increased metabolism of biogenic amines (option D) would reduce their concentration, contradicting the patient's symptoms. Increased presynaptic receptor activation (option E) could potentially explain the patient's symptoms, but this is not the known mechanism of action of amphetamines. Therefore, based on the known pharmacological action of amphetamines and the patient's symptoms, the consensus among the AI responses is that the most likely cause of this patient's symptoms is the increased release of biogenic amines (option F). This mechanism aligns with the known actions of amphetamines, fits the acute onset of symptoms, and explains the overactivity of the sympathetic nervous system observed in the patient.
