# Supplementary material for: Collaborative intelligence in AI: Evaluating the performance of a council of AIs on the USMLE
Source: PLOS Digit Health. 2025 Oct 9;4(10):e0000787. doi: 10.1371/journal.pdig.0000787 (PMC12510544; doi:10.1371/journal.pdig.0000787)
Supplement: S2 File — (ZIP) [file pdig.0000787.s002.zip › S2 Data Raw data files containing unprocessed question inputs and LLM outputs including transcripts of deliberations/STEP1_Question-70-1_Type-MC-NJ.docx]

**Facilitator** **[Round 1]:** A 5-year-old girl is brought to the office by her mother because of a 6-hour history of bloody diarrhea. She is interactive and in no acute distress. Her blood pressure is 90/55 mm Hg. Abdominal examination shows normoactive bowel sounds. Stool cultures are obtained, and the patient's mother is advised to give the girl plenty of fluids. Five days later, the patient develops decreased urine output and is brought back to the office. Her blood pressure is now 135/88 mm Hg. Physical examination shows pallor. Laboratory studies show: Hemoglobin 8.5 g/dL (N=11–15) Hematocrit 26% (N=28%–45%) Platelet count 45,000/mm³ (N=150,000–400,000) Serum creatinine 3.3 mg/dL (N=0.3–0.7) Which of the following infectious agents is the most likely cause of these findings? (A) Campylobacter jejuni (B) Escherichia coli (C) Rotavirus (D) Salmonella enterica serovar enteritidis (E) Yersinia pestis

**AI_C:** (1) RESTATING THE QUESTION: The question is asking the most likely infectious agent that caused the patient's symptoms and lab findings. The patient, a 5-year-old girl, presented with initial symptoms of bloody diarrhea. After 5 days, she developed decreased urine output and hypertension. Her labs also indicate anemia, thrombocytopenia, and increased serum creatinine suggesting a possible hemolytic uremic syndrome due to an infection. (2) PLANNING: The infectious agents provided as options can cause gastrointestinal symptoms, however, the one associated with bloody diarrhea and hemolytic uremic syndrome (HUS), as assumed in this case, should be chosen. HUS comprises of microangiopathic hemolytic anemia, thrombocytopenia, and acute kidney injury which correlate with the lab results. E.coli, particularly E.coli O157:H7 strain, has been commonly associated with HUS. (3) CLINICAL REASONING: The patient's progression of symptoms from bloody diarrhea to decreased urine output, hypertension, and abnormal lab results is potentially indicative of HUS secondary to a gastrointestinal bacterial infection. In context, E.coli O157:H7 specifically has elements known as Shiga-like toxins that injure endothelial cells in the glomeruli leading to platelet activation, thrombosis & eventual microangiopathic hemolytic anemia, thrombocytopenia along with acute kidney injury. (4) CONCLUDING: E.coli exhibits pathogenesis that aligns with the patient's clinical presentation and abnormal lab findings. While other infectious agents can cause gastrointestinal symptoms, none are as strongly associated with bloody diarrhea and HUS as E.coli O157:H7 strain. (5) RESPONSE: Hence, the most likely causative infectious agent in this case is Escherichia coli (Option B).

**AI_E:** RESTATING THE QUESTION: The question is asking for the most likely causative infectious agent in a 5-year-old girl who initially presented with bloody diarrhea and has subsequently developed decrease in urine output, increased blood pressure, pallor, and abnormal laboratory findings including decreased hemoglobin, hematocrit, platelet count, and increased serum creatinine. The choices given are all potential infectious agents with gastrointestinal manifestation. PLANNING: The initial presentation of bloody diarrhea, followed by a subsequent development of decreased urine output, increased blood pressure, pallor, and laboratory results indicative of anemia, thrombocytopenia and kidney damage, suggests a syndrome known as hemolytic uremic syndrome (HUS). HUS is typically triggered by an infection with a specific form of the bacterium Escherichia coli known as E. coli O157:H7. CLINICAL REASONING: The girl’s initial symptom of bloody diarrhea is consistent with an infection of the gastrointestinal tract. The subsequent developments of decreased urine output, high blood pressure, pallor, and the laboratory findings are consistent with Hemolytic Uremic Syndrome (HUS), which is characterized by hemolytic anemia, thrombocytopenia (low platelet count), and acute renal injury. Among the choices given, the bacterium most commonly associated with HUS is Escherichia coli O157:H7, a strain of E. coli that produces a Shiga toxin. The other pathogens listed may cause gastroenteritis, but they are far less likely to cause HUS. CONCLUDING: Given the patient's presentation and laboratory results alongside the typical pathogenesis and pathophysiology of Hemolytic Uremic Syndrome, we can conclude that the bacterium Escherichia coli is the most likely causative agent of these symptoms. RESPONSE: The most likely cause of these findings is (B) Escherichia coli.

**AI_A:** RESTATING THE QUESTION: A 5-year-old girl who previously presented with bloody diarrhea and was advised to maintain hydration has now returned with decreased urine output, high blood pressure, and signs of anemia plus a decreased platelet count. These symptoms along with increased serum creatinine levels suggest kidney involvement. We've been asked to identify the probable infectious agent from a list that includes Campylobacter jejuni, Escherichia coli, Rotavirus, Salmonella enterica serovar enteritidis, and Yersinia pestis. PLANNING: This scenario suggests a condition named Hemolytic Uremic Syndrome (HUS), which is commonly preceded by a diarrheal illness. HUS causes hemolysis, thrombocytopenia, and acute kidney injury. Among different infectious agents causing diarrhea, the most common organisms associated with post-diarrheal HUS are Shiga toxin-producing strains of Escherichia coli. CLINICAL REASONING: Now let's examine the patient's symptoms in a step-by-step manner. Firstly, the girl had bloody diarrhea which may signal an infection by an organism that can cause damage to the intestinal wall. Her recent urine output reduction and increased creatinine levels indicate acute kidney injury. Moreover, the presence of pallor, low hemoglobin, and hematocrit reveals anemia, and the reduced platelet count implies thrombocytopenia. Such a combined presentation forms the triad of HUS. Shiga toxin-producing Escherichia coli (STEC) commonly precedes the development of HUS. CONCLUDING: Shiga toxin from STEC bacterium can disrupt protein synthesis in cells lining the capillaries in the kidney and the gastrointestinal tract, leading to cell death and exposure of thrombogenic subendothelium. This sequence of events subsequently results in platelet aggregation and thrombosis, reduced platelet count, hemolysis (leading to anemia) and can eventually cause acute kidney injury. RESPONSE: Considering the presented signs, symptoms, and laboratory findings, and correlating them with the medical pathology, the most likely causative agent in this case is (B) Escherichia coli.

**AI_B:** RESTATING THE QUESTION: The query is about a 5-year-old girl with an initial presentation of bloody diarrhea and later showing decreased urine output, hypertension, pallor, anemia, thrombocytopenia, and elevated creatinine levels. The purpose is to identify the most probable infectious agent causing these symptoms and lab findings. PLANNING: The enumerated symptoms point towards Hemolytic Uremic Syndrome (HUS), a triad of microangiopathic hemolytic anemia, thrombocytopenia, and acute kidney injury. Each assumption will suggest a distinct causative infectious organism, however, the symptom complex is most often associated with a certain strain of E. coli. But, it is still imperative to review all hypotheses and assumptions. CLINICAL REASONING: Starting from the top: A. Campylobacter jejuni: This bacterium can lead to Gastroenteritis and is a common cause of Guillain-Barré Syndrome, but it's not typically seen in patients with HUS. B. Escherichia coli: Particularly, the O157: H7 strain of E.coli is a common cause for HUS seen in children after episodes of bloody diarrhea. This is backed up by epidemiological studies and clinical evidence. C. Rotavirus: Although rotavirus can cause serious diarrhea, it typically doesn't result in HUS. D. Salmonella: Salmonella enteritidis can cause Gastroenteritis, Typhoid fever and non-typhoidal Salmonellosis but rarely leads to HUS. E. Yersinia pestis: This is the causative agent of plague and does not typically present as HUS. Considering these findings, a number of assumptions can be made but, Escherichia coli especially strain O157:H7 seems to account for both the bloody diarrhea and then the HUS, utilizing the toxin Shiga that can lead to the illness in question. CONCLUDING: Judging from the clinical history, signs and symptoms, and laboratory investigations the young girl appears to be suffering from Hemolytic Uremic Syndrome caused by an infection with Escherichia coli. RESPONSE: The most likely cause of these findings in the 5-year-old girl is (B) Escherichia coli.

**AI_D:** RESTATING THE QUESTION: This case involves a young girl who initially presented with symptoms of a gastrointestinal infection, namely, bloody diarrhea. After several days, her condition evolved into a state suggestive of kidney dysfunction. The clinical cues here are her decreased urine output, increased blood pressure, and the elevated serum creatinine level, all of which are indicators of renal malfunction. Her lab results also reveal a decreased level of hemoglobin, hematocrit, and platelets, indicating possible complications related to blood cells. The question seeks to identify which infectious agent in the provided list might have resulted in these symptoms and findings. PLANNING: Each organism listed is capable of causing gastrointestinal issues. However, we must determine which can potentially lead to the combination of symptoms and laboratory abnormalities seen in this case. The assumptions under examination here relate to the pathological signatures of each listed pathogen. Hemolytic-uremic syndrome (HUS) is a condition worth considering as it presents with symptoms of hemolytic anemia (low hemoglobin and hematocrit), thrombocytopenia (low platelet count), and renal injury (increased serum creatinine, hypertension, oliguria), akin to those seen in this patient. HUS is often associated with a particular serotype of Escherichia coli, E. coli O157:H7. CLINICAL REASONING: HUS typically begins with a prodromal phase demonstrating symptoms of acute gastroenteritis with bloody diarrhea, which is often caused by E. coli O157:H7. The other organisms on the list (Campylobacter jejuni, Rotavirus, Salmonella enterica serovar enteritidis, and Yersinia pestis), while they are indeed capable of causing gastroenteritis with diarrhea, are not typically associated with the development of HUS. CONCLUDING: Given the patient’s clinical presentation and subsequent development of renal impairment and hematological abnormalities, our line of reasoning points us towards a diagnosis of Hemolytic Uremic Syndrome (HUS). This syndrome is typically associated with an infection caused by a specific serotype of Escherichia coli (E. coli O157:H7), which is known to produce Shiga toxins that play a crucial role in the pathogenesis of HUS. RESPONSE: Based on the clinical course and the laboratory findings, the infectious agent most likely responsible for this clinical presentation is (B) Escherichia coli.

**Facilitator** The consensus statement synthesizes the responses of AI_A, AI_B, AI_C, AI_D, and AI_E as follows: The case involves a 5-year-old girl who initially presented with bloody diarrhea and later developed decreased urine output, high blood pressure, pallor, and abnormal laboratory findings indicative of anemia, thrombocytopenia, and increased serum creatinine. These symptoms and lab results suggest a condition known as Hemolytic Uremic Syndrome (HUS), characterized by a triad of microangiopathic hemolytic anemia, thrombocytopenia, and acute kidney injury. The infectious agents provided as options can all cause gastrointestinal symptoms, but the one associated with bloody diarrhea and HUS should be chosen. Among the choices given, Escherichia coli, particularly the O157:H7 strain, is most commonly associated with HUS. This strain of E.coli produces Shiga-like toxins that injure endothelial cells in the glomeruli, leading to platelet activation, thrombosis, and eventual microangiopathic hemolytic anemia, thrombocytopenia, and acute kidney injury. While other infectious agents like Campylobacter jejuni, Rotavirus, Salmonella enterica serovar enteritidis, and Yersinia pestis can cause gastroenteritis, they are not typically associated with the development of HUS. Therefore, considering the patient's clinical presentation, the progression of symptoms, and the laboratory findings, the consensus is that the most likely causative infectious agent in this case is Escherichia coli (Option B).
