# Supplementary material for: Collaborative intelligence in AI: Evaluating the performance of a council of AIs on the USMLE
Source: PLOS Digit Health. 2025 Oct 9;4(10):e0000787. doi: 10.1371/journal.pdig.0000787 (PMC12510544; doi:10.1371/journal.pdig.0000787)
Supplement: S2 File — (ZIP) [file pdig.0000787.s002.zip › S2 Data Raw data files containing unprocessed question inputs and LLM outputs including transcripts of deliberations/STEP1_Question-71-1_Type-MC-NJ.docx]

**Facilitator** **[Round 1]:** A 78-year-old woman is admitted to the hospital because of a 1-week history of jaundice. CT scan of the abdomen shows a mass suggestive of pancreatic cancer. Three hours later, the on-call physician enters the patient's room to discuss the prognosis and obtain consent for a biopsy scheduled for the next morning. On entering the room, the physician greets the patient and her husband. The physician then learns that the patient speaks only Mandarin. Her husband is fluent in Mandarin and English. The hospital interpreter is not available until tomorrow morning. The patient's husband appears anxious and insists that the physician speaks to him and allows him to serve as an interpreter for his wife. Which of the following is the most appropriate next step in management? (A) Allow the patient's husband to serve as an interpreter (B) Consult the hospital ethics committee (C) Explain to the husband that information cannot be provided until the hospital interpreter arrives in the morning (D) Use a telephone interpreter service

**AI_B:** RESTATING THE QUESTION: This scenario depicts a clinical situation where the treating physician must provide critical information and obtain informed consent from a patient with a likely diagnosis of pancreatic cancer. However, there is a language barrier as the patient speaks only Mandarin. A professional hospital interpreter is unavailable until the next day, and the patient's husband has offered to translate. The question asks us to determine the most suitable course of action in this situation. PLANNING: The assumptions here are that the patient's husband could accurately and objectively interpret medical information or that waiting until the next day when the hospital interpreter is available is the best option. An alternate assumption could be the use of a telephone interpreter service or referral to the hospital ethics committee. In the context of a patient's right to full and accurate information, we must clinically prioritize assuming the responsibility of ensuring that the patient gets correct and unbiased medical information. CLINICAL REASONING: Allowing the patient's husband to serve as an interpreter may introduce biases or inaccuracies in the transmission of medical information. Although inviting the hospital ethics committee seems reasonable, this process may be time-consuming and potentially delay crucial medical procedures. Concerning the option of waiting until the hospital interpreter arrives the next morning, it may leave the patient uninformed and anxious overnight. On the other hand, using a telephone interpreter service could provide a good bridge for effective communication. Such services deliver professional interpretation, helping to maintain patient autonomy and their right to make informed decisions. CONCLUDING: Therefore, in such a situation where timely access to professional and unbiased interpretation facilities is critical, resorting to a telephone interpreting service should be the best course of action. RESPONSE: So, the most appropriate next step in management would be (D) Use a telephone interpreter service.

**AI_C:** RESTATING THE QUESTION: A 78-year-old woman with a suspected pancreatic mass is in a hospital, and the physician needs to discuss prognosis and obtain the woman's consent for a scheduled biopsy. However, the elderly woman understands only Mandarin, and her English-Mandarin speaking husband wishes to provide interpretation. In this scenario, it's asked what is the most suitable next step for the physician to maintain proper communication and continue with the management. PLANNING: The assumption here is that effective and clear communication is crucial in relaying information about prognosis and obtaining informed consent, and this can only be provided if the language barrier is overcome. Several alternative assumptions have been proposed, such as allowing a relative to provide interpretation, consulting the hospital ethics committee, waiting for a professional interpreter, or using a telephonic interpreter service. After evaluating each assumption, it seems clinically prudent to first and foremost ensure effective patient-doctor communication, which could result from immediate interpretation assistance. CLINICAL REASONING: While it may seem reasonable for the husband to act as an interpreter, there are numerous disadvantages to utilizing untrained interpreters - emotional involvement may lead to distortion or withholding of information, medical terminology comprehension is not guaranteed, and confidentiality could be compromised. Waiting for the hospital interpreter to be available might result in unnecessary anxiety and delays in consent and management. On the other hand, involving the hospital ethics committee may be more appropriate for ongoing complex decision-making situations rather than immediate language barrier issues. As such, employing a telephonic interpreter service would provide immediate access to trained, professional interpreters uninvolved in the patient’s care, potentially leading to a more objective and effective communication avenue. CONCLUDING: For this particular case, the most appropriate next step is to utilize a telephone interpreter service. This decision is based on the immediate necessity of addressing the patient's language barrier to discuss her prognosis and secure informed consent for her biopsy. RESPONSE: (D) Use a telephone interpreter service.

**AI_D:** RESTATING THE QUESTION: The situation we're discussing here revolves around a 78-year-old woman who has been diagnosed with findings implying potential pancreatic cancer. The physician is required to communicate this prognosis and obtain consent for a biopsy from the patient, who unfortunately, only communicates in Mandarin. Her English-speaking husband is willing to act as the interpreter, however, the hospital's actual interpreter is unavailable until the next morning. The query we're handling is what should be the most appropriate next step in dealing with this scenario: whether to allow her husband to interpret, consult the ethics committee, wait for the hospital interpreter tomorrow, or use a telephone interpreter service. PLANNING: The key considerations here would revolve around the necessity of clear and accurate communication to the patient, the urgency with which the consent is needed, the implications of language mismatches, ethical considerations of patient confidentality, and the practicality of waiting for the arrival of the hospital interpreter or use of an alternative interpretation service. CLINICAL REASONING: In clinical practice, accurate communication and comprehension are key in delivering health information and requiring consent for interventions. We have an underlying assumption that the husband's anxiety may affect his ability to accurately interpret the medical information to his wife, which may lead to misunderstanding. As patient confidentiality is highly valued, having a family member as an interpreter may breach this principle. While waiting for the hospital interpreter could eliminate this possibility, we must consider the schedule of the diagnosis process. Thus, considering language accuracy and ethical clauses, it seems reasonable to hypothesize that using a telephone interpreter service could suffice as the most convenient and efficient solution. CONCLUDING: In summary, to balance both the ethical requirements and the need for urgency in the case at hand, we must find an intervention that provides an immediate, accurate and unbiased interpretation. If the husband is allowed to interpret, we could risk the patient's confidentiality and skew the message due to his anxiety. On the other hand, waiting for the hospital interpreter might delay the crucial biopsy procedure. Consultation with the ethics committee might not provide an immediate solution given the strict timeline. RESPONSE: Therefore, considering the need for urgent, clear, and accurate communication that also respects the patient's confidentiality: (D) the use of a telephone interpretation service appears to be the most appropriate next step in this situation.

**AI_A:** RESTATING THE QUESTION: The predicament in this case involves an elderly female patient suffering from pancreatic cancer who only speaks Mandarin. Even though her husband is bilingual, the issue is whether it's suitable to use him as the interpreter for medical discussions. The choices are: (A) allowing the husband to act as an interpreter, (B) consultation with the hospital's ethics committee, (C) postponing giving information until the arrival of the hospital interpreter, or (D) using a telephone interpreting service. PLANNING: The assumptions here are that the husband can adequately translate complex medical jargon and emotions may not interfere with communication. However, one can also question these assumptions. Technical language can be hard to translate without proper training, and emotions could cloud his interpretation. Also, confidentiality and impartiality might be compromised. Therefore, the initial assumption may be incorrect. With patient understanding and communication playing a critical role in the consent process, perhaps seeking a professional interpreter is the optimal assumption to follow. CLINICAL REASONING: Medical interpretation is more than just language translation. It demands understanding, conveying the right tone, maintaining confidentiality and being neutral, things that might be challenging for a distraught family member. The husband might struggle to accurately translate medical terminologies or could unintentionally alter information due to anxiety or a desire to protect his wife. A professional medical interpreter is trained to handle these complexities, facilitating unbiased and precise communication. Thus, relying on the patient's husband might pose a risk to effective communication and patient consent. CONCLUDING: Given the importance of precise communication, especially where clinical prognosis and consent are concerned, it's important to ensure that doctors and patients understand each other fully. When a professional, hospital-based interpreter isn't immediately available, utilizing resources such as a telephone interpreter service is a more appropriate action plan than relying on a family member for interpretation. RESPONSE: Therefore, the most appropriate next step in management in this case would be (D) Use a telephone interpreter service. This ensures that the patient receives accurate information and can give informed consent for the pending biopsy.

**AI_E:** (1) RESTATING THE QUESTION: This scenario refers to a 78-year-old woman who's been admitted to the hospital with a suspected pancreatic cancer. Since she only speaks Mandarin, her husband who is bilingual, insists that he be allowed to serve as her interpreter in communicating with the physician. The question is, what the best subsequent step for the physician should be given the communication barrier and the husband's insistence. (2) PLANNING: To address this situation we should first consider the factors in play: the unique communication needs of this patient, the husband's anxiety and willingness to act as interpreter, and the options available (using the husband as interpreter, waiting for the hospital interpreter, consulting the hospital ethics committee, or using a telephone interpreter service). The main assumption here could be that using the husband as interpreter is not the most ethically or clinically appropriate decision due to potential bias, emotional involvement, and lack of professional training. (3) CLINICAL REASONING: Though it may seem an acceptable solution to allow the husband to interpret, this could affect the quality of the communication due to his emotional involvement. Key medical information might be deliberately withheld or unintentionally misinterpreted due to stress. Also, using a family member as an interpreter could potentially violate patient privacy rights. Hence, it is generally discouraged in clinical settings. It is also possible that complex medical terminologies may not be effectively translated by a layman, thereby leading to misunderstanding and misinformation. Consequently, it's essential to utilize professional interpretation services where possible to ensure the quality and accuracy of communication, respecting the patient’s rights. (4) CONCLUDING: Given the above reasoning, the most logical and ethically appropriate decision would be to use professional services that ensure accurate communication while respecting the patient rights. In this scenario, the immediate availability of a telephone interpreter service would be optimal. (5) RESPONSE: Consequently, the most appropriate next step in management is to (D) Use a telephone interpreter service. This respects the patient's rights and ensures accurate and unbiased communication.

**Facilitator** The consensus statement synthesizes the responses of AI_A, AI_B, AI_C, AI_D, and AI_E as follows: The situation involves a 78-year-old woman with a suspected pancreatic mass who only speaks Mandarin. Her husband, who is bilingual, offers to act as an interpreter. The hospital's professional interpreter is unavailable until the next day. The question is what the most appropriate next step in management should be, given the options of allowing the husband to interpret, consulting the hospital ethics committee, waiting for the hospital interpreter, or using a telephone interpreter service. The assumptions here are that the husband could accurately and objectively interpret medical information, or that waiting for the hospital interpreter is the best option. However, these assumptions may not be entirely correct. The husband's emotional involvement could potentially lead to distortion or withholding of information, and his understanding of complex medical terminologies is not guaranteed. Furthermore, patient confidentiality could be compromised. Waiting for the hospital interpreter might result in unnecessary anxiety and delays in consent and management. Consulting the hospital ethics committee may be more appropriate for ongoing complex decision-making situations rather than immediate language barrier issues. Given the importance of precise communication, especially where clinical prognosis and consent are concerned, it's important to ensure that doctors and patients understand each other fully. In this scenario, the immediate availability of a telephone interpreter service would be optimal. This service provides professional interpretation, helping to maintain patient autonomy and their right to make informed decisions. It also respects the patient's rights and ensures accurate and unbiased communication. Therefore, the most appropriate next step in management in this case would be (D) Use a telephone interpreter service. This ensures that the patient receives accurate information and can give informed consent for the pending biopsy.
