# Supplementary material for: Collaborative intelligence in AI: Evaluating the performance of a council of AIs on the USMLE
Source: PLOS Digit Health. 2025 Oct 9;4(10):e0000787. doi: 10.1371/journal.pdig.0000787 (PMC12510544; doi:10.1371/journal.pdig.0000787)
Supplement: S2 File — (ZIP) [file pdig.0000787.s002.zip › S2 Data Raw data files containing unprocessed question inputs and LLM outputs including transcripts of deliberations/STEP1_Question-72-1_Type-MC-NJ.docx]

**Facilitator** **[Round 1]:** A 4-year-old boy develops fever 3 weeks after being admitted to the hospital for induction chemotherapy for treatment of acute lymphoblastic leukemia. Chemotherapy medications are L-asparaginase, dexamethasone, doxorubicin, and vincristine. His temperature is 38.2°C (100.8°F), pulse is 122/min, and respirations are 24/min. Physical examination shows pallor, alopecia, and ulcerations over the gums. A central venous catheter with entry site in the right upper chest is present but has no surrounding erythema. A blood culture grows gram-negative rods after 36 hours. Which of the following underlying mechanisms is the most likely cause of this patient's susceptibility to infection? (A) Deficiency of terminal complement (B) Hypogammaglobulinemia (C) Impaired T-lymphocyte function (D) Inhibition of tumor necrosis factor α function (E) Neutropenia

**AI_E:** RESTATING THE QUESTION: A young boy who is being treated for acute lymphoblastic leukemia with several chemotherapy drugs has developed a fever. He has symptoms consistent with this type of cancer and its treatment. An infection with gram-negative bacteria has been identified. We need to identify the most probable mechanism causing his increased susceptibility to this infection. PLANNING: Five options have been proposed. These are: 1) Deficiency of terminal complement, which is part of the immune system that helps with the destruction of pathogens directly or by marking them for destruction. 2) Hypogammaglobulinemia, which is a decrease of gamma globulins including antibodies like IgG, IgM, and IgA in the blood, making the patient more vulnerable to infections. 3) Impaired T-lymphocyte function, which would compromise the cell-mediated immunity and overall immune response. 4) Inhibition of tumor necrosis factor α function, which is a cytokine involved in systemic inflammation and the acute phase reaction. 5) Neutropenia, this is a deficiency of neutrophils, white blood cells that are essential for innate immunity especially against bacterial infections. CLINICAL REASONING: The patient is undergoing chemotherapy, which is known to cause a decrease in the absolute neutrophil count, a condition known as neutropenia. This decrease of neutrophils makes patients to be more at risk of infection, particularly bacterial and fungal infections. Neutrophils are the body's primary defense against bacterial infections; their reduction compromises the body's ability to fight off bacteria, leading to an increased susceptibility to infections. The observed gram negative bacteremia in our patient is consistent with this vulnerability. CONCLUDING: The boy's susceptibility to gram-negative bacterial infection is most likely the result of neutropenia. This likely stems from his chemotherapy treatment, which is known to cause a decrease in neutrophil counts. RESPONSE: Therefore, the most likely underlying mechanism causing this patient's increased susceptibility to infection is (E) Neutropenia.

**AI_C:** (1) RESTATING THE QUESTION: The inquiry revolves around identifying the primary reason for this four-year-old patient's increased vulnerability to infection. The boy, under chemotherapy for acute lymphoblastic leukemia, shows a fever and signs pointing towards infection. Terminal complement deficiency, hypogammaglobulinemia, impaired T-lymphocyte function, inhibition of tumor necrosis factor α function, or neutropenia could potentially be underlying mechanisms. (2) PLANNING: To navigate this problem, we need to take into account the age of the patient, medical history, the clinical manifestations, and the results from the laboratory tests, especially the blood culture. The key aspect to focus on is the relationship between the chemotherapy administered for acute lymphoblastic leukemia and its role in the susceptibility to an infection. (3) CLINICAL REASONING: Chemotherapy, particularly the drugs mentioned in the question - L-asparaginase, dexamethasone, doxorubicin, and vincristine, are known to cause myelosuppression. Myelosuppression results in a decreased production of blood cells including neutrophils, leading to neutropenia - a significant reduction in the number of neutrophils in the blood. Neutrophils play a crucial role in the body's immune response as they rapidly respond to signs of infections, especially bacterial and fungal. A decrease in these cells can hence make the patient more susceptible to infections, particularly by bacteria. Given the physical examination findings and positive blood culture for gram-negative rods, it is clear the patient has developed a bacterial infection. While options A to D can cause immunodeficiency, neutropenia is a common side effect of chemotherapy and is the most likely cause given the acute setting. (4) CONCLUDING: Analyzing the clues provided in the case scenario, the patient's leukemia treatment most likely induced neutropenia, significantly increasing the likelihood of developing bacterial infections. (5) RESPONSE: Hence, the most likely underlying mechanism causing this patient's susceptibility to infection in this particular scenario is (E) Neutropenia.

**AI_A:** RESTATING THE QUESTION: A four-year-old male patient with acute lymphoblastic leukemia who is receiving induction chemotherapy has developed a fever. Along with typical chemotherapy side effects like alopecia and ulcerations over the gums, his blood culture shows presence of gram-negative rods, suggestive of an infection. The query is what biological mechanism underlies his predisposition to infection, with possibilities being deficit of terminal complement, hypogammaglobulinemia, impaired T-lymphocyte function, suppressed TNF-α function, or neutropenia. PLANNING: First assumption: The chemotherapy might have affected his T-lymphocyte function, which could make him susceptible to infections. Second assumption: Neutropenia, a common side effect of chemotherapy, may be the cause for his current susceptibility to infections, particularly bacterial ones with gram-negative rods. Alternate assumptions: Deficiency of terminal complements, hypogammaglobulinemia, or inhibited TNF-α function could also be reasons for susceptibility to infections, though they often lead to specific kinds of infections which differ from the current presentation. CLINICAL REASONING: The provided chemotherapy regimen (L-asparaginase, dexamethasone, doxorubicin, and vincristine) is broadly myelosuppressive which typically contributes to neutropenia. This is particularly significant because neutrophils play a crucial role in limiting bacterial and fungal infections by mediating microbial killing. Gram-negative rods, as cultures have shown here, are a presenting feature of neutropenic patients and this indicates that the current cause should be neutropenia. Other options like terminal complement deficiency or hypogammaglobulinemia have infection patterns that usually are against encapsulated bacteria, and these patterns do not correspond with the clinical scenario. T-lymphocyte impairment or TNF-α inhibition primarily increases susceptibility to viral, fungal, and mycobacterial infections which also do not agree with this presentation. CONCLUDING: Considering the boy's clinical presentation, the nature of the infection, and the potential side effects of the chemotherapy regimen, neutropenia stands as the most plausible cause of his susceptibility to infection. RESPONSE: (E) Neutropenia is the probable mechanism responsible for this patient's susceptibility to infection.

**AI_B:** RESTATING THE QUESTION: This question concerns a young child who has been receiving treatment for acute lymphoblastic leukemia using chemotherapy agents. Some weeks after starting this treatment, he develops a fever and a blood culture later reveals the presence of gram-negative rods. The question asks us to determine the most likely underlying reason why this child has proven so susceptible to infection. The options offered for this include deficits in terminal complement, hypogammaglobulinemia, issues with T-lymphocyte function, inhibition of tumor necrosis factor α function, or neutropenia. PLANNING: Before we attempt to answer this question, we need to understand that an organism's susceptibility to infections can get heightened by factors such as congenital deficiencies in the immune system or drug-induced immune suppression. When cancer patients undergo chemotherapy, it negatively influences the immune cells, thereby increasing the risk of infection. Hence, it's now essential to deliberate about which exact aspect of the immune system these chemotherapy drugs impact and result in this heightened susceptibility. CLINICAL REASONING: Leukemia and the medications being used for its treatment, namely L-asparaginase, dexamethasone, doxorubicin, and vincristine, could all conceivably affect the immune system causing the patient to be more susceptible to infections. The leukemic cells themselves can crowd out normal immune cells in the bone marrow, leading to a decrease in normal immune function. The chemotherapy agents, on the other hand, are cytotoxic and can suppress bone marrow function, leading to decreases in all cell lines, including immune cells. However, one cell line that is particularly affected by these agents is the myeloid line, leading to neutropenia, a low count of neutrophils, which are crucial for combating bacterial infections, especially the gram-negative type represented in this case. CONCLUDING: Based on the analysis, the underlying mechanism most likely causing this child's increased susceptibility to infection is the neutropenia caused by his leukemia and the chemotherapy treatment he is receiving. Neutropenia is an abnormally low concentration of neutrophils, a type of white blood cell that plays a crucial role in warding off infections, especially bacterial ones. RESPONSE: Therefore, my answer would be, (E) Neutropenia.

**AI_D:** RESTATING THE QUESTION: The query here is about the probable underlying reason that made this 4-year-old boy more prone to infection. This boy has been diagnosed with acute lymphoblastic leukemia and has been undergoing chemotherapy. The chemotherapeutic drugs he is being administered are L-asparaginase, dexamethasone, doxorubicin, and vincristine. Presently, he displays a fever and other symptoms like pallor, hair loss, and mouth ulcers. An inserted catheter, possibly for chemotherapy administration, shows no infection signs. His blood culture shows the presence of gram-negative rods. PLANNING: Several factors could influence and increase susceptibility to infections in this case: chemotherapy-induced immunosuppression; possible nutritional deficiencies; the presence of a catheter. Assumptions include the possibility of each underlying mechanism being the most likely. However, considering the definition, characteristic, and roles of each mechanism in infection susceptibility—terminal complement deficiency primarily resulting in recurrent neisserial infections; hypogammaglobulinemia leading to recurrent bacterial and viral infections; impaired T-lymphocyte function primarily resulting in viral, fungal, opportunistic infections; TNF-alpha function inhibition affecting macrophage function and resulting in increased susceptibility to tuberculosis; and neutropenia leading to bacterial and fungal infections—the most probable alternate assumption to pursue is neutropenia. CLINICAL REASONING: Neutropenia, characterized by low levels of neutrophils, is a common side effect of chemotherapy, particularly the regimen outlined here. Neutrophils play a key role in the immune response, particularly against bacterial and fungal infections. Their reduction leaves the patient vulnerable to such infectious agents. Post-chemotherapy patients typically reveal symptoms such as fatigue, pallor, and possibly alopecia; while gum ulcers and the positive culture for gram-negative rods indicate an active bacterial infection in his oral mucosa. The absence of erythema or infection signs around the catheter entry point could suggest that the infection didn’t start from the catheter-skin interface, but from natural body defenses being compromised—supporting the idea of neutropenia. CONCLUDING: Considering these factors, the chemotherapy-induced neutropenia is the most likely cause of this young boy's vulnerability to infection because it would compromise his immune system's ability to effectively fight off bacteria, leading to an overgrowth of gram-negative rods. RESPONSE: (E) Neutropenia could be the most plausible cause of this patient's susceptibility to infection due to chemotherapy-induced reduction in neutrophil levels.

**Facilitator** The consensus statement synthesizes the responses of AI_A, AI_B, AI_C, AI_D, and AI_E as follows: The question pertains to a four-year-old male patient undergoing induction chemotherapy for acute lymphoblastic leukemia who has developed a fever and signs of infection, including the presence of gram-negative rods in his blood culture. The potential underlying mechanisms for his increased susceptibility to infection include deficiency of terminal complement, hypogammaglobulinemia, impaired T-lymphocyte function, inhibition of tumor necrosis factor α function, or neutropenia. The chemotherapy regimen, which includes L-asparaginase, dexamethasone, doxorubicin, and vincristine, is known to be myelosuppressive, leading to a decrease in the production of blood cells, including neutrophils. This condition, known as neutropenia, is a common side effect of chemotherapy. Neutrophils play a crucial role in the body's immune response, particularly against bacterial and fungal infections. A decrease in these cells can make the patient more susceptible to infections, especially bacterial ones, as indicated by the presence of gram-negative rods in the patient's blood culture. While other options like terminal complement deficiency, hypogammaglobulinemia, impaired T-lymphocyte function, or inhibition of tumor necrosis factor α function could also lead to increased susceptibility to infections, they often result in specific types of infections that do not align with the patient's current presentation. Therefore, considering the patient's clinical presentation, the nature of the infection, and the potential side effects of the chemotherapy regimen, the consensus is that neutropenia is the most likely underlying mechanism causing this patient's susceptibility to infection. Hence, the response to the prompt is (E) Neutropenia.
