# Supplementary material for: Collaborative intelligence in AI: Evaluating the performance of a council of AIs on the USMLE
Source: PLOS Digit Health. 2025 Oct 9;4(10):e0000787. doi: 10.1371/journal.pdig.0000787 (PMC12510544; doi:10.1371/journal.pdig.0000787)
Supplement: S2 File — (ZIP) [file pdig.0000787.s002.zip › S2 Data Raw data files containing unprocessed question inputs and LLM outputs including transcripts of deliberations/STEP1_Question-73-1_Type-MC-NJ.docx]

**Facilitator** **[Round 1]:** A 65-year-old woman comes to the office for a follow-up examination 1 year after she underwent operative resection of the right colon and chemotherapy for stage III colon cancer. She reports fatigue. Physical examination shows no abnormalities. A staging CT scan of the chest and abdomen shows five new 2- to 3-cm masses in the liver and both lungs. This patient's cancer most likely spread to the lungs via which of the following structures? (A) Inferior mesenteric vein (B) Inferior vena cava (C) Left colic vein (D) Middle colic artery (E) Pulmonary vein (F) Superior mesenteric artery (G) Superior vena cava

**AI_D:** (1) RESTATING THE QUESTION: The query at hand concerns the possible route of metastasis of this patient's colon cancer to the lungs. That is, via which vessel did the cancer cells most likely travel from her colon to her lungs? Understanding the specific anatomy and physiology of the vasculature of these organs is crucial to addressing the question. (2) PLANNING: Here, the pivotal assumption is the route that colon cancer cells in this situation would take to reach the lungs. One can deduce from the knowledge of anatomy that a possible route is through the inferior vena cava, as the liver (where the metastasis is also found) efficiently drains into it. However, this assumption is not certain. It's crucial to remember how colon cancer commonly metastasizes. The process often involves penetration of the tumor into the intestinal wall, invasion of the local lymph nodes, and subsequent entry into the bloodstream. (3) CLINICAL REASONING: In the clinical scenario presented, the cancer has most likely followed the path of the venous drainage system of the colon. Understanding this standard route can help eliminate certain options. The right colon primarily drains into the superior mesenteric vein, which then goes to the portal vein. From the portal vein, the blood flow continues into the liver, making it a common site of colon cancer metastasis. Ultimately, the blood in the liver is channeled into the inferior vena cava, which then leads to the heart and the lungs. (4) CONCLUDING: The extensive involvement of the liver and lungs and understanding the blood circulation suggest that the cancer cells most likely reached the lungs via the liver. The only choice that fits the travel pattern of the colon cancer cells, from the colon to the liver and hence to the lungs, is the Inferior Vena Cava. The tumor likely used the host's blood to vascularize, primarily migrating through the systemic blood supply since the lung is a secondary site of hematogenous metastasis. (5) RESPONSE: Therefore, considering the anatomy of the vessels involved, the tumor cells from the colon cancer in this patient most likely spread to the lungs via the Inferior Vena Cava (Option B).

**AI_B:** RESTATING THE QUESTION: The patient in this scenario was initially diagnosed with stage III colon cancer and underwent the relevant surgical and adjuvant therapeutic interventions. One year out, she is presenting with fatigue. No abnormalities were identified on physical examination, but her staging CT scan shows that she has developed several new liver and lung masses. We are being asked to deduce the most viable pathway via which the colon cancer metastasized to her lungs. PLANNING: To answer this question, we need to understand the path of colon cancer metastasis. Typically, colon cancer metastasizes via hematogenous (blood) or lymphatic routes. Here we know the cancer has spread to the liver and lungs, which implies a blood-borne route via the portal vein for liver involvement and the systemic circulation for lung spread. Our job is to decide which of the multiple blood vessels listed in the options was pivotal for the lung involvement. CLINICAL REASONING: Based on the anatomy of the vascular system, the colon cancer cells would spread to the liver first via the portal venous system. From the liver, they would enter the systemic circulation through the hepatic veins, which drain into the inferior vena cava (IVC). The IVC then delivers blood to the right atrium, subsequently directing the flow to the lungs via the right ventricle and pulmonary arteries. CONCLUDING: The directionality of blood flow indicates that from the liver (the primary extra-colonic site of metastasis in this case), the next possible pathway for metastasis would be through the hepatic veins into the IVC, subsequently leading to the right side of the heart and then onto the lungs. The other vascular structures listed as options either do not directly link the colon or liver to the lungs or operate in a direction counter to blood flow (e.g., veins returning blood from the lungs to the left side of the heart). RESPONSE: Therefore, the colon cancer in this patient most likely metastasized to the lungs via the (B) Inferior vena cava.

**AI_E:** 1) RESTATING THE QUESTION: The query pertains to the spread or metastasis of colon cancer in a 65-year-old woman, who presented with fatigue. Despite her prior surgical intervention and chemotherapy treatment for stage III colon cancer, recent CT Scan discoveries exhibit growths (or metastasis) in the liver and lungs. Given these developments, a determination of the most likely anatomical pathway for cancer spread to the lungs is sought. 2) PLANNING: The posed question is largely based on the understanding of the circulatory system, the physiological process of metastasis, and the common disease progression of colon cancer. Possible assumptions include that the cancer cells travelled from the colon to the lungs through the blood or lymphatic system. Moreover, it is possible to challenge the assumption that the liver and lung metastases developed from the same primary tumor. 3) CLINICAL REASONING: Colorectal cancer typically spreads via direct extension, lymphatic spread, or hematogenous spread. The liver is a common site of metastasis due to the portal venous system, which drains the gastrointestinal tract. The lungs, on the other hand, are commonly affected secondary to the spread of tumor cells via the systemic circulation. Given the presence of metastases in both the liver and lungs, it is reasonable to hypothesize that the metastasis primarily occurred via the blood vascular system rather than the lymphatic system. 4) CONCLUDING: From the provided options, the most probable route for the spread of this patient's colon cancer cells to the lungs is the flow through the inferior vena cava. The progression can be stated as follows: colon cancer cells drain into the portal venous system, then spread to the liver, subsequently entering the systemic circulation via the hepatic veins. They can then traverse to the inferior vena cava, from where they can reach the lungs via pulmonary arteries. 5) RESPONSE: In the case of the 65-year-old woman who has developed metastases in both the liver and lungs, the most probable route of metastasis to the lungs, in this case, is 'B' the inferior vena cava.

**AI_C:** RESTATING THE QUESTION: The query posed inquires about the likely anatomical route through which a 65-year old woman's colon cancer metastasized to her liver and lungs. To be specific, it wants to know what blood vessel was the most probable route for the cancer cells to travel from the colon to her lungs. PLANNING: This question requires understanding about how colon cancer metastasizes, the intricacies of the circulatory system, as well as the role of veins and arteries in blood flow. Pathologically, most cancers, including colon cancer, metastasize via systemic circulation. It is assumed that the cancer cells break off from the primary tumor, enter the bloodstream and then spread to other parts of the body, like lungs in this case, where they form new tumors. CLINICAL REASONING: If we consider the anatomy of the circulatory system, blood flows from the colon to the liver via the portal circulation. This includes both the superior mesenteric and inferior mesenteric veins, which collect blood from the colon and carry it to the liver. The artery system isn't the primary path of metastasis since arteries carry oxygenated blood from the heart to other parts of the body. Therefore, it can be ruled out in this case. On the other hand, veins like the left colic vein and the inferior mesenteric vein lead to the portal vein, making them potential paths of spread to the liver but not the lungs. After the liver, blood enters the systemic circulation via the inferior vena cava, which connects to the heart. From there, it's pumped into the lungs via the pulmonary artery (not vein, which brings oxygenated blood from the lungs back towards the heart). Therefore, the route of metastasis from the colon to the lungs is likely through the inferior vena cava. CONCLUDING: Given the fact that liver and lung metastasis occurs predominantly through blood vessels that carry blood away from the affected colon towards these organs, the most feasible avenue would be blood vessels involved in circulatory motion away from the colon i.e., veins and not arteries. RESPONSE: Considering these facts, the answer to this question is most definitely, the (B) Inferior vena cava. It is through this large vein that the cancerous cells were carried from the colon to the liver and lungs.

**AI_A:** (1) RESTATING THE QUESTION: The query in consideration is regarding a 65-year-old patient with a history of stage III colon cancer and recent presentation of new masses in both the liver and lungs. The suspected route of cancer spread to the lungs needs to be identified from the following given structures: (A) Inferior mesenteric vein (B) Inferior vena cava (C) Left colic vein (D) Middle colic artery (E) Pulmonary vein (F) Superior mesenteric artery (G) Superior vena cava These are all blood vessels, and understanding their role in cancer spread requires basic understanding of oncology and anatomy. (2) PLANNING: To frame an answer, an assumption has to be made about the route colon cancer commonly takes to metastasize to the liver and lungs. Metastasis typically occurs through two pathways: directly into the surrounding tissues or via hematogenous (bloodborne) or lymphatic routes. However, hematogenous spread is the major pathway for colon cancer. Appreciating the venous drainage of the colon and the systemic circulatory pathway colon cancer cells would take to reach the liver and then the lungs will enable to identify the most likely vessel from the given choices. (3) CLINICAL REASONING: The colonic venous blood, carrying potential cancer cells, drains via the superior and inferior mesenteric veins. The right colon, specifically, primarily drains into the superior mesenteric vein. This vein then merges with the splenic vein to form the portal vein, which takes blood directly to the liver. In the liver, the blood is filtered, and any remaining cells continue into the hepatic veins, which then drain into the inferior vena cava. From the inferior vena cava, the blood (and any cancer cells) are transported into the right side of the heart and subsequently into the pulmonary arteries, which channel the blood into the lungs. (4) CONCLUDING: So among the choices listed, the superior mesenteric vein is mainly responsible for venous drainage of the right colon, where the primary cancer was located, but it is not among the options. However, the consideration of the entire circulatory route the cancer cells had to take to reach the lungs reveals that the inferior vena cava plays a critical part in this process. (5) RESPONSE: Therefore, the patient's cancer most likely spread to the lungs via the structure (B) Inferior vena cava.

**Facilitator** The consensus statement synthesizing the responses of AI_A, AI_B, AI_C, AI_D, and AI_E is as follows: The question at hand pertains to the most probable anatomical pathway through which a 65-year-old woman's stage III colon cancer metastasized to her liver and lungs. To answer this, an understanding of the circulatory system, the physiological process of metastasis, and the common disease progression of colon cancer is required. Colon cancer typically spreads via direct extension, lymphatic spread, or hematogenous (bloodborne) spread. In this case, given the presence of metastases in both the liver and lungs, it is reasonable to hypothesize that the metastasis primarily occurred via the blood vascular system. The colonic venous blood, carrying potential cancer cells, drains via the superior and inferior mesenteric veins. The right colon, specifically, primarily drains into the superior mesenteric vein. This vein then merges with the splenic vein to form the portal vein, which takes blood directly to the liver. In the liver, the blood is filtered, and any remaining cells continue into the hepatic veins, which then drain into the inferior vena cava. From the inferior vena cava, the blood (and any cancer cells) are transported into the right side of the heart and subsequently into the pulmonary arteries, which channel the blood into the lungs. Given the fact that liver and lung metastasis occurs predominantly through blood vessels that carry blood away from the affected colon towards these organs, the most feasible avenue would be blood vessels involved in circulatory motion away from the colon i.e., veins and not arteries. Therefore, considering the anatomy of the vessels involved, the tumor cells from the colon cancer in this patient most likely spread to the lungs via the Inferior Vena Cava (Option B).
