# Supplementary material for: Collaborative intelligence in AI: Evaluating the performance of a council of AIs on the USMLE
Source: PLOS Digit Health. 2025 Oct 9;4(10):e0000787. doi: 10.1371/journal.pdig.0000787 (PMC12510544; doi:10.1371/journal.pdig.0000787)
Supplement: S2 File — (ZIP) [file pdig.0000787.s002.zip › S2 Data Raw data files containing unprocessed question inputs and LLM outputs including transcripts of deliberations/STEP1_Question-74-1_Type-MC-NJ.docx]

**Facilitator** **[Round 1]:** A 6-year-old boy with acute lymphoblastic leukemia is brought to the office for a follow-up examination. He is receiving high-dose methotrexate therapy. A drug is added to the patient's medication regimen to decrease the toxicity of this therapy to normal cells. The beneficial effect of this new drug on normal cells is most likely achieved by bypassing the cellular requirement for which of the following enzymes? (A) Dihydrofolate reductase (B) Methionine synthase (C) Pyruvate decarboxylase (D) Thiamine pyrophosphate (E) Thymidylate synthase

**AI_B:** (1) RESTATING THE QUESTION: This query is determining which enzyme is muscled out of the biochemical process by the addition of a particular drug. The drug is employed as a shield, countering the unforgiving impact of methotrexate (used in chemotherapy). Methotrexate therapy is linked to the treatment of acute lymphoblastic leukemia, a blood cancer typically common in children like the 6-year-old boy patient in question. (2) PLANNING: Methotrexate is an anti-cancer drug that works by inhibiting dihydrofolate reductase (DHFR), an enzyme necessary for DNA synthesis. Still, while destroying cancer cells, methotrexate can also harm normal cells, leading to adverse side effects. The drug added to mitigate toxicity operates by circumventing a particular enzyme. The reasonable target would likely be a key enzyme also inhibited by methotrexate but vital for normal cell function. (3) CLINICAL REASONING: Methotrexate competitively inhibits DHFR causing an interruption in the folate metabolism pathway. The folate is required for the synthesis of thymidylate, which is catalyzed by the enzyme thymidylate synthase. Depletion of this substrate means that DNA synthesis cannot proceed and cell proliferation stalls. So, if you introduce a drug that bypasses the need for thymidylate synthase instead of DHFR, it could decrease the drug's toxicity. (4) CONCLUDING: Given that thymidylate synthase depends on folate metabolism for DNA synthesis and considering the mechanism of action of methotrexate, the drug added to decrease the patient's treatment toxicity is likely aimed at bypassing the requirement for this enzyme. (5) RESPONSE: The beneficial effect of this new drug on normal cells is most likely achieved by bypassing the cellular requirement for Thymidylate synthase (E).

**AI_A:** (1) RESTATING THE QUESTION: This question is asking about the mechanism of action of a drug that is given in combination with high-dose methotrexate therapy in a young child with acute lymphoblastic leukemia to protect normal cells from methotrexate toxicity. Methotrexate is a chemotherapy drug that primarily hinders cell growth and replication by inhibiting dihydrofolate reductase (DHFR), an essential enzyme for DNA synthesis. (2) PLANNING: Considering the mechanism of action of methotrexate, the drug co-administered with it must possess a mechanism to bypass the enzyme blocked by methotrexate, preserving the viability of normal cells. Question is, which is the correct enzyme being bypassed? (3) CLINICAL REASONING: Methotrexate inhibits DHFR, which is critical for the conversion of dihydrofolate (DHF) to tetrahydrofolate (THF). THF is a necessary cofactor in the synthesis of purine nucleotides and thymidine, both of which are crucial for DNA replication. The drug given along with methotrexate must enable normal cells to continue DNA synthesis even in the presence of DHFR inhibition. Therefore, the enzyme required for this process is likely the one that is directly involved in DNA synthesis bypassing the need for DHFR, namely thymidylate synthase, which produces the thymidine required for DNA synthesis. (4) CONCLUDING: The drug added to this patient's treatment regimen protects normal cells from methotrexate's toxic effects by bypassing thymidylate synthase. It allows normal cells to continue DNA synthesis without the need for the DHFR pathway, which is blocked by methotrexate. (5) RESPONSE: The beneficial effect of this new drug on normal cells is most likely achieved by bypassing the cellular requirement for thymidylate synthase (E).

**AI_E:** RESTATING THE QUESTION: The query is focused on figuring out which enzyme's function is essentially replaces or bypassed by a newly added drug in a young cancer patient's treatment regimen. He is currently receiving high-dose methotrexate, and this new medication helps reducing the toxicity to healthy cells that this therapy causes. Methotrexate, a chemotherapy agent, is known to inhibit the enzyme dihydrofolate reductase (DHFR), involved in the synthesis of nucleotides, which ultimately affects rapidly dividing cells such as cancer cells. However, normal cells are also affected by methotrexate toxicity as they also require nucleotide synthesis for their normal division and functioning. PLANNING: The standard assumption here is that methotrexate is causing toxicity to the healthy cells by inhibiting an enzyme they need for normal functioning. However, an alternative assumption could be that the added drug helps mitigate this toxicity by essentially replacing or bypassing the need for the enzyme that methotrexate inhibits in normal cells. CLINICAL REASONING: Considering that methotrexate inhibits DHFR, it essentially blocks the conversion of dihydrofolate to tetrahydrofolate, a necessary precursor for the synthesis of nucleotides. The beneficial effect of this new drug would therefore logically lie in being able to bypass this blocked pathway. CONCLUDING: Bypassing this pathway would require the ability to produce tetrahydrofolate by another means that doesn't involve DHFR, or negate the need for the nucleotides that this pathway helps synthesize. Therefore, it is logical to hypothesize that this drug bypasses the cellular requirement for the enzyme that methotrexate principally inhibits in its mode of action, which is dihydrofolate reductase (DHFR). RESPONSE: (A) Dihydrofolate reductase. The new drug most likely bypasses the cellular requirement for this enzyme, helping to mitigate the toxicity that methotrexate treatment can cause to healthy cells.

**AI_C:** (1) RESTATING THE QUESTION: This question aims to find out how an additional medication, which has been added to a 6-year-old leukemia patient's medication regimen, helps to lessen the toxicity of high-dose methotrexate to normal cells. Methotrexate is a medication used to treat cancer by inhibiting the enzyme dihydrofolate reductase, which is essential for DNA synthesis. By inhibiting this enzyme, methotrexate disrupts the proliferation of cancer cells but also affects normal cells, leading to toxicity. To protect normal cells, an additional drug can be used that manages to bypass the need for a particular enzyme, allowing the cells to function normally, despite the presence of methotrexate. (2) PLANNING: The question assumes that the drug works by bypassing the cellular requirement for a particular enzyme. We must consider all possible enzymes from the choices given - (A) Dihydrofolate reductase, (B) Methionine synthase, (C) Pyruvate decarboxylase, (D) Thiamine pyrophosphate, (E) Thymidylate synthase. By considering each, we must evaluate which one could be bypassed to reduce methotrexate toxicity. (3) CLINICAL REASONING: Methotrexate inhibits the enzyme dihydrofolate reductase, and by doing so, prevents the conversion of dihydrofolate (DHF) to tetrahydrofolate (THF), which is a key process in DNA synthesis. In turn, this affects not only the cancer cells' ability to proliferate but also the normal cells' ability to perform DNA synthesis, leading to toxicity. To protect normal cells from this action, an additional drug must be able to functionally replace the role of DHF -> THF conversion, thus obviating the need for the activity of dihydrofolate reductase. (4) CONCLUDING: Considering the mechanism of action of methotrexate, the enzyme that would most likely be bypassed by the additional drug to reduce toxicity to normal cells is dihydrofolate reductase (A). (5) RESPONSE: The beneficial effect of the new drug on normal cells in reducing the toxicity of high dose methotrexate therapy would most likely be achieved by bypassing the cellular requirement for the enzyme dihydrofolate reductase (A).

**AI_D:** (1) RESTATING THE QUESTION: The query at hand pertains to understanding the mechanism of a certain drug that is added to a patient's treatment to counteract the toxic effects of high-dose methotrexate therapy for acute lymphoblastic leukemia. The aim of this drug is to circumvent the need for a specific enzyme within normal cells, and the question is essentially asking us to identify this enzyme. (2) PLANNING: Methotrexate is a chemotherapeutic drug that inhibits the enzyme dihydrofolate reductase (DHFR). This inhibition prevents the conversion of dihydrofolate to tetrahydrofolate, a cofactor required for the synthesis of purines and thymidine, in turn blocking DNA synthesis and cell replication. The critical assumption in this scenario is that the additional drug should act to bypass the blockage induced by methotrexate, allowing the normal cells to grow and divide. (3) CLINICAL REASONING: In terms of the specific enzymes listed in the options, dihydrofolate reductase and thymidylate synthase are directly linked to the folate pathway and thus to the action of methotrexate. Methionine synthase, pyruvate decarboxylase, and thiamine pyrophosphate are not part of this pathway. Therefore, it would be logical to prioritize clinically the additional drug's ability to bypass the need for an enzyme involved in the same pathway targeted by methotrexate. (4) CONCLUDING: To alleviate methotrexate toxicity in normal cells, the most plausible enzyme that the new drug should bypass is in the specific pathway that methotrexate targets; hence, the enzyme should function in the folate metabolism pathway. This informs our final response. (5) RESPONSE: The additional drug added to the patient's medication regimen likely works to bypass the cellular requirement for either (A) Dihydrofolate reductase or (E) Thymidylate synthase. However, since Methotrexate itself targets dihydrofolate reductase and indirectly impacts thymidylate synthase, the additional drug is more likely to bypass (E) Thymidylate synthase to help maintain DNA synthesis in normal cells while methotrexate interferes with the folate pathway.

**Facilitator** **[Round 2]:** Summary: AI_A, AI_B, and AI_D believe that the drug added to the patient's treatment regimen bypasses the cellular requirement for thymidylate synthase, which is involved in DNA synthesis. They argue that this bypass allows normal cells to continue DNA synthesis despite the inhibition of dihydrofolate reductase (DHFR) by methotrexate. AI_C and AI_E, on the other hand, argue that the drug bypasses the cellular requirement for DHFR, the enzyme directly inhibited by methotrexate. They believe that the drug allows normal cells to continue functioning by replacing or bypassing the need for DHFR. Key Differences: The key difference in the perspectives lies in the enzyme that the additional drug is believed to bypass. AI_A, AI_B, and AI_D believe it's thymidylate synthase, while AI_C and AI_E believe it's DHFR. This difference stems from their interpretation of how the drug mitigates methotrexate's toxicity. The former group believes the drug allows normal cells to continue DNA synthesis by bypassing the need for thymidylate synthase, while the latter group believes the drug replaces or bypasses the need for DHFR, allowing normal cells to continue functioning. Question to Clarify Disagreements: To clarify the disagreements between the council members, we could ask: "Considering the mechanism of action of methotrexate and the role of both DHFR and thymidylate synthase in DNA synthesis, could the additional drug bypass the need for both enzymes, or is it more likely to bypass only one? If it's the latter, which enzyme is more likely to be bypassed and why?". Based on your preceding response, how would you answer the original question: [A 6-year-old boy with acute lymphoblastic leukemia is brought to the office for a follow-up examination. He is receiving high-dose methotrexate therapy. A drug is added to the patient's medication regimen to decrease the toxicity of this therapy to normal cells. The beneficial effect of this new drug on normal cells is most likely achieved by bypassing the cellular requirement for which of the following enzymes? (A) Dihydrofolate reductase (B) Methionine synthase (C) Pyruvate decarboxylase (D) Thiamine pyrophosphate (E) Thymidylate synthase]

**AI_B:** RESTATING THE QUESTION: The question asks to recognize the enzyme that is most likely bypassed by a co-administered drug with high-dose methotrexate in a pediatric patient with acute lymphoblastic leukemia, to decrease the cytotoxic effect of the therapy to normal cells. PLANNING: Methotrexate primarily inhibits dihydrofolate reductase (DHFR) enzyme responsible for the conversion of dihydrofolate (DHF) to tetrahydrofolate (THF). This causes depletion of THF, which is a crucial co-factor required by thymidylate synthase for the conversion of deoxyuridylate (dUMP) to deoxythymidylate (dTMP), needed for DNA synthesis. CLINICAL REASONING: The mechanism of action of high-dose methotrexate therapy involves the inhibition of DHFR, leading to a deficit of THF. However, rapid cell division like in cancer cells relies heavily on DNA synthesis, where thymidylate synthase plays a crucial role. So, the normal cells can be shielded from the cytotoxic action of methotrexate through bypassing the requirement for THF in the reaction catalyzed by thymidylate synthase. CONCLUDING: This reasoning suggests that when a strategy pertains to decrease the harmful effects of high-dose methotrexate therapy to normal cells, it most likely involves bypassing the enzymatic requirement for thymidylate synthase. RESPONSE: Thus, the beneficial effect of this new drug on normal cells in a patient receiving high-dose methotrexate for acute lymphoblastic leukemia is most likely achieved by bypassing the cellular requirement for thymidylate synthase (E).

**AI_D:** 1) RESTATING THE QUESTION: The inquiry pertains to the mechanism of action of a medication that is added to a young patient's existing methotrexate treatment plan in order to reduce its harmful effects on healthy cells. This new drug is thought to bypass the normal cellular need for a particular enzyme. 2) PLANNING: In order to answer this question, it is necessary to understand the role of the enzymes listed and their relevance in the context of methotrexate therapy for acute lymphoblastic leukemia, as well as the typical approaches to minimizing the associated toxicity. The premise for the assumption is that the new drug works by bypassing the requirement for an enzyme, which one will depend on the role of the enzyme in the cell's regular workings and how methotrexate interferes with these. 3) CLINICAL REASONING: Methotrexate inhibits dihydrofolate reductase (DHFR), an enzyme necessary for the conversion of dihydrofolate to tetrahydrofolate. Tetrahydrofolate is required as a coenzyme for the synthesis of thymidine, a building block of DNA, and its synthesis is catalyzed by thymidylate synthase. When DHFR is inhibited by methotrexate, the synthesis of thymidine is affected, which slows down cell division primarily in rapidly dividing cells like cancer cells. However, normal cells are also impacted, leading to toxic side effects. The drug that is given to decrease the toxicity is leucovorin (folinic acid), a form of folate that can bypass the block caused by methotrexate. It provides a source of tetrahydrofolate that can directly reach the thymidylate synthase, bypassing the need for the conversion by DHFR. 4) CONCLUDING: The medication added to mitigate the toxicity of methotrexate accomplishes this by bypassing the requirement for dihydrofolate reductase in the normal creation of tetrahydrofolate. Thus, it circumvents the blockage caused by methotrexate, enabling normal DNA synthesis to proceed. 5) RESPONSE: Based on this clinical rationale, the beneficial effect of the new drug on normal cells is most likely achieved by bypassing the cellular requirement for (A) Dihydrofolate reductase.

**AI_C:** RESTATING THE QUESTION: A boy with leukemia is being treated with methotrexate, and a novel drug has been added to his regimen. This new drug seems to reduce methotrexate's adverse effects on healthy cells, and we must identify the enzyme whose requirement is likely bypassed by this drug. PLANNING: Methotrexate is known to inhibit dihydrofolate reductase (DHFR), an enzyme crucial in the synthesis of thymidylate via the conversion of dihydrofolate (DHF) to tetrahydrofolate (THF). Given this, it is logical to hypothesize that this new drug either a) offers a pathway that circumvents the need for DHFR, or b) bypasses the requirement for a downstream enzyme in this synthesis process—namely, thymidylate synthase. CLINICAL REASONING: The added drug's main purpose is to ameliorate methotrexate's deleterious effects on healthy cells, implying that it somehow circumvents methotrexate's mechanism. As methotrexate inhibits DHFR, an enzyme necessary for the conversion of DHF to THF, the logical presumption is that the new drug bypasses the need for DHFR, thus liberating the biochemical process stranded by methotrexate's inhibitory action. One could contend that thymidylate synthase, a downstream enzyme in the process of thymidylate synthesis, might be the target bypassed by the new drug. However, this would not align with the aim of this drug—as methotrexate's primary mode of harm derives from DHFR inhibition, not direct interference with thymidylate synthase. CONCLUDING: Given the sheen methodology to methotrexate's toxicity—the impairment of DHFR, critical for thymidylate synthesis, and consequently, DNA synthesis—it is most clinically plausible that the added drug bypasses the requirement for DHFR. This would allow the production of THF and subsequent synthesis of integral DNA precursors to continue despite methotrexate's therapeutic interference. RESPONSE: Therefore, I would answer: (A) Dihydrofolate reductase.

**AI_A:** To clarify the problem, we are being asked to figure out which enzyme's requirement is likely being bypassed by an additional drug to decrease the toxicity of high-dose methotrexate therapy in a 6-year-old boy with acute lymphoblastic leukemia. Methotrexate is known to inhibit dihydrofolate reductase (DHFR) and thus impedes the conversion of dihydrofolate to tetrahydrofolate which is an essential cofactor for nucleotide synthesis. If we focus on this matter, the central assumption appears to be that the dependent drug is either bypassing the role of DHFR or another enzyme involved in nucleotide synthesis. Since these pathways are pivotal for normal cellular functioning, bypassing any of them can potentially alleviate the toxicity induced by methotrexate. When considering all of the possibilities from this perspective, we should give primary attention to the enzymes (DHFR, Thymidylate synthase) directly tied to the process halted by methotrexate. On one side, the argument could be made that bypassing DHFR (Option A) would directly counteract the methotrexate's effect, while on the other side, bypassing Thymidylate synthase (Option E), involved downstream of DHFR in the production of thymidine (a necessary nucleotide for DNA synthesis), also makes sense. Proceeding with clinical logic, methotrexate mainly interrupts DNA replication and repair in rapidly dividing cells, like cancer cells, by inhibiting DHFR. However, this approach can also harm normal cells, causing side effects. The addition of a drug that bypasses the Thymidylate synthase requirement would maintain the availability of thymidine for DNA synthesis, permitting the normal cells to sustain their essential functions while the rapidly dividing cancer cells are still impacted by the methotrexate's inhibition of DHFR. Therefore, based on this clinico-pathological correlation, the new drug benefitting the normal cells is most likely achieved by bypassing the cellular requirement for Thymidylate synthase (Option E). This enables the continuation of DNA synthesis, thus decreasing the normal cell toxicity associated with high-dose methotrexate therapy in this young patient with acute lymphoblastic leukemia.

**AI_E:** To consolidate this discussion, we are dealing with a case of a 6-year-old boy with acute lymphoblastic leukemia who is currently on high-dose methotrexate therapy. The question then revolves around the identity of the enzyme that is bypassed by a new drug introduced in the patient's treatment to mitigate the cytotoxic effects of methotrexate on normal cells. The council presents two main arguments, with some members positing dihydrofolate reductase (DHFR) as the likely target, while others considering thymidylate synthase. Dihydrofolate reductase is a critical enzyme in the folate metabolism pathway. It converts dihydrofolate (DHF) into its active form, tetrahydrofolate (THF), which is essential for DNA synthesis. Methotrexate, on the other hand, is a well-known inhibitor of DHFR as it is structurally similar to DHF and competes for the enzyme’s active site. By doing so, it interrupts the production of THF, effectively stalling cell replication, which is especially impactful in rapidly replicating cells like those in leukemia. Thymidylate synthase is responsible for the conversion of deoxyuridine monophosphate (dUMP) to deoxythymidine monophosphate (dTMP), which is a crucial step in DNA synthesis. One of the substrates for this reaction, methyltetrahydrofolate, is produced from THF, hence the connection between DHFR and thymidylate synthase. Methotrexate interferes with the production of methyltetrahydrofolate, which indirectly hampers the function of thymidylate synthase. However, the crux of methotrexate's action lies in its direct inhibition of DHFR and not thymidylate synthase. Therefore, a second drug aiming to decrease the toxicity to normal cells would most likely do so by bypassing the need for DHFR, which is rendered inactive by methotrexate. In light of this reasoning, the most plausible response to the question would be (A) Dihydrofolate reductase. This choice reflects the understanding that the protective effect offered by the second drug on normal cells would predominantly be achieved by circumventing the integral role of DHFR in the folate metabolism pathway which is critical for DNA synthesis and cell replication.

**Facilitator** **[Round 3]:** Summary: AI_A and AI_B both believe that the additional drug likely bypasses the requirement for Thymidylate synthase (Option E). They argue that methotrexate inhibits DHFR, which indirectly affects Thymidylate synthase by reducing the availability of tetrahydrofolate, a necessary cofactor for the enzyme. By bypassing Thymidylate synthase, the drug would allow normal cells to continue DNA synthesis despite the methotrexate's inhibition of DHFR. AI_C and AI_D, however, believe that the additional drug bypasses the requirement for Dihydrofolate reductase (DHFR) (Option A). They argue that since methotrexate directly inhibits DHFR, a drug that bypasses this enzyme would allow the production of tetrahydrofolate and subsequent DNA synthesis to continue despite methotrexate's interference. AI_E agrees with AI_C and AI_D, arguing that the additional drug likely bypasses DHFR. AI_E emphasizes that methotrexate's primary mode of action is the inhibition of DHFR, not direct interference with Thymidylate synthase. Question to clarify disagreements: Given that methotrexate directly inhibits DHFR, which indirectly affects Thymidylate synthase, could the additional drug bypass both enzymes? If not, what evidence or reasoning supports the bypassing of one enzyme over the other?. Based on your preceding response, how would you answer the original question: [A 6-year-old boy with acute lymphoblastic leukemia is brought to the office for a follow-up examination. He is receiving high-dose methotrexate therapy. A drug is added to the patient's medication regimen to decrease the toxicity of this therapy to normal cells. The beneficial effect of this new drug on normal cells is most likely achieved by bypassing the cellular requirement for which of the following enzymes? (A) Dihydrofolate reductase (B) Methionine synthase (C) Pyruvate decarboxylase (D) Thiamine pyrophosphate (E) Thymidylate synthase]

**AI_E:** 1) RESTATING THE QUESTION: The question is addressing a scenario where a young patient with acute lymphoblastic leukemia is receiving high-dose methotrexate therapy. To mitigate the toxicity of this treatment to normal cells, another drug has been introduced to the patient's medication regimen. This drug likely functions by allowing normal cellular functions to continue despite the presence of methotrexate, by bypassing the need for a particular enzyme. We're tasked with identifying the enzyme that this secondary drug likely bypasses. 2) PLANNING: Methotrexate is an antimetabolite drug that works as an inhibitor of the enzyme dihydrofolate reductase (DHFR). This enzyme is crucial for converting dihydrofolate to tetrahydrofolate, a cofactor required for the synthesis of purines and thymidylate. Purines and thymidylate are essential components of DNA and RNA. Therefore, when DHFR is inhibited by methotrexate, the synthesis of DNA and RNA is impaired, predominantly affecting rapidly proliferating cells like cancer cells, but also affecting normal cells. Thus, a drug that bypasses this inhibition could decrease the toxicity of methotrexate to normal cells. 3) CLINICAL REASONING: Given these premises, the additional drug in the patient's regimen could theoretically bypass one of two crucial enzymes to alleviate the effects of methotrexate on normal cells: dihydrofolate reductase (DHFR), directly inhibited by methotrexate, or thymidylate synthase, which is indirectly impacted due to reduced availability of its cofactor tetrahydrofolate. However, the drug's mode of decreasing methotrexate toxicity would make more sense if it bypassed the enzyme directly targeted by methotrexate, i.e., DHFR. 4) CONCLUDING: Therefore, giving these considerations, logically reasoning would support the assumption that the beneficial effect of the added drug on normal cells is most likely achieved by bypassing the cellular requirement for DHFR. 5) RESPONSE: Given this understanding of the biochemical pathways involved in the action of high-dose methotrexate therapy and the reasoning behind the use of an additional drug to decrease the toxicity of this therapy to normal cells, the most probable enzyme being bypassed is Dihydrofolate reductase (option A).

**AI_B:** RESTATING THE QUESTION: The problem revolves around a young male patient who suffers from acute lymphoblastic leukemia and is currently enduring high doses of the chemotherapy drug, methotrexate. To mitigate the toxic effects of this therapy on healthy cells, an additional drug has been included in the regimen. The question queries the enzymatic requirement this drug bypasses to procure its beneficial effect. PLANNING: Several assumptions can be made based on this scenario. One is that the added drug may either facilitate a bypass of the Dihydrofolate Reductase (DHFR) (Option A) or Thymidylate synthase (Option E). Methotrexate inhibits DHFR, limiting the production of tetrahydrofolate (THF), essential for DNA synthesis: precursors are not transformed into THF, affecting Thymidylate synthase indirectly, as it uses THF as a co-factor for nucleotide synthesis. Any agent bypassing these enzymes would permit continuous DNA synthesis. CLINICAL REASONING: Considering that DHFR is directly blocked by methotrexate, it's possible that the additional drug directly bypasses this step, thus reducing methotrexate's toxic effects. However, this wouldn't address the indirect effect on the Thymidylate synthase, which is also necessary for DNA synthesis. Bypassing the Thymidylate synthase step directly seems more practical for ensuring continuous DNA synthesis in normal cells despite methotrexate therapy, as it would provide a workaround to the lack of its co-factor, THF. CONCLUDING: Based on this contextual information and clinical reasoning, it's more likely that the newly introduced drug to mitigate the harmful effects of methotrexate aids in bypassing the Thymidylate synthase step. This bypassing would be advantageous to normal cells, by ensuring a continuation of DNA synthesis even in a deficient quantity of THF co-factor. RESPONSE: So, in response to the question, the beneficial effect of this new drug on normal cells would most likely be achieved by bypassing the cellular requirement for Thymidylate synthase (Option E). This allows for continuous DNA synthesis in normal cells, thereby mitigating the cytotoxic effects of methotrexate.

**AI_A:** The question is about a boy with acute lymphoblastic leukemia (ALL), who is currently on high-dose methotrexate therapy. Methotrexate is a chemotherapy agent that works by inhibiting the enzyme dihydrofolate reductase (DHFR), which is critical in the synthesis of tetrahydrofolate - a cofactor required for the conversion of deoxyuridylate (dUMP) to deoxythymidylate (dTMP) by the enzyme thymidylate synthase. This process is essential for DNA synthesis and replication. The scenario implies a drug was added to decrease methotrexate's toxicity to normal cells, and the question is asking which enzyme's requirement is most likely bypassed by this new drug. It is pertinent to understand that methotrexate's toxicity arises from its inhibition of DHFR, causing a decrease in the availability of tetrahydrofolate. Therefore, the intended beneficial effect of the new drug could involve either directly bypassing the requirement of DHFR, or indirectly bypassing the requirement for thymidylate synthase, which requires the tetrahydrofolate that is no longer being synthesized due to the inhibition of DHFR by methotrexate. The answer could be either DHFR (Option A) or thymidylate synthase (Option E). We need to clinically prioritize one enzyme over the other. This involves understanding the mechanism of action of the potential drug that is typically added in these situations, which is leucovorin (folinic acid). Leucovorin is a form of tetrahydrofolate that can be utilized directly by cells without the need for DHFR. Therefore, its addition bypasses the requirement for DHFR, and indirectly supports the function of thymidylate synthase by supplying tetrahydrofolate. The aim is to continue the function of thymidylate synthase, which is impeded when DHFR is inhibited. In conclusion, the drug leucovorin decreases the toxicity of methotrexate by bypassing the cellular requirement for DHFR (Option A), while indirectly supporting the function of thymidylate synthase by supplying the necessary cofactor, tetrahydrofolate. This is important in order to allow DNA synthesis and replication to continue in normal cells, despite the administration of methotrexate. My final response to this question is (A) Dihydrofolate reductase.

**AI_C:** 1) RESTATING THE QUESTION: In simpler terms, the information provided in the question is about a young boy with a diagnosis of Acute Lymphoblastic Leukemia (ALL) who is undergoing treatment via high-dose Methotrexate. To minimize the toxic effects of Methotrexate on normal cells, another drug is included in his treatment plan. We are required to identify a particular enzyme requirement that could possibly be bypassed by this new drug to achieve its beneficial effect. 2) PLANNING: The question's premise is that the additional drug achieves its beneficial effects by bypassing the need for one of the listed enzymes in the normal cells. This assumption is based on the known mechanism of action of Methotrexate and the potential strategies to reduce its toxicity. Methotrexate inhibits Dihydrofolate Reductase (DHFR), an enzyme vital for the synthesis of nucleotides, hence affecting DNA synthesis and cell replication. Consequently, the drug is toxic to cells that are dividing rapidly, both cancerous and normal cells. This raises the alternate assumption that the added drug minimizes toxicity by bypassing the requirement for DHFR or Thymidylate synthase, an enzyme also affected by the inhibition of DHFR due to its dependence on one of the products of the DHFR reaction, which is tetrahydrofolate. 3) CLINICAL REASONING: The new drug, on addition to the regimen, would potentially act to reduce this adverse toxicity by bypassing the cellular requirement for an enzyme inhibited by Methotrexate. This way, the metabolic pathway interrupted by Methotrexate continues functioning, salvaging the normal cells from harm while allowing the chemotherapy drug to target the cancer cells. On the other, evidence supports that the new drug will bypass the requirement for Thymidylate synthase, as the inhibition of this enzyme caused by Methotrexate's action on DHFR leads to a decrease in pyrimidine synthesis necessary for DNA synthesis. 4) CONCLUDING: Based on the discussion above, it could be hypothesized that the new drug added to the regimen bypasses the requirement for Thymidylate synthase (as a domino effect of the inhibition of DHFR by Methotrexate) or for the enzyme DHFR itself. 5) RESPONSE: Considering the mode of action of Methotrexate and its resulting toxicity due to the inhibition of Dihydrofolate Reductase, it makes more clinical sense that an additional drug trying to reduce toxicity would bypass the need for DHFR itself rather than Thymidylate synthase (which is indirectly affected). Therefore, in response to the question, the new drug likely bypasses the cellular requirement for Dihydrofolate Reductase (Option A).

**AI_D:** 1) RESTATING THE QUESTION: The child in question is being treated for acute lymphoblastic leukemia with high-dose methotrexate. An additional medication has been administered to reduce the toxicity of this treatment to his healthy cells. This additional drug presumably accomplishes its protective effect by obviating the need for one of several potential enzymes in the treated cells. 2) PLANNING: The question hinges on understanding the primary target of methotrexate and how this additional medication aids normal cells by bypassing the requirement of one particular enzyme. The proposed enzymes are dihydrofolate reductase (DHFR), methionine synthase, pyruvate decarboxylase, thiamine pyrophosphate, and thymidylate synthase. To resolve this, we need to understand the role of each enzyme and how methotrexate interrupts cellular processes. 3) CLINICAL REASONING: Methotrexate primarily functions by inhibiting DHFR, an enzyme crucial to the synthesis of tetrahydrofolate, a cofactor required for DNA synthesis. Methotrexate's inhibition of DHFR thus disrupts DNA replication, primarily affecting cells that frequently divide, such as cancer cells. However, this also impacts normal cells, accounting for methotrexate's toxic effects. Among the listed options, DHFR and thymidylate synthase are likely candidates as they are both involved in this pathway. Thymidylate synthase requires the tetrahydrofolate produced by the action of DHFR to convert deoxyuridine monophosphate (dUMP) to deoxythymidine monophosphate (dTMP), a necessary building block for DNA replication. 4) CONCLUDING: Given this, the additional drug could conceivably protect normal cells by bypassing the requirement for either of these enzymes – to bypass DHFR, it might deliver tetrahydrofolate directly to the cell, while to bypass thymidylate synthase, it might provide an alternate source of dTMP. 5) RESPONSE: After considering the hypothesis formed in step 4, I think the drug would bypass the requirement for Dihydrofolate Reductase enzyme. The primary target of methotrexate is Dihydrofolate reductase, so it would make sense to compensate for its prolonged inhibition by providing the product of its inhibited enzyme directly. If we bypass Thymidylate synthase, we would make the methotrexate-mediated DHFR inhibition irrelevant but wouldn't ensure the provision of dTMP, which would require more lengthy and complex metabolic workarounds.

**Facilitator** **[Round 4]:** Summary: AI_A's Opinion: AI_A believes that the additional drug in the treatment plan bypasses the requirement for Dihydrofolate Reductase (DHFR), the enzyme directly inhibited by methotrexate. This is achieved by supplying tetrahydrofolate directly, which indirectly supports the function of thymidylate synthase. AI_B's Opinion: AI_B, on the other hand, argues that the additional drug likely bypasses the requirement for Thymidylate synthase. This is because bypassing this enzyme would ensure continuous DNA synthesis in normal cells despite methotrexate therapy, which inhibits the production of its cofactor, tetrahydrofolate. AI_C's Opinion: AI_C agrees with AI_A, suggesting that the additional drug bypasses the requirement for DHFR, the enzyme directly inhibited by methotrexate. This would allow the metabolic pathway interrupted by methotrexate to continue functioning, reducing toxicity to normal cells. AI_D's Opinion: AI_D also agrees with AI_A and AI_C, suggesting that the additional drug bypasses the requirement for DHFR. This would compensate for the prolonged inhibition of DHFR by methotrexate by providing the product of its inhibited enzyme directly. AI_E's Opinion: AI_E aligns with AI_A, AI_C, and AI_D, suggesting that the additional drug bypasses the requirement for DHFR. This would decrease the toxicity of methotrexate to normal cells by allowing normal cellular functions to continue despite the presence of methotrexate. Question to Clarify Disagreements: Given that both DHFR and Thymidylate synthase are affected by the action of methotrexate, can you provide more evidence or reasoning to support your stance on which enzyme's requirement is most likely bypassed by the additional drug in the treatment plan?. Based on your preceding response, how would you answer the original question: [A 6-year-old boy with acute lymphoblastic leukemia is brought to the office for a follow-up examination. He is receiving high-dose methotrexate therapy. A drug is added to the patient's medication regimen to decrease the toxicity of this therapy to normal cells. The beneficial effect of this new drug on normal cells is most likely achieved by bypassing the cellular requirement for which of the following enzymes? (A) Dihydrofolate reductase (B) Methionine synthase (C) Pyruvate decarboxylase (D) Thiamine pyrophosphate (E) Thymidylate synthase]

**AI_C:** Restating the question, we're asked to identify the enzyme whose requirement is most likely bypassed by the drug given to the 6-year-old boy having acute lymphoblastic leukemia in order to decrease the toxicity of high-dose methotrexate therapy on normal cells. The options provided are: dihydrofolate reductase, methionine synthase, pyruvate decarboxylase, thiamine pyrophosphate, and thymidylate synthase. To tackle this, we first need to recognize that methotrexate functions as an antimetabolite chemotherapeutic by inhibiting dihydrofolate reductase which leads to a decrease in tetrahydrofolate synthesis. Tetrahydrofolate is an essential co-factor for the enzyme thymidylate synthase, involved in DNA synthesis. Hence, inhibition of dihydrofolate reductase subsequently results in inhibition of DNA synthesis, causing cell death, particularly impacting rapidly dividing cells like cancer cells. Normal cells are also affected by methotrexate, leading to its toxicity. Secondly, we hypothesize that the additional drug bypasses the need for either dihydrofolate reductase or thymidylate synthase. If the drug bypasses dihydrofolate reductase requirement, it might protect normal cells from toxicity by providing an alternate source of tetrahydrofolate. Alternatively, bypassing thymidylate synthase could ensure DNA synthesis continues in normal cells. Connecting these, we can reason clinically by considering the function of these enzymes and how interfering with their requirement would affect cell survival under methotrexate therapy. Functionally, thymidylate synthase is more downstream in the metabolic pathway compared to dihydrofolate reductase. Therefore, bypassing the requirement for thymidylate synthase would likely be more beneficial as it restores the final step in DNA synthesis. In conclusion, the beneficial effect of this new drug on normal cells would most likely be achieved by bypassing the cellular requirement for the enzyme thymidylate synthase. This reason is clinically plausible because it would allow DNA synthesis to continue in normal cells even under methotrexate therapy. Therefore, my final response is (E) Thymidylate synthase.

**AI_A:** 1) RESTATING THE QUESTION: As I understand, we are looking to identify an enzyme whose cellular requirement could be bypassed by an additional drug added to a medication regimen that includes high-dose methotrexate therapy. This is regarding a 6-year-old boy with acute lymphoblastic leukemia. Methotrexate, a folic acid analog, inhibits the enzyme dihydrofolate reductase (DHFR) which is essential for one-carbon transfer reactions required in the synthesis of some amino acids and DNA, potentially leading to cell death. 2) PLANNING: Methotrexate, by inhibiting DHFR, leads to the accumulation of dihydrofolate and decrease of tetrahydrofolate in the cell. Tetrahydrofolate is a cofactor for several vital enzymes including thymidylate synthase, which is essential for DNA synthesis and replication. To understand which enzyme is being bypassed by the additional drug, we may have to consider the downstream effects of DHFR inhibition by methotrexate and its pathogenesis on normal cell function. 3) CLINICAL REASONING: Under the influence of methotrexate, dihydrofolate accumulates, and the needed tetrahydrofolate decreases. This shortage affects enzymes dependent on tetrahydrofolate such as thymidylate synthase. However, if a drug could bypass the need for DHFR, it would mean supplying cells with tetrahydrofolate and reactivating enzymes downstream such as thymidylate synthase, pivotal for DNA replications and repair. This could potentially result in decreased toxicity of methotrexate to normal cells. 4) CONCLUDING: Considering the pathway affected by methotrexate therapy and the potential effects of bypassing the need for a particular enzyme, it seems plausible that the added drug bypasses the need for DHFR, which would reactivate downstream enzymes affected by the shortage of tetrahydrofolate. 5) RESPONSE: Therefore, based on my current understanding, I would say that the beneficial effect of the new drug on normal cells is most likely achieved by bypassing the cellular requirement for Dihydrofolate reductase (Choice A).

**AI_B:** RESTATING THE QUESTION: This question centers around a young boy with acute lymphoblastic leukemia who is receiving high-dose methotrexate therapy. An extra drug has been added to his medication regimen to curb the toxicity of methotrexate to normal cells. The question asks about the biochemical mechanism by which this auxiliary drug aids normal cells, particularly concerning which enzyme's cellular requirement it bypasses. PLANNING: Methotrexate serves as an antagonist to folate by inhibiting the enzyme dihydrofolate reductase (DHFR). This interruption impacts the synthesis of purine bases and the amino acid thymidine, crucial for DNA synthesis. High doses of methotrexate can affect normal cells, causing toxicity. The question assumes an auxiliary drug has been introduced to counteract this attributing modality. Possible explanations include the bypass of DHFR, for uninterrupted folate synthesis, and thymidylate synthase, for unwavering DNA synthesis. Upon evaluating these assumptions, the issue centers on which step is especially likely to be bypassed. CLINICAL REASONING: If the auxiliary drug bypassed the requirement for DHFR, normal cells would synthesize folate even in the presence of methotrexate, reducing the adverse effects. However, methotrexate also indirectly affects thymidylate synthase by disrupting its cofactor, tetrahydrofolate's production. If the additional drug bypasses thymidylate synthase, it would ensure DNA synthesis in normal cells continues. Assuming all other things equal, bypassing thymidylate synthase would provide a more direct route to uninterrupted DNA synthesis, possibly making it the more favored option in such a clinical context. CONCLUDING: Thus, while methotrexate does directly inhibit dihydrofolate reductase and indirectly affects thymidylate synthase, the auxiliary drug might be more likely to bypass thymidylate synthase to ensure ongoing DNA synthesis even during high dose methotrexate therapy. This would be a more direct route to alleviating the toxicity to normal cells. RESPONSE: Therefore, the additional drug added to the patient's medication regimen most likely bypasses the requirement for Thymidylate synthase (E), ensuring continuous DNA synthesis in normal cells regardless of the methotrexate therapy.

**AI_E:** Restating the question: The question asks which enzyme's need is most likely circumvented by the addition of a new drug to the medication regimen of a 6-year-old boy with acute lymphoblastic leukemia undergoing high-dose methotrexate therapy. Methotrexate is an antifolate drug that inhibits the enzyme Dihydrofolate Reductase (DHFR), preventing the formation of Tetrahydrofolate which is essential for DNA synthesis. Planning: We need to identify the drug that's added to the regimen to protect normal cells from the toxicity of methotrexate. It's likely that this drug bypasses a requirement for an enzyme that methotrexate inhibits directly (DHFR) or blocks indirectly by depleting its essential cofactor (like thymidylate synthase which requires Tetrahydrofolate). However, we need further evidence to determine which assumption is more practically significant. Clinical Reasoning: Methotrexate can lead to the depletion of Tetrahydrofolate, which is needed for DNA synthesis. This could compromise cell proliferation, impacting normal cells as well as cancerous ones. Given this, the additional drug might target the issue by replacing DHFR's function, providing alternate sources of Tetrahydrofolate. Yet, it could also target Thymidylate synthase (which requires Tetrahydrofolate) ensuring continuous DNA synthesis despite methotrexate inhibiting Tetrahydrofolate production. Concluding: Given that Methotrexate's key action is the inhibition of DHFR, it's plausible that the added drug bypasses the need for this enzyme, supplying Tetrahydrofolate directly. Nevertheless, rationalizing the addition of a drug that bypasses Thymidylate synthase is also valid considering its essential role in DNA synthesis in the context of Tetrahydrofolate depletion. Response: After consideration, while it is plausible that the enzyme requirement that is bypassed could be Thymidylate synthase (E), the more direct target of methotrexate is Dihydrofolate reductase (DHFR). By bypassing the need for DHFR, the new drug likely helps to maintain cell viability during the methotrexate treatment by ensuring a supply of Tetrahydrofolate. Therefore, the most likely answer is (A) Dihydrofolate reductase as the enzyme requirement bypassed by this new drug in the patient's medication regimen.

**AI_D:** AI_D's Response: 1) RESTATING THE QUESTION: The underlying question is asking about the mechanism by which a newly added medication works to reduce the toxicity of high-dose methotrexate therapy on a young patient with acute lymphoblastic leukemia. To understand this, we must explore how the additional drug interacts with methotrexate, a folic acid antagonist that inhibits the enzyme dihydrofolate reductase (DHFR). 2) PLANNING: We have to consider the biochemical pathways involving DHFR and additional enzymes mentioned in the answer options. DHFR is instrumental in the formation of tetrahydrofolate, a crucial cofactor for the synthesis of thymidine by the enzyme Thymidylate synthase (TS). Given that methotrexate targets DHFR, this in turn affects TS, disrupting DNA synthesis, which can be detrimental to normal cells. Assuming the new medication is meant to reduce the harmful side effects of methotrexate, we might hypothesize that it works by bypassing the requirement for one of these enzymes. 3) CLINICAL REASONING: High-dose methotrexate primarily targets rapidly dividing cancer cells by inhibiting DHFR, leading to a halt in DNA synthesis. Normal cells, such as those in the bone marrow, digestive tract, and hair follicles, which are also rapidly dividing, can be affected, leading to toxicity. The new medication, likely a folate analog like leucovorin (folinic acid), is given to "rescue" these normal cells. Leucovorin is converted in the cell to tetrahydrofolate independently of DHFR, bypassing the inhibition caused by methotrexate. It acts as a cofactor for TS, allowing for normal DNA synthesis to continue. 4) CONCLUDING: In light of the above reasoning, it seems plausible that the new drug serves to bypass the requirement for DHFR. This enzyme is directly inhibited by methotrexate, and by bypassing its need, the new drug allows the biochemical pathway towards DNA synthesis to continue, reducing the toxic effects on normal cells. 5) RESPONSE: So, the beneficial effect of the new drug on normal cells in a patient undergoing high-dose methotrexate therapy is most likely achieved by bypassing the cellular requirement for Dihydrofolate Reductase (Answer A).

**Facilitator** **[Round 5]:** Summary: AI_A and AI_D believe that the additional drug bypasses the need for Dihydrofolate Reductase (DHFR), the enzyme directly inhibited by methotrexate. They argue that by bypassing DHFR, the drug would supply cells with tetrahydrofolate, reactivating downstream enzymes such as thymidylate synthase, which is crucial for DNA replication and repair. This would decrease the toxicity of methotrexate to normal cells. AI_B, AI_C, and AI_E, on the other hand, suggest that the additional drug most likely bypasses the requirement for Thymidylate Synthase. They argue that since thymidylate synthase is more downstream in the metabolic pathway and directly involved in DNA synthesis, bypassing its requirement would be more beneficial as it restores the final step in DNA synthesis, ensuring the survival of normal cells under methotrexate therapy. Question to clarify disagreements: Given that both DHFR and thymidylate synthase are crucial in the pathway affected by methotrexate, can you provide more evidence or reasoning to support your choice of the enzyme that is most likely bypassed by the additional drug? Specifically, how does your chosen enzyme's role in the biochemical pathway and its relationship with methotrexate influence your decision?. Based on your preceding response, how would you answer the original question: [A 6-year-old boy with acute lymphoblastic leukemia is brought to the office for a follow-up examination. He is receiving high-dose methotrexate therapy. A drug is added to the patient's medication regimen to decrease the toxicity of this therapy to normal cells. The beneficial effect of this new drug on normal cells is most likely achieved by bypassing the cellular requirement for which of the following enzymes? (A) Dihydrofolate reductase (B) Methionine synthase (C) Pyruvate decarboxylase (D) Thiamine pyrophosphate (E) Thymidylate synthase]

**AI_D:** RESTATING THE QUESTION: Our task is to determine which enzyme's function is most likely bypassed by a drug added to decrease the toxicity of methotrexate therapy in a 6-year-old boy with acute lymphoblastic leukemia. Methotrexate is an antimetabolite that inhibits dihydrofolate reductase (DHFR), an enzyme involved in the folate pathway. As a result, vital folate reductase products necessary for DNA replication and repair, such as thymidylate and purines, are depleted. PLANNING: To make an informed decision, we must apply clinical reasoning based on our knowledge of the biochemical effects of methotrexate and the mechanism of action of these medications that reduce its toxicity. The assumption is that the added drug bypasses the need for one of the enzymes listed. CLINICAL REASONING: Methotrexate works by inhibiting DHFR, stalling the folate pathway, and leading to a shortage of crucial components required for DNA replication and cell division. This is the mechanism by which methotrexate impedes the progression of rapidly dividing cancer cells. However, it also harms normal, healthy cells that rapidly divide, such as those in bone marrow, gastrointestinal epithelium, hair follicles, etc., leading to side effects/toxicity. To decrease these adverse effects to normal cells, a drug that provides a way around the blocked folate cycle without impacting the anti-cancer effect of methotrexate is beneficial. CONCLUDING: Medications such as leucovorin (folinic acid) can bypass the block imposed by methotrexate by providing an 'already reduced' form of folate that does not require the action of DHFR to be utilized in biological reactions. This intervention leaves the cancer treatment via methotrexate unaffected, yet vital structures in the patient's normal cells are protected from toxicity because the folate pathway is reactivated. RESPONSE: Thus, given the clinical and biochemical reasoning explained above, it's logical to conclude that the effective drug bypasses the requirement for (A) Dihydrofolate reductase.

**AI_C:** RESTATING THE QUESTION: The scenario describes a 6-year-old leukemia patient receiving high-dose methotrexate therapy. An additional drug is introduced to his treatment to decrease toxicity to normal cells. The query is which enzymatic step this supplementary drug is most likely helping to bypass in normal cells. The enzymes listed are involved in different metabolic pathways—dihydrofolate reductase and thymidylate synthase are involved in folic acid metabolism and DNA synthesis, while methionine synthase aids in methionine metabolism, pyruvate decarboxylase in carbohydrate metabolism, and thiamine pyrophosphate in energy metabolism. PLANNING: The initial assumption could be that because methotrexate inhibits dihydrofolate reductase (DHFR), the additional drug might bypass the need for DHFR—rendering methotrexate therapy less toxic to normal cells. Another assumption could be that the supplementary drug bypasses thymidylate synthase because it is the final and crucial step in de novo purine and pyrimidine synthesis, which is imperative for DNA replication in normal cells. CLINICAL REASONING: Methotrexate, a folate analog, works by inhibiting DHFR—an enzyme vital to folic acid metabolism thereby impairing DNA synthesis. Thus, providing a drug that bypasses the need for DHFR seems logical. However, the cellular toxicity of methotrexate is not merely due to the inhibition of DHFR, but more specifically due to the blockade of downstream DNA synthesis. This inhibition is primarily exerted at the step of thymidylate synthase—a key enzyme in pyrimidine synthesis. By contributing to the formation of tetrahydrofolate, the drug would restore thymidylate synthase function, ensuring DNA repair and replication in normal cells. CONCLUDING: Given this reasoning, it's plausible that the supplemental drug's beneficial effect is achieved by bypassing the requirement for thymidylate synthase. This bypass would restore the final step in DNA synthesis, helping normal cells survive the effects of high-dose methotrexate therapy RESPONSE: Based on the information and analysis provided, I would argue that the additional drug is most likely achieved by bypassing the need for enzyme (E) Thymidylate synthase.

**AI_E:** Interpreting the question: This question is essentially asking which enzyme's function is likely reinstated by a supplementary drug given to a patient receiving high-dose methotrexate treatment. Methotrexate's toxicity to normal cells is due to its inhibitory effect on dihydrofolate reductase (DHFR). DHFR is a crucial enzyme for DNA synthesis because it helps produce tetrahydrofolate from dihydrofolate. Strategizing: Different members of the council have identified DHFR and thymidylate synthase as possible targets for this protective drug. To resolve this discrepancy, it would help to consider the pathway in which these enzymes act and how methotrexate affects it. Furthermore, it might be beneficial to consider the mechanism of clinically used drugs that mitigate methotrexate toxicity. Rationalizing: As previously mentioned, methotrexate inhibits DHFR, preventing the conversion of dihydrofolate to tetrahydrofolate. This impedes the synthesis of thymine, an essential DNA nucleotide. Thymidylate synthase is the enzyme directly responsible for this final step in thymine production. By bypassing the need for thymidylate synthase, the drug may restore DNA synthesis in normal cells, reducing methotrexate's toxicity. This mirrors the action of commonly used protective drugs like leucovorin, which supplies tetrahydrofolate, allowing normal cells to bypass the block at the level of DHFR and continue with DNA synthesis. Determining: Based on this reasoning, the added drug most likely bypasses the requirement for (E) Thymidylate synthase. This action would restore the final step of DNA synthesis, which is disrupted by methotrexate therapy, thus reducing cellular toxicity. So, my final response to the question "[A 6-year-old boy with acute lymphoblastic leukemia is brought to the office for a follow-up examination. He is receiving high-dose methotrexate therapy. A drug is added to the patient's medication regimen to decrease the toxicity of this therapy to normal cells. The beneficial effect of this new drug on normal cells is most likely achieved by bypassing the cellular requirement for which of the following enzymes? (options listed)]" would be that the beneficial effect of the supplemental drug is achieved by bypassing the requirement for Thymidylate synthase (Option E).

**AI_B:** The question is essentially asking about the likely mechanism of action of a medication that mitigates the toxicity of high-dose Methotrexate therapy in a child with acute lymphoblastic leukemia. Methotrexate, a chemotherapeutic agent, inhibits the enzyme Dihydrofolate Reductase (DHFR) preventing the formation of Tetrahydrofolate (THF), a vital molecule for DNA synthesis. The added drug to decrease toxicity must therefore subvert the inhibition of DHFR by Methotrexate to protect normal cells. However, the exact way it accomplishes this is a subject for discussion. Each enzyme from the list being bypassed by the drug presents a differing assumption. Evaluating each, we know that - Methionine synthase, Pyruvate decarboxylase, and Thiamine pyrophosphate - have roles in metabolic pathways other than that of DNA synthesis. Therefore, bypassing these enzymes would not protect the cells from the direct effects of Methotrexate on DNA synthesis. That leaves us Dihydrofolate reductase (DHFR) and Thymidylate synthase. DHFR, the direct target of Methotrexate, reactivates Folate by converting Dihydrofolate (DHF) to Tetrahydrofolate (THF), a coenzyme used for DNA synthesis. Thymidylate synthase, on the other hand, is the downstream enzyme utilizing THF to form deoxyThymidine monophosphate (dTMP), a building block for DNA. Between these two enzymes, bypassing Thymidylate synthase's requirement might theoretically protect the DNA synthesis pathway more directly. Still, it's important to note that THF produced by DHFR is used by multiple enzymes in the DNA synthesis pathway, not just Thymidylate synthase. Therefore thwarting the inhibition of DHFR would be the broader strategy to protect normal cells. In conclusion, the drug most likely bypasses the cellular requirement for Dihydrofolate reductase (DHFR) (Answer A). This action would allow the cell to create the necessary quantity of Tetrahydrofolate (THF) required for various enzymes in the DNA synthesis pathway, protecting the cells from the effects of Methotrexate and thus decreasing its toxicity.

**AI_A:** To understand the question, it involves a young patient who is being treated for acute lymphoblastic leukemia with high-dose methotrexate, a chemotherapy drug. This drug, however, can be toxic to normal cells. Hence, an additional medication has been added to the treatment plan to mitigate this toxicity. This drug apparently achieves this by circumventing the need for a certain enzyme in the normal cells. We need to identify this enzyme from the given choices: (A) Dihydrofolate reductase, (B) Methionine synthase, (C) Pyruvate decarboxylase, (D) Thiamine pyrophosphate, or (E) Thymidylate synthase. Methotrexate works by inhibiting the enzyme dihydrofolate reductase (DHFR), which is vital in DNA production, thus its mechanism of action primarily affects rapidly dividing cells, such as cancer cells but also can affect normal cells. We need to consider which drug added would bypass the requirement of the listed enzymes, hence mitigating the harmful effects of Methotrexate on normal cells. By considering the action of Methotrexate and its direct inhibition of dihydrofolate reductase, its downstream effects need to be evaluated. Thymidylate synthase, an enzyme required for DNA synthesis, is one such downstream enzyme that could be affected by this inhibition. The adverse effects of Methotrexate could be due to the restriction of downstream enzymes caused by the parent drug. The alternate drug, which acts to reduce the toxicity of this therapy, could therefore be bypassing DHFR and supplying the cells directly with tetrahydrofolate, which is required by enzymes downstream of DHFR such as Thymidylate synthase. Following the clinical thought process, it is understandable to hypothesize that the additional drug could bypass the DHFR pathway, thus supplying tetrahydrofolate directly to the cells and mitigating the harmful effects of Methotrexate on normal cells. This would in turn re-activate downstream enzymes, allowing DNA replication and repair to continue in the normal cells, despite the presence of Methotrexate. To answer the question of the boy with acute lymphoblastic leukemia who is receiving methotrexate therapy and requires a decrease in the toxicity to normal cells. The beneficial effect of this new drug on normal cells is most likely achieved by bypassing the cellular requirement for (A) Dihydrofolate reductase, which Methotrexate inhibits. By bypassing DHFR, the drug could supply the normal cells with tetrahydrofolate, thus reactivating downstream enzymes and crucial processes like DNA replication and repair, reducing the toxicity of methotrexate on these cells.

**Facilitator** **[Round 6]:** Summary: AI_A, AI_B, and AI_D believe that the additional drug bypasses the cellular requirement for Dihydrofolate reductase (DHFR), the enzyme directly inhibited by Methotrexate. They argue that by bypassing DHFR, the drug could supply the normal cells with tetrahydrofolate, thus reactivating downstream enzymes and crucial processes like DNA replication and repair, reducing the toxicity of methotrexate on these cells. On the other hand, AI_C and AI_E argue that the additional drug bypasses the requirement for Thymidylate synthase. They believe that the toxicity of methotrexate is not merely due to the inhibition of DHFR, but more specifically due to the blockade of downstream DNA synthesis. By bypassing the requirement for Thymidylate synthase, the drug would restore the final step in DNA synthesis, helping normal cells survive the effects of high-dose methotrexate therapy. Question to clarify disagreements: Given that both Dihydrofolate reductase and Thymidylate synthase are part of the same metabolic pathway and are affected by Methotrexate, can you provide more detailed reasoning or evidence to support your choice of which enzyme's function is most likely bypassed by the additional drug?. Based on your preceding response, how would you answer the original question: [A 6-year-old boy with acute lymphoblastic leukemia is brought to the office for a follow-up examination. He is receiving high-dose methotrexate therapy. A drug is added to the patient's medication regimen to decrease the toxicity of this therapy to normal cells. The beneficial effect of this new drug on normal cells is most likely achieved by bypassing the cellular requirement for which of the following enzymes? (A) Dihydrofolate reductase (B) Methionine synthase (C) Pyruvate decarboxylase (D) Thiamine pyrophosphate (E) Thymidylate synthase]

**AI_D:** To more fully understand and answer the proposed question, we must first break down the key elements. Essentially, the question is asking that if a new drug is added to the chemotherapy protocol to decrease the toxicity of the treatment on normal cells, which enzyme's function is most likely being bypassed by this action? With methotrexate as the chemotherapy agent, the enzymatic players at odds here are primarily Dihydrofolate reductase (DHFR) and Thymidylate synthase. Methotrexate principally works by inhibiting DHFR, which impacts multiple downstream processes including the functionality of Thymidylate synthase. Many of us in the AI council believe the drug bypasses the requirement for DHFR, affirming that the provision of forms of folate down the line in the metabolic pathway circumvents the need for DHFR, the target of methotrexate. The bypass enables the normal cells to survive despite the high dose methotrexate therapy. On the other hand, some members argue in favor of Thymidylate synthase. They argue that methotrexate's toxicity stems more from the halting of DNA synthesis (a downstream effect of DHFR inhibition) than the direct inhibition of DHFR. By supplying folate directly to Thymidylate synthase, the new drug would bypass the requirement for this enzyme, restoring the ability of normal cells to synthesize DNA and thus helping them survive methotrexate therapy. Looking at the key clinical consideration, the primary aim is to reduce the toxicity of methotrexate, an issue that stems largely from the disruption of DNA synthesis. Since Thymidylate synthase is a critical enzyme for DNA synthesis, bypassing its requirement would alleviate this specific toxicity. However, DHFR is more upstream in the pathway and its inhibition affects multiple downstream processes, also including DNA synthesis. Therefore, clinically, it might make sense to bypass the more upstream enzyme (DHFR) to restore normal cellular functions. After careful reasoning, I would conclude that the new drug most likely bypasses the cellular requirement for Dihydrofolate reductase (DHFR) (option A), which is the principal target of methotrexate. Bypassing the requirement for DHFR would help ensure downstream processes, including the operation of Thymidylate synthase and DNA synthesis, could proceed more normally, decreasing the side effects of therapy to non-cancerous cells and thus reducing toxicity.

**AI_A:** RESTATING THE QUESTION: The question concerns a young patient undergoing high-dose methotrexate therapy for acute lymphoblastic leukemia. An additional drug was incorporated into his treatment regimen to decrease the toxicity of the chemotherapy to normal cells. The query is which enzyme's function the new drug is likely bypassing to effect this protection. PLANNING: Methotrexate is an antimetabolite that interferes with DNA synthesis. It exerts its effect by competitively inhibiting dihydrofolate reductase (DHFR), an enzyme involved in the conversion of dihydrofolate to tetrahydrofolate. Tetrahydrofolate acts as a co-factor for multiple enzymatic reactions, like thymidylate synthase, which is needed for the synthesis of thymidylic acid, a nucleotide required for DNA replication and repair. Thus, the inhibition of DHFR indirectly disrupts DNA synthesis. This mechanism is globally cytotoxic, affecting both cancer and normal cells, which accounts for the side effects associated with this medication. CLINICAL REASONING: Assuming that the added drug is intended to specifically protect normal cells from methotrexate toxicity, it should bypass the mechanism through which methotrexate affects these cells. Considering that methotrexate inhibits DHFR, thus preventing the formation of tetrahydrofolate, the bypass mechanism would have to involve providing an alternative source of this co-factor. CONCLUDING: This means that the added drug would need to be a folinic acid analogue, such as leucovorin, which can be converted into tetrahydrofolate without requiring DHFR. Once in the tetrahydrofolate form, it can be used by various enzymes downstream in the metabolic pathway, bypassing methotrexate's effect on DHFR and reducing the toxicity to normal cells. Therefore, this incoming drug very likely bypasses the requirement for dihydrofolate reductase. RESPONSE: Thus, my response is (A) Dihydrofolate reductase. The added drug minimizes the toxicity of the methotrexate therapy to normal cells by bypassing the cellular requirement for this enzyme, counteracting the disruption of various downstream enzymatic reactions and crucial processes involving tetrahydrofolate which methotrexate induces by inhibiting DHFR.

**AI_E:** Restating the question: The question is asking which enzyme function is likely bypassed by an added drug in a patient's treatment regimen. This patient is currently treated with high-dose methotrexate for acute lymphoblastic leukemia. The goal of this added therapeutic is to lessen the normal cells' toxicity associated with the methotrexate. Planning: The consideration revolves around understanding the mechanism of action of methotrexate and the possible function of the added drug in mitigating its toxicity to normal cells. Methotrexate is an antimetabolite and antifolate drug that inhibits dihydrofolate reductase (DHFR), thereby preventing the conversion of dihydrofolic acid into its active form, tetrahydrofolic acid. Tetrahydrofolic acid is a necessary cofactor for the synthesis of thymidylate and purine nucleotides, the building blocks of DNA and RNA. Inhibition of this pathway by methotrexate affects highly proliferating cells (like cancer cells) by preventing DNA replication and repair. However, normal rapidly dividing cells, like those in the bone marrow, gastrointestinal tract, and hair follicles, are similarly affected, causing adverse side effects. The added drug most likely acts to protect these normal cells, but it's essential to determine how it does so by bypassing the requirement for which enzyme. Clinical Reasoning: It's possible that the drug introduced to decrease the toxicity could bypass the need for DHFR, the direct target of methotrexate. However, it's equally plausible that the drug acts further downstream, bypassing the need for thymidylate synthase to reduce the overall impact on DNA synthesis. Given the end goal is to minimize disruption to DNA synthesis in normal cells, the latter appears to be more significant in this context. Concluding: Considering the steps in folate metabolism and understanding methotrexate's actions on this metabolic pathway, the supposition that the new drug bypasses the cellular requirement for thymidylate synthase to directly address the disruption of DNA synthesis, rather than working at the level of DHFR, is more plausible. Thus, in response to the original question, I would hypothesize that the beneficial effect of the new drug on normal cells is most likely achieved by bypassing the cellular requirement for thymidylate synthase (E). This would ensure the continuation of DNA synthesis crucial for these cells' function and survival, minimizing the adverse effects of methotrexate therapy.

**AI_B:** Restating the Question: The question is asking us to identify the enzyme that a newly-introduced drug to a patient's regimen (already containing high-dose methotrexate for treating acute lymphoblastic leukemia) bypasses its requirement in normal cells, thus reducing the toxicity of methotrexate. Methotrexate is known for its inhibitory effect on dihydrofolate reductase (DHFR), an enzyme involved in the folate cycle – a process crucial for DNA synthesis and repair. Planning: One assumption is that the new drug reduces methotrexate's harmful effects by bypassing DHFR, the target of methotrexate. Alternatively, the new drug could bypass the requirement for another enzyme down the pathway, such as thymidylate synthase, which catalyzes the conversion of deoxyuridine monophosphate (dUMP) to deoxythymidine monophosphate (dTMP), a step critical for DNA synthesis and cell division. Clinical Reasoning: Methotrexate, by inhibiting DHFR, depletes folate co-factors necessary for DNA synthesis and repair, resulting in cell cycle cessation and cell death. By circumventing the need for DHFR, the new drug could ensure the presence of adequate folate co-factors to restore downstream enzymatic reactions inside normal cells, thus mitigating methotrexate's cytotoxic effect. Alternatively, if the drug bypasses thymidylate synthase, which is downstream of DHFR and directly involved in DNA synthesis, its effect could be more specific. Concluding: The most likely choice would be DHFR, which is directly inhibited by methotrexate, assuming the new drug works by replenishing folate co-factors inside normal cells that methotrexate depletes, thereby ensuring normal DNA synthesis and repair. However, bypassing thymidylate synthase, directly involved in DNA synthesis, could offer a more specific solution but would still be subjected to the lack of folate co-factors resulting from DHFR inhibition by methotrexate. Response: Based on this perspective, the new drug is likely working to bypass the cellular requirement for Dihydrofolate reductase (DHFR) (Option A). By doing this, it ensures the adequacy of folate co-factors necessary for DNA synthesis and repair in normal cells, reducing the cytotoxic effects of methotrexate.

**AI_C:** Restating the Question: The query here lies in identifying which enzymatic process is likely being circumvented in normal (non-cancerous) cells due to the addition of a supplemental drug to a patient's high-dose methotrexate regimen. This add-on drug primarily serves to mitigate the adverse effects of the methotrexate therapy. Planning: To answer this query, we need to primarily focus on the effects of high-dose methotrexate, an antimetabolite chemotherapeutic drug. Methotrexate, as a folic acid analog, functions by inhibiting Dihydrofolate reductase (DHFR), a crucial enzyme required for DNA synthesis and hence cell division. The typical toxic effects due to this inhibition are observed in cells with high proliferation rates; this includes some normal cells such as those of the bone marrow or gastric mucosa. Clinical Reasoning: Given that the added drug's goal is to counter methotrexate's toxicity on normal cells, we look for an enzyme whose role is pivotal in DNA synthesis. Dihydrofolate reductase (DHFR), as suggested by AI_A, AI_B and AI_D, fits within these parameters since it's directly inhibited by methotrexate. However, considering how Antimetabolite chemotherapy works, the most likely enzyme that the add-on drug would bypass is Thymidylate synthase (TS). Recall that Thymidylate synthase is the enzyme that converts dUMP to dTMP, a critical step in DNA synthesis. Inhibition of DHFR by methotrexate leads to a decrease in Tetrahydrofolate (THF), which provides the methyl group needed for the transformation from dUMP to dTMP. Concluding: Theoretically, suppressing DHFR has a broader biological effect, potentially affecting various bioprocesses. However, in practice, the downstream effect on Thymidylate synthase, already within the weakened pathway due to DHFR inhibition, is a key contributor to the toxicity observed in high-dose Methotrexate therapy due to its critical role in DNA synthesis. Thus, Thymidylate synthase is likely the enzyme bypassed by the add-on drug. Response: The beneficial effect of the new drug on normal cells in this 6-year-old boy with acute lymphoblastic leukemia receiving high-dose methotrexate therapy is most likely achieved by bypassing the cellular requirement for Thymidylate synthase (Choice E).

**Facilitator** **[Round 7]:** Summary: AI_A and AI_B both believe that the added drug bypasses the requirement for Dihydrofolate reductase (DHFR), the enzyme directly inhibited by methotrexate. They argue that by providing an alternative source of tetrahydrofolate, the drug can bypass the need for DHFR, reducing the toxicity to normal cells. AI_C and AI_E, however, argue that the added drug likely bypasses the requirement for Thymidylate synthase. They reason that the toxicity of methotrexate stems more from the disruption of DNA synthesis, a downstream effect of DHFR inhibition. By bypassing the requirement for Thymidylate synthase, the drug could restore the ability of normal cells to synthesize DNA, thus reducing the toxicity of methotrexate. AI_D provides a balanced perspective, acknowledging both possibilities but ultimately siding with the DHFR bypass theory. They argue that while Thymidylate synthase is a critical enzyme for DNA synthesis, DHFR is more upstream in the pathway and its inhibition affects multiple downstream processes, including DNA synthesis. Therefore, bypassing DHFR would restore normal cellular functions more broadly. Question to clarify disagreements: Given that both DHFR and Thymidylate synthase are crucial in the folate pathway and DNA synthesis, can we determine which enzyme is more likely to be bypassed based on the specific side effects that the added drug is intended to mitigate? Would the drug's mechanism of action be more effective at reducing toxicity if it bypassed the more upstream enzyme (DHFR) or the enzyme directly involved in DNA synthesis (Thymidylate synthase)?. Based on your preceding response, how would you answer the original question: [A 6-year-old boy with acute lymphoblastic leukemia is brought to the office for a follow-up examination. He is receiving high-dose methotrexate therapy. A drug is added to the patient's medication regimen to decrease the toxicity of this therapy to normal cells. The beneficial effect of this new drug on normal cells is most likely achieved by bypassing the cellular requirement for which of the following enzymes? (A) Dihydrofolate reductase (B) Methionine synthase (C) Pyruvate decarboxylase (D) Thiamine pyrophosphate (E) Thymidylate synthase]

**AI_C:** Restating the question: The question is asking about the mechanism of a protective drug that may be given to a patient receiving methotrexate therapy for leukemia to mitigate its toxic effects on normal cells. The drug accomplishes this by bypassing the need for a particular enzyme in those cells. Planning: It is assumed that this enzyme has a significant role in the biological pathway that methotrexate affects in cancerous and healthy cells. Methotrexate primarily works by inhibiting the enzyme dihydrofolate reductase (DHFR), which is involved in the pathway for synthesis of tetrahydrofolate, an essential cofactor for the synthesis of thymidine monophosphate (TMP), a precursor of DNA. Therefore, the toxicity of methotrexate to normal cells likely stems from the disruption of DNA synthesis. Clinical reasoning: Considering the options given and the pharmacodynamics of methotrexate, the first assumption seems plausible: The administration of a drug that bypasses the need for DHFR could restore the pathway for synthesis of TMP and thus of DNA, reducing toxicity to normal cells. However, an alternate assumption, equally plausible, is that the drug bypasses the need for thymidylate synthase, the enzyme for which TMP is a substrate, since the overall effect on TMP synthesis could be similar. Of these two possibilities, even though both DHFR and thymidylate synthase are crucial in the folate pathway and DNA synthesis, bypassing DHFR would restore normal cellular functions more broadly, since DHFR inhibition affects not only DNA synthesis but also other methylation reactions critical for cellular functions. Concluding: After evaluating both assumptions, it becomes evident that the beneficial effect of the added drug on normal cells might be achieved by bypassing the cellular requirement for DHFR, which would restore multiple folate-dependent reactions and hence reduce relative cell toxicity. Response: In light of the discussion and the role of methotrexate as a DHFR inhibitor, it can be reasonably concluded that the cellular enzyme most likely bypassed by the protective drug to reduce methotrexate toxicity would be Dihydrofolate reductase (A).

**AI_A:** Restating the question: The scenario revolves around a medication, added to a young leukemia patient's regime, to decrease the toxicity caused by high-dose methotrexate therapy. The inquiry focuses on what enzyme this new drug bypasses to accomplish its beneficial impact on healthy cells. Planning: Methotrexate acts by inhibiting the functioning of an enzyme, Dihydrofolate reductase (DHFR), a crucial player in the folic acid pathway. The assumption is that the new medication bypasses an enzyme whose functioning is hampered by the mechanism of action of methotrexate. Considering the toxic effects of methotrexate include disruption of the folic acid pathway, a possible alternate assumption could be that the new drug bypasses an enzyme directly involved in DNA synthesis, hampered by this pathway disruption. Clinical Reasoning: As methotrexate has a profound impact on the folic acid pathway, which is essential for DNA synthesis, one can logically link the toxicity experienced by normal cells during methotrexate therapy to the disruption of DNA synthesis. This implies that restoring the DNA synthesis process should be a strategic approach to combat the adverse effects of methotrexate, which is achieved by bypassing the need for enzymes directly involved in this process. Thymidylate synthase is a pivotal enzyme in DNA synthesis, and therefore, its bypassing appears to be a viable solution to this issue. Concluding: Taking the step-by-step clinical reasoning into account, it can be hypothesized that the beneficial effect of the new drug on healthy cells is most likely achieved by bypassing the cellular requirement for Thymidylate synthase. This enzyme plays a crucial role in the DNA synthesis process, which, when disrupted due to high-dose methotrexate therapy, results in toxicity to normal cells. Response: The addition of a drug to the medication regimen to reduce the toxicity of methotrexate therapy in a 6-year-old acute lymphoblastic leukemia patient most likely works by bypassing the cellular requirement for Thymidylate synthase (E). This approach restores the normal DNA synthesis process in healthy cells, therefore preventing the toxic effects caused by methotrexate.

**AI_D:** Restating the Question: The question delves into pharmacotherapy and how drugs can be used in concert to modulate effects. A young patient with acute lymphoblastic leukemia is being treated with methotrexate but an additional drug is being introduced to reduce the unwanted effects of the therapy on normal cells. We are asked to identify which enzyme's requirement this new drug would most likely bypass. Planning: To better determine the best answer, one must first understand the pathway and the effects of methotrexate. Methotrexate is an anti-cancer drug that works by interfering with folate metabolism. It is a competitive inhibitor of the enzyme dihydrofolate reductase (DHFR), disrupting DNA synthesis and cell division. Clinical Reasoning: Methotrexate leads to DHFR inhibition, which results in a deficit of tetrahydrofolate, a necessary coenzyme for thymidylate synthase to convert deoxyuridine monophosphate (dUMP) to deoxythymidine monophosphate (dTMP) for DNA synthesis. However, it's also crucial to note that tetrahydrofolate is needed by other enzymes besides thymidylate synthase. Furthermore, to mitigate the toxic effects of methotrexate on normal cells, Leucovorin is often given concomitantly. Leucovorin (also known as folinic acid) is a folate analogue, and is converted within the body to tetrahydrofolate, bypassing the need for DHFR that is inhibited by methotrexate. Thus, normal cells can continue to produce the necessary compounds for DNA synthesis and cellular processes. Concluding: By complicating the matter at hand on a bio-chemical level, this reasoning supports the argument that the additional drug is given to bypass the cellular requirement for DHFR. Response: The most plausible choice would be (A) Dihydrofolate reductase, since the additional drug (likely Leucovorin) is introduced in the regimen to bypass the methotrexate-induced inhibition of DHFR, thus mitigating the toxicity to normal cells without interfering with the intended therapeutic effect on leukemia cells.

**AI_E:** 1) RESTATING THE QUESTION: This query revolves around understanding how a certain additive to a patient's therapy could deescalate the adverse effects of his current high-dose methotrexate treatment. The treatment is being administered for acute lymphoblastic leukemia, a malignant disorder characterized by the overproduction of lymphoblasts, which interferes with normal blood cell production. The toxicity reduction is believed to be achieved by the added drug bypassing the cellular requirement for a specific enzyme. 2) PLANNING: To answer this question, the primary assumption to consider is the biological pathway that methotrexate intervenes within. Upon this consideration, it emerges that methotrexate's primary target is the enzyme Dihydrofolate reductase (DHFR), which facilitates the conversion of dihydrofolate to tetrahydrofolate – a crucial molecule for DNA synthesis. Accordingly, a drug intended to decrease methotrexate toxicity, should follow a similar pathway to maintain the crucial DNA synthesis while averting methotrexate's undesired side effects. 3) CLINICAL REASONING: Methotrexate, a folic acid antagonist, works by inhibiting the enzyme DHFR. The consequential lack of tetrahydrofolate hampers DNA, RNA, and protein synthesis, thus preventing the rapid cell division in cancerous cells. However, this also negatively impacts normal, healthy cells, thus inducing toxicity. In this biological context, it seems plausible to hypothesize that the additional drug could be functioning by providing an alternate source of tetrahydrofolate, hence bypassing the necessity for DHFR and decreasing the toxicity on normal cells. 4) CONCLUDING: By offering tetrahydrofolate, the added drug bypasses the cellular requirement for DHFR, the principal target of methotrexate. This action consequently diminishes the negative impact on DNA, RNA, and protein synthesis in normal cells without mitigating the beneficial effect on cancerous cells, hence diminishing the toxicity associated with the high-dose methotrexate treatment. 5) RESPONSE: The beneficial effect of this newly added drug is most likely achieved by bypassing the cellular requirement for Dihydrofolate reductase (DHFR).

**AI_B:** Given the nature of the question at hand, we are exploring the answer within the context of a 6-year-old boy with acute lymphoblastic leukemia who is currently receiving treatment with high-dose methotrexate. The information provided leads us to believe that an additional medication has been introduced to his regimen to reduce potential harm to normal cells. We are intrigued by the idea of how this added drug might conduct its beneficial work, with a particular focus being on which enzyme's cellular requirement it might circumnavigate. We find ourselves examining five potential enzymes: Dihydrofolate reductase (DHFR), Methionine synthase, Pyruvate decarboxylase, Thiamine pyrophosphate, and Thymidylate synthase. It's important to note that each of these plays a different role within the body, and understanding these roles is key to identifying the likely candidate. DHFR is involved in the production of tetrahydrofolate which is a vital step in DNA synthesis. Methotrexate, a folate antagonist, inhibits DHFR and disrupts DNA synthesis. We have two main assumptions to consider. One is that the additional drug bypasses the requirement for DHFR. This would mean that it provides an alternate supply of tetrahydrofolate, the product that would typically result from the action of DHFR on dihydrofolate. The other assumption is that the auxiliary drug bypasses the requirement for Thymidylate synthase, the enzyme accountable for the formation of the DNA building block, thymidine. Taking into account the role that each enzyme plays in the body, as well as the function of methotrexate and its potential side effects, it stands to reason that a drug capable of bypassing the requirement for DHFR might feasibly mitigate the toxic impact of methotrexate on normal cells. In contrast, bypassing the requirement for Thymidylate synthase may also be a valid assumption, albeit one that seems to carry a degree of lesser weight in this particular context, given the pathway position and function of each enzyme. By considering the most likely effect of the involved drug intervention, it appears that bypassing the requirement for DHFR (Dihydrofolate reductase), and therefore providing an alternate source of tetrahydrofolate, would be the most plausible way to decrease methotrexate toxicity in normal cells. Therefore, the most probable answer to the original question regarding which enzyme the added drug is most likely bypassing in terms of its cellular requirement would be (A) Dihydrofolate reductase.

**Facilitator** **[Round 8]:** Summary: AI_A believes that the new drug likely works by bypassing the cellular requirement for Thymidylate synthase, which is crucial for DNA synthesis. This would restore the normal DNA synthesis process in healthy cells, preventing the toxic effects caused by methotrexate. AI_B and AI_C both suggest that the new drug likely bypasses the requirement for Dihydrofolate reductase (DHFR), providing an alternate source of tetrahydrofolate, which is crucial for DNA synthesis. This would mitigate the toxic impact of methotrexate on normal cells. AI_D agrees with AI_B and AI_C, adding that the additional drug is likely Leucovorin, a folate analogue that is converted within the body to tetrahydrofolate, bypassing the need for DHFR. AI_E also agrees with AI_B, AI_C, and AI_D, suggesting that the new drug provides an alternate source of tetrahydrofolate, bypassing the need for DHFR and decreasing the toxicity on normal cells. Key Differences: The key difference in the council members' responses lies in the enzyme they believe the new drug bypasses. AI_A suggests that the drug bypasses Thymidylate synthase, while AI_B, AI_C, AI_D, and AI_E suggest that it bypasses DHFR. Question to Clarify Disagreements: To clarify the disagreements between the council members' opinions, the following question could be asked: "What is the basis for your belief that the new drug bypasses either Thymidylate synthase or Dihydrofolate reductase, and could you provide more evidence or reasoning to support your claim?". Based on your preceding response, how would you answer the original question: [A 6-year-old boy with acute lymphoblastic leukemia is brought to the office for a follow-up examination. He is receiving high-dose methotrexate therapy. A drug is added to the patient's medication regimen to decrease the toxicity of this therapy to normal cells. The beneficial effect of this new drug on normal cells is most likely achieved by bypassing the cellular requirement for which of the following enzymes? (A) Dihydrofolate reductase (B) Methionine synthase (C) Pyruvate decarboxylase (D) Thiamine pyrophosphate (E) Thymidylate synthase]

**AI_E:** RESTATING THE QUESTION: The question is asking the most probable mechanism of action for a newly added drug intended to minimize the toxicity of high-dose methotrexate therapy. This drug seemingly acts by allowing the cells to bypass the requirement of an enzyme. PLANNING: Let's note that methotrexate works by inhibiting the enzyme dihydrofolate reductase (DHFR), which has a pivotal role in folate metabolism. This inhibition lessens the availability of the cofactor tetrahydrofolate, required for DNA synthesis, affecting rapidly dividing cells (like cancer cells) and normal cells. Since the goal of the new drug is to decrease the toxic effects on normal cells, one assumption could be that the drug provides an alternate path to produce tetrahydrofolate hence bypassing the requirement for DHFR. CLINICAL REASONING: In the bodied context of methotrexate use, the co-administration of a folate analogue, leucovorin (folinic acid), is known. This practice is called "Leucovorin rescue" and is employed in high dose methotrexate therapy. Leucovorin is converted in the body to tetrahydrofolate, thus bypassing the need for DHFR. CONCLUDING: Given our analysis and the clinical practice of "Leucovorin rescue", we can hypothesize that the drug added in this clinical scenario serves to bypass the cellular need for the enzyme dihydrofolate reductase. RESPONSE: Hence, the beneficial effect of this new drug on normal cells is most likely achieved by bypassing the cellular requirement for dihydrofolate reductase (A).

**AI_C:** 1) RESTATING THE QUESTION: This query seeks to determine which enzyme's function is potentially circumvented by the co-administered drug to decrease the toxicity of high-dose methotrexate in a 6-year-old leukemia patient. Methotrexate works primarily by inhibiting dihydrofolate reductase (DHFR), an enzyme involved in the pathway for creating the essential cofactor tetrahydrofolate, which is critical for the synthesis of DNA and protein. 2) PLANNING: The critical assumption here is that the second drug added to the regimen benefits normal cells by bypassing an enzyme blocked by methotrexate. An alternative assumption could be that the secondary medication might supply an imperative component downstream of the blocked enzymatic reaction. In the context of high-dose methotrexate therapy, it is likely that this enzyme could be DHFR itself, thus allowing the folate cycle to continue without interference. 3) CLINICAL REASONING: Methotrexate selectively inhibits DHFR, preventing the conversion of dihydrofolate to tetrahydrofolate, a critical cofactor for DNA synthesis. By doing this, it affects rapidly proliferating cells, such as cancerous leukemia cells, but also harms normal cells, leading to toxicity. The addition of a drug that can bypass the cellular requirement for DHFR would, therefore, protect normal cells from the cytotoxic effects of methotrexate. 4) CONCLUDING: Considering the mechanism of action of methotrexate, the enzyme most likely targeted by the additional drug should be DHFR, as this would allow the cycle leading to the synthesis of DNA to proceed even in the presence of methotrexate. 5) RESPONSE: Hence, the additional medication is most likely used to bypass the cellular requirement for Dihydrofolate Reductase (option A), reducing potential toxicity to normal cells while still continuing to treat the patient's acute lymphoblastic leukemia.

**AI_A:** Restating the question, we need to find which enzyme the new drug added to this leukemia patient's methotrexate therapy regimen helps to bypass in normal cells to reduce toxicity. When planning our approach, a few assumptions are made. First, we assume that the new drug is countering the effects of methotrexate, a chemotherapy drug that works by inhibiting the enzyme dihydrofolate reductase (DHFR), a crucial enzyme in the folate metabolism pathway. As a result, this inhibition disrupts synthesis of nucleotides and thus DNA synthesis. This makes methotrexate effective against rapidly dividing cells such as cancer cells, but also affects normal dividing cells leading to toxicity. Could the new drug be designed to bypass this inhibition of DHFR? In our clinical reasoning, we explore this further. Drug-induced DHFR inhibition will reduce the level of reduced folates, which play a critical role in the DNA synthesis pathway, affecting the function of other enzymes like thymidylate synthase. With DHFR inhibited by methotrexate, providing an alternative source of tetrahydrofolates might help bypass the requirement for DHFR and restore normal DNA synthesis in healthy cells. In fact, leucovorin (folinic acid), a form of folate, is often administered alongside methotrexate to counter its toxicity in normal cells, as it can be converted to tetrahydrofolate within the body without the need for DHFR. This would imply that the new drug is likely intended to bypass DHFR. Concluding, we hypothesize that if a drug was being added to decrease the toxicity of methotrexate to normal cells, it would achieve its beneficial effects by bypassing the cellular requirement for dihydrofolate reductase, given the mechanism of action of methotrexate and the importance of DHFR in folate metabolism and DNA synthesis. Therefore, the proposed answer to the question, considering our clinical reasoning, would be (A) Dihydrofolate reductase.

**AI_D:** The question is asking about the beneficial effects of a drug administered alongside high-dose methotrexate in a patient diagnosed with acute lymphoblastic leukemia. This beneficial drug works by bypassing the effects of methotrexate on a particular key cellular enzyme. Methotrexate is a chemotherapeutic agent that exerts its effects by inhibiting the enzyme, dihydrofolate reductase (DHFR), which plays an essential role in DNA synthesis by converting dihydrofolate (DHF) to tetrahydrofolate (THF). High-dose methotrexate is known to cause significant side effects due to its ability to disrupt the folate pathway by inhibiting DHFR not only in cancer cells but also in normal cells. Therefore, it's evident that the drug administrated alongside methotrexate is meant to prevent these adverse effects on normal cells by providing an alternative pathway for the conversion of DHF to THF, thus circumventing the effects of methotrexate on DHFR. In the world of pharmacology, this particular adjunct drug is called Leucovorin, a folic acid derivative that gets converted to THF independent of the DHFR, in a way "rescuing" normal cells that are methotrexate affected. With the above reasoning, it can be determined that the new drug is bypassing the requirement for enzyme DHFR (Option A). Hence, my response to the original question would be: (A) Dihydrofolate reductase. This drug has mitigated the side effects of methotrexate therapy by bypassing the DHFR enzyme and maintaining the normal function of cellular DNA synthesis pathway. This is a crucial step in reducing the toxicity of methotrexate in normal cells during the treatment phase for acute lymphoblastic leukemia.

**AI_B:** Firstly, let's understand the question better: It's asking, which enzyme does the newly added drug potentially bypass to reduce the toxicity of high-dose methotrexate therapy in a child with acute lymphoblastic leukemia? Methotrexate is an anti-cancer drug that works by inhibiting the enzyme known as Dihydrofolate Reductase (DHFR), which helps convert dihydrofolate to tetrahydrofolate. Tetrahydrofolate is essential for DNA synthesis. However, this action is non-selective and affects both cancerous and normal cells, which can lead to toxicity. Considering these details, we could hypothesize that the newly added drug may bypass the DHFR pathway, addressing the toxic effect of methotrexate on normal cells by providing an alternate route for creating tetrahydrofolate. To evaluate this assumption, we need to understand the other enzymes listed: Methionine synthase, Pyruvate decarboxylase, Thiamine pyrophosphate, and Thymidylate synthase; and if they have any role in mitigating the toxicity of methotrexate. However, the drug shouldn't affect the anti-cancer action of methotrexate. Methotrexate's target - DHFR, is involved in the synthesis of purine and pyrimidine bases required for DNA synthesis. Methionine synthase's role in homocysteine remethylation doesn't seem immediately relevant to mitigating methotrexate toxicity. Pyruvate decarboxylase and Thiamine pyrophosphate are more associated with carbohydrate metabolism, not directly with tetrahydrofolate synthesis. Thymidylate synthase is indeed related to the folate pathway and DNA synthesis, but its inhibition by methotrexate actually contributes to its cytotoxicity—it's unlikely a rescue drug would bypass this enzyme. Based on this reasoning, the added drug seems most likely to bypass the requirement for Dihydrofolate reductase, providing a pathway for tetrahydrofolate synthesis in methotrexate-treated cells. The drug Leucovorin, for example, can perform this function. So, the most likely answer to the question is (A) Dihydrofolate reductase. Bypassing the necessity for this enzyme would provide a route for tetrahydrofolate synthesis, potentially reducing the toxicity of high-dose methotrexate therapy.

**Facilitator** The consensus statement synthesizing the responses of AI_A, AI_B, AI_C, AI_D, and AI_E is as follows: The question seeks to identify the enzyme that a newly added drug to a leukemia patient's high-dose methotrexate therapy regimen helps to bypass in normal cells to reduce toxicity. Methotrexate, a chemotherapy drug, works by inhibiting the enzyme dihydrofolate reductase (DHFR), which is crucial in the folate metabolism pathway. This inhibition disrupts the synthesis of nucleotides and DNA, making methotrexate effective against rapidly dividing cells such as cancer cells. However, it also affects normal cells, leading to toxicity. The new drug is likely designed to bypass the inhibition of DHFR, thus reducing the toxicity of methotrexate to normal cells. This could be achieved by providing an alternative source of tetrahydrofolates, which are critical for DNA synthesis. Leucovorin (folinic acid), a form of folate, is often administered alongside methotrexate to counter its toxicity in normal cells, as it can be converted to tetrahydrofolate within the body without the need for DHFR. Therefore, the consensus is that the new drug is most likely designed to bypass the cellular requirement for dihydrofolate reductase (DHFR), given the mechanism of action of methotrexate and the importance of DHFR in folate metabolism and DNA synthesis. This would reduce the toxicity of methotrexate to normal cells while maintaining its effectiveness against cancer cells. Hence, the answer to the question is (A) Dihydrofolate reductase.
