# Supplementary material for: Collaborative intelligence in AI: Evaluating the performance of a council of AIs on the USMLE
Source: PLOS Digit Health. 2025 Oct 9;4(10):e0000787. doi: 10.1371/journal.pdig.0000787 (PMC12510544; doi:10.1371/journal.pdig.0000787)
Supplement: S2 File — (ZIP) [file pdig.0000787.s002.zip › S2 Data Raw data files containing unprocessed question inputs and LLM outputs including transcripts of deliberations/STEP1_Question-75-1_Type-MC-NJ.docx]

**Facilitator** **[Round 1]:** A 4082-g (9-lb) male newborn is delivered in the hospital at 40 weeks' gestation to a 28-year-old primigravid woman. Pregnancy and delivery were uncomplicated. Apgar scores were 8 and 9 at 1 and 5 minutes, respectively. Examination of the newborn's tongue shows a small blind pit in the midline located near the intersection of the anterior two-thirds and the posterior one-third. Ultrasonography of the neck shows a mass attached to the hyoid bone. Cells in the mass produce a hormone that binds to which of the following? (A) G protein-linked receptors (B) Ligand-gated ion channels (C) Nuclear receptors (D) Receptor tyrosine kinases (E) σ Receptors

**AI_D:** RESTATING THE QUESTION: The question presents a case of a newborn with a potential thyroid pathology suggested by the evidence of a neck mass attached to the hyoid bone, along with the unique finding on the tongue. It is asked which type of receptors this hormone produced by the mass binds. PLANNING: We need to assume that the cells in the mass are functioning in terms of hormone production, even if this is atypical. The hormone in question might be expected to have mechanisms of action that are more commonly associated with thyroid-associated cells. Often thyroid hormones, like thyroxine, bind to nuclear receptors. CLINICAL REASONING: The pit in the tongue, at the intersection of its anterior two-thirds and posterior third, could indicate the presence of a thyroglossal duct cyst, which is a form of ectopic thyroid tissue. The thyroglossal duct is the embryonic precursor of the thyroid gland and is normally obliterated during development. If remnants persist, they may form a cyst, which can be located anywhere along the duct's path—from the foramen cecum at the base of the tongue to the thyroid's normal anatomic location. The mass attached to the hyoid bone by ultrasound could represent an ectopic thyroid gland. It's important to consider that thyroid hormones, such as thyroxine (T4) and triiodothyronine (T3), primarily interact with nuclear receptors. CONCLUDING: Considering the background knowledge and information given in the clinical scenario, it is most likely that the hormone produced by the cells within the mass - likely thyroid tissue, would bind with nuclear receptors. RESPONSE: Therefore, the hormone produced by cells in this neck mass, likely thyroid cells, would bind to nuclear receptors (C).

**AI_E:** RESTATING THE QUESTION: The question inquires which type of receptor the hormone, produced by the mass found in the neck of a newborn male, binds to. The hormone-secreting mass was identified through an ultrasound-assisted examination following an initial finding of a blind pit on the tongue. PLANNING: To answer this question, we should first understand that this scenario likely describes a thyroglossal duct cyst (TGDC). A TGDC results from the persistent portion of the thyroglossal duct, which normally involutes during fetal development as the thyroid gland descends in the neck. As such, the hormone produced by this mass is likely thyroid hormone, which primarily interacts with nuclear receptors. To challenge this assumption, we could consider other hormones that could be produced by the TGDC or other types of receptors that thyroid hormone could bind to. CLINICAL REASONING: Thyroid hormones (T3 and T4) are primarily involved in regulation of metabolic functions. They bind to nuclear receptors in cells and modulate gene transcription to exert their effects. In case of TGDC, though ectopic thyroid tissue may secrete thyroid hormone, primary thyroid gland istypically functional which prevents clinical hypothyroidism. Hence, it would be fair to assume that the hormone produced by such a cyst binds primarily to nuclear receptors. CONCLUDING: The crucial point here is that thyroid hormones (presumably the ones produced by a TGDC) act primarily through nuclear receptors, interacting with the cell's genetic machinery to modulate physiological activities. Other hormones and respective receptors can be ruled out due to the clinical context provided. RESPONSE: The hormone produced by the mass attached to the hyoid bone in the newborn, presumably a thyroglossal duct cyst, primarily binds to (C) Nuclear receptors.

**AI_A:** RESTATING THE QUESTION: The question presents the scenario of a newborn baby with a mass attached to the hyoid bone that secretes hormones. The question aims to determine the type of receptors this hormone would likely bind to. PLANNING: The first assumption is that the pit in the tongue and the mass in the neck are part of the same pathology. However, one alternate assumption could be that they are simply coincident but unrelated findings. It's also assumed that the mass is producing a hormone, but it could potentially be producing other types of signals or molecules. We should prioritize the assumption that these are related and that the mass is producing a hormone, as it fits closely with common pathologies and helps guide the clinical reasoning process. CLINICAL REASONING: The described features lead to a highly probable diagnosis of a thyroglossal duct cyst. The thyroglossal duct is a developmental structure that typically involutes but can occasionally persist, leading to a cyst that often presents as a midline neck mass that moves with swallowing or tongue protrusion. In some cases, ectopic thyroid tissue might be present in the cyst. Thyroid hormones, namely triiodothyronine (T3) and thyroxine (T4), bind to nuclear receptors, which act as transcription factors that regulate gene expression. Therefore, if the hormone produced by the cells in the mass is a thyroid hormone, it would most likely bind to nuclear receptors. CONCLUDING: The combination of the clinical presentation, the presence of the midline pit in the tongue, and the mass attached to the hyoid bone strongly suggest a thyroglossal duct cyst with ectopic thyroid tissue. If this mass is indeed producing thyroid hormone, it would bind to nuclear receptors in target cells. RESPONSE: Therefore, the cells in the mass are producing a hormone that is most likely to bind to nuclear receptors (C).

**AI_B:** (1) RESTATING THE QUESTION: The question presents a scenario of a newborn baby who has a small blind pit on his tongue and a mass in the neck, attached to the hyoid bone. The mass, presumably, is producing a hormone. We're being asked to identify the type of receptor the hormone would bind with. These potential receptors can be G protein-linked receptors, ligand-gated ion channels, nuclear receptors, receptor tyrosine kinases, or σ receptors. (2) PLANNING: The first assumption would be that this child may have a thyroglossal duct cyst: the midline neck mass connected to the base of the tongue (via a small pit) is typical. This often represents the remnants of the tract through which the thyroid gland descends during embryological development. Then, the question of what hormone might be produced and to which receptor it would bind arises. The thyroid gland produces thyroid hormone (TH), which could be the hormone in question but other hormones might be involved too. However, the most logical assumption is to attribute the hormone production to the thyroid origin of the cyst (given the clinical findings suggestive of a thyroglossal duct cyst). (3) CLINICAL REASONING: Thyroid hormone (TH), produced by thyroid gland, exerts its physiological effects by binding to nuclear receptors. TH is a steroid hormone, and like other steroid hormones, it is lipophilic, allowing it to cross the cell membrane, bind to receptors in the cytoplasm, and then move into the nucleus. Here it interacts with DNA and affects gene transcription, influencing various metabolic processes. (4) CONCLUDING: Therefore, given that a thyroglossal duct cyst is likely (given the child's clinical findings), the hormone presumably being produced would probably be thyroid hormone, due to the thyroid tissue remnants that often exist in these cysts. This hormone interacts with nuclear receptors for its physiological effect. (5) RESPONSE: The hormone produced would bind to (C) Nuclear receptors.

**AI_C:** RESTATING THE QUESTION: To rephrase, we are looking at a perfectly healthy newborn with a normal gestational period, birth weight, and Apgar scores. An abnormality was detected during his physical examination relating to a small blind pit on his tongue at a specific anatomical location, which corresponds to a mass found on his hyoid bone via ultrasonography. A crucial inquiry is to identify the type of receptor that the hormone, secreted by the cells within the said mass, would bind to. The choices involve various signaling mechanisms, including G protein-linked receptors, ligand-gated ion channels, nuclear receptors, receptor tyrosine kinases, and σ receptors. PLANNING: Here assumptions include that the mass related to the tongue pit is the one producing the hormone and could be an ectopic tissue. Our alternative assumption, however, is that the mass could be a lingual thyroid representing an error in embryology where the thyroid tissue didn't descend properly. If so, the hormone it produces would be thyroid hormone. CLINICAL REASONING: Embryologically, the thyroid gland descends from the foramen cecum at the base of the tongue down to its position in front of the neck. In some cases, a portion of or the entire thyroid gland can be left behind, creating a lingual thyroid. The primary hormone produced by thyroid cells is thyroid hormone, which plays a critical role in various body functions, including metabolism, growth, and development. Moving to the types of receptors, the thyroid hormone acts in the cells by binding to nuclear receptors, which then directly modulates the transcription of target genes. CONCLUDING: Given the clinical and embryological context of the case, the assumption that the mass could be a remnant of the thyroid gland's embryological descent - a lingual thyroid - seems plausible. If this is the case, then the hormone produced by this ectopic thyroid tissue would likely be thyroid hormone, which is known to act via nuclear receptors within the cells. RESPONSE: Therefore, the hormone produced by the cells in the mass located at the hyoid bone, presumably a lingual thyroid, likely binds to (C) Nuclear receptors.

**Facilitator** The consensus statement synthesizes the responses of AI_A, AI_B, AI_C, AI_D, and AI_E as follows: The question presents a scenario of a newborn baby with a small blind pit on his tongue and a mass in the neck, attached to the hyoid bone. The mass is assumed to be producing a hormone. The clinical features suggest a probable diagnosis of a thyroglossal duct cyst, which often presents as a midline neck mass that moves with swallowing or tongue protrusion. This cyst could contain ectopic thyroid tissue, which would produce thyroid hormones, namely triiodothyronine (T3) and thyroxine (T4). Thyroid hormones exert their physiological effects by binding to nuclear receptors. These hormones are lipophilic, allowing them to cross the cell membrane, bind to receptors in the cytoplasm, and then move into the nucleus. Here they interact with DNA and affect gene transcription, influencing various metabolic processes. Therefore, given the clinical findings suggestive of a thyroglossal duct cyst, the hormone presumably being produced would probably be thyroid hormone. This hormone interacts with nuclear receptors for its physiological effect. Hence, the hormone produced by the cells in the mass located at the hyoid bone, presumably a thyroglossal duct cyst, likely binds to nuclear receptors.
