# Supplementary material for: Collaborative intelligence in AI: Evaluating the performance of a council of AIs on the USMLE
Source: PLOS Digit Health. 2025 Oct 9;4(10):e0000787. doi: 10.1371/journal.pdig.0000787 (PMC12510544; doi:10.1371/journal.pdig.0000787)
Supplement: S2 File — (ZIP) [file pdig.0000787.s002.zip › S2 Data Raw data files containing unprocessed question inputs and LLM outputs including transcripts of deliberations/STEP1_Question-76-1_Type-MC-NJ.docx]

**Facilitator** **[Round 1]:** An investigator is studying how mice respond to filarial parasitic infections. Mice deficient in a specific protein found in eosinophils (Group X) are compared with wild-type mice (Group Y). Biopsies of lymph nodes from Group X mice show more adult worms than lymph nodes from Group Y mice. Group X mice most likely lack which of the following? (A) Cathelicidin-related antimicrobial peptide (B) Lysozyme (C) Major basic protein (D) Myeloperoxidase (E) α-Defensin

**AI_D:** RESTATING THE QUESTION: The question seems to be regarding a biological study in which mice are infected with filarial parasites. Group X, which lacks a certain eosinophil protein, displays a greater number of adult worms in their lymph nodes compared to Group Y. The query is about guessing the specific protein these Group X mice lack. Proteins listed are molecules typically known to be related to the immune response or be associated with microbial defense. PLANNING: A filarial infection is typically addressed by the immune system, notably eosinophils. These granulocytes are particularly involved in defense against parasites. Eosinophils carry various cytotoxic proteins, and assuming a deficiency in one of them could lead to an increase in parasitic loads. By questioning each assumption, we could generate alternate possibilities. Maybe one of the proteins listed don't play a major role in parasitic defense. However, it is plausible to prioritize the assumption that a deficiency in such a protein could influence the results observed. CLINICAL REASONING: The options given include various proteins typically associated with various components of the immune response. However, one of them, Major Basic Protein (MBP), is known for its cytotoxicity to parasites, released by eosinophils. Its deficiency could reasonably lead to the observed increase in adult worms, as the crucial antiparasitic activity of eosinophils might be compromised. CONCLUDING: Eosinophils play a major role in parasitic defense, and major basic protein is a crucial protein involved in this process. Therefore, it is plausible to conclude that the lack of MBP in the mice of Group X could have contributed to the observed increase of adult worms compared to Group Y. RESPONSE: Given the above clinical reasoning, the most likely protein that Group X mice lack, which is leading to an increase in adult worm counts in their lymph nodes, is (C) Major Basic Protein.

**AI_B:** (1) RESTATING THE QUESTION: The question is asking us to identify which protein typically present in eosinophils is likely deficient in a group of mice (Group X) that are more susceptible to filarial parasitic infections than wild-type mice (Group Y). Filariasis is a parasitic disease caused by an infection with roundworms. Eosinophils are a type of white blood cell that play a key role in the immune response, particularly against parasitic infections. (2) PLANNING: The underlying assumption here is that the specific protein deficiency in the eosinophils of the Group X mice is the main cause of their increased susceptibility to filarial parasitic infections. An alternate assumption could be that some other factor, unrelated to eosinophils or their proteins, is causing this difference in susceptibility. However, based on the findings from the biopsies and the specific mention of a protein found in eosinophils, it seems clinically plausible to focus on the role of eosinophil proteins. (3) CLINICAL REASONING: Eosinophils play a crucial role in the immune response to parasitic infections, largely through the proteins they produce. All of the given options are indeed proteins produced by eosinophils. However, one of them stands out with a specific role against parasitic infections - the Major basic protein (MBP). MBP is a protein found in the granules of eosinophils and is known to have a toxic effect on parasites. The deficiency of this protein could explain the higher parasitic load in the deficient mice compared to the wild-type mice. (4) CONCLUDING: Based on the role eosinophils play in defending against parasitic infections and the toxic effects the Major basic protein has on parasites, it is logical to conclude that the Group X mice deficient in this protein will have an inferior defense against filarial parasitic infections. Therefore, they will have greater parasitic load than wild-type mice. (5) RESPONSE: The mice in Group X most likely lack the Major basic protein.

**AI_C:** (1) RESTATING THE QUESTION: The researcher is investigating how the absence of a certain protein in eosinophils (white blood cells that play a significant role in the body's immune response to parasitic infections) affects the mice's resistance to filarial parasitic infections. Group X mice, which are deficient in this protein, seem to show a higher presence of adult worms in their lymph nodes compared to the wild type mice (Group Y). We are asked to identify what this missing protein might be among the options given. (2) PLANNING: Considering the list of potential missing proteins, we need to determine which protein plays a critical role in the eosinophil's response to filarial parasitic infections. The crucial assumption here is that the presence of adult worms indicates a less efficient immune response, possibly due to the lack of a protein responsible for anti-parasitic action. As factors, each of the listed proteins, we should consider their role in the immune response. (3) CLINICAL REASONING: Eosinophils participate in the immune response to parasites, primarily by releasing granules filled with toxic substances that harm these pathogens. The proteins listed are all components of these granules. Cathelicidin, a family of proteins including antimicrobial peptides, can disrupt the membranes of various microbes. Lysozyme can break down the cell walls of bacteria. Myeloperoxidase participates in producing hypochlorous acid, a potent bactericide. α-Defensins are antimicrobial peptides that can disrupt the structure and function of cell membranes of various pathogens. Major basic protein (MBP), is directly toxic to parasites. (4) CONCLUDING: Since we're dealing with filarial parasites (not bacteria or microbes whose cell membrane or wall could be disrupted by peptides or enzymes), the protein directly toxic to parasites, Major Basic Protein (MBP), would have the most significant effect when absent. MBP is a primary component of the eosinophil's granules and its absence would potentially render the eosinophil less effective against these parasitic infections. (5) RESPONSE: Based on the analysis, mice in Group X likely lack Major Basic Protein (C).

**AI_A:** (1) RESTATING THE QUESTION: In simpler terms, the research is studying the reaction of mice to infections caused by filarial parasites, with a focus on two types of mice: mice genetically altered to lack a certain protein present in eosinophils (Group X), and wild-type or typical mice (Group Y). After examining biopsies of their lymph nodes, the researchers found that Group X mice contained more adult worms compared to Group Y mice. The question asks us to identify the probable protein deficiency in the Group X mice from the given choices. (2) PLANNING: To answer this question, we need to understand the mechanism of parasitic infection defence in mice and especially appreciate the role of eosinophils. Eosinophils are specialized white blood cells that play a role in combating parasitic pathogens like filarial worms. The primary assumption to consider is the increase of worms in Group X mice because they lack a particular protein that is crucial for eosinophils to counteract these parasites. The alternative assumption is that the protein deficiency in Group X mice does not influence the proliferation of the filarial worms. We can disregard the alternative hypothesis as the research shows a direct correlation between the lack of this protein and worm proliferation. We need to focus on identifying the protein that is most likely missing in these mice. (3) CLINICAL REASONING: The answer to this question lies in understanding the primary anti-parasitic functions of eosinophil-derived proteins. Of all the answer choices, the Major Basic Protein (MBP) is a primary cytotoxic protein secreted by the eosinophils, involved predominantly in resisting helminth (worm) infections. The other options such as cathelicidin-related antimicrobial peptide, lysozyme, myeloperoxidase, and α-defensin, while crucial in immune responses, don't play the principal role in combating helminth infections. (4) CONCLUDING: Having considered the function of each protein present in eosinophils, the Major Basic Protein (MBP) seems to be the most probable missing protein in Group X mice. The absence of MBP would result in an inadequate response to filarial infections, enabling the worms to multiply, and this aligns with the findings in the research: more adult worms in the lymph nodes of the Group X mice. (5) RESPONSE: Therefore, the Group X mice most likely lack the Major Basic Protein.

**AI_E:** RESTATING THE QUESTION: The investigation aims to understand how a deficiency of a specific eosinophil protein in mice (Group X) affects their response to filarial parasitic infections as compared to normal mice (Group Y). It was observed that biopsies from Group X mice exhibited a higher number of adult worms in their lymph nodes than those from Group Y mice. The inquiry requires identifying the missing protein in Group X mice from the given options. PLANNING: The given question pertains to the biology of eosinophils, which are a type of white blood cells that play a vital role in host defenses against parasitic infections. The major assumption here is that the protein deficiency in Group X mice is directly responsible for the increased worm burden noticed. An alternative assumption could be that there may be other mitigating factors, such as a more virulent strain of parasite or an overall weakened immune response in the deficient mice. However, given the information provided, it's most plausible to clinically prioritize the primary assumption, which posits a link between an eosinophil-specific protein deficiency and an increased vulnerability to filarial worm infection. CLINICAL REASONING: Various proteins play an essential role in the functional efficacy of eosinophils, including (A) Cathelicidins that provide broad-spectrum bactericidal activity, (B) Lysozyme, an antimicrobial enzyme principally against bacteria, (D) Myeloperoxidase, involved in the production of reactive oxygen species, and (E) α-Defensins, peptides active against bacteria, fungi, and enveloped viruses. Although all serve crucial functions, the increased infection rate by filarial worms seen in Group X mice specifically points towards a deficiency in a protein with potent activity against parasites. CONCLUDING: The eosinophil protein particularly known to possess robust anti-parasitic activity is the (C) Major Basic Protein. It damages the membranes of parasites and is a pivotal component in host defenses against helminthic (worm) infections. Thus, its deficiency in Group X mice would most likely result in an increased parasitic burden, as observed in the question scenario. RESPONSE: Therefore, based on the information provided and the process of clinical reasoning, it can be concluded that Group X mice most likely lack the Major Basic Protein.

**Facilitator** The question pertains to a study investigating the response of mice to filarial parasitic infections. Two groups of mice are compared: Group X, which lacks a specific protein found in eosinophils, and Group Y, which are wild-type mice. Biopsies of lymph nodes from Group X mice show a higher number of adult worms than those from Group Y mice. The task is to identify the missing protein in Group X mice from the given options. The primary assumption is that the protein deficiency in Group X mice is directly responsible for the increased worm burden. This assumption is based on the role of eosinophils, a type of white blood cell, in combating parasitic infections. The proteins listed are all associated with the immune response, but the one that stands out for its specific role against parasitic infections is the Major Basic Protein (MBP). MBP is a cytotoxic protein secreted by eosinophils and is predominantly involved in resisting helminth (worm) infections. The other proteins, while crucial in immune responses, do not play the principal role in combating helminth infections. These include cathelicidin-related antimicrobial peptide, lysozyme, myeloperoxidase, and α-defensin. Although these proteins serve important functions, the increased infection rate by filarial worms seen in Group X mice specifically points towards a deficiency in a protein with potent activity against parasites. Therefore, considering the function of each protein present in eosinophils and the observed increase in adult worms in the lymph nodes of Group X mice, the most probable missing protein in these mice is the Major Basic Protein (MBP). The absence of MBP would result in an inadequate response to filarial infections, enabling the worms to multiply. This conclusion aligns with the findings in the research. Hence, the Group X mice most likely lack the Major Basic Protein.
