# Supplementary material for: Collaborative intelligence in AI: Evaluating the performance of a council of AIs on the USMLE
Source: PLOS Digit Health. 2025 Oct 9;4(10):e0000787. doi: 10.1371/journal.pdig.0000787 (PMC12510544; doi:10.1371/journal.pdig.0000787)
Supplement: S2 File — (ZIP) [file pdig.0000787.s002.zip › S2 Data Raw data files containing unprocessed question inputs and LLM outputs including transcripts of deliberations/STEP1_Question-78-1_Type-MC-NJ.docx]

**Facilitator** **[Round 1]:** A 50-year-old man comes to the office because of a 2-month history of increasing daytime somnolence. He has obstructive sleep apnea for which he has only intermittently used a continuous positive airway pressure device. He is 170 cm (5 ft 7 in) tall and weighs 181 kg (400 lb); BMI is 63 kg/m2. His temperature is 37°C (98.6°F), pulse is 100/min, respirations are 12/min, and blood pressure is 135/80 mm Hg. Physical examination shows a gray-blue tinge to the lips, earlobes, and nail beds. Cardiac examination shows no other abnormalities. Arterial blood gas analysis on room air shows a pH of 7.31, PCO2 of 70 mm Hg, and PO2 of 50 mm Hg. Which of the following additional findings would be most likely in this patient? (A) Decreased serum bicarbonate concentration (B) Increased hemoglobin concentration (C) Increased total lung capacity (D) Left ventricular hypertrophy

**AI_C:** (1) RESTATING THE QUESTION: The query ponders about a specific symptom that we might expect from a 50-year-old man with a history of obstruction sleep apnea and intermittent use of a continuous positive airway pressure device, whose physical characteristics and medical readings hint at a potential respiratory disorder. This observation is based on a variety of factors such as the high body mass index (BMI), observations of cyanosis, and abnormal arterial blood gas analysis. (2) PLANNING: Core assumptions we can draw from the available symptoms and data include that the patient's apparent sleep apnea, elevated BMI, and described symptoms like daytime somnolence and cyanosis, could be related to an underlying issue impacting his respiratory health. The alternate assumption could be that he might be suffering from hypoxemia, as a result of hypoventilation due to his obstructive sleep apnea. This assumption would be clinically prioritized due to the strong and apparent connection between his given symptoms and a potential respiratory disorder. (3) CLINICAL REASONING: The findings of gray-blue tinge in certain areas of the body, which suggests cyanosis, hint at a lack of oxygen in the blood. Furthermore, arterial blood gas analysis shows a pH level of 7.31, suggesting a potential respiratory acidosis. With the PCO2 level observed higher than normal, it indicates hypercapnia, and a PO2 level less than expected signifies hypoxemia. These cardiac readings suggest that the patient is retaining CO2, causing acidemia and at the same time is deficient in O2. (4) CONCLUDING: Considering all the signs and symptoms, and connecting them clinically, it comes across that chronic hypoxia due to hypoventilation from obstructive sleep apnea can lead to secondary polycythemia - an increase in the hemoglobin concentration. In response to chronic hypoxia, the body tries to augment oxygen delivery by producing more red blood cells, thus increasing hemoglobin, which carries oxygen. (5) RESPONSE: Therefore, the most likely additional finding in this patient would be (B) Increased hemoglobin concentration.

**AI_B:** RESTATING THE QUESTION: The case involves a 50-year-old man with a history of obstructive sleep apnea and morbid obesity who presents with cyanosis and daytime somnolence. He has hypercapnia and hypoxemia demonstrated by an arterial blood gas analysis. The question seeks to understand the most likely additional finding in this patient given the choice of options comprising a decreased serum bicarbonate concentration, increased hemoglobin concentration, increased total lung capacity, or left ventricular hypertrophy. PLANNING: The assumptions to consider here include the pathological consequences of his underlying obstructive sleep apnea and morbid obesity such as long-term hypoxemia and hypercapnia. It seems relevant to consider that prolonged hypoxia may trigger physiological responses such as increased red blood cell production. Additionally, obstructive sleep apnea can cause pressure changes in the chest, leading to cardiac changes. Given these implications, the alternate assumptions that might be prioritized would be increased hemoglobin concentration and left ventricular hypertrophy. CLINICAL REASONING: Chronic untreated obstructive sleep apnea may result in sustained low oxygen levels or hypoxemia, which is evident from the arterial blood gas results. Hypoxemia typically induces erythropoiesis—production of red blood cells—as a compensatory mechanism to improve oxygen-carrying capacity of the blood, leading to increased serum hemoglobin concentration. At the same time, the chronic intermittent hypoxia and hypercapnia can cause pulmonary hypertension, which subsequently causes an increase in afterload experienced by the left ventricle, potentially leading to left ventricular hypertrophy over time. Decreased bicarbonate concentration and increased total lung capacity are not directly related to the pathophysiology of obstructive sleep apnea, hence, can be deemed less likely findings. CONCLUDING: Taking into account the patient's symptoms, physical findings, and results of arterial blood gas analysis, and understanding the physiological compensatory mechanisms and pathophysiological alterations in response to chronic hypoxia and hypercapnia occurring in untreated or inadequately treated obstructive sleep apnea, it is reasonable to assume that both increased hemoglobin concentration and left ventricular hypertrophy are probable in such a clinical scenario. RESPONSE: Of all given options, (B) increased hemoglobin concentration and (D) left ventricular hypertrophy would be the most likely additional findings in this patient due to long-term hypoxemia and hypercapnia resulting from his insufficiently treated obstructive sleep apnea.

**AI_D:** RESTATING THE QUESTION: To summarize, the question seems to be regarding the additional findings that are likely to occur in this 50-year-old man who presents with symptoms of increasing daytime somnolence, which indicates excessive daytime sleepiness or prolonged nighttime sleep. He has obstructive sleep apnea (OSA), a chronic condition in which there are repeated episodes of complete or partial blockage of upper airways during sleep. The patient is also morbidly obese (BMI 63kg/m2) and shows cyanotic features (blue-tinged lips, earlobes, nails) that point towards chronic hypoxemia, or low oxygen in the blood. Additionally, his ABG values demonstrate respiratory acidosis, marked by decreased pH and elevated PCO2 levels. The options are possible related conditions or impacts: serum bicarbonate concentration (a measure of metabolic component in the body's acid-base balance), hemoglobin concentration (protein in red blood cells that carries oxygen), total lung capacity (maximum amount of air that can fill the lungs), and left ventricular hypertrophy (enlargement and thickening of the wall of the heart's left pumping chamber). PLANNING: As we know, Obstructive Sleep Apnea is caused by recurrent episodes of upper airway obstruction during sleep which often results in decreased oxygen saturation and arousal from sleep. This chronic hypoxemia can lead to various adaptive mechanisms in the body such as an increased production of red blood cells. On the other hand, the patient's obesity also puts an additional chronic stress on the heart leading to left ventricular hypertrophy. Increased bicarbonate concentration not decreased, may be the body's response to compensate for the respiratory acidosis. Also, the patient may have a normal or even decreased lung capacity because the obesity means less space in the thorax for the lungs to expand. CLINICAL REASONING: Considering the entire picture, the patient's chronic hypoxemia caused by OSA would stimulate the kidneys to produce erythropoietin, a hormone that stimulates the production of red blood cells, thereby increasing hemoglobin concentration. This is a compensatory mechanism body employs to improve oxygen-carrying capacity of blood. Additionally, the patient's left ventricular hypertrophy could be another plausible secondary complication occurring due to obesity and chronic hypoxia, as he has to pump against increased systemic vascular resistance and also needs to compensate for the chronic hypoxia. However, the acid-base changes in the patient suggest a chronic process, and the body might have compensated by increasing the serum bicarbonate concentration, not decreasing it. Lung capacity is likely normal or might even be decreased due to obesity, as the extra tissue restricts lung expansion. CONCLUDING: Therefore, the condition that best fits this patient's clinical picture is the one that deals with body's adaptation to chronic hypoxemia caused by obstructive sleep apnea, which is 'increased hemoglobin concentration'. RESPONSE: This patient will most likely present with increased hemoglobin concentration (Option B).

**AI_A:** RESTATING THE QUESTION: The inquiry focuses on a 50-year-old obese man with obstructive sleep apnea (OSA), substantial weight, and significant daytime sleepiness who presents with a grayish-blue tinge in soft tissues like lips, earlobes, and nail beds. An arterial blood gas evaluation reveals his blood has high carbon dioxide (PCO2) and low oxygen (PO2) levels, and is more acidic (pH 7.31) than normal. The question is asking what additional condition or alteration would most likely be discovered in this patient given his current clinical state. The options proposed involve changes in bicarbonate concentration, hemoglobin concentration, total lung capacity, or the presence of left ventricular hypertrophy. PLANNING: Assumptions could be made based on his signs and symptoms along with his medical history: the cyanosis (gray-blue tinge) hints towards hypoxia, and the blood gas analysis confirms this with a decreased PO2. The high PCO2 and low pH suggest chronic hypoventilation, which is common in patients with untreated OSA, especially those who are obese as this patient. This chronic hypoxemia and hypercapnia could lead to compensatory mechanisms in the body, such as polycythemia, which would mean increased hemoglobin concentration. It could also induce pulmonary hypertension, promoting left ventricular hypertrophy. Changes in bicarbonate would be relevant if the body's compensatory mechanisms for acidity changes are considered, but generally, in chronic hypoventilation, bicarbonate levels increase, not decrease. As for total lung capacity, while obesity can limit thoracic expansion, it's unlikely to lead to an actual increase in total lung capacity. CLINICAL REASONING: Chronic hypoventilation marked by an elevated PCO2 and a decreased PO2 is a common sign of obstructive sleep apnea particularly when it is poorly treated. The body attempts to compensate for this abnormal gas exchange situation. Polycythemia, indicated by an increased hemoglobin concentration, has been known to occur as a compensatory response to chronic hypoxemia. Polycythemia improves the blood's oxygen-carrying capacity. The patient's hypoxemia, manifested as cyanosis, points to the existence of this chronic under-oxygenated state, hence raising the probability of polycythemia. CONCLUDING: Accentuating the entire clinical picture and pathophysiological process, it seems logical to postulate polycythemia (reflected as increased hemoglobin concentration) as a possible additional finding in this patient. This statement is situated within a systematic thought framework, associating chronic hypoventilation from obstructive sleep apnea, the patient's indicative signs and symptoms, and known compensatory processes for sustained hypoxemia in the body. RESPONSE: Therefore, among the options given, it would be most likely to find an "(B) Increased hemoglobin concentration" in this patient, reflecting an adaptive response, namely polycythemia, to his chronic hypoxemic state.

**AI_E:** (1) RESTATING THE QUESTION: A 50-year-old man who has been diagnosed with obstructive sleep apnea has been experiencing increasing daytime sleepiness over the past two months. Although he has been prescribed a continuous positive airway pressure (CPAP) device, he only uses it inconsistently. He is also obese, and has a bluish-grey discoloration to his lips, earlobes, and nail beds. Arterial blood gas analysis indicates respiratory acidosis, characterised by a low blood pH, high carbon dioxide levels, and low oxygen levels. We're being asked to identify the most likely associated finding in this patient from a list of potential options: a decrease in serum bicarbonate concentration, an increase in hemoglobin concentration, an increase in total lung capacity, or left ventricular hypertrophy. (2) PLANNING: The clinical characteristics of this patient indicate a chronic state of hypoxia and hypercapnia, results of untreated or ineffectively treated obstructive sleep apnea. Chronic hypoxia and hypercapnia can result in a compensatory response from the body. Typically, the assumptions to consider in such a situation would involve analysing the possible physiological adaptations the body might make. Among these adaptations could be a fall in serum bicarbonate concentration, a rise in hemoglobin concentration, a rise in total lung capacity, or an enlargement of the left ventricle of the heart. The assumption of a fall in serum bicarbonate could result from the body's attempt to correct the acidosis caused by the buildup of carbon dioxide. However, in chronic conditions, the kidneys work to conserve bicarbonate to compensate for respiratory acidosis, so a decrease in serum bicarbonate is less likely. The assumption of a rise in hemoglobin concentration seems more plausible. To counteract chronic hypoxia, the body may increase the production of red blood cells to improve oxygen-carrying capacity. The assumption of an increase in total lung capacity seems less likely as increased lung capacity would not necessarily improve the patient’s underlying pathology of obstructive sleep apnea. Lastly, the assumption of an enlargement of the left ventricle could stem from the interpretation of the elevated blood pressure reading and chronic hypoxia, possibly resulting in the increased workload on the heart. (3) CLINICAL REASONING: Bearing these assumptions in mind, let's begin with the least likely – a decrease in the serum bicarbonate concentration. As mentioned, the chronic state of the body would result in renal compensation retaining bicarbonate to counteract the acidosis. Therefore, bicarbonate levels are typically normal or even slightly increased in respiratory acidosis. Increased total lung capacity seems less likely as obstructive sleep apnea is a problem with airway resistance rather than lung capacity. So increasing the lung capacity would not necessarily alleviate the problem. Also, obesity can actually decrease total lung capacity due to the extra weight compressing the lungs. Left ventricular hypertrophy is a possibility due to prolonged high blood pressure and chronic hypoxia, both of which increase the workload on the heart. However, since the cardiac examination shows no other abnormalities, this option seems less likely. The most probable assumption to consider is the increase in hemoglobin concentration. Chronic hypoxia induces hyperproduction of erythropoietin, a hormone that stimulates the production of red blood cells, leading to an increase in hemoglobin concentration. This is a compensatory mechanism to enhance the oxygen-carrying capacity of the blood. (4) CONCLUDING: So, given all these possibilities, it's crucial to consider the body's most likely response to chronic hypoxia - increased hemoglobin concentration, as this would make the most significant impact in compensating for the lack of oxygen. (5) RESPONSE: Therefore, the most probable finding you would encounter in this patient is an (B) Increased hemoglobin concentration.

**Facilitator** The question presents a case of a 50-year-old man with obstructive sleep apnea (OSA) and morbid obesity, who has been experiencing increasing daytime sleepiness and presents with cyanosis. His arterial blood gas analysis reveals respiratory acidosis, characterized by a low blood pH, high carbon dioxide levels, and low oxygen levels. The question asks for the most likely additional finding in this patient from a list of options: decreased serum bicarbonate concentration, increased hemoglobin concentration, increased total lung capacity, or left ventricular hypertrophy. The consensus among the AI responses is that the most likely additional finding in this patient would be an increased hemoglobin concentration. This conclusion is based on the understanding that chronic hypoxemia, a condition evident in this patient, often triggers a compensatory response in the body to increase the production of red blood cells, thereby increasing the hemoglobin concentration to improve the oxygen-carrying capacity of the blood. This physiological adaptation is known as polycythemia. The possibility of decreased serum bicarbonate concentration is considered less likely, as in chronic conditions like this, the kidneys typically work to conserve bicarbonate to compensate for respiratory acidosis. Therefore, bicarbonate levels are usually normal or even slightly increased in such cases. Increased total lung capacity is also deemed less likely, as OSA is primarily a problem with airway resistance rather than lung capacity. Furthermore, the patient's obesity could potentially decrease total lung capacity due to the extra weight compressing the lungs. While left ventricular hypertrophy could be a possibility due to prolonged high blood pressure and chronic hypoxia, both of which increase the workload on the heart, it is considered less likely in this case as the cardiac examination shows no other abnormalities. Therefore, the consensus response to the question is that the most probable additional finding in this patient would be an increased hemoglobin concentration (Option B).
