# Supplementary material for: Collaborative intelligence in AI: Evaluating the performance of a council of AIs on the USMLE
Source: PLOS Digit Health. 2025 Oct 9;4(10):e0000787. doi: 10.1371/journal.pdig.0000787 (PMC12510544; doi:10.1371/journal.pdig.0000787)
Supplement: S2 File — (ZIP) [file pdig.0000787.s002.zip › S2 Data Raw data files containing unprocessed question inputs and LLM outputs including transcripts of deliberations/STEP1_Question-79-1_Type-MC-NJ.docx]

**Facilitator** **[Round 1]:** An obese 35-year-old man comes to the office for a follow-up examination. He has chronic kidney disease and was told to keep a record of his meals until this follow-up visit. His daily diet consists of chocolate milk and a peanut butter sandwich for breakfast; pizza and cola for lunch; a chocolate bar as a snack; and a foot-long deli meat sandwich for dinner. He is 165 cm (5 ft 5 in) tall and weighs 113 kg (250 lb); BMI is 42 kg/m². His pulse is 100/min, respirations are 23/min, and blood pressure is 155/100 mm Hg. Physical examination shows diaphoresis. Serum studies show a creatinine concentration of 4.1 mg/dL and phosphorus concentration of 6 mg/dL. Which of the following patterns is most likely in this patient? (A) Downregulated parathyroid hormone synthesis, increased serum calcium, increased Serum 1,25- Dihydroxycholecalciferol (B) Downregulated parathyroid hormone synthesis, increased serum calcium, decreased Serum 1,25- Dihydroxycholecalciferol (C) Downregulated parathyroid hormone synthesis, decreased serum calcium, decreased Serum 1,25- Dihydroxycholecalciferol (D) Upregulated parathyroid hormone synthesis, increased serum calcium, increased Serum 1,25- Dihydroxycholecalciferol (E) Upregulated parathyroid hormone synthesis, increased serum calcium, decreased Serum 1,25- Dihydroxycholecalciferol (F) Upregulated parathyroid hormone synthesis, decreased serum calcium, decreased Serum 1,25- Dihydroxycholecalciferol

**AI_D:** RESTATING THE QUESTION: The question presents the case of a 35-year-old obese man with chronic kidney disease. His dietary habits are poor, mainly based on calorie-rich, nutritionally poor foods, and he presents with hypertension and signs of metabolic stress (diaphoresis). The question wants to know how these characteristics and habits are likely to affect his parathyroid hormone (PTH) synthesis, serum calcium levels, and serum 1,25-Dihydroxycholecalciferol (the active form of Vitamin D) levels. PLANNING: Given the patient's diet, obesity, and existing chronic kidney disease, a reasonable assumption would be that the kidney's functionality is compromised. In the context of CKD, altered PTH synthesis and abnormally regulated serum calcium and phosphate concentrations often occur, leading to a condition known as secondary hyperparathyroidism. Additionally, reduced kidney function can also lead to decreased conversion of Vit D to its active form. This is because the final step in the conversion of inactive Vit D to its active form (1, 25-Dihydroxycholecalciferol) primarily takes place in the kidneys. CLINICAL REASONING: With chronic kidney disease, there is reduced excretion of phosphate, leading to high serum phosphate levels (as indicated in the patient). This high phosphate level can bind to free calcium in the blood, leading to hypocalcemia (low serum calcium levels). Hypocalcemia, together with decreased conversion of Vit D to its active form, results in reduced calcium absorption from the gut. This triggers the parathyroid gland to secrete more Parathyroid hormone (secondary hyperparathyroidism), which breaks down bone to increase blood calcium levels. CONCLUDING: In conclusion, a patient with a similar presentation - obesity, poor diet, chronic kidney disease, high serum phosphate levels - is likely to have an upregulated parathyroid hormone synthesis due to hypocalcemia, and therefore decreased serum calcium levels. Additionally, because of the kidneys' compromised functional capacity, this patient would likely have decreased 1,25-Dihydroxycholecalciferol, the active form of Vitamin D. RESPONSE: Therefore, the most likely pattern in this patient is (F) Upregulated parathyroid hormone synthesis, decreased serum calcium, decreased Serum 1,25- Dihydroxycholecalciferol.

**AI_A:** (1) RESTATING THE QUESTION: The question is asking us to identify what metabolic changes related to calcium, parathyroid hormone, and vitamin D would we expect in an obese man with chronic kidney disease consuming a high calorie diet. Parathyroid hormone (PTH) is the hormone that regulates calcium homeostasis in the body, while 1,25-Dihydroxycholecalciferol, also known as calcitriol, is the active form of vitamin D and plays a pivotal role in the absorption and metabolism of calcium and phosphorus in the body. (2) PLANNING: We need to consider the pathophysiological changes happening in chronic kidney disease, specifically, the effect it has on calcium and phosphate metabolism, and how the parathyroid glands respond to this. Chronic kidney disease dystorts the balance of calcium and phosphorus in the body, primarily due to the kidneys’ decreased ability to excrete phosphorus and convert vitamin D into its active form, which is crucial for calcium absorption in the gastrointestinal tract. It is also well-established that the diet of this patient is high in phosphorus and despite the kidneys failure, the gut continues to absorb dietary phosphates, thus resulting in hyperphosphatemia. This excess phosphorus binds to free calcium, lowering free serum calcium level. Low calcium level and the inability to activate vitamin D due to kidney failure serves as a stimulus to the parathyroid gland to secrete excess PTH, a condition known as secondary hyperparathyroidism. (3) CLINICAL REASONING: The patient's chronically low serum calcium levels due to persistent hyperphosphatemia from his diet and impaired kidney function lead to a constant stimulation of the parathyroid gland. As a result, PTH synthesis is upregulated to increase serum calcium levels by promoting bone resorption- release of calcium from the bones into the bloodstream. In addition, PTH also tends to reduce renal excretion of calcium. However, the synthesis of 1,25-Dihydroxycholecalciferol (active Vitamin D) which aids in both GI absorption of calcium and renal reabsorption of calcium is hampered because synthetic conversion of inactive to active vitamin D occurs in the kidneys- which in case of our patient are not optimally functional due to chronic kidney disease. As a consequence, due to the renal dysfunction, one would expect the levels of active Vitamin D to decrease in this patient's system. (4) CONCLUDING: In sum, this patient, in context of his obesity, chronic kidney disease and high-phosphorus diet, would have upregulated parathyroid hormone synthesis due to persistently low calcium levels which the body would attempt to rectify. However, despite these efforts, the active Vitamin D levels would be decreased due to the impaired ability of the kidneys to convert this vitamin to its active form. (5) RESPONSE: Given the information and reasoning above, the most likely pattern in this patient would be (F) Upregulated parathyroid hormone synthesis, decreased serum calcium, and decreased Serum 1,25- Dihydroxycholecalciferol.

**AI_C:** RESTATING THE QUESTION: This clinical scenario presents a 35-year-old obese man with chronic kidney disease (CKD) who has undergone evaluation including physical exam and serum studies. The man also exhibits symptoms of hyperphosphatemia (indicated by a serum phosphorus concentration of 6 mg/dL; normal range: 2.5 - 4.5 mg/dL) and raised creatinine (an indicator of impaired kidney function). Based on this information, we are asked to predict the most likely physiological response pattern relating to parathyroid hormone (PTH), serum calcium, and Serum 1,25- Dihydroxycholecalciferol (also known as calcitriol, the active form of Vitamin D). PLANNING: The basic assumptions of this scenario include the fact that CKD often affects electrolyte balance and the metabolism of Vitamin D and calcium, mediated largely through effects on PTH synthesis. The diet of the patient can also impact this balance, particularly if it's high in phosphorus like the patient's current diet. Alternate assumptions may include potential interference with these processes via obesity, possible hypertension (indicated by blood pressure of155/100 mm Hg), and the effect of the patient's unhealthy diet on his general health and kidney function. In terms of clinical prioritization, the first hypothesis to pursue would be the specific effect of CKD on PTH synthesis, serum calcium, and Serum 1,25- Dihydroxycholecalciferol due to the direct correlation of CKD with these markers. CLINICAL REASONING: CKD typically leads to a decrease in kidney function, reducing its ability to excrete phosphorus and leading to hyperphosphatemia as seen in the patient. The surplus phosphorus binds to free calcium, reducing serum calcium levels. This decrease in serum calcium is registered by the parathyroid glands which consequently upregulate the synthesis and secretion of PTH (secondary hyperparathyroidism) in an attempt to normalize serum calcium levels. Additionally, CKD results in a reduced capacity to convert inactive Vitamin D to its active form (calcitriol), due to loss of renal 1-alpha-hydroxylase activity. CONCLUDING: Given the man’s reduced renal function (reflected by the high creatinine levels), it is most likely that his physiological response would involve upregulated PTH synthesis in response to reduced serum calcium levels. Furthermore, due to the impaired 1-alpha-hydroxylase activity in CKD, there would likely be a decrease in serum 1,25-Dihydroxycholecalciferol (active Vitamin D). RESPONSE: Therefore, the most likely pattern in this patient can be described by option (F) Upregulated parathyroid hormone synthesis, decreased serum calcium, decreased Serum 1,25-Dihydroxycholecalciferol.

**AI_E:** (1) RESTATING THE QUESTION: We are asked to predict the pattern of parathyroid hormone (PTH) synthesis and its effects on serum calcium and Vitamin D3 (1,25-Dihydroxycholecalciferol) metabolism in a 35-year-old obese male with chronic kidney disease. The patient's diet primarily consists of high calorific food with high salt and potentially high phosphorus content. The consequence of this lifestyle has led to obesity, hypertension, and potentially, renal dysfunction as evident by elevated creatinine and phosphorus serum levels. (2) PLANNING: Generally, in healthy individuals, the parathyroid glands secrete PTH in response to low serum calcium levels. This hormone acts to increase serum calcium by promoting bone resorption, increased renal secretion of phosphate (counteracting hyperphosphatemia), and increased activation of Vitamin D3 in the kidney which enhances calcium absorption in the gut. Therefore, under physiological conditions, when serum calcium levels are low, we should see upregulated PTH synthesis, decreased serum phosphate, and increased 1,25-Dihydroxycholecalciferol. However, this patient has chronic kidney disease (CKD), which alters the relationship between PTH, calcium, phosphate, and Vitamin D3. (3) CLINICAL REASONING: The underlying renal dysfunction in CKD leads to diminished renal phosphate excretion, leading to hyperphosphatemia which is reflected by his high serum phosphorus concentration. Phosphate binds to free calcium, leading to a decrease in free serum calcium causing hypocalcemia. Hypocalcemia is a potent stimulus for PTH synthesis and secretion, leading to secondary hyperparathyroidism. However, CKD also leads to a decrease in the kidney's ability to convert inactive Vitamin D into its active form (1,25-Dihydroxycholecalciferol), leading to decreased levels of this active Vitamin D metabolite. Therefore, in this patient, we might expect to see upregulated PTH synthesis, decreased serum calcium, and decreased 1,25-Dihydroxycholecalciferol. (4) CONCLUDING: Given the patient's clinical picture of obesity, hypertension, diaphoresis and his laboratory findings of increased creatinine and phosphorus concentrations indicative of chronic kidney disease, the most relevant assumption would be that his body is in a state of compensatory secondary hyperparathyroidism due to hypocalcemia (caused by hyperphosphatemia due to renal dysfunction) and decreased active vitamin D metabolite production (due to decreased renal conversion). (5) RESPONSE: Therefore, the correct choice is (F) Upregulated parathyroid hormone synthesis, decreased serum calcium, decreased Serum 1,25- Dihydroxycholecalciferol.

**AI_B:** RESTATING THE QUESTION: This query pertains to the pathophysiology of chronic kidney disease (CKD) and how it affects the metabolism of calcium, phosphorus, and parathyroid hormone (PTH) synthesis. This particular patient, an obese middle-aged man, has been diagnosed with CKD and presents with dietary habits that could potentially exacerbate his condition. His high caloric intake, coupled with lack of exercise, could also contribute to the progression of his kidney disease. An elevated creatinine concentration of 4.1 mg/dL and phosphorus concentration of 6 mg/dL are indicative of impaired renal function. The question is asking us to predict the most likely biochemical alterations pertaining to parathyroid hormone, serum calcium, and 1,25-dihydroxycholecalciferol. PLANNING: The pathological changes in CKD often lead to alterations in calcium and phosphorus homeostasis, largely due to decreased renal function. As kidneys fail, they become less efficient at excreting phosphorus and converting vitamin D into its active form (1,25-dihydroxycholecalciferol), leading to hyperphosphatemia and vitamin D deficiency. These two conditions signal the parathyroid gland to increase production of PTH. Increased PTH levels would then promote calcium release from bones and reduce renal excretion of calcium to correct the hypocalcemia that results from decreased active vitamin D. CLINICAL REASONING: Given the information that the patient has an elevated creatinine level indicative of impaired renal function (4.1 mg/dL when normal is below 1.3 mg/dL), and an elevated serum phosphorus level (6mg/dL whereas normal range is 2.5-4.5 mg/dL), we will look for changes that could occur in chronic kidney disease, specifically changes related to the metabolism of calcium, phosphorus, and PTH. PTH synthesis would likely be upregulated due to the decreased ability of diseased kidneys to convert vitamin D to its active form, 1,25-dihydroxycholecalciferol, which leads to decreased calcium absorption in the gut and subsequent hypocalcemia. The upregulation of PTH is a compensatory mechanism to correct hypocalcemia by increasing calcium release from bones, decreasing renal excretion of calcium, and indirectly increasing gut absorption of calcium by promoting the conversion of vitamin D to its active form favoring calcium absorption. CONCLUDING: Therefore, in light of the available evidence and the reasoning stated above, the patient's biochemical alterations are hypothesized to be increased parathyroid hormone synthesis due to decreased vitamin D activation and hypocalcemia, which would lead to decreased serum calcium and decreased 1,25-dihydroxycholecalciferol. RESPONSE: (F) Upregulated parathyroid hormone synthesis, decreased serum calcium, decreased Serum 1,25- Dihydroxycholecalciferol.

**Facilitator** The consensus statement synthesizing the responses of AI_A, AI_B, AI_C, AI_D, and AI_E is as follows: The question presents a 35-year-old obese man with chronic kidney disease (CKD) who has a high-calorie diet rich in phosphorus. Given his condition and lifestyle, we are asked to predict the most likely physiological response pattern relating to parathyroid hormone (PTH), serum calcium, and Serum 1,25- Dihydroxycholecalciferol (the active form of Vitamin D). In CKD, the kidneys' ability to excrete phosphorus and convert vitamin D into its active form is compromised, leading to hyperphosphatemia and vitamin D deficiency. The excess phosphorus binds to free calcium, resulting in hypocalcemia (low serum calcium levels). This decrease in serum calcium, along with the inability to activate vitamin D due to kidney failure, stimulates the parathyroid gland to secrete excess PTH, a condition known as secondary hyperparathyroidism. The upregulation of PTH is a compensatory mechanism to correct hypocalcemia by promoting bone resorption (release of calcium from the bones into the bloodstream) and reducing renal excretion of calcium. However, the synthesis of 1,25-Dihydroxycholecalciferol (active Vitamin D), which aids in both gastrointestinal absorption of calcium and renal reabsorption of calcium, is hampered due to the impaired kidney function. Therefore, in the context of this patient's obesity, chronic kidney disease, and high-phosphorus diet, we would expect upregulated parathyroid hormone synthesis due to persistently low calcium levels. Despite these efforts, the active Vitamin D levels would be decreased due to the impaired ability of the kidneys to convert this vitamin to its active form. In conclusion, the most likely pattern in this patient would be (F) Upregulated parathyroid hormone synthesis, decreased serum calcium, and decreased Serum 1,25- Dihydroxycholecalciferol.
