# Supplementary material for: Collaborative intelligence in AI: Evaluating the performance of a council of AIs on the USMLE
Source: PLOS Digit Health. 2025 Oct 9;4(10):e0000787. doi: 10.1371/journal.pdig.0000787 (PMC12510544; doi:10.1371/journal.pdig.0000787)
Supplement: S2 File — (ZIP) [file pdig.0000787.s002.zip › S2 Data Raw data files containing unprocessed question inputs and LLM outputs including transcripts of deliberations/STEP1_Question-8-1_Type-MC-NJ.docx]

**Facilitator** **[Round 1]:** A 53-year-old man comes to the physician because of a dry scaly rash on his body for the past year. He has had a 15-kg (33-lb) weight loss during the past year. He is 178 cm (5 ft 10 in) tall and now weighs 54 kg (120 lb); BMI is 17 kg/m². His stools have a large volume and float. Which of the following nutrient deficiencies is most likely? (A) Magnesium (B) Vitamin A (C) Vitamin B12 (cobalamin) (D) Vitamin C (E) Zinc

**AI_C:** RESTATING THE QUESTION: A 53-year-old male patient has been facing a persistent dry, scaly rash for the past year during which he also lost a significant amount of weight, making his BMI drastically low. There are remarkable changes in his bowel movements, his stools now having large volumes and floating, all of which lead us to believe there could be a nutrient deficiency involved. The question aims to identify the deficient nutrient from the options given. PLANNING: The assumptions we have to work with include the presence of a nutrient deficiency based on the patient's symptoms. Given the large volume and floating stools, malabsorption could be a likely secondary assumption. This is because large, floating stools are often associated with fat malabsorption, often seen in conditions like celiac disease or pancreatic exocrine insufficiency. With possible malabsorption, it is reasonable to consider fat-soluble vitamin deficiencies (A, D, E, K). CLINICAL REASONING: The patient's symptoms of a dry, scaly rash suggest skin changes that might occur in deficiency of certain nutrients like vitamins A and E. Significant weight loss could be from malnutrition or malabsorption. The detail about large, floating stools supports malabsorption, specifically steatorrhea. This condition commonly arises due to lack of pancreatic enzymes, leading to difficulty in breaking down and absorbing fat. Vitamin A is fat-soluble and needed for maintaining the health of epithelial cells and skin, so its deficiency might lead to the skin changes seen in the patient. CONCLUDING: The possible malabsorption suggested by the patient's stool characteristics combined with skin changes would likely point towards a deficiency in a fat-soluble vitamin that affects skin health. RESPONSE: The nutrient deficiency most likely in this patient, based on clinical presentation and reasoning, is (B) Vitamin A.

**AI_A:** (1) RESTATING THE QUESTION: The given clinical case involves a 53-year-old man who has shown symptoms of a dry, scaly rash and significant weight loss over a year. He also has steatorrhea (passing stools with high fat content, causing them to be bulky and float). The question essentially asks which nutrient deficiency is mainly associated with these clinical features. (2) PLANNING: Assuming the symptoms to be indicative of various potential nutrient deficiencies, we could consider each of these: Magnesium deficiency could present with neuromuscular symptoms, which are not mentioned here; Vitamin A deficiency could lead to night blindness, which is also not mentioned; B12 deficiency usually presents with anemia, glossitis, and neurological symptoms not reported in this case; Vitamin C deficiency leads to scurvy, the symptoms of which include swollen, bleeding gums and anemia, not reported here; Zinc deficiency would fit with dermatitis, alopecia, and diarrhea, but the patient lacks alopecia symptoms. A distinct clinical feature here is steatorrhea—passing stools with high fat content—indicative of malabsorption, which could hint at deficiencies in fat-soluble vitamins, such as vitamin A. (3) CLINICAL REASONING: Considering the conspicuous symptom of steatorrhea, it leads us to inspect the fat-soluble vitamins' deficiencies namely, vitamins A, D, E, and K. Vitamins D, E, and K have not been mentioned in the options but vitamin A has. This patient's symptoms align closer to the deficiency of vitamin A, particularly, the dermatological manifestation—dry and scaly skin, also known as xerosis. Although night blindness is a classical and a much-flaunted symptom of vitamin A deficiency, it does not mean that all patients with said deficiency will invariably present with night blindness. (4) CONCLUDING: The answer here hinges on connecting the patient's symptoms with the most fitting deficiency-induced clinical presentation. The rash, significant weight loss, and steatorrhea combined point us in the direction of vitamin A deficiency, making it the most plausible option among the given alternatives. (5) RESPONSE: The nutrient deficiency most likely present in this case corresponds to (B) Vitamin A.

**AI_B:** (1) RESTATING THE QUESTION: Here, the main concern revolves around a 53-year-old man presenting multiple symptoms - a dry scaly rash, significant weight loss, and large floating stools. These symptoms suggest potential malabsorption or nutrient deficiency, given their chronic nature and connection to dietary factors. (2) PLANNING: The possible causes for these symptoms involve the deficiencies of magnesium, vitamins A, B12, C or zinc. To establish an assumption, it's important to consider which nutrient's deficiency can lead to all the observed signs - dry scaly rash, significant weight loss, and large floating stools. (3) CLINICAL REASONING: Weight loss and floating stools suggest malabsorption. More specifically, floating stools can indicate steatorrhea, which is typically a consequence of fat malabsorption. Such symptoms could stem from deficiencies linked to nutrients that play a significant role in metabolism, maintaining skin health, and fat absorption. (4) CONCLUDING: Looking at our potential options, vitamins A, B12, and C don’t typically result in malabsorption symptoms like steatorrhea. Magnesium and zinc deficiency may result in weight loss, but don’t typically cause skin changes like a dry scaly rash. However, vitamin A is necessary for maintaining healthy skin and mucous membranes. Vitamin A deficiency can lead to skin changes, like a dry scaly rash, but moreover, serve as a major catalyst in causing steatorrhea and weight loss. (5) RESPONSE: Taking into consideration the symptoms and the role of each nutrient deficiency in the body's function, vitamin A (option B) deficiency would be the most likely cause for this patient's symptomatology.

**AI_E:** (1) RESTATING THE QUESTION: In summary, a 53-year-old man suffering from a long-standing dry, flaky rash and significant weight loss over the past year has presented to the doctor. He has been experiencing bulky, buoyant stools as well. Given these clinical features, we need to determine the nutrient deficiency most likely causing his symptoms. (2) PLANNING: When taking into account the details provided, several assumptions are made regarding this man's clinical picture. The dry, scaly skin, coupled with serious weight loss and bulky, floating stools, typically point to a condition known as steatorrhea, which is characterized by the excretion of abnormal quantities of fat in the feces. One potential cause for steatorrhea comes from malabsorption syndromes, which may result from nutrient deficiencies. Now, the nutrient deficiencies that can cause such a syndrome include fat-soluble vitamins such as vitamins A, D, E, and K, and minerals like magnesium and zinc. There is no direct indication in the vignette of any deficiency causing megaloblastic anemia, which is typically seen with B12 deficiency. Similarly, Vitamin A deficiency generally manifests as night blindness or other ophthalmologic issues, which are not mentioned. Vitamin C deficiency primarily presents with symptoms of scurvy, including gum disease and skin changes, but often does not cause steatorrhea or weight loss. Zinc deficiency usually presents with diarrhea, alopecia, and immune dysfunction, none of which is mentioned. (3) CLINICAL REASONING: By the process of elimination, we could possibly rearrange our list of assumptions to focus primarily on the deficiency in fat-soluble vitamins, as they are generally associated with lipid malabsorption syndromes and can explain the dry, scaly skin (by causing deficiency dermatitis) and the steatorrhea. Comparing each of the fat-soluble vitamins, vitamin A deficiency mainly affects vision and is not aligned with his symptoms. Similarly, Vitamin K mainly affects coagulation, which is not in line with the presented case, and Vitamin D deficiency causes bone demineralization, none of which are indicated in the vignette. Vitamin E deficiency, however, has effects on the skin and it is a fat-soluble vitamin, which aligns with the signs of steatorrhea and weight loss. (4) CONCLUDING: Taking into account the chronic dry skin, substantial weight reduction, and steatorrhea, a nutrient deficiency that affects fat-soluble vitamins absorption, particularly Vitamin E, seems the most likely cause behind these clinical manifestations. (5) RESPONSE: The question does not provide an option for Vitamin E deficiency, which is the leading assumption here. Therefore, it might be better to reconsider the assumptions or check the next best option given. Steatorrhea suggests malabsorption syndrome, and looking at the options given, vitamin A is another fat-soluble vitamin that might be decreased due to malabsorption, even though the symptoms align more accurately with Vitamin E deficiency. Thus, the answer is (B) Vitamin A, based on the options provided.

**AI_D:** RESTATING THE QUESTION: The question describes a 53-year-old male patient who has lost significant weight over the past year, and has a dry, scaly rash on his body. His Body Mass Index (BMI) is 17 kg/m², indicating he is underweight. The patient presents with stools that are unusually large in volume and float, a condition known as steatorrhea, typically indicating malabsorption of fat. Our task is to determine which nutrient deficiency this patient is most likely experiencing. PLANNING: Taking into consideration the patient's symptoms, weight loss, rash and stool characteristics, we can make assumptions towards various nutrient deficiencies. Magnesium deficiency can cause symptoms like muscle cramps and arrhythmias but is not associated with our patient's symptoms. Some vitamin A deficiencies can lead to skin problems and poor growth, but they primarily present with night blindness or full blindness and do not commonly lead to steatorrhea. Vitamin B12 (cobalamin) deficiency may cause neurologic symptoms or megaloblastic anemia. Vitamin C deficiency primarily presents with symptoms of scurvy, including swollen gums and joint pain. Zinc deficiency may cause rash and diarrhea, but is frequently associated with poor wound healing and growth retardation, which are not observed in the presented case. From this initial evaluation, none of these deficiencies precisely fit, however, the symptoms closely align with fat-soluble vitamin deficiency that would manifest from fat malabsorption. CLINICAL REASONING: In considering the patient’s symptoms and signs, the weight loss, dry epidermis, and voluminous floating stools are indicative of malabsorption syndrome, specifically regarding fat-soluble vitamins due to the presence of rash together with steatorrhea. Of the different fat-soluble vitamins (A, D, E, K), the deficiency of vitamin A can lead to a scaly rash, yet is commonly present with vision issues. This leaves us with one hypothesis that we had initially discarded but will now revisit given the new clinical findings theoretical modeling process. CONCLUDING: For the symptoms to all logically tie together, if we consider that this patient is suffering from a deficiency in a nutrient not listed in the options, the pieces might fall into place more conclusively. His symptoms are strongly suggesting a deficiency in the fat-soluble vitamin E. The logic behind this is due to the malabsorption syndrome identified by the steatorrhea and drastic weight loss, along with a dry scaly rash which can occur in vitamin E deficiency. RESPONSE: However, given the options provided, we must reevaluate and choose the closest possible answer with acknowledgment of our alternate hypothesis. In this case, vitamin A (Option B) would be the most likely answer as it is fat-soluble and its deficiency may manifest with skin issues. But the absence of visual changes and the presence of steatorrhea make it only a partially fitting answer.

**Facilitator** The consensus statement synthesizes the responses of AI_A, AI_B, AI_C, AI_D, and AI_E as follows: The clinical case involves a 53-year-old man who has been experiencing symptoms of a dry, scaly rash, significant weight loss, and steatorrhea (large, floating stools) over the past year. These symptoms suggest a potential nutrient deficiency, likely related to malabsorption, given their chronic nature and connection to dietary factors. The possible nutrient deficiencies considered include magnesium, vitamins A, B12, C, and zinc. However, none of these deficiencies perfectly align with the patient's symptoms. Magnesium and zinc deficiencies may result in weight loss but don't typically cause skin changes like a dry scaly rash. Vitamin B12 deficiency usually presents with anemia, glossitis, and neurological symptoms, which are not reported in this case. Vitamin C deficiency leads to scurvy, the symptoms of which include swollen, bleeding gums and anemia, not reported here. The patient's symptoms align closer to the deficiency of a fat-soluble vitamin, particularly vitamin A. The dry, scaly rash suggests skin changes that might occur in deficiency of certain nutrients like vitamins A and E. The significant weight loss and steatorrhea support the assumption of malabsorption, specifically of fat-soluble vitamins. Vitamin A is necessary for maintaining healthy skin and mucous membranes, and its deficiency can lead to skin changes, like a dry scaly rash, and serve as a major catalyst in causing steatorrhea and weight loss. However, it's important to note that while vitamin A deficiency is the most plausible option among the given alternatives, the absence of visual changes and the presence of steatorrhea make it only a partially fitting answer. Some responses suggest that the symptoms strongly suggest a deficiency in the fat-soluble vitamin E, which is not listed in the options. In conclusion, given the options provided, the nutrient deficiency most likely present in this case corresponds to (B) Vitamin A, even though the symptoms align more accurately with Vitamin E deficiency.
