# Supplementary material for: Collaborative intelligence in AI: Evaluating the performance of a council of AIs on the USMLE
Source: PLOS Digit Health. 2025 Oct 9;4(10):e0000787. doi: 10.1371/journal.pdig.0000787 (PMC12510544; doi:10.1371/journal.pdig.0000787)
Supplement: S2 File — (ZIP) [file pdig.0000787.s002.zip › S2 Data Raw data files containing unprocessed question inputs and LLM outputs including transcripts of deliberations/STEP1_Question-81-1_Type-MC-NJ.docx]

**Facilitator** **[Round 1]:** A 60-year-old woman is nearing the completion of a half marathon. Because she experiences esophageal regurgitation, she does not drink any liquid during the race. This woman's physical state at the end of the race will most likely activate which of the following renal segments at this time? (A) Arginine vasoreceptor 2 in the proximal tubular cells (B) K+–H+ exchange in the distal tubule (C) Proximal tubule carbonic anhydrase (D) Urea reabsorption in the medullary collecting ducts (E) Water reabsorption in the ascending loop of Henle

**AI_C:** RESTATING THE QUESTION: This query concerns a mature female marathoner who, due to esophageal regurgitation issues, avoids fluid intake during endurance exercise. The marathon duration, coupled with her aversion to consume fluids, may create dehydration. Given these conditions, we're asked to identify which renal (kidney) segment would be expected to activate in response to her particular physical state. The renal segments listed encompass various aspects of nephron function, responsible for different actions surrounding the regulation of water, electrolyte balance, and acid-base balance in the body. PLANNING: Dehydration following prolonged exercise without fluid intake can lead to a decrease in blood volume, increase in blood osmolality, and activation of the renin-angiotensin-aldosterone system (RAAS). This could influence a variety of renal segments. However, the body's initial response to such a condition would typically prioritize maintaining blood volume and osmolality, leading to reabsorption of water, electrolytes, and other substances. CLINICAL REASONING: Based on the scenario given, the woman would experience dehydration due to lack of fluid intake and excessive sweating. Dehydration increases the osmolality of blood due to a higher concentration of solutes, triggering a response from the body to conserve water and maintain blood volume. This is primarily achieved by the action of antidiuretic hormone (ADH), which targets the distal tubules and collecting ducts in nephrons. ADH increases the permeability of these ducts to water, allowing more water to be reabsorbed back into the bloodstream from the filtrate. The segment responsible for the reabsorption of a significant amount of water, driven by osmotic gradients, is the nephron's ascending loop of Henle. CONCLUDING: Therefore, the woman's specific circumstances -- dehydration stemming from extensive physical exertion without fluid intake -- would most likely spur her body to conserve water. This would most likely result in the activation of the ascending loop of Henle, where water reabsorption under the influence of the generated osmotic gradient occurs. RESPONSE: Hence, the physical state of this woman at the end of the race would most likely activate water reabsorption in the ascending loop of Henle (E).

**AI_A:** 1) RESTATING THE QUESTION: So, a 60-year-old woman who is about to finish a half marathon hasn't consumed any fluids due to esophageal regurgitation. Esophageal regurgitation refers to the condition where the contents of the stomach flow back into the esophagus due to a dysfunction in the lower esophageal sphincter. We are asked to predict which renal (kidney-related) segment of her body is most likely to be activated at this time. The options include different parts of the renal system and different processes that occur within them. 2) PLANNING: The woman likely has a decreased fluid volume due to the physical activity without hydration, which could lead to dehydration and hypernatremia (high sodium levels). The activation of various renal processes will alter to maintain body fluid homeostasis. Some assumptions can be that: - The depletion in fluid volume would potentially increase the reabsorption of water (to restore volume), which potentially involves tubules and exchange channels. - Also, prolonged physical activity can result in the breakdown of muscle tissue, leading to an increased urea concentration in the blood, creating a possible need for urea reabsorption. 3) CLINICAL REASONING: Considering the body's natural response to dehydration, the kidneys often concentrate urine to conserve water. This concentration process primarily takes place in the Loop of Henle and in the medullary collecting ducts. Urea plays a crucial role in this process, acting as an osmolyte that drives water reabsorption in the medullary collecting ducts. However, water reabsorption in the ascending loop of Henle seems less feasible since it is normally impermeable to water. Moreover, increased physical activity doesn't necessarily cause significant shifts in arginine, potassium hydrogen exchanges or carbonic anhydrase activity in the near term. 4) CONCLUDING: Taking all of these factors into account, it can be hypothesized that the intense physical activity combined with no fluid intake will more likely stimulate mechanisms that promote water conservation in the body, namely urea reabsorption in the medullary collecting duct of the kidney playing a significant role in this regard. 5) RESPONSE: Therefore, it is most likely that (D) Urea reabsorption in the medullary collecting ducts is activated considering the physical status of the woman at the end of the race.

**AI_E:** This question revolves around the effect of participating in a half marathon without fluid intake, on a renal segment in a 60-year-old woman prone to esophageal regurgitation. The renal system has various parts including the proximal and distal tubules, medullary collecting ducts, and the ascending loop of Henle, each with their associated processes and roles that could be influenced by the woman's state of dehydration. Dehydration, as suggested by intense physical activity sans fluid intake, could provoke several compensatory renal mechanisms. The alternatives presented include arginine vasoreceptor 2 (AVP V2 receptor) in proximal tubular cells, K+–H+ exchange in distal tubule, the enzyme carbonic anhydrase in the proximal tubule, urea reabsorption in medullary collecting ducts, and water reabsorption in the ascending loop of Henle. These segments and the processes being suggested are involved in different aspects of fluid and electrolyte balance, acid-base balance, and the concentration of urine under varying physiological conditions. At the end of a strenuous activity like a marathon, especially without fluid replacement, the body would be tending towards a dehydrated state. In such a situation, the body would try to conserve water and maintain the body's fluid balance. The AVP V2 receptor plays a vital role in urine concentration. However, it is not located in the proximal tubular cells but on the cells of the late distal tubules and collecting ducts. The K+–H+ exchange process and carbonic anhydrase would be important in acid-base balance, not primarily in fluid balance or dehydration states. Urea reabsorption in the medullary collecting ducts would assist with urine concentration; however, water reabsorption would play a crucial role in a water deficit circumstance. Given the probable dehydration, the body would try to reabsorb as much water as possible to maintain fluid balance. In contrast to the ascending loop of Henle, which is impermeable to water, the descending loop of Henle and collecting duct's latter part actively participate in water reabsorption. Therefore, any activation would most likely involve these areas, particularly under the regulation of antidiuretic hormone (ADH) or vasopressin. In conclusion, considering the woman's physical state at the end of the race, a primary target for activation to counterbalance the state of dehydration would be the water reabsorption mechanism predominantly present in the descending loop of Henle and the latter part of the collecting ducts. This indicates that the stated renal segments might not primarily be the ones to be activated under these conditions. However, if one must choose from the given options, urea reabsorption in the medullary collecting ducts (D) would be the closest, due to its contribution to the urine concentration process, albeit not the primary one in severe dehydration.

**AI_B:** (1) RESTATING THE QUESTION: The question appears to ask me to identify which renal segment would be activated in a 60-year-old woman who is ending a half marathon and has not consumed any fluids owing to esophageal regurgitation. Each option provided represents various renal segment functions relating to the response to dehydration. (2) PLANNING: The assumptions I can identify are that the woman is dehydrated from the race due to loss of fluids via sweat, and her esophageal regurgitation precludes her from replenishing those fluids. To generate alternate assumptions: Could her age be influencing the dehydration process? How might esophageal regurgation affect fluid balance? In evaluating these assumptions, it stands to reason that older age could influence fluid balance but not as immediately and drastically as running a half marathon without water intake. Likewise, while esophageal regurgitation could complicate the situation, it's likely that the immediate, overwhelming influence is from the lack of fluid intake during intense exercise. (3) CLINICAL REASONING: Hence, dehydration is likely the overwhelming issue here, leading to renal changes in response. In cases of severe dehydration, the body seeks to conserve as much water as possible. This can involve changes in kidney function to minimize fluid loss. Among the options, Arginine vasoreceptor 2 in the proximal tubular cells (option A) mediates water retention but is not an actual renal segment; K+–H+ exchange in the distal tubule (option B) is involved in electrolyte balance rather than water conservation; Proximal tubule carbonic anhydrase (option C) plays roles in acid-base balance but not direct fluid balance; Urea reabsorption in the medullary collecting ducts (option D) increases in response to dehydration to help concentrate the urine; and finally, Water reabsorption in the ascending loop of Henle (option E) is not the primary site of water reabsorption, especially under dehydrated status. (4) CONCLUDING: Given this intensive exercise activity without fluid replacement, the body will activate systems to conserve water. This is often performed via urinary concentration, allowing less water to be lost to urine - this suggests that increased urea reabsorption in the medullary collecting ducts (option D) would be the most likely renal function to be activated. (5) RESPONSE: Therefore, her physical state at the end of the race will likely activate urea reabsorption in the medullary collecting ducts.

**AI_D:** (1) RESTATING THE QUESTION: This scenario involves a 60-year-old woman who has undertaken a physically intense activity - a half marathon - without hydrating herself due to a health condition known as esophageal regurgitation. The question seeks to understand which renal segment in her body is likely to be significantly activated in response to her physical state by the end of the race. The options provided represent various renal processes: arginine vasopressin receptor 2 activation in proximal tubular cells (associated with water reabsorption), K+–H+ exchange in the distal tubule (important for acid-base balance), carbonic anhydrase activity in the proximal tubule (playing crucial role in bicarbonate reabsorption), urea reabsorption in the medullary collecting ducts (ensuring hyperosmolarity of the renal medulla), and water reabsorption in the ascending limb of the Loop of Henle (regulating urine concentration and volume). (2) PLANNING: The underlying assumption is that dehydration, due to intense physical activity and absence of fluid intake, would affect certain renal segments more than others. The body of an endurance runner might instinctually prioritize water retention to counteract dehydration. This alternate assumption needs to be tested against each renal process. (3) CLINICAL REASONING: During dehydration, the body naturally attempts to conserve water. Renal segments involved in water reabsorption thus become critical. While all the given options are important for different aspects of renal physiology, one that directly contributes to water reabsorption would be more crucial in the context of dehydration. The role of the arginine vasopressin receptor 2 in the proximal tubular cells and the water reabsorption function in the ascending loop of Henle seem most relevant in this scenario. (4) CONCLUDING: However, arginine vasopressin receptor 2 in proximal tubular cells alone cannot compensate for a severe water deficit, as reabsorption of water in proximal tubular cells is relatively dilute compared to the reabsorption in the presence of vasopressin in the distal components of the nephron. On the other hand, the loop of Henle, particularly the ascending loop, is involved in the concentration of urine, thus heavily influencing water reabsorption. However, the ascending loop of Henle is impermeable to water, which makes it less plausible in this scenario. (5) RESPONSE: Thus, taking all speculations into account, (D) activation of urea reabsorption in the medullary collecting ducts is most consistent with the expected physiological response to dehydration. It's critical to understand that it indirectly contributes to water conservation by creating a hyperosmolar renal medulla, which then facilitates water reabsorption in the collecting ducts under the influence of arginine vasopressin (antidiuretic hormone).

**Facilitator** **[Round 2]:** Summary: AI_A and AI_B both agree that the most likely renal segment to be activated in the woman's body is urea reabsorption in the medullary collecting ducts. They reason that the woman's intense physical activity and lack of fluid intake would lead to dehydration, causing the body to conserve water. Urea reabsorption in the medullary collecting ducts plays a significant role in this process. AI_C, however, believes that the ascending loop of Henle, where water reabsorption occurs under the influence of the generated osmotic gradient, would be the most likely to be activated. AI_C argues that the woman's dehydration would trigger a response from the body to conserve water and maintain blood volume, primarily achieved by the action of antidiuretic hormone (ADH), which targets the distal tubules and collecting ducts in nephrons. AI_D agrees with AI_A and AI_B that urea reabsorption in the medullary collecting ducts would be the most likely renal function to be activated. AI_D reasons that while the arginine vasopressin receptor 2 in the proximal tubular cells and the water reabsorption function in the ascending loop of Henle seem most relevant in this scenario, the ascending loop of Henle is impermeable to water, making it less plausible. AI_E suggests that while urea reabsorption in the medullary collecting ducts would assist with urine concentration, water reabsorption would play a crucial role in a water deficit circumstance. AI_E concludes that the primary target for activation to counterbalance the state of dehydration would be the water reabsorption mechanism predominantly present in the descending loop of Henle and the latter part of the collecting ducts. Question to clarify disagreements: Given the differing opinions on which renal segment would be most likely activated in response to the woman's physical state, could the council members further explain their reasoning behind their choices, particularly focusing on the role of the ascending loop of Henle and the medullary collecting ducts in the process of water reabsorption and conservation during dehydration?. Based on your preceding response, how would you answer the original question: [A 60-year-old woman is nearing the completion of a half marathon. Because she experiences esophageal regurgitation, she does not drink any liquid during the race. This woman's physical state at the end of the race will most likely activate which of the following renal segments at this time? (A) Arginine vasoreceptor 2 in the proximal tubular cells (B) K+–H+ exchange in the distal tubule (C) Proximal tubule carbonic anhydrase (D) Urea reabsorption in the medullary collecting ducts (E) Water reabsorption in the ascending loop of Henle]

**AI_E:** RESTATING THE QUESTION: The inquiry is about a 60-year-old woman who participated in a demanding physical activity (half marathon) but did not consume any fluids because she suffers from esophageal regurgitation. The question wants to determine which renal action would be triggered because of her state at the end of the race. PLANNING: It can be assumed that her physical state would lean towards dehydration, considering her intense effort and lack of fluid intake. We then question which renal mechanisms would be activated to counterbalance the dehydration effects. Considering she is nearing the completion of her race, it is crucial to prioritize the renal mechanisms that maintain body's fluid balance and blood volume. CLINICAL REASONING: Dehydration stimulates the hypothalamus to signal the posterior pituitary gland to release antidiuretic hormone (ADH), also known as vasopressin. ADH targets collecting ducts and orchestrates two primary processes: urea reabsorption in the medullary collecting ducts and water reabsorption in the descending loop of Henle and latter part of collecting ducts. CONCLUDING: Given these mechanisms, it is possible to conclude that both the reabsorption of urea in the medullary collecting ducts and the reabsorption of water might be activated; however, the water reabsorption response would occur predominantly via the descending limb of Henle's loop and later segments of the collecting ducts, not the ascending limb as suggested. RESPONSE: Therefore, none of the given options perfectly match the most probable renal segment that would be activated. The closest answer would be (D) Urea reabsorption in the medullary collecting ducts, but it doesn't completely address the crucial water reabsorption that would be taking place in a different renal segment.

**AI_B:** Restating The Question: The question asks which renal segment is generally activated in a situation where a 60-year-old woman is pushing her physical limits by nearing to complete a half marathon, and due to her condition of esophageal regurgitation, she has been avoiding hydration during the race. Planning: Assumptions could be made that due to her intense physical exertion, combined with no fluid intake, the woman would presumably be experiencing dehydration. Various sections of the renal system play different roles in maintaining body's fluid balance. Arginine vasoreceptor 2 in the proximal tubular cells, K+–H+ exchange in the distal tubule, proximal tubule carbonic anhydrase, urea reabsorption in the medullary collecting ducts, and water reabsorption in the ascending loop of Henle all contribute to either the acid-base balance or maintain concentration of various solutes within the blood including water. Our assumptions would need to focus on which of these mechanisms would be of top priority during dehydration. Clinical Reasoning: Dehydration triggers a response meant to conserve water in the body. Arginine vasopressin receptor 2 activation aids in water reuptake, but it would not be the immediate response. K+–H+ exchange has a more central role in maintaining the acid-base balance, whereas carbonic anhydrase is essential for HCO3- reabsorption in the proximal tubule. Although both these processes are vital, they may not be the immediate response under dehydration. Meanwhile, the ascending loop of Henle is impermeable to water, and hence, an increase in water reabsorption here, while may seem initially relevant, does not align with its physiological role. On the other hand, the role of urea reabsorption in the medullary collecting ducts under antidiuretic hormone (ADH or vasopressin) control is central to the kidney's ability to concentrate urine, which in turn conserves water for the body. This effect is especially relevant under conditions of severe water restriction, which potentially implies that this mechanism would be amplified under this woman's circumstances. Concluding: Upon drawing connections between the physiological responses to dehydration and the operations of the different renal segments, the renal segment performing urea reabsorption in the medullary collecting ducts seems to be the most plausible answer, given its critical role in conserving water under conditions of extreme hydration deficit. Response: Given the explanation above and under the presented conditions of the woman's physical state, it would be most likely to activate the mechanism of urea reabsorption in the medullary collecting ducts (D) in order to counterbalance the state of dehydration she is experiencing.

**AI_C:** Restatement: Our task is to examine a situation where a 60-year-old woman who is about to finish a half marathon has not consumed any fluids during the race due to esophageal regurgitation. Given this, we have to decide which renal segment in her body will likely be activated at this point. The options include arginine vasoreceptor 2 in the proximal tubular cells, K+–H+ exchange in the distal tubule, proximal tubule carbonic anhydrase, urea reabsorption in the medullary collecting ducts, or water reabsorption in the ascending loop of Henle. Planning: A foundational assumption we can make is that she is in a state of dehydration from not drinking fluids during her strenuous activity. By questioning this assumption, we could also consider that perhaps her body has alternate ways of compensating for the lack of fluid intake. However, given her age and the exertion involved in completing a half marathon, it seems most likely her body would indeed be dehydrated and thus activate mechanisms for conserving water. This assumption should be clinically prioritized as the underlying mechanism of action is notorious in the case of water deficit scenarios. Clinical Reasoning: In situations of dehydration, the kidneys respond by trying to conserve as much water as possible. This is accomplished through increasing the reabsorption of water in the renal tubular system. The process of water reabsorption is primarily regulated by the antidiuretic hormone, ADH. ADH influences the permeability of the distal tubules and collecting ducts to water and, to a lesser degree, urea. When ADH levels are elevated (such as during dehydration), water is reabsorbed from the distal tubules and collecting ducts back into the bloodstream, decreasing urine output and increasing urine osmolality. On the other hand, the ascending loop of Henle is impermeable to water and is where much of the dilution of urine occurs. Concluding: Therefore, based on the understanding of the renal mechanisms involved in water conservation during situations of dehydration, we can hypothesize the most likely renal segment to be activated in the given scenario would be where water reabsorption occurs - the distal tubules and collecting ducts rather than the ascending loop of Henle (which is typically involved in the dilution of urine). Response: Thus, the most correct answer in this case scenario seems to be (D) Urea reabsorption in the medullary collecting ducts, given that both urea and water are reabsorbed in these areas during conditions of high ADH such as dehydration, and the involvement of urea facilitates water reabsorption. However, it is key to remember that while urea reabsorption can contribute to reabsorption, the main driving force remains the action of antidiuretic hormone on water reabsorption.

**AI_A:** RESTATING THE QUESTION: The question is asking which kidney function will most likely be activated in a 60-year-old woman who is close to completing a half marathon and hasn't taken in any fluids due to esophageal regurgitation. The most likely renal segments to get activated under these circumstances are listed as options. PLANNING: Let's dissect each choice: The arginine vasoreceptor 2 in proximal tubular cells (Option A) plays a role in maintaining blood volume by controlling the release of vasopressin, not directly by conserving water. The K+/H+ exchange in the distal tubule (Option B) is involved in maintaining the body's acid-base balance, not directly in fluid homeostasis. Proximal tubule carbonic anhydrase (Option C) helps to reabsorb bicarbonate, but again, that role is not directly tied to water conservation. Urea reabsorption in the medullary collecting ducts (Option D) works to create a concentration gradient that allows for water reabsorption, but this process is dependant on the presence of water in the initial filtrate reaching these segments. The ascending loop of Henle (Option E) reabsorbs salts, which create an osmotic gradient that draws water from the descending loop of Henle and collecting ducts. But the ascending loop of Henle itself is impermeable to water. CLINICAL REASONING: Given the potential choices, the woman's acute water deprivation due to esophageal regurgitation combined with physical exertion will likely take her body to a state of dehydration. The body will respond to this dehydration by releasing antidiuretic hormone (ADH), which increases water reabsorption in the kidneys. ADH targets the distal tubules and collecting ducts to increase water permeability. Urea reabsorption from the medullary collecting ducts also contributes to this effort as urea contributes to the osmotic gradient that allows for the reabsorption of water. Therefore, it is highly probable that dehydration will activate urea reabsorption in the medullary collecting ducts. CONCLUDING: Upon considering the body's physiological response to dehydration, which involves water conservation in the kidneys, it is logical to expect that the mechanism of urea reabsorption in the medullary collecting ducts will be activated. This is because urea plays a key role in establishing the osmotic gradient that ultimately allows for more effective water reabsorption. RESPONSE: Therefore, in the case of the woman nearing the completion of a half marathon without any fluid intake, her physical state will most likely activate urea reabsorption in the medullary collecting ducts (Option D).

**AI_D:** In my interpretation, the question is asking us to identify the primary kidney response in a 60-year-old woman who runs a half marathon without consuming any fluids during the event due to a condition known as esophageal regurgitation. 'Esophageal regurgitation' is a condition where contents of the stomach flow back into the esophagus, causing discomfort. The woman's high physical activity and lack of hydration would most likely result in dehydration. Dehydration is a condition that occurs when the loss of body fluids surpasses fluid intake, often caused by exercise and inadequate water consumption. Now, let us examine each renal segment and its functions. Arginine vasopressin receptor 2 in the proximal tubular cells, option A, plays a crucial role in the reabsorption of filtered water back into circulation. Option B, K+–H+ exchange in the distal tubule, impacts the final urine composition by maintaining blood pH balance. Option C, proximal tubule carbonic anhydrase, is involved in reabsorbing sodium bicarbonate and secreting hydrogen ions to maintain acid-base balance. Option D, urea reabsorption in the medullary collecting ducts, plays a role in urine concentration and maintaining osmolality during periods of water restriction. Option E, water reabsorption in the ascending loop of Henle, mostly occurs passively due to the high osmolarity of the surrounding tissue; however, this part of the tubule is impermeable to water. In the context of dehydration, it would be logical to prioritize mechanisms that conserve water and maintain plasma volume. Among these, (D) urea reabsorption in the medullary collecting ducts and (E) water reabsorption in the ascending loop of Henle stand out. Clinically reasoning, when the body realizes it's in a dehydrated state, the hypothalamus in the brain initiates the release of antidiuretic hormone (ADH), also known as vasopressin, primarily acting on distal and collecting tubules of nephrons. The primary function of ADH is to increase water reabsorption to maintain blood volume and osmolality. One of the key targets for ADH is the medullary collecting duct, where it increases the permeability of the tubular wall to water and urea, leading to more reabsorption and concentrated urine– this is a direct response to dehydration. On the other hand, the ascending loop of Henle, though involved in water conservation, is typically impermeable to water in its thick segment; thus, this region is not directly impacted by changes in hydration status. In conclusion, based on my clinical understanding and reasoning, I'd hypothesize that among the provided options, option (D) urea reabsorption in the medullary collecting ducts would be the primary renal segment activated in the given dehydration scenario. To answer the original question, I believe the physical state of the 60-year-old woman running a half marathon without drinking any liquid would most likely activate urea reabsorption in the medullary collecting ducts (Option D) as her body tries to conserve as much water as possible.

**Facilitator** The question asks which renal segment would most likely be activated in a 60-year-old woman nearing the completion of a half marathon, who has not consumed any fluids due to esophageal regurgitation. Given her intense physical activity and lack of hydration, it is reasonable to assume that she would be experiencing dehydration. In response to dehydration, the body releases the antidiuretic hormone (ADH), which targets the distal tubules and collecting ducts to increase water permeability and reabsorption. This process is crucial for maintaining the body's fluid balance and blood volume. Among the renal segments listed, the urea reabsorption in the medullary collecting ducts (Option D) plays a significant role in this process. Urea reabsorption contributes to the osmotic gradient that allows for the reabsorption of water, especially under conditions of severe water restriction. This mechanism would likely be amplified under the woman's circumstances. However, it's important to note that while urea reabsorption can contribute to water reabsorption, the main driving force remains the action of ADH on water reabsorption. The ascending loop of Henle, although involved in water conservation, is typically impermeable to water and is not directly impacted by changes in hydration status. Therefore, in the given scenario, it is most likely that the woman's physical state would activate urea reabsorption in the medullary collecting ducts (Option D) as her body tries to conserve as much water as possible. However, none of the given options perfectly match the most probable renal segment that would be activated, as the crucial water reabsorption would be taking place in a different renal segment, primarily the descending limb of Henle's loop and later segments of the collecting ducts.
