# Supplementary material for: Collaborative intelligence in AI: Evaluating the performance of a council of AIs on the USMLE
Source: PLOS Digit Health. 2025 Oct 9;4(10):e0000787. doi: 10.1371/journal.pdig.0000787 (PMC12510544; doi:10.1371/journal.pdig.0000787)
Supplement: S2 File — (ZIP) [file pdig.0000787.s002.zip › S2 Data Raw data files containing unprocessed question inputs and LLM outputs including transcripts of deliberations/STEP1_Question-82-1_Type-MC-NJ.docx]

**Facilitator** **[Round 1]:** An investigator is studying heart development in an experimental animal model. The animal model is genetically modified so that no cardiac neural crest cells develop. This animal model is most likely to have which of the following cardiac abnormalities? (A) Atrial septal defect (B) Coarctation of the aorta (C) Mitral stenosis (D) Persistent truncus arteriosus (E) Transposition of the great arteries

**AI_A:** (1) RESTATING THE QUESTION: The experiment revolves around the modification of an animal model which prevents the development of cardiac neural crest cells. There is an interest in identifying the probable cardiac abnormality that this modification leads to out of the following options: atrial septal defect, coarctation of the aorta, mitral stenosis, persistent truncus arteriosus, or transposition of the great arteries. Cardiac neural crest cells are a group of cells that migrate from the neural crest to the heart during embryological development. These cells contribute significantly to the formation of the cardiac outflow tract, the part of the heart that channels blood into the pulmonary artery and the aorta. (2) PLANNING: Most assumptions can center around what happens when cardiac neural crest cells are absent during heart development. An absence of these cells disrupts the normal formation of the outflow tract resulting in various abnormalities. One major assumption is that the absence of cardiac neural crest cells could lead to atrial septal defect, coarctation of the aorta, mitral stenosis, or transposition of the great arteries. However, a stronger assumption, given our knowledge of embryology, is that the absence of these cells could lead to persistent truncus arteriosus. (3) CLINICAL REASONING: In normal heart development, cardiac neural crest cells contribute to the formation of the spiral septum, which effectively separates the truncus arteriosus into the aorta and pulmonary artery. If cardiac neural crest cells don't develop, this separation process is disrupted. Atrial septal defects, coarctation of the aorta, mitral stenosis, and transposition of the great arteries, while serious heart abnormalities, are not directly related to the development and function of the cardiac neural crest cells. (4) CONCLUDING: As a result of these observations, it is logical to assume that if cardiac neural crest cells do not develop in this genetically modified animal model, the spiral septum would not form correctly, and this would most likely result in a persistent truncus arteriosus. (5) RESPONSE: Therefore, this animal model is most likely to present the cardiac abnormality known as Persistent truncus arteriosus (Choice D).

**AI_D:** (1) RESTATING THE QUESTION: We're asked to predict the cardiac abnormality that would most likely occur in an experimental animal model where the formation of cardiac neural crest cells is inhibited due to genetic modification. Cardiac neural crest cells are distinct set of cells in the embryo that contribute to the development of several cardiac structures, primarily those that form the outflow tract of the heart, including the splitting of the truncus arteriosus into the aorta and pulmonary trunk. (2) PLANNING: To answer this question, we have to take into account two key assumptions: (a) the genetic modification of the animal model has successfully inhibited cardiac neural crest cell formation, and (b) the absence of these cells directly leads to certain cardiac anomalies due to their role in heart development. Let's question these assumptions and hypothesize: Can the absence of cardiac neural crest cells lead to different cardiac anomalies? And if yes, what type of anomalies would be most probable? (3) CLINICAL REASONING: In cardiac embryology, cardiac neural crest cells are majorly responsible for the formation of the septum that divides the truncus arteriosus into the aorta and pulmonary trunk. If these cardiac neural crest cells fail to develop, as it is in our animal model, the division of the truncus arteriosus will be affected leading to its persistence. Persistent truncus arteriosus is a rare heart defect that is present at birth (congenital) in which a single common blood vessel leads out of the heart, instead of the usual two vessels (the main pulmonary artery and aorta). (4) CONCLUDING: Using the understanding of embryological development of heart and pathogenesis of cardiac developmental anomalies, we can conclude that the lack of development of cardiac neural crest cells should result in defects related to structures that these cells typically help form. In this case, their underdevelopment should lead to the persistence of the truncus arteriosus. (5) RESPONSE: Among the given options, the most likely cardiac anomaly to occur due to lack of cardiac neural crest cells in the animal model would be (D) Persistent truncus arteriosus.

**AI_C:** 1) RESTATING THE QUESTION: A scientific study is being conducted, using a genetically modified animal model to examine the development of the heart. Specifically, this model has been altered such that there is no development of cardiac neural crest cells. The question is asking what kind of heart abnormality is most likely to occur in this animal model. Cardiac neural crest cells are a subpopulation of the neural crest, a group of cells that arise from the edge of the neural plate during development. These cells contribute to a number of critical structures in the developing heart, most notably the aorticopulmonary septum, which forms the separation between the aorta and pulmonary artery, and leads to the division of the heart into four chambers. 2) PLANNING: We should examine each of the provided options in the light of the absence of cardiac neural crest cells and determine the most likely outcome. An atrial septal defect would involve a hole in the wall between the two upper chambers of the heart, the atria. A coarctation of the aorta is a narrowing of a part of the aorta. Mitral stenosis involves a narrowing or blockage of the mitral valve in the heart. In persistent truncus arteriosus, the arterial trunk that arises from the developing heart doesn't divide to form the aorta and pulmonary arteries, and there is typically an accompanying ventricular septal defect. Transposition of the great arteries refers to a condition in which the positions of the pulmonary artery and the aorta are reversed. Given the role of cardiac neural crest cells in forming the aorticopulmonary septum, we can hypothesize that a defect in these cells may most directly lead to persistent truncus arteriosus. 3) CLINICAL REASONING: A defect in cardiac neural crest cells means that these cells wouldn't be present to contribute to the aorticopulmonary septum in the developing heart. This would mean that the septum, which normally divides the truncus arteriosus into separate aortic and pulmonary channels, doesn't form. As a result, the truncus arteriosus persists into adulthood, leading to persistent truncus arteriosus. This entails that a single arterial trunk would arise from the heart, and there would be a defect in the ventricular septum, allowing oxygenated and deoxygenated blood to mix. 4) CONCLUDING: In comparison to the other options, none of which rely directly on the presence of cardiac neural crest cells for their formation, persistent truncus arteriosus is the most likely cardiac abnormality to occur in this particular genetically modified animal model. 5) RESPONSE: Therefore, considering what we know about the role of cardiac neural crest cells in heart development, in an experimental animal model where these cells do not develop, the most likely cardiac abnormality to be present would be (D) Persistent truncus arteriosus.

**AI_E:** RESTATING THE QUESTION: The query inquires about a genetic modification in an experimental animal model that prevents the development of cardiac neural crest cells. It then prompts to identify the expected cardiac abnormality that could possibly arise due to this hindrance. Cardiac neural crest cells are a group of cells that migrate from the neural crest to the heart during vertebrate embryogenesis. They're essential for the proper formation of the heart's structures, particularly the outflow tract, which divides and remodels to become two separate arteries leaving the heart: the pulmonary artery and the aorta. PLANNING: Since the genetically modified animal model lacks cardiac neural crest cells, we can hypothesize that defects related to the outflow tract are the most likely outcomes. To further analyze this problem, let's examine each of these cardiac abnormalities to seek correlations with abnormalities from disrupted cardiac neural crest cell functioning: (A) Atrial septal defect: An opening in the wall dividing the two atria of the heart, often caused by a failure in septation (B) Coarctation of the aorta: A narrowing of a part of the aorta, affecting the body’s blood flow. (C) Mitral stenosis: A narrowing of the mitral valve opening, affecting blood flow from the left atrium to the left ventricle. (D) Persistent truncus arteriosus: A rare, congenital (present at birth) heart defect where a single common blood vessel comes out of the heart, instead of normally two (the pulmonary artery and aorta). (E) Transposition of the great arteries: A severe heart defect in which the two main arteries leaving the heart are reversed (transposed). CLINICAL REASONING: Cardiac neural crest cells are critical for heart development, especially in the division of the heart's outflow tract to form the pulmonary artery and the aorta. Abnormalities due to their absence would likely involve the structures that form from the outflow tracts. Looking at the listed abnormalities, "Persistent truncus arteriosus" is an abnormality where instead of splitting into two separate arteries (the pulmonary artery and the aorta), the outflow tract remains as a single vessel. This directly correlates with the function of cardiac neural crest cells, indicating it as the most likely outcome from their absence. Other listed conditions, though being heart defects, don't specifically involve the aberration of formation of outflow tracts or division of truncus arteriosus, making them less likely. CONCLUDING: Therefore, considering the critical role of cardiac neural crest cells in heart development, particularly in the separation of outflow tracts and formation of the pulmonary artery and aorta, and linking this function to the cardiac abnormalities listed, it suggests that the genetic modification causing the absence of these cells in an animal model would likely lead to an abnormality related to these specific areas. Hence, "Persistent truncus arteriosus" seems to be the most probable cardiac abnormality to occur due to this modification. RESPONSE: The animal model genetically modified to have no cardiac neural crest cells develop is most likely to exhibit persistent truncus arteriosus (D).

**AI_B:** RESTATING THE QUESTION: This question revolves around an experiment on heart development using a genetically modified animal model. In this case, the modification entails no development of cardiac neural crest cells. We're asked to identify the cardiac abnormality that will most likely develop. To explain the context better, it's important to understand that cardiac neural crest cells are a group of cells from the neural crest that migrate into the circulatory system & contribute to the formation of the heart. They are essential for the structural development of the heart, particularly the formation of the septum that divides the four heart chambers and the outflow tract. PLANNING: Since cardiac neural crest cells play such important roles in the division of the heart chambers and the outflow tract, the absence of these crest cells would definitely lead to an abnormal development of these structures. We can take a look at the options given: (A) Atrial septal defect: it's a hole in the part of the septum that separates the left and right atria. (B) Coarctation of the aorta: a narrowing of the large blood vessel (aorta) that leads from the heart. (C) Mitral stenosis: narrowing of the heart's mitral valve. (D) Persistent truncus arteriosus: a rare congenital anomaly where the embryological structure known as the truncus arteriosus fails to properly divide into the pulmonary trunk and aorta. (E) Transposition of the great arteries: a serious but rare congenital heart defect in which the two main arteries leaving the heart are reversed. CLINICAL REASONING: Given the functions of cardiac neural crest cells, it can be reasoned that a lack of them would most likely result in developmental abnormalities directly related to their role - that is to say, with the division of the heart chambers and the outflow tract. Atrial septal defect, coarctation of the aorta and mitral stenosis, while they are significant cardiovascular pathologies, do not typically result directly from the absence of cardiac neural crest cells while forming heart structure. Transposition of the great arteries, while a serious heart defect, doesn't stem from neural crest cells malfunction. However, persistent truncus arteriosus is an anomaly that directly relates to the failure of proper division of the heart's outflow tract - a task that cardiac neural crest cells are integral to. CONCLUDING: Based on developmental embryology perspectives, which involves the functions of cardiac neural crest cells and their roles in heart formation and separation of the circulatory elements, it can be inferred that the absence of these cells would likely lead to an abnormal development in those areas they typically influence. Therefore, Persistent truncus arteriosus (option D) would be the most likely cardiac abnormality to occur in an animal model that has been genetically modified to not develop cardiac neural crest cells. RESPONSE: The most likely cardiac abnormality to occur in this genetically modified animal model which lacks cardiac neural crest cell development, would be (D) Persistent truncus arteriosus.

**Facilitator** The consensus statement synthesizes the responses of AI_A, AI_B, AI_C, AI_D, and AI_E as follows: The question pertains to an experimental animal model that has been genetically modified to prevent the development of cardiac neural crest cells. The task is to identify the most probable cardiac abnormality that would result from this modification. Cardiac neural crest cells are crucial for heart development, particularly in the formation of the outflow tract, which divides into the pulmonary artery and the aorta. Given the role of cardiac neural crest cells in heart development, the absence of these cells would likely lead to abnormalities related to the structures they typically help form. The options provided for potential abnormalities are atrial septal defect, coarctation of the aorta, mitral stenosis, persistent truncus arteriosus, and transposition of the great arteries. Upon examination of these options, persistent truncus arteriosus emerges as the most likely outcome. This condition is a congenital heart defect where a single common blood vessel comes out of the heart, instead of the usual two separate vessels (the pulmonary artery and aorta). This abnormality directly correlates with the function of cardiac neural crest cells, making it the most probable result of their absence. The other listed conditions, while serious heart defects, do not directly involve the formation or division of the outflow tracts, which are the primary roles of cardiac neural crest cells in heart development. Therefore, these conditions are less likely to occur due to the absence of these cells. In conclusion, the genetically modified animal model that lacks cardiac neural crest cells is most likely to exhibit the cardiac abnormality known as persistent truncus arteriosus.
