# Supplementary material for: Collaborative intelligence in AI: Evaluating the performance of a council of AIs on the USMLE
Source: PLOS Digit Health. 2025 Oct 9;4(10):e0000787. doi: 10.1371/journal.pdig.0000787 (PMC12510544; doi:10.1371/journal.pdig.0000787)
Supplement: S2 File — (ZIP) [file pdig.0000787.s002.zip › S2 Data Raw data files containing unprocessed question inputs and LLM outputs including transcripts of deliberations/STEP1_Question-83-1_Type-MC-NJ.docx]

**Facilitator** **[Round 1]:** A 78-year-old woman undergoes an outpatient laparoscopic cholecystectomy. During the procedure, ligation of the cystic artery is planned. It is most appropriate to ligate this artery at its junction with which of the following arteries? (A) Gastroduodenal (B) Left gastric (C) Left hepatic (D) Right gastric (E) Right hepatic

**AI_D:** (1) RESTATING THE QUESTION: The question asks about the most appropriate location to ligate the cystic artery, which is a vital step in laparoscopic cholecystectomy—a surgical procedure to remove the gallbladder. The cystic artery is typically the first branch from the right hepatic artery, though its origin can vary. (2) PLANNING: Considering my above definition, we need to identify the assumption that the cystic artery always arises from the right hepatic artery. An alternate assumption could be the potential anatomical variations in the origin of the cystic artery; this helps to hypothesize the clinical priority that knowledge about surgeries' anatomical consistency and potential variations is crucial in preventing intraoperative complications and ensuring the success of the surgery. (3) CLINICAL REASONING: The right hepatic artery usually gives off the cystic artery; this supplies the gallbladder, making it the ideal point of ligation during a cholecystectomy. However, anatomical variations occur in approximately 20% of cases, meaning the cystic artery might not always originate as expected. Despite these potential variations, it remains most likely that the cystic artery will branch from the right hepatic artery, underlining the importance of thorough knowledge of surgical anatomy. (4) CONCLUDING: Even though there are potential anatomical variations, in the majority of cases, the cystic artery originates from the right hepatic artery. Therefore, it is most appropriate to ligate the cystic artery at its junction with the right hepatic artery in this patient undergoing a laparoscopic cholecystectomy. (5) RESPONSE: From the mentioned options, it would be most appropriate to ligate the cystic artery at its junction with the right hepatic artery (E).

**AI_A:** RESTATING THE QUESTION: You are asking about the anatomical connection of the cystic artery where we should carry out ligation during the laparoscopic cholecystectomy procedure. Ligation means surgical tying off of blood vessels to prevent blood flow. The cystic artery, often a branch of the right hepatic artery, provides blood supply to the gallbladder and is usually ligated in a cholecystectomy, which is a surgical removal of the gallbladder. PLANNING: Ligation of the cystic artery is essential during a cholecystectomy to prevent bleeding. The cystic artery usually arises from the right hepatic artery, but the location can vary. We need to understand this anatomy to make an assumption about where it would make the most sense to ligate the artery. CLINICAL REASONING: We know the cystic artery usually arises from the right hepatic artery, but the exact origin can vary. However, in clinical practice, we operate following the most common scenario. The right hepatic is a branch of the common hepatic artery, which is a branch of the celiac trunk that supplies the liver, gallbladder, stomach, and pancreas. Although it might occasionally arise from other vessels, these are relatively rare cases. Therefore, we should stick with the most common assumption. CONCLUDING: Considering the cystic artery's commonly known anatomy, and rarity of variations, it would be most appropriate to ligate the cystic artery at its junction with the right hepatic artery during a laparoscopic cholecystectomy. RESPONSE: Therefore, the answer is (E) Right hepatic.

**AI_E:** (1) RESTATING THE QUESTION: This question revolves around a surgical scenario in which a 78-year-old woman is undergoing a laparoscopic cholecystectomy. In simpler terms, the woman is having her gallbladder removed through minimally invasive surgery. The medical team plans to ligate, or tie off, the cystic artery during the procedure. The question asks us to identify the artery with which the cystic artery generally forms a junction. (2) PLANNING: The cystic artery, usually a branch of the right hepatic artery, supplies the gallbladder and the cystic duct. Remembering that anatomical variations do occur, the common assumption is that the cystic artery originates from the right hepatic artery. However, we must question whether this is always the case, and then evaluate whether there might be another arterial connection that should be prioritized in the context of this specific surgical procedure. (3) CLINICAL REASONING: The gallbladder situated underneath the liver, receives its arterial nourishment mainly from the hepatic artery. The hepatic artery splits into left and right hepatic arteries, and generally, the cystic artery branches off from the right hepatic artery. Thus, if we contemplate the anatomical situation in a majority of cases, the junction of the cystic artery with the right hepatic artery would be the most appropriate ligation point. Bear in mind, the remaining options (gastroduodenal, left gastric, left hepatic, and right gastric arteries) have anatomical routes and responsibilities differing from the cystic artery, suggesting their junctions would be less suitable for ligation. (4) CONCLUDING: By considering the anatomical relationships between the arteries supplying the liver and gallbladder, we can infer that the right hepatic artery provides the most likely point of origin for the cystic artery. (5) RESPONSE: The cystic artery is most appropriately ligated at its junction with the right hepatic artery (Choice E). This junction point should be verified during surgery, as anatomical variations may occur.

**AI_C:** RESTATING THE QUESTION: The question is asking where the cystic artery, which needs to be ligated during a laparoscopic cholecystectomy, commonly originates from. The cystic artery primarily supplies blood to the gallbladder and is typically a branch of the right hepatic artery. PLANNING: The assumptions inferred here is that the cystic artery arises from any of those arteries mentioned in the options (A) Gastroduodenal, (B) Left gastric, (C) Left hepatic, (D) Right gastric, (E) Right hepatic. By the process of anatomical reasoning, we hypothesize that the cystic artery should originate more probably from an artery that is anatomically closer to the gallbladder, an organ residing within the right part of the abdomen. CLINICAL REASONING: The cystic artery commonly arises from the right hepatic artery, which in itself is a branch of the common hepatic artery (a branch of the coeliac trunk). Thus, to reach the gallbladder, which is a right-sided organ, it would seem more physiologically reasonable for the cystic artery to branch from the right hepatic rather than other arteries like the gastroduodenal, left gastric, left hepatic, or right gastric arteries. These have a less direct route and are not typically associated with gallbladder blood supply. CONCLUDING: Thus, applying anatomical logic, the most appropriate location to ligate the cystic artery during a laparoscopic cholecystectomy would be at its junction with the artery that is anatomically closer and logically related to the supply of the gallbladder. Based on typical anatomy, this would be the right hepatic artery. RESPONSE: Thus, during a laparoscopic cholecystectomy, it is most appropriate to ligate the cystic artery at its junction with the right hepatic artery (E).

**AI_B:** (1) RESTATING THE QUESTION: The query involves an elderly woman who is undergoing a surgical procedure called the laparoscopic cholecystectomy, an endoscopic surgery to remove the gallbladder, which is commonly performed due to gallstones. In this operation, an imperative step is the ligation of the cystic artery, basically tying it off to prevent bleeding. The question is inquiring where the best point of ligation for the cystic artery is situated, given a selection of arteries that could be potential junctions. (2) PLANNING: The underlying assumption here is that the cystic artery branches from one of the listed arteries. It's essential to recollect our anatomical knowledge about the arterial supply of the liver and gallbladder, particularly the branches of the celiac trunk and common hepatic artery. These details would assist in determining the source of the cystic artery. If we re-evaluate this assumption and assess each choice separately, based on anatomical facts, we can determine which artery the cystic artery intersects with. (3) CLINICAL REASONING: The cystic artery usually arises from the right hepatic artery, which is a branch of the common hepatic artery, itself a branch of the celiac trunk. The right hepatic artery, after giving off the cystic artery, further divides into anterior and posterior segmental branches supplying the corresponding parts of the liver. On the other hand, the gastroduodenal, left gastric, and right gastric arteries have different courses and territories of supply not directly related to the gallbladder. The left hepatic artery primarily supplies the left lobe of the liver and doesn't generally give rise to the cystic artery. (4) CONCLUDING: Analyzing these anatomic relationships, the cystic artery—a crucial artery ligated during a cholecystectomy—usually originates from the Right Hepatic Artery, rather than any of the others mentioned. This point has significant clinical implications, as inappropriate ligation could potentially lead to ischemic complications. (5) RESPONSE: The most appropriate artery to ligate the cystic artery at its junction is with the Right Hepatic Artery [Option E].

**Facilitator** The question pertains to the most suitable location for ligation of the cystic artery during a laparoscopic cholecystectomy, a minimally invasive surgical procedure to remove the gallbladder. The cystic artery, which primarily supplies blood to the gallbladder, typically originates from the right hepatic artery. However, it's important to note that anatomical variations can occur. The consensus among the AI responses is that, based on common anatomy, the cystic artery usually arises from the right hepatic artery. This artery is a branch of the common hepatic artery, which itself is a branch of the celiac trunk. The right hepatic artery, after giving off the cystic artery, further divides into anterior and posterior segmental branches supplying the corresponding parts of the liver. The other arteries listed - gastroduodenal, left gastric, left hepatic, and right gastric - have different courses and territories of supply not directly related to the gallbladder. Therefore, they are less likely to be the point of origin for the cystic artery. In conclusion, despite potential anatomical variations, it is most appropriate to ligate the cystic artery at its junction with the right hepatic artery during a laparoscopic cholecystectomy. This is based on the typical anatomical relationship between these arteries and the gallbladder. However, surgeons should always verify this junction point during surgery due to the possibility of anatomical variations.
