# Supplementary material for: Collaborative intelligence in AI: Evaluating the performance of a council of AIs on the USMLE
Source: PLOS Digit Health. 2025 Oct 9;4(10):e0000787. doi: 10.1371/journal.pdig.0000787 (PMC12510544; doi:10.1371/journal.pdig.0000787)
Supplement: S2 File — (ZIP) [file pdig.0000787.s002.zip › S2 Data Raw data files containing unprocessed question inputs and LLM outputs including transcripts of deliberations/STEP1_Question-84-1_Type-MC-NJ.docx]

**Facilitator** **[Round 1]:** A 32-year-old man comes to the office because of a 2-week history of fever and throat pain. He is 173 cm (5 ft 8 in) tall and weighs 63 kg (140 lb); BMI is 21 kg/m2. His pulse is 110/min, respirations are 16/min, and blood pressure is 98/68 mm Hg. Physical examination shows scattered 2- to 4-cm lymph nodes in the neck, axillae, and inguinal regions. There is a bilateral tonsillar exudate but no ulcerations. Results of laboratory studies are shown: Hemoglobin 9.6 g/dL Hematocrit 29% Leukocyte count 1500/mm³ Platelet count 60,000/mm³ A heterophile antibody test result is negative. Which of the following is the most likely diagnosis? (A)  Epstein-Barr virus infection (B)  Gonococcal pharyngitis (C)  HIV infection (D)  Lymphogranuloma venereum infection (E)  Streptococcal pharyngitis

**AI_E:** RESTATING THE QUESTION: The query describes a 32-year-old male who has been experiencing fever and throat pain for the past two weeks. His signs include nodes spread across the neck, axillae, and inguinal regions. Blood tests reveal anemia, leukopenia, and thrombocytopenia. A negative heterophile antibody test intends to rule out infectious mononucleosis, often caused by the Epstein-Barr virus. The final diagnosis needs to be determined from the provided choices, which include several infectious diseases. PLANNING: The data points to consider carefully are the patient's fever and throat pain, the large, scattered lymph nodes and tonsillar exudate indicating a probable infection, and the significant cytopenia in complete blood count suggesting a condition with bone marrow involvement. An assumption could be that the patient is suffering from a simple bacterial throat infection, but the leukopenia and thrombocytopenia suggest otherwise. An alternate assumption, with integrated blood test results and a negative heterophile antibody test, points towards HIV infection as the culprit. CLINICAL REASONING: Starting with the assumption that this could be a simple bacterial infection such as Streptococcus, the patient should have shown leukocytosis in response to the infection, not leukopenia. Furthermore, the hematological findings, along with the presence of generalized lymphadenopathy, suggest a systemic condition like HIV. HIV infection often presents with lymphadenopathy, fever, and pharyngitis. The virus can also cause bone marrow suppression leading to cytopenias. The Epstein-Barr virus, while also causing similar symptoms, is unlikely since the heterophile antibody test was negative. Gonococcal pharyngitis generally presents with more localized symptoms and without generalized lymphadenopathy, making it less likely. Lymphogranuloma venereum infection, caused by specific serovars of Chlamydia trachomatis, leads to potentially painful inguinal lymphadenopathy and often has a more proliferative or pustular presentation. CONCLUDING: Given the bilateral tonsillar exudate, generalized lymphadenopathy, fever, and significant cytopenia, the most likely diagnosis is a systemic infection that affects multiple body systems and can manifest even without unique signs of the infection, such as HIV. The negative heterophile antibody test further confirms the diminished likelihood of an EBV infection. RESPONSE: Therefore, the most probable diagnosis in this case is (C) HIV infection.

**AI_B:** RESTATING THE QUESTION: The presented scenario is about a 32-year-old man who is suffering from fever and throat pain for the past two weeks. He is of average height and weight. An examination revealed lymphadenopathy and tonsillar exudate. Laboratory results indicate anemia, leukopenia, and thrombocytopenia. The heterophile antibody test, used to detect infectious mononucleosis commonly caused by Epstein-Barr virus, is negative. We are asked to diagnose his condition from given options. PLANNING: The heterophile antibody test being negative rules out Epstein-Barr virus infection (option A). Similarly, Streptococcal or Gonococcal pharyngitis (options E and B respectively) wouldn't typically present with significant lymphadenopathy or such profound hematological alterations. Lymphogranuloma venereum (option D) caused by Chlamydia trachomatis primarily affects the genital and anorectal regions, which doesn't match our patient's symptoms. That leaves us with HIV infection (option C) as a possibility. CLINICAL REASONING: The clinical features suggestive of HIV infection include fever, sore throat, and generalized lymphadenopathy - all of which match our patient's presentation. Importantly, the hematological alterations (anemia, leukopenia, thrombocytopenia) seen in this patient reflect bone marrow suppression frequently encountered in advanced HIV infection (AIDS - Acquired Immunodeficiency Syndrome). While patients often report symptoms resembling mononucleosis (caused by the Epstein-Barr virus), testing negative for heterophile antibodies steers us away from this diagnosis. CONCLUDING: In light of the clinical features (fever, sore throat, generalized lymphadenopathy), profound hematological changes indicative of bone marrow suppression, and the negative heterophile antibody test result, HIV infection emerges as the most compatible diagnosis among the given options. RESPONSE: The most likely diagnosis for this 32-year-old man is HIV infection (C). This aligns with his clinical presentation and laboratory findings.

**AI_C:** 1) RESTATING THE QUESTION: This clinical scenario is about a 32-year-old man who presented with a 2-week history of fever and throat pain, accompanied by enlarged lymph nodes in different regions and some abnormal lab results. His hemoglobin is low (suggesting anemia), he has thrombocytopenia (low platelet count), and his leukocyte count, which includes white blood cells, is also low, indicating a decreased immune defense. The heterophile antibody test, which is used primarily for the diagnosis of mononucleosis typically caused by Epstein-Barr virus (EBV), is negative in this case. The question queries for the most likely diagnosis from a given set of options. 2) PLANNING: Possible diagnoses linked with these clinical findings could be viral or bacterial infections, or hematological disorder. Considering leukopenia, anemia, thrombocytopenia, and a negative heterophile antibody test, the most likely assumption becomes a systemic viral infection, specifically HIV. Alternately, the patient could be suffering from a bacterial infection like streptococcal or gonococcal pharyngitis, which could be accompanied by lymphadenopathy, but they usually do not cause such severe hematologic abnormalities. EBV infection could be ruled out because a negative heterophile antibody test significantly reduces the probability of Epstein-Barr virus mononucleosis. 3) CLINICAL REASONING: The clinical assumption leads one towards HIV infection. HIV can affect the immune system and cause generalized lymphadenopathy, sore throat, and systemic symptoms like fever. Furthermore, HIV can induce hematological abnormalities, resulting in anemia, thrombocytopenia, and leukopenia as we see in this patient. Other causes of pharyngitis usually do not cause such hematological changes and are thus less likely. 4) CONCLUDING: From clinical reasoning, HIV infection seems to fit best with the given presentation, although a confirmation with specialized HIV testing would be required. While other presented diagnoses could potentially cause some of the symptoms, none except HIV correlates well with the profound hematologic abnormalities. 5) RESPONSE: Therefore, out of the given options, HIV infection (C) is the most probable diagnosis for the presented clinical scenario.

**AI_D:** RESTATING THE QUESTION: The question describes a 32-year-old male patient who has come in with a 2-week history of fever and throat pain. He has a normal BMI and physical examination reveals significant lymph node enlargement and tonsillar exudate in the absence of ulcerations. His lab reports show anemia, a significant decrease in leukocytes and platelet count. A test for heterophile antibody, commonly associated with mononucleosis, is negative. The purpose of the question is to determine the most likely disease condition he could have, with options that include Epstein-Barr virus infection, Gonococcal pharyngitis, HIV infection, Lymphogranuloma venereum infection, Streptococcal pharyngitis. PLANNING: Let's identify the assumptions. Firstly, considering the symptoms of fever, throat pain, and lymph node enlargement along with the tonsillar exudate, there's an assumption that the patient has some infectious disease. Secondly, the cytopenia (low count of blood cells) might indicate a condition that affects the bone marrow or systemic disease. Thirdly, we are assuming the heterophile antibody test (negative in this case) is to exclude Infectious Mononucleosis predominantly caused by Epstein-Barr virus infection. Thus, the immediate assumption could be the patient may not suffer from that. Now, let's consider alternate assumptions by questioning each assumption: 1) Is every infectious disease associated with these signs and symptoms? 2) Can cytopenia occur in non-marrow or non-systemic diseases? 3) Is a heterophile antibody test exclusively indicative of Epstein-Barr virus infection? Evaluating all these, we may hypothesize the patient could be suffering from a condition other than infectious mononucleosis. CLINICAL REASONING: To start with, a negative heterophile antibody test does rule out Infectious Mononucleosis caused by Epstein-Barr virus (Option A). However, it can be seen in up to 25% of adolescents and young adults during the first week of illness. The presence of significant lymphadenopathy favors a systemic infection. Gonococcal pharyngitis and Streptococcal pharyngitis (Options B & E) typically do not present with generalized lymphadenopathy and neither do they result in cytopenia. On the other hand, HIV infection (Option C) often presents with generalized lymphadenopathy and fever and can cause cytopenias due to bone marrow suppression. This could explain the decreased hematocrit, leukocyte, and platelet count. Transmission could have been through unprotected sex, intravenous drug use, etc. Lastly, Lymphogranuloma venereum (Option D), caused by Chlamydia trachomatis, usually presents with genital ulcers and/or swollen lymph nodes around the genital region, which does not align promptly with this patient's presentation. CONCLUDING: In conclusion, using our clinical thought process and step-by-step explanation, HIV infection seems to be a most probable cause. This is primarily due to the presence of generalized lymphadenopathy and cytopenia in the patient. RESPONSE: Therefore, the most likely diagnosis for this 32-year-old man is (C) HIV Infection.

**AI_A:** RESTATING THE QUESTION: A 32-year-old man has been suffering from fever and throat pain for 2 weeks. He has several enlarged lymph nodes all over his body and his tonsils are inflamed, but not ulcerated. His lab results indicate low hemoglobin and hematocrit values, as well as a considerable reduction in his leukocyte and platelet counts. Although he has some symptoms that could suggest infectious mononucleosis, such as the lymphadenopathy, no heterophile antibodies were detected in his blood. The goal is to identify his most likely medical condition from the options of Epstein-Barr virus infection, Gonococcal pharyngitis, HIV infection, Lymphogranuloma venereum infection, or Streptococcal pharyngitis. PLANNING: It is necessary to assess the features of the provided options in relation to the patient's symptoms, sign, and laboratory results. Epstein-Barr virus (EBV) infection, typically diagnosed by a positive heterophile antibody test, can cause symptoms like fever, sore throat, and lymphadenopathy, which our patient has, but his test was negative. Gonococcal pharyngitis, caused by Neisseria gonorrhoeae, usually presents with sore throat and could induce lymphadenopathy, but doesn't typically cause fever and it doesn't explain the abnormal blood counts. HIV infection can cause symptoms similar to the patient’s, specifically the generalized lymphadenopathy. The altered blood levels could also be due to the bone marrow suppression caused by HIV, which can lead to anemia, leukopenia, and thrombocytopenia. Lymphogranuloma venereum infection, caused by Chlamydia trachomatis, is often asymptomatic in its early stages and then leads to enlarged lymph nodes in the groin, not typically generalized lymphadenopathy. Streptococcal pharyngitis, caused by Streptococcus pyogenes, produces a sore throat, fever, and sometimes lymphadenopathy, but it doesn’t cause the abnormal blood count findings in this case. CLINICAL REASONING: The patient's symptoms of fever, sore throat, and generalized lymphadenopathy initially point towards a systemic infection. Considering the negative heterophile antibody test, an Epstein-Barr virus infection is unlikely. Most bacterial throat infections like Gonorrhea and Streptococcus would not typically result in such marked cytopenias (leukocytopenia, anemia, and thrombocytopenia), suggesting an alternate cause for these changes. HIV infection, however, can lead to the aforementioned systemic symptoms as well as the observed cytopenias, due to bone marrow suppression and infection of leukocytes. It should, however, also be noted that despite generalized lymphadenopathy being somewhat common in advanced HIV, it’s not a defining characteristic, as it is often caused by secondary infections rather than the virus itself. CONCLUDING: Considering the narrative, there's no evidence for ulcerative or localized physical findings that'd suggest sexually transmitted diseases like Gonococcal pharyngitis or Lymphogranuloma venereum. Streptococcal pharyngitis could explain the throat pain and fever but is less likely given the generalized lymphadenopathy and cytopenias. Similarly, while infectious mononucleosis could explain the symptoms experienced by this patient, a negative heterophile antibody test suggests other etiologies. Weighing the evidence, HIV infection seems to best align with our patient's symptoms and findings on examination and bloodwork due to collaboration of asthenia, immunosuppression, lymphadenopathy, and abnormal laboratory data. RESPONSE: The most likely diagnosis for this patient appears to be (C) HIV infection.

**Facilitator** The consensus statement synthesizes the responses of AI_A, AI_B, AI_C, AI_D, and AI_E as follows: The question presents a 32-year-old man with a 2-week history of fever and throat pain, generalized lymphadenopathy, and significant cytopenia (anemia, leukopenia, and thrombocytopenia). A negative heterophile antibody test, typically used to diagnose infectious mononucleosis caused by the Epstein-Barr virus, is also noted. The task is to identify the most likely diagnosis from the given options: Epstein-Barr virus infection, Gonococcal pharyngitis, HIV infection, Lymphogranuloma venereum infection, or Streptococcal pharyngitis. The negative heterophile antibody test makes an Epstein-Barr virus infection unlikely. Gonococcal pharyngitis and Streptococcal pharyngitis, while they could explain the throat pain and fever, do not typically result in generalized lymphadenopathy or significant cytopenia. Lymphogranuloma venereum infection, primarily affecting the genital and anorectal regions, does not align with the patient's symptoms. HIV infection, however, presents a plausible diagnosis. It can cause systemic symptoms like fever and sore throat, generalized lymphadenopathy, and significant cytopenia due to bone marrow suppression. The patient's symptoms, physical findings, and laboratory results align well with this diagnosis, making HIV infection the most likely condition among the given options. Therefore, the consensus among the AI responses is that the most probable diagnosis for this 32-year-old man is HIV infection (Option C). This conclusion is based on the patient's clinical presentation, the profound hematological changes indicative of bone marrow suppression, and the negative heterophile antibody test result. However, it should be noted that a definitive diagnosis would require further specialized HIV testing.
