# Supplementary material for: Collaborative intelligence in AI: Evaluating the performance of a council of AIs on the USMLE
Source: PLOS Digit Health. 2025 Oct 9;4(10):e0000787. doi: 10.1371/journal.pdig.0000787 (PMC12510544; doi:10.1371/journal.pdig.0000787)
Supplement: S2 File — (ZIP) [file pdig.0000787.s002.zip › S2 Data Raw data files containing unprocessed question inputs and LLM outputs including transcripts of deliberations/STEP1_Question-86-1_Type-MC-NJ.docx]

**Facilitator** **[Round 1]:** A 51-year-old man with a 10-year history of gastroesophageal reflux and suspected Barrett esophagus comes to the office because his omeprazole dose ""doesn't work around the Christmas holidays."" He states that he prides himself on having a large appetite and ""holding his liquor"" during the holidays. He currently takes the maximum dose of omeprazole. Which of the following is the most appropriate initial action by the physician? (A) Ask the patient how much he is eating and drinking during the holidays (B) Explain the hazards of untreated reflux in the presence of Barrett esophagus (C) Order an upper endoscopy (D) Refer the patient to a gastroenterologist (E) Switch the omeprazole to pantoprazole

**AI_D:** 1) RESTATING THE QUESTION: The query seeks to ascertain the appropriate initial step a doctor should take given the following scenario: A 51-year-old male patient with a decade-long history of gastroesophageal reflux and suspected Barrett esophagus has become unresponsive to his omeprazole medication during the holiday season. This is possibly due to his increased intake of food and alcohol during this period. Gastroesophageal reflux disease (GERD) is a chronic condition where stomach acid flows back into the esophagus causing irritation and damage. Barrett Esophagus is a complication of GERD where the lining of the esophagus changes to resemble intestinal lining, increasing the risk of esophageal adenocarcinoma. Omeprazole is a proton pump inhibitor used to decrease the production of stomach acid. 2) PLANNING: Some possible assumptions are that the patient's change in diet and alcohol consumption is increasing his acid reflux symptoms, the patient's Barrett esophagus is worsening, or his treatment is ineffective. However, before making any assumptions, the physician should seek to understand the degree of change in the patient's intake of food and alcohol during the holidays. 3) CLINICAL REASONING: Overeating and alcohol increase gastric acid production and decrease the lower esophageal sphincter tone, exacerbating GERD symptoms. Thus, it is essential to assess the extent to which the patient changes his diet and alcohol consumption during the holidays before considering alternate treatment plans. Furthermore, if his increased symptoms are only temporary and linked to his holiday behaviors, it might not warrant changing his overall treatment plan. 4) CONCLUDING: Hence, before making any changes to his treatment, it would be more prudent to first evaluate how much his intake changes around the holidays. 5) RESPONSE: Therefore, the most appropriate initial action by the physician may be to ask the patient how much he is eating and drinking during the holidays (Option A).

**AI_E:** RESTATING THE QUESTION: The query pertains to a man in his early fifties with a history of gastroesophageal reflux disease (GERD) and a possible diagnosis of Barrett esophagus. The issue arises during the holiday season when he consumes larger volumes of food and alcohol, making his standard medication (omeprazole) less effective. The question asks for the most appropriate initial response from the physician given these circumstances. PLANNING: Notable assumptions to be considered are: the patient's reflux symptoms are solely due to increased intake during the festive season; the patient understands the gravity of untreated reflux with Barrett esophagus; an upper endoscopy or referral to a gastroenterologist will yield significant findings; and a change in medication will ameliorate the symptoms. The priority assumption to address is the patient's lifestyle during the holidays, as this period coincides with the deterioration of his symptom control. CLINICAL REASONING: When considering the pharmacology of omeprazole, we know it's a proton pump inhibitor (PPI) and works by reducing stomach acid. However, this action can be overwhelmed by excess food and alcohol intake, which are known to increase stomach acid. Also, alcohol might compromise the protective mechanisms of the gastroesophageal junction, worsening reflux symptoms. Having Barrett esophagus, an epithelial change caused by chronic GERD, puts the patient at a higher risk of esophageal cancer. This understanding suggests the need to control his GERD symptoms effectively. The change in this patient's dietary habits during the holidays appears to be the leading factor that precipitates the failure of his medication, indicating that it should be assessed. The other alternate steps, although clinically relevant, are not the primary interventions. CONCLUDING: Having considered all these elements, the patient's increased food and alcohol consumption during the holiday season is likely to contribute significantly to his symptoms. Hence, it would be beneficial for the physician to inquire about the specifics of his holiday diet. This information will guide dietary advice to mitigate his reflux symptoms. RESPONSE: Thus, the most appropriate initial action by the physician would be to (A) Ask the patient how much he is eating and drinking during the holidays.

**AI_C:** (1) RESTATING THE QUESTION: The patient, a 51-year-old man, has a longstanding history of gastroesophageal reflux disease (GERD) which has led to a suspicion of Barrett esophagus, a complication where normal tissue lining the esophagus changes to tissue resembling the lining of the intestine. Even though he is on maximum dose of omeprazole, a proton pump inhibitor (PPI) that helps decrease the amount of acid the stomach makes, he experiences exacerbated symptoms around the holidays. This happens when he indulges in excessive eating and drinking. He has arrived at the office, and now we need to determine the next appropriate step by his physician. (2) PLANNING: The assumptions here are that the patient's excessive indulgences during holidays are contributing to symptom exacerbation, the current medication is ineffective, and the suspicion of Barrett esophagus is confirmed. Alternate assumptions could be that he might not be adhering strictly to his medication regime during the holidays, or his large meal sizes and alcohol could be complicating his symptoms, or there is a possibility that his condition might have progressed. Based on these, clinical prioritization could lead us to focus on first understanding lifestyle factors contributing to the exacerbation of his symptoms. (3) CLINICAL REASONING: Before proceeding with investigations or referrals, it's prudent to gather more information about the patient's lifestyle, particularly during the holiday period. Excessive alcohol consumption and large meal sizes can exacerbate GERD symptoms by increasing gastric acid production and inducing relaxation of the lower esophageal sphincter, leading to acid reflux. It's also important to establish that the patient is sticking to his medication schedule, as irregularities could also trigger symptoms. (4) CONCLUDING: The patient's holiday behaviors and practices, especially his food and drink intake, likely play a key role in his GERD symptoms resurfacing or intensifying. Thus, the most immediate step is to probe further into these habits. (5) RESPONSE: The most appropriate initial action by the physician should be to (A) Ask the patient how much he is eating and drinking during the holidays, as these are most likely aggravating his GERD symptoms, despite him being on the maximum dose of omeprazole. This will act as a starting point to educate him on lifestyle modifications that could aid in better symptom control.

**AI_B:** RESTATING THE QUESTION: This clinical scenario presents a 51-year-old man with a significant history of gastroesophageal reflux disorder and a suspicion of Barrett esophagus. Despite taking the maximum dose of omeprazole, a proton pump inhibitor used to reduce stomach acid, he complains that his medication doesn't seem effective during the holidays. He admits to consuming larger amounts of food and alcohol during this period. The question is asking us to identify the most appropriate initial action for the physician to take in this situation. PLANNING: The existing assumptions are that his increased culinary indulgence and alcohol intake might exacerbate his symptoms and that his current dose of omeprazole isn't effective in controlling his symptoms during this period. However, it's also possible that his Barrett esophagus may have progressed, warranting an assessment of his esophagus' condition, or that alternative treatment options may need exploring. The physician could prioritize getting more information about his holiday consumption habits, explain the risks of untreated reflux in Barrett esophagus, order an endoscopy to inspect his esophagus directly, refer him to a gastroenterologist for specialist advice, or switch his current medication to another proton pump inhibitor. Despite these alternatives, the initial action should ideally address the immediate cause of his exacerbated symptoms. CLINICAL REASONING: To begin, it would be medically sound to first ascertain his holiday eating and drinking habits. As he himself admits to overindulgence, this could be the source of his increased discomfort during this time. The answer to this may also provide information about the triggers of his symptoms and clarify whether his increased symptoms are due to overeating, increased alcohol consumption, or both. It is important to note that both overeating and excessive alcohol consumption can exacerbate gastroesophageal reflux symptoms, which can even potentiate the development of Barrett's esophagus, a precancerous condition. In light of these considerations, this approach may yield immediate insights into the management of his condition. CONCLUDING: Therefore, before considering the advancement of his esophageal condition or altering his treatment, understanding his eating and drinking behaviors during the holidays will provide valuable context for his symptom exacerbation. By identifying potential contributing factors to his illness during the holidays, it could lead to behavioral modifications or dietary changes that could reduce his discomfort without necessarily changing his medication or undertaking further diagnostic procedures. RESPONSE: So, the most appropriate initial action by the physician would be to (A) Ask the patient how much he is eating and drinking during the holidays.

**AI_A:** (1) RESTATING THE QUESTION: The patient in this case is a 51-year-old man with a history of gastroesophageal reflux disease ('GERD'), which is a chronic condition where stomach acid frequently flows back into the tube connecting the mouth and stomach. He is also suspected of having Barrett's esophagus, a potentially serious complication of GERD where the normal tissue lining the esophagus changes to tissue resembling the lining of the intestine. Currently, his symptoms are not managed effectively with omeprazole, a type of medication used to reduce the production of stomach acid. This is more pronounced during the holidays when he eats more and drinks alcohol excessively. As a healthcare provider, we need to decide the next best step in managing his care. (2) PLANNING: There are various assumptions one could make. The patient's increased food and alcohol intake during the holidays could exacerbate his symptoms, implying that lifestyle modification could be beneficial. Alternatively, the adequacy and compliance with the existing medication could be examined. The seriousness of untreated reflux in the presence of Barrett's esophagus, an increased risk for esophageal cancer, may need to be emphasized. Finally, the need for further assessment via upper endoscopy or referral to a specialist might be considered, or a change in medication to pantoprazole, another proton pump inhibitor, could be examined. (3) CLINICAL REASONING: The patient's symptoms flare-up during the holidays, and he admits to overeating and heavy drinking during this period. Connecting these pieces of information, it seems likely that his symptoms are tied to his lifestyle choices, particularly during these festive periods. Proton pump inhibitors such as omeprazole are generally effective at controlling GERD symptoms, so it's unusual for them to suddenly lose their efficacy at specific times. However, achieving adequate symptom control can be challenging if the patient engages in behavior that promotes acid reflux, such as overeating and consuming alcohol in excess. Although it could be argued that other actions such as ordering an upper endoscopy or referring to a gastroenterologist might be beneficial, these strategies should ideally follow initial attempts to address modifiable risk factors for symptom exacerbation i.e., overeating and alcohol consumption. Warning about the potential hazards of untreated reflux, such as developing esophageal cancer, is also a relevant action, but it may not directly address the immediate issue of failure to control symptoms. The same can also be said of swapping omeprazole to pantoprazole, which basically works in a similar way. (4) CONCLUDING: Considering everything, the key argument points toward addressing lifestyle modifications as the initial step in managing this patient's uncontrolled symptoms. As such, it's crucial to gather information on the patient's dietary habits during the holidays, primarily how much he eats and drinks, before making any further decisions on management. (5) RESPONSE: Based on the clinical reasoning, the most appropriate initial action by the physician is to (A) ask the patient how much he is eating and drinking during the holidays.

**Facilitator** The consensus statement synthesizes the responses of AI_A, AI_B, AI_C, AI_D, and AI_E as follows: The patient in question is a 51-year-old man with a history of gastroesophageal reflux disease (GERD) and suspected Barrett's esophagus. Despite being on the maximum dose of omeprazole, a proton pump inhibitor used to reduce stomach acid, his symptoms are not effectively managed, particularly during the holiday season when he admits to overeating and consuming alcohol excessively. The key assumptions are that the patient's lifestyle choices during the holidays, specifically his increased food and alcohol intake, exacerbate his symptoms, and that his current medication is not effective during this period. It is also possible that his Barrett's esophagus may have progressed, which could warrant further assessment or a change in medication. However, before considering these alternatives, it is crucial to first understand the patient's lifestyle, particularly his eating and drinking habits during the holidays. Overeating and excessive alcohol consumption can increase gastric acid production and induce relaxation of the lower esophageal sphincter, leading to acid reflux. These behaviors are likely contributing to the exacerbation of his GERD symptoms, despite him being on the maximum dose of omeprazole. Therefore, the most appropriate initial action by the physician should be to ask the patient how much he is eating and drinking during the holidays (Option A). This will provide valuable context for his symptom exacerbation and could lead to behavioral modifications or dietary changes that could reduce his discomfort without necessarily changing his medication or undertaking further diagnostic procedures.
