# Supplementary material for: Collaborative intelligence in AI: Evaluating the performance of a council of AIs on the USMLE
Source: PLOS Digit Health. 2025 Oct 9;4(10):e0000787. doi: 10.1371/journal.pdig.0000787 (PMC12510544; doi:10.1371/journal.pdig.0000787)
Supplement: S2 File — (ZIP) [file pdig.0000787.s002.zip › S2 Data Raw data files containing unprocessed question inputs and LLM outputs including transcripts of deliberations/STEP1_Question-87-1_Type-MC-NJ.docx]

**Facilitator** **[Round 1]:** A 60-year-old man comes to the office because of weakness, tingling of his hands and feet, irritability, and forgetfulness for 4 months. Physical examination shows pallor, weakness, and spasticity. Deep tendon reflexes are increased. Sensation to vibration is absent in the lower extremities. Laboratory studies show megaloblastic anemia, serum antiparietal cell antibodies, and increased serum concentrations of methylmalonic acid and total homocyst(e)ine. The synthesis of which of the following amino acids is most likely impaired in this patient? (A) Cysteine (B) Glutamine (C) Methionine (D) Phenylalanine (E) Tyrosine

**AI_D:** (1) RESTATING THE QUESTION: The query is requesting us to identify the amino acid synthesis most likely to be adversely affected in a patient who exhibits symptoms of weakness, paresthesia (tingling) of the hands and feet, mood changes such as irritability, memory difficulties, pallor, muscle weakness, hypertonia (spasticity), hyperreflexia (increased tendon reflexes), and diminished vibration sensation. Lab findings show megaloblastic anemia, anti-parietal antibodies, and elevated concentrations of methylmalonic acid and homocysteine. (2) PLANNING: The symptoms, physical examination findings, and laboratory data suggest a deficiency in vitamin B12. Low B12 status has been linked to pernicious anemia (as indicated by anti-parietal cell antibodies) and neurological issues. Moreover, vitamin B12 is crucial in the conversion of homocysteine to methionine, which is why an elevation in homocysteine levels might point toward a B12 deficiency. The context indirectly asks which amino acid is synthesized using vitamin B12 as cofactor. (3) CLINICAL REASONING: Methionine synthesis requires vitamin B12 as a cofactor. Pernicious anemia factors into this patient's symptom profile. This autoimmune disorder involves atrophic gastritis and leads to destruction of parietal cells in the stomach, which are responsible for secreting intrinsic factor necessary for B12 absorption. The loss of B12 input affects various biological pathways, including methionine synthesis from homocysteine. This disrupted pathway can be supported by the observed elevated levels of homocysteine, a substrate in the reaction, and methymalonic acid, a downstream product of an alternate pathway. (4) CONCLUDING: Based on the presentation, the patient is likely deficient in vitamin B12, which disrupts metabolic pathways, including the one converting homocysteine to methionine. This disruption potentially results in the buildup of homocysteine and a decrease in methionine synthesis. (5) RESPONSE: The synthesis of the amino acid methionine is most likely impaired in this patient (Option C: Methionine).

**AI_A:** RESTATING THE QUESTION: The question refers to a 60-year-old male showing symptoms such as weakness, tingling in extremities, irritability, and forgetfulness. In addition to these, physical examination has revealed more symptoms such as pallor, spasticity, heightened deep tendon reflexes and vibration sensation absent in his lower limbs. His laboratory studies have revealed megaloblastic anemia, serum antiparietal cell antibodies and an elevated level of methylmalonic acid as well as total homocysteine in serum. The question asks us to identify which amino acid's synthesis is most probably disrupted in this patient from the options provided, namely Cysteine, Glutamine, Methionine, Phenylalanine and Tyrosine. PLANNING: We begin by identifying the underlying pathology this patient likely exhibits, given his clinical presentation and lab findings. The symptoms, lab findings, and presence of serum antiparietal cell antibodies suggest intrinsic factor deficiency leading to Vitamin B12 deficiency - a condition commonly known as pernicious anemia. The role of Vitamin B12 in amino acid synthesis should be examined, with emphasis on the options provided. CLINICAL REASONING: Vitamin B12 is essential for the transformation of methylmalonyl-CoA to succinyl-CoA, and homocysteine to methionine. Clearly, in a deficiency state, these reactions will be impaired leading to a rise in methylmalonic acid and homocysteine levels, which is what we notice in this patient's lab findings. Resultantly, there is a noteworthy decrease in the production of Methionine due to an inefficient conversion of Homocysteine to Methionine, which seems to align well with the clinical scenario given. CONCLUDING: Given the impact Vitamin B12 has on the metabolic pathways, it would seem that in the presence of antiparietal cell antibodies (suggesting a deprivation of Vitamin B12), homocysteine isn't efficiently converted to Methionine. The observed presence of an excess in Homocysteine, an intermediate, aligns with this conclusion. Hence, the impaired synthesis is likely to be Methionine. RESPONSE: Given the constellation of signs, symptoms and lab findings, the most likely amino acid that has impaired synthesis in this patient would be Methionine (Option C).

**AI_B:** RESTATING THE QUESTION: The question is asking us to identify which among the mentioned amino acids (Cysteine, Glutamine, Methionine, Phenylalanine, Tyrosine) is most likely having its synthesis impaired in a 60-year-old man who is showing symptoms such as weakness, tingling of hands and feet, irritability, forgetfulness, loss of sensation to vibration in the lower extremities, and physical signs like pallor and spasticity. The man's lab tests further reveal megaloblastic anemia, serum antiparietal cell antibodies, and increased serum concentrations of methylmalonic acid and total homocysteine. PLANNING: The man's symptoms and lab findings suggest a condition known as pernicious anemia, caused by a deficiency in vitamin B12. Vitamin B12 is essential for two metabolic processes: the synthesis of methionine from homocysteine and the conversion of methylmalonyl-CoA to succinyl-CoA. The elevated levels of methylmalonic acid and homocysteine in this man's serum support this assumption. It's also worth noting that vitamin B12 deficiency leads to megaloblastic anemia. Given these findings, we should focus on the role of vitamin B12 in the synthesis of the mentioned amino acids to zero in on the correct answer. CLINICAL REASONING: Vitamin B12 deficiency can lead to impaired methionine synthesis. In the presence of adequate vitamin B12, homocysteine is methylated to form methionine, an essential amino acid. This reaction also requires a methyl donor, which is methyl-tetrahydrofolate. This is an important point because without vitamin B12, this reaction will not occur, leading to an increase in serum homocysteine levels, as seen in our patient. CONCLUDING: Given the patient's clinical and lab findings, as well as our understanding of the metabolic roles of vitamin B12, we can confidently conclude that the synthesis of methionine is likely impaired in this patient. RESPONSE: The synthesis of the amino acid Methionine (C) is most likely impaired in this patient.

**AI_E:** RESTATING THE QUESTION: We have a 60-year-old male patient presenting with weakness, tingling extremities, irritability, forgetfulness, pallor, and physical signs of spasticity with increased deep tendon reflexes and lack of vibratory sense in the lower limbs. These symptoms suggest a neurological disorder. Additionally, laboratory results indicate megaloblastic anemia and the presence of serum antiparietal cell antibodies, raised levels of methylmalonic acid and total homocyst(e)ine. The question queries which amino acid synthesis process is most likely disrupted in this individual. PLANNING: We need first to consider how the symptoms and lab findings in this patient connect to the impairment of an amino acid synthesis. The emphasized symptoms and lab findings suggest a condition related to vitamin B12 deficiency (e.g., pernicious anemia), given the neurological manifestations (e.g., weakness, tingling extremities) and hematologic alterations (e.g., megaloblastic anemia), along with the finding of antiparietal cell antibodies (marker of autoimmune destruction of parietal cells that produce intrinsic factor necessary for B12 absorption). Vitamin B12 plays an essential role in the conversion of homocysteine to methionine. When B12 is deficient, this conversion process is impaired, concurrent with the noted increase in serum levels of homocysteine and methylmalonic acid (a byproduct of the alternate pathway). Therefore, the amino acid synthesis affected in this case, based on the clinical scenario and pathophysiology, would likely be that of methionine. CLINICAL REASONING: In pernicious anemia caused by a deficiency of vitamin B12, synthesis of methionine is impaired because vitamin B12 is a necessary coenzyme for the conversion of homocysteine to methionine. This impaired conversion should cause increased levels of homocysteine and methylmalonic acid (when homocysteine is shunted into the other metabolically active yet less preferred pathway), mirroring the lab findings in the patient. CONCLUDING: After restating the patient's clinical and laboratory findings in the context of impaired amino acid synthesis, and after carefully weighing each assumption against the anatomical, physiological, and biochemical knowledge, I come to a rational conclusion that the patient's clinical scenario indicates a vitamin B12 deficiency, likely due to pernicious anemia. This would impair methionine synthesis due to the necessary role of vitamin B12 in the conversion process from homocysteine. RESPONSE: The synthesis of the amino acid Methionine (C) is most likely impaired in this patient.

**AI_C:** RESTATING THE QUESTION: The presented case involves a 60-year-old man presenting with multiple symptoms including weakness, tingling, increased reflexes, and memory issues. Relevant clinical signs and lab results provide significant clues towards a probable medical condition. Clinically, he has pallor, increased deep tendon reflexes, and an absence of vibration sensation in the lower limbs. His lab results show megaloblastic anemia and the presence of antiparietal cell antibodies in the serum. Furthermore, he also has elevated serum methylmalonic acid and total homocysteine. We are tasked with identifying the amino acid whose synthesis might be impaired in this patient out of the listed ones: cysteine, glutamine, methionine, phenylalanine, or tyrosine. PLANNING: From the symptom profile and the lab results, it seems very likely that this patient might have pernicious anemia—a specific type of vitamin B12 deficiency. Parietal cell antibodies indicate an autoimmune destruction of cells in the stomach that normally produce intrinsic factor, necessary for vitamin B12 absorption. Vitamin B12 is crucial in the synthesis of DNA, the metabolism of fatty acids and amino acids. Specifically, it acts as a cofactor for the conversion of homocysteine to methionine. The key point in relating these symptoms to amino acid synthesis is to understand the role of vitamin B12 in the metabolism of certain amino acids. CLINICAL REASONING: One significant feature of the patient's labs was an elevation in homocysteine levels. An excess of homocysteine can occur because of defects in its metabolism, often related to folate or vitamin B12 deficiencies. Given the signs of possible vitamin B12 deficiency in our patient (megaloblastic anemia, antiparietal cell antibodies, serum methylmalonic acid), it would be reasonable to infer that his high homocysteine levels are a result of impaired conversion to methionine, which requires vitamin B12 as a cofactor. CONCLUDING: Homocysteine's conversion to methionine is a crucial part of amino acid metabolism, specifically requiring the cofactor vitamin B12. Given the patient's likely vitamin B12 deficiency, as evidenced by positive antiparietal cell antibodies and megaloblastic anemia, the presence of elevated homocysteine levels suggests that this reaction is disrupted in the patient. Thus, the synthesis of methionine from homocysteine is likely impaired. RESPONSE: In light of the patient's symptomatology and laboratory findings, it is plausible that his homocysteine to methionine conversion pathway is impaired due to a potential vitamin B12 deficiency. Therefore, the synthesis of the amino acid likely to be most affected in this patient is methionine (C).

**Facilitator** The consensus statement synthesizes the responses of AI_A, AI_B, AI_C, AI_D, and AI_E as follows: The question presents a 60-year-old male patient with symptoms such as weakness, tingling in extremities, irritability, forgetfulness, pallor, spasticity, heightened deep tendon reflexes, and absence of vibration sensation in his lower limbs. Laboratory studies reveal megaloblastic anemia, serum antiparietal cell antibodies, and elevated levels of methylmalonic acid and total homocysteine. The task is to identify which amino acid's synthesis is most likely disrupted in this patient from the options provided: Cysteine, Glutamine, Methionine, Phenylalanine, and Tyrosine. The patient's symptoms and lab findings suggest a condition known as pernicious anemia, caused by a deficiency in vitamin B12. Vitamin B12 is essential for two metabolic processes: the synthesis of methionine from homocysteine and the conversion of methylmalonyl-CoA to succinyl-CoA. The elevated levels of methylmalonic acid and homocysteine in this man's serum support this assumption. Vitamin B12 deficiency can lead to impaired methionine synthesis. In the presence of adequate vitamin B12, homocysteine is methylated to form methionine, an essential amino acid. This reaction also requires a methyl donor, which is methyl-tetrahydrofolate. Without vitamin B12, this reaction will not occur, leading to an increase in serum homocysteine levels, as seen in our patient. Given the patient's clinical and lab findings, as well as our understanding of the metabolic roles of vitamin B12, we can confidently conclude that the synthesis of methionine is likely impaired in this patient. Therefore, the synthesis of the amino acid most likely to be affected in this patient is methionine (Option C).
