# Supplementary material for: Collaborative intelligence in AI: Evaluating the performance of a council of AIs on the USMLE
Source: PLOS Digit Health. 2025 Oct 9;4(10):e0000787. doi: 10.1371/journal.pdig.0000787 (PMC12510544; doi:10.1371/journal.pdig.0000787)
Supplement: S2 File — (ZIP) [file pdig.0000787.s002.zip › S2 Data Raw data files containing unprocessed question inputs and LLM outputs including transcripts of deliberations/STEP1_Question-9-1_Type-MC-NJ.docx]

**Facilitator** **[Round 1]:** In screening for prostate cancer, the current serum prostate-specific antigen concentration at which biopsy is recommended is 4.1 ng/mL. If the threshold for recommending a biopsy procedure were lowered to 2.0 ng/mL, which of the following changes would occur to the positive and negative predictive values of the test? (A) Positive Predictive Value and Negative Predictive Value increase (B) Positive Predictive Value increase and Negative Predictive Value no change (C) Positive Predictive Value increase and Negative Predictive Value decrease (D) Positive Predictive Value no change and Negative Predictive Value increase (E) Positive Predictive Value and Negative Predictive Value no change (F) Positive Predictive Value no change and Negative Predictive Value decrease (G) Positive Predictive Value decrease and Negative Predictive Value increase (H) Positive Predictive Value decrease and Negative Predictive Value no change (I) Positive Predictive Value and Negative Predictive Value decrease

**AI_A:** (1) RESTATING THE QUESTION: The question is asking about the potential influence of changing the threshold at which a biopsy for prostate cancer is recommended, based on serum prostate-specific antigen (PSA) concentration. As it stands, the threshold is 4.1 ng/mL. The question poses a hypothetical scenario where this threshold is lowered to 2.0 ng/mL. In effect, a larger proportion of men would be recommended for biopsy, potentially leading to higher detection rates. We're asked to determine how this change would impact the positive predictive value (the proportion of positive tests that are true positives) and the negative predictive value (the proportion of negative tests that are true negatives) of the test. (2) PLANNING: The assumption here is that lowering the biopsy threshold would lead to more positive PSA tests. This could be true, but it could also lead to more false positives (people without prostate cancer who test positive) because more people are being tested and the threshold for a positive test is lower. Therefore, an alternative assumption could be that lowering the biopsy threshold could increase the rate of false positives, which would actually decrease the positive predictive value. (3) CLINICAL REASONING: If the threshold for recommending a biopsy is lowered, this essentially broadens the pool of individuals that qualifies for the test. This means that you are likely going to catch more individuals who actually do have prostate cancer (true positives), but it also means you might catch more individuals who do not have prostate cancer (false positives). The positive predictive value (PPV) is calculated as the number of true positive results divided by the total number of positive results (true positives + false positives). Therefore, an increase in false positives would lead to a decrease in PPV. Further, the negative predictive value (NPV) takes into account those who have a negative result and really do not have the disease (true negatives). By testing more individuals, there is the possibility of fewer true negatives, leading to a decrease in NPV. (4) CONCLUDING: Therefore, by lowering the threshold for a PSA test from 4.1 ng/mL to 2.0 ng/mL, we know two things: a) there will likely be more positive tests (both true positives and false positives) and b) there might be fewer true negatives. This should lead to a decrease in both positive predictive value (because the number of false positives increase) and negative predictive value (because the number of true negatives decrease) of the test. (5) RESPONSE: Therefore, the correct answer should be (I) Positive Predictive Value and Negative Predictive Value decrease.

**AI_C:** RESTATING THE QUESTION: The question is asking about the impact of lowering the prostate-specific antigen (PSA) concentration threshold for recommending a biopsy on positive and negative predictive values (PPV and NPV) in prostate cancer screening. PPV is the probability that a patient with a positive test truly has the disease, while NPV is the probability that a patient with a negative test truly doesn't have it. PLANNING: The current threshold for biopsy recommendation is 4.1 ng/mL, if we lower it to 2.0 ng/mL, more men will qualify for a biopsy. We should consider that not everyone in this newly considered group will have prostate cancer. This means we essentially increase the chance of getting more false positives and true positives tests in the screening procedure. In other words, we are testing a broader population, including many individuals who do not have the disease, and hence, we increase the chance of having a positive test in the absence of disease. CLINICAL REASONING: A decrease in the threshold means that more patients will test positive (true positives and false positives increase). Since PPV is calculated as the number of true positives divided by the total number of positive test results (true positives + false positives), an increase in false positives may decrease the PPV, because the denominator of the fraction gets larger with numerous false positives. On the other hand, lower biopsy thresholds would allow more true cases to be detected (higher sensitivity) and hence fewer false negatives, which can increase NPV, calculated as the number of true negatives divided by the total number of negative test results (true negatives + false negatives). CONCLUDING: The clinical reasoning leads to believe that with lowering the threshold of PSA concentration for recommending a biopsy, the PPV might decrease because more false positive cases will be added thus reducing the overall proportion of true positive cases among all positive cases. At the same time, the NPV might increase because the reduced biopsy threshold means fewer false negative cases, therefore increasing the overall proportion of true negative cases among all negative cases. RESPONSE: The clinical reasoning indicates that the most probable scenario is(G) Positive Predictive Value decreases and Negative Predictive Value increases.

**AI_E:** RESTATING THE QUESTION: The question asks if, in prostate cancer screening, the threshold for recommending a biopsy is reduced from a serum prostate-specific antigen (PSA) concentration of 4.1 ng/mL to 2.0 ng/mL, how would this change the positive predictive value (PPV) and negative predictive value (NPV) of the test? PPV refers to the probability that someone who has a positive test result truly has the disease, while NPV is the probability that someone who has a negative test result truly does not have the disease. PLANNING: Under the current threshold, if the PSA is above 4.1 ng/mL, a biopsy is recommended. The test is positive and the probability that the person has prostate cancer is high (i.e., high PPV). If the PSA is lower than 4.1 ng/mL, the test is negative and the probability that the person doesn't have prostate cancer is high (i.e., high NPV). Should the threshold be lowered to 2.0 ng/mL, more patients will be recommended for biopsy, meaning more positive tests. If not all these additional tested patients have prostate cancer, the PPV (probability that a positive test actually indicates disease presence) would decrease. CLINICAL REASONING: Predictive values are dependent on the disease prevalence in the population tested. When the PSA threshold is lowered to 2.0 ng/mL, more people will test positive and be recommended for biopsy, leading to a larger number of false positives. The PPV of the PSA screening test will therefore decrease as the number of false positives increases relative to true positives. On the other hand, NPV will increase because with a lower threshold more people who do not have the disease will be correctly identified as not having the disease. Hence, the NPV (probability that a negative test result means disease absence) would increase. CONCLUDING: Altering the PSA threshold from 4.1 ng/mL to 2.0 ng/mL for recommending a biopsy in prostate cancer screening would decrease the PPV and increase the NPV of the test. RESPONSE: Consequently, the answer to the question is (G) Positive Predictive Value decrease and Negative Predictive Value increase.

**AI_D:** (1) RESTATING THE QUESTION: The inquiry at hand asks what would happen to the positive and negative predictive values if we reduced the prostate-specific antigen (PSA) cutoff for recommending a prostate biopsy from 4.1 ng/mL to 2.0 ng/mL. Positive predictive value (PPV) is the probability that someone with a positive test result genuinely has the disease, whereas negative predictive value (NPV) is the likelihood that someone with a negative result is truly disease-free. (2) PLANNING: The key assumptions here are: a) lowering the PSA threshold would lead to more prostate biopsies, b) increased biopsies could potentially include more individuals who don't have prostate cancer, and c) these additional biopsies may or may not identify more individuals with prostate cancer. Each assumption bears clinical significance, but the one we'll prioritize is that the increased number of biopsies could potentially lead to more false positives and hence affect the PPV and NPV values. (3) CLINICAL REASONING: By reducing the cutoff for biopsy recommendation, we would certainly increase the number of positive test results. Hence, the pool of subjects who get a biopsy would be larger. However, this expansion would primarily come from the pool of subjects without the disease (since we're conducting biopsies earlier at a lower threshold, where typically more non-diseased individuals will fall). Consequently, while the number of true positives (TP) might increase to some extent, the increment in false positives (FP) will be larger, leading to a decrease in PPV (PPV=TP/[TP+FP]). Conversely, the number of subjects with negative PSA test results who don’t get a biopsy (true negatives, TN) would decrease, but the number of subjects falsely reassured by a negative PSA test without having a biopsy (false negatives, FN) would remain the same. Hence, the NPV (NPV=TN/[TN+FN]) would decrease. (4) CONCLUDING: If the threshold of PSA for recommending a prostate biopsy were lowered, the outcome would be an increase in both the number of subjects getting a biopsy and the number of false positives. This would subsequently decrease the positive predictive value. However, negative predictive value would also decline due to a decrease in true negatives. (5) RESPONSE: This reasoning deduces that the effect of lowering the PSA threshold for recommending a biopsy would be a decrease in both the positive and negative predictive value. The correct answer to the question would hence be choice (I) "Positive Predictive Value and Negative Predictive Value decrease".

**AI_B:** RESTATING THE QUESTION: This question is about the implications of lowering the threshold of serum prostate-specific antigen (PSA) concentration in the screening for prostate cancer. Currently, a biopsy is recommended when PSA concentration is at 4.1 ng/mL or higher. What the query wants to know is the effect on the test's predictive values if the threshold for recommending biopsy were lowered to 2.0 ng/mL. So, it is essentially about how this change would affect the test's Positive Predictive Value (PPV - the probability that subjects with a positive screening test truly have the disease) and Negative Predictive Value (NPV - the probability that subjects with a negative screening test truly do not have the disease). PLANNING: Assumptions can be made that the prevalence of prostate cancer doesn't change significantly and that all other factors remain constant. However, by questioning these assumptions, we could generate an alternative hypothesis that the alteration of the cut-off value may affect the number of false positives and false negatives, thereby changing the PPV and NPV. Considering the importance of accuracy in clinical decision-making, this situation with direct implications on patient management should be explored further. CLINICAL REASONING: When a threshold for a test is lowered, more people test positive because the criteria to be considered having the disease (in this case prostate cancer) is less stringent. Consequently, there will be more true positives and false positives. This means an increase in sensitivity of the test, but at the cost of increased false positive rate. Now, the PPV (which is the probability that a person who tests positive truly has the disease) will decrease because there are more false positives. In contrast, lowering the threshold means fewer people will test negative, which will result in fewer false negatives, implying an increase in NPV. CONCLUDING: By connecting the clinical reasoning step by step, it can be concluded that lowering the biopsy recommendation threshold from 4.1 ng/mL to 2.0 ng/mL would increase the number of people who test positive (both true and false positives). This, in turn, decreases the PPV since this value depends on the true positives in the positive test results. Additionally, with the decrease in false negatives (as fewer people test negative), the NPV would increase. RESPONSE: Therefore, the expected change in the test's positive and negative predictive values when the threshold is lowered would be that the Positive Predictive Value decreases, and the Negative Predictive Value increases. So, the answer would be (G) Positive Predictive Value decrease and Negative Predictive Value increase.

**Facilitator** **[Round 2]:** Summary: AI_A and AI_D believe that lowering the threshold for a PSA test from 4.1 ng/mL to 2.0 ng/mL would lead to an increase in both true positives and false positives, resulting in a decrease in the positive predictive value (PPV). They also suggest that there might be fewer true negatives, leading to a decrease in the negative predictive value (NPV). Therefore, they conclude that both PPV and NPV would decrease. AI_B, AI_C, and AI_E, on the other hand, agree that lowering the threshold would increase both true positives and false positives, leading to a decrease in PPV. However, they argue that this would result in fewer false negatives, leading to an increase in NPV. Therefore, they conclude that PPV would decrease and NPV would increase. Question to clarify disagreements: The key disagreement between the council members lies in the impact of lowering the PSA threshold on the NPV. Could you please elaborate on why you believe the NPV would decrease or increase when the threshold for a PSA test is lowered from 4.1 ng/mL to 2.0 ng/mL?. Based on your preceding response, how would you answer the original question: [In screening for prostate cancer, the current serum prostate-specific antigen concentration at which biopsy is recommended is 4.1 ng/mL. If the threshold for recommending a biopsy procedure were lowered to 2.0 ng/mL, which of the following changes would occur to the positive and negative predictive values of the test? (A) Positive Predictive Value and Negative Predictive Value increase (B) Positive Predictive Value increase and Negative Predictive Value no change (C) Positive Predictive Value increase and Negative Predictive Value decrease (D) Positive Predictive Value no change and Negative Predictive Value increase (E) Positive Predictive Value and Negative Predictive Value no change (F) Positive Predictive Value no change and Negative Predictive Value decrease (G) Positive Predictive Value decrease and Negative Predictive Value increase (H) Positive Predictive Value decrease and Negative Predictive Value no change (I) Positive Predictive Value and Negative Predictive Value decrease]

**AI_D:** Restating the question: The query at hand is mainly concerned with how changing the threshold for performing a biopsy based on the serum prostate-specific antigen (PSA) concentration from 4.1 ng/mL down to 2.0 ng/mL would impact the positive and negative predictive values (PPV and NPV respectively) of the test. The positive predictive value illustrates the probability that subjects with a positive screening test truly have the disease, while the negative predictive value represents the probability that subjects with a negative screening test truly do not have the disease. Planning: Now, lowering the threshold means more men will qualify for a biopsy, implying one would detect more true positive cases (those with prostate cancer) and increase false positives (those without the disease but test suggests otherwise). This increase in false positives would lower the PPV. As for the NPV, it's contingent on the number of true negatives (men without the disease accurately identified by the test) and false negatives (men with the disease that the test incorrectly reassures). Lowering the threshold, thereby increasing the number of tests, arguably would reduce the false negatives (more prostate cancer cases detected) and possibly reduce true negatives (men without disease but tested due to lower cut-off). Clinical Reasoning: As we're widening our testing net by lowering the PSA threshold, we're essentially catching more cases of prostate cancer (true positives), but we're also misidentifying more men without prostate cancer (false positives). Our PPV, therefore, decreases as the abundance of false positives are lowering the proportion of true positives in all positive results. For NPV, the increased testing reduces the chances of missing out on diagnosing men with prostate cancer (false negatives decrease), but we're also less definitively identifying men free of the disease (possible reduction in true negatives). If true negatives decrease significantly compared to the decrease in false negatives, NPV could decrease as well. But we're assuming a significant reduction in true negatives. Concluding: Thus, if we adjust the threshold for recommending a prostate biopsy based on a PSA concentration from 4.1 ng/mL to 2.0 ng/mL, it's likely we will see an increase in true positives and false positives, therefore decreasing the PPV. Depending on the changes in true negatives (possibly decreasing) and false negatives (decreasing) with a significant drop in true negatives assumed, NPV could decrease too. Response: Based on these considerations, the best answer to the question would be, "Positive Predictive Value and Negative Predictive Value decrease" (I).

**AI_E:** Firstly, to explain this from a technical perspective, let's define what positive predictive value (PPV) and negative predictive value (NPV) entail in the context of medical diagnostics. PPV refers to the probability that patients with a positive screening test truly do have the disease. NPV, conversely, refers to the probability that patients with a negative screening test truly do not have the disease. Now let's unpack this question. We're trying to predict the effect of lowering the threshold for the prostate-specific antigen (PSA) test from 4.1 ng/mL to 2.0 ng/mL on the PPV and NPV. As a fundamental premise, we can extrapolate that lowering the threshold would lead to an increase in the number of positive test results. It's crucial to note that increasing the number of positive test results would not only escalate the true positive results but also inflate the rate of false positive results. Let's reason clinically now. An elevated number of false positives will, quite logically, reduce the PPV because the positive test results' veracity will drop with more false positive instances. Therefore, the PPV decreases as the threshold lowers. Examining NPV requires scrutinising the true negatives and false negatives. With a lower threshold, we reduce the likelihood of false negatives as individuals are more likely to test positive even at lower PSA levels. With reduced false negatives, the NPV would conceivably increase, as a negative test result becomes a more reliable predictor of not having the disease. Given this step-by-step explanation, my final response is that if the threshold for biopsy were lowered from 4.1 ng/mL to 2.0 ng/mL, the Positive Predictive Value would decrease, and the Negative Predictive Value would increase. This aligns with the option (G) from the list initially given. In summary, a decreased serum PSA threshold would increase NPV by reducing the false negatives and decrease PPV due to a relative increase in the false positives.

**AI_C:** Given the input, let me rephrase: The question is asking how changes in the threshold of prostate-specific antigen (PSA) concentration from 4.1 ng/mL to 2.0 ng/mL impacts the positive predictive value (PPV) and negative predictive value (NPV) of the PSA test. PSA is a substance that is naturally produced by the prostate gland and can be found in increased amounts in the blood of men with prostate cancer. By itself, a PSA test can't tell if someone has prostate cancer; higher levels only indicate a greater chance that prostate cancer is present, and a lower-cut off value would presumably catch more true positives (those with cancer) and false positives (those without cancer but with increased PSA). PPV is the probability that subjects with a positive screening test truly have the disease. NPV is the probability that subjects with a negative screening test truly don't have the disease. I'll now generate alternate assumptions based on the proposed changes to the PSA threshold. Firstly, the assumption could be made that lowering the limit for PSA would result in more positive tests, thereby catching more true positives, as well as unfortunately raising the number of false positives. This could potentially decrease the PPV since more patients without the disease, albeit with an increased PSA, would test positive. Moreover, the number of false negatives would presumably be reduced, raising the NPV, as those previously under the radar due to the higher threshold are now highlighted. Secondly, another assumption could be that while the number of positives increase, it does not necessarily result in an increase in true positives, leading to a decrease in PPV. Similarly, if the number of false negatives do not significantly decrease, the NPV could potentially remain the same. After weighing these scenarios, the clinically relevant approach would be assuming a decrease in PPV due to an increase in false positives. On the other hand, given that more individuals previously lying below the threshold may be captured, NPV might likely increase. Clinically, this would mean that while more people would be screened, the percentage of those without the disease testing positive (false positives) would increase, potentially leading to more biopsies. However, the number of people with the disease testing negative would decrease, consequently reducing missed diagnoses. Considering these scenarios and thinking through analytically, in light of a lower PSA threshold, I'd argue that the Positive Predictive Value would decrease due to an increase in false positives, while the Negative Predictive Value would increase because of the reduction in false negatives. Using this reasoning, and answering the question provided, I would opt for (G) Positive Predictive Value decrease and Negative Predictive Value increase. Conclusively, one must bear in mind that while adjusting these thresholds could potentially cause more positive, often misleading, detections, it might also aid in providing earlier diagnoses.

**AI_B:** 1) RESTATING THE QUESTION: This question is inquiring about the effects on positive and negative predictive values of a prostate-specific antigen (PSA) test in the scenario wherein we lower biopsy recommending standards. Prostate-specific antigen is a protein produced exclusively by cells in the prostate gland, and elevated levels of the substance can suggest the presence of prostate cancer. The Positive Predictive Value (PPV) and Negative Predictive Value (NPV) are fundamental concepts in the field of epidemiology and biostatistics. PPV is the probability that a subject with a positive screening test truly has the disease. NPV is the probability that subjects with a negative screening test truly are disease-free. 2) PLANNING: The fundamental assumption here is that lowering the threshold for recommending a biopsy would alter the PPV and NPV of the test. Critically examining this assumption, we could consider if a lower threshold may lead to more positive test results (both true and false positives). Also, it might lead to fewer negative results, properly referring to fewer true negatives and false negatives. By evaluating this approach, a clinically prioritized assumption can be that lowering the threshold should likely decrease PPV (due to an increase in false positives) and increase NPV (due to a reduction in false negatives). 3) CLINICAL REASONING: When the threshold of PSA is lowered, more individuals will be classified as having positive results, including some who are truly diseased (true positives) and some who are not actually diseased but have higher PSA (false positives). The increase in false positives leads to a decrease in PPV because PPV = TP/(TP+FP). Meanwhile, lowering the threshold means fewer people will be classified as negative, including those who truly do not have the disease (true negatives) and, significantly, fewer people who have the disease but are falsely classified as not having it (false negatives). The decreased incidence of false negatives improves the NPV because NPV = TN/(TN+FN). 4) CONCLUDING: Following a meticulous approach delving into the epidemiology and biostatistics of screening test interpretation combined with an understanding of the influence of test thresholds, one can propose that if the threshold of the PSA test for recommending a biopsy is lowered, this could potentially decrease the PPV due to an increased incidence of false positives and increase the NPV owing to a decreased frequency of false negatives. 5) RESPONSE: Thus, under the scenario of lowering the biopsy threshold from 4.1 ng/mL to 2.0 ng/mL for the serum prostate-specific antigen concentration, I surmise that the Positive Predictive Value would decrease while the Negative Predictive Value would increase. In terms of the proposed options, this aligns the situation with option (G).

**AI_A:** 1) RESTATING THE QUESTION: The question asks how lowering the threshold for a prostate-specific antigen (PSA) test, currently at 4.1 ng/mL, to 2.0 ng/mL, impacts the positive and negative predictive values of the test. The positive predictive value (PPV) is the probability that subjects with a positive screening test truly have the disease, while the negative predictive value (NPV) is the probability that subjects with a negative screening test truly do not have the disease. 2) PLANNING: A change in threshold would affect who is considered for biopsy and could impact both true positives (TP), true negatives (TN), false positives (FP), and false negatives (FN). Lowering the threshold could increase the number of TPs and FPs, potentially affecting the PPV. It could also affect the number of TNs and FNs, altering the NPV. But to which direction each value would change, and why, needs further clinical reasoning. 3) CLINICAL REASONING: Lowering the threshold would identify more people for biopsy, possibly catching the disease earlier in some, but also including some without the disease, leading to more TPs and FPs equally. As the denominator of PPV is (TP+FP), and both are increasing, there might not be a significant change in PPV if the ratio stays the same. However, as more people without the disease are also being selected (FP), the FP might increase disproportionately to TP, leading to a decrease in PPV. In terms of NPV, with a decrease in FP, the proportion of TNs in all negatives (TN+FN) might increase, leading to an increased NPV. 4) CONCLUDING: Based on the clinical reasoning, we can hypothesize that lowering the threshold for PSA might decrease the PPV due to an increase in false positives and increase the NPV from a decrease in false negatives. 5) RESPONSE: The answer to the question "[In screening for prostate cancer, the current serum prostate-specific antigen concentration at which biopsy is recommended is 4.1 ng/mL. If the threshold for recommending a biopsy procedure were lowered to 2.0 ng/mL, which of the following changes would occur to the positive and negative predictive values of the test?]" is likely (G) Positive Predictive Value decrease and Negative Predictive Value increase.

**Facilitator** **[Round 3]:** Summary: AI_A, AI_B, AI_C, and AI_E all agree that lowering the threshold for a prostate-specific antigen (PSA) test from 4.1 ng/mL to 2.0 ng/mL would likely decrease the Positive Predictive Value (PPV) due to an increase in false positives and increase the Negative Predictive Value (NPV) due to a decrease in false negatives. They all base their reasoning on the assumption that lowering the threshold would lead to more positive test results, including both true positives and false positives, and fewer negative results, including fewer false negatives. AI_D, however, suggests that while the PPV would likely decrease due to an increase in false positives, the NPV could potentially decrease as well. This is based on the assumption that lowering the threshold could lead to a significant decrease in true negatives, which could outweigh the decrease in false negatives and thus lower the NPV. Question to clarify disagreements: To AI_D, could you elaborate on why you believe that lowering the threshold could lead to a significant decrease in true negatives, and how this could potentially outweigh the decrease in false negatives to lower the NPV?. Based on your preceding response, how would you answer the original question: [In screening for prostate cancer, the current serum prostate-specific antigen concentration at which biopsy is recommended is 4.1 ng/mL. If the threshold for recommending a biopsy procedure were lowered to 2.0 ng/mL, which of the following changes would occur to the positive and negative predictive values of the test? (A) Positive Predictive Value and Negative Predictive Value increase (B) Positive Predictive Value increase and Negative Predictive Value no change (C) Positive Predictive Value increase and Negative Predictive Value decrease (D) Positive Predictive Value no change and Negative Predictive Value increase (E) Positive Predictive Value and Negative Predictive Value no change (F) Positive Predictive Value no change and Negative Predictive Value decrease (G) Positive Predictive Value decrease and Negative Predictive Value increase (H) Positive Predictive Value decrease and Negative Predictive Value no change (I) Positive Predictive Value and Negative Predictive Value decrease]

**AI_D:** You've asked me to elaborate on the potential impact of lowering the PSA test threshold on the PPV and NPV. The PPV is the probability that subjects with a positive screening test truly have the disease. If we decrease the threshold, more individuals who don't have prostate cancer may end up with a positive PSA test (false positives), diluting the pool of true positives, and thus reducing the PPV. As for NPV, it is the probability that subjects with a negative screening result truly do not have the disease. Conventionally, lowering the threshold may indeed decrease the number of false negatives, which may increase the NPV. However, it's plausible that a reduction in threshold might also decrease the number of true negatives (i.e., healthy individuals correctly identified as not having prostate cancer), given that the lower threshold may incorrectly categorize some of these as 'positive'. This could in theory cause a decrease in the NPV as well, although judging the probability of this occurrence would require more specific data. Given these observations, based on the potential effects on false and true positives as well as false and true negatives, the most likely outcome of lowering the prostate-specific antigen concentration threshold from 4.1 ng/mL to 2.0 ng/mL is a decrease in both Positive Predictive Value and Negative Predictive Value due to an increase in false positives and possibly due to an increase in false negatives. Therefore, the choice consistent with this conclusion is (I) Positive Predictive Value and Negative Predictive Value decrease.

**AI_C:** In a clinical context, the question regards the impact of lowering the threshold concentration for a prostate-specific antigen (PSA) test, which is used in screening for prostate cancer. The present concentration required to recommend a biopsy is 4.1 ng/mL, and the hypothetical scenario proposes lowering this to 2.0 ng/mL, seeking an understanding of the subsequent effects on the positive predictive value (PPV) and negative predictive value (NPV) of the test. It's important to define these terms. PPV refers to the probability that people with a positive screening test truly have the disease. NPV is the probability that people with a negative screening test genuinely don't have the disease. Changes in these values have a significant impact on the test's predictive capacity. Our assumptions center on the effects of reducing the threshold for the PSA test. The prevailing assumption is that reducing the threshold will yield more positive results, including true positives and false positives. Meanwhile, fewer negative results will occur, with a specific decrease in false negatives. This interplay between true and false positives and negatives is what will determine the ultimate shifts in predictive values. From a clinical reasoning standpoint, lowering the detection threshold means more patients will return a positive test, including those without the disease (false positives). Consequently, this increase in false positives would decrease the PPV of the test. Conversely, If fewer patients with prostate cancer are delivering negative tests because of the lowered threshold (fewer false negatives), it will increase the NPV. This reasoning would lead us to conclude that if the threshold for recommending a biopsy procedure were lowered to 2.0 ng/mL, the positive predictive value of the test would likely decrease due to the increase in false positives. Simultaneously, the negative predictive value would increase due to the decrease in false negatives. Therefore, the response to this query would correspondingly be Choice (G) Positive Predictive Value decrease, and Negative Predictive Value increase.

**AI_A:** RESTATING THE QUESTION: The question asks us to predict the effect of lowering the threshold for recommending a prostate biopsy based on serum prostate-specific antigen (PSA) concentration from 4.1 ng/mL to 2.0 ng/mL on the Positive Predictive Value (PPV) and Negative Predictive Value (NPV) of the test. PPV is the probability that subjects with a positive test truly have the disease, and NPV is the probability that subjects with a negative test truly do not have the disease. PLANNING: Assumptions include that lowering the threshold would result in more positive test results, including both true positives and false positives, leading to a decrease in PPV. Furthermore, this lower threshold would result in fewer negative results, including fewer false negatives, leading to an increase of the NPV. However, it's also important to consider that a lowered threshold could lead to a decrease in true negatives - unaffected individuals who correctly test negative - thus potentially lowering the NPV. CLINICAL REASONING: Lowering the threshold for PSA could lead to more positive test results, as those who were previously 'borderline' now meet the criteria for a positive result. This increase in the total number of positive tests, which includes both true positives (individuals with prostate cancer) and a surge in false positives (individuals without prostate cancer but with a test result above the lower threshold), reduces the PPV as the proportion of true positives in the expanded positive group decreases. In terms of NPV, the total number of negative test results could decrease, consisting of fewer false negatives (individuals with prostate cancer but a test result below the lower threshold), increasing the NPV. However, potential reduction in true negatives may attenuate or even offset this NPV increase. CONCLUDING: When the threshold of a screening test is lowered, it generally increases the sensitivity and decreases the specificity of the test. As a result, more patients are identified as positive, both those with the disease (true positives) and those without the disease (false positives). This leads to a decreased PPV because a lower proportion of individuals with positive test results truly have the disease. Conversely, the NPV may increase because there are fewer false negatives, but this increase can be mitigated and even reversed if the incrase in false positives is significant. RESPONSE: Based on the above discussion, the conclusion is (G) Positive Predictive Value decreases and Negative Predictive Value increases.

**AI_E:** RESTATING THE QUESTION: The inquiry is about the changes in Positive Predictive Value (PPV) and Negative Predictive Value (NPV) of a prostate-specific antigen (PSA) screening test for prostate cancer, if the threshold for recommending a biopsy is lowered from the current 4.1 ng/mL to 2.0 ng/mL. PPV is the probability that subjects with a positive screening test truly have the disease. NPV is the probability that subjects with a negative screening test truly do not have the disease. PLANNING: A critical assumption here is the relationship between the PSA test threshold and the quintessential screening variables, such as true positive, false positive, true negative, and false negative. Lowering the threshold for conducting a biopsy can lead to identifying more disease-positive cases (both true positive and false positive) and fewer disease-negative cases, which could be reflected as higher sensitivity but lower specificity. However, the actual changes in PPV and NPV would depend on the prevalence of prostate cancer in the population being screened. CLINICAL REASONING: If the threshold for recommending a biopsy is lowered, it is probable there would be more positive test results. This includes more true positives (people who have prostate cancer and test positive) but also more false positives (people who do not have prostate cancer but still test positive). This increase in false positives would lower the PPV. Meanwhile, by capturing more true positive cases, the number of false negatives (people who have prostate cancer but test negative) would decrease, theoretically improving NPV. Nevertheless, the absolute changes in PPV and NPV would also relate to other factors such as disease prevalence rate. CONCLUDING: The lowering of biopsy recommendation threshold for PSA test could result in a greater number of patients being biopsied. This might lead to an increase in both true-positive and false-positive results, decreasing PPV. As fewer prostate cancer cases would be missed, the number of false negatives would decrease and hence, theoretically, the NPV should increase. RESPONSE: Therefore, my answer would be (G): Positive Predictive Value (PPV) would decrease and Negative Predictive Value (NPV) would increase.

**AI_B:** (RESTATING THE QUESTION) The inquiry is considering the impact of lowering the threshold for recommending a prostate biopsy from the current PSA level of 4.1 ng/mL to 2.0 ng/mL. Specifically, it asks how this change would affect the Positive Predictive Value (PPV) and Negative Predictive Value (NPV) of the test. PPV is the likelihood that subjects with a positive screening test truly have the disease. In contrast, NPV is the probability that subjects with a negative screening test genuinely do not have the disease. (PLANNING) In approaching this, one must understand that decreasing the threshold for a test increases the sensitivity and the number of positive tests (both true positives and false positives). This can usually result in a reduction of the PPV due to a higher rate of false-positive results. The NPV, on the other hand, is influenced by the prevalence of the disease and the specificity of the test. (CLINICAL REASONING) Lowering the PSA threshold to 2.0 ng/mL would likely result in more men being classified as test-positive, including some who do not have prostate cancer (false positives). As a consequence, while the true positive rate might rise, the rise in the false-positive rate could cause the PPV to decrease. On the other hand, with a lower threshold, fewer men will be mistakenly identified as free of the disease (false negatives), likely increasing the NPV. However, this is dependent on the specificity of the test and the prevalence of the disease - if there were a significant number of true negatives that became false positives due to the lower threshold, it could potentially lower the NPV. (CONCLUDING) As such, both the Positive Predictive Value and the Negative Predictive Value are impacted, but in different directions. The PPV likely decreases due to the increased number of false positives, while the NPV may either increase or decrease, influenced by the prevalence of the disease and the specificity of the test. (RESPONSE) So, the most probable answer to the inquiry would be either "Positive Predictive Value decrease and Negative Predictive Value increase" (G) or "Positive Predictive Value decrease and Negative Predictive Value no change" (H), depending upon the specific demographics of the patient population and the prevalence of prostate cancer in that population, along with the specificity of the test.

**Facilitator** **[Round 4]:** Summary: AI_A, AI_B, AI_C, and AI_E all agree that lowering the threshold for recommending a prostate biopsy based on serum prostate-specific antigen (PSA) concentration would likely result in an increase in both true positives and false positives, leading to a decrease in the Positive Predictive Value (PPV). They also agree that this would likely result in fewer false negatives, potentially leading to an increase in the Negative Predictive Value (NPV). However, they also note that a decrease in true negatives could potentially offset this increase in NPV. AI_D, on the other hand, suggests that lowering the threshold could potentially lead to a decrease in both PPV and NPV due to an increase in false positives and possibly an increase in false negatives. Question to clarify disagreements: To clarify the disagreements between the council members' opinions, we could ask: "Could you provide more detail on how the decrease in true negatives could potentially impact the NPV, and under what circumstances might we see an increase in false negatives that could lead to a decrease in both PPV and NPV?". Based on your preceding response, how would you answer the original question: [In screening for prostate cancer, the current serum prostate-specific antigen concentration at which biopsy is recommended is 4.1 ng/mL. If the threshold for recommending a biopsy procedure were lowered to 2.0 ng/mL, which of the following changes would occur to the positive and negative predictive values of the test? (A) Positive Predictive Value and Negative Predictive Value increase (B) Positive Predictive Value increase and Negative Predictive Value no change (C) Positive Predictive Value increase and Negative Predictive Value decrease (D) Positive Predictive Value no change and Negative Predictive Value increase (E) Positive Predictive Value and Negative Predictive Value no change (F) Positive Predictive Value no change and Negative Predictive Value decrease (G) Positive Predictive Value decrease and Negative Predictive Value increase (H) Positive Predictive Value decrease and Negative Predictive Value no change (I) Positive Predictive Value and Negative Predictive Value decrease]

**AI_E:** To tackle this question about what would happen if the threshold for recommending a prostate cancer biopsy were lowered from 4.1 ng/mL to 2.0 ng/mL in terms of the test's positive and negative predictive values, we have to initially understand these two concepts. Positive Predictive Value (PPV) is the probability that subjects with a positive screening test truly have the disease. Negative Predictive Value (NPV) is the probability that subjects with a negative screening test truly do not have the disease. Assuming the prevalence of prostate cancer does not change with the lowered threshold, we have to understand how this change affects the PPV and NPV. Lowering the threshold for a biopsy would increase sensitivity (more true positives – capturing more people who have the disease) but might decrease specificity (more false positives – people marked as having the disease when they do not). An increase in the number of true positives and false positives may lead to a change in the PPV of the test. Given that PPV is calculated as the number of true positives divided by the sum of true positives and false positives, an increase in both true positives and false positives doesn't necessarily lead to an increase in PPV. The specific change in PPV would depend on the relative increase in these two groups. On the other hand, lowering the threshold should theoretically increase the NPV due to fewer false negative results, assuming the increase in false positives is not significant enough to offset this improvement. By examining these relationships between test sensitivity, specificity, positive and negative predictive values, and the prevalence of the disease, we hypothesize that the most likely outcome should the biopsy recommendation threshold be lowered is a change in both predictive values, with a decrease in PPV and an increase in NPV. Connecting all these clinical reasoning aspects to generate a final response, we conclude that the most likely answer is option (G) Positive Predictive Value decreases and Negative Predictive Value increases. This agrees with the core principles of medical testing, where a compromise between sensitivity and specificity is frequently encountered, and the balance of predictive values may shift as the threshold for positive results changes.

**AI_C:** (1) RESTATING THE QUESTION: The question asks about the potential implications of lowering the threshold for recommending a biopsy for prostate cancer screening based on serum prostate-specific antigen (PSA) concentration from 4.1 ng/mL to 2.0 ng/mL. The focus here is on the effect such a change might have on the positive predictive value (PPV) and negative predictive value (NPV) of the screening test. To understand this, let's define these terms first. PPV is the probability that subjects with a positive screening test truly have the disease, while NPV is the probability that subjects with a negative screening test truly don't have the disease. (2) PLANNING: Lowering the threshold for a positive test result means that more individuals will test positive, both those with the disease (true positives) and without the disease (false positives). This produces the assumption that more positive test results would increase the pool of both true positive and false positive, potentially decreasing PPV. For the NPV, in theory, having fewer false negatives might increase it, but the change in true negatives needs to be considered. If the lower test threshold reduces the number of true negatives, it could offset the increase in NPV brought by fewer false negatives. (3) CLINICAL REASONING: False negatives would decrease following lower biopsy thresholds because less severe cases, previously slipping through the net at a higher threshold, would now be picked up. This results in a higher true negative rate, which increases NPV. However, lowering the threshold increases the number of positive results—including both true and false positives—which inflates the denominator of the PPV calculation decreasing the PPV. (4) CONCLUDING: Taking into account not just the simple binary outcome of positive or negative but also the weight each of these categories carries (true or false), we hypothesise that lowering the biopsy threshold from 4.1 ng/mL to 2.0 ng/mL is likely to inflate the denominator of the PPV and NPV calculations in different ways. This increases the likelihood of true positives but also false positives, possibly decreasing PPV, while decreasing the likelihood of false negatives, potentially increasing NPV. (5) RESPONSE: I would answer the question as follows: If the threshold for recommending a biopsy procedure were lowered to 2.0 ng/mL, there would likely be an increase in true positives and decrease in false negatives, leading to a decrease in Positive Predictive Value (G) Positive Predictive Value decrease and Negative Predictive Value increase.

**AI_A:** (1) RESTATING THE QUESTION: The question is asking us what would happen to the positive predictive value (PPV) and negative predictive value (NPV) of the prostate-specific antigen (PSA) test for prostate cancer if the threshold for recommending a biopsy based on PSA concentration were lowered from 4.1 ng/mL to 2.0 ng/mL. The PPV is the probability that subjects with a positive screening test truly have the disease. NPV is the probability that subjects with a negative screening test truly don't have the disease. (2) PLANNING: We can make two assumptions here: Assuming the disease prevalence remains unchanged, decreasing the cut-off value would result in more individuals qualifying for a biopsy which means more individuals would be labelled as disease-positive (increase in true positives and false positives). This could decrease the PPV (as we have more false positives) and potentially increase the NPV (as we have fewer false negatives). (3) CLINICAL REASONING: By lowering by threshold, more individuals will fall within the positive disease group (those recommended for a biopsy), this will increase the number of true positives (those who have the disease and are correctly identified by the test) and false positives (those who do not have the disease but are incorrectly identified as such by the test). Since PPV is calculated as true positives/(true positives + false positives), an increase in both the numerator and the denominator of this equation might lead to a decrease in the overall PPV value. On the other hand, NPV is calculated as true negatives/(true negatives + false negatives), since lowering the threshold would tend to reduce the number of false negatives (those who have the disease, but are incorrectly identified by the test), the NPV might increase. (4) CONCLUDING: If we lower the threshold for PSA concentration to recommend a biopsy procedure, we would potentially lower the PPV due to an accumulation of false positives. However, we could increase the NPV because we are catching more true positives and reducing the count of false negatives. (5) RESPONSE: Therefore, based on our reasoning process, the most likely outcome is that the Positive Predictive Value decreases, and Negative Predictive Value increases. So the answer would be (G) Positive Predictive Value decrease and Negative Predictive Value increase.

**AI_D:** RESTATING THE QUESTION: The matter under discussion pertains to the effect of lowering the PSA level threshold for recommending a biopsy in the screening for prostate cancer. We are currently functioning at a threshold of 4.1 ng/mL, and wish to explore the possible consequences of a fall to 2.0 ng/mL on the Positive Predictive Value (PPV) and Negative Predictive Value (NPV) of the test. PPV refers to the proportion of positive test results that are truly positive (leading to correct diagnoses), while NPV is the proportion of negative results that are truly negative (correctly ruling out diagnoses). PLANNING: Here, there are two basic assumptions to consider (i) Lowering the threshold to 2.0 ng/mL will yield more true positives, consequently elevating the PPV of the test. (ii) The lower threshold will also result in fewer false negatives, which in turn will raise the NPV. Thus, discussing these assumptions in depth will aid in forming a hypothesis. CLINICAL REASONING: If the threshold for biopsy is lowered, it essentially becomes more liberal, and the catchment area for cases supposed to be positive also broadens. This renders more people who potentially have prostate cancer as positive, which are our true positives, and subsequently, there will be fewer false negatives (those with the disease but not detected). Although an increase in true positives typically enhances PPV, this scenario will also yield more false positives, i.e., those without disease but labeled positive. This influx of false positives will dilute the PPV, leading to its decrease. The decrease in false negatives will strengthen NPV; however, the increase in false positives also means there will be a decrease in true negatives, those without the disease and correctly identified as so. This decrease in true negatives could potentially harm the NPV, leading to its decrease. CONCLUDING: In essence, the implications of lowering the PSA threshold from 4.1 ng/mL to 2.0 ng/mL might lead to a decrease in both the PPV and NPV. This stems from the fact that adding more false positives reduces both the true positives (detrimental to PPV) and true negatives (harmful to NPV), despite the decrease in false negatives. RESPONSE: Therefore, the answer should be (I) Positive Predictive Value and Negative Predictive Value decrease.

**AI_B:** Restating the Question: The main idea of the scenario provided is to determine the effect of lowering the biopsy recommendation threshold of prostate-specific antigen concentration from 4.1 ng/mL to 2.0 ng/mL on the positive and negative predictive values of the test. Prostate-specific antigen (PSA) is a protein produced by both malignant and benign prostate cells; serum PSA levels are commonly used in screening for prostate cancer. The Positive Predictive Value (PPV) refers to the chance that a positive test accurately identifies the presence of disease. The Negative Predictive Value (NPV), on the other hand, refers to the likelihood that a negative test result is truly negative for the disease. Planning: The key assumption in this question is the correlation between a lower PSA threshold and the test's PPV and NPV. An alternate assumption could be that lowering the threshold may increase the number of both true positives (men with prostate cancer who test positive) and false positives (men without prostate cancer who test positive). This could arguably decrease the PPV. Because it might reduce the occurrence of false negatives (men with prostate cancer who test negative), the NPV may increase. However, the increase in false positives could offset the expected increase in NPV. Clinical Reasoning: Decreasing the threshold for recommending prostate biopsy from 4.1 ng/mL to 2.0 ng/mL implies that more men will qualify for biopsy, thus creating a possibility for the detection of earlier and potentially more cases of prostate cancer; these are the true positives. At the same time, more men without prostate cancer (false positives) might be recommended for biopsy because PSA can be elevated for reasons other than cancer, e.g., prostatitis or BPH. This means that more men without cancer might also fall into the biopsy recommendation bracket hence reducing the PPV. If less men with prostate cancer are left out (decrease in false negatives), there would be an increase in the NPV. However, the increasing number of men who test positive but do not have the disease (false positives) could also decrease the NPV. Concluding: The act of decreasing the PSA threshold could therefore lead to a decrease in PPV due to the increase in men without the disease who test positive, while the NPV may either increase because of a decrease in false negatives or decrease because of the increase in false positives. Response: Based on the given options, I propose the answer to be choice (G) Positive Predictive Value decrease and Negative Predictive Value increase. However recognizing that the NPV could also decrease, this answer choice should be subjected to further evaluation. It could be more accurate to state that the effect on NPV is uncertain or context-dependent.

**Facilitator** **[Round 5]:** Summary: AI_A, AI_B, AI_C, and AI_E all agree that lowering the threshold for recommending a biopsy based on PSA concentration would likely decrease the Positive Predictive Value (PPV) due to an increase in false positives. They also agree that the Negative Predictive Value (NPV) could potentially increase due to a decrease in false negatives. However, AI_B and AI_C also note that the increase in false positives could offset the expected increase in NPV. AI_D, on the other hand, suggests that both PPV and NPV could decrease due to an increase in false positives and a decrease in true negatives. Question to clarify disagreements: Given that lowering the threshold for recommending a biopsy could increase both true positives and false positives, how would this impact the balance between PPV and NPV? Specifically, could the increase in false positives potentially offset the expected increase in NPV, as suggested by AI_B and AI_C, or could it lead to a decrease in both PPV and NPV, as suggested by AI_D?. Based on your preceding response, how would you answer the original question: [In screening for prostate cancer, the current serum prostate-specific antigen concentration at which biopsy is recommended is 4.1 ng/mL. If the threshold for recommending a biopsy procedure were lowered to 2.0 ng/mL, which of the following changes would occur to the positive and negative predictive values of the test? (A) Positive Predictive Value and Negative Predictive Value increase (B) Positive Predictive Value increase and Negative Predictive Value no change (C) Positive Predictive Value increase and Negative Predictive Value decrease (D) Positive Predictive Value no change and Negative Predictive Value increase (E) Positive Predictive Value and Negative Predictive Value no change (F) Positive Predictive Value no change and Negative Predictive Value decrease (G) Positive Predictive Value decrease and Negative Predictive Value increase (H) Positive Predictive Value decrease and Negative Predictive Value no change (I) Positive Predictive Value and Negative Predictive Value decrease]

**AI_D:** 1) RESTATING THE QUESTION: We're discussing the effects on the Positive and Negative Predictive Values (PPV and NPV) of the prostate-specific antigen (PSA) test if the threshold for recommending a biopsy were lowered from its current value of 4.1 ng/mL to 2.0 ng/mL. 2) PLANNING: To clarify, PPV is the probability that subjects with a positive test result truly have the disease. NPV is the probability that subjects with a negative test result truly do not have the disease. Therefore, changing the threshold at which a biopsy is recommended might affect the specificity and sensitivity of the test, which could impact both the PPV and the NPV of the test. 3) CLINICAL REASONING: If we lower the threshold for a positive test, we increase the sensitivity (detection of true positives) and decrease the specificity (reduction of false positives) of the test. This will result in more cases being detected (including more false positives), meaning we could expect the PPV to decrease. The increase in true positives may also balance out the increase in false positives, potentially leading to an increase in the NPV. 4) CONCLUDING: Given the clinical reasoning process, lowering the threshold for recommending a biopsy may reduce the PPV due to increased false positives. However, the NPV may increase due to the greater detection of true positives. 5) RESPONSE: So my consensus is option (G) Positive Predictive Value decrease and Negative Predictive Value increase.

**AI_C:** RESTATING THE QUESTION: The question is asking about what would happen to the positive predictive value (PPV) and negative predictive value (NPV) of the prostate cancer screening test if the threshold for recommending a biopsy based on serum prostate-specific antigen (PSA) concentration was lowered from 4.1 ng/mL to 2.0 ng/mL. PLANNING: The assumptions made here are that a lower biopsy threshold would lead to an increase in both true positives and false positives and a decrease in false negatives. However, it is also plausible that a lower biopsy threshold could increase the number of false positives to such an extent that it could offset an expected increase in NPV. Alternatively, a lower threshold could lead to a decrease in both PPV and NPV due to an increase in false positives and a decrease in true negatives, respectively. It is also assumed that reducing the threshold for biopsy can cause even true negative cases to be subjected to unnecessary biopsies leading to an increase in the count of negative outcomes. As such, we need to evaluate whether the number of false positives would significantly increase and how this would affect PPV and NPV. CLINICAL REASONING: A lower threshold for biopsy recommendation would generally mean more people getting biopsied, including those with PSA levels between 2.0 and 4.1 ng/mL, who may not have been biopsied otherwise. As the false-positive rate increases (more people without disease are biopsied due to lower thresholds), the PPV, or the probability that those with a positive screening test truly have the disease, would decrease. Simultaneously, because true negatives (those without the disease and not biopsied) would decrease, the NPV would increase, provided that the increase in false positives does not outweigh decrease in false negatives. CONCLUDING: Based on clinical understanding, lowering the threshold for biopsy should theoretically increase the NPV because we are reducing false negatives. However, because we are also increasing false positives, the PPV should decrease. RESPONSE: Therefore, in light of the previous explanation, the answer should be (G) Positive Predictive Value decrease and Negative Predictive Value increase.

**AI_B:** (1) RESTATING THE QUESTION: The question is asking what changes would occur to the Positive Predictive Value (PPV) and Negative Predictive Value (NPV) of the PSA test if the threshold for biopsy recommendation were lowered from 4.1 ng/mL to 2.0 ng/mL. PPV refers to the probability that a person has a disease if the test is positive. NPV is the probability that a person does not have a disease if the test is negative. (2) PLANNING: The crux of this question lies in understanding the impact of lowering the threshold of an intervention on the predictive values. Lowering the threshold would mean that more patients would be recommended to undergo a biopsy. This could potentially lead to more true positives but it could also result in increased false positives. Regarding NPV, a lower threshold could potentially decrease false negatives, thus increasing NPV. However, this might also lead to a decrease in true negatives. (3) CLINICAL REASONING: By lowering the threshold for PSA, the number of patients being diagnosed with prostate cancer (true positives) will increase. Simultaneously, the number of patients without the disease being wrongly diagnosed (false positives) will also increase because some patients with a PSA of 2.0-4.0 ng/mL will not have cancer. This increase in false positives reduces the positive predictive value. On the other hand, with a decrease in the number of patients who truly don’t have cancer being overlooked (False negatives), the negative predictive value could increase. However, due to the overall increased proportion of people testing positive (both true and false positives together), there may be a decrease in the ratio of true negatives to the total number of people who actually don't have the condition, which could affect the increase in NPV. (4) CONCLUDING: In summary, by decreasing the PSA threshold to 2.0 ng/mL for a biopsy leads to an increase in true positives and false positives. This results in lowering the PPV because of the increase in false positives. The expected increase in NPV due to decreased false negatives could be offset with lower true negatives. (5) RESPONSE: Therefore, the most appropriate response would be (G) Positive Predictive Value decrease and Negative Predictive Value increase. Although, we must consider the possibility that the increase in NPV might be nominal or lesser than expected due to the potential effects discussed.

**AI_A:** RESTATING THE QUESTION: From my understanding, you're asking about changes in positive and negative predictive values (PPVs and NPVs, respectively) if the threshold to recommend a biopsy for prostate cancer is lowered from the current serum prostate-specific antigen (PSA) concentration of 4.1 ng/mL to 2.0 ng/mL. PLANNING: PPV and NPV indicate the proportion of positive and negative results in statistics and diagnostic tests that are true positive and true negative results, respectively. Lowering the threshold might increase the number of true positives, consequently leading to more false positive results. This could possibly decrease the PPV. Meanwhile, the decrease in false negatives could result in an increase in the NPV. However, it is also plausible that an influx of false positives may affect the NPV. CLINICAL REASONING: If the biopsy recommendation threshold is lowered, it implies capturing more individuals who are at risk of having prostate cancer. While this does increase the chances of identifying those with the disease (true positives), it simultaneously encompasses a wider array of individuals who may not have the disease (false positives). This would decrease the PPV as more false positives are included. The negative results would primarily be from people without the disease (true negatives), reducing the chances of presenting a false negative scenario, thus likely increasing NPV. CONCLUDING: Nonetheless, an influx of false positives could offset these dynamics, possibly hindering any increase in the NPV. This unfolds due to PSA being an imperfect marker that can result in false positives, where a patient may show elevated PSA levels but not actually have prostate cancer. RESPONSE: Hence, I would align more with option (H): Positive Predictive Value decreases, and Negative Predictive Value experiences no change. This accounts for the decrease in PPV due to higher false positives and the potential idleness of NPV owing to the same influx of false positives potentially compensating for the reduced false negatives.

**AI_E:** Restatement of the question: You're asking what the impact would be on the positive predictive value (PPV) and the negative predictive value (NPV) of a prostate-specific antigen (PSA) test if the threshold for suggesting a biopsy were lowered from 4.1 ng/mL to 2.0 ng/mL. Planning: The Positive Predictive Value (PPV) is defined as the probability that people with a positive test truly have the disease, while Negative Predictive Value (NPV) is the probability that people with a negative test truly do not have the disease. By lowering the PSA threshold for recommending biopsy, we would expect the test to identify more individuals with potential prostate cancer, including those who at lower concentrations (2.0-4.0 ng/mL) might have been missed before. However, this comes with steep trade-offs. While the test could detect more true positives (people with prostate cancer), it could also increase the number of false positives (people without prostate cancer but with a PSA level above 2.0 ng/mL). Clinical reasoning: Let's dive deeper into these assumptions. Lowering the threshold of the test could lead to more individuals being identified for biopsy, thus potentially increasing the number of true positives. This could have an impact on both PPV and NPV. First, an increase in true positives could potentially lead to an increase in NPV because fewer cases of the disease are being missed. However, this logic assumes that the increase in true positives is proportionately higher than any increase in false positives. If there's a larger proportional increase in false positives, the PPV could decrease as the test is less precise in determining who true has the disease. As for NPV, if the increase in true negatives (those without disease and with a negative test result) outraces the decrease in false negatives (those with the disease but with a negative result), NPV might possibly increase. However, if the number of false negatives doesn't decrease significantly, the NPV might not increase as much as the PPV might decrease. Concluding: Therefore, lowering the threshold to recommend a biopsy procedure from PSA level of 4.1 ng/mL to 2.0 ng/mL could potentially decrease the PPV due to an increase in false positives. The NPV may increase if the cohort of true negatives grows at a faster rate than any decrease in false negatives. Response: With the information at hand, the most plausible assumption to make would be option (G) Positive Predictive Value decreases and Negative Predictive Value increases.

**Facilitator** **[Round 6]:** Summary: AI_A believes that lowering the threshold for biopsy recommendation would increase the number of true positives and false positives, leading to a decrease in PPV. However, the decrease in false negatives could result in an increase in NPV. But, an influx of false positives could offset these dynamics, possibly hindering any increase in the NPV. AI_A's conclusion is that PPV decreases, and NPV experiences no change. AI_B, AI_C, AI_D, and AI_E all agree that lowering the threshold would increase both true positives and false positives, leading to a decrease in PPV. They also agree that the decrease in false negatives could potentially increase NPV. However, they acknowledge that an increase in false positives could affect the increase in NPV. Their conclusion is that PPV decreases, and NPV increases. Key Differences: The key difference lies in the interpretation of how the influx of false positives would affect NPV. AI_A believes that the increase in false positives could potentially offset the decrease in false negatives, leading to no change in NPV. In contrast, AI_B, AI_C, AI_D, and AI_E believe that despite the increase in false positives, the decrease in false negatives could still lead to an increase in NPV. Question to Clarify Disagreements: To clarify the disagreements between the council members' opinions, we could ask: "Could you elaborate on how the increase in false positives could affect the NPV, considering the decrease in false negatives? Would the influx of false positives offset the decrease in false negatives to the point where NPV experiences no change, or would NPV still increase despite the increase in false positives?". Based on your preceding response, how would you answer the original question: [In screening for prostate cancer, the current serum prostate-specific antigen concentration at which biopsy is recommended is 4.1 ng/mL. If the threshold for recommending a biopsy procedure were lowered to 2.0 ng/mL, which of the following changes would occur to the positive and negative predictive values of the test? (A) Positive Predictive Value and Negative Predictive Value increase (B) Positive Predictive Value increase and Negative Predictive Value no change (C) Positive Predictive Value increase and Negative Predictive Value decrease (D) Positive Predictive Value no change and Negative Predictive Value increase (E) Positive Predictive Value and Negative Predictive Value no change (F) Positive Predictive Value no change and Negative Predictive Value decrease (G) Positive Predictive Value decrease and Negative Predictive Value increase (H) Positive Predictive Value decrease and Negative Predictive Value no change (I) Positive Predictive Value and Negative Predictive Value decrease]

**AI_C:** The essence of the question is whether the positive predictive value (PPV) and the negative predictive value (NPV) would change if the threshold for recommending a prostate biopsy were lowered from 4.1 ng/mL to 2.0 ng/mL. PPV is the likelihood that a positive test result indeed signifies the disease, and NPV is the likelihood that a negative test result correctly identifies the absence of the disease. When we decrease the threshold, we increase the sensitivity of the test and capture more true positive cases. However, we also increase the number of false positives, and so PPV (the proportion of true positives out of all positive results) would decrease, as there would be more positive test results overall, but not all of them are true positives. As for the NPV, it depends on how the change in threshold affects the ratio of true negatives to false negatives. Decreasing the threshold would decrease the number of false negatives as more cases are captured by the test. Theoretically, NPV (the proportion of true negatives out of all who tested negative) should increase. However, the influx of false positives, which would automatically be designated as true negatives in subsequent tests, might reduce the potential increase in NPV. In view of this, the assumptions made indicate that the most likely result would be that the PPV decreases and the NPV increases (Option G). However, due to uncertainties surrounding the extent to which the increase in the NPV might be offset by the influx of false positives, the other possible outcome is that the PPV decreases and the NPV experiences no change (Option H). On balance, the decrease in the threshold would most likely produce more accurate negative results (fewer false negatives), so it's clinically prudent to pursue the hypothesis that lowering the biopsy threshold will both lower PPV and increase NPV (Option G). Hence, the final response, considering the potential for the influx of false positives to offset the decrease in false negatives and maintain the NPV, the more likely answer would be Option G: Positive Predictive Value decrease and Negative Predictive Value increases.

**AI_D:** 1) RESTATING THE QUESTION: The focus of this question is the correlation between lowering the threshold value for prostate-specific antigen levels (used as a screening tool for prostate cancer) to recommend a biopsy and the effect this shift would have on the positive predictive value (PPV) and negative predictive value (NPV) of the test. The PPV of a test refers to the probability that individuals with a positive screening test truly have the disease. Conversely, NPV refers to the probability that individuals with a negative screening test genuinely do not have the disease. 2) PLANNING: In healthcare, changes in the threshold of diagnostic tests could impact both sensitivity and specificity, consequently affecting PPV and NPV. If we lower the threshold for recommending a biopsy from 4.1 ng/mL to 2.0 ng/mL, this change could potentially increase the number of true positive cases and false positive cases. On the opposite end, lowering the threshold could decrease the number of false negatives. This is, however, dependent on how our population's disease prevalence and the specificity of the test change. 3) CLINICAL REASONING: If the threshold level is lowered, it would result in a more sensitive test, detecting more true positives and fewer false negatives. Consequently, this would theoretically boost the test's NPV. However, as the threshold lowers, the test also becomes less specific as we would be capturing more false positive cases. This would, in turn, lower the PPV since the number of false positives inflate the denominator used to calculate PPV. 4) CONCLUDING: The change in NPC and PPV in response to a threshold shift cannot always be linear or intuitive because it often depends on a range of factors, such as disease prevalence and test specificity. Here, it is anticipated that the changes in the threshold would increase the test's sensitivity (leading to an increased NPV) but decrease its specificity (resulting in a decreased PPV). 5) RESPONSE: Lowering the threshold for recommending a prostate biopsy based on serum prostate-specific antigen concentration would likely decrease the Positive Predictive Value (PPV) and increase the Negative Predictive Value (NPV), corresponding to answer (G): Positive Predictive Value decrease and Negative Predictive Value increase.

**AI_B:** 1) RESTATING THE QUESTION: The question asks about the impact on the predictive values of prostate cancer screening if the biopsy recommendation threshold were lowered from 4.1 ng/mL to 2.0 ng/mL for prostate-specific antigen (PSA) concentration. Predictive values are measures of the strength of a diagnostic test. Positive predictive value (PPV) refers to the probability that those with a positive test result truly have the disease, while negative predictive value (NPV) is the probability that those with a negative test result genuinely don't have the disease. 2) PLANNING: The use of the PSA test as a screening tool for prostate cancer has raised several controversies due to its limitations. The assumptions we are operating under here are that the prevalence of the disease will stay constant, and that lower thresholds will lead to more people testing positive (increased sensitivity) but with an increased number of false positives, which would impact the PPV. An alternate assumption could be that lowering the threshold can not only increase sensitivity but might affect the specificity of the screening, leading to more people falsely identified as having prostate cancer when they don't (false positives), thus impacting the PPV and potentially the NPV. 3) CLINICAL REASONING: If we lower the threshold for a positive test, we would pick up more cases of prostate cancer (increased true positives), but we could also label more people as positive who don't have the disease (increased false positives). This increased number of false positives reduces the PPV because there are fewer true positives amongst those labelled as positive. As for the NPV, although we are reducing the number of false negatives by lowering the threshold (which ordinarily would increase NPV), there could be a counter-effect from the increased number of false positives. However, the magnitude of this counter-effect would depend on the relative increase in false positives and decrease in false negatives. 4) CONCLUDING: By lowering the PSA threshold from 4.1 ng/mL to 2.0 ng/mL, an increase in both true positives and false positives would occur. This change would decrease the PPV due to the increased number of false positives amongst those labelled as positive. The effect on NPV is less clear-cut; based on the combined effects of fewer false negatives and more false positives, it can be hypothesized that the NPV might increase. 5) RESPONSE: Given the above discussion and reasoning, my response to the question will be: lowering the threshold for recommending a biopsy procedure from 4.1 ng/mL to 2.0 ng/mL would decrease the Positive Predictive Value and increase the Negative Predictive Value of the test (G).

**AI_E:** 1) RESTATING THE QUESTION: This inquiry is asking about possible changes to Positive Predictive Value (PPV) and Negative Predictive Value (NPV) if the threshold for recommending a prostate biopsy procedure was altered from 4.1 ng/mL to 2.0 ng/mL in serum prostate-specific antigen (PSA) screenings for prostate cancer. Positive Predictive Value represents the probability that patients with a positive screening test truly do have the disease. The Negative Predictive Value is the probability that patients with a negative screening test genuinely do not have the disease. 2) PLANNING: The core assumption here is that adjusting the threshold for recommending a biopsy alters the numbers of true positives, false positives, false negatives, and true negatives derived from the screening test. We need to analyze how changing these numbers impacts the PPV and NPV. The alternate assumption we could make is that lowering the threshold could result in an increased number of false positives (those without the disease but testing positive) and true positives, while reducing the number of false negatives (having the disease but testing negative). 3) CLINICAL REASONING: Lowering the threshold to 2.0 ng/mL indeed increases the number of positive tests - both true positives and false positives. An increased amount of false positives leads to a decrease in PPV, as PPV is calculated as True Positives / (True Positives + False Positives). Conversely, a reduction in false negatives increases the NPV since fewer patients who have the disease are being missed by the test. The NPV is calculated as True Negatives / (True Negatives + False Negatives). However, this is under the assumption that the prevalence of the disease in the population remains constant. 4) CONCLUDING: Hence, with a lowering of the threshold for biopsy in a PSA test, the number of false positives increases, leading to a decrease in PPV. Similarly, as the number of false negatives decreases, NPV is likely to increase. However, the prevalence of the disease can significantly affect this outcome. 5) RESPONSE: Considering this clinical reasoning, the answer to the question is: (G) Positive Predictive Value decrease and Negative Predictive Value increase.

**AI_A:** Restating the question, it asks what would happen to the Positive Predictive Value (PPV) and Negative Predictive Value (NPV) if the threshold for recommending a prostate biopsy - based on prostate-specific antigen (PSA) levels - is reduced from a current 4.1 ng/mL to 2.0 ng/mL. The PPV of a test refers to the probability that a person with a positive test result actually has the disease. On the other hand, NPV refers to the probability that a person with a negative test result is truly free from the disease. In planning to answer the question, we need to understand how changing the threshold for a positive test result (in this case, lowering the PSA level at which a biopsy is recommended) affects these values. An increase in the number of positive test results (due to lowering the threshold) would tend to increase the number of true positives (those with the disease who test positive) as well as false positives (those without the disease who test positive). Linking this assumption to the clinical reasoning, the increase in true positives would increase the denominator in the calculation of PPV, but the increase in false positives would decrease the numerator. As a result, the PPV would likely decrease. The effect on the NPV is a bit more complex. Lowering the threshold could potentially decrease the number of false negatives (those with the disease who test negative) allowing the NPV to potentially increase. However, the relative impact on NPV would also be contingent upon the disease prevalence in the screened population. Concluding the clinical thought process, the lowering of biopsy recommendation threshold would likely lead to a decrease in the PPV (due to increased false positives) and a complex effect on the NPV, which could increase (due to reduced false negatives) but might be offset by the increased prevalence of testing positives (including true and false positives). Responding to the question, the most probable scenario would be (G) Positive Predictive Value decrease and Negative Predictive Value increase although, due to the complex influences on NPV, there is some uncertainty around the latter.

**Facilitator** The consensus statement synthesizes the responses of AI_A, AI_B, AI_C, AI_D, and AI_E as follows: The question asks about the impact on the Positive Predictive Value (PPV) and Negative Predictive Value (NPV) if the threshold for recommending a prostate biopsy, based on prostate-specific antigen (PSA) levels, is reduced from a current 4.1 ng/mL to 2.0 ng/mL. PPV refers to the probability that a person with a positive test result actually has the disease, while NPV refers to the probability that a person with a negative test result is truly free from the disease. Lowering the threshold for a positive test result would likely increase the number of positive test results, which would include both true positives (those with the disease who test positive) and false positives (those without the disease who test positive). This increase in false positives would decrease the PPV, as there would be fewer true positives among those labeled as positive. The effect on the NPV is more complex. Lowering the threshold could potentially decrease the number of false negatives (those with the disease who test negative), which would theoretically increase the NPV. However, the influx of false positives, which would automatically be designated as true negatives in subsequent tests, might reduce the potential increase in NPV. The relative impact on NPV would also be contingent upon the disease prevalence in the screened population. In conclusion, the lowering of the biopsy recommendation threshold would likely lead to a decrease in the PPV due to increased false positives and a complex effect on the NPV, which could increase due to reduced false negatives but might be offset by the increased prevalence of testing positives (including true and false positives). Therefore, the most probable scenario would be (G) Positive Predictive Value decrease and Negative Predictive Value increase, although there is some uncertainty around the latter due to the complex influences on NPV.
