# Supplementary material for: Collaborative intelligence in AI: Evaluating the performance of a council of AIs on the USMLE
Source: PLOS Digit Health. 2025 Oct 9;4(10):e0000787. doi: 10.1371/journal.pdig.0000787 (PMC12510544; doi:10.1371/journal.pdig.0000787)
Supplement: S2 File — (ZIP) [file pdig.0000787.s002.zip › S2 Data Raw data files containing unprocessed question inputs and LLM outputs including transcripts of deliberations/STEP1_Question-90-1_Type-MC-NJ.docx]

**Facilitator** **[Round 1]:** A 12-year-old girl is brought to the office because of a 2-day history of chest pain. She also had a 3-day history of fever that has since resolved. When she lies down, the pain worsens and she has shortness of breath. Her temperature is 37°C (98.6°F). A friction rub is heard. Which of the following is the most likely causal infectious agent? (A) Coxsackievirus (B) Mumps virus (C) Staphylococcus aureus (D) Staphylococcus epidermidis (E) Viridans streptococcus

**AI_A:** (1) RESTATING THE QUESTION: A 12-year-old girl is presented with a medical history of chest pain of 2 days' duration, a recently resolved fever which lasted for 3 days, and the symptom of worsened chest pain and shortness of breath while lying down. Her body temperature appears to be normal at present. A friction rub sound, suggestive of margin rubbing together, is noted during physical examination. The query looks into identifying the infectious agent most likely responsible for her current condition. (2) PLANNING: Based on the clinical signs and symptoms, one can infer that the underlying condition could potentially be pericarditis, an inflammation of the pericardium (outer layer of the heart). A friction rub, characteristic of this condition, results from the inflamed pericardial layers rubbing against each other. Infectious pericarditis, specifically, is often a result of a viral infection. However, it is important to consider bacterial pericarditis, although less common, which may be due to Staphylococcus aureus, Staphylococcus epidermidis, or Viridans streptococcus. Plan-wise, one must prioritize potential infectious agents that are commonly associated with pericarditis. (3) CLINICAL REASONING: The symptoms and signs presented are characteristic of pericarditis. The presence of a fever suggests an infectious cause. While many agents can cause pericarditis, viral causes, particularly Coxsackievirus, are the most common in the pediatric population. Bacterial pericarditis is relatively rare and tends to occur secondary to spread from another site of infection. In contrast, Coxsackievirus is known to cause acute pericarditis, often following an upper respiratory tract infection. (4) CONCLUDING: Based on the primary assumption of pericarditis and the common causative agents for such conditions in children, and also considering the patient's specific symptoms and likely exposure to viral agents, it can be inferred that the patient's condition may result from the Coxsackievirus. (5) RESPONSE: Thus, the most likely infectious agent causing the clinical presentation in this 12-year-old girl is (A) Coxsackievirus.

**AI_B:** RESTATING THE QUESTION: This clinical situation describes a 12-year-old girl who is presenting with a 2-day history of chest pain, worsening when she lies down, and is accompanied by shortness of breath. A friction rub is an abnormal sound that can be heard during medical examination, indicating inflammation of the layers of the pericardium, the sac enclosing the heart. The primary question here is to identify the most likely microorganism that's causing this pericarditis condition. PLANNING: The given choices are (A) Coxsackievirus, (B) Mumps virus, (C) Staphylococcus aureus, (D) Staphylococcus epidermidis, and (E) Viridans streptococcus. Each of these are known to cause certain types of infections. We are to evaluate the patient's symptoms, in conjunction with the history of a recent fever, to consider an infection that may lead to pericarditis. Of course, we have to assume that the given organisms are the only ones we can choose from for the source of this infection. CLINICAL REASONING: Infections by some of these microorganisms might lead to pericarditis either directly, or as a secondary reaction to the infection. Mumps virus, although known to cause viral diseases along with parotitis, meningitis, and orchitis, isn't significantly linked to pericarditis. Staphylococcus aureus and Staphylococcus epidermidis are bacteria commonly associated with skin infections, endocarditis, sepsis, and device-related infections, but not commonly seen in pericarditis in otherwise healthy children. Viridans streptococci, although linked with endocarditis, especially in patients with poor dental hygiene, is also less common in cases of pericarditis. Coxsackievirus, however, is a known cause of pericarditis and myocarditis. This virus usually presents with a recent history of upper respiratory tract infection or gastrointestinal symptoms, preceding cardiac symptoms. CONCLUDING: Given the girl's symptoms, especially the chest pain worsening when lying down and improvement upon sitting up, along with her recent history of fever, it highly suggests a case of acute pericarditis. RESPONSE: Hence, the most likely cause of infection leading to pericarditis in this case would be the Coxsackievirus (A).

**AI_C:** (1) RESTATING THE QUESTION: In this case, we're presented with a 12-year-old girl who has recently experienced chest pain and a fever, which has since abated. She experiences more severe chest pain and shortness of breath when she assumes a supine position. Her body temperature is now normal, but a distinctive sound (a friction rub) is audible. We are to identify the most likely infectious agent underlying these symptoms. A friction rub is usually indicative of inflammation in the protective layers (pleura) of the lungs, which could be due to infections, autoimmune diseases or following a heart attack (myocardial infarction). The infectious agents presented as options here range from viruses to bacteria. (2) PLANNING: To make a reasoned judgment, we will examine each infectious agent's typical presentation, relating such presentation with the clinical information provided. (A) Coxsackievirus: a type of Enterovirus often associated with hand, foot, and mouth disease, myocarditis, and pleurodynia. (B) Mumps virus: usually presents with parotitis, orchitis, and oophoritis. (C) Staphylococcus aureus: a common bacterial infection, usually associated with skin infections, on rarer occasions can cause dangerous conditions like pneumonia and endocarditis. (D) Staphylococcus epidermidis: typically associated with infections in people with weakened immune systems. (E) Viridans streptococci: mostly associated with dental caries and can cause endocarditis, usually following dental surgery. (3) CLINICAL REASONING: The girl's recent fever suggests an acute infection. The friction rub and her symptoms of chest pain and shortness of breath when lying down suggest pleuritic involvement, which could mean pericarditis or pleurisy. Post-viral pericarditis can commonly follow an upper respiratory tract infection (URTI) and it is usually characterized by chest pain that worsens when lying flat and improves with sitting up and leaning forward. This seems in line with the girl's presentation. The Coxsackievirus, an enterovirus, is known to spread through the respiratory route and is a common cause of viral pericarditis and myocarditis, especially in children. (4) CONCLUDING: Comparing the aforementioned infectious agents' presentations with the clinical data available from this case, the clinical features closely align with a post-viral pericarditis condition, often associated with Coxsackievirus infection. (5) RESPONSE: (A) Coxsackievirus would thus be the most probable causal agent in this clinical scenario.

**AI_E:** RESTATING THE QUESTION: The question asks for the most likely cause of a preteen girl's symptoms that include shortness of breath, chest pain that worsens when laying down, a history of recent fever, and a chest exam finding of a friction rub. These conditions are associated with pericarditis, an inflammation of the sac-like covering around the heart (pericardium), which can be caused by a variety of infectious agents. PLANNING: It's critical to consider each potential infectious agent's propensity to cause pericarditis. Coxsackievirus and Staphylococcus aureus are causes of pericarditis, while Mumps, Staphylococcus epidermidis, and Viridans Streptococci are less commonly associated. Therefore, further evaluation should prioritize the assumption that the cause is either Coxsackievirus or Staphylococcus aureus. CLINICAL REASONING: When evaluating a case of possible pericarditis, it's useful to look at the patient's symptoms: chest pain worsened by lying down, shortness of breath and a fever that has since resolved. Also, a noticeable friction rub on the chest exam points towards pericarditis. The culmination of these symptoms suggests an infection has spread to or directly infected the pericardium. Now we have two main causes to consider: Coxsackievirus and Staphylococcus aureus. Considering the patient's age and clinical profile, a viral etiology is more likely. Staphylococcus aureus pericarditis commonly occur secondary to spread from a contiguous focus or in the setting of bacteremia. This girl doesn't have any signs of bacterial infection currently (i.e., no ongoing fever or obvious source of infection), and since her fever had resolved, the most likely causal infectious agent is Coxsackievirus. CONCLUDING: While both Coxsackievirus and Staphylococcus aureus are potential causes of pericarditis, given her age, recent fever which is now resolved, chest pain worsened by laying down, the presence of a friction rub, and lack of signs of bacterial infection, her symptoms are most consistent with a viral infection, specifically Coxsackievirus, as the cause. RESPONSE: Therefore, the most likely infectious agent in this case is (A) Coxsackievirus.

**AI_D:** RESTATING THE QUESTION: A young female patient has been brought in due to chest discomfort over a couple of days. She has also had a recent fever, now resolved, and shows signs of breathlessness that get worse upon lying down. She has a normal body temperature at present. One of the findings during her physical examination is a friction rub, which is a scraping or grating noise heard from the heart region generally associated with inflammation of the pericardium or the outer layer of the heart. Our task is to identify the most probable infective organism that could be causing these symptoms. PLANNING: Examining her symptoms of fever, chest pain, exacerbated pain when lying down, and presence of a friction rub, pericarditis comes to mind first, which is inflammation of the pericardium. The fever indicates an infection, which in the case of pericarditis is commonly viral. The agents listed as options however include both viruses and bacteria. We should also consider other diseases that can present with these symptoms, such as pneumonia and other respiratory tract infections, especially as they can also exhibit chest pain and difficulty in breathing. However, these do not typically produce a friction rub. CLINICAL REASONING: Shortness of breath and chest pain that gets worse on lying down are classical signs of pericarditis. The friction rub also points toward a pericardial disease. Pericarditis usually follows a respiratory tract infection, and although it can be caused by many infectious agents, viral pathogens are the most common. Bacterial pericarditis is rarer and typically occurs as a complication of systemic infection (Staphylococcus aureus, Staphylococcus epidermidis, and Viridans streptococcus). Nevertheless, in the case of a previously healthy child, a viral etiology is more likely, often following an upper respiratory infection caused initially by a virus. CONCLUDING: Given the history, symptoms, and examination findings, this child appears to have a viral pericarditis following a recent fever (indicative of a preceding general infection). With this in mind, the most likely causal agent could be a virus. RESPONSE: Therefore, the most likely infectious agent causing these symptoms is (A) Coxsackievirus, a known common cause of viral pericarditis. The fever is seen in the prodromal phase of the disease, while chest pain and a friction rub are classic for pericarditis. When viral in origin, this condition often follows a non-specific febrile illness similar to the 3-day history of fever the patient had. Of the possible choices, the Coxsackievirus is considered a common etiological agent for pericarditis in children and adolescents.

**Facilitator** The consensus statement synthesizes the responses of AI_A, AI_B, AI_C, AI_D, and AI_E as follows: The case presents a 12-year-old girl with a 2-day history of chest pain, worsened by lying down, and shortness of breath. She also had a recent 3-day fever that has since resolved. Her current body temperature is normal. A friction rub, indicative of inflammation of the pericardium, is noted during her physical examination. The task is to identify the most likely infectious agent responsible for her symptoms. The symptoms and signs, including the friction rub and the chest pain that worsens when lying down, are characteristic of pericarditis, an inflammation of the pericardium. The recent fever suggests an infectious cause. While many agents can cause pericarditis, viral causes are the most common, particularly in the pediatric population. Bacterial pericarditis is relatively rare and tends to occur secondary to spread from another site of infection. The infectious agents presented as options include both viruses and bacteria. Among these, Coxsackievirus, a type of Enterovirus, is known to cause pericarditis and myocarditis and often presents with a recent history of upper respiratory tract infection or gastrointestinal symptoms, preceding cardiac symptoms. The other agents, including Mumps virus, Staphylococcus aureus, Staphylococcus epidermidis, and Viridans streptococcus, are less commonly associated with pericarditis. Given the girl's symptoms, especially the chest pain worsening when lying down and improvement upon sitting up, along with her recent history of fever, it highly suggests a case of acute pericarditis. Considering the patient's age, clinical profile, and the common causative agents for such conditions in children, the most likely infectious agent causing the clinical presentation in this 12-year-old girl is Coxsackievirus. Therefore, the consensus response to the prompt is (A) Coxsackievirus.
