# Supplementary material for: Collaborative intelligence in AI: Evaluating the performance of a council of AIs on the USMLE
Source: PLOS Digit Health. 2025 Oct 9;4(10):e0000787. doi: 10.1371/journal.pdig.0000787 (PMC12510544; doi:10.1371/journal.pdig.0000787)
Supplement: S2 File — (ZIP) [file pdig.0000787.s002.zip › S2 Data Raw data files containing unprocessed question inputs and LLM outputs including transcripts of deliberations/STEP1_Question-91-1_Type-MC-NJ.docx]

**Facilitator** **[Round 1]:** A 32-year-old man comes to the office because of a 1-day history of cough productive of small amounts of blood and a 2-day history of shortness of breath and swelling of his ankles. He also has a 2-week history of progressive fatigue and episodes of dark urine. He has no history of major medical illness and takes no medications. His temperature is 37°C (98.6°F), pulse is 90/min, respirations are 18/min, and blood pressure is 175/110 mm Hg. Pulse oximetry on room air shows an oxygen saturation of 91%. Diffuse inspiratory crackles are heard over all lung bases. There is 2+ pitting edema of both ankles. Results of laboratory studies are shown: Hemoglobin 8.9 g/dL Hematocrit 27% Serum Urea nitrogen 55 mg/dL Creatinine 2.9 mg/dL Urine RBC 20–40/hpf Urinalysis also shows some dysmorphic RBCs and rare RBC casts. Examination of a kidney biopsy specimen shows crescentic glomerulonephritis and linear deposition of IgG along the glomerular capillaries. This patient most likely has antibodies directed against which of the following antigens? (A)  Collagen (B)  Double-stranded DNA (C)  Nucleolar protein (D)  Phospholipid (E)  Proteins in neutrophil cytoplasm

**AI_E:** RESTATING THE QUESTION: The question pertains to a 32-year-old male presenting with hemoptysis, dyspnea, ankle swelling, fatigue, and dark urine. The clinical and laboratory findings hint towards renal pathology with the presence of anemia, elevated blood urea nitrogen and creatinine values, abnormal urine sediment with RBCs and RBC casts, and signs of hypertension. A kidney biopsy revealed crescentic glomerulonephritis with a linear deposition of IgG along the glomerular capillaries. Based on these findings, we are to identify the most likely antigen against which the patient's immune system has produced antibodies. PLANNING: Understanding the patient's disease process involves several assumptions. The patient appears to have a renal disease featuring inflammatory processes, given the presence of glomerulonephritis demonstrated on biopsy. This assumption could be broadened to specify that the patient's condition might be an autoimmune disease, given the presence of IgG deposition, usually characteristic of such conditions. Another assumption that could be surmised from the patient's symptoms is that the condition may have systemic manifestations, encompassing the respiratory and circulatory systems. CLINICAL REASONING: The physical and laboratory findings, particularly the kidney biopsy demonstrating crescentic glomerulonephritis and linear IgG deposition, hint towards an autoimmune disease targeting the kidney predominantly. Goodpasture's syndrome, a form of autoimmune disease characterized by antibodies against the basement membrane in both the kidneys and lungs, is a suitable hypothesis given the clinical picture. These antibodies are directed against the non-collagenous domain of the α3 chain of type IV collagen found in the basement membrane. CONCLUDING: Therefore, considering the systemic nature of the symptoms, the renal pathology, and the pattern of IgG deposition found in the kidney biopsy, the clinical scenario is indicative of Goodpasture's syndrome. This condition is associated with circulating autoantibodies against type IV collagen, a key component of the basement membrane in the lungs and kidneys. RESPONSE: So, this patient most likely has antibodies directed against (A)  Collagen.

**AI_D:** 1) RESTATING THE QUESTION: The current scenario presents a young man who exhibits a range of troubling symptoms such as coughing up blood, shortness of breath, ankle swelling, fatigue, and dark urine. His vital signs and physical examination reveal hypertension, lowered oxygen saturation, lung crackles, and ankle edema. Lab studies reveal anemia, renal impairment, and erythrocyturia. His kidney biopsy displays crescentic glomerulonephritis and IgG deposits on glomerular capillaries. We are asked to identify the most likely target of this man's presumed autoantibodies. 2) PLANNING: Looking at the clinical scenario and lab findings, this patient seems to be suffering from a systemic autoimmune condition that involves kidney and lungs, one might think of diseases like lupus, Goodpasture's disease, or vasculitis. The linear deposition of IgG along the glomerular capillaries are a significant clue. 3) CLINICAL REASONING: A linear pattern of IgG deposition along the glomerular capillaries typically signifies Goodpasture's syndrome (also known as anti-glomerular basement membrane/anti-GBM disease). This autoimmune disorder leads to the body producing antibodies against a particular antigen found in the basement membrane of both the glomeruli and alveoli. This can cause both glomerulonephritis (crescentic in nature, as in this case) manifested as hematuria, proteinuria, and renal failure and pulmonary hemorrhage manifested as hemoptysis or presence of blood in the cough. This can lead to resultant anemia and could also be causing the patient's fatigue. 4) CONCLUDING: The primary antigen targeted in Goodpasture's syndrome is actually a type of collagen (type IV) located in the basement membranes, causing the symptoms and lab findings seen in this case. 5) RESPONSE: Therefore, in the light of this patient's clinical presentation and laboratory findings, it is highly likely that he has antibodies directed against Collagen. So, the correct answer is (A) Collagen.

**AI_B:** 1) RESTATING THE QUESTION: The patient in this scenario is a 32-year-old male who presents with coughing up small amounts of blood, shortness of breath, swelling of his ankles, fatigue, and dark urine. His vitals are slightly off with a higher blood pressure, and his oxygen saturation is a little lower than normal. Physical and laboratory examinations show lung crackles, pitting edema, anemia, and kidney issues. His kidney biopsy revealed crescentic glomerulonephritis and IgG along the glomerular capillaries. Given these findings, the question is trying to determine the likely pathogenesis, specifically which antigen the patient's antibodies are most likely targeting. 2) PLANNING: To answer this, we need to consider the symptoms, physical and laboratory findings, and the pathology findings from the kidney biopsy. Crescentic glomerulonephritis with linear IgG deposition hints towards a type of autoimmune disease, as it showcases an abnormal immune response occurring. The target of that response, i.e., the antigen causing this condition, could be a variety of things depending on the exact disease at hand. We need to consider the types of antigens listed and how they could relate to the patient's condition. 3) CLINICAL REASONING: There are several conditions associated with kidney damage and lung issues as part of their manifestation. For example, systemic lupus erythematosus (SLE) primarily targets double-stranded DNA (option B), but it typically presents with a full-house immunofluorescence pattern, which is not described in this scenario. Instead, this patient's pattern of linear IgG deposits seen in the setting of a rapidly progressive glomerulonephritis (manifested by hematuria, proteinuria, and renal failure) and pulmonary hemorrhage represents a classic presentation of Goodpasture syndrome (anti-glomerular basement membrane [anti-GBM] disease). The immune system in this disease primarily creates antibodies against an antigen present in the basement membrane of glomerular and alveolar capillaries, which is a type III collagen (option A). 4) CONCLUDING: Therefore, considering the clinical presentation, laboratory, and biopsy findings, we can conclude that the cause of this patient's symptoms is most likely Goodpasture syndrome, with the immune system creating antibodies against type III collagen. 5) RESPONSE: The patient's antibodies are most likely directed against (A) Collagen.

**AI_A:** 1) RESTATING THE QUESTION: A 32-year old male patient has come into the office with symptoms of coughing up small amounts of blood, difficulty breathing, ankle swelling, fatigue, and dark urine over the past couple weeks. He has no prior significant medical history. On examination, his body temperature, pulse, and respiration rate are normal but his blood pressure is elevated and he has a slightly decreased oxygen saturation. He has pitting edema of his ankles and coarse lung sounds. His laboratory findings indicate anemia, the presence of red blood cells and RBC casts in his urine, as well as elevated levels of urea and creatinine, which suggest kidney dysfunction. The findings in his kidney biopsy suggest a type of kidney disease known as crescentic glomerulonephritis, characterized by crescent-shaped lesions and linear deposition of IgG along the glomerular capillaries. We are required to identify the possible antigen that the patient has developed antibodies against from a list of options. 2) PLANNING: The main assumption would usually involve considering primary pulmonary and cardiac conditions based on his symptom of coughing blood and shortness of breath. However, the laboratory findings and kidney biopsy results along with the systemic symptoms he presents point towards involvement of the kidney in the underlying disease. We can reconsider our primary assumption and look into conditions that can lead to kidney damage, to help identify the specific antibodies in question. 3) CLINICAL REASONING: The patient's symptoms and findings seem characteristic of a condition known as Goodpasture syndrome, a rare autoimmune disease where antibodies attack the basement membrane in the lungs and kidneys, leading to glomerulonephritis and often pulmonary hemorrhage. The presence of crescentic glomerulonephritis and linear IgG deposition are particularly suggestive of this condition. The antibodies in Goodpasture syndrome are usually directed against a specific antigen present in the basement membrane of both the kidneys and the lungs. 4) CONCLUDING: Given the systemic manifestations involving both lungs and kidneys, it is reasonable to suspect an autoimmune mechanism like Goodpasture syndrome. The conditions correlating to other listed antigens: double-stranded DNA (systemic lupus erythematosus), nucleolar protein (scleroderma), phospholipid (antiphospholipid syndrome), and proteins in neutrophil cytoplasm (vasculitides like Wegener’s granulomatosis) do not fit as well with the clinical scenario compared to Goodpasture syndrome. 5) RESPONSE: The patient most likely has antibodies directed against Collagen (Option A), specifically the alpha-3 chain of type IV collagen, which is a component of the basement membrane in the glomeruli of the kidney and the alveoli of the lungs, both of which are affected in Goodpasture syndrome.

**AI_C:** RESTATING THE QUESTION: The patient is a 32-year-old male presenting with coughing up blood, shortness of breath, swollen ankles, fatigue, and dark urine. He has no significant past medical history. Blood work shows anemia, and his kidney function appears compromised. Urinalysis detected considerable red blood cells, including abnormal ones, and red blood cell casts - a notable sign of kidney injury. A kidney biopsy reveals crescentic glomerulonephritis and IgG deposition along the kidney filters' capillaries. The question here is about identifying the most likely targeted antigen in this patient's autoimmune response, given the provided options of collagen, double-stranded DNA, nucleolar protein, phospholipid, or proteins in neutrophil cytoplasm. PLANNING: Crimson urine, edema, and elevated creatinine levels usually suggest renal pathology. Notably, the presence of crescentic glomerulonephritis, dysmorphic RBCs, and RBC casts points towards an aggressive kidney disease, possibly an autoimmune one. If we presume this condition is an autoimmune renal disease, we need to consider the type. Glomerulonephritis with linear IgG deposition suggests Goodpasture syndrome, which is caused by autoantibodies directed against the glomerular basal membrane's antigens. So, we need to identify which of the suggested antigens exists on the glomerular basement membrane. CLINICAL REASONING: As we suspect Goodpasture syndrome, we recognize that it's an autoimmune disease where the body produces antibodies against the glomerular basement membrane (GBM) and, sometimes, the alveolar basement membranes in the lungs too - which would account for his respiratory symptoms. This disease targets a collagenous type-IV protein in these membranes. This presents with rapid, progressive glomerulonephritis, featuring hematuria, edema, and often dyspnea and hemoptysis due to simultaneous lung involvement. The designation of crescentic refers to the visual shape of epithelial cell proliferation in the glomerulus secondary to damage. Similarly, linear IgG deposition, seen on immunofluorescence microscopy, is diagnostic of anti-GBM disease, like Goodpasture syndrome. CONCLUDING: Given these insights, we can connect the clinical presentation with the laboratory and pathology findings, linking them with Goodpasture syndrome's common clinical manifestation. These pieces of evidence include glomerulonephritis, elevated creatinine, red blood cell presence in urine, coupled with respiratory symptoms. As the suspected antibodies in Goodpasture syndrome targets type-IV collagen found in the glomerular and alveolar basement membranes, we can conclude that Collagen (A) should be the correct answer. RESPONSE: Goodpasture syndrome most likely prompts this patient's symptoms, given his clinical and histological findings. Antibodies in this condition target an antigen found in the basement membranes of both the kidneys and lungs, specifically type-IV collagen. Therefore the patient has antibodies directed against (A) Collagen.

**Facilitator** The 32-year-old male patient presents with symptoms of hemoptysis, dyspnea, ankle swelling, fatigue, and dark urine. His physical examination and laboratory findings indicate anemia, hypertension, decreased oxygen saturation, and kidney dysfunction. The kidney biopsy reveals crescentic glomerulonephritis and linear IgG deposition along the glomerular capillaries. These findings suggest an autoimmune disease, specifically Goodpasture syndrome, which is characterized by the body producing antibodies against the basement membrane in both the kidneys and lungs. This condition typically targets a type of collagen (type IV) located in the basement membranes, leading to the symptoms and lab findings observed in this case. Therefore, it is highly likely that the patient's antibodies are directed against (A) Collagen. Other conditions associated with the listed antigens, such as systemic lupus erythematosus (double-stranded DNA), scleroderma (nucleolar protein), antiphospholipid syndrome (phospholipid), and vasculitides like Wegener’s granulomatosis (proteins in neutrophil cytoplasm), do not align as well with the patient's clinical presentation.
