# Supplementary material for: Collaborative intelligence in AI: Evaluating the performance of a council of AIs on the USMLE
Source: PLOS Digit Health. 2025 Oct 9;4(10):e0000787. doi: 10.1371/journal.pdig.0000787 (PMC12510544; doi:10.1371/journal.pdig.0000787)
Supplement: S2 File — (ZIP) [file pdig.0000787.s002.zip › S2 Data Raw data files containing unprocessed question inputs and LLM outputs including transcripts of deliberations/STEP1_Question-92-1_Type-MC-NJ.docx]

**Facilitator** **[Round 1]:** A 58-year-old man with chronic obstructive pulmonary disease comes to the clinic with his wife for a follow-up examination. He has smoked one pack of cigarettes daily for 35 years. He has tried to quit smoking twice but was unsuccessful both times. At today’s visit, when the physician asks the patient about smoking cessation, he says he is not ready to do so. The patient’s wife states her husband’s smoking makes her cough and gives her chest tightness. Which of the following is the most appropriate physician statement? (A) ""Are there any reasons why you might want to quit smoking?"" (B) ""Are you aware that your lung condition is chronic at this point?"" (C) ""I'm sure you don't want your wife to suffer as a result of your smoking."" (D) ""The majority of your health issues would improve if you quit smoking."" (E) ""Why haven't you been able to stay off cigarettes?""

**AI_A:** RESTATING THE QUESTION: This scenario depicts a chronic smoker with COPD who is currently unprepared for smoking cessation. The situation asks for the most appropriate physician response. The options provided include various communication methods, including motivating readiness for change, information giving, expressing concern for another person's health impacted by smoking, signalling the benefits of quitting smoking, and enquiring about the challenges of quitting smoking. PLANNING: It's key to remember that medical ethics prioritize respect of the patient's autonomy and preference in terms of their treatment. Here, the patient has clearly stated he is not ready to quit smoking. Directly trying to push the patient towards smoking cessation (option B and D) might not respect the patient's autonomy. However, enhancing the patient's motivation toward change through motivational interviewing can be a beneficial approach (option A, C, and E). CLINICAL REASONING: Motivational interviewing is a conversational approach designed to help people discover their own interest in contemplating and making a change in their life (like quitting smoking). It includes open-ended questions, affirmation of patient's autonomy, reflective listening, and summarizing the conversation. Among the choices, option A, which asks about potential reasons to quit smoking, is the best representation of this technique. This can help the patient start realizing the reasons to consider smoking cessation. CONCLUDING: While all statements could be suitable depending on the exact context and the physician-patient relationship, the most universally appropriate approach in this particular situation would be motivational interviewing. The open-ended question about reasons to quit smoking respects the patient’s autonomy while inviting him to reflect on his situation and contemplate a positive change regarding his smoking habits. RESPONSE: Thus, the most appropriate physician statement is (A) "Are there any reasons why you might want to quit smoking?"

**AI_B:** Restating the Question: A 58-year-old man suffering from chronic obstructive pulmonary disease (COPD), a long-term lung disease that blocks airflow causing difficulty in breathing, has come to the clinic for a regular examination with his spouse. He has had a prolonged history of heavy smoking, and despite two attempts, he has been unsuccessful in his attempts to quit smoking. When his physician broaches the topic of smoking cessation at the current visit, the patient is resistant and asserts that he's not ready to quit. His wife mentions her own health complaints related to his smoking, which include cough and tightness in her chest. Given this scenario, the question requires us to identify the most suitable statement the physician can make. Planning: Here, the assumptions are that the patient's resistance to smoking cessation might be related to lack of motivation, denial of health effects, addiction, or lack of awareness of the impact of his smoking on his wife's health. The physician's role is to assess the patient's readiness to change, his understanding of his condition, and effect on the people around him especially his wife in this case, his motivation to quit, and understanding of barriers to cessation. The clinically prioritized assumption will be the patient's lack of motivation to quit smoking, as motivation is a key factor in successful cessation. Clinical Reasoning: Issues like COPD are generally linked to individual behavior and lifestyles, with smoking being one of the leading causes. The COPD patient’s wife is also showing symptoms, which may very likely be due to secondhand smoke exposure, a concern that needs to be addressed but without blaming the patient. Among the options, 'A' seems to align with the principle of Motivational Interviewing (MI). MI engages clients in conversations about their motivations and commitment to change with a focus on building intrinsic motivation and considering patients' autonomy. Concluding: In this situation, when dealing with a patient not yet ready to quit smoking, the physician should employ a motivational interviewing-based approach that increases the patient's motivation to quit. Such approach focuses on giving the patient autonomy rather than giving direct advice. Response: Therefore, the question "Are there any reasons why you might want to quit smoking?" (Answer option A) is the most appropriate physician's statement in this context. It helps explore the patient's perspective, aids in recognizing personal reasons that might motivate him to quit smoking and provides him a sense of autonomy to make his own decisions, thereby improving his intrinsic motivation towards smoking cessation.

**AI_E:** 1) RESTATING THE QUESTION: The issue at hand involves a 58-year-old man with chronic obstructive pulmonary disease (COPD), a long-term lung condition characterized by obstruction of airflow, which has a direct link with long-term tobacco smoking. Despite his condition and multiple attempts to quit smoking, the patient is unwilling to cease tobacco use at his current stage. His habit evidently impacts his wife negatively, inducing cough and chest tightness. The question asks what the most fitting physician statement would be in addressing the patient's smoking cessation reluctance given the options provided. 2) PLANNING: Here, the significant assumptions include the man’s knowledge level regarding his health condition and its relation to smoking, and his motivation towards quitting, knowing that his smoking habit is affecting not only his health but also his wife's. The possible assumption to evaluate critically regards how effectively a particular method of addressing his reluctance to quit smoking might motivate him towards cessation. 3) CLINICAL REASONING: Considering the principles of motivational interviewing—a patient-centered method for enhancing intrinsic motivation to change through exploring and resolving ambivalence—and the patient's autonomy, reiterating the negative consequences of smoking or blaming the patient for his inability to quit might not be the most effective approach. Option (A), which asks if there are reasons the patient might want to stop smoking, allows space for a personalized approach to smoking cessation. Through this approach, the physician can enable the patient to accentuate the pros versus cons of quitting, enhancing motivation for change driven by the patient's values and perspectives rather than imposed by the physician. 4) CONCLUDING: Thus, creating an open dialog that fosters self-motivation could potentially induce a positive attitude towards smoking cessation. This approach could also help tailor a cessation plan that aligns with the patient's personal motivations, potentially improving its efficacy. 5) RESPONSE: Therefore, based on evaluating the options present, the most suitable physician statement at this point would be: (A) "Are there any reasons why you might want to quit smoking?" as it opens a patient-centered, empathetic, and non-judgemental conversation that could potentially enhance the patient's intrinsic motivation to quit smoking.

**AI_C:** (1) RESTATING THE QUESTION: We are given a scenario involving a 58-year-old man with chronic obstructive pulmonary disease (COPD), which is a type of obstructive lung disease known to be caused by long-term exposure to irritating gases, most often from cigarette smoke. He has a long history of smoking, and previous attempts to quit have not been successful. During a recent clinical visit, when asked about his intentions to quit, he affirms he is not ready. His wife also indicates that his smoking habit is causing her discomfort, manifesting as cough and chest tightness. The question is to determine which statement from the physician would be the most appropriate in light of these circumstances. (2) PLANNING: Several assumptions could be made in this situation: a) the patient does not see any reasons to quit smoking b) the patient is not aware of the chronic aspect of his lung condition c) the patient does not consider his wife's discomfort seriously enough to quit smoking d) the patient doesn’t fully comprehend the overall health benefits of quitting smoking e) the patient does not understand why his attempts to quit smoking in the past have been unsuccessful. Upon evaluating these assumptions, it seems the most clinically important assumption to address would be related to the patient's motivation to quit smoking (option A), given that motivation plays a significant role in smoking cessation. (3) CLINICAL REASONING: When discussing smoking cessation, it is crucial to approach the conversation with empathy and open-mindedness. Directly challenging the patient about his decisions can often lead to resistance and disengagement. Furthermore, while the negative health implications of smoking are essential for the patient to understand, the physician should also encourage the patient to recognize personally relevant reasons to quit smoking as these can serve as powerful motivators. (4) CONCLUDING: Therefore, introducing the discussion in a manner that allows the patient to discover his own reasons to quit, rather than imposing external considerations, may prove more effective. This strategy aims to foster a sense of autonomy and enhance internal motivation. (5) RESPONSE: Considering these points, the most appropriate physician statement would be (A) "Are there any reasons why you might want to quit smoking?" This question allows the patient to contemplate his personal motivations for smoking cessation.

**AI_D:** RESTATING THE QUESTION: In essence, following the follow-up examination of a 58-year-old male patient who has chronic obstructive pulmonary disease (COPD) and has been a pack-a-day smoker for 35 years, we are asked to find the best response for the physician to give when the patient demonstrates reluctance to quit smoking. Despite the fact that his habit negatively affects his wife's health, he remains resistant to smoking cessation. PLANNING: The main topic here seems to be about an effective physician-patient communication which undoubtedly is vital in patient management, particularly as it pertains smoking cessation. The goal is to motivate, and empower the patient without passing on judgment. It's abundantly clear that smoking cessation would significantly benefit the patient's health both directly (improvement in lung function) and indirectly (reducing passive smoking hazards to his wife). Considering these facts, it's important to determine the right approach to discuss the issue with the patient so that communication lines remain open and the right kind of influence can be exercised. CLINICAL REASONING: Option A is about the doctor asking the patient if he has any personal reasons for wanting to quit, which opens up the conversation for identifying motivational factors, which could be very helpful. Option B emphasizes the chronic nature of his lung condition, though this fact is likely knowledge he already possesses due to his COPD diagnosis. Option C, however, may come across as blaming the patient for his wife's health problems, which may put him on the defensive and stop productive conversations. Option D emphasizes the benefits of quitting smoking but can come across as lecturing without taking into consideration the patient's readiness to quit. Lastly, option E points to past unsuccessful attempts and can be discouraging rather than encouraging. CONCLUDING: Looking at all the options, it seems important to take an approach that creates dialogue about the patient's interests and motivations in a non-accusing, non-judgmental way. This protects the patient-doctor relationship and opens the door for further discussion on smoking cessation. Discouraging or blaming a patient for failed attempts could deter him from engaging in these discussions or even seeking help in the future. RESPONSE: Therefore, the most appropriate physician statement would be, "Are there any reasons why you might want to quit smoking?" (Option A) as it encourages the patient to reflect on his own motivations and reasons for cessation, opening up the conversation for possible motivational interviewing therapy, a proven effective tool in aiding smoking cessation.

**Facilitator** The consensus among the AI responses is that the most appropriate physician statement in this scenario is (A) "Are there any reasons why you might want to quit smoking?" This approach aligns with the principles of motivational interviewing, a patient-centered method designed to enhance intrinsic motivation for change. It respects the patient's autonomy and invites him to reflect on his personal reasons for considering smoking cessation. This open-ended question avoids blaming the patient for his past unsuccessful attempts to quit or for the health issues of his wife, which could potentially lead to resistance or disengagement. Instead, it fosters a non-judgmental conversation that could potentially enhance the patient's intrinsic motivation to quit smoking. This approach also allows for a personalized plan for smoking cessation that aligns with the patient's values and perspectives, potentially improving its efficacy.
