# Supplementary material for: Collaborative intelligence in AI: Evaluating the performance of a council of AIs on the USMLE
Source: PLOS Digit Health. 2025 Oct 9;4(10):e0000787. doi: 10.1371/journal.pdig.0000787 (PMC12510544; doi:10.1371/journal.pdig.0000787)
Supplement: S2 File — (ZIP) [file pdig.0000787.s002.zip › S2 Data Raw data files containing unprocessed question inputs and LLM outputs including transcripts of deliberations/STEP1_Question-93-1_Type-MC-NJ.docx]

**Facilitator** **[Round 1]:** A 26-year-old man comes to the office because of a 1-week history of increased urinary frequency accompanied by excessive thirst. He says he has been urinating hourly. Physical examination shows no abnormalities. Serum chemistry studies are within the reference ranges. Urine osmolality is 50 mOsmol/kg H2O. After administration of ADH (vasopressin), his urine osmolality is within the reference range. The most likely cause of this patient's symptoms is dysfunction of which of the following structures? (A) Anterior pituitary gland (B) Bowman capsule (C) Glomerulus (D) Hypophysial portal system (E) Loop of Henle (F) Supraoptic nucleus

**AI_B:** RESTATING THE QUESTION: The young man concerned is experiencing polyuria (increased urinary frequency) and polydipsia (excessive thirst). His laboratory results show a clear delineation between low urine osmolality initially, which normalizes after administration of ADH. We are to identify the organ or structure most likely to be dysfunctional causing these symptoms. PLANNING: The main assumption is that there is a functional defect leading to improper urine concentration. Notably, while distal parts of the nephron like the Loop of Henle, the Bowman capsule, or the glomerulus are vital in urine concentration, it's important to note the utility of the hormone ADH in this process. ADH increases water reabsorption in the nephron, leading to more concentrated, less voluminous urine. Thus, a dysfunction in structures involved in ADH production or release could potentially explain the patient's symptoms. CLINICAL REASONING: The Anterior Pituitary gland is responsible for regulating a wide array of bodily functions, but it doesn't directly produce or release ADH. The Hypophysial Portal System plays a role in conveying hormones from the hypothalamus to the anterior pituitary - while dysfunction could lead to a variety of symptoms, it's not directly linked to ADH function in this context. The Supraoptic Nucleus, however, is a part of the hypothalamus that directly produces ADH. Dysfunction here could thus lead to decreased ADH and symptoms as described in the scenario. CONCLUDING: Therefore, assuming deficient ADH is the key to this patient's symptomology, and given the structures listed, we can reasonably conclude that dysfunction of the Supraoptic Nucleus in the hypothalamus (which produces ADH) is a plausible cause for the observed polyuria and polydipsia. RESPONSE: The most likely cause of this patient's symptoms is dysfunction of the (F) Supraoptic nucleus.

**AI_C:** (1) RESTATING THE QUESTION: A young adult male has been experiencing polyuria and polydipsia—increased urination and thirst—for a week, with no other abnormal physical findings. His serum chemistry is normal but his urine concentration is low. However, after receiving ADH, also known as vasopressin, his urine concentration becomes normal. We need to identify which structure is most likely malfunctioning causing his symptoms. (2) PLANNING: The assumptions are that the structures involved in ADH synthesis, secretion, and target organ response are functioning correctly. An alternate assumption is that one of these systems is dysfunctional. By questioning the assumption that the patient's ADH system is normal, although his urine osmolality became normal after receiving external ADH, indicates a possible deficiency or abnormality in his own production or release of ADH. (3) CLINICAL REASONING: Vasopressin, or ADH, primarily regulates the body's retention of water by increasing water permeability in the collecting ducts within the kidneys, allowing more water resorption and leading to more concentrated urine. ADH is synthesized in the hypothalamic neurons, specifically in the supraoptic and paraventricular nuclei, and transported to the posterior pituitary for storage and release. Normal response to administered vasopressin indicates the target kidney structures, including the loop of Henle, glomerulus, and Bowman's capsule are functioning normally. (4) CONCLUDING: Therefore, the problem is more likely related to the production or secretion of ADH rather than its target organ response. If there's a dysfunction in the organs involved in producing or releasing ADH, it could lead to conditions like central diabetes insipidus, where the body can't retain water, causing excessive thirst and frequent urination. (5) RESPONSE: (F) Supraoptic nucleus is the most plausible dysfunctioning structure in this scenario as it is one of the areas responsible for ADH synthesis and transport. Its dysfunction would lead to inadequate production or release of ADH, causing symptoms similar to those described by the patient, but the kidney's normal response to administered ADH, as indicated by normalization of urine osmolality post-administration.

**AI_D:** (1) RESTATING THE QUESTION: Essentially, the question is asking about the possible malfunctioning or dysfunctional structure in a 26-year-old patient who has been experiencing frequent urination and an increased thirst for a week. The relevant test results indicate that his urine osmolality is low but returns to normal after administration of ADH (antidiuretic hormone), also known as vasopressin. ADH is a hormone that controls the amount of water reabsorbed by the kidneys, which can impact urine concentration. (2) PLANNING: The assumptions surrounding this case include the potential dysfunction of structures involved in the production, transportation, or effect of ADH. These structures include the hypothalamus, where ADH is synthesized; the posterior pituitary gland, which stores and releases ADH; the kidneys, which respond to ADH to concentrate urine; and the supraoptic and paraventricular nuclei of the hypothalamus that produces ADH. As the issue resolves after administration of exogenous ADH, it suggests an issue in the production or release of the hormone. (3) CLINICAL REASONING: Let's assess each of these structures and their relevance to the symptoms: (A) The anterior pituitary gland produces various hormones but not ADH, so it seems less likely a contributor to the problem. (B) The Bowman's capsule is part of the kidney structure involved in urine formation, but it doesn't respond directly to ADH, so it's also unlikely. (C) The glomerulus is another part of the kidney involved in the initiation of urine formation, but its function is normal as indicated by the normal serum chemistry studies. (D) The hypophysial portal system is involved in the transportation of hormones from the hypothalamus to the anterior pituitary, but it does not pertain to ADH action, ruling it out as a cause. (E) The loop of Henle does respond to ADH, but if it were dysfunctional, administration of exogenous ADH wouldn't normalize the urine osmolality. (F) The supraoptic nucleus of the hypothalamus produces ADH. Dysfunction here would decrease ADH output, leading to the symptoms and the response to exogenous ADH. (4) CONCLUDING: Upon assessing each option, the supraoptic nucleus is the most plausible origin of dysfunction. It's responsible for ADH synthesis, and disruption in its function would account for the symptoms and their response to exogenous ADH. (5) RESPONSE: The most likely cause of this patient's symptoms is a dysfunction in the Supraoptic nucleus (F).

**AI_E:** RESTATING THE QUESTION: The patient in this case, a 26-year-old male, has been experiencing an increase in urination frequency along with excessive thirst for the past week. His physical examination does not indicate any abnormal findings, and his serum tests appear normal. His urine osmolality, however, is unusually low, at only 50 mOsmol/kg H2O, which improves to within the normal range following ADH administration. This scenario seeks to identify which part of the man's body is most likely malfunctioning to cause his symptoms. The terminology used here refers to various body structures involved in the regulation and secretion of urine, and ADH or vasopressin, a hormone concentrated in the hypothalamus that modulates water balance in the body by influencing the kidneys. PLANNING: I should question if this patient's symptoms can be explained by a dysfunction of the structures provided in the choices: anterior pituitary gland, Bowman capsule, glomerulus, hypophysial portal system, loop of Henle, or supra-optic nucleus. I should also consider the fact that ADH administration normalized the patient's urine osmolality and what effect this has on the potential source of dysfunction. My conclusion will rely heavily on understanding the roles of these structures in urine formation and water balance. CLINICAL REASONING: The anterior pituitary gland controls a variety of endocrine functions but doesn't directly influence urine concentration. The Bowman capsule and the glomerulus are involved in urine formation, but they should not respond to ADH by increasing urine osmolality. Dysfunction of the hypophysial portal system would impact communication between the hypothalamus and pituitary, but wouldn't directly improve with ADH administration. The loop of Henle is part of the kidney nephron and plays a pivotal role in establishing a concentration gradient to enable urine concentration, but again, wouldn't directly respond to ADH administration. On the other hand, the supraoptic nucleus of the hypothalamus produces ADH, which then travels through axons to the posterior pituitary where it is stored and stimulated for release. ADH acts on the kidneys to increase water reabsorption, which results in more concentrated urine. CONCLUDING: Taking into consideration all the information about the patient's condition and response to ADH, the symptoms presented, increased urination, and excessive thirst, are indicative of diabetes insipidus, a condition characterized by insufficient ADH. This is reinforced by the low urine osmolality and its normalization following ADH administration. Thus, a dysfunction in the supraoptic nucleus, which is the area where ADH production occurs, would most plausible cause these symptoms. RESPONSE: (F) Supraoptic nucleus would most likely be the dysfunctional structure causing this 26-year-old man's excessive urination and thirst symptoms, given the low urine osmolality that returns to normal ranges following the administration of ADH.

**AI_A:** 1) RESTATING THE QUESTION: The query at hand requires the identification of an anatomical dysfunction that would most likely explain the symptoms presented by a young man with a history of increased urinary frequency, excessive thirst, and abnormally low urine osmolality that normalizes following administration of a hormone, vasopressin. Vasopressin, also referred to as antidiuretic hormone (ADH), is primarily responsible for water retention in the body and its increased secretion or action can cause urine to concentrate, that is its osmolality to increase, thus, reducing urinary frequency. 2) PLANNING: Keeping in mind the options for answer, we need to prioritize the structures that are most correlated with the function of ADH in the body. A few of the listed options are associated with the kidneys (Bowman capsule, glomerulus, and loop of Henle), which indeed significantly play their part as ADH acts on the renal tubules to promote water reabsorption. We, initially, can't rule out these options. As for the other options, we have the anterior pituitary gland, the hypophysial portal system, and the supraoptic nucleus. The supraoptic nucleus in the hypothalamus synthesizes ADH that is then transported to and stored in the posterior pituitary gland for release. However, it seems the patient responded to exogenous ADH, eliminating ADH deficiency as the potential cause. Therefore, the pathology may be hypothesized to exist at a peripheral site where ADH acts, rather than where it is produced. 3) CLINICAL REASONING: The supraoptic nucleus and the anterior pituitary gland are responsible for releasing hormones that regulate body functions, however, the associated disorder from their dysfunction would most likely not react to vasopressin administration. As for the renal structures, the loop of Henle can be an attractive answer because ADH acts on the collecting ducts and the distal part of the loop of Henle, making these structures very crucial for its effect. However, the glomerulus and the Bowman's capsule would have less direct interaction with this hormone thus we can rule out these options. 4) CONCLUDING: Based on our clinical reasoning, it stands to reason that the major dysfunction leading to the patient's symptoms is likely to be present in the nephron's structure most involved with ADH action. 5) RESPONSE: Therefore the most likely cause of this patient's symptoms is dysfunction of the (E) Loop of Henle.

**Facilitator** **[Round 2]:** Summary: AI_A believes that the patient's symptoms are due to a dysfunction in the Loop of Henle, a part of the kidney where ADH acts to promote water reabsorption. AI_A argues that since the patient responded to exogenous ADH, the dysfunction is likely at a peripheral site where ADH acts, rather than where it is produced. AI_B, AI_C, AI_D, and AI_E all believe that the dysfunction is in the Supraoptic nucleus, a part of the hypothalamus that produces ADH. They argue that the patient's response to exogenous ADH suggests a problem with the production or release of the hormone, rather than its action at the kidneys. Key Differences: The key difference in the perspectives lies in the interpretation of the patient's response to exogenous ADH. AI_A interprets this as evidence that the dysfunction is in the kidney, where ADH acts, while AI_B, AI_C, AI_D, and AI_E interpret it as evidence that the dysfunction is in the hypothalamus, where ADH is produced. Question for Council Members: Given the patient's response to exogenous ADH, how can we definitively determine whether the dysfunction is in the production/release of ADH (in the hypothalamus) or in the action of ADH (in the kidneys)?. Based on your preceding response, how would you answer the original question: [A 26-year-old man comes to the office because of a 1-week history of increased urinary frequency accompanied by excessive thirst. He says he has been urinating hourly. Physical examination shows no abnormalities. Serum chemistry studies are within the reference ranges. Urine osmolality is 50 mOsmol/kg H2O. After administration of ADH (vasopressin), his urine osmolality is within the reference range. The most likely cause of this patient's symptoms is dysfunction of which of the following structures? (A) Anterior pituitary gland (B) Bowman capsule (C) Glomerulus (D) Hypophysial portal system (E) Loop of Henle (F) Supraoptic nucleus]

**AI_E:** RESTATING THE QUESTION: This question presents a case of a 26-year-old male suffering from polyuria (frequent urination) and polydipsia (excessive thirst), typical symptoms of diabetes insipidus. The urine tests reveal a low osmolality and serum chemistry studies are normal. Upon administration of vasopressin (also known as ADH or antidiuretic hormone), his urine osmolality normalizes. We are asked to determine the source of dysfunction leading to these symptoms based on given options. PLANNING: The question revolves around understanding the pathophysiology of diabetes insipidus and the role of ADH in water regulation by kidneys. Understanding how ADH works and where it's produced and secreted can provide insight into the structural dysfunction. This involves questioning the assumption that the problem is with ADH production/secretion vs its action in the kidneys. Since the administration of ADH normalized the osmolality, the defect likely lies with the production or release of ADH, indicating a central origin. CLINICAL REASONING: ADH is synthesized in the hypothalamus, specifically the supraoptic nucleus, and transported to the posterior pituitary gland for storage and secretion. ADH acts on the kidneys to increase water reabsorption, preserving body water while excreting solutes. If there is a problem with the action of ADH at the kidneys, exogenous ADH wouldn't have corrected the urine osmolality. Since it did, it suggests the problem lies with the production or secretion of ADH, which takes place in the hypothalamus. CONCLUDING: Incorporating aforementioned clinical reasoning and medical knowledge about the role and actions of ADH, the most likely underlying dysfunction must reside in the site of ADH production or release, favoring the hypothalamus, particularly the supraoptic nucleus. RESPONSE: The most likely dysfunction leading to this patient's manifestation of symptoms (i.e., polyuria and polydipsia, low urine osmolality that corrects with exogeneous ADH) is likely to lie in the Supraoptic nucleus (F).

**AI_C:** Restating the Question: The question asks about the likely source of a 26-year-old man's symptoms of increased urinary frequency and extreme thirst. In other words, these are symptoms of polyuria and polydipsia. His urine osmolality is lower than usual, indicating diluted urine. However, when given vasopressin (also known as antidiuretic hormone or ADH), his urine osmolality normalizes. The question wants to identify which anatomical structure's dysfunction would best explain these symptoms. Planning: We need to consider the role of each of the given options in urine production and volume maintenance. The anterior pituitary gland, for instance, releases hormones but not ADH. The Bowman's capsule is involved in initial filtration in the kidney, but not directly in concentration or dilution of urine. The Glomerulus primarily filters blood to form urine. The Hypophysial portal system carries hormones from the hypothalamus to the anterior pituitary. The Loop of Henle is responsible for reabsorption of water and electrolytes to concentrate the urine. The Supraoptic nucleus produces ADH, which promotes water reabsorption in the kidney. Clinical Reasoning: The fact that the patient's urine osmolality normalizes after the administration of vasopressin suggests that the renal response to vasopressin is intact. This implies that his symptoms could be due to deficient ADH production or release rather than a problem with ADH action at the kidney (so the problem is likely upstream from the Loop of Henle, Bowman's Capsule, Glomerulus). Given that the available options include the hypothalamic nucleus (the Supraoptic nucleus), which is involved in ADH production, this seems like the most plausible source of the problem. Concluding: Considering that vasopressin acts at the level of the kidney and its exogenous administration illuminated an improvement in patient’s condition, it can be inferred that the problem is likely not at the level of the kidney where ADH functions but at the level where it is produced. Response: Therefore, I would hypothesize that the primary dysfunction is likely in the supraoptic nucleus (F). This part of the hypothalamus produces ADH and a dysfunction would lead to decreased production or release of this hormone, subsequently causing symptoms of diabetes insipidus being polyuria and polydipsia that improved upon administration of exogenous vasopressin.

**AI_B:** Restating the Question: To put it simply, this 26-year-old man has been experiencing frequent urination and thirst for the past week. His exams and lab results do not show any abnormalities, except for a lower urine osmolality. Still, his urine osmolality returns to normal after he is given ADH. We are asked to identify what part of his body is not working correctly, causing these symptoms. Planning: It may be assumed that the frequent urination and excess thirst possibly hint at diabetes insipidus, a condition characterized by a deficiency in or resistance to vasopressin (also known as ADH). The choice of the most plausible assumption will hinge on interpretation of key symptom: the patient’s response to administration of ADH. The reactions to this exogenous ADH can imply either dysfunction in ADH production or a problem at the site of its action. Clinical Reasoning: Considering that the patient responded favorably to the administered ADH, it seems plausible that the issue lies in the production or secretion of the hormone instead of its action at the kidneys. If the abnormality was within the kidneys, such as the Loop of Henle (where ADH acts to increase permeability and allow water reabsorption from urine), administering exogenous ADH may not have corrected his urine osmolality. Nevertheless, the normal functioning of this system requires adequate synthesis and release of ADH from the hypothalamus and posterior pituitary. These steps involve two structures; the hypothalamic supraoptic nucleus (which synthesizes ADH) and the hypophysial portal system (which aids in transportation of the hormone). In this case, it is likely that ADH secretion from the supraoptic nucleus is impaired. Concluding: Based on the evidence at hand, we can conclude that the dysfunction most likely lies in the supraoptic nucleus, a part of the hypothalamus responsible for producing ADH. This would support the existence of a central defect leading to the diabetes insipidus-like symptoms. Response: Hence, in response to the original question, I would say that the most probable cause of this patient's symptoms is dysfunction of the supraoptic nucleus (F), where ADH is produced.

**AI_A:** (1) RESTATING THE QUESTION: This 26-year-old man has symptoms of increased urination and excessive thirst, symptoms commonly seen in conditions with impaired water balance. Following ADH administration, his urine osmolality becomes normal. The question asks which structure is most likely causing his symptoms. When addressing this question, we might consider whether the problem is in the production or the action of ADH. (2) PLANNING: The initial assumption might be that there is a dysfunction in the ADH production system because the patient's symptoms improve with ADH administration. This implies a deficiency in endogenous ADH. However, another assumption could be a peripheral resistance to ADH, which when overwhelmed by a high dose of exogenous ADH, allows the system to respond appropriately. (3) CLINICAL REASONING: If the problem were in the production of ADH, we would expect the supraoptic nucleus (F), where ADH is synthesized, or the hypothalamic-hypophyseal system (D), where ADH is transported and released, to be the dysfunctional structures. However, given that the man's urine becomes concentrated after ADH administration, we can deduce that there is some ability for ADH to have an effect, suggesting that there isn't a complete absence of the hormone. On the other hand, if the issue were peripheral resistance to ADH, then the Loop of Henle (E) might be the problematic structure, being a primary site of ADH action where it promotes water reabsorption to concentrate the urine. Because the patient's urine is dilute despite his dehydration, it suggests that the kidney (specifically the Loop of Henle) isn't responding properly to circulating ADH, leading to excessive water loss. (4) CONCLUDING: Weighing these two possibilities, it seems more likely that the problem lies with the action, not the production, of ADH. The fact that the patient's urine osmolality becomes normal after ADH administration suggests that there isn't a complete inability to produce ADH. Instead, there might be a degree of resistance to its action that can be overcome with high enough doses. (5) RESPONSE: Therefore, the most likely cause of this patient's symptoms is dysfunction of the Loop of Henle (E), where there may be an impaired response to ADH causing the presenting symptoms of excessive urination and thirst.

**AI_D:** 1) RESTATING THE QUESTION: Essentially, we are trying to determine what structure is most likely responsible for causing a young man's continuous thirst and hourly urination. His urine's osmolality is 50 mOsmol/kg H2O, but the osmolality falls within the reference range after vasopressin has been administered. Vasopressin, also known as antidiuretic hormone (ADH), is a hormone responsible for fluid balance in the body. Furthermore, osmolality is a measure of solute concentration. An unusually low osmolality would imply a failure to concentrate urine due to dysfunctions in either ADH production or its action. 2) PLANNING: Several assumptions could be made from this case. One assumption is that the problem could be in the production of ADH, which occurs in the supraoptic nucleus and is then stored and released by the posterior pituitary gland. The kidney, specifically the Loop of Henle (part of renal tubular system), where ADH acts to promote water reabsorption could also be defective. In this case, the failure of response to endogenous ADH but positive response to exogenous ADH leads to the hypothesis that the primary problem may lie within the production and release of ADH, rather than in its renal action. 3) CLINICAL REASONING: Reviewing the symptomatology and biochemical findings, the patient has polyuria and polydipsia. His urine is highly diluted as evidenced by abnormally low urinary osmolality. Logically, if an imbalance in ADH were the issue, then problems with the structures involved in the production, storage, and regulation of ADH should be evaluated first. As the patient responded to exogenous ADH, but the signs and symptoms of ADH deficiency persisted previously, it is reasonable to assume an issue with the production or release of ADH. 4) CONCLUDING: A step-by-step clinical thought process points towards the Supraoptic nucleus. This structure in the hypothalamus is responsible for the synthesis of ADH. ADH is then transported and stored in the posterior pituitary gland from where it is released as needed. As the patient's condition improved significantly with the administration of exogenous ADH, it is suggestive of a dysfunction in the Supraoptic nucleus where ADH is initially produced, making the system unable to respond adequately to endogenous production demand. 5) RESPONSE: Therefore, from the clinical findings and anatomical and physiological exploration, the most likely cause of this patient's symptoms is dysfunction of the Supraoptic nucleus (F). Additionally, ADH is not produced or regulated by the anterior pituitary gland, Bowman capsule, glomerulus, hypophysial portal system, or Loop of Henle, thus eliminating options A, B, C, D, and E.

**Facilitator** **[Round 3]:** Summary: AI_A believes that the patient's symptoms are due to a dysfunction in the Loop of Henle, where there may be an impaired response to ADH. This is based on the assumption that the problem lies with the action, not the production, of ADH, as the patient's urine osmolality becomes normal after ADH administration. AI_B, on the other hand, suggests that the dysfunction most likely lies in the supraoptic nucleus, a part of the hypothalamus responsible for producing ADH. This is based on the patient's favorable response to the administered ADH, implying a problem with the production or secretion of the hormone. AI_C also hypothesizes that the primary dysfunction is likely in the supraoptic nucleus. This is based on the fact that the patient's urine osmolality normalizes after the administration of vasopressin, suggesting that the renal response to vasopressin is intact and the problem is likely upstream from the Loop of Henle. AI_D agrees with AI_B and AI_C, suggesting that the most likely cause of the patient's symptoms is dysfunction of the Supraoptic nucleus, where ADH is initially produced. This is based on the patient's significant improvement with the administration of exogenous ADH. AI_E concurs with AI_B, AI_C, and AI_D, stating that the most likely dysfunction leading to the patient's symptoms is in the Supraoptic nucleus. This is based on the fact that exogenous ADH corrected the urine osmolality, suggesting a problem with the production or secretion of ADH. Question to clarify disagreements: Given that the patient's urine osmolality normalizes after the administration of ADH, how can we definitively determine whether the problem lies in the production or the action of ADH? What additional tests or information would help us confirm whether the dysfunction is in the Supraoptic nucleus or the Loop of Henle?. Based on your preceding response, how would you answer the original question: [A 26-year-old man comes to the office because of a 1-week history of increased urinary frequency accompanied by excessive thirst. He says he has been urinating hourly. Physical examination shows no abnormalities. Serum chemistry studies are within the reference ranges. Urine osmolality is 50 mOsmol/kg H2O. After administration of ADH (vasopressin), his urine osmolality is within the reference range. The most likely cause of this patient's symptoms is dysfunction of which of the following structures? (A) Anterior pituitary gland (B) Bowman capsule (C) Glomerulus (D) Hypophysial portal system (E) Loop of Henle (F) Supraoptic nucleus]

**AI_D:** Since this patient has been facing excessive urination and thirst for a week, it is significant to highlight that such symptoms translate clinically to polyuria and polydipsia. Polyuria and polydipsia could often be symptoms of diabetes insipidus, a condition marked by a disturbance in the body's water homeostasis mechanism. The subject's urine osmolality, which is lower than normal, substantiates this idea, as does the fact that following administration of vasopressin (or anti-diuretic hormone-ADH), his urine osmolality is regularized. In essence, these symptoms decipher to inadequate water reabsorption, leading to a higher urine output and subsequent compensatory increased water intake. When investigating such a case, it is crucial to consider two major types of diabetes insipidus: Central Diabetes Insipidus, where there is a defect in the production or secretion of ADH; and Nephrogenic Diabetes Insipidus, where the kidneys poorly respond to ADH. Therefore, it becomes necessary to re-evaluate whether the malfunction is in ADH's production or its action to correctly diagnose the patient's condition. The detail that when administered with ADH, the patient's urine osmolality improved and normalized could be indicative of the problem with the production or secretion of ADH. If the kidneys could respond to exogenous vasopressin (ADH), thus readjusting urine concentration, it explains that the kidneys have the capacity to respond appropriately to ADH, ruling out nephrogenic diabetes insipidus. Arguably, the main question here revolves around the site of ADH production. To refresh ourselves, ADH is primarily synthesized by the supraoptic and paraventricular nuclei in the hypothalamus. It then gets transported to the posterior pituitary gland from where it is released. Thus, a dysfunction in the supraoptic nucleus can lead to inadequate ADH production and consequently, central diabetes insipidus. In conclusion, taking into account the clinical context from aforementioned reasoning, the symptoms of the presented patient could be suggestive of a dysfunction in the Supraoptic nucleus (choice F). This rationale is supported by noticing the patient's lower urine osmolality normalizing after the administration of ADH, pointing towards an issue in the ADH pathway upstream of the kidney.

**AI_C:** Restating the question: The patient presents with a week-long history of increased frequency of urination and an uncommonly excessive thirst. These are symptoms of polyuria and polydipsia, respectively. Despite hourly urination and abnormal thirst, the patient's physical examination and serum chemistry studies are normal. However, his urine osmolality, which defines the concentration of solutes in the urine, is significantly low at 50 mOsmol/kg H2O. After getting ADH, the patient's urine osmolality corrects itself and becomes normal. We are trying to identify the structure that is not functioning correctly as the most probable source of the patient's symptoms. Planning: Possible assumptions could revolve around the involvement of the anterior pituitary gland, which secretes ADH in response to a signal from the hypothalamus; the Bowman capsule or glomerulus, which are involved in urine formation; the hypophysial portal system, the communication between the hypothalamus and the anterior pituitary; the loop of Henle, where urine is concentrated under the influence of ADH; or finally the supraoptic nucleus, which is part of the hypothalamus and involved in the synthesis of ADH. Clinical Reasoning: The patient's symptoms of polyuria and polydipsia are hallmarks of diabetes insipidus, an illness characterized by the inability to appropriately concentrate urine. Central diabetes insipidus, which is caused by a lack of ADH, is differentiated from nephrogenic diabetes insipidus, which results from renal resistance to ADH, by the patient's ability to concentrate urine after being administered ADH. In this case, the patient's normalization of urine osmolality after ADH administration implies that the dysfunction is upstream of the action of ADH, potentially in the area of ADH synthesis or secretion. Concluding: The problem seems to be the capacity to produce or secrete ADH rather than the function or physiology of the kidneys. Considering this, it would be reasonable to conclude that the patient's supraoptic nucleus, the site for ADH synthesis in the hypothalamus, is the most likely dysfunctional structure. Response: The most probable cause of this patient's symptoms is dysfunction of the supraoptic nucleus (F). The patient's ability to normalize urine osmolality with exogenous ADH points to the issue being with ADH production or secretion rather than its action in the nephron. ADH is synthesized in the supraoptic nucleus and is then transported to and released from the posterior pituitary gland.

**AI_A:** RESTATING THE QUESTION: The issue at hand has to do with a young man who's been experiencing a frequent urge to urinate coupled with extreme thirst. Given that an increase in the urine's concentration occurred after administration of vasopressin, we want to pinpoint where the likely dysfunction is located. Multiple options have been provided, which include Anterior pituitary gland, Bowman capsule, Glomerulus, Hypophysial portal system, Loop of Henle and Supraoptic nucleus. PLANNING: Several assumptions can be made from the given information. If we consider that the urine osmolality was initially low but changes to the normal range after the administration of ADH, it is safe to assume that the ADH production or secretion might be the underlying problem, not so much of the ADH action. Alternatively, we can presume that the patient's kidneys are responding well to the administered vasopressin (a form of ADH). This supports the possible dysfunction of a structure involved in ADH production rather than a disruption in the urinary system structure responding to this hormone. CLINICAL REASONING: ADH is synthesized in the hypothalamic supraoptic and paraventricular nuclei (option F), and then transported to the posterior pituitary gland for storage and release. In the healthy state, low plasma osmolality suppresses the secretion of ADH, leading to the production of dilute urine (low osmolality), while high plasma osmolality stimulates the release of ADH, resulting in the conservation of water and the production of concentrated urine (high osmolality). So, this patient's initial presentation of increased urinary frequency with low urine osmolality suggests that he might have impaired ADH action until exogenous ADH was administered, and the urine osmolality became normal. This shows that ADH can still work effectively to concentrate the urine, therefore, the problem could be with the synthesis or secretion of ADH, implying a hypothalamic or posterior pituitary gland disorder. CONCLUDING: Considering the evidence provided and drawing from the foundational theories, we can conclude that the dysregulation has a high likelihood of being localized in the supraoptic nucleus (option F). This node is principally involved in the production of the Antidiuretic Hormone. Given that the patient's symptoms improved with the administration of Vasopressin (synthetic ADH), this suggests that the root of the man's problem could be tied to deficient ADH production or secretion from the hypothalamus or posterior pituitary. RESPONSE: Consequently, the dysfunction leading to this patient's symptoms of constant urination and excessive thirst, which ameliorate with the administration of ADH, is likely in the Supraoptic nucleus (F) where the hormone is produced.

**AI_E:** Restating the question and defining terms: The question is centered on a patient presenting with polyuria and polydipsia, but normal serum chemistry and the ability to concentrate his urine upon administration of vasopressin. The question seeks to identify which anatomical structure is most likely dysfunctional in this scenario. Vasopressin or Antidiuretic hormone (ADH) is a neurohormone synthesized in the hypothalamus (specifically the supraoptic nucleus) and transported via the hypothalamohypophyseal tract to the posterior pituitary from where it is released into the bloodstream. This hormone primarily targets renal tubules (principally the loop of Henle and the distal and collecting tubules) to promote water reabsorption. Planning: The fundamental assumption to be established here is whether the issue lies in production, secretion, or function of vasopressin. Given the normalization of urine osmolality after administration of ADH, the kidneys appear to be responding to the hormone appropriately. This would imply the problem lies with ADH production or secretion rather than renal response. Clinical Reasoning: If the issue were in the renal response, the Loop of Henle or other renal structures like Bowman's capsule or glomerulus would be suspect. However, given the response to administered vasopressin, it seems unlikely that these structures are the primary problem. The supraoptic nucleus, where ADH is produced, could be suspect if there was an issue in ADH production. On the other hand, if the issue was with the secretion of ADH from the posterior pituitary to the blood, hypothalamic-pituitary connections like the hypophysial portal system would be implicated. Concluding: Given the patient's presentation with symptoms of diabetes insipidus, that corrects with administration of vasopressin, it suggests that either ADH production (Supraoptic nucleus) or ADH secretion (Hypophysial portal system) is impaired. The anterior pituitary gland is less likely to be dysfunctioning here, as it does not secrete ADH. However, the supraoptic nucleus and hypophysial portal system are anatomically close, and both function in the production and release of ADH. Response: Based on the patient's response to the administration of exogenous ADH, it appears there is a deficiency in endogenous ADH production or release, rather than an inability of the kidneys to respond to ADH. This would implicate the structures involved in the production and release of ADH which includes the Supraoptic nucleus and Hypophysial portal system. Given that the latter is involved primarily in the transportation of hormones from the hypothalamus to the anterior pituitary, and not directly in ADH transport to the posterior pituitary, the Supraoptic nucleus (F) would be the most likely choice.

**AI_B:** The question is inquiring about the most probable source of the malfunctions causing the symptoms in a 26-year-old male patient. The symptoms include increased frequency of urination marked by approximately hourly voiding and excessive thirst, occurring over a week. Normal physical examination and serum chemistry were found, while the urine osmolality is observed to be 50 mOsmol/kg H2O. Surprisingly, normal urine osmolality returns after the administration of vasopressin or ADH. We are asked to determine the dysfunctional structure responsible for these symptoms. The options provided are the anterior pituitary gland, Bowman capsule, glomerulus, hypophysial portal system, Loop of Henle, and supraoptic nucleus. One assumption here is that the problem may lie with the storing and secretion of ADH (vasopressin), a hormone that plays a crucial role in maintaining fluid homeostasis in the body. The low urine osmolality confirms the presence of dilute urine which, in normal circumstances, should have triggered the secretion of ADH to conserve water. However, after the exogenous administration of ADH, the urine osmolality improved, suggesting that ADH's action site, the renal tubules – particularly the Loop of Henle and the distal and collecting ducts – are responsive to ADH. This is another assumption: if the miscommunication, so to speak, occurred at the level of the kidney or where ADH acts (like the Loop of Henle), the exogenously administered ADH would not have resolved the problem. While the dysfunction of the Loop of Henle could lead to polyuria and polydipsia (increased thirst), this would most likely happen in conditions like renal tubular acidosis or nephrogenic diabetes insipidus – but in both conditions, the kidneys don't respond to ADH, and the urine osmolality wouldn't normalize after ADH administration, as it did in this case. So, the condition must involve a structure that has a role in ADH production or secretion. Considering this, the supraoptic nucleus, a structure located in the hypothalamus that synthesizes ADH, becomes a significant consideration. A malfunction here could impair the production or secretion of ADH, causing symptoms like polyuria and polydipsia as seen in conditions like neurogenic or central diabetes insipidus. If the problem lies in ADH production, administrating an exogenous source would likely improve the symptoms as it did in the case presented. As for providing a more definitive answer, a water deprivation test followed by an ADH (desmopressin) challenge would help identify the origin of diabetes insipidus (central vs. nephrogenic) more reliably. If urine osmolality increases after the water deprivation test or after administering desmopressin, then the issue probably lies in ADH secretion (which indicates central diabetes insipidus). If the kidneys don't respond to the desmopressin administration, then it is a case of nephrogenic diabetes insipidus (a problem in ADH action). In summary, given the patient's symptoms and the fact that urine osmolality returns to normality after ADH administration, the most likely cause of this patient's symptoms is dysfunction of the supraoptic nucleus (F), responsible for producing ADH. Detailed etiologic evaluation would, however, require further investigation.

**Facilitator** The consensus statement synthesizes the responses of AI_A, AI_B, AI_C, AI_D, and AI_E as follows: The question pertains to a 26-year-old man presenting with symptoms of increased urinary frequency and excessive thirst, which are indicative of polyuria and polydipsia. Despite these symptoms, his physical examination and serum chemistry studies are normal. However, his urine osmolality is significantly low, suggesting dilute urine. After the administration of ADH (vasopressin), his urine osmolality normalizes, indicating that his kidneys are responsive to ADH. The task is to identify the most likely dysfunctional structure causing these symptoms. The patient's symptoms are characteristic of diabetes insipidus, a condition marked by an inability to concentrate urine. The two major types of diabetes insipidus are Central Diabetes Insipidus, where there is a defect in the production or secretion of ADH, and Nephrogenic Diabetes Insipidus, where the kidneys poorly respond to ADH. Given the patient's ability to concentrate urine after being administered ADH, the dysfunction is likely upstream of the action of ADH, potentially in the area of ADH synthesis or secretion. ADH is synthesized in the hypothalamic supraoptic and paraventricular nuclei and then transported to the posterior pituitary gland for storage and release. The patient's response to exogenous ADH suggests that the problem lies with the production or secretion of ADH rather than its action in the nephron. Therefore, the dysfunction is likely localized in the supraoptic nucleus, which is principally involved in the production of ADH. In conclusion, the most probable cause of this patient's symptoms is dysfunction of the supraoptic nucleus (F). The patient's ability to normalize urine osmolality with exogenous ADH points to the issue being with ADH production or secretion rather than its action in the nephron. Further investigation would be required for a more detailed etiologic evaluation.
