# Supplementary material for: Collaborative intelligence in AI: Evaluating the performance of a council of AIs on the USMLE
Source: PLOS Digit Health. 2025 Oct 9;4(10):e0000787. doi: 10.1371/journal.pdig.0000787 (PMC12510544; doi:10.1371/journal.pdig.0000787)
Supplement: S2 File — (ZIP) [file pdig.0000787.s002.zip › S2 Data Raw data files containing unprocessed question inputs and LLM outputs including transcripts of deliberations/STEP1_Question-94-1_Type-MC-NJ.docx]

**Facilitator** **[Round 1]:** A 19-year-old man who is in the US Army is brought to the emergency department 45 minutes after he sustained a knife wound to the right side of his chest during an altercation. He has no history of major medical illness and takes no medications. His temperature is 36.9°C (98.4°F), pulse is 110/min, respirations are 24/min, and blood pressure is 114/76 mm Hg. Pulse oximetry on room air shows an oxygen saturation of 94%. On physical examination, the trachea appears to be shifted to the left. Pulmonary examination of the right chest is most likely to show which of the following findings? (A) Decreased Fremitus, dull Percussion, decreased breath sounds (B) Decreased Fremitus, hyperresonant Percussion, decreased breath sounds (C) Decreased Fremitus, hyperresonant Percussion, dull breath sounds (D) Increased Fremitus, dull Percussion, bronchial breath sounds (E) Increased Fremitus, dull Percussion, decreased breath sounds

**AI_D:** RESTATING THE QUESTION: The question is asking for what you'd expect to find on a pulmonary examination given the physical symptoms of a 19-year-old army man who has presented to the emergency department because of a knife wound to his right chest. Let's define some terms: Fremitus refers to the palpable vibration transmitted through the lungs and chest wall when the victim speaks. Percussion is a diagnostic technique used primarily to detect abnormalities associated with the lungs and chest wall. Breath sounds indicate the pattern of air movement in the airways. PLANNING: The victim's symptoms and signs such as tracheal shift to the left, a knife wound to the right chest, and slightly low oxygen saturation suggest there may be buildup of air within the pleural cavity, a condition known as pneumothorax. This buildup of air exerts pressure on the lung leading to its collapse. CLINICAL REASONING: In a pneumothorax, the lung on the affected side of the chest collapses, leading to a decrease in breath sounds on auscultation, due to the lack of air movement through these regions. Upon palpation, fremitus is also decreased as there is no lung tissue with which the conducted sound vibrations can resonate. Percussion is hyper resonant due to trapped air in the pleural space. CONCLUDING: Given the clinical context, above physiological reasoning, and the potential for a pneumothorax following a knife injury to the chest, the correct answer would be consistent with the findings of a pneumothorax. RESPONSE: Therefore, in this scenario, the pulmonary examination on the right chest most likely to show: (B) Decreased Fremitus, hyperresonant Percussion, decreased breath sounds. This combination of findings is indicative of pneumothorax.

**AI_B:** RESTATING THE QUESTION: The enigma unfolds around a young, seemingly healthy serviceman who has sustained a stab wound to the right chest. The salient clinical observation is a shift of the trachea to the left, which implies some significant alteration in the normal right lung anatomic and functional state. Now, the question is related to the expected findings on a physical pulmonary examination of the injured chest side. PLANNING: To decipher the proper answer, an understanding of chest trauma, and specifically the potential pathology related to a stabbing wound is necessary. Expected consequences from such trauma might include hemothorax, pneumothorax, or even both (hemopneumothorax). So, the assumptions would be that any of these conditions would explain the observed tracheal deviation, the vital signs, and poor oxygenation, and should also correlate with specific findings on pulmonary examination. CLINICAL REASONING: The trachea shifts away from the side of a total lung collapse or large amounts of air or fluid in the pleural space. A tension pneumothorax (which can cause tracheal shift) would typically present with shock, which this person does not have. That leaves a simple pneumothorax as one of the most plausible underlying causes for the observed clinical findings. CONCLUDING: A simple pneumothorax on the right side would indeed shift the trachea to the left. It would result in increased intra-thoracic pressure on the affected side, causing collapse of the lung. This should transpire on physical examination as diminished tactile fremitus (due to the presence of air between the lung and chest wall), hyperresonance on percussion (due to increased air), and diminished breath sounds (as air does not transmit sound well). RESPONSE: Based on the whole line of reasoning, the most plausible finding on pulmonary examination of the right chest (considering the stab wound injury and the subsequent tracheal deviation to the left) would be: Decreased Fremitus, hyperresonant Percussion, and decreased breath sounds. Consequently, the best-choice answer is (B).

**AI_C:** (1) RESTATING THE QUESTION: The question asks what physical findings would be present on a pulmonary examination of the right chest in a 19-year-old healthy male who has sustained a knife wound to the right side of his chest. His trachea is noted to be shifted to the left on examination, and he has a slightly low oxygen saturation. In this context, fremitus refers to the vibratory sensation felt on the chest wall due to speech or cough, percussion involves assessing the resonance produced by striking the chest wall, while breath sounds refer to the noises heard on auscultation of the lungs with a stethoscope. (2) PLANNING: The primary assumption here is that the knife wound has affected the structure or function of the lungs, causing alterations in breath sounds, percussion, and fremitus. An alternate assumption could be that there are other factors causing these changes. However, given the acute presentation, timing, and location of the injury, it is more likely that the knife wound has directly affected the lungs. Thus, pathogenesis and pathophysiology related to lung injuries should be prioritized. (3) CLINICAL REASONING: Knife wounds to the chest can potentially result in pneumothorax, a condition characterized by the presence of air in the pleural space. This can cause lung collapse, leading to a lack of breath sounds on the affected side. Pneumothorax also typically presents with a shift in the trachea away from the affected side, as seen in this patient, due to increased pressure in the pleural space pushing the mediastinal structures away. The presence of air in the pleural space would also make the chest wall more resonant (i.e., hyperresonant) when percussed. Lastly, tactile fremitus would be decreased, as the vibrations generated by speech or cough would not be transmitted as efficiently through the air-filled pleural space and collapsed lung. (4) CONCLUDING: From this step-by-step analysis, it can be concluded that the patient's right knife wound has likely resulted in a pneumothorax. (5) RESPONSE: Therefore, on pulmonary examination of the right chest, findings are likely to include (B) Decreased fremitus, hyperresonant percussion, and decreased breath sounds.

**AI_A:** 1) RESTATING THE QUESTION: The question is asking what would be the most likely physical findings on a pulmonary exam of a 19-year-old man who has received a knife wound to the right side of his chest, and whose trachea has shifted to the left as a result. The physical findings mentioned involve fremitus (vibration perceived on palpation, typically referring to tactile vocal fremitus), percussion (a technique used in physical examination to determine the underlying structure of a specific area of the body), and quality of breath sounds. 2) PLANNING: The current clinical picture suggests that the patient could potentially have a pneumothorax, which can commonly result from a penetrating chest injury like a knife wound. A pneumothorax is when air becomes trapped in the pleural space, causing the lung to collapse. This would lead to a decrease in breath sounds and fremitus. Percussion would be hyperresonant since air is trapped. 3) CLINICAL REASONING: The knife wound to the chest has most likely caused a disruption in the pleural space, which ordinarily maintains a negative pressure that aids in lung expansion. This disruption may have led to air entering into the pleural space (pneumothorax), causing the lung to collapse on the affected side. This would decrease the transmission of vibrations (i.e., reduced fremitus) and reduce breath sounds. When percussed, a pneumothorax will resonate more since air is less dense than lung tissue — a condition described clinically as "hyperresonant" to percussion. Additionally, the trachea's deviation to the left further suggests a tension pneumothorax, suggesting a significant accumulation of air within the right pleural space that's causing a mediastinal shift. 4) CONCLUDING: Given the evidence and clinical reasoning, the most probable clinical presentation includes decreased fremitus, hyperresonant percussion, and decreased breath sounds. This aligns with the physical findings typically found in a pneumothorax, which likely resulted from the patient's knife wound to the chest. 5) RESPONSE: So, the correct answer is likely (B) Decreased Fremitus, hyperresonant Percussion, decreased breath sounds. This is because this combination of findings aligns with a pneumothorax, which would likely result from a penetrating knife wound to this patient's chest.

**AI_E:** RESTATING THE QUESTION: In more simple terms, we are dealing with a young army man, who suffered a stab wound to his right chest. His overall health appears to be stable with satisfactory vital signs, apart from a slightly elevated heart rate which could be because of stress or compensatory measures taken by the body in response to the trauma. His oxygen saturation is slightly below the normal limit, and a noted abnormality is the shift of the trachea to the left. The question is asking what findings could we probably observe if this man's right chest was subject to a pulmonary exam, considering his symptoms and presentation. The options given talk about fremitus (which refers to the vibratory sensations felt on the chest wall during speech), percussion (a method to determine the characteristic of underlying tissues or organs in the chest) and breath sounds (the noises produced by the structures of the lungs during breathing). PLANNING: The man's injury scenario, and more essentially the trachea deviation, indicates a possible tension pneumothorax, which happens when air gets trapped in the pleural cavity (the space between the lung and the chest wall), and it can't escape, causing the lung on the affected side to collapse and the trachea and mediastinal structures to deviate toward the opposite side. Each of the given options will be evaluated for their likelihood in relation to this assumption. CLINICAL REASONING: In case of a tension pneumothorax, the lung on the side of the wound collapses, so there is no lung tissue that could vibrate during patient's speech, thus fremitus would be decreased. During percussion, the presence of air in the pleural space would give a hyperresonant sound, because air is a better conductor of sound as compared to the lung tissue. And since the lung has collapsed, it is less capable, if at all, of transmitting sound from the airways to the chest wall. This will result in decreased breath sounds. CONCLUDING: Looking at the options provided, the most fitting symptoms of tension pneumothorax would be decreased fremitus, hyperresonant percussion, and decreased breath sounds due to the presence of trapped air in the pleural cavity, a collapsed lung causing lack of transmission of vibrations during speech, and finally a decrease in breath sounds due to the lack of airflow inside the collapsed lung. RESPONSE: The most probable physical findings in this case of suspected tension pneumothorax, produced by a knife wound to the right side of the chest, causing trachea deviation to the left, would be decreased fremitus, hyperresonant percussion, and decreased breath sounds (Choice B).

**Facilitator** The consensus statement synthesizing the responses of AI_A, AI_B, AI_C, AI_D, and AI_E is as follows: The question pertains to the expected physical findings on a pulmonary examination of a 19-year-old man who has sustained a knife wound to the right side of his chest, resulting in a shift of his trachea to the left. The physical findings in question involve fremitus (vibrations felt on the chest wall during speech), percussion (a diagnostic technique used to assess the characteristics of underlying tissues or organs in the chest), and breath sounds (noises produced by the structures of the lungs during breathing). The clinical picture suggests that the patient may have a pneumothorax, a condition characterized by the presence of air in the pleural space, which can cause lung collapse. This condition is a common result of penetrating chest injuries like a knife wound. The trachea's deviation to the left further suggests a significant accumulation of air within the right pleural space, causing a mediastinal shift. In the case of a pneumothorax, the lung on the affected side collapses, leading to a decrease in breath sounds due to the lack of air movement through these regions. Fremitus would also be decreased as there is no lung tissue with which the conducted sound vibrations can resonate. Percussion would be hyperresonant due to the trapped air in the pleural space. Therefore, the most probable physical findings on a pulmonary examination of the right chest in this case would be decreased fremitus, hyperresonant percussion, and decreased breath sounds. This combination of findings aligns with the physical findings typically found in a pneumothorax, which likely resulted from the patient's knife wound to the chest. Hence, the correct answer to the question is (B) Decreased Fremitus, hyperresonant Percussion, decreased breath sounds.
