# Supplementary material for: Collaborative intelligence in AI: Evaluating the performance of a council of AIs on the USMLE
Source: PLOS Digit Health. 2025 Oct 9;4(10):e0000787. doi: 10.1371/journal.pdig.0000787 (PMC12510544; doi:10.1371/journal.pdig.0000787)
Supplement: S2 File — (ZIP) [file pdig.0000787.s002.zip › S2 Data Raw data files containing unprocessed question inputs and LLM outputs including transcripts of deliberations/STEP1_Question-95-1_Type-MC-NJ.docx]

**Facilitator** **[Round 1]:** A 45-year-old man comes to the office for a follow-up examination 1 month after routine urinalysis showed an increased protein concentration. He has a 15-year history of type 2 diabetes mellitus and a 10-year history of hypertension. Current medications include hydrochlorothiazide, insulin, and metformin. His blood pressure is 150/80 mm Hg. Physical examination shows no other abnormalities. Daily lisinopril therapy is started. Which of the following sets of changes in glomerular hemodynamics is most likely to occur in this patient as a result of this pharmacotherapy? (A) increased renal blood flow, increased ultrafiltration pressure, decreased glomerular filtration rate (B) increased renal blood flow, decreased ultrafiltration pressure, increased glomerular filtration rate (C) increased renal blood flow, decreased ultrafiltration pressure, decreased glomerular filtration rate (D) decreased renal blood flow, increased ultrafiltration pressure, decreased glomerular filtration rate (E) decreased renal blood flow, decreased ultrafiltration pressure, increased glomerular filtration rate (F) decreased renal blood flow, decreased ultrafiltration pressure, decreased glomerular filtration rate

**AI_A:** 1) RESTATING THE QUESTION: The provided clinical scenario presents a 45-year-old gentleman with established hypertension and type 2 diabetes mellitus who's been found to have protein in his urine (proteinuria) during routine testing. In addition to his ongoing antihypertensive and antidiabetic regimes, his healthcare provider initiated him on lisinopril, a drug belonging to the angiotensin-converting enzyme (ACE) inhibitors class. The query at hand is about the expected modifications in renal physiology as a result of commencing this particular treatment; specifically, changes in renal blood flow, ultrafiltration pressure, and glomerular filtration rate (GFR). 2) PLANNING: Assuming the patient's proteinuria is secondary to diabetic nephropathy, common in long-term diabetics like him, the introduction of lisinopril would assist in slowing down renal disease progression primarily by modifying specific parameters of renal blood flow and filtration. Let's consider the physiology and pathophysiology associated with the action of lisinopril. 3) CLINICAL REASONING: Lisinopril belongs to ACE inhibitors group, which primarily block the conversion of angiotensin I to angiotensin II. This results in dilation of the efferent arterioles (that are dominantly affected by angiotensin II) in the kidneys, thereby decreasing the ultrafiltration pressure - the force that drives filtration across the renal glomerular capillary bed. By decreasing this pressure, ACE inhibitors reduce proteinuria in patients. As efferent arteriolar pressure decreases, it also decreases the glomerular filtration pressure, because the difference in hydrostatic pressures across the glomerular capillaries is reduced. So, it's logical to expect a decrease in GFR. However, ACE inhibitors' action on efferent arterioles also decreases the resistance to renal blood flow, which might increase renal blood flow to some extent. 4) CONCLUDING: Combining these concepts, we understand that lisinopril use could result in increased renal blood flow (due to a decrease in efferent arteriolar resistance), decreased ultrafiltration pressure (because of efferent arteriolar dilation), and possibly decreased glomerular filtration rate (secondary to a decrease in the difference of hydrostatic pressures across the glomerular capillaries). 5) RESPONSE: Thus, the more clinically relevant and logical assumption about the glomerular hemodynamic changes in this patient following the initiation of lisinopril would be (C) increased renal blood flow, decreased ultrafiltration pressure, decreased glomerular filtration rate.

**AI_D:** RESTATING THE QUESTION: A 45-year-old man with a history of diabetes and hypertension comes in for a follow-up, due to increased protein concentration in his urine as revealed by a recent urinalysis. He has been prescribed, among other medications, lisinopril. The concern is what changes in glomerular hemodynamics are likely to take place as a result of this therapy. To better understand this, glomerular hemodynamics include renal blood flow (the quantity of blood delivered to the kidneys per unit time), ultrafiltration pressure (the force driving the formation of glomerular filtrate), and glomerular filtration rate (GFR - the volume of fluid filtered from the glomerular capillaries into the Bowman's capsule per unit time). PLANNING: It's necessary to first acknowledge that the patient is presenting signs of nephropathy (increased protein concentration in urine) possibly due to his long-standing diabetes and hypertension. Given this understanding, it's crucial to reflect on how ACE inhibitors such as Lisinopril affect glomerular hemodynamics. Its function is to inhibit the conversion of angiotensin I to angiotensin II, thus resulting in dilation of efferent arterioles, decreased angiotensin-induced aldosterone release, and subsequently reduced sodium and fluid retention. CLINICAL REASONING: Considering Lisinopril's function, it's plausible to predict how each of the glomerular hemodynamic parameters would be influenced. First, we need to evaluate renal blood flow; Lisinopril reduces angiotensin II levels causing dilation of efferent arterioles. This dilation means reduced afferent-efferent arteriolar pressure gradient and a decreased overall resistance allowing for increased renal blood flow. The decreased afferent-efferent arteriolar gradient would lead to decreased ultrafiltration pressure. Finally, the decreased ultrafiltration pressure would result in a decreased glomerular filtration rate because of the negative effect on the driving force for filtration. CONCLUDING: Therefore, connecting each sentence with a clinical thought process, lisinopril in this diabetic and hypertensive patient, would likely lead to an increased renal blood flow, decreased ultrafiltration pressure, and decreased glomerular filtration rate. It is important to note that these changes, although seemingly negative, in reality, help to protect the kidneys from injury by reducing proteinuria and high filtration pressures often seen in conditions like diabetes, thus slowing the progression of kidney diseases. RESPONSE: So, this patient, being treated with lisinopril, would most likely exhibit the set of changes corresponding to option (C) increased renal blood flow, decreased ultrafiltration pressure, decreased glomerular filtration rate.

**AI_E:** 1) RESTATING THE QUESTION: The clinical scenario provided involves a 45-year-old patient with a history of diabetes and hypertension, who has recently been found with increased protein in the urine. Glomerular hemodynamics is essentially the blood flow dynamics within the kidneys’ glomerular structure, which play a critical role in kidney filtration functions. The patient is started on lisinopril, a drug used to treat high blood pressure and heart failure, that primarily works by blocking the angiotensin-converting enzyme (ACE), resulting in decreased production of angiotensin II, a potent vasoconstrictor. As a result, this leads to vasodilation, which reduces blood pressure and alleviates strain on the kidneys. 2) PLANNING: One fundamental assumption, in this case, would be that lisinopril's mode of action should alter the patient's glomerular hemodynamics in a specific way. An alternate assumption would be: if lisinopril induces systemic vasodilation, it could increase renal blood flow, decrease ultrafiltration pressure, and hence affect glomerular filtration rate (GFR). This necessitates evaluating the detailed interaction of lisinopril with kidney hemodynamics with the aim to prioritize an assumption that would lead to optimal clinical management of the patient. 3) CLINICAL REASONING: Lisinopril is an ACE inhibitor, and within the kidneys, ACE inhibitors decrease the production of angiotensin II. The lowered angiotensin II leads to a reduction in efferent arteriolar vasoconstriction, which helps to protect glomerular capillaries by decreasing the intraglomerular pressure, hence reducing ultrafiltration pressure. However, the systemic vasodilation effect (due to less angiotensin II) might increase the renal blood flow. Reduction in ultrafiltration pressure tends to decrease the GFR. 4) CONCLUDING: By providing a step-by-step explanation, we can conclude that the use of lisinopril in this patient will most likely lead to increased renal blood flow due to systemic vasodilation, decreased ultrafiltration pressure due to diminished efferent arteriolar vasoconstriction, and decreased GFR as a result of reduced ultrafiltration pressure. 5) RESPONSE: Based on the integrated clinical thought process, the choice (C) increased renal blood flow, decreased ultrafiltration pressure, decreased glomerular filtration rate aligns with the clinical alterations expected with the initiation of lisinopril therapy in this patient.

**AI_C:** 1) RESTATING THE QUESTION: This scenario presents a 45-year-old male with a medical history of hypertension and type 2 diabetes mellitus. A proteinuria has been detected and his current antihypertensive and antidiabetic drug regimen consists of hydrochlorothiazide, insulin, and metformin. Despite these measures, his blood pressure remains elevated and an angiotensin-converting enzyme (ACE) inhibitor, lisinopril, is prescribed. The question is asking which changes in the renal blood flow, glomerular ultrafiltration pressure, and glomerular filtration rate would most likely arise from the introduction of lisinopril to his pharmacotherapy. 2) PLANNING: The assumptions here are that the patient's proteinuria is due to diabetic nephropathy, a complication of diabetes, and that his hypertension may be contributing to or exacerbating this condition. Lisinopril, an ACE inhibitor, can affect renal function, especially in the context of hypertension and diabetes. An alternate assumption would be to consider the pharmacodynamics of lisinopril and its effect on the kidneys - it should lower blood pressure, decrease glomerular hyperfiltration and relieve pressure on glomeruli thus altering the renal blood flow, ultrafiltration pressure and ultimately the glomerular filtration rate. 3) CLINICAL REASONING: Lisinopril decreases the production of angiotensin II, which normally causes arterial constriction and stimulates the release of aldosterone, therefore increasing blood pressure. A reduction in angiotensin II leads to arterial dilation, and hence increased renal blood flow. The decrease in endogenous angiotensin II will also reduce aldosterone release which indirectly reduces sodium reabsorption and water retention by the kidneys, thereby decreasing the filtrate/fluid volume and the glomerular filtration pressure. Over time, this should protect the glomeruli from the high pressure and high filtrate volume that could damage their walls, leading to albuminuria/proteinuria, which is what one sees in diabetic nephropathy. Therefore, it is likely that the glomerular filtration rate would decrease as a result of the lower pressure, and thus the lower volume, being filtered from the blood into the glomeruli. 4) CONCLUDING: By examining the effects of an ACE inhibitor like lisinopril on renal and glomerular function, it can be reasonably expected that the use of this medication in the patient would increase renal blood flow, reduce ultrafiltration pressure and ultimately decrease the glomerular filtration rate. 5) RESPONSE: With these elements in consideration, the most likely changes in glomerular hemodynamics that will occur in the patient as a result of lisinopril pharmacotherapy would be the set characterized by increased renal blood flow, decreased ultrafiltration pressure and decreased glomerular filtration rate. Ergo, the most accurate answer to the question posed is (C) "increased renal blood flow, decreased ultrafiltration pressure, decreased glomerular filtration rate".

**AI_B:** RESTATING THE QUESTION: This case presents a 45-year-old male with an elevated protein level in his urine, as well as a longstanding history of type 2 diabetes mellitus and hypertension. He is currently on hydrochlorothiazide, insulin, and metformin. His blood pressure is still elevated, and he is being started on daily lisinopril medication. The question is asking which set of changes relating to the blood flow, ultrafiltration pressure, and filtration rate in the kidney's glomeruli are most likely to occur following this addition of lisinopril to his therapeutic regimen. PLANNING: First, it is important to understand the normal and pathological mechanisms of renal glomerular hemodynamics. The glomerulus is an essential component in the kidney responsible for the filtration of the blood. Renal blood flow refers to the volume of blood passing through the kidneys per unit time. Ultrafiltration pressure is the force that drives fluid and solutes out of the capillaries into the bowman's capsule in the glomerulus to be filtered, whereas the glomerular filtration rate (GFR) measures the total amount of filtrate formed by all renal corpuscles per minute. Key assumptions: Lisinopril, an angiotensin-converting enzyme (ACE) inhibitor, should beneficially impact renal hemodynamics in this patient with type 2 diabetes and hypertension, both of which can harm kidney function. Interacting assumptions: Lisinopril suppresses the conversion of angiotensin I to angiotensin II, reducing its vasoconstrictive effect and thus potentially altering renal blood flow. Furthermore, it should theoretically lessen the ultrafiltration pressure and protect the glomerular filtration rate in this patient with increased proteinuria, a sign of possible diabetic nephropathy. CLINICAL REASONING: The renin-angiotensin-aldosterone system, which ACE inhibitors like lisinopril affect, plays a critical role in maintaining blood pressure and fluid balance in the body. In this case, prevalent hyperglycemia from uncontrolled diabetes and high blood pressures have likely caused damage to the renal capillaries, leading to proteinuria. Lisinopril affects this system by inhibiting the conversion of angiotensin I to angiotensin II. Angiotensin II is a potent vasoconstrictor which, when reduced, leads to vasodilation and a subsequent increase in renal blood flow. The lessening of angiotensin II also reduces the efferent arteriolar resistance, causing a decrease in the ultrafiltration pressure within the glomerulus. The combined effects aid in maintaining the glomerular filtration rate by preventing the excessive protein flux and thereby limiting further kidney damage. CONCLUDING: Therefore, considering the impact of lisinopril on the renin-angiotensin-aldosterone system, the use of lisinopril in this patient should increase renal blood flow due to vasodilation, decrease ultrafiltration pressure because of reduced efferent arteriolar resistance, and maintain or potentially improve the GFR, thereby mitigating the progression of kidney damage. RESPONSE: The answer is (B) increased renal blood flow, decreased ultrafiltration pressure, increased glomerular filtration rate.

**Facilitator** **[Round 2]:** Summary: AI_A, AI_C, AI_D, and AI_E all agree that the introduction of lisinopril in the patient's treatment would result in increased renal blood flow, decreased ultrafiltration pressure, and decreased glomerular filtration rate. They all base their reasoning on the fact that lisinopril, an ACE inhibitor, blocks the conversion of angiotensin I to angiotensin II, leading to dilation of efferent arterioles, decreased ultrafiltration pressure, and potentially decreased GFR due to reduced hydrostatic pressure across the glomerular capillaries. AI_B, however, believes that the introduction of lisinopril would result in increased renal blood flow, decreased ultrafiltration pressure, but an increased glomerular filtration rate. AI_B argues that the reduction of angiotensin II leads to vasodilation and increased renal blood flow, decreased ultrafiltration pressure due to reduced efferent arteriolar resistance, and maintenance or potential improvement of the GFR by preventing excessive protein flux and limiting further kidney damage. Key Differences: The key difference in the perspectives lies in the expected effect of lisinopril on the glomerular filtration rate (GFR). While AI_A, AI_C, AI_D, and AI_E predict a decrease in GFR due to reduced ultrafiltration pressure, AI_B anticipates an increase or maintenance of GFR due to the prevention of excessive protein flux and further kidney damage. Question to Clarify Disagreements: Can the council members provide more insight into the mechanisms by which lisinopril could potentially increase the glomerular filtration rate, as suggested by AI_B, despite the decrease in ultrafiltration pressure?. Based on your preceding response, how would you answer the original question: [A 45-year-old man comes to the office for a follow-up examination 1 month after routine urinalysis showed an increased protein concentration. He has a 15-year history of type 2 diabetes mellitus and a 10-year history of hypertension. Current medications include hydrochlorothiazide, insulin, and metformin. His blood pressure is 150/80 mm Hg. Physical examination shows no other abnormalities. Daily lisinopril therapy is started. Which of the following sets of changes in glomerular hemodynamics is most likely to occur in this patient as a result of this pharmacotherapy? (A) increased renal blood flow, increased ultrafiltration pressure, decreased glomerular filtration rate (B) increased renal blood flow, decreased ultrafiltration pressure, increased glomerular filtration rate (C) increased renal blood flow, decreased ultrafiltration pressure, decreased glomerular filtration rate (D) decreased renal blood flow, increased ultrafiltration pressure, decreased glomerular filtration rate (E) decreased renal blood flow, decreased ultrafiltration pressure, increased glomerular filtration rate (F) decreased renal blood flow, decreased ultrafiltration pressure, decreased glomerular filtration rate]

**AI_A:** 1) RESTATING THE QUESTION: The query is about the effect of daily lisinopril treatment on a 45-year-old man's glomerular hemodynamics. The patient has type 2 diabetes, hypertension, and a recent increase in urinary protein concentration, his current medications are hydrochlorothiazide, insulin, and metformin. 2) PLANNING: Lisinopril is an Angiotensin Converting Enzyme (ACE) inhibitor, which inhibits the conversion of angiotensin I into angiotensin II. Given its function, it's reasonable to postulate that it will impact renal blood flow, ultrafiltration pressure, and the glomerular filtration rate, as these are all influenced by the effects of angiotensin II. 3) CLINICAL REASONING: The normal function of angiotensin II is to constrict the efferent arterioles, which increases ultrafiltration pressure and thus the Glomerular Filtration Rate (GFR). It also decreases renal blood flow due to this constriction. However, because lisinopril inhibits the formation of angiotensin II, it should cause dilation of the efferent arterioles, which should lead to decreased ultrafiltration pressure. This should result in a decrease in the GFR. Furthermore, the dilation of the efferent arterioles should increase renal blood flow. 4) CONCLUDING: The lisinopril therapy will likely lead to an increased renal blood flow, decreased ultrafiltration pressure, and decreased glomerular filtration rate. 5) RESPONSE: Therefore, the most likely scenario after starting lisinopril therapy in the patient is (C) increased renal blood flow, decreased ultrafiltration pressure, decreased glomerular filtration rate.

**AI_E:** Restating the question: The query pertains to understanding the likely changes in certain bodily functions related to the kidney (glomerular hemodynamics) of a middle-aged man with a history of diabetes and high blood pressure (hypertension), after beginning a course of lisinopril, a type of drug called an ACE inhibitor. The parameters to be considered are renal blood flow, ultrafiltration pressure, and glomerular filtration rate. Planning: The main assumption here involves the effects of lisinopril on renal blood flow, ultrafiltration pressure, and the glomerular filtration rate. To generate alternate assumptions, we need to question how lisinopril modifies each of these parameters individually. Lisinopril, as an ACE inhibitor, blocks the conversion of angiotensin I to angiotensin II, which leads to a series of changes in the kidney. The primary clinical priority here is to understand how this change in angiotensin levels impacts kidney function, specifically in the context of glomerular hemodynamics. Clinical reasoning: Lisinopril, by virtue of its inhibition of angiotensin II, causes dilation of efferent arterioles. This dilation typically results in an increased renal blood flow. In addition, the ultrafiltration pressure usually decreases due to this dilation as less pressure is built up in the Bowman's capsule due to the blood flow mechanics. The glomerular filtration rate (GFR), which is the volume of fluid filtered from the renal (kidney) glomerular capillaries into Bowman's capsule per unit time, could theoretically decrease as a result of this decreased ultrafiltration pressure, although that relationship is influenced by many factors, including the status of one's kidney health. Concluding: Considering this sequence of renal changes in relation to the inhibition of angiotensin II production, it would seem plausible that the introduction of lisinopril to this patient's regimen would likely result in increased renal blood flow and decreased ultrafiltration pressure. The change in GFR could potentially be a decrease resulting from the reduced ultrafiltration pressure. Response: Given these reasonings, the most likely set of changes in this patient's glomerular hemodynamics due to this pharmacotherapy would be: increased renal blood flow, decreased ultrafiltration pressure, and decreased glomerular filtration rate (Option C).

**AI_C:** RESTATING THE QUESTION: The question relates to a 45-year-old man with type 2 diabetes and hypertension who is started on lisinopril, an ACE inhibitor, after urinalysis showed increased protein concentration. We are asked to determine the likely changes in glomerular hemodynamics - renal blood flow, ultrafiltration pressure, and the glomerular filtration rate (GFR) as a result of this treatment. PLANNING: The main assumption here is that the administration of lisinopril will alter the patient's renal blood flow, ultrafiltration pressure, and glomerular filtration rate as a result of modification of the renin-angiotensin-aldosterone system (RAAS), which controls blood pressure and fluid balance. CLINICAL REASONING: Lisinopril, being an ACE inhibitor, interrupts the renin-angiotensin-aldosterone system by preventing the conversion of angiotensin I to angiotensin II, thus decreasing angiotensin II-mediated vasoconstriction and aldosterone secretion. The decreased angiotensin II leads to dilation of efferent arterioles, which result in increased renal blood flow and decreased ultrafiltration pressure. Generally, a decrease in ultrafiltration pressure will lead to a decrease in glomerular filtration rate since the driving force for the filtration process is reduced. CONCLUDING: So, considering the mechanism of action of lisinopril, the assumption would be that this drug would lead to increased renal blood flow, decreased ultrafiltration pressure, and decreased glomerular filtration rate due to decreased intraglomerular hydraulic pressure. RESPONSE: Based on the understanding of the pathophysiological alterations caused by lisinopril, the most likely set of changes to occur in this patient's glomerular hemodynamics is increased renal blood flow, decreased ultrafiltration pressure, and decreased glomerular filtration rate (Option C).

**AI_B:** In restating the question, the inquiry revolves around the effects of Lisinopril therapy in the glomerular hemodynamics of a patient with type 2 diabetes and hypertension. Lisinopril is an Angiotensin-Converting Enzyme (ACE) inhibitor. It prevents the conversion of Angiotensin I to Angiotensin II, which is a powerful vasoconstrictor. As more Angiotensin II is blocked, vasoconstriction decreases leading to decreased blood pressure. The options presented in the query are possible changes in renal blood flow, ultrafiltration pressure, and glomerular filtration rate (GFR) upon Lisinopril administration. The underlying assumption here is that Lisinopril, as an ACE inhibitor, will affect glomerular hemodynamics. However, what needs questioning is how exactly it will affect the renal blood flow, ultrafiltration pressure, and particularly the glomerular filtration rate. The physiology of the kidney plays a significant role in better understanding this case. The constriction or dilation of afferent and efferent arterioles highly affects the GFR and renal plasma flow. In hypertensive patients, Angiotensin II normally causes efferent arteriolar constriction, thus maintaining GFR despite the elevated systemic blood pressure. However, with ACE inhibitors like Lisinopril, the effect of Angiotensin II is blocked; leading to dilation of efferent arterioles, increased renal blood flow, decreased ultrafiltration pressure (due to reduced pressure gradient across glomerular capillaries), and ultimately decreased GFR. Leaning towards this clinical reasoning, the conclusion I form is that Lisinopril, an ACE inhibitor, would most likely bring about an increased renal blood flow, decreased ultrafiltration pressure, and decreased glomerular filtration rate in the given patient. Therefore, my response would be choice (C): increased renal blood flow, decreased ultrafiltration pressure, decreased glomerular filtration rate. This choice reflects the pathophysiological changes in the kidneys upon Lisinopril administration – it increases renal blood flow by dilating efferent arterioles, reduces ultrafiltration pressure, and ultimately decreases the glomerular filtration rate due to the reduced pressure gradient across the glomerular capillaries. This response is especially relevant considering the patient's history of hypertension and type 2 diabetes.

**AI_D:** Restating the question: The question asks what would be the likely changes in this patient's renal hemodynamics after starting lisinopril therapy, taking into account his history of diabetes, hypertension, and proteinuria. Lisinopril is an ACE inhibitor, which blocks the production of angiotensin II, a potent vasoconstrictor that also stimulates the release of aldosterone - hence, it affects blood flow within the kidney, ultrafiltration pressure, and the rate at which the kidney filters blood (glomerular filtration rate, GFR). Planning: The assumptions underlying this case are that the increased protein concentration in the patient's urine is due to diabetic nephropathy, caused by long-term damage to the kidneys from the effects of high blood sugar levels and hypertension; this damage can affect the kidney's filtration process. Lisinopril affects this process, which will result in changes in renal blood flow, ultrafiltration pressure, and GFR. Alternate assumptions could be that the proteinuria is due to some other cause, or that lisinopril would not have a significant effect on this patient's renal hemodynamics. Clinical reasoning: Lisinopril, by inhibiting angiotensin II production, will reduce the constriction of both afferent and predominantly efferent arterioles within the kidney, leading to an increase in renal blood flow. It also decreases ultrafiltration pressure as a result of reduced resistance in the efferent arteriole. Given that GFR is partly dependent on this pressure gradient, a reduction in ultrafiltration pressure could potentially lead to decreased GFR. However, lisinopril is often used in patients with diabetic nephropathy precisely because it can help to reduce proteinuria and potentially slow the progression of the disease. Concluding: Considering all of these factors, the most likely changes to occur in this patient's renal hemodynamics as a result of starting lisinopril are increased renal blood flow due to dilatation of renal arterioles, reduced ultrafiltration pressure due to decreased resistance in the efferent arteriole, and possibly decreased GFR due to the reduced pressure gradient, despite the potential protective effects of lisinopril. Response: Therefore, the correct answer would be (C) increased renal blood flow, decreased ultrafiltration pressure, decreased glomerular filtration rate.

**Facilitator** The consensus statement synthesizing the responses of AI_A, AI_B, AI_C, AI_D, and AI_E is as follows: The question pertains to the likely changes in glomerular hemodynamics of a 45-year-old man with a history of type 2 diabetes and hypertension, after starting lisinopril therapy. Lisinopril is an Angiotensin-Converting Enzyme (ACE) inhibitor that blocks the conversion of angiotensin I to angiotensin II, a potent vasoconstrictor. This inhibition is expected to affect renal blood flow, ultrafiltration pressure, and the glomerular filtration rate (GFR). The underlying assumption is that lisinopril will alter these parameters due to its impact on the renin-angiotensin-aldosterone system (RAAS), which controls blood pressure and fluid balance. The clinical reasoning is that the decreased angiotensin II due to lisinopril leads to dilation of efferent arterioles, resulting in increased renal blood flow and decreased ultrafiltration pressure. A decrease in ultrafiltration pressure generally leads to a decrease in GFR since the driving force for the filtration process is reduced. Therefore, the consensus among the AI responses is that the most likely changes in this patient's glomerular hemodynamics as a result of starting lisinopril therapy are increased renal blood flow, decreased ultrafiltration pressure, and decreased glomerular filtration rate. This conclusion aligns with option (C) in the given choices.
