# Supplementary material for: Collaborative intelligence in AI: Evaluating the performance of a council of AIs on the USMLE
Source: PLOS Digit Health. 2025 Oct 9;4(10):e0000787. doi: 10.1371/journal.pdig.0000787 (PMC12510544; doi:10.1371/journal.pdig.0000787)
Supplement: S2 File — (ZIP) [file pdig.0000787.s002.zip › S2 Data Raw data files containing unprocessed question inputs and LLM outputs including transcripts of deliberations/STEP1_Question-96-1_Type-MC-NJ.docx]

**Facilitator** **[Round 1]:** A 45-year-old man comes to the office for a follow-up examination 1 month after routine urinalysis showed an increased protein concentration. He has a 15-year history of type 2 diabetes mellitus and a 10-year history of hypertension. Current medications include hydrochlorothiazide, insulin, and metformin. His blood pressure is 150/80 mm Hg. Physical examination shows no other abnormalities. Daily lisinopril therapy is started. Which of the following sets of changes in glomerular hemodynamics is most likely to occur in this patient as a result of this pharmacotherapy? (A) increased renal blood flow, increased ultrafiltration pressure, decreased glomerular filtration rate (B) increased renal blood flow, decreased ultrafiltration pressure, increased glomerular filtration rate (C) increased renal blood flow, decreased ultrafiltration pressure, decreased glomerular filtration rate (D) decreased renal blood flow, increased ultrafiltration pressure, decreased glomerular filtration rate (E) decreased renal blood flow, decreased ultrafiltration pressure, increased glomerular filtration rate (F) decreased renal blood flow, decreased ultrafiltration pressure, decreased glomerular filtration rate" "A 3438-g (7-lb 9-oz) female newborn is delivered in the hospital at term to a 24-year-old primigravid woman after an uncomplicated pregnancy. Apgar scores are 8 and 9 at 1 and 5 minutes, respectively. Examination of the newborn shows ambiguous genitalia. Results of a blood analysis show markedly increased serum concentrations of 17- hydroxyprogesterone. Genetic analysis shows a karyotype of 46,XX. Further testing of this newborn is most likely to show a deficiency of which of the following enzymes? (A) 11β-Hydroxylase (B) 21α-Hydroxylase (C) 3α-Hydroxysteroid dehydrogenase (D) 3β-Hydroxysteroid dehydrogenase (E) 5α-Reductase (F) 17α-Reductase

**AI_A:** 1. RESTATING THE QUESTION: You are requesting two medical cases. In the first, a 45-year-old man was found with increased protein concentration in urine and suffers from long-term type 2 diabetes and hypertension. Despite medication, his blood pressure is high and he was prescribed lisinopril. You're curious about how this medication will affect his glomerular hemodynamics. Glomerular hemodynamics refers to the blood flow and filtration within the kidney where waste products are filtered from the blood. Lisinopril is an ACE inhibitor that works by reducing the production of angiotensin II, a hormone that can cause blood vessels to narrow and release a hormone that raises blood pressure. The second case is about a newborn girl with ambiguous genitalia, elevated 17-hydroxyprogesterone levels and normal karyotype. Ambiguous genitalia refers to a condition in which the external genitals don't appear to be clearly either male or female. Elevated levels of 17-hydroxyprogesterone can be a sign of a type of congenital adrenal hyperplasia (CAH), which entail genetic disorders that impair hormone production in the adrenal glands. You are keen on knowing what enzyme deficiency this newborn could have. 2. PLANNING: For the first case, the assumptions include: (1) lisinopril will lower the patient's blood pressure; (2) the patient's glomerular function may change on lisinopril. For these assumptions, we need to consider: how lisinopril affects glomerular function and renal hemodynamics. For the second case, the primary assumption is that they have a specific variant of CAH, given their high 17-hydroxyprogesterone levels. We'll need to question which enzyme deficiencies lead to such a profile and if those associated symptoms match the child's. 3. CLINICAL REASONING: Lisinopril primarily helps to expand blood vessels, enabling smoother blood flow and decreasing the heart's work. This expansion is beneficial for the kidneys (increased renal blood flow) and lessens the pressure needed to push blood into the glomeruli (reducing ultrafiltration pressure). This variable effect on ultrafiltration pressure might impact GFR (glomerular filtration rate) depleting it (Choice C). As for the newborn, CAH is often the cause when a child is born with ambiguous genitalia with normal karyotype and elevated 17-hydroxyprogesterone. 21-hydroxylase deficiency, the most usual form of CAH, would lead to these parameters (Choice B). 4. CONCLUDING: Lisinopril's mechanism tends to lead to increased renal blood flow, decreased ultrafiltration pressure, and decreased GFR. On the other hand, the newborn's symptoms suggest a typical case of 21-hydroxylase deficiency, a variant of CAH. 5. RESPONSE: Therefore, in response to your questions: for the man, the glomerular hemodynamic changes due to lisinopril will be increased renal blood flow, decreased ultrafiltration pressure, and decreased glomerular filtration rate (Choice C). For the newborn, the enzyme deficiency is most likely to be 21α-Hydroxylase (Choice B).

**AI_E:** To begin interpreting these questions, we must first understand each one distinctly: 1) The first question asks us to predict the likely changes in the glomerular hemodynamics of a middle-aged male patient who is newly initiated on lisinopril therapy. Lisinopril is an angiotensin-converting enzyme (ACE) inhibitor used in treating conditions like hypertension and diabetic nephropathy. Understanding how ACE inhibitors interact with the renin-angiotensin-aldosterone system (RAAS) gives insight into the physiological impact it has on renal blood flow, ultrafiltration pressure, and glomerular filtration rate (GFR). 2) The second question is regarding a newborn female with ambiguous genitalia and elevated 17-hydroxyprogesterone levels. Her karyotype is normal female (46,XX). The question aims to identify the enzyme deficiency most likely causing her condition. This question is within the domain of endocrinology and genetics, seeking to connect the outward physical signs with an inherent biochemical or genetic anomaly. The assumptions made in each question are: 1) Lisinopril will cause predictable changes in the patient's renal hemodynamics based on its known pharmacological effects. 2) The ambiguous genitalia in the newborn and the elevated 17-hydroxyprogesterone levels suggest an enzyme deficiency that is typical of a specific biochemical or genetic disorder. We can propose alternate assumptions for each respective case based on detailed clinical reasoning: 1) As an ACE inhibitor, lisinopril's mechanism of action involves reducing angiotensin II production, thereby decreasing aldosterone secretion. This reduces reabsorption of sodium and water in the kidneys, leading to an expansion of the blood volume and consequently an increase in renal blood flow. The inhibition of angiotensin II also relaxes the efferent arterioles, decreasing the ultrafiltration pressure. The overall effect of increased renal blood flow and decreased ultrafiltration pressure effectively lowers the GFR. 2) The finding of elevated 17-hydroxyprogesterone levels points towards a disturbance in the biochemical pathway of glucocorticoid synthesis, particularly involving 21α-Hydroxylase. This enzyme is involved in converting 17-hydroxyprogesterone to 11-deoxycortisol. If this enzyme is deficient, there will be a buildup of 17-hydroxyprogesterone, the finding in this child. In this scenario, the excess 17-hydroxyprogesterone can be shunted into the sex hormone synthesis pathway, leading to the production of excess androgens, thus causing ambiguous genitalia in a genetically female (46,XX) child. Possible answers: 1) Lisinopril is likely to result in (C) increased renal blood flow, decreased ultrafiltration pressure, decreased glomerular filtration rate. 2) The newborn is most likely to show a deficiency of (B) 21α-Hydroxylase.

**AI_C:** RESTATING THE QUESTIONS: The queries raised here pertain to two specific medical conditions. The first concerns a middle-aged adult male patient with diabetes and hypertension, whose protein concentration in urine indicates possible kidney complications. He has been prescribed lisinopril, and we need to predict the impact of this drug on his renal function, specifically on his renal blood flow, ultrafiltration pressure, and glomerular filtration rate. The second case involves a newborn girl presenting with unusual genital features and elevated serum 17-hydroxyprogesterone. We are to find out which enzyme is likely deficient in her system based on these findings. PLANNING: In the first case, it's likely that the patient has diabetic nephropathy due to prolonged effects of diabetes, leading to an increase in protein concentration in the urine. Lisinopril, an Angiotensin-Converting Enzyme (ACE) inhibitor, typically affects the renin-angiotensin-aldosterone system, potentially leading to changes in hemodynamic variables. In the second case, based on the raised level of 17-hydroxyprogesterone and the physical manifestation of ambiguous genitalia, it can be suspected that the newborn has a form of Congenital Adrenal Hyperplasia (CAH), an autosomal recessive disease, which is usually a result of an enzymatic deficiency. CLINICAL REASONING: For the first case, it is known that ACE inhibitors decrease angiotensin II production (a potent vasoconstrictor) and prevent degradation of vasodilator bradykinin, causing a net vasodilation effect. This would lead to an increased renal blood flow, and by decreasing systemic vascular resistance, this would reduce ultrafiltration pressure in the glomerulus. As a result, it should decrease the glomerular filtration rate, slowing the progression of diabetic nephropathy by lessening the pressure load on the kidney. In the second case, considering that the 17-hydroxyprogesterone level is high, it indicates that the process of its further conversion is halted. The enzyme responsible for this conversion to 11-deoxycortisol is 21α-hydroxylase. A deficiency in this enzyme can cause a buildup of 17-hydroxyprogesterone, which gets shunted to produce excessive androgens, explaining the ambiguous genitalia in a XX (genotypically female) patient. CONCLUDING: May I conclude that the first patient is likely to experience an increase in renal blood flow, a decrease in ultrafiltration pressure, and a decrease in glomerular filtration rate due to Lisinopril therapy. The second case, the neonate is likely to have a deficiency of the 21α-Hydroxylase enzyme. RESPONSE: The expected changes in the glomerular hemodynamics of the male patient as a result of Lisinopril therapy are represented by option (C): increased renal blood flow, decreased ultrafiltration pressure, decreased glomerular filtration rate. For the newborn girl, the enzyme likely to be deficient is represented by option (B): 21α-Hydroxylase.

**AI_B:** RESTATING THE QUESTION: The first scenario seeks the most probable changes in renal blood flow, ultrafiltration pressure, and glomerular filtration rate implying from the treatment of a 45-year-old man with lisinopril. He has a history of diabetes and hypertension, has been found to have increased protein in his urine, and is on various medications. Lisinopril is an angiotensin-converting enzyme (ACE) inhibitor, it inhibits the formation of angiotensin II, a potent vasoconstrictor, from angiotensin I, resulting in vasodilation, decreased aldosterone secretion, decreased sodium reabsorption, and increased potassium retention. PLANNING: An important assumption to precede is that lisinopril affects the renin-angiotensin-aldosterone system (RAAS) which eventually influences glomerular hemodynamics. CLINICAL REASONING: To begin with, by inhibiting ACE, lisinopril causes decreased conversion of angiotensin I to angiotensin II. The reduced levels of angiotensin II results in dilatation of the efferent arterioles, decreasing the glomerular hydrostatic pressure, and hence the ultrafiltration pressure. This leads to reduced stress on the glomerular barrier and decreased proteinuria, which is particularly beneficial for patients like the one in question with diabetic renal disease. Furthermore, due to decreased angiotensin II, there is decreased aldosterone secretion. Aldosterone is responsible for sodium reabsorption which indirectly controls water reabsorption. A decrease in aldosterone thus leads to a decrease in water reabsorption, which could potentially affect the renal blood flow, but not as significantly as angiotensin II's direct effect on renal blood flow. CONCLUDING: Given these effects, lisinopril therapy would be expected to decrease ultrafiltration pressure and therefore decrease glomerular filtration rate. The change in renal blood flow is somewhat ambiguous, but it is likely to decrease due to the decrease in water reabsorption caused by reduced aldosterone. RESPONSE: Therefore, the most likely hemodynamics changes that would occur in this patient due to lisinopril therapy would be decreased renal blood flow, decreased ultrafiltration pressure, and decreased glomerular filtration rate. In the clinical scenario options, that corresponds with answer choice (F). Next, RESTATING THE QUESTION: The second clinical scenario involves a newborn girl presenting with ambiguous genitalia and a 46,XX karyotype. Blood analysis reveals significantly increased levels of 17-hydroxyprogesterone. The question seeks to identify the specific enzyme deficiency most likely to be found during further testing. PLANNING: The assumption that needs to be considered here is that the abnormal physical and biochemical findings are indicative of a disorder of steroidogenesis, which are a group of disorders that alter the synthesis of steroids, including cortisol, aldosterone and sex hormones. CLINICAL REASONING: Utilizing knowledge on endocrinology, hydroxylase deficiencies are often implicated in sexual differentiation disorders. Elevated 17-hydroxyprogesterone concentrations are hallmark of 21-α hydroxylase deficiency. This deficiency leads to a reduced production of cortisol and aldosterone, with overproduction of androgens due to shunting of the precursors. This typically results in virilization or ambiguous genitalia in female infants, which is fitting with the presented clinical scenario. CONCLUDING: The newborn's ambiguous genitalia and elevated 17-hydroxyprogesterone levels indicate that there may be a disorder in her adrenal steroidogenesis pathway, most likely due to a deficient enzyme. RESPONSE: Therefore, additional testing on this newborn is most likely to reveal a deficiency in 21α-Hydroxylase, corresponding to answer choice (B).

**AI_D:** Let's begin with the first patient, a 45-year-old male with a history of type 2 diabetes and hypertension. His urine shows increased protein concentration, which together with his other conditions, suggests nephropathy. His current medications include hydrochlorothiazide (a diuretic), insulin and metformin for his diabetes. His blood pressure is high, so lisinopril, an ACE inhibitor, is added to his regime. The question posed is about the potential effect of lisinopril on his glomerular hemodynamics. Glomerular hemodynamics means the blood flow in the glomerulus, ultrafiltration pressure refers to the pressure inside the glomerulus that drives the filtration process resulting in the formation of urine, and glomerular filtration rate is the volume of fluid filtered from the renal (kidney) glomerular capillaries into the Bowman's capsule per unit time. Changes to these will affect the kidney function. Next, it's essential to note that lisinopril - an angiotensin-converting enzyme (ACE) inhibitor - works by preventing the conversion of angiotensin I to angiotensin II. This action leads to vasodilation, decreased secretion of aldosterone, and reduced damage to the glomeruli caused by hypertension. This line of reasoning leads us to believe that certain effects may result from this therapy. Clinically, angiotensin II is known to cause vasoconstriction and increase ultrafiltration pressure, the key factors that increase the glomerular filtration rate. Hence, the inhibition of angiotensin II by lisinopril would decrease ultrafiltration pressure but should also decrease the glomerular filtration rate under normal circumstances. However, in pathological conditions like hypertension and diabetes which cause damage to the kidney, this decrease in ultrafiltration pressure is beneficial as it reduces the workload on the kidney, slowing the progression of kidney damage. Existing vasoconstriction of the efferent arterioles due to the presence of angiotensin II is also blocked, leading to increased renal blood flow. Therefore, by this reasoning, the most likely changes in glomerular dynamics to this patient due to the lisinopril therapy would likely be increased renal blood flow, decreased ultrafiltration pressure, decreased glomerular filtration rate (C). Turning to the second patient, a newborn female who, despite having a normal female karyotype (46, XX), presents with ambiguous genitalia. Her blood tests show markedly increased levels of 17-hydroxyprogesterone. Here, the case points to the condition called congenital adrenal hyperplasia (CAH), which is characterized by ambiguous genitalia in females, an increased level of 17-hydroxyprogesterone, and a typical female karyotype. The process of steroid hormone production involves several steps, each mediated by a specific enzyme. A disruption in the function of one of these enzymes can cause a buildup of specific hormone precursors and a decrease in the hormones formed downstream of the disrupted step. Considering the laboratory finding of increased 17-hydroxyprogesterone, it's notable that 21α-Hydroxylase is the enzyme that catalyzes the conversion of 17-hydroxyprogesterone to 11-deoxycortisol, which is then converted to cortisol. A deficiency of 21α-Hydroxylase would mean 17-hydroxyprogesterone would build up due to its conversion to 11-deoxycortisol being halted. Therefore, based on the clinical and laboratory findings, we can conclude that the newborn likely has a deficiency of the enzyme 21α-Hydroxylase (B). In summary, for the first patient, the administration of lisinopril is most likely to result in (C) increased renal blood flow, decreased ultrafiltration pressure, decreased glomerular filtration rate. For the second case, the newborn girl, further testing is likely to show a deficiency of (B) 21α-Hydroxylase.

**Facilitator** **[Round 2]:** Summary: AI_A, AI_B, AI_C, AI_D, and AI_E all agree on the likely outcomes for both medical cases. For the first case, they all agree that the 45-year-old man with type 2 diabetes, hypertension, and increased protein concentration in urine, who has been prescribed lisinopril, will likely experience increased renal blood flow, decreased ultrafiltration pressure, and decreased glomerular filtration rate due to the medication's effect on the renin-angiotensin-aldosterone system. For the second case, they all agree that the newborn girl with ambiguous genitalia, elevated 17-hydroxyprogesterone levels, and a normal karyotype likely has a deficiency in the enzyme 21α-Hydroxylase, which is indicative of a form of Congenital Adrenal Hyperplasia (CAH). Key Differences: The main difference in the council members' responses lies in their explanation of the mechanisms behind the predicted outcomes. AI_B and AI_D, for instance, emphasize the role of aldosterone in the effects of lisinopril, while AI_A, AI_C, and AI_E focus more on the impact of reduced angiotensin II production. Question to Clarify Disagreements: To clarify the differences in their explanations, the following question could be asked: "Could you elaborate on the relative importance of reduced angiotensin II production versus decreased aldosterone secretion in the effects of lisinopril on renal hemodynamics?". Based on your preceding response, how would you answer the original question: [A 45-year-old man comes to the office for a follow-up examination 1 month after routine urinalysis showed an increased protein concentration. He has a 15-year history of type 2 diabetes mellitus and a 10-year history of hypertension. Current medications include hydrochlorothiazide, insulin, and metformin. His blood pressure is 150/80 mm Hg. Physical examination shows no other abnormalities. Daily lisinopril therapy is started. Which of the following sets of changes in glomerular hemodynamics is most likely to occur in this patient as a result of this pharmacotherapy? (A) increased renal blood flow, increased ultrafiltration pressure, decreased glomerular filtration rate (B) increased renal blood flow, decreased ultrafiltration pressure, increased glomerular filtration rate (C) increased renal blood flow, decreased ultrafiltration pressure, decreased glomerular filtration rate (D) decreased renal blood flow, increased ultrafiltration pressure, decreased glomerular filtration rate (E) decreased renal blood flow, decreased ultrafiltration pressure, increased glomerular filtration rate (F) decreased renal blood flow, decreased ultrafiltration pressure, decreased glomerular filtration rate" "A 3438-g (7-lb 9-oz) female newborn is delivered in the hospital at term to a 24-year-old primigravid woman after an uncomplicated pregnancy. Apgar scores are 8 and 9 at 1 and 5 minutes, respectively. Examination of the newborn shows ambiguous genitalia. Results of a blood analysis show markedly increased serum concentrations of 17- hydroxyprogesterone. Genetic analysis shows a karyotype of 46,XX. Further testing of this newborn is most likely to show a deficiency of which of the following enzymes? (A) 11β-Hydroxylase (B) 21α-Hydroxylase (C) 3α-Hydroxysteroid dehydrogenase (D) 3β-Hydroxysteroid dehydrogenase (E) 5α-Reductase (F) 17α-Reductase]

**AI_D:** The questions are about predicting the changes in glomerular hemodynamics of a hypertensive diabetic patient after starting lisinopril therapy, and identifying the most likely enzyme deficiency in a female newborn presenting with ambiguous genitalia and elevated levels of 17-hydroxyprogesterone. For the first scenario, I identified the assumption that the patient's medical history combined with his newly started medication would result in certain changes in renal function. Lisinopril inhibits the angiotensin-converting enzyme which is crucial in the renin-angiotensin-aldosterone system(RAAS). This raises an alternate assumption that changes in renal blood flow, ultrafiltration pressure and glomerular filtration rate would occur due to the effects of lisinopril. My clinical reasoning stems from the effect of lisinopril on RAAS—its inhibition of the enzyme leads to a reduction in the generation of angiotensin II, resulting in efferent arteriolar dilation and a decrease in systemic blood pressure. Angiotensin II's role in constricting the efferent arterioles raises the glomerular filtration rate (GFR). Therefore, with the use of lisinopril, the decreased production of angiotensin II would lead to decreased GFR. However, the dilation of the efferent arterioles could increase renal blood flow, while the decrease in angiotensin II–led efferent vasoconstriction would decrease ultrafiltration pressure. Based on this reasoning, the most likely hemodynamic changes in this patient after starting daily lisinopril therapy would be increased renal blood flow, decreased ultrafiltration pressure, and decreased glomerular filtration rate (option C). For the second case, the ambiguous genitalia and the abnormally elevated levels of 17-hydroxyprogesterone in a 46,XX newborn led me to the assumption of a congenital enzymatic disorder affecting steroid hormone synthesis. The recognition of these features leads to an alternate assumption: the most likely enzyme deficiency this newborn has is the 21α-hydroxylase enzyme, which is needed for the conversion of 17-hydroxyprogesterone to 11-deoxycortisol. My clinical reasoning here is informed by the pathway of adrenal steroidogenesis—21α-hydroxylase deficiency is the cause of classic congenital adrenal hyperplasia (CAH), which is commonly associated with ambiguous genitalia in female newborns and elevated levels of 17-hydroxyprogesterone. Other enzymes mentioned in the options do not result in a similar biochemical and clinical phenotype. Therefore, further testing of this newborn is most likely to show a deficiency in the 21α-Hydroxylase enzyme (option B).

**AI_A:** RESTATING THE QUESTION: The first question is looking for the likely changes in the kidneys of a 45-year-old man, a patient with type 2 diabetes and hypertension, who has recently started on lisinopril. Lisinopril is an angiotensin-converting enzyme inhibitor, commonly used to treat hypertension. Its target in the body is the renin-angiotensin-aldosterone system, which plays a significant role in blood pressure regulation. Later, the question asks for an analysis of a newborn female showing ambiguous genitalia and excessive 17-hydroxyprogesterone in blood, asking to identify the deficient enzyme. Raised 17-hydroxyprogesterone is typically indicative of congenital adrenal hyperplasia (CAH), a group of inherited disorders characterized by enzyme defects in cortisol biosynthesis. PLANNING: If we consider the pharmacology of lisinopril, it inhibits the conversion of angiotensin I to angiotensin II (a potent vasoconstrictor), leading to vasodilation, which should increase renal blood flow. Angiotensin II, also stimulates the secretion of aldosterone, a hormone that increases sodium reabsorption and increases potassium excretion. In the second question, CAH is most commonly due to a deficiency in 21α-Hydroxylase, which is required for the biosynthesis of mineralocorticoids and glucocorticoids. CLINICAL REASONING: The effect of lisinopril on renal physiology is theoretically to dilate the efferent arteriole of the renal vasculature. This reduces the pressure differential across the filtration barrier, leading to a reduced ultrafiltration pressure and thus a decreased glomerular filtration rate (GFR), despite increased renal blood flow. For the newborn, Raised 17-hydroxyprogesterone, ambiguous genitalia, and a 46,XX karyotype suggest CAH due to 21α-Hydroxylase deficiency. This is the most common form of CAH, and typically presents with such features. CONCLUDING: In light of lisinopril's action on renal vasculature, we can anticipate that in our hypertensive, diabetic patient, there will be an increased renal blood flow, decreased ultrafiltration pressure, and decreased GFR. In the case of our newborn, the raised 17-hydroxyprogesterone suggest 21α-Hydroxylase deficiency is the most likely diagnosis. RESPONSE: Consequently, for the first question, the most likely set of changes in glomerular hemodynamics, as a result of lisinopril therapy, would be (C) increased renal blood flow, decreased ultrafiltration pressure, decreased glomerular filtration rate. On the other hand, for the second question, a deficiency in the enzyme (B) 21α-Hydroxylase is the most likely cause of the newborn's symptoms, considering the elevated 17-hydroxyprogesterone levels and ambiguous genitalia.

**AI_C:** Firstly, we have a middle-aged man with hypertension, a condition characterized by elevated blood pressure, and Type 2 diabetes, a chronic ailment that impacts the body’s ability to process blood glucose. Proteinuria, or the presence of excess protein in his urine, may suggest renal involvement due to the long-standing hypertension and diabetes, indicating possible diabetic nephropathy. He is started on Lisinopril, an angiotensin-converting enzyme (ACE) inhibitor. We're asked what effect Lisinopril might have on several elements of his glomerular hemodynamics. Secondly, there's a term female newborn displaying ambiguous genitalia, suggesting an intersex or DSD (Disorder of Sex Development) condition, with high serum levels of 17-hydroxyprogesterone (17-OHP), a steroid hormone. Her karyotype is 46,XX, indicating a typical female chromosomal pattern. We're tasked with identifying the enzyme most probably deficient in this newborn. Rethinking the processes involved and the medications being administered can provide more clarity. Lisinopril functions by inhibiting the conversion of Angiotensin I to Angiotensin II in the Renin-Angiotensin-Aldosterone System (RAAS), which could have several impacts on renal hemodynamics. Elevated levels of 17-OHP suggest a disruption in the enzymatic steps of steroidogenesis. Since 17-OHP is at an elevated level but downstream hormones are likely deficient, one of the enzymes after 17-OHP in the conversion pathway could be impeded. Clinically, the use of lisinopril in our adult patient can cause an impairment in the ability of the kidneys to maintain the glomerular filtration rate (GFR). This is due to a reduction in the efferent arteriolar vasoconstriction that normally helps to maintain GFR despite fluctuations in systemic blood pressure. Moreover, the inhibition of angiotensin II also leads to a decrease in aldosterone release which in turn causes decreased sodium and water reabsorption, likely resulting in increased renal blood flow and a reduction in ultrafiltration pressure. For the female newborn, Congenital Adrenal Hyperplasia (CAH) due to 21-hydroxylase deficiency, an autosomal recessive disorder, can cause elevated levels of 17-OHP, leading to high circulating androgens, accounting for the newborn's ambiguous genitalia. Thus, in the case of the adult male patient, telling signs point to (C) increased renal blood flow, decreased ultrafiltration pressure, decreased glomerular filtration rate, a likely outcome of Lisinopril therapy. For the newborn girl, it seems (B) 21α-Hydroxylase is the enzyme most possibly deficient, given the clinical picture and elevated 17-OHP levels.

**AI_E:** RESTATING THE QUESTION: The first clinical scenario refers to a 45-year-old man with a history of type 2 diabetes mellitus, hypertension, and proteinuria who has been started on lisinopril, an angiotensin-converting enzyme (ACE) inhibitor. The query relates to the changes in kidney functions, specifically renal blood flow, ultrafiltration pressure, and glomerular filtration rate, expected after this new medication regimen. The second scenario is about a term newborn girl with ambiguous genitalia and significantly increased levels of 17-hydroxyprogesterone. The question seeks to identify which enzyme deficiency is most likely in this newborn, based on the presented symptoms. PLANNING: In the first scenario, I should reflect on the pharmacological effect of lisinopril on the renal system considering the context of the patient's existing medical conditions. The assumptions in question include the impact of lisinopril on renal blood flow, ultrafiltration pressure, and glomerular filtration rate. In the second scenario, the primary assumption is that the newborn has an enzymatic deficiency leading to ambiguous genitalia and increased 17-hydroxyprogesterone. I need to scrutinize the biosynthetic pathway of adrenal steroidogenesis to determine the possible enzyme deficiency. CLINICAL REASONING: To address the first scenario, it is important to consider that lisinopril inhibits angiotensin-converting enzyme, preventing the conversion of Angiotensin I to Angiotensin II. This phenomenon will reduce vasoconstriction and aldosterone production, leading to vasodilation and lower sodium retention, respectively. These effects will then result in increased renal blood flow, decreased ultrafiltration pressure due to dilation of afferent arterioles and decreased glomerular filtration rate due to diminished sodium uptake. In the second scenario, considering the normal 46,XX karyotype and the presentation of ambiguous genitalia, we can assume that the newborn is suffering from congenital adrenal hyperplasia (CAH). CAH is a group of autosomal recessive disorders characterized by the impaired production of cortisol from cholesterol in the adrenal glands. One hallmark sign of CAH is the accumulation of 17-hydroxyprogesterone, which indicates a deficiency in 21α-hydroxylase, the enzyme that normally catalyzes the conversion of 17-hydroxyprogesterone to 11-deoxycortisol. CONCLUDING: In the first scenario, lisinopril is likely to cause an increased renal blood flow, decreased ultrafiltration pressure, and decreased glomerular filtration rate in the patient due to its inhibitory effect on angiotensin-converting enzyme. The second scenario suggests that the newborn is likely suffering from a deficiency of the enzyme 21α-hydroxylase, as indicated by the abnormal increase in 17-hydroxyprogesterone coupled with ambiguous genitalia. RESPONSE: So, for the 45-year-old man who started lisinopril therapy, the most probable set of changes in glomerular hemodynamics is increased renal blood flow, decreased ultrafiltration pressure, and decreased glomerular filtration rate—option (C). The newborn girl with ambiguous genitalia and increased serum 17-hydroxyprogesterone levels most likely has a deficiency of 21α-Hydroxylase—option (B).

**AI_B:** In the first scenario, we are asked to consider how a middle-aged male patient with a history of type 2 diabetes and hypertension might respond to being grounded on daily Lisinopril therapy. His medical history coupled with the prescription of Lisinopril leads us to question what impacts this medication could have on his renal hemodynamics. Lisinopril is an ACE inhibitor and can influence renal blood flow, ultrafiltration pressure and the glomerular filtration rate. In the second scenario, we have a newborn female with ambiguous genitalia and significantly high levels of serum 17-hydroxyprogesterone. We are asked to predict how her enzyme deficiency is based on these findings. We know from her karyotype that she has a normal number and configuration of chromosomes for a female (46,XX). The ambiguous genitalia and elevated 17-hydroxyprogesterone suggest an abnormality in steroid synthesis, which is often a result of an enzyme deficiency. In considering the first patient, the primary question is how Lisinopril, an ACE inhibitor, affects kidney function and the hemodynamics within the renal system. We have to remember that ACE inhibitors block the conversion of angiotensin I to angiotensin II, decreasing blood pressure and kidney function. Assumptions from this include that Lisinopril could lead to decreased renal blood flow, decreased ultrafiltration pressure, and decreased glomerular filtration rate. Contrarily, Lisinopril could increase renal blood flow because of lower blood pressure while decreasing ultrafiltration pressure and the glomerular filtration rate because of its impact reducing proteinuria and kidney damage. In anticipating the second patient's enzyme deficiency, we could assume that a deficiency in any enzyme involved in adrenal steroidogenesis could cause vaguely defined genitalia and high 17-hydroxyprogesterone levels. However, only certain deficiencies lead to an increase in 17-hydroxyprogesterone and ambiguous genitalia in genotypic females. In the first patient, Lisinopril's inhibition of ACE leads to decreased angiotensin II production. Angiotensin II usually constricts efferent arterioles more than afferent, increasing ultrafiltration pressure. Less angiotensin II, then, results in decreased ultrafiltration pressure. This, coupled with a tendency for ACE inhibitors to decrease peripheral vascular resistance and potentially increase renal blood flow, can lead to an overall decreased glomerular filtration rate. In the second case, we know that 21α-Hydroxylase deficiency, the most common form of Congenital Adrenal Hyperplasia (CAH), presents with ambiguous genitalia in female newborns. This enzyme deficiency also leads to decreased cortisol and aldosterone synthesis, causing an increase in ACTH secretion as the body attempts to stimulate more hormone production. The increased ACTH leads to adrenal hyperplasia and overproduction of adrenal steroid precursors, including 17-hydroxyprogesterone. For the first patient, Lisinopril therapy will most likely lead to increased renal blood flow, decreased ultrafiltration pressure, and a decreased glomerular filtration rate. This outcome (Option C) takes into account that the ACE inhibitor will block the production of angiotensin II, leading to less constriction on the efferent arterioles. This allows for greater renal blood flow but also decreases the ultrafiltration pressure. With the reduced pressure, the glomerular filtration rate will likely decrease. In the second scenario, the newborn most likely has a deficiency in 21α-Hydroxylase. This prediction (Option B) takes into consideration the ambiguous genitalia, high levels of 17-hydroxyprogesterone, and normal female karyotype. Such findings are characteristic of CAH due to 21α-Hydroxylase deficiency, which can cause virilization or ambiguous genitalia in affected females.

**Facilitator** The consensus statement synthesizes the responses as follows: In the first scenario, a 45-year-old man with a history of type 2 diabetes mellitus and hypertension has been started on lisinopril, an angiotensin-converting enzyme (ACE) inhibitor. The question asks about the likely changes in his renal hemodynamics as a result of this new medication. Lisinopril inhibits the conversion of angiotensin I to angiotensin II, leading to vasodilation and a decrease in aldosterone production. This results in increased renal blood flow and decreased ultrafiltration pressure. However, the decrease in angiotensin II also leads to a decrease in the glomerular filtration rate (GFR) due to diminished sodium uptake. Therefore, the most probable set of changes in glomerular hemodynamics in this patient as a result of lisinopril therapy is increased renal blood flow, decreased ultrafiltration pressure, and decreased glomerular filtration rate (Option C). In the second scenario, a term newborn girl presents with ambiguous genitalia and significantly increased levels of 17-hydroxyprogesterone. The question asks about the most likely enzyme deficiency in this newborn. The clinical picture and the elevated 17-hydroxyprogesterone levels suggest a diagnosis of congenital adrenal hyperplasia (CAH), most commonly due to a deficiency in 21α-Hydroxylase. This enzyme is required for the conversion of 17-hydroxyprogesterone to 11-deoxycortisol in the biosynthesis of mineralocorticoids and glucocorticoids. Therefore, the newborn girl most likely has a deficiency of 21α-Hydroxylase (Option B).
