# Supplementary material for: Collaborative intelligence in AI: Evaluating the performance of a council of AIs on the USMLE
Source: PLOS Digit Health. 2025 Oct 9;4(10):e0000787. doi: 10.1371/journal.pdig.0000787 (PMC12510544; doi:10.1371/journal.pdig.0000787)
Supplement: S2 File — (ZIP) [file pdig.0000787.s002.zip › S2 Data Raw data files containing unprocessed question inputs and LLM outputs including transcripts of deliberations/STEP1_Question-97-1_Type-MC-NJ.docx]

**Facilitator** **[Round 1]:** A 27-year-old woman comes to the emergency department because of a 1-hour history of severe shortness of breath. She has just returned from a cross-country flight. She has a history of borderline hypertension. Her temperature is 36.9°C (98.5°F), pulse is 113/min, respirations are 28/min, and blood pressure is 138/85 mm Hg. Physical examination shows that the right calf has an increased circumference compared with the left calf, and there is tenderness behind the right knee. Which of the following is the most likely underlying cause of this patient's condition? (A) Antithrombin III deficiency (B) Factor V Leiden mutation (C) Glanzmann thrombasthenia (D) Protein C deficiency (E) von Willebrand disease

**AI_A:** RESTATING THE QUESTION: So, we have a 27-year-old woman who presented at the emergency with a shorteness of breath which began 1 hour ago. She had a cross-country flight and has a history of borderline hypertension. Apart from her vital signs, there's a physical finding of an enlarged right calf compared to the left one along with tenderness behind the right knee. The question is asking for the likely underlying cause of the patient's symptoms. PLANNING: Now, we need to consider what might be leading to her symptoms. All the possible causes listed are related to abnormalities in the body's clotting system. An Antithrombin III deficiency, Factor V Leiden mutation, and Protein C deficiency could lead to an increased risk of developing blood clots. Glanzmann thrombasthenia and von Willebrand disease, on the other hand, are conditions that impair the body's ability to form clots – typically presenting with a tendency to bleed, not clot. The patient's symptoms of shortness of breath and the physical examination findings seem suggestive of a venous thromboembolism which can potentially block the blood flow to the lungs causing pulmonary embolism. CLINICAL REASONING: Given the history of recent prolonged immobilization (long cross-country flight), the presence of borderline hypertension, and the physical examination findings indicating a deep vein thrombosis (DVT), it becomes highly probable that her shortness of breath is being caused by a pulmonary embolism. The latter consequences are potentially life-threatening complications of conditions causing hypercoagulability, such as Antithrombin III deficiency, Factor V Leiden mutation, or a Protein C deficiency, rather than conditions leading to bleeding tendencies. CONCLUDING: Comparing all the alternatives in the context of our clinical suspicion, a genetic mutation would be an expected underlying cause in someone her age, rather than deficiencies typically acquired over a longer period of time. Among the given options, the Factor V Leiden mutation is the most common inherited thrombophilia in populations of European descent, making it the most likely cause in a patient presenting with a DVT and pulmonary embolism. RESPONSE: From her symptoms and the results obtained from her physical examination, it can be deduced that the most likely underlying cause of her condition is (B) Factor V Leiden mutation.

**AI_D:** RESTATING THE QUESTION: This is a clinical scenario involving a young woman presenting with acute shortness of breath, borderline hypertension, and evidence of unilateral lower limb edema and tenderness post a period of prolonged immobility (cross-country flight). She may have a predisposing factor increasing the likelihood of a thrombus formation. The task here is to determine the most probable underlying clinical cause for her symptoms among the given conditions, which are different types of coagulation disorders or deficiencies of various elements in the clotting cascade. PLANNING: Let's consider each possible underlying cause one by one. Antithrombin III deficiency, Factor V Leiden mutation, and Protein C deficiency are all conditions that increase the risk of forming abnormal blood clots in veins, which could explain the calf swelling, tenderness, and possibly the woman's shortness of breath if a clot has moved to her lungs (pulmonary embolism). Glanzmann's thrombasthenia and von Willebrand disease, on the other hand, usually cause bleeding problems, not clotting problems, which doesn't fit this clinical picture. CLINICAL REASONING: Given the acute history of profound shortness of breath and recent cross-country flight along with the physical finding of unilateral calf swelling and tenderness, a likely clinical diagnosis is pulmonary embolism (PE), resultant from a deep vein thrombosis (DVT) in the right calve. The listed potential causes are all diseases of coagulation. Antithrombin III deficiency, Protein C deficiency, and Factor V Leiden mutation are all hypercoagulable states that can predispose to PE. However, Factor V Leiden mutation is a more common inherited hypercoagulable disorder. It results in section resistance to activated Protein C leading to high chances of DVT. Glanzmann Thrombasthenia is a rare genetic disorder that mostly manifests as mucocutaneous bleeding, not DVT while von Willebrand disease is a bleeding disorder with manifestations including mucocutaneous bleeds rather than thromboembolic events. CONCLUDING: By correlating the presenting signs and symptoms with the understanding of these potential pathological conditions, it can be analyzed that, among the listed items, only Antithrombin III deficiency, Factor V Leiden mutation, and Protein C deficiency could predispose to DVT. Out of these, the most common cause in the general population is Factor V Leiden mutation, making it the most likely underlying cause. RESPONSE: The most likely underlying cause of this patient's condition is (B) Factor V Leiden mutation.

**AI_E:** Restating the question, a young woman has presented in the emergency department with sudden shortness of breath following a long flight. She has borderline hypertension and physical examination reveals possible signs of deep vein thrombosis (DVT) in her right leg. The query is about the probable underlying cause of her symptoms, with potential answers being several clotting disorders. To plan our clinical reasoning, we should consider a few points. First, we know that she has a leg that is swollen and tender, most likely a DVT. Second, we know she's short of breath, which can be a sign of a pulmonary embolism (PE), which can happen when a blood clot travels to the lungs. We need to look at a condition that primarily causes excessive blood clot formation or inhibits the body's natural anticoagulant mechanisms, keeping in mind our patient's clinical history and demographic. Let's start our clinical reasoning by excluding conditions that don't fit her clinical signs and symptoms. (A) Antithrombin III deficiency or (D) Protein C deficiency both increase clotting, but these are very rare conditions not likely to happen in an otherwise healthy young woman. Moreover, they appear early in life. (C) Glanzmann thrombasthenia and (E) von Willebrand disease both lead to a reduction in normal blood clot formation and her signs and symptoms of a likely DVT does not fit with these disorders. (B) Factor V Leiden mutation is a genetic disorder that results in hypercoagulability (a propensity to form abnormal blood clots in blood vessels). This mutation is the most common inherited hypercoagulability disorder amongst the Caucasian population. The mutation causes an increase in clotting as it makes factor V resistant to the anticoagulant effects of activated Protein C. Long periods of immobility like sitting during cross-country flight increase the risk of DVT in patients with mutation. Hence, the patient's shortness of breath, tenderness behind knee, swollen calf, and recent history of prolonged immobility (long flight) are all suggestive of a PE secondary to DVT, which could likely be secondary to (B) Factor V Leiden mutation in this clinical scenario. So, the most likely underlying cause of this patient's condition would be the Factor V Leiden mutation.

**AI_C:** RESTATING THE QUESTION: The patient, a 27-year-old female, presented with severe shortness of breath that started an hour prior to arriving in the ED. This symptom developed shortly after a long plane trip. Upon physical examination, an increase in circumference in the right calf, as well as tenderness behind that knee, was observed. She has borderline hypertension, and her vital signs include an elevated heart rate and respiration rate. The question is asking what the most likely root cause of these symptoms is, with options relating to different thrombotic disorders or deficiencies. PLANNING: In this phase, the assumptions are that the symptoms exhibited by the patient are due to thrombotic disorder possibly caused by prolonged immobility on a long flight, which induces venous stasis, a risk factor for deep vein thrombosis (DVT), and possibly followed by pulmonary embolism (PE). This assumption links well with the patient's history, her physical examination findings, and her elevated heart and respiration rates, which could be a physiological response to poor oxygen perfusion in the body due to a possible PE. We question each assumption and alternate them with the consideration of different thrombotic disorders which could be the underlying causes. CLINICAL REASONING: The pathogenesis of DVT, caused primarily by venous stasis (from the long flight), endothelial damage, and hypercoagulability can link with each of the listed conditions (A-E), which all predispose to thrombosis. However, it's worth noting the age of the patient and the absence of other significant medical history, which suggests this could be her first episode. Hence, it's likely to be due to a genetic predisposition. Glanzmann thrombasthenia (C) and von Willebrand disease (E) are characterized by bleeding disorders rather than thrombosis. While deficiencies of Antithrombin III (A) and Protein C (D) yield a hypercoagulable state leading to recurrent venous thromboembolism (VTE), the most common genetic thrombophilia is Factor V Leiden mutation (B), causing resistance to activated Protein C. CONCLUDING: Considering the provided information and the patient's background, it's reasonable to assume that the symptoms may be due to pulmonary embolism following deep vein thrombosis, a clinical condition that could result from Factor V Leiden mutation - a common inherited cause of hypercoagulability. RESPONSE: Based on the clinical context and reasoning, the most likely underlying condition for this patient's symptoms would be (B) Factor V Leiden mutation.

**AI_B:** (1) RESTATING THE QUESTION: So, we're trying to understand the most probable underlying cause of a 27-year-old woman's distress, which includes severe shortness of breath, recently traveled long-distance, borderline hypertension, and significant differences in her calves in terms of size and tenderness behind the right knee. The stated options are all related to blood coagulation disorders. Let's dissect these briefly: Antithrombin III deficiency is an inherited disorder that reduces the amount of this protein, leading to increasing the blood's tendency to clot. Factor V Leiden mutation makes clotting more likely due to a hyperactive factor V. Glanzmann thrombasthenia is a rare blood disorder in which the blood does not clot properly due to defects in platelet function. Protein C deficiency is a disorder that increases the risk of developing abnormal blood clots due to a lack of protein C, a protein that prevents clotting. Lastly, von Willebrand disease is a condition that affects blood clot due to a lack of von Willebrand factor. (2) PLANNING: The common premise among all these disorders is that they predispose to blood clotting. The notable symptoms in our patient can be related to a clot, such as shortness of breath (potentially due to a pulmonary embolism) and tenderness of the right calf (potentially a deep vein thrombosis, DVT). What we should then examine in this case is the clotting predisposition of each disorder under the circumstances provided by the patient's information. (3) CLINICAL REASONING: Important to note is that our patient has recently traveled on a cross-country flight, which increases the risk of DVT due to prolonged immobilization known as an economy class syndrome. Also, her borderline hypertension could be a contributing factor, but it would not necessarily increase her risk of DVT. Her symptoms of acute shortness of breath alongside the physical findings of unequal calf circumference and tenderness point towards a DVT which might have dislodged, leading to a pulmonary embolism. Looking into the offered options, von Willebrand disease, Glanzmann thrombasthenia are associated with bleeding tendencies rather than thrombosis, so we can rule out these disorders. Antithrombin III deficiency and Protein C deficiency have significant consequences leading to increased risk of thrombosis but appear more rarely and usually involve extensive familial history of unusual blood clots, which we do not have any information about this. Factor V Leiden, however, is a common genetic disorder in Caucasians, leading to activated protein C resistance. This condition could potentially lead to the formation of abnormal blood clots within blood vessels, causing DVT which subsequently can lead to pulmonary embolism especially after long periods of inactivity such as long flights. (4) CONCLUDING: After going through the process, Factor V Leiden appears to be the most substantial assumption for the patient's clinical picture among the given options, although confirmatory tests should definitely be conducted. (5) RESPONSE: Based on the provided clinical scenario and my clinical reasoning, I would suggest (B) Factor V Leiden mutation as the most likely underlying cause of this patient's condition.

**Facilitator** The consensus statement synthesizes the responses of AI_A, AI_B, AI_C, AI_D, and AI_E as follows: The patient, a 27-year-old woman, presented with severe shortness of breath following a cross-country flight. She has a history of borderline hypertension and physical examination reveals an enlarged right calf compared to the left one, along with tenderness behind the right knee. These symptoms suggest a possible deep vein thrombosis (DVT) and subsequent pulmonary embolism (PE). The question asks for the most likely underlying cause of her symptoms, with options related to various clotting disorders. The possible causes listed are all related to abnormalities in the body's clotting system. Antithrombin III deficiency, Factor V Leiden mutation, and Protein C deficiency could lead to an increased risk of developing blood clots. Glanzmann thrombasthenia and von Willebrand disease, on the other hand, are conditions that impair the body's ability to form clots, typically presenting with a tendency to bleed, not clot. Given the patient's history of recent prolonged immobilization (long cross-country flight), the presence of borderline hypertension, and the physical examination findings indicating a DVT, it becomes highly probable that her shortness of breath is being caused by a PE. These are potentially life-threatening complications of conditions causing hypercoagulability, such as Antithrombin III deficiency, Factor V Leiden mutation, or a Protein C deficiency, rather than conditions leading to bleeding tendencies. Comparing all the alternatives in the context of our clinical suspicion, a genetic mutation would be an expected underlying cause in someone her age, rather than deficiencies typically acquired over a longer period of time. Among the given options, the Factor V Leiden mutation is the most common inherited thrombophilia in populations of European descent, making it the most likely cause in a patient presenting with a DVT and PE. Therefore, based on the clinical context and reasoning, the most likely underlying cause of this patient's condition is the Factor V Leiden mutation.
