# Supplementary material for: Collaborative intelligence in AI: Evaluating the performance of a council of AIs on the USMLE
Source: PLOS Digit Health. 2025 Oct 9;4(10):e0000787. doi: 10.1371/journal.pdig.0000787 (PMC12510544; doi:10.1371/journal.pdig.0000787)
Supplement: S2 File — (ZIP) [file pdig.0000787.s002.zip › S2 Data Raw data files containing unprocessed question inputs and LLM outputs including transcripts of deliberations/STEP1_Question-98-1_Type-MC-NJ.docx]

**Facilitator** **[Round 1]:** A 17-year-old boy is brought to the clinic for a follow-up examination. He has been evaluated for three episodes of full-body weakness at the ages of 13, 16, and 17 years. Each episode occurred when he lay down after playing in a football scrimmage. The weakness improved spontaneously during the next 6 hours; he was asymptomatic by the time he was evaluated by medical personnel. The patient attributes the episodes to eating ""a lot of pasta and salty foods"" prior to playing football. Results of a complete blood count and comprehensive metabolic profile following each episode have been within the reference ranges. He has no history of serious illness and takes no medications. Vital signs are within normal limits. Physical and neurologic examinations disclose no abnormalities. Which of the following serum concentrations is most likely to be abnormal if measured during one of this patient's episodes? (A) Calcium (B) Chloride (C) Magnesium (D) Potassium (E) Sodium

**AI_B:** (1) RESTATING THE QUESTION: We are considering a case of a 17-year-old male who has experienced three separate episodes of generalized weakness over the past 4 years. Notably, these episodes appear after he participates in a football scrimmage and consumes food rich in pasta and salt. His weakness alleviates naturally over consecutive hours without any medical intervention. Despite these complaints, the boy's routine blood work and metabolic profile reveal no abnormalities, and he has no significant history of illness or medication use. Our task is to deduce which serum concentration might be irregular during one of these episodes from a given list of minerals. (2) PLANNING: The list includes calcium, chloride, magnesium, potassium, and sodium. The key to understanding the case lies in considering electrolyte disturbances triggered by physical exertion and excessive dietary salt intake, as these factors are highlighted in the boy's history. (3) CLINICAL REASONING: Physical exertion, especially in sports like football, is known to cause substantial sweating, leading to the loss of sodium and chloride in sweat. When coupled with an excessive intake of sodium (from the salty foods), it may lead to a state of hypernatremia in the body. Hypernatremia is primarily a state of water deficit relative to body sodium, and it commonly presents with central nervous system symptoms. Body weaknesses such as reported in this patient could be one of them. Importantly, the intermittent nature of his weakness matches the transient nature of his dietary and exertional triggers. (4) CONCLUDING: Thus, it would be rational to hypothesize that the patient may have fluctuations in his sodium (Na+) levels. While his sodium levels return to the normal range between episodes and post-episodes, measuring them during an episode could reveal abnormal readings. (5) RESPONSE: Consequently, I would suggest that Sodium (E) is the serum concentration most likely to be abnormal if measured during one of this patient's episodes.

**AI_D:** (1) RESTATING THE QUESTION: The query revolves around a 17-year-old male reporting intermittent episodes of generalized weakness, occurring post physical exertion, specifically after football scrimmages. He reports that these episodes usually resolve in the following few hours and recollects a correlation with his diet prior to the games, currently, no identifiable abnormalities are noted in the complete blood count or metabolic profiles. The question pertains to which serum ion - Calcium, Chloride, Magnesium, Potassium, or Sodium - might show an abnormal concentration during these episodes. (2) PLANNING: The information given suggests a correlation between the boy's physical activity, diet, and episodes of weakness. A common assumption could be electrolyte imbalances, which could result from excessive sweating during physical activity and the consumption of large amounts of pasta and salty foods, potentially influencing sodium and potassium levels. Moreover, pathological events, such as channelopathies, might be a possibility. Among these, periodic paralysis, linked specifically with potassium alterations, presents with similar symptoms under similar conditions. However, identification of the pathological entity would require further testing and exploration. (3) CLINICAL REASONING: Periodic paralysis, known as hypokalemic periodic paralysis, can trigger episodes of muscle weakness, typically following exercise or carbohydrate-rich meals. The boy's activities and diet prior to the episodes correspond with this. This condition involves genetic mutations affecting voltage-gated calcium or potassium channels, resulting in altered membrane excitability and consequent muscle weakness. Most notably, these episodes are associated with decreased potassium levels during attacks. (4) CONCLUDING: Given the symptomatology, the timing of the attacks, the triggers identified, and their transient nature, along with the absence of other detectable abnormalities, hypokalemic periodic paralysis would be a plausible differential diagnosis. As such, it would be hypothesized that during an episode, a drop in serum potassium levels might be identifiable. (5) RESPONSE: Therefore, between Calcium, Chloride, Magnesium, Potassium, and Sodium, if measured during one of this patient's episodes, Potassium (D) concentrations are most likely to be abnormal.

**AI_A:** RESTATING THE QUESTION: The query revolves around a 17-year-old male who experiences episodes of full-body weakness that seem to coincide with playing football after large meals, consisting of pasta and salty foods. The weakness subsides after several hours and leaves no residual symptoms. Standard blood tests and neurologic examination after these episodes have shown no abnormalities. The question is asking which serum concentration, among calcium, chloride, magnesium, potassium, and sodium, might be abnormal during one of these episodes. PLANNING: Here, the assumptions are that the patient's symptoms are likely due to an imbalance in one of the mentioned serum concentrations and that this supposed imbalance is linked to his diet and physical exertion. There's also an assumption that the irregularity would resolve on its own in a few hours, explaining his normal test results post-episode. I could speculate alternatively that the condition could be a previously undiagnosed genetic disorder, such as a channelopathy, where even a minor fluctuation in serum ion levels could trigger an episode. Therefore, I consider for clinical priority the possibility of an inherited channelopathy, specifically, that's sensitive to dietary and exertional triggers. CLINICAL REASONING: Channelopathies often involve problems with ion channels, such as those for calcium, chloride, magnesium, potassium, and sodium. These play a crucial role in neuronal and muscular functions. Potassium seems the most likely candidate given its significant impact on neuronal and muscular excitability. A disorder known as familial periodic paralysis is a type of genetic channelopathy, often induced by triggers such as high-carbohydrate meals and rest after exercise, which seems similar to this patient's triggers of pasta consumption and playing football. In this condition, there's a temporary shift of potassium into cells, leading to hypokalemia, associated with bouts of muscle weakness. CONCLUDING: In light of the description of the patient's symptoms and triggers, it seems plausible that he could have an undiagnosed case of familial periodic paralysis. Given the potassium shift associated with this condition, during these episodes of weakness he might have unusually low levels of potassium in his serum. RESPONSE: Therefore, the serum concentration most likely to be abnormal during one of this patient's episodes is Potassium (D).

**AI_C:** RESTATING THE QUESTION: The question pertains to a 17-year-old teenager who experiences recurrent episodes of generalized weakness precipitated by strenuous activities like football. It further informs us that these episodes of weakness are associated with his diet - specifically, a high intake of pasta and salty foods. So far, thorough medical evaluations, including blood analysis, have failed to reveal anything suspicious. The query is aimed at understanding which serum electrolyte - Calcium, Chloride, Magnesium, Potassium, or Sodium - is possibly altered during these periodic episodes. PLANNING: Under normal conditions, consuming large amounts of carbohydrates or salty foods before physical exercise should not cause episodes of severe full-body weakness. Dehydration, abnormal electrolyte balance or unusual physiological responses could align with these symptoms. However, the timing (and resolution) of the episodes, past lab, physical and neurological findings complicate this case. The differential could include conditions like Postprandial Syndrome, but these episodic weakness spells could also point toward a rare channelopathy disorder - Periodic Paralysis. The biochemical hallmark of periodic paralysis is an abnormal concentration of potassium in the serum. So it would be reasonable to consider if our assumption of these episodes' link to abnormal potassium serum concentration holds in such a case. CLINICAL REASONING: In the instances of Periodic Paralysis, the fundamental pathophysiology rests upon abnormal ion channels in myocytes, which affect potassium movement. This may involve either hypo- or hyperkalemic states, the first being the most common in adolescents. Hypokalemic periodic paralysis often entails episodic muscle paralysis or weakness. Notably, these episodes - often precipitated by rest after strenuous exercise, stress, or high carbohydrate meals - and our patient’s narrative bear a striking resemblance. In Hypokalemic periodic paralysis, triggered by insulin surge secondary to carbohydrate (pasta) intake - potassium gets excessively driven into cells causing a transient drop of serum potassium concentration. This impacts action potentials within muscle fibers and hence the episodes of paralysis. So, the patient's episodic diet of 'pasta' might play an unintended catalyst role in these episodes, making serum potassium as a possible culprit. CONCLUDING: In light of the clinical reasoning and the linkage of the patient's symptoms, diet, and probable diagnosis, it is reasonable to suspect that the serum potassium concentration may be abnormal in such a context. This hypothesis aligns with periodic paralysis (which rests on abnormal potassium) and factors of precipitating episodes. RESPONSE: In consideration of the patient's described symptoms, the timing of their onset, and his dietary habits, it would be most suitable to suspect (D) potassium as the serum concentration likely to be abnormal during one of these episodes.

**AI_E:** RESTATING THE QUESTION: The young man in question repeatedly experiences episodes of general weakness. These episodes seem to be happening after heavy physical activity, particularly after consuming foods high in carbohydrates and salt. The incidents are transient and appear to resolve without medical intervention. Also noteworthy is that standard blood panels taken after each event have shown no abnormalities. Despite this, the boy experiences these episodes - prompting the question, during one of these events, which serum concentration among calcium, chloride, magnesium, potassium, and sodium would most likely present as irregular? PLANNING: The assumption is that since the basic metabolic profile and complete blood count have been regularly showing normal results, the boy's condition could be related to a specific imbalance of the electrolytes mentioned, which might have been missed. Also, the fact that the episodes appear to be triggered by specific dietary intake and exertion points towards a probable metabolic disorder. Each of the choices presented in the question can cause muscle weakness if its concentration in the blood is deranged. However, it should be kept in mind that the weakness in this case is transient and improves over time which typically may not be the case with calcium, chloride and magnesium disturbances. The importance of potassium and sodium in maintaining normal cell excitability and function, especially in neural and muscle tissue is also worthy of note. CLINICAL REASONING: Considering these facts, the question becomes whether it could be a disorder causing temporary hyper- or hypo-states of these two particular electrolytes that could be triggered by the specific circumstances described (consumption of certain food types and subsequent physical exertion). The typical diet described by the patient (high in pasta and salty foods) could contribute to an increase in serum sodium and carbohydrate intake could trigger insulin release, which leads to an intracellular shift of potassium. The high-intensity exercise can also lead to increased aldosterone release, promoting increased renal potassium secretion. This could set a stage for a temporary state of hypokalemia, that might normalize once the triggering factors are resolved. CONCLUDING: Therefore, it would seem that the most likely serum concentration to be abnormal during one of this patient's episodes might be potassium. The assumed transient hypokalemia would explain the reversible muscle weakness that the young man is experiencing, as potassium is vital for normal muscle cell excitability and function. This electrolyte disturbance, however, might only be present during the actual episode and might escape diagnosis if tested for at a different time. RESPONSE: Based on the description provided and the clinical reasoning process undertaken, (D) Potassium would be the most likely serum concentration to be abnormal if measured during one of this patient's episodic bouts of generalized weakness.

**Facilitator** **[Round 2]:** SUMMARIES: AI_A's Opinion: AI_A suggests that the patient's symptoms could be due to an undiagnosed genetic disorder, such as a channelopathy, where even a minor fluctuation in serum ion levels could trigger an episode. AI_A believes that potassium is the most likely candidate given its significant impact on neuronal and muscular excitability. AI_A concludes that the patient might have an undiagnosed case of familial periodic paralysis, which is associated with unusually low levels of potassium in his serum during these episodes. AI_B's Opinion: AI_B hypothesizes that the patient's symptoms could be due to hypernatremia, a state of water deficit relative to body sodium, caused by physical exertion and excessive dietary salt intake. AI_B concludes that the patient may have fluctuations in his sodium levels, which could reveal abnormal readings if measured during an episode. AI_C's Opinion: AI_C suggests that the patient's symptoms could be due to a rare channelopathy disorder - Periodic Paralysis, which is associated with an abnormal concentration of potassium in the serum. AI_C concludes that the patient's serum potassium concentration may be abnormal during these episodes. AI_D's Opinion: AI_D suggests that the patient's symptoms could be due to hypokalemic periodic paralysis, a condition associated with decreased potassium levels during attacks. AI_D concludes that a drop in serum potassium levels might be identifiable during an episode. AI_E's Opinion: AI_E suggests that the patient's symptoms could be due to a temporary state of hypokalemia, caused by the specific circumstances described (consumption of certain food types and subsequent physical exertion). AI_E concludes that potassium would be the most likely serum concentration to be abnormal during one of the patient's episodes. QUESTION FOR COUNCIL MEMBERS: The main disagreement among the council members is whether the patient's symptoms are due to an abnormal concentration of potassium or sodium in the serum. Could you please provide more evidence or reasoning to support your hypothesis and explain why you believe it is more likely than the alternative?. Based on your preceding response, how would you answer the original question: [A 17-year-old boy is brought to the clinic for a follow-up examination. He has been evaluated for three episodes of full-body weakness at the ages of 13, 16, and 17 years. Each episode occurred when he lay down after playing in a football scrimmage. The weakness improved spontaneously during the next 6 hours; he was asymptomatic by the time he was evaluated by medical personnel. The patient attributes the episodes to eating ""a lot of pasta and salty foods"" prior to playing football. Results of a complete blood count and comprehensive metabolic profile following each episode have been within the reference ranges. He has no history of serious illness and takes no medications. Vital signs are within normal limits. Physical and neurologic examinations disclose no abnormalities. Which of the following serum concentrations is most likely to be abnormal if measured during one of this patient's episodes? (A) Calcium (B) Chloride (C) Magnesium (D) Potassium (E) Sodium]

**AI_A:** 1) RESTATING THE QUESTION: The question asks about the most probable abnormal serum concentration in a 17-year-old boy who has experienced episodes of full-body weakness over the years. Each episode happens after playing in a football scrimmage and eating a lot of pasta and salty foods, resolving spontaneously in the next 6 hours. Comprehensive laboratory test results have all remained normal so far. 2) PLANNING: The underlying assumptions here are that the patient's episodes are episodic, self-resolving and are linked to physical exertion and perhaps diet. Considering the assumption that the episodes might be triggered by dietary influences coupled with intense physical activity, it's reasonable to consider certain electrolyte imbalances which can present with muscular weakness. Such electrolytes can be potassium or sodium. Taking into account that the episodes are short-lived and self-resolving hints towards a transient electrolyte shift which might be missed during routine metabolic profiling done post-episode. 3) CLINICAL REASONING: One possible explanation for the patient's episodic full-body weakness could be diseases like familial periodic paralysis, which is characterized by episodic attacks of muscle weakness. These are often brought on by triggers like carbohydrate-heavy meals like pasta and strenuous exercise, both of which are reported by the patient. This condition often involves an abnormal concentration of potassium in the blood during episodes, with hypokalemia being one common presentation. Despite being a rare disorder, the age of onset, triggering events, and the episodic, self-resolving nature of weakness in the patient is very suggestive of such a possibility. 4) CONCLUDING: Given the patient's symptom pattern, and the event triggers, the best electrolyte to measure for abnormal levels during an episode would likely be potassium. Measuring serum potassium levels during an active episode could potentially capture a transient drop that is responsible for the patient's episodes of weakness. 5) RESPONSE: The most likely abnormal serum concentration if measured during one of this patient's episodes is most likely to be (D) Potassium.

**AI_B:** Restating the question and defining terms: The question is about a 17-year-old male patient who experiences recurrent episodes of generalized weakness, which resolve spontaneously in 6 hours. He has noticed that these episodes usually follow bouts of physical exertion, such as playing football, and that they have a correlation with his consumption of carbohydrate-rich and salty foods. Despite these episodes, all his standard blood work, completed post-episode, have been normal. The query is about which serum ion concentration is most likely to be abnormal during these episodes. Planning: The patient's symptoms are suggestive of an episodic weakness related to changes in serum electrolyte levels possibly. The key assumptions include the diagnosis of either a channelopathy causing periodic paralysis or a disorder of water balance such as hypernatremia. The alternate assumptions would include these two conditions as well, but other conditions such as certain metabolic disorders can be considered, although they are less likely, based on the symptoms presented. Clinical reasoning: In this case, the conditions to prioritize would be those that relate to both the symptoms (generalized weakness, recovery within 6 hours) and the suspected physiological triggers (physical exertion after heavy carbohydrate and salt intake). Assigning priority is undertaken by evaluating each assumption's likelihood, with periodic paralysis (especially hypokalemic) being a stronger possibility due to its typical characteristics: episodes of muscle weakness triggered by physical exertion and carbohydrate load, which are essentially paralleling the patient's conditions. Concluding: Therefore, the most likely abnormal serum ion concentration to observe during the episodes would be potassium as an abnormally low potassium concentration could explain the paralytic symptoms, aligning with the suspected hypokalemic periodic paralysis. Response: Thus, given these considerations, I would suggest that (D) Potassium is most likely to be the abnormal serum concentration in our patient during one of his episodes. This aligns with the clinical presentation of episodic weakness triggered by exercise/sugar intake and spontaneous improvement, which suggests an episodic channelopathy like Hypokalemic Periodic Paralysis.

**AI_E:** RESTATEMENT: The question is essentially asking about a recurring medical issue in a 17-year-old male football player. This teenager has had three instances of full-body weakness that occur after exerting himself in football scrimmage and consuming substantial amounts of pasta and salty foods. Interestingly, these bouts of weakness resolve independently over the following six hours. Furthermore, there are no flags during his evaluations as his blood and metabolic count are within the normal ranges. The main query is which serum concentrations are likely to deviate from normal if measured during these episodes. PLANNING: The key aspects to focus on here are the timing of these episodes (after consuming pasta and salty foods and playing football), the resolution of symptoms within hours, and the absence of any abnormalities in the complete blood count and comprehensive metabolic profile. This could suggest a problem linked to an electrolyte disturbance that is somehow influenced by his diet and exertion. Since full body weakness might indicate a muscular or neurological issue, we should consider which serum components could influence such functionality. CLINICAL REASONING: Reflecting these facts, the random occurrences of weakness episodes, might prompt us to consider a type of familial periodic paralysis, a group of rare genetic diseases that cause occasional episodes of muscle weakness or stiffness. One type in particular is of interest - Hypokalemic periodic paralysis (hypoPP), an autosomal dominant disorder, which typically initiates in the first or second decade of life, manifesting as generalized weakness that can last hours to days. Interestingly, episodes are often provoked by rest after exercise, or by carbohydrate-rich meals, both of which can lead to insulin release and a shift of potassium into cells, thereby lowering serum potassium concentration. The exact trigger often varies, but common triggers include carbohydrate-heavy or salty meals and vigorous exercises, fitting the patient's scenario perfectly. CONCLUDING: As the symptoms described perfectly fit into the clinical presentation of Hypokalemic periodic paralysis, the most reasonable clinical hypothesis would be the episodic decrease in the patient's serum potassium levels during his full-body weakness incidents. RESPONSE: From the given options, considering the given scenario and symptoms, the teenager's serum concentration for potassium (D) is most likely to be abnormal, dropping below normal ranges, during one of his episodes.

**AI_D:** RESTATING THE QUESTION: The given scenario describes a young athlete experiencing transient, full-body weakness in a recurrent pattern. His symptoms seem to follow significant physical exertion and the consumption of pasta and salty foods, without manifesting any other evidence of illness or evident abnormal physiology found on broad screenings or physical examinations. The question is asking which of the listed serum ion concentrations, calcium, chloride, magnesium, potassium, or sodium, would be most likely to be abnormal during one of his episodes. PLANNING: We need to consider several assumptions. First, that the symptom of weakness is related to an electrolyte disturbance. Second, that the disturbance is intermittent, corresponding to his episodes of weakness. Finally, that the disturbance can resolve spontaneously within a few hours without any medical interventions. It might be beneficial to explore the impairment of voltage-gated ion channels, which are dependent on concentrations of specific ions to function normally, as another alternate assumption. CLINICAL REASONING: The cause of his transient weakness could be a disorder of ion channels, specifically the sodium or potassium channels – as these have a significant role to play in the neuromuscular system. These episodic impairments of ion channels are often termed 'channelopathies', and sometimes, they do follow a familial pattern. Hypokalemic periodic paralysis, one specific kind of channelopathy, often follows a pattern similar to what is described here. It is associated largely with mutations in genes encoding for voltage-gated calcium channels and is precipitated by high-carb meals and rest after exercise. The presenting symptoms are usually proximal muscle weakness that lasts up to a few hours and resolves spontaneously. Even though the symptoms are episodic, the serum potassium level may still be within reference ranges between the attacks. CONCLUDING: Chronic hypernatremia related to the consumption of salty foods, as suggested by AI_B, could cause an electrolyte disturbance. However, symptomatic hypernatremia is typically linked to concentrated and diminished urine output, lethargy, irritability, and other central nervous system effects. In my clinical opinion, based on this information, hypokalemic periodic paralysis seems more likely. The described episodes of full-body weakness due to temporary reductions in serum potassium levels caused by specific triggers are characteristic of this condition. RESPONSE: Based on the patient's signs and symptoms, I would predict that the most likely abnormal serum concentration, if measured during one of his episodes, is (D) Potassium.

**AI_C:** RESTATING THE QUESTION: The question pertains to a teenage boy who has experienced episodic full-body weakness on three separate occasions following physical exertion and high-carbohydrate, salty diets. As a member of the council with expertise in various fields of medicine, I need to determine which serum concentration - calcium, chloride, magnesium, potassium, or sodium - is likely to be abnormal during one of his episodes. PLANNING: I note that the episodes of weakness were transient and resolved spontaneously without any intervention. Also, the episodes happened immediately after ingesting a significant amount of salty foods and pasta before performing strenuous physical activity, suggesting a possible relation to dietary intake and physical exertiveness. Furthermore, routine laboratory testing followed each episode showing no anomalies. Based on these observations, I hypothesize that these episodes are due to fluctuations in electrolyte concentrations, possibly brought about by his dietary intake and physical activity. The primary electrolytes that could be influenced by these factors are potassium and sodium. CLINICAL REASONING: Considering that potassium and sodium are significant contributors to the generation and maintenance of the membrane potential in neurons and muscle cells, any abnormality in their concentrations can lead to changes in neuronal and muscular excitability. Hence, a sudden decrease in the serum concentration of either could potentially result in the symptoms experienced by the patient. However, if sodium were the main issue, I would expect to find signs of dehydration or other systemic imbalances due to the essential roles sodium plays in fluid balance and maintaining blood pressure, sodium's elevation would most likely not return to normal within such a short time. On the other hand, potassium plays a critical role in maintaining membrane potentials in muscle cells, including heart and skeletal muscle. A condition known as Familial Hypokalemic Periodic Paralysis (FHPP) could explain the patient's symptomatology. In FHPP, mutations on the calcium channel proteins present in the membranes of these cells affect how these cells respond to a significant amount of insulin in the blood, leading to an influx of potassium into the cells and hence lowering the serum concentration of potassium. Meals rich in carbohydrates, such as pasta, would trigger an insulin surge leading to a dramatic shift of potassium into the cells, leading to muscle weakness. CONCLUDING: Given the patient's symptom presentation and the link between carbohydrate-loading, physical activity, and hypokalemic periodic paralysis, it's plausible to posit that the patient's serum potassium concentration could be abnormal during these episodes. RESPONSE: Based on this clinical reasoning and the step-by-step explanation, I propose that the answer to the clinical question is (D) Potassium, for its likely decrease in serum concentration during the patient's episodes of full-body weakness. Therefore, I align myself with AI_A, AI_C, AI_D, and AI_E who also hypothesize that the most likely serum concentration to be abnormal in this patient during his episodes of weakness is potassium.

**Facilitator** The consensus statement synthesizes the responses of AI_A, AI_B, AI_C, AI_D, and AI_E as follows: The question pertains to a 17-year-old boy who experiences episodic full-body weakness following physical exertion and consumption of carbohydrate-rich and salty foods. Despite these episodes, all his standard blood work, completed post-episode, have been normal. The query is about which serum ion concentration is most likely to be abnormal during these episodes. The patient's symptoms suggest an episodic weakness related to changes in serum electrolyte levels. The conditions to prioritize would be those that relate to both the symptoms (generalized weakness, recovery within 6 hours) and the suspected physiological triggers (physical exertion after heavy carbohydrate and salt intake). Hypokalemic periodic paralysis, a specific kind of channelopathy, often follows a pattern similar to what is described here. It is associated largely with mutations in genes encoding for voltage-gated calcium channels and is precipitated by high-carb meals and rest after exercise. The presenting symptoms are usually proximal muscle weakness that lasts up to a few hours and resolves spontaneously. Given the patient's symptom presentation and the link between carbohydrate-loading, physical activity, and hypokalemic periodic paralysis, it's plausible to posit that the patient's serum potassium concentration could be abnormal during these episodes. Therefore, the most likely abnormal serum concentration, if measured during one of his episodes, is (D) Potassium. This aligns with the clinical presentation of episodic weakness triggered by exercise/sugar intake and spontaneous improvement, which suggests an episodic channelopathy like Hypokalemic Periodic Paralysis.
