# Supplementary material for: Collaborative intelligence in AI: Evaluating the performance of a council of AIs on the USMLE
Source: PLOS Digit Health. 2025 Oct 9;4(10):e0000787. doi: 10.1371/journal.pdig.0000787 (PMC12510544; doi:10.1371/journal.pdig.0000787)
Supplement: S2 File — (ZIP) [file pdig.0000787.s002.zip › S2 Data Raw data files containing unprocessed question inputs and LLM outputs including transcripts of deliberations/STEP1_Question-99-1_Type-MC-NJ.docx]

**Facilitator** **[Round 1]:** A 20-year-old woman is brought to the urgent care center because of a 2-month history of progressive weakness of her arms. She also has a 1-week history of moderate back pain and headache. Her only medication is ibuprofen as needed for pain. Muscle strength is 3/5 in the upper extremities. Sensation to pinprick is decreased over the upper extremities. MRI of the spine shows a central syrinx in the cervical spinal cord. It is most appropriate to obtain specific additional history regarding which of the following in this patient? (A) Diet (B) Family illness (C) Recent travel (D) Trauma (E) Unintended weight loss

**AI_A:** RESTATING THE QUESTION: The query is seeking to identify what additional information should be pursued in the medical history of a female patient, in her early adulthood, who is experienced progressive loss of strength in her arms over a 2-month duration, has also been having back pain and headache for a week. Despite utilizing Ibuprofen for pain relief, she has continued symptoms. Further examination revealed reduced muscle strength in upper extremities and decreased sensation to pinprick stimulus over the same area. An MRI revealed the presence of a Syrinx in the central portion of her cervical spinal cord. A Syrinx is a fluid-filled cavity within the spinal cord, a condition often resulting from trauma or a birth defect. PLANNING: Assumptions could be the condition might be due to diet or nutrient deficiency, a genetic or familial disorder, exposure from a recent travel, a history of trauma, or unintended weight loss. Questioning these individually might give alternate assumptions. However, considering the clinical presentation with a syrinx in the spine, traumatic history could be the most clinically relevant to examine. CLINICAL REASONING: Progressive weakness in the patient's arms, decreased sensation to pinprick alongside a syrinx in the cervical spinal cord strongly indicate some kind of neurological damage. Besides congenital conditions, syrinxes commonly develop due to injury or trauma to the spinal cord which leads to disruption of the normal flow of cerebrospinal fluid (CSF) resulting in a fluid-filled cavity or syrinx. Hence, even a minor trauma, often overlooked, can potentially be the culprit. CONCLUDING: Although all aspects would generally be covered in a thorough clinical history, given the presence of a syrinx and the neurological deficits, it is especially pertinent to probe further into the patient's history for episodes of any trauma, even if seemingly minor and possibly overlooked by the patient. RESPONSE: Hence the additional specific history that should ideally be obtained in this scenario would pertain to any traumatic events she may have experienced (Option D).

**AI_E:** RESTATING THE QUESTION: The query refers to a young woman displaying progressive upper limb weakness, along with new-onset back pain and headache. Her medical imaging reveals a syrinx within her cervical spinal cord. She is currently using ibuprofen as required for pain. A syrinx is a fluid-filled cavity that develops within the spinal cord. This ailment has variable presentation, but can be associated with symptoms such as pain, muscle weakness, and loss of sensation, fitting our patient’s clinical picture. PLANNING: A closer examination of her history is needed to pinpoint potential factors contributing to her condition. Possible factors within the scope of the provided choices include dietary intake (A), any family disease history (B), recent travel (C), any preceding trauma (D), or unexpected weight loss (E). Each assumption will be analyzed for clinical relevance. CLINICAL REASONING: (A) Diet: While nutrition can impact overall health, it does not often directly lead to spinal cord syrinx formation. (B) Family illness: Certain inherited conditions may contribute to spinal abnormalities, though they do not typically manifest as syrinxes specifically. (C) Recent travel: While travel can expose individuals to infections or parasites, it doesn't directly correlate with spinal cord syrinx development. (D) Trauma: Trauma to the spine can lead to the formation of a syrinx. For instance, post-traumatic syringomyelia can occur after incidents that cause spinal cord injury. (E) Unintended weight loss: While significant unintentional weight loss could signify systemic disease, it does not typically directly lead to a spinal cord syrinx. CONCLUDING: Our patient's MRI findings reveal a syrinx in the cervical region of her spinal cord, which likely underlies her progressive arm weakness and decreased pinprick sensation. Out of the options, a history of trauma (D) seems clinically most significant, as past incidents of spinal trauma can lead to syrinx formation. RESPONSE: Hence, the most suitable action is exploring any past experience of trauma (D). This information may shed light on potential causes of her spinal cord syrinx, which has likely been contributing to her symptoms. In addition, it may guide the course of her management.

**AI_D:** RESTATING THE QUESTION: We're evaluating a young woman who has been experiencing progressive weakness in her arms over the past two months, along with back pain and headaches in the last week. An MRI of her spine has revealed a syrinx, or a fluid-filled cavity, in the central region of her cervical spinal cord. "Syrinx" is a medical term that refers to a fluid-filled cavity within the spinal cord or brain stem. Given these symptoms and findings, we need to determine what additional history would be most helpful to obtain. PLANNING: With the diagnosis of a syrinx or syringomyelia in her cervical spinal cord, the potential considerations can be trauma, inflammatory conditions, tumors, hemorrhage, or congenital malformations like Chiari malformations. It thus seems prudent to prioritize the exploration of trauma (Option D) to her cervical spine, which can prompt the development of a syrinx. For instance, some patients might not recall or might underestimate the severity of past traumas. CLINICAL REASONING: Let's consider trauma or injury as our alternate assumption - this is based on the phenomenon that the development of a syrinx can be post-traumatic. The syrinx can form as a result of inflammation and scarring following spinal cord injury. This scarring can block the normal flow of cerebrospinal fluid, causing it to accumulate in the central canal of the spinal cord, leading to the formation of a syrinx. CONCLUDING: Linking this line of reasoning to our case, the patient is suffering from a loss of sensory and motor functions (muscle strength is 3/5) in her upper extremities. This might be due to damage to the anterior horn cells and lateral spinothalamic tract, commonly seen with a syrinx in the cervical region. Asking for a history of trauma could help us determine whether an injury was the underlying cause of the syrinx and is contributing to her symptoms. So, the most crucial aspect of her history to probe further into is whether she has had any trauma (Option D). RESPONSE: It's highly recommended to inquire about any history of trauma. This is because trauma could lead to the conditions that might have caused the development of a syrinx in her cervical spinal cord, resulting in her symptoms of progressive arm weakness, back pain, and headache.

**AI_C:** RESTATING THE QUESTION: The task at hand asks for the identification of additional historical information that should be gathered from this young female patient. The patient presents with progressive weakness in her arms, accompanied by back pain and headaches. The medical imaging reveals a syrinx, which refers to a fluid-filled cavity in her cervical spinal cord. PLANNING: In the process of diagnosing, several assumptions can be made. First, the symptoms could be due to a family-related illness or some inherited condition. Second, recent trauma could result in post-traumatic syringomyelia. Third, an unusual diet or unintended weight loss could indicate an underlying nutritional condition leading to neurological symptoms. Fourth, recent travel may expose her to infectious or environmental factors that trigger neurological manifestations. Alternately, each assumption should be evaluated concerning the key aspect: the presence of a syrinx which is likely to link to an underlying neurological condition. CLINICAL REASONING: The syrinx detected in the patient's cervical spinal cord is likely responsible for the progressive weakness and decreased sensation in the upper extremities, symptoms which are consistent with syringomyelia, a condition characterized by the formation of a cyst or cavity within the spinal cord that damages the surrounding neural tissue. This condition often results in muscle weakness and atrophy, pain, and disruptions in temperature and pain sensation below the level of the cyst. CONCLUDING: Syringomyelia can be congenital, often linked to Chiari type I malformation, or could be secondary, often resulting from trauma, meningitis, neoplasms, or arachnoiditis. Given these potential origins, it is pertinent to investigate those historical areas that could lead to the discovery of the primary cause. While all the options provided [i.e., diet, family illness, recent travel, trauma, unintended weight loss] could theoretically be relevant in a general clinical scenario, based on the patient's presentation and differential diagnoses, some areas are more likely relevant than others. RESPONSE: In this context, given the notable presence of a central syrinx as shown on an MRI, the most appropriate additional history would be related to Trauma (D). This is because trauma to the spinal cord can lead to post-traumatic syringomyelia, a form of syringomyelia resulting from the injury that can develop months or even years after the initial event. Any past spinal cord injury, ranging from minor falls to significant accidents, can be crucial to understanding her current condition.

**AI_B:** 1) RESTATING THE QUESTION: The clinical scenario plays out for a 20-year-old female patient with progressive arm weakness over a period of 2 months, accompanied by recent onset of back pain and headaches. She hasn't been exposed to any medications except for incidental use of ibuprofen for pain. The neurological examination confirmed muscle strength deficiency and sensory impairment in terms of a decrease in pinprick sensation over her upper extremities. Based on her MRI findings showing a syrinx in her cervical spinal cord, the query is about the most beneficial area of her history to further explore. Syrinx refers to a fluid-filled cavity within the spinal cord. 2) PLANNING: The possibilities include exploring her dietary history, understanding any family illness, her recent travel history, any incidents of trauma, and unexplained weight loss. Given her MRI finding of a syrinx in the cervical spinal cord, this opens up a differential diagnosis of conditions that could lead to a similar finding, notably syringomyelia, trauma, tumors, and inflammatory conditions. Each of these diagnoses can be associated with some of the provided options in different ways – a familial neurological disorder in the case of 'family illness', incidents of trauma to the spine or neck, and neuroinflammatory or neoplastic conditions often causing 'unintended weight loss'. 3) CLINICAL REASONING: Syringomyelia is a disorder in which a cyst or syrinx forms within the spinal cord. This cyst can expand and elongate over time, destroying part of the spinal cord. Symptoms usually begin between ages 25 and 40, most commonly involving muscle weakness and atrophy, often accompanied by dysesthesias and dissociated sensory loss. The symptoms are typically insidious in onset, pointing away from trauma and hinting more toward a chronic process like a tumor or an inflammatory condition. Understanding the underlying cause of the syrinx formation would require contemplating each available response option independently. While family illness (B) might open avenues exploring genetic disorders, recent travel (C) would be unlikely to be influencing our clinical scenario. Unintended weight loss (E) might indicate an insidious process like a tumor or a systemic condition. 4) CONCLUDING: However, given the MRI findings and the clinical picture, the immediate history that might be most impactful in changing our patient's management would perhaps be any recent or past traumatic incidences (D). Such events could have led to post-traumatic syringomyelia, one of the significant causes of syrinx formation in the cervical spinal cord. 5) RESPONSE: So, while other aspects of the history should not be overlooked, in our quest to elaborate upon our clinical diagnosis derived from acute presentation, recent MRI findings, and manifestation, I would prioritize obtaining additional history regarding '(D) Trauma'.

**Facilitator** The consensus statement synthesizes the responses of AI_A, AI_B, AI_C, AI_D, and AI_E regarding the additional history that should be obtained from a 20-year-old female patient presenting with progressive arm weakness, back pain, and headache. The patient's MRI reveals a syrinx in her cervical spinal cord, a fluid-filled cavity that can cause neurological symptoms. The AI responses suggest that while various aspects of the patient's history could be relevant, including diet, family illness, recent travel, and unintended weight loss, the most pertinent area to explore further is trauma (Option D). This is due to the fact that trauma can lead to conditions such as post-traumatic syringomyelia, which can cause the formation of a syrinx in the spinal cord. The patient's symptoms of progressive arm weakness and decreased sensation, along with the MRI findings, strongly indicate neurological damage, which can be caused by trauma to the spinal cord. Therefore, even minor traumatic events that the patient may have overlooked could be crucial in understanding her current condition. In conclusion, while all aspects of the patient's history should be considered in a thorough clinical examination, given the presence of a syrinx and the neurological symptoms, it is especially important to probe further into the patient's history for any episodes of trauma.
