# Supplementary material for: Collaborative intelligence in AI: Evaluating the performance of a council of AIs on the USMLE
Source: PLOS Digit Health. 2025 Oct 9;4(10):e0000787. doi: 10.1371/journal.pdig.0000787 (PMC12510544; doi:10.1371/journal.pdig.0000787)
Supplement: S2 File — (ZIP) [file pdig.0000787.s002.zip › S2 Data Raw data files containing unprocessed question inputs and LLM outputs including transcripts of deliberations/STEP2_Question-1-1_Type-MC-NJ.docx]

**Facilitator** **[Round 1]:** A 68-year-old man is brought to the emergency department because of a 2-day history of progressive lethargy and tremors. He has alcoholism and opiate, cocaine, and amphetamine use disorder, but he has abstained from alcohol and illicit substances during the past 2 weeks. He also has alcoholic cirrhosis. His only medication is a daily multivitamin. He previously drank six 12-oz beers daily. He appears chronically ill and is somnolent. Temperature is 36.8°C (98.2°F), pulse is 100/min, respirations are 18/min, and blood pressure is 100/60 mm Hg. Pulse oximetry on room air shows an oxygen saturation of 94%. Examination shows conjunctival icterus, ascites, and 2+ edema of the lower extremities. Cranial nerves are intact. Muscle strength is 4/5 throughout. When he is awake, there is a postural and action tremor of the forearms and hands that is accompanied by a sudden, brief loss of muscle strength. His speech is normal, and he is able to follow simple commands. Serum total bilirubin concentration is 3 mg/dL. Blood and urine cultures grow no pathogens. Which of the following is most likely to improve this patient’s cognition and movement disorder? (A) Intravenous vancomycin and cefepime therapy (B) Intravenous vitamin B1 (thiamine) therapy (C) Oral lactulose therapy (D) Oral lorazepam therapy

**AI_B:** RESTATING THE QUESTION: The inquiry seeks to identify the most feasible treatment plan to improve the cognitive function and movement disorder in a 68-year-old patient. He has a history of alcoholism and substance abuse, and is currently experiencing symptoms often associated with these conditions such as progressive lethargy and tremors, as well as features of chronic liver disease (alcoholic cirrhosis) such as conjunctival icterus, ascites, and edema of the lower extremities. PLANNING: Several assumptions can be drawn from the case presented. One, the patient's neurological signs and symptoms could be related to his past substance abuse, suggesting a possible withdrawal syndrome. Alternatively, the neurological manifestations might be a result of another condition entirely, possibly another metabolic or neurological disorder. A second assumption could be that the patient's cognitive and movement disorder might stem from a complication of his liver disease, like hepatic encephalopathy. Given these assumptions, a careful examination of the case details and application of clinical reasoning should be utilized to guide which assumption should be pursued. CLINICAL REASONING: The patient's earlier history of alcohol and substance abuse might make one consider withdrawal syndromes as the cause for his cognitive and motor signs. However, his claimed abstinence from alcohol and illicit substances during the last two weeks and the absence of typically associated symptoms like hallucinations and seizures makes this assumption less likely. On the other hand, the patient exhibits numerous clues suggesting chronic liver disease such as ascites, 2+ edema, and elevated bilirubin. These findings, in conjunction with his neurological signs, could hold a clue to the diagnosis. Considering these, a more plausible condition to consider could be Wernicke's encephalopathy, a neurological disorder caused by thiamine (vitamin B1) deficiency. It is common among individuals with chronic alcoholism due to malnourishment and impaired thiamine absorption. Its classical triad includes mental status changes, ophthalmoplegia, and ataxia; however, it may present atypically as in this case. This patient's cognitive and movement disorders can be ascribed to Wernicke's encephalopathy. CONCLUDING: To treat this patient's cognitive and motor issues, it is important to provide immediate thiamine replacement. Thiamine is a key co-enzyme in carbohydrate metabolism. Its deficiency affects the brain primarily, manifesting cognitive and motor disorders. Thiamine replacement can reverse these symptoms. RESPONSE: The most appropriate treatment to improve this patient's cognition and movement disorder would be (B) Intravenous vitamin B1 (thiamine) therapy.

**AI_A:** RESTATING THE QUESTION: The team is dealing with a 68-year-old male patient who was brought in with a 2-day history of increasing lethargy and tremors. His past medical history is significant for substance use disorder, including alcohol, opiate, cocaine, and amphetamines, but he has been sober from these substances for approximately 2 weeks. He has alcoholic cirrhosis. His daily medication only consists of a multivitamin. He appears to be chronically ill and is currently somewhat mentally clouded. His examination findings are consistent with those normally found in advanced liver disease, including conjunctival icterus, ascites, and 2+ edema of the lower extremities. Interestingly, he has both resting and action tremors and shows signs of weakened muscle strength. His blood and urine cultures grow no pathogens, which rules out an infectious etiology. We are to identify the most suitable therapeutic intervention among intravenous vancomycin and cefepime, intravenous vitamin B1 (thiamine), oral lactulose, or oral lorazepam. PLANNING: The key components in this situation are the chronicity of the patient’s substance use disorder, the manifestation of symptoms typically linked with alcoholic cirrhosis, and his recent improvement in substance use but corresponding decline in mental status and motor functions. Alcoholic cirrhosis obstructs the liver from performing its normal physiological functions, including metabolism of nutrients. His recent abstinence from alcohol (a form of malnutrition) may have led to a deficiency of essential vitamins like thiamine. Thiamine plays a crucial role in glucose metabolism and its deficiency can lead to disorders like Wernicke's encephalopathy, which is linked with confusion, ataxia and abnormal eye movements, Korsakoff's psychosis, or simply nutritional deficiency-associated encephalopathy. CLINICAL REASONING: A dearth of thiamine may induce central nervous system disruptions that may underlie this patient's cognitive and motor deficits. Acknowledging the patient's history of chronic alcoholism, it would be reasonable to propose that the most effective intervention for ameliorating his cognition and movement disorder would be initiating thiamine repletion therapy. The other options do not address the patient's suspected thiamine deficiency: intravenous vancomycin and cefepime are antibiotics, and the patient does not currently have a pathogenic infection; oral lactulose is used to treat hepatic encephalopathy associated with advanced liver disease by reducing serum ammonia levels, but it does not treat the nutrient deficiencies that alcoholics commonly face; oral lorazepam is a benzodiazepine used to manage alcohol withdrawal syndrome, but the patient is not currently displaying symptoms of withdrawal. CONCLUDING: Utilizing a clinical approach that takes into account the patient's history of alcoholic cirrhosis, recent abstinence from alcohol and illicit substances, and presentation with lethargy, tremors, and slight weakness, it would be reasonable to conclude that the most likely therapeutic intervention to improve the patient's cognition and movement disorder would be the replenishment of essential nutrients, specifically thiamine. RESPONSE: Based on these reasons, the most beneficial treatment for this patient, likely to improve his cognitive impairment and movement disorder, would probably be (B) Intravenous vitamin B1 (thiamine) therapy. The replenishment of thiamine would help address the likely nutritional deficiencies associated with his history of chronic alcoholism.

**AI_C:** RESTATING THE QUESTION: The query deals with a man of 68 years old, with a history of substance abuse, currently experiencing lethargy and tremors for the past 2 days. Despite abstaining from alcohol and drugs for the past two weeks, he has a history of alcoholic cirrhosis. Currently, his only medication is a multivitamin and showed typical symptoms of alcoholic cirrhosis during examination. Given his condition, the query requires an optimal strategy to improve his cognitive function and reduce his movement disorder. PLANNING: The patient’s symptoms include cognitive impairment and movement disorders, common in conditions such as Alcoholic Hepatic Encephalopathy and Wernicke-Korsakoff syndrome, both prevalent in heavy alcohol users. There is a necessity to differentiate between these possibilities. Alcoholic Hepatic Encephalopathy is typically treated with oral lactulose while Wernicke-Korsakoff syndrome requires intravenous thiamine therapy. No cause for infection is identified, making the use of antibiotics like vancomycin and cefepime less likely. Lorazepam, a benzodiazepine, might be used for withdrawal symptoms, but the patient has already been abstinent for two weeks and is not showing signs of withdrawal. CLINICAL REASONING: The patient's presentation and history suggests a deficiency of certain nutrients common among those with chronic alcoholism. The presence of tremors, the recent onset of lethargy (suggesting a change in mental status), and his history of alcohol use disorder make a alcohol-related nutritional deficiency like Wernicke-Korsakoff syndrome a likely diagnosis. This condition, a result of chronic thiamine (Vitamin B1) deficiency, could present with such symptoms. The absence of common Hepatic Encephalopathy symptoms (like asterixis) and the negative cultures, along with the primary symptoms and man’s medical history, support leaning towards a diagnosis of Wernicke-Korsakoff syndrome. CONCLUDING: Between all the options, thiamine supplementation could be the potential treatment to improve this person's cognition and movement disorder, as it would be a step towards addressing the likely cause of his condition: Wernicke-Korsakoff syndrome. It is important to note that while lactulose is a treatment for hepatic encephalopathy related to liver disorders, the patient’s symptoms and history do not support prioritizing it as a treatment option at this time. RESPONSE: Therefore, the most beneficial course of action to improve this patient's cognitive and movement disorder is likely be (B) Intravenous vitamin B1 (thiamine) therapy.

**AI_E:** RESTATING THE QUESTION: The situation presented articulates about a 68-year-old man who has been suffering from increased lethargy and tremors for the past 2 days. He has a history of multiple substance use disorders, including alcoholism, but has been abstinent for the past two weeks. In addition, he suffers from alcoholic cirrhosis. Currently, he is only taking daily multivitamins. His physical examination shows signs of chronic illness and associated findings point to liver disease - icterus, ascites, and lower extremity edema. Despite some weakness and an action and postural tremor, his neurologic examination is fairly intact. His blood cultures reveal no infection. His cognition and motor disorders need to be improved and the question pertains to the most probable therapeutic intervention to bring about such changes. PLANNING: The possible assumptions that can be made from this scenario include: 1. His movement disorder and cognitive dysfunction may be related to his history of substance use, including alcohol. Also, his status of abstinence for the past two weeks can be tied in with the manifestations. 2. Since he suffers from chronic liver disease, these symptoms might as well be repercussions of hepatic encephalopathy. Now, it is crucial to evaluate which assumption carries more probable clinical significance. CLINICAL REASONING: The aforementioned symptoms - lethargy and tremors - are not typical for hepatic encephalopathy. Rather, such a condition would lead to signs of altered mental status and asterixis. On the contrary, Wernicke encephalopathy, which stems from thiamine (vitamin B1) deficiency, is commonly seen in heavy alcohol users, and matches the presenting symptoms. Chronic alcoholism often leads to poor nutrition and consequently such deficiency states. Characteristically, Wernicke encephalopathy presents with the clinical triad of altered mental status, oculomotor dysfunction, and ataxia. Although the complete triad is not always present, any suspicion of the syndrome warrants urgent treatment with thiamine to prevent progression to Korsakoff syndrome, which is irreversible and characterized by severe anterograde and retrograde amnesia. CONCLUDING: Given the clinical presentation and the patient's history of significant alcohol use, the most likely diagnosis is Wernicke encephalopathy due to deficiency of vitamin B1 (thiamine). As such, addressing the thiamine deficiency should normalize his mental status and improve the action and postural tremors, thereby improving his overall cognition and movement. RESPONSE: Therefore, the most effective plan to improve this patient’s cognition and movement disorder is (B) Intravenous vitamin B1 (thiamine) therapy.

**AI_D:** RESTATING THE QUESTION: This patient, a 68-year-old male with a history of alcoholism and illicit substance use, is experiencing lethargy and movement disorders marked by tremors. Despite abstaining from alcohol and other substances over the past 2 weeks, his condition has worsened, with symptoms such as chronic ill appearance, somnolence, conjunctival icterus, ascites, 2+ lower extremity edema, and a reduction in muscle strength. While the forefront differential diagnostic considerations include alcohol withdrawal, post-withdrawal syndrome, cirrhosis-related complications, and infectious etiologies, the lack of fever, systemic symptoms, or pathogens isolated in cultures seem to argue against an infectious etiology. The question is asking for the best clinical intervention to improve this patient's cognition and movement disorder. PLANNING: This situation implies Wernicke's encephalopathy, hepatic encephalopathy, withdrawal syndromes, and infection as differential considerations. Each assumption is scrutinized in the context of the presentation. Alcoholic cirrhosis could cause hepatic encephalopathy, but the absence of asterixis, coupled with bilirubin levels and absence of fever, leans against this. Wernicke's encephalopathy caused by thiamine (vitamin B1) deficiency—common in patients with chronic alcohol use due to poor diet and malabsorption—can present with altered mental status, and the patient's tremor might reflect weakness rather than asterixis. This suggests thiamine therapy could improve the patient's cognition and tremor. Withdrawal syndromes seem less likely given the time elapsed since cessation of use and would typically improve with sedative medications like lorazepam, not necessarily thiamine. CLINICAL REASONING: The presentation typifies someone with a chronic alcohol use disorder, abstaining, and presenting with lethargy and tremors. Let's work backward from understood pathology. Alcohol misuse can cause malnutrition leading to deficiencies, with one of the most important in this context being thiamine. Thiamine deficiency can precipitate Wernicke's encephalopathy, characterized by altered mental status, ophthalmoplegia, and ataxia. While his presentation isn't classically described as such, these 'classic' signs might not always be present. There is a postural-action tremor and a sudden, brief loss of muscle strength when he's awake, suggesting underlying neuronal insult. Cultures are negative, making an infectious source seem less likely. This makes thiamine deficiency, leading to Wernicke's encephalopathy, a leading diagnostic consideration. CONCLUDING: The most likely diagnosis for this patient's presentation is Wernicke's encephalopathy secondary to thiamine deficiency, a condition often seen in chronic alcoholics. This conclusion is based on the patient's social history, clinical presentation, and ruling out other common causes in this demographic. Therefore, treatment should target this deficiency to potentially improve the patient's cognitive and movement abnormality. RESPONSE: The best therapy to improve this patient’s cognition and movement disorder would be (B) Intravenous vitamin B1 (thiamine) therapy. This treatment targets the presumed cause of his deterioration — namely, Wernicke's encephalopathy due to thiamine deficiency. Treatment of this condition can lead to rapid clinical improvement if initiated early. While the other options may be considered in different scenarios, they do not appear to directly address the likely cause of this patient's symptoms.

**Facilitator** Consensus Statement: The case presents a 68-year-old male patient with a history of substance use disorder, including alcoholism, and alcoholic cirrhosis. He has been abstinent from alcohol and illicit substances for the past two weeks. His current symptoms include increasing lethargy, tremors, and signs of advanced liver disease. However, his blood and urine cultures show no signs of infection. The question seeks to identify the most effective therapeutic intervention to improve his cognitive function and movement disorder. Given the patient's history of chronic alcoholism and his recent abstinence, it is plausible that his symptoms are due to a deficiency of essential nutrients, specifically thiamine (vitamin B1). Thiamine plays a crucial role in glucose metabolism, and its deficiency can lead to disorders like Wernicke's encephalopathy, which is associated with confusion, ataxia, and abnormal eye movements. The patient's cognitive and motor deficits could be a result of such a deficiency. The other treatment options do not directly address the patient's suspected thiamine deficiency. Intravenous vancomycin and cefepime are antibiotics, which are not suitable as the patient does not currently have a pathogenic infection. Oral lactulose is used to treat hepatic encephalopathy associated with advanced liver disease, but it does not treat the nutrient deficiencies that alcoholics commonly face. Oral lorazepam is a benzodiazepine used to manage alcohol withdrawal syndrome, but the patient is not currently displaying symptoms of withdrawal. Therefore, the consensus among the responses is that the most beneficial treatment for this patient, likely to improve his cognitive impairment and movement disorder, would be intravenous vitamin B1 (thiamine) therapy. This treatment would address the likely nutritional deficiencies associated with his history of chronic alcoholism.
