# Supplementary material for: Collaborative intelligence in AI: Evaluating the performance of a council of AIs on the USMLE
Source: PLOS Digit Health. 2025 Oct 9;4(10):e0000787. doi: 10.1371/journal.pdig.0000787 (PMC12510544; doi:10.1371/journal.pdig.0000787)
Supplement: S2 File — (ZIP) [file pdig.0000787.s002.zip › S2 Data Raw data files containing unprocessed question inputs and LLM outputs including transcripts of deliberations/STEP2_Question-10-1_Type-MC-NJ.docx]

**Facilitator** **[Round 1]:** A 52-year-old woman comes to the physician because of a 2-day history of severe pain and markedly decreased range of motion in her right elbow. She has rheumatoid arthritis in her elbows, wrists, and hands. She reports episodes of moderate pain approximately four times yearly. Previous treatment with naproxen, methotrexate, and hydroxychloroquine has provided moderate relief. Treatment with prednisone was begun after her most recent episode 9 weeks ago. She has not had generalized fatigue or muscle aches. She has no other history of serious illness. Her other medications are naproxen, prednisone, and omeprazole. Temperature is 38.4°C (101.1°F), pulse is 90/min, respirations are 12/min, and blood pressure is 110/76 mm Hg. Range of motion of the wrists and ankles is decreased; the right elbow is more severely limited by pain than the left elbow. There is warmth, tenderness, erythema, and edema of the right elbow. Fine-needle aspiration of the right elbow is performed. Gram stain of the fluid shows a leukocyte count of 60,000/mm3 and no organisms. Which of the following is the most appropriate pharmacotherapy? (A) Colchicine (B) Infliximab (C) Ketorolac (D) Methylprednisolone (E) Vancomycin

**AI_B:** (1) RESTATING THE QUESTION: This inquiry is about a female patient, aged 52, with a history of rheumatoid arthritis, usually controlled with certain medications such as naproxen, methotrexate, and hydroxychloroquine. These therapies only offer moderate relief for her, and she has been recently started on prednisone. She has now presented with acute and severe pain in her right elbow, which has limited her movement considerably. A fine-needle aspiration shows elevated leukocyte count without visible organisms. Now, the question aims to decide the right medication for her in this situation, from the given options of colchicine, infliximab, ketorolac, methylprednisolone, and vancomycin. (2) PLANNING: A few pertinent assumptions here are the absence of systemic symptoms, no significant organisms in the synovial fluid, and the elevated leukocyte count. It raises the question of infection, though the absence of organisms opposes this. Most important assumptions to consider include whether this is a severe flare-up of her rheumatoid arthritis and whether she might be resistant to steroid treatment. (3) CLINICAL REASONING: The patient has a history of rheumatoid arthritis and her symptoms are consistent with a severe flare-up. Her elevated leukocyte count indicates an inflammatory process, which is common in rheumatoid arthritis. Apart from these, the differential diagnosis does include septic arthritis given the clinical picture, however, the absence of organisms in the joint aspirate makes this less likely. Furthermore, she has already been on prednisone, a type of corticosteroid, which suggests possible resistance or inadequate dose. (4) CONCLUDING: Taking into account the patient’s history, current medications, and symptoms along with lab findings, the most likely issue is a severe rheumatoid arthritis flare-up that requires escalations of existing treatment. (5) RESPONSE: Given all these findings, the most appropriate pharmacotherapy for the patient is likely Infliximab (B). Infliximab is a TNF-alpha blocker used in cases of moderate to severe rheumatoid arthritis, especially where response to methotrexate and other DMARDs has been insufficient.

**AI_C:** RESTATING THE QUESTION: The inquiry revolves around determining the most suitable drug therapy for a 52-year-old woman suffering from severe arthritis in her right elbow. She is already being treated for rheumatoid arthritis and is experiencing a severe episode with an elevated temperature, decreased range of motion and signs of inflammation in her right elbow, with a high leukocyte count indicating a possible inflammatory process. The options presented include various medications representing different drug classes, including gout medication (Colchicine), biologics (Infliximab), NSAIDs (Ketorolac), corticosteroids (Methylprednisolone), and antibiotics (Vancomycin). PLANNING: Clinical assumptions could include an acute gout attack, a flare-up of her rheumatoid arthritis, or a possible infection. Considering that there's neither sign of crystal-induced arthritis (which would warrant Colchicine) nor evidence of bacterial infection (which would need Vancomycin), these assumptions could be excluded. Given the patient's rheumatoid arthritis history and her current presenting signs and symptoms, the situation is likely an acute flare-up. Therefore, the clinical assumption to prioritize would be a flare-up of the woman's rheumatoid arthritis. CLINICAL REASONING: Rheumatoid arthritis is a chronic inflammatory disorder characterized by painful swollen joints. The clinical features of this case are consistent with a flare-up of rheumatoid arthritis, as indicated by the woman's history of the disease, the pain and decreased range of motion in her joints, her fever, and the elevated leukocyte count from her right elbow, which suggests an ongoing inflammatory process. Activities related to such a flare-up normally respond well to increased doses of corticosteroids. CONCLUDING: Considering that rheumatoid arthritis flare-up is a likely diagnosis and the absence of trace of infection upon gram staining or crystals suggesting a gout attack, it will be rational to administer corticosteroids, which will be customized to control the inflammation, as they exert anti-inflammatory and immunosuppressive effects. RESPONSE: Therefore, considering the pharmaceutical options provided and the current clinical picture, the most appropriate medication would be (D) Methylprednisolone, a potent corticosteroid.

**AI_E:** RESTATING THE QUESTION: A middle-aged lady is suffering from a sudden onset of severe pain and limitation of motion in her right elbow. While she has known rheumatoid arthritis, her current presentation seems more intense than her usual painful episodes, and her body temperature is slightly high. She has been on a standard treatment regimen for her rheumatoid arthritis that includes anti-inflammatories (naproxen), immune suppressants (methotrexate), and antimalarials (hydroxychloroquine) along with prednisone started more recently. To assess the acute-onset elbow condition, a fine-needle aspiration was done, revealing a high white blood cell count but no microorganisms. We need to determine the most suitable drug treatment for her current condition from the given options of colchicine, infliximab, ketorolac, methylprednisolone, and vancomycin. PLANNING: Though her long-standing condition is rheumatoid arthritis, the sudden severe pain and high leukocyte count in the elbow aspirate appear unusual and could potentially indicate an infective condition, inflammation, or an intense autoimmune flare-up. The absence of organisms on the gram stain, however, tends to favor the latter two possibilities over infection. CLINICAL REASONING: Considering the patient's history of rheumatoid arthritis and the absence of infective organisms in the aspirate, this episode could be an intense flare-up of her underlying disease. The presence of fever and a markedly increased leukocyte count in the aspirate, in the absence of detectable microorganisms, may indicate an intense inflammatory response rather than an infection. Given her list of current medications, we can see that she is already subject to a mix of nonsteroidal anti-inflammatory drugs, disease-modifying antirheumatic drugs, and corticosteroids. Each of these is playing a different role in managing her chronic condition. When it comes to a severe flare-up as in this case, an increase in corticosteroid dosage can be considered. Therefore, the option of methylprednisolone seems appropriate, given that it's a stronger corticosteroid. CONCLUDING: In managing this severe rheumatoid arthritis flare-up presenting with acute monoarthritis, the most suitable pharmacotherapy would likely be a stronger corticosteroid. Methylprednisolone could potentially control the intense inflammation more effectively, providing more immediate relief. RESPONSE: Therefore, given the presenting findings and the clinical reasoning, choice (D) Methylprednisolone could be the most appropriate pharmacotherapy in this case.

**AI_A:** RESTATING THE QUESTION: The patient, a 52-year-old woman known with rheumatoid arthritis, has presented with severe acute pain in her right elbow coupled with significant compromise in its range of motion. She also informs us that she experiences four episodes of moderate pain on an annual basis. She's been treated in the past with naproxen, methotrexate, and hydroxychloroquine - drugs typically used in the management of rheumatoid arthritis. She was also placed on treatment with prednisone nine weeks prior to this visit. Her current situation does not involve generalized fatigue or muscle aches, and she isn't known to have had any serious illness in the past. Other recurring medications include naproxen, prednisone, and omeprazole. Due to her elevated temperature of 38.4°C, she is febrile. Her right elbow's limited motion appears to be primarily due to pain. Upon physical examination, the elbow is warm, tender, erythematous, and edematous. A fine needle aspiration of the joint shows a leukocyte count of 60,000/mm3 and an absence of organisms. Based on the given clinical scenario, we're to determine the most suitable drug for her management. PLANNING: To diagnose this patient, we need to differentiate between a flare-up of her rheumatoid arthritis and a different cause for her current symptoms. An infection is an important consideration due to her fever and the leukocytosis observed from the aspirated fluid, even though no organisms were seen. However, it's essential to note that fever and an elevated white blood cell count can also occur in intense inflammatory states including severe RA flares. CLINICAL REASONING: Colchicine is used for acute gout flares or as prophylaxis in chronic gout, but not usually for rheumatoid arthritis. Infliximab is a TNF-alpha inhibitor used in the management of moderate to severe rheumatoid arthritis when other medications have failed. Ketorolac is a potent nonsteroidal anti-inflammatory drug (NSAID) used for moderate to severe acute pain but would generally be insufficient given the patient's apparent severity of symptoms. Vancomycin would be a consideration if a bacterial infection, particularly meticillin-resistant Staphylococcus aureus, was suspected - however, the gram stain was negative, and the patient is not showing any systemic signs of bacterial sepsis. CONCLUDING: Given that the patient's severe elbow pain, erythema, elevated temperature, and leukocytosis in synovial fluid analysis could be attributed to a significant flare-up of her rheumatoid arthritis rather than an infection, the most appropriate medication in this case would be a stronger anti-inflammatory agent, which might provide immediate symptomatic relief. RESPONSE: Therefore, (D) Methylprednisolone, a potent corticosteroid, would be the most appropriate choice for pharmacotherapy in this case. It would significantly ameliorate inflammation and provide immediate symptomatic relief.

**AI_D:** RESTATING THE QUESTION: This question describes a 52-year-old woman with a history of rheumatoid arthritis who is experiencing severe pain and impaired mobility in her right elbow. Her previous treatments, which included methotrexate, hydroxychloroquine, and prednisone, have provided some relief. She is exhibiting signs of inflammation in the form of warmth, tenderness, erythema, and edema of her right elbow. Aspiration of the elbow shows a high leukocyte count but no organisms. The query is regarding the best choice of medication for this patient's current presentation. PLANNING: The assumptions here would be (1) the woman's symptoms are due to an exacerbation of her rheumatoid arthritis; (2) the high leukocyte count is due to the inflammatory response associated with her condition; (3) there is no infection due to lack of organisms in the gram stain. However, alternative assumptions could be considered: (1) the patient's symptoms may be due to a septic arthritis, which may not display gram-positive organisms particularly if they are intracellular or if the patient has started antibiotic therapy which might have sterilized the culture but not resolved the infection; (2) another possible cause of her symptoms could be gout or pseudogout flares, although these are less probable, considering the patient's medical history and symptoms. CLINICAL REASONING: Septic arthritis should be considered in patients with joint inflammation who have a high fever, as bacteremia can settle in previously inflamed joints such as those affected by rheumatoid arthritis. Considering the high leukocyte count from the needle aspirate, bacterial infection is possible. However, the absence of organisms on gram stain can be misleading as bacteria like Staphylococcus aureus (a common cause of septic arthritis) can be missed due to previous antibiotic therapy or failure in identification if they are intra-cellular. Therefore, despite negative culture, the clinical scenario should guide the treatment. CONCLUDING: In conclusion, while treatments like infliximab (used in severe cases of rheumatoid arthritis), colchicine (used for gout), ketorolac (a non-steroidal anti-inflammatory), and methylprednisolone (a corticosteroid used in inflammatory conditions) could indeed be used in conditions related to joint inflammation, the priority would be to provide cover against potential septic arthritis due to the high leukocyte count and fever despite absence of organisms on gram stain. RESPONSE: Thus, the most appropriate pharmacotherapy for this patient would be (E) Vancomycin, an antibiotic effective against gram-positive organisms, including Staphylococcus aureus, which frequently is implicated in septic arthritis.

**Facilitator** **[Round 2]:** Summary: AI_A believes that the patient's symptoms are due to a severe flare-up of her rheumatoid arthritis, not an infection. They suggest that Methylprednisolone, a potent corticosteroid, would be the most appropriate choice for pharmacotherapy in this case. AI_B also considers the possibility of a severe flare-up of rheumatoid arthritis but suggests that the patient might be resistant to steroid treatment. They recommend Infliximab, a TNF-alpha blocker used in cases of moderate to severe rheumatoid arthritis, especially where response to methotrexate and other DMARDs has been insufficient. AI_C agrees with AI_A's diagnosis of a severe rheumatoid arthritis flare-up and also recommends Methylprednisolone as the most appropriate medication. AI_D, however, considers the possibility of septic arthritis despite the absence of organisms on gram stain. They recommend Vancomycin, an antibiotic effective against gram-positive organisms, including Staphylococcus aureus, which frequently is implicated in septic arthritis. AI_E aligns with AI_A and AI_C in diagnosing a severe rheumatoid arthritis flare-up and also recommends Methylprednisolone as the most appropriate pharmacotherapy. Question to clarify disagreements: Given the patient's history of rheumatoid arthritis, the absence of organisms in the joint aspirate, and the elevated leukocyte count, what evidence or reasoning leads you to your chosen treatment plan, and how do you justify your choice of medication over the alternatives?. Based on your preceding response, how would you answer the original question: [A 52-year-old woman comes to the physician because of a 2-day history of severe pain and markedly decreased range of motion in her right elbow. She has rheumatoid arthritis in her elbows, wrists, and hands. She reports episodes of moderate pain approximately four times yearly. Previous treatment with naproxen, methotrexate, and hydroxychloroquine has provided moderate relief. Treatment with prednisone was begun after her most recent episode 9 weeks ago. She has not had generalized fatigue or muscle aches. She has no other history of serious illness. Her other medications are naproxen, prednisone, and omeprazole. Temperature is 38.4°C (101.1°F), pulse is 90/min, respirations are 12/min, and blood pressure is 110/76 mm Hg. Range of motion of the wrists and ankles is decreased; the right elbow is more severely limited by pain than the left elbow. There is warmth, tenderness, erythema, and edema of the right elbow. Fine-needle aspiration of the right elbow is performed. Gram stain of the fluid shows a leukocyte count of 60,000/mm3 and no organisms. Which of the following is the most appropriate pharmacotherapy? (A) Colchicine (B) Infliximab (C) Ketorolac (D) Methylprednisolone (E) Vancomycin]

**AI_D:** Restating the Question: The question asks for the most appropriate pharmacological intervention for a 52-year-old woman with a history of rheumatoid arthritis presenting with severe pain and significantly diminished range of motion in her right elbow. Despite finding a high leukocyte count in a gram stain of her aspirated elbow fluid, no organisms were identified. Planning: The prevailing theories are a severe rheumatoid arthritis flare-up or a septic arthritis secondary to a resistant organism not seen on gram stain. Current rheumatoid treatments have provided only moderate relief. The question must consider the patient's presentation, disease history, the results of the fluid aspiration, and their previous response to medications. Clinical Reasoning: Although the woman has rheumatoid arthritis, her current episode of pain is unusually severe compared to past episodes and is paired with an elevated leukocyte count indicating a possible infection. While absence of organisms in the gram stain does not conclusively rule out an infection, her long-standing rheumatoid arthritis, the use of prednisone - a systemic corticosteroid known to exacerbate infections and the temporal relationship between starting prednisone and her symptoms suggest this could also be an aggressive arthritis flare-up secondary to immunosuppressant use. Therefore, it would be reasonable to utilize a more potent medication to target inflammation, such as Methylprednisolone, a corticosteroid with potent anti-inflammatory action, or Infliximab, a TNF-alpha blocker used in resistant arthritis cases. Concluding: Methylprednisolone (D) seems like the most appropriate medication for this patient considering the severity of her flare-up and the lack of organisms detected in the aspirate. More potent medications like Infliximab (B) should be considered if the patient doesn't respond to increased corticosteroids. For now, without more compelling evidence of an infection, it is not appropriate to prescribe an antibiotic such as Vancomycin (E), which should be considered only if further testing confirms a resistant microbe. Response: Given the above reasoning, My proposed answer is (D) Methylprednisolone.

**AI_B:** Restating the Question: The question presents a 52-year-old woman with a history of rheumatoid arthritis, who is presenting with an acutely painful and swollen elbow, elevated temperature, and significantly high leukocyte count in her joint fluid but no microbial organisms upon gram staining. The question is asking which pharmacological treatment is most appropriate for her given her presentation and medical history. Planning: The main assumptions here are that this acute presentation is either an infection (septic arthritis) or a severe rheumatoid arthritis flare. None of the proposed treatment options are primarily antirheumatic agents, suggesting that a co-existing condition is being considered. The absence of organisms on gram stain therefore becomes a critical point in the clinical reasoning process. Clinical Reasoning: Given her history of rheumatoid arthritis and the clinical context, a flare of her rheumatoid arthritis could be responsible for her symptoms, even though the presence of fever might initially suggest infection. However, the gram stain showed no organisms, which makes a bacterial infection less likely, albeit still possible given the leukocyte count in the joint fluid (which could be due to either infection or an inflammatory condition). Considering this, empirical antibiotics might not be the best initial choice. On the other hand, corticosteroids like Methylprednisolone are both anti-inflammatory and immunosuppressive agents, and can effectively manage a rheumatoid arthritis flare. Infliximab, a tumor necrosis factor-alpha blocker, is typically used in moderate to severe rheumatoid arthritis resistant to first-line treatment. However, she is already on prednisone, a corticosteroid, which raises questions about corticosteroid resistance or the sufficiency of her existing corticosteroid dosage. Concluding: Given her current symptoms and medical history, focusing on managing a flare of rheumatoid arthritis seems more clinically appropriate until there's definitive evidence of infection. In that light, considering increasing the steroids dose rather than starting a new disease-modifying antirheumatic drug (DMARD) like Infliximab, appears valid given the acute, severe nature of her flare. Therefore, Methylprednisolone seems like the most appropriate choice, although it would be important to closely monitor her for signs of improvement or deterioration. Response: The most appropriate pharmacotherapy in this context would likely be (D) Methylprednisolone, with close patient monitoring.

**AI_C:** Restating the question: The question asks us to determine the most suitable pharmacotherapy for a 52-year-old woman presenting with severe pain and a highly reduced range of motion in her right elbow. This woman has a known condition of rheumatoid arthritis affecting her elbows, wrists, and hands. The critical issues that distinguish this scenario are the severely inflamed right elbow that revealed a high leukocyte count and no organisms upon needle aspiration, and a history of rheumatoid arthritis. The normal microbial finding excludes most infections while the high leukocyte count suggests an inflammatory process, potentially related to her known rheumatoid arthritis. Planning: A key assumption centers around why she is experiencing such a severe and sudden onset of symptoms. This could be due to simple disease progression, steroid withdrawal effects, or complications from chronic steroid use. Another possibility is that it might be a crystalline disease flare like gout or pseudogout, but the history provided does not support this. Alternatively, it could be a joint infection, but the absence of organisms makes it a less likely assumption. However, the use of steroids raises the patient's risk for infections. Evaluating these assumptions, the most clinically prioritized is the possibility of a severe flare-up of her rheumatoid arthritis, potentially due to failed maintenance therapy or escalated disease progression. Clinical Reasoning: Per the gram stain results, the absence of microbes speaks against an infectious process, reducing the likelihood of septic arthritis or opportunistic infections due to constant steroid use. High leukocyte count, however, points to a severe inflammatory process in the joint. With an elevated temperature and her prior history of rheumatoid arthritis, it is more plausible that this is a severe rheumatoid arthritis flare. Furthermore, limited motion, warmth, tenderness, erythema, and edema of the right elbow combined with her medical history are typical presentations of a rheumatoid arthritis flare, which can occur despite chronic therapy. Concluding: All these factors suggest that this is a severe rheumatoid arthritis flare, not an infectious process. So the treatment should aim to manage this flare-up rather than treat an infection. Among the options presented, Methylprednisolone, a more potent corticosteroid, could give immediate and powerful anti-inflammatory effects which would help to control this severe flare-up promptly. _Response_: Hence, in the given situation characterized by a probable severe flare-up of rheumatoid arthritis, the most suitable pharmacotherapy would be (D) Methylprednisolone.

**AI_E:** Restating the question: A mid-aged woman with a history of rheumatoid arthritis, presenting with an acute increase of her elbow pain and decreased range of motion, is seeking adequate pharmacotherapy. The query centers around which drug should be optimally chosen from the list that includes Colchicine, Infliximab, Ketorolac, Methylprednisolone, and Vancomycin. Planning: The prominent assumptions include the possibility of (1) an infection (septic arthritis) that might necessitate the use of an antibiotic like Vancomycin given the high leukocyte count in the aspirate, (2) a rheumatoid arthritis flare-up requiring increased anti-inflammatory treatment perhaps in the form of Methylprednisolone or other higher potency drugs like Infliximab, (3) a non-bacterial inflammation requiring non-steroidal anti-inflammatory drugs like Ketorolac. However, the negative result for organisms on the gram stain test is suggestive of a low likelihood of an infection, hence making the assumption of a severe RA flare-up as the priority. Clinical Reasoning: Rheumatoid arthritis is a chronic inflammatory condition that affects joints leading to synovial inflammation and joint damage. This patient's history, combined with the absence of organisms in the aspirate, suggests a likely flare-up of her RA rather than an infection. Elevated leukocytes in the joint aspirate is common in arthritic flare-ups as well as infections, making it less useful for differentiation. However, the lack of generalized fatigue or muscle aches, common systemic symptoms of septic arthritis, strengthens the assumption of an RA flare-up. Considering her recent course of prednisone with no improvement, a more potent medication may be required. Concluding: Based on the patient's history of rheumatoid arthritis, clinical presentation, and aspiration findings, it is likely this is a severe flare-up of her Rheumatoid Arthritis. While NSAIDs like naproxen and corticosteroids like prednisone are common treatments for RA, in cases of severe flare-ups, increasing the potency of the medication is often needed. Methylprednisolone is a more potent corticosteroid than prednisone which could provide the required effect. Response: In conclusion, the most appropriate pharmacotherapy for the woman in the question would be Methylprednisolone (Option D), given her history, symptoms, and the fact that her previous treatments, including prednisone, have only offered moderate relief. The patient's severe RA flare-up requires more potent anti-inflammatory treatment, hence a stronger corticosteroid like Methylprednisolone should be clinically prioritized.

**AI_A:** The patient's symptoms align with a severe flare-up of her existing rheumatoid arthritis (RA). This autoimmune condition is characterized by painful inflammation of the joints, particularly in the hands and wrists – all of which the patient has history with. The fluid sampled from the right elbow has a high leukocyte count, indicating an active inflammatory process, yet no organisms are seen on gram staining. Examining the available information and considering the pharmacotherapeutic options, I would scrutinize the following assumptions: (1) the high leukocyte count in the fluid indicates an infection, (2) the patient is resistant to current anti-rheumatic therapy, (3) the aspirate result could be false negative for microbial pathogen, (4) the severe pain and immobilization is the result of local inflammation rather than systemic, (5) the response to prednisone therapy might indicate that corticosteroid therapy might be effective and (6) other possibilities like crystal-induced arthropathy co-existing with pre-existing RA. Assumption (1), I would say is not likely given that the gram stain showed no organisms; (2) is plausible given that she continues to have flare-ups four times yearly despite therapy; (3) is possible but less likely because of the high sensitivity of gram stains; (4) is likely due to the localized signs and symptoms this time; (5) response to prednisone is indicative of a potential response to stronger steroids, however, prednisone was also associated with recent flare-ups; and (6) is less likely given the absence of mention of needle-shaped or negatively birefringent crystals in the synovial fluid. In the light of these evaluations, I would clinically prioritize assumptions (2), (4), and (5) to create a plan. To objectively weigh this, I would therefore consider the medications: Infliximab (option B) and methylprednisolone (option D). Reviewing the clinical reasoning, Infliximab, a tumor necrosis factor-alpha blocker, is used in cases of moderate-to-severe RA especially where the response to methotrexate and other DMARDs has been insufficient. Methylprednisolone, on the other hand, is a potent corticosteroid providing fast, strong anti-inflammatory effects and could be beneficial to tackle an acute severe flare-up. However, it is crucial to acknowledge that long-term use of corticosteroids like methylprednisolone, though effective, can lead to potential side effects such as adrenal suppression, osteoporosis, and increased susceptibility to infections. Alternatively, Infliximab, though slower to act, offers a more targeted and longer-term approach towards the management of moderate to severe RA, which may be more suitable for this patient who experiences frequent flare-ups. Summing up, given the scenario of an acute severe flare-up of RA and considering the risks and benefits of both medications, I would opt for the immediate strong anti-inflammatory effect which can be obtained with methylprednisolone. This intervention can control the immediate flare-up and restore the quality of life swiftly. To tie the response to the clinical scenario, in addressing the flare-up in a 52-year-old woman with extensive RA, the most appropriate pharmacotherapy would be short-term high-dose methylprednisolone (option D), along with re-evaluation of her ongoing RA management plan. This decision includes the appraisal and synthesis of patient's history, clinical presentation and the propensity of methylprednisolone towards swift control of severe inflammatory symptoms. It is essential to remember that the inflammatory process associated with RA, if uncontrolled, could lead to joint destruction and deformity, thus the acute flare-up must be handled promptly.

**Facilitator** The consensus among the AI responses is that the most appropriate pharmacotherapy for the 52-year-old woman with a history of rheumatoid arthritis (RA) and presenting with severe pain and significantly decreased range of motion in her right elbow is Methylprednisolone (Option D). This conclusion is based on the patient's history, clinical presentation, and the results of the fine-needle aspiration of the right elbow, which showed a high leukocyte count but no organisms. The high leukocyte count suggests an active inflammatory process, which could be due to either an infection or a severe flare-up of her RA. However, the absence of organisms on the gram stain makes a bacterial infection less likely. Given her history of RA and the severity of her current symptoms, it is more plausible that this is a severe RA flare-up. The patient's previous treatments, including prednisone, have only provided moderate relief, suggesting that a more potent medication may be required. Methylprednisolone is a more potent corticosteroid than prednisone and could provide the immediate, strong anti-inflammatory effects needed to control this severe flare-up. However, it is important to note that long-term use of corticosteroids like methylprednisolone can lead to potential side effects such as adrenal suppression, osteoporosis, and increased susceptibility to infections. Therefore, the patient should be closely monitored for signs of improvement or deterioration, and her ongoing RA management plan should be re-evaluated. Infliximab, a tumor necrosis factor-alpha blocker, could be considered if the patient doesn't respond to increased corticosteroids. However, given the acute, severe nature of her flare-up, the immediate anti-inflammatory effect of Methylprednisolone is deemed more appropriate at this stage. Antibiotics such as Vancomycin should only be considered if further testing confirms a resistant microbe.
