# Supplementary material for: Collaborative intelligence in AI: Evaluating the performance of a council of AIs on the USMLE
Source: PLOS Digit Health. 2025 Oct 9;4(10):e0000787. doi: 10.1371/journal.pdig.0000787 (PMC12510544; doi:10.1371/journal.pdig.0000787)
Supplement: S2 File — (ZIP) [file pdig.0000787.s002.zip › S2 Data Raw data files containing unprocessed question inputs and LLM outputs including transcripts of deliberations/STEP2_Question-100-1_Type-MC-NJ.docx]

**Facilitator** **[Round 1]:** An 18-year-old man is being prepared to undergo left temporal craniotomy for meningioma. General anesthesia has been administered. The surgeon reviews the patient's most recent MRI findings, which are hanging on a view box in the operating room, and prepares to clip the hair from the patient's right scalp. The circulating nurse is concerned because the signed operative consent form indicates a left temporal craniotomy. Which of the following is the most appropriate course of action for the circulating nurse? (A) No action is required (B) Review the MRI to determine the correct operative site (C) Ask the surgeon to continue the operation only after the discrepancy is resolved (D) Change the operative consent to match the surgeon's actions (E) Call the waiting room to confirm the correct operative site with the patient's parents (F) Write a postoperative incident report only

**AI_C:** 1) RESTATING THE QUESTION: The question presents a scenario where there is confusion concerning the surgical site for an 18-year-old male who is due for a craniotomy. A craniotomy is a surgical procedure in which a portion of the skull is removed to access the brain. Here, the patient's consent form indicates a left temporal craniotomy, whereas the surgeon is preparing the right side of the patient's scalp, suggesting an apparent discrepancy. The question asks for the best course of action by the circulating nurse, who has noticed this inconsistency. 2) PLANNING: The underlying assumption in this case is that the discrepancy between the signed consent form and the surgeon's action is an error. We could hypothesize that this is a mistake on either the surgeon's part or the consent form, or even misinterpretation of the MRI scan. Therefore, the critical question is which assumption should be prioritized to ensure patient safety and adherence to ethical and legal guidelines. 3) CLINICAL REASONING: Errors regarding the surgical site are serious incidents that can have substantial implications. Therefore, any discrepancy should be resolved before proceeding with the surgery. The consent form is a vital legal document that sanctions the surgeon to perform the procedure at the specified site only. Performing an intervention inconsistent with the consent form without proper resolution could potentially be considered as assault. Therefore, the nurse should raise the issue before the operation begins. 4) CONCLUDING: In clinical practice, the guiding principle during a situation of incongruence is "safety first". This principle mandates resolution of any discrepancy before initiating any procedure. Therefore, the most appropriate action on the part of the nurse would be to bring the issue to the surgeon's attention, suggesting that the surgery should only proceed after the inconsistency is counterchecked and corrected if necessary. 5) RESPONSE: Accordingly, the most appropriate choice here would be (C) Ask the surgeon to continue the operation only after the discrepancy is resolved.

**AI_B:** (1) RESTATING THE QUESTION: The query put forward is related to the course of action a circulating nurse should adopt when they notice a discrepancy between the surgical site stated in the operative consent form and the site being prepared by the surgeon for operation. The patient, an 18-year-old man, is scheduled for a left temporal craniotomy due to a condition called meningioma - a typically benign tumor that arises from the meninges, the membranes that surround the brain and spinal cord. Despite the consent form for a left temporal craniotomy, the surgeon is preparing the right side of the patient's scalp, which has initialized the nurse's concern. (2) PLANNING: The circulating nurse can take different approaches for addressing the discrepancy, including not taking any action, reviewing the MRI, discussing the issue with the surgeon, altering the operative consent to match the surgeon's actions, confirming the surgical site with the patient's family, or simply documenting the issue in a postoperative report. Each of these assumptions present ethical, medical, and legal implications. (3) CLINICAL REASONING: If the nurse takes no action, this would disregard the importance of patient safety and double-checking essential factors before surgery. Reviewing the MRI may not provide the necessary clarity as the nurse may not have the expertise to interpret radiological images. Although changing the operative consent might seem to solve the immediate issue, this presents ethical and legal problems, as the signed consent belongs to the patient who is now under anesthesia. Contacting the patient's family could potentially create unnecessary confusion and distress unless it's a last resort situation. Writing a postoperative incident report would mean the nurse witnessed a potential mistake but didn't act on it promptly. (4) CONCLUDING: Ultimately, the nurse should strive to ensure patient safety while upholding ethical and legal standards. Therefore, bringing the discrepancy to the surgeon's attention seems to be the most appropriate and immediate course of action. this will require the situation to be clarified and any potential harm to the patient be avoided. (5) RESPONSE: The circulating nurse should (C) ask the surgeon to continue the operation only after the discrepancy is resolved. In maintaining patient safety and ensuring proper procedure is implemented, it is vital that the discrepancy is rectified before the procedure continues.

**AI_D:** (1) RESTATING THE QUESTION: The question is asking what the best action would be for a circulating nurse who notices a potential mistake about to occur in the operating room. Here, an 18-year-old male is set to undergo a left temporal craniotomy for a meningioma, a tumor that forms on the membranes that cover the brain and spinal cord just inside the skull. However, the surgeon appears to be preparing for the operation on the wrong side (the right side) of the patient's head, which contradicts the surgical consent form signed by the patient indicating a left temporal craniotomy. (2) PLANNING: The assumption here is that the surgeon may be about to make a significant surgical error. Given the potential dangers involved in this, a critical evaluation of this assumption dictates immediate action. Alternate assumptions such as the surgeon acting deliberately for some known reason are too dangerous to test under these circumstances. The consensus would therefore lean towards addressing the observed discrepancy in a direct, respectful manner, prioritizing patient safety. (3) CLINICAL REASONING: In the clinical environment, addressing and resolving potential errors involves clear communication and fast problem-solving. The circulating nurse has a vital role in maintaining surgical safety through active participation in confirming operative plans and setups. This includes verification of key details such as the correct patient identity, procedure, and operative site. (4) CONCLUDING: When any discrepancy such as the one described in the scenario is observed, it needs to be addressed explicitly and immediately, regardless of any hierarchical dynamics that could potentially discourage such action. The potential for catastrophic harm to the patient far outweighs any hesitations to communicate within the team. (5) RESPONSE: The most appropriate choice would be option (C) Ask the surgeon to continue the operation only after the discrepancy is resolved. This directly addresses the issue at hand, provides a clear expectation for resolution before proceeding, and prioritizes patient safety in the face of a potential surgical error. Safety should always come above all else in such situations to prevent mistakes like wrong-site surgeries.

**AI_E:** RESTATING THE QUESTION: The scenario presents a case where an 18-year-old is about to undergo a left temporal craniotomy due to a meningioma, which is a type of brain tumor. He has been put under general anesthesia, and his recent MRI scans have been reviewed by the surgeon. However, there's been an inconsistency noted - the surgeon is preparing to operate on the right side, while the signed consent form indicates a left temporal craniotomy. The circulating nurse is now faced with a quandary about the best course of action in such a circumstance. This can potentially be a critical issue as surgeries must be carried out strictly following the patient's consent to avoid any breach in ethical considerations and professional standards. PLANNING: The basic assumption is that the surgeon's preparation on the right side is a mistake, as the consent form communicates left side craniotomy. However, just as it is possible that the surgeon has made a mistake, it could also be a possibility that there is an error in the consent form. Another assumption could be that the surgeon has consciously changed the side for some clinical reasons after obtaining the consent, and it has not been communicated properly. I will now evaluate these assumptions based on clinical and ethical reasoning. CLINICAL REASONING: To begin with, it is important to resolve this discrepancy because operating on the wrong side could lead to serious medical complications and legal consequences. Although reviewing the MRI could help validate the actual side of the tumor, it should not solely form the basis to resolve the discrepancy, as the crucial factor here is the patient's consented operative site. It wouldn’t be appropriate to change the operative consent to match the surgeon's actions as it lacks patient or his legal guardian's agreement for the same. Furthermore, calling the waiting room to confirm with the patient's parents may not be a proper approach because they might not be capable of accurately determining the operative site based on medical images. Instead, the most ethical and logical course of action would be to bring this discrepancy to the attention of the surgeon. CONCLUDING: Therefore, the circulating nurse should halt the operation and ask the surgeon to continue only after the site discrepancy is resolved. It maintains the integrity of the preoperative planning and respects the consent obtained from the patient or his legal guardian. RESPONSE: Hence, the answer is (C) Ask the surgeon to continue the operation only after the discrepancy is resolved. This will ensure that the appropriate surgical site per the patient's signed consent is respected and acted upon. It also safeguards the patient's rights and contributes to safe surgical practices.

**AI_A:** RESTATING THE QUESTION: The query here relates to an 18-year-old patient scheduled for a left craniotomy procedure due to a meningioma diagnosis. He has been given general anesthesia for the operation: the process of inducing a state of controlled unconsciousness for surgical procedures. The surgeon is reviewing the MRI (Magnetic Resonance Imaging) results in the operating theater and appears to be prepared to perform the operation on the right side, contrary to the indicated left side on the signed consent form. The circulating nurse notices this discrepancy and is unsure what to do. The question is asking about the most appropriate course of action for the nurse. PLANNING: My assumptions are that the surgeon is mistaken about the side on which to operate and that the consent form is accurate & that the operation site must be the left side as per the given consent. These assumptions can be challenged considering a) the surgeon could also be correct and the document might have a typo error, or b) the patient might be positioned in a manner that the right side appears as left to the surgeon. After evaluating these assumptions, it seems clinically more relevant to prioritize addressing the discrepancy between the surgeon's actions and signed consent form, thus ensuring patient safety and adherence to medical protocols. CLINICAL REASONING: The clinical thought process here involves a focus on patient safety and respect for patient autonomy. Ensuring that the operation happens on the correct side is paramount. If the operation occurs on the wrong side due to a misunderstanding or miscommunication, it can lead to a regrettable medical error. Mistaken site surgery is a preventable error, often due to lack of proper communication and confirmation protocols in the operative team. Therefore, it's crucial for the nurse to assertively clear this confusion before the operation proceeds. CONCLUDING: The circulating nurse should not assume one party is correct and act based on that assumption. Neither should the nurse unilaterally change the consent form nor disregard the issue. Instead, clear and open communication within the operative team is essential. The surgeon should be made aware of the discrepancy, allowing the team to resolve the confusion and ensure accurate compliance with the consent form. Therefore, the action to be taken should involve active communication and collaborative problem-solving rather than modifying documents or ignoring the issue. RESPONSE: Hence, the nurse should (C) Ask the surgeon to continue the operation only after the discrepancy is resolved.

**Facilitator** The consensus statement synthesizes the responses of AI_A, AI_B, AI_C, AI_D, and AI_E as follows: The question presents a scenario where an 18-year-old male patient is prepared for a left temporal craniotomy due to a meningioma diagnosis. However, the surgeon appears to be preparing for the operation on the right side of the patient's head, contradicting the signed surgical consent form indicating a left temporal craniotomy. The circulating nurse notices this discrepancy and is unsure of the appropriate course of action. The underlying assumption is that there is an error either on the surgeon's part or in the consent form. This discrepancy could also be due to a misinterpretation of the MRI scan. Regardless of the cause, the discrepancy needs to be addressed to ensure patient safety and adherence to ethical and legal guidelines. Errors regarding the surgical site are serious incidents that can have substantial implications, including potential harm to the patient and legal consequences. The circulating nurse has a vital role in maintaining surgical safety through active participation in confirming operative plans and setups. This includes verification of key details such as the correct patient identity, procedure, and operative site. Therefore, any discrepancy should be resolved before proceeding with the surgery. The consent form is a vital legal document that sanctions the surgeon to perform the procedure at the specified site only. Performing an intervention inconsistent with the consent form without proper resolution could potentially be considered as assault. In clinical practice, the guiding principle during a situation of incongruence is "safety first". This principle mandates resolution of any discrepancy before initiating any procedure. Therefore, the most appropriate action on the part of the nurse would be to bring the issue to the surgeon's attention, suggesting that the surgery should only proceed after the inconsistency is counterchecked and corrected if necessary. In conclusion, the circulating nurse should (C) Ask the surgeon to continue the operation only after the discrepancy is resolved. This directly addresses the issue at hand, provides a clear expectation for resolution before proceeding, and prioritizes patient safety in the face of a potential surgical error. Safety should always come above all else in such situations to prevent mistakes like wrong-site surgeries.
