# Supplementary material for: Collaborative intelligence in AI: Evaluating the performance of a council of AIs on the USMLE
Source: PLOS Digit Health. 2025 Oct 9;4(10):e0000787. doi: 10.1371/journal.pdig.0000787 (PMC12510544; doi:10.1371/journal.pdig.0000787)
Supplement: S2 File — (ZIP) [file pdig.0000787.s002.zip › S2 Data Raw data files containing unprocessed question inputs and LLM outputs including transcripts of deliberations/STEP2_Question-101-1_Type-MC-NJ.docx]

**Facilitator** **[Round 1]:** A 52-year-old man is brought to the emergency department because of a 2-day history of severe, progressive shortness of breath associated with exacerbation of his chronic cough. He has a 6-week history of pneumonia, which has responded slowly to treatment. Prior to the onset of his current symptoms, he had minimally productive cough and mild shortness of breath after walking one or two blocks. He also has had a low-grade fever for 6 days. He has no other history of serious illness. Current medications are erythromycin and an over-the-counter cough suppressant. He has smoked one pack of cigarettes daily for 20 years. On arrival, he appears ill. He is diaphoretic and in severe respiratory distress. Temperature is 38.1°C (100.6°F), pulse is 110/min, respirations are 36/min and shallow, and blood pressure is 70/50 mm Hg. Pulse oximetry on room air shows an oxygen saturation of 83%. Examination shows perioral cyanosis. There is no jugular venous distention. Diffuse crackles and wheezes are heard throughout the lungs. Heart sounds are normal. While a blood sample is being taken, the patient's blood pressure decreases, and he stops breathing. He is intubated, and mechanical ventilation is begun. Laboratory studies show: Hematocrit: 42% Leukocyte count: 16,000/mm³ Segmented neutrophils: 85% Lymphocytes: 15% Platelet count: 200,000/mm³ Arterial blood gas analysis on an FIO2 of 1.0 shows: pH: 7.35 PCO₂: 42 mm Hg PO₂: 55 mm Hg Chest x-ray shows diffuse alveolar infiltrates bilaterally and no cardiomegaly. Following intravenous administration of fluids, his blood pressure increases to 118/80 mm Hg. Which of the following is the most appropriate next step in management? (A) Continuation of mechanical ventilation at the current setting (B) Administration of cyclophosphamide (C) Administration of dexamethasone (D) Extracorporeal membrane oxygenation (E) Application of positive end-expiratory pressure

**AI_D:** RESTATING THE QUESTION: The scenario here describes how a 52-year-old man with a history of pneumonia that responded slowly to treatment and a chronic cough was admitted for severe, progressive shortness of breath. He had vital signs and symptoms suggestive of hypoxia, as well as lab data suggesting infection and an imaging study indicating diffuse alveolar infiltrates in the lungs. His oxygen saturation improved, and his blood pressure normalized with mechanical ventilation and intravenous fluids. The query posed is regarding the optimal next step in managing this patient. PLANNING: 1. The patient is hypoxic, acutely deteriorating, and showing signs of sepsis, suggesting a possibly severe infectious process. 2. The option to continue mechanical ventilation at its current setting presumes that the patient's current treatment regimen is adequate, but given the ongoing signs of respiratory distress and hypoxia, this could be challenged. 3. The administration of cyclophosphamide or dexamethasone assumes that the underlying pathology is immune-mediated or inflammatory. However, this is unlikely given the chronicity of the pneumonia, the patient's acute decompensation, and the presence of a risk factor (smoking). 4. The use of extracorporeal membrane oxygenation (ECMO) might be an appropriate intervention for severe cases, but in this scenario, it might not be the first-line treatment. 5. The application of positive end-expiratory pressure (PEEP) could improve alveolar recruitment and oxygenation, potentially alleviating the hypoxia and dyspnea. CLINICAL REASONING: Considering the patient's clinical picture, it seems that the pneumonia may have progressed into acute respiratory distress syndrome (ARDS). ARDS presents as severe dyspnea, hypoxia, and bilateral infiltrates not fully explained by cardiac failure or fluid overload, as seen in this patient. The application of PEEP, a form of positive pressure ventilation, can help improve oxygenation by keeping the alveoli open during expiration, increasing functional residual capacity, and reversing hypoxemia caused by alveolar collapse or fluid-filled alveoli. CONCLUDING: With the consideration of ARDS at hand and the patient's progression into a critical state, the best intervention would be one that could relieve his symptoms and potentially reverse the underlying pathological process. Indeed, management of ARDS includes optimizing oxygenation and reducing ventilator-associated lung injury. The application of PEEP could indeed work toward achieving these goals. RESPONSE: So evaluating the above points, the most appropriate next step in management would be (E) Application of positive end-expiratory pressure.

**AI_E:** RESTATE: This scenario involves a 52-year-old male patient with a history of smoking and recent pneumonia, who is experiencing severe and worsening respiratory distress. He has been intubated and mechanical ventilation has been initiated. The aim is to identify what is the most appropriate next step in his management from five provided options including maintaining the current mechanical ventilation setting, prescribing cyclophosphamide, dexamethasone, applying extracorporeal membrane oxygenation (ECMO), or positive end-expiratory pressure (PEEP). PLANNING: A few assumptions can be drawn from the clinical presentation and lab results. Firstly, the patient's history of smoking and pneumonia combined with persistent symptoms, abnormal laboratory findings, and chest X-ray results, all suggestive of severe respiratory illness, possibly Acute Respiratory Distress Syndrome (ARDS), rather than sole pneumonia. Additionally, despite being ventilated on 100% oxygen (FIO2 of 1.0), the patient's oxygen level (PO2) remains low, suggesting severe pulmonary disease and hypoxemia. ARDS, which results from damage to the alveoli, can occur secondary to several scenarios like sepsis, pneumonia, or inhalation injury, and may be exacerbated by smoking. The efficacy of the treatment options must be evaluated against this backdrop. CLINICAL REASONING: Given this potential diagnosis of ARDS, we need to consider the most effective intervention for improving oxygenation. While the continuation of mechanical ventilation at the current setting is important (Option A), it might not be enough to optimize oxygenation given PO2 remains low despite FIO2 of 1.0. Cyclophosphamide (Option B) is a chemotherapy drug with no role in this case. Dexamethasone (Option C) may assist if inflammation was a predominant issue, but the problem here is more related to the damage to alveolar structure and permeability causing poor oxygen exchange. ECMO (Option D) might become necessary in severe refractory cases, but that's often seen as the last resort. PEEP (Option E) works by preventing alveolar collapse during expiration, increasing the functional residual capacity and thereby improving oxygenation. Thus, PEEP seems to target the underlining pathological process in ARDS more directly. CONCLUDING: Based on the assumption of ARDS due to bilateral diffuse alveolar infiltrates, hypoxemia despite being on 100% oxygen, and the history of persistent pneumonia in a smoker, utilizing an intervention that could improve oxygenation by addressing the issues of alveolar collapse and increased permeability would be the most appropriate next step in this case. RESPONSE: Given that, the most appropriate next step in the management of this patient would be (E) the application of positive end-expiratory pressure.

**AI_C:** RESTATING THE QUESTION: To rephrase, a middle-aged man with recent history of pneumonia, chronic cough and a prolonged history of cigarette smoking has been brought into emergency with worsening shortness of breath and chronic cough. Despite administering erythromycin and a cough suppressant, his conditions has deteriorated, leading to severe respiratory distress, hypotension and hypoxemia, necessitating immediate intubation and mechanical ventilation. His blood work shows signs of an ongoing infection and his chest X-ray shows bilateral alveolar infiltrates. Given his clinical picture, the question presents several possible next courses of action and asks which one is most appropriate. PLANNING: We need to underline a few assumptions in this case to address the next step of management. First, the assumption that the patient's condition is worsening despite antibiotic administration could suggest drug-resistant bacteria, side effects of medication, inappropriate medication or non-bacterial infections. Secondly, the presence of diffuse bilateral alveolar infiltrates might hint towards conditions such as acute respiratory distress syndrome (ARDS), pulmonary edema, or diffuse pneumonia. Lastly, the assumption that the patient has a normal heart sound might negate heart failure as a cause of his symptoms. By re-evaluating these assumptions, we can deduce the potential cause of his exacerbating symptoms and establish a treatment plan. CLINICAL REASONING: The severe respiratory distress and hypoxemia combined with a history of pneumonia not responding effectively to antibiotics, while exhibiting signs of ongoing inflammation (raised leukocyte and neutrophil count), and diffuse alveolar infiltrates on chest X-ray align with the clinical picture of ARDS. ARDS is a severe form of lung injury caused by different aetiologies; in this case, it most likely resulted from pneumonia (commonly bacterial in origin, which matches with his raised neutrophil count). Sepsis, resulting from bacterial pneumonia, is known to trigger ARDS as systemic inflammatory response leads to increased capillary permeability and fluid accumulation in alveoli, disrupting gas exchange. Therefore, from this synthesis, it is hypothesized that the patient might have developed ARDS, which could be the key reason for his drastically worsened respiratory state. CONCLUDING: One of the mainstays of management in ARDS is to improve oxygenation and prevent further lung injury. This can be achieved via a lung-protection strategy employing mechanical ventilation with low tidal volumes and the application of positive end-expiratory pressure (PEEP). PEEP counters the tendency of diseased alveoli to collapse, maintaining their patency, improving gas exchange and reducing hypoxemia. The higher oxygen concentration provided with PEEP would also counteract on hypoxemia. RESPONSE: Therefore, given that the patient is already intubated and on mechanical ventilation, but remains hypoxemic and in respiratory distress, the administration of PEEP (Option E) looks to be the most appropriate course of action. This would aim at improving oxygenation and avoiding further injury to the lungs. It is always best to review the settings if the patient's condition is not improving with the current setting of mechanical ventilation.

**AI_A:** RESTATING THE QUESTION: The question is about managing a 52-year-old male patient who arrived at the emergency department with severe respiratory distress and was diagnosed with pneumonia that responded slowly to treatment. The patient’s conditions worsened over time; his blood pressure dropped, and he stopped breathing, which subsequently led to the initiation of mechanical ventilation. Following a boost in fluid administration, his blood pressure increased. Now, the council's task is to decide on the most suitable next step in the patient's management, selecting from options that include continuing with the mechanical ventilation at the current setting, administering cyclophosphamide, administering dexamethasone, applying extracorporeal membrane oxygenation (ECMO), or applying Positive End-Expiratory Pressure (PEEP). PLANNING: The following assumptions can be made: the patient's condition is related to his long-term cigarette smoking, and he is suffering from a pulmonary disease (possibly Chronic Obstructive Pulmonary Disease (COPD)) which got complicated by pneumonia. The slow response to the treatment and exacerbation of symptoms despite the ongoing treatment indicate a severe infection or another overlapping condition. The need for intubation indicates the severity of respiratory failure. We will also consider the possibility of an autoimmune condition, like Goodpasture's syndrome, necessitating the use of cyclophosphamide, or a severe inflammatory response to infection necessitating the use of dexamethasone. ECMO might be needed if conventional ventilatory support is not effective. CLINICAL REASONING: The absence of serious illness history, the presence of chronic cough and smoking history point toward COPD. The overlap with pneumonia could have led to Acute Respiratory Distress Syndrome (ARDS), which might explain the alveolar infiltrates on the chest x-ray, the severe respiratory distress, and the necessity for ventilation. ARDS can cause hypoxemia refractory to oxygen therapy, as indicated by the lab value of PO2: 55 mm Hg despite the high FIO2 of 1.0. Considering all this, we need to optimize the ventilatory support while the underlying conditions - pneumonia and COPD - are managed. CONCLUDING: Based on our reasoning, the application of Positive End-Expiratory Pressure (PEEP) would be beneficial. PEEP is a method employed in mechanical ventilation to improve oxygenation by increasing the alveolar recruitment and reducing the intrapulmonary shunt, i.e., it helps keep the alveoli open during expiration. This is expected to improve oxygenation in patients with ARDS, such as our case here. RESPONSE: The most appropriate next step in management should be the application of positive end-expiratory pressure (E).

**AI_B:** RESTATING THE QUESTION: The query pertains to the optimal next step in the medical management of a 52-year-old male. This patient presents with serious respiratory distress, perioral cyanosis, and a medical history of six-week long pneumonia, chronic cough, smoking, and more recently developed low-grade fever. He's been mechanically ventilated due to cessation in breathing and has laboratory results indicating a significant decrease in blood-oxygen levels, increased white blood cells, diffuse alveolar infiltrates in both lungs, and no cardiomegaly. In line with the clinical scenario and the ongoing management, the alternatives suggest further medical support via the continuation of current mechanical ventilation setting, administration of drugs like cyclophosphamide and dexamethasone, or employment of medical procedures like extracorporeal membrane oxygenation and positive end-expiratory pressure application. PLANNING: The assumptions about this case include that the patient's pneumonia is the primary cause of his severe respiratory distress, that his pneumonia and history of smoking have led to a chronic lung disease (such as chronic obstructive pulmonary disease, or COPD). Another assumption is that his current treatment (erythromycin and a cough suppressant) and mechanical ventilation are enough to effectively manage his condition. By critically questioning these assumptions, alternate considerations could be that his respiratory distress is due to a severe respiratory infection that may now be complicated by an acute respiratory distress syndrome (ARDS), that mechanical ventilation settings might need adjustment to manage such a condition, or that positive end-expiratory pressure (PEEP) or steroids may aid in lung function. CLINICAL REASONING: His history of pneumonia responding slowly to treatment, chronic cough, smoking, current severe respiratory distress, perioral cyanosis, and recent low-grade fever suggests a severe infection, probably bacterial. Moreover, the elevated leukocyte count indicates an ongoing infection. Furthermore, the diffuse alveolar infiltrates seen bilaterally on the chest X-ray and severe hypoxemia on arterial-blood gas analysis point to a possible complication of ARDS. As such, given the clinical scenario and after questioning our assumptions and alternate assumption, the most appropriate management option will be one that addresses this clinical suspicion of ARDS. CONCLUDING: ARDS often requires specific ventilatory settings. Here, the PO₂ of 55 mm Hg on 100% FiO₂ indicates severe hypoxemia despite full supplemental oxygenation. This indicates that the current mechanical ventilation settings might be insufficient. For ARDS management, the utilization of low tidal volumes is commonly accepted, but the addition of positive end-expiratory pressure (PEEP) is also necessary. PEEP helps to keep the alveoli open and improve oxygenation. RESPONSE: In this context and taking into account the condition of possible ARDS, the best choice amongst these options is (E) Application of positive end-expiratory pressure (PEEP).

**Facilitator** The consensus statement synthesizes the responses of AI_A, AI_B, AI_C, AI_D, and AI_E regarding the management of a 52-year-old male patient with severe respiratory distress, a history of pneumonia, and chronic cough. The patient's condition has worsened despite treatment, leading to hypotension, hypoxemia, and the need for mechanical ventilation. The patient's history of smoking and the slow response to pneumonia treatment suggest a severe infection or an overlapping condition such as Chronic Obstructive Pulmonary Disease (COPD) or Acute Respiratory Distress Syndrome (ARDS). The patient's clinical picture, including the presence of diffuse alveolar infiltrates on the chest X-ray and severe hypoxemia despite high FIO2, aligns with ARDS, a severe form of lung injury often triggered by conditions like sepsis or pneumonia. ARDS disrupts gas exchange due to increased capillary permeability and fluid accumulation in the alveoli. Given the potential diagnosis of ARDS, the most effective intervention would be one that improves oxygenation and prevents further lung injury. Among the options provided, the application of Positive End-Expiratory Pressure (PEEP) appears to be the most suitable next step in the patient's management. PEEP, a method employed in mechanical ventilation, improves oxygenation by keeping the alveoli open during expiration, increasing functional residual capacity, and reversing hypoxemia caused by alveolar collapse or fluid-filled alveoli. Therefore, the consensus among the AI responses is that the most appropriate next step in the management of this patient would be the application of positive end-expiratory pressure (Option E). This intervention aims to improve oxygenation and avoid further injury to the lungs, addressing the underlying pathological process in ARDS more directly.
