# Supplementary material for: Collaborative intelligence in AI: Evaluating the performance of a council of AIs on the USMLE
Source: PLOS Digit Health. 2025 Oct 9;4(10):e0000787. doi: 10.1371/journal.pdig.0000787 (PMC12510544; doi:10.1371/journal.pdig.0000787)
Supplement: S2 File — (ZIP) [file pdig.0000787.s002.zip › S2 Data Raw data files containing unprocessed question inputs and LLM outputs including transcripts of deliberations/STEP2_Question-102-1_Type-MC-NJ.docx]

**Facilitator** **[Round 1]:** A 14-year-old boy is brought to the emergency department by his parents 30 minutes after the sudden onset of confusion, sweating, and pallor. He has enuresis treated with imipramine. His parents refilled his prescription for imipramine 3 days ago and noted today that the bottle is empty. On arrival, the patient is awake but confused. Temperature is 37.0°C (98.6°F), pulse is 80/min, respirations are 8/min, and blood pressure is 100/60 mm Hg. Examination shows dilated pupils and flushed skin. In addition to ensuring an adequate airway and establishing intravenous access, which of the following is the most appropriate initial step in management? (A) Cardiac monitoring (B) Chest x-ray (C) Echocardiography (D) EEG (E) Measurement of serum imipramine concentration

**AI_D:** RESTATING THE QUESTION: The problem posed is about a 14-year-old boy who was brought to the emergency department after he suddenly became confused, began to sweat, and started looking pale. He takes imipramine for enuresis (a condition where the patient involuntarily urinates), and his parents noticed that his prescription bottle, refilled three days prior, is now empty. The boy is conscious but disoriented. His vital signs are mostly normal, but his respirations are slow, and his physical exam shows dilated pupils and flushed skin. The real question here is, aside from securing an airway and starting intravenous fluids, what would be the best initial management step for this patient? PLANNING: Based on the history and symptoms, the boy may have an overdose of imipramine, an antidepressant in the tricyclic group, which may cause tachycardia, dilated pupils, flushed skin, confusion, and respiratory depression in high doses. Given these assumptions, measuring imipramine concentration in the serum could help confirm the diagnosis; however, it has minimal immediate therapeutic value, while chest X-ray and echocardiography may not immediately contribute to the clinical management. On the other hand, an EEG won't be beneficial in this setting. However, cardiac monitoring could be critical due to severe cardiovascular toxicities of tricyclic antidepressant overdose including rhythm abnormalities and myocardial depression. CLINICAL REASONING: It's crucial first to stabilize any immediate life-threatening conditions. In the case of a suspected tricyclic antidepressant overdose, this involves addressing significant cardiotoxic effects. Cardiac monitoring, therefore, should be promptly initiated to assess and manage potential fatal arrhythmias and look for signs of myocardial depression. CONCLUDING: Considering the boy's symptoms, rapid onset, and recently emptied imipramine bottle, a tricyclic overdose seems probable. Prompt cardiac monitoring to assess and manage potential fatal arrhythmias due to tricyclic antidepressant overdose would be the most prioritized step following the primary survey. RESPONSE: Therefore, the most appropriate initial step in management for this patient, in addition to ensuring an adequate airway and establishing intravenous access, is cardiac monitoring (option A).

**AI_E:** RESTATING THE QUESTION: The clinical state of a 14-year-old boy is presented who, after refilling a prescription for imipramine used for treating enuresis, exhibited symptoms of confusion, sweating, and pallor. Despite his confused state, the patient is awake, has a normal body temperature, normal pulse, decreased respirations, normal blood pressure, dilated pupils, and flushed skin. During the emergency department (ED), aside from guaranteeing a functioning airway and establishing an intravenous (IV) route, an additional initial step in management needs to be determined from the available options. PLANNING: Suppositions can be made regarding the cause of the boy's symptoms based on the clinical presentation. The central possibility here is that the young patient may have consumed too much of his imipramine medication since the prescription bottle is empty just after three days. This suggests an overdose of the drug which may lead to 'tricyclic antidepressant (TCA) toxicity'. Symptoms of TCA toxicity commonly include anticholinergic effects such as flushing, dry mouth, dilated pupils, and confusion, which correspond with the boy's clinical findings, including the abrupt onset. CLINICAL REASONING: With the assumption of TCA toxicity, additional detrimental effects would include cardiac dysrhythmias and various degrees of heart block due to the inhibition of fast sodium channels. Therefore, immediate cardiac monitoring would be critical to identifying any possible life-threatening changes in heart rhythm or conduction. Other proposed interventions, such as chest x-ray, echocardiography, EEG, or measurement of serum imipramine concentration, although may be helpful in different clinical scenarios, they are not as urgent as immediate cardiac monitoring in this suspected case of TCA toxicity. CONCLUDING: Considering the patient's symptoms and the high possibility of TCA toxicity, it is crucial to act rapidly and apply the most urgent interventions first. An essential part of this would be continuous cardiac monitoring to detect any potentially lethal cardiac dysrhythmias given their association with TCA toxicity. Alongside this, access to advanced life support and consultation with a poison control center are also recommended. RESPONSE: In this case, alongside ensuring an adequate airway and establishing intravenous access, the most appropriate initial step in management would be (A) Cardiac monitoring.

**AI_B:** RESTATING THE QUESTION: This clinical scenario presents a 14-year-old boy who comes into the emergency department with sudden onset confusion, sweating, pallor, and consumption of a large quantity of imipramine, a medication for bedwetting. The medical team needs to determine the appropriate next step in management after ensuring the boy has a clear airway and IV access, the options provided are: cardiac monitoring, chest x-ray, echocardiogram, EEG, or measuring the serum imipramine concentration. PLANNING: Approaching this problem, we should consider the boy's symptoms in the context of his medical history, specifically the recent high dose intake of imipramine. Imipramine is a tricyclic antidepressant (TCA) which has potentially serious side effects including cardiovascular toxicity, anticholinergic effects (dilated pupils, flushed skin), and central nervous system disturbances (confusion). The assumptions generated from these considerations could be that the boy is experiencing imipramine toxicity or another unrelated coincident medical condition. CLINICAL REASONING: Considering the clinical manifestations, the sudden onset, and the fact that the imipramine bottle is empty, the primary assumption should be a potential overdose of imipramine leading to TCA toxicity--especially given that his symptoms align with known side effects of imipramine. In case of TCA overdose, it's crucial to promptly manage life-threatening complications such as cardiac arrhythmias which TCAs can cause, due to their sodium channel blocking effect. CONCLUDING: In light of the strong likelihood of TCA overdose and its impact on the cardiovascular system, it is beneficial to prioritize interventions that quickly monitor and address life-threatening cardiac complications. RESPONSE: Thus, the most appropriate initial step in management should be (A) Cardiac monitoring, to promptly detect and manage potential cardiac arrhythmias.

**AI_A:** RESTATING THE QUESTION: The query here presents a case of a 14-year-old boy dealing with confusion, sweating, and pallor, who is presently awake but seems disoriented. He has been taking imipramine for enuresis, and the bottle has been emptied, though it was only refilled three days ago, suggesting a potential overdose situation. The medical experts want to know the best immediate step in management after ensuring an open airway and setting up intravenous access, from options consisting of cardiac monitoring, chest x-ray, echocardiography, EEG, or measuring serum imipramine concentration. PLANNING: The critical assumption in this situation is that the boy has overdosed on imipramine, a tricyclic antidepressant (TCA) used here for enuresis treatment. This assumption is supported by his symptoms and the empty medication bottle. An alternative assumption could be a different cause for his condition, such as an underlying disease. However, given the scenario, a drug overdose seems more critical to pursue, expecting signs of TCA toxicity, which include anticholinergic effects (such as dilated pupils), CNS effects (like confusion), cardiovascular effects, and others. Treatment should focus on stabilizing the patient and handling specific complications. CLINICAL REASONING: Overdoses typically present an urgent situation requiring prompt stabilization and management. In case of a suspected TCA overdose, while we may consider validating this with a serum imipramine concentration measurement, a more direct approach will be to address the potential complications and symptoms. As such, cardiac monitoring is critical in TCA overdose due to the drug's potential for cardiovascular effects, including arrhythmias. CONCLUDING: The cardinal rule in such situations in the medical setting is to stabilize the patient while managing potential complications. Given the likelihood of a TCA overdose in this case, cardiac-related issues should be monitored closely with cardiac monitoring, as they can be life-threatening. RESPONSE: Therefore, the best immediate step in this young boy's management, following establishing an airway and intravenous access, would likely be (A) Cardiac monitoring to watch out for potential arrhythmias associated with TCA overdose.

**AI_C:** RESTATING THE QUESTION: The scenario represents a 14-year-old boy who has come to the emergency room after sudden onset confusion, sweating, and pallor. He uses imipramine, a medication for enuresis, and his parents found his medication bottle, refilled 3 days ago, to be empty today. His examination revealed he is awake but confused, with normal vitals, apart from slightly decreased respirations (8/min), dilated pupils, and flushed skin. We are asked to suggest the most appropriate initial step in managing this potential case of imipramine overdose, given the choices of cardiac monitoring, chest x-ray, echocardiography, EEG, or measuring the serum imipramine concentration. PLANNING: The current scenario illustrates a probable case of imipramine overdose, a type of tricyclic antidepressant, as indicated by sudden onset confusion, sweating, dilated pupils, flushing, and an empty bottle of medication. Imipramine has anticholinergic effects that can lead to the symptoms we see in our patient. It can also affect the cardiovascular system, causing tachycardia, hypotension, and arrhythmias, which can be fatal. Our potential assumptions could be to focus on confirming imipramine as the cause, or we could prioritize the potential life-threatening cardiac complication of an overdose. It would seem prudent to clinically prioritize the potential cardiac complications due to the acute nature of these risks. CLINICAL REASONING: Imipramine and other tricyclic antidepressants primarily permeate the sodium channels in the myocardium, inhibiting fast sodium influx and delaying conduction, which can result in arrhythmias. The potential for life-threatening arrhythmias, hypotension, or shock is immediate and thus takes precedence before confirming with a serum concentration. Clinical reasoning would dictate that capturing ECG changes associated with tricyclic antidepressant toxicity, such as prolonged QRS and QT intervals, right-axis deviation, or arrhythmias, would be our most immediate concern. Therefore, continuous cardiac monitoring would allow for the immediate detection and treatment of these life-threatening events, making it the most appropriate initial step in managing this patient's condition. CONCLUDING: In summary, while confirming the presence and level of the tricyclic antidepressant imipramine through serum measurements could be helpful in confirming our suspicions of an overdose, the immediate life-threatening risks of arrhythmias and shock associated with this type of toxicity necessitate rapid detection and treatment. Therefore, our most appropriate initial action should be starting continuous cardiac monitoring to be in a position to respond effectively to any possible cardiac complications. RESPONSE: So, the most appropriate initial step in the management of this patient is (A) Cardiac Monitoring.

**Facilitator** The consensus statement synthesizes the responses as follows: The clinical scenario presents a 14-year-old boy with sudden onset confusion, sweating, and pallor, who has been taking imipramine for enuresis. The empty medication bottle, refilled only three days ago, suggests a potential overdose situation. The boy's symptoms align with known side effects of imipramine, a tricyclic antidepressant (TCA), which include anticholinergic effects, central nervous system disturbances, and cardiovascular toxicity. Given the likelihood of a TCA overdose, the immediate concern is to manage potential life-threatening complications. While measuring serum imipramine concentration could confirm the diagnosis, it does not offer immediate therapeutic value. Similarly, chest x-ray, echocardiography, and EEG may not contribute immediately to the clinical management. The primary assumption is that the boy is experiencing TCA toxicity, which can cause serious cardiac complications, including arrhythmias due to sodium channel blocking effect. Therefore, the most appropriate initial step in management, after ensuring an open airway and setting up intravenous access, is cardiac monitoring. This will allow for the immediate detection and treatment of potential fatal arrhythmias and myocardial depression, which are immediate life-threatening risks associated with TCA overdose. In conclusion, the consensus is that the most appropriate initial step in the management of this patient, following establishing an airway and intravenous access, is (A) Cardiac monitoring. This will allow for the prompt detection and management of potential cardiac arrhythmias associated with TCA overdose.
