# Supplementary material for: Collaborative intelligence in AI: Evaluating the performance of a council of AIs on the USMLE
Source: PLOS Digit Health. 2025 Oct 9;4(10):e0000787. doi: 10.1371/journal.pdig.0000787 (PMC12510544; doi:10.1371/journal.pdig.0000787)
Supplement: S2 File — (ZIP) [file pdig.0000787.s002.zip › S2 Data Raw data files containing unprocessed question inputs and LLM outputs including transcripts of deliberations/STEP2_Question-103-1_Type-MC-NJ.docx]

**Facilitator** **[Round 1]:** A 65-year-old man with mild hypertension comes to the veterans’ administration outpatient clinic because he is concerned about having an abdominal aortic aneurysm. He says he feels well. His medications are metoprolol and lisinopril. His older brother, who was ""seemingly in good health,"" recently underwent emergency repair of a ruptured abdominal aortic aneurysm that had not been diagnosed previously. The patient retired from the military 15 years ago. He smoked one-half pack of cigarettes daily for 20 years but quit 20 years ago. He is 183 cm (6 ft 0 in) tall and weighs 84 kg (185 lb); BMI is 25 kg/m². Pulse is 72/min and regular, and blood pressure is 140/85 mm Hg. Abdominal examination shows no organomegaly or tenderness to palpation. The aortic pulsation is estimated at 3 cm in width. A midepigastric bruit is heard. The remainder of the examination shows no abnormalities. Which of the following is the most appropriate next step in diagnosis? (A) CT scan of the abdomen and pelvis (B) Digital angiography of the aorta (C) Duplex ultrasonography of the abdomen (D) X-ray of the abdomen (E) No diagnostic imaging is indicated

**AI_A:** (1) RESTATING THE QUESTION: We have a concerned 65-year-old gentleman with mild hypertension, a former half a pack a day smoker for 20 years, and a family history of a previously undiagnosed ruptured abdominal aortic aneurysm (AAA) in his brother. Despite feeling well, he is worried about having an AAA due to his family history. His examination, including abdominal palpation, provides no positive signs of aneurysm, although a midepigastric bruit is heard. (2) PLANNING: An AAA is abnormal dilation of the abdominal aorta, a condition that frequently has no symptoms until rupture, which is a life-threatening event. Screening is advised for individuals who have risk factors such as advanced age, male, history of smoking, hypertension, and family history of AAA. Here the assumptions are that the patient may have AAA due to his risk factor and that the best way to screen it should be a cost-effective, available, and reliable method without increasing risk or discomfort for the patient. (3) CLINICAL REASONING: Considering our assumption of AAA, we can rule out the option of 'no diagnostic imaging' (option E). From among the remaining options of CT scan, digital angiography, duplex ultrasonography, and X-ray of the abdomen, we have to consider the sensitivity, specificity and invasiveness of each procedure. A CT scan of the abdomen and pelvis (option A) is highly sensitive, but it exposes the patient to a high dose of radiation. Digital angiography of the aorta (option B) is invasive and carries the risk of complications. An X-ray of the abdomen (option D) is not very effective in diagnosing AAA. This leaves us with duplex ultrasonography of the abdomen (option C) which is non-invasive, cost-effective, and highly sensitive and specific for AAA. (4) CONCLUDING: Given the patient's risk factors and his concern about having an AAA, the best diagnostic approach in this case would be to start with a test that is non invasive, highly sensitive and specific, and cost-effective. (5) RESPONSE: Therefore, the most appropriate next step in the diagnosis is (C) Duplex ultrasonography of the abdomen.

**AI_D:** (1) RESTATING THE QUESTION: In simpler terms, a 65-year-old man, who has mild hypertension, quit smoking 20 years ago, has a family history of abdominal aortic aneurysm (AAA), and is taking metoprolol and lisinopril, is concerned about the possibility of having AAA. His older brother had a ruptured AAA that was previously undiagnosed. The patient's medical examination did not reveal any abnormalities except for a midepigastric bruit and an aortic pulsation of 3 cm. The question here is, what should be the next step in confirming or ruling out an AAA in this patient? (2) PLANNING: An assumption here could be that because of the family history of AAA and present symptoms, some kind of diagnostic imaging to visualize the aorta would need to be performed to either confirm or rule out AAA. Another assumption might suggest 'no diagnostic imaging is needed' if we believe that physical examination and patient history are enough to make a diagnosis. The conventional clinical priority, in this case, would be to pursue diagnostic imaging given his risk factors and concerns. (3) CLINICAL REASONING: Analyzing his risk factors, he is a male above the age of 65, a history of smoking, and has a family history of AAA (his brother), which are all high-risk factors for AAA. Considering that his brother's aneurysm was undiagnosed until it ruptured, our patient's concern is warranted, and diagnostic imaging should be pursued. The presence of a midepigastric bruit, although non-specific, could potentially indicate turbulent blood flow from an AAA. (4) CONCLUDING: In clinical practice, there are a few different imaging tests that can help diagnose an AAA -- CT scan, digital angiography, duplex ultrasonography, and an X-ray of the abdomen. The choice between these would depend upon their advantages and drawbacks to the patient's history and clinical picture. (5) RESPONSE: Duplex ultrasonography of the abdomen (C) stands out as the most appropriate next step for several reasons. First, duplex ultrasonography is less invasive and does not involve radiation exposure, unlike a CT scan and digital angiography. Second, it provides detailed pictures of the aorta allowing measurement of its size and examination of the blood flow, which can help identify an AAA. Lastly, the accuracy of abdominal ultrasound for detection of AAA is quite high (94%-100%). Consequently, duplex ultrasonography of the abdomen is the most appropriate next step in diagnosis.

**AI_E:** RESTATING THE QUESTION: The question wants to discern the next best step in diagnosing a possible abdominal aortic aneurysm (AAA) for a 65-year-old hypertensive male. The patient has a family history of undiagnosed ruptured AAA (in his brother), has a history of smoking, and currently presents with a midepigastric bruit and an aortic pulsation of 3cm in width. PLANNING: We should first consider the most reliable and efficient diagnostic methods for detecting AAAs. The patient's history and physical exam findings could suggest an AAA—the presence of aortic pulsation and midepigastric bruit along with the family history of AAA all make this a possibility. However, we must also remember that he lacks any symptoms suggesting AAA rupture, such as severe sudden-onset abdominal pain or syncope. With this in mind, let's explore potential diagnostic measures, including (A) CT scan of the abdomen and pelvis, (B) Digital angiography of the aorta, (C) Duplex Ultrasonography of the abdomen, (D) X-ray of the abdomen, and (E) No diagnostic imaging. CLINICAL REASONING: To begin, the presence of a midepigastric bruit and a relatively larger aortic pulsation strongly suggests that there might be an abdominal aortic aneurysm. With this suspicion and the patient's high-risk profile due to family history and smoking history, it is crucial not to dismiss the need for further diagnostic imaging (option E). While a CT scan (option A) and digital angiography (option B) can undoubtedly visualize the aorta, these methods are more invasive, costlier, and involve higher radiation exposure. Usually, they are reserved for situations where more precise anatomical detail is required, such as pre-surgical planning. An abdominal x-ray (option D) has low sensitivity and specificity for AAA and, though non-invasive, is not a reliable first-line option. Therefore, a Duplex ultrasonography of the abdomen (option C) should be the most appropriate choice. It is non-invasive, low-cost, and has high sensitivity and specificity in detecting AAAs while providing information regarding its size and extent, meaning it has both diagnostic and monitoring uses. CONCLUDING: Based on the clinical context, our reasoning, and assessment of each diagnostic option, Duplex ultrasonography of the abdomen should be the most appropriate next step in the diagnosis of this patient's suspected abdominal aortic aneurysm. RESPONSE: (C) Duplex ultrasonography of the abdomen.

**AI_C:** RESTATING THE QUESTION: The query is about a 65-year-old man who fears he might have an abdominal aortic aneurysm (AAA). He has symptoms like mild hypertension and former tobacco usage, and his older brother had an emergent surgery due to a ruptured AAA that was not previously diagnosed. Despite feeling well, with his physical examination generally unremarkable, an audible mid-epigastric bruit and a substantial aortic pulsation of 3 cm width raises his concern. The crux of the question is to discern the most relevant diagnostic step to confirm or deny the presence of AAA in the patient. PLANNING: The key assumptions here are that the patient's family history, age, and history of smoking place him at a higher risk for AAA, and the physical examination finding of an aortic pulsation of 3 cm and a mid-epigastric bruit further raise suspicion. Given these factors, it is reasonable to investigate further to either confirm or rule out the presence of an AAA. We could question whether more invasive or less invasive testing would be more appropriate in terms of risk, patient burden, and diagnostic accuracy, such as CT scans, angiography, duplex ultrasonography, x-rays or no testing at all. CLINICAL REASONING: Age, male gender, hypertension, smoking history, and a sibling with AAA place the patient in a higher-risk category for AAA. While AAA could be asymptomatic, physical examination findings such as a palpable abdominal aortic pulsation of 3 cm or greater perpendicular width and an audible bruit could be indicative. Given that the severity of an untreated AAA can be life-threatening if it ruptures, it's crucial to make a prompt and accurate diagnosis. Comparing the diagnostic methods, Duplex ultrasonography has a sensitivity and specificity rate of more than 95%, providing superior visualization of the aorta's length and can assess the velocity of blood flow indicative of possible turbulent flow or occlusion. CT and angiography would be more sensitive and specific but are invasive, have more risk from radiation, and are more expensive. An X-ray may not provide definitive AAA details unless it shows a calcified aortic wall which isn't always present. CONCLUDING: Therefore, considering the high accuracy, relatively low risk, cost-effective method, and its non-invasive nature, duplex ultrasonography stands out as the most suitable step for this patient's diagnosis that connects clinical suspicion to potentially definitive findings. RESPONSE: (C) Duplex ultrasonography of the abdomen

**AI_B:** (1) RESTATING THE QUESTION: The query presented asks what would be the best diagnostic step in assessing a 65-year-old man, demonstrating no clear signs of distress, with a family history of abdominal aortic aneurysm (AAA). An AAA is a local enlargement of the abdominal aorta, occurring when the wall of the aorta weakens. It's both a genetic and lifestyle associated condition and given the patient's history of smoking, even though he quit 20 years ago, he still maintains a risk factor for AAA. (2) PLANNING: I notice certain assumptions here. The first one, based on clinical examination, is that the patient seems to have a relatively quiet clinical presentation. However, the midepigastric bruit and the presence of an abdominal aortic pulsation of 3 cm gives us a reason to double-check this assumption. Secondly, the patient has a significant family history of AAA, prompting an earlier detection and surveillance. The third assumption is based on his history of smoking which could have contributed to the development of an AAA despite cessation 20 years ago. Considering these, I hypothesize that although he is not presenting with severe symptoms, the potential risk of AAA in this patient should be further evaluated given the risk factors associated. (3) CLINICAL REASONING: From my knowledge, AAA is most frequently asymptomatic until rupture, one of the deadliest surgical emergencies. Therefore, early detection and monitoring of AAA are vital, especially in patients with relevant risk factors like advanced age, male gender, history of smoking, hypertension, and family history of AAA – as is the case for this patient. Although his current signs and symptoms do not overtly point to an aortic aneurysm, the clinical suspicion remains due to these risk factors. An enlarged, palpable abdominal aorta (>3 cm) and presence of a midepigastric bruit further fuel this suspicion. To confirm whether he has an AAA, the most effective next step would involve a form of imaging. Here, the options include a CT scan, digital angiography, duplex ultrasonography, or an X-ray. (4) CONCLUDING: Considering the balance between diagnostic accuracy, invasiveness, and cost-effectiveness, duplex ultrasonography stands out. It's noninvasive, cost-effective, and generally preferred for initial diagnosis and follow-up of AAA. CT or MRI can indeed provide excellent anatomic detail, but they are mostly reserved for preoperative planning rather than initial diagnosis due to their higher cost and exposure to radiation or contrast dye. A plain abdominal x-ray is not sensitive or specific enough, and digital angiography of the aorta is too invasive for a first-line diagnostic tool. (5) RESPONSE: Consequently, the most appropriate next step in diagnosis for this patient would be (C) Duplex ultrasonography of the abdomen. It balances patient safety, cost-effectiveness, and diagnostic utility in this context. It will allow us to further evaluate the patient's abdominal aorta and monitor for any potential growth or complications should an AAA be present.

**Facilitator** The consensus among the AI responses is that the most appropriate next step in diagnosing a potential abdominal aortic aneurysm (AAA) in the 65-year-old patient is (C) Duplex ultrasonography of the abdomen. This conclusion is based on the patient's risk factors, including his age, history of smoking, mild hypertension, and a family history of AAA. Despite the patient's lack of overt symptoms, the presence of a midepigastric bruit and a substantial aortic pulsation of 3 cm width raises suspicion of AAA. The AI responses agree that the patient's risk factors and physical examination findings warrant further investigation through diagnostic imaging, ruling out the option of 'no diagnostic imaging'. Among the imaging options, a CT scan and digital angiography, while highly sensitive, are invasive, costly, and expose the patient to high doses of radiation. An X-ray of the abdomen is not very effective in diagnosing AAA. Duplex ultrasonography of the abdomen, on the other hand, is non-invasive, cost-effective, and highly sensitive and specific for AAA. It provides detailed images of the aorta, allowing for the measurement of its size and examination of the blood flow, which can help identify an AAA. Therefore, given the patient's risk factors, clinical presentation, and the need for a safe, cost-effective, and reliable diagnostic method, the consensus is that duplex ultrasonography of the abdomen is the most appropriate next step in the diagnosis.
