# Supplementary material for: Collaborative intelligence in AI: Evaluating the performance of a council of AIs on the USMLE
Source: PLOS Digit Health. 2025 Oct 9;4(10):e0000787. doi: 10.1371/journal.pdig.0000787 (PMC12510544; doi:10.1371/journal.pdig.0000787)
Supplement: S2 File — (ZIP) [file pdig.0000787.s002.zip › S2 Data Raw data files containing unprocessed question inputs and LLM outputs including transcripts of deliberations/STEP2_Question-104-1_Type-MC-NJ.docx]

**Facilitator** **[Round 1]:** A 57-year-old man comes to the physician because of a 1-month history of fatigue and moderate low back pain. He also has had a mildly decreased appetite resulting in a 1.8-kg (4-lb) weight loss during this period. He has a 1-week history of headache. He has had no fever, sweating, or chills. He has hypertension well controlled with triamterene-hydrochlorothiazide. Other medications include 81-mg aspirin, ibuprofen, and a daily multivitamin. He appears tired. Examination shows slightly pale conjunctivae. A grade 2/6 systolic flow murmur is heard best at the lower left sternal border. The remainder of the examination shows no abnormalities. Test of the stool for occult blood is negative. Laboratory studies show: Hematocrit: 32% Mean corpuscular volume: 102 μm³ Leukocyte count :9200/mm³ Segmented neutrophils: 66% Bands: 3% Lymphocytes: 31% Platelet count: 325,000/mm3 Red cell distribution width: 15.6% (N=13%–15%) Serum Ca²⁺: 10.8 mg/dL Urea nitrogen: 18 mg/dL Glucose: 108 mg/dL Creatinine: 1.5 mg/dL Albumin: 3 g/dL X-rays of the lumbosacral spine show mild osteopenia. Which of the following is the most appropriate next step in diagnosis? (A) Serum protein electrophoresis (B) Echocardiography (C) Bone scan (D) Colonoscopy (E) Esophagogastroduodenoscopy

**AI_E:** This clinical scenario presents a 57-year-old man with symptoms including fatigue, back pain, decreased appetite, weight loss, and headaches. He also exhibits slightly pale conjunctivae and a systolic flow murmur. His laboratory studies reveal a slight decrease in hematocrit and indications of macrocytosis (indicated by an increased Mean Corpuscular Volume and a higher Red Cell Distribution Width). Pale conjunctivae can be a symptom of anemia, which can be determined by low hematocrit. His X-ray shows mild osteopenia, which is a condition where bone mineral density is lower than normal. When we consider these, we can identify anemia and potential bone weakness as the primary assumptions drawn from his clinical presentation. Alternative hypotheses may include gastrointestinal maladies, cardiovascular disease, or protein abnormalities. The patient's symptoms include both general and systemic manifestations - the fatigue, weight loss, and pallor could point to a systemic problem, and should therefore be given higher clinical priority. Focusing on this perspective, we can reason out the unique combination of anemia, osteopenia, and back pain. Anemia, signaled by the low hematocrit and larger than normal red blood cells (macrocytosis), can be caused by certain deficiencies (like vitamin B12 or folic acid), kidney disease, or a chronic disease. The elevated Mean Corpuscular Volume (MCV) suggests that the anemia may be due to a deficiency of vitamin B12 or folic acid, which are needed for red blood cell formation. Coupled with other symptoms like mild osteopenia and back pain, a potential cause could be multiple myeloma, a cancer of plasma cells that can cause bone damage and anemia. The absence of gastrointestinal symptoms and negative stool occult blood test make a gastrointestinal cause of macrocytic anemia less likely. We can conclude that our clinical priority should be investigating a protein abnormality consistent with multiple myeloma. Given the overall clinical presentation and laboratory results, we want to detect any potential monoclonal gammopathy which can indicate multiple myeloma. Out of the given diagnostic options, Serum Protein Electrophoresis is used to identify abnormal protein populations in the blood, hence it can be useful in diagnosing cases like multiple myeloma. Thus, my comprehensive response to the question as to the appropriate next step in diagnosis for the given patient would be choice (A) Serum Protein Electrophoresis.

**AI_D:** RESTATING THE QUESTION: The query details a 57-year-old man who has been experiencing fatigue, low back pain, a mild decrease in appetite, weight loss, and headaches for about a month. Despite no indication of fever, he appears visibly tired, has slightly pale conjunctivae, and a grade 2/6 systolic murmur at the lower left sternal border. Lab results reveal, among other things, anemia with high mean corpuscular volume, slightly elevated serum calcium and creatinine levels, and mild osteopenia on lumbosacral X-ray. The question asks which diagnostic test or procedure should be performed next based on these observations. PLANNING: We have the assumptions that the patient's presentation could indicate several conditions, including malnutrition from reduced appetite, anemia from various causes, possible cardiac involvement from the murmur, and kidney dysfunction from the elevated creatinine. However, the more notable findings are the microcytic anemia (i.e., characterized by a high mean corpuscular volume or MCV), slightly elevated serum calcium, and back pain with radiographic evidence of mild osteopenia—findings that suggest a possible hematological malignancy like multiple myeloma. This condition could explain most of the patient's symptoms and abnormalities; hence, it should be clinically prioritized to pursue. CLINICAL REASONING: Multiple myeloma is a type of bone marrow cancer that can lead to anemia, bone pain, and kidney dysfunction. It often presents in older adults with fatigue, back pain, and anemia, as observed in this patient. In addition, multiple myeloma often causes increased serum calcium levels due to bone resorption, which seems to be indicated in this patient as well. The disease can also lead to elevated creatinine levels because the abnormal proteins produced in multiple myeloma can damage the kidneys. To confirm the presence of the abnormal proteins characteristic of multiple myeloma, a serum protein electrophoresis can be done. CONCLUDING: As per clinical reasoning process, given the patient's symptoms of fatigue, back pain, anemia, slightly increased serum calcium and creatinine levels, and mild osteopenia, the most likely medical issue is multiple myeloma, a type of bone marrow cancer. To identify the abnormal proteins produced in multiple myeloma that are responsible for the patient's symptoms, serum protein electrophoresis is the most appropriate next step in diagnosis. RESPONSE: The most appropriate next step in diagnosis for this patient is (A) Serum protein electrophoresis.

**AI_C:** (1) RESTATING THE QUESTION: Our goal is to determine the next steps in diagnosing a patient that has an array of symptoms spanning fatigue, low back pain, slightly decreased appetite leading to weight loss, headache, and hypertension. The patient’s examination and laboratory results indicate anemia, more specifically, a slightly macrocytic anemia as suggested by an increased Mean Corpuscular Volume (MCV) and hematocrit of 32%, which is on the lower side. The patient also has slight pale conjunctiva, which is consistent with anemia, and mild osteopenia as shown by x-rays of the lumbosacral spine. Despite this, stool testing for occult blood is negative, which indicates that GI bleeding is not the cause of his anemia. (2) PLANNING: Given the evidence, we should consider causes of macrocytic anemia, which includes vitamin B12 and folate deficiency, liver disease, hypothyroidism, and myelodysplastic syndromes. However, given the array of symptoms that the patient has presented with, early multiple myeloma, a type of hematological malignancy, could be a possibility. This is characterized by cause intermittent bone pain (e.g., back pain), fatigue, and mild anemia. The next step would be to verify if this assumption could explain the patient's presentation and exlude the other differentials. (3) CLINICAL REASONING: Given the collective evidence, it may be more likely that our patient is suffering from an early form of multiple myeloma. This could explain his fatigue, back pain, and anemia. Multiple myeloma often causes a type of anemia that is normocytic or slightly macrocytic, which aligns with the patient's MCV. However, this generally does not lead to significant weight loss or loss of appetite as seen in our patient. Anemia may also result in fatigue, but the decrease in appetite and weight loss might suggest a malignancy. The eventual bone pain that multiple myeloma is known for might be the source of the patient's back pain and headache. Moreover, the patient's high calcium level could be the result of bone breakdown due to the malignancy, which would cause hypercalcemia. Mild osteopenia seen on X-rays is an indication of bone loss, which supports this. (4) CONCLUDING: Taking these factors into account, we need to conduct further tests that would confirm or rule out multiple myeloma. A serum protein electrophoresis (SPEP) would be most appropriate as it would detect any excessive monoclonal antibodies (M protein) produced by malignant plasma cells, which is a characteristic feature of multiple myeloma. (5) RESPONSE: The most appropriate next step in diagnosis for this case, given the symptoms and test results, would be to conduct a Serum protein electrophoresis (Option A). This test would be useful to detect any excessive production of monoclonal antibodies, which is a feature indicative of multiple myeloma.

**AI_B:** RESTATING THE QUESTION: The patient in discussion, a 57-year-old male, has been presenting symptoms such as fatigue, loss of appetite, weight loss, headache and back pain over varying periods of time. In addition to these, he has slightly pale conjunctiva and a mild systolic flow murmur. Laboratory studies reveal some abnormalities like reduced hematocrit, increased mean corpuscular volume and red cell distribution width, along with a mild osteopenia observed in the X-rays. We are asked to identify the most appropriate test to proceed with for further diagnosis. Orienting on the test options: serum protein electrophoresis is used to separate proteins based on size and charge and is often used to diagnose conditions such as multiple myeloma; Echocardiography is primarily used for assessing heart function; Bone scan is used to identify abnormalities in bones; Colonoscopy and Esophagogastroduodenoscopy are diagnostic procedures used in gastrointestinal tract evaluation. PLANNING: The key assumptions based on symptoms and current lab results are that patient could be suffering from anemia due to various reasons such as vitamin B12 deficiency or chronic disease or could also have a benign stenosis, heart disease, osteoporosis, kidney disease, or even multiple myeloma. Evaluating these assumptions, it can be identified that the patient's symptoms, the laboratory results with increased MCV and RDW, and lack of gastrointestinal bleeding point primarily towards a hematological problem, which requires to be prioritized. CLINICAL REASONING: The presence of fatigue and the paleness of the conjunctivae along with a low hematocrit value suggest that the patient might be suffering from anemia. The increased Mean Corpuscular Volume (MCV) suggests that this could be a macrocytic anemia, a condition where red blood cells are larger than normal. This could be due to Vitamin B12/folate deficiency or due to conditions that affect the bone marrow like myelodysplastic syndrome or leukemia. Getting a complete profile of serum proteins may provide further information, helping in the differential diagnosis of conditions affecting protein levels like multiple myeloma or chronic kidney disease. CONCLUDING: Considering the patient's symptoms which feature fatigue and a pale conjunctiva, suggesting anemia, along with laboratory results including an increased MCV and RDW, it is safe to infer that a hematological problem is the leading suspected clinical presentation. The next most appropriate step to confirm this should involve a procedure that could potentially identify any such conditions, hence Serum protein electrophoresis should be the most suitable choice. RESPONSE: Based on the patient's symptoms and test results, which suggest a possible hematological issue, the most appropriate next step in his diagnosis would be to conduct a Serum protein electrophoresis (A).

**AI_A:** 1) RESTATING THE QUESTION: This patient, a 57-year-old male, presents with a set of symptoms and findings that include fatigue, low back pain, minor weight loss due to diminished appetite, a week-long history of headache, controlled hypertension, and general tiredness. Lab findings indicate anemia with raised mean corpuscular volume, along with slightly pale conjunctivae. Despite negative results for occult blood in the stool, the patient has mild osteopenia as per lumbosacral spine X-Ray results. Among the given options (serum protein electrophoresis, echocardiography, bone scan, colonoscopy, esophagogastroduodenoscopy), the task at hand is to determine the most appropriate next step in the diagnosis. 2) PLANNING: An anemic picture with a high mean corpuscular volume indicates macrocytic anemia, which can be either megaloblastic (due to vitamin B12 or folate deficiency, or a pathologic state that interferes with DNA synthesis) or non-megaloblastic (caused by conditions that distend the red cell membrane, such as reticulocytosis, certain drugs, alcoholism, or liver disease). The patient's fatigue, loss of appetite, and weight loss could suggest an underlying systemic illness, though the results show no direct evidence of gastrointestinal bleeding or cancer (which could explain the weight loss and anemia). The mildly elevated Calcium level and slight osteopenia on the x-ray of spine could hint towards a paraneoplastic syndrome or a primary bone pathology or could be incidental. While it's important to diagnose the specific type of anemia to address the patient's fatigue, the cause of the elevated Calcium should also be explored. 3) CLINICAL REASONING: Given the constellation of symptoms and findings, including fatigue, anemia with raised mean corpuscular volume, slightly raised calcium level, and low back pain combined with osteopenia, these could be connected to form a picture of a potential pathological state suggestive of a paraneoplastic syndrome due to an underlying malignancy. In such a scenario, the mildly raised calcium level could point towards a malignancy producing a parathyroid hormone-related peptide (PTHrP) causing hypercalcemia. Looking at the lab results, it seems that there is no evidence of renal disease (despite a slight increase in creatinine levels), which could otherwise explain the hypercalcemia. Another consideration could be multiple myeloma, a plasma cell dyscrasia often seen older patients; it can cause anemia, hypercalcemia, and bone pain due to osteolytic lesions and osteopenia. Osteolytic lesions might not always be evident on a regular x-ray and osteopenia could be the only clue. 4) CONCLUDING: It will be crucial to firstly rule out the possibility of a plasma cell dyscrasia such as multiple myeloma due to its association with elevated calcium levels, anemia, and the age-group of the patient. These factors make it a high-priority consideration, aligning with the clinical prioritization strategy in diagnosis. The presence of a possible underlying malignancy calls for an urgency over determining the exact cause of a macrocytic anemia since it could also be due to an underlying marrow involvement in malignancy. 5) RESPONSE: Considering the reasonings and conclusion above, the most appropriate next step in diagnosis should be (A) Serum protein electrophoresis. This test can detect paraproteins or M protein, which are seen in disorders like multiple myeloma, thereby can help identify this pathological condition contributing to the patient's symptoms.

**Facilitator** The consensus statement synthesizes the responses of AI_A, AI_B, AI_C, AI_D, and AI_E as follows: The patient, a 57-year-old male, presents with symptoms including fatigue, low back pain, minor weight loss due to diminished appetite, a week-long history of headache, controlled hypertension, and general tiredness. Laboratory findings indicate anemia with raised mean corpuscular volume, slightly pale conjunctivae, and mild osteopenia as per lumbosacral spine X-Ray results. Despite negative results for occult blood in the stool, the patient has slightly elevated serum calcium and creatinine levels. The patient's symptoms and lab results suggest a potential systemic illness, possibly a hematological problem. The anemia, indicated by a high mean corpuscular volume, could be macrocytic, which can be caused by vitamin B12 or folate deficiency, or conditions that interfere with DNA synthesis or distend the red cell membrane. However, the patient's fatigue, loss of appetite, weight loss, slightly raised calcium level, and low back pain combined with osteopenia could be indicative of a paraneoplastic syndrome due to an underlying malignancy, such as multiple myeloma. Multiple myeloma, a plasma cell dyscrasia often seen in older patients, can cause anemia, hypercalcemia, and bone pain due to osteolytic lesions and osteopenia. The mildly elevated calcium level could point towards a malignancy producing a parathyroid hormone-related peptide (PTHrP) causing hypercalcemia. The absence of gastrointestinal symptoms and negative stool occult blood test make a gastrointestinal cause of macrocytic anemia less likely. Given the patient's symptoms and test results, the most appropriate next step in diagnosis would be to conduct a Serum protein electrophoresis (Option A). This test can detect paraproteins or M protein, which are seen in disorders like multiple myeloma, thereby can help identify this pathological condition contributing to the patient's symptoms. This aligns with the clinical prioritization strategy in diagnosis, which suggests that the presence of a possible underlying malignancy calls for urgency over determining the exact cause of a macrocytic anemia.
