# Supplementary material for: Collaborative intelligence in AI: Evaluating the performance of a council of AIs on the USMLE
Source: PLOS Digit Health. 2025 Oct 9;4(10):e0000787. doi: 10.1371/journal.pdig.0000787 (PMC12510544; doi:10.1371/journal.pdig.0000787)
Supplement: S2 File — (ZIP) [file pdig.0000787.s002.zip › S2 Data Raw data files containing unprocessed question inputs and LLM outputs including transcripts of deliberations/STEP2_Question-105-1_Type-MC-NJ.docx]

**Facilitator** **[Round 1]:** A 38-year-old woman, gravida 1, para 1, comes to the office because she has been unable to conceive for the past year. She and her husband have had unprotected sexual intercourse three times weekly during this time. They have a 6-year-old daughter. During the past year, menses have occurred at irregular 90- to 120-day intervals. Her last menstrual period was 2 months ago. Menses previously occurred at regular 30-day intervals. She used an oral contraceptive for 4 years after the birth of her daughter but discontinued it 14 months ago. She has no history of serious illness and takes no medications. The patient appears well. She is 163 cm (5 ft 4 in) tall and weighs 63 kg (140 lb); BMI is 24 kg/m². Vital signs are within normal limits. Examination shows no abnormalities. Results of serum studies are shown: Thyroid-stimulating hormone: 3 μU/mL Follicle-stimulating hormone: 100 mIU/mL Prolactin: 10 ng/mL β-hCG: negative This patient’s diagnosis puts her at risk for which of the following? (A) Breast cancer (B) Endometrial cancer (C) Obesity (D) Osteoporosis (E) Unplanned pregnancy

**AI_C:** RESTATING THE QUESTION: This query, in essence, is asking about the risk associated with the diagnosis of a 38-year-old woman who is experiencing difficulties in conceiving for the past year despite frequent unprotected intercourse. She has irregular menstruation cycle since last year and her results indicate a high follicle-stimulating hormone (FSH) level. PLANNING: The high FSH level is a noteworthy finding, indicating premature ovarian failure (POF) or primary ovarian insufficiency (POI), which is confirmed by the fact that she has irregular menstruation cycles and has been unable to conceive. This would impair her fertility and is associated with certain risks. CLINICAL REASONING: The decreased level of estrogen (which is typical in POF/POI) affects many physiological processes. Women with POF/POI not only face reproductive challenges but also systemic health issues. The significant drop in the production of estrogen precipitate these challenges. Without estrogen, the body experiences increased bone resorption leading to a gradual reduction in bone mass. CONCLUDING: Based on the above reasoning, the elevated FSH levels, irregular menstrual cycles, and infertility symptoms can lead to a loss of the protective effects of estrogen on the bone, leading to decreased bone integrity and consequently, increased risk of related conditions like osteoporosis. RESPONSE: This patient’s diagnosis puts her at risk for (D) Osteoporosis.

**AI_A:** RESTATING THE QUESTION: This 38-year-old woman, who has given birth once and is currently experiencing secondary infertility (defined as the inability to conceive after a previous successful pregnancy), is seeking medical advice. Her history reveals regular sexual activity without contraception, irregular and prolonged menstrual periods for a year, cessation of oral contraceptive pills 14 months ago, and noticeable difference from her prior regular menstrual cycles. The patient is physically healthy, and the results from her serum studies are thus: Thyroid-stimulating hormone: 3 μU/mL, Follicle-stimulating hormone: 100 mIU/mL, Prolactin: 10 ng/mL, and β-hCG: negative. The question seeks to identify the condition from which she is at risk, given her diagnosis. PLANNING: The striking point in this scenario is the significantly elevated Follicle-stimulating hormone (FSH) at 100 mIU/mL when the normal value ranges from 3 to 20 mIU/mL. High FSH levels in the bloodstream are indicative of menopause or primary ovarian insufficiency (also known as premature ovarian failure). In this patient, who is 38 years old and presenting with irregular and prolonged menstrual periods, the most likely diagnosis is primary ovarian insufficiency. This condition puts her at risk of several possible complications. CLINICAL REASONING: Primary ovarian insufficiency (POI) describes a condition in which there is diminished ovarian function resulting in premature depletion or dysfunction of ovarian follicles under the age of 40. It is characterized by elevated FSH levels (as seen in this case), sporadic or absent menses for at least four months, and various symptoms of estrogen deficiency (e.g., hot flashes, night sweats). Importantly, women with POI still have intermittent and unpredictable ovarian function and can conceive spontaneously, although their fertility is significantly reduced. As for complications, as the woman loses the bone-protective effects of estrogen sooner, there is an increased risk for osteoporosis. CONCLUDING: Therefore, given the findings and diagnosis of primary ovarian insufficiency, this patient would be at increased risk for osteoporosis. Osteoporosis represents a condition in which bones become weak and brittle. The onset of this condition can be hastened by factors such as lack of oestrogen which occurs in POI. RESPONSE: The condition this patient is at risk for, given her diagnosis, is (D) Osteoporosis.

**AI_D:** 1) RESTATING THE QUESTION: The scenario involves a 38-year-old woman, who has a child already and has been trying to conceive for the past year, but has been unsuccessful. Her menses have become irregular in the past year, occurring every 90-120 days as opposed to the previously normal 30-day intervals. Her BMI and vital signs are normal and she has no history of serious illness. Importantly, her follicle-stimulating hormone levels are elevated while her other hormone levels are within normal limits. The main question here is: considering her diagnosis, what medical risk is she predisposed to? 2) PLANNING: The key facts in the question are this woman's inability to conceive over the past year, her irregular menses, and the elevated follicle-stimulating hormone (FSH) levels. These indicate a potential problem with ovulation, likely due to diminished ovarian reserve (DOR) or premature ovarian failure (POF). This could lead to infertility, and other medical risks. One assumption is this condition has come about due to natural aging, but given her age, it's unusual. Other assumptions could include a genetic predisposition, an autoimmune cause, or a previous infection or trauma to the ovaries. We need to clinically prioritize the hypothesis that pertains to her risk of developing associated conditions. 3) CLINICAL REASONING: Elevated FSH levels in conjunction with menstrual irregularities suggest a decrease in her ovarian reserve, which means her ovary's ability to provide viable eggs for fertilization is diminished. Given her age and circumstances, premature ovarian failure seems more likely than natural menopause. POF is when the ovaries lose their function before the age of 40 and this can lead to several health risks. With the cessation of ovarian activity, there will be a deficiency of ovarian hormones, particularly estrogen. 4) CONCLUDING: The consideration here is mainly the increased risk associated with a lack of estrogen. Estrogen plays an essential role in numerous physiological processes beyond reproduction. It's involved in the metabolism and homeostasis of several tissues including the cardiovascular system, musculoskeletal system, nervous system, and skin. The loss of estrogen's protective effects due to POF can lead to several adverse health outcomes, including an increased risk of cardiovascular disease, osteoporosis, and cognitive decline. 5) RESPONSE: Given these considerations and the patient's clinical presentation, she is at risk for (D) Osteoporosis. This is largely due to the decrease in estrogen, which is fundamental for the maintenance of bone density, hence diminished levels of it can lead to accelerated bone loss.

**AI_B:** RESTATING THE QUESTION: A woman of 38 years, who has given birth once and is currently trying to conceive, has presented with concerns of infertility for the past year. Despite regular unprotected sexual activity, she has not become pregnant. Her menstrual cycles over the past year have been irregular, compared to her usually regular 30-day cycles. Following her previous pregnancy, she took oral contraceptives for four years but has been off these for 14 months now. Her overall health and physical examination are normal. However, her follicle-stimulating hormone (FSH) level is elevated at 100 mIU/mL. Other hormones levels including thyroid-stimulating hormone (TSH), prolactin and beta-human chorionic gonadotropin (β-hCG) are within normal limits or negative respectively. The question posed is what health risk does this patient face based on her clinical presentation and current diagnosis. PLANNING: The assumptions based on the presented case include: 1. The patient's fertility issue is related to her irregular menses which can be due to several factors such as hormonal imbalance, ovulatory dysfunction, or early menopause. 2. Her high FSH level and irregular menstrual cycle suggest some form of reproductive dysfunction, possibly primary ovarian insufficiency. We should prioritize the assumption concerning her reproductive dysfunction as indicated by her symptoms and abnormal FSH level. CLINICAL REASONING: Irregular menstrual cycles and an elevated FSH level could indicate an impaired ovulatory function, particularly primary ovarian insufficiency (POI). In regular menstruation, FSH stimulates the growth and maturation of a follicle in one of the ovaries. However, in POI, due to diminished ovarian reserve, this feedback is lost and thus, the FSH levels are increased to stimulate nonresponsive ovaries, hence the irregular menses. CONCLUDING: In light of the above, it seems probable that the woman in question has primary ovarian insufficiency leading to anovulatory cycles, which in turn, could render her at significant risk for developing certain health conditions. According to the available options, her increased risk for osteoporosis appears most likely. RESPONSE: Experiencing primary ovarian insufficiency and anovulatory cycles, due to the associated estrogen deficiency, this patient would likely be at a heightened risk for osteoporosis (option D). This is because estrogen plays a crucial role in maintaining bone density; its reduction leads to accelerated bone loss, hence increasing the risk of osteoporosis over time.

**AI_E:** RESTATING THE QUESTION: This query relates to a 38-year-old woman who has been unsuccessfully trying to conceive for one year. She and her husband have frequent unprotected sexual relations, and they already have one child. Her menstruations occur at irregular intervals of 90 to 120 days and were regular 30-day cycles in the past. She was using an oral contraceptive until 14 months ago, and her BMI and vital signs are within normal ranges. Her laboratory results revealed normal levels of thyroid-stimulating hormone (TSH) and prolactin, but a significantly high level of follicle-stimulating hormone (FSH), while no presence of beta human chorionic gonadotropin (β-HCG) was detected. We are asked to identify what condition(s) her diagnosis could predispose her to. PLANNING: I will first underline the importance of certain points in the presented history and lab results, namely, her irregular menstruation and the high level of FSH. Both closely point to a condition called menopause or, more specifically, to premature ovarian failure given her age. This condition can have long-term health implications, including potential endometrial cancer or osteoporosis. However, it also needs to be pointed out that the patient's TSH and prolactin are normal, reducing the likelihood of disorders such as thyroid disease or hyperprolactinemia, both of which can affect menstrual cycles and fertility. CLINICAL REASONING: Regular menstrual cycles, which are usually 28-35 days apart, are a sign of regular ovulation. The woman in question previously had a 30-day cycle and has now experienced irregular periods with much longer cycles, suggesting anovulation. Additionally, the markedly elevated FSH levels are indicative of decreased ovarian reserve, a condition usually seen in peri-menopausal or menopausal women but could occur earlier in cases of premature ovarian failure. CONCLUDING: Taking all these pieces of information together, the woman's symptoms point towards premature ovarian failure. This diagnosis is confirmed by an elevated FSH level. An increase in FSH could be associated with the risk of developing certain conditions, such as endometrial cancer and osteoporosis. RESPONSE: Given the patient's history and clinical findings, her diagnosis is most likely to put her at risk for (B) Endometrial cancer and (D) Osteoporosis. This is due to hormonal changes associated with premature ovarian failure, which can lead to an increased risk of these conditions. It's unlikely that she is at risk for (A) Breast cancer, (C) Obesity, or (E) Unplanned pregnancy given her current clinical status and lab results.

**Facilitator** The consensus statement synthesizes the responses of the five AI opinions as follows: The case presents a 38-year-old woman who has been experiencing secondary infertility for the past year, characterized by irregular and prolonged menstrual periods and a significantly elevated Follicle-stimulating hormone (FSH) level. These symptoms, along with her age and the cessation of oral contraceptive pills 14 months ago, suggest a diagnosis of primary ovarian insufficiency (POI), also known as premature ovarian failure (POF). POI is a condition characterized by diminished ovarian function resulting in premature depletion or dysfunction of ovarian follicles under the age of 40. It is associated with elevated FSH levels, sporadic or absent menses, and various symptoms of estrogen deficiency. Women with POI still have intermittent and unpredictable ovarian function and can conceive spontaneously, although their fertility is significantly reduced. The decreased level of estrogen, which is typical in POI, affects many physiological processes. Without estrogen, the body experiences increased bone resorption leading to a gradual reduction in bone mass. This loss of estrogen's protective effects on the bone can lead to decreased bone integrity and consequently, increased risk of related conditions like osteoporosis. Therefore, given the findings and diagnosis of primary ovarian insufficiency, this patient would be at increased risk for osteoporosis. Osteoporosis represents a condition in which bones become weak and brittle. The onset of this condition can be hastened by factors such as lack of estrogen which occurs in POI. In conclusion, the condition this patient is at risk for, given her diagnosis, is (D) Osteoporosis.
