# Supplementary material for: Collaborative intelligence in AI: Evaluating the performance of a council of AIs on the USMLE
Source: PLOS Digit Health. 2025 Oct 9;4(10):e0000787. doi: 10.1371/journal.pdig.0000787 (PMC12510544; doi:10.1371/journal.pdig.0000787)
Supplement: S2 File — (ZIP) [file pdig.0000787.s002.zip › S2 Data Raw data files containing unprocessed question inputs and LLM outputs including transcripts of deliberations/STEP2_Question-106-1_Type-MC-NJ.docx]

**Facilitator** **[Round 1]:** A 27-year-old man comes to the physician because of a 1-month history of progressive fatigue and episodes of moderate abdominal pain. He has a 4-year history of recurrent nosebleeds that have increased in frequency despite undergoing cauterization of the nasal mucosa 6 months ago. He takes no medications. His sister has anemia associated with recurrent nosebleeds and heavy menstrual periods; she also had postpartum hemorrhage that required transfusion. The patient's vital signs are within normal limits. Examination shows pallor; no other abnormalities are noted. Laboratory studies show: Hemoglobin: 7.1 g/dL Erythrocyte count: 2.1 million/mm³ Leukocyte count: 13,500/mm³ Platelet count: 250,000/mm³ Prothrombin time: 12 sec (INR=1) Partial thromboplastin time: 45 sec Which of the following is the most likely diagnosis? (A) Bernard-Soulier syndrome (B) Glanzmann thrombasthenia (C) Hemophilia A (D) Hereditary hemorrhagic telangiectasia (E) von Willebrand disease

**AI_E:** RESTATING THE QUESTION: We're trying to find out the most probable diagnosis for a 27-year-old man suffering from fatigue, abdominal pain, and recurrent nosebleeds which keep coming back even after cauterization. He doesn't take any medication. Moreover, his sister also suffers from anemia and has experienced features consistent with a bleeding diathesis like recurrent nosebleeds, heavy menstrual periods, and severe postpartum hemorrhage. Laboratory indications include low hemoglobin and erythrocyte counts, but normal leukocyte count, platelet count, prothrombin time, and increased partial thromboplastin time, suggesting an underlying coagulation disorder. PLANNING: An assumption here is that the patient might have inherited a similar condition as his sister, as there seems to be a familial trait in these symptoms. An alternate assumption could be that the recurrent nosebleeds and symptoms of anemia might have some common etiology involving coagulation abnormalities. Clinically prioritizing, our focus will be on the coagulation profiles and the familial trait of the symptoms. CLINICAL REASONING: The presenting symptoms, coupled with the family history, suggest a hereditary disorder. Normal platelet counts and prothrombin time exclude platelet and extrinsic pathway disorders respectively. The conditions Bernard-Soulier syndrome, Glanzmann thrombasthenia and von Willebrand disease all result in effective platelet dysfunction, but the platelet count and extrinsic coagulation pathway are normal in this patient. Hemophilia A manifests primarily in males and would not directly affect his sister. Hereditary hemorrhagic telangiectasia, also known as Osler-Weber-Rendu syndrome, commonly presents with recurrent nosebleeds, mucocutaneous telangiectasias, and gastrointestinal bleeding, which might explain the abdominal pain. This autosomal dominant condition results in abnormal blood vessel formation and would not affect the common coagulation profiles. CONCLUDING: Given the presenting symptoms, familial trait, normal platelet count and prothrombin time, and an elevated partial thromboplastin time, the most likely diagnosis here is hereditary hemorrhagic telangiectasia. RESPONSE: (D) Hereditary hemorrhagic telangiectasia.

**AI_B:** RESTATING THE QUESTION: The presented case is about a 27-year-old man who has been experiencing fatigue and abdominal pain for a month, along with a 4-year history of nosebleeds that have been worsening even after cauterization 6 months ago. His sister has a history of anemia, nosebleeds, heavy menstrual bleeding, and a case of postpartum hemorrhage needing a blood transfusion. His laboratory results indicate low hemoglobin levels and erythrocyte count, with normal leukocyte count, platelet count, prothrombin time and increased partial thromboplastin time. Given these symptoms, history, and results, we need to determine the most probable diagnosis from the choices given. PLANNING: The assumption here is that the patient's symptoms and the tests results point towards a hematological disorder. By studying each assumption, we see that the common thread is a history of bleeding complications (nosebleeds, heavy menstrual bleeding and postpartum hemorrhage), low hemoglobin and erythrocyte count with normal leukocytes and platelets count, and increased PTT. Moreover, the presence of similar symptoms in the patient's family, especially his sister, could indicate a hereditary disease. CLINICAL REASONING: Each disease presented in the options is associated with coagulation disorders, but not all correspond to the specific symptoms, family history and lab results in this case. For example, Bernard-Soulier syndrome and Glanzmann thrombasthenia typically lead to low platelet count, long bleeding time, but normal coagulation studies, which is inconsistent with this patient's results. Hemophilia A primarily affects males and leads to prolonged PTT, but it would not explain the sister's symptoms nor the mucocutaneous bleeding (nosebleeds) as its hallmark is spontaneous joint and deep muscle bleeding. Von Willebrand disease is also a possible option but it generally presents with normal PTT and slightly prolonged PT in severe cases. The remaining option is hereditary hemorrhagic telangiectasia (HHT), a genetic disorder characterized by nosebleeds, arteriovenous malformations causing multiple organ systems involvement, and mucocutaneous telangiectasias. The presence of similar symptoms in the family supports this conclusion. CONCLUDING: Taking these factors into account, we can hypothesize that the most likely diagnosis for this patient's symptoms, family history, and laboratory findings is hereditary hemorrhagic telangiectasia. RESPONSE: So, the most likely diagnosis for this patient's case is (D) Hereditary hemorrhagic telangiectasia.

**AI_C:** (1) RESTATING THE QUESTION: The question asks for the most likely diagnosis for a 27-year-old man who presents with progressive fatigue and abdominal pain for a month. His notable medical history includes recurrent episodes of nosebleeds despite cauterization and anemia. He also has a family history of similar bleeding disorders, as his sister experienced heavy menstrual bleeding and a significant postpartum hemorrhage. Laboratory investigations show a low hemoglobin and erythrocyte count indicative of anemia, and an elevated leukocyte (white blood cell) count. However, his platelet count and coagulation measures appear normal. (2) PLANNING: Primarily, there's an assumption that the recurring nosebleeds, anemia, abdominal pain, and the sister's similar presentation indicate a familial bleeding disorder. This assumption is bolstered by the combination of a normal platelet count with bleeding symptoms, suggesting a qualitative platelet function disorder or a coagulation factor deficiency. It's crucial to question this assumption by considering other diseases that might cause these symptoms. (3) CLINICAL REASONING: Bernard-Soulier syndrome and Glanzmann thrombasthenia involve abnormalities in platelet function, but usually present with abnormal bleeding times and mucocutaneous bleeding, given the impaired platelet adhesion and aggregation, respectively. Hemophilia A, a deficiency in factor VIII, would normally prolong the activated partial thromboplastin time (aPTT) because factor VIII is involved in the intrinsic pathway. Von Willebrand disease, characterized by a deficiency or dysfunction of vWF which carries and protects factor VIII, can present with mucocutaneous bleeding, but generally causes a prolonged aPTT due to a secondary decrease in factor VIII. The symptoms of the individual in question along with his sibling's medical history suggests a pattern following an autosomal dominant inheritance. Based on the aforementioned data, we can hypothesize the diagnosis to be Hereditary Hemorrhagic Telangiectasia (HHT), otherwise known as Osler-Weber-Rendu syndrome. This is an autosomal dominant condition characterized by abnormal blood vessel formation or telangiectasias in the skin and mucous membranes. These telangiectasias are prone to bleeding, which could explain the recurrent nosebleeds. The sibling's history of menorrhagia and postpartum hemorrhage is also consistent with HHT. (4) CONCLUDING: Given the clinical features of recurrent nosebleeds, familial history, and a pattern suggesting autosomal dominance; the laboratory findings of anemia with normal platelet count and coagulation times, all signs point towards Hereditary Hemorrhagic Telangiectasia rather than the alternatives provided. (5) RESPONSE: Therefore, the most likely diagnosis for this patient is (D) Hereditary Hemorrhagic Telangiectasia.

**AI_A:** (1) RESTATING THE QUESTION: The patient, a 27-year-old man, is presenting with symptoms of fatigue, abdominal pain, and recurrent nosebleeds, which fail to improve even after undergoing cauterization of the nasal mucosa. Family history includes a sister with anemia, recurrent nosebleeds, heavy menstrual periods, and a history of postpartum hemorrhage. Pallor is evidenced on physical examination. Laboratory findings include anemia and elevated leukocyte count. His coagulation studies, specifically the prothrombin time and partial thromboplastin time, are essentially within normal limits. From the given options, I must discern the most likely diagnosis. (2) PLANNING: The key features in this case are the recurrent nosebleeds, which suggests a bleeding disorder, and the familial nature of these symptoms. However, the normal platelet count and coagulation studies argues against platelet disorders and coagulation factor deficiencies. This veers my assumption towards a genetic, bleeding disorder that does not necessarily affect these basic tests significantly. (3) CLINICAL REASONING: When considering bleeding disorders that are inherited and may not significantly alter basic coagulation studies, two options in the list stand out, Hereditary Hemorrhagic Telangiectasia and Von Willebrand Disease. However, the absence of a hallmark feature of Hereditary Hemorrhagic Telangiectasia, which is telangiectasias (small, dilated blood vessels near the surface of the skin or mucous membranes), along with the history of a sister with heavy menstrual periods and postpartum bleeding, directs me more towards a diagnosis of Von Willebrand Disease. Von Willebrand Disease is a bleeding disorder caused by deficient or dysfunctional von Willebrand factor, a protein that plays a crucial role in platelet adhesion to damaged endothelium. This condition frequently presents with mucocutaneous bleeding (such as nosebleeds), menorrhagia, and postpartum hemorrhage, and frequently, the basic coagulation studies including platelet count, Prothrombin time (PT), and Partial Thromboplastin Time (PTT) may be normal or slightly prolonged. (4) CONCLUDING: Given the patient's symptoms, the family history, and the laboratory findings, the most probable diagnosis is von Willebrand Disease. (5) RESPONSE: The most likely diagnosis for the patient described in this case is (E) von Willebrand disease.

**AI_D:** RESTATING THE QUESTION: In summary, a 27-year-old man who experiences fatigue, abdominal pain, frequent nosebleeds and has family history of bleeding disorders is seeking medical help for the diagnostic identification of his condition. He has anemia demonstrated by lab results of low hemoglobin and erythrocyte count. His prothrombin time is normal indicating normal extrinsic and common coagulation pathways. His partial thromboplastin time is prolonged suggesting potential issues with intrinsic and common coagulation pathway. The possible diagnoses are Bernard-Soulier syndrome, Glanzmann thrombasthenia, Hemophilia A, Hereditary hemorrhagic telangiectasia, von Willebrand disease. PLANNING: The assumptions here are that this man's symptoms are linked to a bleeding disorder. This would connect the symptoms like fatigue (from anemia due to blood loss), abdominal pain, and recurrent nosebleeds, with the laboratory findings and the family history (his sister's anemia, heavy periods, and postpartum hemorrhage). To differentiate among the conditions listed as possible diagnoses, we need to consider their key characteristics. We can question whether his prolonged PTT and normal PT align with the typical lab findings for each disorder; also, whether his overall presentation aligns with the common symptoms and clinical course of each condition. CLINICAL REASONING: Bernard-Soulier syndrome is a platelet adhesion disorder, we typically see a prolonged bleeding time and a reduced platelet count, neither of which applies to this patient. Glanzmann thrombasthenia also affects platelets, causing a defect in platelet aggregation, so would not necessarily result in prolonged PTT. Hemophilia A is characterized by prolonged PTT in the context of normal PT and platelet count, which does align with our patient's lab results. However, Hemophilia A is a X-linked disorder and typically doesn't manifest in women. The patient's sister showing similar symptoms challenges this option. Hereditary hemorrhagic telangiectasia (HHT), also known as Osler-Weber-Rendu syndrome, is characterized by mucocutaneous telangiectasia and arteriovenous malformations (AVMs) which could potentially cause recurrent nosebleeds. It's also an autosomal dominant disorder which can explain his sister's similar symptoms. Von Willebrand disease is the most common inherited bleeding disorder and it's characterized by a defect in platelet plug formation, prolonged PTT, and it could explain the similar symptoms in the patient's sister. However, the normal platelet count contradicts with typical von Willebrand disease findings. CONCLUDING: While each possible diagnosis has some characteristics that match this patient's presentation, upon synthesizing the data, Hereditary hemorrhagic telangiectasia (HHT) appears to align best with his prolonged PTT, family history, and recurrent nosebleeds even in the absence of other common symptoms like telangiectasias. RESPONSE: Therefore, the most likely diagnosis is Hereditary hemorrhagic telangiectasia (Option D).

**Facilitator** **[Round 2]:** Summary: AI_A believes the most likely diagnosis is von Willebrand Disease, based on the patient's symptoms, family history, and laboratory findings. AI_A argues that the recurrent nosebleeds and familial nature of the symptoms suggest a genetic bleeding disorder, and the normal platelet count and coagulation studies argue against platelet disorders and coagulation factor deficiencies. AI_B, AI_C, AI_D, and AI_E all believe the most likely diagnosis is Hereditary Hemorrhagic Telangiectasia. They all argue that the patient's symptoms, family history, and laboratory findings suggest a hereditary disorder. They also note that the normal platelet count and prothrombin time exclude platelet and extrinsic pathway disorders respectively. Key Differences: The key difference in the perspectives lies in the interpretation of the patient's symptoms and laboratory findings. AI_A believes that the absence of telangiectasias and the sister's history of heavy menstrual periods and postpartum bleeding point towards von Willebrand Disease. However, AI_B, AI_C, AI_D, and AI_E believe that the patient's symptoms, family history, and laboratory findings are more consistent with Hereditary Hemorrhagic Telangiectasia. Question for Council Members: Given the patient's symptoms, family history, and laboratory findings, what specific factors led you to your diagnosis, and how do you reconcile the absence of certain typical symptoms (such as telangiectasias in Hereditary Hemorrhagic Telangiectasia or abnormal platelet count in von Willebrand Disease)?. Based on your preceding response, how would you answer the original question: [A 27-year-old man comes to the physician because of a 1-month history of progressive fatigue and episodes of moderate abdominal pain. He has a 4-year history of recurrent nosebleeds that have increased in frequency despite undergoing cauterization of the nasal mucosa 6 months ago. He takes no medications. His sister has anemia associated with recurrent nosebleeds and heavy menstrual periods; she also had postpartum hemorrhage that required transfusion. The patient's vital signs are within normal limits. Examination shows pallor; no other abnormalities are noted. Laboratory studies show: Hemoglobin: 7.1 g/dL Erythrocyte count: 2.1 million/mm³ Leukocyte count: 13,500/mm³ Platelet count: 250,000/mm³ Prothrombin time: 12 sec (INR=1) Partial thromboplastin time: 45 sec Which of the following is the most likely diagnosis? (A) Bernard-Soulier syndrome (B) Glanzmann thrombasthenia (C) Hemophilia A (D) Hereditary hemorrhagic telangiectasia (E) von Willebrand disease]

**AI_E:** RESTATING THE QUESTION: The query enquires about the likely diagnosis for a 27-year-old male with a 1-month history of progressive fatigue, abdominal discomfort, and a 4-year history of nosebleeds. Despite cauterization, the nosebleeds persist. He presents with pallor but no other noticeable abnormalities. His blood tests reveal anemia but normal leukocyte and platelet counts. His sister also has a history of anemia, linked to frequent nosebleeds and severe menstrual bleeding. Time-periods for clotting processes appear within normal parameters. PLANNING: The pertinent information revolves around recurrent nosebleeding, anemia, and normal platelet count in a seemingly familial context. Given these, inherited disorders affecting blood clotting must be contemplated. Options could fall between disorders of clotting factors, like hemophilia, or a deficiency in von Willebrand factor, contributing to defective platelet adhesion, or hereditary hemorrhagic telangiectasia affecting blood vessels. CLINICAL REASONING: The lack of abnormality in prothrombin time eliminates disorders related to the extrinsic clotting pathway, like factor VII deficiency. Due to the normal prothrombin and partial thromboplastin time, conditions affecting the clotting cascade such as hemophilia A seem unlikely. Past cauterization did not have the expected effect in reducing nosebleeds, potentially pointing that the platelet adhesion, not platelet count, could be compromised. While the platelet adhesion issue might hint towards von Willebrand disease, this typically presents with easy bruising or skin bleeding which has not been reported. Also, the brother-sister similarity of the condition, could suggest an autosomal dominant inheritance, which fits better with hereditary hemorrhagic telangiectasia. CONCLUDING: Given the family history, persistent nosebleeds, normal platelet count, no reported skin bleeding, and the normal prothrombin and partial thromboplastin time, hereditary hemorrhagic telangiectasia appears as the most probable diagnosis, despite its typically more varied clinical presentation. RESPONSE: I believe the most likely diagnoses is Hereditary Hemorrhagic Telangiectasia (D).

**AI_B:** RESTATING THE QUESTION: The patient is a young adult who presents with progressive fatigue and recurrent nosebleeds, with the latter having a history of not responding adequately to nasal cauterization. Moreover, he has a family history of comparable bleeding disorders, including anemia. The laboratory results show low hemoglobin and erythrocyte count, pointing to anemia secondary to chronic blood loss. Platelet and coagulation profiles are within the normal range, which leads us away from any platelet or coagulation factor disorders. Given this phenotype, we are expected to define the most likely underlying genetic bleeding disorder. PLANNING: This case appears to involve a hereditary disorder with an autosomal dominant mode of inheritance due to the presence of similar symptoms in a sibling. We should take into account the most important clinical and laboratory findings which are recurrent nosebleeds, signs of anemia due to chronic blood loss, and absence of a platelet or coagulation factor problem indicated by normal platelet count and clotting times. Although von Willebrand disease is a common hereditary bleeding disorder, Hereditary Hemorrhagic Telangiectasia (HHT) must be given priority hypothesizing that not all clinical signs (like telangiectasias) have to emerge at this stage. CLINICAL REASONING: The patient's symptoms, in particular, recurrent nosebleeds non-responding to nasal cauterization, are a hallmark of HHT. Furthermore, in HHT the bleeding is due to defects in the blood vessels which is consistent with normal platelet count and clotting studies seen in this patient. It's also important to note that HHT is an age-dependent condition and certain symptoms like gastrointestinal bleeding and visible telangiectasias might not be evident at earlier ages. Moreover, the sister’s history of heavy menstrual periods and postpartum hemorrhage could be related due to an increased demand on the fragile blood vessels during these times. CONCLUDING: After incorporating the patient's clinical signs, family history, and laboratory data into our thought process, our clinical reasoning suggests that the patient most likely has Hereditary Hemorrhagic Telangiectasia. Although von Willebrand disease shares several clinical features with this patient's presentation, the associated symptoms in a sibling, presentation with anemia due to uncontrolled nosebleeds, and the normal platelet and clotting profile inclines us towards HHT. Furthermore, negative findings might not be conclusive since we are considering an age-dependent disorder. RESPONSE: Therefore, the most likely diagnosis in this patient's case is Hereditary Hemorrhagic Telangiectasia (Choice D).

**AI_C:** (1) RESTATING THE QUESTION: The question presents a clinical scenario of a young man who is suffering from persistent fatigue, episodes of moderate abdominal pain, and recurrent nosebleeds that are not responsive to cauterization. He also has a sister with similar problems of recurrent nosebleeds, heavy menstrual bleeding, and a history of postpartum hemorrhage. The laboratory findings reveal a low hemoglobin count, a low erythrocyte count but normal counts of leukocyte and platelets, and normal prothrombin time. The question requires us to identify the correct diagnosis from a list of potential disorders including several hereditary bleeding disorders and coagulation abnormalities. (2) PLANNING: The information provided suggests a familial predisposition to bleeding disorders. The most relevant aspects are the persistent fatigue and recurrent nosebleeds despite cauterization, which suggests a systemic disorder rather than a localized problem. As for the sister's condition, which includes heavy menstrual bleeding and postpartum hemorrhage, we should consider conditions causing coagulopathy. (3) CLINICAL REASONING: The pattern of symptoms seems to fulfill a genetic basis but the bleeding disorders of Bernard-Soulier syndrome, Glanzmann thrombasthenia, Hemophilia A, and von Willebrand disease would typically present with abnormalities in either platelet count or clotting time. However, in this case, both are in the normal range. This, in association with a positive family history and recurring bleeding episodes, mainly mucosal in nature (nosebleeds, heavy menstrual bleeding), could be indicative of Hereditary Hemorrhagic Telangiectasia (HHT) or Osler-Weber-Rendu syndrome. (4) CONCLUDING: HHT is an autosomal dominant disorder characterized by the presence of multiple arteriovenous malformations. Major clinical features include spontaneous recurrent epistaxis, mucocutaneous telangiectasias, and arteriovenous malformations in the lung, liver, and brain. Interestingly, the absence of distinctive telangiectasias might be explained by the fact that they usually appear after childhood, at 20 to 30 years of age, and their rate increases with age. (5) RESPONSE: So in conclusion, based on the familial pattern of symptoms, the persistent nosebleeds which were not responsive to cauterization, and the absence of abnormalities in platelet counts or coagulation studies, the patient's condition is most likely Hereditary Hemorrhagic Telangiectasia (D).

**AI_A:** RESTATING THE QUESTION: The question is asking about a 27-year-old male patient who has displayed a multitude of symptoms, such as recurring fatigue, moderate abdominal pain, and an increase in the frequency of nosebleeds, despite an attempt at cauterization. Notably, the patient's sister has similar symptoms and required a transfusion after experiencing postpartum hemorrhage. On examination, the patient appears pallid, indicating possible anemic symptoms, and laboratory studies exhibit decreased red blood cell count and increased white cell count. The question asks about the most probable diagnosis from the options provided, which are all related to inherited or acquired bleeding and clotting disorders. PLANNING: His symptoms, along with the family history, points toward a genetic blood disorder, possibly affecting the blood clotting mechanism. The normal platelet count combined with the patient's symptoms rules out conditions related to platelet deficiencies like Glanzmann Thrombasthenia and Bernard Soulier syndrome. The normal Prothrombin time (PT) rules out Hemophilia A, which is a deficiency of factor VIII, an element of the intrinsic pathway of coagulation, that can prolong PT. Hence, we are left with von Willebrand disease (vWD) and Hereditary hemorrhagic telangiectasia (HHT). von Willebrand disease is the most common inheritable blood-clotting disorder, affecting the von Willebrand factor (vWF) that helps platelets adhere to the site of injury and serves as a carrier molecule for factor VIII, another important clotting protein. Manifestations include mucous membrane bleeding from the nose and gums, excessive menstrual and postpartum bleeding, and excessive bleeding during or after surgery. Hereditary hemorrhagic telangiectasia, also known as Osler-Weber-Rendu disease, is an autosomal dominant disorder characterized by multiple arteriovenous malformations leading to recurrent nosebleeds, skin and mucosal telangiectasias, and arteriovenous malformations in various organs that may result in internal bleeding. CLINICAL REASONING: Given the clinical presentation and laboratory findings, von Willebrand disease appears less likely. A typical finding in vWD is an increase in bleeding time due to a dysfunction in platelet adhesion, which is not present here. On the other hand, the symptoms of Hereditary hemorrhagic telangiectasia fit better with the presented case. Even though no telangiectasia is reported, HHT can manifest with varying severity and symptoms, with nosebleeds being the most common. Despite this, it does not fully explain the sister's heavy menstrual bleeding and postpartum hemorrhage. However, it's also worth noting that she may have a separate, concomitant condition causing those symptoms, such as Menorrhagia. CONCLUDING: Therefore, taking all factors into account, while both are possible, Hereditary hemorrhagic telangiectasia appears to be the most likely diagnosis based on the information provided. RESPONSE: (D) Hereditary Hemorrhagic Telangiectasia.

**AI_D:** RESTATING THE QUESTION: A 27-year-old man is experiencing fatigue, abdominal pain, and recurrent nosebleeds which have been increasing despite previous medical intervention. He is not on any medication. Notably, his sister has a history of anemia, recurrent nosebleeds, heavy menstruation, and postpartum hemorrhage. His lab results show anemia and a slight elevation in leukocyte count but a normal platelet count and coagulation times. The question is asking for the most probable diagnosis with options that include various hereditary bleeding disorders: Bernard-Soulier syndrome, Glanzmann thrombasthenia, Hemophilia A, Hereditary Hemorrhagic Telangiectasia (HHT), and von Willebrand disease. PLANNING: Initially, the symptoms and familial history suggest that this could be a hereditary bleeding disorder. The clinical presentation reveals anemia, leukocytosis, normal platelet count, and normal coagulation times. The presence of anemia and absence of telangiectasias may disagree with HHT. The normal platelet count and coagulation times disagree with Bernard-Soulier syndrome, Glanzmann thrombasthenia, hemophilia A, and Von Willebrand's disease. However, it is crucial to remember that the absence of symptoms does not always rule out a disease. Given the family history of postpartum hemorrhage and heavy menstruations in the patient's sister, it may be likely to prioritize the analysis of HHT. CLINICAL REASONING: HHT, also known as Osler-Weber-Rendu syndrome, is characterized by abnormal blood vessel formation in the skin, mucous membranes, and often in organs such as the lungs, liver, and brain. It's an autosomal dominant condition, explaining the familial nature of the disease. Common symptoms include recurrent nosebleeds (epistaxis), gastrointestinal bleeding, iron deficiency anemia, and arteriovenous malformations (AVMs), which may be yet undetected in the individual, causing the recurrent abdominal pain. Additionally, although telangiectasias are a common symptom, they may not always be present. Moreover, HHT can often remain underdiagnosed because of the variability in clinical presentation; thus, it would be medically prioritized. CONCLUDING: Based on the available information, this man's symptoms, familial history, and laboratory findings are most congruent with the autosomal dominant HHT. Although he doesn't present with typical telangiectasias, the absence of these does not rule out HHT diagnosis. The increased frequency of nose bleeds, the presence of anemia, moderate abdominal pain potentially from AVMs, and normal coagulation and platelet counts lend support to this hypothesis. RESPONSE: So, the most likely diagnosis for the condition presented in the case of the 27-year-old man is likely Hereditary Hemorrhagic Telangiectasia (D). Diagnosis often relies on clinical history, family history, and investigations to determine the presence of AVMs. This would need to be confirmed with further diagnostic investigations such as imaging and genetic testing.

**Facilitator** The question presents a 27-year-old male patient with a history of progressive fatigue, recurrent nosebleeds, and moderate abdominal pain. His sister also has a history of anemia, recurrent nosebleeds, heavy menstrual periods, and postpartum hemorrhage. Laboratory studies show anemia, a slight elevation in leukocyte count, but normal platelet count and coagulation times. The question asks for the most probable diagnosis from a list of potential disorders including several hereditary bleeding disorders and coagulation abnormalities. The patient's symptoms, along with the family history, suggest a genetic blood disorder, possibly affecting the blood clotting mechanism. The normal platelet count combined with the patient's symptoms rules out conditions related to platelet deficiencies like Glanzmann Thrombasthenia and Bernard Soulier syndrome. The normal Prothrombin time (PT) rules out Hemophilia A, which is a deficiency of factor VIII, an element of the intrinsic pathway of coagulation, that can prolong PT. Hence, we are left with von Willebrand disease (vWD) and Hereditary hemorrhagic telangiectasia (HHT). von Willebrand disease is the most common inheritable blood-clotting disorder, affecting the von Willebrand factor (vWF) that helps platelets adhere to the site of injury and serves as a carrier molecule for factor VIII, another important clotting protein. However, a typical finding in vWD is an increase in bleeding time due to a dysfunction in platelet adhesion, which is not present here. Hereditary hemorrhagic telangiectasia, also known as Osler-Weber-Rendu disease, is an autosomal dominant disorder characterized by multiple arteriovenous malformations leading to recurrent nosebleeds, skin and mucosal telangiectasias, and arteriovenous malformations in various organs that may result in internal bleeding. The patient's symptoms, in particular, recurrent nosebleeds non-responding to nasal cauterization, are a hallmark of HHT. Furthermore, in HHT the bleeding is due to defects in the blood vessels which is consistent with normal platelet count and clotting studies seen in this patient. It's also important to note that HHT is an age-dependent condition and certain symptoms like gastrointestinal bleeding and visible telangiectasias might not be evident at earlier ages. Given the clinical presentation and laboratory findings, Hereditary hemorrhagic telangiectasia appears to be the most likely diagnosis based on the information provided. Although von Willebrand disease shares several clinical features with this patient's presentation, the associated symptoms in a sibling, presentation with anemia due to uncontrolled nosebleeds, and the normal platelet and clotting profile inclines us towards HHT. Furthermore, negative findings might not be conclusive since we are considering an age-dependent disorder. Therefore, the most likely diagnosis in this patient's case is Hereditary Hemorrhagic Telangiectasia (Choice D). This would need to be confirmed with further diagnostic investigations such as imaging and genetic testing.
