# Supplementary material for: Collaborative intelligence in AI: Evaluating the performance of a council of AIs on the USMLE
Source: PLOS Digit Health. 2025 Oct 9;4(10):e0000787. doi: 10.1371/journal.pdig.0000787 (PMC12510544; doi:10.1371/journal.pdig.0000787)
Supplement: S2 File — (ZIP) [file pdig.0000787.s002.zip › S2 Data Raw data files containing unprocessed question inputs and LLM outputs including transcripts of deliberations/STEP2_Question-108-1_Type-MC-NJ.docx]

**Facilitator** **[Round 1]:** A 37-year-old woman comes to the office for a follow-up examination 3 weeks after testing confirmed a mass in her left breast to be benign. She says that despite the test results, she has had anxiety, insomnia, and a preoccupation with cancer since noticing the lump. Two weeks ago, she had a 20-minute episode of markedly intense anxiety characterized by heart palpitations and the fear that she was dying. She says she has always been a tense, nervous person and is uncomfortable in social situations. She says she frequently worries and is fearful of the future and apprehensive of dying. She has no other history of panic episodes, traumatic events, or serious illness and takes no medications. She does not drink alcohol or use illicit drugs. Physical examination shows a 2-cm mass in the left breast. On mental status examination, she is cooperative but worried. Her thought processes are linear, and content focuses on possible negative eventualities. Which of the following is the most likely diagnosis? (A) Avoidant personality disorder (B) Generalized anxiety disorder (C) Illness anxiety disorder (hypochondriasis) (D) Persistent depressive disorder (dysthymia) (E) Social anxiety disorder (social phobia)

**AI_D:** RESTATING THE QUESTION: The patient is a 37-year-old female who has been experiencing anxiety, insomnia, and fixation on the possibility of having cancer, despite being cleared of any malignant mass in her breast. Her worries seem incessant and focus on disastrous outcomes, even though she has no history of severe illness, trauma, or panic attacks. She reveals being consistently anxious and uncomfortable in social situations and has not been using any medication, alcohol, or drugs illicitly. Clinicians must identify the best-fitting diagnosis given her symptoms and history. PLANNING: It's necessary to define the potential diagnoses listed. 'Avoidant personality disorder' typically involves social inhibition, feelings of inadequacy, and hypersensitivity to criticism. 'Generalized anxiety disorder' refers to excessive anxiety or worrying about numerous things most days for at least six months. 'Illness anxiety disorder' (previously called hypochondriasis) features excessive worry about having a serious illness despite having no or only mild symptoms. 'Persistent depressive disorder' represents a chronic form of depression, also known as dysthymia, where the person's mood remains consistently low for at least two years. 'Social anxiety disorder' is characterized by intense fear and avoidance of certain social situations that might involve scrutiny or judgement by others. CLINICAL REASONING: The patient's chronic and generalized worry, frequent tension, and fearfulness about her future and death points towards a generalized anxiety disorder (GAD). Although she did show distress about her health, it does not have the specific focus on personal health that characterizes illness anxiety disorder. Her worry is pervasive and not confined solely to social scenarios, making social anxiety disorder less suitable. The symptoms do not align with persistent depressive disorder or avoidant personality disorder either, as they lack their defining traits of long-term low mood and avoidance due to feelings of inadequacy and fear of criticism. CONCLUDING: Following a sequence of clinical reasoning processes, considering her reported excessive and wide-ranging worries which are longstanding and not centered on a single theme such as health or social interactions, a diagnosis of generalized anxiety disorder is most appropriate. It suits the description of excessive, prolonged worry about numerous issues, coupled with physical anxiety symptoms like insomnia and palpitations. RESPONSE: Therefore, the most suitable answer would be (B) Generalized Anxiety Disorder.

**AI_E:** 1) RESTATING THE QUESTION: This 37-year-old woman has a benign mass in her left breast, but despite this benign result, she has had considerable anxiety, insomnia, and a preoccupation with cancer. Recently, she had an intense episode of anxiety, and she consistently describes being particularly nervous in nature. Physical and mental examinations demonstrate a tense demeanor and a predisposition towards the consideration of negative outcomes. Her history otherwise lacks any other panic attacks or severe illnesses and she isn't on any medications nor has she been involved in drug abuse. The inquiry lies in determining the most probable psychiatric diagnosis for her. The options are: (A) Avoidant personality disorder - a long-term pattern of extreme shyness, feeling inadequate, and hyper-sensitivity to rejection. (B) Generalized anxiety disorder (GAD) - a common disorder characterized by long-lasting anxiety that is not focused on any one object or situation. (C) Illness anxiety disorder (hypochondriasis) - a chronic condition where a person worries excessively that they are or may become seriously ill. (D) Persistent depressive disorder (earlier called dysthymia) - a low mood occurring for at least two years, along with at least two other symptoms of depression. (E) Social anxiety disorder (social phobia) - an intense fear of being humiliated or embarrassed in social situations. 2) PLANNING: The woman here has had a persistent history of excessive worry, anxiety, and fearfulness for at least the last three weeks. This could be potentially due to the uncertainty over her physical health, indicative of hypochondriasis. Simultaneously, her chronic nature of being a tense, nervous person could be a sign of generalized anxiety disorder. The proposition here would then be to determine between the two aforementioned potential disorders. 3) CLINICAL REASONING: Hypochondriasis, now known as illness anxiety disorder, is characterized by an exaggerated worry about having a serious illness. This diagnosis might then be relevant, given the woman's excessive worry about her benign lump and its potential to be cancerous. Her specific fear of dying also supports this diagnosis. However, illness anxiety disorder doesn't usually include symptoms of general apprehensiveness and anxiety about the future. On the other hand, generalized anxiety disorder (GAD) is characterized by chronic, excessive worry and fearfulness about numerous things, even when there is little or nothing to provoke it. The symptomatology of the woman reflects this definition; although her fear of having cancer may have catalyzed her anxiety, her 'always been a tense person,' 'uncomfortable in social situations,' and 'worrying about possible negative eventualities' tend to lean towards GAD. 4) CONCLUDING: Though her concern for her benign lump could point towards illness anxiety disorder, her broader and consistent pattern of worrying and anticipated fear points more towards a diagnosis of generalized anxiety disorder. 5) RESPONSE: The most likely diagnosis for the symptoms exhibited by this woman is Generalized Anxiety Disorder (B).

**AI_B:** (1) RESTATING THE QUESTION: This query pertains to a 37-year-old woman who has been experiencing persistent anxiety, sleep disruption, and a preoccupation with her health, particularly cancer, despite being assured that her left breast mass was not cancerous. In addition to an episode of acute anxiety with palpitations and fear of dying, the patient reveals a tendency towards chronic nervousness, discomfort in social situations, worry, and fear about the future. Moreover, the woman exhibits no history of panic outbreaks, traumatic events or grave illnesses. The query seeks to determine the most suitable diagnosis for her condition from a selection of prevalent mental health disorders, including Avoidant Personality Disorder, Generalized Anxiety Disorder, Illness Anxiety Disorder (formerly known as hypochondriasis), Persistent Depressive Disorder (dysthymia), and Social Anxiety Disorder. (2) PLANNING: In this stage, I will assess each possible diagnosis for consistency in the patient's history and behavior. Assuming the symptoms accurately describe her condition, my thought process will prioritize clinical markers that fit the disease definition. (3) CLINICAL REASONING: Let's analyze each assumption: A) Avoidant Personality Disorder is typified by long-standing feelings of inadequacy and hypersensitivity to criticism, which anticipates rejection and leads to social isolation. Our patient is anxious and uncomfortable in social situations but doesn't provide any evidence of fearing criticism or suffering from feelings of inadequacy. B) Generalized Anxiety Disorder (GAD) can be considered as it generally includes constant, excessive worry about multiple aspects of life and persistent feelings of tension. Indeed, the patient shows prolonged periods of severe anxiety, extreme apprehension about the future, frequent worrying and insomnia. C) Illness Anxiety Disorder (IAD), previously called hypochondriasis, may be considered, as the patient exhibits preoccupation with having a serious illness despite having negative findings. In this case, her fear seems focused on her benign breast mass. D) Persistent Depressive Disorder (Dysthymia) typically involves a chronically low mood for at least two years; our patient doesn't mention such a continuous mood disorder. E) Social Anxiety Disorder typically involves extreme fear in social settings that might lead to negative judgments. The information provided does not suggest the patient's discomfort in social situations leads to such severe fear. (4) CONCLUDING: Following the evaluation of all assumptions, Generalized Anxiety Disorder (GAD) remains the most compatible diagnosis, even though elements from other potential diagnoses like social discomfort and fear of illness are present. (5) RESPONSE: Based on the persistent tension, worrying, fear about future, and recurrent bouts of severe anxiety such as the one accompanied by heart palpitations, the clinical phenomena best correspond with Generalized Anxiety Disorder. A significant clue that supports GAD is the comprehensive array of patient's fears and anxieties, rather than a singular focus on health (Illness Anxiety Disorder) or social situations (Social Anxiety Disorder).

**AI_A:** RESTATING THE QUESTION: The case is centered around a 37-year-old woman who has presented with persistent anxiety, insomnia, and a preoccupation with developing cancer - despite tests confirming a benign mass. The patient experienced a severe episode of anxiety recently, characterized by heart palpitations and thoughts of impending death. Though she has no history of panic episodes or serious illness, she admitted to having a personality marked by consistent tension, discomfort in social situations, and constant worry about the future. The question requires me to determine the most fitting diagnosis for this patient, from the listed options: Avoidant personality disorder, Generalized anxiety disorder, Illness anxiety disorder (previously known as hypochondriasis), Persistent depressive disorder, or Social Anxiety disorder. PLANNING: Diagnostically, the woman's symptoms would need to match with criteria from the DSM-5 (Diagnostic and Statistical Manual of Mental Disorders, 5th Edition) for a specific disorder to be diagnosed. The primary assumptions are that the patient's long-standing and varied anxiety symptoms indicate a generalized anxiety-based disorder. However, attention should be given to the alternate assumption: Could this be a somatoform disorder, particularly illness anxiety disorder? This is primarily due to both her preoccupation with having cancer and physical symptoms (a benign mass) with no current confirmed physical illness. I would clinically prioritize evaluating these assumptions based on her symptoms' correspondence with the DSM-5 criteria for these disorders. CLINICAL REASONING: Let us begin with the assumption that the woman has a generalized anxiety disorder. For diagnosing someone with GAD, they should exhibit excessive worry about multiple events or activities occurring more days than not for at least six months. Additionally, the patient should find it hard to control the worry and have at least three associated symptoms: restlessness or feeling on edge, being easily fatigued, irritability, muscle tension, or sleep disturbance. In this patient, the symptoms of restlessness, worry about future events, and sleep disturbances (insomnia) are present. However, the six-month criterion is not explicitly mentioned. Additionally, the level of fear or concern about a single issue (cancer) appears to surpass the generalized nature of the disorder. This isn’t characteristic of GAD, which is typically more unfocused and pervasive. Now let's examine the alternate hypothesis - illness anxiety disorder (previously known as hypochondriasis). The woman worries excessively about having a serious illness despite medical testing and reassurances, which aligns with this disorder's primary diagnostic criterion. She also seems to excessively research her symptoms and the diseases she fears she might have (excessive reassurance-seeking or health-related behaviors). It's worth noting that her 20-minute intense anxiety episode could be a panic attack, but since it was an isolated event without history, it cannot confirm a panic disorder. Based on the evidence, illness anxiety disorder appears to be a more fitting diagnosis. CONCLUDING: The patient's persistent preoccupation with having a serious illness (cancer), despite medical reassurances and benign test results, aligns with the primary diagnostic criterion for illness anxiety disorder. The presence of her physical symptoms (the benign mass) without any confirmed physical disease also aligns with this disorder's characteristic feature. Her anxious behaviors are compelling and excessive, focused on one specific fear (cancer), which differentiates it from the more generalized nature of GAD. RESPONSE: Based on the analysis, the most likely diagnosis for this 37-year-old woman would be (C) Illness anxiety disorder (hypochondriasis).

**AI_C:** RESTATING THE QUESTION: As I understand it, you're seeking a diagnosis for a 37-year-old woman who was shown to have a benign mass in her left breast. Despite the benign nature of the mass, she's been experiencing enduring anxiety, insomnia, and fears related to cancer. She had a particularly intense episode of anxiety a fortnight ago, characterized by racing heart and an intense fear of death. She states that she has always been anxious, appears nervous, and is uncomfortable in social situations. She portrays constant worrying and fear about her future and the prospect of death. She has no prior medical history suggesting panic episodes, traumas, or other severe illnesses. She does not use any medications or substances like alcohol and illicit drugs. During the physical examination, a 2 cm mass was discovered in her left breast. Her mental state examination indicated that although she is cooperative, she is constantly worried and her thoughts mainly revolve around potential negative outcomes. We are tasked to identify her condition out of the provided options - Avoidant personality disorder, Generalized anxiety disorder, Illness anxiety disorder (formerly hypochondriasis), Persistent depressive disorder (or dysthymia), and Social anxiety disorder (also known as social phobia). PLANNING: Differentiating the options provided to us requires making key assumptions, which can be contrasted and evaluated through the woman's clinical presentation. Dysthymia, for instance, largely concerns a chronically low mood spanning at least two years, which doesn't seem to mirror the patient's anxiety-focused symptoms. Social phobia, while consistent with the patient's social unease, typically leads to avoidance of social situations rather than overwhelming worry about health or future. Avoidant personality disorder also centres around social situations, wherein individuals avoid them due to fears of criticism or rejection, which also doesn't align closely with the lady's presentation. Illness anxiety disorder, earlier referred to as hypochondriasis, is characterized by a preoccupation with having a serious illness despite appropriate medical evaluation and reassurance, which very much could apply to the patient's focus on her benign lump and fears associated with cancer. Lastly, generalized anxiety disorder is marked by excessive anxiety and worry about a number of events or activities for a majority of days over a span of at least six months, which seems applicable given the patient's general nervous disposition, excessive worry, and fear. CLINICAL REASONING: In step with the lady's signs and symptoms, generalized anxiety disorder seems to be the foremost fit. Though she has a certain hypervigilance regarding her health, particularly her benign lump and fears of cancer, which somewhat aligns with illness anxiety disorder, such a disorder would require that her somatic symptoms or fears can't be better explained by another disorder - which isn't accurate in this case as generalized anxiety disorder fits her symptomatology even more aptly. Furthermore, and importantly, her generalized excessive worry isn't just confined to her health, but also includes her future and dying. CONCLUDING: Considering all this evidence together, the diagnosis of a generalized anxiety disorder appears to be the most probable. This condition is characterized by excessive anxiety about several different aspects of one's life, like work, health, family troubles, and financial matters - which she has. It manifests with restlessness, fatigue, concentration problems, irritability, muscle tension, and sleep disturbances, which again is largely in tune with her presentation. Though severe anxiety may be occasioned by triggers, people with GAD may be anxious nearly all the time, which seems to fit the persistent worry and feared future the patient has reported. RESPONSE: Thus, given her excessive worry about not just her health but also other aspects of life, along with her general anxious disposition, the most likely diagnosis for this 37-year-old woman would be generalized anxiety disorder (choice B).

**Facilitator** **[Round 2]:** Summary: AI_A believes the woman's symptoms align most closely with Illness Anxiety Disorder (previously known as hypochondriasis). This is due to her persistent preoccupation with having a serious illness (cancer), despite medical reassurances and benign test results. AI_A also notes that her anxious behaviors are compelling and excessive, focused on one specific fear (cancer), which differentiates it from the more generalized nature of GAD. AI_B, AI_C, AI_D, and AI_E all diagnose the woman with Generalized Anxiety Disorder (GAD). They argue that while the woman does exhibit some symptoms of Illness Anxiety Disorder, her anxiety is not solely focused on her health but extends to other aspects of her life as well. They also note that her symptoms of restlessness, worry about future events, and sleep disturbances are characteristic of GAD. Question for Council Members: The main disagreement between council members seems to be whether the woman's anxiety is primarily focused on her health (Illness Anxiety Disorder) or more generalized (Generalized Anxiety Disorder). Could you please clarify how you differentiate between these two disorders in this case, and why you believe one diagnosis is more fitting than the other?. Based on your preceding response, how would you answer the original question: [A 37-year-old woman comes to the office for a follow-up examination 3 weeks after testing confirmed a mass in her left breast to be benign. She says that despite the test results, she has had anxiety, insomnia, and a preoccupation with cancer since noticing the lump. Two weeks ago, she had a 20-minute episode of markedly intense anxiety characterized by heart palpitations and the fear that she was dying. She says she has always been a tense, nervous person and is uncomfortable in social situations. She says she frequently worries and is fearful of the future and apprehensive of dying. She has no other history of panic episodes, traumatic events, or serious illness and takes no medications. She does not drink alcohol or use illicit drugs. Physical examination shows a 2-cm mass in the left breast. On mental status examination, she is cooperative but worried. Her thought processes are linear, and content focuses on possible negative eventualities. Which of the following is the most likely diagnosis? (A) Avoidant personality disorder (B) Generalized anxiety disorder (C) Illness anxiety disorder (hypochondriasis) (D) Persistent depressive disorder (dysthymia) (E) Social anxiety disorder (social phobia) ]

**AI_E:** RESTATING THE QUESTION: A 37-years-old woman is experiencing chronic anxiety and fears regarding her health, specifically relating to cancer, despite being medically reassured that the lump in her breast is benign. In addition to this, she displays noticeable anxiety regarding the future and death, and this has extended to her experiencing insomnia. The task at hand is to diagnose her condition from the following options: Avoidant Personality Disorder, Generalized Anxiety Disorder, Illness Anxiety Disorder (previously known as hypochondriasis), Persistent Depressive Disorder (dysthymia), and Social Anxiety Disorder (social phobia). PLANNING: Primary concerns include addressing the woman's specific fixation on her benign lump and fear of cancer, the generalized expressions of anxiety about future events and death, and also considering her nervousness in social settings. Because these symptoms appear to be long-term, we need to evaluate whether they meet the criteria for the chronic conditions listed in the choices, including Generalized Anxiety Disorder and Persistent Depressive Disorder. CLINICAL REASONING: Although she is focused on a possible health problem (the benign breast lump), the woman's fears extend to general worries about the future and death, and she mentions being typically anxious and nervous, all of which are not solely fixated on her health. These symptoms meet the criteria for Generalized Anxiety Disorder (GAD), a disorder characterized by chronic and excessive worry and tension, often without a clear cause. This is differentiated from Illness Anxiety Disorder (hypochondriasis), which would primarily focus on concerns about having a serious illness despite medical reassurances. CONCLUDING: Therefore, given the chronic symptoms of excessive and generalized worrying, restlessness, and insomnia met within the DSM-5 criteria for GAD, and considering the specific fixation on her health does not overly dominate her presented anxieties, the diagnosis of Generalized Anxiety Disorder (GAD) would be more fitting than Illness Anxiety Disorder. RESPONSE: Amongst the choices given, the most likely diagnosis for this woman is Generalized Anxiety Disorder.

**AI_B:** (1) RESTATING THE QUESTION: The question presents a 37-year-old woman who is struggling with anxiety, insomnia, and an intense fear of cancer, even though the mass in her breast has been confirmed as benign. Her anxiety is not limited to her health but extends to her general life and future. She has experienced one bout of intense anxiety, and while she has always been a 'tense, nervous person', this appears to be a new extreme. Our task is to identify the most likely diagnosis from Avoidant Personality Disorder, Generalized Anxiety Disorder, Illness Anxiety Disorder (earlier known as hypochondriasis), Persistent Depressive Disorder (dysthymia), and Social Anxiety Disorder (also known as social phobia). (2) PLANNING: To determine the diagnosis, we need to identify the primary symptoms and their severity, duration, and impact on her life. The main assumption is that her symptoms stem from a psychological disorder. However, it's equally important to rule out physiological causes such as an underlying endocrine issue, for example, hyperthyroidism, which can mimic certain mental disorders. For the sake of this scenario, it's considered that physiological reasons have been ruled out. (3) CLINICAL REASONING: The endocrine system, which plays a role in physiological stress responses, does not seem to be at the root of her symptoms here, given the information provided. Somatic symptoms, such as the benign breast lump, can trigger and exacerbate mental conditions. Given her history of social discomfort and her general worries about the future and fear of dying, we can infer that her anxiety goes beyond just a focus on her physical health which leans more towards a generalized anxiety disorder. (4) CONCLUDING: From the available details, the woman's worry about her health is only one aspect of her broader, persistent anxiety. Her case is characterized by excessive worry about multiple events or activities and few physiological symptoms, taking into account that she always has been a tense, nervous person, these have been exacerbated by her benign breast lump discovery. (5) RESPONSE: The woman's symptoms suggest she's experiencing Generalized Anxiety Disorder (Option B). This includes her persistent anxiety, feelings of unrest, preoccupations about the future and potential death. Her anxiety seems to extend beyond just the concern for her health, making the diagnosis of Illness Anxiety Disorder less likely.

**AI_A:** RESTATING THE QUESTION: This question involves a 37-year-old woman who has displayed continuous anxiety related to a benign lump in her breast and a general fear of cancer. Other symptoms include a single panic attack, general nervousness, discomfort in social situations, excessive worry about the future, and the fear of death. She has no history of other panic attacks or major illness. Her physical and mental examinations are consistent with her stated symptoms. We are asked to identify the most likely diagnosis out of the following options: Avoidant Personality Disorder, Generalized Anxiety Disorder, Illness Anxiety Disorder (formerly Hypochondriasis), Persistent Depressive Disorder (Dysthymia), Social Anxiety Disorder (Social Phobia). 2) PLANNING: Considering these symptoms, most likely diagnoses include Illness Anxiety Disorder and Generalized Anxiety Disorder. Avoidant Personality Disorder and Social Anxiety Disorder may be suggested by social discomfort, however, her symptoms seem to pertain more to a fear of illness than social situations. Persistent Depressive Disorder doesn't align with her described symptoms. Alternate assumptions include: the symptoms are due to the woman's natural concern about her health – particularly following the discovery of a lump, even if it's benign; also, her social discomfort and worries might be usual traits rather than signs of a disorder. 3) CLINICAL REASONING: Distinguishing between Illness Anxiety Disorder and Generalized Anxiety Disorder in this case is key. In IAD, the anxiety and worry primarily concern having a serious disease, despite medical reassurance. For this woman, her apprehension appears to concentrate intensely on her health status and fear of cancer. In GAD, the worry is excessive and concerns a number of events or activities, not just health. Despite the patient saying that she has always been tense and nervous, her focal point seems to be the fear of illness. 4) CONCLUDING: Based on the stated symptoms, it seems more fitting to diagnose the woman with Illness Anxiety Disorder. Despite the lack of other symptoms often associated with this disorder, such as frequent doctor visits or unnecessary medical procedures, her intense preoccupation with cancer and general fear of serious illness outweigh her other mentioned worries. 5) RESPONSE: The most likely diagnosis for this woman is Illness Anxiety Disorder (C), given her continued anxiety about having a serious illness, despite the absence of severe physical symptoms and the evidence to the contrary. This is what sets Illness Anxiety Disorder apart from Generalized Anxiety Disorder, in which the fear and worry is not specifically centered on health but a variety of different factors. Even though she admits to being a naturally tense person, her focus on the fear of cancer and dying indicate a manifest health concern overriding other anxieties.

**AI_C:** What we are being asked here is, essentially, to determine which type of anxiety disorder most accurately explains the 37-year-old woman's symptoms. She has a benign breast mass, but despite reassurance from the test results, she continues to worry about potential health issues. She reports past incidents of intense anxiety and recurring worries about future events and dying. It is also essential to understand these medical terms and what differentiates each disorder: 1. Avoidant Personality Disorder: characterized by feelings of extreme social inhibition, inadequacy, and sensitivity to negative criticism and rejection. This doesn't seem to fit as she has had no rejections or traumatic events. 2. Generalized Anxiety Disorder (GAD): characterized by excessive, long-lasting anxiety and worries about nonspecific life events, objects, and situations. It seems to fit the woman's condition considering her excessive worry about future events and dying without a specific reason like a traumatic experience. 3. Illness Anxiety Disorder (IAD): it involves excessive worry about having a serious illness, despite having no (or only mild) symptoms, and negative test results. IAD could fit considering the woman's excessive worry about having cancer. 4. Persistent Depressive Disorder: characterized by longstanding depression, usually two years or longer, but the woman does not show depressive symptoms. 5. Social Anxiety Disorder: characterized by fear, anxiety, and avoidance that interfere with routine life, occupational, academic, or social functioning. But the woman seems to function well in her daily life. Observing these, the most fitting assumption would be that the woman could either be suffering from Generalized Anxiety Disorder or Illness Anxiety Disorder. This is due to her excessive worry that doesn't seem coincided with any social or depressive disorder. Because her worries extend to future events and dying, not just illness, GAD should be prioritized for evaluation over IAD. With this in mind, if we rule out any related past traumatic events or medical history, and also consider her continuous worry of getting a serious illness despite having negative results and no severe symptoms, the diagnostic indicator tips more towards GAD. The symptoms such as restlessness, sleep disturbances, and worry about future events are more characteristic of Generalized Anxiety Disorder. Therefore, given all the possible diagnostic options, her reported symptoms of persistent and pervasive restlessness, anxious behaviors, coupled with her excessive worry about future eventualities, the woman's condition best fits the diagnostic criteria for Generalized Anxiety Disorder (B). The conclusion we can derive here is that she may benefit from therapies targeting GAD, such as Cognitive Behavioural Therapy and if needed, pharmacotherapies like SSRIs for its management. This diagnosis should help her get the target based therapeutic assistance she needs. Thus, Concluding, based on the clinical reasoning and evaluated assumptions, Generalized Anxiety Disorder seems to be the most likely diagnosis for the woman based on her reported symptoms of pervasive anxiety, worries, and restless behavior. She should seek the necessary treatment for managing GAD to improve her quality of life.

**AI_D:** To start with, the patient is a 37-year-old woman who has been experiencing consistent anxiety, insomnia, and preoccupation with having cancer, despite testing confirming the benign nature of her breast mass. Besides the health concerns, her anxiety appears to permeate various other aspects of life. She describes herself as a naturally tense and nervous person, uncomfortable in social situations. Her worries circulate around future potentialities and the prospect of dying. Importantly, she has a single episode of intense anxiety with heart palpitations, further intensifying her fears. We need to select the most fitting diagnosis from a list that includes avoidant personality disorder, generalized anxiety disorder, illness anxiety disorder (previously hypochondriasis), persistent depressive disorder (dysthymia), and social anxiety disorder (social phobia). A closer look at each assumption should aid us: Avoidant personality disorder is typically characterized by a pattern of social inhibition, feelings of inadequacy, and hypersensitivity to negative evaluation, all of which are not primarily reported in the patient's history. Though social discomfort is mentioned, it does not present as the main issue. Generalized Anxiety Disorder (GAD) could indeed be a possibility. It is characterized by excessive worry across multiple domains of life, which the patient appears to exhibit – indicating more than just health-related anxieties. Additionally, the symptoms of restlessness, insomnia, and tension present in this patient are commonly found in GAD. Illness Anxiety Disorder (IAD), previously known as hypochondriasis, focuses on the fear of having a serious disease despite medical reassurances. The patient's preoccupation with cancer could point towards IAD, but her anxiety goes beyond her health concerns. Persistent depressive disorder (or dysthymia) is a chronic form of depression, characterized by a depressed mood for at least two years. Although the patient has anxiety and worry, she does not appear to show signs of a persistently depressed mood. Social anxiety disorder, also known as social phobia, manifests as a fear of social or performance situations. While the patient does mention discomfort in social settings, it does not seem to be a significant or primary problem for her. Given this information, GAD seems to be the most fitting diagnosis. It accounts for both her health anxieties and the other areas of concern she mentions – fitting better than Illness Anxiety Disorder which would mainly focus on health-related fears. Thus, in answering the question - the 37-year-old woman who has had pervasive anxiety, insomnia, and worry about potential future risks, despite medical reassurances about her health, is most likely suffering from Generalized Anxiety Disorder (B).

**Facilitator** **[Round 3]:** Summary: AI_A believes the woman's symptoms align most closely with Illness Anxiety Disorder (IAD), given her intense preoccupation with cancer and general fear of serious illness, despite the absence of severe physical symptoms and evidence to the contrary. AI_B, AI_C, AI_D, and AI_E all diagnose the woman with Generalized Anxiety Disorder (GAD). They argue that her anxiety extends beyond just health concerns to include worries about the future and death. They believe her symptoms of restlessness, insomnia, and excessive worry about multiple events or activities are more characteristic of GAD than IAD. Key Differences: The key difference in the council members' perspectives lies in their interpretation of the woman's primary source of anxiety. AI_A believes her intense fear of cancer and serious illness, despite medical reassurances, is indicative of IAD. In contrast, AI_B, AI_C, AI_D, and AI_E argue that her anxiety is more generalized, extending beyond health concerns to worries about the future and death, which is characteristic of GAD. Question to Clarify Disagreements: To what extent does the woman's intense fear of cancer and serious illness, despite medical reassurances, outweigh her generalized worries about the future and death in determining a diagnosis of Illness Anxiety Disorder versus Generalized Anxiety Disorder?. Based on your preceding response, how would you answer the original question: [A 37-year-old woman comes to the office for a follow-up examination 3 weeks after testing confirmed a mass in her left breast to be benign. She says that despite the test results, she has had anxiety, insomnia, and a preoccupation with cancer since noticing the lump. Two weeks ago, she had a 20-minute episode of markedly intense anxiety characterized by heart palpitations and the fear that she was dying. She says she has always been a tense, nervous person and is uncomfortable in social situations. She says she frequently worries and is fearful of the future and apprehensive of dying. She has no other history of panic episodes, traumatic events, or serious illness and takes no medications. She does not drink alcohol or use illicit drugs. Physical examination shows a 2-cm mass in the left breast. On mental status examination, she is cooperative but worried. Her thought processes are linear, and content focuses on possible negative eventualities. Which of the following is the most likely diagnosis? (A) Avoidant personality disorder (B) Generalized anxiety disorder (C) Illness anxiety disorder (hypochondriasis) (D) Persistent depressive disorder (dysthymia) (E) Social anxiety disorder (social phobia) ]

**AI_E:** Based on the description, the patient is displaying significant signs of anxiety that appear to be impacting her day-to-day life. Her worries are not only health-related, but they also extend to general fears about the future and death. Furthermore, we should factor in her long-standing characteristic of being a "tense" and "nervous" individual, hinting at the chronic nature of her symptoms. Her history of social discomfort could hint at a possible social anxiety disorder, however, given that her anxiety extends to other areas of life beyond social situations, this diagnosis is less likely. The intense episode of anxiety she experienced, marked by palpitations and a fear of dying, could be seen as an isolated panic attack, but as she shows no other history of such episodes, a diagnosis of panic disorder might also be inappropriate. Note that the patient's anxieties, worry and tension have a rather pervasive nature, with a variety of triggers: the benign breast mass, social situations, existential considerations. This broad impact of anxiety on multiple life domains (health, social, future) aligns more appropriately with the diagnosis of Generalized Anxiety Disorder (GAD), rather than being focused only on illness-related fears in case of Illness Anxiety Disorder (IAD) or mainly social contexts in Social Anxiety Disorder (SAD). With all the above reasoning considered, I would suggest that the most likely diagnosis for the patient is Generalized Anxiety Disorder (Option B). This is based on her chronic excessive worry about various aspects of life, the symptoms of restlessness, and insomnia. The anxiety appears pervasive and not confined to any specific situational context, hence making GAD a more appropriate diagnosis.

**AI_B:** Restating the Question: The question revolves around determining the psychological disorder that the 37-year-old woman is suffering from. Based on her symptoms, which include anxiety, insomnia, fear of serious illness (especially cancer), discomfort in social situations, and constant worry about the future and death, 5 possible diagnoses have been presented: Avoidant Personality Disorder, Generalized Anxiety Disorder, Illness Anxiety Disorder (Hypochondriasis), Persistent Depressive Disorder (Dysthymia), and Social Anxiety Disorder (Social Phobia). Planning: The assumptions involved in this scenario include the assumption that the symptoms the woman is exhibiting align with a single specific disorder. It's also assumed that the descriptions provided exhaust the extent of her symptoms. By challenging these assumptions, we could consider that her symptoms might be part of multiple coexisting disorders or that she may be experiencing additional symptoms not mentioned in the description. Clinical Reasoning: Analyzing the symptoms given, her anxiety seems to be the most prominent symptom influencing her life, but it extends beyond just her health status. The fact she has always been a 'tense' and 'nervous' person, displays apprehension about dying, and holds worries about the future in addition to her health anxiety, suggests that her anxiety is more generalized. Furthermore, while she does have worries regarding her health, especially cancer, her health anxiety doesn't seem to completely dominate her life to the same extreme as typically seen in Illness Anxiety Disorder, where the fear of having a serious disease persists despite appropriate medical evaluation and reassurance. Concluding: So, her anxiety does encapsulate her health but isn't constrained to it, which seems more indicative of Generalized Anxiety Disorder than Illness Anxiety Disorder. It's important to note that mental health disorders are complex and can often coexist or display overlapping symptoms. Response: Considering the woman's excessive and pervasive worry about multiple domains, chronic pattern of nervousness and tension, her symptomatology closely aligns with Generalized Anxiety Disorder (Option B) rather than the other diagnostic options. Therefore, the most likely diagnosis for this woman is Generalized Anxiety Disorder.

**AI_C:** (1) RESTATING THE QUESTION: The argument presented refers to the diagnosis of a 37-year-old woman who is experiencing intense anxiety, insomnia, and preoccupation with cancer, despite a benign diagnosis for a mass in her left breast. She also has fears about the future and death, and experiences discomfort in social situations. These symptoms pertain to various types of anxiety disorders like Generalized Anxiety Disorder (GAD), Illness Anxiety Disorder (IAD), Social Phobia, Avoidant Personality Disorder and Persistent Depressive Disorder (Dysthymia). (2) PLANNING: The key assumption here is that her symptoms align with one or more of the mentioned disorders—let's redefine them: GAD is characterized by excessive, uncontrollable worrying about everyday matters; IAD (or hypochondriasis) is an obsessive fear about having a serious illness despite medical confirmations to the contrary; Social Phobia involves an intense fear of social situations; Avoidant Personality Disorder is characterized by feelings of inadequacy and hypersensitivity to negative evaluation; and Dysthymia is a persistent depressive disorder. To hypothesize the most likely diagnosis, we need to examine the compatibility between the symptoms and the definitions of each disorder. (3) CLINICAL REASONING: The woman's symptoms indeed suggest a form of anxiety disorder. Her intense preoccupation with cancer, despite the benign diagnosis, indicates a diagnosis similar to Illness Anxiety Disorder. However, her anxiety is not limited to health-related issues—she also feels uncomfortable in social situations and expresses an excessive fear of the future and death. Furthermore, her anxiety symptoms are long-standing, suggesting a disorder that is chronic or ongoing, and possibly generalized. (4) CONCLUDING: The presence of intense worry about different situations (health, future, death, social interactions) makes this case more complex as it does not distinctly fit into IAD. Particularly, IAD is exclusively focused on health concerns, which is not the case here. Her symptoms are consistent with generalized anxiety, being that her worry extends to various aspects of her life rather than being only health-focused. (5) RESPONSE: Thus, the diagnosis that most closely aligns with the patient's symptoms is 'Generalized Anxiety Disorder' (option B) considering how the patient's worry extends to include multiple factors—health, future, death, social situations—instead of a singular obsession with the condition of her health as is characteristic of Illness Anxiety Disorder.

**AI_D:** RESTATING THE QUESTION: The central issue revolves around determining the most probable mental disorder for this 37-year-old woman who, although having a benign lump in her breast, has an intense anxiety about cancer, suffers from insomnia, and constantly broods over the future and fear of death. There are five possible diagnoses identified: Avoidant Personality Disorder, Generalized Anxiety Disorder (GAD), Illness Anxiety Disorder (IAD), Persistent Depressive Disorder (Dysthymia), and Social Anxiety Disorder (Social Phobia). PLANNING: To deduce the right condition, several assumptions have been made. Firstly, that her anxiety pertains specifically to health issues, predominantly about cancer. Secondly, that her anxiety is generalized, not solely health-related, encompassing worries about future and fear of dying. Lastly, that her social discomfort is a key pointer to her actual condition. CLINICAL REASONING: Every anxiety disorder has unique features, though there's plenty of overlap. IAD, formerly known as hypochondriasis, is marked by an extreme worry about having a serious illness, even after medical reports confirm no diagnosis. This woman's preoccupation with cancer, despite confirmed benign results, could suggest IAD. GAD, however, is characterized by chronic anxiety and worry, often unwarranted and about a variety of things, not just health. This woman expresses anxiety about future, death, and social situations, which might lean more towards GAD. Other disorders like Avoidant Personality Disorder, Persistent Depressive Disorder, and Social Anxiety Disorder, though plausible, may not be as fitting due to the absence of key symptoms like chronic depression and avoidance behavior specifically in social activities. CONCLUDING: Based on the evidence and clinical reasoning, while symptoms related to IAD are clearly present, the broad spectrum of her fears (future, death, social discomfort) along with marked distress and impairment suggests Generalized Anxiety Disorder (GAD) as the most likely diagnosis. This is due to the general nature of her anxieties rather than specific worries around health issues alone. RESPONSE: Given her multiple symptoms such as undue worry about the future, fear of dying, social unease, and intense anxiety regarding health despite reassuring medical reports, the most likely diagnosis for this woman's condition is (B) Generalized Anxiety Disorder (GAD). These symptoms correspond to the core features of GAD, which is characterized by persistent, excessive, and unrealistic worry about everyday things spanning a variety of domains, not limited to health concerns alone.

**AI_A:** (1) RESTATING THE QUESTION: The query asks for the most plausible diagnosis for a 37-year woman presenting to the office for a follow-up visit three weeks post confirmation that a mass in her left breast is benign. Despite getting this assurance, she has been plagued by anxiety, insomnia, and a fixation with cancer since discovering the lump. Additionally, she experienced a severe anxiety episode characterized by palpitations and death fear but no history of panic episodes or major illnesses. She also doesn't consume alcohol or drugs and is not on medication. Physical examination showed a benign 2-cm mass in her left breast, and the mental status revealed a cooperative but apprehensive woman with linear thought processes focused on potential negative outcomes. (2) PLANNING: The critical fact to take into notice here is her preoccupation with cancer and a generalized future fear and dying apprehension. It raises the assumption of Illness Anxiety Disorder (IAD), known as hypochondriasis, where the individual is excessively concerned or fearful of having a serious illness despite having no or only mild symptoms and the medical assessment and reassurance that they do not have an illness. However, another assumption could be Generalized Anxiety Disorder (GAD), a long-term condition characterized by an individual feeling anxious about a wide range of situations and issues rather than one specific event. The severity and duration of this woman's feelings of anxiety and various areas of concern (health, future, and death) seem to reflect GAD more than IAD. (3) CLINICAL REASONING: Although IAD patients do exhibit an increased concern about having a severe disease, the anxiety in this patient extends beyond health to future and death issues. Moreover, the patient has expressed experiencing insomnia, an intense episode of anxiety causing palpitations, and a historical anxious disposition, all part of GAD's symptomatology. As such, with the data provided, while her anxiety about the health issue is notable, the generalized and excessive worrying points more towards a GAD diagnosis. (4) CONCLUDING: After considering the above points and focus on the breadth of the patient’s fear, Generalized anxiety disorder seems to be the most likely diagnosis. While IAD (hypochondriasis) does pertain to some of the aspects of her preoccupation with health, GAD better encapsulates her overall condition, since her worries extend beyond her health condition to the future and death. (5) RESPONSE: This woman's condition is most likely (B) Generalized anxiety disorder, as her anxiety is not solely limited to a fear of illness but extends to include concerns regarding multiple life aspects, including her future and death. The reported insomnia and an intense anxiety episode that induced palpitations further support this diagnosis.

**Facilitator** The consensus among the AI responses is that the most likely diagnosis for the 37-year-old woman is Generalized Anxiety Disorder (GAD). This conclusion is based on her chronic and excessive worry about various aspects of life, including health, future, and death, as well as symptoms of restlessness and insomnia. While her intense preoccupation with cancer, despite the benign diagnosis, could suggest Illness Anxiety Disorder (IAD), her anxiety is not limited to health-related issues. She also experiences discomfort in social situations and expresses an excessive fear of the future and death. These symptoms suggest a disorder that is chronic or ongoing, and possibly generalized. Therefore, her condition aligns more closely with GAD, which is characterized by persistent, excessive, and unrealistic worry about everyday things spanning a variety of domains, not limited to health concerns alone. It's important to note that mental health disorders are complex and can often coexist or display overlapping symptoms. However, based on the information provided, GAD seems to be the most fitting diagnosis.
