# Supplementary material for: Collaborative intelligence in AI: Evaluating the performance of a council of AIs on the USMLE
Source: PLOS Digit Health. 2025 Oct 9;4(10):e0000787. doi: 10.1371/journal.pdig.0000787 (PMC12510544; doi:10.1371/journal.pdig.0000787)
Supplement: S2 File — (ZIP) [file pdig.0000787.s002.zip › S2 Data Raw data files containing unprocessed question inputs and LLM outputs including transcripts of deliberations/STEP2_Question-109-1_Type-MC-NJ.docx]

**Facilitator** **[Round 1]:** A 55-year-old man comes to the emergency department because of a 6-hour history of diffuse abdominal pain and subjective fever. His most recent bowel movement 4 hours ago was loose but not bloody. He reports no chills or urinary symptoms. Medical history is remarkable for gastroesophageal reflux disease and alcoholic cirrhosis. Medications are furosemide, spironolactone, lactulose, and pantoprazole. The patient appears uncomfortable. Temperature is 37.8°C (100.0°F), pulse is 88/min, respirations are 14/min, and blood pressure is 104/66 mm Hg. Physical examination shows conjunctival icterus and abdominal distention. Bowel sounds are normal. There is moderate diffuse abdominal tenderness with mild guarding but no rebound tenderness. Results of laboratory studies are shown: Serum ALT: 70 U/L AST: 85 U/L Alkaline phosphatase: 66 U/L Total bilirubin: 3.5 mg/dL Creatinine: 0.6 mg/dL Blood Hematocrit: 39% Hemoglobin: 13.0 g/dL Leukocyte count: 11,300/mm³ Platelet count: 66,000/mm³ Partial thromboplastin time: 39 seconds Prothrombin time: 17.2 seconds Abdominal paracentesis is done, and analysis of the ascitic fluid shows a segmented neutrophil cell count of 288 cells/mm³. Results of blood and ascitic fluid cultures are pending. Which of the following is the most appropriate pharmacotherapy at this time? (A) Cefotaxime (B) Clindamycin (C) Metronidazole (D) Oxacillin (E) Vancomycin (F) No pharmacotherapy is indicated at this time

**AI_D:** RESTATING THE QUESTION: A 55-year-old male patient who has a history of gastroesophageal reflux disease and alcoholic cirrhosis is in the emergency department experiencing a fever and diffuse abdominal pain. He is noticeably uncomfortable and displays physical indications such as conjunctival icterus and abdominal distention. After undergoing an abdominal paracentesis, it was found that his ascitic fluid contains a significant neutrophil count. We are asked to determine what would be the most suitable drug regimen in this situation before the results of his blood and ascitic fluid cultures arrive. PLANNING: The assumption here is that his symptoms, combined with his medical history and the findings from the abdominal paracentesis, might be indicative of spontaneous bacterial peritonitis (SBP). SBP commonly occurs in patients with ascites, especially those with cirrhosis like this patient. His ascitic fluid analysis shows a segmented neutrophil count higher than 250 cells/mm³ which is a typical indication of SBP. CLINICAL REASONING: In the context of SBP, patients often present with diffuse abdominal pain, tenderness, and fever, which aligns with our patient's reported symptoms. The absence of specific microbial culture results makes clinical judgement crucial in this case. Despite the delay, empirical antibiotic therapy is recommended for potential SBP due to the considerable mortality risk associated with this condition. Of the antibiotics listed, cefotaxime stands out as a likely option. CONCLUDING: Cefotaxime is a third-generation cephalosporin and penetrates ascitic fluid well. It covers a wide range of Gram-negative and Gram-positive organisms, making it a suitable empirical therapy for potential SBP in cirrhotic patients. While culture results would help tailor therapy, prompt empirical treatment should not be delayed. RESPONSE: So, considering the patient's current condition and circumstances, the most appropriate pharmacotherapy at this time would be (A) Cefotaxime.

**AI_E:** RESTATING THE QUESTION: A 55-year-old male with a history of gastroesophageal reflux disease and alcoholic cirrhosis presents with diffuse abdominal pain and fever. He has symptoms of gastrointestinal distress, physical signs of jaundice, and abdominal distention. Laboratory findings show elevations in his liver enzymes and bilirubin, low platelet count, and prolonged coagulation times, which are characteristic of liver dysfunction. The abdominal paracentesis reveals a high segmented neutrophil count in his ascitic fluid. As the results of the cultures are still pending, we need to decide upon the most appropriate antimicrobial agent to initiate at this point. PLANNING: The elevated neutrophil count in the ascitic fluid (>250 cells/mm³) strongly suggests spontaneous bacterial peritonitis (SBP). It is a common and life-threatening complication seen in patients with severe cirrhosis and ascites. The underlying assumption here is that the bacteria causing SBP typically originate from the patient's own gut flora, often gram-negative organisms like E.coli or Klebsiella species, and also gram-positive streptococcal species. Antimicrobial treatment must be initiated promptly even before confirmation by culture results, as mortality rates are high. CLINICAL REASONING: Hepatic insufficiency in cirrhotic patients results in a compromised immune response leaving them susceptible to bacterial translocation from the gut. This underlies the pathogenesis of SBP. The presented symptoms, ascitic fluid analysis and the patient's background of cirrhosis all signal towards a likely SBP. Now, the initial empirical antibiotic choice needs to be one that is effective against both gram-negative and positive organisms. CONCLUDING: Taking into account the previous assumptions and the necessity of swiftly starting broad-spectrum antibiotic therapy, cefotaxime, a third-generation cephalosporin, is a strong choice. It is commonly recommended in clinical guidelines for the initial treatment of SBP as it provides excellent coverage against E.coli, Klebsiella, and Streptococcal species. RESPONSE: The most appropriate pharmacotherapy for this patient at this time is (A) Cefotaxime.

**AI_C:** RESTATING THE QUESTION: The patient in question, a 55-year-old male, has come to the emergency department because of 6 hours of diffuse abdominal pain and a subjective fever. His most recent bowel movement was loose, but not bloody. He has a medical history of gastroesophageal reflux disease and alcoholic cirrhosis but shows no urinary symptoms. His current medications are furosemide, spironolactone, lactulose, and pantoprazole. After an abdominal paracentesis, a segment neutrophil cell count of 288 cells/mm³ was found in his ascitic fluid. The results of blood and ascitic fluid cultures are yet to come. Given these findings, we need to identify the most appropriate pharmacotherapy to administer. PLANNING: Two major assumptions can be made from the given symptoms and history. One, this could be a case of spontaneous bacterial peritonitis or SBP (a bacterial infection of the ascites). Two, it might be a different type of bacterial infection affecting the abdomen. The prior diagnosis is more likely, considering his alcoholic cirrhosis, abdominal pain, fever, and ascitic fluid neutrophil count >250 cells/mm³. SBP typically occurs in cirrhotic patients and has symptoms that overlap with the patient's. It's clinically prioritized because if left untreated SBP has a high mortality rate. CLINICAL REASONING: Since alcoholic cirrhosis makes the patient's ascites prone to bacterial colonization, and SBP presents with symptoms such as the fever and abdominal pain indicated here, we need to consider an antibiotic treatment covering most common pathogens in SBP. If other diagnosis were true, then yes, we should consider broader coverage. But based on the triad of cirrhosis, abdominal discomfort, and neutrophilic ascites, SBP is more likely. CONCLUDING: Cirrhosis patients with SBP often present with fever, abdominal pain, and ascitic fluid neutrophil count >250 cells/mm³. Empiric antibiotic therapy is recommended in these cases while still waiting for culture results. The choice of antibiotic should cover the most common pathogens of SBP, primarily gram-negative bacteria, especially Escherichia coli, and some gram-positive cocci. RESPONSE: Given the signs and symptoms that are suggestive of SBP in this patient with a history of alcoholic cirrhosis, Cefotaxime (A), a third-generation cephalosporin, is the most appropriate pharmacotherapy. It has broad-spectrum coverage against gram-negative organisms and some coverage against gram-positive ones. This therapeutic choice improves survival rate while culture results are pending.

**AI_A:** (1) RESTATING THE QUESTION: The clinical scenario describes a 55-year-old man with a known history of gastroesophageal reflux disease and alcoholic cirrhosis. He presents with symptoms of diffuse abdominal pain and subjective fever. His bowel movements have been loose, but not bloody. On physical examination, he exhibits conjunctival icterus, abdominal distension, and moderate diffuse abdominal tenderness with mild guarding. Laboratory reports show abnormal liver enzyme levels, suggestive of liver dysfunction, and a decreased platelet count. Following the abdominal paracentesis, the ascitic fluid neutrophil count is found to be elevated. Pending the results of blood and ascitic fluid cultures, we are supposed to decide on a suitable pharmacotherapy for this patient among the given options. (2) PLANNING: First, we need to consider the patient's symptoms in the context of his past medical history and the lab findings; then, pare down the plausible causes and devise treatment options. The history of alcoholic cirrhosis, the presentation of abdominal pain and fever, along with the finding of increased neutrophils in the ascitic fluid makes spontaneous bacterial peritonitis (SBP) a reasonable assumption. SBP usually occurs in patients where the first line defense mechanisms of their body are compromised, such as in hepatic cirrhosis. The standard initial therapy for SBP is empiric antibiotic treatment, which encompasses gram-negative and gram-positive organisms (mainly enteric pathogens), pending culture results. (3) CLINICAL REASONING: This patient's abdominal discomfort, jaundice, diminished platelet count, and elevated prothrombin time are all consistent with the diagnosis of alcoholic cirrhosis. Furthermore, the raised ascitic fluid neutrophil count raises the suspicion of spontaneous bacterial peritonitis (SBP). SBP typically occurs due to the translocation of gut bacteria across a compromised intestinal barrier in patients with advanced liver disease, like cirrhosis, leading to infection in the ascitic fluid within the peritoneal cavity. The first-line therapy for SBP is empiric antibiotics to cover common enteric pathogens like E. coli, Klebsiella species, and Streptococcus species. Hence, we need to select an antibiotic that has coverage against these bacteria. (4) CONCLUDING: Given the clinical scenario and the potential pathogens involved, we are looking for an antibiotic from the cephalosporin class like cefotaxime or a fluoroquinolone. These antibiotics have broad spectrum coverage against both gram positive and gram negative bacteria, which are responsible for most cases of SBP. (5) RESPONSE: The most appropriate pharmacotherapy at this time is (A) Cefotaxime. This third-generation cephalosporin covers a broad spectrum of bacterial pathogens, including those most commonly seen in SBP. Prompt initiation of antibiotics in this setting has been shown to improve patients' outcomes.

**AI_B:** RESTATING THE QUESTION: This clinical scenario involves a 55-year-old man who has reported to the emergency department with a history of diffuse abdominal pain, subjective fever, and a recent bowel movement that was loose but not bloody. His medical records indicate gastroesophageal reflux disease and alcoholic cirrhosis. His ongoing medications entail furosemide, spironolactone, lactulose, and pantoprazole. Physical examination demonstrates conjunctival icterus and abdominal distention with mild guarding. Laboratory tests reveal elevated liver enzymes and total bilirubin, with normal kidney function. In addition, his hematocrit, hemoglobin, and leukocyte count are within normal limits but the platelet count is low, and his coagulation profile is abnormal. Furthermore, paracentesis of the abdomen has been performed and the segmented neutrophil cell count in the ascitic fluid is 288 cells/mm³, suggestive of a possible infection in the ascitic fluid. The question addresses a decision point in the clinical management of this patient: What is the most appropriate pharmacotherapy at this time? PLANNING: This patient suffers from cirrhosis which is demonstrated by his clinical history, physical examination results, and laboratory findings including elevated liver enzymes, abnormal coagulation profile, low platelet count, and the presence of ascitic fluid. However, the most critical aspect is the neutrophil cell count in the ascitic fluid. Normally, neutrophil cell count in the ascitic fluid should be less than 250 cells/mm³. An elevated neutrophil cell count in the ascitic fluid likely suggests spontaneous bacterial peritonitis (SBP), a life-threatening condition especially in patients with cirrhosis. Therefore, broad-spectrum antibiotics should be initiated immediately even before the culture results become available. If we assume that this patient’s abdominal symptoms and ascites neutrophilia are due to SBP, the preferred empiric antibiotic to cover for the bacterial spectrum that usually causes SBP is a third-generation cephalosporin. CLINICAL REASONING: Spontaneous bacterial peritonitis (SBP), per international guidelines, should be suspected in critically ill patients with cirrhosis and ascites and symptoms such as fever, abdominal pain, or change in mental status. The diagnosis is based on ascitic fluid analysis revealing a segmented neutrophil cell count of >250/mm³ in the absence of a secondary cause for infection such as appendicitis, diverticulitis, or intestinal perforation. The occurrence of SBP in patients with cirrhosis is mainly due to translocation of gut bacteria into the ascitic fluid. Hence, the pathogens usually include gram-negative bacilli like E. coli. Gram-positive cocci, such as streptococci are also commonly isolated. Empirical antibiotic treatment should be initiated immediately while awaiting culture results due to the serious nature of this condition; a third-generation cephalosporin like cefotaxime is most preferred due to broad coverage of both gram-negative and gram-positive organisms. CONCLUDING: Considering the patient’s history of cirrhosis and ascites, presenting symptoms of abdominal pain and fever, the presence of ascites neutrophilia of >250/mm³, the likely diagnosis is spontaneous bacterial peritonitis. Prompt initiation of empirical antibiotic therapy is crucial in the management of this condition due to its potentially lethal consequence. Therefore, cefotaxime, a third-generation cephalosporin, would be the most appropriate pharmacotherapy at this time. RESPONSE: (A) Cefotaxime.

**Facilitator** The consensus among the AI responses is that the 55-year-old male patient, who has a history of gastroesophageal reflux disease and alcoholic cirrhosis, is likely suffering from spontaneous bacterial peritonitis (SBP). This diagnosis is based on his symptoms of diffuse abdominal pain and fever, his medical history, and the elevated neutrophil count in his ascitic fluid. SBP is a common and potentially life-threatening complication in patients with severe cirrhosis and ascites, often resulting from the translocation of gut bacteria into the ascitic fluid. Given the severity of this condition, it is crucial to initiate empirical antibiotic treatment promptly, even before culture results are available. The antibiotic of choice should cover both gram-negative and gram-positive organisms, as these are the most common pathogens involved in SBP. The consensus among the AI responses is that Cefotaxime, a third-generation cephalosporin, is the most appropriate pharmacotherapy in this case. It provides broad-spectrum coverage against the bacterial pathogens most commonly seen in SBP, including E. coli, Klebsiella species, and Streptococcus species. Therefore, the most appropriate pharmacotherapy for this patient at this time is (A) Cefotaxime.
