# Supplementary material for: Collaborative intelligence in AI: Evaluating the performance of a council of AIs on the USMLE
Source: PLOS Digit Health. 2025 Oct 9;4(10):e0000787. doi: 10.1371/journal.pdig.0000787 (PMC12510544; doi:10.1371/journal.pdig.0000787)
Supplement: S2 File — (ZIP) [file pdig.0000787.s002.zip › S2 Data Raw data files containing unprocessed question inputs and LLM outputs including transcripts of deliberations/STEP2_Question-11-1_Type-MC-NJ.docx]

**Facilitator** **[Round 1]:** Abstract: Objective: In patients with cirrhosis and acute bleeding esophageal varices, how do endoscopic sclerotherapy and emergency portacaval shunt compare for control of bleeding and survival? Methods Design: Randomized controlled trial (San Diego Bleeding Esophageal Varices Study). ClinicalTrials.gov NCT00690027. Allocation: Concealed. Blinding: Blinded (gastroenterologist who evaluated patients for portal-systemic encephalopathy). Follow-up period: Up to 17 years. Setting: University of California San Diego Medical Center. Patients: 211 patients (mean age 49 years, 77% men) with acute bleeding esophageal varices resulting from cirrhosis, who required a transfusion of ≥ 2 units of blood and, for patients transferred from other hospitals, observation of upper gastrointestinal bleeding within 48 hours of transfer. Exclusion criterion was > 1 previous session of endoscopic sclerotherapy. Intervention: Endoscopic sclerotherapy (n = 106) or emergency portacaval shunt (n = 105). Emergency portacaval shunt comprised a direct side-to-side or direct end-to-side portacaval shunt done within 8 hours of initial contact. Outcomes: Control of bleeding at > 30 days, survival, readmissions for variceal or nonvariceal bleeding requiring transfusion of packed red blood cells, and recurrent portal-systemic encephalopathy. Patient follow-up: 100% (minimum follow-up until death or 9.4 years). Main results 15-year survival was lower with endoscopic sclerotherapy than with emergency portacaval shunt (10/106 vs 48/105, relative benefit reduction 79%, 95% CI 62 to 89; number needed to harm 3, CI 2 to 4). Other main results are shown in the Table. Endoscopic sclerotherapy (EST) vs emergency portacaval shunt (EPCS) in patients with cirrhosis and acute bleeding esophageal varices Outcomes | Child-Pugh risk class | EST | EPCS | P value | ———————————————————————————————————— Control of bleeding at > 30 days* | | 20% | 100% | <.001 | Median survival (years) | A | 4.62 | 10.43 | .003. | | B | 2.61 | 6.19 | <.001 | |. C | 0.58 | 5.30 | .005. | Mean number of readmissions for variceal bleeding requiring packed red blood cell transfusion | | 6.8 | 0.4 | <.001 | Recurrent portal-systemic encephalopathy† | | 35% | 15% | < .001 | ——————————————————————————————————— *Excluding indeterminate deaths at 14 days from nonbleeding causes. †In patients who survived 30 days and left hospital. ——————————————————————————————————— Conclusion In patients with cirrhosis and acute bleeding esophageal varices, emergency portacaval shunt was better than endoscopic sclerotherapy for control of bleeding, recurrent encephalopathy, and survival. Sources of funding: National Institutes of Health and Surgical Education and Research Foundation. Structured abstract based on: Orloff MJ, Isenberg JI, Wheeler HO, et al. Randomized trial of emergency endoscopic sclerotherapy versus emergency portacaval shunt for acutely bleeding esophageal varices in cirrhosis. J Am Coll Surg. 2009;209:25-40. 19651060 A 52-year-old man with hepatic cirrhosis comes to the emergency department because of a 3-hour history of vomiting blood. Esophagogastroduodenoscopy confirms actively bleeding esophageal varices. Based on the abstract shown, the physician is considering an emergency portacaval shunt (EPCS) procedure rather than endoscopic sclerotherapy (EST). According to the results in the abstract, approximately how many patients must be treated with EPCS rather than EST to prevent one case of recurrent portal-systemic encephalopathy? (A) 1 (B) 3 (C) 5 (D) 10 (E) 16

**AI_C:** Restating the question, we are asked to determine the number of patients that would need to receive the emergency portacaval shunt (EPCS) treatment instead of endoscopic sclerotherapy (EST) to prevent a single case of recurrent portal-systemic encephalopathy. This is effectively asking for the 'number needed to treat' (NNT) to prevent one case of recurrent portal-systemic encephalopathy. The 'number needed to treat' (NNT) is a valuable tool used in evidence-based decision-making to compare the effectiveness of different treatments. It represents the number of patients that need to be treated to prevent one additional bad outcome. We calculate it as the reciprocal of the absolute risk reduction (ARR), or the difference in the outcome rate for a treatment and control group. In the given randomized controlled trial, the outcome of interest is recurrent portal-systemic encephalopathy, which occurred in 35% of patients who received EST and 15% of patients who received EPCS. Therefore, the ARR, which is: ARR = Risk in Control Group - Risk in Treatment Group = 35% (EST) - 15% (EPCS) = 20%. To find the number needed to treat (NNT), we calculate the reciprocal of the ARR. This gives us: NNT = 1/ARR = 1/0.20 = 5. Therefore, we would need to treat approximately 5 patients with EPCS rather than EST to prevent a single case of recurrent portal-systemic encephalopathy. To this end, our response is (C) 5.

**AI_D:** RESTATING THE QUESTION: The case presented is of a 52-year-old man with hepatic cirrhosis who has been brought to the emergency department due to a history of vomiting blood for the past 3 hours. Upon investigation, esophagogastroduodenoscopy confirms that the source of the bleeding is active esophageal varices. In deciding the most effective treatment plan for the patient, the attending physician is considering an emergency portacaval shunt (EPCS) over endoscopic sclerotherapy (EST) and is using the evidence from a recent randomized trial for guidance. The critical question here concerns the comparative effectiveness of these two treatments: Specifically, how many patients need to undergo the EPCS procedure rather than EST to avert one case of recurrent portal-systemic encephalopathy? PLANNING: Portal-systemic encephalopathy is a significant complication of cirrhosis and variceal bleeding, and its prevention is a clear clinical priority. To answer the question at hand, we need to determine the number needed to treat (NNT) with EPCS rather than EST to prevent one case of recurrent portal-systemic encephalopathy. CLINICAL REASONING: The number needed to treat (NNT) is a measure that indicates how many patients need to undergo a specific treatment to prevent one negative event. It is calculated as the inverse of the absolute risk reduction (ARR), which in this case, is the difference in the proportion of recurrent portal-systemic encephalopathy between EST and EPCS. From the abstract, we know that 35% of patients treated with EST experienced recurrent episodes of portal-systemic encephalopathy, whereas only 15% of those treated with EPCS experienced the same. This gives us an ARR of 20% (35% - 15%). CONCLUDING: So, to prevent one case of recurrent portal-systemic encephalopathy, we would need to treat 1/0.20 patients with EPCS rather than EST. RESPONSE: Thus, the number needed to treat (NNT) for preventing one case of recurrent portal-systemic encephalopathy with EPCS compared to EST is 5. Therefore, the answer is (C) 5.

**AI_B:** RESTATING THE QUESTION: The question asks for us to identify the number of patients that would need to receive an emergency portacaval shunt (EPCS), a procedure to relieve high blood pressure in the portal vein, instead of endoscopic sclerotherapy (EST), a procedure to halt bleeding varices, in order to prevent one case of recurrent portal-systemic encephalopathy (PSE). PSE is a condition that causes confusion and other mental changes due to the buildup of toxins in the bloodstream that are ordinarily removed from the liver. PLANNING: This question requires the computation of the number needed to treat (NNT), a metric that describes the number of patients that need to be treated in order to prevent one negative outcome. To calculate NNT, we use the formula: NNT = 1/ Absolute Risk Reduction (ARR), where ARR is the difference in the proportions of negative outcomes (recurrent PSE) in the EST and EPCS groups (ARR = PEST - PEPCS). The rates of PSE in the abstract are given as 35% and 15% for the EST and EPCS groups, respectively. CLINICAL REASONING: Given the rates of PSE in the EST and EPCS groups, calculating the absolute risk reduction (ARR) produces an ARR of 20%. This ARR value then inputs to the NNT formula to give the number of patients that need to be treated with EPCS instead of EST to prevent one case of recurrent PSE. CONCLUDING: By carefully following the clinical reasoning process, which involves understanding the medical concepts, drawing from the information given in the abstract, and performing the necessary computations, we can confidently arrive at the final answer. RESPONSE: To calculate the number needed to treat (NNT), first calculate the absolute risk reduction (ARR) as follows: ARR = PEST - PEPCS = 35% - 15% = 20%. Then compute NNT = 1/ ARR = 1 / 20% = 1 / 0.20 = 5. Therefore, approximately 5 patients must be treated with an EPCS rather than an EST to prevent one case of recurrent PSE. The best choice is (C) 5.

**AI_A:** (1) RESTATING THE QUESTION: This scenario describes a 52-year-old man with hepatic cirrhosis and actively bleeding esophageal varices, conditions associated with advanced liver disease. The question is based on study results depicting treatment efficacy of two procedures - endoscopic sclerotherapy (EST) and emergency portacaval shunt (EPCS). The question essentially asks us to calculate the number needed to treat (NNT) - an epidemiological measure that describes the number of patients that need to be treated to prevent one additional bad outcome (in this case, the prevention of recurrent portal-systemic encephalopathy) using EPCS over EST. (2) PLANNING: To derive the answer, we need to understand the concept of NNT and how it is calculated. The NNT is the reciprocal of the absolute risk reduction (ARR). ARR is calculated as the difference between the event rate in the control group and the event rate in the treatment group. From the abstract, it can be inferred that the control group (EST) has a 35% rate of recurrent portal-systemic encephalopathy, whereas the treatment group (EPCS) has a 15% rate. (3) CLINICAL REASONING: As described earlier, the ARR is calculated by subtracting the event rate of the treatment group from the event rate of the control group. This gives us the ARR for recurrent portal-systemic encephalopathy being (35% - 15%) = 20%. The NNT is then calculated by taking the reciprocal of the ARR, keeping in mind that the values should correspond - meaning, if the ARR is in percentage, the result of NNT will be in 'x' patients out of 100 patients, and if the ARR is in decimal values, the NNT will correspond to the absolute number of patients. (4) CONCLUDING: Using the ARR value of 20%, calculated from the event rates provided in the abstract, the NNT would be 100 divided by ARR, which is 100 divided by 20, results in NNT=5. (5) RESPONSE: Therefore, approximately 5 patients must be treated with the emergency portacaval shunt rather than endoscopic sclerotherapy to prevent one case of recurrent portal-systemic encephalopathy. So the correct answer is (C) 5.

**AI_E:** RESTATING THE QUESTION: The query essentially requires me to calculate the number needed to treat (NNT) with the emergency portacaval shunt (EPCS) rather than endoscopic sclerotherapy (EST) in order to thwart one recurrence of portal-systemic encephalopathy given the data provided in the abstract. The abstract summarizes a clinical trial comparing EPCS with EST, with numerous outcomes observed like control of bleeding, survival, readmissions for bleeding requiring transfusion, and recurrence of portal-systemic encephalopathy, among patients with cirrhosis experiencing acute bleeding esophageal varices. Importantly, portal-systemic encephalopathy can develop in these patients due to shunting of blood around the liver leading to buildup of toxins affecting brain function. PLANNING: Firstly, I need to understand the concept of number needed to treat (NNT). It is a metric giving the average number of patients who need to be treated to prevent one additional bad outcome, and it’s calculated as the inverse of absolute risk reduction (ARR). ARR calculates the difference in outcomes between the control group and the treated group. Looking at the given data on recurrent portal-systemic encephalopathy with EST (35%) and EPCS (15%), our main assumption is that this difference is due to the treatment alone. This assumption is supported by the study design, a randomized controlled trial, which helps balance out other variables that could influence outcome. It's always important to consider if there could be other contributing factors, but in this case it seems logical to attribute the difference in encephalopathy recurrence directly to the treatment modalities. CLINICAL REASONING: Recurrent encephalopathy percentage for EST is 35% and for EPCS it is 15%. Consequently, the ARR would be 35-15=20%. To find the NNT, we take the inverse of this ARR (converting the percentage to a proportion first): 1/(0.20)=5. Therefore, on average, 5 patients would need to undergo EPCS instead of EST to prevent one case of recurrent portal-systemic encephalopathy. Since this is an average, there will likely be some variability around this number in the real patient population. CONCLUDING: Given the data in this study, the number needed to treat (NNT) with Emergency Portacaval Shunt (EPCS), versus Endoscopic Sclerotherapy (EST), to prevent one case of recurrent portal-systemic encephalopathy in patients with cirrhosis and acute bleeding esophageal varices is approximately 5. RESPONSE: The approximate amount of patients that need to be treated with EPCS rather than EST to prevent one case of recurrent portal-systemic encephalopathy is 5 (Option C).

**Facilitator** The question asks for the calculation of the number needed to treat (NNT) with the emergency portacaval shunt (EPCS) rather than endoscopic sclerotherapy (EST) to prevent one case of recurrent portal-systemic encephalopathy. The NNT is a measure that indicates how many patients need to undergo a specific treatment to prevent one negative event. It is calculated as the inverse of the absolute risk reduction (ARR), which is the difference in the proportion of recurrent portal-systemic encephalopathy between EST and EPCS. From the abstract, we know that 35% of patients treated with EST experienced recurrent episodes of portal-systemic encephalopathy, whereas only 15% of those treated with EPCS experienced the same. This gives us an ARR of 20% (35% - 15%). Therefore, to prevent one case of recurrent portal-systemic encephalopathy, we would need to treat 1/0.20 patients with EPCS rather than EST. Thus, the number needed to treat (NNT) for preventing one case of recurrent portal-systemic encephalopathy with EPCS compared to EST is 5. Therefore, the answer is (C) 5.
