# Supplementary material for: Collaborative intelligence in AI: Evaluating the performance of a council of AIs on the USMLE
Source: PLOS Digit Health. 2025 Oct 9;4(10):e0000787. doi: 10.1371/journal.pdig.0000787 (PMC12510544; doi:10.1371/journal.pdig.0000787)
Supplement: S2 File — (ZIP) [file pdig.0000787.s002.zip › S2 Data Raw data files containing unprocessed question inputs and LLM outputs including transcripts of deliberations/STEP2_Question-110-1_Type-MC-NJ.docx]

**Facilitator** **[Round 1]:** A 45-year-old man is brought to the emergency department because of moderate chest pain after a generalized tonic-clonic seizure 30 minutes ago. He has seizure disorder for which he has taken carbamazepine and phenobarbital for the past 20 years. X-ray of the chest shows generalized osteopenia with several rib fractures. Which of the following is the most likely nutritional deficiency? (A) Folic acid (B) Iron (C) Magnesium (D) Vitamin B₂ (riboflavin) (E) Vitamin D (F) Zinc

**AI_D:** Restating the Question: The question is about a 45-year-old male who has had a seizure disorder for the last 20 years. He has taken carbamazepine and phenobarbital to manage his condition. He was brought to emergency following a generalized tonic-clonic seizure and chest pain. He has osteopenia and several rib fractures as evident on a chest X-ray. The question is asking about the most probable nutritional deficiency exhibited by this patient. 'Osteopenia' means a condition characterized by lower bone density than normal, while 'Generalized tonic-clonic seizures' are seizures involving convulsions and muscle rigidity all over the body. Planning: While there could be several nutritional deficiencies in this case, we can link the seizure disorder, long-term use of mentioned antiepileptics, and the observed osteopenia to a deficiency of a particular nutrient. Given the options, multiple deficiencies can present with bone issues, but we must prioritize looking at those directly influenced by the medications used and the associated pathophysiology. Clinical Reasoning: Carbamazepine and phenobarbital, two commonly used medications for seizure treatment, can induce certain deficiencies. These medications are known inducers of certain liver enzymes (cytochrome P450 enzymes), which can increase the metabolism of vitamin D leading to its deficiency. Vitamin D plays a critical role in the body's ability to absorb calcium and phosphate, crucial for bone health. If vitamin D is deficient, it can lead to a decrease in bone mineral density, leading to conditions like osteopenia. Concluding: In conclusion, vitamin D deficiency seems the most likely given the patient's prolonged use of carbamazepine and phenobarbital, both of which can induce its metabolism and render lower levels in the body, contributing to bone-related disorders like osteopenia and fractures. Response: Therefore, the most likely nutritional deficiency in this patient is (E) Vitamin D.

**AI_A:** RESTATING THE QUESTION: We're looking at a male patient in mid-life who presented at the emergency department complaining of chest pain. The chest pain came on following a generalised tonic-clonic seizure. His medical history reveals a seizure disorder and he's been taking two antiepileptic drugs; carbamazepine and phenobarbital for two decades. New findings include an X-Ray of his chest which shows a condition of decreased bone density called generalized osteopenia, along with a few rib fractures. We're asked to identify the most probable nutrient deficiency he may be suffering from, with a range of options given. PLANNING: An important assumption we seem to be working with is that the patient's longstanding use of antiepileptic drugs and the emergence of bone disorders are correlated. This insight would warrant an examination of the long-term effects of carbamazepine and phenobarbital. By questioning these assumptions, alternate standpoints emerge, such as the patient's nutritional habits and the potential presence of underlying pathologies that might be independently contributing to his physiological status. CLINICAL REASONING: In chronic antiepileptic users like our patient, the drug-mediated induction of hepatic enzymes has been observed to escalate the metabolic degradation of certain vitamins like Vitamin D. This heightened metabolism hampers the absorption of calcium, leading to a compounded negative effect on bone health and hence promoting conditions like osteopenia. Further, fractures could baffle the patient's chest pain, post-seizure. CONCLUDING: Putting the pieces together, we associate the patient's prolonged use of carbamazepine and phenobarbital to his bone health status. The possible depletion of Vitamin D owing to the accelerated metabolic degradation induced by these drugs might be compromising calcium absorption, worsening bone health, and predisposing to conditions like osteopenia and fractures. RESPONSE: Therefore, the most likely nutritional deficiency in this case is Vitamin D (E).

**AI_C:** RESTATING THE QUESTION: We are dealing with a 45-year-old male patient who presented to the emergency department with moderate chest pain after experiencing a generalized tonic-clonic seizure. This patient has a long-standing history of seizure disorder, managed with carbamazepine and phenobarbital for two decades. A chest X-ray reveals generalized osteopenia and multiple rib fractures. We are tasked with identifying the most probable nutritional deficiency in this patient from a list of options that includes folic acid, iron, magnesium, vitamin B2 (riboflavin), vitamin D, and zinc. PLANNING: Given the long history of seizure and the pharmacological treatments, the osteopenia, the rib fractures, and the specific options provided, this patient most likely suffers from a deficiency associated with bone health. Both vitamin D and magnesium may have implications for bone health, but we also need to keep in mind the side effects of the medications he has been on for a long period. While carbamazepine and phenobarbital can cause various nutritional deficiencies, vitamin D deficiency stands out due to its vital role in calcium absorption and bone health. CLINICAL REASONING: Pharmacologically-induced vitamin D deficiency is a well-document side effect of both carbamazepine and phenobarbital. These medications are Cytochrome P450 enzyme inducers, accelerating the metabolic clearance of Vitamin D and thereby causing its deficiency. Vitamin D plays an integral role in promoting calcium absorption in the gut and maintaining adequate calcium and phosphate concentrations in the body to support bone health. With prolonged vitamin D deficiency, there may be an insufficient supply of calcium for the remodeling and mineralization of bones, causing osteopenia (a state of decreased bone density) and associated fractures. CONCLUDING: Factoring in the linkage between the patient's medication, his symptoms of chest pain, the visualized osteopenia, the rib fractures, and the potential options, the most probable nutritional deficiency would be Vitamin D. RESPONSE: The most likely nutritional deficiency in the given scenario is Vitamin D (E).

**AI_B:** 1) RESTATING THE QUESTION: The query revolves around a man of 45 years, who has been long-term management on carbamazepine and phenobarbital for his seizure disorder, and has now been admitted to the ER due to chest pain post-seizure. His chest X-ray reveals generalized osteopenia and multiple rib fractures. The implication here is to identify the most probable nutrient deficiency considering his clinical presentation. 2) PLANNING: Assumptions: This case assumes that the patient's osteopenia and fractures would be related to a nutrient deficiency, that the nutrient deficiency is caused by medication or the disease, and that the most likely cause of osteopenia and fractures is a nutritional deficiency. Alternate assumptions may consider: Are there any other underlying medical conditions or medications that could lead to these symptoms? Can long-term use of carbamazepine and phenobarbital cause nutrient deficiencies? Evaluating the assumptions: The symptoms and conditions suggest that a long-term deficiency of a specific nutrient that's important for bone health might be present. Certain medications like carbamazepine and phenobarbital can indeed lead to nutritional deficiencies. 3) CLINICAL REASONING: A nutrient that plays a crucial role in maintaining bone health and whose deficiency can cause osteopenia and increased bone fracture risk is Vitamin D. It aids in the absorption of calcium and phosphate, two nutrients critical for bone mineralization. A lack of Vitamin D can ultimately lead to a decrease in bone mineral density (osteopenia), resulting in fragility and a higher chance of fractures. Long-term use of certain medications, including seizure medications like phenobarbital and carbamazepine, can interfere with the body's vitamin D metabolism resulting in deficiency. 4) CONCLUDING: Based on the patient's long-term medication history and symptoms, it's plausible that the use of carbamazepine and phenobarbital for 20 years could have led to a deficiency in Vitamin D. This, in turn, could have resulted in osteopenia and a higher risk of bone fractures. 5) RESPONSE: Hence, the most probable nutritional deficiency in this patient would be (E) Vitamin D.

**AI_E:** RESTATING THE QUESTION: The scenario presents a 45-year-old male with a history of seizure disorder who is currently on Carbamazepine and Phenobarbital and is now presenting with chest pain following a generalized tonic-clonic seizure. The results of a chest X-ray reveal generalized osteopenia along with multiple rib fractures. Given these findings, we are asked to deduce the most likely nutritional deficiency this patient might have. Nutritional deficiencies are issues caused by a lack of essential nutrients in a person's diet which consequently affect various body functions and structures. PLANNING: Assumptions made from the scenario can be: 1) The seizure led to the chest pain and rib fractures. 2) The long-term use of Carbamazepine and Phenobarbital may have caused the generalized osteopenia. To challenge these, we can propose alternate assumptions that the chest pain and rib fractures were caused by the osteopenia, and the generalized osteopenia could be a result of a nutritional deficiency. Evaluating these alternates, it may be clinically more pertinent to pursue the nutritional deficiency assumption as it offers a more comprehensive explanation of the long-term manifestation of the patient's symptoms and findings. CLINICAL REASONING: Nutritional deficiencies can lead to various physiological dysfunctions. In this case, osteopenia suggests a decrease in bone density, generally associated with a deficiency in Vitamin D and/or Calcium, crucial for bone health. It's important to note that Carbamazepine and Phenobarbital, anticonvulsants, induce liver enzymes leading to increased metabolism of Vitamin D, which could result in its deficiency. A deficiency of Vitamin D perturbs the balance of calcium homeostasis, causing decreased bone mineralization, which leads to osteopenia; this likely contributed to the ease with which the patient's ribs got fractured. CONCLUDING: Therefore, anticonvulsant usage along with the present osteopenia, points towards a Vitamin D deficiency caused by enzyme induction, leading to rapid metabolism of Vitamin D. The seizure would have caused increased stress on an already compromised skeleton, leading to the fractures and consequent chest pain. RESPONSE: (E) Vitamin D is the most likely nutritional deficiency in this 45-year-old man who has been taking Carbamazepine and Phenobarbital for his seizure disorder for the past 20 years resulting in osteopenia and multiple rib fractures.

**Facilitator** The consensus statement synthesizes the responses of AI_A, AI_B, AI_C, AI_D, and AI_E as follows: The question pertains to a 45-year-old male patient with a history of seizure disorder, managed with carbamazepine and phenobarbital for two decades. He presented to the emergency department with chest pain following a generalized tonic-clonic seizure. A chest X-ray revealed generalized osteopenia and multiple rib fractures. The task is to identify the most probable nutritional deficiency in this patient. The assumptions made in this case are that the patient's osteopenia and fractures are related to a nutrient deficiency, and that this deficiency is likely caused by the long-term use of his antiepileptic medications. An alternate assumption could be that other underlying medical conditions or medications could lead to these symptoms. However, the symptoms and conditions suggest a long-term deficiency of a specific nutrient crucial for bone health. Both carbamazepine and phenobarbital are known to induce certain liver enzymes (cytochrome P450 enzymes), which can increase the metabolism of vitamin D, leading to its deficiency. Vitamin D plays a critical role in the body's ability to absorb calcium and phosphate, which are essential for bone health. A deficiency in Vitamin D can lead to a decrease in bone mineral density, resulting in conditions like osteopenia and an increased risk of fractures. Therefore, considering the patient's long-term medication history, his symptoms, and the visualized osteopenia and rib fractures, the most probable nutritional deficiency in this patient is Vitamin D. This deficiency could be due to the accelerated metabolic degradation induced by the long-term use of carbamazepine and phenobarbital, which might be compromising calcium absorption, worsening bone health, and predisposing to conditions like osteopenia and fractures.
