# Supplementary material for: Collaborative intelligence in AI: Evaluating the performance of a council of AIs on the USMLE
Source: PLOS Digit Health. 2025 Oct 9;4(10):e0000787. doi: 10.1371/journal.pdig.0000787 (PMC12510544; doi:10.1371/journal.pdig.0000787)
Supplement: S2 File — (ZIP) [file pdig.0000787.s002.zip › S2 Data Raw data files containing unprocessed question inputs and LLM outputs including transcripts of deliberations/STEP2_Question-111-1_Type-MC-NJ.docx]

**Facilitator** **[Round 1]:** A 32-year-old man comes to the office because of a 2-day history of redness of the right eye. He has not had sensitivity to light or the sensation of a foreign body in the eye. He has no history of serious illness and takes no medications. Examination of the right eye shows erythema and clear discharge. Examination of the left eye shows no abnormalities. The physician prescribes ketorolac ophthalmic solution. Which of the following is the most appropriate way for the physician to write the prescription? (A) Ketorolac 0.5% solution, 1 drop in right eye, four times daily (B) Ketorolac .5% solution, 1 drop in right eye QID (C) Ketorolac 0.5% solution, 1 drop OD four times daily (D) Ketorolac 0.5% solution, 1 drop OD, QID

**AI_A:** RESTATING THE QUESTION: A 32-year-old gentleman is presenting with symptoms of unilateral (right) ocular redness and clear discharge, without photophobia or feeling of foreign body presence in his eye. He's a generally healthy person and takes no medications. An examination only reveals abnormalities in the right eye. He's prescribed ketorolac ophthalmic solution by the attending physician. We're then asked to identify the proper way from the options given for the physician to write down this prescription. PLANNING: The Clinical context here is related to ophthalmology and pharmacology. The assumption is that the patient's symptoms are suggestive of an ocular condition that could benefit from the anti-inflammatory effects of ketorolac. Ketorolac is a non-steroidal anti-inflammatory drug (NSAID). The prescription writing could be judged based on the precision of the terminology and the clear definition of the eye requiring treatment. The Latin abbreviations could cause some confusion here. CLINICAL REASONING: When looking at the prescription options, we must consider that "OD" in medical terminology stands for "Oculus Dexter," referring to the right eye. In contrast, "QID" stands for "Quater In Die," which translates to "four times a day". While both "four times daily" and "QID" mean the same in terms of frequency of administration, the abbreviation QID is more concise and typically used in prescriptions. CONCLUDING: Based on the clinical reasoning, we should look for an option that correctly uses the term "OD" for the right eye and denotes the frequency of administration as QID. RESPONSE: The correct way to write this prescription would be (D) Ketorolac 0.5% solution, 1 drop OD, QID. This uses the accurate acronym for the right eye and provides the distinct frequency for drug administration.

**AI_C:** RESTATING THE QUESTION: The scenario presented here revolves around a 32-year-old man with inflammation of the right eye, which is displayed by redness and clear discharge. The physician has opted to prescribe Ketorolac, an anti-inflammatory drug often used to relieve pain, in an ophthalmic solution form. We are asked to determine which amongst the following prescriptions is given in the most appropriate way. The potential answers all involve the same medication but differ in the prescription abbreviations and formats used. PLANNING: It's crucial to identify the abbreviations and formats used: "QID" (quater in die) means "four times a day," while "OD" (oculus dexter) means "right eye." However, the abbreviation "OD" can often be confused with "once daily." Consequently, it presents a potential risk for medication errors and is no longer recommended in the medical field. CLINICAL REASONING: Patient safety and clarity are paramount in medication prescription to safeguard against any confusion or misuse. An effective prescription should be legible, clear, and avoid ambiguous abbreviations. Although all presented prescriptions imply similar dosage, using "OD" can be misleading and potentially result in medication errors. Thus, it's best to avoid using it when writing prescriptions. CONCLUDING: Given the outlined situation and considering the patient's condition, an optimal prescription would eliminate potential misinterpretation due to ambiguous abbreviations. The prescription should clearly state the medication name, concentration, administration route, and dosing frequency. RESPONSE: Hence, the most likely prescriptions that fulfil the above requirements are either option (A) Ketorolac 0.5% solution, 1 drop in right eye, four times daily, or option (B) Ketorolac .5% solution, 1 drop in right eye QID. Both options avoid the ambiguous "OD" and represent the same prescription, but option (A) is slightly more explicit in the dosing frequency by spelling out "four times daily".

**AI_B:** RESTATING THE QUESTION: The issue under consideration revolves around the optimal way for a physician to write a prescription for a ketorolac ophthalmic solution. Ketorolac is a non-steroidal anti-inflammatory drug (NSAID) typically used in ophthalmology to alleviate symptoms of conjunctivitis, such as eye redness. The given options include variations in the indication of dosage frequency — "four times daily" or "QID", which is an abbreviation for the Latin term "quater in die", meaning four times a day. A further differentiation is made between the phrases "right eye" or "OD", which is derived from the Latin term "oculus dexter" referring to the right eye. PLANNING: The assumption is that these expressions — either in English or Latin abbreviations — should be interchangeable in a medical prescription, as long as they effectively communicate the necessary instructions to the patient. By questioning this presumption however, we should consider the potential implications of incorporating more or less technical language into patient instructions. The priority in such cases would generally be to ensure patient comprehension and adherence to the medication regimen. CLINICAL REASONING: When comparing options A and B, the only variation lies in how the frequency is indicated - "four times daily" vs "QID". These both clearly depict the same instruction. However, "four times daily" might be more self-evident to a patient unfamiliar with medical abbreviations. Considering options C and D, these employ the term "OD" to specify the right eye. While this could seem concise to medical professionals, it may confuse a patient not familiar with Latin medical terminology. CONCLUDING: Drawing from the previous reasoning, we should prioritize patient comprehension and adherence to the medication regimen. Therefore, clear and simple instructions would be preferable. Specialized abbreviations may appear efficient or concise in professional communication, but they can be a barrier to patient understanding in practical implementation. RESPONSE: Therefore, among the given options, the most appropriate way for the physician to write the prescription would be: "Ketorolac 0.5% solution, 1 drop in right eye, four times daily" (Option A). This option maintains clear and unequivocal instructions that minimize the risk for misunderstanding or incorrect use by the patient.

**AI_D:** 1) RESTATING THE QUESTION: The issue relates to a man experiencing right eye redness for two days, with a subsequent exam revealing eye erythema and clear discharge. Following this, the practitioner planned to prescribe ketorolac ophthalmic solution. The question at hand consists of determining the most appropriate prescription manner for the patient's presentation. Ketorolac ophthalmic solution is a nonsteroidal anti-inflammatory drug (NSAID) often used to relieve eye inflammation caused by many conditions. QID and OD are abbreviations typically used in medical terminology; where QID means 'four times a day,' and OD stands for 'right eye.' 2) PLANNING: Assumptions include the appropriate concentration of the medication and the correct terminology for dosage and eye administration. By considering these assumptions, we establish various alternatives: 1) The patient might need four doses daily in his right eye; 2) More or less than 0.5% concentration could be necessary; or 3) There might be a need for administration in both eyes. In this case, it is of clinical importance to prioritize the assumption that the ketorolac must be administered to the right eye four times daily, due to the symptoms present solely in that eye. 3) CLINICAL REASONING: The patient is experiencing symptoms in his right eye, not his left, consequently directing drug administration to the right eye. We'll consider options that include QID, which means 'four times daily' for effective treatment, and we'll look particularly at options stating the medication should be administered specifically to the patient's right eye (OD). 4) CONCLUDING: Reviewing the options with the criteria in mind from the clinical reasoning, we can see that not all of them mention administering the drug to the right eye, which is a necessity given this patient's symptoms. We know from the information given that the concentration of ketorolac should be at 0.5%, it needs to be administered four times daily (notated as QID), and should be administered particularly to the right eye (notated as OD). 5) RESPONSE: The most suitable way for the physician to write the prescription would be: "Ketorolac 0.5% solution, 1 drop OD, QID" because it correctly states the drug name, concentration, dosage, and administration frequency specifically in the ailing right eye. This means that option D matches our criteria best.

**AI_E:** RESTATING THE QUESTION: The case describes a 32-year-old man who's experiencing inflammation in his right eye, apparent from signs of redness and clear discharge, but no light sensitivity or sensation of a foreign body in the eye. He's in overall good health, taking no medications. The doctor has chosen to prescribe ketorolac, a nonsteroidal anti-inflammatory drug (NSAID), to help manage his symptoms. The question now is about which prescription writing format would be most appropriate to administer this medication. PLANNING: There seems to be no difference in the dose suggested across all options, but varying ways of indicating how frequently the patient should apply the drops, and specifically which eye to apply them to. The assumptions here are that certain terms in this context have certain meanings, namely "four times daily" and "QID" both indicate frequency, whereas "right eye" and "OD" both indicate where to apply the drops. We can question whether these assumptions are correct in this context. As we know, in clinical contexts, terms are typically coded in some way to reduce ambiguity and prevent medication errors. CLINICAL REASONING: Diving into these assumptions, we can hopefully arrive at the correct choice. "Four times daily" and "QID" (from the Latin "quater in die"), both straightforwardly indicate that the drop should be applied four times throughout the day. However, "OD" can have a bit of a double meaning. Ordinarily, "OD" would stand for "oculus dexter", which translates to "right eye". However, "OD" can also stand for "once daily", which could add some confusion in this context. As for "right eye", it's unambiguous but maybe less space-effective. CONCLUDING: The potential for confusion with "OD"'s meaning makes options (C) and (D) less ideal in this context. The difference between options (A) and (B) seems primarily to be predicated on the use of "four times daily" versus "QID". RESPONSE: Although "QID" takes up less space and is a more common term in prescriptions, "four times daily" is more patient-friendly because it explicitly states the frequency. Therefore, the physician should write the prescription as (A) Ketorolac 0.5% solution, 1 drop in right eye, four times daily.

**Facilitator** **[Round 2]:** Summary: AI_A believes the prescription should be written as "Ketorolac 0.5% solution, 1 drop OD, QID" because it uses accurate medical terminology and provides a clear frequency for drug administration. AI_B, on the other hand, argues for "Ketorolac 0.5% solution, 1 drop in right eye, four times daily" to ensure patient comprehension and adherence to the medication regimen. AI_C also supports this option, emphasizing the need to avoid ambiguous abbreviations like "OD" to prevent medication errors. AI_D agrees with AI_A, stating that "Ketorolac 0.5% solution, 1 drop OD, QID" is the most suitable prescription as it correctly states the drug name, concentration, dosage, and administration frequency. AI_E, like AI_B and AI_C, prefers "Ketorolac 0.5% solution, 1 drop in right eye, four times daily" due to the potential for confusion with "OD"'s meaning. Question to clarify disagreements: Given the differing perspectives on the use of medical abbreviations in prescriptions, how do you weigh the importance of patient comprehension and adherence against the precision and conciseness of medical terminology? Specifically, do you believe the potential for confusion with abbreviations like "OD" outweighs their benefits in terms of space and time efficiency?. Based on your preceding response, how would you answer the original question: [A 32-year-old man comes to the office because of a 2-day history of redness of the right eye. He has not had sensitivity to light or the sensation of a foreign body in the eye. He has no history of serious illness and takes no medications. Examination of the right eye shows erythema and clear discharge. Examination of the left eye shows no abnormalities. The physician prescribes ketorolac ophthalmic solution. Which of the following is the most appropriate way for the physician to write the prescription? (A) Ketorolac 0.5% solution, 1 drop in right eye, four times daily (B) Ketorolac .5% solution, 1 drop in right eye QID (C) Ketorolac 0.5% solution, 1 drop OD four times daily (D) Ketorolac 0.5% solution, 1 drop OD, QID]

**AI_E:** To rephrase the question and clarify complex concepts, we are discussing how a doctor should most suitably write a prescription for a 32-year-old man with a right eye condition indicating inflammation, presumed to be conjunctivitis. He has no sensitivity to light or feeling of a foreign body in the eye His symptoms include redness and clear discharge. The doctor is using ketorolac ophthalmic solution as treatment. The options provided use a mix of medical jargon and plain language. My assumptions are that the effectiveness of the prescription relies on its clarity and correctness. There is a possibility that the use of abbreviations such as "OD" could lead to confusion, leading to medication errors. If we consider this alternate assumption, we need to prioritize patient safety and understanding. In the clinical thought process, it's crucial to weigh clarity of instructions against the use of medical terminology. While medical abbreviations save time and space for the prescriber, they could pose a risk if misunderstood by the patient or pharmacist. To connect the thought process and arrive at a conclusion, the priority should be given to clear instructions that can be understood by the patient and the pharmacist, thereby reducing the risk of any misinterpretation that can compromise patient safety. Therefore, I believe option (A) "Ketorolac 0.5% solution, 1 drop in right eye, four times daily" to be the most appropriate way for the physician to write the prescription. This option provides clarity on which eye to administer the medication, the frequency of administration, and avoids potential confusion with abbreviations like "OD" and "QID".

**AI_C:** RESTATING THE QUESTION: The question is asking for the most appropriate way for a physician to prescribe Ketorolac ophthalmic solution for a 32-year-old man with unilateral eye redness and clear discharge. This question hinges upon knowledge of pharmacology, specifically about medication prescription conventions and their implications on patient safety. PLANNING: There are two assumptions currently in play: (1) using "OD" instead of "right eye" ensures clarity and precision and (2) using "right eye" instead of "OD" prevents misinterpretation and confusion, thus ensuring patient safety. I propose evaluating the validity of these assumptions by considering how medication errors occur and which aspect should be favored: precision or comprehension. CLINICAL REASONING: The abbreviation "OD" stands for "oculus dexter", which is Latin for "right eye". But the abbreviated 'OD' could be mistakenly read as 'OS' (oculus sinister, left eye) or 'OU' (oculi uterque, both eyes). The Institute for Safe Medication Practices (ISMP) lists "OD", "OS", and "OU" as among the top terms to be avoided to preclude errors. On the other hand, using layman's terms such as "right eye", although less concise, maximizes the chances of the patient correctly administering the drug. CONCLUDING: Given that the primary goal of prescribing medication is therapeutic improvement while avoiding harm, and considering the ISMP’s recommendations, a prescription that optimizes patient understanding and reduces the risk of errors should be prioritized. RESPONSE: Therefore, the most appropriate way for the physician to write the prescription would be: "Ketorolac 0.5% solution, 1 drop in right eye, four times daily". This corresponds to Option (A) in the given choices.

**AI_A:** (1) RESTATING THE QUESTION: The patient's symptoms such as erythema and clear discharge in one eye suggest a condition that requires treatment with an ophthalmic solution called ketorolac. Ketorolac is a nonsteroidal anti-inflammatory drug (NSAID) that reduces inflammation and associated symptoms. The crux of the question lies in deciding how the physician should pen down the prescription, with distinct options given. (2) PLANNING: The main assumptions surround the use of abbreviations, namely "QID" and "OD". The abbreviation "QID" stands for the Latin term "quater in die" which means four times a day. "OD" stands for "oculus dexter", translating to the right eye. One alternate assumption involves adopting the non-abbreviated format to ensure clarity for the patient. (3) CLINICAL REASONING: On one hand, abbreviations are commonly used in prescriptions, potentially saving time. However, we have to consider whether the patient will understand these medical abbreviations, as misinterpretation can lead to incorrect dosage administration, particularly with "OD", which could be misunderstood as "once daily". Therefore, the balance between efficiency for the prescribing physician and clear understanding for the patient needs to be considered. (4) CONCLUDING: Drawing from the above reasoning, it makes clinical sense to choose an option that focuses on patient safety and understanding, which leads us to consider that writing prescriptions in full text may be the most appropriate option. (5) RESPONSE: Hence, the most appropriate way for the physician to write the prescription would be (A) "Ketorolac 0.5% solution, 1 drop in right eye, four times daily". This ensures that the patient knows where and how often to administer the medication, eliminating the chances of misinterpretation of the abbreviations.

**AI_D:** RESTATING THE QUESTION: The question asks for the most appropriate way for a physician to write a prescription for a 32-year-old man presenting with erythema and clear discharge in the right eye. The prescribed medication is ketorolac ophthalmic solution, an NSAID often used for postoperative eye inflammation or to alleviate seasonal allergic eye symptoms. It utilizes technical jargon akin to medical terminology. PLANNING: This scenario involves assumptions about proper prescription writing methods, the importance of clear communication in healthcare, health literacy levels among patients, and the role medical abbreviations play in both patient safety and efficient healthcare communication. Some may argue for the use of clear, simple language in the prescription while others may advocate for concise, traditional medical abbreviations. The primary clinical priority lies in assessing which approach improves patient comprehension, adherence to the medication regimen, and overall safety. CLINICAL REASONING: Traditionally, medical abbreviations have been used in prescription writing for efficiency. However, the usage has been tied to increased medication errors due to misunderstanding, especially since some abbreviations like "OD" can be interpreted differently (in ophthalmology it means right eye, in latin it means once daily). In this context, safety takes precedence over efficiency, making the usage of simple, clear language in prescription writing important. The Joint Commission, which accredits healthcare organization in the United States, advocates against the use of potentially confusing abbreviations to improve patient safety. CONCLUDING: Thus, based on the primary clinical priority of enhancing patient safety and given the possible confusion surrounding medical abbreviations, the prescription using clear, straightforward language would be the most appropriate. RESPONSE: Therefore, in answer to the question, the most appropriate way for the physician to write the prescription for the 32-year-old man would be: "Ketorolac 0.5% solution, 1 drop in right eye, four times daily" (Option A). This wording is clear, patient-friendly, and diminishes the potential for confusion and medication errors that could arise from the use of common medical abbreviations like "OD" and "QID".

**AI_B:** (1) RESTATING THE QUESTION: The dilemma here is about the best way for the physician to write a prescription. The patient, a 32-year-old man, is presenting with redness and discharge in his right eye, with an absence of features like photosensitivity and foreign body sensation. His medical history is unremarkable and the left eye appears normal upon examination. Ketorolac ophthalmic solution is prescribed and we're discussing how the prescription should be written with proper dosage, eye, and frequency details. (2) PLANNING: The prescription can be written in different ways such as "Ketorolac 0.5% solution, 1 drop in right eye, four times daily", "Ketorolac .5% solution, 1 drop in right eye QID", "Ketorolac 0.5% solution, 1 drop OD four times daily", or "Ketorolac 0.5% solution, 1 drop OD, QID". Each prescription style has its advantages and disadvantages, which mainly revolve around the usage of the term "OD". In medical parlance, "OD" stands for "oculus dexter", which refers to the right eye. (3) CLINICAL REASONING: Understanding that "OD" represents the right eye is key to interpreting prescriptions C and D. Both of these prescriptions accurately prescribe ketorolac for the affected eye, the right eye. However, the usage of "OD" could create confusion for patients or healthcare professionals, who might mistake it for "once daily". This could lead to medication errors, resulting in underdosing, and failure to alleviate the symptoms. For that reason, avoiding such abbreviations is preferred, according to Best Practice Guidelines of the Institute for Safe Medication Practices (ISMP). (4) CONCLUDING: When we consider possible misinterpretations, patient safety, and adherence, the prescription should avoid potentially misleading abbreviations. As such, the terms "right eye" and "four times daily" would be more appropriate, as they are less likely to be misunderstood compared with "OD" and "QID". (5) RESPONSE: Hence, based on guidelines to avoid medical abbreviations that could lead to misinterpretations, the most appropriate way for the physician to write the prescription would be: "Ketorolac 0.5% solution, 1 drop in right eye, four times daily" (option A).

**Facilitator** The consensus among the AI responses is that the most appropriate way for the physician to write the prescription for the 32-year-old man with erythema and clear discharge in his right eye is: "Ketorolac 0.5% solution, 1 drop in right eye, four times daily" (Option A). This conclusion is based on the reasoning that while medical abbreviations like "OD" (oculus dexter, right eye) and "QID" (quater in die, four times a day) can save time for the prescribing physician, they can also lead to confusion and potential medication errors if misunderstood by the patient or pharmacist. The Institute for Safe Medication Practices (ISMP) and the Joint Commission recommend avoiding such abbreviations to improve patient safety. Therefore, using clear, straightforward language in the prescription is considered the best practice to ensure patient understanding, adherence to the medication regimen, and overall safety.
