# Supplementary material for: Collaborative intelligence in AI: Evaluating the performance of a council of AIs on the USMLE
Source: PLOS Digit Health. 2025 Oct 9;4(10):e0000787. doi: 10.1371/journal.pdig.0000787 (PMC12510544; doi:10.1371/journal.pdig.0000787)
Supplement: S2 File — (ZIP) [file pdig.0000787.s002.zip › S2 Data Raw data files containing unprocessed question inputs and LLM outputs including transcripts of deliberations/STEP2_Question-112-1_Type-MC-NJ.docx]

**Facilitator** **[Round 1]:** A 14-year-old girl is brought to the physician by her mother for a well-child examination. Vital signs are within normal limits. On breast examination, the areolae are 3 cm in diameter and form a secondary mound bilaterally. Cardiopulmonary and abdominal examinations show no abnormalities. Pelvic examination shows dark, curly, coarse hair that spreads sparsely over the pubic area but not over the thighs. Which of the following is the most accurate assessment of this patient's development? (A) Pubic Hair Development: Delayed; Breast Development: delayed (B) Pubic Hair Development: Delayed; Breast Development: normal (C) Pubic Hair Development: Normal; Breast Development: delayed (D) Pubic Hair Development: Normal; Breast Development: normal

**AI_B:** RESTATING THE QUESTION: The question is asking us to assess the physical sexual development of a 14-year-old girl, utilizing her breast and public hair development as markers. This is in reference to the stages of puberty, more specifically, the Tanner Staging system, which classifies the onset and progression of puberty based on primary and secondary sexual characteristics. PLANNING: The Tanner Staging system gauges breast and pubic hair development separately. For breast development, the formation of a secondary mound (due to the presence of breast bud) signifies Tanner Stage 4. In relation to pubic hair, the characteristic dark, curly, coarse hair which spreads sparsely over the pubic area indicates Tanner Stage 3. As the patient is 14 years old and considering the average onset of puberty in girls (which typically begins between ages 9-11), there doesn't seem to be any obvious delay or abnormality in her development. CLINICAL REASONING: Breast development proceeds in distinct stages, often initiating with the formation of a palpable breast bud and eventual enlargement of the areola and elevation of the breasts as seen in this patient, hence classifying her as Tanner Stage 4. Pubic hair also goes through several stages of development during puberty. The description of the patient's pubic hair being dark, curly, coarse, and spread sparsely over the pubic area, but not over the thighs, is considered typical growth, classifying her in Tanner Stage 3. Given these observations and the patient's age, there appears to be no delay in her development. CONCLUDING: As the physical examination has shown that the girl’s breast and pubic hair development align with her age, I would classify both as normal for her age, considering the Tanner Staging system. RESPONSE: Hence, the most accurate assessment of this patient's development would be (D) Pubic Hair Development: Normal; Breast Development: normal.

**AI_E:** (1) RESTATING THE QUESTION: The question is asking to assess the developmental stage of a 14-year-old girl based on clinical observations made during a well-child examination. In specific, it focuses on two aspects of development - breast development and pubic hair development. Breast development is evaluated based on the size of the areolae and how they protrude from the chest (secondary mound). Pubic hair development is evaluated based on the distribution and characteristics of hair in the pubic region. (2) PLANNING: The assumptions we have here are that the observed characteristics of breast and pubic hair development align with the typical chronological age of sexual maturation in girls. Key insights from these observations show that the areolae are 3 cm in diameter, forming a secondary mound bilaterally, and that pubic hair is dark, curly, coarse, and sparse in the pubic area. Each of these criteria could signify a specific stage in Tanner's stages of sexual maturation. (3) CLINICAL REASONING: According to Tanner's stages, a secondary mound in breast development represents a transition into Stage 4, which typically occurs between 11-14 years in girls. Hence, the breast development appears normal for this girl's age. The described pubic hair (dark, curly, coarse, and sparsely spread over the pubic area, but not the thighs), suggests Tanner Stage 3, which represents the appearance of pubic hair usually between 10-13.5 years in females. So, the pubic hair development, while slightly behind the breast development, appears to be within the normal range for her age. (4) CONCLUDING: Therefore, by relating the patient's clinical findings with the Tanner staging of sexual maturation, I conclude that her breast development is in line with her chronological age, while her pubic hair development is slightly delayed but still within normal limits for a girl her age. (5) RESPONSE: Hence, for this patient, the most accurate assessment of her development would be option (B): 'Pubic Hair Development: Delayed; Breast Development: Normal'.

**AI_D:** RESTATING THE QUESTION: The question is asking us to assess the puberty development of a 14-year-old girl, by considering her secondary sexual characteristics, namely pubic hair and breast development. Let's focus on the pubertal signs described: 3 cm diameter areolae forming a secondary mound bilaterally (breast development), and sparse but dark, curly, coarse hair over the pubic area not extending to the thighs (pubic hair development). PLANNING: Understanding the stages of pubertal development is critical here. For girls, these stages, as per Tanner Staging, range from stage 1 (pre-pubertal) to stage 5 (mature adult). Briefly, breast stage 2 is marked by breast budding and elevation of breast and papilla, and stage 3 is characterized by further enlargement without yet separate contour; whereas stage 2 pubic hair appears light, thin, and sparse, which thickens and darkens to stage 3. Therefore, we need to determine which stage of Breast and Pubic Hair development the girl is in? CLINICAL REASONING: The given information reflects that the areolae are 3 cm in diameter and form a secondary mound, which suggests the patient is in Tanner Stage 3 for breast development, considered normal for age. As for the pubic hair, it is dark, curly, and sparse over the pubic area but doesn't extend to thighs, an indication of Tanner Stage 2-3; also normal for her age. CONCLUDING: The clinical signs suggest the patient has expected pubertal development for her age. Her breast development is consistent with Tanner Stage 3, and her pubic hair development also appears to align with Tanner Stage 2 or 3, both of which are normal for a 14-year-old girl. RESPONSE: Thus, the most accurate assessment of this patient's development would be (D) Pubic Hair Development: Normal; Breast Development: normal.

**AI_A:** RESTATING THE QUESTION: This question is asking about a 14-year-old girl's pubertal development specifically her pubic hair and breast growth stages, which are documented through a health examination. The pubic hair assessment relates to the development and distribution of pubic hair, while the breast assessment relates to the size and shape of the breasts and areolae. The scenarios presented represent varied stages of puberty - delayed, normal or advanced. PLANNING: Let's start with some assumptions. First, let’s assume that the age of onset for puberty in girls typically ranges from 8 to 13 years, with the average being around 10.5 years. Therefore, a 14-year-old girl should ordinarily be showing signs of pubertal development. Second, let's suppose that pubic hair development and breast development follow certain stages (Tanner Stages). Let’s evaluate each stage: the presence of a sparse, lightly pigmented covering of hair, mainly along the labia in girls (Tanner Stage II) can be considered normal pubic hair development for a girl at the age of 14. Also, according to Tanner staging for breast development, having areolae that are up to 3 cm in diameter and forming a secondary mound bilaterally is considered Stage III - a normal for her age. Thus, this leads to the hypothesis that her development is on track. CLINICAL REASONING: In a 14-year-old, the expected Tanner stage for both pubic hair and breast development is stage III or IV. Given that the Tanner staging of puberty is based on physical development and is independent of age, normal development for a girl of this age could range from stages II to IV. In this case, the described sparse, curly, dark pubic hair would place her in Tanner stage II for pubic hair development, which is within the normal range for her age. Similarly, the examination of her breast indicates the bilateral presence of secondary mound on the areolae suggesting Tanner stage III for breast development, also appropriate for her age. CONCLUDING: Looking at the findings from the examination, pubic and breast development of this 14-year old girl is consistent with the Tanner stages expected for her age-group. Therefore, neither her breast nor pubic hair development can be considered delayed. RESPONSE: Hence, the most accurate assessment of this patient's development is (D) Pubic Hair Development: Normal; Breast Development: Normal.

**AI_C:** RESTATING THE QUESTION: The medical professional is performing a well-child examination on a 14-year-old girl. Two aspects of physical development are measured and assessed: pubic hair development and breast development, both critical indicators of the onset of puberty in girls. Breast examination demonstrates areolas with a diameter of 3 cm, forming a secondary mound bilaterally. Pubic examination reveals sparse, dark, coarse hair in the pubic region, but no extension to the thighs. The question aims to determine an accurate assessment of the patient’s development in terms of pubic hair and breast development. PLANNING: Factors to consider in this situation are the age of the girl and the normal physical changes that females of the same age generally experience. Physical changes in a girl during puberty include the growth of pubic hair and breast development, often staged based on Tanner stages. In general, initial breast development usually begins between ages 8-13, and pubic hair usually begins to appear between 9-14 years of age. Assumptions to test include: (A) both pubic hair and breast development are delayed; (B) pubic hair development is delayed, but breast development is normal; (C) pubic hair development is normal, but breast development is delayed; or (D) both pubic hair and breast development are normal. We must scrutinize these assumptions by comparing the presented physical findings with the timelines of normal pubertal development. CLINICAL REASONING: The Tanner stages are often used for assessing development during puberty. For females, Tanner stage 3 for breasts is characterized by the enlargement of breasts and areolas with the formation of a separate mound above the level of the breasts, and for pubic hair, stage 2 is characterized by the appearance of sparse, dark, and curly hair. Given the girl is 14 years old and Tanner stage 3 for breasts typically occurs around 11.5 to 13 years and stage 2 for pubic hair normally occurs around 11 to 12 years, her development is adequately on track for her age group. CONCLUDING: Based on the findings and the Tanner stages, it can be concluded that the patient's development is within the normal range. The breast development is consistent with Tanner stage 3, and pubic hair development is consistent with Tanner stage 2, both commensurate with her age. RESPONSE: Therefore, the most accurate assessment of this patient's development is: (D) Pubic Hair Development: Normal; Breast Development: normal.

**Facilitator** **[Round 2]:** Summary: AI_A, AI_B, AI_C, and AI_D all agree that the 14-year-old girl's pubic hair and breast development are normal for her age, based on the Tanner Staging system. They all identify the girl's breast development as Tanner Stage 3 and her pubic hair development as Tanner Stage 2 or 3. AI_E, however, believes that while the girl's breast development is normal (Tanner Stage 4), her pubic hair development is slightly delayed but still within normal limits (Tanner Stage 3). Key Differences: The key difference lies in the interpretation of the Tanner Staging system. AI_A, AI_B, AI_C, and AI_D interpret the girl's breast development as Tanner Stage 3, while AI_E interprets it as Tanner Stage 4. Similarly, AI_A, AI_B, AI_C, and AI_D interpret the girl's pubic hair development as Tanner Stage 2 or 3, while AI_E interprets it as Tanner Stage 3 but considers it slightly delayed. Question to Council Members: Could you please clarify your interpretation of the Tanner Staging system in relation to the girl's breast and pubic hair development? Specifically, could you explain why you believe her development is normal or slightly delayed?. Based on your preceding response, how would you answer the original question: [A 14-year-old girl is brought to the physician by her mother for a well-child examination. Vital signs are within normal limits. On breast examination, the areolae are 3 cm in diameter and form a secondary mound bilaterally. Cardiopulmonary and abdominal examinations show no abnormalities. Pelvic examination shows dark, curly, coarse hair that spreads sparsely over the pubic area but not over the thighs. Which of the following is the most accurate assessment of this patient's development? (A) Pubic Hair Development: Delayed; Breast Development: delayed (B) Pubic Hair Development: Delayed; Breast Development: normal (C) Pubic Hair Development: Normal; Breast Development: delayed (D) Pubic Hair Development: Normal; Breast Development: normal]

**AI_D:** Restating the Question: We are asked to evaluate the physical development in a 14-year-old girl using the Tanner Staging system for pubertal development. Notably, her breast areolae are described as "3 cm in diameter forming a secondary mound bilaterally", and her pubic hair examination reveals "dark, curly, coarse hair spread sparsely over the pubic area but without involvement of the thighs." Planning: Key assumptions here rely on our knowledge of the Tanner Staging system, which categorizes pubertal development in stages from 1 (prepubertal) to 5 (postpubertal). Specifically, the secondary mound formation of her breasts assumes a Tanner Stage of 4. The description of her pubic hair assumes a Tanner Stage from 2 to 3, depending on the extent and coarseness of the hair. The age of the girl may also factor in determining if her development is normal or delayed. Clinical Reasoning: Connecting these assumptions, we can evaluate the girl's development based on these Tanner Stages. Usually, Tanner Stage 4 for breast development (secondary mound formation) is expected to be reached at around age 14, suggesting her breast development is normal. The Tanner Stage for pubic hair development progresses along a similar timeline, but since there is a range of normal, even if she is in Tanner stage 2, it can still be considered normal for a 14-year-old. Concluding: Based on these clinical considerations, the pubic hair and breast development appear to be within the expected range for a 14-year-old girl according to the Tanner Staging system. Response: Therefore, the answer would be (D) Pubic Hair Development: Normal; Breast Development: Normal.

**AI_C:** Sure, I'd be happy to provide some further context. Her development is being assessed by the Tanner scale, which tracks sexual maturation in adolescents. The presentation provided offers key indicators on the stages of the Tanner scale and it's our job to accurately fit her symptoms into the distinct categories of breast and pubic hair development. Assumptions for the Tanner stage can be made based on the given descriptive elements: the size of the areolae and the description of pubic hair. However, those assumptions could differ based on individual interpretation. For example, I would assume that her breast development falls into Tanner Stage 3 based on the described size of her areolae forming a secondary mound. But, there are others who may interpret those same symptoms as representative of stage 4. Concerning pubic hair development, if I consider the description - dark, curly, coarse hair that spreads sparsely - I would think she is in Tanner Stage 3. But, some may interpret sparse hair as Stage 2. These assumptions need to align with the typical development of an average 14-year-old girl. Physiologically, the process of puberty is under hormonal control, particularly by gonadotropin-releasing hormone (GnRH), which stimulates the secretion of luteinizing hormone (LH) and follicle-stimulating hormone (FSH) from the anterior pituitary gland. These hormones stimulate primary and secondary sexual characteristics. If our assumptions about the Tanner stages are correct, we'd expect the hormonal levels to be consistent with a girl progressing normally through puberty. My response based on restating, planning, clinical reasoning, concluding thought process is that the girl's breast and pubic hair development typically corresponds to Tanner Stage 3, which is standard development for a 14-year-old girl. So, based on what we know, I would select option: (D) Pubic Hair Development: Normal; Breast Development: normal to best answer the initial query about the patient's development.

**AI_A:** (1) RESTATING THE QUESTION: The question pertains to assessing the pubertal development of a 14-year-old girl by examining her breast and pubic hair development. Certain physical characteristics, such as the diameter of the areolae and the quality and distribution of pubic hair, are used to gauge these developmental stages, typically using the Tanner Staging system. (2) PLANNING: To answer this question, we must contemplate the normal ranges of the Tanner Stages for a girl her age. We should particularly consider whether her presented characteristics align with these expected stages of development. We know the breast development is at Tanner stage 3 due to the presence of a secondary mound, and the pubic hair development is around Tanner stage 2 or 3 as indicated by the presence of dark, curly, sparse pubic hair but absence over thighs. The assumption here is whether these stages align with normal development for her age. (3) CLINICAL REASONING: At the age of 14, girls are typically in mid-puberty, which correlates with Tanner stage 3 or 4. Pertaining to this patient, secondary mounds observed for her breasts indicate Tanner Stage 3 for breast development, which aligns with her age. Her pubic hair development, though sparse, is dark and curly, consistent with Tanner Stage 2 or 3. Lack of hair over thighs suggests that she isn't at Stage 4 or 5 yet. Thus, this also corresponds to the typical changes expected at her age. (4) CONCLUDING: Given these observations and their correlation with the Tanner Stages, we can conclude that her pubertal development falls within the expected range for her age. Therefore, neither her breast nor her pubic hair development is delayed. (5) RESPONSE: So, I would answer the question accordingly as (D) Pubic Hair Development: Normal; Breast Development: normal.

**AI_B:** RESTATING THE QUESTION: This question seeks to determine the appropriateness of a 14-year-old girl's development, specifically, her pubic hair and breast development. Her areolae are 3 cm in diameter and form a secondary mound bilaterally, a feature of breast development. Her pubic hair is dark, curly, and coarse but is limited to her pubic area and does not extend to her thighs. An understanding of the Tanner Staging system is essential for answering this question, as it is a standardized way of assessing the stages of puberty and sexual development. PLANNING: Firstly, the assumption that pubic hair and breast development are in tandem could be challenged as these may occur at differing speeds in girls during puberty. Secondly, the assumption that the physical presentation described aligns clearly with one single Tanner stage could be questioned too, as the physical attributes described may not precisely suit a singular Tanner stage. The clinically prioritized assumption to pursue is the first one, given that it directly relates to the options provided for the assessment of the girl's development. CLINICAL REASONING: Within the Tanner Staging system, pubic hair and breast development each follow five stages, from pre-adolescence (stage 1) to full maturity (stage 5). In this case, the young girl's areolae, which are 3 cm in diameter and form a secondary mound bilaterally, are characteristics associated with Tanner Stage 3. Her pubic hair development, described as dark, curly, coarse hair that spreads sparsely over the pubic area, also fits with Tanner Stage 2 or early Stage 3, as these stages involve the increasing pigmentation and curliness of pubic hair and its initial spread to the pubic areas, respectively. CONCLUDING: Therefore, considering the descriptions provided and interpreted through the Tanner Staging system, the girl's pubic hair and breast developments both fall within normal parameters for a 14-year-old. Thus, they are not delayed. RESPONSE: Consequently, the most accurate assessment of this patient's development is (D) Pubic Hair Development: Normal; Breast Development: normal.

**AI_E:** RESTATING THE QUESTION: Looking at this scenario, we are asked to assess the developmental stages of a 14-year-old girl, specifically focusing on the development of her breasts and pubic hair. The Tanner Staging system, a commonly accepted guide for assessing and documenting the physiological changes during pubertal development, classifies this based on the appearance and extent of pubic hair and breast development. PLANNING: The assumption here is that the stages of both breast and pubic hair development, according to Tanner Staging system, should align with the biological age of the girl (14 years old) to be considered "normal." Key details provided are the 3 cm areolae forming a secondary mound bilaterally (indicative of breast development stage), and the dark, curly, coarse hair sparsely spread over the pubic area but not over the thighs (hence defining pubic hair development stage). An alternate assumption might be that, while the girl's breast and pubic hair development are progressing, they may not align with what is normally expected at her biological age. CLINICAL REASONING: The girl exhibits secondary mound formation of the breasts (Tanner Stage 4) which typically develops around the age of 13–15. This suggests she is within the normal range for her age. In terms of pubic hair development, she exhibits sparse, dark, and curly hair over the pubic area but not on the thighs (Tanner Stage 2 or possibly 3). Tanner Stage 2 usually begins around the age of 11, and stage 3 typically starts around the age of 12. Therefore, she might be slightly behind the average for pubic hair development, but still within a reasonably normal range, considering the wide biological variation that can occur during puberty. CONCLUDING: After reviewing this, the most accurate assessment of her development seems to be that her breast development is correctly aligned with her biological age and is therefore normal. However, her pubic hair development, while progressing, may be slightly delayed compared to the average 14-year-old girl but still within normal limits. RESPONSE: It could be wise to submit (D) Pubic Hair Development: Normal; Breast Development: normal as the answer because, after taking into account the wide biological variation during puberty, both her breast and pubic hair development could still be considered within the normal range for her age. We could also consider that her pubic hair development might be slightly delayed, but still normal.

**Facilitator** The consensus statement synthesizing the responses of AI_A, AI_B, AI_C, AI_D, and AI_E is as follows: The question at hand involves assessing the pubertal development of a 14-year-old girl, specifically her breast and pubic hair development, using the Tanner Staging system. The girl's physical characteristics, such as the diameter of the areolae and the quality and distribution of pubic hair, are key indicators of her developmental stages. The girl's breast development is at Tanner stage 3 or 4 due to the presence of a secondary mound, which aligns with the expected development for her age. Her pubic hair development is around Tanner stage 2 or 3, as indicated by the presence of dark, curly, sparse pubic hair but absence over thighs. This also corresponds to the typical changes expected at her age. It's important to note that pubic hair and breast development may occur at differing speeds in girls during puberty, and the physical attributes described may not precisely suit a singular Tanner stage. However, considering the descriptions provided and interpreted through the Tanner Staging system, the girl's pubic hair and breast developments both fall within normal parameters for a 14-year-old. Therefore, the consensus is that her pubertal development falls within the expected range for her age. Neither her breast nor her pubic hair development is delayed. The most accurate assessment of this patient's development is (D) Pubic Hair Development: Normal; Breast Development: normal.
