# Supplementary material for: Collaborative intelligence in AI: Evaluating the performance of a council of AIs on the USMLE
Source: PLOS Digit Health. 2025 Oct 9;4(10):e0000787. doi: 10.1371/journal.pdig.0000787 (PMC12510544; doi:10.1371/journal.pdig.0000787)
Supplement: S2 File — (ZIP) [file pdig.0000787.s002.zip › S2 Data Raw data files containing unprocessed question inputs and LLM outputs including transcripts of deliberations/STEP2_Question-113-1_Type-MC-NJ.docx]

**Facilitator** **[Round 1]:** A healthy 19-year-old woman comes to the clinic requesting advice regarding contraception. Menses occur at regular 28-day intervals with heavy flow. She is sexually active and has had three lifetime male partners. She has smoked one-half pack of cigarettes daily for 2 years. She lives in a college dormitory and says that she often does not sleep in her own room and has trouble remembering to take medications regularly. Her only medication is a daily multivitamin. She is 163 cm (5 ft 4 in) tall and weighs 58 kg (128 lb); BMI is 22 kg/m². Vital signs are within normal limits. Physical examination shows no abnormalities. Which of the following is the most appropriate recommendation regarding contraception for this patient? (A) Condoms with spermicidal foam (B) Diaphragm with spermicidal foam (C) Oral contraceptive therapy (D) Subdermal contraceptive implant (E) Vaginal contraceptive ring

**AI_D:** RESTATING THE QUESTION: The question is asking for the best form of contraception for a sexually active 19-year-old female student who smokes, has a regular cycle with heavy flow, and has trouble remembering to take medications. The options are condoms with spermicidal foam, a diaphragm with spermicidal foam, oral contraceptive therapy, a subdermal contraceptive implant, and a vaginal contraceptive ring. PLANNING: The question's assumptions are that all methods of contraception listed are appropriate for this patient. However, we need to consider her lifestyle, tobacco use, and trouble with medication adherence which may affect the effectiveness and safety of certain contraceptive methods. Oral contraceptive therapy requires daily adherence which might be problematic due to her reported trouble with regular medication intake. Smoking can increase the risk of cardiovascular events with hormonal contraceptives. Therefore, the subdermal contraceptive implant may be the best option to avoid reliance on daily adherence and minimize the cardiovascular risk associated with smoking and hormonal contraceptives. CLINICAL REASONING: This patient's lifestyle and forgetfulness may lead to skipped doses if prescribed oral contraceptives, hence making it less effective. Furthermore, the combination of smoking and hormonal contraception (like oral contraceptive pills and vaginal contraceptive ring) increases the risk of thrombotic events, hence making them less suitable for this patient. The use of barrier methods such as condoms or diaphragms might not suit her lifestyle and has a higher failure rate than hormonal methods. In contrast, a subdermal contraceptive implant would provide continuous protection without the need for daily adherence or depend on correct and consistent use as barrier methods do. CONCLUDING: Given her lifestyle, smoking habit, irregular daily routine, and forgetfulness with medication, the subdermal implant seems to be the best option as it provides reliable contraception without requiring daily adherence and minimizes the risk associated with smoking and hormonal contraceptives. RESPONSE: Thus, the most suitable contraceptive method for this patient is the subdermal contraceptive implant (D).

**AI_E:** RESTATING THE QUESTION: A young woman, who is sexually active with a history of multiple partners, is seeking advice on contraception. She mentioned irregular sleeping habits and difficulty in remembering to take medication regularly. She also smokes half a pack of cigarettes daily. Her physicals are normal and she currently takes a daily multivitamin. She is asking what the best contraception option is for her in terms of condoms with spermicidal foam, diaphragm with spermicidal foam, oral contraceptive therapy, subdermal contraceptive implant, or vaginal contraceptive ring. PLANNING: We know that our patient struggles with regular medication compliance and has an active social life that may hinder the effectiveness of certain methods of contraception. These are key considerations we need to factor in. An assumption here is that regular methods like oral contraceptives, though popular, may not be the best for her given her irregular routine and multiple partners. CLINICAL REASONING: Given the patient’s lifestyle and choices, we need to assess the methods that will provide her with a suitable level of contraceptive protection while not being overly dependent on her remembering to take them or apply them correctly each time. Oral contraceptives, although commonly prescribed, require daily use and her habit of forgetting to take her medication regularly points towards this not being a viable choice for her. Barrier methods such as condoms and diaphragms can be great for contraception but do require the woman to have them on hand and correctly use them each time she has intercourse. The vaginal contraceptive ring requires monthly placement, so it's not a suitable choice either. CONCLUDING: Based on her lifestyle, smoking habits, and forgetfulness in taking daily medication, the subdermal contraceptive implant seems to be the most suitable option. This is a progestin-only contraceptive that is placed under the skin of the upper arm and works for up to 3 years. Additionally, it doesn't require daily or per-coital administration, which fits our patient's needs. RESPONSE: (D) Subdermal contraceptive implant could be the most appropriate recommendation for this patient given their lifestyle and habits. It offers long-term contraception without the need for consistent daily adherence or activity-based planning. A healthcare professional should discuss this option thoroughly with the patient as well as the potential risks and side-effects associated with it.

**AI_C:** RESTATING THE QUESTION: A young woman, who leads a sexually active lifestyle and sometimes struggles with remembering to take her medications consistently, is seeking advice on contraception methods. She is a regular cigarette smoker and has a normal physical examination and vital signs. There are a few contraception options laid out: condoms with spermicidal foam, a diaphragm with spermicidal foam, oral contraceptive therapy, a subdermal contraceptive implant, or a vaginal contraceptive ring. PLANNING: The main factors to consider here are the ideal contraception method to suit the lifestyle of the woman, her smoking habit, her heavy menstrual flow, and her irregular sleeping arrangements. Ideally, her method of contraception should be highly effective, convenient, and safe for a smoker. CLINICAL REASONING: Daily oral contraceptives may not be suitable in this case because the patient has reported having trouble remembering to take medications regularly. Condoms or diaphragms with spermicidal foam can be effective but require effort to use correctly every time before sex, which can be inconvenient. Additionally, spermicides can increase risk of urinary tract infections in women. The subdermal implant (a small rod implanted under the skin that releases hormones to prevent pregnancy) and the vaginal ring are both long-acting reversible contraceptives. Among these, the subdermal implant is not user-dependent and its efficacy is not influenced by the user’s behaviors, such as forgetting to replace the ring on time, and it is safer for smokers under 35. CONCLUDING: Considering her lifestyle, forgetfulness with medication, and smoking habit, a subdermal contraceptive implant would be the most practical and effective form of contraception for the patient. This implant is a small device that is inserted under the skin and prevents pregnancy by releasing hormones. It does not require daily attention, making it ideal for someone with her lifestyle. RESPONSE: The most suitable contraceptive method for this patient is (D) a subdermal contraceptive implant.

**AI_A:** (1) RESTATING THE QUESTION: A 19-year-old woman who is consistently sexually active and has had multiple partners has asked for advice on contraception. She has regular heavy periods every 28 days. However, she has a habit of smoking half a pack of cigarettes daily and has said she has difficulty remembering to take medications consistently. She has declared living in a college dormitory and has an irregular sleep pattern. Her health appears to be normal, with regular vital signs, a normal physical examination, and a healthy body mass index (BMI). The question posits which contraceptive method would be best suited for her. (2) PLANNING: Important factors in the patient's case that should guide our recommendation are her sexual activity, multiple partners, heavy menstruation, smoking habit, irregular medication intake, and her living situation. Here, I’m considering her forgetting to take medications regularly, her smoking habit, and her multiple partners. The options given are all types of contraception — condoms with spermicidal foam, diaphragm with spermicidal foam, oral contraceptive therapy, subdermal contraceptive implant, and a vaginal contraceptive ring. The analysis assumes that each has its own advantages and disadvantages considering the patient's characteristics. (3) CLINICAL REASONING: Condoms could provide some protection against sexually transmitted infections but might not be reliable for someone who has multiple partners and irregular usage can lead to contraception failure. Given her irregular sleep habits and trouble with medication adherence, it would be unsafe to assume she could consistently use a diaphragm or remember to take oral contraceptives. Considering her smoking habit, oral contraceptive pills are not recommended due to increased risk of thromboembolism. Also, the vaginal contraceptive ring requires regular replacement that the patient might struggle with due to her forgetfullness. (4) CONCLUDING: The best approach in this case, considering the regular sexual activity, multiple partners, smoking habits, and forgetfulness with medication, appears to be the subdermal contraceptive implant. Unlike condoms, diaphragms, or vaginal rings it does not require regular use maintenance, and unlike oral therapy, it is not associated with increased thromboembolism risk with smoking. (5) RESPONSE: The most appropriate recommendation regarding contraception for this patient would be a subdermal contraceptive implant (D).

**AI_B:** RESTATING THE QUESTION: A young, sexually active woman who regularly smokes cigarettes is seeking advice on contraception. She lives in a dormitory, often doesn't sleep in her own room, and has difficulties remembering to take medication regularly. She has a normal BMI, vital signs, and no abnormalities post-physical examination. The query is about which contraception method is most appropriate for her condition from the following options: condoms with spermicidal foam, diaphragm with spermicidal foam, oral contraceptive therapy, subdermal contraceptive implant, or a vaginal contraceptive ring. PLANNING: Key assumptions in this question include: her ability to remember to use contraception consistently, the health risks associated with her smoking, her lifestyle, and the effectiveness and convenience of different contraceptive methods. However, we need to question these assumptions, particularly her ability to use contraception effectively and consistently and the risks associated with smoking. Because smoking is a contraindication for some contraception methods, such as combined oral contraceptives due to an increased risk of thromboembolic events, this assumption should be clinically prioritized. CLINICAL REASONING: When choosing a contraceptive method, factors such as lifestyle, comfort, individual preferences, and medical history should be taken into account. In this scenario, we must remember that the patient has difficulty remembering to take medicines regularly, therefore, a method that requires daily or even weekly application (like oral contraceptives or a vaginal ring) may not be appropriate. Furthermore, due to her smoking habits, combined hormonal contraception like oral contraceptives and the vaginal ring are less recommended as they have the potential to increase the risk of cardiovascular events. CONCLUDING: With precluding the use of hormonal oral contraceptives and vaginal contraceptive rings due to the patient's smoking history as well as her noted difficulty with remembering to take daily medication, a long-acting, low-maintenance contraceptive is most appropriate. Also considering the patient's lifestyle that involves multiple sexual partners and living in a communal environment, we need to be cognizant of sexually transmitted infections. Thus, a contraceptive method that offers continued protection without daily interference would seem to be the most fitting choice. RESPONSE: Given the circumstances, the appropriate recommendation for this 19-year-old woman is option (D), a Subdermal contraceptive implant. It is a long-acting reversible contraceptive method that reduces the risk of user error and offers reliable contraception for several years without the need for the patient to remember to take or apply it regularly. However, it is important to remember that it does not guard against sexually transmitted diseases. Therefore, the use of condoms during sexual intercourse would still be necessary in order to reduce her risk of contracting an STI.

**Facilitator** The consensus among the AI responses is that the most appropriate recommendation for contraception for this 19-year-old woman is a subdermal contraceptive implant (D). This conclusion is based on several factors. Firstly, the woman's lifestyle, which includes regular sexual activity with multiple partners, living in a college dormitory, and irregular sleep patterns, suggests that a long-acting, low-maintenance contraceptive method would be most suitable. Secondly, her habit of smoking half a pack of cigarettes daily makes certain hormonal contraceptives, such as oral contraceptive therapy and the vaginal contraceptive ring, less recommended due to an increased risk of thromboembolic events. Thirdly, her difficulty in remembering to take medications regularly makes daily or weekly contraceptive methods less reliable. Therefore, the subdermal contraceptive implant, which provides continuous protection without the need for daily adherence and minimizes the risk associated with smoking and hormonal contraceptives, is the most suitable option. However, it is important to note that while the subdermal implant is effective for contraception, it does not protect against sexually transmitted infections, so the use of condoms during sexual intercourse would still be necessary to reduce her risk of contracting an STI.
