# Supplementary material for: Collaborative intelligence in AI: Evaluating the performance of a council of AIs on the USMLE
Source: PLOS Digit Health. 2025 Oct 9;4(10):e0000787. doi: 10.1371/journal.pdig.0000787 (PMC12510544; doi:10.1371/journal.pdig.0000787)
Supplement: S2 File — (ZIP) [file pdig.0000787.s002.zip › S2 Data Raw data files containing unprocessed question inputs and LLM outputs including transcripts of deliberations/STEP2_Question-115-1_Type-MC-NJ.docx]

**Facilitator** **[Round 1]:** A previously healthy 17-year-old girl comes to the emergency department because of a 3-day history of moderate right lower quadrant pain, fever, and loss of appetite. She has not had abnormal vaginal discharge. Four months ago, she was treated with oral antibiotics for cystitis. She currently takes no medications. Menses occur at regular 28-day intervals. Her last menstrual period was 10 days ago. She has been sexually active with two male partners during the past 6 months and uses condoms inconsistently. Temperature is 38.5°C (101.3°F), pulse is 105/min, respirations are 14/min, and blood pressure is 127/82 mm Hg. Abdominal examination shows tenderness to palpation of the lower quadrants with rebound in the right lower quadrant; there is no distention. Bowel sounds are decreased. Pelvic examination shows blood-tinged discharge from the cervical os and cervical motion tenderness. There is a 5-cm mass in the right adnexal region. Which of the following is the most likely diagnosis? (A) Appendicitis (B) Ectopic pregnancy (C) Ovarian torsion (D) Pyelonephritis (E) Tubo-ovarian abscess

**AI_E:** RESTATING THE QUESTION: A 17-year-old female with all the important hallmarks of health before is now undergoing abdominal troubles. Symptoms include moderate right lower quadrant pain, fever, and loss of appetite. She displays symptoms indicative of a pelvic inflammatory disease, confirmed by the presence of a notable mass in the right adnexal region. We're tasked with identifying the probable medical condition based on this clinical presentation. PLANNING: Given the symptoms, we could consider a few gynecological conditions. Appendicitis is an inflammation of the appendix, often marked by severe abdominal pain. An ectopic pregnancy involves a fertilized egg implanting outside the uterus, often causing lower abdominal pain and complications. Ovarian torsion is another possibility where the ovary twists around its supporting tissues. Pyelonephritis is a serious urinary tract infection that starts in the urethra or bladder and travels up to one or both of the kidneys. Lastly, we have a tubo-ovarian abscess, which can occur as a complication of pelvic inflammatory disease, causing lower abdominal pain and a pelvic mass. CLINICAL REASONING: The patient's symptoms don't lean heavily towards appendicitis or Pyelonephritis since these conditions don't conventionally present with a pelvic mass. An ectopic pregnancy could be a possible diagnosis but the patient's regular menstrual cycle and a time-stamped menstrual period contradict its likelihood. Ovarian torsion typically presents with sudden severe pain and is less likely with a presentation of right lower quadrant pain for the past 3 days. Considering all the symptoms and her sexual history, the presence of a 5-cm mass in the adnexal region falls in-line with the presentation of a tubo-ovarian abscess—a complication of pelvic inflammatory disease, usually resulting from sexually transmitted bacteria. CONCLUDING: In light of her symptoms including lower quadrant pain, fever, decreased appetite, blood-tinged discharge from the cervical os, cervical motion tenderness, as well as a palpable adnexal mass, and the patient's sexual history, the most likely diagnosis here would be a tubo-ovarian abscess. RESPONSE: The most likely diagnosis is (E) Tubo-ovarian abscess.

**AI_D:** (1) RESTATING THE QUESTION: The question involves a 17-year-old girl who appears to be in overall good health. She has been experiencing certain symptoms – pain in her right lower abdomen, fever, and a lack of appetite – for the past three days. She has a history of cystitis, for which she took antibiotics. She is regularly menstruating, had her last menstrual period ten days ago, and has been sexually active, albeit with inconsistent use of condoms. She has a somewhat elevated heart rate and body heat, and exhibits tenderness when the lower parts of her abdomen are palpated, especially the right lower quandrant. Upon further examination, there is evidence of a blood-tinged discharge from the cervical os, cervical motion tenderness, and a 5 cm mass in the right adnexal region. We have to identify a condition that corresponds with the combination of these manifestations. (2) PLANNING: I am considering five potential diagnoses: appendicitis, ectopic pregnancy, ovarian torsion, pyelonephritis, and tubo-ovarian abscess. This situation requires focusing on symptoms and signs that differentiate between these conditions. (3) CLINICAL REASONING: Appendicitis typically presents with right lower quadrant pain, but the evidence of a mass in the adnexal region and cervical motion tenderness is not common. An ectopic pregnancy is plausible given her sexual history and irregular condom usage, but her recent menstrual period and lack of abnormal vaginal discharge make this less likely. Ovarian torsion could present with an adnexal mass and right lower quadrant pain, but it doesn’t typically present with fever. Pyelonephritis, a urinary tract infection, could cause fever but doesn't usually results in an adnexal mass or cervical motion tenderness. Thus, these conditions are less likely. (4) CONCLUDING: Based on the symptoms, the most plausible diagnosis is that of a tubo-ovarian abscess. The right quadrant pain, fever, and adnexal mass align with the characteristics of this condition. Moreover, the prior case of cystitis (urinary tract infection) could have ascended to the reproductive tract, and the inconsistent usage of condoms raises the risk of sexually transmitted infections, which can cause pelvic inflammatory disease ultimately leading to tubo-ovarian abscess. (5) RESPONSE: The most likely diagnosis given the symptoms and conditions is (E) Tubo-ovarian abscess.

**AI_B:** RESTATING THE QUESTION: A girl, aged 17, in good health, recently presented at the emergency department with history of moderate right lower quadrant pain, fever, and diminished appetite over the past 3 days. Apart from a cystitis treated with oral antibiotics four months ago, her medical history doesn't show any abnormalities. The girl had her last period 10 days ago, which came at a regular 28-day interval as is typical. She has been sexually active with two partners in the last 6 months and does not always use protection. Her vital signs are as follows: a fever of 38.5°C (101.3°F), a slightly elevated pulse rate of 105/min, normal respirations at 14/min, and blood pressure of 127/82 mm Hg. A physical examination reveals tenderness in the lower abdominal quadrants which is more pronounced in the right lower quadrant accompanied by rebound tenderness. She has decreased bowel sounds, a blood-tinged discharge from the cervical os, and cervical motion tenderness. There's also a notable 5-cm mass found in the right adnexal region. Based on the symptoms, what is the most probable medical condition: Appendicitis, Ectopic pregnancy, Ovarian torsion, Pyelonephritis, or Tubo-ovarian abscess? PLANNING: Appendicitis typically presents with periumbilical pain migrating to the right lower quadrant, which is not exactly the case here. An ectopic pregnancy could potentially contribute to the symptoms however, she had her last menstrual period 10 days ago indicating no pregnancy. Ovarian torsion could be considered since the patient has a palpable mass in the adnexal region, yet it typically presents with severe, sudden onset pain, contrary to the mild, progressive pain of this case. Pyelonephritis usually presents with flank pain and fever, but there is usually no adnexal mass, making this less likely. Tubo-ovarian abscess, however, could potentially explain the symptoms as it may follow an episode of pelvic inflammatory disease and the patient admits to engage in unprotected sex. CLINICAL REASONING: This patient’s fever, right lower quadrant abdominal pain, and cervical motion tenderness suggest a diagnosis of pelvic inflammatory disease (PID), which is common in sexually active young women and frequently undiagnosed. In PID, inflammation may extend to the ovaries and Fallopian tubes with formation of the tubo-ovarian abscess, indicated by the right adnexal mass in this case. CONCLUDING: Given the right lower quadrant pain, fever, history of unprotected sex, and a palpable mass in the right adnexal region, the patient's most likely diagnosis is a tubo-ovarian abscess, a complication of pelvic inflammatory disease. RESPONSE: (E) Tubo-ovarian abscess.

**AI_A:** 1) RESTATING THE QUESTION: The clinical scenario describes a healthy 17-year-old girl with a 3-day history of fever, right lower quadrant pain, and decreased appetite. With a history of cystitis treated with antibiotics continuity of menses, and inconsistent use of protection during sexual activity, she presents with physical signs including tenderness and rebound in right lower quadrant, blood-stained cervical discharge, and a palpable mass in the right adnexal region. The question involves correctly identifying her condition from the five given options: appendicitis, ectopic pregnancy, ovarian torsion, pyelonephritis, and tubo-ovarian abscess. 2) PLANNING: The classical symptoms of abdominal pain and fever indicate some form of intra-abdominal pathology. Decreased bowel sounds hint towards peritonitis or inflammatory causes. The presence of a mass in the adnexal region could signify gynecological pathology. Absence of distention rules out some causes of lower abdominal pain such as bowel obstruction. Additionally, the sexual history and inconsistent condom use raises the possibility of a sexually transmitted infection or reproductive complication. 3) CLINICAL REASONING: Appendicitis generally presents with a migratory pain to the right lower quadrant, likely accompanied by nausea, vomiting, and fever. Physical signs would include rebound tenderness at McBurney's point but unlikely to cause a palpable adnexal mass. An ectopic pregnancy could present with lower abdominal pain, irregular bleeding, and a palpable adnexal mass; however, the patient's regular menstrual cycle and timing of the last period make this less likely. Ovarian torsion would present with a sudden onset of severe lower abdominal pain but less likely to cause a fever. Pyelonephritis typically presents with flank pain, fever, urinary symptoms; the patient's history of cystitis could potentially link to this but the presence of a palpable mass is inconsistent. Lastly, a tubo-ovarian abscess from PID can occur due to sexually transmitted infections and could explain the lower quadrant pain, fever, and the adnexal mass. 4) CONCLUDING: After doing a comprehensive clinical reasoning, the likely diagnosis with the most consistent findings would be a Tubo-ovarian abscess. The sexual history, inconsistency in condom use, presence of blood-stained cervical discharge, fever along with localized lower abdominal pain, and the palpable adnexal mass all point towards a gynecological infection such as a tubo-ovarian abscess, which could be a complication of pelvic inflammatory disease. 5) RESPONSE: The symptoms, signs, and sexual behaviour of the patient are indicative of a tubo-ovarian abscess (option E). I would recommend further diagnostic steps such as a transvaginal ultrasound and prompt initiation of broad-spectrum antibiotics for this severe form of pelvic inflammatory disease.

**AI_C:** RESTATING THE QUESTION: A 17-year-old girl, who was otherwise healthy, has appeared in the ER with a three-day history of right lower quadrant abdominal pain, fever, and anorexia. She had been treated for cystitis with antibiotics some four months back. At present, she is not on any medication. Her periods occur every 28 days, and her last period was 10 days ago. She has been sexually active in the past 6 months, with inconsistent condom use. Her physical exam indicates an elevated temperature, rapid pulse, normal respirations, and high-normal blood pressure. On abdominal examination, tenderness is noted in the lower quadrants with a rebound in the right lower quadrant. Bowel sounds are reduced. Pelvic examination reveals some bloody discharge from the cervix and tenderness during cervix movement. There is also a 5 cm mass in the right adnexal area. The purpose here is to identify the most probable diagnosis from a list of options including appendicitis, ectopic pregnancy, ovarian torsion, pyelonephritis, and a tubo-ovarian abscess. PLANNING: Several assumptions can be made from this brief history. One, given her age and the abdominal pain and tenderness, she may have a gynecological condition. However, the recent history of cystitis and subsequent antibiotic treatment may also point to some urinary condition. Since she is sexually active and has been inconsistent with contraceptive use, she is at risk of STIs and consequentially pelvic inflammatory disease, which can cause lower abdominal pain, cervicitis (indicated by cervical motion tenderness), and a tubo-ovarian abscess. The presence of a mass in the adnexal region can be associated with several conditions like an ovarian cyst, ectopic pregnancy, or spontaneous ovarian torsion. An alternate assumption could be an ectopic pregnancy. This can result from unprotected sex, and while her last period occurred just 10 days ago, ectopic pregnancies can sometimes still present with vaginal bleeding. This scenario might also be explained by appendicitis. However, the presence of a cervical discharge, cervical motion tenderness, and an adnexal mass does not typically align with a diagnosis of appendicitis. CLINICAL REASONING: This patient shows signs of a lower abdominal infection, such as fever and a right lower quadrant pain along with rebound tenderness. This could suggest appendicitis, but her cervical motion tenderness and adnexal mass imply a gynecological origin. The blood-tinged discharge, adnexal mass, and cervical motion tenderness make a strong case for a tubo-ovarian abscess, which is a complication of pelvic inflammatory disease (PID). PID is a risk in sexually active women, particularly with multiple partners and inconsistent condom use. The finding of an adnexal mass also suggests a possible ovarian cyst or torsion, but they usually don't present with a cervical discharge or fever. Similarly, an ectopic pregnancy could explain the adnexal mass, but the absence of abnormal vaginal discharge and her recent menstruation make this less likely. Pyelonephritis might still contribute to her right abdominal pain and fever, but don't typically exhibit findings such as cervical motion tenderness or adnexal mass. CONCLUDING: Upon assessing her complete subjective and objective data, it's crucial to prioritize the clinical factors that fit into a consistent pattern of presentation. The patient’s combination of abdominal pain, cervical motion tenderness, right adnexal mass, and sexual behavior history align most compellingly with a diagnosis of a tubo-ovarian abscess, which is an advanced form of PID. RESPONSE: The most likely diagnosis, given the presented symptoms and clinical findings, is a tubo-ovarian abscess (Choice E).

**Facilitator** The consensus statement synthesizes the responses of AI_A, AI_B, AI_C, AI_D, and AI_E as follows: The clinical scenario describes a 17-year-old girl who presents with a 3-day history of right lower quadrant pain, fever, and decreased appetite. She has a history of cystitis treated with antibiotics, regular menstrual cycles, and inconsistent use of protection during sexual activity. Physical examination reveals tenderness and rebound in the right lower quadrant, blood-stained cervical discharge, and a palpable mass in the right adnexal region. The task is to identify her condition from the given options: appendicitis, ectopic pregnancy, ovarian torsion, pyelonephritis, and tubo-ovarian abscess. The symptoms of abdominal pain and fever suggest some form of intra-abdominal pathology. The presence of a mass in the adnexal region could signify gynecological pathology. The sexual history and inconsistent condom use raise the possibility of a sexually transmitted infection or reproductive complication. Appendicitis generally presents with migratory pain to the right lower quadrant, nausea, vomiting, and fever, but it is unlikely to cause a palpable adnexal mass. An ectopic pregnancy could present with lower abdominal pain, irregular bleeding, and a palpable adnexal mass; however, the patient's regular menstrual cycle and timing of the last period make this less likely. Ovarian torsion would present with a sudden onset of severe lower abdominal pain but is less likely to cause a fever. Pyelonephritis typically presents with flank pain, fever, urinary symptoms; the patient's history of cystitis could potentially link to this but the presence of a palpable mass is inconsistent. The most consistent findings point towards a Tubo-ovarian abscess. The sexual history, inconsistency in condom use, presence of blood-stained cervical discharge, fever along with localized lower abdominal pain, and the palpable adnexal mass all suggest a gynecological infection such as a tubo-ovarian abscess, which could be a complication of pelvic inflammatory disease. Therefore, the consensus among the AI responses is that the most likely diagnosis, given the presented symptoms and clinical findings, is a tubo-ovarian abscess (Choice E). Further diagnostic steps such as a transvaginal ultrasound and prompt initiation of broad-spectrum antibiotics are recommended for this severe form of pelvic inflammatory disease.
