# Supplementary material for: Collaborative intelligence in AI: Evaluating the performance of a council of AIs on the USMLE
Source: PLOS Digit Health. 2025 Oct 9;4(10):e0000787. doi: 10.1371/journal.pdig.0000787 (PMC12510544; doi:10.1371/journal.pdig.0000787)
Supplement: S2 File — (ZIP) [file pdig.0000787.s002.zip › S2 Data Raw data files containing unprocessed question inputs and LLM outputs including transcripts of deliberations/STEP2_Question-116-1_Type-MC-NJ.docx]

**Facilitator** **[Round 1]:** A 15-year-old girl is brought to the office because she has never had a menstrual period. She has no history of serious illness and receives no medications. Growth and development are appropriate for age. Breast development began at the age of 10 years. She has never been sexually active. She is at the 90th percentile for height, 75th percentile for weight, and 69th percentile for BMI. Sexual maturity rating is 4 for breast development. Pelvic examination shows a normal-appearing vagina; the cervix and uterus cannot be palpated. Her serum testosterone concentration is 340 ng/dL (N=5–40). Serum concentrations of luteinizing hormone, follicle-stimulating hormone, thyroid-stimulating hormone, 17α-hydroxyprogesterone, and prolactin are within the reference ranges. Serum β-hCG testing is negative. Pelvic ultrasonography shows no uterus. Her karyotype is 46,XY. Which of the following is the most appropriate next step in management? (A) Estrogen and progestin therapy (B) Gonadectomy (C) Gonadotropin-releasing hormone agonist therapy (D) Progestin therapy only (E) No further management is indicated

**AI_B:** RESTATING THE QUESTION: To summarize the case, this 15-year-old girl, despite having normal female phenotypic characteristics, has neither experienced menstruation nor palpable uterine structures. She has an elevated testosterone level and a male karyotype - 46,XY - but lacks any other significant hormonal abnormalities. This information brings up the question of what should be the management for this patient. PLANNING: This case seems to be a form of sexual development condition known as Androgen Insensitivity Syndrome (AIS), where there's a partial or complete inability of cells to respond to androgens. This leads to the development of female secondary sexual characteristics with a male (46,XY) karyotype, and the presence of testes since Mullerian inhibiting factor is secreted during embryogenesis, leading to the absence of uterus and fallopian tubes. However, the elevated testosterone level could potentially be transformed peripherally into estradiol responsible for breast development. The main concerns for this patient are the risk of malignancy from undescended testes and psychological implications. CLINICAL REASONING: Primary amenorrhea due to the absence of uterus despite normal sexual maturity, coupled with a 46,XY karyotype, suggests androgen insensitivity syndrome. Although she has developed as a phenotypic female, the high testosterone level originating from the testes highlights the presence of gonadal tissue that is typically undescended in this syndrome. The potential for malignancy arising from these undescended testes makes gonadectomy an essential consideration. CONCLUDING: Thus, while managing AIS, the primary concern's to mitigate the risk of gonadal malignancy and provide necessary psychological support. Hormone replacement therapy (like estrogen and progestin or progestin-only) is necessary post-gonadectomy to maintain secondary sexual characteristics and bone health, notwithstanding it's not the immediate step. Gonadotropin-releasing hormone agonist therapy is not suitable since there are no ovaries, and follicular development or ovulation can't be induced. Therefore, the immediate step for this patient is to proceed with gonadectomy. RESPONSE: Based on her clinical presentation and test results, the most appropriate next step in management would be a gonadectomy (B).

**AI_C:** RESTATING THE QUESTION: A 15-year-old girl with a history of delayed menarche - that is, she is yet to have her first menstrual period - has come for a medical evaluation. Key points in her case include normal growth and development for her age, a advanced sexual maturity rating for breast development, higher serum testosterone concentration than average, absence of a visible cervix and uterus in a pelvic examination and ultrasound, and a 46,XY karyotype, which is typically male. This poses the question, what should be the most appropriate next step in her management among the given options. PLANNING: We know several things from the case. The girl has normal growth patterns and her sexual maturation as evidenced by breast development is advanced. However, her menstrual cycle hasn't begun yet, and both physical examination and imaging studies show an absence of uterus and cervix. She also has elevated testosterone levels and her karyotype is 46,XY which is typically seen in males. The key assumption here is that she might have Androgen Insensitivity Syndrome (AIS) or another form of intersex disorder given the absence of internal female genitalia, elevated testosterone levels, and 46,XY karyotype. For these conditions, various treatment options exist including hormone therapy or surgery. CLINICAL REASONING: In the absence of uterus and with male karyotype, her age-appropriate development and high serum testosterone concentration support the diagnosis of Androgen Insensitivity Syndrome (AIS). In AIS, individuals possess a 46,XY karyotype but are phenotypically female due to a mutation in the androgen receptor gene, leading to end-organ resistance to androgens. Despite the high testosterone levels, these individuals develop as females, because estrogen, produced peripherally from adrenal androgens, drives the development of secondary sexual characteristics. However, the intra-abdominal gonads are at an increased risk for malignancy, especially after puberty, warranting consideration for removal. CONCLUDING: Given the clinical findings and clinical reasoning, the most appropriate management for this patient would be to pursue gonadectomy or removal of intra-abdominal gonads. This is primarily due to the risk of potential malignant transformation. RESPONSE: (B) Gonadectomy is the most appropriate next step in management for this patient.

**AI_E:** The situation here involves a 15-year-old girl, who despite her normal growth, development, and breast development, has not started menstruating. The organs typically associated with menstruation, the cervix and uterus, are not palpable, and ultrasonography does not show a uterus. Despite these findings, she has typically female secondary sexual characteristics such as breast development. Serum testosterone levels are high, but other hormone levels are normal. Incredibly, her genetic makeup is 46,XY, which is typically found in males. In essence, we are trying to determine the most appropriate next step in managing a case of a girl who is anatomically intersex but phenotypically female. Let's consider our assumptions. One is that she has an atypical presentation of a disorder of sexual development, and the other is that this is the outcome of a normal variant of sexual differentiation. The first premise suggests an inherent biological issue with her sex chromosomes or hormone receptors, possibly indistinguishable from a complete or partial androgen insensitivity syndrome. The second assumption suggests that this could represent an extreme end of the typical spectrum of female development, although this is less likely given the presence of XY chromosomes and absent uterus. Clinical reasoning suggests that with biologically male chromosomes but phenotypically female characteristics - such as breast development and a normal-appearing vagina - the girl may have androgen insensitivity syndrome. This condition, caused by mutations in the androgen receptor gene, results in one's inability to respond to androgens (male hormones). Consequently, this leads to the development of female secondary sexual characteristics like breasts. Without androgens, the Müllerian ducts (which normally form a female's Fallopian tubes, uterus, cervix, and upper vagina) cannot be suppressed and thus disappear, which is signified by the absence of a uterus and cervix. The high levels of serum testosterone is a clue telling us there is a source of androgen production that would typically lead to male secondary sexual characteristics in an individual sensitive to such hormones. Given that this patient has female development and a male karyotype, it can be inferred that the testosterone is likely being produced from internal testes that are not seen on imaging. In terms of management, the next appropriate step would be gonadectomy – the removal of the internal testes. Despite their insensitivity to androgens, there's a risk that these tissues could become cancerous and ought to be removed. The other treatments listed: estrogen and progestin therapy, gonadotropin-releasing hormone agonist therapy, progestin therapy only, or no further management, do not address the root issue and potential future risks. Therefore, the correct answer is, (B) Gonadectomy.

**AI_A:** RESTATING THE QUESTION: A teenager who has not yet menstruated is being assessed. Despite hitting usual growth and development markers, there are some anomalies. Her uterus is absent, and she has testosterone levels well above the average range for females, but her other hormone levels are normal. Interestingly, her genetic makeup suggests that she is biologically male (46,XY). PLANNING: Typically, puberty in girls involves menstruation, which hasn't happened in this case, making us consider that she might have a condition that prevents her from having periods, such as Mullerian agenesis or Androgen Insensitivity Syndrome. Mullerian agenesis (also known as Mayer-Rokitansky-Küster-Hauser (MRKH) syndrome) is a disease that causes the uterus and part of the vagina to be absent in females. Androgen Insensitivity Syndrome (AIS) leads the body to not respond properly to male hormones (androgens), and thus external female genitalia develop but without a uterus, as seen in this case. Furthermore, elevated testosterone levels and 46,XY karyotype point towards AIS as it's consistent with individuals having testicles. CLINICAL REASONING: In patients with Androgen Insensitivity Syndrome (AIS), the body does not recognize androgens. This means individuals with AIS are genetically male with testes but do not develop typically male characteristics due to the lack of androgen response. Breast development occurs as the patient's testes produce testosterone, which is converted to estrogen through the process of aromatization, causing breast growth. However, since the body cannot respond to testosterone, internal male organs (such as the prostate and seminal vesicles) do not develop. Without the development of a uterus and due to the absence of Mullerian structures, menstruation cannot occur in these individuals. Identification of AIS in patients is crucial because they have testes that, after puberty, have an increased risk of becoming cancerous. CONCLUDING: The combination of her physical exam, lab results (high testosterone, normal female hormones), absence of a uterus, and a 46,XY karyotype ultimately leads to the diagnosis of AIS. RESPONSE: Considering the risk of developing malignancy in the undescended testes after puberty in AIS patients, it is vital to mitigate this risk by surgically removing the testes (gonadectomy) once the patient's breast development (happening due to the conversion of testosterone to estrogen) completes, hence the answer is (B) Gonadectomy.

**AI_D:** RESTATING THE QUESTION: A female aged 15 is being evaluated due to the fact that she has never reached menarche. Although her physical development seems progressing appropriately, a physical examination revealed an absence of the cervix and uterus, despite normal vaginal anatomy. Furthermore, her serum testosterone level is significantly elevated, while other hormones remain within the typical range. A pelvic ultrasound confirmed the absence of a uterus and her karyotype revealed that she is genotypically male (46, XY). The question requires determining the most suitable next step in her management. PLANNING: The key findings of this case are amenorrhea, elevated testosterone levels, absence of uterus and cervix, and male karyotype (46, XY). These findings suggest a condition called Complete Androgen Insensitivity Syndrome (CAIS), a disorder that causes genetic males to exhibit physical characteristics typical of females due to their bodies' inability to respond to androgens. The defining features of CAIS include female secondary sexual characteristics such as breasts, absence of a uterus and cervix, elevated testosterone, and a 46,XY karyotype. Coming up with alternate assumptions or hypotheses is a bit constrained in this scenario as the unambiguous combination of clinical and laboratory findings bounded us to place bet onto CAIS. Given this information, we must identify which one of the proposed treatments is considered the standard of care for CAIS patients. CLINICAL REASONING: In CAIS, individuals have a male karyotype (46, XY), but due to a mutation in the Androgen receptor gene, their bodies can't respond to androgenic hormones. This leads to feminization of the exterior phenotype, but the unaffected development of the intra-abdominal gonadal tissue as testes rather than ovaries. These intra-abdominal testes also produce testosterone (explaining the elevated levels), which gets converted to estrogen, thus leading to the development of secondary female characteristics like breast development. However, since this individual is genetically male, Mullerian inhibitory substance is produced causing the regression of internal female structures(i.e., uterus and cervix) leading to primary amenorrhea. This is where the preferred management option comes in. The next step in treating CAIS individuals is prophylactic gonadectomy after puberty to prevent the development of malignant tumors that may arise from the undescended testes. CONCLUDING: Considering the clinical, hormonal, and genetic data — a very high testosterone level, absence of internal female reproductive organs, and a 46,XY karyotype, the girl seems to have Complete Androgen Insensitivity Syndrome (CAIS). If this condition is present, the typical practice is to carry out gonadectomy after puberty to prevent malignancy but also to avoid the psychosocial distress of a gender role change at an early age. RESPONSE: Therefore, the most appropriate next step in the management of this patient is (B) Gonadectomy.

**Facilitator** The case involves a 15-year-old girl who has not yet menstruated despite normal growth and development, including breast development. A physical examination and ultrasound reveal the absence of a uterus, and her testosterone levels are significantly elevated. Her karyotype is 46,XY, typically associated with males. These findings suggest a condition known as Androgen Insensitivity Syndrome (AIS), where the body is unable to respond to androgens, leading to the development of female secondary sexual characteristics despite a male karyotype. The elevated testosterone levels are likely due to the presence of internal testes, which are not visible on imaging. Given the risk of these tissues becoming cancerous, the consensus among the AI responses is that the most appropriate next step in management is a gonadectomy (B), the removal of the internal testes. Other treatments such as hormone therapy do not address the root issue and potential future risks.
