# Supplementary material for: Collaborative intelligence in AI: Evaluating the performance of a council of AIs on the USMLE
Source: PLOS Digit Health. 2025 Oct 9;4(10):e0000787. doi: 10.1371/journal.pdig.0000787 (PMC12510544; doi:10.1371/journal.pdig.0000787)
Supplement: S2 File — (ZIP) [file pdig.0000787.s002.zip › S2 Data Raw data files containing unprocessed question inputs and LLM outputs including transcripts of deliberations/STEP2_Question-117-1_Type-MC-NJ.docx]

**Facilitator** **[Round 1]:** A 50-year-old man returns to the office for a follow-up examination. During the past 2 months, he has had increased blood pressure readings during several checks at the office and at home. He says he has felt well. He has major depressive disorder and gout. Medications are fluoxetine and allopurinol. His maternal grandfather had congestive heart failure and the patient's older brother has hypertension. The patient has smoked one-half pack of cigarettes daily for 32 years. He is 180 cm (5 ft 11 in) tall and weighs 95 kg (210 lb); BMI is 29 kg/m². Pulse is 70/min and blood pressure is 152/90 mm Hg. Physical examination discloses no abnormalities. Which of the following medications is contraindicated in this patient? (A) Amlodipine (B) Doxazosin (C) Hydrochlorothiazide (D) Lisinopril (E) Metoprolol

**AI_C:** (1) RESTATING THE QUESTION: The query involves deciding which antihypertensive medication would not be appropriate to prescribe for a 50-year-old man presenting with increased blood pressure readings. He has a history of gout and major depressive disorder and is currently taking fluoxetine for his depression and allopurinol for his gout. (2) PLANNING: The assumption here is that all listed drugs (Amlodipine, Doxazosin, Hydrochlorothiazide, Lisinopril, Metoprolol) are generally used as antihypertensive medications. However, one must bear in mind that a specific medication may be contraindicated within the context of the patient’s particular comorbidities, medical history, and other medications. We need to consider the pharmacological effects of these drugs, their potential interactions, and side-effects relative to the patient's medical background. (3) CLINICAL REASONING: All of the stated medications are widely used in the treatment of hypertension. However, they each have different mechanisms of action. Hydrochlorothiazide is a diuretic and can worsen gout, which this patient has, because it reduces uric acid secretion. Fluoxetine, his current medication for major depressive disorder, wouldn't interfere directly with any of the meds chosen. His family medical history and smoking habit, while important for risk stratification, doesn't directly influence the choice of medication. (4) CONCLUDING: So, given the patient's history of gout and the fact he's already medicated with allopurinol for this condition, hydrochlorothiazide, which can worsen gout by reducing uric acid excretion, would be best avoided. (5) RESPONSE: So it can be inferred that among the listed options, Hydrochlorothiazide (C) is contraindicated in this patient due to his history of gout.

**AI_D:** RESTATING THE QUESTION: The clinical case involves a 50-year-old man who has been experiencing elevated blood pressure readings over the last 2 months. Other aspects of his medical history include major depressive disorder, gout, a family history of congestive heart failure and hypertension, long-term smoking habit, and a somewhat elevated body mass index (BMI). His current medications are fluoxetine, which is a selective serotonin reuptake inhibitor (SSRI) used to manage depression, and allopurinol, a xanthine oxidase inhibitor used to prevent gout attacks. The question requires the identification of a medication from the given options that is contraindicated for this patient. PLANNING: To answer this question, it is crucial to analyse the given-specific information about the patient's health status, predisposing factors, and current medication, then relate that to the pharmacological profiles of the drugs listed in the options. Bearing those factors in mind, an evaluation of the antihypertensive medication options that could potentially negatively interact with the patient's current medication or worsen his existing health conditions should be conducted. CLINICAL REASONING: The medication options include Amlodipine (calcium channel blocker), Doxazosin (alpha-1 blocker), Hydrochlorothiazide (diuretic), Lisinopril (Angiotensin-Converting Enzyme inhibitor), and Metoprolol (beta blocker). Given the patient's depressive disorder and current usage of fluoxetine, a key factor is understanding that SSRIs can interact with other drugs. Beta-blockers interact by potentially leading to an additive impact on slowing the heart rate and may potentiate depressive symptoms. Metoprolol, a type of beta-blocker, would, therefore, be contraindicated as it may exacerbate the patient's depressive disorder and slow the heart rate excessively when combined with fluoxetine. CONCLUDING: After a detailed clinical reasoning process, it can be concluded that the combination of the SSRI fluoxetine and a beta-blocker such as metoprolol could potentially exacerbate the patient's existing depressive symptoms while negatively affecting his cardiovascular function. RESPONSE: Therefore, the contraindicated medication for this patient is Metoprolol (E).

**AI_E:** (1) RESTATING THE QUESTION: The question presents a 50-year-old man who has been experiencing elevated blood pressure levels for the past two months. He has a history of major depressive disorder, which is being treated with fluoxetine, and gout, which is being treated with allopurinol. He also has a family history of congestive heart failure and hypertension. The question is asking which of the listed medications would not be suitable for this patient's management due to contraindications moving forward. The medications listed are Amlodipine, Doxazosin, Hydrochlorothiazide, Lisinopril, and Metoprolol, primarily used for managing cardiovascular issues including hypertension. (2) PLANNING: The assumptions here include that one of the listed medications would be contraindicated in managing the patient's condition due to their current medical background and prescriptions. The potential is considered for unknown contraindications between these medicines and the fluoxetine and allopurinol he is currently taking, or due to his other medical conditions and lifestyle (e.g., smoking). These alternate assumptions will be evaluated when determining the most appropriate medication. (3) CLINICAL REASONING: Fluoxetine belongs to a class of drugs known as selective serotonin reuptake inhibitors (SSRIs). Doxazosin, which falls under the alpha-blocker category, carries a risk when combined with SSRIs like fluoxetine because of potential interactions that can lead to significant postural hypotension. Our patient’s current use of fluoxetine and his history of hypertension needs to be considered, particularly with the choice of a new antihypertensive where the propensity to cause significant postural hypotension would be unfavorable. (4) CONCLUDING: Given the man's current medication, major depressive disorder, hypertension, family history of cardiovascular disease, and lifestyle choices (smoking), selecting an antihypertensive for this patient requires careful consideration of all these factors. Notably, the combination of fluoxetine and Doxazosin poses a risk due to the possibility of postural hypotension. (5) RESPONSE: Therefore, among Amlodipine, Doxazosin, Hydrochlorothiazide, Lisinopril, and Metoprolol, Doxazosin (B) represents the option that would be contraindicated in this patient based on his current medication regimen and overall health status.

**AI_A:** (1) RESTATING THE QUESTION: I am being asked to determine which of the listed medications is contraindicated for this patient. A 50-year-old man displaying indications of hypertension with persistent high blood pressure readings over the past 2 months. There's a medical history of major depressive disorder and gout, and consumes fluoxetine and allopurinol. Family history reveals congestive heart failure and hypertension. Personal habits include chronic smoking for over 32 years. Despite high blood pressure readings, his physical examination is generally normal. A contraindication is a factor that serves as a reason to withhold a certain medical treatment due to the harm that it would cause the patient. (2) PLANNING: The clinical approach to this question would involve considering the patient's current medications (fluoxetine and allopurinol), pre-existing conditions (Major Depressive Disorder - MDD and gout), and ongoing habits (smoking). We need to cross-match these conditions with possible side effects or counter-interactions of the listed medications. All the listed medications are used in the treatment of hypertension: Amlodipine (Calcium Channel Blocker), Doxazosin (Alpha 1 Blocker), Hydrochlorothiazide (Diuretic), Lisinopril (ACE inhibitor), and Metoprolol (Beta-1 Blocker). However, it should be noted that some could exacerbate existing conditions or interact negatively with currently consumed medications. (3) CLINICAL REASONING: Hydrochlorothiazide would not be contraindicated as it can assist in controlling the patient's high blood pressure while also helping to manage gout. Amlodipine, Lisinopril, and Metoprolol are all useful in treating hypertension. However, Doxazosin—an alpha-1 receptor antagonist—could potentially worsen the patient's existing depressive disorder. The antidepressant fluoxetine can cause orthostatic hypotension as a side effect. When combined with Doxazosin, which itself can induce hypotension, the risk of a significant drop in blood pressure is enhanced. This might potentially lead to more severe episodes of orthostatic hypotension, increasing the risk for falls and related injuries. (4) CONCLUDING: Taking into consideration the patient's individual health profile, it is reasonable to conclude that his current conditions and medications present a contraindication for the use of Doxazosin, given its potential to exacerbate orthostatic hypotension when used concurrently with fluoxetine. (5) RESPONSE: Among the given choices, (B) Doxazosin is the medication most contraindicated in this patient.

**AI_B:** (1) RESTATING THE QUESTION: The question involves a 50-year-old man who is dealing with increased blood pressure readings, a diagnosed major depressive disorder, and gout. His current treatment regimen includes fluoxetine (a serotonin reuptake inhibitor used to treat depression) and allopurinol (a medication used to lower high blood uric acid levels to treat gout). Family history involves congestive heart failure and hypertension. He has a long history of moderate smoking. His body mass index indicates that he is overweight. The task is to identify a medication among the listed alternatives, Amlodipine, Doxazosin, Hydrochlorothiazide, Lisinopril, and Metoprolol, that would be contraindicated for this patient. (2) PLANNING: The primary assumption here is that one of the listed drugs would potentially interfere with his health status or compromise his current treatment plan. By questioning each drug's potential interactions or adverse effects with his current health status, we could identify the contraindicated medication. (3) CLINICAL REASONING: Amlodipine is a calcium channel blocker used to manage hypertension. Considering the patient's elevated blood pressure, introducing amlodipine would not pose a significant risk. Doxazosin is an alpha blocker used to treat hypertension. It has potential side effects of dizziness and faintness; however, the patient's physical examination didn't reveal related issues. Hydrochlorothiazide is a thiazide diuretic used to treat hypertension, and it can increase uric acid levels, potentially worsening the patient's gout. Lisinopril is an ACE inhibitor used to manage hypertension, heart failure, and is given to patients post-heart attacks. Its usage is also not relative to the patient's health status, and hence does not pose significant risks. Metoprolol, a beta blocker, can be used to treat high blood pressure and heart failure, and it also does not pose any significant contraindications for this patient. (4) CONCLUDING: Following a step-by-step clinical examination of these drugs in relation to the patient's medical conditions and the medications he is already on, the only drug that can potentially aggravate a current condition is hydrochlorothiazide because it can worsen the patient's gout condition by increasing uric acid levels. (5) RESPONSE: Therefore, it can be concluded that among the given options, Hydrochlorothiazide (C) is contraindicated in this patient due to its potential to elevate uric acid levels, worsening the patient's existing gout condition.

**Facilitator** **[Round 2]:** Summary: AI_A's opinion: The council member believes that Doxazosin is contraindicated for the patient due to its potential to exacerbate orthostatic hypotension when used concurrently with fluoxetine, a medication the patient is currently taking. AI_B's opinion: This council member concludes that Hydrochlorothiazide is contraindicated for the patient because it can worsen the patient's gout condition by increasing uric acid levels. AI_C's opinion: Similar to AI_B, AI_C also believes that Hydrochlorothiazide is contraindicated due to the patient's history of gout, as it can reduce uric acid excretion and worsen the condition. AI_D's opinion: The council member concludes that Metoprolol is contraindicated for the patient as it may exacerbate the patient's depressive disorder and slow the heart rate excessively when combined with fluoxetine. AI_E's opinion: Like AI_A, AI_E also believes that Doxazosin is contraindicated for the patient due to the risk of significant postural hypotension when combined with fluoxetine. Key premises and logic that distinguish differing perspectives: The key premises that distinguish the differing perspectives are the potential interactions of the listed medications with the patient's current medications and conditions. AI_A and AI_E focus on the potential interaction between Doxazosin and fluoxetine, leading to significant postural hypotension. AI_B and AI_C focus on the potential of Hydrochlorothiazide to worsen the patient's gout by increasing uric acid levels. AI_D focuses on the potential interaction between Metoprolol and fluoxetine, which could exacerbate the patient's depressive disorder and slow the heart rate excessively. Question to clarify disagreements: Given the patient's current medications and conditions, how do we prioritize the potential risks associated with each medication (Doxazosin, Hydrochlorothiazide, and Metoprolol) and determine which one is most contraindicated?. Based on your preceding response, how would you answer the original question: [A 50-year-old man returns to the office for a follow-up examination. During the past 2 months, he has had increased blood pressure readings during several checks at the office and at home. He says he has felt well. He has major depressive disorder and gout. Medications are fluoxetine and allopurinol. His maternal grandfather had congestive heart failure and the patient's older brother has hypertension. The patient has smoked one-half pack of cigarettes daily for 32 years. He is 180 cm (5 ft 11 in) tall and weighs 95 kg (210 lb); BMI is 29 kg/m². Pulse is 70/min and blood pressure is 152/90 mm Hg. Physical examination discloses no abnormalities. Which of the following medications is contraindicated in this patient? (A) Amlodipine (B) Doxazosin (C) Hydrochlorothiazide (D) Lisinopril (E) Metoprolol]

**AI_E:** To understand the question, we need to consider the patient's clinical condition, current medications, and potential safety concerns regarding the addition of new medications. The patient has a history of increased blood pressure readings over the past two months and poses risk factors for cardiovascular diseases, such as obesity, smoking, and family history of hypertension and heart failure. The patient is also diagnosed with major depressive disorder and gout, and his current medications include fluoxetine for depression and allopurinol for gout. Planning would involve assessing the proposed medications in relation to the patient's conditions and current therapeutic regimen. We'll review (A) Amlodipine, (B) Doxazosin, (C) Hydrochlorothiazide, (D) Lisinopril, and (E) Metoprolol, checking each for potential adverse effects or interactions with the patient's existing medications and clinical picture. The process of clinical reasoning involves a comprehensive evaluation of the patient's medical history, assessment, diagnosis, and therapy. In this scenario, it would be crucial to consider potential drug-drug interactions and the exacerbation of existing conditions. As we examine the proposed medications, it becomes clear that Hydrochlorothiazide (C) is the contraindicated medication for the patient. Hydrochlorothiazide is a diuretic often used to treat hypertension. However, it can increase uric acid levels in the body, potentially exacerbating gout symptoms. As the patient is already diagnosed with gout, and is on allopurinol-a drug used to decrease uric acid levels, adding a medication that increases uric acid appears contradictory and potentially harmful. My conclusion then is that among the listed medications, Hydrochlorothiazide (C) is contraindicated in this patient due to its potential to increase uric acid levels and exacerbate the patient's gouty symptoms. To manage his hypocalemia, we must ensure a balanced and careful selection of medications. The aim should be to control the patient's blood pressure, while considering his overall health and comorbidities. Therefore, the best answer for this case is option (C) Hydrochlorothiazide.

**AI_B:** Restating the question and defining technical terms and concepts, we're asked to identify which of the given options (Amlodipine, Doxazosin, Hydrochlorothiazide, Lisinopril, Metoprolol) is contraindicated in a patient with elevated blood pressure, major depressive disorder managed with fluoxetine, gout managed with allopurinol, and a significant smoking history. To plan, we need to consider the potential adverse effects and interactions each medication could have with the patient's existing conditions and medications. When generating alternate assumptions, we should question the impact and likelihood of these adverse effects and interactions. However, clinically, the assumption that should be prioritized is the one involving a medication that poses significant risks to the patient's existing health condition. The clinical reasoning necessitates a knowledge of the pharmacokinetics and pharmacodynamics of each medication. Fluoxetine, an SSRI, can interact with certain medications to cause serious side effects. Similarly, certain anti-hypertensive drugs can exacerbate gout, and some beta-blockers may worsen depression. After rationalizing these considerations, it can be noted that hydrochlorothiazide (Choice C), a thiazide diuretic commonly used to treat hypertension, is contraindicated in this patient. It is known that thiazides can increase uric acid levels in the body, thereby exacerbating the symptoms of gout, a condition the patient is known to have. Moreover, the patient is on allopurinol, a medication to reduce uric acid levels, creating a contradictory scenario where one medication increases and the other decreases uric acid levels. In conclusion, upon connecting the clinical reasoning with the drugs in question, we can ascertain that hydrochlorothiazide, which can exacerbate the patient's gout condition, is contraindicated for this patient. Hence in response to the question: Which of the following medications is contraindicated in this patient - Amlodipine, Doxazosin, Hydrochlorothiazide, Lisinopril or Metoprolol, the answer would be Hydrochlorothiazide (Choice C).

**AI_D:** RESTATING THE QUESTION: We are essentially asked to determine the proper antihypertensive for a 50-year-old male who has several medical conditions including major depressive disorder and gout. He is currently taking fluoxetine a selective serotonin reuptake inhibitor for his depression, and allopurinol for his gout. The patient also has a history of tobacco use and a family history of cardiovascular disease. The choices presented to us are Amlodipine, Doxazosin, Hydrochlorothiazide, Lisinopril, and Metoprolol. PLANNING: Given the patient's current medications and medical history, we can speculate that certain antihypertensives could exacerbate his existing conditions, interact negatively with his current medications, or pose additional health risks based on his family history. We should prioritize the patient's overall health status in choosing the right antihypertensive, focusing on those that might worsen his depressive disorder or gout, or induce harmful interactions with fluoxetine or allopurinol. CLINICAL REASONING: Considering the patient's current medications and medical history, Doxazosin, an α1 blocker, could potentially worsen orthostatic hypotension if combined with fluoxetine. Hydrochlorothiazide, a diuretic, can aggravate gout by increasing uric acid levels in the bloodstream. Meanwhile, Metoprolol, a β1 selective blocker, might exacerbate depressive symptoms and slow down the heart rate excessively, especially alongside fluoxetine. Amlodipine, a calcium channel blocker, and Lisinopril, an ACE inhibitor, have the least potential drug interactions and contraindications considering the patient's context. CONCLUDING: In prioritizing the patient's overall health status and minimizing risk of exacerbating existing conditions or provoking potential drug interactions, we might consider Metoprolol as the most contraindicated medication. It could potentially worsen the patient's major depressive disorder and slow the heart rate excessively, especially with concurrent use of fluoxetine. RESPONSE: Hence, based on the information given and considering the multiple factors involved, including the potential exacerbation of the patient's existing conditions and the interactions with his current medications, Metoprolol (option E) seems to be the most contraindicated medication in this patient. However, a holistic approach and careful monitoring are required if any antihypertensive is used, keeping in mind that individual patient response might vary.

**AI_A:** Responding to the question, we're trying to determine the most inadequate drug for a patient who has a history of increased blood pressure, major depressive disorder, and gout while taking fluoxetine and allopurinol. His personal and family history also includes smoking, obesity, and a genetic predisposition to heart disease and hypertension. The crucial assumptions for each medication can be summarized as follows: 1. Amlodipine: It's a calcium channel blocker used for treating hypertension, yet doesn't appear to conflict with any of the patient's conditions or current medications. 2. Doxazosin: This alpha-1 antagonist can help control hypertension but may exacerbate orthostatic hypotension when mixed with fluoxetine. 3. Hydrochlorothiazide: It's used as a diuretic and treatment for hypertension. However, it could worsen gout by encouraging urate accumulation. 4. Lisinopril: An ACE inhibitor that helps manage hypertension with no direct contradictions with patient's present medication or condition. 5. Metoprolol: As a beta-blocker, it is used for managing hypertension, but, it may worsen depression symptoms and possibly produce bradycardia in combination with fluoxetine. By evaluating these assumptions, a critical factor to consider is whether the complications related to drug interactions, gout exacerbation or worsening of depression symptoms would likely occur and impact the patient severely. The process of clinical reasoning implicates that while amlodipine and lisinopril don't seem to counteract with any of the patient's existing conditions or his current medications, doxazosin, hydrochlorothiazide, and metoprolol do present some risks. Particularly, doxazosin may exacerbate orthostatic hypotension, hydrochlorothiazide may exacerbate gout, and metoprolol may exacerbate depressive symptoms. Weighing the clinical consequences, it seems reasonable to prioritize the avoidance of depressive symptom exacerbation due to the immediate impact on patient's mental health, but the other risks associated with doxazosin and hydrochlorothiazide should also be considered, as they can contribute to patient discomfort or potentially severe physiological changes. In conclusion, my final response would be metoprolol as the contraindicated medication for this patient. The likelihood of exacerbating depressive symptoms and potential for bradycardia seem more immediately concerning for this patient's well-being. Managing the patient's gout or potential orthostatic hypotension could be addressed with careful monitoring and adjustment of his treatment, while managing exacerbated depressive symptoms could be more challenging. Therefore, given the patient's current presentation, I hypothesize that metoprolol should be contraindicated.

**AI_C:** The discussion is based on the question that has been presented, seeking to identify the medication contraindicated for a 50-year-old male with high blood pressure, history of gout and major depressive disorder. He is currently taking fluoxetine, an antidepressant, and allopurinol for gout. He also has a smoking habit and a family history of hypertension and heart failure. The diverse thoughts from the council derive from weighing the potential interactions of the prospective medications with the patient's current medical conditions and treatments. Those medications include Doxazosin, Hydrochlorothiazide, and Metoprolol. The critical question to consider here is how one can prioritize the relative risks posed by each of these proposed medications, given the patient's current medical situation and his prescribed protocol of medicines. This line of enquiry further influences the direction of the final response towards the posed question. In understanding each concern: Doxazosin, an alpha blocker, has the potential to provoke pronounced hypotension, especially when compounded with fluoxetine. This reaction could result in orthostatic hypotension, whereby sudden positional changes - such as rising from a seated position - might induce fainting or dizziness. This risk imposes a limitation, given that the patient is already on fluoxetine. Hydrochlorothiazide, a diuretic, could potentially exacerbate gout. This is because it decreases uric acid excretion, leading to increased levels of uric acid in the body, which could enhance gout symptoms. Considering the patient’s history of gout, and ongoing treatment with allopurinol, this risk should be factored significantly. Lastly, Metoprolol, a beta blocker, could possibly aggravate the patient's depressive symptoms given its side effect profile. Additionally, its interaction with fluoxetine might lead to a significant decrease in heart rate. Identifying the most contraindicated drug would be reliant on the potential severity and impact of these interactions on the individual's health. Gout flare-ups, resulting from increased uric acid levels due to hydrochlorothiazide, may cause significant discomfort and complications; however, they are typically manageable and reversible upon cessation of the drug. The management of cardiovascular effects such as orthostatic hypotension or decreased heart rate due to interactions between the proposed drug and fluoxetine might involve more complex risk management, potentially impacting the individual's quality of life significantly. Concluding, given the patient's clinical picture and the assessment of potential risks, hydrochlorothiazide may not be the most contraindicated drug when compared to Doxazosin or Metoprolol. The side effects of these drugs in conjunction with fluoxetine may pose greater inherent risks. Between Doxazosin and Metoprolol, the latter's potential to exacerbate depression and significantly lower heart rate might impose severe implications, thus I would lean towards Metoprolol (E) as the contraindicated drug in this case. This is, of course, considering that the depressive symptoms are indeed major and well-controlled under current medication and that the patient's current heart rate is within the normal range. A final diagnosis would greatly benefit from more precise information about the severity of the patient's depressive disorder and the effectiveness of his current treatment. This is my response to the scenario at present, with the understanding that prescribing medications is a clinical decision that must take into account individual-specific factors and be based on a comprehensive evaluation of potential benefits and risks. The dialogue among healthcare providers and the patient should ensure that the medication protocol established is best suitable for the patient.

**Facilitator** **[Round 3]:** Summary: AI_A believes that Metoprolol is the most contraindicated medication for the patient due to its potential to exacerbate depressive symptoms and cause bradycardia when combined with fluoxetine. AI_B, on the other hand, identifies Hydrochlorothiazide as the contraindicated medication because it can increase uric acid levels, thereby exacerbating gout. AI_C agrees with AI_A, suggesting Metoprolol as the contraindicated drug due to its potential to worsen depression and significantly lower heart rate. AI_D also concurs with AI_A and AI_C, identifying Metoprolol as the most contraindicated medication due to its potential to worsen the patient's major depressive disorder and slow the heart rate excessively. AI_E, however, aligns with AI_B, identifying Hydrochlorothiazide as the contraindicated medication due to its potential to increase uric acid levels and exacerbate gout. Question to clarify disagreements: Given the patient's medical history and current medications, how do you prioritize the potential risks associated with each medication? Specifically, how do you weigh the risk of exacerbating depressive symptoms and causing bradycardia with Metoprolol against the risk of exacerbating gout with Hydrochlorothiazide?. Based on your preceding response, how would you answer the original question: [A 50-year-old man returns to the office for a follow-up examination. During the past 2 months, he has had increased blood pressure readings during several checks at the office and at home. He says he has felt well. He has major depressive disorder and gout. Medications are fluoxetine and allopurinol. His maternal grandfather had congestive heart failure and the patient's older brother has hypertension. The patient has smoked one-half pack of cigarettes daily for 32 years. He is 180 cm (5 ft 11 in) tall and weighs 95 kg (210 lb); BMI is 29 kg/m². Pulse is 70/min and blood pressure is 152/90 mm Hg. Physical examination discloses no abnormalities. Which of the following medications is contraindicated in this patient? (A) Amlodipine (B) Doxazosin (C) Hydrochlorothiazide (D) Lisinopril (E) Metoprolol]

**AI_E:** 1) RESTATING THE QUESTION: The case presents a 50-year-old man with a history of major depressive disorder and gout, who's been having increased blood pressure readings for 2 months. Current medications include fluoxetine (a selective serotonin reuptake inhibitor (SSRI) for depression) and allopurinol (a urate-lowering agent for gout). Given these facts, the question asks which proposed antihypertensive (blood pressure-lowering) medication is most contraindicated for this patient. 2) PLANNING: The key facts to consider are the patient's existing conditions and medications. We have two main assumptions related to these - one related to the potential interaction of antihypertensives with fluoxetine, potentially exacerbating depressive symptoms, and another related to exacerbating gout. The clinical priority should be determined by the severity of potential complications and the probability of these occurring. 3) CLINICAL REASONING: Fluoxetine, an SSRI, can increase the concentration of beta-blockers like Metoprolol in the blood, potentially leading to an increased risk of bradycardia and exacerbating depressive symptoms. However, these side effects can be managed by close monitoring and adjusting Metoprolol's dosage. On the other hand, Hydrochlorothiazide, a diuretic class medication, can increase serum uric acid levels, which may exacerbate existing gout. Gout flare-ups can be severely painful and debilitating. 4) CONCLUDING: The negative synergistic effect of Hydrochlorothiazide with allopurinol and the potential to exacerbate the patient's gout condition potentially outweighs the risk associated with Metoprolol. 5) RESPONSE: Since the potential for Hydrochlorothiazide to exacerbate the patient's gout may lead to more acute complications than the risks associated with Metoprolol, the contraindicated medication for this patient is Hydrochlorothiazide (Option C).

**AI_D:** The question asks which antihypertensive medication is contraindicated in a 50-year-old man who is suffering from raised blood pressure, gout, and major depressive disorder, and is currently on fluoxetine for depression and allopurinol for gout. His lifestyle factors include cigarette smoke exposure, a family history of congestive heart failure and hypertension, and an elevated BMI categorizing him as overweight. Given this context, it would be prudent to evaluate each medication for its potential to exacerbate any of the patient's current conditions or interact negatively with the current medications. To reiterate and clarify the standpoints of each AI on the council: - AI_A and AI_C raised the concern that Metoprolol, a beta-blocker, might exacerbate the patient's depressive symptoms, given the known central adverse effects of beta-blockers, and potentially bradycardia due to the additive effects with fluoxetine. - AI_B and AI_E highlighted that Hydrochlorothiazide, a thiazide diuretic, could exacerbate the patient's gout by increasing uric acid levels. If we dig deeper into the clinical reasoning of these concerns, the use of Metoprolol might be preferred to delta over Hydrochlorothiazide. Because while exacerbation of depressive symptoms is a known potential side effect of beta-blockers, it is less common than the urate-raising effects of thiazides, which are well-established and frequent. Furthermore, the patient is already on a therapeutic regime for depression with fluoxetine; the synergistic bradycardic effects could be clinically monitored and managed, but sudden flares of gout might be more detrimental to the overall health of the patient. To conclude, weighing the potential risks of both medications, neither is ideal but considering the patient's current conditions and medications, Hydrochlorothiazide seems a bit more contraindicated. It's essential for the clinician to closely monitor whichever medication is chosen, for potential side effects and adverse reactions. In response to the question, hence, (C) Hydrochlorothiazide appears to be the most contraindicated drug for this patient.

**AI_C:** To summarize the clinical dilemma, we are deciding between two potential contraindicated medicines in a 50-year-old male patient who has high blood pressure, gout, major depressive disorder, and a smoking history. Either Hydrochlorothiazide, due to its uric acid-raising properties, or Metoprolol, due to its potential interaction with fluoxetine to exacerbate depressive symptoms and induce bradycardia, could be deemed unsuitable. These decisions require balancing different risks and evaluating their respective clinical priorities. Assumptions made here include: (1) that the elevation in uric acid levels from hydrochlorothiazide will be significant enough to exacerbate the patient's gout, considering he's already on allopurinol; and (2) that metoprolol will unduly lower the patient's heart rate and worsen his depression, considering he is already on fluoxetine. Coming to the clinical reasoning, it needs to be noted that fluoxetine, a widely used selective serotonin reuptake inhibitor antidepressant, can occasionally cause a dose-dependent lengthening of the QT interval, leading to ventricular arrhythmias. Metoprolol, a beta-blocker, is also known to cause bradycardia and could potentially exacerbate any underlying arrhythmias, causing a significant clinical issue. Depression is a common side effect of beta-blockers like metoprolol, hence its use in a patient with a major depressive disorder may lead to aggravation of the condition. On the other hand, Hydrochlorothiazide is a diuretic that may elevate the serum uric acid level by decreasing its renal excretion, potentially exacerbating gout. However, the patient is already on allopurinol, which lowers blood uric acid levels, likely mitigating this potential side effect of hydrochlorothiazide. To conclude, based on the evidence available, there is valid concern over both Hydrochlorothiazide and Metoprolol. However, taking into account the patient's current medications and pathophysiological state, it seems that the risks associated with Metoprolol (the possibility of worsening depression and inducing bradycardia and potentially life-threatening arrhythmias) could be more clinically significant than the potential gout exacerbation from Hydrochlorothiazide, given that the patient is currently treated with allopurinol. Therefore, the response would be in favor of Metoprolol (E) as the medication most contraindicated for this patient.

**AI_A:** The question pertains to the selection of an anti-hypertensive medication for a 50-year-old man presenting with increased blood pressure, history of major depressive disorder, gout, and smoking. The patient is currently taking fluoxetine for depression and allopurinol for gout. This scenario requires an understanding of drug interactions, factors complicating hypertension, and contraindications of several medications including amlodipine, doxazosin, hydrochlorothiazide, lisinopril, and metoprolol. Given the patient's medical history and conditions, one must carefully consider each medication's potential effects. Metoprolol, a beta-blocker, has a known side effect of potentially worsening depressive symptoms. This poses a risk as the patient is currently managing major depressive disorder with fluoxetine. Hydrochlorothiazide, a diuretic, while effective in treating hypertension, can increase uric acid levels in the body potentially exacerbating gout, a condition this patient is also managing with allopurinol. For clinical prioritization, the consideration hinges on two factors: the potential risk of exacerbation of major depressive disorder with Metoprolol, and the possible worsening of gout with Hydrochlorothiazide. Given that both conditions are currently managed with medications (fluoxetine and allopurinol), an evaluation of the efficiency of these medications and the severity of each condition may help elucidate which risk should be prioritized. Considering these factors, engaging in a thorough patient-centered approach, and reviewing recent literature or guidelines may support clinical decision-making. Moreover, considering the patient's lifestyle, psychosocial factors can further influence the choice of medication. Smoking cessation should be addressed as it's a modifiable factor contributing to hypertension. Finally, it is important to mention that the use of Lisinopril, Amlodipine, and Doxazosin could be valid options in this case; nevertheless, these options require a comprehensive analysis of their side effects and contraindications in the context of the patient's clinical picture. Based on the given data and clinical prioritization, my response would be to avoid prescribing a medication that could exacerbate either of the patient's existing conditions (major depressive disorder and gout). Given that the major depressive disorder could be potentially life-threatening due to suicide risk, it might be prudent to avoid Metoprolol due to its potential to worsen depressive symptoms. However, this decision should be personalized depending on the patient's condition, the effectiveness of his current medications, and a thorough risk-benefit analysis.

**AI_B:** (1) REPHRASING THE QUESTION: The question involves determining which blood pressure medication is not suitable for an overweight, slightly hypertensive 50-year old smoker with a family history of high blood pressure and heart disease. He also suffers from major depressive disorder and gout and is on respective treatment. Five possible medications are listed for consideration. (2) PLANNING: We need to look at the patient's medical history, his current medications, and any possible contraindications with the proposed medications. Assuming the medication with the greatest impact on the patient's existing condition(s) is the most contraindicated, we should consider the effects of the listed drugs on depression and gout. For alternate assumptions, we might consider each drug's effects on smoking, hypertension, or general cardiovascular health. However, based on the available information, our initial assumption seems most reasonable. (3) CLINICAL REASONING: Amlodipine, a calcium channel blocker, is typically well-tolerated and doesn't seem to have any significant interaction with fluoxetine or allopurinol. Doxazosin, an alpha-blocker, has a relatively low side-effect profile but may occasionally cause hypotension or light-headedness when taking fluoxetine. But this is unlikely to be a significant issue for the patient. Hydrochlorothiazide is a thiazide diuretic, which can increase uric acid levels and possibly worsen gout. Considering the fact that the patient is already on allopurinol for gout, adding Hydrochlorothiazide may increase the frequency and severity of gout attacks. Lisinopril, an ACE inhibitor, is a good choice for hypertensive patients, and it doesn't seem to have any significant interaction with fluoxetine or allopurinol. Metoprolol, a beta-blocker, can potentially exacerbate depression. Given the patient's major depressive disorder and concurrent use of fluoxetine, adding Metoprolol into the mix may deteriorate the patient's mental health condition. (4) CONCLUDING: According to this reasoning, the decision seems to be between Hydrochlorothiazide and Metoprolol, both having potential risks. However, the question specifically asked for the contraindicated medicine in this case, not the preferred one for hypertension. Even though Metoprolol could worsen depression, its use is not strictly contraindicated in patients with depression. While on the other hand, it's a well-known fact that thiazide diuretics, like Hydrochlorothiazide, can worsen gout, and hence, their use is generally avoided in patients with a history of gout. (5) RESPONSE: Therefore, considering these factors, the answer is (C) Hydrochlorothiazide. This medication can exacerbate the patient's gout, making it the most contraindicated medication listed.

**Facilitator** **[Round 4]:** Summary: AI_A's opinion: The council member suggests avoiding medications that could exacerbate the patient's existing conditions (major depressive disorder and gout). They lean towards avoiding Metoprolol due to its potential to worsen depressive symptoms, but emphasize the need for a personalized decision based on the patient's condition and a thorough risk-benefit analysis. AI_B's opinion: This council member concludes that Hydrochlorothiazide is the most contraindicated medication due to its potential to exacerbate the patient's gout. They argue that while Metoprolol could worsen depression, its use is not strictly contraindicated in patients with depression. AI_C's opinion: This council member suggests that Metoprolol is the most contraindicated medication due to its potential to worsen depression and induce bradycardia and potentially life-threatening arrhythmias. They argue that the risks associated with Metoprolol could be more clinically significant than the potential gout exacerbation from Hydrochlorothiazide. AI_D's opinion: This council member concludes that Hydrochlorothiazide is the most contraindicated medication due to its potential to exacerbate the patient's gout. They argue that while Metoprolol might exacerbate depressive symptoms, this is less common than the urate-raising effects of thiazides. AI_E's opinion: This council member also concludes that Hydrochlorothiazide is the most contraindicated medication due to its potential to exacerbate the patient's gout. They argue that the negative synergistic effect of Hydrochlorothiazide with allopurinol potentially outweighs the risk associated with Metoprolol. Question to clarify disagreements: Given the differing perspectives on the potential risks of Metoprolol and Hydrochlorothiazide, how would each council member weigh the potential for Metoprolol to exacerbate depressive symptoms and induce bradycardia against the potential for Hydrochlorothiazide to exacerbate gout? Would the patient's current medications (fluoxetine for depression and allopurinol for gout) and their effectiveness factor into this decision?. Based on your preceding response, how would you answer the original question: [A 50-year-old man returns to the office for a follow-up examination. During the past 2 months, he has had increased blood pressure readings during several checks at the office and at home. He says he has felt well. He has major depressive disorder and gout. Medications are fluoxetine and allopurinol. His maternal grandfather had congestive heart failure and the patient's older brother has hypertension. The patient has smoked one-half pack of cigarettes daily for 32 years. He is 180 cm (5 ft 11 in) tall and weighs 95 kg (210 lb); BMI is 29 kg/m². Pulse is 70/min and blood pressure is 152/90 mm Hg. Physical examination discloses no abnormalities. Which of the following medications is contraindicated in this patient? (A) Amlodipine (B) Doxazosin (C) Hydrochlorothiazide (D) Lisinopril (E) Metoprolol]

**AI_C:** Restating the question: This inquiry seeks to determine the most contraindicated hypertension treatment for a 50-year-old male patient with multiple concurrent conditions and risk factors, including major depressive disorder, gout, family history of cardiovascular conditions, and significant tobacco use. Planning: This would involve weighing the risk-benefit ratio of each potential medication against this patient's specific medical profile. Major considerations would include the potential of certain medications to exacerbate his co-occurring conditions and the potential harmful interactions with his current medications (fluoxetine and allopurinol). Clinical Reasoning: Considering these factors critically prompts closer inspection of each medication: (A) Amlodipine is a calcium channel blocker with limited side effects, infrequently exacerbating depression or gout. (B) Doxazosin is an alpha-1 antagonist which presents minimal risk towards existing depressive disorder or gout. (C) Hydrochlorothiazide, a thiazide diuretic, could exacerbate gout due to its uric-acid-boosting properties, despite being commonly used for hypertension. (D) Lisinopril is an ACEI with negligible risks for depression or gout. (E) Metoprolol, a beta blocker, could worsen the depressive disorder – yet, this is not strictly contraindicated for use in individuals with depression. Concluding: Clinical guidelines and the detailed risk-benefit ratio support the use of any of these medications in his hypertension management, albeit with vigilant monitoring of depressive symptoms if Metoprolol is chosen or of gout if Hydrochlorothiazide is selected. Response: Despite the potential risks of Metoprolol contributing to depressive symptoms and Hydrochlorothiazide exacerbating gout, their contraindication isn't absolute. It's usually centered around patient-specific contexts. Here, the risk of Hydrochlorothiazide worsening gout appears more significant due to its negative synergy with allopurinol. Therefore, considering the patient's circumstance, Hydrochlorothiazide would likely be the most contraindicated agent (Option C).

**AI_A:** RESTATING THE QUESTION: A 50-year-old male patient with depression and gout, and a family history of hypertension and heart failure, has consistently high blood pressure readings. He takes fluoxetine for depression and allopurinol for gout. He's also a smoker. There are no physical abnormalities, and it needs to be decided what medication should not be prescribed to him out of: Amlodipine, Doxazosin, Hydrochlorothiazide, Lisinopril, and Metoprolol. PLANNING: The key assumptions here are that the prescribed medication should not exacerbate the patient's present conditions and should not interact adversely with his existing medications. The patient's smoking habit also needs to be considered as this can have implications for cardiovascular health. Each of the listed drugs has potential side effects and contraindications that might not be suitable for this patient. CLINICAL REASONING: Amlodipine, a calcium channel blocker, and Lisinopril, an ACE inhibitor, are unlikely to negatively interact with the patient's conditions or medicines. Doxazosin, an alpha-blocker, has a low risk of depression, and gout could be a concern. Hydrochlorothiazide, a thiazide diuretic, could exacerbate gout as it promotes uric acid retention. Metoprolol, a beta-blocker, could worsen depressive symptoms, slowing down the heart rate, and also has potential interactions with smoking. CONCLUDING: There are two foreseeable negative interactions among the proposed drugs: Hydrochlorothiazide's potential to exacerbate gout, and Metoprolol's potential to worsen depression. Both conditions are being currently managed through medications, but this doesn't negate the possible negative impacts. RESPONSE: Weighing the relative risk and benefits of each potential medication, the council would likely advise against the use of Hydrochlorothiazide (option C) in this patient. While Metoprolol could potentially exacerbate depressive symptoms, the increase in uric acid levels associated with Hydrochlorothiazide usage presents a more defined risk - particularly as the patient has a diagnosed history of gout.

**AI_E:** The question is asking which medication from a list of antihypertensives is contraindicated for a middle-aged man with a history of increased blood pressure, major depressive disorder, gout, and significant familial history for cardiovascular diseases. The patient is on fluoxetine for depression and allopurinol for gout. There has been a disagreement among the council members regarding whether Hydrochlorothiazide or Metoprolol is more contraindicated. Examining these positions in light of the clinical data, there are two key considerations. Firstly, Metoprolol, a beta-blocker, has been associated with potential depressive symptoms. However, these effects remain less substantiated and inconsistent across studies. Beta-blockers can be used in patients with depression, but with caution. As our patient is on fluoxetine and reports feeling well, his depressive disorder could be considered well managed. The second issue lies with Hydrochlorothiazide, a diuretic associated with increased serum urate level, which could exacerbate gout. This patient is already on allopurinol, a medication used for gout by inhibiting uric acid synthesis. Considering the patient's well-managed major depressive disorder and progressive gout condition, which of the two choices - Metoprolol or Hydrochlorothiazide, can be seen as more harmful? Clinically, bothersome symptoms like gout flare-ups might outweigh the potential risk of beta-blockers on depressive symptoms. Given that the patient's depression appears to be controlled with fluoxetine, we do not necessarily expect exacerbation of depression with the addition of Metoprolol. Thus, integrating this information, I believe Hydrochlorothiazide (Choice C) is contraindicated for this patient due to the potential exacerbation of gout, even when the patient is on allopurinol. The risk of worsening gout may outweigh the potential risk of Metoprolol exacerbating depression, particularly as the latter's depressive effects are inconsistently demonstrated and the patient's depressive condition appears stable under fluoxetine.

**AI_B:** In comprehending the case, we are tasked to identify the most contraindicated medication in a 50-year-old man who has hypertension, history of major depressive disorder (MDD) and gout. His current medications include fluoxetine, for his MDD, and allopurinol, for his gout. The choice of medications are Amlodipine, Doxazosin, Hydrochlorothiazide, Lisinopril, and Metoprolol. The term "contraindicated" refers to a condition or factor that increases the risks of a particular medical treatment and would usually suggest that the treatment should not be used. In planning, the assumptions I make are that each medication communicates a set of significant, individualized risks to this patient. I need to weigh these different risks against each other. One alternate assumption would be to focus on the risks posed by each medication on the existing diseases (MDD and gout). Engaging in clinical reasoning, when considering the patient's gout history, Hydrochlorothiazide is a thiazide diuretic that is known to increase uric acid levels, potentially triggering a gout attack in this patient. Concerning his depression, amongst the medications listed, Metoprolol, a β1-selective β-blocker, is known to potentially exacerbate the symptoms of depression. Nonetheless, it is important to note that modern guidelines do not strictly contraindicate the use of β-blockers in patients with depression. Thus, when concluding, I believe Hydrochlorothiazide would be the most contraindicated medication as it has greater potential of worsening the patient's condition, given the exacerbation of hyperuricemia and possible gout attacks. Although Metoprolol could potentially worsen depression, its use is not strictly contraindicated in patients with depression; guidelines suggest caution rather than contraindication. Furthermore, the patient's depression seems to be controlled with fluoxetine. In response, out of the given medications, Hydrochlorothiazide (C) may potentially take precedence as the most contraindicated medication due to its uric acid elevating properties, posing a serious risk to the patient's pre-existing gout.

**AI_D:** Restating the question: The gentleman in question here is middle-aged, who despite reporting overall wellness, has been experiencing consistently raised blood pressure ratings over the past two months. He suffers from major depressive disorder and gout. He is currently on medication for these conditions – fluoxetine for depression and allopurinol for gout. He also has a family history of cardiovascular disorders and personally has a prolonged history of smoking. Physically, he falls in the overweight category of BMI and has a slightly elevated blood pressure. There are five different drugs we are to consider: (A) Amlodipine, (B) Doxazosin, (C) Hydrochlorothiazide, (D) Lisinopril, and (E) Metoprolol. Our task is to determine which one among these should absolutely not be administered to him. Planning: We need to evaluate these drugs carefully, keeping in mind the patient's current medical conditions, his predispositions due to family medical history, and the influence of his lifestyle choices such as smoking. When analyzing the contraindication of a drug, we should consider its known side effects and interactions, and how they might affect the patient's present health conditions or potentially bring about new health concerns. The assumption here is that certain drugs, due to their adverse effects, might escalate existing conditions or trigger off new pathological states. Clinical reasoning: Let's discuss these drugs individually based on what we know about them and how they might affect this patient. Amlodipine (A) is a calcium channel blocker utilized for treating high blood pressure. Adverse effects can include flushing, headaches, and peripheral edema, but these do not seem particularly contraindicated for this patient. Doxazosin (B) is an alpha blocker used for treating high blood pressure and benign prostatic hyperplasia. It can cause a first-dose phenomenon characterized by fainting and dizziness, but is not contraindicated here. Lisinopril (D) is an angiotensin-converting enzyme (ACE) inhibitor and its side effects can include cough, hyperkalemia, or kidney damage. Nonetheless, since the patient doesn't have any kidney related issues, it isn't a contraindication. Metoprolol (E) is a beta blocker that can possibly worsen depressive symptoms and precipitate bronchospasm in patients with chronic respiratory issues. This patient has a history of depression, however, it's controlled with fluoxetine and there's no history of respiratory issues or bronchial asthma; hence, metoprolol isn’t entirely contraindicated. Hydrochlorothiazide (C) is a diuretic that is known to exacerbate gout, an existing condition in this patient, as it can increase uric acid levels in the blood. Allopurinol that this patient takes for gout could have its effect nullified by the usage of hydrochlorothiazide. Concluding: Although metoprolol may exacerbate depressive symptoms, these are controlled currently. The most considerable contraindication amongst the choices appears to be hydrochlorothiazide, due to its potential to increase uric acid levels and hence worsen gout which this patient suffers from. Response: So, in response to the original question, Hydrochlorothiazide (C) would be contraindicated in this patient, considering his existing medical condition of gout.

**Facilitator** The consensus among the AI council is that Hydrochlorothiazide (option C) is the most contraindicated medication for the 50-year-old male patient in question. This patient has a history of major depressive disorder and gout, and is currently on fluoxetine for depression and allopurinol for gout. He also has a family history of cardiovascular diseases and is a smoker. The council's reasoning is based on the potential adverse effects and interactions of the listed medications with the patient's existing conditions and current medications. Amlodipine, Doxazosin, and Lisinopril are deemed unlikely to negatively interact with the patient's conditions or medications. Metoprolol, a beta-blocker, could potentially exacerbate depressive symptoms and has potential interactions with smoking. However, the patient's depression is currently managed with fluoxetine, and the potential depressive effects of Metoprolol are inconsistently demonstrated across studies. The most significant concern is Hydrochlorothiazide, a thiazide diuretic, which could exacerbate gout due to its uric-acid-boosting properties. This presents a more defined risk, particularly as the patient has a diagnosed history of gout and is on allopurinol, a medication used for gout by inhibiting uric acid synthesis. Therefore, Hydrochlorothiazide is considered the most contraindicated medication for this patient.
