# Supplementary material for: Collaborative intelligence in AI: Evaluating the performance of a council of AIs on the USMLE
Source: PLOS Digit Health. 2025 Oct 9;4(10):e0000787. doi: 10.1371/journal.pdig.0000787 (PMC12510544; doi:10.1371/journal.pdig.0000787)
Supplement: S2 File — (ZIP) [file pdig.0000787.s002.zip › S2 Data Raw data files containing unprocessed question inputs and LLM outputs including transcripts of deliberations/STEP2_Question-118-1_Type-MC-NJ.docx]

**Facilitator** **[Round 1]:** A 23-year-old woman is brought to the emergency department by her friends 30 minutes after the sudden onset of nausea, facial flushing, and chest pressure while eating at a Thai restaurant. She does not have any known allergies to foods or medications. She had asthma during childhood but has not required treatment for 5 years. On arrival, she is diaphoretic and appears anxious. Temperature is 37.1°C (98.8°F), pulse is 92/min, respirations are 20/min, and blood pressure is 126/68 mm Hg. Pulse oximetry on room air shows an oxygen saturation of 99%. Examination of the neck shows erythema; no masses or nodules are palpated. Cardiopulmonary examination shows no abnormalities. Which of the following is the most likely diagnosis? (A) Asthma exacerbation (B) Carcinoid syndrome (C) Panic attack (D) Reaction to food additive (E) Systemic mastocytosis (F) Thyrotoxicosis

**AI_B:** RESTATING THE QUESTION: A 23-year-old female is experiencing sudden nausea, facial flushing, and chest pressure after eating at a Thai restaurant. Some childhood history of asthma is mentioned but no occurrence for the past 5 years. She appears anxious and is perspiring excessively. Vital sign indicates her temperature, pulse, respirations, blood pressure, and oxygen saturation are normal. There is some redness on her neck but no palpable masses or nodules. The question asks which of the provided conditions would explain her symptoms: asthma exacerbation, carcinoid syndrome, panic attack, reaction to food additive, systemic mastocytosis, or thyrotoxicosis. PLANNING: Assuming her symptoms relate to what she ate, her symptoms appear aligned with an allergic reaction, despite no known history. We could hypothesize a sensitivity or allergy to an unencountered foreign agent, for example, found in Thai cuisine. A reaction to a food additive (D) could be a logical assumption. CLINICAL REASONING: Reaction to food additive makes sense given the acute onset of her symptoms immediately after consuming food from a Thai restaurant which might have contained an additive to which she never had exposure before. The symptoms such as facial flushing and chest pressure suggest a systemic reaction possibly due to a release of histamines in her body. Although she has a previous history of asthma, her current symptoms do not support an asthma exacerbation as there was no mention of wheezing or breathing difficulty. Also, her oxygen saturation is normal and her cardiopulmonary examination doesn't show any abnormalities, ruling out conditions such as a panic attack or thyrotoxicosis which would have different presentation. Carcinoid syndrome and systemic mastocytosis are chronic conditions and would not likely manifest suddenly after a meal. CONCLUDING: Her symptoms likely point to a systemic reaction triggered by a consumed substance; specifically, the sudden onset suggests it could be a reaction to a food additive, which is commonly reported in individuals with no prior knowledge of such allergy or sensitivity. RESPONSE: The most likely diagnosis for this 23-year-old woman's condition, considering her symptoms, history, behavior and the temporal relationship with her meal is a Reaction to a Food Additive (D).

**AI_C:** RESTATING THE QUESTION: This 23-year-old female patient is experiencing sudden nausea, flushing of the face, and chest pressure after eating at a Thai restaurant. She has no known food or medication allergies and had childhood asthma, which has not necessitated treatment for the past five years. Upon her arrival in the emergency department, she appears anxious and diaphoretic, with signs of neck erythema but no palpable neck masses or nodules. Her vitals including temperature, pulse, respiration, blood pressure, and oxygen saturation are within normal range. The question asks for the most likely diagnosis given these symptoms and medical history. PLANNING: Some possible diagnoses could include asthma exacerbation, carcinoid syndrome, panic attack, reaction to food additives, systemic mastocytosis, and thyrotoxicosis. Each of those conditions does present with some of the symptoms our patient is exhibiting; however, we need to look at the entire clinical picture to determine which diagnosis is most likely. We should pay attention to the fact that these symptoms developed suddenly after having a meal. CLINICAL REASONING: Asthma exacerbation could be possible given her history, but patients usually present shortness of breath, wheeze, or cough, which are not reported here. Carcinoid syndrome could cause flushing but it's usually chronic and associated with diarrhea, which is not the case here. Panic attack may cause anxiety and chest pressure but is not likely connected to food intake. Thyrotoxicosis usually presents with signs of hyper metabolism like weight loss, heat intolerance, and thyromegaly, which are not present in this patient. Systemic mastocytosis is a rare disease characterized by an excessive accumulation of mast cells, and patients usually have recurrent episodes of flushing, pruritus, hives, diarrhea, and anaphylaxis. However, her immediate symptoms after eating Thai food may indicate a reaction to a specific food additive. Thai food often contains monosodium glutamate (MSG), a flavor enhancer that can trigger these symptoms, termed "Chinese Restaurant Syndrome" or MSG symptom complex. CONCLUDING: Taking into account her symptoms’ chronology and the lack of other physical findings which would be predicted with the other conditions, the most likely diagnosis is a reaction to a food additive, more specifically MSG. RESPONSE: (D) Reaction to food additive.

**AI_E:** (1) RESTATING THE QUESTION: The case presents a 23-year-old female who demonstrated symptoms of nausea, facial flushing, and chest discomfort following a meal at a Thai restaurant. After arrival at the emergency department, she was observed to be diaphoretic and anxious, with an erythematous neck but otherwise normal physical examination findings. The key question is to identify the most probable diagnosis from the provided options which include: asthma exacerbation, carcinoid syndrome, panic attack, reaction to a food additive, systemic mastocytosis, and thyrotoxicosis. (2) PLANNING: The immediate onset of symptoms after a meal could suggest a reaction to a food additive like monosodium glutamate often used in Thai food. However, allergic reactions would typically involve more systemic symptoms including skin rash and respiratory compromise. Further, this woman has no known food or medication allergies. An asthma exacerbation is less likely without reported wheezing or respiratory discomfort. Panic attacks typically present with palpitations, shortness of breath, or feelings of impending doom, which aren't clearly stated here. Systemic mastocytosis, carcinoid syndrome, and thyrotoxicosis are chronic illnesses that would likely have presented prior to this visible acute episode. Each has distinguishing features not apparent in this case. (3) CLINICAL REASONING: The sudden onset of nausea, facial flushing, and chest pressure associated with eating at a Thai restaurant seems to point towards a reaction triggered by something ingested during that meal. Given she has no known food allergies, a reaction to a food additive would be more likely than an asthma exacerbation, which usually presents with wheezing and breathing difficulty. Furthermore, her symptoms are not fully congruous with a panic attack. Chronic conditions like systemic mastocytosis, carcinoid syndrome, and thyrotoxicosis are less likely in an acute setting without previous manifestations and specific signs (e.g., chronic diarrhea in carcinoid syndrome, thyroid enlargement in thyrotoxicosis). (4) CONCLUDING: The provided evidence leads to the suggestion that the most likely diagnosis following clinical reasoning is a reaction to a food additive. This is congruous with the presentation of a sudden onset of symptoms after eating Thai food, a cuisine type known to prominently use food additives not commonly found in other food. (5) RESPONSE: Therefore, the most probable diagnosis in this case is (D) Reaction to a food additive.

**AI_D:** (1) RESTATING THE QUESTION: The clinical scenario presents a 23-year-old woman with sudden symptoms of nausea, facial flushing, and chest pressure occurring while having a meal at a Thai restaurant. Her past medical history is relevant of childhood asthma which has been stable for the past five years. She has no known food or drug allergies. Current visual examination reveals anxiousness, sweating (diaphoresis), and neck redness (erythema), however, there are no palpable neck masses or nodules. Her vital signs and oxygen saturation are within normal limits. We are tasked to come up with the most likely diagnosis based on these facts. (2) PLANNING: Evaluating the symptoms, timing, and setting might help us deduce the cause of her symptoms. Is it possible that she had an asthmatic reaction? But she hasn't needed treatment for years and her oxygen saturation is normal. Could it be carcinoid syndrome? Generally, it involves multiple symptoms over prolonged periods, and we don't see any vegetative signs here. A panic attack? Usually, it is associated with a trigger or stressor, which is not clearly present here. A reaction to a food additive? Thai cuisine uses various additives, which might have triggered an allergic response in her. Systemic mastocytosis? It is a very rare disorder involving various body systems over a long time period. Lastly, thyrotoxicosis? It can manifest with a myriad symptoms but her vitals do not strongly point to this diagnosis. Given the sudden onset, a reaction to a food additive seems the most fitting hypothesis. (3) CLINICAL REASONING: The presentation includes nausea, facial flushing, chest pressure, and erythema, all pointing towards a hypersensitivity reaction. Food allergies or sensitivities to specific food additives, for instance - monosodium glutamate (MSG) which is commonly used in Asian cuisine, have been known to elicit such reactions. This presentation often occurs shortly after ingestion—typically within minutes to a few hours—which aligns with the sudden onset here. Other symptoms often associated with MSG sensitivity includes rapid, fluttering heartbeats (palpitations), facial pressure or tightness, and sweating - features that mirror this patient's presentation. This condition is often termed the 'Chinese restaurant syndrome'. (4) CONCLUDING: Considering the timing of symptoms, their association with the ingestion of food, along with the absence of any major abnormalities upon physical examination, a certain food additive is most likely the culprit agent driving the patient's symptoms. (5) RESPONSE: Hence, the most likely diagnosis is (D) Reaction to food additive.

**AI_A:** RESTATING THE QUESTION: This scenario details the urgent case of a 23-year-old woman who presents with acute onset of nausea, facial redness, and chest discomfort while consuming food at a Thai restaurant. However, she doesn't have known allergies to foods or drugs. She experienced asthma during her childhood, but has not needed treatment for the past 5 years. Upon arrival at the hospital, she is sweating profusely and seems anxious. Her vitals include a normal temperature and slightly elevated heart rate, with normal breathing and blood pressure. Blood oxygen level is normal as well. A physical examination shows redness of her neck but no observable nodules or masses. Her heart and lungs examination is also reported to be normal. The question at hand is to determine the most probable condition she may be suffering from among the given choices, which include an asthma exacerbation, carcinoid syndrome, panic attack, reaction to food additive, systemic mastocytosis, and thyrotoxicosis. PLANNING: First, let us break down our assumptions. On an apparent level, given the context of her having been at a restaurant, it would be easy to jump to the conclusion that her symptoms are food-related, such as an allergy or a reaction to a food additive. However, her history of not having known allergies to food or medication challenges this assumption. She also has a history of childhood asthma which has been inactive for years, and her current symptoms may seem suggestive of an asthma attack, but her perfectly normal Cardiopulmonary examination contradicts this. The symptoms could also be the manifestation of a panic attack, but this assumption could be challenged by the sudden onset and the specific location (i.e., a restaurant). Conditions like carcinoid syndrome, systemic mastocytosis, and thyrotoxicosis also need to be considered, albeit less likely due to the acute onset of symptoms and lack of other pathognomonic signs. The strongest assumptions to take forward are a reaction to a food additive and panic attack. CLINICAL REASONING: Let's reason through these assumptions. A reaction to a food additive implies she might have consumed something that her body negatively reacted to. MSG (Monosodium Glutamate) is commonly used in Thai and other Asian cuisines, and some people are known to have an intolerance to this food additive, hence causing a syndrome known as "Chinese Restaurant Syndrome." Symptoms are similar to what this woman is currently experiencing: flushing, palpitations (suggestive by her high pulse rate), and chest pressure. Moreover, her symptom onset quickly occurred while eating at a restaurant, further supporting this assumption. In contrast, a panic attack is indeed plausible given the appearance of anxiety and the diaphoresis, however, panic attacks usually don't cause skin flushing, and the sudden onset of symptoms at a Thai restaurant suggests a more direct trigger than a mental health scenario. CONCLUDING: In conclusion, while it seems, the symptoms may be explained through several diagnostic lenses, given the abrupt onset of symptoms specifically while eating in a Thai restaurant, in a woman with no prior food allergies or related histories suggests, quite strongly, a reaction to a food additive might be the underlying cause. RESPONSE: The most likely diagnosis for this 23-year-old woman's sudden onset of nausea, facial flushing, and chest pressure, along with elevated heart rate and sweating, is (D) Reaction to food additive, specifically MSG which is commonly found in Thai cuisine and can cause such symptoms.

**Facilitator** The consensus among the AI responses is that the most likely diagnosis for the 23-year-old woman's sudden onset of nausea, facial flushing, and chest pressure, along with elevated heart rate and sweating, is a reaction to a food additive, specifically monosodium glutamate (MSG). This conclusion is based on the immediate onset of symptoms after consuming food at a Thai restaurant, a cuisine known to use MSG, and the absence of other symptoms that would suggest alternative diagnoses such as asthma exacerbation, carcinoid syndrome, panic attack, systemic mastocytosis, or thyrotoxicosis. The woman's symptoms align with those commonly associated with MSG sensitivity, also known as "Chinese Restaurant Syndrome." Despite her history of childhood asthma, the normal cardiopulmonary examination and lack of respiratory distress make an asthma exacerbation unlikely. Similarly, the absence of specific signs and symptoms related to the other conditions, along with the acute onset of her symptoms, make them less probable. Therefore, the most probable diagnosis is (D) Reaction to food additive.
