# Supplementary material for: Collaborative intelligence in AI: Evaluating the performance of a council of AIs on the USMLE
Source: PLOS Digit Health. 2025 Oct 9;4(10):e0000787. doi: 10.1371/journal.pdig.0000787 (PMC12510544; doi:10.1371/journal.pdig.0000787)
Supplement: S2 File — (ZIP) [file pdig.0000787.s002.zip › S2 Data Raw data files containing unprocessed question inputs and LLM outputs including transcripts of deliberations/STEP2_Question-119-1_Type-MC-NJ.docx]

**Facilitator** **[Round 1]:** A 45-year-old woman returns to the office to discuss results of a fine-needle aspiration biopsy of a 0.5-cm right breast mass obtained 1 week ago. She is unaccompanied. Medical history is unremarkable and she takes no medications. Her mother and maternal aunt were diagnosed with breast cancer at the ages of 45 and 52 years, respectively. Physical examination discloses no palpable axillary nodes. The biopsy specimen showed malignant cells. The patient is counseled that she has an excellent chance of cure with lumpectomy and radiation therapy. She adamantly refuses this treatment plan and states that she will try natural therapies, including megavitamins and herbal remedies instead. After exploring the reasons behind the patient's decision, which of the following is the most appropriate response? (A) Advise the patient that there is no proof that these alternative therapies work and that she is essentially throwing her life away (B) Agree to the alternative therapies and schedule a follow-up visit in 6 months (C) Ask the patient if she would be willing to return to the office in 2 weeks to discuss treatment options again (D) Inform the patient that she will be referred to another physician based on her treatment decision

**AI_E:** RESTATING THE QUESTION: The case discusses a 45-year-old woman who has recently been diagnosed with breast cancer after a biopsy of a 0.5-cm mass in her right breast. Despite being advised that she has a high chance of cure with lumpectomy and radiation therapy, she rejects this treatment, choosing instead to use alternative therapies like megavitamins and herbal remedies. The question asks for the most appropriate response to this decision. PLANNING: The assumptions are that based on scientific evidence lumpectomy and radiation therapy are viable and effective treatment options for her condition. An alternative assumption would be that the patient has autonomy and the ability to make informed decisions about her health. It's also assumed that the patient fully understands the implications of her choice. We could therefore surmise that the patient's choice might not be fully informed, or she might have fears or misconceptions about the recommended treatment. CLINICAL REASONING: By considering the patient's express desire not to undergo lumpectomy and radiation therapy, we respect her autonomy, a pivotal principle in medical ethics. However, ensuring that the patient is making an informed decision is also crucial. It might be that the patient's decision stems from erroneous information or fears. It's crucial to explore these possibilities and ensure that the patient truly comprehends the implications of forgoing the recommended treatment for alternative remedies. CONCLUDING: A balanced response would be ensuring that the patient has fully understood the consequence of her decision and provide an additional opportunity to discuss and possibly reconsider her stance. RESPONSE: Thus, the most appropriate response would be (C): Ask the patient if she would be willing to return to the office in 2 weeks to discuss treatment options again. This approach respects the patient's autonomy while also allowing time for further digestion of the information and ensuring that she fully understands the decision she is making.

**AI_B:** RESTATING THE QUESTION: A woman of 45 years with a familial history of breast cancer revealed a malignant breast mass via a fine-needle aspiration biopsy. After being informed of a promising treatment plan involving lumpectomy and radiation therapy, she decided against it opting for natural therapies. After understanding the reasons for her decision, we need to determine the best clinical response. PLANNING: Assumptions that can be identified from the question include the patient's understanding of her disease, the risk and benefits of both conventional and alternative treatments, and her capacity to make well-informed decisions about her health. An alternate assumption could be that the patient might be unsuitable to comprehend her situation completely, which has led her to prefer an unconventional method of treatment. Another assumption could be that she is afraid of the side effects and potential complications of conventional treatments. Evaluating the assumptions, it seems crucial to focus on her understanding of the disease and its implications, and how well she comprehends the proposed treatment plan. CLINICAL REASONING: A patient's comprehension of her disease and treatment varies, especially in a stressful situation like cancer with a poor prognosis. Her fears and beliefs need to be addressed, and more information about her perception of the situation should be obtained. If her understanding is incomplete, incorrect, or overwhelmed by anxiety, it may influence her decision-making capacity. Hence, she should be given time to process this diagnosis as it is something significant and life-altering. CONCLUDING: The patient's capacity to make a well-informed decision about her health needs to be examined and respected. Simultaneously, the physician's role is to provide accurate information and guidance, create a therapeutic alliance, and not coerce her into any treatment decision. RESPONSE: (C) Ask the patient if she would be willing to return to the office in 2 weeks to discuss treatment options again. This response would incorporate respect for patient autonomy and support constant responsible medical consultation, promoting a shared decision-making process, rather than forcing a decision on the patient. As a provider, it's important to offer patient-centered care that weighs the patient's wishes, even if they diverge from conventional practices.

**AI_C:** 1) RESTATING THE QUESTION: This scenario presents a 45-year-old woman diagnosed with breast cancer through a fine-needle aspiration biopsy. She has family history of breast cancer but is otherwise healthy, with non-detectable axillary nodes. After being advised of a good prognosis following lumpectomy and radiation therapy, she rejects this option in favor of natural therapies. The question asks how best to respond to her decision. 2) PLANNING: The alternate assumptions to her choice are that she perhaps lacks complete understanding of her condition, her prognosis and the potential efficacy of her chosen therapies. Or, she may have her personal reasons, fears or beliefs that led her to her decision, or she may even be in a state of denial, requiring further psychological evaluation. Evaluating these, the most clinically relevant issue to address first should be her understanding and acceptance of her condition and its implications. 3) CLINICAL REASONING: Given her family history and the fact that she is unaccompanied, the impact of her diagnosis and what it could entail for her personally might indeed have been hard to fully comprehend. A responsible clinical approach would be to ensure she first fully understands her condition, its implications, the recommended treatment options and the lack of evidence-based efficacy of her chosen alternative treatments. 4) CONCLUDING: It's crucial to approach this from an educational and understanding standpoint first. Before decisions that could affect her survival and wellbeing significantly are made, she should have all possible information, inputs and support. Therefore, it is best for her to return to the office for another round of discussion about these aspects. 5) RESPONSE: The most appropriate response in this situation is (C): Ask the patient if she would be willing to return to the office in 2 weeks to discuss treatment options again. This would allow her to be adequately informed and supported in processing her diagnosis and proposed treatment options. It also fosters patient autonomy and involvement in decision-making about her own health, a fundamental principle in medicine.

**AI_A:** RESTATING THE QUESTION: A middle-aged woman with a strong family history of breast cancer and a positive breast biopsy for malignancy rejects the suggested medical treatment comprising a lumpectomy and radiation therapy. In favor of her decision, she opts for natural therapies such as megavitamins and herbals remedies. The question prompts the most suitable response from the available options after understanding her rationale for this decision. PLANNING: The assumptions here are that the patient realizes the gravity of her diagnosis, she understands the proven efficacy of the recommended treatment in extending survival and minimizing recurrence, and she acknowledges the lack of scientific support for the efficacy of the alternative therapies she prefers. The alternate assumption could be that she may harbor fear or mistrust toward mainstream medical treatments, or simply be uninformed or misinformed about the gravity of her disease and the benefits and risks of the respective treatments. The chosen assumption to explore further would be her potential lack of complete understanding of her condition and its treatments. CLINICAL REASONING: As a rule of thumb in clinical practice, respect for patients' autonomy is critical. They have a right to refuse treatment. However, it's also important for healthcare professionals to ensure that a patient's decision is informed. This implies the patient has a complete understanding of her medical condition, the potential benefits and risks of recommended treatments, as well as those of the alternative therapies she prefers, and still, she chooses to continue with her decision. CONCLUDING: Based on the respect for patients' autonomy, the need to make sure her decision is informed, and the possibility that she may not fully understand her medical situation and treatments, it's appropriate to engage her in further conversation about her disease and treatments. RESPONSE: Hence, option (C) - "Ask the patient if she would be willing to return to the office in 2 weeks to discuss treatment options again" would be the most suitable response. This approach provides an opportunity for further education and discussion which may lead to an informed decision being made by the patient.

**AI_D:** RESTATING THE QUESTION: A middle-aged woman, with family history of breast cancer, has recently been diagnosed with a 0.5-cm right breast mass which on biopsy showed malignancy, is refusing the proposed plan of treatment (lumpectomy and radiation therapy). Instead, she's opting for untested, natural remedies including herbal treatments and megavitamins. In light of this, we have to decide the most suitable response to her decision. PLANNING: Upon hearing her refusal, several assumptions could be made. First, she may be scared or have misconceptions about the proposed line of treatment. Second, she might believe that the natural therapies, including megavitamins and herbal remedies could cure her without having to experience the side effects associated with surgery and radiation. Now, examining these assumptions, we need to figure out if there's any validity in her chosen path or if our medical expertise should reign supreme. CLINICAL REASONING: Breast cancer is a serious and deadly disease if not treated properly. Therefore, it is important to approach the patient's treatment with surgery and radiation, which have proven efficacy and survival benefits. While alternative therapies might alleviate symptoms, they do not cure or treat underlying diseases. The patient's refusal of treatment might be influenced by fear, lack of knowledge, misunderstanding, or cultural beliefs. We should not disregard or belittle the patient's choices, instead, we must respect her values and concerns and attempt to guide her towards an informed and balanced decision. Alternative therapies might worsen her condition without proper treatment. CONCLUDING: Considering all this information, the main priority is to communicate with the patient, respect her decision but also provide more insights, knowledge, and guidance about her condition, as her prognosis with proven therapeutic methods is excellent. It would not be beneficial to abruptly inform her that her decision is equivalent to "throwing her life away", nor to postpone her follow-up for 6 months as her condition might deteriorate. Similarly, referring the patient to another physician based on her treatment decision might give the impression that we're abandoning her. RESPONSE: The most suitable approach would be to ask the patient if she will return to the office in two weeks to discuss these options once again (Option C). This ensures her autonomy, provides her with a window for reflection, and allows for more in-depth dialogue about her health.

**Facilitator** The consensus statement synthesizes the responses as follows: The scenario presents a 45-year-old woman with a strong family history of breast cancer who has been diagnosed with a malignant breast mass. Despite being advised of a promising prognosis with lumpectomy and radiation therapy, she rejects this treatment plan in favor of natural therapies. The question asks for the most appropriate response to her decision. Several assumptions can be made about her decision. She may fully understand her diagnosis and the efficacy of the recommended treatment, but prefers alternative therapies due to personal beliefs or fears. Alternatively, she may lack a complete understanding of her condition and the potential benefits and risks of the respective treatments. It's also possible that she may be in a state of denial or have misconceptions about the proposed treatment. Respect for patient autonomy is a fundamental principle in medical ethics. Therefore, her decision to refuse treatment should be respected. However, it's also crucial to ensure that her decision is informed. This means she should have a complete understanding of her medical condition, the potential benefits and risks of recommended treatments, as well as those of the alternative therapies she prefers. Given the gravity of her diagnosis and the potential implications of her decision, it's important to engage her in further conversation about her disease and treatments. This approach provides an opportunity for further education and discussion, which may lead to an informed decision being made by the patient. Therefore, the most appropriate response is to ask the patient if she would be willing to return to the office in 2 weeks to discuss treatment options again (Option C). This response respects her autonomy, provides her with a window for reflection, and allows for more in-depth dialogue about her health. It also fosters patient autonomy and involvement in decision-making about her own health, a fundamental principle in medicine.
