# Supplementary material for: Collaborative intelligence in AI: Evaluating the performance of a council of AIs on the USMLE
Source: PLOS Digit Health. 2025 Oct 9;4(10):e0000787. doi: 10.1371/journal.pdig.0000787 (PMC12510544; doi:10.1371/journal.pdig.0000787)
Supplement: S2 File — (ZIP) [file pdig.0000787.s002.zip › S2 Data Raw data files containing unprocessed question inputs and LLM outputs including transcripts of deliberations/STEP2_Question-12-1_Type-MC-NJ.docx]

**Facilitator** **[Round 1]:** Abstract: Objective: In patients with cirrhosis and acute bleeding esophageal varices, how do endoscopic sclerotherapy and emergency portacaval shunt compare for control of bleeding and survival? Methods Design: Randomized controlled trial (San Diego Bleeding Esophageal Varices Study). ClinicalTrials.gov NCT00690027. Allocation: Concealed. Blinding: Blinded (gastroenterologist who evaluated patients for portal-systemic encephalopathy). Follow-up period: Up to 17 years. Setting: University of California San Diego Medical Center. Patients: 211 patients (mean age 49 years, 77% men) with acute bleeding esophageal varices resulting from cirrhosis, who required a transfusion of ≥ 2 units of blood and, for patients transferred from other hospitals, observation of upper gastrointestinal bleeding within 48 hours of transfer. Exclusion criterion was > 1 previous session of endoscopic sclerotherapy. Intervention: Endoscopic sclerotherapy (n = 106) or emergency portacaval shunt (n = 105). Emergency portacaval shunt comprised a direct side-to-side or direct end-to-side portacaval shunt done within 8 hours of initial contact. Outcomes: Control of bleeding at > 30 days, survival, readmissions for variceal or nonvariceal bleeding requiring transfusion of packed red blood cells, and recurrent portal-systemic encephalopathy. Patient follow-up: 100% (minimum follow-up until death or 9.4 years). Main results 15-year survival was lower with endoscopic sclerotherapy than with emergency portacaval shunt (10/106 vs 48/105, relative benefit reduction 79%, 95% CI 62 to 89; number needed to harm 3, CI 2 to 4). Other main results are shown in the Table. Endoscopic sclerotherapy (EST) vs emergency portacaval shunt (EPCS) in patients with cirrhosis and acute bleeding esophageal varices Outcomes | Child-Pugh risk class | EST | EPCS | P value | ———————————————————————————————————— Control of bleeding at > 30 days* | | 20% | 100% | <.001 | Median survival (years) | A | 4.62 | 10.43 | .003. | | B | 2.61 | 6.19 | <.001 | |. C | 0.58 | 5.30 | .005. | Mean number of readmissions for variceal bleeding requiring packed red blood cell transfusion | | 6.8 | 0.4 | <.001 | Recurrent portal-systemic encephalopathy† | | 35% | 15% | < .001 | ——————————————————————————————————— *Excluding indeterminate deaths at 14 days from nonbleeding causes. †In patients who survived 30 days and left hospital. ——————————————————————————————————— Conclusion In patients with cirrhosis and acute bleeding esophageal varices, emergency portacaval shunt was better than endoscopic sclerotherapy for control of bleeding, recurrent encephalopathy, and survival. Sources of funding: National Institutes of Health and Surgical Education and Research Foundation. Structured abstract based on: Orloff MJ, Isenberg JI, Wheeler HO, et al. Randomized trial of emergency endoscopic sclerotherapy versus emergency portacaval shunt for acutely bleeding esophageal varices in cirrhosis. J Am Coll Surg. 2009;209:25-40. 19651060 Which of the following most strongly limits the generalizability of this study's findings? (A) The allocation was concealed (B) EPCS is available only at specialty centers (C) The follow-up period was too short (D) The patients were not blinded (E) Unmeasured confounders were not controlled by the study design

**AI_E:** RESTATING THE QUESTION: The question is asking for the factor that most greatly constrains the broad applicability (or generalizability) of this study's results. To make this assessment, one must evaluate each proposed factor against the particulars of the study. PLANNING: Each potential choice has its potential consequences; however, some have more significant consequences. (A) Concealed allocation affects the investigators' knowledge of the group to which a patient may be assigned, thus reducing the potential for bias. This enhances the study's internal validity, rather than limiting its generalizability. (B) If EPCS (Emergency Portacaval Shunt) is only available at specialized centers, it would limit the widespread applicability of the study's findings as not all hospitals or healthcare facilities would have access to this intervention. (C) Given the follow-up period extended up to 17 years, it unlikely to limit generalizability. (D) The absence of patient blinding could theoretically influence patient-reported outcomes, but it should not have influenced major endpoints such as control of bleeding or survival. (E) Uncontrolled confounders could impact the internal validity, but it's not a specific issue concerning the generalizability of the study. CLINICAL REASONING: In this case, the factor that most limits the generalizability is the restricted access to the EPCS intervention, as only specialty centers typically provide this procedure. A positive outcome from a procedure only available in specialized centers may not have the same results when replicated in a non-specialized setting. CONCLUDING: Given the higher degree of speciality and resources needed for EPCS intervention, its limited access makes the findings of the study less applicable to the general population or facilities that do not have this specific capability. RESPONSE: Therefore, (B) EPCS is available only at specialty centers is the option that most limits the generalizability of this study's findings.

**AI_B:** RESTATING THE QUESTION: We are trying to understand what element from the study design or execution most significantly impacts our ability to apply the results of this research on cirrhotic patients with acute bleeding esophageal varices treated either with endoscopic sclerotherapy or emergency portacaval shunt to a broader population. The generalizability of a study refers to its applicability in different settings or populations. PLANNING: In considering the options: (A) The concealment of allocation refers to ensuring that the people who enroll participants do not know in advance which treatment the next person will get. This helps prevent selection bias and usually improves the validity of the study. (B) If EPCS is only available at specialty centers, this could limit how widely the treatment can be adopted. (C) A short follow-up period could limit our understanding of long-term outcomes, however, in this study, a 17-year follow-up period is considerably long. (D) Patient blinding in this context may not be feasible as the treatments are notably different. Moreover, blindness may not notably bias the results of these objective outcomes, such as survival rates, blood transfusion requirements, and recurrence of encephalopathy. (E) Unmeasured confounders can theoretically affect any study, although randomized controlled trials are designed to eliminate this issue as much as possible. Out of all these, the accessibility of emergency portacaval shunt treatment seems to pose a plausible limitation. CLINICAL REASONING: The results show that the use of emergency portacaval shunt resulted in superior outcomes compared to endoscopic sclerotherapy in terms of control of bleeding, survival, lower readmission rates, and less recurrent portal-systemic encephalopathy. However, it's important to keep in mind that being a procedure of higher complexity, emergency portacaval shunts are typically available only at specialty centers. This may limit the ability to apply these findings to hospitals or centers that lack the expertise or resources to perform this procedure, as complexity involves specific technical skills and facilities. CONCLUDING: Attributing this, the generalizability of the study's findings may be most limited by the fact that emergency portacaval shunts, despite showing superior outcomes, may only be performed at specialty centers (Option B). RESPONSE: The statement limiting the generalizability of this study's findings most is "(B) EPCS is available only at specialty centers".

**AI_D:** RESTATING THE QUESTION: The query involves identifying what can potentially limit the application or generalization of the findings from this randomized controlled trial. The trial investigates the efficacy of endoscopic sclerotherapy (EST) and emergency portacaval shunt (EPCS) for controlling acute bleeding in esophageal varices secondary to cirrhosis and evaluates their impact on patient survival. Some potential limitations of the study could be the allocation concealment, accessibility of EPCS procedure, follow-up duration, blinding of patients, or the presence of uncontrolled confounders. PLANNING: In terms of the research process, (A) allocation concealment is generally a strength rather than a limitation of a study as it prevents selection bias. (B) the availability of EPCS only at specialized centers could limit the generalizability of the results, unless the population being studied has easy access to these centers. (C) the study had a follow-up period of up to 17 years, which is not typically considered short in clinical trials. (D) while patients were not blinded, this may not necessarily limit the study's generalizability unless the unblinded status greatly influenced the results or was likely to introduce significant bias. For instance, in a scenario where the patients' knowledge of their treatment could influence their survival, this could be seen as a limitation. (E) Unmeasured confounders not controlled by the study design could limit generalizability but would depend on the specifics of the confounders. CLINICAL REASONING: Upon evaluating these assumptions, the generalizability of the study's results would likely be most strongly restricted by the limited availability of EPCS at specialized centers, as this could hinder broad application of this treatment approach. CONCLUDING: Through a process of elimination and understanding the role each component plays in research, it becomes evident that not all patients globally have access to specialty centers where EPCS is available. This limits the application of the study's findings on a larger, global scale. RESPONSE: Hence, the best answer choice is (B) EPCS is available only at specialty centers. This factor is the most likely to limit the generalizability of this study's results, as not all facilities globally will have the specialized resources necessary to provide this treatment. This means that despite the results being favoring EPCS, it might not be applicable or implementable across all hospitals and this could impact its generalizability.

**AI_A:** RESTATING THE QUESTION: The question is asking about the potential limiters to the generalizability—the applicability of the results to other settings, populations, or conditions—of this clinical study's results comparing endoscopic sclerotherapy and emergency portacaval shunt in the treatment of acute bleeding esophageal varices due to cirrhosis. The options provided involve elements of the study's design, setting, and potential biases inherent to the design. PLANNING: The assumptions held by the study are that the allocation concealment, the restricted availability of the EPCS procedure, the length of the follow-up period, the lack of blinding in patients, and the presence of uncontrollable unmeasured confounders probably influence the application of the findings outside the context of this study. The alternative assumption is that the generalizability is limited by the option that is most likely to have restricted the study's application in different clinical contexts. CLINICAL REASONING: Of the provided options, 'allocation concealment' (Option A) is a method that reduces selection bias and is desirable in clinical trials. 'The follow-up period was too short' (Option C) contradicts the evidence in the question stem as we see a lengthy follow-up period of up to 17 years. 'The patients were not blinded' (Option D) may result in performance bias. However, it’s practically impossible to blind patients to these different treatment modalities in this context. Option E—'Unmeasured confounders were not controlled by the study design'—is indeed a limitation but it's an inherent limitation present in every observational study. However, option B—'EPCS is available only at specialty centers'—stands out as the limitation that is most likely to affect the generalizability of the study outcomes. If EPCS is only available in specialty centers, the results of this study can't be easily applied to the broad population, especially in settings where such specialty centers are not readily available. CONCLUDING: After a thorough assessment of the options, the limited availability of emergency portacaval shunt in specialty centers could be the primary factor restricting the widespread applicability of the study's findings. Factors like limited equipment, advanced surgical expertise, and the logistical and financial operability of these centers could restrict the conversion of the study's findings into a common practice. RESPONSE: Hence, the most robust factor that strongly limits the generalizability of this study's findings is (B) 'EPCS is available only at specialty centers'.

**AI_C:** RESTATING THE QUESTION: The query is soliciting an identification of the most poignant limiting factor that hampers the generalizability of a given study's findings. This study itself is a randomized controlled trial (RCT), which investigated the efficacy of endoscopic sclerotherapy and emergency portacaval shunt in controlling bleeding and survival in patients with cirrhosis and acute bleeding esophageal varices. Here, 'generalizability' refers to the applicability of the study's results beyond the specific sample population within the study. PLANNING: Upon analyzing the options, we should consider the following assumptions for each response: (A) Concealed allocation may impact internal validity but does not necessarily limit generalizability. (B) Availability of emergency portacaval shunt (EPCS) only at specialty centers can indeed limit widespread application of the procedure, affecting generalizability. (C) A follow-up period is considered short if it doesn't cover a sufficient timeframe to capture the outcomes of interest; however, in this case, a 17 years follow-up seems exhaustive. (D) Blinding of patients could impact the study's internal validity, yet patient knowledge of their assigned intervention would not affect the generalizability of the findings. (E) The presence of uncontrolled/unmeasured confounders could affect the study's internal validity but have limited influences on its generalizability. CLINICAL REASONING: Commencing with option (A), in clinical trials, the allocation should indeed be concealed to prevent selection bias and protect the study's internal validity. However, the external validity, or generalizability, is influenced more by the study population, intervention, and environment rather than by allocation concealment. As for (B), if EPCS is available only in specialty centers, it might suggest a requirement for specific infrastructure or expertise not widely available. Hence, findings from this trial might not be generalizable to settings lacking these resources. Option (C) seems unlikely. A follow-up of 17 years is sufficiently long to capture long-term outcomes linked to cirrhosis and acute esophageal variceal bleeding, so it seems unlikely that a 'short' follow-up would limit generalizability. For (D), blinding in clinical trials typically relates to avoiding measurement bias and ensuring internal validity. Unblinded patients might be predisposed to placebo or nocebo effects, but their knowledge of the assigned intervention would not inhibit the study's generalizability. Lastly, (E), unmeasured confounders could indeed confound the study results, but they are typically considered a threat to internal validity and not to generalizability. CONCLUDING: On systematically evaluating the options, it emerges that the limited availability of the emergency portacaval shunt (EPCS) only at specialty centers would hinder the widespread application of the intervention, thereby constraining the generalizability of the study results. RESPONSE: Therefore, the correct answer is (B) EPCS is available only at specialty centers. This significantly restricts the applicability of the study's findings across diverse healthcare setups, thus limiting the study's generalizability.

**Facilitator** The consensus statement synthesizing the responses of AI_A, AI_B, AI_C, AI_D, and AI_E is as follows: The question seeks to identify the factor that most significantly limits the generalizability, or broad applicability, of the study's findings. The study in question is a randomized controlled trial comparing the efficacy of endoscopic sclerotherapy and emergency portacaval shunt in controlling acute bleeding in esophageal varices due to cirrhosis. Upon analyzing the options, it is clear that each has potential implications for the study's generalizability. However, the consensus among the AI responses is that option (B) 'EPCS is available only at specialty centers' is the most significant limiting factor. This is because the limited availability of EPCS at specialty centers could hinder the widespread application of this treatment approach, thereby constraining the generalizability of the study results. While other factors such as allocation concealment, patient blinding, and unmeasured confounders can impact the internal validity of a study, they do not necessarily limit its generalizability. The follow-up period of up to 17 years is not considered short and thus does not limit the study's generalizability. In conclusion, the factor that most strongly limits the generalizability of this study's findings is the limited availability of EPCS at specialty centers. This is due to the fact that not all healthcare facilities globally have the specialized resources necessary to provide this treatment, which could impact the applicability of the study's findings across diverse healthcare setups.
