# Supplementary material for: Collaborative intelligence in AI: Evaluating the performance of a council of AIs on the USMLE
Source: PLOS Digit Health. 2025 Oct 9;4(10):e0000787. doi: 10.1371/journal.pdig.0000787 (PMC12510544; doi:10.1371/journal.pdig.0000787)
Supplement: S2 File — (ZIP) [file pdig.0000787.s002.zip › S2 Data Raw data files containing unprocessed question inputs and LLM outputs including transcripts of deliberations/STEP2_Question-120-1_Type-MC-NJ.docx]

**Facilitator** **[Round 1]:** A 47-year-old woman comes to the office because of a 2-month history of progressive weakness and numbness of her right leg. She has a 3-year history of type 2 diabetes mellitus treated with metformin. She has worked as a stock person in a storeroom for 12 years; she often moves and lifts heavy objects and sometimes crawls into tight spaces. On neurologic examination, muscle strength is 5−/5 in the hip extensor, thigh abductor, hamstring, and gastrocnemius muscles on the right and intact on the left. Muscle strength is intact in the iliopsoas, thigh adductor, quadriceps, and tibial anterior muscles bilaterally. Deep tendon reflexes are 1+ in the right ankle and 2+ in the left ankle. Sensation to light touch is mildly decreased over the lateral and inferior aspects of the right foot. Sensation otherwise is intact. Which of the following is the most likely diagnosis? (A) Common peroneal neuropathy (B) Femoral neuropathy (C) L2 radiculopathy (D) L4 radiculopathy (E) Obturator neuropathy (F) S1 radiculopathy

**AI_B:** RESTATING THE QUESTION: This question describes a 47-year-old woman with a history of type 2 diabetes mellitus who presents with weakness and numbness in her right leg for 2 months. Key findings in her examination include 5/5 muscle strength in multiple right leg muscles, 1+ deep tendon reflex in her right ankle and mild decrease in sensation in her right foot. The question asks which of the following options is the most likely diagnosis given the symptoms and results from her examination. PLANNING: Pathological conditions of the nervous system often present with sensory or motor deficit symptoms, and the location of these deficits can often help in localising the level of neural compromise. We will need to consider the distribution of sensory and motor deficits, any specific actions that the patient has difficulty with, and any signs of lower motor or upper motor neuron symptoms. There are common peroneal neuropathy, femoral neuropathy, L2 radiculopathy, L4 radiculopathy, obturator neuropathy, and S1 radiculopathy options. CLINICAL REASONING: The clinical picture and neurological examination findings are most consistent with an isolated peripheral nerve lesion. Particular attention should be paid to the weakened foot dorsiflexion, decreased sensation over the right foot, and decreased right ankle reflex, which suggest the peripheral nerve involved is more likely distal. Among the options, common peroneal neuropathy more closely fits the presentation. This neuropathy can occur due to type 2 diabetes mellitus, which our patient has. It can also occur due to injury or compression of the nerve, typically in situations where the nerve's superficial location at the fibular head puts it at risk. This risk may be increased by the patient's occupation, which involves moving heavy objects and occasionally crawling. CONCLUDING: Given that the woman's symptoms include progressive weakness and numbness of her right leg, along with an isolated decrease in sensation along her right foot and slight decrease in her right ankle reflex, it's most likely that she's experiencing common peroneal neuropathy. This is further supported by her occupation and existing health conditions. RESPONSE: Therefore, based on the patient's history, examination findings, and our clinical reasoning, the most likely diagnosis is (A) Common peroneal neuropathy.

**AI_D:** (1) RESTATING THE QUESTION: This clinical situation poses a diagnostic question about a middle-aged woman experiencing progressive weakness and numbness in her right lower limb. This woman has a history of type 2 diabetes mellitus, and her occupation involves considerable physical activity, including lifting and shifting heavy objects. Neurologic examination reveals mildly decreased right foot sensation and a slight disparity between deep tendon reflexes in the ankles. The decision needs to be made between radiculopathy at varying lumbar nerve root levels and specific peripheral neuropathies. (2) PLANNING: The diagnosis could be radiculopathy involving the L2, L4, or S1 nerve roots or neuropathy involving common peroneal, femoral, or obturator nerves. Radiculopathy refers to a condition where the nerve roots leading away from the spine get compressed or irritated, resulting in symptoms at the locations innervated by the affected nerve(s). Peripheral neuropathy involves damage to the peripheral nerves causing weakness, numbness, and pain. An essential assumption is whether there is evidence of nerve compression in the spine or in peripheral regions. (3) CLINICAL REASONING: Key pieces of information from the neurologic exam include intact muscle strength in the iliopsoas, thigh adductor, quadriceps, and tibialis anterior muscles on both sides; relatively decreased deep tendon reflex in the right ankle compared to the left; and a mild decrease in light touch sensation involving the lateral and inferior aspects of the right foot. With bilateral intact muscle strengths coupled with unilateral sensory impairment, this suggests focus on peripheral neuropathy as the clinical priority. Also, the specific description of sensory impairment in the lateral and inferior aspects of the right foot provides a clue. (4) CONCLUDING: The accumulated evidence leans towards a peripheral neuropathy possibly due to her occupational activities and long-term disease state which can potentially damage peripheral nerves. As the clinical features mentioned do not fit femoral, obturator, L2, L4, or S1 radiculopathy, diagnosis option A best explains the condition. (5) RESPONSE: The most likely diagnosis, given the clinical presentation and neurologic examination findings, is common peroneal neuropathy (A), which is consistent with the pattern of impairment of light touch sensation along the lateral and inferior aspects of the right foot.

**AI_C:** (1) RESTATING THE QUESTION: The query revolves around a 47-year-old female who has reported to the hospital with a two-month history of increasing leg weakness and numbness exclusively on the right side. She has a past medical history of type 2 diabetes mellitus that has been managed with metformin for the past three years. Her profession is physically demanding, often requiring considerable manual labor. Her neurological examination shows predominant muscle strength on the right side but noticeably diminished deep tendon reflexes and reduced perception to light touch on the right foot's lateral and inferior aspects. We have to identify the most likely diagnosis from the options provided. (2) PLANNING: There's an array of assumptions to work with. The initial assumption looks at the correlation between diabetes and progressive weakness and numbness. Another assumption would be considering the lady's occupation as a possible factor contributing to her symptoms. Her job demands might cause excessive strain, leading to certain types of neuropathies or radiculopathies. The examination findings of 1+ deep tendon reflexes and reduced sensory touch in the right foot's lateral and inferior aspects can lead us to an alternate assumption, whereby these findings could be indicative of localized neurological damage rather than systemic. (3) CLINICAL REASONING: Each assumption brings forth an explanation. The lady's progressive weakness and numbness could be a result of deteriorating peripheral neuropathy, a common complication associated with chronic diabetes. However, her symptoms are rather localized and not bilateral, as often seen in diabetic neuropathy. Then considering her demanding job aspect, this could lead to certain types of neurological damages, but again not selective to one side. The notion of localized neurological damage directs our attention to specific anatomical paths correlating to her neurological findings. The reduced sensation in the lateral aspects of the right foot indicates an impairment in the function of Myelin P2 protein, a component of the myelin sheaths of peripheral nerves, often impacted in 'Common Peroneal Neuropathy'. And her 1+ deep tendon reflex in the right ankle further points towards this localized damage. (4) CONCLUDING: By progressively reasoning out the cause behind her symptoms, it is highly likely that this woman could be dealing with Common Peroneal Neuropathy. It aligns with her localized symptoms and precise neurological examination findings. This type of peripheral neuropathy occurs due to damage to the common peroneal nerve, leading to paresis or paralysis of the muscles it innervates, numbness, and decreased sensation. (5) RESPONSE: Hence, based on an in-depth analysis of the patient's symptoms, history, and clinical findings, the most likely diagnosis for this 47-year-old woman is (A) Common peroneal neuropathy.

**AI_A:** (1) RESTATING THE QUESTION: A middle-aged woman presents to the clinic with a 2-month history of progressive weakness and numbness in her right leg. She has type 2 diabetes mellitus which is managed with metformin and works in a physically demanding job. Her neurological exam reveals slightly weakened muscle strength and diminished sensation in the right leg, particularly in the lateral and inferior aspects of the right foot. The question asks us to determine the most likely diagnosis from a list of neurological conditions based on these clinical findings. In general, neuropathy refers to any disease or injury to nerves. Radiculopathy specifically refers to a condition where a single or several nerves are affected by compression or irritation of nerve roots. The common peroneal nerve is a branch of the sciatic nerve that innervates the anterior muscles of the leg and the skin of the lower leg and foot. The femoral nerve, obturator nerve, and L2, L4, S1 nerve roots also have specific responsibilities in innervating various muscles and areas of the leg. (2) PLANNING: Based on the presentation and findings, the assumptions could be that the woman's symptomatology is a result of her diabetes, her physically demanding job, or another independent neurological condition. I'll generate alternate assumptions and evaluate the most likely one to pursue clinically. Considering the woman's type 2 diabetes, diabetic neuropathy could be considered, but this typically presents as distal symmetric polyneuropathy, which doesn't quite fit her presentation. Looking at the physically demanding nature of her job, a musculoskeletal injury or nerve entrapment could cause her symptoms. (3) CLINICAL REASONING: Keeping these assumptions in mind, we can start examining the possibilities from the list. Given the decreased sensation over the lateral foot, associated with weakness of hip extensor, thigh abductor, hamstring, and gastrocnemius muscles, the pathology seems to be linked to the nerve network serving these areas. Due to the nature of her work, this could potentially be caused by direct nerve damage resulting from physical trauma or repetitive strain. However, the localized, asymmetric pattern of her symptoms suggests a specific nerve or nerve root involvement. (4) CONCLUDING: Looking at the specific nerves and roots mentioned, the closest match to her symptoms is the S1 nerve root, as it serves many of the areas where she is experiencing symptoms, including the gastrocnemius muscle and the lateral foot (which is innervated by the sural nerve, a branch of the sciatic nerve, which roots from L4, L5, and S1, S2). The decrease in reflexes in the right ankle also points towards S1 radiculopathy, since ankle reflex mostly tests the S1 nerve root. (5) RESPONSE: Therefore, based on the clinical findings, the most likely diagnosis in this patient is 'S1 radiculopathy' (Option F).

**AI_E:** RESTATING THE QUESTION: The patient is a 47-year-old woman who has persistently been experiencing progressive weakness and numbness in her right leg for the past two months. She's been managing type 2 diabetes with metformin for 3 years. She has a physically demanding job that involves lifting heavy loads and squeezing into cramped spaces. The neurologic examination reveals that her muscle strength is slightly less on the right side of her body compared to her left side, particularly in the hip extensor, thigh abductor, hamstring, and gastrocnemius muscles. Additionally, her right ankle reflex is a bit weaker than her left, and she has mildly decreased sensation to light touch in the lateral and inferior aspects of her right foot. Given these clinical features, the question asks us to consider a number of diagnoses related to nerve damage or neuropathy. PLANNING: Each option (A to F) represents a different form of neuropathy or radiculopathy. Neuropathy refers to the damage to peripheral nerves, which carry information to and from the brain and spinal cord. Radiculopathy, on the other hand, involves the compression or irritation of nerve roots exiting the spine, causing symptoms in the areas the nerve provides sensation. The assumptions are that this patient's symptoms align with one of these conditions. By comparing her symptoms and the affected muscles with the nerves that innervate these muscles, we can potentially identify the affected nerve or nerves. If her symptoms don't align with one of these conditions, it would imply a different diagnosis altogether. CLINICAL REASONING: Considering the patient's clinical manifestations, her symptoms are consistent with a neuropathy or radiculopathy that would involve the periphery of her right lower limb. She has weakness and mildly reduced sensation in the lateral and inferior aspects of her foot. Furthermore, her diminished ankle reflex on the right corresponds to the nerves typically associated with these regions, reinforcing the likelihood of a peripheral nerve lesion. The common peroneal nerve, a branch of the sciatic nerve, innervates the short head of the biceps femoris, tibialis anterior, and extensor digitorum longus. Damage to this nerve may lead to foot drop and sensory defects on the dorsal surface of the foot and the lateral part of the leg. However, in this patient, the weakness was localized to the hip extensor, thigh abductor, hamstring, and gastrocnemius muscles and not the muscles innervated by the common peroneal nerve. Femoral neuropathy, which is characterized by impaired quadriceps function (leading to difficulties in leg extension and knee flexion) and decreased sensation of the medial leg and foot, also doesn't align with her symptomatology, as her quadriceps function was normal. L2 radiculopathy presents with weakness in hip flexion (due to iliopsoas muscle weakness) and the patient may have decreased sensation on the anterior thigh, which isn't evident here. Obturator neuropathy, characterized by weakness in the thigh adductor muscles and decreased sensation over the medial thigh doesn't match her presentation, as her thigh adductor muscle strength was normal. On the other hand, L4 and S1 radiculopathies both involve the nerve roots supplying the lower limbs. The L4 radiculopathy often leads to weaknesses in the quadriceps and tibialis anterior muscles and a reduction in the knee jerk reflex, but here, her quadriceps and tibialis anterior function and knee jerk reflex were normal. Lastly, S1 radiculopathy, can cause weaknesses in the gastrocnemius muscle and depress the ankle jerk reflex, as well as causing sensory loss in the lateral and plantar foot - which matches the patient's symptoms. CONCLUDING: All things considered, an S1 radiculopathy appears to be the most likely cause of this patient's observed clinical manifestations. Her decreased muscle strength in the gastrocnemius muscle, weakened ankle reflex, and decreased sensation over the lateral and inferior aspects of the foot align well with the expected presentation of this condition. RESPONSE: The most probable diagnosis for the patient's clinical condition is (F) S1 radiculopathy.

**Facilitator** **[Round 2]:** Summary: AI_A believes the woman's symptoms are most likely due to S1 radiculopathy. This conclusion is based on the woman's decreased sensation over the lateral foot, associated with weakness of hip extensor, thigh abductor, hamstring, and gastrocnemius muscles, and the decrease in reflexes in the right ankle. AI_B, AI_C, and AI_D all conclude that the woman's symptoms are most likely due to common peroneal neuropathy. They base their conclusions on the woman's weakened foot dorsiflexion, decreased sensation over the right foot, and decreased right ankle reflex, which suggest the peripheral nerve involved is more likely distal. AI_E also concludes that the woman's symptoms are most likely due to S1 radiculopathy. This conclusion is based on the woman's decreased muscle strength in the gastrocnemius muscle, weakened ankle reflex, and decreased sensation over the lateral and inferior aspects of the foot. Key Differences: The key differences in the council members' perspectives lie in their interpretation of the woman's symptoms and their understanding of the different types of neuropathies and radiculopathies. AI_A and AI_E focus on the woman's decreased sensation over the lateral foot and the decrease in reflexes in the right ankle, which they believe point to S1 radiculopathy. On the other hand, AI_B, AI_C, and AI_D focus on the woman's weakened foot dorsiflexion and decreased sensation over the right foot, which they believe point to common peroneal neuropathy. Question for Council Members: To clarify the disagreements between your opinions, could you please explain why you believe the woman's symptoms point to either S1 radiculopathy or common peroneal neuropathy, and why you do not believe the other condition is the most likely diagnosis?. Based on your preceding response, how would you answer the original question: [A 47-year-old woman comes to the office because of a 2-month history of progressive weakness and numbness of her right leg. She has a 3-year history of type 2 diabetes mellitus treated with metformin. She has worked as a stock person in a storeroom for 12 years; she often moves and lifts heavy objects and sometimes crawls into tight spaces. On neurologic examination, muscle strength is 5−/5 in the hip extensor, thigh abductor, hamstring, and gastrocnemius muscles on the right and intact on the left. Muscle strength is intact in the iliopsoas, thigh adductor, quadriceps, and tibial anterior muscles bilaterally. Deep tendon reflexes are 1+ in the right ankle and 2+ in the left ankle. Sensation to light touch is mildly decreased over the lateral and inferior aspects of the right foot. Sensation otherwise is intact. Which of the following is the most likely diagnosis? (A) Common peroneal neuropathy (B) Femoral neuropathy (C) L2 radiculopathy (D) L4 radiculopathy (E) Obturator neuropathy (F) S1 radiculopathy]

**AI_B:** RESTATING THE QUESTION: A 47-year-old woman with type 2 diabetes mellitus, working in a physically demanding job, has been experiencing progressive weakness and numbness in her right leg for two months. She exhibits decreased sensation to light touch on her right foot, particularly over the lateral and inferior aspects, and slightly reduced muscle strength and deep tendon reflex in the same leg, especially in the muscles around the hip, thigh, and calf. We need to figure out the most likely diagnosis for her condition, with the options being: common peroneal neuropathy, femoral neuropathy, L2 radiculopathy, L4 radiculopathy, obturator neuropathy, or S1 radiculopathy. PLANNING: The woman's symptomatology indicates a potential problem with her nervous system, possibly of peripheral nerve or radicular origin. To arrive at a diagnosis, we must consider the exact muscles that are weak, the sensation areas that are affected and the reflexes that are diminished. Her diabetic status could point towards peripheral neuropathies, but we must also consider her physically strenuous work, which could put her at risk for mechanical nerve issues such as radiculopathies. These are all potential assumptions that require careful evaluation. CLINICAL REASONING: Radicular pain, or radiculopathy, is caused by compression, inflammation and/or injury to a spinal nerve root. The presented symptomatology does not clearly point towards a specific spinal level. The affected muscles span a range of innervation levels from the spinal cord, not pointing clearly to the involvement of a single root, such as L2, L4 or S1. On the other hand, common peroneal neuropathy, a type of peripheral neuropathy, results from damage to the peroneal nerve and can manifest as weakness in foot dorsiflexion, numbness on the lateral side of the foot, and decreased ankle reflex, symptoms correlating well with those presented by the patient. This neuropathy can be caused by direct pressure on the nerve, an important risk factor being frequent crossing of the legs or pressure at the fibular head during certain physical activities. The patient's job, which involves moving and lifting heavy objects and sometimes crawling into tight spaces, might put sustained pressure on this nerve, making this assumption plausible. Furthermore, her diabetic status is a well-known risk factor for peripheral neuropathies. CONCLUDING: Given the woman's presentation of weakness of the hip, thigh, and calf muscles, decreased reflexes at her ankle, along with reduced sensation over the lateral and inferior aspects of her foot, together with her work history and diabetic status, the most likely diagnosis appears to be common peroneal neuropathy. The symptoms do not clearly point towards a single root radiculopathy, but rather suggest peripheral nerve involvement. RESPONSE: Based on the considerations above, the most likely diagnosis for the woman's presentation and history is (A) Common peroneal neuropathy.

**AI_D:** RESTATING THE QUESTION: The scenario describes a 47-year-old woman with type 2 diabetes mellitus who has been experiencing progressive weakness and numbness in her right leg for two months. She has been working in a stockroom for 12 years, often moving heavy objects and sometimes crawling into tight spaces. A physical examination has revealed certain neurologic anomalies. Although her muscle strength is mostly preserved, she has slightly diminished sensation on the lateral and inferior aspects of her right foot and a reduction in the ankle reflex on the right side. The question requires a determination of the most probable diagnosis from the listed options. PLANNING: The possible diagnoses include various types of neuropathies and radiculopathies. Neuropathies refer to disorders that affect the peripheral nerves, while radiculopathies pertain to conditions where a nerve root in the spine becomes disrupted. For specificity, common peroneal neuropathy relates to lower motor neuron syndrome involving the common peroneal nerve, typically resulting in foot drop. Femoral neuropathy is a mononeuropathy of the femoral nerve that can lead to muscle weakness and sensory disturbances in the thigh and leg. L2, L4, and S1 radiculopathies refer to issues with specific nerve roots in the lumbar and sacral region which could potentially cause lower limb motor and sensory deficits. And obturator neuropathy describes a disorder of the obturator nerve causing weakness in hip adduction and internal rotation. CLINICAL REASONING: The presentation is consistent with a peripheral nerve disorder due to the decreased sensation over the right foot and diminished right ankle reflex, both of which suggest a distal peripheral nerve involvemenIn general, the combination of weakness in the hip extensor, thigh abductor, hamstring, and gastrocnemius muscles, as well as the sensory loss in the lateral and inferior aspects of the right foot, specifically fits with the distribution of the common peroneal nerve. CONCLUDING: While S1 radiculopathy could also potentially cause similar deficits, the lack of more proximal symptoms (i.e., back pain, symptoms in the buttock and posterior thigh), the absence of radicular pain, and the lack of a sensory level argue against this diagnosis. Also, the involvement of toe and foot dorsiflexion, which are innervated by the deep peroneal nerve, a branch of the common peroneal nerve, supports the diagnosis of common peroneal neuropathy. RESPONSE: Given the woman's occupation – which often involves moving heavy objects and sometimes crawling into tight spaces – the risk of an affliction like common peroneal neuropathy increases. Combining the clinical findings and occupational history, the most likely diagnosis is (A) common peroneal neuropathy.

**AI_A:** We are presented with a 47-year-old woman who is experiencing progressive weakness and numbness in her right leg for the past two months. Her medical history includes type 2 diabetes mellitus which has been treated with metformin. Her profession as a stock person in a storeroom involves strenuous physical activity such as moving and lifting heavy objects as well as crawling into tight spaces. Her neurological examination revealed a slightly reduced muscle strength in the hip extensor, thigh abductor, hamstring, and gastrocnemius muscles on the right side, while maintaining intact strength on the left side. Her deep tendon reflexes are diminished on the right ankle compared to the left. Additionally, she experiences a slight decrease in sensation to light touch over the lateral and inferior aspects of the right foot. The task at hand is to diagnose the most likely cause of her symptoms. Alternate assumptions involve each of the options provided, which include common peroneal neuropathy, femoral neuropathy, L2 radiculopathy, L4 radiculopathy, obturator neuropathy and S1 radiculopathy. Each of these conditions can cause leg weakness and altered sensation - symptoms the patient is presenting with. The clinical reasoning then involves understanding the typical clinical presentations of each of these conditions and comparing them with the patient's symptoms. Common peroneal neuropathy typically presents with foot drop and sensory loss over the lateral lower leg and dorsum of the foot. Femoral neuropathy commonly presents with weakness in hip flexion and knee extension and sensory loss in the anteromedial thigh. L2 radiculopathy typically presents with weakness in hip flexion and sensory loss in the anteromedial thigh and medial lower leg. L4 radiculopathy presents with weakness in knee extension and foot inversion, and sensory loss over the medial lower leg and medial aspect of the foot. Obturator neuropathy presents with weakness in thigh adduction and negligible sensory symptoms. On the other hand, S1 radiculopathy commonly presents with weakness in plantar flexion (calf muscles), loss of the ankle reflex, and sensory loss over the lateral foot and posterior lower leg. Given the patient's clinical presentation, S1 radiculopathy seems to be the most probable diagnosis. Even though the patient's muscle strength in the hip extensor, thigh abductor, hamstring, and gastrocnemius muscles on the right side is slightly compromised, it remains at 5−/5 which is considered normal. However, there is a diminished deep tendon reflexes on the right ankle and slight sensory loss over the lateral and inferior aspects of the right foot. These findings are most consistent with S1 radiculopathy. In conclusion, after assessing and analyzing various assumptions and their corresponding clinical manifestations, it's plausible to conclude that the woman most likely has S1 radiculopathy. This conclusion is primarily based on her symptoms - weak ankle reflex on the right side and mildly decreased sensation on the lateral and inferior aspects of the right foot - which are in line with typical S1 radiculopathy presentations.

**AI_E:** RESTATING THE QUESTION: This question presents a clinical scenario of a 47-year-old woman seeking medical consultancy due to progressive weakness and numbness in her right leg over a period of 2 months. Her medical history includes type 2 diabetes mellitus for 3 years, managed with metformin. Her occupational history comprises of physical labor involving heavy lifting and crawling into constricted spaces. The findings of her neurological examination are reported, including muscle strength ratings, deep tendon reflexes, and sensory evaluation. The question asks us to identify the most probable diagnosis from a given list of nerve-related disorders, including common peroneal neuropathy, femoral neuropathy, L2 and L4 radiculopathy, obturator neuropathy, and S1 radiculopathy. PLANNING: The primary assumptions I am making here include: 1. The symptoms of progressive weakness and numbness in the right leg over a period of 2 months represent a neurological disorder. 2. The occupational history of the woman, involving heavy physical labor and sometimes squeezing herself into cramped spaces, might have contributed to these symptoms. 3. The physical examination findings, notably muscle strength, reflexes, and sensory analysis, will provide the key to diagnosing the specific neurological disorder. Alternate assumptions generated by questioning these primary assumptions include: A. Could the progressive weakness and numbness of the right leg be due to causes other than neurological disorders, such as orthopedic or vascular issues? B. Can her diabetes or metformin use contribute to her symptoms, separate to or in aggregation with her job demands? C. Do other components of the physical examination, or even laboratory and imaging studies, need to be considered for an accurate diagnosis? After evaluating these alternative assumptions, the primary assumptions should be prioritized, given the specific details provided in the question stem. CLINICAL REASONING: Firstly, it’s crucial to determine which section of the nervous system – central (e.g. radiculopathy) or peripheral (e.g. neuropathy) – is most likely implicated based on her reported clinical and neurological examination findings. With only the right leg affected, a localized process is more probable. The decreased sensation over the lateral and inferior aspects of the right foot suggests involvement of the common peroneal nerve, which supplies these areas. The normal strength of the muscles innervated by the iliopsoas, thigh adductor, quadriceps, and tibialis anterior (all supplied by the femoral and obturator nerves and higher lumbar roots) further support involvement of the common peroneal nerve (branch of sciatic nerve) or the corresponding root (S1). The normal muscle strength in the hip extensor, thigh abductor, hamstring, and gastrocnemius muscles, which are commonly affected in S1 radiculopathy, is inconsistent with this diagnosis. Moreover, S1 radiculopathy would also typically manifest with decreased ankle jerk reflex and plantar flexion, which aren't reported here. The woman's findings better match common peroneal neuropathy, characterized by sensory changes in the nerve distribution and relative preservation of muscle strength. This peripheral nerve may get compressed at the fibular head level, possibly triggered by her reported professional activities. CONCLUDING: The clinical reasoning process suggests that the reported symptoms and findings of the woman make common peroneal neuropathy the most fitting diagnosis. Her occupation may have contributed to this condition, likely due to nerve compression in the leg. RESPONSE: The woman's clinical presentation, consisting of progressive right leg weakness, numbness especially over the lateral and inferior aspects of the foot, coupled with physical findings of decreased sensation and slightly reduced deep tendon reflex are indicative of a neurological disorder and, given her occupational history, can be localized to the common peroneal nerve. Hence, her most probable diagnosis is 'Common peroneal neuropathy' (option A).

**AI_C:** RESTATING THE QUESTION: Our objective is to find the most plausible explanation for a 47-year-old woman's 2-month history of progressing weakness and numbness in her right leg. This woman has a 3-year history of type 2 diabetes mellitus, which she has been managing with metformin. Her occupation involves frequent heavy lifting and maneuvering through confined spaces, which could potentially have impacts on her musculoskeletal system. Upon conducting a neurologic examination, we found that her right-sided hip extensor, thigh abductor, hamstring, and gastrocnemius muscles are slightly weak (rated at 5-/5), but other muscles in her body function normally. Deep tendon reflexes in her right ankle are weaker (1+) compared to her left (2+), and her sensation to light touch is slightly decreased over the lateral and inferior aspects of her right foot, although her other sensory functions are intact. Our task is to determine the most likely diagnosis for her condition, choosing from: Common peroneal neuropathy, Femoral neuropathy, L2 radiculopathy, L4 radiculopathy, Obturator neuropathy, and S1 radiculopathy. PLANNING: Based on this patient's symptoms and history, we can make several assumptions 1) her leg symptoms are due to nerve issues and not directly due to muscle problems because the weakness and numbness suggest a neurological component. 2) The nervous system issue resulting in her symptoms is likely localized in her lower body because her symptoms are isolated to her right leg. We can generate alternative hypotheses by questioning each of these assumptions; for instance, could the symptoms be due to an issue in a different part of the body affecting her leg? Is there a systemic condition, e.g., her Type 2 diabetes, which could cause these symptoms as a complication? But for this case, the focus is more so on the neurolocalization than the etiology, hence, it's clinically crucial to prioritize these anatomically pin-pointed deductions and assumptions over more general disease situations. CLINICAL REASONING: Let's clinically reason about these assumptions: 1) Deficit in muscle strength in the face of neurological symptoms led us to consider nerve issues. Numbness further supports a sensory nerve problem. 2) Her symptoms are concentrated to her right leg (in particular the foot area), which tells us that the problem likely lies in the nerves serving that part of the body rather definitively. Now let's evaluate the offered diagnostic options: (A) Common peroneal neuropathy: This usually presents with foot drop and sensory deficits in the dorsum of the foot. (B) Femoral neuropathy: This often presents with weakness in hip flexion and knee extension, with sensory loss over the anterior thigh and medial lower leg. (C) L2 radiculopathy: This condition would likely cause weakness in hip flexion, with sensory loss over the anterior thigh. (D) L4 radiculopathy: This could lead to weakness in quadriceps (knee extension) with sensory loss over the medial lower leg and foot. (E) Obturator neuropathy: This would typically result in weakness in hip adduction, with sensory loss over the medial thigh. (F) S1 radiculopathy: Typically it presents with weakness in plantar flexion (gastrocnemius and soleus muscles), ankle jerk would be diminished, with sensory loss over the posterior aspect of the thigh, and/or the lateral aspect of the foot. CONCLUDING: Given that this patient's muscle weakness is localized to the hip extensor, thigh abductor, hamstring, and gastrocnemius muscles, her symptoms seem to be more in line with an issue that would affect the common peroneal nerve or S1 nerve root. Specifically, her mildly decreased sensation over the lateral and inferior aspects of her right foot points towards a possible problem with the common peroneal nerve or S1 nerve root. RESPONSE: Therefore, considering the patient's symptoms most closely align with those typical of common peroneal neuropathy (A) and S1 radiculopathy (F), the final answer should be more specific towards one of them. However, there is no mention of foot drop, which is a more common symptom in common peroneal neuropathy, so that sidelines option A a bit. But her decreased sensation to light touch in the lateral and inferior aspects of her foot and right ankle reflex being diminished perfectly aligns it to S1 radiculopathy (which provides sensation to the lateral and plantar foot). Most importantly given the nature of her work involving much of lifting and crawling; there seems to have a likely possibility of the issue at S1 nerve root level due to significant mechanical strain/pressure that her working condition imposes on her spine, mainly the lumbosacral region. So, in response to the original question, "Which of the following is the most likely diagnosis?" my answer would lean towards "S1 radiculopathy" (F).

**Facilitator** **[Round 3]:** Summary: AI_A believes the woman's symptoms align most closely with S1 radiculopathy, based on her diminished deep tendon reflexes on the right ankle and slight sensory loss over the lateral and inferior aspects of the right foot. AI_B, AI_D, and AI_E, however, argue that the woman's symptoms are more indicative of common peroneal neuropathy, given her work history, diabetic status, and the specific areas of weakness and sensory loss. AI_C suggests that the symptoms could be due to either common peroneal neuropathy or S1 radiculopathy, but leans towards S1 radiculopathy due to the nature of her work and the specific symptoms presented. Question for Council Members: Given the disagreement between the diagnoses of common peroneal neuropathy and S1 radiculopathy, could you elaborate on the key distinguishing factors between these two conditions that led you to your respective conclusions?. Based on your preceding response, how would you answer the original question: [A 47-year-old woman comes to the office because of a 2-month history of progressive weakness and numbness of her right leg. She has a 3-year history of type 2 diabetes mellitus treated with metformin. She has worked as a stock person in a storeroom for 12 years; she often moves and lifts heavy objects and sometimes crawls into tight spaces. On neurologic examination, muscle strength is 5−/5 in the hip extensor, thigh abductor, hamstring, and gastrocnemius muscles on the right and intact on the left. Muscle strength is intact in the iliopsoas, thigh adductor, quadriceps, and tibial anterior muscles bilaterally. Deep tendon reflexes are 1+ in the right ankle and 2+ in the left ankle. Sensation to light touch is mildly decreased over the lateral and inferior aspects of the right foot. Sensation otherwise is intact. Which of the following is the most likely diagnosis? (A) Common peroneal neuropathy (B) Femoral neuropathy (C) L2 radiculopathy (D) L4 radiculopathy (E) Obturator neuropathy (F) S1 radiculopathy]

**AI_B:** RESTATING THE QUESTION: We are considering the appropriate clinical diagnosis for this 47-year old woman who is presenting with a 2-month history of progressive weakness and numbness in her right leg. She has a 3-year medical history of type 2 diabetes mellitus, which is treated with metformin. She also has a physically strenuous job in a storeroom, which involves lifting heavy objects and crawling into tight spaces. The neurological examination revealed that she has full strength in most major leg muscle groups but diminished deep tendon reflexes (1+ in the right ankle compared to 2+ in the left), and mildly decreased touch sensitivity on the lateral and inferior aspects of her right foot. PLANNING: Our diagnosis should primarily consider conditions that can cause unilateral leg weakness and altered sensation, worsened by physical stress, and potentially linked with diabetes. The options provided are Common peroneal neuropathy, Femoral neuropathy, L2 radiculopathy, L4 radiculopathy, Obturator neuropathy, and S1 radiculopathy. CLINICAL REASONING: Common peroneal neuropathy tends to present with foot drop and sensory deficits over the dorsal foot which this patient doesn't seem to have. Femoral neuropathy would likely affect the quadriceps muscle causing difficulty with stair climbing and rising from a chair, but the patient's quadriceps strength is intact. L2 and L4 radiculopathy involve the hip muscles and quadriceps, respectively, which are also normal in this patient. Obturator neuropathy primarily affects the thigh adductors which are shown as normal in the patient. The diminished Achilles reflex and sensory loss over the lateral and inferior foot point towards an S1 radiculopathy. CONCLUDING: The S1 nerve root in the spine innervates the gastrocnemius muscle, enables the Achilles reflex, and provides sensation to the lateral and inferior aspects of the foot. This matches our patient's presentation. RESPONSE: Based on the clinical presentation and physical examination findings consistent with impairment of the S1 nerve root, the most likely diagnosis for this woman is S1 radiculopathy (F).

**AI_C:** RESTATING THE QUESTION: This woman's case involves a progressive weakening and numbness in her right leg over two months. She also has a history of type 2 diabetes, managed with metformin. Her job requires physical activity including heavy lifting and maneuvering in tight spaces. Her neurological examination shows almost normal muscle strength, except for slightly decreased reflex in the right ankle compared to the left, and mildly decreased sensation to light touch on the lateral and inferior aspects of the right foot. The task here is to find the most likely diagnosis among common peroneal neuropathy; femoral, L2, L4, and S1 radiculopathy; and obturator neuropathy. PLANNING: The muscle strength in the affected areas suggests the motor function of nerves innervating these muscles (e.g., sciatic, femoral, obturator) is not highly compromised, suggesting the problem may lie in the roots of corresponding spinal nerves (radiculopathy). Decreased ankle reflex suggests the issue may lie with the S1 root, which controls ankle reflex, versus the common peroneal nerve, which controls sensation to the lateral and inferior aspects of the foot. Given the patient's physical work history, spinal nerve root compression seems plausible. CLINICAL REASONING: This is a complex case with elements pointing to multiple possible diagnoses. However, the decreased sensation over specific areas of the foot, the slight loss of ankle reflex, the lack of severe muscle weakness, and the patient's physical labor suggest the problem is more likely localized at the S1 spinal root level, rather than involving the peripheral common peroneal nerve directly. CONCLUDING: While both S1 radiculopathy and common peroneal neuropathy could theoretically generate similar symptoms, the specific pattern in the presentation of this case, combined with the patient's physical job history, leads towards a diagnosis of S1 radiculopathy. RESPONSE: Given her symptoms, including the slight decrease in her right ankle deep tendon reflex and mild decrease in sensation over the lateral and inferior aspects of her right foot, in conjunction with her employment involving physical labor and maneuvering in tight spaces, the most likely diagnosis is S1 radiculopathy (F).

**AI_E:** 1) RESTATING THE QUESTION: The question pertains to a 47-year-old female patient who has been experiencing increasing weakness and numbness in her right leg over the past two months. This woman has a history of type 2 diabetes, which she manages with metformin. She maintains a physically demanding job that involves heavy lifting and moving into tight areas, which may contribute to her pain. The physical assessment showed some muscle strength discrepancies and minor sensory loss in her right foot. The task is to diagnose her condition based on the given symptoms and medical history. 2) PLANNING: The majority of AI council members are torn between a diagnosis of common peroneal neuropathy versus S1 radiculopathy. Assumptions related to common peroneal neuropathy include potential localized compression of the nerve at the fibular head due to her physically demanding work, while those related to potential S1 radiculopathy include suspecting radicular compression related to heavy lifting and movements. 3) CLINICAL REASONING: The common peroneal nerve, when compressed or damaged, can cause weakness in foot and ankle, and a decrease in sensation in the affected areas. The S1 nerve root, on the other hand, when compressed, can cause symptoms like diminished Achilles reflex and sensory loss along the back of the thigh, calf, and outer foot - which are all consistent with the patient's symptoms. 4) CONCLUDING: While both potential diagnoses have similar symptoms that correspond with the patient's manifestations, the absence of weakness in gastrocnemius muscles and hamstrings, typically involved in S1 radiculopathy, leans the diagnosis more towards common peroneal neuropathy. 5) RESPONSE: Based upon the clinical symptomology, the medical history, and the physical examination findings, my diagnosis would tend to favor common peroneal neuropathy (Complex A), although S1 radiculopathy (Complex F) remains a secondary possibility pooling all the information about the patient. Both these possibilities should be confirmed using appropriate diagnostic tools like Electromyography (EMG) and Nerve Conduction Studies (NCS) for further diagnosis and management.

**AI_A:** (1) RESTATING THE QUESTION: The key question here is about diagnosing a 47-year-old woman with progressive neurological symptoms in her right lower limb. Her long-term employment in a physical job, her 3-year history of type 2 diabetes being treated with metformin, and her lowered deep tendon reflexes, including a mildly decreased sensation to light touch over the lateral and inferior aspects of her right foot, are the salient aspects of this case. We need to consider these factors as we ponder the potential diagnoses of common peroneal neuropathy, femoral neuropathy, L2 radiculopathy, L4 radiculopathy, obturator neuropathy, and S1 radiculopathy. (2) PLANNING: A key assumption to consider is that the woman's symptoms originate from a single peripheral nerve or nerve root disorder, which might not always be the case especially in a multi-morbid patient such as this one. If we question this assumption, an alternate one could be that her symptoms could be due to systemic issues related to her diabetes or her physically demanding work. Consequently, we should consider her diabetes, lifestyle, and physical exertions in our differential diagnoses. (3) CLINICAL REASONING: The presented clinical features are suggestive of radiculopathy, which relates to a lesion on a spinal nerve root, including diminished reflexes and sensory loss. The mentioned symptoms usually line up with S1 radiculopathy. The deep peroneal nerve, in contrast, originates from the common peroneal nerve, and primarily affects the sensation to the first web space and motor function of the tibialis anterior, extensor hallucis longus, and extensor digitorum longus muscles, none of which appears to be significantly affected in this case. (4) CONCLUDING: Given the insufficient evidence for systemic pathology and the precise constellation of symptoms presented, it is less likely that the symptoms reflect a systemic issue related to her job or diabetes. Subsequently, taking into account the diminished reflexes and sensory loss, S1 radiculopathy seems to be the most probable diagnosis pathologically. (5) RESPONSE: Therefore, given the evidence, the characteristics of the woman's clinical presentation point more towards S1 radiculopathy (Option F) as the most likely diagnosis.

**AI_D:** 1) RESTATING THE QUESTION: This question is about a 47-year-old woman with type 2 diabetes who's been experiencing progressive weakness and numbness in her right leg for two months. Her job as a stock person involves frequent heavy lifting and crawling into tight spaces. Her neurological examination shows some reduction in right ankle deep tendon reflexes and sensation on the right foot. We are asked to diagnose her condition from the following options: Common peroneal neuropathy, Femoral neuropathy, L2 radiculopathy, L4 radiculopathy, Obturator neuropathy or S1 radiculopathy. 2) PLANNING: It is essential to consider the physical aspects of her job, her diabetic status, her age, and the specific pattern of sensory and muscle strength findings in her examination. While all options could theoretically result in weakness and numbness in the leg, they each affect different parts of the nervous system and would result in distinct signs and symptoms. A critical assumption is that her symptoms are due to a localized issue in the peripheral or central nervous system, and we should confirm or validate this based on her history and examination findings. 3) CLINICAL REASONING: Common peroneal neuropathy, also known as foot drop, might arise from compression or injury to the common peroneal nerve that runs near the surface of the skin at the lateral aspect of the knee. Signs and symptoms would typically include weakness in foot and toe lift and, in some cases, numbness or decreased sensation on the upper part of the foot and the skin between the first and second toe. However, the primary findings in the presented case, which are weakness in the right ankle reflex and lateral and inferior aspects of the right foot, align with the S1 nerve root distribution. This makes S1 radiculopathy a more likely diagnosis. 4) CONCLUDING: Despite her strenuous work, her symptomatology does not fully align with common peroneal neuropathy; there is an absence of foot drop usually seen in this condition. While the diabetes status could make her more prone to peripheral neuropathies, the pattern noted here fits more with a radiculopathy, which is pathology located at the nerve root level, specifically at S1 level due to the sensory and reflex findings on her right leg and foot. 5) RESPONSE: Therefore, given the considerations mentioned above, I would propose “S1 radiculopathy" (F) as the most likely diagnosis for this patient's current presentation.

**Facilitator** **[Round 4]:** Summary: AI_A, AI_B, AI_C, and AI_D all agree that the most likely diagnosis for the woman's symptoms is S1 radiculopathy. They all note the diminished reflexes and sensory loss in the right foot, which align with the S1 nerve root distribution. They also consider the woman's physically demanding job and diabetes, but conclude that these factors do not fully explain her symptoms. AI_E, however, leans towards a diagnosis of common peroneal neuropathy, noting that the woman's physically demanding job could have led to localized compression of the nerve. AI_E also suggests that S1 radiculopathy remains a secondary possibility. Key Differences: The key difference in the perspectives lies in the interpretation of the woman's symptoms and medical history. AI_A, AI_B, AI_C, and AI_D focus on the diminished reflexes and sensory loss in the right foot, which they believe align more with S1 radiculopathy. They also consider the woman's physically demanding job and diabetes, but do not believe these factors fully explain her symptoms. AI_E, on the other hand, believes that the woman's physically demanding job could have led to localized compression of the common peroneal nerve, leading to common peroneal neuropathy. AI_E also suggests that S1 radiculopathy remains a secondary possibility. Question to Clarify Disagreements: Given the woman's physically demanding job and diabetes, how do these factors influence the likelihood of common peroneal neuropathy versus S1 radiculopathy? Could her symptoms be a result of a combination of both conditions, and if so, how would this affect our approach to diagnosis and treatment?. Based on your preceding response, how would you answer the original question: [A 47-year-old woman comes to the office because of a 2-month history of progressive weakness and numbness of her right leg. She has a 3-year history of type 2 diabetes mellitus treated with metformin. She has worked as a stock person in a storeroom for 12 years; she often moves and lifts heavy objects and sometimes crawls into tight spaces. On neurologic examination, muscle strength is 5−/5 in the hip extensor, thigh abductor, hamstring, and gastrocnemius muscles on the right and intact on the left. Muscle strength is intact in the iliopsoas, thigh adductor, quadriceps, and tibial anterior muscles bilaterally. Deep tendon reflexes are 1+ in the right ankle and 2+ in the left ankle. Sensation to light touch is mildly decreased over the lateral and inferior aspects of the right foot. Sensation otherwise is intact. Which of the following is the most likely diagnosis? (A) Common peroneal neuropathy (B) Femoral neuropathy (C) L2 radiculopathy (D) L4 radiculopathy (E) Obturator neuropathy (F) S1 radiculopathy]

**AI_E:** The question at hand is to determine the most likely diagnosis for a 47-year-old woman, who has been experiencing progressive weakness and numbness in her right leg over the past two months. She also has a known history of type 2 diabetes mellitus, for which she is on metformin. Additionally, her job involves a lot of physical labor including lifting heavy items and sometimes having to squeeze into tight spaces. Her neurological examination shows decreased reflexes and slight decrease in sensation to touch on the lateral and inferior aspects of her right foot. The potential diagnoses that have been suggested include common peroneal neuropathy, femoral neuropathy, L2 radiculopathy, L4 radiculopathy, obturator neuropathy, and S1 radiculopathy. First, let's identify the assumptions: the symptoms arise from a single nerve disorder, it is related to her diabetes, or it is an occupational injury. Given the history of diabetes and heavy lifting, we should consider whether these could lead to mononeuropathy (diabetic peripheral neuropathy), peripheral nerve compression, or radiculopathy (nerve root compression). When evaluating the assumptions, multiple nerve issues seem unlikely given the localized symptoms. Peripheral nerve compression would be more likely with specific occupational risks. As for radiculopathy, diabetes increases the risk but there are no consistent back pain or sciatica, which are common in radiculopathy. In the process of clinical reasoning, the decreased ankle reflex and decreased sensation to light touch on the lateral and inferior aspects of her foot correlate with common peroneal nerve and S1 nerve root distributions. However, the intact muscle strength contradicts the common peroneal neuropathy as it would have affected the foot and toe dorsiflexors. In conclusion, while this case suggests a nerve issue in the distribution of the common peroneal nerve and S1 nerve root, the intact dorsiflexion strength points more towards an S1 radiculopathy rather than a common peroneal neuropathy. Thus, my response is that the most likely diagnosis in this case is S1 radiculopathy (Choice F). Other potential diagnoses such as common peroneal neuropathy and L2, L4, or obturator radiculopathy can be considered but are less likely given the specific findings on the neurological examination.

**AI_A:** Question Interpretation: A middle-aged woman with a history of type 2 diabetes has been experiencing progressive weakness and numbness in her right leg over the past two months. Despite the physically demanding nature of her job in a storeroom and having to often lift heavy objects, her muscle strength remains largely intact, with some decrease in sensation to light touch in her right foot. The question asks us to determine the most likely cause of her symptoms from the following list: common peroneal neuropathy, femoral neuropathy, L2 radiculopathy, L4 radiculopathy, obturator neuropathy, and S1 radiculopathy. Planning: First, we must understand the nature of each potential diagnosis. Radiculopathies involve the compression of a nerve root, which could result from several factors including heavy lifting and crawling into tight spaces (both behaviors reported by the patient). Meanwhile, neuropathies refer to nerve damage, which is a common complication of diabetes mellitus. Clinical Reasoning: Given the patient's type 2 diabetes, neuropathy should certainly be considered. Diabetic neuropathy often leads to weakness, numbness and pain (usually in the hands or feet). However, it usually affects both sides, but in this case, the left is spared. On the other hand, common peroneal neuropathy usually results from leg trauma, including habitually crossing the legs, or prolonged bed rest, neither of which were reported here. Considering her age cluster, a localized issue seems more probable, hinting towards an S1 radiculopathy that is likely due to the nature of her work (heavy lifting and tight spaces), which can contribute to disc herniation. The patient's report of decreased sensation is also in line with S1 distributions. Compared to her energetic work that could predispose her to disc herniation to cause radiculopathy, diabetes seems less likely to be the primary cause here given its bilateral pattern. Conclusion: Based upon the patient's reported symptoms, her physically demanding work, and her history of diabetes (which seems less likely due to the characteristic bilateral involvement in case of peripheral neuropathy), the most likely diagnosis would be (F) S1 radiculopathy. Response: The most clinically consistent diagnosis for this 47-year-old woman, with right-sided lower extremity weakness and sensory loss, history of diabetes mellitus type 2, and physically demanding job is (F) S1 radiculopathy.

**AI_D:** RESTATING THE QUESTION: This is about a 47-year-old woman who has been experiencing progressive weakness and numbness in her right leg for the past two months. This woman is also a type 2 diabetes mellitus patient who has been on metformin for the past 3 years. From the neurological examination, there is a mildly decreased sensation to light touch over the lateral and inferior aspects of the right foot. The rest is intact. The main query is to determine which, among the given options, is the most probable medical condition this patient is dealing with. PLANNING: Among the possible explanations are common peroneal neuropathy, femoral neuropathy, radiculopathies of L2 and L4, obturator neuropathy, and S1 radiculopathy. Considering her diabetes history, diabetic neuropathy is a potential diagnosis, however, this patient presents with a localized weakness and sensory loss suggesting a neurologic lesion. Therefore, it's critical to examine each option through the lens of her symptoms to determine the most likely diagnosis. CLINICAL REASONING: Common peroneal neuropathy generally manifests as foot drop and involves the lateral leg and dorsum of the foot, which do not align with this woman's symptoms. Femoral neuropathy presents with quadriceps weakness and loss of sensation over the anterior thigh and medial leg, which is inconsistent with her reported symptoms. As for the radiculopathies, L2 radiculopathy usually impacts hip flexors, while L4 radiculopathy presents with tibialis anterior and quadriceps weakness, neither of which aligns with this patient's clinical presentation. Obturator neuropathy would lead to a loss of adduction of the thigh, which this patient does not exhibit. On the other hand, S1 radiculopathy presents with weakness in the gastrocnemius, decreased ankle reflexes, and sensory loss over the lateral foot: characteristics that fit with this patient's symptom presentation. CONCLUDING: The step by step clinical reasoning process based on symptom presentation and examination findings, along with an understanding of each probable condition leads me to conclude that the most likely diagnosis would be S1 radiculopathy. RESPONSE: Therefore, the most likely diagnosis for this 47-year-old woman, with the current presentation and history, is (F) S1 radiculopathy.

**AI_B:** (1) RESTATING THE QUESTION: The clinical scenario describes a middle-aged woman with type 2 diabetes and a physically demanding job, who presents with progressive weakness and numbness in one leg. Examination shows full muscle strength in all muscles except mild sensory loss in the lateral and inferior aspects of the right foot, along with mildly diminished ankle reflex on the right. We need to identify the most likely clinical diagnosis, looking at options that include different types of neuropathies and radiculopathies - conditions that affect nerve function due to damage or compression. (2) PLANNING: Assuming that the signs and symptoms could be caused by neuropathy or radiculopathy, they appear localized to certain areas consistent with specific nerve root or peripheral nerve distributions. The differential diagnosis includes common peroneal neuropathy (affecting the nerve that runs along the outside of the calf and inner foot), S1 radiculopathy (involving the S1 nerve root), or other radiculopathies involving the L2, L4, or L5 nerve roots, as well as femoral or obturator neuropathy. To propose the most likely diagnosis, we should consider individually whether her signs and symptoms correlate with each of these assumed conditions. (3) CLINICAL REASONING: The woman's symptoms of progressive lower extremity weakness and numbness, along with reduced right ankle reflex and mild sensory loss in the lateral and inferior aspects of the right foot, suggest damage to a peripheral nerve or nerve root. The specific distribution of her sensory loss and the involvement of the ankle reflex suggest both could be linked to the S1 nerve root, as this root supplies the sensation to the lateral foot and heel, and also contributes to the ankle reflex. Neuropathies typically occur from conditions like diabetes that affect the nerves, but her diabetes is well controlled with metformin, and the sensory loss pattern doesn't fit with diabetic peripheral neuropathy which usually has a "stocking-glove" pattern. Other kinds of neuropathies like peroneal or femoral don't perfectly fit with her neurologic findings. Peroneal neuropathy, for instance, would present with foot drop, and femoral neuropathy with weakening of the quadriceps. Radiculopathy at L2 or L4 would affect different muscles and sensation areas compared to her symptoms, making them unlikely. (4) CONCLUDING: Taking into account my clinical reasoning, I believe the most suitable explanation for her symptoms is S1 radiculopathy. Her sensory loss pattern, diminished ankle reflex, and lack of significant muscle weakness despite her complaints, strongly suggest a lesion at the S1 level. (5) RESPONSE: Based on the clinical presentation and my reasoning, the most likely diagnosis in this case is (F) S1 radiculopathy.

**AI_C:** (1) RESTATING THE QUESTION: The patient is a 47-year-old woman who presents with a 2-month history of progressive weakness and numbness in her right leg. She has type 2 diabetes mellitus, which she manages with metformin. She has a physically demanding job, which often requires moving and lifting heavy items. Her neurologic examination presents with slight decreases in specific reflexes and light touch sensations in certain areas of the right foot. From the given choices, we are asked to identify the most likely diagnosis for her condition. (2) PLANNING: We can start by identifying the crucial assumptions. Here, the assumptions center around the diagnosis aligning with the specific patterns of reflex, sensory, and muscular functioning shown in the patient's presentation. Another assumption would be considering the impact of her diabetes and job nature. An alternate assumption could be that the reason for her symptoms is not a single entity but a result of multiple factors, including her underlying diabetes, job profile, and perhaps an additional neuropathic or radiculopathic condition. After evaluation, our clinical priority should be placed on the assumption that the diagnosis ought to be an entity in the peripheral nervous system that aligns with her symptoms while factoring in her history and clinical examination. (3) CLINICAL REASONING: The patient's examination findings of diminished ankle reflex, weakness, and decreased sensation in the right foot are indicative of a neuropathy or radiculopathy affecting the lower limb's periphery. The 5-/5 muscle strength in the right hip extensor, thigh abductor, hamstring, and gastrocnemius muscles, along with decreased sensation and diminished ankle reflex in the lower periphery, would suggest lumbar radiculopathy or peripheral neuropathy. (4) CONCLUDING: Given that the patient's diminished sensation is around the lateral and inferior aspects of the right foot and that the ankle reflex, controlled in part by the S1 nerve root, is less on the right compared to the left, S1 radiculopathy seems most likely. If this were an isolated peripheral neuropathy such as common peroneal neuropathy, we would expect more widespread weakness amongst the muscles it innervates. Additionally, diabetes tends to cause more symmetric peripheral neuropathies rather than isolated, unilateral deficits. (5) RESPONSE: In light of the patient's clinical presentation and findings on examination, the most likely diagnosis is S1 radiculopathy (F). These findings are best attributed to damage or irritation at the level of the S1 nerve root, which innervates the regions where impairments are observed. Other options, such as common peroneal neuropathy (A), might lead to similar deficits but are less likely given the relatively isolated sensory loss and the nature of the patient's reflex impairments.

**Facilitator** The consensus statement synthesizes the responses of AI_A, AI_B, AI_C, AI_D, and AI_E as follows: The patient in question is a 47-year-old woman with a history of type 2 diabetes mellitus, who has been experiencing progressive weakness and numbness in her right leg over the past two months. Her job involves physically demanding tasks, including lifting heavy objects and occasionally crawling into tight spaces. Her neurological examination reveals a mildly decreased sensation to light touch over the lateral and inferior aspects of the right foot, but muscle strength remains largely intact. Given the patient's symptoms and history, the potential diagnoses include common peroneal neuropathy, femoral neuropathy, L2 radiculopathy, L4 radiculopathy, obturator neuropathy, and S1 radiculopathy. However, the patient's symptoms do not align with the typical presentations of common peroneal neuropathy, femoral neuropathy, L2 radiculopathy, L4 radiculopathy, or obturator neuropathy. On the other hand, the patient's symptoms of decreased sensation in the lateral and inferior aspects of the right foot, diminished right ankle reflex, and lack of significant muscle weakness despite her complaints, strongly suggest a lesion at the S1 level. This aligns with the presentation of S1 radiculopathy, which involves the S1 nerve root and can result from physically demanding activities such as heavy lifting and crawling into tight spaces. Therefore, the consensus among the AI responses is that the most likely diagnosis for this patient, given her clinical presentation and history, is S1 radiculopathy.
