# Supplementary material for: Collaborative intelligence in AI: Evaluating the performance of a council of AIs on the USMLE
Source: PLOS Digit Health. 2025 Oct 9;4(10):e0000787. doi: 10.1371/journal.pdig.0000787 (PMC12510544; doi:10.1371/journal.pdig.0000787)
Supplement: S2 File — (ZIP) [file pdig.0000787.s002.zip › S2 Data Raw data files containing unprocessed question inputs and LLM outputs including transcripts of deliberations/STEP2_Question-13-1_Type-MC-NJ.docx]

**Facilitator** **[Round 1]:** Abstract: Objective: In patients with cirrhosis and acute bleeding esophageal varices, how do endoscopic sclerotherapy and emergency portacaval shunt compare for control of bleeding and survival? Methods Design: Randomized controlled trial (San Diego Bleeding Esophageal Varices Study). ClinicalTrials.gov NCT00690027. Allocation: Concealed. Blinding: Blinded (gastroenterologist who evaluated patients for portal-systemic encephalopathy). Follow-up period: Up to 17 years. Setting: University of California San Diego Medical Center. Patients: 211 patients (mean age 49 years, 77% men) with acute bleeding esophageal varices resulting from cirrhosis, who required a transfusion of ≥ 2 units of blood and, for patients transferred from other hospitals, observation of upper gastrointestinal bleeding within 48 hours of transfer. Exclusion criterion was > 1 previous session of endoscopic sclerotherapy. Intervention: Endoscopic sclerotherapy (n = 106) or emergency portacaval shunt (n = 105). Emergency portacaval shunt comprised a direct side-to-side or direct end-to-side portacaval shunt done within 8 hours of initial contact. Outcomes: Control of bleeding at > 30 days, survival, readmissions for variceal or nonvariceal bleeding requiring transfusion of packed red blood cells, and recurrent portal-systemic encephalopathy. Patient follow-up: 100% (minimum follow-up until death or 9.4 years). Main results 15-year survival was lower with endoscopic sclerotherapy than with emergency portacaval shunt (10/106 vs 48/105, relative benefit reduction 79%, 95% CI 62 to 89; number needed to harm 3, CI 2 to 4). Other main results are shown in the Table. Endoscopic sclerotherapy (EST) vs emergency portacaval shunt (EPCS) in patients with cirrhosis and acute bleeding esophageal varices Outcomes | Child-Pugh risk class | EST | EPCS | P value | ———————————————————————————————————— Control of bleeding at > 30 days* | | 20% | 100% | <.001 | Median survival (years) | A | 4.62 | 10.43 | .003. | | B | 2.61 | 6.19 | <.001 | |. C | 0.58 | 5.30 | .005. | Mean number of readmissions for variceal bleeding requiring packed red blood cell transfusion | | 6.8 | 0.4 | <.001 | Recurrent portal-systemic encephalopathy† | | 35% | 15% | < .001 | ——————————————————————————————————— *Excluding indeterminate deaths at 14 days from nonbleeding causes. †In patients who survived 30 days and left hospital. ——————————————————————————————————— Conclusion In patients with cirrhosis and acute bleeding esophageal varices, emergency portacaval shunt was better than endoscopic sclerotherapy for control of bleeding, recurrent encephalopathy, and survival. Sources of funding: National Institutes of Health and Surgical Education and Research Foundation. Structured abstract based on: Orloff MJ, Isenberg JI, Wheeler HO, et al. Randomized trial of emergency endoscopic sclerotherapy versus emergency portacaval shunt for acutely bleeding esophageal varices in cirrhosis. J Am Coll Surg. 2009;209:25-40. 19651060 Which of the following conclusions is most appropriate based on the results presented in the table? (A) The 95% confidence interval for the difference in survival between EPCS and EST for Child-Pugh class A patients includes 0 years (B) EPCS is more effective than EST in decreasing hospital readmissions for variceal bleeding requiring transfusion (C) The median survival after EPCS is statistically significantly less for Child-Pugh class C than for Child-Pugh class B (D) The randomization procedure was ineffective in decreasing bias in this study

**AI_A:** RESTATING THE QUESTION: The assessment is asking us to choose the most suitable conclusion based on the presented clinical study data. This study compared the effectiveness of two procedures—endoscopic sclerotherapy (EST) and emergency portacaval shunt (EPCS)—in managing patients with cirrhosis and acute bleeding esophageal varices. The data provided includes varied clinical outcomes and the corresponding statistical significances. PLANNING: When examining the outcome parameters, including the control of bleeding at >30 days, median survival, readmissions for variceal bleeding requiring blood transfusion, and recurrent portal-systemic encephalopathy, we notice that the EPCS outperforms EST markedly. However, we should note that these are aggregate data and do not take into account the finer ramifications of individual Child-Pugh class groups. This may lead to incorrect conclusions if not carefully examined. CLINICAL REASONING: Examining each of the potential conclusions in detail we find that: (A) The 95% confidence interval for survival difference between EPCS and EST is not provided, so we can't confirm this. (B) The data does show a significant reduction in mean number of hospital readmissions for variceal bleeding requiring transfusion with EPCS as compared to EST. (C) The median survival data does not provide a comparison of survival between Child-Pugh class B and C patients following EPCS, so a comparison can't be confirmed. (D) No specific details are provided about the effectiveness of the randomization process. CONCLUDING: Based on the data provided and the reasoning applied in interpreting this data, one can say that the conclusion that matches best is that EPCS is more effective than EST in decreasing hospital readmissions for variceal bleeding requiring transfusion. RESPONSE: Therefore, the most appropriate conclusion based on the table is (B) EPCS is more effective than EST in decreasing hospital readmissions for variceal bleeding requiring transfusion.

**AI_B:** RESTATING THE QUESTION: The question asks us to select an accurate conclusion from the data presented in the table of a study done to compare the effectiveness of endoscopic sclerotherapy (EST) and emergency portacaval shunt (EPCS) for the management of acute bleeding esophageal varices in patients with cirrhosis. The options provided are all different interpretations of the study data. PLANNING: To answer the question accurately, the critical assumptions I must make are: 1) The table data represent the study results truthfully and 2) Each response option makes a valid assessment or interpretation of the data. Now, let's evaluate each answer option. (A) asserts that the 95% confidence interval for the difference in survival between EPCS and EST for Child-Pugh class A patients includes 0 years - but the table doesn't provide concrete enough information about the confidence interval for this group's survival. (B) suggests that EPCS is more effective than EST in decreasing hospital readmissions for variceal bleeding requiring a transfusion, which is supported by the mean number of readmissions data in the table. (C) says that the median survival after EPCS in Child-Pugh class C patients is significantly less than that of class B patients, but the table does not provide enough information to determine a significant difference in these two groups alone. (D) argues that this study's randomization procedure was ineffective in reducing bias, but we are not provided with information on procedure specifics or any bias detection in this abstract to substantiate this claim. CLINICAL REASONING: Given the available evidence, (A) and (D) can be eliminated, as the table lacks the necessary data to support these claims. Comparing Child-Pugh class B and C median survival times for EPCS, option (C) could be correct, but it is also not definitive without statistical testing data. On the other hand, the assertion in (B) aligns with the provided data. CONCLUDING: From these four options, (B) seems to be the best supported by the current data and should be chosen. RESPONSE: (B) EPCS is more effective than EST in decreasing hospital readmissions for variceal bleeding requiring transfusion.

**AI_D:** (1) RESTATING THE QUESTION: We're being asked to choose the most fitting conclusion based on the results revealed by the study. The possibilities mentioned are: (A) For Child-Pugh class A patients, the 95% confidence interval for the difference in survival between Emergency Portacaval Shunt (EPCS) and Endoscopic Sclerotherapy (EST) includes 0 years; (B) EPCS is more effective than EST in reducing hospital readmissions for variceal bleeding necessitating transfusion; (C) the median survival post-EPCS is significantly less for Child-Pugh class C patients than for Child-Pugh class B patients; and (D) the randomization method employed in this study failed to decrease bias. (2) PLANNING: The primary assumption here is that the available options are valid conclusions based on the study results. However, by scrutinizing each proposition, it will be possible to challenge or substantiate those assumptions. The Child Pugh Score is a method to group patients with liver disease into severity classes, and the table provides survival data for each class. (3) CLINICAL REASONING: The details in the table do not point to 0 years inclusion in the 95% confidence interval for survival difference between EPCS and EST in Child-Pugh class A patients, which negates option (A). Similarly, it is not indicated that the median survival time for Child-Pugh class C is statistically significantly less than that of class B post-EPCS treatment, which negates option (C). Information about the effectiveness of the randomization procedure in minimizing bias is not directly provided, which raises doubts about option (D). However, option (B), which suggests that EPCS is more efficient than EST at reducing hospital readmissions attributable to variceal bleeding necessitating transfusion, aligns perfectly with what is stated in the results: mean number of readmissions for variceal bleeding requiring packed red blood cell transfusion was 6.8 for EST and 0.4 for EPCS; P value < .001. (4) CONCLUDING: A step by step review of each possible conclusion based on the study result points solely to conclusion (B) being the valid one. It fits with the results presented and doesn't make assumptions or interpretations beyond what has been explicitly provided. (5) RESPONSE: Based on the results presented, the most appropriate conclusion is that "EPCS is more effective than EST in decreasing hospital readmissions for variceal bleeding requiring transfusion" (B).

**AI_E:** (1) RESTATING THE QUESTION: The question asks us to analyze the result of a study and determine the most valid conclusion. Endoscopic sclerotherapy (EST) and emergency portacaval shunt (EPCS) were compared for their efficacy in controlling bleeding and improving survival in patients with cirrhosis and acute bleeding esophageal varices. This efficacy is measured based on controlled bleeding, survival, hospital readmission for bleeding that requires blood transfusion, and recurrence of portal-systemic encephalopathy. (2) PLANNING: A clinical study was conducted that used a random controlled trial design and included 211 patients with cirrhosis and acute bleeding esophageal varices. Endoscopic sclerotherapy (EST) and emergency portacaval shunt (EPCS) were compared. EPCS involves diverting blood flow from the portal vein directly to the inferior vena cava to relieve pressure in the portal system, thus reducing the risk of variceal bleeding. On the other hand, EST involves using a flexible endoscope to inject a sclerosing agent into the esophageal veins to promote clotting and scar formation, thus stopping the bleeding. Let's evaluate the presented assumptions: (A) The 95% confidence interval for the difference in survival between EPCS and EST for Child-Pugh class A patients includes 0 years: This assumption is not observable from the data presented. Thus, it needs further evidence. (B) EPCS is more effective than EST in decreasing hospital readmissions for variceal bleeding requiring transfusion: The table provides enough evidence to make this assumption, showing less readmission with EPCS (0.4) than with EST (6.8). (C) The median survival after EPCS is statistically significantly less for Child-Pugh class C than for Child-Pugh class B: From the table, we can't conclude this as we need further statistical testing to determine the significance of the difference within EPCS treatment. (D) The randomization procedure was ineffective in decreasing bias in this study: This is a methodological assumption, and no concrete evidence has been provided to suggest randomization bias. After analyzing these assumptions, assumption 'B' seems to be clinically prioritized as it directly reflects the clinical efficacy of EPCS and EST treatments. (3) CLINICAL REASONING: Child-Pugh classification, used to assess the prognosis of chronic liver disease, classifies patients into risk classes A, B, or C. This classification provides useful information to evaluate the therapeutic response to EST and EPCS in different severity levels of liver disease. The data presented reveal that EPCS provides better control over bleeding, longer median survival in Child-Pugh classes A, B, and C, fewer readmissions for variceal bleeding requiring transfusion, and less recurrent portal-systemic encephalopathy. Thus, EPCS shows overall improved clinical performance in comparison to EST. (4) CONCLUDING: From the provided data, we can conclude that EPCS is more effective than EST in controlling bleeding and improving survival in cirrhotic patients with acute bleeding esophageal varices. This conclusion is further reinforced by the lower rates of hospital readmission for variceal bleeding requiring transfusion in patients treated with EPCS. (5) RESPONSE: Option (B) EPCS is more effective than EST in decreasing hospital readmissions for variceal bleeding requiring transfusion is the most appropriate conclusion based on the results presented in the table.

**AI_C:** 1) RESTATING THE QUESTION: In simpler terms, we are tasked with determining the most relevant conclusion drawn from this study which compares endoscopic sclerotherapy (EST) and emergency portacaval shunt (EPCS) in the treatment of patients with cirrhosis and acute bleeding esophageal varices. A reference to Child-Pugh class introduces the stratification of cirrhosis severity where class A indicates a relatively mild form of the disease, class B a more serious form, and class C the most severe form. Key aspects to be evaluated include bleeding control, hospital readmissions for bleeding, survival rates, and the potential bias in the study's conduct. 2) PLANNING: Reasonable assumptions made in this study include uniform application of therapies, comparable patient populations, valid and reliable measure of outcomes, and equitable follow-up. Evaluating these assumptions, we should also consider factors such as differences in intervention timings and techniques, clinical characteristics of patients that could influence outcomes (e.g., age, cirrhosis severity, comorbidities), measurement inaccuracies, and follow-up limitations. The results highlight key differential features: significant survival benefit and fewer readmissions with EPCS when compared to EST, across all Child-Pugh classes. 3) CLINICAL REASONING: Upon analyzing the table, we note several critical points. The control of bleeding at >30 days post-intervention was significantly higher in EPCS (100%) compared to EST (20%). Survival rates in years across all Child-Pugh risk classes are higher for EPCS compared to EST, and these differences are statistically significant. Notably, the median survival for Child-Pugh class C patients with EPCS is lower than that for Child-Pugh class B, but significantly higher than that of class C patients with EST. The mean number of readmissions due to variceal bleeding requiring transfusion of packed red blood cells is significantly lower with EPCS (0.4) compared to EST (6.8). The frequency of recurrent portal-systemic encephalopathy was lower in patients who survived 30 days and left the hospital with EPCS (15%) compared to EST (35%). 4) CONCLUDING: The primary endpoint in this study is a comparison of the survival rates and control of bleeding between patients treated with EST and EPCS. In each outcome, EPCS outperforms EST. No information in the provided text directly supports the conclusion that the 95% confidence interval for the difference in survival for Child-Pugh class A patients includes 0 years (option A), or that the randomization procedure was ineffective in decreasing bias in this study (option D). As the median survival for Child-Pugh class C patients is lower in EPCS than in class B, this does not negate the observation that EPCS still offers a better survival advantage compared to EST for the same class of patients (option C). 5) RESPONSE: Based on the provided table, the most reasonable conclusion is that (B) EPCS is more effective than EST in decreasing hospital readmissions for variceal bleeding requiring transfusion. The survival advantages of EPCS over EST, as evidenced by superior control of bleeding, dials down the necessity for readmissions due to rebleeding. This is concretely supported by the significantly lower mean number of hospital readmissions for variceal bleeding requiring transfusion for EPCS-treated patients across all Child-Pugh classes as given in the table.

**Facilitator** The consensus statement synthesizing the responses of AI_A, AI_B, AI_C, AI_D, and AI_E is as follows: The question at hand asks us to determine the most appropriate conclusion based on the results of a clinical study comparing the effectiveness of endoscopic sclerotherapy (EST) and emergency portacaval shunt (EPCS) in managing patients with cirrhosis and acute bleeding esophageal varices. The data provided includes various clinical outcomes and their corresponding statistical significances. Upon examining the data and potential conclusions, it is clear that the 95% confidence interval for the difference in survival between EPCS and EST for Child-Pugh class A patients is not provided, thus we cannot confirm option (A). Similarly, the data does not provide a comparison of survival between Child-Pugh class B and C patients following EPCS, so option (C) cannot be confirmed. There are no specific details provided about the effectiveness of the randomization process, making it impossible to confirm option (D). However, the data does show a significant reduction in the mean number of hospital readmissions for variceal bleeding requiring transfusion with EPCS as compared to EST, supporting option (B). This conclusion aligns with the provided data and does not make assumptions or interpretations beyond what has been explicitly provided. Therefore, based on the data provided and the reasoning applied in interpreting this data, the most appropriate conclusion is that EPCS is more effective than EST in decreasing hospital readmissions for variceal bleeding requiring transfusion (option B). This conclusion is further reinforced by the lower rates of hospital readmission for variceal bleeding requiring transfusion in patients treated with EPCS, as evidenced in the table.
