# Supplementary material for: Collaborative intelligence in AI: Evaluating the performance of a council of AIs on the USMLE
Source: PLOS Digit Health. 2025 Oct 9;4(10):e0000787. doi: 10.1371/journal.pdig.0000787 (PMC12510544; doi:10.1371/journal.pdig.0000787)
Supplement: S2 File — (ZIP) [file pdig.0000787.s002.zip › S2 Data Raw data files containing unprocessed question inputs and LLM outputs including transcripts of deliberations/STEP2_Question-14-1_Type-MC-NJ.docx]

**Facilitator** **[Round 1]:** A 42-year-old man comes to the office as a new patient. Three weeks ago, he was hospitalized for treatment of community-acquired pneumonia and diagnosed with HIV infection. He has no other history of serious illness and takes no medications. Vital signs are within normal limits. Examination shows no abnormalities. CD4+ T-lymphocyte count is 490/mm³, and plasma HIV viral load is 45,000 copies/mL. Results of other laboratory studies are within the reference ranges. Test results for syphilis and hepatitis B and C are negative. The patient agrees to begin antiretroviral therapy. He asks what his long-term prognosis will be. With adherence to the medication regimen, this patient is at greatest risk of death from which of the following? (A) Cardiovascular disease (B) Chronic kidney disease (C) HIV-related malignancy (D) Non–HIV-related malignancy (E) Opportunistic infection

**AI_B:** RESTATING THE QUESTION: The query pertains to a 42-year-old male patient newly diagnosed with HIV infection and is agreeing to undergo antiretroviral therapy. He is interested in understanding what his long-term prognosis might be, and particularly, what condition he is more likely to die from amidst cardiovascular disease, chronic kidney disease, HIV-related malignancies, Non-HIV-related malignancies, and opportunistic infections. PLANNING: The assumptions underlying this query are that the patient's CD4+ T-lymphocyte count and HIV viral load are associated with his survival and that his long-term prognosis is linked to his vulnerability to the listed conditions. Alternate assumptions could be that the patient's overall health, lifestyle factors, genetic predisposition, and effectiveness of antiretroviral medication also play a role in his long-term prognosis. However, the assumption that should be clinically prioritized should pertain to the effect of antiretroviral therapy on these listed conditions. CLINICAL REASONING: When looking at HIV-infected patients, disruptions of the immune system can lead to opportunistic infections which used to be the leading cause of death. But with the advent of antiretroviral therapy (ART), the immune function has been able to be restored, reducing their occurrence. As for malignancies, while HIV patients can be more predisposed to certain cancers due to their impaired immunity, therapy again reduces this risk. Nowadays, the typical causes of death shifted, and chronic non-communicable diseases became the leading cause of death in HIV patients on ART. They are more likely to die from illnesses commonly found in the aging population, such as cardiovascular disease, which is often increased by the long-term use of the antiretroviral medications. CONCLUDING: The patient's overall health, and in particular his cardiovascular system, will play a significant role in this prognosis. As such, ongoing management of potential cardiovascular risk factors is an important part of treating HIV patients. RESPONSE: Given his adherence to the antiretroviral medication regimen, this 42-year-old patient is at greatest risk of death from Cardiovascular disease (A). This risk arises due to the long-term use of antiretroviral medications, which can increase cardiovascular risks, and the overall shift in causes of mortality seen in patients effectively treated for HIV. As the patient ages further, careful management of cardiovascular health would be an integral part of his HIV treatment plan.

**AI_A:** RESTATING THE QUESTION: The question is asking about the most significant long-term health risk for a 42-year-old man who has recently been diagnosed with HIV. He has agreed to begin antiretroviral therapy and is currently in a relatively healthy condition with no other serious health issues or abnormalities in physical examination or laboratory tests. PLANNING: In forming an understanding of the prognosis for this patient, we should consider that antiretroviral therapy has significantly improved the life expectancy of people with HIV. These medications work to maintain a suppressed viral load, thus preserving CD4 T-lymphocyte count, preventing progression to AIDS, and substantially reducing the risk for opportunistic infections, HIV-related malignancies and progression of the disease to AIDS. However, patients with HIV are at a higher risk for some diseases than the general population, even when the virus is well-controlled. The options given for the question include cardiovascular disease, chronic kidney disease, HIV-related malignancy, non-HIV-related malignancy and opportunistic infections. CLINICAL REASONING: The evolution of HIV treatment has significantly reduced the risk of death from opportunistic infections and HIV-related malignancies, turning the disease into a chronic condition with which patients can live for many years. However, with increased life expectancy, other health risks such as diseases typically associated with aging have become more prevalent and fatal. Among these, cardiovascular disease has been identified as a particularly significant risk for people with HIV, which would also explain why option (D) Non–HIV-related malignancy, for example, is less likely. Because with effective viral suppression from antiretroviral therapy, rates of HIV-related malignancies have declined dramatically, but the rates of non-HIV-related malignancies are not significant enough to make them the primary cause of death. Similarly, while antiretroviral therapy can potentially lead to chronic kidney disease due to toxicity, it's not the leading cause of death among those with HIV. CONCLUDING: By process of elimination, combining our understanding of the historical risks of HIV infection along with the impact of antiretroviral therapy, the most significant long-term health risk for this patient is likely to be cardiovascular disease. This is due to the fact that HIV itself can cause inflammation and immune activation throughout the body, which can damage the blood vessels increasing the risk of cardiovascular disease, as well as the fact that some antiretroviral medications can contribute to elevated cholesterol and triglyceride levels, further increasing this risk. RESPONSE: With adherence to the medication regimen, this patient is at greatest risk of death from (A) Cardiovascular disease.

**AI_D:** RESTATING THE QUESTION: The query is with regards to a male patient, mid-aged with a recent HIV diagnosis who hasn't any past episodes of severe illness. His laboratory measures including his CD4+ T-lymphocyte count, HIV viral load and test results for syphilis, and hepatitis B and C are given. Despite being infected with HIV, his vitals and physical check are standard and he is about to commence antiretroviral therapy. The patient's question is on his long-term prognosis and what his highest risk of death would be assuming he adheres to his medication regimen. The options provided are cardiovascular disease, chronic kidney disease, HIV-related malignancy, non-HIV related malignancy, and opportunistic infection. PLANNING: In this scenario, the main assumption is that HIV primarily predisposes the patient to severe, life-threatening infections and malignancies which are known as AIDS-defining illnesses. Nevertheless, alternate assumptions here are that, with effective and sustained antiretroviral therapy (ART), HIV patients can witness substantial suppression of the viral load, CD4+ T-lymphocyte recovery, and a significant reduction in the risk of developing AIDS-defining illnesses. As a result, the life expectancy of people living with HIV has considerably increased over the past two decades, and non-AIDS-related diseases are an emerging cause of morbidity and mortality in this population. CLINICAL REASONING: In recent years, with wider availability and improved effectiveness of ART, there has been a shift in the causes of mortality among HIV-infected individuals. Instead of opportunistic infections and AIDS-defining cancers that were once commonly seen, there is increasing recognition of the role of non-AIDS-related conditions such as cardiovascular disease and non–HIV-related malignancies. This shift is because ART effectively suppresses HIV replication, enabling recovery of the immune system, and significantly reduces the risk of AIDS-defining illnesses. However, persons living with HIV continue to have a chronic inflammatory state even when HIV replication is adequately controlled, which may increase their risk of chronic diseases. CONCLUDING: The patient's CD4+ T-lymphocyte count (490/mm³) is not severely low, meaning he is not currently at high risk for opportunistic infections or AIDS-defining cancers. Instead, with effective antiretroviral therapy, the greatest burden of disease and risk of mortality for this patient are likely to shift to non–HIV-related chronic conditions or diseases. RESPONSE: Therefore, with adherence to the medication regimen, this patient is at greatest risk of death from (A) Cardiovascular disease.

**AI_E:** (1) RESTATING THE QUESTION: The inquiry in question is interested in determining what the greatest risk of mortality would be for a 42-year-old man who has recently been diagnosed with HIV infection, received treatment for community-acquired pneumonia, but otherwise has no history of significant illness. His CD4+ T-lymphocyte count is 490/mm³ and his HIV viral load is 45,000 copies/mL. He has agreed to start antiretroviral therapy. The options provided include cardiovascular disease, chronic kidney disease, HIV-related malignancy, non–HIV-related malignancy and opportunistic infections. (2) PLANNING: Our assumptions include that treatment for HIV includes highly active antiretroviral therapy (HAART), which significantly reduces the viral load and increases the CD4 count. Subsequently reducing the risk of opportunistic infections. Assuming patient's adherence to the antiretroviral therapy as stated in the question, the likelihood of opportunistic infections is relatively low. Therefore, the question aims to gauge whether the most significant long-term risk arises from cardiovascular disease, chronic kidney disease, or malignancies (both HIV-related and non-HIV-related). (3) CLINICAL REASONING: The patient's viral load and CD4 count, though indicative of an active HIV infection, are not dire. With proper adherence to antiretroviral therapy, his chances of developing AIDS and subsequent opportunistic infections should diminish significantly. While HIV infection can lead to various comorbid conditions like cardiovascular or kidney disease, or increase the risk of malignancies, research has demonstrated that the primary cause of death among virally suppressed patients on antiretroviral therapy is now increasingly due to non-HIV related conditions, including cardiovascular disease. (4) CONCLUDING: Consequently, given the known impact of HIV, the associated chronic inflammation, the metabolic effects of antiretroviral therapy, and factoring in the possible lifestyle attributes with age, it is quite reasonable to determine that in the current era of effective antiretroviral therapy, cardiovascular disease could be the leading cause of mortality in patients with well-controlled HIV. (5) RESPONSE: Therefore, despite the circumstances specific to this patient, when considering the likely risks associated with long-term HIV management, and assuming adequate viral control and reduced risk of opportunistic infections with antiretroviral therapy, the man is at greatest risk of succumbing to (A) Cardiovascular disease.

**AI_C:** (1) RESTATING THE QUESTION: The patient, a 42-year-old man, recently got diagnosed with HIV following a bout of community-acquired pneumonia. His infection is not advanced, considering the CD4+ T-cell count of 490/mm³, and he has a significant HIV viral load (45,000 copies/ml). He doesn't suffer from any other severe illnesses, he doesn't use any medicine, and his physical examination and lab results are within the normal range. His tests for Hepatitis B, C, and syphilis came back negative. Considering that he will adhere to the antiretroviral therapy, he inquires about his long term prognosis, focusing on his highest risk of mortality. (2) PLANNING: The fundamental assumption is that the primary cause of death for an HIV patient is due to HIV-related complications such as opportunistic infections or HIV-related malignancies. However, it's essential to question this assumption, as the situation for HIV-positive individuals has significantly changed with the introduction of effective antiretroviral therapy (ART). With the use of ART, many HIV positive individuals are living long enough to face chronic conditions not directly associated with HIV, a condition known as non-AIDS defining illnesses. Hence, an alternate assumption that should be clinically prioritized to pursue is that the patient's highest risk of death might not necessarily be from HIV-related malignancies or opportunistic infections, but from a chronic illness including cardiovascular disease or chronic kidney disease or non-HIV related malignancies. (3) CLINICAL REASONING: With effective antiretroviral therapy, the life expectancy for those living with HIV has dramatically improved, closely resembling that of the general population. This increased lifespan results in an increased prevalence of chronic diseases commonly associated with aging, such as cardiovascular disease, chronic kidney disease, and non-HIV related malignancies. Yet, the risk of these diseases is elevated in the HIV population compared to the general population, possibly due to the lingering inflammatory effects of HIV infection and/or the side effects of antiretroviral medication. Among these, cardiovascular disease has been identified as a common cause of death in people with well-controlled HIV infection. As such, the patient's prognosis depends not only on their HIV status but also on other possible, non-HIV related complications they may face in the long run. (4) CONCLUDING: With consistent adherence to the medication regimen, this patient, while indeed at some risk of HIV-related malignancies and opportunistic infections, faces a more urgent threat from long-term, chronic diseases associated with age and partially, the HIV infection itself. (5) RESPONSE: Given that the patient is responding favorably to antiretroviral therapy, the long-term prognosis would show that he is at greatest risk of death from (A) Cardiovascular disease.

**Facilitator** The consensus statement synthesizes the responses of AI_A, AI_B, AI_C, AI_D, and AI_E as follows: The question pertains to a 42-year-old man recently diagnosed with HIV, who is otherwise in good health and has agreed to begin antiretroviral therapy. He is interested in understanding his long-term prognosis and the most significant health risk he faces. The options provided include cardiovascular disease, chronic kidney disease, HIV-related malignancy, non-HIV-related malignancy, and opportunistic infections. The advent of antiretroviral therapy has significantly improved the life expectancy of people with HIV by suppressing the viral load, preserving CD4 T-lymphocyte count, and reducing the risk of progression to AIDS. This has led to a decrease in the risk of opportunistic infections and HIV-related malignancies, which were once the leading causes of death in HIV patients. However, even with well-controlled HIV, patients are at a higher risk for some diseases than the general population. With increased life expectancy, diseases typically associated with aging, such as cardiovascular disease, have become more prevalent and fatal in HIV patients. This is due to the chronic inflammatory state caused by HIV and the side effects of some antiretroviral medications, which can contribute to elevated cholesterol and triglyceride levels, further increasing the risk of cardiovascular disease. While antiretroviral therapy can potentially lead to chronic kidney disease due to toxicity, and HIV patients can be more predisposed to certain cancers due to their impaired immunity, these are not the leading causes of death among those with HIV. Therefore, with adherence to the medication regimen, the most significant long-term health risk for this patient is likely to be cardiovascular disease. As the patient ages further, careful management of cardiovascular health would be an integral part of his HIV treatment plan.
