# Supplementary material for: Collaborative intelligence in AI: Evaluating the performance of a council of AIs on the USMLE
Source: PLOS Digit Health. 2025 Oct 9;4(10):e0000787. doi: 10.1371/journal.pdig.0000787 (PMC12510544; doi:10.1371/journal.pdig.0000787)
Supplement: S2 File — (ZIP) [file pdig.0000787.s002.zip › S2 Data Raw data files containing unprocessed question inputs and LLM outputs including transcripts of deliberations/STEP2_Question-15-1_Type-MC-NJ.docx]

**Facilitator** **[Round 1]:** A 40-year-old divorced woman comes to the office because of a 1-year history of difficulty sleeping. She says she sometimes does not fall asleep before 2 AM and does not feel rested at 6:30 AM when she has to get up and get her children ready for school. Sometimes she wakes after 1 to 2 hours of sleep and cannot fall back to sleep. On these occasions, she typically gets up for about 30 minutes, does some housework, and smokes a cigarette. She smokes one pack of cigarettes every three to four days. She works in a customer service call center and says she enjoys helping people resolve their problems. She now finds her job much harder and less enjoyable because she feels tired and sleepy. She says she would take a nap if she could, but she picks her children up as soon as she leaves work and spends the evening doing homework with them, making their dinner, and preparing lunches for the following day. By 9 PM, the children are in bed so she eats her own dinner and does some housework. She says that after 9 PM is when she smokes most of her cigarettes. She has not had depressed mood, excessive worry, or change in weight. Vital signs are within normal limits. Examination shows dark circles under the eyes and injected conjunctivae. Which of the following is the most appropriate initial step in management? (A) Education on sleep hygiene (B) Mirtazapine therapy (C) Sertraline therapy (D) Setting a date for smoking cessation (E) Zolpidem therapy

**AI_E:** RESTATING THE QUESTION: We are faced with a clinical scenario involving a 40-year-old woman who is experiencing ongoing issues with her sleep. She describes patterns that suggest delayed sleep onset, interrupted sleep, and overall non-restorative sleep. Despite her everyday obligations, she finds her sleep issues are affecting both her job and personal life. These symptoms are not accompanied by mood fluctuations, changes in weight, or elements of anxiety. Vital signs are within a normal range, but physical examination does show dark under-eye circles and injected conjunctivae. The question is asking which of the given actions would be most effective as the first step in managing her situation. PLANNING: We can make assumptions from the information given to us. Considering her sedentary lifestyle, her smoking habit, and her nighttime behaviors that suggest poor sleep hygiene, there are various factors that might be contributing to her sleep difficulties. These factors may be categorized into lifestyle behaviors that are inhibiting her ability to sleep (poor sleep hygiene and nicotine use), physiological changes (possible insomnia disorder), and potential co-existing conditions (even though she doesn't manifest signs of depression or anxiety, these cannot be completely ruled out). Each assumption could lead to a different management approach, therefore it's important to prioritize which factor might be most immediately influential to her sleep issues. CLINICAL REASONING: One could argue that smoking cessation could be prioritized due to the stimulating effect of nicotine and the possibility of nicotine withdrawal contributing to her interrupted sleep. However, her main smoking habit is done primarily after 9 PM, which directly impacts her ability to fall asleep, thus suggesting poor sleep hygiene. Additionally, sleep disruptions due to smoking would not account for the entirety of her sleep disturbances described. Although her delayed bedtime of 2 AM indicates a potential for a delayed sleep-wake disorder (DSWD), it's important to note that possibly her delay is due to poor sleep habits rather than a physiological rhythm disorder. Lastly, while we can't rule out potential comorbid disorders (like depression or anxiety), she does not exhibit clear signs or symptoms of these conditions. Hence, a psychoactive drug that targets these conditions might not be the first line of action. CONCLUDING: In evaluating these considerations, it appears reasonable to prioritize addressing her nocturnal behaviors that inhibit healthy sleep practices, categorized as poor sleep hygiene. This can potentially provide an immediate influence on her sleep if she is able to make modifications to her bedtime routine and late-night activities. This identified, the intervention that fits most appropriately would be to provide her with education on sleep hygiene. RESPONSE: The most appropriate initial step in management of this woman's sleep difficulties is (A) Education on sleep hygiene.

**AI_C:** RESTATING THE QUESTION: This scenario describes a 40-year-old woman who is experiencing chronic insomnia, which is characterized by difficulty falling asleep, not feeling rested despite sleeping, and struggling to return to sleep once woken up. Insomnia can profoundly affect a person's daily functioning, as it seems to be doing in this case. Her work productivity, mood, and overall quality of life seem to be impacted. It's also important to note that she smokes cigarettes, more so at night, which can contribute to her insomnia. We are asked to identify the most appropriate initial step in managing her condition from the following options: advice about sleep hygiene, mirtazapine therapy, sertraline therapy, setting a date for smoking cessation, and zolpidem therapy. PLANNING: It is crucial to consider the woman's nighttime habits when choosing the best management approach. Since she mentions smoking substantially more cigarettes after 9 PM, it is possible that nicotine - a stimulant - may be contributing to her difficulty falling into and maintaining sleep. Furthermore, tasks like doing housework at night can lead to arousal that impedes the initiation of sleep. The assumptions here are that the first steps to manage her condition would be either related to her smoking habits or the medications she could be prescribed to help her sleep or potentially manage any undiagnosed mood disorders. Given the habits described, an alternative assumption would be that improving her sleep hygiene could be prioritized as the initial management step. CLINICAL REASONING: Sleep hygiene is a behavioral approach which involves adjustments to lifestyle and environmental factors that influence sleep. It is among the first-line treatment strategies for insomnia because it addresses habits, such as nighttime smoking and engaging in activating tasks before bed, that can exacerbate sleep problems. Medications, while beneficial, should typically be considered if non-pharmacological interventions are unsuccessful or if the insomnia is severe. The woman in this case has not shown severity that necessitates immediate pharmacological intervention, or symptoms of depression or anxiety that would suggest a mood disorder that requires separate treatment (e.g., with mirtazapine, a primarily anti-depressant medication, or sertraline, another medication used for mood disorders). Therefore, it would be reasonable to focus initially on altering her sleep habits before considering the need for more advanced interventions such as medication or a concentrated effort to cease smoking. CONCLUDING: The woman's habit of smoking more cigarettes after 9 PM, engaging in housework before bedtime, and her problems with not feeling rested even after sleep, points towards poor sleep hygiene as a potential exacerbating factor for her insomnia. Given that she doesn't display symptoms requiring immediate pharmacological intervention, the initial focus should be on improving her sleep hygiene before considering more advanced therapeutic measures. RESPONSE: So, the most appropriate initial step in managing this patient's insomnia would be (A) Education on sleep hygiene.

**AI_D:** RESTATING THE QUESTION: This query pertains to a 40-year-old woman who has been experiencing insomnia for the past year. Despite displaying symptoms like dark circles under the eyes and red eyes, her vital signs are normal. She doesn't display symptoms of depression, anxiety, or significant weight changes, but it is mentioned that she smokes cigarettes, mainly in the late evening. Given this context, we are asked to identify the most suitable initial step in managing her condition. PLANNING: The available options propose either drug therapies such as Mirtazapine (an antidepressant), Sertraline (SSRI, used for anxiety/depression), and Zolpidem (a sedative-hypnotic used for insomnia); or behavioral approaches such as education on sleep hygiene or setting a date for smoking cessation. It's crucial to recognize that her insomnia symptoms might be related to a number of factors - stress (as a working mother, divorcee, customer service worker), lifestyle habits (late-night activities, smoking), or could potentially be a symptom of a psychological disorder (despite the lack of depression or anxiety symptoms). As the first step, we would like to address the issue that is most reversible and least likely to cause harm or side effects. CLINICAL REASONING: While ending cigarette smoking is indeed useful for general health, it may not immediately alleviate her insomnia and could actually trigger withdrawal symptoms leading to exacerbation of insomnia. Medicinal interventions may help with immediate symptom relief but they do not address the underlying behavioral factors that may be causing her insomnia and can also have side effects. Upon inspection of her daily routine, it appears that the patient has poor sleep hygiene habits - she stays up late doing housework and smokes cigarettes, known stimulants, in the evening before bed. Poor sleep hygiene is a common cause of insomnia and is reversible by altering behaviors and establishing patterns conducive to better sleep. CONCLUDING: Proper sleep hygiene education could help her implement changes like setting a regular sleep and wake schedule, creating a sleep-friendly environment, limiting exposure to screens before bed, limiting evening consumption of stimulants like nicotine and engaging in relaxing pre-sleep activities. These strategies can have a significant positive impact on insomnia without the side effects associated with medication. RESPONSE: (A) Education on sleep hygiene - This would likely be the most appropriate initial step in managing her symptoms. This approach takes into account the factors that may be directly contributing to her insomnia, such as her tendency to smoke late at night and maintain a busy schedule prior to attempting sleep. By ensuring that the patient is educated about beneficial sleep habits and behaviors, she can potentially reduce her sleep disturbances without resorting to pharmacological treatments.

**AI_B:** 1) RESTATING THE QUESTION: This case presents a 40-year-old woman who is experiencing difficulty sleeping for about a year now. Her sleep issues seem to be causing her significant distress, as they affect her daily duties and work. She does not fall asleep until late at night, wakes abruptly after a short period of sleep, and suffers from an inability to return to sleep afterwards. She presents no signs of affective disorders such as depression or anxiety. She has a habit of smoking, mostly after 9 PM as stated. The physical examination reveals signs of sleep deprivation. The inquiry here is about the most appropriate initial management step in this case. 2) PLANNING: We can identify a number of assumptions in the case. She might be suffering from insomnia or a sleep disorder, her late-night smoking habit could be contributing to her inability to sleep, or her job could be causing her stress leading to sleep problems. From these assumptions, we can generate possible interventions such as pharmacological treatment for insomnia, smoking cessation, or stress management techniques. Nevertheless, the absence of affective symptoms or any physical illness signs, together with her smoking habit, suggest the assumption that poor sleep hygiene might be the main factor causing her sleep issues. Therefore, it should be clinically prioritized to pursue. 3) CLINICAL REASONING: Insomnia is mainly classified into primary and secondary types. The primary type is not associated with any underlying health condition, while secondary is related to a health problem. In this case, there are no clear signs of any underlying health issues such as depression, anxiety, or a general medical disorder. Therefore, primary insomnia is suggested in this scenario. Primary insomnia includes poor sleep habits, which include irregular sleep schedule, activities that cause excitement, tension or anxiety near bedtime, and an unhealthy lifestyle including heavy smoking. In the clinical context, the patient's late-night smoking and irregular sleep schedule might be contributing to her struggle with sleep. 4) CONCLUDING: Given the suspected case of primary insomnia driven by poor sleep habits, it makes sense to initially focus on non-pharmacological measures. A key part of managing primary insomnia is to improve one's sleep hygiene, which means habits and behaviors that promote a good sleep. This approach can be the first line treatment before considering pharmacological interventions. This includes advice on establishing a regular sleep schedule, avoiding stimulants like nicotine near bedtime, and creating a suitable sleep environment. 5) RESPONSE: Given that the woman's sleeping difficulties appear to be caused, at least in part, by poor sleep hygiene such as smoking late at night and lacking a regular sleeping schedule, the first step should be to educate the patient about the benefits of good sleep hygiene. This would involve sharing information and suggestions to improve bedtime habits and sleep environment, hence the best answer is (A) Education on sleep hygiene.

**AI_A:** RESTATING THE QUESTION: The 40-year-old woman is experiencing chronic sleep disturbances, which are impacting her job and daily routine. She smokes a pack of cigarettes every three to four days and most of her smoking is done after 9 PM. She's under stress due to her work, parenting responsibilities, and the lack of adequate sleep, although she hasn't experienced any significant mood changes, anxiety, or weight change. Physical examination indicates signs of sleep deprivation. The inquiry is about the best initial plan for managing her case, given the options of sleep hygiene education, mirtazapine therapy, sertraline therapy, setting a date for smoking cessation, or zolpidem therapy. PLANNING: The assumptions in this case are that this woman's sleep disturbances are tied solely to her lifestyle and habit of smoking in the evening, and that a medicinal intervention may immediately alleviate her symptoms. An alternate assumption would be that these sleep disorders are multi-factorial, which could include lifestyle, psychological stress, and perhaps even undiagnosed medical conditions. Therefore, it seems reasonable to focus on non-pharmacological interventions as an initial step, allowing for a potentially more diverse and encompassing solution. CLINICAL REASONING: Considering her lifestyle, the woman's bedtime routine seems to include activities that can interfere with sleep onset and quality. The woman is parenting, working, and performing house chores till late night. These activities might increase her alertness levels and reduce her sleep pressure by the time she decides to sleep. Moreover, she smokes cigarettes mostly during night hours. Nicotine, the primary active substance in cigarettes is a stimulant that can affect sleep induction. This overall lifestyle likely contributes to the woman's delayed sleep onset. It would be beneficial to educate her on sleep hygiene, highlighting the importance of a calm and sleep-facilitating environment prior to bedtime, the avoidance of stimulants such as caffeine and nicotine, particularly before one's planned sleep time. CONCLUDING: Rather than directly resorting to medicinal interventions, which come with their own side effects and complications, non-pharmacological measures are often a safer and more sustainable first step. A healthy bedtime routine as part of sleep hygiene can help create an environment conducive for sleep and appropriately time sleep according to one's circadian rhythm. RESPONSE: The most appropriate initial step in management is to provide education on sleep hygiene (Choice A). This includes creating a sleep schedule, creating a restful environment, limiting exposure to bright lights in the evenings, and practicing a relaxing bedtime routine. It would be also beneficial to recommend steps to gradually decrease her nicotine intake, primarily before bedtime as an initial part of her sleep hygiene education. The impact of these will be assessed before deciding on other interventions.

**Facilitator** The consensus statement synthesizes the responses of AI_A, AI_B, AI_C, AI_D, and AI_E as follows: The case presents a 40-year-old woman experiencing chronic sleep disturbances, which are impacting her daily routine and job. She smokes a pack of cigarettes every three to four days, primarily after 9 PM. Despite her sleep issues, she hasn't experienced any significant mood changes, anxiety, or weight change. Physical examination indicates signs of sleep deprivation. The question asks for the best initial plan for managing her case. The woman's sleep disturbances could be tied to her lifestyle, including her habit of smoking in the evening, and her work and parenting responsibilities. It's also possible that these sleep disorders are multi-factorial, potentially including undiagnosed medical conditions. However, given her lifestyle and the absence of affective symptoms or any physical illness signs, it seems reasonable to focus on non-pharmacological interventions as an initial step. Her bedtime routine includes activities that can interfere with sleep onset and quality, such as parenting, working, and performing house chores till late night. These activities might increase her alertness levels and reduce her sleep pressure by the time she decides to sleep. Moreover, she smokes cigarettes mostly during night hours. Nicotine, the primary active substance in cigarettes, is a stimulant that can affect sleep induction. This overall lifestyle likely contributes to the woman's delayed sleep onset. Given the suspected case of primary insomnia driven by poor sleep habits, it makes sense to initially focus on non-pharmacological measures. A key part of managing primary insomnia is to improve one's sleep hygiene, which means habits and behaviors that promote good sleep. This approach can be the first line treatment before considering pharmacological interventions. This includes advice on establishing a regular sleep schedule, avoiding stimulants like nicotine near bedtime, and creating a suitable sleep environment. Therefore, the most appropriate initial step in managing this patient's insomnia would be to provide education on sleep hygiene. This includes creating a sleep schedule, creating a restful environment, limiting exposure to bright lights in the evenings, and practicing a relaxing bedtime routine. It would also be beneficial to recommend steps to gradually decrease her nicotine intake, primarily before bedtime as an initial part of her sleep hygiene education. The impact of these will be assessed before deciding on other interventions.
