# Supplementary material for: Collaborative intelligence in AI: Evaluating the performance of a council of AIs on the USMLE
Source: PLOS Digit Health. 2025 Oct 9;4(10):e0000787. doi: 10.1371/journal.pdig.0000787 (PMC12510544; doi:10.1371/journal.pdig.0000787)
Supplement: S2 File — (ZIP) [file pdig.0000787.s002.zip › S2 Data Raw data files containing unprocessed question inputs and LLM outputs including transcripts of deliberations/STEP2_Question-16-1_Type-MC-NJ.docx]

**Facilitator** **[Round 1]:** A 58-year-old woman is brought to the office by her husband because of a 6-week history of increasing confusion. Her husband says that, initially, she had difficulty doing “normal” things, such as preparing meals. Since then, she has become progressively less able to care for herself or even use a telephone. During this time, she also has had increased anxiety and restlessness. She has type 2 diabetes mellitus well controlled with an oral hypoglycemic agent. She is alert and appears anxious. She is oriented to person but not to place or time. She startles when the door to the examination room is slammed shut. Her temperature is 36.8°C (98.2°F), pulse is 78/min and regular, and blood pressure is 132/94 mm Hg. Physical examination shows no other abnormalities. On neurologic examination, she can repeat three objects immediately but is unable to recall them after 3 minutes. She is unable to copy a drawing of a cube. Results of a complete blood count and serum electrolyte concentrations are within the reference ranges. Her serum glucose concentration is 155 mg/dL. CT scan of the head shows no abnormalities. Which of the following is the most appropriate next step in diagnosis? (A) Determination of hemoglobin A1c (B) Measurement of cerebrospinal fluid 14-3-3 protein concentration (C) Measurement of cerebrospinal fluid beta amyloid concentration (D) Measurement of cerebrospinal fluid methylmalonic acid concentration (E) Measurement of serum angiotensin-converting enzyme activity (F) Serum protein electrophoresis

**AI_D:** RESTATING THE QUESTION: The query revolves around a 58-year-old female patient who has progressively worsening confusion, restlessness, and anxiety over a period of six weeks. Despite suffering from well-controlled Type 2 diabetes, she shows no other physical abnormalities except her inability to perform tasks of daily living, recall objects after a short delay, and draw a simple figure. All her lab results, including blood count, electrolyte levels, and serum glucose, are normal, as is her CT scan. We are asked to suggest the next best step in the further diagnosis of her condition. PLANNING: Given the patient's development of cognitive deficits, our assumptions should focus on diseases affecting cognitive abilities. Factors to consider are her impressively rapid onset of symptoms and well-controlled diabetes, which isn't generally associated with such acute cognitive decline. Alzheimer's disease, Creutzfeldt-Jakob disease, diabetic dementia, vitamin B12 deficiency, and granulomatous disease are potentially relevant conditions, and we might evaluate each one based on her symptoms, history, and lab results. CLINICAL REASONING: Alzheimer's is a common cause of dementia but usually manifests over a longer period. Diabetic encephalopathy may initially seem relevant due to her known condition, but her well-controlled diabetes and rapidly progressing symptoms argue against it. Vitamin B12 deficiency could also cause cognitive decline, but other symptoms (like anemia and peripheral neuropathy) and abnormal blood results aren't present. Granulomatous diseases aren't consistent with her presentation either. This leaves us witThe query revolves around a 58-year-old female patient who has progressively worsening confusion, restlessness, and anxiety over a period of six weeks.h Creutzfeldt-Jakob disease, a rapidly progressing neurodegenerative disorder. It causes rapidly progressing dementia, myoclonus, and often psychotic symptoms such as anxiety, which match this patient's presentation. Furthermore, its diagnosis is supported by the detection of protein 14-3-3 in cerebrospinal fluid. CONCLUDING: Considering the pattern and rapid progression of cognitive deficits, her anxiety, the lack of abnormalities on physical examination or lab tests, and the exclusion of other likely conditions, the patient's condition is suggestive of Creutzfeldt-Jakob disease. The next most appropriate diagnostic step would be the measurement of cerebrospinal fluid 14-3-3 protein concentration, a marker for this disease. RESPONSE: Therefore, the most appropriate next step in diagnosis for this patient would be (B) Measurement of cerebrospinal fluid 14-3-3 protein concentration.

**AI_C:** RESTATING THE QUESTION: The scenario given explain about a 58-year-old woman experiencing a 6-week trajectory of increasing confusion, difficulty doing daily tasks, heightened anxiety, and restlessness. Moreover, she has type 2 diabetes which is well-controlled with oral medication. Her neurologic examination reveals troubles in recalling and spatial orientation despite being alert and oriented to people. The current problem is identifying the next most appropriate step in diagnosis, given the choices focused on different laboratory investigations. PLANNING: This woman's condition has rapidly developed over six weeks, leading to disorientation and cognitive impairment. Given that her metabolic profile, complete blood count and head CT scan do not show significant abnormality, we need to consider alternate diagnoses such as neurodegenerative disorders. We can question if she has a rapid-onset type of dementia, such as Creutzfeldt-Jakob disease. This condition could be indicated through the detection of 14-3-3 protein in cerebrospinal fluid, a protein associated with rapid neurodegenerative conditions. On the other hand, slower types of dementia, like Alzheimer's disease, would not present so rapidly and would be typically associated with higher CSF beta amyloid. CLINICAL REASONING: Clinically, the woman's rapidly progressing symptoms, significant cognitive impairment (unable to recall objects after 3 minutes, disorientation to time and place, difficulty copying a simple cube), and increased anxiety and startle response are suggestive of a rapidly progressing neurodegenerative disorder. Her diabetes is well-controlled, and metabolic causes for confusion such as hypoglycemia or electrolyte imbalances can be ruled out based on lab results. Central nervous system infection or malignancy are less likely given the lack of fever, head CT scan abnormalities, or other typical signs of these conditions. In this situation, diseases such as Alzheimer's would not likely develop so quickly (within 6 weeks) and typically, the pattern of memory loss in Alzheimer's disease is insidious and gradual. Thus, the next logical step would be to look for markers of a rapidly progressing neurodegenerative disorder, such as Creutzfeldt-Jakob disease. CONCLUDING: Regarding the laboratory investigations presented, the measurement of cerebrospinal fluid 14-3-3 protein concentration will allow us to investigate a potential Creutzfeldt-Jakob disease, which is in tune with the patient's rapidly increasing and high levels of cognitive impairment. RESPONSE: Therefore, the most appropriate next step in diagnosing this woman's situation would be option (B) Measurement of cerebrospinal fluid 14-3-3 protein concentration.

**AI_E:** Restating the question in simpler terms, a 58-year-old woman is exhibiting progressive symptoms of cognitive decline, encompassing memory loss and impaired executive functions. In addition, she exhibits increased anxiety, restlessness, and seems to startle easily. Despite her well-controlled type 2 diabetes mellitus and normal physical examination, she struggles with spatial and temporal orientation and has difficulty with tasks requiring memory and cognitive reasoning. An initial battery of tests, including a head CT scan, complete blood count, and serum electrolyte concentration tests, shed no light on her condition. We are asked to identify the best next step in diagnostic procedures. The options suggest various clinical entities that could explain her clinical presentation. Measurement of cerebrospinal fluid 14-3-3 protein concentration and beta amyloid concentration relate to conditions like Creutzfeldt-Jakob disease and Alzheimer's, respectively. Determination of hemoglobin A1c and serum glucose level would dive into her diabetes control, while serum angiotensin converting enzyme activity speaks towards sarcoidosis. Lastly, measuring cerebrospinal fluid methylmalonic acid concentration and performing serum protein electrophoresis are used to detect conditions like vitamin B12 deficiency, and disorders of protein metabolism such as multiple myeloma, respectively. Reviewing the options and assessing the clinical profile of our patient, we can formulate an alternate assumption: assuming Alzheimer's disease seems plausible due to the presented cognitive and memory issues. However, these symptoms also overlap with vitamin B12 deficiency, which can induce subacute combined degeneration. Despite the diabetes, her glucose levels seem well controlled and no neuropathic symptoms have been mentioned, making this an unlikely priority at this point. Anxiety and restlessness, too, are seen with both B12 deficiency and Alzheimer's disease. We proceed to connect symptoms to conditions. The patient presents with rapid cognitive decline, difficulty with tasks that require complex motor planning (drawing a cube), impaired recall and, importantly, an absence of clear abnormalities on brain CT. This pattern is consistent with metabolic or prion diseases. Hyperintensity in the basal ganglia may be seen only in about two-thirds of sporadic Creutzfeldt-Jakob disease cases; normal imaging does not exclude this diagnosis. In contrast, Alzheimer's, although a common reason for dementia, usually happens gradually over years. Concluding this reasoning process, cognitive decline over weeks to months in an anxious and neurologically intact patient points to prion disease, for instance Creutzfeldt-Jakob disease, rather than a metabolic or Alzheimer's disease. The most appropriate next step in diagnosis would thus be (B) Measurement of cerebrospinal fluid 14-3-3 protein concentration in pursuit of identifying prion diseases like Creutzfeldt-Jakob disease.

**AI_B:** RESTATING THE QUESTION: There is a 58-year-old woman who has been experiencing a decline in her cognitive function over the past 6 weeks, which is causing her to have difficulty with everyday tasks, such as meal preparation and telephone use. Additionally, she seems increasingly anxious and restless. Despite having type 2 diabetes, it's well-managed with an oral hypoglycemic medication. Her vital signs are within normal ranges, and physical examination does not show any abnormalities. Her memory is significantly affected as she can't recall three objects after being able to repeat them immediately, and she also has difficulty with visuospatial tasks shown by her inability to copy a cube drawing. Routine blood tests and a CT scan of the head have not shown any abnormalities. The question is asking what would be the best next step to help diagnose her condition. PLANNING: Each option presents a different diagnostic pathway which implies a different underlying cause. Hemoglobin A1c (HbA1c) measures long-term blood glucose control, but her diabetes is already known and seems well controlled. 14-3-3 protein in cerebrospinal fluid (CSF) is associated with Creutzfeldt-Jacob disease, a type of prion disease causing rapid cognitive decline. CSF beta amyloid and CSF methylmalonic acid levels are associated with Alzheimer's disease and vitamin B12 deficiency, respectively, both of which can cause cognitive impairment. Serum angiotensin-converting enzyme (ACE) activity is associated with sarcoidosis, and serum protein electrophoresis can help diagnose multiple myeloma and other protein abnormalities. CLINICAL REASONING: Considering the clinical presentation, an important consideration would be to rule out rapidly progressive dementia conditions, as the symptoms came on relatively quickly (over 6 weeks). Alzheimer's disease (beta amyloid) usually doesn't present with rapidly progressive symptoms and is less likely. Her diabetes management (depicted by HbA1c) doesn't seem relevant to her cognitive symptoms. Vitamin B12 deficiency (methylmalonic acid) could potentially cause cognitive symptoms but would typically have additional symptoms like megaloblastic anemia, which is not present here. Serum ACE activity is checked when we suspect sarcoidosis, but there is no evidence to suggest that condition here. Changes in serum proteins often indicate systemic diseases like multiple myeloma, but again, there are no symptoms suggesting this. This leaves us with CSF 14-3-3 protein concentration as the most clinically prioritized test, implying a suspicion of Creutzfeldt-Jakob disease, the most common of the rapidly progressive dementias, which presents with memory problems, behavior changes, disorientation, and visual disturbances. CONCLUDING: Given the rapid onset of symptoms, the cognitive difficulties including memory problems and visuospatial difficulties, the anxiety, and the startle response, these are suggestive of a rapidly progressive dementia. Among the options presented, the most appropriate next step would be testing for CSF 14-3-3 protein concentration, notably suggestive of a prion disease like Creutzfeldt-Jakob disease. RESPONSE: The most appropriate next step in diagnosis for this patient would be ${(B) Measurement of cerebrospinal fluid 14-3-3 protein concentration} because of its association with rapidly progressive dementias like Creutzfeldt-Jakob disease, which aligns with the patient's clinical presentation.

**AI_A:** RESTATING THE QUESTION: A middle-aged woman has been experiencing escalating symptoms of confusion, anxiety, and a decrease in her ability to perform everyday tasks over the past six weeks. Her past medical history includes well-controlled Type 2 diabetes. Though she is alert and knows who she is, she does not know where she is or the current time. Her physical and basic laboratory findings are normal, including a CT scan of the head. The question is asking what the best next step would be in diagnosing her condition from a range of tests including measuring hemoglobin A1C, cerebrospinal fluid protein or amyloid levels or methylmalonic acid concentration, serum angiotensin-converting enzyme activity, or carrying out serum protein electrophoresis. PLANNING: The initial assumptions diagnose this woman's symptoms as being the result of her well-controlled diabetes or possibly a new neurological condition. Testing her A1C levels would check the former, while measuring her cerebrospinal fluid (CSF) protein or amyloid concentrations would investigate the latter. Another assumption would be that her condition is the result of a hematological or endocrine disturbance, which can be probed by measuring her CSF methylmalonic acid concentration, serum angiotensin-converting enzyme activity, or serum protein. It is crucial to discern whether her condition is due to a typical complication of diabetes, a new neurological disease, or another systemic illness, in order to determine the most appropriate next step for her diagnosis. CLINICAL REASONING: Given that her diabetes is well-controlled and serum glucose levels are only slightly elevated, it is moderately unlikely that her symptoms are the result of her diabetes. We are not informed of any hereditary illnesses, therefore the option of measuring her beta-amyloid concentrations becomes less likely as most hallmark diseases related with increased beta-amyloid proteins (like Alzheimer's disease) are of late onset and of a genetic predisposition. There are no indications of vascular or connective tissue diseases, thus measuring serum angiotensin-converting enzyme activities or carrying out serum protein electrophoresis seem to be unnecessary. However, these symptoms may be indicative of a prion disease, like Creutzfeldt-Jakob disease which is associated with elevated CSF 14-3-3 protein concentrations. So, potentially, measuring the levels of CSF 14-3-3 protein concentration could better facilitate our diagnosis. CONCLUDING: Upon inspection of her symptoms and the clinical tests available, the most effective course of action seems to be measuring her cerebrospinal fluid 14-3-3 protein concentration. This protein is often elevated in prion diseases like Creutzfeldt-Jakob disease, which could explain her symptoms such as rapid onset dementia. RESPONSE: Based on our patient’s symptoms and the missing link to connect her medical engine, the most appropriate next step in diagnosis would be measuring her cerebrospinal fluid 14-3-3 protein concentration (B), as this could potentially indicate a prion disease like Creutzfeldt-Jakob disease.

**Facilitator** The consensus among the AI responses is that the most appropriate next step in diagnosing the 58-year-old woman's condition would be to measure her cerebrospinal fluid 14-3-3 protein concentration. This conclusion is based on her rapidly progressing cognitive decline, anxiety, and restlessness over a six-week period, despite her well-controlled Type 2 diabetes and normal physical examination results. The reasoning behind this decision is that her symptoms align with a rapidly progressive neurodegenerative disorder, such as Creutzfeldt-Jakob disease, which is associated with elevated levels of 14-3-3 protein in the cerebrospinal fluid. Other potential conditions, such as Alzheimer's disease, vitamin B12 deficiency, and diabetic encephalopathy, were considered less likely due to the rapid onset of her symptoms and the absence of other typical signs of these conditions. Therefore, the consensus is that the measurement of cerebrospinal fluid 14-3-3 protein concentration is the most appropriate next step in diagnosing this patient's condition.
