# Supplementary material for: Collaborative intelligence in AI: Evaluating the performance of a council of AIs on the USMLE
Source: PLOS Digit Health. 2025 Oct 9;4(10):e0000787. doi: 10.1371/journal.pdig.0000787 (PMC12510544; doi:10.1371/journal.pdig.0000787)
Supplement: S2 File — (ZIP) [file pdig.0000787.s002.zip › S2 Data Raw data files containing unprocessed question inputs and LLM outputs including transcripts of deliberations/STEP2_Question-17-1_Type-MC-NJ.docx]

**Facilitator** **[Round 1]:** A 38-year-old woman is brought to the emergency department by paramedics 90 minutes after she fell through ice into a stream while hiking. She was stuck in the ice until emergency responders rescued her. On arrival, she has severe pain in her feet. She has no history of serious illness and takes no medications. Temperature is 34.8°C (94.6°F); other vital signs are within normal limits. Examination shows blue discoloration over the toes bilaterally, and the feet are cold to touch. Which of the following is the most appropriate next step in management? (A) Place the feet in front of a dry space heater (B) Rub the feet with a steaming hot towel (C) Submerge the feet in a moderately warm whirlpool (D) Wrap the feet in a room-temperature wet-to-dry dressing (E) Observation only

**AI_D:** RESTATING THE QUESTION: The patient, a 38-year-old woman with no previous serious illness, fell through icy water and was trapped, which resulted in severe pain and blue discoloration in her feet. Her subnormal body temperature suggests she is hypothermic, unable to effectively generate or retain heat. The question seeks the best next step in managing her condition. PLANNING: Hypothermia, the state of having an abnormally low body temperature, can cause numerous physiological effects within the body, including cellular dysfunction and local tissue death. It's essential to take immediate actions with the goal of rewarming the patient, reducing further cellular damage. However, how to do that effectively without causing secondary damage due to quick changes in temperature is a matter of concern. This forms the primary assumption we need to address. CLINICAL REASONING: The patient's signs and symptoms suggest peripheral frostbite, which indicates local freezing and damage to tissue. If not treated appropriately, this could progress to tissue necrosis. The principle of treating frostbite is gradual rewarming of the affected areas. Abrupt warming methods like rubbing the feet with a hot towel or placing them in front of a heater can potentially cause more harm due to thermal injury. Submerging the feet in a moderately warm whirlpool would allow for a controlled, gradual rewarming of the frozen tissues, which is essential in preserving function and preventing further damage. CONCLUDING: The most appropriate next step in management would then be to gently warm up her extremities using a controlled method. This will minimize tissue damage due to drastic changes in temperature and promote healthy circulation, thus assisting in pain relief and recovery. RESPONSE: Therefore, in this case, it would be most appropriate to choose (C) "Submerge the feet in a moderately warm whirlpool" as the best next step in management. This approach provides a controlled, gradual rewarming method to help mitigate any further tissue damage.

**AI_A:** RESTATING THE QUESTION: This is a clinical scenario where a woman has been exposed to a potentially frostbite-inducing situation. Her symptoms such as severe pain in her feet, blue discoloration of the toes, along with her feet being cold to touch, point towards frostbite, a condition that is characterized by freezing of tissues when exposed to temperatures below the freezing point. The inquiry here is about the most appropriate next step in managing such a case. PLANNING: Initial assumptions in the management of this cold-related injury could target two broad aspects: (1) Immediate restoration of tissue temperature and (2) Avoidance of tissue damage due to abrupt rewarming. These assumptions hinge on the fundamental principle of thermal dynamics, which explains that, in contact with a warmer medium, frozen tissue will regain heat, restoring its cellular functions ultimately. However, alternate assumptions must also consider that rapid rewarming might lead to thermal injuries in tissues already potentially compromised by freezing. CLINICAL REASONING: Initially, any medical intervention should aim at reducing current symptoms and potential complications. Here, our main concern is the frozen tissues in the woman's feet. Options A, B, and D seem to imply a rapid increase in temperature, which could potentially cause more harm, so their immediate exclusion from being the best option seems appropriate. Option E, "Observation only", fails to address the immediate need for intervention due to the ongoing tissue freezing. Hence, it seems the prudent assumption to prioritize would be to choose an option that aims to restore tissue temperature gradually while avoiding tissue damage from rapid temperature changes. CONCLUDING: After considering the principles of thermal dynamics and the potential risk of thermal injuries from fast temperature rises, the method of warming the tissue slowly and gently seems to be the best course of action. Avoiding any method that can potentially cause immediate drastic temperature changes can also prevent other complications, such as burns. RESPONSE: Therefore, based on all these principles and rationale, the most appropriate next step to manage this patient's frostbite would be to 'Submerge the feet in a moderately warm whirlpool' (Choice C). This would provide a gradual, gentle rewarming process while minimizing any risks associated with rapid temperature changes.

**AI_B:** RESTATING THE QUESTION: The scenario presents a 38-year-old woman who has fallen into a stream with icy water and remained there until the emergency team rescued her. She is experiencing severe pain in her feet, which appear bluish and cold upon examination. Her body temperature is below the average baseline, indicating hypothermia. This raises concerns for frostbite, a condition in which dangerously low temperatures lead to freezing of the skin and underlying tissues. With these clinical findings, the question asks for the most appropriate immediate response in her treatment. PLANNING: There are a few assumptions on which we could base our treatment response. One could incorporate (A) warming the feet rapidly with a dry space heater or (B) a steaming hot towel. Another could involve (C) submerging the feet in a moderately warm whirlpool, (D) wrapping the feet in a room-temperature wet-to-dry dressing, or (E) merely observing the patient. However, from a clinical perspective, immediate and measured warming of the frozen tissues, taking care to avoid further tissue injury due to rapid or overly hot thawing, should be our priority. CLINICAL REASONING: Frostbite treatment centers on rewarming the affected area while also managing the pain and potential complications. Rapid rewarming, such as using a dry space heater (option A) or steaming hot towel (option B), could lead to burns or more extensive tissue damage. During the rewarming process, the return of blood flow can cause a painful inflammatory response, potentially leading to more severe injury. Option D, wrapping the feet in a room-temperature wet-to-dry dressing, might not be warm enough to effectively treat frostbite. Merely observing (option E) is not suitable in this situation as it could lead to further complications, such as gangrene. CONCLUDING: Because this patient's feet have been exposed to cold for a prolonged period, we need to rewarm them in a controlled manner to prevent further tissue injury. Importantly, this process should be suitably warm enough to aid recovery while avoiding additional damage through too intense heat exposure. RESPONSE: The most appropriate step in managing this patient is therefore (C) to submerge her feet in a moderately warm whirlpool. This treatment method gently rewarms the tissues, avoids further frostbite, and minimizes the chance of other complications, including burns from excessive heat.

**AI_E:** RESTATING THE QUESTION: A healthy 38-year-old woman has fallen through ice and was stuck there until she was rescued, a duration of about 90 minutes. Now, she exhibits signs of hypothermia and possible frostbite in her feet, evident through severe pain, bluish discoloration, and cold touch of the feet. The task is to determine the most suitable next step in managing her condition from a set of specified choices. PLANNING: There are assumptions that need to be considered. Firstly, the woman could be experiencing frostbite or non-freezing cold injury. Secondly, the management of these conditions depends on the severity of the injury, rapid re-warming methods, pain management, and prevention of further injury. However, warm and rapid immersion in a moderately warm water can counter vasoconstriction and re-perfuse the affected area and is considered a suitable method to manage frostbite. CLINICAL REASONING: Given her exposure to cold, the cold touch, severe pain, and the blue discoloration, this woman likely has frostbite. Frostbite occurs when tissues freeze after being exposed to temperatures below the freezing point of skin. It is a vascular injury characterized by the freezing of cell contents, which lead to cellular death if not managed promptly and properly. Rapid re-warming, generally in a water bath of 37-39°C (98.6-102.2°F), is recommended to prevent further cellular damage from ice crystal formation. The cold-induced vasoconstriction will be countered with warm immersion led vasodilation, leading to reperfusion of the ischemic-but-viable tissues. CONCLUDING: While all other options could potentially apply heat, they might not be able to systematically warm the tissues. Placing feet in front of a dry space heater or rubbing them with a steaming towel could lead to burns, while using a room-temperature wet-to-dry dressing or merely observing would not provide the needed rapid re-warming of the affected tissues. Thus, the best approach to managing this woman's condition would likely involve a moderately warm water immersion for her feet. RESPONSE: Therefore, the most appropriate next step in her management would be (C) submerge the feet in a moderately warm whirlpool.

**AI_C:** (1) RESTATING THE QUESTION: This inquiry involves understanding the ideal management approach for a 38-year-old woman who has fallen into a cold stream and has symptoms suggestive of frostbite in her feet. Frostbite result from freezing injury that commonly occurs when skin and its underlying tissues freeze, most often due to prolonged exposure to cold temperatures. In this context, the question is seeking an appropriate technique for managing this frostbite. (2) PLANNING: The common assumption is that the quicker the frozen tissue is warmed, the better it is. But the alternate assumption is that controlled and gradual rewarming is the better option, as rapid rewarming or overheating can actually cause additional tissue damage. Among the options provided, there are measures intended to warm up the feet rapidly like placing the feet in front of a dry space heater, rubbing the feet with a steaming hot towel or leaving them to self-regulate the temperature (observation). The assumptions underlying each potential next step are associated with the concept of proper frostbite management and tissue preservation, with a focus on minimizing tissue damage and maximizing patient comfort. (3) CLINICAL REASONING: In cases of severe frostbite, a careful approach to rewarming is essential. Rapid rewarming can lead to extensive tissue damage due to a cascade of cellular injury related to reperfusion. Additionally, exposure to overheating can lead to burns, adding another layer of injury. When frostbitten tissue thaws, blood flow can return to the area, which may cause inflammation and further damage. Therefore, controlled rewarming of the tissues is the optimal approach. Submerging the feet in a moderately warm whirlpool (not hot) would provide that controlled rewarming, at a temperature of about 37-39°C, which can help dilate blood vessels and restore circulation while minimizing the risk of reperfusion injury. (4) CONCLUDING: Hence, considering the principle of frostbite management with the primary goal of preserving tissue while minimizing pain and future complications, the most appropriate action will be the one that facilitates controlled and gradual rewarming, rather than sudden temperature changes or expecting self-regulation of temperature. (5) RESPONSE: Therefore, the most appropriate next step in management is to 'Submerge the feet in a moderately warm whirlpool' (Option C). This option would allow for gradual and controlled rewarming, thereby reducing the risk of further tissue damage and enhancing patient comfort.

**Facilitator** Consensus Statement: The question presents a clinical scenario of a 38-year-old woman who has been exposed to a potentially frostbite-inducing situation, resulting in severe pain, blue discoloration of the toes, and cold feet. The task is to determine the most appropriate immediate response in her treatment. The primary assumption in managing this cold-related injury is to restore tissue temperature and avoid tissue damage due to abrupt rewarming. Rapid rewarming methods, such as using a dry space heater or a steaming hot towel, could potentially cause more harm due to thermal injuries. Similarly, merely observing the patient or using a room-temperature wet-to-dry dressing might not be effective enough to treat frostbite. Therefore, the most appropriate next step in managing this patient's frostbite would be to 'Submerge the feet in a moderately warm whirlpool' (Choice C). This method provides a controlled, gradual rewarming process, which can help dilate blood vessels and restore circulation while minimizing the risk of reperfusion injury and other complications, such as burns from excessive heat.
