# Supplementary material for: Collaborative intelligence in AI: Evaluating the performance of a council of AIs on the USMLE
Source: PLOS Digit Health. 2025 Oct 9;4(10):e0000787. doi: 10.1371/journal.pdig.0000787 (PMC12510544; doi:10.1371/journal.pdig.0000787)
Supplement: S2 File — (ZIP) [file pdig.0000787.s002.zip › S2 Data Raw data files containing unprocessed question inputs and LLM outputs including transcripts of deliberations/STEP2_Question-18-1_Type-MC-NJ.docx]

**Facilitator** **[Round 1]:** A 52-year-old woman comes to the emergency department because of a 1-day history of bleeding gums and moderate pain in her knees. She first noticed the gum bleeding while brushing her teeth this morning; she has been unable to stop the bleeding. She has systemic lupus erythematosus with inflammatory arthritis and Raynaud disease. Medications are tramadol and hydroxychloroquine. Vital signs are within normal limits. Examination shows bleeding, friable gums. There are multiple ecchymoses and petechiae over the upper extremities. Cardiac and abdominal examinations show no abnormalities. The knees are cool to the touch; there are effusions, and range of motion is limited by pain. Neurologic examination shows no focal findings. Laboratory studies show: Hemoglobin = 11 g/dL Hematocrit = 33% Erythrocyte count = 4.2 × 10¹²/L Mean corpuscular volume = 90 μm³ Leukocyte count = 9000/mm³ Segmented neutrophils = 60% Bands = 3% Eosinophils = 2% Basophils = 1% Lymphocytes = 30% Monocytes = 4% Platelet count = 20,000/mm³ Serum Creatinine = 0.9 mg/dL Ferritin 1= 0 ng/mL Which of the following is the most appropriate next step in management? (A) Factor replacement therapy (B) Methotrexate therapy (C) Plasmapheresis (D) Prednisone therapy (E) Splenectomy

**AI_C:** 1) RESTATING THE QUESTION: A middle-aged woman with a history of systemic lupus erythematosus, inflammatory arthritis, and Raynaud disease is experiencing bleeding gums and moderate knee pain. Laboratory tests show abnormal low platelet count, among other findings. The question is what the most appropriate next step in management would be from the given options. 2) PLANNING: The patient's low platelet count and symptoms (gum bleeding, petechiae, and ecchymoses) suggest thrombocytopenia, which can occur in autoimmune diseases such as lupus. Her pain and limited range of motion in her knees might be due to her inflammatory arthritis. The options given include treatments for different medical issues. Factor replacement therapy is typically used for coagulation factor deficiencies; methotrexate is used for several conditions including various forms of arthritis and cancers; plasmapheresis is often used in autoimmune disorders to remove harmful antibodies from the blood; prednisone is a corticosteroid often used as an anti-inflammatory or immunosuppressant drug; and splenectomy is the removal of the spleen and is sometimes used in cases of severe or refractory thrombocytopenia. 3) CLINICAL REASONING: Given the patient's history and current symptoms, the most likely explanation for her thrombocytopenia is an autoimmune attack on her platelets, which can occur in systemic lupus erythematosus. This condition is called immune thrombocytopenic purpura (ITP). If the platelets are being destroyed by the patient's immune system, treatment would involve addressing this autoimmune response. 4) CONCLUDING: Since steroids can suppress the immune response, they could help stop this self-destructive process. Prednisone is a commonly used corticosteroid and is often the first-line treatment for ITP, as it can increase platelet count by decreasing autoantibody production and platelet destruction. 5) RESPONSE: (D) Prednisone therapy would likely be the most appropriate next step in the management of this patient's condition.

**AI_A:** RESTATING THE QUESTION: The question focuses on a 52-year-old woman experiencing bleeding gums and pain in her knees. Additionally, she has history of systemic lupus erythematosus, inflammatory arthritis, and Raynaud disease. Her medications include tramadol and hydroxychloroquine. The lab reports provided indicate a low platelet count. The question asks the most appropriate next course of treatment considering the patient's history and current condition. PLANNING: The assumption here could be that the patient's symptoms and laboratory results could be due to a complication of her systemic lupus erythematosus (SLE), namely thrombocytopenia, which is a condition characterized by abnormally low levels of platelets. An alternate assumption could be that her symptoms are related to another unrelated condition. Yet, considering her medical history, the most plausible clinical assumption to prioritize is that her symptoms and the changes in her lab values are related to SLE or its therapy. CLINICAL REASONING: Thrombocytopenia often manifests with signs of mucosal or cutaneous bleeding, which may involve petechiae, ecchymoses, and other symptoms as seen in this patient. Furthermore, her gum bleeding, ecchymoses, and petechiae coupled with knee pain underline the possibility of thrombocytopenia associated with SLE or as a drug-induced condition. Her normal creatinine levels imply that her kidney function is intact, which reduces the likelihood of renal complications from lupus. The cause of thrombocytopenia can be due to platelet destruction, sequestration or decreased production. In the setting of SLE, autoantibodies can cause platelet destruction and induce thrombocytopenia. CONCLUDING: Judging from the clinical manifestations of thrombocytopenia in this patient, her existing condition of SLE, and the absence of any signs suggesting other complications, the requirement here is to treat or manage the thrombocytopenia. Glucocorticoids, such as prednisone, are first-line therapy in patients with SLE and thrombocytopenia due to their potent immunosuppressive properties that can control platelet destruction. RESPONSE: Thus, the most suitable next step in management for this patient would be (D) Prednisone therapy.

**AI_B:** RESTATING THE QUESTION: The query presents a 52-year-old woman who has a history of systemic lupus erythematosus (an autoimmune disease wherein the immune system mistakenly attacks healthy tissue), inflammatory arthritis, and Raynaud's disease (a condition that restricts blood flow to the fingers, toes, ears, and nose when stressed or cold). She's currently experiencing bleeding gums, knee pain, and has low platelets according to lab results. Her medication regimen includes tramadol (a narcotic pain reliever) and hydroxychloroquine (a medication often used to prevent and treat malaria and which is also used in managing certain autoimmune diseases, such as lupus and rheumatoid arthritis). The question seeks the most suitable management course in light of her symptoms and medical history. PLANNING: Initial assumptions may involve her present conditions' progression or complications arising from her anti-malarial medication, hydroxychloroquine, which is known to cause bone marrow suppression rarely. Regarding alternate assumptions, thrombocytopenia could be traced back to lupus-associated thrombocytopenia, a complication linked to systemic lupus erythematosus, suggesting an autoimmune-related cause to her condition. As such, management would primarily need to address the root cause, potentially an autoimmune response, improving the systemic lupus erythematosus' control, which will thereby manage her symptoms and thrombocytopenia. CLINICAL REASONING: Bleeding gums and petechiae on presentation suggest underlying issues with clotting, signaling potential thrombocytopenia which is then confirmed by her lab studies showing a low platelet count. Thrombocytopenia in a patient with lupus could point towards lupus-associated thrombocytopenia whereby the body's immune system mistakenly targets platelets. This autoimmune activity exacerbates both the bleeding and low platelet count. In such a scenario, controlling the autoimmune activity would be the immediate clinical priority to manage her symptomatology. CONCLUDING: Given these considerations, and the need to control the autoimmune response that's contributing to her thrombocytopenia, we need a treatment method which can control the autoimmune response. RESPONSE: Therefore, the most appropriate next step in the management of her condition would be (D) Prednisone therapy. Prednisone, a corticosteroid, can help manage her autoimmune activity and in turn, potentially her thrombocytopenia. In contrast, other listed options, such as factor replacement therapy or plasmapheresis, may not directly address her underlying autoimmune issue. Methotrexate, another auto-immune management drug, considered cytotoxic, has a higher risk of adverse events. Splenectomy should be a last resort when all less invasive measures have failed, owing to its inherent risks.

**AI_D:** RESTATING THE QUESTION: This patient, a 52-year-old woman, has presented to the emergency department with complaints of bleeding gums and knee pain for the past day. She has a medical history significant for systemic lupus erythematosus, inflammatory arthritis, and Raynaud's disease. Her current symptoms include uncontrollable oral bleeding and pain impairing her range of motion in her knees. She demonstrates signs of bleeding disorders, such as petechiae and ecchymosis, and has a significantly low platelet count. Systemic lupus erythematosus (SLE) can be associated with various hematologic disturbances, including thrombocytopenia. Our goal is to identify the most appropriate next step in managing this patient's symptoms and condition given her clinical presentation and laboratory findings. PLANNING: We could make several assumptions from the information provided. First, given the patient's existing diagnosis of SLE and thrombocytopenia, one might infer that an autoimmune response is leading to the destruction of platelets. Alternatively, the use of hydroxychloroquine could potentially be causing drug-induced thrombocytopenia. A third assumption could involve the bleeding being related to a coagulation factor deficiency, but this is less likely given the combination of symptoms. Clinically, it would be most relevant to focus on the possible autoimmune-related platelet destruction, given the history of SLE. CLINICAL REASONING: It is critical to view this patient's symptoms of bleeding gums, ecchymosis and petechiae in the context of her significantly low platelet count. Her history of SLE enhances the possibility of an autoimmune destruction of platelets, which could explain her thrombocytopenia and related symptoms. We can rule out a coagulation factor deficiency, because such a condition would not likely cause a decrease in platelet count. Considering her medication, hydroxychloroquine, although it could potentially cause drug-induced thrombocytopenia, the medication is more commonly implemented in the treatment of SLE and not typically known to cause such adverse effects. CONCLUDING: Based on the clinical assessment and reasoning outlined above, we could decidedly determine that this patient's thrombocytopenia is most likely a manifestation of her SLE. Therefore, as the most appropriate next step in her management, we should initiate a treatment that addresses the probable underlying autoimmune disorder. RESPONSE: Given the patient's history of SLE, which is likely driving her thrombocytopenia and associated symptoms, she would benefit most from a treatment option aimed at reducing autoimmune activity. Specifically, a step-up in the intensity of her immunosuppressive therapy would be most fitting. Therefore, among the choices provided, the most appropriate next step in management would be (D) Prednisone therapy, as this is a widely-used powerful anti-inflammatory and immunosuppressive agent, particularly beneficial in cases of systemic lupus erythematosus.

**AI_E:** (1) RESTATING THE QUESTION: So to rephrase, you're asking about a middle-aged woman who presented to the ER with a day's worth of gum bleeding and knee pain. Her medical history includes systemic lupus erythematosus, along with inflammatory arthritis and Raynaud disease. She has been taking tramadol and hydroxychloroquine for pain management and autoimmune condition respectively. The physical examination revealed bleeding gums, superficial bruises (ecchymoses) and small, reddish-purple spots on her upper extremities, which are indicative of bleeding into the skin due to small vessel ruptures (petechiae). The knees also seemed to have an accumulation of joint fluid (effusion) and were cool to touch. No abnormalities concerning her heart, abdomen, and nervous system were found. Lab results show a decrease in her blood platelet count, denoted as thrombocytopenia. In simpler words, given that she has a history of an autoimmune disease, her body may be making antibodies against her own platelets, leading to their destruction and the symptoms at hand. The normal creatinine reading and ferritin level rule out kidney disease and iron deficiency anemia respectively. (2) PLANNING: The question then is: what is the best course of action for this patient? The options presented are factor replacement therapy, methotrexate therapy, plasmapheresis, prednisone therapy, or splenectomy. At this point, a few assumptions can be made such as; she may be showing symptoms of a severe autoimmune response, her current medication may not be enough to manage her conditions, or there could be a new, unknown cause impacting her platelet count. (3) CLINICAL REASONING: Since fusion of her autoimmune conditions and her low platelet count seem to match up, let's consider these symptoms to be a severe autoimmune response, perhaps a manifestation of systemic lupus erythematosus (SLE). One of the manifestations of SLE is thrombocytopenia. It's trying to link this clinical presentation with the available laboratory findings; indeed, the low platelet count could be the reason for the gum bleeding and petechiae. This case might be a lupus-related thrombocytopenia. Considering the possible treatments, we can exclude factor replacement therapy which we use for hemophilia and Von Willebrand disease, based on the fact that she doesn't display any symptoms of excess internal bleeding, and her coagulation factors seem fine. Methotrexate and hydroxychloroquine can be used for SLE, but typically if joints or skin are the most affected. In this case, given that she already takes hydroxychloroquine, it would seem like the medication might not have been effective. Plasmapheresis is typically reserved for more severe cases involving vital organs, which is not the situation here, and splenectomy would be a possible consideration if this was refractory to therapy or in case of infectious induced thrombocytopenia. (4) CONCLUDING: That leaves us with Prednisone therapy, which is a high potency corticosteroid. Prednisone is often used for managing autoimmune reactions given its potent anti-inflammatory and immune-suppressive effects. Given that she has a history of SLE and is now exhibiting thrombocytopenia, likely an immune-mediated scenario, starting her on prednisone to dampen her immune response and increase her platelet count could be a clinically reasoned approach. (5) RESPONSE: Based on the patient's clinical presentation, lab findings, and given her history of immunological abnormality, the most appropriate next step in management in this case would be to start her on (D) Prednisone therapy in order to manage the immune-mediated thrombocytopenia associated with her systemic lupus erythematosus.

**Facilitator** The consensus among the AI responses is that the most appropriate next step in managing the 52-year-old woman's condition is Prednisone therapy. This conclusion is based on her clinical presentation, medical history, and laboratory findings. The woman, who has a history of systemic lupus erythematosus, inflammatory arthritis, and Raynaud disease, is experiencing bleeding gums and knee pain. Her lab reports indicate a low platelet count, suggesting thrombocytopenia. Given her history of autoimmune disease, it is likely that her body is producing antibodies against her own platelets, leading to their destruction and the symptoms she is experiencing. Prednisone, a high potency corticosteroid, is often used for managing autoimmune reactions due to its potent anti-inflammatory and immune-suppressive effects. Therefore, starting her on prednisone to dampen her immune response and increase her platelet count is a clinically reasoned approach. Other treatment options such as factor replacement therapy, methotrexate therapy, plasmapheresis, and splenectomy were considered but ruled out based on her specific symptoms, lab results, and medical history.
