# Supplementary material for: Collaborative intelligence in AI: Evaluating the performance of a council of AIs on the USMLE
Source: PLOS Digit Health. 2025 Oct 9;4(10):e0000787. doi: 10.1371/journal.pdig.0000787 (PMC12510544; doi:10.1371/journal.pdig.0000787)
Supplement: S2 File — (ZIP) [file pdig.0000787.s002.zip › S2 Data Raw data files containing unprocessed question inputs and LLM outputs including transcripts of deliberations/STEP2_Question-19-1_Type-MC-NJ.docx]

**Facilitator** **[Round 1]:** A 13-year-old boy is brought to the emergency department because of a 3-day history of progressive fatigue, shortness of breath, and difficulty walking up stairs. He had been well until 1 week ago, when he developed rhinorrhea, cough, and a sore throat. These symptoms resolved spontaneously 2 days ago. He has no history of serious illness and takes no medications. On arrival, he is pale and in moderate respiratory distress. Pulse is 120/min, respirations are 25/min, and blood pressure is 80/40 mm Hg. Pulse oximetry on room air shows an oxygen saturation of 91%. On pulmonary examination, crackles are heard throughout the lung fields. On cardiac examination, a gallop is heard. S1 is soft. A grade 2/6, high-pitched holosystolic murmur is heard best at the apex. The remainder of the examination shows no abnormalities. Which of the following is the most likely diagnosis? (A) Glomerulonephritis (B) Myocarditis (C) Pneumonia (D) Rheumatic fever (E) Systemic lupus erythematosus

**AI_D:** 1) RESTATING THE QUESTION: In consideration of the aforementioned symptoms and examination findings of a 13-year-old boy, we must determine the most likely diagnosis from a multitude of possible diseases. To assist in such determination, let's understand the terms mentioned. Rhinorrhea means a runny nose, and holosystolic refers to a murmur that lasts throughout the entirety of systole, the phase of the heartbeat when the heart muscle contracts and pumps blood from the chambers into the arteries. 2) PLANNING: The boy's symptoms point towards respiratory distress and cardiovascular complications. It might also be noteworthy to consider his recent history of rhinorrhea, cough, and sore throat, which suggests a preceding respiratory tract infection that resolved spontaneously. Several diagnoses on our list could potentially result from such an antecedent event, including myocarditis and pneumonia. 3) CLINICAL REASONING: Myocarditis characteristically presents with symptoms of heart failure following a viral illness, in this case presented as fatigue, breathlessness, and difficulty walking upstairs due to reduced cardiac output. Both glomerulonephritis and rheumatic fever often follow streptococcal infections and could theoretically present with similar symptoms. However, the gallop rhythm, soft S1, and holosystolic murmur heard in the cardiac examination more directly point to myocardial dysfunction seen in myocarditis. Pneumonia could potentially explain the respiratory symptoms and lung crackles but would be unlikely to cause the cardiac findings. Systemic lupus erythematosus (SLE) is typically accompanied by other systemic symptoms, like fever, arthritis, malar rash, and renal involvement, none of which are documented here. 4) CONCLUDING: Upon considering all the available data, the most viable assumption seems to be that the boy's recent viral upper respiratory tract infection has led to myocarditis, causing cardiac insufficiency and the correlating symptoms as a result 5) RESPONSE: Thus, considering his symptoms, signs, and preceding illness, the most likely diagnosis is (B) Myocarditis.

**AI_B:** RESTATING THE QUESTION: The query presents a clinical case of a 13-year-old boy who initially had upper respiratory symptoms, which spontaneously cleared, followed by signs of fatigue, breathlessness, and difficulty walking up stairs. His current state includes tachycardia, tachypnea, hypotension, oxygen desaturation, lung crackles, cardiac gallop, soft S1, and a grade 2/6 holosystolic murmur. From this, we are to deduce the most likely diagnosis from the given alternatives. PLANNING: Assumptions to consider are: the initial respiratory infection may have caused secondary issues; his cardiovascular symptoms may be related to the primary problem; his clear past health argues against a chronic condition; and the rapid onset of these symptoms suggests an acute pathological process. Recasting these assumptions, we might question if the primary issue might be cardiovascular and the initial respiratory symptoms were coincidental. Could there be a chronic condition that just hasn't manifest until now or is this an acute issue? It's worth investigating whether there could be an immunologic reaction because of the respiratory symptoms. CLINICAL REASONING: If his current symptoms were due to the respiratory infection, we'd expect elevated temperature and persistent respiratory symptoms, which is inconsistent with this case, arguing against pneumonia. The demographics, symptom duration, and the lack of joint pain and renal involvement do not match with glomerulonephritis or systemic lupus erythematosus. In addition, the absence of recent streptococcal infection and joint inflammation makes rheumatic fever less likely. The symptom onset, immediately following a respiratory infection, together with signs of cardiovascular compromise, suggests an issue that initially appeared as a common cold, but subsequently affected the heart. Myocarditis fits into this narrative. It is an inflammatory condition of the myocardium, often triggered by a viral infection, like the common cold. Myocarditis could lead to dilated cardiomyopathy, congestive heart failure symptoms such as fatigue and difficulty in handling physical exertion, and a fall in blood pressure which is noted in this patient. CONCLUDING: Therefore, the most clinically prioritised assumption to pursue appears to be an inflammation of the heart muscle secondary to a possible viral infection. RESPONSE: So, based on this young patient's symptomatology and sequence of events, the most likely diagnosis seems to be (B) Myocarditis.

**AI_E:** (1) RESTATING THE QUESTION: We're tasked with diagnosing a 13-year-old boy who has presented to the emergency room with fatigue, difficulty breathing, especially on exertion, and has recently recovered from upper respiratory infection-like symptoms. His vital signs raise concerns about cardiovascular status and his physical examination suggests both lung and heart involvement. The multiple-choice options suggest we are looking for a diagnosis within cardiac, pulmonary, renal, and auto-immune diseases. (2) PLANNING: The major assumptions made here are that the recent respiratory illness is connected to the current symptoms and that the physical findings are due to the same underlying process. Alternate assumptions could include, for example, the possibility that the boy's physical signs are an amalgamation of unrelated diseases. However, for clinical prioritization, it would be more practical to assume a single, systemic issue. (3) CLINICAL REASONING: The primary symptoms--fatigue, breathlessness, difficulty in walking up stairs--indicate a systemic issue, more in line with a cardiac abnormality. His previous upper respiratory infection-like symptoms and current pulmonary examination findings might suggest a post-infection outcome like myocarditis. Low BP, tachycardia, moderate respiratory distress, abnormal cardiac sounds (like a gallop rhythm and a holosystolic murmur heard best at apex), and decreased oxygen saturation strengthens the worry for a cardiovascular problem, possibly heart failure. Notably, these symptoms can occur in myocarditis, which can occur following a viral respiratory tract infection. On the other hand, symptoms of Rheumatic fever usually appear around 2-4 weeks after a streptococcal throat infection, making it less likely. Glomerulonephritis, pneumonia, and systemic lupus erythematosus do not align as closely with the provided history and clinical presentation. (4) CONCLUDING: Using a step-by-step reasoning process, taking into account the boy's previous infection, presentation of systemic symptoms, and crucially the coexistence of pulmonary and cardiac signs, a single unifying diagnosis would be a post-infectious complication affecting the heart. Myocarditis, an inflammation of the heart muscle that can occur after a viral infection, fits this pattern. (5) RESPONSE: Considering symptom onset, clinical features, and the line of reasoning outlined above, the most probable diagnosis in this scenario is (B) Myocarditis.

**AI_A:** (1) RESTATING THE QUESTION: A young teenager has been experiencing a worsening condition over the past three days that includes fatigue, shortness of breath, and difficulty climbing stairs. He also had episodes of rhinorrhea (runny nose), coughing, and a painful throat a week ago, which disappeared by themselves a couple of days back. No history of serious diseases is present and he doesn't take any medicines. He's found to be pale, with considerable trouble in respiration, an elevated pulse and low blood pressure, along with reduced oxygen saturation upon his arrival. On examination, abnormal sounds are heard both in his lungs and his heart, there's even a murmur detected at the apex. There are no other abnormalities found. Given these facts, we need to determine the most likely medical condition affecting him. (2) PLANNING: Let's look at the provided options. (A) Glomerulonephritis is a type of kidney disease caused by inflammation of the glomeruli, but the current symptom profile doesn't indicate any renal involvement. (B) Myocarditis is inflammation of the heart muscle often associated with viral infections, which may present with the signs and symptoms seen here. (C) Pneumonia can cause respiratory distress and abnormal lung sounds but doesn't usually present with heart murmurs. (D) Rheumatic fever is a complication of streptococcal infections that can affect the heart, but it usually has a longer latency period. (E) Systemic lupus erythematosus is a chronic autoimmune diseases that can affect multiple systems in the body but would usually present with additional symptoms. (3) CLINICAL REASONING: Given the recent history of an upper respiratory tract infection, the presentation of fatigue, shortness of breath, difficulty with climbing stairs can all be indicative of an issue with the heart. The cardiovascular examination reveals a gallop rhythm and a soft S1, findings that suggest possible acute heart failure. This could be due to myocardial dysfunction, which can also lower systemic blood pressure. A high pitched holosystolic murmur at the apex could be a functional mitral regurgitation due to the dilation of ventricle. Considering the viral prodrome, the clinical picture leans towards an acute viral myocarditis. (4) CONCLUDING: Considering the presence of an antecedent viral illness, and his present symptoms along with the findings of the physical examination like raised tachycardia, low blood pressure, poor oxygen saturation, abnormal lung and heart sounds, the condition seems to be affecting the heart's functioning majorly. Among the given options, the most probable cause seems to be myocarditis. (5) RESPONSE: (B) Myocarditis is the most likely diagnosis for this 13-year-old boy.

**AI_C:** RESTATING THE QUESTION: A 13-year-old boy is presented with symptoms of increasing fatigue, difficulty breathing, and struggle to ascend stairs for the past three days. Prior to this, he was healthy but had a brief episodes of runny nose, cough, and a sore throat that lasted roughly a week but have since resolved. He does not have a history of serious disease and is not currently taking any medications. Upon admission, pale appearance and moderate breathing distress are noted, with elevated pulse and respiration rates, as well as lowered blood pressure and oxygen saturation noted. Lung examination reveals crackling sounds, and the heart shows signs of a rapid rhythm of triple of four beats, a soft first heart sound (S1), and a holosystolic (occurring through the entire ventricular systole) murmur highest at the apex. All other physical exams return normal results. We're asked to identify the most likely diagnosis among glomerulonephritis, myocarditis, pneumonia, rheumatic fever, systemic lupus erythematosus. PLANNING: The assumptions that emerge from this young boy's symptomatology include potential heart-related conditions (reflected in his fatigue, respiratory distress, difficulty in walking upstairs, fast pulse, low blood pressure, abnormal heart sounds) and potential respiratory conditions (indicated from the rhinorrhea, cough, sore throat, pale appearance, abnormal lung sounds). The reported symptoms indicate a possible systemic illness that affects both heart and lungs. Each assumption should be evaluated against each of the potential diagnoses. For example, does rheumatic fever typically present in this manner? Is this a common presentation of systemic lupus erythematosus in a child of this age? Does the presentation align with that of glomerulonephritis, myocarditis or pneumonia? Based on my clinical knowledge, the assumption that should be prioritized for evaluation is whether this could be a case of myocarditis. CLINICAL REASONING: Myocarditis is typically associated with symptoms such as fatigue, difficulty breathing and walking upstairs, fast pulse, lower blood pressure, abnormal heart sounds – all of which have been observed in this patient. Additionally, myocarditis is known to occur as a complication of viral infections, which aligns with the patient's past week of rhinorrhea, coughing, and a sore throat. The resolution of these symptoms could point towards the post-viral phase where myocarditis may occur. The presented manifestation might not align entirely with glomerulonephritis, pneumonia, rheumatic fever, or systemic lupus erythematosus in their typical presentations. CONCLUDING: Taking into account the symptoms, the history of a recent viral-type illness, and the lack of alignment with the other provided possible diagnoses, a primary assumption is that this could be a case of myocarditis. RESPONSE: Therefore, based on the presented symptoms, and in comparison with other possible illnesses provided, this young boy's condition aligns most likely with (B) Myocarditis.

**Facilitator** The consensus statement synthesizes the responses of AI_A, AI_B, AI_C, AI_D, and AI_E as follows: The case presents a 13-year-old boy with a recent history of upper respiratory infection-like symptoms, followed by progressive fatigue, shortness of breath, and difficulty walking up stairs. His current condition includes tachycardia, tachypnea, hypotension, oxygen desaturation, lung crackles, cardiac gallop, soft S1, and a grade 2/6 holosystolic murmur. The primary assumptions are that the recent respiratory illness is connected to the current symptoms and that the physical findings are due to the same underlying process. The boy's symptoms point towards respiratory distress and cardiovascular complications. The major symptoms--fatigue, breathlessness, difficulty in walking up stairs--indicate a systemic issue, more in line with a cardiac abnormality. His previous upper respiratory infection-like symptoms and current pulmonary examination findings might suggest a post-infection outcome like myocarditis. Myocarditis characteristically presents with symptoms of heart failure following a viral illness. It is an inflammatory condition of the myocardium, often triggered by a viral infection, like the common cold. Myocarditis could lead to dilated cardiomyopathy, congestive heart failure symptoms such as fatigue and difficulty in handling physical exertion, and a fall in blood pressure which is noted in this patient. The other potential diagnoses provided, including glomerulonephritis, pneumonia, rheumatic fever, and systemic lupus erythematosus, do not align as closely with the provided history and clinical presentation. Therefore, considering the boy's symptoms, signs, preceding illness, and the line of reasoning outlined above, the most probable diagnosis in this scenario is (B) Myocarditis.
