# Supplementary material for: Collaborative intelligence in AI: Evaluating the performance of a council of AIs on the USMLE
Source: PLOS Digit Health. 2025 Oct 9;4(10):e0000787. doi: 10.1371/journal.pdig.0000787 (PMC12510544; doi:10.1371/journal.pdig.0000787)
Supplement: S2 File — (ZIP) [file pdig.0000787.s002.zip › S2 Data Raw data files containing unprocessed question inputs and LLM outputs including transcripts of deliberations/STEP2_Question-2-1_Type-MC-NJ.docx]

**Facilitator** **[Round 1]:** A 45-year-old woman comes to the emergency department because of a 3-day history of progressive shortness of breath, cough productive of blood-tinged sputum, and fever. She also has a 3-month history of numbness of her left leg and tingling in her right arm. She has not had chills. She was diagnosed with asthma 6 months ago; it is well controlled with inhaled budesonide and albuterol as needed. She has no other history of serious illness and takes no other medications. She appears to be in mild respiratory distress. Temperature is 38.1°C (100.6°F), pulse is 105/min and regular, respirations are 24/min, and blood pressure is 145/95 mm Hg. Pulse oximetry on room air shows an oxygen saturation of 92%. Crackles are heard at the right lung base, and there are occasional diffuse wheezes. Cardiac examination shows no abnormalities. On neurologic examination, sensation to pinprick is decreased over the lateral aspect of the left lower extremity and right upper extremity. Muscle strength is 5/5 throughout. Babinski sign is absent. Deep tendon reflexes are 2+ and symmetric. Which of the following is the most appropriate next step in diagnosis? (A) CT angiography of the chest (B) Determination of serum angiotensin-converting enzyme activity (C) Measurement of serum IgE concentration (D) Mesenteric angiography (E) Serum antiglomerular basement membrane assay (F) Serum antineutrophil cytoplasmic autoantibody assay (G) Sweat chloride testing

**AI_A:** (1) RESTATING THE QUESTION: The query is seeking to identify the best next diagnostic step for a 45-year-old woman experiencing respiratory distress, featuring fever and blood-tinged sputum, along with a history of numbness in her left leg and tingling in her right arm. She has a diagnosed condition of asthma, but also presents with decreased sensation to pinprick over the lateral aspects of the left lower extremity and right upper extremity. Crackles, a characteristic sound suggesting the presence of fluid in the lungs, are heard at the right lung base and occasional diffuse wheezes are also observed. No other signs of major illness are present. (2) PLANNING: The diverse clinical symptoms presented, particularly the respiratory distress, fever, blood-tinged sputum, and altered sensations in extremities suggest an underlying systemic disease which could affect multiple organ systems. While the initial assumption could be an escalated state of asthma, the patient's condition is well controlled with inhaled budesonide and albuterol, indicating the presence of another pathogenesis. Thus, this scenario would benefit from an exploration of systemic, autoimmune or vasculitis diseases that might cause both respiratory and neurological symptoms. (3) CLINICAL REASONING: Based on the symptoms, diseases such as Wegener's granulomatosis (now known as Granulomatosis with polyangiitis, GPA) and Goodpasture syndrome stand as possibilities. GPA is a small vessel vasculitis that often manifests with pulmonary, renal, and upper respiratory tract (including sinus) symptoms. Her fever, cough, shortness of breath, and presence of crackles in her lungs could be attributed to pulmonary involvement. Goodpasture syndrome, typically presents with an aggressive form of glomerulonephritis and lung disease, but the patient's neurological symptoms do not align. (4) CONCLUDING: Although the clinical picture is not completely clear, the lung involvement mixed with odd neurological symptoms might be indicative of systemic vasculitis such as GPA. In such a case, the standard method of diagnosing GPA would be the measure of serum antineutrophil cytoplasmic autoantibodies (ANCAs), which are often found in patients with this condition. (5) RESPONSE: Given the symptoms, I would recommend the Serum antineutrophil cytoplasmic autoantibody assay (option F) as the most appropriate next step in diagnosis. If positive, it can support the suspected diagnosis of GPA, guiding the choice of subsequent diagnostic procedures and therapeutic interventions.

**AI_D:** 1) RESTATING THE QUESTION: The question asks what the best next step would be to diagnose a woman who has presented with a 3-day history of shortness of breath, a cough producing blood-tinted sputum, fever, along with a three-month history of numbness in her left leg and tingling in her right arm. She has been diagnosed with asthma 6 months ago, however her symptoms seem to have intensified and additional symptoms have become apparent. 2) PLANNING: The woman's primary complaint suggests a respiratory illness, potentially a secondary infection. However, the co-occurring neurological symptoms are concerning and unusual for a respiratory infection. The major assumption here is that the respiratory and neurological signs and symptoms are linked and not separate entities. Therefore, both should be investigated together. 3) CLINICAL REASONING: Since there is evidence of a lung infection (fever, blood-tinged sputum, crackles at the right lung base), it would be reasonable to investigate for a pulmonary cause first. A CT angiography of the chest (A) could be done, but it would primarily look for pulmonary embolism, which does not connect to her neurological symptoms. Determination of Serum IgE concentration (C), Antiglomerular basement membrane assay (E), and Sweat chloride test (G) could be helpful in specific conditions but again there is no clear link to her neurological symptoms. Serum angiotensin-converting enzyme activity (B) could be measured when suspecting sarcoidosis which may present with systemic and neurological symptoms. Mesenteric angiography (D) is unrelated as it investigates blood flow in the abdomen. Meanwhile, the antineutrophil cytoplasmic autoantibody (ANCA) assay (F) is used to identify autoimmune vasculitis, which can affect both the lungs and central nervous system, hence accounting for both respiratory and neurological symptoms. 4) CONCLUDING: Based on her symptoms and the range of potential diagnoses, it appears that an autoimmune process may be at play. Vasculitis is one such condition that could impact both the lungs and peripheral nervous system, aligning with the patient’s symptoms. An ANCA assay would thus be the most targeted way to confirm or rule out this potential diagnosis. 5) RESPONSE: Therefore, the most appropriate next step in diagnosing this 45-year-old woman's condition is Serum antineutrophil cytoplasmic autoantibody assay (F).

**AI_B:** RESTATING THE QUESTION: This 45-year-old woman has come to the ER presenting several symptoms: over the last three days, she has experienced progressive shortness of breath, a cough that produces blood-tinged sputum, and a fever. This is further complicated by a 3-month history of numbness in the left leg and tingling sensation in the right arm. These are unusual symptoms that may suggest an underlying condition beyond her diagnosed asthma, which is managed with inhaled budesonide and albuterol. The combination of respiratory and neurological symptoms demands further investigation to determine the most accurate diagnosis and appropriate treatment. PLANNING: Let's consider each potential next step. (A) CT angiography of the chest can help visualize the heart and lungs for signs of disease. (B) Determination of serum angiotensin-converting enzyme (ACE) activity may be a possible next step in suspected cases of sarcoidosis. (C) Measurement of serum IgE concentration is used in diagnosing allergic reactions. (D) Mesenteric angiography helps visualize the blood flow in the mesenteric arteries that supply the intestines. (E) Serum antiglomerular basement membrane (anti-GBM) assay is used to detect antibodies that target the GBM in patients with suspected Goodpasture syndrome. (F) Serologic testing for antineutrophil cytoplasmic antibodies (ANCA) would be critical for diagnosing conditions like vasculitis and Wegener’s granulomatosis. (G) Sweat chloride testing is typically used to diagnose cystic fibrosis. CLINICAL REASONING: The symptoms this patient presents with may suggest a pathological process affecting both the lungs and the nervous system. While her numbness and tingling are largely isolated to the left leg and right arm, the shortness of breath, productive cough, and fever suggest a potential respiratory issue. The mentioned tests are all useful tools for diagnosing various conditions, but the key is to find a test that could be most relevant and appropriate based on her symptoms. The fact that our patient has both lung and nerve symptoms and no other signs of systemic illness narrows our focus. In this case, the presence of hemoptysis (coughing blood), fever, reduced oxygen saturation, and decreased sensation to pinprick over the lateral aspect of the left lower extremity and right upper extremity suggests an autoimmune or inflammatory condition. CONCLUDING: This woman's combination of respiratory and neurological symptoms—especially the presence of hemoptysis—have more congruity with diseases like Wegener's granulomatosis or Goodpasture syndrome. To detect the possibility of these conditions, tests for specific antibodies linked to them would be the most pertinent diagnostic procedures. RESPONSE: Therefore, based on these lines of reasoning, I would suggest (F) Serum antineutrophil cytoplasmic autoantibody assay as the most appropriate next step in diagnosis. The presence of these antibodies can help confirm vasculitis, such as Wegener's granulomatosis, which could explain this patient's systemic symptoms.

**AI_C:** RESTATING THE QUESTION: The lady in question is a 45-year-old who has presented with respiratory and neurological symptoms that have been progressing over different timescales. Her respiratory symptoms (shortness of breath, a cough with blood-tinged sputum, fever, and wheezing) have been present for three days. These could indicate a lung disease such as pneumonia or a pulmonary embolism. Her neurological symptoms (numbness in one leg and tingling in the opposite arm) have been present for three months and could be indicative of a neurological disorder such as multiple sclerosis, a brain tumor, or a stroke. Moreover, the patient was diagnosed with asthma six months ago and is under treatment. We are asked to identify the next best diagnostic step based on her clinical presentation, which includes these differentials: CT angiography; determining serum angiotensin-converting enzyme (ACE) activity; measuring serum IgE; mesenteric angiography; serum anti-GBM assay; serum ANCA assay; or sweat chloride test. PLANNING: Given the two sets of symptoms (respiratory and neurological), there is an assumption that they could be interconnected and explained by a single underlying condition, particularly given the relatively short timeframe. On the other hand, they may also represent two separate conditions. It is beneficial to generate alternate assumptions for each potential cause. The assumption that the respiratory symptoms are due to an infection such as pneumonia could be questioned as she has no chills and the fever is relatively mild for such an acute infection. Similarly, the assumption that her neurological symptoms are due to a stroke could be questioned given the long duration of symptoms and the lack of other classic stroke symptoms. CLINICAL REASONING: Given her respiratory symptoms, a differential diagnosis could include an infection or a vasculitis affecting the lungs. The presence of haemoptysis could suggest a more serious condition, such as lung cancer or vasculitis. On the neurological side, long-term numbness and paresthesia could suggest a chronic condition, such as a demyelinating disease (e.g., multiple sclerosis), peripheral neuropathy, or another form of neurological disorder. The absence of other neurological signs makes a central cause such as stroke less likely. When the respiratory and neurological symptoms are considered together, the possibility of a systemic cause becomes more likely. One potential unified diagnosis would be vasculitis, such as Granulomatosis with Polyangiitis (GPA - formerly Wegener’s), which can affect both the lungs and peripheral nerves. CONCLUDING: Assessing a patient with both respiratory and neurological symptoms poses a diagnostic challenge. The focus should be on identifying potentially serious conditions that could unify both sets of symptoms. Given the combination of symptoms and their progression, one plausible diagnosis could be a form of vasculitis like GPA. Consequently, a relevant diagnostic test could be ANCA testing, which can be positive in vasculitides like GPA. RESPONSE: The most appropriate next step in the diagnosis, based on her presenting blood-tinged sputum, fever, numbness in one leg, and tingling in the opposite arm, would be (F) Serum antineutrophil cytoplasmic autoantibody assay.

**AI_E:** RESTATING THE QUESTION: A 45-year-old woman with a past medical history of asthma presents to the emergency department (ED) with a 3-day history of progressive dyspnea, haemoptysis, and fever. She also complains of numbness in her left leg and tingling sensation in her right arm which has been present for 3 months. Despite the fever, chills are absent. Her asthma is well-managed with inhaled budesonide (a corticosteroid) and as-needed albuterol (a beta-2 agonist). She doesn't have any other significant medical history or take any other medications. Clinical examination showed signs of mild respiratory distress and decreased sensation to pinprick over the lateral aspect of the left lower extremity and right upper extremity but normal muscle strength and deep-tendon reflexes. It is being asked which among the listed investigations would be the most logical next step in establishing a diagnosis? PLANNING: The question is based on identifying the right diagnostic test to confirm or refute the underlying cause for her current illness - considering also her reported neurological symptoms. Some of the listed tests are related to different diseases such as (A) pulmonary embolism, (B) sarcoidosis, (C) allergic asthma, (D) mesenteric ischemia, (E) Goodpasture syndrome, (F) vasculitis, or (G) cystic fibrosis. To select the most suitable test, I should consider the clinical manifestations that are most noticeable and/or characteristic that could help to narrow down to one condition. CLINICAL REASONING: The patient has described a combination of respiratory and neurological symptoms. Respiratory symptoms include cough, bloody sputum, fever, and shortness of breath, in addition to abnormal findings on lung auscultation such as crackles and wheezes. Her sensation to pinprick is decreased over the lateral aspect of the left lower extremity and right upper extremity. Pathologies like pulmonary embolism, sarcoidosis, Goodpasture syndrome, and vasculitis can potentially result in lung symptoms. However, looking at the vascular nature of her neurological symptoms (with sensory loss in a non-dermatomal distribution), vasculitis involving small vessels presents as a likely underlying cause. In this case, Wegener's granulomatosis (which has been renamed as granulomatosis with polyangiitis) or microscopic polyangiitis might be the cause. Both these conditions are associated with antineutrophil cytoplasmic autoantibodies (ANCA). Furthermore, she is negative for Babinski's sign (which often indicates central nervous system pathology). Given these factors, a serum ANCA test might be the most appropriate next step, as it would confirm or refute the potential diagnosis of small-vessel vasculitis. CONCLUDING: The patient's presenting symptoms, her physical examination findings, and clinical picture lead to the suspicion of a systemic disease like small-vessel vasculitis, namely granulomatosis with polyangiitis or microscopic polyangiitis. In order to confirm this, a serum antineutrophil cytoplasmic autoantibody assay (option F), a pertinent diagnostic step for these conditions, would be the most suitable next action. RESPONSE: Therefore, the most appropriate next step in the investigation is (F) Serum antineutrophil cytoplasmic autoantibody assay.

**Facilitator** The consensus among the AI responses is that the most appropriate next step in diagnosing the 45-year-old woman's condition is the Serum antineutrophil cytoplasmic autoantibody assay (option F). This conclusion is based on her presenting symptoms, which include respiratory distress, fever, blood-tinged sputum, and altered sensations in her extremities. These symptoms suggest an underlying systemic disease that could affect multiple organ systems. While her diagnosed asthma could be a contributing factor, it is well controlled, indicating the presence of another pathogenesis. The combination of respiratory and neurological symptoms, particularly the presence of hemoptysis, aligns more with diseases like Wegener's granulomatosis (now known as Granulomatosis with polyangiitis, GPA) or Goodpasture syndrome. However, the patient's neurological symptoms do not align with Goodpasture syndrome, making GPA a more likely possibility. The standard method of diagnosing GPA is the measure of serum antineutrophil cytoplasmic autoantibodies (ANCAs), which are often found in patients with this condition. Therefore, the Serum antineutrophil cytoplasmic autoantibody assay is recommended as the most appropriate next step in diagnosis. If positive, it can support the suspected diagnosis of GPA, guiding the choice of subsequent diagnostic procedures and therapeutic interventions.
