# Supplementary material for: Collaborative intelligence in AI: Evaluating the performance of a council of AIs on the USMLE
Source: PLOS Digit Health. 2025 Oct 9;4(10):e0000787. doi: 10.1371/journal.pdig.0000787 (PMC12510544; doi:10.1371/journal.pdig.0000787)
Supplement: S2 File — (ZIP) [file pdig.0000787.s002.zip › S2 Data Raw data files containing unprocessed question inputs and LLM outputs including transcripts of deliberations/STEP2_Question-20-1_Type-MC-NJ.docx]

**Facilitator** **[Round 1]:** A 10-day-old female newborn is brought to the emergency department because of a 2-day history of bilious vomiting. She was born at term following an uncomplicated pregnancy and spontaneous vaginal delivery. She was 50.8 cm (20 in) in length and weighed 3260 g (7 lb 3 oz) at birth. Examination following delivery showed no abnormalities, and she was discharged at the age of 2 days. She is exclusively breast-fed. She appears ill. Today, she is 50.8 cm (20 in) in length and weighs 3062 g (6 lb 12 oz). Temperature is 38.3°C (100.9°F), pulse is 180/min, and respirations are 52/min. The abdomen is distended; the patient cries on palpation of the abdomen. Which of the following is the most likely diagnosis? (A) Duodenal atresia (B) Intussusception (C) Meconium ileus (D) Necrotizing enterocolitis (E) Volvulus

**AI_D:** Restating the question, we are trying to establish a diagnosis for a 10-day-old full-term female infant who has been presenting with bilious vomiting for the past 2 days. She was perfectly healthy at birth and has been exclusively breast-fed. However, she currently presents as unwell, with a fever, tachycardia, tachypnea, and abdominal distension; she also reacts negatively to abdominal palpation. Planning, we consider the provided differential diagnoses: Duodenal atresia, intussusception, meconium ileus, necrotizing enterocolitis, and volvulus. Duodenal atresia is a congenital condition that restricts the flow of substances through the digestive tract, whereas intussusception refers to the intestine folding into itself. Meconium ileus also hinders the passage of contents through the digestive system due to sticky, thick meconium, necrotizing enterocolitis refers to severe inflammation and damage to the intestine, and volvulus refers to the twisting of a part of the digestive tract, cutting off blood supply potentially. The assumptions here are that all conditions present themselves the same way, and they occur at the same frequency in newborns. However, that's not the case considering clinical presentations and age-specific rates of occurrence. For instance, duodenal atresia usually presents earlier, and meconium ileus is connected to cystic fibrosis, which is not indicated here. Clinical Reasoning, weighing the symptoms and considering the age of the newborn, it seems necrotizing enterocolitis (NEC) should be given priority. NEC is common in infants, especially premature ones (though, in this case, baby is full-term). NEC typically presents with the symptoms this baby has: bilious vomiting, abdominal distension, fever, and irritability on abdominal palpation. Also, since NEC can rapidly worsen and be life-threatening, suspicion should be high considering the baby's apparent severe illness. Concluding, using the presented symptoms and the most matching clinical picture, the likely diagnosis is necrotizing enterocolitis. Response: The most likely diagnosis in this scenario is option (D) Necrotizing enterocolitis.

**AI_B:** RESTATING THE QUESTION: The question asks for a presumptive diagnosis based on the given clinical presentation of a 10-day-old female newborn displaying features such as bilious vomiting, weight loss, elevated temperature, increased pulse and respiration rates, and abdominal distension accompanied by discomfort on palpation. PLANNING: Assumptions include that she is suffering from a condition impacting her digestive tract, like (A) Duodenal atresia — congenital obstruction of duodenum; (B) Intussusception — telescoping of one part of the intestine into another, leading to obstruction and impaired blood flow; (C) Meconium ileus — obstruction of the ileum by thick meconium in the newborn, typically associated with cystic fibrosis; (D) Necrotizing enterocolitis (NEC) — an illness of the bowel that affects mainly premature infants, characterized by necrosis of intestinal tissue; or (E) Volvulus — twisting of the intestine on its mesenteric axis, leading to obstruction and ischemia. However, we can also assume that bilious vomiting and rapid weight loss in a newborn are indicative of a congenital intestinal obstruction. CLINICAL REASONING: The presence of bilious vomiting strongly indicates an obstruction beyond the level of the ampulla of Vater, which is usually located in a proximal part of the small intestine. Weight loss and physical signs of distress suggest that this obstruction is significant enough to disrupt normal development. In the absence of other signs or symptoms pointing towards specific diseases like cystic fibrosis (Meconium ileus) or necrotizing enterocolitis (more common in premature newborns or those who have had a period of oxygen deprivation), we should consider congenital malformation as a cause. Duodenal atresia often presents with bilious vomiting but is usually diagnosed due to characteristic radiographic findings of double bubble sign (two air-filled spaces in the abdomen seen on a radiograph). On the other hand, Volvulus often presents early with bilious vomiting and may lead to rapid decompensation, including symptoms such as abdominal distension and signs of shock. CONCLUDING: Based on clinical features, rapid decompensation, and the age of the patient, a diagnosis of Volvulus is most likely. Duodenal atresia would not likely lead to rapid decompensation, and other diagnoses have less supportive features given the history and the clinical presentation. RESPONSE: Therefore, the most likely diagnosis for the newborn's condition is (E) Volvulus.

**AI_A:** RESTATING THE QUESTION: You're asking for a diagnosis for a 10-day old infant who is displaying ill symptoms, notably bilious vomiting, weight loss, a fever, a high pulse rate, and rapid breathing. The infant's abdomen is also distended and palpation causes discomfort. The proposed diagnoses include duodenal atresia, intussusception, meconium ileus, necrotizing enterocolitis, and volvulus. Each of these refers to potentially life-threatening gastrointestinal disorders: duodenal atresia is a blockage in the duodenum, the first part of the small intestine; intussusception occurs when a portion of the intestine folds into another section; meconium ileus is a disorder where thick, sticky meconium blocks the ileum; necrotizing enterocolitis involves severe inflammation and infection of the bowel; and volvulus is the twisting of the intestine. PLANNING: The symptoms of distended abdomen, vomiting, fever, and discomfort can indicate any of these conditions but taking all the information into account will help narrow the diagnosis. The fact that the baby was born after a full-term, uncomplicated pregnancy and showed no abnormalities suggests that conditions that often show symptoms immediately at birth - like duodenal atresia or meconium ileus - might be less likely. However, the bilious vomiting and fever could potentially be associated with all the conditions, more so with necrotizing enterocolitis and volvulus. The baby's noticeable weight loss could also suggest a severe issue like these. CLINICAL REASONING: Normal bilious vomiting at this age could indicate a lower digestive tract obstruction. The baby's drastic weight loss - 198 grams in eight days - is a very concerning indicator of a severe GI dysfunction. Additionally, the elevated fever and heart rate are likely signs of a serious infection. Among the diseases listed, the one that most regularly encompasses these symptoms, as well as typically develops in the first week to ten days of life, primarily in full-term infants, is necrotizing enterocolitis. CONCLUDING: Taking all this into account, based on the information provided and the severity of the symptoms -- particularly the rapid weight loss, distended abdomen, and the onset timing -- necrotizing enterocolitis would be the most likely diagnosis. RESPONSE: Thus, the most probable diagnosis for the symptoms exhibited by the 10-day-old infant would be (D) Necrotizing enterocolitis.

**AI_E:** RESTATING THE QUESTION: A female infant, who is just over a week old, has been brought to the ER due to bilious vomiting which has been occurring for the past two days. All details of the birth were standard, with the baby having normal vitals and measurements at birth, and was released after two days. She is exclusively drinking breast milk, and currently appears unwell. Her weight has decreased, and she has a slightly elevated body temperature, tachycardia, and tachypnea. Upon physically examining her abdomen, it was observed to be distended and she exhibited discomfort. We need to discern most likely diagnosis from the given options: duodenal atresia, intussusception, meconium ileus, necrotizing enterocolitis, and volvulus. PLANNING: We know that duodenal atresia manifests right after birth with bilious vomiting and the classic radiological "double bubble" sign. Intussusception is rare in newborns and usually starts with intermittent severe abdominal pain. Meconium ileus typically causes abdominal distension and failure to pass stool immediately after birth, often in infants with cystic fibrosis. Necrotizing enterocolitis is more common in preterm infants and usually associated with bloody stools and pneumatosis intestinalis on radiographic imaging. Volvulus typically presents in the first month of life, with symptoms such as bilious vomiting, poor feeding, lethargy, and bloody stools. CLINICAL REASONING: In the scenario presented, this 10- day old neonate, previously healthy, now demonstrates signs such as bilious vomiting, abdominal distension and systemic ill-health which points to an acute deterioration. These symptoms, coupled with weight loss and the acute presentation of the symptoms, align most closely with the presentation seen with a volvulus, specifically a malrotation with midgut volvulus. This condition is a surgical emergency that often manifests in the first month of life when the midgut, which normally undergoes a 270-degree counterclockwise rotation around the superior mesenteric artery during embryologic development, fails to complete rotation and fixation, leaving the intestines prone to rotation and subsequent ischemia. Unlike the other options, it often presents after a few days of normal health, and does not necessarily need any associated conditions or risk factors to occur. CONCLUDING: Based on the infant’s condition, the sudden onset of symptoms, time frame of the symptom presentation, the absence of notable birth history, weight loss, and the physical examination findings, the most likely diagnosis would be volvulus. RESPONSE: The most likely diagnosis, given the patient's clinical picture, is (E) Volvulus.

**AI_C:** 1) RESTATING THE QUESTION: The query essentially relates to a diagnosis of a 10-day-old female newborn experiencing bilious vomiting for 2 days and appears ill. She was born at term with no abnormalities but now shows symptoms that include distended abdomen, fever, rapid heart rate, and fast breathing. Let me define a few terms and concepts for clarity; bilious vomiting is vomiting greenish yellow bile, indicating a possible blockage in the small intestine. Duodenal atresia is a congenital defect where the duodenum is not developed properly, causing a blockage. Intussusception is a condition where one part of the intestine slides into another part, commonly seen in older infants and children. Meconium ileus is a bowel obstruction in newborns caused by thick meconium. Necrotizing enterocolitis involves damage and infection or necrosis of the intestinal tissue, primarily seen in premature infants. Volvulus refers to a twisting of the intestine, leading to intestinal obstruction. 2) PLANNING: The clinical features include bilious vomiting, rapid weight loss, distended abdomen, and the patient's critical condition. The girl was born without any troubles, and the symptoms have appeared after 10 days, suggesting a potential pathological issue surfaced post-birth rather than a congenital defect. Considering these details, we have to weigh the possibilities among duodenal atresia, intussusception, meconium ileus, necrotizing enterocolitis, and volvulus. 3) CLINICAL REASONING: Duodenal atresia or meconium ileus usually present with issues soon after birth, not after a week. Furthermore, they are often associated with other congenital anomalies or cystic fibrosis, and the girl was born without abnormalities. Intussusception is more common in older infants and children, making it less likely. Necrotizing enterocolitis predominantly occurs in premature infants, and this child was born at term. Considering her being fed, the bilious vomiting, and an episode of illness starting after more than one week of life, the clinical picture suggests volvulus, which is a condition manifested by a twisting of a bowel loop around its mesentery, leading to bowel obstruction and possible ischemia. 4) CONCLUDING: Based on the clinical presentation and reasoning, a condition that emerges after the newborn period, can lead to bowel obstruction with bilious vomiting, and affects term infants who are fed is likely to be the cause. Volvulus, particularly malrotation with volvulus, matches this description. 5) RESPONSE: Hence, the most probable diagnosis in this case is (E) Volvulus.

**Facilitator** **[Round 2]:** Summary: AI_A believes the most probable diagnosis for the infant's symptoms is necrotizing enterocolitis. This conclusion is based on the severity of the symptoms, the rapid weight loss, distended abdomen, and the onset timing. AI_B, on the other hand, suggests that the most likely diagnosis is volvulus. This conclusion is based on the clinical features, rapid decompensation, and the age of the patient. AI_C also concludes that the most probable diagnosis is volvulus, based on the clinical presentation and reasoning, and the fact that the condition emerges after the newborn period, can lead to bowel obstruction with bilious vomiting, and affects term infants who are fed. AI_D believes the most likely diagnosis is necrotizing enterocolitis, based on the presented symptoms and the most matching clinical picture. AI_E concludes that the most likely diagnosis is volvulus, based on the infant’s condition, the sudden onset of symptoms, time frame of the symptom presentation, the absence of notable birth history, weight loss, and the physical examination findings. Question to clarify disagreements: Given that the council members have differing opinions on whether the most likely diagnosis is necrotizing enterocolitis or volvulus, could each member provide more detailed reasoning behind their chosen diagnosis, specifically addressing why they believe their diagnosis is more likely than the alternative?. Based on your preceding response, how would you answer the original question: [A 10-day-old female newborn is brought to the emergency department because of a 2-day history of bilious vomiting. She was born at term following an uncomplicated pregnancy and spontaneous vaginal delivery. She was 50.8 cm (20 in) in length and weighed 3260 g (7 lb 3 oz) at birth. Examination following delivery showed no abnormalities, and she was discharged at the age of 2 days. She is exclusively breast-fed. She appears ill. Today, she is 50.8 cm (20 in) in length and weighs 3062 g (6 lb 12 oz). Temperature is 38.3°C (100.9°F), pulse is 180/min, and respirations are 52/min. The abdomen is distended; the patient cries on palpation of the abdomen. Which of the following is the most likely diagnosis? (A) Duodenal atresia (B) Intussusception (C) Meconium ileus (D) Necrotizing enterocolitis (E) Volvulus]

**AI_A:** Restated Question: The inquiry is about a newborn female, who's just ten days old. She was born in term without any complications during the pregnancy. A recent 2-day history of bilious vomiting, significant weight loss, ill appearance, distension of abdomen and associated pain, recorded fever, and elevated pulse and respirations are reported. Our task is to identify the most probable diagnosis given the options - Duodenal Atresia, Intussusception, Meconium Ileus, Necrotizing Enterocolitis, and Volvulus. Plan: Let's analyze our assumptions: (1) The bilious vomiting suggests some type of bowel obstruction; (2) The significant weight loss implies severe illness or malabsorption; (3) The fever and elevated vital signs could indicate an infection, inflammation, or systemic response to the obstruction or underlying disease. It's crucial to evaluate which of the five proposed diagnoses would cause these symptoms in a newborn. Clinical reasoning: Duodenal atresia usually presents right after birth with bilious vomiting but the newborn wouldn't have been discharged in this scenario. Intussusception generally occurs later in infancy, between 4 to 10 months of age. Meconium ileus, associated with cystic fibrosis, usually presents right after birth with a failure to pass meconium and doesn't match the clinical history. Necrotizing enterocolitis, a condition seen in premature infants, typically shows signs of sickness and intestinal injury. However, this infant was full-term and initially healthy before developing symptoms, which is less typical for this condition. Finally, volvulus, specifically malrotation with volvulus, can lead to a bowel obstruction with bilious vomiting, often presents after the newborn period, and can result in rapid decompensation, which seems to fit the patient’s profile best. Conclusion: Given this detailed explanation and the clinical presentation, a clinical picture best fitting this patient's condition and symptoms is Volvulus. Response: Therefore, the most likely diagnosis, considering the symptoms, clinical history, and age of the newborn, is (E) Volvulus.

**AI_E:** Restating the question: We're being asked to determine the most probable cause for a 10-day-old newborn's rapid weight loss, high temperature, tachycardia, tachypnea, and particularly, biolous vomiting and abdominal distension with tenderness. These symptoms and signs are highly suggestive of a severe gastrointestinal pathology. Planning: Understanding crucial points like the fact that she was born at term with no anomalies, appeared healthy till 8 days post-birth, is exclusively breast-fed, and presented suddenly ill are instrumental in hypothesizing diagnoses. We need to explore, are these symptoms due to a congenital obstruction, acquired obstruction, or is it a consequence of bowel ischemia? Clinical Reasoning: This neonate presented acutely sick at 10 days of life. Congenital causes like Duodenal atresia and Meconium ileus are less likely as they typically present immediately after birth. Intussusception typically presents beyond the neonatal age group. Necrotizing enterocolitis (NEC) is a plausible diagnosis; NEC usually affects preterm neonates that are just started on feeds and typically present with bloody stools which is absent in our case. Volvulus, on the other hand, characteristically presents after the first week of life, especially in previously healthy term neonates. Predisposing risk factors include the presence of a malrotation of the midgut; however, this finding is not apparent in the provided history. Moreover, abdominal distension, bilious vomiting, rapid weight loss, and a significant systemic response as depicted by our patient's condition are frequent presentations. Concluding: Taking into account the age of presentation, severe systemic response, gastrointestinal symptoms, and the exclusive breast-feeding history, the most probable diagnosis is volvulus. Response: Based on my clinical reasoning process, I conclude that the most likely diagnosis for this newborn is volvulus (E).

**AI_D:** Restating the question: The newborn has been experiencing bilious vomiting for 2 days. The child is systemically unwell, with a fever, tachycardia, tachypnea and distress. She shows signs of abdominal distension and tenderness, which suggests an abdominal pathology. The possible diagnoses provided for consideration are all conditions that produce intestinal obstruction in newborns. We are asked to determine the most likely diagnosis based on the clinical presentation. Planning: Each of the proposed diagnosis has their characteristic clinical presentations and risk factors. Duodenal atresia usually presents with vomiting right after birth, and is often associated with Down syndrome. Intussusception is rare in neonates and more common in older infants or toddlers, with a typical presentation of intermittent abdominal pain and currant-jelly stool. Meconium ileus typically occurs in newborns with cystic fibrosis and presents with failure to pass stool in first few days of life. Necrotizing enterocolitis tends to occur primarily in premature infants, featuring feeding intolerance, bloody stools and systemic signs of infection. And finally, volvulus which occurs due to malrotation of the intestine, can lead to an acute abdomen in neonates due to volvulus and obstruction, which fits well with our case. After careful evaluation, volvulus stands out as the most clinically prioritized assumption to pursue. Clinical Reasoning: The infant was well until 8 days of age, implying an acute development of symptoms rather than congenital conditions that would present earlier. Bilious vomiting suggests an obstruction distal to the ampulla of Vater. The systemic symptoms and rapid weight loss indicates a serious underlying condition causing malnutrition and dehydration. The acute, severe abdominal pain and distension points towards a twisting or obstruction, as seen in cases of volvulus. Considering volvulus usually presents within the first month of life and can rapidly deteriorate, resulting in bowel necrosis, this diagnosis fits well with our clinical picture. Concluding: Therefore, given the acute onset of bilious vomiting, distension, tenderness, and rapid clinical deterioration in a newborn, as well an absence of any obvious predisposing factors (prematurity, cystic fibrosis, Down syndrome), the most likely diagnosis is a high-grade small-bowel obstruction secondary to midgut volvulus secondary to malrotation. Response: Based on this, the most probable diagnosis for the patient in the presented case is (E) Volvulus.

**AI_C:** Regarding the condition of this 10-day-old newborn girl, she has a 2-day history of bilious vomiting, rapid weight loss, fever, tachycardia, tachypnea, abdominal distention, and she appears ill. Let's explore these symptoms more. In terms of anatomy, the duodenum is the first part of the small intestine immediately beyond the stomach, and atresia refers to the congenital absence or closure of a natural passage in the body. Intussusception is a serious condition in which part of the intestine slides into an adjacent part, and meconium ileus is a disorder occurring in newborns where the meconium, the newborn's first feces, is abnormally thick and causes a blockage in the ileum, the final section of the small intestine. Necrotizing enterocolitis is a medical condition where a portion of the bowel dies, more common in premature babies. Volvulus is a condition of abnormal twisting of part of the stomach or bowels that may cause their closure. I will begin by assuming that each possible answer to the question represents a plausible explanation for this newborn's symptoms and each assumes that this infant is suffering from some form of digestive tract issue due to the bilious vomiting and abdominal distention. For duodenal atresia, I question whether this onset is too late for a congenital condition that might typically be identified sooner after birth. Meconium ileus is often associated with cystic fibrosis, and there's no stated reason to suspect that condition in this infant. While intussusception typically affects older infants and children. Necrotizing enterocolitis most commonly affects premature infants, and this infant was full-term. This leads me to suspect that volvulus might be the current condition causing the infant's symptoms. Volvulus can lead to bowel obstruction, which aligns with the reported bilious vomiting which is associated with intestinal obstruction. The abdominal distention adds further support to this possibility, and the infant's discomfort upon abdominal palpation is consistent with a bowel disorder. Crucially, volvulus can emerge after the initial newborn period, providing a reason why the infant was discharged without complications but then developed symptoms. Based on my reasoning, the most likely diagnosis for this 10-day-old female newborn based on her presenting symptoms and clinical history would be Volvulus. Therefore, my answer to the original question would be (E) Volvulus.

**AI_B:** The council has been tasked to identify the most likely diagnosis for a 10-day-old female newborn with symptoms of multiple episodes of bilious vomiting for the past 2 days, consistent rapid weight loss, significant physical distress exhibited by crying on abdominal palpation, coupled with a distended abdomen. Bilious vomiting is the regurgitation of bile through the stomach to the mouth. This indicates some form of obstruction in the gastrointestinal tract, which is common in certain pediatric conditions such as duodenal atresia, intussusception, meconium ileus, necrotizing enterocolitis, and volvulus. The clinical symptoms and history shared have to be weighed against each other to identify potential assumptions in the diagnostic process. Key assumptions could be centered on whether the child has a pathological process related to bowel obstruction, ischemia, or sepsis. However, each of these assumptions also require scrutinizing to identify alternate assumptions such as whether the baby has an inherent anatomical defect, bowel movement or peristalsis defect, or a severe infection affecting the intestinal wall. After cross-examining these possibilities, clinically, the priority would be to pursue the assumption related to bowel obstruction or severe infection because of the urgency attached to these conditions. The process of identifying the most likely condition will be based on the key features of each mentioned diagnostic possibility. Duodenal atresia typically is associated with polyhydramnios (too much amniotic fluid) and Down syndrome, and usually presents earlier than 10 days of age with non-bilious vomiting. Intussusception usually presents in older infants and children with intermittent abdominal pain, often described as colicky. Meconium ileus is often associated with cystic fibrosis and presents immediately after birth with obstruction. The newborn's pretty quick decompensation, significant weight loss (more than 10% of birth weight), high-grade fever, tachycardia, tachypnea, and bilious vomiting heavily point towards a severe infection or ischemic pathology such as necrotizing enterocolitis or volvulus. Necrotizing enterocolitis is a severe bacterial infection affecting the wall of the intestines, more commonly seen in pre-term infants, and it presents with pneumatosis intestinalis on imaging. On the other hand, volvulus, or twisting of the bowel, results in an acute bowel obstruction with risk of bowel necrosis that quickly leads to severe manifestations. While the newborn's condition would be grave with either necrotizing enterocolitis or volvulus, the bilious vomiting, which suggests obstruction, and the typical age of occurrence, tips the scale in favor of malrotation with midgut volvulus. Hence, based on the presented case of the newborn girl with rapid decompensation and severe symptoms of infection, ischemia, and a high suspicion of bowel obstruction, I would conclude that the most probable diagnosis is volvulus (option E). A diagnosis of volvulus is severe and requires immediate surgical intervention to prevent life-threatening complications such as bowel necrosis.

**Facilitator** The consensus among the AI responses is that the most probable diagnosis for the 10-day-old female newborn, who presents with a 2-day history of bilious vomiting, rapid weight loss, fever, tachycardia, tachypnea, and abdominal distension, is Volvulus (E). This conclusion is based on the following reasoning: 1. The bilious vomiting and abdominal distension suggest a form of bowel obstruction. 2. The rapid weight loss, fever, and elevated vital signs indicate a severe illness or systemic response. 3. The onset of symptoms after the newborn period and the rapid decompensation align with the typical presentation of volvulus. 4. Other potential diagnoses were considered less likely due to various factors: Duodenal atresia and Meconium ileus typically present immediately after birth; Intussusception generally occurs later in infancy; Necrotizing enterocolitis is more common in premature infants and often presents with bloody stools, which is not reported in this case. Therefore, given the clinical presentation and the age of the newborn, the most likely diagnosis is Volvulus (E). This condition requires immediate surgical intervention to prevent life-threatening complications such as bowel necrosis.
