# Supplementary material for: Collaborative intelligence in AI: Evaluating the performance of a council of AIs on the USMLE
Source: PLOS Digit Health. 2025 Oct 9;4(10):e0000787. doi: 10.1371/journal.pdig.0000787 (PMC12510544; doi:10.1371/journal.pdig.0000787)
Supplement: S2 File — (ZIP) [file pdig.0000787.s002.zip › S2 Data Raw data files containing unprocessed question inputs and LLM outputs including transcripts of deliberations/STEP2_Question-21-1_Type-MC-NJ.docx]

**Facilitator** **[Round 1]:** An 85-year-old man with severe dementia, Alzheimer type, is admitted to the hospital because of a nonhealing ulcer on his right second toe. He lives in a nursing care facility and has been bedridden for the past 3 years. He has type 2 diabetes mellitus, hypertension, and peripheral vascular disease. His medications are metformin, amlodipine, and aspirin. Noninvasive vascular studies of the lower extremities show marked bilateral vascular disease. Subsequent peripheral angiography shows severe stenoses of the right superficial femoral and the left common iliac arteries. The patient undergoes uncomplicated placement of two sequential vascular stents in the superficial femoral artery and placement of an additional stent in the left common iliac artery. The type of stents used was recently approved by the US Food and Drug Administration and has been heavily marketed as a long-lasting solution to restenosis. Each stent costs the hospital $15,000. Alternatives to this approach include amputation of the toe, balloon angioplasty, and use of less expensive stents. Which of the following best describes the public policy implications of this scenario? (A) Absence of resource stewardship (B) Ageism (C) Health care disparities (D) Health care fraud and abuse (E) Lack of access to care

**AI_E:** RESTATING THE QUESTION: The scenario outlines a complex health situation involving an elderly man with multiple conditions including dementia, diabetes mellitus, and peripheral vascular disease, among other ailments. His recent hospital stay has required the placement of costly vascular stents to address issues related to his vascular disease. Despite the existence of cheaper alternatives, these expensive treatments were chosen. We are asked to scrutinize this situation for its public policy implications, essentially asking us to think about how the utilization of resources, patient demographics, and treatment choices illustrate broader concerns about the healthcare system. PLANNING: The assumptions here are that the high-cost treatment was necessary, and was the best option for this patient's health. It's also assumed that there's no financial inappropriateness involved in selecting such a costly solution. To question these assumptions, we could consider whether less costly treatments could have been as effective, or whether the man's age or dementia condition were factors in the decision to use the most expensive treatment. We could also explore whether there were any concerns about limited access to care or health care disparities. Following a thorough assessment, we would hypothesize that the main concern here is the absence of resource stewardship given the expensive treatments used in the face of cheaper alternatives. CLINICAL REASONING: The man has multiple conditions that complicate his health, but his recent hospital stay centered on the issue of a nonhealing ulcer and his peripheral vascular disease. Various diagnostic methods revealed the severity of his vascular disease and prompted the use of vascular stents as a treatment solution, despite the existence of other possible methods. Moreover, it's important to note that the stents were not only used, but they were of a type that's notably more expensive. CONCLUDING: Given the assortment of cheaper alternatives and the selection of more costly treatments, this case portrays the absence of resource stewardship. All patients deserve a high standard of care, but the use of resources should be mindful of cost-effectiveness, especially when other options are available that can achieve similar outcomes for less expense. RESPONSE: Thus, this scenario best illustrates (A) Absence of resource stewardship.

**AI_C:** RESTATING THE QUESTION: The scenario describes a medically complex, elderly, bedridden patient with nonhealing toe ulcers and severe peripheral vascular disease. The patient received stent placements in both femoral artery and common iliac artery as a preferred treatment to reduce restenosis, instead of opting for less expensive alternatives like toe amputation, balloon angioplasty, or less costly stents. The question seeks to identify the public policy implication of choosing the expensive stents, considering other available alternatives. PLANNING: The underlying assumptions here are that; the chosen stenting technique is the best treatment option for this patient irrespective of the cost, the price is an accurate reflection of the stent's efficacy and longevity, and the hospital has the financial resources to repeatedly bear such costs. If we question any of these assumptions, we might conclude that using such high-cost procedures without considering less expensive alternatives might not be the most efficient use of resources. Therefore, the priority assumption to evaluate here would be the efficient use of health resources. CLINICAL REASONING: The healing of wounds is mainly dependent on the circulation which is compromised in this patient due to peripheral vascular disease. The best option could be a vascular stent to restore blood flow, even though it may come at a steep cost. Alternatives like amputation, balloon angioplasty, or cheaper stents are viable, but each has its limitations in terms of patient comfort, likelihood of success, and recurrence rates. However, the priority must be given to efficiently using health resources. If the chosen procedure is significantly more expensive, yet provides comparable outcomes to less costly alternatives, it suggests that there may be a lack of resource stewardship. CONCLUDING: In light of the above reasoning and reflections on costs associated with health resources, specifically using high-priced stents in this case where other alternatives are available that could offer relatively similar outcomes, it appears that there might be an absence of judicious use of resources. RESPONSE: Thus, given the fact that the patient was treated with a high-cost procedure when less expensive alternatives were available, the scenario best reflects an (A) Absence of resource stewardship.

**AI_A:** 1) RESTATING THE QUESTION: The question wants us to consider the public policy implications of an 85-year-old man with several comorbid conditions (dementia, diabetes, hypertension, and peripheral vascular disease) receiving costly stent surgeries. There are several alternative treatment approaches available that are less expensive. Public policy implications often revolve around resource allocation, ethical considerations, equality, or fraud, as suggested by the answer choices. 2) PLANNING: The various assumptions behind this scenario include: that the patient's care is being optimally managed, that the cost of treatment is justified by the outcome, that the resources being used are not being wasted, that all patients have access to the same care, and that there is no abuse or fraud in the healthcare system. By questioning these assumptions, we can generate alternate assumptions such as: the patient's care may not be optimally managed given the costs involved and the availability of cheaper alternatives, resources may be being wasted, not all patients have access to the same care, or there could be abuse in the system - perhaps in the form of overuse of expensive procedures. 3) CLINICAL REASONING: This elderly patient, who has severe dementia and multiple comorbid conditions, resides in a nursing care facility and is bedridden. He was presented with a complication (ulcer on his toe) associated with his peripheral vascular disease and diabetes mellitus. His hospital course included expensive procedures that proved to be successful, but alternative, less expensive procedures were available. The placement of costly stents, especially given the patient's age and prognosis, can be questioned in terms of resource stewardship. 4) CONCLUDING: Considering the alternate assumption of waste of resources, the cost of the stents, the age and general health status of the patient, and the presence of less expensive alternative procedures, it's plausible to prioritize concerns about resource stewardship. 5) RESPONSE: Thus, the public policy implication best exhibited in this scenario is (A) Absence of resource stewardship, given the above clinical reasoning and conclusion.

**AI_B:** RESTATING THE QUESTION: The clinical scenario presents an elderly, comorbid patient with severe dementia of the Alzheimer's type who has been admitted due to a nonhealing ulcer on his right second toe. The patient was subjected to cost-intensive treatments, such as placement of expensive stents instead of lower-cost alternatives such as amputation of the toe, balloon angioplasty, or using less expensive stents. The question asks to identify the public policy ramifications of this situation from a given set of choices: lack of resource stewardship, ageism, health care disparities, health care fraud and abuse, and lack of access to care. PLANNING: Assumptions could include that the best available treatment was chosen without consideration of cost, that all available treatment options were considered and the decision was made based on the best medical outcome for the patient, or that the medical professionals were influenced by the marketing of the recently approved stents. It could also be assumed that the patient or the patient's legal representative was appropriately informed and involved in the decision-making process. However, not all these assumptions directly lead us to the fatuity of such expenses under all circumstances. One key assumption to clinically prioritize is whether the most appropriate treatment considering the patient's overall condition, prognosis, and the costs involved was selected. CLINICAL REASONING: Considering the patient has several comorbidities, severe dementia, and a nonhealing ulcer, it is important to treat his condition effectively while also considering the ethical aspects, which include cost-effectiveness and the patient's quality of life. Given the presence of more cost-effective alternatives to the expensive stents, it seems that the hospital favored a high-cost procedure potentially influenced by aggressive marketing of the new stents. CONCLUDING: Linking clinical reasoning and planning, the public policy implications could be interpreted as an unnecessary use of expensive resources when effective, less pricey options are available. From a wider social perspective, such decisions can contribute to an increased economic burden on the healthcare system. RESPONSE: Thus, this scenario best describes the Absence of resource stewardship (A), where resource allocation decisions do not reflect an optimal or rational use of available resources.

**AI_D:** RESTATING THE QUESTION: This query involves the scenario of an elderly patient with severe dementia (Alzheimer type) admitted to the hospital for the management of a nonhealing ulcer on his second toe. The patient has underlying comorbid conditions like type 2 diabetes, hypertension, and peripheral vascular disease. Significantly, the patient goes through the expensive process of vascular stent placements rather than choosing amongst potentially less costly alternatives like toe amputation, balloon angioplasty, or usage of cheaper stents. The context requests an examination of how this healthcare situation correlates with various public policy implications. PLANNING: This case involves a critically ill elderly patient receiving highly expensive healthcare treatment. Key assumptions include: the financial cost healthcare providers incur through the utilization of the expensive stents; the decision-making process behind choosing this expensive option over less costly ones, especially considering the patient's age and condition; and the larger implications these underline for healthcare delivery and public policy. Alternate assumptions could question whether the stent placements were the best treatment option for the patient or if other approaches were considered, what factors influenced the choice of such an expensive treatment, and how this scenario might exemplify a larger trend in healthcare delivery. The assumption to prioritize would evaluate if this situation pertains to resource stewardship in healthcare delivery. CLINICAL REASONING: With the key assumption suggesting an illustration of economic resource allocation in the healthcare system, we can navigate the path towards better understanding. This elderly patient was administered an expensive treatment strategy, involving stent placements that have a higher upfront cost, even though there exist other significantly cheaper alternatives. Given the financial implications and the patient's age and condition, there arises the question of why such an expensive option was chosen instead of other less costly options. One aspect to consider would be the recent approval and marketing of the stents used, suggesting a potential influence on the decision to employ them. This highlights the question of if more cost-effective, appropriate options were adequately considered or ignored. CONCLUDING: Based on the clinical reasoning thus explained, this scenario appears to represent a case where more expensive medical resources were used when less expensive options were available. This calls into question the stewardship of resources in healthcare, specifically with regards to economic healthcare delivery decisions. RESPONSE: Therefore, the best answer would be (A) Absence of resource stewardship. This case underscores potential concerns about the efficient use of healthcare resources, especially where more cost-effective alternatives are available. The high cost of the chosen treatment raises questions about the justifiable allocation of healthcare resources and hence, portrays an absence of resource stewardship.

**Facilitator** The consensus statement synthesizes the responses of AI_A, AI_B, AI_C, AI_D, and AI_E as follows: The scenario presents an 85-year-old man with severe dementia and multiple comorbid conditions, including diabetes, hypertension, and peripheral vascular disease. He was admitted to the hospital due to a nonhealing ulcer on his toe, a complication associated with his peripheral vascular disease and diabetes. Despite the availability of less expensive treatment alternatives, the patient underwent costly stent surgeries. The underlying assumptions in this scenario include the belief that the patient's care is being optimally managed, that the cost of treatment is justified by the outcome, and that the resources being used are not being wasted. However, these assumptions can be questioned, leading to alternate assumptions such as the possibility of resource wastage, unequal access to care, or potential abuse in the healthcare system. Given the patient's age, general health status, and the availability of cheaper alternatives, it is plausible to prioritize concerns about resource stewardship. The decision to use high-cost procedures when effective, less pricey options are available can contribute to an increased economic burden on the healthcare system. This scenario raises questions about the efficient use of healthcare resources, especially where more cost-effective alternatives are available. Therefore, the public policy implication best exhibited in this scenario is the absence of resource stewardship. This case underscores potential concerns about the efficient use of healthcare resources, particularly when more cost-effective alternatives are available. The high cost of the chosen treatment raises questions about the justifiable allocation of healthcare resources and hence, portrays an absence of resource stewardship.
