# Supplementary material for: Collaborative intelligence in AI: Evaluating the performance of a council of AIs on the USMLE
Source: PLOS Digit Health. 2025 Oct 9;4(10):e0000787. doi: 10.1371/journal.pdig.0000787 (PMC12510544; doi:10.1371/journal.pdig.0000787)
Supplement: S2 File — (ZIP) [file pdig.0000787.s002.zip › S2 Data Raw data files containing unprocessed question inputs and LLM outputs including transcripts of deliberations/STEP2_Question-22-1_Type-MC-NJ.docx]

**Facilitator** **[Round 1]:** A 62-year-old woman with myasthenia gravis comes to the office because of a 2-week history of falling. She says her legs ""give out"" once daily; she falls to the ground but does not hit her head. She took pyridostigmine until 2 weeks ago when she decided that it no longer worked. Respirations are 30/min. Examination shows a hypophonic nasal voice and ptosis bilaterally. Muscle strength is 2/5 in all extremities. The patient is told that she is in imminent danger of respiratory compromise and that she should be admitted to the hospital. She says she knows her breathing could pose a problem, but she hates hospitals and does not want to undergo more tests or therapy. She asks if she can have a prescription for her former medication and if she can go home. Which of the following is the most appropriate next step in management? (A) Assessment of medical decision-making capacity (B) Electromyography and nerve conduction studies (C) Involuntary hospital admission (D) Oxygen therapy by nasal canula (E) Pulmonary function testing

**AI_C:** 1) RESTATING THE QUESTION: A patient with myasthenia gravis has been experiencing frequent falls due to muscle weakness, and recently stopped taking her prescribed treatment, pyridostigmine. She presents with respiratory compromise and was advised to be admitted to hospital, but she refuses and asks to continue with her former medication instead. We need to determine the best next step in managing her case. 2) PLANNING: It seems the patient not only has physical health issues but is also demonstrating potentially harmful decision-making. Key assumptions here are that she understands her medical condition, can make competent decisions, and that she's aware of the severity of her situation. Alternatively, the patient might lack the capacity to make such decisions. Evaluating which assumption to prioritize clinically leans toward assessing her medical decision-making capacity. 3) CLINICAL REASONING: Myasthenia gravis causes muscle weakness that can progress to severe disability and even be life-threatening, especially when it involves breathing muscles. Her clinical findings are consistent with a myasthenic crisis, perhaps precipitated by discontinuation of the pyridostigmine. She has been advised to be admitted due to imminent danger of respiratory failure, but she's refusing, indicating possible impairment of her decision-making capacity. Evaluating her capacity will confirm whether she understands the consequences and can make a safe and appropriate choice. 4) CONCLUDING: Given the clinical picture of a myasthenic crisis, the seriousness of her physical condition, her refusal of hospitalization and potential misunderstanding of the situation, we need to first clarify if she's capable of making sound medical decisions. 5) RESPONSE: Therefore, the most appropriate next step in management should be (A) Assessment of medical decision-making capacity. This will help determine whether the patient has the mental capacity to understand the health information and consequences of her decisions, hence providing a basis for further interventions.

**AI_A:** RESTATING THE QUESTION: The question describes a 62-year-old woman who has advanced myasthenia gravis (a chronic auto-immune disorder that causes muscle weakness). Despite worsening symptoms such as frequent falling, weakened voice, rapid respiratory rate, and overt muscle weakness, she chooses to discontinue her medication. She understands her condition could escalate into a grave respiratory crisis, yet she prefers avoiding hospitalization. The question is what should be the best approach to manage her condition from the presented options. PLANNING: The initial assumption in this case might be that she requires urgent medical intervention, given her worsened state, which could lead to involuntary hospital admission or be rushed on an oxygen therapy regime. However, these actions violate her autonomous right to refuse treatment (if competent). Therefore, an alternate assumption would prioritize the assessment of her capacity to make medical decisions. This means evaluating her ability to understand the medical condition, the risks and benefits of various options, and the ability to convey her decision appropriately. Only once this is done can it be safely decided if she can be allowed to return home with medication, needs to be admitted despite her will, or if further testing is immediately necessary. CLINICAL REASONING: As a physician, while one of the immediate concerns would be to uplift her dangerously decreased respiratory state and recent muscular weakness likely driven by her ceasing pyridostigmine; it should be remembered that overriding a patient's wishes (including the decision to refuse care) would be ethical acceptable only if the patient lacks medical decision-making capacity. In this clinical setting, with worsening myasthenia gravis, decision-making capacity can be affected; therefore, it is critical to determine capacity before deciding about her management plan. CONCLUDING: The patient has demonstrated understanding by acknowledging her respiratory risk. Besides, she dislikes hospitals and needs to avoid further diagnostic measures. Importantly, she has even requested to resume previous medication. Despite the severity of her disease, these signs indicate an appreciable level of understanding and reasoning about her health situation. Therefore, it is important to ascertain her decision-making capacity before any forced intervention is considered. RESPONSE: Based on the outlined problem and thought process, the most appropriate next step in management would be (A) Assessment of her medical decision-making capacity. Once her capacity is verified, the decision between hospitalization, home medication, or further testing would respect her autonomy and be better informed by her desires and medical necessity.

**AI_E:** RESTATING THE QUESTION: The query pertains to a 62-year-old female patient who has myasthenia gravis and has stopped taking her medication pyridostigmine. This has resulted in her falling frequently due to weakness in her legs. She exhibits symptoms of myasthenic crisis like increased respiratory rate, weakness in her voice, saggy eyelids (ptosis), and diminished strength in her limbs. Her condition is severe enough to necessitate hospitalization due to potential impending respiratory failure. Nonetheless, she does not wish to be hospitalized or undergo further tests. The question asks what the most suitable next step should be in managing her condition. PLANNING: Under normal care, her critical condition would have led to hospitalization. However, her refusal brings another aspect into play: evaluation of the patient's capability to make informed health decisions. This different assumption would evaluate her cognitive ability and understanding of the situation (Option A: Assessment of medical decision-making capacity). On the other hand, more diagnostic tests and therapies could further elucidate her condition or help manage her symptoms, but those would be against her wishes (Option B: Electromyography and nerve conduction studies; Option E: Pulmonary function testing; Option D: Oxygen therapy by nasal canula). Lastly, overriding her wishes due to the severity of her condition is a possible course (Option C: Involuntary hospital admission), but this would only be justified if her cognitive faculties didn't allow her to make informed decisions. CLINICAL REASONING: With the clinical assumption being that the patient has stopped her medication leading to severe myasthenic manifestations, the symptoms reported are very consistent with this. Her increased communications and muscle weakness signify myasthenic crisis, a state that can lead to life-threatening respiratory failure. Therefore, immediate hospitalization and treatment are paramount to prevent fatal outcomes. However, given her lack of compliance and refusal to be hospitalized, it becomes essential to assess whether she is fully aware of the gravity of her situation and possible outcomes. If she isn't, it may be justifiable to hospitalize her involuntarily. CONCLUDING: Factoring in all these components, the priority should be given to assessing the patient's medical decision-making capacity. This will allow us to understand her cognitive ability to comprehend the gravity of her situation. As patient autonomy is paramount, her wishes can only be overridden if it's determined that she lacks the cognitive capacity to make informed decisions regarding her health. RESPONSE: The most appropriate next step in managing this patient is (A) Assessment of medical decision-making capacity. This assessment will guide whether we can respect the patient's wishes or need to act against those due to her deteriorated cognitive capacity to comprehend her medical situation.

**AI_D:** RESTATING THE QUESTION: This case involves a 62-year-old woman who has myasthenia gravis, a chronic autoimmune disease characterized by muscular weakness and fatigue. She stopped taking her medication, pyridostigmine, two weeks ago because she believed that it was no longer effective. Recently, she has been experienced falls as her legs unexpectedly fail her once every day. Her physical examination indicates signs of advanced myasthenia gravis including a weakened voice (hypophonic nasal voice), drooping eyelids (ptosis), and reduced muscle strength. Her increased respirations suggest possible respiratory compromise due to respiratory muscle weakness. Despite being informed of the risk to her respiratory health, she refuses hospitalization and asks if she can return home with a prescription for her previous medication. I'm required to identify the most appropriate next step in managing her case, with options including the assessment of her capacity to make sound medical decisions, running electromyography and nerve conduction studies, forcing hospital admission, providing oxygen therapy, or conducting pulmonary function tests. PLANNING: With the assumption that this patient has the ability to make sound medical decisions due to her refusal for hospitalization, it could be plausible to consider other options such as her desire for continuing with the same medication. Nevertheless, we could also consider developing an alternate assumption as it might be crucial to question whether she is truly capable of making sound medical decisions considering her disregard for her severe condition and advising her to go to the hospital. Thus, we could hypothesize that the clinical priority in this instance is to verify her decision-making ability. CLINICAL REASONING: The clinical thought process here is that the patient's respiratory rate is elevated, indicative of the fact that the problem has escalated and requires immediate medical attention. Her muscular strength, voice strength, and eyelid droop severity also point towards the progression of her condition - myasthenia gravis. The refusal of the patient to seek hospitalization despite the critical nature of her condition highlights the need to assess her ability to make medical decisions. This necessity arises as the patient's life is in imminent danger due to the potential risk of respiratory failure, and her willingness to reject necessary care raises valid concerns about her capacity to understand these risks and make appropriate decisions. CONCLUDING: By connecting all these points, the patient's elevated respiratory rate as well as her other symptoms indicate a severe stage of myasthenia gravis. Despite recognizing her breathing problems, the patient refuses further testing and therapy, opting instead for a prescription of her previous medication and a return home. Given her disregard for the risks associated with her condition, as evidenced by her refusal to seek hospital care, an assessment of her medical decision-making capacity is warranted. RESPONSE: Thus, the most appropriate next step in management of this patient's case is (A) assessment of medical decision-making capacity. Her refusal to seek hospitalization despite the critical nature of her condition raises valid concerns about her understanding of these risks and her capacity to make appropriate decisions.

**AI_B:** RESTATING THE QUESTION: We are dealing with a 62-year-old woman who has myasthenia gravis, an autoimmune neuromuscular disease leading to fluctuating muscle weakness and fatigue. She's been struggling with falls due to muscle weakness, and has stopped taking her medication, pyridostigmine, an acetylcholinesterase inhibitor used to treat myasthenia gravis. She has high respiratory rate, hypophonic nasal voice, bilateral ptosis which suggest increased severity. She has low muscle strength, which indicates significant muscle weakness. Despite the urgent advice she was given due to danger of respiratory compromise, she despises hospitals and holds preference against undergoing further tests or therapies. She wants a prescription for her prior medication (pyridostigmine) and prefers to go home. The question requires us to determine the most appropriate next step in her management. PLANNING: Medical decision-making capacity, electromyography and nerve conduction studies, involuntary hospital admission, oxygen therapy via nasal canula, and pulmonary function testing are the proposed management plans. But it would be prudent to consider aspects such as: Can the patient make sound health decisions since she abruptly stopped her medication on her own? Is there a need for immediate diagnostic testing to assess extent of neuromuscular compromise? If hospital admission is opposed, what other mechanisms can be used to ensure her safety? Will oxygen supplementation suffice in managing the expected respiratory compromise? Will pulmonary function testing yield any beneficial information that can alter management? CLINICAL REASONING: The clinical picture of increased respiratory rate, nasal dysphonia, ptosis, and profound muscle weakness indicates a potential crisis in myasthenia gravis. Abrupt discontinuation of pyridostigmine might have worsened the situation, but the patient's wish not to be hospitalized or tested further implies a potential impairment in her medical decision-making capacity considering the imminent danger of respiratory compromise. Electromyography and nerve conduction studies might provide an assessment of the neuromuscular compromise, but they wouldn't alter the immediate management of a potential myasthenic crisis. Involuntary hospital admission is only considered once it's decided that the patient lacks medical decision-making capacity. Oxygen therapy by nasal cannula or pulmonary function testing would not directly address the issue at hand as they are neither curative nor fully diagnostic in this scenario. CONCLUDING: The first urgent step would be to evaluate the patient's medical decision-making capacity. A patient can refuse treatment only if she fully understands the medical information given, appreciates the consequences of her situation, reasoning the information rationally in the context of her values and is able to express a choice that is stable over time. RESPONSE: Given the potential risk of respiratory crisis and reluctance to hospitalisation despite understanding the risk, it is crucial to evaluate the patient's decision-making capacity (Option A). If the patient is found to lack capacity, surrogate decision makers or legal instruments (living will, power of attorney for health care) might be used to aid in decision making. If none of these are available, emergency involuntary hospitalization may be necessary. In case the patient has decision-making capacity and continues to refuse hospitalization, management should be aimed at the most effective treatment that can be administered outside the hospital setting, which might include resuming pyridostigmine, close outpatient supervision and home health support.

**Facilitator** The consensus statement synthesizes the responses of AI_A, AI_B, AI_C, AI_D, and AI_E as follows: The case involves a 62-year-old woman with advanced myasthenia gravis who has discontinued her medication, leading to worsening symptoms such as frequent falls, weakened voice, rapid respiratory rate, and overt muscle weakness. Despite understanding the potential risk of a severe respiratory crisis, she refuses hospitalization and further testing, expressing a preference to return home with her previous medication. The primary concern is her deteriorating health, which could necessitate urgent medical intervention, including involuntary hospital admission or oxygen therapy. However, these actions could infringe on her autonomous right to refuse treatment, assuming she is competent to make such decisions. Therefore, it is crucial to assess her medical decision-making capacity, which involves evaluating her understanding of her medical condition, the risks and benefits of various options, and her ability to communicate her decision appropriately. Her refusal to seek hospital care despite the severity of her condition raises valid concerns about her understanding of these risks and her capacity to make appropriate decisions. Therefore, the most appropriate next step in managing her condition would be to assess her medical decision-making capacity (Option A). This assessment will guide whether her wishes can be respected or if action against her wishes is necessary due to her potentially impaired cognitive capacity to comprehend her medical situation. If she is found to lack capacity, surrogate decision makers or legal instruments might be used to aid in decision making. If none of these are available, emergency involuntary hospitalization may be necessary. If the patient has decision-making capacity and continues to refuse hospitalization, management should be aimed at the most effective treatment that can be administered outside the hospital setting, which might include resuming pyridostigmine, close outpatient supervision, and home health support.
