# Supplementary material for: Collaborative intelligence in AI: Evaluating the performance of a council of AIs on the USMLE
Source: PLOS Digit Health. 2025 Oct 9;4(10):e0000787. doi: 10.1371/journal.pdig.0000787 (PMC12510544; doi:10.1371/journal.pdig.0000787)
Supplement: S2 File — (ZIP) [file pdig.0000787.s002.zip › S2 Data Raw data files containing unprocessed question inputs and LLM outputs including transcripts of deliberations/STEP2_Question-23-1_Type-MC-NJ.docx]

**Facilitator** **[Round 1]:** A 55-year-old man is brought to the emergency department by paramedics 45 minutes after his daughter found him unresponsive in his apartment. Paramedics initiated intravenous fluid warming and resuscitation at the scene. The patient has major depressive disorder, hypertension, and alcoholism. His medications are chlordiazepoxide, fluoxetine, and lisinopril. On arrival, he is arousable but confused. Temperature is 34.0°C (93.2°F), pulse is 64/min, respirations are 14/min, and blood pressure is 94/62 mm Hg. Physical examination shows no sign of trauma or prolonged immobilization. On mental status examination, he is oriented to person but not to place or time. His speech is verbose and nonsensical. Serum glucose concentration is 44 mg/dL, and blood alcohol concentration is 312 mg/dL. Urine toxicology screening is positive for alcohol and benzodiazepines. The most appropriate next step in management is administration of which of the following? (A) 50% Dextrose (B) Flumazenil (C) Lorazepam (D) Naloxone (E) Vitamin B1 (thiamine)

**AI_D:** Sure, let's decipher this clinical scenario. RESTATING THE QUESTION: A middle-aged man with a history of major depressive disorder, hypertension, and alcoholism was found unresponsive and is now confused and disoriented, with lab results indicating hypoglycemia, high blood alcohol concentration, and benzodiazepine positivity. The question wants to know which treatment should be administered next. 1) PLANNING: We need to identify and question the assumptions in this case. We could assume that the patient's confusion and disorientation might be due to his high blood alcohol concentration, hypoglycemia or benzodiazepine ingestion. However, because the patient is a chronic alcoholic, he might also be prone to Wernicke's encephalopathy, a condition caused by vitamin B1 (thiamine) deficiency. 2) CLINICAL REASONING: Chronic alcoholism can lead to poor nutrition which can result in vitamin B1 deficiency. Wernicke's encephalopathy, a specific syndrome caused by B1 deficiency, is characterized by confusion, ataxia, and nystagmus, which seems to align with the presenting symptoms of this patient. Intravenous dextrose without thiamine administration could exacerbate the condition due to increased utilization of thiamine in glucose metabolism. Also, the patient’s high blood alcohol concentration might have suggested administering naloxone, however, this is an opioid reversal agent, and there’s no indication of opioid ingestion. Benzos counteracts the activity of a specific brain chemical (GABA), and disorientation, confusion and nonsensical speech might suggest considering an antidote like flumazenil. But benzodiazepine withdrawal syndrome can also exhibit similar symptoms and thus, administering flumazenil might cause severe reactions. 3) CONCLUDING: The clinical reasoning indicates towards treating the possible Wernicke's encephalopathy first due to chronic alcoholism and symptoms presented. 4) RESPONSE: Regarding the next management step for this patient, the most appropriate intervention would be to administer Vitamin B1 (thiamine) to address the potential Wernicke's encephalopathy resulting from chronic alcoholism and malnutrition (E).

**AI_E:** RESTATING THE QUESTION: The question presents the case of a 55-year-old man who has been found unresponsive in his apartment. He has a past medical history of major depressive disorder, hypertension, and alcoholism, and is currently on chlordiazepoxide, fluoxetine, and lisinopril. Clinical findings show that he is disoriented and speaking nonsensical sentences, his blood glucose is low while blood alcohol level is high, and urine tests positive for alcohol and benzodiazepines. The question asks for the most suitable immediate treatment for this patient from the given choices, which include 50% dextrose, flumazenil, lorazepam, naloxone, and vitamin B1 (thiamine). PLANNING: Asssumptions here include low blood glucose and high blood alcohol levels playing a role in the man's current state. There is also an indication of benzodiazepine use, either due to his prescribed chlordiazepoxide or potentially from misuse. Moreover, the patient could be suffering from hypothermia due to the low body temperature, which may result from alcohol intake. Indeed, chronic alcoholism may lead to nutritional deficiency - particularly of thiamine, Wernicke's encephalopathy - which can cause disorientation and nonsensical speech. Since multiple factors are at play, it can be complex deciding which to prioritize. CLINICAL REASONING: Considering the patient's low serum glucose, administering 50% Dextrose could correct the hypoglycemia. But treating only the hypoglycemia might not address an underlying brain disorder resulting from vitamin deficiency, which is called Wernicke-Korsakoff syndrome. Administering Flumazenil would antagonize the effects of benzodiazepines, possibly improving his consciousness. Still, this can lead to seizures in patients with a long history of benzodiazepine use, thereby outweighing the potential benefits. Administering Lorazepam or Naloxone wouldn't aid in this situation as there's no evidence of an opioid overdose, and additional benzodiazepines could potentially harm more than help. CONCLUDING: While it's clear that the low blood glucose level and benzodiazepine and alcohol use contribute to his current state, considering his alcoholism, a chronic nutritional deficiency could be inducing the observed mental status changes. Hence, it would be essential to address this condition, specifically Wernicke-Korsakoff syndrome, due to thiamine (vitamin B1) deficiency. RESPONSE: Thus, the most appropriate next step in the management of this patient would be to administer Vitamin B1 (thiamine) (Option E), to address possible Wernicke-Korsakoff syndrome due to chronic alcohol use.

**AI_B:** 1) RESTATING THE QUESTION: The question is asking what the best next step is in managing a 55-year-old male who was found unresponsive. The man has a history of depression, high blood pressure, and alcoholism. His medications include chlordiazepoxide (a benzodiazepine), fluoxetine (an SSRI), and lisinopril (an ACE inhibitor). He presents confused and disoriented, with low body temperature and low blood pressure. His blood work showed low glucose and a high alcohol level, and his urine tested positive for alcohol and benzodiazepines. 2) PLANNING: The next step in management has to take into account both his glucose levels and alcohol concentration. Hypoglycemia could impair consciousness, but the extensively high alcohol level is likely to contribute to his symptoms. The concurrent benzodiazepine usage might elevate the risk for benzodiazepine tolerance and dependence, however, in this context, benzodiazepine withdrawal syndrome is an unlikely possibility due to his ongoing use. Therefore, the options for treatment could be 50% Dextrose, Flumazenil, Lorazepam, Naloxone, or Vitamin B1 (thiamine). 3) CLINICAL REASONING: While reversing hypoglycemia with 50% dextrose is essential in managing any patient with significantly low glucose, this patient's alcohol level and probable benzodiazepine dependence cannot be overlooked. Flumazenil could potentially reverse the effects of benzodiazepine overdose, but it's contraindicated in long-term benzodiazepine users due to the risk of precipitating seizures. Lorazepam would not be useful in this scenario, as the patient already exhibits signs of benzodiazepine intoxication. Naloxone is used for reversing opioid overdose, not alcohol or benzodiazepine toxicity. That leaves us with vitamin B1 (thiamine). 4) CONCLUDING: Thiamine is crucial in carbohydrate metabolism. A rapid infusion of dextrose without first administering thiamine could precipitate Wernicke's encephalopathy in a malnourished, chronic alcoholic patient. Wernicke's encephalopathy presents with confusion, oculomotor dysfunction, and ataxia, all of which can be consequently worsened in a patient with acute alcohol intoxication. Therefore, thiamine should be administered before dextrose, to prevent the onset of Wernicke's encephalopathy in alcohol-dependent patients. 5) RESPONSE: Accordingly, the most appropriate next step in management would be the administration of vitamin B1 (thiamine) (option E).

**AI_C:** RESTATING THE QUESTION: In simpler words, this is a case of a middle-aged man with a history of depression, hypertension, and alcoholism. He was found unresponsive at home and brought to the ER where his current physical examination reveals low body temperature, proper heartbeat rate, normal respirations, and a rather low blood pressure. His mental status examination shows confusion and disorientation. His laboratory tests demonstrate hypoglycemia, a very high blood alcohol concentration, and evidence of benzodiazepine use. The query requires us to decide the best immediate intervention from a medical treatment list that includes 50% dextrose, flumazenil, lorazepam, naloxone, and Vitamin B1 (thiamine). PLANNING: Some of the assumptions that can be made from this case, based on the patient's symptoms and laboratory findings, are possible hypoglycemia, alcohol intoxication, and benzodiazepine use. In terms of hypoglycemia (low blood sugar), the patient's glucose levels are indeed very low, which could contribute to his unresponsiveness and confusion. The significant blood alcohol concentration may explain his altered mental status, and the benzodiazepine use could also contribute to his current state. We should also examine the possibility of Wernicke's encephalopathy, a condition caused by thiamine (Vitamin B1) deficiency commonly seen in chronic alcoholics, which is characterized by confusion, ataxia, and ophthalmoplegia. CLINICAL REASONING: Let's examine each alternative treatment option one by one. 50% dextrose could be a treatment option if the patient's symptoms were solely due to hypoglycemia. However, his condition doesn't seem to be strictly due to low blood sugar as he also has a significant blood alcohol concentration and evidence of benzodiazepine use. Flumazenil is a benzodiazepine antagonist and could reverse the effects of benzodiazepines but is typically reserved for cases of benzodiazepine overdose and not indicated here due to the risk of precipitating seizures in a chronic alcohol abuser. Lorazepam, a benzodiazepine, is not indicated given the patient's positive urine toxicology for benzodiazepines and his clinical picture. Naloxone is an opioid antagonist used in opioid overdoses, which is not the case here. Finally, thiamine (Vitamin B1) is typically given to alcoholics before glucose administration, as glucose administration in a thiamine-deficient state can precipitate Wernicke's encephalopathy. CONCLUDING: Though the 50% dextrose is essential to correct the patient's hypoglycemia, it's not the most appropriate first step in this patient's management. It's critical to precede the administration of dextrose with thiamine in a suspected alcoholic to prevent worsening of thiamine deficiency, thereby preventing the precipitation of Wernicke's encephalopathy. RESPONSE: Accordingly, the most appropriate next step in the management of this patient is administration of Vitamin B1 (thiamine) (Choice E).

**AI_A:** RESTATING THE QUESTION: The scenario presents a 55-year-old man who was found unresponsive and is confused upon arrival at the emergency department. He has a history of major depressive disorder, hypertension, and alcoholism, and is on chlordiazepoxide, fluoxetine, and lisinopril. His vital signs and physical exam are significant for hypothermia and low blood pressure, but no signs of trauma or prolonged immobilization. His speech is nonsensical and he's disoriented to place and time. Blood tests show hypoglycemia and a high blood alcohol concentration; urine test is positive for alcohol and benzodiazepines. The question asks for the most appropriate next step in our management plan considering the given patient conditions and drug options, namely, 50% Dextrose, Flumazenil, Lorazepam, Naloxone, and Vitamin B1. PLANNING: Firstly, it's imperative for us to observe that the patient is overall hypothermic, hypoglycemic (serum glucose concentration is critically low at 44 mg/dL), and under the influence of both alcohol and benzodiazepines. The apparent confusion and disorientation could result from any of these conditions separately or in combination. His ongoing medications chlordiazepoxide (a benzodiazepine) and fluoxetine (an antidepressant) might influence his mental status. Despite a high blood alcohol concentration, the absence of violent behavior or withdrawal symptoms reduces the chance of alcohol withdrawal being a major issue at this moment. CLINICAL REASONING: Here, among the given choices, 50% Dextrose and Vitamin B1(thiamine) are the two frontrunners. Dextrose is a quick remedy for hypoglycemia and Thiamine prevents Wernicke's encephalopathy in chronic alcoholics. Still, the best order to administer them is crucial. A rapid correction of hypoglycemia with Dextrose in a chronically alcohol-dependent individual before Thiamine supplementation is a well-established risk factor for precipitating Wernicke's encephalopathy, a condition characterized by confusion, ophthalmoplegia, and ataxia due to thiamine depletion. Considering this scenario, prioritizing thiamine supplementation becomes the safe clinical approach. CONCLUDING: In summary, it is critical to prioritize patient safety and effective linking of clinical knowledge and reasoning. A comprehensive understanding of the risks and benefits attached to each medication under consideration is crucial to ensure that our approach optimally benefits the patient and mitigates as much risk as possible. RESPONSE: Given all the above, the most appropriate next step in the management of this patient would be the administration of Vitamin B1 (thiamine) (Option E). The Hallmark triad of Wernicke's Encephalopathy—confusion, ophthalmoplegia, and ataxia—possesses an overlap with our patient's presentation. Giving thiamine before dextrose can help prevent exacerbating or precipitating Wernicke's Encephalopathy. After administration of thiamine, which rapidly corrects possible thiamine deficiency, the patient's hypoglycemia should be corrected with dextrose.

**Facilitator** The consensus statement synthesizes the responses as follows: The question presents a 55-year-old man with a history of major depressive disorder, hypertension, and alcoholism, who was found unresponsive and is now confused and disoriented. His lab results indicate hypoglycemia, high blood alcohol concentration, and benzodiazepine positivity. The question asks for the most suitable immediate treatment for this patient from the given choices, which include 50% dextrose, flumazenil, lorazepam, naloxone, and vitamin B1 (thiamine). The patient's confusion and disorientation could be due to his high blood alcohol concentration, hypoglycemia, or benzodiazepine ingestion. However, because the patient is a chronic alcoholic, he might also be prone to Wernicke's encephalopathy, a condition caused by vitamin B1 (thiamine) deficiency. This condition is characterized by confusion, ataxia, and nystagmus, which aligns with the presenting symptoms of this patient. While reversing hypoglycemia with 50% dextrose is essential in managing any patient with significantly low glucose, this patient's alcohol level and probable benzodiazepine dependence cannot be overlooked. Flumazenil could potentially reverse the effects of benzodiazepine overdose, but it's contraindicated in long-term benzodiazepine users due to the risk of precipitating seizures. Lorazepam would not be useful in this scenario, as the patient already exhibits signs of benzodiazepine intoxication. Naloxone is used for reversing opioid overdose, not alcohol or benzodiazepine toxicity. Therefore, the most appropriate next step in the management of this patient would be the administration of vitamin B1 (thiamine). Thiamine is crucial in carbohydrate metabolism. A rapid infusion of dextrose without first administering thiamine could precipitate Wernicke's encephalopathy in a malnourished, chronic alcoholic patient. Wernicke's encephalopathy presents with confusion, oculomotor dysfunction, and ataxia, all of which can be consequently worsened in a patient with acute alcohol intoxication. Therefore, thiamine should be administered before dextrose, to prevent the onset of Wernicke's encephalopathy in alcohol-dependent patients.
