# Supplementary material for: Collaborative intelligence in AI: Evaluating the performance of a council of AIs on the USMLE
Source: PLOS Digit Health. 2025 Oct 9;4(10):e0000787. doi: 10.1371/journal.pdig.0000787 (PMC12510544; doi:10.1371/journal.pdig.0000787)
Supplement: S2 File — (ZIP) [file pdig.0000787.s002.zip › S2 Data Raw data files containing unprocessed question inputs and LLM outputs including transcripts of deliberations/STEP2_Question-24-1_Type-MC-NJ.docx]

**Facilitator** **[Round 1]:** A 16-year-old boy is brought to the emergency department because of a 6-hour history of severe right-sided scrotal pain. He has not had penile discharge, fever, abdominal pain, vomiting, or diarrhea. He has no history of similar pain or any trauma to the region. He is sexually active with one female partner and uses condoms inconsistently. Vital signs are within normal limits. Abdominal examination shows no abnormalities. Genitourinary examination shows moderate edema and diffuse tenderness to palpation of the right scrotum; there is mild erythema. Lifting the right testicle does not relieve the pain. The left testicle is lower than the right testicle. Cremasteric reflex is present on the left and absent on the right. Which of the following is the most likely diagnosis? (A) Epididymitis (B) Testicular torsion (C) Testicular tumor (D) Torsion of the appendix testis (E) Urinary tract infection (F) Varicocele

**AI_D:** RESTATING THE QUESTION: The patient, a 16-year-old male, presents with severe pain in his right scrotum persisting for the last 6 hours. Despite not experiencing any discharge, fever, abdominal discomfort, vomiting, or diarrhea, he is showing symptoms of moderate edema, and diffuse tenderness in his right scrotum with mild erythema. Notably, manipulation of the right testicle does not alleviate the pain, and it's observed to be higher compared to the left one. The cremasteric reflex is absent on the right, but present on the left. PLANNING: Based on the symptoms presented, possible diagnoses could be epididymitis, testicular torsion, a testicular tumor, torsion of the appendix testis, urinary tract infection, or varicocele. CLINICAL REASONING: The patient's symptoms, such as the abrupt onset of severe testicular pain, testicle at a higher position, and absent cremasteric reflex on the painful side, closely match the typical clinical picture of testicular torsion. This condition results from the twisting of the spermatic cord, leading to ischemia. The Prehn sign, pain relief upon lifting the testicle, is not seen in this case. Epididymitis, although it can cause scrotal edema and pain, typically presents with an elevated scrotum rather than ascending, urinary symptoms or systemic signs of infection, which this patient lacks. Similarly, testicular tumors are usually asymptomatic and discovered as a painless testicular mass, which is inconsistent with the acute pain in this case. Torsion of the appendix testis typically presents with "blue dot" sign, which is absent in this case. Urinary tract infections generally have systemic symptoms such as fever and dysuria, and varicoceles aren't typically painful unless there's acute thrombosis. CONCLUDING: After considering the clinical manifestations, their alignment with the characteristic features of the potential diagnoses, and the absence of some specific signs for the alternative diagnoses, it is inferred that testicular torsion is the most likely diagnosis in this case. RESPONSE: The most likely diagnosis is (B) Testicular Torsion.

**AI_B:** RESTATING THE QUESTION: A 16-year-old boy with severe right-sided scrotal pain for six hours is being evaluated. His medical history, symptoms, and clinical signs do not suggest any systemic or infectious condition, trauma or penile pathology. His sexual activity also does not seem to relate directly to his current condition. However, his genitourinary examination shows swelling and diffuse pain on palpation of the right scrotum with mild skin redness, and absence of right-sided cremasteric reflex - a reflex that pulls up the testicles when the inner part of upper thigh is stroked. Notably, the right testicle is at a higher position compared to the left. The question now is, what is the most likely diagnosis considering these facts? PLANNING: To come up with the correct diagnosis, we will focus on the findings from the genitourinary examination. We will have to consider the significance of the following features: severe pain, edema and erythema of the right scotum, high-riding right testicle, the absence of right-sided cremasteric reflex, and absence of relief upon lifting the right testicle. These conditions might suggest possible torsional, inflammatory, neoplastic, or vascular disorders of the right testicle. CLINICAL REASONING: Severe scrotal pain with edema and erythema could initially suggest an inflammatory or infectious process, such as epididymitis or a urinary tract infection. However, the absence of systemic signs like fever, penile discharge, or relevant history reduce the likelihood of an infection. Testicular tumors usually do not present with such acute onset of severe pain and are often painless, so this option can also be ruled out. Varicocele, a swelling of the veins that drain the testicle, is usually associated with left-sided scrotal pain or discomfort, and it commonly presents as a lower-hanging, 'bag-of-worms' feeling on examination, which doesn't match this case. Torsion of the appendix testis can present similar symptoms including abrupt-onset pain, but it is generally less severe, and often associated with 'blue-dot' sign on physical examination, which is not mentioned here. The absence of the right-sided cremasteric reflex and the high-riding position of the right testicle, along with severe pain unrelieved by lifting the testicle (negative Prehn’s sign), point towards a specific condition - testicular torsion, a sudden rotational twisting of the testicle that cuts off its blood flow. This condition is known for its acuity, severity of pain, and loss of cremasteric reflex. CONCLUDING: Putting together all the restated facts, symptoms, clinical findings, and reasoning based on medical knowledge, we can deduce that this case is highly suggestive of testicular torsion. RESPONSE: (B) Testicular torsion is most likely the correct diagnosis for this 16-year-old boy.

**AI_E:** RESTATING THE QUESTION: The case describes a 16-year-old male with severe, right-sided scrotal pain lasting for 6 hours. The patient doesn't evidence any typical urinary symptoms like penile discharge, fever, abdominal pain, vomiting, or diarrhea. It's also clear that there's no history of trauma to the region. The patient is sexually active, but uses condoms inconsistively. His vital signs are normal. Upon physical examination, there's noticed edema and tenderness in the right scrotum with mild erythema; lifting the right testicle doesn't alleviate the pain. His left testicle is lower than the right one. His cremasteric reflex is intact on the left but not on the right. Based on these findings, we need to determine the most likely diagnosis. The options are epididymitis, testicular torsion, testicular tumor, torsion of the appendix testis, urinary tract infection, and varicocele. PLANNING: The likely diagnoses for severe scrotal pain in this age group could be situations involving the testes or epididymis directly (such as testicular torsion or epididymitis) or related to other structures in the scrotum (such as torsion of the appendix testis). Non-testicular causes of scrotal pain like varicocele and urinary tract infection also merit consideration, but these are typically accompanied by other symptoms, making them less likely. Testicular tumor would also usually present with a mass rather than acute pain. These assumptions need verification through the patient's clinical presentation and examination details. CLINICAL REASONING: Although different conditions can cause scrotal pain, the pain's acute onset, combined with the elevated position of the testicle and absent cremasteric reflex on the right side, suggest a severe, surgical emergency such as testicular torsion. Testicular torsion is caused by the twisting of the spermatic cord, which cuts off the blood supply to the testicle. The severe pain is due to ischemia. The high position of the right testicle (termed "bell-clapper" deformity) and the absence of the ipsilateral cremasteric reflex are common signs associated with this diagnosis. CONCLUDING: Given the severity, sudden onset of symptoms, physical findings suggestive of visceral involvement (acute testicular pain, high-riding testicle, absent cremasteric reflex), the working diagnosis would be a testicular torsion, which requires immediate surgical intervention to restore blood flow and prevent testicular necrosis. RESPONSE: Therefore, option (B) Testicular torsion is the most likely diagnosis in this scenario.

**AI_C:** 1) RESTATING THE QUESTION: The question pertains to diagnosing a 16-year-old male patient who has been experiencing intense right-sided scrotal pain for the past 6 hours. While his vital signs remain normal, physical examination reveals moderate edema, diffuse tenderness and mild erythema of the right scrotum. Additional significant findings include the higher placement of the right testicle when compared to the left, and an absent Cremasteric reflex on the right. The Cremasteric reflex is a superficial reflex where upward movement of the testicle in males is observed in response to stroking of the inner thigh. It, along with other symptoms, can aid in diagnosing the underlying health complaint. 2) PLANNING: Several potential diagnoses arise on examining the patient's sexual history, overall health, and physical symptoms. These include Epididymitis, Testicular torsion, Testicular tumor, Torsion of the appendix testis, Urinary tract infection, and Varicocele. Each possible condition will be evaluated by its probability given the symptoms reported. 3) CLINICAL REASONING: The significant factors hinting towards 'Testicular torsion' include the severe, acute onset of pain, increased edema and tenderness of the right testicle, along with the high-riding of the testicle and absent Cremasteric reflex on the affected side. Epididymitis could generally cause similar symptoms but it is usually associated with urinary symptoms and the scrotal pain is relieved by elevating the testicle (Prehn's sign), which is not the case here. In the case of a testicular tumor, the patient usually presents with a painless testicular mass and systemic symptoms if metastasis occurs, which are not observed in this case. 'Torsion of the appendix testis' could cause acute scrotal pain but it is usually less severe and is associated with a "blue dot sign" (blue discoloration at the top of the testis). Urinary tract infection, although it can cause discomfort, does not typically result in such acute and severe localized scrotal pain without any urinary symptoms. Lastly, a varicocele generally causes unilateral scrotal swelling but it does not typically cause acute, severe pain. 4) CONCLUDING: Therefore, given the acute onset of severe scrotal pain, the increased edema, and tenderness of the scrotum, as well as the high ride of the affected testicle and absent Cremasteric reflex, the most likely diagnosis is 'Testicular Torsion'. This condition is a medical emergency that requires immediate surgical attention as it can lead to testicular necrosis due to the cut-off of blood supply to the testicle. 5) RESPONSE: Based on the detailed clinical analysis and reasoning, (B) Testicular torsion is the most likely diagnosis in this case.

**AI_A:** (1) RESTATING THE QUESTION: We are presented here with a clinical case of a 16-year-old boy who came to the emergency room because he has been experiencing an intense pain in the right part of his scrotum for the past 6 hours. He hasn't manifested any additional symptoms such as discharge from the penis, fever, abdominal pain, vomiting or diarrhea. There's been no past record of similar pain episodes or trauma to the region. Regarding his sexual behavior, he has one female partner and does not always use condoms. His vital signs are normal. The physical examination hasn't found anything wrong with his abdomen, but his genitourinary exam revealed moderate swelling and general pain when touching the right scrotum, as well as a slight redness. When the right testicle was lifted, it did not alleviate his pain. The left testicle is positioned lower than the right one. The cremasteric reflex is normal on the left but not observable on the right side of his body. We are asked to identify the most probable diagnosis from the following options: Epididymitis, Testicular torsion, Testicular tumor, Torsion of the appendix testis, Urinary tract infection, or Varicocele. (2) PLANNING: To reach a conclusion, we need to first weigh the information provided. The boy's age, the acute onset of severe unilateral scrotal pain, the duration of the symptoms, lack of relief with testicle elevation (negative Prehn's sign), and the absence of right-sided cremasteric reflex in the context of an otherwise normal physical examination, all together indicate a plausible condition affecting the testis structure rather than an infection or systemic disease. (3) CLINICAL REASONING: In the context of this clinical scenario, the prominent symptoms of scrotal pain, edema and tissue erythema are common features of acute epididymitis, testicular torsion, and torsion of the appendix testis. However, the absence of fever, urinary symptoms, and normal testicular elevation (Prehn's sign) make epididymitis less likely, as this condition usually presents with these symptoms along with a positive Prehn's sign. Another differential diagnosis to consider is a testicular tumor, but this normally presents as a painless scrotal mass and is therefore also unlikely. The absence of urinary tract symptoms makes urinary tract infection (UTI) improbable, and varicocele usually presents with a "bag of worms" appearance and is often asymptomatic, which does not align with our case here. (4) CONCLUDING: What mostly fits the clinical picture here is a manifestation of testicular torsion. Testicular torsion presents with a sudden onset of severe unilateral testicular pain, often with associated nausea and vomiting, as the testicular blood supply is compromised by the twisting of the spermatic cord. An absent cremasteric reflex (which is a reflexive raising of the testicle upon stroking the upper inner thigh) can be a sign of testicular torsion and is a clinical emergency to prevent testicular necrosis. (5) RESPONSE: Therefore, considering the symptomatology and the patient’s age, the most likely diagnosis in this case is (B) Testicular torsion. An urgent surgical intervention (detorsion and orchidopexy) might be necessary to save the affected testicle. Further diagnostic investigations such as ultrasound with Doppler might give additional confirmatory information. Despite the patient's sexual activity, his use of condoms, even if inconsistently, and the lack of typical symptoms, make Sexually Transmitted Infections less likely, helping us to focus on structural causes like testicular torsion.

**Facilitator** The consensus statement synthesizes the responses of AI_A, AI_B, AI_C, AI_D, and AI_E as follows: The case presents a 16-year-old male experiencing severe right-sided scrotal pain for the past 6 hours. His vital signs are normal, and he has no additional symptoms such as penile discharge, fever, abdominal pain, vomiting, or diarrhea. The patient's sexual history reveals inconsistent condom use with one female partner. Physical examination reveals moderate edema, diffuse tenderness, and mild erythema of the right scrotum. The right testicle is positioned higher than the left, and lifting it does not alleviate the pain. The cremasteric reflex is absent on the right side but present on the left. Given these symptoms and findings, the possible diagnoses include epididymitis, testicular torsion, testicular tumor, torsion of the appendix testis, urinary tract infection, and varicocele. However, the acute onset of severe pain, the high-riding position of the right testicle, and the absence of the right-sided cremasteric reflex strongly suggest testicular torsion. This condition, caused by the twisting of the spermatic cord, leads to ischemia and severe pain due to the cut-off of blood supply to the testicle. Other conditions such as epididymitis, testicular tumor, torsion of the appendix testis, urinary tract infection, and varicocele are less likely given the absence of their typical symptoms or signs in this case. For instance, epididymitis usually presents with urinary symptoms and relief of pain with testicle elevation (Prehn's sign), which are not observed here. Testicular tumors are often asymptomatic and present as a painless mass, which is inconsistent with the acute pain in this case. Torsion of the appendix testis typically presents with a "blue dot sign", which is not mentioned here. Urinary tract infections generally have systemic symptoms such as fever and dysuria, and varicoceles aren't typically painful unless there's acute thrombosis. Therefore, the consensus among the AI responses is that the most likely diagnosis for this patient is (B) Testicular torsion. This condition is a medical emergency that requires immediate surgical intervention to prevent testicular necrosis. Further diagnostic investigations such as ultrasound with Doppler might provide additional confirmatory information.
