# Supplementary material for: Collaborative intelligence in AI: Evaluating the performance of a council of AIs on the USMLE
Source: PLOS Digit Health. 2025 Oct 9;4(10):e0000787. doi: 10.1371/journal.pdig.0000787 (PMC12510544; doi:10.1371/journal.pdig.0000787)
Supplement: S2 File — (ZIP) [file pdig.0000787.s002.zip › S2 Data Raw data files containing unprocessed question inputs and LLM outputs including transcripts of deliberations/STEP2_Question-27-1_Type-MC-NJ.docx]

**Facilitator** **[Round 1]:** A 64-year-old man comes to the clinic because of a 2-week history of severe pain and generalized muscle weakness. He has hypercholesteremia and hypertension. Three months ago, he had a myocardial infarction. At that time, he began atorvastatin therapy. Other medications are clopidogrel, lisinopril, and amiodarone. He is 178 cm (5 ft 10 in) tall and weighs 82 kg (180 lb); BMI is 26 kg/m² Pulse is 80/min and blood pressure is 125/67 mm Hg. Muscle strength is 4/5 on abduction of the shoulders and flexion of the hips. Deep tendon reflexes are 2+ throughout. No other abnormalities are noted. Measurement of which of the following is the most appropriate initial step in diagnosis? (A) Erythrocyte sedimentation rate (B) Serum ALT activity (C) Serum calcium concentration (D) Serum C-reactive protein concentration (E) Serum creatine kinase activity

**AI_A:** RESTATING THE QUESTION: To dissect the given question, we are presented with a 64-year-old male patient who has chronic conditions including hypertension and hypercholesteremia. He has recently suffered from myocardial infarction for which he has been placed on statin therapy among other treatments. He presents us with severe pain and muscle weakness. Some possible diagnoses come to mind immediately; however, determining which biochemical parameters we should measure initially to diagnose his condition is the focus. The options are checking Erythrocyte sedimentation rate (ESR), Serum ALT activity, Serum calcium concentration, Serum C-reactive protein concentration (CRP), and Serum creatine kinase (CK) activity. PLANNING: Assumptions could be drawn from the patient's recent change in medication (statin) and new findings of muscle weakness with pain, even after accounting for his primary illnesses (hypertension, hypercholesteremia). An alternate assumption might be that the statin therapy could be linked to this new onset of symptoms. Evaluating this, given his history and the known adverse effects of statins, could be of clinical priority. CLINICAL REASONING: Statin therapy, specifically atorvastatin, was started after the patient's myocardial infarction. This drug class is known for reducing cholesterol levels in the blood, which helps in preventing heart disease and stroke. However, they can occasionally have side effects including muscle-related complications - statin-associated muscle symptoms (SAMS), in particular. These can manifest as muscle pain and weakness. CONCLUDING: In light of the patient's history and the known side effects of statins, it would seem clinically prudent to investigate a potential link between the statins and the new-onset muscle problems. By assessing the level of serum creatine kinase, a muscle enzyme that becomes elevated in cases of muscle damage (including statin-induced), we could establish a diagnosis or guide further investigations. RESPONSE: So, in order to initiate the diagnosis process for this patient's symptoms, the most appropriate step would be to measure the Serum creatine kinase activity (E).

**AI_C:** RESTATING THE QUESTION: Given the scenario presented, an elderly man has visited the clinic experiencing severe pain and troubling muscle weakness for two weeks. He suffers from high cholesterol and high blood pressure and suffered a myocardial infarction three months ago. Following the myocardial infarction, he was put on atorvastatin, and his other medications include clopidogrel, lisinopril, and amiodarone. We are looking to identify what would be the most fitting initial diagnostic step to gain a clearer understanding of his condition. The options provided include measures of inflammation (Erythrocyte sedimentation rate and C-reactive protein concentration), liver function (ALT activity), electrolyte imbalance (Serum calcium concentration), and muscle damage (Creatine kinase activity). PLANNING: The primary assumption might be that the patient's symptoms are a reaction to one of his many medications. A secondary assumption might be that the patient has developed an unrelated condition that is causing these new symptoms. From these assumptions, priority must be given to the assumption that suggests a reaction to medication since the patient's condition developed soon after new pharmacological treatments were introduced. Atorvastatin, a cholesterol-lowering medication, can potentially cause muscle pain and weakness - a condition known as statin-induced myopathy. CLINICAL REASONING: The symptoms of severe pain and generalized muscle weakness that the man is experiencing, in conjunction with his history of beginning atorvastatin therapy, point toward a likely case of statin-induced myopathy. Atorvastatin, which is a statin, is known to potentially cause this condition. Symptoms of this condition include muscle pain, tenderness, or weakness. These symptoms may develop anytime during therapy but will usually appear after initiation of therapy. Statin-induced myopathy is diagnosed by an elevation in serum creatine kinase (CK), an enzyme that is released into the bloodstream when muscle tissue is damaged. CONCLUDING: Given the possible link between atorvastatin use and muscle symptoms, checking for signs of muscle damage is a sensible step. Creatine kinase (CK) levels would be a relevant and appropriate choice for an initial diagnostic step, given CK’s role in detecting muscle damage. Higher than usual CK levels could indicate a muscle disorder like statin-induced myopathy. RESPONSE: Therefore, in this case, the most appropriate initial step for diagnosis would be the measurement of serum creatine kinase activity (E).

**AI_E:** 1) RESTATING THE QUESTION: The query presents the case of a 64-year-old male patient who is complaining of severe pain and muscle weakness that started two weeks ago. He has a known medical history of hypercholesteremia and hypertension, along with a recent myocardial infarction three months prior. He is currently on atorvastatin, clopidogrel, lisinopril, and amiodarone. The physical examination indicates 4/5 muscle strength with shoulder abduction and hip flexion, and 2+ deep tendon reflexes throughout. The question seeks the most suitable diagnostic measurement to take initially. 2) PLANNING: The medical history indicates hypercholesteremia for which atorvastatin, a statin drug, was prescribed. From this arises the assumption that the patient's muscle weakness and pain could potentially be related to the atorvastatin use, as this drug has known side effects including myopathy (muscle weakness) and myalgias (muscle pain). Another assumption could be that another disease process, such as infection or autoimmune disease, might be causing his symptoms. Some assumptions like hypercalcemia might be considered, but based on the symptom's onset following atorvastatin therapy, statin-associated muscle symptoms (SAMS) is the most likely cause. 3) CLINICAL REASONING: Statins are commonly prescribed to manage high cholesterol levels. They work by inhibiting the enzyme HMG-CoA reductase, which plays a vital role in cholesterol synthesis in the liver. Though generally well-tolerated, statins have been associated with side effects such as muscle symptoms (myalgias, weakness), which is consistent with the patient's symptoms. Other causes need to be considered, but their likelihood may be low due to the temporal relation between the initiation of statin therapy and the onset of these symptoms. 4) CONCLUDING: Considering the clinical indications of muscle weakness and pain described, the potential association with atorvastatin use, and the patient's overall medical history, the most plausible initial diagnostic measurement is to check for an enzyme which would indicate muscle breakdown — creatine kinase. 5) RESPONSE: Therefore, the most appropriate initial step in diagnosis for this patient would be to test for 'Serum creatine kinase activity' (Choice E). This will help determine if the patient's muscle weakness and pain are resulting from a myopathic process related to his use of statins.

**AI_B:** (1) RESTATING THE QUESTION: The question describes a case of a 64-year-old man presenting with severe pain and generalized muscle weakness for two weeks. He has histories of hypercholesteremia, hypertension, and myocardial infarction. His medications include atorvastatin, clopidogrel, lisinopril, and amiodarone. The key question is which diagnostic test should be performed first to identify the cause of his symptoms. (2) PLANNING: There are different assumptions that can be made from the given information: (i) The patient's symptoms may be a side effect from one of his medications. Since he commenced taking atorvastatin around the time the symptoms began, the muscle pain and weakness may be related to a condition known as statin-induced myopathy. (ii) Alternatively, considering his cardiovascular history, his symptoms may stem from a new or progressing cardiovascular issue such as congestive heart failure. (iii) Other possibilities could be erythrocyte sedimentation rate (ESR) to rule out inflammatory diseases, Serum ALT could suggest liver diseases, Serum Calcium could suggest an electrolyte disturbance, Serum CRP could suggest inflammation, and lastly, Serum creatine kinase could suggest muscle damage. Considering the timing of symptom onset with the initiation of atorvastatin, it seems most probable that the patient might be facing a side effect of this medication, making assumption (i) the immediate priority to address. (3) CLINICAL REASONING: Atorvastatin belongs to a class of drugs called statins, which are commonly used to treat hypercholesteremia. A known adverse effect of statins is myopathy, which can manifest as muscle pain and weakness. While this class of drug is typically well-tolerated, in rare cases, it can cause potentially severe muscle damage known as rhabdomyolysis. In this condition, muscle tissue breaks down rapidly, leading to muscle pain, weakness, and the release of muscle cell contents into the bloodstream. The measurement of serum creatine kinase (CK) would be the most appropriate initial step in diagnosis, as it is elevated in conditions of muscle damage such as rhabdomyolysis, and it is associated with statin-induced myopathy. (4) CONCLUDING: In summary, given the patient's recent start of atorvastatin therapy followed by the appearance of muscle pain and weakness, it would be prudent to examine for a potential statin-induced myopathy. The initial test to undertake in this scenario would be to measure the patient's serum creatine kinase levels, as it would help ascertain any muscle damage which is a key characteristic of this condition. (5) RESPONSE: Therefore, in the current scenario, the most appropriate initial diagnostic step would be to measure the patient's Serum creatine kinase activity (E).

**AI_D:** (1) RESTATING THE QUESTION: The scenario presents a 64-year-old male with hypercholesteremia (high cholesterol levels) and hypertension (high blood pressure), who 3 months ago experienced a myocardial infarction (heart attack). After his heart attack, he began using atorvastatin (a medication used to lower cholesterol levels), as well as clopidogrel (an antiplatelet drug), lisinopril (an angiotensin converting enzyme inhibitor that treats high blood pressure), and amiodarone (an antiarrhythmic drug). Now, he has come to the clinic with a 2-week history of severe pain and widespread muscle weakness. He has 4/5 muscle strength during shoulder abduction and hip flexion, and no other abnormalities have been found. The question is asking which measurement would be the most suitable first step in diagnosing his current condition. (2) PLANNING: An implicit assumption in the question is that the patient's current symptoms are connected to his recent medical history or the medications that he's been taking, particularly atorvastatin, which is known to cause myopathy (muscle inflammation leading to pain and weakness) as a side effect. Alternate assumptions could be that his symptoms are due to a systemic inflammatory condition (which could be suggested by an increased erythrocyte sedimentation rate or C-reactive protein), a liver condition (suggested by increased serum ALT activity), or hypercalcemia (excess calcium in the blood, suggested by an elevated serum calcium concentration). However, given that the patient's symptoms are consistent with myopathy and his exposure to atorvastatin, the assumption that myopathy due to atorvastatin is the cause of the patient's pain and weakness is the most clinically relevant one to pursue. (3) CLINICAL REASONING: After assessing the patient’s medical history, medications, and presenting symptoms, the next step in the diagnostic process would be to confirm the hypothesis of myopathy due to atorvastatin. Clinical reasoning suggests using a laboratory test that would evaluate muscle damage. An increase in the serum creatine kinase (CK) level is often the initial laboratory sign of muscle damage as CK is an enzyme found primarily in skeletal muscle, cardiac muscle, and the brain and can be released into the bloodstream upon the damage of any of these tissues. As such, a serum CK concentration measurement becomes a rational next step in the diagnostic workup of the patient. It's also worth noting that other lab measurements laid out as options do not directly relate to muscle damage. (4) CONCLUDING: After evaluating background and initial clinical findings, and considering the known side effects of atorvastatin, an assessment of serum creatine kinase activity offers a beneficial first step in the diagnostic approach to this patient. This would verify if there's ongoing muscle damage that might be consistent with atorvastatin-induced myopathy. (5) RESPONSE: Serum creatine kinase activity (Option E) represents the most suitable initial diagnostic step to be executed in this patient's case, in light of his medical history, symptoms, and medication use.

**Facilitator** The consensus statement synthesizes the responses of AI_A, AI_B, AI_C, AI_D, and AI_E as follows: The question presents a 64-year-old male patient with a history of hypercholesteremia, hypertension, and a recent myocardial infarction. He has been on atorvastatin therapy since his myocardial infarction and is currently experiencing severe pain and generalized muscle weakness. The key question is to identify the most appropriate initial diagnostic step to understand his condition. Given the patient's medical history and the timing of the onset of his symptoms, the primary assumption is that the patient's symptoms could be a side effect of his atorvastatin medication, specifically statin-induced myopathy. This condition is a known adverse effect of statins and can manifest as muscle pain and weakness. Other assumptions, such as a systemic inflammatory condition, liver disease, or electrolyte imbalance, are considered less likely due to the temporal relation between the initiation of statin therapy and the onset of these symptoms. In light of the patient's history and the known side effects of statins, it would seem clinically prudent to investigate a potential link between the statins and the new-onset muscle problems. The most appropriate initial diagnostic step would be to measure the patient's serum creatine kinase (CK) activity. CK is an enzyme that becomes elevated in cases of muscle damage, including statin-induced myopathy. Therefore, an elevated CK level could indicate a muscle disorder like statin-induced myopathy and guide further investigations. In conclusion, given the patient's recent start of atorvastatin therapy followed by the appearance of muscle pain and weakness, it would be prudent to examine for a potential statin-induced myopathy. The initial test to undertake in this scenario would be to measure the patient's serum creatine kinase levels. This would help ascertain any muscle damage which is a key characteristic of this condition. Therefore, in this case, the most appropriate initial step for diagnosis would be the measurement of serum creatine kinase activity (E).
