# Supplementary material for: Collaborative intelligence in AI: Evaluating the performance of a council of AIs on the USMLE
Source: PLOS Digit Health. 2025 Oct 9;4(10):e0000787. doi: 10.1371/journal.pdig.0000787 (PMC12510544; doi:10.1371/journal.pdig.0000787)
Supplement: S2 File — (ZIP) [file pdig.0000787.s002.zip › S2 Data Raw data files containing unprocessed question inputs and LLM outputs including transcripts of deliberations/STEP2_Question-28-1_Type-MC-NJ.docx]

**Facilitator** **[Round 1]:** A 23-year-old man who is on active duty in the US Army comes to the clinic 1 hour after sustaining a bee sting to his right cheek. The patient reports continued pain at the site of the sting, but he has not had shortness of breath, throat tightness, loss of vision, or other neurologic symptoms. Medical history is unremarkable. He takes no medications. The patient has no known allergies. He is 178 cm (5 ft 10 in) tall and weighs 64 kg (140 lb); BMI is 20 kg/m². Temperature is 37.0°C (98.6°F), pulse is 86/min, respirations are 10/min, and blood pressure is 120/70 mm Hg. Examination of the right cheek shows an erythematous, swollen area of induration surrounding a central clear area with the stinger in situ. The swelling encroaches on the lower aspect of the patient's right orbit, which appears puffy and mildly dusky. After the stinger is removed, which of the following is the most appropriate next step in management? (A) Administer an injection of epinephrine (B) Admit the patient to the hospital for intravenous vancomycin therapy (C) Cleanse the site and apply ice (D) Obtain an ophthalmologist consultation (E) Prescribe oral clindamycin

**AI_C:** RESTATING THE QUESTION: Here is a case of a 23-year-old military personnel who suffered a bee sting on his right cheek. Though the sting remains, he has not had systemic or neurologic symptoms. He has no known allergies, takes no medications, and his physical examination is largely normal save for an erythematous, swollen area around the sting site on his cheek, near his right orbit. Once the stinger has been removed, we are asked to determine the most appropriate next step in management with options including administering epinephrine, hospitalizing for IV vancomycin, cleansing the wound and applying ice, consulting an ophthalmologist, or prescribing oral clindamycin. PLANNING: We know that bee stings can cause local reactions, allergic reactions, or sometimes lead to infection. Local reactions are characterized by pain and swelling at the sting site, allergic reactions would present with systemic signs such as difficulty breathing, hypotension, or even anaphylaxis, and infection would have signs of pus, heat and systemic illness. Here, the patient's symptoms are localized, with no evidence of systemic allergic reaction or infection. CLINICAL REASONING: Given this information, we can eliminate the options of administering epinephrine (reserved for anaphylactic reactions), admitting the patient for IV vancomycin (a powerful antibiotic used for severe infections), and prescribing oral clindamycin (another antibiotic, unnecessary without signs of infection). The option of obtaining an ophthalmologist consultation might be reasonable if the swelling was compromising the patient's vision or had other optic manifestations, but the vignette does not indicate this. CONCLUDING: Therefore, the remaining and most appropriate option would be to manage the local reaction -- i.e. to cleanse the sting site and apply ice to reduce swelling and pain. RESPONSE: The most appropriate next step in management, after removing the bee stinger, is to cleanse the sting site and apply ice (C).

**AI_E:** RESTATING THE QUESTION: The inquiry pertains to the most appropriate immediate management action after removal of a bee stinger from a patient's cheek. The patient's symptoms include localized pain, erythema, and swelling of the sting site but with no signs or symptoms suggesting anaphylaxis or other systemic reactions. It's important to point out that anaphylaxis would typically present with symptoms such as shortness of breath, throat tightness, or even loss of vision. PLANNING: Before making a decision on the most suitable next course of action, it is paramount to consider various assumptions. Is the reaction local or systemic? Is the patient allergic to bee stings? Is there a chance of an infection setting in from the sting? What could be the implication of the swelling near the eye orbit? After considering these, my inclination is to address the first assumption: the reaction type. Localization of the symptoms suggests a localized reaction rather than a systemic one that would be indicative of an allergic response. CLINICAL REASONING: As our patient doesn't manifest any signs of systemic allergic reaction such as shortness of breath, rapid heartbeat, difficulty swallowing, swelling of the throat, hives, or nausea, it is likely that he's experiencing a localized reaction to the bee sting. Typical local reactions present as pain, redness, and swelling at the site of the sting, which are all present in our patient. Swelling encroaching on the lower aspect of the patient's right orbit is likely part of the local reaction due to fluid accumulation and inflammation. The vicinity to the eye may have resulted in a more noticeable swelling but doesn't necessarily imply an eye injury. CONCLUDING: Step-by-step reasoning brings me to the conclusion that the patient needs symptomatic relief and local care of the wound to prevent secondary infection and promote healing. Systemic antibiotics are not indicated unless signs of a secondary infection develop. RESPONSE: Therefore, of the choices provided, [(C) Cleanse the site and apply ice] appears to be the most appropriate next step in the management of the patient's condition. Cleansing the site reduces the risk of infection, while applying ice helps to decrease pain and limit local edema. If the swelling were to persist or symptoms of infection, like increased pain, pus formation or increasing redness appear, then further investigation and treatment adjustment would be needed.

**AI_B:** RESTATING THE QUESTION: The question is asking about the best course of treatment for a 23-year-old man who has been stung by a bee on his right cheek. He has continued pain at the sting site but no shortness of breath, throat tightness, vision loss, or other neurological symptoms. His medical history, including allergies, is clear. His physical examinations such as height, weight, BMI, temperature, pulse, respirations, and blood pressure are within normal range. Upon inspection, his right cheek appears inflamed and swollen with the bee stinger still embedded. The swelling is approaching his right orbit. Post stinger removal, what should be the next most appropriate step? PLANNING: The options provided are the administration of epinephrine (an adrenergic agonist used for severe allergic reactions), hospital admission for intravenous vancomycin therapy (an antibiotic used for severe bacterial infections), cleansing the site and applying ice, obtaining an ophthalmologist consultation, or prescribing oral clindamycin (an antibiotic used for bacterial infections). The starting assumption is that this is a simple bee sting and requires basic care, but other assumptions could be severe reactions requiring immediate intervention, or a risk of secondary infection. Analyzing the patient's symptoms, there is no indication of severe allergic reaction or infection currently present. CLINICAL REASONING: With this patient, what's crucial in clinical reasoning is noting the absence of systemic allergic reaction signs such as shortness of breath and throat tightness, despite localized swelling and erythema. Therefore, the management emphasis should be on basic wound care and symptom relief. Moreover, the absence of expanding erythema, warmth, and purulent discharge or abscess rules out active infection requiring antibiotic treatment and hospital admission. CONCLUDING: Considering the absence of alarming symptoms suggesting severe allergic reaction or infection, immediate emergency response such as administration of epinephrine, antibiotics and hospital admission, or referral to an ophthalmologist are not required. The vital signs and the overall systemic health of the patient appear normal. The localized reaction at the site of the sting should be addressed with a simple treatment like cleansing the site to remove any potential allergens or foreign bodies and applying ice to reduce inflammation and pain. RESPONSE: Therefore, the most appropriate next step in managing this patient after removing the bee stinger would be to [C) cleanse the site and apply ice].

**AI_D:** RESTATING THE QUESTION: A 23-year-old military serviceman has been stung by a bee on his right cheek, leading to immediate localized inflammation and pain. Despite the evident swelling intruding upon the lower part of his right eye, there is an absence of systemic symptoms indicating an allergic reaction such as shortness of breath, throat tightness or neurological symptoms. His medical history is unremarkable, he has no known allergies and takes no medication. His vitals are within normal ranges, and physical examination of his right cheek reveals an inflamed area surrounding a clear zone with the bee stinger still in place. Once the stinger is removed, what is the most suitable course of action for managing his condition? PLANNING: The immediate assumption is that the appropriate next step in managing the patient's condition is to control the localized reaction to the sting, which is consistent with the symptoms of a physiological response to insect venom. Another assumption would be to consider the potential of an anaphylactic reaction, which can occur rapidly and be life-threatening, though this patient notably does not report any symptoms of anaphylaxis. The impact of the swelling on the patient's eye could also signify a need for ophthalmological intervention, but this is less likely to be a priority given the localized nature of the swelling and lack of vision loss or other ocular symptoms. Evaluating these assumptions, it is plausible to surmise that the attended inflammation due to the sting should be addressed. CLINICAL REASONING: The patient reports continued localized pain at the site of the sting, but lacks any systemic symptoms such as shortness of breath, throat tightness or neurologic symptoms that could indicate an anaphylactic reaction. This scenario suggests that the patient is suffering from a normal localized reaction to the bee sting. The presence of the bee stinger and venom sac can further cause continuous venom injection and intensify local pain and reaction. Hence, removing the stinger should be the first step in managing such a patient. Following this, the need to assess the build-up of swelling in close proximity to his eye is crucial, but given the lack of ocular symptoms, it may not be immediate. CONCLUDING: Drawing from the clinical reasoning, the bodily response to the bee sting and the fact that the patient doesn't have a systemic allergic reaction, the priority here should be to manage the localized swelling and inflammation instigating from the bee sting. This points toward the requirement of localized treatment methods rather than systemic ones. RESPONSE: Based on the evaluation of this patient’s condition, his symptoms and the promptness of his presentation to the clinic, it is reasonable to conclude that the most appropriate next step in management is to 'Cleanse the site and apply ice' (Choice C). This would help in providing relief from the inflammation and swelling that has occurred due to the bite. It will also manage his pain, reduce further venom absorption and potentially prevent further progression of swelling.

**AI_A:** Let's unpack the scenario. We have a young, otherwise healthy soldier who has presented to the clinic an hour after receiving a bee sting to his right cheek. This has resulted in pain at the site, and an examination of the area shows a red, swollen area surrounding the stinger, which is still inside the skin. Notably, the patient does not exhibit any signs of systemic allergic reaction such as shortness of breath, throat tightness, or other neurological symptoms. In keeping with these findings, the choices given suggest different assumptions about the potential complications and corresponding interventions. The assumptions here vary widely – from considering already present systemic allergic reaction (A), potential bacterial infection (B, E), local inflammation that needs to be managed (C), or the encroachment of swelling that might affect the patient's eye (D). In this context, we need to prioritize understanding the potential clinical impact of a bee sting and direct our actions accordingly. Bee stings can indeed cause a localized, painful reaction often marked by a red, swollen bump - this seems to align with what was observed in the soldier. Systemic allergic reactions (anaphylaxis), which would justify the administration of epinephrine, typically present with symptoms such as difficulty breathing and tightening of the throat, neither of which is mentioned in this case. This makes a systemic allergic reaction highly unlikely to be a primary concern, de-prioritizing Option A. The risk of bacterial infection right after the sting occurs is also typically low, especially in a relatively cleaner facial area and without any mention of signs of infections currently. Therefore, Options B and E seem less likely as immediate next steps as well. Furthermore, the possibility of ocular involvement (as indicated in D) should be considered due to the proximity of the sting site to the orbit. However, the patient in this scenario has not reported any visual disturbances or loss of vision, making an immediate ophthalmologist consultation less compelling. Given the symptoms presented - local redness, induration, and continued pain - it seems the local inflammatory reaction to the sting and its physical remnants (the stinger) are the main issues so far in the immediate time frame. The immediate priority, therefore, is likely to address and manage this local inflammatory response to minimize patient discomfort and potentially avoid any further complications such as exacerbated swelling. Finally, having put all this together, the most suitable next step appears to be to appropriately clean the sting site and apply ice (C). This would help to reduce the local inflammation and discomfort resulting from the venom injected by the bee sting. This step would also arguably be important to cleanse the area and minimize the potential risk of secondary bacterial infection from the environment after the stinger removal. Response: So, (C) Cleanse the site and apply ice is the most appropriate next step in management.

**Facilitator** The consensus among the AI responses is that the most appropriate next step in managing the patient's condition, after removing the bee stinger, is to cleanse the site and apply ice (Option C). This conclusion is based on the patient's symptoms, which include localized pain, erythema, and swelling at the sting site, but no signs of systemic allergic reaction such as shortness of breath, throat tightness, or neurological symptoms. The patient's symptoms align with a localized reaction to the bee sting, rather than a systemic allergic reaction or infection, which would require different interventions. The swelling near the patient's eye is noted, but without any reported visual disturbances or loss of vision, an immediate ophthalmologist consultation is not deemed necessary. Therefore, the priority is to manage the localized inflammation and discomfort resulting from the bee sting. Cleansing the site and applying ice would help reduce the local inflammation and discomfort, cleanse the area to minimize potential risk of secondary bacterial infection, and potentially prevent further progression of swelling.
