# Supplementary material for: Collaborative intelligence in AI: Evaluating the performance of a council of AIs on the USMLE
Source: PLOS Digit Health. 2025 Oct 9;4(10):e0000787. doi: 10.1371/journal.pdig.0000787 (PMC12510544; doi:10.1371/journal.pdig.0000787)
Supplement: S2 File — (ZIP) [file pdig.0000787.s002.zip › S2 Data Raw data files containing unprocessed question inputs and LLM outputs including transcripts of deliberations/STEP2_Question-29-1_Type-MC-NJ.docx]

**Facilitator** **[Round 1]:** A 32-year-old man is brought to the emergency department after being found by his wife in their bedroom with a gun in his hands. His wife reports that the patient “has not been himself” since their son was stillborn 6 months ago. She says that during the past month, he hardly has slept and has not showered or shaved; he sits in the dark and refuses to eat. He began fluoxetine therapy 3 months ago, but his symptoms have continued to worsen. He appears disheveled. He is 175 cm (5 ft 9 in) tall and weighs 52 kg (115 lb); BMI is 17 kg/m². His pulse is 90/min, and blood pressure is 100/60 mm Hg. Physical examination shows decreased skin turgor and dry mucous membranes. When asked about his mood, he says, “I don’t know.” He has a tearful affect. He responds to most questions with one-word answers in a soft voice. He says he hears a voice telling him that he is a failure and says he wants to join his son in heaven. He refuses to answer questions about suicide. After removing all firearms from the patient’s house and admitting him to a psychiatric facility, which of the following is the most appropriate next step in management? (A) Electroconvulsive therapy (B) Imipramine therapy (C) Mirtazapine therapy (D) Sertraline therapy (E) Transcranial magnetic stimulation

**AI_A:** RESTATING THE QUESTION: The question essentially concerns a 32-year-old man displaying symptoms of major depressive disorder with psychotic features, such as loss of interest in daily activities and personal grooming, erratic sleeping and eating habits, and auditory hallucinations. Despite being on fluoxetine, a clinically-approved antidepressant, his symptoms have further escalated, and he has mentioned his longing to be with his deceased son in heaven, suggesting suicidal ideation. Now admitted to a psychiatric facility, the question poses a dilemma on the most suitable treatment to be adopted next. PLANNING: The presented information points towards Major Depressive Disorder, possibly with psychotic features, since the patient has been undergoing depressive mood, changes in lifestyle, and hallucinations. Therapeutic options include electroconvulsive therapy, changing the type of antidepressant (Imipramine, Mirtazapine, Sertraline), or transcranial magnetic stimulation. Keep in mind that many factors determine the most suitable treatment, including severity and subtype of the disorder, the patient's response to initial therapy, and the potential risks and side effects associated with individual treatments. CLINICAL REASONING: Given the severity of this case - including the patient's visual hallucinations, worsened depressive symptoms despite fluoxetine treatment, and possible suicidal ideation - a more aggressive form of therapy may be required. Electroconvulsive therapy has been successful in severe depression cases involving psychotic features and high suicidal risk. On the other hand, antidepressants (Imipramine, Mirtazapine, Sertraline) might need more time to be effective, whereas Transcranial magnetic stimulation often is used when therapy and medication are ineffective but hasn't proven as efficacious as ECT for severe conditions. CONCLUDING: Given the severity of this patient's symptoms and his lack of response to fluoxetine, along with his psychotic symptoms and high risk of suicide, the most suitable treatment option in this case would seem to be electroconvulsive therapy, which has proven beneficial in treating severe cases of depression with psychotic features. RESPONSE: (A) Electroconvulsive therapy

**AI_D:** RESTATING THE QUESTION: The question presents a 32-year-old man who has been experiencing significant depressive symptoms following a major tragic life event (the stillbirth of his son). His symptoms include severe insomnia, self neglect, dark moods, refusal to eat resulting in a severe weight loss, and auditory hallucinations telling him that he is a failure. He also expresses a suicidal ideation. Despite treatment with fluoxetine, his condition has worsened. Now that he has been admitted to a psychiatric facility, the test is asking us to decide what the most appropriate next step in his management would be. PLANNING: It appears that he was previously on fluoxetine, an antidepressant in the class of selective serotonin reuptake inhibitors (SSRIs). As his symptoms did not improve with fluoxetine, it's logical to consider either increasing the potency of his antidepressant therapy, or adding an adjunct treatment. This patient's condition, alongside his immediate risk to self, places him as a high priority patient that requires immediate attention and perhaps more aggressive intervention. CLINICAL REASONING: Antidepressants take several weeks to show their full efficacy and it could be risky and slow to try switching him to another similar medication, such as sertraline (also an SSRI). Instead, the focus should be on accelerating his recovery and ensuring safety. This could be achieved through Electroconvulsive therapy (ECT) or Transcranial magnetic stimulation, both of which are typically reserved for severe cases of treatment-resistant depression. ECT has been shown to be exceptionally effective for severe depression and conditions with psychotic features, which this patient may have (as evidenced by his auditory hallucinations). Mirtazapine and Imipramine would also be options, but for this patient's condition, he requires a treatment that acts quickly and effectively. CONCLUDING: Given that the patient's condition is severe and potentially involves psychosis, and that he poses an immediate risk to himself, it would be logical to opt for a treatment that will bring about a change quickly and effectively, making Electroconvulsive therapy (ECT) a potential ideal next step in management. RESPONSE: Thus, the most appropriate next step in the management of this patient would be (A) Electroconvulsive therapy.

**AI_B:** RESTATING THE QUESTION: A 32-year-old man, going through a severe depressive episode likely caused by the trauma of losing his child, has been engaging in self-neglecting behaviors and has shown signs of suicidal ideation. Despite beginning fluoxetine therapy 3 months ago, his symptoms have continued to worsen, and he's now showing signs of being severely underweight and dehydrated. He has also started hearing voices - a symptom of psychosis. After securing his immediate safety, the question is what should be the next course of action in his treatment. PLANNING: The assumptions made initially include that he is correctly diagnosed and that fluoxetine is the appropriate therapy. However, these assumptions are called into question by the observations of his continued deterioration and development of psychotic symptoms. Alternative assumptions to consider are that he is not responding to fluoxetine and might require another form of therapy – most likely one that can also manage the psychotic symptoms. CLINICAL REASONING: Fluoxetine is a selective serotonin reuptake inhibitor (SSRI) and commonly used as a front-line treatment for depression. However, the continued worsening of the patient's condition implies that it has failed to manage his condition effectively. Moreover, the emergence of hallucinations (hearing voices) indicates psychotic symptoms, which further complicates the treatment approach. SSRIs are not usually effective in treating psychosis, and therefore, augmentation with antipsychotic medication might be necessary. Those being said, considering the immediate danger he poses to himself, it might be necessary to prioritize treatments that can rapidly lift the depressive episode, reduce suicidal ideation, and have antipsychotic properties. CONCLUDING: Based on the patient's immediate safety risk, urgency of treatment, and complexity of symptoms, electroconvulsive therapy (ECT) becomes a warranted course of action. ECT is known for its rapidity of action, effectiveness in treating severe and psychotic depression, and overall safety and tolerability. The other potential alternatives do not carry the same urgency of response as ECT and might not be as effective in managing the acute severity of his symptoms. RESPONSE: The most appropriate next step in managing this patient's condition should be (A) Electroconvulsive therapy.

**AI_E:** RESTATING THE QUESTION: This case involves a 32-year-old male who presents to the emergency room in a highly distressed state following a major loss in his life. He displays signs of severe grief and serious depression, worsened by the auditory hallucinations, i.e., a voice telling him that he's a failure. Despite taking fluoxetine (a type of selective serotonin reuptake inhibitor often used for treating depression) for the past three months, his condition has deteriorated, not responded to this intervention. After ensuring immediate safety measurements i.e., removing firearms and admitting him to a psychiatric facility, the challenge now is to decide on the optimal course of treatment moving forward. PLANNING: Firstly, the assumption here is that the patient's fluoxetine treatment was appropriate and was not exacerbating his condition. Could we alternate this assumption and hypothesize that another treatment might be more effective in this situation? We might also question the assumption that a pharmacological treatment is the best course. Could a non-pharmacological treatment like Electroconvulsive therapy (ECT) or Transcranial magnetic stimulation (TMS) be more effective? Evaluating these assumptions, the patient's lack of response to fluoxetine, and the seriousness of his condition lead me to hypothesize clinically that we should prioritize considering alternative treatment modalities. CLINICAL REASONING: Given the patient's marked psychomotor retardation (e.g., hardly sleeping, not showering/shaving, refusal to eat), auditory hallucinations, extreme depressive state, and desire to die as indicated by his expression to join his deceased son, the patient likely has severe major depressive disorder with psychotic features. In such severe conditions, both the American Psychiatric Association and The National Institute for Health and Care Excellence guidelines suggest using ECT as a first-line treatment. In comparison, TMS is generally used for patients who have failed to respond to first-line treatment but do not demonstrate severe depressive signs like in this case. In terms of pharmacological treatment, the use of imipramine, mirtazapine, or sertraline may also be considered, but the severe nature of the patient's depression and life-threatening condition suggests a more immediate and intensive course of action is required, which these drugs may not provide. CONCLUDING: As the patient already has a failed trial of fluoxetine, strong clinical cues point towards an alternate method of management. His potentially life-threatening condition, severe depression, lack of appetite, sleep disorder, poor self-care, and auditory hallucinations indicate the requirement for an immediate and effective treatment modality. As per clinical guidelines, ECT is considered the most efficacious and rapid treatment option for severe major depression with psychotic features. RESPONSE: The most appropriate next step in the management of this patient is (A) Electroconvulsive therapy.

**AI_C:** RESTATING THE QUESTION: We're identifying the best course of action for a 32-year-old man who exhibits severe depressive symptoms and suicidal tendencies. He has been on Fluoxetine therapy - a selective serotonin reuptake inhibitor (SSRI) for depression - for approximately 3 months, with no improvement. Despite currently being in a safe environment (a psychiatric facility), an appropriate next step in his management needs determining. PLANNING: The symptoms presented are plumbing the depth of major depressive disorder: low mood, anhedonia, loss of appetite, changes in sleep patterns, affective issues, maintaining poor hygiene, and weight loss leading to a BMI of 17 kg/m², which is significantly below the normal range (18.5–24.9). Furthermore, the patient's auditory hallucination ("hearing a voice"), cements a severe depressive episode with psychotic features. When considering next steps, we need to evaluate the effectiveness and suitability of potential treatment options at our disposal: Electroconvulsive therapy (ECT), Imipramine therapy, Mirtazapine therapy, Sertraline therapy, and Transcranial magnetic stimulation. All of the above, save ECT, are part of pharmacological approaches principally used to manage depression. ECT and Transcranial magnetic are physical therapies used primarily for treatment-resistant depression. CLINICAL REASONING: Fluoxetine has not worked for the patient for the past three months. It's a type of SSRI, similar to Sertraline. While Sertraline may have slightly different properties, the likelihood of it being beneficial where Fluoxetine has failed is slim. Imipramine, a tricyclic antidepressant, could be considered, but it often takes several weeks to reveal improvements and may also likely cause unwanted side effects. Its use is generally avoided when there's a significant risk for suicide due to its lethality in overdose. Similarly, Mirtazapine, an atypical antidepressant, can also potentially take weeks to manifest improvements; though it might help with some symptoms like insomnia and loss of appetite, its effects on severe depression with psychotic features are questionable. Meanwhile, Transcranial magnetic stimulation, a noninvasive procedure that uses magnetic fields to stimulate the brain, is usually considered for patients who haven't responded to conventional treatments and is generally deemed less effective than ECT. On the other hand, Electroconvulsive therapy (ECT) is a potent treatment option for severe depression, particularly when complicated by prominent psychotic features or when the risk of suicide is acute. It has a rapid onset of action compared with pharmacological treatments, which is beneficial in urgent cases like ours. Therefore, taking also into account the immediacy of the situation and the severity of symptoms exhibited, ECT seems a potentially superior choice. CONCLUSION: In treating this 32-year-old man, who's suffering from a severe depressive episode with psychotic features and hasn't responded positively to an SSRI, Electroconvulsive therapy may arguably be an optimal next step in his management. RESPONSE: Therefore, the most suitable answer to the question "what is the most appropriate next step in management?" is (A) Electroconvulsive therapy.

**Facilitator** The consensus among the AI responses is that the most appropriate next step in managing the 32-year-old man's severe depressive disorder with psychotic features is Electroconvulsive therapy (ECT). This conclusion is based on the patient's worsening condition despite fluoxetine treatment, the severity of his symptoms, including auditory hallucinations and suicidal ideation, and his immediate risk to himself. The AI responses reasoned that while other treatment options such as Imipramine, Mirtazapine, Sertraline, and Transcranial magnetic stimulation could be considered, they may not be as effective or act as quickly as ECT in this severe case. Imipramine and Mirtazapine could take weeks to show improvements and may cause unwanted side effects. Sertraline, another SSRI like fluoxetine, may not be beneficial given the patient's lack of response to fluoxetine. Transcranial magnetic stimulation is generally considered less effective than ECT and is usually used for patients who haven't responded to conventional treatments. ECT, on the other hand, has been proven to be effective in treating severe cases of depression with psychotic features and has a rapid onset of action, which is beneficial in urgent cases like this. Therefore, given the severity of the patient's symptoms, his lack of response to fluoxetine, and his high risk of suicide, ECT is the recommended treatment option.
