# Supplementary material for: Collaborative intelligence in AI: Evaluating the performance of a council of AIs on the USMLE
Source: PLOS Digit Health. 2025 Oct 9;4(10):e0000787. doi: 10.1371/journal.pdig.0000787 (PMC12510544; doi:10.1371/journal.pdig.0000787)
Supplement: S2 File — (ZIP) [file pdig.0000787.s002.zip › S2 Data Raw data files containing unprocessed question inputs and LLM outputs including transcripts of deliberations/STEP2_Question-3-1_Type-MC-NJ.docx]

**Facilitator** **[Round 1]:** An 87-year-old woman, gravida 6, para 6, is brought to the emergency department because of a 24-hour history of severe abdominal pain and an inability to void. She notes that for the past 2 months, she has had difficulty emptying her bladder, especially at the end of the day. Twenty years ago, she underwent mastectomy for breast cancer; she has been cancer-free since then. She has a 10-year history of osteoporosis. All of her children were delivered vaginally without complications. Her only medication is alendronate. Examination shows the cervix protruding 7.5 cm from the vagina. After the cervix is replaced into the vagina, a urinary catheter drains 700 mL of urine. Urinalysis shows no abnormalities. Which of the following is the most likely diagnosis? (A) Overactive bladder (B) Overflow incontinence (C) Stress incontinence (D) Urinary retention (E) Urinary tract infection

**AI_A:** RESTATING THE QUESTION: The elderly woman in question has been having trouble voiding her bladder for the past two months, but the problem has recently escalated to severe abdominal pain and an inability to void for 24 hours. Following a physical examination that revealed her cervix was protruding from her vagina and the drainage of 700 mL of urine, we are asked to determine the most likely diagnosis out of a list that includes overactive bladder, overflow incontinence, stress incontinence, urinary retention, and urinary tract infection. PLANNING: We are dealing with a case of urinary issues in an elderly woman. The assumptions we could make are that her symptoms are related to her age, past childbirth history, osteoporosis or the medication she's taking. An alternative assumption we could make is that her urinary symptoms are connected to the physical finding of the cervix protruding from her vagina. Clinically, it would be most valuable to prioritize this assumption, as this presentation can be a cause of her symptoms. CLINICAL REASONING: If the cervix is protruding from the vagina, it suggests a condition called 'uterine prolapse'. This condition is common in multiparous (having multiple pregnancies) women and results in a downward displacement or protrusion of the uterus into the vaginal canal. This mechanical displacement could be causing an 'obstructive' type of urinary issue, making it difficult for the woman to fully empty her bladder. Such an obstruction can cause 'overflow incontinence', which is the involuntary release of urine due to a full bladder. CONCLUDING: Given her age, history of multiple pregnancies, the physical finding of her cervix protruding from the vagina, and her symptoms, the most likely cause of this woman's inability to void is due to uterine prolapse leading to overflow incontinence. RESPONSE: Therefore, out of the given options, the most likely diagnosis for this woman's symptoms is (B) Overflow incontinence.

**AI_D:** RESTATING THE QUESTION: The council is presented with an elderly woman, who has given birth six times (indicated by the terms gravida 6, para 6), experiencing severe abdominal pain and difficulty voiding. Though she had battled breast cancer and has been living with osteoporosis for a decade, she has been otherwise healthy. Her only medication is Alendronate, which is used to treat osteoporosis. An abnormal physical finding is her cervix protruding significantly from her vagina. After replacing it and catheterizing her, 700 mL of urine is drained. Despite these symptoms, her urinalysis is normal. The objective is to determine this woman's most likely diagnosis from the given list. PLANNING: The data suggests an issue either with capacity (indicated by the inability to completely empty bladder and the large amount of urine drained) or control (evidenced by cervix's protrusion from the vagina). Underlying factors to consider include her age, history of vaginal births, and osteoporosis. Each of these has implications relating to muscle weakness and pelvic floor dysfunction, hence affecting both bladder and reproductive organs' function. CLINICAL REASONING: The woman's advanced age, history of numerous vaginal births, and osteoporosis are likely to have weakened her pelvic floor muscles. This weakness could compromise her bladder control and result in prolapse of the uterus (as suggested by the protruding cervix). This prolapse could exert pressure on the urinary bladder, causing the retention of urine that she can't fully expel, leading to symptoms characteristic of overflow incontinence. CONCLUDING: The evidence points towards a diagnosis of overflow incontinence (Option B). The advanced age, multiple vaginal births, and osteoporosis collectively contribute to weakened pelvic floor muscles, ultimately leading to uterine prolapse and bladder compression. This leads to difficulty in bladder emptying, resulting in overflow incontinence despite a normal urinalysis. RESPONSE: The most likely diagnosis for this 87-year-old woman, who has difficulty emptying her bladder with a history of multiple vaginal deliveries and pelvic organ (uterine) prolapse, is Overflow incontinence (Option B).

**AI_E:** RESTATING THE QUESTION: The question is asking for a diagnosis for an elderly woman with a history of childbirth and breast cancer who presents with severe abdominal pain and difficulty voiding (emptying her bladder), among other symptoms. Voiding difficulties can often be caused by various urinary tract conditions. The examination results reveal that her cervix protrudes significantly from her vagina, and a large volume of urine is drained via a catheter. Nevertheless, her urinalysis is normal. From the options provided, we need to figure out which condition is most likely responsible for her symptoms. PLANNING: One important information is the patient's inability to void, along with prior reported challenges in emptying her bladder. This could represent conditions such as urinary retention, overflow incontinence, or other bladder dysfunctions. She also has a cervix that's protruding which could indicate a form of pelvic organ prolapse. The replacement of the cervix followed by significant urine output might suggest a physical obstruction contributing to her urinary symptoms. The presence of a normal urinalysis should also lead us to rule out conditions that would typically demonstrate abnormal findings, such as a urinary tract infection. CLINICAL REASONING: Let's start with her inability to void despite a full bladder. This aligns well with urinary retention. However, urinary retention alone doesn't explain why the patient's cervix is protruding. Pelvic organ prolapse is a condition prevalent in women with multiple childbirths or advanced age, both of which apply to this patient. More notably, one symptom of this condition is difficulty with urination or even urinary retention, as the prolapsed organ can obstruct the urethra. Her symptoms improved with the replacement of the cervix, further suggesting the likelihood of this condition. Also, prolapse can cause kinking of the urethra leading to urinary retention with overflow and incomplete bladder emptying, meaning the bladder is essentially "overflowing." CONCLUDING: Keeping the above factors in mind, the combination of prolapsed cervix and her symptoms can explain an overflow of incontinence caused by a physical blockage (cervix) obstructing the urethral opening. RESPONSE: Therefore, the most likely diagnosis for this patient is (B) Overflow incontinence. This diagnosis aligns with the clinical picture because it explains the reported issues with bladder emptying given the anatomical obstruction caused by the prolapsed cervix.

**AI_C:** RESTATING THE QUESTION: This 87-year-old multiparous woman, who previously had breast cancer and is currently on alendronate for osteoporosis, has presented with severe abdominal pain, difficulty in voiding, protrusion of the cervix from the vagina, and is found to have retained a significant volume of urine in her bladder. The differential diagnoses include conditions such as overactive bladder, overflow incontinence, stress incontinence, urinary retention, and urinary tract infection. PLANNING: Let's consider each of the options. Overactive bladder is characterized by a sudden, compelling desire to urinate that is difficult to suppress, which does not align well with her symptoms. Urinary tract infection typically presents with symptoms like painful urination, increased frequency, and urgency, which she does not have. Stress incontinence is typically characterized by the involuntary loss of urine during events that increase intra-abdominal pressure such as laughing, sneezing, or lifting weights, which is not consistent with her signs and symptoms. Urinary retention is the inability to empty the bladder, but her symptoms are not entirely consistent with this option. Overflow incontinence refers to involuntary urine leakage due to pressure from an overly-filled bladder, usually caused by bladder outlet obstruction or impaired detrusor contractility, which seems to fit her case. CLINICAL REASONING: The scenario describes an older woman with a history of multiple vaginal deliveries who cannot fully empty her bladder and has a cervix protruding from the vagina. This suggests some degree of pelvic organ prolapse, which is not uncommon among women with multiple pregnancies and deliveries. The presence of pelvic organ prolapse can lead to kinking or obstruction of the urethra, causing urinary symptoms such as incomplete bladder emptying and, consequently, to overflow incontinence, which is consistent with this patient's presentation. Overflow incontinence is also often associated with large post-void residual volumes and chronic urinary retention, as seen here, with the catheterization draining a significant amount of urine. CONCLUDING: Thus, considering the history of multiple vaginal deliveries, the presence of a cervix protruding from the vagina indicating pelvic organ prolapse as well as the incomplete bladder emptying and resultant large post-void residual volume, the diagnosis of overflow incontinence is most likely in this patient. RESPONSE: The most likely diagnosis in this situation is (B) Overflow incontinence.

**AI_B:** 1) RESTATING THE QUESTION: The question centers around an 87-year-old woman with a gynecologic history of six pregnancies and deliveries and a past medical history of breast cancer treated with mastectomy, and osteoporosis managed with alendronate. The woman presents with severe abdominal pain and the inability to void, expressing that she's been having trouble completely emptying her bladder for two months. An examination revealed a protruding cervix, which once repositioned, allowed a catheterization to drain a significant amount of urine. There was no abnormal urinalysis. Given these specifics, the aim is to identify the most likely diagnosis from the given options: overactive bladder, overflow incontinence, stress incontinence, urinary retention, or urinary tract infection. 2) PLANNING: The main assumptions include the large volume of residual urine drained, the lack of urinalysis abnormalities, the cervix's protrusion from the vagina, and her history of vaginal deliveries. We could hypothesize that the cervix's protrusion – a likely sign of pelvic organ prolapse due to the woman's age and obstetric history – may be mechanistically linked to her urinary symptoms. It's also worthy to consider the lack of urinalysis abnormalities, suggesting an absence of infection or other urinary pathology as the primary issue. 3) CLINICAL REASONING: In light of these assumptions, the high residual urine volume after catheterization and the reported difficulty in completely emptying the bladder, coupled with abdominal pain, suggest urinary retention. This would be consistent with overflow incontinence, where the bladder becomes overly filled and leaks urine. However, given the evidence of her cervix protruding from the vagina, it'd appear that she has a pelvic organ prolapse, likely a uterine prolapse. This is a condition often seen in multiparous women, which our patient is, and can cause a kinking or obstruction of the urethra, leading to retention and subsequent overflow incontinence. 4) CONCLUDING: Therefore, the chronic strain on the pelvic floor muscles from multiple vaginal births, combined with age and possibly exacerbated by the osteoporosis, could cause a uterine prolapse. This, in turn, could obstruct the urethra, leading to difficulty voiding and subsequent overflow incontinence marked by bladder distention, abdominal pain, and eventual leakage of urine once the bladder's capacity is surpassed. 5) RESPONSE: Given these considerations, the most likely diagnosis is Overflow incontinence (B).

**Facilitator** Consensus Statement: The question presents an 87-year-old woman with a history of multiple vaginal deliveries, breast cancer, and osteoporosis, who is currently experiencing severe abdominal pain and difficulty voiding. She has been unable to void for 24 hours and has had trouble emptying her bladder for the past two months. A physical examination reveals her cervix protruding from her vagina, and upon repositioning the cervix, a significant amount of urine is drained via catheterization. Her urinalysis is normal. The task is to determine the most likely diagnosis from the given options: overactive bladder, overflow incontinence, stress incontinence, urinary retention, and urinary tract infection. The woman's age, history of multiple vaginal deliveries, and osteoporosis are likely contributing to weakened pelvic floor muscles, which could lead to a condition known as uterine prolapse. This is suggested by the physical finding of her cervix protruding from her vagina. Uterine prolapse can cause a mechanical obstruction, making it difficult for the woman to fully empty her bladder. This obstruction can lead to urinary retention and subsequent overflow incontinence, where the bladder becomes overly filled and leaks urine. The large volume of residual urine drained after catheterization and the reported difficulty in completely emptying the bladder, coupled with abdominal pain, further support this diagnosis. The normal urinalysis suggests an absence of infection or other urinary pathology as the primary issue. Therefore, considering the woman's age, history of multiple pregnancies, the physical finding of her cervix protruding from the vagina, the large volume of residual urine, and her symptoms, the most likely diagnosis is Overflow incontinence (Option B). This diagnosis aligns with the clinical picture because it explains the reported issues with bladder emptying given the anatomical obstruction caused by the prolapsed cervix.
