# Supplementary material for: Collaborative intelligence in AI: Evaluating the performance of a council of AIs on the USMLE
Source: PLOS Digit Health. 2025 Oct 9;4(10):e0000787. doi: 10.1371/journal.pdig.0000787 (PMC12510544; doi:10.1371/journal.pdig.0000787)
Supplement: S2 File — (ZIP) [file pdig.0000787.s002.zip › S2 Data Raw data files containing unprocessed question inputs and LLM outputs including transcripts of deliberations/STEP2_Question-30-1_Type-MC-NJ.docx]

**Facilitator** **[Round 1]:** A 60-year-old woman comes to the office as a new patient. She recently moved to the area. She feels well. She has hypertension treated with amlodipine and asthma well controlled with inhaled fluticasone and inhaled albuterol as needed. According to the medical records she brought with her, a mammography 6 months ago showed no abnormalities, results of laboratory studies 1 year ago were within the reference ranges, and colonoscopy 5 years ago showed no abnormalities. Her past three Pap smears have shown no abnormalities; the most recent Pap smear was 1 year ago. Her mother has type 2 diabetes mellitus, and her maternal aunt had breast cancer at the age of 55 years. The patient does not smoke cigarettes. She drinks two glasses of wine weekly. She works as a nurse. She has been monogamous with her husband for 30 years. Pulse is 76/min, respirations are 16/min, and blood pressure is 132/86 mm Hg. Examination shows no abnormalities. Screening for which of the following is the most appropriate next step in management? (A) Abdominal aortic aneurysm (B) Breast cancer (C) Chlamydia trachomatis infection (D) Colon cancer (E) Hepatitis C

**AI_B:** RESTATING THE QUESTION: The question is asking which medical screening should be prioritized for a 60-year-old female patient who has been following regular medical check-ups and found to be in good health. She has a history of hypertension and asthma, which are under control. Her medical tests so far have shown no abnormalities, and she has no notable lifestyle risk factors, except moderate alcohol consumption. The genetic history consists of type 2 diabetes in her mother and breast cancer in her maternal aunt. PLANNING: We should take into account her age, her lifestyle habits, her health condition, family history, and her exposure risk. In this circumstance, the assumptions that all prior screening tools used were precise and that her life conditions have remained more or less the same must hold. We should examine these assumptions under variables such as age progression, lifestyle changes, or genetics that might have influenced her disease vulnerability. CLINICAL REASONING: The assumption here is that because the woman is aged 60, does not smoke, and has a stable sexual relationship, her likelihood of developing a sexually transmitted infection like Chlamydia trachomatis, or a hepatitis virus related to lifestyle risk factors like intravenous drug usage, is low. The chances of her having an abdominal aortic aneurysm are also low as she does not smoke and has her hypertension under control. Breast cancer and colon cancer screenings have been done within recommended time frames and show no abnormalities. However, due to her mother's history of diabetes and her increasing age, there may be an elevated risk of diabetes. CONCLUDING: Considering the age, overall health status, immediate family having a history of diabetes, and genetic predisposition, our focus should be towards evaluating her for type 2 diabetes. RESPONSE: However, this does not imply she should not be screened for the other conditions, rather, this would take clinical priority at this time. Therefore, none of the suggested options (Abdominal aortic aneurysm, Breast Cancer, Chlamydia trachomatis, Colon Cancer, Hepatitis C) are appropriate. Instead, screening for type 2 diabetes would be the proper next step in managing her health.

**AI_C:** RESTATING THE QUESTION: The question asks for the most reasonable next step in management or monitoring of a 60-year-old woman's health, one who has a history of hypertension and asthma, maternal family history of diabetes and breast cancer, and shows no significant findings in medical examinations, including a recent mammography, Pap smear, and colonoscopy. PLANNING: Potentially, screening for any health conditions related to her hypertension, asthma, or family medical history could be considered. Furthermore, given her occupation as a nurse, exposure to communicable diseases is plausible. However, one should consider what the guidelines recommend for routine screening according to her age and family history. CLINICAL REASONING: The clinical guidelines recommend mammography every 1 to 2 years for a woman over the age of 50. She had a normal mammogram 6 months ago, so she does not require breast cancer screening at this stage (B). Given her sexual history and age, Chlamydia trachomatis infection (C) is less likely, and she has regular PAP smear tests. She had a colonoscopy 5 years ago with no abnormalities; colon cancer screening is indicated every 10 years by colonoscopy in individuals with no additional risk factors (D). She doesn't have a history of drug use or other risk factors for Hepatitis C (E). However, for older adults, particularly men and smokers, a one-time screening for abdominal aortic aneurysm (AAA) with an ultrasound is indicated between the ages of 65 to 75, especially in those with a history of smoking. Our patient is an adult woman who does not smoke, hence is less likely to have AAA (A). CONCLUDING: Through step-by-step elimination using age-specific and risk-based recommendations, it seems none of the options listed are dictated by either current guidelines or her individual risk factors at present. RESPONSE: Given the moderate hypertension, family history, and lifestyle, monitoring and treatment of her blood pressure, and counseling for maintaining healthy lifestyle, perhaps in addition to a screen for diabetic risk due to family history of Type 2 diabetes, are appropriate next steps rather than the choices given. However if I have to choose from the list I choose, (B) Breast cancer, as she is of age and has family history of breast cancer, though she recently had her mammogram 6 months ago.

**AI_D:** (1) RESTATING THE QUESTION: The 60-year-old patient is well on the whole, with some controlled chronic conditions. She recently relocated and is a new patient. She has regular screening tests, which have so far revealed no issues. Her mother suffered from type 2 diabetes, and her maternal aunt had breast cancer at age 55. The patient does not smoke and moderately consumes alcohol. She worked as a nurse and has had a monogamous relationship with her husband for years. The patient is physically stable and presents no anomalies upon examination. The question at hand is, given all this, what should be the next step in her clinical management in terms of preventive health screening? (2) PLANNING: The assumptions made are all valid given that they are based on the patient's documented medical history as well as current health status. She does not present any symptoms suggestive of a specific condition. Therefore, preventive health screening should be based on age-based guidelines and the patient's risk factors, which include controlled hypertension, asthma, and family history of type 2 diabetes and breast cancer. An alternate assumption could be made based on her age, as certain health screenings are recommended for females over 60. (3) CLINICAL REASONING: Looking at the available choices, an abdominal aortic aneurysm is normally screened for in men between the ages of 65 to 75 who have smoked, which does not apply to our patient. For breast cancer, the patient had a mammogram six months ago, which was clear. Chlamydia trachomatis infection is unlikely due to her monogamous relationship and no symptoms of a sexually transmitted infection. Colon cancer is regularly screened for in adults aged 50 and older, but the patient had a normal colonoscopy 5 years ago. Hepatitis C screening could be considered, as the CDC and USPSTF recommend one-time testing for all adults aged 18 years and older. (4) CONCLUDING: Based on the routine age-based recommendations, her recent normal screenings, and her outlined risk factors, the next appropriate screening for this patient would therefore be Hepatitis C, as recommended by clinical guidelines and despite her not having specific risk factors other than age. (5) RESPONSE: The most appropriate next step in management for this new patient is to screen her for Hepatitis C (E). It is recommended as a one-time screening test for adults of her age, regardless of specific risk factors.

**AI_A:** RESTATING THE QUESTION: The case revolves around a 60-year-old female patient who has a history of hypertension and asthma, both of which are well managed with medication. She recently moved to the current location and has brought with her medical records, which display no abnormal health conditions based on tests done thus far, including mammography and colonoscopy. The patient's genetic history indicates her mother has type 2 diabetes mellitus and her aunt had breast cancer. Without any specific health complaints, the question is about determining which screening test would be the most appropriate for her next step in managing her health. PLANNING: Several assumptions can be made based on the information provided. Among these are that her hypertension and asthma are currently well controlled, she doesn't have any evident high risk for specific diseases such as diabetes, breast cancer, and colon cancer based on her current conditions and family history. Furthermore, she lives a moderate lifestyle with no cigarette smoking and moderate alcohol intake. But considering her age and disease risk factors, we should examine the patient's need for certain screening tests. The assumptions made allow for alternate assumptions such as whether a screening for abdominal aortic aneurysm, chlamydia trachomatis infection, or hepatitis C should be pursued. CLINICAL REASONING: Progressing through and considering these alternate assumptions, we follow a clinical thought process. We carefully vote out screening for (C) Chlamydia Trachomatis infection and (E) Hepatitis C because there is no indicated high risk for these diseases in her. Her sexual history is described as monogamous with her spouse, and there is no mention of intravenous drug use that could raise the suspicion for hepatitis C. The patient's medical record doesn’t indicate any digestive symptoms or apparent abnormalities and a colonoscopy 5 years ago showed no abnormalities, so screening for (D) Colon Cancer might not be the highest priority currently. Her history of hypertension and her age could potentially raise the concern for (A) Abdominal Aortic Aneurysm. (B) Breast cancer screening might be considered, but a mammography 6 months ago showed no abnormalities and can typically be performed every 1-2 years depending on her risk factors. CONCLUDING: By the clinical thought process and information from the medical history, and following the normal schedule of tests in women her age and risk, (A) Abdominal Aortic Aneurysm seems to be the most appropriate next step in management. This is a common condition affecting women over the age of 60, especially those who have a history of hypertension, and often has few symptoms until it becomes acute. RESPONSE: Given her age, medical history of hypertension, and absence of recent screening for such a condition, I propose the next step in her healthcare management should involve screening for an (A) Abdominal Aortic Aneurysm.

**AI_E:** RESTATING THE QUESTION: This scenario presents a 60-year-old woman who has known hypertension and asthma. She has had regular screenings for breast cancer, cervical cancer, and colon cancer, all of which have so far shown no abnormalities. This patient has a family history of type 2 diabetes mellitus and breast cancer. She does not smoke and drinks moderately. The question asks what should be the most appropriate next step in preventive health measures for this patient. PLANNING: Considering the patient's age, health status, family history, and lifestyle habits, various screening options could be considered: abdominal aortic aneurysm, breast cancer, Chlamydia trachomatis infection, colon cancer, and Hepatitis C. However, we need to consider the risk stratification, guidelines, cost, benefits, potential harms, and patient preferences in identifying the most clinically appropriate screening test. CLINICAL REASONING: Abdominal aortic aneurysm (AAA) screening is generally recommended for male smokers aged 65-75, and this patient does not fit into that demographic. She has had recent breast cancer and colon cancer screenings, and given her age, the frequency for these would usually be one to two years for mammography and ten years for colonoscopy if the previous exams were normal, as per guidelines. Additionally, Chlamydia trachomatis infection screening would typically be carried out in sexually active young women or those with risk factors for sexually transmitted infections (e.g., multiple sexual partners), not applicable in this case based on the information provided. Hepatitis C screening would usually be considered for those with risk factors including intravenous drug use or receiving a blood transfusion prior to 1992, which hasn't been mentioned in the patient's history. CONCLUDING SENTENCE: Given the patient's health, her demographics, and the screening she has recently undergone, none of the traditional screening options seem necessary at the given moment, which leads us to question which is the best option for her given her specific circumstances. RESPONSE: Taking into account all the information provided, the most appropriate next step to manage this woman's health would be (A) screening for abdominal aortic aneurysm. Despite this typically being recommended for male smokers aged 65-75, it is reasonable to consider this test given that the other options are not clinically indicated based on her history and recent screenings. The USPSTF recommends one-time screening for AAA by ultrasound in men ages 65-75 who have ever smoked and also considers it for those who have never smoked. Although the guideline doesn't specifically mention women, it may be beneficial in certain cases of women aged 65 or older who have cardiovascular risk factors.

**Facilitator** **[Round 2]:** Summary: AI_A suggests that the patient should be screened for an Abdominal Aortic Aneurysm (AAA) due to her age and history of hypertension. AI_B, however, believes that the patient should be screened for type 2 diabetes due to her mother's history of the disease and her increasing age. AI_C suggests that none of the options listed are necessary at present, but if forced to choose, would opt for breast cancer screening due to the patient's age and family history. AI_D recommends screening for Hepatitis C, as it is a one-time screening test recommended for all adults aged 18 years and older. AI_E, like AI_A, suggests screening for AAA, despite the patient not fitting the typical demographic for this screening, as the other options are not clinically indicated based on her history and recent screenings. Question for Council Members: Given the differing opinions, could you please clarify the basis of your clinical reasoning, particularly how you prioritized the patient's age, family history, and lifestyle habits in determining the most appropriate screening test? Additionally, could you elaborate on how you interpreted and applied the screening guidelines in this specific case?. Based on your preceding response, how would you answer the original question: [A 60-year-old woman comes to the office as a new patient. She recently moved to the area. She feels well. She has hypertension treated with amlodipine and asthma well controlled with inhaled fluticasone and inhaled albuterol as needed. According to the medical records she brought with her, a mammography 6 months ago showed no abnormalities, results of laboratory studies 1 year ago were within the reference ranges, and colonoscopy 5 years ago showed no abnormalities. Her past three Pap smears have shown no abnormalities; the most recent Pap smear was 1 year ago. Her mother has type 2 diabetes mellitus, and her maternal aunt had breast cancer at the age of 55 years. The patient does not smoke cigarettes. She drinks two glasses of wine weekly. She works as a nurse. She has been monogamous with her husband for 30 years. Pulse is 76/min, respirations are 16/min, and blood pressure is 132/86 mm Hg. Examination shows no abnormalities. Screening for which of the following is the most appropriate next step in management? (A) Abdominal aortic aneurysm (B) Breast cancer (C) Chlamydia trachomatis infection (D) Colon cancer (E) Hepatitis C]

**AI_A:** (1) RESTATING THE QUESTION: To begin with, we have an otherwise healthy 60-year-old female patient with controlled hypertension and asthma. Her clinical background, including regular screenings, shows no identified issues. Her family history includes type 2 diabetes and breast cancer. In view of this, the question that arises is which particular screening procedure would be beneficial for this patient? (2) PLANNING: Analysing the family history and the patient's health conditions, various assumptions can be made. The presence of hypertension may enhance the risk for cardiovascular diseases, such as an Abdominal Aortic Aneurysm (AAA). Furthermore, her family's medical history of diabetes and breast cancer may also expose her to a higher risk for these diseases. Oppositely, evaluation of her lifestyle - namely her moderate alcohol consumption, nonsmoking status, stable sexual relationship, and occupation as a nurse - does not suggest she might be at increased risk of Chlamydia trachomatis infection or Hepatitis C. Additionally, her normal recent mammogram, Pap smears, and colonoscopy suggest no immediate concern for breast or colon cancer. Thus, the most pertinent screening to be pursued could be for either AAA or diabetes. (3) CLINICAL REASONING: AAA is generally screened in men of age 65-75 who have ever smoked. There is no consensus on screening guidelines for women, though some recommend screening for women over the age of 65 who have ever smoked or have a family history of AAA; this patient, however, doesn't fit the category. With regards to diabetes screening, the American Diabetes Association recommends screening for type 2 diabetes mellitus in adults who are overweight or obese and have one or more additional risk factors for diabetes. While our patient's mother had type 2 diabetes mellitus, the patient in question is not described as being overweight or obese and has no other noted risk factors. Still, considering her age and family history, a glucose tolerance test may be useful. (4) CONCLUDING: Therefore, upon balancing potential health risks and patient circumstances, a primary approach to diabetes screening seems most justifiable, even if guidelines do not overtly advocate for this in her scenario. This is validated by her age and familial predisposition for the disease, outweighing ambiguities in her risk profile. (5) RESPONSE: To answer the original question, none of the provided options (AAA, Breast cancer, Chlamydia trachomatis infection, Colon cancer, Hepatitis C) are the most appropriate next steps in management for this patient. Nonetheless, considering her family history and age, a test for type 2 Diabetes Mellitus would be advisable, although it is not among the available options.

**AI_B:** In order to address this scenario, let's break down the details provided. This woman is 60 years old with health conditions such as hypertension and asthma, meticulously controlled by medications. Her medical history suggests regular health maintenance screenings like mammography and colonoscopy, which were unremarkable. The most recent Pap smear was taken a year back and had no alarming findings. Family history is reflective of her mother being a type-2 diabetic. Lastly, she leads a life abstaining from smoking, has a moderate consumption of alcohol, and has been engaging in a monogamous relationship. The question at hand is to identify the most optimal next step for screening this patient's health. Given the facts presented, we can put forth several assumptions: some obvious are pertaining to the need for screening for abdominal aortic aneurysm (AAA), breast cancer, Chlamydia trachomatis infection, colon cancer, or hepatitis C. Generating alternate assumptions opens up new screening possibilities like diabetes, given the familial history, or continued monitoring of hypertension and asthma. Evaluating the assumptions, we should consider this woman's homeostasis further. Given her age, we can rule out the necessity for Chlamydia trachomatis infection screening as this woman is not part of the high-risk demographic for this sexually transmitted infection. The normal results from the latest Pap smear further support this conclusion. Her records indicate she has been regularly screened for breast and colon cancers with normal results each time, making immediate rescreening for these unlikely to yield new insights. While she does have a family history of breast cancer, the recent normal mammography results would argue against immediate rescreening. Screening for hepatitis C could be considered. However, she does not seem to have known risk factors for this disease, such as intravenous drug use. Thus, we come to screening for AAA and while it's true that the combination of this woman's age and hypertensive condition increase the risk of AAA, AAA screening is generally recommended for men aged 65-75 who have ever smoked. Therefore, none of the options provided seem like the perfect match. However, for the purpose of answering the question, we are pushed towards AAA, given that none of the other conditions align with the patient's profile. Therefore, resultingly, the most appropriate next step in management could be the ""screening for abdominal aortic aneurysm" - Option (A). Even though the patient isn't the typical candidate for such a screening (as she does not possess all the risk factors), it represents the most viable choice out of the provided options.

**AI_C:** Restating the Question: The practicing physician is seeking guidance on the most appropriate health screening to administer next for her 60-year-old female patient. The client has a history of hypertension and asthma, and her family has a history of diabetes and breast cancer. All her recent screenings showed no abnormalities. It's critical to understand health screenings, which aim to detect diseases early in asymptomatic individuals, ideally before symptoms appear. These measures can help to improve health outcomes through early interventions. Planning: The question revolves around the clinical guidance on screenings based on the patient's family history, age, lifestyle habits, and current medical status. To decide which is the most appropriate next step in management, we need to consider each option's clinical relevance in light of the patient's medical, familial, and personal considerations. The suggested screenings include: Abdominal aortic aneurysm (AAA), Breast cancer, Chlamydia trachomatis, Colon cancer, and Hepatitis C; we need to evaluate the risk factors, benefits, and guidelines for each screening test. Clinical Reasoning: AAA screening is usually recommended for men aged 65-75 who've had a history of smoking, not primarily targeted at women or hypertensive patients. Breast cancer screening through mammography is typically done every one to two years for women aged 50-74; our patient already had a mammogram six months ago, with no abnormalities. Chlamydia Trachomatis screening is generally suggested for sexually active women under the age of 24, or older women with risk factors like new or multiple sex partners; the patient's age and monogamous relationship reduce the need for this screening. Colon cancer screening, such as colonoscopy, is typically performed every 10 years for individuals aged 50-75; being only five years out from her last colonoscopy, our patient does not currently need another. Hepatitis C screening is recommended as a one-time measure for all individuals born between 1945 and 1965. Concluding: Bearing in mind the patient's age, medical history, family history, lifestyle habits and recent test results, none of the proposed screening tests seem imperative at this moment. However, since the patient falls into the birth cohort for Hepatitis C, she may benefit from that one-time screening, although it is not directly indicated by her medical or family history. Response: Given the patient's detailed case, and the identified assumption regarding the timing of screenings, existing guidelines, risk factors, and her specific medical history, the most appropriate screening test would likely be for Hepatitis C (E), as it is a recommended one-time screening for her age group, even though it is not directly indicated by her personal or family medical history.

**AI_E:** 1) RESTATING THE QUESTION: The query requires us to identify the most suitable screening procedure for a 60-year-old woman, given her medical history and current health status. According to her health records, she has hypertension managed with amlodipine, well-controlled asthma, no history of sexual diseases, and no recent abnormal results on mammograms, Pap smears, or colonoscopies. Her mother had type 2 diabetes and an aunt had breast cancer at 55 years. The screenings proposed are for abdominal aortic aneurysm, breast cancer, Chlamydia trachomatis infection, colon cancer, and Hepatitis C. 2) PLANNING: We must decide which among the potential health risks needs prioritizing. First, let's evaluate her risk for each condition. Given her age and history of hypertension, she may be at risk for an abdominal aortic aneurysm. However, screening for AAA is typically recommended for men aged 65-75 who have ever smoked, and our patient is a non-smoker. Breast cancer screening, while appropriate given her maternal aunt's history, was recently done 6 months ago with no abnormalities found. The risk of a Chlamydia trachomatis infection is very low given her stable monogamous relationship, negative Pap smear, and her profession as a nurse likely implies awareness of safe sexual practices. As for Hepatitis C, there's no mention of her being at an increased risk. Her colonoscopy 5 years ago showed no abnormalities, but its frequency is every 10 years. Therefore, it's unlikely to be the next step. Considering all the data and the patient's demographic, the potential for type 2 diabetes seems to be the most substantial. 3) CLINICAL REASONING: Although the patient does not appear to be a typical individual with significant risk factors for any of the given conditions, we must also consider the regularity of screenings. Breast, colon, and cervical cancer screenings have recently been conducted. Regarding the remaining potential health concerns, Hepatitis C screening could be seen as appropriate given recent CDC guidelines suggesting one-time Hepatitis C testing for all adults aged 18 and above. However, the patient's mother's history of type 2 diabetes can't be overlooked. 4) CONCLUDING: Applying clinical thought, I reasoned that the conditions for which the patient has currently been screened, such as breast and colon cancer, and Chlamydia trachomatis infection, are unlikely to need repeat testing in such a short period. Meanwhile, screening for an Abdominal Aortic Aneurysm (AAA) typically targets a different demographic group. Considering her family history in conjunction with recent updates from the U.S. Preventive Services Task Force guidelines, I conclude that diabetes screening may be a suitable consideration, even though it's not listed in the given choices. 5) RESPONSE: Among the presented options, Hepatitis C screening might be the most relevant considering the CDC's recent guidelines. However, outside of these choices, screening for type 2 diabetes may be a more immediate priority, guided by her age and family history. Clinical decision-making often requires weighing the balance between guidelines, patient's specific risk factors, and disease prevalence in the general population.

**AI_D:** 1) RESTATING THE QUESTION: The patient, a 60-year-old woman with a medical history of hypertension and asthma, both well-controlled, has presented for a routine check-up. She has no apparent symptoms and her vital signs are within normal limits. Her previous screenings including mammograms, laboratory studies, colonoscopies, and Pap smears have shown no abnormalities. Her family history includes a mother with type 2 diabetes and an aunt who had breast cancer. She doesn't smoke, drinks a moderate amount of alcohol, and is in a stable, monogamous relationship. She works as a nurse. We are to decide the most appropriate next step regarding screening her for potential health issues based on her current health status and history. 2) PLANNING: There are five possible directions we could go in with regards to screening - abdominal aortic aneurysm (AAA), breast cancer, C.trachomatis infection, colon cancer, and Hepatitis C. The patient has recently had a clean mammogram and is regularly screened for cervical issues via Pap smears. Meanwhile, her colonoscopy five years ago revealed no abnormalities. We will need to consider the current guidelines for these screenings in the context of the patient's age, her family's medical history, and her own medical history and lifestyle choices. 3) CLINICAL REASONING: We know that AAA screening is typically recommended for males between the ages of 55-75 who have a history of smoking. Given the patient's gender and non-smoking status, this patient does not meet these prerequisites, making this option the least plausible. C.trachomatis infection is sexually transmitted and our patient maintains a single sexual partner for the past 30 years and had regular Pap smears, making the chances for this unlikely. Breast and colon cancer screenings are already up-to-date. Considering Hepatitis C, although there is a recommendation for one-time screening of all adults over 18, it is particularly directed towards people with certain risk factors such as previous/present injection drug use, birth between 1945-1965, long-term hemodialysis, etc. Adding to this, the patient's profession as a nurse might put her at a risk of potential exposure to blood-borne pathogens, considered a risk of occupational exposure. 4) CONCLUDING: Thus, based on a step-by-step evaluation of each potential area of concern, considering the patient's health and lifestyle, and applying the existing screening guidelines, we conclude that screening for Hepatitis C is the most appropriate next step. 5) RESPONSE: It would be most appropriate to next screen this patient for Hepatitis C (Option E), given the prevalent hepatitis C screening recommendations, in addition to her healthcare profession which may expose her to a higher risk of bloodborne pathogen transmission. This decisison was made after a thorough assessment of her medical history, family history, and the frequency of her current screenings.

**Facilitator** **[Round 3]:** Summary: AI_A believes that the most appropriate screening for the patient would be for type 2 diabetes, given her age and family history, despite it not being among the provided options. AI_B, on the other hand, suggests that the most appropriate next step in management could be screening for an abdominal aortic aneurysm (AAA), as it is the most viable choice out of the provided options, even though the patient doesn't possess all the risk factors. AI_C recommends a one-time screening for Hepatitis C, as it is a recommended screening for her age group, even though it is not directly indicated by her personal or family medical history. AI_D also suggests screening for Hepatitis C, considering the prevalent hepatitis C screening recommendations and her healthcare profession which may expose her to a higher risk of bloodborne pathogen transmission. AI_E, like AI_A, believes that screening for type 2 diabetes may be a more immediate priority, guided by her age and family history, but among the presented options, Hepatitis C screening might be the most relevant considering the CDC's recent guidelines. Question: Given the differing opinions, could the council members elaborate on the reasoning behind their chosen screening procedure, particularly focusing on how they weighed the patient's personal and family medical history, her lifestyle, and the current guidelines for each screening?. Based on your preceding response, how would you answer the original question: [A 60-year-old woman comes to the office as a new patient. She recently moved to the area. She feels well. She has hypertension treated with amlodipine and asthma well controlled with inhaled fluticasone and inhaled albuterol as needed. According to the medical records she brought with her, a mammography 6 months ago showed no abnormalities, results of laboratory studies 1 year ago were within the reference ranges, and colonoscopy 5 years ago showed no abnormalities. Her past three Pap smears have shown no abnormalities; the most recent Pap smear was 1 year ago. Her mother has type 2 diabetes mellitus, and her maternal aunt had breast cancer at the age of 55 years. The patient does not smoke cigarettes. She drinks two glasses of wine weekly. She works as a nurse. She has been monogamous with her husband for 30 years. Pulse is 76/min, respirations are 16/min, and blood pressure is 132/86 mm Hg. Examination shows no abnormalities. Screening for which of the following is the most appropriate next step in management? (A) Abdominal aortic aneurysm (B) Breast cancer (C) Chlamydia trachomatis infection (D) Colon cancer (E) Hepatitis C]

**AI_A:** RESTATING THE QUESTION: To perform a thorough analysis, we must consider the presented query, which involves a 60-year-old woman whose medical history includes hypertension presently under control with amlodipine and well-managed asthma with the use of fluticasone and albuterol as needed. Her most recent mammography, laboratory tests, and colonoscopy returned no abnormalities, and she has had three consecutive normal Pap smears. She has a family history of type 2 diabetes mellitus and breast cancer. The woman doesn't smoke, consumes two glasses of wine per week, and has been monogamous with her husband for three decades. Her vital signs and physical examination are within normal ranges. The question is: which is the most appropriate next step in management for this patient among the given options - abdominal aortic aneurysm, breast cancer, Chlamydia trachomatis infection, colon cancer, or Hepatitis C screening? PLANNING: To respond to this query, we will need to focus primarily on the patient's age, current health status, family medical history, and lifestyle factors. We would also contemplate the frequency and recent guidelines related to each screening test. CLINICAL REASONING: For abdominal aortic aneurysm (AAA) screening, risk factors typically include a history of smoking, being male, and older age, especially those between 65 and 75, none of which she possesses. Therefore, this may not be the most suitable screening choice in this case. Breast cancer screening, having recently been conducted (six months ago), is not due for another 18 months typically. Chlamydia trachomatis infection is unlikely due to her monogamous relationship status. Colon cancer screening would typically be repeated every ten years, so she would not be due for another five years since her past colonoscopy showed no abnormalities. Lastly, the CDC recommends one-time Hepatitis C testing for individuals born between 1945 and 1965. As she was likely born in that timeframe, this screening makes sense. Additionally, being a nurse, she may be at a higher risk of bloodborne pathogen transmission. CONCLUDING: Consequently, contemplating all the facts and variables, including the patient's personal and family medical history, lifestyle behaviors, recent preventive health screenings, and appropriate guidelines, Hepatitis C screening appears to be the most suitable next step in her management. RESPONSE: Based on the information at hand, Hepatitis C screening is the most suitable choice for the next step for the 60-year-old woman's management as her age cloud be a risk factor for the disease, along with her profession as a nurse that potentially exposes her to bloodborne pathogens.

**AI_C:** Restating the question: The objective here is to decide upon the most suitable subsequent step in managing this new, asymptomatic, 60-year-old patient with well-controlled hypertension and asthma. This woman, who has a family history of diabetes and breast cancer, possesses normal physical examination results, along with recent and non-remarkable medical and laboratory records, including mammography and pap smear. The choices indicate potential screenings for abdominal aortic aneurysm, breast cancer, Chlamydia trachomatis infection, colon cancer, and Hepatitis C. Planning: Key factors to consider here are the patient's age, past medical history, family medical history, and lifestyle choices. Certain assumptions that can be made are that she is currently not manifesting any signs or symptoms of the conditions listed for possible screening. However, her family history of diabetes and breast cancer, as well as her age, could indicate a potential predisposition for certain disorders. Exploring alternate assumptions, it could be hypothesized that her occupation as a nurse might enhance her risk to certain infections like Hepatitis C. Clinical Reasoning: Starting from the first choice (A): an abdominal aortic aneurysm screening is recommended for men aged 65-75 who have ever smoked. However, our patient does not fulfill these criteria. Option (B): breast cancer screening has recently been done and did not show any abnormalities. A repeat mammography is usually done every 1 to 2 years, so it might not be the next immediate step in management. Choice (C): Chlamydia trachomatis infection is unlikely, given the patient's monogamous relationship. For choice (D): colon cancer screening, although the stating risk factor is age above 50, she already had a colonoscopy 5 years ago with no abnormalities detected. Finally, choice (E): Hepatitis C screening; according to CDC recommendation, all adults aged 18 years and older should be screened at least once for Hepatitis C. Concluding: Connecting the dots from our previous steps, as we factored in the patient's personal information, occupational risk, and recent guidelines, the evidence points towards hepatitis C screening as the most appropriate next step. Response: Therefore, the answer to the question is (E) Hepatitis C. This is because, despite the patient's recent health screenings and absence of relevant symptoms, recommendations indicate hepatitis C screening for all adults at least once, regardless of their risk factors or lifestyle habits. The patient's occupation as a nurse also puts her at higher risk for this infection due to potential exposure to this bloodborne pathogen. Therefore, it would be prudent to screen for Hepatitis C as the most appropriate next step in her management.

**AI_E:** Restate: The question is asking for the most suitable screening test for a 60-year old woman who has no symptoms of disease and has a medical history of hypertension and asthma, both well controlled. She has no personal history of malignancies or infections, and her family history is noteworthy for type 2 diabetes mellitus and breast cancer. Despite her occupation as a nurse, which may expose her to communicable diseases, she does not have a history of bloodborne pathogen transmission. We ought to consider the overall health status of the patient, her family medical history, and her lifestyle when deciding on the most appropriate screening test. Plan: We could assume that since the patient's cardiovascular status and respiratory conditions are well managed and she has regular appropriate cancer screenings, we might opt to focus on the silent diseases that she might be at risk for based on her family history and lifestyle. Another line of reasoning might assume the importance of occupational exposure, hence looking at her risk of communicable diseases. We need to consider the prevalence, morbidity, and mortality of diseases involved, the reliability and validity of the screening tests available, and the cost and potential harm associated with screening. Clinical Reasoning: The patient has a family history of type 2 diabetes, but given the absence of this option, we excluded that from our list. Her maternal aunt had breast cancer; however, her most recent mammogram was negative. The patient neither smokes nor has multiple sexual partners, and since there is no suggestion of a communicable disease in her history, Chlamydia trachomatis or Hepatitis C infection wouldn't be the immediate assumption. However, considering her occupational exposure, we might prioritize Hepatitis C as an assumption under specific circumstances, such as a potential for increased risk in her workplace. AAA may affect her due to her age, and the consequences are fatal if untreated; however, she doesn't have the high-risk factors such as being a male or a smoker. Concluding: To determine the most appropriate next step in management, the most clinically relevant factor is understanding the risks associated with each option versus the benefits gained through early detection. The patient's odds seem to lean towards the presence of a silent disease that may result from her family history, lifestyle, or occupational exposure. Response: Based on all the above reasoning, the most appropriate next step in management for this patient, among the given options, is likely to be screening for Hepatitis C (E). Although not directly indicated by her personal or family medical history, the CDC recommends one-time Hepatitis C screening for all adults aged 18 years and older. Given the patient's profession and age, this might be the most clinically sensible route.

**AI_D:** RESTATING THE QUESTION: The patient is a 60-year-old woman with controlled hypertension, well-managed asthma, a significant family history of type 2 diabetes and breast cancer, and no reported personal history of infectious diseases. She's a nurse who's been in a monogamous relationship for many years and does not smoke but drinks moderately. She has stayed current with the recommended age appropriate screenings and all have been normal. However, she has recently moved to the area and keen on maintaining her health, so we are exploring what would be the most appropriate screening test for her at this point given the options provided: abdominal aortic aneurysm (AAA), breast cancer, Chlamydia trachomatis infection, colon cancer, or Hepatitis C. PLANNING: Each option carries a certain risk based on the patient's age, lifestyle, occupation, and family history. For example, it's known that increasing age, tobacco use, and family history are key risk factors for AAA; Breast cancer risks include increasing age, family history, as well as several lifestyle factors like alcohol use; Chlamydia trachomatis infection would normally be considered for individuals with multiple sexual partners and who are sexually active; Colon cancer risks include age, family history, and lifestyle factors like diet; Lastly, Hepatitis C screening is recommended for individuals born between 1945 and 1965, and high-risk individuals like healthcare workers. CLINICAL REASONING: Examining the provided options and keeping our patient's characteristics in mind, several prioritizations could be made. AAA screening, while a potential consideration, could be weighed lower as she has no history of smoking. Breast cancer screening seems up-to-date with a recent normal mammogram. Screening for Chlamydia trachomatis infection might not be indispensable given her long term monogamous relationship. The patient's colonoscopy was normal five years ago, so a routine check-up is not yet due according to the 10-year interval recommended for individuals with normal results. This leaves us with Hepatitis C screening, which is pertinent not only because she was born between 1945 and 1965 but also because she's a healthcare worker exposed to larger risks of bloodborne pathogens. CONCLUDING: Hypothetically, if type 2 diabetes were on the list considering her family history and age, it could have been a priority. However, among the listed options and integrating the guidelines, Hepatitis C screening would be the most suitable test to conduct. RESPONSE: The most appropriate next step in management, specifically in terms of screening, would be for Hepatitis C (E), given her age and occupation which places her in a higher risk category.

**AI_B:** 1. RESTATING THE QUESTION: This question is asking about the most appropriate next step specifically in terms of screening procedures for a healthy 60-year-old woman presenting with controlled hypertension and asthma, and who has a family history of type 2 diabetes and breast cancer. Among the available options are screenings for abdominal aortic aneurysm, breast cancer, Chlamydia trachomatis infection, colon cancer, and hepatitis C. The most appropriate choice should reflect consideration of her overall health, previous findings, family history, lifestyle and current screening guidelines. 2. PLANNING: I would first lay down assumptions regarding the necessity, relevance, and frequency of each provided option. A routine screening for abdominal aortic aneurysm is usually performed on men who smoke between 65-75 years, hence it might not be the first priority for our non-smoking female patient. Breast cancer screening is also an option, but her recent mammography showed no abnormalities. Screening for Chlamydia trachomatis infection might not be necessary given her long stable monogamous relationship. Colon cancer screening, although usually started at 50 years old, the patient's previous colonoscopy was normal. Finally, while screening for Hepatitis C is recommended in every individuals born between 1945-1965, the patient doesn't have an approved risk factor, such as intravenous drug use or a history of blood transfusion. 3. CLINICAL REASONING: Considering the patient’s family history, the likely predisposing factors for illness in her lifetime are type 2 diabetes mellitus and breast cancer. However, the most recent mammogram, a year ago, didn't show any abnormalities suggesting the risk for breast cancer is minimal for now. In regards to type 2 diabetes mellitus, there are routine tests that can be conducted during her upcoming regular medical testing. Among the given options, a one-time screening for Hepatitis C could be conducted because it's a part of universal screening recommendations for individuals born between 1945 and 1965. 4. CONCLUDING: Weighing the patient's personal and family medical history, lifestyle preferences, her regular tests and the current guidelines for each disease, even though the patient does not have direct risk factors for Hepatitis C, a one-time screening seems justifiable and aligns with the current Centers for Disease Control and Prevention (CDC) recommendations. 5. RESPONSE: In conclusion, among the given options (A) Abdominal aortic aneurysm, (B) Breast cancer, (C) Chlamydia trachomatis infection, (D) Colon cancer, (E) Hepatitis C, I would suggest (E) Hepatitis C to be the most appropriate next step in management, based on universal screening recommendations for people born between 1945 and 1965.

**Facilitator** Restating the question: The task is to determine the most appropriate next step in management for a 60-year-old woman with a medical history of well-controlled hypertension and asthma, and a family history of type 2 diabetes and breast cancer. She is a non-smoker, drinks moderately, and has been in a monogamous relationship for three decades. Her recent health screenings, including mammography, laboratory tests, and colonoscopy, have shown no abnormalities. The options for the next step in management include screenings for abdominal aortic aneurysm, breast cancer, Chlamydia trachomatis infection, colon cancer, and Hepatitis C. Planning: The decision should be based on the patient's age, current health status, family medical history, lifestyle factors, and the frequency and guidelines related to each screening test. The patient's occupation as a nurse, which may expose her to bloodborne pathogens, should also be considered. Clinical Reasoning: The patient does not possess the typical risk factors for abdominal aortic aneurysm, such as a history of smoking, being male, and being between the ages of 65 and 75. Her recent mammography showed no abnormalities, and she is not due for another breast cancer screening for another 18 months. Given her monogamous relationship, a Chlamydia trachomatis infection is unlikely. Her last colonoscopy showed no abnormalities, and she is not due for another screening for five years. The CDC recommends one-time Hepatitis C testing for individuals born between 1945 and 1965, which likely includes the patient. Additionally, her occupation as a nurse may put her at a higher risk of bloodborne pathogen transmission. Concluding: After considering all the facts and variables, including the patient's personal and family medical history, lifestyle behaviors, recent preventive health screenings, and appropriate guidelines, Hepatitis C screening appears to be the most suitable next step in her management. Response: Therefore, among the given options, Hepatitis C screening is the most appropriate next step in management for this 60-year-old woman. This recommendation is based on her age, which could be a risk factor for the disease, and her profession as a nurse, which potentially exposes her to bloodborne pathogens. Despite the patient's recent health screenings and absence of relevant symptoms, recommendations indicate Hepatitis C screening for all adults at least once, regardless of their risk factors or lifestyle habits.
