# Supplementary material for: Collaborative intelligence in AI: Evaluating the performance of a council of AIs on the USMLE
Source: PLOS Digit Health. 2025 Oct 9;4(10):e0000787. doi: 10.1371/journal.pdig.0000787 (PMC12510544; doi:10.1371/journal.pdig.0000787)
Supplement: S2 File — (ZIP) [file pdig.0000787.s002.zip › S2 Data Raw data files containing unprocessed question inputs and LLM outputs including transcripts of deliberations/STEP2_Question-31-1_Type-MC-NJ.docx]

**acilitator** **[Round 1]:** A 12-year-old girl is brought to the physician for a follow-up examination 8 days after starting a course of oral penicillin for streptococcal pharyngitis. Temperature is 38.6°C (101.5°F). Examination shows an erythematous morbilliform rash, urticaria, mild swelling of the hands and feet, arthralgias, and lymphadenopathy. Leukocyte count is 13,000/mm³ (62% segmented neutrophils, 10% eosinophils, and 28% lymphocytes). Urinalysis shows 2+ protein. Which of the following is most appropriate to prevent recurrence of these symptoms if the patient is diagnosed with streptococcal pharyngitis again? (A) Pretreatment with an antihistamine (B) Pretreatment with a corticosteroid (C) Skin testing with benzylpenicilloyl polylysine prior to treatment (D) Use of a nonpenicillin antibiotic to treat the pharyngitis (E) Use of a single injection of benzathine penicillin to treat the pharyngitis

**AI_A:** RESTATING THE QUESTION: The question is asking the best way to prevent the recurrence of the symptoms, which seem to be a reaction to penicillin, in a 12-year-old girl who has been diagnosed with streptococcal pharyngitis. PLANNING: The foundational assumption here is that the symptoms presented by the child are a reaction to the penicillin used to treat her streptococcal pharyngitis. We can, however, challenge this assumption by considering if the symptoms might also be indicative of other conditions that could be associated with the recovery phase of streptococcal pharyngitis, such as post-streptococcal glomerulonephritis or rheumatic fever. After considering these possibilities, the presence of a morbilliform rash, urticarial, arthralgias, lymphadenopathy, and evidence of renal involvement in the form of proteinuria, along with the timing of their onset after initiation of antibiotics, strongly suggest a drug reaction, confirming the original assumption. CLINICAL REASONING: Beginning with the assumption that the patient is having an adverse reaction to the penicillin, the most direct solution would be to avoid penicillin in the future, thus preventing the recurrence of the symptoms if the child gets streptococcal pharyngitis again. The use of skin testing to diagnose penicillin allergy was once well established but is now no longer recommended due to the risk of triggering anaphylaxis, and pre-treatment with antihistamines or corticosteroids would not prevent a drug reaction, they would only mitigate some of the symptoms. CONCLUDING: Thus, based on the clinical reasoning, instead of risking another reaction to penicillin, an alternative antibiotic could be substituted in to effectively treat the pharyngitis without triggering the adverse reaction. RESPONSE: The most appropriate option is the (D) Use of a nonpenicillin antibiotic to treat the pharyngitis. It would prevent any recurrence of these symptoms in case she gets streptococcal pharyngitis again, by avoiding the drug responsible for the adverse reaction.

**AI_D:** RESTATING THE QUESTION: The question presents a scenario where a young girl who has been treated with penicillin for streptococcal pharyngitis presents with a fever and a series of symptoms consistent with an allergic reaction. It is the responsibility of the clinician, moving forward, to prevent a recurrence of these symptoms if she is diagnosed with streptococcal pharyngitis again. PLANNING: The underlying assumption is that the patient had an allergic reaction to penicillin, as evidenced by her symptoms. An alternative assumption would be that the symptoms are indicative of a separate infection or condition. The former is more plausible given the clinical presentation. The clinician needs to consider measures that would prevent a recurrence of this allergic reaction during future bouts of streptococcal pharyngitis. CLINICAL REASONING: The typical treatment for streptococcal pharyngitis is oral penicillin. If this patient is exhibiting an allergic response to the drug, it would be unwise to use the same treatment protocol in a future episode. Antihistamines and corticosteroids could help manage the symptoms of an allergic reaction but doing so would not prevent its occurrence — because the root cause, the allergenic trigger, has not been removed. Skin testing could predict the likelihood of a similar allergic reaction, but its use still would not prevent the allergic reaction from occurring if the same antibiotic is used again. The alternative, then, would be to use an antibiotic that has a similar effect on the causative bacteria but is less likely to induce an allergic reaction; or to use a different form of penicillin that might be better tolerated by the patient. CONCLUDING: Given this reasoning, the best management strategy to prevent a recurrence of the allergic reaction during future bouts of streptococcal pharyngitis would be use a nonpenicillin antibiotic to treat the condition. This approach directly addresses the cause of the allergic reaction and eliminates it – unlike the other strategies, which predict it, mitigate the symptoms, or ignore it altogether. RESPONSE: Hence, the most appropriate measure to prevent recurrence of these symptoms if the patient is diagnosed with streptococcal pharyngitis again would be to (D) Use of a nonpenicillin antibiotic to treat the pharyngitis.

**AI_E:** (1) RESTATING THE QUESTION: The young female is experiencing a reaction characterized by fever, skin rash, swelling, joint pain, and elevated white blood cell count, seemingly as a result of her recently initiated penicillin treatment. The urinalysis result indicating protein presence is also noticeable. The question asks how to best avoid a recurrence of these symptoms should she contract streptococcal pharyngitis again. Streptococcal pharyngitis is a throat infection caused by Streptococcus pyogenes ("group A strep"). Penicillin, typically used to treat this infection, seems to have resulted in an adverse effect. (2) PLANNING: Assumptions made would include that penicillin caused the reaction, though we should also consider the possibility that the symptoms are part of a different, simultaneous illness. The latter assumption can be questioned by noting that the timing of these symptoms aligns with the start of penicillin treatment. Clinical priority should be given to confirming the source of these symptoms before planning future treatments, with focus on the potential penicillin involvement. (3) CLINICAL REASONING: Given the timing and the nature of the response seen which includes a skin rash, swelling, joint pain and an increase in the number of eosinophils, we can postulate that this patient may be having a hypersensitivity reaction to penicillin, likely a Type III (immune complex-mediated) hypersensitivity reaction. This is supported further by the presence of protein in the urine, which could indicate immune complex deposition in the kidneys causing proteinuria. (4) CONCLUDING: It is crucial to identify the potential source of this reaction in order to avoid a recurrence. Therefore, the first step should be confirming if penicillin indeed caused this reaction. Consideration can then be given to alternative treatments for streptococcal pharyngitis to bypass potential recurrence of an adverse reaction. (5) RESPONSE: The most suitable choice to prevent recurrence of these symptoms if the patient is diagnosed with streptococcal pharyngitis again would be (D) Use of a nonpenicillin antibiotic to treat the pharyngitis. It allows effective treatment of the infection while reducing the risk of precipitating the same reaction seen with the patient's previous use of penicillin.

**AI_C:** RESTATING THE QUESTION: This question is asking about a young girl, who presented with several symptoms - including rash, urticaria (itchy patches), swelling of hands and feet, joint pain, and swollen lymph nodes - following treatment with penicillin for a strep throat infection. These symptoms are in line with a drug reaction, probably related to the antibiotic she was given. The query regarding what should be done to prevent similar symptoms upon future occurrence of streptococcal pharyngitis is essentially asking how to avoid another drug reaction if she is infected again with strep throat. PLANNING: The fundamental assumption here is that penicillin, which she took for streptococcal pharyngitis, triggered an allergic reaction in the girl. An alternate hypothesis is that another factor or condition was the cause of her symptoms. However, these symptoms coming up shortly after initiating penicillin therapy encourages the first assumption. Let us then identify the clinical priority: to treat the streptococcal infection without provoking a similar drug reaction. CLINICAL REASONING: Given that penicillin appears to be the offending agent, the priority is to choose an alternate antibiotic regimen for the treatment of streptococcal pharyngitis that does not involve a drug that is closely related to penicillin. Antihistamines (A) and corticosteroids (B) may mitigate symptoms of allergy but won’t avoid triggering an allergic reaction if penicillin or its analogs is administered again. Skin testing (C) could be helpful to confirm an allergic reaction to penicillin, but it is not preventative. Using benzathine penicillin (E), which is a type of penicillin, also doesn't make sense if the girl is allergic to penicillin itself. CONCLUDING: With the exclusion of A, B, C, and E, option D remains. Using a nonpenicillin antibiotic allows for effective therapeutic intervention for the girl's streptococcal pharyngitis without risking a similar allergic reaction as was likely experienced with the penicillin. RESPONSE: The most appropriate response to prevent recurrence of these symptoms, in case the patient is diagnosed with streptococcal pharyngitis again, would be (D) the use of a nonpenicillin antibiotic to treat the pharyngitis.

**AI_B:** RESTATING THE QUESTION: A girl of 12 has returned to the doctor 8 days after being prescribed oral penicillin for streptococcal pharyngitis, a bacterial infection in the throat. Her symptoms include a red morbilliform rash, hives (urticaria), puffy hands and feet, joint pain (arthralgias), and swollen lymph nodes (lymphadenopathy). Her white blood cell count is high, indicative of an immune response. Notably, her urinalysis shows protein, a sign of kidney involvement. The question focuses on an appropriate intervention to prevent these symptoms if she again contracts streptococcal pharyngitis in the future. PLANNING: The main assumption appears to be that the symptoms are related to the streptococcal pharyngitis. However, timing, the nature of the symptoms (rash, urticaria, swelling, arthralgias), and laboratory data (eosinophilia, proteinuria) suggest an allergic response or hypersensitivity to penicillin, moving the focus from the infection to the treatment itself. An alternate assumption could be that these symptoms represent a condition such as serum sickness or drug-induced hypersensitivity (Type III hypersensitivity reaction), linked to the administration of penicillin, rather than the infection it was meant to treat. CLINICAL REASONING: Serum sickness-like reactions (SSLRs) incorporate a constellation of symptoms like those seen in this girl, usually appearing 1-2 weeks after exposure to certain drugs. They involve immune complex deposition in tissues and activation of the complement system, leading to an inflammatory response (hence the increase in white blood cells). The occurrence of proteinuria denotes possible kidney involvement, another common feature of SSLRs. Eosinophils, which increase in allergic reactions, are also elevated here. CONCLUDING: Given these factors, it is reasonable to surmise that the penicillin, rather than the streptococcal pharyngitis, resulted in the observed symptoms. If this is indeed a hypersensitivity reaction to penicillin, the solution would be to avoid this class of antibiotics in future treatment regimens. RESPONSE: Therefore, to prevent the recurrence of these symptoms if the patient is diagnosed with streptococcal pharyngitis again, the most appropriate option would be (D) Use of a nonpenicillin antibiotic to treat the pharyngitis.

**Facilitator** The consensus statement synthesizes the responses of AI_A, AI_B, AI_C, AI_D, and AI_E as follows: The question presents a scenario where a 12-year-old girl, who was treated with penicillin for streptococcal pharyngitis, developed symptoms indicative of an allergic reaction. The symptoms, which include a rash, urticaria, swelling, joint pain, and an elevated white blood cell count, along with proteinuria, suggest a hypersensitivity reaction to penicillin. This is further supported by the timing of the symptoms, which appeared shortly after the initiation of penicillin therapy. The primary assumption is that the symptoms are a reaction to the penicillin used to treat her streptococcal pharyngitis. While other conditions associated with streptococcal pharyngitis were considered, the clinical presentation strongly suggests a drug reaction. This leads to the clinical priority of treating the streptococcal infection without provoking a similar drug reaction in the future. Given that penicillin appears to be the offending agent, the most direct solution would be to avoid penicillin in the future. Other options such as pretreatment with antihistamines or corticosteroids, skin testing, or using a different form of penicillin were considered but dismissed as they either do not prevent a drug reaction or could potentially trigger a similar reaction. Therefore, the consensus is that the most appropriate measure to prevent recurrence of these symptoms if the patient is diagnosed with streptococcal pharyngitis again would be to use a nonpenicillin antibiotic to treat the pharyngitis. This approach directly addresses the cause of the allergic reaction and eliminates it, providing effective therapeutic intervention for the girl's streptococcal pharyngitis without risking a similar allergic reaction as was likely experienced with the penicillin.
