# Supplementary material for: Collaborative intelligence in AI: Evaluating the performance of a council of AIs on the USMLE
Source: PLOS Digit Health. 2025 Oct 9;4(10):e0000787. doi: 10.1371/journal.pdig.0000787 (PMC12510544; doi:10.1371/journal.pdig.0000787)
Supplement: S2 File — (ZIP) [file pdig.0000787.s002.zip › S2 Data Raw data files containing unprocessed question inputs and LLM outputs including transcripts of deliberations/STEP2_Question-32-1_Type-MC-NJ.docx]

**Facilitator** **[Round 1]:** A 45-year-old woman is hospitalized for management of Staphylococcus aureus endocarditis with persistent bacteremia. The patient is discussed during interdisciplinary rounds, which includes physicians, nurses, pharmacists, and social workers. During rounds, a pharmacy student notices that the patient missed two doses of her scheduled antibiotic last week but is unsure why. The physician and nurse are unaware of these missed doses, and the student does not mention her observation. Which of the following measures is most likely to improve communication within this interdisciplinary health care team? (A) Conduct interdisciplinary rounds in a quieter location (B) Encourage questions from all team members (C) Implement a checklist for standardizing patient rounds (D) Use computers during rounds to review medications

**AI_A:** (1) RESTATING THE QUESTION: So, the issue at hand involves a 45-year-old woman hospitalized with Staphylococcus aureus endocarditis and persistent bacteremia. In the interdisciplinary team handling her case, a gap in communication was identified when a pharmacy student noticed the patient had missed two doses of her antibiotic regime but did not share this observation. The question aims to identify the best measure to improve this communication gap within the team. (2) PLANNING: The assumptions may include that (A) conducting rounds in a quieter location will lead to better communication, (B) encouraging questions from all team members will facilitate better interaction, (C) standardizing patient rounds using a checklist will improve communication, or (D) the use of computers during rounds will automatically enhance information sharing. However, we need to look deeper into the nature of the problem. Is it environmental distractions causing communication disruption, reticence from team members, lack of a structured system for rounds, or inadequate usage of technology for information access? (3) CLINICAL REASONING: The stated challenge revolves around a potentially critical piece of patient information not being communicated. A quieter location (A) might reduce distractions but doesn't necessarily encourage open communication. Similarly, using computers (D) might help with reviewing medications but will not necessarily promote sharing comments or concerns. A checklist (C) can serve as a guide, but it might limit discussions to only items on the list, excluding spontaneous observations. Encouraging questions from everyone (B), on the other hand, fosters an open communication culture where team members are confident to share or ask about patient-specific information. (4) CONCLUDING: The issue isn't primarily environmental distractions, nor limited access to patient information via technology or lack of a structured system, but more pertaining to a work culture that doesn't encourage enough open communication. Hence, active encouragement to ask questions and share observations should yield the most significant improvement in this scenario. (5) RESPONSE: Therefore, answer choice (B), encouraging questions from all team members, is the most likely measure to improve communication within this interdisciplinary health care team. This encourages a culture of open communication, promoting participative care and improved patient outcomes.

**AI_D:** RESTATING THE QUESTION: This is a scenario of a middle-aged woman who is dealing with a serious bacterial infection known as Staphylococcus aureus endocarditis which is associated with persistent bacteremia. Bacteremia refers to the presence of bacteria in the blood, a condition typically associated with severe infections. This woman's care involving multiple healthcare professionals including physicians, nurses, pharmacists, and social workers. A pharmacy student in the team noticed that she missed two doses of her important antibiotic treatment last week, but did not communicate this observation. The attending physician and nurse also seem unaware of the missed doses. Now, the question is what measure can be introduced to enhance the communication within this interdisciplinary team? PLANNING: The underlying assumptions here are that the environment of interdisciplinary rounds could be distracting, team members might feel hesitant to ask questions, there might be a lack of standard procedures during patient rounds, and the use of technology may improve medication review. To improve communication, we need to create an environment where everyone feels comfortable enough to speak up about their observations, have a structured approach during patient rounds to ensure nothing is overlooked, and leverage technology to keep track of medication histories. CLINICAL REASONING: Considering this situation, the lack of communication and the missed doses of antibiotics could lead to a deterioration in the patient's condition, hence it is crucial that all findings are shared amongst the team. Changing the location of the rounds won't necessarily promote better communication. The use of computers to review medications could potentially improve the situation, but it doesn't solve the broader issue of undercommunication across different disciplines. Implementing a standardized checklist for patient rounds could definitely improve the overall process, but it may not necessarily encourage open discussions. In this particular scenario, students, residents, or staff might still feel hesitant to share their observations due to hierarchical structures or to avoid confrontation. CONCLUDING: In light of all the above points, it would be critically essential to encourage the sharing of all observations and questions from all members of the team. An open and collaborative atmosphere would promote dialogue and discussions, thereby enhancing both the quality of care and patient safety. RESPONSE: Thus, the measure most likely to improve communication within this interdisciplinary health care team would be (B) Encourage questions from all team members. This would ensure that any irregularities like the missed doses of antibiotics would be identified and discussed promptly for better patient outcomes.

**AI_E:** (1) RESTATING THE QUESTION: This scenario is about a patient in a hospital suffering from a Staphylococcus aureus endocarditis and perpetual bacteremia. They've missed two doses of their necessary antibiotics, a fact discovered by a pharmacy student during interdisciplinary rounds including doctors, nurses, pharmacists and social workers. However, the student doesn't communicate this finding, and the rest of team remains unaware. The question centers around identifying the most effective action to be undertaken to enhance communication within the interdisciplinary health care team. (2) PLANNING: While all options might have some impact on communication, each one carries its own assumptions. For instance, conducting rounds in a quieter location (A) implicitly assumes that noise and interruptions are the primary barriers to communication. Encouraging questions from all team members (B) assumes that team members are hesitant to ask questions. Implementing a checklist for standardizing patient rounds (C) presumes that the current system of rounds is unstandardized and chaotic, leading to missed information. Using computers to review medications (D) assumes that medication errors occur due to not utilizing technology. Given the information, the assumption that team members might be hesitant to share observations or ask questions seems the most likely. (3) CLINICAL REASONING: In this scenario, the communication breakdown occurred when the pharmacy student noticed important information about the patient’s care (i.e., missed antibiotics doses) but did not share this observation with the larger team. This seems to imply that the issue here isn't so much about noisy environments, lack of standardization, or insufficient technology but rather hesitance or an unclear communication hierarchy. (4) CONCLUDING: Evaluating our hypothesized assumptions and correlating them with clinical reasoning, it suggests that undermining the culture that promotes silence over speaking up and reinforcing a culture of open communication would likely have the greatest impact. (5) RESPONSE: Therefore, the optimal measure to improve communication within this interdisciplinary health care team would be to Encourage questions from all team members (B). This seems the best course of action because it can make all team members feel comfortable to highlight potential issues or provide important information about the patient's care. This pathway not only promotes proper information dissemination but also accelerates learning and fosters a more inclusive and effective care environment.

**AI_C:** (1) RESTATING THE QUESTION: We're examining the scenario of a middle-aged woman in the hospital, who is being treated for a serious bacterial infection affecting her heart (Staphylococcus aureus endocarditis) and has bacteria in her blood (persistent bacteremia). During interdisciplinary rounds (meetings where the patient's progress and care are discussed by various healthcare professionals), a pharmacy student notices that the patient has missed two doses of her antibiotic medication. No one else on the team is aware of these missed doses, and the student doesn't share this information. The question is asking for the most effective strategy to improve communication within this diverse healthcare team. (2) PLANNING: Here are the assumptions: we are supposing that the existing communication method isn't adequately effective (as demonstrated by the missed medication doses information wasn't properly communicated), and that the primary objective is to improve communication. The alternatives are whether to change the location of rounds, to encourage questions from all team members, to implement a checklist for rounds, or to use computers during rounds to review medications. The clinically high prioritized action should be the one that directly ensures that important information like missed doses of medication is properly relayed to all members of the care team. (3) CLINICAL REASONING: Let's analyze each option: (A) Conducting rounds in a quieter location might decrease distractions, but this does not guarantee better communication, especially regarding specific missed medications which is the key issue discussed here. (B) Encouraging questions could lead to more open communication, but it doesn't guarantee that all important information will be brought up or noticed. (C) Implementing a checklist for standardizing patient rounds could potentially ensure that all relevant topics are covered during the discussion, hence could help members to systematically bring up issues like medication adherence. (D) Using computers during rounds to review medications ensures the team takes a look at the medication administration record, but it relies on the presumption that the missing doses were correctly documented and might not still directly aid in communication of these crucial details. (4) CONCLUDING: Based on the clinical thought process, it appears that the best way to ensure that all critical information, such as missed medication doses, gets communicated across all team members would be to implement a systematic approach to patient rounds. Hence, a checklist would be the most effective measure to improve communication. (5) RESPONSE: Consequently, answer (C) Implement a checklist for standardizing patient rounds is the most appropriate strategy to improve communication within this interdisciplinary health care team. It enables the care team to systematically discuss each aspect of a patient's care, making it less likely that essential information will be overlooked or remain unmentioned.

**AI_B:** (1) RESTATING THE QUESTION: The query revolves around a patient suffering from Staphylococcus aureus endocarditis, a serious infection of the heart's endocardium often caused by the Staphylococcus aureus bacterium. There seems to be a communication issue in the interdisciplinary health care team managing her as a pharmacy student noticed that two doses of her antibiotic regimen were missed and did not convey this observation. The team was not aware of these missed doses either. The question now is which among the given strategies could improve the communication within this multidisciplinary team. Technical terms/concepts: - Staphylococcus aureus endocarditis refers to a bacterial infection of the inner lining of the heart (endocardium). - Persistent bacteremia is the ongoing presence of bacteria in the blood, which can lead to serious complications if not addressed. - Interdisciplinary rounds involve multiple health care professionals from different disciplines/disciplines discussing and coordinating the care of a patient. - Antibiotic treatment is crucial in managing bacterial infections like endocarditis, with missed doses potentially leading to treatment failure or complications. (2) PLANNING: The issue at hand appears to revolve around the communication lapse within the interdisciplinary health care team. Suggested measures for improvement include changing the location of rounds, fostering an open environment for questions, implementation of patient rounds' checklist, and technological aid during rounds. Each assumption needs to be evaluated for potential clinical application: - Assumption#1: Changing the location will improve communication. - Assumption#2: Encouraging questions will foster better communication. - Assumption#3: Standardizing patient rounds with a checklist enhances communication. - Assumption#4: Using computers during rounds will improve medication review. Hypothesis: Considering the presented scenario, Assumption#2 may be the most relevant, as it would empower the pharmacy student to voice their observations, resulting in improved team awareness and patient care. (3) CLINICAL REASONING: Let's analyze how each assumption could potentially impact the clinical scenario. - Assumption#1: Conducting rounds in a quieter location might limit distractions, but it doesn't necessarily enhance communication among team members (especially those less comfortable voicing their concerns). - Assumption#2: Encouraging questions can foster an environment where each team member, irrespective of their role, feels comfortable sharing their observations, leading to all team members having a more comprehensive understanding of the patient's condition and treatment. This contributes directly to the case presented, where the pharmacy student noticed an important observation but did not communicate it. - Assumption#3: Implementing a checklist could help standardize patient rounds, ensure all important facets of patient care are reviewed, and potentially limit error. Still, it doesn't necessarily promote open communication and might not address the observation of missed antibiotic doses if it isn't part of the checklist. - Assumption#4: Using computers for medication review can help keep track of medication administration, but it doesn't actively improve open communication within the team. (4) CONCLUDING: An open communication environment where all members feel comfortable sharing their observations and asking questions is pivotal for comprehensive and effective patient care. In the context of the scenario presented, this approach would have allowed the pharmacy student to communicate their observation, alerting the team to the missed antibiotic doses, which are critical for the successful treatment of bacterial endocarditis. (5) RESPONSE: Therefore, the measure most likely to improve communication within this interdisciplinary health care team would be to (B) Encourage questions from all team members. Not only would this directly address the communication issue highlighted in this scenario, but it would also foster an environment of shared responsibility and collaboration, leading to comprehensive patient management and improved health outcomes.

**Facilitator** **[Round 2]:** Summary: AI_A and AI_B both believe that encouraging questions from all team members (B) is the best way to improve communication within the interdisciplinary health care team. They argue that the issue is not primarily environmental distractions, lack of a structured system, or inadequate usage of technology, but rather a work culture that doesn't encourage open communication. AI_C, however, believes that implementing a checklist for standardizing patient rounds (C) is the most effective measure to improve communication. They argue that a systematic approach to patient rounds would ensure that all critical information, such as missed medication doses, gets communicated across all team members. AI_D agrees with AI_A and AI_B that encouraging questions from all team members (B) is the best way to improve communication. They argue that an open and collaborative atmosphere would promote dialogue and discussions, thereby enhancing both the quality of care and patient safety. AI_E also agrees with AI_A, AI_B, and AI_D that encouraging questions from all team members (B) is the best way to improve communication. They argue that this would make all team members feel comfortable to highlight potential issues or provide important information about the patient's care. Question to clarify disagreements: AI_C, you suggested that implementing a checklist for standardizing patient rounds is the most effective measure to improve communication. However, AI_A, AI_B, AI_D, and AI_E believe that encouraging questions from all team members is the best way to improve communication. Can you explain why you believe a checklist would be more effective than encouraging open communication?. Based on your preceding response, how would you answer the original question: [A 45-year-old woman is hospitalized for management of Staphylococcus aureus endocarditis with persistent bacteremia. The patient is discussed during interdisciplinary rounds, which includes physicians, nurses, pharmacists, and social workers. During rounds, a pharmacy student notices that the patient missed two doses of her scheduled antibiotic last week but is unsure why. The physician and nurse are unaware of these missed doses, and the student does not mention her observation. Which of the following measures is most likely to improve communication within this interdisciplinary health care team? (A) Conduct interdisciplinary rounds in a quieter location (B) Encourage questions from all team members (C) Implement a checklist for standardizing patient rounds (D) Use computers during rounds to review medications]

**AI_A:** Restating the question: The question is asking for the most suitable measure to help improve the communication in an interdisciplinary health care team. The problem raised is linked with the missed awareness and communication about the patient's significant missed antibiotic doses, which are detrimental for an ongoing bacterial infection, specifically Staphylococcus aureus endocarditis, a dangerous infection of the interior part of the heart (endocardium) by the bacterium Staphylococcus aureus. Planning: The assumptions here are that (A) the location of the rounds is noisy, (B) all team members do not feel comfortable asking questions, (C) the patient rounds are not standardized, and (D) computers are not being used currently to review medications. Now, we could hypothesize that assuming rounds are conducted in a quieter location, or a checklist is made or computers are used, or people are encouraged to ask more questions, communication within the health care team can be improved. But among these assumptions, we should prioritize the one about facilitating open communication as it stands foundational in any collaborative medical decision-making. Clinical Reasoning: When analyzing the scenario, the primary issue lies in the pharmacy student's hesitance to voice an important concern about the patient's missed medication doses. The reasons might be her not feeling comfortable, knowledgeable, or authorized enough to interact over a critical care aspect. Irrespective of the location where rounds are conducted, or the tools used to review medications, or even the existence of a standard checklist, if individual team members do not feel empowered to raise questions or concerns, critical information may still be missed. Concluding: Addressing the root cause in terms of promoting an open and supportive culture that facilitates a dialogue from all members, regardless of their hierarchy or role, should be targeted for improvement. It encourages not just outward communication but also inward assimilation in decision-making. Response: Therefore, the most likely measure to improve communication within this interdisciplinary health care-team is to "Encourage questions from all team members" (Option B). It would foster a more open and engaging environment, bridging communication gaps, and thereby enhancing comprehensive patient care.

**AI_B:** Restating the question: This question is about enhancing healthcare communication, within an interdisciplinary team providing care to a 45-year-old woman with endocarditis due to Staphylococcus aureus. During patient rounds, a pharmacy student notices an important issue where the patient missed two doses of her antibiotic. However, the student doesn't share her observations with the team, leaving the physician and nurse uninformed about this critical medication adherence issue. The question poses four potential strategies for improving communication in this context and asks for the most likely to be effective. Planning: Assumptions include that the student didn't communicate because either she felt her voice wasn't important, she was unsure about her observations, or there was no structured system to communicate such findings. Alternatives would be that she was simply forgetful or lacked the confidence to voice her observation. After evaluating these, it is important to prioritize the change that most directly addresses the root cause of the missed communication, i.e., the culture within the healthcare team and the common protocols in place during patient rounds. Clinical Reasoning: An open team culture and an organized system both have important contributions to effective communication. However, it seems that the root cause of the missed communication event leaned more towards a culture issue, where the pharmacy student didn't feel empowered or comfortable to voice her observations. Therefore, while a checklist might help to standardize procedures, it might not directly solve the problem if the students or junior team members still feel hesitant to speak up. Concluding: The most successful approach would likely combine a cultural change that encourages every member to voice their observations and concerns, alongside an organized system that ensures every important piece of information is addressed. This way, the team dynamics would positively impact patient care by promoting open communication and accountability. Response: Based on this reasoning approach, encouraging questions from all team members (B) should be the most likely measure to improve communication within this interdisciplinary health care team. This would promote an open atmosphere where everyone's contributions are recognized as important, potentially leading to enhanced patient care quality and safety. Concurrently, implementing a more structured communication system such as a checklist (C) or utilizing technological aids (D) could also be considered to complement this culture change.

**AI_E:** RESTATING THE QUESTION: The question is asking for the most effective way to improve communication among an interdisciplinary health care team consisting of physicians, nurses, pharmacists, and social workers. The issue presented is that a pharmacy student noticed a patient had missed two doses of her antibiotic last week and did not communicate this information to the rest of the team. PLANNING: To answer this question, we should consider the dynamics of the health care team, the implications of missed medication doses, the nature of the health care environment, strategies for effective communication in such a setting, and the potential benefits and challenges of each of the proposed solutions. CLINICAL REASONING: The primary problem here seems to be a lack of open communication within the health care team. The pharmacy student did not feel comfortable or deemed it necessary to share her observation. This might indicate an issue with the team culture that does not foster an environment of asking questions and sharing information freely, irrespective of roles or hierarchies. This issue could be remedied by encouraging questions from all team members, which would likely enhance collaboration, promote a learning environment, and ultimately improve patient care and safety. Implementing a checklist for standardizing patient rounds or using computers during rounds to review medications might help ensure consistent information sharing, but these measures may not necessarily instill a culture of open communication. As for conducting rounds in a quieter location, it wouldn't necessarily solve the problem of poor communication if the issue at heart is one of power-dynamic or confidence to speak up. CONCLUDING: Though all proposed measures might contribute to improving the overall care of patients to varying extents, based on the given scenario, the most effective measure to improve communication within this interdisciplinary health care team seems to be to encourage questions from all team members (B) to foster an open and communicative culture. RESPONSE: Encouraging questions from all team members (B) would ensure that all pertinent aspects of patient care, including missed doses of medication, are thoroughly discussed and addressed during the patient rounds, thus promoting patient safety and effectiveness of medical care.

**AI_D:** RESTATING THE QUESTION: The question is asking for the most effective method to improve communication within a multidisciplinary healthcare team taking care of a 45-year-old woman with Staphylococcus aureus endocarditis. Endocarditis is an inflammation of the inner layer of the heart, which can be caused by bacteria such as Staphylococcus aureus, resulting in persistent bacteremia or presence of bacteria in the bloodstream. PLANNING: I would argue for the clinical priority of (B) Encouraging questions from all team members. Here, we are assuming that the primary issue is not the lack of system and process (such as a checklist) or the use of technology or environmental factors but rather the communication culture within the team itself. CLINICAL REASONING: First, it is evident that the pharmacy student has observed a critical issue related to the patient's treatment plan - two missed doses of a schedule antibiotic. However, the student does not communicate this observation. The physician and nurse are not aware of these missed doses, which could have serious implications for the patient's health, particularly considering the patient's given condition of Staphylococcus aureus endocarditis. Secondly, the lack of communication from the student may stem from a culture that does not encourage open information sharing and discussion. Encouraging questions from all team members, irrespective of their roles at the hierarchy, can create a safe space for open dialogue that in turn can improve the quality of care and even patient safety. An environment where everyone is encouraged to share observations, ask questions, and participate in discussions creates a more well-rounded perspective for patient care planning and execution, facilitating informed decision-making. CONCLUDING: The failure to communicate the missed doses could be attributed to a lack of inclusive and open communication atmosphere where individuals may not feel safe or confident to talk about their observations, voicing their concerns or opinions, or asking critical questions. RESPONSE: Therefore, I believe that the most effective measure to improve communication within this interdisciplinary healthcare team is to (B) Encourage questions from all team members. Through fostering a climate of inclusivity, respect, and openness, every team member's observations and insights can contribute meaningfully to the patient's care.

**AI_C:** Restating the question: The scenario describes an interdisciplinary healthcare team caring for a hospitalized 45-year-old woman with Staphylococcus aureus endocarditis, a condition where the heart's inner lining is infected. The patient had missed two doses of her antibiotics unbeknownst to part of her care team, and it's unclear why the pharmacy student who noticed this did not communicate it to the rest of the team. The question is thus seeking the best strategy for improving communication among the team members, to prevent such instances from reoccurring. Planning: Here, an assumption is that there isn't an effective enough system in place for the multidisciplinary team to communicate about critical elements of patient care. Alternately, the problem might be a cultural one within the team, where not all members feel empowered to share their Observations or voice concerns. Both options have potential merit, but the clinical priority would be to ensure all critical patient information is known by all involved in the patient's care. Clinical Reasoning: For example, if a system such as a checklist was in place, this could provide a structure ensuring detailed communication about crucial elements of a patient's care, including medication administration. This would likely have led to the missed medication doses being identified and addressed promptly. Alternatively, fostering a culture that encourages questions and active participation from all team members could have prompted the pharmacy student to bring up the medication issue during the team's discussions. Concluding: Hence, it is a complex issue involving both the structure of their care rounds and the team's culture. However, considering clinical prioritization and the urgency of the patient's condition, the action that needs immediate implementation would arguably be the system that ensures vital information does not get missed. Response: Therefore, based on my domain knowledge and the process outlined above, I would favor Option C: "Implement a checklist for standardizing patient rounds". This systematic approach would encourage and ensure communication about critical elements of patient care, which in the given scenario is evidently lacking and thus causing the most immediate potential harm to the patient. Of course, it would also be beneficial in the longer term to work on fostering an encouraging team culture as per Option B.

**Facilitator** **[Round 3]:** Summary: AI_A believes that the most effective measure to improve communication within the interdisciplinary health care team is to encourage questions from all team members. They argue that the root cause of the problem is the pharmacy student's hesitance to voice an important concern, which could be due to not feeling comfortable, knowledgeable, or authorized enough to interact over a critical care aspect. AI_B also supports encouraging questions from all team members as the most effective measure. They argue that the root cause of the missed communication event leaned more towards a culture issue, where the pharmacy student didn't feel empowered or comfortable to voice her observations. AI_C, however, favors implementing a checklist for standardizing patient rounds. They argue that the immediate implementation of a system that ensures vital information does not get missed is crucial, considering the urgency of the patient's condition. AI_D agrees with AI_A and AI_B, stating that the most effective measure is to encourage questions from all team members. They argue that the failure to communicate the missed doses could be attributed to a lack of inclusive and open communication atmosphere. AI_E also supports the measure of encouraging questions from all team members. They argue that the primary problem is a lack of open communication within the health care team, which could be remedied by encouraging questions from all team members. Question to clarify disagreements: The main disagreement lies between AI_C and the rest of the council members. AI_C believes that implementing a checklist for standardizing patient rounds is the most effective measure, while the others believe that encouraging questions from all team members is the most effective measure. To clarify this disagreement, we could ask: "How do you weigh the importance of implementing a structured system like a checklist against fostering an open communication culture in the healthcare team? Could these two approaches be combined for a more effective solution?". Based on your preceding response, how would you answer the original question: [A 45-year-old woman is hospitalized for management of Staphylococcus aureus endocarditis with persistent bacteremia. The patient is discussed during interdisciplinary rounds, which includes physicians, nurses, pharmacists, and social workers. During rounds, a pharmacy student notices that the patient missed two doses of her scheduled antibiotic last week but is unsure why. The physician and nurse are unaware of these missed doses, and the student does not mention her observation. Which of the following measures is most likely to improve communication within this interdisciplinary health care team? (A) Conduct interdisciplinary rounds in a quieter location (B) Encourage questions from all team members (C) Implement a checklist for standardizing patient rounds (D) Use computers during rounds to review medications]

**AI_E:** Restating the Question: This question seeks to best solve the problem of improving communication within an interdisciplinary healthcare team treating a 45-year old woman for Staphylococcus aureus endocarditis with persistent bacteremia. The issue is that crucial information, in this case, missed antibiotic doses, is not adequately relayed through the team. Planning: Several assumptions are embedded here. One major assumption is that the communication problem lies in team members' fears or hesitations to ask or relay questions/information. Another assumption is implementation of a standardized checklist during patient rounds would aid better communication. Yet another possibility is the idea that a quieter location or use of computers to review medications could improve communication. These assumptions are based on the idea that the environment or protocols of the interdisciplinary team are the solution. However, for the purposes of this situation, the clinical priority seems to be fostering an atmosphere of open communication where all members, including students, feel comfortable voicing their observations. Clinical Reasoning: There is no evidence indicating that noise, lack of structure in the rounds, or absence of computer-mediated medication reviews are the core reasons for the missed communication of the pharmacy student's observation. On the other hand, the available information suggests the pharmacy student felt unsure about revealing her observations. This indicates a potential problem with the team's communication culture rather than its organizational structure, location, or technological aids. Concluding: Thus, after considering the various assumptions and aligning the most fitting one with the provided situational information, fostering open communication within the healthcare team through encouraging the voicing of questions or observations by all team members is the priority. Response: Therefore, the measure most likely to improve communication within this interdisciplinary health care team is (B) Encouraging questions from all team members.

**AI_B:** The question outlines a communication flaw within an interdisciplinary health care team dealing with a critical case of a patient with Staphylococcus aureus endocarditis, marked by persistent bacteremia. The problem emerged when the pharmacy student observed significant, inadequate adherence to the antibiotic regimen but didn't communicate her observations. Given the held professional responsibility of the interdisciplinary health care team, this gap in communication could affect the progress of the patient's infection management. There are multiple assumptions to consider here: the cause of the pharmacy student's reluctance to voice an observation, the efficiency of the current system of rounds, and the implication of the missed doses on the patient’s condition. However, it would be crucial to prioritize the assumption that the pharmacy student hesitated to speak up due to a lack of communication transparency and inclusivity. Medical rounds involve a structured interaction between various care providers to ensure the efficiency of care. The development of rapport, mutual respect, and inclusive communication become vital to this setting. An environment that encourages questioning potentially from every team member secures an open platform for sharing observations, thoughts, and concerns. Consequently, this intervention can be hypothesized to tackle the root cause of the issue that led to the lapse in communication. It is safe to presume that communication obstacles diminish when an environment facilitates transparency and inclusivity, promoting problem-solving and enriching the delivery of care. Therefore a step-by-step deduction leads me to the conclusion that encouraging questions from all team members (option B) will likely improve communication within this interdisciplinary health care team. By fostering an open culture that values questions and input from all team members, the student would feel included and more comfortable voicing concerns, leading to better communication and, potentially, improved patient outcomes.

**AI_C:** 1) RESTATING THE QUESTION: The question is asking which measure would most likely improve the communication within a multidisciplinary health care team in a situation where a pharmacy student noticed that a patient with Staphylococcus aureus endocarditis and persistent bacteremia had missed two doses of her scheduled antibiotic but did not mention her observation. 2) PLANNING: The issue in this scenario is that potentially relevant clinical information wasn't communicated within the team. Assumptions at play could be that every team member feels comfortable speaking up about their observations or that existing procedures are efficient in covering all relevant patient information. An alternate assumption could be that there is a lack of open communication and an environment that encourages mutual respect and knowledge sharing amongst various members of the team, which could be prioritized to rectify. 3) CLINICAL REASONING: The missed communication about the antibiotic doses is likely a symptom of a larger issue – a communication gap in the healthcare team. The input from pharmacy students or any other junior member should be valued as it could have significant patient care implications. The significance of these two missed doses in this case could be high, as it could contribute to the resistance of Staphylococcus aureus to the antibiotic, worsening the patient's condition. 4) CONCLUDING: Considering these issues, the measure to be taken should address all communication bottlenecks and encourage inputs from all members. Any hierarchy should not create barriers to communication. Therefore, measures that encourage open communication, mutual respect, and shared decision-making should be preferred. 5) RESPONSE: With this reasoning, encouraging questions from all team members (option B) appears to be the most effective measure. It fosters an environment that values inputs from all, irrespective of their position in the team, which can lead to more comprehensive and improved patient care. This approach not only would have potentially prevented the communication gap in the current scenario but would also likely lessen the chances of similar instances happening in the future, improving overall team communication.

**AI_D:** (1) RESTATING THE QUESTION: In essence, the inquiry is centered around identifying the most efficacious strategy for bolstering intercommunication among a healthcare team consisting of various professionals and students. This is grounded in a scenario where a pharmacy student observed that a patient didn't receive two doses of her expected antibiotic regimen for Staphylococcus aureus endocarditis with ongoing bacteremia, but failed to disclose this information. Endocarditis is an inflammation of the endocardium (inner layer of the heart), often caused by bacterial infection, and bacteremia refers to the presence of bacteria in the bloodstream. (2) PLANNING: Given these circumstances, the assumptions are: a) this information withholding may have resulted from the student's belief that they lacked sufficient authority or qualifications to bring up this issue; b) perhaps the student did not recognize the clinical significance of the missed doses; or c) there could be a lack of a systemized method to report such discoveries, leading to some information falling through the cracks in a dynamic and busy patient rounds. I would prioritize exploring the assumption that the pharmacy student felt they may not have the proper authority or qualifications to broach this topic. (3) CLINICAL REASONING: Generally, a culture of open communication in healthcare settings promotes better patient outcomes by minimizing potential harm due to medication errors or miscommunications. In this instance, the student, despite being part of the interdisciplinary team, hesitated to share a critical piece of patient information. This suggests there could be a lack of a safe communication culture wherein every team member, irrespective of their hierarchy or role, feels comfortable voicing their concerns or observations. (4) CONCLUDING: As such, focusing on cultivating an environment that encourages questions and discussions from all team members could vastly improve communication. People in such cultures often feel valued, respected, and become more effectively engaged in patient care. (5) RESPONSE: Therefore, an appropriate way to improve communication within this interdisciplinary health team would be to "Encourage questions from all team members" (Option B). This would empower every individual, including students, to participate actively during rounds, sharing observations that may be crucial to patient care.

**AI_A:** Restating the question: The question asks for the most appropriate measure to enhance communication within an interdisciplinary health care team who are responsible for a patient with Staphylococcus aureus endocarditis and persistent bacteremia. The team is composed of physicians, nurses, pharmacists, and social workers. The problem presented involves a pharmacy student who noticed the patient missed two doses of her antibiotic treatment. However, the student didn't share this observation with the team due to an unspecified reason. Planning: A few assumptions could be considered here. Primarily, it could be assumed that the pharmacy student was either not comfortable, not authoritative, or not knowledgeable enough to mention her observation. Alternatively, maybe the pharmacy student was willing to share the information but didn't have the appropriate setting or channel to do so, perhaps due to the lack of structure in the rounds. Clinical Reasoning: In addressing the first assumption, one must understand that an interdisciplinary team functions best when every member is encouraged to voice their thoughts openly. This is particularly paramount in a setting where patient's life could be at risk, such as in this case, where a serious infection's treatment regimen gets unintentionally affected. Therefore, fostering a culture that encourages all team members, regardless of their role or seniority, to ask questions and share observations can bridge the communication gap. Evaluating the second assumption, the need for a more structured conduct during the rounds, such as implementing a checklist, seems crucial to avoid the omission of important details. A checklist may ensure that every aspect of patient care, from diagnosis to medication administration, gets systematically addressed. Conclusions: Balancing both alternate assumptions, it seems plausible that generating an open and inclusive communication culture should take precedence over immediate structural changes. This is because a constructive culture can enhance the usability and effectiveness of any system subsequently imposed. However, once the open culture is in place, adopting a standardized checklist during patient rounds seems beneficial too. Response: Therefore, in response to the original question, the most likely measure to improve communication within this interdisciplinary health care team would be to 'Encourage questions from all team members' (Option B). Creating this culture would allow important observations to be openly communicated and discussed, which can potentially optimise patient care. It would enable the pharmacy student, in this case, to share her observation, highlighting a crucial issue relating to the patient's treatment regimen.

**Facilitator** The consensus statement synthesizes the responses of AI_A, AI_B, AI_C, AI_D, and AI_E as follows: The question at hand involves identifying the most effective measure to improve communication within an interdisciplinary healthcare team treating a patient with Staphylococcus aureus endocarditis and persistent bacteremia. The issue arose when a pharmacy student noticed the patient missed two doses of her antibiotic treatment but did not share this observation with the team. Several assumptions were considered, including the student's comfort level in sharing observations, the efficiency of the current system of rounds, and the potential impact of the missed doses on the patient's condition. However, the consensus among the AI responses is that the primary issue lies in the communication culture within the team. The interdisciplinary team's function is optimized when all members, regardless of their role or seniority, are encouraged to voice their thoughts openly. This is particularly crucial in a setting where a patient's life could be at risk, such as in this case, where a serious infection's treatment regimen is unintentionally affected. Therefore, fostering a culture that encourages all team members to ask questions and share observations can bridge the communication gap. While implementing a checklist for standardizing patient rounds or using computers during rounds to review medications could be beneficial, these structural changes should follow the establishment of an open and inclusive communication culture. This is because a constructive culture can enhance the usability and effectiveness of any system subsequently imposed. Therefore, the consensus is that the most likely measure to improve communication within this interdisciplinary health care team would be to 'Encourage questions from all team members' (Option B). This approach would allow important observations to be openly communicated and discussed, which can potentially optimize patient care. It would enable the pharmacy student, in this case, to share her observation, highlighting a crucial issue relating to the patient's treatment regimen.
