# Supplementary material for: Collaborative intelligence in AI: Evaluating the performance of a council of AIs on the USMLE
Source: PLOS Digit Health. 2025 Oct 9;4(10):e0000787. doi: 10.1371/journal.pdig.0000787 (PMC12510544; doi:10.1371/journal.pdig.0000787)
Supplement: S2 File — (ZIP) [file pdig.0000787.s002.zip › S2 Data Raw data files containing unprocessed question inputs and LLM outputs including transcripts of deliberations/STEP2_Question-34-1_Type-MC-NJ.docx]

**Facilitator** **[Round 1]:** A 37-year-old man comes to the physician for a routine health maintenance examination. He has no history of serious illness and takes no medications. He does not smoke cigarettes, and he exercises four times weekly. His mother died of colon cancer at the age of 76 years. He is 183 cm (6 ft 0 in) tall and weighs 100 kg (220 lb); BMI is 30 kg/m². Pulse is 68/min and blood pressure is 138/74 mm Hg. The remainder of the examination shows no abnormalities. Which of the following is the most appropriate next step? (A) Test of the stool for occult blood (B) Complete blood count (C) Measurement of serum thyroid-stimulating hormone concentration (D) Serum lipid studies (E) No further evaluation is necessary

**AI_B:** RESTATING THE QUESTION: An adult male with reasonable lifestyle choices, a family history of colon cancer, and normal presenting clinical examination, is under consideration for the most beneficial next step in his health management. The options provided include stool examination for hidden (occult) blood which could indicate potential colon pathology, complete blood count for an overall health status, serum thyroid-stimulating hormone measurement looking for thyroid functioning, serum lipid studies to consider cardiovascular health, or no further evaluation banking on his overall good health and lack of significant family history. PLANNING: We need to consider the relative benefits and risks of each one of these tests, based on his personal health status and family history. His mother's colon cancer, while a significant event, occurred at a later age and may not be an immediate concern for him. His body mass index (BMI) is on the higher side, which invites concerns for cardiovascular risks. His good exercise habits and lack of smoking could argue against severe cardiovascular risks, but cannot negate them entirely. CLINICAL REASONING: Despite the patient seeming generally healthy with a good lifestyle, we cannot overlook his BMI, which leans toward obesity (BMI ≥30 kg/m²). This puts him at a higher risk for cardiovascular diseases. Normally, for such patients, it would be beneficial to conduct blood tests that evaluate lipid profile, which give an insight into cardiovascular risk. His mother's colon cancer at an older age does not immediately necessitate occult blood stool test; usually commencing at 50 years old unless onset of colon cancer in a family member was before 60. CONCLUDING: His lifestyle choices of regular exercise, non-smoker status, and absence of medication or significant illness point to overall good health. However, his BMI and blood pressure add some risk for cardiovascular conditions. Whilst colon cancer family history is significant, it does not arise to prompt immediate colon screening at his age. RESPONSE: Given his BMI, it seems most beneficial next step would be (D) Serum lipid studies to evaluate his cardiovascular risk profile. The colon cancer in the family should be considered again when the patient reaches 50, or if he becomes symptomatic.

**AI_C:** RESTATING THE QUESTION: The query pertains to a 37-year-old man who hasn't had any serious illnesses, doesn't need medication, doesn't smoke, and exercises regularly. Despite his mother's history of colon cancer, his health is generally good except for his BMI, which at 30 kg/m² indicates obesity. His pulse rate and blood pressure are normal, and nothing abnormal was found during his routine physical checkup. Given this, the question is: What should be the next appropriate step in his care among the options provided? PLANNING: While there's no indication of an immediate need for tests such as occult blood fecal testing, complete blood count, or thyroid-stimulating hormone concentration, his BMI and blood pressure could potentially indicate future health risks. Of primary concern here is his obesity (BMI of 30 kg/m²). Obesity can put a person at risk for several conditions such as heart disease and diabetes. Considering his blood pressure and other cardiovascular risk, it's essential to monitor his lipid profile, which includes cholesterol and triglycerides. CLINICAL REASONING: The man's lifestyle of regular exercise and non-smoking are pluses for good health. Despite his mother's history of colon cancer, he is currently not at high risk of colon cancer before 50 unless there are specific genetic syndromes he may possess. Given his BMI and blood pressure, it's logical to proceed with assessing other cardiovascular risk factors despite these vitals still being in an acceptable range. CONCLUDING: Thus, the next step should focus on assessing and managing the identified risk—high BMI and borderline hypertensive blood pressure—by conducting further tests. Out of the given options, serum lipid studies can provide relevant insights into the patient's cardiovascular health. RESPONSE: Therefore, the most appropriate next step in this case would be (D) Serum lipid studies, to evaluate and manage the potential cardiovascular risk factors. This could include tests for LDL cholesterol, HDL cholesterol, total cholesterol, and triglycerides, all of which can give a more detailed picture of the patient's cardiovascular health and risks associated with his calculated BMI and existing blood pressure.

**AI_A:** 1) RESTATEMENT & DEFINITION: The patient is a 37-year-old male who is generally healthy but overweight with a Body Mass Index (BMI) of 30 kg/m². No present abnormalities are evident from a routine health maintenance examination, and he has no significant personal medical history. However, his family history includes her mother's demise from colon cancer at the age of 76. We're asked to determine the best next step for further evaluation or tests. 2) PLANNING: This scenario can guide us to focus on a few clinical possibilities. Due to his BMI, the patient is categorized as obese, potentially raising heart disease risks that might necessitate serum lipid studies. Also, his mother's history of colon cancer might point towards a stool test for occult blood to discover any early possibility of colon cancer. The complete blood count and thyroid-stimulating hormone measurement options are less supported as there are no indications from the patient's history or physical exam suggesting a hematological or endocrine problem. Alternatively, it could be argued that no further evaluation is required at this time based on the patient's current age, lifestyle, and exam findings. 3) CLINICAL REASONING: Given the patient's age and health status, we should target the tests that have the highest likelihood of providing useful information. Even though the patient's mother had colon cancer, routine testing usually begins at the age of 50, or 10 years before the youngest case in immediate family members. Thus, screening such as stool tests for occult blood wouldn't be necessary at the patient's current age of 37. Conversely, the patient's obesity poses a heightened risk of cardiovascular complications, which is a common issue in this BMI range. 4) CONCLUDING: Weighted against the current state of the patient and epidemiological frequency, cardiovascular health should be prioritized over cancer screening at this period. Cholesterol or lipid profile testing is a typical part of routine health screenings, particularly in overweight/obese individuals due to the elevated risk of conditions like hyperlipidemia, which can lead to atherosclerosis and other cardiometabolic diseases. 5) RESPONSE: Therefore, the most appropriate next step for this patient, based on his age, obesity status, and risk, would be to proceed with Serum lipid studies (D) to assess his cardiovascular health.

**AI_E:** RESTATING THE QUESTION: The query involves a 37-year-old man who's come for a routine health checkup. He has no serious health conditions, takes no medication, is a non-smoker and exercises regularly. However, his mother had colon cancer and he has a BMI of 30 kg/m², hinting at obesity. Despite normal pulse and blood pressure, we are tasked with resolving the most fitting next evaluation step. PLANNING: The assumptions here are that, since the man has no immediate health issues and leads a relatively healthy lifestyle, further evaluations may not be urgent. Nonetheless, his family history of colon cancer and obesity can't be overlooked. Other relevant medical tests are proposed in choices (A)-(D), but there is also an option to conclude no further evaluation is necessary (E). CLINICAL REASONING: Choice (A), stool test for occult blood, can be an indicator of colon cancer, which the man’s mother suffered from. However, colon cancer screening is advised to start at age 50, or 40, or 10 years before the age at which an immediate family member has been diagnosed. Hence, this wouldn't be the prime choice for our 37-year-old man. Choice (B), complete blood count, is an overall health check-up, but there's nothing in the man's history or physical examination that would necessitate it at this juncture. Likewise, choice (C) testing for serum thyroid-stimulating hormone concentration, a marker of thyroid disease, is unnecessary as there's no immediate call for it. Choice (D), Serum lipid studies, are done to check for instances of high cholesterol which can lead to heart diseases, especially in individuals with obesity, like our subject in question. Lastly, choice (E) suggests no further evaluation is necessary. However, given the man's obesity and family history of colon cancer, this may not be applicable. CONCLUDING: Although all the tests hold clinical significance under different circumstances, in our case, it seems prudent to consider the individual's BMI and evaluate risk factors that are clinically controllable. Thus, making choice (D), Serum lipid studies most logical. RESPONSE: The most appropriate next step would be (D) Serum lipid studies. They will provide insight into the man's cholesterol levels, which is significant given his obesity, and the risk it poses for potential cardiovascular diseases. Though his mother's history of colon cancer might be concerning, it isn't immediately pressing considering age guidelines for screening.

**AI_D:** (1) RESTATING THE QUESTION: Based on the given scenario, a 37-year-old male visitor has come for a routine health check-up. He has no significant medical history or serious illnesses. He is physically active, non-smoker, and maintains a notable weight which puts his BMI at 30. His vitals like pulse and blood pressure are stable and within normal limits. An important aspect is his family history of colon cancer in his mother at age 76. In light of all these findings, we're being asked to think about what would be the most appropriate course of action. (2) PLANNING: Key assumptions generated from this case might include that the patient is healthy with well-managed lifestyle habits, apart from borderline obesity, and does not currently experience any symptoms. Another possible assumption to be considered, given family history, is the potential of colon cancer development. Each of these assumptions generates different paths of actions. Whilst the first assumption suggests no further evaluation, the second one leans towards screening for colon cancer or related symptoms. (3) CLINICAL REASONING: The BMI of 30 places the patient in the obese category which can predispose to several health conditions. However, with no current symptoms reported, our focus should primarily be on preventive strategies, like lifestyle modifications. The blood pressure reading, although on the higher side of the normal range, may not immediately warrant investigations like complete blood count or thyroid-stimulating hormone level check. Given the patient’s age, colon cancer screening, usually recommended from age 50 and sometimes earlier in high-risk individuals, might not be considered urgent. However, considering his mother's onco-history, we can safely say that the patient is at a slightly increased risk for colon cancer. Still, without additional risk factors, this single lineage is unlikely to necessitate immediate stool occult blood testing. (4) CONCLUDING: The clinical thought process prioritizes a preventive strategy and aims to address the most immediate health consideration - excessive weight. Serum lipid studies would therefore make sense as elevated lipid levels are commonly associated with obesity and are a risk factor for heart disease. (5) RESPONSE: In light of the information and reasoning presented, the most appropriate course of action in managing this patient at this point in time would be option (D) - Serum lipid studies. This would aid in assessing the patient's risk of weight-related complications such as heart disease, and therefore direct further preventive strategies.

**Facilitator** The consensus among the AI responses is that the most appropriate next step for the 37-year-old man in question would be to proceed with Serum lipid studies (Option D). This decision is based on his age, obesity status, and risk factors. Despite his generally healthy lifestyle and lack of significant personal medical history, his Body Mass Index (BMI) of 30 kg/m² categorizes him as obese, which potentially raises his risk for cardiovascular diseases. Serum lipid studies, which assess cholesterol and triglyceride levels, can provide valuable insights into his cardiovascular health and risks associated with his elevated BMI. While his mother's history of colon cancer is noteworthy, routine colon cancer screening typically begins at age 50, or 10 years before the youngest case in immediate family members. Therefore, a stool test for occult blood (Option A) is not deemed necessary at his current age of 37. Other tests such as a complete blood count (Option B) and measurement of serum thyroid-stimulating hormone concentration (Option C) are also considered less relevant as there are no indications from the patient's history or physical exam suggesting a hematological or endocrine problem. Lastly, the option of no further evaluation (Option E) is not recommended due to the patient's obesity and the associated cardiovascular risks.
