# Supplementary material for: Collaborative intelligence in AI: Evaluating the performance of a council of AIs on the USMLE
Source: PLOS Digit Health. 2025 Oct 9;4(10):e0000787. doi: 10.1371/journal.pdig.0000787 (PMC12510544; doi:10.1371/journal.pdig.0000787)
Supplement: S2 File — (ZIP) [file pdig.0000787.s002.zip › S2 Data Raw data files containing unprocessed question inputs and LLM outputs including transcripts of deliberations/STEP2_Question-35-1_Type-MC-NJ.docx]

**Facilitator** **[Round 1]:** A 35-year-old woman is evaluated for a persistent fever 4 days after admission to the hospital for treatment of pyelonephritis. She has type 2 diabetes mellitus poorly controlled with insulin. She has received ciprofloxacin since admission. Results of initial blood cultures are pending. Temperature is 40.0°C (104.0°F), pulse is 110/min, respirations are 20/min, and blood pressure is 100/60 mm Hg. Examination shows marked right costovertebral angle tenderness. Which of the following is the most appropriate next step in management? (A) Addition of clindamycin to the medication regimen (B) Addition of vancomycin to the medication regimen (C) CT scan of the abdomen (D) Doppler ultrasonography of the lower extremities (E) Repeat blood cultures and fungal cultures

**AI_A:** RESTATING THE QUESTION: The concern here is about a 35-year-old woman who has been suffering from high fever for 4 days following her hospitalization for pyelonephritis. She also has poorly controlled type 2 diabetes and has been using insulin for the same. She has been on a medication regimen including ciprofloxacin since her admission to the hospital. Her initial blood culture reports are awaited. PUpon examination, significant tenderness was noted at the right costovertebral angle. The main query pertains to deciding the most ideal next step in her clinical management. PLANNING: Certain assumptions can be made from the available information. Her body temperature has remained elevated indicating a possible ongoing infection which has not responded to ciprofloxacin, an antibiotic commonly used to treat urinary tract infections like pyelonephritis. The right costovertebral angle tenderness may suggest that the initial urinary infection has not been fully resolved, or that there may be a complication such as an abscess. The persistence of fever despite the antibiotic treatment could signify a resistant bacteria, an abscess, or an inappropriate initial treatment selection due to incorrect diagnosis or complications related to her diabetes. CLINICAL REASONING: Considering the alternate assumptions, a resistant organism can be easily dealt with by adjusting the antibiotics based on culture and sensitivity results, while a complication like abscess would require imaging such as a CT scan for detection, and might necessitate a different approach to management. Therefore, generating a diagnostic hypothesis that assumes a complication like an abscess as a cause of persistent fever seems to be the more feasible approach. CONCLUDING: Resistant bacteria can usually be managed by tailoring antibiotic therapy according to the culture and sensitivity results. However, with an ongoing fever and marked tenderness in the right costovertebral angle, these findings may suggest a possible complication such as an abscess prevailing. This could be a consequence of the initial infection, which might not have been completely resolved. Therefore, further imaging may be necessary to either confirm or rule out this possibility. RESPONSE: Based on my clinical reasoning, the most appropriate next step in management would be to proceed with a CT scan of the abdomen (option C). This would facilitate the detection of any prevailing complications, such as abscesses, that possibly might have arisen from the initial infection and could be causing the persisting fever despite antibiotic therapy.

**AI_E:** RESTATING THE QUESTION: The clinical scenario describes a 35-year-old female who has been admitted to a healthcare facility for pyelonephritis, which is an inflammation of the kidneys typically caused by a bacterial infection. Even after four days of treatment with ciprofloxacin, an antibiotic effective against the most common causative organisms of pyelonephritis, her fever persists. Preliminary blood cultures are pending. Her physical examination reveals increased tenderness in the right costovertebral area, indicating kidney involvement. The question requires an appropriate next step in her clinical management, amongst the listed possibilities. PLANNING: The assumption here is that the antibiotic therapy is not efficacious, as suggested by the persisting fever and marked right costovertebral angle tenderness. Clinical clues point towards complications of pyelonephritis like renal/perinephric abscess, emphysematous pyelonephritis, or even antibiotic resistance; thus her management plan needs reconsideration. We can consider alternate hypotheses, such as ciprofloxacin resistance, fungal infection, lower-extremity thrombosis (clotting), or bacterial infection not eradicated by ciprofloxacin. CLINICAL REASONING: The patient did not demonstrate clinical improvement even after ciprofloxacin treatment, bringing into question its effectiveness. The persistence of right costovertebral angle tenderness despite antibiotic treatment is an alarming sign of possible complications, such as a renal or perinephric abscess. If an abscess is present, it can be a source of continued fever due to poor antibiotic penetration. The patient’s diabetes may also predispose her to more severe or complicated infections. These complications can be assessed by imaging studies of the abdomen, specifically a CT scan. CONCLUDING: Thus, with clinical reasoning, the most plausible cause of continued fever and marked right costovertebral angle tenderness, despite the antibiotic therapy, appears to be a complication of pyelonephritis. A CT scan of the abdomen should be the priority, as it would provide details on the severity and source of the infection, hence assisting in optimizing her clinical management. RESPONSE: (C) CT scan of the abdomen is the most appropriate next step in this case. It will help rule out complications of pyelonephritis, and aid in tailoring her treatment strategy.

**AI_B:** RESTATING THE QUESTION: A 35-year-old female patient presented with persistent fever four days following hospital admission for pyelonephritis treatment. She's also a poorly managed Type 2 Diabetic. Ciprofloxacin formed her primary treatment regimen since her admission and initial blood cultures' results are still awaited. Findings from the physical examination suggest an ailing right kidney. Now, with the patient's condition in mind, we ought to ascertain the next best course of action. PLANNING: Basics of clinical assumption: The persistent fever in a patient under treatment for pyelonephritis might be due to an unresolved infection. It suggests that the ongoing treatment is unfruitful, perhaps due to antibiotic resistance or an abscess that the antibiotics can't penetrate. Alternate assumption 1: The infection persists due an abscess or other obstruction, investigated by a CT scan of the abdomen (Option C). Alternate assumption 2: The infection is resistant to current antibiotic therapy. We can introduce another antibiotic - either clindamycin (Option A) or vancomycin (Option B) - arguing upon the specific antibiotic choice. Alternate assumption 3: Blood cultures could be performed again to identify potential resistant organisms or check for fungemia (Option E). Alternate assumption 4: A Doppler Ultrasonography of the lower extremities (Option D) assumes embolism or vascular conditions could be causing fever which is less likely given the context and the marked renal tenderness. CLINICAL REASONING: This patient's clinical presentation is consistent with the continuation of a kidney infection despite treatment, which implies either resistance to administered antibiotics or the formation of an abscess. Thus, the best step is to revise the diagnosis. In such cases, a definitive imaging study like a CT Scan for the abdomen (Option C) would allow for a comprehensive evaluation of the kidneys and adjoining tissues. It would detect potential abscesses or obstruction that might be the cause of continued infection. CONCLUDING: Adding another antibiotic into the regimen like Clindamycin or Vancomycin (Options A and B respectively) should only be considered after having identified a microorganism resistant to the current antibiotic (Ciprofloxacin). Similarly, repeating blood cultures and conducting fungal cultures (Option E) might not be the first choice unless there are indicators of systemic fungal infection, which the case does not suggest. Lastly, the Doppler Ultrasonography (Option D) does not align with the clinical picture as there are no signs of lower extremity deep vein thrombosis. RESPONSE: Therefore, based on the above assumptions and clinical reasoning, the most appropriate next step in this patient's management would be to order a CT scan of the abdomen (Option C). This will evaluate the possibility of abscess or obstruction, providing a better direction for subsequent treatment.

**AI_D:** RESTATING THE QUESTION: The scenario describes a 35-year-old woman who, despite being treated for pyelonephritis (an infection of the kidney), is still experiencing a fever. She also has inadequately managed type 2 diabetes and is using ciprofloxacin for her treatment. Her persistent fever can indicate an ongoing infection or abscess formation. The query here is about the best next step in managing this case. PLANNING: In regards to the assumptions, if the woman's condition hasn't improved despite receiving antibiotics, the source of infection might not have been eliminated or there could be an antibiotic-resistant pathogen. There may be a complication related to the infection such as an abscess, necrosis, or sepsis. The woman may also have an undetected infection elsewhere, considering her diabetic status. To confirm the issues, we should consider conducting further diagnostic procedures in order to rule out complications, detect any resistant organisms, or discover an unidentified infection. CLINICAL REASONING: Considering the continued high fever and marked right costovertebral angle tenderness, a renal or perinephric abscess might be the complication preventing recovery. Pyelonephritis, along with diabetes, could potentially facilitate an abscess's development. A CT scan of the abdomen (Option C) can reveal the presence of an abscess, which can then be appropriately managed. Meanwhile, since initial blood cultures are pending, repeating them (Option E) at this time would be premature. Moreover, the lack of improvement does not necessarily indicate a fungal infection, making fungal cultures less necessary. A persistent fever in pyelonephritis doesn't directly suggest a thrombotic complication, making a Doppler ultrasonography for the lower extremities (Option D), less critical. Also, changing or adding an antibiotic (Options A and B) to her regimen should not be done without clear evidence of antibiotic failure or toxicity, drug resistance, or an additional infection, none of which has been established yet. CONCLUDING: After a thorough examination of different assumptions and diagnosis possibilities, it appears that the most likely etiology for the persistent fever might be a complication of the primary infection. Thus, it would be reasonable to identify any potential abscess or complication caused by the infection through a CT scan of the abdomen. RESPONSE: So, the most appropriate next step in management should be a CT scan of the abdomen (Option C). This could potentially reveal a renal or perinephric abscess, which might explain the persistence of the fever and marked costovertebral angle tenderness despite antibiotic therapy.

**AI_C:** (1) RESTATING THE QUESTION: A 35-year-old woman with poorly managed type 2 diabetes is being examined for a lingering fever, which has been present for 4 days after she was admitted to the hospital to receive treatment for a kidney infection, also called pyelonephritis. She has been taking ciprofloxacin since she got admitted. Her blood cultures, which were ordered when she got admitted, are still awaited. She is feverish at 40°C (104°F), her pulse rate is 110/min, her respiratory rate is 20/min, and her blood pressure is 100/60 mm Hg. A physical exam reveals severe tenderness on the right side of her mid to lower back (the costovertebral angle), a common symptom of pyelonephritis. We're asked to determine which of the options (A) Further prescription of clindamycin; (B) Further prescription of vancomycin; (C) CT abdomen; (D) Doppler ultrasound of the lower legs; or (E) Request for repeat blood and fungus cultures would be the best next step in managing her condition. (2) PLANNING: Key assumptions here include the persistence of her symptoms despite treatment indicating potential unresolved infection source or an associated complication. Each of these assumptions needs evaluation. For example, the decision to add another antibiotic like clindamycin or vancomycin would assume that the causative bacteria are resistant to ciprofloxacin, getting a CT scan would assume an anatomical abnormality or complication as the cause, a Doppler ultrasound would assume a thromboembolic event, and repeating the blood and fungal cultures would assume the possibility of a secondary infection or an atypical primary agent. Given the patient's prolonged fever even after receiving antibiotics and her localized physical symptom of costovertebral angle tenderness, an associated complication should be clinically prioritized. (3) CLINICAL REASONING: Patients with pyelonephritis typically vastly improve within 48-72 hours of being administered appropriate antibiotics. This patient, however, continues to maintain a high fever even 96 hours (4 days) post-treatment. Simultaneously, she continues to experience costovertebral angle tenderness, indicating that the source of the infection is still extant. Provided these clinical considerations, it would be prudent to look for a persistent source of infection or an associated complication with a CT scan of the abdomen rather than modifying the antibiotic regimen, looking for a completely new source of infection with new cultures, or anticipating a thromboembolic event with Doppler ultrasound. (4) CONCLUDING: Given the patient's continuing symptoms and their lack of improvement with antibiotic treatment, it's necessary to investigate potential complications or sources of persistent infection. Among the presented options, a CT scan of the abdomen is the best tool to visualize any renal abnormalities such as abscess, obstruction, or anatomical abnormalities, which can contribute to an unresolving infection. (5) RESPONSE: Therefore, the most appropriate next step in this patient's management would be to conduct a CT scan of the abdomen (Option C).

**Facilitator** The consensus among the AI responses is that the most appropriate next step in managing the 35-year-old woman's condition is to conduct a CT scan of the abdomen (Option C). This decision is based on the assumption that her persistent fever and marked right costovertebral angle tenderness, despite antibiotic treatment, may indicate a complication such as a renal or perinephric abscess. The CT scan would help identify any such complications, providing a clearer direction for subsequent treatment. The addition of other antibiotics or repeating blood cultures should only be considered after the results of the CT scan and pending blood cultures are available. Doppler ultrasonography of the lower extremities is not considered a priority as there are no signs of lower extremity deep vein thrombosis.
