# Supplementary material for: Collaborative intelligence in AI: Evaluating the performance of a council of AIs on the USMLE
Source: PLOS Digit Health. 2025 Oct 9;4(10):e0000787. doi: 10.1371/journal.pdig.0000787 (PMC12510544; doi:10.1371/journal.pdig.0000787)
Supplement: S2 File — (ZIP) [file pdig.0000787.s002.zip › S2 Data Raw data files containing unprocessed question inputs and LLM outputs including transcripts of deliberations/STEP2_Question-36-1_Type-MC-NJ.docx]

**Facilitator** **[Round 1]:** A 23-year-old man is being evaluated in the intensive care unit because of steadily decreasing blood pressure since he was admitted 2 hours ago for treatment of septic shock caused by pneumonia. Medical history is remarkable for cystic fibrosis and severe obstructive lung disease. In the emergency department, the patient was intubated, had three peripheral intravenous catheters placed, and was treated empirically with broad-spectrum antibiotics, bronchodilators, a continuous infusion of propofol and fentanyl, and 4 L of 0.9% saline. Immediately after administration of fluids, the patient's pulse was 105/min and blood pressure was 100/50 mm Hg. The patient is 173 cm (5 ft 8 in) tall and weighs 59 kg (130 lb); BMI is 20 kg/m². Pulse is 135/min and blood pressure is 79/43 mm Hg. Auscultation of the lungs discloses diffuse rhonchi and inspiratory crackles with low-pitched bilateral expiratory wheezes. Cardiac examination discloses no murmurs, rubs, or gallops. Bedside ultrasonography of the abdomen shows the inferior vena cava is 1.8 cm in diameter with minimum respirophasic collapsibility, and cardiac ultrasonography shows that the left ventricular ejection fraction is approximately 0.65. Results of laboratory studies obtained on admission and now are shown: On Admission, the White Blood Cell count is 21,000/mm³ and Blood Lactic acid is 5.2 mg/dL. Now the patient's White Blood Cell count is 19,000/mm³ and Blood Lactic acid is 4.0 mg/dL Which of the following is the most appropriate next step in management? (A) Administer an additional 1-L bolus of 0.9% saline (B) Administer intravenous hydrocortisone (C) Administer intravenous voriconazole (D) Begin a continuous infusion of norepinephrine (E) Order CT scan of the head

**AI_D:** RESTATING THE QUESTION: This is a clinical decision problem involving a critically ill 23-year-old man who has been hospitalized with pneumonia and subsequent septic shock. In spite of aggressive management, his blood pressure has been declining steadily. His medical history includes cystic fibrosis and severe obstructive lung disease. He has been treated with multiple interventions including mechanical ventilation, intravenous antibiotics and fluids, bronchodilators, and pain medications. He also has an abnormal lung examination. Despite the administration of 4 liters of normal saline, his vital signs and lab results indicate continued disease progression. The question asks for the most appropriate next step in managing this patient's septic shock. PLANNING: Critical consideration here is the patient's continued hemodynamic instability (decreasing blood pressure) despite aggressive fluid resuscitation and broad-spectrum antibiotics, indicative of persistent shock. The decrease in blood pressure could be due to worsening sepsis or other causes. The presence of a normal-sized Inferior Vena Cava (IVC) with minimal respirophasic collapsibility on ultrasound argues against hypovolemia as a cause of the hypotension. The absence of cardiac murmurs, rubs, or gallops combined with an ejection fraction of 65% implies a normal cardiac function, thus cardiac dysfunction seems less likely. This leaves us with inadequate end organ perfusion due to sepsis-induced vasodilation as the likely main contributor to the patient's hypotension. CLINICAL REASONING: Treatment in such a scenario involves the use of vasopressors, drugs that constrict blood vessels and increase blood pressure. Vasopressors, specifically norepinephrine, is the clinical recommendation for patients who are in septic shock upon failing an initial adequate fluid resuscitation. This agent will help to restore perfusion and sustain life-supporting blood pressures. CONCLUDING: In assessing the available choices, we find that administer an additional 1-L bolus of 0.9% saline (Choice A) could be an unnecessary intervention given that the patient has already received a substantial fluid resuscitation and does not have clinical or ultrasound evidence of hypovolemia. Administering intravenous hydrocortisone (Choice B) could be considered in septic shock patients who are not responsive to fluid and vasopressor therapy, not in the early stages. Intravenous voriconazole (Choice C) isn't appropriate because there is no evidence or suspicion of a fungal infection in this case. Last, a CT scan of the head (Choice E) is not indicated as there are no clinical signs or reported symptoms suggesting a neurological issue. RESPONSE: Therefore, the most appropriate next step in management based on the clinical reasoning process established above would be to begin a continuous infusion of norepinephrine (Choice D), which is consistent with recommended treatment guidelines for septic shock that is unresponsive to initial fluid resuscitation.

**AI_C:** (1) RESTATING THE QUESTION: You're essentially asking me to help manage a young man with a history of cystic fibrosis, currently in the ICU due to septic shock from pneumonia. His blood pressure is progressively dropping despite being given fluids and started on broad-spectrum antibiotics. The options you provided: more saline, adding hydrocortisone, adding an antifungal medication (voriconazole), starting norepinephrine, or ordering a head CT. (2) PLANNING: Let's evaluate the assumptions for each option. Option (A) assumes the patient needs more volume expansion. However, his inferior vena cava is not significantly collapsible, which likely indicates adequate volume status and may not benefit from additional fluid. Option (B) suggests adding hydrocortisone, which would be useful if the patient had refractory shock due to adrenal insufficiency. However, no data points to adrenal insufficiency. Option (C) involves adding voriconazole, suggesting a fungal infection. Given the patient's rapid deterioration and known bacterial infection (pneumonia), a fungal infection seems less likely at this point. Option (D) implies the patient might benefit from a vasopressor like norepinephrine, commonly used in septic shock when blood pressure can't be maintained despite adequate fluid resuscitation. Option (E), a head CT, would be useful if the patient showed neurological symptoms or concerning head injury. (3) CLINICAL REASONING: Considering the information, the patient has a known bacterial infection (pneumonia), signs of shock (decreasing blood pressure, high pulse) despite volume repletion (seen by the non-collapsible Inferior Vena Cava). There's no indication of adrenal insufficiency or fungal infection. There has been a slight decrease in lactic acid—used as a marker of tissue hypoperfusion—indicating some response to treatment. No neurological symptoms indicating a need for a head CT. Broad-spectrum antibiotics cover most probable pathogens, including Pseudomonas, common in cystic fibrosis patients. Therefore, the assumption of needing vasoactive support (Increasing the tone of the blood vessels) is high. (4) CONCLUDING: Given these findings, it would be plausible to assert that the patient's ongoing septic shock needs reinforcement of the supportive measures. The failure to maintain his blood pressure despite adequate fluid resuscitation and blood lactic acid (although decreasing but still elevated) indicate that his tissues are not receiving enough perfusion. Starting a drug like norepinephrine, a vasopressor, would help increase vascular tone and improve his blood pressure. (5) RESPONSE: Therefore, in this clinical context, and from our clinical reasoning process, the most appropriate next step in this patient's management would be to "begin a continuous infusion of norepinephrine" (Option D).

**AI_E:** RESTATING THE QUESTION: In simpler terms, a young adult male with a history of cystic fibrosis and severe obstructive lung disease has been admitted to the hospital with septic shock resulting from pneumonia. Despite initial treatment attempts, his blood pressure has been decreasing, suggesting that his condition is not improving. His physical and ultrasound examination elucidate certain features, such as inspiratory crackles, rhonchi, wheezes as well as certain laboratory readings such as white blood cell count and lactic acid levels which have decreased slightly since admission. The issue at hand is determining the most effective next step in his treatment protocol from among the given options. PLANNING: The question hinges on understanding the disease process occurring and the principles of managing septic shock, a severe condition where infection leads to life-threatening low blood pressure. The underlying disease state may contribute to an altered response to the infection (e.g., cystic fibrosis may alter lung structure and immune response). The decrease in blood pressure despite fluid resuscitation is concerning and may suggest refractory septic shock. The slightly decreased white blood cell count and lactic acid may indicate a response to treatment but do not necessarily correlate with the overall severity of the patient's condition. Therefore, the decision of next steps in management should prioritize restoring the patient's blood pressure and maintaining vital organ perfusion. CLINICAL REASONING: Hypotension in septic shock is mainly due to peripheral vasodilation, intravascular volume depletion, and myocardial depression. The patient has already received significant fluid resuscitation (4L of 0.9% saline) without sustained improvement, hence, simply adding more fluid (Option A) may not resolve the ongoing shock. The patient's inferior vena cava diameter and minimum collapsibility suggest he is not significantly volume depleted. While hydrocortisone (Option B) can be beneficial in refractory septic shock, it is usually considered after vasopressor therapy and there is no mention of any adrenal insufficiency which it is primarily used for. Voriconazole is an antifungal medication (Option C), not typically indicated in suspected bacterial pneumonia unless there is concern for an invasive fungal infection, which is not demonstrated in this case at this time. A CT scan of the head (Option E) seems irrelevant and does not address the ongoing hypotension. CONCLUDING: Based on the clinical scenario, one plausible explanation is that the patient may be in refractory septic shock, where his hypotension is not responding to initial fluid resuscitation. With the use of broad-spectrum antibiotics which should cover the suspected bacterial pneumonia, and his ultrasound findings revealing that the patient is not necessarily volume depleted, it suggests that the decreased blood pressure may be mainly due to peripheral vasodilation and/or myocardial depression. RESPONSE: Therefore, the most appropriate next step in this management would be (D) to begin a continuous infusion of norepinephrine. It constitutes a first-line vasopressor in septic shock to restore vascular tone and thus increase blood pressure, improving perfusion to vital organs.

**AI_A:** RESTATING THE QUESTION: This query centers on a 23-year-old male patient in the intensive care unit (ICU) who has a history of cystic fibrosis and severe obstructive lung disease and is currently admitted for septic shock precipitated by pneumonia. His blood pressure has been progressively dropping since being admitted 2 hours ago. Despite receiving treatment including broad-spectrum antibiotics, bronchodilators, continuous propofol and fentanyl infusion, and a 4-L 0.9% saline fluid infusion, his vitals indicate a lower blood pressure and increased pulse rate suggestive of persistent shock. His diagnostic tests, including bedside ultrasonography and laboratory studies, have ruled out cardiac dysfunction as the underlying reason for his shock state and indicate a likely role of septic shock due to his pneumonia. The question is soliciting the most proper step in his management considering his current clinical state. PLANNING: The assumptions that can be gathered from the scenario include: (1) the patient's septic shock state isn't improving despite initial treatment; (2) a mere volume issue isn't the sole contributor given he's already received a substantial fluid bolus with minimal improvement; (3) his cardiac function seems preserved, ruling out cardiogenic causes of shock. Alternative assumptions include: (1) the possibility of the patient being in a state of vasodilatory shock, not just septic; (2) the potential need for vasopressor drugs to constrict his blood vessels and increase systemic vascular resistance, thereby markedly improving his blood pressure. CLINICAL REASONING: Septic shock, a kind of distributive shock, is characterized by massive systemic vasodilation leading to tissue hypoperfusion, despite a normal or increased cardiac output. This is why, along with fluid resuscitation, the use of vasoactive substances is key in the management, and hence the first-line drug of choice in septic shock is norepinephrine. The patient's persistent low blood pressure, tachycardia, and the ultrasonographic findings of an inferior vena cava of 1.8 cm diameter with a minuscule collapse during inspiration (suggesting a well-filled intravascular volume) pushes one to evaluate whether a vasodilator state could be contributing to his unstable hemodynamics. CONCLUDING: Given the continued hemodynamic instability and the absence of improving response from the considerable fluid resuscitation, along with the ultrasonographic evidence of adequate filling of the inferior vena cava and the absence of impaired cardiac function, it would be plausible to deduce that his septic shock might have progressed to a vasodilatory state. Hence, the optimal next approach would be to start the patient on a vasopressor drug to increase his systemic vascular resistance and thereby improve his blood pressure. RESPONSE: So, the most appropriate next step in this patient's management would be to begin a continuous infusion of norepinephrine (Choice D).

**AI_B:** RESTATING THE QUESTION: This 23-year-old male patient is presenting with decreasing blood pressure, despite having been admitted for septic shock treatment triggered by pneumonia. He has pronounced cystic fibrosis and severe obstructive lung disease from his medical history. After being administered a variety of treatments, including antibiotics, bronchodilators, sedatives, analgesics, and fluid resuscitation, there is still evidence of hypoperfusion with increased heart rate and decreased blood pressure. His lung sounds are abnormal, but his heart sounds are normal. Additionally, abdominal ultrasound shows an inferior vena cava diameter of 1.8 cm with minimal collapsibility, and cardiac ultrasound reveals a left ventricular ejection fraction of approximately 0.65. His lab values show a white blood cell count of 19,000/mm³ and a lactic acid level of 4.0 mg/dL, both of which are improved compared to the time of admission. The patient's current condition begs the question: what should be our next step in his treatment? PLANNING: We are making several assumptions, with the primary one being that the septic shock resulting from pneumonia has stabilized due to the treatment administered so far; this assertion is supported by the decrease in the patient's white cell count and lactic acid levels, both of which are markers of infection and a stress response. However, it's important to question this belief because ongoing hypotension (indicated by the low blood pressure of 79/43 mm Hg) and tachycardia (evidenced by the high pulse rate) may imply that the infection has not adequately resided or there is ongoing hypoperfusion - inadequate blood supply to the organs. This concerning pattern necessitates a potential revision or enhancement of the current treatment strategy. CLINICAL REASONING: Especially in cases of septic shock, the key treatment goals aim at treating the infection (antibiotics) and restoring adequate circulatory volume and perfusion (fluids and vasopressors). Although our patient has been given broad-spectrum antibiotics and initial fluid resuscitation of 4 L of 0.9% saline, his persistent hypotension and elevated heart rate suggest ongoing hypoperfusion. Furthermore, the ultrasound findings of an inferior vena cava diameter of 1.8 cm with minimal collapsibility indicate adequate fluid resuscitation or possible fluid overload, ruling out further aggressive fluid resuscitation (option A) as a beneficial next step. Hydrocortisone (option B) can sometimes be included if the septic shock is refractory to fluid and vasopressor therapy, but this is usually considered after vasopressors have been implemented, and that is not the case yet here. The use of voriconazole (option C) would be more indicated if there was a suspicion of a refractory fungal infection, but there's no suggestion of that in this case. CONCLUDING: Given the aforementioned rationales and indications, the most suitable action would be to initiate vasopressor therapy. Norepinephrine (option D) is typically the first-line vasopressor in septic shock and is used to maintain adequate blood pressure and perfusion to the vital organs. A CT scan of the head (option E) would be an unnecessary measure at this juncture, without any indication of neurological involvement. RESPONSE: Thus, the most appropriate next step in management is to begin a continuous infusion of norepinephrine (Option D).

**Facilitator** The consensus statement synthesizes the responses of AI_A, AI_B, AI_C, AI_D, and AI_E as follows: The question pertains to a 23-year-old male patient with a history of cystic fibrosis and severe obstructive lung disease, who is currently in the ICU due to septic shock caused by pneumonia. Despite receiving broad-spectrum antibiotics, bronchodilators, continuous propofol and fentanyl infusion, and a 4-L 0.9% saline fluid infusion, his blood pressure has been progressively dropping, indicating persistent shock. His diagnostic tests, including bedside ultrasonography and laboratory studies, have ruled out cardiac dysfunction as the underlying reason for his shock state and indicate a likely role of septic shock due to his pneumonia. The patient's persistent low blood pressure, tachycardia, and the ultrasonographic findings of an inferior vena cava of 1.8 cm diameter with a minuscule collapse during inspiration suggest that his septic shock might have progressed to a vasodilatory state. Given the continued hemodynamic instability and the absence of improving response from the considerable fluid resuscitation, it would be plausible to deduce that the optimal next approach would be to start the patient on a vasopressor drug to increase his systemic vascular resistance and thereby improve his blood pressure. Considering the available choices, administering an additional 1-L bolus of 0.9% saline, intravenous hydrocortisone, intravenous voriconazole, or ordering a CT scan of the head are not appropriate next steps in this patient's management. The patient has already received significant fluid resuscitation without sustained improvement, there is no evidence of adrenal insufficiency or fungal infection, and there are no clinical signs or reported symptoms suggesting a neurological issue. Therefore, the consensus among the AI responses is that the most appropriate next step in this patient's management would be to begin a continuous infusion of norepinephrine (Choice D). This is consistent with recommended treatment guidelines for septic shock that is unresponsive to initial fluid resuscitation. Norepinephrine is typically the first-line vasopressor in septic shock and is used to maintain adequate blood pressure and perfusion to the vital organs.
