# Supplementary material for: Collaborative intelligence in AI: Evaluating the performance of a council of AIs on the USMLE
Source: PLOS Digit Health. 2025 Oct 9;4(10):e0000787. doi: 10.1371/journal.pdig.0000787 (PMC12510544; doi:10.1371/journal.pdig.0000787)
Supplement: S2 File — (ZIP) [file pdig.0000787.s002.zip › S2 Data Raw data files containing unprocessed question inputs and LLM outputs including transcripts of deliberations/STEP2_Question-37-1_Type-MC-NJ.docx]

**Facilitator** **[Round 1]:** A 32-year-old nulligravid woman comes to the office for counseling prior to conception. Menses occur at irregular 40- and 60-day intervals. She has impaired glucose tolerance controlled by diet. She takes no medications. She is 165 cm (5 ft 5 cm) tall and weighs 84 kg (185 lb); BMI is 31 kg/m². Vital signs are within normal limits. Examination shows no abnormalities. Her fasting serum glucose concentration is 120 mg/dL. This patient is at greatest risk for developing which of the following? (A) Gestational diabetes (B) Hypothyroidism (C) Intrauterine growth restriction (D) Placenta previa (E) Preterm labor

**AI_D:** RESTATING THE QUESTION: Here, a 32-year-old woman with no prior pregnancies (nulligravid) seeks counseling before she plans to conceive. She has irregular menstrual cycles, occurring between 40 to 60 days apart, and impaired glucose tolerance managed by diet control. Her height is 165 cm, and she weighs 84 kg, giving her a Body Mass Index (BMI) of 31 kg/m², placing her in the obese category. All her vital signs are normal, and her physical examination shows no abnormalities. But, her fasting blood glucose level is 120 mg/dL, which, although high, is still within the prediabetes range. We are to evaluate which among gestational diabetes, hypothyroidism, intrauterine growth restriction, placenta previa, and preterm labor she is at the greatest risk for developing. PLANNING: This requires us to understand her risk factors and how they can impact pregnancy. The risk factors are her irregular menstrual cycles, obesity, and impaired glucose tolerance. Irregular menstrual periods could indicate a variety of potential hormonal imbalances, one of them being Polycystic Ovary Syndrome (PCOS). Obesity is a risk factor for many health issues including gestational diabetes and preterm labor. And impaired glucose tolerance, even when controlled by diet, can indicate a predisposition to gestational diabetes. CLINICAL REASONING: This woman's irregular menses could be a sign of anovulation, potentially due to PCOS. Women with PCOS frequently have insulin resistance, which is exacerbated by obesity. Weight is a critical factor in glucose regulation and insulin resistance. Her impaired glucose tolerance, even though controlled by diet, indicates a certain degree of insulin resistance already at baseline. Pregnancy itself further exacerbates insulin resistance, so any predilection for hyperglycemia would be heightened during gestation, especially in light of her obesity. This increases her risk of gestational diabetes. CONCLUDING: Although she has risk factors for other complications, such as preterm labor due to her obesity, the primary concern in this woman’s case, given her poor glucose control indicated by impaired glucose tolerance and given that hyperglycemia is exacerbated during pregnancy, is gestational diabetes. Hypothyroidism, intrauterine growth restriction, placenta previa do not appear to have any direct link with her present health profile. RESPONSE: Given the patient's impaired glucose tolerance, irregular menstrual periods, and obesity, she is at the greatest risk for developing Gestational diabetes (Option A).

**AI_B:** RESTATING THE QUESTION: We are asked to assess the future disease risk of a 32-year-old woman. She has never been pregnant (nulligravid) and wants to conceive. Irregular menstrual cycles are present, occurring every 40 to 60 days. She has been diagnosed with impaired glucose tolerance that she controls with her diet alone, without medications. Her body mass index (BMI) is high at 31 kg/m², making her obese. She measured 120 mg/dL on her fasting glucose test. Using this information, we are tasked with identifying which condition she is most likely to develop. PLANNING: We need to consider several pieces of information. The patient's age, nulligravid status, irregular menstrual cycles, impaired glucose tolerance, obesity, and her diet control are all factors. Several assumptions can be made, for example, the assumption that her irregular menstrual cycle and obesity could indicate Polycystic Ovarian Syndrome (PCOS), a common endocrinopathy marked by anovulation, androgen excess and polycystic ovaries. PCOS can possibly lead to gestational diabetes due to insulin resistance. Another assumption could be that her high BMI, impaired glucose intolerance, and age might predispose her to hypothyroidism. However, this might be less probable, since no clinical signs or symptoms of hypothyroidism are mentioned. CLINICAL REASONING: Hermes occur outside the normal range of 28 days, suggesting a possible hormonal imbalance. A common cause is Polycystic Ovarian Syndrome (PCOS), which is characterized by anovulation, androgen excess, and multiple small ovarian cysts. This endocrinopathy is associated with insulin resistance and hyperinsulinemia, suggesting a link with her impaired glucose tolerance. Obesity is a common feature in women with PCOS, and further exacerbates insulin resistance. Insulin resistance increases the likelihood of gestational diabetes during pregnancy. The continuous glucose monitoring of gestational diabetes is necessary because uncontrolled blood glucose can have deleterious effects on both mother and fetus. CONCLUDING: Using the information provided, the most likely assumption would be that the patient's risk factors, including an increased BMI, impaired glucose tolerance coupled with an irregular menstrual cycle, point more towards developing gestational diabetes (Option A), particularly considering her desire to conceive. RESPONSE: The patient is at greatest risk for developing gestational diabetes (Option A).

**AI_E:** (1)RESTATING THE QUESTION: So, the query here is about a 32-year-old woman, who is nulligravid (hasn't been pregnant before), is seeking advice before trying to conceive. She has irregular menstrual cycles oscillating between 40 and 60 days. This woman also has impaired glucose tolerance, which she manages through diet alone. Physically she is observed to be overweight, with a BMI of 31 kg/m² indicating obesity. There's no sign of any abnormalities during her examination, and her vital signs are also normal. Her fasting serum glucose concentration is slightly elevated at 120 mg/dL (normal range 70-100 mg/dL). The discussion is centred around what condition this patient is at the highest risk of developing from the given options, which include gestational diabetes, hypothyroidism, intrauterine growth restriction, placenta previa, and preterm labor. (2)PLANNING: Firstly, considering her irregular menstrual cycles, which might indicate an endocrine disorder such as Polycystic Ovary Syndrome (PCOS), this condition is linked with insulin resistance and would explain her impaired glucose tolerance. Also, her obesity is another contributing factor to developing insulin resistance, as excess fat can make cells less responsive to insulin. The fact that she's planning on conceiving with these conditions puts her at a higher risk of gestational diabetes. The other conditions, while they can occur in any pregnancy, don't seem directly linked to her existing conditions like gestational diabetes would be. (3) CLINICAL REASONING: The clinical reasoning here suggests that this patient's risk of developing gestational diabetes is higher due to her existing conditions. Indications of this are seen from her impaired glucose tolerance and obesity. Insulin resistance is common in both these conditions, and this resistance can worsen with pregnancy. As gestational diabetes is characterized by high blood glucose levels during pregnancy in women who weren't diabetic prior to pregnancy, the combination of risk factors makes gestational diabetes a probable condition she might develop. (4) CONCLUDING: After considering her medical history and pre-existing conditions, it leads to the conclusion that the patient is at a higher risk for developing gestational diabetes. She already has impaired glucose tolerance and is obese, both of which are risk factors for gestational diabetes. And as she is planning to conceive, her body's demand for insulin will increase during pregnancy, possibly worsening the insulin resistance. This reasoning is specifically linked to gestational diabetes compared to other conditions listed here. (5) RESPONSE: Therefore, based on the patient's current health status, she is at the greatest risk for developing (A) Gestational diabetes.

**AI_C:** RESTATING THE QUESTION: A 32-year-old woman who has never been pregnant is seeking advice before trying to conceive. Her periods happen at irregular intervals of 40 to 60 days rather than the typical 28-day cycle. She has an issue metabolizing glucose, implying a degree of insulin resistance, which she manages by watching her diet. No medications are being used at the moment. Her height is 165cm and she weighs 84kg, giving her a BMI of 31 and placing her in the "obese" category. These might be contributing factors to her irregular menstrual cycles. All her vital signs (such as blood pressure, heart rate, temperature) are within normal limits, showing no signs of systemic illness. There are no detected anomalies upon physical examination. However, a fasting blood glucose level of 120 mg/dL indicates slightly elevated glucose levels. PLANNING: In assessing this case, current key assumptions involve her obesity, irregular menstrual cycles, and impaired glucose tolerance. Recognizing that these could contribute to various pregnancy-related complications, such as gestational diabetes, thyroid disorders, fetal growth restriction, problems with the placenta, or preterm labor, it's important to address which she is at highest risk of developing. To generate alternate assumptions, we could question whether her obesity or impaired glucose metabolism are the leading factors, or whether her menstrual irregularity could be indicative of another underlying condition, like Polycystic Ovary Syndrome (PCOS), which could increase her risk for certain complications. CLINICAL REASONING: It's viable to hypothesize that due to her obesity, impaired glucose tolerance, and menstrual irregularity - possible signs of PCOS, a condition commonly associated with insulin resistance - this patient could be at higher risk for gestational diabetes. Gestational diabetes refers to high blood sugar levels that develop during pregnancy in women who didn't have diabetes before their pregnancy. Obesity and insulin resistance are known risk factors for developing gestational diabetes, and this patient exhibits both. CONCLUDING: Given the underlying insulin resistance (evident from her impaired glucose tolerance and possibly from the speculated PCOS) and obesity, the woman's risk of developing gestational diabetes should be clinically prioritized due to the strong connection between obesity, insulin resistance, and the incidence of gestational diabetes. RESPONSE: This patient falls under one of the high-risk categories for developing (A) Gestational diabetes due to her pre-existing insulin resistance (impaired glucose tolerance) and obesity (BMI 31 kg/m²). Therefore, her becoming pregnant could increase the chance of developing gestational diabetes.

**AI_A:** (1) RESTATING THE QUESTION: A woman who has never been pregnant is thinking about becoming pregnant and has come in for pre-pregnancy counseling. She has irregular menstrual cycles, an impaired ability to metabolize glucose, and is overweight with a body mass index of 31. She is currently not taking any medication, has a normal physical examination, and her fasting blood sugar is slightly elevated. The query is about the health risk she is most likely to face once she gets pregnant. (2) PLANNING: The assumptions in this case include (a) the patient's irregular menstrual cycles may suggest a hormone imbalance; (b) her impaired glucose tolerance and elevated BMI suggest possible pre-existing conditions, such as polycystic ovary syndrome (PCOS) or pre-diabetes; (c) these conditions could affect her pregnancy, with the most significant risks being gestational diabetes, intrauterine growth restriction, and preterm labor. Hypothyroidism and placenta previa are less likely given the patient's current symptoms and history. We must clinically prioritize the assumption that her pre-existing conditions, specifically impaired glucose tolerance and elevated BMI, could lead to complications in pregnancy. (3) CLINICAL REASONING: Irregular menses and high BMI can sometimes be associated with PCOS, a condition that arises due to hyperinsulinemia stimulating excessive ovarian androgen production. High insulin levels also decrease synthesis of sex hormone binding globulin, leading to more free androgens. This situation interferes with the ovulation process and maintains higher levels of plasma glucose, which is why impaired glucose tolerance is associated with PCOS. If this patient becomes pregnant, her pre-existing impaired glucose tolerance can be exacerbated by the insulin-resistant state of pregnancy, leading to higher chances of gestational diabetes. (4) CONCLUDING: The risks to this patient's pregnancy are amplified due to her impaired glucose tolerance, high BMI, and potentially untreated PCOS. While the other conditions mentioned such as hypothyroidism, intrauterine growth restriction, placenta previa, and preterm labor do occur in pregnancy, they are not the most probable ones based on her current health status. Therefore, we conclude that the most likely risk she stands to face is gestational diabetes. (5) RESPONSE: This 32-year-old woman with impaired glucose tolerance and a high BMI is at greatest risk for developing gestational diabetes during her pregnancy.

**Facilitator** The consensus statement synthesizes the responses of AI_A, AI_B, AI_C, AI_D, and AI_E as follows: The case in question involves a 32-year-old nulligravid woman seeking pre-conception counseling. She presents with irregular menstrual cycles, impaired glucose tolerance managed by diet, and obesity, as indicated by a BMI of 31 kg/m². Her physical examination shows no abnormalities, and her vital signs are within normal limits. However, her fasting serum glucose concentration is slightly elevated at 120 mg/dL. The key assumptions in this case revolve around her irregular menstrual cycles, impaired glucose tolerance, and obesity. These factors suggest possible pre-existing conditions such as Polycystic Ovary Syndrome (PCOS) or pre-diabetes, which could potentially affect her pregnancy. The irregular menstrual cycles could indicate a hormonal imbalance, possibly due to PCOS, a condition often associated with insulin resistance. Her impaired glucose tolerance and obesity further exacerbate this insulin resistance, which could be heightened during pregnancy. Given these factors, the woman's risk of developing gestational diabetes should be clinically prioritized. Gestational diabetes refers to high blood sugar levels that develop during pregnancy in women who didn't have diabetes before their pregnancy. Obesity and insulin resistance are known risk factors for developing gestational diabetes, and this patient exhibits both. While other conditions such as hypothyroidism, intrauterine growth restriction, placenta previa, and preterm labor can occur in pregnancy, they are not the most probable ones based on her current health status. Therefore, the consensus is that the patient is at the greatest risk for developing gestational diabetes.
