# Supplementary material for: Collaborative intelligence in AI: Evaluating the performance of a council of AIs on the USMLE
Source: PLOS Digit Health. 2025 Oct 9;4(10):e0000787. doi: 10.1371/journal.pdig.0000787 (PMC12510544; doi:10.1371/journal.pdig.0000787)
Supplement: S2 File — (ZIP) [file pdig.0000787.s002.zip › S2 Data Raw data files containing unprocessed question inputs and LLM outputs including transcripts of deliberations/STEP2_Question-38-1_Type-MC-NJ.docx]

**Facilitator** **[Round 1]:** A 17-year-old girl is brought to the physician by her parents because they are concerned she is using illicit drugs. They report that, “she just isn’t herself anymore.” She has no history of serious illness and takes no medications. She is upset about being “tricked” to come to the doctor. Physical examination shows no abnormalities. On mental status examination, she has an angry mood and anxious affect. The patient’s parents privately request a toxicology screening for their daughter but ask that she be told that her blood will be taken to “check her thyroid gland.” After discussing the parents’ wish for secrecy, which of the following is the most appropriate next step in management? (A) Comply with the parents’ wishes (B) Inform the parents that everything the patient tells the physician is confidential (C) Inform the parents that if the patient’s toxicology screening is positive, it must be reported to the police (D) Inform the parents that the physician cannot deceive the patient

**AI_D:** (1) RESTATING THE QUESTION: This question is asking about ethical principles that govern patient care and physician duties. A healthy 17-year-old female has been brought in by her parents who are worried that she is using drugs, though her physical examination is normal. Her parents privately ask for her to undergo a toxicology screen under false pretenses. The question is asking what the correct next step would be for the physician to take following this unusual request from the parents to perform a drug test without the patient's knowledge or consent. (2) PLANNING: We'll assume that the patient is indeed the parents' legitimate concern regarding their daughter's possible drug use. This requires us to understand the physician's role in handling this situation ethically and the potential legal implications of the four suggested responses. (3) CLINICAL REASONING: In health care, the principle of autonomy is shown by respecting the patient’s rights to self-determination. In the context of this scenario, autonomy involves clinician's honesty and transparency towards the patient, who is of an age where she has a right to know about any medical tests that are to be performed on her. Importantly, it is not left to the parents to decide what their children should understand about their health, especially for a teenager who is nearly an adult. (4) CONCLUDING: There are clear ethical principles which dictate that patient autonomy, privacy, and consent are to be respected. These principles apply to all patients, including adolescents. Deception or withholding information about diagnostic tests is not ethically appropriate. (5) RESPONSE: The most appropriate choice, according to medical ethics and respecting patient autonomy, is (D) Inform the parents that the physician cannot deceive the patient.

**AI_E:** (1) RESTATING THE QUESTION: The question here involves a 17-year-old girl who was brought to the physician by her parents, as they suspect she's possibly using illicit drugs. They notice a change in her and she is irritable and anxious but has no notable physical findings. The parents request a drug test but want this information to be hidden from their daughter, requesting that she be told her blood is being drawn to test for thyroid issues. The question asks what next step is most suitable in managing this case. (2) PLANNING: Various assumptions are presented here. One, the parents suspect their daughter of drug use and want a test conducted in secrecy. Two, the physician has a responsibility towards the parents as caregivers of the 17-year-old but also a duty of honesty and respect for the autonomy of the adolescent patient. Lastly, the physician might want to consider the ethics surrounding deceit, confidentiality and mandatory reporting of certain findings (if applicable). (3) CLINICAL REASONING: The Ethics of clinical practice involves respecting the patient’s autonomy while balancing this with potential greatest benefit and least harm to the patient. In this scenario, respecting the autonomy of the patient would involve being honest with her about her care and any tests that are being ordered. While the parents wish to keep the testing secret, they can be informed that physician-patient confidentiality will be maintained, as well as being told that they cannot deceive their daughter about the reason for drawing blood. Confidentiality is a fundamental part of the patient-doctor relationship and any breach of it should be considered only under exceptional circumstances (in most jurisdictions, this would be if the patient poses a significant risk of harm to themselves or others). (4) CONCLUDING: It's crucial to communicate openly with adolescent patients about their health and treatment options, respecting their autonomy. The best way to handle the situation in question would be to explain why honesty in care is important and that any information shared with the physician will be treated confidentially. (5) RESPONSE: Thus, the most appropriate next step would be to inform the parents that the physician cannot deceive the patient (option D). It's essential to underscore that deception in patient care is unethical and can harm the trust between the doctor and patient. Being upfront and honest will aid in keeping the patient-physician relationship strong and foster better health outcomes.

**AI_C:** RESTATING THE QUESTION: The scenario presents a 17-year-old female who demonstrates an irate mood and anxious demeanor, leading to her parents' concerns about potential drug abuse. The parents request a toxicology screen under the guise of a thyroid check, thus aspiring to keep this information concealed from their daughter. The question prompts us to ascertain the proper course of action for the physician, given the posed ethical and legal conundrum. PLANNING: There are several assumptions to consider in this context. These comprise the relatives' concerns indeed indicating a substance abuse issue, the viability of a secret drug test, the requirement of obligatory reporting to the police, and acknowledging the importance of patient autonomy and honesty in the doctor-patient relationship. However, the key assumption of pivotal clinical importance revolve around the fundamental ethical principles of medical practice which underscore the necessity for transparency and respect for patient autonomy. CLINICAL REASONING: Patient autonomy is key in healthcare and the preservation of truth-telling is paramount to foster trust in the physician-patient relationship. Conversely, it's important to acknowledge the parents' concerns and it’s necessary to assess the patient for possible substance abuse due to her change in behavior. However, deception could potentially harm the patient and erode trust. It’s unethical to conduct a test without consent or by misleading the patient about the intent of the test.​CONCLUDING:​In light of the ethical considerations and respect for patient autonomy, it's crucial the physician not deceive the patient. The next appropriate step would be to inform the parents that deception is not permissible. Additionally, the patient should be approached and assessed for her capacity to make health decisions and her concerns should be addressed. Drug screening should only be proceeded with, if consent is obtained upon disclosing the true intentions of the test. RESPONSE: Thus, the most appropriate next course of action would be (D) Inform the parents that the physician cannot deceive the patient. Patient autonomy and transparency must be respected in all aspects of healthcare.

**AI_A:** (1) RESTATING THE QUESTION: In this scenario, we have a 17-year-old girl who has been brought to the doctor by her worried parents, suspecting that she might be using illegal drugs. The parents want to conduct a toxicology test on their daughter without disclosing the real purpose of the test to her, and are asking the doctor to lie to her and tell her it's for a thyroid function check. The query here is: what should the doctor's next course of action be? (2) PLANNING: The decision here revolves around ethical and legal considerations. One option is to accede to the parents' request and carry out the test without telling the patient. However, this might be construed as a breach of the patient's right to informed consent. Another option is to inform the parents that all information shared between the physician and the patient is confidential. While this may reinforce trust between the doctor and patient, it could make the parents feel dismissed. The third option is to emphasize mandatory reporting in the event of a positive toxicological screen. This approach might give the impression that the physician is more interested in legalities rather than the patient's wellbeing. The last option involves expressing to the parents that the doctor cannot deceive the patient, which upholds the principle of truth-telling in medicine but might also create tension with the parents. (3) CLINICAL REASONING: Ethical principles play a key role here. The principle of autonomy, which is central to medical ethics, necessitates that the individual has the right to make decisions about their own health and medical conditions. As a minor, certain decisions can be made by her parents, but mature minors have a unique status and have the often have the capability to make their own health decisions. This girl appears to be an angry and anxious but competent minor, where more emphasis is placed on her input. Bearing this in mind, proceeding with the test without her informed consent is infringing her rights. Reporting a positive toxicology screening to the police might not automatically be the appropriate path to take, especially since a positive result may warrant a therapeutic intervention instead of a legal one. Medical confidentiality is crucial, but relaying solely on this fact does not address the whole issue. (4) CONCLUDING: Based on applying the principle of autonomy and considering the teen's ability to provide informed consent, it appears more acceptable to uphold her rights by not deceiving her about why the test is being done. (5) RESPONSE: Therefore, the most appropriate step in the management of this case would be (D) Inform the parents that the physician cannot deceive the patient. It's important to maintain the girl's trust in her caregiver, as it's pivotal in establishing a successful doctor-patient relationship which directly influences the quality of care she receives.

**AI_B:** (1) RESTATING THE QUESTION: The situation presented involves a 17-year-old female whose parents are worried that she may be using illegal substances, as indicated by changes in her behavior. The parents urge a toxicology screening while also wanting to keep this proposal secretive, requesting that the girl is told her blood is being tested for thyroid function instead. The predicament here is determining the best step in managing this parental request, evaluating whether the physician should comply with the deception, uphold confidentiality rules, disclose positive toxicology results to law enforcement, or outright refuse to misdirect the patient. (2) PLANNING: To plan our response, we must assess the assumptions that may be implicit in the parental request. First, it's assumed that it's ethically appropriate for the doctor to deceive the patient to satisfy the parents' curiosity. Secondly, it is assumed that everything the patient shares with the doctor is confidential, concerning the patient's autonomy and right to privacy. Thirdly, it's assumed that if the results turn out positive, the physician is obliged to report to the police. Lastly, it assumes that the doctor cannot lie to the patient. Through evaluating each, we can hypothesize that in line with ethical medical conduct, the priority is to respect patient autonomy and uphold honesty and trust in the doctor-patient relationship. (3) CLINICAL REASONING: Intentionally deceiving a patient is a violation of their autonomy and trust, even when a minor's health is at risk. Maintaining a sound therapeutic relationship underlies clinical practices, thereby making parents' preference less optimal. Informing parents about the confidentiality principle is correct but doesn't directly address the issue at hand - their wish for deception, not the information the girl would share. Patients' privacy should be respected unless issues of life-threatening scenarios or legal obligations forcing disclosure arise. However, the assumption that positive toxicology results must be reported to the police isn't typically required unless there are county or state stipulations regarding mandatory reporting of certain illegal drug use detected. Hence, this would be the least prioritized approach. In comparison, informing the parents that the healthcare provider cannot lie to the patient signifies the duty to preserve the patient's right to autonomous decision-making, integrity in communication, and the importance of the patient-healthcare provider trust. (4) CONCLUDING: Upon evaluating each assumption, we can conclude that the most decent response, grounded in medical ethics principles, is to respect the patient’s right to truth and trust in the healthcare setting. (5) RESPONSE: The finest course of action in this predicament would be to (D) Inform the parents that the physician cannot deceive the patient. Rather, parental concerns should be addressed collaborating with the adolescent within the bounds of ethical practice, promoting open conversation on substance use and potential necessity of screening if agreed upon by the minor.

**Facilitator** The consensus among the AI responses is that the most appropriate next step in managing this case would be to inform the parents that the physician cannot deceive the patient (option D). This conclusion is based on the principle of patient autonomy, which is central to medical ethics and necessitates that the individual has the right to make decisions about their own health and medical conditions. Deceiving the patient about the purpose of the test would infringe upon her rights and potentially harm the trust in the physician-patient relationship. While the parents' concerns are valid, it is crucial to maintain open and honest communication with the patient. The physician should respect the patient's autonomy and uphold honesty and trust in the doctor-patient relationship, even when dealing with minors. It's also important to note that medical confidentiality is a fundamental part of the patient-doctor relationship and any breach of it should be considered only under exceptional circumstances. Therefore, the physician should inform the parents that they cannot deceive their daughter about the reason for drawing blood.
