# Supplementary material for: Collaborative intelligence in AI: Evaluating the performance of a council of AIs on the USMLE
Source: PLOS Digit Health. 2025 Oct 9;4(10):e0000787. doi: 10.1371/journal.pdig.0000787 (PMC12510544; doi:10.1371/journal.pdig.0000787)
Supplement: S2 File — (ZIP) [file pdig.0000787.s002.zip › S2 Data Raw data files containing unprocessed question inputs and LLM outputs including transcripts of deliberations/STEP2_Question-39-1_Type-MC-NJ.docx]

**Facilitator** **[Round 1]:** A 52-year-old woman with type 2 diabetes mellitus comes to the office for a follow-up examination. Two weeks ago, she was treated with amoxicillin for a urinary tract infection. At that time, her blood pressure was 165/98 mm Hg, and serum creatinine concentration was 1.2 mg/dL; lisinopril therapy was also begun. During the past week, she has had increased swelling of the lower extremities and shortness of breath. Her dysuria has resolved. She appears anxious. Temperature is 36.8°C (98.2°F), pulse is 110/min, respirations are 20/min, and blood pressure is 185/105 mm Hg. Diffuse bilateral crackles are heard on pulmonary examination. An S3 gallop is heard. There is 3+ pitting bilateral pretibial edema. The laboratory studies showed that the levels for Serum Urea nitrogen is 45 mg/dL and Serum Creatinine is 4 mg/dL. As for the urine, its specific gravity is 1.010, no blood, and has moderate numbers of Hyaline casts. Which of the following is the most likely diagnosis? (A) Acute glomerulonephritis (B) Acute interstitial nephritis (C) Acute kidney injury (D) Acute obstructive uropathy (E) Intravascular volume depletion

**AI_A:** RESTATING THE QUESTION: The patient in question is a 52-year-old woman with a history of type 2 diabetes mellitus. She was recently treated with amoxicillin for a urinary tract infection and lisinopril therapy was initiated due to elevated blood pressure. Currently, she is experiencing swelling in the lower extremities, shortness of breath, and anxiety. Upon physical examination, she displays diffuse crackles heard on pulmonary examination, an S3 gallop, and 3+ pitting bilateral pretibial edema. Her lab studies showed high levels of serum urea nitrogen, serum creatinine and the presence of hyaline casts in her urine. We are asked to identify the likely diagnosis from the given options. PLANNING: The patient's symptoms suggest an impairment in kidney function as indicated by the increase in serum creatinine and urea and the presence of hyaline casts in the urine. Given her past history of hypertension and type 2 diabetes mellitus, plus initiation of lisinopril therapy, it is plausible to assume that the elevated blood pressure and/or the use of lisinopril could have led to kidney issues. It is furthermore critical to consider her other symptoms: bilateral edema and pulmonary crackles, which may hint towards a fluid overload issue (possible heart failure or kidney dysfunction), and the increase in pulse rate which could be due to the ongoing stress on the body or due to inadequate fluid management. CLINICAL REASONING: Given her clinical presentation and the recent medication history, the increase in serum creatinine concentration from 1.2 to 4 mg/dL suggests an acute kidney impairment. This deterioration could have been caused by the angiotensin-converting enzyme inhibitor, lisinopril, which she started two weeks ago. Lisinopril can lead to 'acute kidney failure,' particularly in patients with bilateral renal artery stenosis, heart failure, or volume depletion. It is important to note that her blood pressure is high, she has symptoms of fluid overload (crackles, S3 gallop, edema), and her urine sediment is bland which is more suggestive of pre-renal causes of acute kidney injury. CONCLUDING: In light of the information presented and considering the patient's symptoms, recent medication history, and physical examination (crackles, S3 gallop, edema) along with the laboratory findings (increased creatinine and urea, hyaline casts), it is almost certain that this patient has suffered an acute kidney injury. RESPONSE: So, the most likely diagnosis for this patient from the options provided is: (C) Acute kidney injury.

**AI_E:** (1) RESTATING THE QUESTION Given is a case of a 52-year-old woman with a history of type 2 diabetes, who was treated for a urinary tract infection with amoxicillin and had high blood pressure for which lisinopril was prescribed. Following this, she has presenting symptoms consistent with fluid overload, particularly peripheral edema and pulmonary crackles, as well as increased heart rate. Her laboratory studies also show a significant increase in serum urea nitrogen and creatinine levels, indicative of decreased kidney function. The urinary examination reveals normal specific gravity and no blood, but the presence of hyaline casts. The objective is to diagnose her condition from the given options. (2) PLANNING Assumptions under consideration are: -It could be an acute glomerulonephritis that typically presents with edema and hypertension but is usually accompanied by hematuria, which is absent in this case. -Another assumption could be the onset of acute interstitial nephritis, a condition sometimes associated with certain drugs, including antibiotics like amoxicillin. -Considering her symptoms and raised kidney function indicators, acute kidney injury cannot be ruled out. -Acute obstructive uropathy typically presents with lower urinary symptoms and pain, which does not match this clinical scenario. -Lastly, intravascular volume depletion seems unlikely as the symptoms suggest volume overload. Given the recent introduction of lisinopril, the assumption of drug-induced kidney damage due to angiotensin-converting enzyme (ACE) inhibitor stands out as a priority. (3) CLINICAL REASONING Lisinopril, a type of ACE inhibitor, is commonly used to reduce blood pressure and protect the kidneys, particularly in patients with diabetes. However, in certain instances, particularly in cases when renal perfusion is compromised, ACE inhibitors can decrease the glomerular filtration rate (GFR) and cause acute kidney injury. The increase in serum urea and creatinine levels points to decreased renal function. The presence of moderate hyaline casts could be a result of protein reabsorption in the kidney tubules due to decreased GFR. The lack of blood in the urine makes glomerular damage less likely. The fluid overload symptoms including bilateral crackles, S3 gallop, and peripheral edema point to the inability to excrete water and sodium, suggesting renal dysfunction. (4) CONCLUDING With the given history, changes in blood pressure, the recent introduction of lisinopril, symptomatic presentation, and lab findings, the clinical scenario most closely fits with acute kidney injury. (5) RESPONSE The most likely diagnosis in this case is (C) Acute kidney injury.

**AI_C:** (1) RESTATING THE QUESTION: The given patient is a middle-aged woman with a history of type 2 diabetes mellitus and recently treated urinary tract infection. She has been experiencing swelling in her lower extremities and shortness of breath. Recent laboratory studies show elevated levels of Serum Urea nitrogen and Serum Creatinine, and urinalysis shows hyaline casts with no blood. From five given options, the task is to correctly diagnose her condition. (2) PLANNING: To give the correct diagnosis, we will have to consider the symptoms, physical examination findings, medical history, and test results of the patient. Based on those, Acute glomerulonephritis, Acute interstitial nephritis, and Acute kidney injury are possible diagnoses due to the elevation of Serum Urea nitrogen and Serum Creatinine. However, in Acute glomerulonephritis, we might expect to see proteinuria or hematuria, which isn't the case with the patient. Similarly, Acute interstitial nephritis is typically associated with the use of certain medications or immunological reactions, none of which are mentioned in regard to the patient. It seems reasonable to hypothesize that the patient's condition might be an Acute kidney injury, given the acute elevation in serum creatinine, the edema, and the shortness of breath which could be due to fluid overload. (3) CLINICAL REASONING: Lisinopril, a type of angiotensin-converting enzyme (ACE) inhibitor, is known to decrease glomerular filtration rate (GFR) and hence can potentially cause Acute kidney injury, particularly in patients with risk factors such as older age, hypertension, diabetes mellitus, and possible baseline renal insufficiency. Additionally, the patient's elevated blood pressure despite being on Lisinopril suggests a renal pathology. The bilateral crackles heard on pulmonary examination and the S3 gallop indicate possible pulmonary edema due to fluid overload, a clinical manifestation of kidney injury. The elevated serum urea nitrogen and creatinine levels are clear indications of a decline in kidney function. Hyaline casts, found in the urine, are characteristic of tubular damage, which is consistent with acute tubular necrosis, the most common cause of Acute kidney injury. (4) CONCLUDING: Through an integrated consideration of the patient's clinical symptoms, medical history, drug therapy, and laboratory findings, we can reasonably hypothesize that she might be experiencing Acute kidney injury. (5) RESPONSE: Therefore, the most likely diagnosis for this patient is (C) Acute kidney injury. The combination of her history, physical examination, and laboratory findings including hyaline casts, increased Serum Urea Nitrogen and Creatinine levels, suggest that the patient is suffering from Acute Kidney Injury, possibly induced by lisinopril treatment.

**AI_B:** RESTATING THE QUESTION: This scenario is about a 52-year-old woman with a history of type 2 diabetes who was treated for a Urinary Tract Infection with amoxicillin two weeks ago. She started lisinopril due to increased blood pressure, and her serum creatinine concentration was 1.2 mg/dL then. She presents now with shortness of breath, increased leg swelling, and increased heart rate and blood pressure. Her laboratory findings show an increase in serum creatinine and urea. The urine exam has no blood or infection signs but shows moderate hyaline casts. We are tasked with making a diagnosis based on this information. PLANNING: The primary assumptions from this case are that she could either be having acute renal disease or complications from the medications. Some factors to consider include her recent antibiotic use, the start of lisinopril, and her history of diabetes. A high priority should be targeted at reasoning out if her renal function has been acutely compromised, leading to an increase in creatinine and urea, and symptoms of fluid overload as evident from her edemas and crackles in the lungs. CLINICAL REASONING: Our patient's symptoms of lower extremity edema, shortness of breath, and bilateral crackles suggest fluid overload, while lab studies support renal dysfunction. Hyaline casts in the urine are usually associated with diseases that decrease the GFR, such as prerenal azotemia or acute tubular necrosis (ATN). Yet, her history of recent antibiotic treatment and hypertension control using lisinopril make Acute Interstitial Nephritis (AIN) and Angiotensin Converting Enzyme (ACE) inhibitors induced renal dysfunction possibilities. However, AIN usually presents with fever, rash, eosinophilia or hematuria, which she does not have. As for ACE inhibitors, they cause renal dysfunction by lowering intraglomerular pressure, especially in patients with renal artery stenosis. It typically occurs within days of starting the medication, and the creatinine increases less than 30% of baseline. But her creatinine level shot up to 4 mg/dL, which supposes a more than 30% increase from baseline. CONCLUDING: From all these findings, it would be educate to diagnose her with an acute renal disorder. Acute kidney injury (AKI) may occur for various reasons, but this patient's clinical presentation and sudden significant creatinine rise point to acute tubular necrosis (ATN) as a likely subtype of this. It occurs following severe illness, toxic damage, or ischemia. The hyaline casts - usually seen in prerenal azotemia and ATN - further support this conclusion. RESPONSE: This suggests that the most likely diagnosis for her condition is acute kidney injury (Option C).

**AI_D:** RESTATING THE QUESTION: We're analysing a case of a woman who is 52 years old with a known medical history of type 2 diabetes mellitus. She has been showing symptoms like swelling of the lower extremities, shortness of breath, and she seems anxious. The tests show a spike in her blood pressure, an elevated level of serum creatinine, and hyaline casts in her urine. On physical examination, an S3 gallop along with bilateral crackles in lungs and pitting edema are noticed. Given this context, we're asked to identify the most likely diagnosis from an offered list that includes Acute glomerulonephritis, Acute interstitial nephritis, Acute kidney injury, Acute obstructive uropathy, and Intravascular volume depletion. PLANNING: Each assumption includes a unique set of processes and effects: (A) Acute glomerulonephritis typically presents with decreased urine output, and possibly hypertension and fluid overload. However, it typically also includes hematuria, which this patient does not have. (B) Acute interstitial nephritis can result from certain medications, and might include symptoms like fever, rash, and arthralgia, none of which are present in this case. (C) Acute kidney injury (AKI) could occur due to various reasons, including conditions that may harm the kidneys like certain medications, a sudden drop in blood flow to the kidneys, or blockage of urine from the kidneys. Patients with AKI may have several clinical features such as decreased urine output, fluid retention leading to swelling in the lower extremities, and shortness of breath. (D) Acute obstructive uropathy would usually present with symptoms like abdominal pain or difficulty urinating, which this patient does not exhibit. (E) Intravascular volume depletion involves a decrease in the volume of blood in the body, which can result from severe dehydration or bleeding. Symptoms might include dizziness, fainting, dry mouth, which this patient does not show. Therefore, given the clinical signs, lab results, history of the patient and her symptoms, it would appear that Acute Kidney Injury might the most reasonable diagnosis. CLINICAL REASONING: Earlier, the patient's blood pressure was 165/98 mm Hg, and serum creatinine concentration was 1.2 mg/dL. Currently, the blood pressure has risen to 185/105 mm Hg and the serum creatinine has elevated to 4 mg/dL. This indicates deteriorating kidney function over time. The increase in Serum Urea Nitrogen along with a rise in serum creatinine suggest decreased renal perfusion and filtering capacity. Moreover, the patient has developed edema and symptoms of fluid overload, such as shortness of breath and bilateral crackles in the lungs, indicating fluid accumulation due to diminished urine production and fluid clearance, a key feature of Acute Kidney Injury (AKI). Although patients with diabetes are often at risk of glomerulonephritis, the absence of hematuria and the presence of hyaline casts (typically suggestive of pre-renal etiologies of acute kidney injury) rather than red or white cell casts argue against Acute Glomerulonephritis. The administration of amoxicillin might suggest Acute Interstitial Nephritis, but the lack of rash, fever, or eosinophilia, common symptoms associated with this condition, makes it less likely. Acute Obstructive Uropathy commonly presents with abdominal pain, which the patient doesn’t have. Finally, while Intravascular Volume Depletion could initially result in a lowered urine output, our patient’s raised blood pressure argues against this as a primary diagnosis. CONCLUSION: Based on a thorough evaluation of the patient's medical history, presenting symptoms, and lab findings, the evidence points more conclusively to Acute Kidney Injury. This conclusion is largely supported by her presenting signs of fluid overload, higher blood pressure, sharply increased serum creatinine and presence of hyaline casts in the urine. RESPONSE: Taking all factors into account, the most likely diagnosis for this patient with elevated blood pressure, swollen lower extremities, shortness of breath, and signs of fluid overload, together with laboratory findings suggestive of declining kidney function, is (C) Acute Kidney Injury.

**Facilitator** The consensus statement synthesizes the responses of AI_A, AI_B, AI_C, AI_D, and AI_E as follows: The patient in question is a 52-year-old woman with a history of type 2 diabetes mellitus. She was recently treated with amoxicillin for a urinary tract infection and lisinopril therapy was initiated due to elevated blood pressure. Currently, she is experiencing swelling in the lower extremities, shortness of breath, and anxiety. Upon physical examination, she displays diffuse crackles heard on pulmonary examination, an S3 gallop, and 3+ pitting bilateral pretibial edema. Her lab studies showed high levels of serum urea nitrogen, serum creatinine and the presence of hyaline casts in her urine. The patient's symptoms suggest an impairment in kidney function as indicated by the increase in serum creatinine and urea and the presence of hyaline casts in the urine. Given her past history of hypertension and type 2 diabetes mellitus, plus initiation of lisinopril therapy, it is plausible to assume that the elevated blood pressure and/or the use of lisinopril could have led to kidney issues. It is furthermore critical to consider her other symptoms: bilateral edema and pulmonary crackles, which may hint towards a fluid overload issue (possible heart failure or kidney dysfunction), and the increase in pulse rate which could be due to the ongoing stress on the body or due to inadequate fluid management. Given her clinical presentation and the recent medication history, the increase in serum creatinine concentration from 1.2 to 4 mg/dL suggests an acute kidney impairment. This deterioration could have been caused by the angiotensin-converting enzyme inhibitor, lisinopril, which she started two weeks ago. Lisinopril can lead to 'acute kidney failure,' particularly in patients with bilateral renal artery stenosis, heart failure, or volume depletion. It is important to note that her blood pressure is high, she has symptoms of fluid overload (crackles, S3 gallop, edema), and her urine sediment is bland which is more suggestive of pre-renal causes of acute kidney injury. In light of the information presented and considering the patient's symptoms, recent medication history, and physical examination (crackles, S3 gallop, edema) along with the laboratory findings (increased creatinine and urea, hyaline casts), it is almost certain that this patient has suffered an acute kidney injury. Therefore, the most likely diagnosis for this patient from the options provided is: (C) Acute kidney injury.
