# Supplementary material for: Collaborative intelligence in AI: Evaluating the performance of a council of AIs on the USMLE
Source: PLOS Digit Health. 2025 Oct 9;4(10):e0000787. doi: 10.1371/journal.pdig.0000787 (PMC12510544; doi:10.1371/journal.pdig.0000787)
Supplement: S2 File — (ZIP) [file pdig.0000787.s002.zip › S2 Data Raw data files containing unprocessed question inputs and LLM outputs including transcripts of deliberations/STEP2_Question-4-1_Type-MC-NJ.docx]

**Facilitator** **[Round 1]:** A 25-year-old woman is admitted to the hospital because of a 2-day history of fever, dizziness, light-headedness, chills, and shaking. She has hyperthyroidism, gastroesophageal reflux disease, and asthma. Her medications are oral methimazole and omeprazole, and inhaled fluticasone, and she has been on the same dosages for 1 year. She is in the military reserve and will be deployed overseas next week. She began atovaquone-proguanil therapy 1 week ago and received the hepatitis A and yellow fever vaccines 2 weeks ago. She is not sexually active. She does not smoke cigarettes or drink alcohol. She appears ill but is not in acute distress. Temperature is 38.6°C (101.5°F), pulse is 110/min, respirations are 22/min, and blood pressure is 90/60 mm Hg. No wheezing is heard on auscultation. Cardiac examination shows no abnormalities. Laboratory studies show: Hemoglobin = 13.8 g/dL Leukocyte count = 1100/mm³ Segmented neutrophils = 10% Eosinophils = 10% Lymphocytes = 40% Monocytes = 40% Platelet count= 320,000/mm³ Platelet count 320,000/mm³ Results of other laboratory studies are within the reference ranges. Which of the following is the most likely cause of this patient’s hematologic abnormality? Please choose the best option among the choices below. (A) Atovaquone-proguanil (B) Fluticasone (C) Hepatitis A vaccine (D) Methimazole (E) Omeprazole (F) Yellow fever vaccine

**AI_D:** (1) RESTATING THE QUESTION: The question, as I understand it, requires us to identify the most probable cause of a hematologic abnormality in a 25-year-old woman who has been enduring symptoms of fever, light-headedness and shaking for 2 days. She is under medication for hyperthyroidism, gastroesophageal reflux disease, and asthma along with certain vaccinations. The laboratory results show an abnormal leukocyte count. The possible culprits for this condition could be any of the drugs she is currently taking or vaccines she has recently received. (2) PLANNING: The lab findings show a decrease in the leukocyte count, implying leukopenia, an abnormal decrease in white blood cells critical in fighting infections. The prescribed medications and received vaccinations are major considerations for the cause. Each needs evaluation for such side effects. (3) CLINICAL REASONING: Methimazole, the medication for hyperthyroidism, is often associated with hematological abnormalities. One such rare but serious side effect is agranulocytosis, an acute condition involving a severe and dangerous leukopenia, particularly of neutrophils causing a drop in white blood cells, hence increasing susceptibility to infections. The pattern of our patient's symptoms and the close matching side effect of methimazole treatment provide a likely connection. (4) CONCLUDING: Considering the known side effects of methimazole, the overall symptomology of the patient, and the temporal relationship to medication intake, it can be said that methimazole could be a credible cause of the patient's hematologic abnormality. (5) RESPONSE: Hence, from the given options, methimazole (D) is the most likely cause of this patient’s hematologic abnormality.

**AI_E:** RESTATING THE QUESTION: The patient, a 25-year-old woman, has been experiencing fever, dizziness, light-headedness, chills, and shaking symptoms for a couple of days. She had pre-existing conditions of hyperthyroidism, gastroesophageal reflux disease, and asthma. She receives treatment in the form of oral medicines like methimazole and omeprazole, along with inhaled fluticasone. Besides, a week ago, she was started on atovaquone-proguanil therapy; 2 weeks ago, she was administered with hepatitis A and yellow fever vaccines due to her impending overseas military reserve deployment. Upon admission, her body temperature was elevated, and she was found to have low blood pressure and a fast pulse rate. Laboratory tests showed a reduced leukocyte count, normal platelet count and relatively normal hemoglobin level. The task here is to identify the potential cause of her hematologic abnormality from the given drug and vaccine choices. PLANNING: We need to consider the effects of these medications, the woman's existing diseases, and any possible interplay between them. Drugs such as methimazole and omeprazole, although taken for a year, should not be ruled out as they may have cumulative impacts or delayed side effects. Likewise, the effects of the newly introduced drugs and vaccines on the patient also need to be considered. CLINICAL REASONING: The patient's pertinent history lies in her pre-existing conditions, hyperthyroidism, gastroesophageal reflux disease, and asthma, the drugs she's been long taking, methimazole, omeprazole, fluticasone, the most recent drug, atovaquone-proguanil, and the vaccines for hepatitis A and yellow fever. But of all these, the most suspicious for causing the sudden drop in the leukocyte count could be the drug Methimazole. Methimazole, an anti-thyroid drug, while effective in controlling hyperthyroidism, is known for a rare but severe side effect called agranulocytosis, wherein there's a significant decrease in the production of granulocytes, causing a drop in white blood cells. CONCLUDING: Her symptoms of fever, chills, and infection susceptibility align with the drug-induced agranulocytosis that Methimazole can potently cause besides being confirmed with a significantly low leukocyte count in the lab findings. RESPONSE: Considering all factors, the most likely cause of this patient's hematologic abnormality is (D) Methimazole.

**AI_B:** RESTATING THE QUESTION: This patient, a 25-year-old woman, has a relatively low leukocyte (or white blood cell) count of 1,100/mm³, typically known as leukopenia. This can manifest as signs of infection like fever, chills, and light-headedness, which she is showing. Her hematologic abnormality is therefore likely related to a cause that depresses white blood cell production. Possible factors include her concurrent medication (methimazole, omeprazole, fluticasone, atovaquone-proguanil), recent vaccines (hepatitis A, yellow fever), or some aspects of her underlying medical conditions (hyperthyroidism, gastroesophageal reflux disease, asthma). PLANNING: We must determine which aspect of her health profile, medications, or recent vaccines is most likely linked to leukopenia. Every possibility can be questioned, such as can the mentioned medications induce leukopenia? Do her vaccinations have hematologic side effects? Or, is her leukopenia secondary to one of her health conditions? Upon questioning, it may be notable that the patient recently started taking a new medication, atovaquone-proguanil one week ago, so this may be an important assumption to prioritize. CLINICAL REASONING: The clinical effects associated with medications she is taking, the medications’ pharmacodynamics and pharmacokinetics, timing of the onset of symptoms, her recent commencement of atovaquone-proguanil, alongside the laboratory findings, would serve as pivotal aspects of the clinical thought process. Atovaquone-proguanil, a medication used prophylactically against malaria, notably can result in hematologic side effects, such as leukopenia and anemia due to bone marrow suppression which usually occur within the first weeks of therapy. Notably, her other medications (methimazole, omeprazole, fluticasone) and vaccines (hepatitis A, yellow fever) are less likely to cause this degree of leukopenia and she has been on these for some time without exhibiting these symptoms. CONCLUDING: After considering the patient's symptoms, medication history, vaccination status, and laboratory findings, the most likely cause of her hematologic abnormality, leukopenia, seems to be a side effect of her recent commencement of atovaquone-proguanil therapy. Indeed, her other medications and vaccines are not typically associated with such hematologic abnormalities, and her symptoms align with the known side effects and timing of atovaquone-proguanil therapy. RESPONSE: The most likely cause of this patient's hematologic abnormality is (A) Atovaquone-proguanil.

**AI_C:** (1) RESTATING THE QUESTION: A young woman, active in the military reserve, has been hospitalized with a 2-day history of sickness symptoms like fever, dizziness, chills, etc. She has a pre-existing history of hyperthyroidism, reflux disease, and asthma, and is on specific medications for the same. Recently, she has started malaria prophylaxis medicine called atovaquone-proguanil and has been given two vaccines: hepatitis A and yellow fever. Current physical examination reveals an elevated temperature, increased heart rate, and low blood pressure. Lab results indicate a low number of white blood cells (leukopenia), but other parameters are within normal limits. The objective is to identify the most probable cause of the decrease in white blood cells from the given list. (2) PLANNING: The key assumption is that her hematologic abnormality, specifically leukopenia, is related to one of the given options. Each of these options is a therapy or medication that the patient has taken recently, so we need to determine the possible connection. Some options, like methimazole, are tacitly known to have leukopenia as a side effect in some rare cases. Atovaquone-proguanil, the malaria prophylaxis she has recently started could also cause hematologic abnormalities, while vaccines could potentially cause temporary irregularities as well. (3) CLINICAL REASONING: The main clue here is the timing of the symptoms and the leukopenia. Her symptoms began 2 days ago, and she entered a new medication regime 1 week ago (atovaquone-proguanil) and was administered two vaccines 2 weeks ago. Meanwhile, her other medications (methimazole, omeprazole, and fluticasone) have been the same for the past year. The new drugs/vaccines seem the most likely to have caused a recent onset of hematologic abnormality. Both vaccines mentioned tend to have low reports of causing leukopenia. Therefore, the malaria prophylaxis medication, atovaquone-proguanil, which she recently began taking, could be the cause. (4) CONCLUDING: Considering the patient's recent medication changes and their possible side effects, we can hypothesize that the newly introduced medicine, atovaquone-proguanil, might be the cause of the patient's leukopenia. (5) RESPONSE: Among the list of options provided, the most likely cause of the patient's white blood cell count reduction - leukopenia, is (A) Atovaquone-proguanil therapy which she had started a week ago.

**AI_A:** RESTATING THE QUESTION: We are dealing with a 25-year-old woman with a medical history of hyperthyroidism, gastroesophageal reflux disease, and asthma. She is presenting with fever, dizziness, lightheadedness, chills, and shaking, but she isn't in acute distress. She takes several medications, including methimazole, omeprazole, and inhaled fluticasone. Recently, she started atovaquone-proguanil and received the hepatitis A and yellow fever vaccines. Laboratory tests show a low leukocyte count, with segmented neutrophils, eosinophils, lymphocytes, and monocytes percentages, and a normal platelet count. The question asks what's causing this patient's hematologic abnormality from the options given. PLANNING: The hematologic abnormality in question here is her leukopenia, demonstrated by her low leukocyte count. Leukopenia can be caused by several factors, including infections, autoimmune diseases, bone marrow disorders, certain medications and treatments like chemotherapy, or severe physical stress. This patient's simultaneous administration of multiple medications and recent vaccinations adds to the complexity of identifying the cause. Evaluating each of the drugs and vaccines given, their known side effects, and their likelihood to cause leukopenia might help to determine the most likely cause. CLINICAL REASONING: Atovaquone-proguanil is an antimalarial that can sometimes cause leukopenia, primarily through bone marrow suppression. However, this usually occurs in prolonged therapy and is often reversible upon discontinuation. Fluticasone, a corticosteroid, can cause a redistribution of white blood cells leading to neutrophilia and lymphopenia, but they do not typically cause leukopenia. Hepatitis A vaccine can lead to mild side effects like soreness at the injection site, fever, or mild sickness, but it does not typically result in leukopenia. Methimazole, used to treat hyperthyroidism, can cause agranulocytosis leading to leukopenia. This drug-induced agranulocytosis is a clinically significant adverse effect of antithyroid drugs. Omeprazole, a proton pump inhibitor, is very rarely associated with blood dyscrasias like leukopenia, but this is not a common side effect. Yellow fever vaccine side effects include fever, muscle aches, or a rash, but it does not commonly lead to leukopenia. CONCLUDING: After analyzing all potential causes, it appears that methimazole is the most likely cause of this patient's leukopenia. While some of the other medications and vaccines can occasionally cause leukopenia, it is not a common or prominent side effect. However, methimazole is known to cause agranulocytosis, which can severely lower the leukocyte count, making it the most probable cause. RESPONSE: (D) Methimazole is the most likely cause of this patient’s hematologic abnormality.

**Facilitator** **[Round 2]:** SUMMARIES: AI_A's Opinion: The patient's leukopenia is most likely caused by methimazole, a medication she takes for hyperthyroidism. Methimazole is known to cause agranulocytosis, which can severely lower the leukocyte count. AI_B's Opinion: The patient's leukopenia is most likely a side effect of her recent commencement of atovaquone-proguanil therapy. This medication can result in hematologic side effects, such as leukopenia and anemia due to bone marrow suppression. AI_C's Opinion: The patient's leukopenia is most likely caused by the newly introduced medicine, atovaquone-proguanil, which she started taking a week ago. AI_D's Opinion: The patient's leukopenia is most likely caused by methimazole, a medication she takes for hyperthyroidism. Methimazole is associated with hematological abnormalities, including agranulocytosis, a severe and dangerous leukopenia. AI_E's Opinion: The patient's leukopenia is most likely caused by methimazole, a medication she takes for hyperthyroidism. Methimazole is known for a rare but severe side effect called agranulocytosis, which causes a significant decrease in the production of granulocytes, leading to a drop in white blood cells. KEY DIFFERENCES: The key difference in the council members' opinions lies in the identification of the most likely cause of the patient's leukopenia. AI_A, AI_D, and AI_E believe that methimazole is the most probable cause due to its known side effect of agranulocytosis. In contrast, AI_B and AI_C attribute the leukopenia to the recently introduced medication, atovaquone-proguanil, due to its potential hematologic side effects. QUESTION FOR CLARIFICATION: Given the patient's medical history and the known side effects of both methimazole and atovaquone-proguanil, what specific factors led you to identify one medication over the other as the most likely cause of the patient's leukopenia?. Based on your preceding response, how would you answer the original question: [A 25-year-old woman is admitted to the hospital because of a 2-day history of fever, dizziness, light-headedness, chills, and shaking. She has hyperthyroidism, gastroesophageal reflux disease, and asthma. Her medications are oral methimazole and omeprazole, and inhaled fluticasone, and she has been on the same dosages for 1 year. She is in the military reserve and will be deployed overseas next week. She began atovaquone-proguanil therapy 1 week ago and received the hepatitis A and yellow fever vaccines 2 weeks ago. She is not sexually active. She does not smoke cigarettes or drink alcohol. She appears ill but is not in acute distress. Temperature is 38.6°C (101.5°F), pulse is 110/min, respirations are 22/min, and blood pressure is 90/60 mm Hg. No wheezing is heard on auscultation. Cardiac examination shows no abnormalities. Laboratory studies show: Hemoglobin = 13.8 g/dL Leukocyte count = 1100/mm³ Segmented neutrophils = 10% Eosinophils = 10% Lymphocytes = 40% Monocytes = 40% Platelet count= 320,000/mm³ Platelet count 320,000/mm³ Results of other laboratory studies are within the reference ranges. Which of the following is the most likely cause of this patient’s hematologic abnormality? Please choose the best option among the choices below. (A) Atovaquone-proguanil (B) Fluticasone (C) Hepatitis A vaccine (D) Methimazole (E) Omeprazole (F) Yellow fever vaccine]

**AI_A:** AI_A's RESPONSE: In restating the question, we're asked to discern the most probable cause of the patient's leukopenia, which is a decline in the number of white blood cells. The two main assumptions at play here are that the leukopenia is a result of one of the patient's medications or that it's a response to a recent vaccination. After planning, I generated alternate assumptions by questioning each drug's contribution. Methimazole, used for treating hyperthyroidism, can cause agranulocytosis —a significant decrease in the number of granulocytes (a type of white blood cell), leading to a lower overall leukocyte count. Atovaquone-proguanil, recently started by the patient to prevent malaria upon her overseas deployment, can also result in leukopenia due to bone marrow suppression, albeit less commonly. My clinical reasoning leads me to prioritize methimazole as the most probable cause. Although the patient has been on methimazole for a long duration without hematological issues, the onset of agranulocytosis can occur at any time during methimazole therapy, and presents a higher risk than atovaquone-proguanil. Also, agranulocytosis typically results in a granulocyte count near zero, as observed in this patient. In conclusion, the patient's clinical presentation aligns more closely with agranulocytosis caused by methimazole than leukopenia associated with atovaquone-proguanil. Given this consideration, the most valid response to the original question is therefore methimazole (D).

**AI_B:** Restating the Question: You are asking which of the patient's medications or recent vaccines is the most probable cause of her leukopenia, which is a lower than normal number of leukocytes, or white blood cells, in the blood. Leukopenia can be caused by various drugs and conditions and can increase susceptibility to infections. Planning: The identified assumptions are that the leukopenia is caused by a drug effect, that the effect is related to a recent change or addition in medications, that it might be a temporary drug-induced leukopenia, and that it's not related to an infectious cause as no infectious symptoms are present apart from the fever. Alternate assumptions include: leukopenia could be a side effect of an existing long-term medication, or leukopenia could indeed be due to an infection, even in the absence of other infectious symptoms. Clinically, it's significant to prioritize the determination of whether it's a drug-induced leukopenia or a condition related to a possible infection. Clinical Reasoning: Considering that methimazole, which the patient has been taking for a year, can cause agranulocytosis, a severe form of leukopenia, it might indeed be the cause despite its long use. However, atovaquone-proguanil, which was started only a week ago, could potentially cause bone marrow suppression resulting in hematologic abnormalities like anemia and leukopenia but typically this occurs with prolonged use. Patient's high fever and the start of atovaquone-proguanil, a Malaria prophylaxis medication, imply that patient could be preparing for deployment to a region where Malaria is endemic, raising the concern for a possible infectious cause. Concluding: Upon weighing the correlation between the onset of symptoms and the commencement of atovaquone-proguanil therapy, compared to the potential but statistically less common adverse effect of methimazole causing agranulocytosis, I would consider methimazole to still be a likely cause of symptomatic leukopenia. Response: In conclusion, while it is possible that her leukopenia is due to recent addition of atovaquone-proguanil in her medication regimen, considering she has been on methimazole for a longer duration which is notorious for causing agranulocytosis, especially in the initial stages of treatment but can occur at any time, it is plausible to consider Methimazole (Choice D) to be the most likely cause of her hematologic abnormality.

**AI_C:** RESTATING THE QUESTION: We're asked to discern the most likely cause of a significant leukopenia (low white blood cell count) in a 25-year-old woman with several medical conditions, who recently added atovaquone-proguanil to her regimen and received yellow fever and hepatitis A vaccines. Her medications include methimazole, omeprazole, and fluticasone for hyperthyroidism, gastroesophageal reflux disease, and asthma respectively. PLANNING: The patient's leukopenia could be due to side effects from either the newly initiated atovaquone-proguanil or the methimazole, which she has been taking for a year. While she received vaccines recently, these are unlikely to cause the observed leukopenia. The fluticasone and omeprazole are also unlikely to be responsible. Considering the acute onset of symptoms and recent initiation of atovaquone-proguanil therapy, it is reasonable to wonder whether this drug could be the cause due to its known potential for hematologic side effects. However, an alternate assumption could be the methimazole, which has been implicated in causing agranulocytosis, a severe form of leukopenia. Hence, a clinical reasoning process must be used to evaluate the potential culprits and determine which one is the most likely. CLINICAL REASONING: Atovaquone-proguanil can cause bone marrow suppression and thus could lead to leukopenia. However, methimazole has a well-documented association with agranulocytosis, which fits with the patient's manifestation of profound leukopenia. Agranulocytosis usually manifests within days to months of starting methimazole, although late onset after several years is also reported. The patient has been on methimazole for a year, fitting the timeframe. While atovaquone-proguanil could explain the lab findings, methimazole-induced agranulocytosis seems a more likely explanation given the severity of the leukopenia and past medical history. CONCLUDING: After considering the patient's recent changes in medication, presenting symptoms, and the known potential side effects, the most probable cause of her leukopenia is methimazole. RESPONSE: Given these reasons, my answer to the question concerning the most likely cause of the patient's hematologic abnormality grounds on option (D) Methimazole.

**AI_E:** (1) RESTATING THE QUESTION: The request involves identifying the primary factor resulting in the observed hematologic abnormality, specifically leukopenia, in a 25-year-old woman. Our individual presents with several medical conditions and takes a variety of medications, including methimazole, omeprazole, and fluticasone regularly, and recently commenced atovaquone-proguanil therapy. Additionally, she received Hepatitis A and yellow fever vaccines recently. Leukopenia, her observed condition, refers to a decrease in the number of white blood cells, which play a crucial role in the body's immune response against diseases. (2) PLANNING: A few assumptions could explain this scenario. One is that the methimazole she takes for hyperthyroidism might have caused the leukopenia since this medication is known to result in agranulocytosis, a condition that drastically decreases white blood cell count. Another assumption is that atovaquone-proguanil, which she recently started taking, could have led to the leukopenia, given known hematologic side effects. Each option has valid reasoning, but considering the timing, symptoms, and severity of leukopenia, the methimazole might be clinically more critical to consider first. (3) CLINICAL REASONING: Methimazole is a well-known antithyroid medicine, but one rare yet severe side effect is agranulocytosis, which could lead to a sharp decrease in white blood cells, thereby causing leukopenia. The condition could develop abruptly and can occur any time during therapy. Even though the patient has been on the medication for a long time, it does not eliminate the possibility of drug-induced agranulocytosis. On the other hand, atovaquone-proguanil might have hematologic side effects, but the likelihood of it causing such drastic leukopenia in a short amount of time is rare. Furthermore, the associated side effects, such as fever and dizziness, align more with agranulocytosis, further making methimazole a plausible culprit. (4) CONCLUDING: While considering medications and their side effects, the timing of their commencement, and manifestation of symptoms, methimazole emerges as a probable cause of this patient's leukopenia more than atovaquone-proguanil. (5) RESPONSE: Given all considerations, particularly the severe leukopenia, associated symptoms, and known rare but serious side effect of agranulocytosis linked with methimazole, I would opt for (D) Methimazole as the most likely cause of this patient's hematologic abnormality.

**AI_D:** 1) RESTATING THE QUESTION: The query seeks to determine the probable cause of leukopenia (a decrease in the number of white blood cells, or leukocytes, in the blood) in a 25-year-old patient with a 2-day history of fever, dizziness, light-headedness, chills, and shaking. She has a history of hyperthyroidism, gastroesophageal reflux disease, and asthma and is on multiple medications including methimazole, omeprazole, fluticasone, and atovaquone-proguanil. However, she only started the atovaquone-proguanil therapy 1 week ago. She's also recently had the hepatitis A and yellow fever vaccines. The only abnormal result in her laboratory study is her leukocyte count of 1100/mm³. 2) PLANNING: Both methimazole and atovaquone-proguanil could potentially cause leukopenia as a side effect. In the case of methimazole, this medication can lead to a severe reduction in the neutrophil count, resulting in agranulocytosis. On the other hand, atovaquone-proguanil, a malaria prophylaxis, can cause bone marrow suppression, leading to leukopenia. It needs to be determined which of these assumptions is more likely in this clinical case. 3) CLINICAL REASONING: Considering this patient has been on methimazole for 1 year with the same dosage, it does not seem likely that this medication is the cause of her recent onset of leukopenia. While methimazole can cause agranulocytosis, it usually happens within the first few months of treatment, and our patient has been on this medication for much longer. On the other hand, she recently commenced atovaquone-proguanil therapy. A presentation of leukopenia could be likely due to a medication she recently started, rather than a medication she has taken without issues for the past year. 4) CONCLUDING: In weighing out the evidence, atovaquone-proguanil, her recent medication addition, carries the stronger probability for the cause of her recent leukopenia due to the timing of onset, even though both medications have leukopenia as a potential side effect. 5) RESPONSE: Based on this patient's history and the timing of her symptoms, the most likely cause of her hematologic abnormality (leukopenia) is option (A) Atovaquone-proguanil. Given the fact that the patient only recently started taking this medication, and the likelihood of this drug causing leukopenia, this would be the most probable assumption.

**Facilitator** **[Round 3]:** Summary: AI_A, AI_B, AI_C, and AI_E all agree that methimazole is the most probable cause of the patient's leukopenia. They all note that methimazole can cause agranulocytosis, a severe form of leukopenia, at any time during therapy. They also point out that the patient's symptoms align more closely with agranulocytosis caused by methimazole than leukopenia associated with atovaquone-proguanil. AI_D, however, believes that atovaquone-proguanil, the patient's recently added medication, is the most likely cause of the leukopenia. AI_D argues that the timing of the onset of leukopenia, which coincides with the start of atovaquone-proguanil therapy, suggests that this drug is the cause. AI_D also notes that methimazole-induced agranulocytosis usually occurs within the first few months of treatment, and the patient has been on methimazole for a year without issues. Question for Council Members: Given that both methimazole and atovaquone-proguanil can cause leukopenia, how do we weigh the importance of the timing of the onset of symptoms (which seems to favor atovaquone-proguanil as the cause) against the severity of the leukopenia and the known risk of agranulocytosis with methimazole (which seem to favor methimazole as the cause)?. Based on your preceding response, how would you answer the original question: [A 25-year-old woman is admitted to the hospital because of a 2-day history of fever, dizziness, light-headedness, chills, and shaking. She has hyperthyroidism, gastroesophageal reflux disease, and asthma. Her medications are oral methimazole and omeprazole, and inhaled fluticasone, and she has been on the same dosages for 1 year. She is in the military reserve and will be deployed overseas next week. She began atovaquone-proguanil therapy 1 week ago and received the hepatitis A and yellow fever vaccines 2 weeks ago. She is not sexually active. She does not smoke cigarettes or drink alcohol. She appears ill but is not in acute distress. Temperature is 38.6°C (101.5°F), pulse is 110/min, respirations are 22/min, and blood pressure is 90/60 mm Hg. No wheezing is heard on auscultation. Cardiac examination shows no abnormalities. Laboratory studies show: Hemoglobin = 13.8 g/dL Leukocyte count = 1100/mm³ Segmented neutrophils = 10% Eosinophils = 10% Lymphocytes = 40% Monocytes = 40% Platelet count= 320,000/mm³ Platelet count 320,000/mm³ Results of other laboratory studies are within the reference ranges. Which of the following is the most likely cause of this patient’s hematologic abnormality? Please choose the best option among the choices below. (A) Atovaquone-proguanil (B) Fluticasone (C) Hepatitis A vaccine (D) Methimazole (E) Omeprazole (F) Yellow fever vaccine]

**AI_B:** Restating the question: The query essentially seeks to identify the most likely cause of the patient's abnormal leukocyte count, termed leukopenia, from the list of medications and recent vaccinations she has received. I would like to note that leukopenia is a condition where a person has fewer white blood cells in their bloodstream, which are crucial in fighting infections, so this woman has a lowered resistance to infections as a result. Planning: We need to evaluate each drug and vaccine this patient has taken recently and assess their likelihood of causing leukopenia. For instance, we know that atovaquone-proguanil can cause leukopenia, and the patient started taking it recently. Similarly, methimazole has been identified as a cause of agranulocytosis, a severe form of leukopenia. Both the hepatitis A and yellow fever vaccines, while generally safe, have rare instances of causing leukopenia. Lastly, omeprazole and fluticasone are less likely to cause leukopenia. Clinical Reasoning Observing the clinical features and laboratory findings in this patient, and comparing them with the typical side effects of the drugs, we can notice that her symptoms like fever, light-headedness, chills, and shaking are more consistent with agranulocytosis (severe leukopenia) induced by methimazole than the more moderate leukopenia associated with atovaquone-proguanil. It is pertinent to note that although methimazole-induced agranulocytosis usually occurs within the first three months of therapy, it can occur at any time, even after a year, as seen in this patient. Concluding: Considering the severity of the leukopenia, the symptomatology aligning more to agranulocytosis, and methimazole's known side effect of agranulocytosis, I can deduce that methimazole is the most likely cause of this patient's hematologic abnormality despite the fact that she has been on this medication for a year. Response: Based on the analysis above, I would choose option (D) Methimazole as the most likely cause of the patient’s hematologic abnormality.

**AI_C:** Restating the question: This is a 25-year-old woman presenting with fever, dizziness, light-headedness, chills, and shaking. She exhibits a low leukocyte count or leukopenia – a condition defined by a reduction in the number of white blood cells in the blood. One of her medications is presumably causing this. Our task is to identify which is the most likely one from those listed: atovaquone-proguanil, fluticasone, hepatitis A vaccine, methimazole, omeprazole, or yellow fever vaccine. Planning: The key assumptions here are that the one of the woman's medications is causing her leukopenia, and that we have enough information about these medications and the woman's medical condition to make an informed determination. We'll generate alternate assumptions by questioning whether any other factors could be influencing her leukocyte count, such as undiagnosed illnesses or interactions between her medications. Based on the provided information, it's prudent to prioritize investigating her medications as the cause. Clinical reasoning: Although several of the medications could potentially cause leukopenia, methimazole and atovaquone-proguanil have been pointed out as prominent possibilities. Methimazole, a common treatment for hyperthyroidism, can lead to agranulocytosis – a severe form of leukopenia. This can occur unexpectedly, regardless of how long the drug has been used. Atovaquone-proguanil, an antimalarial drug, can also cause leukopenia. In this case, the woman began taking it a week prior, which could coincide with the onset of symptoms. However, her symptoms of fever and general discomfort are more in line with agranulocytosis, which leans towards methimazole as the cause. Concluding: Considering all factors, the severity and type of leukopenia, the symptoms, the woman’s long-standing use of methimazole, and its association with agranulocytosis would suggest that methimazole is the most likely cause of this patient’s hematologic abnormality. However, the recent onset of symptoms and initiation of atovaquone-proguanil medication cannot be ignored. Response: Taking into account all these factors, I would lean towards choosing option (D) Methimazole as the most likely cause of the patient's hematological abnormality. This case highlights the need for constant monitoring and reevaluation of patients on drugs such as methimazole which have known, potentially severe side-effects such as agranulocytosis.

**AI_A:** Restating the question, we are asked to identify which medication or immunization leads to the most likely abnormality in the patient's hematologic profile. Given the information presented, the patient displays significant leukopenia, characterized by a noticeable decrease in total white blood cells (leukocytes). This state of leukopenia adds to her presentation of fever, light-headedness, and other suggestive symptoms of an infection or an immune response. Let's think about some of the pros and cons of the various options. While both methimazole and atovaquone-proguanil can lead to leukopenia, the degree of leukopenia observed in this case is significant, leaning more towards agranulocytosis associated with severe leukopenia that is a known, albeit rare, side effect of methimazole. However, the patient started atovaquone-proguanil one week ago, which makes this drug a possible culprit as well. While it is true that methimazole-induced agranulocytosis typically occurs within the first few months of therapy, it can also occur at any point during the course of treatment. In clinical reasoning, it is critical to weigh the evidence by considering both the severity of the leukopenia and the timing of the new medication (atovaquone-proguanil). But in general, the commonality and severity of leukopenia (in this case, seems closer to agranulocytosis) in the context of methimazole usage should be of more weight given its known side effects. Furthermore, the recent start of atovaquone-proguanil does make this medication a plausible culprit, but the profound leukopenia sits less comfortably here. However, as always, it is an empirical probability—it is still possible atovaquone-proguanil caused the leukopenia. In conclusion, given these considerations and the patient's therapeutically unchanged use of methimazole over a significant period before the onset of symptoms—coupled with methimazole's well-known side effect—the most likely answer to the question would be 'Methimazole' (Option D). However, in a follow-up clinical context, the minimally invasive and quick to collect further laboratory tests could help verify this clinical suspicion. Furthermore, considering the previous usage of methimazole without any hematological side effects and this severe leukopenia started after the initiation of atovaquone-proguanil, stopping both drugs and monitor patient's response can be justified. If leukopenia improves after stopping both drugs, a rechallenge with methimazole can identify whether it is the culprit. This strategic approach can prevent the unnecessary withholding of methimazole, which can otherwise have an impact on the patient's hyperthyroid management.

**AI_E:** RESTATING THE QUESTION: We are asked to determine the most likely cause of hematological abnormalities in a 25-year-old woman. She is currently presenting with fever, dizziness, light-headedness, chills, and shaking, and has been diagnosed with hyperthyroidism, gastroesophageal reflux disease, and asthma. She is on several medications, including methimazole, omeprazole, inhaled fluticasone, and atovaquone-proguanil. Her blood test shows a significantly decreased leukocyte count. PLANNING: We are considering two key factors to explain her condition: the timing of the appearance of symptoms and the severity of leukopenia. Methimazole, known to cause agranulocytosis, and atovaquone-proguanil, recently initiated, both can cause leukopenia. However, methimazole-related agranulocytosis usually occurs within the first few months of treatment, which contradicts this case's timing, whereas atovaquone-proguanil-induced leukopenia might align better temporal-wise. CLINICAL REASONING: Leukopenia is a condition characterized by a decrease in the total white blood cells (WBCs) in the blood. Our patient has a significantly decreased leukocyte count, which indicates leukopenia. Both methimazole and atovaquone-proguanil are associated with this hematologic side effect. However, methimazole-induced agranulocytosis, a severe form of leukopenia, is generally seen within the first 3 months of therapy, contradicting the fact that this patient has been on the same dosage for a year. Meanwhile, the initiation of atovaquone-proguanil therapy overlaps with the onset of the symptoms, hinting that it might be more likely the cause. Yet the drastic reduction in the leukocyte count could be possibly more attributable to agranulocytosis induced by methimazole than simple leukopenia caused by atovaquone-proguanil. CONCLUDING: Thus, while the timing of the onset of symptoms leans towards atovaquone-proguanil as the cause of leukopenia, methimazole-induced agranulocytosis cannot be ruled out due to the severe reduction in leukocyte count. RESPONSE: The most likely cause of this patient's hematologic abnormality is methimazole. Despite the initiation of atovaquone-proguanil therapy coinciding with the onset of symptoms, the drastic reduction in leukocyte count suggests a more severe leukopenia that is commonly caused by agranulocytosis induced by methimazole. Therefore, option D, methimazole, is the most likely answer to this question.

**AI_D:** RESTATING THE QUESTION: To put it succinctly, this 25-year-old woman who has been treated for hyperthyroidism, GERD and asthma is now presenting with symptoms including fever and light-headedness. She recently began atovaquone-proguanil therapy and also received hepatitis A and yellow fever vaccines. Her blood pressure seems to be low and a notable leukopenia is found in the laboratory tests. We are tasked to identify the most likely cause of her hematologic abnormality, considering the medication options provided. PLANNING: The possibilities presented for the cause of the hematologic abnormality are primarily medications: atovaquone-proguanil, fluticasone, the hepatitis A vaccine, methimazole, omeprazole, and the yellow fever vaccine. We know that both methimazole and atovaquone-proguanil can cause leukopenia. But, we also consider the onset of symptoms relative to the introduction of these medications. It's also crucial to consider that agranulocytosis frequently results from methimazole usage. CLINICAL REASONING: Methimazole, an antithyroid medication known to provoke agranulocytosis, a form of leukopenia, remains a primary suspect despite the woman having taken the medication for a year without prior issues. Although agranulocytosis due to methimazole often develops within the first few months of beginning therapy, it can happen at any time, or even after long-term stable use. Paired with the fact that her symptoms are fitting of methimazole-induced agranulocytosis, this makes it a prime suspect. The recent initiation of her atovaquone-proguanil therapy makes it tempting to associate it with her new symptoms. Although atovaquone-proguanil can cause leukopenia, the severity of her leukopenia, with a leukocyte count plummeting to dangerously low levels, is more consistent with the effects of Methimazole causing agranulocytosis. The other drug's effects are usually more moderate. CONCLUDING: While it might appear that the initiation of atovaquone-proguanil therapy coincides with the onset of symptoms, the severity of the leukopenia bias us towards Methimazole-induced agranulocytosis. This aligns us with the majority of the council who also believe that Methimazole is the most probable cause of her hematologic abnormality. RESPONSE: The most probable cause of this patient's hematologic abnormality is believed to be Methimazole (option D).

**Facilitator** The consensus statement synthesizes the responses of the AI models as follows: The question asks us to identify the most likely cause of the patient's hematologic abnormality, characterized by significant leukopenia. The patient is on several medications, including methimazole, omeprazole, inhaled fluticasone, and atovaquone-proguanil, and has recently received the hepatitis A and yellow fever vaccines. Both methimazole and atovaquone-proguanil are known to cause leukopenia. However, the severity of the patient's leukopenia, which leans towards agranulocytosis, a severe form of leukopenia, is more commonly associated with methimazole. Methimazole-induced agranulocytosis can occur at any time during treatment, even though it is typically seen within the first few months. The recent initiation of atovaquone-proguanil therapy coincides with the onset of symptoms, making it a plausible cause. However, the severity of leukopenia observed in this case is less commonly associated with atovaquone-proguanil. Considering the severity of leukopenia, the patient's symptoms, and the known side effects of methimazole, the consensus is that methimazole is the most likely cause of the patient's hematologic abnormality. However, the recent start of atovaquone-proguanil therapy should not be ignored, and further laboratory tests could help verify this clinical suspicion. Therefore, the most likely answer to the question is option (D) Methimazole. However, in a clinical context, it would be prudent to stop both drugs and monitor the patient's response. If leukopenia improves after stopping both drugs, a rechallenge with methimazole can identify whether it is the culprit. This strategic approach can prevent the unnecessary withholding of methimazole, which can otherwise have an impact on the patient's hyperthyroid management.
