# Supplementary material for: Collaborative intelligence in AI: Evaluating the performance of a council of AIs on the USMLE
Source: PLOS Digit Health. 2025 Oct 9;4(10):e0000787. doi: 10.1371/journal.pdig.0000787 (PMC12510544; doi:10.1371/journal.pdig.0000787)
Supplement: S2 File — (ZIP) [file pdig.0000787.s002.zip › S2 Data Raw data files containing unprocessed question inputs and LLM outputs including transcripts of deliberations/STEP2_Question-40-1_Type-MC-NJ.docx]

**Facilitator** **[Round 1]:** A 15-year-old girl is brought to the emergency department by her father because of a 2-hour history of right leg pain that began after she fell from her bicycle. Medical history is remarkable for bilateral hearing loss, a fracture of the humerus at age 8 years, and multiple rib fractures. She takes no medications. Family history is remarkable for kyphoscoliosis, hearing loss, and early onset osteoporosis in her mother; the mother died from complications related to a hip fracture at age 44 years. The patient is a refugee who has recently immigrated. The patient appears to be in distress. She is below the 5th percentile for height, at the 70th percentile for weight, and above the 95th percentile for BMI. Her temperature is 37.0°C (98.6°F), pulse is 118/min, respirations are 12/min, and blood pressure is 130/68 mm Hg. Examination shows kyphosis and hypermobile joints of the hands, wrists, and feet. Examination of the right lower extremity shows superficial abrasions and ecchymoses; there is tenderness to palpation and movement of the extremity. Which of the following is the most likely underlying diagnosis? (A) Idiopathic juvenile osteoporosis (B) Juvenile Paget disease (C) Osteogenesis imperfecta (D) Osteomalacia (E) Rickets

**AI_B:** RESTATING THE QUESTION: The main query here is the accurate diagnosis for an adolescent female refugee presenting with a recent right leg injury from falling off her bicycle and a notable medical history of bilateral hearing loss and broken bones. She is short for her age, overweight, has high blood pressure, and clinical evidences of bone and joint abnormalities. Her mother had kyphoscoliosis, hearing loss, and was osteoporotic at a young age. PLANNING: Here we have a young patient with multiple fractures, hearing loss, kyphosis, and hypermobility of joints, all of which reflect bone fragility and compromised structural integrity. In light of these findings, we weigh the probabilities of several bone diseases: Idiopathic juvenile osteoporosis, Juvenile Paget disease, Osteogenesis imperfecta, Osteomalacia, and Rickets. CLINICAL REASONING: To start, her short stature, limb and rib fractures, kyphosis, hearing loss, and her mother's early-onset osteoporosis immediately rules out Idiopathic juvenile osteoporosis, which usually affects preteens and adolescents with no distinct symptoms until a fracture occurs, and also Juvenile Paget disease, a very rare bone disorder characterized by rapid bone turnover that usually presents in infancy. Rickets and Osteomalacia share similar symptoms of bone softening and deformities, but the former occurs in children and the latter in adults. Moreover, these conditions typically present with a history of inadequate sunlight exposure or absorption issues of calcium and phosphate, none of which applies to our patient. Therefore, the plausible diagnosis is Osteogenesis imperfecta, a genetic disorder causing brittle bones that break easily. It also explains the hearing loss, considered a less common symptom caused by abnormalities in the middle ear bones. CONCLUDING: Upon clinical reasoning, while the patient shares some symptoms with the other conditions, only Osteogenesis imperfecta comprehensively explains her symptoms and family history of early onset osteoporosis, hearing loss, and kyphoscoliosis. RESPONSE: The most likely underlying diagnosis is (C) Osteogenesis imperfecta.

**AI_C:** (1) RESTATING THE QUESTION: Our patient is a 15-year-old girl with a history of various bone fractures and bilateral hearing loss, presenting with acute right leg pain after falling from her bicycle. Her family history includes kyphoscoliosis, hearing loss, and early onset osteoporosis. She appears distressed, is of short stature with a high BMI, her vital signs are within normal range, and she has physical findings of kyphosis and hypermobile joints. The focus is to find out what is the most probable diagnosis considering all these factors. (2) PLANNING: In context, it is important to consider the relation of her symptomatology to various elements that contribute to her situation. The patient's short stature, high BMI, recurrent fractures, and hypermobility point towards a possible connective tissue disorder. The family history of early-onset osteoporosis, kyphoscoliosis, and hearing loss further signifies a genetic component. Therefore, a genetic connective tissue disorder affecting bone density and strength is the assumption that we should clinically prioritize. (3) CLINICAL REASONING: Our patient presents features of frequent fractures, hearing impairment and family history reminiscent of early-onset osteoporosis. Osteogenesis imperfecta (OI) should be considered, as it is a genetic disorder characterized by increased bone fragility and hearing loss, often noticeable from childhood. The two types of OI, Type I and Type IV, can be associated with hearing loss, blue sclera, dentinogenesis imperfecta and history of multiple fractures, which could explain our patient's symptoms and history. (4) CONCLUDING: After considering all the aforementioned factors, OI seems to be the most likely diagnosis. However, it is important to note that OI has a wide clinical variability, ranging from perinatal lethality to mild forms with few fractures. In their milder forms, these conditions can go undiagnosed until adulthood. (5) RESPONSE: Hence, among the options provided, (C) Osteogenesis imperfecta seems to be the most likely underlying diagnosis in this patient.

**AI_E:** (1) RESTATING THE QUESTION: This patient is a 15-year-old girl who presented to the emergency department after falling from her bicycle. She has a history of recurrent bone fractures, hearing loss, and her mother had kyphoscoliosis, hearing loss, and early onset osteoporosis. She is undersized (below the 5th percentile for height), overweight (70th percentile for weight), and has a high Body Mass Index (BMI, above the 95th percentile). Physical exam showed kyphosis (an exaggerated rounding of the back) and hypermobile joints. She is in distress and her right leg is tender and bruised. The question is asking for the most probable underlying diagnosis. (2) PLANNING: Key assumptions to generate include she may have a bone disorder, given her multiple fractures and the family history - primarily her mother's early onset osteoporosis. Her hearing loss, short stature, and kyphosis add more credence to a potential connective tissue disorder. Options provided for diagnosis include idiopathic juvenile osteoporosis, juvenile Paget disease, osteogenesis imperfecta, osteomalacia, and rickets. Each of these diagnoses will be evaluated for their clinical fit to the history and physical exam findings. (3) CLINICAL REASONING: The first hypothesis could be Idiopathic juvenile osteoporosis. However, this condition is typically not associated with hearing loss or a family history of osteoporosis. Juvenile Paget disease is a second alternative, but it would usually present with more skeletal deformities, and the symptoms of hearing loss don't align well. The third hypothesis could be Osteogenesis imperfecta which is characterized by brittle bones, leading to frequent fractures, and it can also cause bluish sclera, short stature, dentinogenesis imperfecta, and hearing loss. Her existent fractures, hearing loss, family history and short stature fit this diagnosis. Fourth alternative is osteomalacia, but it would be expected to present with deficiency of Vitamin D, calcium, or phosphate. The final hypothesis, Rickets, also primarily affects children who are deficient in Vitamin D. Considering the above reasoning, Osteogenesis imperfecta seems the most clinically proper assumption to diagnose. (4) CONCLUDING: Based on the familial pattern of hearing loss and the presence of multiple bone fractures, which could indicate bone fragility, along with the physical findings like kyphosis and hypermobile joints, Osteogenesis imperfecta—an inherited disorder primarily affecting the connective tissue—nudges out the other options. (5) RESPONSE: Therefore, the most likely diagnosis for this young girl's underlying condition is Osteogenesis imperfecta (C).

**AI_D:** RESTATING THE QUESTION: This patient is a young girl presenting with a right leg pain that started after falling from her bicycle. Her previous medical history includes bilateral hearing loss, fractures of the humerus and ribs. In addition, her family history is significant for kyphoscoliosis, hearing loss, and early-onset osteoporosis in her mother, who passed away due to hip fracture complications. The patient recently immigrated as a refugee, presenting with distress and physical characteristics such as low height, high BMI, and hypermobile joints. At this point, the task at hand is to determine her most likely underlying diagnosis from options including idiopathic juvenile osteoporosis, juvenile Paget disease, osteogenesis imperfecta, osteomalacia, and rickets. PLANNING: Analyzing the question, we note that the conditions listed as possible diagnoses are all related to bone health and structure. To conduct a proper evaluation, it's crucial to understand each of the conditions well, including their defining aspects and manifestations. Juvenile osteoporosis refers to decreased bone density often without a clear cause; juvenile Paget's disease involves excessive breakdown and formation of bones, leading to abnormal bone structure; osteogenesis imperfecta (OI), also known as brittle bone disease, is a genetic disorder characterized by fragile bones that break easily; osteomalacia refers to weakening of bones due to severe vitamin D deficiency; and rickets, primarily seen in children, is also associated with severe vitamin D deficiency leading to soft and weak bones. CLINICAL REASONING: Beginning with the provided history, the patient's multiple fractures, hearing loss, and kyphoscoliosis (spinal deformity), we note her family history of early-onset osteoporosis and her mother's fatal complications from a hip fracture. Given these facts, and the patient's additional symptom of hypermobile joints, the signs point more towards a genetic condition rather than a nutritional deficiency or an idiopathic cause. This makes idiopathic juvenile osteoporosis, osteomalacia, and rickets less likely. Juvenile Paget's disease usually presents with an enlarged skull and bowed long bones, which does not align with this patient's presented symptoms. Osteogenesis imperfecta, a genetic disorder that causes defective collagen leading to easy fracturing of bones, kyphoscoliosis, hearing loss due to abnormalities of the ossicles, and hypermobile joints due to ligamentous laxity, appears to be the most clinically appropriate priority. CONCLUDING: By following a step-wise clinical approach, it becomes evident that this girl's symptoms coincide more closely with the clinical manifestations of osteogenesis imperfecta than the other given conditions. Aside from her turbulent medical history, osteogenesis imperfecta would also explain her relatively short stature and hypermobile joints, which are often seen in individuals suffering from this condition. RESPONSE: Given the patient's symptomatology and corresponding clinical characteristics, the most probable underlying diagnosis is firmly believed to be (C) Osteogenesis imperfecta.

**AI_A:** RESTATING THE QUESTION: A 15-year-old female patient has a history of fractures, hearing loss, and is abnormally short for her age. Her mother had early onset osteoporosis, hearing loss, and kyphoscoliosis. The girl currently suffers from right leg pain which has occurred after a fall from her bicycle. This patient's physical exam revealed hypermobility on the joints of her hands, wrists, and feet, as well as kyphosis. We are tasked with diagnosing her condition from a range of five options related to bone health disorders, of which the potential candidates are (A) Idiopathic juvenile osteoporosis, (B) Juvenile Paget disease, (C) Osteogenesis imperfecta, (D) Osteomalacia, and (E) Rickets. PLANNING: From the patients' history and presentation, it's reasonable to assume that the patient may have a bone-related disorder. Her history of fractures, mother's early onset osteoporosis, and current pain after a fall, could all point towards a bone health disorder. We could consider the possibility of juvenile osteoporosis, given the family history and multiple fractures in the patient's history. Juvenile Paget disease may also be a possibility, as it involves bone remodeling disorder, however, it's not typically associated with hearing loss. Osteogenesis imperfecta certainly fits with this patient's presentation, as it relates to a genetic disorder causing fragile bones, short stature, hearing loss, and is associated with hypermobility and kyphosis. Another assumption could be osteomalacia, a softening of the bones usually caused by a deficiency in vitamin D, but this isn't typically associated with hearing loss or hypermobility of the joints. Rickets could also be considered, but this usually presents in infancy or early childhood with delayed growth and bone pain. CLINICAL REASONING: Osteogenesis imperfecta, also known as brittle bone disease, is a collagen synthesis disorder that results in weak bones, multiple fractures, blue sclerae, hearing loss, and occasionally dental abnormalities. The patient's hearing loss and history of multiple fractures hint strongly towards OI. Other hallmark symptoms of OI, such as blue sclerae and dental abnormalities aren't mentioned, but their absence doesn't rule out the condition. On the other hand, findings such as kyphosis can happen in this condition. Although other conditions such as juvenile osteoporosis, Paget disease, and osteomalacia can cause weak bones and fractures, they are less likely to cause hearing loss, kyphosis, or joint hypermobility, all of which this patient exhibits. CONCLUDING: Given both the patient's hearing loss and the presence of multiple fractures from seemingly minor trauma, as well as the physical characteristics of joint hypermobility and kyphosis, despite the lack of certain distinguishing features of this condition like blue sclerae and dental abnormalities, the most likely diagnosis for our 15-year-old patient is osteogenesis imperfecta. RESPONSE: (C) Osteogenesis imperfecta is the most likely underlying diagnosis.

**Facilitator** The consensus among the AI responses is that the most likely diagnosis for the 15-year-old female patient is Osteogenesis Imperfecta (C). This conclusion is based on her history of multiple fractures, hearing loss, and her mother's early onset osteoporosis. Her physical exam revealed hypermobility on the joints of her hands, wrists, and feet, as well as kyphosis. These symptoms align with Osteogenesis Imperfecta, a genetic disorder causing fragile bones, short stature, hearing loss, and is associated with hypermobility and kyphosis. Other conditions such as juvenile osteoporosis, Paget disease, and osteomalacia can cause weak bones and fractures, but they are less likely to cause hearing loss, kyphosis, or joint hypermobility, all of which this patient exhibits. Therefore, the consensus is that Osteogenesis Imperfecta is the most likely underlying diagnosis.
