# Supplementary material for: Collaborative intelligence in AI: Evaluating the performance of a council of AIs on the USMLE
Source: PLOS Digit Health. 2025 Oct 9;4(10):e0000787. doi: 10.1371/journal.pdig.0000787 (PMC12510544; doi:10.1371/journal.pdig.0000787)
Supplement: S2 File — (ZIP) [file pdig.0000787.s002.zip › S2 Data Raw data files containing unprocessed question inputs and LLM outputs including transcripts of deliberations/STEP2_Question-41-1_Type-MC-NJ.docx]

**Facilitator** **[Round 1]:** A 22-year-old primigravid woman at 10 weeks’ gestation comes to the emergency department (ED) because of a 3- day history of persistent vomiting. She is treated with intravenous hydration, pyridoxine, and doxylamine. As the resident physician prepares the patient for discharge, he asks who will be picking her up from the ED. She says her husband will be arriving soon and asks if the hospital staff can refer to her symptoms as a “stomach flu.” She does not want anyone to mention to her husband that she is pregnant because the pregnancy resulted from an oral contraceptive failure and “he is not expecting this.” Which of the following is the most appropriate next question to ask this patient? (A) “Are finances tight at home?” (B) “How do you think he will react?” (C) “Is there somebody else who can pick you up?” (D) “Was there a problem with your birth control?” (E) “Will he try to force you to do something you aren’t comfortable with?”

**AI_D:** (1) RESTATING THE QUESTION: A 22-year-old pregnant woman attended the Emergency Department due to continuous vomiting for the past 3 days. She was treated and prepped to be discharged. She requested that her pregnancy be kept a secret from her husband who came to pick her up, stating that her pregnancy was an unexpected result of oral contraceptive failure. We now need to determine the most relevant question to ask her next - about her financial situation, her husband's potential reaction, alternative transport options, possible problems with her birth control, or concerns about her husband forcing her to do something she is uncomfortable with. (2) PLANNING: These assumptions considering her request may be due to a variety of reasons including financial distress, fear of her husband's reaction, lack of support, contraceptive mismanagement, or fear of coercion. Each of these assumptions leads to different questions. It is of course critical to assess all aspects, but in a priority sequence that places the patient's safety and wellbeing first, the question addressing potential domestic abuse or coercion should take precedence. (3) CLINICAL REASONING: When a patient requests concealment of health information from their partner, particularly regarding significant matters such as pregnancy, it signals potential social or personal problems. While financial stress (suggestion A) or contraceptive complications (suggestion D) might be valid concerns, these can be addressed later or in other settings. If there is a potential for coercion or abuse (suggestion E), this is an immediate risk and must be addressed first in the name of patient safety. (4) CONCLUDING: Considering patient safety and well-being, the need to identify any potential coercion or abuse, and the critical nature of such situations, it is vital to first address whether the patient feels safe and comfortable in her relationship. (5) RESPONSE: Hence, the most suitable next question to ask the patient would be, "Will he try to force you to do something you aren’t comfortable with?" (E).

**AI_C:** 1) RESTATING THE QUESTION: The query involves a 22-year-old pregnant woman, first-time mother, at 10 weeks of gestation who visited the ED due to persistent vomiting for three days. She was treated with intravenous hydration, pyridoxine, and doxylamine. As she is prepared for discharge, she asks that her symptoms be referred to as a "stomach flu" because she does not want hospital staff to mention her pregnancy to her husband. The question is to identify the most suitable query to ask the patient after understanding her situation. 2) PLANNING: Some underlying assumptions can be drawn from the situation: a) the patient may fear her husband's reaction to the news, b) she might want to avoid the topic until she finds a suitable time to share the news on her own, c) there could be financial or marital issues they are dealing with so she doesn't want to stress her husband with more news. Each alternate assumption can lead to a different approach to handle the situation, but the most empathetic and professional tactic would be to try and understand her fear or anticipation of her husband's reaction (Assumption a) and thereby assist her with any guidance or support she might need. 3) CLINICAL REASONING: From a clinical standpoint, the physician should always prioritize patient's autonomy, confidentiality, and comfort. Though other aspects like knowing if there was an issue with her birth control or if there are financial concerns at home might be relevant in different contexts, the immediate concern here might be the patient’s emotional well-being. Her request suggests a level of anxieties about her husband's reaction to her pregnancy, which will be of more immediate concern. 4) CONCLUDING: Based on the patient's request to not disclose her pregnancy status to her husband, it appears that her primary concern relates to her husband's reaction. While it's crucial to ensure the patient's safety and well-being, in this context, it might be more beneficial and ethical to ask about her anticipations about her husband's reaction to the pregnancy news. 5) RESPONSE: Hence, the most appropriate next question to ask this patient would be (B) "How do you think he will react?"

**AI_E:** (1) RESTATING THE QUESTION: From the query, it appears that a young first-time pregnant woman is at the hospital with symptoms of persistent vomiting, which may indicate hyperemesis gravidarum due to her being at 10 weeks gestation. She requested not to disclose her pregnancy results to her husband, who will be picking her up. The question revolves around determining the best approach to support the patient's request given the circumstances. (2) PLANNING: Several assumptions can be made from the information provided: She's fearful of her husband's reaction to the news, she may not want anyone else but her husband to pick her up, her contraceptive failure may be a result of incorrect usage or due to an issue with the contraceptive itself, she might be experiencing financial difficulties, or she anticipates her husband might pressure her into something unpleasant. Each of these assumptions, while valid, should be evaluated critically: the focus of questioning now should not be about contraceptive failure or financial issues but the patient's present emotional and psychological status and the nature of her relationship with her husband. The underlying concern that needs to be clinically prioritized is the potential for emotional or psychological distress and fear, possibly indicating intimate partner violence. (3) CLINICAL REASONING: The vomiting could be attributed to her pregnancy. In the clinical context of this scenario, it's important to keep the patient’s safety and confidentiality in mind. Her request not to disclose her pregnancy to her husband raises concerns about the dynamics of their relationship. (4) CONCLUDING: Therefore, based on the details given, it can be determined that the primary concerns should be the patient's comfort, safety, and welfare. Understanding her fear of her husband's reaction is critical; it can inform the physician about her emotional state and the potential dynamics in her relationship. (5) RESPONSE: Therefore the most appropriate next question to ask this patient, given her reluctance to divulge her condition to her husband, should focus on understanding her expectation of her husband's reaction. The best choice, in this case, is (B) "How do you think he will react?" This question not only recognizes her fear regarding the disclosure but also provides insight into her relationship with her spouse, which may help evaluate her safety and emotional well-being.

**AI_A:** RESTATING THE QUESTION: The situation presented here is about a pregnant woman, specifically, a 22-year-old woman experiencing her first pregnancy. She sought medical attention due to a three-day history of persistent vomiting and is presented at a gestational age of 10 weeks. When nearing discharge after treatment, the woman requested her pregnancy to be undisclosed - especially to her husband - as it was an unintended result of contraceptive failure. We are asked to choose an appropriate subsequent question from options focused on financial issues, her husband's potential reaction, alternative pick-up options, problems with her birth control, and potential coercion from her husband. PLANNING: There are assumptions that we can identify from this case which could form the basis for the next question. First, there's the assumption that the husband is unaware of the pregnancy and his reaction might be negative, hence why the woman is fearful of telling him. Secondly, it is also assumed that alternative pick-up options are available. Thirdly, we could assume that there might have been a problem with her birth control leading to the unintended pregnancy. Lastly, based on her fear, it's possible that there could be a power imbalance in their relationship, leading to possible coercion from the husband. CLINICAL REASONING: Given the information presented, the most significant aspect to explore further is the patient's fear of revealing her pregnancy to her husband because of potentially negative reactions. Issues about birth control failures or financial challenges, while important, are secondary to the immediate emotional well-being and safety of the patient. While financial constraints or contraceptive failures could be discussed at a later time, understanding the patient's fear and assessing the husband's potential reaction should be considered urgently. CONCLUDING: The aim here should be to ensure the patient's emotional welfare and safety, and the most direct way to do so would be by asking her to elaborate on her concerns about her husband's reaction. This will offer insight into whether she anticipates a violent reaction or simply an unfavorable one. Any indication of potential violence would warrant an entirely different line of questioning and immediate resources to protect her. The answers to questions about financials and contraceptive failure can be sought out during follow-ups, but the immediate priority should be the woman's safety. RESPONSE: Given that, the answer would be (B) "How do you think he will react?" Understanding her perspective on her husband's reaction could unravel far more about their relationship dynamic and her security, and comes before other considerations in this scenario.

**AI_B:** (1) RESTATING THE QUESTION: In this case, we are presented with a 22-year-old pregnant woman who is in her early first trimester. She has visited the ED due to persistent vomiting, which was managed with hydration and medications. As discharge procedures are being wrapped up, she asks that her pregnancy be kept a secret from her husband, who is on his way to pick her up. The pregnancy resulted from an unexpected failure of her oral contraceptive. We are required to identify the most appropriate next question to ask her. Two main issues seem to be at play here, one medical and one relating to her personal relationships and life situation. The medical issue pertains to her recent history of persistent vomiting, a common early pregnancy symptom known as 'morning sickness' that can be severe in some cases (hyperemesis gravidarum). On the personal front, there appears to be some form of communication or relationship stress with her husband. (2) PLANNING: The question puts forward 5 potential routes of inquiry. These could all be based on different assumptions: the first asks about financial issues, the second is about her husband's potential reaction, the third about her ride home, the fourth looks into the contraceptive failure and the fifth queries potential coercion or domestic issues. All are valid questions, but if we prioritize the clinical vignette, the primary concern should not only focus on the matter of her medical condition, but also take into account the psychological or social factors that might play significant roles in her health. It is important to understand how the unexpected pregnancy could impact her relationship, specifically her husband's reaction. (3) CLINICAL REASONING: The first step is to consider the immediate situation. The woman is symptomatic but has been successfully treated, with the primary concern being undisclosed pregnancy. This reveals a potential anxiety about her husband's reaction to the pregnancy. Therefore, it would be important to understand her expectation regarding his reaction. The second step is to factor in the medical history, which suggests that the contraceptive failure leading to this pregnancy was unexpected. This could understandably lead to some distress and anxiety. Asking about finances or potential coercion might be relevant in certain situations, but they don't seem directly related in this case scenario. The more pressing concern is undoubtedly how the husband might react, given that he is unaware of the pregnancy. (4) CONCLUDING: In clinical practice, understanding a patient's feelings and perceptions about a situation and helping to address those feelings, can be as important as diagnosing and treating the physical ailment. This contextually relevant issue seems to be the fear or anxiety she is experiencing regarding her husband's knowledge and reaction. (5) RESPONSE: As such, the most appropriate next question to ask this patient would be "How do you think he will react?" (Option B). Understanding her fear or anxiety about her husband's reaction can guide the physician in providing her with the appropriate support and advice.

**Facilitator** **[Round 2]:** Summary: AI_A, AI_B, AI_C, and AI_E all agree that the most appropriate subsequent question to ask the patient is "How do you think he will react?" They all believe that understanding the patient's fear and assessing her husband's potential reaction should be the immediate priority. They argue that issues about birth control failures or financial challenges, while important, are secondary to the immediate emotional well-being and safety of the patient. AI_D, however, believes that the most suitable next question to ask the patient would be, "Will he try to force you to do something you aren’t comfortable with?" AI_D argues that when a patient requests concealment of health information from their partner, it signals potential social or personal problems, and if there is a potential for coercion or abuse, this is an immediate risk and must be addressed first in the name of patient safety. Question for Council Members: Could you please elaborate on why you believe your chosen question is the most immediate and critical to ask, especially in light of the differing opinion that potential coercion or abuse should be the primary concern?. Based on your preceding response, how would you answer the original question: [A 22-year-old primigravid woman at 10 weeks’ gestation comes to the emergency department (ED) because of a 3- day history of persistent vomiting. She is treated with intravenous hydration, pyridoxine, and doxylamine. As the resident physician prepares the patient for discharge, he asks who will be picking her up from the ED. She says her husband will be arriving soon and asks if the hospital staff can refer to her symptoms as a “stomach flu.” She does not want anyone to mention to her husband that she is pregnant because the pregnancy resulted from an oral contraceptive failure and “he is not expecting this.” Which of the following is the most appropriate next question to ask this patient? (A) “Are finances tight at home?” (B) “How do you think he will react?” (C) “Is there somebody else who can pick you up?” (D) “Was there a problem with your birth control?” (E) “Will he try to force you to do something you aren’t comfortable with?”]

**AI_A:** 1) RESTATING THE QUESTION: The situation presents a 22-year-old pregnant woman who is about to be discharged from the hospital but is hesitant to let her husband know about her pregnancy. The question seeks to identify the most appropriate next line of questioning that the resident physician should take to support her emotional wellbeing and safety. 2) PLANNING: In evaluating the situation, a range of assumptions is made - that she is afraid of her husband's reaction, that she possibly doesn't want to continue the pregnancy, she might be facing financial issues, or that she may have concerns about birth control failure. On analyzing these, the crucial step is to prioritize addressing her immediate psychological well-being and safety. 3) CLINICAL REASONING: The clinical thought process suggests that when a patient requests for concealment of health information from their partner, it could signal potential personal or social problems. However, being speculative of coercion or abuse without concrete evidence can cause unnecessary distress and could infringe the patient's autonomy. The more convenient approach would be to understand her concerns about her husband's potential reaction first. If the response hints at the risk of coercion, then question (E) can be pursued subsequently. 4) CONCLUDING: A step-by-step explanation suggests that addressing the patient's immediate emotional wellbeing must take precedence. Given the circumstance, understanding her fear about her husband’s potential reaction should be the immediate priority over questioning other aspects at this point of time. 5) RESPONSE: The most appropriate next question to ask this patient is “How do you think he will react?” (Option B).

**AI_C:** 1) RESTATING THE QUESTION: We are asked to determine the most suitable question to ask a 22-year-old woman who does not wish to reveal her pregnancy to her husband due to it being an unexpected result of oral contraceptive failure. 2) PLANNING: The information provided in the question points to a sensitive situation. The woman has asked healthcare providers not to mention her pregnancy, indicating she is yet to disclose it to her husband. Being primigravid, her fear and stress might be escalated due to the unfamiliarity with pregnancy and childbirth. The key assumptions here are that she is anxious or fearful about her husband's reaction and that she might be in a complicated personal situation. However, an alternate assumption is the potential for coercion or abuse that might be present, and that should be clinically prioritized for the patient's safety. 3) CLINICAL REASONING: Understanding the patient's fears or concerns and assessing her husband's potential reaction can provide valuable insight into her mental and emotional state, which could be impacting her physical health, hence option (B) is clinically justified. However, it is equally important to evaluate her safety in her environment, which makes option (E) relevant. 4) CONCLUDING: After comparing potential options, the safety of the patient should always be prioritized in clinical settings. However, without any explicit indication of a threat, it may be premature to directly inquire about potential coercion or force. Therefore, diplomatically asking about the husband's potential reaction might be a more respectful way of understanding her exact fears and circumstances. 5) RESPONSE: Based on this, the most appropriate next question to ask this patient might be "How do you think he will react?" (B). This respects the patient's current request for privacy, aims to understand her concerns, and could potentially reveal further information about her situation, including safety aspects.

**AI_E:** Restating the Question: The case presents a 22-year-old pregnant woman who hasn't yet disclosed her pregnancy to her husband. The woman wants the hospital staff to refer to her symptoms as a 'stomach flu' to avoid revealing the pregnancy to her husband. The question focuses on determining the most appropriate next question for the physician to ask this woman. Planning: The case raises concerns about her emotional, social, and personal safety. However, to better understand the specific situation, I feel it's crucial to identify her fears. Why is she anxious about revealing her pregnancy? Does she expect a negative reaction, financial problems, relationship conflicts, or is there a risk of coercion or abuse? Each assumption should be evaluated based on her responses and clinical priority should be given to the immediate safety and well-being of the patient. Clinical Reasoning: Patient-centred care promotes open communication, allowing healthcare providers to understand the patient's concerns and emotional distress. Understanding her fears would help identify any immediate threats to her well-being. If her concern is regarding an anticipated negative reaction from her husband, addressing this fear could provide emotional support. Questions relating to issues with birth control or financial problems may also provide insight but don't seem to be directly related to her immediate distress. Concluding: Based on this thought process and placing clinical priority on her immediate emotional well-being and safety, it seems pertinent to first address her fears about her husband's reaction to the pregnancy. Response: Therefore, the most appropriate next question to ask this patient would be: "How do you think he will react?" (B). This question provides an opportunity to address her immediate distress and further evaluate her situation, contributing to patient-centred care. Furthermore, this question helps connect the patient's personal context to her healthcare, aiding in effective clinical decision-making.

**AI_D:** Restating the Question: The question is asking which is the most suitable inquiry to make from the given options, given the patient's personal situation of concealing her pregnancy from her partner, which resulted unexpectedly due to oral contraceptive failure, after she has sought treatment for persistent vomiting during her early pregnancy. Planning: We need to understand why this woman does not want her husband to know about her pregnancy. The reasons could range from personal, social, or financial. The assumptions that can be made are: 1) She's afraid of her husband's reaction, 2) There may be financial difficulties, 3) There is a problem with the efficacy of birth control, 4) She's unable to secure a safe and supportive ride home, 5) She might be subject to forceful behaviors. Each of these assumptions has to be considered, scrutinized, and prioritized. Given that our immediate goal would be to ensure the patient's safety and well-being, exploring potential risk from her husband would be a priority. Clinical Reasoning: The primary concern is to figure out why she doesn't want her husband to know about the pregnancy and if there is a potential risk involved to her. It could be emotionally distressing for her and indirectly affect the pregnancy. If the reason is financial instability or birth control failure then it could be handled with social services or a more in-depth discussion about contraceptives respectively. If she lacks a proper support system, arrangements could be made for her. However, if she's under the risk of forceful behavior, immediate measures have to be undertaken to support her. Concluding: Understanding the husband's potential reaction, which seems to be causing distress in the patient, should be the immediate concern. This helps us assess the immediate emotional well-being and safety of the patient Response: Therefore, the most appropriate next question to this patient would be: “How do you think he will react?” (Option B). This query will help to gauge her understanding of her husband’s possible reaction and determine whether additional support or interventions are necessary for her safety.

**AI_B:** 1) RESTATING THE QUESTION: The question asks for the most suitable, immediate question to ask a pregnant woman who, because of specific circumstances, does not want her husband to know about her pregnancy. The woman is dealing with unexpected pregnancy and her request implies she may have concerns about her husband's reaction. 2) PLANNING: The assumptions here could be that the woman fears her husband's reaction, she's concerned about societal or personal repercussions or she worries about her partner's potential coercive or abusive behavior. The woman's request is indirectly indicating her potential fear about her husband's reaction, which could potentially be harmful or damaging. It's also assumed that she is safe and is not in immediate danger; however, this assumption may need to be explored further for patient safety reasons. 3) CLINICAL REASONING: Given that the woman is requesting to conceal her health condition, it suggests that she may be dealing with possible social or personal issues that warrant further exploration. It's essential to understand her main concern before proceeding with any further action or advice. While it's imperative to address potential health risks and clear any misconceptions regarding birth control or pregnancy, addressing her immediate fear, i.e., her husband's reaction, should be prioritized for her emotional well-being and safety. However, if there is an apparent or even an implied threat to her safety, it should be the primary concern in this context. 4) CONCLUDING: The clinical thought process should ideally aim at addressing the immediate concern of the patient, which seems to be her fear of the husband's reaction. Concurrently, it's crucial to identify if there is a risk or fear of coercion or abuse which could put her at immediate risk and would thus require immediate attention and action. 5) RESPONSE: Based upon the above reasoning, the question ‘How do you think he will react?’ (B) should be asked next to understand her fears and apprehensions better. However, it is also important to remain alert for any indication of a safety concern, which may require asking question E (‘Will he try to force you to do something you aren’t comfortable with?’) if her responses suggest potential coercion or abuse.

**Facilitator** The consensus among the AI responses is that the most appropriate next question to ask the 22-year-old pregnant woman who does not want her husband to know about her pregnancy is "How do you think he will react?" (Option B). This question is deemed most suitable as it directly addresses the woman's immediate emotional wellbeing and safety, which is the primary concern in this situation. The question aims to understand her fears and apprehensions about her husband's potential reaction to the pregnancy news. It respects her request for privacy and could potentially reveal further information about her situation, including any safety concerns. However, the AI responses also suggest that the physician should remain alert for any indication of a safety concern, which may require asking question E ("Will he try to force you to do something you aren’t comfortable with?") if her responses suggest potential coercion or abuse.
