# Supplementary material for: Collaborative intelligence in AI: Evaluating the performance of a council of AIs on the USMLE
Source: PLOS Digit Health. 2025 Oct 9;4(10):e0000787. doi: 10.1371/journal.pdig.0000787 (PMC12510544; doi:10.1371/journal.pdig.0000787)
Supplement: S2 File — (ZIP) [file pdig.0000787.s002.zip › S2 Data Raw data files containing unprocessed question inputs and LLM outputs including transcripts of deliberations/STEP2_Question-42-1_Type-MC-NJ.docx]

**Facilitator** **[Round 1]:** Five days after admission to the hospital for management of an ST-elevation myocardial infarction, a 59-year-old man has the sudden onset of severe abdominal and bilateral lower extremity pain, cyanosis of the lower body, and paresthesias. On hospital days 1 and 2, he received vasopressor and inotropic therapy. On hospital day 2, echocardiography showed extensive anterolateral akinesis of the left ventricle. He has no history of serious illness. His medications are metoprolol, lisinopril, simvastatin, and dalteparin. He appears to be in acute distress. Pulse is 105/min and regular, respirations are 24/min, and blood pressure is 80/60 mm Hg. Examination shows lower body cyanosis to the level of the costal margins. Crackles are heard over the lower lung fields bilaterally. Distant heart sounds but no murmurs are heard. The abdomen is nondistended and nontender. Pulses are 1+ in the upper extremities and absent in the lower extremities. Muscle strength is 3/5 in the lower extremities. Sensation to touch and temperature is decreased over the lower extremities. ECG shows sinus tachycardia. Which of the following is the most likely explanation for this patient’s clinical findings? (A) Aortic dissection (B) Aortic embolism (C) Pericardial tamponade (D) Right ventricular failure (E) Ventricular rupture

**AI_B:** (1) RESTATING THE QUESTION: We're looking at a 59-year-old man who was admitted to the hospital due to an ST-elevation myocardial infarction (a complete blockage of a coronary artery, depriving heart muscle of oxygen and nutrients). Five days later, he's developed severe abdominal pain, bilateral lower extremity pain, lower body cyanosis (a blue-ish complexion implying poor perfusion or oxygenation), and paresthesias (abnormal sensations). His echocardiogram revealed extensive anterolateral akinesis of the left ventricle (meaning a large portion of his left heart muscle isn't contracting effectively). His pulse is fast, his blood pressure is low, and he has decreased sensation and pulses in his lower extremities. He's on medications for heart failure and to prevent blood clotting. The aim is to identify the most likely explanation for these symptoms. (2) PLANNING: The possible explanations in the options are all largely cardiovascular and could plausibly lead to poor perfusion of the lower body. However, specific symptoms and signs point more to one over the others. For instance, the sudden onset of the symptoms, lower body cyanosis, abdominal and lower extremity pain, increased heart rate, and reduced strength and sensations in the lower limbs may indicate an aortic embolism. This would also fit in with his akinesis of the left ventricle which could cause blood stasis and clot formation. (3) CLINICAL REASONING: Aortic dissection classically presents with tearing chest pain and discrepancies in upper limb blood pressures, which this patient does not have. Pericardial tamponade typically involves impaired diastolic filling due to the pericardium's restriction on the heart, leading to global hypoperfusion and not isolated to the lower body. Right ventricular failure usually presents with systemic fluid retention and peripheral edema, not sudden pain and cyanosis. Ventricular rupture is a catastrophic event that leads to profound circulatory collapse and usually death – it would not selectively affect the lower body as seen in this patient. (4) CONCLUDING: Based on our clinical reasoning and the patient's signs and symptoms which include severe abdominal and bilateral lower extremity pain, lower body cyanosis, decreased pulses and sensation in the lower extremities, the most likely diagnosis would point towards an aortic embolism. (5) RESPONSE: The most likely explanation for this patient’s clinical findings is (B) Aortic embolism.

**AI_A:** RESTATING THE QUESTION: This inquiry aims to determine the underlying cause of the sudden and severe abdominal and bilateral lower extremity pain, lower body cyanosis, and paresthesia experienced by a 59-year-old male patient who was admitted five days prior for management of an ST-elevation myocardial infarction (STEMI). The patient received vasopressor and inotropic therapy and a subsequent echocardiogram showed significant anterolateral akinesis of the left ventricle. His meds include metoprolol, lisinopril, simvastatin, and dalteparin. An examination revealed lower body cyanosis, distant heart sounds, and physical signs suggesting poor circulation to the lower extremities. To clarify terms, STEMI is a severe heart attack caused by the occlusion of a coronary artery, anterolateral akinesis indicates lack of movement in the front and side walls of the left ventricle, and the medications mentioned primarily focus on managing heart conditions and preventing blood clot formation. PLANNING: The initial assumption could be that these symptoms are further manifestations of his recently managed STEMI. Hence, taking into account this patient's recent myocardial infarction and the extensive anterolateral akinesis that was noted, we can think in terms of embolization from a left ventricular thrombus as an alternate assumption. This poses the question: Could the current manifestation be related to an embolic event? CLINICAL REASONING: To justify this assumption emphasis should be given to the recent STEMI and the anterolateral akinesis of the left ventricle which creates a functional reason for emboli to accumulate. The patient's lower body cyanosis, decreased peripheral pulses, and the altered sensation in his lower extremities all suggest the possibility of an embolus occluding the aorta and impairing blood flow to these regions. Additionally, the use of inotropic agents and vasopressors could have potentially dislodged a thrombus that formed in the non-functioning myocardial region leading to an embolic event. CONCLUDING: Given the circumstances and considering clinical facts, the severe abdominal and lower extremity pain, lower extremity cyanosis, and the paresthesias, all develop as a coherent clinical picture pointing towards the direction of an Aortic Embolism originating from the left ventricle with dead walls following STEMI, and being dislodged possibly due to the use of vasopressors and inotropics. RESPONSE: The most likely explanation for this patient’s clinical findings is an Aortic Embolism (Option B).

**AI_D:** RESTATING THE QUESTION: The scenario presents a 59-year-old man who had an ST-elevation myocardial infarction. As part of his treatment, he was given vasopressor and inotropic therapy and he is on several medications like metoprolol, lisinopril, simvastatin, and dalteparin. He later develops symptoms that include severe abdominal pain, bilaterally lower extremity pain, cyanosis of the lower body, and paresthesias. His examination results include distant heart sounds, a nondistended, non-tender abdomen, weak pulses in the upper extremities, absent pulses in the lower extremities, reduced lower extremity muscle strength, decreased sensation in the lower extremity, and ECG shows sinus tachycardia. The question seeks the most likely explanation for these symptoms and clinical findings. PLANNING: Dealing with the main question involves making certain assumptions. The main one being that the cause of the patient's symptoms is attributed to some form of cardiovascular pathology because of his recent myocardial infarction history. Potential alternative assumptions could include other complications like infections, medication side effects, or electrolyte imbalances. Let's focus on the cardiovascular-related possibilities, as they're more relevant to the patient's recent medical history. CLINICAL REASONING: The patient's symptoms of abdominal and lower extremity pain, cyanosis, and paresthesias indicate a severe reduction in blood supply to these areas. However, there is an absence of murmurs which would be expected in conditions such as aortic dissection and ventricular rupture. The clinical findings and the ECG showing sinus tachycardia along with his history of myocardial infarction point towards a potential issue involving blood clot formation. Specifically, a large embolus that can originate from the left ventricle due to wall akinesis caused by the myocardial infarction. This embolus can lead to sudden-onset of abdominal and lower extremity symptoms following its migration to the aorta. CONCLUDING: Given the above reasoning, it can be concluded that this is a case of aortic embolism where a clot has likely formed at the part of the left ventricle that had myocardial infarction. This has subsequently been dislodged and travelled to the abdominal aorta blocking it. This causes the abdominal pain and affects the blood flow to the lower extremities leading to lower extremity pain, cyanosis, and paresthesias witnessed in this case. RESPONSE: Therefore, the most likely explanation for this patient’s clinical findings is (B) Aortic embolism.

**AI_E:** RESTATING THE QUESTION: This question concerns a male patient who, five days post-admission for ST-elevation myocardial infarction (ST-Elevation MI), exhibits symptoms indicative of severe abdominal and bilateral lower extremity pain, cyanosis of the lower body, and paresthesias. His hospital treatment has included vasopressor and inotropic therapy, and an echocardiogram has shown significant anterolateral akinesis of the left ventricle. Furthermore, the patient’s medications consist of metoprolol, lisinopril, simvastatin, and dalteparin. Now, given the patient's symptoms of distress, low blood pressure, decreased sensation to touch and temperature over the lower extremities, as well as an ECG showing sinus tachycardia, we must identify the most likely cause of these clinical findings. PLANNING: We'll first need to consider the possible conditions that could manifest the patient's latest symptoms. The options presented are: aortic dissection, aortic embolism, pericardial tamponade, right ventricular failure, and ventricular rupture. Hierarchically, we prioritize embolic complications because they may cause sudden and severe symptoms in the extremities (pain, cyanosis) and abdomen, as described in the scenario. Likewise, the patient's medical history, especially the event of an ST-elevation myocardial infarction and the presence of extensive left ventricular akinesis, both increase the risk of thrombosis and embolism. CLINICAL REASONING: The symptoms that are most indicative of an embolic event, and which don't align well with the other given options, are the severe pain and cyanosis in the lower extremities, coupled with decreased sensation. These point strongly toward an arterial blood flow disruption, which an embolic event would cause. If we consider the other options: (A) aortic dissection typically presents with a sharp, severe, tearing chest and back pain, which does not seem to align with the patient's symptoms; (C) pericardial tamponade would likely present with distended neck veins, hypotension, and muffled heart sounds (known as Beck's triad), which is not the case; (D) right ventricular failure primarily presents with systemic congestion, including hepatic congestion, ascites, peripheral edema and jugular venous distension, but this profile does not fit the current scenario; (E) ventricular rupture would rapidly lead to cardiogenic shock or cardiac arrest, likely with more pronounced hypotension and an irregular pulse, making it less likely given the patient's stable, albeit tachycardic, pulse. CONCLUDING: In light of the clinical picture presented — ST-elevation myocardial infarction, extensive anterolateral akinesis of the left ventricle, severe abdominal and bilateral lower extremity pain, lower body cyanosis — coupled with acute presentations, responsiveness to pain stimuli, and reduced pulses, our conclusion would lean more heavily to embolic events, an occurrence that aligns with this patient's recent cardiovascular compromise. RESPONSE: Based on the evidence and clinical reasoning, it would seem that (B) Aortic embolism is the most likely explanation for this patient’s clinical findings.

**AI_C:** RESTATING THE QUESTION: The given patient, a 59-year-old male who has been in the hospital for five days due to ST-elevation myocardial infarction, is exhibiting severe abdominal and bilateral lower extremity pain, body cyanosis, and paresthesias. The patient is in evident distress, suffering fast pulse rate, decreased blood pressure, lower body cyanosis up to the costal margins, bilateral lower lung crackles, distant heart sounds, weakened pulses, weakened lower extremity muscle strength, and lowered sensitivity in the lower extremities. His ECG indicates sinus tachycardia. We are to ascertain the most probable cause from the options provided: aortic dissection, aortic embolism, pericardial tamponade, right ventricular failure, or ventricular rupture. PLANNING: Let's begin by defining some of these terms to better understand the potential diagnoses: - Aortic dissection refers to a serious condition where the inner layer of the aorta tears, causing blood to surge into the wall of the aorta. - Aortic embolism is when an embolus (a blood clot or other particle) travels to the aorta and obstructs blood flow. - Pericardial tamponade is a serious condition in which fluid accumulates in the pericardium and creates pressure on the heart. - Right ventricular failure refers to a condition where the right side of the heart is unable to pump blood properly. - Ventricular rupture means a tear in the wall of the ventricles of the heart. Considering the clinical findings together with the available diagnosis options, our main assumption should be that the patient's condition has resulted from a complication related to his ST-elevation myocardial infarction. CLINICAL REASONING: Given this patient's recent history of ST-elevation myocardial infarction accompanied by hypothesized severe abdominal and lower extremity pain, cyanosis, and paresthesias, these symptoms suggest some form of arterial compromise. The absent pulses and decreased sensation further hint at ischemia in the lower part of his body. Since the onset of these signs is sudden, it could likely be due to an acute obstruction in the blood flow to the lower body. Now, reviewing the potential causes, pericardial tamponade and right ventricular failure could explain the hypotension and other symptoms, but they are less likely to cause isolated lower body ischemia as seen in this patient. On the other hand, ventricular rupture would most likely result in hemodynamic collapse and is typically associated with chest pain and signs of shock. Hence, these can be reasonably ruled out. An aortic dissection could indeed cause such symptoms, but it is primarily associated with severe chest or back pain which is not seen here. This leaves us with an aortic embolism which fits the clinical picture best, as it could obstruct blood flow to the lower body, causing ischemia and resultant pain, cyanosis, and absent pulses in the lower extremities. CONCLUSION: The patient's ST-elevation myocardial infarction indicates a high possibility for a blood clot to form and consequently release emboli. Such an embolus could have lodged in the aorta, causing an aortic embolism and therefore resulting in the acute distress and lower body symptoms we observe in this patient. RESPONSE: The most probable explanation for this patient's clinical findings, based on the reasoning provided and the symptoms portrayed, is therefore (B) Aortic Embolism.

**Facilitator** The consensus statement synthesizes the responses of AI_A, AI_B, AI_C, AI_D, and AI_E as follows: The question pertains to a 59-year-old male patient who was admitted to the hospital for an ST-elevation myocardial infarction (STEMI) and is now experiencing severe abdominal and bilateral lower extremity pain, lower body cyanosis, and paresthesias. His treatment included vasopressor and inotropic therapy, and his medications include metoprolol, lisinopril, simvastatin, and dalteparin. An echocardiogram revealed extensive anterolateral akinesis of the left ventricle. Given the patient's recent history of STEMI and the symptoms he is presenting, the consensus among the AI responses is that the most likely explanation for his clinical findings is an aortic embolism. This conclusion is based on the patient's symptoms of severe abdominal and lower extremity pain, cyanosis, and paresthesias, which suggest a severe reduction in blood supply to these areas, likely due to an acute obstruction in blood flow. The extensive anterolateral akinesis of the left ventricle, a result of the STEMI, creates a functional reason for emboli to accumulate. The use of inotropic agents and vasopressors could have potentially dislodged a thrombus that formed in the non-functioning myocardial region, leading to an embolic event. The other options provided, including aortic dissection, pericardial tamponade, right ventricular failure, and ventricular rupture, were considered but ultimately ruled out due to the specific symptoms and clinical findings of the patient. Therefore, the consensus is that the most likely explanation for this patient’s clinical findings is an Aortic Embolism (Option B).
