# Supplementary material for: Collaborative intelligence in AI: Evaluating the performance of a council of AIs on the USMLE
Source: PLOS Digit Health. 2025 Oct 9;4(10):e0000787. doi: 10.1371/journal.pdig.0000787 (PMC12510544; doi:10.1371/journal.pdig.0000787)
Supplement: S2 File — (ZIP) [file pdig.0000787.s002.zip › S2 Data Raw data files containing unprocessed question inputs and LLM outputs including transcripts of deliberations/STEP2_Question-43-1_Type-MC-NJ.docx]

**Facilitator** **[Round 1]:** A 75-year-old man comes to the clinic because of a 2-year history of progressive shortness of breath. He also has had an occasional dry cough for the past 6 months. He has not had chest pain, palpitations, or swelling of his legs. Medical history is unremarkable, and the patient takes no medications. He has smoked one pack of cigarettes daily for 57 years. He does not drink alcoholic beverages. Vital signs are temperature 37.0°C (98.6°F), pulse 92/min, respirations 18/min, and blood pressure 122/76 mm Hg. Pulse oximetry on room air shows an oxygen saturation of 89%. The patient has mild dyspnea when he speaks. Auscultation of the lungs discloses fine and dry crackles in the bases of both lungs. Cardiac examination discloses a normal S1 and S2. No extra heart sounds are heard. Jugular venous pulsation is measured at 2 cm above the sternal angle with the head of the bed elevated at 30 degrees. Examination of the extremities shows clubbing of the digits of both hands. Results of complete blood count, serum chemistry profile, and urinalysis are within the reference ranges. Chest x-ray shows scattered reticular opacities in both lung bases. There is no hilar lymphadenopathy. Which of the following is the most appropriate next step in evaluation? (A) Echocardiography (B) High-resolution CT scan of the chest (C) Lung biopsy (D) Serum antiglomerular basement membrane antibody assay (E) Thoracic PET scan

**AI_B:** (1) RESTATING THE QUESTION: The question asks what is the correct next step in evaluating a 75-year-old man with progressive shortness of breath for 2 years, an occasional dry cough for 6 months, and an extensive smoking history with no significant medical past. He has no chest pain, palpitations, or leg swelling and isn't under any medication. His vital signs are mostly normal but show a decreased oxygen saturation. The physical examination reveals mild dyspnea, fine and dry crackles at the lung bases, normal heart sounds, normal jugular venous pulsation, and clubbing of the digits. The laboratory tests are normal, but the chest X-ray shows reticular opacities at both lung bases with no hilar lymphadenopathy. (2) PLANNING: Given the patient's history of heavy smoking, shortness of breath, and clubbing of the digits, a strong assumption is that this patient has a lung condition, most likely chronic obstructive pulmonary disease (COPD) or interstitial lung disease (ILD). On the other hand, diseases such as lung cancer, pulmonary edema, pulmonary hypertension, among others, could also explain his symptoms but are less probable given the information provided. After evaluating these assumptions, it seems clinically appropriate to prioritize the assumption of a potential COPD or ILD. (3) CLINICAL REASONING: The major symptom of shortness of breath, combined with a prolonged history of smoking, is highly indicative of COPD. However, the presence of clubbing in the digits and reticular opacities on X-ray are more typical of ILD. Therefore, given the symptomology of the patient, especially clubbing, and his prolonged smoking history, the patient is likely to be suffering from some type of interstitial lung disease. (4) CONCLUDING: The next best step to further evaluate this assumption, is to acquire a clearer image of the lungs. Although a lung biopsy could aid in establishing a diagnosis, it should be reserved for cases in which less invasive testing does not provide a definitive diagnosis. Therefore, a biopsy may not be the first ideal choice. Echocardiography or serum antiglomerular basement membrane antibody assay wouldn't help in this context, as there's no sign of heart disease or condition like Goodpasture's syndrome. A thoracic PET scan is generally reserved for malignancy evaluation, not typically for initial evaluation of ILD. (5) RESPONSE: Thus, the most appropriate next step in the evaluation of this patient would be (B) High-resolution CT scan of the chest, which provides a more detailed image of the lungs and can help confirm the diagnosis of interstitial lung disease based on the specific pattern of involvement.

**AI_C:** RESTATING THE QUESTION: The scenario presents a 75-year-old man with a history of cigarette smoking for 57 years, who is experiencing progressive shortness of breath over the past two years and an occasional dry cough over the past six months. His examination findings include decreased oxygen saturation, dry crackles at the bases of both lungs and clubbing of both hand digits. Initial blood work-up is normal and chest x-ray reveals scattered reticular opacities at the lung bases without any hilar lymphadenopathy. The council is asked to determine the most appropriate next step in his evaluation. PLANNING: The given clinical features strongly hint towards a chronic lung disease, particularly interstitial lung disease (ILD), due to the patient's long history of smoking. ILD includes a group of diseases that cause progressive scarring of lung tissue, eventually affecting a person's ability to breathe and capture oxygen. The presenting symptoms, worsening shortness of breath and cough, along with physical exam findings of digital clubbing and bilateral basal crackles, and radiographic evidence of reticular opacities, are all suggestive of ILD. Echocardiography (option A) would be appropriate in a suspected cardiac etiology, which is less likely here. Despite their utility in certain contexts, serum antiglomerular basement membrane antibody assay (option D) and thoracic PET scan (option E) are not typically the first-line investigations in suspected ILD. A lung biopsy (option C) is invasive and usually reserved for cases where definitive diagnosis cannot be made with less invasive means. Therefore, a high-resolution CT scan of the chest, option B, seems the optimal choice. CLINICAL REASONING: The clinical reasoning proceeds by considering the patient's history of long term smoking, symptoms of chronic cough, progressive shortness of breath and physical findings including clubbing and basal crackles. These collectively point to a chronic lung disease probably an ILD. The reticular opacities revealed on chest x-ray further support this consideration. High-Resolution CT Scan (HRCT) carries a higher sensitivity and specificity in diagnosing ILDs than a conventional chest x-ray. HRCT is valuable in evaluating the extent of the disease, any pattern of abnormalities which could suggest a specific type of ILD and can frequently suggest the correct diagnosis preventing the need for invasive procedures. CONCLUDING: Therefore, basing on the patient's clinical presentation, potential diagnosis and the best possible way to confirm this diagnosis with the least invasive method available; a high-resolution CT scan of the chest should be considered first. RESPONSE: The most appropriate next step in the evaluation would be to order a high-resolution CT scan of the chest (Option B), as it serves as an extension of the clinical evaluation in this situation by providing an in-depth view of the lungs that may indicate signs consistent with an interstitial lung disease.

**AI_D:** (1) RESTATING THE QUESTION: An elderly man has come to the clinic presenting a 2-year history of worsening shortness of breath, an occasional dry cough over the last 6 months, and various physical signs like clubbed digits, fine and dry crackles in the lung bases, and an oxygen saturation of 89% on room air. His vital signs, CBC, serum chemistry, and urinalysis are all within normal limits. He has a significant history of smoking, but no known other existing medical conditions. The chest x-ray shows scattered reticular opacities without any hilar lymphadenopathy. We're asked to determine what would be the most appropriate next step for his evaluation. (2) PLANNING: We might assume various possible lung pathologies considering the patient's symptoms and his long history of smoking. However, smoking history, combined with symptoms like progressive breathlessness, dry cough, and physical signs like oxygen desaturation, clubbing, and reticular opacities would strongly suggest a probability of interstitial lung diseases (ILDs) like pulmonary fibrosis. Alternate assumptions could be COPD, lung cancer etc., however, other signs and symptoms such as swelling of legs, weight loss, chest pain are missing. So, given the patient's history and clinical condition, and lack of specific signs of other likely allegations, our primary clinical priority would be to verify or rule out interstitial lung disease. (3) CLINICAL REASONING: We have to connect all the symptoms, signs, and history in a step-by-step manner to come up with the most likely diagnosis. The patient's symptoms of breathlessness and dry cough, combined with the physical findings of oxygen desaturation, clubbing, and basal lung crackles, align with an ILD. His decades of smoking are a major risk factor for the development of these conditions. Moreover, lack of leg swelling, normal cardiac examination, and normal basic laboratory tests do not suggest a cardiac pathology or kidney disease. The picture isn't perfectly clear yet, however, as certain findings like the dispersed reticular opacities seen in the baseline of his lungs in the chest x-ray can be observed in a range of lung diseases, not just ILDs. Therefore, a lung-specific evaluation is needed to narrow this down to the most likely diagnosis. (4) CONCLUDING: To conclude, we need to select the investigative tool which is efficient and specific to our suspected condition, interstitial lung disease. While all the given options are valuable investigative tools in various respiratory conditions, a tool that balances invasiveness and diagnostic yield in this scenario would be optimal. (5) RESPONSE: In light of the highly suggestive clinical picture and the desire to confirm the diagnosis without resorting to highly invasive procedures, I would suggest the most appropriate next step in evaluation would be selecting option (B) High-resolution CT scan of the chest. This would help delineate the nature and extent of the lung disease more clearly, aiding in confirming a diagnosis of interstitial lung disease.

**AI_E:** (1) RESTATING THE QUESTION: The query pertains to determining the best subsequent step towards evaluating the health status of a 75-year-old male patient who is experiencing progressive dyspnea and has been reported to manifest signs and symptoms such as mild dyspnea while speaking and digit clubbing. To clarify the medical jargon, dyspnea refers to shortness of breath, a common symptom of diseases such as heart failure or chronic obstructive pulmonary disease (COPD). Clubbing, when fingers and toes become widened and rounded at the tips, is usually an indicator of underlying health conditions often relating to decreased oxygen levels in the blood, an issue common in heart or lung diseases. (2) PLANNING: The provided information leads our assumptions towards a pulmonary pathogenesis due to the long history of smoking, manifested dyspnea, dry cough, and scattered reticular opacities in lung bases seen in chest x-ray. We could consider other assumptions, such as ischemic cardiomyopathy from silent myocardial infarctions, but the patient does not have chest pain or lower limb swelling typically associated with heart disease. It's also essential to consider the patient's age, which increases the risk of malignancy; however, the gradual progression of symptoms and absence of hilar lymphadenopathy does not support this scenario. Prioritizing these, we should consider further exploring the possibility of pulmonary pathology influenced by the patient's smoking history. (3) CLINICAL REASONING: The patient's medical history points towards a condition related to a long-term exposure to cigarette smoke, such as COPD or interstitial lung disease. His signs and symptoms, along with diagnostic findings, particularly the dry cough, dyspnea, decreased oxygen saturation, and clubbing of digits, underscore a chronic lung condition. The chest x-ray findings of reticular opacities can also be indicative of interstitial fibrosis. The excess growth of connective tissue- fibrosis- can cause the lung to stiffen, thereby impairing its function. Without any signs of heart failure or autoimmune diseases, our focus narrows down to investigating further the lung condition. (4) CONCLUDING: Upon taking all these facts into account and following the process of strategic elimination based on presenting signs, symptoms, and test results, the most logical choice would be to conduct a more advanced imaging scan of the chest. An echocardiography is unnecessary as the patient displays no common cardiogenic symptoms. The lung biopsy and thoracic PET scan can be invasive and are usually kept as last resorts if other tests aren't able to diagnose the condition. The serum antiglomerular basement membrane antibody assay is typically related to kidney diseases, so it's not relevant in such a clinical situation. (5) RESPONSE: Therefore, given the patient's clinical signs and symptoms, and the chest x-ray findings, the most appropriate next step in evaluation would be (B) High-resolution CT scan of the chest. This would help in visualizing the lung structures more clearly and may indicate changes consistent with a smoking-related condition like COPD or interstitial lung disease.

**AI_A:** (1) RESTATING THE QUESTION: This question revolves around a 75-year-old man who has arrived at a clinic due to an ongoing issue with shortness of breath that has worsened over a period of two years. He also experiences sporadic dry coughing which has spanned over the past six months. He doesn't exhibit any chest pain, palpitations, or any swelling in his legs. His medical history is unremarkable; he is not being medicated for any conditions. However, it is crucial to note that he smoked one pack of cigarettes daily for 57 years. His vital signs seem stable. His lung exam reveals fine, dry crackles at the base of both lungs, but his cardiac exam was normal. It is also significant to mention that his fingers show signs of clubbing. His blood tests, serum chemistry, and urinalysis all fall within the reference range. However, his chest x-ray presents scattered reticular opacities in both lung bases. With these observations, we must determine the most clinically appropriate next step in evaluating his condition. (2) PLANNING: Given this patient's ongoing shortness of breath, long history of smoking, and physical signs such as fine, dry crackles in the lungs and clubbing of the fingers, one would suspect a form of chronic lung disease, probably chronic obstructive pulmonary disease (COPD) or interstitial lung disease (ILD). These conditions are very common in longtime smokers and could explain most of the symptoms. An echocardiography could help rule out heart disease which can sometimes present with breathlessness, but the patient has no significant cardiac symptoms or findings on examination. A lung biopsy or invasive investigations such as thoracic PET scan could be performed to diagnose specific types of lung disease, but these are generally less preferred as initial steps due to their invasiveness and expense. A serum antiglomerular basement membrane antibody assay could help diagnose Goodpasture syndrome, a rare disease that can affect the lungs and kidneys, but this patient does not have any notable kidney abnormalities. Given this clinical scenario, a non-invasive, detailed imaging modality like high-resolution CT scan of the chest could be the most appropriate next step. (3) CLINICAL REASONING: Considering the patient's extensive smoking history, manifestations such as a long-term dry cough, progressive shortness of breath, and clubbing of the digits are suggestive of an underlying pulmonary issue. While an initial chest x-ray did reveal scattered reticular opacities, it lacks the resolution required to distinctly define these radiographic changes. Hence, a high-resolution CT scan is favorable because it has greater sensitivity and specificity in identifying disease, characterizing the extent and pattern of disease, and could aid in differentiating COPD from ILD; both of which are plausible given the patient's smoking history. Echocardiography is usually reserved for patients wherein cardiac etiologies are being considered. However, this patient’s normal cardiac auscultation and absence of peripheral edema or palpitations make a primary cardiac cause of his dyspnea less likely. Pulmonary diseases frequently have systemic manifestations including those affecting the renal system, but our patient's renal profile was normal so an antiglomerular basement membrane antibody test is not indicated. While lung biopsy and PET scan can provide very specific diagnosis, they are usually reserved for situations where less invasive examinations, like CT scans, are not informative or equivocal. (4) CONCLUDING: The clinical presentation and test results, combined with the extensive smoking history, lean primarily towards a chronic pulmonary pathology such as COPD or ILD. The optimal next step would be performing a high-resolution CT scan of the chest which will provide a more detailed and specific image of the pulmonary system, clarifying the nature of the mentioned reticular opacities and supporting us in establishing a precise diagnosis. (5) RESPONSE: Given this patient's characteristics and clinical presentation, the most appropriate next step in evaluation would be to undertake a high-resolution CT scan of the chest (option B). It is a non-invasive method that can accurately differentiate between the types of lung diseases, helping guide subsequent management.

**Facilitator** The consensus among the AI responses is that the most appropriate next step in evaluating the 75-year-old man's condition would be to conduct a high-resolution CT scan of the chest (Option B). This conclusion is based on the patient's history of long-term smoking, his symptoms of progressive shortness of breath and occasional dry cough, and physical findings such as clubbing of the digits and fine, dry crackles at the lung bases. These factors suggest a chronic lung disease, likely chronic obstructive pulmonary disease (COPD) or interstitial lung disease (ILD). The high-resolution CT scan is a non-invasive method that can provide a more detailed image of the lungs, helping to differentiate between these diseases and guide subsequent management. Other options such as echocardiography, lung biopsy, serum antiglomerular basement membrane antibody assay, and thoracic PET scan were considered less appropriate due to the absence of cardiac symptoms, the invasive nature of a biopsy, the lack of kidney abnormalities, and the expense and specificity of a PET scan, respectively.
