# Supplementary material for: Collaborative intelligence in AI: Evaluating the performance of a council of AIs on the USMLE
Source: PLOS Digit Health. 2025 Oct 9;4(10):e0000787. doi: 10.1371/journal.pdig.0000787 (PMC12510544; doi:10.1371/journal.pdig.0000787)
Supplement: S2 File — (ZIP) [file pdig.0000787.s002.zip › S2 Data Raw data files containing unprocessed question inputs and LLM outputs including transcripts of deliberations/STEP2_Question-44-1_Type-MC-NJ.docx]

**Facilitator** **[Round 1]:** A previously healthy 20-year-old woman, who is a sophomore in college, is brought to the emergency department by her roommates because of an 8-hour history of weakness and vomiting blood and a 2-day history of dizziness. She has no history of similar symptoms. She says she overeats in response to the stress of her classes. She induces vomiting after consuming a significant amount of food because she feels ""too full"" and ""out of control."" She now vomits almost daily. She feels she is slightly overweight. She is physically active and exercises moderately two to three times weekly. She does not use laxatives or diuretics. She has many friends and enjoys going out socially. She takes no medications. She drinks three to four beers on weekends and does not use illicit drugs. She appears uncomfortable and is tearful and mildly diaphoretic. She is 157 cm (5 ft 2 in) tall and weighs 59 kg (130 lb); BMI is 24 kg/m². Temperature is 37.5°C (99.5°F), pulse is 115/min and regular, and blood pressure is 100/68 mm Hg. Examination shows tender parotid glands bilaterally and poor dentition. Results of laboratory studies are most likely to show which of the following in this patient? (A) K+ is Decreased, Cl⁻ is decreased, HCO³⁻ is decreased (B) K+ is Decreased, Cl⁻ is decreased, HCO³⁻ is increased (C) K+ is Decreased, Cl⁻ is increased, HCO³⁻ is decreased (D) K+ is Increased, Cl⁻ is decreased, HCO³⁻ is increased (E) K+ is Increased, Cl⁻ is increased, HCO³⁻ is decreased (F) K+ is Increased, Cl⁻ is increased, HCO³⁻ is increased

**AI_B:** RESTATING THE QUESTION: The case presents a 20-year-old college student with blood vomiting (hematemesis) and weakness, accompanied by daily self-induced vomiting after overeating due to stress. She is physically active and socially involved but appears stressed and uncomfortable, with signs such as tender parotid glands and poor dentition. This situation prompts the need to know which set of blood parameters - potassium (K+), chloride (Cl⁻), and bicarbonate (HCO³⁻) levels - is most likely manifest in her current condition. PLANNING: Given the student's self-induced vomiting, one central assumption is that she could be manifesting signs of an eating disorder, such as bulimia nervosa, which is characterized by cycles of binge eating followed by purging behaviors like vomiting. This repetitive vomiting can lead to various electrolyte disturbances. These disturbances might involve reduced levels of potassium and chloride due to loss in vomitus and increased bicarbonate due to compensatory metabolic alkalosis. CLINICAL REASONING: In our patient's case, the routine vomiting can lead to loss of gastric acid (hydrochloric acid), basically composed of hydrogen (H+) and chloride (Cl⁻) ions. The loss of acid (HCl) triggers hypochloremia (low level of Cl⁻). It also creates a state of metabolic alkalosis characterized by a decrease in the hydrogen ion concentration (or increase in pH), leading to compensatory renal conservation and generation of bicarbonates (HCO³⁻), resulting in hyperbicarbonatemia (increased level of HCO³⁻). Simultaneously, during vomiting, in addition to the loss of acid, there is also loss of potassium ions (K+) in the gastric secretions, leading to hypokalemia (low level of K+). CONCLUDING: Given her self-induced vomiting history, which loses gastric secretions rich in hydrochloric acid and potassium, the most plausible scenario would be the patient having decreased levels of potassium and chloride, and an increased level of bicarbonate due to compensatory changes by the kideny to correct increasing pH as a result of continual loss of stomach acid. RESPONSE: Therefore, the most probable laboratory study results for this patient would be (B) K+ is Decreased, Cl⁻ is decreased, HCO³⁻ is increased.

**AI_E:** (1) RESTATING THE QUESTION: The scenario presents a 20-year-old woman exhibiting symptoms of prolonged, self-induced vomiting, weakness, dizziness, and bloody vomiting. It further notes regular overeating due to stress, followed by induced vomiting due to feeling excessively full and out of control. The question requests a prediction in this case for the most likely laboratory findings related to serum potassium (K+), chloride (Cl⁻), and bicarbonate (HCO³⁻) levels. (2) PLANNING: To answer the question, it's necessary to remember that vomiting purges the body of gastric secretions, which are high in hydrogen (H+) and Cl⁻ ions. This loss can lead to a contraction alkalosis, a type of metabolic alkalosis that occurs when the body loses fluid and electrolytes. Since the kidneys work to balance serum levels of electrolytes, it's plausible to hypothesize that a prolonged history of vomiting could lead to lower serum K+ levels and higher bicarbonate (HCO³⁻) levels. Chloride (Cl⁻) could also be decreased due to its exclusion in vomitus. (3) CLINICAL REASONING: She's been vomiting frequently, almost daily, which would lead to losses of gastric H+ ions and Cl⁻. When hydrogen ions are lost, this could potentially lead to metabolic alkalosis, seen as an elevation in serum HCO³⁻. Loss of K+ in vomitus could lead to hypokalemia, and excessive renal compensation for the alkalosis could further enhance K+ losses. Although Cl⁻ is lost in vomitus, renal compensation includes increased Cl⁻ reabsorption which might prevent blood level depletion. However, given her prolonged and frequent vomiting episodes, it's more likely that Cl⁻ serum levels would also be diminished. (4) CONCLUDING: Thus, with prolonged and repeated vomiting, we'd typically see losses of K+ and Cl⁻ ions along with an increase in blood bicarbonate concentration due to metabolic alkalosis as a compensatory mechanism for lost H+ ions. (5) RESPONSE: Therefore, the answer would be (B) K+ is Decreased, Cl⁻ is decreased, HCO³⁻ is increased.

**AI_D:** (1) RESTATING THE QUESTION: The patient in this case seems to present with eating disorder behavior, most likely bulimia, characterized by her overeating and subsequent self-induced vomiting. She has symptoms like weakness, vomiting blood, dizziness, which can be suggestive of potential disturbances in her electrolyte levels. The question asks us to predict what her laboratory results would most likely show, in relation to potassium (K+), chloride (Cl-), and bicarbonate (HCO3-) levels. (2) PLANNING: Possible assumptions can be: a) Due to her regular vomiting, the woman might have metabolic disturbances. b) The vomiting could result in a loss of stomach acid and create electrolyte imbalances. c) Since she is not taking any medications, laxatives, or diuretics, the stated symptoms might be related to her eating disorder. (3) CLINICAL REASONING: Frequent self-induced vomiting, an integral part of bulimia, can cause multiple electrolyte imbalances, leading to the retention of bicarbonate as a compensatory mechanism. Loss of gastric fluids through vomiting causes a loss of hydrochloric acid, which leads to metabolic alkalosis. Metabolic alkalosis is a condition in which the body's pH is higher than normal (increased HCO3-), often due to the loss of acid from vomiting. The loss of chloride ions with acid in vomitus leads to low blood chloride (hypochloremia), and consequently, hypokalemia (low K+) due to increased urinary excretion as the kidneys reabsorb K+ in exchange for excess H+. (4) CONCLUDING: That means an individual with bulimia who frequently induces vomiting would find herself in a state of metabolic alkalosis with hypokalemia and hypochloremia. (5) RESPONSE: So, the answer would most likely be (B) K+ is Decreased, Cl- is decreased, HCO3- is increased, best encapsulating the patient's probable metabolic status, given her pattern of self-induced vomiting. Although this clinical pattern correlates with bulimia, one must obtain further information to firmly establish the diagnosis. Once a clinician encounters symptoms reminiscent of an eating disorder, it is crucial to approach the subject delicately, given the high potential for denial and manipulation. Comprehensive treatment including nutritional rehabilitation, psychotherapy, and possibly medication might be required. Additionally, in the acute phase, rehydrating and correcting metabolic anomalies is of utmost importance to prevent severe complications, such as cardiac arrhythmias, which can prove fatal.

**AI_C:** The question at hand pertains to the expected results of laboratory studies focusing on concentrations of potassium (K+), chloride (Cl⁻), and bicarbonate (HCO³⁻) in a young woman exhibiting symptoms of regular vomiting, weakness, dizziness, and a habit of overeating followed by self-induced vomiting. Potassium (K+) is an essential mineral and electrolyte vital for cell function including nerve signal communication, muscle contractions, fluid balance, and various chemical reactions. Chloride (Cl⁻) mainly helps maintain fluid balance and pH in the body. Bicarbonate (HCO³⁻) is a component of the body's pH buffering system, contributing to the acid/base balance essential for normal cellular functions. It could be hypothesized then, that the regular vomiting the patient is experiencing could be changing these concentration levels. Primarily, frequent vomiting typically results in the loss of significant amounts of gastric acid, which is high in hydrochloric acid (HCl), leading to loss of chloride ions (Cl⁻). Chronic vomiting can also reduce body potassium (K+) levels due to its presence in the gastric secretions, leading to hypokalemia. As a compensatory mechanism, the kidneys will attempt to conserve H+ ions (acid) to balance the loss, leading to increased bicarbonate reabsorption (HCO³⁻). This process is the body's way of counteracting the metabolic alkalosis caused by the initial loss of gastric acid via vomiting. To connect these understandings to the case at hand, given her symptoms of frequent self-induced vomiting, it is highly plausible that the patient's levels of K+ and Cl⁻ will be decreased, and her HCO³⁻ may be increased. These clinical insights suggest that her laboratory results would most likely show option (B) K+ is Decreased, Cl⁻ is Decreased, HCO³⁻ is Increased, considering the impact her daily vomiting could be having on her electrolyte and acid/base balance. Therefore, to answer the question, the most expected result of the laboratory studies in this patient's case would be (B): Potassium (K+) and Chloride (Cl⁻) levels are decreased and Bicarbonate (HCO³⁻) level is increased. However, a definitive diagnosis should be confirmed through actual laboratory testing, considering the patient's complete clinical picture. This conclusion also indicates a need for psychological treatment for her abnormal eating behavior along with the necessary medical treatment to correct her electrolyte and acid-base imbalances.

**AI_A:** RESTATING THE QUESTION: The clinical case describes a young woman, who despite appearing healthy, has been displaying severe symptoms such as weakness, vomiting blood and dizziness. She has a history of overeating due to stress, and inducing vomiting due to a feeling of fullness and loss of control, which is occurring daily now. Despite feeling slightly overweight, she leads an active lifestyle, is sociable, and does not use laxatives, diuretics, illicit drugs or any prescribed medication. She does, however, consume alcohol moderately on weekends. Upon clinical examination, tender parotid glands were found along with poor dentition. The question requires us to predict the likely results of her laboratory tests, focusing specifically on her potassium (K+), chloride (Cl-) and bicarbonate (HCO3-) levels. PLANNING: The phenomenon of consistent vomiting can lead to certain physiological changes in the body, affecting electrolytes like potassium, chloride and bicarbonate. Given her lifestyle, and particularly her self-induced vomiting, it's plausible to think that her electrolyte balance might be affected. However, we need to consider each electrolyte separately: 1. Potassium (K+) is an important electrolyte, and its balance is crucial for proper body function. However, vomiting, especially chronic, can lead to a loss of potassium, leading to hypokalemia (low potassium levels). 2. Chloride (Cl-) levels might also be affected. With vomiting, gastric acid, which contains a significant amount of chloride, is lost, possibly leading to hypochloremia (low chloride levels). 3. The effect on bicarbonate (HCO3-) levels is more complex. The loss of stomach acid due to vomiting can lead to a state of metabolic alkalosis as the body responds by producing more bicarbonate, thus possibly leading to increased levels of HCO3- in the blood. CLINICAL REASONING: Considering the clinical history presented, the patient is likely suffering from Bulimia nervosa, a psychological eating disorder characterized by binge eating and subsequent inappropriate compensatory behaviors to prevent weight gain, such as self-induced vomiting. Repeated vomiting results in loss of gastric and intestinal fluids high in hydrogen (H+), potassium (K+), and chloride (Cl-) ions. This loss can lead to metabolic alkalosis (due to a loss of H+) and hypokalemia (needs to be confirmed by decrease in K+ levels). As a compensatory mechanism to the alkalosis (a pH above the body's normal range of ~7.35-7.45), the kidneys excrete bicarbonate (HCO3-) in urine. However, because of repeated episodes of vomiting and limited interval for kidney compensation, this patient might still present with high HCO3- level in the blood tests initially. CONCLUDING: As a result, considering the nature of the ischemic vomiting, it's expected to find a decrease in K+ and Cl- due to the chemical loss incurred during vomiting. However, a heightened level of HCO3- might persist initially due to body's compensatory response to metabolic alkalosis. RESPONSE: It seems reasonable to expect an increase in the levels of bicarbonate (due to metabolic alkalosis), along with a decrease in potassium and chloride levels (as a result of losses through vomiting). So, the most likely scenario in this case would be (B) K+ Decreased, Cl- Decreased, HCO3- Increased.

**Facilitator** The consensus statement synthesizes the responses of AI_A, AI_B, AI_C, AI_D, and AI_E as follows: The case presents a 20-year-old woman who exhibits symptoms of regular self-induced vomiting, weakness, and dizziness. She has a habit of overeating due to stress, followed by self-induced vomiting due to feelings of fullness and loss of control. Given her symptoms and behaviors, it is plausible to hypothesize that she may be suffering from an eating disorder, likely bulimia nervosa. The frequent self-induced vomiting can lead to significant physiological changes in the body, particularly affecting electrolyte balance. The vomiting results in the loss of gastric secretions, which are rich in hydrochloric acid (HCl), leading to a loss of hydrogen (H+) and chloride (Cl-) ions. This loss can trigger a state of metabolic alkalosis, characterized by a decrease in the hydrogen ion concentration (or increase in pH). As a compensatory mechanism, the kidneys attempt to conserve H+ ions (acid) to balance the loss, leading to increased bicarbonate reabsorption (HCO3-). This process is the body's way of counteracting the metabolic alkalosis caused by the initial loss of gastric acid via vomiting. Simultaneously, during vomiting, there is also a loss of potassium ions (K+) in the gastric secretions, leading to hypokalemia (low level of K+). The loss of chloride ions with acid in vomitus leads to low blood chloride (hypochloremia). Therefore, given her symptoms of frequent self-induced vomiting, it is highly plausible that the patient's levels of K+ and Cl- will be decreased, and her HCO3- may be increased. These clinical insights suggest that her laboratory results would most likely show option (B) K+ is Decreased, Cl- is Decreased, HCO3- is Increased, considering the impact her daily vomiting could be having on her electrolyte and acid/base balance. However, a definitive diagnosis should be confirmed through actual laboratory testing, considering the patient's complete clinical picture. This conclusion also indicates a need for psychological treatment for her abnormal eating behavior along with the necessary medical treatment to correct her electrolyte and acid-base imbalances.
