# Supplementary material for: Collaborative intelligence in AI: Evaluating the performance of a council of AIs on the USMLE
Source: PLOS Digit Health. 2025 Oct 9;4(10):e0000787. doi: 10.1371/journal.pdig.0000787 (PMC12510544; doi:10.1371/journal.pdig.0000787)
Supplement: S2 File — (ZIP) [file pdig.0000787.s002.zip › S2 Data Raw data files containing unprocessed question inputs and LLM outputs including transcripts of deliberations/STEP2_Question-45-1_Type-MC-NJ.docx]

**Facilitator** **[Round 1]:** A 58-year-old man comes to the clinic for a follow-up examination 3 months after beginning warfarin therapy for deep venous thrombosis. He noted blood in his urine 1 week ago but has not seen any since that time. He has not had pain, fever, or increased urinary frequency. He has not sustained any recent trauma. His prothrombin time and INR have been within the therapeutic ranges during his previous measurements. He has hypertension and benign prostatic hyperplasia. Medications are warfarin and hydrochlorothiazide. He smoked one pack of cigarettes daily for 40 years but quit when diagnosed with deep venous thrombosis. Temperature is 35.9°C (96.6°F), pulse is 88/min, respirations are 18/min, and blood pressure is 135/87 mm Hg. The prostate is moderately enlarged; no nodules are noted. Laboratory studies showed that the Prothrombin time is 19 sec (INR=2.5), serum Urea nitrogen is 18 mg/dL, serum Creatinine is 1 mg/dL, and Prostate-specific antigen is 3.8 ng/mL (N<4). The laboratory result on urine showed the following: blood is 3+, protein is 1+, RBC is too numerous to count, WBC is 2/hpf, no casts, and negative for Nitrites and Leukocyte esterase. In addition to a CT scan of the abdomen, which of the following is the most appropriate next step in management? (A) Adjustment of the warfarin dosage to maintain an INR between 1.5 and 2.0 (B) Ciprofloxacin therapy (C) Cystoscopy (D) Discontinuation of hydrochlorothiazide therapy (E) Finasteride therapy

**AI_D:** 1) RESTATING THE QUESTION: The question is about a 58-year-old man who has been on warfarin for deep venous thrombosis (DVT) and noticed he had blood in his urine a week ago, but it hasn't reoccurred since then. Despite his use of warfarin and hydrochlorothiazide, his prothrombin time (PT) and International Normalized Ratio (INR) have been within therapeutic ranges. He also has a history of hypertension and benign prostatic hyperplasia (BPH). Preliminary lab tests reveal an abundance of red blood cells in his urine, but no nitrites or leukocyte esterase, which are markers for urinary tract infections (UTIs). Besides scheduling a CT scan, we're asked to decide on the next best step in management from the listed options. 2) PLANNING: Some of the central assumptions here are that his bleeding is related to the warfarin, a blood thinner, and his INR and PT levels are indicative of the drug's effect. Another assumption is that an infection could have caused the hematuria, hence the option of the antibiotic. However, the absence of nitrites and leukocyte esterase in his urine makes this less likely. The condition could also be associated with his current medication, hydrochlorothiazide, or his BPH, hence the choice of discontinuing this medication or using finasteride, which treats BPH. Given his symptoms, the most clinically prioritized assumption to pursue would likely be investigating urological issues. 3) CLINICAL REASONING: Since the man in question does not exhibit any signs of infection or side effects of his antihypertensive medication, the bleeding is most likely associated or caused by a urological issue. This could include kidney or bladder issues, which may not necessarily be related to his medication. The man's history of smoking also places him at risk for bladder cancer, which increases the need for a thorough urological examination. 4) CONCLUDING: The clinical reasoning process indicates that a more thorough examination of his urological situation is necessary. A cystoscopy provides visualization of the urinary tract and helps in diagnosing diseases of the bladder, urethra, and prostate, potentially identifying the source of the hematuria. 5) RESPONSE: Given this man's clinical presentation and laboratory findings, the most appropriate next step in management would be (C) Cystoscopy. It will allow a direct visualization of the urinary tract, potentially identifying the source of the hematuria without jumping into adjusting pharmacological treatments until the root cause is confirmed.

**AI_B:** RESTATING THE QUESTION: This query is about a 58-year-old man who has been on warfarin for the treatment of deep venous thrombosis for the past three months. He noticed blood in his urine a week ago, but that symptom has not recurred. He hasn't experienced any pain, fever, increased urinary frequency, or recent trauma. The patient suffers from hypertension and benign prostatic hyperplasia, with his medications including warfarin and hydrochlorothiazide. Lab tests reveal therapeutic prothrombin time and INR values, normal BUN, Creatinine, and PSA within normal range, but urinalysis showing significant blood, mild proteinuria, and many red blood cells. The question asks for the most appropriate next step in management, besides a CT scan of the abdomen. PLANNING: The patient's symptoms indicate hematuria but we need to explore whether it's a result of his existing conditions (warfarin therapy, hypertension, or benign prostatic hyperplasia) or something else. The assumptions we could make based on this are: (1) the warfarin dosage might be too high causing bleeding, despite the INR being within the therapeutic range, (2) there might be a urinary tract infection, albeit without classical symptoms, (3) There could be an issue within his bladder like bladder cancer or stones, or (4) the hydrochlorothiazide or benign prostatic hyperplasia might be causing urinary abnormalities. Out these, issues related to bladder (assumption 3) should be clinically prioritized because of the absence of other specific symptoms pointing to the other conditions and the presence of significant red blood cells in the urine. CLINICAL REASONING: Although the patient is being treated with warfarin, his INR and prothrombin time are within therapeutic ranges so it's less likely that this is the cause of the hematuria. Further, the patient's symptoms don't point to a urinary tract infection as he lacks classic UTI symptoms like fever, itching, or burning during urination as well as negative nitrites and leukocyte esterase on the urine test. Hydrochlorothiazide, a diuretic, could theoretically lead to abnormal urination, but it’s not particularly associated with hematuria. Thus, ruling these options out makes it more likely that the issue is with the bladder. Given this exploration, performing an endoscopic examination of the urinary bladder (cystoscopy) would be the best next step for detecting any abnormalities or lesions that could be causing the hematuria. CONCLUDING: Despite the patient's medications and existing conditions, the symptoms and lab results most likely point to an issue within the bladder. Therefore, our clinical reasoning leads us to prioritize a comprehensive diagnostic approach focused on visualizing the interior of the bladder. RESPONSE: The most appropriate next step in management, in addition to a CT scan of the abdomen, is (C) Cystoscopy.

**AI_E:** RESTATING THE QUESTION: A 58-year-old man who started warfarin treatment for deep venous thrombosis 3 months ago and has been managing hypertension and benign prostatic hyperplasia is reporting a week-old episode of hematuria. There are no traumatic, feverish, painful, or urinary discomfort associated with the blood within his urine. His prothrombin time and INR measurements remained within the therapeutic range. Notably, he used to be a heavy smoker. His vitals are within normal limits, and a physical examination reveals an enlarged prostate. Various laboratory tests including blood and urine analysis are conducted. There might seem to be an inclination to consider this as a possible adverse reaction to his treatment, however, the question concludes by asking, apart from a CT scan, which of the given options would be the best management step? PLANNING: First, let's assess the assumptions based on the case. It might first be assumed that due to the use of warfarin, an anticoagulant, the bleeding is a side effect. Second, given the patient's presentation and findings, it could be assumed that the patient's benign prostatic hyperplasia is exacerbating. Then, we could consider the assumption that the patient's condition may have evolved into something worse, like bladder cancer, because of prolonged smoking history and hematuria. We could generate alternate assumptions by questioning the initial assumptions: Could the bleeding be brought on by something other than the anticoagulation therapy? Is the benign prostatic hyperplasia playing a primary role in the bleeding? Is it possible that there is a developing pathology beyond the BPH, such as bladder cancer? The clinically prioritized assumption would be that there might be an underlying pathology causing the bloody urine, given the patient's history of heavy smoking. CLINICAL REASONING: Before reaching the final decision, let's go by each assumption. First, the prothrombin time and INR of the patient have been consistently within the therapeutic range, undermining the argument that the warfarin is causing bleeding as an overdose would be more likely to result in excessive bleeding. Second, the patient's benign prostatic hyperplasia would not generally result in hematuria, making that an unlikely cause. The third assumption then remains, which involves insight into the patient's long history of smoking – a leading cause of bladder cancer. The painless hematuria further suggests the possibility of bladder cancer since this is a typical initial sign of the disease. Therefore, it becomes essential to inspect the bladder to verify or rule out the presence of bladder cancer. CONCLUDING: Based on the patient's clinical history and presentation, an appraisal is that an occult malignancy, such as bladder cancer, could be the source of the hematuria. Given the patient's long smoking history, seeming absence of infection, no recent trauma or overdose of anticoagulation therapy, together with painless hematuria, a potential bladder cancer diagnosis cannot be ignored. Therefore, a more thorough examination of the bladder is warranted. RESPONSE: Given the clinical scenario, the next appropriate management step would be (C) Cystoscopy. The patient's medical history, including his long smoking history, and the manifestation of painless hematuria, necessitate ruling out bladder cancer, which a cystoscopy would facilitate.

**AI_C:** RESTATING THE QUESTION: A 58-year-old male patient, who has been on warfarin treatment for deep venous thrombosis and also has a history of hypertension and benign prostatic hyperplasia, reported about a week ago having seen blood in his urine. He has no other urinary symptoms or recent trauma. His medications include warfarin and hydrochlorothiazide, and his INR has always been within the therapeutic range. His laboratory results show that his prothrombin time is 19 sec (INR=2.5), the urine test found a high number of blood cells and a little protein, but it was negative for nitrites and leukocyte esterase. The patient is also a former long-term smoker. The question here is about the most appropriate next clinical step, considering also an abdominal CT scan. PLANNING: The primary assumption here could be a urinary tract infection (UTI), considering the hematuria, but the lack of nitrites and leukocyte esterase, which are typically present in UTIs, questions this assumption. The second assumption is that the warfarin is causing the bleeding, but the prothrombin and INR results do not suggest over-anticoagulation. The third assumption could be benign prostatic hyperplasia (BPH), a common condition in men his age, but BPH doesn't usually cause visible blood in urine. The fourth assumption could be a urologic malignancy, one that can also present with intermittent, painless gross hematuria. Considering the age of patient and history of long-term smoking, the presence of a urologic malignancy seems the most plausible assumption which could explain the symptoms. CLINICAL REASONING: The high number of red blood cells and the three-plus blood in the patient’s urine test suggest significant hematuria. In patients at high risk for malignancy, such as those over 35 years old with a smoking history and painless hematuria, a full workup is required to rule out urological malignancies. This could be bladder cancer, which is strongly associated with smoking and presents as painless gross intermittent hematuria. Hence, more than a UTI, over-anticoagulation, or BPH, urological malignancy seems to be a priority to rule out. CONCLUDING: Although warfarin can cause hematuria, the INR is in the therapeutic range. UTI would usually be suggested by positive nitrites or leukocyte esterase in the patient's urine analysis. The lack of these labs indicates against UTI. BPH can cause microscopic hematuria but rarely results in gross hematuria. After considering all these factors, the clinical presentation, including the patient's age, smoking history, and the symptomatic gross hematuria, the possibility of a urinary tract malignancy should be evaluated. RESPONSE: Therefore, option (C) Cystoscopy, a diagnostic procedure that allows direct visualization of the urinary tract, is the most appropriate next step in management. This procedure would be the best course of action to identify any malignancies in this patient's urinary tract.

**AI_A:** (1) RESTATING THE QUESTION: This is a case of a 58-year-old man who is diagnosed with deep venous thrombosis and currently on warfarin medication. He has made observations of hematuria (presence of blood in urine), however, exhibits no pain, fever, or any urinary frequency irregularities. No recent trauma has been sustained by him and laboratory analyses such as prothrombin time and international normalized ratio (INR) are within therapeutic ranges. He also has a background of hypertension and benign prostatic hyperplasia, and is on hydrochlorothiazide medication. Despite having quit smoking recently, his smoking history includes 40 daily packs for 40 years. He reveals a slightly high blood pressure, but other vital signs are within normal parameters. His physical examination shows an enlarged prostate but no nodules, and his laboratory studies show a high RBC count in urine, among other results. (2) PLANNING: The assumed problem at hand here could potentially be due to the side effects of the man's medications (warfarin leading to hematuria), bleeding from benign prostatic hyperplasia, or another unknown urinary-related issue. However, given the high number of red blood cells in his urine along with the lack of urinary symptoms or recent trauma, an alternate hypothesis may involve urological malignancy. The absence of infection indicators in the urine analysis (negative Nitrites and Leukocyte esterase) along with stable vitals, including the therapeutic INR, direct us towards non-infective and non-anticoagulant-related causes. Therefore, in a man of his age with a significant smoking history, malignancy should be highly considered. (3) CLINICAL REASONING: The presence of hematuria is often a symptom of either urinary tract infection (UTI), kidney disease, or malignancy in the urinary tract. The absence of dysuria and a negative nitrite and leukocyte esterase test, as well as the normal serum creatinine in this case, significantly reduces the likelihood of a UTI or a kidney disease. Given his age and significant smoking history - a well-known risk factor for bladder cancer - along with the physical examination indicating an enlarged prostate suggestive of benign prostatic hyperplasia (BPH), the risk for malignancy, particularly bladder cancer, should be considered. (4) CONCLUDING: Based on the clinical reasoning and the man's profile, conditions related to infection or renal causes seem unlikely. Therefore, the most appropriate next step in management would be to investigate the possibility of malignancy. (5) RESPONSE: Given the absence of infectious signs, stable condition of the patient, and the ongoing concern for a possible malignant condition, the most appropriate next step then would be to conduct a cystoscopy (Option C) in order to inspect the interior of the urinary bladder and possibly obtain a biopsy if a suspicious area is identified.

**Facilitator** The consensus among the AI responses is that the most appropriate next step in management for the 58-year-old man, who has been on warfarin for deep venous thrombosis and has noticed blood in his urine, is to conduct a cystoscopy (Option C). This conclusion is based on the following reasoning: 1. The patient's prothrombin time and INR are within therapeutic ranges, suggesting that the warfarin dosage is not causing the hematuria. 2. The absence of nitrites and leukocyte esterase in the urine, along with the lack of typical urinary tract infection symptoms, makes a urinary tract infection unlikely. 3. The patient's benign prostatic hyperplasia is not typically associated with visible blood in urine, making it an unlikely cause of the hematuria. 4. The patient's long history of smoking, coupled with the presence of painless hematuria, raises the possibility of a urological malignancy, such as bladder cancer. Given these considerations, a cystoscopy is recommended as it allows for direct visualization of the urinary tract, potentially identifying any malignancies that could be causing the hematuria.
