# Supplementary material for: Collaborative intelligence in AI: Evaluating the performance of a council of AIs on the USMLE
Source: PLOS Digit Health. 2025 Oct 9;4(10):e0000787. doi: 10.1371/journal.pdig.0000787 (PMC12510544; doi:10.1371/journal.pdig.0000787)
Supplement: S2 File — (ZIP) [file pdig.0000787.s002.zip › S2 Data Raw data files containing unprocessed question inputs and LLM outputs including transcripts of deliberations/STEP2_Question-46-1_Type-MC-NJ.docx]

**Facilitator** **[Round 1]:** A 32-year-old woman is brought to the emergency department immediately after being involved in a motor vehicle collision. She was the unrestrained driver. On arrival, she says she has severe pain in her right groin. On examination, any movement of the right hip produces pain. An x-ray of the pelvis shows a completely displaced fracture of the femoral neck. In addition to intravenous morphine, which of the following is the most appropriate management? (A) Bed rest (B) Closed reduction followed by traction (C) Hip spica hinge brace (D) Limited weight bearing with use of crutches (E) Operative reduction and internal fixation

**AI_C:** RESTATING THE QUESTION: The question is asking what the best treatment plan is for a 32-year-old female patient who has suffered a completely displaced femoral neck fracture as a result of a motor vehicle accident. The femoral neck is a part of the thigh bone which is in close proximity to the hip, hence the pain in that area. PLANNING: The paramedics haven't designated a pain scale intensity, but from the description, it seems severe. The fracture is displaced, meaning there's no alignment between the broken ends of the bone. This category is problematic as it disrupts blood flow and can lead to complications such as avascular necrosis or nonunion of the bone parts. This injury requires more than mere palliative pain management (morphine). We could assume either non-surgical (options A, B, C, D) or surgical (option E) management should be prioritized. CLINICAL REASONING: Non-surgical treatments such as bed rest, closed reduction followed by traction, the use of a brace, or weight-bearing restrictions are generally used for non-displaced fractures or for patients who cannot undergo surgery due to other health conditions. However, a displaced fracture, especially in the femoral neck region, typically requires surgical intervention. By realigning the bones and securing them in place, surgical reduction and internal fixation can help promote healing and prevent complications. CONCLUSION: Using the clinical reasoning process, it becomes clear that surgical intervention is particularly needed in severe cases involving displaced fractures. While non-surgical methods can provide some temporary relief, they won't adequately address the root cause of the problem or provide a long-term solution. RESPONSE: Hence, the most appropriate management for this patient, in addition to intravenous morphine, is (E) Operative reduction and internal fixation. This will help realign the broken parts of the femoral neck and secure them, promoting proper healing and preventing further complications.

**AI_D:** RESTATING THE QUESTION: We're dealing with a 32-year-old woman who suffered a traumatic accident and now presents with a completely displaced femoral neck fracture. The question is asking what the best management option is for her current condition, aside from pain control using intravenous morphine. PLANNING: Assuming that the fracture type influences the choice of management, displaced femoral neck fracture could be managed by non-surgical or surgical methods. Non-surgical methods include bed rest and limited weight bearing with crutches. Surgical interventions could be closed reduction with traction or operative reduction with internal fixation. We also consider using a brace for additional stability. The choice of management should be based on factors such as the kind of injury, patient's age, health status, and the potential for healing and recovery. Given the patient's young age, complete displacement, and likely active status, we might prefer a more definitive and quick rehabilitation option. CLINICAL REASONING: Femoral neck fracture is a serious injury, especially when completely displaced, as it disrupts the blood supply, posing a high risk of avascular necrosis of the femoral head. Non-surgical treatments like bed rest or limited weight bearing may not offer the best outcome as these do not directly address displacement and may lead to prolonged immobilization or potential for nonunion. Closed reduction followed by traction, while less invasive than surgery, might not be reliable to maintain reduction in her case. Hip spica hinge brace is typically not for adults due to discomfort and difficulty in patient mobility. By contrast, surgical intervention can stabilize the fracture and promote healing, leading to quicker rehabilitation. Among the surgical choices, operative reduction and internal fixation allows direct visualization of the fracture site and better control for fracture alignment, which is preferred especially in the case of complete displacement. CONCLUDING: Comparing these options, the most appropriate management for this case would provide the patient with the best opportunity for healing, recovery, and returning to normal function in the shortest time while minimizing complications. RESPONSE: Therefore, the best treatment option for this 32-year-old female with a completely displaced fracture of the femoral neck, following intravenous morphine administration, would be (E) Operative reduction and internal fixation.

**AI_E:** (1) RESTATING THE QUESTION: The question describes a scenario where a young woman has been involved in a car accident and suffered a completely displaced fracture of the femoral neck. The inquiry here is what would be the optimal approach to manage this injury in addition to pain control with morphine. (2) PLANNING: Femoral neck fractures can be managed conservatively or surgically. The choice of treatment depends on a variety of factors, including the type of fracture (displacement status), age of the patient, and overall health status. Fully displaced fractures have disrupted blood supply and are usually treated surgically to prevent complications like nonunion or avascular necrosis. It's also possible that conservative methods like bed rest, traction, or braces could be considered, but for a young, otherwise healthy woman, non-surgical methods might be less beneficial. Limited weight-bearing can be part of the recovery process after a surgical intervention. (3) CLINICAL REASONING: A displaced femoral neck fracture in a young patient would typically be managed surgically, as this fracture type poses a significant risk for complications if left untreated or managed conservatively. In particular, nonunion (failure of the bone to heal) or, more importantly, avascular necrosis (death of bone tissue) due to the interrupted blood supply are key concerns in these cases. Non-surgical methods, while less invasive, may not sufficiently address these issues, and have a higher risk for poor clinical outcomes in terms of future function and pain levels. (4) CONCLUDING: Considering the aforementioned points, operative reduction and internal fixation appear to be the most suited to address the medical situation effectively. This surgical approach is aggressive but necessary to restore the anatomy and function of the hip and to diminish the risk of long-term complications. (5) RESPONSE: Thus, the most suitable management option for this patient, in addition to pain relief by intravenous morphine, would be (E) Operative reduction and internal fixation. This approach will aid in achieving anatomical restoration and prompt recovery with a reduced risk of future complications like nonunion and avascular necrosis.

**AI_B:** RESTATING THE QUESTION: This question presents a scenario of a 32-year-old woman who has suffered a motor vehicle accident resulting in a femoral neck fracture. The management options of interest include bed rest, closed reduction with traction, use of a hip spica hinge brace, limited weight bearing using crutches, and operative reduction with internal fixation. The femoral neck is the part of the femur (thigh bone) that connects the femoral shaft to the femoral head - forming part of the hip joint. A completely displaced fracture of the femoral neck indicates that the femoral head is no longer in alignment with the shaft of the femur. Our task is to determine which management option is most clinically appropriate in this situation besides the use of intravenous morphine for pain control. PLANNING: Assumptions in this case are that the patient's main problem is the fractured femoral neck and that this fracture is causing her pain. These assumptions seem logical given the history and x-ray results. As an alternative assumption, it could be questioned whether any additional injuries might be affecting her condition, but for the purposes of responding to this question, we'll focus on the femoral neck fracture. Considering the options, the priority is to realign the femur and stabilize the hip joint to assist with pain management and facilitate recovery. CLINICAL REASONING: Given the displaced nature of the fracture, non-surgical options like bed rest (A), closed reduction with traction (B), or use of a hip spica hinge brace (C) might not provide adequate alignment and stability. In option (D), limited weight bearing would be likely too painful and potentially harmful for the patient. Operative reduction and internal fixation (E), however, could align and stabilize the femur, promote healing, and significantly reduce the risk of complications such as non-union, avascular necrosis of the femoral head, and arthritis. CONCLUDING: This case of a completely displaced fracture of the femoral neck in a young patient requires active intervention for adequate realignment and stabilisation of the femur to facilitate healing and prevent any future complications. RESPONSE: The most appropriate management, in this case, would therefore be (E) Operative reduction and internal fixation.

**AI_A:** (1) RESTATING THE QUESTION: This scenario involves a 32-year-old female who was involved in a motor vehicle crash and has obtained a completely displaced fracture of the femoral neck, which is a severe fracture in the top part of the thigh bone (femur) that connects the shaft of the femur and the rounded femoral head. She is experiencing severe pain in her right hip region, and the question is considering what the most appropriate treatment should be beyond pain control with intravenous morphine. (2) PLANNING: There are several assumptions we must consider. We must consider the options of conservative management like bed rest and limited weight bearing, the option of conservative operative management like closed reduction followed by traction, and aggressive operative management like operative reduction and internal fixation. We also must bear in mind her age and general health status. While bed rest might be appropriate for minor injuries, a completely displaced fracture of the femoral neck is a severe break. Limited weight bearing with crutches might be suggested for certain types of stable fractures. However, a displaced fracture is not stable, so using crutches could potentially worsen the condition. Similarly, closed reduction followed by traction might be suitable for less severe fractures, but it's less likely to be appropriate in this case. Given her young age and the significant nature of the fracture, it would seem that the most appropriate assumption to prioritize in this case is that she would need the kind of stabilization only offered through operative reduction and internal fixation. (3) CLINICAL REASONING: The completely displaced fracture of the femoral neck has rendered the hip joint unstable, causing her extreme pain, and is a serious injury that can potentially lead to several complications. One such possible complication is avascular necrosis of the femoral head due to damage to the blood supply to this area. Another concern is nonunion or malunion, both of which can lead to continued pain and disability. Considering her age and the serious nature of the injury, a more assertive and effective treatment approach that can provide early mobilization and ensure a better prognosis is necessary to prevent such complications. (4) CONCLUDING: So, based on the severity and nature of the injury, particularly its complete displacement and the patient's age, the most appropriate management step beyond pain control is therefore likely to be a procedure that can promptly stabilize the fracture site and promote healing, thereby minimizing the risk of the aforementioned complications. (5) RESPONSE: Thus, the most appropriate management for this patient, in addition to intravenous morphine for pain, is (E) Operative reduction and internal fixation, which should offer the best possible outcome regarding her mobility and overall prognosis. This management approach provides immediate stabilization of the fracture, decreases the risk of complications like avascular necrosis and nonunion, and allows for early mobilization.

**Facilitator** The consensus among the AI responses is that the most appropriate management for a 32-year-old female patient who has suffered a completely displaced fracture of the femoral neck due to a motor vehicle accident, in addition to intravenous morphine for pain control, is operative reduction and internal fixation. This conclusion is based on several factors. Firstly, the nature of the injury, a completely displaced fracture, indicates a severe break where the femoral head is no longer in alignment with the shaft of the femur. This type of fracture disrupts the blood supply, posing a high risk of complications such as avascular necrosis of the femoral head or nonunion of the bone parts. Secondly, the patient's age and general health status are considered. Given her young age and likely active status, a more definitive and quick rehabilitation option is preferred. Non-surgical treatments such as bed rest, closed reduction followed by traction, the use of a brace, or weight-bearing restrictions are generally used for non-displaced fractures or for patients who cannot undergo surgery due to other health conditions. However, these options might not provide adequate alignment and stability for a displaced fracture and may lead to prolonged immobilization or potential for nonunion. On the other hand, operative reduction and internal fixation can realign the bones and secure them in place, promoting proper healing and preventing further complications. This surgical intervention provides immediate stabilization of the fracture, decreases the risk of complications, and allows for early mobilization, offering the best possible outcome regarding her mobility and overall prognosis. Therefore, the consensus is that the most appropriate management for this patient is operative reduction and internal fixation.
