# Supplementary material for: Collaborative intelligence in AI: Evaluating the performance of a council of AIs on the USMLE
Source: PLOS Digit Health. 2025 Oct 9;4(10):e0000787. doi: 10.1371/journal.pdig.0000787 (PMC12510544; doi:10.1371/journal.pdig.0000787)
Supplement: S2 File — (ZIP) [file pdig.0000787.s002.zip › S2 Data Raw data files containing unprocessed question inputs and LLM outputs including transcripts of deliberations/STEP2_Question-47-1_Type-MC-NJ.docx]

**Facilitator** **[Round 1]:** A 57-year-old man comes to the physician 1 week after he had a 45-minute episode of slurred speech and clumsiness of his right hand. The episode occurred during his daily walk; the symptoms resolved during the next 24 hours. He has no history of similar symptoms. He has a 20-year history of hypertension and a 15-year history of hypercholesterolemia controlled with a low-cholesterol diet and daily exercise. He takes no medications. He has smoked one pack of cigarettes daily for 40 years and drinks one to two glasses of wine nightly with dinner. He is alert and fully oriented. He is 178 cm (5 ft 10 in) tall and weighs 86 kg (190 lb); BMI is 27 kg/m². His temperature is 37.0°C (98.6°F), pulse is 88/min, respirations are 10/min, and blood pressure is 170/90 mm Hg. Examination, including neurologic examination, shows no other abnormalities. Daily enteric-coated aspirin therapy is begun. Which of the following is the most appropriate next step to decrease this patient’s risk for subsequent cerebral infarction? (A) Alcohol cessation (B) Smoking cessation (C) Weight loss program (D) Antihypertensive therapy (E) Cholesterol-lowering therapy

**AI_B:** 1) RESTATING THE QUESTION: The question aims to identify the best management option to reduce the risk of another cerebral infarction for a 57-year-old man. The patient recently experienced an episode of slurred speech and clumsiness in his right hand, indicative of a transient ischemic attack (TIA). The patient has a past medical history significant for hypertension, hypercholesterolemia, long-term smoking, and moderate alcohol consumption. 2) PLANNING: The assumptions here include considering the factors that increase cerebrovascular risk. These include hypertension, hypercholesterolemia, smoking, and obesity. The clinical assumption that needs to be prioritized is the patient's hypertension, given its significant association with the risk of cerebral infarctions and the fact that the patient's blood pressure reading is elevated at 170/90 mm Hg. 3) CLINICAL REASONING: While all the listed options (weight loss, smoking cessation, alcohol cessation, and cholesterol-lowering therapy) can contribute to overall health improvement and possible stroke risk reduction, addressing the patient's hypertension should be the priority. Hypertension is one of the most common and treatable risk factors for cerebrovascular disease. Furthermore, the patient's current blood pressure of 170/90 mm Hg is far above the target range, even for individuals with hypertension. Control of blood pressure has been associated with a substantial decrease in the risk of recurrent stroke. 4) CONCLUDING: Despite other existing risk factors, tackling the patient's uncontrolled hypertension offers the most immediate and impactful reduction in this patient's risk of a subsequent cerebral infarction. 5) RESPONSE: Consequently, the most appropriate next step to decrease this patient’s risk for subsequent cerebral infarction would be (D) Antihypertensive therapy.

**AI_E:** RESTATING THE QUESTION: A 57-year-old man experienced a brief episode of neurological deficits, namely slurred speech, and clumsiness of one hand. His symptoms resolved within a day. Notably, he has risk factors of hypertension for 20 years and has maintained hypercholesterolemia control via diet and exercise. Borderline obesity, daily smoking habit, and moderate alcohol consumption are other contributory factors. Despite the absence of a similar episode before, and no current neurological abnormalities, his episode and risk factors suggest he had a transient ischemic attack (TIA). Considering these, we are asked to select the best intervention which will reduce his risk of a future brain infarction or stroke. PLANNING: Several assumptions that could be made based on his history: His TIA may be due to hypercholesterolemia, hypertension, obesity, smoking, or alcohol consumerism, or a combination of his unfavourable lifestyle habits and medical conditions. Among these, the control of hypertension could be prioritized, as it stands as a dominant risk factor for stroke. Hypertension causes damage to the blood vessels and leads to different forms of cerebrovascular diseases. CLINICAL REASONING: The patient's episode of temporary slurred speech and clumsiness of the right hand resolved within the next 24 hours, which is characteristic of a TIA. A TIA is often a precursor to a more severe stroke, and the patient's existing cardiovascular risk factors such as a 20-year history of hypertension, hypercholesterolemia, smoking, and moderate alcohol use increase his likelihood. Among these, hypertension stands out as the most significant risk factor for cerebrovascular diseases, including TIA and stroke. Controlling this would have a strong impact on preventing future events. CONCLUDING: Instituting an intervention to manage the patient's hypertension would, therefore, be a significant step in reducing his risk of a future stroke or cerebral infarction. Enteric-coated aspirin has already been initiated for its antithrombotic properties which helps in stroke prevention. What this patient needs alongside, however, is antihypertensive therapy for optimal cerebrovascular protection. RESPONSE: The most appropriate next step to decrease this patient’s risk for subsequent cerebral infarction would be to initiate antihypertensive therapy (D).

**AI_A:** (1) RESTATING THE QUESTION: A 57-year-old man who experienced an episode of transient neurological symptoms and has chronic hypertension as well as hypercholesterolemia, now managed by lifestyle choices, informs he's a smoker and has a moderate alcohol consumption. He has no active symptoms during the examination, but is given daily aspirin therapy. The question is the most suitable intervention to reduce his risk of suffering a future stroke, with the choices being stopping alcohol, discontinuing smoking, starting a weight loss program, commencing antihypertensive therapy, or beginning a cholesterol-lowering treatment. (2) PLANNING: The primary causes that contribute to his risk factors include hypertension, hypercholesterolemia, smoking, alcohol and overweight. But among these, hypertension stands out due to it being a significant, modifiable risk factor for stroke, especially if it's long-standing and poorly controlled. Given the patient's stated blood pressure level of 170/90 mm Hg, it appears very elevated. (3) CLINICAL REASONING: Cerebral infarction, also known as ischemic stroke, can be caused by different risk factors such as hypertension, smoking, alcohol abuse, obesity, and hypercholesterolemia. Each patient's individual profile should be considered before deciding on the most suitable intervention. This patient's hypertension is markedly poor with a reading of 170/90 – a high risk factor for stroke, especially one that has been persistently uncontrolled, despite the patient's active adjustments to their lifestyle. The episode the patient reported can be considered a transient ischemic attack (TIA), which can be a precursor to a full-blown stroke. The single largest modifiable risk factor for stroke is hypertension, particularly when poorly controlled. Therefore, managing this hypertension effectively may be seen as an essential priority. (4) CONCLUDING: From the given context and considering the patient's history and current condition, it's necessary to manage his uncontrolled hypertension effectively. Aggressive management of this condition is pivotal in reducing the risk of a subsequent stroke. Therefore, initiating a therapy to manage hypertension can be prioritized. (5) RESPONSE: (D) Antihypertensive therapy is the most appropriate next step to decrease this patient's risk for subsequent cerebral infarction.

**AI_C:** RESTATING THE QUESTION: The patient is a 57-year-old man who experienced a short-lived episode of slurred speech and clumsiness in his right hand during his daily walk. It is crucial to note that he doesn't have a history of similar symptoms. He has hypertension, high cholesterol levels, and other underlying health conditions like a history of smoking and minimal alcohol consumption. Despite these, he shows no neurological abnormalities, but has started daily aspirin therapy. The question seeks to identify the most effective next action to reduce the risk of him experiencing another cerebral infarction. PLANNING: The options provided include alcohol cessation, smoking cessation, weight loss program, antihypertensive therapy, and cholesterol-lowering therapy. Cerebral infarction, often associated with stroke, could be due to several risk factors like smoking, high alcohol consumption, obesity, hypertension, and high cholesterol levels. Here, we reckon with the assumptions that all risk factors may contribute equally to future cerebral infarctions, but this may not necessarily hold true. Therefore, examining the relative impact of each risk factor on cerebral infarction may present a different priority of management. CLINICAL REASONING: Beginning with alcohol cessation, although excessive consumption of alcohol is linked to an increased risk of stroke, the patient’s current consumption can be considered moderate. For the weight loss program, his BMI falls within the overweight category, but it isn't significantly high so as to pose a severe risk for a cerebrovascular event. Regarding smoking cessation, extensive research has demonstrated a strong and direct relationship between smoking and stroke, which increases the relative risk by a significant amount compared to the other factors. This is due, in part, to the increased coagulability and unfavorable changes in lipid profiles associated with smoking. In terms of hypertension, it is well established that uncontrolled hypertension significantly increases the risk of cerebral vascular events. Lastly, even though the patient has hypercholesterolemia, it seems to be under control with diet and exercise. CONCLUDING: Based on the information provided and the clinical reasoning process, it is plausible to posit that the highest risk factors for this patient, given the current information, are his smoking habit and his hypertension. RESPONSE: The patient’s risk for subsequent cerebral infarction could be significantly reduced by addressing his most potent risk factors. Therefore, the most appropriate next step to decrease this patient’s risk for subsequent cerebral infarction is (D) Antihypertensive therapy. Treatment of hypertension is crucial in this scenario as his blood pressure readings are consistently high. Smoking cessation is another critical measure; however, the hypertensive condition needs immediate attention due to its robust correlation with cerebrovascular events.

**AI_D:** (1) RESTATING THE QUESTION: This query revolves around a 57-year-old male patient who experienced a brief episode of speech impairment and right-handed clumsiness. These symptoms, which transpired during his daily walk, dissipated within 24 hours. Besides, the patient has a history of long-standing hypertension and hypercholesterolemia, both of which he manages through diet and exercise. Despite this, he doesn't use any medications but has been a smoker for the past four decades and consumes wine moderately. Further evaluation identifies no current abnormalities, but given his symptoms, a prophylactic treatment with daily aspirin therapy was initiated. The issue at hand is figuring out the most ideal next intervention to mitigate his risk for a future cerebral infarction or stroke. (2) PLANNING: When considering the assumption that the patient's transient neurological symptoms could suggest a transient ischemic attack (TIA), a precursor to stroke, pathogenesis typically lies in vascular insufficiency to a given brain region due to either embolization or local thrombosis. Coronary artery disease and cerebrovascular disease share many similar risk factors, such as hypertension, hypercholesterolemia, smoking, obesity, and alcohol use. So, it's essential to factor in these conditions and health behaviors while strategizing the next steps. (3) CLINICAL REASONING: Each of the proposed interventions (cessation of alcohol, quitting smoking, starting a weight loss program, initiation of antihypertensive therapy, and cholesterol-lowering treatment) has potential benefits but should ideally be placed in the context of the existing risk factors and their relative risk for ischemic stroke. Even though this individual does consume alcohol, it is in moderation and it isn't considered a recognized strong risk factor for ischemic stroke. Similarly, despite being overweight, the patient's BMI is not in the obesity range, which typically raises the risk of stroke. On the other hand, his untreated hypertension, smoking habit, and hypercholesterolemia (even if managed with lifestyle) are all well-established, significant risk factors for stroke. (4) CONCLUDING: To best address the patient's stroke risk, it would be ideal to tackle the strongest, modifiable risk factors first, then proceed to less influential elements. Antihypertensive therapy is vital due to the patient's long-standing history of hypertension, and it's a leading cause of cerebrovascular disease. Furthermore, smoking cessation should also be strived for due to a substantial attributable risk for cerebrovascular disease. (5) RESPONSE: Therefore, while all of the proposed next steps have merits, the priority should be given to initiating antihypertensive therapy (D) to control his high blood pressure, followed by encouraging him to quit smoking (B). These interventions could significantly decrease his chances of experiencing a cerebral infarction in the future.

**Facilitator** The consensus among the AI responses is that the most appropriate next step to decrease the risk of subsequent cerebral infarction for the 57-year-old man, who recently experienced transient neurological symptoms indicative of a transient ischemic attack (TIA), is to initiate antihypertensive therapy. This conclusion is based on the patient's history of chronic hypertension, which is currently uncontrolled as indicated by his elevated blood pressure reading of 170/90 mm Hg. Hypertension is a significant, modifiable risk factor for stroke, especially when poorly controlled. While other risk factors such as hypercholesterolemia, smoking, moderate alcohol consumption, and being overweight are present, the consensus is that managing the patient's hypertension should be prioritized due to its strong association with the risk of cerebral infarctions. Therefore, the most suitable intervention to reduce his risk of suffering a future stroke is (D) Antihypertensive therapy.
