# Supplementary material for: Collaborative intelligence in AI: Evaluating the performance of a council of AIs on the USMLE
Source: PLOS Digit Health. 2025 Oct 9;4(10):e0000787. doi: 10.1371/journal.pdig.0000787 (PMC12510544; doi:10.1371/journal.pdig.0000787)
Supplement: S2 File — (ZIP) [file pdig.0000787.s002.zip › S2 Data Raw data files containing unprocessed question inputs and LLM outputs including transcripts of deliberations/STEP2_Question-48-1_Type-MC-NJ.docx]

**Facilitator** **[Round 1]:** A 27-year-old primigravid woman at 21 weeks’ gestation comes to the emergency department because of a 2-day history of moderate headache, shortness of breath, nausea, vomiting, muscle aches, and malaise. She also has had cough occasionally productive of sputum. Pregnancy had been uncomplicated. She has no history of serious illness, and her only medication is a prenatal vitamin. She immigrated to the USA from India 6 months ago. Temperature is 38.3°C (100.9°F), pulse is 100/min, respirations are 18/min, and blood pressure is 100/60 mm Hg. On examination, breath sounds are decreased at the right lung base. Fundal height is 22 cm. Fetal heart rate is 160/min. Chest x-ray shows right-sided interstitial infiltrates. Which of the following is the most likely infectious agent? (A) Haemophilus influenzae (B) Influenza A virus (C) Legionella pneumophila (D) Mycobacterium tuberculosis (E) Pseudomonas aeruginosa (F) Streptococcus pneumoniae

**AI_A:** RESTATING THE QUESTION: A 27-year-old pregnant woman who has recently immigrated to the USA from India, presents with symptoms such as headache, respiratory discomfort, and malaise. Her physical exam findings indicate a possible lung infection, and she has been asked to identify the most likely infectious agent from a given list. The listed agents are various bacteria and viruses known to cause respiratory tract infections. PLANNING: Given her symptoms, recent immigration from India, and the finding of interstitial infiltrates on chest x-ray, we should consider possible agents that would be common in her demographic and that could cause a similar clinical picture. While all the listed agents can affect the respiratory tract, not all of them align with her recent travel history and specific symptoms. CLINICAL REASONING: The patient's symptoms include systemic signs of infection (fever, malaise, muscle aches) along with a productive cough and respiratory findings, suggesting a lung infection. She also immigrated from India just six months ago, which contributes to a higher likelihood of exposure to certain infectious agents. Among the listed infectious agents, Mycobacterium tuberculosis (D) is a common pathogen in India and should be highly suspected given her immigration history. Additionally, the presence of systemic symptoms and right-sided interstitial infiltrates on chest x-ray are consistent with tuberculosis. CONCLUDING: Upon synthesizing the patient's symptomatology, clinical presentation, imaging findings, and travel history, the clinical picture appears to be most strongly suggestive of Mycobacterium tuberculosis infection. RESPONSE: Therefore, Mycobacterium tuberculosis (D) is the most likely infectious agent in this 27-year-old primigravid woman.

**AI_E:** (1) RESTATING THE QUESTION: This question presents a 27-year-old pregnant woman presenting with symptoms such as headache, shortness of breath, nausea, vomiting, muscle aches, coughing up sputum, and malaise. The clinical indicators combined with her recent immigration from India and a chest X-ray showing right-sided interstitial infiltrates suggest a possible respiratory infection. The question requires identification of the most likely causative pathogen in this clinical scenario. (2) PLANNING: Here, the key points are the symptoms, the chest radiography findings, and the patient's recent immigration from India. The interstitial infiltrates seen on chest radiograph imply respiratory infection, and the infectious agents listed could all, under certain circumstances, result in these signs and symptoms. However, the inclusion of Mycobacterium tuberculosis (M. tuberculosis), a common bacterium in India, stimulates consideration of tuberculosis as a potential cause. (3) CLINICAL REASONING: In choosing the most likely infectious agent, it's important to prioritize assumptions based on the patient's state of health and history. Her recent arrival from a country where tuberculosis is common (India), the prolonged duration (2 days) of her systemic symptoms (malaise, nausea, vomiting, and headaches), and her radiographic findings favor tuberculosis. Tuberculosis often presents with systemic symptoms and can affect pregnant women, particularly those with reduced immune status. The other organisms presented, while capable of causing respiratory infections, do not align as closely with this patient's history and clinical picture. (4) CONCLUDING: Comparing all these findings, the clinical picture aligns most closely with tuberculosis. So, taking into account the patient's individual history, the epidemiology of the disease, and the symptoms presented, M. tuberculosis is the most likely infectious agent. (5) RESPONSE: The most likely infectious agent in this scenario is (D) Mycobacterium tuberculosis.

**AI_D:** (1) RESTATING THE QUESTION: A 27-year-old pregnant woman initially from India presents with systemic symptoms including headache, shortness of breath, and nausea, along with a productive cough and fever. She has decreased breath sounds and right-sided interstitial infiltrates as per the chest X-ray. The query here is to identify the most probable infectious agent causing her symptoms from those listed. (2) PLANNING: The main assumption would be that these symptoms are caused by an infectious disease, given her fever and lung findings. One approach would be to consider each infectious agent individually. We must also factor in her recent immigration status from India. (3) CLINICAL REASONING: With a right-sided interstitial infiltrate as per the chest X-ray and symptoms, a pulmonary infection seems likely. (A) Haemophilus influenzae and (F) Streptococcus pneumoniae are common causes of pneumonia. However, they typically cause lobar pneumonia, not interstitial infiltrates. (B) Influenza A virus could cause systemic symptoms and lung infiltrates, but the clinical presentation is somewhat atypical. (C) Legionella pneumophila often presents as interstitial pneumonia, but usual risk factors such as recent travel or exposure to contaminated water appear not to be present. (E) Pseudomonas aeruginosa is not a common causative agent of pneumonia in healthy individuals and is usually associated with hospital-acquired infections. (D) Mycobacterium tuberculosis is a significant concern because of her recent immigration from India where TB is endemic, and it typically presents as an interstitial lung disease with varying systemic symptoms, fits her presentation well. (4) CONCLUDING: Therefore, considering the interstitial infiltrate seen on chest X-ray, her symptoms, and epidemiological factors (recent immigration from India), Mycobacterium tuberculosis should be highly suspected as the likely infectious agent. (5) RESPONSE: Given these considerations, (D) Mycobacterium tuberculosis is the most likely infectious agent in this clinical scenario.

**AI_B:** RESTATING THE QUESTION: The clinical case presents a 27-year-old pregnant woman in her second trimester, who is experiencing symptoms such as headache, shortness of breath, nausea, vomiting, muscle aches, and a productive cough. She also has fever and an abnormal finding in her chest x-ray that shows a right-sided interstitial infiltrate. Her recent immigration from India is noteworthy. We are tasked with identifying the most probable infectious agent associated with her condition from a list of potential bacterial and viral pathogens. PLANNING: The assumption made in this case is that the woman's symptoms are due to an infectious disease, with the nature of her symptoms and chest x-ray findings suggesting a pulmonary infection. A key point to scrutinize further is her travel history, considering that different regions may have distinctive endemic diseases. Should we factor in her travel history and consider tuberculosis (Mycobacterium tuberculosis) as a possible infectious agent? Or should we consider other common respiratory infectious agents, such as Streptococcus pneumoniae, Haemophilus influenzae, or respiratory viruses like Influenza A? CLINICAL REASONING: The woman's symptoms—headache, shortness of breath, cough productive of sputum and malaise—along with the chest x-ray findings could indicate a pulmonary infection. In a healthy young woman, community-acquired pneumonia (CAP) pathogens, such as Streptococcus pneumoniae (F), would normally be considered. However, we should not ignore her recent immigration from India, a country with a high incidence of tuberculosis (TB). TB, caused by Mycobacterium tuberculosis (D), is characterized by chronic symptoms like cough, fever, night sweats, and weight loss, but it can also present acutely, especially in pregnant women due to the immunological changes occurring during pregnancy. Thus, her history and clinical presentation align more convincingly with a tuberculosis infection. CONCLUDING: Although community-acquired pathogens are common causes of pneumonia, the patient's immigration from a TB-endemic country, together with her symptoms and the chest x-ray finding of right-sided interstitial infiltrates, suggest tuberculosis as a more likely diagnosis in this instance. RESPONSE: Thus, the most likely infectious agent in this case is (D) Mycobacterium tuberculosis.

**AI_C:** 1) RESTATING THE QUESTION: We are given a case of a pregnant, immunocompetent woman hailing from India recently immigrated to the USA, presenting with fever, cough with sputum, breathlessness, general discomfort, headache, and right-sided pulmonary infiltrates on chest imaging. The question aims to identify the most probable pathogenic organism causing her sickness from the choices given. 2) PLANNING: Our main assumptions focus on her recent immigration from India, her pregnancy, and her clinical and radiographic symptoms, which suggest a respiratory infection. We must consider that each of the infectious agents provided can cause pulmonary infections, but their probability depends on various factors such as geographical prevalence of the disease, status of the patient's immune system, course of the disease, and radiographic findings. 3) CLINICAL REASONING: Her recent immigration from India flags Mycobacterium tuberculosis as the differential because India is a high endemic area for tuberculosis (TB), and TB has been known to reactivate under stress or changes in immune system. However, TB usually presents with chronic symptoms over weeks, and her presentation seems acute. Legionella pneumophila, Pseudomonas aeruginosa usually affect people with weak immune systems or chronic lung conditions, which she lacks. Her pregnancy might change her immune status, but not significantly to predispose to these infections. Streptococcus pneumoniae and Haemophilus influenzae also cause community-acquired pneumonia, but she lacks the typical findings of lobar pneumonia associated with them. Influenza A virus though can cause her symptoms, most notably muscle aches, headache, and pulmonary infiltrates. 4) CONCLUDING: So, considering the acute presentation of symptoms, we can cross off Mycobacterium tuberculosis. Her immune system's conditions do not favor infection by Legionella pneumophila and Pseudomonas aeruginosa. The lack of lobar pneumonia findings in radiographs moves us away from Streptococcus pneumoniae and Haemophilus influenzae, hence, Influenza A virus seems to be the most likely infectious agent. 5) RESPONSE: As such, the most probable infectious agent causing her illness seems to be (B) Influenza A virus.

**Facilitator** **[Round 2]:** Summary: AI_A and AI_B both agree that the most likely infectious agent is Mycobacterium tuberculosis (D), given the patient's symptoms, recent immigration from India, and the presence of interstitial infiltrates on her chest x-ray. They both emphasize the importance of considering the patient's travel history and the prevalence of tuberculosis in India. AI_C, however, believes that the most probable infectious agent is Influenza A virus (B). AI_C argues that the patient's acute presentation of symptoms and the lack of typical findings of lobar pneumonia associated with other potential pathogens, such as Streptococcus pneumoniae and Haemophilus influenzae, point towards Influenza A virus as the most likely cause. AI_D agrees with AI_A and AI_B, concluding that Mycobacterium tuberculosis (D) is the most likely infectious agent. AI_D considers the patient's recent immigration from India, the interstitial infiltrate seen on chest X-ray, and the patient's symptoms as key factors leading to this conclusion. AI_E also agrees with AI_A, AI_B, and AI_D, identifying Mycobacterium tuberculosis (D) as the most likely infectious agent. AI_E emphasizes the patient's recent immigration from India, the duration of her systemic symptoms, and her radiographic findings as the main reasons for this conclusion. Question to clarify disagreements: Given that AI_C has identified Influenza A virus as the most likely infectious agent, while AI_A, AI_B, AI_D, and AI_E have identified Mycobacterium tuberculosis, could AI_C elaborate on why the acute presentation of symptoms is more indicative of Influenza A virus, despite the patient's recent immigration from a country where tuberculosis is common? Similarly, could AI_A, AI_B, AI_D, and AI_E explain why they believe the patient's immigration history and systemic symptoms outweigh the acute presentation in their diagnosis of tuberculosis?. Based on your preceding response, how would you answer the original question: [A 27-year-old primigravid woman at 21 weeks’ gestation comes to the emergency department because of a 2-day history of moderate headache, shortness of breath, nausea, vomiting, muscle aches, and malaise. She also has had cough occasionally productive of sputum. Pregnancy had been uncomplicated. She has no history of serious illness, and her only medication is a prenatal vitamin. She immigrated to the USA from India 6 months ago. Temperature is 38.3°C (100.9°F), pulse is 100/min, respirations are 18/min, and blood pressure is 100/60 mm Hg. On examination, breath sounds are decreased at the right lung base. Fundal height is 22 cm. Fetal heart rate is 160/min. Chest x-ray shows right-sided interstitial infiltrates. Which of the following is the most likely infectious agent? (A) Haemophilus influenzae (B) Influenza A virus (C) Legionella pneumophila (D) Mycobacterium tuberculosis (E) Pseudomonas aeruginosa (F) Streptococcus pneumoniae]

**AI_D:** To answer the question, it can be restated like this: given the patient's symptoms, recent immigration from India, and the radiological finding of right-sided interstitial infiltrates, what is the most likely infectious agent causing her symptoms? From the list of possible infectious agents provided, there are a few assumptions. We are assuming that the symptoms are indeed caused by an infectious agent, and we are also assuming that the infectious agent is among the ones listed. We should alternate these assumptions with possibilities that the symptoms might not be infectious in nature, or that the infectious agent could come from outside the listed options. After considering these factors, the assumption that her symptoms are due to an infectious disease and that the infectious agent should be among the given list is the most practical in a clinical setting. Pursuing the prioritized assumption, we should consider the information given. Symptoms such as fever, cough with possible productive sputum, and interstitial infiltrates seen on the x-ray, along with her recent immigration from India, hint towards a particular disease. India has a high prevalence of tuberculosis, and the symptoms along with the radiological findings match that of tuberculosis. Right-sided interstitial infiltrates on the chest X-ray are a characteristic finding in tuberculosis patients. Mycobacterium tuberculosis, which causes tuberculosis, also lines up with the chronicity of her systemic symptoms which include malaise, headache, muscle aches, and nausea. Considering all the findings given, and comparing them to other potential infectious diseases, it could be concluded that Mycobacterium tuberculosis is the most likely infectious agent causing her symptoms. As a response to the question, I would conclude that the most likely infectious agent causing this 27-year-old primigravid woman's symptoms is Mycobacterium tuberculosis (D).

**AI_B:** 1) RESTATING THE QUESTION: This question presents a 27-year-old pregnant woman from India, who is 21 weeks into her first pregnancy. Over the past two days, she's experienced a number of symptoms: moderate headache, shortness of breath, nausea, vomiting, muscle aches, and malaise. She's also had a cough that occasionally produces sputum. Her pregnancy has been uncomplicated, she takes only prenatal vitamins, she has no history of serious illness, and she's been in the USA for six months. Given these details, we are asked to identify the most likely infectious agent causing her condition. 2) PLANNING: Based on the question, we can assume that the woman's symptoms and recent history point toward one of the presented agents. An alternative assumption could be that an agent not listed is responsible. However, given the detail provided, the former should be pursued. We can prioritize finding an agent that fits the woman's profile and the symptoms she presents. 3) CLINICAL REASONING: The woman's presenting symptoms suggest an infection of the respiratory system. The interstitial infiltrates revealed on her chest x-ray supports this diagnosis. The details that she recently immigrated from India and the primarily unilateral presentation (reduced breath sounds and infiltrates on just one side) suggest a specific type of respiratory infection, more particularly tuberculosis which is caused by (D) Mycobacterium tuberculosis. This assumption is further solidified by the fact that tuberculosis is endemic in India and immigrants from such areas are at higher risk. 4) CONCLUDING: Given the symptoms, investigation results and the patient's history of recent immigration from India where tuberculosis is prevalent, the patient most likely has an infection with the Mycobacterium tuberculosis. 5) RESPONSE: Therefore, based on the woman's symptoms, the chest x-ray findings, and her history, Mycobacterium tuberculosis (D) is the most likely infectious agent in this case.

**AI_E:** The question seeks to find out the most probable infectious agent causing a 27-year-old pregnant woman's symptoms, considering her immigration from India, as well as her clinical and radiographical findings. Tuberculosis, caused by Mycobacterium tuberculosis, is endemic in India, making this pathogen a significant consideration. Other potential pathogens listed include Haemophilus influenzae, Influenza A virus, Legionella pneumophila, Pseudomonas aeruginosa, and Streptococcus pneumoniae. Multiple assumptions can be made: one assumption could be that based on her symptoms and x-ray findings, the woman has pneumonia. Another assumption is that because she recently immigrated from India, a region with high TB prevalence, she might be suffering from TB. A third assumption could be that given her current physiological state of pregnancy, she might be more susceptible to typical respiratory pathogens. Prioritizing one assumption over the others requires clinical judgment that takes into account primarily her immigration history, her symptoms, and x-ray findings. The symptoms she presents with - moderate headache, shortness of breath, nausea, vomiting, muscle aches, malaise, and productive cough - can be interpreted in two ways. One way is to see these as systemic symptoms caused by an infection, which including fever, are signs of a systemic response to infection, pointing more towards pathogens causing systemic disease, like Mycobacterium tuberculosis. Alternatively, focusing on the respiratory symptoms alone, one might consider typical respiratory pathogens, like the influenza virus. However, the fact that her x-ray shows right-sided interstitial infiltrates should guide our clinical reasoning. This finding is more indicative of certain types of pneumonia rather than an acute respiratory disease like influenza. Interstitial infiltrate can be caused by multiple pathogens, including Mycobacterium tuberculosis, which is known to be a cause of interstitial pneumonia. Given this line of reasoning, while the acute onset of symptoms may lean some to postulate influenza, the chest radiograph's specific findings coupled with her recent immigration from India six months ago propels Mycobacterium tuberculosis as the most likely infectious agent. Responding to the question directly, while Influenza A virus (B) could be a potential cause considering the acuity of symptom onset, Mycobacterium tuberculosis (D) is the most likely infectious agent in this case, considering her recent travel history, systemic symptoms, and the interstitial infiltrate on her chest x-ray.

**AI_A:** RESTATING THE QUESTION: We're being asked to diagnose which of the listed infectious agents is most likely responsible for the symptoms of a pregnant woman presenting with headache, shortness of breath, nausea, vomiting, muscle aches, malaise, and cough. She immigrated from India six months ago and her symptoms have developed over the past two days. The physical examination and her chest x-ray suggests an infection in her right lung. PLANNING: The key assumptions here are that her symptoms are due to a single infectious agent and that this agent is one of the options listed. The woman's recent immigration from India, where tuberculosis remains a health challenge, and the finding of right-sided interstitial infiltrates on chest x-ray suggest possible tuberculosis [Mycobacterium tuberculosis (D)]. However, her symptoms developed acutely in just two days, which is more characteristic of a rapidly-progressing viral infection, such as influenza [Influenza A virus (B)]. The presence of productive cough, fever, and reduced breath sounds are suggestive of bacterial pneumonia, which could indicate agents such as Streptococcus pneumoniae or Legionella pneumophila. CLINICAL REASONING: Despite the acuteness of her symptoms, the other clinical details make tuberculosis a more probable diagnosis. Mycobacterium tuberculosis (D) is responsible for tuberculosis, a disease characterized by insidious onset and presents with systemic symptoms such as malaise, weight loss, and shortness of breath. Interstitial infiltrates observed in the chest x-ray are consistent with a tuberculosis infection. Furthermore, the patient's history of recent immigration from a country with a high burden of tuberculosis significantly increases the chances of this diagnosis. While influenza virus could be responsible for the acute symptoms, it doesn't usually cause reduced breath sounds or localized interstitial infiltrates seen here. CONCLUDING: Clinically prioritizing an infectious agent will depend on a combination of patient history and characteristic symptoms. In this pregnant woman with constitutional symptoms, sudden onset, recent immigration from India, decreased breath sounds, and right-sided interstitial infiltrates on chest radiography, Mycobacterium tuberculosis appears to be the most likely infectious agent. RESPONSE: Therefore, the most likely infectious agent involved, given the patient's presenting features, is Mycobacterium tuberculosis (D).

**AI_C:** Restating the Question & Definitions: We're attempting to determine the most probable cause of this pregnant patient's illness, given her recent immigration from India, her symptoms, and the findings on physical and radiological exam. A word of caution: each infectious agent presents with different sets of signs and symptoms. Let's expand on them: Mycobacterium tuberculosis often causes chronic cough, night sweats, fever, and weight loss; Influenza A virus can cause acute onset of fever, myalgia, and cough; Legionella pneumophila often causes headache, myalgia, cough, and gastrointestinal symptoms; Streptococcus pneumoniae and Haemophilus influenzae often cause acute lobar pneumonia; Pseudomonas aeruginosa rarely causes pneumonia in healthy individuals and is more associated with pneumonia in hospital settings or patients with cystic fibrosis. Planning: The key assumptions for this case include consideration of the patient's immigration history, her pregnancy status, and the fact that she's presenting with an acute onset of systemic symptoms alongside an abnormal chest x-ray. To generate alternate assumptions, we question the timeline of onset for tuberculosis and influenza. While tuberculosis generally has a slower progression, influenza tends to have a rapid onset. Also, the patient has traveled from a region where tuberculosis is endemic, which increases the likelihood of this diagnosis. Conversely, could the predominance of systemic symptoms, such as myalgia, headache, and malaise over respiratory complaints, point towards influenza? Clinical Reasoning: Given the chest x-ray finding of interstitial infiltrates, the differential diagnosis leans toward diseases that usually present with such a pattern – Mycobacterium tuberculosis and viral infections including Influenza A. Both these organisms could present with symptoms of systemic illness. The fact that our patient recently immigrated from India, where TB is prevalent, further supports tuberculosis. This said it is crucial not to rule out influenza owing to its high incidence and the acute onset of illness. Other agents are less likely given the clinical history and x-ray findings. Concluding & Response: Taking into account the patient's travel history from a high TB prevalence area, her symptoms (many of which, such as muscle aches and malaise, could be part of a systemic inflammatory response), the chest x-ray findings, and the overall clinical picture points towards a common cause of interstitial infiltrates on chest x-ray, Mycobacterium tuberculosis (D) could be the most likely infectious agent in this case. However, pending further diagnostic results, definitive determination and treatment will rely on additional testing to confirm the presence of the TB bacteria.

**Facilitator** The consensus among the AI responses is that the most likely infectious agent causing the symptoms in the 27-year-old pregnant woman, who recently immigrated from India, is Mycobacterium tuberculosis (D). This conclusion is based on several factors. Firstly, the woman's symptoms, including headache, shortness of breath, nausea, vomiting, muscle aches, malaise, and productive cough, are indicative of a systemic infection. Secondly, the chest x-ray findings of right-sided interstitial infiltrates are characteristic of tuberculosis. Lastly, the woman's recent immigration from India, a country with a high prevalence of tuberculosis, significantly increases the likelihood of this diagnosis. While the acute onset of symptoms could suggest a viral infection such as influenza, the specific radiological findings and the patient's immigration history make tuberculosis a more probable diagnosis. Therefore, the consensus is that Mycobacterium tuberculosis (D) is the most likely infectious agent in this case.
