# Supplementary material for: Collaborative intelligence in AI: Evaluating the performance of a council of AIs on the USMLE
Source: PLOS Digit Health. 2025 Oct 9;4(10):e0000787. doi: 10.1371/journal.pdig.0000787 (PMC12510544; doi:10.1371/journal.pdig.0000787)
Supplement: S2 File — (ZIP) [file pdig.0000787.s002.zip › S2 Data Raw data files containing unprocessed question inputs and LLM outputs including transcripts of deliberations/STEP2_Question-49-1_Type-MC-NJ.docx]

**Facilitator** **[Round 1]:** A 7-year-old boy is brought to the physician because of a 1-year history of poor performance in school. His parents say that he is bright, has many friends, and seems to want to do well in school. His teachers report that he seems frustrated with his own progress and his inability to meet the expectations of his parents. Speech fluency and articulation and motor skills are appropriate for age. Physical examination shows no abnormalities. When asked to read during the examination, he has significant difficulty sounding out words he is unfamiliar with. Visual acuity test and audiometry show no abnormalities. Which of the following is the most likely diagnosis? (A) Attention-deficit/hyperactivity disorder (B) Expressive language disorder (C) Intellectual developmental disorder (D) Learning disorder (E) Social anxiety disorder (social phobia) (F) Normal behavior

**AI_C:** RESTATING THE QUESTION: So, a 7-year-old boy presents with complaints of poor academic performance over the past year. While his social skills seem normal, he has difficulty meeting academic expectations. His motor coordination, articulation, and speech fluency are appropriate for his age, and he doesn’t have any abnormalities upon physical examination. Notably, he struggles with sounding out unfamiliar words during reading. Both his visual acuity and auditory tests turned out normal. We need to decide the most viable diagnosis for his condition out of the options given. PLANNING: Considering the provided options, one might first think of attention-deficit/hyperactivity disorder (ADHD), expressive language disorder, intellectual developmental disorder, learning disorder, social anxiety disorder, or normal behavior. The boy's difficulty with reading unfamiliar words (though having normal visual acuity and auditory ability), without exhibiting symptoms of inattention or hyperactivity, trouble with general communication, global cognitive deficits, socially-induced anxieties, or any abnormal findings in physical examination, raises suspicion towards a specific learning disorder. CLINICAL REASONING: The fact that the child is reported to have the desire to do well in school and is frustrated with his lack of progress implies adequate intellect and commitment. The absence of hyperactivity, inattention, or social anxieties along with appropriate speech and motor skills for his age eliminates possibilities like ADHD and social phobia. This directs our focus towards disorders more specifically related to academics, particularly reading. The boy struggles with sounding out unfamiliar words, a key characteristic of dyslexia – a type of learning disorder. CONCLUDING: Therefore, given the description of the child's situation where he is struggling specifically with reading unfamiliar words, with other domains of his life and health seeming normal, this aligns more closely with the case of a specific learning disorder, possibly dyslexia, rather than the other listed options. RESPONSE: The most likely diagnosis for this 7-year-old boy is (D) Learning disorder.

**AI_E:** RESTATING THE QUESTION: The presented scenario features a 7-year-old youngster who is underperforming at school and seems dissatisfied with his own progress. He experiences difficulty pronouncing unfamiliar words, despite having normal vision, hearing, speech fluency, articulation, and motor skills. The question requires me to identify his most likely condition from the given alternatives, which encompass various developmental and behavioral disorders as well as normal behavior. PLANNING: A primary assumption made here is that since the boy's struggles seem to revolve around school performance and reading unfamiliar words, and given that physical exams and vision and hearing checks show no abnormalities, the issue likely lies within a domain related to learning and cognition. However, this does not disregard other alternatives. This boy's condition could potentially result from psychological or behavioral issues (like ADHD or social anxiety), although such disorders often come with additional signs not reported in the presented case. So, we should prioritize exploring the assumption that a learning-related issue is causing the boy's difficulties. CLINICAL REASONING: Addressing the assumption mentioned in the planning step, we notice that the boy's difficulties specifically revolve around reading and sounding out unfamiliar words. The rest of his performance, including social behavior, aspiration for good performance, and age-adequate articulation, speech fluency, and motor skills, seems to be typical. This indicates that his issues are narrowly focused on reading. Released from the constraints of physical or intellectual defects, as the examination and testing outcomes indicate, this could likely point towards a specific learning disorder - Dyslexia. CONCLUDING: In conclusion, analyzing the presented case and the boy's specific difficulties with reading, together with the results of physical examination and testing, point towards a conclusion that he may have a specific learning disorder such as Dyslexia. Dyslexia is marked by difficulties with accurate and fluent reading and poor spelling and decoding abilities, keeping in alignment with the boy's difficulties sounding out unfamiliar words. The boy's otherwise normal development and social behaviour is also a characteristic feature of specific learning disorders. RESPONSE: The most likely diagnosis for this 7-year-old boy is (D) Learning disorder. It's important to note that early identification and adequate support and coping strategies can help the boy continue to progress in school and other aspects of life.

**AI_D:** (1) RESTATING THE QUESTION: In this scenario, a 7-year-old boy is performing poorly at school, despite indications from his parents that he is intelligent and shows a desire to do well. He experiences frustration due to his inability to fulfill his parents' expectations. He has no trouble with speech fluency, articulation, or motor skills and there are no abnormal physical findings. During a reading test, he struggles with unfamiliar words. However, testing rules out visual and hearing impairments. The task here is to identify the condition that best explains the boy's symptoms, given the options of ADHD, expressive language disorder, intellectual developmental disorder, learning disorder, social anxiety disorder, or normal behavior. (2) PLANNING: Considering the boy's difficulty in reading unfamiliar words coupled with normal physical, visual, and auditory examination results, this would suggest a specific learning difficulty. Let's evaluate that assumption by looking at the provided options: (A) ADHD typically involves problems with inattentiveness, impulsivity, and hyperactivity, none of which are reported here. (B) An expressive language disorder means difficulty expressing thoughts, feelings, or ideas verbally, which doesn't seem to be the case here. (C) Intellectual developmental disorder would manifest as significantly below average intellectual and adaptive functioning; however, the boy in question is described as bright. (D) A learning disorder usually involves difficulty learning and using academic skills, which seems to be the case here. (E) Social anxiety disorder is characterized by extreme fear of social or performance situations, which doesn't seem to fit this case. (F) Normal behavior wouldn't account for the boy's struggle with reading. (3) CLINICAL REASONING: Although all these assumptions are plausible, the assumption of a learning disorder seems most congruent with the given information. Considering this child's struggle with reading unfamiliar words, alongside his average abilities in other areas and his lack of sensory problems, a specific learning disorder seems most likely. This is further supported by the fact that the child's social abilities and behavior are reported as normal. (4) CONCLUDING: Thus, by systematically examining the clinical information and comparing it with characteristics of the listed conditions, a specific learning disorder - which is characterized by a difficulty learning or using an academic skill that is not accounted for by intellectual disabilities, sensory problems, or unadjusted environmental situations - fits the boy's symptoms best. (5) RESPONSE: Given these points, the most likely diagnosis for this 7-year-old boy's academic difficulties would be (D) Learning disorder.

**AI_B:** RESTATING THE QUESTION: This query revolves around a 7-year-old boy who has been struggling in school despite having a desire to perform well, exhibiting normal social skills, and lacking any physical abnormalities. Notably, he has difficulty reading and sounding out unknown words, but his visual and auditory tests are normal. The task at hand is to figure out the most accurate diagnosis from the given options: Attention-deficit/hyperactivity disorder, Expressive language disorder, Intellectual developmental disorder, Learning disorder, Social anxiety disorder (social phobia), or Normal behavior. PLANNING: In this context, it is necessary to question each potential diagnostic assumption regarding its relevance to the presented symptoms. Attention-deficit/hyperactivity disorder typically includes symptoms of impulsivity, inattention, and hyperactivity, none of which are highlighted in the description of the child. Expressive language disorder generally involves difficulty expressing oneself through speech, whereas this child shows appropriate speech fluency and articulation. An intellectual developmental disorder suggests below-average intellectual functioning and adaptive ability, but the child described here is considered bright by his parents. Social anxiety disorder would usually present with fear or anxiety about social situations, which is not the case with this boy, as he has many friends. Normal behavior does not seem likely given the child's difficulty with reading unfamiliar words which is causing distress and a year-long poor academic performance - this surpasses what can be expected from typical developmental variations. Upon reconsidering the options against the described symptoms, the assumption that this boy may have a Learning disorder seems the most promising. This condition can cause problems with reading, writing, and spelling, which aligns with the case description. CLINICAL REASONING: A Learning disorder (LD) is characterized by performance in reading, writing, or mathematics that is significantly below the expected level for a person's age, schooling, and level of intelligence. While this is a broad category, there is a subtype of Learning disorder called Dyslexia (a reading disorder), which can be characterized by difficulties with accurate or fluent word recognition, poor decoding abilities, and weak spelling skills. Children with dyslexia often struggle in school despite having at least average intelligence, as seems to be the case with the boy in the scenario. It's important to note that LDs are not due to sensory (audio or visual) defects, intellectual disabilities, or a lack of educational opportunities, making this a likely diagnosis given the presented clinical picture and the elimination of other plausible conditions. CONCLUDING: The patient’s normal visual and auditory function, combined with appropriate speech fluency, articulation, and motor skills for his age, point away from intellectual and sensory impediments as the cause for his academic struggles. His difficulty sounding out unfamiliar words and his frustration with his academic progress - despite having the drive to succeed - strongly suggest a specific learning disorder, likely dyslexia. RESPONSE: Given the aforementioned reasoning process, the most probable diagnosis for the 7-year-old boy would be (D) Learning disorder.

**AI_A:** RESTATING THE QUESTION: The scenario describes a 7-year-old boy who appears to have good social skills and ambition to do well academically but has been displaying a pattern of poor school performance for a year. Despite good speech and motor skills and no physical abnormalities, he has a significant difficulty in reading especially words he is not familiar with. All evaluations such as vision and hearing tests are typical. The task is to identify the most plausible diagnosis for this child from a list of options that includes Attention-deficit/hyperactivity disorder, Expressive language disorder, Intellectual developmental disorder, Learning disorder, Social anxiety disorder or simply normal behavior. PLANNING: In this case it's important to consider the specific query - which is the most likely diagnosis, with focus on the fact that the boy has difficulty in reading unfamiliar words but speech fluency, articulation and motor skills are appropriate for age. This could suggest a learning disorder, specifically a reading disorder ('dyslexia'). Others conditions like ADHD, expressive language disorder, intellectual developmental disorder, social anxiety disorder or normal behavior might be less likely based on the provided information. CLINICAL REASONING: Let's explore each assumption in context of the conditions listed: 1) Attention-deficit/hyperactivity disorder might be an assumption because it can cause poor academic performance. However, kids with ADHD typically have difficulty focusing, are impulsive, and might have hyperactivity - symptoms not explicitly presented in this case. 2) Expressive language disorder might be assumed due to the reading difficulty described. But in this disorder, a child has trouble with verbal expression and not with reading or understanding language. 3) Intellectual developmental disorder could be considered because of the poor academic performance. However, these children typically have deficiency in both intellectual functioning and adaptive behavior, not just difficulty in reading. 4) Learning disorder might be the assumption because learning disorders are neurodevelopmental in nature that affect cognitive processes related to learning, specifically in reading, writing, or mathematics. 5) Social anxiety disorder might be an assumption because the child might be anxious about his academic performance. However, there's no report of the child showing anxiety in social situations or avoidance. 6) Normal behavior might be an assumption since children can temporarily struggle academically. However, 'normal behavior' wouldn't typically involve a one-year-long, consistent difficulty in a specific skill area like reading. CONCLUDING: After considering each condition in the list and applying clinical reasoning, while also considering the criteria for each disorder, the most likely diagnosis in this case is a learning disorder. The child does not exhibit signs of ADHD, has no difficulties with verbal expression that would suggest expressive language disorder, has not have a general intellectual deficiency as indicated by intellectual developmental disorder, presents no signs of social anxiety, and the behavior described is not typicially associated with 'normal' behavior fluctuations. RESPONSE: So, the most likely diagnosis should be (D) Learning disorder. This is because the main struggle for this child is with sounding out unfamiliar words (a skill related to decoding, which is required for reading), while his other cognitive, motor and social skills appear typical for his age.

**Facilitator** The consensus among the AI responses is that the most likely diagnosis for the 7-year-old boy described in the scenario is a Learning Disorder (D). This conclusion is based on the boy's specific difficulty with reading unfamiliar words, despite having normal vision, hearing, speech fluency, articulation, and motor skills. Other potential diagnoses such as Attention-deficit/hyperactivity disorder, Expressive language disorder, Intellectual developmental disorder, Social anxiety disorder, and Normal behavior were considered less likely due to the absence of their typical symptoms in the boy's presentation. The boy's struggle with reading, coupled with his desire to do well in school and frustration with his progress, aligns with the characteristics of a specific learning disorder, possibly dyslexia. It's important to note that early identification and appropriate support can help the boy continue to progress in school and other aspects of life.
