# Supplementary material for: Collaborative intelligence in AI: Evaluating the performance of a council of AIs on the USMLE
Source: PLOS Digit Health. 2025 Oct 9;4(10):e0000787. doi: 10.1371/journal.pdig.0000787 (PMC12510544; doi:10.1371/journal.pdig.0000787)
Supplement: S2 File — (ZIP) [file pdig.0000787.s002.zip › S2 Data Raw data files containing unprocessed question inputs and LLM outputs including transcripts of deliberations/STEP2_Question-5-1_Type-MC-NJ.docx]

**Facilitator** **[Round 1]:** A 16-year-old boy is brought to the emergency department because of a 2-day history of fever, nausea, vomiting, headache, chills, and fatigue. He has not had any sick contacts. He underwent splenectomy for traumatic injury at the age of 13 years. He has no other history of serious illness and takes no medications. He appears ill. His temperature is 39.2°C (102.5°F), pulse is 130/min, respirations are 14/min, and blood pressure is 110/60 mm Hg. On pulmonary examination, scattered crackles are heard bilaterally. Abdominal examination shows a well-healed midline scar and mild, diffuse tenderness to palpation. Which of the following is the most appropriate next step in management? (A) Antibiotic therapy (B) Antiemetic therapy (C) CT scan of the chest (D) X-ray of the abdomen (E) Reassurance

**AI_C:** RESTATING THE QUESTION: The question is about a 16-year-old male patient who is brought to the emergency department due to a 2-day history of fever, nausea, vomiting, headache, chills, and fatigue. The patient had a splenectomy at the age of 13 due to a traumatic injury and appears to be currently unwell. Clinical examinations indicate elevated temperature and pulse, normal respirations and blood pressure, presence of crackles in the lungs, and a tender abdomen with a scar from the splenectomy. The question seeks the most appropriate management for this patient given these symptoms and medical history. PLANNING: The symptoms displayed by the patient suggest an infection which could be localized or systemic. The absence of the spleen, an essential organ in the immune response, leaves him vulnerable to encapsulated bacteria, such as Streptococcus pneumoniae, Haemophilus influenzae, and Neisseria meningitidis. Antibiotic therapy would be logical if an infection is assumed. However, antiemetic therapy might be considered based on the presenting symptoms of nausea and vomiting. CT scan of the chest could evaluate lung involvement due to reported crackles. An X-ray of the abdomen could rule out post-surgical complications. Reassurance would be an unlikely approach given the patient's serious symptoms. CLINICAL REASONING: While other investigations like CT scan of the chest and X-ray of the abdomen can provide further diagnostic information, considering that the patient is febrile, has pulmonary symptoms, and is post-splenectomy, starting antibiotic therapy might be crucial due to the increased risk of severe bacterial infections in asplenic patients. The scattered crackles in lungs might suggest pneumonia, which can be caused by encapsulated bacteria. Again, antiemetic therapy might help in alleviating vomiting, but doesn't address the underlying issue. CONCLUDING: The boy's symptoms of high fever, nausea, vomiting, headache, chills, and fatigue suggest an infectious process, possibly pneumonia. His history of splenectomy is significant because the spleen plays a substantial role in fighting encapsulated bacteria, which asplenic individuals have a higher risk of acquiring septicemia from. Therefore, it seems important to start antibiotic therapy immediately, potentially targeting potential encapsulated bacteria, even before results from further diagnostic tests are available. RESPONSE: The most appropriate next step in the management of this patient would be to start (A) Antibiotic therapy.

**AI_B:** (1) RESTATING THE QUESTION: The inquiry is essentially asking the next appropriate step in managing a febrile 16-year-old boy who recently underwent a splenectomy three years ago and is presenting symptoms suggestive of infection, such as fever, nausea, vomiting, headache, chills, and fatigue, alongside physical findings of ill appearance, and evidence of pulmonary involvement like scattered bilateral crackles. Note that a splenectomy is a surgical removal of the spleen, an organ that plays an integral part in immune response, particularly in the clearance of encapsulated bacteria. (2) PLANNING: There are several assumptions that we can make based on this presentation. We could hypothesize that the cause of his illness could be an infection, dietary or environmental exposure, or a post-surgical complication, among others. However, given his history of splenectomy, the likelihood of infection, particularly with encapsulated bacteria, should be prioritized. This assumption is supported by his symptoms, physical examination findings, and his pronounced post-splenectomy status, which makes him more susceptible to infections. (3) CLINICAL REASONING: In individuals who have had a splenectomy, there is an increased risk of infections, especially those caused by encapsulated bacteria such as Streptococcus pneumoniae, Neisseria meningitidis, and Haemophilus influenzae. The spleen plays a crucial role in the immune system's response to such organisms. Since this young man has multiple symptoms indicative of a systemic infection, including fever, nausea, vomiting, headache, chills, and fatigue, the symptoms, combined with his history and current examination findings, strongly suggest an infectious etiology. The presence of scattered bilateral crackles on lung auscultation further supports a potential respiratory infection. (4) CONCLUDING: Considering his history of splenectomy, critical role in handling encapsulated bacteria, and the potential severity of systemic bacterial infections, empiric antibiotic treatment should be promptly initiated while further diagnostic work-up, such as blood cultures or chest imaging, is ongoing. Delaying antibiotic treatment could have serious, potentially life-threatening consequences, considering his increased susceptibility to overwhelming postsplenectomy infections (OPSI). (5) RESPONSE: Therefore, the most appropriate next step in the management of this patient would be (A) Antibiotic therapy. This should be performed with urgency given the patient's vulnerability to serious systemic infections due to his asplenic state.

**AI_E:** (1) RESTATING THE QUESTION: You're seeking advice on how to manage a 16-year-old boy presenting with symptoms of fever, nausea, vomiting, headache, chills, and fatigue. He looks unwell and has a history of splenectomy. His vital signs indicate tachycardia and fever, and an examination reveals scattered pulmonary crackles and mild abdominal tenderness. The key options for management seem to include initiating antibiotic or antiemetic therapies, performing imaging studies or simply reassurance. (2) PLANNING: Key assumptions here involve the boy's symptoms and his history of splenectomy. Assumptions might be that his symptoms may be related to an infection, which could be systemic or located in specific organs (e.g., chest or abdomen) since he displays both scattered crackles (often associated with pulmonary infections) and abdominal tenderness. However, another assumption relates to his history of splenectomy, which may increase his susceptibility to certain infections due to altered immune function. When we consider alternative assumptions, we can also evaluate his symptoms as possibly non-infective, perhaps related to another systemic illness or condition. Prioritizing clinically, we need to address the immediacy of managing a potential infection versus procedural diagnostics (i.e., imaging studies). (3) CLINICAL REASONING: Taking onboard this boy's history of splenectomy, he's likely immunocompromised in a particular way; the spleen plays a crucial role in fighting certain bacterial infections especially encapsulated bacteria such as Streptococcus pneumoniae, Haemophilus influenzae, and Neisseria meningitidis. Consequently, his symptoms may well indicate an infection caused by these bacteria. The presence of fever and tachycardia supports this notion, as does the presence of scattered crackles on chest exam, which could indicate a pulmonary infection (e.g., pneumonia). Antibiotic therapy should be initiated as quickly as possible given these signs and his immunocompromised state, especially before blood cultures arise if sepsis is suspected. (4) CONCLUDING: While other diagnostic tests could be useful in further delineating the exact disease process, given the urgent need to treat what is highly likely to be a bacterial infection in this asplenic patient, the next appropriate step should be to begin empiric antibiotic therapy targeting the potential causative pathogens. (5) RESPONSE: Based on the clinical history and current presentation, the most appropriate next step would be to provide (A) Antibiotic therapy to manage the potential bacterial infection.

**AI_A:** (1) RESTATING THE QUESTION: We are presented with a 16-year-old male who has been experiencing several non-specific symptoms including fever, nausea, vomiting, headache, chills, and fatigue for the past two days. These symptoms, especially when combined with his elevated pulse, indicate a systemic disease process that is mounting significant stress on his body. It is critical to note that he underwent a splenectomy following a traumatic injury three years ago. The spleen is an organ that filters blood and helps the body fight infections, especially those caused by certain types of bacteria like Streptococcus pneumoniae, Haemophilus influenzae, and Neisseria meningitidis. Its absence might be compromising his immune function, making him more susceptible to these types of infections. Furthermore, scattered crackles heard on pulmonary examination suggests a potential infectious process in the lungs. We are asked to identify the most appropriate next step in his management. (2) PLANNING: The main assumption in this scenario is that his symptoms are due to an infection, possibly bacterial, given his history of splenectomy. An alternate assumption could be that his symptoms are a result of another systemic process, such as an autoimmune disease or malignancy, although these would be less common in his age group and might not align with the result of his pulmonary exam. Considering the severity of his symptoms and his immune-compromised state due to the absence of the spleen, it is essential to prioritize managing the likely infection first. (3) CLINICAL REASONING: This patient's history of splenectomy makes him significantly more prone to specific types of bacterial infections, including those that could lead to conditions like pneumonia or meningitis - both which can present with fever, chills, fatigue, and other systemic symptoms seen in this patient. The presence of bilateral scattered crackles on pulmonary examination could signify a potential bacterial pneumonia, a common complication in asplenic individuals. Furthermore, the absence of medications reduces the likelihood of his symptoms being side-effects and redirects the diagnostic strategy towards a bacterial cause. (4) CONCLUDING: Hence, based on the clinical picture, the underlying history of splenectomy, and the physiological functions of the spleen, there is a high probability that this patient is suffering from a bacterial infection. His clinical status, including his high temperature and tachycardia, suggest this infection is severe, and it possibly involves his lungs given the physical examination findings. Therefore, prompt initiation of antibiotic therapy is crucial. (5) RESPONSE: With that in mind, the most appropriate next step in the management of this patient would be (A) Antibiotic therapy. Other diagnostic tests or therapies should not delay the initiation of antibiotics given the patient's precarious immunological state and severity of symptoms.

**AI_D:** (1) RESTATING THE QUESTION: A 16-year-old male presents to the emergency department with signs of an infection, including fever and bilateral pulmonary crackles. He's also experiencing nausea, vomiting, headache, chills, and fatigue. He had his spleen removed as a result of traumatic injury at the age of 13 and doesn't take any medications. His clinical indications require a subsequent step in management. The spleen's function involves filtering antigens, producing antibodies, and storing white blood cells, all of which are critical in controlling infections. (2) PLANNING: There are several assumptions in play here. The first is that his symptoms are due to an infection that has taken place - potentially a severe one, given his asplenic state. Another assumption could be that his symptoms are just viral and self-limiting, not requiring any specific treatment. Lastly, we can assume that his symptoms might be due to a non-infectious cause such as a metabolic, auto-inflammatory, or neurological disorder. Given that he is post-splenectomy and demonstrating multiple signs of systemic infection, including fever and alterations of his pulmonary examination (crackles), we should primarily consider the infection hypothesis. (3) CLINICAL REASONING: The high fever and systemic symptoms, in conjunction with the unusual sonorous, rattling sounds heard during the lung examination, symbolize a probable infection. Since the patient does not have a functioning spleen, his immunological response to combat this potential infection may be altered, leading to an accelerated and more serious disease process. Patients without a functioning spleen are specifically at risk for overwhelming postsplenectomy infection (OPSI), which is often caused by encapsulated bacteria like Streptococcus pneumoniae, Haemophilus influenzae, and Neisseria meningitidis. This scenario typically presents as a sudden-onset, severe systemic infectious disease and can quickly progress to sepsis if not properly managed. Therefore, starting him on antibiotic therapy immediately would be the most appropriate next step to handle such a potentially life-threatening condition. Reviewing the other options, anti-emetic would tackle one of his symptoms (vomiting), but it wouldn't address the possible infection. A CT scan or an abdominal x-ray could help visualize the lungs or abdomen but would delay potentially life-saving treatment. Finally, reassurance would be inappropriate given the high likelihood of a serious infection. (4) CONCLUDING: Based on his clinical features and the fact that he has had a splenectomy, the most probable diagnosis is a severe systemic infection, which may progress to OPSI if not promptly treated. Therefore initiating broad-spectrum antibiotics, with coverage for organisms that often lead to OPSI in splenectomy patients, is the most appropriate subsequent step in his management, while further diagnostic testing to isolate the causal organism is being pursued. (5) RESPONSE: The most appropriate next step in the management of this patient is (A) Antibiotic therapy. This is due to the clinical suspicion of a severe systemic infection, which could progress rapidly, especially considering his asplenic condition. Thus, immediate empirical antibiotics are warranted.

**Facilitator** The consensus among the AI responses is that the most appropriate next step in the management of the 16-year-old male patient, who presents with fever, nausea, vomiting, headache, chills, and fatigue, is to initiate antibiotic therapy. This decision is based on the patient's history of splenectomy, which makes him more susceptible to infections, particularly from encapsulated bacteria such as Streptococcus pneumoniae, Haemophilus influenzae, and Neisseria meningitidis. His symptoms, combined with physical examination findings of scattered bilateral crackles in the lungs, suggest a systemic bacterial infection, possibly pneumonia. Given the severity of his symptoms and his compromised immune state due to the absence of the spleen, it is crucial to manage the likely infection promptly. While further diagnostic tests could provide additional information, they should not delay the initiation of antibiotics due to the potential severity of the infection and the patient's increased risk of overwhelming postsplenectomy infections. Therefore, the consensus is that the most appropriate next step in the management of this patient is (A) Antibiotic therapy.
