# Supplementary material for: Collaborative intelligence in AI: Evaluating the performance of a council of AIs on the USMLE
Source: PLOS Digit Health. 2025 Oct 9;4(10):e0000787. doi: 10.1371/journal.pdig.0000787 (PMC12510544; doi:10.1371/journal.pdig.0000787)
Supplement: S2 File — (ZIP) [file pdig.0000787.s002.zip › S2 Data Raw data files containing unprocessed question inputs and LLM outputs including transcripts of deliberations/STEP2_Question-50-1_Type-MC-NJ.docx]

**Facilitator** **[Round 1]:** A 23-year-old woman who is an active-duty enlisted airman in the US Air Force comes to the base clinic because of a 4-week history of an itchy sensation in her right ear. She has not had ringing in her ears or hearing loss. She works as a fuel specialist and at first attributed her symptom to the earplugs she uses when she is on the flight line. The symptom has not improved despite the patient's wearing headphones instead of the earplugs during the past 2 weeks. She has no history of serious illness and takes no medications. Vital signs are within normal limits. During examination, pulling of the right pinna inferiorly to examine the ear canal produces pain. The right ear canal appears erythematous and edematous. The tympanic membrane cannot be fully visualized because of the presence of cerumen. Examination of the left ear shows no abnormalities. Which of the following is the most likely diagnosis? (A) Cerumen impaction (B) Ear canal trauma (C) Otitis externa (D) Polychondritis (E) Seborrheic dermatitis

**AI_B:** RESTATING THE QUESTION: A 23-year-old healthy woman has been experiencing an itch in her right ear for the past 4 weeks. The sensation has not improved even though she substituted her earplugs with headphones. No other symptoms, like ringing or hearing loss, accompany this irritation. Vital signs are normal, and her medical history doesn't note any serious illnesses or medications. On examination, her right ear appears inflamed, and the movement of her ear causes her pain. Her tympanic membrane isn't clearly visible due to the presence of earwax. Her left ear seems normal. We're asked to consider the most plausible diagnosis from a given list. PLANNING: Looking at the options given, we have cerumen impaction, ear canal trauma, otitis externa, polychondritis, and seborrheic dermatitis. We need to consider how the patient's symptoms align or do not align with each possible condition. CLINICAL REASONING: Cerumen impaction could be a possibility since there was mention of cerumen obstructing the view of the tympanic membrane. Nevertheless, this condition doesn't typically cause an inflamed ear canal. Ear canal trauma could potentially cause inflammation and pain, but the patient's symptoms have persisted for 4 weeks, which seems long for trauma-related symptoms, provided there would be no ongoing cause of injury. Otitis externa characteristically causes ear canal inflammation and discomfort, which can be exacerbated by moving the ear or pinna. Polychondritis involves the inflammation of cartilage throughout the body and usually has other accompanying symptoms, such as joint pain or nose inflammation, which are not present in this patient. Finally, seborrheic dermatitis is a condition that causes flaky, itchy skin, but it's not specific to the ear and wouldn't selectively affect only one ear without affecting other areas. CONCLUDING: With the information available, it seems otitis externa aligns most closely with the patient's symptoms. It accounts for the persisting itch, the inflamed ear canal, and the enhanced pain when the ear is moved. RESPONSE: Based on the clinical history, examination, and symptom duration, the most likely diagnosis for this active-duty Air Force servicewoman is otitis externa (Choice C).

**AI_D:** RESTATING THE QUESTION: In question, a young, otherwise healthy woman, who serves in the US Air Force, experiences an itchy sensation in her right ear for roughly a month, without any hearing impairment or tinnitus. She initially linked these symptoms to wearing earplugs at her job as a fuel specialist. Changing to headphones did not provide any substantial relief. Examination revealed a painful and inflamed ear canal with a cerumen buildup that obstructed complete visualization of the tympanic membrane. The possible diagnoses are: cerumen impaction, ear canal trauma, otitis externa, polychondritis, and seborrheic dermatitis. PLANNING: Cerumen impaction could cause an itchy sensation but it is not usually associated with an erythematous, painful, and swollen ear canal. Ear canal trauma typically follows a history of direct injury or invasive procedures, neither of which are present in this case. Otitis externa, inflammation of the outer ear and ear canal, can be caused by prolonged water exposure or by trauma. Polychondritis, a rare disease involving cartilage inflammation, typically impacts multiple sites and shows other systemic symptoms. Lastly, seborrheic dermatitis presents with greasy, yellowish scales and typically affects areas rich in sebaceous glands, not solely the ear canal. CLINICAL REASONING: Given the patient's profession, she likely relies on hearing protection measures such as headphones or earplugs. The change from earplugs to headphones did not improve the symptoms, which implies the source of her discomfort is not solely due to foreign bodies in the ear canal. The pain, erythema, and edema suggest inflammation and infection rather than mere cerumen impaction. The absence of systemic illness or multiple site involvement makes conditions like polychondritis unlikely. Her presentation isn't typically associated with the distribution or presentation of seborrheic dermatitis. CONCLUDING: Taking all this into account, otitis externa seems to be the most probable diagnosis. This condition could result from frequent usage of earplugs or headphones that could create a moist environment promoting bacterial growth or cause minor trauma allowing pathogen entry. RESPONSE: The most likely diagnosis for the patient is (C) Otitis externa.

**AI_E:** 1) RESTATING THE QUESTION: The clinical vignette describes a 23-year-old woman, who is a fuel specialist for the US Air Force, presenting with a 4-week history of an itchy sensation in her right ear. Although she has been using earplugs when she is on the flight line, she switched to wearing headphones for the previous 2 weeks, but this did not improve her symptoms. She has no history of serious illnesses and does not take any medications. She experiences pain when the right pinna is pulled inferiorly for examination, and the right ear canal appears erythematous and edematous. The tympanic membrane is not fully visible due to the presence of cerumen. The left ear appears normal. The question is asking which of the following potential diagnoses listed is the most likely explanation for her symptoms and findings. 2) PLANNING: We need to consider ear-related conditions that could cause itching and inflammation. The possible diagnoses given are cerumen impaction (accumulation of earwax), ear canal trauma, otitis externa (inflammation of the outer ear and ear canal), polychondritis (an autoimmune disorder where cartilage in the body becomes inflamed), and seborrheic dermatitis (a common skin disease that mainly affects the scalp, causing itchy, sore skin with white to yellowish scales). We should bear in mind her occupational exposure, particularly use of earplugs, as potential contributors to her condition. 3) CLINICAL REASONING: The patient's main symptom is itching of the ear, which continued despite switching from earplugs to headphones, suggesting an alternate cause. The physical examination findings suggest inflammation of the ear canal (redness and swelling). The symptoms and examination results do not suggest otologic trauma or hearing loss, making ear canal trauma and cerumen impaction less likely. Polychondritis and seborrheic dermatitis typically present with other symptoms – joint pain and skin changes in other areas, respectively – which are not mentioned in this case. 4) CONCLUDING: Under the circumstances and considering the patient's occupational exposure to potential irritants due to the use of earplugs, the symptoms of ear itching along with the physical examination findings of an erythematous and edematous ear canal suggest a diagnosis of otitis externa, an inflammation or infection of the outer ear and ear canal, commonly associated with prolonged wetness in the ear or skin breakdown due to various causes (e.g., earplug use, swimming). 5) RESPONSE: Therefore, the most likely diagnosis for this patient's condition is (C) Otitis externa.

**AI_C:** RESTATING THE QUESTION: A 23-year-old military woman has come to the clinic due to an ongoing itchiness in her right ear that has persisted for about a month. She doesn't have any hearing loss or ringing in her ears but does experience pain when her ear is manipulated for examination. Her job role involves frequent use of earplugs, which she initially thought was the cause of her discomfort. Regardless of changing her ear protection to headphones, the symptoms have not improved. Signs such as erythema and edema are noticeable in the right ear upon examination, and there is cerumen obstructing the full view of the tympanic membrane, while her left ear is normal. We need to determine the most suitable diagnosis from the options provided: cerumen impaction, ear canal trauma, otitis externa, polychondritis, or seborrheic dermatitis. PLANNING: The likely assumptions are that her symptoms could be due to the frequent use of ear protection devices such as earplugs and headphones as they can traumatize the ear canal, cause cerumen impaction, or trigger otitis externa. Alternatively, her symptoms might be related to her job as a fuel specialist, exposing her to chemicals and allergens which could contribute to skin conditions such as seborrheic dermatitis or polychondritis. CLINICAL REASONING: Cerumen impaction typically presents with hearing loss, which this patient is not experiencing. Moreover, pulling on the pinna causing pain, accompanied by erythema and edema, suggests inflammation beyond just cerumen impaction. Ear canal trauma might seem likely at first glance given the patient's use of earplugs and headphones. However, such trauma usually causes direct lesions or damage as opposed to symptoms that persist for four weeks. Polychondritis, a multisytem inflammatory disorder, usually presents with inflammation of various cartilaginous structures, notably in the ears, nose, joints, and respiratory tract. But this patient's symptoms are localized. Seborrheic dermatitis typically involves the scalp, face, or chest and is characterized by redness and scaly patches, which are absent in this case. Otitis externa (swimmer's ear), on the other hand, presents with itching, redness, and swelling of the external ear canal, often with pain exacerbated by tugging on the outer ear or pressing on the tragus, and can be associated with exposure to water or mechanical trauma (e.g., from earplugs or hearphones). CONCLUDING: Based on her symptomatology, exposure history, and physical examination findings (especially since her pain is exacerbated when pulling the pinna, with presence of erythema and edema), an assumption of otitis externa would be clinically sound to pursue. RESPONSE: The most likely diagnosis for the young airwoman suffering from an itchy sensation and persisting discomfort in her right ear, accompanied by redness and swelling of her external ear canal, is otitis externa (C).

**AI_A:** (1) RESTATING THE QUESTION: A 23-year-old woman has been experiencing an itchy sensation in her right ear for the past four weeks. The suspect for the culprit of her discomfort was her earplugs, but even after ditching them for headphones, she still experienced the discomfort. Examining her ear showed inflammation and the presence of excess earwax, also known as cerumen. What could be causing this recurrent discomfort? When discussing ear-related health issues, there are several possibilities that need to be noted: (A) Cerumen impaction refers to the blockage of the ear canal due to an excessive build-up of earwax. (B) Ear canal trauma refers to any injury to the ear canal, such as from inserting a foreign object. (C) Otitis externa, also known as swimmer's ear, is an inflammation of the outer ear and ear canal. (D) Polychondritis is an uncommon, multi-system inflammatory disorder characterized by recurrent episodes of inflammation and deterioration of cartilage. The pinna, or outer ear, can be affected. (E) Seborrheic dermatitis is a common skin condition that primarily affects the sebaceous gland-rich areas of the body, such as the scalp, face, and trunk. (2) PLANNING: Initially, it would be easy to conclude that the patient's symptoms are due to the usage of earplugs or headphones leading to cerumen impaction. However, other symptoms don't line up. Though cerumen impaction might prevent full visualization of the tympanic membrane, the patient's report of pain when moving the pinna and the characteristic appearance (erythematous and edematous) of the ear canal upon examination make it necessary to rethink our assumptions. In reevaluating the assumptions, we need to consider the inflammation, pain, and cerumen build up as part and parcel of another condition rather than unrelated symptoms. (3) CLINICAL REASONING: Given the patient's profession, an active-duty airman, it could be assumed that she might be exposed to various environmental factors that could impact her health. Her profession requires her to be outdoors frequently, and she may be exposed to foreign particles, weather conditions, and pollutants that could affect her ears. The other cause could be related to her ear plug usage as she initially thought. However, switching to headphones and experiencing no relief, we should consider another pathogenetic factor. Inflammatory conditions of the ear, such as otitis externa, also raise the possibility here. The symptom pattern of erythema and edema both suggests inflammation, as does the patient's localized pain when moving the pinna. (4) CONCLUDING: An inflammation-related diagnosis could account for both the painful condition of the pinna and the blocked view of the tympanic membrane. Occupationally related conditions such as cerumen impaction and ear canal trauma seem less likely, as they wouldn't necessarily cause the same degree of inflammation. Polychondritis is rare and typically presents with other symptoms beyond the scope of ear signs, and seborrheic dermatitis, while associated with inflammation, usually affects broader skin areas and wouldn't explain the cerumen build-up. (5) RESPONSE: Based on the findings and considerations, it seems the most likely diagnosis for the patient's ear discomfort is Otitis Externa (C). This condition would explain the combination of cerumen accumulation, inflammation, and pain, particularly in light of the fact that other assumptions do not fully explain all presented symptoms.

**Facilitator** The consensus among the AI responses is that the most likely diagnosis for the 23-year-old active-duty airman experiencing a persistent itchy sensation in her right ear, along with inflammation and pain upon manipulation of the pinna, is Otitis Externa (Choice C). This conclusion is reached after considering and ruling out other potential diagnoses such as cerumen impaction, ear canal trauma, polychondritis, and seborrheic dermatitis. Cerumen impaction and ear canal trauma were considered less likely due to the absence of hearing loss and direct injury or invasive procedures, respectively. Polychondritis, a multisystem inflammatory disorder, typically presents with inflammation of various cartilaginous structures beyond the ear, which is not the case here. Seborrheic dermatitis, while associated with inflammation, usually affects broader skin areas and wouldn't explain the cerumen build-up. Otitis externa, also known as swimmer's ear, is an inflammation of the outer ear and ear canal. It aligns with the patient's symptoms of itching, redness, and swelling of the external ear canal, often with pain exacerbated by tugging on the outer ear. The condition could be associated with exposure to water or mechanical trauma, such as from earplugs or headphones, which fits with the patient's occupational exposure. Therefore, Otitis Externa is the most plausible diagnosis given the patient's symptoms, occupational exposure, and physical examination findings.
