# Supplementary material for: Collaborative intelligence in AI: Evaluating the performance of a council of AIs on the USMLE
Source: PLOS Digit Health. 2025 Oct 9;4(10):e0000787. doi: 10.1371/journal.pdig.0000787 (PMC12510544; doi:10.1371/journal.pdig.0000787)
Supplement: S2 File — (ZIP) [file pdig.0000787.s002.zip › S2 Data Raw data files containing unprocessed question inputs and LLM outputs including transcripts of deliberations/STEP2_Question-51-1_Type-MC-NJ.docx]

**Facilitator** **[Round 1]:** A 34-year-old woman who is on active duty in the US Army comes to the military urgent care clinic because of a 4- day history of headache, purulent nasal discharge, and left facial pain. She has not had fever or chills. She has chronic bilateral knee pain, which she attributes to multiple parachute jumps while she was assigned to an airborne unit 7 years ago. The knee pain is exacerbated by long-distance running on pavement. Her only medication is occasional ibuprofen for the knee pain. The patient has no known medication allergies. Temperature is 37.1°C (98.8°F), pulse is 60/min, respirations are 14/min, and blood pressure is 122/80 mm Hg. Cervical lymph nodes are not palpable. There is tenderness to palpation over the left maxillary sinus. Nasal examination shows inflamed turbinates and purulent discharge. The patient needs to blow her nose twice during the examination. Bilateral otoscopic examination discloses no abnormalities. Examination of the oropharynx shows scant purulent discharge posteriorly. The remainder of the examination discloses no abnormalities. Which of the following is the most appropriate next step in management? (A) CT scan of the sinuses (B) Gram stain and culture of nasal discharge (C) Ibuprofen and decongestant therapy (D) Trimethoprim-sulfamethoxazole therapy (E) X-ray of the sinuses

**AI_E:** (1) RESTATING THE QUESTION: The patient, a 34-year old female active military member, is dealing with symptoms including a headache, purulent nasal discharge, and left facial pain which have been ongoing for 4 days. She also has a history of chronic bilateral knee pain attributed to previous parachute jumps and exacerbated by long-distance runs on pavement. Given her current state, what constitutes the best approach in managing her symptoms? (2) PLANNING: To address this clinical problem effectively, we need to hypothesize what could be the root cause of her symptoms. The key assumptions are that these symptoms could be indicative of a sinusitis, sinus infection, or related issue based on the described clinical manifestations. Alternately, her symptoms could be due to a non-sinusitis related issue, but the clinical presentations suggest that this is unlikely. The assumption about sinusitis needs to be clinically prioritized for further investigation as this is the most likely scenario based on the patient's symptoms. (3) CLINICAL REASONING: Sinusitis typically presents with a purulent nasal discharge, headache, and face pain due to the inflammation and infection in the sinus cavities. This matches the patient's complaints. As such, it would be reasonable to begin managing her condition as sinusitis. Antibiotics may be necessary if it's a bacterial infection, but these are typically reserved for severe cases or when sinusitis lasts more than 7-10 days since viral sinusitis is more common and it would resolve on its own. (4) CONCLUDING: Given the patient's symptoms and the clinical picture, it appears that she likely has sinusitis. Treatment usually begins conservatively with decongestants to relieve congestion and NSAIDs like Ibuprofen for pain and inflammation. (5) RESPONSE: Therefore, the most appropriate next step in the management of this patient would be (C) Ibuprofen and decongestant therapy, as it would address her symptoms and possibly resolve the issue if it is a case of viral sinusitis. It also avoids unnecessary investigations and treatments at the current stage of her illness.

**AI_B:** 1) RESTATING THE QUESTION: The clinical scenario you presented relates to a 34-year-old woman, who is a solider, with recent episodic symptoms suggestive of a sinus-related issue - demonstrated by her headache, nasal discharge, and facial pain. She also has chronic knee pain from her previous military activities. The question seeks to identify the most suitable next step for managing the patient's condition. 2) PLANNING: We need to ascertain if the symptoms are indicative of acute sinusitis or another pathological condition, and how best to manage the situation. The options indicate exploratory steps (CT scan and Gram stain/culture), pharmacological treatments (Ibuprofen/decongestants or Trimethoprim-sulfamethoxazole), or further diagnostic measures (X-ray of the sinuses). 3) CLINICAL REASONING: Acute rhinosinusitis is a clinical diagnosis; the patient's purulent nasal discharge, facial pain, particularly over the maxillary sinus, in addition to headache, are classic symptoms. Although sometimes a viral infection, bacterial involvement is suggested when symptoms persist over several days, as is the case here. Causative bacterial agents could include Streptococcus pneumoniae, Haemophilus influenzae, or Moraxella catarrhalis. However, her vital signs are stable and she does not have a fever, indicating that her body may be effectively combating the infection and avoiding it spreading systemically. Imaging studies (CT scan, X-ray) would typically be reserved for recurrent or chronic cases or if complications are suspected, which doesn't seem to be the case here. Therefore, CT scan and X-ray can be considered as less favourable options. Gram stain and culture of nasal discharge, on the other hand, are rarely necessary in uncomplicated acute bacterial rhinosinusitis. 4) CONCLUDING: Consequently, the best course of action seems to be symptomatic treatment - addressing the pain and reducing the congestion in the sinus, which would alleviate her irritation and potentially expedite the recovery process. Though antibiotic therapy using Trimethoprim-sulfamethoxazole is theoretically plausible, it's worth noting that many cases of acute sinusitis resolve spontaneously, and liberal antibiotic use contributes to bacterial resistance. 5) RESPONSE: Based on all the clinical reasoning and understanding of acute rhinosinusitis management, the most appropriate next step in her management is likely (C) Ibuprofen and decongestant therapy, which would offer symptomatic relief and potentially aid in natural resolution of the infection. This option assumes a careful watch-and-wait approach, with escalation to antibiotics if the condition persists or worsens.

**AI_D:** RESTATING THE QUESTION: This scenario is centered around a 34-year-old woman presenting to a military urgent care clinic due to a 4-day history of headache, purulent nasal discharge, and left facial pain. Neither fever nor chills are noted, but she does have a history of chronic bilateral knee pain due to her military service activities. She uses ibuprofen occasionally for the knee pain. During the physical examination, inflammation and discharge have been observed in the nasal area, and there is also tenderness over the left maxillary sinus. The question here asks for the most appropriate next step in the management of this patient. PLANNING: Severity and duration of her symptoms as well as clinical examination findings suggest a possible acute bacterial sinusitis. While a CT scan of the sinuses (choice A) or an X-ray of the sinuses (choice E) would provide a more detailed visualization of the sinuses, they may not be immediately necessary as the primary diagnosis can often be made based on clinical findings. The options of Gram stain and culture of nasal discharge (choice B) or the prescription of Trimethoprim-sulfamethoxazole therapy (choice D) could potentially be valid, they might not be the first-line approach in management. CLINICAL REASONING: In managing sinusitis, the first-line approach often involves the prescription of analgesics for pain and decongestants to aid in the drainage of the sinuses. In this case, since the patient already uses ibuprofen (an analgesic), combining it with a decongestant could provide symptomatic relief. Antibiotics, such as Trimethoprim-sulfamethoxazole mentioned in choice D, are generally saved for cases where symptoms are severe, have lasted more than 7 to 10 days, or are associated with high fever (>39°C, 102.2°F), purulent nasal discharge, or facial pain. CONCLUDING: In light of the above, the most appropriate next step for managing this patient would likely involve symptomatic relief via the continued use of ibuprofen for the associated pain and initiation of decongestant therapy to aid in the drainage of the sinus. RESPONSE: (C) Ibuprofen and decongestant therapy.

**AI_C:** (1) RESTATING THE QUESTION: The patient is a 34-year-old woman who is experiencing symptoms of headache, purulent nasal discharge, and left facial pain over the last 4 days. She also has a history of chronic bilateral knee pain. The patient has just normal body temperature and no signs of systemic illness. Her exam is remarkable for tenderness over the left maxillary sinus, inflamed turbinates, and purulent nasal discharge. Her other physical examination results are normal. In this context, purulent refers to the presence of pus, which indicates an infection. The question is asking: what is the most appropriate next course of action given her current symptoms and findings? (2) PLANNING: In this instance, the main assumption would be that the patient is suffering from a type of sinusitis, given her symptoms of headache, purulent discharge, facial pain and tenderness over the maxillary sinus. Sinusitis is an inflammation or swelling of the tissue lining the sinuses. However, another possible assumption could be that these symptoms are due to a localized infection in the nasal passage or other structures in the respiratory tract, though this seems less likely given her specific symptomatology. The clinical priority in this case is to manage her current symptoms and prevent any potential complications that may arise from her presumed sinusitis. (3) CLINICAL REASONING: Considering the patient's symptoms and physical examination results, it seems most likely that she is suffering from acute sinusitis. The symptoms and clinical findings are quite characteristic, with sinus tenderness, purulent nasal discharge, and facial pain. The absence of systemic signs of illness, like fever or increased heart rate, suggest that it is an uncomplicated case of sinusitis, which is usually of viral etiology. Sinusitis is often caused by the same viruses that cause common cold, but can also be caused by bacteria. However, bacterial sinusitis is less common and typically follows a viral upper respiratory tract infection. (4) CONCLUDING: Given the likely diagnosis of uncomplicated viral sinusitis, antibiotics are not indicated at this stage. According to current management guidelines, supportive care is the first-line treatment which includes over-the-counter analgesics for pain and fever, and intranasal corticosteroids for nasal symptoms. Additional imaging like CT scans or X-rays are not generally needed, unless there are signs of complications or if the symptoms do not improve after initial management. (5) RESPONSE: Therefore, the most appropriate next step in management is to initiate symptomatic treatment for the presumed viral sinusitis. This would primarily involve over-the-counter pain management and decongestant therapy to alleviate her symptoms, making option (C) Ibuprofen and decongestant therapy the best choice.

**AI_A:** RESTATING THE QUESTION: The situation presented in the question details a 34-year-old woman on military duty who has been experiencing headache, facial pain, and purulent nasal discharge for four days. This woman also has a history of chronic bilateral knee pain due to multiple parachute jumps, the symptoms of which are worsened by prolonged running on hard surfaces. She only takes ibuprofen occasionally to manage her knee pain. Despite her symptoms, she doesn't present with any fever or chills. The physical examination reveals inflammation in the nasal cavity, tenderness over the left maxillary sinus, and some purulent discharge in the oropharynx. Now, we are tasked to decide the best management approach from the given options. PLANNING: Her symptoms seem to suggest a sinusitis, an inflammation of the sinus cavities commonly caused by a viral or bacterial infection. The assumptions we can generate include (A) imaging scan which could help identify the extent and severity of the sinus infection; (B) lab testing of the nasal discharge for definitive diagnosis; (C) symptomatic treatment with anti-inflammatory drugs and decongestants; (D) immediate antibiotic treatment; and (E) an X-ray scan of the sinuses for diagnosis. CLINICAL REASONING: Assuming that the patient has acute sinusitis based on her signs and symptoms, we can follow the logic that acute bacterial sinusitis is often preceded by a viral upper respiratory infection, which is usually self-limiting and resolves without antibiotics. Antibiotics should ideally be reserved for severe, persistent, or recurrent cases of sinusitis. The choice between symptomatic treatment and antibiotic therapy hinges on the duration of illness and severity of symptoms. Likewise, the choice between laboratory testing and imaging relies on the clinical appearance and the potential severity of the outcomes. Performing an expensive and invasive test such as a CT scan or even an X-ray scan of the sinuses may not be warranted in mild to moderate cases without complications. Moreover, a gram stain and culture could help identify the specific causative agent, but may not be necessary for an uncomplicated acute sinusitis. CONCLUDING: Adopting a step-by-step approach, the first line of management should target symptom relief, monitoring the patient's condition before moving on to more invasive diagnostic and therapeutic methods if the symptoms persist, worsen, or recur. RESPONSE: So, the most suitable next step in managing this patient's condition would be (C) Ibuprofen and decongestant therapy. This approach can relieve the discomforting symptoms heralded by acute sinusitis, and allows time for the infection (if present) to resolve on its own.

**Facilitator** The patient, a 34-year-old woman on active military duty, presents with a 4-day history of headache, purulent nasal discharge, and left facial pain, symptoms indicative of sinusitis. She also has a history of chronic bilateral knee pain due to multiple parachute jumps. Despite her symptoms, she doesn't present with any fever or chills, suggesting that her body may be effectively combating the infection and avoiding it spreading systemically. The physical examination reveals inflammation in the nasal cavity, tenderness over the left maxillary sinus, and some purulent discharge in the oropharynx. Given her symptoms and clinical findings, it seems most likely that she is suffering from acute sinusitis. The symptoms and clinical findings are quite characteristic, with sinus tenderness, purulent nasal discharge, and facial pain. The absence of systemic signs of illness, like fever or increased heart rate, suggest that it is an uncomplicated case of sinusitis, which is usually of viral etiology. Sinusitis is often caused by the same viruses that cause common cold, but can also be caused by bacteria. However, bacterial sinusitis is less common and typically follows a viral upper respiratory tract infection. Considering the patient's symptoms and physical examination results, it seems most likely that she is suffering from acute sinusitis. The symptoms and clinical findings are quite characteristic, with sinus tenderness, purulent nasal discharge, and facial pain. The absence of systemic signs of illness, like fever or increased heart rate, suggest that it is an uncomplicated case of sinusitis, which is usually of viral etiology. Sinusitis is often caused by the same viruses that cause common cold, but can also be caused by bacteria. However, bacterial sinusitis is less common and typically follows a viral upper respiratory tract infection. Given the likely diagnosis of uncomplicated viral sinusitis, antibiotics are not indicated at this stage. According to current management guidelines, supportive care is the first-line treatment which includes over-the-counter analgesics for pain and fever, and intranasal corticosteroids for nasal symptoms. Additional imaging like CT scans or X-rays are not generally needed, unless there are signs of complications or if the symptoms do not improve after initial management. Therefore, the most appropriate next step in management is to initiate symptomatic treatment for the presumed viral sinusitis. This would primarily involve over-the-counter pain management and decongestant therapy to alleviate her symptoms, making option (C) Ibuprofen and decongestant therapy the best choice. This approach can relieve the discomforting symptoms heralded by acute sinusitis, and allows time for the infection (if present) to resolve on its own. This option assumes a careful watch-and-wait approach, with escalation to antibiotics if the condition persists or worsens.
