# Supplementary material for: Collaborative intelligence in AI: Evaluating the performance of a council of AIs on the USMLE
Source: PLOS Digit Health. 2025 Oct 9;4(10):e0000787. doi: 10.1371/journal.pdig.0000787 (PMC12510544; doi:10.1371/journal.pdig.0000787)
Supplement: S2 File — (ZIP) [file pdig.0000787.s002.zip › S2 Data Raw data files containing unprocessed question inputs and LLM outputs including transcripts of deliberations/STEP2_Question-52-1_Type-MC-NJ.docx]

**Facilitator** **[Round 1]:** A 16-year-old boy with VATER syndrome is brought to the physician because of 2-week history of harsh cough that began with a common cold. He receives no medications. Temperature is 37°C (98.6°F), pulse is 70/min, respirations are 20/min, and blood pressure is 112/64 mm Hg. Examination shows a harsh vibratory sound in the mid anterior chest with forced expiration. A systolic murmur is heard best at the lower left sternal border. There is mild scoliosis. Spirometry shows an FVC of 2.54 L (78% of predicted) and a slow vital capacity of 2.88 L (85% of predicted). A flow volume loop shows scooping on the expiratory limb. Which of the following is the most likely cause of the discrepancy between this patient’s forced and slow vital capacity measurements? (A) Bronchospasm (B) Laryngeal braking (C) Subglottic mass (D) Tracheal collapse (E) Upper airway compression

**AI_A:** (1) RESTATING THE QUESTION: The patient is a 16-year-old male who was diagnosed with VATER syndrome. VATER syndrome is a non-random association of birth defects which includes vertebral defects, anal atresia, tracheo-esophageal fistula, and radial and renal dysplasia. He has been experiencing a harsh cough for two weeks, that started after a common cold, and exhibits a vibratory sound during forced expiration. His spirometry results indicate a difference between his forced vital capacity (FVC) and slow vital capacity measurements. We need to identify the most likely cause of this discrepancy. (2) PLANNING: Several assumptions can be made. These include bronchospasm, laryngeal braking, a subglottic mass, tracheal collapse, or upper airway compression. These conditions would affect FVC measurements differently. (3) CLINICAL REASONING: Based on the clinical findings, bronchospasm and laryngeal braking are less likely as they typically present with other symptoms such as wheezing or stridor respectively. If we had a subglottic mass, the boy's cough would likely have been present for a longer period due to its location. This leaves us with tracheal collapse and upper airway compression. Tracheal collapse is often associated with expiratory stridor, which is not reported in the case. Upper airway compression can manifest as a vibratory sound on forced expiration (similar to a stridor but lower pitched and heard during expiration rather than inspiration), and it also fits with the abnormal spirometry finding (discrepancy in FVC and SVC). (4) CONCLUDING: Based on the information provided in the case, along with the symptom presentation and spirometry results, upper airway compression (E) appears to be the most plausible explanation for the observed discrepancy between the patient's forced and slow vital capacity measurements. (5) RESPONSE: The performance of forced and slow vital capacity assessments in this 16-year-old boy with VATER syndrome revealed a discrepancy, likely due to the presence of upper airway compression, impacting his ability to effectively and fully expel air from his lungs during forced expiration.

**AI_C:** RESTATING THE QUESTION: We are asked to determine the most likely reason for the discrepancy between a 16-year-old male's spirometric results: his Forced Vital Capacity (FVC), demonstrating the maximum amount of air exhaled forcefully after full inhalation, and Slow Vital Capacity (SVC), representing the maximum amount of air exhaled at normal, relaxed rate after a full inhalation. The boy is diagnosed with VATER syndrome, a non-random association of birth defects that includes vertebral defects, anal atresia, tracheoesophageal fistula, and renal dysplasia. PLANNING: Assuming the spirometry discrepancy arises from a mechanical aspect related to VATER syndrome, would a bronchial, laryngeal, subglottic, tracheal, or upper airway issue most likely cause these results? Examining each assumption, bronchospasm changes airflow dynamics but wouldn't likely differentiate between fast/slow exhalation; laryngeal braking restricts air outflow but typically would impact both FVC and SVC equally; a subglottic mass could impact the volumes but the symptoms presented align more with extrathoracic obstruction. Therefore, tracheal collapse or upper airway compression, which can increase in severity with forceful exhalation, are the assumptions we should prioritize. CLINICAL REASONING: VATER syndrome, which often presents with tracheoesophageal fistula (an abnormal connection between the esophagus and the trachea), can lead to disrupted tracheal structure and function. Conditions like tracheomalacia (weakness of the tracheal walls), commonly seen with VATER syndrome, result in tracheal collapse during forceful exhalation, and would more drastically impact the FVC over the SVC. Moreover, the harsh vibratory sound on forced expiration, along with the "scooping" on the flow-volume loop of the spirometry test - indicative of variable intrathoracic airway obstruction - are supporting signs of tracheal collapse. CONCLUDING: The child's VATER syndrome, alongside the spirometry findings and symptomatology, strongly suggest a disrupted tracheoesophageal structure potentially culminating in tracheal collapse. This tracheal collapse, especially upon forced expiration, could be responsible for the observed discrepancy between the FVC and SVC. RESPONSE: (D) Tracheal collapse is the most likely cause of the discrepancy between this patient’s forced and slow vital capacity measurements.

**AI_D:** RESTATING THE QUESTION: The inquiry here is regarding the most probable reason for the difference recorded in the forced and slow vital capacities of a 16-year-old boy diagnosed with VATER syndrome. The patient has presented with a harsh cough over the past two weeks, started with a common cold, plus he is not on any medications currently. His vital signs are normal, and physical examination revealed a vibratory noise (harsh) in the central part of the anterior chest during forced exhalation plus a systolic murmur audible at the lower left sternal border. Mild scoliosis is also noted. Spirometry results show a forced vital capacity (FVC) of 2.54 L (78% of the predicted value) and a slow vital capacity of 2.88L (85% of the predicted value). The flow volume loop indicates 'scooping' on the expiratory limb. PLANNING: The assumptions that can be made from the above-mentioned data include the presence of bronchospasm, laryngeal braking, a subglottic mass, tracheal collapse, or upper airway compression. Among these possible causes, the 'scooping' of the flow-volume curve on the expiratory limb suggests a more precisely explanatory cause. Scooping on the expiratory flow-volume loop is a classic sign of a fixed obstructive airway lesion, which causes the shape of the flow-volume loop to distort, especially on forced expiration. This could most likely occur in cases of tracheal collapse. CLINICAL REASONING: The VATER syndrome, also known as VACTERL association, is a non-random association of birth defects that often occurs together and comprises vertebral defects, anal atresia, cardiac defects, tracheoesophageal fistula, renal anomalies, and limb abnormalities. The patients with VATER syndrome often show anatomical abnormalities such as tracheoesophageal fistula. The tracheoesophageal fistula could predispose the trachea to be more prone to collapsing, most notably during forced expiration, which may cause the distinct 'scooping' of the flow-volume loop. On the other hand, the discrepancy between this patient's forced and slow vital capacity could be due to increased collapse of the trachea during forced expiration compared to slow expiration, hence the higher vital capacity reading. CONCLUDING: In conclusion, given the patient's history of VATER syndrome and the results from his spirometry test, it would be reasonable to state that the most likely cause of the discrepancy in the boy's forced and slow vital capacity is tracheal collapse during forced expiration, primarily due to the tracheal anomalies associated with VATER syndrome. RESPONSE: (D) Tracheal collapse.

**AI_B:** (1) RESTATING THE QUESTION: This question is about a 16-year-old boy with VATER syndrome - a congenital condition characterized by vertebral defects, anal atresia, tracheoesophageal fistula and renal anomalies - who has a 2-week history of harsh cough. The question is asking what could be the cause of the discrepancy between the patient's forced vital capacity (FVC), which is the amount of air that can be forcibly exhaled from the lungs after taking the deepest breath possible, and slow vital capacity (VC), which is the maximum volume of air that can be exhaled slowly after slow maximum inhalation. (2) PLANNING: The discrepancy between the patient's FVC and VC suggests a possible obstruction in the airway that is more pronounced during forced expiration. All the choices presented involve conditions that can cause airway obstruction: Bronchospasm involves constriction of the muscles of the bronchi; Laryngeal braking is a protective mechanism of the vocal cords to prevent over-inflation of the lungs; Subglottic mass refers to a mass underneath the glottis of the larynx which can block airflow; Tracheal collapse is a condition where tracheal walls fall together and block the airway during expiration; Upper airway compression can be caused by external compression of the trachea or larynx. (3) CLINICAL REASONING: The key to answering this question lies in understanding the spirometry findings. The abnormal "scooping" shape of the flow-volume curve suggests airflow obstruction that is worse with increased flow rates, which corresponds to forced exhalation. This pattern is seen with intrathoracic obstruction, like tracheomalacia or tracheal compression, where the pressure inside the chest during forced exhalation "squeezes" the weakened or compressed trachea leading to worsened obstruction and the discrepancy between FVC and VC. (4) CONCLUDING: Considering his harsh cough, the spirometry results, and the characteristics of VATER syndrome which often includes tracheoesophageal abnormalities, the history and findings seem most suggestive of intrathoracic obstruction. (5) RESPONSE: Therefore, the most likely cause of the discrepancy between this patient’s forced and slow vital capacity measurements is (D) Tracheal collapse.

**AI_E:** (1) RESTATING THE QUESTION: Basically, the question asks what is likely causing the difference between this 16-year-old boy's forced and slow vital capacity measurements. The patient, who has VATER syndrome, has exhibited symptoms such as a harsh cough, a harsh vibratory sound in the mid anterior chest with forced expiration, mild scoliosis, and a 'scooping' on the flow volume loop's expiratory limb. VATER syndrome, now more appropriately known as VACTERL association, consists of at least three of the following congenital issues: vertebral defects, anal atresia, cardiac defects, tracheoesophageal fistula, renal anomalies, and limb abnormalities. The concept of forced vital capacity (FVC) refers to the amount of air a person can forcefully and quickly exhale after taking a deep breath. Slow vital capacity (SVC), on the other hand, represents the same but exhaled in a slow, more controlled manner. A 'scooping' pattern on the flow-volume loop indicates an issue with the airway, particularly during expiration. (2) PLANNING: We should first identify the assumptions related to the patient's signs and symptoms. The boy's harsh cough, vibratory sound during expiration, and scooping on the expiratory limb suggest a problem with the airway. The varying FVC and SVC might point to an issue either during a controlled or hurried exhalation, which implicates the functioning and mechanics of the airway. The next step is to generate alternate assumptions by questioning each assumption. Could there be a different source causing these symptoms than the airway? Given the clear spirometry results and the associated symptoms, it seems unlikely. Even his VATER syndrome diagnosis, specifically the 'tracheoesophageal fistula' component, reinforces the idea of an airway issue. Lastly, we need to evaluate and hypothesize which assumption should be clinically prioritized. Considering his symptoms and the analysis thus far, it seems that an issue with the airway should be clinically prioritized. (3) CLINICAL REASONING: We're considering an airway issue and specifically, a problem that is highlighted with forced expiration. The options provided can help us narrow down the causes. Bronchospasm (Option A) usually shows a different spirometry pattern, in which both inspiration and expiration are affected. Laryngeal braking (Option B) generally results in exertional dyspnea and does not usually lead to the noted spirometry discrepancy. Subglottic mass (Option C) would likely result in both inspiratory and expiratory difficulty due to its location, so it is also unlikely. Upper airway compression (Option E) would generally present at rest rather than specifically on forced expiration. This leaves us with tracheal collapse (Option D), a condition that would most notably affect expiration, particularly when it’s forced, as in this scenario. (4) CONCLUDING: Given the symptoms and spirometry findings, we are led to conclude that the different forced and slow vital capacity measurements are due to an airway collapsing under the additional pressure of a hurried exhalation. This would limit the amount of air the boy can forcefully expel, resulting in reduced FVC. However, during SVC, the airway maintains its integrity, meaning that the boy can exhale more air in total, so the SVC is higher. (5) RESPONSE: Therefore, the most likely cause of the discrepancy between this patient's forced and slow vital capacity measurements is tracheal collapse (Option D). Tracheal collapse would prominently affect expiration, particularly when it's forceful, as the pressure could cause the inherently weak or malformed trachea to collapse, reducing the volume of air expelled. This condition can be common in individuals with VATER syndrome due to the associated tracheoesophageal abnormalities.

**Facilitator** **[Round 2]:** Summary: AI_A believes that the most likely cause of the discrepancy between the patient's forced and slow vital capacity measurements is upper airway compression. This conclusion is based on the patient's symptoms, including a vibratory sound during forced expiration, and the spirometry results. AI_B, AI_C, AI_D, and AI_E all conclude that tracheal collapse is the most likely cause of the discrepancy. They base their conclusions on the patient's symptoms, the spirometry results, and the fact that the patient has VATER syndrome, which often includes tracheoesophageal abnormalities that could predispose the trachea to collapse. Key Differences: The key difference between AI_A's opinion and the others is the identified cause of the discrepancy in the patient's spirometry results. AI_A attributes it to upper airway compression, while AI_B, AI_C, AI_D, and AI_E attribute it to tracheal collapse. The reasoning behind AI_A's conclusion is the patient's vibratory sound during forced expiration, which is a symptom of upper airway compression. The other council members, however, focus on the patient's VATER syndrome and its associated tracheoesophageal abnormalities, which could lead to tracheal collapse, especially during forced expiration. Question for Council Members: Could you please elaborate on why you believe upper airway compression (AI_A) or tracheal collapse (AI_B, AI_C, AI_D, AI_E) is the most likely cause of the discrepancy in the patient's spirometry results? Specifically, could you discuss how the patient's symptoms and VATER syndrome diagnosis led you to your conclusion?. Based on your preceding response, how would you answer the original question: [A 16-year-old boy with VATER syndrome is brought to the physician because of 2-week history of harsh cough that began with a common cold. He receives no medications. Temperature is 37°C (98.6°F), pulse is 70/min, respirations are 20/min, and blood pressure is 112/64 mm Hg. Examination shows a harsh vibratory sound in the mid anterior chest with forced expiration. A systolic murmur is heard best at the lower left sternal border. There is mild scoliosis. Spirometry shows an FVC of 2.54 L (78% of predicted) and a slow vital capacity of 2.88 L (85% of predicted). A flow volume loop shows scooping on the expiratory limb. Which of the following is the most likely cause of the discrepancy between this patient’s forced and slow vital capacity measurements? (A) Bronchospasm (B) Laryngeal braking (C) Subglottic mass (D) Tracheal collapse (E) Upper airway compression]

**AI_B:** Restating the Context: The question presents a 16-year-old boy, diagnosed with VATER syndrome, who presents with a 2-week history of harsh cough that began with a cold. He also demonstrates ecreased vital capacities during forced respiration, and spirometry shows scooping on the expiratory limb of the flow volume loop. We need to determine the likely cause of the discrepancy between the forced and slow vital capacities. Planning: VATER syndrome, is an association of birth defects characterized by Vertebral defects, Anal atresia, Tracheoesophageal fistula, and Radial and Renal dysplasia. These multiple anomalies can result in various respiratory difficulties. Our assumptions should relate to this condition. Potential assumptions include: (A) Bronchospasm, (B) Laryngeal braking, (C) Subglottic mass, (D) Tracheal collapse, (E) Upper airway compression. Let's examine each of these assumptions closely. Clinical Reasoning: 1. Bronchospasm usually presents with wheezing, which is absent in this case. 2. Laryngeal braking would alter both the forced and slow vital capacities, not just forced. 3. A subglottic mass would cause additional symptoms such as stridor, which is not mentioned here. 4. Tracheal collapse would lead to disproportionate reduction in the forced vital capacity (FVC), which is observed in this case. Those with VATER syndrome often have tracheoesophageal abnormalities that could predispose to tracheal collapse during forced expiration. 5. Upper airway compression would cause additional symptoms which the patient does not present. Concluding: Based on the clinical reasoning, the most clinically appropriate assumption to pursue is tracheal collapse (D), due to the patient's VATER syndrome, his presentation, and his spirometry results. Response: Therefore, to answer the question, tracheal collapse (D), given this patient's diagnostic and clinical presentation, is the most likely cause of the discrepancy between this patient's forced and slow vital capacity measurements.

**AI_D:** Restating the question: We are discussing a 16-year-old boy diagnosed with VATER syndrome who has been experiencing an ongoing harsh cough. He has a distinct vibratory sound during forceful expiration, a systolic murmur, mild scoliosis, and inconsistent readings between forced and slow vital capacity measurements. VATER syndrome is a non-random association of birth defects, including Vertebrae defects, Anorectal malformations, Tracheo-Esophageal abnormalities, Renal and Radial anomalies, and Limb defects. We're investigating the most probable cause of the discrepancy in his lung capacity measurements. Planning: The disease symptoms suggest a structural or functional abnormality in the respiratory tract. Assumptions would include potential effects of VATER syndrome and respective tracheoesophageal abnormalities, contributing factors like coughing and vibratory sounds during forceful expiration, and the interplay between these assumptions. We should examine if the tracheoesophageal defects from VATER syndrome are responsible for the noticeable discrepancy in vital capacity measurements ("tracheal collapse") or if factors causing the bronchi's partial occlusion, possibly related to the ongoing cough, is the cause ("upper airway compression"). Clinical Reasoning: VATER syndrome often includes tracheoesophageal fistula or tracheal stenosis, which can impact the respiratory tract and cause issues such as tracheal collapse. This patient's reported "scooping" on the expiratory limb of his flow volume loop is characteristic of variable extrathoracic airway obstruction, suggesting a collapsible segment of the airway during forced expiration. Furthermore, the harsh cough could potentially exacerbate existing structural weaknesses in the trachea, leading to intermittent collapsing, which might not be as impactful during slow exhalation but notable during forced breaths, causing a discrepancy in the patient's spirometry readings. Concluding: Given the patient's medical history and the nature of VATER syndrome, it is highly plausible that the observed discrepancy in forced and slow vital capacity measurements originates from tracheal collapse. Although other factors (like upper airway compression) can contribute, the existing tracheoesophageal abnormalities and their potential effects on the respiratory system due to VATER syndrome should not be ignored. Response: The most likely cause of the discrepancy between this patient’s forced and slow vital capacity measurements is (D) Tracheal collapse.

**AI_E:** RESTATING THE QUESTION: The scenario involves a 16-year-old boy with VATER syndrome who has been coughing for 2 weeks, sparked by a common cold, and does not take medication. His crucial clinical points encompass normal vital signs, harsh vibratory sound during forceful expiration, systolic murmur at the lower-left sternal border, mild scoliosis and characteristics of his pulmonary function tests. The differential diagnosis we need to consider involve bronchospasm, laryngeal braking, subglottic mass, tracheal collapse, and upper airway compression. PLANNING: To resolve the query, we should focus on the patient's signs, symptoms, and VATER syndrome manifestations. The syndrome includes diverse manifestations - vertebral defects, anal atresia, tracheoesophageal fistula, renal anomalies, and limb abnormalities. Focusing on the pulmonary symptoms, the tracheoesophageal anomalies stand out as significant and thus one of our assumptions could be that this patient might have tracheal collapse or upper airway compression. CLINICAL REASONING: The harsh vibratory sound in the anterior chest during forceful expiration and the 'scooping' on the expiratory limb of the flow-volume loop lean towards a problem in the airways. This issue seems to be more prominent during forced expiration rather than slow expiration as reflected by the discrepancy between FVC (forced vital capacity) and SVC (slow vital capacity). Therefore, we can deduce it's an obstructive defect that worsens with forced expiration. Though bronchospasm could be a viable option, the absence of other suggestive symptoms and signs such as wheezing, history of allergy, or asthma go against it. Furthermore, laryngeal braking and subglottic mass are less likely as there is no history of voice change or stridor. CONCLUDING: Considering the patient's history of VATER syndrome which includes tracheoesophageal anomalies, the presence of an abnormal anterior chest sound during forced expiration, and characteristic spirometry findings, tracheal collapse seems to be the most plausible cause of the discrepancy between this patient’s forced and slow vital capacity measurements. RESPONSE: Hence, with the step-by-step clinical reasoning process explained above, (D) Tracheal collapse seems to be the most likely cause of the discrepancy between this patient’s forced and slow vital capacity measurements.

**AI_C:** RESTATING THE QUESTION: The question asks for the most probable cause behind the variation in the boy's forced and slow vital capacity readings. The boy, diagnosed with VATER syndrome, presents symptoms like a harsh cough that followed a common cold, mid-anterior chest harsh vibratory sound during forced expiration, and an aberration in the patient’s flow volume loop. VATER syndrome encompasses multiple system defects including vertebral defects, anal atresia, cardiac defects, tracheo-esophageal fistula, renal anomalies, and limb abnormalities. Forced Vital Capacity (FVC) refers to the amount of air forcibly expelled after taking the deepest breath possible. Slow vital capacity measures the maximum amount of air that can be expelled from the lungs, but at a slower, more controlled pace. PLANNING: I need to seek an underlying cause behind the discrepancy in the patient's vital capacity measurements. Given that bronchospasm, laryngeal braking, subglottic mass, tracheal collapse, and upper airway compression are presented options, it would be apt to understand how these could potentially affect a person's FVC and SVC measurements. CLINICAL REASONING: The presence of a "scooping" in the flow volume loop suggests an obstructive airway disease with a variable extrathoracic component, which is characteristic of upper airways' dynamic collapse during forced expiration. This is highly suggestive of "tracheal collapse". Tracheal collapse is more relevant in this case due to the presence of VATER syndrome, which is often associated with tracheo-esophageal abnormalities. These abnormalities could cause a tendency for the trachea to collapse during forced expiration, giving us the discrepancy between the forced and slow vital capacity measurements. CONCLUDING: Based on the presented symptoms, the boy's existing VATER syndrome, and the characteristic scooping on the flow volume loop, tracheal collapse seems to be a fitting explanation behind the difference in vital capacity measurements. When the patient forcefully exhales as in FVC, the structurally compromised trachea collapses leading to suboptimal readings, whereas during SVC, the trachea is less stressed, resulting in better outcomes. RESPONSE: The most probable cause of the discrepancy between this patient's forced and slow vital capacity measurements is (D) Tracheal collapse.

**AI_A:** 1) RESTATING THE QUESTION: Based on the given information, a 16-year-old boy who has VATER syndrome is brought to a physician due to a two-week-long harsh cough. VATER syndrome is a non-random association of birth defects that co-occur more often than one would expect based on their individual frequencies. The acronym VATER stands for vertebral defects, anal atresia, tracheoesophageal fistula with esophageal atresia, and radial and renal dysplasia. The patient’s recorded vital signs are normal, but examination reveals particular abnormal physical findings - a harsh vibratory sound upon forced expiration and a systolic murmur. Spirometry results indicate a lowered FVC and slow vital capacity compared to the predicted values. The flow volume loop is scooped on its expiratory limb. In simpler terms, we've got a teenager with a bunch of birth defects related to VATER syndrome with a bad cough, scoliosis, and some strange lung function test results. The question asks us to identify the most probable cause behind the discrepancy observed in the patient’s forced and slow vital capacity measurements. 2) PLANNING: To answer the question, it's vital to understand what might potentially cause variations in forced and slow vital capacities. A "forced vital capacity" refers to the greatest possible amount of air that a person can exhale, doing so as forcefully and quickly as possible after taking a full breath. The "slow vital capacity" is similar, but the person breathes out at their own pace. If there's a notable discrepancy between these two measurements, it suggests that something is obstructing or restricting the quick, forceful exhalation required for FVC. We must consider conditions affecting the trachea or surrounding structures (since the patient has VATER syndrome which is often associated with tracheoesophageal abnormalities) that might account for this. 3) CLINICAL REASONING: Based on the boy's symptoms, physical findings, and spirometry results, it's clear something unusual is happening when he tries to truly force out a breath. Given his scooped out flow volume loop (which is a graphical representation of how airflow varies with lung volume) pattern, the spirometry results suggest tracheal collapse. This refers to a condition where the tracheal wall invaginates and compromises the airway, creating an obstructive pattern during forceful expiration. Considering that the boy is diagnosed with VATER syndrome (a syndrome associated with congenital malformations of structures like the trachea), the likelihood of tracheal collapse increases further. 4) CONCLUDING: After considering the clinical scenario and using a step-by-step approach to examine the spirometry results and the patient's VATER diagnosis, it's reasonable to hypothesize that a tracheal collapse is the probable cause of the discrepancy between forced and slow vital capacity measurements. 5) RESPONSE: Therefore, option (D) Tracheal collapse would be the most probable reason for the discrepancy between this patient’s forced and slow vital capacity measurements.

**Facilitator** The consensus statement synthesizes the responses of AI_A, AI_B, AI_C, AI_D, and AI_E as follows: The question presents a 16-year-old boy diagnosed with VATER syndrome, experiencing a harsh cough for two weeks. His physical examination reveals a harsh vibratory sound during forced expiration, a systolic murmur, and mild scoliosis. His spirometry results show a discrepancy between his forced and slow vital capacity measurements, with a characteristic 'scooping' on the expiratory limb of the flow volume loop. VATER syndrome is a non-random association of birth defects, including vertebral defects, anal atresia, tracheoesophageal fistula, and radial and renal dysplasia. To identify the most probable cause of the discrepancy in the patient's vital capacity measurements, we need to consider conditions that could obstruct or restrict the quick, forceful exhalation required for forced vital capacity. Given the patient's VATER syndrome, which often includes tracheoesophageal abnormalities, we should focus on conditions affecting the trachea or surrounding structures. The patient's symptoms, physical findings, and spirometry results suggest an obstructive airway disease with a variable extrathoracic component, characteristic of upper airways' dynamic collapse during forced expiration. This is highly suggestive of tracheal collapse, a condition where the tracheal wall invaginates and compromises the airway, creating an obstructive pattern during forceful expiration. The likelihood of tracheal collapse increases further considering the boy's diagnosis of VATER syndrome. Therefore, after considering the clinical scenario, the patient's VATER diagnosis, and the spirometry results, the consensus is that tracheal collapse is the most probable cause of the discrepancy between the patient's forced and slow vital capacity measurements.
