# Supplementary material for: Collaborative intelligence in AI: Evaluating the performance of a council of AIs on the USMLE
Source: PLOS Digit Health. 2025 Oct 9;4(10):e0000787. doi: 10.1371/journal.pdig.0000787 (PMC12510544; doi:10.1371/journal.pdig.0000787)
Supplement: S2 File — (ZIP) [file pdig.0000787.s002.zip › S2 Data Raw data files containing unprocessed question inputs and LLM outputs including transcripts of deliberations/STEP2_Question-55-1_Type-MC-NJ.docx]

**Facilitator** **[Round 1]:** A chief medical officer at a hospital has received several reports that one of her colleagues, a 64-year-old male surgeon, has “not been acting like himself” recently. An administrative assistant says that during the past 6 months, the surgeon’s dictations have become hard to follow and appear to be missing information; previously, they were comprehensive and easy to transcribe. The assistant adds that after the surgeon missed three meetings in a row during the past 2 months, he asked her to send him a text message 5 minutes before the next meeting to let him know its location. Previously, he was always in attendance and on time for meetings. A senior resident also has noted unusual behavior by the surgeon; the resident says that while on rounds 3 days ago, the surgeon listed incorrect indications for a specific surgical procedure. A scrub nurse reported that the surgeon used unsterile practices three times during a 2-hour procedure today. The chief medical officer meets with the surgeon and asks him about these reported incidents. The surgeon says that “nothing is wrong” and the people who reported him are angry with him for various reasons and “are trying to stir up trouble.” Which of the following is the most appropriate action for the chief medical officer to take? (A) Accept the surgeon’s answer and only pursue further action if there is another complaint (B) Determine whether other staff have noticed anything different about the surgeon’s behavior (C) Inform the surgeon that he should consider retirement (D) Instruct the surgeon to take a temporary leave of absence and undergo a medical evaluation

**AI_B:** 1) RESTATING THE QUESTION: The chief medical officer is dealing with a situation involving a 64-year-old male surgeon who has been behaving unusually, making it harder for the staff working with him. Despite his denials, multiple reports testify to issues such as memory loss, inaccurate medical information, and unsterile practices. The officer is looking to determine a proper course of action, which may include dismissing the surgeon's assertions and waiting for any further complaints, seeking out more staff testimony, suggesting the surgeon consider retirement, or directing him to take a temporary leave of absence while getting a medical evaluation. 2) PLANNING: The assumption here is that the surgeon's behavior is abnormal, and that it is negatively affecting his work and the hospital's functioning. Alternative assumptions could be that the surgeon is under exceptional stress or that he has a medical condition that might be affecting his cognitive function. Given the reported drastic change in behavior and potentially dangerous inaccuracies in surgical practices, it seems critical, from a health and safety perspective, to validate the assumption that the surgeon might indeed be undergoing a medical issue. 3) CLINICAL REASONING: A sudden change in behavior like this can often be indicative of a medical condition, particularly given that the surgeon is now in his mid-60s. His forgetfulness might be a sign of age-related cognitive decline or a neurological disorder. Potentially, it could also stem from stress or depression. While these conditions are not uncommon in the elderly, they could be managed or treated once diagnosed. The safety of patients is paramount and erring on the side of caution seems to be a justified course for the Chief Medical Officer. 4) CONCLUDING: A logical progression from the clinical reasoning would be to have the surgeon temporarily step back from his duties until a thorough medical evaluation can be conducted. This safeguards patient safety and facilities while also showing concern for the well-being of the surgeon. This approach validates the observations of the staff and reassures them of their safety and well-being too. 5) RESPONSE: The most appropriate action for the Chief Medical Officer to take is (D) instruct the surgeon to take a temporary leave of absence and undergo a medical evaluation. This ensures the hospital's prudence and conscientiousness while also incorporating the possibility that the surgeon might be dealing with a medical condition that is affecting his performance.

**AI_D:** RESTATING THE QUESTION: The situation revolves around a senior surgeon who has demonstrated substantial changes in his professional behavior and cognitive performance. The focus lies on the chief medical officer's approach to handling this issue. The options provided range from accepting the surgeon's explanation to compelling him to take a leave of absence for medical investigation. PLANNING: The assumptions include: (1) the surgeon is perfectly healthy and his colleagues are fabricating stories out of resentment; (2) a decline in the surgeon’s cognitive abilities or health is being mistaken for other issues; (3) the surgeon should consider retirement based on these reports; (4) the surgeon may have an undetected health problem that needs medical attention. Considering all aspects, it can be assumed that the most important assumption to evaluate would be whether the behavioral changes observed in this surgeon could be linked to a medical condition that can affect his expertise and patient safety—an assumption that can be verified through a medical evaluation. CLINICAL REASONING: Changes in cognitive abilities can often be subtle and manifest as changes in behavior or performance, especially in individuals involved in highly skilled tasks such as surgery. The multiple examples cited here — difficulty with dictations, losing track of meeting schedules, making procedural errors, and non-sterile practices — suggest the possibility of cognitive impairment. Cognitive impairment in adults can be caused by many medical conditions including neurodegenerative diseases, stroke, and metabolic conditions. The surgeon’s dismissal of concern can be considered as unawareness which is common in people with cognitive impairment. Thus, it is crucial to determine whether these changes are due to an underlying medical condition that needs intervention, or if other factors are affecting the surgeon's abilities. CONCLUDING: It is the responsibility of the chief medical officer to ensure the safety and well-being of not only the patients but also the healthcare providers. The evidence of behavioral change and cognitive slip-ups exhibited by the surgeon point towards the potential presence of a medical condition leading to impaired functioning. Therefore, medical evaluation would allow correct identification, treatment, and management of any underlying conditions, and thereby ensure patient safety and the surgeon's well-being as well. RESPONSE: (D) Instruct the surgeon to take a temporary leave of absence and undergo a medical evaluation is the most appropriate action for the chief medical officer to take. This course of action would bear the utmost regard for the ongoing health concerns, professionally ethical conduct, and patient safety.

**AI_E:** (1) RESTATING THE QUESTION: The question posits a scenario where a senior surgeon's behaviour at a hospital has drastically changed, arousing concern among his colleagues. Several instances of recently unusual behaviour have been reported: difficulty in understanding his dictations, forgetfulness, incorrect clinical judgments, and violation of sterile surgical practices. Despite these alarming indicators, the surgeon is in denial, blaming personal conflict for the complaints. The chief medical officer, who has been alerted to these issues, is now faced with determining the next best course of action. (2) PLANNING: Various potential causes could be underlying the surgeon's change in behaviour. These include possible cognitive impairment due to age-related conditions such as dementia or neurodegenerative disorders, psychological stress, or other medical conditions like an undiagnosed metabolic or hormonal imbalance that can affect cognition. It's also possible that the surgeon may be experiencing side effects from medications or substances. Another assumption could be that the surgeon is experiencing burnout or work-related stress. Clinical prioritization thus requires ruling out any potential health issues that could be compromising his ability to perform his duties. It's essential not to hastily conclude retirement as the ideal solution, or merely relying on further complaints, as these neither address the root cause nor assure patient safety in the interim. (3) CLINICAL REASONING: In such a case where potentially dangerous clinical errors are being reported, it's critical to assess whether the surgeon is medically fit to continue work - especially before the situation escalates to harm patients or himself. These behavioural changes could be indicative of mental or physical health issues, which necessitates immediate medical evaluation. Furthermore, placing the surgeon on temporary leave minimizes patient safety risk and allows the individual to seek the necessary care. (4) CONCLUDING: Given the pattern of change in behaviour and professional conduct, probable cognitive impairment or another significant medical issue cannot be ruled out. The chief medical officer has a responsibility towards the safety of the patients, the wellbeing of the staff, and the surgeon himself. Therefore, the surgeon should not continue working without being evaluated for potential health issues. (5) RESPONSE: Thus, the chief medical officer should go with option (D): Instruct the surgeon to take a temporary leave of absence and undergo a medical evaluation. By choosing this action, she will address the specific concern while also ensuring the safety of the hospital's patients during this interim period.

**AI_C:** RESTATING THE QUESTION: The Chief Medical Officer (CMO) at a hospital has received several reports about a 64-year-old male surgeon who has been behaving unusually in recent times. This unusual behavior includes hard-to-follow dictations, missing meetings, stating incorrect surgical indications, and reportedly using unsterile practices. When the CMO questioned the surgeon, he claimed that the reports were made out of spite and denied any issues. Given these circumstances, the CMO is considering her options: to accept the surgeon's answer until more complaints come in, inquire if other staff members have noticed peculiarities in the surgeon's behavior, suggest retirement to the surgeon, or command him to take a leave of absence and get medically evaluated. PLANNING: The primary assumptions here are that the surgeon is experiencing some medical or psychological condition causing cognitive impairment or there might be interpersonal conflicts leading to false reports. An alternate assumption, though less likely could be that there is a systematic issue in the hospital's working environment. These assumptions must be clinically prioritized given their potential ramifications on patient safety. CLINICAL REASONING: The age of the surgeon (64 years), coupled with the reported signs such as declined cognitive abilities (difficulty with dictations, forgetting meeting timings, incorrect indications), and breaches in standard practice (unsterile practices) can imply onset of a cognitive disorder like dementia. Contrariwise, if the reports are being falsified due to interpersonal distress, then it might be a separate administrative issue. However, the gravity of complaints, especially those regarding patient safety, should be addressed immediately. Moreover, the surgeon's denial of the problem could be a coping mechanism if he is dealing with a cognitive issue, adding further complexity. CONCLUDING: By considering the above facts, the immediate step should be towards assessing the surgeon's health. While communicating with staff about their experiences could be valuable, it does not directly address the safety of patients nor the surgeon's well-being. Retirement suggestion or waiting for more complaints does not actively deal with the potential problem and could jeopardize patient safety. RESPONSE: The most appropriate action for the Chief Medical Officer is to instruct the surgeon to take a temporary leave of absence and undergo a medical evaluation (Choice D). This will both ensure immediate patient safety and possibly confirm or rule out a medical condition that may be causing the surgeon's reported behaviors.

**AI_A:** (1) RESTATING THE QUESTION: This question describes a situation where a 64-year-old male surgeon has recently been showing a change in his routine behavior. His dictations have become hard to follow, he is missing meetings which he previously never did, and his medical practices at times are improper. Additionally, a senior resident reports that the surgeon has provided incorrect indications for a surgical procedure. Upon confronting him, he denies all allegations, stating that those who reported him are simply stirring up trouble. The problem at hand is to identify the most suitable course of action for the chief medical officer, out of the suggested options, to handle this situation. (2) PLANNING: The first assumption could be that the surgeon is indeed under some sort of stress which is affecting his performance and professionalism, despite his insistence that nothing is wrong. Alternatively, his decline in performance and behavioral change could be attributed to a medical issue, for example neurologic conditions such as Alzheimer's or a brain tumor. Also, his responses suggest a voluntary denial of the complaints against him, which adds another layer of complexity to this case. It is unknown at this point whether this is a coping mechanism or part of his personality. (3) CLINICAL REASONING: Considering both assumptions, focusing only on interpersonal issues as the surgeon suggests could lead to overlooking a possible underlying medical condition that requires diagnosis and treatment. Symptoms such as forgetfulness, disorientation, difficulty following intricate processes, all point toward possible cognitive impairment while actions such as missed meetings, failing to follow sterile protocols, and incorrect indications for a surgical procedure raise concerns over his professional competency. The decline in the surgeon's abilities combined with his age suggest that there might be an underlying issue, potentially medical. (4) CONCLUDING: The chief medical officer has a responsibility not only towards the surgeon in question but also for patients' safety and quality of health services of the hospital. Given the assumption that a medical condition could be a possibility, ignoring these signs could lead to grave consequences affecting patients' health and safety. It is crucial to rule out any physical or mental health issues that might be causing these reported changes in the surgeon’s behavior. (5) RESPONSE: Therefore, given the variety and severity of the reported incidents, the most appropriate course of action would be for the chief medical officer to '(D) Instruct the surgeon to take a temporary leave of absence and undergo a medical evaluation'. It is critical in ensuring the care and safety of patients, as well as the well-being of the surgeon. Conducting a full medical evaluation will enable a better understanding of the surgeon's sudden behavioural changes and can provide a foundation for determining the next steps accordingly.

**Facilitator** The consensus among the AI responses is that the most appropriate course of action for the chief medical officer, given the reported changes in the 64-year-old male surgeon's behavior and professional conduct, is to instruct the surgeon to take a temporary leave of absence and undergo a medical evaluation. This decision is based on the assumption that the surgeon's behavioral changes could be indicative of a potential medical or cognitive issue, such as age-related cognitive decline, a neurological disorder, or other health conditions that might affect his cognitive function. The surgeon's denial of the problem could be a coping mechanism if he is dealing with a cognitive issue, adding further complexity. The chief medical officer has a responsibility towards the safety of the patients, the wellbeing of the staff, and the surgeon himself. Therefore, the surgeon should not continue working without being evaluated for potential health issues. This course of action would bear the utmost regard for the ongoing health concerns, professionally ethical conduct, and patient safety.
