# Supplementary material for: Collaborative intelligence in AI: Evaluating the performance of a council of AIs on the USMLE
Source: PLOS Digit Health. 2025 Oct 9;4(10):e0000787. doi: 10.1371/journal.pdig.0000787 (PMC12510544; doi:10.1371/journal.pdig.0000787)
Supplement: S2 File — (ZIP) [file pdig.0000787.s002.zip › S2 Data Raw data files containing unprocessed question inputs and LLM outputs including transcripts of deliberations/STEP2_Question-56-1_Type-MC-NJ.docx]

**Facilitator** **[Round 1]:** A 33-year-old woman is admitted to the hospital because of visual field loss following a 48-hour history of acute eye pain. She also has a 3-week history of numbness and a feeling of ""heaviness"" in both legs. She has no history of serious illness and takes no medications. Six weeks ago, she returned from a hiking expedition in the northeastern USA; she has no recent history of international travel. Vital signs are within normal limits. Muscle strength is 5/5 in the upper extremities, 3/5 in the hip flexors and knee extensors, and 4/5 in the knee flexors and ankle dorsiflexors. Patellar and ankle reflexes are 3+ bilaterally, and there is nonsustained ankle clonus bilaterally. Sensation to pinprick is absent throughout the lower extremities. Ophthalmologic examination discloses optic neuritis and reconfirms the visual field loss. Which of the following studies is most likely to confirm the diagnosis? (A) CT scan of the cervical spine (B) Electromyography and nerve conduction studies (C) Lumbar puncture for examination of cerebrospinal fluid (D) Measurement of serum Lyme (Borrelia burgdorferi) antibody concentration (E) MRI of the brain

**AI_A:** RESTATING THE QUESTION: The question presents a case where a 33-year-old woman experiences a number of symptoms including acute eye pain with visual field loss, leg numbness and heaviness, and absent sensation to pinprick in the lower extremities. An ophthalmologic examination also reveals that she has optic neuritis. These signs and symptoms could indicate a neurological disorder. The question then asks which of the listed studies is most likely to confirm the diagnosis. PLANNING: The symptoms indicate a potential neurological disorder, possibly involving both the central and peripheral nervous system. The presence of optic neuritis specifically, along with the described symptoms affecting the lower limbs, can suggest a condition like Multiple Sclerosis. The patient's recent travel history to the northeastern USA and her symptoms might suggest Lyme disease as a possibility as well. However, the presence of optic neuritis and the distribution of weakness and numbness suggest a disorder affecting the central nervous system more than a peripheral nervous disorder, which is typical in Lyme disease. CLINICAL REASONING: Multiple Sclerosis commonly presents with episodes of neurological dysfunctions, like the optic neuritis and the lower limb sensory and motor symptoms in this case. It is a condition characterized by demyelination in the central nervous system (CNS). Neurological examination findings and a patient's symptoms can be suggestive, but confirmation frequently requires imaging to visualize the characteristic demyelinated areas in the CNS. CONCLUDING: Considering the described symptoms and the physical examination findings which suggest a CNS disorder, along with optic neuritis which is a common feature in Multiple Sclerosis, performing an MRI of the brain would be the most appropriate next step in this diagnostic process. RESPONSE: Therefore, the most likely study to confirm the diagnosis in this scenario would be (E) MRI of the brain.

**AI_C:** RESTATING THE QUESTION: The question is asking us to determine, from the given options, the most suitable diagnostic test to confirm the diagnosis for a 33-year-old woman. She presents with a myriad of symptoms including visual field loss, acute eye pain diagnosed as optic neuritis, heaviness and numbness in both legs, abnormal muscle strength, and abnormal reflexes. PLANNING: An assumption in this case could be that the combination of optic neuritis, abnormal muscle strength, altered reflexes, and altered sensation are pointing to a pathological process that is systemic or affecting multiple areas of the nervous system rather than a localized one. An opposite assumption might be that the symptoms are the result of a more localized issue. However, the assortment of neurologic findings, paired with optic neuritis, raises suspicion for a disease involving the central nervous system, likely multiple sclerosis (MS) because MS is a demyelinating disease that can present with this variety of symptoms. CLINICAL REASONING: The clinical presentation of this woman suggests a demyelinating disease such as multiple sclerosis. The optic neuritis is a classic symptom of multiple sclerosis, often presenting as pain with eye movement and visual loss. The sensory and motor symptoms in the legs could be related to spinal cord involvement, which causes weakness, increased reflexes and clonus from upper motor neuron damage, and loss of sensation due to damage to the sensory tracts. Multiple sclerosis is classically episodic, with symptoms appearing and disappearing, which could explain the woman's acute eye symptoms on top of her ongoing leg symptoms. CONCLUDING: Given the aforementioned clinical reasoning, a diagnostic study that would allow us to visualize potential multiple areas of demyelination in the brain and spinal cord would be the most beneficial in confirming the diagnosis. RESPONSE: The recommendation would be (E) MRI of the brain, recognisable as the best tool for visualizing changes indicative of multiple sclerosis, such as demyelinated lesions.

**AI_B:** RESTATING THE QUESTION: Our task is to identify the most suitable diagnostic study for a 33-year-old woman who presents with symptoms including visual field loss, acute eye pain, numbness, a feeling of "heaviness" in both legs, abnormal muscle strength and reflexes, and absence of sensation to pinprick throughout the lower extremities. Her ophthalmologic examination reveals optic neuritis. These symptoms suggest some form of neurological issue. PLANNING: The different choices available point to various assumptions about the possible diagnosis. Options (A) and (E) suggest some form of central nervous system pathology. Option (B) would suggest a peripheral nervous system disorder. Option (C) would indicate a possible infectious or autoimmune process. Option (D) points to Lyme disease (a tick-borne illness common in the northeastern USA). Given the woman's history of neurological symptoms, recent travel to an area endemic for Lyme disease, eye pain indicating optic neuritis and change in lower limb strength and sensation, a disorder affecting the central nervous system seems apparent. CLINICAL REASONING: Optic neuritis, which is an inflammation of the optic nerve, often occurs in the context of multiple sclerosis (MS), an autoimmune disorder of the central nervous system. MS could also present with limb numbness and motor weakness, as seen in this patient. The positive travel history to the northeastern USA could suggest Lyme Disease; however, the absence of typical early manifestations such as erythema migrans (EM) rash, and the presence of central nervous system features such as optic neuritis, suggest a more likely diagnosis of MS. CONCLUDING: While both multiple sclerosis and neurologic Lyme disease could present with neurological symptoms, the occurrence of optic neuritis is more commonly associated with MS than with Lyme disease. Thus, a test revealing demyelinating lesions (a hallmark of MS) would be expected to confirm the most likely diagnosis. RESPONSE: Therefore, an (E) MRI of the brain would be the most appropriate study to confirm the diagnosis in this case, as it can reveal multiple demyelinating lesions, a typical feature in the brain of a person with multiple sclerosis.

**AI_D:** 1) RESTATING THE QUESTION: The question asks for the best investigation to confirm the diagnosis for the presented patient's situation: she's a 33-year-old female who recently embarked on a hiking trip in the northeastern USA, and now presents with visual field loss, acute eye pain (specifically optic neuritis), absence of pinprick sensation in the lower extremities, and both numbness/heaviness in and reflex abnormalities of both legs. Anatomically, her optic neuritis involves the optic nerve, a part of the CNS. Physiologically, the clonus (involuntary muscle contractions) and abnormal reflexes indicate possible hyper-reflexia, pointing toward an upper motor neuron lesion also within the CNS. Concurrently, she exhibits loss of sensation and weakness of leg muscles, which signify lower motor neuron involvement, yet not necessarily rooted in the CNS. Notably, she doesn't have a history of serious illnesses or medication use, but she was on a hiking trip in a region synonymous with Lyme disease, a condition caused by the bacterium Borrelia burgdorferi. 2) PLANNING: As such, the syndromal presentation points towards a CNS pathology involving both upper and lower motor neurons. We should ponder whether a single illness such as Multiple Sclerosis or neurologic Lyme disease could be responsible, or if multiple, concurrent pathologies could explain her symptoms, such as Lyme disease alongside a separate CNS pathology. 3) CLINICAL REASONING: One alternate assumption is Multiple Sclerosis (MS), a pathogenesis involving immune-mediated inflammation and demyelination. It tends to affect young adults and may present as optic neuritis and lower motor neuron involvement - both observed in this patient. If MS is the clinical priority, MRI of the brain (option E), with its capability to visualize white matter lesions associated with MS, becomes the testing priority. Alternatively, neurologic Lyme disease could account for her symptoms, especially given her recent hiking expedition. However, her bilateral hyperactive reflexes and clonus are less typical of Lyme disease. In this case, serum Lyme antibody concentration measurement (option D) would be the fitting assessment. 4) CONCLUDING: Whereas Lyme disease could explain some symptoms, her clinical picture fits more comprehensively with an immune-mediated CNS disorder like MS. Her symptoms are not confined to peripheral nerves that Lyme disease typically affects, but instead imply both central and peripheral nervous system pathology, best encapsulated by MS. Thus, option E, MRI of the brain, should be the prioritized diagnostic study. 5) RESPONSE: To confirm the diagnosis in this patient who presents signs indicative of Multiple Sclerosis, an MRI of the brain (E) is the most appropriate course of action.

**AI_E:** RESTATING THE QUESTION: The clinical scenario is of a 33-year-old woman who came to the hospital due to visual loss and acute eye pain. She also experiences numbness and feelings of heaviness in the legs, with no history of significant past illness or use of medications. Her travel history includes having returned from a hiking expedition in the northeastern USA six weeks ago but no recent international travel. Her muscle strength varies between the upper and lower extremities, with decreased sensation in the lower extremities. Her ophthalmologic examination revealed optic neuritis and confirmed visual field loss. We are asked to identify the most appropriate diagnostic test to confirm her diagnosis. PLANNING: This woman has signs and symptoms suggestive of neurological or neuro-ophthalmologic disorders, including visual loss, acute eye pain, numbness, and heaviness in both legs. On neurological examination, she presents with a decrease in muscle strength in the lower extremities, altered reflexes, nonsustained ankle clonus, and absent sensation to pinprick in the lower extremities. Optic neuritis is also observed. Various diagnostic tests can help identify the potential cause of this syndrome, such as a CT scan of the cervical spine, electromyography and nerve conduction studies, lumbar puncture for CSF examination, antibody testing for Lyme (Borrelia burgdorferi), and MRI of the brain. CLINICAL REASONING: The clinical features including visual field loss from optic neuritis, disturbances in upper motor neuron functions (evidenced by muscle strength differences, increased reflexes, unsustained ankle clonus), and sensory changes indicate a central nervous system lesion. The presentation is concerning for multiple sclerosis (MS), which often manifests as optic neuritis, spinal cord symptoms (such as the paresthesias and strength changes in the legs seen in this patient), and symptoms disseminated in time and space. MS is commonly diagnosed in women in their third or fourth decade of life, aligning with this patient's demographics. While a CT scan of the cervical spine could show an abnormality indicative of MS, such as lesion or demyelinating plaque, MS plaques in the brain may be missed. EMG and nerve conduction studies are typically more useful in peripheral nerve disease but not as useful in central demyelinating disorders like MS. Lumbar puncture can reveal important signs of MS, including oligoclonal bands and increased IgG index, but it is not the initial diagnostic test of choice. Lyme disease can sometimes mimic MS symptomatically, but her clinical picture doesn't strongly suggest Lyme disease. CONCLUDING: While other diagnostic tests may provide useful information, an MRI of the brain is the best initial test in the context suggestive of multiple sclerosis, hence, it is the diagnostic modality that is most likely to confirm the diagnosis in this scenario. RESPONSE: (E) MRI of the brain.

**Facilitator** The consensus statement synthesizes the responses of AI_A, AI_B, AI_C, AI_D, and AI_E as follows: The question presents a case of a 33-year-old woman who has been experiencing visual field loss, acute eye pain, numbness, and a feeling of "heaviness" in both legs. She has no history of serious illness and takes no medications. Her recent travel history includes a hiking expedition in the northeastern USA. Her ophthalmologic examination reveals optic neuritis and confirms the visual field loss. The question asks which of the listed studies is most likely to confirm the diagnosis. The symptoms and signs presented by the woman suggest a neurological disorder, possibly involving both the central and peripheral nervous system. The presence of optic neuritis, along with the described symptoms affecting the lower limbs, can suggest a condition like Multiple Sclerosis (MS). The patient's recent travel history to the northeastern USA might suggest Lyme disease as a possibility as well. However, the presence of optic neuritis and the distribution of weakness and numbness suggest a disorder affecting the central nervous system more than a peripheral nervous disorder, which is typical in Lyme disease. Multiple Sclerosis commonly presents with episodes of neurological dysfunctions, like the optic neuritis and the lower limb sensory and motor symptoms in this case. It is a condition characterized by demyelination in the central nervous system (CNS). Neurological examination findings and a patient's symptoms can be suggestive, but confirmation frequently requires imaging to visualize the characteristic demyelinated areas in the CNS. While both multiple sclerosis and neurologic Lyme disease could present with neurological symptoms, the occurrence of optic neuritis is more commonly associated with MS than with Lyme disease. Thus, a test revealing demyelinating lesions (a hallmark of MS) would be expected to confirm the most likely diagnosis. Therefore, considering the described symptoms and the physical examination findings which suggest a CNS disorder, along with optic neuritis which is a common feature in Multiple Sclerosis, performing an MRI of the brain would be the most appropriate next step in this diagnostic process. Hence, the most likely study to confirm the diagnosis in this scenario would be (E) MRI of the brain.
