# Supplementary material for: Collaborative intelligence in AI: Evaluating the performance of a council of AIs on the USMLE
Source: PLOS Digit Health. 2025 Oct 9;4(10):e0000787. doi: 10.1371/journal.pdig.0000787 (PMC12510544; doi:10.1371/journal.pdig.0000787)
Supplement: S2 File — (ZIP) [file pdig.0000787.s002.zip › S2 Data Raw data files containing unprocessed question inputs and LLM outputs including transcripts of deliberations/STEP2_Question-57-1_Type-MC-NJ.docx]

**Facilitator** **[Round 1]:** A 68-year-old woman comes to the office because she is concerned about developing Alzheimer disease. She says she has had increasing difficulty recalling names of people and objects during the past 6 months. She has not had difficulty with her activities of daily living, finances, or shopping. She teaches part-time at a local community college. She has no history of serious illness and takes no medications. She does not smoke cigarettes. She drinks one 5-oz glass of wine daily. Her mother developed dementia, Alzheimer type, in her mid 70s. Physical and neurologic examinations of the patient show no abnormalities. On cognitive testing, she has delayed recall of three of five words. She is able to perform serial sevens, knows similarities, is fully oriented, and has intact executive, visuospatial, and attention abilities. Which of the following is the most appropriate next step in management? (A) Amyloid plaque imaging (B) Determination of ApoE allele status (C) Measurement of beta-amyloid in the cerebrospinal fluid (D) MRI of the brain with volumetric analysis (E) No additional diagnostic studies are indicated

**AI_D:** Restating the question, a 68-year-old woman who is worried about her risk of developing Alzheimer's disease, given her mother's history, presents with minor memory difficulties despite preserving her daily living functions, professional responsibilities, and cognitive abilities. In such a context, we are asked which next step in her management would be most appropriate. The options include imaging and genetic methods to detect the presence of Alzheimer's markers, MRI of the brain for structural changes, and the possibility of no further diagnostic studies. The woman's mild difficulties recalling people's names and objects, paired with her family history of Alzheimer's disease in her mother, lead to the assumption that she might be experiencing early signs of Alzheimer's. It would also seem logical to assume that the woman's consumption of wine might be a contributing factor to potential cognitive decline. However, an alternate assumption would be to recognize that she is maintaining her executive, visuospatial, and attention abilities and has no difficulty managing her everyday activities, suggesting her cognitive functions are still largely intact. Furthermore, because her mother developed Alzheimer's in her mid-seventies, and this woman is only 68, it is worth questioning whether an inherited predisposition through genetic factors is definitively applicable in this case. If we prioritize the alternate assumption, we look at the woman's situation through a clinical lens. From a clinical perspective, it is typical to notice some cognitive decline as one ages, and mild memory difficulty, especially recall of names, is a common complaint among older adults. Furthermore, several tests suggest that this woman's cognitive function, except for the slight issue with recall, is well within the normal range. She also retains the ability to perform her day-to-day tasks and professional responsibilities, which further indicates that her memory concerns may not be symptomatic of a significant pathological process like Alzheimer's disease at present. In conclusion, with her current cognitive testing outcomes, lack of significant functional impairment, and absence of other risk factors apart from her mother's history of Alzheimer's, it would be reasonable to consider her as having age-associated memory impairment rather than proceeding immediately towards extensive testing for Alzheimer's disease. Thus, the most appropriate next step in her management is to avoid additional diagnostic studies at this point (E) until there's a significant change in her cognitive abilities or daily functioning, keeping in mind that a close follow-up is necessary in her case given her family history of Alzheimer's disease. This approach is coherent with the principle of primum non nocere - "First, do no harm", avoiding unnecessary invasive testing in a patient where the clinical judgment doesn't suggest any definite pathology.

**AI_B:** RESTATING THE QUESTION: A 68-year-old woman has demonstrated some cognitive declines, such as occasional difficulty recalling names of people and objects over the past 6 months. Despite this, she has not experienced issues with daily activities, finances, or shopping, and continues to teach part-time. Her past health history is largely unremarkable, with no significant illnesses and no current medications. She has a positive family history of Alzheimer's disease from her mother who developed it in her mid-70s. However, a physical and neurologic examination revealed no abnormalities, and her cognitive testing had mostly positive results except for the delayed recall of three out of five words. We need to decide on the best course of action in terms of management and further investigations. PLANNING: The assumptions here are that her symptoms may be indicative of early Alzheimer's disease, especially given her positive family history, or that these could simply be signs of age-related cognitive decline. However, her preservation of executive, visuospatial, and attention abilities suggests the latter. It's also important to consider whether her symptoms are significant enough to warrant further diagnostic testing. CLINICAL REASONING: Alzheimer's disease is characterized by the impairment in daily activities and cognitive functions including language, praxis, gnosis, and executive functions, which this woman currently does not demonstrate. Her symptoms could also be attributed to Mild Cognitive Impairment, a state of cognitive function between normal aging and dementia, which doesn't affect activities of daily living. This condition may or may not progress to Alzheimer's disease and is characterized by a predominant memory impairment, which aligns more with her primary issue of recalling names. Notably, while advanced diagnostic techniques exist, their use isn't generally recommended unless there's a specific clinical reason such as an unclear diagnosis, exceptionally fast progression of the disease, or patients of younger than usual age. CONCLUDING: In conclusion, this woman's symptoms align more with Mild Cognitive Impairment rather than Alzheimer's. Given that her symptoms are not significantly impacting her daily living or cognitive functions outside of recall, and considering the risks, costs and psychological implications of further testing, it may not be necessary to undergo these tests. RESPONSE: Therefore, the most appropriate next step in management is (E): No additional diagnostic studies are indicated. The patient's cognitive issues do not significantly impede her daily life and further diagnostic testing may not change the current management or provide significant additional information. It would be important to monitor her cognitive status over time and provide information about healthy brain aging strategies.

**AI_A:** RESTATING THE QUESTION: The query in question concerns a 68-year-old female patient who is worried about potential signs of Alzheimer's disease. Her symptoms include increasing difficulty in remembering the names of people and objects during the last half year. Despite these memory issues, she has no difficulty with daily living tasks, finances or shopping. Her past medical history is unremarkable and she is currently not on any medications. She doesn't smoke and consumes moderate amounts of alcohol. There is a family history of Alzheimer's disease given that her mother developed it during her 70s. Physical and neurologic examinations appear normal. Cognitive testing revealed delayed recall of three out of five words. However, she was competent in performing serial sevens, knew similarities, was fully conscious, and showed no impairment in her executive, visuospatial, or attention abilities. The query asks for the most appropriate next step in her management from a list of options. PLANNING: In examining the current situation, we could make the assumption that the patient's symptoms already suggest a diagnosis of Alzheimer's disease and that further testing is required. However, let's generate an alternate assumption that her symptoms could be a normal part of aging and are not indicative of a disease process. This alternate assumption seems to be clinically relevant given the absence of impairment in her daily activities, apart from her difficulty in recalling names. CLINICAL REASONING: Following this line of reasoning, we work on the assumption that the patient's signs are part of aging rather than indicative of Alzheimer's disease. Her ability to handle daily life activities, finances and shopping tasks suggests that her cognitive function is largely preserved. Her cognitive testing results further support this view as aside from a delayed recall of three words, she shows remarkable cognitive abilities in other areas. The family history of Alzheimer's disease in her mother is important to consider but does not automatically mean she will develop the disease. CONCLUDING: Based on the alternate assumption and the evidence provided, the most appropriate next step in management seems to be monitoring her status without the need for further diagnostic studies. RESPONSE: Hence, the response to the question, "Which of the following is the most appropriate next step in management?" would be (E) No additional diagnostic studies are indicated.

**AI_E:** RESTATING THE QUESTION: The scenario involves a 68-year-old woman who has been experiencing difficulties in recalling names of people and objects for the past 6 months. She is anxious about the possibility of developing Alzheimer's disease, particularly as her mother was diagnosed with Alzheimer's in her 70s. Her cognitive function remains relatively intact, as evidenced by stable performance with daily activities and tasks that require executive, visuospatial, attention abilities, and knowledge of similarities. Despite possessing a delayed recall of three out of five words, no abnormalities have emerged in the patient's physical and neurological examinations. The management plan necessitates selectivity from options including amyloid plaque imaging, measurement of beta-amyloid in cerebrospinal fluid, determination of ApoE allele status, an MRI of the brain with volumetric analysis, or no additions to the diagnostic studies. PLANNING: From her self-reported memory problems concerning recalling names to her difficulty in remembering three of five words, an examination of her memory issues is paramount. However, as her mother developed Alzheimer's disease, it takes us to the assumption of a genetic predisposition. CLINICAL REASONING: When considering Alzheimer's disease, the primary focus tends to be placed on beta-amyloid plaques and tau tangles in the brain. While options A, B, and C are targeted towards identifying these Alzheimer disease markers, they are typically reserved for clinical research or ambiguous diagnostic cases. Critical to note is the distinct difference between normal aging and early Alzheimer's disease. Memory lapses, such as occasionally forgetting names or words, are common as individuals age and do not necessarily indicate Alzheimer's disease; however, these symptoms can foster anxiety, especially in those with a family history of dementia. In this patient's scenario, despite her mild memory complaint, she exhibits no substantive cognitive impairment, determined from her intact abilities in working memory, visual perception, attention skills, and cognitive flexibility. CONCLUDING: Bearing in mind the above-discussed aspects, it could be inferred that the symptoms presented by the patient don't necessarily coincide with those of Alzheimer's disease. Instead, they could be manifestations of normal aging-induced forgetfulness. Anxiety, significantly driven by her mother's medical history of Alzheimer's, might have exacerbated these forgetful incidents. RESPONSE: Hence, the choice (E), 'No additional diagnostic studies are indicated', would be the most appropriate next step in her management. The patient's worry about her memory should be addressed, and it might be beneficial to provide reassurance that her cognitive abilities are within the range of normal aging. However, if cognitive complaints persist or worsen, they warrant reassessment.

**AI_C:** (1) RESTATING THE QUESTION: A 68-year-old woman has been experiencing increasing struggle with recalling names of people and objects over the past six months, which has led to her concern over possibly developing Alzheimer's disease. Alzheimer's disease is a neurodegenerative condition characterized by memory loss and impaired cognitive functions. She has been able to manage her daily activities, finances and shopping, and remains intellectually active through part-time teaching. She has no serious medical history and does not take medications. Regular alcohol use, in the form of one daily glass of wine, is her lifestyle. A family history of Alzheimer's disease is present, with her mother developing the condition in her mid-seventies. From medical examinations, there are no observable physical or neurological abnormalities. Cognitive testing, however, shows delay in recall, although all other cognitive functions are intact. The question, therefore, is what would be the best next step in managing her case. (2) PLANNING: The five given possibilities link to our primary assumption that this patient is having the precursor symptoms of Alzheimer's disease. However, the alternative assumption would be that her memory symptoms could be age-related or due to other non-Alzheimer's causes. Evaluating these assumptions, one should prioritize determining if the symptoms are indicative of early Alzheimer's disease or not. (3) CLINICAL REASONING: The woman's symptoms, delayed recall but with other cognitive functions intact, suggests mild cognitive impairment (MCI), specifically, the amnestic type. Amnestic MCI presents with memory problems more severe than normally expected for the patient's age, but not severe enough to interfere with daily life or independent function - just as this woman's case. Therefore, onset Alzheimer's is a possibility, as the majority of MCI cases progress to Alzheimer's disease. However, it's also important to note that not all do, and some might even remain stable or return to normal over time. (4) CONCLUDING: The recent guidelines advise against routine use of genetic (such as ApoE status), or biomarker measures (such as measurement of beta-amyloid in CSF or amyloid plaque imaging) for Alzheimer's disease. In mild cognitive impairment, volumetric brain MRIs, while could aid in our understanding of disease progression, are not typically indicated. The absence of physical and neurological abnormalities, coupled with her ability to maintain her daily activities successfully, despite her forgetfulness, does not call for further diagnostic measures just yet. (5) RESPONSE: Therefore, from the available options, the most appropriate next step in managing the woman's case would be 'E'. No additional diagnostic studies are indicated at this time. Her situation should be monitored and reassessed periodically to spot any changes in her condition.

**Facilitator** The consensus statement synthesizes the responses of AI_A, AI_B, AI_C, AI_D, and AI_E as follows: The question pertains to a 68-year-old woman who has been experiencing increasing difficulty in recalling names of people and objects over the past six months. Despite these memory issues, she has no difficulty with daily living tasks, finances, or shopping. Her past medical history is unremarkable, and she is currently not on any medications. She doesn't smoke and consumes moderate amounts of alcohol. There is a family history of Alzheimer's disease given that her mother developed it during her 70s. Physical and neurologic examinations appear normal. Cognitive testing revealed delayed recall of three out of five words. However, she was competent in performing serial sevens, knew similarities, was fully conscious, and showed no impairment in her executive, visuospatial, or attention abilities. The primary assumption is that the patient's symptoms suggest a diagnosis of Alzheimer's disease and that further testing is required. However, an alternate assumption is that her symptoms could be a normal part of aging and are not indicative of a disease process. This alternate assumption seems to be clinically relevant given the absence of impairment in her daily activities, apart from her difficulty in recalling names. Her ability to handle daily life activities, finances, and shopping tasks suggests that her cognitive function is largely preserved. Her cognitive testing results further support this view as aside from a delayed recall of three words, she shows remarkable cognitive abilities in other areas. The family history of Alzheimer's disease in her mother is important to consider but does not automatically mean she will develop the disease. Her symptoms could also be attributed to Mild Cognitive Impairment, a state of cognitive function between normal aging and dementia, which doesn't affect activities of daily living. This condition may or may not progress to Alzheimer's disease and is characterized by a predominant memory impairment, which aligns more with her primary issue of recalling names. Given her current cognitive testing outcomes, lack of significant functional impairment, and absence of other risk factors apart from her mother's history of Alzheimer's, it would be reasonable to consider her as having age-associated memory impairment rather than proceeding immediately towards extensive testing for Alzheimer's disease. Therefore, based on the alternate assumption and the evidence provided, the most appropriate next step in management seems to be monitoring her status without the need for further diagnostic studies. Hence, the response to the question, "Which of the following is the most appropriate next step in management?" would be (E) No additional diagnostic studies are indicated. The patient's cognitive issues do not significantly impede her daily life and further diagnostic testing may not change the current management or provide significant additional information. It would be important to monitor her cognitive status over time and provide information about healthy brain aging strategies.
