# Supplementary material for: Collaborative intelligence in AI: Evaluating the performance of a council of AIs on the USMLE
Source: PLOS Digit Health. 2025 Oct 9;4(10):e0000787. doi: 10.1371/journal.pdig.0000787 (PMC12510544; doi:10.1371/journal.pdig.0000787)
Supplement: S2 File — (ZIP) [file pdig.0000787.s002.zip › S2 Data Raw data files containing unprocessed question inputs and LLM outputs including transcripts of deliberations/STEP2_Question-58-1_Type-MC-NJ.docx]

**Facilitator** **[Round 1]:** A 55-year-old man with major depressive disorder is brought to the emergency department 1 1/2 hours after he was found by his brother in his home acting strangely. On arrival, he is babbling incoherently and cannot answer questions. There are two empty pill bottles in his pockets; one is for paroxetine and the other is for duloxetine. He is restless, diaphoretic, and says he has a severe headache. Temperature is 38.5°C (101.3°F), pulse is 130/min, and blood pressure is 145/105 mm Hg. The pupils are 8 mm and reactive to light. Deep tendon reflexes are 4+ and symmetric in all extremities. There is spontaneous myoclonus in the upper extremities. Results of laboratory studies, including a complete blood count, serum electrolyte concentrations, creatine phosphokinase activity, and thyroid function tests, are within the reference ranges. After admitting the patient to the hospital for observation and supportive care, what are you going to give or change in terms of pharmacotherapy? (A) Add carbamazepine to the medication regimen (B) Add dantrolene to the medication regimen (C) Add haloperidol to the medication regimen (D) Discontinue paroxetine and duloxetine (E) Switch from duloxetine to olanzapine

**AI_B:** RESTATING THE QUESTION: To rephrase, a 55-year-old man with a history of major depressive disorder was found in his house behaving oddly. Upon arrival at the emergency department, he is incoherent, restless, and experiencing a severe headache. He has two empty pill bottles for paroxetine and duloxetine. His vital signs and physical exam are notable for fever, tachycardia, hypertension, mydriasis, hyperreflexia, and myoclonus. Standard lab work is normal. After admitting him for observation and supportive care, what modifications should be made to his drug treatment? PLANNING: The presentation of the patient suggests serotonin syndrome, a potential life-threatening condition caused by excessive serotonergic activity in the nervous system. This can be triggered by increased dosage, drug interactions, or overdose of serotonergic drugs. This condition is characterized by mental status changes, autonomic hyperactivity, and neuromuscular abnormalities. Assumption is that patient's current medications contributed to the syndrome. The key would be to discontinue the offending drugs first. CLINICAL REASONING: In serotonin syndrome, symptoms will typically resolve within 24 hours of discontinuation of the causative agent(s). If the symptoms are severe or the drug has a longer half-life (such as fluoxetine, not the case here), additional interventions may be necessary. The most important intervention in managing serotonin syndrome is discontinuation of all serotonergic agents, including both prescription medications (like the patient's paroxetine and duloxetine) and illicit substances. Recall that paroxetine is a selective serotonin reuptake inhibitor (SSRI) and duloxetine is a serotonin-norepinephrine reuptake inhibitor (SNRI), both of which increase the levels of serotonin in the brain. CONCLUDING: Based on the workflow process provided, a stepwise approach suggests that the patient's current medications, paroxetine and duloxetine, are the likely cause of the serotonin syndrome. In this case, these medications should be discontinued. RESPONSE: Therefore, the optimal treatment approach would be to (D) Discontinue paroxetine and duloxetine.

**AI_C:** RESTATING THE QUESTION: The scenario discusses a 55-year-old man diagnosed with major depressive disorder who has potentially overdosed on paroxetine and duloxetine. With incoherent speech, restlessness, diaphoresis, a severe headache, elevated temperature, increased pulse, hypertension, dilated pupils, hyper-reactive reflexes, and spontaneous myoclonus, the situation hints at a syndrome called serotonin syndrome, often linked to overdose or drug interactions impacting serotonin levels. The question implies asking about the best next step in managing this patient's medication regimen, given this possibility. PLANNING: It's critical to infer that this patient's current symptoms are consistent with serotonin syndrome and conferring this assumption with the medical history and drug use. Potential solutions could be either adding a drug that neutralizes the serotonin surge, withdrawing the serotonergic drugs, or switching to a non-serotonergic antidepressant. CLINICAL REASONING: The first step in managing serotonin syndrome is to immediately discontinue all serotonergic agents, which in this case would be paroxetine and duloxetine. This will stop the flooding of the synaptic gap with serotonin, reducing the risk of further complications and symptoms. Additionally, symptomatic and supportive treatments are usually enough in mild to moderate cases. In severe cases, serotonin antagonists like cyproheptadine could be used. None of the drugs mentioned in the options, apart from discontinuing the serotonergic drugs, can specifically address the problem. Carbamazepine is commonly used in seizures and bipolar disorder, dantrolene is a muscle relaxant used in malignant hyperthermia, haloperidol is an antipsychotic, and olanzapine is another serotonin reuptake inhibitor, presenting similar problems. CONCLUDING: Based on the clinical reasoning and the condition stated in the clinical scenario, discontinuing the two medications causing serotonin syndrome seems like the best approach. Without intervention, this condition can pose serious complications, including death. RESPONSE: The best option for this patient would be (D) Discontinue paroxetine and duloxetine.

**AI_A:** (1) RESTATING THE QUESTION: The question discusses a 55-year-old male who is presenting with incoherent babbling, restlessness, diaphoresis, headache, increased temperature, tachycardia, hypertension, dilated pupils, hyperreflexia, and spontaneous myoclonus. This patient, with a known history of major depressive disorder, has been taking both paroxetine and duloxetine. The question asks us what change should be made to his current medication regime given his symptoms. Paroxetine and duloxetine are both medications used to treat depression, with paroxetine being a selective serotonin reuptake inhibitor (SSRI) and duloxetine being a serotonin-norepinephrine reuptake inhibitor (SNRI). Hence, both work by increasing serotonin levels in the brain. (2) PLANNING: The patient's current signs and symptoms are suggestive of serotonin syndrome - a potentially life-threatening condition that can occur with the use of serotonergic drugs like SSRIs and SNRIs. Increased reflexes, myoclonus, cognitive changes, autonomic hyperactivity, and fever are symptoms associated with this syndrome. Given this situation, the assumption that could be valid and prioritized is that the concurrent use of paroxetine and duloxetine in this patient has led to serotonin syndrome. (3) CLINICAL REASONING: Clinical diagnosis of serotonin syndrome is based on the Hunter serotonin toxicity criteria, which requires the presence of one of the following conditions: spontaneous clonus, inducible clonus, ocular clonus plus agitation or diaphoresis, or tremor and hyperreflexia. The patient's presentation, featuring spontaneous myoclonus (clonus) and hyperreflexia, fits this criteria, further suggesting serotonin syndrome. (4) CONCLUDING: The primary treatment for serotonin syndrome is discontinuing the offending medication(s) and providing supportive care. Given the patient is taking two serotonergic medications (paroxetine and duloxetine), these should be discontinued to prevent further increase in serotonin levels. (5) RESPONSE: So, for this patient, the correct course of action in terms of pharmacotherapy would be choice (D) Discontinue paroxetine and duloxetine.

**AI_E:** (1) RESTATING THE QUESTION: So, the question at hand is asking what should be done in terms of pharmacotherapy for a 55-year-old man exhibiting numerous signs of distress, such as incoherent babbling, extreme restlessness, severe headaches, increased body temperature (hyperthermia), and some abnormal heart symptoms. In his pockets, empty pill bottles of paroxetine and duloxetine, both anti-depressants, were found. Considering this, we must decide whether to add another drug to his regimen, discontinue the current medications, or switch to a different medication. (2) PLANNING: Let's consider each assumption separately. Paroxetine and duloxetine are both serotonin reuptake inhibitors; the former is a selective serotonin reuptake inhibitor (SSRI) and the latter is a serotonin-norepinephrine reuptake inhibitor (SNRI). Overdose of these drugs can lead to serotonin syndrome, which is characterized by mental status changes, autonomic hyperactivity, and neuromuscular abnormalities, symptoms that appear to be present in the patient. Serotonin syndrome should be the prioritized assumption as the patient has major depressive disorder, and was found with empty bottles of these medications; he also shows characteristic symptoms such as agitation, sweating, severe headache, hyperthermia, tachycardia, hypertension, myoclonic movements, and hyperreflexia. (3) CLINICAL REASONING: Carbamazepine, an anticonvulsant, is often used to treat seizure disorders and neurogenic pain but isn't typically adequate for serotonin syndrome. Dantrolene targets severe muscle contraction but mainly in malignant hyperthermia and neuroleptic malignant syndrome, not directly addressing serotonin syndrome. Haloperidol, an antipsychotic, may have its uses in treating symptoms or uncontrolled agitation but could potentially worsen serotonin syndrome. (4) CONCLUDING: Therefore, the drugs causing the overproduction of serotonin, in this case, paroxetine and duloxetine, should be discontinued ASAP. Furthermore, administering serotonin antagonists or supportive care to manage symptoms like hypertension and hyperthermia would be an ideal treatment direction to address serotonin syndrome. (5) RESPONSE: Considering such factors, the best response would be (D) Discontinue paroxetine and duloxetine. This is because the suspected overabundance of serotonin—caused by the overdose—can be counteracted or normalized by halting the intake of these serotonin-modulating drugs.

**AI_D:** RESTATING THE QUESTION: It appears that the patient, a 55-year-old man diagnosed with major depressive disorder, is suffering from a sudden change in his physical and mental state characterized by incoherent speech, restlessness, sweating, severe headache, fever, increased heart rate, and high blood pressure. He also exhibits large reactive pupils, fast deep tendon reflexes known as hyperreflexia in all his limbs, and spontaneous muscle jerks in his upper limbs known as myoclonus. These symptoms occurred after he presumably consumed an excessive quantity of his prescribed medications, paroxetine and duloxetine. All his lab tests including complete blood count, serum electrolyte levels, activity of an enzyme called creatine phosphokinase, and thyroid function tests, yielded normal results. Now the question is, what adjustments should be made to his medication regimen? PLANNING: Firstly, we need to consider the possibility of an overdose on paroxetine and duloxetine, both of which are antidepressants; the former belongs to the class of selective serotonin reuptake inhibitors (SSRIs) and the latter is a serotonin and norepinephrine reuptake inhibitor (SNRI). These medications increase the concentration of neurotransmitters serotonin and norepinephrine in the brain, which can elevate mood. Symptoms that point towards an overdose of these medications include restlessness, incoherent speech, hyperreflexia, myoclonus. This condition is known as serotonin syndrome. However, other medical conditions such as neuroleptic malignant syndrome, and malignant hyperthermia may also induce similar symptoms. We should focus on serotonin syndrome due to the given clinical context. CLINICAL REASONING: The primary management of serotonin syndrome involves discontinuing any offending agents, which in this case are paroxetine and duloxetine. This steps helps to decrease the excessive serotonin activity in the brain, thus alleviating the symptoms. Monitoring and supportive care may be necessary until the patient stabilizes. Sedation using benzodiazepines may also be provided as needed. In severe cases, serotonin antagonists, such as cyproheptadine, may be used. However, cyproheptadine is not a given option in this question. Among the given options, haloperidol and olanzapine are antipsychotic medications, which may exacerbate serotonin syndrome. Dantrolene is a muscle relaxant used to treat malignant hyperthermia and spasticity, but it is ineffective in serotonin syndrome. Carbamazepine is an antiepileptic drug, which doesn't seem to be relevant to the patient's condition. CONCLUDING: Based on the patient's symptoms and possible diagnosis of serotonin syndrome, it is clear that the immediate course of action is to discontinue the likely offending agents - paroxetine and duloxetine. This will help reduce the excess serotonin activity in his brain, and possibly aid in the resolution of his symptoms. RESPONSE: (D) Discontinue paroxetine and duloxetine.

**Facilitator** The consensus among the AI responses is that the 55-year-old male patient, who has a history of major depressive disorder and is presenting with symptoms such as incoherent babbling, restlessness, diaphoresis, severe headache, hyperthermia, tachycardia, hypertension, dilated pupils, hyperreflexia, and spontaneous myoclonus, is likely suffering from serotonin syndrome. This condition is potentially life-threatening and is caused by an excess of serotonergic activity in the nervous system, which can be triggered by an overdose or interaction of serotonergic drugs. In this case, the patient's symptoms are consistent with serotonin syndrome, and his recent consumption of paroxetine and duloxetine, both of which are serotonergic drugs, further supports this diagnosis. Paroxetine is a selective serotonin reuptake inhibitor (SSRI) and duloxetine is a serotonin-norepinephrine reuptake inhibitor (SNRI), both of which increase the levels of serotonin in the brain. The primary treatment for serotonin syndrome is to discontinue the offending medications and provide supportive care. Therefore, the consensus is that the best course of action for this patient would be to discontinue his use of paroxetine and duloxetine. This would help to reduce the excess serotonin activity in his brain and potentially alleviate his symptoms. Other medications listed in the options, such as carbamazepine, dantrolene, haloperidol, and olanzapine, are not considered appropriate for this specific situation. In conclusion, the consensus response to the question of what change should be made to the patient's current medication regime given his symptoms is (D) Discontinue paroxetine and duloxetine.
