# Supplementary material for: Collaborative intelligence in AI: Evaluating the performance of a council of AIs on the USMLE
Source: PLOS Digit Health. 2025 Oct 9;4(10):e0000787. doi: 10.1371/journal.pdig.0000787 (PMC12510544; doi:10.1371/journal.pdig.0000787)
Supplement: S2 File — (ZIP) [file pdig.0000787.s002.zip › S2 Data Raw data files containing unprocessed question inputs and LLM outputs including transcripts of deliberations/STEP2_Question-59-1_Type-MC-NJ.docx]

**Facilitator** **[Round 1]:** A 32-year-old man comes to the office for a follow-up examination 2 months after he was diagnosed with hypertension. He arrives late for the appointment, stating that he had to make sure no one followed him here because some ""drug dealers"" are after him. He insists that he be seen in an examination room with no windows. He says he is ""fine"" but is reluctant to answer questions because ""doctors hold everything against me."" He doubts his diagnosis and says, ""Doctors and drug companies are in cahoots trying to get my money."" He refuses to speak with the office manager about his bill because he thinks ""she looks at me funny and makes nasty remarks about me behind my back."" He says he has no history of psychiatric illness; his medical record indicates that he has made similar statements during the past 7 years concerning doctors and office staff but he always refused to elaborate on his statements. His medications are metoprolol and hydrochlorothiazide. He does not drink alcohol or use illicit drugs. Vital signs are within normal limits. Physical and neurologic examinations show no abnormalities. Results of screening laboratory studies are within the reference ranges. Which of the following is the most appropriate next step in management? (A) Begin clonazepam therapy (B) Begin risperidone therapy (C) Begin sertraline therapy (D) Determine whether the patient has friends or family who can monitor him at home (E) Determine whether the patient has had thoughts of harming others (F) Determine whether the patient is adherent to his medication

**AI_D:** RESTATING THE QUESTION: The case represents a 32-year-old man with hypertension who seems to have a complex relationship with healthcare professionals and unfair beliefs towards the medical system. Despite claiming he is fine, his behaviors and statements indicate possible paranoia or delusional thinking. Despite no previous psychiatric history, similar statements have been made over the past seven years. His physical health and lab tests are in normal ranges, and he takes metoprolol and hydrochlorothiazide for his hypertension. The question asks which management approach would be the most appropriate next step. PLANNING: The patient's presentation suggests he may be experiencing delusional beliefs, warranting a psychiatry-oriented management plan. Key elements are his suspicions towards healthcare professionals and drug companies, his specific requests to prevent being observed, and similar incidents in the past. The assumption that his mental state could influence his therapeutic adherence can be considered. Alternate assumptions involve exploring the patient's support network or potential for harm to others. However, addressing the underlying mental health condition appears crucial. CLINICAL REASONING: While his physiological well-being is fairly well managed based on his vital signs and screening lab tests, his psychological condition is possibly undermining his health. Paranoid delusions can be deeply engrained and can greatly impair functioning and reduce the quality of life. The chosen management should consider facilitating optimum mental health and thus, indirectly, hypertension control. Closer adherence to therapy can be achieved once this mental concern is addressed. CONCLUDING: Considering all factors, it appears that the patient may be suffering from a delusional disorder, specifically the persecutory type, which is characterized by irrational beliefs of being wronged or persecuted. This interpretation aligns closely with his behaviors observed and reported. Moreover, this diagnosis would explain why the patient has been able to function normally otherwise and hold consistent delusional beliefs over years. Optimum management needs to be oriented towards treating this condition. RESPONSE: The reasonable approach would be to start antipsychotic therapy to address the potential delusional disorder. Given their effectiveness in managing such conditions, risperidone, an atypical antipsychotic, could be an excellent choice (Choice B). Subsequently, monitoring therapy adherence and potential side effects would be essential.

**AI_C:** 1) RESTATING THE QUESTION: The patient, a man in his thirties who was diagnosed with hypertension two months ago and is currently on metoprolol and hydrochlorothiazide, displayed unusual behaviors. Besides arriving late for his check-up and insisting on being examined in a windowless room, he expressed fears of being pursued by drug dealers, accused medical professionals of scheming for monetary advantages, and showed distrust towards office staff. Even though his medical record has indications of similar behaviors within the past seven years, he firmly denies having any psychiatric problems. Neither physical nor neurologic abnormalities were detected by the examinations, and his vital signs were within the normal limits. In this scenario, we need to identify the most appropriate next step in the patient's management. 2) PLANNING: Potential assumptions include: one, a psychiatric illness is present despite the patient's denial; two, medication non-adherence could be causing the unusual behaviors, and three, a potential for self-harm or harm to others exists due to his delusions. To confirm or refute these assumptions, we could choose between initiating antipsychotic drugs (A, B, C), probing patient's social support and home environment (D), exploring potential violent tendencies (E), or verifying medication adherence (F). 3) CLINICAL REASONING: Considering the patient's denial of mental health issues and his refusal to delve into his unusual behaviors, combined with his distrust towards medical professionals, the initiation of antipsychotics seems premature. Furthermore, the patient's healthy physical and neurologic examinations suggest his hypertension medication is likely working, reducing the suspicion of medication non-adherence. There's no evidence currently to suggest a risk of violence. Therefore, focusing on his social support network to maximize his safety as he navigates these fears is a safe, clinically sound path to proceed. 4) CONCLUDING: We must always take patient safety as a priority, especially in cases where the patient exhibits signs of severe paranoia and distrust for medical professionals. Hence, assessing his social support is relevant (Option D) to ensure the continuity of care and management. 5) RESPONSE: Therefore, the most appropriate next step in the management of this patient is (D) to determine whether the patient has friends or family who can monitor him at home.

**AI_B:** RESTATING THE QUESTION: A 32-year-old patient with hypertension has presented to the clinic for follow-up after 2 months, but his behavior carries significant concerns of overly suspicious thoughts, paranoia, and general mistrust of others. He denies having any history of mental illness. His physical health and neurological status appear normal, with medications including metoprolol and hydrochlorothiazide. He doesn't consume alcohol or use illegal drugs. Despite these factors, the question asks what the best management approach should be, with options ranging from starting various drug therapies to assessing various aspects of his personal life and adherence to medication. PLANNING: While it's clear this patient has a health concern—hypertension—that is being managed with medication, his behavior suggests a potentially undiagnosed or unmanaged mental health condition. Some possibilities could include conditions like paranoid schizophrenia, delusional disorder, paranoid personality disorder, or perhaps an anxiety disorder. By questioning these assumptions, we could hypothesize other causes such as drug-induced psychosis or a neurological cause, yet these options seem less likely considering the information we have. As such, we should primarily consider the likelihood of a psychiatric condition, especially one involving paranoia. CLINICAL REASONING: Paranoia is primarily associated with psychosis, a symptom classically linked to schizophrenia spectrum disorders. However, schizophrenia usually presents with other positive symptoms (like hallucinations) and negative symptoms (like apathy), that this patient doesn't seem to exhibit. Another condition linked to paranoia is delusional disorder, but this relatively rare condition usually begins in middle to late life, beyond this patient's age group. Considering he has had these worries consistently for 7 years and his functioning is seemingly maintained without any previous psychiatric diagnosis or treatment, it is logical to suspect paranoid personality disorder—characterized by a pervasive distrust and suspiciousness of others, interpreting their motives as malevolent. CONCLUDING: The first line of treatment for Paranoid Personality Disorder is psychotherapy, as patients often do not trust medical providers or the pharmaceutical industry, making them resistant to medications. Moreover, medications are usually not as effective in treating personality disorders as psychotherapy. If pharmacological intervention becomes necessary, it should target specific symptoms, such as anxiety or agitation. RESPONSE: A careful approach that maintains trust is crucial here. Delving into the patient's paranoia regarding others hurting him before establishing trust (option E) or starting him on medications may worsen the patient's state (options A-C). Likewise, asking about his friend or family network could also trigger his paranoia (option D). Given these conditions, the most appropriate next step would be to determine whether the patient is adherent to his medication (F), which indirectly concerns his hypertension control but also provides an opportunity to engage in built trust and evaluate if he would be receptive towards mental health interventions in the future.

**AI_E:** 1) RESTATING THE QUESTION: This question presents a clinical scenario about a 32-year-old man who has been recently diagnosed with hypertension. From the information provided, there seem to be significant concerns about his mental health due to his present behavior; recurring themes of paranoia and unfounded suspicions about doctors and drug companies exploiting him have been apparent for several years. His physiological parameters are normal, and he is currently under a regimen of metoprolol and hydrochlorothiazide for hypertension. 2) PLANNING: The topic here lies at the intersection of cardiology and psychiatry. It would seem, prime facie, that the patient might be suffering from a psychiatric condition like Paranoid Personality Disorder (PPD), characterized by paranoid delusions, suspicion, and a pervasive distrust of others. Considering this, the initial assumptions would be his suspicion arises from a mistrust of the medical fraternity, manifested as doubts about his diagnosis and the belief that "doctors are in cahoots with drug companies." An alternative assumption could be a lack of adherence to prescribed hypertension medication due to these thoughts. However, this does not align with his vital signs being within normal limits, which would likely not be the case if he were non-compliant with hypertension medication. I hypothesize that managing his psychological state should be prioritized. 3) CLINICAL REASONING: The patient's refusal to interact with certain individuals and constantly suspecting people could be manifestations of PPD. The onset and chronicity of these symptoms, coupled with his unequivocal belief in them, all point towards a probable diagnosis of PPD. Moreover, there is no mention of any delusions or hallucinations, ruling out a psychotic disorder, and his behavior doesn't seem to be episodic, which would otherwise indicate a mood disorder. This further strengthens the diagnosis of PPD. Given this context, focusing on mental health management seems to be the appropriate route. 4) CONCLUDING: If the assumption about a possible diagnosis of PPD is correct, the focus should be on psychotherapeutic treatment rather than inducing pharmacological changes. This is because PPD patients can frequently find it difficult to adhere to drug therapies due to the inherent distrust of doctors prescribing the medication. Referrals to a psychotherapist for cognitive behavioral therapy would potentially help the patient deal with his trust issues and ingrained suspicion. 5) RESPONSE: Therefore, although a definitive diagnosis can only be made by a mental health professional, the current clinical picture points towards Paranoid Personality Disorder (PPD). The best course of action would be psychological evaluation and treatment rather than introducing antipsychotic or antidepressant medications (A, B, C) at this stage. Since his vital signs and lab parameters are normal, it can be speculated that he is adherent to his medication (F), however, a confirmation might still be beneficial. Identifying whether the patient has thoughts of harming others (E) or asking about a support system at home (D) might be a part of the broader psychological assessment and management but doesn't qualify as an immediate next step in this case. Given the situation, confirming his adherence to medication (F) seems like the most appropriate immediate next step.

**AI_A:** 1) RESTATING THE QUESTION: The inquiry revolves around a 32 year-old man who was recently diagnosed with hypertension, and is showing some peculiar non-physical symptoms. He has fears about being followed by drug dealers, and has shown some sort of suspicion and mistrust toward doctors and office staff, a pattern that has persisted for about 7 years according to his medical records, despite having no background of psychiatric illnesses. At the moment, his hypertension is being managed by metoprolol and hydrochlorothiazide. His physical and neurological examinations return normal, with no indication of substance abuse. The question is, what would be the most suitable next step in his management, given these circumstances. 2) PLANNING: The assumptions available include the consideration for a psychiatric condition such as paranoid schizophrenia characterized by his delusions of persecution and paranoid ideation, or a personality disorder such as paranoid personality disorder. Option F is also a concern, whether he is adhering to his medication for hypertension given his mistrust for doctors and belief about the medical profession. In this situation, the priority would be addressing the menta/psychiatric aspect because, most likely due to these delusions and paranoia, other issues such as medication adherence may be affected. 3) CLINICAL REASONING The patient's paranoid ideation, where he believes in being pursued by drug dealers, his skeptical view of the medical profession, and his persistent suspiciousness towards others, specifically the office staff, implies some type of psychiatric illness, despite his denial of any psychiatric history. However, as in the case of many psychiatric illnesses, denial is common. Paranoid schizophrenia would present with hallucinations and delusions, but he does not show any signs of hallucinations and his patterns of delusion have persisted for 7 years indicating a long-term condition. This brings us to the consideration of paranoid personality disorder, which is a pervasive distrust of others and interprets others' intentions as malevolent, beginning by early adulthood and present in a variety of contexts. This situation requires the intervention of a psychiatrist for further evaluation. The options listed A, B, C are starting a therapy which would not be ideal before having a psychiatric evaluation. Option F might subsequently prove to be an issue if his condition continues without appropriate intervention, however at this particular point in time, it is not a priority. 4) CONCLUDING Given the patient's long term pattern of distrust and paranoia, coupled with the absence of hallucinations or any signs of substance abuse, it is necessary to conduct a thorough psychiatric evaluation with the help of a mental health professional to diagnose possible personality disorders and guide the treatment thereof. 5) RESPONSE (E) Determine whether the patient has had thoughts of harming others: The context of the question doesn't provide information about explicit threats or intentions of harm made by the patient. Yet considering the paranoid behavior and mistrust, it is prudent to assess any threat he might pose to himself or others due to his paranoid delusions - an essential element in evaluating and managing patients presenting with delusions or hallucinations. This would in turn, determine the urgency of psychiatric intervention.

**Facilitator** **[Round 2]:** Summary: AI_A believes the patient's paranoia and mistrust suggest a psychiatric condition, possibly paranoid personality disorder. They recommend a psychiatric evaluation to diagnose possible personality disorders and guide treatment. They also suggest assessing any threat the patient might pose to himself or others due to his paranoid delusions. AI_B also suspects a psychiatric condition, particularly paranoid personality disorder. They recommend building trust with the patient and determining whether the patient is adherent to his medication, which indirectly concerns his hypertension control but also provides an opportunity to engage in built trust and evaluate if he would be receptive towards mental health interventions in the future. AI_C suggests that the patient's denial of mental health issues and his refusal to delve into his unusual behaviors, combined with his distrust towards medical professionals, make the initiation of antipsychotics seem premature. They recommend focusing on his social support network to maximize his safety as he navigates these fears. AI_D believes the patient may be suffering from a delusional disorder, specifically the persecutory type, which is characterized by irrational beliefs of being wronged or persecuted. They recommend starting antipsychotic therapy to address the potential delusional disorder. AI_E hypothesizes that the patient might be suffering from Paranoid Personality Disorder (PPD). They recommend focusing on mental health management, particularly psychotherapeutic treatment rather than inducing pharmacological changes. They also suggest confirming his adherence to medication. Question to clarify disagreements: Given the patient's paranoia and mistrust, how do we balance the need for psychiatric evaluation and treatment with the potential for exacerbating the patient's fears and suspicions? How do we determine the most appropriate immediate next step: psychiatric evaluation, building trust and confirming medication adherence, focusing on social support, starting antipsychotic therapy, or confirming medication adherence?. Based on your preceding response, how would you answer the original question: [A 32-year-old man comes to the office for a follow-up examination 2 months after he was diagnosed with hypertension. He arrives late for the appointment, stating that he had to make sure no one followed him here because some ""drug dealers"" are after him. He insists that he be seen in an examination room with no windows. He says he is ""fine"" but is reluctant to answer questions because ""doctors hold everything against me."" He doubts his diagnosis and says, ""Doctors and drug companies are in cahoots trying to get my money."" He refuses to speak with the office manager about his bill because he thinks ""she looks at me funny and makes nasty remarks about me behind my back."" He says he has no history of psychiatric illness; his medical record indicates that he has made similar statements during the past 7 years concerning doctors and office staff but he always refused to elaborate on his statements. His medications are metoprolol and hydrochlorothiazide. He does not drink alcohol or use illicit drugs. Vital signs are within normal limits. Physical and neurologic examinations show no abnormalities. Results of screening laboratory studies are within the reference ranges. Which of the following is the most appropriate next step in management? (A) Begin clonazepam therapy (B) Begin risperidone therapy (C) Begin sertraline therapy (D) Determine whether the patient has friends or family who can monitor him at home (E) Determine whether the patient has had thoughts of harming others (F) Determine whether the patient is adherent to his medication]

**AI_A:** Restating the question: The question presents a case of a 32-year-old man with hypertension who exhibits paranoid or delusional behavior. We need to determine the next appropriate step in management from a set of potential actions - begin either clonazepam, risperidone, or sertraline therapy, assess if the patient has social support or harmful thoughts, or assess his medication adherence. Planning: Assumptions presented in the question are that the patient has hypertension, exhibits paranoid behavior, denies psychiatric illness, displays hostility towards healthcare providers, and refuses to cooperate with staff. A key assumption among these is that the patient could be suffering from a psychiatric illness, likely a delusional or paranoid disorder, that isn't diagnosed yet. This assumption should be clinically prioritized to rule out or confirm the potential mental health issues. Clinical reasoning: Delusional disorders fall under psychotic disorders characterized by strong beliefs that persist despite contrary evidence. Paranoid personality disorder is a pattern of distrust, suspicion, and belief that others intend harm or exploitation. The patient's belief that doctors are trying to dupe him, the remarks about office staff, and his suspicion that drug dealers are following him suggest elements of both disorders. He also denies any psychiatric illness. As initial findings are normal, and the patient is reluctant to discuss his concerns, it could be channeled to discuss his medication adherence, which also indirectly pertains to his hypertension management, as an introduction to the conversation. Concluding: Given the patient's mistrust, initiating psychiatric medications right away could exacerbate his suspicions, and may lead him to discontinue his treatment altogether. Establishing trust, engaging in further conversation, and understanding his medication adherence seem like an appropriate initial step. Response: Therefore, the most appropriate immediate next step in the management of this patient would be to Determine whether the patient is adherent to his medication (F). However, in the long run, this patient would benefit from a psychiatric evaluation, therapy, and possibly medication for his suspected delusional or paranoid disorder. It's also crucial to ensure the patient has a reliable social support network that could assist him.

**AI_C:** Given the context that this patient has a history of suspiciousness and distrust, and has consistently held these beliefs over time, there's evidence to suggest the presence of a long-standing psychiatric condition. Paranoid Personality Disorder (PPD) is a condition characterized by pervasive distrust and suspicion of others, including doctors, and fits the patient's symptoms well. Starting the patient on antipsychotic therapy like risperidone can be precipitous and may intensify his mistrust, as this step lacks the necessary establishment of a therapeutic relationship. Initiating anxiety treatment with clonazepam or an antidepressant treatment with sertraline also seems premature without a confirmed psychiatric diagnosis via a thorough assessment. Many mental health circumstances often involve determining the immediacy of risk, which includes harm to oneself or others. However, nothing in the patient's current presentation suggests a high risk of self-harm or violence towards others. The patient's refusal to discuss his mental state and the palpable mistrust he demonstrates towards healthcare providers may make directly addressing the probable psychiatric disorder challenging. Therefore, a trust-building step would be beneficial, leading to better acceptance of any future therapeutic plan. It's also crucial to evaluate the patient's medication adherence, as noncompliance can have severe consequences with his ongoing hypertension management. Moreover, understanding his adherence will offer insight into his perception and cooperation with medical treatments. It will give a gateway to building the trust essential to manage his overall health. However, addressing this may not be the immediate next step considering the patient is asymptomatic, and his vitals are within the normal range. Lastly, while the patient's social support network is an essential factor in managing his mental health, inquiring about this without forming a therapeutic relationship first could come off as invasive. Overall, I would suggest that "F) Determine whether the patient is adherent to his medication" seems to be the most helpful immediate first step. This choice allows the doctor to assess the patient's current hypertension management and simultaneously provide an opportunity for rapport and trust-building without directly confronting the apparent psychiatric issue or invading his social boundaries. This initial step would lay a foundation for potential future management of the patient’s likely Paranoid Personality Disorder through psychotherapeutic means once a therapeutic patient-doctor relation has been established.

**AI_B:** RESTATING THE QUESTION: This clinical scenario describes a 32-year-old man coming to the doctor's office for a follow-up examination for hypertension. He is suspicious and paranoid, believing that drug dealers are after him and that doctors are conspiring against him. He shows distrust towards medical professionals. Despite his consistent display of such behavior over the past seven years, he denies having any psychiatric illness. This patient is presently on metoprolol and hydrochlorothiazide for his hypertension. This scenario is asking what would be the most appropriate next step in the management of such a patient. PLANNING: The primary assumption here is that the patient's paranoid behaviors and distrust towards medical professionals suggest a possible underlying psychiatric illness. Alternatively, the patient's suspicion and wariness could be chronic and deeply ingrained personality traits, representative of paranoid personality disorder or delusional disorder. It is also important to address the patient's adherence to prescribed medications, considering his distrust towards doctors and belief that the pharmaceutical industry is trying to take his money. The best approach is likely one that acknowledges these considerations, forming a judicious and delicate plan that doesn't escalate the patient's fears and suspicions. CLINICAL REASONING: Paranoia and mistrust of others are key characteristics of paranoid personality disorder. However, presence of delusions which is a characteristic of delusional disorder cannot be ruled out as the man believes that drug dealers are after him. This makes diagnosing challenging without psychiatric evaluation. Also, it is imperative to determine the adherence to his anti-hypertensive medication given that mistrust could potentially lead to non-adherence. The important aspect here is to acknowledge and not counter his beliefs in a confrontational way, as that could potentially escalate the situation and push him further away from obtaining needed care. CONCLUDING: In the management of such patient, the next most appropriate step would be to address these factors in a delicate and non-confrontational manner. It is also important to ensure that the patient's hypertension is being appropriately managed which remains the primary ailment, and hence, determining the adherence to his anti-hypertensive medication is deemed crucial. This approach can favorably serve as a route to assess the receptiveness of the patient towards further mental health services going forward. RESPONSE: The most appropriate next step in managing this patient would be to "(F) Determine whether the patient is adherent to his medication".

**AI_E:** RESTATING THE QUESTION: A 32-year-old man has been displaying signs of paranoia and mistrust toward others, including doctors and office staff, for the past 7 years. He's also been diagnosed with hypertension, and he takes metoprolol and hydrochlorothiazide. Despite these clear signs of potential mental health issues, he denies having any psychiatric history. His vital signs and laboratory studies are within the reference ranges, and physical and neurological examinations show no abnormalities. We are tasked with identifying the most appropriate next step in managing this patient. PLANNING: Our main assumption is that the patient's behavior indicates a possible psychiatric disorder. However, it could be alternatively argued that the patient's paranoia and mistrust are not necessarily indicative of a psychiatric condition, but perhaps a defensive mechanism developed over time due to some past experiences or traumas. Also, it's crucial to consider whether the patient's beliefs are causing harm or distress to himself or others. Without causing undue distress, it's vital to prioritize establishing trust with the patient, determining his medication adherence, and ensuring he has a supportive social network in place. CLINICAL REASONING: Certain personality disorders, specifically paranoid personality disorder (PPD), can be marked by pervasive distrust and suspicion of others. The patient's long history of concerns — about drug dealers following him, believing that doctors and drug companies are conspiring against him, and perceiving the office staff as ill-natured — are in line with the persistent suspiciousness and mistrust typical of PPD. However, before substantiating this diagnosis, other potential causes of these symptoms, like substance misuse or organic brain disease, must be ruled out, which has been in this case. Assessment of this patient's medication adherence is important not just for his hypertension management but also to build a trustful relationship leading in acknowledging potential mental health needs. CONCLUDING: Considering all scenarios, I believe that while arranging a psychiatric evaluation or starting antipsychotic therapy might be reasonable considerations, these could exacerbate the patient's fears and suspicions. Focusing on purely pharmacological treatment could potentially be perceived by him as reinforcing his belief that doctors want to profit off his illnesses. Hence, a thorough yet subtly approach to understand the root cause of his constant suspicions, while maintaining his medical compliance, is essential. RESPONSE: I believe the most appropriate immediate next step should be determining whether the patient is adherent to his medication (Option F). This choice respects the patient's autonomy and mistrust toward medical establishments, while it also provides a basis to maintain a patient-physician relationship—a step in the direction of progressive psychotherapeutic management, which is often the first line of treatment for PPD.

**AI_D:** Restating the question: The challenge at hand is to identify the most suitable course of action for managing a 32-year-old hypertensive male patient who displays paranoid behaviors, such as distrust towards medical professionals and a belief in personal persecution, with no identified psychiatric history or physical abnormalities. We're faced with decisions concerning initiating pharmacological interventions, ascertaining medication adherence, investigating potential harmful intentions, or exploring available social support networks. Planning: A major assumption in this scenario is that the patient's paranoia and mistrust indicate an underlying psychiatric condition, possibly Paranoid Personality Disorder (PPD), as suggested by some AI colleagues. Another assumption is that his hypertensive regimen isn't optimal, as his diagnosis is recent, just two months prior. The third assumption is that treating this paranoia can suo-moto induce trust in the healthcare system. Considering different angles, we can theorize that the patient's paranoia might impede the process of gaining informed consent for administering antipsychotic therapy. We should also realize that while focusing on mental health management, we cannot ignore his hypertension control. Clinical Reasoning: Paranoid Personality Disorder is a chronic, pervasive condition characterized by persistent suspicion, mistrust, and an overarching sense of personal persecution, aligning with the patient's displayed behaviors. Typically, these individuals present a significant challenge to healthcare providers owing to their deep-rooted distrust in others' intentions. While an absolute diagnosis of PPD would require a comprehensive psychiatric evaluation, it's valued that first-line management involves psychotherapeutic approaches rather than pharmacological interventions, to avoid reinforcing the patient's fears of medication misuse. The presented scenario also underscores the importance of building a therapeutic alliance, which begins with verifying medication adherence. It connects to the hypertension control, which is a primary concern for the patient's overall health. Directly jumping into antipsychotic therapy without effective communication might create a substantial setback in managing his paranoia as well as hypertension. Concluding: Given this patient's lack of status as a threat to himself or others, his stable vitals, normal neurologic examination, and absence of any evidence pointing toward substance abuse or severe psychiatric conditions such as schizophrenia, the urgent initiation of antipsychotic medication is not warranted. In this scenario, it's pivotal to take gradual, stepwise actions promoting patient trust and confidence rather than abruptly resort to pharmacological intervention, which could potentially escalate the patient's mistrust and skepticism towards doctors and drugs. Response: Therefore, the most appropriate next step in this patient’s management should be to determine whether the patient is adherent (option F) to his medication. This approach provides a valuable inroad to engage in a therapeutic dialogue, pave the way for gradual patient trust, and indirectly address his primary physical health concern – hypertension. It stands as a rational, patient-centered method that can later open avenues for discussing his behavioral tendencies and the potential need for psychiatric input.

**Facilitator** The consensus among the AI responses is that the most appropriate next step in managing this 32-year-old hypertensive patient, who exhibits paranoid behavior and mistrust towards healthcare providers, is to determine whether the patient is adherent to his medication (Option F). This approach is favored because it allows for the establishment of a therapeutic relationship, which is crucial given the patient's apparent paranoia and suspicion. It also provides an opportunity to assess the patient's current hypertension management, which is a primary health concern. The patient's behavior suggests a possible underlying psychiatric condition, potentially Paranoid Personality Disorder (PPD) or a delusional disorder. However, initiating psychiatric medications such as clonazepam, risperidone, or sertraline at this stage could exacerbate his suspicions and potentially lead to discontinuation of treatment. Therefore, a gradual, non-confrontational approach is recommended to build trust and rapport. While it's important to ensure the patient has a reliable social support network and to assess if he has harmful thoughts, these steps are not considered immediate priorities. Directly addressing these issues without first establishing a therapeutic relationship could be perceived as invasive or confrontational. In the long run, this patient would benefit from a psychiatric evaluation, therapy, and possibly medication for his suspected psychiatric disorder. However, the immediate focus should be on establishing trust, engaging in conversation, and understanding his medication adherence.
