# Supplementary material for: Collaborative intelligence in AI: Evaluating the performance of a council of AIs on the USMLE
Source: PLOS Digit Health. 2025 Oct 9;4(10):e0000787. doi: 10.1371/journal.pdig.0000787 (PMC12510544; doi:10.1371/journal.pdig.0000787)
Supplement: S2 File — (ZIP) [file pdig.0000787.s002.zip › S2 Data Raw data files containing unprocessed question inputs and LLM outputs including transcripts of deliberations/STEP2_Question-6-1_Type-MC-NJ.docx]

**Facilitator** **[Round 1]:** A previously healthy 29-year-old woman is admitted to the hospital because of a 1-day history of moderate headache, fever, and lethargy. Two days ago, she underwent wisdom tooth extraction. She takes no medications. She cannot remember her vaccination history. She does not smoke cigarettes, drink alcohol, or use illicit drugs. She is a college student, lives in an apartment complex with other students, and eats all her meals in the campus dining hall. She says her study partner had a “really bad fever” during the past week. The patient appears sleepy. Temperature is 40.0°C (104.0°F), pulse is 140/min, and blood pressure is 90/40 mm Hg. Examination shows a diffuse purpuric rash over the trunk and all extremities. There is nuchal rigidity. Lungs are clear to auscultation. Heart sounds are normal. Neurologic examination shows no other focal findings. Which of the following is most likely to have prevented this patient’s current condition? (A) Adherence to immunization guidelines (B) Avoidance of eating meals in the campus dining hall (C) Consistent hand washing (D) Isolation of other students on campus with febrile illness (E) Prophylactic antibiotic therapy

**AI_A:** RESTATING THE QUESTION: We're being asked to determine which action would have most likely prevented the current health condition of a 29-year-old woman who recently had a wisdom tooth extraction. She's showing signs of a high fever, lethargy, a diffuse purpuric rash, nuchal rigidity, and hypotension. A "purpuric rash" refers to a type of skin rash that results from bleeding into the skin or mucous membrane. "Nuchal rigidity" refers to a stiff neck, often associated with conditions like meningitis. PLANNING: One possible assumption could be that her medical condition might be related to a lack of immunization, hence a possible answer could be (A) adherence to immunization guidelines. However, this initial assumption requires us to look at other key components such as her environmental factors and recent dental surgery. It would not be inappropriate to hypothesize that her wisdom tooth extraction played a role as suggested in option (E), prophylactic antibiotic therapy. Or maybe, it could be related to the hygiene practices as in option (C), consistent hand washing. CLINICAL REASONING: The woman’s symptoms are consistent with meningococcal meningitis. Meningococcal meningitis often presents with headache, nuchal rigidity, and purpura. The patient's recent history of close contact with a sick person, her living in a close quarters with other students, and her high fever with purpura strongly suggests this diagnosis. Meningococcus (Neisseria meningitidis) is a bacteria that causes meningitis, and it is commonly transmitted through respiratory droplets. CONCLUDING: Also, this bug is known to be common in crowded living conditions like college dormitories. Meningococcal meningitis can be largely prevented through vaccination. Therefore, it's reasonable to assume that (A) adherence to immunization guidelines could have helped prevent her current condition. RESPONSE: As per my clinical reasoning, and given the clinical features of the patient, the most likely preventive measure that could have prevented this patient’s current condition would have been (A) Adherence to immunization guidelines.

**AI_E:** RESTATING THE QUESTION: The scenario involves a 29-year-old woman who recently underwent a dental procedure and is now presenting with symptoms suggestive of a systemic infection (e.g., headache, fever, lethargy) accompanied by a purpuric rash and nuchal rigidity. The query revolves around what intervention might have helped to prevent her current ailment. PLANNING: The question assumes that this woman's condition could have been prevented. The options provided range from immunizations, eating habits, personal and community hygiene, to preventative antibiotic therapy. Based on the symptomology, this could indicate a bacterial or viral illness, possibly meningococcal meningitis. Assumptions to challenge could include her vaccination history, the possible source of the bacteria (including her recent dental procedure or her campus dining or living environment), the likelihood of it being a contagious disease (given her study partner's recent fever), and the role of antibiotics both as prophylaxis for dental procedures and as a treatment for a viral illness. CLINICAL REASONING: A key symptom in this scenario is the presence of nuchal rigidity and a purpuric rash, which are classic signs of meningitis. Meningitis is typically bacterial or viral; if it's bacterial, Neisseria meningitidis, which causes meningococcal meningitis, is a likely culprit. This bacterium usually spreads via respiratory droplets and is not commonly associated with food consumption or lack of hand hygiene. Therefore, avoiding meals at the dining hall or practicing consistent hand washing might not have made a difference in this context. Isolation could potentially have prevented contact if her feverish study partner was the carrier, but it's not the most reliable measure. The use of prophylactic antibiotics following her dental procedure might have been useful if her current condition was due to a bacterial infection relevant to the oral cavity, but less so if her illness is viral or due to N. meningitidis, which is not commonly found in oral flora. However, adherence to immunization guidelines could explain the potential prevention strategy. Vaccination against N. meningitidis is part of routine immunization in many regions, especially for individuals living in closed and semi-closed communities, such as college dorms, military barracks, etc., which predispose to the rapid spread of this bacterium. CONCLUDING: Based on the symptomology, which corresponds to meningococcal meningitis, adherence to immunization guidelines could have arguably provided the greatest chance of preventing this disease in the first place. RESPONSE: Most likely, the intervention that could have prevented this patient’s current condition is (A) Adherence to immunization guidelines.

**AI_D:** (1) RESTATING THE QUESTION: The question asks for the most effective measure that could have potentially prevented the current condition of a 29-year-old female patient. She came to the hospital presenting a set of symptoms including fever, lethargy, diffuse purpuric rash, and nuchal rigidity. She underwent a wisdom tooth extraction two days ago and has an unknown vaccination history. She has a close contact who recently had a fever, and she resides and has meals in a communal setting of a campus. The possible preventive actions proposed in the question are: (A) adherence to immunization guidelines, (B) not eating in the campus dining hall, (C) washing hands routinely, (D) isolation of febrile students on campus, and (E) use of preventative antibiotics. (2) PLANNING: The main assumptions here pertain to the likely cause of this patient's condition, which, given her symptoms and circumstances, is typically characteristic of a severe systemic bacterial infection, perhaps due to Neisseria meningitidis. Other factors, such as her recent dental procedure, her communal living situation, and her contact with a person of similar symptoms are also important considerations. We need to prioritize the assumption that associates with the most common and clinically relevan pathway of the disease, which in this case, is the airborne transmission of N. meningitidis. (3) CLINICAL REASONING: Clinical manifestation given as fever, headache, lethargy, purpuric rash and nuchal rigidity are indicative of bacterial meningitis and the rash specifically suggests meningococcemia, a severe systemic infection caused by N. meningitidis. The bacterium is transmitted through respiratory droplets and is prevalent in close-knit communities like college dorms. It should be noted that adherence to immunization guidelines could potentially prevent an infection like this. Vaccines for N. meningitidis are recommended for college students, especially those living in dormitories, due to their elevated risk of infection. On the other hand, eating in the campus dining hall, consistently washing hands, isolating other students with fever, or prophylactic antibiotic therapy are generally suggested measures but may not be as directly effective in preventing a meningococcal illness. (4) CONCLUDING: While the other options could offer protection against general infections, N. meningitidis is more specifically prevented through adherence to immunization guidelines since there is a vaccine available against 4 common strains of N. meningitidis. (5) RESPONSE: Therefore, the most likely prevention of this patient’s current condition would have been (A) Adherence to immunization guidelines.

**AI_C:** RESTATING THE QUESTION: The query entails determining the most viable preventative measure this 29-year-old woman, currently in hospital due to symptoms indicative of a possible infection, could have taken to avoid her current condition. Her symptoms include a high fever, headache, lethargy, diffuse purpuric rash, and nuchal rigidity (indicators of meningitis). She lives in close quarters with others, recently underwent dental surgery, her vaccination history is uncertain, and a friend was recently unwell with a fever. PLANNING: The first assumption could be that the woman may have contracted a form of meningitis, most likely bacterial, based on the symptoms provided. Alternate assumptions might question whether the source of infection could be linked to her recent dental surgery or her contact with ill individuals around her, specifically her study partner who had a fever. It can also be questioned whether her unidentified vaccination status contributed to susceptibility to disease. These assumptions could be clinically prioritized towards investigating the nature of her infection, any potential exposure sources, and her vaccination history. CLINICAL REASONING: Bacterial meningitis, characterized by symptoms such as fever, nuchal rigidity, and rash, can be prevented through adherence to immunization guidelines, particularly vaccines against Neisseria meningitidis, Streptococcus pneumoniae, and Haemophilus influenzae type b. Living in close proximity to others, such as in campus dorms, may increase her exposure risk. Additionally, her recent oral surgery might have enabled bacterial entry into her bloodstream, though the onset of symptoms is a bit later for a typical oral-flora pathogen. The fact that her study partner had a fever could suggest a possible contagion as well. CONCLUDING: All considered, although consistent hand washing, isolation of sick individuals, and proper food hygiene can help prevent some types of infections, the symptoms displayed by this patient, combined with her living situation and close contact with a sick individual, suggest a form of meningitis. Prophylactic antibiotics could have been given before dental procedure, but given the short time frame it is an unlikely source of her current condition. Therefore, the most probable measure to have prevented her condition would be adherence to immunization guidelines, assuming she was unvaccinated against the meningitis-causing bacteria. RESPONSE: Hence, (A) Adherence to immunization guidelines is most likely to have prevented this patient’s current condition.

**AI_B:** RESTATING THE QUESTION: The patient, a 29-year-old woman with no other health issues, has been admitted with symptoms such as a moderate headache, lethargy, and fever, which started a day prior. She had a wisdom tooth extraction two days ago and does not take any medication. It's unknown whether she has received any vaccinations as she doesn't remember. She's a nonsmoker, doesn't consume alcohol, and doesn't use illicit drugs. She is a university student who lives with other students and eats in the campus dining hall. She reports that her study partner was significantly ill with a fever the previous week. She appears drowsy and has a temperature of 40.0°C, a pulse of 140/min, and a blood pressure of 90/40 mm Hg. Physical examination reveals a widespread purpuric rash over her trunk and all limbs. She also has nuchal rigidity but no other specific neurological findings. Options for the prevention of her present condition are proposed like following immunization guidelines, not eating in the campus dining hall, frequent hand washing, isolating other feverish students, and taking prophylactic antibiotics. PLANNING: Several clinical assumptions are being considered based on the given information: 1) the patient's present condition could have been prevented by adherence to vaccination protocols, 2) the condition may be linked to consumption of meals in the university dining area, 3) a lack of proper hygiene could have contributed to this condition, 4) it is also possible that the illness could be infectious and that the patient got infected from a fellow student whose illness was not properly managed, and 5) the patient's dental procedure might have exposed her to an infection that could have been prevented with prophylactic antibiotics. CLINICAL REASONING: Given the patient's high temperature, lethargy, nuchal rigidity, and a purpuric rash, we ought to consider bacterial meningitis, which is usually caused by the Neisseria meningitidis or Streptococcus pneumoniae. In this case, her recent dental surgery and reported exposure to a febrile illness suggest possible routes of infection. Vaccination against meningococcal and pneumococcal bacteria could have provided some protection against these diseases depending on the prior immunization status. Eating in the communal dining hall is unlikely to directly contribute to the condition unless there were specifically contaminated food items. Hand hygiene can indeed reduce the risk of many infections, but its effectiveness for prevention of specific conditions such as bacterial meningitis is limited. Isolation of febrile individuals reduces the spread of many infections, but its effectiveness would largely depend on the nature and infectivity of the underlying pathogen. Lastly, prophylactic antibiotics prior to wisdom tooth extraction can prevent some infections but typically target bacteria such as viridans group streptococci and are not as effective against meningococcal or pneumococcal bacteria. CONCLUDING: In the context of the patient’s symptoms suggestive of meningitis, among the given options, adherence to immunization guidelines is logically the most effective preventive measure against bacterial meningitis, especially due to Neisseria meningitidis and Streptococcus pneumoniae, common pathogens in this case. RESPONSE: Therefore, (A) Adherence to immunization guidelines is most likely to have prevented this patient’s current condition.

**Facilitator** The consensus among the AI responses is that the most likely preventative measure that could have prevented the current condition of the 29-year-old woman is (A) Adherence to immunization guidelines. The woman's symptoms, including high fever, lethargy, a diffuse purpuric rash, nuchal rigidity, and hypotension, are consistent with meningococcal meningitis. This condition is often caused by the Neisseria meningitidis bacteria, which is commonly transmitted through respiratory droplets and is prevalent in crowded living conditions like college dormitories. Vaccination against meningococcal and pneumococcal bacteria could have provided protection against these diseases. While other measures such as consistent hand washing, isolation of sick individuals, and prophylactic antibiotics can help prevent some types of infections, they are not as directly effective in preventing a meningococcal illness. Therefore, given the patient's symptoms and living situation, adherence to immunization guidelines is the most effective preventive measure against bacterial meningitis.
