# Supplementary material for: Collaborative intelligence in AI: Evaluating the performance of a council of AIs on the USMLE
Source: PLOS Digit Health. 2025 Oct 9;4(10):e0000787. doi: 10.1371/journal.pdig.0000787 (PMC12510544; doi:10.1371/journal.pdig.0000787)
Supplement: S2 File — (ZIP) [file pdig.0000787.s002.zip › S2 Data Raw data files containing unprocessed question inputs and LLM outputs including transcripts of deliberations/STEP2_Question-61-1_Type-MC-NJ.docx]

**Facilitator** **[Round 1]:** An 87-year-old woman, gravida 2, para 2, is brought to the emergency department from a skilled nursing care facility because of profuse vaginal bleeding for 2 days. At the age of 41 years, she underwent a cold knife cone biopsy for cervical dysplasia. Annual follow-up Pap smears until the age of 65 years showed no abnormalities. She is in moderate distress. Temperature is 36.1°C (97.0°F), pulse is 84/min, respirations are 30/min, and blood pressure is 140/90 mm Hg. Abdominal examination shows mild distention and tenderness; no masses are palpated. Pelvic examination shows a large verrucous lesion on the perineum partially covering the anal verge. Speculum examination shows atrophic changes on the vagina and cervix. Which of the following is the most likely explanation for these findings? (A) Cervical carcinoma (B) External hemorrhoids (C) Rectal carcinoma (D) Rectal polyps (E) Vulvar carcinoma

**AI_D:** 1) RESTATING THE QUESTION: The patient, who is an 87-year-old woman, is experiencing profuse vaginal bleeding for 2 days and a physical examination reveals a large verrucous lesion on her perineum as well as atrophic changes on the vagina and cervix. We have to determine the most plausible cause from the given options which include cervical carcinoma, external hemorrhoids, rectal carcinoma, rectal polyps, or vulvar carcinoma. 2) PLANNING: To make a well-informed decision, we need to consider the patient's age, her symptoms, her history of cervical dysplasia, the normal results of her Pap smears, the presence of the verrucous lesion, the atrophic changes noted, and the absence of palpable masses in the abdominal region. With those, the suspected conditions could be determined. 3) CLINICAL REASONING: An older female with a verrucous lesion on the perineum and atrophic changes in the vagina and cervix with a past history of cervical dysplasia might be expected to have a predisposition toward neoplastic conditions, but her regular Pap smears revealed no abnormalities which make cervical carcinoma less likely. External hemorrhoids typically present as painful swelling or lumps around the anus and don't generally cause vaginal bleeding. Rectal carcinoma and rectal polyps would be unusual to cause vaginal bleeding and don't commonly present as verrucous lesions. On the other hand, vulvar carcinoma often presents as a verrucous, cauliflower-like mass and can be associated with vaginal bleeding. 4) CONCLUDING: Given the symptomatology revolving around vaginal bleeding and the presence of a verrucous lesion on the perineum, coupled with the process of elimination which excludes rectal carcinoma, rectal polyps, external hemorrhoids and cervical carcinoma, the diagnosis most logical and supportive of the clinical presentation seems to be vulvar carcinoma. 5) RESPONSE: Therefore, the most probable cause of these findings would be vulvar carcinoma, denoted by answer option (E).

**AI_C:** RESTATING THE QUESTION: We are being asked to deduce the most likely cause of an elderly woman's profuse vaginal bleeding given a clinical scenario. Factors to consider include her previous history of cervical dysplasia treated with biopsy, consecutive normal Pap smears, and presence of a verrucous lesion on the perineum. A verrucous lesion is a type of growth that presents with a rough, warty surface. PLANNING: One assumption made in the scenario is that the verrucous lesion and the profuse vaginal bleeding are correlated. Another is that the previous history of cervical dysplasia has no impact on the present case due to the consecutive normal Pap smears. However, each of these assumptions should be questioned, especially given the array of clinical features. Clinically, it would make sense to prioritize the relationship between the bleeding and the verrucous lesion. CLINICAL REASONING: The verrucous lesion partially covering the anal verge could logically be associated with the observed vaginal bleeding, especially given the absence of abnormalities in the vagina and cervix. While hemorrhoids or rectal conditions could result in bleeding, this would most likely manifest as rectal, not vaginal, bleeding. The sites and features of the noted lesion make vulvar carcinoma a likely cause. Vulvar carcinoma can indeed present as a cauliflower-like, verrucous lesion. Its advanced stages can lead to bleeding, fitting the clinical presentation in this case. CONCLUDING: In light of the aforementioned information, despite the woman's past history of cervical dysplasia and a normal Pap smear history, the findings align more with vulvar, rather than cervical, carcinoma. RESPONSE: The most likely explanation for these findings, taking into account the clinical presentation and physical examination findings, is (E) Vulvar carcinoma.

**AI_A:** (1) RESTATING THE QUESTION: The patient, an 87-year-old woman, presents with vaginal bleeding. She has a history of cervical dysplasia for which she had a cold knife cone biopsy. Her Pap smears were normal till age 65. Her current examination reveals an abnormal lesion on the perineum, with signs of atrophy in the vagina and cervix. She does not have any palpable abdominal masses. The question seeks the most likely cause of her current symptoms. (2) PLANNING: Key information in the patient's presentation help us make assumptions. She's had cervical dysplasia and bleeding is a common sign of malignancies. However, her normal Pap smears until 65 suggest against cervical carcinoma. The presence of a verrucous lesion on the perineum is significant. Possible etiologies include cervical carcinoma (A), external hemorrhoids (B), rectal carcinoma (C), rectal polyps (D), and vulvar carcinoma (E). (3) CLINICAL REASONING: Given the location of the lesion and the presenting symptom of vaginal bleeding, a gynecologic origin of the lesion is more likely. Cervical cancer could cause vaginal bleeding but her long history of normal Pap smears makes it less likely. External hemorrhoids or rectal polyps would not typically present with vaginal bleeding nor produce a perineal lesion. Rectal carcinoma could potentially produce a large local lesion, but it would more commonly cause rectal bleeding, not vaginal bleeding. Thus, vulvar carcinoma (E), which could present with a vaginal bleeding and a large verrucous lesion on the perineum, seems the most likely choice. (4) CONCLUDING: Based on the clinical presentation, location, and nature of the lesion combined with the symptom of vaginal bleeding, it is rational to suggest Vulvar carcinoma (E) as the most likely explanation for this patient's condition. The patient's history of cervical dysplasia increases the overall risk for HPV-related malignancies, although Pap smears were normal until the age of 65. (5) RESPONSE: (E) Vulvar carcinoma is the most likely explanation for these findings. The presence of the verrucous lesion on the perineum and the patient's symptom of vaginal bleeding suggest a gynecological malignancy, specifically of the vulva.

**AI_B:** (1) RESTATING THE QUESTION: We're discussing an 87-year-old woman who has been experiencing vaginal bleeding for two days. She has a previous history of cervical dysplasia for which she underwent a cold knife cone biopsy at 41 years. Her pap smears till 65 years showed no abnormalities. She is currently distressed with mild abdominal distension and tenderness. A pelvic examination unearthed a large verrucous (wart-like) lesion on the perineum partially covering the anal verge. The speculum examination showed atrophic changes on her vagina and cervix. Atrophic vaginal changes can be frequently seen in post-menopausal women due to reduced estrogen levels. The concern is to determine what might be causing these signs and symptoms. (2) PLANNING: Assumptions we can consider include cervical carcinoma (due to previous dysplasia), external hemorrhoids (this could explain the lesion but not the bleeding or the atrophic vaginal changes), rectal carcinoma or polyps (could explain the verrucous lesion but not the vaginal bleeding), and vulvar carcinoma (could explain the lesion and possibly the bleeding). Given the age of the patient, previous pathology, as well as the vaginal bleeding and verrucous lesion, it is clinically prudent to prioritize consideration of various forms of carcinoma over the benign option of hemorrhoids. (3) CLINICAL REASONING: The patient's history provides certain cues that may help us rule out some of the diagnosis. The annual follow-up Pap smears showed no abnormalities, making cervical carcinoma less likely as such persistent infection is typically associated with human papilloma virus which would've been picked up in pap smear. Additionally, her symptoms of vaginal bleeding are a common sign for gynecologic malignancy, but the location of the verrucous lesion on the perineum and near anal verge suggests the carcinoma may be vulvar more than rectal or cervical considering the anatomical position of the structures involved. (4) CONCLUDING: Taking into account the patient's age, distinct clinical signs, previous medical history and the position of the verrucous lesion, the case would lean more towards a type of carcinoma, more specifically vulvar carcinoma, rather than the options of external hemorrhoids, rectal carcinoma or polyps, or cervical carcinoma. (5) RESPONSE: Given all these factors, the most plausible explanation for the findings in this patient appears to be (E) Vulvar carcinoma.

**AI_E:** RESTATING THE QUESTION: An 87-year-old woman with a history of cold knife cone biopsy for cervical dysplasia and no subsequent abnormalities on Pap smears has been experiencing significant vaginal bleeding. She is in mild distress and presents with an unremarkable abdominal examination but has a physical finding of a large verrucous lesion at the perineal region near the anus. Vaginal and cervical changes are characterized as atrophic upon speculum examination. We need to identify what condition is most likely causing her symptoms and findings. PLANNING: The woman's age and symptoms raise suspicion for malignancy. Considering her history of cervical dysplasia and the presence of a verrucous lesion on her perineum, possible malignancies include cervical carcinomas and vulvar carcinomas. It's also possible that the lesion could be a manifestation of something benign -- for example external hemorrhoids or rectal polyps. Another concern could be rectal carcinoma, given her age and the location of the lesion. CLINICAL REASONING: It's crucial to consider the patient's prior history of cervical dysplasia and the benign status of subsequent Pap smears in the context of her current symptoms. Cervical carcinoma (option A) could theoretically arise from a history of dysplasia, but regular screening and treatment would likely have caught it before it progressed this far, making it less likely. Similarly, the changes observed in her vagina and cervix being termed 'atrophic' doesn't strongly support a diagnosis of cervical carcinoma. Rectal carcinoma (option C) and rectal polyps (option D) could potentially cause bleeding, but they would be less likely to present with the verrucous perineal lesion described here. External hemorrhoids (option B) could potentially cause both bleeding and a perineal growth, but hemorrhoids typically don't produce the kind of profuse bleeding described here. That leaves us with vulvar carcinoma (option E) as the most likely diagnosis. The presence of a verrucous growth and severe bleeding are both consistent with this form of cancer. The fact that the lesion is observed on the perineum, which is part of the vulva, also makes vulvar carcinoma more likely than any other locations. CONCLUDING: Based on the symptom of profuse vaginal bleeding, the past medical history of cervical dysplasia, and the presence of an unusual lesion on the perineum and considering all the potential causes, the overall clinical presentation seems to point towards Vulvar carcinoma. RESPONSE: The most likely explanation for these findings is (E) Vulvar carcinoma.

**Facilitator** The consensus among the AI responses is that the most likely explanation for the symptoms and findings in the 87-year-old woman is Vulvar carcinoma (E). This conclusion is based on several factors. Firstly, the patient's age and the presence of a verrucous lesion on the perineum, which is part of the vulva, suggest a gynecological malignancy. Secondly, the symptom of profuse vaginal bleeding is consistent with this form of cancer. Thirdly, while the patient's history of cervical dysplasia could theoretically lead to cervical carcinoma, her regular Pap smears until the age of 65 showed no abnormalities, making this less likely. Lastly, other potential causes such as external hemorrhoids, rectal carcinoma, and rectal polyps were considered less likely due to the nature of the lesion and the type of bleeding. Therefore, taking into account the patient's clinical presentation, past medical history, and physical examination findings, Vulvar carcinoma (E) is the most plausible explanation for these findings.
