# Supplementary material for: Collaborative intelligence in AI: Evaluating the performance of a council of AIs on the USMLE
Source: PLOS Digit Health. 2025 Oct 9;4(10):e0000787. doi: 10.1371/journal.pdig.0000787 (PMC12510544; doi:10.1371/journal.pdig.0000787)
Supplement: S2 File — (ZIP) [file pdig.0000787.s002.zip › S2 Data Raw data files containing unprocessed question inputs and LLM outputs including transcripts of deliberations/STEP2_Question-62-1_Type-MC-NJ.docx]

**Facilitator** **[Round 1]:** A 47-year-old woman comes to the office for a routine health maintenance examination. She feels well and has no history of serious illness. Examination shows a 2-cm, soft, nontender nodule in the right lobe of the thyroid gland. There is no lymphadenopathy. Her serum thyroid-stimulating hormone concentration is within the reference range. Which of the following is the most appropriate initial step in management? (A) CT scan of the neck (B) Fine-needle aspiration biopsy (C) Reexamination in 3 months (D) Surgical excision (E) Ultrasonography of the thyroid gland

**AI_D:** (1) RESTATING THE QUESTION: The clinical scenario presents a 47-year-old woman who, during a routine health examination, is found to have a 2-cm, soft, painless lump in the right lobe of her thyroid gland. No lymph nodes are enlarged, and the hormone responsible for thyroid gland stimulation is at normal levels in her blood. The inquiry is about what would be the most clinically relevant first step to manage this finding. (2) PLANNING: The major assumption here is that the woman has a thyroid nodule. Given this finding, the initial approach should be to assess if the nodule is benign or malignant, still keeping in mind that the majority of thyroid nodules are often benign. This assessment can be achieved through various methods such as a CT scan, a biopsy, periodic examination, surgical removal, or an ultrasound of the thyroid gland. (3) CLINICAL REASONING: The presence of a thyroid nodule in the absence of any other significant clinical symptoms, as well as normal levels of thyroid-stimulating hormone, could indicate a benign neoplasm. In such cases, a CT scan would likely yield inconclusive information and exposes the patient to unnecessary radiation. Fine-needle aspiration biopsy is invasive and should be reserved for when there's a high suspicion of malignancy. Surgery is a definitive but highly invasive option that should only be considered if malignancy is confirmed or strongly suspected. Although periodic examination might seem reasonable, a thyroid nodule of the given size is a significant finding that requires more immediate evaluation. An ultrasound, therefore, presents the least invasive and most informative procedure to assess the characteristics of the nodule, which would aid in determining if further interventions, such as a fine-needle aspiration biopsy or surgical excision, are necessary. (4) CONCLUDING: Given the size of the nodule and the need for identification of its nature, performing an ultrasound of the thyroid gland would be the best initial step in management, as it provides necessary information without subjecting the patient to undue risk or discomfort. (5) RESPONSE: Therefore, the most appropriate initial step in the management of this 47-year-old woman with a 2-cm thyroid nodule is (E) Ultrasonography of the thyroid gland.

**AI_E:** 1) RESTATING THE QUESTION: The question is asking for the most suitable initial step in managing a middle-aged woman who, during a routine health check, was identified to have a 2-cm nodule in the right lobe of her thyroid. The nodule is soft and isn't causing her any discomfort. Additionally, she has no previous history of severe illness and her serum thyroid-stimulating hormone concentration is within the reference range (normal). 2) PLANNING: An unsuspected thyroid nodule can be a benign condition, such as a colloid nodule or follicular adenoma, or it can be malignant. Risk factors for malignancy include a history of radiation exposure, male gender, age less than 20 or over 60, and nodule size above 4 cm. Given its size and the patient's lack of symptoms and history, malignancy is less likely, but cannot be definitively ruled out without further diagnostic information. On the other hand, although the patient's TSH levels are normal, pseudonormal levels may not exclude a functioning nodule (toxic adenoma or Plummer disease). So, an approach that helps disclose the nature and functional status of the nodule should be chosen. 3) CLINICAL REASONING: A CT scan of the neck won't provide functional information, so is not the best initial step. Fine-needle aspiration biopsy can reveal cytological information, but it might not be necessary if ultrasonography shows the nodule to be benign, syndicating it won't be the initial step. Reexamination in 3 months could monitor potential growth. Yet if the nodule is toxic, it might require antithyroid medications or radioactive iodine earlier than 3 months to prevent hyperthyroidism symptoms. Surgical excision may be too invasive for the initial step and is usually reserved for definitive treatment upon clear indication. 4) CONCLUDING: Starting with thyroid ultrasonography seems to be the logical initial step. It can identify benign features (e.g., spongiform or cystic), calculate the nodule size for follow-up, and check for suspicious features (irregular margins, intra-nodular vascular spots, microcalcifications) that may increase the suspicion of malignancy and thus indicate further evaluation by fine-needle aspiration. 5) RESPONSE: Therefore, the most appropriate initial step for this patient in managing her incidental finding of the thyroid nodule would be (E) Ultrasonography of the thyroid gland.

**AI_B:** 1) RESTATING THE QUESTION: The question concerns a 47-year-old woman with no history of any major sickness who came in for a routine check-up. While generally feeling well, the examination revealed a nontender 2-cm, soft nodule on the right lobe of her thyroid gland. No lymph node enlargement was noted, and her thyroid-stimulating hormone (TSH) levels are within the normal range. The query seeks to determine the most suitable first step in management given these circumstances. 2) PLANNING: This scenario involves an asymptomatic woman with an incidental finding of a thyroid nodule. The main assumption here is that the nodule could be benign or malignant, hence it needs appropriate investigation. Another assumption could be that the nodule is just a thyroid cyst, which is a potential benign clinical scenario. An alternate assumption is that the nodule has no clinical significance because the patient is asymptomatic and her TSH levels are normal. However, it is important that we rule out malignancy due to the implications it would have on the patient's prognosis and management. Therefore, based on the risk-benefit evaluation, the thyroid nodule should be evaluated further to rule out malignancy. 3) CLINICAL REASONING: Thyroid nodules, irrespective of the presence of symptoms, need to be appropriately investigated to determine their nature – whether benign or malignant. While her normal TSH levels and absence of lymphadenopathy may point towards a benign process, it is crucial to note that absence of specific signs or symptoms does not entirely rule out malignancy. A fine-needle aspiration biopsy (option B) could certainly yield valuable information about the cytology of the nodule but it is generally not the first step in investigating such nodules. Surgical excision (option D), is also an incongruous first step as it may not be warranted if the nodule is benign, leading to potential unnecessary harm to the patient. 4) CONCLUDING: Given the importance of ruling out malignancy but also considering the patient's asymptomatic status and normal TSH levels, a stepwise diagnostic approach is crucial. This involves initially performing a less invasive but effective testing that can efficiently separate benign from potentially malignant nodules, to ensure further investigation is only carried on those which truly need it or are suspicious. 5) RESPONSE: Ultrasonography of the thyroid gland (option E) fulfills this purpose. It allows for a detailed visualization of the nodule and assessment of features such as size, borders, presence of calcification, vascularity, among others, providing clues regarding its nature. Hence, it is the most appropriate initial step in management of this case.

**AI_C:** (1) RESTATING THE QUESTION: The question involves the management approach to a seemingly asymptomatic 47-year-old woman who incidentally discovered a 2-cm thyroid nodule during a routine check-up. Thyroid nodules are abnormal growths or lumps in the thyroid gland, and lymphadenopathy (enlargement of the lymph nodes), which is not present in this case, could indicate an invasive process or malignancy. The woman's thyroid-stimulating hormone (TSH) level is within the normal reference range, suggesting that the nodule is not functioning autonomously to produce excess thyroid hormones. (2) PLANNING: Assumptions include: the nodule could be benign or malignant, its nature can be determined clinically, or that a definitive diagnosis can only be achieved through invasive procedures. The alternative assumption that needs to be clinically prioritized is whether this nodule, even in the absence of any symptoms or lymphadenopathy, could be harboring malignancy, and what would be the most appropriate initial step in this context. (3) CLINICAL REASONING: If the nodule were indeed malignant, she would be asymptomatic given the nodule's relatively small size and the absence of spread to surrounding lymph nodes. However, malignancy cannot be definitively ruled out on clinical grounds alone. Because of these considerations, an initial diagnostic approach to a thyroid nodule typically involves a non-invasive imaging modality to assess the nodule's characteristics. (4) CONCLUDING: A computed tomography (CT) scan of the neck provides a general assessment of the neck area but doesn't specifically evaluate the internal structures or characteristics of a thyroid nodule. A fine-needle aspiration biopsy provides a definitive diagnosis but is invasive and typically reserved for nodules with specific characteristics suggestive of malignancy on imaging. Reexamination in 3 months can miss an opportunity for early diagnosis if the nodule is indeed malignant. Surgical excision, besides being invasive, is not the initial step for an asymptomatic nodule, but a last resort for symptomatic or confirmed malignant cases. Ultrasonography of the thyroid gland provides a detailed evaluation of the nodule's characteristics and is non-invasive, thus, making it the most appropriate initial diagnostic step. (5) RESPONSE: Thus, the most appropriate initial management step for this woman with an asymptomatic thyroid nodule is 'Ultrasonography of the thyroid gland' (Option E).

**AI_A:** (1) RESTATING THE QUESTION: The issue in this scenario revolves around the detection of a 2-cm nodule in the right lobe of the thyroid gland in a 47-year-old woman, who has no history of serious illness, and is currently asymptomatic. She doesn't have lymphadenopathy, and her thyroid-stimulating hormone (TSH) level is within the normal range. The question is asking what should be the most appropriate first step in managing her condition: CT scan of the neck, fine-needle aspiration biopsy, reexamination in 3 months, surgical excision, or ultrasonography of the thyroid gland. (2) PLANNING: First and foremost, the benign nature of the nodule is assumed due to its characteristics (soft, nontender) and the absence of lymphadenopathy. However, this doesn't exclude the possibility of malignancy. Another assumption could be that the thyroid function isn't affected, given the normal TSH levels. However, TSH alone doesn't provide a complete assessment of thyroid function. Thirdly, it's assumed that previous examinations revealed no thyroid nodules, hence the advice to reexamine after 3 months, but the information is not there. Among these assumptions, the possibility of malignancy should be clinically prioritized as it has serious implications. (3) CLINICAL REASONING: Confirming the nature of the nodule is crucial, whether it's malignant or benign, and this is usually achieved by biopsy, more specifically, fine-needle aspiration (FNA) biopsy. However, before going for an invasive procedure like FNA, a non-invasive procedure, such as a radiological imaging, can be used. While CT scan helps in detailed imaging of the neck, it may not provide sufficient resolution for a small thyroid nodule. Hence, the most appropriate imaging modality for thyroid nodules is ultrasonography (USG). Given the size of the nodule (2-cm), which is relatively large, makes ultrasonography a sensible choice. (4) CONCLUDING: Although the clinical features of the nodule may suggest a benign nature, we must not ignore the fact that it also could be malignant. Hence, initially, a non-invasive ultrasonography should be performed to assess the nodule's traits and further risk stratification. Depending on this result, a fine-needle aspiration biopsy could be performed subsequently, if necessary. (5) RESPONSE: I would go with the option 'E'. Ultrasonography of the thyroid gland should be the most appropriate initial step in management of this asymptomatic woman with a thyroid nodule, normal TSH levels, and no history of serious illness or lymphadenopathy.

**Facilitator** The consensus among the AI responses is that the most appropriate initial step in managing the 47-year-old woman's 2-cm thyroid nodule is to perform an ultrasonography of the thyroid gland. This decision is based on the need to determine the nature of the nodule, which could be either benign or malignant. Despite the woman's asymptomatic status and normal thyroid-stimulating hormone levels, the possibility of malignancy cannot be ruled out based solely on these factors. Ultrasonography is a non-invasive procedure that provides a detailed evaluation of the nodule's characteristics, making it a sensible first step. It can identify benign features, calculate the nodule size for follow-up, and check for suspicious features that may increase the suspicion of malignancy. This approach allows for a stepwise diagnostic process, ensuring further invasive procedures like fine-needle aspiration biopsy or surgical excision are only carried out if necessary based on the ultrasound findings. Therefore, the consensus response to the prompt is (E) Ultrasonography of the thyroid gland.
