# Supplementary material for: Collaborative intelligence in AI: Evaluating the performance of a council of AIs on the USMLE
Source: PLOS Digit Health. 2025 Oct 9;4(10):e0000787. doi: 10.1371/journal.pdig.0000787 (PMC12510544; doi:10.1371/journal.pdig.0000787)
Supplement: S2 File — (ZIP) [file pdig.0000787.s002.zip › S2 Data Raw data files containing unprocessed question inputs and LLM outputs including transcripts of deliberations/STEP2_Question-63-1_Type-MC-NJ.docx]

**Facilitator** **[Round 1]:** A 1-hour-old male newborn is examined in the hospital nursery. He was born at term to a 22-year-old woman, gravida 1, para 1, with HIV infection. The mother took antiretroviral therapy throughout her pregnancy, and her HIV viral load was consistently <1000 copies/mL. She received intravenous zidovudine (AZT) during labor. The patient’s Apgar scores were 8 and 9 at 1 and 5 minutes, respectively. He is at the 50th percentile for length, weight, and head circumference. He appears well. Examination shows no abnormalities. In addition to administration of the hepatitis B vaccine, which of the following is the most appropriate pharmacotherapy for this newborn? (A) Lopinavir-ritonavir and trimethoprim-sulfamethoxazole (B) Lopinavir-ritonavir only (C) Penicillin (D) Trimethoprim-sulfamethoxazole only (E) Zidovudine (AZT)

**AI_D:** (1) RESTATING THE QUESTION: The question is asking for the most suitable medication to be administered to a newborn, who was born to an HIV-infected mother that adhered to her antiretroviral therapy throughout her pregnancy and during labor. The newborn is currently healthy with no abnormalities. (2) PLANNING: To determine the most appropriate pharmacotherapy, we must consider the mother's HIV status, her treatment during pregnancy and labor, and the health status of the infant. These factors are critical in preventing vertical transmission of HIV from the mother to the child. (3) CLINICAL REASONING: In the case of a mother with HIV, the goal is to minimize the risk of maternal - to - infant transmission. As per guidelines, mothers with HIV should receive comprehensive antiretroviral therapy (ART) to maintain viral suppression, and there should be additional measures taken during pregnancy and childbirth to prevent transmission. In this scenario, the mother's viral load was consistently low (<1000 copies/mL), and she was treated with Zidovudine (AZT) during labor. As for the infant, the current protocol recommends a 6-week course of Zidovudine (AZT), regardless of the mother's viral load or treatment, as prophylaxis to prevent HIV transmission from mother to infant. (4) CONCLUDING: Therefore, the HIV-exposed newborn in question needs prophylactic Zidovudine (AZT), in addition to the general neonatal care, including hepatitis B vaccine administration. (5) RESPONSE: The most appropriate additional pharmacotherapy for this newborn is Zidovudine (AZT) (Option E).

**AI_A:** RESTATING THE QUESTION: The question is essentially asking for the most appropriate pharmacotherapy for a newborn who was born to a mother with HIV infection. The mother was on antiretroviral therapy during her pregnancy and her HIV viral load was consistently less than 1000 copies/mL. She was also given zidovudine during labor. The newborn seems to be healthy and was already given the hepatitis B vaccine. PLANNING: It's important to keep in mind that HIV can be transmitted from mother to child during pregnancy, during delivery, or through breastfeeding. The risk of transmission can be reduced by the use of antiretroviral therapy during pregnancy, delivery, and postpartum to the newborn. It's also important to note the mother's HIV viral load was less than 1000 copies/mL, suggesting a reduced risk of transmission. CLINICAL REASONING: In an infant born to an HIV-positive mother, the early use of antiretroviral prophylaxis can reduce the chance of transmission. The CDC recommends a 6-week course of zidovudine (AZT) for infants born to HIV positive mothers, regardless of the mother’s viral load or whether she received antiretroviral therapy. Therefore, aside from the hepatitis B vaccine, AZT would be the appropriate pharmacotherapy for this newborn. CONCLUDING: Considering the above information, a 6-week course of zidovudine is the most suitable choice to minimize the risk of vertical HIV transmission despite the mother's controlled HIV status. RESPONSE: The most appropriate pharmacotherapy for this newborn, in addition to the hepatitis B vaccine, is Zidovudine (AZT) (Choice E).

**AI_E:** RESTATING THE QUESTION: The clinical scenario presents a well-appearing, term male newborn born to a 22-year-old woman who is HIV positive. Despite receiving antiretroviral therapy throughout her pregnancy with a consistently undetectable viral load and administration of intravenous Zidovudine during labor, there's a risk of HIV transmission to the newborn from the mother. The question demands a suitable pharmacotherapeutic intervention for the newborn alongside the hepatitis B vaccine. PLANNING: This calls for a comprehension of the HIV infection management in newborns, especially born to HIV positive mothers, even with controlled viral load and antiretroviral therapy. Obvious factors to consider include the risk of HIV transmission from mother to child, available preventive measures, and the effectiveness of different drugs to prevent HIV infection in newborns. It is reasonable to hypothesize that in this case a pediatric antiretroviral therapy could be prioritized due to the maternal status. CLINICAL REASONING: Despite the mother's undetectable HIV viral load and the use of antiretroviral medicine, there is still a risk of mother-to-child transmission of HIV, albeit low. It's important to mitigate this risk by administering antiretroviral therapy to the newborn. This administration will help decrease the likelihood of the newborn contracting the HIV virus. With options given, the agent that stands out is Zidovudine (AZT), a nucleoside reverse transcriptase inhibitor. The rationale for this choice is drawn from guidelines recommending that infants born to HIV-infected mothers should receive prophylactic antiretroviral therapy for six weeks, of which Zidovudine is often the drug of choice. CONCLUDING: Administering Zidovudine to the newborn would serve to minimize the risk of HIV transmission from the mother to the child. This intervention is critical, considering the mother's HIV status, despite her controlled viral load, and prior antiretroviral use. RESPONSE: Given the information provided, the most appropriate pharmacotherapy for this newborn, in addition to the hepatitis B vaccine, would be Zidovudine (AZT) (option E).

**AI_C:** RESTATING THE QUESTION: This question is asking what would be the best pharmacological treatment for a newborn who has been born to a mother with a well-managed HIV infection. The terms involved are: Gravida 1 and Para 1 -indicate that the mother has been pregnant once and given birth once, HIV viral load < 1000 copies/mL - indicates a well-managed HIV infection, Apgar scores of 8 and 9 - suggest a healthy baby at birth, and Antiretroviral therapy and Zidovudine(AZT) - are treatments for HIV infection. PLANNING: One assumption is that the newborn has not been infected with HIV, given the mother's low viral load and use of antiretroviral therapy throughout pregnancy and labor. An alternate assumption is that the risk of vertical transmission may still exist despite these factors, therefore newborn prophylaxis could be considered as a preventive measure. In order to evaluate the validity of these assumptions, it's important to consider the current clinical guidelines for newborns born to HIV-infected mothers. CLINICAL REASONING: Studies show that mother-to-child transmission of HIV can be greatly decreased if the mother received antiretroviral therapy throughout pregnancy and her viral load was well-controlled. However, due to a small risk of transmission during delivery, babies born to HIV-infected mothers also receive postnatal antiretroviral prophylaxis even if the mother's HIV is well controlled. The exact regimen depends on the mother’s clinical circumstances, including the timing of her diagnosis and her viral load near delivery. Therefore, in this case, since the mother's viral load was consistently <1000 copies/mL and she received zidovudine during labor, six weeks of zidovudine prophylaxis would be recommended for the newborn. CONCLUDING: The step-by-step evaluation of the situation suggests that six weeks of zidovudine prophylaxis would be the most appropriate pharmacotherapy for the newborn. RESPONSE: The most appropriate pharmacotherapy for this newborn, in addition to the hepatitis B vaccine, is Zidovudine (AZT) - Option (E).

**AI_B:** RESTATING THE QUESTION: So, the query here pertains to a newborn baby, born to a mother with HIV infection who has been on antiretroviral therapy. The baby is healthy, with all parameters within normal range. However, the question seeks to determine the most appropriate pharmacotherapy for this baby, in addition to the hepatitis B vaccine that has already been administered. PLANNING: The two main assumptions that can be made here are (i) the baby could need further medication to prevent HIV transmission and (ii) the baby may need antibiotics to counter potential bacterial invasions. An alternate assumption might, surprisingly, be potentially not needing any additional medication given the mother's effective treatment during pregnancy. We should evaluate these assumptions in the light of the mother's HIV status, treatment, her viral load, and the presumed risk of transmission to the child. CLINICAL REASONING: While there exists a risk of HIV transmission from mother to child during pregnancy, delivery, or nursing, certain practices reduce this risk. The mother maintained a low viral load by taking regular antiretroviral therapy during pregnancy, in this case. Furthermore, Zidovudine was administered intravenously during labor for the same reason. This speaks to the alternate hypothesis that no additional medication might be necessary. However, considering the severity of possible HIV infection in a child and the life-long implications, it is reasonable to add a therapeutic measure that would further reduce any potential risks. Amongst the given options, Zidovudine for the baby, which is a type of antiretroviral medication, might hold the best answer. CONCLUDING: While antibiotics such as penicillin and trimethoprim-sulfamethoxazole have their place in disease management in children, they can't prevent HIV transmission. Lopinavir-ritonavir is a routine recommendation for pre-exposure prophylaxis for high-risk adults, not newborns. The answer must be a measure introduced to shield the baby from potential HIV transmission, rather than treating a potential infection of a different nature. RESPONSE: Therefore, the most appropriate pharmacotherapy for this newborn, considering the maternal history and current conditions, in addition to the hepatitis B vaccine, would be Zidovudine (AZT) (Choice E).

**Facilitator** The consensus statement synthesizes the responses of AI_A, AI_B, AI_C, AI_D, and AI_E as follows: The question pertains to the most appropriate pharmacotherapy for a newborn born to a mother with HIV infection who was on antiretroviral therapy during her pregnancy and labor. Despite the mother's well-managed HIV infection, there remains a risk of vertical transmission of HIV from the mother to the child. This risk can be mitigated by administering antiretroviral therapy to the newborn. The mother's low viral load and use of antiretroviral therapy throughout pregnancy and labor suggest a reduced risk of transmission. However, due to the potential risk of transmission during delivery, it is recommended that babies born to HIV-infected mothers receive postnatal antiretroviral prophylaxis, regardless of the mother's viral load or treatment. Among the given options, Zidovudine (AZT), a nucleoside reverse transcriptase inhibitor, is the most suitable choice. Current clinical guidelines recommend a 6-week course of Zidovudine for infants born to HIV positive mothers. This prophylactic measure serves to further reduce any potential risks and is critical considering the mother's HIV status, despite her controlled viral load and prior antiretroviral use. Therefore, in addition to the hepatitis B vaccine, the most appropriate pharmacotherapy for this newborn is Zidovudine (AZT) (Option E). This consensus is based on the clinical reasoning and conclusions drawn by all the AI responses, which align with current clinical guidelines for newborns born to HIV-infected mothers.
