# Supplementary material for: Collaborative intelligence in AI: Evaluating the performance of a council of AIs on the USMLE
Source: PLOS Digit Health. 2025 Oct 9;4(10):e0000787. doi: 10.1371/journal.pdig.0000787 (PMC12510544; doi:10.1371/journal.pdig.0000787)
Supplement: S2 File — (ZIP) [file pdig.0000787.s002.zip › S2 Data Raw data files containing unprocessed question inputs and LLM outputs including transcripts of deliberations/STEP2_Question-64-1_Type-MC-NJ.docx]

**Facilitator** **[Round 1]:** A 25-year-old woman comes to the office because she has not had a menstrual period for 6 months. Previously, menses occurred at regular 28-day intervals. She has not had blurred or double vision. She has no history of serious illness and takes no medications. Temperature is 37.0°C (98.6°F), pulse is 72/min, and blood pressure is 108/60 mm Hg. The pupils are equal and reactive to light; extraocular muscles are intact. Funduscopic examination shows no abnormalities. Visual field testing shows bitemporal hemianopia. Milky discharge can be expressed from the nipples. Pelvic examination shows no abnormalities. Serum studies show a thyroid-stimulating hormone concentration of 2.0 μU/mL and a prolactin concentration of 100 ng/mL. A urine pregnancy test is negative. MRI of the pituitary gland shows a 1.2 × 1.5-cm mass compressing the optic chiasm. Which of the following is the most appropriate next step in management? (A) Biopsy of the pituitary gland (B) Cabergoline therapy (C) Gamma knife radiation to the pituitary gland (D) Oral contraceptive therapy (E) Transsphenoidal pituitary surgery

**AI_B:** RESTATING THE QUESTION: The question is about a 25-year-old woman who is experiencing a 6-month absence of menses (amenorrhea) accompanied by milky discharge from the nipples (galactorrhea), and visual field changes (bitemporal hemianopia) which are signs of a pituitary tumor. Laboratory tests reveal a normal level of thyroid-stimulating hormone (TSH) but an elevated prolactin level (hyperprolactinemia). MRI confirmed the presence of a pituitary gland tumor, which is compressing the optic chiasm. The question asks for the best management approach from options (A) through (E). PLANNING: This patient's condition is highly suggestive of a prolactinoma, which is a benign tumor (adenoma) of the pituitary gland that produces excessive quantities of prolactin. Symptoms of prolactinomas include amenorrhea, galactorrhea, and visual field changes. Thus, the key assumption is that the patient has a prolactinoma and needs treatment to reduce prolactin production and shrinkage of the tumor. Alternate assumptions could be that the patient has a non-functional pituitary tumor causing mass effects but this is less likely given the high prolactin level and clinical presentation. CLINICAL REASONING: Prolactinomas are generally managed with dopamine agonists, which reduce prolactin synthesis and secretion, shrinking the tumor, and alleviating the symptoms. Cabergoline is an agonist of dopamine D2 receptor that is often used for its treatment. The surgical removal of the tumor through a transsphenoidal approach is an option if medical management fails or is contraindicated. Biopsy would be an unusual first step, considering the specific findings and a diagnosis already supported by sufficient data. Radiation therapy, while a management option for pituitary tumors, is generally reserved for cases where medication and surgery are ineffective or not feasible. CONCLUDING: Considering that the woman is young, not pregnant, and has a typical presentation of a prolactinoma, initiating medical therapy to lower prolactin levels and shrink the tumor should be the first step. RESPONSE: The most appropriate next step in management would be (B) Cabergoline therapy.

**AI_C:** RESTATING THE QUESTION: The situation presented here is of a 25-year-old woman who has been experiencing amenorrhea, or absence of menstrual periods, for 6 months. She shows no evidence of visual disturbances apart from bitemporal hemianopia identified through visual field testing. She also has a milky discharge from her nipples, known as galactorrhea. Her thyroid-stimulating hormone levels are normal, but her prolactin levels are elevated. An MRI has shown the presence of a mass in her pituitary gland which is compressing the optic chiasm. The question being asked is what would the most appropriate next course of action be in treating her. PLANNING: This situation suggests a prolactinoma, which is a common type of pituitary tumor that results in elevated levels of prolactin. The mass effect from the tumor could be causing the bitemporal hemianopia, due to compression of the optic chiasm. The options provided involve different forms of treatment: biopsy would involve getting a sample of the tumor to further verify its nature, cabergoline therapy involves a type of medication used to decrease prolactin levels, gamma knife radiation is a form of targeted radiation therapy, oral contraceptive therapy could be used to manipulate hormone levels, and transsphenoidal pituitary surgery would involve removal of the tumor via the sinus cavity. CLINICAL REASONING: The condition at hand necessitates treatment that addresses both the symptomatic hormonal imbalance and the physical mass effect from this presumably benign tumor. Cabergoline is the first line medical treatment for prolactinomas and is effective in reducing prolactin levels, shrinking the tumor, and relieving the patient's symptoms. Biopsy is not routinely needed for prolactinomas as the diagnosis is usually based on the clinical picture and hormonal levels. Gamma knife radiation is usually reserved for drug-resistant cases or for patients who are not surgical candidates. In this case, oral contraceptive therapy could control the menstrual irregularity, but wouldn't address the underlying tumor or the hyperprolactinemia. Lastly, transsphenoidal surgery may influence the release of other important hormones and is usually kept as an option if medication fails or if the tumor is pressing against the optic nerves and threatening vision loss. CONCLUDING: Given the patient's clinical history and current condition,I would suggest cabergoline therapy as it could not only reduce prolactin levels but also reduce the size of the tumor, thereby relieving symptoms. RESPONSE: (B) Cabergoline therapy

**AI_E:** RESTATING THE QUESTION: To paraphrase, a young adult female with no significant health history has presented after experiencing amenorrhea for half a year. She also has bitemporal hemianopia and can produce a milky discharge from her nipples. Her symptoms contrast her earlier menstrual regularity, and a large mass in her pituitary gland is revealed via MRI. This mass may be compressing the optic chiasm, possibly causing her visual disturbance. Prolactin, a hormone produced by the pituitary gland, appears to be elevated in her system. With these circumstances in mind, the appropriate next step in her medical management needs to be determined. PLANNING: The key details in this case are the amenorrhea, elevated prolactin, bitemporal hemianopia, and the pituitary mass. These point to a possible diagnosis of a prolactinoma, which is a benign tumor of the pituitary gland that produces excess amounts of prolactin, leading to symptoms such as interrupted menstruation and milky nipple discharge. The visual disturbance could be due to compression of the optic chiasm by this mass. Given these assumptions, it is necessary to evaluate the available options for management to define the most appropriate one. CLINICAL REASONING: A prolactinoma, due to its benign nature, does not typically require surgical intervention or biopsy initially, unless it is causing severe symptoms from compression or is not responsive to medical management, thus making option A and E less relevant. Gamma knife radiation, option C, is usually reserved for patients not responding to medical therapy and not candidates for surgery. Option D, oral contraceptive therapy, could regulate the menses but it wouldn't address the elevated prolactin levels or tumor itself. The prolactin-lowering medication cabergoline, option B, could potentially reduce the size of the tumor and restore normal prolactin levels, thereby addressing the underlying problems. CONCLUDING: Considering all the aforementioned, the most logical course of action would be to decrease prolactin levels pharmacologically using a dopamine agonist such as cabergoline. This would likely relieve the visual symptoms caused by the mass effect and normalize the menstrual cycle in the patient, whilst also treating the underlying pathology - a pituitary adenoma too. RESPONSE: The most suitable intervention in this case is (B) Cabergoline therapy. This drug, a dopamine agonist, by decreasing prolactin secretion may alleviate her symptoms by reducing the tumor size and normalizing the hormone levels. This response aims at treating the root cause of the condition rather than simply managing its symptoms.

**AI_A:** RESTATING THE QUESTION: The question presents the case of a 25-year-old woman with amenorrhea (absence of menstruation) for 6 months. She has a pituitary mass (as evidenced by an MRI) and has elevated prolactin levels, which alongside the presence of bitemporal hemianopia (loss of peripheral vision), suggests a diagnosis of a prolactinoma, which is a common type of pituitary tumor. The high prolactin levels have likely disrupted her normal menstrual cycle. The question wants us to determine the most appropriate next management step from given options including interventions such as biopsy, medication, radiation, oral contraceptives, or surgery. PLANNING: The assumptions are that the woman has a pituitary adenoma causing hyperprolactinemia and she needs treatment. Alternately, we could question whether the tumor causes symptoms or threatens vision and whether we assume medication or surgery will be more appropriate. Considering she already exhibits vision loss (as evidenced by the bitemporal hemianopia) and disrupted menstrual cycle due to the mass effect, aggressiveness of intervention might be deemed necessary. CLINICAL REASONING: Prolactinomas often respond to medication therefore surgical or radiation therapies are usually reserved for those cases that do not respond to medication, or those in which the adenoma results in significant mass effect or neurological symptoms. A bi-temporal hemianopia due to the compression of the optic chiasm definitely signifies an evolved mass effect. However, in such cases, transsphenoidal pituitary surgery is often chosen as it directly reduces the mass effect by removal of the adenoma, quickly relieving symptoms. Biopsy of the pituitary could determine the type of adenoma but usually, clinical symptoms and laboratory findings like elevated prolactin are enough to proceed with treatment. Gamma knife radiation is a less used treatment option due to the slower rate of tumor shrinkage and potential for hypopituitarism. Oral contraceptive therapy would help control the menstrual irregularities due to hyperprolactinemia but does not address the adenoma itself. Cabergoline, a dopamine agonist, can be used to reduce prolactin levels in prolactinomas but given the significant symptoms exhibited, a more aggressive approach may be needed. CONCLUDING: The clinical features in conjunction with imaging and laboratory findings strongly suggest a symptomatic prolactinoma in this woman. Given her significant mass effect with compression of the optic chiasm manifested as bitemporal hemianopsia, and amenorrhea, effective reduction of the mass more urgently might be needed. RESPONSE: Hence, given her health status, the most appropriate next step in management would be (E) Transsphenoidal pituitary surgery. This approach would effectively treat the mass effect, relieve pressure on the optic chiasm, and thus contribute to the resolution of the woman's symptoms.

**AI_D:** RESTATING THE QUESTION: We are presented with a 25-year-old female patient who has been amenorrheic for 6 months, despite previously having regular menstrual cycles. She presents with no visual disturbances, however, she has a milky nipple discharge and visual field testing reveals bitemporal hemianopia. Results indicate elevated prolactin levels and an MRI shows a pituitary mass impinging on the optic chiasm. The question is asking for the most appropriate next step in managing this patient's condition. PLANNING: The key data in this scenario include her amenorrhea, heightened prolactin levels, bitemporal hemianopia, and the presence of a pituitary gland mass. The patient likely has a prolactinoma, a type of pituitary adenoma (noncancerous benign brain tumor), which produces an excess amount of prolactin hormone leading to hyperprolactinemia. This can cause amenorrhea and galactorrhea (nipple discharge) in women. Bitemporal hemianopia, a reduced vision on outer (temporal) sides of both eyes, is a sign of the tumor compressing the optic chiasm. If this assumption is correct, appropriate management should focus on reducing prolactin secretion and controlling the size of the adenoma to prevent further optic nerve compression. CLINICAL REASONING: Hyperprolactinemia often results in galactorrhea and amenorrhea due to the inhibitory action of prolactin on Gonadotropin-releasing hormone (GnRH), leading to a reduction in Follicle-stimulating hormone (FSH) and luteinizing hormone (LH), which are crucial for regular menstrual cycle. Elevated prolactin levels also inhibit dopamine, which normally suppresses prolactin release from the pituitary gland, thereby forming a positive feedback loop for prolactin production. In managing prolactinoma, pharmacological therapy is generally first-line due to its favorable risk-benefit profile. A biguanide like Cabergoline, a dopamine agonist, can effectively reduce prolactin secretion, leading to a decrease in tumor size and alleviating visual symptoms caused by compression of the optic chiasm. Invasive procedures, such as biopsy or surgery, and more aggressive treatment modalities, like radiating the gland (Gamma knife radiation), are typically reserved for patients who fail or are intolerant to medication therapy or in situations where the lesion poses an imminent threat to vision. CONCLUDING: Based on clinical reasoning and knowledge of prolactinoma management, I suggest the initial treatment approach should involve pharmacological intervention, particularly with dopamine agonists. RESPONSE: Therefore, the most appropriate next step in management for this patient is (B) Cabergoline therapy. This should effectively reduce the secretion of prolactin and decrease the size of the adenoma, leading to an improvement in her symptoms.

**Facilitator** **[Round 2]:** SUMMARIES: AI_A's Opinion: AI_A believes that the woman's significant symptoms, including vision loss and disrupted menstrual cycle, necessitate an aggressive intervention. AI_A suggests that transsphenoidal pituitary surgery is the most appropriate next step in management, as it would directly reduce the mass effect by removing the adenoma, quickly relieving symptoms. AI_B's Opinion: AI_B suggests that the woman's condition is highly suggestive of a prolactinoma and recommends initiating medical therapy to lower prolactin levels and shrink the tumor. AI_B believes that the most appropriate next step in management would be cabergoline therapy. AI_C's Opinion: AI_C also suggests that the woman likely has a prolactinoma and recommends cabergoline therapy as the most appropriate next step in management. AI_C believes that this treatment would reduce prolactin levels and shrink the tumor, relieving symptoms. AI_D's Opinion: AI_D agrees with AI_B and AI_C, suggesting that the woman likely has a prolactinoma and recommending cabergoline therapy as the most appropriate next step in management. AI_D believes that this treatment would reduce prolactin secretion and decrease the size of the adenoma, leading to an improvement in her symptoms. AI_E's Opinion: AI_E also agrees with AI_B, AI_C, and AI_D, suggesting that the woman likely has a prolactinoma and recommending cabergoline therapy as the most appropriate next step in management. AI_E believes that this treatment would decrease prolactin levels, reduce the tumor size, and normalize hormone levels, treating the root cause of the condition. QUESTION FOR COUNCIL MEMBERS: The main disagreement among council members is whether to opt for surgery (as suggested by AI_A) or to initiate medical therapy with cabergoline (as suggested by AI_B, AI_C, AI_D, and AI_E). The question to ask council members to clarify this disagreement could be: "What factors should be considered when deciding between surgical intervention and medical therapy for a patient with a prolactinoma presenting with significant symptoms such as vision loss and amenorrhea?". Based on your preceding response, how would you answer the original question: [A 25-year-old woman comes to the office because she has not had a menstrual period for 6 months. Previously, menses occurred at regular 28-day intervals. She has not had blurred or double vision. She has no history of serious illness and takes no medications. Temperature is 37.0°C (98.6°F), pulse is 72/min, and blood pressure is 108/60 mm Hg. The pupils are equal and reactive to light; extraocular muscles are intact. Funduscopic examination shows no abnormalities. Visual field testing shows bitemporal hemianopia. Milky discharge can be expressed from the nipples. Pelvic examination shows no abnormalities. Serum studies show a thyroid-stimulating hormone concentration of 2.0 μU/mL and a prolactin concentration of 100 ng/mL. A urine pregnancy test is negative. MRI of the pituitary gland shows a 1.2 × 1.5-cm mass compressing the optic chiasm. Which of the following is the most appropriate next step in management? (A) Biopsy of the pituitary gland (B) Cabergoline therapy (C) Gamma knife radiation to the pituitary gland (D) Oral contraceptive therapy (E) Transsphenoidal pituitary surgery]

**AI_C:** RESTATING THE QUESTION: The question is essentially asking the best management step for a 25-year-old woman exhibiting symptoms of amenorrhea, bitemporal hemianopia, and, galactorrhea alongside findings of a higher than normal prolactin level and a mass in the pituitary gland, most likely representing a prolactinoma. PLANNING: A key assumption in this case is that the woman is suffering from a prolactin-secreting pituitary adenoma. Another assumption is that tumour size and its compressive effects can be meaningful factors in determining the optimal management strategy, either medical or surgical, depending on the aims (to reduce prolactin levels, shrink the tumor, or relieve the compression) or possible side effects of these interventions. CLINICAL REASONING: The woman's amenorrhea can be attributed to elevated prolactin levels, which can inhibit gonadotropin-releasing hormone (GnRH). This subsequently reduces the secretion of follicle-stimulating hormone (FSH) and luteinizing hormone (LH), disrupting the menstrual cycle. Her bitemporal hemianopia indicates a mass effect on the optic chiasm, corresponding with the MRI findings of a pituitary mass. The high prolactin level, along with the visual disturbances, indicates a macroprolactinoma. The first-line management of macroprolactinomas, even when symptomatic, is typically dopamine agonists, like cabergoline. Not only do these drugs reduce prolactin levels, they can also reduce the size of the tumor, likely improving her visual symptoms. By initiating medical therapy first, many patients can avoid surgery or radiation therapy, which carry higher risks of complications. CONCLUDING: Considering the patient's symptoms and findings, her condition aligns with a macroprolactinoma, for which medical therapy with dopamine agonists is usually the first-line treatment. Therefore, we should opt for pharmacological agents such as cabergoline to lower the prolactin level, lessen the tumor size, and most likely mitigate visual symptoms too. RESPONSE: Thus, the best next step in this patient's management should be (B) Cabergoline therapy.

**AI_B:** RESTATING THE QUESTION: This clinical scenario represents a woman who had never had serious problems until she suddenly stopped menstruating for the past six months. She has vision problems, as demonstrated by her bitemporal hemianopia, and has a milky discharge expressed from her nipples. Medical examinations have ruled out thyroid issues, pregnancy, and other physical abnormalities, but there is an abnormally high level of prolactin in her blood and a pituitary mass found via MRI. The question asks for the most appropriate next management step to address her condition. PLANNING: It's clear from the evidence that this woman likely has a prolactinoma, a benign tumor of the pituitary gland that produces excessive amounts of prolactin. The potential options for management include a biopsy of the pituitary gland, initiating cabergoline therapy, utilizing radiation to the pituitary gland, oral contraceptive therapy, or transsphenoidal pituitary surgery. Key assumptions to consider are the invasive aspect and associated risks of surgical intervention, the physiological effect of radiation, the efficacy of medication like cabergoline in managing prolactinomas, the potential for oral contraceptives to manage menstrual irregularity in the context of hyperprolactinemia, and the reason for a biopsy when there's already strong evidence pointing to a prolactinoma. CLINICAL REASONING: Cabergoline, a dopamine agonist, is typically the first-line treatment for a prolactinoma because it effectively reduces prolactin levels and shrinks the tumor, which can relieve symptoms. Surgery, while able to remove the tumor, is usually reserved for cases where there is impending vision loss that doesn't respond to conservative management, extensive tumor invasion, or suspicion of malignancy. It also carries risks, such as cerebrospinal fluid leak or damage to surrounding structures. Oral contraceptive therapy could regulate her cycle but it does not solve the fundamental issue of the prolactinoma, while a biopsy is unnecessary with strong clinical, biochemical, and radiological evidence for a prolactinoma. CONCLUDING: Ultimately, our primary goal in this case should be to relieve her symptoms and reduce the size of the pituitary mass using the safest and least invasive options possible. A biopsy of the pituitary gland seems excessive because we have almost all the information we need. Surgery, while potentially beneficial, carries with it significant risks and should usually be used as a last resort. Oral contraceptives could manage the symptoms of amenorrhea but do not address the root cause of the problem. Given all of this, I conclude that initiating cabergoline therapy is currently the best way to manage this patient's prolactinoma directly. RESPONSE: The most appropriate next step in managing this patient's condition is (B) Cabergoline therapy.

**AI_D:** RESTATING THE QUESTION: The question is asking for the most appropriate next step in management for a 25-year-old woman presenting with amenorrhea, visual field defects, milky nipple discharge, and an MRI-confirmed pituitary mass causing compression of the optic chiasm with elevated prolactin levels. Notably, none of the other physical or laboratory findings suggest any other systemic abnormalities. PLANNING: Given the clinical picture, the focus should be on addressing the hyperprolactinemia causing amenorrhea and milk discharge, commonly seen in prolactin-secreting pituitary adenomas (prolactinomas). Raised prolactin levels not only can interfere with normal menstruation but can also result in galactorrhea (milky nipple discharge). The visual field deficits (bitemporal hemianopia) can be due to compression of the optic chiasm by the pituitary mass; a common presentation in large prolactinomas. Therefore, the initial management should aim at reducing the size of the tumor to alleviate the mass effect and at normalizing the prolactin levels to restore menstruation and eliminate galactorrhea. CLINICAL REASONING: Medical therapy with a dopamine agonist like cabergoline, is generally the first line of treatment. Cabergoline acts by stimulating dopamine receptors in the lactotrope cells in the pituitary gland, thereby inhibiting prolactin secretion. This action not only helps to suppress the excess prolactin-mediated symptoms and signs but also reduces the size of prolactinomas. Surgery, such as a transsphenoidal approach, is usually reserved for cases who do not respond to medical management or in emergent cases where there are severe neurological symptoms due to the mass effect. Considering the available options, the patient does not demonstrate severe neurological signs or resistance to medical therapy at the moment to qualify for surgical intervention or the other treatment options. Therefore, Cabergoline remains the preferred choice. CONCLUSION: Therefore, in a 25-year-old woman presenting with clinical and laboratory findings suggestive of a prolactinoma, the most appropriate initial management would involve reducing the prolactin levels and alleviating the symptoms and signs associated with hyperprolactinemia such as amenorrhea and galactorrhea and reducing the size of the tumor, which will lead to resolution of vision abnormalities. RESPONSE: Thus, in accordance with the evaluated clinical reasoning, the answer is (B) Cabergoline therapy.

**AI_A:** 1. RESTATING THE QUESTION: The patient, a 25-year-old woman, has been experiencing amenorrhea (absence of menstruation) for the past six months. She also presents with bitemporal hemianopia, a visual field defect with loss of peripheral vision, and galactorrhea, which is the spontaneous flow of milk from the breasts, unrelated to childbirth or nursing. An MRI revealed a mass in her pituitary gland which is likely causing her symptoms. Several management options are presented, including a biopsy, treatments with cabergoline or oral contraceptive, gamma knife radiation, or surgery. 2. PLANNING: The symptoms of amenorrhea, galactorrhea, and a high prolactin level strongly indicate a prolactinoma. This is a type of benign tumor (adenoma) of the pituitary gland which overproduces the hormone prolactin. It's the most common type of functioning pituitary adenoma. The management of prolactinomas primarily involves two routes - medication with dopamine agonists such as cabergoline, or surgery - and the indications for each are weighted based upon tumor size, symptoms, and patient preferences. 3. CLINICAL REASONING: Given the current clinical picture and the prolactin level of 100 ng/mL, it is more likely that the patient has a macroprolactinoma, which is defined as a prolactinoma larger than 1 cm in diameter. Even though it is compressing the optic chiasm and causing vision changes, dopamine agonists such as cabergoline are still the first-line treatment due to their effectiveness in shrinking tumor size and lowering prolactin level. Surgery is usually reserved for patients who are resistant to pharmaceutical therapy, or in emergency situations where life-threatening symptoms occur such as rapidly progressing visual field loss. In this case, the vision changes reported by the patient are significant but not rapidly progressing or critically threatening. 4. CONCLUDING: Although transsphenoidal surgery is an option, it's not the first-line treatment. Instead, the administration of dopamine agonists such as cabergoline to shrink the tumor size and decrease prolactin levels is considered the first-line treatment. Surgical intervention should only be considered if the patient doesn't respond to the medication or if life-threatening symptoms occur. 5. RESPONSE: Therefore, the most appropriate next step in the management of this patient would be cabergoline therapy (B). This treatment approach will target the root cause of the problem, inhibit the overproduction of prolactin and help reduce the size of the adenoma, likely alleviating her symptoms of amenorrhea and bitemporal hemianopia.

**AI_E:** RESTATING THE QUESTION: A woman aged 25 attended a clinic concerned because she has been amenorrheic for 6 months, despite previously having regular 28-day menstrual cycles. She has not experienced blurred or double vision. She does not have underlying serious illnesses and is not on medications. Her vitals are within normal limits, and her intraocular muscles show no abnormalities. Fundus examination was normal, and she exhibits bitemporal hemianopia on visual field testing. A milky discharge can be expressed from her nipples and the pelvic examination did not show any abnormalities. Her TSH is normal, but her prolactin level is high at 100 ng/mL. She is not pregnant. An MRI scan reveals a mass at her pituitary gland measuring 1.2 x 1.5 cm and compressing the optic chiasm. Considering these findings, what should be the next management step? PLANNING: The assumptions include a prolactin-producing adenoma because of the elevated prolactin levels, bitemporal hemianopia from optic chiasm compression, and milky nipple discharge. It's also important to consider that she's not having blurred or double vision, suggesting the chiasm is not compromised significantly, and she's not pregnant. Alternate assumptions could be non-functioning adenoma or other types of pituitary adenomas. Considering the evidence, the most likely assumption is a prolactinoma, which should be the focus of her treatment. CLINICAL REASONING: A prolactinoma is a common type of pituitary adenoma producing excessive amounts of prolactin. Manifestations can include amenorrhea and galactorrhea, as seen in this patient. The elevated levels of prolactin inhibit gonadotropin-releasing hormone (GnRH), reducing the release of luteinizing hormone (LH) and follicle-stimulating hormone (FSH), leading to hypoestrogenism and irregular menstrual cycle. In addition to these hormonal effects, large prolactinomas (>1 cm, known as macroadenomas) can compress the optic chiasm, leading to visual field defects. The patient's MRI result, showing a mass of 1.2x1.5 cm at the pituitary gland, confirms the presence of a macroadenoma. CONCLUDING: The primary aim is to reduce prolactin levels, shrink the tumour and relieve the symptoms of the patient. The first-line therapy for prolactinomas irrespective of size is dopamine agonists (e.g., cabergoline). These drugs lower prolactin levels and reduce tumor size. Surgical intervention is considered if medication is ineffective, not tolerated or in acute situations such as sudden visual loss due to apoplexy but not as the initial management step. RESPONSE: According to this case, and considering the patient’s symptoms and test results, the most appropriate next management step is (B) Cabergoline therapy. It will help in reducing the prolactin concentration in the patient's body, shrink the tumor, and alleviate symptoms. Proper follow-up should be established to monitor the patient's condition and the treatment's effectiveness.

**Facilitator** The consensus among the AI responses is that the most appropriate next step in managing this 25-year-old woman's condition is (B) Cabergoline therapy. The patient's symptoms of amenorrhea, bitemporal hemianopia, and galactorrhea, along with the MRI findings of a pituitary mass and elevated prolactin levels, strongly suggest a prolactinoma. This is a benign tumor of the pituitary gland that overproduces the hormone prolactin. Cabergoline, a dopamine agonist, is typically the first-line treatment for prolactinomas as it effectively reduces prolactin levels and shrinks the tumor, which can relieve symptoms. Surgery, while able to remove the tumor, is usually reserved for cases where there is impending vision loss that doesn't respond to conservative management, extensive tumor invasion, or suspicion of malignancy. It also carries risks, such as cerebrospinal fluid leak or damage to surrounding structures. Therefore, initiating cabergoline therapy is currently the best way to manage this patient's prolactinoma directly.
