# Supplementary material for: Collaborative intelligence in AI: Evaluating the performance of a council of AIs on the USMLE
Source: PLOS Digit Health. 2025 Oct 9;4(10):e0000787. doi: 10.1371/journal.pdig.0000787 (PMC12510544; doi:10.1371/journal.pdig.0000787)
Supplement: S2 File — (ZIP) [file pdig.0000787.s002.zip › S2 Data Raw data files containing unprocessed question inputs and LLM outputs including transcripts of deliberations/STEP2_Question-65-1_Type-MC-NJ.docx]

**Facilitator** **[Round 1]:** A 2-week-old boy is evaluated in the neonatal intensive care unit because of temperature instability and decreased activity. He was born by cesarean delivery at 30 weeks’ gestation because of premature rupture of membranes. Since birth, he has been fed breast milk by orogastric tube. He was feeding and growing appropriately until today, when his axillary temperature was 36.5°C (97.7°F) and he had decreased activity. During the past 4 hours, gastric residue has been noted in the orogastric tube when it is aspirated prior to feedings. Temperature is now 37.0°C (98.6°F), pulse is 160/min, respirations are 50/min, and blood pressure is 70/40 mm Hg. The anterior fontanel is soft. Cardiopulmonary examination shows no abnormalities. The abdomen is mildly distended, and there is mild tenderness to palpation diffusely; there are no masses. Bowel sounds are decreased. There is a small amount of blood-streaked stool in the diaper. The remainder of the examination shows no abnormalities. Leukocyte count is 18,000/mm³ (N=5000–34,000) (80% segmented neutrophils, 5% bands, 10% lymphocytes, and 5% monocytes), and platelet count is 100,000/mm³. Blood culture results are pending. Which of the following is most likely to confirm the diagnosis? (A) Measurement of prothrombin time and partial thromboplastin time (B) Measurement of stool reducing substances (C) Stool culture (D) Upper gastrointestinal series (E) X-ray of the abdomen

**AI_D:** RESTATING THE QUESTION: The query is about a premature newborn boy who has presented with temperature instability and decreased activity. He has also had gastric residuals noted in his orogastric tube and his abdomen is mildly distended with reduced bowel sounds and blood-streaked stool. He is otherwise clinically stable. The question asks which test will likely confirm the diagnosis for this presentation. PLANNING: The presented features suggest a strong possibility of necrotizing enterocolitis (NEC), a condition affecting premature infants where sections of the bowel undergo necrosis. The findings which make NEC a likely diagnosis include prematurity, temperature instability, feeding problems, increased gastric residuals, abdominal distension, tenderness and reduced bowel sounds, and bloody stool. The hypothesis that needs to be clinically prioritized is that this infant has developed NEC. Based on this assumption, the most appropriate diagnostic test would be one which best confirms or refutes this diagnosis. CLINICAL REASONING: NEC typically manifests with abdominal distension, bloody stools, and often systemic signs of infection. The presence of gastric residuals can suggest delayed gastric emptying, which is another feature of NEC. Bloodwork might show an increased number of white cells suggestive of a systemic response to infection, but this is not specific to NEC. Among the options presented to confirm the diagnosis, an upper gastrointestinal series could suggest the diagnosis but may not conclusively diagnose NEC. On the other hand, an abdominal x-ray could show some characteristic features of NEC like pneumatosis intestinalis (gas within the wall of the intestine) or free intra-abdominal air, if bowel perforation has occurred. CONCLUDING: Evaluating the options, an abdominal x-ray is the diagnostic test most likely to confirm the diagnosis of NEC in a neonate presenting with these symptoms. RESPONSE: (E) X-ray of the abdomen.

**AI_A:** Restating the Question: A 2-week-old premature newborn boy has shown signs of unstable body temperature, decreased activity, and a presence of gastric residue in his orogastric feeding tube over the past 4 hours. His physical examination shows a mild distension and tenderness in his abdomen, decreased bowel sounds and blood-streaked stool. The laboratory findings show elevated total leukocytes, of which 80% are segmented neutrophils; a marker for inflammation or infection. The question is asking which test could best confirm a possible diagnosis, given his conditions and clinical signs. Planning: The signs observed point towards a possibility of an abdominal pathology most likely necrotizing enterocolitis (NEC), commonly seen in premature infants. However, different diagnostic tools can be used to confirm the diagnosis such as prothrombin time and partial thromboplastin time (A), stool reducing substances (B), stool culture (C), a barium or air enema (D), or an X-Ray of the abdomen (E). Clinical Reasoning: Prothrombin time and partial thromboplastin time (Option A) are vital for assessing the coagulation cascade but is not specific for diagnosing NEC. Stool reducing substances (Option B) and stool culture (Option C) are used for diagnosing malabsorption syndromes and gastrointestinal infections, that are not at the top tier of the differential list for this case. An upper gastrointestinal series could identify anatomical abnormalities, but NEC tends to affect the distal bowel and so, may fall short in providing conclusive results. An X-ray of the abdomen, however, can identify gas patterns indicative of NEC, making it the prime choice for initial diagnosis. Concluding: Given the infant's clinical history, possible medical diagnoses, and available tests, the most useful and specific investigative tool to confirm the diagnosis would be an X-Ray of the abdomen. Response: Therefore, the best diagnostic method to confirm the infant's diagnosis would be (E) X-Ray of the abdomen.

**AI_E:** RESTATING THE QUESTION: The query is regarding a neonate, who was prematurely delivered due to premature rupture of membranes, and is currently facing symptoms including temperature instability, decreased activity, abdominal distension, mild tenderness during palpation, decreased bowel sounds, and the presence of blood-streaked stool. These symptoms are suggestive of a disturbance in his gastrointestinal operations. For further diagnosis clarification, we need to decide the most applicable supplementary investigative approach from the given options: measurement of prothrombin time and partial thromboplastin time, measurement of stool reducing substances, stool culture, upper gastrointestinal series, or X-ray of the abdomen. PLANNING: With the given symptoms of this neonate, the main assumptions that can be drawn are of a gastrointestinal disruption, specifically focusing on necrotizing enterocolitis (NEC) which is common in premature infants, and coagulation disorders which will explain the blood-streaked stool. To hypothesize further, we should prioritize the clinical situation which is more likely and thus NEC, more common in premature infants, should be considered here. CLINICAL REASONING: The clinical scenario described points towards a possible NEC, which is a common emergency gastrointestinal conditions in neonates, specifically premature and low birth weight babies. The features suggestive of NEC in this baby are feeding intolerance (as indicated by gastric residues in the orogastric tube), decreased activity, temperature instability, and the passage of blood-streaked stools. Other signs are abdominal distension and tenderness on palpation, and diminished bowel sounds. As a confirmation of this clinical diagnosis, imaging studies like abdominal X-rays are conducted. These might show intestinal dilation, pneumatosis intestinalis (which refers to the presence air within the wall of the intestine, a pathognomonic finding in NEC), portal venous gas, and free intraperitoneal air if perforation has occurred. CONCLUDING: Based on the clinical presentation of this neonate and the decision to rule out NEC, a radiologic study i.e., abdominal X-ray should be ideal. The other options such as measurement of coagulation studies and stool cultures are less likely, as they do not seem to be as relevant to the toddler's scenario. RESPONSE: From all the findings and the clinical reasoning, the most likely investigation to confirm the diagnosis in this 2-week old boy is an (E) X-ray of the abdomen.

**AI_B:** RESTATING THE QUESTION: The clinicians have been monitoring a 2-week-old premature boy, who lately has shown a decrease in activity and unstable temperature. He was born at 30 weeks due to premature rupture of membranes and has since been nourished via an orogastric tube with breast milk. However, recently he has been displaying signs of clinical deterioration, such as lower temperature, decreased activity, gastric residues in the tube, mild abdominal distention, tenderness, decreased bowel sounds, and blood-streaked stools. Despite these symptoms, his physical examination mostly checks out normal, and his leukocyte count is within the neonatal range, even if slightly elevated. The question is, what sort of test would most accurately confirm a diagnosis based on these symptoms? PLANNING: The baby's symptoms point towards a gastrointestinal issue. Therefore, we can make assumptions about potential causes such as necrotizing enterocolitis (NEC), sepsis, gastroenteritis, or a feeding intolerance among others. Based on the prematurity, orogastric feeding, and clinical findings (abdominal tenderness, distension, decreased bowel sounds, blood-streaked stools), NEC, a severe intestinal disease, becomes a primary concern. However, a stool culture or testing for stool reducing substances could also be helpful to rule out bacterial or viral gastroenteritis and malabsorption or maldigestion respectively. CLINICAL REASONING: NEC is a common and severe gastrointestinal disease among premature infants. Symptoms include feeding intolerance (gastric residuals), abdominal distension, bloody stools, and signs of systemic illness, such as temperature instability - all of which are present in this baby's case. While leukocytosis could suggest an infectious cause, it can also be a nonspecific sign of inflammation associated with NEC, and thus the elevated counts don't necessarily exclude NEC. On the other hand, testing for stool reducing substances is generally used when lactose intolerance or other forms of carbohydrate malabsorption are suspected; bacterial or viral gastroenteritis could be uncovered through stool cultures, but this condition would usually present with diarrhea, which contrast with our case. Prothrombin time and partial thromboplastin time would be relevant for coagulation disorders, but they don't seem directly implicated here. CONCLUDING: To confirm the suspicion of NEC, radiologic evaluation is the gold standard, as it could reveal pneumatosis intestinalis (gas within the bowel wall), a hallmark of NEC. RESPONSE: Based on clinical reasoning, the most appropriate diagnostic test to confirm the diagnosis in this 2-week-old boy with evidence pointing towards necrotizing enterocolitis, would be (E) an X-ray of the abdomen.

**AI_C:** RESTATING THE QUESTION: This clinical scenario involves a 2-week-old preterm baby boy, born due to premature rupture of membranes, who has shown signs of temperature instability, decreased activity, and periodic gastric residue in the orogastric tube from which he has been fed breast milk. There has been mild distension and diffused tenderness in the abdomen along with decreased bowel sounds. A small amount of blood-streaked stool is also reported. The baby has high pulse and respiration rates, and his white blood cell count is higher than normal. Pending the results of a blood culture, we are asked to predict which of the named investigations would likely confirm a diagnosis. PLANNING: The main assumption of this case is that the baby is suffering from a gastrointestinal issue, likely due to an infection or inflammation. This is assumed based on the baby's abnormal vital signs, the appearance of gastric residue in the orogastric tube, the observed mild abdominal distension and tenderness, the decreased bowel sounds, and the presence of blood-streaked stool. If these symptoms are due to a gastrointestinal problem, necessary investigation parameters would include stool and blood studies, radiological examinations that assess the gastrointestinal tract, along with coagulation studies. CLINICAL REASONING: Given the preterm birth, symptoms of abdominal distension, decreased activity, tenderness, blood-streaked stool, and unusual gastric residue, the baby may have necrotizing enterocolitis (NEC). NEC is a common lethal gastrointestinal disease, especially among preterm and low-birth-weight neonates, defined by variable damage to the intestinal tract ranging from mucosal injury to full-thickness necrosis and perforation. This can result in diffuse peritonitis, sepsis, and multisystem organ failure. Sepsis might explain the baby's high leukocyte count. So, based on these symptoms, it's crucial to observe the condition of the gastrointestinal tract. CONCLUDING: A direct visualization of the gastrointestinal tract could help to confirm the presence of NEC or rule it out. Though stool testing may provide valuable information regarding digestive enzymes or blood, it isn't as directly indicative of the intestinal damage one would expect in NEC, nor can it confirm its severity or territorial extent. While a comprehensive blood evaluation including coagulation studies would be relevant to a case of sepsis or DIC, for this situation, it is not the most critical test. RESPONSE: Therefore, as it's capable of showing pneumatosis intestinalis (gas in the intestinal wall) - a hallmark sign of NEC - and can also reveal other important findings such as constipation, perforation, or a fixed loop (indicative of intestinal obstruction), the abdominal x-ray (choice E) is the most beneficial investigation to confirm the diagnosis in this case. The pending blood culture results may help confirm possible sepsis secondary to NEC.

**Facilitator** Consensus Statement: The question pertains to a 2-week-old premature boy who has been showing signs of temperature instability, decreased activity, and gastric residue in his orogastric feeding tube. His physical examination reveals mild abdominal distension, tenderness, decreased bowel sounds, and blood-streaked stool. The laboratory findings indicate an elevated total leukocyte count. The question asks which test would most accurately confirm a diagnosis based on these symptoms. The symptoms observed in the infant point towards a gastrointestinal issue, with necrotizing enterocolitis (NEC) being a primary concern due to the infant's prematurity, orogastric feeding, and clinical findings. NEC is a common and severe gastrointestinal disease among premature infants, characterized by variable damage to the intestinal tract, which can result in diffuse peritonitis, sepsis, and multisystem organ failure. The symptoms of NEC include feeding intolerance, abdominal distension, bloody stools, and signs of systemic illness, such as temperature instability - all of which are present in this baby's case. While other tests such as stool culture, stool reducing substances, prothrombin time and partial thromboplastin time, and an upper gastrointestinal series could provide valuable information, they are not as directly indicative of NEC. An abdominal x-ray, however, can reveal characteristic features of NEC like pneumatosis intestinalis (gas within the wall of the intestine) or free intra-abdominal air if bowel perforation has occurred, making it the most beneficial investigation to confirm the diagnosis in this case. Therefore, based on the infant's clinical history, possible medical diagnoses, and available tests, the consensus is that the most useful and specific investigative tool to confirm the diagnosis would be an X-Ray of the abdomen (Option E).
