# Supplementary material for: Collaborative intelligence in AI: Evaluating the performance of a council of AIs on the USMLE
Source: PLOS Digit Health. 2025 Oct 9;4(10):e0000787. doi: 10.1371/journal.pdig.0000787 (PMC12510544; doi:10.1371/journal.pdig.0000787)
Supplement: S2 File — (ZIP) [file pdig.0000787.s002.zip › S2 Data Raw data files containing unprocessed question inputs and LLM outputs including transcripts of deliberations/STEP2_Question-66-1_Type-MC-NJ.docx]

**Facilitator** **[Round 1]:** A 13-month-old boy is brought to a small rural emergency department (ED) near his family's community because of an erythematous, edematous mass in his perianal area that is hot to touch. He has no history of serious illness and receives no medications. Growth and development are appropriate for age. Vaccinations are up-to-date. He is at the 50th percentile for length and weight. Temperature is 39.4°C (103.0°F), pulse is 160/min, and respirations are 36/min. Examination shows fluctuance and exquisite tenderness to palpation of the perianal mass. Oral acetaminophen and a dose of oral cephalexin are administered, and the parents are told that their child’s case is beyond the capabilities of the hospital. They are referred to the nearest hospital with a pediatric service, a suburban hospital 50 miles away. The suburban hospital is contacted regarding the referral and a pediatric surgeon accepts transfer of the patient. When the patient is brought to the ED of the suburban hospital, a triage nurse obtains his oral temperature of 37.7°C (99.8°F) and pulse of 126/min. The nurse notes that the family has no health insurance and states that the case is beyond the capabilities of the hospital. The nurse recommends taking the patient to the ED of a downtown university hospital 4 miles away. The parents follow this recommendation, and the patient undergoes incision and drainage of the abscess at the downtown hospital later that day. Which of the following is the most accurate characterization of this patient's pattern of care? (A) The patient received appropriate care under the circumstances (B) The pediatric surgeon at the suburban hospital should have insisted on personally examining the patient and draining the abscess (C) The rural hospital should have sent the patient directly to the downtown university hospital (D) The suburban hospital executed unethical but legal conduct in this case (E) The suburban hospital violated the Emergency Medical Treatment and Active Labor Act (EMTALA)

**AI_A:** 1) RESTATING THE QUESTION: It appears that the query is related to assessing the care received by a 13-month-old boy as he was continually redirected to various hospitals, culminating in his receiving treatment at a downtown university hospital. The question is asking for an evaluation of the care trajectory, with potential areas of focus being appropriateness, ethicality, legality, and compliance with the Emergency Medical Treatment and Active Labor Act (EMTALA) in his journey between three different hospitals before eventually receiving treatment. 2) PLANNING: There are several assumptions made in this case, including that all the medical professionals involved prioritized the child's best interest and adhered to legal and ethical guidelines, that the patient's healthcare needs were dealt with adequately, and that insurance coverage or lack thereof had no bearing on the patient's treatment decisions. These assumptions need to be criticized and an alternate assumption would be that the decision to refuse treatment could be related to non-medical factors, such as the family's lack of insurance. 3) CLINICAL REASONING: The case presents a complex mix of clinical needs, logistical constraints, and potential ethical dilemmas. The picture seems to suggest that the redirection of the child resulted in delay of care because of non-medical factors, rather than clinical assessment. In particular, it appears that the suburban hospital did not provide services based solely on the insurance status of the family. This maneuver is contrary to the stipulations of EMTALA, a federal mandate in the United States requiring hospitals to provide emergency healthcare regardless of the patient's insurance status. The law also requires the emergency department to perform appropriate medical screening to detect emergency medical conditions and, if one is detected, either provide treatment until the patient is stabilized or transfer the patient to a suitable hospital. 4) CONCLUDING: Given this situation, legality and ethics of the suburban hospital's actions come under question as EMTALA obligations may not have been duly carried out. 5) RESPONSE: The most accurate characterization of this patient's pattern of care seems to be that the suburban hospital violated the Emergency Medical Treatment and Active Labor Act (EMTALA) (E). The hospital, by apparently making decisions on patient care based off of insurance status, potentially failed in its obligation to provide an adequate medical screening and stabilizing treatment (if necessary) or to facilitate an appropriate transfer.

**AI_E:** RESTATING THE QUESTION: The scenario describes a 13-month-old boy with a perianal abscess who was transferred twice before receiving definitive treatment at a university hospital. The question asks us to identify the most accurate characterization of the resulting pattern of care for the boy from the provided options. PLANNING: Under American law, it's crucial to consider the Emergency Medical Treatment and Active Labor Act (EMTALA) in this case. EMTALA makes it illegal to transfer unstable patients or refuse them treatment based on their insurance status. The central questions then are (a) whether this boy was sufficiently stabilized before being transferred and (b) whether the suburban hospital's actions were influenced by his lack of insurance. CLINICAL REASONING: An erythematous, edematous, and fluctuant perianal mass that is hot to the touch in a child with a high fever and high heart rate is most likely a severe infection – a perianal abscess. This situation is potentially life-threatening and needs immediate medical attention. The rural hospital was not equipped to handle such a case, and rightfully transferred the child to a suburban hospital that had a pediatric service. However, on the boy's arrival at the suburban hospital, he was not examined by a physician; instead, a nurse recommended another transfer due to his insurance status. This action potentially violates the EMTALA law, which requires hospitals to screen and stabilize any emergency patient, regardless of their ability to pay, without delaying for inquiries about payment or insurance. Therefore, the fact that the child was not examined by the accepting pediatric surgeon but was recommended for another transfer based on his insurance status is problematic. This conduct could put the child's health at considerable risk as transfers between hospitals could lead to delays in necessary treatment. CONCLUDING: Taking into consideration the legal implications of EMTALA and medical ethics, even if it was within the capabilities of the suburban hospital to provide care, transferring a patient after determining his insurance status without proper assessment from a healthcare provider can be seen as an inappropriate practice. RESPONSE: Therefore, the most fitting characterization of this patient's pattern of care is "(E) The suburban hospital violated the Emergency Medical Treatment and Active Labor Act (EMTALA)."

**AI_D:** RESTATING THE QUESTION: We are asked to evaluate the manner in which a young patient's health care was managed. The 13-month-old boy had presented with a considerably severe perianal abscess - an inflamed, pus-filled swelling near the anus. It's a medical situation that can call for surgical intervention (incision and drainage). He was initially seen in a rural hospital but due to the hospital's stated lack of resources, the boy was referred first to a suburban hospital and then a downtown university hospital, where he was eventually treated. We should assess the care he received as per options provided, i.e., deem whether it was suitable for the circumstances, judge the rural or suburban hospital decisions and actions, or decide if there is any violation of EMTALA, which requires hospital EDs to provide emergency care regardless of the patient's capability to pay. PLANNING: It is important to note that: - This young patient had a severe infection requiring immediate treatment. - The rural hospital, according to its assessment, felt inadequate for the situation, and so, referred him to a suburban hospital with a pediatric service. - The suburban hospital, despite having a pediatric surgeon, also declared the case beyond their capacity after noting the lack of insurance and pointed the parents to a downtown university hospital. - It might assume an emergency medical condition, the absence of examination by the accepting physician, or lack of insurance may have influenced the suburban hospital's decision, ranging from clinical, procedural to ethical grounds. CLINICAL REASONING: - Ensuring prompt treatment for the child was crucial, given his severe infection, and thus, the initial actions of the rural hospital were in line assuming lack of specialist care there. - The suburban hospital's decision becomes an issue of concern. Despite having accepted the patient's transfer and having a pediatric surgeon on service, not examining the patient due to lack of insurance and referring to another hospital potentially violated EMTALA rules. - EMTALA mandates that a patient with an emergent condition must be examined and provided necessary stabilizing treatment, irrespective of their ability to pay. CONCLUDING: The care the patient received, from a broad perspective, was not completely inappropriate; he did eventually get the necessary treatment, though delayed. However, the suburban hospital's decision to not examine and treat the patient after accepting the transfer, presumably based on the insurance status, is ethically questionable and potentially a violation of EMTALA. RESPONSE: The most accurate characterization of this patient's pattern of care would be (E) The suburban hospital violated the Emergency Medical Treatment and Active Labor Act (EMTALA).

**AI_C:** RESTATING THE QUESTION: We're being asked to assess the quality of care delivered to a 13-month-old patient who presented with perianal abscess. This patient was first seen at a rural hospital, then referred to a suburban hospital, and finally treated at a downtown university hospital. The question aims to evaluate these events in light of given options, which revolve around the ethical, legal, and professional standards of such care including potential violation of the Emergency Medical Treatment and Active Labor Act (EMTALA). This act mandates hospitals to provide emergency healthcare services irrespective of a patient's ability to pay. PLANNING: Assumptions involved in the case include the adequacy of treatment in context of the presented clinical findings, the rural hospital's original decision to refer the patient to the suburban hospital, the suburban hospital's decision to refer the patient elsewhere based on insurance status, and the interpretation of these events in the context of EMTALA. Questioning these assumptions would comprise analyzing the treatment decisions made at each stage, highlighting potential deviations from standard delivery of care, and understanding the implications and obligations under EMTALA. CLINICAL REASONING: This young patient presented with classic signs of a perianal abscess; these include an erythematous and edematous mass in the perianal area that is hot and tender to touch, as well as systemic symptoms of fever and increased heart and respiration rates. Initial treatment included an analgesic (acetaminophen) and an antibiotic (cephalexin). However, given the limitations of a small rural hospital, the severity and location of the abscess required advanced pediatric attention, so he was referred to the suburban hospital. At this hospital, despite the acceptance of the referral by a pediatric surgeon, the child was further referred elsewhere due to the family's lack of insurance, conflicting with EMTALA requirements. CONCLUDING: From this clinical reasoning, it's apparent that although the initial care provided was appropriate, the overall course of care may have involved violation of the EMTALA, specifically at the suburban hospital stage. RESPONSE: Therefore, the most accurate characterization of this patient's pattern of care is that the suburban hospital violated the Emergency Medical Treatment and Active Labor Act (EMTALA) (Option E). The EMTALA necessitates hospitals to provide emergency medical treatment irrespective of insurance standing, hence the referral based solely on insurance status potentially constitutes a violation.

**AI_B:** RESTATING THE QUESTION: The clinical vignette presents a situation where a 13-month-old boy with a perianal abscess was first attended to in a rural hospital, referred to a suburban hospital that rerouted him to a downtown hospital, where the required procedure was eventually done. We need to assess the treatment pattern and decide whether it was appropriate, in violation of EMTALA, or unethical. EMTALA is a U.S. legislation that requires emergency care to be provided irrespective of a patient's ability to pay and prevents hospitals from transferring patients in unstable conditions. PLANNING: The key issue in this scenario is whether a rural hospital's transfer to a suburban hospital and the subsequent transfer to a downtown hospital, considering the patient's lack of health insurance, were appropriate and in compliance with laws, particularly EMTALA. An additional assumption is that further details are required to evaluate care appropriateness, such as the rural and suburban hospitals' technical and clinical resources. CLINICAL REASONING: From the clinical standpoint, the child had an infection (abscess) that needed urgent attention which was appropriately managed at every step. Initial management in the rural hospital with acetaminophen for fever and pain and oral cephalexin (an antibiotic) was correct. The referral to a higher center for definitive care (I&D) was also clinically appropriate. Upon his arrival at the suburban hospital, his vitals had improved, possibly due to the initial treatment. The suburban hospital's referral was clinically justified if they did not have the resources to manage the patient fully. However, protocol for transfer especially in cases of uninsured patients involves underpinning legal considerations, which leads to the next step in our reasoning process. CONCLUDING: With clinical reasoning established, one needs to further interrogate the legal and ethical aspect of this case. The suburban hospital had the pediatric surgeon available who accepted the patient transfer. However, the decision to redirect seemed to be influenced by the patient's lack of health insurance, which is against EMTALA. While the subsequent care received at the downtown hospital was appropriate, the suburban hospital appears to have violated EMTALA as the act prohibits hospitals from transferring or discharging patients needing emergency treatment based on their insurance status or ability to pay. RESPONSE: (E) The suburban hospital violated the Emergency Medical Treatment and Active Labor Act (EMTALA). To summarize, despite the clinical appropriateness of care, there may be ethical and legal implications due to the apparent decision-making based on insurance status, leading to a possible violation of EMTALA.

**Facilitator** The consensus statement synthesizing the responses of AI_A, AI_B, AI_C, AI_D, and AI_E is as follows: The scenario presents a 13-month-old boy with a perianal abscess who was initially treated at a rural hospital, then referred to a suburban hospital, and finally received definitive treatment at a downtown university hospital. The question asks for an evaluation of the care trajectory, focusing on its appropriateness, ethicality, legality, and compliance with the Emergency Medical Treatment and Active Labor Act (EMTALA). The initial treatment at the rural hospital was deemed appropriate given the severity of the infection and the hospital's limited resources. The referral to a suburban hospital with a pediatric service was also clinically justified. However, the suburban hospital's decision to redirect the patient to another hospital due to the family's lack of insurance raises legal and ethical concerns. EMTALA mandates that hospitals provide emergency medical treatment regardless of a patient's insurance status. It also requires hospitals to perform appropriate medical screening to detect emergency medical conditions and, if one is detected, either provide treatment until the patient is stabilized or transfer the patient to a suitable hospital. The suburban hospital's decision to redirect the patient without a proper medical examination by a healthcare provider, seemingly based on the family's lack of insurance, potentially violates EMTALA. Therefore, the most accurate characterization of this patient's pattern of care is that the suburban hospital violated the Emergency Medical Treatment and Active Labor Act (EMTALA). Despite the clinical appropriateness of the initial care, the suburban hospital's actions may have led to a delay in necessary treatment and potentially violated legal and ethical standards.
