# Supplementary material for: Collaborative intelligence in AI: Evaluating the performance of a council of AIs on the USMLE
Source: PLOS Digit Health. 2025 Oct 9;4(10):e0000787. doi: 10.1371/journal.pdig.0000787 (PMC12510544; doi:10.1371/journal.pdig.0000787)
Supplement: S2 File — (ZIP) [file pdig.0000787.s002.zip › S2 Data Raw data files containing unprocessed question inputs and LLM outputs including transcripts of deliberations/STEP2_Question-67-1_Type-MC-NJ.docx]

**Facilitator** **[Round 1]:** A 25-year-old woman is brought to the emergency department by paramedics because of a seizure that began 45 minutes ago at home. Intravenous antiepileptic therapy begun en route to the hospital has not stopped her seizure. Her parents report that she initially began crying, then fell to the floor and exhibited asymmetric kicking and thrashing of all extremities. Fifteen years ago, she was diagnosed with a seizure disorder. Her seizures had been well controlled with oral antiepileptic therapy until this morning. Today, while the physician is in the patient’s room, the movements of her extremities gradually subside. The patient then sits up and asks the physician what happened. She is fully oriented. Vital signs are within normal limits. Examination shows no evidence of tongue biting, urinary incontinence, or other abnormalities. Which of the following is most likely to confirm this patient’s diagnosis? (A) Interview following amobarbital administration (B) Measurement of serum antiepileptic concentration (C) Measurement of serum creatine kinase activity (D) Neuropsychological testing (E) Video EEG monitoring

**AI_B:** RESTATING THE QUESTION: To paraphrase, a young woman with a long-standing history of seizures that had been well-managed, suffers a prolonged event. Despite pre-hospital interventions to terminate the seizure, no resolution occurred until she was at the hospital. Once the event terminated, she was fully alert and oriented without evident physical harm from the seizure. The key issue is to discern the best step to accurately diagnose her condition. PLANNING: The assumptions are that she suffered a seizure, her long-time seizure disorder is the cause, and that IV antiepileptic medication could not control the incident. These can be challenged by exploring whether the event was an actual seizure, whether her known seizure condition was responsible, and if the IV medication failure indicates a worsening situation. The clinical priority is diagnosis verification, which could potentially uncover a condition other than her known disorder as causative. CLINICAL REASONING: If it was not an actual seizure, it might have been nonepileptic psychogenic seizures (PNES), physical manifestation of psychological distress, sharing similar characteristics with epileptic seizures. The lack of common physical outcomes (tongue biting or incontinence) and the immediate recovery with orientation fits well with PNES. PNES being psychological and not biological in nature, would render antiepileptic medication ineffective. CONCLUDING: While presence of a known seizure disorder can cause an assumption bias, the unusual presentation pushes towards an exploration of an alternate or additional diagnosis. In PNES, there is no abnormal electrical activity in the brain, which video EEG monitoring could reveal, differentiating it from epileptic seizures. RESPONSE: Given this context and reasoning, the most appropriate measure to confirm this patient’s diagnosis would be (E) Video EEG monitoring. This procedure will help distinguish between epileptic seizures and nonepileptic psychogenic seizures, the latter being a strong potential in this case due to the atypical presentation and immediate post-event recovery.

**AI_E:** Restating the question, the clinical scenario involves a 25-year-old woman who experienced a prolonged seizure, which remained unresponsive to intravenous antiepileptic treatment. Her seizure behavior included her initially crying, later followed by asymmetric kicking and thrashing of all extremities. However, it's important to notice that despite this episode, the individual regained consciousness without the typical postictal symptoms of tongue biting or urinary incontinence. Thus, the question asks, what would be the most appropriate diagnostic method to confirm the patient's condition based on her abnormal seizure characteristics? Moving to the planning phase, it would be beneficial to think about the differences in presentation between epileptic seizures and nonepileptic seizures, also known as psychogenic non-epileptic seizures (PNES). The absence of postictal confusion, and absence of tongue bite or urinary incontinence can arguably point towards PNES. In such a case, it would be vital to confirm the diagnosis with testing that would capture the event and allow differentiation between these two conditions. For clinical reasoning, let's understand few things. An EEG is usually the preferred testing modality for evaluating seizures. The primary advantage of video EEG over EEG alone is the ability to correlate the observed behavior of the patient with the changes seen on the EEG. The Preservation of awareness and lack of postictal state clues to the possibility of a Psychogenic Non- epileptic Seizure (PNES), which is a psychological (not neurologic) disorder. PNES may exhibit hyperkinetic features, with thrashing and violent movements, often asymmetrical and asynchronous, which is inconsistent with epileptic seizures. Therefore, diagnosis of PNES is often achieved by capturing the typical events with video-EEG. The EEG would show normal brain electrical activity during the apparent 'seizure' event. To conclude, considering the circumstances, and the need for evidence supporting either epileptic seizures or PNES, the most suitable method would be video EEG monitoring (Choice E). This approach would provide visuals of the patient's behavior during the episode, whilst offering insights into her brain's electrical activity, thus allowing healthcare professionals to definitively identify the nature of her seizures. Response: (E) Video EEG monitoring

**AI_C:** RESTATING THE QUESTION: A 25-year-old woman who has a history of seizure disorder has had a seizure that was not controlled by intravenous antiepileptic therapy. The seizure was characterized by asymmetric kicking and thrashing movements. After the seizure has ended, she appeared fully oriented and examination did not reveal typical post-seizure symptoms such as tongue biting or urinary incontinence. PLANNING: Assuming the type of seizure the patient has is an epileptic seizure, I will consider options such as measurement of serum antiepileptic concentration to look into the potential problems with the antiepileptic agent's efficacy. On the other hand, if I assume that she had a nonepileptic seizure, techniques such as an interview following amobarbital administration, neuropsychological testing, or video EEG monitoring might be beneficial. As for measurement of serum creatine kinase activity, it could be used to evaluate if the patient had muscle damage due to prolonged seizure activity. To decide upon the priority, I would evaluate the distinct nature of this seizure episode and its apparent discrepancy from typical epileptic episodes. CLINICAL REASONING: The fact that the patient's seizure was not controlled by intravenous antiepileptic therapy brings up the question of whether she had a type of seizure that is resistant to this therapy. However, upon closer inspection, the discrepancies in the symptoms and aftermath (asymmetric movements, quick recovery, lack of typical post-seizure signs) seem to suggest that this might not have been an epileptic seizure. Additionally, the absence of abnormalities on examination makes it less likely that a serum antiepileptic concentration or serum creatine kinase activity test would provide any useful information. CONCLUDING: Considering the unique characteristics of this seizure episode and the immediate recovery without typical postictal symptoms, it seems more plausible that this seizure might have been a nonepileptic seizure. Nonepileptic seizures (NES), also known as psychogenic non-epileptic seizures (PNES), are events that clinically resemble epileptic seizures but are not associated with the typical electrical discharges seen in epilepsy. The best diagnostic tool for NES is a video EEG monitoring. RESPONSE: Therefore, to confirm this patient's diagnosis, the most likely test would be (E) Video EEG monitoring. This test would allow for visual observation of her actions during a seizure and simultaneous electroencephalographic monitoring. In case of epileptic seizure, there would be corresponding abnormal electrical activity; in case of a nonepileptic seizure, there would be no such changes. This would definitively classify her seizure type and inform appropriate treatment strategies.

**AI_D:** To rephrase the question, a 25-year-old woman known with a seizure disorder is brought into the ER due to a prolonged seizure incident. Despite administration of intravenous antiepileptic drugs, the seizure persists. She initially exhibited crying, followed by falling to the ground, and asymmetrically thrashing and kicking with all of her limbs. Her seizures, which have been present for 15 years, have always been well-managed by oral antiepileptic drugs until the current event. Once in the hospital, her physical movements eventually ceased and she was fully alert and oriented, with no evident signs of injuries typical for epileptic seizure patients. The question is now, what would be the best method to definitively diagnose this patient's condition? The given options include inducing an interview following amobarbital administration, measuring the concentration of antiepileptic drugs in her bloodstream, checking for serum creatine kinase activity, carrying out a neuropsychological testing, or conducting a video EEG monitoring. To ponder upon this, we need to understand that the patient's symptoms involved an quite unusual progression of events and a quick return to orientation. These signs might suggest a condition other than a true seizure disorder, despite her past diagnosis. It's also relevant that the intravenous antiepileptic drugs administered did not have an apparent effect. In regards to clinical reasoning, we should consider that the uniqueness of her condition and response to medication suggests a possible Non-epileptic seizure, which are psychological in nature as opposed to stemming from abnormal electrical brain activity of epileptic seizures. Non-epileptic seizures can resemble epileptic seizures, but they have distinct differences. For example, their sudden onset, prolonged duration, resistance to antiepileptic drugs, fast recovery, and absence of postictal symptoms, such as tongue bites and urinary incontinence, which were noted in the patient's case. Given the convergence of these observations, to confirm whether these are Non-epileptic seizures (psychogenic in nature) or epileptic seizures, the best option available is Video EEG monitoring (option E) as it can capture the brain's electrical activity during these symptoms. Epileptic seizures will have abnormal brain electrical activity, while Non-epileptic seizures functionally won't. Therefore, to answer your question, my recommendation for the most effective method to confirm this patient's diagnosis is Video EEG monitoring. It is because of the mixed features (asymmetry, rapid recovery, no visible injuries) of her condition that do not typically associate with epileptic seizures, which urges the reassessment of her past diagnosis using this test.

**AI_A:** RESTATING THE QUESTION: In simpler terms, a 25-year-old woman presenting with a history of seizure disorder suffered what seemed to be a prolonged seizure episode. Despite the use of intravenous antiepileptic medication, the seizure continued until she was in the emergency department. Suddenly, her movements ceased and upon recovery, seemed disoriented and unaware of what had transpired. Upon physical examination, there was no evidence of common indicators of a typical seizure such as tongue biting and urinary incontinence. The question seeks to identify the best diagnostic tool or procedure that can provide a conclusive diagnosis for her present condition. PLANNING: The immediate assumption could be that she's had a breakthrough seizure despite being on antiepileptic medication for years. This could be due to numerous factors, such as medication noncompliance, metabolic disturbances, or new onset pathology. However, the absence of postictal confusion, tongue bite, or incontinence, and the immediate transition from the seizure to normal consciousness, could lead us towards an alternate assumption: a pseudoseizure or psychogenic non-epileptic seizure (PNES). With this alternate assumption, we can hypothesize that a tool or test that can distinguish between the electrical activity within the brain during a true seizure and the lack thereof during a pseudoseizure would be the more clinically significant avenue to pursue. CLINICAL REASONING: Pseudoseizures or psychogenic non-epileptic seizures (PNES) are physical manifestations or changes that can mimic seizures but are not associated with abnormal electrical activity in the brain. A definitive diagnosis of PNES generally requires video electroencephalography (Video EEG) monitoring, which can demonstrate normal brain electrical activity during an episode that appears clinically to be a seizure - unlike an epileptic seizure, which shows abnormal electrical activity. CONCLUDING: Taking into account the patient's history, the clinical course of the event, and the absence of postictal signs, the most plausible working diagnosis is a pseudoseizure or psychogenic non-epileptic seizure (PNES). The most effective method of confirming this would be by demonstrating a lack of abnormal brain electrical activity during one of these episodes. RESPONSE: Therefore, the best course of action to confirm this patient’s diagnosis is (E) Video EEG monitoring.

**Facilitator** The consensus statement synthesizing the responses of AI_A, AI_B, AI_C, AI_D, and AI_E is as follows: The question presents a 25-year-old woman with a history of seizure disorder who experienced a prolonged seizure episode that was unresponsive to intravenous antiepileptic medication. Despite the seizure, she regained full consciousness without the typical post-seizure symptoms such as tongue biting or urinary incontinence. The question seeks to identify the most appropriate diagnostic tool to confirm her condition. The immediate assumption could be that she experienced a breakthrough seizure due to factors such as medication noncompliance, metabolic disturbances, or new onset pathology. However, the absence of postictal confusion, tongue bite, or incontinence, and the immediate transition from the seizure to normal consciousness, could suggest an alternate diagnosis: a pseudoseizure or psychogenic non-epileptic seizure (PNES). PNES are physical manifestations that can mimic seizures but are not associated with abnormal electrical activity in the brain. The definitive diagnosis of PNES generally requires video electroencephalography (Video EEG) monitoring, which can demonstrate normal brain electrical activity during an episode that appears clinically to be a seizure. This contrasts with an epileptic seizure, which shows abnormal electrical activity. Given the patient's history, the clinical course of the event, and the absence of postictal signs, the most plausible working diagnosis is a pseudoseizure or PNES. The most effective method of confirming this would be by demonstrating a lack of abnormal brain electrical activity during one of these episodes. Therefore, the consensus among the AI responses is that the best course of action to confirm this patient’s diagnosis is Video EEG monitoring. This procedure will help distinguish between epileptic seizures and nonepileptic psychogenic seizures, the latter being a strong potential in this case due to the atypical presentation and immediate post-event recovery.
