# Supplementary material for: Collaborative intelligence in AI: Evaluating the performance of a council of AIs on the USMLE
Source: PLOS Digit Health. 2025 Oct 9;4(10):e0000787. doi: 10.1371/journal.pdig.0000787 (PMC12510544; doi:10.1371/journal.pdig.0000787)
Supplement: S2 File — (ZIP) [file pdig.0000787.s002.zip › S2 Data Raw data files containing unprocessed question inputs and LLM outputs including transcripts of deliberations/STEP2_Question-68-1_Type-MC-NJ.docx]

**Facilitator** **[Round 1]:** A 32-year-old woman comes to the emergency department because of a 1-day history of sharp, right-sided chest pain that worsens with coughing and sneezing. Four days ago, she had a mild sore throat and runny nose followed by nonproductive cough 1 day later. Over-the-counter decongestant and aspirin mildly relieved the symptoms. She has not had shortness of breath, blood-tinged sputum, fever, or chills. She has a long-standing history of recurrent aphthous ulcers. Her only medication is an oral contraceptive. Temperature is 37.2°C (99.0°F), pulse is 65/min, and respirations are 14/min. Pulse oximetry on room air shows an oxygen saturation of 99%. Splinting is observed over the right hemithorax with deep breathing. On cardiac examination, no abnormalities are heard. The remainder of the examination shows no abnormalities. Chest x-ray shows no abnormalities. Which of the following is the most appropriate next step in management? (A) Azithromycin therapy (B) CT angiography (C) Electrocardiography (D) Ibuprofen therapy (E) Prednisone therapy (F) Transthoracic echocardiography

**AI_A:** RESTATING THE QUESTION: The patient is a 32-year-old woman who has been dealing with a sharp chest pain, primarily on the right side which gets worsened by coughing and sneezing. The pain started a day ago and was preceded by an episode of sore throat, runny nose, and nonproductive cough. She also has a history of recurrent aphthous ulcers. She has been taking an oral contraceptive. Her vital signs appear to be normal and a chest x-ray shows no abnormalities. Now we need to decide on her next steps in management, which could range from medications like Azithromycin, Ibuprofen, Prednisone, to diagnostic procedures like CT angiography, Electrocardiography, or Transthoracic echocardiography. PLANNING: I need to consider several assumptions. 1) The chest pain is linked to the recent upper respiratory symptoms. 2) The chest pain could be unrelated to the respiratory symptoms. 3) The pain might be originating from the cardiovascular system due to her use of oral contraceptives. After reviewing these assumptions, I hypothesize that the most clinically relevant is that the chest pain is related to the recent upper respiratory symptoms and represents a form of pleuritic chest pain. CLINICAL REASONING: The clinical reasoning behind the hypothesis that the chest pain is a form of pleuritic chest pain stems from the fact that her pain is sharp and worsens with coughing and sneezing. Pleuritic chest pain tends to be worsened by movements or actions that stretch the pleura, like deep breaths, coughing, or sneezing. Also, the onset of chest pain followed an episode of upper respiratory symptoms which could have led to viral pleurisy—a type of pleuritic chest pain caused by viral infections. The tendency for the patient to show splinting on the right side, and the absence of any abnormal findings on the chest X-ray or cardiac examination, furthers my suspicion of pleuritic chest pain. CONCLUDING: Pleuritic chest pain tends to be a benign and self-limiting condition and does not necessitate diagnostic imaging such as CT angiography or transthoracic echocardiography. Electrocardiography is also not required since the patient's heart rate, oxygen saturation, and cardiac examination are normal, and there's no indication of any cardiovascular involvement. While prednisone and azithromycin could be used to treat different types of chest pain, they are not the best choices for pleuritic chest pain resulting from viral infections. RESPONSE: Thus, the most appropriate next step in management would be (D) Ibuprofen therapy. This nonsteroidal anti-inflammatory drug can be used to reduce both pain and inflammation associated with pleuritic chest pain.

**AI_E:** RESTATING THE QUESTION: The case relates to a 32-year-old woman experiencing chest pain that becomes more severe with coughing and sneezing. She has a recent history of upper respiratory tract symptoms (such as a sore throat and runny nose), along with a nonproductive cough. Other symptoms have been somewhat mitigated by over-the-counter decongestants and aspirin. The patient has no other troubling symptoms, such as shortness of breath, blood in the sputum, fever, or chills. She has a past history of recurrent aphthous ulcers and is currently only on oral contraceptives. Despite the pain during deep breaths (indicated by "splinting"), her vitals are generally normal and physical examination has come back clear, including a chest x-ray. The question requires us to determine the most appropriate next step in managing this case. PLANNING: Among the options provided, we can make several assumptions. (A) suggests that this could be a bacterial infection requiring antibiotics treatment. (B) implies this could be a condition requiring a more detailed view of the chest, such as a pulmonary embolism. (C) suggests the possibility of a cardiac issue. (D) implies that this might be a case of inflammation or mild pain requiring nonsteroidal anti-inflammatory drugs. (E) suggests a possible requirement for steroid treatment and (F) implies a more in-depth cardiac analysis is required. Reviewing her condition and symptoms leads me to question the need for antibiotics, CT angiography, and ECG, given no signs of infection, pulmonary embolism, or cardiac distress are present. Given her pain that increases with cough and sneeze, oral contraceptive usage, and recent respiratory tract infection, the most likely diagnosis is pleurisy/pleuritic chest pain, likely due to viral infection. Hence, I would lean towards the assumption that first-line treatment for symptomatic relief, such as analgesics (nonsteroidal anti-inflammatory drug like Ibuprofen), should be a priority. CLINICAL REASONING: Pleurisy often follows an upper respiratory infection and is characterized by sharp, unilateral chest pain that worsens with coughing, sneezing, or deep breathing. The pain is due to inflammation of the pleura- the membranes surrounding the lungs. A viral infection is the most common cause of pleurisy. There is no specific treatment for viral pleurisy except for symptomatic relief. Nonsteroidal anti-inflammatory drugs (NSAIDs) like ibuprofen are commonly used to manage the pain and inflammation. CONCLUDING: A detailed clinical analysis and examination have ruled out potential cardiac issues, pulmonary embolism, or bacterial infection. Given the symptomology and recent respiratory tract infection, a clinically reasoned diagnosis of pleuritis becomes the most likely. Therefore, symptomatic relief using NSAIDs should be the first-line treatment approach. RESPONSE: The most appropriate next step in the management of this woman would be (D) Ibuprofen therapy.

**AI_B:** 1) RESTATING THE QUESTION: The query requires the identification of the best management step for a 32-year-old woman exhibiting symptoms of sharp right-sided chest pain that intensifies with coughing and sneezing. The woman had a mild sore throat and runny nose four days ago, followed by a dry cough. Her condition slightly improved with over-the-counter decongestants and aspirin. She furthermore has a history of reoccurring aphthous ulcers, does not take any medication aside from oral contraceptives, and exhibits no breath shortness, blood-tinted sputum, fever, chills, or cardiac abnormalities. Her physical examination was otherwise normal. Sharp, right-sided chest pain is often indicative of conditions such as pleurisy, pneumonia, pulmonary embolism, chest trauma, and costochondritis, among others. 2) PLANNING: The primary assumption is that the chest pain may be due to an infectious disease (given her recent sore throat and non-productive cough), prompting the choice of azithromycin treatment. This presumption questions if the patient has respiratory symptoms corresponding to an infectious disease. Interestingly, instead of an infection, her symptoms may be due to pleuritic pain related to her previous upper respiratory tract symptoms. If the pain is pleuritic, it would worsen with coughing, sneezing, and deep breathing, which matches the patient's symptoms. 3) CLINICAL REASONING: If the patient had pneumonia or another infectious process, the chest X-ray would likely show findings indicative of infection which: fever, hypoxia, tachypnea, or increased work of breathing. However, these symptoms are absent in this case. Therefore, it's necessary to rethink the possibility of an infectious disease. Pleuritis, an inflammation of the tissues that line the lungs and chest cavity (pleura), often results from viral upper respiratory tract infections and presents with sharp, stabbing chest pain. The pain is often worse with deep respiration, coughing, or sneezing; exactly what our patient presents with. 4) CONCLUDING: From a clinical reasoning viewpoint, and tracing back to the respiration-aggravated sharp right-sided chest pain, the inflammation of the pleura, pleuritis, seems to be a plausible cause of the patient's symptoms. It typically develops following upper respiratory tract infections, as seen in the history of this patient. 5) RESPONSE: Consequently, the use of nonsteroidal anti-inflammatory drugs (NSAIDs) like Ibuprofen (Option D) should be ideally effective in combating pleuritic chest pain by mitigating the inflammation hence reducing the pain. Antibiotics like azithromycin (Option A) will not be the best line of management as we are not dealing with a bacterial infection. CT angiography (Option B), electrocardiography (Option C), prednisone therapy (Option E), and transthoracic echocardiography (Option F) are valid considerations, but there is currently no concrete evidence to necessitate these diagnostic tests or therapies. Therefore, 'Ibuprofen therapy' (Option D) is the most appropriate next step in the patient's management.

**AI_D:** RESTATING THE QUESTION: This scenario describes a 32-year-old woman with a prior history of aphthous ulcers, who has presented with a sudden onset of sharp, right-sided chest pain, which escalates when she coughs or sneezes. A few days back, she had signs of an upper respiratory tract infection (i.e., sore throat and runny nose, followed by a cough), but the general symptoms (such as fever, chills, blood-tinged sputum or shortness of breath) are not observed. The pain is notably relieved by a commercially obtained decongestant and aspirin. On examination, she shows signs of splinting over the right side of her chest when taking deep breaths. However, no relevant signs are picked up on her cardiac examination, and both her chest x-ray and physical examination are otherwise normal. Based on these findings, we have to decide on the most appropriate next step in terms of management, out of the options provided: Azithromycin therapy, CT angiography, Electrocardiography, Ibuprofen therapy, Prednisone therapy, or Transthoracic echocardiography. PLANNING: One plausible assumption from this patient's history and examination could be that she is suffering from a condition related to her previous upper respiratory tract infection, such as pleurisy or a viral pneumonitis, given her chest pain and history of a cough, sore throat and runny nose. Another possibility is that the symptoms are a flare up of her chronic aphthous ulcers, although this would be unusual given the chest pain and other symptoms. Lastly, her use of oral contraceptives might predispose her to conditions such as pulmonary embolism, although this is less likely given her otherwise normal medical exam. Hence, we should lean towards a possibility that also correlates with her normal clinical and radiological findings, while addressing her most distressing symptom - the chest pain. CLINICAL REASONING: Pleurisy, also referred to as pleuritis, is an inflammation of the pleura, which can often present as sharp, localized chest pain that worsens with deep breaths or coughing - a symptom the patient is reporting. A common cause of pleurisy is a viral infection, which aligns with the patient's history of a recent upper respiratory tract infection. Bearing in mind patient's completely normal physical, cardiac, and radiological examination, her symptoms fit with this diagnostic possibility. Her discomfort on the right side of her chest during deep inhalation, known as splinting, can also support this diagnosis. While her history of recurrent aphthous ulcers and use of oral contraceptives call for clinical attention, they don't directly link with her current presentation, and hence cannot be prioritized. CONCLUDING: Based on this clinical reasoning, the most appropriate next step in management would be to address the chest pain from the underlying inflammation. RESPONSE: Given that this patient's presentation is consistent with pleurisy or inflammation of the pleura likely secondary to a viral infection, the most suitable next step would be administration of a non-steroidal anti-inflammatory drug to alleviate the inflammation and associated pain. Therefore, (D) Ibuprofen therapy would be the correct answer.

**AI_C:** (1) RESTATING THE QUESTION: We have a 32-year-old woman with recurrent aphthous ulcers who has a history of sore throat, runny nose, and a nonproductive cough that is now associated with right-sided chest pain exacerbated by coughing and sneezing. With no other symptoms such as fever, chills, or shortness of breath and a clear chest x-ray, we are asked to determine the most appropriate next step in her management from a selection of options including antibiotic (azithromycin) therapy, diagnostic imaging (CT angiography, electrocardiography, transthoracic echocardiography), and symptomatic treatment strategies (ibuprofen, prednisone). (2) PLANNING: Given the clinical scenario and the options provided, the relevant assumptions are that the patient's symptoms are related to either a respiratory or cardiac condition. However, given her symptoms and history, another assumption is that the condition could relate to her recurrent aphthous ulcers which could suggest a systemic condition such as Behçet's disease. These assumptions need definite diagnostic or therapeutic strategies potentially involving antibiotics, pain relief, inflammation reduction, or further diagnostic imaging. (3) CLINICAL REASONING: This patient's upper respiratory tract infection symptoms followed by chest pain worsening with deep breath, or coughing/sneezing, a normal cardiopulmonary examination, normal oxygen saturation, and normal chest x-ray suggest a non-cardiac, non-infectious cause. The chest pain she is experiencing could be secondary to inflammation of the pleura due to a viral infection or possibly an autoimmune disorder such as Behçet's disease, given her history of recurrent aphthous ulcers. If it is pleuritic chest pain secondary to an inflammatory condition, anti-inflammatory medication would be the most appropriate management, rather than antibiotics or further diagnostic imaging. (4) CONCLUDING: Taking into account the assumptions used in the Clinical Reasoning, a clinical step-by-step conclusion is that while the infection-like symptoms are concerning, her symptoms do not include fever, chills, or shortness of breath which often indicate a bacterial infection. Her chest x-ray results are also normal. This suggests that there isn't an active bacterial infection requiring antibiotic therapy or further imaging to diagnose pulmonary embolism, making options A and B less likely. Electrocardiography and echocardiography would be useful if a cardiac etiology was suspected, but the absence of cardiac symptoms and normal cardiac examination make C and F unlikely. Therefore, the best next step should involve relieve her symptoms and reduce inflammation. (5) RESPONSE: In conclusion, the most appropriate next step in management for this patient is to provide (D) Ibuprofen therapy to alleviate her symptoms and reduce inflammation.

**Facilitator** The consensus among the AI responses is that the most appropriate next step in managing the 32-year-old woman's condition is to administer Ibuprofen therapy. This conclusion is based on the patient's symptoms, which include sharp, right-sided chest pain that worsens with coughing and sneezing, following a recent upper respiratory tract infection. The pain is likely due to inflammation of the pleura, a condition known as pleurisy or pleuritic chest pain, which is often caused by a viral infection. Given the absence of symptoms such as fever, chills, or shortness of breath, and normal findings on the chest x-ray, there is no indication of an active bacterial infection requiring antibiotic therapy or further imaging to diagnose pulmonary embolism. Similarly, the absence of cardiac symptoms and normal cardiac examination make it unlikely that the pain is due to a cardiac issue. Therefore, the use of a nonsteroidal anti-inflammatory drug like Ibuprofen is recommended to alleviate the inflammation and associated pain.
