# Supplementary material for: Collaborative intelligence in AI: Evaluating the performance of a council of AIs on the USMLE
Source: PLOS Digit Health. 2025 Oct 9;4(10):e0000787. doi: 10.1371/journal.pdig.0000787 (PMC12510544; doi:10.1371/journal.pdig.0000787)
Supplement: S2 File — (ZIP) [file pdig.0000787.s002.zip › S2 Data Raw data files containing unprocessed question inputs and LLM outputs including transcripts of deliberations/STEP2_Question-69-1_Type-MC-NJ.docx]

**Facilitator** **[Round 1]:** A 30-year-old nulligravid woman, who is a military veteran, comes to the office because she has not had a menstrual period for the past year. She has not had nausea, vomiting, or night sweats. Menses previously occurred at regular 28-day intervals. She is sexually active with one male partner and uses condoms consistently. Three weeks ago, she returned from a 1-year deployment in the Middle East, where she worked as an army combat medic. She resumed work as a teacher 2 weeks ago. She appears tearful but otherwise in no distress. Temperature is 37.1°C (98.7°F), pulse is 88/min, respirations are 16/min, and blood pressure is 116/72 mm Hg. Physical examination, including pelvic examination, shows no abnormalities. Serum studies show: Thyroid-stimulating hormone: 2 μU/mL Follicle-stimulating hormone: 7 mIU/mL Luteinizing hormone: 10 mIU/mL Prolactin: 10 ng/mL β-hCG: negative Which of the following is the most likely cause of this patient’s amenorrhea? (A) Gonadal dysgenesis 45,X (Turner syndrome) (B) Hypothalamic hypogonadism (C) Polycystic ovarian syndrome (D) Premature ovarian failure (E) Prolactinoma

**AI_D:** RESTATING THE QUESTION: This 30-year-old patient is presenting with amenorrhea, i.e., absence of menstrual period for a full year, in the absence of pregnancy (as indicated by negative β-hCG). She has not been experiencing nausea, vomiting, night sweats, and her hormone levels are normal. The question seeks to identify the most probable cause of her condition from the choices given. PLANNING: Based on available information, her hormone levels do not suggest known conditions such as Turner syndrome (abnormally low level of sex hormones), Polycystic Ovarian Syndrome (high androgen levels), Premature Ovarian Failure (elevated FSH), or prolactinoma (elevated prolactin). However, Hypothalamic Hypogonadism (HH) has not been eliminated as it's characterized by a failure of the hypothalamus to secrete releasing hormones, leading to lower levels of pituitary and gonadal hormones, and manifests as amenorrhea. The underlying cause can often be stress, extreme weight change, or intense physical activity, which is congruent with being a combat medic in a warzone. CLINICAL RECORDING: Given her recent experiences and lifestyle, I would lean towards hypothesizing hypothalamic. The HPG (Hypothalamic-Pituitary-Gonadal) axis controls most bodily functions related to sexual development and fertility. Stressful situations can cause this axis to malfunction. The intense physical activity and stress that she might have faced during her deployment could be a contributing factor causing the hypothalamus to lower its production of Gonadotropin-Releasing Hormone (GnRH), which would lower LH and FSH levels, resulting in amenorrhea. CONCLUDING: To ascertain this diagnosis, further examination would be needed to rule out other possibilities. However, given the hormone levels, her recent emotionally strenuous work situation, absence of other symptomatology, and undetectable growths or tumors, Hypothalamic Hypogonadism caused by stress is a plausible diagnosis. RESPONSE: The most likely cause of this patient’s amenorrhea is (B) Hypothalamic hypogonadism.

**AI_B:** RESTATING THE QUESTION: The query is directed at understanding the underlying cause of amenorrhea in a 30-year-old nulliparous woman, who has been regular previously but has not experienced a menstrual cycle for the past year. Her hormonal levels seem to be within normal limits with no other significant symptoms or physical anomalies. Her β-hCG is also negative, confirming she isn't pregnant. The potential causes proposed include Turner syndrome, hypoactive hypothalamic gonadotropin-releasing system, Polycystic ovarian syndrome (PCOS), premature ovarian failure, and hyperprolactinemia due to a prolactinoma. PLANNING: We need to consider the clinical presentation and serum studies' results in this woman. While Turner syndrome, PCOS, premature ovarian failure, and prolactinoma may cause amenorrhea, the patient's age, background, presentation, and negative physical findings make these etiologies less likely. Hypothalamic hypogonadism is a plausible cause due to a potential impact of her recent stressful deployment and emotional state on her hypothalamic-pituitary-gonadal (HPG) axis. CLINICAL REASONING: The hypothalamus, a part of the brain, signals the pituitary gland to produce hormones like follicle-stimulating hormone (FSH) and luteinizing hormone (LH), which further stimulate the ovaries to produce estrogen and progesterone, leading to normal menstrual cycles. Stress, such as the one experienced by this patient due to her military deployment and her emotional state, can potentially disrupt this signaling and cause functional hypothalamic amenorrhea. Her hormonal levels being within the normal range and a lack of other physical findings can support this idea. CONCLUDING: The other options mentioned, although possible causes of amenorrhea, do not comfortably fit into the clinical picture presented by the patient. Turner syndrome usually presents earlier in life with stunted growth and primary amenorrhea. PCOS typically shows signs including hirsutism, obesity, and menstrual irregularities. Premature ovarian failure would present with elevated FSH levels, and prolactinoma would involve increased prolactin levels. Therefore, considering the overall clinical picture, stress-induced hypothalamic hypogonadism seems to be the most logical diagnosis. RESPONSE: Thus, the most likely cause of this patient’s amenorrhea is hypothalamic hypogonadism (Option B). This condition is typically due to stress or significant changes in weight, exercise, or nutrition, which leads to suppression of the hypothalamic-pituitary-ovarian axis, thus causing loss of menstrual cycles.

**AI_A:** RESTATING THE QUESTION: A 30-year-old adult woman, who has never been pregnant and has served in the military, has not experienced menstrual periods for one year. She does not experience nausea, vomiting, or night sweats, was sexually active and used protective measures, and also recently returned from deployment in the Middle East. Her physical state appears normal except for an emotionally distressed state. Her thyroid-stimulating hormone, follicle-stimulating hormone, luteinizing hormone, and prolactin are within normal limits and there's no indication of pregnancy. The query is to identify the most probable cause from options including Turner syndrome, hypothalamic hypogonadism, polycystic ovarian syndrome, premature ovarian failure, or prolactinoma. PLANNING: The assumptions we have are based on her reported symptoms and the clinical readings, which indicate normal hormonal levels and absence of pregnancy. One should question, could this amenorrhea be related to physiological stress or is it due to an inherent issue like the options provided? Further consideration should be given to her recent deployment and drastic lifestyle changes, her emotional state of being tearful, and the stressors associated with her work roles. This leads to an alternate hypothesis that her amenorrhea is a result of functional hypothalamic amenorrhea (FHA), which is not one of the provided answers but certainly a viable explanation clinically. CLINICAL REASONING: FHA often occurs in women who are under severe emotional or physiological stress. Stress can cause disturbances in the normal functioning of the hypothalamus, which can disrupt the release of gonadotropin-releasing hormone (GnRH), leading to decreased release of luteinizing hormone (LH) and follicle-stimulating hormone (FSH) from the anterior pituitary, which in turn impairs ovarian follicle development and can result in amenorrhea. The patient's occupation, recent deployment, and emotionally distressed appearance indeed suggest a high-stress lifestyle. Additionally, her hormonal readings fall within normal limits and no physical abnormalities were detected in the reproductive tract; this also aligns with a diagnosis of FHA. The five options provided in the clinical vignette correlate with various causes of ovarian or hormonal dysregulation, but all of these conditions would likely show abnormalities in the hormonal profile or physical examination - none of which has been observed in this patient. CONCLUDING: Stress-induced FHA is a more likely cause for her amenorrhea than the provided options, considering her lifestyle, absence of notable hormonal imbalance or physical pathology, and the well-documented impact of stress on the hypothalamic-pituitary-ovarian axis. However, our response requires selection from the provided options. RESPONSE: The closest fit in the given options for this adult woman's amenorrhea is (B) Hypothalamic hypogonadism, considering her recent high-stress deployment, the health data provided and the lack of indicative signs or distinct clinical abnormalities for the other choices. However, remember this option is an umbrella term under which stress-induced FHA--the more precise diagnosis--falls.

**AI_E:** RESTATING THE QUESTION: The case presented involves a 30-year-old woman who has never been pregnant. She has not had a menstrual cycle for a full year. In her recent history, she spent a year deployed in the Middle East and has recently resumed work as a teacher. She does not have any physical symptoms or findings apart from seeming emotionally upset. Her hormonal profile reveals a euthyroid state, normal pituitary hormone levels, negative pregnancy status, and standard reproductive hormone levels. The question asks me to find the most likely reason behind her amenorrhea, that is, the absence of a menstrual period for more than six months. PLANNING: There are various causes of amenorrhea, such as Turner syndrome, hypothalamic hypogonadism, polycystic ovarian syndrome, premature ovarian failure, and prolactinoma. Each condition can be identified by distinct symptoms, physical examination findings, and hormonal profiles. Evaluating these aspects will facilitate the accurate diagnosis. Most importantly, accurately determining the levels of the hormones involved in menstruation, such as follicle-stimulating hormone (FSH), luteinizing hormone (LH), prolactin, and thyroid-stimulating hormone (TSH) will be paramount. CLINICAL REASONING: Gonadal dysgenesis or Turner syndrome is unlikely in this case since it usually presents with short stature and other physical abnormalities, which are not reported in this case. Hypothalamic hypogonadism is characterized by low levels of FSH and LH due to decreased secretion of gonadotropin-releasing hormone (GnRH) from the hypothalamus, which is not mentioned in the lab report. Polycystic ovarian syndrome (PCOS) usually presents with symptoms of androgen excess such as hirsutism, acne, and obesity, which our patient does not have. A prolactinoma would show an increased level of prolactin, and it is also normal for our patient. Lastly, premature ovarian failure would present with high levels of FSH, indicating a primary ovarian problem, which is not the case here either as FSH levels are within normal limits. CONCLUDING: The woman's normal hormonal status and the absence of physical signs of an organic disease lead us to consider a possible psychogenic cause of her amenorrhea. It should be noted that significant emotional distress, such as that potentially caused by deployment to a war zone or other stressful life events, can lead to hypothalamic dysfunction. This can manifest as amenorrhea even in the presence of normal hormonal levels, a condition known as functional hypothalamic amenorrhea. RESPONSE: Bearing in mind all the details about this patient's case, the most likely cause of her amenorrhea seems to be (B) hypothalamic hypogonadism. Despite having normal hormonal levels, the psychological stress she might've undergone during deployment could've led to this diagnosis. It's often referred to as functional hypothalamic amenorrhea.

**AI_C:** RESTATING THE QUESTION: This case involves a 30-year-old woman, who has never been pregnant (nulligravid), and is currently seeing a physician due to her complete absence of menstruation (amenorrhea) for the past year. Previously, her menses were regular and occurred every 28 days. She does not exhibit symptoms of pregnancy or conditions that might induce similar symptoms, such as gastrointestinal disorders or tuberculosis. She was recently on deployment as an army combat medic and has just returned to her job as a teacher. Despite appearing emotionally distressed, her vital signs are within normal limits. Her physical examination, specifically her pelvic examination, was unremarkable. Moreover, despite her amenorrhea, her hormone levels, including Thyroid-stimulating hormone (TSH), Prolactin, Follicle-stimulating hormone (FSH), and Luteinizing hormone (LH), are within the normal range. Importantly, she tested negative for human chorionic gonadotropin (β-hCG), a hormone produced during pregnancy. The question asks for the identification of the most probable cause of her amenorrhea from several possible options. PLANNING: To correctly answer the question at hand, it's crucial to go over each provided option and evaluate their potential as the cause of the patient's symptoms. First, Turner syndrome (monosomy X, 45,X), involves a number of physical abnormalities that are typically present from birth, making it a less likely option for a 30-year-old woman with regular menses up until a year ago. Hypothalamic hypogonadism typically refers to the insufficient production of gonadotrophins (FSH and LH) by the hypothalamus, usually attributed to extreme physical or psychological stress. This option should be considered, as the patient's recent military deployment may have caused significant stress. Polycystic ovarian syndrome (PCOS) leads to menstrual irregularities, elevated androgens, and polycystic ovaries. This doesn't match the patient's presentation either, as her menses were regular before amenorrhea, and there are no signs of androgen excess. Premature ovarian failure or primary ovarian insufficiency involves the cessation of ovarian function before the age of 40, often leading to high FSH levels and symptoms of menopause. The woman's FSH levels are normal, making this unlikely. Finally, a prolactinoma is a skilled guard tumor that can lead to hyperprolactinemia and amenorrhea - but the woman's prolactin level is normal. CLINICAL REASONING: Taking the woman's history and presenting symptoms into consideration, the most likely cause of her amenorrhea is hypothalamic hypogonadism. The stressors of her recent military deployment could have triggered a physical and emotional response that led to the suppression of her hypothalamic-pituitary-ovarian axis, resulting in insufficient gonadotropin-releasing hormone (GnRH) and subsequently, amenorrhea. This condition is also known as functional hypothalamic amenorrhea and is typically associated with severe stress, strenuous exercise, or significant weight loss, all of which could reasonably be related to her recent deployment. CONCLUDING: Keeping in mind the patient's history, symptom presentation, and test results, hypothalamic hypogonadism due to the high levels of stress and physical exertion associated with her military deployment is the most clinically plausible explanation for her condition. RESPONSE: (B) Hypothalamic hypogonadism is the most probable cause of this patient's amenorrhea.

**Facilitator** The consensus among the AI responses is that the most probable cause of the patient's amenorrhea is hypothalamic hypogonadism (Option B). This conclusion is based on the patient's history, her normal hormonal levels, and the absence of physical signs of other conditions. The patient's recent military deployment and return to work as a teacher could have induced significant stress, which can disrupt the hypothalamic-pituitary-gonadal (HPG) axis and lead to functional hypothalamic amenorrhea (FHA). FHA often occurs in women under severe emotional or physiological stress, which can disrupt the release of gonadotropin-releasing hormone (GnRH), leading to decreased release of luteinizing hormone (LH) and follicle-stimulating hormone (FSH) from the anterior pituitary, impairing ovarian follicle development and resulting in amenorrhea. The other options provided, such as Turner syndrome, polycystic ovarian syndrome, premature ovarian failure, and prolactinoma, are less likely given the patient's age, background, presentation, and negative physical findings. These conditions would typically present with abnormalities in the hormonal profile or physical examination, which are not observed in this patient. Therefore, hypothalamic hypogonadism due to stress is the most plausible diagnosis for this patient's amenorrhea.
