# Supplementary material for: Collaborative intelligence in AI: Evaluating the performance of a council of AIs on the USMLE
Source: PLOS Digit Health. 2025 Oct 9;4(10):e0000787. doi: 10.1371/journal.pdig.0000787 (PMC12510544; doi:10.1371/journal.pdig.0000787)
Supplement: S2 File — (ZIP) [file pdig.0000787.s002.zip › S2 Data Raw data files containing unprocessed question inputs and LLM outputs including transcripts of deliberations/STEP2_Question-7-1_Type-MC-NJ.docx]

**Facilitator** **[Round 1]:** A 27-year-old primigravid woman comes to the physician for her first prenatal visit 10 weeks after a home pregnancy test was positive. She feels well, and her pregnancy has been uncomplicated. She previously used an oral contraceptive but discontinued it 3 months ago. She has no history of sexually transmitted diseases. She is sexually active in a monogamous relationship with a male partner who is the father. She has had five lifetime sexual partners. She has a pet cat. Examination shows a uterus consistent in size with a 10-week gestation. Testing for which of the following infectious conditions is the most appropriate recommendation for this patient? (A) Bacterial vaginosis (B) Cytomegalovirus (C) Epstein-Barr virus (D) HIV (E) Human papillomavirus

**AI_E:** RESTATING THE QUESTION: The inquiry revolves around a pregnant woman who is making her initial prenatal visit after confirming her pregnancy at home. She has been in reproductive good health with no history of sexually transmitted diseases, yet she's been sexually active with multiple partners over time. Besides, she owns a house cat. Given her history, the question seeks to identify which potential infection should be the primary focus during her testing. PLANNING: In the light of these factors- the woman's sexual history, her pregnancy, and her exposure to a pet cat, common infections that can have an impact on pregnancy need to be considered. Diseases like bacterial vaginosis, cytomegalovirus, Epstein-Barr virus, HIV, and human papillomavirus can all be sexually transmitted and may cause complications during pregnancy. However, we need to also consider in our hypothesis potential infections that this woman could have picked from her pet cat. CLINICAL REASONING: Upon analyzing each alternative individually, bacterial vaginosis, although common, is usually symptomatic and diagnosed when symptoms occur. Cytomegalovirus, Epstein-Barr Virus, and HPV screenings are either not routinely performed due to widespread exposure or are screened before conception. One unique feature in her history is the presence of a pet cat, which can expose her to Toxoplasma gondii, a parasite that can cause severe fetal complications if a woman gets infected during pregnancy. However, no option aligns with this scenario in the answer list. CONCLUDING: When infected with HIV, pregnant women can pass the virus to their children during pregnancy, delivery or breastfeeding. Given the high impact in case of transmission and the silent course of the disease, HIV needs to be correctly identified and managed to prevent mother-to-child transmission. Therefore, women with risk factors, such as having multiple sexual partners, should be screened during pregnancy. RESPONSE: Therefore, the most appropriate infectious condition to test this woman for, given her history, would be HIV (D), which can be screened during pregnancy to prevent mother-to-child transmission.

**AI_A:** RESTATING THE QUESTION: The query inquires about the most suitable infectious condition screening to be recommended for a 27-year-old first-time pregnant woman who is 10 weeks along, in a monogamous relationship, with a history of multiple sexual partners but no sexually transmitted diseases past, and who owns a pet cat. PLANNING: Assumptions can be made that the woman is at a higher risk for diverse infections due to her active sexual history and pet ownership. The conditions listed encompass bacterial, viral, and sexually transmitted infections. However, we must prioritize a test that best aligns with her lifestyle and potential risks. CLINICAL REASONING: 'Primigravid' refers to a woman in her first pregnancy. Generally, routine screening for parturient females includes checks for infectious diseases that may adversely impact the mother or the fetus. Bacterial vaginosis, although common, is typically screened if symptoms present. Cytomegalovirus and Epstein-Barr virus are widespread infections usually encountered during childhood; adults often have antibodies against them, making testing unnecessary. HIV should ideally be screened in all pregnant women; however, the patient doesn't possess risk factors like IV drug use or multiple current sexual partners. Human papillomavirus, despite its prevalence, does not generally trouble pregnancies, and regular screening is not routinely performed in pregnant women unless abnormal cervical cells are noted. CONCLUDING: Given the patient's cat ownership, a vital concern is the possibility of transmission of Toxoplasma gondii, a parasite that cats often carry and humans can contract by touching infected cat feces. Toxoplasma infection in pregnant women presents significant hazards, potentially causing serious complications like blindness, mental disabilities, or preterm birth. As such, it is necessary to account for pet-associated zoonotic diseases in her testing. RESPONSE: None of the choices listed directly applies to this patient's circumstances. However, given her features and pet cat ownership, she should ideally be tested for Toxoplasma gondii exposure. If, however, I must pick one of the given alternatives, it would be appropriate to screen for HIV (Option D) due to the patient's sexual history, as part of routine prenatal care despite her low associated risk profile.

**AI_B:** RESTATING THE QUESTION: The query focuses on a pregnant woman going through her first prenatal visit. Factors about her sexual history, contraceptive use, and pet ownership are noted. In view of all this, we are asked to identify which among the listed infectious conditions she should be tested for: bacterial vaginosis, cytomegalovirus, Epstein-Barr virus, HIV, or human papillomavirus. PLANNING: Here, the underlying assumptions are that some or all these infectious conditions could potentially affect her pregnancy due to her sexual history, pet ownership, or prior contraceptive usage. An alternate assumption could be that her risk for these conditions is based more on the prevalence of these infections in the population and guidelines for prenatal care rather than her specific circumstances. Considering these factors, we should prioritize the clinical assumption around routine prenatal care guidelines. CLINICAL REASONING: Let's take a stepwise approach here. In routine prenatal care, all expectant mothers are recommended to undertake certain screenings irrespective of specific risk factors. Although her sexual history and pet ownership could potentially increase her risk of contracting STDs or infections like toxoplasmosis, the information given doesn't signify any immediate risk factors except for being sexually active, which is indeed quite common. Among the listed conditions, HIV is the one for which routine prenatal screening is universally recommended, regardless of individual risk factors. Early detection and treatment of HIV can significantly reduce the risk of maternal-to-child transmission. In contrast, testing for conditions like bacterial vaginosis and human papillomavirus are usually based on symptoms, cytomegalovirus is not routinely screened for in pregnant women, and Epstein-Barr virus is less of a concern in pregnancy. CONCLUDING: Adopting a logic that connects individual sentences, it becomes clear that regardless of the specifics of this patient's sexual history or the fact she owns a cat, routine prenatal testing should not deviate. Efficient standard screenings, including HIV, serve their purpose well in such cases. RESPONSE: Therefore, the most appropriate recommended test for the patient, a 27-year-old primigravid woman, would be an HIV test (Option D).

**AI_D:** RESTATING THE QUESTION: This question is about a healthy, sexually active 27-year-old pregnant woman. The woman has not been previously diagnosed with any sexually transmitted diseases, and she recently discontinued her use of oral contraceptives. She is in a monogamous relationship and her pregnancy seems to progressing normally. The query in this context is asking for which infectious condition the woman should be screened for, given her circumstances. PLANNING: The base assumption here is that pregnancy brings additional health risks for women, including potential infections. Given the options in this scenario, the woman could potentially contract bacterial vaginosis (a common vaginal infection in women of reproductive age), cytomegalovirus (a common virus that can be problematic in pregnant women), Epstein-Barr virus (a common and usually mild virus), HIV (a virus that attacks cells that help the body fight infection), or human papillomavirus (a common sexually transmitted infection). Looking at past medical and sexual history, it could be pertinent to consider all of these as potential risks. CLINICAL REASONING: However, it's necessary to prioritize which condition presents the highest risk to the patient, both mother and baby, in the clinical context of pregnancy. Considering her circumstances, one might hypothesize that HIV should be a priority, despite her currently monogamous relationship. This is because the disease can be transferred to the unborn baby, causing serious health issues - even if the mother herself is asymptomatic. So, HIV testing should be made part of standard prenatal care. CONCLUDING: Consequently, given the potential severe impact on the fetus, the relatively high incidence in sexually active populations, and the fact that transmission can occur from mother to child during gestation, delivery, or breastfeeding, it would be most fitting to recommend an HIV test. RESPONSE: (D) HIV testing is the most appropriate recommendation for this patient, as given the severe health impact on the unborn baby, it should be part of standard prenatal care irrespective of perceived risk. This would help in early detection and intervention to prevent mother-to-child transmission if the mother is indeed HIV positive.

**AI_C:** RESTATING THE QUESTION: The patient in question is a 27-year-old primigravida, meaning it's her first pregnancy, currently at 10 weeks gestation based on the size of her uterus. She has a history of oral contraceptive use and has not had any sexually transmitted diseases in the past. Currently, she's in a monogamous relationship with the baby's father. This woman also has had five lifetime sexual partners and owns a pet cat. We're being asked to determine the most suitable infectious disease to test this patient for. PLANNING: The diseases offered as possibilities are bacterial vaginosis (a common vaginal infection), cytomegalovirus (CMV, a common virus that can lead to serious disease in unborn babies when a pregnant woman contracts it for the first time), Epstein-Barr virus (EBV, cause of mononucleosis and several types of cancer), HIV (human immunodeficiency virus, a virus that causes AIDS), and human papillomavirus (HPV, the most common sexually transmitted infection). It's important to consider that a number of factors can influence the risk of these diseases, such as sexual behavior, prior immune status, and environmental exposures. It's worthwhile to question, however, which among these diseases could have severe implications for an unborn child, and would thus necessitate prenatal screening. CLINICAL REASONING: Bacterial vaginosis, although common, is often symptomatic and thus unlikely to be present if the woman feels well. In any case, while it can have implications for pregnancy, it doesn't necessitate routine prenatal screening. CMV is certainly a consideration given its potential for severe disease in unborn babies, yet routine prenatal screening isn't recommended due to limited treatment options and the high prevalence of previous exposure in adults, which offers protection to the fetus. EBV also isn't routinely screened for during pregnancy, since primary infection is typically asymptomatic in adults and it's not associated with adverse pregnancy outcomes. HIV, on the other hand, can have severe implications for a fetus and newborn if not managed properly and is thus tested for in pregnant women regardless of risk factors. Similarly, while HPV is also sexually transmitted, routine prenatal screening isn't performed due to its lack of immediate implications for the fetus. It should be noted that abnormal pap tests in pregnancy can be managed conservatively until after the delivery in most cases. CONCLUDING: Given the assumed prevalence and potential implications for the unborn child, HIV would likely be the most appropriate condition to test for in this scenario. Although this woman belongs to a relatively low-risk group (e.g., monogamous relationship, no history of STDs), all pregnant women are typically screened for HIV. RESPONSE: Considering the potential risks and severity of outcomes for the unborn child, HIV (D) would be the most appropriate condition to recommend testing for in this patient.

**Facilitator** The consensus among the AI responses is that the most appropriate infectious condition to test for in the case of the 27-year-old primigravid woman, who is 10 weeks pregnant, sexually active, and owns a pet cat, would be HIV. This recommendation is based on the potential severe impact on the fetus, the relatively high incidence in sexually active populations, and the fact that transmission can occur from mother to child during gestation, delivery, or breastfeeding. Despite the woman's monogamous relationship and no history of sexually transmitted diseases, all pregnant women are typically screened for HIV as part of standard prenatal care. This is due to the silent course of the disease and the necessity for early detection and management to prevent mother-to-child transmission. While the woman's pet cat ownership raises concerns about potential exposure to Toxoplasma gondii, this is not among the listed options. Other conditions like bacterial vaginosis, cytomegalovirus, Epstein-Barr virus, and human papillomavirus are either not routinely screened for during pregnancy or are screened based on symptoms or prior to conception. Therefore, HIV testing (Option D) is the most appropriate recommendation for this patient.
