# Supplementary material for: Collaborative intelligence in AI: Evaluating the performance of a council of AIs on the USMLE
Source: PLOS Digit Health. 2025 Oct 9;4(10):e0000787. doi: 10.1371/journal.pdig.0000787 (PMC12510544; doi:10.1371/journal.pdig.0000787)
Supplement: S2 File — (ZIP) [file pdig.0000787.s002.zip › S2 Data Raw data files containing unprocessed question inputs and LLM outputs including transcripts of deliberations/STEP2_Question-70-1_Type-MC-NJ.docx]

**Facilitator** **[Round 1]:** A 45-year-old woman comes to the office because she has not had a menstrual period for 1 year. Menses previously occurred at regular 30-day intervals. She feels well, has no history of serious illness, and takes no medications. She has smoked one-half pack of cigarettes daily for 20 years. She is sexually active with one male partner and uses condoms consistently. She is 163 cm (5 ft 4 in) tall and weighs 63 kg (140 lb); BMI is 24 kg/m². Vital signs are within normal limits. Physical examination, including pelvic examination, shows no abnormalities. Measurement of which of the following serum concentrations is most likely to confirm the diagnosis? (A) Estradiol (B) Follicle-stimulating hormone (C) β-hCG (D) Luteinizing hormone (E) Progesterone

**AI_D:** RESTATING THE QUESTION: A woman, who is 45 years old, reports that she hasn't had her menstrual period for a year, even though previously she had regular menstrual cycles. Besides, she is a long-term smoker, isn't taking any medication, is sexually active, and uses prophylactic contraceptive method, specifically, condoms. Her physical examinations including a pelvic examination and vital signs are normal. We are asked to determine which serum concentrations would be the most helpful in diagnosing her condition. PLANNING: The key assumptions here include menopause as the leading prospect due to her age and absent menstrual periods for a year. Also, she is not pregnant as her β-hCG levels are likely normal given her use of condoms. Given these assumptions, we could think about elevations in follicle-stimulating hormone (FSH) or luteinizing hormone (LH), which increase with menopause, or decreased estradiol or progesterone, which also occur with menopause. But the single testing value most likely to confirm the diagnosis of menopause is the FSH concentration. CLINICAL REASONING: Menopause is defined by an absence of menstrual cycles for one year and is a diagnosis of exclusion. It is usually confirmed by the clinical picture of a woman in the perimenopausal age group (average age is 51 in the United States) in concert with associated symptoms. However, testing for serum FSH can help in confirming the diagnosis especially when the clinical picture is uncertain. FSH levels rise in menopause because the negative feedback inhibition from ovarian hormones (estradiol and inhibin) decreases with the loss of ovarian follicles. CONCLUDING: In conclusion, considering the patient's age and the duration of her amenorrhea, her smoking history which can lead to earlier onset of menopause, the normal physical examination, and her consistent use of condoms ruling out pregnancy, the most probable diagnosis is menopause. Therefore, the most helpful serum concentration to confirm this diagnosis would be that of follicle-stimulating hormone. RESPONSE: (B) Follicle-stimulating hormone.

**AI_A:** RESTATING THE QUESTION: A middle-aged woman who has not experienced a menstrual cycle for a year, despite having regular menses in the past, and who has no major health issues or use of medication is seeking a diagnosis. She smokes a moderate amount of cigarettes, is sexually active with consistent condom use, has a healthy body mass index, and shows no physical abnormalities. We're asked to determine which serum concentrations would most likely confirm her diagnosis. The choices include Estradiol (a form of estrogen), Follicle-stimulating hormone (FSH), β-hCG (a hormone produced during pregnancy), Luteinizing hormone (LH), and Progesterone (a steroid hormone involved in the menstrual cycle). PLANNING: Assuming that the woman's symptoms relate to menopause, common for women her age, we should focus on hormone levels associated with this period of life. I would question the need to measure β-hCG, given her use of condoms and the absence of other signs of pregnancy. Instead, I would prioritize verifying the concentration of hormones indicative of menopause. CLINICAL REASONING: After the woman's last menstrual cycle, the ovaries no longer release eggs and our body produces fewer hormones like estrogen and progesterone. Reduced estrogen levels are typical for menopause, so measuring estradiol (Choice A) might seem reasonable. However, measuring Follicle-stimulating hormone (FSH) (Choice B) might be more indicative. FSH regulates the growth and recruitment of immature ovarian follicles in the ovary and is produced in larger quantities when the ovaries start to fail - as seen in menopause. CONCLUDING: Therefore, in this woman's case, measuring FSH levels would be more likely to confirm menopause, taking into account her age, cessation of menses, the absence of serious illness, and normal physical examination. RESPONSE: (B) Follicle-stimulating hormone. Verification of increased levels of FSH in her serum is the most probable serum measurement that could confirm the diagnosis of menopause in this woman.

**AI_C:** RESTATING THE QUESTION: The clinician is seeking the most appropriate lab test to confirm the diagnosis of a 45-year-old woman who hasn't menstruated for a year but is otherwise in good health. The potential tests include measuring serum concentrations of estradiol, follicle-stimulating hormone (FSH), β-hCG, luteinizing hormone (LH), or progesterone. PLANNING: If we look at our assumptions: - The woman could be experiencing natural menopause, which is defined by the absence of menstrual periods for 12 months in the absence of other pathological or physiological causes. Natural menopause is typically confirmed by elevated levels of FSH. - However, other conditions could also stop menstruation, such as pregnancy, which would show elevated β-hCG. - Chronic conditions, stress, extreme exercise, and certain lifestyle factors like smoking can also disrupt menstrual cycles. In this situation, the most likely clinical scenario is menopause. CLINICAL REASONING: This patient is 45 years old, which is somewhat early but within the expected age range for menopause. On average, women reach menopause between the ages of 48 and 52, with some variation based on genetics, lifestyle, and other factors. In menopause, the depletion of ovarian follicles leads to a decrease in estradiol production and an increase in FSH due to decreased feedback inhibition. As most of the ovarian follicles have been consumed by this age, estrogen production drops markedly, thus removing the primary source of negative feedback on the hypothalamic-pituitary axis. As a result, FSH and LH levels are typically elevated in menopause. CONCLUDING: The best measurement to confirm her diagnosis would be a serum FSH concentration. This would be consistent with menopause due to increased levels in response to the decline in estradiol. However, a single measurement of serum FSH isn't sufficient to diagnose menopause. As FSH levels can fluctuate during the perimenopausal period, an elevated FSH on at least two occasions, roughly one month apart, would add additional support to the diagnosis of menopause. RESPONSE: So, measurement of the 'Follicle-stimulating hormone' (FSH) serum concentrations (option B) is most likely to confirm the diagnosis in this 45-year-old woman experiencing cessation of menstruation for one year. This aligns with the clinical picture of natural menopause. A high FSH level indicates that the ovaries are no longer responding to FSH stimulation to produce estrogen, thus resulting in the symptoms and cessation of menses observed in menopausal women.

**AI_E:** RESTATING THE QUESTION: In essence, the question is seeking to identify the best way to confirm a possible diagnosis for a 45-year-old woman, with no menstrual period for 1 year. She is otherwise in good health and her physical examination shows no abnormalities. She takes no medication and her only notable lifestyle habit is smoking. We are expected to choose from a range of hormones involved in the female reproductive cycle for the one that is most likely to confirm the diagnosis. PLANNING: There are several important aspects to consider here. Firstly, the woman's age, absence of menstruation for a year, and the regularity of her previous menstrual cycles suggest that she may be experiencing menopause, which typically occurs in women between the ages of 45 and 55. Secondly, the hormones listed—Estradiol, Follicle-stimulating hormone (FSH), β-hCG, Luteinizing hormone (LH), and Progesterone, are all involved in the menstrual cycle to varying degrees. With this in mind, it seems reasonable to hypothesize that the hormone levels likely to confirm menopause would be those that experience significant alterations during this stage in a woman's life. CLINICAL REASONING: Menopause is marked by the end of a woman's reproductive ability, which coincides with cessation of the menstrual cycle due to the depletion of ovarian follicles. Ovarian follicles, when active, respond to FSH and LH, producing Estradiol and Progesterone. When these follicles are depleted during menopause, there is a decline in Estradiol and Progesterone and a rise in the levels of FSH and LH due to lack of feedback inhibition. β-hCG, on the other hand, is a hormone associated with pregnancy and in this context, would not be of great diagnostic value. CONCLUDING: Thus, considering that menopause is most likely in this case—a diagnosis characterized by a rise in Follicle-stimulating hormone and Luteinizing hormone due to lack of feedback inhibition from dwindling levels of Estradiol and Progesterone—it follows that measurement of the concentrations of either FSH or LH could confirm this diagnosis. RESPONSE: Therefore, in this context, a measurement most likely to confirm the diagnosis is of serum concentrations of the Follicle-stimulating hormone (B). The FSH level is typically chosen because it generally has a larger magnitude of change compared to LH during menopause.

**AI_B:** RESTATING THE QUESTION: The provided case is of a woman aged 45 years who presents with the absence of menstrual periods for 1 year. Her menses were previously regular and observed every 30 days. She has no significant past history of illness and leads a generally healthy lifestyle, although she does have a smoking history of 20 years. The woman's vital signs are within the normal range, and there's nothing remarkable about her physical exam, including the pelvic examination. The inquiry here is essentially which blood test can best help confirm the presumptive diagnosis in this case - the options being testing for Estradiol, Follicle-stimulating hormone (FSH), β-hCG, Luteinizing hormone (LH), or Progesterone. PLANNING: Let's first consider the patient's clinical picture. She is 45 years old, and her menstruation has ceased for a year. Considering her age and the symptoms presented, the initial and most probable assumption would be that she is experiencing menopause, which generally occurs in women within the age range from 45 to 55 years and is characterized by the absence of menses for 12 consecutive months. Now, each of the blood tests corresponds to different conditions and stages of the menstrual cycle or pregnancy. 1. Estradiol and progesterone levels are usually reduced in menopause due to the cessation of ovarian activity. However, measuring their levels wouldn't specifically confirm menopause. 2. The β-hCG test is primarily used to verify pregnancy, which doesn't seem to be our case. 3. FSH and LH levels typically rise after the onset of menopause due to loss of the negative feedback from declining estrogen levels, which leads us to prioritize FSH as the serum concentration test to confirm menopause—this hypothesis of menopause stands valid until we have any contradictory signs or symptoms. CLINICAL REASONING: It's important to note that in a woman presenting with amenorrhea (absence of menstruation), various possible conditions could be causing it. The most common cause is pregnancy, yet here, the patient's consistent usage of condoms and lack of pregnancy symptoms makes it very unlikely. Other reasons could include stress, sudden weight loss, or endocrine disorders, but these don't seem to apply given the case details. Given the patient's age and the duration of amenorrhea, physiological menopause appears to be the most plausible explanation. Menopause is a normal age-related process in women, and usually occurs between ages 45 and 55. It is diagnosed clinically by the absence of menses for one year. During menopause, the ovarian production of estrogen and progesterone decreases, and as a result, the hypothalamic-pituitary axis tries to stimulate the ovaries by increasing secretion of LH and FSH, leading to elevated serum levels of the hormones. This forms the rationale of checking serum FSH levels, as they help confirm menopause. CONCLUDING: Given the clinical scenario, our leading assumption is that this 45-year-old woman, presenting with amenorrhea for 1 year, is likely going through menopause. To support this diagnosis, it would be reasonable to measure the serum concentration of the Follicle-stimulating hormone (FSH), as it's known to be elevated after menopause due to increased pituitary secretion in response to a decline in ovarian estrogen. Other hormone levels, though also influenced by menopause, are less specific or not relevant to the suspected diagnosis. RESPONSE: Therefore, in this case, the serum concentration most likely to confirm the diagnosis is (B) the Follicle-stimulating hormone (FSH).

**Facilitator** The consensus among the AI responses is that the most likely diagnosis for the 45-year-old woman who has not had a menstrual period for a year, despite previously regular cycles, is menopause. This is based on her age, the duration of her amenorrhea, her healthy lifestyle, and the absence of any significant health issues or abnormalities in her physical examination. The hormone levels that would most likely confirm this diagnosis are those that undergo significant changes during menopause. While estradiol and progesterone levels decrease, the levels of follicle-stimulating hormone (FSH) and luteinizing hormone (LH) increase due to the lack of feedback inhibition from the declining levels of ovarian hormones. However, the consensus is that FSH is the hormone that should be measured to confirm the diagnosis, as it generally shows a larger magnitude of change compared to LH during menopause. Therefore, the serum concentration most likely to confirm the diagnosis in this case is the follicle-stimulating hormone (FSH) (Option B). It's important to note that while a single measurement of FSH can support the diagnosis, fluctuations can occur during the perimenopausal period, so elevated FSH levels on at least two occasions, roughly one month apart, would provide additional confirmation. The β-hCG hormone, associated with pregnancy, is not considered relevant in this case due to the woman's consistent use of condoms and the absence of other signs of pregnancy.
