# Supplementary material for: Collaborative intelligence in AI: Evaluating the performance of a council of AIs on the USMLE
Source: PLOS Digit Health. 2025 Oct 9;4(10):e0000787. doi: 10.1371/journal.pdig.0000787 (PMC12510544; doi:10.1371/journal.pdig.0000787)
Supplement: S2 File — (ZIP) [file pdig.0000787.s002.zip › S2 Data Raw data files containing unprocessed question inputs and LLM outputs including transcripts of deliberations/STEP2_Question-71-1_Type-MC-NJ.docx]

**Facilitator** **[Round 1]:** A 2-month-old girl is brought to the office by her mother for a routine examination. Newborn screening electrophoresis showed hemoglobin FS. The mother says her daughter has not had fever or pallor. The patient is feeding well and gaining weight appropriately. She receives no medications. She appears well. She is at the 35th percentile for length and weight. Temperature is 37.0°C (98.6°F), pulse is 158/min, and respirations are 28/min. Examination shows no abnormalities. All routine immunizations are administered. In addition to routine anticipatory guidance about feeding and development, the mother is instructed to observe the patient for pallor and fever and to palpate for an enlarged spleen. Which of the following is most appropriate to prevent complications in this patient? (A) Amoxicillin as needed for febrile illness (B) Instructions for the parents to palpate the long bones routinely (C) Instructions for the parents to take and record the patient’s pulse daily (D) Low-dose aspirin therapy (E) Oral prophylactic penicillin therapy

**AI_A:** RESTATING THE QUESTION: The question involves a 2-month-old baby girl whose newborn screening electrophoresis revealed an FS hemoglobin type. The mother hasn't noticed any signs of fever or paleness in her infant, who's feeding well, gaining weight appropriately, and shows no abnormal findings on examination. Regular vaccinations have been given. The mother has been instructed to be vigilant about the baby's complexion and to check for an enlarged spleen. The question asks about the best approach to prevent potential health complications related to this child's hemoglobin variant. PLANNING: Hemoglobin FS is indicative of sickle cell disease (SCD), an autosomal recessive blood disorder caused by a mutation in the β-globin chain of hemoglobin. This mutation makes hemoglobin molecules 'sickle' in low oxygen conditions, leading to microvascular occlusions and predisposing to infections, especially by encapsulated organisms such as Streptococcus pneumoniae. Key assumptions here are that the child does have SCD and is symptom-free at present. However, we should consider an alternate assumption that complications can arise in the future despite the current asymptomatic status. Therefore, preventive measures should be undertaken. CLINICAL REASONING: In the context of SCD, the child is potentially at risk of invasive pneumococcal disease. This is because the disease can cause splenic infarcts that lead to autosplenectomy, reducing the immunity against encapsulated organisms. We can link this to the need for prophylactic penicillin therapy, which is recommended for children with SCD until they're five years old. CONCLUDING: From the understanding of the pathophysiology and potential complications of SCD, it becomes clear that prophylactic measures to prevent pneumococcal infections should be a priority. Hence, the use of penicillin as a prophylactic measure is the most suitable in this case. Among the given options, (E) Oral prophylactic penicillin therapy seems most appropriate. RESPONSE: The most appropriate measure to prevent complications in this patient, who has been identified with SCD via newborn screening, would be (E) Oral prophylactic penicillin therapy.

**AI_B:** 1) RESTATING THE QUESTION: The inquiry is about a 2-month-old girl who has been tested during a routine examination and found to possess hemoglobin FS. Hemoglobin FS refers to sickle cell disease, a genetic condition where there are abnormal hemoglobin types, which can cause complications such as anemia and infection. There are specific precautions to consider to prevent or manage potential complications from this condition. 2) PLANNING: The assumptions are that the patient may suffer from complications related to her condition. These complications could be infections, pain episodes, or even stroke. Challenging this assumption, it could be considered that she may not develop severe complications. Still, it is not advisable to overlook such a potential threat in a patient with sickle cell disease, hence preventative measures should be taken. 3) CLINICAL REASONING: Patients with sickle cell disease are at increased risk of various infections, especially encapsulated organisms such as Streptococcus pneumoniae and Haemophilus influenzae, due to functional asplenia. The prevalence of asplenia grows with age in these patients, and it has severe consequences because of the spleen’s role in curtailing infections. Children with a functional or anatomical asplenia or hyposplenia are recommended to receive prophylactic penicillin until at least their fifth birthday, to prevent such infections. 4) CONCLUDING: It becomes apparent from the patient's well-being that no severe complications are currently being experienced. Standing on a preventative ground, it would be valid to institute prophylaxis against potential infections that, if this patient contracted, could have serious consequences due to their sickle cell disease. Children with this condition, in their first years of life especially, should receive preventative treatment against infections, pneumonia particularly. 5) RESPONSE: Therefore, in light of preventing complications in this patient who has sickle-cell disease, the most appropriate measure would be (E) Oral prophylactic penicillin therapy. This is intended to keep potential serious infections related to her condition at bay.

**AI_C:** (1) RESTATING THE QUESTION: The query here revolves around an infant girl who on screening was found to have Hemoglobin FS, indicating a condition known as Sickle Cell Disease (SCD). This condition arises due to a genetic anomaly leading to misshapen RBCs that restrict blood flow. The mother noticed no symptoms, such as fever or pallor in her child, who is also faring well on other health parameters. The infant appears healthy, with her height and weight advancing as per expected growth curve, and no anomalies revealed during the physical examination. Given this context, the parent is seeking advice on preventive measures to avert any potential complications arising from SCD in her child. The question invites suggestions for the optimal preventive approach/categories of measures in this case. (2) PLANNING: To address this situation, several assumptions can be made. Primary among these assumptions are – the child will remain asymptomatic, infections could present a significant risk to the child, routine palpation of bones or recording of pulse will be useful arms of preventive care, aspirin therapy could have a role, or that oral penicillin prophylaxis could be potentially beneficial. Reviewing these assumptions, the prime risk factor for persons with SCD is acquiring serious infections. It is therefore logical to prioritize infection prevention. (3) CLINICAL REASONING: With the assumption of infection prevention made, a clinical approach would entail implementing strategies reducing the risk of infections, specifically encapsulated bacteria such as Streptococcus pneumoniae. While amoxicillin can treat such infections, preventive strategies are typically more beneficial. Monitoring fever or palpating for an enlarged spleen can help detect possible infection, but doesn't constitute as a preventive approach. Furthermore, neither routine bone palpation or pulse recording, nor low-dose aspirin which is typically more related to cardiovascular diseases, hold relevance in preventing infections in SCD. (4) CONCLUDING: By connecting the details provided about SCD, its potential complications, and the interventions proposed, the focus should ideally be on preventing infections. As SCD patients are asplenic, they have a heightened risk for infections, notably from encapsulated bacteria such as Streptococcus pneumoniae for which penicillin is known to be effective. (5) RESPONSE: Therefore, the most appropriate step to prevent complications in this patient would be to start oral prophylactic penicillin therapy (E) to prevent serious infections.

**AI_D:** (1) RESTATING THE QUESTION: The request for evaluation is regarding a 2-month-old female infant who is overall well and without symptoms. The important finding is the presence of hemoglobin FS as per her newborn screening. Hemoglobin FS indicates say the presence of both hemoglobin F (fetal hemoglobin) and S. Hemoglobin S is typical of sickle cell disease, a genetic condition that affects the red blood cells' shape and function, reducing their oxygen-carrying capacity. The question is about identifying the most suitable prophylaxis measure to prevent complications for this infant. (2) PLANNING: There are certain crucial assumptions to understand here. First, we hypothesize that the presence of hemoglobin FS could suggest that the infant has sickle cell disease. Second, due to her probable condition, she might be at risk of specific complications like infections (especially from encapsulated organisms), painful vaso-occlusive crises, acute chest syndrome, and splenic sequestration, among others. We must focus on preventing these complications to ensure her well-being. Going against the potential assumption that the child might not require any intervention because she is asymptomatic, the necessity for prophylactic measures against the possible complications should be prioritized. (3) CLINICAL REASONING: In sickle cell disease, regular prophylaxis against infections - especially those caused by encapsulated organisms (like Streptococcus pneumoniae and Haemophilus influenzae type B) - plays a critical role. This is essential because these patients are particularly susceptible to these organisms due to autosplenectomy from repeated splenic infarctions. Thus, measures such as pneumococcal and Hib vaccines are routinely administered. However, for very young patients like our 2-month-old girl, daily oral prophylaxis against these organisms with an antibiotic like penicillin is paramount until they are old enough for immunization. These measures—vaccination and prophylactic antibiotics—are more relevant than pallor, fever monitoring, or long bone palpation, as they directly prevent the common complications. (4) CONCLUDING: Based on the clinical reasoning presented, the most suitable intervention for preventing complications in this sickle cell disease patient appears to be the initiation of prophylactic antibiotics. (5) RESPONSE: Therefore, the best step towards preventing complications in this infant, who is likely to have sickle cell disease, would be (E) Oral prophylactic penicillin therapy. This intervention prevents severe infections, particularly from encapsulated organisms, until the child is older and gets the comprehensive range of immunizations.

**AI_E:** (1) RESTATING THE QUESTION: The query involves the case of a 2-month old female infant who was brought in for a regular check-up. The mother reports that the child is feeding and growing well, and currently shows no signs of illness such as fever or pallor. Importantly, it was noted from her newborn screening electrophoresis that her hemoglobin type is FS, which is linked to the genetic condition of Sickle Cell Disease (SCD). With this disease, individuals have abnormal hemoglobin molecules called hemoglobin S, which can distort red blood cells into a sickle, or crescent shape. This condition can lead to various health complications which could be potentially prevented with specific intervention measures. The question asks which proposed intervention (from the available options) is most appropriate to prevent complications in this patient. (2) PLANNING: The key assumption underpinning this scenario is that the right preventive measure can avert or mitigate potential health risks in the infant. Some alternate assumptions could be that the baby may not show signs of sickness associated with SCD, or that the parents may not have sufficient knowledge or resources to implement the preventative measures. However, based on the available evidence, the assumption to clinically prioritize is the need for the right preventive measure. This is because, despite the current healthy state of the child, the hemoglobin FS detected in her blood indicates a higher than average risk of developing complications related to SCD. (3) CLINICAL REASONING: Among the options presented, those related to pharmacological prevention (Amoxicillin as needed for febrile illness, low-dose aspirin therapy, and oral prophylactic penicillin therapy), palpating of long bones, and daily pulse measurement are suggested. From a clinical perspective, it's important to assess the benefits and drawbacks of each preventive measure, considering the age of the patient, the nature of the condition (SCD), and current guidelines on SCD management in infants. (4) CONCLUDING: In the case of SCD in infants, the most endorsed preventive measures include vaccinations and oral penicillin prophylaxis, which are usually administered to infants diagnosed with the condition from the age of 2 months to 5 years. The use of amoxicillin as needed for febrile illness, the palpation of the long bones or daily pulse recording by parents, and the administration of aspirin don't align with current medical guidelines or expert suggestions on managing infants with SCD. (5) RESPONSE: Therefore, based on the presented clinical scenario and existing guidelines on managing infants with Sickle Cell Disease, the most appropriate preventive measure for this patient would be Oral prophylactic penicillin therapy (E). The therapy is recommended to protect against pneumococcal infection, which individuals with SCD are particularly susceptible to due to the increased risk of splenic dysfunction related to their condition.

**Facilitator** The question pertains to a 2-month-old girl who, despite appearing healthy and showing no signs of illness, has been identified with Hemoglobin FS, indicative of Sickle Cell Disease (SCD), through newborn screening. SCD is a genetic condition that can lead to various health complications, including an increased risk of infections, particularly from encapsulated organisms such as Streptococcus pneumoniae. Given the potential risks associated with SCD, the question seeks the most appropriate preventive measure to avert these complications. The consensus among the responses is that the most suitable preventive measure in this case is oral prophylactic penicillin therapy. This recommendation is based on the understanding that SCD can lead to functional asplenia, making the child more susceptible to infections, especially from encapsulated bacteria. Prophylactic penicillin therapy is typically recommended for children with SCD from the age of 2 months to 5 years to prevent such infections. This measure is considered more beneficial and aligned with current medical guidelines than other options such as amoxicillin as needed for febrile illness, routine palpation of long bones, daily pulse recording, or low-dose aspirin therapy. Therefore, despite the child's current asymptomatic status, the initiation of oral prophylactic penicillin therapy is deemed the most appropriate step to prevent potential complications related to SCD.
