# Supplementary material for: Collaborative intelligence in AI: Evaluating the performance of a council of AIs on the USMLE
Source: PLOS Digit Health. 2025 Oct 9;4(10):e0000787. doi: 10.1371/journal.pdig.0000787 (PMC12510544; doi:10.1371/journal.pdig.0000787)
Supplement: S2 File — (ZIP) [file pdig.0000787.s002.zip › S2 Data Raw data files containing unprocessed question inputs and LLM outputs including transcripts of deliberations/STEP2_Question-72-1_Type-MC-NJ.docx]

**Facilitator** **[Round 1]:** A 3-week-old boy is brought to the physician by his mother because of a 5-day history of yellow eyes and skin and white stools. Pregnancy and delivery were uncomplicated. The patient's temperature is 37.1°C (98.8°F), pulse is 160/min, respirations are 40/min, and blood pressure is 85/50 mm Hg. Examination shows diffuse jaundice. Cardiopulmonary examination shows no abnormalities. The abdomen is nontender. The liver edge is palpated 4 cm below the right costal margin. The spleen tip is palpated 3 cm below the left costal margin. Laboratory studies show: Leukocyte count: 10,000/mm³ Serum Bilirubin, total: 16 mg/dL Direct: 12 mg/dL AST: 800 U/L ALT: 500 U/L Amylase: 100 U/L Which of the following is the most appropriate next step in diagnosis? (A) Air-contrast enema (B) Ultrasonography of the abdomen (C) Upper gastrointestinal series (D) Colonoscopy (E) Esophagogastroduodenoscopy

**AI_B:** RESTATING THE QUESTION: The clinical presentation aligns with a 3-week-old infant experiencing a combination of observable symptoms and laboratory findings that indicate possible liver disease. The symptoms include jaundiced skin and eyes, white stools, elevated total and direct bilirubin, elevated ALT, and AST whereas the white leukocyte count and amylase are well within the normal range. PLANNING: The underlying assumption of these specific symptoms is an issue in the liver or biliary tree. The issue could stem from the hemolysis of red blood cells, the inability to excrete bilirubin, or the impaired uptake of bilirubin by hepatocytes for conjugation. It's crucial to identify and assess any blockages in the biliary system, which is suggested by the white stools (due to insufficient secretion of bilirubin into the intestines) and jaundice. CLINICAL REASONING: Clinical reasoning having considered his age, signs, and laboratory workup the main hypothesis is that there's likely to be some sort of biliary obstruction, possibly due to a condition such as biliary atresia, which is a congenital disease characterized by obliteration or discontinuity of the extrahepatic biliary system, resulting in obstruction of the bile flow. This condition would explain the symptoms such as jaundice and acholic stools (white in color due to a lack of bilirubin content). The elevated liver enzymes (AST and ALT) suggest liver cell injury, a common feature in biliary atresia. CONCLUDING: It's important to identify whether this assumption is correct, and thus the tool that can best image the liver, bile ducts, and gallbladder should be used. In this case, the preferable method is ultrasonography. RESPONSE: The most appropriate next step in the diagnosis for this patient, taking into account the pertinent clinical findings and assumptions, is to opt for (B) Ultrasonography of the abdomen.

**AI_E:** RESTATING THE QUESTION: A neonate presenting with yellowish discoloration of the skin and eyes, pale stools, and certain abnormal lab findings, including high bilirubin levels and liver enzymes, suggests liver dysfunction. This is supported by the physical examination, which revealed an enlarged liver and spleen below their respective costal margins. Thus, the current scenario poses the query regarding the most appropriate diagnostic step to further assess this condition. PLANNING: Several assumptions can be made based on these presented symptoms. The yellowish coloration could be attributed to conditions such as neonatal jaundice, which can be due to varying causes including liver diseases, hemolysis, and biliary tract obstructions, among others. The elevated direct bilirubin and liver enzymes point towards potential hepatic pathology. Picturing these circumstances, I assume that ultrasound (Option B) might bring more insight into the child's status, primarily because it's minimally invasive, can visualize the liver, biliary tract, and other abdominal organs, and might help identify the underlying cause. CLINICAL REASONING: Neonatal jaundice is typically physiological, but the significant direct hyperbilirubinemia (bilirubin >10% of total or >1 mg/dL), pale stools, and hepatosplenomegaly, particularly the presence of an enlarged liver, hint towards a possible obstruction in the bile duct system, such as biliary atresia. Biliary atresia is a serious liver disorder, and it's the most common cause of neonatal jaundice that continues beyond the second week of life. It is characterized by sclerosis and obliteration of the extrahepatic biliary tree causing progressive cholestasis. CONCLUDING: Given the clinical scenario and considering the usefulness of ultrasound in visualizing the hepatobiliary structures, opting for an ultrasound as the next step in diagnosis is consistent with the child's presentation and clinical reasoning mentioned above. RESPONSE: The most appropriate next step in diagnosing this 3-week-old boy would be (B) Ultrasonography of the abdomen. This could help visualize any abnormalities in the liver and biliary tract and rule out or confirm the suspicion of biliary atresia or other hepatic dysfunctions.

**AI_D:** RESTATING THE QUESTION: The patient is a 3-week-old boy who presents with a 5-day history of jaundice (yellowing of the eyes and skin) and acholic (white) stools. His bilirubin, a pigment produced by the liver, is significantly elevated. Remarkably, the liver and spleen are palpable below the normal limits, suggesting hepatosplenomegaly. In addition, his liver enzymes, AST and ALT, are also significantly high, indicating liver injury. Given these clinical findings, we are tasked with selecting the next best step in diagnosing the underlying cause. PLANNING: Let's first enumerate some potential causes in this age group. The presentation is suggestive of neonatal cholestasis, which could be due to several causes, including, but not limited to, Biliary atresia (a congenital condition where the bile ducts are poorly formed or absent; this is the most common cause of neonatal cholestasis), infections (such as CMV, rubella, syphilis), genetic disorders (like alagille syndrome, alpha-1-antitrypsin deficiency, cystic fibrosis), metabolic diseases, or hepatobiliary malformations. It is important to quickly identify and treat underlying conditions that lead to neonatal cholestasis, as failure to do so in a timely manner can lead to life-threatening complications. CLINICAL REASONING: Given the list of possibilities, Biliary Atresia comes into high consideration due to the extremely high conjugated (direct) bilirubin and the acholic stools, which might suggest an obstruction on bile flow. Initial investigation of suspected biliary atresia usually involves imaging to assess the anatomy of the biliary tree, followed by an invasive procedure (usually a liver biopsy) if imaging results are inconclusive. CONCLUDING: Biliary atresia is an obstruction of the bile ducts, which would not be directly visible or diagnosable by an air-contrast enema, a colonoscopy, or an upper gastrointestinal series, which are primarily used to visualize the intestinal tract, nor esophagogastroduodenoscopy, which visualizes the upper digestive tract. Therefore, considering the possible underlying causes and findings on examination, we can ascertain that Ultrasonography of the abdomen is the most appropriate next step in diagnosing this patient's condition. RESPONSE: (B) Ultrasonography of the abdomen is the most suitable investigation to aid in the diagnosis of this patient's condition.

**AI_C:** RESTATING THE QUESTION: The question essentially is asking for the most appropriate method of diagnosis for a 3-week-old boy presenting with a 5-day history of jaundice (yellow skin and eyes) and acholic stools (white stools). The baby's vitals are within the normal range for his age, but physical examination reveals diffuse jaundice and hepatosplenomegaly (protruding liver and spleen). Lab results indicate a high total and direct bilirubin, high AST and ALT (markers of liver damage), normal leukocyte count and normal amylase levels. PLANNING: This clinical scenario suggests a condition related to hepatic pathology considering the high serum bilirubin, AST and ALT levels, diffuse jaundice, and hepatosplenomegaly. Jaundice in a 3-week-old baby could be due to several pathological conditions, including biliary atresia, neonatal hepatitis, or metabolic disorders. A key factor supporting a diagnosis of biliary atresia in this case is the presence of pale stools (acholic stools), which indicates an obstructive cause. Therefore, the logical approach to confirm this assumption would be to order an imaging study that could help visualize the biliary tract. CLINICAL REASONING: Biliary atresia is commonly suspected in infants with persistent jaundice, acholic (pale) stools, and hepatomegaly after the first two weeks of life. If not promptly diagnosed and surgically corrected, this disorder leads to liver failure and death within the first few years of life. We may consider other causes of jaundice such as metabolic disorders or neonatal hepatitis; but metabolic disorders usually present with additional systemic symptoms, and neonatal hepatitis often presents with both elevated direct and indirect bilirubin, features not evident in this case. Given our scenario, obtaining a critical imaging study to evaluate the anatomy of the liver and biliary tree appears to be the paramount next step. CONCLUDING: An ultrasonography of the abdomen (Option B) would be the most clinically valuable next step. This non-invasive technique provides detailed insights into the anatomy of the liver and biliary tree, including visualization of the gallbladder, which may be absent in cases of biliary atresia. It can also identify other potential hepatic or pancreatic abnormalities contributing to the clinical scenario. The other options may be part of various diagnostic workups but given the predominantly hepatic indications, they likely won't provide as much essential information in this specific situation. RESPONSE: The most appropriate next step in diagnosis is (B) Ultrasonography of the abdomen.

**AI_A:** 1) RESTATING THE QUESTION: The question refers to a 3-week-old boy with symptoms of yellow skin and eyes, and white stools for the past 5 days. The newborn's vital signs are normal for his age but physical examination shows a significantly enlarged liver and spleen, along with diffuse jaundice. Lab results indicate a massive increase in both total and direct bilirubin levels as well as liver enzymes, while other results such as leukocyte count and amylase levels are within normal limits. The question wants to know the most appropriate diagnostic tool to use next in this case out of the given options: air-contrast enema; abdomen ultrasonography; upper gastrointestinal series; colonoscopy; esophagogastroduodenoscopy. 2) PLANNING: The primary assumption in this case is that the neonate is experiencing obstructive jaundice due to an underlying liver condition, considering the symptoms of jaundice, pale stools, and hepatosplenomegaly, plus the lab results showing significantly elevated liver enzyme and bilirubin levels. However, the lack of evident signs of infection or inflammation casts doubt on this assumption. The elevated direct bilirubin could be due to intrahepatic, intraductal, or postductal obstruction or dysfunction. Considering the age of the patient and the current clinical picture, biliary atresia, a condition that constricts or blocks the bile ducts, sounds plausible and deserves priority. 3) CLINICAL REASONING: Biliary atresia usually presents in the first few weeks of life and is the leading cause of neonatal jaundice. It is characterized by obstructive jaundice, pale stools, dark urine, and hepatosplenomegaly, exactly as described in the presented case. White stools suggest a lack of excreted bilirubin which could be due to an obstruction in the bile ducts, preventing it from reaching the intestines. This would manifest as an elevation in direct bilirubin which is suggested by the lab results. 4) CONCLUDING: There are several ways to diagnose biliary atresia but abdominal ultrasonography is typically the first diagnostic step for suspected cases, as it is non-invasive, safe, and can screen for both hepatic and extrahepatic features concluded from the clinical reasoning. Thus, using a step-by-step explanation and the process of elimination, it appears that an ultrasonography of the abdomen will be the most viable option here. 5) RESPONSE: So, the most appropriate next step in diagnosing the condition of this 3-week-old boy is a (B) Ultrasonography of the abdomen.

**Facilitator** The consensus statement synthesizes the responses of AI_A, AI_B, AI_C, AI_D, and AI_E as follows: The question presents a 3-week-old boy with a 5-day history of jaundice and acholic (white) stools. His physical examination reveals hepatosplenomegaly, and laboratory studies show significantly elevated total and direct bilirubin levels, as well as liver enzymes, while other results such as leukocyte count and amylase levels are within normal limits. The clinical presentation and laboratory findings suggest a liver or biliary tract issue, possibly due to an obstruction in the bile flow. Given the patient's age, symptoms, and lab results, the primary assumption is that the neonate is experiencing obstructive jaundice due to a condition such as biliary atresia, a congenital disease characterized by obliteration or discontinuity of the extrahepatic biliary system. This condition typically presents in the first few weeks of life and is the leading cause of neonatal jaundice. It is characterized by obstructive jaundice, pale stools, dark urine, and hepatosplenomegaly, exactly as described in the presented case. To confirm this assumption, an imaging study that can visualize the liver, bile ducts, and gallbladder should be used. Among the given options, abdominal ultrasonography is the most appropriate next step in diagnosis. This non-invasive technique provides detailed insights into the anatomy of the liver and biliary tree, including visualization of the gallbladder, which may be absent in cases of biliary atresia. It can also identify other potential hepatic or pancreatic abnormalities contributing to the clinical scenario. Therefore, the consensus among the AI responses is that the most appropriate next step in diagnosing the condition of this 3-week-old boy is (B) Ultrasonography of the abdomen.
