# Supplementary material for: Collaborative intelligence in AI: Evaluating the performance of a council of AIs on the USMLE
Source: PLOS Digit Health. 2025 Oct 9;4(10):e0000787. doi: 10.1371/journal.pdig.0000787 (PMC12510544; doi:10.1371/journal.pdig.0000787)
Supplement: S2 File — (ZIP) [file pdig.0000787.s002.zip › S2 Data Raw data files containing unprocessed question inputs and LLM outputs including transcripts of deliberations/STEP2_Question-73-1_Type-MC-NJ.docx]

**Facilitator** **[Round 1]:** A 52-year-old woman comes to the physician for a follow-up examination. She received the diagnosis of hypertension 3 weeks ago, and lisinopril therapy was begun at that time. She has had a constant, nonproductive, hacking cough for 2 weeks but has not had any other problems. She has no history of serious illness and takes no other medications. She does not smoke. She drinks one to two glasses of wine daily. She does aerobic exercise for 30 minutes four to five times weekly. Her blood pressure has decreased from 164/92 mm Hg 3 weeks ago to 140/84 mm Hg today. Lungs are clear to auscultation. Which of the following is the most appropriate next step in management of this patient's cough? (A) Pulmonary function tests (B) CT scan of the chest (C) Course of prednisone (D) Discontinuation of lisinopril (E) Use of a cough suppressant (F) No change in management is indicated at this time

**AI_A:** RESTATING THE QUESTION: The query centers around a 52-year-old woman who has been diagnosed with hypertension and started on lisinopril three weeks ago. Since then, she has developed a persistent, nonproductive cough. Her medical background is otherwise unremarkable, and her lifestyle is fairly healthy. She doesn't take any other drugs and her blood pressure is relatively controlled. The question is interested in the most appropriate future management of her cough in this setting. PLANNING: There's an assumption that her cough may be related to her recent medication change (starting lisinopril). This could be questioned: could it be an unrelated new issue? However, given the temporal relationship and the known side effects of lisinopril, it seems reasonable to prioritize the assumption that the lisinopril may be causing her cough. Thus, examining the actions that could be done if this assumption is correct will be the main plan. CLINICAL REASONING: Lisinopril, an angiotensin-converting enzyme (ACE) inhibitor, is commonly known to cause cough in patients. This happens due to accumulation of bradykinin in the respiratory tract lining. Initial management typically involves discontinuing the ACE inhibitor and starting an alternate blood pressure medication. Considering the patient's symptoms and their occurrence following lisinopril initiation, her clear lung auscultation and lack of other potential cough causes, this management strategy seems reasonable. CONCLUDING: Upon evaluation, the signs point toward lisinopril-induced cough given the relative timing of medication initiation and symptom onset, along with the absence of other apparent causes. Thus, the lisinopril should be discontinued. RESPONSE: The most suitable next step in managing this patient's cough would be (D) discontinuation of lisinopril.

**AI_D:** RESTATING THE QUESTION: This scenario requires us to determine the best next step in managing a 52-year-old woman with a constant, nonproductive, hacking cough since being diagnosed with hypertension and starting therapy with lisinopril three weeks prior. The patient's blood pressure has improved, and no other major health issues or medications are noted. PLANNING: In examining these symptoms, the first assumption might be that the cough is due to an unrelated respiratory condition. However, a deeper consideration of these facts suggests an alternate assumption: the cough could be a side effect of the lisinopril treatment, which is an angiotensin-converting enzyme (ACE) inhibitor. This type of drug can sometimes cause a dry, persistent cough in some people due to build-up of bradykinin in the respiratory tract. CLINICAL REASONING: When comparing these assumptions with the patient's clinical history, there's several points to observe. First, the cough presented after the initiation of the lisinopril treatment. Second, the nature of the cough (dry, hacking, persistent) corroborates a side effect of ACE inhibitors. Third, the patient's lungs are clear upon auscultation, implying an absence of an active pulmonary disease, and her healthy habits like non-smoking and regular exercising reduce the risk of chronic respiratory conditions. Finally, the improvement in her hypertension supports that the lisinopril is having its desired effect. CONCLUDING: Thus, by correlating clinical facts with possibilities, it's suggested that the most likely cause for her cough is the initiation of lisinopril. It's important to address this side effect as it may affect the patient's quality of life and compliance with the hypertension treatment. RESPONSE: Therefore, the appropriate next step in the management of this patient's cough would be option D, discontinuation of lisinopril. A suitable alternative antihypertensive from the ARBs (Angiotensin II Receptor Blockers) category may then be considered, which tend not to induce cough as lisinopril does.

**AI_B:** RESTATING THE QUESTION: The scenario presents a 52-year-old woman who has recently been diagnosed with hypertension, for which she was given lisinopril. She has developed a persistent non-productive cough over the past two weeks but has no further health issues. She has a healthy lifestyle and is not on any other medication. Given her treatment progress, we're asked to suggest the best next step to manage her cough from the provided options. PLANNING: The key assumptions are that her persistent cough is related to her hypertension treatment, i.e., lisinopril usage, and not due to any other conditions like respiratory disease. Another assumption would be that the cough, being non-productive and accompanied by clear lung auscultation, does not need any severe interventions. Let's evaluate these assumptions in the light of her medical history, current health status and lifestyle habits while factoring in the side-effects profile of lisinopril. CLINICAL REASONING: Lisinopril belongs to a class of drugs called angiotensin-converting enzyme (ACE) inhibitors which are widely used for hypertension treatment. One well-recognized side effect of ACE inhibitors is a dry, persistent, nonproductive cough. Considering her cough started after beginning lisinopril therapy and her lung examination is normal, her symptom can be attributed to the drug usage and not any underlying pulmonary disorder. Furthermore, pulmonary function tests, CT scans or administration of prednisone may not be beneficial in this situation as they are usually considered in symptoms indicative of severe respiratory disorders (like difficulty in breathing, wheezing, chest pain, blood in phlegm) which she evidently lacks. CONCLUDING: As per the clinical reasoning and evaluation, the cough appears as a side effect of ACE inhibitor therapy. In such a case, a logical course of action would be to replace this drug with another antihypertensive medication that does not produce similar side-effects, instead of enduring or suppressing the discomforting side effect. RESPONSE: Therefore, the most appropriate next step in the management of this patient's cough is the discontinuation of lisinopril (Choice D). The physician can consider substituting it with another class of antihypertensive, for example, a Calcium Channel Blocker or an ARB (Angiotensin II Receptor Blocker), depending upon her overall clinical profile.

**AI_C:** RESTATING THE QUESTION: A 52-year-old female patient who was recently diagnosed with hypertension is currently under treatment with lisinopril. Despite having no history of serious illness or other medications, the patient presents with a nonproductive, hacking cough that she's had for 2 weeks. The patient is a non-smoker, consumes moderate levels of alcohol, and regularly engages in aerobic exercise. Her blood pressure level, previously at 164/92 mm Hg, has since decreased to140/84 mm Hg as of her most recent examination, and her lungs clear upon auscultation. The question asks what the most appropriate next step in treating her cough would be. PLANNING: The patient's cough started after she began her treatment with lisinopril, a drug used to control her hypertension. We need to consider the possibility that the cough is a side effect of the medication. The other options—pulmonary function tests, a CT scan of the chest, a course of prednisone, use of a cough suppressant, or no change in management—are potentially valid approaches, but clearly, stopping the medication that could be causing the cough in the first place should be considered. CLINICAL REASONING: Lisinopril is an angiotensin-converting enzyme (ACE) inhibitor, a category of medications well-known for causing a persistent, dry cough in some patients. This side effect is due to the accumulation of bradykinin, a peptide that promotes dilation of blood vessels and that can also stimulate cough receptors in the lungs. The timing of the onset of this patient's cough coincides with the initiation of her treatment with lisinopril, making this a more likely cause than any new onset lung disease given the patient's history of not smoking and her clear lung exam. CONCLUDING: Upon reviewing medical history, vital signs, and presenting symptoms, as well as considering the potential side effects of current medications, it would be reasonable to hypothesize that the lisinopril is the likely cause of this patient's dry cough. Confirmation of this hypothesis can be achieved by discontinuing the medication and observing whether the cough subsides. RESPONSE: The most appropriate next step in the management of this patient's cough would be (D) Discontinuation of lisinopril. If her cough subsides after discontinuation, it would serve to confirms that the ACE inhibitor was indeed the culprit. An alternative antihypertensive medication that doesn't have this side effect, such as a calcium channel blocker or an angiotensin II receptor blocker (ARB), could then be prescribed.

**AI_E:** 1) RESTATING THE QUESTION: We are presented with an adult female patient who was diagnosed with hypertension three weeks ago. She was started on lisinopril, an angiotensin-converting enzyme (ACE) inhibitor used to lower blood pressure. Recently, the woman has developed a persistent, nonproductive cough, which began two weeks following the initiation of lisinopril therapy. She has no history of serious illness, doesn't smoke, enjoys moderate alcohol consumption, and exercises regularly. Her hypertension is under good control, with her blood pressure dropping from 164/92 mm Hg to 140/84 mm Hg. Despite the cough, lung examination is normal. We are asked to identify the next appropriate step in managing her cough given the options of pulmonary function tests, chest CT scan, prednisone course, discontinuation of lisinopril, a cough suppressant, or no change in management. 2) PLANNING: The patient's cough could logically be attributed to several possible causes. Being a non-smoker, less probability can be given to lung pathology like COPD or lung cancer. In addition, her clear lung examination further argues against these and similar pulmonary illnesses. Therefore, a pulmonary function test (A) or a chest CT scan (B) might not be immediately necessary. The patient is not described as having any symptoms of allergy or inflammation that would warrant a course of prednisone (C), a corticosteroid. The use of a cough suppressant (E) would only temporarily mitigate the symptom without addressing the underlying cause. With no other apparent causes given, the question remains whether the cough could be a side effect of her recent medical management which primarily included the initiation of lisinopril for her hypertension. Lisinopril is an ACE inhibitor which are known to potentially induce a cough as a side effect in some patients. No change in management is indicated at this time (F) seems inappropriate given the onset and persistency of her symptoms. 3) CLINICAL REASONING: The cough began a week after beginning lisinopril therapy. Lisinopril is an ACE inhibitor, a class of medication known to cause a persistent, dry cough as a common side effect in some patients due to increase in bradykinin levels. This makes it a plausible cause of this patient's symptoms. Because it occurs as a side effect, it would persist as long as the patient continued to take the medication, supporting the time course described. While the drug has successfully lowered the patient's blood pressure as intended, an alternative antihypertensive medication may need to be considered if the cough is negatively impacting her quality of life. 4) CONCLUDING: Given the timing of the onset of her cough, which coincides with the start of her lisinopril therapy, the nature of her cough, which is consistent with this common side effect of ACE inhibitors, and the absence of any other potential causes, it seems plausible that the most appropriate next step would be to address the potential source of her cough, the lisinopril. 5) RESPONSE: So, given the evidence, the most appropriate next step in the management of this patient should be (D) Discontinuation of lisinopril. This is because the cough is a common side effect of ACE inhibitor like lisinopril, and stopping the drug may alleviate her symptom. Her hypertension control will need to continue, of course, but an alternative antihypertensive could be used to replace the lisinopril.

**Facilitator** The consensus among the AI responses is that the most appropriate next step in managing the 52-year-old woman's persistent, nonproductive cough is to discontinue her lisinopril treatment. This conclusion is based on the following reasoning: 1. The patient's cough began after the initiation of lisinopril therapy for her hypertension. Lisinopril is an angiotensin-converting enzyme (ACE) inhibitor, a class of medication known to cause a persistent, dry cough as a common side effect in some patients due to an increase in bradykinin levels. This makes it a plausible cause of this patient's symptoms. 2. The patient's clear lung examination and healthy lifestyle habits, including non-smoking and regular exercise, reduce the likelihood of a pulmonary disease being the cause of her cough. Therefore, pulmonary function tests or a chest CT scan might not be immediately necessary. 3. The patient is not described as having any symptoms of allergy or inflammation that would warrant a course of prednisone, a corticosteroid. The use of a cough suppressant would only temporarily mitigate the symptom without addressing the underlying cause. 4. The patient's hypertension has been well controlled with lisinopril, but if the cough is negatively impacting her quality of life, an alternative antihypertensive medication may need to be considered. Therefore, the consensus is that the most appropriate next step in the management of this patient's cough is the discontinuation of lisinopril. An alternative antihypertensive medication that doesn't have this side effect, such as a calcium channel blocker or an angiotensin II receptor blocker (ARB), could then be prescribed.
