# Supplementary material for: Collaborative intelligence in AI: Evaluating the performance of a council of AIs on the USMLE
Source: PLOS Digit Health. 2025 Oct 9;4(10):e0000787. doi: 10.1371/journal.pdig.0000787 (PMC12510544; doi:10.1371/journal.pdig.0000787)
Supplement: S2 File — (ZIP) [file pdig.0000787.s002.zip › S2 Data Raw data files containing unprocessed question inputs and LLM outputs including transcripts of deliberations/STEP2_Question-74-1_Type-MC-NJ.docx]

**Facilitator** **[Round 1]:** A 27-year-old primigravid woman at 41 weeks' gestation is admitted to the hospital for induction of labor. She has received cervical ripening agents and is now receiving oxytocin. The cervix is 9 cm dilated and 100% effaced; the vertex is at 0 station. Fetal monitoring shows contractions every 3 minutes and a reassuring heart rate. She requests pain medication. Which of the following is the most likely adverse effect of an intravenous opioid in this patient? (A) Maternal fever (B) Maternal tachycardia (C) Neonatal acidosis (D) Neonatal arrhythmia (E) Neonatal respiratory depression

**AI_E:** RESTATING THE QUESTION: To rephrase the question: what is the most likely complication a 27-year-old first-time mother may experience if given intravenous opioid pain relief during labor? The options provided include maternal fever, maternal tachycardia, neonatal acidosis, neonatal arrhythmia, or neonatal respiratory depression. PLANNING: In such scenarios, we should remember that opioids as analgesics during labor can cross the placenta and consequently affect the newborn. The assumptions could be as follows: (1) opioids can cause maternal fever, (2) opioids can cause maternal tachycardia, (3) Opioids can lead to neonatal acidosis. (4) opioids can lead to neonatal arrhythmia (5) opioids can lead to neonatal respiratory depression. After analyzing these assumptions, the most critical is the potential for opioids to cause neonatal respiratory depression, given their well-documented effects on the respiratory centers of the brain. CLINICAL REASONING: Opioids have been shown to decrease respiratory drive, leading to hypoventilation, hypercarbia, and eventually respiratory depression. This is especially true in neonates, whose respiratory centers are still developing and who may not be able to compensate as effectively for the suppressive effects of opioids. CONCLUDING: After completing the clinical thought process, it is clear that while opioids could theoretically have several impacts on the mother and baby, the most critical potential complication is neonatal respiratory depression. This is due to the opioid's ability to cross the placenta, its known effects on the respiratory centers of the brain, and the neonate's limited ability to compensate for these effects. RESPONSE: Therefore, considering the ability of opioids to suppress respiratory drive in neonates, the most likely adverse effect of administering an intravenous opioid in this patient would be 'Neonatal respiratory depression' (E).

**AI_B:** (1) RESTATING THE QUESTION: The clinical scenario outlined here refers to a 27-year-old woman who is having her first child and is currently in labor at 41 weeks of gestation. She is receiving labor induction treatment, including cervical ripening agents and oxytocin. The labor progress is indicated by 9 cm cervical dilation, 100% effacement, and the vertex at 0 station. The woman now desires pain relief in the form of intravenous opioids. The question is asking about the primary potential adverse effect of such opioid administration in this case. (2) PLANNING: I'll now try to identify possible assumptions based on my knowledge: opioids can induce various adverse effects, including fever and tachycardia in the mother, and acidosis, arrhythmia, or respiratory depression in the neonate. A primary assumption could be that the effect will manifest in the mother since she is the one receiving the opioid. Conversely, the alternate assumption is that the neonate might be affected, given opioids can cross the placenta. Given the clinical context, this second assumption relating to neonatal consequences might be more significant to consider. (3) CLINICAL REASONING: While maternal side-effects such as fever or tachycardia are possible with opioid use, they are less common and less significant compared to potential neonatal risks. Opioids, including those used for pain relief during labor, can cross the placental barrier, exposing the fetus to the drug. This exposure can indeed lead to various neonatal effects, including neonatal acidosis from prolonged labor, neonatal arrhythmia, or neonatal respiratory depression, which is a more common and more immediate clinical concern related to the opioid's action affecting the respiratory centers in the neonate's brainstem. (4) CONCLUDING: Among the given options and based on the comprehensive analysis offered above, both maternal and neonatal potential adverse effects can occur upon intravenous opioid administration for labor pain management in the specific case presented. (5) RESPONSE: However, the most likely significant adverse effect is '(E) Neonatal respiratory depression'. This is primarily due to opioids crossing the placental barrier and acting on the neonate's central nervous system, leading to the temporary suppression of the respiratory centers within their brainstem.

**AI_A:** Restating the Question: This woman nearing childbirth is being admitted to a hospital for labor induction. Her cervix is almost completely dilated and effaced, indicating that delivery is imminent. Despite being under the influence of cervical ripening agents and oxytocic medication, the woman is experiencing pain and requests medication for relief. Given this, we need to determine what the most probable adverse effect might be from an opioid given intravenously to this patient. The options we have are: maternal fever, maternal tachycardia, neonatal acidosis, neonatal arrhythmia, or neonatal respiratory depression. Planning: The inherent assumption here is that considering the use of opioids during labor can have multiple effects, some of which might be harmful either to the mother or the newborn. For the mother, these include an elevated body temperature (fever) or increased heart rate (tachycardia). For the neonate, effects might include a disrupted acid/base balance in the body (acidosis), irregular heartbeat patterns (arrhythmia), or suppressed breathing (respiratory depression). To choose the best course, we need to thoroughly understand and assess the risks and benefits of these options, keeping in mind that the patient's safety and comfort, as well as the baby's safety, is of paramount importance. Clinical Reasoning: Opioids are known to act on the central nervous system, suppressing the pain signals. However, they also suppress other autonomic functions like respiration which may lead to respiratory depression. Applied to our situation, we realize that the fetus, intrauterine, is dependent on maternal oxygenation for its own oxygen supply. If an opioid causes maternal respiratory depression, the direct consequence in the fetus would be hypoxia, potentially causing acidosis and depressed neonatal respiratory efforts after delivery. Concluding: Overall, while administering an opioid might provide the requested relief from pain, it could potentially result in neonatal respiratory depression. Even though risks like maternal fever, maternal tachycardia, neonatal acidosis, and neonatal arrhythmia are feasible, considering the autonomous depressant aspects of opioids, the danger of neonatal respiratory depression should be a primary clinical concern. Response: Therefore, in this clinical scenario, the most probable adverse effect of an intravenous opioid in this patient is (E) Neonatal respiratory depression.

**AI_D:** (1) RESTATING THE QUESTION: The presented scenario is about a 27-year-old woman who is in labor. She's past the usual term of pregnancy (41 weeks) and is receiving medication to stimulate labor. In terms of labor progression, she's heavily dilated, and the baby's position suggests labor is well-advanced. The baby's condition is stable, as indicated by fetal monitoring. She's asking for pain relief, and the question being asked is: what is the most likely harmful effect of providing intravenous pain relief via an opioid on this patient? Opioids are a class of drugs often used in pain management, although with potential side effects. Intravenous (IV) opioids in this scenario can potentially impact both the mother and the newborn. (2) PLANNING: Opioids are known to have certain side effects, such as fever, tachycardia, and respiratory depression. If given to a patient in labor, the drug can cross the placenta and affect the baby, potentially causing conditions such as acidosis and arrhythmia. In thinking about the most likely adverse effect, we should focus on the characteristics of opioids and their well-established side effects. Of the listed options, neonatal respiratory depression stands out as a well-known possible harmful effect. (3) CLINICAL REASONING: Opioids work by binding to opioid receptors in the brain and other parts of the body, they suppress the sensation of pain, but they also affect the brain's control of respiration. This action becomes particularly crucial in a neonate whose respiratory system is just beginning to function independently. During labor, administered drugs can cross the placenta and reach the fetus. When opioids enter the neonate's system, they can cause a decrease in the respiratory drive, leading to respiratory depression. This is especially concerning due to the immaturity of a neonate's respiratory center, such that it may not compensate quickly enough to overcome the depressant effect of the opioid. (4) CONCLUDING: Given the nature of opioids, the phase of the pregnancy, and the fact that opioids can cross the placenta, it seems plausible to expect that neonatal respiratory depression is the most likely adverse effect of using an intravenous opioid for managing labor pain in the described patient. (5) RESPONSE: Therefore, in response to the question, the most likely adverse effect of an intravenous opioid in this patient is (E) Neonatal respiratory depression.

**AI_C:** (1) RESTATING THE QUESTION: We are asked what the most probable negative consequence of administering an intravenous opioid to a 27-year-old woman in labor would be. The patient is in her first pregnancy, currently at 41 weeks, and is in the late stages of labor with her cervix being 9 cm dilated and 100% effaced. Her child's head (vertex) is at the starting position for birth (0 station). Labor has been induced with cervical ripening agents and oxytocin, and contractions are occurring every three minutes with fetal monitoring showing a normal heart rate. (2) PLANNING: To answer this question, we would need to consider the effects of intravenous opioids on both the mother and the newborn. Opioids can have various effects, such as inducing fever or tachycardia in the mother, or causing acidosis, arrhythmia, or respiratory depression in the newborn. Looking at the alternatives, we can eliminate Maternal fever (A) and Maternal tachycardia (B) as they are less common opioids side effects. Between Neonatal acidosis (C), Neonatal arrhythmia (D), and Neonatal respiratory depression (E), neonatal respiratory depression stands out because opioids affect the central nervous system, which includes the respiratory center in the brain. (3) CLINICAL REASONING: Opioids work by binding to and activating opioid receptors in the brain, spinal cord, and other organs in the body. Once activated, these receptors can reduce the perception of pain. However, one of the areas in the brain that opioids affect is the medulla, which controls the body's respiratory rate. Thus, opioids can slow breathing. This effect can be amplified in a neonate, whose respiratory system might not have fully matured yet. Therefore, considering the options, opioids would more likely cause Neonatal respiratory depression (E) than the other options provided, as the baby's respiratory system may not be able to combat the effects of the opioids as effectively as the mother's. (4) CONCLUDING: Intravenous opioids can depress the central nervous system, including the respiratory center in the brain. While opioids can cause other adverse effects, in neonates, the effect on the respiratory system can be particularly dangerous due to the system's immaturity. (5) RESPONSE: Thus, among the choices provided, it is most likely that the administration of an intravenous opioid in a laboring patient would lead to Neonatal respiratory depression (E).

**Facilitator** The consensus statement synthesizes the responses as follows: The question at hand pertains to a 27-year-old woman in her first pregnancy, who is at 41 weeks' gestation and is in the advanced stages of labor. She has been administered cervical ripening agents and oxytocin to induce labor. Her cervix is 9 cm dilated and 100% effaced, indicating that delivery is imminent. Despite these interventions, she is experiencing pain and requests medication for relief. The question asks us to identify the most probable adverse effect of administering an intravenous opioid to this patient. The options provided include maternal fever, maternal tachycardia, neonatal acidosis, neonatal arrhythmia, and neonatal respiratory depression. Opioids are known to have various effects on both the mother and the newborn. In the mother, these can include fever or tachycardia, while in the newborn, they can cause acidosis, arrhythmia, or respiratory depression. However, the most significant concern is the potential for opioids to cause neonatal respiratory depression. This is due to the fact that opioids can cross the placenta and affect the newborn. Opioids work by binding to and activating opioid receptors in the brain, spinal cord, and other organs in the body, reducing the perception of pain. However, they also affect the medulla, which controls the body's respiratory rate, potentially slowing breathing. This effect can be particularly pronounced in a neonate, whose respiratory system might not have fully matured yet. Therefore, while opioids can cause other adverse effects, in neonates, the effect on the respiratory system can be particularly dangerous due to the system's immaturity. Given the nature of opioids, the phase of the pregnancy, and the fact that opioids can cross the placenta, it seems plausible to expect that neonatal respiratory depression is the most likely adverse effect of using an intravenous opioid for managing labor pain in the described patient. Thus, among the choices provided, it is most likely that the administration of an intravenous opioid in a laboring patient would lead to Neonatal respiratory depression (E).
