# Supplementary material for: Collaborative intelligence in AI: Evaluating the performance of a council of AIs on the USMLE
Source: PLOS Digit Health. 2025 Oct 9;4(10):e0000787. doi: 10.1371/journal.pdig.0000787 (PMC12510544; doi:10.1371/journal.pdig.0000787)
Supplement: S2 File — (ZIP) [file pdig.0000787.s002.zip › S2 Data Raw data files containing unprocessed question inputs and LLM outputs including transcripts of deliberations/STEP2_Question-75-1_Type-MC-NJ.docx]

**Facilitator** **[Round 1]:** A 7-year-old girl is brought to the clinic by her father because of behavior problems at school since she started second grade 6 months ago. Her teacher is concerned because she cannot sit still or complete tasks and is not learning at the same pace as her classmates. Psychoeducational testing shows normal intelligence. The father says his daughter’s behavior problems have worsened since last year. She snores at night and wets the bed several nights weekly. She has no history of serious illness and receives no medications. She has been at the 25th percentile for height and weight for the past 3 years. Pulse is 105/min and regular, respirations are 22/min, and blood pressure is 105/65 mm Hg. The tonsils are 3+. Cardiopulmonary and abdominal examinations show no abnormalities. On neurologic examination, cranial nerves are intact. Deep tendon reflexes are 2+ in all extremities. Finger-nose testing shows no abnormalities. Romberg sign is absent. Her gait is normal. Which of the following is the most appropriate next step in diagnosis? (A) EEG (B) Karyotype analysis (C) MRI of the brain (D) Polysomnography (E) Thyroid function tests

**AI_D:** RESTATING THE QUESTION: The question asks which diagnostic test should be the next step for a 7-year-old girl who is showing signs of behavioral issues and academic struggles at school, along with snoring, bedwetting, and large tonsils, but has normal neurological tests and normal intelligence. Here, EEG refers to electroencephalography - a test used to find abnormalities related to electrical activity of the brain. A karyotype analysis studies an individual's chromosome structure which can help diagnose genetic diseases. MRI of the brain gives detailed images of distinct structures within the brain, helping to diagnose abnormalities or diseases. Polysomnography is a sleep study that evaluates for sleep disorders. Thyroid function tests evaluate the function of the thyroid gland, which regulates metabolism. PLANNING: The symptoms described, including the inability to concentrate, restlessness, poor school performance, snoring, and bedwetting, along with the physical finding of enlarged tonsils may suggest the possibility of a sleep disorder specifically Obstructive Sleep Apnea (OSA). Alternate assumptions can be behavioral or neurological disorders, or even metabolic conditions. However, further neurologic disorders are less likely due to the normal intelligence and normal neurological examination findings in the child. Similarly, metabolic conditions like hypothyroidism (which could result in poor school performance and growth retardation) are less probable due to the lack of specific symptoms and physical examination findings leaning towards those conditions. CLINICAL REASONING: The 3+ tonsils can obstruct the upper airway during sleep, leading to apneic episodes. This would disrupt the child's sleep cycle, resulting in daytime sleepiness, inattentiveness, hyperactivity, and poor academic performance, which are key symptoms of attention-deficit/hyperactivity disorder (ADHD). Furthermore, OSA can result in enuresis (bedwetting), likely due to increased nocturnal urine production resulting from the secretion of atrial natriuretic peptide secondary to the increased intrathoracic pressure that occurs with apneic episodes. CONCLUDING: Given the significant findings of enlarged tonsils, along with the constellation of symptoms consistent with possible OSA, this condition should be prioritized and examined further due to its potential to adversely affect the child's behavior, sleep, and schooling. RESPONSE: Therefore, a Polysomnography (Choice D) is the most appropriate next step in the diagnosis. It is the gold standard for diagnosing Obstructive Sleep Apnea. The other tests may not identify or rule out this highly probable condition based on her symptoms.

**AI_C:** (1) RESTATING THE QUESTION: A 7-year-old girl, who has been demonstrating increased behavior problems and difficulty with task completion at school, is being assessed to identify possible causes. She is also noted to snore at night and have episodes of bedwetting. She is of normal intelligence, has no major health issues, and is not on any medication. Her growth pattern is consistent, falling within the 25th percentile for height and weight. Routine clinical examination has not shown any significant findings, and her neurologic examination is also normal. The question is asking for the most suitable method to further investigate her condition from five given options. (2) PLANNING: The key assumptions here are that the girl's problems could be linked to a neurological or behavioral condition, genetic abnormalities, a structural brain problem, a sleep disorder, or a thyroid condition. We can question these assumptions individually: Does her behavior imply any central nervous system disorder? Is her snoring indicative of sleep-disordered breathing that could be affecting her behavior and school performance? Bridging the assumptions, we can hypothesize that her issues are related to a sleep disorder, given that she snores and wets the bed several nights a week. (3) CLINICAL REASONING: The absence of abnormal findings in the physical examination (including neurological testing) makes it less likely that the girl's issues are due to a severe neurological issue or genetic abnormalities. Therefore, running an EEG or karyotyping may not yield much insight. An MRI of the brain is typically done when there are signs of neurological abnormalities, which are absent in this case, hence making it a less priority option. Though she has normal height and weight percentiles, it wouldn't hurt to check thyroid function, but given that her primary complaint is behavioral, they are of less relevance for now. Her reported symptoms of snoring and bedwetting are indicative of a possible sleep disorder, which could cause daytime behavior problems in children due to sleep deprivation. These symptoms, combined with her enlarged tonsils (3+), point towards obstructive sleep apnea (OSA), a condition where the upper airway becomes blocked during sleep, leading to interrupted breathing and consequently poor sleep quality. (4) CONCLUDING: Given the sleep related issues depicted in her history, and the likelihood that they could be affecting her daytime behavior, identifying and then addressing any sleep disorders might be a critical first step in her diagnosis and management. (5) RESPONSE: Thus, the most appropriate next step in the diagnosis for the young girl would be (D) Polysomnography. This diagnostic tool could help in detecting obstructive sleep apnea or another sleep disorder, which may be the underlying cause of her behavioral problems.

**AI_A:** RESTATING THE QUESTION: The patient, a 7-year-old girl with behavior problems at school, inability to sit still, conducting tasks, and learning at a slower rate, has normal intelligence based on psychoeducational testing. Her behavioral issues escalated over the past year. She exhibits snoring and bedwetting several times weekly. Despite lacking any serious illness history, she has remained at the 25th percentile for height and weight for the past three years. She has a pulse of 105/min and regular, a respirations of 22/min, a blood pressure of 105/65 mm Hg, and her tonsils are 3+. Cardio, pulmonary, abdominal, and neurological examinations were normal. The question is asking for the best subsequent procedure for diagnosis given these symptoms. PLANNING: To evaluate the patient's symptoms and step up a diagnosis based on her conditions, one must weigh the assumptions made surrounding the symptoms. One assumption could be that the behavior problems and the inability to focus can be attributed to an attention disorder. Another assumption could be that the bedwetting is a symptom of anxiety caused by her challenging experiences at school. A third consideration might involve the girl's increased tonsil size, which might be obstructing her airway leading to snoring, and could also potentially explain the bedwetting, arising from sleep apnea or sleep disturbances. The consideration of the snoring, bedwetting, and problems at school seem to correlate with each other considering the history of the girl. CLINICAL REASONING: Following the logic stated above, it would be important to ascertain whether there's an obstruction of the airway which leads to sleep disturbances. Considering the girl's symptoms in correspondence with the enlargement of her tonsils, we could reason towards the direction of sleep apnea. The disturbed sleep due to sleep apnea could catalyze cognitive difficulties, hence the problems at school. The correlation between the symptoms gives us a clear path to follow, one that can be addressed by a specific test. CONCLUDING: In lieu of the previous analysis, the ideal following step of the diagnosis would tilt towards a means of assessing the girl's sleep. This mode of arriving to a conclusion has not attributed a direct link to the other initial assumptions like attention disorders or anxiety disorders, rather it places emphasis on a series of interconnected problems that dar needs a targeted line of action. RESPONSE: The most appropriate next step in the diagnosis would be to conduct a polysomnography (D). This would offer the most valuable information pertaining to the possibility of sleep apnea, which could explain the range of symptoms the girl is experiencing.

**AI_B:** RESTATING THE QUESTION: We're discussing a 7-year-old girl presenting with behavioral issues, sluggish learning progress, excessive snoring, bed-wetting, and normal intelligence. Physical examination suggests enlarged tonsils without evident neurological, cardiopulmonary, or abdominal anomalies. She is on the smaller side (25th percentile), but consistently so over the past years. Possible diagnostic evaluations include an electroencephalography (EEG); a karyotype analysis to look at her chromosomes; a magnetic resonance imaging (MRI) scan of the brain; a polysomnography, which is a sleep study that records brain waves, oxygen levels, heart rate, and eye and leg movement; or thyroid function tests to assess her thyroid hormone levels. PLANNING: The initial assumption from these symptoms could be symptoms related to neurologic, genetic, endocrine, or sleep disorders. However, since she has normal intelligence, no abnormalities in the cranial nerves, reflexes, gait, Romberg sign, or fingertip-nose test, the neurologic assumption (hence EEG and MRI) seems less credible. Looking at her consistent stature percentile, an endocrine issue (prompting for thyroid function tests) becomes less probable. There's no supportive evidence presented to direct towards a genetic disorder (karyotype analysis), particularly when she has normal intelligence and no manifest phenotypic abnormalities. Also, her behavioral problems have been escalating over the past year and correlating with her start of grade 2. The predominant alternate assumption to investigate is whether these issues are instigated due to poor sleep, emanating from sleep-disordered breathing considering the presence of enlarged tonsils, nocturnal snoring, and enuresis (bed-wetting). CLINICAL REASONING: Enlarged tonsils can obstruct airways, especially in a recumbent position during sleep. This sleep-disordered breathing can cause restless (or poor-quality) sleep, which explains her inability to sit still, complete tasks, and slower learning progress at school. Also, it commonly presents with secondary enuresis through mechanisms relating to increased urine production due to increased venous return induced by ineffective respiration or awakenings related to apneas. Constant poor sleep quality (sleep deprivation) is related to cognitive and behavior problems, attention deficits, and consequently, academic issues. CONCLUDING: The girl's clinical presentation suggests the possibility of sleep-disordered breathing caused by enlarged tonsils. The absence of neurological abnormalities on examination and normal intelligence diminishes the probability of neurological disorder, and her constant growth percentiles do not favor an endocrine problem. So, the most appropriate diagnostic test would be one that can confirm or rule out a sleep disorder. RESPONSE: So, based on the discussion above, I would suggest the most appropriate next step in this 7-year-old girl's diagnosis would be (D) Polysomnography, which will assess her brain waves, heart rate, respiratory effort, oxygen saturation, and eye and leg movements as she sleeps, and confirm if sleep-disordered breathing is the underlying cause for her behavioral issues and academic struggles.

**AI_E:** RESTATING THE QUESTION: The question provides a case of a 7-year-old girl who presents with behavior problems at school including inability to sit still or complete tasks. These issues have worsened over the past year and are now accompanied by snoring at night and bedwetting multiple times a week. Despite these symptoms, psychoeducational testing shows normal intelligence and she has no history of serious illness or medication usage. Her physical examination is relatively normal apart from enlarged tonsils (3+). The question asks what should be the next step in diagnosing her condition. The options include conducting an EEG, carrying out a karyotype analysis, performing an MRI of the brain, undertaking a polysomnography, or testing thyroid function. PLANNING: In this step, several assumptions could be made from the clinical presentation. The girl could have a neurologic condition that affects behavior, suggested by behavioral problems at school and normal intelligence. The snoring at night could indicate a sleep disorder, possibly because of the enlarged tonsils obstructing the airway. The symptom of bedwetting could suggest either a systemic condition such as diabetes insipidus, a urinary tract disorder, stress or potentially an obstructive sleep apnea (OSA) affecting her sleep pattern. Considering these assumptions, I will hypothesize that a sleep disorder such as OSA might be the underlying problem given the presentation of snoring and enlarged tonsils. CLINICAL REASONING: OSA is a condition characterized by episodes of complete or partial airway obstruction during sleep, leading to disruptive sleep patterns. It may present in children as learning problems, hyperactive behavior, and nocturnal enuresis (bedwetting), among other symptoms. The girl's behavior problems at school (hyperactivity, inability to sit still or complete tasks), normal intelligence, and enlarged tonsils point towards this condition. If OSA is indeed the cause, the intermittent hypoxia that occurs during sleep may result in neural deficits causing behavioral problems. The snoring at night and wetting the bed several nights weekly further support this. In children, the most common cause of OSA is chronic adenotonsillar hypertrophy, with the girl's exam showing enlarged tonsils. Therefore, this warrants further investigation with an overnight sleep study to determine the presence, severity, and effects of OSA. CONCLUDING: The symptoms presentation, especially the nocturnal symptoms of snoring and bedwetting, coupled with behavioral issues at school point towards the possibility of OSA. Since the most common cause of OSA in children is chronic adenotonsillar hypertrophy, and looking at the enlarged tonsils from the physical exam, the most clinically prioritized step should be to investigate OSA. RESPONSE: In considering the girl's symptoms, the most appropriate next step to take in this diagnostic process would be (D) Polysomnography. This is the most definitive test for diagnosing OSA, which seems to correlate with this girl's symptoms. It monitors various body functions including brain (EEG), eye movements, muscle activity or skeletal muscle activation, and heart rhythm during sleep, and can detect episodes of apnoea and hypopnoea.

**Facilitator** The consensus among the AI responses is that the most appropriate next step in diagnosing the 7-year-old girl's condition would be to conduct a polysomnography (Option D). This conclusion is based on the girl's symptoms, which include behavioral problems at school, snoring, bedwetting, and enlarged tonsils. These symptoms suggest the possibility of a sleep disorder, specifically Obstructive Sleep Apnea (OSA), which could be causing her behavioral issues and academic struggles. OSA is characterized by episodes of complete or partial airway obstruction during sleep, often due to enlarged tonsils in children, leading to disruptive sleep patterns. This can result in daytime symptoms such as hyperactivity, inability to sit still, and learning difficulties, which align with the girl's presentation. Polysomnography, a sleep study that records brain waves, oxygen levels, heart rate, and eye and leg movement, is the gold standard for diagnosing OSA. The other diagnostic options, including EEG, karyotype analysis, MRI of the brain, and thyroid function tests, are considered less likely to yield useful information given the girl's specific symptoms and normal intelligence.
