# Supplementary material for: Collaborative intelligence in AI: Evaluating the performance of a council of AIs on the USMLE
Source: PLOS Digit Health. 2025 Oct 9;4(10):e0000787. doi: 10.1371/journal.pdig.0000787 (PMC12510544; doi:10.1371/journal.pdig.0000787)
Supplement: S2 File — (ZIP) [file pdig.0000787.s002.zip › S2 Data Raw data files containing unprocessed question inputs and LLM outputs including transcripts of deliberations/STEP2_Question-77-1_Type-MC-NJ.docx]

**Facilitator** **[Round 1]:** An 8-year-old boy is brought to the emergency department (ED) by ambulance 45 minutes after he was struck by a car while riding his bicycle. He did not lose consciousness but has multiple rib fractures with depression of the left anterior chest wall. His parents have been notified and are on their way to the hospital. On the patient's arrival to the ED, his temperature is 37.2°C (99.0°F), pulse is 180/min, respirations are 36/min, and blood pressure is 65/30 mm Hg. The abdomen is distended with exquisite tenderness to palpation. A rapid intravenous infusion of 0.9% saline is begun. CT scans are quickly obtained and show a disrupted spleen and an abdominal cavity filled with free fluid. The parents arrive at the hospital and are informed that their son needs an emergency laparotomy and splenectomy to control the internal bleeding. A saline infusion is continued to support the patient's blood pressure. The parents provide consent to proceed with the operation but refuse to consent to transfusion of blood or blood products, regardless of consequences, because of their religious beliefs. As the patient is transferred to the operating room, his pulse is 210/min and blood pressure is 50/20 mm Hg. Hemoglobin concentration is 3.8 g/dL. The surgeon agrees with the anesthesiologist that the patient will die without transfusion of blood and plasma. Which of the following is the most appropriate action? (A) Contact the national organization of the parents’ religion and insist that the parents speak with a representative on the phone regarding the matter (B) Contact the parents’ religious advisor to request help in acquiring consent for blood transfusion (C) Proceed with the blood transfusion while obtaining an emergency telephone court order (D) Proceed with the operation and abide by the parents’ wishes that no blood products be used (E) Transfer the patient by ambulance immediately to a bloodless surgical center

**AI_A:** To summarize the scenario, an 8-year-old boy was severely harmed in a vehicular accident and has sustained multiple injuries, including a damaged spleen leading to significant internal bleeding. Despite urgent need for a blood transfusion due to his critical state, the parents, while giving consent for surgery, have refused the usage of blood or blood products based on religious grounds. The medical staff, however, are of the opinion that without a transfusion, the boy's life is in grave danger. From this situation, a number of considerations come to mind, primarily the child's critical health status and potential for recovery, the parents' religious beliefs, the capacity (and responsibility) of healthcare professionals to save lives, and the role of law and ethics in decision-making. Variations like involving representative of religion, getting help from religious advisor, securing an emergency court order for the transfusion, performing surgery without blood products, or transferring the patient to a hospital specialized in bloodless surgery could be explored, but the prevailing priority must be towards the emergent need to save the boy's life. Looking at it from a clinical perspective, the boy has sustained significant injuries. His vital signs are highly concerning with a high pulse rate, low blood pressure, and severe decrease in hemoglobin concentration suggesting significant blood loss and shock. This coupled with evidence of a ruptured spleen and free fluid in his abdomen on the CT scan implies that immediate corrective action, including a blood transfusion, is vital to save his life. In conclusion, while respect for the parents' religious beliefs is crucial, the urgency of the situation commands an immediate life-saving decision. Therefore, the need to save the boy's life should take precedence over other considerations. Thus, proceeding with the blood transfraction while obtaining an emergency telephone court order (Option C) appears to be the most appropriate and ethically sound action. This approach respects the parents' religious beliefs but recognizes the healthcare professionals' duty to preserve life, consequently seeking legal authority to proceed with a necessary intervention.

**AI_D:** RESTATING THE QUESTION: The case pertains to an 8-year-old boy who was severely injured in a car accident. Medical examination and CT scans revealed multiple rib fractures, a disrupted spleen, and an abdominal cavity filled with free fluid, suggesting internal bleeding. A laparotomy and splenectomy have been recommended to control the bleeding. However, the parents, asserting their religious beliefs, have refused to provide consent for blood transfusion, which the medical team deems critical. The clinical scenario asks for the most appropriate action given the circumstances, by either contacting relevant religious authorities, proceeding with blood transfusion while requesting an emergency court order, meeting the parents' wishes of not using any blood products, or transferring the patient to a bloodless surgical center. PLANNING: The presented case brings up important ethical and medical dilemmas involving parents' religious beliefs and a child's health. The assumption here is about the acceptance of blood transfusion by the parents, which they are currently denying based on their religious beliefs. An alternative assumption could be the legal and ethical responsibility of the healthcare providers to prioritize the child's best interests and immediate health needs over parental consent. This assumption must be critically examined and prioritized in light of the child's deteriorating health. CLINICAL REASONING: Medical ethics encompasses autonomy, beneficence, non-maleficence, justice, and in a pediatric context, best interests for the child. Autonomy signifies the right of patients or guardians to make informed decisions, yet a child's life being at risk due to parental refusal of potentially life-saving treatment challenges this. When imminent risk of death is present, physicians may intervene without consent. Given the parents' refusal to consent to transfusion, these ethical princples conflict. Interpreting the urgency and severity of the case, it would be in the child's best interest for healthcare providers to intervene, maximizing beneficence and minimizing non-maleficence. CONCLUDING: Based on the clinical reasoning presented, considering the acute, life-threatening conditions, it would be most appropriate to prioritize the child's best interests. This involves a rapid decision-making process that emphasizes life preservation and minimizes harm, effectively negating the refusal of blood transfusion by the parents. RESPONSE: Therefore, the most appropriate action is (C) Proceed with the blood transfusion while obtaining an emergency telephone court order. This response prioritizes the child's life and ensures immediate care, while also respecting the legal process and the conflict between medical intervention and the parents' religious beliefs.

**AI_E:** RESTATING THE QUESTION: An 8-year-old boy has been involved in a serious bike accident, sustaining injuries including multiple rib fractures and spleen disruption. His urgent condition necessitates a life-saving operation that customarily requires blood transfusion; however, his parents refuse any blood products because of their religious beliefs. The health care team must now decide on the best course of action, which potentially involves: contacting the parents' religious organization or adviser (A and B), obtaining a court order to proceed with blood transfusion (C), respecting the parents' preferences and not transfuse blood (D), or transfer the patient to a bloodless surgery center (E). PLANNING: This scenario revolves around the ethics of medical practice, which involve the embodiment of principles of beneficence and respect for autonomy. Beneficence, in this case, would be the necessity of a blood transfusion for the child's survival. Autonomy is expressed through the parents' refusal of blood products based on religious beliefs. However, there is also the medical-legal principle that protects minors and dependent adults in life-threatening situations, known as parens patriae, which permits the state to advocate in the child’s best interests, even overriding parental wishes. CLINICAL REASONING: Given the severity of the child's injuries and his vital signs, there seems to be hypovolemic shock caused by internal bleeding, a condition that typically necessitates blood transfusion to replace lost circulating volume. Hypovolemic shock could lead to inadequate tissue perfusion and multi-organ failure, which are life-threatening. His hemoglobin level is critically low at 3.8 g/dL, which points to a significant blood loss and immediate replenishment necessity. This child's life-threatening situation needs an urgent decision, addressing both medical needs and ethical concerns. CONCLUDING: Therefore, given the severity of the situation and the urgency required, it would likely be most appropriate to act in line with the doctrine of parens patriae. Even though the parents refuse due to religious beliefs, the child's health and potential survival are more urgent and necessitate appropriate treatment. RESPONSE: The most appropriate action to take in this situation would be (C) Proceed with the blood transfusion while obtaining an emergency telephone court order. This approach acknowledges the parental refusal due to religious beliefs yet proceeds with actions necessary for the child's survival. In addition, it appropriately escalates the issue to legal authority to protect the medical team's decisions and child's best interest.

**AI_C:** RESTATING THE QUESTION: The predicament here revolves around the dilemma created between respecting the religious beliefs of the parents, that prevent the use of blood transfusion, and ensuring the best clinical care for a minor patient who has been involved in an accident. The patient is in a critical condition due to severe internal bleeding resulting from multiple injuries, including disrupted spleen, multiple rib fractures, and an abdominal cavity filled with fluid, causing a dangerously low blood pressure and hemoglobin concentration. The question seeks the most appropriate action to take in this situation under the given circumstances. PLANNING: This situation involves certain assumptions. Firstly, it's assumed that the medical team is obligated to respect the parents' religious beliefs to not use blood transfusion during the surgical procedures. Secondly, it is also assumed that the child could die without a blood transfusion. We need to question both these assumptions. For one, is it appropriate to prioritize the parents' beliefs over the child's life? Also, is there possibly an alternative to blood transfusion that might ensure the child's survival? In terms of clinical priority, the immediate goal is to ensure the child's survival. Once his condition is stabilized, other long-term goals can be pursued. CLINICAL REASONING: The parents have the legal right to give or withhold consent for most medical treatments for their children, but their refusal might place the child at substantial risk, even death in this case. This crisis happens when the obligation to safeguard a child's health overrides the right to parental autonomy. Ethically speaking, for minor patients in life-threatening situations, providers need to seek protective custody and a court order permitting lifesaving treatment despite parental objections. Moreover, other options like contacting the national organization of the parents’ religion or their religious advisor for consent might delay prompt medical care, which is not advisable in such an emergent situation. CONCLUDING: Thus, considering the immediate need to save the child's life, it would be justifiable to override parental refusal of treatment when a life-threatening situation exists. From my reasoning, getting an emergency court order to proceed with the transfusion seems to be the most appropriate course, as it ensures the child's survival while also dealing with the legal aspects involved in overriding parental authority. RESPONSE: Based on the gravity of the situation and prioritizing the life of the minor patient, the most appropriate action would be to '(C) Proceed with the blood transfusion while obtaining an emergency telephone court order'. This option takes into cognizance the urgency, legality, and medical necessity of the situation.

**AI_B:** RESTATING THE QUESTION: Essentially, the question presents a serious ethical and legal dilemma: a critically injured child is in urgent need of a blood transfusion in addition to surgery after a severe accident. His condition is rapidly deteriorating, and medical professionals believe he will die without a blood transfusion. However, the parents have refused to consent to the use of blood or blood products due to their religious beliefs. The question asks us to identify the most suitable course of action from the given options. PLANNING: This question hinges on several assumptions. Firstly, it is assumed that the parents' religious objections overrule the immediate medical needs of the child. Secondly, the question is presuming that the parents cannot be persuaded or overruled in this critical situation. Another assumption is that the health professionals are not conducting any response that would violate the parents' rights or beliefs. The alternate assumption could be that the child's right to life and survival may supersede the parents' religious objections in such a critical situation. Among these assumptions, it should be clinically prioritized to determine whether the patient's right to life should take precedence over the parents' religious objections. CLINICAL REASONING: As medical professionals, we must strive to respect patients and their families' wishes, especially concerning religious beliefs. However, when it involves life-threatening situations, particularly in children, it is crucial to balance these rights against the child's right to life and health. Despite the parents' refusal, it could be ethically and legally justified to provide the necessary treatment, including a blood transfaction, to save the young patient's life. With the patient's hemoglobin at 3.8 g/dL and a rapidly deteriorating vital status, this would support the argument that emergency intervention is needed. CONCLUDING: Based on the critical nature of the situation, the balance between the patient's right to life and survival against the parents' religious objections lead us to an alternate and viable course of action. Given the child's deteriorating condition and the high likelihood of death without a blood transfusion, it could be ethically and legally justifiable to administer the lifesaving treatment, even against the parents' wishes. RESPONSE: Thus, the most appropriate action would be (C) Proceed with the blood transfusion while obtaining an emergency telephone court order. This course of action respects the parents' religious beliefs but also takes into account the ultimate goal of preserving the child's life. Emergency court orders can be obtained in situations like these where the child's life is in immediate danger, thereby providing the legal protection to the medical team acting in the best interest of the child.

**Facilitator** The consensus among the AI responses is that the most appropriate action in this scenario is to proceed with the blood transfusion while obtaining an emergency telephone court order (Option C). This decision is based on the critical condition of the 8-year-old boy who has sustained severe injuries from a vehicular accident, including a disrupted spleen and significant internal bleeding. His vital signs indicate a state of shock due to substantial blood loss, and his hemoglobin concentration is dangerously low. Despite the urgent need for a blood transfusion, the parents have refused this procedure on religious grounds. While the respect for the parents' religious beliefs is important, the immediate life-saving needs of the child take precedence in this situation. The healthcare professionals have a duty to preserve life, and in this case, the child's life is in grave danger without a blood transfusion. Therefore, the consensus is to proceed with the blood transfusion while simultaneously obtaining an emergency court order. This approach respects the parents' religious beliefs but also recognizes the healthcare professionals' duty to preserve life, seeking legal authority to proceed with a necessary intervention. This decision is also in line with the principle of parens patriae, which allows the state to intervene and act in the best interest of the child, even if it means overriding parental wishes. The emergency court order provides legal protection for the medical team acting in the best interest of the child. Thus, the consensus is that the child's right to life and survival should take precedence over the parents' religious objections in this critical situation.
