# Supplementary material for: Collaborative intelligence in AI: Evaluating the performance of a council of AIs on the USMLE
Source: PLOS Digit Health. 2025 Oct 9;4(10):e0000787. doi: 10.1371/journal.pdig.0000787 (PMC12510544; doi:10.1371/journal.pdig.0000787)
Supplement: S2 File — (ZIP) [file pdig.0000787.s002.zip › S2 Data Raw data files containing unprocessed question inputs and LLM outputs including transcripts of deliberations/STEP2_Question-78-1_Type-MC-NJ.docx]

**Facilitator** **[Round 1]:** A female newborn has the onset of tachypnea 4 hours after birth. She was delivered at term to a 24-year-old woman following an uncomplicated pregnancy and delivery. Apgar scores were 9 and 9 at 1 and 5 minutes, respectively. Her parents had refused prenatal screening. The patient's temperature is 37.2°C (99.0°F), pulse is 150/min, respirations are 60/min, and blood pressure is 55/35 mm Hg. On cardiac examination, a single, loud S₂ is heard. The liver edge is palpated 3 cm below the right costal margin. Capillary refill time is 6 seconds. Peripheral pulses are difficult to palpate in all extremities. Arterial blood gas analysis on room air shows: pH: 7.24 PCO₂: 35 mm Hg (N=27–40) PO₂: 68 mm Hg HCO₃⁻: 15 mEq/L Chest x-ray shows increased pulmonary haziness and vascular markings suggestive of bilateral pulmonary edema. The most appropriate next step in management is administration of which of the following? (A) Bolus of 0.9% saline (B) Bolus of sodium bicarbonate (C) Intravenous alprostadil (prostaglandin E₁) (D) Intravenous digoxin (E) Intravenous dopamine

**AI_D:** RESTATING THE QUESTION: The clinical scenario describe a term female newborn with the onset of tachypnea 4 hours after birth. She has an elevated respiratory rate, single loud S2, increased liver size, poor peripheral perfusion, and acid-base imbalance. The chest X-ray shows signs of bilateral pulmonary edema. The question asks for the most effective next step in management among saline infusion, sodium bicarbonate, prostaglandin E1, digoxin, and dopamine. PLANNING: Key findings in this infant include the sudden onset of breathlessness after birth, a single loud S2, signs of poor perfusion (delayed capillary refill, hard to palpate pulses, and an enlarged liver), metabolic acidosis and chest X-ray findings suggestive of fluid overload. These findings point towards some form of congenital cardiac lesion. A single loud S2 could represent closure of aortic and pulmonary valves at the same time, which commonly happens in condition like persistent of patent ductus arteriosus (PDA). With the limited option provided, we could consider that the baby might be suffering from cardiogenic shock due to a congenital heart defect such as PDA, hence, the use of prostaglandin E1 to maintain the patency of PDA can be considered. CLINICAL REASONING: We have assumed the baby is in cardiogenic shock from a potential PDA. In a normal fetus, the PDA allows blood to bypass the lungs. After birth, it's expected to close. If it remains open, it can strain the heart and increase blood pressure in the lung arteries. Notably, the clinical manifestations of a significant PDA depend on the size of the ductus and the direction of shunting, which in turn is influenced by the relative resistances in the systemic and pulmonary vascular beds. The administration of prostaglandin E1 would maintain the patency of ductus arteriosus and aids in shunting of blood from the aorta to the pulmonary artery, which would decrease the pressure overload on the left side of the heart and improve peripheral circulations until definitive surgical treatment can be done. CONCLUDING: Considering the infant's signs of congestive heart failure and potential PDA, improving circulatory dynamics is critical. Prostaglandin E1, which keeps the ductus arteriosus patent until corrective surgery can be done, would be most beneficial amongst the options provided. RESPONSE: The most appropriate next step in management is administration of Intravenous alprostadil (prostaglandin E₁).

**AI_B:** (1) RESTATING THE QUESTION: We are asked to determine the best immediate intervention for a neonate who is experiencing respiratory distress, has a single, loud S2 heart sound, poor peripheral perfusion and evidence of pulmonary edema on chest X-ray. The neonate also presents with an arterial blood gas showing metabolic acidosis (`pH: 7.24` and `HCO₃⁻: 15 mEq/L` are lower than the normal range, indicating acidosis). The possible interventions given are a bolus of 0.9% saline, a bolus of sodium bicarbonate, Intravenous alprostadil, Intravenous digoxin, and Intravenous dopamine. (2) PLANNING: One assumption is that the symptoms are due to a simple deficiency of fluids, in which case a saline bolus (Option A) would correct the issue. But the severe symptoms including tachypnea, metabolic acidosis, and pulmonary edema would imply more than simple dehydration. Another assumption is that the baby's symptoms are due to metabolic acidosis itself, which sodium bicarbonate (Option B) could correct. However, sodium bicarbonate is usually reserved for severe and life-threatening acidosis, which may not be the immediate situation here. A third assumption is that these symptoms could be related to a congenital heart defect that prevents oxygen-rich blood from reaching the body tissues effectively, such as patent ductus arteriosus (PDA), which IV alprostadil (Option C) could address. Options D (digoxin) and E (dopamine) are commonly used for heart failure, and while the baby has pulmonary edema, there's no direct evidence of heart failure. (3) CLINICAL REASONING: After reviewing the symptoms and blood gas results, and considering the neonate's uncomplicated birth history and the new onset of these symptoms, I'm leaning more towards a cardiac-related issue. The single, loud S2 heart sound, poor peripheral perfusion, and metabolic acidosis could indicate a problem with oxygenated blood circulation. An untreated congenital heart defect such as PDA could lead to pulmonary edema, as noted in the X-ray. Alprostadil is a type of prostaglandin used to keep the ductus arteriosus open in infants with certain heart defects, to allow for better blood flow. (4) CONCLUDING: Given the aforementioned clinical features and reasoning, a congenital cardiac condition like patent ductus arteriosus seems to be the most plausible diagnosis here. Therefore, treatment should aim at management of this condition. (5) RESPONSE: The most appropriate next step in management of this neonate is administration of Intravenous alprostadil (prostaglandin E₁) (Option C), to keep the ductus arteriosus open and improve her blood flow.

**AI_A:** RESTATING THE QUESTION: A full-term newborn girl, sourced from a 24-year-old healthy pregnancy, demonstrates sudden tachypnea after four hours of life. No prenatal screenings were conducted due to parental refusal. Additionally, her clinical signs include tachycardia, hypotension, lowered oxygen levels, and metabolic acidosis, as well as a single, pronounced second heart sound (S₂). A prolonged capillary refill time, a liver physiologically 3cm below the ribcage, and attenuated peripheral pulses are also verifiable. A chest x-ray reveals bilateral pulmonary edema. Now we ask, what would be the ideal next step for her management? The answer choices are: a bolus of 0.9% saline, a bolus of sodium bicarbonate, alprostadil (prostaglandin E₁) administered intravenously, intravenous digoxin, or dopamine via an IV. PLANNING: Let's make some assumptions. The newborn's clinical manifestations direct towards congenital heart disease. An isolated single, loud S₂ hints at patent ductus arteriosus (PDA). PDAs tend to manifest soon after birth, causing cogwheel pulse, widened pulse pressure, and continuous machinery murmur. However, our case doesn't match these signs. Another assumption would be that the newborn is suffering from transient tachypnea of the newborn (TTN). However, her symptoms do not consist of a normal-Apgar score, bilateral pulmonary edema, and poor peripheral perfusion. With these contradictions in mind, it could be possible that the newborn has ductal-dependent congenital heart disease. In this case, the ductus arteriosus may be serving the necessary function of providing systemic circulation. Subsequent closure of the duct might lead to all the symptoms seen here. CLINICAL REASONING: Closing the ductus arteriosus can result in decreased blood flow to the lower body and organs, leading to hypotension, metabolic acidosis due to poor perfusion of the tissues, decreased peripheral pulses, and hepatomegaly. At the same time, the increased afterload on the left side of the heart, owing to the closure of duct, could precipitate pulmonary edema, which could be responsible for the tachypnea and chest x-ray findings. CONCLUDING: With the above considerations, this newborn girl might be suffering from a ductus-dependent congenital heart disease. Restoring patency of the ductus arteriosus should be the management priority to ensure survival. RESPONSE: The most appropriate next step in management is administration of '(C) Intravenous alprostadil (prostaglandin E₁)'. Prostaglandin E1 (alprostadil) maintains the patency of the ductus arteriosus and can improve systemic blood flow in babies with ductal-dependent congenital heart diseases until surgical correction is possible.

**AI_C:** RESTATING THE QUESTION: The query presents us with a case of a female newborn who commenced tachypnea four hours after her birth. Born to a 24-year-old woman at term without any troubles during pregnancy or delivery, her Apgar scores were normal. The baby's vitals indicate an elevated pulse of 150/min and a high respiratory rate of 60/min. Upon a physical examination, abnormal findings such as a single loud S2 heart sound, an enlarged liver, and decreased peripheral perfusion were found. Arterial blood gas analysis and a chest x-ray suggest a situation of acidosis and bilateral pulmonary edema. Given this scenario, we are asked to suggest the best immediate treatment. PLANNING: The chief assumption in this case links the newborn's symptoms to a pathophysiological condition, possibly related to her cardiovascular system. By questioning this assumption, we could consider that her pathology might be due to a typical neonatal condition such as transient tachypnea of the newborn (TTN), meconium aspiration, pneumonia, or a congenital heart disease. Evaluating these assumptions, a critical takeaway is the single loud S2 sound and the signs of poor peripheral perfusion. These findings could be indicative of a cardiovascular issue like persistent pulmonary hypertension of the newborn (PPHN), or closure of the patent ductus arteriosus (PDA) as would be more likely in this case, leading us to hypothesize that this is congenital heart disease like a duct-dependent congenital heart lesion, possibly a condition such as hypoplastic left heart syndrome (HLHS) or interrupted aortic arch (IAA), and not a pulmonary-born problem. CLINICAL REASONING: Let's elucidate this hypothesis further. The infant presented with tachypnea and signs of poor systemic perfusion as evidenced by the prolonged capillary refill time, weak peripheral pulses, and lower than normal blood pressure. Her arterial blood gas shows metabolic acidosis, indicating acidemia likely due to poor tissue perfusion. The chest x-ray suggests bilateral pulmonary edema, which might result from left-sided heart failure, frequently seen in duct-dependent lesions as the heart attempts to pump blood against an outflow tract obstruction. The infant's heart sounds include a single loud S2 (single second heart sound), which may indicate the absence of the aortic component due to disrupted or absent aortic flow, a key feature of lesions like HLHS or IAA. The liver edge palpated 3 cm below the right costal margin is an indication of hepatomegaly, which can occur due to heart failure. These clinical features all point to a duct-dependent congenital heart disease. CONCLUDING: As parts of the baby's heart are reliant on the ductus arteriosus for oxygenated blood in conditions like HLHS or IAA, the closure of this duct after birth can lead to cardiorespiratory distress. Prostaglandin E1, also known as alprostadil, is capable of dilating and maintaining the openness of the ductus arteriosus until definitive surgical intervention can be performed. They permit shunting and thus circulation of blood, making it the appropriate immediate management plan for this baby given the emergent nature of these conditions. RESPONSE: Considering the signs and findings, the most appropriate next step in management in this neonate would be the administration of (C) Intravenous alprostadil (prostaglandin E₁).

**AI_E:** (1) RESTATING THE QUESTION: To demonstrate our understanding, we are interpreting this scenario involving a term female newborn displaying breathing troubles or tachypnea 4 hours post-delivery. The mother's pregnancy and the child's birth were complication-free. The baby had healthy Apgar scores but now presents with specific symptoms including a quickened heartbeat, rapid respirations, low blood pressure, a single, loud S₂ on cardiac examination, an enlarged liver, delayed capillary refill time, and poor peripheral pulses. An arterial blood gas analysis reveals a low pH (acidemia), normocapnia (a normal PCO₂), a low PO₂ (hypoxemia), and a low bicarbonate level (metabolic acidosis). A chest X-ray suggests fluid in the lungs or pulmonary edema. The question is which therapy should be administered next: (A) A bolus of normal saline (0.9%), (B) A bolus of sodium bicarbonate, (C) Alprostadil (prostaglandin E₁) intravenously, (D) Digoxin intravenously, or (E) Dopamine intravenously. (2) PLANNING: The history and examination suggest that this newborn is suffering from heart failure and the likely underlying pathology could be a condition resulting from the child's inability to transition normally from fetal circulation to neonatal circulation after birth. Hypoxemia, acidosis, and symptoms suggestive of heart failure, such as pulmonary edema and hepatomegaly, indicate a condition such as persistent pulmonary hypertension or closure of a significant fetal blood vessel, like the ductus arteriosus. A single, loud S₂, as presented in this case, might be a clue to the latter. Questioning the assumption about the bolus of 0.9% saline, would any newborn in heart failure be helped by it? Questioning the assumption about sodium bicarbonate: Will it resolve the underlying pathology? It might correct the acidosis. Questioning the assumption about alprostadil: Given everything we know, could this help keep the severely-important-for-life ductus arteriosus open? Questioning digoxin: Can it resolve the underlying pathology? It's used for heart failure, but is it the best option in this circumstance? And finally, dopamine: Can it resolve the underlying pathology? I am hypnotizing that alprostadil should be clinically prioritized in this situation as it helps keep the ductus arteriosus open, providing a temporary bypass for the blood to mix while receiving definitive therapy for the condition. (3) CLINICAL REASONING: In newborns, the transition from fetal to postnatal circulation usually involves closure of the ductus arteriosus which shunts blood from the pulmonary artery to the aorta. This ductus arteriosus may, however, fail to close, leading to a condition known as Patent Ductus Arteriosus (PDA). Symptoms typically develop hours after birth, as in this case, and are associated with acidosis, hypoxemia, features of heart failure, including pulmonary edema and hepatomegaly. Ductal closure is supported by high oxygen tension and low prostaglandin E₁ levels, while it is kept patent/open by low oxygen tension and high prostaglandin levels. This is potentially the reason for the hypoxemia and acidemia we notice in the baby. With regards to the single loud S₂, delivered at term, this may be indicative of a patent ductus arteriosus or possibly a right-to-left shunt, typically seen in conditions like Tetralogy of Fallot. However, the clinical picture here aligns more with a patent ductus arteriosus. (4) CONCLUDING: This baby has findings supportive of a patent ductus arteriosus, and the unique management aspect of this condition revolves around maintaining the patency of the ductus arteriosus to prevent worsening cardiac output and respiratory distress. The standard first-line approach is intravenous administration of prostaglandin E₁, known as alprostadil, which promotes the dilation of the ductus arteriosus. (5) RESPONSE: In the case of this newborn, the most appropriate next step in management is the administration of intravenous alprostadil (prostaglandin e1), which would keep the ductus arteriosus patent until surgical or catheter-based intervention could be arranged to address the patent ductus arteriosus definitively.

**Facilitator** The consensus statement synthesizes the responses of AI_A, AI_B, AI_C, AI_D, and AI_E as follows: The clinical scenario presents a full-term newborn girl who, four hours after birth, exhibits sudden tachypnea, tachycardia, hypotension, lowered oxygen levels, and metabolic acidosis. Additional signs include a single, pronounced second heart sound (S₂), prolonged capillary refill time, a liver physiologically 3cm below the ribcage, attenuated peripheral pulses, and bilateral pulmonary edema as revealed by a chest x-ray. The clinical manifestations suggest a congenital heart disease, possibly a ductal-dependent congenital heart disease. The single, loud S₂ could hint at patent ductus arteriosus (PDA), a condition where the ductus arteriosus fails to close after birth. However, the absence of other typical signs of PDA, such as cogwheel pulse, widened pulse pressure, and continuous machinery murmur, makes this diagnosis less likely. The symptoms also do not align with transient tachypnea of the newborn (TTN), as the newborn's condition includes poor peripheral perfusion and bilateral pulmonary edema. Therefore, the most plausible diagnosis is a ductal-dependent congenital heart disease, where the ductus arteriosus is providing necessary systemic circulation. Closure of the duct could lead to the observed symptoms. The closure of the ductus arteriosus can result in decreased blood flow to the lower body and organs, leading to hypotension, metabolic acidosis due to poor perfusion of the tissues, decreased peripheral pulses, and hepatomegaly. Concurrently, the increased afterload on the left side of the heart, due to the closure of the duct, could precipitate pulmonary edema, which could be responsible for the tachypnea and chest x-ray findings. Given these considerations, the most appropriate next step in management is the administration of intravenous alprostadil (prostaglandin E₁). Alprostadil maintains the patency of the ductus arteriosus and can improve systemic blood flow in babies with ductal-dependent congenital heart diseases until surgical correction is possible. This treatment approach is prioritized over other options such as a bolus of 0.9% saline, a bolus of sodium bicarbonate, intravenous digoxin, or intravenous dopamine, which may not address the underlying pathology effectively.
