# Supplementary material for: Collaborative intelligence in AI: Evaluating the performance of a council of AIs on the USMLE
Source: PLOS Digit Health. 2025 Oct 9;4(10):e0000787. doi: 10.1371/journal.pdig.0000787 (PMC12510544; doi:10.1371/journal.pdig.0000787)
Supplement: S2 File — (ZIP) [file pdig.0000787.s002.zip › S2 Data Raw data files containing unprocessed question inputs and LLM outputs including transcripts of deliberations/STEP2_Question-79-1_Type-MC-NJ.docx]

**Facilitator** **[Round 1]:** A 43-year-old woman comes to the office for a follow-up examination 1 week after she was discharged from the hospital following an overnight admission for an episode of crushing, aching chest pain. The pain was located behind her sternum and radiated to her left jaw and shoulder. She also had shortness of breath, palpitations, and nausea. Initial ECG obtained on admission showed ST-segment depressions in leads V₃ through V₆. Nitroglycerin was administered, and the chest pain resolved. Follow-up serial ECGs disclosed no abnormalities and serum troponin concentrations were within the reference ranges. Cardiac catheterization obtained the next morning showed no substantial narrowing of the coronary arteries. At discharge, the patient declined medication to prevent recurrence of pain. The patient reports that since discharge, she has had two episodes of chest discomfort lasting 2 to 4 minutes, similar in location and quality to the pain precipitating her hospitalization. Medical history otherwise is unremarkable and she takes no routine medications. Vital signs are within normal limits. Physical examination discloses no abnormalities. Which of the following is the most appropriate pharmacotherapy to prescribe at this time? (A) Diltiazem (B) Lisinopril (C) Propranolol (D) Warfarin (E) No pharmacotherapy is indicated at this time

**AI_B:** RESTATING THE QUESTION: The presentation suggests that this 43-year-old lady has experienced episodes of chest pain since being discharged from the hospital, where she was admitted for a similar type of pain. Importantly, her ECG during the pain episode showed ischemic changes (ST-segment depression), but her serum troponin levels and cardiac catheterization were normal. Since her discharge, she has experienced further episodes without obvious trigger, but she is currently not on any medication for this condition. Now, we are asked to determine the most appropriate pharmacotherapy for her. PLANNING: In analyzing this, we can make assumptions. One assumption could be that the woman's chest pains are due to coronary artery disease (CAD). However, the absence of significant narrowing of the coronary arteries on cardiac catheterization questions this assumption. An alternate assumption can be that the chest pain is due to variant angina, which is characterized by transient ST-segment changes, chest pain not necessarily associated with exertion, and normal coronary arteries on angiography. This assumption seems to be more consistent with her clinical presentation and is therefore clinically prioritized. CLINICAL REASONING: Variant angina, or Prinzmetal's angina, is caused by coronary artery spasm. ECG abnormalities are usually transient and occur during episodes of pain. The coronary angiogram is typically normal between episodes. The fact that our patient's chest pain also responds to nitroglycerin further supports this diagnosis, as nitroglycerin can relieve coronary artery spasm. However, a preventive strategy is needed as the pain episodes continue to occur. CONCLUDING: As in the case of our patient, in variant angina, the treatment primarily aims to prevent the coronary artery spasm. Medications commonly used for this include coronary vasodilators like calcium channel blockers and nitrates. RESPONSE: Therefore, the optimal pharmacotherapy for our patient at this point would be a calcium channel blocker, which inhibits the influx of calcium ions into the vascular smooth muscle, causing relaxation and preventing spasm. So, "Diltiazem" (option A) would be the most appropriate medication to prescribe for her, as it is a calcium channel blocker.

**AI_E:** 1) RESTATING THE QUESTION: The patient is a middle-aged woman who presented with classic symptoms of angina (crushing, aching chest pain radiating to the jaw and shoulder) along with ST-segment depressions on her initial EKG. Following treatment with nitroglycerin, her symptoms resolved and later tests, including cardiac catheterization, were negative for significant coronary artery disease. Her serum troponin levels remained normal, and follow-up EKGs did not indicate any noticeable changes or abnormalities. Interestingly, since her discharge, she experienced two more instances of similar chest discomfort, yet she has declined any regular medications to prevent recurrence. The question thus is about which pharmacological treatment, if any, would be the most prudent to prescribe at this stage. 2) PLANNING: Some assumptions to consider are: (i) her symptoms are indicative of cardiac angina, (ii) her symptoms are not related to significant coronary artery disease as per her cardiac catheterization results, (iii) although her symptoms resolved with nitroglycerin, they have recurred, suggesting some ongoing pathological process in her heart. 3) CLINICAL REASONING: Connecting these assumptions, one might consider that typical angina is often relieved by nitroglycerin and is caused by reduced oxygen supply to the heart muscles, usually because of a blockage or narrowing in the coronary arteries. However, in this patient’s case, the normal cardiac catheterization report essentially rules out any critical coronary artery disease. Instead, the patient's recurring chest discomfort despite the absence of coronary artery stenosis suggests a diagnosis of vasospastic (or Prinzmetal's) angina. This condition is caused by the constriction of coronary artery smooth muscle that leads to myocardial ischemia, and symptoms tend to occur at rest and not necessarily during exertion. 4) CONCLUDING: In terms of pharmacotherapy, calcium channel blockers like Diltiazem are often used as first-line treatment for vasospastic angina. They cause relaxation of the coronary vascular smooth muscle, which can help prevent spasm. 5) RESPONSE: Given this clinical picture, the most appropriate pharmacotherapy to prescribe for this patient at this time would be (A) Diltiazem.

**AI_A:** (1) RESTATING THE QUESTION: The inquiry presents a case of a 43-year-old woman who experienced a severe episode of chest pain, exhibited indicative ECG changes, but subsequent tests did not confirm narrowing of coronary arteries. Despite relief from the initial episode with nitroglycerin, she continues having short episodes of chest pain. She has no regular medications and her overall health appears within a normal range. The question asks to determine the optimal pharmacotherapy in this situation. (2) PLANNING: The key aspects of the case include: chest pain alleviated with nitroglycerin, ST-segment depressions, normal coronary arteries, recurrent episodes of chest pain, and a patient not currently on any medication. It's essential to consider the possible conditions causing these signs and symptoms and the patient's reluctance to take medication. Prinzmetal's Angina that involves spasm of the coronary arteries is a likely consideration. This could explain the transient ST-segment depressions, lack of coronary artery disease, and resolution of symptoms with nitroglycerin. (3) CLINICAL REASONING: Clinically, the presentation of chest pain, combined with ECG changes and symptom relief following nitroglycerin administration is suggestive of angina. However, typical angina pain is caused by a fixed obstruction (such as atherosclerosis), which usually does not completely resolve and is thus accompanied by elevations in serum cardiac markers like troponin. The absence of such changes, along with clear coronary arteries, set a different path for consideration. Prinzmetal's Angina or variant angina is characterized by focal spasm of the coronary arteries, which could be triggered by exposure to cold or stress. This condition frequently manifests with chest pain at rest as opposed to during exertion and may cause transient ST-segment depression or elevation. The pain in Prinzmetal's Angina generally responds well to nitroglycerin and calcium-channel blockers. These insights warrant a review of the proposed medication options. (4) CONCLUDING: Based on the clinical reasoning process above, the preferred drug should ideally act to prevent coronary vasospasm, without necessarily being dependent on continued patient adherence. This narrows the options down to Diltiazem, which is a calcium-channel blocker and commonly used in the management of Prinzmetal's Angina. (5) RESPONSE: Given the patient's clinical scenario and the reasoning explained, the most appropriate pharmacotherapy to prescribe at this time would be Diltiazem (A).

**AI_C:** RESTATING THE QUESTION: This question involves a 43-year-old woman who had been hospitalized for chest pain that was radiating to the left jaw and shoulder and accompanied by shortness of breath, palpitations, and nausea. Although her pain resolved with nitroglycerin and tests including ECG and cardiac catheterization did not reveal major abnormalities, she experienced two episodes of similar pain since her discharge. We are tasked with determining the most appropriate medication to prescribe in her case from the options provided which include Diltiazem, Lisinopril, Propranolol, Warfarin, or no pharmacotherapy. PLANNING: Let's review our assumptions. We can assume that the patient's symptoms point to angina, considering the nature of the pain, its response to nitroglycerin, the ST-segment depression on ECG, and absence of evidence for substantial narrowing of the coronary arteries which would indicate heart attack. An alternate assumption could be that she suffers from another cardiac condition, like arrhythmia or valvular disease. However, considering the pattern of her symptoms and response to nitroglycerin, angina seems more plausible. CLINICAL REASONING: Now, in angina, especially Prinzmetal's (variant) angina which is suggested by the ST depressions in the precordial leads symptomatic relief can be provided by medications that reduce myocardial oxygen demand or improve supply. Nitroglycerin, which she responded to, is one such medication which acts by dilating the coronary arteries, improving oxygen supply. Diltiazem, a calcium channel blocker, is also used in managing Prinzmetal's angina as it relaxes coronary vascular smooth muscle and suppresses coronary artery spasm, improving oxygen supply. Lisinopril is an ACE inhibitor primarily used to treat high blood pressure and heart failure. Propranolol, a beta-blocker, is generally used to treat hypertension, arrhythmias, and conditions where reducing heart rate and contractility will be beneficial. Warfarin is an anticoagulant used to prevent blood clots. CONCLUDING: Given these considerations, among the options provided, Diltiazem would be most suitable as it would help prevent coronary artery spasm and provide symptomatic relief in case of recurring Prinzmetal's or variant angina. ANSWER: (A) Diltiazem.

**AI_D:** RESTATING THE QUESTION: We are essentially being asked to determine the most appropriate medication for a 43-year-old woman who has experienced recurrent episodes of chest pain. These episodes mimic the symptoms of angina pectoris, a condition characterized by chest discomfort due to insufficient blood flow to the heart muscle. However, tests including ECGs, troponin levels, and cardiac catheterization have revealed no abnormalities in the function or structure of her heart. PLANNING: First, we should consider some assumptions: (1) her symptoms indicate a cardiac origin; (2) the lack of abnormalities in testing means she has no heart damage; (3) her chest discomfort episodes will continue; (4) pharmacotherapy is necessary and will alleviate her symptoms. We can question each of these assumptions. A possible alternate assumption is that her symptoms, though resembling angina, may have a non-cardiac origin. The normal test results support this idea, and thus, a non-cardiac cause should be at least considered and prioritized for further investigation. CLINICAL REASONING: Although her presenting symptoms such as crushing chest pain, shortness of breath, and nausea can mirror those of conditions like angina, we need to remember that the tests showed no actual heart damage or abnormal function. This kind of presentation is common with a condition known as Prinzmetal's Angina (or variant angina), a disorder characterized by recurrent episodes of chest discomfort due to a spasm in a coronary artery. Unlike traditional angina, Prinzmetal's is not associated with atherosclerosis or clot formation, so cardiac catheterization would appear normal. The absence of high troponin levels also suggests that there's no myocardial cell death. Prinzmetal's Angina usually responds well to calcium-channel blockers like Diltiazem. These drugs help by relaxing the coronary artery smooth muscle. CONCLUDING: Considering that our patient's symptoms are suggestive of a coronary artery spasm, despite the lack of abnormalities in testing, it would be reasonable to think she might have a condition like Prinzmetal's Angina. Since there are no cardiac catheterization findings of atherosclerosis or high troponin levels indicating myocardial cell death, this supports the likelihood of a diagnosis of Prinzmetal's Angina, which typically responds to calcium-channel blockers. RESPONSE: Therefore, taking into consideration the recurring nature of her symptoms, lack of apparent heart damage, and the possibility of a non-atherosclerotic cause such as coronary artery spasm, the most appropriate pharmacotherapy to prescribe for this patient at this time would be Diltiazem (A).

**Facilitator** The consensus statement synthesizes the responses of AI_A, AI_B, AI_C, AI_D, and AI_E as follows: The case presents a 43-year-old woman who experienced severe chest pain, indicative ECG changes, but subsequent tests did not confirm narrowing of coronary arteries. Despite relief from the initial episode with nitroglycerin, she continues having short episodes of chest pain. She has no regular medications and her overall health appears within a normal range. The key aspects of the case include chest pain alleviated with nitroglycerin, ST-segment depressions, normal coronary arteries, recurrent episodes of chest pain, and a patient not currently on any medication. Clinically, the presentation of chest pain, combined with ECG changes and symptom relief following nitroglycerin administration is suggestive of angina. However, typical angina pain is caused by a fixed obstruction (such as atherosclerosis), which usually does not completely resolve and is thus accompanied by elevations in serum cardiac markers like troponin. The absence of such changes, along with clear coronary arteries, set a different path for consideration. Prinzmetal's Angina or variant angina is characterized by focal spasm of the coronary arteries, which could be triggered by exposure to cold or stress. This condition frequently manifests with chest pain at rest as opposed to during exertion and may cause transient ST-segment depression or elevation. The pain in Prinzmetal's Angina generally responds well to nitroglycerin and calcium-channel blockers. Given the patient's clinical scenario and the reasoning explained, the most appropriate pharmacotherapy to prescribe at this time would be Diltiazem (A). Diltiazem is a calcium-channel blocker and commonly used in the management of Prinzmetal's Angina. It acts to prevent coronary vasospasm, without necessarily being dependent on continued patient adherence. This medication inhibits the influx of calcium ions into the vascular smooth muscle, causing relaxation and preventing spasm. Therefore, Diltiazem would be most suitable as it would help prevent coronary artery spasm and provide symptomatic relief in case of recurring Prinzmetal's or variant angina.
