# Supplementary material for: Collaborative intelligence in AI: Evaluating the performance of a council of AIs on the USMLE
Source: PLOS Digit Health. 2025 Oct 9;4(10):e0000787. doi: 10.1371/journal.pdig.0000787 (PMC12510544; doi:10.1371/journal.pdig.0000787)
Supplement: S2 File — (ZIP) [file pdig.0000787.s002.zip › S2 Data Raw data files containing unprocessed question inputs and LLM outputs including transcripts of deliberations/STEP2_Question-8-1_Type-MC-NJ.docx]

**Facilitator** **[Round 1]:** An investigator would like to decrease the incidence of type 2 diabetes mellitus among adult patients with risk factors including obesity, decreased physical activity, and family history of diabetes mellitus. A total of 200 men and women aged 30 to 64 years will be enrolled in a regular exercise program for 6 months. A nurse reviews the details of the study with each participant, and each participant signs an informed consent form. One month after the study begins, one of the participants asks to drop out of the study. Which of the following represents the most appropriate next step by this study participant? (A) Complete the study because she signed the consent form (B) Discuss withdrawal with the institutional review board (C) Negotiate her withdrawal with the principal investigator (D) Wait to withdraw until after the investigator consults with an ethics team (E) Withdraw from the study now

**AI_C:** RESTATING THE QUESTION: A study is conducted to see if a regular exercise program can decrease the incidence of type 2 diabetes among adults at risk due to obesity, lack of physical activity, and family history. After signing informed consent, one participant wants to withdraw one month in. What should be the next appropriate step? PLANNING: The first assumption is that having signed the consent form obliges the subject to complete the study. However, an alternate assumption is that informed consent includes the right to withdraw at any point without penalty. The principle of autonomy, a cornerstone of biomedical ethics, supports this assumption. Decisions regarding participation in research should be voluntary and uncoerced and participants should be free to reconsider their decision to participate at any time. CLINICAL REASONING: Informed consent is an ongoing process. It is based on three elements: comprehension, disclosure, and voluntariness. It obliges the researchers to make sure participants understand the study's purpose, risks, benefits, and alternatives, and their right to refuse participation or to withdraw from the study at any point—without facing any negative consequences. The principle of autonomy or respect for individuals ensures the participants' rights to make an informed decision about their participation in any activity, including research. This tenet stands, even after they've given initial consent and joined the study. CONCLUDING: Based on this ethical principle of autonomy in biomedical research, it is clear that even after joining the study and signing the consent form, the participant holds the right to withdraw at any point and without bringing any detriment upon themselves. Conversations with the review board, principal investigator, or waiting for the ethics team are not necessary prerequisites to withdraw. RESPONSE: Thus, the most appropriate next step for the participant who wishes to withdraw from the study would be (E) Withdraw from the study now.

**AI_B:** (1) RESTATING THE QUESTION: The question is asking what the most appropriate course of action is for a participant who wants to drop out of a clinical study after it has already commenced. It's centered on the concept of participants' rights and ethical considerations in clinical research. An 'informed consent form' is a document that participants sign before engaging in a study, which indicates they understand the risks, benefits, and details of the study, and that their participation is voluntary. (2) PLANNING: The underlying assumption in some of the options presented may be that the participant is bound to the agreement because of the signed consent form. However, there is an alternate assumption we should consider which could be more ethical and respectful of participants' rights: that despite signing the consent form, participants in a study maintain the right to leave the study at any point should they choose to do so. This aligns with commonly accepted ethical considerations in patient autonomy. (3) CLINICAL REASONING: A clinical research study must always take into consideration the ethical rights of the participants, primarily informed consent and autonomy. Informed consent isn't only about obtaining approval from participants before the start of the study, but also about maintaining their understanding and voluntary participation throughout the process. Options such as waiting for consultation with an ethics team or negotiating withdrawal with the principal investigator seem to undermine autonomy by giving the impression of a fettered decision. (4) CONCLUDING: Therefore, the appropriate step should not impose undue limitations on the participant's ability to exit the study. It must be recognized that autonomy and voluntariness remain paramount throughout the study, despite having originally agreed to participate. (5) RESPONSE: Based on these considerations, (E) 'Withdraw from the study now' is the most ethically appropriate choice. This decision respects the participant's autonomy and reflects the fundamental principle of voluntary participation in clinical research, which can be modified or withdrawn at any time without penalty or need to provide a reason.

**AI_E:** RESTATING THE QUESTION: In this situation, an investigator has established a study that aims to reduce the incidence of type 2 diabetes mellitus in adults who are at high risk due to obesity, inactive lifestyle, and a family history of diabetes. Participants, who are 200 men and women between the ages of 30 and 64, embark on a regular exercise regimen that's supposed to last for 6 months. Details of the study, including the participants' rights, expectations and responsibilities, are provided to them through a nurse, securing their informed consent before the study kicks off. However, a month into the study, a participant wishes to exit the study. The question demands identification of the most suitable next action for this participant to take from the provided options. PLANNING: This situation's main issue revolves around the participant's right to withdraw from a research study, which is a principle in research ethics. The alternate assumption here could be that despite the signing of an informed consent form, a participant retains the right to exit a research study at any point, without any need for further consultation or negotiation. The options provided can be evaluated in line with these principles. CLINICAL REASONING: In recognizing the ethics of clinical trials, respect for autonomy is critical. It demands that each research participant's decision-making capacity is honored, allowing them to make informed decisions about their participation without undue coercion or manipulation. This includes the right to withdraw from the study at any point, irrespective of the consent given at its start. It isn't obligatory for a participant to complete the study (Option A) merely because they signed the consent form. Equally, they don't need approval from the institutional review board (Option B) or negotiation with the principal investigator (Option C) before withdrawing. Finally, the participant doesn't need to wait for ethics team consultation (Option D) to withdraw. CONCLUDING: Drawing from the above clinical reasoning and the principle of respect for autonomy in research ethics, the participant, regardless of having initially consented, retains the unencumbered right to withdraw from the study whenever they choose without needing any negotiation or approval from any party. RESPONSE: Therefore, the answer is Option (E) Withdraw from the study now.

**AI_D:** RESTATING THE QUESTION: The question is essentially asking for the most appropriate action for a participant who wishes to withdraw from a clinical study, one month after its commencement, considering she already signed the informed consent form. The informed consent form is a document that explains the study procedures, potential risks and benefits, and the participant's rights, which include the right to withdraw from the trial anytime, regardless of the reason. PLANNING: Now, we'll scrutinize some assumptions that could guide the participant's next step. She could assume, based on signing the consent form (A), that she has to complete the study; or that she needs to undergo formal discussions with the institutional review board (B) or principal investigator (C) before exiting; or she could presume she has to wait until after ethical consultation (D). Lastly, she could simply assume that she can withdraw from the study immediately (E). CLINICAL REASONING: When the process of obtaining informed consent is correctly executed, it ensures that the participant fully comprehends the nature of the study, the risks involved, the benefits and the freedom to willingly participate or withdraw at any time. Thereby, once the participant gives informed consent, it does not create an obligation (A) to stay in the study until its end. In many clinical studies, it's not necessarily required for a participant to directly discuss withdrawal with the institutional review board (B) or negotiate their withdrawal with the principal investigator (C). Also, waiting to withdraw until after the investigator consults with an ethics team (D) seems too formal and time-consuming. The incisive intent and principle of autonomy in informed consent ensures choice remains with the participant, and she should feel able to withdraw at any time, without giving a reason. CONCLUDING: Therefore, based on our understanding of informed consent and its foundational principles which primarily focus on the autonomy of the participant, the most viable option would prioritize the participant's freedom and decision-making capacity in their involvement within the research study. RESPONSE: The study participant should (E) Withdraw from the study now. This option best embraces the authenticity of the autonomy principle in the informed consent process and acknowledges the participant's freedom to decide without the need for further discussion, negotiation, or waiting.

**AI_A:** RESTATING THE QUESTION: The question posits a hypothetical situation where an investigator is conducting a clinical study to reduce the incidence of type 2 diabetes mellitus among patients with specific risk factors. The study involves regular physical exercise regimen among 200 participants between the ages of 30 and 64 who signed an informed consent form. Above a month into the study, one participant wishes to withdraw from the study. The question seeks to determine the most suitable action by the participant given the five options provided. PLANNING: This situation primarily relates to the ethical aspects of clinical trials participation and patient autonomy. The principles of patient autonomy and the ethical conduct surrounding clinical trials necessitate that a participant has the right to withdraw from a study at any point, regardless of the signed consent form. The signed consent form does not bind the participant to complete the study; it simply acknowledges that the participant was informed about the study's purpose, procedures, potential benefits, and risks. CLINICAL REASONING: The function of the institutional review board in a clinical trial is to protect the rights and welfare of the participants. It approves the protocol of the study, ensuring it is ethical and the risk/benefit ratio is acceptable. However, it’s not directly involved with the participants' decisions to withdraw. Similarly, consulting an ethics team or negotiating with the principal investigator should not be required for a participant to exercise their right. These considerations lean towards the option of a participant being able to withdraw from the study without needing further discussions or permissions. CONCLUDING: The fundamental premise in clinical research is the voluntary participation of subjects and respect for their autonomy. The function of an informed consent form is to provide subjects with enough information about the clinical trial to allow for an informed decision about their participation. It doesn't contractually bind them to complete the trial nor takes away their right to voluntarily exit at any point during the study. RESPONSE: Therefore, (E) Withdraw from the study now is the most appropriate next step by the participant since the principles of autonomy grant them the right to withdraw at any point, without the requirement of any further discussions or permissions.

**Facilitator** The consensus statement is as follows: The question presents a scenario where a participant in a clinical study, aimed at reducing the incidence of type 2 diabetes mellitus among adults with risk factors, wishes to withdraw a month after the study begins. The participant had previously signed an informed consent form. The question asks for the most appropriate next step for this participant. The key issue here revolves around the participant's right to withdraw from a research study, which is a fundamental principle in research ethics. Despite the signing of an informed consent form, a participant retains the right to exit a research study at any point, without any need for further consultation or negotiation. This right is grounded in the principle of autonomy, which respects each research participant's decision-making capacity, allowing them to make informed decisions about their participation without undue coercion or manipulation. This includes the right to withdraw from the study at any point, irrespective of the consent given at its start. Therefore, the participant does not need to complete the study merely because they signed the consent form, nor do they need approval from the institutional review board or negotiation with the principal investigator before withdrawing. Finally, the participant doesn't need to wait for ethics team consultation to withdraw. Thus, the most appropriate next step for the participant who wishes to withdraw from the study is to withdraw immediately. This decision respects the participant's autonomy and reflects the fundamental principle of voluntary participation in clinical research, which can be modified or withdrawn at any time without penalty or need to provide a reason.
