# Supplementary material for: Collaborative intelligence in AI: Evaluating the performance of a council of AIs on the USMLE
Source: PLOS Digit Health. 2025 Oct 9;4(10):e0000787. doi: 10.1371/journal.pdig.0000787 (PMC12510544; doi:10.1371/journal.pdig.0000787)
Supplement: S2 File — (ZIP) [file pdig.0000787.s002.zip › S2 Data Raw data files containing unprocessed question inputs and LLM outputs including transcripts of deliberations/STEP2_Question-81-1_Type-MC-NJ.docx]

**Facilitator** **[Round 1]:** A 35-year-old woman is brought to the emergency department by police after they found her wandering the streets and appearing intoxicated. On arrival, she is tremulous and appears distressed. She says she moved out of her abusive boyfriend’s apartment 1 week ago because she was afraid he would kill her. She has no financial or emotional family support. She worked at a grocery store until 1 month ago when she was fired for missing four shifts during the course of 1 week. During the past week, she has slept less than 4 hours nightly because she has slept on the streets or in shelters. She has panic disorder treated with fluoxetine. She takes no other medications. Ten years ago, she underwent 6 months of psychotherapy for dependent personality disorder. During the past year, she has drunk four or more glasses of wine and other alcoholic beverages nightly. She tried to quit drinking alcohol twice during the past year but was unsuccessful. Her last alcoholic drink was 24 hours ago. Pulse is 90/min and blood pressure is 140/90 mm Hg. Physical examination shows resting hand tremors and delayed reflexes. Mental status examination shows slurred speech. She has an irritable mood and anxious affect. Blood alcohol concentration is 140 mg/dL. Which of the following is the strongest predisposing risk factor for suicide in this patient? (A) Adjustment disorder with depressed mood (B) Alcohol use disorder (C) Dependent personality disorder (D) History of physical abuse (E) Panic disorder

**AI_A:** RESTATING THE QUESTION: In this question, we are presented with a 35-year-old woman who has a history of multiple conditions including panic disorder, dependent personality disorder, and alcohol use disorder. She also has a history of physical abuse and recently lost her job and home. Our objective is to identify which among these risk factors is the most potent predisposing factor for suicide in this particular patient. PLANNING: Assumptions could be made that any of her current conditions (i.e., alcohol use disorder, panic disorder) or historically significant factors (dependent personality disorder, history of physical abuse) could predispose her to suicidal tendencies. It is also plausible that her recent bouts of stress and instability (job loss, homelessness, and relationship breakdown) could contribute to her risks. However, while each factor carries a degree of risk, we need to prioritize the one that has the strongest clinical correlation with suicide. CLINICAL REASONING: It is known that affective (mood) disorders, substance use disorders, paranoid ideation, and a history of nonfatal attempts are strongly correlated to suicide risks. This patient presents multiple risk factors but her report of drinking four or more alcoholic beverages nightly along with her unsuccessful attempts to quit drinking suggest she is suffering from alcohol use disorder, and this is strongly associated with suicide, and could be considered the most potent risk factor. CONCLUDING: A multidisciplinary approach to treat the identified risk factor (alcohol use disorder) and other associated conditions (panic disorder, dependent personality disorder, etc.), including medical treatment and psychotherapy or counseling, may lessen the suicidal risk associated with these conditions. RESPONSE: Hence, I believe the strongest predisposing risk factor for suicide in this patient is (B) Alcohol use disorder.

**AI_E:** (1) RESTATING THE QUESTION: The patient is a 35-year-old woman exhibiting symptoms of chronic stress, mental and physical health issues, and a history of physical abuse, who has been presented to the emergency department. She demonstrates symptoms consistent with alcohol intoxication and withdrawal. The clinical question is asking for the most prominent risk factor, from the given options, that predisposes her to suicide. (2) PLANNING: The options given are Adjustment disorder with a depressed mood (A), Alcohol use disorder (B), Dependent personality disorder (C), History of physical abuse (D) and Panic disorder (E). A review of her case reveals that she has a history of physical abuse, has tried to quit drinking but was unsuccessful thus suggesting alcohol use disorder, she carries a diagnosis of dependent personality disorder, she shows signs of extreme stress, and has a preexisting diagnosis of panic disorder managed with Fluoxetine. The task here is to consider which has the greatest association with increased suicide risk. (3) CLINICAL REASONING: While all the given options can incrementally increase the risk for suicide to various degrees, the option with the most significant weight is Alcohol Use Disorder (B). Alcohol use disorder has a strong correlation with suicide, this is due to its psycho-biological impacts which can exacerbate depression, anxiety, impulsiveness, and disrupt interpersonal relationships. Alcohol also impairs judgment and increases the likelihood of impulsive suicide attempts. In addition, the physiological effects of alcohol on the body such as tremors and poor sleep could further add to the distress. (4) CONCLUDING: While other factors like the history of physical abuse, dependent personality disorder, or panic disorder do contribute to suicide risk, they are not as significantly correlated as alcohol use disorder - particularly in the presence of unsuccessful quit attempts. Therefore, the strongest predisposing risk factor for suicide in this patient is her Alcohol Use Disorder. (5) RESPONSE: The strongest predisposing risk factor for suicide in this patient is (B) Alcohol use disorder.

**AI_B:** RESTATING THE QUESTION: This question involves a 35-year-old woman with multiple psychiatric and lifestyle issues, including abusive relationship, alcohol use disorder, panic disorder, dependent personality disorder, homelessness, and unemployment. We need to identify which among these conditions listed in the answer choices has the strongest association with suicide risk. PLANNING: It is significant to note that all the given answer choices may increase the risk for suicide. However, the task is to recognize the option that stands out as most influential. The restated question will lead us to evaluate the risk of suicide linked with (A) Adjustment disorder with depressed mood, (B) Alcohol use disorder, (C) Dependent personality disorder, (D) History of physical abuse, and (E) Panic disorder. CLINICAL REASONING: Patient's numerous risk factors can be divided into psychosocial (physical abuse, lack of financial and emotional support), psychiatric (panic disorder, dependent personality disorder, potential for adjustment disorder with depressed mood given recent life changes), and lifestyle (alcohol use disorder, unemployment, homelessness). Studies suggest that psychiatric conditions, particularly mood disorders, and substance use disorders are particularly strong risk factors for suicide. Among the options, alcohol use disorders are found to have a high co-morbidity with suicide, especially when combined with psychosocial and lifestyle stressors. People with alcohol use disorders have a higher risk of suicide than those with other psychiatric disorders, including major depression and anxiety disorders. On the other hand, panic disorder, although linked to increased suicide risk, it is not as closely linked as alcohol use disorder. Similarly, dependent personality disorder and adjustment disorder, although they could increase the risk for suicide, they are associated with a relatively lower risk compared to alcohol use disorders. Lastly, while the history of physical abuse can contribute to mental, emotional, and psychological distress which might predispose to suicide, the risk associated is less strong than that for alcohol use disorder. CONCLUDING: I understand the inquiry is asking for the strongest predisposing risk factor for suicide within the context of this patient's personal, clinical, and life situation. Given the patient's alcohol use disorder with failed attempts at cessation, coupled with her psychosocial and lifestyle stressors, alcohol use disorder appears to stand out as the most significant suicide risk factor according to the literature. RESPONSE: Therefore, (B) Alcohol use disorder is the strongest predisposing risk factor for suicide in this patient's case. This is due to the strong association between alcohol use disorders and suicide, particularly in patients with additional psychosocial stressors like this patient.

**AI_D:** 1) RESTATING THE QUESTION: The situation we're examining concerns a 35-year-old woman who was found wandering the streets, appearing intoxicated. Upon arrival at the emergency department, it was observed that she was tremulous and distressed. She has shared a history of physical abuse, lack of financial and emotional support, unstable employment and housing, a panic disorder being managed with fluoxetine, alcohol use disorder, and a history of therapy for dependent personality disorder. Her most recent alcoholic drink was 24 hours ago and her current blood alcohol concentration is 140 mg/dL. Our task is to identify which among the options (Adjustment disorder with depressed mood, Alcohol use disorder, Dependent personality disorder, History of physical abuse, Panic disorder) is the strongest predisposing risk factor for suicide in this patient. 2) PLANNING: There are several assumptions about this patient's situation, one of which that her current state of distress and tremulousness is primarily due to alcohol withdrawal. Another assumption is that her past history of abuse, unstable employment and housing, and mental health conditions (i.e., panic disorder and dependent personality disorder) are contributing to her current state. In terms of suicide risk, we could assume any or all of these factors influence her risk. To generate an alternate assumption, we might question whether the alcohol use disorder itself, separate from these other conditions, is the major predisposing risk factor. 3) CLINICAL REASONING: Chronic alcohol use disorder leads to neuroadaptation which manifests as physical dependency. This can result in intense cravings and withdrawal symptoms when alcohol intake ceases or reduces. This patient's insomnia, anxiety, and tremulousness suggest that she's experiencing these effects. Engaging in repeated unsuccessful attempts to stop drinking is a strong indication of alcohol use disorder. Alcohol use disorder and its consequent life destabilization can lead to hopelessness, lowered inhibitions, and impaired judgment, all of which are associated with an increased risk of suicide. 4) CONCLUDING: While the patient's unstable life circumstances and mental health conditions undoubtedly contribute to her condition, it stands to reason that her chronic, uncontrolled alcohol use is a major contributing factor to her unstable life situation and possibly her mental health condition(s). Thus, this chronic, severe alcohol use is likely the strongest risk factor for suicide in her case, both as a direct result of its physiological effects and as an aggravator of her other risk factors. 5) RESPONSE: The strongest predisposing risk factor for suicide in this patient is (B) Alcohol use disorder.

**AI_C:** RESTATING THE QUESTION: The question refers to a female subject who was discovered on the streets exhibiting signs of alcohol intoxication. She ended an abusive relationship recently, has no familial or financial support, is homeless, and has a history of multiple psychiatric disorders including panic disorder and dependent personality disorder. She is also a chronic alcohol user. The woman displays physical signs of alcohol withdrawal. We need to identify which condition among the patient's issues would make her most susceptible to suicidal tendencies. PLANNING: We have multiple assumptions to consider here. (A) Her current situation of disrupted living environment and emotional distress might instigate depressive tendencies, leading to adjustment disorder with depressed mood that might drive her to suicide; (B) Chronic alcohol usage and her unsuccessful attempts to quit could have led to an alcohol use disorder, which also correlates with suicidal risk; (C) Her history of dependent personality disorder might make her vulnerable to the risk of suicide, particularly considering her current abrupt loss of a relationship; (D) Recent physical abuse might have made her more susceptible to suicide due to emotional trauma; (E) Lastly, her existing panic disorder might increase her likelihood of suicide due to chronic anxiety and fear. However, among these potential reasons, the chronic alcohol use or alcohol use disorder emerges as the most potent risk factor due to the strong established correlation between alcohol dependency and suicide in several studies. CLINICAL REASONING: The patient's current emotional state does point towards an adjustment disorder, but adjustment disorders are generally a temporary and immediate response to a stressor, making the direct connection to suicidal tendencies less clear-cut. While dependent personality disorder might make her vulnerable to suicidal thoughts, particularly after the detachment from a close relationship, the current crisis doesn't seem to be caused directly by this personality trait. Past physical abuse, while undoubtedly traumatic, is not the immediate cause of the current crisis. Her panic disorder, although treated with medication, might exacerbate her stress levels but does not directly lead to suicide. Therefore, taking into consideration her chronic alcohol use, visible physical withdrawal symptoms, and her failed attempts at sobriety, it's the alcohol use disorder that may have the greatest impact on increasing her suicide risk. CONCLUDING: The patient's clinical scenario with tremors, anxious behavior, raised blood pressure, and evidentiary blood alcohol concentration indicates chronic alcohol consumption and dependence, which is prominently linked to an escalated suicidal risk. While all listed factors may contribute to an augmented suicide risk in some way, it's the alcohol use disorder that stands out the most. RESPONSE: The strongest predisposing risk factor for suicide in this patient, given all the information, would most likely be (B) Alcohol use disorder.

**Facilitator** The consensus statement synthesizing the responses of AI_A, AI_B, AI_C, AI_D, and AI_E is as follows: The question presents a 35-year-old woman with a complex history of physical abuse, mental health disorders, and lifestyle issues, including alcohol use disorder, panic disorder, dependent personality disorder, homelessness, and unemployment. She is currently experiencing distress and physical symptoms of alcohol withdrawal. All these factors could potentially increase her risk for suicide. However, the task is to identify the most potent predisposing risk factor for suicide among these conditions. While adjustment disorder with depressed mood, dependent personality disorder, history of physical abuse, and panic disorder could contribute to an increased risk of suicide, they are not as strongly associated with suicide as alcohol use disorder. The patient's chronic alcohol use, unsuccessful attempts to quit, and physical withdrawal symptoms suggest a severe alcohol use disorder. This condition is known to have a strong correlation with suicide due to its psycho-biological impacts, which can exacerbate depression, anxiety, impulsiveness, and disrupt interpersonal relationships. Alcohol also impairs judgment and increases the likelihood of impulsive suicide attempts. Therefore, despite the presence of multiple risk factors in this patient's case, the strongest predisposing risk factor for suicide is her alcohol use disorder. A multidisciplinary approach to treat this identified risk factor and other associated conditions, including medical treatment and psychotherapy or counseling, may lessen the suicidal risk associated with these conditions.
