# Supplementary material for: Collaborative intelligence in AI: Evaluating the performance of a council of AIs on the USMLE
Source: PLOS Digit Health. 2025 Oct 9;4(10):e0000787. doi: 10.1371/journal.pdig.0000787 (PMC12510544; doi:10.1371/journal.pdig.0000787)
Supplement: S2 File — (ZIP) [file pdig.0000787.s002.zip › S2 Data Raw data files containing unprocessed question inputs and LLM outputs including transcripts of deliberations/STEP2_Question-82-1_Type-MC-NJ.docx]

**Facilitator** **[Round 1]:** A 76-year-old woman, who is receiving home hospice care because of end-stage metastatic lung cancer diagnosed 1 month ago, is examined at home at the request of her family because of a 3-hour history of labored breathing despite receiving 100% oxygen at 5 L/min via face mask. Pulse is 92/min, respirations are 18/min, and blood pressure is 105/62 mm Hg. Pulse oximetry on 100% oxygen via face mask shows an oxygen saturation of 85%. The patient appears cachectic and is in mild distress, showing increased work of breathing with prominent sternocleidomastoid muscles and intermittent agonal respirations. Which of the following is the most appropriate next step in management? (A) Administer intravenous morphine (B) Admit the patient to the hospital for further evaluation (C) Initiate a propofol infusion (D) Transition the patient to noninvasive positive pressure ventilation (E) No further action is indicated

**AI_B:** RESTATING THE QUESTION: This scenario revolves around a 76-year-old woman with end-stage metastatic lung cancer. Despite receiving 100% oxygen via face mask at home in a hospice regime, she displays labored breathing and her oxygen saturation is only 85%. Her vital signs suggest that she's distressed, and physical signs such as prominent sternocleidomastoid muscles and intermittent agonal respirations provide further evidence of respiratory struggle. The question asks us to choose the most appropriate subsequent management option. PLANNING: The options given can be classified into the categories of symptomatic relief (intravenous morphine), hospital evaluation (admission for further evaluation), sedation (propofol infusion), respiratory aid (noninvasive positive pressure ventilation), or no operation (no further action). The primary assumption is that her current management plan is insufficient as evidenced by the continuing respiratory distress. Therefore, her clinical status needs reevaluation and potential alteration. The alternative assumption argues the need for prioritization of symptom relief and palliation at this stage. CLINICAL REASONING: In terms of her general condition, this patient is receiving hospice care, which indicates that her disease has progressed to a point where curative treatments are no longer being pursued. Rather, emphasis is placed on symptom management and the maintenance of her comfort. Due to the palliative nature of her treatment, hospital admission, additional diagnostic assessments, or invasive interventions like noninvasive positive pressure ventilation may not align with her overall care goals. Therefore, these options may not be valuable. CONCLUDING: In the context of hospice and palliative care, intravenous morphine is frequently employed to alleviate symptoms such as pain and dyspnea. Morphine functions to depress the respiratory center in the brain, thereby minimizing the distress associated with the perception of breathlessness at the end of life, making this a feasible option. Propofol, conversely, is a potent sedative used primarily in the context of anesthesia and is normally reserved for more invasive procedures, rendering it potentially inappropriate in this setting. RESPONSE: So, connecting all, I would advocate for (A) Administer intravenous morphine as the most suitable action in this scenario, aligned with the goal of maximizing comfort and quality of life.

**AI_C:** RESTATING THE QUESTION: A 76-year-old woman with a terminal stage of metastatic lung cancer is having difficulty breathing despite being on high-flow oxygen. Her vital signs are relatively stable, but she appears undernourished and is laboring to breathe. The question asks what should be the best course of action under these circumstances: Should she be administered morphine intravenously, admitted to the hospital for further evaluation, initiated on a propofol infusion, supported with noninvasive positive pressure ventilation, or should no further action be taken considering her current state of health? PLANNING: The assumptions in this situation include the understanding that the woman's labored breathing is primarily due to her metastatic lung cancer and the possibility that her current oxygenation support might not be enough. Alternate assumptions could revolve around the consideration of potential side effects caused by her current medications, or the possibility of a new or secondary condition causing distress. However, considering her terminal illness and the aim of providing comfort in end-of-life care, the assumption to prioritize is how to best manage her breathing difficulty. CLINICAL REASONING: This patient has a terminal illness and is likely in the end stages of her life due to the metastatic lung cancer. The data presented suggests she is having difficulty in maintaining adequate oxygen saturation despite receiving high-flow oxygen, causing her to have labored breathing. Agonal respiration, a form of labored breathing characterizing end-of-life and characterized by gasping, labored breathing, is also described. This combination can be distressing to both the patient and her loved ones. CONCLUDING: In terms of her management, the objective at this stage is mainly to provide comfort and reduce her distress. Administering intravenous morphine can achieve this as it will not only help to control any existing pain but can also reduce respiratory distress by decreasing the perception of breathlessness without significantly affecting oxygen saturation. This approach maintains the core principles of end-of-life care by focusing on the comfort and dignity of the patient. RESPONSE: Therefore, the most appropriate next step in managing this patient's condition would indeed be to administer intravenous morphine (A) to relieve both her physical discomfort and emotional distress.

**AI_D:** RESTATING THE QUESTION: The presented situation of this elderly female patient with end-stage metastatic lung cancer is complex. Despite being on oxygen therapy, she is observed to have labored breathing and hypoxia. To clarify, hypoxia refers to insufficient oxygen at tissue level, while agonal respirations are abnormal pattern of breathing characterized by gasping, labored breathing, accompanied by strange vocalizations, and myoclonus. This could indicate an ongoing crisis in her health status. We are tasked with determining the most suitable next step in her treatment. PLANNING: Assumptions here could include: 1. The patient's breathing difficulties need immediate management. 2. The patient is experiencing distress due to severe dyspnea. 3. Palliative care principles should guide our decision as the patient is in a home hospice setting. Given these, I will question whether relieving her distress and dyspnea should be the primary focus in this setting. CLINICAL REASONING: Her pulse oximetry reading indicates hypoxia, which can be due to a combination of factors such as progressive disease or even a superadded infection. Dyspnea and anxiety in end-of-life care can be treated effectively with opioids like morphine through its effects on central perception of breathlessness and anxiety but, that in itself doesn't improve the oxygen saturation. Noninvasive ventilation could potentially improve oxygen saturation but could lead to discomfort and may not necessarily relieve the sensation of breathlessness. The goal in a terminally ill patient's care is to make the patient comfortable, not to increase lifespan. CONCLUDING: Thus, based on her condition, the principle of palliative care, and the potential effects and limitations of the suggested treatments, it seems our priority should be to relieve the patient's distress and labored breathing symptomatically. RESPONSE: Therefore, the most appropriate next step in management for this 76-year-old woman in home hospice care with labored breathing and hypoxia, despite oxygen therapy, would be to (A) Administer intravenous morphine. It would help alleviate her sensation of breathlessness and reduce anxiety, which aligns with palliative care principles.

**AI_E:** 1) RESTATING THE QUESTION: A 76-year-old woman, who has been diagnosed with end-stage metastatic lung cancer and is currently under home hospice care, is showing signs of labored breathing that have been going on for the last 3 hours despite being on supplementary oxygen via face mask. She appears in a weakened, thin condition and is mildly distressed, with visible effort seen in her breathing and occasional gasping respirations. We are asked to determine the most suitable next steps for her medical management given her condition. 2) PLANNING: We have several assumptions to consider in this question. Assumption [A] is to administer intravenous morphine which works by suppressing the respiratory centers in the brain thereby reducing the sensation of breathlessness. Assumption [B] is to admit the patient to the hospital for further evaluation which could help diagnose any new complications, but may not align with the patient's end-of-life wishes. Assumption [C] is to initiate a propofol infusion, an anesthetic used for sedation, but its use can be associated with respiratory depression. Assumption [D] is to transition the patient to noninvasive positive pressure ventilation which helps by providing breathing support but might not be comfortable for the patient. Assumption [E] is to take no further action, however, the patient's symptoms indicate that she is in distress and needs relief. 3) CLINICAL REASONING: Based on the clinical presentation, the patient is suffering from dyspnea secondary to end-stage metastatic lung cancer. The patient's need for oxygen, effort to breathe, and low oxygen saturation levels indicate respiratory distress. This is a common and distressing symptom in end-of-life care, particularly for patients with lung cancer. Given the patient's condition and the goal of hospice care to maintain comfort, a solution addressing symptom management, rather than aggressive treatment, aligns better with her care plan. 4) CONCLUDING: In hospice care, the main goal is to provide comfort and symptom relief rather than aggressive curative measures. Of all options, administering intravenous morphine would be the most appropriate as it helps to alleviate the sensation of breathlessness in end-stage respiratory disease patients. Hospital admission, propofol infusion, and noninvasive positive pressure ventilation seem aggressive and may disturb the patient's peace, which is not the aim in a hospice setting. 5) RESPONSE: Hence, we are led to conclude that the most appropriate next step in the management of this patient would be to "Administer intravenous morphine" (A) to provide symptom relief, maintain the patient's comfort and follow the patient's wishes with respect to end-of-life care.

**AI_A:** RESTATING THE QUESTION: The question pertains to a 76-year-old female patient in home hospice care for the terminal stage of metastatic lung cancer. For the past three hours, she has been experiencing difficulty in breathing, despite being administered 100% oxygen at a rate of 5 liters per minute. Her heart rate is slightly elevated (normal is 60-100 beats/min), and her pulse oximetry shows her oxygen saturation is lower than normal (>95% is considered normal). The patient looks malnourished, shows signs of increased effort in breathing with visibly contracting neck muscles, and sporadic agonal respiration. We are tasked to decide the most appropriate succeeding step to manage her condition. Agonal respiration, to put it simply, is an abnormal pattern of breathing marked by gasping, labored breaths, and possibly gurgling sounds, often occurring during severe illnesses like metastatic lung cancer or near death. PLANNING: Our assumptions here are that her labored breathing and hypoxia are caused by her end-stage lung cancer, the effects of cachexia, and possibly a progression or comorbidity, possibly involving the heart. We also need to remember the goals of hospice care, which primarily prioritizes the patient's comfort above all. By considering these factors, we can evaluate the options: (A) administering morphine, (B) admitting her to the hospital for further evaluation, (C) starting a propofol infusion, (D) transitioning to noninvasive positive pressure ventilation, or (E) not taking any further action. CLINICAL REASONING: Morphine (option A), a type of opioid, can actually decrease respiratory center sensitivity to carbon dioxide, reduce the work of breathing, and mitigate the distress associated with breathlessness, despite possibly reducing the respiratory rate. Hence, using it in a patient experiencing respiratory distress due to end-stage lung cancer might be beneficial and aligns with the goals of hospice care. Admitting her to the hospital (option B) might be unnecessary since the focus at this stage of disease (hospice) is mostly comfort, not aggressive investigative or therapeutic interventions. Furthermore, hospital environments often add stress that impairs the well-being of a terminally ill patient. Regarding propofol (option C), this medication would generally be unnecessary and potentially risky since it involves inducing general anesthesia. There are significant potential side effects such as hypotension, respiratory depression or even cessation, which are unneeded and could potentially exacerbate her current condition. Transitioning her to noninvasive positive pressure ventilation (option D) might temporarily relieve her respiratory distress, but it could also cause discomfort, distress and known risks, which might be inappropriate for a hospice patient. Choosing no further action (option E) contradicts the principle of hospice palliative care, which is to provide comfort and palliate symptoms. CONCLUDING: Upon reviewing and analyzing each option with clinical reasoning and within the context of a patient in home hospice care dealing with end-stage metastatic lung cancer and current symptoms, I lean towards the administration of morphine as being the most suitable course of action. RESPONSE: So, given the clinical setting and the clear aim of prioritizing the comfort of a patient in home hospice care, the most appropriate next step in managing this patient's distress and labored breathing due to her end-stage metastatic lung cancer is (A) Administer intravenous morphine.

**Facilitator** The consensus statement synthesizes the responses of AI_A, AI_B, AI_C, AI_D, and AI_E regarding the management of a 76-year-old woman with end-stage metastatic lung cancer who is experiencing labored breathing despite receiving 100% oxygen. The patient is in home hospice care, indicating a focus on comfort and symptom management rather than aggressive curative measures. The options for her management include administering intravenous morphine, admitting her to the hospital for further evaluation, initiating a propofol infusion, transitioning her to noninvasive positive pressure ventilation, or taking no further action. The consensus among the AI responses is that the most appropriate next step in managing this patient's condition is to administer intravenous morphine. This option aligns with the goals of hospice care, which prioritize the patient's comfort. Morphine can decrease the sensitivity of the respiratory center to carbon dioxide, reduce the work of breathing, and alleviate the distress associated with breathlessness. Admitting her to the hospital for further evaluation, initiating a propofol infusion, or transitioning her to noninvasive positive pressure ventilation may not align with the patient's overall care goals and could potentially exacerbate her current condition. Taking no further action contradicts the principle of hospice palliative care, which is to provide comfort and palliate symptoms. Therefore, given the clinical setting and the clear aim of prioritizing the comfort of a patient in home hospice care, the consensus is that the most appropriate next step in managing this patient's distress and labored breathing due to her end-stage metastatic lung cancer is to administer intravenous morphine.
