# Supplementary material for: Collaborative intelligence in AI: Evaluating the performance of a council of AIs on the USMLE
Source: PLOS Digit Health. 2025 Oct 9;4(10):e0000787. doi: 10.1371/journal.pdig.0000787 (PMC12510544; doi:10.1371/journal.pdig.0000787)
Supplement: S2 File — (ZIP) [file pdig.0000787.s002.zip › S2 Data Raw data files containing unprocessed question inputs and LLM outputs including transcripts of deliberations/STEP2_Question-83-1_Type-MC-NJ.docx]

**Facilitator** **[Round 1]:** A 42-year-old woman comes to the physician because of a 1-month history of moderate constant abdominal pain. She was evaluated in the emergency department 6 weeks ago for renal calculi and was told that her serum calcium concentration was increased. She has labile hypertension treated with hydrochlorothiazide and enalapril. Two brothers have hyperparathyroidism, and one sister has an adrenal tumor. The patient's temperature is 37.0°C (98.6°F), pulse is 80/min, respirations are 12/min, and blood pressure is 182/98 mm Hg. Funduscopic examination shows arteriovenous nicking. There is mild, diffuse tenderness to palpation of the abdomen. The remainder of the examination shows no abnormalities. Serum calcium concentration is 13.2 mg/dL. Urine metanephrine concentration is 4000 μg/24 h (N=140–785). This patient is most likely at risk for which of the following conditions? (A) Carcinoid syndrome (B) Chronic autoimmune (Hashimoto) thyroiditis (C) Familial hypocalciuric hypercalcemia (D) Medullary thyroid carcinoma (E) Parathyroid carcinoma

**AI_E:** RESTATING THE QUESTION: Our 42-year old female patient presents with chronic abdominal pain associated with a high calcium level in her serum. She is diagnosed with labile hypertension and has a family history of hyperparathyroidism and adrenal tumors. Clinical findings indicate high blood pressure, arteriovenous nicking revealed through funduscopy, and abdominal tenderness. She also has an elevated urine metanephrine level. Given these factors, we are asked to identify the high-risk condition she might be susceptible to among a list including Carcinoid syndrome, Chronic autoimmune (Hashimoto) thyroiditis, Familial hypocalciuric hypercalcemia, Medullary thyroid carcinoma, and Parathyroid carcinoma. PLANNING: Assumptions that can be made include that her hypercalcemia is due to hyperparathyroidism, given her family history and the fact that it's the most common cause of hypercalcemia. Her labile hypertension and high urine metanephrine levels suggest a problem with her adrenal gland, perhaps pheochromocytoma. If we combine these assumptions, an alternative assumption could be that she has Multiple Endocrine Neoplasia type1 or 2 (MEN 1 or MEN 2). MEN 1 involves parathyroid tumors, pituitary tumors and pancreatic tumors, while MEN 2 involves medullary thyroid carcinoma, pheochromocytoma, and parathyroid tumors. Of these two, MEN 2 is a better hypothesis due to the presence of high urine metanephrine, a marker for pheochromocytoma. CLINICAL REASONING: If she had MEN 1, we would expect to find signs of the other two types of tumors typical in this condition i.e., pituitary and pancreatic which have not been confirmed in the examination. On the other hand, elevated serum calcium and metanephrine levels, combined with a family history of hyperparathyroidism, support the diagnosis of MEN 2. The adrenal tumor in her family history might explain the elevated metanephrine levels due to pheochromocytoma, a tumor of the adrenal medulla that often occurs in MEN 2. CONCLUDING: If the patient has MEN 2, we could expect to find evidence of medullary thyroid carcinoma and/or parathyroid adenoma, in addition to the pheochromocytoma her presented symptoms suggest. Therefore, out of the choices presented, it is medullary thyroid carcinoma (D) that is a component of MEN 2, and thus it seems to fit the patient's clinical picture the best. RESPONSE: The patient is most likely at risk for option D, Medullary thyroid carcinoma.

**AI_C:** RESTATING THE QUESTION: This 42-year-old woman is experiencing abdominal pain, along with elevated serum calcium and urine metanephrine levels. She also has a family history of hyperparathyroidism and adrenal tumors. Our task is to identify the most likely condition she is at risk for, based on these clinical findings and familial history. PLANNING: The primary assumptive cause of abdominal pain, hypercalcemia, and increased urine metanephrines in the setting of a familial history of hyperparathyroidism and adrenal tumors could be a genetic disorder. We could also hypothesize endocrine or parathyroid related conditions due to the high level of serum calcium and familial history of hyperparathyroidism. However, another alternative assumption in this situation could be a multiple endocrine neoplasia syndrome, considering the family history of both hyperparathyroidism and adrenal tumors. CLINICAL REASONING: A critical element here is the elevated serum calcium and urine metanephrine concentration, suggesting an overactive endocrine system. Additionally, the patient's family history of hyperparathyroidism and adrenal tumors points towards a genetic predisposition. In particular, Multiple Endocrine Neoplasia (MEN) syndromes, which are genetic disorders leading to tumors in various endocrine glands, could explain these symptoms. For instance, MEN type 2 is associated with medullary thyroid carcinoma, pheochromocytoma (adrenal gland tumor), and parathyroid adenoma or hyperplasia. The elevated metanephrines in this patient's urine could result from a pheochromocytoma, a tumor of the adrenal medulla that secretes catecholamines, including metanephrines. Meanwhile, hyperparathyroidism could result from parathyroid adenomas, leading to an overproduction of parathyroid hormone and subsequent hypercalcemia. CONCLUDING: The various factors, such as the family history of hyperparathyroidism and adrenal tumors, elevated serum calcium levels, and increased urine metanephrine levels, lead us to consider a diagnosis related to multiple endocrine neoplasia. RESPONSE: Out of the options given, Medullary Thyroid Carcinoma (D) fits the symptoms and family history best, as it is commonly associated with MEN type 2, which takes into account the patient's familial predispositions and clinical presentations.

**AI_B:** In essence, a 42-year-old woman with a history of abdominal pain, renal calculi, elevated serum calcium, labile hypertension, and a family history of hyperparathyroidism and adrenal tumors, presents with hypertension and increased urinary metanephrine. She seems to be inquiring about the health risk that she has concerning some specified conditions, including Carcinoid syndrome, Chronic autoimmune (Hashimoto) thyroiditis, Familial hypocalciuric hypercalcemia, Medullary thyroid carcinoma, and Parathyroid carcinoma. Metanephrines are the metabolites of catecholamines (adrenaline and noradrenaline), and their increased level in urine signifies an excess release of these hormones, usually indicative of an adrenal tumor such as pheochromocytoma. Thus, it would be reasonable to investigate this patient's condition from the perspective of hormonal abnormalities, considering her significant family history. To evaluate which clinical condition might be probable, one pathway to pursue is the link between her symptoms, the elevated serum calcium, and familial hyperparathyroidism. Another is her labile hypertension and elevated urinary metanephrines, which is typical of pheochromocytoma. The final potential pathway is familial clustering of these tumors - her brothers' hyperparathyroidism and sister's adrenal tumor might suggest a hereditary syndrome. A clinical thought process needs to consider the possibility of multiple endocrine neoplasia or MEN, characterized genetically by an autosomal dominant inheritance and clinically by the occurrence of tumors involving two or more endocrine glands. In this case, MEN type 2a should be considered given the finding of hyperparathyroidism and adrenal tumor in the family, as it often consists of medullary thyroid carcinoma, pheochromocytoma, and parathyroid hyperplasia. In conclusion, this patient, considering her familial history, physical examination findings, and elevated laboratory markers, would most likely be at risk for Medullary thyroid carcinoma (MEN 2A). This condition, due to RET proto-oncogene mutations, leads to cancer formation in different endocrine glands such as the thyroid (where medullary carcinoma is common), adrenal glands (pheochromocytoma), and even the parathyroid glands. Therefore, the woman in question is most likely at risk for Medullary thyroid carcinoma (option D). Her labile hypertension along with elevated urinary metanephrines points towards a pheochromocytoma. Moreover, hypercalcemia and her family history denotes a possible parathyroid disorder. The combined evidence leads to a suspicion of multiple endocrine neoplasia (MEN) type 2A, which includes medullary thyroid carcinoma as its prominent feature.

**AI_A:** RESTATING THE QUESTION: The 42-year-old female presents with moderate persistent abdominal pain for a month, a history of high serum calcium concentrations, fluctuating hypertension that she controls with hydrochlorothiazide and enalapril, a familial history of hyperparathyroidism in two brothers and adrenal tumor in a sister, now observed with arteriovenous nicking in funduscopic examination, mild generalized tenderness upon abdominal palpation, a serum calcium concentration of 13.2 mg/dL and significantly high urine metanephrine levels. Given all this, the question looks for the condition this patient is most susceptible to. PLANNING: Previous hyperparathyroidism cases in the family, remarkably increased serum calcium levels, drastic fluctuations in blood pressure, and elevated urine metanephrine concentrations suggest the possibility of either a parathyroid or adrenal abnormality. Considering patient's genetic predisposition and her clinical and laboratory findings, one should prioritize hypotheses of disorders that occur due to inherited factors affecting the parathyroid glands or the adrenal glands. CLINICAL REASONING: Hyperparathyroidism, characterized by high concentrations of serum calcium, could explain the renal calculi. However, the drastic changes in blood pressure, manifesting as labile hypertension and arteriovenous nicking observed during funduscopic examination, point out potential adrenal abnormalities. Furthermore, the elevated levels of urine metanephrine coincide with an adrenal tumor's typical manifestation which often secretes catecholamines, a group of hormones that includes metanephrines. Also, because the patient's sister had an adrenal tumor, it's plausible this patient may have a similar disorder. While hyperparathyroidism and adrenal tumors aren't typically linked, genetically inherited syndromes, such as Multiple Endocrine Neoplasia (MEN), may explain the coincidence of these two phenomena in the patient's familial history. CONCLUDING: Given the genetic history, the fluctuating hypertension, and the increased urine metanephrine levels, the woman appears to be suffering from an adrenal gland condition that secretes catecholamines. Moreover, the presence of hyperparathyroidism in the family and her high serum calcium level suggest the existence of an inherited syndrome affecting multiple endocrine glands. RESPONSE: This patient's clinical picture aligns most closely with Multiple Endocrine Neoplasia type 2A or 2B (MEN 2A or MEN 2B), where medullary thyroid carcinoma is the predominant risk. Medullary thyroid carcinoma affects practically all individuals with MEN 2B and about 95% of those with MEN 2A. Therefore, the most probable answer is (D) Medullary thyroid carcinoma.

**AI_D:** RESTATING THE QUESTION: The information provided relates to a 42-year-old woman who is experiencing persistent abdominal pain and has a history of renal calculi. The patient presents with elevated serum calcium and labile hypertension. She has familial antecedents of hyperparathyroidism and adrenal tumor. Vital signs are mostly within normal limits, except for the elevated blood pressure. The abnormal funduscopic examination shows arteriovenous nicking. The patient's heightened serum calcium concentration and significantly increased urine metanephrine concentration raise suspicion of a specific underlying condition. Our task, thus, is to surmise from these findings what condition she is most likely at risk for. PLANNING: The first assumption that can be deduced from the provided information is that the ongoing abdominal pain, high serum calcium, and family history of hyperparathyroidism and adrenal tumor, could suggest some defect in the parathyroid glands or adrenal glands, particularly with the known familial traits. Alternatively, the increased urine metanephrine levels could indicate a catecholamine-secreting tumor like a pheochromocytoma. The patient's labile hypertension, arteriovenous nicking, and family history further support this assumption. It would be clinically rational to prioritize examining the relationships between all these symptoms and conditions, specifically focusing on disorders that encompass both parathyroid and adrenal gland anomalies. CLINICAL REASONING: Renal calculi, hypercalcemia, and a history of hyperparathyroidism among relatives imply a likelihood of parathyroid gland disorder. Parathyroid disorders are also known to cause labile hypertension. However, the addition of elevated urine metanephrine and a family history of an adrenal tumor also suggest a catecholamine-secreting tumor, such as pheochromocytoma, which is an adrenal gland disorder known to cause labile hypertension and present with elevated urine metanephrine levels. Notably, a genetic disorder that includes both parathyroid gland and adrenal gland anomalies is Multiple Endocrine Neoplasia type 1 (MEN1). CONCLUDING: A key clinical consideration in this case is the constellation of symptoms and signs related to both the parathyroid and adrenal glands, along with the familial history of disorders in these glands. The presence of the elevated serum calcium, high urine metanephrine, labile hypertension, and arteriovenous nicking are all indicative of a condition that affects multiple endocrine organs. RESPONSE: Considering all the given patient details, the most likely condition this patient is at risk for is Familial hypocalciuric hypercalcemia (Choice C) that might be part of a broader syndrome such as Multiple Endocrine Neoplasia (not listed in the answer options). Familial hypocalciuric hypercalcemia is characterized by mild to moderate hypercalcemia, along with low-normal or decreased urinary calcium excretion. The condition is usually detected in families with abnormalities associated with multiple endocrine glands, such as the parathyroids and adrenal gland here.

**Facilitator** **[Round 2]:** Summary: AI_A, AI_B, AI_C, and AI_E all agree that the patient's symptoms, family history, and lab results suggest Multiple Endocrine Neoplasia type 2A or 2B (MEN 2A or MEN 2B), with Medullary thyroid carcinoma being the most likely risk. They all highlight the patient's elevated serum calcium and urine metanephrine levels, labile hypertension, and family history of hyperparathyroidism and adrenal tumors as indicative of this condition. AI_D, however, suggests that the patient might be at risk for Familial hypocalciuric hypercalcemia, which could be part of a broader syndrome such as Multiple Endocrine Neoplasia. AI_D emphasizes the symptoms related to both the parathyroid and adrenal glands, along with the familial history of disorders in these glands. Key Differences: The key difference lies in the interpretation of the patient's symptoms and family history. While AI_A, AI_B, AI_C, and AI_E focus on the combination of symptoms to suggest MEN 2A or 2B, AI_D considers the possibility of Familial hypocalciuric hypercalcemia as part of a broader syndrome like MEN. Question for Council Members: Could you elaborate on why you believe the patient's symptoms and family history suggest Multiple Endocrine Neoplasia type 2A or 2B (MEN 2A or MEN 2B) versus Familial hypocalciuric hypercalcemia as part of a broader syndrome like MEN? What specific factors led you to your conclusion?. Based on your preceding response, how would you answer the original question: [A 42-year-old woman comes to the physician because of a 1-month history of moderate constant abdominal pain. She was evaluated in the emergency department 6 weeks ago for renal calculi and was told that her serum calcium concentration was increased. She has labile hypertension treated with hydrochlorothiazide and enalapril. Two brothers have hyperparathyroidism, and one sister has an adrenal tumor. The patient's temperature is 37.0°C (98.6°F), pulse is 80/min, respirations are 12/min, and blood pressure is 182/98 mm Hg. Funduscopic examination shows arteriovenous nicking. There is mild, diffuse tenderness to palpation of the abdomen. The remainder of the examination shows no abnormalities. Serum calcium concentration is 13.2 mg/dL. Urine metanephrine concentration is 4000 μg/24 h (N=140–785). This patient is most likely at risk for which of the following conditions? (A) Carcinoid syndrome (B) Chronic autoimmune (Hashimoto) thyroiditis (C) Familial hypocalciuric hypercalcemia (D) Medullary thyroid carcinoma (E) Parathyroid carcinoma]

**AI_E:** RESTATING THE QUESTION: The physician is asked to identify the medical risk most likely faced by this 42-year-old woman, who has presented with constant abdominal pain, renal calculi, high serum calcium and urine metanephrine levels, and a family history of hyperparathyroidism and adipose tumor. PLANNING: A noteworthy facet of her health situation is her elevated serum calcium and urine metanephrine levels, linked to the function of the parathyroid and adrenal glands under normal conditions. Her familial history adds a potential hereditary component to the evaluation. Both of these factors point toward a systemic, rather than isolated, issue affecting multiple endocrine organs. CLINICAL REASONING: This collaborative dynamic between elevated serum calcium and heightened urine metanephrine, besides labile hypertension, hints at a condition known as Multiple Endocrine Neoplasia (MEN). MEN is a group of rare, inherited disorders leading to tumors in several endocrine glands. Individual types of MEN (1, 2A, 2B, and 4) are associated with different constellations of neoplastic disorders. CONCLUDING: The specific form of MEN suggested by the patient's symptoms is type 2A or 2B, as these types manifest as medullary thyroid carcinoma, pheochromocytomas, and hyperparathyroidism. These symptoms reflect the patient's clinical picture involving abnormal parathyroid and adrenal function (evidenced by high calcium and metanephrine levels and hypertensive state) combined with her family history. RESPONSE: This brings us to the conclusion that the patient is most likely at risk for Medullary Thyroid Carcinoma (option D) as this is a typical condition associated with MEN types 2A and 2B.

**AI_C:** The question presented pertains to the most likely risk for a 42-year-old woman with moderate, constant abdominal pain, notable lab and clinical findings, and a family history of endocrine disorders. I'll break down the information provided. Firstly, she's previously been evaluated for kidney stones (renal calculi) and was informed of an elevated serum calcium level. An elevated calcium concentration often suggests a dysfunction in the calcium homeostasis in the body, which is regulated by the parathyroid glands. This could mean a problem in the parathyroid glands themselves or in the calcium/vitamin D intake/absorption/balance. Secondly, her hypertension, specifically the labile type - meaning it fluctuates - prompts an investigation into the adrenal glands, as they regulate blood pressure through hormones such as aldosterone and adrenaline. Her urine metanephrine concentration being significantly high further points to an adrenal gland dysfunction, specifically, a pheochromocytoma, which is a rare tumor of the adrenal medulla that secretes metanephrines. Combining this with the family history of both hyperparathyroidism and adrenal tumors, the picture suggests the presence of a familial syndromic condition. One such condition that fits is Multiple Endocrine Neoplasia (MEN), a group of disorders that affect the body's endocrine system. MEN type 2A and 2B both involve medullary thyroid carcinoma, pheochromocytomas, and parathyroid tumors which explain the lab and clinical findings. However, medullary thyroid carcinoma (D), would be a specific condition that the patient is at risk for under the broader umbrella of MEN. Therefore, after a careful investigation into each assumption, my concluding response, based on the clinical reasoning, is that the patient is most likely at risk for Medullary thyroid carcinoma (D), due to the suspicion of her having Multiple Endocrine Neoplasia. It's essential to remember that although other options such as Parathyroid carcinoma (E) might seem viable, the elevation in urine metanephrines is pointing to a pheochromocytoma which is not a feature of parathyroid carcinoma, but is for Medullary thyroid carcinoma under the context of MEN.

**AI_D:** (1) RESTATING THE QUESTION: The scenario revolves around a 42-year-old woman who presented with abdominal pain, evaluated six weeks ago for kidney stones, was informed about elevated calcium levels, and treated for labile hypertension. She also has a familial history of hyperparathyroidism and adrenal tumors. Her tests showed elevations in both serum calcium and urine metanephrine, suggestive of hyperparathyroidism and pheochromocytoma respectively. The key question is to identify the condition she is most likely at risk for. (2) PLANNING: Her elevations in serum calcium and urine metanephrine parallel with symptoms of abdominal pain could suggest a single condition causing these manifestations, rather than several standalone conditions. A widespread issue could be from hereditary syndromes like Multiple Endocrine Neoplasia (MEN), where multiple organs are involved concurrently. On another thought, could the previously detected renal calculi, labile hypertension, and family history favor a syndrome such as Familial hypocalciuric hypercalcemia (FHH), which typically doesn't involve adrenal glands but does produce elevated serum calcium levels and could increase kidney stone risk? Analyzing these assumptions should shed light on the most suitable answer. (3) CLINICAL REASONING: MEN has subtypes (2A, 2B) which could present with hyperparathyroidism and pheochromocytoma concurrently, in addition to the possibility of medullary thyroid carcinoma (MTC), explaining the combination of symptoms from parathyroid and adrenal glands. On the other hand, FHH is more limited in its manifestations and doesn't typically involve adrenal issues. A prevalence of elevated urine metanephrines isn't usually associated with FHH, but more closely resembles adrenal gland issues, or pheochromocytoma in MEN 2 types. Therefore, even though this patient could theoretically have FHH given her serum calcium levels and renal stones, it's less likely because she also exhibits features indicative of pheochromocytoma. (4) CONCLUDING: Comparing the MEN syndrome, particularly MEN 2 types, with FHH, the former is more suited to her presentation, considering the additional symptoms like hypertension, increased urine metanephrine levels (suggesting functioning adrenal tumor), which aren't typically expected in FHH. Additionally, the family histories of hyperparathyroidism in two brothers and adrenal tumor in one sister could suggest the hereditary nature potentially seen in syndromes like MEN. (5) RESPONSE: Considering the comprehensive clinical picture, this patient is most likely at risk for Medullary thyroid carcinoma (choice D), which is associated with MEN 2A and MEN 2B, syndromes that could simultaneously explain hyperparathyroidism (reflected by her elevated calcium levels) and pheochromocytoma (suggested by her increased metanephrine level).

**AI_A:** RESTATING THE QUESTION: The question is asking which condition a 42-year-old woman is most likely at risk for, given her symptoms, medical history, and lab results. She has a 1-month history of moderate constant abdominal pain, was diagnosed with renal calculi six weeks ago, and has an increased serum calcium concentration. She also has labile hypertension treated with hydrochlorothiazide and enalapril. Her family history includes two brothers with hyperparathyroidism and a sister with an adrenal tumor. She is showing arteiovenous nicking on funduscopic exam, mild diffuse tenderness across her abdomen, a serum calcium concentration of 13.2 mg/dL, and increased urine metanephrine concentrations. From these symptoms, we are given several conditions to consider. PLANNING: 1. First assumption: Our initial assumption, given her familial history of hyperparathyroidism and the presence of increased serum calcium levels on lab evaluation, could lead us to believe that this patient may have a parathyroid-related disorder like hyperparathyroidism. 2. Alternate assumption: However, considering her other clinical features, like the presence of labile hypertension, an adrenal tumor in the family, and the elevated urine metanephrine levels, another assumption could be that this patient might have a more complex syndrome like Multiple Endocrine Neoplasia (MEN). 3. Evaluation/ Hypothesized Assumption: Upon hypothesizing as well as recognizing the pattern of her clinical presentation, genetics, and maternal family history of hyperparathyroidism, we can lean towards an increased risk for a syndrome-like MEN. CLINICAL REASONING: Labile hypertension, adrenal tumor in family, and increased metanephrine levels point towards an adrenal disorder (specifically, pheochromocytoma, an adrenal medulla tumor characterized by high metanephrine). Furthermore, hyperparathyroidism in the family along with her raised serum calcium levels hint towards a parathyroid disorder. These combined symptoms are suggestive of a form of MEN syndrome. MEN 2A, for instance, presents with medullary thyroid carcinoma, pheochromocytoma, and hyperparathyroidism. Elevated calcium levels indicate parathyroid involvement and elevated metanephrine levels are indicative of a pheochromocytoma (adrenal medulla tumor). CONCLUDING: After analyzing all the given information, the patient's presentation aligns closely with Multiple Endocrine Neoplasia (MEN) 2A. Moving beyond just the presence of hyperparathyroidism, one has to also consider the pheochromocytoma presentation. RESPONSE: Among the provided options, the patient is most likely at risk for (D) Medullary thyroid carcinoma, a component of MEN 2A.

**AI_B:** Rephrasing the question, this 42-year-old woman is experiencing abdominal pain, hypercalcemia diagnosed during a previous visit for kidney stones, labile hypertension, and a family history of hyperparathyroidism and adrenal tumors. Her recent laboratory results indicate high levels of calcium and urine metanephrine. We need to identify which medical condition she is at highest risk for, based on her symptoms and family history. The options are Carcinoid syndrome, Chronic autoimmune (Hashimoto) thyroiditis, Familial hypocalciuric hypercalcemia, Medullary thyroid carcinoma, and Parathyroid carcinoma. When dealing with this case, it is important to consider its key aspects: a familial background of hyperparathyroidism and adrenal tumor; a contemporary history of hypercalcemia; clinical findings of hypertension, and labile, hypertensive changes in fundus examination; and significantly elevated urine metanephrine level. These factors would narrow down our focus towards conditions that commonly present with such symptoms and fall within a hereditary context. One significant risk to keep in mind is that parathyroid disorders such as hyperparathyroidism and parathyroid carcinoma might lead to hypercalcemia. Since the familial pattern is suggestive, it is plausible to consider Familial hypocalciuric hypercalcemia. Medullary thyroid carcinoma, another familial condition, is typically connected with MEN type 2 syndromes and could also show hypercalcemia as a secondary feature due to the production of hormonal substances causing an excessive release of calcium into the bloodstream. With this patient's elevated urine metanephrine levels, the prospect of a carcinoid syndrome, in which serotonin-producing tumors lead to elevated urinary 5-HIAA levels, becomes less likely. Bringing these considerations together, I would hypothesize that Multiple Endocrine Neoplasia, either type 2A or 2B, appears most likely in this case. This condition can incorporate adrenal, parathyroid, and thyroid pathologies – which mimic the patient's symptoms. To create a step-by-step clinical rationale, I would first link the family history to the possible presence of a hereditary condition. Genetic factors play a significant role in conditions like hyperparathyroidism and adrenal tumors. Second, the presence of hypercalcemia and hypertension, along with elevated serum metanephrines, point towards involvement of multiple endocrine glands, guiding us to consider Multiple Endocrine Neoplasia. Third, recalling that Medullary Thyroid Carcinoma can be part of the MEN syndrome, it aligns well with the presentation and family history of this patient. Considering these factors, my final conclusion leads me to believe that this patient is most likely at risk of developing Medullary Thyroid Carcinoma (D), associated with Multiple Endocrine Neoplasia (type 2A or 2B), given the combination of her symptoms and family history.

**Facilitator** The question pertains to a 42-year-old woman with a history of moderate constant abdominal pain, renal calculi, elevated serum calcium concentration, labile hypertension, and a family history of hyperparathyroidism and adrenal tumors. Her lab results show increased serum calcium and urine metanephrine concentrations. The task is to identify the condition she is most likely at risk for. The woman's symptoms, lab results, and family history suggest a systemic issue affecting multiple endocrine organs, rather than isolated conditions. Her elevated serum calcium levels could indicate a parathyroid-related disorder, such as hyperparathyroidism. However, her labile hypertension, family history of adrenal tumors, and elevated urine metanephrine levels suggest an adrenal disorder, specifically pheochromocytoma, a tumor of the adrenal medulla that secretes metanephrines. Given the combination of these symptoms, the patient's condition aligns closely with Multiple Endocrine Neoplasia (MEN), a group of inherited disorders leading to tumors in several endocrine glands. Specifically, her symptoms suggest MEN type 2A or 2B, which manifest as medullary thyroid carcinoma, pheochromocytomas, and hyperparathyroidism. These symptoms reflect the patient's clinical picture involving abnormal parathyroid and adrenal function, combined with her family history. Therefore, the consensus among the responses is that the patient is most likely at risk for Medullary Thyroid Carcinoma (option D), a condition associated with MEN types 2A and 2B. This conclusion is based on her elevated serum calcium and urine metanephrine levels, labile hypertension, and family history of hyperparathyroidism and adrenal tumors.
