# Supplementary material for: Collaborative intelligence in AI: Evaluating the performance of a council of AIs on the USMLE
Source: PLOS Digit Health. 2025 Oct 9;4(10):e0000787. doi: 10.1371/journal.pdig.0000787 (PMC12510544; doi:10.1371/journal.pdig.0000787)
Supplement: S2 File — (ZIP) [file pdig.0000787.s002.zip › S2 Data Raw data files containing unprocessed question inputs and LLM outputs including transcripts of deliberations/STEP2_Question-84-1_Type-MC-NJ.docx]

**Facilitator** **[Round 1]:** A 26-year-old woman with common variable immunodeficiency comes to the office because of a 2-month history of night sweats. She also has had a 10-kg (22-lb) weight loss during the past 3 months despite no change in appetite. She has a 15-year history of recurrent sinusitis and otitis media. Her only medication is intravenous immune globulin. She is 163 cm (5 ft 4 in) tall and weighs 54 kg (120 lb); BMI is 21 kg/m². Vital signs are within normal limits. Examination shows cervical and epitrochlear lymphadenopathy. Which of the following is the most appropriate next step in management? (A) Lymph node biopsy (B) Methylprednisolone therapy (C) Penicillin therapy (D) Plasmapheresis (E) Trimethoprim-sulfamethoxazole therapy

**AI_E:** (1) RESTATING THE QUESTION: The question is about a 26-year-old woman who presents with a number of symptoms including night sweats, significant weight loss, and lymphadenopathy. She has a diagnosis of common variable immunodeficiency (CVID), characterized by hypogammaglobulinemia leading to recurrent infections. Her past medical history is notable for recurrent sinusitis and otitis media, consistent with her immunodeficiency. The question is asking for the most appropriate next step in managing her current condition. (2) PLANNING: The main assumption here could be that the night sweats, weight loss, and lymphadenopathy are due to an ongoing infection due to her immunodeficiency. However, given the chronicity and severity of her symptoms, another underlying condition such as malignancy should be considered. Therefore, the alternate assumption would be that her symptoms indicate a possible malignancy. (3) CLINICAL REASONING: In CVID patients, chronicity and severity of symptoms suggest the possibility of lymphoma, which is more common in these patients than in the general population. Night sweats, weight loss, and lymphadenopathy are part of the systemic 'B' symptoms often seen in lymphoma, further supporting this assumption. Her history of recurrent sinusitis and otitis media are standard for CVID but do not account for these new findings. (4) CONCLUDING: Given these considerations, the first step in managing this patient would be to investigate the possibility of lymphoma, as this would drastically alter her treatment plan and prognosis. (5) RESPONSE: Therefore, among the listed options, the most appropriate next step in management would be (A) Lymph node biopsy. This would allow for histopathological examination of the lymph node, potentially identifying if a malignancy such as lymphoma is present.

**AI_D:** RESTATING THE QUESTION: The patient is presenting with some worrisome symptoms including weight loss, night sweats and lymphadenopathy. These can often be associated with malignancy such as lymphoma, which is more common among patients with common variable immunodeficiency (CVID). CVID is a condition characterized by low levels of most or all of the immunoglobulin (Ig) classes which leads to an increased susceptibility to infections. Intravenous immune globulin (IVIG) is a treatment for CVID that provides the antibodies the immune system is not making on its own. PLANNING: My initial assumption is that her symptoms are likely related to an infection, considering her immunodeficient condition. However, I need to consider alternative assumptions as well. One could be that her symptoms might be due to an underlying malignancy, hence examining the lymph nodes could provide us with useful information. Another possibility is that her symptoms might merely be a flare-up of her underlying condition, in which case therapy with corticosteroids might prove beneficial. It is equally important to consider opportunistic infections considering her immunodeficient state. The decision to choose which alternate assumption to pursue clinically is a matter of balancing these possibilities. CLINICAL REASONING: Her weight loss and night sweats are not typical of a simple infection, even in an immunosuppressed individual, and the presence of lymphadenopathy further indicates something more serious. This suggests that the clinical picture points more towards a lymphoproliferative disorder, such as a lymphoma, than an uncontrolled infection. Although she’s receiving IVIG which helps with antibody deficiencies - it does not prevent lymphoid malignancies, which are common in CVID patients. CONCLUDING: As such, it becomes necessary to rule out this serious condition and therefore a lymph node biopsy would be the most appropriate next step. The biopsy would either confirm or rule out lymphoma and guide further management accordingly. RESPONSE: Thus, in the case of a 26-year-old woman with CVID presenting with night sweats, weight loss, and lymphadenopathy, the most appropriate next step in management would be (A) Lymph node biopsy.

**AI_B:** RESTATING THE QUESTION: The clinical scenario describes a 26-year-old woman who has common variable immunodeficiency (CVID), a disorder that impairs the immune system. She's been suffering from night sweats and significant weight loss without any apparent reasons. She has a history of recurrent sinusitis and otitis media, which are common complications of CVID due to her reduced immunity. She's currently on intravenous immune globulin, a standard therapy for CVID. A physical examination shows cervical and epitrochlear lymphadenopathy. Now we need to decide the next step in her management, from these options - (A) undertaking a lymph node biopsy, (B) starting methylprednisolone therapy, (C) starting penicillin therapy, (D) initiating a process called plasmapheresis, or (E) beginning trimethoprim-sulfamethoxazole therapy. PLANNING: Given the symptoms and signs, the priority is to determine the cause of her generalized symptoms such as night sweat and the unexpected weight loss, and the newly appeared lymphadenopathy. Other frequent infections are related to her CVID condition and seem already managed by immune globulin therapy. Therefore, the assumptions include a possible progress of her immunodeficiency or a new pathological process causing these symptoms and signs. CLINICAL REASONING: The progressive symptoms and new lymphadenopathy in a patient with immunodeficiency can suggest possibilities like an opportunistic infections or malignancy, especially lymphoma which is a recognized risk in patients with CVID. Infections should usually improve with her immunoglobulin therapy, and if they were the cause of her systemic symptoms and lymphadenopathy, she might have other signs of severe infection which are absent in her case currently. That leads us to suspect a potential malignancy, mainly lymphoma, as a more probable explanation of her condition. CONCLUDING: Considering the patient's new occurrence of weight loss, night sweats, and lymphadenopathy, in conjunction with her existing history of CVID which makes her more prone to developing lymphoma, it is reasonably appropriate to consider taking a lymph node biopsy for accurate diagnosis. Examination of the living tissue under a microscope would allow for an accurate diagnosis and guide subsequent management. RESPONSE: Thus, the most appropriate next step in management for this patient is (A) a lymph node biopsy. This would help ascertain if her symptoms are due to the suspected lymphoma or any other underlying condition, enabling targeted management accordingly.

**AI_C:** (1) RESTATING THE QUESTION: The presented scenario is of a 26-year-old woman diagnosed with common variable immunodeficiency (CVID). This immunodeficiency condition is known for the reduction or absence of certain immunoglobulins, leading to an inability of the immune system to efficiently defend against pathogens. This causes recurrent infections including sinusitis and otitis media, as seen in our patient. The patient is expressing concerns about experiencing night sweats and significant weight loss over the past few months, despite no changes in her appetite. On physical examination, she manifests lymphadenopathy, swelling of the lymph nodes in the neck and elbow region. The query is regarding the most suitable further action to manage her current condition. (2) PLANNING: Current symptoms of night sweats and weight loss, in combination with lymphadenopathy, might suggest systemic disease progression such as malignancies like lymphoma or generalized infection. Therefore, it’s critical to evaluate these potential developments. While therapeutic alternatives like methylprednisolone, penicillin, plasmapheresis, or trimethoprim-sulfamethoxazole might be beneficial in certain circumstances, these pharmacotherapies should be based on the specific underlying condition. Therefore, determining the exact cause of her new symptoms is paramount and will likely require further evaluation. (3) CLINICAL REASONING: Individuals with common variable immunodeficiency are at risk for several complications, including autoimmune disorders, granulomatous disease, and malignancies such as lymphoma. The symptoms of lymphoma often include fever, night sweats, and unexplained weight loss – often referred to as B symptoms – along with lymphadenopathy. Based on the clinical picture, these symptoms along with her history of CVID and the identified lymphadenopathy suggest the possibility of a lymphoproliferative disorder like lymphoma. Immediate treatment without a specific diagnosis could mask the symptoms or even have detrimental effects if the chosen treatment doesn't align with the actual condition. (4) CONCLUDING: Given her symptoms and clinical history, a lymph node biopsy seems to be the most appropriate next action. This diagnostic test will provide valuable information about possible malignancy such as lymphoma or other systemic disorders that may be causing these symptoms. Once we have a clearer understanding of her condition, a more directed treatment plan can be developed. (5) RESPONSE: Therefore, the most appropriate next step in management for this patient should be a lymph node biopsy (Choice A), to provide a definite diagnosis and guide future treatment decisions. Methylprednisolone therapy (Choice B), Penicillin therapy (Choice C), Plasmapheresis (Choice D), or Trimethoprim-sulfamethoxazole therapy (Choice E) might be potential steps following a confirmed diagnosis but are not appropriate at this point without proper identification of the patient's condition.

**AI_A:** RESTATING THE QUESTION: The query pertains to a 26-year-old female patient diagnosed with common variable immunodeficiency, who has been experiencing night sweats and significant weight loss over the last 2-3 months without any appetite changes. She has a medical history of recurrent sinusitis and otitis media. Her current therapy includes intravenous immune globulin. Upon examination, cervical and epitrochlear lymphadenopathy were discovered. In this context, we are being asked to identify the most appropriate next course of action, among options including lymph node biopsy, methylprednisolone therapy, penicillin therapy, plasmapheresis, and trimethoprim-sulfamethoxazole therapy. PLANNING: Typically, persistent night sweats and significant weight loss are signs associated with systemic diseases, infections, malignancies or autoimmune diseases. Among those, some would be more unlikely with this patient due to her specific condition of common variable immunodeficiency (CVID). Given her history of recurrent infections and detected lymphadenopathy, it might indicate either recurrent, chronic infections or development of a malignancy, such as lymphoma. It is critical to rule out a potential malignancy due to the patient's immunodeficient state. So, performing a lymph node biopsy can be crucial. Looking into the other possible choices, methylprednisolone, penicillin, plasmapheresis, and trimethoprim-sulfamethoxazole don't seem to be the priority in this case without further diagnostic investigation. CLINICAL REASONING: The systemic symptoms (night sweats and weight loss) along with existing lymphadenopathy, and her immunodeficient status need to be thoroughly investigated. The current vital signs make an active infection less likely. A lymph node biopsy would help in identifying the cause of the lymphadenopathy - be it a neoplastic condition (such as lymphoma, which is higher in CVID patients) or a chronic granulomatous disease. Methylprednisolone and plasmapheresis are therapies used in severe phases of autoimmune diseases or organ transplant rejections, which are not evident in this case. Penicillin and Trimethoprim-sulfamethoxazole therapies are specific antimicrobial treatments usually administered for known bacterial infections. However, the presented systemic signs, weight loss and night sweats, are generally not prominent with localized infections and the patient doesn't present other signs of active infections. Hence, these choices would come later based on the biopsy results, if an infection source is identified. CONCLUDING: It is crucial to recognize the potential red flags of systemic disease or malignancy in this patient with common variable immunodeficiency, who presents with night sweats, significant weight loss, and lymphadenopathy without signs of active infection. The priority lies in diagnosing the cause, rather than presumptively initiating antimicrobial or immunosuppressive therapy. The risks of potential scenarios, the patient's history, and the suspicious physical examination findings, all point towards the necessity of a lymph node biopsy. RESPONSE: Therefore, the most appropriate next step in the management of this patient would be to perform a lymph node biopsy (Choice A).

**Facilitator** The consensus among the AI responses is that the most appropriate next step in managing the 26-year-old woman with common variable immunodeficiency (CVID) who presents with night sweats, significant weight loss, and lymphadenopathy, is to perform a lymph node biopsy (Choice A). This decision is based on the patient's symptoms and clinical history, which suggest the possibility of a systemic disease or malignancy, such as lymphoma, a condition more common in patients with CVID. The systemic symptoms, including night sweats and weight loss, along with the detected lymphadenopathy, warrant thorough investigation. A lymph node biopsy would provide valuable information about possible malignancy or other systemic disorders causing these symptoms. Once a clearer understanding of her condition is obtained, a more directed treatment plan can be developed. Other therapeutic options like methylprednisolone, penicillin, plasmapheresis, or trimethoprim-sulfamethoxazole might be beneficial in certain circumstances, but these should be based on the specific underlying condition identified through the biopsy.
