# Supplementary material for: Collaborative intelligence in AI: Evaluating the performance of a council of AIs on the USMLE
Source: PLOS Digit Health. 2025 Oct 9;4(10):e0000787. doi: 10.1371/journal.pdig.0000787 (PMC12510544; doi:10.1371/journal.pdig.0000787)
Supplement: S2 File — (ZIP) [file pdig.0000787.s002.zip › S2 Data Raw data files containing unprocessed question inputs and LLM outputs including transcripts of deliberations/STEP2_Question-85-1_Type-MC-NJ.docx]

**Facilitator** **[Round 1]:** A 9-month-old boy is brought to the office by his mother because of a 6-hour history of persistent fussiness and decreased appetite. Two weeks ago, he was diagnosed with an upper respiratory tract infection. The symptoms have since resolved. He lives with his parents and a pet lizard. His diet consists of cow milk-based formula, baby food, and some solid foods. He has no history of serious illness and receives no medications. Immunizations are up-to-date. Today, he is inconsolable. He is at the 50th percentile for length and weight. Examination shows multiple flaccid bullae on an erythematous base over the buttocks. The mother says she had no knowledge of these findings. Which of the following is the most appropriate next step in management? (A) Abdominal ultrasonography (B) Anti–Saccharomyces cerevisiae antibody testing (C) Nitroblue tetrazolium testing (D) Rectal swab for group B streptococcus (E) Skeletal survey (F) Stool culture for Salmonella enteritidis

**AI_C:** 1) RESTATING THE QUESTION: The problem is about a 9-month-old boy who was brought in by his mother due to fussiness and decreased appetite for the past 6 hours. He had an upper respiratory tract infection that was treated 2 weeks ago, but now he has flaccid bullae on an erythematous base over his buttocks. Given his clinical presentation and history, we need to decide what would be the most appropriate next step in management. 2) PLANNING: The boy doesn't seem to have a history of a serious illness or medication use, and he lives with a pet lizard. Given the sudden intercourse of symptoms and the occurrence of flaccid bullae on the buttocks, differential diagnosis might include bacterial or viral infections. The clinical test that will help identify the cause of the symptoms will vary depending on the assumed causes. For instance, abdominal ultrasonography would help with abdominal issues; Anti-Saccharomyces cerevisiae antibodies test is done to confirm Crohn's disease, Nitroblue tetrazolium testing is done in case of Chronic granulomatous disease, a rectal swab will show if there is Group B Streptococcus (GBS), a skeletal survey may show signs of physical abuse or systemic disease, and a stool culture for Salmonella Enteritidis would reveal if salmonellosis is an issue. 3) CLINICAL REASONING: Now this case is indeed perplexing but if we connect the dots, there is a unique clue here that he was living with a pet lizard. Reptiles are known carriers of Salmonella spp., and children are more susceptible to Salmonella infection. The presentation of fussiness and poor appetite can be associated with Salmonella gastroenteritis. The appearance of flaccid bullae could be extraintestinal manifestations of Salmonella infection, more specifically recognised as bullous impetigo which is a skin infection. Therefore, the history of recent exposure to a pet lizard offers a plausible link to potential salmonella infection. 4) CONCLUDING: The combination of symptoms and environmental exposure makes Salmonella gastroenteritis a strong potential diagnosis in this case. Identifying the specific strain of Salmonella that could potentially be causing the infection could help in developing the most effective treatment plan. 5) RESPONSE: The most suitable next step in management, given these symptoms and background, would be Stool culture for Salmonella enteritidis (F).

**AI_D:** Firstly, let's interpret the question: A 9-month-old boy is brought in by his mother due to persistent fussiness and a decreased appetite over the past 6 hours. Recently, he was diagnosed with an upper respiratory tract infection, yet those symptoms have since cleared. The boy lives at home with his family and a pet lizard, his diet comprises a combination of cow milk-based formula, baby food and some solid food. There is no prior history of severe illnesses and he takes no drugs. His immunizations are all current. He was upset during today's visit and further physical examination shows multiple soft fluid-filled blisters (flaccid bullae) on a red base over his buttocks. The mother reported she was unaware of this skin issue. The main task is to determine the most appropriate next step in the management of this case. When we assess the information given, we're drawn to examine several assumptions. Firstly, the recent upper respiratory tract infection, although mentioned, was also noted to have resolved and thus may not be directly connected to his current behaviour and presentation. Secondly, the presence of the pet lizard in his environment could be a potential source of infection. Thirdly, his diet might be a source of potential allergenic reactions or microbial infections. Fourthly, the mother's unawareness of the skin condition does not rule out the potential that it is linked to the boy's fussiness and reduced appetite. Now, let's hypothesize based on these assumptions: If the blisters are an allergic reaction, they would likely have been presented in other areas of skin exposure and not solely the buttocks. If we consider the pet lizard, reptiles are known to harbor Salmonella species, which can cause salmonellosis in humans, typically presenting with gastrointestinal symptoms, fever and may occasionally show extra-intestinal signs such as skin manifestations. Fussiness and decreased appetite could be explained by an underlying infection as well. With these assumptions and speculation, we need to evaluate which path to take. Given the connection between pet reptiles and salmonellosis, combined with the child's gastrointestinal symptoms, it would be worthwhile to consider this as the primary assumption. The skin blisters may be indicative of disseminated infection which can occur in children due to their immature immune system. So, let's formulate a response based on this clinical reasoning: Given the symptoms of persistent fussiness, decreased appetite, and the presence of flaccid bullae on an erythematous base over the buttocks - along with the known risk of Salmonella infection from reptiles including lizards, the most appropriate next step in management would be to confirm or rule out Salmonella infection. The definitive response to the question, therefore, is: (F) Stool culture for Salmonella enteritidis. This would enable us to determine if Salmonella is the underlying culprit in this case, guiding us towards the correct treatment.

**AI_A:** RESTATING THE QUESTION: A 9-month-old infant with an upper respiratory tract infection that resolved two weeks ago is brought to a healthcare facility due to an ongoing fuss and diminished appetite over the past six hours. The child, who lives with his parents and a pet lizard, feeds mostly on cow milk-based formula, infant foods, and some solid meals. Without a history of any severe illness, the kid isn't on medications and his immunizations are up-to-date. The baby who is inconsolable and physically normal with regards to weight and height percentile, exhibits multiple loose blisters over an erythematous base on his buttocks- a condition the mother claims to be unaware of. The query seeks to ascertain the best next step to manage this situation. PLANNING: Let's examine the assumptions made here. The first assumption is that the symptoms may be related to a newly developed dermatological issue, given the recent discovery of "flaccid bullae on an erythematous base over the buttocks." The ailment might also be linked to his recent infection, his diet, a condition related to the pet lizard at home, or possibly an undiagnosed immunological factor. We could generate alternate assumptions by questioning each assumption, and then evaluate and hypothesize which assumption should be clinically prioritized to pursue. CLINICAL REASONING: The recent respiratory infection seems less likely to be the cause of the current symptoms, especially since these symptoms have been resolved. The food he consumes, including cow milk-based formula, could potentially alter his gut flora, but it does not directly explain the dermatological findings. Among the options, a stool culture for Salmonella Enteritidis (choice F) would be an appropriate test to consider, given that contact with reptiles has been associated with Salmonella infection in humans. Incorrect handling or cleaning of these pets can lead to fecal-oral contamination. Moreover, Salmonella infection can manifest as irritability and decreased appetite in infants, along with gastroenteritis and occasionally, a rash. CONCLUDING: The child’s pet lizard might have transmitted the Salmonella bacteria, making the stool culture test for Salmonella enteritidis a beneficial diagnostic process. It might help confirm or refute the assumption that the pet reptile is the root cause of the child's condition. In return, it will guide the clinician towards the correct therapy course, minimizing the risk of complications. RESPONSE: Therefore, the most appropriate next step in managing this situation is to conduct a stool culture for Salmonella enteritidis (choice F).

**AI_E:** RESTATING THE QUESTION: The symptoms of a 9-month-old male infant have been observed by his mother including fussiness, a lack of appetite, and flaccid bullae (blisters) on an inflamed base on his buttocks. He had a recent upper respiratory tract infection and lives with a pet lizard. He feeds on cow milk formula, baby food, and solid foods. The child has an up-to-date immunization status. The question is asking what the most appropriate subsequent step in the treatment of this infant would be. PLANNING: The child's symptoms might be due to a variety of causes including infections, inflammation, metabolic or immunological disorders. The detail about his pet lizard and his recent infection may be relevant. Thus, we must reconsider each testing option relative to the child's potential condition and the significance of each test. 1. Abdominal ultrasonography - generally used to visualize organs within the abdomen to identify problems such as appendicitis, pancreatitis, liver disease, and kidney disease. 2. Anti-Saccharomyces cerevisiae antibody testing – used in identifying inflammatory bowel disease (Crohn's disease). 3. Nitroblue tetrazolium testing - used to diagnose chronic granulomatous disease, a rare immunodeficiency disorder. 4. Rectal swab for group B streptococcus - regularly performed on pregnant women near the time of delivery but could be used in cases where invasive streptococcal disease is suspected. 5. Skeletal survey - used to evaluate for fractures or other signs of non-accidental trauma. 6. Stool culture for Salmonella enteritidis - the main investigation for Salmonella species, which is a common cause of foodborne illness. CLINICAL REASONING: The baby had a recent upper respiratory infection that has resolved but is now showing new symptoms in the form of fussiness, decreased appetite, and rashes. The presence of the pet lizard indicates a possibility of salmonella infection since pet reptiles are known carriers of Salmonella spp. Salmonellosis often presents with nonspecific symptoms such as fever, fussiness, and decreased appetite in infants. Moreover, any product of animal origin, including cow milk formula, presents a risk for Salmonella contamination. Further, the description of the rash - multiple flaccid bullae on an erythematous base, may hint at a secondary staphylococcal or streptococcal skin infection, which can follow a primary viral infection or occur due to diaper dermatitis in infants. However, this seems less likely because usually the primary dermatitis or impetigo would have been noticed by the mother before. CONCLUDING: Putting all these considerations together, the upper respiratory tract infection that resolved, the new nonspecific symptoms, the pet lizard, and the diet that includes cow milk-based formula all contribute to raising suspicion for salmonellosis. Therefore, the most clinically appropriate next step would be to perform a stool culture for Salmonella enteritidis to either confirm or rule out this diagnosis. RESPONSE: (F) Stool culture for Salmonella enteritidis is the most appropriate next step in management.

**AI_B:** RESTATING THE QUESTION: The query concerns a 9-month-old boy who recently had an upper respiratory tract infection but has now developed an unrelated condition characterized by multiple blisters (flaccid bullae) on an inflamed (erythematous) base around the buttocks area. He hasn't been very well today with signs of fussiness and diminished appetite, although his previous symptoms related to the respiratory infection have resolved. The mother wants to know what the next step in the child's management should be, referring to various testing and diagnostic options. PLANNING: Clearly, there are a couple of assumptions that we need to consider here: one, the cause of the present symptoms could be related to the recent infection, or it might be entirely new. Two, the cause could be connected to his environment, including his diet or exposure to the family pet. By questioning each of these assumptions and considering the presentation, especially the characteristic presence of flaccid bullae on an erythematous base, we could deduce that the condition is most likely caused by a bacterial infection. We should hypothesize that the immediate step should be to identify the causative organism and see whether there might be additional medical concerns associated with the condition. CLINICAL REASONING: Looking at the provided choices, we can evaluate each one in terms of its relevance to the child's symptoms and our hypothesis. Let's consider the following: - (A) Abdominal ultrasonography: This imaging study is used to visualize the structures within the abdomen and might be useful when suspecting abdominal disorders. In this case, however, there is no reason to believe the child's symptoms are related to his abdominal organs. - (B) Anti-Saccharomyces cerevisiae antibodies (ASCA): This is an important marker in inflammatory bowel diseases like Crohn's disease but doesn't seem related to the observed skin changes around the buttocks. - (C) Nitroblue tetrazolium test: This is used to diagnose chronic granulomatous disease, an inherited immune system disorder that leads to severe, recurrent infections. Although it fits with the hypothesis of infection, the patient lacks other typical signs, like lymphadenopathy or hepatosplenomegaly. - (D) Rectal swab for group B streptococcus: Given that Group B Streptococcus is a common neonatal pathogen, this option might seem relevant. However, it doesn't typically cause skin changes as described, and most cases occur in the first week after birth. - (E) Skeletal survey: Is used in cases of suspected physical abuse or metabolic bone diseases, both of which are inconsistent with this case. - (F) Stool culture for Salmonella enteritidis: This fits well with the presentation, especially as the child lives with a pet lizard. Reptiles are often asymptomatic carriers of Salmonella species, and Salmonella enteritidis can cause gastroenteritis as well as skin infections, which may explain the child's symptoms. CONCLUDING: Given the clinical reasoning, we can conclude that a stool culture for Salmonella enteritidis (Option F) is likely the most appropriate next step in management, considering the patient's exposure to a pet lizard and the presence of cutaneous manifestations supportive of a Salmonella infection. RESPONSE: Considering the presence of flaccid bullae on an erythematous base and the recent history of decreased appetite and fussiness, combined with the patient's exposure to a pet lizard, it is most appropriate to conduct a stool culture for Salmonella enteritidis (Option F) as the next step in management.

**Facilitator** The consensus statement synthesizes the responses of the five AI opinions as follows: The question pertains to a 9-month-old boy who has been experiencing fussiness and a decreased appetite for the past six hours. He had an upper respiratory tract infection two weeks ago, which has since resolved. The boy lives with his parents and a pet lizard, and his diet includes cow milk-based formula, baby food, and some solid foods. He has no history of serious illness, takes no medications, and his immunizations are up-to-date. Upon examination, multiple flaccid bullae on an erythematous base were found on his buttocks, a condition his mother was unaware of. The task is to determine the most appropriate next step in managing this situation. The boy's symptoms could be due to a variety of causes, including infections, inflammation, metabolic or immunological disorders. The detail about his pet lizard and his recent infection may be relevant. The presence of the pet lizard indicates a possibility of salmonella infection since pet reptiles are known carriers of Salmonella spp. Salmonellosis often presents with nonspecific symptoms such as fever, fussiness, and decreased appetite in infants. Moreover, any product of animal origin, including cow milk formula, presents a risk for Salmonella contamination. Given the symptoms of persistent fussiness, decreased appetite, and the presence of flaccid bullae on an erythematous base over the buttocks - along with the known risk of Salmonella infection from reptiles including lizards, the most appropriate next step in management would be to confirm or rule out Salmonella infection. Therefore, the consensus among the AI opinions is that the most appropriate next step in managing this situation is to conduct a stool culture for Salmonella enteritidis.
