# Supplementary material for: Collaborative intelligence in AI: Evaluating the performance of a council of AIs on the USMLE
Source: PLOS Digit Health. 2025 Oct 9;4(10):e0000787. doi: 10.1371/journal.pdig.0000787 (PMC12510544; doi:10.1371/journal.pdig.0000787)
Supplement: S2 File — (ZIP) [file pdig.0000787.s002.zip › S2 Data Raw data files containing unprocessed question inputs and LLM outputs including transcripts of deliberations/STEP2_Question-86-1_Type-MC-NJ.docx]

**Facilitator** **[Round 1]:** A 62-year-old woman is admitted to the hospital because of an 8-hour history of severe right upper abdominal pain, nausea, and fever that began after she ate fried fish and potatoes. During the past 3 years, she has had 12 episodes of similar pain after meals, but it has not lasted as long or been as severe as this episode. She has hypertension, hyperlipidemia, and alcohol use disorder. Her medications are amlodipine, benazepril, and simvastatin. She drinks one 12-oz beer daily. She is 163 cm (5 ft 4 in) tall and weighs 86 kg (190 lb); BMI is 33 kg/m². Temperature is 38.5°C (101.3°F), pulse is 120/min, respirations are 28/min, and blood pressure is 100/68 mm Hg. Pulse oximetry on room air shows an oxygen saturation of 98%. On abdominal examination, there is diffuse tenderness to palpation, rebound, and guarding. Bowel sounds are decreased. Laboratory studies show: Hematocrit: 35% Leukocyte count: 16,000/mm³ Serum Ca²⁺: 7.9 mg/dL Urea nitrogen: 26 mg/dL Glucose: 200 mg/dL Creatinine: 2 mg/dL Triglycerides: 180 mg/dL AST: 162 U/L ALT: 98 U/L Amylase: 682 U/L Lipase: 515 U/L (N=14–280) Serum electrolyte concentrations and lactate dehydrogenase activities are within the reference ranges. Which of the Following is the most appropriate next step in diagnosis? (A) CT scan of the abdomen (B) Endoscopic retrograde cholangiopancreatography (C) MR cholangiopancreatography (D) MRI of the pancreas (E) Ultrasonography of the right upper abdominal quadrant

**AI_C:** RESTATING THE QUESTION: This elderly woman has been admitted to the hospital with a history of severe upper abdominal pain, nausea, fever, and prior episodes of similar pain after meals. She has medical history significant for hypertension, hyperlipidemia, and alcohol use disorder. Upon examination, her abdomen is tender, and her lab results show elevated white blood count and certain enzymes indicative of a possible pancreatic problem. We are being asked about the next most appropriate diagnostic step, among a CT scan of the abdomen, endoscopic retrograde cholangiopancreatography (ERCP), MR cholangiopancreatography (MRCP), MRI of the pancreas, or an ultrasonography of the right upper abdominal quadrant. PLANNING: Before diagnosing, let's focus on the clinical scenario: she has recurrent right upper quadrant pain, especially after meals, along with nausea, and fever. This often points to a gallbladder issue, likely cholecystitis or biliary colic. Interestingly, she also has elevated amylase and lipase levels, indicative of acute pancreatitis. It could also suggest an episode of acute cholecystitis complicated by gallstone pancreatitis, possibly due to a stone obstructing the common bile duct. These possibilities must be clarified, as the treatment varies widely. CLINICAL REASONING: Given her symptoms and lab results, she likely has acute pancreatitis, frequently caused by gallstones or alcohol abuse, both of her risk factors. Ultrasound of the right upper quadrant is typically the first-line imaging test for suspected gallstone disease due to its high sensitivity and specificity for gallbladder stones, as well as lower cost and lack of radiation exposure. On the other hand, a CT scan would give much broader information regarding other abdominal structures but it's less sensitive than ultrasound in gallstone diagnosis. ERCP and MRCP can both visualize the biliary and pancreatic ducts, detect gallstones, and even allow therapeutic interventions. However, they are typically reserved for cases where obstruction (e.g., gallstone) is strongly suspected. MRI of the pancreas alone won't provide adequate visualization of the gallbladder or other biliary structures. CONCLUDING: Therefore, an appropriate clinical strategy would involve first imaging the gallbladder to find the potential source of the problem. Focusing on the pancreas itself or the general abdomen might not be the most efficient strategy given the suspicion of gallstones. RESPONSE: Given this line of reasoning and her clinical picture, the most appropriate next step would be choice (E), ultrasonography of the right upper abdominal quadrant.

**AI_D:** RESTATING THE QUESTION: The case presented involves a 62-year-old woman who has been admitted to the hospital with severe right upper abdominal pain, nausea, and fever that began after a meal of fried fish and potatoes. She has had similar episodes in the past, though not as severe, and her medical history includes hypertension, hyperlipidemia, and alcohol use disorder. Lab results indicate elevated leukocyte count, glucose, and pancreatic enzymes (amylase and lipase), all of which might suggest inflammation or infection in the pancreas or biliary system. The query is asking for the most suitable next diagnostic procedure to be undertaken from options including abdominal CT, endoscopic retrograde cholangiopancreatography (ERCP), MR cholangiopancreatography (MRCP), an MRI of the pancreas, and ultrasonography of the right upper abdominal quadrant. PLANNING: Her chronic history of abdominal pain post-meals suggests the possibility of gallstone disease, and the recent intensification of her symptoms, together with the fever and leukocytosis, may indicate acute cholecystitis or acute biliary pancreatitis. Acute cholecystitis, suggesting inflammation of the gallbladder, is typically diagnosed via ultrasonography, which would show gallstones and possible signs of inflammation. Acute biliary pancreatitis, suggesting a gallstone blockage causing inflammation of the pancreas, is typically identified based on elevated amylase and lipase levels, abdominal pain, and possible imaging evidence of gallstones or bile duct dilation. The woman's drinking habits may be relevant, as alcohol is a common cause of chronic pancreatitis, which could exacerbate given triggering factors like a fatty meal. CLINICAL REASONING: Given her symptoms and history, her lab results indicating elevated amylase and lipase likely suggest acute pancreatitis resulting from biliary disease. A fatty meal can trigger gallstone migration, leading to ductal obstruction and pancreatitis. An imaging test round in the right upper quadrant would possibly show gallstones and signs of inflammation. Yet still, it’s crucial to affirm if the suspected location of pathology is indeed right, which will determine which imaging modality to proceed with. CONCLUDING: The situation calls for imaging the suspected region of pathology. Ultrasound would be a feasible option as it is non-invasive, relatively cheap, widely accessible, and particularly sensitive for gallstones. In contrast, CT scan, ERCP, MRCP, and MRI are more appropriate when there are complications or other differentials expected, which is not the current case considering the available evidences. RESPONSE: Given the patient's symptoms and the likelihood of gallstone disease leading to biliary pancreatitis, the most appropriate next step in diagnosis would be (E) Ultrasonography of the right upper abdominal quadrant.

**AI_E:** RESTATING THE QUESTION: The scenario presents a 62-year-old female patient who has been admitted to the hospital due to severe right upper abdominal pain, nausea, and fever, which started after she consumed fried fish and potatoes. This patient experienced 12 episodes of similar pain post meals over the past three years, but the current episode is longer and more severe. She has a medical history of hypertension, hyperlipidemia, and alcohol use disorder, and she is on amlodipine, benazepril, and simvastatin medications. Her physical examination revealed a BMI of 33 kg/m², a temperature of 38.5°C (101.3°F), a pulse rate of 120/min, a respiration rate of 28/min, and a blood pressure of 100/68 mm Hg. Her lab results showed an elevated leukocyte count, urea nitrogen, glucose, creatinine, triglycerides, AST, ALT, amylase, and lipase. The task is to determine the most appropriate next diagnostic step. PLANNING: This patient presents with severe right upper abdominal pain, nausea, and fever, all symptoms indicative of an acute inflammatory disease in the upper abdomen such as cholecystitis, choledocholithiasis, or pancreatitis. However, this patient's elevated amylase and lipase levels suggest acute pancreatitis, which is often associated with gallstones and heavy alcohol use. The recurrent episodes of pain post meals might indicate gallstone disease, but the intensity and duration of this episode points more towards an acute pancreatitis due to underlying gallstone disease. This hypothesis should be prioritized and pursued. CLINICAL REASONING: In cases of suspected acute pancreatitis secondary to gallstone disease, the first step in the diagnostic process should ideally be imaging studies, specifically ultrasonography as it is highly sensitive for gallstone disease and can also reveal signs of pancreatitis. However, if the ultrasound does not provide definitive results, a CT scan of the abdomen may be pursued as a second choice as it is very sensitive to detect pancreatitis. MR cholangiopancreatography (MRCP) and endoscopic retrograde cholangiopancreatography (ERCP) are typically reserved for cases where there is suspicion of common bile duct stones or neoplasms, which is not specifically indicated by this patient's presentation. CONCLUDING: Given the acute presentation and the strongly suggestive laboratory findings indicating an acute pancreatitis, it seems most plausible that this condition is secondary to gallstone disease, that would also explain the recurrent episodes of postprandial pain. Thus, an imaging technique that could diagnose both conditions should be the next step, which makes ultrasonography of the right upper abdominal quadrant our first choice. RESPONSE: (E) Ultrasonography of the right upper abdominal quadrant is the most appropriate next step in diagnosis.

**AI_B:** RESTATING THE QUESTION: The patient is a 62-year-old woman with a history of recurring abdominal pain after meals, which has currently escalated to severe right upper abdominal pain, nausea and fever. It appears as though these symptoms are most severe and long lasting after her recent meal of fried fish and potatoes. Her current medication regimen consists of amlodipine, benazepril, and simvastatin. Additionally, she has hypertension, hyperlipidemia, alcohol use disorder, and clinical obesity (BMI 33 kg/m²). Her lab results indicate abnormal levels of calcium, urea nitrogen, glucose, creatinine, triglycerides, AST, ALT, amylase, and lipase. The question asks for the most appropriate next step in diagnosing the cause of her symptoms, given the options of: a CT scan of the abdomen, endoscopic retrograde cholangiopancreatography (ERCP), MR cholangiopancreatography (MRCP), MRI of the pancreas, and ultrasonography of the right upper abdominal quadrant. PLANNING: The significant elevations in lipase and amylase, along with the right upper abdominal pain point towards pancreatitis. The frequent episodes of pain after meals suggests a possibility of gallbladder disease leading to biliary pancreatitis, while her increased lab values may indicate an inflammatory or infectious process. We need to consider the best diagnostic modality that would accurately and safely evaluate the main conditions her presentation suggests. CLINICAL REASONING: While a CT scan (option A) is often used in cases of abdominal pain, it may not be the first-line diagnostic tool in this scenario due to radiation exposure, especially considering the patient might have biliary-related pancreatitis. ERCP (option B) is more of a therapeutic procedure and involves a significant risk, including the risk of causing pancreatitis; thus, it isn't the first-line diagnostic tool in this patient. MRI of the pancreas (option D) is useful for chronic pancreatitis and tumor detection but does not offer much information about gallstones. Ultrasonography of the right upper abdominal quadrant (option E) is the modality of choice for gallstone detection, which is a common cause of her probable pancreatitis. Thus, it should be prioritized in this patient with possible biliary pancreatitis. CONCLUDING: Given her presenting symptoms and medical history, the most probable culprits for her symptoms are a gallstone and acute pancreatitis. The most superior diagnostic modality would be the one able to visualize the gallbladder and assess for gallstones, as a gallstone blockage of the common bile duct can lead to biliary pancreatitis. RESPONSE: The most appropriate next step in diagnosis is (E) Ultrasonography of the right upper abdominal quadrant.

**AI_A:** 1) RESTATING THE QUESTION: We're asked to evaluate the best next step in diagnosing a 62-year-old woman who is experiencing severe right upper abdominal pain, nausea, and fever after eating a meal of fried fish and potatoes. This episode is more severe but similar to 12 others she's had in the past three years after meals. She has a medical history of hypertension, hyperlipidemia, and alcohol use disorder, and is taking amlodipine, benazepril, and simvastatin. The patient drinks one beer daily, is overweight, and is showing clinical signs of systemic inflammatory response and abdominal distress. Additionally, her labs show an elevated leukocyte count (indicating infection or inflammation), elevated serum glucose, creatinine, and urea nitrogen levels (indicating a possible compromised renal function), as well as significantly elevated amylase and lipase levels (indicating possible pancreatitis). We need to decide whether a CT scan of the abdomen, endoscopic retrograde cholangiopancreatography (ERCP), MR cholangiopancreatography (MRCP), MRI of the pancreas, or ultrasonography of the right upper abdominal quadrant will be best for this diagnosis. 2) PLANNING: The main assumption here would be that her symptoms, history, and lab results suggest a disorder related to the liver, gallbladder, or pancreas, likely gallstone disease or pancreatitis (given her elevated amylase and lipase levels). However, we may need to consider other possibilities given her alcohol use and obesity, such as alcoholic or non-alcoholic fatty liver disease, and how these may impact her presentation and thus the best diagnostic strategy. 3) CLINICAL REASONING: Given the episode's trigger by a fatty meal, the recurrent nature of previous episodes, and the patient's alcohol use and obesity (risk factors for gallstone formation), cholelithiasis leading to gallstone pancreatitis is a very likely diagnosis. An obstructing gallstone in the bile duct, evident by the severe increase in amylase and lipase, leads to a backup of pancreatic enzymes and inflammation of the pancreas. While a CT scan of the abdomen or MRI of the pancreas may show inflammation, they won't be most beneficial to visualize the common bile duct to identify the presence of a gallstone. ERCP, while diagnostic and therapeutic (allowing for removal of the stone), carries more risk and is usually reserved for cases where less invasive measures fail. Therefore, ultrasound (option E), a less invasive procedure that can still visualize the gallbladder and bile ducts, would be a clinically appropriate next step in this diagnosis. 4) CONCLUDING: According to the patient's symptoms, history, and lab results, an obstructing gallstone leading to gallstone pancreatitis seems likely. While we could consider other conditions due to the patient's history of alcohol use and obesity, her presentation aligns most suggestively with gallstone disease in the context of pancreatitis. An ultrasound would be the best next step to visualize the gallbladder and bile ducts for possible gallstones, while being less risky than ERCP. 5) RESPONSE: Therefore, the most appropriate next step in diagnosing this patient is option E, ultrasonography of the right upper abdominal quadrant.

**Facilitator** The consensus among the AI responses is that the most appropriate next step in diagnosing the 62-year-old woman's condition is ultrasonography of the right upper abdominal quadrant. This conclusion is based on her symptoms of severe right upper abdominal pain, nausea, and fever, which began after a meal and are more severe than similar episodes she's had in the past. Her medical history of hypertension, hyperlipidemia, and alcohol use disorder, along with her daily alcohol consumption and obesity, are also taken into account. Her lab results show elevated leukocyte count, glucose, creatinine, urea nitrogen, and significantly elevated amylase and lipase levels, suggesting possible pancreatitis. The recurrent nature of her symptoms, particularly after meals, and her risk factors of alcohol use and obesity, suggest a likely diagnosis of gallstone disease leading to gallstone pancreatitis. While other diagnostic procedures like a CT scan of the abdomen, endoscopic retrograde cholangiopancreatography (ERCP), MR cholangiopancreatography (MRCP), or MRI of the pancreas could provide useful information, they are either less sensitive in diagnosing gallstones, carry more risk, or are typically reserved for cases where less invasive measures fail. Ultrasonography, on the other hand, is a less invasive procedure that can effectively visualize the gallbladder and bile ducts for possible gallstones. Therefore, given the patient's symptoms, history, and lab results, the consensus is that ultrasonography of the right upper abdominal quadrant is the most appropriate next step in diagnosis.
