# Supplementary material for: Collaborative intelligence in AI: Evaluating the performance of a council of AIs on the USMLE
Source: PLOS Digit Health. 2025 Oct 9;4(10):e0000787. doi: 10.1371/journal.pdig.0000787 (PMC12510544; doi:10.1371/journal.pdig.0000787)
Supplement: S2 File — (ZIP) [file pdig.0000787.s002.zip › S2 Data Raw data files containing unprocessed question inputs and LLM outputs including transcripts of deliberations/STEP2_Question-88-1_Type-MC-NJ.docx]

**Facilitator** **[Round 1]:** A 63-year-old man with chronic obstructive pulmonary disease comes to the urgent treatment center because of a 4- day history of worsening shortness of breath. Use of his albuterol rescue inhaler has provided no improvement in his symptoms. Medical history otherwise is unremarkable. Additional medications are formoterol, budesonide, and tiotropium. The patient smoked two packs of cigarettes daily for 45 years but currently smokes only three to four cigarettes daily and says he is trying hard to quit. He appears anxious. He is 175 cm (5 ft 9 in) tall and weighs 100 kg (220 lb); BMI is 33 kg/m². Temperature is 37.2°C (99.0°F), pulse is 96/min, respirations are 26/min, and blood pressure is 128/76 mm Hg. Pulse oximetry on room air discloses an oxygen saturation of 85%. Mild cyanosis is noted, but there is no clubbing or edema of the extremities. Auscultation of the lungs discloses diffuse expiratory wheezes. Cardiac examination discloses distant but regular heart sounds. Results of arterial blood gas analysis on room air are shown: PO2: 48 mm Hg PCO2: 55 mm Hg pH: 7.36 After an albuterol nebulizer treatment is administered, the patient reports feeling somewhat better but says he is feeling ""jittery"" and is starting to have a mild tremor. In addition to providing smoking cessation counseling and prescribing antibiotic and corticosteroid therapy, which of the following is the most appropriate next step in management? (A) Continuous nebulized albuterol therapy (B) CT scan of the chest without contrast (C) Intravenous aminophylline therapy (D) Pulmonary function testing (E) Pulmonary rehabilitation (F) Titrated oxygen therapy

**AI_B:** RESTATING THE QUESTION: The clinical challenge here involves a 63-year-old male patient who is a long-term smoker and has chronic obstructive pulmonary disease (COPD). His worsening shortness of breath, despite using his albuterol rescue inhaler, and the fact that his supplemental oxygen saturation is only 85% despite albuterol nebulizer treatment, indicates an exacerbation of his COPD. His arterial blood gas analysis shows hypoxemia (low oxygen supply) and hypercapnia (excessive carbon dioxide). We need to figure out the most appropriate next step in management, after smoking cessation counseling and prescribing antibiotic and corticosteroid therapy. PLANNING: Multiple assumptions could be made given the clinical situation. One might infer that a more aggressive approach such as continuous nebulized albuterol therapy may be needed given the inadequate response to existing required regimen. Another assumption could be that supplemental oxygen might be beneficial considering his low oxygen saturation. These assumptions need to be verified with clinical reasoning and connected to appropriate evidence in the medical literature. CLINICAL REASONING: The patient's symptoms and lab values are characteristic of an acute COPD exacerbation, usually precipitated by infections or environmental pollutants. Standard treatment includes bronchodilators, corticosteroids, and antibiotics based on risk factors. Continuous nebulized albuterol may cause more harms such jitters than benefit. A CT scan of the chest won't change the immediate management. Aminophylline has more side-effects and no better efficacy than other bronchodilators. Pulmonary function tests wouldn't be feasible or useful in an acute exacerbation and rehabilitation is a part of long-term patient management. Therefore, taking into account his low oxygen saturation level and the results of the arterial blood gas study, supplemental oxygen would be the best immediate treatment. CONCLUDING: Given this patient's COPD exacerbation, elevated PCO2, and low oxygen levels, in addition to systemic therapy with corticosteroids and antibiotics, the most medically appropriate next step in the treatment would most likely be to initiate supplemental oxygen to correct the hypoxemia. The goal of oxygen therapy in COPD patients is to increase the oxygen level in the blood without suppressing the respiratory drive. RESPONSE: The most appropriate next step in management of this patient's increased COPD symptoms would likely be (F) Titrated oxygen therapy. This will address his hypoxemia, which is likely causing his dyspnea, and improve his overall physiologic status.

**AI_E:** RESTATING THE QUESTION: The case presents a 63-year-old man with chronic obstructive pulmonary disease (COPD), who is currently experiencing worsening shortness of breath, despite using his albuterol rescue inhaler. He has a history of heavy smoking, which is a known risk factor for COPD. His current oxygen saturation reveals a low level, and arterial blood gases suggest the presence of hypoxemia (lowered oxygen concentration in the blood) and hypercapnia (increased concentration of carbon dioxide in the blood). After administering an albuterol nebulizer, he feels mildly better but also reports feeling jittery and has developed a mild tremor. In addition to ongoing treatment measures, including smoking cessation counseling and prescription of antibiotic and corticosteroid therapy, we need to determine the most appropriate next management step. PLANNING: Current assumptions include that the patient's presentation is due to worsening COPD, and the measures taken so far along with his current medication regimen are not sufficiently managing his condition. Looking at the options presented, we can question whether continuing with nebulized albuterol therapy might help manage his COPD, or whether a different approach might be more beneficial, such as titrated oxygen therapy, pulmonary function testing, pulmonary rehabilitation, a CT scan of the chest, or the administration of intravenous aminophylline therapy. The choice should prioritize managing his COPD symptoms promptly. CLINICAL REASONING: COPD is characterized by persistent respiratory symptoms and airflow limitation due to airway and/or alveolar abnormalities often caused by significant exposure to harmful particles or gases, like cigarette smoke. This patient shows features of an acute exacerbation of his COPD, as shown by increased shortness of breath and worsening blood gas parameters (low oxygen saturation and higher CO2 levels). The primary goal of therapy in acute exacerbations of COPD is to minimize the impact of the current episode and to prevent the development of future episodes. Given the patient's hypoxemia, administering titrated oxygen therapy should be prioritized to correct the low oxygen levels in his blood. CONCLUDING: Although options like continued nebulized albuterol therapy can offer symptomatic relief, in this case, the patient is already showing side effects from the albuterol treatment (jitteriness and tremors). As such, focusing on correcting his low oxygen levels emerges as the priority, as persistent low oxygen levels can lead to organ damage. A CT scan, intravenous aminophylline therapy, pulmonary function testing, or pulmonary rehabilitation do not immediately address this acute issue. RESPONSE: The most appropriate next step in managing this patient's condition would be the administration of titrated oxygen therapy (Option F). This aims to improve the hypoxemia seen in the current exacerbation and minimize the possibility of further complications.

**AI_D:** RESTATING THE QUESTION: This scenario presents a 63-year-old man with a long history of smoking, who is now dealing with worsening shortness of breath due to chronic obstructive pulmonary disease (COPD). Albuterol inhaler, which he has been using, did not improve his symptoms. He is currently on three other medications for COPD; formoterol, budesonide, and tiotropium. His oxygen saturation is low, indicating inadequacy of oxygen supply. He displays signs of anxiety and minor tremors after the use of an albuterol nebulizer. The findings of arterial blood gas analysis show hypoxemia (low oxygen levels) and hypercapnia (high carbon dioxide levels). The question asks for the most appropriate next step in his management, after smoking cessation counseling, and antibiotic and corticosteroid administration. PLANNING: In structurally defining the focus of the question; we need to assess and select efficacious medical interventions that would alleviate the patient's symptoms and improve his oxygenation level, while also considering the side effects and potential complications. CLINICAL REASONING: Continuous nebulized albuterol therapy (Option A) would certainly help, but the patient is already experiencing side effects (jittery, mild tremor) from the albuterol nebulizer treatment and this may exacerbate these symptoms. A CT scan of the chest without contrast (Option B) would not benefit in determining the acute management of this patient. Intravenous aminophylline therapy (Option C) can be used as a bronchodilator but it has several side effects. Pulmonary function testing (Option D) is not an acute management option and is not beneficial in a patient with an acute exacerbation. Pulmonary rehabilitation (Option E) is a long-term management plan in stable patients, not an acute measure. Titrated oxygen therapy (Option F) becomes a viable assumption to prioritize as it can provide immediate relief from hypoxia and might be the most appropriate next step in management, considering the patient's condition. High flow oxygen is avoided in COPD patients due to the risk of hypercapnia and suppression of respiratory drive (known as the hypoxic drive theory). Therefore, controlled or titrated oxygen is beneficial in maintaining oxygenation without causing hypercapnia. CONCLUDING: As a consequential process of elimination, while utilizing the relevant information from the presented case and the principles of clinical medicine, it is reasonable to conclude that Titrated oxygen therapy (F) seems more fitting as the next step in this patient's management, considering the urgency to relieve his hypoxia, the characteristic pathophysiology of COPD, and taking into account the potential risk of hypercapnia. RESPONSE: In this scenario, the most appropriate next step in management would be titrated oxygen therapy (F).

**AI_C:** The question entails deciding on the most suitable next step of management for a 63-year-old man with chronic obstructive pulmonary disease (COPD), who presents with a worsening shortness of breath for the past 4 days. The man has a long smoking history. His current medications (the albuterol rescue inhaler, formoterol, budesonide, and tiotropium) have not alleviated his symptoms. He also displays anxious behavior, mild cyanosis, and diffuse expiratory wheezes, and his arterial blood gas analysis shows a low PO2 and high PCO2 with mild acidosis, indicating respiratory failure. After receiving an albuterol nebulizer treatment, the man felt somewhat better but reported feeling jittery and having a mild tremor. Considering the man's condition, let's evaluate the assumptions that the available options for the next step in management imply. Option A, continuous nebulized albuterol therapy, assumes urgent need for bronchodilation. Option B, Chest CT without contrast, hypothesizes that there might be an under-diagnosed underlying pulmonary pathology. Option C, intravenous aminophylline therapy, signifies the need for bronchodilation. Option D, pulmonary function testing, indicates the reassessment of lung function. Option E, pulmonary rehabilitation, postulates the need for comprehensive long-term management. Option F, titrated oxygen therapy, presumes oxygen insufficiency that needs to be rectified promptly. From a clinical perspective, the key features in this scenario are: exacerbated COPD symptoms, failure of current medication regimen, mild cyanosis, diffuse expiratory wheezes, changes in arterial blood gas analysis suggesting respiratory failure, and side effects (jitters and tremors) post albuterol nebulizer treatment. The manifestation of these features suggests the need to address two concerns urgently: the inadequate control of COPD symptoms and the low oxygen saturation indicating a state of hypoxia. Analyzing through clinical reasoning, addressing the state of hypoxia should be of primary concern since hypoxia can quickly deteriorate into life-threatening complications. Secondly, considering that the patient's symptoms did not improve significantly with his current medications, alternative treatment strategies could be considered but are less urgent at this stage. Therefore, the assumption that should be clinically prioritized to pursue is that the oxygen deficit needs to be promptly corrected to prevent further complications. In conclusion, a step-by-step explanation would start with the identification of the exacerbation of COPD symptoms and respiratory failure in the patient, followed by an understanding of the failed response to current treatment, and the side effects observed post the use of an albuterol nebulizer. Subsequently, it recognizes the hypoxic status of the patient, which if not attended immediately can lead to severe detrimental outcomes. Hence, the primary concern to be addressed would be the rectification of hypoxia. To respond to the question, the most appropriate next step in management would be (F) Titrated oxygen therapy. Care should be ensured to avoid overly aggressive oxygen therapy, as excess oxygen might suppress the respiratory drive in some patients with COPD. This strategy directly addresses the patient's immediate need - correcting hypoxia. Other options can be considered once the patient's condition stabilizes.

**AI_A:** (1) RESTATING THE QUESTION: A 63-year-old man diagnosed with chronic obstructive pulmonary disease (COPD) is having an exacerbation of his disease with poor symptom control even after using an albuterol inhaler. The disease is primarily caused by long-term tobacco smoking, as seen in this patient who smoked for 45 years, causing chronic inflammation and damage to the lung's bronchial tree. By interpretation of the patient's blood gas analysis, he is experiencing hypoxemia (low oxygen levels in the blood) and hypercapnia (high carbon dioxide levels in the blood), showing that his lungs are incapable of proper gas exchange due to COPD. After inhaled albuterol therapy, he experiences mild side effects like feeling jittery. The question asked is what is the most suitable next therapeutic strategy, focusing on these options: Continuous nebulized albuterol therapy, CT scan of the chest without contrast, Intravenous aminophylline therapy, Pulmonary function testing, Pulmonary rehabilitation, or Titrated oxygen therapy. (2) PLANNING: The core assumptions are: The patient is suffering from ongoing COPD exacerbation implying an inflammatory reaction in his lungs that has increased hindering appropriate gas exchange; The exacerbation is severe enough to cause acute hypoxemia and hypercapnia; Albuterol therapy provided only partial relief with emergence of side effects. Thus, a possible alternate plan of action should involve addressing the ongoing inflammation, ensuring optimal gas exchange, and mitigating side effects of previous therapy. By contemplating on these clinical assumptions, titrated oxygen therapy might be reasonable to clinically prioritize due to its potential to instantly correct the blood oxygen levels and also reduce patient discomfort caused by shortness of breath. (3) CLINICAL REASONING: COPD exacerbations are hallmarked by worsening of symptoms, including shortness of breath, which can lead to increased work of breathing due to the increased obstruction and inflammation. This is often accompanied by hypoxemia and, in severe cases, hypercapnia - both of which can cause an increased respiratory drive and aggravate symptoms. Titrating supplemental oxygen based on the individual patient's needs can immediately rectify hypoxia and offer symptom alleviation. Furthermore, its performance does not rely on the patient's inspiratory effort, as is the case with inhaled medications, which could be suboptimal when the patient is breathless. Considering the aggravation of the patient's condition and the acute episode, management should focus on immediate stabilization and symptom control. This argument lends support to the justification of titrated oxygen therapy being the most appropriate next step. (4) CONCLUDING: The response to the query posed should indicate a prompt and efficient approach to managing a critical situation. In this case, the patient's increased obstruction and pulmonary inflammation leading to an exacerbation of COPD symptoms requires an intervention that can quickly improve his symptomatology as well as the underlying hypoxia and hypercapnia. Therefore, considering the patient's acute condition with the potential for significant morbidity, a treatment approach that can promptly correct these pathophysiological changes is essential. (5) RESPONSE: Consequently, with the clinical thought process and planning outlined above, (F) Titrated oxygen therapy represents the most appropriate next step in managing this patient's COPD exacerbation.

**Facilitator** The question presents a 63-year-old man with chronic obstructive pulmonary disease (COPD), who is experiencing worsening shortness of breath despite using his albuterol rescue inhaler. His arterial blood gas analysis shows hypoxemia (low oxygen levels) and hypercapnia (high carbon dioxide levels), indicating that his lungs are not performing proper gas exchange. After using an albuterol nebulizer, he reports feeling somewhat better but also experiences side effects like feeling jittery and having a mild tremor. The question asks for the most appropriate next step in his management. The patient's symptoms and lab values are characteristic of an acute COPD exacerbation, usually precipitated by infections or environmental pollutants. The primary goal of therapy in acute exacerbations of COPD is to minimize the impact of the current episode and to prevent the development of future episodes. Given the patient's hypoxemia, administering titrated oxygen therapy should be prioritized to correct the low oxygen levels in his blood. Although options like continued nebulized albuterol therapy can offer symptomatic relief, in this case, the patient is already showing side effects from the albuterol treatment (jitteriness and tremors). As such, focusing on correcting his low oxygen levels emerges as the priority, as persistent low oxygen levels can lead to organ damage. Other options such as a CT scan, intravenous aminophylline therapy, pulmonary function testing, or pulmonary rehabilitation do not immediately address this acute issue. Therefore, the most appropriate next step in managing this patient's condition would be the administration of titrated oxygen therapy. This aims to improve the hypoxemia seen in the current exacerbation and minimize the possibility of further complications.
