# Supplementary material for: Collaborative intelligence in AI: Evaluating the performance of a council of AIs on the USMLE
Source: PLOS Digit Health. 2025 Oct 9;4(10):e0000787. doi: 10.1371/journal.pdig.0000787 (PMC12510544; doi:10.1371/journal.pdig.0000787)
Supplement: S2 File — (ZIP) [file pdig.0000787.s002.zip › S2 Data Raw data files containing unprocessed question inputs and LLM outputs including transcripts of deliberations/STEP2_Question-89-1_Type-MC-NJ.docx]

**Facilitator** **[Round 1]:** A 49-year-old man returns to the office for follow-up 2 weeks after he was evaluated for a 4-month history of intermittent diarrhea associated with cramping abdominal pain, urgency, and fecal incontinence. Since the onset of these symptoms, he has had four to five bowel movements daily that contain blood and mucus. Endoscopy done 1 week ago showed erythematous, inflamed mucosa of the rectum and sigmoid colon; there were no abnormalities of the ileum. He also had an episode of uveitis treated with intraocular corticosteroids 3 months ago. He takes no routine medications. He is 185 cm (6 ft 1 in) tall and weighs 77 kg (170 lb); BMI is 22 kg/m². Vital signs are temperature 37.3°C (99.2°F), pulse 88/min, respirations 18/min, and blood pressure 128/80 mm Hg. Skin examination shows raised, tender nodules that are 2 to 3 cm in diameter on the anterior surfaces of both lower extremities. Palpation of the abdomen discloses mild tenderness. Rectal examination discloses no fissures or ulcers. Test of the stool for occult blood is trace positive. This patient should be counseled that he is at greatest risk for which of the following? (A) Adenocarcinoma of the colon (B) Cholelithiasis (C) Nephrolithiasis (D) Primary biliary cirrhosis (E) Spontaneous bacterial peritonitis

**AI_E:** 1) RESTATING THE QUESTION: The question asks the possible risks that this 49-year-old man with a history of intermittent diarrhea, bloody and mucoid stools, uveitis treated with corticosteroids, and inflammation of the rectum and sigmoid colon, is exposed to. The patient has pain in the abdomen, tenderness in the lower extremities associated with raised tender nodules (which may indicate erythema nodosum), and he was tested positive for blood in the stool. It's important to understand that these signs and symptoms could be correlating to an underlying systemic disease. 2) PLANNING: One of the assumptions is that these symptoms could be secondary to inflammatory bowel disease (IBD). Another could be a standalone condition like colorectal cancer, but that doesn't account for uveitis and erythema nodosum. By questioning these assumptions, we generate an alternate assumption that these symptoms can be due to Ulcerative Colitis (UC), a type of IBD often associated with complications beyond the digestive tract, like uveitis or erythema nodosum. UC also tends to prolong the inflammation of the digestive tract, increasing the possibility of colorectal cancers. 3) CLINICAL REASONING: As we previously noted, UC is often associated with extraintestinal manifestations, notably uveitis and skin conditions such as erythema nodosum. The prolonged inflammation of the colon in UC enhances the risk of colon cancer, with the risk increasing with the extent and duration of disease. Given the patient's history and symptoms - bloody diarrhea, inflamed mucosa of the rectum and sigmoid colon on endoscopy, uveitis, and skin lesions - UC could be a leading diagnosis. 4) CONCLUDING: Based on UC's tendency to increase the possibility of colorectal cancer, the lack of abnormalities in ileum (which is usually associated with Crohn's), and the presence of other systemic symptoms that could be attributed to UC, we're led towards the risk of adenocarcinoma of the colon. 5) RESPONSE: Therefore, this patient should be counseled that he is at greatest risk for Adenocarcinoma of the colon (option A), owing to the manifestations and diagnosis of Ulcerative Colitis.

**AI_C:** RESTATING THE QUESTION: The patient, a 49-year-old man, is presenting with a history of ongoing diarrhea, blood and mucus in his stool, cramping abdominal pain, urgency, and incontinence. Furthermore, he has inflamed mucosa in his rectum and sigmoid colon, an episode of uveitis in the past and tender skin nodules on his lower extremities, all pointing towards some chronic inflammatory condition which might be systemic given the involvement of various organ systems. He has been asked about the potential risks he faces, with the focus on these conditions: Adenocarcinoma of the colon, Cholelithiasis (gallstones), Nephrolithiasis (kidney stones), Primary biliary cirrhosis, and Spontaneous bacterial peritonitis. PLANNING: The relevant assumption here is that his chronic symptoms and findings are related to an underlying inflammatory condition. Alternate assumptions can be obtained by exploring each option and identifying the probability based on the risk factors presented. For example, the primary risk factor for adenocarcinoma of the colon involves a history of chronic intestinal diseases like ulcerative colitis or Crohn's. Gallstones would typically present with biliary colic, right upper quadrant pain, or related complications. Primary biliary cirrhosis and spontaneous bacterial peritonitis are associated more with liver disorder or failure, while kidney stones would present with flank pain, hematuria, or urinary abnormalities. CLINICAL REASONING: The most significant clue connecting his symptoms to a potential risk is his history of an inflammatory bowel disease with rectal bleeding and mucus in the stool alongside skin involvement and episcleritis. This seems to indicate a chronic, systemic inflammatory disease such as Inflammatory Bowel Disease (like Crohn's disease or ulcerative colitis). Long-term inflammatory conditions of the gastrointestinal system, particularly those involving the colon, can predispose patients to developing cancer - especially adenocarcinoma. CONCLUDING: After reviewing his symptoms and medical history and considering their possible implications, I concluded that the patient is most at risk for developing adenocarcinoma of the colon. This conclusion is based on his history of long-term inflammatory bowel disease symptoms - a key risk factor for this type of cancer. RESPONSE: Given the patient's prolonged history of inflammatory bowel disease symptoms - including bloody and mucus-filled stools and inflamed colonic mucosa - along with his age, he should be counseled on the increased risk of Adenocarcinoma of the colon (Answer choice A). This type of cancer may occur through the dysplasia-carcinoma sequence due to persistent mucosal inflammation. It is important to have regular follow-ups for early detection and prevention.

**AI_D:** RESTATING THE QUESTION: This query outlines a clinical case of a 49-year-old male, presenting with several symptoms like repeated diarrhea associated with abdominal cramping, urgency, bowel incontinence, and bloody, mucus-filled stools. His endoscopy revealed inflamed mucosa but only in the rectum and sigmoid colon, while his ileum was unscathed. He also previously had uveitis, and there are raised, tender nodules on his lower extremities. With these symptoms and findings in mind, we are tasked with identifying the medical condition this patient is at greatest risk for from the provided list. PLANNING: To plan our approach, we need to make a few important assumptions. Firstly, it would be crucial to examine the correlation between the patient's symptoms and the possibilities listed. For instance, could the blood and mucus in the stool, uveitis, and lower extremity nodules be indicators of one of the potential conditions? Secondly, we could further scrutinize the lower extremities’ nodules and the uveitis - could these be extraintestinal manifestations of a particular intestinal disorder? Also, could these factors point towards a chronic inflammatory condition of the gastrointestinal tract? CLINICAL REASONING: Given the range of symptoms including chronic diarrhea, abdominal cramping, rectal bleeding, stool mucus, and inflammation of the rectum and sigmoid colon, as well as the occurrence of uveitis and dermal nodules - a form of skin condition often associated as an extraintestinal manifestation, we could infer that this patient suffers from a form of chronic inflammatory bowel disease (IBD). Diseases like ulcerative colitis or Crohn's disease are chronic, incurable inflammatory bowel diseases, and they're associated with a variety of extraintestinal symptoms and conditions. However, based on the findings that the inflammation is limited to the rectum and sigmoid colon and hasn't affected the ileum, the patient's condition is more likely to be an ulcerative colitis (UC) case. UC's complications have been associated with a higher risk of colorectal cancer, i.e., adenocarcinoma of the colon, compared to the general population. CONCLUDING: Cramping abdominal pain, bloody stools, inflamed rectum, and sigmoid colon, past uveitis, the presence of skin nodules, and an absence of ileum involvement suggest the patient's greatest risk might lie in the region of (A) Adenocarcinoma of the colon, a complication related to chronic inflammatory conditions like UC in this clinical scenario. RESPONSE: Given his clinical picture and the choices provided, this patient should be counseled that he is at greatest risk for (A) Adenocarcinoma of the colon.

**AI_A:** RESTATING THE QUESTION: The 49-year-old patient is coming for a follow-up visit after being evaluated for issues including intermittent diarrhea, abdominal pain, fecal incontinence, urgency, and frequent bloody and mucus-filled bowel movements over the past four months. His recent colonoscopy showed inflammation of the rectum and sigmoid colon, indicating a kind of inflammatory bowel disease. He also had an occurrence of uveitis that was treated with corticosteroids. Moreover, he has several tender nodules on his lower legs and has mild abdominal tenderness. There are no routine medicines he takes. Considering all these factors, we're tasked to find out what medical condition he is most at risk of developing. PLANNING: First, let's identify the assumptions. The key assumption here is that all his symptoms are interconnected and possibly point towards one major diagnostic threat. This assumption can be contested. What if the symptoms are independent? What disease conditions could be related to each symptom, individually? For example, bloody diarrhea and abdominal pain could point towards several pathological conditions like intestinal infection or malignancy. By questioning this assumption, we generate multiple possibilities. Evaluating these alternatives, the inflammation of the rectum and sigmoid colon (a symptom of ulcerative colitis), uveitis, and the nodules on legs (a symptom of erythema nodosum), all guide us to consider looking at autoimmune disease-related risks. Thus, among disease risks like adenocarcinoma of the colon, cholelithiasis, nephrolithiasis, primary biliary cirrhosis, and spontaneous bacterial peritonitis, we should clinically prioritize colon adenocarcinoma due its connection with long-term instances of ulcerative colitis. CLINICAL REASONING: Hemorrhagic diarrhea and abdominal pain, coupled with inflamed mucosa of the rectum and sigmoid colon, point towards an inflammatory bowel disease condition such as ulcerative colitis. Moreover, non-specific inflammatory conditions like uveitis and erythema nodosum (implied by tender nodules on the legs) can be associated systemic manifestations of this disease. While a range of conditions can arise due to underlying chronic systemic inflammation, the specific presentation in the lower digestive tract, primarily in the rectum and sigmoid colon, draws attention towards colorectal carcinoma, specifically adenocarcinoma, as a potential risk. CONCLUDING: Based on the cluster of symptoms presented, and the systemic complications latent in inflammatory bowel disease, it can be concluded that this patient's symptoms are interconnected and suggestive of a risk for longer-term issues. With the clinical focus on the inflammatory pattern in the lower GI tract and associated uveitis and erythema nodosum, adenocarcinoma of the colon becomes a notable risk due to the chronic presence of ulcerative colitis. RESPONSE: Therefore, given the above discussion, this patient is at the greatest risk for developing Adenocarcinoma of the colon (answer A).

**AI_B:** Restating the question, this patient, who is a 49-year-old male, came in for a follow-up session after being evaluated around two weeks ago due to symptoms which included intermittent diarrhea, abdominal pain that was associated with cramping, urgency, and fecal incontinence that has been going on for four months. Since these symptoms began, he has been having bowel movements around four to five times in a day, which have the presence of blood and mucus. An endoscopy executed around a week ago revealed erythematous, inflamed mucosa in the rectum and sigmoid colon with no issues found in the ileum. Previously, an episode of uveitis was treated with intraocular corticosteroids around three months ago. The patient is currently not on any medications. Physically, he is relatively tall, weighing 77 kilograms with a BMI of 22 kg/m². His vital signs show a body temperature of 37.3°C, a pulse of 88/min, 18/min respiratory rates, and a blood pressure of 128/80 mm Hg. Upon skin examination, raised, tender nodules that measure from 2 to 3 cm were found on the anterior surfaces of his lower extremities. The palpation of his abdomen disclosed mild tenderness. A rectal exam showed there were no ulcers or fissures and the stool test for occult blood turned up trace positive. The question at hand is what this patient is at most risk for regarding their health condition. We can identify several assumptions based on the patient's presentation. For instance, the erythematous, inflamed mucosa of the rectum and sigmoid colon on endoscopy, coupled with frequent bloody stools, suggests colonic inflammation. Combined with signs of systemic inflammation (uveitis and tender nodules on the anterior surfaces of his lower extremities called erythema nodosum), the clinical picture strongly suggests the presence of an inflammatory bowel disease (IBD), most likely ulcerative colitis (UC). These findings can predispose the patient to certain risks. Other assumptions would be possible complications from steroid treatments or a completely unrelated condition manifesting simultaneously. Given these facts, I'm questioning a reasonable assumption. Which condition appears to correlate most with his symptoms and would likely be a significant risk for this patient? Could it be adenocarcinoma of the colon, nephrolithiasis, primary biliary cirrhosis, cholelithiasis, or spontaneous bacterial peritonitis? Clinically reasoning here, IBD, particularly UC, increases the risk of colon cancer due to the long-lasting inflammatory state. Frequent bloody stools, erythematous inflamed mucosa, and systemic inflammatory signs are highly indicative of moderate to severe UC, although definitive pathogenesis can only be confirmed by biopsy. UC specifically affects the rectum and may extend proximally to involve other parts of the colon, sparing the ileum, consistent with this patient's endoscopy findings. Cholelithiasis, nephrolithiasis, primary biliary cirrhosis, and spontaneous bacterial peritonitis are generally unrelated to the pathophysiology of UC, making them less likely in terms of risk analysis. Therefore, considering given information and using a step-by-step clinical thought process, it can be concluded that, while this possible UC patient would be at risk for multiple health issues, the most significant one in this case would likely be adenocarcinoma of the colon due to the direct links established between colon inflammation and the development of this type of cancer. In response to the question posed, this patient should be counseled that he is at greatest risk for developing adenocarcinoma of the colon (Option A).

**Facilitator** Consensus Statement: The question presents a 49-year-old male patient with a four-month history of intermittent diarrhea, abdominal pain, urgency, fecal incontinence, and bloody, mucus-filled bowel movements. His recent endoscopy revealed inflamed mucosa in the rectum and sigmoid colon, and he had a past episode of uveitis treated with corticosteroids. He also has tender nodules on his lower legs and mild abdominal tenderness. The patient is not on any routine medications. The key assumption here is that all his symptoms are interconnected and possibly point towards one major diagnostic threat. This assumption can be contested. What if the symptoms are independent? What disease conditions could be related to each symptom, individually? For example, bloody diarrhea and abdominal pain could point towards several pathological conditions like intestinal infection or malignancy. By questioning this assumption, we generate multiple possibilities. Evaluating these alternatives, the inflammation of the rectum and sigmoid colon (a symptom of ulcerative colitis), uveitis, and the nodules on legs (a symptom of erythema nodosum), all guide us to consider looking at autoimmune disease-related risks. Given the range of symptoms including chronic diarrhea, abdominal cramping, rectal bleeding, stool mucus, and inflammation of the rectum and sigmoid colon, as well as the occurrence of uveitis and dermal nodules - a form of skin condition often associated as an extraintestinal manifestation, we could infer that this patient suffers from a form of chronic inflammatory bowel disease (IBD). Diseases like ulcerative colitis or Crohn's disease are chronic, incurable inflammatory bowel diseases, and they're associated with a variety of extraintestinal symptoms and conditions. However, based on the findings that the inflammation is limited to the rectum and sigmoid colon and hasn't affected the ileum, the patient's condition is more likely to be an ulcerative colitis (UC) case. UC's complications have been associated with a higher risk of colorectal cancer, i.e., adenocarcinoma of the colon, compared to the general population. Therefore, considering given information and using a step-by-step clinical thought process, it can be concluded that, while this possible UC patient would be at risk for multiple health issues, the most significant one in this case would likely be adenocarcinoma of the colon due to the direct links established between colon inflammation and the development of this type of cancer. Given his clinical picture and the choices provided, this patient should be counseled that he is at greatest risk for (A) Adenocarcinoma of the colon.
