# Supplementary material for: Collaborative intelligence in AI: Evaluating the performance of a council of AIs on the USMLE
Source: PLOS Digit Health. 2025 Oct 9;4(10):e0000787. doi: 10.1371/journal.pdig.0000787 (PMC12510544; doi:10.1371/journal.pdig.0000787)
Supplement: S2 File — (ZIP) [file pdig.0000787.s002.zip › S2 Data Raw data files containing unprocessed question inputs and LLM outputs including transcripts of deliberations/STEP2_Question-9-1_Type-MC-NJ.docx]

**Facilitator** **[Round 1]:** A 70-year-old man comes to the clinic because of a 3-day history of fatigue, dark urine, decreased urine output, mild pain with urination, and a low-grade fever. He has gout, hypertension, and osteoarthritis. His medications are allopurinol, hydrochlorothiazide, and acetaminophen. Temperature is 37.9°C (100.2°F), pulse is 88/min, respirations are 24/min, and blood pressure is 160/95 mm Hg. Pulse oximetry on room air shows an oxygen saturation of 97%. Jugular venous pressure is 7 cm H2O (N=5–9). Cardiopulmonary examination shows no abnormalities. Which of the following is the most appropriate next step in management? (A) Discontinuation of acetaminophen therapy (B) Intravenous administration of fluids (C) Measurement of postvoid residual volume (D) Oral trimethoprim-sulfamethoxazole therapy (E) Renal biopsy

**AI_A:** (1) RESTATING THE QUESTION: You're essentially asking about the best course of action for a 70-year-old male patient who has come to the clinic presenting symptoms like fatigue, dark urine, minimized urine output, slight pain during urination, and a low-grade fever. He has a history of gout, hypertension, and osteoarthritis, and is currently on a medication regimen consisting of allopurinol, hydrochlorothiazide, and acetaminophen. His vital signs reveal a mildly increased temperature and elevated blood pressure, but show a normal pulse and oxygen saturation levels. His jugular venous pressure is within the normal range and there do not seem to be any issues when examining his heart and lungs. (2) PLANNING: The key assumptions I'd make based on this information would be that the symptoms might stem from dehydration or an infection in the urinary tract. Pain during urination, dark urine, and a low-grade fever are classic symptoms of a urinary tract infection (UTI), potentially made worse by dehydration due to the hydrochlorothiazide, which increases urine output. A second assumption is that his symptoms could be due to the side effects of his ongoing medication, particularly the allopurinol which is known to cause such symptoms. (3) CLINICAL REASONING: Considering these assumptions, I'll explore the UTI hypothesis first. UTIs are quite prevalent in older population due to immunosenescence, or the gradual decrease in immune function that comes with age. Given the mild elevation in body temperature and his urine-related symptoms, an infection seems probable. The dark urine could be explained by concentrated urine if the patient is dehydrated, but it may also be indicative of an infection or inflammation. The decreased urine output and mild pain with urination are consistent with a UTI. If the UTI hypothesis is correct, appropriate management would involve initiating an antibiotic therapy, in this case, Oral trimethoprim-sulfamethoxazole therapy (Option D). Alternatively, the reaction to allopurinol could be a possibility, but it seems less likely given the patient's symptoms and the popularity of the drug as a treatment for gout. (4) CONCLUDING: It's essential to establish the cause of the patient's symptoms before proceeding. However, in the given clinical scenario, the most appropriate and immediate next step in management seems to be starting treatment for a probable UTI, while simultaneously monitor the patient's response and adjusting treatment as clinically indicated. (5) RESPONSE: Therefore, Oral trimethoprim-sulfamethoxazole therapy (Option D) would be the most appropriate next step in this patient's management.

**AI_E:** RESTATING THE QUESTION: The question presents a 70-year-old man with gout, hypertension, and osteoarthritis who has been experiencing symptoms of fatigue, dark urine, decreased urine output, pain with urination, and a mild fever for the past 3 days. He is currently taking allopurinol, hydrochlorothiazide, and acetaminophen. His blood pressure is slightly high, but his general examination and vital signs are generally unremarkable. Given his recent symptoms, we're being asked to determine the best next step in clinical management. PLANNING: The primary assumptions here seem to revolve around the man's symptoms potentially being related to renal pathology or urinary tract pathology, potentially resulting from medication, infection, obstruction, or underlying renal disease. It's important to consider the effects of his current medications, any potential infections, and other common causes of his symptoms such as dehydration. However, considering the symptoms and medical history, an infection—the presentation of which can worsen by hydrochlorothiazide—a diuretic medication could lead to complications like volume depletion encouraging infections. CLINICAL REASONING: The combination of dark urine, decreased urine output, and pain with urination suggests a urinary tract issue. Given his age and use of a diuretic (hydrochlorothiazide), we should consider urinary tract infection (UTI) which can be common in older adults using diuretics that can cause dehydration and urinary stasis. His fever likely signifies an active infection. Also, his medications—including allopurinol, acetaminophen, and hydrochlorothiazide—all can have renal side effects including interstitial nephritis, renal tubular necrosis and reduced renal blood flow, but these are less likely considering the acute onset of his symptoms. CONCLUDING: Given the man's symptoms, plus his medication regimen and medical history, it seems most appropriate to consider a urinary tract infection as a plausible explanation for his symptoms. As such, the priority should be to initiate appropriate antibiotic therapy while the clinical situation awaits more confirmatory tests. RESPONSE: The most appropriate next step in management is (D) Oral trimethoprim-sulfamethoxazole therapy. Because this patient's clinical picture is suggestive of a urinary tract infection, the primary task should be to start an empiric oral antibiotic (e.g., trimethoprim-sulfamethoxazole) in accordance with typical treatment guidelines.

**AI_B:** RESTATING THE QUESTION: The presented case is about a 70-year-old man who has symptoms of fatigue, dark urine, lower urine output, pain during urination, and mild fever for three days. His medical history reveals gout, hypertension, and osteoarthritis. He is on medication, including allopurinol for gout, hydrochlorothiazide for hypertension, and acetaminophen for pain related to osteoarthritis. His vital signs are also indicated, including increased temperature and blood pressure. His oxygen saturation level is within normal limits, and there are no abnormalities observed in his cardiopulmonary examination. The question posed is regarding the next most appropriate step to managing his case based on the given choices. PLANNING: The first assumption might be that the patient's symptoms indicate a urinary tract infection (UTI), given the presence of fever, dysuria (painful urination), and dark urine. An alternate assumption, considering the use of hydrochlorothiazide, a diuretic, may be that the patient is experiencing dehydration. The symptoms also suggest the possibility of acute kidney injury or failure, particularly given the presence of decreased urine output, dark-colored urine, and fatigue. The patient's medical history and age are also factors that might increase his risk for kidney dysfunction. CLINICAL REASONING: In discussing the possible assumptions mentioned above, the patient's symptoms do suggest a UTI. However, there are missing clinical signs to firmly establish it, such as urgency, frequency, and pyuria (pus in the urine). Although the raised temperature indicates the presence of an infection, UTI is usually accompanied by a higher grade fever than presented. Further, considering the relation between allopurinol, gout, and renal failure, which could manifest as fatigue, decreased urine output, and dark-colored urine, the possibility of acute kidney injury appears to be more likely. Allopurinol is known to potentially trigger acute or chronic kidney failure. Hydrochlorothiazide, a diuretic, may aggravate dehydration, which may have also contributed to acute kidney injury. CONCLUDING: In this case, the next step would be to evaluate the renal function, specifically assessing the rate of filtration happening in the kidney to identify if a renal injury has occurred and its severity. But it's noteworthy that the options presented do not directly include any investigations related to kidney function. Given the absence of typical UTI symptoms, an alternate way could be to rule out UTI for now and take care of the hydration status since dehydration impairs kidney function further. RESPONSE: The most appropriate next step in management would be (B) Intravenous administration of fluids. This would help correct any fluid deficits, which may be contributing to renal dysfunction and triggering the observed symptoms. Further investigations related to kidney function should follow the fluid replenishment to more accurately diagnose the underlying condition.

**AI_D:** RESTATING THE QUESTION: The case presented is of a 70-year-old man who has been dealing with urinary symptoms (dark urine, decreased urine output, and mild pain during urination), fatigue, and a mild fever for the past three days. He has a past medical history of gout, hypertension, and osteoarthritis, and he is currently taking allopurinol, hydrochlorothiazide, and acetaminophen. His vitals raise a few concerns, mainly his high blood pressure and low-grade fever. A cardiac and pulmonary evaluation did not reveal any problems. The question background suggests the need to identify the best immediate action to manage his condition. PLANNING: The patient's fever, pain during urination, fatigue, and dark, reduced urine output all suggest the possibility of an infection, more specifically, a urinary tract infection (UTI). However, given his age, comorbidities and medications, we must also consider other causes like dehydration (due to hydrochlorothiazide use), nephrolithiasis, a side effect of allopurinol (interstitial nephritis), acetaminophen toxicity, or even a result of worsening hypertension. Maintaining a balanced assumption is critical because misjudging the situation may lead to inappropriate management, which could exacerbate the patient's condition. CLINICAL REASONING: Starting with the first suspicion of a UTI, this assumption is backed up by the patient's symptoms of dark urine, decreased urine output, mild pain with urination, and low-grade fever, which are indicative of this infection. Evaluating the relevance of patient's medication for this case, allopurinol is typically prescribed to prevent gout attacks, acetaminophen for pain (possibly secondary to the osteoarthritis), and hydrochlorothiazide for hypertension. The role of these medications in the current clinical picture is multifactorial. Firstly, Allopurinol can cause acute interstitial nephritis, which can present with similar symptoms, but it is a rare side effect. Secondly, if acetaminophen is consumed in large amounts, it can lead to toxicity, which can result in metabolic acidosis and acute liver injury. However, the absence of hepatic signs weakens this possibility. Lastly, hydrochlorothiazide, a diuretic, can lead to volume depletion due to excessive fluid loss, presenting as thirst, light-headedness, or weakness, which may cause hemoconcentration and dark urine. But, judging by his normal pulse, absence of orthostatic hypotension, and no signs of dry mucosa, volume depletion seems less likely. CONCLUDING: Therefore, considering the patient's age, symptomology, medication history, and the lack of cardiopulmonary abnormalities, the possibility of a urinary tract infection seems most plausible. Hence, the treatment of choice would be an antibiotic that covers common UTI pathogens, so administering oral trimethoprim-sulfamethoxazole therapy would be the most appropriate next step in management. RESPONSE: The patient's condition aligns with classic symptoms of a urinary tract infection; thus, the best immediate action is to commence antibiotics appropriate for treatment of this infection. Oral trimethoprim-sulfamethoxazole therapy (choice D), a commonly used antibiotic for UTIs, would be the ideal path to follow in terms of management.

**AI_C:** Restating the Question: To paraphrase, a 70-year-old man has been experiencing some troubling symptoms for the past few days. These include fatigue, dark urine, decreased urine output, mild urination pain, and a slight fever. He has a history of gout, hypertension, and osteoarthritis and is currently taking allopurinol, hydrochlorothiazide, and acetaminophen. His temperature, pulse, respiration, and blood pressure are somewhat elevated, but his oxygen saturation is normal, and the jugular venous pressure isn't worrisome. A thorough cardiopulmonary examination doesn't reveal any issues. The question requires determining the best strategy for managing his symptoms given his current conditions and treatment regimen. Planning: Key facts to consider in this case are the presence of urinary symptoms such as dark urine, decreased urine output, and a mild pain during urination, along with a fever. Theses findings are suggestive of a potential urinary tract infection (UTI). Allopurinol, taken for gout treatment, has been linked to increased risk of UTIs. Hydrochlorothiazide, a diuretic used for hypertension, can potentiate concentration of urine, promoting bacterial growth. Additionally, involutional changes of the urinary tract as seen in aging may predispose to UTIs as well. Thus, one should consider the infection-related assumption first in the clinical context. Another possible assumption could be related to acetaminophen-induced nephrotoxicity due to the strong implication of acetaminophen in kidney damages, but it is less likely as this usually present with acute kidney injury and not exactly with UTI symptoms. Clinical Reasoning: Given the presented symptoms and the patient's current medication, it would be reasonable to consider a UTI as the primary working hypothesis. His symptoms and physical findings including fever, dark urine, decreased output, and mild pain during urination are all indicative of a potential UTI. Notably, allopurinol, which this patient takes for his gout, has been linked with an increased risk of UTIs. Coupled with the fact that he is on hydrochlorothiazide, a diuretic medication that can potentially increase the concentration of urine and subsequently bacterial growth, the chances of UTI occurring increases. Age-related changes in the urinary tract that compromise its defense mechanism can further increase susceptibility to UTIs. A UTI caused by a common bacteria such as Escherichia Coli could be suspected. If this is the case, initiating an antibiotic therapy (like trimethoprim-sulfamethoxazole which is a commonly used antibiotic for UTIs) would likely be the next appropriate step in management. Concluding: The pattern of the symptoms and the likelihood of a urinary tract infection being the cause, especially considering the patient's age and medication, lead to this direction. Additionally, rapid initiation of appropriate antibiotic therapy upon suspicion is crucial to prevent any urinary tract infection from ascending any further, potentially causing a more serious condition, such as a kidney infection (pyelonephritis). Therefore, instead of discontinuing medications or attempting more invasive or non-specific interventions (like renal biopsy, IV fluids, or measurement of postvoid residual volume), initiating oral trimethoprim-sulfamethoxazole therapy would be more appropriate to directly address the possible UTI and prevent it from progressing further. Response: Given this context, the most likely cause of symptoms in the presented patient would be a urinary tract infection. Considering the most direct and efficient approach would be to initiate therapy against the probable cause, "Oral trimethoprim-sulfamethoxazole therapy" (Choice D) would be the most appropriate next step in managing the condition of the patient.

**Facilitator** **[Round 2]:** Summary: AI_A, AI_C, AI_D, and AI_E all agree that the patient's symptoms suggest a urinary tract infection (UTI), possibly exacerbated by the patient's medication and age. They all recommend initiating oral trimethoprim-sulfamethoxazole therapy as the most appropriate next step. AI_B, however, suggests that the symptoms might indicate acute kidney injury, possibly due to the patient's medication and dehydration. AI_B recommends intravenous administration of fluids as the most appropriate next step, followed by further investigations related to kidney function. Key Differences: The key difference in the perspectives lies in the interpretation of the patient's symptoms. AI_A, AI_C, AI_D, and AI_E focus on the symptoms indicative of a UTI, such as dark urine, decreased urine output, and mild pain during urination, and recommend initiating antibiotic therapy. AI_B, on the other hand, considers the possibility of acute kidney injury due to the patient's medication and dehydration, and recommends fluid replenishment as the first step in management. Question for Council Members: Given the patient's symptoms and medical history, what specific factors led you to your conclusion about the most likely diagnosis and the most appropriate next step in management? How would you prioritize the need for immediate symptom relief versus further diagnostic investigations?. Based on your preceding response, how would you answer the original question: [A 70-year-old man comes to the clinic because of a 3-day history of fatigue, dark urine, decreased urine output, mild pain with urination, and a low-grade fever. He has gout, hypertension, and osteoarthritis. His medications are allopurinol, hydrochlorothiazide, and acetaminophen. Temperature is 37.9°C (100.2°F), pulse is 88/min, respirations are 24/min, and blood pressure is 160/95 mm Hg. Pulse oximetry on room air shows an oxygen saturation of 97%. Jugular venous pressure is 7 cm H2O (N=5–9). Cardiopulmonary examination shows no abnormalities. Which of the following is the most appropriate next step in management? (A) Discontinuation of acetaminophen therapy (B) Intravenous administration of fluids (C) Measurement of postvoid residual volume (D) Oral trimethoprim-sulfamethoxazole therapy (E) Renal biopsy]

**AI_E:** Restating the question: The query revolves around a clinical case of a 70-year-old male with a medical history of gout, hypertension, and osteoarthritis, who presents with a 3-day long episode of fatigue, dark urine, decreased urine output, mild urination pain, and a slight fever. He's currently on allopurinol, hydrochlorothiazide, and acetaminophen. The task pertains to identifying the most suitable immediate next step in the patient's management. Planning: The symptoms point toward a problem related to the urinary system, possibly a urinary tract infection (UTI). Dark urine could be indicative of a UTI or dehydration while reduced urine output could suggest kidney impairment. The pain during urination and fever also correspond with a UTI. His medications are also important to consider, hydrochlorothiazide, a diuretic, could exacerbate dehydration while allopurinol is often associated with kidney issues. Clinical reasoning: Given the patient's age and his mild fever along with urination-related symptoms, UTI leads as a prime probable cause. Dark urine and decreased output may be secondary to decreased kidney function due to aging, drug side effects, or due to the cause of the UTI itself - dehydration or urinary obstruction. However, hypertension could also contribute to kidney impairments over time. Additionally, frequent UTIs could lead to chronic kidney disease. The pain during urination is usually indicative of inflammation along the urinary tract, typically caused by an infection. Concluding: Keeping the clinical reasoning in view, I am led to think that the patient might have a UTI, perhaps complicated by mild kidney function abnormality. The immediate management step should focus on addressing the UTI, as well as considering the patient's kidney function. Response: Given these considerations, the most appropriate next step in management would be oral trimethoprim-sulfamethoxazole therapy (D). It offers a direct and immediate address to treat the presumed UTI. Further downstream, kidney function tests might be needed to assess the precise state of renal function given the patient's age, history, and complaints of decreased urine output.

**AI_C:** Restatement: The patient, a 70-year old male, presents with symptoms suggestive of a urinary tract infection such as fatigue, dark urine, reduced urine output, slight pain while urinating, and a low-grade fever. This man also has a medical history of gout, hypertension, and osteoarthritis for which he's taking allopurinol, hydrochlorothiazide, and acetaminophen respectively. His vital signs are generally within normal limits except for a slightly elevated temperature and high blood pressure. The question aims at identifying the most accurate next step in managing this patient's situation. Planning: The possible assumptions to consider here include the ongoing use of the patient's medications, the factor of dehydration, or presence of urinary tract infection (UTI), and acute kidney injury (AKI). Each assumption could lead to slightly different strategies for management. Clinical Reasoning: Key in this patient's history is the dark urine, decreased urine output, and pain during urination – these are indicative of a urinary tract issue, most likely a UTI. His low-grade fever supports this diagnosis. Moreover, he is at a higher risk for UTIs given his age and use of allopurinol that can cause UTIs. However, the decreased urine output and dark urine could also suggest an AKI, which could be brought on by dehydration (increased risk due to hydrochlorothiazide usage) or directly by his medications (acetaminophen, if used inappropriately). Concluding: The management strategies could include: antibiotic therapy if we consider UTI to be the likely cause; administering fluids intravenously if dehydration is considered the main issue; or modifying his medication regime in view of a potential AKI. However, considering the strong indications of a UTI, initiating antibiotic therapy seems the most justifiable at this stage, with oral trimethoprim-sulfamethoxazole therapy being a common choice. Response: (D) Oral trimethoprim-sulfamethoxazole therapy is the most appropriate next step in management for this patient, following the assumption that the main cause of his symptoms is a urinary tract infection. If the symptoms persist or worsen, further investigation for potential kidney damage will be needed. It is also crucial to monitor the patient's use of allopurinol and acetaminophen to reduce potential adverse effects.

**AI_B:** Restating the Question: The query presents the case of a 70-year-old gentleman with a history of gout, hypertension, and osteoarthritis, experiencing a sequence of unpleasant symptoms like exhaustion, dark urine, reduced urine output, mild urination pain, with mild fever for three days. The issue seeks to identify the most appropriate next step in management given his clinical presentation. Planning: The assumptions involved in this case are that these symptoms could indicate a urinary tract infection (UTI), acute kidney injury (AKI), or adverse effects of his medications. These assumptions can be questioned by considering the timing and combination of symptoms, any history of similar symptoms, and the potential side effects of his medications. Hypothetically, if the symptoms indicate a UTI, initiating antibiotic treatment would be advisable. However, if the symptoms are due to AKI, then rehydration may be needed, potentially adjusting medications if they are contributing. Clinical Reasoning: The symphony of symptoms this patient is presenting with, including fatigue, dark urine, decreased urine output, and mild discomfort during urination, suggests a urinary tract infection, potentially pyelonephritis considering there is low-grade fever. Hydrochlorothiazide can cause volume depletion which may worsen UTI symptoms. Allopurinol can sometimes cause hypersensitivity, causing fever, rash and renal impairment, but since there are no rashes, this can be a lesser possibility. Acute kidney injury may first present as decreased urine output and fatigue but it usually comes with a more significant fever and pain. Concluding: Based on the patient's symptoms and medical history, and also considering his medication, the likely cause of his symptoms is a UTI possibly secondary to volume depletion induced by hydrochlorothiazide. Antibiotic therapy could resolve the UTI symptoms, but also assessing his volume status and considering the adjustment of hydrochlorothiazide is important. It should be noted that a urinary tract infection in an elderly patient can sometimes present atypically, and it could potentially be a severe condition like urosepsis which might need intravenous fluid and antibiotics, not just oral ones. Response: Taking all the above considerations into account, the most appropriate next step in management would be (D) Oral trimethoprim-sulfamethoxazole therapy for potential urinary tract infection, however, assessing volume status and consideration for intravenous therapy if necessary should also be considered.

**AI_A:** 1) RESTATING THE QUESTION: The patient, a 70-year-old male, is presenting with fatigue, dark urine, decreased urine output, mild pain during urination, and a low-grade fever. These symptoms, in conjunction with his medication use (allopurinol, hydrochlorothiazide, and acetaminophen) and his existing medical conditions (gout, hypertension, and osteoarthritis), lead us to consider which intervention will be most appropriate to manage his symptoms, including discontinuation of medication, initiation of fluid therapy, measurement of postvoid residual volume, antibiotic therapy, or a renal biopsy. 2) PLANNING: Considering the patient’s symptoms, current medications, and underlying conditions, one assumption could be that he has developed a urinary tract infection (UTI) due to his narrowed urethra. Another assumption could be that he's experiencing acute kidney injury, likely related to his medication regimen and possible dehydration states. Another possibility is that he is experiencing urinary retention, due to his medication or urethral stricture, which might require measuring the postvoid residual volume. I believe considering a urinary tract infection or acute kidney injury should be clinically prioritized, given the provided symptoms and medical history. 3) CLINICAL REASONING: The patient has presented with fatigue, darkened urine, reduced urination, pain during urination, and a mild fever—all symptoms that can be indicative of a UTI. In addition, his use of medications like allopurinol and hydrochlorothiazide may expose him to UTIs. It is also possible that he has developed acute nephritis or an acute kidney injury, considering his medication use or possibly due to dehydration with or without infection. The latter, however, might be less likely given the absence of flank pain or edema and no indications of fluid overload (such as elevated jugular venous pressure or cardiopulmonary abnormalities). 4) CONCLUDING: After considering the patient's symptoms, drug regimen, and underlying conditions, I believe the most appropriate next step in management would be to treat the suspected urinary tract infection. 5) RESPONSE: Therefore, amongst the given options, starting oral trimethoprim-sulfamethoxazole therapy (D) seems to be the most appropriate course of action. This antibiotic is frequently used to treat UTIs. Further diagnostic investigations, such as measuring postvoid residual volume or doing a renal biopsy, might be necessary depending on the patient's response to treatment and evolution of symptoms. Hence, the original question reduces to a clinical decision between addressing the evident symptoms now or initiating further investigations.

**AI_D:** Restating the Question: The scenario describes a 70-year-old man with a few underlying medical conditions such as gout, hypertension, and osteoarthritis, who presents to the clinic with fatigue, dark urine, reduced urine output, mild pain during urination, and a low-grade fever. These symptoms could be indicative of a urinary tract infection (UTI), considering the dysuria and fever, or could be symptoms of renal impairment due to the decreased urine output and hypertension. The question seeks to identify the best next step in management, which requires evaluating the options provided with reference to the patient's symptoms and existing medical history. Planning: In understanding the right approach to this patient, it is crucial to consider his underlying conditions and medications. Allopurinol is known for treating gout, hydrochlorothiazide is a diuretic commonly used for hypertension, and acetaminophen is a pain reliever. However, all these medications can potentially influence kidney function. The patient's clinical presentation indicates a possible UTI or a renal disorder. Thus, the provided options should be evaluated based on their potential for diagnosing or treating these conditions. The options include: discontinuation of acetaminophen (which might be causing renal dysfunction), initiation of fluids intravenously (for potential dehydration), measurement of postvoid residual volume (to evaluate bladder function), administration of trimethoprim-sulfamethoxazole (a common UTI treatment), or renal biopsy (to directly assess kidney tissue). Clinical Reasoning: Given the patient's symptoms, it is wise to initially target the most likely diagnosis. In this case, the patient's mild pain with urination, fever, and dark urine suggest a UTI. Also, considering the patient's age, urinary tract infections are quite common. Therefore, management should initially aim at treating this condition, and the condition most likely to provide immediate symptom relief and potentially resolve the condition is the initiation of antibiotics, specifically trimethoprim-sulfamethoxazole. However, we must also keep in mind the patient's decreased urine output might be a sign of dehydration or potential kidney injury due to his medications and hypertension. This warrants the consideration of fluid administration and evaluation of kidney function. However, prioritizing symptom relief and the most likely diagnosis would favor the initiation of antibiotic therapy. Concluding: In order to manage this patient's condition effectively, we should first address his symptoms, which highly suggest a UTI. This aligns with a step-by-step approach where UTI symptoms are first managed, and if symptoms persist or worsen, a subsequent evaluation for possible kidney injury can be considered. If the antibiotic treatment is ineffective, intravenous fluid administration may be considered. Response: Therefore, option (D) Oral trimethoprim-sulfamethoxazole therapy is the most appropriate next step in this patient's management, as it offers immediate symptomatic relief and addresses the most likely cause, a urinary tract infection.

**Facilitator** The consensus among the AI responses is that the 70-year-old male patient's symptoms of fatigue, dark urine, decreased urine output, mild pain during urination, and a low-grade fever, in conjunction with his medication use (allopurinol, hydrochlorothiazide, and acetaminophen) and his existing medical conditions (gout, hypertension, and osteoarthritis), suggest a urinary tract infection (UTI) as the most likely diagnosis. The patient's age, symptoms, and medication use, particularly allopurinol, increase his risk for UTIs. However, the possibility of acute kidney injury (AKI) due to medication use or dehydration should not be overlooked. Given the patient's symptoms and medical history, the most appropriate next step in management would be to treat the suspected UTI. Therefore, initiating oral trimethoprim-sulfamethoxazole therapy (option D) is considered the most appropriate course of action. This antibiotic is frequently used to treat UTIs and offers immediate symptomatic relief. However, it is also important to monitor the patient's response to treatment and evolution of symptoms. If symptoms persist or worsen, further diagnostic investigations, such as measuring postvoid residual volume or conducting a renal biopsy, might be necessary. Additionally, the patient's volume status should be assessed, and consideration for intravenous therapy if necessary should also be considered. It is also crucial to monitor the patient's use of allopurinol and acetaminophen to reduce potential adverse effects.
