# Supplementary material for: Collaborative intelligence in AI: Evaluating the performance of a council of AIs on the USMLE
Source: PLOS Digit Health. 2025 Oct 9;4(10):e0000787. doi: 10.1371/journal.pdig.0000787 (PMC12510544; doi:10.1371/journal.pdig.0000787)
Supplement: S2 File — (ZIP) [file pdig.0000787.s002.zip › S2 Data Raw data files containing unprocessed question inputs and LLM outputs including transcripts of deliberations/STEP2_Question-90-1_Type-MC-NJ.docx]

**Facilitator** **[Round 1]:** A 27-year-old man is examined in the intensive care unit 1 day after he sustained a closed head injury, liver laceration, and pelvic fracture in a motor vehicle collision. He is intubated and mechanically ventilated. He is receiving intravenous midazolam and 5% dextrose in lactated Ringer solution. Temperature is 37.8°C (100.0°F), pulse is 95/min, and blood pressure is 100/70 mm Hg. Cardiopulmonary examination shows no abnormalities. The abdomen is soft. He withdraws appropriately to painful stimuli. Laboratory studies show: Hematocrit: 28% Leukocyte count: 13,000/mm³ Serum Na⁺: 138 mEq/L K⁺: 3.5 mEq/L Cl⁻: 102 mEq/L HCO₃⁻: 24 mEq/L Urea nitrogen: 20 mg/dL Glucose: 220 mg/dL Creatinine: 1 mg/dL Which of the following is the most likely mechanism of these laboratory findings? (A) Decreased uptake of glucose by insulin-sensitive tissues (B) Exogenous production of glucose by bacteria (C) Impaired excretion of glucose by the kidneys (D) Increased infusion of intravenous glucose (E) Insulin deficiency

**AI_C:** RESTATING THE QUESTION: A 27-year-old male, post-accident, is in the ICU and has been intubated and mechanically ventilated. He is currently on intravenous midazolam (a sedative and muscle relaxant) and 5% dextrose in lactated Ringer solution (a fluid and electrolyte replenisher). His temperature, pulse, blood pressure are within near-normal ranges and there's no apparent cardiopulmonary abnormalities. Laboratory findings show an elevated glucose level of 220 mg/dL, along with other slightly deviating parameters. The inquiry here is about the most plausible cause for these laboratory results. PLANNING: Given the administration of 5% dextrose in lactated Ringer solution, it can be assumed that there is an external source of glucose. However, elevated blood glucose can also come from endogenous production, decreased utilization or uptake, or impaired excretion. Our task lies in evaluating how these alternatives might be clinically prioritized given our patient's situation. CLINICAL REASONING: As defined, glucose is administered externally in our case through infusion, an important point. Other aspects to consider are that the body's reaction to stress, such as a severe accident, often involves increased release of stress hormones like cortisol and glucagon that stimulate gluconeogenesis, adding to the glucose levels. Further, amid critical illness and given the likely sedation-induced immobilization in this patient, decreased uptake of glucose by insulin-sensitive tissues is plausible. Cells likely aren't demanding as much energy, leading to less glucose usage. Kidney function, evidenced by normal creatinine, should be adequate, making impaired excretion less likely. CONCLUDING: Connecting all these elements, I'd hypothesize that the most likely mechanism contributing to this patient's elevated glucose level is the infusion of intravenous glucose. RESPONSE: Therefore, my selection would be (D) Increased infusion of intravenous glucose. This, combined with the physiological reactions to stress and sedation, makes it a more probable reason for this gentleman's hyperglycemia over other options.

**AI_A:** RESTATING THE QUESTION: The clinical case implies a post-accident status of a young man who's in the ICU after experiencing a car crash causing multiple injuries, including a head injury, liver laceration, and pelvic fracture. He's been on mechanical ventilation and is receiving a midazolam sedative and lactated Ringer solution, which contains a 5% concentration of dextrose. His vitals are stable but he presents abnormal hematocrit, leukocyte count and particularly high blood glucose levels (220mg/dL). We are asked to consider the most likely cause for these abnormal findings among options that include decreased glucose uptake by sensitive tissues, bacterial glucose production, deficient kidney excretion of glucose, excessive IV glucose infusion, or an insulin deficiency. PLANNING: We naturally assume that the patient's high blood glucose level is due to insulin-related issues. However, let's revisit this assumption, remembering that he's been administered a solution containing 5% dextrose while having otherwise normal kidney function (indicated by his creatinine level). CLINICAL REASONING: Under normal physiological conditions, glucose supplied from intravenous fluids is metabolized and used up by the body's tissues, and any excess sugar is excreted by the kidneys. Insulin helps the body's cells take up the glucose they need, and any deficit in its activity due to resistance or low production might lead to high glucose levels. However, the patient's kidneys are functioning normally and should be able to excrete the extra glucose. Also, while he may have a minor infection or inflammation indicated by the slightly elevated leukocyte count, this does not seem to be severe enough to cause such a rise in glucose through enhanced glycolysis or gluconeogenesis. CONCLUDING: We, therefore, need to consider an underlying condition that actually matches the observations. The patient is receiving a solution that includes a 5% concentration of dextrose. This constant supply could cause high blood glucose levels, especially in a patient like ours who's largely immobile and therefore unlikely to burn off this extra glucose supply. RESPONSE: Thus, the most likely reason for the observed high glucose levels in this ICU patient who is receiving a 5% dextrose solution intravenously is (D) Increased infusion of intravenous glucose.

**AI_D:** RESTATING THE QUESTION: The question is asking what is the most likely cause for the laboratory findings in a 27-year-old man who is in the intensive care unit after suffering various injuries in a car accident. Specifically, the focus is on the high glucose reading of 220 mg/dL. The potential causes presented suggest problems with glucose uptake, glucose production, glucose excretion, glucose infusion, or insulin. PLANNING: Assumptions underlying these answer choices include: The patient's high glucose reading is due to decreased uptake of glucose by insulin-sensitive tissues (A), metabolism by bacteria is causing increased glucose (B), the kidneys are unable to adequately excrete glucose (C), the infusion he is receiving is delivering too much glucose (D), or he lacks sufficient insulin (E). Questioning these assumptions, let's consider: has the patient been diagnosed with insulin resistance or diabetes mellitus that would affect glucose uptake (A)? Is there any infection reported leading to bacterial glucose production (B)? Are there any renal abnormalities reported that may compromise glucose excretion (C)? Is there any evidence suggesting a high glucose infusion rate (D)? Lastly, are there any signs of insulin deficiency such as diabetic ketoacidosis or hyperglycemic hyperosmolar state (E)? Clinically, priority should be given to the most immediate and acute variables, which in this case would be his immediate treatment in the ICU, specifically the IV treatment with 5% dextrose in lactated Ringer solution. CLINICAL REASONING: The patient is in the ICU having sustained severe injuries. Critical care settings often involve the administration of various intravenous treatments, with the effects of such treatments reflected in lab findings, particularly in glucose levels, since it is frequently included as an energy source for patients unable to feed themselves. The administration of 5% dextrose means the patient is receiving a glucose-rich infusion, which can cause elevated blood glucose levels. CONCLUDING: When evaluated collectively, the given clinical information and test results point to a scenario where the high glucose reading in this patient is likely due to the infusion of glucose through his IV treatment. RESPONSE: (D) Increased infusion of intravenous glucose is the most likely cause of the elevated glucose reading in this patient's laboratory findings.

**AI_E:** (1) RESTATING THE QUESTION: The subject of our discussion is a 27-year-old man who is currently in the intensive care unit due to a car accident injury involving his head, liver, and pelvis. He's currently intubated, undergoing mechanical ventilation, and receiving treatments like intravenous midazolam and 5% dextrose in lactated Ringer solution. His vital signs seem normal but his labs indicate a high serum glucose level of 220 mg/dL. We are tasked with determining the probable cause of this high blood glucose level from the options (A) Decreased uptake of glucose by insulin-sensitive tissues; (B) Exogenous production of glucose by bacteria; (C) Impaired excretion of glucose by the kidneys; (D) Increased infusion of intravenous glucose; (E) Insulin deficiency. (2) PLANNING: On the surface, each option appears to be a plausible assumption as they can all potentially contribute to hyperglycemia. However, in order to provide a clinical prioritization, we have to consider each aspect of the patient's medical context. 'A' requires insulin resistance which we have no evidence for in this case. 'B' can be ruled out as there is no evidence of a bacterial infection. 'C' is unlikely as his kidney function represented by the creatinine level is normal. Option 'E' – insulin deficiency – there is no evidence to suggest he was diabetic or there was an issue with insulin production. The only remaining assumption is 'D' - the patient is indeed receiving an intravenous glucose infusion. (3) CLINICAL REASONING: Hyperglycemia or elevated blood sugar often occurs in critically ill patients, even those without a history of diabetes. Elevated blood glucose can result from a complex interplay of hormonal and metabolic changes triggered by illness or stress, known as "stress hyperglycemia." In this patient's case, it's noteworthy that he is receiving 5% dextrose in Ringer's lactate solution. The glucose provision is designed to meet basal caloric needs but its administration might also raise plasma glucose levels. (4) CONCLUDING: Based on the patient's situation and the availability of various medical facets, the most plausible cause of the patient's hyperglycemia can be attributed to an increase in infused intravenous glucose. (5) RESPONSE: Therefore, the most likely mechanism for these laboratory findings is (D) Increased infusion of intravenous glucose.

**AI_B:** RESTATING THE QUESTION: This patient has experienced major trauma and is currently in the Intensive Care Unit (ICU). He has suffered a head injury, injury to the liver and a pelvic fracture from a car accident and is put on mechanical ventilation. He's also infused with intravenous 5% dextrose and midazolam. Despite his injuries, he seems to show no major abnormalities upon examination. However, the laboratory results are concerning, showing a high blood glucose level (hyperglycemia) of 220 mg/dL. The question suggests that we need to identify the most likely reason for his hyperglycemia from the given options. PLANNING: Considering the patient's circumstances, it's plausible that the hyperglycemia could be attributed to a few factors: (1) intravenous glucose dextrose; (2) stress-induced state following the trauma; or (3) impairment in glucose metabolism due to injury. The other findings include a lowered hematocrit, which aligns with the physical trauma the patient experienced, and an increased leukocyte count, suggesting a potential immune response to the trauma. CLINICAL REASONING: The dextrose infusion could contribute to hyperglycemia. However, as he's been observed for a day post-accident, there's the chance that it's stress-induced hyperglycemia, which is more common in critical care patients. Stress induces a hyper metabolic state which could result in increased glucose production and decreased peripheral uptake, hence causing the patient's high glucose level. Additionally, the hypothesis of insulin deficiency is contradicting the typical physiology of stress response. In a stress response, your body typically ramps up its production of counterregulatory hormones (including glucagon, cortisol, and catecholamines) and these hormones individually have insulin-antagonistic effects, further fueling gluconeogenesis, glycogenolysis, and impairing glucose uptake by muscles and fat. CONCLUDING: Although there are several possibilities that could explain the patient's high glucose level, the most likely reason is the body's response to stress. In dire situations like critical illness, the body naturally increases its glucose production and decreases uptake to provide the system with additional energy. This physiological response can often lead to hyperglycemia even in patients without a history of diabetes. An insulin deficiency state as in scenario (E) is less likely, given that acute stress response usually enhances insulin counter regulatory hormones rather than causing insulin deficiency. RESPONSE: So, in this case, the most probable cause for these laboratory findings is (A) Decreased uptake of glucose by insulin-sensitive tissues, which is induced by the stress response following the trauma.

**Facilitator** **[Round 2]:** Summary: AI_A, AI_C, AI_D, and AI_E all agree that the most likely cause for the patient's high glucose levels is the increased infusion of intravenous glucose (D). They argue that the patient's normal kidney function should allow for the excretion of excess glucose, and there's no evidence of a severe infection or insulin deficiency that could explain the high glucose levels. The constant supply of glucose through the 5% dextrose solution, especially given the patient's immobility, is seen as the most probable cause. AI_B, however, believes that the most likely cause is decreased uptake of glucose by insulin-sensitive tissues (A), induced by the stress response following the trauma. AI_B argues that stress-induced hyperglycemia is common in critical care patients and that the body's response to stress can lead to increased glucose production and decreased uptake. AI_B also notes that the stress response typically enhances insulin counter regulatory hormones, making an insulin deficiency less likely. Question for Council Members: Given that stress-induced hyperglycemia is common in critical care patients, how do you weigh the impact of the stress response and the infusion of the 5% dextrose solution on the patient's high glucose levels? Could these factors be working in tandem, and if so, how would you determine which is the primary cause?. Based on your preceding response, how would you answer the original question: [A 27-year-old man is examined in the intensive care unit 1 day after he sustained a closed head injury, liver laceration, and pelvic fracture in a motor vehicle collision. He is intubated and mechanically ventilated. He is receiving intravenous midazolam and 5% dextrose in lactated Ringer solution. Temperature is 37.8°C (100.0°F), pulse is 95/min, and blood pressure is 100/70 mm Hg. Cardiopulmonary examination shows no abnormalities. The abdomen is soft. He withdraws appropriately to painful stimuli. Laboratory studies show: Hematocrit: 28% Leukocyte count: 13,000/mm³ Serum Na⁺: 138 mEq/L K⁺: 3.5 mEq/L Cl⁻: 102 mEq/L HCO₃⁻: 24 mEq/L Urea nitrogen: 20 mg/dL Glucose: 220 mg/dL Creatinine: 1 mg/dL Which of the following is the most likely mechanism of these laboratory findings? (A) Decreased uptake of glucose by insulin-sensitive tissues (B) Exogenous production of glucose by bacteria (C) Impaired excretion of glucose by the kidneys (D) Increased infusion of intravenous glucose (E) Insulin deficiency]

**AI_C:** Restating the Question: The question highlights a critically ill patient who has suffered multiple traumas and is currently under intensive care with the administration of a 5% dextrose in lactated Ringer solution, among other treatments. The medical tests show that he has high glucose levels, and we are asked to identify the most likely cause of this condition from a given set of options. Planning: The five medical conditions provided as choices involve different aspects of blood glucose regulation, i.e., glucose absorption by tissues, bacterial glucose production, renal glucose excretion, intravenous glucose administration, and deficiency of insulin. Normally, our body regulates the blood glucose levels by a complex interplay of insulin and counter-regulatory hormones. Clinical Reasoning: In a critically ill patient like ours, who has experienced severe trauma, a likely assumption could be the development of stress hyperglycemia, which is characterized by increased glucose production and decreased uptake by muscles due to high levels of stress hormones like cortisol, glucagon, growth hormone, and catecholamines. Yet, the patient's existing renal functionality doesn't point towards impaired excretion of glucose by the kidneys. The patient's leucocyte count does not indicate an ongoing severe bacterial infection which could potentially contribute to blood glucose through bacterial metabolism, making option B less likely. Meanwhile, the patient is receiving a continuous infusion of 5% dextrose, which might contribute to the observed hyperglycemia. While an insulin deficiency could be a cause for high glucose levels, the patient's traumatic and physiological condition indicates no evidence of pre-existing insulin deficiency or diabetes. Concluding: In a clinical scenario such as this, elevated blood glucose levels could be expected due to both the stress response associated with severe trauma and illness, as well as the continuous infusion of glucose-containing solutions, posing a dilemma. Response: Effectively, despite this patient likely undergoing a 'stress response', it's essential to note that he is concurrently receiving a 5% dextrose infusion. Such intravenous dextrose administration doesn't just get 'used up' by the body's need for energy especially when the patient is non-ambulatory. This could explain the hyperglycemia evident in the laboratory results to a reasonable degree. Therefore, the answer should be (D) Increased infusion of intravenous glucose.

**AI_A:** RESTATING THE QUESTION: The question is asking us to identify which choice is the most likely reason for the laboratory findings in a specific clinical scenario. The patient is a 27-year-old male who is currently in the ICU due to multiple injuries from a motor vehicle collision. He is receiving intravenous midazolam and a 5 percent dextrose lactated Ringer solution. His temperature is slightly elevated, his heart rate is on the higher normal side, and blood pressure is on the low end of normal. Most notably, his laboratory results show a high glucose level. We are asked to determine the most likely cause for this elevated glucose level. PLANNING: We need to consider various factors such as his injury, medications, infusion, and body response to stress. Option A suggests higher glucose due to decreased uptake by insulin-sensitive tissues. Option B considers an exogenous (from outside) source of glucose production. Option C questions the kidney's capability to excrete glucose, resulting in a higher level. Option D considers the possibility of the direct increase of glucose supply from the infusions. Lastly, option E suggests the presence of an insulin deficiency that could be causing the high glucose level. We should logically analyze each assumption against the patient's presentation and lab reports before confirming our hypothesis. CLINICAL REASONING: This patient has multiple injuries and is being taken care of in the ICU, indicating a high-stress situation. The glucose infusion through the 5 percent dextrose lactated Ringer solution could be contributing to his elevated glucose level. However, a stress-induced hormonal response could also result in high glucose levels by increasing glucose production and decreasing uptake (option A). He has slightly elevated leukocytes, suggesting bodily stress, but it does not necessarily imply infection to support option B. His creatinine level is normal, indicating the kidneys' functional potential, which reduces the likelihood of option C. Significant insulin deficiency (option E) would likely result in even higher levels of glucose. CONCLUDING: By meticulously analyzing each of the options against the patient's clinical features and the understanding of the body's physiological response to stress and injury, we can hypothesize that the high glucose is most likely the combination of intravenous glucose infusion and the body's stress response due to the trauma and ICU environment. RESPONSE: The above critical thinking process leads to the conclusion that the mechanism behind the elevated glucose level in this injured and ICU-admitted 27-year-old male is a combination of intravenous glucose infusion (D) and the body's stress response to injury/a high-stress environment through decreased uptake of glucose by insulin-sensitive tissues (A).

**AI_B:** RESTATING THE QUESTION: The problem at hand involves determining the most probable mechanism behind the lab findings of a 27-year-old man who is in intensive care after experiencing multiple injuries due to a traffic accident. The patient's current lab results include an elevated glucose level of 220 mg/dL. PLANNING: The underlying assumption here is that the elevated glucose level can be explained by one of these mechanisms: reduced glucose uptake by tissues that are sensitive to insulin, glucose production by bacteria, disrupted glucose excretion by the kidneys, increased administration of glucose intravenously, or a deficiency of insulin. CLINICAL REASONING: When considering trauma patients in ICU settings, it is not uncommon to observe hyperglycemia. This typically results from a physiological stress response that increases glucose production and reduces uptake. In this context, the patient is experiencing multiple sources of stress due to his head injury, liver laceration, and pelvic fracture. Furthermore, termed as "stress hyperglycemia", this transient state of hyperglycemia is not caused by an absolute insulin deficiency, diminishing the likelihood of option (E). It's important to look at the complete picture and not solely focus on the glucose element. Evaluating the patient's renal function is crucial, especially in deciding whether the kidneys can adequately excrete glucose. Two markers are typically indicative of proper renal function: creatinine and urea nitrogen levels. In this case, both values are within the normal range, suggesting normal renal function. Consequently, option (C) is unlikely due to the kidneys' ability to excrete glucose. Infected ICU patients can indeed experience glucose production due to bacteria. Yet, while our patient has leukocytosis, there are no other supporting signs of infection (e.g., fever, presence of a pathogenic organism), making choice (B) unlikely. The patient is being given a 5% dextrose solution, which increases the level of circulating glucose. However, and of utmost importance, is that the stress response following the trauma would have led to higher levels of glucose in the blood regardless of said glucose infusion. CONCLUDING: Given the patient's traumatic injuries and normal renal function, and in light of the context formed by the available lab results, it is reasonable to conclude that the elevated glucose level is due to not only to the dextrose infusion but also a physiological response to stress that increases glucose production and minimizes uptake by insulin-sensitive tissues. RESPONSE: Hence, option (A) decreased uptake of glucose by insulin-sensitive tissues due to stress hyperglycemia appears to be the most likely mechanism for these clinical findings, albeit the effect of the increased glucose infusion also contributes to this state of hyperglycemia.

**AI_E:** Restating the Question: Our task is to identify the most plausible cause for the patient's elevated glucose levels, given the context of his health condition, treatment, and bloodwork. The potential causes for examination are decreased uptake of glucose by insulin-sensitive tissues, exogenous production of glucose by bacteria, impaired excretion of glucose by the kidneys, increased infusion of intravenous glucose, or insulin deficiency. Planning: Consider the patient's condition, particularly his recent head injury and liver laceration. Notably, the patient is receiving intravenous midazolam and a 5% dextrose solution. His temperature is slightly elevated and his glucose level is high. Leukocyte count also suggests some level of inflammation, which could be due to the injuries. The assumptions on the table are that either the increased infusion of intravenous glucose or decreased uptake of glucose by insulin-sensitive tissues due to stress response are responsible for the elevated glucose levels. These are substantiated by existing clinical knowledge that both stress response and influx of intravenous glucose can cause hyperglycemia. Clinical Reasoning: It's not improbable that both increased intravenous glucose ingestion and the body's stress response are jointly contributing to the patient's hyperglycemia. Nevertheless, it is critical to differentiate which mechanism is primarily accountable in this situation. To do this, we should consider the physiological response following significant trauma. In such situations, the systemic inflammatory response typically initiates cortisol release, triggering a rise in hepatic glucose production alongside decreased glucose uptake in peripheral tissues, resulting in hyperglycemia. In critical illness, cortisol and other stress hormones can oppose insulin's action (a state known as insulin resistance), leading to hyperglycemia. But unlike people with diabetes, most critically ill patients have functioning pancreatic β cells that increase insulin output to match the rise in insulin resistance. Concerning intravenous glucose ingress, while a 5% dextrose solution does supply the patient with glucose, this effect would be modulated by the patient's insulin response rendering an extreme rise in glucose levels unlikely. Concluding: Given the complex interplay between stress response and the constant influx of intravenous glucose, it's challenging to assign a single primary cause for the patient's elevated glucose levels. However, considering physiological response post-trauma and the body's compensatory mechanisms against hyperglycemia, it seems reasonable to assume that the primary mechanism underlying these readings is the decreased uptake of glucose by insulin-sensitive tissues as a result of stress response. Response: Based on the reasoning above, the most likely mechanism of these laboratory findings is likely: (A) Decreased uptake of glucose by insulin-sensitive tissues.

**AI_D:** Restate: This query seeks to investigate the most probable explanation for a 27-year-old male's elevated glucose levels, evident from laboratory test results, after sustaining significant traumatic injuries. The patient is currently in an Intensive Care Unit (ICU), intubated, mechanically ventilated, and receiving intravenous midazolam alongside 5% dextrose in lactated Ringer's. Glucose levels are evidently high at 220 mg/dL. The options provided propose various mechanisms for this anomaly, including decreased glucose uptake by tissues sensitive to insulin, glucose production by bacteria (potentially due to infection), impaired renal glucose excretion, an increased infusion of glucose directly, or insulin deficiency. Plan: In order to identify the most likely explanation, we need to evaluate each proposed mechanism in the context of the patient's current situation and available laboratory results. Therefore, we need to consider whether the patient exhibits any signs of significant infection, whether the intravenous supplements being administered could contribute to increased glucose levels, assess renal function, and also evaluate any evidence leading to the possibility of insulin deficiency. Clinical Reasoning: Given the patient's current state, it is crucial to note the absence of infection indicators such as fever, an elevated leukocyte count, or other signs of sepsis. This makes the possibility of glucose production by bacteria (option B) fairly unlikely. In regards to renal function, creatinine levels are normal, suggesting that glucose excretion should be functioning correctly (therefore option C, impaired excretion, appears unlikely). Similarly, without concrete evidence of insulin deficiency present (like known history or clinical findings), option E becomes a less probable choice. This narrows down our options to A and D, namely, decreased glucose uptake due to tissues being insulin-sensitive and increased infusion of intravenous glucose. Concluding: Considering the fact that the patient is receiving 5% dextrose in lactated Ringer solution, this means he is getting a steady supply of glucose intravenously. The patient, currently in the ICU and likely immobile, is not expending much energy and therefore his cells might not require as much glucose, leading to a rise in blood glucose levels. This seems to be a more probable cause than a sudden decrease in glucose uptake by insulin-sensitive tissues (option A), as the latter would typically result in symptoms of insulin resistance, of which there is no indication. Finishing Response: Therefore, upon considering the above, the most likely cause of the patient's elevated blood glucose levels is an increased infusion of intravenous glucose (Option D). The receipt of direct and plentiful glucose supply through 5% dextrose in lactated Ringer solution, in the context of the patient's immobility (and thus lower energy expenditure), most logically accounts for the hyperglycemia noted in the lab results.

**Facilitator** **[Round 3]:** Summary: AI_A and AI_B both believe that the elevated glucose level in the patient is due to a combination of the body's stress response (decreased uptake of glucose by insulin-sensitive tissues) and the intravenous glucose infusion. AI_C and AI_D, however, believe that the primary cause is the increased infusion of intravenous glucose. AI_E agrees with AI_A and AI_B but emphasizes the role of the stress response more than the glucose infusion. Key Differences: The key differences in the council members' opinions lie in the weight they assign to the two potential causes: the body's stress response and the intravenous glucose infusion. AI_A and AI_B see these two factors as equally contributing to the patient's elevated glucose levels, while AI_C and AI_D believe the glucose infusion is the primary cause. AI_E, on the other hand, believes that the stress response is the primary cause, with the glucose infusion playing a secondary role. Question for Council Members: To clarify the disagreements between your opinions, could you please elaborate on why you believe one factor (the body's stress response or the intravenous glucose infusion) contributes more significantly to the patient's elevated glucose levels than the other?. Based on your preceding response, how would you answer the original question: [A 27-year-old man is examined in the intensive care unit 1 day after he sustained a closed head injury, liver laceration, and pelvic fracture in a motor vehicle collision. He is intubated and mechanically ventilated. He is receiving intravenous midazolam and 5% dextrose in lactated Ringer solution. Temperature is 37.8°C (100.0°F), pulse is 95/min, and blood pressure is 100/70 mm Hg. Cardiopulmonary examination shows no abnormalities. The abdomen is soft. He withdraws appropriately to painful stimuli. Laboratory studies show: Hematocrit: 28% Leukocyte count: 13,000/mm³ Serum Na⁺: 138 mEq/L K⁺: 3.5 mEq/L Cl⁻: 102 mEq/L HCO₃⁻: 24 mEq/L Urea nitrogen: 20 mg/dL Glucose: 220 mg/dL Creatinine: 1 mg/dL Which of the following is the most likely mechanism of these laboratory findings? (A) Decreased uptake of glucose by insulin-sensitive tissues (B) Exogenous production of glucose by bacteria (C) Impaired excretion of glucose by the kidneys (D) Increased infusion of intravenous glucose (E) Insulin deficiency]

**AI_A:** RESTATING THE QUESTION: The question is asking us to explain the likely cause of the patient's lab findings who happens to be a 27-year-old male suffering from multiple injuries due to a motor vehicle accident. His lab results show an elevated serum glucose level of 220 mg/dL. The options provided all are related to different mechanisms affecting glucose metabolism and balancing within the body. PLANNING: Looking at the information provided, one might make an assumption that his elevated glucose level could be as a result of the infused 5% dextrose and lactated Ringer solution he is receiving intravenously. This solution contains glucose which could raise his serum glucose level. However, a critical look at his situation brings the assumption that there could be another contributor to his elevated glucose. The patient is under a significant amount of stress from his injuries, which could lead to a stress response mediated by hormones like cortisol and catecholamines. These hormones reduce the effectiveness of insulin in its action of promoting glucose uptake into cells, thus leading to an increase in blood glucose levels. CLINICAL REASONING: Under our initial assumption that the glucose infusion is causing the hyperglycemia, we can justify the phrasing of the question. Observations were made '1 day after' his accident, which means the patient has been receiving IV dextrose potentially for the duration of this time. However, under the alternate assumption of a stress response being a key factor, it's important to consider the role of cortisol and catecholamines. They are major stress hormones that increase during times of physical stress, like following a trauma as experienced by the patient. They promote gluconeogenesis and glycogenolysis in the liver, increasing blood glucose levels. Additionally, they antagonize insulin's effects, reducing the amount of glucose taken up by peripheral tissues. CONCLUDING: In the clinical context, it may be more practical to attribute an exaggerated blood glucose concentration to an aggravated stress response than it is to the infusion of glucose, especially considering the severity of the patient's condition. Therefore, the glucose infusion might contribute to the hyperglycemia, but not as a primary cause. RESPONSE: I would answer the original question as (A) Decreased uptake of glucose by insulin-sensitive tissues, considering it as a result of stress response causing reduced insulin effectiveness.

**AI_D:** RESTATING THE QUESTION: The question is asking about the most likely explanation behind the laboratory findings of a 27-year-old male who was involved in a vehicle accident. The patient is currently being managed in the ICU where he is intubated and ventilated. The patient, who sustained multiple injuries in the incident, is receiving IV midazolam and a 5% dextrose solution, among other treatments. The laboratory results show that several values are abnormal, notably his hematocrit is low, leukocyte count is high, and his glucose level is elevated. From the options given, the most applicable for his condition needs to be determined. PLANNING: We could assume that the patient's elevated glucose level is due to: decreased uptake of glucose by insulin-sensitive tissues (option A), exogenous glucose production by bacteria (option B), impaired excretion of glucose by kidneys (option C), increased intravenous infusion of glucose (option D), or insulin deficiency (option E). CLINICAL REASONING: The body responds to stress by increasing the production of counter-regulatory hormones such as glucagon, epinephrine, cortisol, and growth hormone. These hormones work against the action of insulin, thereby increasing the glucose level in the bloodstream, a phenomenon known as stress hyperglycemia. However, the patient is also undergoing treatment with an infusion of 5% dextrose, which is directly adding glucose into the bloodstream. The infusion of glucose is a controlled factor, and it can be closely monitored, whereas the stress response is a physiological response that is not within our immediate control. CONCLUDING: Based on the information provided, and understanding the impact of stress and controlled infusion on the glucose levels, it is necessary to deliberate whether one has a more significant involvement than the other. RESPONSE: Considering that the patient is in a state of trauma and stress and is receiving a controlled dextrose infusion, both factors contribute to increased blood glucose levels. However, as the glucose infusion can be more closely clinically controlled, and we know the patient is in a state of considerable trauma, it is currently more plausible to assume that the glucose elevation is primarily due to decreased uptake of glucose by insulin-sensitive tissues (A) due to the stress response. The intravenous infusion of glucose likely is contributing, but its involvement can be more controlled, thus leading to the conclusion that it is not the primary cause of these laboratory findings.

**AI_E:** RESTATING THE QUESTION: The question is asking about the cause of elevated glucose levels in a patient who has been in a major accident and is receiving intravenous dextrose among other treatments. Five potential causes have been provided: decreased uptake of glucose by tissues due to increased insulin resistance (option A), glucose production by bacteria (option B), kidney's failure to excrete glucose (option C), excessive infusion of glucose (option D), and a shortage of insulin (option E). The stress from the accident can increase insulin resistance, the intravenous dextrose solution contains glucose and can increase blood glucose level. PLANNING: It is known that both acute stress (as in severe physical injury) and a continuous supply of intravenous glucose can contribute to hyperglycemia. It is also known that the body's response to stress is a priority over metabolic reactions. The assumption here, then, is between whether the body's stress-induced insulin resistance (option A) is contributing more to the hyperglycemia than an increased infusion of intravenous glucose (option D). CLINICAL REASONING: After a major trauma like this, the body's initial response is to release stress hormones such as cortisol and epinephrine. These hormones promote gluconeogenesis and glycogenolysis to increase the blood glucose level and oppose the action of insulin, leading to insulin resistance and subsequent hyperglycemia. Alongside this, the patient is receiving 5% dextrose in lactated Ringer solution, which adds to the blood glucose. However, this hyperglycemia is likely more attributable to the stress-induced insulin resistance, as the body's response to massive stress is a physiological priority over metabolic reactions linked to glucose infusion. CONCLUDING: Looking at the patient's conditions, it can be summarized that the patient is critically ill after a high-impact accident, which leads to a stress response resulting in increased insulin resistance, and the patient is also receiving a dextrose infusion. The glucose in the dextrose infusion unquestionably contributes to the hyperglycemia, but its role is likely secondary to the stress-induced increase in insulin resistance resulting from the body's response to the patient's critical condition. RESPONSE: Therefore, the most likely mechanism of these laboratory findings is decreased uptake of glucose by insulin-sensitive tissues (Option A), due to a stress-induced increase in insulin resistance.

**AI_C:** Restating the question: The clinical scenario describes a 27-year-old male who sustained multiple injuries in a motor vehicle collision and is currently in the intensive care unit. He is intubated, mechanically ventilated, and receiving intravenous fluids including midazolam (a sedative) and 5% dextrose in lactated Ringer solution. His lab results show an elevated glucose level. We are asked to identify the most likely cause of the elevated blood glucose given the options provided. Planning: To establish the cause of the elevated glucose level, we need to examine each possible option and how it relates to this patient's current condition and treatments. Given that the patient is receiving intravenous dextrose solution and is under significant stress due to his injuries and hospitalization, both could contribute to increased glucose. Moreover, other factors such as decreased tissue uptake of glucose, or insulin deficiency due to the stress response may also play a role. Clinical Reasoning: Severe stress can induce a state similar to type 2 diabetes, in which there is insulin resistance leading to increased blood glucose levels (Option A). However, increased blood glucose could also be secondary to infusion of intravenous glucose (Option D). Although insulin deficiency may be part of the stress response, it's not the primary cause in this situation as we expect insulin levels to be increased under stress to try to maintain glucose homeostasis (Option E). Concluding: While exogenous production of glucose by bacteria (Option B) could cause hyperglycemia, there's no evidence to suggest bacterial infection in this case. Impaired excretion of glucose by the kidneys (Option C) would present with a higher renal threshold for glucose, which also doesn't appear to be the case. Response: Considering all of these factors, decreased uptake of glucose by insulin-sensitive tissues (Option A) and increased infusion of intravenous glucose (Option D) both seem to be contributing to the observed hyperglycemia. However, given the fact that the patient is receiving an intravenous dextrose infusion, it seems reasonable to prioritize Option D (increased infusion of intravenous glucose) as the most likely cause of the elevated glucose levels. Nonetheless, it's important to understand that this is a complex physiological response, and multiple factors could be contributing at the same time.

**AI_B:** (1) RESTATING THE QUESTION: The medical team is attempting to understand the underlying mechanism causing the laboratory findings in a 27-year-old man with multiple injuries from a motor vehicle collision. The patient is in intensive care and is receiving respiratory support and intravenous medications, including midazolam and lactated Ringer solution containing 5% dextrose. He presents with a high glucose level and we are looking for the most plausible explanation for this elevation. (2) PLANNING: The various assumptions we could make about this patient's high glucose include decreased uptake of glucose by insulin-sensitive tissues, exogenous production of glucose by bacteria in the body, impaired excretion of glucose by the kidneys, increased infusion of intravenous glucose, or insulin deficiency. However, given the patient's traumatic injuries and the clinical settings, the most likely assumptions that should be clinically prioritized are decreased uptake of glucose by insulin-sensitive tissues and increased infusion of intravenous glucose. Decreased uptake of glucose by insulin-sensitive tissues could be driven by the patient's body stress response due to the injuries sustained. This stress increases stress hormones such as catecholamines and cortisol, which contribute to insulin resistance. On the other hand, increased infusion of intravenous glucose could be directly related to the fluid he is receiving intravenously (5% dextrose in lactated Ringer solution). This solution could be contributing to the high glucose levels in his serum. (3) CLINICAL REASONING: We know that traumatic events such as the ones this patient has sustained can result in a stress response involving increased levels of certain hormones (like catecholamines and cortisol) that induce a state of insulin resistance, resulting in less glucose being taken up by insulin-sensitive tissues. This is a biological mechanism to conserve resources during acute stress. In addition, the presence of a glucose-infusing solution could lead to a state where there's an influx of glucose into the bloodstream, further raising the patient's glucose levels. (4) CONCLUDING: Based on these clinical reasonings and current treatment measures, the elevated glucose levels in this patient could be attributed to both decreased uptake of glucose by insulin-sensitive tissues and increased infusion of intravenous glucose. In the absence of more detailed information, it would be unlikely to attribute the high glucose levels to the other options. (5) RESPONSE: For the question, the most likely mechanism of these laboratory findings in this patient would be (A) decreased uptake of glucose by insulin-sensitive tissues and potentially in combination with (D) increased infusion of intravenous glucose. However, given the explicit options to select one, we weigh the clinical importance of the body's stress response leading to decreased uptake of glucose by insulin-sensitive tissues, most aligned with option (A), as the most critical responsible mechanism.

**Facilitator** **[Round 4]:** Summary: AI_A believes that the patient's elevated glucose levels are primarily due to a stress response causing reduced insulin effectiveness, leading to decreased uptake of glucose by insulin-sensitive tissues. While the glucose infusion might contribute to the hyperglycemia, it is not seen as the primary cause. AI_B agrees with AI_A that decreased uptake of glucose by insulin-sensitive tissues is a significant factor due to the body's stress response. However, AI_B also considers the increased infusion of intravenous glucose as a potential contributing factor, but ultimately prioritizes the stress response as the most critical mechanism. AI_C suggests that both decreased uptake of glucose by insulin-sensitive tissues and increased infusion of intravenous glucose are contributing to the hyperglycemia. However, AI_C prioritizes the infusion of glucose as the most likely cause, given the patient's current treatment. AI_D, like AI_A and AI_B, believes that the primary cause of the elevated glucose levels is decreased uptake of glucose by insulin-sensitive tissues due to the stress response. The infusion of glucose is seen as a contributing factor, but not the primary cause. AI_E concurs with AI_A, AI_B, and AI_D that the stress-induced increase in insulin resistance, leading to decreased uptake of glucose by insulin-sensitive tissues, is the most likely cause of the elevated glucose levels. The glucose infusion is seen as a secondary contributing factor. Question to clarify disagreements: Given that all council members agree that both stress-induced insulin resistance and glucose infusion contribute to the patient's hyperglycemia, how can we determine which factor is the primary cause? What additional information or tests would help us better understand the relative contributions of these two factors to the patient's elevated glucose levels?. Based on your preceding response, how would you answer the original question: [A 27-year-old man is examined in the intensive care unit 1 day after he sustained a closed head injury, liver laceration, and pelvic fracture in a motor vehicle collision. He is intubated and mechanically ventilated. He is receiving intravenous midazolam and 5% dextrose in lactated Ringer solution. Temperature is 37.8°C (100.0°F), pulse is 95/min, and blood pressure is 100/70 mm Hg. Cardiopulmonary examination shows no abnormalities. The abdomen is soft. He withdraws appropriately to painful stimuli. Laboratory studies show: Hematocrit: 28% Leukocyte count: 13,000/mm³ Serum Na⁺: 138 mEq/L K⁺: 3.5 mEq/L Cl⁻: 102 mEq/L HCO₃⁻: 24 mEq/L Urea nitrogen: 20 mg/dL Glucose: 220 mg/dL Creatinine: 1 mg/dL Which of the following is the most likely mechanism of these laboratory findings? (A) Decreased uptake of glucose by insulin-sensitive tissues (B) Exogenous production of glucose by bacteria (C) Impaired excretion of glucose by the kidneys (D) Increased infusion of intravenous glucose (E) Insulin deficiency]

**AI_C:** RESTATING THE QUESTION: This question concerns a patient with multiple injuries, who is in a state of high-stress and receiving intravenous drugs and fluids, including a glucose solution. The high glucose observed in his lab results could be due to various mechanisms which are listed in the multiple-choice options. PLANNING: The assumptions regarding the possible mechanisms for his elevated glucose include decreased tissue uptake of glucose due to insulin resistance, exogenous bacterial glucose production, impaired renal glucose excretion, increased i.v. glucose infusion, and insulin deficiency. We need to question each assumption and evaluate based on this patient's clinical picture. CLINICAL REASONING: In the context of the patient's critical illness and head injury, a stress response would be anticipated. This could potentially lead to increased levels of counter-regulatory hormones (cortisol, catecholamines, glucagon, growth hormone), which would decrease the uptake of glucose by insulin-sensitive tissues. As the patient is receiving i.v. dextrose, this could also contribute to the elevated glucose levels. The other options provided (B bacteria-induced glucose production, C impaired renal excretion, and E insulin deficiency) are less likely in this context. CONCLUDING: Based on the patient's clinical situation and the given laboratory data, it can be concluded that the stress response to his critical illness, resulting in a decrease in the uptake of glucose by insulin-sensitive tissues, is primarily responsible for his hyperglycemia. Increased glucose infusion is a contributing factor but is not the primary cause. RESPONSE: The most likely mechanism of the raised glucose levels is decreased uptake of glucose by insulin-sensitive tissues (Option A).

**AI_D:** In order to assess these discordances, I would suggest looking at blood insulin levels to determine the extent of the patient's physiological response to stress and to assess the adequacy of their insulin production in response to hyperglycemia. In the scenario of decreased uptake of glucose by insulin-sensitive tissues, the body should be producing more insulin to compensate for elevated blood glucose levels. If blood insulin levels are elevated, this could support the notion that decreased uptake of glucose by insulin-sensitive tissues due to stress-induced insulin resistance is a primary factor in the patient's hyperglycemia. However, examining the rate and volume of the 5% dextrose infusion can also provide insight. If the volume is significant and administered in a rapid manner, one can argue that this is the contributing factor to the hyperglycemia. In addition, the investigation of potential difficulties in the renal excretion of glucose, as well as hyperglycemia associated with sepsis or other infectious processes, could provide a broader understanding of the situation. In the case of this 27-year-old man who sustained multiple injuries from a motor vehicle collision and is currently in the ICU, multiple factors are contributing to his lab findings. The critical condition the patient is in, initiates a 'stress response', leading to increased catecholamine, cortisol and glucagon levels. These hormones yield a counter-regulatory effect against insulin, leading to insulin resistance and subsequent hyperglycemia due to a decreased uptake of glucose by insulin-sensitive tissues (Answer A). In addition to the physiological stress response, receiving an intravenous infusion of a 5% dextrose-containing solution would also contribute to the patient's hyperglycemia (Answer D). However, given the existing stress conditions, this intravenous glucose infusion would contribute less to his hyperglycemic state. Hence, in this case, while several mechanisms could contribute to the patient's hyperglycemia, the most impactful one would be the decreased uptake of glucose by insulin-sensitive tissues due to hormonal changes during a stress response (Answer A). Therefore, I agree with AI_A, AI_B, AI_D, and AI_E and differ in perspective from AI_C.

**AI_A:** 1) RESTATING THE QUESTION: The question is asking what could be the most likely reason for the changes observed in this patient's laboratory results, specifically the elevated glucose level. Some of the potential reasons given are: decreased tissue sensitivity to insulin (option A), glucose produced by bacteria (option B), decreased renal glucose excretion (option C), increased glucose infusion (option D), and a deficiency of insulin (option E). 2) PLANNING: Let's consider each of these possibilities. Insulin-deficiency, bacteria-produced glucose, and impaired renal glucose excretion seem less likely given the current clinical scenario of a patient in a post-injury stress state. However, both decreased glucose uptake by tissues and increased glucose infusion are viable possibilities in this case. The patient's acute stress and injury response can lead to a state of peripheral insulin resistance, resulting in decreased glucose uptake (option A). At the same time, he's receiving 5% dextrose solution intravenously that is directly increasing his blood glucose (Option D). 3) CLINICAL REASONING: After analyzing the question, it becomes evident that there are two equally possible causes: the stress-induced insulin resistance (decreased glucose uptake by tissues) and the intravenous infusion of dextrose (increased glucose input). However, considering the severe extent of the patient's injuries and the intensity of post-traumatic stress, a reasonable hypothesis is that the stress response might be playing a bigger role here than the glucose infusion. 4) CONCLUDING: In conclusion, although both decreased uptake of glucose by insulin-sensitive tissues (due to stress-induced insulin resistance) and increased glucose infusion (from the 5% dextrose in lactated Ringer solution) are contributing to the hyperglycemia, the stress response's impact could be even more significant given the severity of patient's injuries. Therefore, it is crucial to understand that while both factors play a role in the patient's hyperglycemia, the decimated tissue sensitivity to insulin due to the stress response may be a more predominant mechanism. 5) RESPONSE: Answer choice (A), Decreased uptake of glucose by insulin-sensitive tissues, might be the most likely mechanism causing hyperglycemia in this patient. However, it's worth noting that medical management should aim at controlling both contributing factors to manage the patient's elevated blood glucose levels effectively.

**AI_B:** Restating the question: This question pertains to a 27-year-old male patient in the intensive care unit (ICU) who has sustained injuries from a motor vehicle collision. He is mechanically ventilated and is receiving medications and intravenous fluids, one of which includes glucose. Despite an absence of physical signs of infection or other abnormalities, his laboratory results reveal an above-average glucose level, coupled with a reduced hematocrit and an elevated leukocyte count. The question asks us to identify which among the given possible mechanisms is the most likely cause behind these laboratory results. Planning: We could consider several assumptions, including: 1) Hyperglycemia, arising from a decreased uptake of glucose by insulin-sensitive tissues due to a body's physiological response to stress. 2) There could be an exogenous production of glucose by bacteria, suggesting a severe bacterial infection. 3) The kidneys may not be sufficiently excreting glucose. 4) Hyperglycemia may result from the increased infusion of intravenous glucose supplementing the patient's fluid intake. 5) An insulin deficiency might be causing the elevated glucose levels. We should discard exogenous production of glucose by bacteria, impaired glucose excretion by the kidneys, and insulin deficiency, respectively, due to absence of clinical signs of infection, normal kidney function tests, and the acute onset of hyperglycemia without a prior history of diabetes mellitus. Therefore, we should focus on the possibility of physiological stress and hyperglycemia from increased infusion of intravenous glucose. Clinical reasoning: The process of clinical reasoning should aid us in distinguishing between these two assumptions. The body's stress response following a life-threatening traumatic event such as this often leads to a surge of counterregulatory hormones (cortisol, catecholamines, glucagon, and growth hormone). This surge induces insulin resistance, thereby limiting glucose uptake by insulin-sensitive tissues, resulting in hyperglycemia. On the other hand, while dextrose solutions indeed deliver glucose into the bloodstream, the glucose concentration (5%) in the provided lactated Ringer's solution shouldn't be enough to independently cause such high glucose levels, unless given in incredibly high volumes or in a patient with disrupted glucose homeostasis. Concluding: Given this information and the sequence of reasoning applied, the overall clinical picture suggests that the trauma-induced stress response causing insulin resistance leading to decreased uptake of glucose by insulin-sensitive tissues contributes more substantially to the patient's hyperglycemia than the glucose being delivered via the intravenous fluids. Response: The clinical, laboratory, and supportive data imply that the most likely cause for these findings is (A) Decreased uptake of glucose by insulin-sensitive tissues.

**AI_E:** In summarizing the presented case, we have a 27-year-old man who is in the intensive care unit due to a closed head injury, liver laceration, and pelvic fracture from a motor vehicle collision. His treatment includes mechanical ventilation, intravenous midazolam (a sedative), and a solution of 5% dextrose in lactated Ringer's solution (which contains electrolytes and is frequently used for fluid replacement), which could contribute to increased glucose levels in his blood. His vital signs are stable despite his severe injuries. His bloodwork, however, reveals an increased level of glucose, indicative of hyperglycemia. The differential diagnosis revolves around this hyperglycemia, considering factors such as decreased glucose uptake, exogenous glucose production, impaired glucose excretion, excessive glucose infusion, or insulin deficiency. In revisiting the alternatives, both the patient's crisis and ongoing management regimes should be considered. Intense stress from the accident could induce a physiological response (Catecholamine surge) that inhibits insulin release (option E), boosting glucose levels in the bloodstream. Simultaneously, infusion of 5% dextrose in his fluid replacement regime could elevate the rate of glucose introduction into the bloodstream (Option D). The available evidence doesn't suggest the patient's kidney is unable to excrete glucose (Option C), nor does it indicate bacterial glucose production. Additionally, there's no sign of an inherent insulin deficiency (Option E) given the scenario; the hyperglycemia and incident would instead point to an issue of insulin resistance from stress rather than a deficiency itself. Therefore, the situation points towards decreased glucose uptake due to stress (Option A) as well as increased glucose infusion (Option D) influencing the patient’s hyperglycemia. During the clinical reasoning process, we must acknowledge the relationship between stress and blood glucose levels. Biological responses to severe physiological stress, such as in this case, could cause an increased blood glucose level due to the body’s fight or flight response, marked by the release of stress hormones such as cortisol and catecholamines. These hormones counter insulin's effects and promote hepatic glucose production, leading to increased blood glucose levels. Consequently, hyperglycemia can be a common finding in critically ill patients. Furthermore, the administration of 5% dextrose could cause an artificial increase in blood glucose measurements taken from the central venous line. In conclusion, considering the patient's severe injuries, it's reasonable to attribute the hyperglycemia to both decreased glucose uptake due to the intense stress of his situation and the additional glucose introduced through the 5% dextrose infusion. However, given the severity of the patient’s physical trauma and its physiological responses, including a possible catecholamine surge that prompts insulin resistance and increased hepatic glucose production, the most likely mechanism for the observed hyperglycemia would be decreased uptake of glucose by insulin-sensitive tissues (Option A). This reasoning is supported by understanding the body's response to traumatic injury and stress, importantly, how the stress response affects glucose regulation. Therefore, (A) Decreased uptake of glucose by insulin-sensitive tissues is the most likely mechanism responsible for these laboratory findings, with his clinical situation primarily contributing to this occurrence.

**Facilitator** **[Round 5]:** Summary: AI_A, AI_B, AI_C, and AI_E all agree that the most likely cause of the patient's hyperglycemia is decreased uptake of glucose by insulin-sensitive tissues due to stress-induced insulin resistance (Option A). They all acknowledge that the patient's severe injuries and the resulting stress response could lead to a state of peripheral insulin resistance, resulting in decreased glucose uptake. They also agree that the intravenous infusion of dextrose (Option D) is contributing to the hyperglycemia, but they believe that the stress response's impact could be more significant given the severity of the patient's injuries. AI_D, on the other hand, suggests that while decreased uptake of glucose by insulin-sensitive tissues due to stress-induced insulin resistance is a primary factor in the patient's hyperglycemia, the rate and volume of the 5% dextrose infusion should also be considered. AI_D believes that if the volume is significant and administered rapidly, it could be a contributing factor to the hyperglycemia. AI_D also suggests looking at blood insulin levels to determine the extent of the patient's physiological response to stress and to assess the adequacy of their insulin production in response to hyperglycemia. Question for Council Members: Given that all council members agree on the role of stress-induced insulin resistance in causing hyperglycemia, how significant do you believe the contribution of the 5% dextrose infusion is to the patient's hyperglycemia? Could the volume and rate of the infusion be a more significant factor than currently considered?. Based on your preceding response, how would you answer the original question: [A 27-year-old man is examined in the intensive care unit 1 day after he sustained a closed head injury, liver laceration, and pelvic fracture in a motor vehicle collision. He is intubated and mechanically ventilated. He is receiving intravenous midazolam and 5% dextrose in lactated Ringer solution. Temperature is 37.8°C (100.0°F), pulse is 95/min, and blood pressure is 100/70 mm Hg. Cardiopulmonary examination shows no abnormalities. The abdomen is soft. He withdraws appropriately to painful stimuli. Laboratory studies show: Hematocrit: 28% Leukocyte count: 13,000/mm³ Serum Na⁺: 138 mEq/L K⁺: 3.5 mEq/L Cl⁻: 102 mEq/L HCO₃⁻: 24 mEq/L Urea nitrogen: 20 mg/dL Glucose: 220 mg/dL Creatinine: 1 mg/dL Which of the following is the most likely mechanism of these laboratory findings? (A) Decreased uptake of glucose by insulin-sensitive tissues (B) Exogenous production of glucose by bacteria (C) Impaired excretion of glucose by the kidneys (D) Increased infusion of intravenous glucose (E) Insulin deficiency]

**AI_D:** Restating the question: We are tasked with determining the most likely cause of an elevated blood glucose level (hyperglycemia) in a severely injured patient. Specifically, we are considering various options including decreased tissue uptake of glucose, bacterial production of glucose, impaired glucose excretion, increased infusion of intravenous glucose, and lack of insulin. Planning: Assuming hyperglycemia is the consequence of stress-induced insulin resistance, the body's physiological response to severe injury often includes a state of hyperglycemia due to insulin resistance in peripheral tissues. This mechanism (Option A) could potentially be the most clinically relevant. Other sources of glucose, such as a 5% dextrose infusion (Option D), could also contribute to hyperglycemia in this context. However, the contributions of other factors such as bacterial glucose production (Option B), impaired kidney function (Option C), or insulin deficiency (Option E) seem less plausible under the current circumstances. Clinical Reasoning: Insulin resistance alters glucose homeostasis by reducing glucose uptake into insulin-sensitive tissues, which contributes to hyperglycemia. This is a common response to stress, including severe injury such as that experienced by this patient. In addition, the patient is receiving a 5% dextrose infusion, which is adding exogenous glucose to his system, potentially exacerbating hyperglycemia. However, given the severity of the patient's injuries and the physiological stress reaction, the contribution of the glucose infusion to the current hyperglycemia state might be less significant compared to the insulin resistance. Concluding: Given the above considerations, the option "Decreased uptake of glucose by insulin-sensitive tissues" likely presents the most suitable explanation for the observed laboratory findings, including the hyperglycemia. This highlights the fact that although the patient is receiving a glucose infusion that may be contributing to the hyperglycemia, the stress-induced insulin resistance seems to be a more significant factor in the observed state of high blood glucose. Response: Based on available data and physiological understanding, the most likely mechanism of these laboratory findings is decreased uptake of glucose by insulin-sensitive tissues (Option A), primarily due to stress-induced insulin resistance following severe trauma.

**AI_E:** Restating the Question: The question requires us to determine the most likely cause or mechanism behind the elevated blood glucose levels (hyperglycemia) seen in a severely injured patient who is currently in an intensive care unit, receiving treatments such as mechanical ventilation and intravenous infusions of midazolam (a benzodiazepine used for sedation) and 5% dextrose in lactated Ringer solution (a fluid replenishing and energy providing solution). Planning: The sustained injuries, as well as subsequent surgical interventions and ongoing intensive care, can trigger a significant stress response in the body. This stress response can involve multiple hormonal pathways, leading to changes in various metabolic processes, including glucose metabolism. The options provided all relate to mechanisms associated with glucose metabolism, including the role of insulin in facilitating glucose uptake by body tissues (Option A), the possibility of bacterial glucose production (Option B), kidney function in glucose excretion (Option C), the effects of intravenous glucose administration (Option D), and insulin deficiency (Option E). Each of these assumptions needs to be evaluated in the context of the patient's clinical presentation and the physiological reactions to such severe injuries. Clinical Reasoning: Elevated glucose levels in this setting are typically a response to the stress of the injury and subsequent medical intervention. The body responds to the stress by producing counter-regulatory hormones, such as cortisol and catecholamines, which create a state of insulin resistance, meaning that tissues do not take up glucose as effectively as they usually would (Option A). This mechanism primarily contributes to hyperglycemia in acutely ill patients. While the patient is receiving 5% dextrose intravenously, which would lead to increased blood glucose levels (Option D), the stress response-induced insulin resistance likely plays a more significant role in hyperglycemia in this setting. Concluding: Although multiple factors could contribute to this patient's elevated blood glucose levels, it is most likely that insulin resistance induced by the stress response to injury and subsequent medical intervention is the primary cause. Insulin resistance leads to decreased glucose uptake by body tissues, resulting in hyperglycemia. Simultaneously, the effect of receiving 5% dextrose intravenously likely has a comparatively lesser but contributory role in the hyperglycemia. Response: Therefore, considering the clinical context and physiological processes involved, the most probable mechanism for the hyperglycemia seen in this patient is decreased uptake of glucose by insulin-sensitive tissues due to stress-induced insulin resistance (Option A), with contribution from the continued intravenous administration of 5% dextrose.

**AI_A:** RESTATING THE QUESTION: The council is being asked to discuss the laboratory findings of a 27-year-old man who suffered multiple injuries in a car collision. He is currently in an intensive care unit (ICU) post trauma, intubated, ventilated, and receiving treatment with midazolam and lactated Ringer solution, containing 5% dextrose. His laboratory studies reveal an elevated glucose level of 220 mg/dL. The council is asked to identify the most likely mechanism for this hyperglycemia among listed options. PLANNING: This scenario is a clinical setting involving major trauma and intensive care. Post trauma, stress responses such as elevated glucose, are expected. Furthermore, the patient is receiving intravenous dextrose, which could directly add to blood glucose. Assumptions include: (a) Hyperglycemic response is due to stress; (b) dextrose infusion contributes to hyperglycemia; (c) damaged organs could cause disrupted glucose metabolism, or; (d) the sedative drug being administered could influence glucose levels. Of these, the first two assumptions seem to have the highest plausibility based on typical physiological responses to ICU stay, trauma, and therapies received. CLINICAL REASONING: In the setting of trauma and stress, the body usually reacts by increasing production of counter-regulatory hormones including cortisol, glucagon, catecholamines such as adrenaline and noradrenaline, and growth hormone. This hormonal surge triggers a state of insulin resistance, which impedes glucose entry into insulin-dependent tissues such as muscle, promoting hyperglycemia. This is a typical 'fight or flight' response. Additionally, the 5% dextrose given intravenously provides an additional, more direct, source of circulating glucose. CONCLUDING: Based on this discussion, it seems that both the stress response to trauma (causing peripheral insulin resistance and decreased glucose uptake in tissues) and the intravenous administration of 5% dextrose in lactated Ringer solution are contributing significantly to the patients' hyperglycemia. The patient's condition and the treatment he is receiving can contribute both directly (intravenous glucose) and indirectly (stress-induced insulin resistance) to the elevated blood glucose values observed in the laboratory findings. RESPONSE: In response to the original question, "Which of the following is the most likely mechanism of these laboratory findings?", I would support option (A) "Decreased uptake of glucose by insulin-sensitive tissues" as the most significant contributor to the patient's hyperglycemia, while acknowledging the contributory role of option (D) "Increased infusion of intravenous glucose". The patient's intensive care status, the severity of his trauma and the associated stress response is more likely to induce a significant physiological change causing insulin resistance and thus decreased glucose uptake into tissues, than the amount of glucose contributed by the infusion. In a typical intensive care setting with a trauma patient, the stress-induced physiological response would take precedence. Although the 5% dextrose would add to the glucose levels, the body’s response to stress with hormonal changes and insulin resistance leads to option (A) being more clinically relevant.

**AI_C:** RESTATING THE QUESTION: The inquiry requires us to discern the primary factor behind the laboratory results for a 27-year-old male ICU patient, specifically focusing on his elevated blood glucose level. His injuries and current medical treatment, along with the lab results, need to be analyzed to figure out the primary cause. We have five potential reasons: (A) reduced glucose absorption by insulin-reactive tissues, (B) glucose production by external bacteria, (C) hindered glucose elimination by the kidneys, (D) an upsurge of intravenous glucose infusion, or (E) insulin shortage. PLANNING: The initial supposition is that the patient's hyperglycemia is primarily due to stress-induced insulin resistance leading to a lower intake of glucose by insulin-sensitive tissues (Option A). This presumption is based on the understanding that severe trauma can induce a stress response that leads to insulin resistance. Another thought could be that the continuous infusion of 5% dextrose in lactated Ringer's solution is contributing immensely to the patient's hyperglycemia (Option D). This alternate hypothesis could be evaluated by assessing the infusion rate and volume. CLINICAL REASONING: Stress-induced insulin resistance, as a result of the patient's injuries, can indeed lead to heightened blood glucose levels as the capacity of insulin-sensitive tissues to uptake glucose diminishes. Additionally, the continuous infusion of dextrose would contribute to increasing serum glucose. Nonetheless, the extent of the patient's hyperglycemia suggests more than just an external supply of glucose; hence, the presence of stress-induced insulin resistance seems a more plausible explanation. As for whether the dextrose infusion plays a more significant role, this would largely depend on the infusion's pulse and volume. However, considering the patient's condition, it seems more likely that the physiological stress response is the primary factor. CONCLUDING: The primary contributor to the patient's hyperglycemia, most likely, is the decrease in glucose uptake by insulin-sensitive tissues due to the stress response brought about by his severe injuries (Option A), rather than an increase in glucose infusion (Option D). Despite the continuous infusion of dextrose potentially contributing to increased serum glucose, given the severity of the patient's condition and the resulting physiological responses, it's more plausible to consider stress-induced insulin resistance as the main factor. RESPONSE: Given the provided information, decreased uptake of glucose by insulin-sensitive tissues (Option A) seems most likely to be the primary mechanism behind these laboratory findings. The grave physiological stress from his injuries likely induced insulin resistance leading to higher glucose levels. The infusion of 5% dextrose in lactated Ringer's solution, though contributing to hyperglycemia, wouldn't solely be responsible for such an excessive rise in blood glucose levels.

**AI_B:** Restating the question: In this clinical scenario, we aim to determine the most likely reason for the laboratory findings of a 27-year-old man who recently went through a significant trauma, resulting in a closed head injury, liver lacerations, and pelvic fracture. The patient's glucose level is notably high at 220 mg/dL, which is the primary finding of interest. We are considering five options: decreased glucose uptake by insulin-sensitive tissues, glucose production by bacteria, impaired glucose excretion by kidneys, increased glucose infusion intravenously, and insulin deficiency. Planning: The patient's hyperglycemia could be due to stress-induced insulin resistance (option A). This is often a response to severe trauma, surgery, or critical illness, where increased levels of counter-regulatory hormones like glucagon, cortisol, catecholamines, and growth hormone can cause increased hepatic glucose production and decreased peripheral tissue glucose uptake. Systemic infections or sepsis could lead to exogenous production of glucose by bacteria (option B), but the patient has no indication of an infection. Impaired excretion of glucose by the kidney (option C) could result in hyperglycemia. However, renal glucose handling generally doesn't affect serum glucose levels appreciably unless there is renal failure or a condition of renal glycosuria– neither of which are suggested in our patient. The hyperglycemia can be from the increased infusion of glucose (option D by way of the 5% dextrose in lactated Ringer solution the patient is receiving. However, this alone is unlikely to cause such a significant rise in glucose levels without other contributing factors. Option E suggests insulin deficiency, but acute severe stress like the trauma our patient has experienced typically leads to an increase, not decrease, in insulin release as a physiological response to hyperglycemia caused by the stress response. Clinical Reasoning: Inflammation and systemic stress responses lead to an increase in hormone levels that counteract insulin's effects, promoting gluconeogenesis and glycogenolysis while reducing peripheral tissue glucose uptake. This physiological response to critical illness, which we can refer to as stress-induced insulin resistance, is primarily responsible for the patient's hyperglycemia. The 5% dextrose he is receiving might further exacerbate the hyperglycemia but is unlikely to be the main causative factor. The patient does not currently exhibit signs of active infection or sepsis to suggest bacterial glucose production, there's no kidney-related disease present that would affect glucose excretion, and there's no indication of insulin deficiency. Concluding: From the available information, we can infer that the patient's hyperglycemia is likely a product of enhanced gluconeogenesis, glycogenolysis, and reduced peripheral glucose use caused by a surge in counter-regulatory hormones as part of the stress-induced response to the trauma he experienced. The dextrose infusion could be contributing to the hyperglycemia but is unlikely to be the primary cause. Response: The most likely mechanism behind these laboratory findings is decreased uptake of glucose by insulin-sensitive tissues (option A).

**Facilitator** The consensus among the AI responses is that the most likely mechanism for the elevated glucose levels in the 27-year-old man's laboratory findings is decreased uptake of glucose by insulin-sensitive tissues (Option A). This is primarily due to stress-induced insulin resistance, a common physiological response to severe trauma and intensive care. The body's stress response to the patient's injuries and subsequent medical intervention leads to the production of counter-regulatory hormones, which create a state of insulin resistance. This means that tissues do not take up glucose as effectively as they usually would, resulting in hyperglycemia. While the patient is also receiving 5% dextrose intravenously, which would contribute to increased blood glucose levels (Option D), this is considered to play a secondary, albeit contributory, role to the hyperglycemia. The other options, such as exogenous production of glucose by bacteria (Option B), impaired excretion of glucose by the kidneys (Option C), and insulin deficiency (Option E), are deemed less plausible given the patient's clinical presentation and the physiological reactions to his severe injuries.
