# Supplementary material for: Collaborative intelligence in AI: Evaluating the performance of a council of AIs on the USMLE
Source: PLOS Digit Health. 2025 Oct 9;4(10):e0000787. doi: 10.1371/journal.pdig.0000787 (PMC12510544; doi:10.1371/journal.pdig.0000787)
Supplement: S2 File — (ZIP) [file pdig.0000787.s002.zip › S2 Data Raw data files containing unprocessed question inputs and LLM outputs including transcripts of deliberations/STEP2_Question-91-1_Type-MC-NJ.docx]

**Facilitator** **[Round 1]:** A 72-year-old woman is evaluated in the acute rehabilitation facility where she has been receiving physical therapy since sustaining a stroke 2 weeks ago that resulted in moderate left hemiparesis. A swallowing study obtained on admission to the facility showed no abnormalities and she has been progressing well with her rehabilitation. She is able to walk with the aid of a walker and assistant, has no speech impairment, and tolerates a regular diet. Medical history also is notable for well-controlled hypertension and hyperlipidemia. Medications are lisinopril, simvastatin, and daily aspirin. Temperature is 37.0°C (98.6°F), pulse is 72/min and regular, respirations are 12/min, and blood pressure is 130/80 mm Hg. Cardiopulmonary examination shows no abnormalities. Cranial nerves are intact. Muscle strength in the extremities is 4/5 on the left and 5/5 on the right. Sensation is intact bilaterally. Standardized assessment of cognitive function shows no abnormalities. For the past 2 days, the patient has insisted that she is well enough to go home despite not being fully cleared to do so by her physical therapist and physiatrist, who want her to stay for 1 more week to try to advance from the walker to a cane. The patient lives with her daughter, son-in-law, and grandson, and has a bedroom on the main floor of the house with no need to use stairs. The daughter works as a stay-at-home mother. Which of the following is the most appropriate next step in management? (A) Discharge the patient with a plan for home health care and physical therapy (B) Inform the patient that she would need to sign out against medical advice (C) Inform the patient that transfer to a long-term care facility is most suitable for her (D) Request psychiatric evaluation of the patient's decision-making capacity

**AI_B:** RESTATING THE QUESTION: An elderly woman who has been in rehab following her stroke two weeks ago has improved notably and insists on returning home, even though her therapists recommend staying for another week to shift from a walker to a cane. It's important to determine the best next step in managing her situation. PLANNING: There are a few assumptions here. First, the belief that more rehabilitation will result in more improvement in her mobility. Second, the contention that being at home might not provide the same level of care or recovery. Third, the assertion that patient's insistence on going home might be due to her inability to rationally decide what's in her best interest. CLINICAL REASONING: Being at a rehabilitation facility guarantees a level of care that might not be available at home. However, the patient has shown significant improvement, managing to walk with the help of a walker and assistant and doesn't require a special diet. Moreover, the patient lives in a house with family who can support her daily needs. Another important aspect to consider is the patient's mental health. Since there are no cognitive abnormalities, the desire to transition from a hospital setting to a homely environment might help in faster recovery by improving her mental wellbeing. CONCLUDING: In connecting this train of thought, option A i.e., sending the patient home with a plan for home health care and physical therapy seems the most reasonable choice. It balances the patient's desire to get back to her regular life and still maintain her therapy routine. RESPONSE: Hence, the answer would be (A) Discharge the patient with a plan for home health care and physical therapy. This would allow her to continue her recovery in a familiar and comfortable environment while ensuring she receives the necessary medical care.

**AI_E:** RESTATING THE QUESTION: In essence, the query pertains to the best course of action for managing a 72-year-old woman recovering from a stroke that has been insisting she is well enough to go home, though her therapists don't think she's quite ready yet. They wish for her to stay another week to advance from a walker to a cane. The patient lives with her family in a one-story home and her medical history is well managed. Technical terms mentioned include hemiparesis which refers to weakness on one side of the body, often as a result of a stroke and physiatrist, who is a doctor specializing in physical rehabilitation. PLANNING: The alternatives proposed include discharging the patient with a plan for home health care and physical therapy, informing the patient that she'll need to sign out against medical advice if she leaves, transferring her to a long-term care facility, or requesting a psychiatric evaluation for her decision-making capacity. The important assumptions to consider include the patient's physical capabilities as well as her decision-making capacity and ability to perform certain tasks independently. CLINICAL REASONING: We can hypothesize the patient's readiness to be discharged based on her physical improvements and her cognitive function reports. She has decent muscle strength, no speech impairment, tolerates a regular diet and is insistent on going home. Moreover, she has supportive family at home in a setup that doesn't require her to climb stairs. Given these factors, it seems unlikely that a transfer to a long-term care facility or a psychiatric evaluation for decision-making capacity is necessary. CONCLUDING: Evaluation of her physical state and home support infrastructure seems to suggest that she may manage well with home health care and physical therapy. However, her physiatrist and physical therapist haven't approved of her readiness to shift from using a walker to a cane, indicating some level of physical rehabilitation is still needed. Discharging a patient against medical advice may have negative impacts on patient outcomes and should be avoided if possible. RESPONSE: Taking all these considerations into account, the most suitable option to pursue would likely be (A) Discharge the patient with a plan for home health care and physical therapy. This option would let the patient recover at home, which she is eager to do, while still ensuring that she continues to receive the care and rehabilitation she needs to recover fully from her stroke.

**AI_A:** 1. RESTATING THE QUESTION: The presented scenario talks about a 72-year-old woman who has been in a rehabilitation program for the past two weeks following a stroke that caused moderate left hemiparesis. Although she has not been cleared to go home yet by her healthcare providers, the patient is insisting she is ready to leave the facility despite not being fully rehabilitated - she still needs the use of a walker. Her living conditions appear to be well-suited to her physical state, as she has a willing caregiver and a bedroom located on the first floor of the house, negating the need for stair use. Therefore, the challenge here is to decide the appropriate next step in this scenario: whether to discharge her home with a plan for home physical therapy (A), inform her that she'd need to leave against medical advice (B), discuss with her about a transfer to a long-term care facility (C), or request a psychiatric evaluation for her decision-making capacity (D). 2. PLANNING: I will begin by questioning each presented option while considering the current health status of the patient, her rehabilitation progress, and her living conditions. 3. CLINICAL REASONING: Given that the patient's health status is stable, her rehabilitation progress is promising, and she has a safe home environment with supportive family caregiving, an alternate assumption that might be considered is that it could be beneficial to her recovery to discharge her home with a planned home health care and physical therapy (Option A). This will allow for her to continue her rehabilitation in a more comfortable, familiar environment. On the contrary, making her stay against her will (Option B) might negatively affect her motivation and progress. Option C seems inappropriate given her good recovery progress and a safe and supportive home environment. Option D could be considered if there was any evidence of cognitive abnormalities affecting her decision-making capacity, but assessment showed no such abnormalities. 4. CONCLUDING: Therefore, It seems reasonable to discharge the patient home with continued rehabilitation at home whilst constantly monitoring the situation. It is important to ensure her safety and to continue her therapy to potentially advance from the need of a walker to a cane. 5. RESPONSE: The most appropriate next step in management in this scenario, considering all the given information and possible outcomes, would be to discharge the patient with a plan for home health care and physical therapy (Option A) as this allows her to continue her therapy in a comfortable environment while also taking into consideration her wishes and her family's supportive role in her recovery.

**AI_C:** (1) RESTATING THE QUESTION: This scenario presents a case of an elderly woman who suffered a stroke two weeks ago which has caused her moderate left hemiparesis. Despite the persisting weakness affecting her left side, her swallowing, speech, cognitive function, and sensation have all been accounted for and are unimpaired. She also has well-managed hypertension and hyperlipidemia. Her recovery is progressing well and while she is not yet fully cleared by her therapy team, she insists that she could continue her recovery at home. At home, she lives with family in a setting that appears to be supportive and accessible. The core question here is to determine the best approach to her insistence of going home – should she be discharged with at-home care and physical therapy, asked to sign against medical advice, transferred to long-term care, or evaluated for a potentially compromised decision-making capacity? (2) PLANNING: There are several assumptions in this scenario. One is that further progress in physical therapy would not be possible at home. Another is that the patient is able to make sound decisions about her healthcare. Thirdly there is an assumption that the home environment is suitable for continuing her rehabilitation. Looking at these assumptions, we could hypothesize that the first and third can be clinically prioritized. If her home environment is suitable for her recovery (due to the presence of her family who can offer support, and the accessibility, as she has no need to climb stairs) and the provision of home health services, she could potentially continue her progress at home. (3) CLINICAL REASONING: It is acknowledged she hasn't yet fully regained her strength (as evidenced by muscle strength of 4/5 on the left), but considering her encouraging progress and motivation, outpatient physical therapy with the assistance of home health care might be possible. In rehabilitation medicine, patient motivation and a supportive environment can significantly contribute to recovery. This is especially true if the patient's healthcare providers endorse their confidence in their recovery potential, which is seemingly the case here. (4) CONCLUDING: I would lean more towards the possibility that patient can indeed proceed with the recovery at home considering the provided information about her condition, progress, home environment, and motivation. It's also significant to note that discharging her might lessen her exposure to hospital-associated complications such as infections. A plan for outpatient physical therapy and appropriate home healthcare should be devised for the patient that would serve her rehabilitative needs while being in the comfort of her own home. (5) RESPONSE: So, in light of this discussion, (A) Discharging the patient with a plan for home health care and physical therapy seems to be the most appropriate next step in management. The benefits of continuing recovery at home, coupled with the provision of professional physical therapy and healthcare at home, along with her personal motivation, could indeed contribute positively to the patient's recovery process.

**AI_D:** RESTATING THE QUESTION: The clinical question concerns a 72-year-old woman who recently experienced a stroke resulting in moderate left hemiparesis or weakness of the left side of her body. Despite showing good progress in her rehabilitation program, her medical team, including a physical therapist and a physiatrist, believe she should continue her in-facility rehabilitation for another week. However, she feels ready to return home where her daughter, who stays at home, son-in-law, and grandson live. The home environment suits her current physical capabilities. The question is asking for the appropriate next step in managing her situation. Should she be discharged with a home health care and physical therapy plan (Option A), should she be informed that she would need to sign out against medical advice (Option B), should she be informed that a transfer to a long-term care facility might be best for her (Option C), or should a psychiatric evaluation of her decision-making capacity be requested (Option D)? PLANNING: One of the main assumptions underlying the question is that the patient needs more therapy. This assumption is based on the physical therapist and physiatrist's recommendations and the fact that the patient still requires assistance to walk. However, an alternate hypothesis might be that the patient can continue her rehabilitation at home with her family's support. This alternative assumption hinges on the patient's health status, her home environment, and the availability of home health care services. CLINICAL REASONING: The patient exhibits good progress in her recovery, as evidenced by her ability to walk with the aid of a walker and her intact cognitive function. Despite her hemiparesis, she can tolerate a regular diet and has no speech impairment. Moreover, her home environment is suitable for her condition - she does not need to use stairs and her daughter stays at home. Considering these observations, she might be able to continue her rehabilitation at home, minimizing the disruption to her usual life and promoting her independence. This approach, however, requires the availability and adequacy of home health care services, including physical therapy. CONCLUDING: Taking into account the multidisciplinary evaluation involving physical and cognitive assessment, along with household environmental assessment, a decision can be made in favor of the patient's preference for home-based care. RESPONSE: Given her progress, her home environment, and her family support, it may be most appropriate to discharge the patient with a plan for home health care and physical therapy (Option A). This allows her to continue her rehabilitation in a comfortable and supportive environment. It also respects her autonomy, a significant principle in managing patient care. Therefore, option A seems to be the most clinically appropriate next step in management.

**Facilitator** The consensus among the AI responses is that the most appropriate next step in managing the 72-year-old woman's situation is to discharge her with a plan for home health care and physical therapy (Option A). This conclusion is based on several factors. Firstly, the patient has shown significant progress in her rehabilitation, as evidenced by her ability to walk with the aid of a walker and her intact cognitive function. Secondly, her home environment is suitable for her condition, with a supportive family and no need to use stairs. Thirdly, her motivation to continue her recovery at home, in a familiar and comfortable environment, is considered beneficial for her mental wellbeing and overall recovery. Lastly, the availability of home health care services, including physical therapy, allows her to continue her rehabilitation at home. Therefore, discharging the patient with a plan for home health care and physical therapy respects her autonomy, minimizes disruption to her usual life, and promotes her independence while ensuring she receives the necessary medical care.
