# Supplementary material for: Collaborative intelligence in AI: Evaluating the performance of a council of AIs on the USMLE
Source: PLOS Digit Health. 2025 Oct 9;4(10):e0000787. doi: 10.1371/journal.pdig.0000787 (PMC12510544; doi:10.1371/journal.pdig.0000787)
Supplement: S2 File — (ZIP) [file pdig.0000787.s002.zip › S2 Data Raw data files containing unprocessed question inputs and LLM outputs including transcripts of deliberations/STEP2_Question-92-1_Type-MC-NJ.docx]

**Facilitator** **[Round 1]:** A 46-year-old woman comes to the office for an annual health maintenance examination. She reports a 6-week history of fatigue, which she attributes to working full time and caring for her three children. Her blood pressure has been consistently high as measured at home with her husband's automated cuff, with many readings greater than 150/90 mm Hg. Medical history is unremarkable and routine preventive screenings, including mammography and Pap smear, have shown no abnormalities. Her only medications are a daily multivitamin and ibuprofen as needed. She is 165 cm (5 ft 5 in) tall and weighs 72 kg (158 lb); BMI is 26 kg/m². Pulse is 84/min, and blood pressure is 152/98 mm Hg. Physical examination shows trace edema of the ankles but no other abnormalities. Results of serum studies are shown: Urea nitrogen: 12 mg/dL Creatinine: 0.9 mg/dL Na⁺: 141 mEq/L K⁺: 3.1 mEq/L Cl⁻: 100 mEq/L HCO₃⁻: 23 mEq/L Which of the following is the most likely explanation for this patient's increased blood pressure? (A) Cushing syndrome (B) Fibromuscular dysplasia (C) Hyperaldosteronism (D) Hypothyroidism (E) Obstructive sleep apnea

**AI_E:** RESTATING THE QUESTION: The question asks that among the listed potential disorders including Cushing syndrome, fibromuscular dysplasia, hyperaldosteronism, hypothyroidism, and obstructive sleep apnea, which is most likely causing a 46-year-old woman's increased blood pressure. The woman has been experiencing consistent high blood pressure readings above 150/90 mm Hg, traces of ankle edema, low serum potassium (K⁺: 3.1 mEq/L), and reports of fatigue over a 6-week period. PLANNING: It is necessary to assess the medical conditions specified, considering the links to high blood pressure, fatigue, and low potassium (hypokalemia). Cushing syndrome can lead to hypertension and hypokalemia due to the excessive cortisol, however, it would also typically present with more specific symptoms such as central obesity, facial rounding, and striae, which are not observed in this patient. Fibromuscular dysplasia, a cause of secondary hypertension, primarily affects younger women and would not typically cause isolated hypokalemia. Hypothyroidism can lead to fatigue, however, it usually manifests with other symptoms such as weight gain, cold intolerance and contains no direct linkage to hypokalemia. Obstructive sleep apnea can also cause hypertension, but it's commonly associated with obesity and often presents with symptoms of disturbed sleep and daytime somnolence which this patient does not report. CLINICAL REASONING: The condition that attracts notable attention in this instance is hyperaldosteronism. Aldosterone promotes sodium (and water) reabsorption and potassium excretion, contributing to both hypertension and hypokalemia. Fatigue can stem from hypokalemia, although it's a nonspecific symptom. This aligns more compellingly with the patient’s lab findings and needs to be clinically prioritized. CONCLUDING: Employing the assumption that specific mysteries of hypertension are often found in the balance and distribution of electrolytes, and considering the trace edema noted, the hypokalemia and the high blood pressure, this potentially points towards hyperaldosteronism. RESPONSE: Thus, the most likely explanation for this patient's increased blood pressure relative to other possibilities listed, considering her results and symptoms, would be (C) Hyperaldosteronism.

**AI_A:** RESTATING THE QUESTION: This situation requires determining the likely reason for a 46-year-old woman's elevated blood pressure. The woman has been handling fatigue for 6 weeks and her blood pressure measurements, taken at home, mostly surpass 150/90 mm Hg. Even with an unremarkable medical history and no abnormal preventive test results. Current physical examination discloses ankle edema while serum studies results are generally normal apart from potassium levels which are fairly low. We're asked to consider a number of conditions that could cause high blood pressure: Cushing syndrome, fibromuscular dysplasia, hyperaldosteronism, hypothyroidism, obstructive sleep apnea. PLANNING: To determine the most likely cause, it's crucial to consider the key symptoms: her fatigue, high blood pressure, trace ankle edema and low potassium levels. These symptoms might overlap with some conditions. However, the pattern of the symptoms is significant. She doesn't have clear symptoms that belong to Cushing syndrome (e.g., central obesity, 'moon' face, and purple striae) and hypothyroidism (cold intolerance, constipation and weight gain). Even though both conditions can cause hypertension, they are less likely due to absence of specific symptoms. Fibromuscular dysplasia is usually characterized by bruits and is generally found in younger women. Obstructive sleep apnea is often associated with snoring, observed apneas and excessive daytime sleepiness. Their absence makes these options less likely. Thus, given the remaining symptoms and the absence of the others, Hyperaldosteronism seems to be the most plausible option. CLINICAL REASONING: In hyperaldosteronism, overproduction of aldosterone leads to increased sodium and water reabsorption, producing hypertension and hypokalemia (due to exchange with hydrogen ions in the collecting tubule to prevent acidosis). This explains her high blood pressure and the observed low potassium levels. Furthermore, Aldosterone can cause mild edema due to its sodium retaining properties which could explain the ankle edema. The fatigue our patient experiences could be a general symptom associated with high blood pressure and hypokalemia. CONCLUDING: After carefully considering the symptoms of the patient and how they match with the various conditions, and correlating her symptoms with the physiological implications of these conditions, I am inclined towards identifying Hyperaldosteronism as the cause of her increased blood pressure. RESPONSE: Thus, the most likely cause for this patient's increased blood pressure is found in Choice (C) Hyperaldosteronism.

**AI_B:** RESTATING THE QUESTION: The medical scenario presents a 46-year-old woman showing consistent high blood pressure above 150/90 mm Hg, a 6-week history of fatigue and trace edema of the ankles. Laboratory results show normal creatinine and urea nitrogen (indicating intact renal function), but the patient has a low potassium level (K+: 3.1 mEq/L). There is a need to determine the probable cause of her hypertension, and for this we have five options: Cushing syndrome, fibromuscular dysplasia, hyperaldosteronism, hypothyroidism, and obstructive sleep apnea. PLANNING: To start with, Cushing syndrome is an endocrine disorder typically caused by an excess of cortisol, which can result in hypertension. Fibromuscular dysplasia is a non-atherosclerotic, non-inflammatory vascular disease that affects the renal arteries and can cause renovascular hypertension. Hyperaldosteronism is an endocrine disorder characterized by an excessive secretion of aldosterone, leading to volume overload and hypertension. Hypothyroidism on the other hand, can cause low heart rate and elevated cholesterol levels but is typically associated with normal or low blood pressure. Lastly, Obstructive sleep apnea can cause hypertension due to recurrent nocturnal hypoxia, hypercapnia, and arousals that activate the sympathetic nervous system. CLINICAL REASONING: Let's explore the assumptions. The absence of clinical signs typical of Cushing syndrome such as weight gain, thinning skin, easy bruising, or striae makes it a less likely cause of her hypertension. Similarly, there is no finding in the case (such as a bruit over the renal arteries, or a young female patient with severe hypertension) that strongly suggests fibromuscular dysplasia. Hypothyroidism is unlikely as it does not typically present with hypertension, and obstructive sleep apnea seems less probable as it usually presents with obesity, daytime sleepiness, and loud snoring, symptoms that are not reported here. On the other hand, hyperaldosteronism could explain the hypertension, the fatigue (due to muscle weakness from hypokalemia), and the trace edema. Further, the low potassium level in our patient can be a sign of hyperaldosteronism because aldosterone promotes renal sodium reabsorption and potassium secretion, resulting in potential hypokalemia. CONCLUDING: Based on our step-by-step clinical reasoning, and while we should consider all possibilities, the most likely explanation for this patient's increased blood pressure, given the symptoms and laboratory findings, would be hyperaldosteronism. More tests would be needed to confirm this diagnosis. RESPONSE: Therefore, the most likely explanation for this patient's increased blood pressure is (C) Hyperaldosteronism.

**AI_C:** 1) RESTATING THE QUESTION: A 46-year-old female has come for her annual checkup. She mentions being fatigued for the past 6 weeks, which she thinks is due to her employment and child care responsibilities. She's been recording high blood pressure readings at home and now, her blood pressure is found to be elevated during the clinical exam as well. Her medical history is otherwise unremarkable. She takes a daily multivitamin and ibuprofen as needed. She is slightly overweight according to her Body Mass Index (BMI). The clinical examination revealed mild ankle edema but no other abnormalities. Despite her serum electrolytes being in the normal reference range, her potassium level is on the lower side. She doesn't have any other clinical signs or symptoms. 2) PLANNING: Considering her persistent high blood pressure, fatigue, and slightly low potassium, the following clinical assumptions can be drawn: A) She could have Cushing syndrome, characterized by overproduction of glucocorticoids that can cause hypertension and fatigue; B) She could be suffering from Fibromuscular dysplasia, a condition that affects medium-sized arteries and can cause hypertension; C) The low potassium might indicate Hyperaldosteronism, as aldosterone excretion can lead to potassium loss, hypertension, and sometimes fatigue; D) Fatigue could be a sign of Hypothyroidism, though hypertension is not a common symptom; E) Obstructive Sleep Apnea often presents with fatigue and hypertension due to nocturnal hypoxiation. 3) CLINICAL REASONING: We can first rule out Cushing Syndrome as there are no symptoms of weight gain, facial puffiness, or purple stretch marks, which are typically associated with the condition. Fibromuscular dysplasia can potentially be ruled out too, as it more commonly affects young women and can often cause headaches and tinnitus, which are not reported here. The patient also lacks typical symptoms of hypothyroidism such as weight gain, cold intolerance, constipation and dry skin. While Obstructive Sleep Apnea is a reasonable consideration, it typically comes with symptoms such as snoring, apnea episodes, and daytime sleepiness, which the patient has not reported. On the other hand, the presentation of hypertension combined with low potassium and fatigue could quite reasonably be due to Hyperaldosteronism. Primary hyperaldosteronism is an autonomous overproduction of aldosterone from the adrenal gland, often due to an adenoma (ie, Conn syndrome) or bilateral adrenal hyperplasia. Chronic overproduction of aldosterone results in increased sodium reabsorption with concomitant potassium and hydrogen ion excretion at the cortical collecting duct. 4) CONCLUDING: Increased sodium reabsorption expands plasma volume, which increases blood pressure, whereas increased potassium loss leads to hypokalemia, helping to confirm hyperaldosteronism. Increased hydrogen ion excretion can lead to metabolic alkalosis, but the patient's bicarbonate level is not high, which may be due to her kidneys compensating for the disorder to maintain the acid-base balance. 5) RESPONSE: Thus, the most likely explanation for this patient's increased blood pressure is Hyperaldosteronism (C).

**AI_D:** RESTATING THE QUESTION: The patient, a 46-year-old woman, presented at the clinic for her regular annual check-up. Over the past six weeks, she has been experiencing fatigue, attributing it to her work and childcare responsibilities. Lately, her blood pressure readings have been consistently high, exceeding 150/90 mm Hg. The rest of her medical history is unexceptional, and she has no notable results from regular screenings. Her medication consists of a daily multivitamin and occasional ibuprofen. As per her physical examination, her vital signs included a pulse of 84/bpm and a blood pressure of 152/98 mm Hg. Moreover, trace edema was observed in her ankles. Her serum study results show slightly decreased potassium levels. The question is seeking the most plausible reason behind her elevated blood pressure from a selection of medical conditions. PLANNING: To answer this question, we can look at each listed condition and evaluate if it could raise blood pressure. It's important to consider the symptoms, signs, and laboratory tests that can be associated with each condition, as well as how they might present in a patient. While all these conditions could potentially cause hypertension, we need to identify which is most likely given her specific symptoms and laboratory data. CLINICAL REASONING: We can start by evaluating each condition in light of the patient's presentation: A) Cushing syndrome often presents with obesity, especially in the trunk and face, thin skin, stretch marks, and other distinct physical changes. This patient doesn't have these manifestations. B) Fibromuscular dysplasia is a disease that causes abnormal growth or ‘dysplasia’ within the wall of an artery. It's commonly seen in medium-sized arteries and can lead to hypertension, but it usually occurs in younger women and often presents with other symptoms like abdominal pain or pulsatile tinnitus, none of which this patient has. D) Hypothyroidism could potentially cause fatigue and elevated cholesterol levels, leading to hypertension. However, the lack of other typical symptoms like weight gain, dry skin, or cold intolerance makes this less likely. E) Obstructive sleep apnea could lead to hypertension, but it's often accompanied by symptoms like snoring, daytime sleepiness, morning headaches, and often seen in obese individuals. This patient has none of these symptoms and her BMI is just slightly above normal. This leaves us with C) Hyperaldosteronism. Primary hyperaldosteronism, otherwise known as Conn's syndrome, can lead to hypertension due to the excess production of aldosterone which causes sodium and water retention and potassium excretion. This should be highly suspected in a patient with hypertension and hypokalemia, exactly what our patient has. CONCLUDING: The patient presents with secondary hypertension and hypokalemia, which suggests excess aldosterone activity. While several causes can lead to these clinical features, their presence with no other specific localized symptoms or laboratory findings that point to a single cause makes hyperaldosteronism the most likely explanation for this patient's increased blood pressure. RESPONSE: Therefore, the most likely explanation for this patient's increased blood pressure is Hyperaldosteronism (C).

**Facilitator** Consensus Statement: The question presents a 46-year-old woman who has been experiencing fatigue for the past six weeks and consistently high blood pressure readings at home, often exceeding 150/90 mm Hg. Her medical history is unremarkable, and her only medications are a daily multivitamin and ibuprofen as needed. Physical examination reveals trace edema of the ankles, and serum studies show slightly low potassium levels. The task is to identify the most likely cause of her elevated blood pressure from a list of potential conditions: Cushing syndrome, fibromuscular dysplasia, hyperaldosteronism, hypothyroidism, and obstructive sleep apnea. Upon evaluating each condition, Cushing syndrome and hypothyroidism are less likely due to the absence of their specific symptoms such as central obesity, 'moon' face, and purple striae for Cushing syndrome, and cold intolerance, constipation, and weight gain for hypothyroidism. Fibromuscular dysplasia, typically found in younger women and characterized by bruits, is also less probable. Obstructive sleep apnea, often associated with snoring, observed apneas, and excessive daytime sleepiness, is less likely due to the absence of these symptoms. The remaining condition, hyperaldosteronism, aligns most closely with the patient's symptoms. In hyperaldosteronism, overproduction of aldosterone leads to increased sodium and water reabsorption, producing hypertension and hypokalemia. This explains her high blood pressure and the observed low potassium levels. Aldosterone can also cause mild edema due to its sodium retaining properties, which could explain the ankle edema. The fatigue our patient experiences could be a general symptom associated with high blood pressure and hypokalemia. Therefore, after carefully considering the symptoms of the patient and how they match with the various conditions, and correlating her symptoms with the physiological implications of these conditions, the consensus is that hyperaldosteronism is the most likely cause of her increased blood pressure.
