# Supplementary material for: Collaborative intelligence in AI: Evaluating the performance of a council of AIs on the USMLE
Source: PLOS Digit Health. 2025 Oct 9;4(10):e0000787. doi: 10.1371/journal.pdig.0000787 (PMC12510544; doi:10.1371/journal.pdig.0000787)
Supplement: S2 File — (ZIP) [file pdig.0000787.s002.zip › S2 Data Raw data files containing unprocessed question inputs and LLM outputs including transcripts of deliberations/STEP2_Question-93-1_Type-MC-NJ.docx]

**Facilitator** **[Round 1]:** A 50-year-old woman is brought to the emergency department because of a 3-day history of severely painful lesions over her right leg and temperatures to 38.7°C (101.7°F). She has a 12-year history of type 2 diabetes mellitus. Her only medication is insulin. On arrival, temperature is 39.3°C (102.8°F). Examination of the right lower extremity shows exquisite tenderness to palpation, edema, and warmth. The extremity appears shiny and diffusely erythematous without sharp margins. The subcutaneous tissue is firm, and there are areas of notable skin breakdown with bullae containing thick purple fluid. Examination of the left lower extremity shows no abnormalities. Which of the following is the most likely diagnosis? (A) Cellulitis (B) Dermatophytosis (C) Erysipelas (D) Erythema nodosum (E) Herpes zoster (F) Necrotizing fasciitis

**AI_E:** RESTATING THE QUESTION: A 50-year-old woman with a history of type 2 diabetes mellitus has been brought to the emergency department. Presenting with severely painful lesions over her right leg accompanied by fever, upon examination we find symptoms of swelling and heat in the right leg, tender to touch, shiny and diffused redness without defined margins, hardened subcutaneous tissue, and significant skin damage areas with fluid-filled blisters containing thick purple substance. No abnormalities are noted on the left leg. We are asked to provide a likely diagnosis from the alternatives given. PLANNING: The symptoms presented can be attributed to inflammation and infection of skin and underlying tissues, potentially caused by a bacterial infection. This patient's risk may be heightened by her long-standing diabetes, which can compromise the immune system and make her more susceptible to infections. We must categorize her symptoms, and associate them with potential causes to hypothesize the most reasonable explanation. CLINICAL REASONING: Severe pain, erythema, edema, warmth, and bullae filled with purple fluid all suggest a severe skin and soft tissue infection. The presence of a severe, progressive infection is further suggested by high fever and symptomatic localization to one extremity. Given the patient's history of diabetes, her risk for infections, including severe ones, is increased. The lack of sharp margins of the erythema and the systemic symptoms such as fever support a deeper spreading infection in contrast to a superficial one. CONCLUDING: After initial evaluation and analysis, we understand that our patient presents severe signs of infection localized in one extremity, the hardened subcutaneous tissue and the lack of sharp margins of the erythema indicating a potentially deeper skin and soft tissue infection, which could indicate necrotizing fasciitis, a severe type of infection that rapidly destroys skin, fat, and muscle tissue. RESPONSE: In light of the patient's symptoms and examination findings, and considering her predisposed condition -- type 2 diabetes, the most likely diagnosis for the presented case is choice (F) Necrotizing fasciitis. This condition is a rapidly progressive inflammatory infection of the fascia, with secondary necrosis of the subcutaneous tissues. The toxins produced by the bacteria can destroy the surrounding tissues quickly, which matches what has been described in the patient's case.

**AI_D:** RESTATING THE QUESTION: The patient, a 50-year-old woman with a history of type 2 diabetes mellitus, is presenting with severe and painful lesions on her right leg, along with a high fever. These lesions are characterized by tenderness, edema, warmth, a diffusely reddened and shiny appearance without clear boundaries, firm subcutaneous tissue, and areas of skin breakdown presenting with bullae filled with a thick purple fluid. Given these characteristics, we are asked to identify the most probable diagnosis from a list of options: Cellulitis, Dermatophytosis, Erysipelas, Erythema nodosum, Herpes Zoster, or Necrotizing fasciitis. PLANNING: The potential diagnoses are all conditions with distinct clinical pictures. We'll need to rule out each based on the patient's symptoms and history. Diabetes mellitus can complicate soft tissue infections, rendering the patient more susceptible to severe forms like necrotizing fasciitis. CLINICAL REASONING: Although the patient's symptoms initially seem consistent with conditions such as cellulitis (A), erysipelas (C), or erythema nodosum (D), the presence of bullae filled with thick purple fluid suggests a more serious infection. Bullae, or fluid-filled blisters, can indicate deep tissue involvement. Erysipelas typically has clear, raised boundaries, which is inconsistent with the diffuse erythema observed here. Dermatophytosis (B), caused by fungi, generally does not lead to the systemic symptoms observed in this patient such as high fever. Herpes Zoster (E) would usually present with a unilateral, dermatomal rash, quite unlike the one described here. CONCLUDING: Considering the patient's symptoms and her history of diabetes, Necrotizing fasciitis (F) seems to be the most urgent diagnosis to consider. RESPONSE: Given the described symptoms - bullae, systemic symptoms of fever, and expansive soft tissue involvement with edema and erythema, all in a patient with a history of diabetes, the most likely diagnosis would be (F) Necrotizing fasciitis.

**AI_C:** RESTATING THE QUESTION: The patient, who is a 50-year-old woman with a medical history of type 2 diabetes mellitus, presented to the emergency department due to severe pain from lesions on her right leg, along with a high temperature for the past three days. Physical exploration of the affected area exhibits a shiny, diffusely erythematous surface with no clear boundaries. The subcutaneous region is firm with evident skin breakdown, containing bullae filled with a purplish thick fluid. She does not show similar symptoms on her left leg. The question seeks to identify the most appropriate diagnosis for her condition from a list of potential options. PLANNING: From the provided options, this could be Cellulitis as it's characterized by warmth, erythema, and tenderness. However, it rarely leads to bullae formation and the presentation is unusually severe. Erysipelas generally shows clear margins unlike our patient's symptoms. Dermatophytosis mainly has a superficial impact, while Erythema nodosum primarily affects the pretibial area. Herpes zoster follows a dermatomal pattern which is not apparent in this case. Necrotizing fasciitis seems to have a better correlation with the patient's symptoms as it is a severe deep skin infection causing pain, erythema, edema, and tissue necrosis leading to blisters. The presence of diabetes further increases the possibility due to immunocompromised state. CLINICAL REASONING: Given the patient's history of diabetes, her body's ability to resist infections is significantly compromised. This susceptibility, combined with her symptoms of fever, severe pain, erythema, edema, and bullae filled with purple fluid, could be indicative of a profound, aggressive infection involving not just the skin but also deeper tissues. This pattern matches with Necrotizing Fasciitis which involves the subcutaneous layer resulting in necrosis. CONCLUDING: The clinical picture exhibits a deep infection that is severe, acute in onset, and associated with systemic symptoms such as fever. The examination findings of erythema, warmth, edema, and notably, bullae with thick purple fluid suggest involvement below the cutaneous layer. These clinical cues, coupled with a history of diabetes, point towards a severe skin and subcutaneous tissue infection which could be due to Necrotizing fasciitis. RESPONSE: Considering the clinical manifestations, her medical history, and examination findings, this patient most probably has Necrotizing fasciitis (F).

**AI_A:** RESTATING THE QUESTION: The patient is a 50-year-old woman with a history of type 2 diabetes mellitus. She presents with very painful lesions on her right leg that have been present for 3 days. She also reports having a fever. On examination, her right limb appears swollen, tender, warm, and reddish without clear borders. The tissues underneath her skin feel hard, and the skin itself shows evidence of breakdown and blistering (or bullae) filled with dark purple fluid. Given these findings, we must determine what the most likely diagnosis is, considering a list of possible conditions: Cellulitis, Dermatophytosis, Erysipelas, Erythema nodosum, Herpes zoster, or Necrotizing fasciitis. PLANNING: Our assumptions could include that the woman's type 2 diabetes mellitus is an underlying factor, contributing to her susceptibility to various infections, and that the presentation is characteristic of a serious bacterial or viral infection. However, each of these proposed diagnoses have unique characteristics that need to be taken into account. For instance, is the location and type of tissue involvement typical for these diagnoses? CLINICAL REASONING: Let's analyze each condition. Cellulitis is a deeper subcutaneous infection, which usually presents with erythema, warmth, and edema but may lack the severe pain the patient is experiencing. Dermatophytosis refers to fungal infections, but these usually affect the upper layer of skin and don't typically cause a fever. Erysipelas is a skin infection that typically presents as a fiery red, painful rash with well-demarcated edges, which does not align with our case. Erythema Nodosum is an inflammatory condition causing red, painful nodules, and often has systemic symptoms but doesn't exhibit the localized signs this patient has. Herpes Zoster, while painful and sometimes associated with fever, typically follows a dermatomal pattern and doesn't cause the diffuse inflammation seen in the patient. Lastly, Necrotizing fasciitis is a rapidly progressive infection involving the subcutaneous tissue, often associated with severe pain. Diabetic patients are at increased risk, and the presence of bullae filled with dark fluid is strongly suggestive. CONCLUDING: The presentation in this case, with severe pain, rapid progression of symptoms, bullae with dark fluid, and systemic signs like fever, is most suggestive of Necrotizing fasciitis, a serious infection of the skin and underlying tissues. RESPONSE: According to the patient's clinical picture, the most likely diagnosis is (F) Necrotizing fasciitis.

**AI_B:** RESTATING THE QUESTION: A 50-year-old woman suffering from type 2 diabetes for 12 years visits the emergency department due to severe pain in several skin lesions on her right leg for the past three days along with a high body temperature. Her only medical treatment is insulin. Upon arrival, her body temperature has risen higher. Physical examination reveals that her right lower leg is markedly tender to touch, swollen, and warm. The skin appears shiny and red all over, without clear borders. The tissue underneath her skin feels stiff, and some of the skin has notable sign of damage with blisters filled with a thick, purple-colored liquid. Her left lower leg appears normal. Given these observations, what can be regarded as the most probable diagnosis out of these options: Cellulitis, Dermatophytosis, Erysipelas, Erythema nodosum, Herpes zoster, or Necrotizing fasciitis? PLANNING: From the clues given in the symptoms and signs, it's assumed that the woman is likely dealing with an infection, considering her history of diabetes, increased temperatures, localized symptoms on one leg, and the presence of bullae with purple fluid. Questioning this assumption, could there be a non-infectious etiology for these symptoms? While this is theoretically possible, given that inflammation and localized skin changes can occur due to immune-mediated, vascular or malignant conditions among others, the combination of systemic symptoms such as fever, the dramatic local signs and symptoms, and the presence of fluid-filled blisters highly suggest an infectious etiology. Therefore, the clinically indicated assumption should be to prioritize infectious causes, particularly those that commonly affect the lower extremity in patients with diabetes. CLINICAL REASONING: Now the goal is to determine which infectious condition best fits the described symptoms among the listed options. Condition A, Cellulitis, is an infection of the dermis and subcutaneous tissue, commonly presenting with tenderness, erythema, and warmth, but not typically characterized by distinct lesions or the described bullae with purplish fluid. Condition B, Dermatophytosis, is a fungal infection typically involving only the superficial layers of the skin and not associated with such severe system and local symptoms. Condition C, Erysipelas, is also a skin infection but usually presents with a clearly demarcated, raised, fiery red lesion, which doesn't align with the diffusely erythematous presentation without sharp margins mentioned in the question. Condition D, Erythema nodosum, is an immune-mediated condition presenting with inflamed nodules typically on the shins and is thus not a good fit. Condition E, Herpes Zoster, is a viral infection that usually follows a dermatomal distribution, which is not mentioned in the case and it doesn't typically produce such dramatic local inflammation. Ultimately, Condition F, Necrotizing fasciitis, aligns best with the symptoms described here. This is a deep-seated infection of the subcutaneous tissue that is often associated with severe systemic symptoms, exquisite local tenderness and can produce bullae and skin necrosis. The marked tenderness out of proportion to physical findings is a key feature of this disease, and it can frequently occur in patients with diabetes, who have a suppressed immune response. CONCLUDING: Thus, the patient's symptoms seem to best align with Necrotizing fasciitis, a serious deep-seated infection of the skin and subcutaneous tissues that results in severe local and systemic symptoms, particularly noted in this case by the shiny, firm skin and the bullae containing purple fluid. RESPONSE: Given these findings, the most likely diagnosis for this 50-year-old woman is (F) Necrotizing fasciitis.

**Facilitator** The patient, a 50-year-old woman with a history of type 2 diabetes mellitus, presents with severe pain in lesions on her right leg, accompanied by a high fever. The physical examination reveals a swollen, tender, and warm right leg with a shiny, diffusely erythematous appearance without clear boundaries. The subcutaneous tissue is firm, and there are areas of skin breakdown with bullae filled with a thick, purple fluid. No abnormalities are noted on the left leg. Given these symptoms, we are asked to identify the most probable diagnosis from a list of options: Cellulitis, Dermatophytosis, Erysipelas, Erythema nodosum, Herpes Zoster, or Necrotizing fasciitis. The patient's symptoms suggest a severe skin and soft tissue infection, potentially caused by a bacterial infection. Her risk may be heightened by her long-standing diabetes, which can compromise the immune system and make her more susceptible to infections. The severe pain, erythema, edema, warmth, and bullae filled with purple fluid all suggest a severe skin and soft tissue infection. The presence of a severe, progressive infection is further suggested by high fever and symptomatic localization to one extremity. Upon analyzing each potential diagnosis, we find that the symptoms do not align with Cellulitis, Dermatophytosis, Erysipelas, Erythema nodosum, or Herpes Zoster. These conditions either do not typically present with the severe symptoms observed in this patient or do not match the pattern of symptoms. However, the symptoms align closely with Necrotizing fasciitis, a severe type of infection that rapidly destroys skin, fat, and muscle tissue. This condition is often associated with severe systemic symptoms, exquisite local tenderness, and can produce bullae and skin necrosis. The marked tenderness out of proportion to physical findings is a key feature of this disease, and it can frequently occur in patients with diabetes, who have a suppressed immune response. In conclusion, given the patient's symptoms, her history of diabetes, and the severity and rapid progression of her condition, the most likely diagnosis is Necrotizing fasciitis. This diagnosis aligns with the patient's severe signs of infection localized in one extremity, the hardened subcutaneous tissue, and the lack of sharp margins of the erythema indicating a potentially deeper skin and soft tissue infection.
