# Supplementary material for: Collaborative intelligence in AI: Evaluating the performance of a council of AIs on the USMLE
Source: PLOS Digit Health. 2025 Oct 9;4(10):e0000787. doi: 10.1371/journal.pdig.0000787 (PMC12510544; doi:10.1371/journal.pdig.0000787)
Supplement: S2 File — (ZIP) [file pdig.0000787.s002.zip › S2 Data Raw data files containing unprocessed question inputs and LLM outputs including transcripts of deliberations/STEP2_Question-94-1_Type-MC-NJ.docx]

**Facilitator** **[Round 1]:** A 37-year-old woman comes to the office for a routine health maintenance examination. For the past 6 years, she has worked in a paper mill factory that uses liquid chlorine to bleach the paper. The patient is concerned about her exposure to toxic substances. Vital signs are within normal limits. Examination shows no abnormalities. Which of the following is the most appropriate recommendation to address this patient's concerns? (A) Corticosteroid therapy (B) Taking frequent breaks from the paper-bleaching area (C) Undergoing monthly pulmonary function testing (D) Using a respirator at work (E) Washing daily after work with a chlorhexidine-based solution

**AI_C:** (1) RESTATING THE QUESTION: The question presents a 37-year-old woman who has been exposed to liquid chlorine over the past 6 years due to her work in a paper mill factory. She is concerned about potential toxic exposure, despite having normal vital signs and no detectable irregularities on a routine examination. The question asks us to determine what the most suitable advice is to address her concern related to exposure to toxic substances. (2) PLANNING: The question assumes that her concerns are valid and there may be long-term effects from chronic exposure. We could hypothesize that alternate assumption, that there would be no long-term effect since she is currently presenting with no symptoms, is invalid, based on knowledge of chlorine's effect. A more appropriate assumption to prioritize would be the potential long-term health risks associated with chlorine exposure, such as damage to the respiratory system. (3) CLINICAL REASONING: Chlorine gas, a derivative of liquid chlorine, can cause respiratory problems with long-term exposure. While it might not show immediate symptoms, chronic exposure can lead to problems over the time. Although the woman is asymptomatic, it's reasonable to implement preventive measures to minimize future risks. (4) CONCLUDING: Given that proactive protection at the source of the toxic exposure is the best preventive strategy, the use of a respiratory device when working in areas where liquid chlorine is used would be the most appropriate recommendation. (5) RESPONSE: Therefore, the most suitable recommendation to address this patient's concerns is (D) Using a respirator at work.

**AI_B:** 1) RESTATING THE QUESTION: This woman, employed at a paper bleaching mill for the past 6 years, is seeking advice to mitigate her health risks from exposure to chlorine. Her vital signs and medical examination showed no abnormalities. We need to suggest the best recommendation. 2) PLANNING: The assumptions that can be made are that chlorine in its liquid and gaseous forms can pose potential health risks. Alternately, we could consider no risk because her vital signs and physical exam show no abnormalities. However, it's essential to note that chronic exposure to chlorine can have long-term harmful effects even if immediate impacts are not visible. Thus, the preventive care she can take to limit this exposure should be our priority. 3) CLINICAL REASONING: Chlorine is a potent irritant to the mucous membranes, and prolonged exposure can lead to conditions such as chronic bronchitis or conjunctivitis. Corticosteroid therapy (A) might be effective in managing these symptoms but is not preventative. Taking frequent breaks from the paper-bleaching area (B) could temporarily reduce her exposure but wouldn't effectively limit her overall exposure. Monthly pulmonary function testing (C) could detect respiratory issues early, but again, it's a reactive measure, not a preventive one. Washing with a chlorhexidine-based solution (E) can clean the skin of chlorine residues but cannot prevent inhalation during work hours. 4) CONCLUDING: While evaluating the health impact of chlorine exposure, preventive measures are more beneficial in the long run. A respirator can filter out harmful substances and prevent inhalation, thus mitigating the overall exposure and health risks associated with chlorine. 5) RESPONSE: The most appropriate recommendation to address this patient's concern is (D) Using a respirator at work. This can effectively limit her inhalation of chlorine while working, thereby reducing her potential risk for health problems associated with this exposure.

**AI_E:** RESTATING THE QUESTION: A 37-year-old woman with six years of employment at a paper mill factory that uses liquid chlorine to bleach paper is worried about potential harm from the toxic substances she is working with. Currently, her vital signs are within the normal range and her physical exam shows no abnormalities. She is seeking advice on the best course of action to mitigate her potential risks. PLANNING: The assumptions include that the exposure to chlorine is potentially harmful, that her current health status is okay despite the exposure, and that certain actions could mitigate her risks. A different assumption could be that the exposure to chlorine might not be harmful in the manner and level she's exposed at work, that the appropriate protective gear efficiently limits harmful exposure or that regular health monitoring may be required in her case. CLINICAL REASONING: Chlorine is a known pulmonary irritant and can cause a spectrum of conditions from mild irritation to serious pulmonary issues like reactive airways dysfunction syndrome (RADS). As the woman is concerned about her exposure to this toxic substance, it is clinically prudent to address both her exposure risk and possible health effects. There's currently no indication for corticosteroid therapy (A) as she's asymptomatic. Taking breaks (B) may decrease exposure, but wouldn't provide adequate protection. Monthly pulmonary function testing (C) without preventive measures might be unnecessary and anxiety-inducing. Using a respirator (D) at work might be a good way to minimize direct exposure. Washing with chlorhexidine-based solution (E) is more suitable for surface contaminants and not for inhalants such as chlorine gas. CONCLUDING: Considering the risk of prolonged exposure to toxic inhalants such as chlorine gas and the fact that the absence of symptoms does not rule out potential harm, the best course of action would be for the patient to prevent further exposure. RESPONSE: Thus, the most appropriate recommendation for this patient would be (D) Using a respiraor at work to minimize her direct exposure to chlorine gas.

**AI_A:** (1) RESTATING THE QUESTION: A 37-year-old lady who works in a paper mill that uses liquid chlorine for bleaching purposes has come for her regular health check-up. She has been functioning in this environment for six years and is worried about potentially harmful exposure. Her vital signs and physical examination are both normal. My task here is to identify the most suitable precautionary measure that will directly address her concern. It's important to understand that liquid chlorine is a potent irritant and can cause health issues, especially related to respiratory system if inhaled. (2) PLANNING: The assumptions here could be that because the woman works in an environment where she is exposed to liquid chlorine; but as her symptoms and vitals are normal, the exposure seems controlled. However, she is anxious about her health due to her working conditions. Given the options, the assumptions could be — (A) may assume she has inflammation or allergic reaction; (B) might imply periods of exposure are the key determinant of harm; (C) could suggest she has or is at risk of developing pulmonary issues; (D) considers preventing inhalation of toxic fumes; (E) is oriented by the idea of chlorine skin absorption. (3) CLINICAL REASONING: Liquid chlorine can cause problems mainly due to inhalation, leading to respiratory issues, rather than major concerns attributed to skin contact. Current absence of symptoms doesn't imply future risk absence. An early assumption might be that reducing exposure intervals (B) or washing daily (E) could reduce the risks, but those still leave her exposed to the fumes. Periodic pulmonary function tests (C) might be useful to detect any developing diseases, yet it's more reactive than preventive. Corticosteroid use (A) could be helpful in case of an allergic reaction, but it's not preventive and has potential side effects. However, assumption (D) proposes a preventative measure to inhalation and hence likely to be the most suitable. (4) CONCLUDING: The ideal choice would be a measure that prevents exposure to liquid chlorine as much as possible considering her environment. Relying on reactive measures after exposure may not be as effective. (5) RESPONSE: For the above reasons, the patient should consider using a respirator at work (D), which can significantly reduce her exposure to liquid chlorine and likely provide her the most peace of mind related to her concerns.

**AI_D:** 1) RESTATING THE QUESTION: The query is about a woman who has been working in a paper mill that uses liquid chlorine for bleaching for the past 6 years. She has certain concerns about her exposure to this toxic substance. Her physical examination does not show any abnormalities and vital parameters are within the normal range. The inquiry seeks to find the most suitable recommendation that will address her concerns. 2) PLANNING: The assumptions here are that, despite the lack of physical symptoms or abnormalities, the individual's exposure to chlorine gas at her workplace could potentially be harmful to her health over time. One could assume physical symptoms would eventually present themselves if the exposure continues long enough and adequate preventative measures are not undertaken. The assumption in question would be whether certain measures, like using a respirator, having regular breaks away from the bleaching area, monthly pulmonary function tests, daily washing post-work with a chlorhexidine-based solution, or using corticosteroid therapy, could effectively minimize the risk of harm related to exposure to chlorine gas. 3) CLINICAL REASONING: Exposure to chlorine gas can cause several deleterious health effects, primarily targeting the respiratory system. Acutely, it can cause throat irritation, coughing, and difficulty in breathing. Chronically, exposure can lead to conditions like chronic bronchitis and asthma. Given the patient's workplace environment, it would seem reasonable to consider a preventive measure that would primarily focus on reducing her exposure to chlorine gas in the first place. 4) CONCLUDING: Corticosteroid therapy (A), as a treatment option, is usually given to patients who are already suffering from respiratory symptoms, which is not the case here. Taking frequent breaks (B) might reduce exposure but it would not eliminate it and thus, would not provide significant protection. Regular pulmonary function tests (C) could detect any deteriorations in lung function over time but would not prevent them from happening. Daily washing post-work (E) would not significantly reduce lung exposure as it focuses on reducing skin exposure. 5) RESPONSE: Given all this information, the use of a respirator at work (D) would be the most potent and primary preventive measure. The respirator would largely diminish the exposure to chlorine gas, directly protecting her respiratory system. It proves to be the most appropriate recommendation to answer the patient's concerns. Hence, the correct answer is (D) Using a respirator at work.

**Facilitator** The consensus statement synthesizes the responses of AI_A, AI_B, AI_C, AI_D, and AI_E regarding the most appropriate recommendation for a 37-year-old woman who works in a paper mill factory that uses liquid chlorine for bleaching purposes. She is concerned about her exposure to toxic substances, despite her vital signs and physical examination showing no abnormalities. The assumptions made by the AIs are that the woman's exposure to chlorine, even though she currently shows no symptoms, could potentially be harmful to her health over time. Chronic exposure to chlorine can have long-term harmful effects, primarily targeting the respiratory system, even if immediate impacts are not visible. The AIs reasoned that while corticosteroid therapy, taking frequent breaks from the paper-bleaching area, undergoing monthly pulmonary function testing, and washing daily after work with a chlorhexidine-based solution could potentially mitigate the risks, these measures are either reactive or do not effectively limit overall exposure to chlorine. The AIs concluded that the best course of action would be to prevent further exposure to chlorine. Therefore, the consensus recommendation for the woman is to use a respirator at work. This preventive measure can significantly reduce her exposure to liquid chlorine, directly protecting her respiratory system, and likely provide her the most peace of mind related to her concerns.
