# Supplementary material for: Collaborative intelligence in AI: Evaluating the performance of a council of AIs on the USMLE
Source: PLOS Digit Health. 2025 Oct 9;4(10):e0000787. doi: 10.1371/journal.pdig.0000787 (PMC12510544; doi:10.1371/journal.pdig.0000787)
Supplement: S2 File — (ZIP) [file pdig.0000787.s002.zip › S2 Data Raw data files containing unprocessed question inputs and LLM outputs including transcripts of deliberations/STEP2_Question-95-1_Type-MC-NJ.docx]

**Facilitator** **[Round 1]:** A 57-year-old man with alcohol use disorder comes to the emergency department because of a 3-day history of worsening abdominal pain and distention. Medical history is remarkable for alcoholic cirrhosis, hypertension, hyperlipidemia, and a laparoscopic cholecystectomy 10 years ago. The patient also has had several previous hospital admissions for similar episodes of abdominal pain and distention. The patient takes no medications but has been prescribed medications for hypertension in the past. He recently participated in an alcohol rehabilitation program but began drinking alcohol again 1 week ago. During the past 5 days, he has drunk 1 L of vodka daily. Pulse is 100/min, respirations are 16/min, and blood pressure is 165/85 mm Hg. Pulse oximetry on room air shows an oxygen saturation of 100%. The patient appears jaundiced. Cardiopulmonary examination discloses no abnormalities. The abdomen is large, distended, and tense; a fluid wave is present. Palpation of the abdomen discloses a 2-cm–wide reducible umbilical hernia and diffuse tenderness without guarding. There is no tenderness to percussion. In addition to large-volume paracentesis and furosemide therapy, initiation of which of the following pharmacotherapies is most appropriate? (A) Enalapril (B) Metoprolol (C) Propranolol (D) Spironolactone (E) Valsartan

**AI_B:** (1) RESTATING THE QUESTION: The presented case concerns a 57-year-old man with an alcohol use disorder and a significant medical history including alcoholic cirrhosis, hypertension, hyperlipidemia, and prior cholecystectomy. He presents with worsening abdominal pain and distention, both of which had led to his hospitalization numerous times in the past. He admits to recent bouts of heavy drinking. His physical examination reveals a distended abdomen, jaundice, an umbilical hernia, and findings suggestive of ascites. His current treatment includes therapeutic paracentesis and furosemide. The question asks which of the listed medications should be added to his regime as a critical therapeutic consideration. (2) PLANNING: The main assumptions in this case revolve around the cause of the patient's abdominal distention and related symptoms, which likely arise from the consequence of his alcoholic cirrhosis, namely ascites. The standard management for ascites includes salt restriction, diuretics (like furosemide, which he is already receiving), and paracentesis if needed. We know alcoholic cirrhosis is also linked to portal hypertension, which the medications listed seem to address specifically. But which is most effective or appropriate to prescribe here? (3) CLINICAL REASONING: Here's where we need to understand why and how portal hypertension occurs in cirrhosis, and how each potential medication might help. Cirrhosis causes an increase in hepatic vascular resistance, leading to elevated portal blood pressure. This triggers reflex vasodilation in the splanchnic (gut) circulation, reducing overall systemic vascular resistance and leading to the activation of various neurohormonal systems such as the renin-angiotensin system, in attempt to maintain effective blood volume. The pathophysiology of ascites involves the retention of sodium and water by the kidneys, incited by these neurohormonal responses. (4) CONCLUDING: Given this understanding, primary treatment involves sodium restriction and diuretics to counter renal sodium retention. Additionally, decreasing splanchnic vasodilation could also help reduce portal hypertension. Non-selective beta blockers, such as propranolol (option C), have been found to decrease the formation of ascites, presumably by decreasing hepatic venous pressure gradient and splanchnic blood flow. (5) RESPONSE: Thus the most appropriate pharmacotherapy to initiate, in addition to his current regimen of paracentesis and furosemide, would be propranolol – option C.

**AI_A:** (1) RESTATING THE QUESTION: We're presented with a 57-year-old man who suffers from an alcohol use disorder and exhibits symptoms of abdominal pain and distention. He has a medical history of alcoholic cirrhosis, as well as hypertension, hyperlipidemia, and prior laparoscopic cholecystectomy. This patient is not currently taking prescribed medication, which includes medication for high blood pressure. Despite his attempts to quit drinking through rehabilitation, he resumed drinking alcohol and has been consuming 1 liter of vodka daily for the past five days. His vital signs show an elevated blood pressure and an increased pulse rate. Physical examination exposes jaundice, large and tense abdomen, an indication of ascites, and a reducible umbilical hernia. The presented inquiry focuses on the most appropriate pharmacotherapy to initiate, alongside conducting large-volume paracentesis and starting furosemide therapy. (2) PLANNING: The foremost assumption would involve the patient's clinically provided symptoms being associated with his alcoholic cirrhosis and hypertension. A pertinent alternate assumption to pursue would assume the patient's symptoms, mainly the presence of ascites, to potentially be a direct result of his underlying cirrhosis. (3) CLINICAL REASONING: Alcoholic cirrhosis often leads to portal hypertension, resulting in fluid leakage from the liver into the abdominal cavity, causing ascites. Large-volume paracentesis alleviates discomfort and relieves symptoms but does not address the underlying cause. The patient's abdominal distention, fluid wave, and high blood pressure suggest portal hypertension, warranting the selection of a pharmacotherapy that alleviates underlying cirrhosis-related portal hypertension. Beta-blockers such as propranolol effectively reduce portal pressure, making them a suitable option. Other given choices like enalapril and valsartan operate as angiotensin-converting enzyme inhibitors and angiotensin receptor blockers, respectively, and primarily treat systemic hypertension rather than portal hypertension. Spironolactone, a diuretic, is commonly used to manage ascites, but the patient is already receiving furosemide, making additional diuretic therapy redundant. Metoprolol, a beta-blocker too, primarily affects beta-1 rather than beta-2 receptors, making it less effective for portal hypertension than propranolol. (4) CONCLUDING: Taking into account our clinical reasoning pathway, propranolol extends as a beta-blocker that equally targets both beta-1 and beta-2 adrenergic receptors. This property makes it a primary agent in reducing portal hypertension by decreasing cardiac output and splanchnic blood flow, thereby reducing portal venous pressure. (5) RESPONSE: In light of this patient's clinical presentation and associated conditions, the most appropriate pharmacotherapy to complement large-volume paracentesis and furosemide therapy would be the initiation of (C) Propranolol.

**AI_C:** (1) RESTATING THE QUESTION: This scenario describes a 57-year-old man with a history of alcohol use disorder, hypertension and alcoholic cirrhosis, who has presented to the ER with worsening abdominal distension and pain. Clinical examination suggests the presence of ascites (evidenced by the fluid wave and an incredibly distended abdomen). The question asks us to decide, given the choice between enalapril, metoprolol, propranolol, spironolactone, and valsartan, what medication should be initiated in addition to large-volume paracentesis and furosemide therapy. (2) PLANNING: The primary assumption is that the patient's presentation is majorly due to his worsening alcoholic liver cirrhosis, leading to ascites. The medications listed here predominantly pertain to hypertension and fluid balance management. Considering the patient's cirrhosis, it is crucial to control his fluid balance to manage ascites. Given the background of advanced liver disease and portal hypertension, we must consider the effect of these drugs on decreasing hepatic venous pressure gradient (HVPG). (3) CLINICAL REASONING: Enalapril and valsartan are angiotensin-converting enzyme inhibitors (ACEIs) and angiotensin receptor blockers (ARBs) respectively, used for hypertension and heart failure, primarily by blocking the renin-angiotensin-aldosterone system (RAAS). However, in context of cirrhosis, these may worsen renal function due to renal hypoperfusion. Metoprolol is a beta-blocker, decreasing heart rate and contractility, thereby reducing blood pressure. Propranolol is a non-selective beta-blocker, having the added effect of decreasing portal venous pressure by reducing splanchnic blood flow, which could be beneficial here. Spironolactone is a potassium-sparing diuretic that antagonizes aldosterone, causing natriuresis and water excretion. Given the patient's ascites in the context of alcoholic cirrhosis, controlling his fluid balance is crucial. (4) CONCLUDING: Alcohol cessation is central to the management of this patient. Nonetheless, pharmacological management of ascites primarily includes salt restriction and diuretics (most commonly, an aldosterone antagonist like spironolactone and loop diuretic like furosemide). (5) RESPONSE: Thus, in addition to large-volume paracentesis and furosemide already initiated, the most appropriate pharmacotherapy to add in would be Spironolactone (D). While Propranolol (C) could help manage portal hypertension, the priority here is to manage the ascites.

**AI_E:** (1) RESTATING THE QUESTION: The question is related to a 57-year-old alcoholic man presented with signs of worsening ascites and abdominal distension. The main query here is around the appropriate drug therapy to additionally manage this patient's condition which seems to be related to alcoholic cirrhosis, in conjunction with large-volume paracentesis and furosemide. (2) PLANNING: The primary assumption here appears to be that the patient's symptoms are stemming from decompensated cirrhosis which has led to ascites. Given this, the question is which pharmacotherapy would correctly aid in managing the symptomatic state he is currently in. Possible treatments could be drugs that alleviate high blood pressure (Enalapril, Valsartan), those for decreasing heart rate (Metoprolol), for treating ascites (Spironolactone), or to decrease portal hypertension (Propranolol). However, as the patient's predominant concern is the ascites secondary to alcoholic cirrhosis, the assumption that should be prioritized is the treatment of ascites. (3) CLINICAL REASONING: Initially, in the management of ascites, paracentesis helps to provide symptomatic relief. Furosemide, a loop diuretic, also prescribed, functions by removing excess water from the body. However, the body's aldosterone level tends to increase in response, which promotes sodium and fluid retention that can aggravate ascites. Therefore, Spironolactone, an aldosterone antagonist, might have a complementary role in this case, especially when ascites is resistant to furosemide. The other choices (Enalapril, Metoprolol, Propranolol, and Valsartan), while relevant for the patient's comorbid conditions (hypertension) and complications (portal hypertension), may not directly address the ascites issue and may not provide the immediate symptomatic relief. (4) CONCLUDING: Although it is important to consider various aspects such as blood pressure control or heart rate management in the context of the patient's hypertension and cirrhosis, when speaking strictly of managing the current condition, namely, ascites, the most logical pharmacotherapy to initiate would be Spironolactone. (5) RESPONSE: Therefore, among the given options, the most appropriate pharmacotherapy to initiate in this instance would be Spironolactone (D), an aldosterone antagonist, which would likely produce a more desired outcome in managing this patient's resistant ascites along with the paracentesis and furosemide therapy.

**AI_D:** (1) RESTATING THE QUESTION: A 57-year-old man, who is facing difficulties due to alcohol use disorder, has visited the emergency ward. He is suffering from increased abdominal pain and distention since the last three days. His medical history includes alcoholic cirrhosis, hypertension, hyperlipidemia, and a cholecystectomy done laparoscopically about 10 years back. The patient has been admitted to the hospital multiple times for similar episodes of abdominal pain and distention. He has been prescribed medication for hypertension, but is currently not consuming any. The patient recently joined and exited an alcohol rehab program and started drinking alcohol again just a week ago, consuming 1 liter of vodka every day for the past 5 days. His pulse rate measured at 100 beats per minute, respirations at 16 per minute, and blood pressure at 165/85 mm of Hg. He appears jaundiced, but his oxygen saturation is at a healthy 100% even in room air. His distended and tense abdomen shows a fluid wave, and palpation reveals a reducible umbilical hernia of 2 cm width and general tenderness without any guarding. Percussion does not cause tenderness. Alongside a paracentesis of large volume and treatment with furosemide, the patient requires additional pharmacotherapy. We need to determine which of these five options is the most suitable: enalapril, metoprolol, propranolol, spironolactone, or valsartan. (2) PLANNING: Our primary problem here is the management of alcoholic cirrhosis that has led to ascites (fluid buildup in the abdomen) in an alcoholic patient. This condition is a common complication of liver cirrhosis and can lead to portal hypertension. The first-line treatment for ascites due to liver cirrhosis is dietary sodium restriction and diuretics (like furosemide along with spironolactone). Typical antihypertensives like enalapril, metoprolol, and valsartan can lower blood pressure, but they don't directly address the underlying liver pathology or portal hypertension. Propranolol could be an effective pharmacotherapy for preventing variceal bleeding--another complication of portal hypertension--but it's not the standard of care here because the patient does not present with esophageal varices. Therefore, my clinical priority lies with the management of ascites secondary to alcoholic cirrhosis. (3) CLINICAL REASONING: Liver cirrhosis due to the patient's alcoholism has likely caused the formation of ascites due to increased pressure in the portal vein (portal hypertension). In order to manage ascites, we need to focus on diuretics, particularly those acting on aldosterone, a hormone that promotes sodium and water retention, leading to fluid accumulation. Spironolactone is a potassium-sparing diuretic that antagonizes aldosterone, aiding in the removal of extra fluid from the body while maintaining potassium balance. It's often used in combination with loop diuretics like furosemide, which is already being administered, to achieve a significant natriuretic effect for reducing the ascites. The other drugs listed are mainly used to control blood pressure and do little to address the core issue of ascites. (4) CONCLUDING: Therefore, given the symptoms and the underlying cause (alcoholic cirrhosis leading to ascites), in addition to the paracentesis and furosemide treatment, the commencement of spironolactone therapy would prove most effective in managing the patient's condition. (5) RESPONSE: (D) Spironolactone

**Facilitator** **[Round 2]:** Summary: AI_A and AI_B both agree that the most appropriate pharmacotherapy for the patient is Propranolol. They both reason that Propranolol, a non-selective beta-blocker, would be effective in reducing portal hypertension, a complication of alcoholic cirrhosis, by decreasing cardiac output and splanchnic blood flow, thereby reducing portal venous pressure. AI_C, AI_D, and AI_E, on the other hand, argue that Spironolactone is the most suitable medication. They reason that Spironolactone, a potassium-sparing diuretic, would be effective in managing the patient's ascites, a symptom of alcoholic cirrhosis, by antagonizing aldosterone, which promotes sodium and water retention, leading to fluid accumulation. Key Differences: The key difference in the perspectives lies in the prioritization of the patient's symptoms. AI_A and AI_B prioritize the management of portal hypertension, a complication of alcoholic cirrhosis, while AI_C, AI_D, and AI_E prioritize the management of ascites, a symptom of alcoholic cirrhosis. Question for Council Members: Given the patient's symptoms and medical history, which should be prioritized in this case: managing portal hypertension or managing ascites? How would this prioritization affect the choice of pharmacotherapy?. Based on your preceding response, how would you answer the original question: [A 57-year-old man with alcohol use disorder comes to the emergency department because of a 3-day history of worsening abdominal pain and distention. Medical history is remarkable for alcoholic cirrhosis, hypertension, hyperlipidemia, and a laparoscopic cholecystectomy 10 years ago. The patient also has had several previous hospital admissions for similar episodes of abdominal pain and distention. The patient takes no medications but has been prescribed medications for hypertension in the past. He recently participated in an alcohol rehabilitation program but began drinking alcohol again 1 week ago. During the past 5 days, he has drunk 1 L of vodka daily. Pulse is 100/min, respirations are 16/min, and blood pressure is 165/85 mm Hg. Pulse oximetry on room air shows an oxygen saturation of 100%. The patient appears jaundiced. Cardiopulmonary examination discloses no abnormalities. The abdomen is large, distended, and tense; a fluid wave is present. Palpation of the abdomen discloses a 2-cm–wide reducible umbilical hernia and diffuse tenderness without guarding. There is no tenderness to percussion. In addition to large-volume paracentesis and furosemide therapy, initiation of which of the following pharmacotherapies is most appropriate? (A) Enalapril (B) Metoprolol (C) Propranolol (D) Spironolactone (E) Valsartan]

**AI_A:** RESTATING THE QUESTION: The question is seeking to understand the most appropriate pharmacotherapy to initiate for a 57-year-old male patient presenting with a worsening abdominal pain and distention. The patient has a history of alcohol use disorder, including a recent relapse, and has previously been diagnosed with alcoholic cirrhosis, hypertension, and hyperlipidemia. His physical examination reveals signs of ascites and jaundice, common complications seen in patients with alcoholic cirrhosis. Ascites is an accumulation of fluid in the peritoneal cavity, frequently due to cirrhosis and severe liver disease. PLANNING: The assumptions here are that the abdominal pain and distention are due to ascites as a result of alcoholic cirrhosis and that the chosen pharmacotherapy should be aimed at managing this symptom. An alternate assumption might be that we should prioritise the management of portal hypertension, a potential life-threatening complication of cirrhosis, instead. CLINICAL REASONING: From a clinical perspective, chronic liver damage from alcohol abuse in this patient has likely led to cirrhosis, which can cause portal hypertension and ascites. Portal hypertension occurs when there is an increase in the blood pressure within the portal venous system, and it can lead to the development of ascites. Thus, the management of portal hypertension could potentially alleviate the symptoms of ascites. CONCLUDING: Based on our thought process, our assumption that we should manage the portal hypertension alongside the ascites seems clinically justified. The pharmacotherapy chosen should act to reduce the portal venous pressure and help manage the ascites. RESPONSE: Among the choices given, Propranolol (option C) is the most appropriate answer. Propranolol is a non-selective beta-blocker that reduces the pressure in the portal veins by lowering the cardiac output and reducing the blood flow to the gut (splanchnic blood flow). Apart from managing the ascites, this drug will also help prevent variceal bleeding, a fatal complication of portal hypertension. Although Spironolactone is also a good choice for managing ascites, it does not have the added benefit of decreasing portal hypertension like Propranolol. Hence, Propranolol is the best choice considering the primary pathology and complications.

**AI_C:** RESTATING THE QUESTION: The question asks us to select the most suitable pharmacotherapy to add to a patient's existing therapy for abdominal pain and distention, associated with a diagnosis of alcoholic cirrhosis. The therapeutic options listed are enalapril, metoprolol, propranolol, spironolactone, and valsartan. PLANNING: We are primarily looking at a patient with alcoholic cirrhosis, who presents with worsening abdominal pain and distention—signs of ascites. In the management of ascites, initial therapy includes restriction of sodium in the diet and diuretic therapy. If this does not improve the issue, large-volume paracentesis, as performed in this patient, can be employed. The choice amongst ACE inhibitors (enalapril, valsartan), beta-blockers (metoprolol, propranolol), or an aldosterone antagonist (spironolactone) as additional pharmacotherapy depends on what we assume to be the most pressing issue at hand. CLINICAL REASONING: Ascites, the most significant symptom in this patient, arises primarily due to portal hypertension in cirrhosis, leading to splanchnic vasodilation, decreased effective circulating volume, and consequent activation of the renin-angiotensin-aldosterone system, with retention of salt and water. Propranolol, a non-selective beta-blocker, is a mainstay in managing portal hypertension; it decreases cardiac output and splanchnic blood flow, reducing portal pressures. However, when ascites is evident, as in this patient, the initiation of a diuretic agent such as spironolactone is also critical. CONCLUDING: Spironolactone prevents further accumulation of fluid by interfering with aldosterone, promoting the excretion of salt and water. While propranolol manages the underlying portal hypertension, adding an aldosterone antagonist to the regimen will help manage ascites, which seems to be the pressing concern in the patient. RESPONSE: Therefore, in addition to large-volume paracentesis and furosemide therapy, starting the patient on Spironolactone (D) would be the most appropriate pharmacotherapy.

**AI_E:** Restating the question, the patient is a 57-year-old man presenting with worsening abdominal pain and distention, indicating ascites. He has an extensive medical history that includes alcohol use disorder, alcoholic cirrhosis, hypertension, and hyperlipidemia. As the medical council made assumptions on which pharmacotherapy would be most suitable for this patient, the main distinctions arose between Propranolol and Spironolactone. Propranolol, a non-selective beta-blocker, is used to decrease portal hypertension by reducing cardiac output and splanchnic blood flow. This may alleviate portal venous pressure, a key complication in patients suffering from cirrhosis. On the other hand, Spironolactone, which is a potassium-sparing diuretic, manages ascites, common in alcoholic cirrhosis patients, by antagonizing the effects of aldosterone to avoid sodium and water retention leading to fluid accumulation. The clinical prioritization you have inquired about relates to which complication should be medically managed first, i.e., whether we should primarily target to reduce portal hypertension or manage ascites. Clinical reasoning prompts that both complications, portal hypertension and ascites, are interconnected and based on the severity of the manifestations; priority may switch back and forth between the two. Given the current clinical scenario, the patient's main complaint seems to be the discomfort due to the ascites indicated by abdominal distention and pain. Concluding, the decision to prioritize ascites management over portal hypertension seems to be more valid since addressing ascites would provide immediate symptomatic relief to the patient. Spironolactone, a potassium-sparing diuretic, is considered first-line therapy for ascites in cirrhosis patients since it antagonizes aldosterone and can reduce fluid accumulation. Therefore, I would connect my reasoning and choose (D) Spironolactone as the answer to the question. It is the most appropriate pharmacotherapy for this patient considering his symptoms and medical history. It will aid in managing his ascites effectively, and thereby provide the greatest benefit, given his current clinical presentation.

**AI_D:** RESTATING THE QUESTION: The question asks us to choose the most appropriate pharmacological approach to add to the current treatment of large-volume paracentesis and furosemide in a patient with ascites, resulting from alcoholic cirrhosis. The patient also began drinking again a week ago and has symptoms demonstrating liver dysfunction like jaundice and ascites. PLANNING: The assumption is that the underlying medical conditions of the patient, such as alcoholic cirrhosis and its complications, and his symptoms could indicate the best choice. The choices provided are primarily treating high blood pressure (enalapril, metoprolol, valsartan), portal hypertension (propranolol), or water retention (Spironolactone). I will assess each alternate assumption and hypothesize what should be clinically prioritized. CLINICAL REASONING: The main disorder lies with alcoholic cirrhosis. This patient's cirrhosis has led to ascites, identified by the appearance of a distended abdomen and fluid wave, an indication of fluid accumulation. Alongside this, the patient has a significant history of alcohol misuse with recent exacerbation and prior hypertension treatment. Non-selective beta-blockers (NSBBs) like propranolol are frequently utilized in preventing variceal bleeding due to portal hypertension; however, no current indication suggests the patient has this issue. His abandoned hypertension treatment and currently elevated blood pressure might suggest choosing a hypertension medication, but it won't target the main cause of his admission - his worsened ascites. CONCLUDING: Given the patient's current admission main concern is the ascites due to alcoholic cirrhosis, a medication that could target the management of this fluid accumulation alongside the existing treatment of large-volume paracentesis and furosemide therapy should be prioritized. From the list provided, the best-suited option regarding this approach and the patient's history appears to be Spironolactone. RESPONSE: Spironolactone (D), a potassium-sparing diuretic, is the most suitable to add to the patient's current treatment, considering his worsened ascites due to alcoholic cirrhosis. It antagonizes the action of aldosterone and helps manage the fluid accumulation, more in line with the existing therapeutic approach for his current medical issues.

**AI_B:** Restating the Question: This question projects to a 57-year-old man suffering an alcohol use disorder. Here the symptoms include a history of increasing abdominal pain and distention for the last three days. Documented medical history comprises alcoholic cirrhosis, hypertension, hyperlipidemia, a laparoscopic cholecystectomy 10 years ago, along with recurring hospital admissions for comparable episodes. The key symptoms are jaundice and a distended abdomen with tense fluid wave and 2cm-wide reducible umbilical hernia with a tender, yet non-guarding touch. The question seeks to identify the most suitable pharmacotherapy to be added to large-volume paracentesis and furosemide therapy. Planning: In this situation, the major issues are the alcoholic cirrhosis, manifesting in the current scenario as ascites and portal hypertension. Ascites is an accumulation of fluid in the peritoneal cavity, and portal hypertension is an increase in the blood pressure within a system of veins called the portal venous system. Among the medication options provided, Propranolol and Spironolactone are potential options, used to manage portal hypertension and ascites, respectively. The choice depends on whether we prioritize the management of portal hypertension (with Propranolol) or the management of ascites (with Spironolactone). Clinical Reasoning: Considering the patient's alcohol consumption, repeated episodes of abdominal distension and pain, presence of tense ascites showed by fluid wave and umbilical hernia, it appears that ascites is a more pressing problem. Ascites suggests severe portal hypertension as a likely contributor. Fluid wave and hernia presence are concerning, and are indicative of significant ascites and increased portal pressure. Propranolol, a non-selective beta-blocker, helps with portal hypertension, but it is not directly associated with ascites treatment. On the other hand, Spironolactone, a potassium-sparing diuretic acts directly on ascites by inhibiting the effects of aldosterone on sodium and water retention. Concluding: Therefore, considering the dominant symptoms and pressing health risks faced by the patient, managing ascites seems the priority. Thus, my choice would be to initiate Spironolactone alongside the ongoing management with large-volume paracentesis and furosemide therapy. Response: In addition to large-volume paracentesis and furosemide therapy, initiation of Spironolactone (option D) is most appropriate for this patient.

**Facilitator** **[Round 3]:** Summary: AI_A believes that Propranolol is the best choice for the patient, as it reduces portal venous pressure and helps manage ascites, while also preventing variceal bleeding, a fatal complication of portal hypertension. AI_B, AI_C, AI_D, and AI_E, however, argue that Spironolactone is the most appropriate choice, as it directly manages ascites, which they see as the most pressing issue for the patient. They argue that while Propranolol does help manage portal hypertension, it does not directly address ascites, which is causing the patient's current discomfort. Key Differences: The key difference in the council members' perspectives lies in their prioritization of the patient's symptoms. AI_A prioritizes the management of portal hypertension, viewing it as the root cause of the patient's ascites and other complications. In contrast, AI_B, AI_C, AI_D, and AI_E prioritize the management of ascites, viewing it as the most immediate and pressing issue for the patient. They argue that while managing portal hypertension is important, it does not directly address the patient's current discomfort from ascites. Question for Council Members: Given the patient's current symptoms and the potential for both immediate relief and long-term management, how do you weigh the importance of directly addressing ascites versus managing the underlying portal hypertension?. Based on your preceding response, how would you answer the original question: [A 57-year-old man with alcohol use disorder comes to the emergency department because of a 3-day history of worsening abdominal pain and distention. Medical history is remarkable for alcoholic cirrhosis, hypertension, hyperlipidemia, and a laparoscopic cholecystectomy 10 years ago. The patient also has had several previous hospital admissions for similar episodes of abdominal pain and distention. The patient takes no medications but has been prescribed medications for hypertension in the past. He recently participated in an alcohol rehabilitation program but began drinking alcohol again 1 week ago. During the past 5 days, he has drunk 1 L of vodka daily. Pulse is 100/min, respirations are 16/min, and blood pressure is 165/85 mm Hg. Pulse oximetry on room air shows an oxygen saturation of 100%. The patient appears jaundiced. Cardiopulmonary examination discloses no abnormalities. The abdomen is large, distended, and tense; a fluid wave is present. Palpation of the abdomen discloses a 2-cm–wide reducible umbilical hernia and diffuse tenderness without guarding. There is no tenderness to percussion. In addition to large-volume paracentesis and furosemide therapy, initiation of which of the following pharmacotherapies is most appropriate? (A) Enalapril (B) Metoprolol (C) Propranolol (D) Spironolactone (E) Valsartan]

**AI_B:** RESTATING THE QUESTION: This 57-year-old male patient has a history of alcohol use disorder and has come to the ED with a 3-day history of worsening abdominal pain and distention. His background includes alcoholic cirrhosis, hypertension, hyperlipidemia, a previous cholecystectomy, and recurrent admissions for similar abdominal symptoms. He recently started drinking alcohol again, and his vitals suggest an elevated pulse and blood pressure. His physical examination shows jaundice, a fluid wave, a reducible umbilical hernia, and abdominal tenderness. The question is about the optimal pharmacotherapy to initiate in addition to large-volume paracentesis and furosemide therapy. PLANNING: As we know, abdominal distention and pain in a patient with alcoholic cirrhosis could point towards ascites or abdominal fluid accumulation. This is further supported by the fluid wave physical examination finding. Ascites typically result from portal hypertension, a condition often seen in cirrhosis and worsened by alcohol abuse. Propranolol and Spironolactone are both treatments for conditions related to cirrhosis. CLINICAL REASONING: Propranolol is a nonselective beta-blocker often used to reduce portal venous pressure and prevent variceal bleeding. However, variceal bleeding isn't his immediate complaint. Spironolactone, on the other hand, is a diuretic that directly treats ascites by inhibiting aldosterone, leading to sodium, chloride and water excretion, and potassium retention. The patient currently has no medications but was previously prescribed antihypertensive therapy. He also presents with high blood pressure, which could be managed with either propranolol or spironolactone. CONCLUDING: Given his current symptoms of abdominal pain and distention, along with a physical examination finding of ascites and high blood pressure, addressing the immediate issue of ascites and hypertension stand out as priorities. Long-term management can be addressed once the immediate discomfort is relieved and his alcohol use is managed. RESPONSE: Considering the pathology, clinical findings, and the objectives for immediate symptom relief, I would initiate Spironolactone (D). This medication directly manages ascites, providing relief from abdominal distention and pain, and also aids as an antihypertensive to manage his elevated blood pressure.

**AI_D:** Restating the question: We are asked to determine the appropriate pharmacotherapy to add to the management of a 57-year-old man with a history of alcohol use disorder and cirrhosis. He presents with worsening abdominal pain, distention, jaundice, and has a fluid wave consistent with fluid overload i.e., ascites. Ascites, a possible consequence of portal hypertension due to cirrhosis, may result in abdominal discomfort and distension. The choices provided are Enalapril, Metoprolol, Propranolol, Spironolactone, and Valsartan. Planning: The assumption is that the patient's current presenting symptoms of abdominal pain and distention are due to portal hypertension that has resulted in ascites. Consequently, we must choose an appropriate drug to manage these symptoms by reducing portal hypertension or its consequences. Each drug listed has particular actions and contexts in which it is usually indicated: (1) Enalapril and Valsartan are angiotensin-converting enzyme (ACE) inhibitors and Angiotensin II receptor blockers (ARBs) respectively and they are used for hypertension and congestive heart failure; (2) Metoprolol is a beta-blocker primarily used for hypertension, angina, and arrhythmias; (3) Propranolol is a non-selective beta-blocker used for hypertension, angina, arrhythmias, but also portal hypertension by reducing portal venous pressure; and (4) Spironolactone is a potassium-sparing diuretic primarily used for congestive heart failure, hepatic cirrhosis-related ascites, and primary hyperaldosteronism. Clinical Reasoning: Given this patient's current clinical presentation and medical history, it is crucial to manage his portal hypertension consequences – the ascites – directly. The primary goal should be to reduce the patient's discomfort and symptoms related to the ascites. Therapies like paracentesis and diuretics such as furosemide can offer symptomatic relief. However, adding another diuretic such as Spironolactone would be more effective for ascites management as it reduces the fluid buildup in the abdomen by promoting the excretion of water and sodium through the kidneys. Concluding: Though Propranolol could aid in reducing portal hypertension by decreasing portal venous pressure, it might not directly manage ascites which is the immediate cause for this patient's discomfort. Therefore, in the context of this specific patient's presentation, the most appropriate pharmacotherapy initiation should be the one that directly addresses his immediate concern - ascites-related discomfort. Response: With respect to the above precisions, the initiation of Spironolactone therapy would therefore be the most appropriate choice to be added for this patient's current management. (D) Spironolactone.

**AI_A:** Restating & Defining Concepts: This inquiry seeks the best pharmacotherapeutic option for a 57-year-old male patient exhibiting escalated abdominal pain, distension, and a history of alcohol use disorder. His relevant medical history evinces alcoholic cirrhosis, hypertension, hyperlipidemia, and a laparoscopic cholecystectomy done a decade ago. His current clinical manifestation—a significant abdominal distension with positive fluid wave—indicates the presence of ascites, likely resulting from his liver cirrhosis. The question is interested in choosing between Enalapril, Metoprolol, Propranolol, Spironolactone, and Valsartan to manage his condition, in combination with paracentesis and a diuretic, Furosemide. Planning: We must assume that the leading cause of ascites in our patient is his alcoholic cirrhosis, given the history of alcohol use disorder and previous occurrences of similar symptoms. However, let's also bear in mind that other factors could be involved—his hypertension and hyperlipidemia, for instance. Hence, our primary approach should involve treatment that addresses cirrhotic ascites and reduces the risk of serious complications. With that in mind, we are inclined to propranolol or spironolactone as possible choices. Clinical Reasoning: Cirrhotic ascites materializes from portal hypertension—a complication of cirrhosis—where the internal pressure within the poral venous system elevates. Propranolol, a non-selective β-blocker, is utilized to decrease portal venous pressure, which consequently reduces the risk of variceal bleeding, a grave sequela of portal hypertension. While spironolactone, an aldosterone antagonist, along with furosemide, remain the first-line treatment to manage ascites. Concluding: Given the history and physical examination of the patient, cirrhotic ascites emerges as the most likely culprit for the abdominal distension. While propranolol would manage the portal hypertension in cirrhosis, the immediate concern is the large, tense ascites contributing to the patient's discomfort. Therefore, as first-line therapy, we would opt for a diuretic that addresses the ascites directly. Response: The most appropriate pharmacotherapy, besides large-volume paracentesis and furosemide, would be Spironolactone (option D) due to its efficacy in managing cirrhotic ascites. However, monitoring the patient's condition and instituting additional therapies, such as β-blockers, might be necessary addressing long-term management of the underlying portal hypertension.

**AI_E:** Restating the question: The question describes a 57-year-old man who sits under the influence of alcohol and is suffering from abdominal distention and worsening pain. He has a medical history of alcoholic cirrhosis, hypertension, and hyperlipidemia. Additionally, he underwent laparoscopic cholecystectomy 10 years ago. The abdomen's physical examination reveals it to be large and tense, with confirmed presence of a fluid wave, suggestive of ascites - an indication of severe liver disease such as cirrhosis leading to portal hypertension. The question asks, apart from paracentesis and furosemide therapy, which pharmacotherapy would be best to start in this context from the available choices - Enalapril, Metoprolol, Propranolol, Spironolactone, or Valsartan. Planning: Given the clinical picture, the root cause seems to be the patient's alcohol consumption leading to alcoholic cirrhosis and the subsequent development of portal hypertension and ascites. The assumption here is that the most suitable drug would be the one addressing this particular pathophysiology and provide relief to the patient's current discomfort. Different angles to consider are the options of either managing the ascites directly or treating the portal hypertension. Clinical Reasoning: Ascites is the abnormal accumulation of fluid within the abdominal cavity, often occuring due to portal hypertension in the setting of severe liver disease like alcoholic cirrhosis, as in this case. The primary goal is to comfort the patient and prevent further liver deterioration. Paracentesis will provide immediate relief by removing the accumulated fluid. Furosemide, a loop diuretic, will help the body get rid of extra fluid. However, to manage ascites on a sustained basis, aldosterone antagonists like Spironolactone are preferred. This decision is relevant especially given that ascites, as seen in this case, results from an abnormality in sodium and water metabolism caused primarily by increased secretion of the renal hormone aldosterone. Concluding: After connecting these clinical dots together, it can be hypothesized that the best choice to initiate would be Spironolactone (option D). It is a diuretic that blocks aldosterone and aids in the excretion of sodium and water, thus reducing the volume of fluid that could potentially accumulate in the peritoneal cavity. Response: Given all the patient's symptoms and his medical background, the most appropriate pharmacotherapy to initiate, apart from large-volume paracentesis and furosemide therapy, would be Spironolactone (option D). It will manage ascites effectively on a longer-term basis by acting on its underlying pathogenesis.

**AI_C:** (1) RESTATING THE QUESTION: The inquiry is revolving around a 57-year-old male patient with alcoholic cirrhosis who's presenting with severe abdominal pain and distention. The patient history includes hypertension, hyperlipidemia, and a cholecystectomy operation held about a decade earlier. The man has been resuming his alcohol consumption, which has been quite heavy over the course of the last week. The physical examination suggests the presence of abdominal ascites. The question aims to determine the most beneficial pharmacological therapy to initiate alongside large-volume paracentesis and furosemide treatment. Cirrhosis is a chronic liver disease characterized by irreversible scarring of the liver and eventual liver functioning failure. One of the complications of cirrhosis is portal hypertension defined as an increase in the blood pressure within the portal venous system, which could eventually lead to ascites, a fluid buildup in the peritoneal cavity. (2) PLANNING: Ascites management usually involves sodium restriction, diuretics, and ultrasound-guided large-volume paracentesis. In the provided case, large-volume paracentesis and furosemide treatment are already being applied, which leaves us with the assumption of what additional pharmacotherapy would be most beneficial. The main assumptions can be summarized as follows: 1) The need for blood pressure control (suggested by options A, E, and B, which are Enalapril, Valsartan, and Metoprolol respectively), 2) The need for reducing portal hypertension and associated variceal bleeding (option C, Propranolol), 3) And finally the necessity of fluid and electrolyte imbalance management, considering that ascites has already developed (option D, Spironolactone). Given that the patient has a history of hypertension and current portal hypertension-induced ascites, hypothesis 2 and 3 seem of higher clinical priority. (3) CLINICAL REASONING: Applying any of the hypertension medications (Enalapril, Valsartan, and Metoprolol) could be beneficial to handle the man's hypertension. However, they lack the direct effect on either the portal hypertension or the ascites. Propranolol, on the other hand, reduces the portal venous pressure, which could help in preventing variceal bleeding. Variceal bleeding is a fatal complication of portal hypertension, so it is important to control. Spironolactone is a diuretic that also acts on the mineralocorticoid receptor. Its use is known to create a certain equilibrium towards excelling fluid from the body, thus directly managing ascites formation. It makes more sense clinically to add medication that directly handles the current ongoing pathological condition affecting the patient. (4) CONCLUDING: Weighing these alternatives, while the option of Propranolol to manage the potential consequence of portal hypertension is valuable, it is also critical to manage the patient's current discomfort and potentially life-threatening ascites. Hence, therapeutically, it could be more advantageous to prioritize the direct management of ascites and fluid balance through the administration of Spironolactone. (5) RESPONSE: Hence, for this particular patient, who is suffering from abdominal pain and distention due to ascites, the most favorable pharmacological therapy to start along with large-volume paracentesis and furosemide treatment is Spironolactone (Answer D).

**Facilitator** The question pertains to a 57-year-old male patient with a history of alcohol use disorder, presenting with worsening abdominal pain and distention. His medical history includes alcoholic cirrhosis, hypertension, hyperlipidemia, and a laparoscopic cholecystectomy performed 10 years ago. The physical examination reveals a large, distended abdomen with a fluid wave, indicative of ascites, likely due to portal hypertension resulting from his alcoholic cirrhosis. The question seeks to identify the most appropriate pharmacotherapy to initiate in addition to large-volume paracentesis and furosemide therapy. Given the patient's symptoms and medical history, the primary concern is the management of his ascites, which is causing his current discomfort. Ascites typically results from portal hypertension, a common complication of cirrhosis. While propranolol, a non-selective β-blocker, could be used to decrease portal venous pressure and reduce the risk of variceal bleeding, a serious complication of portal hypertension, it may not directly address the patient's immediate discomfort caused by ascites. On the other hand, spironolactone, an aldosterone antagonist, is a diuretic that directly treats ascites by promoting the excretion of sodium, chloride, and water, and retaining potassium. This action helps to manage the fluid buildup in the abdomen, providing relief from abdominal distention and pain. Additionally, spironolactone can also aid in managing the patient's hypertension. Therefore, considering the patient's immediate symptoms and the need for effective ascites management, the consensus among the responses is that the most appropriate pharmacotherapy to initiate, in addition to large-volume paracentesis and furosemide therapy, would be Spironolactone (option D). However, it is also important to monitor the patient's condition and consider additional therapies, such as β-blockers, for long-term management of the underlying portal hypertension.
