# Supplementary material for: Collaborative intelligence in AI: Evaluating the performance of a council of AIs on the USMLE
Source: PLOS Digit Health. 2025 Oct 9;4(10):e0000787. doi: 10.1371/journal.pdig.0000787 (PMC12510544; doi:10.1371/journal.pdig.0000787)
Supplement: S2 File — (ZIP) [file pdig.0000787.s002.zip › S2 Data Raw data files containing unprocessed question inputs and LLM outputs including transcripts of deliberations/STEP2_Question-96-1_Type-MC-NJ.docx]

**Facilitator** **[Round 1]:** A 48-year-old man is admitted to the hospital 2 hours after the onset of severe abdominal pain, nausea, and vomiting. The pain radiates to the back but improves when he bends forward. He has no history of serious illness and takes no medications. He does not smoke cigarettes. He drinks two 12-oz beers weekly. He appears restless. He is 173 cm (5 ft 8 in) tall and weighs 90 kg (198 lb); BMI is 30 kg/m² . Temperature is 38.0°C (100.4°F), pulse is 110/min, respirations are 20/min, and blood pressure is 110/60 mm Hg. On pulmonary examination, decreased breath sounds are heard at the lung bases; there is dullness to percussion. Abdominal examination shows distention and severe tenderness to palpation of the epigastrium and left upper quadrant. Laboratory studies show: Hemoglobin: 13.5 g/dL Serum Urea nitrogen: 30 mg/dL Glucose: 120 mg/dL Creatinine: 1.2 mg/dL Cholesterol: 300 mg/dL Triglycerides: 1500 mg/dL Total bilirubin: 1.1 mg/dL Amylase: 300 U/L Administration of intravenous fluids and analgesics is begun. The patient’s abdominal pain, nausea, and vomiting decrease. Which of the following is the most appropriate next step in management? Choose one among the choices below. (A) Apheresis (B) Ezetimibe therapy (C) Gemfibrozil therapy (D) Metformin therapy (E) Recommendation for weight loss

**AI_E:** (1) RESTATING THE QUESTION: In this clinical scenario, a 48-year-old male patient presenting with severe abdominal pain, nausea, and vomiting was admitted to the hospital. His symptoms seem to be centrally located and seem to alleviate when he leans forward. His test results revealed elevated serum urea nitrogen, glucose, cholesterol, and especially high triglyceride levels. Although he is started on intravenous fluids and analgesics, which reduce his symptoms, we are tasked with identifying the most suitable subsequent step in management. (2) PLANNING: Considering the presenting symptoms and their alleviation by forward-leaning, the significantly high triglyceride levels, and the patient's body mass index and lifestyle, the following clinical assumptions can be made. The symptoms might be indicative of pancreatitis, which could be due to hypertriglyceridemia. It's important to note here that hypertriglyceridemia is a known risk factor for acute pancreatitis when levels exceed 1000 mg/dL, and the dullness heard on percussion at the lung bases can indicate pleural effusions, a complication of pancreatitis. For managing acute pancreatitis resulting from hypertriglyceridemia, options range from symptomatic treatment, weight loss recommendations, lipid-lowering medications to more invasive methods such as apheresis. It's crucial to prioritize immediate management strategies that befit the severity of the condition and decrease the potential for complications. (3) CLINICAL REASONING: When the diagnosis is pancreatitis, symptomatic treatment consists of analgesics for pain relief and intravenous fluids to prevent dehydration from vomiting. These steps have already been undertaken. Next, addressing the likely cause of the pancreatitis, which is hypertriglyceridemia in this case, is pivotal. Apheresis could serve as an immediate strategy to reduce triglyceride levels dramatically, and could thus lower the risk of pancreatic inflammation. This intervention outweighs starting the patient on drugs at first, which can take time to effectively reduce triglyceride levels. (4) CONCLUDING: Although long-term management should incorporate lifestyle modifications and medication adjustments to address the patient's hypertriglyceridemia, immediate intervention requires therapeutic measures that rapidly lower the triglyceride levels to mitigate further inflammatory damage to the pancreas. (5) RESPONSE: As a result, the most appropriate next step in treating this patient would be (A) Apheresis, ensuring a rapid reduction of triglyceride levels that would attenuate the risk of persistent or worsening pancreatitis.

**AI_A:** RESTATING THE QUESTION: The information provided outlines a case about a 48-year-old man who presented at the hospital with symptoms including severe abdominal pain, nausea, and vomiting. Despite no past history of serious illness or medication intake, he shows signs of possible systemic distress, indicated by his feverish symptoms, increased pulse rate, restlessness and decreased breath sounds at the lung bases. There is also severe tenderness upon abdominal palpation around the epigastrium and left upper quadrant. His laboratory results reveal notably high levels of cholesterol and triglycerides. Following administration of intravenous fluids and analgesics, his symptoms have decreased. The question demands an appropriate next step of management from a set of given choices: Apheresis, Ezetimibe therapy, Gemfibrozil therapy, Metformin therapy, or a recommendation for weight loss. PLANNING: To solve this case, we must understand and define our terms. Apheresis is a medical procedure that involves removing whole blood from a patient and separating the blood into individual components so the needed element can be removed. Ezetimibe is a medication used to lower levels of total cholesterol and low-density lipoprotein (LDL, or "bad cholesterol") in the blood. Gemfibrozil is a lipid-regulating medication used to lower high levels of triglycerides in patients who are at risk of pancreatitis (inflammation of the pancreas). Metformin is an oral medication that helps control blood sugar levels and is generally used for patients with type 2 diabetes. Lastly, making a recommendation for weight loss is a non-medical intervention that involves lifestyle modifications to decrease body weight, often used to combat health conditions related to overweight or obesity, such as diabetes, hypertension, and high cholesterol levels. CLINICAL REASONING: Given the findings, it's clear that the patient's symptoms were not random, but likely related to a diagnosable medical issue. The severe abdominal pain that radiates to the back, nausea, vomiting, and elevated levels of amylase and lipids suggest a possible acute pancreatitis. Acute pancreatitis can be triggered by very high levels of blood triglycerides (above 1000 mg/dL). The clinical picture along with the lab findings is highly suggestive of pancreatitis secondary to hypertriglyceridemia. The goal of treatment in this case would be to lower the triglyceride levels rapidly to reduce the risk of further attacks. CONCLUDING: Taking into account the patient's signs, symptoms, physical examination findings, and the laboratory results, as well as the definition of each proposed course of action, it appears necessary to prioritize a treatment plan that directly addresses the alarming triglyceride levels. RESPONSE: Therefore, (C) Gemfibrozil therapy is the most appropriate next step in management. This medication helps decrease triglyceride levels and thus can help mitigate risk of further pancreatitis episodes.

**AI_C:** (1) RESTATING THE QUESTION: A middle-aged man is admitted to the hospital due to sudden severe abdominal pain, nausea, and vomiting. Although he doesn't have a history of serious illnesses, his elevated physiological indicators suggest he may have a health issue. He is slightly overweight at 90 kg with a BMI of 30 kg/m², and his laboratory studies show increased serum urea nitrogen, glucose, creatinine, cholesterol, triglycerides, and amylase. His current treatment has been the administration of intravenous fluids and analgesics, which reduced his symptoms. The query now is to determine the most appropriate next step for the patient's management among apheresis, ezetimibe therapy, gemfibrozil therapy, metformin therapy, or a weight loss regimen. (2) PLANNING: Upon evaluating the patient's case and the provided management choices, it's noticeable that most choices correspond to conditions related to metabolic syndrome and hyperlipidemia. However, the cause of sudden severe abdominal pain, nausea, vomiting, particularly pain radiating towards the back and improving with forward bending, and paired with raised amylase level, the symptoms hint towards acute pancreatitis. High cholesterol and triglycerides, found in the lab results, can correlate directly to acute pancreatitis through pancreatitis secondary to hypertriglyceridemia. (3) CLINICAL REASONING: To support the assumption of acute pancreatitis due to hypertriglyceridemia, the clinical findings must be correlated further. Acute pancreatitis is an inflammatory condition of the pancreas, which usually presents with sudden onset, severe abdominal pain that radiates to the back, nausea, and vomiting - symptoms matching our patient's presentation. On investigating the patient's laboratory results, it's observed that his serum triglyceride levels are vastly elevated (1500 mg/dL). Pancreatitis secondary to hypertriglyceridemia usually occurs when the serum triglyceride levels exceed 1000 mg/dL. Abnormally high triglyceride levels increase the blood's viscosity, promoting sludging and ischemia, and the formation of toxic free fatty acids via the action of pancreatic lipase, which leads to an acute inflammatory response in the pancreas (ie, pancreatitis). (4) CONCLUDING: Conclusively, a severe elevation in the serum triglyceride level (>1000 mg/dL) is a well-recognized cause of acute pancreatitis. Immediate measures to reduce the serum triglyceride levels are necessary to decrease the extent of pancreatitis and prevent occurrence. Weight reduction, although beneficial in the long run, is not an immediate remedy. Antihyperglycemic treatment (metformin) or cholesterol-absorption inhibitor (ezetimibe) are not specific for reducing severe hypertriglyceridemia. (5) RESPONSE: Based on the clinical scenario and the available choices, the most appropriate next step in the management of this patient is to initiate (C) Gemfibrozil therapy. Gemfibrozil is a fibric acid derivative that effectively reduces serum triglyceride levels. Fibric acid derivatives function by upregulating lipoprotein lipase, leading to increased hydrolysis of triglycerides, thereby ultimately reducing the serum triglyceride levels.

**AI_D:** RESTATING THE QUESTION: A 48-year-old man has been admitted to the hospital with acute symptoms of severe abdominal pain, nausea, and vomiting. The abdominal pain radiates to his back but somewhat improves when he bends forward. He has no previous history of a serious illness, takes no medications, doesn't smoke, but consumes beer moderately. He is overweight with a BMI of 30 kg/m² and appears restless. On lung examination, reduced breath sounds are noted, and his abdominal exam reveals distention and severe tenderness in the epigastric region and left upper quadrant. His lab results indicate significantly elevated serum cholesterol and triglycerides levels along with an elevated amylase level. After receiving intravenous fluids and analgesics, his symptoms have improved. The question is seeking the most appropriate next step in managing this patient's case. PLANNING: To interpret these observations and formulate a hypothesis, we should consider the following assumptions: (1) His abdominal pain radiating to the back, worsened by positioning, hints toward a pathology involving the pancreas; (2) Elevated serum triglyceride levels provide a link to his potential case of pancreatitis since hypertriglyceridemia above 1000 mg/dL can induce acute pancreatitis; (3) The patient's BMI indicates obesity, which could contribute to his abnormal lipid profile; (4) The elevated amylase level supports pancreatitis diagnosis; (5) The therapeutic approach should focus on alleviating his acute condition while managing his underlying dyslipidemia. CLINICAL REASONING: Acute pancreatitis is typically a direct result of gallstones or alcohol, but hypertriglyceridemia can also cause it in some patients. Establishing this association is vital as it informs the immediate and long-term management of the condition. The response to treatment thus far – relief of symptoms with intravenous fluids and analgesics – aligns with the standard care for acute pancreatitis. The next step would require managing the underlying trigger. Given the patient's non-existent history of gallstones or significant alcohol intake, the considerably high triglycerides level (1,500 mg/dL) appears to be the most likely precipitant. Understanding this, the next step should focus on rapid reduction of these triglycerides to prevent further bouts of pancreatitis. CONCLUDING: In terms of options, (A) apheresis (a procedure that separates and removes potentially harmful substances from the blood), might not be essential at this stage; (B) Ezetimibe therapy, decreases cholesterol absorption but has little effect on triglycerides; (D) Metformin therapy, is primarily used in the management of type 2 diabetes and not specifically meant for triglyceride reduction. (E) Recommendation for weight loss is a long-term plan that would not help the immediate situation; therefore it can't be considered the most appropriate next step. RESPONSE: Therefore, the choice that appears most suited to address the immediate issue of hypertriglyceridemia is (C) Gemfibrozil therapy, which can effectively lower triglycerides level in the blood and prevent future episodes of hypertriglyceridemia-induced pancreatitis. This should be started along with dietary modifications to manage his dyslipidemia in the long term.

**AI_B:** RESTATING THE QUESTION: A 48-year-old male patient with a BMI of 30 kg/m², presenting a recent episode of severe abdominal pain, nausea, vomiting, and disturbed renal function (elevated serum urea nitrogen and normal creatinine), is under consideration for further treatment steps. Along with pain, his labs show abnormally high levels of cholesterol, triglycerides, and amylase. Given his symptoms have improved after initial intravenous fluids and analgesics, the hospital staff wonders which of the options (apheresis, ezetimibe therapy, gemfibrozil therapy, metformin therapy, or weight loss recommendation) is best suited for this patient's management. PLANNING: Identifiable assumptions are firstly, the cause of the patient's abdominal pain might be due to acute pancreatitis given the nature of the pain and the elevated level of amylase. Secondly, pancreatitis in this patient might be due to hypertriglyceridemia since his triglycerides level is found to be very high. Therefore, managing the underlying hypertriglyceridemia should be a priority and the most relevant management choices would be: (A) Apheresis - normally used for lipid disorders that are resistant to diet and maximum tolerated lipid-lowering medication. (B) Ezetimibe therapy - a cholesterol absorption inhibitor used for managing primary hypercholesterolemia (C) Gemfibrozil therapy - a fibrate, usually used to reduce triglycerides levels in patients with severe hypertriglyceridemia (D) Metformin therapy - generally used for type 2 diabetes treatment (E) Recommendation for weight loss. CLINICAL REASONING: The patient's clinical profile and lab results point towards acute pancreatitis given the severe, radiating abdominal pain that is reduced with leaning forward, the presence of nausea and vomiting, and elevated serum amylase levels. Acute pancreatitis can have several causes, one of them being hypertriglyceridemia. His extraordinarily high triglyceride levels (> 1500 mg/dL) might have precipitated this acute episode. It's reasonable to assume reducing this risk factor should be a priority in his management. This rules out options like (B) Ezetimibe therapy which focuses on cholesterol, not triglycerides and (D) Metformin therapy which is primarily for managing blood glucose levels in diabetes. (E) Recommendation for weight loss, though beneficial for overall health and management of hyperlipidemia, won't offer immediate benefit required in this case. Among the remaining options, (A) Apheresis and (C) Gemfibrozil therapy are both effective in managing hypertriglyceridemia. Apheresis is an invasive procedure, which can quickly reduce the levels of triglycerides but does not prevent their re-accumulation and is not a standard treatment for primary hypertriglyceridemia. Gemfibrozil therapy, on the other hand, is a fibrate which helps reduce serum triglycerides levels and is often used for severe hypertriglyceridemia. CONCLUDING: Given the evidence, it seems logical to opt for the less invasive option that offers control of triglycerides levels, which in turn, will help in managing the suspected acute pancreatitis belived to be because of hypertriglyceridemia in this patient. RESPONSE: Thereby, the most appropriate next step in the management of this patient would likely be (C) Gemfibrozil therapy.

**Facilitator** **[Round 2]:** Summary: AI_A, AI_B, AI_C, and AI_D all agree that the patient's symptoms and lab results suggest acute pancreatitis due to hypertriglyceridemia. They all recommend Gemfibrozil therapy as the most appropriate next step in management, as it directly addresses the high triglyceride levels that likely triggered the pancreatitis. They also agree that other options like Ezetimibe therapy, Metformin therapy, and weight loss recommendation are not suitable immediate interventions for this case. However, AI_E differs in opinion. While AI_E also identifies the patient's condition as acute pancreatitis due to hypertriglyceridemia, they recommend Apheresis as the most appropriate next step. AI_E argues that Apheresis would provide a more immediate reduction in triglyceride levels, thus reducing the risk of further pancreatic inflammation. Key Differences: The key difference in perspectives lies in the recommended immediate intervention for the patient's hypertriglyceridemia. AI_A, AI_B, AI_C, and AI_D advocate for Gemfibrozil therapy, a medication that helps decrease triglyceride levels. They believe this approach is the most suitable given the patient's condition. On the other hand, AI_E recommends Apheresis, a procedure that rapidly reduces triglyceride levels, arguing that this approach would provide a more immediate response to the patient's severe hypertriglyceridemia. Question for Council Members: Given the severity of the patient's hypertriglyceridemia and the risk of further pancreatitis episodes, could you elaborate on why you believe your recommended intervention (Gemfibrozil therapy or Apheresis) is the most effective immediate response?. Based on your preceding response, how would you answer the original question: [A 48-year-old man is admitted to the hospital 2 hours after the onset of severe abdominal pain, nausea, and vomiting. The pain radiates to the back but improves when he bends forward. He has no history of serious illness and takes no medications. He does not smoke cigarettes. He drinks two 12-oz beers weekly. He appears restless. He is 173 cm (5 ft 8 in) tall and weighs 90 kg (198 lb); BMI is 30 kg/m² . Temperature is 38.0°C (100.4°F), pulse is 110/min, respirations are 20/min, and blood pressure is 110/60 mm Hg. On pulmonary examination, decreased breath sounds are heard at the lung bases; there is dullness to percussion. Abdominal examination shows distention and severe tenderness to palpation of the epigastrium and left upper quadrant. Laboratory studies show: Hemoglobin: 13.5 g/dL Serum Urea nitrogen: 30 mg/dL Glucose: 120 mg/dL Creatinine: 1.2 mg/dL Cholesterol: 300 mg/dL Triglycerides: 1500 mg/dL Total bilirubin: 1.1 mg/dL Amylase: 300 U/L Administration of intravenous fluids and analgesics is begun. The patient’s abdominal pain, nausea, and vomiting decrease. Which of the following is the most appropriate next step in management? Choose one among the choices below. (A) Apheresis (B) Ezetimibe therapy (C) Gemfibrozil therapy (D) Metformin therapy (E) Recommendation for weight loss]

**AI_D:** 1) RESTATING THE QUESTION: The query wants to know the most suitable next step in the management of a 48-year old male patient admitted to the hospital with signals indicative of acute pancreatitis and found to have high levels of triglycerides in his blood. Hypertriglyceridemia in this context is an abnormally high concentration of triglycerides in the blood, while acute pancreatitis refers to an inflammation of the pancreas due in part to this patient's elevated triglyceride levels. 2) PLANNING: To address the question appropriately, we need to assume that the patient's high triglyceride levels prompted the acute pancreatitis episode. One plausible alternative assumption is that pancreatitis could have been induced by alcohol consumption or gallstone disease. However, it makes sense to prioritize the hypertriglyceridemia assumption clinically given this patient's majorly elevated triglyceride levels (1500 mg/dL) seen in his lab results. 3) CLINICAL REASONING: Beginning with this assumption of high triglycerides as the cause, our goal would be to abate those levels. This would steer the pancreas away from the potential damaging effects of these fatty substances in the blood, thus curbing any further inflammation. Options like Metformin, a glucose-lowering medication for type 2 diabetes and Ezetimibe, a cholesterol-lowering medication, wouldn’t be most suitable in this case as they target different aspects not specifically tied to the elevated triglyceride levels. 4) CONCLUDING: Gemfibrozil therapy is a better choice for immediate management, since it is a type of fibrate that directly helps lower blood triglyceride levels. Although weight loss is also advisable for the patient in the long run due to his BMI of 30 kg/m², it wouldn't provide an immediate intervention to the acute issue of hypertriglyceridemia-induced acute pancreatitis. Apheresis, a procedure that purifies the blood, is usually reserved for cases with extreme triglyceride levels (>2000 mg/dL) and remains a last resort measure for immediate reduction of triglycerides. 5) RESPONSE: Based on the clinical reasoning above, the most appropriate next step in management of this case should be (C) Gemfibrozil Therapy. This treatment could provide a direct response to the patient's high triglyceride levels, believed to be the underlying cause of his acute pancreatitis.

**AI_A:** RESTATING THE QUESTION: The question concerns a 48-year-old male patient with severe abdominal pain, nausea, and vomiting which seems to result from hypertriglyceridemia-induced acute pancreatitis. To manage his condition, we are given several choices: apheresis, ezetimibe therapy, gemfibrozil therapy, metformin therapy, and recommendation for weight loss. The task is to decide between these options and identify the most appropriate immediate intervention. PLANNING: First, we need to properly consider the situation. The patient's abdominal pain, plus the elevated levels of triglycerides and amylase, converge to indicate acute pancreatitis. His condition’s most likely cause is hypertriglyceridemia, considering that triglyceride levels beyond 1000 mg/dL can precipitate acute pancreatitis. All interventions listed are related to managing triglyceride levels except for the recommendation for weight loss, which addresses his obesity. Apheresis is a procedure to rapidly remove triglycerides from the blood. However, it's not typically the first-line treatment for hypertriglyceridemia. Ezetimibe is a cholesterol absorption inhibitor, and Metformin is an antidiabetic drug - neither are front line drugs for elevated triglycerides. Gemfibrozil, a fibrate, is used primarily to lower triglyceride levels. CLINICAL REASONING: Fibrates are often first-line treatment for patients with very high triglyceride levels, as it can reduce triglycerides by as much as 50%. This makes gemfibrozil an attractive therapeutic option in this situation. If the patient does not respond to pharmacotherapy (i.e., gemfibrozil), or if he has another episode of pancreatitis, the use of plasmapheresis to rapidly reduce the triglyceride level could then be considered. CONCLUDING: Given the emergency nature of acute pancreatitis and the very high triglyceride levels, the immediate goal of any intervention should focus on rapidly lowering triglycerides to prevent further bouts of pancreatitis, as well as managing the current episode. In this context, the recommended intervention (Gemfibrozil therapy) appears to be the most suitable choice, considering its ability to significantly reduce triglyceride levels. RESPONSE: I would choose (C) Gemfibrozil therapy as the most appropriate immediate intervention to lower the patient's very high triglyceride levels and manage his acute pancreatitis. This is notwithstanding the potential consideration of apheresis should the patient not respond to pharmacotherapy or if a recurrence of pancreatitis occurs. Alternatively, recommending weight loss, while valuable in the long term, is not an immediate solution to the immediate problem. Similarly, ezetimibe and metformin therapies, while useful in other contexts, may not be the most effective in aggressively lowering triglycerides in this situation.

**AI_E:** 1) RESTATING THE QUESTION: The question pertains to the management of a 48-year-old man who has been admitted to the hospital with severe abdominal pain, nausea, and vomiting. Data from his physical examination and laboratory results indicate acute pancreatitis, probably attributed to his significantly elevated serum triglycerides, indicated by a level of 1500 mg/dL (normal range is under 150 mg/dL). The question requires us to choose the most appropriate next step in management. 'Apheresis' refers to a medical procedure that involves removing certain substances from the blood. 'Ezetimibe' is a medication used to lower levels of certain fats (like cholesterol) in the blood. 'Gemfibrozil' is another medication used principally to treat dyslipidemia, particularly hypertriglyceridemia. 'Metformin' is a medication used primarily for type 2 diabetes. The final option, 'weight loss', is self-explanatory but it's generally considered as a lifestyle management strategy. 2) PLANNING: One assumption we could make is that the underlying cause of pancreatitis could and should be managed in the long-term without an immediate intervention on the triglyceride levels (examples include considering Gemfibrozil therapy, Ezetimibe therapy, Metformin therapy or weight loss). Alternatively, we could reason that the significantly high triglyceride levels should be corrected promptly during this acute episode (which could be executed via Apheresis). The latter option seems reasonable, considering the high level of the patient's triglycerides might worsen the ongoing inflammation of his pancreas or even provoke more episodes in the immediate future. 3) CLINICAL REASONING: Acute pancreatitis can be caused by several factors, including gallstones, alcohol, hypertriglyceridemia, and others. However, this patient's symptoms, along with the elevated levels of triglycerides and amylase (an enzyme secreted by the pancreas), suggest hypertriglyceridemia-induced acute pancreatitis. Although the other options listed in the management choices could help lower the serum triglyceride or cholesterol levels, only Apheresis can acutely lower the triglyceride levels, potentially alleviating the ongoing pancreatic inflammation. Interventions such as Gemfibrozil therapy, Ezetimibe therapy, Metformin therapy or weight loss are generally long-term strategies. 4) CONCLUDING: Given the patient's current immediate crisis of severe hypertriglyceridemia leading to acute pancreatitis, the need for timely intervention that could quickly reduce serum triglycerides becomes evident. Apheresis can provide such an acute intervention by rapidly decreasing the triglyceride levels, which then could potentially reduce ongoing pancreatic damage. 5) RESPONSE: In this scenario where the patient is suffering from acute pancreatitis likely due to severe hypertriglyceridemia, the most appropriate next step in management would be to significantly decrease the triglyceride levels without any delay. Considering the potential of Apheresis to reduce serum triglycerides rapidly in this acute scenario, I'd determine that the best choice among the listed options is 'Apheresis' (Option A).

**AI_C:** Restating the question: The patient is a 48-year-old male, presenting with severe abdominal pain, nausea, vomiting, and elevated fever. The pain improves when he bends forward, and he has been admitted to the hospital for treatment. Based on the laboratory findings, he has elevated serum triglyceride levels (1500 mg/dL) and amylase (300 U/L), suggesting acute pancreatitis. His symptoms improved after the administration of intravenous fluids and analgesics, and now the question poses the most appropriate next management step, with choices as: Apheresis; Ezetimibe therapy; Gemfibrozil therapy; Metformin therapy; or a weight loss recommendation. Planning: The assumptions begin with the patient's presenting symptoms and laboratory findings consistent with acute pancreatitis, most probably triggered by his high serum triglyceride levels. Acute pancreatitis is a severe inflammatory condition of the pancreas, commonly caused by gallstones, alcohol, and hypertriglyceridemia. Elevated serum amylase, along with his symptoms, aids the diagnosis. Management often starts with fluid therapy, analgesia, and identifying and treating the underlying cause. This patient has been given fluids and analgesics, which have improved his condition. The next step would be handling the profound hypertriglyceridemia. The choices provided in the management question propose various means of modifying his lipid profile or addressing his overweight condition. Clinical Reasoning: Hypertriglyceridemia initiates pancreatitis by leading to the production of free fatty acids in the pancreatic capillaries. This causes capillary plugging, ischemia, and inflammation of the pancreas. Therefore, one should deal with the elevated triglycerides to stop the progression of the disease. Gemfibrozil is a fibrate class medication commonly used for hypertriglyceridemia. It works by decreasing triglyceride synthesis in the liver. Therefore, it takes time to act. Apheresis, on the other hand, is a procedure that could swiftly decrease the triglycerides by mechanically extricating them from the blood. Although effective, it's usually reserved for severe or refractory cases mainly due to its intrusive nature. The other choices, Ezetimibe and Metformin, while effective for dyslipidemia and diabetes, respectively, do not directly lower triglycerides. Weight loss is a long-term strategy and impractical for immediate implementation in the current acute scenario. Concluding: Considering the urgency to reduce the triglyceride level, the most immediate and effective method would be gemfibrozil therapy. Even though apheresis may offer quicker results, it's considered a second-line or salvage therapy and may not be the first choice in this setting. Moreover, the patient's condition is stabilizing with current management, suggesting that the need for aggressive intervention like apheresis might be unwarranted at this point. Weight loss, the remedy to address his overweight status, is more of a long-term management approach and would not have an immediate impact on his triglyceride levels. Response: Therefore, with supported reasoning, the most appropriate next step in the management of this patient would be (C) Gemfibrozil therapy.

**AI_B:** Restating the question: We're discussing a 48-year-old male patient that presented with acute abdominal pain, nausea, and vomiting. The pain radiates to his back and is described as severe. His abdominal examination revealed distention and extreme tenderness in the epigastric area and the left upper quadrant. Results of laboratory tests showed that his serum triglyceride levels are significantly elevated. After the initial management with intravenous fluids and analgesics, his symptoms have reduced. Now, we're tasked with determining the most appropriate next step in the patient's management among five options: Apheresis, Ezetimibe therapy, Gemfibrozil therapy, Metformin therapy, and weight loss. Planning: The patient's symptoms, physical examination findings, and laboratory results suggest acute pancreatitis likely due to severe hypertriglyceridemia. Upon evaluating the presented options for further management, we need to consider the need to rapidly decrease serum triglyceride levels for avoiding further damage to the pancreas and prevent other triglyceride-related complications. Clinical Reasoning: Given the severity of the patient's hypertriglyceridemia (triglyceride levels are 1500 mg/dL), immediate action to reduce these levels is needed. While all options can potentially contribute to a decrease in triglyceride levels, not all may offer the required immediacy or are applicable given the patient's current state. Apheresis is an invasive procedure used to rapidly decrease the concentration of triglycerides. However, it requires specialized equipment and trained personnel, may have potential complications, and is not generally the first course of action for managing hypertriglyceridemia-induced pancreatitis. Ezetimibe and Metformin help control high cholesterol and glucose levels, respectively, and their impact on triglyceride levels is not as potent or direct. Recommending weight loss, while a long-term solution, would not result in an immediate decrease in triglyceride levels. Gemfibrozil is a fibrate, drugs that are known to significantly lower triglyceride levels. They can result in a rapid decrease in triglyceride levels and are best suited for treating hypertriglyceridemia. Concluding: Given the severity and urgency of the patient's condition (acute pancreatitis due to severe hypertriglyceridemia), immediate action must be undertaken to lower his triglyceride levels. While all presented interventions can contribute towards decreasing triglyceride levels, Gemfibrozil therapy is the most suitable option. This therapy is known to rapidly decrease triglyceride levels, with fewer risks compared to invasive procedures like apheresis. Response: The most appropriate next step in managing this patient would be Gemfibrozil therapy (Option C). This therapeutic approach directly addresses the hypertriglyceridemia, which is likely the cause of acute pancreatitis in this patient, offering a rapid reduction in the patient's triglyceride levels. It also poses fewer risks compared to other interventions, like apheresis. The other options, while valuable in specific contexts, do not offer the immediacy required by this case. Ezetimibe and Metformin do not have a direct or potent impact on triglycerides, and while weight loss is an advisable long-term approach, it won't immediately mitigate the patient's hypertriglyceridemia.

**Facilitator** **[Round 3]:** Summary: AI_A, AI_B, AI_C, and AI_D all agree that Gemfibrozil therapy is the most appropriate immediate intervention for the patient. They argue that this medication, a fibrate, is often the first-line treatment for patients with very high triglyceride levels and can reduce these levels by as much as 50%. They also note that while apheresis can rapidly reduce triglyceride levels, it is typically not the first-line treatment and is reserved for severe or refractory cases. Ezetimibe and Metformin, they argue, are not front-line drugs for elevated triglycerides, and weight loss, while valuable in the long term, is not an immediate solution to the problem. AI_E, however, disagrees and argues that apheresis is the most appropriate immediate intervention. They reason that given the patient's severe hypertriglyceridemia and the resulting acute pancreatitis, an immediate intervention that can quickly reduce serum triglycerides is needed. They argue that apheresis can provide such an intervention by rapidly decreasing the triglyceride levels, potentially reducing ongoing pancreatic damage. Question to clarify disagreements: To AI_E: Given that Gemfibrozil therapy is often the first-line treatment for patients with very high triglyceride levels and can reduce these levels by as much as 50%, why do you believe that apheresis, which is typically not the first-line treatment and is reserved for severe or refractory cases, is the most appropriate immediate intervention in this case?. Based on your preceding response, how would you answer the original question: [A 48-year-old man is admitted to the hospital 2 hours after the onset of severe abdominal pain, nausea, and vomiting. The pain radiates to the back but improves when he bends forward. He has no history of serious illness and takes no medications. He does not smoke cigarettes. He drinks two 12-oz beers weekly. He appears restless. He is 173 cm (5 ft 8 in) tall and weighs 90 kg (198 lb); BMI is 30 kg/m² . Temperature is 38.0°C (100.4°F), pulse is 110/min, respirations are 20/min, and blood pressure is 110/60 mm Hg. On pulmonary examination, decreased breath sounds are heard at the lung bases; there is dullness to percussion. Abdominal examination shows distention and severe tenderness to palpation of the epigastrium and left upper quadrant. Laboratory studies show: Hemoglobin: 13.5 g/dL Serum Urea nitrogen: 30 mg/dL Glucose: 120 mg/dL Creatinine: 1.2 mg/dL Cholesterol: 300 mg/dL Triglycerides: 1500 mg/dL Total bilirubin: 1.1 mg/dL Amylase: 300 U/L Administration of intravenous fluids and analgesics is begun. The patient’s abdominal pain, nausea, and vomiting decrease. Which of the following is the most appropriate next step in management? Choose one among the choices below. (A) Apheresis (B) Ezetimibe therapy (C) Gemfibrozil therapy (D) Metformin therapy (E) Recommendation for weight loss]

**AI_E:** Restating the Question: This scenario presents a 48-year-old man who arrived at the hospital with severe abdominal pain, nausea, and vomiting suggestive of an acute abdominal process. His blood tests show elevated levels of serum triglycerides at 1500 mg/dL, which is an indication of hypertriglyceridemia. Apheresis, Ezetimibe therapy, Gemfibrozil therapy, Metformin therapy, or a recommendation for weight loss are being considered as the next best steps in managing his condition. Planning: While all options proposed have considerations on managing elevated triglyceride levels or contributing factors, our main focus should be on immediate resolution of the presenting serious illness, acute pancreatitis, suspected due to severe epigastric pain, nausea, and elevated amylase levels. Acute pancreatitis in this case is likely secondary to hypertriglyceridemia, a known cause of the disease. Therefore, we need to prioritize therapies that reduce triglyceride levels acutely. Clinical Reasoning: Considering the described methods for managing triglyceride levels, Gemfibrozil therapy, a fibrate, is known to lower triglyceride levels effectively over time and could be a solid consideration for chronic management. However, in this acute setting, a more prompt reduction might be necessary to control the driving cause of his acute pancreatitis. Apheresis can cause a rapid reduction in triglyceride levels and might be more suitable in this acute setting. Other options, although useful in managing dyslipidemia and weight, do not offer the acute benefits needed in this situation. Concluding: Given the acuity of the patient's symptoms and the severity of his hypertriglyceridemia, prompt intervention is essential to prevent further complications from his pancreatitis. Therefore, therapies that offer immediate reduction of triglyceride levels would be more suitable in the initial management of this case. Chronic options can be considered at a later time when the acute crisis is resolved. Response: Given the presented facts, apheresis (choice A) would be the most appropriate next step in managing this patient. It offers the quickest reduction in triglyceride levels which is essential in managing his suspected hypertriglyceridemia-induced acute pancreatitis. This is not negating the potential benefits of other options such as Gemfibrozil therapy, but in the setting of an acute crisis, immediate resolution of the causative factors should take precedence.

**AI_D:** RESTATING THE QUESTION: This query seeks to identify the best course of action in managing a hospitalized 48-year-old male patient who presented with severe upper abdominal pain, vomiting, and nausea. The patient’s discomfort becomes less severe when he bends forward, might be a characteristic of pancreatitis. His lab results show significantly elevated cholesterol and triglyceride levels. PLANNING: The current situation is of a patient suffering from pancreatitis most likely due to hypertriglyceridemia (triglyceride level >1000 mg/dL). Hypertriglyceridemia can cause acute pancreatitis as the triglycerides in the blood are hydrolyzed by pancreatic lipase into toxic free fatty acids. The management depends on the severity of the pancreatitis and the serum triglyceride level. CLINICAL REASONING: Choices (A) Apheresis, (C) Gemfibrozil therapy, (D) Metformin therapy, and (E) Recommendation for weight loss are potential options for hypertriglyceridemia management. However, considering the time frame, immediate intervention, such as pharmaceutical therapy, may be the likely step forward. Apheresis is generally reserved for very severe cases of hypertriglyceridemia that haven't responded to other treatments and is unlikely to be the first choice here without more information. We've administered intravenous fluids and provided pain relief, resulting in decreased symptoms, but haven't yet addressed the root issue: the elevated triglycerides. CONCLUDING: Among the given choices for managing hypertriglyceridemia (elevated blood triglycerides), Ezetimibe, and Metformin therapy mechanisms are mainly concentrated on low-density lipoprotein (LDL) cholesterol reduction and blood sugar control, respectively, not directly on lowering triglycerides. Apheresis is an extracorporeal technique, mainly used in critically ill patients with complications like refractory pancreatitis, where the triglyceride level does not decrease despite conventional management. In light of the immediate need to lower triglyceride levels, Gemfibrozil, a fibrate, appears to be the best option. Fibrates are effective in reducing serum triglyceride levels by increasing the activity of lipoprotein lipase, an enzyme that breaks down triglycerides. RESPONSE: So in this scenario, where a prompt reduction of triglyceride levels is required, 'Gemfibrozil therapy' (Option C) would be the most appropriate next step in management, considering it's a first-line pharmaceutical therapy for high triglyceride levels.

**AI_B:** RESTATING THE QUESTION: The question outlines a clinical scenario presenting a 48-year-old man hospitalized with acute abdominal pain, nausea, and vomiting. The patient has been given intravenous fluids and pain relief and is improving. His significant laboratory findings include high cholesterol and severely elevated triglycerides. The question seeks to identify the most appropriate next step in management. Options presented include apheresis, ezetimibe therapy, gemfibrozil therapy, metformin therapy, or a recommendation for weight loss. PLANNING: Key aspects of this question include the patient's current symptoms, significant laboratory results, and the potential contributions of each potential management option. Given that the patient seems to be suffering from acute pancreatitis due to hypertriglyceridemia (triglyceride level >1000 mg/dL), it is worth considering which amongst the therapeutic options could best lower his triglyceride levels rapidly and prevent further pancreatic injury. CLINICAL REASONING: Acute pancreatitis due to severe hypertriglyceridemia requires rapid reduction in serum triglycerides to prevent ongoing lipotoxic injury to the pancreas. Therefore, the most clinically effective intervention would involve a method proven to quickly lower triglycerides. Fibrates such as gemfibrozil can reduce triglyceride levels rather significantly, but it generally takes several days to start working. Ezetimibe and metformin aren't primarily used for reducing triglycerides. Weight loss, while beneficial in the long term, would have no immediate effects on triglyceride levels. Alternatively, apheresis is a process that mechanically removes triglycerides from the blood and can be an effective treatment in severe or refractory cases. CONCLUDING: Considering the overtly elevated triglyceride levels and the acute presentation of pancreatitis in this patient, the immediate intervention to avoid further pancreatic injury is the key. Gemfibrozil, usually the primary choice for triglyceride reduction, may take days to act, which could lead to sustained pancreatic injury. RESPONSE: Therefore, in this particular clinical scenario, given the urgency to reduce the triglyceride level quickly, the most suitable immediate intervention should be (A) Apheresis. While gemfibrozil may be considered a more common first-line treatment, the severity and acute nature of the patient's condition call for more immediate measures. Apheresis, due to its rapid action, fulfills this requirement. On a parallel path, lifestyle modification like weight loss and long-term medication adjustments could be considered as part of long term management.

**AI_A:** RESTATING THE QUESTION: The case we are examining is a 48-year-old man, with no known serious previous medical history, who has been admitted to the hospital with severe abdominal pain and nausea/vomiting. He has no personal history of smoking or high-risk alcohol consumption. His physical and blood examination findings suggest inflammation, possibly within the pancreas, and very high blood triglyceride levels, along with a high cholesterol level. Our task is to determine the most appropriate next step in the management of this patient's health condition from the listed options. This will involve understanding the patient's symptoms, relaying clinical knowledge on each treatment option, and evaluating the beneficial impact they could have in this particular scenario. PLANNING: Central to this case are our patient's severe abdominal pain, high fever, and high levels of triglycerides in the blood. Looking at these symptoms collectively, they suggest acute pancreatitis, specifically, hypertriglyceridemia-induced pancreatitis. Hypertriglyceridemia refers to a state of abnormally high levels of triglycerides in the blood and can result in the inflammation of the pancreas. To approach his symptoms, we could assume conservatively and treat his high fever and pain first, maintaining hydration and supportive care, and then move on to his hypertriglyceridemia later. Or, we could tackle his very high triglyceride levels head on with a more urgent approach as they're potentially the root of his symptoms. Given the potential life-threatening sequelae of untreated hypertriglyceridemia-induced pancreatitis, the latter alternate assumption that urgent management of hypertriglyceridemia as a priority seems appropriate to pursue. CLINICAL REASONING: Let's relate this to the options. Apheresis, which involves the physical removal of parts of the blood, is an effective way of reducing very high triglyceride levels quickly but it is invasive and generally not the first-line option. Ezetimibe therapy is a drug that helps reduce blood cholesterol levels, not specifically triglyceride levels hence it is not relevant in this case. Gemfibrozil therapy is a treatment option that significantly lowers triglyceride levels, and it is often used in cases like this. Metformin therapy is primarily used for managing diabetes, and it does not address the patient's current needs. Last, recommendation for weight loss is a long-term prevention strategy, helpful for overall management but not the immediate resolution of an acute issue. CONCLUDING: Therefore, considering our patient's current condition and the need for a quick reduction in his triglyceride levels to manage the acute pancreatitis, the administration of Gemfibrozil therapy appears to be the most appropriate immediate next step in this scenario. It offers a balance between a quick response to his condition and a safer, less invasive approach than Apheresis. RESPONSE: (C) Gemfibrozil therapy.

**AI_C:** RESTATING THE QUESTION: The patient, a 48-year-old man, came to the hospital with acute severe abdominal pain, nausea, and vomiting. He shows signs of severe distress, with a high pulse rate and fever. He has physical findings suggestive of a grave digestive tract ailment such as decreased breath sounds, dullness to percussion and tenderness in the upper abdominal quadrants. His laboratory tests are remarkable for elevated urea nitrogen and drastic elevation of triglycerides to 1500 mg/dL, which are suggestive of an acute pancreatitis likely secondary to severe hypertriglyceridemia. Given his symptoms have somewhat subsided following initial supportive drugs and fluids, we need to determine the next best step in management from a list of options, namely, apheresis, ezetimibe therapy, gemfibrozil therapy, metformin therapy, and weight loss recommendation. PLANNING: The alternate assumption would be that his pancreatitis is exacerbated by severe hypertriglyceridemia, which is often a precipitating factor. Considering the triglycerides are drastically high, bringing down the triglycerides levels would be clinically prioritized. The optimal management can be debated between an immediate but infrequently used method like apheresis and a widely accepted first-line drug, namely, a fibrate like gemfibrozil. The options of ezetimibe, metformin, and weight loss do not directly help in acutely lowering the triglycerides and would not be directly of help to the current situation. CLINICAL REASONING: Apheresis is an approach where blood is filtered to remove certain components, often used in situations like drug overdoses or severe lipid abnormalities that are not getting under control with medications. In context, apheresis would rapidly reduce the triglyceride levels in the blood, which might alleviate ongoing pancreatic inflammation resulting from triglyceride metabolism. On the other hand, gemfibrozil, a fibrate, helps lower triglyceride levels directly and is often the first-line management in cases of high triglycerides. Therefore, clinically deciding between the two hinges on whether the potency of the immediate action of apheresis is needed here or whether the more common routine drug therapy is sufficient. CONCLUDING: Given this patient is suffering from acute pancreatitis, most likely instigated by hypertriglyceridemia, rapid control of the triglycerides is paramount to reducing ongoing pancreatic damage. Although gemfibrozil is typically the first line of management for high triglycerides, in this scenario of acute pancreatitis with significant hypertriglyceridemia, an immediate robust reduction in triglycerides may be needed. However, apheresis is a procedure typically used when drug treatments aren't enough. RESPONSE: The case can be evidently argued for the initiation of gemfibrozil therapy (C). While the immediate significant reduction of triglycerides with apheresis seems attractive, gemfibrozil is a well-established, effective first-line medication for high triglycerides, and the patient's pancreatitis has shown improvement with initial management. Further supportive management alongside gemfibrozil therapy can be anticipated to bring stable amelioration and control of the patient's situation. Given the context, the next appropriate step would be gemfibrozil therapy.

**Facilitator** **[Round 4]:** Summary: AI_A, AI_C, and AI_D suggest that Gemfibrozil therapy is the most appropriate next step in managing the patient's condition. They argue that while Apheresis can rapidly reduce triglyceride levels, it is invasive and not typically the first-line option. Gemfibrozil, a fibrate, is often used in cases like this and offers a balance between a quick response to the patient's condition and a safer, less invasive approach. On the other hand, AI_B and AI_E argue that Apheresis is the most suitable immediate intervention. They believe that due to the severity and acute nature of the patient's condition, immediate measures are required. Apheresis, due to its rapid action, fulfills this requirement. Key Differences: The key differences in the council members' perspectives lie in their approach to managing the patient's condition. AI_A, AI_C, and AI_D prioritize a less invasive, first-line treatment option (Gemfibrozil therapy) that is effective in reducing triglyceride levels, albeit not as rapidly as Apheresis. They believe that the patient's condition can be managed effectively with this approach, given that his pancreatitis has shown improvement with initial management. In contrast, AI_B and AI_E emphasize the need for immediate intervention due to the acute nature of the patient's condition. They argue that Apheresis, despite being more invasive, is necessary to prevent further complications from the patient's pancreatitis. Question for Council Members: Given the differing perspectives, a clarifying question could be: "How do you weigh the need for immediate intervention (via Apheresis) against the potential risks and invasiveness of this procedure, compared to a less invasive but slower-acting first-line treatment (Gemfibrozil therapy)?". Based on your preceding response, how would you answer the original question: [A 48-year-old man is admitted to the hospital 2 hours after the onset of severe abdominal pain, nausea, and vomiting. The pain radiates to the back but improves when he bends forward. He has no history of serious illness and takes no medications. He does not smoke cigarettes. He drinks two 12-oz beers weekly. He appears restless. He is 173 cm (5 ft 8 in) tall and weighs 90 kg (198 lb); BMI is 30 kg/m² . Temperature is 38.0°C (100.4°F), pulse is 110/min, respirations are 20/min, and blood pressure is 110/60 mm Hg. On pulmonary examination, decreased breath sounds are heard at the lung bases; there is dullness to percussion. Abdominal examination shows distention and severe tenderness to palpation of the epigastrium and left upper quadrant. Laboratory studies show: Hemoglobin: 13.5 g/dL Serum Urea nitrogen: 30 mg/dL Glucose: 120 mg/dL Creatinine: 1.2 mg/dL Cholesterol: 300 mg/dL Triglycerides: 1500 mg/dL Total bilirubin: 1.1 mg/dL Amylase: 300 U/L Administration of intravenous fluids and analgesics is begun. The patient’s abdominal pain, nausea, and vomiting decrease. Which of the following is the most appropriate next step in management? Choose one among the choices below. (A) Apheresis (B) Ezetimibe therapy (C) Gemfibrozil therapy (D) Metformin therapy (E) Recommendation for weight loss]

**AI_B:** RESTATING THE QUESTION: The inquiry posed is regarding the most suitable next step to take in the management of a 48-year-old physically restless man who presented with severe abdominal pain, nausea, vomiting and decreased breath sounds at the lung bases. After his symptoms declined following the administration of intravenous fluids and analgesics, the next decision concerns the most appropriate treatment path given his laboratory results, specifically, considerable levels of triglycerides. PLANNING: The assumption would be that this patient has pancreatitis inflicted by hypertriglyceridemia due to the clinical scenario. The factors to consider feature pain alleviation, the implementation of apheresis, various types of pharmaceutical interventions (e.g., ezetimibe, gemfibrozil, metformin), as well as a recommendation for weight loss. CLINICAL REASONING: Based on his clinical presentation and laboratory results, the patient is likely suffering from acute pancreatitis due to severe hypertriglyceridemia, suggested by the presence of severe epigastric pain radiating to the back, nausea, vomiting, and elevated serum amylase. The initial priority in management befalls "pain control, fluid resuscitation, electrolyte repletion, and nutritional support". Given the laboratory results, the concentration of triglycerides warrants high attention. While, apheresis is effective in rapidly reducing triglyceride levels, it's usually reserved for when medical therapy fails. In the light of pharmaceutical approaches, gemfibrozil, a fibrate, could be most fitting. It works primarily by reducing hepatic triglyceride production along with a small increase in lipoprotein lipase activity, which accelerates the clearance of triglycerides from the bloodstream. Ezetimibe operates by reducing intestinal cholesterol absorption, but has little to no effect on triglycerides. Metformin is essentially used in managing type 2 diabetes mellitus but does not significantly impact triglyceride concentrations. Lastly, advising weight loss is essential for long-term management, but not as an immediate intervention in this scenario. CONCLUDING: Considering the immediacy and mechanisms of action, the preference leans toward implementing gemfibrozil therapy rather than resorting to an invasive procedure like apheresis or using other therapeutics which lack explicit impacts on the patient's high triglyceride level. RESPONSE: Therefore, the most appropriate next step in management is (C) Gemfibrozil therapy.

**AI_C:** (1) RESTATING THE QUESTION: This query involves determining the best management strategy for a 48-year-old male patient who has been admitted to hospital with severe abdominal pain, nausea and vomiting – symptoms which seem indicative of pancreatitis. The patient's condition has shown some improvement following administration of intravenous fluids and analgesics, but the next step in his treatment plan needs to be determined. Notably, the patient's laboratory studies reveal significantly elevated levels of serum triglycerides and cholesterol, both of which are relevant to the potential treatment options listed. (2) PLANNING: The assumption guiding this question is to identify the treatment plan which can manage the current acute condition and the underlining triglycerides and cholesterol abnormalities. Alternate assumptions would include potentially looking at managing the patient's weight, considering his BMI is 30 kg/m², or considering long-term management of his lipid profile. However, given the acute nature of his condition (presumably acute pancreatitis caused by hypertriglyceridemia), the most crucial approach to prioritize now should be managing the hypertriglyceridemia, which can result in a resolution of the acute pancreatitis. (3) CLINICAL REASONING: The patient's presentation and lab test results strongly suggest acute pancreatitis secondary to hypertriglyceridemia. Hypertriglyceridemia is an established cause of acute pancreatitis when levels are >1000 mg/dL, as in this patient. First-line management is typically fasting, aggressive hydration, and analgesia, all of which this patient has received with some improvement. To manage the hypertriglyceridemia, several treatments are in consideration, including fibrate therapy (gemfibrozil), as they can drastically reduce serum triglycerides by increasing lipoprotein lipase activity and reducing hepatic production of very-low-density lipoprotein (VLDL). Apheresis can rapidly reduce triglycerides but is more invasive and not typically first-line. Ezetimibe blocks intestinal absorption of cholesterol, but this would not significantly affect serum triglycerides. Metformin is used in the management of type 2 diabetes and can have a modest effect on lowering triglycerides, but is not primarily indicated here. Weight loss would be a good long-term goal for this patient, but it would not have an immediate impact on his triglyceride levels or acute pancreatitis. (4) CONCLUDING: After considering the clinical scenario and contemplating various assumptions, the patient's acute pathology should be considered the priority. An approach that aims to rapidly reduce the serum triglycerides would be the best immediate method to manage the patient's current crisis. (5) RESPONSE: Based on the combination of clinical context and the mechanisms of the therapeutics in question, the most suitable next step in managing this patient would likely be the initiation of Gemfibrozil therapy (Choice C). This would aim to rapidly decrease serum triglyceride levels, thereby alleviating the patient's acute pancreatitis symptoms, while also being a less invasive approach compared to apheresis.

**AI_A:** (1) RESTATEMENT: The explained scenario describes a 48-year-old man who has been admitted to the hospital due to severe abdominal pain, nausea, and vomiting. His abdominal pain radiates to the back but improves when he bends forward. This patient has no history of serious illnesses and does not consume any medications. Lab results suggest that he has high triglycerides and cholesterol levels. With the initial management, his abdominal pain has decreased. We are asked to identify the most optimal next steps in his management. (2) PLANNING: The possible assumptions include that the patient's acute abdominal pain is caused by high triglyceride levels, causing acute pancreatitis. An alternative assumption could be a gallstone pancreatitis given the patient's BMI. However, given that the patient has enormously elevated triglyceride levels (1500 mg/dL), which can be the cause for acute pancreatitis, we should prioritize the management of his hypertriglyceridemia acutely. (3) CLINICAL REASONING: The key clinical finding is the patient’s elevated triglyceride level, which may lead us to consider that this patient might be suffering from acute pancreatitis caused by hypertriglyceridemia. Severe hypertriglyceridemia can cause pancreatitis as excessive chylomicron production can lead to obstruction and inflammation of the pancreatic ducts. This results in excessive activation of trypsin leading to auto-digestion of the pancreas by pancreatic enzymes leading to pancreatitis. Pain radiating to the back and an improvement when bending forward are congruent paucity of symptoms seen in pancreatitis, further bolstering the potential diagnosis. The presence of decreased breath sounds and dullness to percussion at the lung bases is reminiscent of pleural effusion, which can occur due to pancreatitis creating an inflammatory exudate that seeps into the pleural space. (4) CONCLUSION: Based on the clinical reasoning of this patient potentially suffering from acute pancreatitis due to hypertriglyceridemia, the aim of the next management step should focus on reducing serum triglyceride levels. (5) RESPONSE: Amongst the choices given, the most appropriate next step in this patient's management would be (C) Gemfibrozil therapy. Gemfibrozil is a fibrate that works primarily by reducing triglyceride levels (up to 50%) by inhibiting lipolysis and reducing the hepatic extraction of free fatty acids, which are the precursors of triglycerides. The other interventions (including Apheresis, Ezetimibe, and Metformin), although they might have some role in managing hypertriglyceridemia and hypercholesterolemia in the long term, are not the best immediate choices. Apheresis has the swiftest action but is invasive and is not the first-line treatment. Ezetimibe has a limited impact on triglyceride levels, and Metformin, while beneficial in managing associated insulin resistance, acts slowly and won't address the acute need to lower triglyceride levels swiftly. Weight loss is of course, mandatory in overall management, but it is a long-term management strategy not suited for acute scenarios. Hence, Gemfibrozil is the most logical and effective immediate next step in management in this scenario.

**AI_D:** RESTATING THE QUESTION: The inquiry calls for an evaluation of the appropriate next step in a patient's management. The patient, a 48-year-old man, presented with severe abdominal pain, nausea, and vomiting. His abdominal discomfort radiates to the back, but bending forward produces relief. The patient has a record of good general health and no medication usage, no smoking history, and moderate alcohol consumption equivalent to two 12-oz beers a week. Clinical findings include restlessness, BMI of 30 kg/m² indicative of obesity, fever, tachycardia, normal blood pressure, decreased breath sounds and dullness to percussion at the lung bases, and marked tenderness at the epigastrium and left upper quadrant. Laboratory results reveal high serum levels of urea nitrogen, cholesterol, triglycerides, and amylase. Following intravenous hydration and pain relief, the patient's clinical condition improved. The query demands an evaluation of whether to institute (A) Apheresis, (B) Ezetimibe therapy, (C) Gemfibrozil therapy, (D) Metformin therapy, or (E) Recommendation for weight loss as a follow-up management step. PLANNING: The primary assumption in this case is that the patient's symptoms and laboratory findings, particularly the elevated amylase levels and severe epigastric pain radiating to the back, suggest acute pancreatitis. The likely triggering factor might be hypertriglyceridemia, as indicated by the patient's significantly high serum triglyceride level. Notably, acute pancreatitis can occur when serum triglyceride levels exceed 1000 mg/dL. However, the possibility that the pain could also arise from gallstone disease should not be dismissed entirely, even though the information provided does not suggest this. An alternate assumption, therefore, is that management should aim to rapidly lower the triglyceride levels while addressing the abdominal pain and associated symptoms. CLINICAL REASONING: The elevated amylase level and the pattern of abdominal pain point towards acute pancreatitis. An important cause of acute pancreatitis – particularly in the context of this patient's high triglyceride level – can be hypertriglyceridemia, which tends to increase the viscosity of the pancreatic juices leading to their accumulation and hence the onset of pancreatitis. It becomes critical then to rapidly lower the triglyceride levels, while managing the pain and other digestive symptoms. Given the patient’s elevated cholesterol and triglycerides, the initiation of a therapy that lowers lipid levels is warranted. In this setting, drug choices like Ezetimibe, Gemfibrozil, or even recommending weight loss could be effective. Ezetimibe prevents the absorption of cholesterol in the small intestine and is commonly utilized to treat hyperlipidemia, but its effect on serum triglyceride levels is not as substantial. Apheresis is a procedure that can rapidly reduce serum triglyceride levels and could be useful in this setting, but it carries a greater risk due to its invasive nature. CONCLUDING: Gemfibrozil, a fibrate, is used mainly to decrease high triglyceride levels. It is typically considered a first-line option due to its effectiveness and safety profile. Given the patient's acute pancreatitis likely triggered by extreme hypertriglyceridemia, rapid reduction of triglycerides is the key to preventing further pancreatic damage. Thus, Gemfibrozil therapy might be the most effective way to achieve this. RESPONSE: In light of the information given and the predominating theoretical approach, the most appropriate next step in managing this patient's condition would be to institute (C) Gemfibrozil therapy, given its efficacy in effectively lowering high triglyceride levels whilst concurrently managing the symptoms of acute pancreatitis.

**AI_E:** Restating the Question: Let's summarize this case. We are presented with a 48-year-old man who arrived at the hospital with severe abdominal pain, nausea, and vomiting that began two hours ago. The discomfort extends to the back but is somewhat alleviated by bending forward. The patient has no serious illness history, appears restless, and with the given height and weight, is classified as obese. His physical examination is remarkable for decreased breath sounds and dullness to percussion at the lung bases, distended abdomen, and palpation-induced severe epigastric and left upper quadrant tenderness. His labs reveal elevated serum urea nitrogen, glucose, cholesterol, triglycerides, and amylase. Clinical management has started with the administration of intravenous fluids and analgesics, leading to a decrease in abdominal pain, nausea, and vomiting. Now, we are tasked with determining the most appropriate next step in this patient's management, given choices on apheresis, ezetimibe therapy, gemfibrozil therapy, metformin therapy, or recommendation for weight loss. Planning: Before proceeding with clinical reasoning, there are several assumptions in this case. The patient's symptom profile, examination findings, and laboratory values suggest an episode of acute pancreatitis, potentially triggered by hypertriglyceridemia- this being suggested by the markedly elevated triglycerides levels. While there are other causes for acute episodes, without history of stones, alcohol abuse, or other known risk factors, triglycerides emerge as the prime suspect. Moreover, the presence of hypertriglyceridemia could lead to severe complications such as chylomicronemia syndrome, which may cause pancreatitis, eruptive xanthomas, lipemia retinalis, and mental status changes. However, this patient may not be at an imminent risk of developing this syndrome, or he may already have it, which has not yet been confirmed in the case. Clinical Reasoning: Given the initial response to the measures taken (administration of IV fluids and analgesics), it suggests that therapeutic modality should firstly act to curb the possibility of further pancreatitis trigger and control the lipid abnormality. Inspecting the provided choices, we have apheresis – a procedure that rapidly removes triglycerides, though highly invasive. Ezetimibe therapy, primarily indicated for lowering low-density lipoprotein cholesterol, might not significantly impact triglycerides. Metformin therapy is widely used for controlling blood glucose levels in type 2 diabetes mellitus, while the patient's glucose level is elevated, it's not significant enough to implicate diabetes and crucially metformin doesn't address the hypertriglyceridemia. Weight loss is always recommended in such patients, but it's more of a long-term goal than an immediate intervention. Concluding: With that said, the choice reducing down to apheresis or gemfibrozil therapy. While apheresis can quickly reduce triglycerides, it is typically reserved for cases where more conservative measures, like medications and lifestyle changes, are ineffective. Gemfibrozil, on the other hand, effectively lowers triglycerides by reducing hepatic lipoprotein synthesis and is less invasive. Response: Given all these factors, I would argue that the most appropriate next step in this patient's management should be (C) Gemfibrozil therapy. This medication could balance the need for relatively rapid reduction of triglycerides and the lowered invasiveness as compared to apheresis. It would mitigate the immediate danger while serving as an initial step towards long-term management of the patient's hypertriglyceridemia.

**Facilitator** The consensus statement synthesizes the responses of AI_A, AI_B, AI_C, AI_D, and AI_E as follows: The case presents a 48-year-old man with severe abdominal pain, nausea, and vomiting, which are indicative of acute pancreatitis. His pain radiates to the back but improves when he bends forward. The patient's lab results reveal significantly elevated levels of serum triglycerides and cholesterol. After initial management with intravenous fluids and analgesics, his symptoms have decreased. The next step in his management needs to be determined. The primary assumption is that the patient's acute abdominal pain is caused by hypertriglyceridemia-induced acute pancreatitis. This is supported by the patient's enormously elevated triglyceride levels, which can cause pancreatitis due to excessive chylomicron production leading to obstruction and inflammation of the pancreatic ducts. This results in excessive activation of trypsin leading to auto-digestion of the pancreas by pancreatic enzymes, causing pancreatitis. Given the patient's acute condition, the immediate management should focus on reducing serum triglyceride levels. Among the choices given, the most appropriate next step in this patient's management would be Gemfibrozil therapy. Gemfibrozil is a fibrate that works primarily by reducing triglyceride levels by inhibiting lipolysis and reducing the hepatic extraction of free fatty acids, which are the precursors of triglycerides. Other interventions such as Apheresis, Ezetimibe, and Metformin, while potentially beneficial in managing hypertriglyceridemia and hypercholesterolemia in the long term, are not the best immediate choices. Apheresis is invasive and not the first-line treatment. Ezetimibe has a limited impact on triglyceride levels, and Metformin, while beneficial in managing associated insulin resistance, acts slowly and won't address the acute need to lower triglyceride levels swiftly. Weight loss is a long-term management strategy not suited for acute scenarios. Therefore, based on the clinical context and the mechanisms of the therapeutics in question, the most suitable next step in managing this patient would likely be the initiation of Gemfibrozil therapy. This would aim to rapidly decrease serum triglyceride levels, thereby alleviating the patient's acute pancreatitis symptoms, while also being a less invasive approach compared to apheresis.

**Facilitator** **[Round 1]:** A 48-year-old man is admitted to the hospital 2 hours after the onset of severe abdominal pain, nausea, and vomiting. The pain radiates to the back but improves when he bends forward. He has no history of serious illness and takes no medications. He does not smoke cigarettes. He drinks two 12-oz beers weekly. He appears restless. He is 173 cm (5 ft 8 in) tall and weighs 90 kg (198 lb); BMI is 30 kg/m² . Temperature is 38.0°C (100.4°F), pulse is 110/min, respirations are 20/min, and blood pressure is 110/60 mm Hg. On pulmonary examination, decreased breath sounds are heard at the lung bases; there is dullness to percussion. Abdominal examination shows distention and severe tenderness to palpation of the epigastrium and left upper quadrant. Laboratory studies show: Hemoglobin: 13.5 g/dL Serum Urea nitrogen: 30 mg/dL Glucose: 120 mg/dL Creatinine: 1.2 mg/dL Cholesterol: 300 mg/dL Triglycerides: 1500 mg/dL Total bilirubin: 1.1 mg/dL Amylase: 300 U/L Administration of intravenous fluids and analgesics is begun. The patient’s abdominal pain, nausea, and vomiting decrease. Which of the following is the most appropriate next step in management? Choose one among the choices below. (A) Apheresis (B) Ezetimibe therapy (C) Gemfibrozil therapy (D) Metformin therapy (E) Recommendation for weight loss

**AI_E:** (1) RESTATING THE QUESTION: In this clinical scenario, a 48-year-old male patient presenting with severe abdominal pain, nausea, and vomiting was admitted to the hospital. His symptoms seem to be centrally located and seem to alleviate when he leans forward. His test results revealed elevated serum urea nitrogen, glucose, cholesterol, and especially high triglyceride levels. Although he is started on intravenous fluids and analgesics, which reduce his symptoms, we are tasked with identifying the most suitable subsequent step in management. (2) PLANNING: Considering the presenting symptoms and their alleviation by forward-leaning, the significantly high triglyceride levels, and the patient's body mass index and lifestyle, the following clinical assumptions can be made. The symptoms might be indicative of pancreatitis, which could be due to hypertriglyceridemia. It's important to note here that hypertriglyceridemia is a known risk factor for acute pancreatitis when levels exceed 1000 mg/dL, and the dullness heard on percussion at the lung bases can indicate pleural effusions, a complication of pancreatitis. For managing acute pancreatitis resulting from hypertriglyceridemia, options range from symptomatic treatment, weight loss recommendations, lipid-lowering medications to more invasive methods such as apheresis. It's crucial to prioritize immediate management strategies that befit the severity of the condition and decrease the potential for complications. (3) CLINICAL REASONING: When the diagnosis is pancreatitis, symptomatic treatment consists of analgesics for pain relief and intravenous fluids to prevent dehydration from vomiting. These steps have already been undertaken. Next, addressing the likely cause of the pancreatitis, which is hypertriglyceridemia in this case, is pivotal. Apheresis could serve as an immediate strategy to reduce triglyceride levels dramatically, and could thus lower the risk of pancreatic inflammation. This intervention outweighs starting the patient on drugs at first, which can take time to effectively reduce triglyceride levels. (4) CONCLUDING: Although long-term management should incorporate lifestyle modifications and medication adjustments to address the patient's hypertriglyceridemia, immediate intervention requires therapeutic measures that rapidly lower the triglyceride levels to mitigate further inflammatory damage to the pancreas. (5) RESPONSE: As a result, the most appropriate next step in treating this patient would be (A) Apheresis, ensuring a rapid reduction of triglyceride levels that would attenuate the risk of persistent or worsening pancreatitis.

**AI_A:** RESTATING THE QUESTION: The information provided outlines a case about a 48-year-old man who presented at the hospital with symptoms including severe abdominal pain, nausea, and vomiting. Despite no past history of serious illness or medication intake, he shows signs of possible systemic distress, indicated by his feverish symptoms, increased pulse rate, restlessness and decreased breath sounds at the lung bases. There is also severe tenderness upon abdominal palpation around the epigastrium and left upper quadrant. His laboratory results reveal notably high levels of cholesterol and triglycerides. Following administration of intravenous fluids and analgesics, his symptoms have decreased. The question demands an appropriate next step of management from a set of given choices: Apheresis, Ezetimibe therapy, Gemfibrozil therapy, Metformin therapy, or a recommendation for weight loss. PLANNING: To solve this case, we must understand and define our terms. Apheresis is a medical procedure that involves removing whole blood from a patient and separating the blood into individual components so the needed element can be removed. Ezetimibe is a medication used to lower levels of total cholesterol and low-density lipoprotein (LDL, or "bad cholesterol") in the blood. Gemfibrozil is a lipid-regulating medication used to lower high levels of triglycerides in patients who are at risk of pancreatitis (inflammation of the pancreas). Metformin is an oral medication that helps control blood sugar levels and is generally used for patients with type 2 diabetes. Lastly, making a recommendation for weight loss is a non-medical intervention that involves lifestyle modifications to decrease body weight, often used to combat health conditions related to overweight or obesity, such as diabetes, hypertension, and high cholesterol levels. CLINICAL REASONING: Given the findings, it's clear that the patient's symptoms were not random, but likely related to a diagnosable medical issue. The severe abdominal pain that radiates to the back, nausea, vomiting, and elevated levels of amylase and lipids suggest a possible acute pancreatitis. Acute pancreatitis can be triggered by very high levels of blood triglycerides (above 1000 mg/dL). The clinical picture along with the lab findings is highly suggestive of pancreatitis secondary to hypertriglyceridemia. The goal of treatment in this case would be to lower the triglyceride levels rapidly to reduce the risk of further attacks. CONCLUDING: Taking into account the patient's signs, symptoms, physical examination findings, and the laboratory results, as well as the definition of each proposed course of action, it appears necessary to prioritize a treatment plan that directly addresses the alarming triglyceride levels. RESPONSE: Therefore, (C) Gemfibrozil therapy is the most appropriate next step in management. This medication helps decrease triglyceride levels and thus can help mitigate risk of further pancreatitis episodes.

**AI_C:** (1) RESTATING THE QUESTION: A middle-aged man is admitted to the hospital due to sudden severe abdominal pain, nausea, and vomiting. Although he doesn't have a history of serious illnesses, his elevated physiological indicators suggest he may have a health issue. He is slightly overweight at 90 kg with a BMI of 30 kg/m², and his laboratory studies show increased serum urea nitrogen, glucose, creatinine, cholesterol, triglycerides, and amylase. His current treatment has been the administration of intravenous fluids and analgesics, which reduced his symptoms. The query now is to determine the most appropriate next step for the patient's management among apheresis, ezetimibe therapy, gemfibrozil therapy, metformin therapy, or a weight loss regimen. (2) PLANNING: Upon evaluating the patient's case and the provided management choices, it's noticeable that most choices correspond to conditions related to metabolic syndrome and hyperlipidemia. However, the cause of sudden severe abdominal pain, nausea, vomiting, particularly pain radiating towards the back and improving with forward bending, and paired with raised amylase level, the symptoms hint towards acute pancreatitis. High cholesterol and triglycerides, found in the lab results, can correlate directly to acute pancreatitis through pancreatitis secondary to hypertriglyceridemia. (3) CLINICAL REASONING: To support the assumption of acute pancreatitis due to hypertriglyceridemia, the clinical findings must be correlated further. Acute pancreatitis is an inflammatory condition of the pancreas, which usually presents with sudden onset, severe abdominal pain that radiates to the back, nausea, and vomiting - symptoms matching our patient's presentation. On investigating the patient's laboratory results, it's observed that his serum triglyceride levels are vastly elevated (1500 mg/dL). Pancreatitis secondary to hypertriglyceridemia usually occurs when the serum triglyceride levels exceed 1000 mg/dL. Abnormally high triglyceride levels increase the blood's viscosity, promoting sludging and ischemia, and the formation of toxic free fatty acids via the action of pancreatic lipase, which leads to an acute inflammatory response in the pancreas (ie, pancreatitis). (4) CONCLUDING: Conclusively, a severe elevation in the serum triglyceride level (>1000 mg/dL) is a well-recognized cause of acute pancreatitis. Immediate measures to reduce the serum triglyceride levels are necessary to decrease the extent of pancreatitis and prevent occurrence. Weight reduction, although beneficial in the long run, is not an immediate remedy. Antihyperglycemic treatment (metformin) or cholesterol-absorption inhibitor (ezetimibe) are not specific for reducing severe hypertriglyceridemia. (5) RESPONSE: Based on the clinical scenario and the available choices, the most appropriate next step in the management of this patient is to initiate (C) Gemfibrozil therapy. Gemfibrozil is a fibric acid derivative that effectively reduces serum triglyceride levels. Fibric acid derivatives function by upregulating lipoprotein lipase, leading to increased hydrolysis of triglycerides, thereby ultimately reducing the serum triglyceride levels.

**AI_D:** RESTATING THE QUESTION: A 48-year-old man has been admitted to the hospital with acute symptoms of severe abdominal pain, nausea, and vomiting. The abdominal pain radiates to his back but somewhat improves when he bends forward. He has no previous history of a serious illness, takes no medications, doesn't smoke, but consumes beer moderately. He is overweight with a BMI of 30 kg/m² and appears restless. On lung examination, reduced breath sounds are noted, and his abdominal exam reveals distention and severe tenderness in the epigastric region and left upper quadrant. His lab results indicate significantly elevated serum cholesterol and triglycerides levels along with an elevated amylase level. After receiving intravenous fluids and analgesics, his symptoms have improved. The question is seeking the most appropriate next step in managing this patient's case. PLANNING: To interpret these observations and formulate a hypothesis, we should consider the following assumptions: (1) His abdominal pain radiating to the back, worsened by positioning, hints toward a pathology involving the pancreas; (2) Elevated serum triglyceride levels provide a link to his potential case of pancreatitis since hypertriglyceridemia above 1000 mg/dL can induce acute pancreatitis; (3) The patient's BMI indicates obesity, which could contribute to his abnormal lipid profile; (4) The elevated amylase level supports pancreatitis diagnosis; (5) The therapeutic approach should focus on alleviating his acute condition while managing his underlying dyslipidemia. CLINICAL REASONING: Acute pancreatitis is typically a direct result of gallstones or alcohol, but hypertriglyceridemia can also cause it in some patients. Establishing this association is vital as it informs the immediate and long-term management of the condition. The response to treatment thus far – relief of symptoms with intravenous fluids and analgesics – aligns with the standard care for acute pancreatitis. The next step would require managing the underlying trigger. Given the patient's non-existent history of gallstones or significant alcohol intake, the considerably high triglycerides level (1,500 mg/dL) appears to be the most likely precipitant. Understanding this, the next step should focus on rapid reduction of these triglycerides to prevent further bouts of pancreatitis. CONCLUDING: In terms of options, (A) apheresis (a procedure that separates and removes potentially harmful substances from the blood), might not be essential at this stage; (B) Ezetimibe therapy, decreases cholesterol absorption but has little effect on triglycerides; (D) Metformin therapy, is primarily used in the management of type 2 diabetes and not specifically meant for triglyceride reduction. (E) Recommendation for weight loss is a long-term plan that would not help the immediate situation; therefore it can't be considered the most appropriate next step. RESPONSE: Therefore, the choice that appears most suited to address the immediate issue of hypertriglyceridemia is (C) Gemfibrozil therapy, which can effectively lower triglycerides level in the blood and prevent future episodes of hypertriglyceridemia-induced pancreatitis. This should be started along with dietary modifications to manage his dyslipidemia in the long term.

**AI_B:** RESTATING THE QUESTION: A 48-year-old male patient with a BMI of 30 kg/m², presenting a recent episode of severe abdominal pain, nausea, vomiting, and disturbed renal function (elevated serum urea nitrogen and normal creatinine), is under consideration for further treatment steps. Along with pain, his labs show abnormally high levels of cholesterol, triglycerides, and amylase. Given his symptoms have improved after initial intravenous fluids and analgesics, the hospital staff wonders which of the options (apheresis, ezetimibe therapy, gemfibrozil therapy, metformin therapy, or weight loss recommendation) is best suited for this patient's management. PLANNING: Identifiable assumptions are firstly, the cause of the patient's abdominal pain might be due to acute pancreatitis given the nature of the pain and the elevated level of amylase. Secondly, pancreatitis in this patient might be due to hypertriglyceridemia since his triglycerides level is found to be very high. Therefore, managing the underlying hypertriglyceridemia should be a priority and the most relevant management choices would be: (A) Apheresis - normally used for lipid disorders that are resistant to diet and maximum tolerated lipid-lowering medication. (B) Ezetimibe therapy - a cholesterol absorption inhibitor used for managing primary hypercholesterolemia (C) Gemfibrozil therapy - a fibrate, usually used to reduce triglycerides levels in patients with severe hypertriglyceridemia (D) Metformin therapy - generally used for type 2 diabetes treatment (E) Recommendation for weight loss. CLINICAL REASONING: The patient's clinical profile and lab results point towards acute pancreatitis given the severe, radiating abdominal pain that is reduced with leaning forward, the presence of nausea and vomiting, and elevated serum amylase levels. Acute pancreatitis can have several causes, one of them being hypertriglyceridemia. His extraordinarily high triglyceride levels (> 1500 mg/dL) might have precipitated this acute episode. It's reasonable to assume reducing this risk factor should be a priority in his management. This rules out options like (B) Ezetimibe therapy which focuses on cholesterol, not triglycerides and (D) Metformin therapy which is primarily for managing blood glucose levels in diabetes. (E) Recommendation for weight loss, though beneficial for overall health and management of hyperlipidemia, won't offer immediate benefit required in this case. Among the remaining options, (A) Apheresis and (C) Gemfibrozil therapy are both effective in managing hypertriglyceridemia. Apheresis is an invasive procedure, which can quickly reduce the levels of triglycerides but does not prevent their re-accumulation and is not a standard treatment for primary hypertriglyceridemia. Gemfibrozil therapy, on the other hand, is a fibrate which helps reduce serum triglycerides levels and is often used for severe hypertriglyceridemia. CONCLUDING: Given the evidence, it seems logical to opt for the less invasive option that offers control of triglycerides levels, which in turn, will help in managing the suspected acute pancreatitis belived to be because of hypertriglyceridemia in this patient. RESPONSE: Thereby, the most appropriate next step in the management of this patient would likely be (C) Gemfibrozil therapy.

**Facilitator** **[Round 2]:** Summary: AI_A, AI_B, AI_C, and AI_D all agree that the patient's symptoms and lab results suggest acute pancreatitis due to hypertriglyceridemia. They all recommend Gemfibrozil therapy as the most appropriate next step in management, as it directly addresses the high triglyceride levels that likely triggered the pancreatitis. They also agree that other options like Ezetimibe therapy, Metformin therapy, and weight loss recommendation are not suitable immediate interventions for this case. However, AI_E differs in opinion. While AI_E also identifies the patient's condition as acute pancreatitis due to hypertriglyceridemia, they recommend Apheresis as the most appropriate next step. AI_E argues that Apheresis would provide a more immediate reduction in triglyceride levels, thus reducing the risk of further pancreatic inflammation. Key Differences: The key difference in perspectives lies in the recommended immediate intervention for the patient's hypertriglyceridemia. AI_A, AI_B, AI_C, and AI_D advocate for Gemfibrozil therapy, a medication that helps decrease triglyceride levels. They believe this approach is the most suitable given the patient's condition. On the other hand, AI_E recommends Apheresis, a procedure that rapidly reduces triglyceride levels, arguing that this approach would provide a more immediate response to the patient's severe hypertriglyceridemia. Question for Council Members: Given the severity of the patient's hypertriglyceridemia and the risk of further pancreatitis episodes, could you elaborate on why you believe your recommended intervention (Gemfibrozil therapy or Apheresis) is the most effective immediate response?. Based on your preceding response, how would you answer the original question: [A 48-year-old man is admitted to the hospital 2 hours after the onset of severe abdominal pain, nausea, and vomiting. The pain radiates to the back but improves when he bends forward. He has no history of serious illness and takes no medications. He does not smoke cigarettes. He drinks two 12-oz beers weekly. He appears restless. He is 173 cm (5 ft 8 in) tall and weighs 90 kg (198 lb); BMI is 30 kg/m² . Temperature is 38.0°C (100.4°F), pulse is 110/min, respirations are 20/min, and blood pressure is 110/60 mm Hg. On pulmonary examination, decreased breath sounds are heard at the lung bases; there is dullness to percussion. Abdominal examination shows distention and severe tenderness to palpation of the epigastrium and left upper quadrant. Laboratory studies show: Hemoglobin: 13.5 g/dL Serum Urea nitrogen: 30 mg/dL Glucose: 120 mg/dL Creatinine: 1.2 mg/dL Cholesterol: 300 mg/dL Triglycerides: 1500 mg/dL Total bilirubin: 1.1 mg/dL Amylase: 300 U/L Administration of intravenous fluids and analgesics is begun. The patient’s abdominal pain, nausea, and vomiting decrease. Which of the following is the most appropriate next step in management? Choose one among the choices below. (A) Apheresis (B) Ezetimibe therapy (C) Gemfibrozil therapy (D) Metformin therapy (E) Recommendation for weight loss]

**AI_D:** 1) RESTATING THE QUESTION: The query wants to know the most suitable next step in the management of a 48-year old male patient admitted to the hospital with signals indicative of acute pancreatitis and found to have high levels of triglycerides in his blood. Hypertriglyceridemia in this context is an abnormally high concentration of triglycerides in the blood, while acute pancreatitis refers to an inflammation of the pancreas due in part to this patient's elevated triglyceride levels. 2) PLANNING: To address the question appropriately, we need to assume that the patient's high triglyceride levels prompted the acute pancreatitis episode. One plausible alternative assumption is that pancreatitis could have been induced by alcohol consumption or gallstone disease. However, it makes sense to prioritize the hypertriglyceridemia assumption clinically given this patient's majorly elevated triglyceride levels (1500 mg/dL) seen in his lab results. 3) CLINICAL REASONING: Beginning with this assumption of high triglycerides as the cause, our goal would be to abate those levels. This would steer the pancreas away from the potential damaging effects of these fatty substances in the blood, thus curbing any further inflammation. Options like Metformin, a glucose-lowering medication for type 2 diabetes and Ezetimibe, a cholesterol-lowering medication, wouldn’t be most suitable in this case as they target different aspects not specifically tied to the elevated triglyceride levels. 4) CONCLUDING: Gemfibrozil therapy is a better choice for immediate management, since it is a type of fibrate that directly helps lower blood triglyceride levels. Although weight loss is also advisable for the patient in the long run due to his BMI of 30 kg/m², it wouldn't provide an immediate intervention to the acute issue of hypertriglyceridemia-induced acute pancreatitis. Apheresis, a procedure that purifies the blood, is usually reserved for cases with extreme triglyceride levels (>2000 mg/dL) and remains a last resort measure for immediate reduction of triglycerides. 5) RESPONSE: Based on the clinical reasoning above, the most appropriate next step in management of this case should be (C) Gemfibrozil Therapy. This treatment could provide a direct response to the patient's high triglyceride levels, believed to be the underlying cause of his acute pancreatitis.

**AI_A:** RESTATING THE QUESTION: The question concerns a 48-year-old male patient with severe abdominal pain, nausea, and vomiting which seems to result from hypertriglyceridemia-induced acute pancreatitis. To manage his condition, we are given several choices: apheresis, ezetimibe therapy, gemfibrozil therapy, metformin therapy, and recommendation for weight loss. The task is to decide between these options and identify the most appropriate immediate intervention. PLANNING: First, we need to properly consider the situation. The patient's abdominal pain, plus the elevated levels of triglycerides and amylase, converge to indicate acute pancreatitis. His condition’s most likely cause is hypertriglyceridemia, considering that triglyceride levels beyond 1000 mg/dL can precipitate acute pancreatitis. All interventions listed are related to managing triglyceride levels except for the recommendation for weight loss, which addresses his obesity. Apheresis is a procedure to rapidly remove triglycerides from the blood. However, it's not typically the first-line treatment for hypertriglyceridemia. Ezetimibe is a cholesterol absorption inhibitor, and Metformin is an antidiabetic drug - neither are front line drugs for elevated triglycerides. Gemfibrozil, a fibrate, is used primarily to lower triglyceride levels. CLINICAL REASONING: Fibrates are often first-line treatment for patients with very high triglyceride levels, as it can reduce triglycerides by as much as 50%. This makes gemfibrozil an attractive therapeutic option in this situation. If the patient does not respond to pharmacotherapy (i.e., gemfibrozil), or if he has another episode of pancreatitis, the use of plasmapheresis to rapidly reduce the triglyceride level could then be considered. CONCLUDING: Given the emergency nature of acute pancreatitis and the very high triglyceride levels, the immediate goal of any intervention should focus on rapidly lowering triglycerides to prevent further bouts of pancreatitis, as well as managing the current episode. In this context, the recommended intervention (Gemfibrozil therapy) appears to be the most suitable choice, considering its ability to significantly reduce triglyceride levels. RESPONSE: I would choose (C) Gemfibrozil therapy as the most appropriate immediate intervention to lower the patient's very high triglyceride levels and manage his acute pancreatitis. This is notwithstanding the potential consideration of apheresis should the patient not respond to pharmacotherapy or if a recurrence of pancreatitis occurs. Alternatively, recommending weight loss, while valuable in the long term, is not an immediate solution to the immediate problem. Similarly, ezetimibe and metformin therapies, while useful in other contexts, may not be the most effective in aggressively lowering triglycerides in this situation.

**AI_E:** 1) RESTATING THE QUESTION: The question pertains to the management of a 48-year-old man who has been admitted to the hospital with severe abdominal pain, nausea, and vomiting. Data from his physical examination and laboratory results indicate acute pancreatitis, probably attributed to his significantly elevated serum triglycerides, indicated by a level of 1500 mg/dL (normal range is under 150 mg/dL). The question requires us to choose the most appropriate next step in management. 'Apheresis' refers to a medical procedure that involves removing certain substances from the blood. 'Ezetimibe' is a medication used to lower levels of certain fats (like cholesterol) in the blood. 'Gemfibrozil' is another medication used principally to treat dyslipidemia, particularly hypertriglyceridemia. 'Metformin' is a medication used primarily for type 2 diabetes. The final option, 'weight loss', is self-explanatory but it's generally considered as a lifestyle management strategy. 2) PLANNING: One assumption we could make is that the underlying cause of pancreatitis could and should be managed in the long-term without an immediate intervention on the triglyceride levels (examples include considering Gemfibrozil therapy, Ezetimibe therapy, Metformin therapy or weight loss). Alternatively, we could reason that the significantly high triglyceride levels should be corrected promptly during this acute episode (which could be executed via Apheresis). The latter option seems reasonable, considering the high level of the patient's triglycerides might worsen the ongoing inflammation of his pancreas or even provoke more episodes in the immediate future. 3) CLINICAL REASONING: Acute pancreatitis can be caused by several factors, including gallstones, alcohol, hypertriglyceridemia, and others. However, this patient's symptoms, along with the elevated levels of triglycerides and amylase (an enzyme secreted by the pancreas), suggest hypertriglyceridemia-induced acute pancreatitis. Although the other options listed in the management choices could help lower the serum triglyceride or cholesterol levels, only Apheresis can acutely lower the triglyceride levels, potentially alleviating the ongoing pancreatic inflammation. Interventions such as Gemfibrozil therapy, Ezetimibe therapy, Metformin therapy or weight loss are generally long-term strategies. 4) CONCLUDING: Given the patient's current immediate crisis of severe hypertriglyceridemia leading to acute pancreatitis, the need for timely intervention that could quickly reduce serum triglycerides becomes evident. Apheresis can provide such an acute intervention by rapidly decreasing the triglyceride levels, which then could potentially reduce ongoing pancreatic damage. 5) RESPONSE: In this scenario where the patient is suffering from acute pancreatitis likely due to severe hypertriglyceridemia, the most appropriate next step in management would be to significantly decrease the triglyceride levels without any delay. Considering the potential of Apheresis to reduce serum triglycerides rapidly in this acute scenario, I'd determine that the best choice among the listed options is 'Apheresis' (Option A).

**AI_C:** Restating the question: The patient is a 48-year-old male, presenting with severe abdominal pain, nausea, vomiting, and elevated fever. The pain improves when he bends forward, and he has been admitted to the hospital for treatment. Based on the laboratory findings, he has elevated serum triglyceride levels (1500 mg/dL) and amylase (300 U/L), suggesting acute pancreatitis. His symptoms improved after the administration of intravenous fluids and analgesics, and now the question poses the most appropriate next management step, with choices as: Apheresis; Ezetimibe therapy; Gemfibrozil therapy; Metformin therapy; or a weight loss recommendation. Planning: The assumptions begin with the patient's presenting symptoms and laboratory findings consistent with acute pancreatitis, most probably triggered by his high serum triglyceride levels. Acute pancreatitis is a severe inflammatory condition of the pancreas, commonly caused by gallstones, alcohol, and hypertriglyceridemia. Elevated serum amylase, along with his symptoms, aids the diagnosis. Management often starts with fluid therapy, analgesia, and identifying and treating the underlying cause. This patient has been given fluids and analgesics, which have improved his condition. The next step would be handling the profound hypertriglyceridemia. The choices provided in the management question propose various means of modifying his lipid profile or addressing his overweight condition. Clinical Reasoning: Hypertriglyceridemia initiates pancreatitis by leading to the production of free fatty acids in the pancreatic capillaries. This causes capillary plugging, ischemia, and inflammation of the pancreas. Therefore, one should deal with the elevated triglycerides to stop the progression of the disease. Gemfibrozil is a fibrate class medication commonly used for hypertriglyceridemia. It works by decreasing triglyceride synthesis in the liver. Therefore, it takes time to act. Apheresis, on the other hand, is a procedure that could swiftly decrease the triglycerides by mechanically extricating them from the blood. Although effective, it's usually reserved for severe or refractory cases mainly due to its intrusive nature. The other choices, Ezetimibe and Metformin, while effective for dyslipidemia and diabetes, respectively, do not directly lower triglycerides. Weight loss is a long-term strategy and impractical for immediate implementation in the current acute scenario. Concluding: Considering the urgency to reduce the triglyceride level, the most immediate and effective method would be gemfibrozil therapy. Even though apheresis may offer quicker results, it's considered a second-line or salvage therapy and may not be the first choice in this setting. Moreover, the patient's condition is stabilizing with current management, suggesting that the need for aggressive intervention like apheresis might be unwarranted at this point. Weight loss, the remedy to address his overweight status, is more of a long-term management approach and would not have an immediate impact on his triglyceride levels. Response: Therefore, with supported reasoning, the most appropriate next step in the management of this patient would be (C) Gemfibrozil therapy.

**AI_B:** Restating the question: We're discussing a 48-year-old male patient that presented with acute abdominal pain, nausea, and vomiting. The pain radiates to his back and is described as severe. His abdominal examination revealed distention and extreme tenderness in the epigastric area and the left upper quadrant. Results of laboratory tests showed that his serum triglyceride levels are significantly elevated. After the initial management with intravenous fluids and analgesics, his symptoms have reduced. Now, we're tasked with determining the most appropriate next step in the patient's management among five options: Apheresis, Ezetimibe therapy, Gemfibrozil therapy, Metformin therapy, and weight loss. Planning: The patient's symptoms, physical examination findings, and laboratory results suggest acute pancreatitis likely due to severe hypertriglyceridemia. Upon evaluating the presented options for further management, we need to consider the need to rapidly decrease serum triglyceride levels for avoiding further damage to the pancreas and prevent other triglyceride-related complications. Clinical Reasoning: Given the severity of the patient's hypertriglyceridemia (triglyceride levels are 1500 mg/dL), immediate action to reduce these levels is needed. While all options can potentially contribute to a decrease in triglyceride levels, not all may offer the required immediacy or are applicable given the patient's current state. Apheresis is an invasive procedure used to rapidly decrease the concentration of triglycerides. However, it requires specialized equipment and trained personnel, may have potential complications, and is not generally the first course of action for managing hypertriglyceridemia-induced pancreatitis. Ezetimibe and Metformin help control high cholesterol and glucose levels, respectively, and their impact on triglyceride levels is not as potent or direct. Recommending weight loss, while a long-term solution, would not result in an immediate decrease in triglyceride levels. Gemfibrozil is a fibrate, drugs that are known to significantly lower triglyceride levels. They can result in a rapid decrease in triglyceride levels and are best suited for treating hypertriglyceridemia. Concluding: Given the severity and urgency of the patient's condition (acute pancreatitis due to severe hypertriglyceridemia), immediate action must be undertaken to lower his triglyceride levels. While all presented interventions can contribute towards decreasing triglyceride levels, Gemfibrozil therapy is the most suitable option. This therapy is known to rapidly decrease triglyceride levels, with fewer risks compared to invasive procedures like apheresis. Response: The most appropriate next step in managing this patient would be Gemfibrozil therapy (Option C). This therapeutic approach directly addresses the hypertriglyceridemia, which is likely the cause of acute pancreatitis in this patient, offering a rapid reduction in the patient's triglyceride levels. It also poses fewer risks compared to other interventions, like apheresis. The other options, while valuable in specific contexts, do not offer the immediacy required by this case. Ezetimibe and Metformin do not have a direct or potent impact on triglycerides, and while weight loss is an advisable long-term approach, it won't immediately mitigate the patient's hypertriglyceridemia.

**Facilitator** **[Round 3]:** Summary: AI_A, AI_B, AI_C, and AI_D all agree that Gemfibrozil therapy is the most appropriate immediate intervention for the patient. They argue that this medication, a fibrate, is often the first-line treatment for patients with very high triglyceride levels and can reduce these levels by as much as 50%. They also note that while apheresis can rapidly reduce triglyceride levels, it is typically not the first-line treatment and is reserved for severe or refractory cases. Ezetimibe and Metformin, they argue, are not front-line drugs for elevated triglycerides, and weight loss, while valuable in the long term, is not an immediate solution to the problem. AI_E, however, disagrees and argues that apheresis is the most appropriate immediate intervention. They reason that given the patient's severe hypertriglyceridemia and the resulting acute pancreatitis, an immediate intervention that can quickly reduce serum triglycerides is needed. They argue that apheresis can provide such an intervention by rapidly decreasing the triglyceride levels, potentially reducing ongoing pancreatic damage. Question to clarify disagreements: To AI_E: Given that Gemfibrozil therapy is often the first-line treatment for patients with very high triglyceride levels and can reduce these levels by as much as 50%, why do you believe that apheresis, which is typically not the first-line treatment and is reserved for severe or refractory cases, is the most appropriate immediate intervention in this case?. Based on your preceding response, how would you answer the original question: [A 48-year-old man is admitted to the hospital 2 hours after the onset of severe abdominal pain, nausea, and vomiting. The pain radiates to the back but improves when he bends forward. He has no history of serious illness and takes no medications. He does not smoke cigarettes. He drinks two 12-oz beers weekly. He appears restless. He is 173 cm (5 ft 8 in) tall and weighs 90 kg (198 lb); BMI is 30 kg/m² . Temperature is 38.0°C (100.4°F), pulse is 110/min, respirations are 20/min, and blood pressure is 110/60 mm Hg. On pulmonary examination, decreased breath sounds are heard at the lung bases; there is dullness to percussion. Abdominal examination shows distention and severe tenderness to palpation of the epigastrium and left upper quadrant. Laboratory studies show: Hemoglobin: 13.5 g/dL Serum Urea nitrogen: 30 mg/dL Glucose: 120 mg/dL Creatinine: 1.2 mg/dL Cholesterol: 300 mg/dL Triglycerides: 1500 mg/dL Total bilirubin: 1.1 mg/dL Amylase: 300 U/L Administration of intravenous fluids and analgesics is begun. The patient’s abdominal pain, nausea, and vomiting decrease. Which of the following is the most appropriate next step in management? Choose one among the choices below. (A) Apheresis (B) Ezetimibe therapy (C) Gemfibrozil therapy (D) Metformin therapy (E) Recommendation for weight loss]

**AI_E:** Restating the Question: This scenario presents a 48-year-old man who arrived at the hospital with severe abdominal pain, nausea, and vomiting suggestive of an acute abdominal process. His blood tests show elevated levels of serum triglycerides at 1500 mg/dL, which is an indication of hypertriglyceridemia. Apheresis, Ezetimibe therapy, Gemfibrozil therapy, Metformin therapy, or a recommendation for weight loss are being considered as the next best steps in managing his condition. Planning: While all options proposed have considerations on managing elevated triglyceride levels or contributing factors, our main focus should be on immediate resolution of the presenting serious illness, acute pancreatitis, suspected due to severe epigastric pain, nausea, and elevated amylase levels. Acute pancreatitis in this case is likely secondary to hypertriglyceridemia, a known cause of the disease. Therefore, we need to prioritize therapies that reduce triglyceride levels acutely. Clinical Reasoning: Considering the described methods for managing triglyceride levels, Gemfibrozil therapy, a fibrate, is known to lower triglyceride levels effectively over time and could be a solid consideration for chronic management. However, in this acute setting, a more prompt reduction might be necessary to control the driving cause of his acute pancreatitis. Apheresis can cause a rapid reduction in triglyceride levels and might be more suitable in this acute setting. Other options, although useful in managing dyslipidemia and weight, do not offer the acute benefits needed in this situation. Concluding: Given the acuity of the patient's symptoms and the severity of his hypertriglyceridemia, prompt intervention is essential to prevent further complications from his pancreatitis. Therefore, therapies that offer immediate reduction of triglyceride levels would be more suitable in the initial management of this case. Chronic options can be considered at a later time when the acute crisis is resolved. Response: Given the presented facts, apheresis (choice A) would be the most appropriate next step in managing this patient. It offers the quickest reduction in triglyceride levels which is essential in managing his suspected hypertriglyceridemia-induced acute pancreatitis. This is not negating the potential benefits of other options such as Gemfibrozil therapy, but in the setting of an acute crisis, immediate resolution of the causative factors should take precedence.

**AI_D:** RESTATING THE QUESTION: This query seeks to identify the best course of action in managing a hospitalized 48-year-old male patient who presented with severe upper abdominal pain, vomiting, and nausea. The patient’s discomfort becomes less severe when he bends forward, might be a characteristic of pancreatitis. His lab results show significantly elevated cholesterol and triglyceride levels. PLANNING: The current situation is of a patient suffering from pancreatitis most likely due to hypertriglyceridemia (triglyceride level >1000 mg/dL). Hypertriglyceridemia can cause acute pancreatitis as the triglycerides in the blood are hydrolyzed by pancreatic lipase into toxic free fatty acids. The management depends on the severity of the pancreatitis and the serum triglyceride level. CLINICAL REASONING: Choices (A) Apheresis, (C) Gemfibrozil therapy, (D) Metformin therapy, and (E) Recommendation for weight loss are potential options for hypertriglyceridemia management. However, considering the time frame, immediate intervention, such as pharmaceutical therapy, may be the likely step forward. Apheresis is generally reserved for very severe cases of hypertriglyceridemia that haven't responded to other treatments and is unlikely to be the first choice here without more information. We've administered intravenous fluids and provided pain relief, resulting in decreased symptoms, but haven't yet addressed the root issue: the elevated triglycerides. CONCLUDING: Among the given choices for managing hypertriglyceridemia (elevated blood triglycerides), Ezetimibe, and Metformin therapy mechanisms are mainly concentrated on low-density lipoprotein (LDL) cholesterol reduction and blood sugar control, respectively, not directly on lowering triglycerides. Apheresis is an extracorporeal technique, mainly used in critically ill patients with complications like refractory pancreatitis, where the triglyceride level does not decrease despite conventional management. In light of the immediate need to lower triglyceride levels, Gemfibrozil, a fibrate, appears to be the best option. Fibrates are effective in reducing serum triglyceride levels by increasing the activity of lipoprotein lipase, an enzyme that breaks down triglycerides. RESPONSE: So in this scenario, where a prompt reduction of triglyceride levels is required, 'Gemfibrozil therapy' (Option C) would be the most appropriate next step in management, considering it's a first-line pharmaceutical therapy for high triglyceride levels.

**AI_B:** RESTATING THE QUESTION: The question outlines a clinical scenario presenting a 48-year-old man hospitalized with acute abdominal pain, nausea, and vomiting. The patient has been given intravenous fluids and pain relief and is improving. His significant laboratory findings include high cholesterol and severely elevated triglycerides. The question seeks to identify the most appropriate next step in management. Options presented include apheresis, ezetimibe therapy, gemfibrozil therapy, metformin therapy, or a recommendation for weight loss. PLANNING: Key aspects of this question include the patient's current symptoms, significant laboratory results, and the potential contributions of each potential management option. Given that the patient seems to be suffering from acute pancreatitis due to hypertriglyceridemia (triglyceride level >1000 mg/dL), it is worth considering which amongst the therapeutic options could best lower his triglyceride levels rapidly and prevent further pancreatic injury. CLINICAL REASONING: Acute pancreatitis due to severe hypertriglyceridemia requires rapid reduction in serum triglycerides to prevent ongoing lipotoxic injury to the pancreas. Therefore, the most clinically effective intervention would involve a method proven to quickly lower triglycerides. Fibrates such as gemfibrozil can reduce triglyceride levels rather significantly, but it generally takes several days to start working. Ezetimibe and metformin aren't primarily used for reducing triglycerides. Weight loss, while beneficial in the long term, would have no immediate effects on triglyceride levels. Alternatively, apheresis is a process that mechanically removes triglycerides from the blood and can be an effective treatment in severe or refractory cases. CONCLUDING: Considering the overtly elevated triglyceride levels and the acute presentation of pancreatitis in this patient, the immediate intervention to avoid further pancreatic injury is the key. Gemfibrozil, usually the primary choice for triglyceride reduction, may take days to act, which could lead to sustained pancreatic injury. RESPONSE: Therefore, in this particular clinical scenario, given the urgency to reduce the triglyceride level quickly, the most suitable immediate intervention should be (A) Apheresis. While gemfibrozil may be considered a more common first-line treatment, the severity and acute nature of the patient's condition call for more immediate measures. Apheresis, due to its rapid action, fulfills this requirement. On a parallel path, lifestyle modification like weight loss and long-term medication adjustments could be considered as part of long term management.

**AI_A:** RESTATING THE QUESTION: The case we are examining is a 48-year-old man, with no known serious previous medical history, who has been admitted to the hospital with severe abdominal pain and nausea/vomiting. He has no personal history of smoking or high-risk alcohol consumption. His physical and blood examination findings suggest inflammation, possibly within the pancreas, and very high blood triglyceride levels, along with a high cholesterol level. Our task is to determine the most appropriate next step in the management of this patient's health condition from the listed options. This will involve understanding the patient's symptoms, relaying clinical knowledge on each treatment option, and evaluating the beneficial impact they could have in this particular scenario. PLANNING: Central to this case are our patient's severe abdominal pain, high fever, and high levels of triglycerides in the blood. Looking at these symptoms collectively, they suggest acute pancreatitis, specifically, hypertriglyceridemia-induced pancreatitis. Hypertriglyceridemia refers to a state of abnormally high levels of triglycerides in the blood and can result in the inflammation of the pancreas. To approach his symptoms, we could assume conservatively and treat his high fever and pain first, maintaining hydration and supportive care, and then move on to his hypertriglyceridemia later. Or, we could tackle his very high triglyceride levels head on with a more urgent approach as they're potentially the root of his symptoms. Given the potential life-threatening sequelae of untreated hypertriglyceridemia-induced pancreatitis, the latter alternate assumption that urgent management of hypertriglyceridemia as a priority seems appropriate to pursue. CLINICAL REASONING: Let's relate this to the options. Apheresis, which involves the physical removal of parts of the blood, is an effective way of reducing very high triglyceride levels quickly but it is invasive and generally not the first-line option. Ezetimibe therapy is a drug that helps reduce blood cholesterol levels, not specifically triglyceride levels hence it is not relevant in this case. Gemfibrozil therapy is a treatment option that significantly lowers triglyceride levels, and it is often used in cases like this. Metformin therapy is primarily used for managing diabetes, and it does not address the patient's current needs. Last, recommendation for weight loss is a long-term prevention strategy, helpful for overall management but not the immediate resolution of an acute issue. CONCLUDING: Therefore, considering our patient's current condition and the need for a quick reduction in his triglyceride levels to manage the acute pancreatitis, the administration of Gemfibrozil therapy appears to be the most appropriate immediate next step in this scenario. It offers a balance between a quick response to his condition and a safer, less invasive approach than Apheresis. RESPONSE: (C) Gemfibrozil therapy.

**AI_C:** RESTATING THE QUESTION: The patient, a 48-year-old man, came to the hospital with acute severe abdominal pain, nausea, and vomiting. He shows signs of severe distress, with a high pulse rate and fever. He has physical findings suggestive of a grave digestive tract ailment such as decreased breath sounds, dullness to percussion and tenderness in the upper abdominal quadrants. His laboratory tests are remarkable for elevated urea nitrogen and drastic elevation of triglycerides to 1500 mg/dL, which are suggestive of an acute pancreatitis likely secondary to severe hypertriglyceridemia. Given his symptoms have somewhat subsided following initial supportive drugs and fluids, we need to determine the next best step in management from a list of options, namely, apheresis, ezetimibe therapy, gemfibrozil therapy, metformin therapy, and weight loss recommendation. PLANNING: The alternate assumption would be that his pancreatitis is exacerbated by severe hypertriglyceridemia, which is often a precipitating factor. Considering the triglycerides are drastically high, bringing down the triglycerides levels would be clinically prioritized. The optimal management can be debated between an immediate but infrequently used method like apheresis and a widely accepted first-line drug, namely, a fibrate like gemfibrozil. The options of ezetimibe, metformin, and weight loss do not directly help in acutely lowering the triglycerides and would not be directly of help to the current situation. CLINICAL REASONING: Apheresis is an approach where blood is filtered to remove certain components, often used in situations like drug overdoses or severe lipid abnormalities that are not getting under control with medications. In context, apheresis would rapidly reduce the triglyceride levels in the blood, which might alleviate ongoing pancreatic inflammation resulting from triglyceride metabolism. On the other hand, gemfibrozil, a fibrate, helps lower triglyceride levels directly and is often the first-line management in cases of high triglycerides. Therefore, clinically deciding between the two hinges on whether the potency of the immediate action of apheresis is needed here or whether the more common routine drug therapy is sufficient. CONCLUDING: Given this patient is suffering from acute pancreatitis, most likely instigated by hypertriglyceridemia, rapid control of the triglycerides is paramount to reducing ongoing pancreatic damage. Although gemfibrozil is typically the first line of management for high triglycerides, in this scenario of acute pancreatitis with significant hypertriglyceridemia, an immediate robust reduction in triglycerides may be needed. However, apheresis is a procedure typically used when drug treatments aren't enough. RESPONSE: The case can be evidently argued for the initiation of gemfibrozil therapy (C). While the immediate significant reduction of triglycerides with apheresis seems attractive, gemfibrozil is a well-established, effective first-line medication for high triglycerides, and the patient's pancreatitis has shown improvement with initial management. Further supportive management alongside gemfibrozil therapy can be anticipated to bring stable amelioration and control of the patient's situation. Given the context, the next appropriate step would be gemfibrozil therapy.

**Facilitator** **[Round 4]:** Summary: AI_A, AI_C, and AI_D suggest that Gemfibrozil therapy is the most appropriate next step in managing the patient's condition. They argue that while Apheresis can rapidly reduce triglyceride levels, it is invasive and not typically the first-line option. Gemfibrozil, a fibrate, is often used in cases like this and offers a balance between a quick response to the patient's condition and a safer, less invasive approach. On the other hand, AI_B and AI_E argue that Apheresis is the most suitable immediate intervention. They believe that due to the severity and acute nature of the patient's condition, immediate measures are required. Apheresis, due to its rapid action, fulfills this requirement. Key Differences: The key differences in the council members' perspectives lie in their approach to managing the patient's condition. AI_A, AI_C, and AI_D prioritize a less invasive, first-line treatment option (Gemfibrozil therapy) that is effective in reducing triglyceride levels, albeit not as rapidly as Apheresis. They believe that the patient's condition can be managed effectively with this approach, given that his pancreatitis has shown improvement with initial management. In contrast, AI_B and AI_E emphasize the need for immediate intervention due to the acute nature of the patient's condition. They argue that Apheresis, despite being more invasive, is necessary to prevent further complications from the patient's pancreatitis. Question for Council Members: Given the differing perspectives, a clarifying question could be: "How do you weigh the need for immediate intervention (via Apheresis) against the potential risks and invasiveness of this procedure, compared to a less invasive but slower-acting first-line treatment (Gemfibrozil therapy)?". Based on your preceding response, how would you answer the original question: [A 48-year-old man is admitted to the hospital 2 hours after the onset of severe abdominal pain, nausea, and vomiting. The pain radiates to the back but improves when he bends forward. He has no history of serious illness and takes no medications. He does not smoke cigarettes. He drinks two 12-oz beers weekly. He appears restless. He is 173 cm (5 ft 8 in) tall and weighs 90 kg (198 lb); BMI is 30 kg/m² . Temperature is 38.0°C (100.4°F), pulse is 110/min, respirations are 20/min, and blood pressure is 110/60 mm Hg. On pulmonary examination, decreased breath sounds are heard at the lung bases; there is dullness to percussion. Abdominal examination shows distention and severe tenderness to palpation of the epigastrium and left upper quadrant. Laboratory studies show: Hemoglobin: 13.5 g/dL Serum Urea nitrogen: 30 mg/dL Glucose: 120 mg/dL Creatinine: 1.2 mg/dL Cholesterol: 300 mg/dL Triglycerides: 1500 mg/dL Total bilirubin: 1.1 mg/dL Amylase: 300 U/L Administration of intravenous fluids and analgesics is begun. The patient’s abdominal pain, nausea, and vomiting decrease. Which of the following is the most appropriate next step in management? Choose one among the choices below. (A) Apheresis (B) Ezetimibe therapy (C) Gemfibrozil therapy (D) Metformin therapy (E) Recommendation for weight loss]

**AI_B:** RESTATING THE QUESTION: The inquiry posed is regarding the most suitable next step to take in the management of a 48-year-old physically restless man who presented with severe abdominal pain, nausea, vomiting and decreased breath sounds at the lung bases. After his symptoms declined following the administration of intravenous fluids and analgesics, the next decision concerns the most appropriate treatment path given his laboratory results, specifically, considerable levels of triglycerides. PLANNING: The assumption would be that this patient has pancreatitis inflicted by hypertriglyceridemia due to the clinical scenario. The factors to consider feature pain alleviation, the implementation of apheresis, various types of pharmaceutical interventions (e.g., ezetimibe, gemfibrozil, metformin), as well as a recommendation for weight loss. CLINICAL REASONING: Based on his clinical presentation and laboratory results, the patient is likely suffering from acute pancreatitis due to severe hypertriglyceridemia, suggested by the presence of severe epigastric pain radiating to the back, nausea, vomiting, and elevated serum amylase. The initial priority in management befalls "pain control, fluid resuscitation, electrolyte repletion, and nutritional support". Given the laboratory results, the concentration of triglycerides warrants high attention. While, apheresis is effective in rapidly reducing triglyceride levels, it's usually reserved for when medical therapy fails. In the light of pharmaceutical approaches, gemfibrozil, a fibrate, could be most fitting. It works primarily by reducing hepatic triglyceride production along with a small increase in lipoprotein lipase activity, which accelerates the clearance of triglycerides from the bloodstream. Ezetimibe operates by reducing intestinal cholesterol absorption, but has little to no effect on triglycerides. Metformin is essentially used in managing type 2 diabetes mellitus but does not significantly impact triglyceride concentrations. Lastly, advising weight loss is essential for long-term management, but not as an immediate intervention in this scenario. CONCLUDING: Considering the immediacy and mechanisms of action, the preference leans toward implementing gemfibrozil therapy rather than resorting to an invasive procedure like apheresis or using other therapeutics which lack explicit impacts on the patient's high triglyceride level. RESPONSE: Therefore, the most appropriate next step in management is (C) Gemfibrozil therapy.

**AI_C:** (1) RESTATING THE QUESTION: This query involves determining the best management strategy for a 48-year-old male patient who has been admitted to hospital with severe abdominal pain, nausea and vomiting – symptoms which seem indicative of pancreatitis. The patient's condition has shown some improvement following administration of intravenous fluids and analgesics, but the next step in his treatment plan needs to be determined. Notably, the patient's laboratory studies reveal significantly elevated levels of serum triglycerides and cholesterol, both of which are relevant to the potential treatment options listed. (2) PLANNING: The assumption guiding this question is to identify the treatment plan which can manage the current acute condition and the underlining triglycerides and cholesterol abnormalities. Alternate assumptions would include potentially looking at managing the patient's weight, considering his BMI is 30 kg/m², or considering long-term management of his lipid profile. However, given the acute nature of his condition (presumably acute pancreatitis caused by hypertriglyceridemia), the most crucial approach to prioritize now should be managing the hypertriglyceridemia, which can result in a resolution of the acute pancreatitis. (3) CLINICAL REASONING: The patient's presentation and lab test results strongly suggest acute pancreatitis secondary to hypertriglyceridemia. Hypertriglyceridemia is an established cause of acute pancreatitis when levels are >1000 mg/dL, as in this patient. First-line management is typically fasting, aggressive hydration, and analgesia, all of which this patient has received with some improvement. To manage the hypertriglyceridemia, several treatments are in consideration, including fibrate therapy (gemfibrozil), as they can drastically reduce serum triglycerides by increasing lipoprotein lipase activity and reducing hepatic production of very-low-density lipoprotein (VLDL). Apheresis can rapidly reduce triglycerides but is more invasive and not typically first-line. Ezetimibe blocks intestinal absorption of cholesterol, but this would not significantly affect serum triglycerides. Metformin is used in the management of type 2 diabetes and can have a modest effect on lowering triglycerides, but is not primarily indicated here. Weight loss would be a good long-term goal for this patient, but it would not have an immediate impact on his triglyceride levels or acute pancreatitis. (4) CONCLUDING: After considering the clinical scenario and contemplating various assumptions, the patient's acute pathology should be considered the priority. An approach that aims to rapidly reduce the serum triglycerides would be the best immediate method to manage the patient's current crisis. (5) RESPONSE: Based on the combination of clinical context and the mechanisms of the therapeutics in question, the most suitable next step in managing this patient would likely be the initiation of Gemfibrozil therapy (Choice C). This would aim to rapidly decrease serum triglyceride levels, thereby alleviating the patient's acute pancreatitis symptoms, while also being a less invasive approach compared to apheresis.

**AI_A:** (1) RESTATEMENT: The explained scenario describes a 48-year-old man who has been admitted to the hospital due to severe abdominal pain, nausea, and vomiting. His abdominal pain radiates to the back but improves when he bends forward. This patient has no history of serious illnesses and does not consume any medications. Lab results suggest that he has high triglycerides and cholesterol levels. With the initial management, his abdominal pain has decreased. We are asked to identify the most optimal next steps in his management. (2) PLANNING: The possible assumptions include that the patient's acute abdominal pain is caused by high triglyceride levels, causing acute pancreatitis. An alternative assumption could be a gallstone pancreatitis given the patient's BMI. However, given that the patient has enormously elevated triglyceride levels (1500 mg/dL), which can be the cause for acute pancreatitis, we should prioritize the management of his hypertriglyceridemia acutely. (3) CLINICAL REASONING: The key clinical finding is the patient’s elevated triglyceride level, which may lead us to consider that this patient might be suffering from acute pancreatitis caused by hypertriglyceridemia. Severe hypertriglyceridemia can cause pancreatitis as excessive chylomicron production can lead to obstruction and inflammation of the pancreatic ducts. This results in excessive activation of trypsin leading to auto-digestion of the pancreas by pancreatic enzymes leading to pancreatitis. Pain radiating to the back and an improvement when bending forward are congruent paucity of symptoms seen in pancreatitis, further bolstering the potential diagnosis. The presence of decreased breath sounds and dullness to percussion at the lung bases is reminiscent of pleural effusion, which can occur due to pancreatitis creating an inflammatory exudate that seeps into the pleural space. (4) CONCLUSION: Based on the clinical reasoning of this patient potentially suffering from acute pancreatitis due to hypertriglyceridemia, the aim of the next management step should focus on reducing serum triglyceride levels. (5) RESPONSE: Amongst the choices given, the most appropriate next step in this patient's management would be (C) Gemfibrozil therapy. Gemfibrozil is a fibrate that works primarily by reducing triglyceride levels (up to 50%) by inhibiting lipolysis and reducing the hepatic extraction of free fatty acids, which are the precursors of triglycerides. The other interventions (including Apheresis, Ezetimibe, and Metformin), although they might have some role in managing hypertriglyceridemia and hypercholesterolemia in the long term, are not the best immediate choices. Apheresis has the swiftest action but is invasive and is not the first-line treatment. Ezetimibe has a limited impact on triglyceride levels, and Metformin, while beneficial in managing associated insulin resistance, acts slowly and won't address the acute need to lower triglyceride levels swiftly. Weight loss is of course, mandatory in overall management, but it is a long-term management strategy not suited for acute scenarios. Hence, Gemfibrozil is the most logical and effective immediate next step in management in this scenario.

**AI_D:** RESTATING THE QUESTION: The inquiry calls for an evaluation of the appropriate next step in a patient's management. The patient, a 48-year-old man, presented with severe abdominal pain, nausea, and vomiting. His abdominal discomfort radiates to the back, but bending forward produces relief. The patient has a record of good general health and no medication usage, no smoking history, and moderate alcohol consumption equivalent to two 12-oz beers a week. Clinical findings include restlessness, BMI of 30 kg/m² indicative of obesity, fever, tachycardia, normal blood pressure, decreased breath sounds and dullness to percussion at the lung bases, and marked tenderness at the epigastrium and left upper quadrant. Laboratory results reveal high serum levels of urea nitrogen, cholesterol, triglycerides, and amylase. Following intravenous hydration and pain relief, the patient's clinical condition improved. The query demands an evaluation of whether to institute (A) Apheresis, (B) Ezetimibe therapy, (C) Gemfibrozil therapy, (D) Metformin therapy, or (E) Recommendation for weight loss as a follow-up management step. PLANNING: The primary assumption in this case is that the patient's symptoms and laboratory findings, particularly the elevated amylase levels and severe epigastric pain radiating to the back, suggest acute pancreatitis. The likely triggering factor might be hypertriglyceridemia, as indicated by the patient's significantly high serum triglyceride level. Notably, acute pancreatitis can occur when serum triglyceride levels exceed 1000 mg/dL. However, the possibility that the pain could also arise from gallstone disease should not be dismissed entirely, even though the information provided does not suggest this. An alternate assumption, therefore, is that management should aim to rapidly lower the triglyceride levels while addressing the abdominal pain and associated symptoms. CLINICAL REASONING: The elevated amylase level and the pattern of abdominal pain point towards acute pancreatitis. An important cause of acute pancreatitis – particularly in the context of this patient's high triglyceride level – can be hypertriglyceridemia, which tends to increase the viscosity of the pancreatic juices leading to their accumulation and hence the onset of pancreatitis. It becomes critical then to rapidly lower the triglyceride levels, while managing the pain and other digestive symptoms. Given the patient’s elevated cholesterol and triglycerides, the initiation of a therapy that lowers lipid levels is warranted. In this setting, drug choices like Ezetimibe, Gemfibrozil, or even recommending weight loss could be effective. Ezetimibe prevents the absorption of cholesterol in the small intestine and is commonly utilized to treat hyperlipidemia, but its effect on serum triglyceride levels is not as substantial. Apheresis is a procedure that can rapidly reduce serum triglyceride levels and could be useful in this setting, but it carries a greater risk due to its invasive nature. CONCLUDING: Gemfibrozil, a fibrate, is used mainly to decrease high triglyceride levels. It is typically considered a first-line option due to its effectiveness and safety profile. Given the patient's acute pancreatitis likely triggered by extreme hypertriglyceridemia, rapid reduction of triglycerides is the key to preventing further pancreatic damage. Thus, Gemfibrozil therapy might be the most effective way to achieve this. RESPONSE: In light of the information given and the predominating theoretical approach, the most appropriate next step in managing this patient's condition would be to institute (C) Gemfibrozil therapy, given its efficacy in effectively lowering high triglyceride levels whilst concurrently managing the symptoms of acute pancreatitis.

**AI_E:** Restating the Question: Let's summarize this case. We are presented with a 48-year-old man who arrived at the hospital with severe abdominal pain, nausea, and vomiting that began two hours ago. The discomfort extends to the back but is somewhat alleviated by bending forward. The patient has no serious illness history, appears restless, and with the given height and weight, is classified as obese. His physical examination is remarkable for decreased breath sounds and dullness to percussion at the lung bases, distended abdomen, and palpation-induced severe epigastric and left upper quadrant tenderness. His labs reveal elevated serum urea nitrogen, glucose, cholesterol, triglycerides, and amylase. Clinical management has started with the administration of intravenous fluids and analgesics, leading to a decrease in abdominal pain, nausea, and vomiting. Now, we are tasked with determining the most appropriate next step in this patient's management, given choices on apheresis, ezetimibe therapy, gemfibrozil therapy, metformin therapy, or recommendation for weight loss. Planning: Before proceeding with clinical reasoning, there are several assumptions in this case. The patient's symptom profile, examination findings, and laboratory values suggest an episode of acute pancreatitis, potentially triggered by hypertriglyceridemia- this being suggested by the markedly elevated triglycerides levels. While there are other causes for acute episodes, without history of stones, alcohol abuse, or other known risk factors, triglycerides emerge as the prime suspect. Moreover, the presence of hypertriglyceridemia could lead to severe complications such as chylomicronemia syndrome, which may cause pancreatitis, eruptive xanthomas, lipemia retinalis, and mental status changes. However, this patient may not be at an imminent risk of developing this syndrome, or he may already have it, which has not yet been confirmed in the case. Clinical Reasoning: Given the initial response to the measures taken (administration of IV fluids and analgesics), it suggests that therapeutic modality should firstly act to curb the possibility of further pancreatitis trigger and control the lipid abnormality. Inspecting the provided choices, we have apheresis – a procedure that rapidly removes triglycerides, though highly invasive. Ezetimibe therapy, primarily indicated for lowering low-density lipoprotein cholesterol, might not significantly impact triglycerides. Metformin therapy is widely used for controlling blood glucose levels in type 2 diabetes mellitus, while the patient's glucose level is elevated, it's not significant enough to implicate diabetes and crucially metformin doesn't address the hypertriglyceridemia. Weight loss is always recommended in such patients, but it's more of a long-term goal than an immediate intervention. Concluding: With that said, the choice reducing down to apheresis or gemfibrozil therapy. While apheresis can quickly reduce triglycerides, it is typically reserved for cases where more conservative measures, like medications and lifestyle changes, are ineffective. Gemfibrozil, on the other hand, effectively lowers triglycerides by reducing hepatic lipoprotein synthesis and is less invasive. Response: Given all these factors, I would argue that the most appropriate next step in this patient's management should be (C) Gemfibrozil therapy. This medication could balance the need for relatively rapid reduction of triglycerides and the lowered invasiveness as compared to apheresis. It would mitigate the immediate danger while serving as an initial step towards long-term management of the patient's hypertriglyceridemia.

**Facilitator** The consensus statement synthesizes the responses of AI_A, AI_B, AI_C, AI_D, and AI_E as follows: The case presents a 48-year-old man with severe abdominal pain, nausea, and vomiting, which are indicative of acute pancreatitis. His pain radiates to the back but improves when he bends forward. The patient's lab results reveal significantly elevated levels of serum triglycerides and cholesterol. After initial management with intravenous fluids and analgesics, his symptoms have decreased. The next step in his management needs to be determined. The primary assumption is that the patient's acute abdominal pain is caused by hypertriglyceridemia-induced acute pancreatitis. This is supported by the patient's enormously elevated triglyceride levels, which can cause pancreatitis due to excessive chylomicron production leading to obstruction and inflammation of the pancreatic ducts. This results in excessive activation of trypsin leading to auto-digestion of the pancreas by pancreatic enzymes, causing pancreatitis. Given the patient's acute condition, the immediate management should focus on reducing serum triglyceride levels. Among the choices given, the most appropriate next step in this patient's management would be Gemfibrozil therapy. Gemfibrozil is a fibrate that works primarily by reducing triglyceride levels by inhibiting lipolysis and reducing the hepatic extraction of free fatty acids, which are the precursors of triglycerides. Other interventions such as Apheresis, Ezetimibe, and Metformin, while potentially beneficial in managing hypertriglyceridemia and hypercholesterolemia in the long term, are not the best immediate choices. Apheresis is invasive and not the first-line treatment. Ezetimibe has a limited impact on triglyceride levels, and Metformin, while beneficial in managing associated insulin resistance, acts slowly and won't address the acute need to lower triglyceride levels swiftly. Weight loss is a long-term management strategy not suited for acute scenarios. Therefore, based on the clinical context and the mechanisms of the therapeutics in question, the most suitable next step in managing this patient would likely be the initiation of Gemfibrozil therapy. This would aim to rapidly decrease serum triglyceride levels, thereby alleviating the patient's acute pancreatitis symptoms, while also being a less invasive approach compared to apheresis.
